# *Dnmt1* links *BCR-ABLp210* to epigenetic tumor stem cell priming in myeloid leukemia

**DOI:** 10.1038/s41375-018-0192-z

**Published:** 2018-06-28

**Authors:** Carolina Vicente-Dueñas, Inés González-Herrero, Lalit Sehgal, Idoia García-Ramírez, Guillermo Rodríguez-Hernández, Belén Pintado, Oscar Blanco, Francisco Javier García Criado, María Begoña García Cenador, Michael R. Green, Isidro Sánchez-García

**Affiliations:** 1 Institute of Biomedical Research of Salamanca (IBSAL), Salamanca, Spain; 2 Experimental Therapeutics and Translational Oncology Program, Instituto de Biología Molecular y Celular del Cáncer, CSIC-USAL, Campus M. de Unamuno s/n, Salamanca, Spain; 3 Department of Lymphoma/Myeloma, University of Texas MD Anderson Cancer Center, Houston, TX USA; 4 Transgenesis Facility CNB-CBMSO, CSIC-UAM, Madrid, Spain; 5 Departamento de Anatomía Patológica, Universidad de Salamanca, Salamanca, Spain; 6 Departamento de Cirugía, Universidad de Salamanca, Salamanca, Spain; 7 Department of Genomic Medicine, University of Texas MD Anderson Cancer Center, Houston, TX USA; 8 Center for Cancer Epigenetics, University of Texas MD Anderson Cancer Center, Houston, TX USA

**Keywords:** Chronic myeloid leukaemia, Cancer stem cells

The clonal nature of cancer evolution dictates that all tumor cells carry the same cancer-initiating genetic lesions. However, the latest findings have shown that the mode of action of oncogenes is not homogeneous throughout the developmental history of the tumor. Studies on different types of hematopoietic and solid tumors have shown that the contribution of some oncogenes to cancer development is mediated through the epigenetic reprogramming of the cancer-initiating target cell [1–4]. Epigenetic reprogramming is therefore a new type of interaction between oncogenes and tumor cells, in which the oncogene primes for cancer development by establishing a new pathological tumor cell identity. The current challenge is to test whether epigenetic remodeling in the absence of the driver oncogene is sufficient for tumorigenesis.

Chronic myeloid leukemia (CML) is a malignancy of hematopoietic stem cells (HSCs) that harbor the Philadelphia (Ph) chromosome [5] and leads to increased abundance of myeloid cells and other non-lymphoid lineages in the blood and bone marrow. The Ph translocation creates the BCR-ABL fusion protein, with nearly all CML patients harboring a breakpoint that results in a 210-kD protein (*BCR-ABLp210*). This oncogene alone is capable of transforming hematopoietic progenitors and inducing CML [5]. The BCR-ABL protein is a constitutively active kinase that can be directly targeted with ABL-specific kinase inhibitors, such as Imatinib. The introduction of these agents that specifically target the cancer-initiating oncogene was a breakthrough in the clinical management of CML. However, treatment with Imatinib or other second-generation ABL kinase inhibitors is not curative and the disease returns upon cessation of the drug or the development of resistance. This is because CML stem cells are not dependent on BCR-ABL activity [6], suggesting that there may be an oncogenic function of *BCR-ABLp210* that can persist following inhibition of ABL kinase activity. In support of this notion, we have previously shown that the transient expression of *BCR-ABLp210* restricted to the hematopoietic stem/progenitor cell (HSPC) compartment of mice is capable of inducing mature myeloid leukemia [7]. In addition, we have also demonstrated that the expression of other oncogenes, such as *Bcl6*, within the HSPC compartment can reprogram HSPCs toward malignancy by fueling epigenetic changes that can be traced from the HSPCs to the malignant cells [1]. These observations led us to hypothesize that *BCR-ABLp210* may function, in part, via epigenetic mechanisms that prime the tumor stem cell for malignant myeloid differentiation.

In order to investigate epigenetic reprogramming by the *BCR-ABLp210* oncogene, we performed reduced-representation bisulfite sequencing to interrogate the DNA methylation landscape in HSPCs (Sca1+Lin−) from wild-type and *Sca1-BCR-ABLp210* transgenic mice. The methods for all experiments are described in detail in supplementary information. This revealed a broad and significant loss of methylation at CpG islands that have a low-to-moderate level of methylation in wild-type HSPCs (Fig. 1a, Table S1). Importantly, this hypomethylation phenotype was conserved in mature myeloid cells from *Sca1-BCR-ABLp210* mice, despite the absence of the *BCR-ABLp210* oncogene in these cells (Fig. 1a, b, Table S2). This shows that transient HSPC-restricted expression of the *BCR-ABLp210* oncogene is capable of inducing significant and lasting changes in DNA methylation that may underlie stem cell reprogramming.

**Fig. 1:**
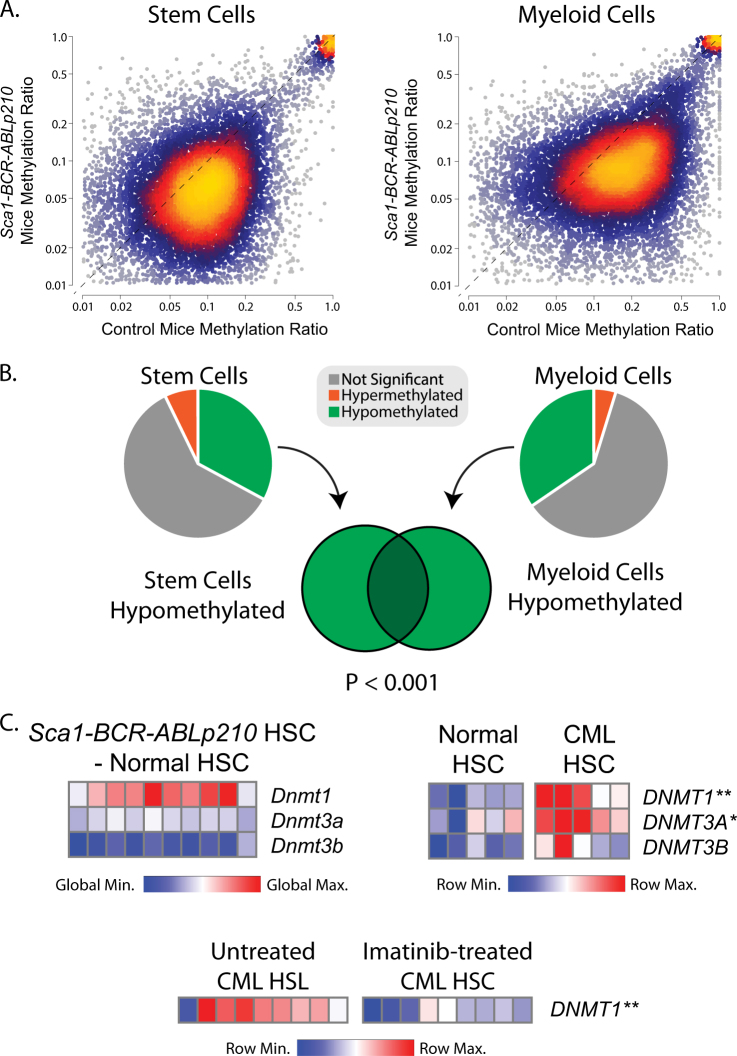
Global hypomethylation and *DNMT1* overexpression associated with BCR-ABL oncogene expression in hematopoietic stem cells (HSCs). **a** Heat scatter plots show RRBS methylation profiling data from the HSPCs (Sca1+Lin−) of wild-type mice compared to those from *Sca1-BCR-ABLp210* transgenic mice (left) and from mature myeloid cells of wild type compared to *Sca1-BCR-ABLp210* mice (right). A significant and global loss of DNA methylation can be observed in the HSPCs and myeloid cells from *Sca1-BCR-ABLp210* mice. **b** Pie graphs and a Venn diagram illustrate that the majority of the changes in DNA methylation in each cellular compartment were hypomethylation, and the regions of hypomethylation significantly overlapped in HSPCs and mature myeloid cells. **c** Heat maps show the expression of DNA methyltransferases in murine and human HSCs. The relative expression of *Dnmt1*, but not other DNA methyltransferases, is higher in HSC from *Sca1-BCR-ABLp210* transgenic mice compared to wild-type mice (top left) and in HSC from CML patients compared to those from healthy donors (top right). The expression of *DNMT1* in HSC from CML patients is significantly reduced by BCR-ABL inhibition (bottom). *P < 0.05, **P < 0.01

Prior studies have shown that DNA methyltransferase (DNMT) genes, including *DNMT1*, are aberrantly overexpressed in myeloid leukemias [8]. In line with this, gene expression profiling (GEP) data from HSPCs of *Sca1-BCR-ABLp210* mice compared to those from wild-type mice [7] showed upregulation of *Dntm1* and downregulation of *Dnmt3b* expression, with no change in *Dnmt3a* expression (Fig. 1c). Higher expression of *DNMT1* was also observed in GEP data from human CML stem cells compared to normal HSCs [9] (*T* test *P* value = 0.003; Fig. 1c). The expression of *DNMT3A* was also significantly higher (*T* test *P* value = 0.012). Furthermore, GEP data from untreated and Imatinib-treated bone marrow cells of CML patients [10] showed that inhibition of BCR-ABL significantly reduces *DNMT1* expression (*T* test *P* value = 0.0004; Fig. 1c). Together these data show that *BCR-ABLp210* expression is associated with overexpression of *DNMT1* in both murine and human HSCs and that its expression may be linked to the activity of this oncogene.

The deregulation of DNA methylation by somatic mutations is one of the hallmarks of acute myeloid leukemia. Mutations of *IDH1*, *IDH2*, or *TET2*, function in the same pathway and inhibit the active and passive recycling of 5-methylcytosine to cytosine thereby inducing global hypermethylation. In contrast, mutations of the de novo methyltransferase, *DNMT3A*, function as dominant negative to inhibit the formation of DNMT3A homotetramers that efficiently methylate cytosine and leads to global hypomethylation. The DNMT3A protein performs its role as a de novo methyltransferase following its recruitment by other transcriptionally repressive complexes such as the polycomb repressor 2 (PRC2) complex [11]. DNMT1, DNMT3A, and DNTM3B have each been shown to interact with a similar region of the catalytic subunit of PRC2, EZH2 [11]. It is therefore plausible that overexpression of DNMT1 may sterically hinder the association between EZH2 and DNMT3A. The loss of only a single allele of *Dnmt3a* is sufficient to promote myeloid leukemia in mice, despite causing only modest changes in DNA methylation [12], demonstrating that slight perturbations in Dnmt function are sufficient for leukemogenesis. Human CML also shows disordered DNA methylation [13], but the mechanism for this has not been defined. Deregulation of this axis is therefore clearly important for myeloid malignancies. But there is still not a clear understanding of specific genes or pathways that contribute to myeloid transformation as a result of perturbations in DNA methylation. Our observations therefore led us to investigate whether *Dnmt1* overexpression was mechanistically linked with perturbed DNA methylation and myeloid leukemogenesis.

Transient expression of *BCR-ABLp210* in HSPCs was sufficient to promote myeloid leukemia, and Imatinib treatment leads to downregulation of *Dnmt1* but does not eradicate the disease. We therefore hypothesized that transient expression of *Dnmt1* would also be sufficient for stem cell reprogramming, and we modeled this by expressing *Dnmt1* under control of the endogenous *Sca1* promoter for HSPC-restricted expression (Figs. S1–2). Notably, the expression of *Dnmt1* resulted in DNA hypomethylation in HSPCs similar to that observed in *Sca1-BCR-ABLp210* mice, with the most notable changes at loci with normally low/moderate levels of methylation (Fig. 2a, Table S3). No significant alterations were observed in the frequencies of major hematologic subsets in the bone marrow of young *Sca1-Dnmt1* mice (3–7 months), but there was a significant expansion of myeloid cells in the peripheral blood compared to wild-type mice (Fig. 2a). Although the DNA hypomethylation phenotype persisted in the HSPCs of older mice (16–24 months, Fig. 2a, Table S4), there was no significant difference in myeloid cells in either the bone marrow or the peripheral blood at this stage. The HSPC-restricted expression of *Dnmt1* is therefore capable of phenocopying the pattern of lasting DNA hypomethylation that was observed in *BCR-ABLp210* mice.

**Fig. 2:**
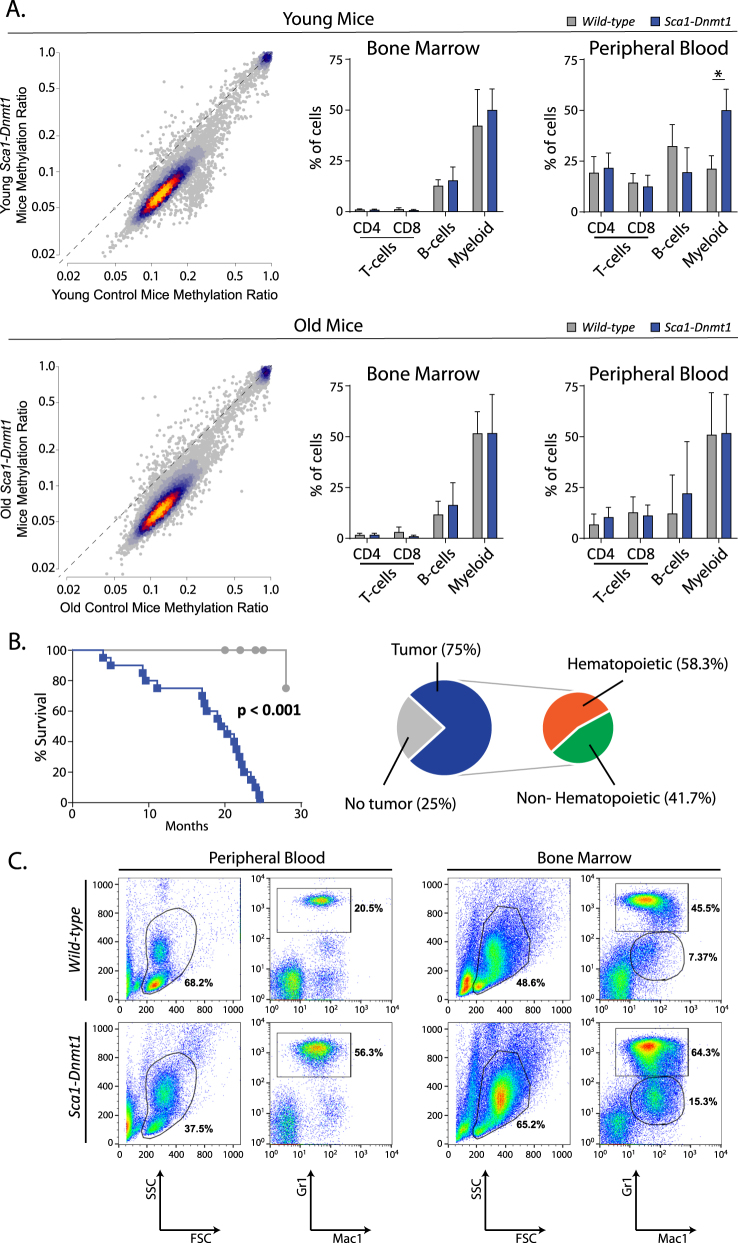
Global hypomethylation and myeloid malignancies induced by *Dnmt1* expression in hematopoietic stem cells. **a** Heat scatter plots show the methylation ratio of CpG islands in the HSPCs from young (top) or old (bottom) wild-type control mice compared to those from young *Sca1-Dnmt1* mice. A clear loss of methylation can be observed in regions with a normally low-to-moderate level of methylation (0.1–0.2) in control mice. Bar graphs summarize the percentage of major hematological populations, interrogated by flow cytometry of bone marrow and peripheral blood of young (top) and old (bottom) wild-type and *Sca1-Dnmt1* mice. No significant differences were observed in the bone marrow at either time point. A significantly increase in the percentage of myeloid cells was observed in the peripheral blood of young disease-free *Sca1-Dnmt1* mice compared to their wild-type counterparts (*T* test *P* value < 0.001), but this was not observed in older mice. **b** Kaplan–Meier plot shows a significant reduction in survival of *Sca1-Dnmt1* mice (blue) compared to their wild-type counterparts (gray). Pie graphs summarize the necropsy results from *Sca1-Dnmt1* mice, with 75% of animals bearing tumors that were primarily of hematopoietic origin. **c** An illustrative example of flow cytometry of diseased *Sca1-Dnmt1* mice compared to age-matched controls shows the expansion of Gr1^+^Mac1^+^ myeloid cells in the blood and bone marrow, as well as the appearance of an abnormal Gr1^low^Mac1^+^ population in the bone marrow

Aging *Sca1-Dnmt1* mice had a shorter lifespan than wild-type mice due to the development of cancer, the majority of which were myeloid malignancies (Fig. 2b). The *Sca1-Dnmt1* mice that developed myeloid malignancies showed marked expansion of Mac1^+^Gr1^+^ granulocytes in the blood and bone marrow (Fig. 2c and S3), as well as the presence of an abnormal myeloid population (Mac1^+^Gr1^low^) in the bone marrow (Fig. 2c). This was also associated with a loss of normal architecture in the spleen (Fig. S4), with tumor-bearing *Sca1-Dnmt1* mice showing atrophic white pulp and hyperplasic red pulp infiltrated by myeloid cells. In the liver, tumor infiltration was accompanied by deposition of an eosinophilic hyaline substance (Fig. S4). A prior study showed that the expression of a hypomorphic *Dnmt1* allele induced global DNA hypomethylation and led to tumors in mice [14], potentially via the promotion of chromosomal instability resulting from the reactivation of endogenous retroviral elements [15]. We did not observe the expression of the *Cdkn2a* (*p19*^*Arf*^*)* gene in bone marrow cells from *Sca1-Dnmt1* mice, indicating the absence of oncogenic stress resulting from chromosome instability (data not shown). Together, these data show that HSPC-restricted expression of *Dnmt1* is sufficient to phenocopy the DNA hypomethylation phenotype induced by *BCR-ABLp210* expression in the same compartment and to promote the development of myeloid malignancies. The deregulation of DNA methylation alone is therefore sufficient to prime HSPCs for the development of myeloid leukemia.

In conclusion, here we have characterized epigenetic reprogramming linked to HSPC-restricted expression of *BCR-ABLp210*, which persists in myeloid cells despite the absence of the oncogene. We identified upregulation of *Dnmt1* as a consequence of *BCR-ABLp210*, and we show that HSPC-restricted expression of *Dnmt1* in transgenic mice is sufficient to phenocopy the *BCR-ABLp210*-associated DNA methylation changes and induce myeloid malignancies. This provides, to our knowledge, the first experimental evidence that epigenetic tumor stem cell reprogramming by itself is sufficient to drive cancer development and establish the tumor cell identity. These observations provide important mechanistic insight into the epigenetic reprogramming of HSC by the *BCR-ABLp210* oncogene and the etiology of CML.

## Electronic supplementary material

Supplementary Methods and Figures

Table S1

Table S2

Table S3

Table S4
