# Supplementary material for: Dnmt1 links BCR-ABLp210 to epigenetic tumor stem cell priming in myeloid leukemia
Source: Leukemia. 2018 Jun 28;33(1):249–78. doi: 10.1038/s41375-018-0192-z (PMC6326950; doi:10.1038/s41375-018-0192-z)
Supplement: Supplementary file 1 — Supplementary Methods and Figures [file 41375_2018_192_MOESM1_ESM.pdf]

Vicente-Dueñas and González-Herrero *et al.*

***Dnmt1* links *BCR-ABLp210* to epigenetic tumor stem cell reprogramming in myeloid leukemia**

SUPPLEMENTARY MATERIALS

Online Methods

Supplementary Figures

Figure S1: Schematic representation of Sca1-Dnmt1 transgene.

Figure S2: Confirmation of Sca1-Dnmt1 mice genotypes

Figure S3: Summary of flow cytometry data for Gr1+ Mac1+ cells in an extended number of Sca1-Dnmt1 mice and age matched controls.

Figure S4: Histology of myeloid tumors in *Sca1-Dnmt1* mice.

Supplementary Tables

Table S1: RRBS methylation changes in HSPCs of *Sca1-BCR-ABLp210* mice compared to wild-type controls.

Table S2: RRBS methylation changes in myeloid cells of *Sca1-BCR-ABLp210* mice compared to wild-type controls.

Table S3: RRBS methylation changes in bone marrow of young *Sca1-Dnmt1* mice compared to wild-type controls.

Table S4: RRBS methylation changes in bone marrow of old *Sca1-Dnmt1* mice compared to wild-type controls.

Online Methods

Generation of *Sca1-BCR-ABLp210* and *Sca1-Dnmt1* transgenic mouse strains

All animal work has been conducted according to relevant national and international guidelines and it has been approved by the Bioethics Committee of University of Salamanca and by the Bioethics Subcommittee of Consejo Superior de Investigaciones Cientificas (CSIC).

The Sca1-Dnmt1 vector was generated by inserting the Dnmt1 cDNA into the ClaI site of the pLy6 vector. The transgene fragment was excised from its vector by restriction digestion with NotI, purified and injected (2 ng/mL) into CBAxC57BL/6J fertilized eggs. Sca1-BCR-ABLp210 transgenic mice have been previously described[1]. Transgenic mice were identified by Southern blot analysis of tail snip DNA after EcoRI digestion, using Dnmt1 and BCR-ABLp210 cDNA respectively to detect the transgene. Upon signs of disease, mice were euthanized and subjected to standard necropsy procedures. All major organs were examined under the dissecting microscope. Tissue samples were taken from homogenous portions of the resected organ and fixed immediately after excision. Samples of each organ were processed into paraffin, sectioned and examined histologically including routinely standard haematoxylin and eosin. Differences in Kaplan-Meier survival plots of transgenic and WT mice were analyzed using the log-rank (Mantel-Cox) test.

Flow cytometry

Nucleated cells were obtained from total mouse bone marrow (flushing from the long bones), peripheral blood, thymus, or spleen. In order to prepare cells for flow cytometry, contaminating red blood cells were lysed with RCLB lysis buffer and the remaining cells were then washed in PBS with 1% FCS. After staining, all cells were washed once in PBS with 1% FCS containing 2 mg/mL propidium iodide (PI) to allow dead cells to be excluded from both analyses and sorting procedures. The samples and the data were acquired in an AccuriC6 Flow Cytometer and analyzed using Flowjo software. Specific fluorescence of FITC, PE, PI and APC excited at 488 nm (0.4 W) and 633 nm (30 mW), respectively, as well as known forward and orthogonal light scattering properties of mouse cells were used to establish gates. Nonspecific antibody binding was suppressed by preincubation of cells with CD16/CD32 Fc-block solution (BD Biosciences). For each analysis, a total of at least 50,000 viable (PI-) cells were assessed.

The following antibodies were used for flow cytometry: anti-B220 (RA3-6B2) (1:100), CD4 (RM4-5) (1:500), CD8a (53-6.7) (1:500), CD11b/Mac1 (M1/70) (1:200), CD19 (1D3) (1:100), CD117/c-Kit (2B8) (1:200), Ly-6G/Gr1 (RB6-8C5) (1:100), IgM (R6-60.2) (1:100) and Sca1/Ly-6A/E (E13-161.7) (1:50) antibodies. Unspecific antibody binding was suppressed by preincubation with CD16/CD32 (2.4G2) (1:100) Fc-block solution. All antibodies were purchased from BD Biosciences.

DNA methylation profiling

EpiQuest library construction. EpiQuest libraries were prepared from 200-500 ng mouse genomic DNA obtained from primary bone marrow cells from Sca1-BCR-ABLp210 and Sca1-Dnmt1 transgenic mice and/or wild-type mice, as performed previously[2]. The DNA was digested with 60 units of TaqI and 30 units of MspI (New England Biolabs) sequentially. Size-selected TaqI-MspI fragments (40-120 bp and 120-350 bp) were filled in and 3'-terminal-A extended, extracted with a DNA Clean & Concentrator kit (Zymo Research). Ligation to pre-annealed adapters containing 5'-methyl-cytosine instead of cytosine was performed using the Illumina DNA preparation kit and protocol. Purified, adaptor ligated fragments were bisulphite-treated using the EZ DNA Methylation-Direct Kit (Zymo Research). Preparative-scale PCR (18 cycles) was performed and purified PCR products were subjected to a final size selection on a 4% NuSieve 3:1 agarose gel. SYBR-green-stained gel slices containing adaptor-ligated fragments of 130-210 bp or 210-460 bp in size were excised. Library material was recovered from the gel using a Zymoclean Gel DNA Recovery Kit (Zymo Research) and sequenced on an GAIIx genome analyzer (Illumina), yielding between 33,461,395 and 61,540,932 total reads for each condition (see table).

Sequence alignments and data analysis. Sequence reads from bisulphite-treated EpiQuest libraries were identified using standard Illumina base-calling software and then analyzed using a Zymo Research proprietary computational pipeline. Residual cytosines (Cs) in each read were first converted to thymines (Ts), with each such conversion noted for subsequent analysis. A reference sequence database was constructed from the 36-bp ends of each computationally predicted MspI-TaqI fragment in the 40-220-bp size range. All Cs in each fragment end were then converted to Ts (only the C-poor strands are sequenced in the RRBS process; The converted reads were aligned to the converted reference by finding all 12-bp perfect matches and then extending to both ends of the treated read, not allowing gaps (reverse complement alignments

were not considered). The number of mismatches in the induced alignment was then counted between the unconverted read and reference, ignoring cases in which a T in the unconverted read is matched to a C in the unconverted reference. For a given read, the best alignment was kept if the second-best alignment had 2 more mismatches; otherwise the read was discarded as non-unique. The mean CpG coverages ranged between 5-11X and total number of unique mapped reads ranged between 1,573,821-21,361,676 for each condition (see table). The methylation level of each sampled cytosine was estimated as the number of reads reporting a C, divided by the total number of reads reporting a C or T. A bioinformatics pipeline was used to score epigenetic alterations according to strength and significance, and links them to potentially affected genes. To that end, we collected a comprehensive set of regions of interest, which includes promoters, CpG islands and repetitive elements. For each of these regions, the number of methylated and unmethylated CpG observations is determined, and a p-value is assigned using Fisher's exact test. Once all p-values are calculated, multiple-testing correction is performed separately for each region type using the q-value method, which controls the false discovery rate to be below a user-specified threshold (typically 10%). The software pipeline is implemented in Python (alignment processing module) and R (statistical analysis module). Unsupervised clustering was performed on methylation ratio data from each condition using Kendall tau rank correlation.

References for Online Methods

1. Perez-Caro M, Cobaleda C, Gonzalez-Herrero I, Vicente-Duenas C, Bermejo-Rodriguez C, Sanchez-Beato M*, et al.* Cancer induction by restriction of oncogene expression to the stem cell compartment. *EMBO J* 2009 Jan 07; **28**(1)**:** 8-20.

2. Green MR, Vicente-Duenas C, Romero-Camarero I, Long Liu C, Dai B, Gonzalez-Herrero I*, et al.* Transient expression of Bcl6 is sufficient for oncogenic function and induction of mature B-cell lymphoma. *Nature communications* 2014; **5:** 3904.

Supplementary Figures

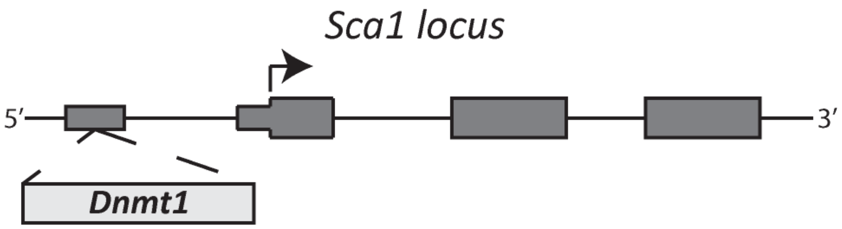

Figure S1: A schematic representation of the *Sca1-Dnmt1* transgene.

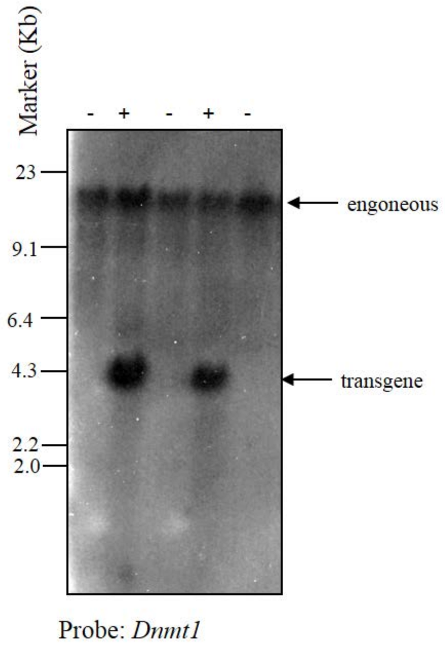

Figure S2: Confirmation of Sca1-Dnmt1 mice genotypes. Identification of the transgenic mice by Southern-blot analysis of tail snip DNA after EcoRI digestion. Sca1-Dnmt1 is indicated. The cDNA of Dnmt1 was used as probe.

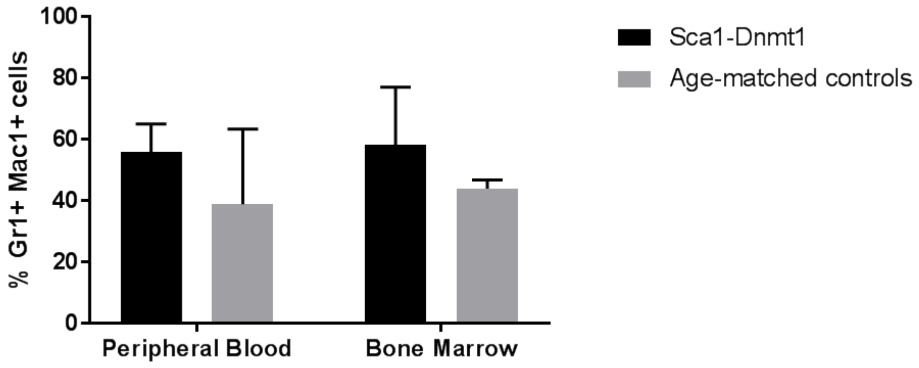

Figure S3: Summary of flow cytometry data for Gr1+ Mac1+ cells in an extended number of Sca1-Dnmt1 mice and age matched controls. Numbers represent the mean of the percentage (+/- SD) of myeloid cells in PB and BM of aged Sca1-Dnmt1 and age-matched control wild-type mice. The Gr1+ Mac1+ population is significantly higher in Sca1-Dnmt1 mice (T-test p-value = 0.0362).

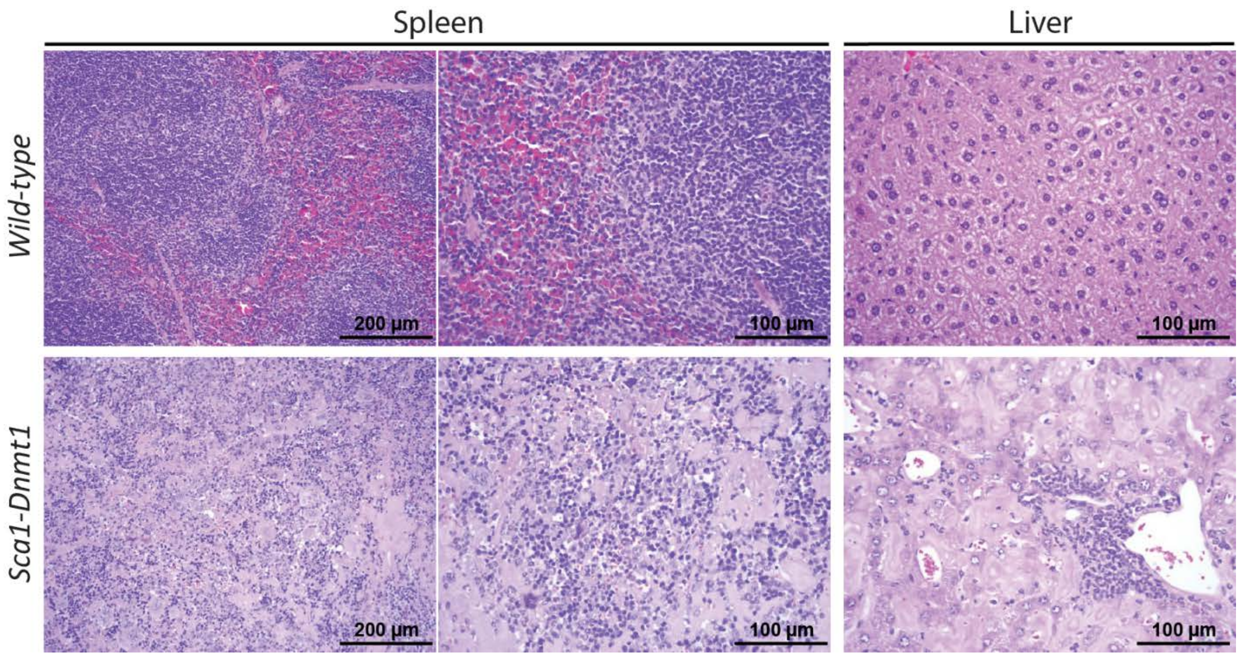

Figure S4: Histology of myeloid tumors in *Sca1-Dnmt1* mice. Illustrative examples of hematoxylin and eosin staining of sections from the spleen and liver of diseased *Sca1-Dnmt1* mice shows a loss of normal architecture resulting from a myeloid tumor. Specifically, atrophic white pulp and hyperplasic red pulp is infiltrated by myeloid cells in the spleen and accompanied by the deposition of an eosinophilic hyaline substance. The liver is also infiltrated by the myeloid tumor formed by mature myeloid cells with a round to oval nucleus that may be flattened on one side and myeloblast with a larger rounder nucleus. Images are representative of 3 replicates. Scale bar represents 200 µm (=200X) and 100 µm (=400X) for inset.
