# Supplementary material for: Dnmt1 links BCR-ABLp210 to epigenetic tumor stem cell priming in myeloid leukemia
Source: Leukemia. 2018 Jun 28;33(1):249–78. doi: 10.1038/s41375-018-0192-z (PMC6326950; doi:10.1038/s41375-018-0192-z)
Supplement: Supplementary file 2 — Table S1 [file 41375_2018_192_MOESM2_ESM.pdf]

Table S1: RRBS methylation changes in HSPCs of Sca1-BCR-ABLp210 mice compared to wild-type controls.

Samples name:	zr151_1	HSPC of wild type mice
	zr151_4	HSPC of Sca1-BCR-ABLp210 mice

chrom	start	end	methDiff (zr151_1.RRBS vs zr151_4)	pValue (zr151_1.RRBS vs zr151_4)	pvalClass (zr151_1.RRBS vs zr151_4)	Gene Symbol	Number of CpGs zr151_1	Number of CpGs zr151_4
chr1	4486494	4488494	-0.32051	0.0000449	hypomethylated	Sox17	13	11
chr1	6203742	6205742	-0.045506	0.0082632	hypomethylated	Rb1cc1	50	50
chr1	7078000	7080000	-0.14345	0.0000842	hypomethylated	Pcmtd1	31	30
chr1	9289811	9291811	-0.30271	0.034474	hypomethylated	Sntg1	9	7
chr1	9537126	9539126	0.51031	0.0021052	stronglyhypermeth	Adhfe1	7	4
chr1	9690290	9692290	-0.11367	0.0000761	hypomethylated	Mybl1	27	20
chr1	9787210	9789210	-0.016957	0.016399	hypomethylated	Sgk3	51	55
chr1	12981216	12983216	-0.3668	0.0032179	stronglyhypometh	Slco5a1	15	11
chr1	16654271	16656271	-0.16502	0.0012302	hypomethylated	Tmem70	31	30
chr1	20810294	20812294	0.22145	0.01821	hypermethylated	Mcm3	3	3
chr1	21069290	21071290	-0.2507	6.87E-22	hypomethylated	Tram2	34	30
chr1	21069306	21071306	-0.26558	4.49E-22	hypomethylated	Tram2	32	28
chr1	25886552	25888552	-0.068914	0.0026321	hypomethylated	Bai3	34	34
chr1	32228650	32230650	-0.20389	0.0000779	hypomethylated	Khdrbs2	26	26
chr1	33964466	33966466	-0.020406	0.033278	hypomethylated	Bend6,Dst	50	50
chr1	34067669	34069669	-0.33185	0.0000288	hypomethylated	Dst	29	27
chr1	34496517	34498517	-0.17832	0.015202	hypomethylated	Ccdc115,Imp4	6	7
chr1	34905803	34907803	-0.15281	0.028523	hypomethylated	Plekhb2	11	10
chr1	36124244	36126244	-0.013906	0.0064231	hypomethylated	Hs6st1	56	55
chr1	36363577	36365577	-0.0030619	0.00063871	hypomethylated	Arid5a	20	20
chr1	36527441	36529441	-0.14103	0.0000071	hypomethylated	Cnnm4	25	24
chr1	36747331	36749331	-0.2915	0.0298	hypomethylated	Cox5b	13	16
chr1	36996372	36998372	-0.10424	0.00000141	hypomethylated	Tmem131	27	28
chr1	37355737	37357737	-0.086022	0.00038149	hypomethylated	Inpp4a	35	30
chr1	37921924	37923924	-0.1009	0.001798	hypomethylated	Tsga10	23	20
chr1	38053854	38055854	-0.037165	8.93E-09	hypomethylated	Eif5b,Txndc9	70	77
chr1	38054053	38056053	-0.03497	9.32E-09	hypomethylated	Eif5b,Txndc9	70	75
chr1	39250116	39252116	-0.066825	0.0077172	hypomethylated	Npas2	50	43
chr1	39956757	39958757	-0.11732	2.01E-09	hypomethylated	Map4k4	73	73
chr1	42751670	42753670	-0.1635	0.00017242	hypomethylated	2610017I09Rik,Pou3f3	28	38
chr1	42907077	42909077	-0.52664	0.00000024	stronglyhypometh	Mrps9	11	11
chr1	43008716	43010716	-0.084307	0.0018019	hypomethylated	Gpr45	27	25
chr1	43220806	43222806	-0.11155	0.021328	hypomethylated	Fhl2	12	10
chr1	43501595	43503595	-0.020314	0.00074621	hypomethylated	Nck2	72	73
chr1	43884553	43886553	-0.25679	1.01E-08	hypomethylated	Uxs1	25	21
chr1	43989851	43991851	-0.13296	0.016253	hypomethylated	Tpp2	26	19
chr1	44175618	44177618	-0.19653	0.021248	hypomethylated	Bivm,Kdelc1	9	4
chr1	44175812	44177812	-0.25486	0.014087	hypomethylated	Bivm,Kdelc1	6	4
chr1	46910354	46912354	-0.019132	0.0019487	hypomethylated	Slc39a10	31	32
chr1	51535243	51537243	-0.15766	0.031938	hypomethylated	Nabp1	11	8
chr1	52557292	52559292	-0.12951	0.012094	hypomethylated	Nab1	15	13
chr1	53352938	53354938	-0.11242	0.0064507	hypomethylated	Ormdl1,Pms1	16	13
chr1	53353840	53355840	-0.11242	0.0064507	hypomethylated	Ormdl1,Pms1	16	13
chr1	53409543	53411543	-0.20443	0.0000892	hypomethylated	Asnsd1	9	9
chr1	53842059	53844059	-0.24437	0.0000302	hypomethylated	Stk17b	14	15
chr1	55293020	55295020	-0.049922	0.0000232	hypomethylated	Mars2	52	53
chr1	57433463	57435463	-0.031447	0.036598	hypomethylated	1700066M21Rik,4930558J18R	32	33
chr1	57463518	57465518	-0.21225	0.020768	hypomethylated	9430016H08Rik,Tyw5	14	7
chr1	58026368	58028368	-0.098722	0.0010071	hypomethylated	Kctd18	37	39
chr1	58026928	58028928	-0.18227	0.0060544	hypomethylated	Kctd18	9	14
chr1	58051817	58053817	-0.035714	0.015393	hypomethylated	Sgol2	7	12
chr1	58642442	58644442	-0.38883	0.0000535	stronglyhypometh	Fam126b,Ndufb3	9	9
chr1	58643177	58645177	-0.38883	0.0000535	stronglyhypometh	Fam126b,Ndufb3	9	9
chr1	59293937	59295937	-0.1437	8.61E-08	hypomethylated	Als2	14	21

chr1	59820480	59822480	-0.04639	0.00042242	hypomethylated	Bmpr2	35	32
chr1	59968849	59970849	-0.082841	0.00019492	hypomethylated	Fam117b	32	34
chr1	60099931	60101931	-0.055972	0.0081564	hypomethylated	Ica1l	36	41
chr1	60154124	60156124	0.1079	0.00029712	hypermethylated	Carf,Wdr12	29	29
chr1	60154751	60156751	0.1079	0.00029712	hypermethylated	Carf,Wdr12	29	29
chr1	60154978	60156978	0.1079	0.00029712	hypermethylated	Carf,Wdr12	29	29
chr1	60399215	60401215	-0.033887	0.0089117	hypomethylated	Cyp20a1	23	26
chr1	60465462	60467462	0.057906	0.0005505	hypermethylated	Abi2	38	30
chr1	60623609	60625609	-0.096004	0.010398	hypomethylated	Raph1	19	24
chr1	61684397	61686397	-0.037297	0.0063384	hypomethylated	Pard3b	49	47
chr1	63160841	63162841	-0.13903	6.98E-08	hypomethylated	Gm11602,Ino80d	11	11
chr1	63222404	63224404	-0.0072731	0.003866	hypomethylated	Eef1b2,Ndufs1	39	40
chr1	63223396	63225396	-0.047781	0.0010595	hypomethylated	Eef1b2,Ndufs1,Snora41	22	22
chr1	63318842	63320842	-0.036917	0.0023032	hypomethylated	Zdbf2	55	54
chr1	64167963	64169963	-0.019446	0.0082428	hypomethylated	Klf7	23	20
chr1	64578377	64580377	-0.047833	0.0000428	hypomethylated	Creb1	66	67
chr1	64663742	64665742	-0.17593	0.0000253	hypomethylated	Mettl21a	9	10
chr1	65110014	65112014	-0.29762	0.043209	hypomethylated	Crygd	3	3
chr1	66220902	66222902	-0.06662	0.0079933	hypomethylated	Map2	21	23
chr1	69732534	69734534	-0.020063	0.0023965	hypomethylated	Ikzf2	36	37
chr1	69872561	69874561	-0.041667	0.012716	hypomethylated	Spag16	10	10
chr1	71602729	71604729	-0.0091868	0.041424	hypomethylated	Atic	24	24
chr1	72258881	72260881	-0.15789	0.0007247	hypomethylated	Mreg	6	6
chr1	72870076	72872076	-0.24399	2.66E-11	hypomethylated	Igfbp2	21	23
chr1	74324173	74326173	-0.12804	0.0085542	hypomethylated	Gpbar1	6	6
chr1	74378183	74380183	-0.0824	0.0022298	hypomethylated	Pnkd	13	14
chr1	74437182	74439182	-0.14295	1.64E-16	hypomethylated	Ctdsp1	57	56
chr1	74551633	74553633	-0.089838	0.0070212	hypomethylated	Rqcd1	31	36
chr1	74900602	74902602	-0.049837	0.01241	hypomethylated	Cdk5r2	58	61
chr1	74947295	74949295	-0.33509	0.0010713	stronglyhypometh	Mir375	13	10
chr1	75138359	75140359	0.16178	0.000011	hypermethylated	Cnppd1,Fam134a	30	30
chr1	75138942	75140942	0.06747	0.00029789	hypermethylated	Cnppd1,Fam134a	25	25
chr1	75164219	75166219	-0.018148	0.00014728	hypomethylated	Zfand2b	25	25
chr1	75164308	75166308	-0.018148	0.00014728	hypomethylated	Zfand2b	25	25
chr1	75206403	75208403	-0.13208	0.01316	hypomethylated	Glb1l,Stk16	14	19
chr1	75207353	75209353	-0.15776	0.0011363	hypomethylated	Glb1l,Stk16	13	18
chr1	75231997	75233997	-0.079725	0.0037355	hypomethylated	Dnajb2	17	17
chr1	75381184	75383184	-0.20962	0.0000619	hypomethylated	Speg	7	10
chr1	75475106	75477106	-0.10223	0.00000109	hypomethylated	Chpf,Tmem198	68	68
chr1	75502651	75504651	-0.080915	0.0000448	hypomethylated	Inha,Obsl1	21	25
chr1	75503027	75505027	-0.35284	0.0029057	stronglyhypometh	Inha,Obsl1	6	9
chr1	77511663	77513663	-0.13036	0.00076071	hypomethylated	Epha4	22	19
chr1	78305920	78307920	-0.12176	0.0036704	hypomethylated	Sgpp2	18	18
chr1	78653399	78655399	-0.048068	0.040842	hypomethylated	Acsl3,Utp14b	37	37
chr1	78654014	78656014	-0.048068	0.040842	hypomethylated	Acsl3,Utp14b	37	37
chr1	79758344	79760344	-0.29138	0.048679	hypomethylated	Wdfy1	9	7
chr1	82312153	82314153	-0.079022	0.0090162	hypomethylated	Rhbdd1	16	14
chr1	82720492	82722492	-0.14416	7.45E-08	hypomethylated	Mff	23	23
chr1	82835057	82837057	-0.053969	0.0000023	hypomethylated	Agfg1	61	60
chr1	84692796	84694796	0.28211	0.005305	hypermethylated	Dner,Mir5126	2	5
chr1	84835415	84837415	-0.049532	0.0000612	hypomethylated	Fbxo36,Trip12	47	48
chr1	84835879	84837879	-0.12775	0.0000175	hypomethylated	Fbxo36,Trip12	22	25
chr1	84931658	84933658	-0.29333	0.002635	hypomethylated	Slc16a14	5	5
chr1	87689021	87691021	-0.075907	3.64E-09	hypomethylated	Cab39	50	37
chr1	87790084	87792084	-0.1616	0.00023104	hypomethylated	Itm2c	16	22
chr1	88198795	88200795	-0.096297	0.027399	hypomethylated	B3gnt7	27	29
chr1	88255148	88257148	0.14088	0.0019042	hypermethylated	C130036L24Rik,Ncl	38	32
chr1	88256030	88258030	0.35225	0.027763	stronglyhypermeth	C130036L24Rik,Ncl	18	12
chr1	88321903	88323903	-0.19444	0.010175	hypomethylated	1700019O17Rik	3	3
chr1	88422310	88424310	-0.050962	0.0015332	hypomethylated	Ptma	85	77
chr1	89159938	89161938	-0.12413	0.0011988	hypomethylated	Efhd1	34	36

chr1	89222572	89224572	-0.1072	0.000000174	hypomethylated	Gigyf2	37	36
chr1	89222603	89224603	-0.1072	0.000000174	hypomethylated	Gigyf2	37	36
chr1	89406937	89408937	-0.063474	0.0089482	hypomethylated	Ngef	17	17
chr1	89515886	89517886	-0.32683	2.84E-08	hypomethylated	Inpp5d	5	5
chr1	89748861	89750861	-0.064826	4.68E-08	hypomethylated	Dgkd	62	62
chr1	90174132	90176132	0.093508	0.01762	hypermethylated	A730008H23Rik,Hjurp	5	5
chr1	90598766	90600766	-0.21598	0.000000172	hypomethylated	Arl4c	16	16
chr1	91350385	91352385	-0.056402	0.00083728	hypomethylated	Agap1	81	94
chr1	92894303	92896303	-0.059039	0.0014092	hypomethylated	Lrrfip1	46	42
chr1	93075398	93077398	-0.063101	0.013127	hypomethylated	Ramp1	12	18
chr1	93145895	93147895	-0.051713	0.00000742	hypomethylated	Ube2f	44	51
chr1	93390244	93392244	-0.065905	0.00051319	hypomethylated	Traf3ip1	44	37
chr1	93697053	93699053	0.095301	0.023534	hypermethylated	Twist2	35	37
chr1	94727262	94729262	-0.079193	0.000000271	hypomethylated	Gpc1	77	78
chr1	94745954	94747954	-0.25	0.031331	hypomethylated	Mir149	4	4
chr1	94806981	94808981	-0.098889	9.14E-14	hypomethylated	Rnpepl1	67	64
chr1	94998442	95000442	-0.10995	0.002007	hypomethylated	Kif1a	12	10
chr1	95030852	95032852	-0.18292	0.0095877	hypomethylated	Agxt	6	6
chr1	95057447	95059447	-0.13637	0.028583	hypomethylated	2310007B03Rik	9	10
chr1	95131473	95133473	-0.073105	0.032898	hypomethylated	Sned1	34	35
chr1	95239221	95241221	-0.053848	0.022302	hypomethylated	Pask,Ppp1r7	28	31
chr1	95239365	95241365	-0.091399	0.00092011	hypomethylated	Pask,Ppp1r7	19	22
chr1	95269472	95271472	-0.30682	0.0036031	hypomethylated	Ano7	8	8
chr1	95651415	95653415	0.087395	0.011258	hypermethylated	Atg4b,Thap4	34	34
chr1	99558595	99560595	-0.10287	1.58E-12	hypomethylated	D1Ertd622e	19	19
chr1	99992209	99994209	-0.11675	0.030857	hypomethylated	Pam	14	10
chr1	107559437	107561437	-0.11146	0.011414	hypomethylated	2310035C23Rik,Pign	30	30
chr1	107560253	107562253	-0.11176	0.017223	hypomethylated	2310035C23Rik,Pign	29	29
chr1	107676299	107678299	-0.082392	0.0000768	hypomethylated	Tnfrsf11a	32	32
chr1	108067445	108069445	-0.025834	0.0010507	hypomethylated	Gm20753,Phlpp1	59	62
chr1	120217419	120219419	-0.4637	0.017883	stronglyhypometh	Mki67ip	15	6
chr1	120284634	120286634	-0.097558	0.047187	hypomethylated	Clasp1	30	31
chr1	120950196	120952196	-0.20252	8.89E-08	hypomethylated	Gli2	27	26
chr1	121318825	121320825	0.067973	0.0039096	hypermethylated	Inhbb	15	15
chr1	121421743	121423743	-0.039596	0.023576	hypomethylated	Tmem185b	30	31
chr1	122016763	122018763	-0.25713	9.81E-08	hypomethylated	3110009E18Rik,Dbi	16	14
chr1	122017496	122019496	-0.37577	5.74E-08	stronglyhypometh	3110009E18Rik,Dbi	16	12
chr1	122017673	122019673	-0.36484	0.0033545	stronglyhypometh	3110009E18Rik,Dbi	10	6
chr1	123224339	123226339	-0.216	0.00083208	hypomethylated	Insig2	18	20
chr1	123326643	123328643	-0.079685	0.0012425	hypomethylated	Ccdc93	17	20
chr1	127456592	127458592	-0.091991	0.000000421	hypomethylated	Slc35f5	22	32
chr1	130139757	130141757	-0.14675	0.0026736	hypomethylated	Ubxn4	22	32
chr1	131168880	131170880	-0.19002	0.0000133	hypomethylated	Thsd7b	24	26
chr1	132612398	132614398	-0.041516	0.0134	hypomethylated	Pfkfb2,Yod1	25	22
chr1	132994120	132996120	-0.10983	0.0000367	hypomethylated	Mapkapk2	26	26
chr1	133806034	133808034	-0.10606	0.00013368	hypomethylated	Nucks1	21	31
chr1	133903181	133905181	-0.06391	0.0035633	hypomethylated	Elk4	33	33
chr1	133964639	133966639	-0.1759	0.0029176	hypomethylated	Mfsd4	7	7
chr1	134087012	134089012	-0.19879	0.023529	hypomethylated	Lemd1	50	39
chr1	134194202	134196202	-0.039728	0.032893	hypomethylated	Klhdc8a	52	50
chr1	134539435	134541435	-0.22225	0.046034	hypomethylated	Nfasc	3	3
chr1	134775931	134777931	-0.052988	0.015884	hypomethylated	Lrrn2	48	46
chr1	134921925	134923925	0.22977	0.011021	hypermethylated	Mdm4	4	4
chr1	135320789	135322789	-0.3373	0.000000584	stronglyhypometh	Sox13	6	6
chr1	135506857	135508857	-0.1715	0.02003	hypomethylated	Snrpe	5	5
chr1	136311043	136313043	-0.060633	0.00080692	hypomethylated	Adipor1	49	54
chr1	136351131	136353131	-0.088725	0.00015211	hypomethylated	Klhl12	13	13
chr1	136390236	136392236	-0.19858	3.06E-10	hypomethylated	Rabif	29	29
chr1	136542257	136544257	-0.057644	0.00066074	hypomethylated	Syt2	22	21
chr1	137661661	137663661	-0.042766	0.0088312	hypomethylated	Phlda3	41	36
chr1	137714174	137716174	-0.27966	0.0000771	hypomethylated	Lad1	26	23

chr1	138242681	138244681	-0.25	0.0033836	hypomethylated	Camsap2	4	4
chr1	138310847	138312847	-0.10585	0.0000292	hypomethylated	9230116N13Rik,Ddx59	27	25
chr1	138312096	138314096	-0.13641	0.00079128	hypomethylated	9230116N13Rik,Ddx59	11	12
chr1	140859285	140861285	-0.11771	0.00000647	hypomethylated	Dennd1b	52	54
chr1	141273653	141275653	-0.37174	0.00614	stronglyhypometh	4933436E23Rik,Crb1	2	2
chr1	148341795	148343795	-0.24508	0.036041	hypomethylated	Fam5c	8	8
chr1	151946253	151948253	-0.36209	0.0014733	stronglyhypometh	7530420F21Rik,Ptgs2	7	7
chr1	151946896	151948896	-0.44615	0.00049644	stronglyhypometh	7530420F21Rik,Ptgs2	4	4
chr1	152840565	152842565	-0.052032	0.00018693	hypomethylated	Hmcn1	9	9
chr1	153190627	153192627	-0.052807	0.0000373	hypomethylated	Ivns1abp	52	51
chr1	153274777	153276777	0.12999	0.00020144	hypermethylated	Swt1,Trmt1l	13	15
chr1	153417502	153419502	-0.020785	0.00029762	hypomethylated	Fam129a	22	13
chr1	154245996	154247996	0.11069	0.010312	hypermethylated	Glt25d2	13	14
chr1	154612671	154614671	-0.057973	1.31E-08	hypomethylated	Arpc5	79	82
chr1	154801230	154803230	-0.07519	0.0028414	hypomethylated	Nmnat2	19	20
chr1	155179916	155181916	-0.0346	0.02472	hypomethylated	Lamc1	25	32
chr1	155586482	155588482	0.076701	0.028089	hypermethylated	Rgs16	14	15
chr1	155746074	155748074	-0.08446	0.00050649	hypomethylated	Glul	51	51
chr1	156946766	156948766	-0.22222	0.0019348	hypomethylated	Ier5	1	1
chr1	157004832	157006832	-0.025566	0.0067182	hypomethylated	Stx6	46	44
chr1	157404249	157406249	0.022898	0.028822	hypermethylated	Acbd6	48	48
chr1	157589157	157591157	-0.046106	0.0099573	hypomethylated	Lhx4	47	40
chr1	157660029	157662029	0.13807	0.00050445	hypermethylated	Qsox1	15	16
chr1	162124390	162126390	-0.14205	0.0057366	hypomethylated	Mrps14	8	10
chr1	162835541	162837541	-0.11324	4.49E-08	hypomethylated	Rc3h1	27	25
chr1	162964296	162966296	0.015795	0.046276	hypermethylated	Gas5,Zbtb37	31	33
chr1	162964390	162966390	0.015795	0.046276	hypermethylated	Gas5,Zbtb37	31	33
chr1	163071842	163073842	-0.48765	0.00099269	stronglyhypometh	Ankrd45	6	9
chr1	163806792	163808792	-0.20414	0.00000796	hypomethylated	Suco	8	10
chr1	163898318	163900318	-0.12607	0.00000144	hypomethylated	4930558K02Rik,Pigc	18	20
chr1	164478476	164480476	-0.056457	0.0091575	hypomethylated	Mettl13	16	11
chr1	165859194	165861194	-0.088761	0.00000516	hypomethylated	Scyl3	42	42
chr1	165924912	165926912	-0.035714	0.033088	hypomethylated	BC055324,Mettl18	11	11
chr1	166178185	166180185	-0.10887	4.59E-08	hypomethylated	Slc19a2	34	31
chr1	166204728	166206728	-0.25325	0.030223	hypomethylated	4930455F23Rik	7	11
chr1	166236805	166238805	-0.24566	0.00034379	hypomethylated	Blzf1,Nme7	15	15
chr1	167124564	167126564	0.17695	0.0020069	hypermethylated	Sft2d2	7	7
chr1	167692910	167694910	-0.27113	0.00000165	hypomethylated	Creg1	22	22
chr1	169215258	169217258	-0.062695	0.042402	hypomethylated	Uck2	10	10
chr1	169279121	169281121	0.042045	0.00033938	hypermethylated	Aldh9a1	22	23
chr1	169618688	169620688	-0.10585	0.00000256	hypomethylated	Lmx1a	43	43
chr1	172105077	172107077	-0.052605	0.013773	hypomethylated	Uap1	20	21
chr1	172237991	172239991	-0.2513	0.018987	hypomethylated	1700015E13Rik	7	7
chr1	172573662	172575662	-0.25121	0.0000123	hypomethylated	Olfml2b	21	21
chr1	172797902	172799902	-0.082937	0.024184	hypomethylated	Atf6	6	5
chr1	173340811	173342811	-0.1782	0.00055476	hypomethylated	Usf1	27	26
chr1	173696262	173698262	-0.35317	0.033824	stronglyhypometh	Slamf1	2	2
chr1	174241537	174243537	0.061111	0.0042081	hypermethylated	Igsf8	11	10
chr1	174305661	174307661	-0.11138	2.74E-11	hypomethylated	Pigm	37	35
chr1	174429376	174431376	-0.0036328	0.01264	hypomethylated	Tagln2	20	16
chr1	177809908	177811908	-0.077307	0.0085682	hypomethylated	Exo1	24	25
chr1	180335011	180337011	-0.082255	0.0086114	hypomethylated	Efcab2	34	30
chr1	181475659	181477659	-0.037059	0.013673	hypomethylated	Cnst,Tfb2m	61	60
chr1	182260680	182262680	-0.083398	0.032752	hypomethylated	Gm5069,Itpkb	48	49
chr1	182498105	182500105	-0.13382	0.012076	hypomethylated	Parp1	17	21
chr1	182655173	182657173	-0.044792	0.025004	hypomethylated	Acbd3	43	33
chr1	182743734	182745734	-0.15598	6.92E-10	hypomethylated	H3f3a	38	26
chr1	182780281	182782281	-0.050035	0.045802	hypomethylated	Sde2	22	22
chr1	184053867	184055867	-0.13824	0.00000023	hypomethylated	Srp9	19	23
chr1	185857877	185859877	-0.2947	0.0030463	hypomethylated	1700056E22Rik,Dusp10	11	11
chr1	186556372	186558372	-0.18128	0.0000565	hypomethylated	Hlx	19	19

chr1	186706915	186708915	-0.098589	0.0060817	hypomethylated	C130074G19Rik	9	9
chr1	186823428	186825428	-0.096338	2.6E-13	hypomethylated	Mark1	69	72
chr1	187277726	187279726	-0.14289	0.0013697	hypomethylated	Gm2061,Slc30a10	31	33
chr1	188790294	188792294	-0.12262	0.0013082	hypomethylated	D1Pas1	23	14
chr1	191167232	191169232	-0.19006	0.018026	hypomethylated	Kcnk2	21	23
chr1	191511965	191513965	-0.072253	0.011151	hypomethylated	Cenpf	10	12
chr1	191551146	191553146	0.078587	0.0000139	hypermethylated	Ptpn14	48	56
chr1	191746167	191748167	-0.29266	0.0043236	hypomethylated	Smyd2	17	22
chr1	191994559	191996559	-0.22196	0.0090369	hypomethylated	Prox1	21	18
chr1	192848229	192850229	-0.044608	0.0093887	hypomethylated	A230020J21Rik,Mfsd7b	40	28
chr1	192885899	192887899	-0.058858	0.00027387	hypomethylated	Nsl1,Tatdn3	18	20
chr1	192886811	192888811	-0.096479	0.00000519	hypomethylated	Nsl1,Tatdn3	15	18
chr1	192921292	192923292	-0.26298	0.00000014	hypomethylated	Batf3	27	23
chr1	193007212	193009212	-0.097471	0.0025945	hypomethylated	Atf3	27	31
chr1	193220920	193222920	-0.0802	0.0075137	hypomethylated	Ppp2r5a	25	25
chr1	193644351	193646351	-0.057204	0.0081263	hypomethylated	Nek2	32	32
chr1	193729659	193731659	-0.10668	7.9E-09	hypomethylated	1700034H15Rik,Slc30a1	53	63
chr1	193731406	193733406	-0.125	0.00076083	hypomethylated	1700034H15Rik,Slc30a1	4	12
chr1	194013753	194015753	-0.1129	2.22E-11	hypomethylated	Kcnh1	39	37
chr1	194861892	194863892	-0.28718	0.0016616	hypomethylated	Syt14	18	14
chr1	194978305	194980305	-0.12504	0.031548	hypomethylated	Irf6	22	22
chr10	3133303	3135303	-0.034157	0.012046	hypomethylated	Cnksr3	34	28
chr10	4423140	4425140	0.18158	0.00085181	hypermethylated	Rgs17	19	19
chr10	5150833	5152833	-0.047646	0.013704	hypomethylated	Syne1	8	8
chr10	5913188	5915188	-0.13137	0.0077117	hypomethylated	1700052N19Rik,Rmnd1	31	37
chr10	5913936	5915936	-0.14232	0.0026264	hypomethylated	1700052N19Rik,Rmnd1	20	20
chr10	5957432	5959432	-0.025142	0.00844	hypomethylated	Zbtb2	56	53
chr10	7308597	7310597	-0.037882	0.000000341	hypomethylated	Lrp11	54	54
chr10	7382168	7384168	-0.11466	0.00036566	hypomethylated	A630066F11Rik,Pcmt1	44	44
chr10	7386301	7388301	-0.26154	0.032566	hypomethylated	Nup43	2	2
chr10	7500715	7502715	0.095454	0.038314	hypermethylated	Ginm1	12	13
chr10	9620838	9622838	-0.042972	0.014902	hypomethylated	Stxbp5	18	20
chr10	12897829	12899829	-0.33333	0.0066019	stronglyhypometh	Zc2hc1b	3	2
chr10	13685184	13687184	-0.31369	8.35E-11	hypomethylated	Hivep2	62	57
chr10	14264842	14266842	-0.15743	0.00017098	hypomethylated	Gpr126	29	28
chr10	17774745	17776745	0.042512	0.0077812	hypermethylated	Reps1	70	74
chr10	17954787	17956787	-0.35714	0.0007094	stronglyhypometh	Ccdc28a	5	5
chr10	18563876	18565876	-0.026245	0.0000161	hypomethylated	Perp	19	19
chr10	19431392	19433392	-0.031406	0.0095878	hypomethylated	Il20ra	18	18
chr10	19653331	19655331	-0.036747	0.020478	hypomethylated	Map3k5	83	84
chr10	20031274	20033274	-0.13759	4.36E-08	hypomethylated	Bclaf1	41	41
chr10	20066624	20068624	-0.15397	0.0071415	hypomethylated	Mtfr2	10	10
chr10	20880790	20882790	-0.093783	0.00000147	hypomethylated	Myb	25	24
chr10	21014784	21016784	-0.28551	9.91E-09	hypomethylated	Hbs1l	25	25
chr10	21877414	21879414	-0.037222	0.017457	hypomethylated	Raet1a	16	20
chr10	21992279	21994279	-0.13654	0.0002466	hypomethylated	H60b	16	19
chr10	22363816	22365816	-0.2527	0.033229	hypomethylated	Slc2a12	12	14
chr10	23069709	23071709	-0.015998	0.041617	hypomethylated	Eya4	33	36
chr10	25078603	25080603	-0.016984	0.020753	hypomethylated	Epb4.1l2	25	21
chr10	25797060	25799060	0.20178	0.00047924	hypermethylated	Gm9767,Tmem200a	35	39
chr10	28862801	28864801	0.080023	0.0019132	hypermethylated	Soga3	12	12
chr10	29920346	29922346	-0.11458	0.016233	hypomethylated	Cenpw	8	7
chr10	31032210	31034210	-0.043425	0.015207	hypomethylated	Hddc2	17	19
chr10	36693349	36695349	-0.032312	0.0055853	hypomethylated	Hdac2	57	59
chr10	38684320	38686320	-0.20143	0.025559	hypomethylated	Lama4	5	5
chr10	38852828	38854828	-0.20381	0.016065	hypomethylated	Fam229b,Tube1	15	13
chr10	40021992	40023992	-0.10737	0.0000232	hypomethylated	Amd1,Amd2	13	12
chr10	40402087	40404087	-0.061156	0.00014063	hypomethylated	Mettl24	30	30
chr10	41238305	41240305	-0.03273	0.0022156	hypomethylated	Cd164	27	19
chr10	42220859	42222859	-0.090881	0.0035192	hypomethylated	Snx3	45	48
chr10	42303394	42305394	-0.13055	0.024703	hypomethylated	Nr2e1	16	15

chr10	42480301	42482301	-0.050861	0.0084728	hypomethylated	Sec63	32	28
chr10	42893509	42895509	-0.10048	0.00014787	hypomethylated	9030612E09Rik,Sobp	58	68
chr10	42894336	42896336	-0.033757	0.015947	hypomethylated	9030612E09Rik,Sobp	24	33
chr10	43197945	43199945	-0.029553	0.0013462	hypomethylated	Bend3	69	74
chr10	43297974	43299974	-0.1021	9.81E-10	hypomethylated	Cd24a	43	48
chr10	43620612	43622612	-0.065411	0.00078127	hypomethylated	Qrsl1,Rtn4ip1	16	15
chr10	45054567	45056567	0.053801	0.037347	hypermethylated	Bves	35	34
chr10	50614456	50616456	-0.13921	0.0003039	hypomethylated	Sim1	38	41
chr10	51741491	51743491	-0.27009	0.00014071	hypomethylated	Vgll2	20	16
chr10	51952424	51954424	-0.017932	0.01972	hypomethylated	Dcbld1	44	45
chr10	52101930	52103930	-0.21726	0.0024683	hypomethylated	Gopc	7	8
chr10	52409306	52411306	-0.0869	0.011594	hypomethylated	Slc35f1	31	31
chr10	57205190	57207190	-0.084334	0.00000196	hypomethylated	4930467K11Rik,Hsf2	46	58
chr10	57785213	57787213	-0.069302	0.0010004	hypomethylated	Lims1	37	47
chr10	58684595	58686595	0.0401	0.043067	hypermethylated	Sept10,Sowahc	19	19
chr10	59568004	59570004	-0.079891	0.0050937	hypomethylated	Spock2	39	39
chr10	60937580	60939580	-0.26885	0.041631	hypomethylated	Lrrc20	16	10
chr10	61110368	61112368	-0.19246	0.01163	hypomethylated	Ppa1	34	30
chr10	61157261	61159261	-0.05346	0.034214	hypomethylated	Tysnd1	47	45
chr10	61693966	61695966	-0.079072	0.0048833	hypomethylated	Tspan15	8	7
chr10	61910907	61912907	0.10256	0.0066706	hypermethylated	4930507D05Rik,Supv3l1	22	21
chr10	62065046	62067046	-0.231	0.0000326	hypomethylated	Ddx21	7	12
chr10	62705544	62707544	-0.11874	1.76E-09	hypomethylated	Herc4	37	34
chr10	62801743	62803743	-0.092785	0.002837	hypomethylated	Sirt1	22	28
chr10	62801780	62803780	-0.092785	0.002837	hypomethylated	Sirt1	22	28
chr10	62801783	62803783	-0.092785	0.002837	hypomethylated	Sirt1	22	28
chr10	66589005	66591005	-0.010221	0.030439	hypomethylated	Jmjd1c	82	83
chr10	68185493	68187493	-0.38931	0.00000177	stronglyhypometh	Tmem26	6	7
chr10	69387279	69389279	-0.34208	0.0037921	stronglyhypometh	Ank3	13	12
chr10	69558868	69560868	-0.035512	0.001527	hypomethylated	Ccdc6	64	57
chr10	70062039	70064039	0.043103	0.032343	hypermethylated	Phyhipl	44	37
chr10	70748010	70750010	-0.16667	0.00096474	hypomethylated	Ube2d1	8	10
chr10	74495298	74497298	-0.10684	0.02761	hypomethylated	Rtdr1	13	13
chr10	74495331	74497331	-0.10684	0.02761	hypomethylated	Rtdr1	13	13
chr10	74522640	74524640	-0.02798	0.0091661	hypomethylated	Bcr	46	55
chr10	74673817	74675817	-0.10567	0.0069178	hypomethylated	Specc1l	25	29
chr10	74674134	74676134	-0.10567	0.0069178	hypomethylated	Specc1l	25	29
chr10	75035337	75037337	-0.12689	0.02229	hypomethylated	Ggt1	4	4
chr10	75261329	75263329	-0.42917	0.010586	stronglyhypometh	Gstt1	4	4
chr10	75355142	75357142	0.085155	0.020761	hypermethylated	Derl3	11	9
chr10	75397317	75399317	-0.036476	0.0075881	hypomethylated	Chchd10	16	16
chr10	75494356	75496356	-0.24682	0.0026178	hypomethylated	Zfp280b	17	16
chr10	75609195	75611195	-0.44792	0.0024338	stronglyhypometh	Slc5a4a	8	8
chr10	75670154	75672154	-0.13333	0.025906	hypomethylated	Mir678	5	5
chr10	75808007	75810007	-0.023129	0.036264	hypomethylated	Dip2a	34	31
chr10	75931859	75933859	0.061342	0.030006	hypermethylated	Mcm3ap,Ybey	12	10
chr10	75993371	75995371	-0.15681	0.000000164	hypomethylated	Lss	11	9
chr10	77084065	77086065	-0.052252	0.002563	hypomethylated	Ube2g2	32	32
chr10	77432617	77434617	0.20745	0.017732	hypermethylated	Trpm2	6	3
chr10	77531112	77533112	-0.042691	0.0054504	hypomethylated	Icosl	31	29
chr10	77707387	77709387	-0.10402	0.016416	hypomethylated	Trappc10	12	12
chr10	77814445	77816445	-0.02903	0.00097422	hypomethylated	Agpat3	47	47
chr10	77927693	77929693	-0.080871	0.048884	hypomethylated	Pdxk	12	12
chr10	79100663	79102663	-0.17188	0.0048233	hypomethylated	Shc2	9	10
chr10	79126318	79128318	-0.2438	0.00028129	hypomethylated	Madcam1	18	18
chr10	79178378	79180378	-0.025042	0.044838	hypomethylated	Hcn2	47	47
chr10	79209326	79211326	-0.13789	0.008681	hypomethylated	Polrmt	3	3
chr10	79229666	79231666	0.24218	0.042786	hypermethylated	Rnf126	17	15
chr10	79229686	79231686	0.26029	0.009154	hypermethylated	Rnf126	16	14
chr10	79239018	79241018	0.038333	0.0097732	hypermethylated	Fstl3	21	19
chr10	79255316	79257316	-0.051806	0.0001033	hypomethylated	Palm	20	20

chr10	79316349	79318349	-0.057305	0.0041423	hypomethylated	E130317F20Rik,Ptbp1	39	39
chr10	79317716	79319716	-0.059504	0.011199	hypomethylated	E130317F20Rik,Ptbp1	31	30
chr10	79371683	79373683	-0.56706	0.0021561	stronglyhypometh	Med16	4	2
chr10	79388816	79390816	-0.094907	0.005014	hypomethylated	Arid3a	51	49
chr10	79450344	79452344	-0.068257	0.030973	hypomethylated	Cnn2	34	34
chr10	79515254	79517254	-0.079058	4.53E-08	hypomethylated	Gpx4	45	44
chr10	79515781	79517781	-0.10409	2.06E-08	hypomethylated	Gpx4	44	43
chr10	79600132	79602132	-0.046787	0.0022228	hypomethylated	Dos	39	37
chr10	79629585	79631585	-0.040392	0.033808	hypomethylated	Cirbp	33	33
chr10	79726735	79728735	-0.05462	0.00024281	hypomethylated	Dazap1	44	44
chr10	79754175	79756175	-0.034707	0.0055639	hypomethylated	Rps15	37	38
chr10	79763564	79765564	-0.32858	0.045121	hypomethylated	Apc2	6	7
chr10	79818203	79820203	-0.13889	0.033803	hypomethylated	Plk5	40	32
chr10	79896398	79898398	0.2066	0.0029941	hypermethylated	Tcf3	8	9
chr10	80040041	80042041	-0.21774	7.49E-10	hypomethylated	Klf16	34	30
chr10	80064624	80066624	0.20875	0.0000145	hypermethylated	Adat3,Scamp4	20	20
chr10	80064626	80066626	0.20875	0.0000145	hypermethylated	Adat3,Scamp4	20	20
chr10	80260479	80262479	-0.12212	0.0000418	hypomethylated	Plekhj1,Sf3a2	63	63
chr10	80488122	80490122	-0.20208	0.0010506	hypomethylated	Diras1	8	13
chr10	80567691	80569691	-0.045077	0.0031628	hypomethylated	Map2k2	30	35
chr10	80729671	80731671	-0.063597	0.00079208	hypomethylated	Apba3,Mrpl54	16	16
chr10	80729916	80731916	-0.12051	0.0011331	hypomethylated	Apba3,Mrpl54	12	12
chr10	80733341	80735341	-0.27418	0.014849	hypomethylated	Mir3057	4	5
chr10	80754012	80756012	-0.086255	0.028736	hypomethylated	Pip5k1c,Tjp3	23	23
chr10	80754716	80756716	-0.16877	0.000000401	hypomethylated	Pip5k1c,Tjp3	36	36
chr10	80782847	80784847	-0.22719	3.25E-08	hypomethylated	Cactin	45	35
chr10	81007791	81009791	-0.021267	0.0024845	hypomethylated	Gna11	22	12
chr10	81021304	81023304	-0.032631	0.00000984	hypomethylated	Aes	40	36
chr10	81037276	81039276	-0.092868	0.037705	hypomethylated	Tle2	12	17
chr10	81704020	81706020	-0.1875	0.023905	hypomethylated	Zfp938	2	3
chr10	82322085	82324085	-0.072288	0.034802	hypomethylated	Txnrd1	30	29
chr10	82821965	82823965	-0.096224	0.000000018	hypomethylated	D10Wsu102e	40	38
chr10	83996633	83998633	-0.13652	0.00012279	hypomethylated	Ckap4	42	44
chr10	84084181	84086181	-0.11861	0.013196	hypomethylated	Polr3b	20	19
chr10	84379360	84381360	0.096631	0.019906	hypermethylated	Ric8b	16	16
chr10	84379400	84381400	0.096631	0.019906	hypermethylated	Ric8b	16	16
chr10	84564371	84566371	-0.1639	4.72E-14	hypomethylated	AI597468,Fhl4	23	26
chr10	84565240	84567240	-0.16727	3.69E-14	hypomethylated	AI597468,Fhl4	23	23
chr10	85060155	85062155	0.2279	0.0069541	hypermethylated	Btbd11	11	12
chr10	85333575	85335575	-0.18125	0.00033992	hypomethylated	Pwp1	16	19
chr10	85762156	85764156	-0.04726	0.015689	hypomethylated	Timp3	18	14
chr10	86167555	86169555	-0.11526	0.0084251	hypomethylated	BC030307,Hsp90b1	17	15
chr10	87819820	87821820	0.25441	0.0054862	hypermethylated	Dram1	5	5
chr10	87841156	87843156	0.051178	0.026928	hypermethylated	Gnptab	48	48
chr10	87921331	87923331	-0.053939	0.042865	hypomethylated	Sycp3	13	13
chr10	89149030	89151030	-0.10741	0.014921	hypomethylated	Scyl2	11	13
chr10	92622620	92624620	-0.032742	0.0000372	hypomethylated	Cdk17,Mir1931	58	51
chr10	94150534	94152534	-0.10068	0.00022605	hypomethylated	4932415G12Rik,Ccdc41	54	53
chr10	94151358	94153358	-0.065508	0.00091414	hypomethylated	4932415G12Rik,Ccdc41	42	42
chr10	94877917	94879917	0.043207	0.0067044	hypermethylated	5730420D15Rik,Socs2	58	59
chr10	94878118	94880118	0.044698	0.0066441	hypermethylated	5730420D15Rik,Socs2	58	58
chr10	94976795	94978795	-0.034167	0.00000187	hypomethylated	Ube2n	60	67
chr10	98376785	98378785	-0.034506	0.0000175	hypomethylated	Atp2b1	111	117
chr10	98568804	98570804	-0.082937	0.0000795	hypomethylated	Galnt4,Poc1b	41	37
chr10	98569768	98571768	-0.094278	0.0010225	hypomethylated	Galnt4,Poc1b	43	42
chr10	106707886	106709886	0.076903	0.0015663	hypermethylated	Lin7a	11	11
chr10	107598455	107600455	-0.081544	0.000054	hypomethylated	Ppp1r12a	58	56
chr10	110356231	110358231	-0.083541	0.0000126	hypomethylated	Csrp2	28	24
chr10	110447122	110449122	0.073112	0.00078728	hypermethylated	Zdhhc17	35	31
chr10	110600857	110602857	-0.041928	0.0074679	hypomethylated	Osbpl8	50	56
chr10	110942341	110944341	-0.11567	0.0034356	hypomethylated	Phlda1	51	52

chr10	111408750	111410750	-0.23484	0.000000108	hypomethylated	Krr1	21	21
chr10	111519409	111521409	-0.14377	0.014661	hypomethylated	Glipr1l2	5	5
chr10	111707178	111709178	-0.40873	0.0059829	stronglyhypometh	Kcnc2	3	6
chr10	115910579	115912579	0.016637	0.00643	hypermethylated	Kcnmb4	33	42
chr10	116585530	116587530	-0.1453	0.003596	hypomethylated	Frs2	13	13
chr10	117147772	117149772	-0.15353	0.0000192	hypomethylated	Mdm2	35	35
chr10	120416386	120418386	-0.095738	0.0000139	hypomethylated	Lemd3	29	29
chr10	120748245	120750245	-0.59921	0.0000139	stronglyhypometh	Tbc1d30	3	3
chr10	121484249	121486249	0.15078	0.011108	hypermethylated	Srgap1	48	48
chr10	122114817	122116817	-0.085648	1.51E-09	hypomethylated	Ppm1h	53	52
chr10	122633979	122635979	-0.042611	0.0035987	hypomethylated	Usp15	19	15
chr10	126414772	126416772	-0.084142	0.00049763	hypomethylated	Ctdsp2	44	43
chr10	126497240	126499240	-0.3025	0.0011583	hypomethylated	March9	11	9
chr10	126499658	126501658	0.11413	0.0017915	hypermethylated	Cdk4	21	23
chr10	126601886	126603886	0.016743	0.0094896	hypermethylated	B4galnt1	57	60
chr10	126626879	126628879	0.19218	0.018156	hypermethylated	Arhgef25	9	10
chr10	126648678	126650678	0.074224	0.045729	hypermethylated	Pip4k2c	14	16
chr10	126702317	126704317	-0.03324	0.010069	hypomethylated	Dctn2	28	29
chr10	126726848	126728848	0.033798	0.0050411	hypermethylated	Ddit3	15	15
chr10	127079041	127081041	-0.069721	0.00081348	hypomethylated	Stat6	16	14
chr10	127175593	127177593	0.019837	0.03837	hypermethylated	Zbtb39	35	35
chr10	127630690	127632690	-0.030841	0.0071751	hypomethylated	Gls2	32	34
chr10	127648850	127650850	-0.25833	0.0014818	hypomethylated	Spryd4	10	10
chr10	127668118	127670118	-0.16118	0.00068363	hypomethylated	Timeless	18	18
chr10	127674121	127676121	-0.37254	0.0045215	stronglyhypometh	Timeless	3	3
chr10	127813179	127815179	-0.088883	0.018793	hypomethylated	Ankrd52	41	38
chr10	127846852	127848852	-0.076176	0.0003015	hypomethylated	Nabp2,Rnf41	14	11
chr10	127984800	127986800	0.24084	0.0010305	hypermethylated	Rpl41,Zc3h10	13	14
chr10	128002990	128004990	-0.20996	0.000000555	hypomethylated	Pa2g4	8	10
chr10	128183934	128185934	-0.037263	0.014086	hypomethylated	Wibg	19	17
chr10	128184577	128186577	-0.031839	0.0089979	hypomethylated	Wibg	21	19
chr10	128241426	128243426	-0.033556	0.0017863	hypomethylated	Dnajc14,Tmem198b	36	34
chr10	128241731	128243731	-0.037093	0.0063558	hypomethylated	Dnajc14,Tmem198b	37	35
chr10	128344974	128346974	-0.20349	0.024394	hypomethylated	Cd63	9	6
chr10	128345921	128347921	-0.13801	0.0000556	hypomethylated	Cd63	39	33
chr10	128369868	128371868	-0.082054	0.0025435	hypomethylated	Itga7	16	16
chr11	3023023	3025023	-0.29048	0.00000161	hypomethylated	Pisd-ps1,Pisd-ps3	5	5
chr11	3023029	3025029	-0.29048	0.00000161	hypomethylated	Pisd-ps1,Pisd-ps3	5	5
chr11	3093466	3095466	-0.096045	1.25E-12	hypomethylated	Sfi1	11	11
chr11	3101355	3103355	0.075035	0.00045257	hypermethylated	Eif4enif1	78	78
chr11	3101585	3103585	0.075035	0.00045257	hypermethylated	Eif4enif1	78	78
chr11	3101997	3103997	0.075035	0.00045257	hypermethylated	Eif4enif1	78	78
chr11	3102021	3104021	0.075035	0.00045257	hypermethylated	Eif4enif1	78	78
chr11	3352328	3354328	-0.20683	0.0000381	hypomethylated	8430429K09Rik,Rnf185	7	10
chr11	3795129	3797129	0.2524	0.0024332	hypermethylated	4921536K21Rik,Dusp18	9	11
chr11	4034162	4036162	-0.29637	1.99E-11	hypomethylated	Rnf215	19	19
chr11	4117253	4119253	-0.058886	0.00000987	hypomethylated	Gatsl3	35	37
chr11	4166097	4168097	-0.24994	0.0068193	hypomethylated	Lif	37	29
chr11	4646781	4648781	0.046035	0.0015228	hypermethylated	Cabp7	38	40
chr11	4848067	4850067	-0.2308	0.0058924	hypomethylated	Ap1b1,Nefh	11	9
chr11	4957130	4959130	-0.046108	0.0000815	hypomethylated	Rasl10a	44	39
chr11	4998440	5000440	-0.10153	0.012373	hypomethylated	Ewsr1,Rhbdd3	36	36
chr11	5344850	5346850	-0.11717	0.017698	hypomethylated	Znrf3	9	9
chr11	5442220	5444220	-0.16691	0.041781	hypomethylated	Ccdc117	3	3
chr11	5661152	5663152	0.44505	0.035423	stronglyhypermeth	2210015D19Rik,Urgcp	8	15
chr11	5687485	5689485	-0.069112	0.0063654	hypomethylated	Dbnl	26	27
chr11	6190599	6192599	-0.065349	0.0026924	hypomethylated	Ogdh	34	34
chr11	6288366	6290366	-0.032291	0.00017648	hypomethylated	Zmiz2	43	38
chr11	6314872	6316872	-0.025297	0.049654	hypomethylated	Ppia	36	36
chr11	6344446	6346446	-0.077952	0.036453	hypomethylated	H2afv	10	10
chr11	6526070	6528070	-0.19258	0.00000219	hypomethylated	Tbrg4	15	15

chr11	9017010	9019010	-0.29583	0.00011116	hypomethylated	Upp1	6	6
chr11	9017105	9019105	-0.29583	0.00011116	hypomethylated	Upp1	6	6
chr11	9017509	9019509	-0.29583	0.00011116	hypomethylated	Upp1	6	6
chr11	11585215	11587215	-0.065351	0.0000393	hypomethylated	Ikzf1	54	67
chr11	11937423	11939423	-0.12015	9.41E-09	hypomethylated	Grb10	41	37
chr11	16651205	16653205	-0.11412	0.00000181	hypomethylated	Egfr	38	32
chr11	17058300	17060300	-0.085257	0.00015437	hypomethylated	Ppp3r1	58	58
chr11	20990326	20992326	-0.093315	0.0000737	hypomethylated	Peli1	33	29
chr11	21270882	21272882	-0.44406	2.46E-11	stronglyhypometh	Ugp2	21	23
chr11	21901654	21903654	-0.0019744	0.0011826	hypomethylated	Otx1	28	31
chr11	22759735	22761735	-0.1613	0.0041675	hypomethylated	B3gnt2	25	19
chr11	22760336	22762336	-0.31173	0.0003833	hypomethylated	B3gnt2	11	6
chr11	22871028	22873028	-0.073359	0.014132	hypomethylated	Zrsr1	32	35
chr11	22882284	22884284	-0.44325	0.0003379	stronglyhypometh	Commd1	8	6
chr11	22889592	22891592	-0.073199	0.00000253	hypomethylated	Cct4	28	28
chr11	23564673	23566673	-0.056533	0.041017	hypomethylated	Pex13,Pus10	18	23
chr11	23564975	23566975	-0.056533	0.041017	hypomethylated	Pex13,Pus10	18	23
chr11	23565935	23567935	-0.054666	0.036795	hypomethylated	Pex13,Pus10	6	5
chr11	23670970	23672970	0.083632	0.00096785	hypermethylated	Rel	26	25
chr11	23979694	23981694	-0.080175	0.00000346	hypomethylated	Bcl11a	25	25
chr11	26110576	26112576	-0.056571	0.04547	hypomethylated	5730522E02Rik	9	9
chr11	29029750	29031750	-0.025803	0.018504	hypomethylated	Pnpt1	15	11
chr11	29071906	29073906	-0.14029	0.0066415	hypomethylated	Smek2	34	31
chr11	31899458	31901458	0.17407	0.0017381	hypermethylated	Nsg2	12	12
chr11	32100279	32102279	-0.38974	0.00011321	stronglyhypometh	Il9r	3	3
chr11	33743585	33745585	-0.39873	0.0000524	stronglyhypometh	Kcnip1	7	5
chr11	35793591	35795591	-0.11226	0.024369	hypomethylated	Wwc1	27	31
chr11	41996432	41998432	-0.325	0.042622	hypomethylated	Gabra1	3	3
chr11	43286845	43288845	-0.11052	0.0063734	hypomethylated	C1qtnf2	18	17
chr11	43341285	43343285	-0.019626	0.0000267	hypomethylated	Ccnjl	39	37
chr11	44430635	44432635	0.024286	0.0083508	hypermethylated	Ebf1	18	26
chr11	45664465	45666465	-0.065846	0.00024876	hypomethylated	Clint1	15	16
chr11	45792811	45794811	-0.071059	0.011761	hypomethylated	Sox30	58	56
chr11	45868488	45870488	-0.16721	0.00014562	hypomethylated	Adam19	16	16
chr11	46520606	46522606	-0.45455	0.0005296	stronglyhypometh	Timd2	2	2
chr11	46623300	46625300	-0.26613	0.010672	hypomethylated	Timd4	4	4
chr11	48630893	48632893	-0.057738	0.048544	hypomethylated	Trim41	12	12
chr11	49422180	49424180	-0.21548	0.007575	hypomethylated	Flt4	35	32
chr11	49659252	49661252	-0.078783	0.000033	hypomethylated	Mapk9	34	34
chr11	49837832	49839832	-0.083983	0.0051995	hypomethylated	Rnf130	11	12
chr11	50190220	50192220	-0.28024	0.0011319	hypomethylated	Hnrnph1	25	29
chr11	51076453	51078453	-0.056499	0.0017628	hypomethylated	BC049762,Clk4	13	13
chr11	51102421	51104421	-0.12819	0.023279	hypomethylated	Col23a1	45	41
chr11	51397258	51399258	-0.19676	0.010596	hypomethylated	Agxt2l2	12	9
chr11	51449398	51451398	-0.18045	5.64E-08	hypomethylated	Rmnd5b	15	15
chr11	51463455	51465455	-0.13293	0.00000394	hypomethylated	D930048N14Rik,N4bp3	35	35
chr11	51502136	51504136	-0.04386	0.017994	hypomethylated	0610009B22Rik	19	18
chr11	51576164	51578164	-0.12238	0.001849	hypomethylated	Sar1b	8	11
chr11	52173616	52175616	-0.053771	0.031906	hypomethylated	Vdac1	38	40
chr11	53137190	53139190	-0.26667	0.020735	hypomethylated	Zcchc10	8	5
chr11	53163268	53165268	-0.014838	0.027022	hypomethylated	Aff4	83	83
chr11	53269706	53271706	-0.051241	0.047825	hypomethylated	Shroom1	29	24
chr11	53582515	53584515	0.1035	0.0001565	hypermethylated	Irf1	23	25
chr11	53582974	53584974	0.1035	0.0001565	hypermethylated	Irf1	23	25
chr11	53705205	53707205	0.085714	0.0036015	hypermethylated	Slc22a5	15	15
chr11	54335348	54337348	-0.16244	0.0068261	hypomethylated	Rapgef6	21	18
chr11	55016841	55018841	0.33318	0.000000235	hypermethylated	Slc36a1	12	13
chr11	55282253	55284253	-0.23503	0.0054671	hypomethylated	G3bp1	29	26
chr11	55421292	55423292	-0.27883	0.0015509	hypomethylated	Glra1	12	7
chr11	57614138	57616138	-0.0049305	0.019838	hypomethylated	Sap30l	70	69
chr11	57645649	57647649	-0.14982	0.025791	hypomethylated	Hand1	17	17

chr11	57821565	57823565	-0.083324	2.72E-15	hypomethylated	Larp1	86	86
chr11	57984155	57986155	-0.091827	0.011642	hypomethylated	Mrpl22	11	11
chr11	58143219	58145219	-0.29025	0.030385	hypomethylated	Sh3bp5l,Zfp672	22	17
chr11	58452966	58454966	-0.16333	0.010947	hypomethylated	Trim58	10	10
chr11	58767186	58769186	-0.080877	0.02757	hypomethylated	Hist3h2a,Hist3h2bb-ps	13	13
chr11	59660574	59662574	-0.080319	0.0011181	hypomethylated	Nt5m	37	37
chr11	59917514	59919514	-0.083784	2.21E-15	hypomethylated	Rai1	58	51
chr11	60165881	60167881	-0.31495	0.00076688	hypomethylated	Lrrc48,Tom1l2	10	15
chr11	60267118	60269118	-0.083288	0.0018159	hypomethylated	Drg2	21	14
chr11	61080629	61082629	-0.20386	0.0075276	hypomethylated	Aldh3a2	11	11
chr11	61307705	61309705	-0.032081	0.0013118	hypomethylated	Mapk7	30	29
chr11	61317673	61319673	-0.11838	0.023986	hypomethylated	B9d1	17	17
chr11	61496911	61498911	-0.12324	0.0000396	hypomethylated	Fam83g	24	33
chr11	62093974	62095974	-0.0040855	0.020605	hypomethylated	Ttc19,Zswim7	63	62
chr11	62094897	62096897	-0.024648	0.00033387	hypomethylated	Ttc19,Zswim7	50	50
chr11	62691991	62693991	-0.19472	0.043902	hypomethylated	Tvp23b	13	17
chr11	63891985	63893985	-0.21415	4.98E-12	hypomethylated	2810001G20Rik,Cox10	16	21
chr11	63892974	63894974	-0.17664	0.0000688	hypomethylated	2810001G20Rik,Cox10	8	13
chr11	64791536	64793536	0.061483	0.046604	hypermethylated	Elac2	16	16
chr11	65619745	65621745	0.037641	0.033311	hypermethylated	Zkscan6	17	17
chr11	68368687	68370687	-0.098512	0.017041	hypomethylated	Mfsd6l	16	8
chr11	68714067	68716067	-0.058362	0.001941	hypomethylated	Gm15772,Rpl26	32	30
chr11	68714090	68716090	-0.058362	0.001941	hypomethylated	Gm15772,Rpl26	32	30
chr11	68912659	68914659	-0.16493	0.0017699	hypomethylated	Per1	13	12
chr11	69208292	69210292	-0.11254	0.0086289	hypomethylated	Cyb5d1,Lsmd1	19	16
chr11	69208848	69210848	-0.046712	0.02851	hypomethylated	Cyb5d1,Lsmd1,Tmem88	15	14
chr11	69211736	69213736	-0.03972	0.0045942	hypomethylated	Tmem88	17	14
chr11	69392826	69394826	-0.095947	0.014731	hypomethylated	Trp53,Wrap53	15	15
chr11	69392860	69394860	-0.08995	0.014704	hypomethylated	Trp53,Wrap53	16	16
chr11	69495472	69497472	-0.4851	0.000095	stronglyhypometh	Senp3	12	11
chr11	69495586	69497586	-0.4851	0.000095	stronglyhypometh	Senp3	12	11
chr11	69571725	69573725	-0.091979	0.0091546	hypomethylated	Polr2a	29	32
chr11	69578413	69580413	-0.14722	0.00040497	hypomethylated	Zbtb4	25	24
chr11	69694487	69696487	0.062073	0.029388	hypermethylated	Kctd11	20	21
chr11	69726693	69728693	-0.16069	1.67E-11	hypomethylated	Gps2	16	22
chr11	69748400	69750400	-0.050637	0.0000163	hypomethylated	Ybx2	70	66
chr11	69777280	69779280	-0.11083	0.0046599	hypomethylated	Cldn7	13	13
chr11	69777922	69779922	-0.074526	0.0027535	hypomethylated	Cldn7	28	28
chr11	69808272	69810272	-0.089443	0.012147	hypomethylated	Phf23	27	27
chr11	69831106	69833106	0.19121	0.0083735	hypermethylated	Dlg4	5	5
chr11	70375382	70377382	-0.050841	0.0046129	hypomethylated	Mink1	26	26
chr11	70513657	70515657	-0.017553	0.0038322	hypomethylated	Inca1,Kif1c	46	46
chr11	70576948	70578948	-0.024641	0.013546	hypomethylated	Zfp3	42	46
chr11	70657264	70659264	-0.10393	3.64E-12	hypomethylated	Rabep1	45	52
chr11	70783464	70785464	-0.2619	0.0074136	hypomethylated	Nup88,Rpain	7	6
chr11	70783475	70785475	-0.2619	0.0074136	hypomethylated	Nup88,Rpain	7	6
chr11	71563204	71565204	-0.053785	0.024351	hypomethylated	Wscd1	36	39
chr11	72016873	72018873	-0.15788	0.0018271	hypomethylated	4933427D14Rik	10	10
chr11	72027639	72029639	0.095937	0.0049224	hypermethylated	4930563E22Rik,Med31	16	17
chr11	72419762	72421762	-0.13427	3.21E-18	hypomethylated	Ube2g1	74	70
chr11	72903096	72905096	-0.49487	0.0012976	stronglyhypometh	Itgae	3	2
chr11	72973031	72975031	-0.083032	0.017376	hypomethylated	P2rx5	21	21
chr11	74403660	74405660	-0.17252	0.0000194	hypomethylated	Rap1gap2	17	17
chr11	74461996	74463996	-0.050408	0.019385	hypomethylated	Cluh	26	26
chr11	74537360	74539360	0.12594	4.21E-08	hypermethylated	Pafah1b1	42	45
chr11	74643425	74645425	-0.11038	0.004931	hypomethylated	Mnt	40	42
chr11	74710581	74712581	-0.10266	0.0064508	hypomethylated	Sgsm2,Tsr1	30	38
chr11	74710582	74712582	-0.10266	0.0064508	hypomethylated	Sgsm2,Tsr1	30	38
chr11	74981654	74983654	-0.041059	0.0023634	hypomethylated	Hic1	58	60
chr11	75006494	75008494	-0.10311	0.00000908	hypomethylated	Rtn4rl1	61	62
chr11	75160934	75162934	0.069333	0.029529	hypermethylated	Rpa1,Smyd4	15	18

chr11	75299278	75301278	-0.11323	0.037198	hypomethylated	Prpf8	14	17
chr11	75344195	75346195	-0.047926	0.00032904	hypomethylated	Slc43a2	46	39
chr11	75344613	75346613	-0.067443	0.00015025	hypomethylated	Slc43a2	41	34
chr11	75400609	75402609	-0.13689	0.0000152	hypomethylated	Pitpna	40	43
chr11	75464010	75466010	-0.039025	0.022919	hypomethylated	Myo1c	18	16
chr11	75464651	75466651	-0.07968	0.035023	hypomethylated	Myo1c	19	20
chr11	75491811	75493811	-0.052384	0.0045959	hypomethylated	Crk	43	39
chr11	75545388	75547388	-0.16893	3.79E-10	hypomethylated	Ywhae	36	40
chr11	76014557	76016557	-0.027404	0.00085908	hypomethylated	Fam57a	43	41
chr11	76056237	76058237	-0.10815	0.0016435	hypomethylated	Glod4,Rnmtl1	29	28
chr11	76057201	76059201	-0.10775	0.011449	hypomethylated	Glod4,Rnmtl1	14	16
chr11	76758157	76760157	-0.11698	0.0000019	hypomethylated	Blmh	26	27
chr11	76811098	76813098	0.15727	0.0021229	hypermethylated	Slc6a4	16	10
chr11	76891674	76893674	-0.061396	0.00013308	hypomethylated	Ccdc55,Mir423	29	28
chr11	76891937	76893937	-0.078927	0.0005185	hypomethylated	Ccdc55,Mir423	20	19
chr11	77276414	77278414	-0.2298	0.0089944	hypomethylated	Coro6	11	9
chr11	77303180	77305180	-0.12099	0.00000135	hypomethylated	Ankrd13b	36	35
chr11	77326775	77328775	0.069623	0.0039861	hypermethylated	Abhd15,Trp53i13	40	39
chr11	77421317	77423317	-0.13766	0.0098861	hypomethylated	Taok1	5	5
chr11	77742790	77744790	-0.12777	0.00000182	hypomethylated	Gm11190,Sez6	40	40
chr11	77743444	77745444	-0.078477	0.0000263	hypomethylated	Gm11190,Sez6	66	66
chr11	77850442	77852442	-0.099101	0.0000162	hypomethylated	Flot2	39	34
chr11	77907174	77909174	0.11696	0.0075925	hypermethylated	Fam222b	7	7
chr11	77990168	77992168	-0.16238	0.00000409	hypomethylated	Nek8,Tlcd1	23	33
chr11	77991267	77993267	-0.10367	0.00047964	hypomethylated	Tlcd1	11	21
chr11	78074255	78076255	-0.026695	0.0025764	hypomethylated	2610507B11Rik	47	47
chr11	78156023	78158023	0.045126	0.038375	hypermethylated	Unc119	20	25
chr11	78325670	78327670	0.15357	0.0013273	hypermethylated	Poldip2,Tmem199	5	5
chr11	78349762	78351762	0.2774	0.0000389	hypermethylated	Ift20,Tnfaip1	17	19
chr11	79068197	79070197	0.37214	0.019532	stronglyhypermeth	Wsb1	4	5
chr11	79775889	79777889	-0.34048	0.0000118	stronglyhypometh	Utp6	7	10
chr11	79805607	79807607	-0.070613	0.00048448	hypomethylated	Suz12	44	43
chr11	79966663	79968663	-0.1851	0.0034608	hypomethylated	Adap2	13	12
chr11	79996373	79998373	-0.038582	0.00023307	hypomethylated	Rnf135	14	14
chr11	80113413	80115413	-0.12072	0.000000359	hypomethylated	Rhbdl3	67	68
chr11	80241116	80243116	-0.048353	0.0031255	hypomethylated	Psmd11	54	54
chr11	80966405	80968405	-0.079327	0.021051	hypomethylated	Asic2	32	33
chr11	81781898	81783898	-0.35595	0.043709	stronglyhypometh	Asic2	6	6
chr11	82684246	82686246	-0.021416	0.021644	hypomethylated	Rffl	34	18
chr11	82764603	82766603	-0.39808	0.00000317	stronglyhypometh	Slfn5	7	8
chr11	83222573	83224573	-0.17322	0.000000834	hypomethylated	Rasl10b	34	37
chr11	83566138	83568138	-0.20232	8.91E-12	hypomethylated	Heatr6	25	27
chr11	83943070	83945070	0.049972	0.042078	hypermethylated	Tada2a	16	14
chr11	84327003	84329003	-0.03466	0.0021132	hypomethylated	Aatf	21	21
chr11	84983597	84985597	-0.28383	0.028908	hypomethylated	1700125H20Rik	10	10
chr11	85047667	85049667	-0.10826	2.26E-08	hypomethylated	Appbp2,D630032N06Rik	70	70
chr11	85123755	85125755	-0.11791	1.57E-08	hypomethylated	Ppm1d	37	31
chr11	85645116	85647116	-0.086211	0.048101	hypomethylated	2610027K06Rik,Tbx2	47	51
chr11	86071070	86073070	-0.34313	0.0094762	stronglyhypometh	Ints2	7	5
chr11	86171027	86173027	-0.093652	0.0040206	hypomethylated	Med13	24	30
chr11	86570994	86572994	-0.029582	0.035991	hypomethylated	Cltc	13	9
chr11	87550066	87552066	-0.061916	0.031476	hypomethylated	Supt4a	14	15
chr11	87800087	87802087	-0.22141	2.44E-12	hypomethylated	Dynll2	34	35
chr11	87860172	87862172	0.047838	0.0155	hypermethylated	Srsf1	32	32
chr11	87911647	87913647	-0.036357	0.000000133	hypomethylated	2210416O15Rik,Cuedc1	59	47
chr11	87982066	87984066	-0.15888	0.0030832	hypomethylated	Cuedc1	16	12
chr11	88579543	88581543	-0.31823	0.000000011	hypomethylated	C030037D09Rik,Msi2	23	20
chr11	90109789	90111789	-0.082095	0.0038927	hypomethylated	Mmd	33	33
chr11	90498497	90500497	-0.21534	0.00000963	hypomethylated	Cox11,Stxbp4	23	14
chr11	90499422	90501422	-0.22248	0.0000275	hypomethylated	Cox11,Stxbp4	22	12
chr11	93746532	93748532	-0.085686	0.00000288	hypomethylated	Mbtd1,Utp18	44	44

chr11	93747080	93749080	-0.09252	0.0000315	hypomethylated	Mbtd1,Utp18	21	15
chr11	93817002	93819002	0.12472	0.00070633	hypermethylated	Nme2	16	14
chr11	93856404	93858404	-0.11415	0.044008	hypomethylated	Spag9	63	55
chr11	94071767	94073767	-0.032055	0.0028522	hypomethylated	Tob1	63	62
chr11	94183225	94185225	-0.27746	0.00000335	hypomethylated	Luc7l3	25	24
chr11	94335512	94337512	-0.075965	0.0000046	hypomethylated	Cacna1g	33	34
chr11	94410521	94412521	-0.21514	0.0042289	hypomethylated	Rsad1	3	4
chr11	94851525	94853525	-0.017519	0.00000197	hypomethylated	Ppp1r9b	54	57
chr11	94938028	94940028	-0.31825	0.00071735	hypomethylated	Itga3	17	13
chr11	95245235	95247235	-0.44548	0.0090037	stronglyhypometh	Slc35b1	11	7
chr11	95375879	95377879	-0.040447	0.0019176	hypomethylated	Nxph3	18	18
chr11	95527270	95529270	-0.31786	0.0077637	hypomethylated	Phb	4	4
chr11	95684813	95686813	-0.015543	0.027073	hypomethylated	Phospho1	41	45
chr11	95776185	95778185	-0.10554	0.00000131	hypomethylated	B4galnt2	13	8
chr11	95867258	95869258	-0.017498	0.01993	hypomethylated	Igf2bp1	50	43
chr11	95936945	95938945	-0.36667	0.0034063	stronglyhypometh	Atp5g1	5	5
chr11	95937008	95939008	-0.36667	0.0034063	stronglyhypometh	Atp5g1	5	5
chr11	96131643	96133643	-0.046076	0.040596	hypomethylated	Hoxb9	33	34
chr11	96146959	96148959	-0.14177	0.0000155	hypomethylated	Hoxb7	43	42
chr11	96163825	96165825	-0.10183	0.0055823	hypomethylated	Hoxb5	14	13
chr11	96226071	96228071	-0.23912	0.013836	hypomethylated	Hoxb1	6	6
chr11	96638869	96640869	-0.029869	0.0024449	hypomethylated	Snx11	14	17
chr11	96649449	96651449	-0.17743	0.011231	hypomethylated	Cbx1	13	12
chr11	96690816	96692816	-0.2522	0.00000383	hypomethylated	Nfe2l1	20	19
chr11	96839002	96841002	-0.18079	0.015734	hypomethylated	Sp2	6	8
chr11	96890265	96892265	-0.20291	0.013453	hypomethylated	Scrn2	10	12
chr11	96911133	96913133	-0.24346	0.00055025	hypomethylated	Osbpl7	8	9
chr11	97011026	97013026	-0.019976	3.61E-08	hypomethylated	Tbkbp1	76	76
chr11	97049206	97051206	-0.069169	1.59E-09	hypomethylated	Kpnb1	41	43
chr11	97436440	97438440	-0.016254	0.0089793	hypomethylated	Srcin1	40	42
chr11	97546265	97548265	-0.37908	5.09E-10	stronglyhypometh	Cisd3	17	17
chr11	97560962	97562962	-0.34383	0.011177	stronglyhypometh	Pcgf2	23	17
chr11	97643080	97645080	-0.25341	0.00000101	hypomethylated	Rpl23,Snora21	24	20
chr11	97847760	97849760	-0.23185	0.0015987	hypomethylated	Plxdc1	13	16
chr11	97887023	97889023	-0.22443	6.61E-11	hypomethylated	Rpl19	24	23
chr11	97887237	97889237	-0.22443	6.61E-11	hypomethylated	Rpl19	24	23
chr11	98010930	98012930	-0.10482	0.00045979	hypomethylated	Fbxl20	30	32
chr11	98411410	98413410	0.081289	0.046365	hypermethylated	Zpbp2	7	7
chr11	98542867	98544867	-0.07367	1.65E-10	hypomethylated	Psmd3	24	30
chr11	98656082	98658082	-0.014253	0.018036	hypomethylated	Msl1	80	81
chr11	98697098	98699098	-0.12358	0.0017411	hypomethylated	Rapgefl1	52	57
chr11	98723911	98725911	-0.21463	0.0020595	hypomethylated	Wipf2	21	11
chr11	98901573	98903573	-0.35546	0.00000762	stronglyhypometh	Igfbp4	21	17
chr11	100180309	100182309	-0.012331	0.0023787	hypomethylated	Eif1	19	25
chr11	100217455	100219455	-0.27456	0.015106	hypomethylated	Hap1	2	2
chr11	100276133	100278133	-0.17011	0.0043075	hypomethylated	Fkbp10,Leprel4	16	14
chr11	100302237	100304237	-0.15447	0.008462	hypomethylated	Klhl10,Nt5c3l	8	6
chr11	100302403	100304403	-0.15447	0.008462	hypomethylated	Klhl10,Nt5c3l	8	6
chr11	100573781	100575781	-0.19454	0.00012466	hypomethylated	Hspb9,Kat2a	18	19
chr11	100574163	100576163	-0.19454	0.00012466	hypomethylated	Hspb9,Kat2a	18	19
chr11	100599444	100601444	0.21222	0.0000663	hypermethylated	Rab5c	9	10
chr11	100621092	100623092	-0.15293	0.01525	hypomethylated	Kcnh4	6	6
chr11	100719664	100721664	0.14125	0.023478	hypermethylated	Stat5a	9	9
chr11	100720797	100722797	0.15806	0.02305	hypermethylated	Stat5a	9	10
chr11	100831931	100833931	0.1587	0.0065168	hypermethylated	Ptrf	7	7
chr11	100869768	100871768	-0.16864	7.04E-10	hypomethylated	Atp6v0a1	22	22
chr11	100956715	100958715	-0.14356	0.010674	hypomethylated	Psmc3ip	13	15
chr11	100981138	100983138	-0.055228	0.043338	hypomethylated	Fam134c,Tubg1	17	19
chr11	101114056	101116056	-0.42828	0.000000023	stronglyhypometh	Vps25	9	9
chr11	101120880	101122880	-0.20858	0.000000046	hypomethylated	Wnk4	36	40
chr11	101176564	101178564	0.049391	0.0011911	hypermethylated	Psme3	27	34

chr11	101285398	101287398	-0.23909	0.00095691	hypomethylated	Ptges3l,Rundc1	24	20
chr11	101302558	101304558	-0.067672	0.00012026	hypomethylated	Rpl27	33	28
chr11	101328651	101330651	-0.067848	0.033206	hypomethylated	Rnd2	24	23
chr11	101488262	101490262	0.18519	0.011295	hypermethylated	Rdm1	6	3
chr11	101525854	101527854	-0.19686	0.024194	hypomethylated	Arl4d	10	9
chr11	101888269	101890269	-0.045833	0.0078667	hypomethylated	Mpp3	12	16
chr11	102046570	102048570	0.33861	0.000000945	stronglyhypermeth	Lsm12	9	11
chr11	102253748	102255748	-0.34758	0.00024478	stronglyhypometh	Rundc3a	13	11
chr11	102525901	102527901	-0.32648	7.46E-08	hypomethylated	Gm1564	36	34
chr11	103128715	103130715	-0.28465	0.0041877	hypomethylated	Map3k14	21	18
chr11	103634488	103636488	-0.0028071	0.0049751	hypomethylated	Wnt3	41	36
chr11	104091749	104093749	-0.17167	0.00000425	hypomethylated	Mapt	25	26
chr11	104410976	104412976	-0.15982	8.99E-08	hypomethylated	Cdc27,Myl4	38	26
chr11	105041843	105043843	-0.020813	0.0014673	hypomethylated	1700052K11Rik,Tlk2	81	71
chr11	105450299	105452299	-0.019766	0.0037411	hypomethylated	Tanc2	47	49
chr11	105805461	105807461	-0.20803	0.000000587	hypomethylated	Cyb561	25	23
chr11	105828260	105830260	-0.3198	0.00011633	hypomethylated	Ace	33	40
chr11	105868516	105870516	0.1486	0.043565	hypermethylated	Kcnh6	11	11
chr11	105897185	105899185	-0.10935	0.0000272	hypomethylated	Dcaf7	38	34
chr11	105945215	105947215	-0.033113	0.0084035	hypomethylated	Map3k3	47	47
chr11	106021456	106023456	-0.118	0.030919	hypomethylated	Limd2	24	29
chr11	106117116	106119116	-0.062462	0.00026607	hypomethylated	Ftsj3,Psmc5	13	12
chr11	106361304	106363304	-0.40676	0.0035359	stronglyhypometh	Snord104	13	11
chr11	106649565	106651565	-0.12394	0.0000289	hypomethylated	Cep95,Ddx5	33	32
chr11	106649808	106651808	-0.14083	0.0000145	hypomethylated	Cep95,Ddx5	29	28
chr11	106888536	106890536	-0.059156	0.00061214	hypomethylated	1810010H24Rik	45	50
chr11	106993236	106995236	-0.041563	0.017399	hypomethylated	Bptf	22	18
chr11	107339841	107341841	-0.37037	0.00093931	stronglyhypometh	Psmd12	9	12
chr11	107655778	107657778	-0.045571	0.0011542	hypomethylated	Cacng4	48	40
chr11	108205202	108207202	-0.19424	1.24E-08	hypomethylated	Prkca	23	23
chr11	108285579	108287579	-0.13794	0.0033015	hypomethylated	Cep112	15	14
chr11	108780662	108782662	-0.050841	0.016048	hypomethylated	Axin2	95	96
chr11	109223107	109225107	-0.0075735	0.014261	hypomethylated	Gm11696,Gna13	59	57
chr11	109286259	109288259	-0.12844	0.0044975	hypomethylated	Amz2	10	13
chr11	109511262	109513262	-0.039481	0.0001947	hypomethylated	Prkar1a	40	49
chr11	113484570	113486570	-0.17708	0.00025688	hypomethylated	Sstr2	10	10
chr11	113509842	113511842	-0.17739	0.00000442	hypomethylated	Cog1	31	32
chr11	113544725	113546725	-0.14406	0.00000394	hypomethylated	D11Wsu47e,Fam104a	38	31
chr11	114657206	114659206	-0.24635	0.000000029	hypomethylated	Btbd17	5	4
chr11	114711845	114713845	-0.098635	0.000067	hypomethylated	Gprc5c	33	38
chr11	114711853	114713853	-0.12458	0.0000358	hypomethylated	Gprc5c	34	38
chr11	114712343	114714343	-0.16594	0.0000168	hypomethylated	Gprc5c	37	34
chr11	114951744	114953744	-0.15956	0.020493	hypomethylated	Rab37	19	14
chr11	115023654	115025654	-0.10192	0.018186	hypomethylated	Slc9a3r1	41	39
chr11	115128557	115130557	-0.11083	0.0041297	hypomethylated	Grin2c	10	10
chr11	115242229	115244229	-0.15566	7.12E-08	hypomethylated	Cdr2l	29	32
chr11	115335990	115337990	0.098364	0.0086447	hypermethylated	Armc7	16	8
chr11	115353128	115355128	-0.20049	0.00024536	hypomethylated	Nt5c	5	5
chr11	115424757	115426757	-0.27273	0.026083	hypomethylated	Nup85	4	4
chr11	115674906	115676906	-0.021048	0.0029223	hypomethylated	Caskin2,Tsen54	55	45
chr11	115971330	115973330	-0.06748	0.033191	hypomethylated	Trim47	32	32
chr11	116141252	116143252	0.26896	0.001508	hypermethylated	Galr2	8	7
chr11	116168052	116170052	-0.16898	0.001297	hypomethylated	Exoc7	6	9
chr11	116294407	116296407	0.058652	0.0014265	hypermethylated	Gm7367,Ubald2	42	46
chr11	116294526	116296526	0.057136	0.001441	hypermethylated	Gm7367,Ubald2	42	47
chr11	116392224	116394224	-0.14761	0.0000507	hypomethylated	Sphk1	25	31
chr11	116392757	116394757	-0.070989	0.027215	hypomethylated	Sphk1	46	52
chr11	116393551	116395551	-0.052582	0.031612	hypomethylated	Sphk1	58	53
chr11	116937096	116939096	-0.28535	9.17E-10	hypomethylated	2810008D09Rik	14	14
chr11	117059974	117061974	-0.13208	0.010387	hypomethylated	Sept9	28	32
chr11	117514602	117516602	0.035748	0.039658	hypermethylated	Tnrc6c	59	54

chr11	117709550	117711550	-0.065618	0.014628	hypomethylated	Birc5	25	25
chr11	118902227	118904227	-0.17576	0.0008159	hypomethylated	Cbx8	39	42
chr11	118947551	118949551	-0.10967	0.00092415	hypomethylated	Cbx4	32	32
chr11	119351660	119353660	-0.04132	0.023854	hypomethylated	Endov	21	17
chr11	119351933	119353933	-0.04132	0.023854	hypomethylated	Endov	21	17
chr11	119409134	119411134	-0.055591	0.0000423	hypomethylated	Gm11762,Nptx1	32	41
chr11	119803405	119805405	-0.037718	0.0000496	hypomethylated	Baiap2	58	57
chr11	119876176	119878176	-0.13925	0.042513	hypomethylated	Mir3065,Mir338	9	11
chr11	120209798	120211798	-0.068506	0.00000662	hypomethylated	0610009L18Rik,Actg1	79	75
chr11	120299014	120301014	-0.8	0.00000324	stronglyhypometh	Nploc4	4	9
chr11	120318442	120320442	-0.091929	0.00064488	hypomethylated	Ccdc137,Oxld1	24	20
chr11	120327948	120329948	-0.040217	0.0206	hypomethylated	Arl16,Hgs	23	21
chr11	120328914	120330914	-0.041068	0.04377	hypomethylated	Arl16,Hgs	15	15
chr11	120352150	120354150	-0.31465	0.00013818	hypomethylated	Slc25a10	22	19
chr11	120459679	120461679	-0.41646	0.0082085	stronglyhypometh	Alyref,Anapc11	13	19
chr11	120575081	120577081	-0.043223	0.01454	hypomethylated	Lrrc45,Stra13	7	7
chr11	120657709	120659709	-0.00064397	0.012916	hypomethylated	Dus1l	14	17
chr11	120852647	120854647	-0.53968	0.00031881	stronglyhypometh	Csnk1d	5	3
chr11	121120300	121122300	-0.042543	0.0001481	hypomethylated	Foxk2	66	70
chr11	121281686	121283686	-0.045186	0.0297	hypomethylated	Fn3krp	18	15
chr11	121380656	121382656	0.18338	7.84E-16	hypermethylated	Zfp750	4	4
chr11	121534465	121536465	-0.17917	0.00031718	hypomethylated	B3gntl1	8	8
chr11	121562740	121564740	-0.10017	0.00000031	hypomethylated	Metrnl	27	27
chr12	3234790	3236790	-0.096431	0.0014249	hypomethylated	1700012B15Rik	22	24
chr12	3364131	3366131	-0.17063	0.0046356	hypomethylated	Kif3c	22	21
chr12	3425883	3427883	-0.04962	0.018236	hypomethylated	1110002L01Rik,Asxl2	37	34
chr12	3426747	3428747	-0.062426	0.00035947	hypomethylated	1110002L01Rik,Asxl2	31	28
chr12	3571390	3573390	0.026196	0.0031879	hypermethylated	Dtnb	44	36
chr12	3572040	3574040	0.0156	0.0063272	hypermethylated	Dtnb	45	36
chr12	3805979	3807979	-0.043321	1.02E-10	hypomethylated	Dnmt3a	129	127
chr12	4848401	4850401	-0.23353	0.037163	hypomethylated	BC068281,Fkbp1b	12	9
chr12	5382307	5384307	-0.055814	0.0050418	hypomethylated	2810032G03Rik,Klhl29	22	22
chr12	9035802	9037802	-0.025801	0.042591	hypomethylated	Ttc32	12	12
chr12	11271691	11273691	-0.080794	0.0001785	hypomethylated	Gen1,Smc6	47	49
chr12	11272593	11274593	-0.081618	0.0010063	hypomethylated	Gen1,Smc6	41	43
chr12	12267944	12269944	0.10371	0.032784	hypermethylated	Fam49a	24	29
chr12	15823591	15825591	-0.022022	0.036636	hypomethylated	Trib2	30	31
chr12	16596576	16598576	-0.36667	0.0038751	stronglyhypometh	Lpin1	10	4
chr12	16816770	16818770	-0.17718	3.87E-09	hypomethylated	E2f6	45	31
chr12	16900783	16902783	-0.083157	0.00014174	hypomethylated	Rock2	64	44
chr12	17331536	17333536	0.35936	0.0000667	stronglyhypermeth	Atp6v1c2	19	18
chr12	17696619	17698619	-0.083593	0.0000448	hypomethylated	Hpcal1	35	35
chr12	18520351	18522351	-0.22287	6.16E-10	hypomethylated	5730507C01Rik	28	4
chr12	21291157	21293157	-0.097353	0.010072	hypomethylated	Cpsf3,Itgb1bp1	16	14
chr12	21379452	21381452	-0.074574	0.0032556	hypomethylated	Adam17	16	16
chr12	22989696	22991696	-0.23665	0.0013494	hypomethylated	2410018L13Rik	18	8
chr12	25182445	25184445	-0.14261	0.0000892	hypomethylated	Taf1b	13	10
chr12	25256151	25258151	-0.0486	0.012254	hypomethylated	Grhl1	71	72
chr12	25335235	25337235	0.097661	0.010898	hypermethylated	Klf11	49	49
chr12	28027583	28029583	-0.33521	0.04801	stronglyhypometh	Sox11	21	11
chr12	29435259	29437259	-0.14539	0.00048011	hypomethylated	Trappc12,Tssc1	16	8
chr12	29435318	29437318	-0.20812	0.0000397	hypomethylated	Trappc12,Tssc1	16	8
chr12	29435692	29437692	-0.1733	0.025222	hypomethylated	Trappc12,Tssc1	14	6
chr12	30621900	30623900	0.14289	0.032486	hypermethylated	Pxdn	18	19
chr12	32184405	32186405	-0.14134	0.00000432	hypomethylated	Cbll1	27	20
chr12	32339691	32341691	0.21773	0.018819	hypermethylated	Cog5,Dus4l	8	16
chr12	33062653	33064653	-0.010067	0.028194	hypomethylated	Ccdc71l	65	68
chr12	33637809	33639809	-0.095381	0.031349	hypomethylated	4933406C10Rik,Sypl	29	18
chr12	33638050	33640050	-0.095381	0.031349	hypomethylated	4933406C10Rik,Sypl	29	18
chr12	33832451	33834451	-0.23121	0.046	hypomethylated	Atxn7l1,F730043M19Rik	16	19
chr12	34641535	34643535	-0.089748	0.011559	hypomethylated	Twist1	52	55

chr12	35730860	35732860	-0.081653	2.01E-10	hypomethylated	Snx13	52	50
chr12	36219661	36221661	-0.16122	0.0011283	hypomethylated	Ahr	7	7
chr12	36769065	36771065	-0.30556	0.0000327	hypomethylated	Tspan13	6	7
chr12	36883412	36885412	-0.339	0.003638	stronglyhypometh	Ankmy2,Bzw2	11	8
chr12	40949398	40951398	-0.074293	0.025991	hypomethylated	Gm7008,Ifrd1,Mir1938	23	23
chr12	40949776	40951776	-0.19242	0.0005285	hypomethylated	Gm7008,Ifrd1,Mir1938	9	9
chr12	41749676	41751676	-0.14174	0.022076	hypomethylated	Immp2l	19	14
chr12	45369140	45371140	-0.027734	0.0014778	hypomethylated	Pnpla8	33	33
chr12	45428871	45430871	-0.1055	0.0067149	hypomethylated	Nrcam	54	50
chr12	52790952	52792952	-0.037994	0.023256	hypomethylated	Ap4s1,Strn3	23	23
chr12	52930523	52932523	-0.058949	0.0000717	hypomethylated	Hectd1	43	38
chr12	55963864	55965864	-0.051578	0.04727	hypomethylated	Cfl2	12	16
chr12	56698903	56700903	0.02521	0.046765	hypermethylated	Insm2	47	52
chr12	57636093	57638093	0.021898	0.034829	hypermethylated	Nkx2-1	28	32
chr12	60113004	60115004	-0.14088	1.17E-08	hypomethylated	Gemin2,Sec23a	16	24
chr12	60113379	60115379	-0.050766	0.00081136	hypomethylated	Gemin2,Sec23a	9	10
chr12	60231439	60233439	-0.081484	8.83E-08	hypomethylated	Ctage5	56	56
chr12	60320470	60322470	0.02999	0.0049236	hypermethylated	Fbxo33	51	51
chr12	68323536	68325536	-0.23274	0.0000362	hypomethylated	Mdga2	8	8
chr12	70285054	70287054	0.043293	0.0072057	hypermethylated	Mgat2,Rpl36al	55	47
chr12	70396667	70398667	-0.060967	0.00015997	hypomethylated	Klhdc2	24	24
chr12	70782839	70784839	-0.04797	0.020555	hypomethylated	Sos2	36	39
chr12	70824948	70826948	-0.045667	0.0015306	hypomethylated	Atp5s,L2hgdh	25	25
chr12	71087989	71089989	-0.028459	0.012266	hypomethylated	Sav1	26	26
chr12	71212912	71214912	0.29079	0.000000118	hypermethylated	Nin	8	8
chr12	72115953	72117953	-0.038856	0.016043	hypomethylated	3110056K07Rik,Arid4a	95	102
chr12	74018752	74020752	0.19048	0.010729	hypermethylated	4930447C04Rik	3	3
chr12	74039931	74041931	-0.23318	0.00000368	hypomethylated	Six6	16	14
chr12	75007853	75009853	-0.11996	0.0027726	hypomethylated	Hif1a	51	48
chr12	75064516	75066516	-0.089202	0.021623	hypomethylated	Snapc1	21	17
chr12	75384262	75386262	-0.059975	0.00018241	hypomethylated	1700086L19Rik	15	16
chr12	75397460	75399460	-0.17537	0.0026879	hypomethylated	Dbpht2	4	4
chr12	76836716	76838716	-0.041667	0.0099373	hypomethylated	Sgpp1	15	16
chr12	77504356	77506356	-0.10673	0.00085848	hypomethylated	Hspa2	26	28
chr12	77865559	77867559	-0.026329	0.028777	hypomethylated	Churc1	17	22
chr12	78063235	78065235	-0.20645	0.00039061	hypomethylated	Max	22	16
chr12	79848933	79850933	-0.08543	0.0037245	hypomethylated	Mpp5	58	53
chr12	80273445	80275445	-0.12376	0.00022716	hypomethylated	Vti1b	22	21
chr12	80397268	80399268	-0.10779	0.00010225	hypomethylated	Rad51b,Zfyve26	11	11
chr12	80397269	80399269	-0.10779	0.00010225	hypomethylated	Rad51b,Zfyve26	11	11
chr12	81233831	81235831	-0.1369	0.020243	hypomethylated	2310015A10Rik	3	3
chr12	81792587	81794587	-0.15212	0.0015431	hypomethylated	0610009B14Rik,Plekhd1	22	30
chr12	82126794	82128794	-0.074341	0.030787	hypomethylated	Smoc1	30	34
chr12	82731355	82733355	-0.11917	0.002525	hypomethylated	Ttc9	29	24
chr12	82882157	82884157	-0.015298	0.025367	hypomethylated	Map3k9	21	16
chr12	83270002	83272002	-0.06972	0.0027009	hypomethylated	Sipa1l1	73	70
chr12	84972183	84974183	-0.085049	0.0074783	hypomethylated	Rbm25	25	25
chr12	85349451	85351451	-0.067448	0.00060314	hypomethylated	Acot1	11	7
chr12	85534965	85536965	-0.30407	0.0017064	hypomethylated	Elmsan1	11	4
chr12	85559831	85561831	-0.072092	0.04104	hypomethylated	Elmsan1	15	15
chr12	85701917	85703917	-0.33281	0.001737	hypomethylated	Coq6,Fam161b	13	9
chr12	85702771	85704771	-0.21011	0.017187	hypomethylated	Coq6,Fam161b	11	7
chr12	85757149	85759149	-0.48026	0.000000239	stronglyhypometh	Rnf113a2	15	9
chr12	85958416	85960416	-0.13481	0.016492	hypomethylated	Abcd4	3	3
chr12	85982845	85984845	-0.18708	0.020542	hypomethylated	Vrtn	10	9
chr12	86450782	86452782	-0.013311	0.022159	hypomethylated	Dlst	46	45
chr12	86518235	86520235	0.070256	0.0010496	hypermethylated	Pgf	9	8
chr12	86939365	86941365	-0.076807	1.79E-08	hypomethylated	Jdp2	54	57
chr12	88488852	88490852	0.22143	0.045682	hypermethylated	Gstz1,Pomt2	10	10
chr12	88783845	88785845	-0.24027	0.02944	hypomethylated	Alkbh1,Nrp,Slirp	12	10
chr12	92638432	92640432	-0.14963	0.0039972	hypomethylated	Tshr	6	7

chr12	92828089	92830089	0.093955	0.007165	hypermethylated	Gtf2a1	41	45
chr12	99816150	99818150	-0.40765	0.0020889	stronglyhypometh	Kcnk10	6	5
chr12	99975615	99977615	-0.31175	0.0000122	hypomethylated	Ptpn21	22	22
chr12	99984177	99986177	-0.02935	0.012912	hypomethylated	Zc3h14	30	39
chr12	102146408	102148408	-0.065357	0.02236	hypomethylated	Gpr68	5	5
chr12	102321912	102323912	0.086905	0.026888	hypermethylated	Smek1	5	10
chr12	103213183	103215183	-0.13352	0.00076673	hypomethylated	Cpsf2	32	32
chr12	103792178	103794178	-0.35596	0.012635	stronglyhypometh	Chga	20	18
chr12	103923230	103925230	-0.16146	0.014182	hypomethylated	Mir1936	4	4
chr12	103995184	103997184	-0.1167	0.0021257	hypomethylated	AK010878,Gm20604,Ubr7	33	42
chr12	103996020	103998020	-0.12911	0.0016737	hypomethylated	AK010878,Gm20604,Ubr7	21	29
chr12	104480356	104482356	-0.034038	0.0025175	hypomethylated	Prima1	23	18
chr12	104552168	104554168	-0.31297	5.49E-11	hypomethylated	Fam181a	15	15
chr12	104769774	104771774	-0.08141	0.003395	hypomethylated	Ppp4r4	50	47
chr12	106269559	106271559	-0.066135	1.53E-12	hypomethylated	Glrx5,Mir3069,Scarna13,Snhg	45	38
chr12	106269898	106271898	-0.05226	9.5E-12	hypomethylated	Glrx5,Mir3069,Scarna13,Snhg	42	34
chr12	106270489	106272489	-0.11386	1.63E-09	hypomethylated	Glrx5,Scarna13,Snhg10	36	25
chr12	106691185	106693185	-0.018171	0.033705	hypomethylated	D430019H16Rik	15	17
chr12	107247472	107249472	-0.055805	0.00022007	hypomethylated	Vrk1	15	18
chr12	109241624	109243624	-0.11483	2.89E-10	hypomethylated	Bcl11b	46	43
chr12	109416947	109418947	-0.054249	0.0000232	hypomethylated	Ccnk,Setd3	60	63
chr12	109417494	109419494	-0.080803	0.000233	hypomethylated	Ccnk,Setd3	18	11
chr12	109660025	109662025	-0.0040389	0.0088683	hypomethylated	Eml1	51	48
chr12	109842975	109844975	-0.30769	0.024588	hypomethylated	Evl	3	2
chr12	109940516	109942516	-0.19192	0.0054148	hypomethylated	Degs2	4	4
chr12	110030520	110032520	-0.037724	0.003549	hypomethylated	Yy1	98	108
chr12	110830055	110832055	-0.17405	0.037421	hypomethylated	6430411K18Rik,Mir127,Mir43	13	13
chr12	110949012	110951012	-0.25642	0.000000366	hypomethylated	Mir1197,Mir299,Mir323,Mir3	9	9
chr12	110949526	110951526	-0.25642	0.000000366	hypomethylated	Mir1197,Mir299,Mir323,Mir3	9	9
chr12	110949717	110951717	-0.25642	0.000000366	hypomethylated	Mir1197,Mir299,Mir323,Mir3	9	9
chr12	110950019	110952019	-0.25642	0.000000366	hypomethylated	Mir1197,Mir323,Mir329,Mir3	9	9
chr12	110950690	110952690	-0.21533	0.00040167	hypomethylated	Mir1197,Mir323,Mir329,Mir3	6	6
chr12	110960667	110962667	-0.44848	0.0013454	stronglyhypometh	Mir300,Mir376a,Mir376b,Mir	3	6
chr12	110960990	110962990	-0.44848	0.0013454	stronglyhypometh	Mir300,Mir376a,Mir376b,Mir	3	6
chr12	110961522	110963522	-0.28312	0.0000328	hypomethylated	Mir300,Mir376a,Mir376b,Mir	7	9
chr12	111975158	111977158	-0.19018	0.000031	hypomethylated	Wdr20a	32	38
chr12	112079149	112081149	-0.45455	0.022222	stronglyhypometh	Stk30	2	2
chr12	112217231	112219231	-0.093393	0.0052131	hypomethylated	Ankrd9	18	20
chr12	112508321	112510321	-0.17621	0.0015113	hypomethylated	Amn	17	20
chr12	112811720	112813720	-0.071996	0.0094698	hypomethylated	2810029C07Rik,Mark3	27	35
chr12	112910549	112912549	-0.19206	0.018316	hypomethylated	Ckb	3	5
chr12	113383418	113385418	-0.0065971	0.032299	hypomethylated	Kif26a	39	31
chr12	113912487	113914487	-0.12661	0.0000299	hypomethylated	Akt1	19	20
chr12	113959384	113961384	-0.038536	0.0028074	hypomethylated	Cep170b	59	59
chr12	114046185	114048185	-0.077146	0.0016398	hypomethylated	BC022687	29	24
chr12	114067600	114069600	-0.10349	0.023562	hypomethylated	Cdca4	22	22
chr12	114167706	114169706	-0.12489	0.017294	hypomethylated	Jag2	50	45
chr12	114213692	114215692	-0.018325	0.0012404	hypomethylated	Btbd6	19	28
chr12	114213773	114215773	-0.018325	0.0012404	hypomethylated	Btbd6	19	28
chr12	114238832	114240832	0.17786	0.002741	hypermethylated	Brf1	21	21
chr12	114251718	114253718	-0.18424	0.0039891	hypomethylated	Pacs2	43	44
chr12	114389222	114391222	0.18692	0.0058005	hypermethylated	Crip1	10	17
chr12	117284934	117286934	-0.18333	0.00087712	hypomethylated	Zfp386	10	13
chr12	117643634	117645634	0.14153	0.035996	hypermethylated	D430020J02Rik,Ncapg2	13	23
chr12	119539913	119541913	-0.050682	0.00011396	hypomethylated	Sp4	37	41
chr12	120083801	120085801	0.045937	0.012624	hypermethylated	Sp8	18	14
chr13	3476548	3478548	-0.13864	0.0000796	hypomethylated	2810429I04Rik	19	17
chr13	9092150	9094150	0.036	0.0021007	hypermethylated	Larp4b	69	69
chr13	9274771	9276771	-0.072017	0.00060525	hypomethylated	Dip2c	36	32
chr13	12657149	12659149	-0.017125	0.027512	hypomethylated	Ero1lb	34	30
chr13	13485891	13487891	-0.13889	0.00088914	hypomethylated	Gpr137b	5	5

chr13	13875805	13877805	0.0011442	0.0050126	hypermethylated	Gng4	26	32
chr13	14045940	14047940	0.2042	0.0000829	hypermethylated	B3galnt2	29	40
chr13	14155058	14157058	-0.030741	0.021012	hypomethylated	Arid4b,Ggps1	73	72
chr13	19039239	19041239	-0.026859	0.023237	hypomethylated	Amph	37	39
chr13	23515735	23517735	0.076299	0.030683	hypermethylated	Abt1	7	7
chr13	23645900	23647900	-0.35666	0.0025974	stronglyhypometh	Hist1h1d	4	5
chr13	23853026	23855026	-0.10563	0.000000557	hypomethylated	Hist1h1a,Hist1h3a,Hist1h4a	24	35
chr13	24922527	24924527	-0.10903	0.00015065	hypomethylated	Acot13,Tdp2	35	42
chr13	24923358	24925358	-0.11398	0.00010087	hypomethylated	Acot13,Tdp2	33	36
chr13	25112248	25114248	-0.24286	9.16E-08	hypomethylated	Mrs2	5	7
chr13	30750964	30752964	-0.056712	0.032023	hypomethylated	Dusp22	27	28
chr13	30750969	30752969	-0.056712	0.032023	hypomethylated	Dusp22	27	28
chr13	32892898	32894898	-0.099794	0.0037631	hypomethylated	Wrnip1	42	42
chr13	34093742	34095742	-0.054097	0.00017097	hypomethylated	Ripk1,Serpinb6a	38	38
chr13	36826323	36828323	0.091286	0.023925	hypermethylated	Nrn1	16	17
chr13	38128163	38130163	-0.10955	0.0014473	hypomethylated	Cage1,Riok1	21	23
chr13	38128806	38130806	-0.07609	0.011153	hypomethylated	Cage1,Riok1	16	18
chr13	38242162	38244162	0.015377	0.00022857	hypermethylated	Dsp	31	22
chr13	38436584	38438584	-0.10423	0.0069387	hypomethylated	Bmp6	44	47
chr13	41095378	41097378	-0.02114	0.002208	hypomethylated	Pak1ip1	23	23
chr13	41344212	41346212	-0.086611	0.02304	hypomethylated	Smim13	21	28
chr13	42146389	42148389	-0.057667	0.00000743	hypomethylated	Hivep1	62	80
chr13	43399541	43401541	0.078301	0.003847	hypermethylated	Gfod1	22	19
chr13	43710165	43712165	-0.066518	0.0024608	hypomethylated	Rnf182	19	17
chr13	45484110	45486110	-0.047533	0.011579	hypomethylated	Mylip	44	39
chr13	45484325	45486325	-0.047533	0.011579	hypomethylated	Mylip	44	39
chr13	46512668	46514668	-0.10379	0.0000244	hypomethylated	Rbm24	36	34
chr13	46596271	46598271	-0.075424	0.0091314	hypomethylated	Cap2	28	29
chr13	46763890	46765890	-0.0077874	0.0077027	hypomethylated	C78339	50	47
chr13	49063197	49065197	0.017976	0.028309	hypermethylated	Fam120a,Fam120aos	82	82
chr13	49243383	49245383	-0.11904	0.029278	hypomethylated	Wnk2	9	9
chr13	49281915	49283915	-0.077305	7.24E-09	hypomethylated	Ninj1	33	33
chr13	49515679	49517679	-0.12588	7.17E-08	hypomethylated	Ippk	43	40
chr13	51195266	51197266	0.0079956	0.012014	hypermethylated	9430083A17Rik,Spin1	79	79
chr13	51265393	51267393	-0.4787	0.00000538	stronglyhypometh	Nxnl2	18	20
chr13	51502986	51504986	0.16447	0.000000431	hypermethylated	S1pr3	24	23
chr13	51941043	51943043	-0.092913	0.0020697	hypomethylated	Gadd45g	41	30
chr13	52677805	52679805	-0.093945	0.00011673	hypomethylated	Syk	32	30
chr13	54166213	54168213	-0.041992	0.022688	hypomethylated	Sfxn1	23	22
chr13	54604165	54606165	-0.13119	0.000000237	hypomethylated	Simc1	42	40
chr13	54690591	54692591	-0.2047	0.0000181	hypomethylated	Higd2a,Nop16	18	18
chr13	54691435	54693435	-0.20744	0.00066608	hypomethylated	Higd2a,Nop16	12	12
chr13	54795321	54797321	-0.076192	0.0002059	hypomethylated	Rnf44	33	36
chr13	54851030	54853030	0.064908	0.0033011	hypermethylated	Gprin1	28	28
chr13	55205669	55207669	0.05367	0.00016278	hypermethylated	Zfp346	28	28
chr13	55253178	55255178	-0.135	0.023014	hypomethylated	Fgfr4	18	21
chr13	55422415	55424415	-0.010884	0.033904	hypomethylated	Prelid1,Rab24	27	32
chr13	55700471	55702471	0.063198	0.031433	hypermethylated	B4galt7	11	11
chr13	55735387	55737387	0.048678	0.00027609	hypermethylated	Ddx46	36	37
chr13	55793484	55795484	-0.027983	0.0084209	hypomethylated	B230219D22Rik	33	33
chr13	55932786	55934786	-0.13383	0.00000146	hypomethylated	Pitx1	20	20
chr13	56803370	56805370	0.050844	0.0000387	hypermethylated	Smad5	67	72
chr13	56803412	56805412	0.050844	0.0000387	hypermethylated	Smad5	67	72
chr13	56804028	56806028	0.06042	0.0005561	hypermethylated	Smad5	61	66
chr13	58229917	58231917	-0.17177	7.57E-09	hypomethylated	Hnrnpa0	53	44
chr13	58502608	58504608	-0.0347	0.0033592	hypomethylated	Hnrnpk,Rmi1	99	89
chr13	58502957	58504957	-0.011156	0.0016422	hypomethylated	Hnrnpk,Rmi1	82	72
chr13	58906956	58908956	0.087477	0.0056606	hypermethylated	Ntrk2	11	8
chr13	59685693	59687693	-0.14251	0.0014847	hypomethylated	A230056J06Rik,Naa35	54	50
chr13	59924508	59926508	-0.15161	0.0029774	hypomethylated	Zcchc6	16	16
chr13	60702307	60704307	0.1446	0.0000235	hypermethylated	Dapk1	61	62

chr13	60702571	60704571	0.1446	0.0000235	hypermethylated	Dapk1	61	62
chr13	64349846	64351846	-0.16857	0.0006939	hypomethylated	1810034E14Rik,Cdc14b	5	7
chr13	67856071	67858071	-0.29677	0.0023458	hypomethylated	Zfp85-rs1	7	5
chr13	70776512	70778512	-0.051362	0.012699	hypomethylated	BC018507	14	14
chr13	72765425	72767425	-0.015519	0.031218	hypomethylated	Gm20554,Irx2	64	58
chr13	72766012	72768012	-0.027665	0.038826	hypomethylated	Gm20554,Irx2	50	55
chr13	73396944	73398944	-0.16226	0.00036447	hypomethylated	Irx4	40	46
chr13	73740748	73742748	-0.05605	0.00015869	hypomethylated	Clptm1l	20	17
chr13	73900144	73902144	-0.085934	0.0001999	hypomethylated	Slc12a7	25	28
chr13	74257962	74259962	-0.25046	0.0093996	hypomethylated	Slc9a3	19	16
chr13	74429757	74431757	-0.2	0.017896	hypomethylated	Ahrr	2	2
chr13	74776319	74778319	0.11488	0.0037994	hypermethylated	Erap1	21	19
chr13	76081272	76083272	-0.11536	1.22E-08	hypomethylated	Rhobtb3	14	14
chr13	78338243	78340243	-0.11774	1.03E-09	hypomethylated	A830082K12Rik,Nr2f1	33	31
chr13	81795804	81797804	-0.069943	0.00055942	hypomethylated	Lysmd3	21	24
chr13	93194923	93196923	-0.062956	0.029752	hypomethylated	Ankrd34b	44	52
chr13	93300765	93302765	-0.16529	2.89E-09	hypomethylated	Zfyve16	16	18
chr13	94443390	94445390	-0.31049	0.00000975	hypomethylated	Bhmt2,Dmgdh	28	28
chr13	94540633	94542633	-0.021301	0.0061465	hypomethylated	Arsb	28	30
chr13	95557897	95559897	-0.11514	1.08E-11	hypomethylated	Tbca	33	30
chr13	97840597	97842597	-0.017036	0.017969	hypomethylated	Fam169a	56	68
chr13	98010059	98012059	-0.018891	0.001599	hypomethylated	Enc1	48	52
chr13	99123199	99125199	-0.035217	0.031023	hypomethylated	Foxd1	54	55
chr13	100876973	100878973	-0.25997	0.0012472	hypomethylated	Serf1	19	22
chr13	101386899	101388899	-0.25366	0.00000127	hypomethylated	Marveld2	5	5
chr13	101386926	101388926	-0.22368	0.0080804	hypomethylated	Marveld2	2	2
chr13	101420297	101422297	-0.060175	0.0000346	hypomethylated	Rad17,Taf9	67	63
chr13	101420561	101422561	-0.061063	0.0000308	hypomethylated	Rad17,Taf9	65	61
chr13	101421014	101423014	-0.061226	0.0014885	hypomethylated	Rad17,Taf9	50	48
chr13	101438435	101440435	-0.12992	0.0042605	hypomethylated	Ccdc125	20	19
chr13	101603382	101605382	-0.16667	0.0045568	hypomethylated	Slc30a5	4	4
chr13	105018034	105020034	-0.01841	0.021672	hypomethylated	Cenpk,Ppwd1	18	18
chr13	105018467	105020467	-0.01841	0.021672	hypomethylated	Cenpk,Ppwd1	18	18
chr13	105018924	105020924	-0.11852	0.0010493	hypomethylated	Cenpk,Ppwd1	9	9
chr13	105606318	105608318	-0.18333	0.0000156	hypomethylated	Cwc27,Srek1ip1	20	22
chr13	105607033	105609033	-0.2098	0.000011	hypomethylated	Cwc27,Srek1ip1	16	16
chr13	107736208	107738208	-0.24556	0.021891	hypomethylated	Dimt1	15	11
chr13	109003597	109005597	-0.033316	0.0039369	hypomethylated	Elovl7	38	37
chr13	109105530	109107530	-0.062137	0.0078977	hypomethylated	Depdc1b	47	46
chr13	110693016	110695016	-0.11357	1.3E-09	hypomethylated	Mir1904	32	20
chr13	112280249	112282249	-0.056976	0.00035342	hypomethylated	Gpbp1	55	53
chr13	112475385	112477385	-0.066829	0.0000129	hypomethylated	Mier3	85	81
chr13	112599191	112601191	-0.084185	0.010801	hypomethylated	Map3k1	53	28
chr13	113717000	113719000	-0.21409	0.001232	hypomethylated	Dhx29,Skiv2l2	26	34
chr13	113717588	113719588	-0.099855	0.048667	hypomethylated	Dhx29,Skiv2l2	18	22
chr13	113777009	113779009	0.14564	0.047094	hypermethylated	Ccno	39	37
chr13	114583715	114585715	-0.059319	0.000000547	hypomethylated	Arl15	35	35
chr13	115607444	115609444	-0.12649	0.0032151	hypomethylated	Mocs2	30	20
chr13	115722249	115724249	0.10505	0.0023638	hypermethylated	Itga2	9	8
chr13	118390126	118392126	-0.13737	0.0041105	hypomethylated	Hcn1	26	41
chr13	119502505	119504505	0.05733	0.0018007	hypermethylated	Fgf10	21	17
chr13	120197818	120199818	-0.018205	0.00000203	hypomethylated	Nnt	12	9
chr13	120216406	120218406	-0.037302	0.0000211	hypomethylated	Paip1	86	88
chr13	120216713	120218713	-0.036153	0.0000216	hypomethylated	Paip1	88	88
chr13	120250565	120252565	-0.25	0.00032702	hypomethylated	4833420G17Rik	16	12
chr14	9045661	9047661	0.019387	0.048154	hypermethylated	Kctd6	61	61
chr14	13115722	13117722	-0.12082	0.023953	hypomethylated	3830406C13Rik	15	17
chr14	13178379	13180379	-0.4166	0.00000111	stronglyhypometh	Fezf2	12	10
chr14	14844004	14846004	-0.029756	0.00000811	hypomethylated	Atxn7	27	32
chr14	19102655	19104655	-0.022872	0.00081595	hypomethylated	Nkiras1,Rpl15	28	39
chr14	19103500	19105500	-0.00052873	0.015026	hypomethylated	Nkiras1,Rpl15	14	25

chr14	20569478	20571478	-0.15409	0.00051655	hypomethylated	Nid2	11	17
chr14	20796471	20798471	0.29293	0.0080689	hypermethylated	Gng2	6	5
chr14	21492542	21494542	-0.096152	0.000000474	hypomethylated	Sec24c	18	18
chr14	21525773	21527773	-0.12388	0.022562	hypomethylated	Zswim8	16	20
chr14	22318075	22320075	-0.12641	0.0000661	hypomethylated	Kat6b	66	67
chr14	22567844	22569844	-0.24381	0.049657	hypomethylated	Dusp13,Samd8	6	5
chr14	22649782	22651782	-0.12844	0.001317	hypomethylated	Vdac2	19	18
chr14	22808823	22810823	-0.19265	0.00000499	hypomethylated	Zfp503	29	22
chr14	22837933	22839933	-0.1092	0.0042069	hypomethylated	1700112E06Rik	17	18
chr14	26277777	26279777	-0.0029706	0.01152	hypomethylated	Zmiz1	55	57
chr14	26352924	26354924	-0.40556	0.029208	stronglyhypometh	Mir3075	3	3
chr14	26588342	26590342	0.10066	0.02987	hypermethylated	Zcchc24	12	8
chr14	27851186	27853186	-0.043601	0.006191	hypomethylated	Il17rd	38	38
chr14	28241032	28243032	-0.07987	0.00065742	hypomethylated	D14Abb1e	40	44
chr14	29317658	29319658	0.10188	0.023437	hypermethylated	Wnt5a	30	31
chr14	30780565	30782565	-0.050483	0.045473	hypomethylated	Selk	23	23
chr14	31166672	31168672	-0.058146	0.014763	hypomethylated	Cacna1d	44	45
chr14	31291750	31293750	0.064519	0.0016889	hypermethylated	Dcp1a	17	20
chr14	31637779	31639779	-0.1238	0.000000832	hypomethylated	Tmem110	22	22
chr14	31763673	31765673	-0.25744	0.0000353	hypomethylated	Nek4	19	12
chr14	31813578	31815578	-0.11814	0.00049858	hypomethylated	Glt8d1,Spcs1	36	34
chr14	31813618	31815618	-0.11814	0.00049858	hypomethylated	Glt8d1,Spcs1	36	34
chr14	31831323	31833323	0.070436	0.012711	hypermethylated	Gnl3,Pbrm1	32	35
chr14	32137082	32139082	-0.15397	0.015696	hypomethylated	Dnahc1	7	7
chr14	32307264	32309264	0.076591	0.032648	hypermethylated	Eaf1,Mettl6	21	22
chr14	32897913	32899913	-0.095613	0.0000475	hypomethylated	Dph3,Oxnad1	23	22
chr14	32972077	32974077	-0.1568	0.014411	hypomethylated	Ncoa4	10	14
chr14	33014156	33016156	-0.19236	9.26E-09	hypomethylated	Parg,Timm23	33	39
chr14	33015077	33017077	-0.22764	1.22E-11	hypomethylated	Parg,Timm23	28	28
chr14	33278036	33280036	-0.083288	0.0045966	hypomethylated	Chat,Slc18a3	26	23
chr14	33412112	33414112	0.028594	0.028579	hypermethylated	Prrxl1	21	26
chr14	34260344	34262344	-0.020175	0.013433	hypomethylated	Mapk8	19	19
chr14	35122912	35124912	-0.053867	0.010817	hypomethylated	Fam35a,Glud1	78	81
chr14	35486113	35488113	-0.083489	0.00000609	hypomethylated	Wapal	58	58
chr14	37948325	37950325	-0.095731	0.022662	hypomethylated	Ghitm	16	16
chr14	37948508	37950508	-0.3125	0.0044955	hypomethylated	Ghitm	4	4
chr14	41780096	41782096	-0.185	0.0094399	hypomethylated	Tspan14	12	12
chr14	45838391	45840391	-0.11082	0.0016097	hypomethylated	Gpr137c,Txndc16	38	32
chr14	45839069	45841069	-0.11602	0.0015005	hypomethylated	Gpr137c,Txndc16	37	34
chr14	45969860	45971860	0.4106	0.00061851	stronglyhypermeth	Styx	11	17
chr14	48091235	48093235	-0.10063	0.0000099	hypomethylated	Fbxo34	38	27
chr14	48739543	48741543	-0.060772	0.0000488	hypomethylated	Peli2	44	46
chr14	49065026	49067026	-0.16524	0.0012219	hypomethylated	6720456H20Rik	39	39
chr14	49685169	49687169	-0.1785	5.56E-10	hypomethylated	Ap5m1,Exoc5	49	53
chr14	49790901	49792901	-0.11736	0.00000411	hypomethylated	Naa30	62	59
chr14	51426121	51428121	0.21333	0.0016429	hypermethylated	Parp2,Rpph1	12	15
chr14	51426620	51428620	0.21333	0.0016429	hypermethylated	Parp2,Rpph1	12	15
chr14	52512284	52514284	-0.23268	0.042831	hypomethylated	Slc39a2	4	4
chr14	52603507	52605507	0.014687	0.012985	hypermethylated	Arhgef40	25	36
chr14	52897820	52899820	-0.09886	0.0092239	hypomethylated	Rab2b,Tox4	13	18
chr14	54979524	54981524	-0.23412	0.023627	hypomethylated	Oxa1l	12	14
chr14	55049440	55051440	-0.15476	0.0021695	hypomethylated	Mmp14	22	14
chr14	55173198	55175198	-0.087878	0.016972	hypomethylated	Haus4	10	10
chr14	55224745	55226745	0.1406	0.00042366	hypermethylated	4931414P19Rik	10	18
chr14	55304007	55306007	-0.15283	0.00069885	hypomethylated	1700123O20Rik,Acin1	25	28
chr14	55482995	55484995	-0.24342	0.028928	hypomethylated	Homez	6	7
chr14	55633290	55635290	-0.20652	0.000000263	hypomethylated	Ngdn	14	15
chr14	55710885	55712885	0.20355	0.017272	hypermethylated	Thtpa,Zfhx2	10	11
chr14	56108929	56110929	-0.1842	0.00000015	hypomethylated	Lrrc16b	39	33
chr14	56177865	56179865	0.062207	0.0012581	hypermethylated	Dcaf11	27	29
chr14	56178759	56180759	0.061543	0.0010939	hypermethylated	Dcaf11	24	26

chr14	56196330	56198330	-0.17779	0.0000245	hypomethylated	Psme1	13	13
chr14	56341013	56343013	-0.11652	0.0000155	hypomethylated	Rabggta	4	5
chr14	56363529	56365529	-0.046862	0.0015485	hypomethylated	Dhrs1,Nop9	23	29
chr14	56364521	56366521	-0.067996	0.0033305	hypomethylated	Dhrs1,Nop9	21	25
chr14	56379760	56381760	0.12272	0.044866	hypermethylated	Ltb4r2	25	26
chr14	56442631	56444631	-0.33453	0.00060689	stronglyhypometh	Nfatc4	14	14
chr14	56471951	56473951	-0.20437	0.015892	hypomethylated	Nynrin	8	9
chr14	57193455	57195455	-0.17793	0.003282	hypomethylated	Parp4	8	6
chr14	57430553	57432553	-0.032282	0.046234	hypomethylated	Zmym5	31	32
chr14	57676782	57678782	-0.059225	0.0000532	hypomethylated	Gja3	22	22
chr14	58041907	58043907	-0.13177	0.014063	hypomethylated	Ift88	27	29
chr14	58283793	58285793	-0.039477	0.0020165	hypomethylated	Xpo4	26	26
chr14	58690522	58692522	-0.14161	1.18E-08	hypomethylated	Fgf9	18	18
chr14	60996122	60998122	-0.11859	0.0000877	hypomethylated	Amer2	44	48
chr14	62301210	62303210	0.016298	0.017953	hypermethylated	Dleu2	27	20
chr14	62949941	62951941	-0.091495	0.00030483	hypomethylated	Rnaseh2b	21	18
chr14	63379949	63381949	-0.11125	0.0080957	hypomethylated	4931440J10Rik,Ints6	19	14
chr14	63455526	63457526	-0.17767	0.0015178	hypomethylated	Wdfy2	27	27
chr14	63796630	63798630	-0.1625	0.0000487	hypomethylated	Fdft1	20	20
chr14	64162790	64164790	-0.18616	0.0053296	hypomethylated	Mtmr9	3	3
chr14	64561542	64563542	-0.0029997	0.045827	hypomethylated	Sox7	39	42
chr14	65208493	65210493	-0.19717	0.0092932	hypomethylated	Mir124a-1,Mir3078	5	5
chr14	65716943	65718943	-0.15431	0.00067072	hypomethylated	Extl3	10	10
chr14	65976512	65978512	-0.036463	0.0054542	hypomethylated	Zfp395	48	52
chr14	66452806	66454806	-0.2	0.007013	hypomethylated	Esco2	3	3
chr14	66455138	66457138	-0.2331	0.000000853	hypomethylated	Ccdc25	27	23
chr14	66572581	66574581	0.22748	0.00039263	hypermethylated	Scara3	13	13
chr14	66586319	66588319	-0.057672	0.017032	hypomethylated	Clu	12	12
chr14	67486686	67488686	-0.15138	0.0035392	hypomethylated	Dpysl2,Gm5464	24	24
chr14	67487437	67489437	-0.15298	0.034394	hypomethylated	Dpysl2,Gm5464	20	20
chr14	67529044	67531044	-0.030961	0.00034673	hypomethylated	Pnma2	33	37
chr14	67851128	67853128	-0.12131	0.008804	hypomethylated	Ebf2	28	29
chr14	68333154	68335154	-0.14888	3.17E-08	hypomethylated	Cdca2,Kctd9	57	54
chr14	68333667	68335667	-0.13746	2.75E-09	hypomethylated	Cdca2,Kctd9	41	38
chr14	68333898	68335898	-0.13716	2.85E-09	hypomethylated	Cdca2,Kctd9	41	37
chr14	68700940	68702940	-0.039153	0.0049485	hypomethylated	Nefl	28	27
chr14	69789075	69791075	-0.018376	0.033047	hypomethylated	1700092C10Rik,Nkx2-6	6	9
chr14	69954207	69956207	0.063278	0.03612	hypermethylated	Entpd4,Gm16677	32	38
chr14	70132377	70134377	-0.062213	0.021658	hypomethylated	Chmp7	16	18
chr14	70689256	70691256	-0.032738	0.0050736	hypomethylated	Ppp3cc	13	13
chr14	70952862	70954862	0.072782	0.014521	hypermethylated	Hr	32	32
chr14	70976653	70978653	0.13852	0.00000433	hypermethylated	Nudt18	26	29
chr14	70999642	71001642	-0.13386	0.0040875	hypomethylated	Fam160b2	6	6
chr14	73541316	73543316	0.057414	0.00068146	hypermethylated	Rcbtb2	34	41
chr14	73541591	73543591	0.057414	0.00068146	hypermethylated	Rcbtb2	34	41
chr14	73908855	73910855	-0.10794	0.0010272	hypomethylated	Med4	15	16
chr14	73951592	73953592	-0.067496	0.00092637	hypomethylated	Sucla2	31	31
chr14	75530690	75532690	-0.088044	0.015007	hypomethylated	Lcp1,Lrrc63	9	12
chr14	75683179	75685179	-0.13287	0.0000114	hypomethylated	Zc3h13	21	21
chr14	76814627	76816627	-0.026685	0.004305	hypomethylated	Tsc22d1	72	64
chr14	76903316	76905316	-0.14247	0.00015922	hypomethylated	Tsc22d1	19	26
chr14	77435578	77437578	-0.2262	0.015662	hypomethylated	Ccdc122,Lacc1	31	29
chr14	77555622	77557622	-0.13425	0.0051118	hypomethylated	Enox1	69	67
chr14	78198775	78200775	-0.32778	0.0024476	hypomethylated	Gm1587	6	5
chr14	80171119	80173119	-0.082432	0.034979	hypomethylated	Pcdh8	12	12
chr14	87540921	87542921	-0.095604	0.0027908	hypomethylated	Diap3	10	16
chr14	99444595	99446595	0.038923	0.012305	hypermethylated	Bora,Mzt1	64	73
chr14	99696909	99698909	-0.030904	0.0021653	hypomethylated	Klf5	44	38
chr14	102052183	102054183	-0.023625	0.00011904	hypomethylated	Uchl3	40	50
chr14	102128144	102130144	-0.085762	0.00036247	hypomethylated	Lmo7	32	29
chr14	118535348	118537348	-0.034471	0.019988	hypomethylated	Gpr180	36	38

chr14	119105441	119107441	-0.062022	0.027832	hypomethylated	Abcc4	22	24
chr14	119253171	119255171	-0.32807	0.0000763	hypomethylated	Cldn10	16	15
chr14	119336153	119338153	-0.069789	0.00031993	hypomethylated	Dnajc3	48	51
chr14	121309415	121311415	-0.095576	0.000000143	hypomethylated	Ipo5	65	63
chr14	121433795	121435795	-0.12089	0.00019944	hypomethylated	Farp1	70	74
chr14	122196956	122198956	-0.15228	0.00085971	hypomethylated	Dock9	50	44
chr14	122864880	122866880	-0.48293	0.0000428	stronglyhypometh	Zic5	6	6
chr14	122873605	122875605	-0.0012897	0.014859	hypomethylated	2610035F20Rik,Zic2	67	70
chr14	123312387	123314387	-0.076761	0.00099096	hypomethylated	Ggact	11	11
chr15	3977406	3979406	0.37857	0.024006	stronglyhypermeth	BC037032,Oxct1	2	2
chr15	5065612	5067612	0.032503	0.043672	hypermethylated	Rpl37,Snord72	28	28
chr15	5092860	5094860	-0.035921	0.0091368	hypomethylated	Prkaa1	61	47
chr15	5134559	5136559	-0.27966	0.0000164	hypomethylated	Ttc33	19	17
chr15	6657380	6659380	-0.084347	0.00021265	hypomethylated	Rictor	70	70
chr15	8058312	8060312	-0.28193	0.0012565	hypomethylated	Nup155	7	4
chr15	8394463	8396463	-0.19579	2.52E-09	hypomethylated	Nipbl	45	45
chr15	9000008	9002008	-0.041522	0.0000433	hypomethylated	Nadkd1	45	47
chr15	10414788	10416788	-0.16049	0.0000102	hypomethylated	Brix1,Rad1	16	19
chr15	10415692	10417692	-0.22318	0.00020417	hypomethylated	Brix1,Rad1	4	7
chr15	10881110	10883110	-0.32517	0.0035102	hypomethylated	C1qtnf3	2	3
chr15	10910510	10912510	0.10315	0.0060771	hypermethylated	Amacr	15	13
chr15	12046605	12048605	-0.11924	0.00000149	hypomethylated	Zfr	79	76
chr15	12753569	12755569	-0.078881	0.0040679	hypomethylated	6030458C11Rik,Drosha	51	53
chr15	12754412	12756412	-0.079332	0.0062756	hypomethylated	6030458C11Rik,Drosha	33	35
chr15	20596505	20598505	-0.52941	0.0000176	stronglyhypometh	Acot10	2	2
chr15	25772018	25774018	-0.13756	0.004033	hypomethylated	Fam134b	26	24
chr15	26237826	26239826	-0.024498	0.0000699	hypomethylated	March11	51	51
chr15	27611253	27613253	-0.23143	0.00015279	hypomethylated	Fam105a	5	5
chr15	31153139	31155139	-0.0029504	0.0045913	hypomethylated	Dap	17	19
chr15	31460792	31462792	-0.04506	0.0000279	hypomethylated	March6	34	24
chr15	31531559	31533559	-0.024107	0.015071	hypomethylated	Cct5,Fam173b	15	16
chr15	34166780	34168780	-0.057744	0.0011884	hypomethylated	Laptm4b	37	39
chr15	34235435	34237435	0.089658	0.030806	hypermethylated	Matn2	14	14
chr15	34608461	34610461	-0.14606	0.0000801	hypomethylated	Nipal2	10	11
chr15	34766135	34768135	-0.11581	0.032736	hypomethylated	Kcns2	31	23
chr15	35224866	35226866	-0.11622	0.0000874	hypomethylated	Osr2	35	35
chr15	35300300	35302300	-0.049	0.016438	hypomethylated	Vps13b	46	48
chr15	36102771	36104771	-0.3258	0.0000402	hypomethylated	Polr2k	10	8
chr15	36108284	36110284	-0.047273	0.0046905	hypomethylated	Spag1	10	10
chr15	36212902	36214902	-0.072755	0.000000371	hypomethylated	Rnf19a	12	12
chr15	36538728	36540728	-0.0073345	0.0013165	hypomethylated	Pabpc1	38	42
chr15	37161790	37163790	-0.16645	0.0065551	hypomethylated	Grhl2	19	16
chr15	38836825	38838825	0.017271	0.0053509	hypermethylated	Fzd6	34	41
chr15	38836878	38838878	0.017271	0.0053509	hypermethylated	Fzd6	34	41
chr15	39028877	39030877	-0.056035	1.45E-12	hypomethylated	Rims2	84	80
chr15	39775303	39777303	-0.024739	0.040579	hypomethylated	Lrp12	28	32
chr15	40485587	40487587	-0.14432	0.0000222	hypomethylated	Zfpm2	48	50
chr15	41278027	41280027	-0.090216	0.047053	hypomethylated	Oxr1	51	53
chr15	41619579	41621579	0.030888	0.013673	hypermethylated	Oxr1	39	42
chr15	41620060	41622060	0.030888	0.013673	hypermethylated	Oxr1	39	42
chr15	44258656	44260656	-0.11981	0.017349	hypomethylated	Eny2,Nudcd1	17	22
chr15	44450186	44452186	-0.10017	0.0052131	hypomethylated	Ebag9	48	51
chr15	44711939	44713939	-0.22186	0.011897	hypomethylated	A930017M01Rik	4	4
chr15	50721587	50723587	-0.028571	0.020976	hypomethylated	Mir1907,Trps1	14	9
chr15	51823306	51825306	0.081573	0.036416	hypermethylated	Rad21	7	7
chr15	53177738	53179738	-0.048698	0.00054453	hypomethylated	Ext1	30	30
chr15	54576482	54578482	-0.0035482	0.00076749	hypomethylated	Nov	11	11
chr15	55387962	55389962	-0.071295	0.02217	hypomethylated	Mrpl13,Mtbp	20	20
chr15	55388867	55390867	-0.071295	0.02217	hypomethylated	Mrpl13,Mtbp	20	20
chr15	57971990	57973990	-0.061533	0.0000176	hypomethylated	Wdyhv1	20	23
chr15	58703203	58705203	-0.17231	0.026571	hypomethylated	Trmt12	31	28

chr15	58719783	58721783	-0.044372	0.030681	hypomethylated	Rnf139	30	30
chr15	59204752	59206752	-0.041252	0.0000563	hypomethylated	E430025E21Rik,Nsmce2	22	17
chr15	60653519	60655519	-0.26053	0.00020515	hypomethylated	9930014A18Rik	25	33
chr15	61815895	61817895	-0.099389	0.0002745	hypomethylated	Myc	25	28
chr15	66117786	66119786	-0.14393	0.00000116	hypomethylated	Kcnq3	31	27
chr15	72919841	72921841	-0.098279	0.0082041	hypomethylated	Chrac1	17	17
chr15	74345625	74347625	-0.079769	0.00000894	hypomethylated	Bai1	32	35
chr15	74538590	74540590	-0.036416	0.034598	hypomethylated	4933427E11Rik,Jrk	37	36
chr15	74784480	74786480	-0.1569	0.00000312	hypomethylated	Ly6e	15	15
chr15	74784500	74786500	-0.1569	0.00000312	hypomethylated	Ly6e	15	15
chr15	74784535	74786535	-0.1569	0.00000312	hypomethylated	Ly6e	15	15
chr15	75739164	75741164	-0.094128	0.0000054	hypomethylated	Eef1d,Tigd5	71	72
chr15	75920443	75922443	-0.046822	0.0067297	hypomethylated	Nrbp2	29	32
chr15	76028639	76030639	-0.10069	0.00057983	hypomethylated	Plec	13	13
chr15	76029836	76031836	-0.44758	0.0093943	stronglyhypometh	Plec	7	7
chr15	76059927	76061927	-0.24069	0.03126	hypomethylated	Plec	5	6
chr15	76123863	76125863	-0.17782	0.014375	hypomethylated	Smpd5	12	15
chr15	76156826	76158826	-0.16815	0.00000907	hypomethylated	Exosc4	25	23
chr15	76180723	76182723	-0.034706	0.003927	hypomethylated	Maf1,Sharpin	39	32
chr15	76198327	76200327	-0.1429	0.0016609	hypomethylated	Fam203a	33	29
chr15	76306874	76308874	-0.033624	0.0044271	hypomethylated	Bop1,Hsf1	51	51
chr15	76352559	76354559	-0.17266	0.0000779	hypomethylated	Scrt1	33	36
chr15	76552603	76554603	0.12523	0.048229	hypermethylated	C030006K11Rik,Lrrc24	52	50
chr15	76554275	76556275	-0.0092666	0.0054305	hypomethylated	C030006K11Rik	25	26
chr15	76733500	76735500	-0.37778	0.0000908	stronglyhypometh	Rpl8	8	9
chr15	78034586	78036586	-0.21042	0.011678	hypomethylated	Pvalb	8	8
chr15	78236141	78238141	-0.14248	0.0017149	hypomethylated	Mpst,Tst	15	9
chr15	78236230	78238230	-0.14345	0.017832	hypomethylated	Mpst,Tst	13	7
chr15	78258057	78260057	0.082839	0.0041817	hypermethylated	Kctd17	20	15
chr15	78548543	78550543	-0.060308	0.024171	hypomethylated	Elfn2	39	42
chr15	78603875	78605875	-0.10869	0.015364	hypomethylated	Mfng	5	5
chr15	78672076	78674076	-0.054187	0.0010454	hypomethylated	Cdc42ep1	22	27
chr15	78743348	78745348	0.053748	0.0010767	hypermethylated	Pdxp	33	43
chr15	78764362	78766362	0.13311	0.00053957	hypermethylated	Nol12	17	22
chr15	78812485	78814485	-0.065935	0.01295	hypomethylated	Triobp	37	39
chr15	78857641	78859641	-0.033252	0.0000192	hypomethylated	H1f0	75	76
chr15	78904652	78906652	-0.25721	0.000000107	hypomethylated	Eif3l	28	16
chr15	78970796	78972796	-0.12736	0.0008905	hypomethylated	1700088E04Rik,Polr2f	19	18
chr15	78994920	78996920	-0.076709	0.020456	hypomethylated	Gm10863,Sox10	20	17
chr15	78995495	78997495	-0.055034	0.042366	hypomethylated	Gm10863,Sox10	21	17
chr15	79115939	79117939	-0.44444	0.0009085	stronglyhypometh	Baiap2l2	3	3
chr15	79177107	79179107	-0.12724	0.0000285	hypomethylated	Maff	42	42
chr15	79233733	79235733	-0.14	0.0013826	hypomethylated	Tmem184b	5	5
chr15	79520325	79522325	-0.025436	0.014848	hypomethylated	Gtpbp1	25	32
chr15	79604897	79606897	-0.3228	0.0035382	hypomethylated	Dnalc4	7	7
chr15	79962583	79964583	-0.079087	0.00067729	hypomethylated	Tab1	25	24
chr15	80003150	80005150	-0.045879	0.0055878	hypomethylated	Mgat3	74	75
chr15	80063509	80065509	0.18667	0.0000605	hypermethylated	Smcr7l	30	17
chr15	81229617	81231617	-0.15329	0.01211	hypomethylated	St13,Xpnpep3	21	24
chr15	81230124	81232124	-0.15919	0.039466	hypomethylated	St13,Xpnpep3	19	19
chr15	81688756	81690756	-0.2128	0.00045424	hypomethylated	Tob2	24	27
chr15	81756643	81758643	-0.16677	0.024587	hypomethylated	Polr3h	12	8
chr15	81957351	81959351	-0.10115	0.0039398	hypomethylated	Ccdc134	24	23
chr15	81976698	81978698	-0.18661	0.00000583	hypomethylated	Srebf2	49	47
chr15	82074766	82076766	0.039871	0.040614	hypermethylated	Cenpm	18	18
chr15	82169256	82171256	-0.37228	0.0015645	stronglyhypometh	Fam109b,Naga	2	2
chr15	82175475	82177475	-0.13432	0.0031395	hypomethylated	1500032L24Rik	25	28
chr15	82728891	82730891	-0.040438	0.0037338	hypomethylated	Gm20324,Tbrg3	69	62
chr15	82979074	82981074	-0.045062	0.0033124	hypomethylated	Poldip3,Rnu12	37	39
chr15	83997245	83999245	-0.17898	0.0000207	hypomethylated	Pnpla3	28	23
chr15	84061472	84063472	-0.010211	0.0062591	hypomethylated	Parvb	25	15

chr15	84549481	84551481	-0.1719	0.00047502	hypomethylated	Arhgap8,Gm20556	14	11
chr15	84549508	84551508	-0.1719	0.00047502	hypomethylated	Arhgap8,Gm20556	14	11
chr15	84961528	84963528	-0.20508	0.00051659	hypomethylated	Ribc2,Smc1b	18	23
chr15	84962387	84964387	-0.12948	0.0031407	hypomethylated	Ribc2,Smc1b	14	16
chr15	85035437	85037437	-0.15063	0.00000127	hypomethylated	Fbln1	24	24
chr15	85408500	85410500	-0.39792	0.014121	stronglyhypometh	Wnt7b	8	8
chr15	85411159	85413159	-0.015368	0.010115	hypomethylated	AU022754,Wnt7b	75	74
chr15	85564993	85566993	-0.11365	0.00000184	hypomethylated	Ppara	54	51
chr15	85565205	85567205	-0.11365	0.00000184	hypomethylated	Ppara	54	51
chr15	85652163	85654163	-0.039155	0.0029397	hypomethylated	Pkdrej	15	15
chr15	85661733	85663733	-0.18706	0.00000011	hypomethylated	Ttc38	18	17
chr15	85887136	85889136	-0.43013	1.48E-09	stronglyhypometh	Gramd4	12	13
chr15	86043888	86045888	-0.089041	0.0097616	hypomethylated	Tbc1d22a	34	25
chr15	87454659	87456659	-0.028028	0.032408	hypomethylated	Fam19a5	71	71
chr15	88581140	88583140	-0.10188	0.000000011	hypomethylated	Zbed4	59	59
chr15	88649075	88651075	-0.039193	0.00017715	hypomethylated	Alg12,Creld2	51	51
chr15	88649748	88651748	-0.039076	0.0011129	hypomethylated	Alg12,Creld2	41	41
chr15	88691623	88693623	-0.025597	0.039569	hypomethylated	Pim3	55	54
chr15	88905493	88907493	-0.081908	0.0000782	hypomethylated	1810021B22Rik,Trabd	42	41
chr15	88918536	88920536	-0.027523	0.00072577	hypomethylated	1300018J18Rik	43	43
chr15	89145742	89147742	-0.27778	0.0029857	hypomethylated	Sbf1	3	3
chr15	89185152	89187152	-0.13589	0.022709	hypomethylated	Lmf2,Ncaph2	26	26
chr15	89204249	89206249	-0.4227	0.00076467	stronglyhypometh	Sco2	16	12
chr15	89207468	89209468	0.079828	0.0053188	hypermethylated	Odf3b,Tymp	14	15
chr15	89283341	89285341	-0.16065	0.019065	hypomethylated	Mapk8ip2	12	11
chr15	90880379	90882379	-0.16034	0.00000324	hypomethylated	Kif21a	16	15
chr15	92426539	92428539	-0.50667	0.000000889	stronglyhypometh	Pdzrn4	5	5
chr15	93227780	93229780	-0.083017	0.017245	hypomethylated	Pphln1,Zcrb1	49	42
chr15	94458615	94460615	-0.049181	0.007544	hypomethylated	Tmem117	34	36
chr15	95620273	95622273	0.090523	0.0071289	hypermethylated	Ano6	44	49
chr15	97076537	97078537	-0.074058	0.00000294	hypomethylated	Amigo2,Pced1b	42	50
chr15	97077718	97079718	-0.066633	0.000000326	hypomethylated	Amigo2,Pced1b	39	47
chr15	97922019	97924019	-0.23689	2.27E-09	hypomethylated	Pfkm,Senp1	18	17
chr15	98126514	98128514	-0.040541	0.00010005	hypomethylated	Zfp641	19	19
chr15	98396754	98398754	-0.16815	4.27E-10	hypomethylated	9330020H09Rik,Ccnt1	39	30
chr15	98398067	98400067	-0.16302	2.25E-08	hypomethylated	9330020H09Rik,Ccnt1	27	26
chr15	98619287	98621287	-0.11991	0.01232	hypomethylated	Wnt1	19	19
chr15	98783932	98785932	-0.41626	3.13E-08	stronglyhypometh	Tuba1a	4	4
chr15	98859321	98861321	0.18149	0.028674	hypermethylated	Tuba1c	18	23
chr15	99054406	99056406	-0.13428	0.014895	hypomethylated	Kcnh3	39	46
chr15	99082392	99084392	-0.10833	0.0020088	hypomethylated	1700120C14Rik,Mcrs1	10	10
chr15	99124839	99126839	-0.15482	0.014212	hypomethylated	Prpf40b	28	30
chr15	99305161	99307161	-0.60054	0.00000739	stronglyhypometh	Bcdin3d	5	5
chr15	99420458	99422458	-0.039556	0.028617	hypomethylated	Aqp5	53	54
chr15	99500148	99502148	-0.074041	0.016129	hypomethylated	Asic1	56	55
chr15	99531717	99533717	-0.084385	9.52E-08	hypomethylated	Smarcd1	41	40
chr15	99802210	99804210	-0.0359	0.009977	hypomethylated	2310068J16Rik,Larp4	28	28
chr15	99868094	99870094	-0.13347	0.0017257	hypomethylated	Dip2b	41	41
chr15	100057289	100059289	-0.18766	0.0000507	hypomethylated	Atf1	61	54
chr15	100253486	100255486	-0.12066	0.0013356	hypomethylated	Gm5475,Slc11a2	21	23
chr15	100298464	100300464	-0.05256	0.008686	hypomethylated	Letmd1	31	32
chr15	100382378	100384378	-0.17809	0.0000901	hypomethylated	Tfcp2	15	15
chr15	100591177	100593177	-0.075468	0.00012281	hypomethylated	Slc4a8	30	30
chr15	100700113	100702113	-0.088671	0.00042116	hypomethylated	Scn8a	61	55
chr15	101003555	101005555	-0.0399	0.00081251	hypomethylated	Acvr1b	55	61
chr15	101053637	101055637	-0.097924	0.0020619	hypomethylated	A330009N23Rik,Grasp	31	34
chr15	101241833	101243833	-0.055679	0.010612	hypomethylated	Krt7	12	15
chr15	101681217	101683217	-0.24333	0.037342	hypomethylated	Krt1	5	3
chr15	101903203	101905203	-0.11364	0.0041019	hypomethylated	Eif4b	11	11
chr15	102076783	102078783	0.16411	0.027311	hypermethylated	Rarg	12	12
chr15	102108886	102110886	-0.14029	0.011608	hypomethylated	Mfsd5	46	39

chr15	102155546	102157546	-0.1429	0.041948	hypomethylated	Pfdn5	17	11
chr15	102235746	102237746	0.018157	0.0092419	hypermethylated	Sp1	41	40
chr15	102300062	102302062	-0.10449	0.000048	hypomethylated	Pcbp2	40	42
chr15	102501478	102503478	0.35	0.000000525	stronglyhypermeth	Atp5g2,Mir688	18	18
chr15	102806462	102808462	-0.10236	0.013745	hypomethylated	Hoxc9	14	12
chr15	102843438	102845438	0.18048	0.014745	hypermethylated	Hoxc5,Mir615	9	9
chr15	102863825	102865825	-0.047817	0.045029	hypomethylated	Hoxc4	16	17
chr15	102993715	102995715	-0.17159	0.00018483	hypomethylated	Smug1	8	8
chr15	103102348	103104348	-0.23993	0.00074114	hypomethylated	Copz1	9	7
chr15	103332728	103334728	-0.095543	0.0019869	hypomethylated	Pde1b	7	7
chr16	3883618	3885618	-0.070948	0.000000502	hypomethylated	Naa60	42	36
chr16	3908008	3910008	0.090493	0.033384	hypermethylated	1700037C18Rik,Cluap1	20	24
chr16	4077810	4079810	-0.14455	0.00047513	hypomethylated	Trap1	9	10
chr16	4213404	4215404	-0.012547	0.01211	hypomethylated	Crebbp	50	55
chr16	4885251	4887251	-0.069076	0.0000242	hypomethylated	Mgrn1	43	41
chr16	4964330	4966330	-0.11146	0.0085971	hypomethylated	4930451G09Rik,Anks3	7	7
chr16	5146201	5148201	-0.21931	0.019252	hypomethylated	Sec14l5	14	17
chr16	5256049	5258049	0.14072	0.039483	hypermethylated	Fam86	7	8
chr16	8636799	8638799	-0.056933	0.0035463	hypomethylated	Pmm2,Tmem186	30	28
chr16	10544479	10546479	-0.41848	0.0014465	stronglyhypometh	Clec16a	31	25
chr16	13255573	13257573	0.038956	0.00064008	hypermethylated	Mkl2	51	55
chr16	13939787	13941787	-0.2199	0.0021282	hypomethylated	Mpv17l	20	20
chr16	14360652	14362652	-0.093004	0.0000165	hypomethylated	Abcc1	27	27
chr16	15636958	15638958	-0.16036	0.00030308	hypomethylated	Mcm4,Prkdc	43	43
chr16	15637493	15639493	-0.069183	0.016096	hypomethylated	Mcm4,Prkdc	28	28
chr16	15863415	15865415	-0.21956	0.0028848	hypomethylated	Mzt2	8	7
chr16	15886378	15888378	-0.031789	0.00000215	hypomethylated	Cebpd	73	70
chr16	16212437	16214437	-0.050311	0.00015405	hypomethylated	Pkp2	24	28
chr16	16302074	16304074	-0.09893	0.037858	hypomethylated	Yars2	19	19
chr16	16869348	16871348	-0.23676	0.0000778	hypomethylated	Top3b,Vpreb1	22	20
chr16	16869983	16871983	-0.24002	0.0000147	hypomethylated	Top3b,Vpreb1	25	23
chr16	16982474	16984474	0.053992	0.021849	hypermethylated	Mapk1	40	42
chr16	17146071	17148071	-0.1478	0.000000656	hypomethylated	Ydjc	25	28
chr16	17207227	17209227	-0.20758	0.00021332	hypomethylated	Rimbp3	28	29
chr16	17232679	17234679	0.0354	0.04901	hypermethylated	Hic2	32	30
chr16	17451079	17453079	0.15554	0.0000144	hypermethylated	Crkl	21	21
chr16	17488783	17490783	-0.077083	0.040994	hypomethylated	Aifm3	24	24
chr16	17758713	17760713	-0.1243	0.012233	hypomethylated	Klhl22	36	40
chr16	17893295	17895295	-0.11828	0.02309	hypomethylated	Tssk1	11	5
chr16	17915152	17917152	-0.092727	0.0086631	hypomethylated	Gsc2	4	4
chr16	18089283	18091283	-0.37435	0.013576	stronglyhypometh	Prodh	13	6
chr16	18126798	18128798	-0.075746	0.000000337	hypomethylated	Rtn4r	50	52
chr16	18235229	18237229	0.084416	0.037464	hypermethylated	Zdhhc8	12	11
chr16	18247975	18249975	-0.02556	0.0086413	hypomethylated	Ranbp1,Trmt2a	42	42
chr16	18289261	18291261	-0.09994	0.014046	hypomethylated	Dgcr8	21	21
chr16	18344025	18346025	-0.14955	0.00087154	hypomethylated	Tango2	9	9
chr16	18875842	18877842	-0.059523	0.0012623	hypomethylated	Hira,Mrpl40	48	53
chr16	18876730	18878730	-0.075494	0.0001016	hypomethylated	Hira,Mrpl40	43	48
chr16	19759326	19761326	-0.052832	0.023399	hypomethylated	B3gnt5	16	22
chr16	20096626	20098626	-0.11814	0.028208	hypomethylated	Klhl24	41	37
chr16	20426467	20428467	-0.037229	0.017111	hypomethylated	Abcc5	15	15
chr16	20497889	20499889	-0.028963	0.01325	hypomethylated	Eif2b5	11	19
chr16	20621351	20623351	0.033076	0.010912	hypermethylated	Camk2n2	35	29
chr16	20716709	20718709	-0.027796	0.021585	hypomethylated	Clcn2,Polr2h	20	16
chr16	20716898	20718898	-0.026124	0.044052	hypomethylated	Clcn2,Polr2h	21	17
chr16	20732199	20734199	-0.025149	0.007519	hypomethylated	Chrd,Thpo	46	46
chr16	21203867	21205867	-0.12691	0.00018497	hypomethylated	Ephb3	37	48
chr16	22439643	22441643	-0.33732	0.00000231	stronglyhypometh	Etv5	22	19
chr16	22856917	22858917	-0.081035	0.0034832	hypomethylated	Dnajb11,Tbccd1	24	24
chr16	22857642	22859642	-0.081035	0.0034832	hypomethylated	Dnajb11,Tbccd1	24	24
chr16	23223834	23225834	-0.033125	0.0054706	hypomethylated	St6gal1	16	15

chr16	23930880	23932880	-0.13892	0.00342	hypomethylated	Rtp2	9	8
chr16	26580790	26582790	0.0024291	0.00054881	hypermethylated	Il1rap	13	19
chr16	27388062	27390062	-0.1429	0.0045606	hypomethylated	Ccdc50	13	13
chr16	28445313	28447313	0.091705	0.030966	hypermethylated	Fgf12	49	53
chr16	28929784	28931784	-0.047708	0.0091342	hypomethylated	Mb21d2	33	34
chr16	30064442	30066442	-0.14874	0.0000173	hypomethylated	Hes1	14	16
chr16	30598808	30600808	-0.11499	3.61E-11	hypomethylated	Fam43a	53	55
chr16	31427838	31429838	0.055526	0.047799	hypermethylated	Bdh1	29	32
chr16	31947631	31949631	-0.071541	0.030963	hypomethylated	0610012G03Rik,Ncbp2	50	50
chr16	32246312	32248312	-0.11691	0.0000242	hypomethylated	Fbxo45,Wdr53	36	43
chr16	32246338	32248338	-0.11509	0.0000112	hypomethylated	Fbxo45,Wdr53	35	42
chr16	32276546	32278546	-0.18555	3.44E-14	hypomethylated	Rnf168	37	32
chr16	32430006	32432006	-0.086582	0.00000147	hypomethylated	Pcyt1a	42	44
chr16	32430105	32432105	-0.093781	0.00000604	hypomethylated	Pcyt1a	41	43
chr16	32430315	32432315	-0.10357	0.00000181	hypomethylated	Pcyt1a	41	42
chr16	32607981	32609981	-0.54167	0.00010505	stronglyhypometh	Tfrc	10	6
chr16	32643728	32645728	-0.060743	0.039958	hypomethylated	Tnk2	37	38
chr16	32913185	32915185	-0.079162	0.0084695	hypomethylated	Lrch3	41	41
chr16	33055538	33057538	-0.21651	0.00000161	hypomethylated	Iqcg,Rpl35a	19	14
chr16	33055567	33057567	-0.21651	0.00000161	hypomethylated	Iqcg,Rpl35a	19	14
chr16	33055599	33057599	-0.21651	0.00000161	hypomethylated	Iqcg,Rpl35a	19	14
chr16	33056272	33058272	-0.26019	5.23E-08	hypomethylated	Iqcg,Rpl35a	15	10
chr16	33061606	33063606	-0.045572	0.0087468	hypomethylated	Lmln	38	38
chr16	33379860	33381860	-0.042357	0.013373	hypomethylated	1700007L15Rik,Zfp148	49	54
chr16	33828750	33830750	-0.035274	0.026755	hypomethylated	Itgb5	35	27
chr16	33967089	33969089	-0.28333	0.0017114	hypomethylated	Umps	4	7
chr16	35769442	35771442	0.041762	0.026106	hypermethylated	Dirc2,Hspbap1	14	12
chr16	35982448	35984448	-0.068755	0.00059485	hypomethylated	Kpna1	62	39
chr16	37010871	37012871	-0.088384	0.043764	hypomethylated	Polq	17	16
chr16	37654454	37656454	-0.54412	0.0033196	stronglyhypometh	Ndufb4	4	4
chr16	37867485	37869485	-0.068377	1.37E-10	hypomethylated	Lrrc58	51	50
chr16	38088086	38090086	-0.18081	0.02745	hypomethylated	BC031361,Gsk3b	41	34
chr16	38346084	38348084	-0.25417	0.030148	hypomethylated	Cox17	8	9
chr16	38741376	38743376	-0.057184	0.00034374	hypomethylated	B4galt4	37	37
chr16	38901457	38903457	0.068629	0.0022062	hypermethylated	Igsf11	37	37
chr16	43889014	43891014	-0.13874	0.00065023	hypomethylated	2610015P09Rik,Qtrtd1	14	16
chr16	43889789	43891789	-0.15624	0.00075903	hypomethylated	2610015P09Rik,Qtrtd1	9	11
chr16	44138921	44140921	-0.074845	0.0058838	hypomethylated	Atp6v1a,Naa50	40	44
chr16	44139132	44141132	-0.074845	0.0058838	hypomethylated	Atp6v1a,Naa50	40	44
chr16	44723411	44725411	-0.095649	0.000000838	hypomethylated	BC027231	40	43
chr16	45157941	45159941	-0.21262	0.00000704	hypomethylated	Atg3,Slc35a5	25	19
chr16	45158786	45160786	-0.05595	0.0014166	hypomethylated	Atg3,Slc35a5	20	19
chr16	46010526	46012526	-0.32	0.00015584	hypomethylated	Plcxd2	6	6
chr16	49854766	49856766	-0.03169	0.0091668	hypomethylated	Cd47	56	52
chr16	55895392	55897392	-0.036075	0.0402	hypomethylated	Nxpe3	20	20
chr16	56074521	56076521	-0.22985	0.00028232	hypomethylated	Senp7	10	9
chr16	57548354	57550354	0.041667	0.016769	hypermethylated	Filip1l	9	9
chr16	58727156	58729156	-0.13158	0.046449	hypomethylated	Cldn25	26	24
chr16	62853159	62855159	0.0573	0.038783	hypermethylated	Pros1	11	11
chr16	65562942	65564942	-0.078373	0.048801	hypomethylated	Chmp2b	6	7
chr16	77328572	77330572	-0.33803	0.0000354	stronglyhypometh	2810055G20Rik	8	6
chr16	81199941	81201941	-0.04331	0.034986	hypomethylated	Ncam2	39	41
chr16	84834368	84836368	-0.072529	0.0000408	hypomethylated	Atp5j,Gabpa	36	49
chr16	84835819	84837819	-0.089904	0.0000779	hypomethylated	Atp5j,Gabpa	17	29
chr16	87698198	87700198	-0.13259	1.77E-20	hypomethylated	Bach1	54	52
chr16	90141481	90143481	-0.20647	0.0000806	hypomethylated	Gm10789	29	25
chr16	90830103	90832103	-0.032171	0.014944	hypomethylated	Eva1c	56	51
chr16	90935094	90937094	-0.09264	0.037108	hypomethylated	1110004E09Rik	20	18
chr16	91010493	91012493	-0.070389	0.0003028	hypomethylated	4930404I05Rik,Synj1	41	49
chr16	91269013	91271013	-0.10572	0.00025141	hypomethylated	Olig1	27	27
chr16	91372027	91374027	-0.028592	0.0038509	hypomethylated	Ifnar2	18	18

chr16	91597925	91599925	0.078385	0.0000902	hypermethylated	Tmem50b	16	20
chr16	92057566	92059566	-0.031848	0.0010741	hypomethylated	Mrps6,Slc5a3	55	60
chr16	92057580	92059580	-0.031848	0.0010741	hypomethylated	Mrps6,Slc5a3	55	60
chr16	92300547	92302547	-0.1254	0.018867	hypomethylated	Smim11	7	6
chr16	93604060	93606060	-0.39826	0.00031426	stronglyhypometh	Setd4	8	10
chr16	93711151	93713151	-0.090868	1.54E-10	hypomethylated	Dopey2	30	31
chr16	93831365	93833365	-0.042633	0.00031517	hypomethylated	Morc3	57	56
chr16	93883145	93885145	0.025595	0.024817	hypermethylated	Chaf1b	38	36
chr16	94084504	94086504	-0.089488	0.000000111	hypomethylated	Sim2	41	47
chr16	94549028	94551028	-0.19628	0.0068425	hypomethylated	Ripply3	14	8
chr16	95923013	95925013	-0.15766	9.42E-08	hypomethylated	Ets2	38	38
chr16	96212510	96214510	0.032025	0.0040105	hypermethylated	Psmg1	14	15
chr16	96349332	96351332	-0.086454	0.00016879	hypomethylated	Hmgn1	27	27
chr16	98072834	98074834	-0.23897	0.0044509	hypomethylated	Prdm15	56	57
chr17	3113971	3115971	-0.044243	0.0069297	hypomethylated	Scaf8	61	59
chr17	5840379	5842379	-0.062501	0.0012225	hypomethylated	Snx9	63	76
chr17	5940279	5942279	-0.046617	0.034518	hypomethylated	Synj2	33	28
chr17	8502967	8504967	-0.21239	0.0000134	hypomethylated	4930506C21Rik,Sft2d1	26	23
chr17	8532270	8534270	-0.043175	0.029701	hypomethylated	Prr18	39	45
chr17	8532603	8534603	-0.035215	0.045017	hypomethylated	Prr18	43	45
chr17	8993609	8995609	-0.053516	0.00036962	hypomethylated	Pde10a	43	46
chr17	10511783	10513783	-0.04924	0.0095393	hypomethylated	B930003M22Rik,Qk	53	48
chr17	10512226	10514226	-0.049962	0.041538	hypomethylated	B930003M22Rik,Qk	39	34
chr17	11032249	11034249	-0.18537	0.0000296	hypomethylated	Pacrg,Park2	12	14
chr17	11033057	11035057	-0.16528	0.0027432	hypomethylated	Pacrg,Park2	10	12
chr17	12511526	12513526	-0.11855	0.00019823	hypomethylated	4732491K20Rik,Map3k4	8	9
chr17	12700570	12702570	-0.32792	0.00000641	hypomethylated	Slc22a3	7	7
chr17	13199704	13201704	0.22306	0.0000132	hypermethylated	Sod2	25	25
chr17	15079188	15081188	-0.092688	0.0064134	hypomethylated	1600012H06Rik,Wdr27	31	27
chr17	15512787	15514787	-0.056726	0.00020997	hypomethylated	Dll1	41	38
chr17	15635240	15637240	0.12636	0.00183	hypermethylated	Psmb1,Tbp	31	28
chr17	15840930	15842930	-0.089963	0.0010184	hypomethylated	Chd1	83	77
chr17	17761453	17763453	-0.014767	0.000004	hypomethylated	Lnpep	43	38
chr17	22098525	22100525	-0.26601	0.000000873	hypomethylated	Zfp942,Zfp943	3	9
chr17	23796489	23798489	-0.28936	0.0015513	hypomethylated	Ccdc64b	13	12
chr17	24278556	24280556	0.10993	0.041633	hypermethylated	Pdpk1	7	7
chr17	24278561	24280561	0.10993	0.041633	hypermethylated	Pdpk1	7	7
chr17	24342507	24344507	-0.11541	0.0018127	hypomethylated	Tbc1d24	33	24
chr17	24487990	24489990	0.076855	0.034491	hypermethylated	Abca3	30	42
chr17	24562627	24564627	-0.1099	0.00080684	hypomethylated	Eci1	17	13
chr17	24606417	24608417	-0.033238	0.0000667	hypomethylated	Pgp	68	63
chr17	24685894	24687894	-0.070836	0.012594	hypomethylated	Pkd1	44	34
chr17	24805696	24807696	-0.011704	0.022852	hypomethylated	Zfp598	34	36
chr17	24826894	24828894	0.20417	0.011117	hypermethylated	Syngr3	4	4
chr17	24986434	24988434	-0.14639	3.53E-10	hypomethylated	Fahd1,Hagh	31	36
chr17	24986611	24988611	-0.17454	3.6E-11	hypomethylated	Fahd1,Hagh	30	35
chr17	24987247	24989247	-0.12336	0.000000451	hypomethylated	Fahd1,Hagh	23	26
chr17	25031064	25033064	-0.16748	0.030037	hypomethylated	Eme2,Mrps34,Nme3	17	20
chr17	25032444	25034444	-0.012121	0.00031315	hypomethylated	Eme2,Mrps34,Nme3	11	11
chr17	25324344	25326344	-0.026987	0.00055003	hypomethylated	Unkl	50	48
chr17	25376114	25378114	-0.028887	0.0070924	hypomethylated	Gnptg,Tsr3	28	22
chr17	25377061	25379061	-0.039515	0.0039225	hypomethylated	Gnptg,Tsr3	21	21
chr17	25707631	25709631	-0.10344	0.0027707	hypomethylated	2810468N07Rik,Sox8	15	11
chr17	25945029	25947029	-0.020675	0.021503	hypomethylated	Fbxl16	47	49
chr17	26011444	26013444	0.22194	0.030783	hypermethylated	0610011F06Rik	9	5
chr17	26056659	26058659	-0.19114	0.00053579	hypomethylated	Rab40c	10	8
chr17	26338295	26340295	-0.17445	0.000000345	hypomethylated	Arhgdig,Rgs11	8	7
chr17	26388854	26390854	-0.13757	0.000000198	hypomethylated	Luc7l	30	21
chr17	26550909	26552909	-0.046087	0.0094625	hypomethylated	Neurl1b	55	46
chr17	26812340	26814340	-0.038787	0.0014201	hypomethylated	Atp6v0e	25	25
chr17	26851594	26853594	-0.143	0.00018724	hypomethylated	Crebrf	50	32

chr17	27053035	27055035	-0.11898	0.0079873	hypomethylated	Kifc5b	8	9
chr17	27076413	27078413	-0.034873	0.0024126	hypomethylated	Cuta	16	15
chr17	27076423	27078423	-0.034873	0.0024126	hypomethylated	Cuta	16	15
chr17	27193248	27195248	-0.07468	0.0090287	hypomethylated	Itpr3	31	35
chr17	27692518	27694518	-0.017945	0.013187	hypomethylated	Hmga1,Hmga1-rs1	79	75
chr17	27692548	27694548	-0.022614	0.0055958	hypomethylated	Hmga1,Hmga1-rs1	77	73
chr17	27692597	27694597	-0.022614	0.0055958	hypomethylated	Hmga1,Hmga1-rs1	77	73
chr17	27772187	27774187	-0.014678	0.0051018	hypomethylated	Rps10	17	16
chr17	27992451	27994451	-0.14859	6.79E-14	hypomethylated	Uhrf1bp1	34	28
chr17	28217529	28219529	-0.34257	0.041901	stronglyhypometh	Tcp11	12	8
chr17	28217584	28219584	-0.50952	0.01945	stronglyhypometh	Tcp11	7	3
chr17	28449474	28451474	0.032298	0.049401	hypermethylated	Fance	23	23
chr17	28487545	28489545	0.074053	0.015251	hypermethylated	Tead3	28	32
chr17	28487750	28489750	0.080215	0.011273	hypermethylated	Tead3	23	24
chr17	28666805	28668805	-0.30161	0.0028774	hypomethylated	Armc12	6	7
chr17	28685431	28687431	-0.23878	0.02018	hypomethylated	Clpsl2	7	7
chr17	28711663	28713663	-0.020147	0.037586	hypomethylated	Lhfpl5	13	13
chr17	29089160	29091160	-0.10988	0.00029919	hypomethylated	Kctd20	28	26
chr17	29484849	29486849	-0.13016	0.0094327	hypomethylated	Mtch1	8	5
chr17	29750775	29752775	-0.34342	0.000000339	stronglyhypometh	Rnf8	33	21
chr17	30141031	30143031	-0.03698	0.0000379	hypomethylated	Zfand3	63	57
chr17	31431427	31433427	-0.052532	0.0000473	hypomethylated	Slc37a1	20	20
chr17	31700717	31702717	0.039544	0.018218	hypermethylated	Pknox1	77	76
chr17	31992737	31994737	-0.1936	0.0098506	hypomethylated	Sik1	26	26
chr17	32171453	32173453	0.12496	0.0086846	hypermethylated	Hsf2bp,Rrp1b	9	9
chr17	32172106	32174106	0.12496	0.0086846	hypermethylated	Hsf2bp,Rrp1b	9	9
chr17	32421068	32423068	-0.046554	0.0016901	hypomethylated	Brd4	55	65
chr17	32524762	32526762	-0.0687	0.019966	hypomethylated	Wiz	20	20
chr17	32525895	32527895	-0.0687	0.019966	hypomethylated	Wiz	20	20
chr17	33101775	33103775	-0.18788	0.011912	hypomethylated	Cyp4f41-ps,Zfp472	17	13
chr17	33102661	33104661	-0.27994	0.0056329	hypomethylated	Cyp4f41-ps,Zfp472	11	7
chr17	33660168	33662168	0.08857	0.0063247	hypermethylated	Adamts10	15	11
chr17	33946467	33948467	-0.026854	0.00050791	hypomethylated	Kank3	9	9
chr17	34076667	34078667	-0.10706	0.0013025	hypomethylated	H2-Ke2,Wdr46	34	37
chr17	34077275	34079275	-0.10706	0.0013025	hypomethylated	H2-Ke2,Wdr46	34	37
chr17	34077288	34079288	-0.10706	0.0013025	hypomethylated	H2-Ke2,Wdr46	34	37
chr17	34091826	34093826	-0.20441	9.49E-12	hypomethylated	Rps18,Vps52	44	46
chr17	34092586	34094586	-0.19572	2.36E-08	hypomethylated	Rps18,Vps52	31	33
chr17	34161625	34163625	-0.019866	0.039451	hypomethylated	Mir219-1,Ring1	43	39
chr17	34258692	34260692	0.15663	0.016638	hypermethylated	Brd2,H2-DMa	17	7
chr17	34271615	34273615	0.1012	0.047042	hypermethylated		10	10
chr17	34323500	34325500	-0.1024	0.012393	hypomethylated	Psmb9,Tap1	14	12
chr17	34324275	34326275	-0.1024	0.012393	hypomethylated	Psmb9,Tap1	14	12
chr17	34340423	34342423	-0.14922	0.0000108	hypomethylated	Tap2	10	10
chr17	34728415	34730415	-0.017684	0.0067152	hypomethylated	Pbx2	39	39
chr17	34764042	34766042	0.14753	0.04828	hypermethylated	Ppt2,Prrt1	28	22
chr17	34783124	34785124	0.17609	0.0095155	hypermethylated	Atf6b	44	46
chr17	34806479	34808479	-0.1674	0.00019327	hypomethylated	Tnxb	17	17
chr17	34972963	34974963	-0.13837	0.00000951	hypomethylated	Dom3z,Stk19	56	61
chr17	34973848	34975848	-0.078381	0.00097286	hypomethylated	Dom3z,Stk19	38	47
chr17	34986335	34988335	-0.14931	8.69E-09	hypomethylated	Nelfe,Skiv2l	40	35
chr17	34987149	34989149	-0.12727	0.00000015	hypomethylated	Nelfe,Skiv2l	39	35
chr17	35067197	35069197	-0.575	0.0041749	stronglyhypometh	Neu1	4	8
chr17	35258392	35260392	-0.086243	0.035711	hypomethylated	Csnk2b,Gpank1	9	6
chr17	35271186	35273186	-0.14244	0.03026	hypomethylated	Bag6	34	35
chr17	35301822	35303822	-0.03919	0.004068	hypomethylated	Prrc2a	18	15
chr17	35330451	35332451	-0.20539	0.012299	hypomethylated	Ltb	5	5
chr17	35377690	35379690	-0.21795	2.02E-13	hypomethylated	Ddx39b	39	32
chr17	35456502	35458502	-0.1766	0.0064281	hypomethylated	H2-Q1	22	21
chr17	35478277	35480277	0.11858	0.00071842	hypermethylated	H2-Q2	18	18
chr17	35688072	35690072	-0.41484	0.000000383	stronglyhypometh	Cdsn	11	11

chr17	35837535	35839535	-0.51623	0.0060882	stronglyhypometh	Ddr1	20	13
chr17	35959301	35961301	-0.06972	0.0000685	hypomethylated	Flot1,Ier3	23	20
chr17	35977442	35979442	0.060659	0.020515	hypermethylated	Mdc1	15	16
chr17	36034323	36036323	-0.28955	0.00000607	hypomethylated	2310061I04Rik	10	10
chr17	36047013	36049013	-0.63636	4.66E-09	stronglyhypometh	Atat1	6	4
chr17	36116770	36118770	-0.049643	0.0052734	hypomethylated	Gnl1,Prr3	56	42
chr17	36130448	36132448	-0.23713	0.0040297	hypomethylated	A930015D03Rik	3	5
chr17	36280962	36282962	-0.090956	0.0000174	hypomethylated	2410017I17Rik	11	11
chr17	36973085	36975085	-0.059974	0.0051034	hypomethylated	Trim26	33	30
chr17	37005518	37007518	-0.15009	0.0014886	hypomethylated	Trim10	7	8
chr17	37094536	37096536	-0.071429	0.039826	hypomethylated	Znrd1,Znrd1as	14	12
chr17	37181910	37183910	0.23181	0.033996	hypermethylated	Gabbr1	27	23
chr17	39980080	39982080	-0.18207	0	hypomethylated	Rn45s	81	92
chr17	43013373	43015373	-0.077441	0.00047489	hypomethylated	Cd2ap	22	23
chr17	43152503	43154503	-0.081608	0.000000891	hypomethylated	Tnfrsf21	35	29
chr17	43937799	43939799	0.019112	0.00010609	hypermethylated	Rcan2	30	31
chr17	44242757	44244757	-0.06658	0.012335	hypomethylated	Enpp4	16	16
chr17	44913119	44915119	-0.072863	0.00000448	hypomethylated	Supt3	30	28
chr17	45570656	45572656	0.25966	1.07E-10	hypermethylated	Cdc5l	17	20
chr17	45700100	45702100	-0.2134	0.018389	hypomethylated	Slc35b2	20	20
chr17	45736552	45738552	-0.0898	0.0052025	hypomethylated	Gm7325,Slc29a1	19	23
chr17	45822320	45824320	-0.092075	0.00037665	hypomethylated	Mrpl14,Tmem63b	45	34
chr17	46338803	46340803	-0.098725	0.049917	hypomethylated	Polh,Xpo5	20	20
chr17	46339574	46341574	-0.098725	0.049917	hypomethylated	Polh,Xpo5	20	20
chr17	46384028	46386028	0.10409	0.00022687	hypermethylated	Polr1c,Yipf3	19	16
chr17	46384994	46386994	0.10652	0.0012047	hypermethylated	Polr1c,Yipf3	20	17
chr17	46390113	46392113	-0.12829	0.0000769	hypomethylated	Lrrc73	26	30
chr17	46433369	46435369	-0.11806	0.00067377	hypomethylated	Dlk2	20	18
chr17	46811204	46813204	-0.1147	0.0010121	hypomethylated	Rrp36	8	8
chr17	46889161	46891161	0.22484	0.00017572	hypermethylated	Cnpy3	10	13
chr17	47276890	47278890	-0.13046	0.033454	hypomethylated	Trerf1	22	26
chr17	47747544	47749544	-0.020073	0.033356	hypomethylated	Bysl,Med20	23	27
chr17	47748441	47750441	-0.039764	0.016019	hypomethylated	Bysl,Med20	14	14
chr17	47831155	47833155	-0.062188	0.00054355	hypomethylated	Frs3	45	42
chr17	47872985	47874985	-0.050388	0.038798	hypomethylated	Tfeb	44	39
chr17	47873879	47875879	-0.052154	0.014051	hypomethylated	Tfeb	61	65
chr17	48548404	48550404	-0.014862	0.016839	hypomethylated	Nfya,Oard1	49	52
chr17	51971972	51973972	0.12705	0.00000122	hypermethylated	Satb1	23	30
chr17	56097609	56099609	-0.0039316	0.03791	hypomethylated	Ccdc94	6	4
chr17	56108904	56110904	0.023109	0.016169	hypermethylated	Shd	18	18
chr17	56175999	56177999	0.047739	0.00017584	hypermethylated	Sh3gl1	15	15
chr17	56279766	56281766	-0.046863	0.034311	hypomethylated	Sema6b	14	15
chr17	56323343	56325343	0.19382	0.040537	hypermethylated	D17Wsu104e	4	3
chr17	56811679	56813679	-0.053508	0.0012075	hypomethylated	Ranbp3	27	32
chr17	56856184	56858184	0.013928	0.012146	hypermethylated	Ndufa11,Vmac	31	20
chr17	56970431	56972431	0.067424	0.0000187	hypermethylated	Rfx2	18	22
chr17	57128686	57130686	0.072777	0.02749	hypermethylated	Clpp	15	15
chr17	57170930	57172930	-0.14192	0.000000254	hypomethylated	Khsrp	9	13
chr17	57200699	57202699	-0.21273	0.0076062	hypomethylated	Crb3	14	9
chr17	57387873	57389873	-0.075	0.047042	hypomethylated	Gpr108,Trip10	13	13
chr17	57907992	57909992	-0.49919	0.0020857	stronglyhypometh	Cntnap5c	4	4
chr17	64949988	64951988	-0.055349	0.021228	hypomethylated	Man2a1	66	68
chr17	65962895	65964895	-0.045345	0.00040028	hypomethylated	Vapa	26	32
chr17	66426386	66428386	-0.070362	0.0000223	hypomethylated	Ankrd12	29	29
chr17	69732317	69734317	-0.018926	0.0061202	hypomethylated	Zbtb14	60	59
chr17	69764786	69766786	-0.16052	0.0051678	hypomethylated	C030034I22Rik	9	13
chr17	69787665	69789665	-0.082944	0.011074	hypomethylated	A330050F15Rik	40	41
chr17	71199130	71201130	-0.2167	0.0018588	hypomethylated	Tgif1	22	21
chr17	71660305	71662305	0.14938	0.0023775	hypermethylated	Emilin2	9	7
chr17	71824683	71826683	-0.043008	0.011964	hypomethylated	Smchd1	36	32
chr17	71900400	71902400	-0.07588	0.0001862	hypomethylated	Spdya	44	39

chr17	71947621	71949621	-0.050901	0.00000126	hypomethylated	Trmt61b	28	25
chr17	71948101	71950101	-0.024113	0.002414	hypomethylated	Trmt61b	15	11
chr17	72130286	72132286	0.046129	0.040541	hypermethylated	Clip4	22	22
chr17	74694203	74696203	-0.15282	2.21E-12	hypomethylated	Memo1	43	41
chr17	74737326	74739326	-0.057493	0.00635	hypomethylated	Spast	46	43
chr17	75116089	75118089	-0.14667	0.0000627	hypomethylated	Ttc27	10	12
chr17	75403868	75405868	-0.06786	0.00013148	hypomethylated	Ltbp1	62	71
chr17	79135900	79137900	-0.18003	0.000000587	hypomethylated	Strn	18	16
chr17	79233855	79235855	0.072563	0.007974	hypermethylated	Gpatch11,Heatr5b	27	26
chr17	79234721	79236721	0.23802	0.000000151	hypermethylated	Gpatch11,Heatr5b	6	11
chr17	80013239	80015239	0.068727	0.048713	hypermethylated	Rmdn2	33	33
chr17	80688551	80690551	-0.10264	0.0031988	hypomethylated	Dhx57,Morn2	17	17
chr17	80879793	80881793	0.37849	0.0000146	stronglyhypermeth	Sos1	9	12
chr17	81342971	81344971	-0.16471	0.0090142	hypomethylated	Tmem178	51	51
chr17	81464425	81466425	0.10908	0.028827	hypermethylated	Thumpd2	12	13
chr17	83613622	83615622	-0.066596	0.00032275	hypomethylated	Pkdcc	66	63
chr17	84031235	84033235	-0.093273	1.67E-09	hypomethylated	Kcng3	40	38
chr17	84910234	84912234	-0.13432	5.12E-14	hypomethylated	Plekhh2	36	31
chr17	85356340	85358340	-0.060788	0.0084189	hypomethylated	1110020A21Rik,Ppm1b	38	37
chr17	85356763	85358763	-0.083008	0.0014738	hypomethylated	1110020A21Rik,Ppm1b	36	35
chr17	85357050	85359050	-0.083008	0.0014738	hypomethylated	1110020A21Rik,Ppm1b	36	35
chr17	86566124	86568124	-0.057622	2.92E-09	hypomethylated	Prkce	76	78
chr17	87361450	87363450	-0.077035	0.00099484	hypomethylated	Rhoq	55	58
chr17	87681225	87683225	0.061653	0.023683	hypermethylated	4833418N02Rik,Ttc7	59	59
chr17	87682154	87684154	0.056065	0.0027956	hypermethylated	4833418N02Rik,Ttc7	33	31
chr17	88197334	88199334	-0.039221	0.0017286	hypomethylated	Kcnk12	23	22
chr17	88839051	88841051	0.031123	0.013474	hypermethylated	Foxn2	59	62
chr17	93597761	93599761	-0.22222	0.011157	hypomethylated	Adcyap1	4	5
chr17	95233760	95235760	-0.091667	0.016735	hypomethylated	2610044O15Rik8,Gm1976	6	6
chr18	3506954	3508954	-0.042637	0.014302	hypomethylated	Bambi	53	55
chr18	5334437	5336437	-0.16147	0.02966	hypomethylated	Zfp438	16	21
chr18	7867855	7869855	-0.030528	0.016445	hypomethylated	Wac	45	51
chr18	9706645	9708645	0.10597	0.0039438	hypermethylated	Colec12	26	26
chr18	11996782	11998782	-0.017998	0.02495	hypomethylated	Cables1,Gm6277,Mir1901	63	77
chr18	12279962	12281962	0.12024	0.018992	hypermethylated	Tmem241	6	6
chr18	12286358	12288358	-0.071566	0.00047203	hypomethylated	Riok3	26	26
chr18	12326238	12328238	-0.062356	0.0014132	hypomethylated	3110002H16Rik	35	32
chr18	12394895	12396895	-0.47236	0.0000014	stronglyhypometh	Npc1	20	21
chr18	12464229	12466229	-0.22857	0.00021143	hypomethylated	Ankrd29	5	5
chr18	12801041	12803041	-0.10877	0.0000025	hypomethylated	Ttc39c	20	20
chr18	14841423	14843423	0.14734	0.012012	hypermethylated	Ss18	13	13
chr18	14940753	14942753	-0.15753	0.00000313	hypomethylated	Taf4b	34	27
chr18	16967558	16969558	0.0083261	0.033238	hypermethylated	Cdh2	36	29
chr18	20904905	20906905	-0.12389	0.042431	hypomethylated	B4galt6	7	8
chr18	21158841	21160841	-0.039592	0.044904	hypomethylated	Rnf138	37	40
chr18	22502589	22504589	0.086795	4.15E-10	hypermethylated	Asxl3	46	44
chr18	23911225	23913225	-0.25	0.036969	hypomethylated	Mapre2	6	7
chr18	23961470	23963470	-0.10323	0.012303	hypomethylated	Mapre2	10	18
chr18	24362844	24364844	-0.015908	0.0055399	hypomethylated	Galnt1	79	75
chr18	24363494	24365494	-0.0050487	0.0065949	hypomethylated	Galnt1	81	77
chr18	24866945	24868945	-0.027678	0.0065164	hypomethylated	Fhod3	57	55
chr18	25326520	25328520	-0.059077	0.023962	hypomethylated	AW554918,Tpgs2	51	51
chr18	25327378	25329378	-0.1149	0.0060037	hypomethylated	AW554918,Tpgs2	29	29
chr18	31793036	31795036	-0.12928	0.010524	hypomethylated	Sap130	28	29
chr18	31962710	31964710	-0.073644	0.0010434	hypomethylated	Wdr33	16	19
chr18	32226387	32228387	-0.085763	0.01213	hypomethylated	Iws1	19	23
chr18	32398984	32400984	-0.1302	0.0034853	hypomethylated	Ercc3	12	16
chr18	33097694	33099694	-0.045179	0.00060926	hypomethylated	Camk4	33	26
chr18	34379637	34381637	-0.017364	0.0000685	hypomethylated	Apc,Gm10548	38	44
chr18	34533069	34535069	-0.19423	0.033616	hypomethylated	Reep5	4	5
chr18	35114005	35116005	-0.099558	3.99E-09	hypomethylated	Hspa9	25	33

chr18	35720811	35722811	-0.42474	6.33E-08	stronglyhypometh	Matr3	32	15
chr18	35930212	35932212	-0.09777	0.0021095	hypomethylated	Ube2d2a	39	51
chr18	36439815	36441815	-0.06703	0.00022576	hypomethylated	Pura	36	38
chr18	36507277	36509277	-0.046361	0.046583	hypomethylated	Cystm1	23	23
chr18	36829965	36831965	0.44494	0.0033486	stronglyhypermeth	Sra1	2	2
chr18	36837868	36839868	-0.031707	0.01857	hypomethylated	Apbb3,Slc35a4	19	13
chr18	36893723	36895723	-0.18617	0.0031534	hypomethylated	Tmco6	16	15
chr18	36918892	36920892	-0.87742	0.017642	stronglyhypometh	Wdr55	2	4
chr18	36960416	36962416	-0.10008	0.018428	hypomethylated	Vaultrc5	8	8
chr18	37453493	37455493	-0.32833	0.025067	hypomethylated	Pcdhb2	5	4
chr18	37643674	37645674	-0.34967	0.0000197	stronglyhypometh	Pcdhb17	5	3
chr18	37884359	37886359	-0.18663	0.0094089	hypomethylated	Pcdhga8	11	11
chr18	37905841	37907841	0.3509	0.026028	stronglyhypermeth	Pcdhga10	10	14
chr18	37973732	37975732	-0.23864	0.0098654	hypomethylated	Pcdhgc4	6	12
chr18	38095065	38097065	-0.18205	0.00024423	hypomethylated	Diap1	13	13
chr18	38129385	38131385	-0.013985	0.0015603	hypomethylated	Fchsd1	12	11
chr18	39151798	39153798	-0.093023	0.00000963	hypomethylated	Arhgap26	52	54
chr18	40379053	40381053	-0.19802	0.00020471	hypomethylated	Yipf5	14	12
chr18	42670140	42672140	-0.068405	0.00032175	hypomethylated	Tcerg1	57	56
chr18	43058643	43060643	0.42616	0.014309	stronglyhypermeth	Ppp2r2b	12	8
chr18	44539153	44541153	-0.12451	0.0000101	hypomethylated	Dcp2	31	36
chr18	44971836	44973836	0.10256	0.047078	hypermethylated	Mcc	6	6
chr18	44987318	44989318	0.10944	0.025734	hypermethylated	Ythdc2	36	29
chr18	46372261	46374261	-0.2958	0.0017129	hypomethylated	Trim36	7	6
chr18	49991666	49993666	0.05996	0.040268	hypermethylated	Dmxl1	34	34
chr18	51276551	51278551	-0.19636	0.00022563	hypomethylated	Prr16	26	21
chr18	52624346	52626346	-0.088282	0.00035307	hypomethylated	Srfbp1	28	24
chr18	53404315	53406315	-0.091091	0.0000421	hypomethylated	Snx24	45	45
chr18	53623199	53625199	-0.031845	0.0004573	hypomethylated	Prdm6	82	81
chr18	54020766	54022766	-0.035515	0.00074642	hypomethylated	Csnk1g3	54	54
chr18	56731524	56733524	0.22367	0.0097352	hypermethylated	Aldh7a1,Phax	28	33
chr18	56732593	56734593	0.3006	0.007704	hypermethylated	Aldh7a1,Phax	23	24
chr18	56866466	56868466	-0.026362	0.00000446	hypomethylated	Lmnb1	43	62
chr18	58037331	58039331	-0.050922	0.000000703	hypomethylated	Slc12a2	85	78
chr18	60684874	60686874	-0.044855	0.017804	hypomethylated	Dctn4	16	15
chr18	60719439	60721439	-0.073457	0.01434	hypomethylated	Rbm22	24	24
chr18	60933249	60935249	0.077699	0.030163	hypermethylated	Rps14	38	38
chr18	61008618	61010618	-0.14545	0.0077013	hypomethylated	Tcof1	4	4
chr18	61070893	61072893	-0.06797	0.012334	hypomethylated	Arsi	23	20
chr18	61336703	61338703	-0.022159	0.040419	hypomethylated	Hmgxb3	10	10
chr18	61336704	61338704	-0.022159	0.040419	hypomethylated	Hmgxb3	10	10
chr18	61371250	61373250	-0.030377	0.030872	hypomethylated	Slc26a2	20	18
chr18	61560085	61562085	0.20413	0.0038096	hypermethylated	Ppargc1b	15	22
chr18	61885043	61887043	-0.10607	0.00014417	hypomethylated	1500015A07Rik,Grpel2	27	23
chr18	62708397	62710397	-0.42917	0.0016533	stronglyhypometh	Fbxo38,Spink10	5	8
chr18	64499017	64501017	-0.034729	0.043178	hypomethylated	Onecut2	62	55
chr18	65589650	65591650	-0.043287	0.013376	hypomethylated	Malt1	43	52
chr18	66032147	66034147	-0.14304	0.028089	hypomethylated	Grp	11	11
chr18	67502217	67504217	-0.38721	0.00053679	stronglyhypometh	B430212C06Rik,Cidea	18	14
chr18	67623502	67625502	-0.14173	0.00000721	hypomethylated	Slmo1	9	14
chr18	67800990	67802990	-0.10408	0.00071094	hypomethylated	Cep76,Psmg2	39	38
chr18	68091910	68093910	-0.090719	1.96E-09	hypomethylated	Ldlrad4	64	62
chr18	69503145	69505145	0.14163	0.0035157	hypermethylated	Tcf4	21	22
chr18	69504374	69506374	-0.092232	0.00017575	hypomethylated	Tcf4	44	44
chr18	70689792	70691792	-0.28421	0.0040211	hypomethylated	Poli	4	4
chr18	70726945	70728945	-0.063693	0.00045535	hypomethylated	Mbd2	60	62
chr18	73731358	73733358	-0.052508	0.000000165	hypomethylated	Mex3c	115	109
chr18	75120917	75122917	-0.050949	0.0028283	hypomethylated	Lipg	13	11
chr18	75159130	75161130	-0.054837	0.0000078	hypomethylated	Rpl17,Snord58b	37	37
chr18	75159721	75161721	-0.054837	0.0000078	hypomethylated	Rpl17,Snord58b	37	37
chr18	75177425	75179425	-0.098702	3.41E-08	hypomethylated	Dym	30	28

chr18	76400578	76402578	-0.032228	0.00061632	hypomethylated	Smad2	96	95
chr18	77302946	77304946	-0.25054	6.24E-10	hypomethylated	Pias2	39	40
chr18	77303418	77305418	-0.21335	1.29E-08	hypomethylated	Pias2	37	40
chr18	77803875	77805875	0.077868	0.013818	hypermethylated	Rnf165	23	27
chr18	78011506	78013506	-0.023302	0.0000058	hypomethylated	Atp5a1	22	24
chr18	78032288	78034288	-0.0748	0.0000792	hypomethylated	Pstpip2	41	42
chr18	79306130	79308130	-0.072205	0.00073565	hypomethylated	Setbp1	50	58
chr18	80451964	80453964	0.11058	0.014145	hypermethylated	Pqlc1	10	8
chr18	80452044	80454044	0.11058	0.014145	hypermethylated	Pqlc1	10	8
chr18	80666406	80668406	-0.15582	0.0058692	hypomethylated	Ctdp1	16	17
chr18	80904912	80906912	0.23719	0.00060439	hypermethylated	Nfatc1	20	20
chr18	80909810	80911810	-0.070813	0.023296	hypomethylated	Nfatc1	19	22
chr18	81183317	81185317	-0.078511	0.0000246	hypomethylated	Sall3	80	85
chr18	82576169	82578169	0.12562	0.038098	hypermethylated	Galr1	9	9
chr18	83079270	83081270	-0.17212	0.0000352	hypomethylated	Zfp516	32	23
chr18	84758896	84760896	0.040265	0.034246	hypermethylated	Zfp407	14	10
chr18	84888633	84890633	-0.05438	0.0010978	hypomethylated	Fam69c	40	40
chr18	85120916	85122916	-0.17097	0.000000156	hypomethylated	Timm21	18	18
chr19	3282046	3284046	-0.41757	0.00000337	stronglyhypometh	Ighmbp2,Mrpl21	16	8
chr19	3283010	3285010	-0.14395	0.00011842	hypomethylated	Ighmbp2,Mrpl21	13	9
chr19	3905230	3907230	-0.22514	0.0084295	hypomethylated	Tcirg1	14	12
chr19	3912717	3914717	-0.2	0.034355	hypomethylated	Ndufs8	3	3
chr19	3934185	3936185	-0.11516	0.0013619	hypomethylated	Unc93b1	6	5
chr19	4099116	4101116	-0.05321	0.00088823	hypomethylated	Pitpnm1	28	32
chr19	4099621	4101621	-0.049105	0.0031366	hypomethylated	Pitpnm1	26	31
chr19	4439424	4441424	-0.18353	0.00055584	hypomethylated	A930001C03Rik,Rhod	18	18
chr19	4710222	4712222	-0.1384	0.00000445	hypomethylated	Sptbn2	34	39
chr19	4755524	4757524	-0.087193	0.00053408	hypomethylated	Rbm4b	19	27
chr19	4877667	4879667	-0.23328	0.000078	hypomethylated	Actn3,Zdhhc24	20	23
chr19	4877689	4879689	-0.23328	0.000078	hypomethylated	Actn3,Zdhhc24	20	23
chr19	4877884	4879884	-0.21526	0.0049898	hypomethylated	Actn3,Zdhhc24	13	16
chr19	4961305	4963305	-0.16435	0.0000416	hypomethylated	Mrpl11	6	6
chr19	5023005	5025005	0.1096	0.047907	hypermethylated	Slc29a2	11	11
chr19	5085477	5087477	0.19388	0.0022822	hypermethylated	Tmem151a	21	21
chr19	5295455	5297455	0.085103	0.014394	hypermethylated	Gal3st3,Sf3b2	18	19
chr19	5365812	5367812	0.032678	0.0011558	hypermethylated	Banf1,Eif1ad	52	48
chr19	5366347	5368347	0.03672	0.0010749	hypermethylated	Banf1,Eif1ad	47	43
chr19	5366645	5368645	0.070925	0.00028722	hypermethylated	Banf1,Eif1ad	27	23
chr19	5387335	5389335	-0.099218	0.0022875	hypomethylated	D330050I16Rik,Sart1	50	46
chr19	5424916	5426916	-0.052101	0.00036945	hypomethylated	AI837181,Drap1	30	34
chr19	5560575	5562575	-0.19355	0.0000854	hypomethylated	Ovol1	27	26
chr19	5636489	5638489	-0.070287	0.0046439	hypomethylated	Rela	50	62
chr19	5688130	5690130	-0.10994	3.89E-11	hypomethylated	Map3k11,Pcnxl3	56	53
chr19	5688908	5690908	-0.10111	0.0000194	hypomethylated	Map3k11,Pcnxl3	42	39
chr19	5845478	5847478	0.30913	0.044328	hypermethylated	Neat1	8	12
chr19	6084096	6086096	-0.11925	0.0000017	hypomethylated	Cdca5,Zfpl1	16	24
chr19	6084891	6086891	-0.13205	0.023316	hypomethylated	Cdca5,Zfpl1	18	26
chr19	6183409	6185409	-0.47597	0.0000335	stronglyhypometh	1700123I01Rik	4	7
chr19	6333978	6335978	-0.4957	1.52E-08	stronglyhypometh	Men1	17	21
chr19	6334012	6336012	-0.4957	1.52E-08	stronglyhypometh	Men1	17	21
chr19	6334038	6336038	-0.4957	1.52E-08	stronglyhypometh	Men1	17	21
chr19	6340249	6342249	-0.08205	0.044482	hypomethylated	Map4k2	29	29
chr19	6996298	6998298	-0.089313	0.023071	hypomethylated	Esrra	20	20
chr19	6999870	7001870	0.089108	0.020983	hypermethylated	Tex40	14	16
chr19	7015344	7017344	-0.13471	0.026677	hypomethylated	Bad,Gpr137	40	41
chr19	7015683	7017683	-0.13471	0.026677	hypomethylated	Bad,Gpr137	40	41
chr19	7054930	7056930	0.097756	0.015814	hypermethylated	Fkbp2	10	10
chr19	7054951	7056951	0.097756	0.015814	hypermethylated	Fkbp2	10	10
chr19	7069620	7071620	-0.0016738	0.0000381	hypomethylated	Nudt22,Trpt1	18	16
chr19	7070527	7072527	-0.13814	0.0000467	hypomethylated	Nudt22,Trpt1	8	10
chr19	7130257	7132257	-0.15207	0.000000291	hypomethylated	Macrod1	16	21

chr19	7567529	7569529	-0.045493	0.040843	hypomethylated	Atl3	29	32
chr19	7567927	7569927	-0.045493	0.040843	hypomethylated	Atl3	29	32
chr19	8847974	8849974	-0.043314	0.00000705	hypomethylated	Gm2518,Tmem179b	51	50
chr19	8848957	8850957	-0.053758	0.000000842	hypomethylated	Gm2518,Tmem179b	46	46
chr19	8966476	8968476	-0.32102	3.96E-08	hypomethylated	Ints5	22	13
chr19	8971600	8973600	-0.17649	0.0000233	hypomethylated	Ganab	24	21
chr19	8993882	8995882	-0.43777	2.71E-11	stronglyhypometh	B3gat3	17	17
chr19	9974023	9976023	-0.11212	0.001943	hypomethylated	Incenp	10	10
chr19	10115037	10117037	-0.080884	0.00000184	hypomethylated	Fads3	32	32
chr19	10175993	10177993	-0.031744	0.010018	hypomethylated	Fads2	25	23
chr19	10256377	10258377	-0.04555	0.035541	hypomethylated	Fads1	45	39
chr19	10599699	10601699	-0.036517	0.00045395	hypomethylated	Cpsf7,Sdhaf2	33	31
chr19	10762304	10764304	-0.055656	0.0092795	hypomethylated	Vps37c	25	25
chr19	11023756	11025756	-0.12437	0.0010079	hypomethylated	Ccdc86	9	9
chr19	11985888	11987888	-0.052932	0.041237	hypomethylated	Patl1	35	32
chr19	12039333	12041333	0.043838	0.00046346	hypermethylated	Osbp	47	46
chr19	14672473	14674473	0.043795	0.045479	hypermethylated	Tle4	30	30
chr19	16206320	16208320	-0.091101	5.76E-17	hypomethylated	Gnaq	97	95
chr19	16509156	16511156	-0.051508	0.045143	hypomethylated	Gna14	24	24
chr19	17467532	17469532	-0.033099	0.0016969	hypomethylated	Rfk	29	19
chr19	18744269	18746269	-0.083943	0.00000124	hypomethylated	2410127L17Rik	42	34
chr19	21726798	21728798	-0.05762	0.0000237	hypomethylated	1110059E24Rik,Fam108b	64	67
chr19	21727281	21729281	-0.043138	0.00075309	hypomethylated	1110059E24Rik,Fam108b	60	63
chr19	21851831	21853831	-0.08422	0.0000023	hypomethylated	Tmem2	41	42
chr19	23760889	23762889	-0.14306	0.00000361	hypomethylated	Ptar1	49	52
chr19	25310691	25312691	-0.19673	0.0003483	hypomethylated	Kank1	38	42
chr19	25579195	25581195	-0.0017827	0.0044675	hypomethylated	Dmrt1	25	28
chr19	27290509	27292509	-0.016807	0.0038118	hypomethylated	Vldlr	52	55
chr19	27503526	27505526	0.08631	0.0064753	hypermethylated	C030016D13Rik,D19Bwg1357	28	29
chr19	29063983	29065983	-0.057159	0.038058	hypomethylated	Cdc37l1	28	35
chr19	29325317	29327317	-0.070519	0.00014372	hypomethylated	Jak2	71	63
chr19	29595771	29597771	-0.056228	0.00000494	hypomethylated	A930007I19Rik,C030046E11Ri	90	95
chr19	29596477	29598477	-0.055131	0.00000358	hypomethylated	A930007I19Rik,C030046E11Ri	86	93
chr19	29880499	29882499	-0.14223	0.0001424	hypomethylated	9930021J03Rik	26	26
chr19	30077279	30079279	-0.20614	0.0038089	hypomethylated	Trpd52l3	3	3
chr19	34265759	34267759	-0.070191	0.00015023	hypomethylated	Stambpl1	20	23
chr19	34952407	34954407	-0.055861	0.00036042	hypomethylated	Pank1	51	54
chr19	34953945	34955945	-0.37424	0.0011375	stronglyhypometh	Pank1	3	5
chr19	34995847	34997847	-0.090043	0.013888	hypomethylated	Kif20b	18	22
chr19	36131850	36133850	-0.0073224	0.0011869	hypomethylated	Htr7	36	28
chr19	36907721	36909721	0.04533	0.0049886	hypermethylated	Tnks2	32	26
chr19	37281034	37283034	0.023366	0.024916	hypermethylated	Cpeb3,March5	66	72
chr19	37281783	37283783	0.039867	0.013104	hypermethylated	Cpeb3,March5	48	54
chr19	37449892	37451892	-0.20023	0.00038274	hypomethylated	Kif11	17	13
chr19	38469469	38471469	0.048522	0.00035918	hypermethylated	Slc35g1	23	23
chr19	40588226	40590226	-0.10456	0.0035737	hypomethylated	Sorbs1	6	6
chr19	40904768	40906768	-0.043352	0.00011958	hypomethylated	Ccnj	66	66
chr19	41985360	41987360	-0.075118	0.0083805	hypomethylated	Pgam1	30	30
chr19	42006961	42008961	-0.034318	0.034873	hypomethylated	Exosc1,Zdhhc16	43	48
chr19	42055252	42057252	-0.07956	0.001916	hypomethylated	Mms19,Ubtd1	66	58
chr19	42055626	42057626	-0.087057	0.0021855	hypomethylated	Mms19,Ubtd1	56	55
chr19	42687296	42689296	-0.39773	0.0010899	stronglyhypometh	Loxl4	10	11
chr19	43599095	43601095	-0.28057	0.0038943	hypomethylated	Got1	8	10
chr19	44144176	44146176	0.064474	0.0031981	hypermethylated	Erlin1	20	20
chr19	44144265	44146265	0.089578	0.0053669	hypermethylated	Erlin1	13	13
chr19	44144275	44146275	0.084222	0.013509	hypermethylated	Erlin1	12	12
chr19	44367165	44369165	-0.063492	0.0016769	hypomethylated	Scd2	13	9
chr19	44406815	44408815	-0.30355	0.047107	hypomethylated	Scd4	2	2
chr19	44482199	44484199	-0.10585	0.037031	hypomethylated	Scd1	6	6
chr19	44629905	44631905	0.50661	0.000018	stronglyhypermeth	Ndufb8	6	6
chr19	44830883	44832883	-0.17446	0.000000136	hypomethylated	Pax2	50	41

chr19	45004608	45006608	-0.13635	0.0000623	hypomethylated	Fam178a	18	20
chr19	45080047	45082047	-0.049291	0.029902	hypomethylated	Mrpl43,Peo1	41	43
chr19	45734683	45736683	-0.04388	0.021916	hypomethylated	Fbxw4	19	20
chr19	46130028	46132028	-0.0033825	0.036251	hypomethylated	Pprc1	34	36
chr19	46149352	46151352	-0.0036096	0.000000178	hypomethylated	Nolc1	36	26
chr19	47141237	47143237	-0.09367	0.00000295	hypomethylated	Taf5	32	28
chr19	47252309	47254309	-0.069422	0.00000609	hypomethylated	Neurl1a	60	63
chr19	47538901	47540901	-0.080628	0.016891	hypomethylated	Sh3pxd2a	22	31
chr19	47911851	47913851	-0.064031	0.025914	hypomethylated	Wdr96	6	8
chr19	47993789	47995789	-0.29542	0.00051214	hypomethylated	Itprip	19	15
chr19	48279514	48281514	-0.12083	0.0000328	hypomethylated	Sorcs3	52	36
chr19	50753102	50755102	-0.20279	0.00024417	hypomethylated	Sorcs1	13	20
chr19	53383995	53385995	-0.083614	0.00050683	hypomethylated	Mxi1	30	26
chr19	53465063	53467063	-0.19176	0.0075839	hypomethylated	Smndc1	17	17
chr19	53750795	53752795	-0.054198	0.0004755	hypomethylated	Rbm20	34	32
chr19	55326858	55328858	-0.0078373	0.043603	hypomethylated	Acsl5	8	15
chr19	58528956	58530956	-0.19006	0.0036146	hypomethylated	Gfra1	30	32
chr19	58833212	58835212	-0.31825	0.0032565	hypomethylated	Pnliprp2	6	6
chr19	58868904	58870904	-0.17403	0.010006	hypomethylated	1700019N19Rik	11	10
chr19	59420270	59422270	-0.049173	0.0066672	hypomethylated	Pdzd8	43	43
chr19	59532179	59534179	-0.12634	0.0026384	hypomethylated	Emx2,Emx2os	55	50
chr19	60019267	60021267	-0.10714	0.0010444	hypomethylated	Rab11fip2	12	12
chr19	60019557	60021557	-0.26923	0.0000234	hypomethylated	Rab11fip2	4	4
chr19	60830889	60832889	-0.054605	0.00000115	hypomethylated	Nanos1	57	52
chr2	3629729	3631729	-0.11423	0.0018833	hypomethylated	Fam107b	28	28
chr2	4637876	4639876	-0.031637	0.0027399	hypomethylated	Bend7	47	47
chr2	4839041	4841041	-0.4459	0.000000195	stronglyhypometh	Phyh	10	11
chr2	4933837	4935837	-0.33095	0.0026676	hypomethylated	Mcm10	2	3
chr2	5057821	5059821	-0.024019	0.049227	hypomethylated	Ccdc3	35	32
chr2	5765079	5767079	-0.47685	0.015704	stronglyhypometh	Cdc123,Nudt5	10	9
chr2	9801872	9803872	-0.093685	0.023583	hypomethylated	4930412O13Rik	13	19
chr2	11093008	11095008	-0.18321	0.0059131	hypomethylated	Prkcq	25	16
chr2	11698379	11700379	-0.11894	0.00025215	hypomethylated	Ankrd16,Fbxo18	18	20
chr2	14975988	14977988	-0.2709	3.58E-12	hypomethylated	Arl5b,Nsun6	39	41
chr2	14976499	14978499	-0.2709	3.58E-12	hypomethylated	Arl5b,Nsun6	39	41
chr2	18593088	18595088	-0.10217	0.0024527	hypomethylated	Commd3	19	19
chr2	18597644	18599644	-0.049196	0.00000329	hypomethylated	Bmi1	85	86
chr2	18918945	18920945	-0.14252	3.68E-08	hypomethylated	4930426L09Rik,Pip4k2a	56	55
chr2	19578688	19580688	-0.052148	0.0048648	hypomethylated	Gm3230,Otud1	72	72
chr2	19579524	19581524	-0.059814	0.047181	hypomethylated	Gm3230,Otud1	61	61
chr2	20440139	20442139	-0.14149	0.0078863	hypomethylated	Etl4	21	21
chr2	20889348	20891348	-0.062749	0.0000491	hypomethylated	Arhgap21,Gm13375	88	84
chr2	20889500	20891500	-0.061637	0.0000689	hypomethylated	Arhgap21,Gm13375	86	84
chr2	22476846	22478846	-0.061985	0.0041264	hypomethylated	Gad2	16	16
chr2	22750041	22752041	0.11646	0.00037705	hypermethylated	Pdss1	28	28
chr2	23011065	23013065	0.093604	0.0000222	hypermethylated	Mastl,Yme1l1	25	23
chr2	23011544	23013544	0.064693	0.002901	hypermethylated	Mastl,Yme1l1	22	18
chr2	24775110	24777110	0.17235	0.018531	hypermethylated	Ehmt1	14	18
chr2	24804321	24806321	-0.062152	0.00000381	hypomethylated	Zmynd19	63	67
chr2	24908898	24910898	-0.062993	0.0071106	hypomethylated	Nsmf	23	23
chr2	25092307	25094307	-0.24487	0.00092372	hypomethylated	Gm757	4	4
chr2	25110934	25112934	-0.15018	0.0152	hypomethylated	Ndor1,Tmem203	23	21
chr2	25117117	25119117	0.10668	0.00086606	hypermethylated	Tprn	32	32
chr2	25144430	25146430	-0.078086	0.014208	hypomethylated	E130003G02Rik,Lrrc26,Tmem2	28	26
chr2	25226840	25228840	0.12884	0.0040672	hypermethylated	Sapcd2	20	25
chr2	25257602	25259602	-0.025044	0.019606	hypomethylated	Npdc1	23	23
chr2	25402414	25404414	0.28791	0.040482	hypermethylated	Traf2	10	10
chr2	25412419	25414419	0.1029	0.00080729	hypermethylated	Edf1	27	26
chr2	25430391	25432391	0.15088	0.015019	hypermethylated	Phpt1	11	15
chr2	25463966	25465966	-0.13364	0.0000965	hypomethylated	Rabl6	13	13
chr2	25718373	25720373	-0.2268	0.00000713	hypomethylated	Kcnt1	12	12

chr2	25877280	25879280	-0.053495	0.0089501	hypomethylated	Ubac1	12	12
chr2	26207630	26209630	-0.38909	0.00000168	stronglyhypometh	Dnlz	4	4
chr2	26300736	26302736	-0.076025	0.0011095	hypomethylated	Sec16a	13	12
chr2	26438383	26440383	-0.40767	0.0019229	stronglyhypometh	Egfl7	2	2
chr2	26494841	26496841	0.14127	0.005288	hypermethylated	Snhg7,Snora17	9	12
chr2	26770940	26772940	-0.39772	7.16E-08	stronglyhypometh	Surf1,Surf2	19	19
chr2	26772050	26774050	-0.39286	0.0000181	stronglyhypometh	Surf1,Surf2	7	8
chr2	27282345	27284345	0.032653	0.039412	hypermethylated	Vav2	22	22
chr2	27331193	27333193	-0.029804	0.021599	hypomethylated	Brd3	24	18
chr2	27369666	27371666	-0.38984	0.00000155	stronglyhypometh	Wdr5	25	25
chr2	27531720	27533720	-0.083927	1.47E-12	hypomethylated	Rxra	71	70
chr2	27740944	27742944	-0.082266	0.0013454	hypomethylated	Col5a1	39	39
chr2	28321844	28323844	-0.088732	0.012983	hypomethylated	1700007K13Rik,Mrps2	29	24
chr2	28322585	28324585	-0.078927	0.022175	hypomethylated	1700007K13Rik,Mrps2	37	32
chr2	28387983	28389983	-0.11623	0.00000289	hypomethylated	Ralgds	22	22
chr2	28694925	28696925	-0.21265	0.0022252	hypomethylated	Ddx31,Gtf3c4	30	25
chr2	28695880	28697880	-0.34271	0.00802	stronglyhypometh	Ddx31,Gtf3c4	20	14
chr2	28771941	28773941	-0.082862	0.048702	hypomethylated	Barhl1	32	34
chr2	28771960	28773960	-0.082862	0.048702	hypomethylated	Barhl1	32	34
chr2	29657199	29659199	-0.034039	0.010876	hypomethylated	Slc27a4	35	31
chr2	29681908	29683908	-0.26776	0.0000938	hypomethylated	Urm1	17	16
chr2	29701247	29703247	-0.16771	3.31E-08	hypomethylated	Mir219-2	34	32
chr2	29724013	29726013	-0.25856	0.00071742	hypomethylated	Cercam	12	13
chr2	29904399	29906399	-0.09474	0.025559	hypomethylated	Wdr34	18	18
chr2	29979974	29981974	-0.077289	0.017251	hypomethylated	Zer1	13	13
chr2	30092288	30094288	-0.12321	0.0000292	hypomethylated	1700084E18Rik,Lrrc8a	37	38
chr2	30093151	30095151	-0.13744	0.0000146	hypomethylated	1700084E18Rik,Lrrc8a	33	33
chr2	30140952	30142952	-0.20633	0.03008	hypomethylated	Dolk,Nup188	15	16
chr2	30270569	30272569	-0.061229	5.48E-08	hypomethylated	Crat,Ppp2r4	44	41
chr2	30807520	30809520	-0.047004	0.012577	hypomethylated	Tor1b	23	21
chr2	31006835	31008835	-0.14229	0.035904	hypomethylated	D330023K18Rik,Gpr107	16	12
chr2	31100442	31102442	-0.085174	0.00000381	hypomethylated	Ncs1	64	66
chr2	31543075	31545075	-0.11418	0.01122	hypomethylated	Abl1	42	40
chr2	31614464	31616464	-0.12278	0.024332	hypomethylated	Abl1	10	10
chr2	31804822	31806822	-0.15122	0.0016534	hypomethylated	Aif1l	29	31
chr2	31828969	31830969	-0.23	0.020488	hypomethylated	Nup214	27	29
chr2	32091202	32093202	0.21255	0.021704	hypermethylated	Pomt1	16	16
chr2	32304976	32306976	-0.036863	0.0034614	hypomethylated	Naif1,Slc25a25	38	37
chr2	32306990	32308990	-0.24241	0.0025109	hypomethylated	Slc25a25	13	13
chr2	32730653	32732653	-0.34445	0.0000164	stronglyhypometh	Fam129b	24	28
chr2	32816231	32818231	-0.13144	0.00021556	hypomethylated	Lrsam1,Rpl12,Snora65	28	28
chr2	32816771	32818771	-0.062915	0.00073427	hypomethylated	Lrsam1,Rpl12,Snora65	24	24
chr2	32837576	32839576	-0.18387	0.011705	hypomethylated	Slc2a8	10	14
chr2	33324052	33326052	-0.32857	8.41E-08	hypomethylated	Zbtb43	7	7
chr2	35056640	35058640	-0.16471	0.0008249	hypomethylated	Rab14	9	9
chr2	35959580	35961580	0.015042	0.017687	hypermethylated	Lhx6	28	27
chr2	35990917	35992917	-0.14185	0.00020754	hypomethylated	Mrrf,Rbm18	16	15
chr2	35992224	35994224	-0.15556	0.00083343	hypomethylated	Mrrf,Rbm18	9	9
chr2	37297804	37299804	-0.14225	0.0087391	hypomethylated	Rabgap1,Zbtb26	7	7
chr2	38142904	38144904	-0.15444	0.04746	hypomethylated	Dennd1a	5	5
chr2	38366216	38368216	0.039212	0.0044868	hypermethylated	Nek6	32	32
chr2	38366395	38368395	0.039212	0.0044868	hypermethylated	Nek6	32	32
chr2	38860651	38862651	-0.10198	0.004117	hypomethylated	Rpl35	22	21
chr2	38862658	38864658	-0.085405	0.0007884	hypomethylated	Arpc5l	37	41
chr2	44965657	44967657	-0.15826	0.0003189	hypomethylated	Gm13476	20	17
chr2	48804027	48806027	-0.20089	0.033482	hypomethylated	Mbd5,Orc4	19	18
chr2	51927356	51929356	-0.077522	6.1E-10	hypomethylated	Rif1	52	57
chr2	54287797	54289797	-0.097574	0.0000012	hypomethylated	Galnt13	59	52
chr2	56976414	56978414	-0.096358	0.00077468	hypomethylated	Nr4a2	29	29
chr2	60046992	60048992	-0.13691	6.17E-09	hypomethylated	March7	64	62
chr2	68698613	68700613	-0.11951	0.00070173	hypomethylated	Cers6	43	39

chr2	69550663	69552663	-0.12048	0.0016216	hypomethylated	Fastkd1	7	7
chr2	69626793	69628793	-0.042051	0.010371	hypomethylated	Ccdc173,Phospho2	19	14
chr2	70311979	70313979	-0.14449	0.0001879	hypomethylated	Sp5	20	23
chr2	70399220	70401220	-0.21241	0.027721	hypomethylated	1500002O10Rik,Gad1	11	10
chr2	70498565	70500565	-0.042623	0.014974	hypomethylated	Gorasp2	42	47
chr2	70892800	70894800	0.053014	0.021066	hypermethylated	Dcaf17,Mettl8	18	17
chr2	70955110	70957110	0.08495	0.0081982	hypermethylated	Cybrd1	20	19
chr2	71226316	71228316	0.060785	0.024481	hypermethylated	Hat1	9	13
chr2	71290394	71292394	-0.063571	0.049134	hypomethylated	Metap1d	16	15
chr2	71366501	71368501	-0.09631	3.58E-08	hypomethylated	Dlx1	34	39
chr2	71624139	71626139	-0.036216	0.000166	hypomethylated	Itga6	57	63
chr2	72313275	72315275	-0.11973	0.042939	hypomethylated	Cdca7	26	20
chr2	73149708	73151708	-0.082337	0.0025118	hypomethylated	Cir1,Scrn3	23	27
chr2	73150649	73152649	-0.064387	0.013657	hypomethylated	Cir1,Scrn3	15	15
chr2	73418363	73420363	-0.29045	0.001828	hypomethylated	Chrna1	3	3
chr2	73730685	73732685	-0.37406	0.00000163	stronglyhypometh	Atf2	5	11
chr2	74505366	74507366	0.026018	0.013845	hypermethylated	Hoxd13	40	47
chr2	74529004	74531004	0.17717	0.0036115	hypermethylated	Hoxd10	33	31
chr2	74600036	74602036	-0.062642	0.000000579	hypomethylated	Hoxd1	53	51
chr2	75496315	75498315	-0.07587	0.012763	hypomethylated	Gm6793,Hnrnpa3	56	51
chr2	75496346	75498346	-0.07587	0.012763	hypomethylated	Gm6793,Hnrnpa3	56	51
chr2	76176710	76178710	-0.18242	0.038935	hypomethylated	Pde11a	11	11
chr2	76486051	76488051	-0.13208	0.0013011	hypomethylated	Dfnb59,Prkra	15	15
chr2	76511155	76513155	-0.019721	0.042937	hypomethylated	Fkbp7,Plekha3	8	10
chr2	80154622	80156622	-0.1982	0.000000273	hypomethylated	Dnajc10	29	26
chr2	80477968	80479968	-0.18073	0.026114	hypomethylated	Nup35	17	13
chr2	80478420	80480420	-0.18073	0.026114	hypomethylated	Nup35	17	13
chr2	83483734	83485734	-0.0060566	0.003045	hypomethylated	Zc3h15	31	33
chr2	83651884	83653884	0.076785	0.041868	hypermethylated	Fam171b	21	24
chr2	84637984	84639984	-0.04799	0.037001	hypomethylated	Ube2l6	5	4
chr2	84726849	84728849	-0.033149	0.014599	hypomethylated	Rtn4rl2	21	21
chr2	84975516	84977516	-0.093197	0.04588	hypomethylated	Aplnr	7	7
chr2	90584526	90586526	-0.047268	0.00034596	hypomethylated	Fnbp4	31	35
chr2	90621900	90623900	-0.12815	0.014312	hypomethylated	Agbl2	21	18
chr2	90686311	90688311	-0.12728	0.022826	hypomethylated	Mtch2	42	41
chr2	90724942	90726942	-0.060026	0.0002531	hypomethylated	C1qtnf4	14	14
chr2	90779614	90781614	-0.017081	0.00022696	hypomethylated	Celf1	54	54
chr2	90910378	90912378	-0.064185	0.00052854	hypomethylated	Slc39a13	19	19
chr2	91023204	91025204	-0.18667	0.022207	hypomethylated	Madd	2	2
chr2	91095969	91097969	-0.084704	0.00026269	hypomethylated	Pacsin3	29	28
chr2	91096485	91098485	-0.073371	0.021534	hypomethylated	Pacsin3	21	18
chr2	91104271	91106271	-0.17673	0.0000876	hypomethylated	Arfgap2	26	25
chr2	91296687	91298687	-0.084484	0.036992	hypomethylated	Lrp4	37	36
chr2	91803720	91805720	-0.11815	0.00000433	hypomethylated	Dgkz	11	11
chr2	92023338	92025338	-0.001946	0.000000482	hypomethylated	Phf21a	53	55
chr2	92024639	92026639	-0.088596	0.0037994	hypomethylated	Phf21a	22	22
chr2	92031133	92033133	-0.36184	0.016788	stronglyhypometh	Mir1955	2	2
chr2	92274226	92276226	-0.054251	0.00085813	hypomethylated	Cry2	11	13
chr2	93026740	93028740	-0.056364	0.0000365	hypomethylated	Trp53i11	54	53
chr2	93481590	93483590	-0.12264	8.87E-16	hypomethylated	Alx4	52	52
chr2	93689933	93691933	0.14903	0.0000162	hypermethylated	Accs	9	9
chr2	101517596	101519596	-0.030289	0.002004	hypomethylated	Traf6	41	42
chr2	102497839	102499839	-0.086494	0.0000898	hypomethylated	Slc1a2	40	37
chr2	103405466	103407466	-0.09572	3.69E-08	hypomethylated	Abtb2	56	52
chr2	103601407	103603407	-0.15254	0.0000338	hypomethylated	Nat10	14	12
chr2	103934957	103936957	-0.26052	0.00054489	hypomethylated	Cd59a	6	8
chr2	103961925	103963925	-0.15012	0.00075191	hypomethylated	A930018P22Rik	33	33
chr2	104250491	104252491	0.11264	0.021862	hypermethylated	D430041D05Rik	13	13
chr2	104429640	104431640	-0.25357	1.29E-09	hypomethylated	Cstf3	14	12
chr2	104656853	104658853	-0.029661	0.033285	hypomethylated	Qser1	28	31
chr2	109119894	109121894	-0.14967	1.09E-15	hypomethylated	Kif18a	14	12

chr2	109531592	109533592	-0.25911	0.00034574	hypomethylated	Bdnf	9	15
chr2	109532719	109534719	-0.01978	0.021968	hypomethylated	Bdnf	38	44
chr2	109730034	109732034	-0.050274	0.0024465	hypomethylated	Lin7c	28	27
chr2	112105470	112107470	-0.12232	0.0000118	hypomethylated	Slc12a6	46	45
chr2	112294181	112296181	-0.17983	0.000000653	hypomethylated	Emc7	19	16
chr2	112332120	112334120	-0.014207	0.0040915	hypomethylated	Aven	56	53
chr2	115890357	115892357	-0.11032	0.0072393	hypomethylated	Meis2	18	14
chr2	115890794	115892794	-0.092381	0.015855	hypomethylated	Meis2	21	15
chr2	118597504	118599504	-0.2712	0.00020742	hypomethylated	Phgr1	4	4
chr2	118638738	118640738	-0.22424	0.00416	hypomethylated	Knstrn	24	20
chr2	118726350	118728350	-0.026599	0.028739	hypomethylated	Bahd1	89	92
chr2	119062095	119064095	-0.054447	0.031606	hypomethylated	Spint1	40	31
chr2	119151933	119153933	-0.074583	0.00095978	hypomethylated	Dll4,Gm14207	33	35
chr2	119175977	119177977	0.12978	0.0072336	hypermethylated	Chac1	17	14
chr2	119372442	119374442	-0.063244	0.00099673	hypomethylated	Chp1,Exd1	38	34
chr2	119499803	119501803	-0.081395	0.0018906	hypomethylated	Rtf1	43	48
chr2	119567072	119569072	-0.070732	0.0049592	hypomethylated	Itpka	67	67
chr2	119624249	119626249	-0.062607	0.000000345	hypomethylated	Tyro3	63	61
chr2	119980311	119982311	-0.1492	0.018227	hypomethylated	Ehd4	8	6
chr2	120434171	120436171	-0.1318	0.00000482	hypomethylated	Haus2,Lrrc57	13	16
chr2	120435051	120437051	-0.17687	0.000000241	hypomethylated	Haus2,Lrrc57	6	9
chr2	120557253	120559253	-0.22744	0.00077659	hypomethylated	Cdan1	10	10
chr2	120801797	120803797	-0.11845	0.0016209	hypomethylated	Tmem62	19	25
chr2	120995941	120997941	-0.029339	0.00021638	hypomethylated	Tubgcp4,Zscan29	37	43
chr2	120996885	120998885	-0.05093	0.0000444	hypomethylated	Tubgcp4,Zscan29	25	25
chr2	121238637	121240637	-0.0668	0.00024382	hypomethylated	Catsper2,Pdia3	41	41
chr2	121239528	121241528	-0.02136	0.030991	hypomethylated	Catsper2,Pdia3	29	29
chr2	121268337	121270337	-0.38345	0.0061723	stronglyhypometh	Ell3	2	4
chr2	121281823	121283823	-0.50192	0.0049394	stronglyhypometh	Hypk,Serinc4	7	9
chr2	121282500	121284500	-0.50192	0.0049394	stronglyhypometh	Hypk,Serinc4	7	9
chr2	121691705	121693705	-0.14068	0.000000647	hypomethylated	Casc4	25	31
chr2	121944122	121946122	0.14545	0.014449	hypermethylated	Spg11	10	11
chr2	122059574	122061574	-0.11948	0.030107	hypomethylated	Sord	12	11
chr2	125332174	125334174	-0.17495	0.00051756	hypomethylated	Fbn1	9	9
chr2	125497835	125499835	-0.21559	0.0023146	hypomethylated	Eid1	10	17
chr2	126377759	126379759	-0.09365	0.00018732	hypomethylated	Slc27a2	26	26
chr2	126895392	126897392	-0.1626	0.0042635	hypomethylated	Blvra	15	15
chr2	127033139	127035139	-0.095094	0.0026616	hypomethylated	1810024B03Rik,Snrnp200	15	15
chr2	127034016	127036016	-0.036508	0.00090283	hypomethylated	1810024B03Rik,Snrnp200	15	15
chr2	127094964	127096964	-0.13844	0.000000474	hypomethylated	Stard7	36	35
chr2	127160894	127162894	-0.026697	0.030231	hypomethylated	Dusp2	38	40
chr2	127188021	127190021	-0.04688	0.0039136	hypomethylated	Adra2b	27	32
chr2	127410413	127412413	-0.10769	0.020953	hypomethylated	Mrps5,Zfp661	5	5
chr2	127614590	127616590	-0.035482	0.002554	hypomethylated	Nphp1	7	7
chr2	127950773	127952773	-0.011788	0.015993	hypomethylated	Bcl2l11	47	46
chr2	128643038	128645038	-0.02701	0.0091558	hypomethylated	Tmem87b	20	18
chr2	128792137	128794137	0.045324	0.017526	hypermethylated	Zc3h6	63	60
chr2	129417574	129419574	0.092021	0.049437	hypermethylated	Sirpa	25	18
chr2	129417930	129419930	0.10494	0.018532	hypermethylated	Sirpa	27	19
chr2	129925129	129927129	-0.14167	0.019166	hypomethylated	AU015228	6	6
chr2	130099147	130101147	-0.063464	0.0089796	hypomethylated	Nop56,Snord110	26	29
chr2	130120674	130122674	-0.089594	0.00000696	hypomethylated	Ebf4	67	68
chr2	130249055	130251055	-0.20278	0.00068373	hypomethylated	Pced1a,Vps16	19	19
chr2	130250024	130252024	-0.24762	0.0034656	hypomethylated	Pced1a,Vps16	14	14
chr2	130275013	130277013	-0.085191	0.00000102	hypomethylated	Ptpra	30	33
chr2	130388492	130390492	-0.10222	0.000034	hypomethylated	4930473A02Rik,Mrps26	30	25
chr2	130492576	130494576	-0.24802	0.00016341	hypomethylated	Itpa	12	9
chr2	131087235	131089235	-0.0614	0.0082842	hypomethylated	Pank2	25	25
chr2	131178628	131180628	-0.16997	0.00010004	hypomethylated	Rnf24	16	12
chr2	132087992	132089992	-0.1267	0.00000164	hypomethylated	Cds2	32	28
chr2	132211183	132213183	-0.1655	0.042268	hypomethylated	Prokr2	7	8

chr2	132606013	132608013	-0.25357	0.0051602	hypomethylated	Chgb	14	12
chr2	132641070	132643070	-0.035974	0.0019236	hypomethylated	Mcm8,Trmt6	19	11
chr2	132641790	132643790	-0.079101	0.0006144	hypomethylated	Mcm8,Trmt6	15	9
chr2	136717142	136719142	-0.15714	0.022131	hypomethylated	Mkks,Slx4ip	10	7
chr2	143370868	143372868	-0.055672	0.0078636	hypomethylated	C630020P19Rik,Pcsk2	40	37
chr2	143740066	143742066	-0.075889	0.0085304	hypomethylated	Dstn	25	26
chr2	144095770	144097770	-0.040046	0.0001149	hypomethylated	Mgme1,Snx5	23	31
chr2	144096308	144098308	-0.11315	0.0043251	hypomethylated	Mgme1,Snx5	17	16
chr2	144157098	144159098	-0.089877	0.0065072	hypomethylated	Ovol2	24	27
chr2	144352480	144354480	-0.069061	0.026362	hypomethylated	Dzank1,Polr3f	19	17
chr2	144353134	144355134	-0.067411	0.032181	hypomethylated	Dzank1,Polr3f	18	16
chr2	145759519	145761519	-0.10527	0.0040317	hypomethylated	4930529M08Rik,Crnkl1	8	13
chr2	146046732	146048732	-0.042717	0.016713	hypomethylated	Insm1	82	75
chr2	146837795	146839795	-0.10859	0.0000029	hypomethylated	Xrn2	44	42
chr2	146911081	146913081	-0.0034343	0.013414	hypomethylated	Nkx2-4	15	15
chr2	147872705	147874705	-0.11808	0.00035737	hypomethylated	Foxa2	14	11
chr2	149655518	149657518	0.063747	0.035244	hypermethylated	Syndig1	34	39
chr2	150573816	150575816	-0.081208	0.0000877	hypomethylated	Entpd6	18	18
chr2	150611531	150613531	-0.066433	0.01049	hypomethylated	Pygb	18	18
chr2	150730467	150732467	-0.071429	0.0063958	hypomethylated	Abhd12	6	6
chr2	151301887	151303887	-0.40196	0.00000112	stronglyhypometh	4921509C19Rik	2	3
chr2	151367234	151369234	-0.028895	0.006041	hypomethylated	Fkbp1a	36	31
chr2	151526743	151528743	-0.031287	0.0031214	hypomethylated	Tmem74b	54	54
chr2	151667662	151669662	0.044815	0.0078708	hypermethylated	Rspo4	25	25
chr2	151906264	151908264	0.064249	0.0020469	hypermethylated	Scrt2	43	38
chr2	151930465	151932465	-0.23281	0.0000332	hypomethylated	Srxn1	40	40
chr2	152118607	152120607	-0.05652	0.0040095	hypomethylated	Tbc1d20	20	25
chr2	152169796	152171796	-0.43476	0.00071509	stronglyhypometh	Gm14164,Trib3	6	3
chr2	152202302	152204302	0.28539	0.0000743	hypermethylated	Nrsn2	7	7
chr2	152223782	152225782	-0.022865	0.031569	hypomethylated	Sox12	23	25
chr2	152672699	152674699	0.14632	0.012088	hypermethylated	Tpx2	6	11
chr2	152736087	152738087	-0.26143	0.023498	hypomethylated	Mylk2	6	7
chr2	152777141	152779141	-0.17469	0.0000404	hypomethylated	Dusp15	17	18
chr2	152777318	152779318	-0.28159	0.00024388	hypomethylated	Dusp15	9	10
chr2	153066267	153068267	-0.032013	0.011437	hypomethylated	Plagl2,Pofut1	33	33
chr2	153067094	153069094	-0.058591	0.0026802	hypomethylated	Plagl2,Pofut1	19	19
chr2	153170874	153172874	-0.075782	0.0026304	hypomethylated	2500004C02Rik,Asxl1	37	44
chr2	153355707	153357707	-0.096826	0.00000998	hypomethylated	8430427H17Rik	37	36
chr2	153474189	153476189	0.13363	0.017943	hypermethylated	Dnmt3b	38	45
chr2	153566022	153568022	-0.1104	0.0010887	hypomethylated	Mapre1	25	25
chr2	154395588	154397588	-0.020062	0.030053	hypomethylated	E2f1	18	18
chr2	154438103	154440103	-0.043701	0.00014374	hypomethylated	Zfp341	55	48
chr2	154900233	154902233	-0.096104	0.034184	hypomethylated	Ahcy	7	10
chr2	154958216	154960216	-0.12914	0.0000367	hypomethylated	Itch	42	40
chr2	155061268	155063268	-0.035	0.0025462	hypomethylated	Dynlrb1	15	12
chr2	155101179	155103179	-0.058573	0.000000293	hypomethylated	Map1lc3a	44	45
chr2	155206591	155208591	-0.027157	0.00046692	hypomethylated	Trp53inp2	27	28
chr2	155781293	155783293	-0.027248	0.0262	hypomethylated	Cep250	24	24
chr2	155889948	155891948	-0.090419	0.018997	hypomethylated	Spag4	26	24
chr2	155968888	155970888	-0.11454	0.00000366	hypomethylated	Nfs1,Romo1	18	28
chr2	155968970	155970970	-0.11454	0.00000366	hypomethylated	Nfs1,Romo1	18	28
chr2	155969079	155971079	-0.11454	0.00000366	hypomethylated	Nfs1,Romo1	18	28
chr2	155969922	155971922	-0.14307	0.00000344	hypomethylated	Nfs1,Romo1	14	19
chr2	156137208	156139208	-0.10004	0.0000127	hypomethylated	Cnbd2,Scand1	36	36
chr2	156245787	156247787	-0.098715	0.0046664	hypomethylated	Epb4.1l1	25	27
chr2	156438440	156440440	-0.037294	0.0068641	hypomethylated	Dlgap4,Gm14169	51	49
chr2	156545720	156547720	-0.056508	0.027015	hypomethylated	4930405A21Rik,Dlgap4	34	39
chr2	156546014	156548014	-0.066804	0.00075904	hypomethylated	4930405A21Rik,Dlgap4	37	42
chr2	156687857	156689857	-0.064417	0.005279	hypomethylated	1110008F13Rik,5430405H02R	50	56
chr2	157030270	157032270	-0.11304	0.0088717	hypomethylated	Rbl1	18	20
chr2	157384845	157386845	0.11416	0.028979	hypermethylated	Nnat	13	13

chr2	158592834	158594834	-0.092846	0.0026255	hypomethylated	Fam83d	23	29
chr2	163245206	163247206	-0.11096	0.022299	hypomethylated	3230401D17Rik	24	23
chr2	163819932	163821932	-0.1351	0.00000139	hypomethylated	Ywhab	31	36
chr2	163898913	163900913	-0.20463	1.87E-08	hypomethylated	Stk4	21	24
chr2	164268688	164270688	-0.13047	0.00000345	hypomethylated	Sdc4	12	16
chr2	164310639	164312639	-0.2164	0.00016939	hypomethylated	Dbndd2	31	30
chr2	164310954	164312954	-0.2857	0.00000632	hypomethylated	Dbndd2	34	33
chr2	164311376	164313376	-0.30374	0.000000521	hypomethylated	Dbndd2	36	33
chr2	164629613	164631613	-0.12549	0.00023501	hypomethylated	Acot8,Zswim3	17	15
chr2	164659096	164661096	0.29983	0.0024551	hypermethylated	Ctsa,Neurl2	10	7
chr2	164792487	164794487	-0.029163	0.014652	hypomethylated	Slc12a5	37	34
chr2	165151979	165153979	-0.16492	0.00018861	hypomethylated	Elmo2	18	17
chr2	165817136	165819136	-0.02745	0.00035726	hypomethylated	Ncoa3	41	46
chr2	166730595	166732595	-0.045863	0.013181	hypomethylated	Cse1l	29	27
chr2	167066037	167068037	-0.7	0.00000299	stronglyhypometh	Ptgis	5	4
chr2	167317144	167319144	-0.14374	3.37E-09	hypomethylated	Rnf114,Spata2	32	36
chr2	167318374	167320374	-0.098117	0.0000984	hypomethylated	Rnf114,Spata2	13	16
chr2	168055879	168057879	-0.095564	0.0000105	hypomethylated	Dpm1,Mocs3	76	79
chr2	168415783	168417783	-0.098136	0.0021172	hypomethylated	Nfatc2	14	16
chr2	168415848	168417848	-0.23527	0.00000669	hypomethylated	Nfatc2	9	11
chr2	170335729	170337729	-0.069805	0.0032035	hypomethylated	Gm16796,Pfdn4	21	23
chr2	172265077	172267077	-0.10182	0.001113	hypomethylated	Rtfdc1	13	12
chr2	172374092	172376092	-0.084808	0.00568	hypomethylated	Tfap2c	20	22
chr2	172375490	172377490	-0.12249	0.0000484	hypomethylated	Tfap2c	30	37
chr2	172846402	172848402	-0.036017	0.016345	hypomethylated	Rbm38	39	34
chr2	173485040	173487040	-0.058978	0.019011	hypomethylated	Ppp4r1l-ps,Rab22a	25	25
chr2	173934851	173936851	-0.075868	0.00045866	hypomethylated	Npepl1	35	46
chr2	174108820	174110820	-0.10649	0.001154	hypomethylated	Gnas	25	31
chr2	174240304	174242304	-0.042984	0.0054248	hypomethylated	Nelfcd	20	14
chr2	179710557	179712557	0.040297	0.0032298	hypermethylated	4921531C22Rik,Taf4a	82	85
chr2	179758691	179760691	-0.0027459	0.0020957	hypomethylated	Lsm14b	50	54
chr2	179776187	179778187	-0.034865	0.001527	hypomethylated	Psma7,Ss18l1	76	82
chr2	179804297	179806297	-0.15548	0.000007	hypomethylated	Gtpbp5	13	15
chr2	179905292	179907292	0.09258	0.0018713	hypermethylated	Adrm1	39	44
chr2	179960564	179962564	0.12258	0.0000534	hypermethylated	Lama5	17	18
chr2	179991083	179993083	0.22317	0.0000073	hypermethylated	Mir3091,Rps21	24	22
chr2	179991240	179993240	0.22317	0.0000073	hypermethylated	Mir3091,Rps21	24	22
chr2	180233680	180235680	0.023965	0.023199	hypermethylated	Ntsr1	18	13
chr2	180700929	180702929	-0.075745	0.00000215	hypomethylated	Arfgap1	25	28
chr2	180701004	180703004	-0.075745	0.00000215	hypomethylated	Arfgap1	25	28
chr2	180921047	180923047	-0.18182	0.038689	hypomethylated	Ppdpf	11	11
chr2	181098635	181100635	0.060084	0.015638	hypermethylated	Arfrp1,Zgpat	20	17
chr2	181194131	181196131	-0.10816	0.0005368	hypomethylated	Zbtb46	32	32
chr2	181327166	181329166	-0.18202	0.000102	hypomethylated	Znf512b	37	37
chr2	181428629	181430629	-0.15754	0.00063764	hypomethylated	Rgs19	15	15
chr3	5217553	5219553	-0.17975	0.040054	hypomethylated	Zfhx4	10	10
chr3	5576150	5578150	-0.3328	0.0000119	hypomethylated	Pex2	19	14
chr3	5576151	5578151	-0.3328	0.0000119	hypomethylated	Pex2	19	14
chr3	5576239	5578239	-0.3981	0.0000339	stronglyhypometh	Pex2	15	10
chr3	7365603	7367603	-0.071978	0.0016476	hypomethylated	Pkia	13	12
chr3	10011605	10013605	-0.15079	0.0013118	hypomethylated	Fabp5	6	6
chr3	14611256	14613256	0.051514	0.011429	hypermethylated	1810022K09Rik	14	14
chr3	14885425	14887425	-0.045068	0.00035974	hypomethylated	Car2	48	44
chr3	17953324	17955324	-0.023334	0.038849	hypomethylated	Bhlhe22	46	46
chr3	19211322	19213322	-0.051021	0.00018093	hypomethylated	Pde7a	46	46
chr3	19956810	19958810	-0.12103	0.035344	hypomethylated	Hltf	19	20
chr3	26052229	26054229	-0.21481	0.022872	hypomethylated	Nlgn1	5	6
chr3	28679232	28681232	-0.055745	0.037979	hypomethylated	Eif5a2	46	45
chr3	30690797	30692797	-0.036547	0.0079969	hypomethylated	Sec62	48	43
chr3	30992982	30994982	-0.010758	0.04648	hypomethylated	Skil	108	103
chr3	31047841	31049841	-0.11118	0.037253	hypomethylated	Cldn11	12	12

chr3	32408471	32410471	-0.028344	0.040736	hypomethylated	Zfp639	29	31
chr3	32408513	32410513	-0.028344	0.040736	hypomethylated	Zfp639	29	31
chr3	32427403	32429403	0.089194	0.02498	hypermethylated	Mfn1	20	19
chr3	32715547	32717547	-0.077734	0.0033872	hypomethylated	Usp13	32	32
chr3	33698116	33700116	0.022527	0.031293	hypermethylated	Ttc14	38	44
chr3	34547926	34549926	-0.069544	0.00027649	hypomethylated	Sox2	46	49
chr3	35652059	35654059	-0.060778	0.00072479	hypomethylated	Atp11b	41	36
chr3	36450527	36452527	0.031818	0.044243	hypermethylated	Exosc9	10	10
chr3	36470918	36472918	-0.17432	0.049392	hypomethylated	Ccna2	14	14
chr3	36961577	36963577	-0.10146	0.014543	hypomethylated	Adad1	18	18
chr3	37210475	37212475	-0.27553	7.03E-08	hypomethylated	Bbs12,Cetn4	9	9
chr3	37211368	37213368	-0.27553	7.03E-08	hypomethylated	Bbs12,Cetn4	9	9
chr3	37246574	37248574	-0.09326	2.45E-09	hypomethylated	Fgf2	33	33
chr3	37537871	37539871	-0.06903	8.07E-08	hypomethylated	Spry1	73	63
chr3	40438688	40440688	-0.090118	0.021829	hypomethylated	Intu	15	11
chr3	40548534	40550534	-0.24503	0.0015585	hypomethylated	Hspa4l	32	21
chr3	40602872	40604872	-0.1456	4.59E-08	hypomethylated	Plk4	29	33
chr3	41366304	41368304	-0.12926	0.003934	hypomethylated	Phf17	8	8
chr3	41366672	41368672	-0.096443	0.0075591	hypomethylated	Phf17	12	11
chr3	41367801	41369801	-0.0706	0.000000183	hypomethylated	Phf17	78	61
chr3	41545539	41547539	-0.053664	0.0091471	hypomethylated	D3Ertd751e,Sclt1	30	26
chr3	51027368	51029368	-0.034966	0.034756	hypomethylated	Ccrn4l	64	59
chr3	51218937	51220937	-0.15022	0.0000388	hypomethylated	Naa15	57	57
chr3	52071258	52073258	-0.026622	0.0013698	hypomethylated	Foxo1	40	40
chr3	52844468	52846468	-0.15985	0.0058879	hypomethylated	Lhfp	21	11
chr3	53461277	53463277	-0.25	0.00078236	hypomethylated	Frem2	4	8
chr3	53959025	53961025	0.27987	0.034826	hypermethylated	Trpc4	4	4
chr3	54915029	54917029	-0.090124	0.0015991	hypomethylated	Spg20	21	23
chr3	54915087	54917087	-0.090124	0.0015991	hypomethylated	Spg20	21	23
chr3	55585431	55587431	-0.083127	0.033686	hypomethylated	Mab21l1	11	11
chr3	55987623	55989623	-0.087681	0.00000249	hypomethylated	Nbea	17	17
chr3	57651679	57653679	-0.08249	0.000000287	hypomethylated	Pfn2	26	26
chr3	58379579	58381579	0.019361	0.023784	hypermethylated	2810407C02Rik	37	37
chr3	58809899	58811899	-0.01579	0.043248	hypomethylated	Med12l	58	58
chr3	60304173	60306173	-0.11848	0.00000214	hypomethylated	Mbnl1	46	46
chr3	60805716	60807716	0.038041	0.012174	hypermethylated	P2ry1	22	19
chr3	62141698	62143698	-0.12392	0.0013971	hypomethylated	Arhgef26	32	33
chr3	63733307	63735307	-0.25443	9.12E-08	hypomethylated	E130311K13Rik	8	8
chr3	65469149	65471149	-0.02524	0.017741	hypomethylated	Lekr1	23	18
chr3	65469156	65471156	-0.02524	0.017741	hypomethylated	Lekr1	23	18
chr3	68297114	68299114	0.043853	0.0034337	hypermethylated	Schip1	41	36
chr3	68672507	68674507	-0.023995	0.00032756	hypomethylated	1110032F04Rik	49	50
chr3	69025340	69027340	0.34821	0.0023907	stronglyhypermeth	Arl14	4	4
chr3	69119839	69121839	-0.017809	0.010765	hypomethylated	Ppm1l	75	79
chr3	69663818	69665818	-0.20833	0.00019528	hypomethylated	Sptssb	8	8
chr3	79371601	79373601	-0.10651	0.0082585	hypomethylated	Fnip2	29	27
chr3	80839337	80841337	-0.069258	0.0086356	hypomethylated	Pdgfc	14	18
chr3	81735537	81737537	-0.19487	0.0021702	hypomethylated	Ctso	10	10
chr3	82160993	82162993	-0.14949	0.0000112	hypomethylated	Map9	32	34
chr3	82858459	82860459	-0.32136	0.011407	hypomethylated	Plrg1	11	5
chr3	83569242	83571242	-0.089137	0.000000539	hypomethylated	Sfrp2	44	39
chr3	83844083	83846083	-0.22095	0.00006	hypomethylated	D930015E06Rik	16	12
chr3	84386547	84388547	-0.09232	0.0068451	hypomethylated	Arfip1	27	30
chr3	84618498	84620498	-0.12104	0.00013771	hypomethylated	Fbxw7	48	54
chr3	86027611	86029611	-0.026824	0.02404	hypomethylated	Lrba	54	61
chr3	87709242	87711242	-0.059365	0.000000263	hypomethylated	Hdgf	42	46
chr3	87733235	87735235	0.05967	0.036762	hypermethylated	Isg20l2,Rrnad1	18	23
chr3	87734117	87736117	0.089011	0.00087995	hypermethylated	Isg20l2,Rrnad1	17	19
chr3	87774014	87776014	-0.057793	0.0000231	hypomethylated	Nes	34	38
chr3	87862417	87864417	-0.16679	0.032225	hypomethylated	Apoa1bp	4	4
chr3	87945316	87947316	-0.056402	0.042564	hypomethylated	Mef2d	48	48

chr3	88100056	88102056	-0.1464	0.04002	hypomethylated	Cct3,Tsacc	9	9
chr3	88100760	88102760	-0.13176	0.043705	hypomethylated	Cct3,Tsacc	10	10
chr3	88138355	88140355	0.037949	0.02577	hypermethylated	Smg5,Tmem79	10	10
chr3	88340304	88342304	-0.48837	0.0078987	stronglyhypometh	Mir1905	7	8
chr3	88356637	88358637	0.042178	0.048568	hypermethylated	Lamtor2,Ubqln4	38	30
chr3	88424027	88426027	0.034092	0.0056662	hypermethylated	Arhgef2	46	40
chr3	88424315	88426315	0.034092	0.0056662	hypermethylated	Arhgef2	46	40
chr3	88488715	88490715	0.24614	0.0030742	hypermethylated	2810403A07Rik	22	22
chr3	88967726	88969726	-0.056913	0.00095896	hypomethylated	Clk2	34	34
chr3	89083567	89085567	-0.20767	0.00000439	hypomethylated	Efna1	19	23
chr3	89153932	89155932	-0.22032	0.00073417	hypomethylated	Adam15	25	21
chr3	89169161	89171161	-0.13857	0.0000244	hypomethylated	Dcst1	5	7
chr3	89222213	89224213	-0.44667	0.0048286	stronglyhypometh	Cks1b,Shc1	6	5
chr3	89323085	89325085	-0.07326	0.0010886	hypomethylated	Kcnn3	10	13
chr3	89576530	89578530	-0.031075	0.016363	hypomethylated	Ube2q1	44	46
chr3	89802608	89804608	0.072656	0.013396	hypermethylated	Gm19710,Hax1	7	7
chr3	89855738	89857738	-0.21503	0.00000349	hypomethylated	4933434E20Rik,Ubap2l	30	23
chr3	90057202	90059202	0.069813	0.0098732	hypermethylated	Crtc2	26	18
chr3	94113053	94115053	-0.085272	0.011578	hypomethylated	Them4	12	8
chr3	94216239	94218239	-0.28816	0.000000557	hypomethylated	Tdrkh	7	7
chr3	94246257	94248257	-0.063491	0.039618	hypomethylated	Mrpl9	13	13
chr3	94910780	94912780	-0.077822	0.023575	hypomethylated	Pip5k1a	5	5
chr3	95021864	95023864	-0.50917	0.00021401	stronglyhypometh	Gabpb2	10	10
chr3	95031701	95033701	0.049124	0.021909	hypermethylated	Cdc42se1,Mllt11	31	31
chr3	95031873	95033873	0.049124	0.021909	hypermethylated	Cdc42se1,Mllt11	31	31
chr3	95032599	95034599	0.075199	0.013041	hypermethylated	Cdc42se1,Mllt11	25	25
chr3	95085856	95087856	0.083263	0.045297	hypermethylated	Fam63a,Prune	17	20
chr3	95085998	95087998	0.083263	0.045297	hypermethylated	Fam63a,Prune	17	20
chr3	95461642	95463642	-0.010165	0.0026534	hypomethylated	Mcl1	83	83
chr3	95491781	95493781	-0.034478	0.02184	hypomethylated	Adamtsl4	19	19
chr3	95907449	95909449	-0.079714	0.000000877	hypomethylated	Otud7b	44	40
chr3	96042999	96044999	-0.070076	0.033272	hypomethylated	Hist2h2aa1,Hist2h2aa2,Hist2h	8	8
chr3	96432850	96434850	-0.49215	0.0076772	stronglyhypometh	6330549D23Rik,Rbm8a	13	8
chr3	96530533	96532533	-0.086361	0.0000241	hypomethylated	Polr3c,Rnf115	23	21
chr3	96531362	96533362	-0.036096	0.00051366	hypomethylated	Polr3c,Rnf115	21	21
chr3	97461134	97463134	-0.064102	0.0033492	hypomethylated	Prkab2	33	32
chr3	97571937	97573937	-0.16733	0.028916	hypomethylated	Pde4dip	7	7
chr3	99965440	99967440	-0.02978	0.0086164	hypomethylated	Gdap2,Wdr3	30	32
chr3	99966326	99968326	-0.060491	0.0076552	hypomethylated	Gdap2,Wdr3	17	19
chr3	100293115	100295115	-0.06391	0.0044518	hypomethylated	Fam46c	32	33
chr3	100489396	100491396	-0.009328	0.0061227	hypomethylated	Man1a2	42	42
chr3	100725415	100727415	-0.019641	0.0378	hypomethylated	Trim45	17	16
chr3	101408580	101410580	-0.063632	0.0045487	hypomethylated	Atp1a1	15	13
chr3	102008616	102010616	-0.362	0.0028398	stronglyhypometh	Vangl1	4	9
chr3	102537692	102539692	-0.094557	0.049385	hypomethylated	2410057H14Rik,Tspan2	26	26
chr3	102740023	102742023	-0.14007	0.0018099	hypomethylated	Sycp1	10	10
chr3	102798662	102800662	0.20433	0.00000991	hypermethylated	Sike1	14	11
chr3	102823468	102825468	-0.13302	0.020185	hypomethylated	Csde1	22	30
chr3	103378203	103380203	-0.17648	0.00000521	hypomethylated	Syt6	29	27
chr3	103595198	103597198	-0.026291	0.046733	hypomethylated	Hipk1	20	20
chr3	103612439	103614439	-0.26899	0.0016922	hypomethylated	Ap4b1,Dclre1b	18	21
chr3	103613310	103615310	-0.34355	0.015187	stronglyhypometh	Ap4b1,Dclre1b	13	14
chr3	103717042	103719042	-0.063529	0.038141	hypomethylated	Rsbn1	43	41
chr3	104315779	104317779	0.2961	0.0448	hypermethylated	Lrig2	11	11
chr3	104441590	104443590	-0.071968	4.79E-08	hypomethylated	Slc16a1	76	75
chr3	105260733	105262733	-0.23737	0.025159	hypomethylated	Kcnd3	6	4
chr3	105506516	105508516	-0.067029	0.0012756	hypomethylated	Fam212b	13	14
chr3	105507375	105509375	-0.067029	0.0012756	hypomethylated	Fam212b	13	14
chr3	105761415	105763415	-0.058699	0.031025	hypomethylated	Atp5f1,Wdr77	26	27
chr3	106349744	106351744	-0.14659	0.0014316	hypomethylated	Cept1,Dram2	32	24
chr3	106838079	106840079	-0.075187	0.0072568	hypomethylated	Kcna3	34	30

chr3	107261816	107263816	-0.0071562	0.022253	hypomethylated	Kcnc4	36	33
chr3	107396948	107398948	-0.075547	0.0000983	hypomethylated	Alx3	45	46
chr3	107988252	107990252	-0.0013531	0.011429	hypomethylated	Amigo1	48	48
chr3	108185721	108187721	-0.11766	0.00017154	hypomethylated	Gm12522,Psrc1	8	8
chr3	108185755	108187755	-0.11766	0.00017154	hypomethylated	Gm12522,Psrc1	8	8
chr3	108373616	108375616	-0.049209	0.00000487	hypomethylated	Taf13	18	15
chr3	108393195	108395195	-0.104	0.00032013	hypomethylated	Wdr47	26	23
chr3	108525217	108527217	-0.40625	0.0043848	stronglyhypometh	Gpsm2	8	8
chr3	108714622	108716622	-0.094232	0.0007445	hypomethylated	Prpf38b	19	19
chr3	110053916	110055916	-0.1672	0.0062431	hypomethylated	Prmt6	7	6
chr3	115709366	115711366	0.1115	0.00015962	hypermethylated	Extl2,Slc30a7	21	23
chr3	115710214	115712214	0.091548	0.024278	hypermethylated	Extl2,Slc30a7	16	18
chr3	115710324	115712324	0.12199	0.0071751	hypermethylated	Extl2,Slc30a7	7	9
chr3	116126950	116128950	-0.5404	0.0020625	stronglyhypometh	Cdc14a	4	4
chr3	116511084	116513084	-0.37798	0.00000286	stronglyhypometh	Agl	6	6
chr3	117277378	117279378	-0.058941	0.04392	hypomethylated	4833424O15Rik	36	36
chr3	118264095	118266095	-0.10794	0.041335	hypomethylated	Dpyd	5	5
chr3	121655243	121657243	-0.068094	0.00056726	hypomethylated	Arhgap29	35	37
chr3	121976331	121978331	0.19383	0.00000294	hypermethylated	Dnttip2	24	23
chr3	122626314	122628314	0.014094	0.014016	hypermethylated	1810037I17Rik,4933405D12Ri	22	25
chr3	122969413	122971413	-0.36679	0.0000178	stronglyhypometh	Sec24d	19	17
chr3	123148830	123150830	-0.069967	0.020219	hypomethylated	Prss12	42	42
chr3	127335062	127337062	-0.1059	0.0046318	hypomethylated	Neurog2	32	32
chr3	127540410	127542410	-0.3017	0.012178	hypomethylated	Ap1ar	8	5
chr3	128901835	128903835	-0.096001	0.022546	hypomethylated	Pitx2	16	17
chr3	128901841	128903841	-0.096001	0.022546	hypomethylated	Pitx2	16	17
chr3	128915854	128917854	-0.087819	0.0090796	hypomethylated	Pitx2	16	10
chr3	129234303	129236303	-0.15649	0.0025536	hypomethylated	Elovl6	9	9
chr3	131226731	131228731	0.13098	0.0000877	hypermethylated	Papss1	29	29
chr3	134490970	134492970	-0.20758	0.024839	hypomethylated	Tacr3	8	7
chr3	135086397	135088397	-0.023096	0.043446	hypomethylated	Cisd2	16	16
chr3	135354511	135356511	0.15402	0.0024733	hypermethylated	Nfkb1	11	13
chr3	136332733	136334733	-0.065849	0.00017379	hypomethylated	Ppp3ca	107	107
chr3	137285635	137287635	-0.22164	0.00036786	hypomethylated	Ddit4l	15	19
chr3	137644513	137646513	-0.2563	0.030443	hypomethylated	Dapp1	7	7
chr3	137806352	137808352	-0.0043313	0.022909	hypomethylated	Mttp,Trmt10a	31	34
chr3	138152346	138154346	-0.13157	0.00010559	hypomethylated	Metap1	25	24
chr3	138188154	138190154	-0.20556	0.00015348	hypomethylated	Eif4e,Mir1956	6	6
chr3	138188384	138190384	-0.040233	0.0033456	hypomethylated	Eif4e,Mir1956	17	22
chr3	138404158	138406158	-0.053977	0.00082663	hypomethylated	Tspan5	55	57
chr3	144232390	144234390	-0.1065	0.0040665	hypomethylated	Hs2st1,Sep15	37	41
chr3	144233180	144235180	-0.16567	0.018654	hypomethylated	Hs2st1,Sep15	20	24
chr3	145238171	145240171	-0.1163	0.003429	hypomethylated	Znhit6	12	13
chr3	145649833	145651833	-0.024327	0.0056228	hypomethylated	Syde2	103	97
chr3	145882924	145884924	-0.037571	0.04943	hypomethylated	Lpar3	42	46
chr3	146112456	146114456	0.072538	0.0050996	hypermethylated	Ctbs	9	8
chr3	146161799	146163799	0.034409	0.0009818	hypermethylated	Gng5,Spata1	61	57
chr3	146162717	146164717	0.055367	0.00000376	hypermethylated	Gng5,Spata1	46	46
chr3	152057973	152059973	-0.015265	0.0099948	hypomethylated	Zzz3	85	85
chr3	152058657	152060657	-0.016699	0.007443	hypomethylated	Zzz3	87	88
chr3	153607396	153609396	0.4068	0.0033021	stronglyhypermeth	Acadm	6	6
chr3	154258975	154260975	-0.074216	0.00000129	hypomethylated	Cryz,Tyw3	22	26
chr3	156223757	156225757	-0.083525	0.000000629	hypomethylated	4930570G19Rik,Negr1	40	44
chr3	156223922	156225922	-0.083525	0.000000629	hypomethylated	4930570G19Rik,Negr1	40	44
chr3	157196360	157198360	-0.043344	0.0013322	hypomethylated	Zranb2	47	49
chr3	157228855	157230855	-0.052982	0.011469	hypomethylated	Ptger3	27	31
chr3	157609429	157611429	-0.0089694	0.00073226	hypomethylated	Ankrd13c	69	68
chr3	157694718	157696718	-0.10849	0.00036345	hypomethylated	Srsf11	7	7
chr4	3604267	3606267	-0.10197	8.19E-08	hypomethylated	Lyn	25	25
chr4	6117251	6119251	-0.10868	0.012117	hypomethylated	Ubxn2b	18	17
chr4	6381418	6383418	-0.47778	0.010195	stronglyhypometh	Nsmaf	5	3

chr4	8461790	8463790	-0.31624	3.26E-12	hypomethylated	Rab2a	31	38
chr4	10800644	10802644	0.026324	0.011851	hypermethylated	2610301B20Rik	48	48
chr4	10934766	10936766	-0.071581	0.0050383	hypomethylated	Plekhf2	15	12
chr4	11082587	11084587	-0.054673	0.0021898	hypomethylated	Trp53inp1	20	17
chr4	11313930	11315930	0.15854	0.00000314	hypermethylated	Esrp1	39	43
chr4	11485118	11487118	-0.10218	0.002909	hypomethylated	Rad54b	14	14
chr4	11630593	11632593	-0.054167	0.027662	hypomethylated	Gem	12	13
chr4	11892860	11894860	-0.086082	9.18E-10	hypomethylated	1700123M08Rik,Pdp1	46	46
chr4	11893597	11895597	-0.20833	0.0032645	hypomethylated	1700123M08Rik,Pdp1	4	4
chr4	12015104	12017104	-0.375	0.00065257	stronglyhypometh	Rbm12b2,Tmem67	2	2
chr4	12015516	12017516	-0.375	0.00065257	stronglyhypometh	Rbm12b2,Tmem67	2	2
chr4	13669448	13671448	-0.1041	3.66E-08	hypomethylated	Runx1t1	48	43
chr4	14790365	14792365	-0.071014	0.013518	hypomethylated	Tmem55a	11	10
chr4	15807410	15809410	-0.034203	0.041065	hypomethylated	Calb1	7	6
chr4	15884113	15886113	-0.10524	1.82E-09	hypomethylated	Nbn	39	39
chr4	16090645	16092645	-0.15639	0.0000179	hypomethylated	A530072M11Rik,Ripk2	26	26
chr4	17779628	17781628	0.16207	0.00000646	hypermethylated	Mmp16	11	17
chr4	19636140	19638140	-0.054783	0.0021742	hypomethylated	Wwp1	24	25
chr4	19968198	19970198	-0.042024	0.037943	hypomethylated	Ggh	25	25
chr4	21857472	21859472	-0.08697	0.000000628	hypomethylated	Faxc	39	41
chr4	22283711	22285711	-0.042835	0.0080807	hypomethylated	Fbxl4	35	34
chr4	24422608	24424608	0.024278	0.010336	hypermethylated	Mms22l	36	40
chr4	24824229	24826229	-0.020265	0.027759	hypomethylated	Ndufaf4	22	19
chr4	26273799	26275799	-0.092857	0.023824	hypomethylated	Manea	7	7
chr4	28739294	28741294	-0.085914	0.026961	hypomethylated	Epha7	28	32
chr4	32050081	32052081	-0.34885	1.19E-12	stronglyhypometh	Map3k7	28	28
chr4	33068972	33070972	-0.0805	0.00034107	hypomethylated	Rragd	50	38
chr4	33294965	33296965	-0.036637	0.036568	hypomethylated	Srsf12	44	37
chr4	34010606	34012606	-0.10423	0.00011858	hypomethylated	Cnr1	49	45
chr4	34562191	34564191	-0.057503	5.44E-09	hypomethylated	Orc3,Rars2	19	26
chr4	36898777	36900777	-0.29459	0.02835	hypomethylated	Lingo2	13	12
chr4	40089297	40091297	-0.052183	0.0027995	hypomethylated	Aco1	24	24
chr4	40419162	40421162	0.34931	0.0000686	stronglyhypermeth	Tmem215	10	5
chr4	40668500	40670500	-0.12063	0.000000219	hypomethylated	Dnaja1,Gm6297,Mir207	43	48
chr4	40668949	40670949	-0.12063	0.000000219	hypomethylated	Dnaja1,Gm6297,Mir207	43	48
chr4	40668954	40670954	-0.12063	0.000000219	hypomethylated	Dnaja1,Gm6297,Mir207	43	48
chr4	40668962	40670962	-0.12063	0.000000219	hypomethylated	Dnaja1,Gm6297,Mir207	43	48
chr4	40894585	40896585	-0.039375	0.00000324	hypomethylated	Bag1,Chmp5	39	39
chr4	40916975	40918975	-0.095611	0.00095044	hypomethylated	Nfx1	28	31
chr4	40995169	40997169	-0.19971	0.031628	hypomethylated	Aqp7	2	2
chr4	41082053	41084053	-0.17885	0.028583	hypomethylated	Ube2r2	49	40
chr4	41295028	41297028	-0.30124	0.0047325	hypomethylated	Ubap1	20	18
chr4	41411180	41413180	-0.19275	0.00014042	hypomethylated	Kif24,Nudt2	23	20
chr4	41411881	41413881	-0.6	0.022474	stronglyhypometh	Kif24,Nudt2	5	2
chr4	41454797	41456797	-0.24285	0.0060851	hypomethylated	1110017D15Rik	7	5
chr4	41720974	41722974	-0.25313	0.00089552	hypomethylated	Ccl27a	3	6
chr4	41721007	41723007	-0.25313	0.00089552	hypomethylated	Ccl27a	3	6
chr4	41721049	41723049	-0.26817	0.0012148	hypomethylated	Ccl27a	3	3
chr4	42929122	42931122	0.019925	0.047141	hypermethylated	N28178	50	50
chr4	43505275	43507275	-0.29923	0.00000138	hypomethylated	Ccdc107,Rmrp	23	12
chr4	43505931	43507931	-0.40856	0.000000135	stronglyhypometh	Ccdc107,Rmrp	17	6
chr4	44085545	44087545	0.15122	0.016309	hypermethylated	Gne	4	4
chr4	44312788	44314788	0.086867	0.038876	hypermethylated	Melk	19	18
chr4	44723312	44725312	0.025277	0.046816	hypermethylated	Pax5	13	19
chr4	44768430	44770430	-0.24718	0.0000377	hypomethylated	Zcchc7	20	20
chr4	44993282	44995282	-0.28437	0.0057318	hypomethylated	Grhpr	19	16
chr4	45810893	45812893	-0.13768	0.028091	hypomethylated	Aldh1b1	12	13
chr4	46356065	46358065	-0.048723	0.0000463	hypomethylated	Foxe1	82	91
chr4	46462988	46464988	-0.080202	0.00000321	hypomethylated	Anp32b	84	77
chr4	47004586	47006586	-0.14541	0.026328	hypomethylated	Gabbr2	20	18
chr4	47070178	47072178	-0.056638	0.048943	hypomethylated	Anks6	18	18

chr4	47103824	47105824	-0.059133	0.0021542	hypomethylated	Galnt12	23	19
chr4	48136790	48138790	0.14403	0.00075121	hypermethylated	Stx17	27	27
chr4	48486294	48488294	-0.039931	0.03936	hypomethylated	Tex10	14	14
chr4	48551817	48553817	0.038487	0.0044956	hypermethylated	Msantd3	54	60
chr4	48551952	48553952	0.038487	0.0044956	hypermethylated	Msantd3	54	60
chr4	48552370	48554370	0.038487	0.0044956	hypermethylated	Msantd3	54	60
chr4	48597064	48599064	-0.053047	0.004964	hypomethylated	Tmeff1	73	69
chr4	49071333	49073333	-0.27039	0.045075	hypomethylated	E130309F12Rik	20	15
chr4	52451120	52453120	-0.043245	0.047305	hypomethylated	2700081L22Rik,Smc2	16	11
chr4	53023795	53025795	-0.043855	0.0090413	hypomethylated	Nipsnap3b	14	10
chr4	53172767	53174767	-0.13836	0.00000543	hypomethylated	Abca1	11	12
chr4	53283104	53285104	-0.20659	0.033476	hypomethylated	AI427809	11	10
chr4	53643342	53645342	-0.28237	2.8E-09	hypomethylated	Fsd1l	28	29
chr4	53726053	53728053	-0.058668	0.043004	hypomethylated	Fktn	18	7
chr4	55361913	55363913	0.049641	0.00000764	hypermethylated	Rad23b	75	74
chr4	55545347	55547347	-0.11238	2.96E-09	hypomethylated	Klf4	34	24
chr4	56814200	56816200	-0.11907	0.0084454	hypomethylated	BC026590,Ikbkap	29	29
chr4	56815203	56817203	-0.19831	0.0067696	hypomethylated	BC026590,Ikbkap	8	8
chr4	59201421	59203421	-0.038543	0.0000266	hypomethylated	Ugcg	43	37
chr4	59593434	59595434	-0.14941	0.00018783	hypomethylated	Hsdl2	22	19
chr4	59638092	59640092	-0.022878	0.0055719	hypomethylated	E130308A19Rik	43	43
chr4	59638198	59640198	-0.022878	0.0055719	hypomethylated	E130308A19Rik	43	43
chr4	59796727	59798727	-0.097368	0.022783	hypomethylated	Inip	6	6
chr4	61946478	61948478	-0.021859	0.031585	hypomethylated	Slc31a2	18	15
chr4	62140100	62142100	-0.087345	0.00028251	hypomethylated	Bspry	7	8
chr4	62875445	62877445	-0.062108	0.021747	hypomethylated	Col27a1	26	37
chr4	63204798	63206798	-0.1026	0.0000129	hypomethylated	Atp6v1g1	9	12
chr4	70071401	70073401	0.15778	0.0000111	hypermethylated	Cdk5rap2	15	10
chr4	71861277	71863277	0.15073	0.0000908	hypermethylated	C630043F03Rik,Tle1	29	30
chr4	73436506	73438506	-0.077511	0.0054252	hypomethylated	Gm11240,Rasef	29	29
chr4	73658506	73660506	0.13195	0.04099	hypermethylated	Frmd3	4	4
chr4	81088709	81090709	-0.1233	0.01969	hypomethylated	Mpdz	14	21
chr4	82151212	82153212	-0.048916	0.0044205	hypomethylated	Nfib	72	77
chr4	83132294	83134294	-0.36234	0.00010475	stronglyhypometh	Psip1	7	7
chr4	84320990	84322990	-0.042686	0.0094533	hypomethylated	Bnc2	33	33
chr4	84529230	84531230	0.081605	0.0055856	hypermethylated	Cntln	43	38
chr4	86503271	86505271	-0.077328	0.00010013	hypomethylated	Rps6	16	16
chr4	87739533	87741533	-0.098715	0.037242	hypomethylated	Focad	14	16
chr4	88940523	88942523	-0.11064	0.00087031	hypomethylated	Cdkn2a	54	57
chr4	91038746	91040746	-0.078864	0.0072987	hypomethylated	Elavl2	10	9
chr4	93002202	93004202	-0.23333	0.0040738	hypomethylated	Tusc1	6	6
chr4	94269938	94271938	-0.081622	0.0016004	hypomethylated	Plaa	12	17
chr4	97251282	97253282	-0.13532	0.022123	hypomethylated	E130114P18Rik	4	4
chr4	98589500	98591500	-0.063897	0.020252	hypomethylated	Usp1	58	57
chr4	99321989	99323989	-0.072553	1.47E-08	hypomethylated	Foxd3	51	49
chr4	99495190	99497190	-0.080952	0.0060712	hypomethylated	Efcab7,Itgb3bp	6	8
chr4	99495809	99497809	-0.080952	0.0060712	hypomethylated	Efcab7,Itgb3bp	6	8
chr4	99601055	99603055	-0.013672	0.0005556	hypomethylated	Pgm2	36	30
chr4	101090893	101092893	-0.039909	0.028929	hypomethylated	Ak4	45	46
chr4	101090916	101092916	-0.039909	0.028929	hypomethylated	Ak4	45	46
chr4	101090925	101092925	-0.039909	0.028929	hypomethylated	Ak4	45	46
chr4	101091307	101093307	-0.039909	0.028929	hypomethylated	Ak4	45	46
chr4	101222198	101224198	-0.1669	0.000000284	hypomethylated	Dnajc6	23	23
chr4	102786069	102788069	-0.020435	0.00000591	hypomethylated	Mier1,Wdr78	46	49
chr4	102786904	102788904	-0.057299	0.0000016	hypomethylated	Mier1,Wdr78	30	31
chr4	102985450	102987450	-0.069444	0.0035698	hypomethylated	Oma1	18	23
chr4	104828951	104830951	-0.012849	0.00043189	hypomethylated	Ppap2b	41	43
chr4	105987817	105989817	-0.081853	0.00040643	hypomethylated	Usp24	65	65
chr4	106289843	106291843	-0.10481	0.0019149	hypomethylated	BC055111	7	6
chr4	106739471	106741471	-0.061869	0.036379	hypomethylated	Mrpl37	12	13
chr4	106925538	106927538	-0.063243	0.0000784	hypomethylated	Hspb11,Lrrc42	26	28

chr4	107473863	107475863	-0.020528	0.015086	hypomethylated	Lrp8	54	54
chr4	107561503	107563503	-0.1348	0.0039137	hypomethylated	0610037L13Rik	19	19
chr4	107837070	107839070	-0.088056	0.00060829	hypomethylated	Echdc2	11	11
chr4	108131030	108133030	-0.16943	0.0056383	hypomethylated	Zcchc11	31	22
chr4	108251058	108253058	-0.13154	0.0084284	hypomethylated	Orc1,Prpf38a	30	23
chr4	108251941	108253941	-0.19025	0.0064982	hypomethylated	Orc1,Prpf38a	19	17
chr4	108506189	108508189	0.14152	0.0034402	hypermethylated	Btf3l4,Txndc12	11	12
chr4	108506282	108508282	0.14152	0.0034402	hypermethylated	Btf3l4,Txndc12	11	12
chr4	108519461	108521461	-0.057889	0.0092587	hypomethylated	Kti12	40	42
chr4	108550674	108552674	-0.21243	0.035282	hypomethylated	Rab3b	11	13
chr4	109337977	109339977	-0.051758	0.00091818	hypomethylated	Cdkn2c	56	49
chr4	109348231	109350231	-0.023508	0.0021182	hypomethylated	Faf1	50	50
chr4	114078328	114080328	-0.11711	0.00000137	hypomethylated	Trabd2b	38	41
chr4	114493324	114495324	-0.15324	0.0016102	hypomethylated	Gm12830	12	16
chr4	114580893	114582893	-0.018798	0.00074169	hypomethylated	9130206I24Rik,Foxd2	64	61
chr4	114659833	114661833	0.066052	0.047702	hypermethylated	Cmpk1	13	13
chr4	114671722	114673722	-0.27708	0.00022493	hypomethylated	Stil	9	3
chr4	115456589	115458589	-0.013277	0.040035	hypomethylated	Atpaf1	17	17
chr4	115499696	115501696	-0.043083	0.00078056	hypomethylated	Mob3c	25	25
chr4	115510850	115512850	-0.008165	0.030616	hypomethylated	Mknk1	9	12
chr4	115690507	115692507	0.13367	0.016333	hypermethylated	Faah	5	5
chr4	115747069	115749069	-0.24409	4.8E-10	hypomethylated	Lrrc41,Uqcrh	36	39
chr4	115747675	115749675	-0.17587	0.00000317	hypomethylated	Lrrc41,Uqcrh	44	47
chr4	115822122	115824122	0.033042	0.045354	hypermethylated	Pomgnt1	46	37
chr4	115893518	115895518	-0.091468	0.007886	hypomethylated	Pik3r3	32	32
chr4	116179153	116181153	-0.074548	0.0011774	hypomethylated	Ipp	23	26
chr4	116228548	116230548	-0.0036359	0.022928	hypomethylated	Gpbp1l1,Tmem69	40	40
chr4	116229331	116231331	-0.016465	0.0096954	hypomethylated	Gpbp1l1,Tmem69	43	47
chr4	116268334	116270334	-0.14676	0.00000534	hypomethylated	C530005A16Rik,Ccdc17	12	12
chr4	116827179	116829179	-0.057366	0.022423	hypomethylated	Rps8,Snord38a,Snord55	23	23
chr4	116923593	116925593	-0.11662	0.0000558	hypomethylated	Gm1661,Tmem53	17	14
chr4	116924522	116926522	-0.070617	0.018702	hypomethylated	Gm1661,Tmem53	11	8
chr4	117506862	117508862	-0.09119	0.0039236	hypomethylated	Slc6a9	30	29
chr4	117545075	117547075	-0.41036	0.00000564	stronglyhypometh	Ccdc24	6	8
chr4	117602368	117604368	0.41667	0.048583	stronglyhypermeth	Artn	3	2
chr4	118080941	118082941	-0.077056	0.010612	hypomethylated	Med8,Szt2	22	19
chr4	118081868	118083868	-0.10238	0.0099049	hypomethylated	Med8,Szt2	18	19
chr4	118216331	118218331	0.41667	0.0025781	stronglyhypermeth	Tmem125	4	4
chr4	118846461	118848461	0.24978	0.040529	hypermethylated	Zfp691	12	7
chr4	118866914	118868914	-0.046361	0.0099257	hypomethylated	Ccdc23	28	26
chr4	118867129	118869129	-0.046361	0.0099257	hypomethylated	Ccdc23	28	26
chr4	118967118	118969118	-0.053606	0.00061486	hypomethylated	Ybx1	25	25
chr4	120419781	120421781	-0.064366	6.03E-08	hypomethylated	Kcnq4	57	63
chr4	120624306	120626306	-0.19705	0.0080184	hypomethylated	Zfp69	16	16
chr4	120711170	120713170	-0.14161	0.0000956	hypomethylated	Col9a2	15	16
chr4	122638431	122640431	-0.089286	0.049416	hypomethylated	Mfsd2a	6	7
chr4	122672341	122674341	-0.10222	0.0017468	hypomethylated	Mycl1	40	40
chr4	122781407	122783407	-0.17993	0.00088162	hypomethylated	Bmp8b	47	42
chr4	122859746	122861746	-0.12681	0.038496	hypomethylated	Hpcal4	22	22
chr4	123427542	123429542	-0.03782	0.0020341	hypomethylated	Akirin1	20	20
chr4	123581255	123583255	-0.025104	0.026452	hypomethylated	Mycbp	34	34
chr4	123593675	123595675	-0.15067	0.0040732	hypomethylated	Rragc	45	45
chr4	124333888	124335888	-0.034225	0.0015704	hypomethylated	Pou3f1	79	92
chr4	124376942	124378942	-0.071646	0.017062	hypomethylated	Fhl3	38	41
chr4	124527002	124529002	-0.053086	0.026516	hypomethylated	1110065P20Rik,Yrdc	67	63
chr4	124742901	124744901	-0.049413	0.027545	hypomethylated	Dnali1,Snip1	41	41
chr4	124742936	124744936	-0.049413	0.027545	hypomethylated	Dnali1,Snip1	41	41
chr4	124761390	124763390	-0.04544	0.012973	hypomethylated	Meaf6	53	54
chr4	125879954	125881954	-0.12396	0.00016319	hypomethylated	Thrap3	14	14
chr4	125938648	125940648	-0.1127	0.00339	hypomethylated	Trappc3	24	25
chr4	126106786	126108786	-0.047475	0.044794	hypomethylated	Ago3	10	10

chr4	126145665	126147665	-0.041667	0.030691	hypomethylated	Ago1	22	20
chr4	126233223	126235223	0.14737	0.0000518	hypermethylated	Clspn	22	28
chr4	126286097	126288097	-0.079582	0.0012226	hypomethylated	5730409E04Rik	17	20
chr4	127648085	127650085	-0.028269	0.030458	hypomethylated	CK137956	9	7
chr4	128364981	128366981	-0.1487	0.020054	hypomethylated	Phc2	3	3
chr4	128483356	128485356	-0.1078	0.00000818	hypomethylated	Zfp362	45	49
chr4	128560383	128562383	-0.079976	0.044117	hypomethylated	Trim62	35	40
chr4	128669508	128671508	-0.068831	0.0039353	hypomethylated	Ak2	13	13
chr4	128669557	128671557	-0.068831	0.0039353	hypomethylated	Ak2	13	13
chr4	128734514	128736514	-0.017577	0.044808	hypomethylated	Rnf19b	69	64
chr4	128781859	128783859	-0.078484	0.00024644	hypomethylated	Tmem54	26	22
chr4	128798535	128800535	-0.14343	0.0022386	hypomethylated	Hpca	16	14
chr4	128866038	128868038	-0.037138	0.013409	hypomethylated	S100pbp,Yars	20	20
chr4	129012614	129014614	-0.15568	0.010374	hypomethylated	Rbbp4,Zbtb8os	13	12
chr4	129219890	129221890	-0.038056	0.04221	hypomethylated	Hdac1	20	20
chr4	129496722	129498722	-0.010686	0.046446	hypomethylated	Ptp4a2	71	57
chr4	129661321	129663321	-0.063933	0.00012962	hypomethylated	Bai2	62	79
chr4	129783799	129785799	0.084956	0.023548	hypermethylated	Pef1	9	9
chr4	129815795	129817795	0.13529	0.00091865	hypermethylated	Hcrtr1	27	19
chr4	129816406	129818406	0.17383	0.01004	hypermethylated	Hcrtr1	11	9
chr4	130036378	130038378	-0.011025	0.040391	hypomethylated	Snrnp40,Zcchc17	14	17
chr4	131428553	131430553	-0.085848	0.00020275	hypomethylated	Srsf4	65	69
chr4	132089574	132091574	0.19801	0.00068802	hypermethylated	Atpif1,Dnajc8	8	8
chr4	132193960	132195960	-0.060925	0.00063237	hypomethylated	Eya3	22	24
chr4	132193970	132195970	-0.060925	0.00063237	hypomethylated	Eya3	22	24
chr4	132288461	132290461	-0.032147	3.57E-12	hypomethylated	Xkr8	12	12
chr4	132440373	132442373	0.15463	0.0012446	hypermethylated	Stx12	20	20
chr4	132566420	132568420	0.036663	0.028542	hypermethylated	Ahdc1	40	40
chr4	132768451	132770451	0.030171	0.022893	hypermethylated	Gpr3	20	15
chr4	133073877	133075877	-0.093389	0.011199	hypomethylated	1810019J16Rik	16	13
chr4	133101942	133103942	-0.2888	0.00000565	hypomethylated	Nudc	5	5
chr4	133843761	133845761	-0.20979	0.0000487	hypomethylated	Pdik1l	10	10
chr4	133928462	133930462	-0.14318	0.0043971	hypomethylated	Extl1	4	4
chr4	134478539	134480539	-0.12146	0.00000151	hypomethylated	D4Wsu53e	41	36
chr4	134485894	134487894	-0.026798	0.00000833	hypomethylated	Syf2	22	22
chr4	135411006	135413006	-0.11036	0.00042869	hypomethylated	Srsf10	60	47
chr4	135698736	135700736	-0.13928	0.0045109	hypomethylated	Id3	14	17
chr4	135865890	135867890	-0.18071	0.00030475	hypomethylated	Hnrnpr	49	57
chr4	135978438	135980438	-0.32633	0.017344	hypomethylated	Htr1d	11	9
chr4	137149103	137151103	-0.15326	0.00010318	hypomethylated	Usp48	28	23
chr4	137236617	137238617	-0.037071	0.006356	hypomethylated	Rap1gap	21	25
chr4	137417151	137419151	-0.098697	0.0022828	hypomethylated	Ece1	29	23
chr4	137548384	137550384	-0.08829	0.000000112	hypomethylated	Eif4g3	65	68
chr4	137771541	137773541	-0.084732	0.000088	hypomethylated	Hp1bp3	72	67
chr4	137772045	137774045	-0.079284	0.00030977	hypomethylated	Hp1bp3	75	70
chr4	137805325	137807325	-0.030504	0.0039645	hypomethylated	Sh2d5	13	8
chr4	137817165	137819165	-0.13796	0.0000143	hypomethylated	Kif17	43	44
chr4	137923870	137925870	-0.10996	0.004217	hypomethylated	Cda	13	11
chr4	137989586	137991586	-0.091195	0.00012354	hypomethylated	Mul1	27	28
chr4	138010062	138012062	-0.062301	3.71E-09	hypomethylated	Camk2n1	65	68
chr4	138280239	138282239	-0.11281	0.043855	hypomethylated	Pla2g2c,Ubxn10	12	10
chr4	138686952	138688952	-0.14085	2.75E-09	hypomethylated	Minos1	11	11
chr4	138687028	138689028	-0.17528	0.00000348	hypomethylated	Minos1	6	6
chr4	138747845	138749845	-0.1401	0.0000253	hypomethylated	Capzb	25	24
chr4	138747893	138749893	-0.1401	0.0000253	hypomethylated	Capzb	25	24
chr4	139208452	139210452	-0.21795	0.010447	hypomethylated	Tas1r2	3	3
chr4	139388883	139390883	-0.091339	0.015675	hypomethylated	Pax7	16	15
chr4	140256387	140258387	-0.064648	0.00010301	hypomethylated	Rcc2	37	47
chr4	140461274	140463274	0.19534	0.018186	hypermethylated	Padi2	4	4
chr4	140516185	140518185	-0.12581	0.00000346	hypomethylated	Sdhb	31	29
chr4	140565338	140567338	-0.18688	0.0000049	hypomethylated	Mfap2	9	10

chr4	140565551	140567551	-0.18872	0.0000506	hypomethylated	Mfap2	10	11
chr4	140634260	140636260	-0.14671	0.0011657	hypomethylated	Necap2	9	9
chr4	140702836	140704836	-0.2253	0.000000416	hypomethylated	Fbxo42	42	38
chr4	140856154	140858154	-0.046643	0.038124	hypomethylated	Epha2	30	29
chr4	140954014	140956014	-0.27143	0.007169	hypomethylated	Clcnka	4	4
chr4	140954621	140956621	-0.29206	0.0016033	hypomethylated	Clcnka	6	6
chr4	140999587	141001587	-0.045054	0.000019	hypomethylated	Zbtb17	45	49
chr4	141094512	141096512	0.09277	0.010988	hypermethylated	Spen	23	29
chr4	141101076	141103076	-0.013342	0.0164	hypomethylated	B330016D10Rik	59	58
chr4	141220030	141222030	-0.20189	4.12E-10	hypomethylated	Plekhm2	22	22
chr4	141279334	141281334	-0.06498	0.0082139	hypomethylated	Ddi2	16	18
chr4	141348526	141350526	-0.074652	0.00034672	hypomethylated	Casp9	15	15
chr4	142938652	142940652	0.037614	0.00014773	hypermethylated	Lrrc38	37	35
chr4	142983343	142985343	-0.066404	0.0042807	hypomethylated	Pramel1	13	13
chr4	147242087	147244087	-0.13942	0.0019526	hypomethylated	Fv1,Miip	17	17
chr4	147310885	147312885	-0.038373	0.01382	hypomethylated	Plod1	12	12
chr4	147314003	147316003	-0.18131	0.000000947	hypomethylated	2510039O18Rik	54	51
chr4	147931535	147933535	0.082909	0.0093299	hypermethylated	Exosc10	26	27
chr4	148177500	148179500	0.11682	0.00000146	hypermethylated	Casz1	43	42
chr4	148681807	148683807	-0.080761	0.020685	hypomethylated	Kif1b	30	30
chr4	148858441	148860441	-0.029296	0.0088422	hypomethylated	Lzic,Nmnat1	32	32
chr4	148859251	148861251	-0.026121	0.032463	hypomethylated	Lzic,Nmnat1	26	26
chr4	148891349	148893349	-0.05235	0.00078632	hypomethylated	Ctnnbip1	13	13
chr4	148959746	148961746	-0.085774	0.0070831	hypomethylated	Clstn1	76	74
chr4	149148376	149150376	-0.22895	1.06E-11	hypomethylated	Slc25a33	11	11
chr4	149655024	149657024	-0.12754	0.0017878	hypomethylated	Rere	30	24
chr4	150284030	150286030	-0.25919	0.001652	hypomethylated	Park7	6	6
chr4	151235877	151237877	-0.11685	0.00000573	hypomethylated	Camta1	36	37
chr4	151381999	151383999	-0.047262	0.00000246	hypomethylated	Klhl21	27	31
chr4	151412435	151414435	-0.15713	0.00011743	hypomethylated	Nol9,Tas1r1	54	54
chr4	151412599	151414599	-0.15713	0.00011743	hypomethylated	Nol9,Tas1r1	54	54
chr4	151647470	151649470	-0.073416	0.0000361	hypomethylated	Gpr153	37	31
chr4	151670459	151672459	-0.079271	0.000054	hypomethylated	Icmt	57	58
chr4	151692734	151694734	-0.18035	0.027981	hypomethylated	Rnf207	7	10
chr4	151851250	151853250	-0.12743	0.0000135	hypomethylated	Kcnab2,Nphp4	32	31
chr4	151851588	151853588	-0.057958	0.00013533	hypomethylated	Kcnab2,Nphp4	27	28
chr4	152856939	152858939	-0.20796	0.0075505	hypomethylated	Ajap1	24	22
chr4	153348669	153350669	0.018129	0.040937	hypermethylated	Cep104,Dffb	33	40
chr4	153349190	153351190	0.016688	0.0012642	hypermethylated	Cep104,Dffb	19	17
chr4	153534793	153536793	-0.21535	0.0066737	hypomethylated	Tprgl	15	10
chr4	154334031	154336031	-0.013091	0.018362	hypomethylated	Hes5	65	67
chr4	154460406	154462406	0.10838	0.010463	hypermethylated	Morn1,Rer1	22	23
chr4	154596644	154598644	-0.052705	0.0060281	hypomethylated	Ski	81	81
chr4	155067580	155069580	-0.099968	0.00000606	hypomethylated	B930041F14Rik	36	51
chr4	155077923	155079923	-0.077515	0.0063047	hypomethylated	Ssu72	31	33
chr4	155185597	155187597	0.13204	0.012479	hypermethylated	Ccnl2	31	31
chr4	155204754	155206754	0.015557	0.017658	hypermethylated	Aurkaip1	20	19
chr4	155212788	155214788	-0.29722	0.048499	hypomethylated	Mxra8	6	6
chr4	155220520	155222520	-0.13944	0.000000431	hypomethylated	Dvl1	59	59
chr4	155242675	155244675	-0.050632	0.0026718	hypomethylated	Cpsf3l,Gltpd1	28	26
chr4	155243549	155245549	-0.061683	0.007308	hypomethylated	Cpsf3l,Gltpd1	25	26
chr4	155264983	155266983	-0.070586	0.000000481	hypomethylated	Acap3,Pusl1	49	50
chr4	155265871	155267871	-0.059615	0.0000382	hypomethylated	Acap3,Pusl1	39	39
chr4	155366022	155368022	-0.070953	0.0011399	hypomethylated	B3galt6,Sdf4	63	62
chr5	3343311	3345311	0.075256	0.0002009	hypermethylated	Cdk6	22	22
chr5	3542832	3544832	-0.038049	0.0000758	hypomethylated	Fam133b	33	32
chr5	3802164	3804164	-0.065126	0.010418	hypomethylated	Ankib1,Krit1	51	57
chr5	3802180	3804180	-0.065126	0.010418	hypomethylated	Ankib1,Krit1	51	57
chr5	3803109	3805109	-0.13847	0.0023087	hypomethylated	Ankib1,Krit1	22	22
chr5	3927185	3929185	-0.047285	0.0005104	hypomethylated	Akap9	34	39
chr5	8421849	8423849	-0.099041	0.014949	hypomethylated	Dbf4,Slc25a40	51	50

chr5	15439508	15441508	-0.094103	0.0046339	hypomethylated	Cacna2d1	62	62
chr5	18731863	18733863	-0.038103	0.038687	hypomethylated	4921504A21Rik,Magi2	21	19
chr5	20561615	20563615	-0.019323	0.011032	hypomethylated	Ptpn12	33	31
chr5	21207163	21209163	-0.17418	0.0044198	hypomethylated	Napepld	22	22
chr5	21290100	21292100	-0.12803	0.00097239	hypomethylated	Dnajc2,Psmc2	35	32
chr5	21290983	21292983	-0.18853	0.033585	hypomethylated	Dnajc2,Psmc2	31	30
chr5	22251010	22253010	-0.032411	0.0000435	hypomethylated	A930003O13Rik,Lhfpl3	23	25
chr5	23669780	23671780	-0.083844	0.0068328	hypomethylated	Nupl2	21	21
chr5	23869636	23871636	-0.40431	0.0000635	stronglyhypometh	Nos3	7	7
chr5	24083285	24085285	-0.2087	0.014728	hypomethylated	Abcf2	7	7
chr5	25004601	25006601	-0.021506	0.0048403	hypomethylated	4831440E17Rik,Mll3	71	72
chr5	25004614	25006614	-0.01606	0.041086	hypomethylated	4831440E17Rik,Mll3	69	70
chr5	28117879	28119879	-0.023414	0.0023676	hypomethylated	Paxip1	18	22
chr5	28491235	28493235	-0.039818	0.0026553	hypomethylated	En2	82	93
chr5	28792523	28794523	-0.092308	0.0071974	hypomethylated	9530036O11Rik,Shh	8	5
chr5	29704930	29706930	-0.18155	0.021328	hypomethylated	Lmbr1	4	4
chr5	30061437	30063437	-0.14052	0.00022296	hypomethylated	Dnajb6,Gm5129	31	34
chr5	30062476	30064476	-0.17033	0.00099142	hypomethylated	Dnajb6,Gm5129	24	24
chr5	30558157	30560157	-0.31699	0.0000405	hypomethylated	Ept1	11	11
chr5	30767449	30769449	-0.13279	0.0015784	hypomethylated	1700001C02Rik	18	17
chr5	30949311	30951311	-0.039369	0.0064678	hypomethylated	Slc35f6	27	27
chr5	31013267	31015267	-0.19359	0.0030345	hypomethylated	Dpysl5	18	9
chr5	31210095	31212095	-0.1155	0.00000102	hypomethylated	Ost4	10	10
chr5	31210161	31212161	-0.1155	0.00000102	hypomethylated	Ost4	10	10
chr5	31223267	31225267	-0.17147	0.037787	hypomethylated	Khk	10	10
chr5	31395900	31397900	-0.13603	0.0000686	hypomethylated	Slc30a3	16	17
chr5	31542290	31544290	-0.04747	0.0000106	hypomethylated	Nrbp1	53	59
chr5	31796133	31798133	-0.087969	0.0003126	hypomethylated	Gpn1	28	29
chr5	31999422	32001422	0.040261	0.0021705	hypermethylated	Bre,Rbks	33	33
chr5	31999983	32001983	0.046657	0.00017255	hypermethylated	Bre,Rbks	28	28
chr5	32912605	32914605	-0.095251	0.00000305	hypomethylated	Yes1	61	68
chr5	33617653	33619653	0.074254	0.015243	hypermethylated	Ctbp1	14	14
chr5	33677220	33679220	-0.056625	0.0017386	hypomethylated	Maea	56	57
chr5	34063372	34065372	-0.065341	0.0000166	hypomethylated	Fgfr3	58	58
chr5	34063408	34065408	-0.044997	0.000045	hypomethylated	Fgfr3	62	62
chr5	34063954	34065954	-0.050397	0.00015926	hypomethylated	Fgfr3	71	68
chr5	34125353	34127353	-0.17104	0.0005757	hypomethylated	Letm1	7	7
chr5	34630973	34632973	-0.0515	0.00032106	hypomethylated	Zfyve28	23	24
chr5	35002797	35004797	-0.014379	0.01482	hypomethylated	Grk4,Nop14	27	29
chr5	35103388	35105388	-0.14665	0.016826	hypomethylated	Htt	29	23
chr5	35291096	35293096	-0.11148	5.31E-08	hypomethylated	Rgs12	29	31
chr5	35448346	35450346	-0.0067857	0.0057922	hypomethylated	Lrpap1	10	10
chr5	35620214	35622214	-0.074696	6.15E-08	hypomethylated	Adra2c	38	35
chr5	35924708	35926708	-0.12209	0.036633	hypomethylated	Acox3	25	21
chr5	36099528	36101528	-0.16642	0.0036615	hypomethylated	Ablim2	23	27
chr5	36826236	36828236	-0.08455	0.00000506	hypomethylated	Ccdc96,Tada2b	57	59
chr5	36826934	36828934	-0.094942	0.00000113	hypomethylated	Ccdc96,Tada2b	34	36
chr5	37258808	37260808	-0.0382	0.000265	hypomethylated	Ppp2r2c	62	65
chr5	37632318	37634318	-0.073601	0.019213	hypomethylated	Crmp1	49	50
chr5	37728120	37730120	-0.19021	0.00000155	hypomethylated	Evc,Evc2	15	13
chr5	38550706	38552706	-0.16251	0.013775	hypomethylated	Nsg1	24	23
chr5	38610720	38612720	-0.1117	0.0021853	hypomethylated	Lyar,Zbtb49	33	31
chr5	38611667	38613667	-0.092809	0.0032326	hypomethylated	Lyar,Zbtb49	21	21
chr5	38667642	38669642	0.11776	0.010313	hypermethylated	Otop1	20	20
chr5	38709747	38711747	-0.24223	0.00065565	hypomethylated	Drd5	22	20
chr5	42099394	42101394	-0.14387	0.0092558	hypomethylated	Rab28	6	7
chr5	42235554	42237554	-0.36825	0.0032018	stronglyhypometh	Bod1l	7	7
chr5	44052684	44054684	-0.27976	0.046703	hypomethylated	Cc2d2a	2	4
chr5	45910467	45912467	-0.20293	0.0000219	hypomethylated	Med28	23	23
chr5	46247791	46249791	-0.078114	2.76E-11	hypomethylated	Lcorl	44	47
chr5	48762630	48764630	-0.27679	0.01846	hypomethylated	Pacrgl	2	2

chr5	53131812	53133812	-0.11151	0.00002	hypomethylated	Pi4k2b	26	31
chr5	53604691	53606691	0.077687	0.0070253	hypermethylated	Sel1l3	14	13
chr5	53657344	53659344	-0.070786	0.010133	hypomethylated	1810013D10Rik	21	23
chr5	53980453	53982453	0.18841	0.007893	hypermethylated	Rbpj	79	80
chr5	58109158	58111158	-0.07078	0.0001078	hypomethylated	4932441J04Rik,Pcdh7	46	51
chr5	64483188	64485188	-0.10386	0.0000215	hypomethylated	Pgm1	35	35
chr5	65360313	65362313	0.026616	0.0064514	hypermethylated	Fam114a1,Mir574	18	18
chr5	65360556	65362556	0.026616	0.0064514	hypermethylated	Fam114a1,Mir574	18	18
chr5	65726878	65728878	-0.13685	0.0000359	hypomethylated	Rfc1	10	9
chr5	65884074	65886074	-0.12244	0.00000109	hypomethylated	Smim14	26	25
chr5	65927499	65929499	-0.053577	0.0020416	hypomethylated	Ube2k	49	46
chr5	66542207	66544207	-0.15318	0.00028467	hypomethylated	Rbm47	6	11
chr5	66650363	66652363	-0.11744	0.00051243	hypomethylated	Nsun7	30	31
chr5	67650890	67652890	-0.19346	0.0018402	hypomethylated	Tmem33	38	35
chr5	67819038	67821038	-0.3732	0.0031264	stronglyhypometh	Bend4	17	27
chr5	67854136	67856136	-0.38966	0.0000471	stronglyhypometh	C330024D21Rik	8	5
chr5	69947180	69949180	-0.047951	0.005097	hypomethylated	Guf1	32	32
chr5	72559422	72561422	-0.16667	0.017839	hypomethylated	Commd8	4	4
chr5	72895447	72897447	-0.24898	0.0036667	hypomethylated	Corin	7	7
chr5	73259687	73261687	-0.083126	0.00045519	hypomethylated	Tec	24	19
chr5	73304555	73306555	-0.035241	0.011354	hypomethylated	Slain2	59	65
chr5	74464436	74466436	-0.12368	5.54E-08	hypomethylated	Usp46	35	33
chr5	74590350	74592350	-0.2545	0.00000338	hypomethylated	Rasl11b	27	25
chr5	75551190	75553190	-0.1565	0.0055414	hypomethylated	Pdgfra	16	16
chr5	75970011	75972011	0.32816	0.012153	hypermethylated	Kit	15	11
chr5	75970015	75972015	0.32816	0.012153	hypermethylated	Kit	15	11
chr5	76611904	76613904	0.026079	0.023499	hypermethylated	Tmem165	43	42
chr5	76733573	76735573	0.030163	0.035099	hypermethylated	Clock	51	51
chr5	77444047	77446047	-0.12299	6.67E-09	hypomethylated	1700023E05Rik	18	21
chr5	77693518	77695518	-0.026717	0.00000833	hypomethylated	Rest	81	78
chr5	77738508	77740508	0.15898	0.033386	hypermethylated	Noa1,Polr2b	39	43
chr5	77837070	77839070	-0.22375	0.0078476	hypomethylated	Igfbp7	13	13
chr5	88982507	88984507	-0.048631	0.030796	hypomethylated	Utp3	30	32
chr5	89011598	89013598	-0.33586	0.0015218	stronglyhypometh	Rufy3	3	3
chr5	89193037	89195037	0.021721	0.011959	hypermethylated	Dck	29	32
chr5	92390907	92392907	-0.13977	0.00012139	hypomethylated	Rchy1,Thap6	28	29
chr5	92392094	92394094	-0.11959	0.02611	hypomethylated	Rchy1,Thap6	18	16
chr5	92472032	92474032	-0.17418	0.044917	hypomethylated	Cdkl2	14	10
chr5	92511943	92513943	0.085207	0.013678	hypermethylated	G3bp2	36	27
chr5	92565963	92567963	-0.077565	0.0000395	hypomethylated	Uso1	51	52
chr5	92707207	92709207	-0.14722	0.0044308	hypomethylated	Naaa	6	6
chr5	92777880	92779880	-0.15278	0.033923	hypomethylated	Cxcl10	3	3
chr5	92983113	92985113	-0.53598	0.00065168	stronglyhypometh		3	4
chr5	93031076	93033076	-0.16449	0.0097853	hypomethylated	Stbd1	11	14
chr5	93237413	93239413	-0.18744	0.01098	hypomethylated	Shroom3	13	13
chr5	93474048	93476048	-0.11657	0.0062038	hypomethylated	Sowahb	22	22
chr5	93635521	93637521	-0.33215	0.037929	hypomethylated	2010109A12Rik,Ccni	6	6
chr5	93695598	93697598	-0.040338	0.011891	hypomethylated	Ccng2	44	41
chr5	96801973	96803973	-0.12465	0.00021548	hypomethylated	Fras1	17	14
chr5	97425707	97427707	0.037429	0.03355	hypermethylated	Bmp2k	63	71
chr5	98757322	98759322	-0.18587	0.0092487	hypomethylated	1700007G11Rik	22	20
chr5	100407957	100409957	-0.14835	1.02E-10	hypomethylated	Hnrnpd	30	36
chr5	100468012	100470012	-0.070173	4.82E-11	hypomethylated	Enoph1,Hnrpdl	58	57
chr5	100468241	100470241	-0.091534	4.24E-09	hypomethylated	Enoph1,Hnrpdl	38	35
chr5	100858554	100860554	-0.063158	0.024911	hypomethylated	5430416N02Rik	13	13
chr5	100927592	100929592	-0.050852	0.0024219	hypomethylated	Lin54	39	42
chr5	101103275	101105275	-0.19649	0.005275	hypomethylated	Coq2	6	6
chr5	102193148	102195148	-0.10157	0.0079421	hypomethylated	Cds1	53	57
chr5	103853210	103855210	-0.09713	0.000000318	hypomethylated	Ptpn13	59	49
chr5	104450815	104452815	0.44694	0.015605	stronglyhypermeth	Hsd17b11	6	3
chr5	104475029	104477029	-0.12074	0.0021778	hypomethylated	Nudt9	27	23

chr5	105843793	105845793	-0.02568	0.048436	hypomethylated	Lrrc8b	31	36
chr5	105947489	105949489	0.10352	1.87E-08	hypermethylated	Lrrc8c	42	39
chr5	106887185	106889185	-0.033691	0.0204	hypomethylated	Barhl2	46	46
chr5	107831531	107833531	-0.084592	0.0093446	hypomethylated	Ephx4	26	28
chr5	107859567	107861567	-0.28949	0.0079385	hypomethylated	Lpcat2b	3	3
chr5	108025398	108027398	-0.09459	0.0000438	hypomethylated	Glmn,Rpap2	18	25
chr5	108025735	108027735	-0.09459	0.0000438	hypomethylated	Glmn,Rpap2	18	25
chr5	108026658	108028658	-0.093955	0.0000477	hypomethylated	Glmn,Rpap2	18	24
chr5	108026907	108028907	-0.16532	0.00014662	hypomethylated	Glmn,Rpap2	6	6
chr5	108696915	108698915	-0.093228	0.029701	hypomethylated	Dr1	38	34
chr5	108889350	108891350	-0.097708	0.0000147	hypomethylated	Pcgf3	28	30
chr5	109057828	109059828	-0.33333	0.012097	hypomethylated	Gak,Tmem175	5	2
chr5	109122247	109124247	-0.07191	0.0033217	hypomethylated	Fgfrl1	57	52
chr5	110659059	110661059	-0.029415	0.004516	hypomethylated	Ankle2	27	27
chr5	110877522	110879522	-0.38847	0.00000328	stronglyhypometh	Fbrsl1	15	8
chr5	111081469	111083469	-0.16504	0.0062607	hypomethylated	Ddx51,Noc4l	42	37
chr5	111082401	111084401	-0.13198	0.00031419	hypomethylated	Ddx51,Noc4l	29	26
chr5	111239100	111241100	-0.19258	0.00039006	hypomethylated	Ulk1	11	12
chr5	111758782	111760782	0.051825	0.0032168	hypermethylated	Pitpnb	29	28
chr5	111846185	111848185	-0.037471	0.00000208	hypomethylated	Mn1	90	93
chr5	112009580	112011580	-0.14038	0.007495	hypomethylated	C130026L21Rik	13	12
chr5	112704726	112706726	-0.077235	0.0000857	hypomethylated	Tpst2	19	21
chr5	112771114	112773114	-0.075926	0.0011426	hypomethylated	Hps4,Srrd	32	36
chr5	112785053	112787053	-0.0098291	0.010108	hypomethylated	Gm6583	3	3
chr5	113006205	113008205	-0.27085	0.0047625	hypomethylated	Sez6l	12	7
chr5	114184790	114186790	-0.032243	0.0057125	hypomethylated	Ficd	23	27
chr5	114221658	114223658	-0.16338	6.33E-08	hypomethylated	Iscu,Sart3	36	33
chr5	114221820	114223820	-0.14927	0.00025989	hypomethylated	Iscu,Sart3	31	28
chr5	114358715	114360715	-0.10093	6.36E-09	hypomethylated	Coro1c	27	27
chr5	114829615	114831615	0.080127	0.0021417	hypermethylated	Kctd10,Ube3b	26	27
chr5	114830514	114832514	0.099982	0.0040292	hypermethylated	Kctd10,Ube3b	16	17
chr5	115156787	115158787	-0.2137	0.00000014	hypomethylated	Tchp	24	23
chr5	115224148	115226148	-0.020544	0.032003	hypomethylated	4930515G01Rik,Ankrd13a,Git:	45	45
chr5	115372248	115374248	-0.3284	0.0010456	hypomethylated	Oasl1	8	8
chr5	115684859	115686859	-0.17627	0.000024	hypomethylated	Pop5	31	29
chr5	115750999	115752999	-0.031902	0.0010409	hypomethylated	Dynll1	13	19
chr5	115790255	115792255	-0.12828	0.003832	hypomethylated	Gatc,Triap1	27	24
chr5	115955710	115957710	-0.063221	0.00023242	hypomethylated	Pxn	23	30
chr5	116080995	116082995	-0.046254	0.00029682	hypomethylated	1110006O24Rik,Rab35	83	82
chr5	116081825	116083825	-0.081994	9.57E-08	hypomethylated	1110006O24Rik,Rab35	59	58
chr5	116738994	116740994	-0.20782	0.00011326	hypomethylated	Ccdc60	11	14
chr5	117737573	117739573	-0.13897	0.0015938	hypomethylated	Pebp1	19	19
chr5	117768274	117770274	-0.12703	5.2E-10	hypomethylated	Vsig10	49	49
chr5	117839032	117841032	-0.10073	0.044296	hypomethylated	Rfc5	14	14
chr5	118618772	118620772	-0.063172	0.00000195	hypomethylated	Hrk	53	57
chr5	120119677	120121677	-0.23486	0.01666	hypomethylated	Tbx3	43	33
chr5	120283671	120285671	-0.15453	0.0063944	hypomethylated	Tbx5	13	12
chr5	120565521	120567521	0.23291	0.022227	hypermethylated	Rbm19	12	21
chr5	121097830	121099830	-0.054015	0.0062295	hypomethylated	Rasal1	36	36
chr5	121641406	121643406	-0.19447	2.36E-11	hypomethylated	Ptpn11	14	14
chr5	121901717	121903717	-0.024424	0.021183	hypomethylated	Erp29,Tmem116	26	28
chr5	122043833	122045833	-0.086349	0.018209	hypomethylated	Aldh2	10	10
chr5	122286810	122288810	0.21162	0.0051591	hypermethylated	Sh2b3	11	7
chr5	122607287	122609287	-0.053949	0.0059157	hypomethylated	Ppp1cc	38	33
chr5	122658745	122660745	-0.10239	0.01779	hypomethylated	Hvcn1	15	14
chr5	122659306	122661306	-0.042316	0.041011	hypomethylated	Hvcn1	11	10
chr5	122821516	122823516	-0.12727	0.0058395	hypomethylated	Fam216a,Gpn3	22	16
chr5	122821972	122823972	-0.12727	0.0058395	hypomethylated	Fam216a,Gpn3	22	16
chr5	122871452	122873452	-0.053339	0.00000191	hypomethylated	Anapc7	30	35
chr5	122952234	122954234	-0.23529	0.000000116	hypomethylated	Atp2a2	17	9
chr5	123156565	123158565	-0.16999	0.0018349	hypomethylated	P2rx4	30	30

chr5	123299196	123301196	-0.32969	0.001131	hypomethylated	Rnf34	26	18
chr5	123438542	123440542	-0.082602	0.038639	hypomethylated	A930024E05Rik,Kdm2b	19	19
chr5	123439101	123441101	-0.075617	0.020364	hypomethylated	A930024E05Rik,Kdm2b	18	18
chr5	123677198	123679198	0.0601	0.010859	hypermethylated	Psmd9	13	13
chr5	123793456	123795456	0.018869	0.023897	hypermethylated	Bcl7a	81	81
chr5	124134300	124136300	-0.036707	0.017935	hypomethylated	Clip1	29	29
chr5	124198734	124200734	-0.095104	0.00099879	hypomethylated	Kntc1,Rsrc2	18	21
chr5	124199421	124201421	-0.12989	0.00015431	hypomethylated	Kntc1,Rsrc2	13	16
chr5	124330029	124332029	0.78544	0.0012556	stronglyhypermeth	Gpr81	3	4
chr5	124777097	124779097	-0.14771	3.64E-09	hypomethylated	2810006K23Rik,Mphosph9	30	27
chr5	124981400	124983400	-0.26027	0.035361	hypomethylated	Rilpl1	7	4
chr5	124989799	124991799	-0.18925	0.013635	hypomethylated	Tmed2	42	31
chr5	125047757	125049757	-0.0094266	0.041013	hypomethylated	Tctn2	14	14
chr5	125342074	125344074	-0.12896	4.69E-12	hypomethylated	Ccdc92,Zfp664	107	121
chr5	125342591	125344591	-0.1412	0.0000209	hypomethylated	Ccdc92,Zfp664	74	85
chr5	125659584	125661584	0.24142	0.00056259	hypermethylated	Ncor2	12	7
chr5	125821444	125823444	-0.372	0.0023102	stronglyhypometh	Scarb1	5	8
chr5	125920937	125922937	-0.059571	0.000000144	hypomethylated	Bri3bp	38	48
chr5	126011787	126013787	-0.027094	0.001186	hypomethylated	Tmem132b	57	51
chr5	127721195	127723195	-0.011146	0.014611	hypomethylated	Tmem132c	44	45
chr5	129105980	129107980	-0.038285	0.0017902	hypomethylated	5930412G12Rik,Fzd10	74	71
chr5	129459237	129461237	-0.30714	0.001421	hypomethylated	Rimbp2	4	4
chr5	130175963	130177963	-0.27143	0.0042618	hypomethylated	Zfp11	3	3
chr5	130229949	130231949	-0.13745	0.000000174	hypomethylated	Gbas	22	30
chr5	130292260	130294260	-0.31441	0.00010041	hypomethylated	Cct6a,Psph	17	14
chr5	130293129	130295129	-0.20914	0.0053643	hypomethylated	Cct6a,Psph	10	10
chr5	130416982	130418982	-0.10778	0.0085025	hypomethylated	Vkorc1l1	27	16
chr5	130646688	130648688	-0.026491	0.0000104	hypomethylated	Rabgef1	37	37
chr5	130697204	130699204	-0.080578	0.0014895	hypomethylated	Tmem248	44	44
chr5	130844327	130846327	-0.29972	0.036963	hypomethylated	Caln1	7	5
chr5	134574617	134576617	-0.16268	0.008463	hypomethylated	Gatsl2	27	27
chr5	134658907	134660907	-0.031378	0.0072835	hypomethylated	Gtf2ird2	31	27
chr5	134790616	134792616	-0.28363	2.14E-10	hypomethylated	Gtf2i	26	26
chr5	134932581	134934581	-0.016257	0.0056816	hypomethylated	Gtf2ird1	14	14
chr5	135057559	135059559	-0.0625	0.00096223	hypomethylated	Rfc2	12	12
chr5	135115198	135117198	-0.060442	0.00032441	hypomethylated	Eif4h	14	14
chr5	135498441	135500441	0.057266	0.00000305	hypermethylated	Stx1a	28	29
chr5	135554136	135556136	-0.31622	8.84E-16	hypomethylated	Vps37d	16	14
chr5	135726917	135728917	-0.072538	0.0072364	hypomethylated	Fzd9	21	27
chr5	135844822	135846822	0.1023	0.049615	hypermethylated	Nsun5	12	13
chr5	136164083	136166083	-0.087611	0.0061767	hypomethylated	Por	23	17
chr5	136220042	136222042	-0.23776	0.0080793	hypomethylated	Tmem120a	11	11
chr5	136410511	136412511	0.067308	0.0441	hypermethylated	Ywhag	8	8
chr5	137461888	137463888	-0.053345	0.0042725	hypomethylated	Plod3,Znhit1	34	41
chr5	137466302	137468302	-0.098168	0.010656	hypomethylated	Mir702	11	10
chr5	137734896	137736896	-0.11727	0.010964	hypomethylated	Ufsp1	22	22
chr5	137755469	137757469	-0.084218	0.014005	hypomethylated	Trip6	14	17
chr5	137790336	137792336	-0.15723	0.0049142	hypomethylated	Ephb4	6	5
chr5	137943657	137945657	-0.039366	0.0023015	hypomethylated	Pop7	22	22
chr5	138042268	138044268	-0.26515	0.0019718	hypomethylated	Mospd3	4	5
chr5	138069350	138071350	-0.15595	0.00040181	hypomethylated	Gm20605,Lrch4	15	15
chr5	138219145	138221145	-0.11157	0.00055681	hypomethylated	Ppp1r35	39	40
chr5	138557132	138559132	-0.018308	0.00025783	hypomethylated	Zscan21	6	9
chr5	138634541	138636541	-0.028535	0.028898	hypomethylated	Mblac1	11	13
chr5	138695709	138697709	-0.032781	0.031952	hypomethylated	Lamtor4	12	7
chr5	139060971	139062971	-0.15079	0.047241	hypomethylated	Zfp68	3	3
chr5	139230034	139232034	-0.0048643	0.0000284	hypomethylated	Fam20c	79	75
chr5	139470907	139472907	-0.038797	0.048864	hypomethylated	Pdgfa	84	85
chr5	139675623	139677623	-0.076764	0.0000828	hypomethylated	Sun1	38	37
chr5	139855620	139857620	-0.086936	0.0011073	hypomethylated	Gpr146	22	22
chr5	140018851	140020851	-0.14837	0.012959	hypomethylated	Uncx	38	42

chr5	140865201	140867201	-0.04456	0.0052606	hypomethylated	Snx8	20	20
chr5	141124985	141126985	-0.15029	0.029079	hypomethylated	Ttyh3	7	9
chr5	141178332	141180332	-0.070661	0.0041126	hypomethylated	Brat1,Iqce	8	8
chr5	141179976	141181976	-0.021907	0.032703	hypomethylated	Brat1	10	10
chr5	143006030	143008030	-0.14969	0.018185	hypomethylated	Papolb	11	11
chr5	143579066	143581066	-0.19277	0.00055333	hypomethylated	Tnrc18	20	22
chr5	143668403	143670403	-0.018664	0.016568	hypomethylated	Actb	11	10
chr5	143721033	143723033	0.05084	0.0027272	hypermethylated	Fscn1	45	46
chr5	143941695	143943695	0.021466	0.00011861	hypermethylated	4933411G11Rik,Rbak	20	23
chr5	143942422	143944422	0.1143	0.00000652	hypermethylated	4933411G11Rik,Rbak	8	10
chr5	144090905	144092905	0.22434	0.000061	hypermethylated	0610040B10Rik,Zdhhc4	12	13
chr5	144118016	144120016	-0.26693	0.00028115	hypomethylated	Grid2ip	9	10
chr5	144224360	144226360	-0.11737	0.000059	hypomethylated	Daglb	29	29
chr5	144288861	144290861	-0.042988	0.0025235	hypomethylated	Rac1	20	26
chr5	144382315	144384315	-0.047933	0.033026	hypomethylated	Cyth3	16	13
chr5	144493149	144495149	-0.028104	0.040241	hypomethylated	Usp42	25	25
chr5	144518222	144520222	-0.32786	6.49E-09	hypomethylated	D130017N08Rik	12	12
chr5	144577660	144579660	-0.24968	0.000000583	hypomethylated	Eif2ak1	8	8
chr5	145118981	145120981	-0.087755	0.032003	hypomethylated	Baiap2l1	10	7
chr5	145305755	145307755	-0.20186	0.0000298	hypomethylated	Nptx2	49	67
chr5	145843737	145845737	-0.11265	0.021936	hypomethylated	Arpc1a	9	12
chr5	145874124	145876124	-0.076415	0.0010443	hypomethylated	Arpc1b	25	25
chr5	145900958	145902958	0.036369	0.0059196	hypermethylated	Bud31,Pdap1	19	14
chr5	145927092	145929092	-0.16644	5.95E-08	hypomethylated	Cpsf4,Ptcd1	34	37
chr5	145927973	145929973	-0.089992	0.0020101	hypomethylated	Cpsf4,Ptcd1	26	28
chr5	145964427	145966427	-0.18578	0.0000126	hypomethylated	Zkscan5	16	16
chr5	147033013	147035013	-0.040305	0.00068311	hypomethylated	Rnf6	12	12
chr5	147042250	147044250	0.13287	0.0084031	hypermethylated	Cdk8	36	32
chr5	148241155	148243155	-0.19625	2.32E-08	hypomethylated	Pan3	78	80
chr5	148537564	148539564	-0.044514	0.00076265	hypomethylated	Flt1	29	29
chr5	148671203	148673203	-0.10091	0.011611	hypomethylated	Pomp	19	13
chr5	148767895	148769895	-0.037149	0.0096399	hypomethylated	Mtus2	44	37
chr5	151397100	151399100	-0.06902	0.0000737	hypomethylated	N4bp2l1	20	18
chr5	152453783	152455783	0.062516	0.0070737	hypermethylated	Rfc3	11	11
chr6	4852319	4854319	-0.050148	0.00026968	hypomethylated	Ppp1r9a	40	36
chr6	8158226	8160226	0.084569	0.011704	hypermethylated	Mios	35	48
chr6	8208287	8210287	-0.15062	0.017434	hypomethylated	Gm16039,Rpa3	22	18
chr6	8209141	8211141	-0.15062	0.017434	hypomethylated	Gm16039,Rpa3	22	18
chr6	11874880	11876880	-0.026049	0.0013138	hypomethylated	Phf14	20	21
chr6	17412956	17414956	-0.0757	0.0014851	hypomethylated	Met	32	22
chr6	17586097	17588097	-0.1141	1.3E-09	hypomethylated	Capza2	53	52
chr6	17980445	17982445	-0.21339	0.00028734	hypomethylated	Wnt2	27	23
chr6	18797634	18799634	-0.041627	0.012142	hypomethylated	Naa38	34	36
chr6	21165108	21167108	-0.092063	0.018932	hypomethylated	Kcnd2	6	7
chr6	21802515	21804515	-0.40114	0.028457	stronglyhypometh	Tspan12	4	4
chr6	21898614	21900614	0.05096	0.026493	hypermethylated	Ing3	15	15
chr6	22306081	22308081	0.041667	0.041733	hypermethylated	Fam3c	22	22
chr6	23198264	23200264	0.17101	6.37E-09	hypermethylated	Fezf1	21	21
chr6	28084369	28086369	-0.061257	0.0000513	hypomethylated	Grm8	22	22
chr6	28211601	28213601	-0.033142	0.0045343	hypomethylated	Zfp800	34	34
chr6	28429347	28431347	-0.068718	0.0058798	hypomethylated	Snd1	20	18
chr6	28781747	28783747	-0.16734	3.15E-10	hypomethylated	Lrrc4	37	36
chr6	29345635	29347635	-0.027985	0.004962	hypomethylated		35	34
chr6	29475732	29477732	-0.062577	0.00073187	hypomethylated	Irf5	49	52
chr6	29643255	29645255	0.041295	0.016392	hypermethylated	Tspan33	21	13
chr6	29866012	29868012	-0.12492	0.032617	hypomethylated	Strip2	32	32
chr6	29996987	29998987	0.05639	0.0090725	hypermethylated	Nrf1	51	58
chr6	30846760	30848760	0.10119	0.035765	hypermethylated	Copg2	12	12
chr6	34303163	34305163	-0.43091	0.00000512	stronglyhypometh	Akr1b8	3	10
chr6	34828065	34830065	-0.076603	0.032366	hypomethylated	3110062M04Rik	12	13
chr6	35126615	35128615	-0.033165	0.024799	hypomethylated	Nup205	50	47

chr6	38204009	38206009	-0.1579	0.0000676	hypomethylated	D630045J12Rik	15	15
chr6	38382924	38384924	-0.096175	4.36E-08	hypomethylated	Ubn2	60	64
chr6	38612068	38614068	-0.036763	0.0018205	hypomethylated	Clec2l	37	44
chr6	39507833	39509833	0.18308	0.0045742	hypermethylated	Dennd2a	8	3
chr6	39541581	39543581	-0.05672	0.038607	hypomethylated	Ndufb2	31	31
chr6	39760935	39762935	-0.094792	0.0028684	hypomethylated	Mrps33	8	8
chr6	40274476	40276476	0.065626	0.038251	hypermethylated	Agk	24	24
chr6	41554480	41556480	-0.13141	0.036885	hypomethylated	Ephb6	21	21
chr6	42194933	42196933	-0.016645	0.031807	hypomethylated	Gstk1	9	17
chr6	42298826	42300826	0.031475	0.017795	hypermethylated	Zyx	52	54
chr6	43259553	43261553	-0.21591	0.033642	hypomethylated	Nobox	2	2
chr6	47403322	47405322	-0.11681	1.22E-09	hypomethylated	Cul1	88	86
chr6	47784659	47786659	0.033848	0.011428	hypermethylated	Zfp398	24	33
chr6	47892173	47894173	-0.26265	0.0011905	hypomethylated	Zfp783	30	21
chr6	48577165	48579165	-0.22668	7.89E-13	hypomethylated	AI854703	14	14
chr6	49164953	49166953	-0.03323	0.017517	hypomethylated	Igf2bp3	24	17
chr6	49771727	49773727	-0.14059	0.0035329	hypomethylated	Npy	20	20
chr6	50515641	50517641	-0.31277	0.00000559	hypomethylated	5430402O13Rik,Cycs	28	16
chr6	51219909	51221909	-0.13254	0.00023982	hypomethylated	Mir148a	38	38
chr6	51962548	51964548	-0.19813	0.000000134	hypomethylated	Skap2	14	14
chr6	52114830	52116830	-0.23492	0.0003078	hypomethylated	Hoxa2	9	9
chr6	52210874	52212874	-0.35293	0.00000151	stronglyhypometh	Hoxa13	8	9
chr6	52262491	52264491	-0.25759	0.0028941	hypomethylated	5730457N03Rik,Evx1	16	21
chr6	53236288	53238288	-0.073412	0.0000621	hypomethylated	9430076C15Rik	57	56
chr6	53988925	53990925	-0.054337	0.00076588	hypomethylated	Chn2	40	40
chr6	54516376	54518376	-0.16863	0.000000996	hypomethylated	Scrn1	7	7
chr6	54543122	54545122	-0.15237	0.0147	hypomethylated	Fkbp14,Plekha8	8	10
chr6	54544104	54546104	-0.075635	0.00000151	hypomethylated	Fkbp14,Plekha8	30	29
chr6	54765909	54767909	-0.027771	0.021058	hypomethylated	Znrf2	72	69
chr6	55400973	55402973	-0.17017	0.0032603	hypomethylated	Adcyap1r1	11	10
chr6	56781052	56783052	-0.070725	0.00054088	hypomethylated	Fkbp9	22	22
chr6	63205850	63207850	-0.13555	0.038684	hypomethylated	Grid2	47	39
chr6	64678139	64680139	-0.093361	0.0073435	hypomethylated	Atoh1	24	30
chr6	64991660	64993660	-0.10238	0.038568	hypomethylated	Smarcad1	41	38
chr6	65727955	65729955	-0.083386	0.0040102	hypomethylated	Prdm5	18	20
chr6	66484461	66486461	-0.094054	0.0011464	hypomethylated	Mad2l1	15	18
chr6	66845390	66847390	-0.08856	0.0000846	hypomethylated	Gng12	36	37
chr6	66845884	66847884	-0.08856	0.0000846	hypomethylated	Gng12	36	37
chr6	70742169	70744169	-0.057021	0.000059	hypomethylated	Rpia	13	13
chr6	71390631	71392631	-0.097306	0.025208	hypomethylated	Rmnd5a	4	4
chr6	71492847	71494847	-0.19947	0.0000887	hypomethylated	Chmp3	25	27
chr6	72296310	72298310	-0.065099	0.0041455	hypomethylated	0610030E20Rik	31	23
chr6	72330462	72332462	-0.26999	0.023326	hypomethylated	Vamp5	10	8
chr6	72389552	72391552	0.066986	0.014667	hypermethylated	4930414L22Rik,Mat2a	14	18
chr6	72566994	72568994	-0.24131	0.003647	hypomethylated	Tgoln1,Tgoln2	7	7
chr6	72849973	72851973	-0.1029	0.00011311	hypomethylated	Kcmf1	24	22
chr6	82983217	82985217	-0.17573	0.00091344	hypomethylated	Dok1,Loxl3	21	21
chr6	82983465	82985465	-0.33548	0.0026064	stronglyhypometh	Dok1,Loxl3	11	11
chr6	83035358	83037358	-0.1848	0.024428	hypomethylated	Lbx2	6	6
chr6	83050509	83052509	-0.14376	0.00000502	hypomethylated	Ccdc142	35	31
chr6	83064499	83066499	-0.04473	0.00061569	hypomethylated	Mogs	44	41
chr6	83071455	83073455	-0.15844	0.00000661	hypomethylated	Wbp1	22	22
chr6	83128582	83130582	-0.28788	0.0011782	hypomethylated	Dctn1	2	2
chr6	83267598	83269598	-0.15568	0.0001837	hypomethylated	Mthfd2	5	5
chr6	83275032	83277032	0.16784	0.0000195	hypermethylated	Mob1a	21	21
chr6	83298477	83300477	-0.057252	0.00022837	hypomethylated	Bola3	24	28
chr6	83390748	83392748	-0.066851	0.014717	hypomethylated	B230319C09Rik,Tet3	32	32
chr6	85136924	85138924	-0.18123	0.0000416	hypomethylated	Emx1	29	23
chr6	85401296	85403296	0.37267	0.017259	stronglyhypermeth	Cct7,Pradc1	24	19
chr6	85463536	85465536	-0.19233	0.034042	hypomethylated	Egr4	22	19
chr6	85864712	85866712	-0.17039	0.001845	hypomethylated	Cml1,Tprkb	18	20

chr6	85865671	85867671	-0.18813	0.0031415	hypomethylated	Cml1,Tprkb	16	17
chr6	86144244	86146244	-0.031198	0.019913	hypomethylated	Tgfa	43	42
chr6	86320533	86322533	-0.058081	0.017573	hypomethylated	Snrpg	11	11
chr6	86476159	86478159	-0.091484	0.00000394	hypomethylated	1600020E01Rik,Pcbp1	44	53
chr6	86619153	86621153	-0.076586	0.00000145	hypomethylated	Mxd1	35	38
chr6	87801100	87803100	0.10606	0.030544	hypermethylated	Cnbp	10	10
chr6	88396533	88398533	-0.26728	0.03488	hypomethylated	Eefsec	15	10
chr6	88468794	88470794	-0.15926	3.32E-10	hypomethylated	Sec61a1	16	19
chr6	88791929	88793929	-0.17801	0.0011044	hypomethylated	Abtb1,Gm15612	34	29
chr6	90568209	90570209	-0.45937	5.49E-11	stronglyhypometh	Slc41a3	6	7
chr6	92040411	92042411	-0.05939	0.00000448	hypomethylated	Nr2c2	75	76
chr6	100237352	100239352	-0.060174	0.0053565	hypomethylated	Rybp	50	52
chr6	101327891	101329891	0.35246	0.002147	stronglyhypermeth	Pdzrn3	8	10
chr6	107478778	107480778	-0.16285	0.039804	hypomethylated	Lrrn1	11	14
chr6	110594591	110596591	-0.1158	0.00032038	hypomethylated	Grm7	16	20
chr6	112439802	112441802	0.042625	0.045374	hypermethylated	Oxtr	8	6
chr6	113186836	113188836	0.1976	0.007064	hypermethylated	Mtmr14	28	31
chr6	113341490	113343490	-0.13428	0.00016792	hypomethylated	Ttll3	17	18
chr6	113391628	113393628	-0.092977	1.29E-08	hypomethylated	Jagn1	21	21
chr6	113480675	113482675	-0.4087	0.023994	stronglyhypometh	Emc3,Fancd2	9	7
chr6	113553765	113555765	-0.14125	0.0012682	hypomethylated	Brk1	23	22
chr6	113646492	113648492	0.03654	0.026961	hypermethylated	Tatdn2	54	53
chr6	114080234	114082234	-0.06707	0.012506	hypomethylated	Slc6a11	8	8
chr6	114346929	114348929	-0.1487	0.0060491	hypomethylated	Hrh1	23	25
chr6	115083919	115085919	-0.062427	0.0000991	hypomethylated	Syn2	31	29
chr6	115310238	115312238	-0.10939	0.030081	hypomethylated	Pparg	30	27
chr6	115626653	115628653	-0.29465	0.0015008	hypomethylated	Raf1	10	10
chr6	115758121	115760121	-0.19855	0.000016	hypomethylated	Rpl32,Snora7a	22	22
chr6	115945023	115947023	-0.0541	0.00000101	hypomethylated	Plxnd1	13	14
chr6	117790259	117792259	-0.039719	0.0013392	hypomethylated	Zfp637	16	18
chr6	117855799	117857799	-0.028011	0.0045125	hypomethylated	Hnrnpf	67	71
chr6	117856821	117858821	-0.0091659	0.01088	hypomethylated	Hnrnpf	75	78
chr6	118147762	118149762	-0.235	0.0033186	hypomethylated	Ret	4	4
chr6	119124270	119126270	-0.10964	0.018384	hypomethylated	Dcp1b	11	11
chr6	119279222	119281222	-0.32308	0.0036885	hypomethylated	Lrtm2	19	9
chr6	119428685	119430685	-0.017776	0.00020833	hypomethylated	Fbxl14	74	82
chr6	119797210	119799210	-0.10032	0.0022929	hypomethylated	3110021A11Rik,Erc1	43	50
chr6	119798168	119800168	-0.077159	0.00044744	hypomethylated	3110021A11Rik,Erc1	19	19
chr6	119851715	119853715	-0.12978	3.48E-14	hypomethylated	Rad52	24	25
chr6	119988673	119990673	0.05625	0.019521	hypermethylated	Wnk1	16	12
chr6	120313116	120315116	-0.07833	1.51E-08	hypomethylated	Ccdc77,Kdm5a	42	42
chr6	120481317	120483317	0.064829	0.043441	hypermethylated	Cecr5	7	5
chr6	120615438	120617438	-0.033941	0.00000702	hypomethylated	Cecr2	107	117
chr6	120866838	120868838	0.17611	0.030697	hypermethylated	Bid	5	5
chr6	121194923	121196923	-0.3043	1.31E-13	hypomethylated	Usp18	13	14
chr6	122436323	122438323	-0.038077	0.0037091	hypomethylated	Rimklb	24	19
chr6	122692763	122694763	-0.015385	0.030259	hypomethylated	Slc2a3	5	6
chr6	122769201	122771201	-0.062039	0.0072103	hypomethylated	Foxj2	36	35
chr6	124662196	124664196	-0.29333	0.040439	hypomethylated	Emg1,Phb2	12	4
chr6	124757958	124759958	-0.067469	0.0028562	hypomethylated	Spsb2	18	21
chr6	124779193	124781193	-0.29808	0.0022651	hypomethylated	Cdca3,Usp5	23	22
chr6	124880405	124882405	-0.036499	0.0000505	hypomethylated	Mlf2	27	25
chr6	124945737	124947737	-0.033333	0.01762	hypomethylated	Pianp	19	22
chr6	124958822	124960822	-0.040291	0.034779	hypomethylated	Zfp384	44	51
chr6	124998944	125000944	-0.10792	0.0067344	hypomethylated	Acrbp	6	6
chr6	125016937	125018937	-0.20942	0.046336	hypomethylated	Lpar5	2	2
chr6	125181878	125183878	-0.37464	0.00046866	stronglyhypometh	E130112N10Rik,Tapbpl	5	4
chr6	125298740	125300740	-0.32397	0.02539	hypomethylated	Tnfrsf1a	12	14
chr6	125330522	125332522	-0.3724	0.000000383	stronglyhypometh	Plekhg6	10	8
chr6	126116762	126118762	-0.21508	0.033526	hypomethylated	Ntf3	4	9
chr6	128093596	128095596	-0.13335	0.014625	hypomethylated	Tspan9	14	10

chr6	128305869	128307869	0.26875	0.00015002	hypermethylated	Tulp3	4	7
chr6	128312011	128314011	-0.062472	0.000013	hypomethylated	Foxm1,Rhno1	35	40
chr6	128312840	128314840	-0.01006	0.019677	hypomethylated	Foxm1,Rhno1	18	22
chr6	128312915	128314915	-0.01006	0.019677	hypomethylated	Foxm1,Rhno1	18	22
chr6	128348783	128350783	-0.084416	0.048029	hypomethylated	Nrip2	7	7
chr6	128387774	128389774	0.025526	0.04628	hypermethylated	Fkbp4,Gm10069	45	59
chr6	133054256	133056256	-0.091503	0.04961	hypomethylated	2700089E24Rik	17	17
chr6	134589957	134591957	-0.39715	6.53E-10	stronglyhypometh	Loh12cr1	24	30
chr6	134779216	134781216	-0.13095	0.0029633	hypomethylated	Crebl2	10	9
chr6	134846978	134848978	0.12792	0.00027751	hypermethylated	2810454H06Rik,Gpr19	24	14
chr6	134869418	134871418	-0.099968	0.0036568	hypomethylated	Cdkn1b	18	33
chr6	135147004	135149004	-0.018276	0.0060637	hypomethylated	8430419L09Rik	15	27
chr6	137118239	137120239	-0.036826	0.032865	hypomethylated	Rerg	7	6
chr6	137199819	137201819	-0.048392	0.0014197	hypomethylated	Ptpro	13	15
chr6	140571209	140573209	-0.25258	0.047953	hypomethylated	Aebp2	38	40
chr6	140571818	140573818	-0.19791	0.0000535	hypomethylated	Aebp2	44	46
chr6	142334762	142336762	-0.030328	0.006497	hypomethylated	Golt1b,Recql	53	50
chr6	142335607	142337607	-0.05144	0.04768	hypomethylated	Golt1b,Recql	33	34
chr6	142519876	142521876	-0.10786	0.003752	hypomethylated	Kcnj8	8	9
chr6	143114749	143116749	-0.11645	6.89E-12	hypomethylated	Etnk1	60	59
chr6	145158653	145160653	-0.27808	0.0000552	hypomethylated	Casc1,Lyrm5	14	15
chr6	145158666	145160666	-0.27808	0.0000552	hypomethylated	Casc1,Lyrm5	14	15
chr6	145159490	145161490	-0.16923	5.18E-09	hypomethylated	Casc1,Lyrm5	10	12
chr6	145198751	145200751	-0.054575	0.0031712	hypomethylated	Gm15706,Kras	25	23
chr6	146525432	146527432	-0.038127	0.014918	hypomethylated	Asun,Fgfr1op2	32	37
chr6	147212607	147214607	-0.084692	0.035132	hypomethylated	Pthlh	16	16
chr6	147423392	147425392	-0.12467	0.0013594	hypomethylated	Ccdc91	32	36
chr6	148844648	148846648	0.10229	0.010013	hypermethylated	Caprin2	9	9
chr7	3217626	3219626	-0.26544	0.0056062	hypomethylated	D7Ertd143e,Mir290,Mir291a,I	14	6
chr7	3217784	3219784	-0.26544	0.0056062	hypomethylated	D7Ertd143e,Mir290,Mir291a,I	14	6
chr7	3217919	3219919	-0.28555	0.0070859	hypomethylated	D7Ertd143e,Mir290,Mir291a,I	12	6
chr7	3218189	3220189	-0.28555	0.0070859	hypomethylated	D7Ertd143e,Mir290,Mir291a,I	12	6
chr7	3218482	3220482	-0.28555	0.0070859	hypomethylated	D7Ertd143e,Mir290,Mir291a,I	12	6
chr7	3595870	3597870	-0.11942	0.0000747	hypomethylated	Cnot3	40	28
chr7	3644211	3646211	-0.1769	0.0000855	hypomethylated	Mboat7,Tsen34	24	29
chr7	4087657	4089657	-0.1024	0.00000216	hypomethylated	D030047H15Rik,Leng8	28	28
chr7	4088528	4090528	-0.1022	0.00000122	hypomethylated	D030047H15Rik,Leng8	26	26
chr7	4474045	4476045	-0.20513	0.010527	hypomethylated	Tnni3	7	8
chr7	4641529	4643529	-0.23698	0.023936	hypomethylated	Brsk1	8	12
chr7	4965855	4967855	-0.017547	0.0042218	hypomethylated	Fiz1,Zfp524	20	30
chr7	4966109	4968109	-0.041455	0.0049685	hypomethylated	Fiz1,Zfp524	19	29
chr7	4966299	4968299	-0.075528	0.00021736	hypomethylated	Fiz1,Zfp524	14	24
chr7	4966330	4968330	-0.075528	0.00021736	hypomethylated	Fiz1,Zfp524	14	24
chr7	5007327	5009327	-0.20045	0.023816	hypomethylated	Ccdc106	7	7
chr7	5012783	5014783	-0.076344	0.0044259	hypomethylated	U2af2	31	14
chr7	5077552	5079552	-0.11918	0.016009	hypomethylated	Rasl2-9	7	8
chr7	6123114	6125114	-0.1138	0.00011607	hypomethylated	Zfp444	25	25
chr7	6237181	6239181	-0.22854	5.6E-14	hypomethylated	Zfp667	15	17
chr7	6682451	6684451	-0.31789	0.0042848	hypomethylated	Peg3,Usp29	5	7
chr7	6929373	6931373	-0.15022	0.019945	hypomethylated	Zim3	11	15
chr7	7231000	7233000	-0.32147	1.14E-08	hypomethylated	Vmn2r29	8	14
chr7	13062653	13064653	-0.21867	6.96E-08	hypomethylated	Zfp606	19	19
chr7	13482163	13484163	-0.058093	0.033952	hypomethylated	Zscan22	9	6
chr7	13550212	13552212	-0.47102	0.022105	stronglyhypometh	Zfp324	10	14
chr7	13623327	13625327	-0.22325	0.000000384	hypomethylated	Ube2m	19	22
chr7	13623619	13625619	-0.16523	0.0074967	hypomethylated	Ube2m	8	8
chr7	13863174	13865174	-0.37871	0.042683	stronglyhypometh	6330408A02Rik,Lig1	24	32
chr7	13863613	13865613	-0.37871	0.042683	stronglyhypometh	6330408A02Rik,Lig1	24	32
chr7	15208415	15210415	-0.19373	0.0000661	hypomethylated	Nlrp5-ps	22	22
chr7	16714648	16716648	-0.031891	0.049833	hypomethylated	Slc8a2	13	12
chr7	16759728	16761728	-0.063451	0.0033106	hypomethylated	Meis3	14	14

chr7	17402081	17404081	0.11592	0.01408	hypermethylated	Fkrp,Strn4	16	16
chr7	17427413	17429413	-0.13355	0.010279	hypomethylated	Prkd2	13	17
chr7	17529030	17531030	-0.21869	3.28E-08	hypomethylated	Pnmal2	21	25
chr7	19406940	19408940	-0.52778	0.00016903	stronglyhypometh	Psg17	3	4
chr7	19575593	19577593	-0.10948	0.0000196	hypomethylated	Mypop	21	20
chr7	19608725	19610725	-0.014217	0.03413	hypomethylated	Foxa3,Sympk	59	50
chr7	19608888	19610888	-0.019066	0.016976	hypomethylated	Foxa3,Sympk	55	49
chr7	19660548	19662548	-0.072996	0.0000695	hypomethylated	Dmwd	44	37
chr7	19734186	19736186	-0.13674	0.019224	hypomethylated	Qpctl,Snrpd2	11	11
chr7	19895394	19897394	-0.17597	0.028291	hypomethylated	Fosb	14	12
chr7	20043843	20045843	-0.088172	0.028161	hypomethylated	Mark4	15	18
chr7	20093077	20095077	-0.009893	0.00087179	hypomethylated	Bloc1s3,Trappc6a	28	22
chr7	20093680	20095680	-0.062451	0.0023913	hypomethylated	Bloc1s3,Trappc6a	20	20
chr7	20161635	20163635	-0.18916	0.0044225	hypomethylated	Zfp296	18	21
chr7	20250379	20252379	0.18222	0.024309	hypermethylated	Clptm1	14	11
chr7	20334922	20336922	-0.30805	0.00039255	hypomethylated	Pvrl2	6	5
chr7	20506492	20508492	-0.14505	0.00083067	hypomethylated	Pvr	7	4
chr7	24865962	24867962	-0.15148	0.03713	hypomethylated	Zfp180	11	9
chr7	25022617	25024617	-0.57407	0.0000851	stronglyhypometh	Zfp109	6	2
chr7	25246518	25248518	-0.089931	0.00095201	hypomethylated	Plaur	8	9
chr7	25457069	25459069	-0.11337	0.015104	hypomethylated	Tex101	6	7
chr7	25668732	25670732	0.005975	0.004157	hypermethylated	Rps19	33	33
chr7	25861223	25863223	-0.063093	0.0029235	hypomethylated	Zfp574	31	26
chr7	25861264	25863264	-0.063093	0.0029235	hypomethylated	Zfp574	31	26
chr7	26090126	26092126	-0.098858	0.043784	hypomethylated	Tmem145	19	23
chr7	26101182	26103182	-0.09566	0.043239	hypomethylated	Megf8	16	15
chr7	26411642	26413642	0.073356	0.037615	hypermethylated	B3gnt8	15	16
chr7	26460185	26462185	-0.15	0.01149	hypomethylated	Tmem91	7	7
chr7	28118667	28120667	-0.074247	0.0000337	hypomethylated	Ltbp4	20	20
chr7	28181217	28183217	0.069769	0.011193	hypermethylated	Sptbn4	9	10
chr7	28181572	28183572	0.059848	0.029713	hypermethylated	Sptbn4	8	10
chr7	28231608	28233608	-0.081689	0.000000172	hypomethylated	Blvrb,Sptbn4	31	36
chr7	28231996	28233996	-0.1441	0.00040652	hypomethylated	Blvrb,Sptbn4	18	23
chr7	28270971	28272971	-0.096642	0.00021073	hypomethylated	Sertad1	34	27
chr7	28437942	28439942	-0.1603	0.001247	hypomethylated	Ttc9b	26	27
chr7	28622602	28624602	0.18395	0.000029	hypermethylated	Zfp59	10	10
chr7	28764176	28766176	0.20426	0.0023592	hypermethylated	Zfp780b	5	5
chr7	28963501	28965501	-0.24308	0.00000195	hypomethylated	Dyrk1b	37	31
chr7	28963512	28965512	-0.24308	0.00000195	hypomethylated	Dyrk1b	37	31
chr7	29123738	29125738	0.035336	0.0078204	hypermethylated	Supt5	7	7
chr7	29157681	29159681	0.016857	0.030108	hypermethylated	Plekhg2	19	14
chr7	29221210	29223210	-0.076136	0.0000269	hypomethylated	Gmfg,Samd4b	20	16
chr7	29221465	29223465	-0.11917	0.0000214	hypomethylated	Gmfg,Samd4b	14	16
chr7	29236256	29238256	-0.032196	0.0090454	hypomethylated	Lrfn1	54	44
chr7	29383203	29385203	-0.20745	0.017853	hypomethylated	Pak4	3	3
chr7	29416034	29418034	-0.38434	0.0029628	stronglyhypometh	C330005M16Rik	4	5
chr7	29526800	29528800	-0.12764	0.040549	hypomethylated	Mrps12,Sars2	16	16
chr7	29594908	29596908	-0.16886	0.018098	hypomethylated	Hnrnpl	26	22
chr7	29766833	29768833	-0.084444	0.0033185	hypomethylated	Eif3k,Map4k1	15	12
chr7	29766872	29768872	-0.094872	0.0022764	hypomethylated	Eif3k,Map4k1	13	10
chr7	30017541	30019541	-0.31839	0.027525	hypomethylated	Kcnk6	11	8
chr7	30033486	30035486	0.28	0.0000223	hypermethylated	2200002D01Rik	6	4
chr7	30073338	30075338	-0.19905	3.06E-08	hypomethylated	Ppp1r14a	32	27
chr7	30600090	30602090	-0.29967	0.038268	hypomethylated	Zfp790	9	7
chr7	31017048	31019048	-0.18713	0.008402	hypomethylated	Polr2i,Tbcb	16	17
chr7	31098834	31100834	-0.37029	1.65E-08	stronglyhypometh	Syne4	10	11
chr7	31147791	31149791	-0.047786	0.021232	hypomethylated	Lrfn3	14	14
chr7	31230580	31232580	-0.21628	0.00025675	hypomethylated	Aplp1	11	10
chr7	31320028	31322028	0.06781	0.0060882	hypermethylated	Arhgap33	9	9
chr7	31347358	31349358	-0.10335	0.024782	hypomethylated	Psenen,U2af1l4	22	15
chr7	31348203	31350203	-0.21195	0.0000366	hypomethylated	Igflr1,Psenen,U2af1l4	32	17

chr7	31571923	31573923	-0.26611	0.0001417	hypomethylated	Krtdap	11	10
chr7	31741822	31743822	0.15336	0.00000777	hypermethylated	Usf2	36	36
chr7	31836473	31838473	-0.18981	0.0034692	hypomethylated	Fxyd7	12	7
chr7	35015324	35017324	-0.094318	0.049437	hypomethylated	Gpi1	5	5
chr7	35903311	35905311	-0.097834	0.0024046	hypomethylated	Cebpa	81	74
chr7	36118255	36120255	-0.065867	0.015776	hypomethylated	Rhpn2	18	11
chr7	36340947	36342947	-0.12771	0.00073731	hypomethylated	Nudt19	3	3
chr7	36539473	36541473	0.089409	0.0020541	hypermethylated	Dpy19l3	13	13
chr7	38554771	38556771	-0.058094	0.0017487	hypomethylated	Zfp536	57	42
chr7	38892509	38894509	-0.061237	0.026127	hypomethylated	Ccne1	44	39
chr7	38967235	38969235	-0.11521	0.0000949	hypomethylated	1600014C10Rik	12	10
chr7	38967805	38969805	-0.24161	0.0000102	hypomethylated	1600014C10Rik	14	10
chr7	48153647	48155647	-0.043945	0.0065708	hypomethylated	A230077H06Rik,Vstm2b	45	40
chr7	49948088	49950088	-0.39683	0.00018225	stronglyhypometh	Gm5595	3	3
chr7	50745393	50747393	0.092211	0.027464	hypermethylated	Iglon5	24	21
chr7	50926400	50928400	-0.30926	0.037827	hypomethylated	Ctu1	6	3
chr7	51491492	51493492	-0.22953	0.014565	hypomethylated	1700028J19Rik	5	5
chr7	51564633	51566633	-0.097458	0.00000302	hypomethylated	Shank1	27	29
chr7	51750957	51752957	-0.079233	0.0039727	hypomethylated	Emc10,Fam71e1	30	28
chr7	51751883	51753883	-0.15989	0.0043865	hypomethylated	Emc10,Fam71e1	18	16
chr7	51780039	51782039	0.19606	0.044708	hypermethylated	Mybpc2	9	8
chr7	51804185	51806185	0.59848	0.0000889	stronglyhypermeth	Pold1	2	2
chr7	51845255	51847255	-0.016249	0.00000925	hypomethylated	Kcnc3	56	59
chr7	52003987	52005987	0.12447	0.0038047	hypermethylated	Vrk3,Zfp473	11	9
chr7	52070739	52072739	0.19034	0.0031747	hypermethylated	Atf5,Nup62,Nup62-il4i1	22	26
chr7	52070789	52072789	0.19034	0.0031747	hypermethylated	Atf5,Nup62,Nup62-il4i1	22	26
chr7	52071028	52073028	0.19034	0.0031747	hypermethylated	Atf5,Nup62,Nup62-il4i1	22	26
chr7	52150448	52152448	-0.19637	0.011107	hypomethylated	Fuz	9	9
chr7	52184860	52186860	0.1259	0.0056431	hypermethylated	Ap2a1	17	12
chr7	52308251	52310251	-0.21545	0.00087876	hypomethylated	Prr12	15	12
chr7	52359192	52361192	-0.61346	0.00000163	stronglyhypometh	Fcgrt	8	4
chr7	52378481	52380481	-0.10544	0.002782	hypomethylated	Rps11,Snord35b	21	21
chr7	52382833	52384833	0.050991	0.017072	hypermethylated	Mir5121,Rpl13a,Snord32a,Snc	18	14
chr7	52384105	52386105	0.098043	0.024854	hypermethylated	Rpl13a	12	8
chr7	52418290	52420290	-0.102	0.0073864	hypomethylated	Slc17a7	20	19
chr7	52467253	52469253	-0.22652	0.00012362	hypomethylated	Dkkl1	6	5
chr7	52675315	52677315	-0.2082	1.82E-08	hypomethylated	Lhb	21	21
chr7	52972440	52974440	-0.13023	0.037059	hypomethylated	Rpl18,Sphk2	36	35
chr7	53014925	53016925	-0.25597	0.0027743	hypomethylated	Sult2b1	7	7
chr7	53288065	53290065	0.073759	0.00077162	hypermethylated	Nomo1	29	31
chr7	54263784	54265784	-0.30769	0.0062688	hypomethylated	Spty2d1	4	4
chr7	56500558	56502558	-0.065324	0.027022	hypomethylated	Nav2	49	48
chr7	57013475	57015475	-0.24118	0.000000284	hypomethylated	Htatip2	21	23
chr7	57013881	57015881	-0.24118	0.000000284	hypomethylated	Htatip2	21	23
chr7	58876199	58878199	0.16662	0.00069695	hypermethylated	Slc17a6	15	13
chr7	63048517	63050517	-0.096528	0.0057067	hypomethylated	Tubgcp5	24	13
chr7	63216900	63218900	-0.027862	0.0051591	hypomethylated	A230056P14Rik,Nipa2	27	27
chr7	64641641	64643641	-0.030816	0.0047583	hypomethylated	Gabrg3,Gm9962	29	27
chr7	64844903	64846903	-0.074277	0.00000715	hypomethylated	Gabrb3	48	52
chr7	64845542	64847542	-0.066381	0.000000148	hypomethylated	Gabrb3	56	61
chr7	65912571	65914571	-0.26814	1.58E-08	hypomethylated	Atp10a	19	27
chr7	69520864	69522864	-0.096088	0.012908	hypomethylated	Magel2	16	16
chr7	71645591	71647591	0.031139	0.018993	hypermethylated	Apba2	50	46
chr7	72301414	72303414	-0.1049	0.044055	hypomethylated	Fam189a1	35	32
chr7	73006021	73008021	-0.14325	0.004604	hypomethylated	Pcsk6	47	49
chr7	73223534	73225534	-0.030608	0.006044	hypomethylated	Vimp	17	26
chr7	73253400	73255400	-0.060219	0.0056413	hypomethylated	Chsy1	81	76
chr7	73983620	73985620	-0.041381	0.004814	hypomethylated	Adamts17	44	43
chr7	74366429	74368429	-0.13095	0.012845	hypomethylated	Lysmd4	10	10
chr7	74366501	74368501	-0.13095	0.012845	hypomethylated	Lysmd4	10	10
chr7	80884192	80886192	-0.035797	0.016697	hypomethylated	Fam174b	15	21

chr7	81699666	81701666	-0.074593	0.032679	hypomethylated	Slco3a1	18	18
chr7	82599419	82601419	-0.024654	0.000000242	hypomethylated	Akap13	30	14
chr7	85927016	85929016	-0.32878	0.0065146	hypomethylated	Mrpl46,Mrps11	16	9
chr7	85992097	85994097	0.090909	0.015073	hypermethylated	Det1	5	5
chr7	86536223	86538223	-0.029254	0.027539	hypomethylated	Fanci	13	10
chr7	86649149	86651149	-0.11228	0.00000911	hypomethylated	Mir9-3	34	34
chr7	86804081	86806081	-0.061742	0.0054912	hypomethylated	Ticrr	22	22
chr7	86859072	86861072	-0.11846	0.0000108	hypomethylated	Kif7	26	25
chr7	87168699	87170699	-0.10663	0.018273	hypomethylated	Zfp710	15	15
chr7	87170113	87172113	-0.091413	0.0026466	hypomethylated	Zfp710	43	45
chr7	87376778	87378778	-0.069231	0.00028623	hypomethylated	Cib1,Gdpgp1	29	29
chr7	87377502	87379502	-0.083699	0.00044736	hypomethylated	Cib1,Gdpgp1	30	30
chr7	87390261	87392261	0.085714	0.032227	hypermethylated	Ttll13	5	5
chr7	87405100	87407100	-0.15089	0.026709	hypomethylated	Ngrn	20	16
chr7	87413540	87415540	-0.12884	0.017825	hypomethylated	Vps33b	19	19
chr7	87469340	87471340	-0.16114	0.0013923	hypomethylated	Rccd1	8	10
chr7	87516012	87518012	-0.043567	0.025987	hypomethylated	Man2a2	40	42
chr7	87948217	87950217	0.42857	0.00038839	stronglyhypermeth	Iqgap1	7	7
chr7	88005993	88007993	-0.2619	0.0085832	hypomethylated	Zscan2	3	3
chr7	88092436	88094436	-0.17857	0.014682	hypomethylated	Sec11a	4	8
chr7	88201485	88203485	-0.23659	0.0003155	hypomethylated	Alpk3	12	8
chr7	89079345	89081345	-0.16801	0.022693	hypomethylated	Hdgfrp3	30	25
chr7	90014842	90016842	-0.099006	0.00034192	hypomethylated	Mex3b	67	66
chr7	91032851	91034851	-0.13783	0.0079612	hypomethylated	Mesdc1	24	26
chr7	91558469	91560469	-0.06335	0.0077638	hypomethylated	Arnt2	14	13
chr7	96551875	96553875	-0.13449	1.39E-08	hypomethylated	Fzd4	44	36
chr7	96666096	96668096	-0.12083	0.0037906	hypomethylated	Prss23	13	9
chr7	97277741	97279741	-0.062337	0.00022399	hypomethylated	2310010J17Rik,Picalm	57	50
chr7	97590290	97592290	0.023564	0.0021861	hypermethylated	Crebzf	62	64
chr7	99889223	99891223	-0.13505	0.0070386	hypomethylated	4632427E13Rik,Rab30	13	12
chr7	99889969	99891969	-0.41898	0.0010364	stronglyhypometh	4632427E13Rik,Rab30	4	3
chr7	104099036	104101036	-0.30648	0.00000225	hypomethylated	Nars2	4	6
chr7	104229260	104231260	-0.070267	0.010493	hypomethylated	Gab2	59	61
chr7	104479832	104481832	-0.18214	0.0000198	hypomethylated	Kctd21	14	9
chr7	104628465	104630465	0.3043	0.00020714	hypermethylated	Ints4	7	4
chr7	105509798	105511798	-0.10128	0.048866	hypomethylated	Tsku	19	14
chr7	105850872	105852872	-0.064362	0.0013778	hypomethylated	Prkrir	48	41
chr7	106289654	106291654	-0.10559	0.026694	hypomethylated	Uvrag	5	5
chr7	106331223	106333223	-0.41274	0.023342	stronglyhypometh	Dgat2	12	12
chr7	106415855	106417855	-0.051146	0.000000161	hypomethylated	Map6	34	34
chr7	106529058	106531058	-0.088784	0.0051085	hypomethylated	Gdpd5	31	30
chr7	106631442	106633442	-0.065422	0.042744	hypomethylated	Rps3,Snord15a	11	8
chr7	107375116	107377116	-0.097117	0.0017795	hypomethylated	Pgm2l1	28	27
chr7	107519742	107521742	-0.13901	8.57E-08	hypomethylated	C2cd3,Ppme1	29	26
chr7	107520406	107522406	-0.15562	0.0059611	hypomethylated	C2cd3,Ppme1	17	18
chr7	107640853	107642853	-0.068944	0.016787	hypomethylated	Ucp2	19	16
chr7	107755099	107757099	-0.09257	0.0014383	hypomethylated	Mrpl48,Rab6a	53	46
chr7	107756510	107758510	-0.11602	0.020917	hypomethylated	Mrpl48,Rab6a	33	32
chr7	107854215	107856215	0.16965	0.00090574	hypermethylated	Fam168a	6	7
chr7	108256288	108258288	-0.1118	0.0078266	hypomethylated	Fchsd2	42	39
chr7	108495582	108497582	-0.020456	0.00000503	hypomethylated	Arap1	19	20
chr7	108541901	108543901	-0.18667	0.0094404	hypomethylated	Arap1	5	5
chr7	108986338	108988338	-0.14563	0.0071649	hypomethylated	Inppl1	22	13
chr7	109043928	109045928	-0.14719	0.0003656	hypomethylated	Anapc15	16	17
chr7	109213226	109215226	-0.18157	0.00000303	hypomethylated	Rnf121,Trpc2	20	19
chr7	109213646	109215646	-0.13586	0.0000473	hypomethylated	Rnf121,Trpc2	19	19
chr7	111366308	111368308	-0.0074675	0.015718	hypomethylated	Trim6	11	7
chr7	112788053	112790053	0.094345	0.01898	hypermethylated	Arfip2,Timm10b	10	8
chr7	112885014	112887014	-0.081631	0.0087535	hypomethylated	Ilk,Rrp8	15	11
chr7	112959601	112961601	0.57197	0.00044128	stronglyhypermeth	Mrpl17	3	6
chr7	114351958	114353958	-0.08554	0.023598	hypomethylated	Rbmxl2	65	70

chr7	114513303	114515303	0.053676	0.00093557	hypermethylated	Syt9	48	48
chr7	114901510	114903510	-0.11607	0.013604	hypomethylated	Cyb5r2	4	4
chr7	116076928	116078928	-0.18022	0.00015986	hypomethylated	Eif3f	9	8
chr7	116153393	116155393	-0.024609	0.0064648	hypomethylated	Tub	37	40
chr7	116582567	116584567	-0.2533	0.0000127	hypomethylated	Stk33	17	16
chr7	116895777	116897777	-0.089372	0.023305	hypomethylated	Tmem9b	24	20
chr7	116925059	116927059	-0.076981	0.023522	hypomethylated	Nrip3	9	15
chr7	117264572	117266572	-0.091712	0.00000183	hypomethylated	Wee1	64	67
chr7	117364216	117366216	0.043509	0.00028613	hypermethylated	Swap70	22	22
chr7	118226544	118228544	-0.048247	0.0022773	hypomethylated	Eif4g2	11	11
chr7	118266189	118268189	-0.10952	0.026074	hypomethylated	1700012D14Rik	7	7
chr7	118923491	118925491	-0.2465	0.000000236	hypomethylated	Galnt18	17	17
chr7	119570219	119572219	0.44744	0.03916	stronglyhypermeth	Parva	3	5
chr7	120512853	120514853	-0.12462	0.00014361	hypomethylated	Btbd10	26	22
chr7	120656375	120658375	-0.091962	0.0015726	hypomethylated	Far1	36	36
chr7	123235893	123237893	-0.016718	0.00017026	hypomethylated	1110004F10Rik,1700003G18R	14	16
chr7	123236663	123238663	-0.016718	0.00017026	hypomethylated	1110004F10Rik,1700003G18R	14	16
chr7	123477704	123479704	-0.080984	0.017879	hypomethylated	Rps13	19	25
chr7	125386420	125388420	-0.054862	0.0000309	hypomethylated	4930583K01Rik,Smg1	42	49
chr7	125387151	125389151	-0.11207	0.031986	hypomethylated	4930583K01Rik,Smg1	13	20
chr7	125999127	126001127	-0.069143	0.0000641	hypomethylated	2310008H09Rik,Iqck	33	28
chr7	126936643	126938643	-0.056479	0.030582	hypomethylated	2610020H08Rik,Eri2	23	17
chr7	126936700	126938700	-0.056479	0.030582	hypomethylated	2610020H08Rik,Eri2	23	17
chr7	127985396	127987396	-0.36131	0.00046279	stronglyhypometh	Eef2k	18	12
chr7	128176958	128178958	-0.06779	0.00027676	hypomethylated	Mettl9	62	62
chr7	128534809	128536809	-0.040748	0.0020237	hypomethylated	Hs3st2	46	46
chr7	129431638	129433638	-0.1054	0.00015107	hypomethylated	Prkcb	24	28
chr7	130266398	130268398	-0.045082	0.016093	hypomethylated	Tnrc6a	19	25
chr7	130520495	130522495	-0.29762	0.00000683	hypomethylated	Lcmt1	8	8
chr7	132587189	132589189	-0.13421	0.0000979	hypomethylated	Kdm8	28	28
chr7	133571280	133573280	-0.09271	0.0000717	hypomethylated	Rabep2	27	29
chr7	133629868	133631868	0.10533	0.000045	hypermethylated	Tufm	17	15
chr7	133646816	133648816	-0.092808	0.000000832	hypomethylated	Atxn2l	32	34
chr7	133791822	133793822	-0.095377	1.88E-08	hypomethylated	Ccdc101	21	29
chr7	133908927	133910927	-0.3881	0.000895	stronglyhypometh	Gdpd3	4	6
chr7	133919488	133921488	-0.037	0.023552	hypomethylated	Ypel3	19	24
chr7	133990066	133992066	-0.063675	0.0052515	hypomethylated	Doc2a	33	37
chr7	134071392	134073392	-0.18095	0.000000992	hypomethylated	Kctd13	38	33
chr7	134118862	134120862	-0.035043	0.049832	hypomethylated	Cdipt	20	19
chr7	134118901	134120901	-0.035043	0.049832	hypomethylated	Cdipt	20	19
chr7	134158108	134160108	-0.16771	0.0012279	hypomethylated	Mvp,Pagr1a	8	10
chr7	134164725	134166725	-0.10453	0.0013887	hypomethylated	Prrt2	11	11
chr7	134233949	134235949	-0.45455	0.017646	stronglyhypometh	AI467606	3	2
chr7	134351982	134353982	0.11094	0.0031189	hypermethylated	Tbc1d10b	16	17
chr7	134387039	134389039	-0.05436	0.00079039	hypomethylated	Zfp771	34	36
chr7	134488828	134490828	-0.068259	0.00050233	hypomethylated	Zfp768	42	46
chr7	134656383	134658383	-0.12612	0.0095955	hypomethylated	1700008J07Rik	8	8
chr7	134984321	134986321	-0.17108	0.0000016	hypomethylated	Stx4a	31	35
chr7	135146891	135148891	-0.35246	0.00029239	stronglyhypometh	Trim72	8	7
chr7	135379870	135381870	-0.074256	0.021349	hypomethylated	9130023H24Rik,Armc5	45	42
chr7	135389384	135391384	0.11111	0.029208	hypermethylated	Tgfb1i1	6	6
chr7	135605027	135607027	-0.081079	0.0037561	hypomethylated	Tial1	31	31
chr7	135887383	135889383	-0.10773	5.7E-12	hypomethylated	Sec23ip	24	21
chr7	136734376	136736376	-0.047751	0.0049766	hypomethylated	Wdr11	23	23
chr7	137663044	137665044	-0.053632	0.00000868	hypomethylated	Ate1	29	29
chr7	137663053	137665053	-0.053632	0.00000868	hypomethylated	Ate1	29	29
chr7	138008423	138010423	-0.042039	0.0000378	hypomethylated	Plekha1	51	51
chr7	138685476	138687476	-0.23254	6.06E-08	hypomethylated	Hmx3	50	57
chr7	139506346	139508346	-0.11481	0.0000758	hypomethylated	Chst15,Gm10584	29	36
chr7	140004879	140006879	-0.023882	0.027517	hypomethylated	Fam53b	39	30
chr7	140049907	140051907	-0.063456	0.021635	hypomethylated	Fam175b	25	25

chr7	140315166	140317166	0.11994	0.0031263	hypermethylated	Ctbp2	47	47
chr7	140828357	140830357	0.022691	0.016163	hypermethylated	2700050L05Rik	16	16
chr7	140900015	140902015	-0.071102	0.0013466	hypomethylated	Bccip,Uros	32	31
chr7	142728506	142730506	0.052901	0.0028773	hypermethylated	Ptpre	30	33
chr7	144506128	144508128	0.054854	0.0000125	hypermethylated	Ebf3	35	35
chr7	146101189	146103189	0.14048	0.0029585	hypermethylated	Bnip3	7	10
chr7	146270899	146272899	-0.092771	0.0064975	hypomethylated	Dpysl4	32	37
chr7	146397886	146399886	0.18398	0.0000173	hypermethylated	Lrrc27,Stk32c	7	12
chr7	146398173	146400173	0.18398	0.0000173	hypermethylated	Lrrc27,Stk32c	7	12
chr7	146433380	146435380	-0.088612	0.000000873	hypomethylated	Pwwp2b	76	80
chr7	147221289	147223289	-0.31726	0.0003436	hypomethylated	Tubgcp2,Zfp511	25	29
chr7	147222249	147224249	-0.37066	0.00030568	stronglyhypometh	Tubgcp2,Zfp511	21	21
chr7	148030463	148032463	-0.45139	0.00023539	stronglyhypometh	C330022C24Rik,Scgb1c1	4	8
chr7	148379028	148381028	-0.12206	5.54E-08	hypomethylated	Hras1,Lrrc56	64	64
chr7	148379903	148381903	-0.23684	2.62E-10	hypomethylated	Hras1,Lrrc56	28	28
chr7	148380041	148382041	-0.25131	1.45E-08	hypomethylated	Hras1,Lrrc56	24	24
chr7	148400758	148402758	-0.080447	0.012801	hypomethylated	1600016N20Rik,Rassf7	21	26
chr7	148407392	148409392	-0.1455	0.00076314	hypomethylated	Mir210	11	11
chr7	148413686	148415686	-0.033959	0.0000119	hypomethylated	Phrf1	25	20
chr7	148600024	148602024	0.17083	0.044174	hypermethylated	Pddc1	8	10
chr7	149683740	149685740	-0.37143	0.03701	stronglyhypometh	Tnnt3	2	2
chr7	149718021	149720021	-0.27073	0.0014945	hypomethylated	Mrpl23	18	22
chr7	149843386	149845386	-0.24355	1.13E-08	hypomethylated	Igf2,Igf2as	38	34
chr7	149844597	149846597	-0.15534	0.000000025	hypomethylated	Igf2,Igf2as	62	60
chr7	149844709	149846709	-0.15534	0.000000025	hypomethylated	Igf2,Igf2as	62	60
chr7	150292158	150294158	0.2709	0.0035091	hypermethylated	Kcnq1	16	17
chr7	150688429	150690429	-0.063142	0.0076215	hypomethylated	Phlda2	14	15
chr7	150785947	150787947	0.17053	0.0047328	hypermethylated	Cars	7	5
chr7	151768341	151770341	-0.22619	0.021753	hypomethylated	Fadd	7	5
chr7	152023516	152025516	-0.10936	0.0000105	hypomethylated	Fgf3	41	48
chr7	152046290	152048290	-0.098048	0.00018175	hypomethylated	Fgf4	48	46
chr7	152100098	152102098	-0.099434	0.00075523	hypomethylated	Oraov1	13	16
chr8	3492137	3494137	-0.098143	3.46E-12	hypomethylated	Zfp358	34	27
chr8	3586449	3588449	-0.080284	0.00087233	hypomethylated	Camsap3	25	27
chr8	3620550	3622550	-0.19837	0.0002501	hypomethylated	Pet100,Xab2	12	14
chr8	3621296	3623296	-0.21605	0.00013595	hypomethylated	Pet100,Xab2	7	7
chr8	3630159	3632159	-0.16503	0.0007674	hypomethylated	Stxbp2	11	10
chr8	3675476	3677476	-0.10414	0.012157	hypomethylated	Trappc5	10	9
chr8	4348587	4350587	-0.23455	0.041161	hypomethylated	Ccl25	2	2
chr8	10928457	10930457	-0.098205	0.043368	hypomethylated	3930402G23Rik	6	6
chr8	11008430	11010430	-0.12714	0.00065681	hypomethylated	9530052E02Rik,Irs2	64	65
chr8	11635754	11637754	0.15769	0.0034213	hypermethylated	Ankrd10	12	13
chr8	11757329	11759329	-0.025411	0.036137	hypomethylated	Arhgef7	35	31
chr8	12756015	12758015	0.037212	0.0020512	hypermethylated	Atp11a	73	75
chr8	13025033	13027033	-0.21259	0.039101	hypomethylated	F7	5	5
chr8	13104720	13106720	-0.10283	0.027042	hypomethylated	Cul4a,Pcid2	69	63
chr8	13105343	13107343	-0.12062	0.014437	hypomethylated	Cul4a,Pcid2	46	40
chr8	13158134	13160134	-0.2	0.044176	hypomethylated	Lamp1	10	9
chr8	13287012	13289012	0.060181	0.028758	hypermethylated	Dcun1d2,Tmco3	37	37
chr8	13338673	13340673	-0.13508	2.15E-11	hypomethylated	Tfdp1	33	44
chr8	13784614	13786614	-0.07603	0.000000121	hypomethylated	Upf3a	42	45
chr8	13906805	13908805	-0.18326	2.33E-09	hypomethylated	Fbxo25	20	26
chr8	14090327	14092327	-0.1702	0.00032551	hypomethylated	Erich1	10	10
chr8	14094874	14096874	-0.08923	0.018095	hypomethylated	Dlgap2	45	52
chr8	15010024	15012024	-0.1078	0.000000932	hypomethylated	Kbtbd11	44	38
chr8	23586171	23588171	-0.024298	0.019365	hypomethylated	AI316807,Slc20a2	54	56
chr8	23916126	23918126	-0.0461	0.022419	hypomethylated	Ap3m2	20	20
chr8	24084353	24086353	-0.040133	0.00000552	hypomethylated	Ank1	67	62
chr8	24318925	24320925	-0.075309	0.00029358	hypomethylated	Agpat6	19	19
chr8	24367552	24369552	-0.34098	0.0026036	stronglyhypometh	Golga7	9	5
chr8	26126682	26128682	-0.10636	0.0010089	hypomethylated	Adam9,Tm2d2	28	38

chr8	26127394	26129394	-0.078502	0.016863	hypomethylated	Adam9,Tm2d2	18	29
chr8	26212283	26214283	-0.078278	0.0000942	hypomethylated	Plekha2	23	26
chr8	26711777	26713777	-0.081471	0.0012329	hypomethylated	Whsc1l1	62	61
chr8	26829519	26831519	-0.11111	0.0011718	hypomethylated	Ppapdc1b	9	6
chr8	26864752	26866752	-0.14921	1.74E-08	hypomethylated	Ddhd2	29	29
chr8	26895062	26897062	-0.058455	0.00030082	hypomethylated	Bag4,Lsm1	42	39
chr8	26895681	26897681	-0.028193	0.012823	hypomethylated	Bag4,Lsm1	29	26
chr8	27267640	27269640	-0.036415	0.016022	hypomethylated	Thap1	45	55
chr8	28133330	28135330	-0.11875	0.00000498	hypomethylated	Erlin2	20	22
chr8	28369798	28371798	0.18383	0.00018108	hypermethylated	Eif4ebp1	23	23
chr8	32221317	32223317	-0.088703	0.017309	hypomethylated	Rnf122	26	29
chr8	32259787	32261787	-0.15193	0.0066578	hypomethylated	Tti2	12	11
chr8	34709092	34711092	-0.054949	0.04302	hypomethylated	Ppp2cb	45	35
chr8	35209793	35211793	-0.12593	0.0027638	hypomethylated	Leprotl1	18	17
chr8	36889613	36891613	-0.076751	0.00043779	hypomethylated	Cldn23	16	16
chr8	38055628	38057628	-0.11779	0.00047065	hypomethylated	AI429214	9	9
chr8	41508168	41510168	-0.066417	0.00098512	hypomethylated	Zdhhc2	53	55
chr8	41946720	41948720	-0.051076	0.04704	hypomethylated	Slc7a2	8	8
chr8	41946746	41948746	-0.051076	0.04704	hypomethylated	Slc7a2	8	8
chr8	42324112	42326112	-0.24314	0.0000208	hypomethylated	Pcm1	23	23
chr8	47380025	47382025	-0.1205	0.016837	hypomethylated	Helt	11	11
chr8	47555395	47557395	-0.049714	0.0042834	hypomethylated	Acsl1	53	53
chr8	47636422	47638422	-0.069755	0.00051004	hypomethylated	Mlf1ip	22	22
chr8	48799285	48801285	-0.046878	0.0015028	hypomethylated	Cdkn2aip	35	38
chr8	59798779	59800779	0.048036	0.011425	hypermethylated	Hand2	20	14
chr8	61390424	61392424	-0.15682	0.041665	hypomethylated	BC030500,Galntl6	20	20
chr8	63110656	63112656	-0.16831	0.0000363	hypomethylated	Mfap3l	36	40
chr8	63462108	63464108	-0.083537	0.0031619	hypomethylated	Clcn3	20	20
chr8	63701967	63703967	-0.077435	0.0033956	hypomethylated	Sh3rf1	48	44
chr8	64405885	64407885	-0.13533	0.020221	hypomethylated	Ddx60	3	3
chr8	65429242	65431242	-0.099333	0.031683	hypomethylated	Spock3	13	18
chr8	67212375	67214375	0.050899	0.044071	hypermethylated	Sc4mol	11	11
chr8	67470083	67472083	-0.034609	0.01254	hypomethylated	Tmem192	39	42
chr8	69211993	69213993	-0.1039	0.047494	hypomethylated	Npy5r	7	7
chr8	69383115	69385115	-0.028225	0.0016654	hypomethylated	Naf1	53	54
chr8	70017773	70019773	-0.078968	0.0090395	hypomethylated	Nat2	11	11
chr8	70017846	70019846	-0.078968	0.0090395	hypomethylated	Nat2	11	11
chr8	71259041	71261041	-0.3141	0.010505	hypomethylated	Csgalnact1	8	5
chr8	72149074	72151074	-0.2	0.0026483	hypomethylated	Zfp868	4	4
chr8	72331585	72333585	-0.18783	0.0000968	hypomethylated	Gmip	17	15
chr8	72425113	72427113	-0.11839	3.56E-12	hypomethylated	Ndufa13,Tssk6	46	46
chr8	72520278	72522278	-0.046628	0.046482	hypomethylated	Gatad2a	13	16
chr8	72654231	72656231	-0.074175	0.0020183	hypomethylated	Nr2c2ap	20	24
chr8	72757124	72759124	-0.0016003	0.0062384	hypomethylated	Armc6,Sugp2	25	25
chr8	72825683	72827683	-0.10014	0.014215	hypomethylated	Cope,Ddx49	14	14
chr8	72963472	72965472	-0.073923	0.014427	hypomethylated	Crtc1	18	12
chr8	73034266	73036266	0.11793	0.0000862	hypermethylated	Uba52	16	14
chr8	73132213	73134213	-0.050417	0.0027792	hypomethylated	Ssbp4	23	22
chr8	73363619	73365619	-0.33033	0.00000304	hypomethylated	Arrdc2	13	9
chr8	73421311	73423311	-0.18084	0.00045646	hypomethylated	Rpl18a	10	11
chr8	73421342	73423342	-0.18084	0.00045646	hypomethylated	Rpl18a	10	11
chr8	73428872	73430872	-0.014122	0.022366	hypomethylated	Map1s	36	31
chr8	73894196	73896196	0.057867	0.0015206	hypermethylated	Ocel1	23	25
chr8	73919700	73921700	0.070451	0.026907	hypermethylated	Babam1,Ushbp1	17	18
chr8	74011001	74013001	-0.3039	0.0036687	hypomethylated	Gtpbp3	11	7
chr8	74091825	74093825	0.089819	0.02916	hypermethylated	Slc27a1	10	11
chr8	74658190	74660190	0.082446	0.0051923	hypermethylated	Tpm4	27	20
chr8	74742628	74744628	0.038377	0.017676	hypermethylated	Fam32a	18	16
chr8	74841960	74843960	-0.04233	0.029678	hypomethylated	Klf2	56	44
chr8	75016513	75018513	-0.11157	0.0000144	hypomethylated	Slc35e1	27	26
chr8	75246186	75248186	-0.36376	0.015864	stronglyhypometh	Sin3b	12	8

chr8	77516601	77518601	-0.077262	0.00000521	hypomethylated	Hmgxb4	41	46
chr8	80960551	80962551	-0.23928	0.0034567	hypomethylated	Pou4f2	17	25
chr8	82162574	82164574	-0.019827	0.015171	hypomethylated	Otud4	71	86
chr8	84926444	84928444	-0.27931	0.045702	hypomethylated	Il15	8	5
chr8	85298025	85300025	-0.125	0.014439	hypomethylated	Zfp330	4	6
chr8	85856385	85858385	-0.055952	0.041579	hypomethylated	Elmod2	6	6
chr8	86131073	86133073	-0.12427	7.89E-09	hypomethylated	Dnajb1	52	51
chr8	86422996	86424996	-0.039791	0.0061513	hypomethylated	Lphn1	84	81
chr8	86478592	86480592	-0.11535	0.00000476	hypomethylated	Asf1b	29	33
chr8	86495876	86497876	-0.046764	0.025741	hypomethylated	Prkaca	40	37
chr8	86535532	86537532	0.14088	0.00031222	hypermethylated	Mir1199	38	32
chr8	86544372	86546372	-0.1857	0.00027573	hypomethylated	Palm3	13	14
chr8	86794259	86796259	-0.2401	0.0017271	hypomethylated	Ccdc130	15	14
chr8	87224355	87226355	-0.14538	0.0000473	hypomethylated	Lyl1	14	18
chr8	87364540	87366540	-0.12061	0.00000188	hypomethylated	Rad23a	5	5
chr8	87431522	87433522	0.011559	0.026191	hypermethylated	Dnase2a	24	28
chr8	87492546	87494546	-0.16976	2.67E-10	hypomethylated	Prdx2	25	25
chr8	87549731	87551731	0.12768	0.023502	hypermethylated	2310036O22Rik,Asna1	19	28
chr8	88015515	88017515	-0.052909	0.0000578	hypomethylated	Gpt2	27	18
chr8	89268597	89270597	0.014316	0.00051865	hypermethylated	Gm10638,Siah1a	78	84
chr8	90641657	90643657	-0.11267	0.0091927	hypomethylated	Cnep1r1	16	14
chr8	90660783	90662783	0.029197	0.015899	hypermethylated	Heatr3	30	29
chr8	90722111	90724111	0.079559	0.0075976	hypermethylated	Papd5	78	88
chr8	90795301	90797301	-0.057438	0.0044523	hypomethylated	Adcy7	24	16
chr8	91044242	91046242	-0.042174	0.0062689	hypomethylated	Nkd1	65	66
chr8	95378248	95380248	-0.055662	0.0066951	hypomethylated	Lpcat2	16	26
chr8	96337455	96339455	-0.15905	0.010828	hypomethylated	4930488L21Rik	5	5
chr8	96560097	96562097	-0.18305	0.0029091	hypomethylated	Nudt21,Ogfod1	19	23
chr8	96737500	96739500	-0.041667	0.0013781	hypomethylated	Nup93	19	24
chr8	96959003	96961003	-0.2	0.0000358	hypomethylated	9330175E14Rik	5	5
chr8	97055927	97057927	-0.034304	0.00000112	hypomethylated	Cpne2	44	45
chr8	97125626	97127626	-0.22303	0.000000823	hypomethylated	Fam192a,Rspry1	19	22
chr8	97189654	97191654	-0.03326	0.027909	hypomethylated	Arl2bp	27	28
chr8	97190005	97192005	-0.03326	0.027909	hypomethylated	Arl2bp	27	28
chr8	97604100	97606100	-0.24167	0.001944	hypomethylated	Katnb1	17	11
chr8	97855183	97857183	-0.078207	0.000000656	hypomethylated	Usb1,Zfp319	55	57
chr8	97855850	97857850	-0.15295	0.0070302	hypomethylated	Usb1,Zfp319	21	23
chr8	97875236	97877236	-0.11968	0.0000826	hypomethylated	Mmp15	53	54
chr8	98012720	98014720	-0.06727	0.00037754	hypomethylated	Csnk2a2	12	14
chr8	98156458	98158458	-0.036933	0.042462	hypomethylated	Gins3	22	26
chr8	106863493	106865493	-0.22299	0.0000429	hypomethylated	Cmtm3	31	30
chr8	107693573	107695573	-0.098556	0.00000116	hypomethylated	Cbfb	71	73
chr8	107792773	107794773	-0.10013	0.0010882	hypomethylated	Hsf4	32	33
chr8	107871157	107873157	-0.064039	0.0026831	hypomethylated	Fhod1,Slc9a5	28	33
chr8	107871870	107873870	-0.052302	0.00064678	hypomethylated	Fhod1,Slc9a5	37	41
chr8	107898280	107900280	0.0023267	0.038863	hypermethylated	Plekhg4	22	24
chr8	108041645	108043645	0.023456	0.048523	hypermethylated	Hsd11b2	22	24
chr8	108159437	108161437	-0.038158	1.42E-08	hypomethylated	Ctcf	109	107
chr8	108224053	108226053	-0.20219	0.0000001	hypomethylated	Acd,Pard6a	31	29
chr8	108224548	108226548	-0.11656	0.000000678	hypomethylated	Acd,Pard6a	28	26
chr8	108224995	108226995	-0.1769	0.00016595	hypomethylated	Acd,Pard6a	20	16
chr8	108231202	108233202	-0.10606	0.0012891	hypomethylated	4933405L10Rik,Enkd1	4	5
chr8	108232068	108234068	-0.012197	0.00013371	hypomethylated	4933405L10Rik,Enkd1	8	8
chr8	108403850	108405850	-0.19443	0.000018	hypomethylated	Edc4	12	12
chr8	108582502	108584502	-0.09456	0.00019572	hypomethylated	Nfatc3	40	39
chr8	108660874	108662874	-0.17231	0.044766	hypomethylated	Esrp2	15	12
chr8	108673298	108675298	-0.13659	0.046393	hypomethylated	Pla2g15	22	21
chr8	108733953	108735953	-0.075523	0.015252	hypomethylated	Prmt7,Slc7a6os	42	42
chr8	109458649	109460649	0.16605	0.00080956	hypermethylated	Sntb2	38	37
chr8	109554225	109556225	-0.045892	0.003993	hypomethylated	Vps4a	50	42
chr8	109579776	109581776	-0.12494	0.0000412	hypomethylated	Cog8,Nip7	62	62

chr8	109580637	109582637	-0.17971	0.0000448	hypomethylated	Cog8,Nip7	35	34
chr8	109673560	109675560	-0.13292	0.00029613	hypomethylated	Cyb5b	27	27
chr8	109948938	109950938	-0.1155	0.015949	hypomethylated	Nob1	16	16
chr8	111237543	111239543	-0.045488	0.00010621	hypomethylated	Zfhx3	65	72
chr8	111459759	111461759	-0.25	0.036765	hypomethylated	Mir3108	3	2
chr8	112228448	112230448	-0.02767	0.010637	hypomethylated	Zfp821	37	36
chr8	112228987	112230987	-0.02767	0.010637	hypomethylated	Zfp821	37	36
chr8	112602633	112604633	-0.088613	0.00088861	hypomethylated	Zfp612	11	13
chr8	112740859	112742859	-0.099519	0.0017644	hypomethylated	Ftsjd1	9	9
chr8	113244383	113246383	-0.095755	0.00058562	hypomethylated	Mtss1l	42	42
chr8	113369923	113371923	-0.13218	0.00038036	hypomethylated	Cog4,Sf3b3	21	15
chr8	113370703	113372703	-0.2369	0.00020583	hypomethylated	Cog4,Sf3b3	6	5
chr8	113551670	113553670	-0.15227	0.0016362	hypomethylated	Ddx19b	4	5
chr8	113579238	113581238	-0.016789	0.019719	hypomethylated	Exosc6	52	52
chr8	113783102	113785102	-0.27433	0.00025892	hypomethylated	Glg1	17	12
chr8	114463617	114465617	-0.08548	0.00067614	hypomethylated	Gabarapl2	25	20
chr8	114534258	114536258	-0.016254	0.00014554	hypomethylated	Kars,Terf2ip	30	31
chr8	114535205	114537205	-0.020976	0.0001414	hypomethylated	Kars,Terf2ip	27	30
chr8	115092942	115094942	-0.22698	0.040554	hypomethylated	Cntnap4	5	5
chr8	116656478	116658478	0.11154	0.0021642	hypermethylated	Nudt7	6	5
chr8	119444639	119446639	-0.13725	0.0012122	hypomethylated	Cenpn,Cmc2	16	22
chr8	119445336	119447336	-0.12729	0.0042971	hypomethylated	Cenpn,Cmc2	14	10
chr8	119466292	119468292	-0.031349	0.043312	hypomethylated	Atmin	41	28
chr8	119681034	119683034	0.036736	0.0094929	hypermethylated	Gan	50	48
chr8	119779918	119781918	-0.066793	0.000000817	hypomethylated	Cmip	72	73
chr8	120806654	120808654	0.059975	0.0061145	hypermethylated	Cdh13	20	20
chr8	121917791	121919791	-0.080261	0.012626	hypomethylated	Mlycd	63	58
chr8	121969618	121971618	-0.015375	0.013887	hypomethylated	Necab2	67	67
chr8	122433751	122435751	-0.13898	0.0023235	hypomethylated	Usp10	49	49
chr8	123112975	123114975	-0.044949	0.0041735	hypomethylated	Gins2	9	10
chr8	123158282	123160282	-0.069573	0.00000812	hypomethylated	1190005I06Rik	17	17
chr8	123191189	123193189	-0.051722	0.00075556	hypomethylated	Cox4i1,Emc8	53	47
chr8	123192012	123194012	-0.13789	0.000000196	hypomethylated	Cox4i1,Emc8	35	30
chr8	123259275	123261275	-0.29795	0.020064	hypomethylated	Irf8	5	5
chr8	123639070	123641070	-0.26529	0.00000632	hypomethylated	Foxc2	47	35
chr8	124175833	124177833	-0.16494	1.62E-08	hypomethylated	Zcchc14	78	93
chr8	124253462	124255462	-0.048885	6.26E-09	hypomethylated	Jph3	77	74
chr8	124430732	124432732	-0.10565	0.0000285	hypomethylated	BC048644,Slc7a5	39	32
chr8	124805040	124807040	-0.044823	0.000000838	hypomethylated	Zfpm1	60	59
chr8	124967322	124969322	0.038688	0.024454	hypermethylated	9330133O14Rik,Mvd	15	11
chr8	124999964	125001964	-0.019905	0.023199	hypomethylated	Ctu2,Rnf166	42	44
chr8	125134525	125136525	-0.05282	0.0000838	hypomethylated	Galns,Trappc2l	12	12
chr8	125135387	125137387	-0.1182	0.0000203	hypomethylated	Galns,Trappc2l	13	12
chr8	125202075	125204075	-0.047673	0.0045952	hypomethylated	Cbfa2t3	52	43
chr8	125565897	125567897	-0.054099	0.000000146	hypomethylated	2810013P06Rik,Ankrd11	76	83
chr8	125896652	125898652	-0.16279	7.75E-09	hypomethylated	Tcf25	27	24
chr8	125896723	125898723	-0.16279	7.75E-09	hypomethylated	Tcf25	27	24
chr8	125896734	125898734	-0.16279	7.75E-09	hypomethylated	Tcf25	27	24
chr8	125934463	125936463	-0.10312	0.022554	hypomethylated	Tubb3	15	9
chr8	126000761	126002761	0.054226	0.042184	hypermethylated	Afg3l1	11	11
chr8	126041734	126043734	-0.14682	0.0017727	hypomethylated	Gas8	14	18
chr8	126473165	126475165	0.15067	0.028853	hypermethylated	Nup133	15	15
chr8	127187269	127189269	-0.33428	0.0051871	stronglyhypometh	2310022B05Rik	8	8
chr8	127473154	127475154	0.044666	0.019177	hypermethylated	Egln1	40	40
chr8	128192717	128194717	-0.066848	0.0068122	hypomethylated	Map10	33	30
chr8	128433349	128435349	-0.24013	0.0000285	hypomethylated	BC021891	57	57
chr8	128821478	128823478	-0.059773	0.0001884	hypomethylated	Slc35f3	36	30
chr8	128945400	128947400	-0.15727	0.00000874	hypomethylated	Coa6	27	28
chr8	129117336	129119336	0.02333	0.0036321	hypermethylated	Irf2bp2	76	85
chr8	129822511	129824511	-0.14739	0.0084192	hypomethylated	Pard3	7	7
chr8	129822551	129824551	-0.14739	0.0084192	hypomethylated	Pard3	7	7

chr8	131208553	131210553	-0.047813	0.00016304	hypomethylated	Itgb1	47	57
chr9	7835255	7837255	-0.13024	0.0031304	hypomethylated	Birc2	17	12
chr9	8004596	8006596	-0.091714	0.00065213	hypomethylated	Yap1	11	11
chr9	8543141	8545141	-0.24126	0.0010121	hypomethylated	Trpc6	11	13
chr9	8898832	8900832	-0.0059524	0.024284	hypomethylated	Pgr	7	6
chr9	9239013	9241013	-0.26758	0.0174	hypomethylated	Arhgap42	18	14
chr9	14419444	14421444	0.18222	0.0064742	hypermethylated	Amotl1	9	11
chr9	14587408	14589408	-0.077296	0.03155	hypomethylated	Ankrd49,Mre11a	14	15
chr9	14588150	14590150	-0.077296	0.03155	hypomethylated	Ankrd49,Mre11a	14	15
chr9	14663697	14665697	-0.19795	0.0011596	hypomethylated	Gpr83	20	20
chr9	15162232	15164232	-0.14722	0.0021489	hypomethylated	5830418K08Rik	6	6
chr9	15513212	15515212	-0.094331	0.042419	hypomethylated	Slc36a4	36	35
chr9	18278003	18280003	0.12609	0.02428	hypermethylated	Zfp558	8	8
chr9	20297190	20299190	-0.045362	0.018572	hypomethylated	Zfp426	7	7
chr9	20325863	20327863	-0.3803	3.81E-08	stronglyhypometh	Zfp266	11	12
chr9	20691618	20693618	-0.080468	0.00000704	hypomethylated	Ppan	23	23
chr9	20781237	20783237	-0.29823	0.026564	hypomethylated	S1pr2	3	3
chr9	20832816	20834816	-0.058619	0.0056189	hypomethylated	Icam4	20	14
chr9	21068742	21070742	-0.029955	0.0063641	hypomethylated	Atg4d	26	26
chr9	21215280	21217280	-0.075988	0.012678	hypomethylated	Gm16853,Qtrt1	16	16
chr9	21228388	21230388	0.25555	0.0015969	hypermethylated	Dnm2	22	22
chr9	21314630	21316630	-0.33	0.0000763	hypomethylated	Tmed1	5	5
chr9	21419612	21421612	-0.075132	0.040648	hypomethylated	Smarca4	20	18
chr9	21657079	21659079	-0.1873	0.0000298	hypomethylated	Dock6	9	15
chr9	21722565	21724565	-0.31737	0.00015717	hypomethylated	Rab3d	11	10
chr9	21806478	21808478	-0.1342	0.000000332	hypomethylated	Ccdc151,Prkcsh	21	20
chr9	21807078	21809078	-0.19564	5.82E-08	hypomethylated	Ccdc151,Prkcsh	16	16
chr9	21875789	21877789	-0.43012	6.13E-09	stronglyhypometh	Gm16845,Zfp653	8	8
chr9	21889826	21891826	-0.2161	0.0000216	hypomethylated	Ecsit	16	10
chr9	21936309	21938309	0.072124	0.045742	hypermethylated	Acp5	9	8
chr9	23026519	23028519	0.036233	0.0000344	hypermethylated	Bmper	59	61
chr9	24307584	24309584	-0.066481	0.0048105	hypomethylated	Dpy19l1	51	49
chr9	25059168	25061168	-0.086711	0.039491	hypomethylated	Sept7	64	72
chr9	26836759	26838759	-0.028117	0.00000813	hypomethylated	Ncapd3,Vps26b	47	37
chr9	26837679	26839679	-0.044919	0.0022873	hypomethylated	Ncapd3,Vps26b	20	17
chr9	26962965	26964965	0.031347	0.016698	hypermethylated	Jam3	10	11
chr9	30749147	30751147	-0.17888	0.000000425	hypomethylated	Adamts8	42	42
chr9	30939384	30941384	0.32837	0.039609	hypermethylated	St14	4	6
chr9	31192776	31194776	-0.048114	0.0012021	hypomethylated	Nfrkb	33	35
chr9	32348953	32350953	-0.10377	0.024461	hypomethylated	Fli1	8	7
chr9	35017787	35019787	-0.12261	0.016067	hypomethylated	Foxred1,Srpr	42	39
chr9	35074465	35076465	-0.31023	0.0000348	hypomethylated	Fam118b,Rpusd4	15	16
chr9	36575163	36577163	-0.20033	0.025996	hypomethylated	Stt3a	11	8
chr9	36604653	36606653	-0.17778	5.45E-12	hypomethylated	Ei24	16	16
chr9	36604978	36606978	-0.17938	0.000000358	hypomethylated	Ei24	5	5
chr9	37295905	37297905	-0.020917	0.040022	hypomethylated	Msantd2	64	63
chr9	39998900	40000900	-0.02172	0.030445	hypomethylated	Zfp202	33	35
chr9	40493046	40495046	0.20242	0.0034799	hypermethylated	Clmp	19	18
chr9	42072383	42074383	-0.098889	0.0080111	hypomethylated	Sc5d	18	15
chr9	42913801	42915801	-0.077202	0.00019642	hypomethylated	Arhgef12	50	45
chr9	43850466	43852466	-0.14438	0.014199	hypomethylated	Thy1	5	4
chr9	43892002	43894002	-0.15025	0.0029869	hypomethylated	Usp2	29	26
chr9	43914327	43916327	0.11593	0.0094709	hypermethylated	C1qtnf5	14	14
chr9	43914353	43916353	0.11593	0.0094709	hypermethylated	C1qtnf5	14	14
chr9	43914391	43916391	0.11593	0.0094709	hypermethylated	C1qtnf5	14	14
chr9	44059547	44061547	-0.14444	0.0021603	hypomethylated	Pdzd3	8	8
chr9	44128365	44130365	-0.14352	0.0075991	hypomethylated	C2cd2l	9	9
chr9	44150464	44152464	-0.071961	0.003481	hypomethylated	Hmbs	17	14
chr9	44214796	44216796	-0.15666	0.0028766	hypomethylated	Rps25,Trappc4	29	31
chr9	44575891	44577891	-0.43	0.002474	stronglyhypometh	Arcn1	5	4
chr9	45713651	45715651	-0.11263	0.000046	hypomethylated	Pcsk7,Rnf214	60	54

chr9	46047926	46049926	-0.26364	0.043796	hypomethylated	Apoa4	4	4
chr9	48644050	48646050	-0.018433	0.0096221	hypomethylated	Zbtb16	25	28
chr9	50411954	50413954	-0.061716	0.0483	hypomethylated	Sdhd,Timm8b	5	10
chr9	50424425	50426425	-0.26633	0.0000956	hypomethylated	AU019823,Pih1d2	29	29
chr9	50536089	50538089	0.063258	0.011368	hypermethylated	Dixdc1	10	10
chr9	50582434	50584434	-0.0061355	0.028047	hypomethylated	Alg9	40	38
chr9	50910183	50912183	-0.25398	4.32E-08	hypomethylated	4833427G06Rik,Mir34b,Mir34	22	20
chr9	50911215	50913215	-0.25112	0.00000278	hypomethylated	Mir34b,Mir34c	22	21
chr9	50911750	50913750	-0.14388	0.00097237	hypomethylated	Mir34b,Mir34c	12	11
chr9	51855045	51857045	-0.12222	0.00000483	hypomethylated	Gm6981,Rdx	66	70
chr9	51856810	51858810	-0.2578	9.22E-08	hypomethylated	Gm6981,Rdx	18	18
chr9	51976216	51978216	-0.20318	0.017915	hypomethylated	Zc3h12c	44	29
chr9	53191127	53193127	-0.12904	0.000000456	hypomethylated	Kdelc2	22	22
chr9	53212390	53214390	-0.26667	0.040992	hypomethylated	4930550C14Rik	3	2
chr9	53344151	53346151	-0.15446	0.0000155	hypomethylated	Atm,Npat	16	21
chr9	53344776	53346776	-0.12821	0.0010828	hypomethylated	Atm,Npat	13	19
chr9	54546365	54548365	-0.15455	0.0069318	hypomethylated	Dnaja4	43	46
chr9	54710561	54712561	-0.05996	0.04805	hypomethylated	Ireb2	26	32
chr9	54996175	54998175	-0.12262	1.53E-13	hypomethylated	Ube2q2	77	72
chr9	55055741	55057741	-0.039335	0.019178	hypomethylated	Fbxo22	20	20
chr9	55387955	55389955	-0.16413	0.0000169	hypomethylated	Isl2	29	30
chr9	56776178	56778178	-0.16667	0.043902	hypomethylated	Snx33	2	1
chr9	56841774	56843774	-0.084336	0.00000511	hypomethylated	Ptpn9	52	54
chr9	56918846	56920846	-0.2074	0.00013494	hypomethylated	Gm10658,Sin3a	19	12
chr9	56977583	56979583	-0.1167	0.0011097	hypomethylated	2410133F24Rik,Man2c1	16	16
chr9	57110406	57112406	-0.38595	0.025305	stronglyhypometh	1700017B05Rik	7	10
chr9	57384334	57386334	-0.25946	0.0000105	hypomethylated	Fam219b	19	21
chr9	57436258	57438258	-0.22489	0.0016428	hypomethylated	Ulk3	11	12
chr9	57544419	57546419	-0.055725	0.020629	hypomethylated	Cyp1a1	5	4
chr9	57682041	57684041	-0.20464	0.00000507	hypomethylated	Arid3b	8	8
chr9	58335409	58337409	-0.095263	0.0003376	hypomethylated	6030419C18Rik	78	80
chr9	58884248	58886248	-0.05825	7.22E-08	hypomethylated	Neo1	47	51
chr9	59138378	59140378	-0.042494	0.00021114	hypomethylated	Adpgk	42	42
chr9	59386473	59388473	0.068149	0.0059734	hypermethylated	Hexa	25	26
chr9	59503414	59505414	-0.095321	0.014086	hypomethylated	Pkm	31	30
chr9	59597980	59599980	-0.25096	0.0029456	hypomethylated	Myo9a,Senp8	33	32
chr9	59598456	59600456	-0.26516	0.00074165	hypomethylated	Myo9a,Senp8	32	31
chr9	61762317	61764317	-0.25194	0.0049174	hypomethylated	Rplp1	6	6
chr9	62129986	62131986	-0.4	0.010761	stronglyhypometh	Spesp1	2	3
chr9	62188149	62190149	-0.079711	0.00000256	hypomethylated	Anp32a	30	31
chr9	62996768	62998768	-0.1508	0.00047059	hypomethylated	Skor1	33	28
chr9	63225659	63227659	0.18681	0.029712	hypermethylated	Map2k5	4	4
chr9	63605801	63607801	-0.27676	8.03E-11	hypomethylated	Smad3	25	27
chr9	63869866	63871866	-0.10744	0.014026	hypomethylated	Smad6	16	16
chr9	64657817	64659817	-0.044439	0.026013	hypomethylated	Dennd4a	35	47
chr9	64807638	64809638	-0.19965	0.000000282	hypomethylated	Vwa9	19	17
chr9	64807743	64809743	-0.19965	0.000000282	hypomethylated	Vwa9	19	17
chr9	65061496	65063496	-0.18935	0.0010252	hypomethylated	Parp16	32	31
chr9	65141101	65143101	0.18661	0.00019885	hypermethylated	Clpx	20	20
chr9	65523354	65525354	-0.20866	0.0032961	hypomethylated	Oaz2	15	11
chr9	65675371	65677371	-0.12612	0.00021996	hypomethylated	Zfp609	24	26
chr9	65906975	65908975	-0.10778	0.00000946	hypomethylated	Ppib	30	30
chr9	66197256	66199256	-0.10537	0.0011191	hypomethylated	Herc1	29	28
chr9	66792924	66794924	-0.073153	0.00049286	hypomethylated	Rps27l	20	20
chr9	67606243	67608243	-0.27042	1.06E-14	hypomethylated	C2cd4b	32	37
chr9	69608747	69610747	0.085048	0.0014593	hypermethylated	B230323A14Rik,Foxb1	10	10
chr9	69836307	69838307	-0.026435	0.011796	hypomethylated	Bnip2	33	41
chr9	70054156	70056156	-0.041385	0.00021596	hypomethylated	Myo1e	16	16
chr9	71062595	71064595	-0.11425	0.0022083	hypomethylated	Aldh1a2	88	81
chr9	71332765	71334765	-0.11749	0.0096896	hypomethylated	Polr2m	23	23
chr9	72379046	72381046	0.022612	0.0052102	hypermethylated	4930509E16Rik,Rfx7	36	35

chr9	72509153	72511153	0.1074	0.000000029	hypermethylated	Nedd4	53	57
chr9	72654080	72656080	-0.21103	9.57E-10	hypomethylated	Prtg	52	53
chr9	72772456	72774456	-0.057327	0.00013663	hypomethylated	Pygo1	56	55
chr9	74708727	74710727	0.046561	0.00056673	hypermethylated	Onecut1	30	23
chr9	74799859	74801859	-0.1563	2.15E-13	hypomethylated	Fam214a	38	34
chr9	74884420	74886420	-0.12905	0.0031902	hypomethylated	Arpp19	28	31
chr9	74884538	74886538	-0.12905	0.0031902	hypomethylated	Arpp19	28	31
chr9	75078820	75080820	-0.088133	0.0055204	hypomethylated	Myo5c	19	21
chr9	75161048	75163048	-0.11562	0.00010593	hypomethylated	Gnb5	28	26
chr9	75257166	75259166	-0.12896	2.37E-08	hypomethylated	4933433G15Rik,Mapk6	39	33
chr9	75257821	75259821	-0.23035	0.000000023	hypomethylated	4933433G15Rik,Mapk6	22	20
chr9	75288330	75290330	0.54545	0.007864	stronglyhypermeth	Leo1	12	11
chr9	76171385	76173385	-0.49455	0.0000918	stronglyhypometh	Hcrtr2	5	4
chr9	77601616	77603616	-0.063662	2.47E-12	hypomethylated	Gclc	42	46
chr9	77764171	77766171	-0.027482	0.005655	hypomethylated	Elovl5	31	33
chr9	82722412	82724412	0.28619	5.43E-10	hypermethylated	Irak1bp1	31	30
chr9	83699849	83701849	-0.038462	0.028494	hypomethylated	Elovl4	13	13
chr9	83699912	83701912	-0.041667	0.013602	hypomethylated	Elovl4	11	11
chr9	85642941	85644941	-0.10257	0.029375	hypomethylated	Ibtk	11	12
chr9	85735458	85737458	0.12406	0.014903	hypermethylated	Tpbg	19	26
chr9	86358523	86360523	-0.24815	0.001906	hypomethylated	Dopey1,Ube2cbp	3	3
chr9	86359760	86361760	-0.034913	0.0033205	hypomethylated	Dopey1	30	18
chr9	86915863	86917863	0.04296	0.0071713	hypermethylated	Cyb5r4	22	22
chr9	87038140	87040140	0.12882	0.014633	hypermethylated	Mrap2	9	14
chr9	88221446	88223446	-0.15557	0.00041891	hypomethylated	Nt5e	30	28
chr9	89948104	89950104	-0.24711	0.0038253	hypomethylated	Ctsh	11	8
chr9	94438500	94440500	0.065283	0.0070179	hypermethylated	1190002N15Rik	40	40
chr9	95757015	95759015	0.029288	0.0000897	hypermethylated	1700065D16Rik,Atr	33	33
chr9	96789841	96791841	-0.16684	0.031141	hypomethylated	Acpl2	32	31
chr9	96918774	96920774	-0.093971	0.012333	hypomethylated	Spsb4	36	36
chr9	98464030	98466030	-0.13731	0.029951	hypomethylated	4930579K19Rik,Copb2	18	13
chr9	98756009	98758009	-0.12615	0.0068025	hypomethylated	Gm6406	11	8
chr9	98885791	98887791	-0.015878	0.021426	hypomethylated	Faim	38	35
chr9	100543041	100545041	-0.053985	0.031263	hypomethylated	Stag1	85	72
chr9	100977450	100979450	-0.070329	0.0063507	hypomethylated	Msl2	28	27
chr9	102407467	102409467	0.09433	0.0046567	hypermethylated	Ky	20	22
chr9	102528454	102530454	-0.1408	0.0087891	hypomethylated	Anapc13,Cep63	22	29
chr9	102909818	102911818	-0.1724	0.036768	hypomethylated	Slco2a1	18	14
chr9	103190627	103192627	-0.35302	0.0071532	stronglyhypometh	1300017J02Rik	4	6
chr9	103903873	103905873	-0.038622	0.00018405	hypomethylated	Nphp3	31	34
chr9	104954597	104956597	0.033333	0.0082282	hypermethylated	Mrpl3	15	8
chr9	106060469	106062469	-0.22956	0.0000206	hypomethylated	Glyctk	11	10
chr9	106072259	106074259	-0.071497	0.00027455	hypomethylated	Wdr82	50	45
chr9	106150285	106152285	-0.31802	0.0000758	hypomethylated	Alas1	11	12
chr9	106269962	106271962	0.10152	0.00000827	hypermethylated	Dusp7	49	53
chr9	106330869	106332869	-0.20141	0.013238	hypomethylated	Rpl29	17	5
chr9	106355187	106357187	-0.060575	0.0000708	hypomethylated	Pcbp4	39	39
chr9	106378639	106380639	-0.081361	0.01408	hypomethylated	Parp3,Rrp9	18	17
chr9	106378982	106380982	-0.081361	0.01408	hypomethylated	Parp3,Rrp9	18	17
chr9	106794269	106796269	0.26438	0.0085562	hypermethylated	Manf	11	11
chr9	107489048	107491048	0.067606	0.011462	hypermethylated	Ifrd2	17	16
chr9	107511572	107513572	-0.18219	0.026033	hypomethylated	Sema3b	3	3
chr9	107808219	107810219	-0.12526	0.0002363	hypomethylated	Mst1r	10	6
chr9	107837250	107839250	0.010857	0.041898	hypermethylated	Camkv	14	14
chr9	107852293	107854293	-0.27597	0.0017109	hypomethylated	Traip	6	7
chr9	107996811	107998811	-0.17089	0.00030354	hypomethylated	Apeh	20	17
chr9	108207535	108209535	-0.014055	0.0088542	hypomethylated	Rhoa,Tcta	57	47
chr9	108208282	108210282	-0.029562	0.00050331	hypomethylated	Rhoa,Tcta	50	43
chr9	108249161	108251161	-0.11535	3.42E-08	hypomethylated	Usp4	46	43
chr9	108294164	108296164	-0.28125	0.0044732	hypomethylated	1700102P08Rik	4	4
chr9	108392006	108394006	0.086673	0.0079375	hypermethylated	Usp19	29	27

chr9	108471222	108473222	-0.066362	0.011459	hypomethylated	Dalrd3,Mir191,Mir425	22	17
chr9	108499931	108501931	-0.066626	0.048842	hypomethylated	P4htm	14	14
chr9	108727650	108729650	-0.14956	1.29E-13	hypomethylated	Celsr3	51	53
chr9	108755373	108757373	-0.091475	0.003665	hypomethylated	Slc26a6	13	13
chr9	108870966	108872966	-0.33457	0.000000187	stronglyhypometh	Mir711	8	7
chr9	108961765	108963765	-0.35	0.010657	stronglyhypometh	Trex1	2	2
chr9	108962237	108964237	-0.35	0.010657	stronglyhypometh	Trex1	2	2
chr9	108976638	108978638	0.2	0.021212	hypermethylated	Atrip	6	5
chr9	108996949	108998949	-0.034087	0.015304	hypomethylated	Plxnb1	58	53
chr9	109777082	109779082	-0.23452	0.0029367	hypomethylated	Cdc25a	40	32
chr9	109833277	109835277	-0.046667	0.0000287	hypomethylated	Map4	42	39
chr9	110033527	110035527	-0.037235	0.0030585	hypomethylated	Smarcc1	36	40
chr9	110206695	110208695	0.32281	0.044738	hypermethylated	Elp6	12	13
chr9	110646190	110648190	-0.20819	0.010156	hypomethylated	Pth1r	14	11
chr9	110728193	110730193	-0.25604	0.0052144	hypomethylated	Prss43	7	7
chr9	111213242	111215242	-0.24175	6.84E-13	hypomethylated	Trank1	48	52
chr9	112088453	112090453	-0.32	0.0000979	hypomethylated	Arpp21	6	10
chr9	112137345	112139345	-0.048065	0.0065072	hypomethylated	2900079G21Rik,Arpp21	25	30
chr9	113839051	113841051	0.091805	0.0013812	hypermethylated	Ubp1	61	61
chr9	113965471	113967471	-0.08808	0.011295	hypomethylated	Susd5	22	22
chr9	114309236	114311236	-0.13635	0.0040931	hypomethylated	Glb1,Tmppe	36	36
chr9	117948616	117950616	-0.079594	0.0071661	hypomethylated	Azi2	30	34
chr9	119010595	119012595	-0.078068	0.00034906	hypomethylated	Dlec1	17	9
chr9	119310618	119312618	-0.098414	0.0000162	hypomethylated	Acvr2b	56	53
chr9	119353040	119355040	0.23314	0.0000129	hypermethylated	Exog	23	23
chr9	119488134	119490134	-0.13562	0.00022147	hypomethylated	Scn5a	26	26
chr9	119845723	119847723	-0.14292	0.000011	hypomethylated	Gorasp1,Ttc21a	15	15
chr9	119846676	119848676	-0.17216	0.0000415	hypomethylated	Gorasp1,Ttc21a	11	11
chr9	120035883	120037883	-0.092713	0.00043718	hypomethylated	Rpsa	33	20
chr9	120212190	120214190	-0.064081	0.0013326	hypomethylated	Myrip	61	70
chr9	120447935	120449935	-0.20751	0.0014155	hypomethylated	Entpd3	7	9
chr9	120479633	120481633	-0.056345	0.0018011	hypomethylated	Rpl14	32	29
chr9	121404807	121406807	-0.57642	0.0000317	stronglyhypometh	Cck	7	5
chr9	121550684	121552684	-0.079684	0.00062838	hypomethylated	Lyzl4,Vipr1	34	35
chr9	121550833	121552833	-0.079684	0.00062838	hypomethylated	Lyzl4,Vipr1	34	35
chr9	121766649	121768649	-0.047899	0.0045608	hypomethylated	Higd1a	35	30
chr9	122259733	122261733	-0.035766	0.0072347	hypomethylated	Abhd5	46	40
chr9	122480618	122482618	-0.077091	0.0000685	hypomethylated	9530059O14Rik	40	43
chr9	123386818	123388818	-0.068505	0.040375	hypomethylated	Limd1	22	26
chrX	5976262	5978262	0.36769	1.53E-10	stronglyhypermeth	Shroom4	28	30
chrX	7149725	7151725	0.15111	0.0052358	hypermethylated	4930524L23Rik,Ppp1r3f	32	28
chrX	7248404	7250404	0.18917	0.0047274	hypermethylated	Plp2	6	5
chrX	7338786	7340786	0.15924	0.0030399	hypermethylated	Tfe3	15	14
chrX	7366120	7368120	-0.52273	0.0051722	stronglyhypometh	Gripap1	5	4
chrX	7417956	7419956	0.23845	4.9E-09	hypermethylated	Otud5	51	65
chrX	7454431	7456431	0.10364	0.0020167	hypermethylated	Pim2	24	20
chrX	7721552	7723552	0.10757	0.023017	hypermethylated	2900002K06Rik,Rbm3	11	8
chrX	7753307	7755307	-0.3416	0.010635	stronglyhypometh	Tbc1d25	5	5
chrX	8468478	8470478	0.012979	0.027591	hypermethylated	B630019K06Rik	33	29
chrX	8776098	8778098	0.15641	0.00000756	hypermethylated	Lancl3	36	40
chrX	10293490	10295490	0.34921	7.04E-09	stronglyhypermeth	Mid1ip1	23	28
chrX	11657679	11659679	0.25838	0.015384	hypermethylated	Bcor	21	20
chrX	12163884	12165884	0.18634	0.00013751	hypermethylated	Atp6ap2	7	7
chrX	12647623	12649623	0.18868	1.03E-08	hypermethylated	Usp9x	34	35
chrX	19635695	19637695	0.21516	0.000000633	hypermethylated	Chst7	48	51
chrX	20193628	20195628	0.42833	0.000000232	stronglyhypermeth	Ndufb11,Rbm10	7	18
chrX	20193884	20195884	0.39368	0.000000628	stronglyhypermeth	Ndufb11,Rbm10	8	19
chrX	20194690	20196690	0.25828	0.0017746	hypermethylated	Ndufb11,Rbm10	4	13
chrX	20539104	20541104	0.34286	0.047388	stronglyhypermeth	A230072C01Rik,Uxt	3	3
chrX	23269242	23271242	0.15505	0.00033109	hypermethylated	Wdr44	25	18
chrX	33427826	33429826	0.26074	0.000000392	hypermethylated	Dock11	30	30

chrX	33651133	33653133	0.22299	0.000000126	hypermethylated	Il13ra1	23	25
chrX	34137219	34139219	0.25544	0.0082859	hypermethylated	Pgrmc1	26	27
chrX	34334646	34336646	0.15173	0.015416	hypermethylated	Slc25a5	21	25
chrX	34413360	34415360	0.33378	0.00070233	stronglyhypermeth	Ube2a	41	52
chrX	34587839	34589839	0.1361	0.031321	hypermethylated	Sowahd	31	25
chrX	34665757	34667757	0.18587	0.000000418	hypermethylated	Nkap	17	22
chrX	34730337	34732337	0.14361	0.0040966	hypermethylated	Ndufa1,Rnf113a1	32	35
chrX	34731233	34733233	0.15153	0.0050239	hypermethylated	Ndufa1,Rnf113a1	27	30
chrX	35541969	35543969	0.20782	0.00056066	hypermethylated	Zbtb33	15	29
chrX	39501588	39503588	0.29384	4.61E-10	hypermethylated	Stag2	53	51
chrX	39502877	39504877	0.25627	0.000000838	hypermethylated	Stag2	36	35
chrX	45264632	45266632	0.20333	0.016602	hypermethylated	Ocrl	5	5
chrX	45609110	45611110	0.41253	0.0013004	stronglyhypermeth	Utp14a	10	13
chrX	46822626	46824626	0.31541	0.00059797	hypermethylated	Arhgap36	9	10
chrX	48193612	48195612	0.24746	0.00096056	hypermethylated	2610018G03Rik	10	14
chrX	48371195	48373195	0.13263	0.00000139	hypermethylated	Rap2c	49	39
chrX	49033654	49035654	0.19137	4.46E-09	hypermethylated	Hs6st2	34	28
chrX	49518100	49520100	0.21384	0.02776	hypermethylated	Gpc4	16	29
chrX	50264442	50266442	0.50433	0.00000119	stronglyhypermeth	Phf6	11	16
chrX	50340254	50342254	0.27519	3.99E-20	hypermethylated	Hprt	28	29
chrX	53862011	53864011	0.19498	0.0027798	hypermethylated	Slc9a6	17	26
chrX	54305791	54307791	0.5976	0.0000128	stronglyhypermeth	Htatsf1	15	17
chrX	55282804	55284804	0.077117	0.0048858	hypermethylated	Zic3	22	26
chrX	57657156	57659156	0.16164	0.004694	hypermethylated	Atp11c	24	39
chrX	65930729	65932729	0.088841	0.0083648	hypermethylated	Fmr1,Gm10474	46	42
chrX	65931574	65933574	0.089058	0.011925	hypermethylated	Fmr1,Gm10474	47	43
chrX	66612505	66614505	0.14746	0.00000329	hypermethylated	Aff2	25	28
chrX	67618260	67620260	0.39116	0.000000392	stronglyhypermeth	Ids	5	6
chrX	68467180	68469180	0.30492	0.015669	hypermethylated	Mtm1	7	7
chrX	68467256	68469256	0.30492	0.015669	hypermethylated	Mtm1	7	7
chrX	68616934	68618934	0.21808	0.0000248	hypermethylated	Mtmr1	23	25
chrX	68808167	68810167	0.11556	0.0053432	hypermethylated	Hmgb3	43	43
chrX	68915941	68917941	0.21223	0.00041377	hypermethylated	Gpr50	29	26
chrX	69207359	69209359	0.17519	0.00072747	hypermethylated	Prrg3	31	31
chrX	70162859	70164859	0.37926	0.010818	stronglyhypermeth	Cetn2,Nsdhl	8	8
chrX	70231677	70233677	0.35806	0.000000529	stronglyhypermeth	Zfp185	17	24
chrX	70586958	70588958	0.30267	0.0095862	hypermethylated	Zfp275	12	13
chrX	70586959	70588959	0.30267	0.0095862	hypermethylated	Zfp275	12	13
chrX	70917471	70919471	0.23604	0.00000171	hypermethylated	Slc6a8	44	45
chrX	71211654	71213654	0.16319	0.00081452	hypermethylated	Hcfc1	13	10
chrX	71499177	71501177	0.15271	0.02489	hypermethylated	Emd	11	12
chrX	71613866	71615866	0.2219	0.0037381	hypermethylated	Ubl4	14	14
chrX	71985265	71987265	-0.35606	0.03156	stronglyhypometh	Gm6890	2	2
chrX	72340192	72342192	-0.59524	0.0054838	stronglyhypometh	Dkc1	4	4
chrX	72660966	72662966	0.27083	0.032705	hypermethylated	Brcc3,Mtcp1	8	8
chrX	73010038	73012038	-0.67879	0.00036027	stronglyhypometh	4933407K13Rik	3	6
chrX	74755565	74757565	0.13284	7.19E-08	hypermethylated	Tbl1x	41	43
chrX	75041299	75043299	0.39353	0.013752	stronglyhypermeth	Prkx	12	11
chrX	90877494	90879494	0.63839	0.004365	stronglyhypermeth	Pola1	7	13
chrX	91077434	91079434	0.25604	0.00086193	hypermethylated	Pdk3	16	8
chrX	91479268	91481268	0.21856	1.26E-11	hypermethylated	Klhl15	62	62
chrX	91611448	91613448	0.21342	0.0098321	hypermethylated	Apoo,Apoo-ps	10	13
chrX	91611462	91613462	0.21342	0.0098321	hypermethylated	Apoo,Apoo-ps	10	13
chrX	91611492	91613492	0.21342	0.0098321	hypermethylated	Apoo,Apoo-ps	10	13
chrX	92906016	92908016	0.45291	0.000000153	stronglyhypermeth	Zc3h12b	8	8
chrX	96132119	96134119	0.27255	0.000000663	hypermethylated	Yipf6	21	24
chrX	96330468	96332468	0.21144	0.0034675	hypermethylated	Efnb1	24	29
chrX	97015407	97017407	0.19984	0.0000442	hypermethylated	Tmem28	58	63
chrX	97169944	97171944	0.29209	0.00000054	hypermethylated	Eda	24	20
chrX	97688629	97690629	0.26999	0.0000204	hypermethylated	Igbp1	13	15
chrX	97820403	97822403	0.24715	0.0000369	hypermethylated	Kif4,Pdzd11	16	23

chrX	97821246	97823246	0.27041	0.00000698	hypermethylated	Kif4,Pdzd11	13	20
chrX	97924185	97926185	0.41688	0.019606	stronglyhypermeth	Gdpd2	5	4
chrX	97962061	97964061	0.45382	0.0020691	stronglyhypermeth	Dlg3	9	9
chrX	97962065	97964065	0.45382	0.0020691	stronglyhypermeth	Dlg3	9	9
chrX	97968833	97970833	0.24877	0.00955	hypermethylated	Dlg3	8	6
chrX	98468429	98470429	0.10256	0.03727	hypermethylated	Med12	21	22
chrX	99043723	99045723	0.13931	0.00020881	hypermethylated	Nhsl2	52	54
chrX	99447108	99449108	0.18223	0.0016603	hypermethylated	Cited1,Gm14858	15	15
chrX	102314734	102316734	0.24015	0.040925	hypermethylated	Magee1	8	11
chrX	102958815	102960815	0.23877	1.9E-11	hypermethylated	Fgf16	40	45
chrX	103381462	103383462	-0.2226	0.0017709	hypomethylated	Pgk1	16	16
chrX	103680568	103682568	0.21405	0.00029808	hypermethylated	Fndc3c1	17	18
chrX	106028816	106030816	0.28463	0.025847	hypermethylated	2810403D21Rik,Brwd3	11	14
chrX	109713134	109715134	0.44312	0.00066076	stronglyhypermeth	Zfp711	5	8
chrX	126283277	126285277	0.30173	0.000000874	hypermethylated	Diap2	29	19
chrX	130592887	130594887	0.21026	9.53E-12	hypermethylated	Cstf2	33	28
chrX	130828763	130830763	0.42286	0.040033	stronglyhypermeth	Tmem35	7	5
chrX	131011029	131013029	-0.42157	0.0032017	stronglyhypometh	Taf7l	4	6
chrX	131076168	131078168	0.030609	0.00037427	hypermethylated	Timm8a1	17	17
chrX	132276230	132278230	0.32235	0.00089823	hypermethylated	Armcx5,Gprasp1	16	16
chrX	136144158	136146158	0.13635	0.015615	hypermethylated	Rnf128	31	42
chrX	138661479	138663479	0.25928	0.0064371	hypermethylated	Nxt2	11	10
chrX	138661735	138663735	0.27815	0.0067342	hypermethylated	Nxt2	10	9
chrX	139114942	139116942	0.27317	4.96E-09	hypermethylated	Tmem164	30	37
chrX	139115268	139117268	0.26778	3.1E-14	hypermethylated	Tmem164	35	45
chrX	143396055	143398055	0.35783	0.0000429	stronglyhypermeth	Htr2c	12	7
chrX	147451836	147453836	0.10125	0.0041255	hypermethylated	Gnl3l	15	13
chrX	147451865	147453865	0.10125	0.0041255	hypermethylated	Gnl3l	15	13
chrX	147954214	147956214	0.10482	0.001308	hypermethylated	Phf8	25	25
chrX	148236824	148238824	0.38835	0.049155	stronglyhypermeth	Huwe1	16	24
chrX	148449970	148451970	0.30014	0.00000872	hypermethylated	Ribc1,Smc1a	15	14
chrX	148450838	148452838	0.30014	0.00000872	hypermethylated	Ribc1,Smc1a	15	14
chrX	148577810	148579810	0.024037	0.0071202	hypermethylated	Iqsec2	32	34
chrX	148666772	148668772	-0.083193	0.02247	hypomethylated	Kdm5c	17	17
chrX	148802654	148804654	-0.29943	0.00020303	hypomethylated	3010001F23Rik,Gpr173	9	10
chrX	149671587	149673587	0.48119	0.00406	stronglyhypermeth	Klf8	6	3
chrX	149931774	149933774	0.13829	0.048705	hypermethylated	Ubqln2	23	12
chrX	151696042	151698042	0.084977	0.043786	hypermethylated	Acot9	10	13
chrX	152057870	152059870	0.24202	0.006517	hypermethylated	Ptchd1	19	28
chrX	153929978	153931978	0.28525	0.0037698	hypermethylated	Sms	9	19
chrX	154006917	154008917	-0.22455	0.00058628	hypomethylated	Yy2	9	9
chrX	154036647	154038647	0.11188	0.023022	hypermethylated	Mbtps2	3	6
chrX	154481042	154483042	0.2629	0.00013565	hypermethylated	Cnksr2	46	32
chrX	155693051	155695051	0.15753	0.011361	hypermethylated	Rps6ka3	24	41
chrX	155851509	155853509	0.11386	0.0025974	hypermethylated	Map7d2	31	33
chrX	155969599	155971599	0.097884	0.029359	hypermethylated	A830080D01Rik	24	24
chrX	156064339	156066339	0.18219	0.0057775	hypermethylated	Sh3kbp1	38	41
chrX	156939097	156941097	0.3018	0.00054484	hypermethylated	Phka2	8	6
chrX	156939365	156941365	0.3018	0.00054484	hypermethylated	Phka2	8	6
chrX	160346091	160348091	0.36282	2.37E-21	stronglyhypermeth	Ap1s2	32	42
chrX	162675874	162677874	0.43727	0.0000345	stronglyhypermeth	Gpm6b	10	10
chrX	162877756	162879756	0.24102	0.00000298	hypermethylated	Ofd1,Trappc2	26	20
chrX	162878636	162880636	0.23102	0.012267	hypermethylated	Ofd1,Trappc2	14	14
chrX	163647150	163649150	0.3446	0.00027607	stronglyhypermeth	Tmsb4x	6	7
chrX	165111834	165113834	0.14273	0.0033839	hypermethylated	Msl3	13	18
chrX	165758275	165760275	0.21055	0.00000667	hypermethylated	Hccs	13	17
