# Supplementary material for: Dnmt1 links BCR-ABLp210 to epigenetic tumor stem cell priming in myeloid leukemia
Source: Leukemia. 2018 Jun 28;33(1):249–78. doi: 10.1038/s41375-018-0192-z (PMC6326950; doi:10.1038/s41375-018-0192-z)
Supplement: Supplementary file 3 — Table S2 [file 41375_2018_192_MOESM3_ESM.pdf]

Table S2: RRBS methylation changes in myeloid cells of Sca1-BCR-ABLp210 mice compared to wild-type controls

Samples name:	zr151_3	Myeloid cells of wild type mice
	zr151_5	Myeloid cells of Sca1-BCR-ABLp210 mice

chrom	start	end	methDiff (zr151_3.RRBS vs zr151_5)	pValue (zr151_3.RRBS vs zr151_5)	pvalClass (zr151_3.RRBS vs zr151_5)	Gene Symbol	Number of CpGs zr151_3	Number of CpGs zr151_5
chr1	4486494	4488494	-0.7574	0.0006816	stronglyhypometh	Sox17	3	13
chr1	4796973	4798973	-0.21876	5.05E-09	hypomethylated	Lypla1	31	30
chr1	4846774	4848774	-0.33088	0.0039378	hypomethylated	Tcea1	21	29
chr1	4847408	4849408	-0.33088	0.0039378	hypomethylated	Tcea1	21	29
chr1	5009507	5011507	-0.16695	0.0062507	hypomethylated	Rgs20	20	23
chr1	6348411	6350411	-0.21111	0.00036862	hypomethylated	Fam150a	15	11
chr1	9690290	9692290	-0.20231	2.17E-10	hypomethylated	Mybl1	12	27
chr1	9837361	9839361	-0.51488	0.004551	stronglyhypometh	Sgk3	12	12
chr1	9897718	9899718	-0.20138	0.006961	hypomethylated	Mcmdc2	11	11
chr1	10027979	10029979	-0.20534	0.0074177	hypomethylated	Cops5,Cspp1	29	28
chr1	10222751	10224751	-0.15294	0.0032367	hypomethylated	Arfgef1	27	32
chr1	10982545	10984545	-0.091218	0.0020011	hypomethylated	Prex2	18	21
chr1	12681510	12683510	-0.21554	0.0031997	hypomethylated	Sulf1	7	7
chr1	13364164	13366164	-0.34178	0.0000588	stronglyhypometh	Ncoa2	15	19
chr1	16509383	16511383	-0.09013	0.0054483	hypomethylated	Stau2	12	12
chr1	16654271	16656271	-0.067875	0.0097787	hypomethylated	Tmem70	30	33
chr1	17134453	17136453	-0.31534	0.0073149	hypomethylated	Gdap1	9	12
chr1	20940706	20942706	-0.91667	0.00042553	stronglyhypometh	Efhc1	2	2
chr1	23390014	23392014	-0.25925	0.00022625	hypomethylated	Ogfrl1	8	14
chr1	23767764	23769764	-0.11667	0.014192	hypomethylated	B3gat2	25	28
chr1	24684382	24686382	-0.56208	0.000000479	stronglyhypometh	Lmbrd1	21	16
chr1	31006555	31008555	-0.079161	0.0027591	hypomethylated	Gm13363,Ptp4a1	29	28
chr1	31006600	31008600	-0.079161	0.0027591	hypomethylated	Gm13363,Ptp4a1	29	28
chr1	33775740	33777740	-0.11965	0.0015488	hypomethylated	Rab23	35	38
chr1	33776263	33778263	-0.11965	0.0015488	hypomethylated	Rab23	35	38
chr1	33964466	33966466	-0.04005	0.0074884	hypomethylated	Bend6,Dst	50	50
chr1	34857566	34859566	-0.12339	0.0033524	hypomethylated	Arhgef4	14	14
chr1	36363577	36365577	-0.30177	0.00047936	hypomethylated	Arid5a	18	20
chr1	36567720	36569720	-0.049831	0.0030019	hypomethylated	Cnnm3	68	65
chr1	36747331	36749331	-0.3019	0.0050996	hypomethylated	Cox5b	12	12
chr1	36996372	36998372	-0.21668	0.0012386	hypomethylated	Tmem131	28	28
chr1	37486948	37488948	-0.51768	0.00012573	stronglyhypometh	Coa5,Unc50	3	11
chr1	37946397	37948397	-0.36097	0.006957	stronglyhypometh	Mitd1,Mrpl30	10	10
chr1	37947256	37949256	-0.36097	0.006957	stronglyhypometh	Mitd1,Mrpl30	10	10
chr1	39423695	39425695	-0.18574	0.000000401	hypomethylated	Rpl31	34	42
chr1	39956757	39958757	0.073911	0.0036005	hypermethylated	Map4k4	66	69
chr1	42752990	42754990	-0.22222	2.29E-09	hypomethylated	Pou3f3	48	69
chr1	43501595	43503595	-0.094868	0.0000173	hypomethylated	Nck2	63	73
chr1	43989851	43991851	-0.080505	0.036239	hypomethylated	Tpp2	14	19
chr1	44159233	44161233	-0.059131	0.0078044	hypomethylated	Tex30	14	17
chr1	44175618	44177618	-0.62949	5.72E-10	stronglyhypometh	Bivm,Kdelc1	11	10
chr1	44175812	44177812	-0.75242	5.87E-10	stronglyhypometh	Bivm,Kdelc1	8	10
chr1	44203588	44205588	0.1892	0.0041647	hypermethylated	Ercc5	12	27
chr1	46122582	46124582	-0.66329	0.0000559	stronglyhypometh	Dnahc7b	6	9
chr1	52175281	52177281	-0.1595	0.00058983	hypomethylated	Stat1	25	26
chr1	53009684	53011684	-0.54701	0.027986	stronglyhypometh	1700019D03Rik	8	11
chr1	53352938	53354938	-0.30285	0.00072411	hypomethylated	Ormdl1,Pms1	11	21
chr1	53353840	53355840	-0.29332	0.00097872	hypomethylated	Ormdl1,Pms1	11	16
chr1	57462391	57464391	-0.33771	0.0000131	stronglyhypometh	9430016H08Rik,Tyw5	29	30
chr1	57463518	57465518	-0.41329	0.00000477	stronglyhypometh	9430016H08Rik,Tyw5	15	14
chr1	57830704	57832704	-0.060079	0.015754	hypomethylated	Spats2l	41	44

chr1	58448979	58450979	-0.016601	0.0036951	hypomethylated	Bzw1	23	46
chr1	58642442	58644442	0.18453	0.04497	hypermethylated	Fam126b,Ndufb3	9	10
chr1	58643177	58645177	0.18453	0.04497	hypermethylated	Fam126b,Ndufb3	9	10
chr1	59176650	59178650	-0.13757	0.0092714	hypomethylated	Tmem237	26	30
chr1	60099931	60101931	-0.04409	0.011863	hypomethylated	Ica1l	35	35
chr1	63160841	63162841	-0.077312	0.0040664	hypomethylated	Gm11602,Ino80d	11	11
chr1	63491477	63493477	-0.12262	2.19E-09	hypomethylated	Adam23	29	39
chr1	64663742	64665742	-0.28619	0.0099555	hypomethylated	Mettl21a	5	5
chr1	66746466	66748466	-0.38597	0.00032687	stronglyhypometh	Rpe	18	18
chr1	69732534	69734534	0.085769	0.028999	hypermethylated	Ikzf2	24	36
chr1	71602729	71604729	-0.32404	0.000000214	hypomethylated	Atic	20	20
chr1	72352994	72354994	0.15873	0.041098	hypermethylated	Xrcc5	5	6
chr1	72870076	72872076	-0.32026	0.00046134	hypomethylated	Igfbp2	21	16
chr1	74551633	74553633	-0.049963	0.000000249	hypomethylated	Rqcd1	24	35
chr1	74647028	74649028	-0.26145	0.000000437	hypomethylated	Rnf25,Stk36	7	8
chr1	74817465	74819465	-0.12133	0.00025515	hypomethylated	Wnt6	24	21
chr1	74900602	74902602	-0.050682	0.01436	hypomethylated	Cdk5r2	52	57
chr1	74947295	74949295	-0.16997	0.0043861	hypomethylated	Mir375	9	10
chr1	74998225	75000225	0.18554	0.033047	hypermethylated	Ihh	8	8
chr1	75138359	75140359	-0.12382	0.00000572	hypomethylated	Cnppd1,Fam134a	31	30
chr1	75138942	75140942	-0.14739	0.00000481	hypomethylated	Cnppd1,Fam134a	26	25
chr1	75188497	75190497	-0.13333	0.006816	hypomethylated	Ankzf1,Atg9a	3	11
chr1	75215828	75217828	-0.080073	0.012621	hypomethylated	Tuba4a	26	27
chr1	75231997	75233997	-0.13366	0.025446	hypomethylated	Dnajb2	15	18
chr1	75232276	75234276	-0.13458	0.042576	hypomethylated	Dnajb2	17	20
chr1	75355918	75357918	0.1161	0.036569	hypermethylated	Des	23	27
chr1	75370871	75372871	-0.095626	0.033721	hypomethylated	Gm15179,Speg	19	23
chr1	75476046	75478046	-0.076739	0.00024031	hypomethylated	Chpf,Tmem198	22	24
chr1	79758344	79760344	-0.52232	0.0065649	stronglyhypometh	Wdfy1	4	9
chr1	82288014	82290014	-0.28537	0.00000435	hypomethylated	Irs1	17	19
chr1	87941437	87943437	0.065549	0.0005626	hypermethylated	2810459M11Rik	52	50
chr1	88050354	88052354	-0.2875	0.015333	hypomethylated	Armc9	15	24
chr1	88050389	88052389	-0.2875	0.015333	hypomethylated	Armc9	15	24
chr1	88255148	88257148	-0.28161	1.82E-08	hypomethylated	C130036L24Rik,Ncl	39	42
chr1	88256030	88258030	-0.49432	1.2E-09	stronglyhypometh	C130036L24Rik,Ncl	21	21
chr1	88482674	88484674	-0.47436	0.0055816	stronglyhypometh	Cops7b	7	7
chr1	89109488	89111488	0.012414	0.03228	hypermethylated	Eif4e2	32	34
chr1	89109513	89111513	0.012414	0.03228	hypermethylated	Eif4e2	32	34
chr1	89159938	89161938	-0.21416	3.82E-11	hypomethylated	Efhd1	29	34
chr1	89406937	89408937	-0.047288	0.013532	hypomethylated	Ngef	17	17
chr1	89651645	89653645	-0.07419	0.0063657	hypomethylated	Atg16l1	42	42
chr1	90174132	90176132	0.32223	0.00017154	hypermethylated	A730008H23Rik,Hjurp	5	5
chr1	90174154	90176154	0.29865	0.006953	hypermethylated	A730008H23Rik,Hjurp	3	3
chr1	90598766	90600766	-0.19054	0.000000124	hypomethylated	Arl4c	16	16
chr1	90966036	90968036	-0.2816	0.00000184	hypomethylated	Sh3bp4	37	42
chr1	92498999	92500999	-0.4344	0.0028064	stronglyhypometh	Cops8	13	18
chr1	92949020	92951020	-0.47151	0.049318	stronglyhypometh	Lrrfip1	6	11
chr1	93390244	93392244	-0.089172	0.0000161	hypomethylated	Traf3ip1	45	45
chr1	93436141	93438141	-0.0852	0.035769	hypomethylated	Asb1	19	20
chr1	93436656	93438656	-0.0852	0.035769	hypomethylated	Asb1	19	20
chr1	94806981	94808981	-0.23309	0.00054195	hypomethylated	Rnpepl1	53	64
chr1	95030852	95032852	0.038084	0.030447	hypermethylated	Agxt	6	6
chr1	95131473	95133473	-0.26372	0.00000588	hypomethylated	Sned1	27	32
chr1	95239221	95241221	-0.18985	0.000000181	hypomethylated	Pask,Ppp1r7	19	24
chr1	95239365	95241365	-0.26112	0.00000437	hypomethylated	Pask,Ppp1r7	14	15
chr1	95374569	95376569	-0.40885	0.00000235	stronglyhypometh	Hdlbp,Sept2	25	35
chr1	95374637	95376637	-0.4255	0.000000158	stronglyhypometh	Hdlbp,Sept2	26	36

chr1	95375385	95377385	-0.4976	2.95E-08	stronglyhypometh	Hdlbp,Sept2	15	17
chr1	97209204	97211204	-0.21601	0.0025692	hypomethylated	Fam174a	14	15
chr1	99558595	99560595	-0.07498	0.0088692	hypomethylated	D1Ertd622e	19	19
chr1	99665755	99667755	-0.29684	0.00057026	hypomethylated	Gin1,Ppip5k2	17	15
chr1	107676299	107678299	-0.090826	0.021846	hypomethylated	Tnfrsf11a	32	32
chr1	107885982	107887982	-0.050904	0.0089295	hypomethylated	Zcchc2	57	63
chr1	108067445	108069445	0.067277	0.0002129	hypermethylated	Gm20753,Phlpp1	61	62
chr1	108693302	108695302	-0.39881	0.015738	stronglyhypometh	Vps4b	2	2
chr1	120284634	120286634	-0.31032	0.0014432	hypomethylated	Clasp1	26	30
chr1	120950196	120952196	-0.085137	0.032987	hypomethylated	Gli2	25	27
chr1	122236158	122238158	-0.28196	0.00058833	hypomethylated	C1ql2	29	33
chr1	123224339	123226339	-0.091654	0.00010699	hypomethylated	Insig2	16	20
chr1	129669740	129671740	-0.16566	3.08E-09	hypomethylated	2900009J06Rik,Ccnt2	33	35
chr1	130256233	130258233	-0.054144	0.015155	hypomethylated	Mcm6	8	9
chr1	130488876	130490876	-0.41423	0.0000202	stronglyhypometh	Cxcr4	10	17
chr1	131168880	131170880	-0.19158	0.022528	hypomethylated	Thsd7b	18	23
chr1	133034811	133036811	-0.44781	0.024023	stronglyhypometh	Dyrk3	2	3
chr1	133141755	133143755	-0.20605	0.048486	hypomethylated	Rassf5	17	19
chr1	133423565	133425565	-0.37726	5.51E-11	stronglyhypometh	Fam72a,Srgap2	29	29
chr1	133423938	133425938	-0.5549	1.95E-12	stronglyhypometh	Fam72a,Srgap2	16	15
chr1	133762853	133764853	-0.19015	0.047613	hypomethylated	Rab7l1	8	19
chr1	133858543	133860543	-0.22539	5.2E-09	hypomethylated	Slc45a3	43	50
chr1	134087012	134089012	-0.16572	0.00028278	hypomethylated	Lemd1	41	50
chr1	134287858	134289858	-0.11008	0.0022283	hypomethylated	Tmcc2	34	36
chr1	134941588	134943588	-0.017413	0.00064298	hypomethylated	Pik3c2b	41	49
chr1	135975732	135977732	-0.24847	0.02344	hypomethylated	Btg2	11	12
chr1	136301566	136303566	0.40979	0.00091065	stronglyhypermeth	Cyb5r1	7	9
chr1	136311043	136313043	-0.016347	0.00586	hypomethylated	Adipor1	49	49
chr1	137042410	137044410	-0.20262	0.0000129	hypomethylated	Arl8a	46	52
chr1	137210314	137212314	0.5642	0.0043841	stronglyhypermeth	Timm17a	5	9
chr1	137481932	137483932	0.735	0.0010522	stronglyhypermeth	Nav1	4	15
chr1	138242681	138244681	0.43506	0.0053336	stronglyhypermeth	Camsap2	4	4
chr1	141317959	141319959	-0.08309	0.0019552	hypomethylated	Zbtb41	27	41
chr1	141350349	141352349	-0.19784	0.00068741	hypomethylated	Aspm	19	23
chr1	142141833	142143833	-0.37607	0.00022166	stronglyhypometh	Kcnt2	8	11
chr1	145585478	145587478	0.18469	0.00068094	hypermethylated	Glrx2	23	24
chr1	145585697	145587697	0.14779	0.0033702	hypermethylated	Glrx2	24	26
chr1	145585744	145587744	0.14779	0.0033702	hypermethylated	Glrx2	24	26
chr1	145586576	145588576	0.13887	0.005212	hypermethylated	Glrx2	23	25
chr1	151946253	151948253	-0.57468	0.0000103	stronglyhypometh	7530420F21Rik,Ptgs2	7	7
chr1	152238967	152240967	-0.0221	0.0069729	hypomethylated	BC003331,Tpr	37	34
chr1	153190627	153192627	0.024702	0.0089413	hypermethylated	Ivns1abp	48	60
chr1	153601503	153603503	-0.28592	0.00000607	hypomethylated	Edem3	30	35
chr1	154245996	154247996	-0.11012	0.016999	hypomethylated	Glt25d2	12	13
chr1	154612671	154614671	-0.11518	0.0062376	hypomethylated	Arpc5	77	78
chr1	154749776	154751776	-0.16063	4.04E-13	hypomethylated	Smg7	31	30
chr1	155499166	155501166	-0.3	0.0061178	hypomethylated	Rgs8	4	5
chr1	155746074	155748074	-0.11856	0.0031322	hypomethylated	Glul	42	54
chr1	157589157	157591157	-0.2738	0.000023	hypomethylated	Lhx4	31	45
chr1	157881793	157883793	-0.30404	9.35E-10	hypomethylated	Tor1aip1,Tor1aip2	22	24
chr1	157881856	157883856	-0.30404	9.35E-10	hypomethylated	Tor1aip1,Tor1aip2	22	24
chr1	157881947	157883947	-0.30404	9.35E-10	hypomethylated	Tor1aip1,Tor1aip2	22	24
chr1	157882165	157884165	-0.30404	9.35E-10	hypomethylated	Tor1aip1,Tor1aip2	22	24
chr1	158233479	158235479	-0.40635	6.59E-12	stronglyhypometh	Tdrd5	13	21
chr1	158487917	158489917	-0.12037	0.0011461	hypomethylated	Abl2	20	34
chr1	158604470	158606470	-0.15862	0.009471	hypomethylated	Tor3a	8	8
chr1	158869518	158871518	-0.33685	0.0014309	stronglyhypometh	Ralgps2	22	23

chr1	159341482	159343482	-0.046367	0.0032579	hypomethylated	2810025M15Rik,Rasal	51	46
chr1	162124390	162126390	-0.28245	0.00000023	hypomethylated	Mrps14	9	11
chr1	162142908	162144908	0.075466	0.017522	hypermethylated	Cacybp	30	31
chr1	162723069	162725069	-0.56818	0.00425	stronglyhypometh	Rabgap1l	8	3
chr1	165924912	165926912	-0.38639	0.0020205	stronglyhypometh	BC055324,Mettl18	8	7
chr1	166178185	166180185	-0.27636	0.0081728	hypomethylated	Slc19a2	23	34
chr1	166236805	166238805	-0.19765	0.022656	hypomethylated	Blzf1,Nme7	15	15
chr1	167124564	167126564	-0.14931	0.016965	hypomethylated	Sft2d2	7	7
chr1	167390338	167392338	-0.098483	0.0097695	hypomethylated	Dcaf6,Mpc2	30	37
chr1	167390594	167392594	-0.15602	0.032129	hypomethylated	Dcaf6,Mpc2	18	25
chr1	167692910	167694910	-0.16602	0.0031177	hypomethylated	Creg1	11	22
chr1	168168875	168170875	0.077381	0.015473	hypermethylated	Mael	5	7
chr1	168183269	168185269	-0.055681	0.013497	hypomethylated	Ildr2	32	35
chr1	168308297	168310297	-0.179	0.00056204	hypomethylated	Tada1	33	35
chr1	169237800	169239800	-0.34616	1.07E-08	stronglyhypometh	Tmco1	20	22
chr1	169279121	169281121	-0.12246	0.00084375	hypomethylated	Aldh9a1	17	18
chr1	172519980	172521980	-0.075499	0.021038	hypomethylated	Nos1ap	12	12
chr1	173274829	173276829	-0.50834	0.00063529	stronglyhypometh	Nit1,Pfdn2	10	11
chr1	173275776	173277776	-0.53876	0.00011057	stronglyhypometh	Nit1,Pfdn2	10	9
chr1	173340811	173342811	-0.020079	0.017623	hypomethylated	Usf1	22	26
chr1	173366691	173368691	-0.2866	0.0053134	hypomethylated	F11r	6	13
chr1	174011659	174013659	-0.26872	1.47E-08	hypomethylated	Copa,Ncstn	14	15
chr1	174012880	174014880	-0.26921	4.34E-08	hypomethylated	Copa,Ncstn	14	14
chr1	174077145	174079145	-0.38686	0.00000813	stronglyhypometh	Dcaf8	22	25
chr1	174429376	174431376	-0.30701	1.15E-08	hypomethylated	Tagln2	18	15
chr1	176430955	176432955	-0.1342	0.018691	hypomethylated	Fmn2	11	9
chr1	177809908	177811908	-0.21307	0.00000473	hypomethylated	Exo1	19	21
chr1	180116547	180118547	-0.2184	0.0079125	hypomethylated	Desi2	9	4
chr1	180267915	180269915	-0.086667	0.0079607	hypomethylated	Hnrnpu	15	15
chr1	180335011	180337011	0.15727	0.022553	hypermethylated	Efcab2	17	35
chr1	180458255	180460255	-0.19717	3.01E-08	hypomethylated	Kif26b	33	48
chr1	181476398	181478398	0.075246	0.020871	hypermethylated	Cnst,Tfb2m	37	46
chr1	181597361	181599361	-0.12974	0.000000616	hypomethylated	Sccpdh	46	48
chr1	182260680	182262680	-0.15417	0.0037839	hypomethylated	Gm5069,Itpkb	30	45
chr1	182655173	182657173	-0.18058	0.0000157	hypomethylated	Acbd3	39	44
chr1	182743734	182745734	-0.051407	0.0019461	hypomethylated	H3f3a	31	30
chr1	182780281	182782281	-0.23636	0.0011476	hypomethylated	Sde2	22	22
chr1	183281758	183283758	-0.1336	0.00013508	hypomethylated	Cnih3	31	31
chr1	183772532	183774532	-0.18473	0.00021654	hypomethylated	Lbr	23	23
chr1	184338297	184340297	-0.35399	0.00000749	stronglyhypometh	Trp53bp2	40	47
chr1	185857339	185859339	-0.42156	1.05E-10	stronglyhypometh	1700056E22Rik,Dusp1	31	43
chr1	185857877	185859877	-0.44548	0.001486	stronglyhypometh	1700056E22Rik,Dusp1	3	11
chr1	186556372	186558372	0.15132	0.044827	hypermethylated	Hlx	11	19
chr1	187155037	187157037	-0.8392	4.85E-11	stronglyhypometh	Bpnt1	8	8
chr1	187185973	187187973	-0.036323	0.03123	hypomethylated	Eprs	20	34
chr1	187277726	187279726	-0.12452	0.02532	hypomethylated	Gm2061,Slc30a10	32	39
chr1	191511965	191513965	-0.19234	0.0054979	hypomethylated	Cenpf	10	8
chr1	191551146	191553146	-0.099821	0.0042961	hypomethylated	Ptpn14	43	52
chr1	191746167	191748167	0.1507	0.019874	hypermethylated	Smyd2	20	17
chr1	192751383	192753383	-0.030197	0.0030089	hypomethylated	Angel2	40	39
chr1	193007212	193009212	-0.1947	0.0000172	hypomethylated	Atf3	25	30
chr1	193220920	193222920	-0.19811	1.29E-10	hypomethylated	Ppp2r5a	25	25
chr1	193398612	193400612	-0.081987	0.019034	hypomethylated	Dtl,Ints7	20	20
chr1	193399413	193401413	-0.21474	0.0013157	hypomethylated	Dtl,Ints7	8	8
chr1	193961669	193963669	-0.27148	6.6E-15	hypomethylated	Gm10516,Rcor3	47	44
chr1	194978305	194980305	-0.15194	0.010875	hypomethylated	Irf6	22	19
chr1	196445022	196447022	-0.17591	0.021997	hypomethylated	Plxna2	39	49

chr1	196957764	196959764	-0.8775	0.021961	stronglyhypometh	Cr1l	2	2
chr10	4423140	4425140	-0.17552	0.00000127	hypomethylated	Rgs17	17	21
chr10	4794848	4796848	-0.24246	0.0087267	hypomethylated	Syne1	9	12
chr10	5913188	5915188	-0.36705	1.66E-12	stronglyhypometh	1700052N19Rik,Rmnd	18	31
chr10	5913936	5915936	-0.35556	3.24E-11	stronglyhypometh	1700052N19Rik,Rmnd	12	20
chr10	5957432	5959432	-0.14553	0.019006	hypomethylated	Zbtb2	52	56
chr10	7382168	7384168	-0.10403	0.0025133	hypomethylated	A630066F11Rik,Pcmt1	39	44
chr10	7500715	7502715	-0.17317	0.032068	hypomethylated	Ginm1	11	12
chr10	7511691	7513691	-0.072333	0.028682	hypomethylated	Ppil4	15	25
chr10	7675921	7677921	-0.022167	0.01948	hypomethylated	Tab2	32	48
chr10	8605868	8607868	-0.3186	0.022548	hypomethylated	Sash1	16	15
chr10	12727255	12729255	-0.25266	0.0000909	hypomethylated	Sf3b5	20	20
chr10	12809593	12811593	-0.51092	1.69E-20	stronglyhypometh	Plagl1	23	29
chr10	13043966	13045966	0.092805	0.00047504	hypermethylated	Phactr2	20	20
chr10	13271712	13273712	-0.46888	0.016751	stronglyhypometh	Adat2,Pex3	19	16
chr10	17442033	17444033	-0.065157	0.00081272	hypomethylated	Cited2	33	44
chr10	17954787	17956787	-0.45509	0.00011028	stronglyhypometh	Ccdc28a	4	4
chr10	18463564	18465564	-0.40564	0.00060747	stronglyhypometh	D10Bwg1379e	6	11
chr10	19867725	19869725	-0.13026	1.04E-08	hypomethylated	Map7	82	85
chr10	20031274	20033274	-0.12137	0.0000048	hypomethylated	Bclaf1	42	41
chr10	20880790	20882790	0.026048	0.018403	hypermethylated	Myb	23	25
chr10	21014784	21016784	-0.48966	0.000016	stronglyhypometh	Hbs1l	19	24
chr10	21711028	21713028	-0.94862	2.85E-11	stronglyhypometh	Sgk1	6	12
chr10	21712695	21714695	-0.10951	0.00024167	hypomethylated	Sgk1	41	39
chr10	21877414	21879414	-0.10179	0.010009	hypomethylated	Raet1a	16	16
chr10	22363816	22365816	0.16358	0.0027669	hypermethylated	Slc2a12	7	12
chr10	31032210	31034210	-0.15255	0.012568	hypomethylated	Hddc2	13	18
chr10	33739422	33741422	-0.31204	0.00000316	hypomethylated	Rwdd1	17	19
chr10	34000995	34002995	-0.11386	0.0011536	hypomethylated	Tspyl1	15	14
chr10	34202205	34204205	-0.18088	0.013438	hypomethylated	Frk	9	10
chr10	36225612	36227612	-0.23525	0.000011	hypomethylated	Hs3st5	24	32
chr10	36693349	36695349	-0.063423	0.00072575	hypomethylated	Hdac2	54	56
chr10	38684320	38686320	-0.25511	0.027192	hypomethylated	Lama4	5	5
chr10	38853701	38855701	-0.23986	0.0018539	hypomethylated	Fam229b,Tube1	10	9
chr10	39088604	39090604	-0.10742	0.00000123	hypomethylated	Fyn	36	48
chr10	39450965	39452965	0.04282	0.045103	hypermethylated	E130307A14Rik,Rev3l	57	67
chr10	40402087	40404087	-0.13816	0.0044485	hypomethylated	Mettl24	30	30
chr10	40602949	40604949	0.079026	0.014999	hypermethylated	Cdc40,Wasf1	60	69
chr10	41529379	41531379	-0.26093	0.0052001	hypomethylated	Cep57l1,Sesn1	25	29
chr10	41529436	41531436	-0.26093	0.0052001	hypomethylated	Cep57l1,Sesn1	25	29
chr10	41996548	41998548	-0.081996	0.015244	hypomethylated	Foxo3	44	64
chr10	42480301	42482301	-0.067541	0.00097322	hypomethylated	Sec63	23	19
chr10	42893509	42895509	-0.092398	0.00058687	hypomethylated	9030612E09Rik,Sobp	59	63
chr10	42940291	42942291	-0.084797	0.049288	hypomethylated	Pdss2	18	17
chr10	43620612	43622612	-0.22045	0.00020972	hypomethylated	Qrsl1,Rtn4ip1	14	14
chr10	43621542	43623542	-0.38889	0.0017891	stronglyhypometh	Qrsl1,Rtn4ip1	4	3
chr10	45008110	45010110	-0.082896	0.0041068	hypomethylated	Popdc3	22	21
chr10	50614456	50616456	0.078637	0.0098819	hypermethylated	Sim1	26	38
chr10	52136352	52138352	-0.23923	0.00000624	hypomethylated	Nus1	21	35
chr10	57205190	57207190	-0.15662	0.004985	hypomethylated	4930467K11Rik,Hsf2	46	42
chr10	57513349	57515349	-0.2408	0.010835	hypomethylated	Smpdl3a	11	17
chr10	57785213	57787213	-0.26482	3.23E-16	hypomethylated	Lims1	40	45
chr10	58683669	58685669	-0.24841	0.0023772	hypomethylated	Sept10,Sowahc	37	55
chr10	58785043	58787043	-0.041759	0.048703	hypomethylated	P4ha1	43	52
chr10	59164382	59166382	-0.56247	0.0000163	stronglyhypometh	Micu1	13	14
chr10	59568004	59570004	-0.23133	0.000000733	hypomethylated	Spock2	33	39
chr10	59739375	59741375	-0.33804	0.013324	stronglyhypometh	Psap	13	13

chr10	60215530	60217530	-0.48846	3.08E-11	stronglyhypometh	Slc29a3	4	5
chr10	60937580	60939580	-0.45591	0.0000101	stronglyhypometh	Lrrc20	10	10
chr10	61110368	61112368	-0.37552	1.77E-11	stronglyhypometh	Ppa1	34	34
chr10	61157261	61159261	-0.074917	0.024517	hypomethylated	Tysnd1	35	46
chr10	61910907	61912907	-0.30205	0.035768	hypomethylated	4930507D05Rik,Supv3	21	17
chr10	61949346	61951346	-0.12884	0.017325	hypomethylated	Vps26a	16	17
chr10	61949553	61951553	-0.18182	0.018553	hypomethylated	Vps26a	11	11
chr10	61970503	61972503	-0.69167	0.011675	stronglyhypometh	Srgn	3	5
chr10	62113946	62115946	-0.41521	0.0020025	stronglyhypometh	Ddx50	11	11
chr10	62408776	62410776	-0.1456	0.029105	hypomethylated	Dna2	20	33
chr10	62561903	62563903	-0.011871	0.023337	hypomethylated	Atoh7	20	21
chr10	62801743	62803743	-0.15596	0.000000181	hypomethylated	Sirt1	23	21
chr10	62801780	62803780	-0.16348	0.000000166	hypomethylated	Sirt1	22	21
chr10	62801783	62803783	-0.16348	0.000000166	hypomethylated	Sirt1	22	21
chr10	62844190	62846190	-0.030572	0.014996	hypomethylated	Dnajc12	5	7
chr10	66559605	66561605	-0.15202	0.0000156	hypomethylated	Reep3	14	14
chr10	66999616	67001616	-0.11154	0.029315	hypomethylated	Egr2	15	15
chr10	68674404	68676404	-0.42593	0.00048679	stronglyhypometh	Rhobtb1	26	42
chr10	69387279	69389279	-0.46095	0.00000317	stronglyhypometh	Ank3	12	13
chr10	69558868	69560868	-0.11632	0.0010613	hypomethylated	Ccdc6	48	64
chr10	69902415	69904415	0.25774	0.0019876	hypermethylated	Fam13c	8	11
chr10	69902657	69904657	0.25774	0.0019876	hypermethylated	Fam13c	8	11
chr10	70809540	70811540	-0.085422	0.00000468	hypomethylated	Ipmk	47	52
chr10	74522640	74524640	0.047385	0.03341	hypermethylated	Bcr	41	63
chr10	74673817	74675817	-0.25899	0.00020045	hypomethylated	Specc1l	24	24
chr10	74674134	74676134	-0.25899	0.00020045	hypomethylated	Specc1l	24	24
chr10	74778687	74780687	-0.17755	0.000000144	hypomethylated	Adora2a	31	35
chr10	75395208	75397208	-0.30761	0.0076744	hypomethylated	Mmp11	5	5
chr10	75397317	75399317	-0.21275	0.045956	hypomethylated	Chchd10	16	16
chr10	75416258	75418258	0.33131	0.040603	hypermethylated	Gm5134	1	6
chr10	75700610	75702610	-0.031954	0.027836	hypomethylated	Prmt2	9	8
chr10	76424692	76426692	-0.20882	0.0027444	hypomethylated	Pcbp3	10	12
chr10	76494483	76496483	-0.14593	0.017196	hypomethylated	Slc19a1	4	8
chr10	76494956	76496956	-0.14593	0.017196	hypomethylated	Slc19a1	4	8
chr10	76629275	76631275	-0.36032	0.0069797	stronglyhypometh	Col18a1	5	5
chr10	76721968	76723968	-0.06227	0.0071208	hypomethylated	Gm10941,Pofut2	21	28
chr10	76880120	76882120	-0.041474	0.0086725	hypomethylated	Adarb1,Gm17769	33	42
chr10	76881018	76883018	-0.13775	0.00000287	hypomethylated	Adarb1,Gm17769	14	16
chr10	77043511	77045511	-0.16667	0.015205	hypomethylated	Pttg1ip	6	9
chr10	77084065	77086065	-0.13141	0.0057479	hypomethylated	Ube2g2	33	33
chr10	77440394	77442394	-0.0030017	0.0010272	hypomethylated	1810043G02Rik	30	33
chr10	77814445	77816445	-0.052621	0.00019011	hypomethylated	Agpat3	44	48
chr10	78054709	78056709	-0.28192	0.015576	hypomethylated	Syde1	7	11
chr10	78242240	78244240	0.20899	0.0073885	hypermethylated	Slc1a6	10	12
chr10	79100663	79102663	-0.074618	0.0054804	hypomethylated	Shc2	12	12
chr10	79131154	79133154	-0.16672	0.00000379	hypomethylated	Tpgs1	36	42
chr10	79166102	79168102	-0.33413	0.0020934	stronglyhypometh	Bsg	7	9
chr10	79255316	79257316	-0.047297	0.00010981	hypomethylated	Palm	20	20
chr10	79388816	79390816	-0.1715	5.58E-15	hypomethylated	Arid3a	32	46
chr10	79450344	79452344	-0.26499	0.00064098	hypomethylated	Cnn2	29	34
chr10	79578280	79580280	-0.242	6.87E-08	hypomethylated	Stk11	38	44
chr10	79600132	79602132	-0.15951	0.00063706	hypomethylated	Dos	37	39
chr10	79604059	79606059	-0.5	0.0000274	stronglyhypometh	Atp5d	8	10
chr10	79610034	79612034	-0.12642	0.00000183	hypomethylated	Midn	34	34
chr10	79688342	79690342	-0.22708	0.01951	hypomethylated	Mum1	15	20
chr10	79711196	79713196	-0.50926	0.0097323	stronglyhypometh	Ndufs7	6	6
chr10	79726735	79728735	-0.28706	9.42E-12	hypomethylated	Dazap1	44	46

chr10	79783293	79785293	-0.22224	0.0034505	hypomethylated	2310011J03Rik	3	8
chr10	79811157	79813157	-0.16687	0.0029326	hypomethylated	Adamtsl5	28	30
chr10	79811191	79813191	-0.17382	0.013072	hypomethylated	Adamtsl5	25	27
chr10	79862224	79864224	-0.18437	0.031951	hypomethylated	Mbd3	7	7
chr10	79869566	79871566	-0.47907	0.0000345	stronglyhypometh	Uqcr11	8	8
chr10	80040041	80042041	-0.23118	0.016895	hypomethylated	Klf16	32	32
chr10	80064624	80066624	-0.37093	1.02E-09	stronglyhypometh	Adat3,Scamp4	9	20
chr10	80064626	80066626	-0.37093	1.02E-09	stronglyhypometh	Adat3,Scamp4	9	20
chr10	80260479	80262479	-0.32555	2.7E-12	hypomethylated	Plekhj1,Sf3a2	60	63
chr10	80261371	80263371	-0.40368	0.0014009	stronglyhypometh	Plekhj1,Sf3a2	26	25
chr10	80290541	80292541	-0.27206	0.0026987	hypomethylated	Mir1982	4	4
chr10	80391835	80393835	-0.22881	0.000000199	hypomethylated	Gadd45b	24	29
chr10	80531827	80533827	-0.6	0.00032287	stronglyhypometh	Thop1	10	10
chr10	80598015	80600015	-0.14324	0.0000315	hypomethylated	Zbtb7a	48	68
chr10	80656435	80658435	-0.27294	0.0000279	hypomethylated	2310050B05Rik	11	11
chr10	80719289	80721289	-0.27942	0.00013079	hypomethylated	Matk	26	30
chr10	80754716	80756716	-0.1148	0.00098023	hypomethylated	Pip5k1c,Tjp3	29	36
chr10	81068052	81070052	-0.48898	0.0086325	stronglyhypometh	BC025920	5	4
chr10	82447241	82449241	-0.122	0.0050681	hypomethylated	Chst11	54	53
chr10	82821965	82823965	-0.15531	0.000000563	hypomethylated	D10Wsu102e	33	40
chr10	84038691	84040691	-0.36606	0.00000022	stronglyhypometh	Tcp11l2	13	17
chr10	84217792	84219792	-0.11559	0.00031808	hypomethylated	Rfx4	27	32
chr10	84564371	84566371	-0.22188	0.000000129	hypomethylated	AI597468,Fhl4	23	24
chr10	84565240	84567240	-0.2196	0.000000141	hypomethylated	AI597468,Fhl4	23	23
chr10	84647799	84649799	-0.17919	5.01E-09	hypomethylated	Cry1	29	28
chr10	84848558	84850558	-0.19918	0.014281	hypomethylated	Btbd11	23	36
chr10	85060155	85062155	0.28764	0.02766	hypermethylated	Btbd11	10	15
chr10	86167555	86169555	-0.38277	0.015468	stronglyhypometh	BC030307,Hsp90b1	13	19
chr10	86168189	86170189	-0.61051	0.014452	stronglyhypometh	BC030307,Hsp90b1	9	6
chr10	86519790	86521790	-0.37462	0.012768	stronglyhypometh	1700113H08Rik	4	5
chr10	88193158	88195158	-0.16532	0.0046878	hypomethylated	Arl1,Gm4925	34	42
chr10	88193846	88195846	-0.16048	0.031681	hypomethylated	Arl1,Gm4925	30	36
chr10	89206735	89208735	-0.18596	1.59E-09	hypomethylated	Uhrf1bp1l	19	18
chr10	90544783	90546783	-0.11096	0.0079962	hypomethylated	Apaf1,Ikbip	36	39
chr10	90545488	90547488	-0.26444	0.0000006	hypomethylated	Apaf1,Ikbip	17	18
chr10	90586708	90588708	-0.42479	0.00027925	stronglyhypometh	Slc25a3	2	6
chr10	92622620	92624620	-0.092335	0.0049681	hypomethylated	Cdk17,Mir1931	42	56
chr10	92915140	92917140	0.13641	0.0017769	hypermethylated	Lta4h	10	10
chr10	93002376	93004376	-0.18101	0.00033197	hypomethylated	Amdhd1,Ccdc38	10	11
chr10	93609675	93611675	-0.20877	0.0007741	hypomethylated	Nr2c1	11	11
chr10	93976601	93978601	-0.23419	0.000000595	hypomethylated	Tmcc3	53	52
chr10	94150534	94152534	-0.21734	1.95E-10	hypomethylated	4932415G12Rik,Ccdc4	52	46
chr10	94151358	94153358	-0.281	2.69E-11	hypomethylated	4932415G12Rik,Ccdc4	41	40
chr10	95026801	95028801	-0.082655	0.00062412	hypomethylated	Nudt4	20	19
chr10	98568804	98570804	-0.20649	0.0000284	hypomethylated	Galnt4,Poc1b	33	37
chr10	98569768	98571768	-0.22154	0.00000112	hypomethylated	Galnt4,Poc1b	38	39
chr10	98724864	98726864	-0.42145	0.00000147	stronglyhypometh	Dusp6	20	24
chr10	102491411	102493411	-0.15649	0.042588	hypomethylated	Alx1	17	17
chr10	106707886	106709886	-0.47729	0.0051481	stronglyhypometh	Lin7a	8	8
chr10	107598455	107600455	-0.11785	0.00010669	hypomethylated	Ppp1r12a	46	59
chr10	110356231	110358231	-0.18253	0.020866	hypomethylated	Csrp2	24	26
chr10	110447122	110449122	-0.15962	0.00066847	hypomethylated	Zdhhc17	29	36
chr10	110600857	110602857	-0.093242	0.007733	hypomethylated	Osbpl8	39	45
chr10	110942341	110944341	-0.073	0.0019216	hypomethylated	Phlda1	39	47
chr10	114821014	114823014	-0.12199	0.000000131	hypomethylated	Thap2,Zfc3h1	55	70
chr10	114821491	114823491	-0.11754	0.000000419	hypomethylated	Thap2,Zfc3h1	55	62
chr10	115454418	115456418	-0.1585	0.046387	hypomethylated	Ptprr	7	7

chr10	116018567	116020567	-0.06936	0.0092952	hypomethylated	Cnot2	38	37
chr10	116585530	116587530	-0.28795	0.014795	hypomethylated	Frs2	13	13
chr10	117283030	117285030	0.36204	0.014706	stronglyhypermeth	Rap1b	14	18
chr10	117577842	117579842	-0.28007	0.0000337	hypomethylated	Mdm1	23	30
chr10	118677111	118679111	-0.1465	0.0020281	hypomethylated	Cand1	21	23
chr10	119644881	119646881	-0.15107	0.00037758	hypomethylated	Tmbim4	15	15
chr10	119913991	119915991	-0.1151	0.0014827	hypomethylated	Hmga2	10	13
chr10	120470059	120472059	-0.49005	1.3E-10	stronglyhypometh	Wif1	13	10
chr10	120801145	120803145	-0.16611	0.00032241	hypomethylated	Gns	19	25
chr10	120913306	120915306	0.15015	0.0084549	hypermethylated	Rassf3	6	14
chr10	121023850	121025850	-0.37222	0.00000823	stronglyhypometh	Tbk1	9	9
chr10	121063372	121065372	-0.38969	0.0056598	stronglyhypometh	Xpot	9	9
chr10	121077756	121079756	-0.10037	0.0000143	hypomethylated	D930020B18Rik	17	37
chr10	122114817	122116817	-0.18456	0.000000139	hypomethylated	Ppm1h	45	52
chr10	122633979	122635979	-0.25563	0.0018206	hypomethylated	Usp15	19	15
chr10	126414772	126416772	-0.30516	0.0000854	hypomethylated	Ctdsp2	38	47
chr10	126477987	126479987	-0.14335	0.0050222	hypomethylated	LOC100504608,Mettl1	30	29
chr10	126499658	126501658	-0.4068	0.0041917	stronglyhypometh	Cdk4	17	21
chr10	126601280	126603280	-0.078389	0.000000157	hypomethylated	B4galnt1	50	56
chr10	126601886	126603886	-0.098683	2.56E-09	hypomethylated	B4galnt1	53	57
chr10	126626879	126628879	-0.099192	0.013506	hypomethylated	Arhgef25	9	10
chr10	126632765	126634765	0.3127	0.0015772	hypermethylated	Dtx3,F420014N23Rik	7	9
chr10	126648678	126650678	-0.13914	0.00090628	hypomethylated	Pip4k2c	12	13
chr10	126725827	126727827	-0.28106	0.015499	hypomethylated	Ddit3,Mbd6	12	15
chr10	126726848	126728848	-0.33614	0.0012316	stronglyhypometh	Ddit3	14	14
chr10	127175593	127177593	0.031532	0.04658	hypermethylated	Zbtb39	35	35
chr10	127495037	127497037	-0.027653	0.027407	hypomethylated	Ptges3	45	50
chr10	127528838	127530838	0.26025	0.0021976	hypermethylated	Baz2a	19	29
chr10	127630690	127632690	-0.33898	3.35E-15	stronglyhypometh	Gls2	26	28
chr10	127668118	127670118	-0.21204	0.0017903	hypomethylated	Timeless	14	14
chr10	127758514	127760514	-0.45449	0.0063932	stronglyhypometh	Cnpy2	5	5
chr10	127773887	127775887	-0.085002	0.00051651	hypomethylated	Cs	27	29
chr10	127846852	127848852	-0.14853	0.025041	hypomethylated	Nabp2,Rnf41	10	13
chr10	127984800	127986800	-0.37248	0.0083424	stronglyhypometh	Rpl41,Zc3h10	13	13
chr10	128002990	128004990	-0.24596	0.022358	hypomethylated	Pa2g4	8	11
chr10	128026557	128028557	-0.027636	0.009707	hypomethylated	Erbb3	45	47
chr10	128110974	128112974	-0.16544	0.0057277	hypomethylated	Suox	7	12
chr10	128241426	128243426	-0.25631	0.0026757	hypomethylated	Dnajc14,Tmem198b	28	34
chr10	128241731	128243731	-0.23636	0.0043872	hypomethylated	Dnajc14,Tmem198b	28	35
chr10	128328774	128330774	-0.38506	0.00060175	stronglyhypometh	Gdf11	9	12
chr10	128369868	128371868	-0.067022	0.035689	hypomethylated	Itga7	16	16
chr11	3351439	3353439	-0.19788	2.31E-09	hypomethylated	8430429K09Rik,Rnf18!	23	24
chr11	3547536	3549536	-0.14634	0.000083	hypomethylated	Morc2a,Tug1	46	50
chr11	3548496	3550496	-0.10859	0.00011173	hypomethylated	Morc2a,Tug1	46	51
chr11	3548811	3550811	-0.12917	0.0000759	hypomethylated	Morc2a,Tug1	33	35
chr11	3794242	3796242	-0.3579	0.0018336	stronglyhypometh	4921536K21Rik,Dusp1	7	9
chr11	3862977	3864977	-0.31111	0.0000113	hypomethylated	Pes1	7	7
chr11	4034162	4036162	0.11608	0.02255	hypermethylated	Rnf215	15	19
chr11	4085835	4087835	-0.23799	0.0019094	hypomethylated	Tbc1d10a	7	12
chr11	4166097	4168097	-0.15404	0.0066941	hypomethylated	Lif	31	29
chr11	4749530	4751530	-0.091207	0.00072558	hypomethylated	Nf2	7	14
chr11	4794345	4796345	-0.098187	0.0014154	hypomethylated	Thoc5	19	19
chr11	5161613	5163613	-0.1534	0.013698	hypomethylated	Kremen1	35	35
chr11	5687485	5689485	-0.011215	0.034797	hypomethylated	Dbnl	19	26
chr11	6190599	6192599	0.11685	0.0011497	hypermethylated	Ogdh	27	34
chr11	6962491	6964491	-0.10507	0.000000531	hypomethylated	Adcy1	39	46
chr11	7113926	7115926	-0.24206	3.34E-08	hypomethylated	Igfbp3	17	20

chr11	8911140	8913140	-0.12165	0.039799	hypomethylated	Hus1	7	10
chr11	11585215	11587215	-0.013134	0.010803	hypomethylated	Ikzf1	62	71
chr11	16651205	16653205	-0.24082	0.000000481	hypomethylated	Egfr	33	38
chr11	16950936	16952936	-0.38366	0.029915	stronglyhypometh	Cnrip1	8	7
chr11	17058300	17060300	-0.13468	0.00026722	hypomethylated	Ppp3r1	48	60
chr11	17111592	17113592	0.27778	0.028879	hypermethylated	Pno1,Wdr92	3	3
chr11	19823444	19825444	-0.14669	0.00073012	hypomethylated	Spred2	15	22
chr11	20012954	20014954	-0.29747	0.00011821	hypomethylated	Actr2	18	20
chr11	20100604	20102604	-0.17701	0.0013993	hypomethylated	Rab1	29	33
chr11	20442255	20444255	-0.1377	0.0011188	hypomethylated	Sertad2	26	37
chr11	20530979	20532979	-0.10162	0.0043856	hypomethylated	Sertad2	35	47
chr11	20731111	20733111	-0.12114	0.0041032	hypomethylated	Lgalsl	9	14
chr11	21137891	21139891	-0.14355	0.00000322	hypomethylated	Vps54	49	49
chr11	21901654	21903654	0.074144	0.024392	hypermethylated	Otx1	30	36
chr11	22411285	22413285	-0.045452	0.0029025	hypomethylated	Tmem17	34	34
chr11	22759735	22761735	-0.18177	0.0053476	hypomethylated	B3gnt2	24	24
chr11	22871028	22873028	-0.29188	0.00058164	hypomethylated	Zrsr1	26	29
chr11	23155040	23157040	-0.55952	0.0000125	stronglyhypometh	Xpo1	6	20
chr11	23670970	23672970	-0.21554	0.040561	hypomethylated	Rel	22	22
chr11	23977055	23979055	-0.29034	6.72E-09	hypomethylated	Bcl11a	31	43
chr11	23979694	23981694	-0.1391	0.00048521	hypomethylated	Bcl11a	15	23
chr11	26110576	26112576	-0.16516	0.0038229	hypomethylated	5730522E02Rik	9	9
chr11	26286083	26288083	-0.70642	0.00000261	stronglyhypometh	Fancl	7	14
chr11	28752204	28754204	-0.5	0.00064817	stronglyhypometh	Efemp1	3	3
chr11	29029750	29031750	-0.32145	0.0000191	hypomethylated	Pnpt1	10	19
chr11	29071906	29073906	-0.070518	0.013253	hypomethylated	Smek2	29	35
chr11	29425456	29427456	-0.026367	0.016608	hypomethylated	Mtif2	12	13
chr11	29591897	29593897	-0.14592	0.00000213	hypomethylated	Rtn4	32	47
chr11	29592773	29594773	-0.21545	1.49E-08	hypomethylated	Rtn4	34	47
chr11	32125504	32127504	-0.11626	0.02735	hypomethylated	Mpg	22	27
chr11	32432265	32434265	-0.13053	0.044441	hypomethylated	Stk10	30	41
chr11	40546143	40548143	0.052794	0.008132	hypermethylated	Hmmr,Nudcd2	15	16
chr11	40546939	40548939	0.052794	0.008132	hypermethylated	Hmmr,Nudcd2	15	16
chr11	43494499	43496499	0.029939	0.018142	hypermethylated	Pwwp2a	38	36
chr11	43649834	43651834	-0.1524	0.036863	hypomethylated	Adra1b	22	21
chr11	48612861	48614861	-0.28281	0.00030566	hypomethylated	Gnb2l1,Snord96a	15	15
chr11	48638639	48640639	-1	1.33E-09	stronglyhypometh	Trim7	8	6
chr11	49422180	49424180	-0.11983	0.0057162	hypomethylated	Flt4	27	27
chr11	49714336	49716336	-0.2332	0.00012115	hypomethylated	Rasgef1c	45	45
chr11	49943861	49945861	-0.40473	3.84E-09	stronglyhypometh	Tbc1d9b	21	27
chr11	49987352	49989352	-0.125	0.0081338	hypomethylated	3010026O09Rik	8	4
chr11	50414586	50416586	-0.081285	0.044467	hypomethylated	Adamts2	75	76
chr11	50663186	50665186	0.059698	0.047857	hypermethylated	Grm6	28	28
chr11	50871758	50873758	-0.28122	0.00074138	hypomethylated	Zfp354a	16	18
chr11	51420383	51422383	-0.29776	0.036359	hypomethylated	Hnrnpab	16	16
chr11	51432274	51434274	-0.10714	0.032907	hypomethylated	Nhp2	8	12
chr11	51449398	51451398	-0.043209	0.037801	hypomethylated	Rmnd5b	13	15
chr11	51463455	51465455	-0.12385	0.040277	hypomethylated	D930048N14Rik,N4bp	29	34
chr11	51502136	51504136	-0.15073	0.00000817	hypomethylated	0610009B22Rik	13	13
chr11	51576164	51578164	-0.11093	0.00022937	hypomethylated	Sar1b	8	9
chr11	51670983	51672983	0.20741	0.0047854	hypermethylated	Phf15	17	18
chr11	51816722	51818722	-0.20105	0.0000509	hypomethylated	Cdkl3	18	23
chr11	51911325	51913325	-0.14536	0.00035246	hypomethylated	Ppp2ca	44	53
chr11	52208929	52210929	-0.32516	0.0000448	hypomethylated	9530068E07Rik	14	18
chr11	52577207	52579207	-0.0046732	0.026093	hypomethylated	Fstl4	41	47
chr11	53379880	53381880	-0.10714	0.001095	hypomethylated	Kif3a	14	14
chr11	53582515	53584515	-0.33472	0.000000193	stronglyhypometh	Irf1	23	27

chr11	53582974	53584974	-0.33472	0.000000193	stronglyhypometh	Irf1	23	27
chr11	53672758	53674758	-0.23571	0.032059	hypomethylated	Gm12216	5	3
chr11	53705205	53707205	-0.094167	0.0010209	hypomethylated	Slc22a5	12	15
chr11	53841592	53843592	-0.21281	4.46E-08	hypomethylated	Slc22a4	23	23
chr11	54769624	54771624	-0.060466	0.031897	hypomethylated		11	11
chr11	55016841	55018841	-0.33428	0.001057	stronglyhypometh	Slc36a1	9	13
chr11	55274640	55276640	-0.045275	0.048672	hypomethylated	Atox1	12	12
chr11	57331166	57333166	0.071688	0.0043031	hypermethylated	Fam114a2,Mfap3	22	28
chr11	57457943	57459943	-0.23584	0.00000203	hypomethylated	Galnt10	22	26
chr11	57614138	57616138	-0.19162	1.01E-10	hypomethylated	Sap30l	63	65
chr11	57645649	57647649	-0.29202	0.00040885	hypomethylated	Hand1	10	10
chr11	57984155	57986155	0.12401	0.015344	hypermethylated	Mrpl22	7	11
chr11	58119570	58121570	-0.21774	0.02428	hypomethylated	Zfp692	28	30
chr11	58143219	58145219	-0.24871	0.033125	hypomethylated	Sh3bp5l,Zfp672	20	18
chr11	58452966	58454966	-0.42857	0.0065369	stronglyhypometh	Trim58	6	7
chr11	58790617	58792617	-0.12631	0.00052401	hypomethylated	Trim11	28	33
chr11	59020862	59022862	-0.79242	0.00028352	stronglyhypometh	2310033P09Rik	11	4
chr11	59041664	59043664	-0.17251	0.00083082	hypomethylated	Arf1	15	16
chr11	59041769	59043769	-0.22668	0.0010959	hypomethylated	Arf1	10	10
chr11	59104253	59106253	0.066976	0.036372	hypermethylated	Wnt3a	24	26
chr11	59474996	59476996	-0.071416	0.0075513	hypomethylated	Mprip	48	54
chr11	59623275	59625275	-0.12535	0.0059553	hypomethylated	Flcn,Gm16062	12	17
chr11	59917514	59919514	-0.081339	0.0000747	hypomethylated	Rai1	47	53
chr11	59952583	59954583	-0.024	0.010364	hypomethylated	Rai1	17	19
chr11	60165881	60167881	-0.38382	0.0042801	stronglyhypometh	Lrrc48,Tom1l2	9	12
chr11	60229646	60231646	-0.24837	0.0000291	hypomethylated	Atpaf2,Gid4	23	28
chr11	60230601	60232601	-0.25054	0.0000214	hypomethylated	Atpaf2,Gid4	23	30
chr11	60512191	60514191	-0.13501	0.0013705	hypomethylated	Llgl1	32	35
chr11	60540723	60542723	-0.2856	0.030431	hypomethylated	Flii,Mir5100,Smcr7	28	26
chr11	60540899	60542899	-0.37628	0.0020912	stronglyhypometh	Flii,Mir5100,Smcr7	22	20
chr11	60744558	60746558	-0.087593	0.0080502	hypomethylated	Gm16516,Map2k3	34	34
chr11	60745369	60747369	-0.045939	0.016445	hypomethylated	Gm16516,Map2k3	30	30
chr11	61393153	61395153	-0.13348	0.022009	hypomethylated	Epn2	9	9
chr11	61668594	61670594	-0.22171	0.00000275	hypomethylated	Ulk2	9	15
chr11	61889598	61891598	-0.25875	0.000000359	hypomethylated	Specc1	17	17
chr11	62061485	62063485	-0.24174	0.0000445	hypomethylated	Adora2b	37	46
chr11	62386987	62388987	-0.39011	0.0028374	stronglyhypometh	Trpv2	4	4
chr11	62461165	62463165	-0.05279	0.019672	hypomethylated	Fam211a,Mmgt2	52	52
chr11	63891985	63893985	-0.18736	0.0000094	hypomethylated	2810001G20Rik,Cox10	14	19
chr11	63892974	63894974	-0.22024	0.00041873	hypomethylated	2810001G20Rik,Cox10	8	8
chr11	66865171	66867171	-0.40434	0.00099469	stronglyhypometh	Adprm,Sco1	18	16
chr11	66866120	66868120	-0.55672	0.035105	stronglyhypometh	Adprm,Sco1	9	10
chr11	67735655	67737655	0.051967	0.02668	hypermethylated	Usp43	25	23
chr11	68244626	68246626	-0.12429	0.027184	hypomethylated	Pik3r5	23	22
chr11	68770360	68772360	0.32533	0.016935	hypermethylated	Arhgef15	2	5
chr11	68780632	68782632	-0.0099879	0.0010474	hypomethylated	Slc25a35	24	30
chr11	68828412	68830412	-0.18628	0.045334	hypomethylated	Ctc1	7	11
chr11	69211736	69213736	-0.12275	0.030233	hypomethylated	Tmem88	18	13
chr11	69445472	69447472	-0.24205	2.38E-13	hypomethylated	Fxr2	42	38
chr11	69571725	69573725	-0.17134	0.033146	hypomethylated	Polr2a	27	27
chr11	69648351	69650351	-0.40641	0.00066061	stronglyhypometh	Nlgn2	5	5
chr11	69659118	69661118	-0.18399	0.00018263	hypomethylated	Plscr3	11	14
chr11	69659145	69661145	-0.18399	0.00018263	hypomethylated	Plscr3	11	14
chr11	69714379	69716379	-0.13732	0.0084765	hypomethylated	2810408A11Rik,Neurl4	31	41
chr11	69714488	69716488	-0.15132	0.0039401	hypomethylated	2810408A11Rik,Neurl4	29	39
chr11	69726693	69728693	-0.41028	1.03E-10	stronglyhypometh	Gps2	22	23
chr11	69748400	69750400	-0.14843	0.00000149	hypomethylated	Ybx2	60	70

chr11	69793669	69795669	-0.43716	0.00000649	stronglyhypometh	Ctdnep1,Elp5	12	25
chr11	69793745	69795745	-0.43716	0.00000649	stronglyhypometh	Ctdnep1,Elp5	12	25
chr11	69794737	69796737	-0.78494	0.0000179	stronglyhypometh	Ctdnep1,Elp5	5	8
chr11	69808272	69810272	-0.025784	0.0077706	hypomethylated	Phf23	16	25
chr11	69831106	69833106	-0.23333	0.034241	hypomethylated	Dlg4	5	5
chr11	70245154	70247154	-0.21791	0.0036618	hypomethylated	Arrb2	13	27
chr11	70272123	70274123	0.15	0.0023118	hypermethylated	Cxcl16,Zmynd15	7	14
chr11	70375382	70377382	-0.019029	0.0023387	hypomethylated	Mink1	27	26
chr11	70657264	70659264	-0.027905	0.0027532	hypomethylated	Rabep1	42	48
chr11	71563204	71565204	-0.1724	0.00034873	hypomethylated	Wscd1	31	45
chr11	71855003	71857003	-0.52361	0.0011472	stronglyhypometh	Fam64a	7	11
chr11	72080106	72082106	-0.56843	0.025237	stronglyhypometh	1700051A21Rik,Slc13a	8	3
chr11	72303406	72305406	0.21705	0.0073968	hypermethylated	Spns2	10	10
chr11	72502503	72504503	-0.05881	0.035158	hypomethylated	Ankfy1	25	29
chr11	72608727	72610727	-0.25812	0.000000885	hypomethylated	Cyb5d2,Zzef1	29	28
chr11	72609341	72611341	-0.13881	0.00041007	hypomethylated	Cyb5d2,Zzef1	22	26
chr11	72773670	72775670	-0.3155	0.035921	hypomethylated	Atp2a3	23	32
chr11	72811646	72813646	-0.66098	0.00021982	stronglyhypometh	P2rx1	4	4
chr11	72973031	72975031	-0.16929	0.038391	hypomethylated	P2rx5	21	21
chr11	74403660	74405660	-0.2499	0.035066	hypomethylated	Rap1gap2	17	17
chr11	74710581	74712581	-0.19368	0.0000115	hypomethylated	Sgsm2,Tsr1	29	36
chr11	74710582	74712582	-0.19368	0.0000115	hypomethylated	Sgsm2,Tsr1	29	36
chr11	74738373	74740373	-0.24856	0.00010088	hypomethylated	Smg6,Srr	20	22
chr11	74739300	74741300	-0.37833	0.0000352	stronglyhypometh	Smg6,Srr	13	13
chr11	75274040	75276040	-0.33908	0.00019232	stronglyhypometh	Mir22hg	10	17
chr11	75344195	75346195	-0.20446	0.0000014	hypomethylated	Slc43a2	36	51
chr11	75344613	75346613	-0.080026	0.00011422	hypomethylated	Slc43a2	31	47
chr11	75464010	75466010	-0.073827	0.049158	hypomethylated	Myo1c	10	15
chr11	75464651	75466651	-0.35269	0.0047089	stronglyhypometh	Myo1c	15	18
chr11	75812413	75814413	-0.59267	0.000000214	stronglyhypometh	1700016K19Rik	8	8
chr11	76014557	76016557	-0.029922	0.010332	hypomethylated	Fam57a	32	38
chr11	76030114	76032114	-0.32623	0.00036365	hypomethylated	Dbil5,Gemin4	12	11
chr11	76056237	76058237	0.096506	0.0081676	hypermethylated	Glod4,Rnmtl1	23	30
chr11	76212643	76214643	-0.063214	0.0075823	hypomethylated	Nxn	10	10
chr11	76219668	76221668	-0.51111	0.00000162	stronglyhypometh	Timm22	18	14
chr11	76758157	76760157	-0.22779	0.015555	hypomethylated	Blmh	23	29
chr11	76811098	76813098	-0.48014	0.0097976	stronglyhypometh	Slc6a4	10	10
chr11	76891674	76893674	-0.21183	0.012633	hypomethylated	Ccdc55,Mir423	30	31
chr11	76891937	76893937	-0.40381	0.0000177	stronglyhypometh	Ccdc55,Mir423	19	20
chr11	77326775	77328775	-0.0082801	0.020797	hypomethylated	Abhd15,Trp53i13	37	35
chr11	77327618	77329618	-0.13402	0.0028256	hypomethylated	Abhd15,Trp53i13	45	47
chr11	77850442	77852442	-0.13009	0.0000166	hypomethylated	Flot2	30	37
chr11	77990168	77992168	-0.23034	0.00000416	hypomethylated	Nek8,Tlcd1	21	25
chr11	78000928	78002928	-0.12454	0.000000205	hypomethylated	Rab34	42	48
chr11	78001249	78003249	-0.12454	0.000000205	hypomethylated	Rab34	42	48
chr11	78156023	78158023	-0.29241	0.003147	hypomethylated	Unc119	23	22
chr11	78325670	78327670	-0.40373	0.0000903	stronglyhypometh	Poldip2,Tmem199	7	7
chr11	78510927	78512927	-0.14375	0.00072325	hypomethylated	Nlk	16	16
chr11	78639096	78641096	-0.29932	0.0402	hypomethylated	Lyrm9	17	19
chr11	79403713	79405713	-0.51351	8.25E-10	stronglyhypometh	Rab11fip4	5	23
chr11	79524470	79526470	-0.17255	0.00012891	hypomethylated	Mir193	50	56
chr11	79894457	79896457	-0.19297	0.0000068	hypomethylated	Crlf3	10	10
chr11	79955655	79957655	-0.37471	0.04608	stronglyhypometh	Tefm	3	7
chr11	80021556	80023556	-0.29327	0.00033065	hypomethylated	Rhot1	18	33
chr11	80241116	80243116	-0.090278	0.00014178	hypomethylated	Psmd11	54	54
chr11	80289547	80291547	-0.09837	0.00011329	hypomethylated	Cdk5r1	55	70
chr11	80966405	80968405	-0.17816	0.003242	hypomethylated	Asic2	28	35

chr11	82201401	82203401	-0.11737	0.0011474	hypomethylated	Tmem132e	42	52
chr11	82721897	82723897	-0.18429	0.00054508	hypomethylated	Nle1,Unc45b	11	9
chr11	82764603	82766603	-0.29148	0.0044268	hypomethylated	Slfn5	8	9
chr11	83104200	83106200	-1	7.81E-14	stronglyhypometh	AA465934,AI450353	6	10
chr11	83222573	83224573	-0.077916	0.00014847	hypomethylated	Rasl10b	31	37
chr11	83285609	83287609	-0.16223	0.036446	hypomethylated	Taf15	25	45
chr11	83566138	83568138	-0.24388	0.000012	hypomethylated	Heatr6	21	27
chr11	83881859	83883859	0.15164	0.019416	hypermethylated	Dusp14	16	20
chr11	83943070	83945070	-0.31327	0.0000933	hypomethylated	Tada2a	16	16
chr11	84327003	84329003	-0.078127	0.00048943	hypomethylated	Aatf	21	21
chr11	84339036	84341036	-0.40872	0.000000212	stronglyhypometh	1500016L03Rik,Lhx1	17	26
chr11	84692721	84694721	-0.13793	0.0000698	hypomethylated	Myo19,Pigw	40	45
chr11	84693697	84695697	-0.17273	0.0039431	hypomethylated	Myo19,Pigw	19	23
chr11	84693787	84695787	-0.2339	0.037113	hypomethylated	Myo19,Pigw	11	15
chr11	84953457	84955457	-0.15337	0.015761	hypomethylated	Usp32	11	11
chr11	85047667	85049667	-0.16558	5.3E-11	hypomethylated	Appbp2,D630032N06F	70	70
chr11	85165665	85167665	-0.35714	0.0042709	stronglyhypometh	Bcas3	4	7
chr11	86171027	86173027	-0.11857	0.001886	hypomethylated	Med13	21	28
chr11	86297158	86299158	-0.072576	0.0080911	hypomethylated	Rnft1	18	18
chr11	86900279	86902279	-0.30523	0.00032301	hypomethylated	Smg8	6	6
chr11	86939578	86941578	-0.34118	9.12E-09	stronglyhypometh	Trim37	30	39
chr11	87172496	87174496	-0.29942	0.017775	hypomethylated	Ppm1e	21	17
chr11	87800087	87802087	-0.13315	0.0000892	hypomethylated	Dynll2	33	23
chr11	87860172	87862172	-0.11847	0.000000494	hypomethylated	Srsf1	29	33
chr11	87911647	87913647	-0.069703	0.0000458	hypomethylated	2210416O15Rik,Cuedc	40	55
chr11	88016919	88018919	-0.097291	0.022853	hypomethylated	Mrps23	15	15
chr11	88579543	88581543	-0.12396	0.0011566	hypomethylated	C030037D09Rik,Msi2	16	20
chr11	88834248	88836248	-0.21655	0.0000672	hypomethylated	2210409E12Rik,Coil	10	14
chr11	89163873	89165873	-0.081247	0.012675	hypomethylated	Nog	29	27
chr11	90498497	90500497	-0.077407	0.0024045	hypomethylated	Cox11,Stxbp4	20	24
chr11	90499422	90501422	-0.086195	0.0031745	hypomethylated	Cox11,Stxbp4	18	22
chr11	90548915	90550915	-0.26879	0.0015084	hypomethylated	Tom1l1	10	13
chr11	92959603	92961603	0.18421	0.0000018	hypermethylated	Car10	30	30
chr11	93746532	93748532	-0.018512	0.037935	hypomethylated	Mbtd1,Utp18	39	42
chr11	93747080	93749080	-0.052639	0.0007937	hypomethylated	Mbtd1,Utp18	16	16
chr11	93817002	93819002	-0.1377	0.0050785	hypomethylated	Nme2	16	18
chr11	94183225	94185225	-0.29499	0.0000109	hypomethylated	Luc7l3	23	24
chr11	94254290	94256290	-0.39644	0.047788	stronglyhypometh	Abcc3	3	4
chr11	94361288	94363288	-0.45545	0.00010441	stronglyhypometh	Epn3	4	10
chr11	94851525	94853525	-0.050125	0.0020144	hypomethylated	Ppp1r9b	53	52
chr11	94980430	94982430	-0.25467	4.23E-10	hypomethylated	Dlx3	33	35
chr11	95197331	95199331	-0.3015	7.07E-12	hypomethylated	Fam117a	33	27
chr11	95245235	95247235	0.20768	0.029522	hypermethylated	Slc35b1	6	7
chr11	95274396	95276396	-0.06415	0.0044536	hypomethylated	Spop	8	11
chr11	95895230	95897230	-0.08831	0.00234	hypomethylated	Snf8	21	21
chr11	96054674	96056674	-0.47383	0.00000073	stronglyhypometh	Hoxb13	13	15
chr11	96125477	96127477	-0.47954	0.0004434	stronglyhypometh	Mir196a-1	5	7
chr11	96131643	96133643	-0.072346	0.00011998	hypomethylated	Hoxb9	34	37
chr11	96146959	96148959	-0.024731	0.0000187	hypomethylated	Hoxb7	38	45
chr11	96710189	96712189	-0.24444	0.0029124	hypomethylated	Copz2,Mir152	15	18
chr11	96710706	96712706	-0.2507	0.0074717	hypomethylated	Copz2,Mir152	14	17
chr11	97011026	97013026	-0.12196	0.00000363	hypomethylated	Tbkbp1	59	74
chr11	97049206	97051206	-0.026144	0.0064982	hypomethylated	Kpnb1	35	38
chr11	97222864	97224864	-0.11032	1.21E-09	hypomethylated	Socs7	58	64
chr11	97523725	97525725	-0.015892	0.0031428	hypomethylated	Mllt6	38	41
chr11	97560698	97562698	-0.13111	0.009818	hypomethylated	Pcgf2	32	40
chr11	97560962	97562962	-0.31069	0.00000112	hypomethylated	Pcgf2	16	24

chr11	97561811	97563811	-0.30494	0.000000933	hypomethylated	Pcgf2,Psmb3	16	14
chr11	97643080	97645080	-0.36165	0.00058607	stronglyhypometh	Rpl23,Snora21	23	23
chr11	97659985	97661985	-0.27539	0.0000305	hypomethylated	Lasp1	17	30
chr11	97887023	97889023	-0.14146	0.046723	hypomethylated	Rpl19	24	21
chr11	97887237	97889237	-0.14146	0.046723	hypomethylated	Rpl19	24	21
chr11	98010930	98012930	-0.19472	0.00000195	hypomethylated	Fbxl20	18	32
chr11	98063618	98065618	-0.055476	0.011564	hypomethylated	Cdk12	10	20
chr11	98246945	98248945	-0.34722	0.0038551	stronglyhypometh	Pnmt	9	9
chr11	98307147	98309147	-0.48978	0.0036814	stronglyhypometh	Grb7	6	10
chr11	98458604	98460604	-0.53333	0.00099937	stronglyhypometh	Gm12	5	3
chr11	98602186	98604186	0.06894	0.013985	hypermethylated	Thra	16	26
chr11	98656082	98658082	-0.16071	1.58E-08	hypomethylated	Msl1	69	85
chr11	98723911	98725911	-0.30755	2.58E-08	hypomethylated	Wipf2	14	19
chr11	98820784	98822784	-0.15019	0.00000142	hypomethylated	Rara	42	43
chr11	100180309	100182309	-0.52731	0.00000241	stronglyhypometh	Eif1	23	25
chr11	100259053	100261053	-0.16832	0.04871	hypomethylated	Jup	4	4
chr11	100276007	100278007	-0.40641	0.00000938	stronglyhypometh	Fkbp10,Leprel4	10	20
chr11	100276133	100278133	-0.51916	0.0000671	stronglyhypometh	Fkbp10,Leprel4	8	16
chr11	100435252	100437252	-0.15138	0.001247	hypomethylated	Cnp	19	19
chr11	100436204	100438204	-0.050401	0.0000906	hypomethylated	Cnp	24	24
chr11	100483268	100485268	-0.25726	0.00000456	hypomethylated	Nkiras2	14	18
chr11	100599444	100601444	-0.52222	0.034957	stronglyhypometh	Rab5c	9	12
chr11	100719664	100721664	-0.46428	0.0072871	stronglyhypometh	Stat5a	9	9
chr11	100720797	100722797	-0.53798	0.00019384	stronglyhypometh	Stat5a	14	9
chr11	100869768	100871768	-0.15292	0.0032062	hypomethylated	Atp6v0a1	19	22
chr11	100942938	100944938	0.14662	0.014993	hypermethylated	Coasy	10	12
chr11	100956715	100958715	-0.15551	0.0024424	hypomethylated	Psmc3ip	10	14
chr11	100980444	100982444	-0.072573	0.0039402	hypomethylated	Fam134c,Tubg1	37	45
chr11	100981138	100983138	-0.017449	0.0021151	hypomethylated	Fam134c,Tubg1	14	20
chr11	101032616	101034616	0.40346	0.0023581	stronglyhypermeth	Plekhh3	11	18
chr11	101106647	101108647	-0.073226	0.024933	hypomethylated	Ramp2	24	27
chr11	101114056	101116056	-0.38645	0.00000436	stronglyhypometh	Vps25	9	9
chr11	101120880	101122880	-0.055434	9.99E-08	hypomethylated	Wnk4	31	37
chr11	101176564	101178564	-0.030044	0.0030328	hypomethylated	Psme3	30	32
chr11	101285398	101287398	-0.092966	0.0000741	hypomethylated	Ptges3l,Rundc1	20	27
chr11	101286647	101288647	-0.26268	0.0000946	hypomethylated	Ptges3l,Rundc1	16	16
chr11	101328651	101330651	-0.28374	0.00031907	hypomethylated	Rnd2	23	24
chr11	101413171	101415171	-0.96241	0.00031898	stronglyhypometh	Brca1,Nbr1	2	7
chr11	101413269	101415269	-0.86842	0.0011257	stronglyhypometh	Brca1,Nbr1	2	2
chr11	101442555	101444555	-0.2269	0.016576	hypomethylated	Tmem106a	8	8
chr11	101646624	101648624	-0.19885	0.00040142	hypomethylated	Etv4	8	9
chr11	101846105	101848105	0.090085	0.007325	hypermethylated	1700006E09Rik,Dusp3	10	10
chr11	102109195	102111195	-0.15159	0.0000398	hypomethylated	BC030867	14	15
chr11	102178601	102180601	-0.094632	0.00021349	hypomethylated	Ubtf	95	112
chr11	102253748	102255748	0.15245	0.016825	hypermethylated	Rundc3a	9	15
chr11	102268831	102270831	-0.088727	0.029112	hypomethylated	Slc25a39	15	24
chr11	102290635	102292635	-0.1487	0.018932	hypomethylated	Grn	11	13
chr11	102559039	102561039	-0.133	0.049919	hypomethylated	Ccdc43	20	23
chr11	102621752	102623752	0.095269	0.0095152	hypermethylated	Adam11	26	31
chr11	102680473	102682473	-0.087886	0.016736	hypomethylated	Gjc1	28	28
chr11	102786438	102788438	0.16182	0.0010878	hypermethylated	Kif18b	11	11
chr11	103128715	103130715	-0.37604	0.039906	stronglyhypometh	Map3k14	13	17
chr11	103611135	103613135	-0.10417	0.0012667	hypomethylated	Wnt9b	6	8
chr11	103634488	103636488	-0.38111	0.000000772	stronglyhypometh	Wnt3	34	36
chr11	104046640	104048640	-0.3344	0.0017207	stronglyhypometh	Sppl2c	4	8
chr11	104303605	104305605	-0.3215	0.000000935	hypomethylated	Kansl1	28	30
chr11	104411934	104413934	-0.48889	0.00096824	stronglyhypometh	Cdc27,Myl4	3	5

chr11	105041843	105043843	-0.022524	0.041494	hypomethylated	1700052K11Rik,Tlk2	66	81
chr11	105152959	105154959	-0.52778	9.95E-08	stronglyhypometh	Mrc2	12	11
chr11	105828260	105830260	-0.39503	0.0000042	stronglyhypometh	Ace	18	32
chr11	105897185	105899185	-0.099385	0.017157	hypomethylated	Dcaf7	32	36
chr11	105945215	105947215	-0.12433	0.011984	hypomethylated	Map3k3	47	46
chr11	106116498	106118498	-0.37079	0.00010177	stronglyhypometh	Ftsj3,Psmc5	12	14
chr11	106117116	106119116	-0.14254	0.012273	hypomethylated	Ftsj3,Psmc5	11	13
chr11	106888536	106890536	-0.10674	0.00000158	hypomethylated	1810010H24Rik	44	41
chr11	107339841	107341841	-0.30405	0.0002312	hypomethylated	Psmd12	4	6
chr11	107408273	107410273	-0.20546	0.0034038	hypomethylated	Helz	28	39
chr11	108205202	108207202	-0.21822	0.000000222	hypomethylated	Prkca	19	23
chr11	109223107	109225107	-0.040005	0.0000731	hypomethylated	Gm11696,Gna13	49	57
chr11	109286259	109288259	-0.21468	0.000092	hypomethylated	Amz2	10	10
chr11	109334543	109336543	-0.076985	0.049621	hypomethylated	Arsg,Slc16a6	33	44
chr11	109334910	109336910	-0.16878	0.017949	hypomethylated	Arsg,Slc16a6	15	18
chr11	113509842	113511842	0.19638	0.019649	hypermethylated	Cog1	28	29
chr11	113927265	113929265	-0.056857	0.0074515	hypomethylated	Sdk2	73	74
chr11	114535781	114537781	-0.29313	0.014598	hypomethylated	Ttyh2	17	12
chr11	114951744	114953744	-0.0762	0.033229	hypomethylated	Rab37	17	19
chr11	115014491	115016491	-0.41473	0.000000242	stronglyhypometh	Rab37	25	18
chr11	115023654	115025654	-0.068879	0.0048831	hypomethylated	Slc9a3r1	28	42
chr11	115047800	115049800	-0.054028	0.012238	hypomethylated	Nat9,Tmem104	13	14
chr11	115048630	115050630	-0.054028	0.012238	hypomethylated	Nat9,Tmem104	13	14
chr11	115195047	115197047	-0.59848	0.0020691	stronglyhypometh	Otop3	4	4
chr11	115229033	115231033	-0.16667	0.00022641	hypomethylated	Hid1	4	6
chr11	115242229	115244229	-0.015593	0.0050622	hypomethylated	Cdr2l	23	29
chr11	115424757	115426757	-0.30806	0.0026636	hypomethylated	Nup85	3	4
chr11	115464464	115466464	-0.20848	0.0011377	hypomethylated	Gga3,Mrps7	47	66
chr11	115465220	115467220	-0.45464	2.14E-12	stronglyhypometh	Gga3,Mrps7	20	34
chr11	115674906	115676906	-0.26224	9.7E-15	hypomethylated	Caskin2,Tsen54	42	48
chr11	115675052	115677052	-0.26405	8.45E-13	hypomethylated	Caskin2,Tsen54	37	43
chr11	115890635	115892635	-0.3047	0.0000306	hypomethylated	Unk	10	12
chr11	116059168	116061168	-0.32767	0.0000345	hypomethylated	Acox1,Ten1	18	19
chr11	116274346	116276346	-0.10839	0.0026867	hypomethylated	Rnf157	19	21
chr11	116351660	116353660	-0.24258	0.0089803	hypomethylated	Prpsap1	11	12
chr11	116393551	116395551	-0.12188	0.011054	hypomethylated	Sphk1	45	59
chr11	116531973	116533973	0.23524	0.025133	hypermethylated	1810032O08Rik,Snord	13	19
chr11	116714328	116716328	-0.11005	0.0062486	hypomethylated	Mfsd11,Srsf2	9	16
chr11	116714408	116716408	-0.11005	0.0062486	hypomethylated	Mfsd11,Srsf2	9	16
chr11	116779176	116781176	-0.063771	0.00023523	hypomethylated	Mgat5b	61	66
chr11	117192028	117194028	-0.42028	0.0000137	stronglyhypometh	Sept9	11	10
chr11	117192626	117194626	-0.27245	0.00010524	hypomethylated	Sept9	11	12
chr11	117514602	117516602	-0.14616	6.85E-10	hypomethylated	Tnrc6c	59	62
chr11	117642610	117644610	-0.54435	0.011264	stronglyhypometh	Tmc6,Tmc8	8	7
chr11	117669980	117671980	-0.067931	0.022557	hypomethylated	Syngr2	23	23
chr11	117709550	117711550	-0.21565	0.017758	hypomethylated	Birc5	22	25
chr11	118109906	118111906	-0.45786	0.00000107	stronglyhypometh	Cyth1	13	13
chr11	118280366	118282366	-0.047164	0.047236	hypomethylated	Cant1	13	13
chr11	118337273	118339273	-0.32837	0.00012852	hypomethylated	Engase	13	14
chr11	118883342	118885342	-0.093128	0.010896	hypomethylated	Cbx2	39	39
chr11	118902227	118904227	-0.24718	0.0010726	hypomethylated	Cbx8	32	39
chr11	119215870	119217870	-0.25678	0.0095249	hypomethylated	Sgsh,Slc26a11	16	26
chr11	119216824	119218824	-0.22551	0.031004	hypomethylated	Sgsh,Slc26a11	14	20
chr11	119250785	119252785	-0.13788	0.015025	hypomethylated	Mir1932	34	34
chr11	119803405	119805405	-0.080185	0.000000164	hypomethylated	Baiap2	59	58
chr11	120209798	120211798	-0.14783	0.000000435	hypomethylated	0610009L18Rik,Actg1	75	76
chr11	120442934	120444934	0.41442	0.000042	stronglyhypermeth	Arhgdia	7	8

chr11	120468790	120470790	-0.81883	0.0000434	stronglyhypometh	Npb	5	10
chr11	120574266	120576266	-0.24759	0.035691	hypomethylated	Lrrc45,Stra13	6	6
chr11	120581781	120583781	-0.15805	0.0045729	hypomethylated	Rac3	35	31
chr11	120644585	120646585	-0.095859	0.046367	hypomethylated	Gps1,Rfng	75	81
chr11	120644828	120646828	-0.095859	0.046367	hypomethylated	Gps1,Rfng	75	81
chr11	120645518	120647518	-0.096084	0.045096	hypomethylated	Gps1,Rfng	59	57
chr11	120685861	120687861	-0.34511	0.0037322	stronglyhypometh	Fasn	16	16
chr11	121097549	121099549	0.091349	0.0067359	hypermethylated	Narf	18	17
chr11	121312262	121314262	-0.26589	0.00050416	hypomethylated	Tbcd	5	11
chr11	121534465	121536465	-0.5625	0.0000279	stronglyhypometh	B3gntl1	8	8
chr11	121690531	121692531	-0.36361	0.032417	stronglyhypometh	Ptchd3	4	9
chr12	3234790	3236790	0.10218	0.0047331	hypermethylated	1700012B15Rik	18	21
chr12	3309969	3311969	-0.26432	0.034747	hypomethylated	Rab10	8	6
chr12	3425883	3427883	-0.14802	0.00012483	hypomethylated	1110002L01Rik,Asxl2	34	38
chr12	3426747	3428747	-0.11874	0.0020703	hypomethylated	1110002L01Rik,Asxl2	28	32
chr12	3890743	3892743	-0.36557	0.000000442	stronglyhypometh	Dnmt3a	7	13
chr12	4132396	4134396	-0.068389	0.0011952	hypomethylated	Adcy3	32	38
chr12	4133152	4135152	-0.01125	0.0052173	hypomethylated	Adcy3	30	41
chr12	4823413	4825413	-0.043255	0.0025805	hypomethylated	0610009D07Rik	17	24
chr12	4914326	4916326	-0.17691	1.64E-08	hypomethylated	Ubxn2a	28	22
chr12	8319238	8321238	-0.1587	0.0083063	hypomethylated	Hs1bp3	13	14
chr12	8506791	8508791	-0.36012	0.0011002	stronglyhypometh	Rhob	5	9
chr12	8679939	8681939	0.016317	0.039936	hypermethylated	Pum2	58	60
chr12	8680064	8682064	0.016317	0.039936	hypermethylated	Pum2	58	60
chr12	8680486	8682486	0.016317	0.039936	hypermethylated	Pum2	58	60
chr12	8777201	8779201	-0.10504	0.0009127	hypomethylated	Sdc1	37	45
chr12	8953734	8955734	-0.072379	0.0031368	hypomethylated	Matn3	21	25
chr12	11157179	11159179	-0.18969	0.011594	hypomethylated	Kcns3	13	11
chr12	11443455	11445455	-0.37819	0.00024537	stronglyhypometh	4930511A02Rik,Vsnl1	9	12
chr12	15823591	15825591	-0.052137	0.039484	hypomethylated	Trib2	21	30
chr12	16807692	16809692	-0.093672	0.035846	hypomethylated	Greb1	7	7
chr12	16900783	16902783	-0.16251	0.00000028	hypomethylated	Rock2	36	46
chr12	17272400	17274400	-0.13676	0.00000625	hypomethylated	Pdia6	36	39
chr12	17550678	17552678	-0.10607	0.0033957	hypomethylated	Odc1	47	44
chr12	17696619	17698619	-0.15189	0.019497	hypomethylated	Hpcal1	26	35
chr12	25182445	25184445	-0.4359	0.00000451	stronglyhypometh	Taf1b	13	9
chr12	25256151	25258151	-0.059306	0.031108	hypomethylated	Grhl1	67	70
chr12	25335235	25337235	-0.14199	0.0009003	hypomethylated	Klf11	38	48
chr12	25780957	25782957	-0.23589	0.0040145	hypomethylated	Id2	10	11
chr12	27153079	27155079	-0.12101	0.0097224	hypomethylated	Cmpk2	20	25
chr12	30621900	30623900	-0.26028	0.0022787	hypomethylated	Pxdn	17	18
chr12	31268307	31270307	-0.36325	0.020785	stronglyhypometh	Tmem18	13	13
chr12	31757832	31759832	-0.32038	0.035316	hypomethylated	Fam110c	14	18
chr12	31949158	31951158	-0.48429	0.020007	stronglyhypometh	Lamb1	10	10
chr12	32184405	32186405	-0.18042	0.011458	hypomethylated	Cbll1	21	23
chr12	32319462	32321462	-0.15301	0.0051284	hypomethylated	Bcap29	21	21
chr12	32319523	32321523	-0.21421	0.0040562	hypomethylated	Bcap29	14	14
chr12	33637809	33639809	-0.11848	0.0066391	hypomethylated	4933406C10Rik,Sypl	36	31
chr12	33638050	33640050	-0.11848	0.0066391	hypomethylated	4933406C10Rik,Sypl	36	31
chr12	33832451	33834451	-0.018783	0.046215	hypomethylated	Atxn7l1,F730043M19F	11	18
chr12	34113488	34115488	-0.24849	0.0000941	hypomethylated	Twistnb	13	19
chr12	35730860	35732860	-0.10075	0.0032906	hypomethylated	Snx13	52	52
chr12	37107105	37109105	-0.25941	5.42E-13	hypomethylated	Ispd	17	21
chr12	40763963	40765963	-0.039147	0.037123	hypomethylated	Arl4a	32	47
chr12	41171639	41173639	-0.22818	8.1E-09	hypomethylated	Dock4,Zfp277	50	51
chr12	41172377	41174377	-0.20973	6.61E-08	hypomethylated	Dock4,Zfp277	46	51
chr12	45428871	45430871	-0.17783	0.0000124	hypomethylated	Nrcam	47	52

chr12	52477566	52479566	-0.10387	0.045008	hypomethylated	Scfd1	8	8
chr12	52792901	52794901	0.056662	0.010584	hypermethylated	Ap4s1,Strn3	20	17
chr12	52930523	52932523	-0.067248	0.00000614	hypomethylated	Hectd1	37	41
chr12	53072308	53074308	-0.726	2.52E-11	stronglyhypometh	Heatr5a	10	10
chr12	55757559	55759559	-0.25903	0.0000689	hypomethylated	Sptssa	15	16
chr12	56402623	56404623	-0.55783	0.0007063	stronglyhypometh	1110008L16Rik,Ppp2r3	9	8
chr12	56593634	56595634	-0.21187	0.0262	hypomethylated	Nfkbia	6	12
chr12	57714271	57716271	-0.33033	0.000000262	hypomethylated	Nkx2-9	16	19
chr12	57795625	57797625	-0.18473	0.020877	hypomethylated	Pax9	9	16
chr12	60113004	60115004	-0.24493	0.018784	hypomethylated	Gemin2,Sec23a	17	20
chr12	66017897	66019897	-0.067027	0.0000517	hypomethylated	Gm527	33	37
chr12	66174925	66176925	-0.045341	0.018748	hypomethylated	Fancm,Fkbp3	40	40
chr12	66175592	66177592	-0.066548	0.018289	hypomethylated	Fancm,Fkbp3	32	33
chr12	66273567	66275567	-0.12934	0.041638	hypomethylated	Mis18bp1	14	16
chr12	70297197	70299197	-0.02945	0.008205	hypomethylated	9330151L19Rik,Dnaaf2	60	66
chr12	70329177	70331177	-0.33667	0.0004914	stronglyhypometh	Pole2	5	4
chr12	70341818	70343818	0.17608	0.0010909	hypermethylated	Klhdc1	14	14
chr12	70472136	70474136	-0.17411	1.39E-12	hypomethylated	Arf6	64	83
chr12	70890330	70892330	-0.25555	0.026171	hypomethylated	4930512B01Rik,Cdkl1	13	16
chr12	70891694	70893694	-0.15253	0.017353	hypomethylated	4930512B01Rik,Cdkl1	11	12
chr12	71212912	71214912	0.31426	0.030436	hypermethylated	Nin	8	10
chr12	72115953	72117953	-0.05589	0.0054835	hypomethylated	3110056K07Rik,Arid4a	68	99
chr12	72237662	72239662	-0.60985	0.0018162	stronglyhypometh	2700049A03Rik,Timm9	3	7
chr12	73186109	73188109	0.081836	0.046628	hypermethylated	Jkamp,L3hypdh	7	14
chr12	73542235	73544235	-0.1215	0.043879	hypomethylated	Lrrc9	14	15
chr12	73861197	73863197	-0.05903	0.0011091	hypomethylated	Ppm1a	41	51
chr12	74214232	74216232	-0.55388	0.0039556	stronglyhypometh	Six4	11	12
chr12	74386841	74388841	-0.038526	0.0028686	hypomethylated	Slc38a6,Trmt5	15	25
chr12	75007853	75009853	-0.077494	0.00053557	hypomethylated	Hif1a	44	50
chr12	75064516	75066516	-0.21777	0.0078539	hypomethylated	Snapc1	21	21
chr12	75097747	75099747	-0.14408	0.0032435	hypomethylated	Syt16	19	20
chr12	76278319	76280319	-0.15579	0.0092953	hypomethylated	Kcnh5	12	11
chr12	76697187	76699187	-0.16015	0.005904	hypomethylated	Ppp2r5e	21	26
chr12	76836716	76838716	-0.12489	0.00000302	hypomethylated	Sgpp1	16	16
chr12	77424877	77426877	-0.17827	0.021819	hypomethylated	Akap5	18	19
chr12	77470451	77472451	0.033195	0.030845	hypermethylated	Zbtb1,Zbtb25	56	70
chr12	77504162	77506162	-0.36346	0.0070552	stronglyhypometh	Hspa2	10	16
chr12	77504356	77506356	-0.23708	0.023405	hypomethylated	Hspa2	14	20
chr12	79326641	79328641	0.074459	0.002357	hypermethylated	Gphn	41	48
chr12	79962058	79964058	-0.10652	0.0018855	hypomethylated	Atp6v1d,Eif2s1	21	28
chr12	79962625	79964625	-0.2649	0.0000344	hypomethylated	Atp6v1d,Eif2s1	9	12
chr12	80007925	80009925	-0.14607	0.033735	hypomethylated	Plek2	3	4
chr12	80129149	80131149	0.092312	0.00041732	hypermethylated	Plekhh1	35	35
chr12	81214000	81216000	-0.49266	4.18E-08	stronglyhypometh	Zfp36l1	26	25
chr12	81618976	81620976	-0.15673	0.00000757	hypomethylated	Galnt16	56	71
chr12	81861702	81863702	-0.19975	0.026636	hypomethylated	Ccdc177	3	3
chr12	82731355	82733355	-0.39499	0.000000163	stronglyhypometh	Ttc9	29	29
chr12	82882157	82884157	-0.12107	0.0000142	hypomethylated	Map3k9	16	21
chr12	84972183	84974183	-0.23627	0.00075808	hypomethylated	Rbm25	19	26
chr12	85028512	85030512	-0.34567	0.022437	stronglyhypometh	Psen1	27	27
chr12	85290557	85292557	-0.10385	0.00055302	hypomethylated	2410016O06Rik	50	61
chr12	85440603	85442603	-0.3489	0.0013301	stronglyhypometh	Acot6	5	8
chr12	85749017	85751017	-0.58436	0.0000762	stronglyhypometh	Ccdc176,Entpd5	8	17
chr12	85757149	85759149	-0.531	0.00000309	stronglyhypometh	Rnf113a2	8	8
chr12	85909801	85911801	-0.24592	0.00020317	hypomethylated	Vsx2	25	25
chr12	86113219	86115219	-0.29835	0.018875	hypomethylated	Isca2,Npc2	14	12
chr12	86114062	86116062	-0.71	0.0015929	stronglyhypometh	Isca2,Npc2	6	5

chr12	87086488	87088488	-0.27698	0.0018008	hypomethylated	Mfsd7c	14	17
chr12	87164899	87166899	-0.22051	0.00047426	hypomethylated	0610007P14Rik,Ttll5	19	20
chr12	87165495	87167495	-0.125	0.004511	hypomethylated	0610007P14Rik,Ttll5	13	13
chr12	87419991	87421991	-0.20458	0.0066319	hypomethylated	Tgfb3	6	6
chr12	87581827	87583827	-0.51533	0.011705	stronglyhypometh	Gpatch2l	7	18
chr12	88286992	88288992	-0.47791	0.000000119	stronglyhypometh	2310044G17Rik	19	28
chr12	88287292	88289292	-0.47791	0.000000119	stronglyhypometh	2310044G17Rik	19	28
chr12	88329626	88331626	0.051598	0.038954	hypermethylated	Zdhhc22	21	21
chr12	89598244	89600244	-0.58257	3.52E-08	stronglyhypometh	Adck1	13	13
chr12	96929435	96931435	-0.36035	1.91E-09	stronglyhypometh	Flrt2	17	20
chr12	101121652	101123652	-0.12251	0.00028896	hypomethylated	Efcab11,Tdp1	14	20
chr12	101121724	101123724	-0.12591	0.00026454	hypomethylated	Efcab11,Tdp1	14	22
chr12	101436750	101438750	-0.054191	0.00087588	hypomethylated	Calm1	36	49
chr12	102267193	102269193	-0.43556	0.00000383	stronglyhypometh	Ccdc88c	18	15
chr12	103196453	103198453	0.067788	0.03247	hypermethylated	Atxn3	10	10
chr12	103366628	103368628	-0.27905	9.77E-09	hypomethylated	Slc24a4	28	35
chr12	103706343	103708343	-0.099487	0.00035206	hypomethylated	Golga5	46	48
chr12	103707119	103709119	-0.10082	0.00049981	hypomethylated	Golga5	45	47
chr12	103792178	103794178	-0.16443	0.00058827	hypomethylated	Chga	12	14
chr12	103980969	103982969	-0.070322	0.00056824	hypomethylated	Moap1,Tmem251	29	33
chr12	103995184	103997184	-0.16849	0.0000539	hypomethylated	AK010878,Gm20604,U	34	38
chr12	103996020	103998020	-0.30981	0.000000693	hypomethylated	AK010878,Gm20604,U	23	25
chr12	105711446	105713446	-0.67328	0.00061464	stronglyhypometh	Gsc	5	9
chr12	106236887	106238887	-0.37083	0.04421	stronglyhypometh	Syne3	3	3
chr12	106269559	106271559	-0.13009	0.0010485	hypomethylated	Glrx5,Mir3069,Scarna1	39	41
chr12	106922561	106924561	-0.21785	0.00000135	hypomethylated	Atg2b,Gskip	46	53
chr12	106923451	106925451	-0.43566	0.0000058	stronglyhypometh	Atg2b,Gskip	20	23
chr12	107685876	107687876	-0.40684	0.0034213	stronglyhypometh	1700121N20Rik	5	9
chr12	109241624	109243624	-0.090442	0.0051782	hypomethylated	Bcl11b	34	42
chr12	109513627	109515627	-0.12627	0.011185	hypomethylated	Ccdc85c	30	36
chr12	109571590	109573590	0.05974	0.0053667	hypermethylated	Cyp46a1	23	29
chr12	109791929	109793929	-0.0039951	0.03077	hypomethylated	Evl,Gm16596	66	73
chr12	109940516	109942516	-0.93557	0.0000443	stronglyhypometh	Degs2	5	7
chr12	110690032	110692032	-0.059508	0.000069	hypomethylated	Dlk1	25	25
chr12	110690664	110692664	-0.088531	0.0000637	hypomethylated	Dlk1	26	27
chr12	111516278	111518278	-0.042434	0.024573	hypomethylated	Dio3,Dio3os,Mir1247	59	64
chr12	111516339	111518339	-0.042434	0.024573	hypomethylated	Dio3,Dio3os,Mir1247	59	64
chr12	111684388	111686388	-0.11854	0.000000482	hypomethylated	Ppp2r5c	25	49
chr12	111828583	111830583	-0.4	0.00049046	stronglyhypometh	B930059L03Rik	10	17
chr12	111975158	111977158	-0.30866	2.69E-08	hypomethylated	Wdr20a	26	25
chr12	112217231	112219231	-0.21706	0.00052374	hypomethylated	Ankrd9	18	18
chr12	112403758	112405758	-0.099877	0.011206	hypomethylated	Traf3	48	48
chr12	112508321	112510321	-0.33256	0.0031177	hypomethylated	Amn	10	14
chr12	112679871	112681871	-0.19074	0.032023	hypomethylated	Tnfaip2	2	18
chr12	112775311	112777311	-0.10469	0.03574	hypomethylated	Eif5	50	64
chr12	112811720	112813720	-0.11324	0.013853	hypomethylated	2810029C07Rik,Mark3	26	33
chr12	112910549	112912549	-0.375	0.014021	stronglyhypometh	Ckb	4	7
chr12	112950479	112952479	-0.098117	0.042361	hypomethylated	Apopt1,Bag5	58	66
chr12	113383418	113385418	-0.090713	0.00091955	hypomethylated	Kif26a	37	39
chr12	113959384	113961384	0.03212	0.034945	hypermethylated	Cep170b	56	56
chr12	114393631	114395631	-0.07813	0.00013187	hypomethylated	4930427A07Rik	36	35
chr12	117284934	117286934	-0.1375	0.010572	hypomethylated	Zfp386	10	10
chr12	117315195	117317195	0.16617	0.0033928	hypermethylated	Vipr2	17	18
chr12	119539913	119541913	-0.099513	0.00063884	hypomethylated	Sp4	30	43
chr13	3536320	3538320	-0.29356	1.67E-10	hypomethylated	Gdi2	42	38
chr13	3632321	3634321	-0.23102	0.016795	hypomethylated	Asb13	23	29
chr13	3632323	3634323	-0.23102	0.016795	hypomethylated	Asb13	23	29

chr13	12350267	12352267	-0.41882	0.00000531	stronglyhypometh	Mtr	17	17
chr13	12486641	12488641	-0.20527	0.00067401	hypomethylated	Heatr1	11	12
chr13	12742662	12744662	-0.115	0.027023	hypomethylated	Gpr137b-ps	5	5
chr13	13485891	13487891	-0.2	0.00082441	hypomethylated	Gpr137b	5	5
chr13	13681675	13683675	-0.14316	0.0095948	hypomethylated	Lyst	29	26
chr13	14045940	14047940	-0.64068	2.99E-18	stronglyhypometh	B3galnt2	24	33
chr13	14155058	14157058	-0.15571	5.61E-13	hypomethylated	Arid4b,Ggps1	65	77
chr13	14155668	14157668	-0.13032	1.3E-09	hypomethylated	Arid4b,Ggps1	54	62
chr13	14704508	14706508	-0.16667	0.017857	hypomethylated	Mrpl32,Psma2	3	5
chr13	15554555	15556555	-0.15138	3.53E-08	hypomethylated	Gli3	77	79
chr13	18036051	18038051	0.26147	0.0053575	hypermethylated	Rala	12	21
chr13	19039239	19041239	-0.12076	0.00049461	hypomethylated	Amph	33	34
chr13	21270827	21272827	-0.074914	0.010604	hypomethylated	Trim27	59	66
chr13	23461071	23463071	-0.1544	0.026955	hypomethylated	Zfp322a	9	14
chr13	23621912	23623912	-0.55417	0.029349	stronglyhypometh	Hist1h4h	6	4
chr13	23853026	23855026	0.06053	0.035668	hypermethylated	Hist1h1a,Hist1h3a,Hist	24	28
chr13	24083083	24085083	-0.55105	0.0035592	stronglyhypometh	Scgn	4	4
chr13	24892525	24894525	-0.11963	0.0090765	hypomethylated	BC005537	33	43
chr13	30750964	30752964	-0.14854	0.044205	hypomethylated	Dusp22	27	27
chr13	30750969	30752969	-0.14854	0.044205	hypomethylated	Dusp22	27	27
chr13	30840126	30842126	-0.52374	0.00000551	stronglyhypometh	Irf4	11	11
chr13	31897514	31899514	-0.097722	0.00044122	hypomethylated	Foxc1	59	77
chr13	32430413	32432413	-0.053946	0.021816	hypomethylated	Gmds	26	26
chr13	32892898	32894898	-0.13792	0.00080867	hypomethylated	Wrnip1	23	49
chr13	34169877	34171877	-0.089459	0.011372	hypomethylated	Tubb2a	11	15
chr13	34744550	34746550	0.054379	0.033928	hypermethylated	Pxdc1	20	28
chr13	36058774	36060774	-0.46305	4.33E-09	stronglyhypometh	Ppp1r3g	12	26
chr13	37916906	37918906	-0.27322	0.032288	hypomethylated	Rreb1	38	39
chr13	37917780	37919780	-0.13288	0.010334	hypomethylated	Rreb1	57	74
chr13	37918261	37920261	-0.079231	0.013537	hypomethylated	Rreb1	53	65
chr13	38128163	38130163	-0.21191	0.0000268	hypomethylated	Cage1,Riok1	29	28
chr13	38436584	38438584	-0.049327	0.0011771	hypomethylated	Bmp6	37	51
chr13	40825812	40827812	-0.16894	0.0022029	hypomethylated	Tfap2a	18	24
chr13	40829192	40831192	-0.066713	0.0066553	hypomethylated	Tfap2a	10	10
chr13	40981534	40983534	-0.65476	1.07E-08	stronglyhypometh	Gcnt2	10	14
chr13	41095378	41097378	-0.18082	0.0010367	hypomethylated	Pak1ip1	18	24
chr13	41110618	41112618	-0.18738	0.00089659	hypomethylated	Tmem14c	22	22
chr13	41344212	41346212	-0.10117	0.0072085	hypomethylated	Smim13	18	21
chr13	43266728	43268728	-0.22101	0.000036	hypomethylated	Tbc1d7	18	19
chr13	44825271	44827271	-0.01842	0.021728	hypomethylated	Jarid2	39	44
chr13	45601837	45603837	-0.29618	0.010498	hypomethylated	Gmpr	17	21
chr13	46060345	46062345	-0.71949	3.4E-09	stronglyhypometh	Atxn1	15	21
chr13	46512668	46514668	-0.095328	0.048844	hypomethylated	Rbm24	20	30
chr13	46596271	46598271	-0.20653	0.028139	hypomethylated	Cap2	22	30
chr13	47025087	47027087	-0.026895	0.031162	hypomethylated	Kif13a	30	31
chr13	47288371	47290371	-0.25893	0.039917	hypomethylated	Rnf144b	7	18
chr13	48757404	48759404	0.089842	0.020455	hypermethylated	Barx1	34	38
chr13	48966254	48968254	-0.080407	0.028149	hypomethylated	Phf2	24	28
chr13	49062480	49064480	-0.088509	0.00079608	hypomethylated	Fam120a,Fam120aos	99	96
chr13	49243383	49245383	-0.048348	0.0479	hypomethylated	Wnk2	9	10
chr13	49311395	49313395	-0.47398	0.0017528	stronglyhypometh	1110007C09Rik	6	10
chr13	49435917	49437917	-0.29102	0.04668	hypomethylated	Bicd2	17	21
chr13	49747718	49749718	-0.17465	0.00029372	hypomethylated	Cenpp,Nol8	20	21
chr13	49748100	49750100	-0.20286	0.00036559	hypomethylated	Cenpp,Nol8	15	15
chr13	51265393	51267393	-0.36827	0.00087056	stronglyhypometh	Nxnl2	22	20
chr13	51662453	51664453	0.080038	0.0032914	hypermethylated	Shc3	30	30
chr13	51889013	51891013	-0.5	0.00000303	stronglyhypometh	Sema4d	5	5

chr13	51941043	51943043	-0.17829	0.017625	hypomethylated	Gadd45g	28	40
chr13	53076408	53078408	-0.078199	0.0010995	hypomethylated	Nfil3	38	38
chr13	54151027	54153027	-0.29167	0.024104	hypomethylated	Drd1a	3	3
chr13	54690591	54692591	-0.058087	0.029444	hypomethylated	Higd2a,Nop16	18	18
chr13	54851030	54853030	-0.098151	0.015776	hypomethylated	Gprin1	26	28
chr13	54889765	54891765	-0.1633	0.0000101	hypomethylated	Tspan17	18	22
chr13	55049792	55051792	-0.12211	0.0038321	hypomethylated	Unc5a	61	64
chr13	55310142	55312142	-0.14728	0.035083	hypomethylated	Nsd1	38	45
chr13	55422415	55424415	-0.3181	0.0033418	hypomethylated	Prelid1,Rab24	21	23
chr13	55423341	55425341	-0.54267	5.63E-08	stronglyhypometh	Prelid1,Rab24	13	15
chr13	55545694	55547694	-0.16482	0.0000145	hypomethylated	Grk6	39	39
chr13	55671336	55673336	-0.14085	0.0056197	hypomethylated	Fam193b	25	33
chr13	55700471	55702471	0.12078	0.0027097	hypermethylated	B4galt7	11	11
chr13	55735387	55737387	-0.3165	0.0000087	hypomethylated	Ddx46	37	34
chr13	55827728	55829728	-0.05345	0.0066506	hypomethylated	Pcbd2	29	30
chr13	56538715	56540715	-0.4903	0.001738	stronglyhypometh	Slc25a48	13	25
chr13	56804028	56806028	-0.075987	0.044837	hypomethylated	Smad5	54	61
chr13	58258009	58260009	-0.60472	0.00096345	stronglyhypometh	Idnk	6	8
chr13	58375549	58377549	-0.15412	0.0018824	hypomethylated	Gkap1	29	35
chr13	58503877	58505877	-0.14255	0.037588	hypomethylated	Hnrnpk,Rmi1	18	32
chr13	59685693	59687693	-0.08202	0.00085873	hypomethylated	A230056J06Rik,Naa35	36	48
chr13	59870326	59872326	-0.32395	0.00000186	hypomethylated	Etohd2,Isca1	24	28
chr13	62568172	62570172	-0.46032	0.00066775	stronglyhypometh	Zfp935	4	4
chr13	64254507	64256507	-0.2824	0.00030737	hypomethylated	Zfp367	14	14
chr13	67032765	67034765	-0.25741	2.36E-14	hypomethylated	Mterfd1,Ptdss1	38	38
chr13	67034008	67036008	-0.32236	2.92E-14	hypomethylated	Mterfd1,Ptdss1	29	29
chr13	69672742	69674742	-0.10833	0.0065178	hypomethylated	Papd7	85	93
chr13	69947759	69949759	-0.125	0.0040781	hypomethylated	Med10	11	12
chr13	72765425	72767425	-0.064097	0.019514	hypomethylated	Gm20554,Irx2	47	70
chr13	72766012	72768012	-0.074472	0.017398	hypomethylated	Gm20554,Irx2	42	59
chr13	73396944	73398944	-0.090289	0.00020285	hypomethylated	Irx4	29	40
chr13	75226434	75228434	-0.23644	0.043626	hypomethylated	Pcsk1	8	12
chr13	76521408	76523408	0.041915	0.037593	hypermethylated	Mctp1	26	38
chr13	78338243	78340243	0.051014	0.0078364	hypermethylated	A830082K12Rik,Nr2f1	31	31
chr13	81849415	81851415	-0.034314	0.022405	hypomethylated	Mblac2,Polr3g	34	41
chr13	81850012	81852012	-0.10114	0.013382	hypomethylated	Mblac2,Polr3g	21	28
chr13	85328081	85330081	-0.50317	0.043686	stronglyhypometh	Ccnh	5	5
chr13	90228271	90230271	-0.17303	0.00022341	hypomethylated	Tmem167,Xrcc4	16	32
chr13	91363592	91365592	0.14605	0.00041682	hypermethylated	Atg10	9	9
chr13	91599701	91601701	-0.11912	0.00026422	hypomethylated	Ssbp2	79	103
chr13	91880125	91882125	-0.36768	3.58E-08	stronglyhypometh	4833422C13Rik,Acot1:	12	19
chr13	93613741	93615741	-0.66667	0.00064023	stronglyhypometh	Mtx3	6	11
chr13	94073449	94075449	-0.12007	0.00000629	hypomethylated	Homer1	47	62
chr13	94269644	94271644	-0.5631	0.000000101	stronglyhypometh	Jmy	14	20
chr13	94444257	94446257	-0.45014	0.0058164	stronglyhypometh	Bhmt2,Dmgdh	8	12
chr13	94540633	94542633	-0.010719	0.023909	hypomethylated	Arsb	26	28
chr13	94826750	94828750	-0.11056	2.12E-09	hypomethylated	Lhfpl2	34	39
chr13	95055236	95057236	-0.072346	0.00091018	hypomethylated	Scamp1	29	30
chr13	95127914	95129914	-0.23706	0.00096642	hypomethylated	Ap3b1,Gm9776	31	30
chr13	95557897	95559897	-0.28667	0.00045802	hypomethylated	Tbca	16	26
chr13	96094191	96096191	-0.29895	0.00010959	hypomethylated	Zbed3	16	24
chr13	96214786	96216786	0.51	0.022676	stronglyhypermeth	Crhbp	5	5
chr13	96295195	96297195	-0.15422	0.0031652	hypomethylated	F2rl1	6	6
chr13	98010059	98012059	-0.043909	0.019129	hypomethylated	Enc1	44	47
chr13	99464786	99466786	-0.08068	0.019386	hypomethylated	Tmem171	12	16
chr13	100874025	100876025	0.098216	0.01759	hypermethylated	Bdp1	14	15
chr13	101420297	101422297	-0.078415	0.0015574	hypomethylated	Rad17,Taf9	60	66

chr13	101420561	101422561	-0.079854	0.0016022	hypomethylated	Rad17,Taf9	58	64
chr13	101421014	101423014	-0.10124	0.00000196	hypomethylated	Rad17,Taf9	43	49
chr13	101603382	101605382	-0.85	0.00000329	stronglyhypometh	Slc30a5	4	4
chr13	102538172	102540172	-0.24629	0.00014938	hypomethylated	Pik3r1	14	26
chr13	104710594	104712594	-0.057159	0.0000437	hypomethylated	Erbb2ip	67	78
chr13	104898869	104900869	-0.25229	0.027993	hypomethylated	Nln,Sgtb	13	23
chr13	104899694	104901694	-0.24744	0.038539	hypomethylated	Nln,Sgtb	7	17
chr13	104968546	104970546	-0.66	0.0022658	stronglyhypometh	Trappc13,Trim23	5	5
chr13	106083094	106085094	-0.20295	0.00082565	hypomethylated	Rnf180	26	29
chr13	107736208	107738208	-0.32873	0.0049575	hypomethylated	Dimt1	12	15
chr13	107812194	107814194	0.035756	0.0041677	hypermethylated	3830408C21Rik,Kif2a	53	61
chr13	108680258	108682258	-0.01341	0.039521	hypomethylated	Zswim6	52	56
chr13	108833667	108835667	-0.49745	2.38E-10	stronglyhypometh	Smim15	21	23
chr13	108948819	108950819	-0.18688	0.029579	hypomethylated	Ercc8,Ndufaf2	11	14
chr13	109003597	109005597	-0.12825	0.0000736	hypomethylated	Elovl7	25	40
chr13	109105530	109107530	-0.23159	7.34E-09	hypomethylated	Depdc1b	33	47
chr13	112280249	112282249	-0.096222	0.00095778	hypomethylated	Gpbp1	49	48
chr13	113589999	113591999	-0.35887	2.64E-15	stronglyhypometh	Ppap2a	25	29
chr13	113590130	113592130	-0.35887	2.64E-15	stronglyhypometh	Ppap2a	25	29
chr13	113717000	113719000	-0.18244	0.036869	hypomethylated	Dhx29,Skiv2l2	20	26
chr13	113777009	113779009	-0.10582	0.000000627	hypomethylated	Ccno	28	37
chr13	113783075	113785075	-0.11529	0.01432	hypomethylated	Mcidas	40	44
chr13	114583715	114585715	-0.16992	0.0090372	hypomethylated	Arl15	31	37
chr13	115248938	115250938	-0.15099	0.00076051	hypomethylated	Fst	35	40
chr13	115880366	115882366	-0.57128	0.000000559	stronglyhypometh	Pelo	7	8
chr13	119502505	119504505	-0.22563	0.00000178	hypomethylated	Fgf10	17	20
chr13	120216406	120218406	-0.21318	0.000000402	hypomethylated	Paip1	64	72
chr13	120216713	120218713	-0.21318	0.000000402	hypomethylated	Paip1	64	72
chr13	120250565	120252565	-0.22374	0.0042126	hypomethylated	4833420G17Rik	9	15
chr13	120275845	120277845	-0.2342	0.00085072	hypomethylated	3110070M22Rik,Gm71	20	35
chr13	120276666	120278666	-0.18764	0.0065341	hypomethylated	3110070M22Rik,Gm71	20	36
chr14	8649470	8651470	-0.44411	0.000000173	stronglyhypometh	Flnb	27	22
chr14	9045661	9047661	0.037177	0.046694	hypermethylated	Kctd6	47	60
chr14	13115722	13117722	-0.13182	0.0011025	hypomethylated	3830406C13Rik	11	21
chr14	13178379	13180379	-0.17795	0.0053888	hypomethylated	Fezf2	17	12
chr14	14117152	14119152	-0.48665	0.036667	stronglyhypometh	Gm5087,Synpr	3	5
chr14	14844004	14846004	-0.14832	1.4E-11	hypomethylated	Atxn7	21	42
chr14	14953418	14955418	-0.41154	0.0071157	stronglyhypometh	Psmd6	2	2
chr14	15534538	15536538	-0.030219	0.0017236	hypomethylated	Slc4a7	65	66
chr14	19103500	19105500	-0.1525	0.00035489	hypomethylated	Nkiras1,Rpl15	24	25
chr14	20643045	20645045	-0.275	0.00025092	hypomethylated	2700060E02Rik	5	8
chr14	21364514	21366514	0.14147	0.00000537	hypermethylated	1810062O18Rik,Ppp3c	41	43
chr14	21492542	21494542	0.20704	0.0020665	hypermethylated	Sec24c	18	18
chr14	21747654	21749654	-0.17929	0.00033167	hypomethylated	Vcl	39	44
chr14	21870854	21872854	-0.29083	0.020711	hypomethylated	Adk,Ap3m1	20	22
chr14	22567844	22569844	-0.1519	0.028838	hypomethylated	Dusp13,Samd8	5	5
chr14	22649782	22651782	-0.27624	0.000000742	hypomethylated	Vdac2	14	23
chr14	22837933	22839933	-0.0013239	0.0046239	hypomethylated	1700112E06Rik	17	17
chr14	25308902	25310902	-0.69278	5.76E-11	stronglyhypometh	Rps24	11	16
chr14	26277777	26279777	-0.12495	0.00029601	hypomethylated	Zmiz1	55	55
chr14	26512655	26514655	-0.22838	0.00074871	hypomethylated	Ppif	27	32
chr14	26660640	26662640	-0.11395	0.017263	hypomethylated	Anxa11	23	31
chr14	27353226	27355226	-0.23319	0.0035988	hypomethylated	Slmap	36	34
chr14	27851186	27853186	-0.027901	0.002562	hypomethylated	Il17rd	34	37
chr14	28241032	28243032	0.072054	0.00010371	hypermethylated	D14Abb1e	40	40
chr14	28434627	28436627	-0.42263	0.00010123	stronglyhypometh	Erc2	14	34
chr14	29317658	29319658	0.1171	0.016133	hypermethylated	Wnt5a	31	33

chr14	30780565	30782565	-0.13805	0.0000547	hypomethylated	Selk	23	23
chr14	30821185	30823185	-0.15929	0.000000594	hypomethylated	Chdh,Il17rb	26	26
chr14	30822082	30824082	-0.083124	0.0070564	hypomethylated	Chdh,Il17rb	18	18
chr14	31166672	31168672	-0.030093	0.010159	hypomethylated	Cacna1d	41	43
chr14	31291750	31293750	-0.2174	0.017668	hypomethylated	Dcp1a	15	17
chr14	31466560	31468560	-0.10102	0.012384	hypomethylated	Rft1	13	14
chr14	31527352	31529352	-0.11997	0.01588	hypomethylated	Sfmbt1	32	58
chr14	31527800	31529800	-0.10542	0.016161	hypomethylated	Sfmbt1	42	78
chr14	31637779	31639779	-0.096621	0.00000067	hypomethylated	Tmem110	22	22
chr14	31763673	31765673	-0.35495	0.0063969	stronglyhypometh	Nek4	13	17
chr14	31813578	31815578	-0.20741	0.002278	hypomethylated	Glt8d1,Spcs1	32	34
chr14	31813618	31815618	-0.20741	0.002278	hypomethylated	Glt8d1,Spcs1	32	34
chr14	31814852	31816852	-0.54029	0.0000475	stronglyhypometh	Glt8d1,Spcs1	11	11
chr14	32307264	32309264	-0.24804	1.65E-12	hypomethylated	Eaf1,Mettl6	21	21
chr14	32308163	32310163	-0.25076	1.01E-08	hypomethylated	Eaf1,Mettl6	19	19
chr14	32453242	32455242	-0.36801	0.0000105	stronglyhypometh	Btd,Hacl1	12	13
chr14	32454151	32456151	-0.36742	0.0000119	stronglyhypometh	Btd,Hacl1	12	12
chr14	32898851	32900851	0.28636	0.013165	hypermethylated	Dph3,Oxnad1	5	11
chr14	32977977	32979977	-0.35193	0.00044467	stronglyhypometh	Ncoa4	11	11
chr14	33014156	33016156	-0.0024334	8.44E-08	hypomethylated	Parg,Timm23	29	38
chr14	33015077	33017077	-0.15174	0.000000143	hypomethylated	Parg,Timm23	24	27
chr14	33278036	33280036	-0.15528	0.0032452	hypomethylated	Chat,Slc18a3	20	20
chr14	33325706	33327706	-0.040103	0.0018185	hypomethylated	Ercc6	20	22
chr14	34260344	34262344	-0.26036	0.016089	hypomethylated	Mapk8	18	19
chr14	34965605	34967605	-0.33125	0.0047603	hypomethylated	Ppyr1	4	4
chr14	35122912	35124912	-0.03252	0.01494	hypomethylated	Fam35a,Glud1	62	78
chr14	35315732	35317732	-0.21537	0.00000346	hypomethylated	Bmpr1a	38	47
chr14	35486113	35488113	-0.15158	0.0066945	hypomethylated	Wapal	42	62
chr14	47450918	47452918	-0.33521	0.00012374	stronglyhypometh	Cgrrf1	13	21
chr14	47809077	47811077	-0.26702	0.0084869	hypomethylated	Gch1	15	15
chr14	47895817	47897817	-0.14695	0.00055644	hypomethylated	Socs4,Wdhd1	15	32
chr14	47896532	47898532	-0.29657	0.0000381	hypomethylated	Socs4,Wdhd1	11	22
chr14	47916988	47918988	-0.20989	0.00000036	hypomethylated	Mapk1ip1l	36	39
chr14	48739543	48741543	-0.18687	5.41E-11	hypomethylated	Peli2	37	48
chr14	48817390	48819390	-0.24873	0.048293	hypomethylated	Gm6498	5	5
chr14	49065026	49067026	-0.20091	0.0000148	hypomethylated	6720456H20Rik	33	33
chr14	49685169	49687169	-0.1371	4.7E-09	hypomethylated	Ap5m1,Exoc5	49	47
chr14	49790901	49792901	-0.12854	0.00066953	hypomethylated	Naa30	50	58
chr14	51543695	51545695	-0.33237	0.00055903	hypomethylated	Apex1,Osgep	7	17
chr14	52503516	52505516	-0.33851	0.00063733	stronglyhypometh	Mettl17	8	13
chr14	52533163	52535163	-0.45544	0.0069408	stronglyhypometh	Ndrg2	10	11
chr14	52603507	52605507	-0.042157	0.0000535	hypomethylated	Arhgef40	24	29
chr14	52816914	52818914	-0.11756	0.001755	hypomethylated	Supt16	6	16
chr14	52897820	52899820	-0.41861	7.63E-13	stronglyhypometh	Rab2b,Tox4	13	20
chr14	52899070	52901070	-0.42966	1.49E-08	stronglyhypometh	Rab2b,Tox4	6	9
chr14	54979524	54981524	-0.49902	0.00000244	stronglyhypometh	Oxa1l	10	13
chr14	55224745	55226745	-0.18785	0.045781	hypomethylated	4931414P19Rik	18	18
chr14	55235622	55237622	-0.30086	8.78E-08	hypomethylated	Mir686,Psmb5	13	13
chr14	55236832	55238832	-0.36757	0.00045682	stronglyhypometh	Psmb5	6	6
chr14	55304007	55306007	-0.27911	0.0000112	hypomethylated	1700123O20Rik,Acin1	28	26
chr14	55501261	55503261	-0.3652	0.00026343	stronglyhypometh	Bcl2l2	13	13
chr14	56177865	56179865	-0.35128	0.0000409	stronglyhypometh	Dcaf11	22	25
chr14	56178759	56180759	-0.35233	0.00047053	stronglyhypometh	Dcaf11	19	22
chr14	56209626	56211626	-0.33925	0.000000102	stronglyhypometh	Psme2,Rnf31	28	26
chr14	56209858	56211858	-0.38882	8.92E-09	stronglyhypometh	Psme2,Rnf31	25	23
chr14	56209938	56211938	-0.38882	8.92E-09	stronglyhypometh	Psme2,Rnf31	25	23
chr14	56331925	56333925	0.19716	0.01108	hypermethylated	Tgm1	8	11

chr14	56363529	56365529	-0.098089	0.042313	hypomethylated	Dhrs1,Nop9	22	29
chr14	56471951	56473951	-0.15467	0.022073	hypomethylated	Nynrin	6	7
chr14	57193455	57195455	-0.28788	0.0023889	hypomethylated	Parp4	11	8
chr14	57430553	57432553	-0.20869	0.0000225	hypomethylated	Zmym5	30	29
chr14	58142665	58144665	-0.0798	2.09E-10	hypomethylated	Il17d	52	53
chr14	58509099	58511099	-0.091852	0.0000896	hypomethylated	Zdhhc20	19	20
chr14	59819064	59821064	-0.052981	0.027804	hypomethylated	Rcbtb1	48	52
chr14	60058817	60060817	-0.15324	0.01732	hypomethylated	Cab39l,Setdb2	14	19
chr14	60059714	60061714	-0.21242	0.015228	hypomethylated	Cab39l,Setdb2	10	15
chr14	60243117	60245117	0.14317	0.0049171	hypermethylated	Shisa2	29	34
chr14	60870215	60872215	-0.46587	0.0000551	stronglyhypometh	Nupl1	5	18
chr14	61252565	61254565	-0.30606	0.048203	hypomethylated	Spata13	5	5
chr14	61402402	61404402	0.080739	0.0026864	hypermethylated	Mipep	20	18
chr14	61756293	61758293	-0.21465	0.00063269	hypomethylated	Sacs	6	6
chr14	62301210	62303210	0.096664	0.010091	hypermethylated	Dleu2	21	18
chr14	63796630	63798630	-0.29805	0.0011299	hypomethylated	Fdft1	16	20
chr14	63812362	63814362	-0.58333	0.00000192	stronglyhypometh	Neil2	9	9
chr14	63864097	63866097	-0.39575	3.45E-08	stronglyhypometh	Gata4	35	29
chr14	64054230	64056230	-0.9	7.26E-11	stronglyhypometh	Fam167a	10	10
chr14	65270367	65272367	-0.085107	0.00063783	hypomethylated	Kif13b	19	24
chr14	65716943	65718943	-0.3913	1.29E-10	stronglyhypometh	Extl3	10	10
chr14	66452806	66454806	-0.46491	0.0044509	stronglyhypometh	Esco2	3	3
chr14	66455138	66457138	-0.085397	0.0029682	hypomethylated	Ccdc25	23	21
chr14	66586319	66588319	-0.32977	0.0000207	hypomethylated	Clu	17	12
chr14	66899889	66901889	-0.14167	0.047297	hypomethylated	Ptk2b	2	2
chr14	66914861	66916861	-0.17305	0.000000107	hypomethylated	1700001G11Rik,Trim3	29	32
chr14	67487437	67489437	-0.084848	0.013468	hypomethylated	Dpysl2,Gm5464	11	21
chr14	67851128	67853128	-0.18587	0.014536	hypomethylated	Ebf2	27	28
chr14	68551629	68553629	-0.22085	1.04E-10	hypomethylated	Dock5	16	20
chr14	69954207	69956207	-0.15392	1.15E-18	hypomethylated	Entpd4,Gm16677	35	36
chr14	70008282	70010282	-0.25367	0.000077	hypomethylated	Loxl2	26	23
chr14	70132377	70134377	-0.1216	0.01959	hypomethylated	Chmp7	16	17
chr14	70166278	70168278	-0.31979	1.62E-13	hypomethylated	Tnfrsf10b	14	16
chr14	70498951	70500951	-0.12921	0.0065316	hypomethylated	Bin3	18	22
chr14	70689256	70691256	-0.24231	3.82E-09	hypomethylated	Ppp3cc	13	13
chr14	70751231	70753231	-0.48174	6.01E-08	stronglyhypometh	Slc39a14	12	11
chr14	70856323	70858323	-0.26872	0.0047222	hypomethylated	Phyhip	10	11
chr14	70929627	70931627	-0.20011	2.22E-11	hypomethylated	Lgi3	12	17
chr14	70999642	71001642	-0.50714	4.43E-10	stronglyhypometh	Fam160b2	6	6
chr14	71166435	71168435	-0.029867	0.036608	hypomethylated	Xpo7	21	24
chr14	71288936	71290936	-0.028687	0.0019583	hypomethylated	Gfra2	16	25
chr14	73541316	73543316	-0.17717	0.0000314	hypomethylated	Rcbtb2	46	41
chr14	73541591	73543591	-0.17717	0.0000314	hypomethylated	Rcbtb2	46	41
chr14	73908855	73910855	-0.19178	0.00027867	hypomethylated	Med4	5	12
chr14	75347684	75349684	-0.63258	0.0000227	stronglyhypometh	Lrch1	5	8
chr14	75530690	75532690	-0.10865	0.020256	hypomethylated	Lcp1,Lrrc63	8	12
chr14	75683179	75685179	-0.31772	0.00070134	hypomethylated	Zc3h13	17	21
chr14	76509697	76511697	-0.4733	0.000000128	stronglyhypometh	1200011I18Rik,Nufip1	28	40
chr14	76510622	76512622	-0.56734	4E-13	stronglyhypometh	1200011I18Rik,Nufip1	13	20
chr14	77555622	77557622	-0.05431	0.0085609	hypomethylated	Enox1	51	65
chr14	78274724	78276724	-0.082639	0.018905	hypomethylated	Dnajc15	8	8
chr14	79124896	79126896	-0.34237	0.015422	stronglyhypometh	Dgkh	14	16
chr14	79247984	79249984	-0.26222	0.00013962	hypomethylated	Vwa8	17	17
chr14	79986497	79988497	-0.32635	1.74E-09	hypomethylated	Sugt1	16	16
chr14	84842369	84844369	-0.23443	0.043838	hypomethylated	Pcdh17	4	8
chr14	94287951	94289951	-0.30635	0.022516	hypomethylated	Pcdh9	4	6
chr14	99444595	99446595	-0.074546	0.024709	hypomethylated	Bora,Mzt1	69	67

chr14	99497651	99499651	-0.10863	0.0075598	hypomethylated	Dis3,Pibf1	25	31
chr14	102128144	102130144	-0.1918	0.0088203	hypomethylated	Lmo7	25	29
chr14	104048459	104050459	-0.071469	0.041967	hypomethylated	Slain1	29	36
chr14	104867216	104869216	-0.051156	0.015059	hypomethylated	Pou4f1	23	35
chr14	106296036	106298036	-0.23701	0.00021501	hypomethylated	Spry2	8	23
chr14	118636252	118638252	-0.64249	0.0044174	stronglyhypometh	Sox21	7	10
chr14	119105441	119107441	-0.30338	0.000000483	hypomethylated	Abcc4	22	22
chr14	119336153	119338153	-0.078578	0.000029	hypomethylated	Dnajc3	43	48
chr14	121309415	121311415	-0.080826	0.000000931	hypomethylated	Ipo5	59	64
chr14	121433795	121435795	-0.068187	0.00072014	hypomethylated	Farp1	51	69
chr14	121778452	121780452	-0.2587	0.0025513	hypomethylated	Stk24	23	19
chr14	122505303	122507303	-0.054531	0.0014427	hypomethylated	A330035P11Rik,Tm9sf	47	55
chr14	122506196	122508196	-0.031926	0.0058201	hypomethylated	A330035P11Rik,Tm9sf	42	47
chr14	122873605	122875605	-0.16154	0.026532	hypomethylated	2610035F20Rik,Zic2	59	71
chr14	122932549	122934549	-0.26505	0.00011332	hypomethylated	Pcca	7	10
chr14	123312387	123314387	-0.064331	0.0113	hypomethylated	Ggact	11	11
chr15	3253526	3255526	0.16667	0.0054586	hypermethylated	Ccdc152	7	7
chr15	5065612	5067612	-0.22517	0.0017589	hypomethylated	Rpl37,Snord72	28	28
chr15	5092860	5094860	-0.20576	0.0000202	hypomethylated	Prkaa1	51	55
chr15	5134559	5136559	-0.12594	0.004124	hypomethylated	Ttc33	17	21
chr15	7348304	7350304	-0.27679	0.0046331	hypomethylated	Egflam	6	11
chr15	9070207	9072207	0.17222	0.02886	hypermethylated	Lmbrd2,Skp2	11	28
chr15	10414788	10416788	-0.16277	0.00000129	hypomethylated	Brix1,Rad1	16	16
chr15	10881110	10883110	0.40774	0.038956	stronglyhypermeth	C1qtnf3	3	2
chr15	10967723	10969723	-0.43939	0.0287	stronglyhypometh	Rxfp3	3	3
chr15	11835429	11837429	0.064688	0.0011026	hypermethylated	Npr3	11	16
chr15	12250250	12252250	-0.10242	0.0010094	hypomethylated	Golph3	40	44
chr15	12753569	12755569	-0.047587	2.2E-09	hypomethylated	6030458C11Rik,Drosha	47	54
chr15	12754412	12756412	-0.048992	0.000000063	hypomethylated	6030458C11Rik,Drosha	30	36
chr15	25551304	25553304	-0.093395	0.00010723	hypomethylated	Myo10	44	54
chr15	26237826	26239826	-0.16269	0.0075079	hypomethylated	March11	50	60
chr15	27395431	27397431	-0.11008	0.0062986	hypomethylated	Ank	47	60
chr15	30101347	30103347	-0.1771	0.00027677	hypomethylated	Ctnnd2	56	59
chr15	31153139	31155139	-0.45004	0.00000127	stronglyhypometh	Dap	14	16
chr15	32173567	32175567	-0.38913	0.0017738	stronglyhypometh	0610007N19Rik,Sema5	11	16
chr15	33011883	33013883	-0.23892	0.019691	hypomethylated	Cpq	7	7
chr15	34424065	34426065	-0.42403	0.0005337	stronglyhypometh	Hrsp12,Pop1	4	13
chr15	34425001	34427001	-0.4177	0.00055388	stronglyhypometh	Hrsp12,Pop1	4	12
chr15	34766135	34768135	-0.28607	0.0013014	hypomethylated	Kcns2	31	28
chr15	35224866	35226866	-0.2786	3.01E-09	hypomethylated	Osr2	35	35
chr15	35300300	35302300	0.0557	0.037653	hypermethylated	Vps13b	36	39
chr15	37161790	37163790	-0.54502	0.0018077	stronglyhypometh	Grhl2	6	17
chr15	38590658	38592658	-0.15543	0.0039183	hypomethylated	Atp6v1c1	22	24
chr15	38943419	38945419	-0.074761	0.047005	hypomethylated	Dcaf13,Slc25a32	23	25
chr15	39028877	39030877	-0.071326	0.010361	hypomethylated	Rims2	85	83
chr15	39775303	39777303	-0.2198	0.023944	hypomethylated	Lrp12	32	33
chr15	40485587	40487587	-0.046646	0.0077086	hypomethylated	Zfpm2	44	51
chr15	43307774	43309774	-0.04338	0.02898	hypomethylated	Emc2,Gm10373	17	18
chr15	54576482	54578482	-0.13268	0.04255	hypomethylated	Nov	8	8
chr15	54943037	54945037	-0.071246	0.037823	hypomethylated	Deptor,Gm9920	28	36
chr15	57525221	57527221	-0.24467	0.000000109	hypomethylated	Zhx2	37	43
chr15	57966637	57968637	-0.1248	0.000000145	hypomethylated	Atad2	14	18
chr15	57971990	57973990	-0.27303	0.0084106	hypomethylated	Wdyhv1	17	20
chr15	58046433	58048433	-0.47642	0.041414	stronglyhypometh	Fbxo32	6	9
chr15	58719783	58721783	-0.20963	0.00098659	hypomethylated	Rnf139	30	30
chr15	58764364	58766364	-0.097071	0.044257	hypomethylated	Ndufb9,Tatdn1	21	27
chr15	58913581	58915581	0.11777	0.002921	hypermethylated	Mtss1	19	25

chr15	59145646	59147646	0.042936	0.010863	hypermethylated	Sqle	30	31
chr15	59204752	59206752	0.040574	0.016667	hypermethylated	E430025E21Rik,Nsmce	22	22
chr15	59479208	59481208	-0.15587	0.000000309	hypomethylated	Trib1	69	76
chr15	60653519	60655519	-0.15287	0.00097101	hypomethylated	9930014A18Rik	15	26
chr15	65617602	65619602	-0.19343	0.000000427	hypomethylated	Efr3a	39	41
chr15	66801203	66803203	-0.21711	0.0037046	hypomethylated	Ndrg1	7	16
chr15	72919841	72921841	-0.27894	1.45E-09	hypomethylated	Chrac1	16	17
chr15	74538590	74540590	-0.048782	0.0097218	hypomethylated	4933427E11Rik,Jrk	31	37
chr15	75446114	75448114	-0.17741	0.00000512	hypomethylated	Zfp41	42	47
chr15	75739164	75741164	-0.10104	0.00016662	hypomethylated	Eef1d,Tigd5	60	71
chr15	75739770	75741770	-0.092417	0.00032516	hypomethylated	Eef1d,Tigd5	39	49
chr15	75798614	75800614	-0.19484	0.0016287	hypomethylated	Zfp707	18	17
chr15	75900160	75902160	-0.060526	0.01474	hypomethylated	Scrib	12	13
chr15	76029836	76031836	0.51757	0.0030835	stronglyhypermeth	Plec	5	7
chr15	76030265	76032265	0.57963	0.00000154	stronglyhypermeth	Plec	4	6
chr15	76036751	76038751	-0.13575	0.024268	hypomethylated	Plec	9	9
chr15	76156826	76158826	-0.081849	0.024648	hypomethylated	Exosc4	27	27
chr15	76160723	76162723	-0.16828	0.0072818	hypomethylated	Gpaa1	23	24
chr15	76172952	76174952	-0.18513	0.0082642	hypomethylated	Cyc1	26	28
chr15	76180723	76182723	-0.079968	0.00075551	hypomethylated	Maf1,Sharpin	34	35
chr15	76181540	76183540	-0.21104	8.68E-08	hypomethylated	Maf1,Sharpin	15	16
chr15	76209942	76211942	-0.31137	0.0000311	hypomethylated	Mroh1	17	12
chr15	76306874	76308874	0.06553	0.020775	hypermethylated	Bop1,Hsf1	45	51
chr15	76342248	76344248	-0.15493	0.0095857	hypomethylated	Dgat1	19	20
chr15	76352559	76354559	-0.19328	0.0072407	hypomethylated	Scrt1	32	30
chr15	76490070	76492070	-0.14851	0.0037617	hypomethylated	Cyhr1,Kifc2	11	16
chr15	76490491	76492491	-0.14851	0.0037617	hypomethylated	Cyhr1,Kifc2	11	16
chr15	76490538	76492538	-0.14851	0.0037617	hypomethylated	Cyhr1,Kifc2	11	16
chr15	76500303	76502303	-0.1433	0.0030587	hypomethylated	Foxh1,Ppp1r16a	24	25
chr15	76501109	76503109	-0.15165	0.0013178	hypomethylated	Foxh1,Ppp1r16a	27	28
chr15	76552603	76554603	-0.11086	0.042581	hypomethylated	C030006K11Rik,Lrrc24	38	46
chr15	77759424	77761424	-0.22163	0.00065538	hypomethylated	Txn2	9	9
chr15	78004538	78006538	-0.73643	0.00028043	stronglyhypometh	Ift27	5	7
chr15	78034586	78036586	-0.59729	0.00071847	stronglyhypometh	Pvalb	8	8
chr15	78258057	78260057	-0.22104	0.0005471	hypomethylated	Kctd17	12	20
chr15	78374775	78376775	-0.29759	0.022178	hypomethylated	Sstr3	4	4
chr15	78672076	78674076	-0.032675	0.0070132	hypomethylated	Cdc42ep1	14	26
chr15	78706619	78708619	-0.19922	0.025325	hypomethylated	Gga1	39	38
chr15	78743348	78745348	0.040643	0.035668	hypermethylated	Pdxp	23	38
chr15	78812485	78814485	-0.16082	0.037462	hypomethylated	Triobp	30	30
chr15	78938412	78940412	0.052523	0.017985	hypermethylated	Micall1	54	60
chr15	78970796	78972796	-0.35137	0.00032975	stronglyhypometh	1700088E04Rik,Polr2f	10	24
chr15	78971681	78973681	-0.37914	0.0011539	stronglyhypometh	1700088E04Rik,Polr2f	5	12
chr15	79572960	79574960	0.084726	0.019861	hypermethylated	Gm16576,Sun2	22	24
chr15	79664763	79666763	-0.076569	0.00036657	hypomethylated	Cbx6,Npcd	7	7
chr15	79913336	79915336	-0.41584	3.49E-10	stronglyhypometh	Rpl3,Snord43	18	14
chr15	79913354	79915354	-0.41584	3.49E-10	stronglyhypometh	Rpl3,Snord43	18	14
chr15	79920763	79922763	-0.015664	0.0053464	hypomethylated	Syngr1	17	22
chr15	80084613	80086613	-0.14927	0.0044643	hypomethylated	Atf4	18	26
chr15	80540742	80542742	-0.11636	0.0011395	hypomethylated	Tnrc6b	43	44
chr15	81020282	81022282	-0.26066	0.000000903	hypomethylated	4930483J18Rik,Mkl1	31	36
chr15	81021187	81023187	-0.45227	0.011186	stronglyhypometh	4930483J18Rik,Mkl1	14	15
chr15	81064928	81066928	-0.64848	0.00071648	stronglyhypometh	Mchr1	5	5
chr15	81415643	81417643	-0.006295	0.00051043	hypomethylated	Ep300	61	66
chr15	81493365	81495365	-0.56281	0.00000222	stronglyhypometh	L3mbtl2	13	6
chr15	82104364	82106364	-0.16862	0.00046749	hypomethylated	Sept3	38	38
chr15	82728891	82730891	-0.092477	0.00045163	hypomethylated	Gm20324,Tbrg3	58	75

chr15	83609452	83611452	-0.021048	0.031376	hypomethylated	Mpped1	51	53
chr15	84061472	84063472	-0.39024	0.0004523	stronglyhypometh	Parvb	12	15
chr15	84510427	84512427	-0.068288	0.043614	hypomethylated	Prr5	49	59
chr15	84686559	84688559	-0.21003	0.00013683	hypomethylated	Phf21b	12	21
chr15	85564993	85566993	0.056666	0.027617	hypermethylated	Ppara	42	47
chr15	85565205	85567205	0.056666	0.027617	hypermethylated	Ppara	42	47
chr15	85652163	85654163	-0.34923	1.41E-20	stronglyhypometh	Pkdrej	10	15
chr15	85864207	85866207	-0.18872	0.00069041	hypomethylated	Celsr1	16	18
chr15	87454659	87456659	0.058851	0.0021904	hypermethylated	Fam19a5	66	71
chr15	88581140	88583140	-0.045991	0.000017	hypomethylated	Zbed4	47	55
chr15	88649075	88651075	-0.035018	0.033313	hypomethylated	Alg12,Creld2	48	51
chr15	88691623	88693623	-0.15894	0.000000588	hypomethylated	Pim3	49	51
chr15	88918536	88920536	-0.11382	0.00025318	hypomethylated	1300018J18Rik	43	43
chr15	88980036	88982036	-0.20972	0.045813	hypomethylated	Mapk11	12	12
chr15	89145742	89147742	-0.42222	0.0038158	stronglyhypometh	Sbf1	3	3
chr15	89185152	89187152	-0.17141	0.014921	hypomethylated	Lmf2,Ncaph2	25	26
chr15	89186090	89188090	-0.22798	0.0068919	hypomethylated	Lmf2,Ncaph2	20	20
chr15	89329287	89331287	-0.20655	0.000000681	hypomethylated	Shank3	62	70
chr15	91880595	91882595	-0.13726	0.0021443	hypomethylated	Cntn1	15	13
chr15	93167366	93169366	-0.19495	0.00049858	hypomethylated	Yaf2	33	33
chr15	94234781	94236781	-0.034552	0.00079067	hypomethylated	Adamts20	20	26
chr15	95620273	95622273	-0.060648	0.0000237	hypomethylated	Ano6	44	50
chr15	97598097	97600097	-0.14996	0.015976	hypomethylated	Rapgef3	17	19
chr15	97613795	97615795	0.067905	0.0077615	hypermethylated	Slc48a1	29	31
chr15	97662102	97664102	-0.012674	0.044654	hypomethylated	Hdac7	26	27
chr15	97835155	97837155	-0.14633	0.0000152	hypomethylated	Col2a1	13	14
chr15	97922019	97924019	-0.21409	0.011529	hypomethylated	Pfkm,Senp1	13	16
chr15	97997236	97999236	-0.3933	0.0088098	stronglyhypometh	AI836003	11	11
chr15	98126514	98128514	-0.21702	1.06E-08	hypomethylated	Zfp641	19	19
chr15	98398067	98400067	-0.076418	0.0000522	hypomethylated	9330020H09Rik,Ccnt1	17	28
chr15	98493320	98495320	-0.16212	0.027773	hypomethylated	Ddx23	9	9
chr15	98619287	98621287	-0.18781	0.00018815	hypomethylated	Wnt1	18	18
chr15	98661939	98663939	-0.081776	0.045266	hypomethylated	Prkag1	4	6
chr15	98701614	98703614	-0.0099182	0.022159	hypomethylated	Mll2	47	55
chr15	98764821	98766821	-0.10319	0.0027	hypomethylated	Tuba1b	21	22
chr15	98859321	98861321	-0.21927	0.000000665	hypomethylated	Tuba1c	21	25
chr15	99082392	99084392	-0.085328	0.024052	hypomethylated	1700120C14Rik,Mcrs1	9	10
chr15	99555048	99557048	-0.11238	0.033493	hypomethylated	Cox14	8	15
chr15	99868094	99870094	-0.084282	0.0018268	hypomethylated	Dip2b	37	45
chr15	100057289	100059289	-0.1486	8.63E-12	hypomethylated	Atf1	46	62
chr15	100253486	100255486	-0.19404	4.57E-14	hypomethylated	Gm5475,Slc11a2	21	21
chr15	100298464	100300464	-0.21947	0.00000524	hypomethylated	Letmd1	29	31
chr15	100467286	100469286	-0.24437	0.00000605	hypomethylated	Smagp	17	17
chr15	100467296	100469296	-0.24437	0.00000605	hypomethylated	Smagp	17	17
chr15	100591177	100593177	-0.13778	0.0055722	hypomethylated	Slc4a8	30	30
chr15	100957967	100959967	-0.15268	0.0041166	hypomethylated	Acvrl1	9	9
chr15	101003555	101005555	-0.013241	0.00016844	hypomethylated	Acvr1b	51	54
chr15	101053637	101055637	0.063381	0.0095846	hypermethylated	A330009N23Rik,Grasp	31	31
chr15	101966646	101968646	-0.68582	0.0000065	stronglyhypometh	Spryd3	13	15
chr15	102108886	102110886	-0.099055	0.018534	hypomethylated	Mfsd5	39	48
chr15	102155546	102157546	-0.21325	0.00000187	hypomethylated	Pfdn5	11	14
chr15	102347435	102349435	-0.18129	0.001229	hypomethylated	Map3k12,Tarbp2	18	23
chr15	102750561	102752561	-0.23821	0.00024177	hypomethylated	Hoxc13	48	49
chr15	102783956	102785956	0.0012115	0.007598	hypermethylated	Hoxc11	30	37
chr15	102796295	102798295	-0.46911	0.0067078	stronglyhypometh	Hoxc10	13	18
chr15	102802780	102804780	-0.34756	0.012792	stronglyhypometh	Mir196a-2	14	14
chr15	102843438	102845438	-0.20425	0.0000167	hypomethylated	Hoxc5,Mir615	9	9

chr15	102844340	102846340	-0.16813	0.000018	hypomethylated	Hoxc5,Mir615	16	16
chr15	102993715	102995715	-0.60625	0.00000614	stronglyhypometh	Smug1	8	8
chr16	3743098	3745098	-0.22791	0.038361	hypomethylated	Zfp263	16	25
chr16	3883618	3885618	-0.10705	0.0012121	hypomethylated	Naa60	31	41
chr16	3908008	3910008	-0.32338	0.0000278	hypomethylated	1700037C18Rik,Cluap1	20	20
chr16	4213404	4215404	-0.061317	0.00029905	hypomethylated	Crebbp	44	58
chr16	4419587	4421587	-0.067946	0.00047815	hypomethylated	Adcy9	63	74
chr16	4879851	4881851	-0.23772	0.0000969	hypomethylated	Ubald1	26	24
chr16	4885251	4887251	-0.27725	0.000000185	hypomethylated	Mgrn1	31	41
chr16	4938110	4940110	0.16253	0.014262	hypermethylated	Gm16861,Nudt16l1	8	8
chr16	4964330	4966330	-0.12181	0.00301	hypomethylated	4930451G09Rik,Anks3	6	7
chr16	5013646	5015646	-0.74877	0.00000272	stronglyhypometh	Rogdi	4	6
chr16	5050003	5052003	-0.018575	0.00068531	hypomethylated	Glyr1,Ubn1	56	56
chr16	5146201	5148201	-0.81593	8.63E-08	stronglyhypometh	Sec14l5	13	15
chr16	5204105	5206105	-0.42857	0.00018898	stronglyhypometh	Nagpa	1	1
chr16	8512521	8514521	-0.16324	0.00020407	hypomethylated	Abat	14	14
chr16	8637794	8639794	-0.14797	0.0000181	hypomethylated	Pmm2,Tmem186	11	11
chr16	8738435	8740435	0.028862	0.034062	hypermethylated	Usp7	29	40
chr16	10543147	10545147	-0.46121	0.03678	stronglyhypometh	Clec16a,Dexi	4	4
chr16	10834151	10836151	-0.084744	0.00097093	hypomethylated	Rmi2	26	27
chr16	11134625	11136625	-0.068824	0.013198	hypomethylated	Txndc11	23	23
chr16	11203385	11205385	-0.66892	0.00050134	stronglyhypometh	2610020C07Rik,Rsl1d1	5	13
chr16	11254418	11256418	-0.31833	0.0014686	hypomethylated	Gspt1,Mir1945	18	20
chr16	11254538	11256538	-0.25833	0.021546	hypomethylated	Gspt1,Mir1945	12	14
chr16	11983205	11985205	-0.28035	0.00095918	hypomethylated	Shisa9	24	35
chr16	13108828	13110828	-0.31694	8.36E-09	hypomethylated	Ercc4	17	20
chr16	13255573	13257573	-0.0061241	0.034274	hypomethylated	Mkl2	47	56
chr16	15863415	15865415	-0.24548	0.015906	hypomethylated	Mzt2	10	9
chr16	15886378	15888378	-0.025627	0.017275	hypomethylated	Cebpd	66	73
chr16	16895561	16897561	-0.25313	0.01691	hypomethylated	Ppm1f	10	7
chr16	16982474	16984474	-0.2067	4.67E-11	hypomethylated	Mapk1	34	40
chr16	17132476	17134476	-0.55976	0.026691	stronglyhypometh	Sdf2l1	9	8
chr16	17645562	17647562	-0.092539	0.045178	hypomethylated	Ccdc74a	23	33
chr16	18089283	18091283	-0.50143	0.0006523	stronglyhypometh	Prodh	6	2
chr16	18347366	18349366	-0.2347	0.000000345	hypomethylated	Arvcf	31	32
chr16	18497860	18499860	-0.40007	0.00059794	stronglyhypometh	Gnb1l	20	18
chr16	18497963	18499963	-0.40007	0.00059794	stronglyhypometh	Gnb1l	20	18
chr16	18630031	18632031	-0.29623	0.049258	hypomethylated	Sept5	18	19
chr16	18775939	18777939	-0.31325	0.00000839	hypomethylated	Cldn5	21	21
chr16	18811386	18813386	-0.50923	0.0076342	stronglyhypometh	Cdc45,Ufd1l	16	16
chr16	18875842	18877842	-0.10177	0.00034791	hypomethylated	Hira,Mrpl40	44	48
chr16	18876730	18878730	-0.094274	0.015439	hypomethylated	Hira,Mrpl40	39	43
chr16	20716709	20718709	-0.37962	0.000000427	stronglyhypometh	Clcn2,Polr2h	9	11
chr16	20716898	20718898	-0.36172	0.0011784	stronglyhypometh	Clcn2,Polr2h	10	12
chr16	20732199	20734199	0.017743	0.042789	hypermethylated	Chrd,Thpo	36	43
chr16	21203867	21205867	0.17392	0.0065252	hypermethylated	Ephb3	38	45
chr16	22008556	22010556	-0.27079	0.0000049	hypomethylated	Senp2	28	31
chr16	22163372	22165372	-0.21111	0.0094504	hypomethylated	Igf2bp2	6	9
chr16	22439643	22441643	-0.28125	0.014657	hypomethylated	Etv5	8	22
chr16	22856917	22858917	-0.1784	0.0000107	hypomethylated	Dnajb11,Tbccd1	19	24
chr16	22857642	22859642	-0.1784	0.0000107	hypomethylated	Dnajb11,Tbccd1	19	24
chr16	23106551	23108551	-0.19924	0.00018973	hypomethylated	Eif4a2,Snord2	46	46
chr16	23108025	23110025	-0.35407	0.000772	stronglyhypometh	Eif4a2,Snora81,Snord2	20	11
chr16	23988698	23990698	-0.22865	3.4E-12	hypomethylated	Bcl6	24	24
chr16	27388062	27390062	0.28621	0.000000129	hypermethylated	Ccdc50	11	11
chr16	30007752	30009752	-0.22932	0.0006216	hypomethylated	4632428C04Rik	19	23
chr16	30388616	30390616	-0.093254	0.0055135	hypomethylated	Atp13a3	27	29

chr16	31427838	31429838	-0.31744	0.000000048	hypomethylated	Bdh1	19	29
chr16	31663124	31665124	-0.17346	0.0000286	hypomethylated	Dlg1	31	49
chr16	31947631	31949631	-0.20966	8.68E-11	hypomethylated	0610012G03Rik,Ncbp2	49	52
chr16	31948607	31950607	-0.37519	0.000000396	stronglyhypometh	0610012G03Rik,Ncbp2	17	20
chr16	32098887	32100887	-0.34686	2.7E-09	stronglyhypometh	Cep19,Pigx	21	25
chr16	32099813	32101813	-0.38377	0.00010198	stronglyhypometh	Cep19,Pigx	9	14
chr16	32246312	32248312	0.2217	0.01445	hypermethylated	Fbxo45,Wdr53	28	32
chr16	32246338	32248338	0.25489	0.0057639	hypermethylated	Fbxo45,Wdr53	27	31
chr16	32331337	32333337	-0.31988	4.94E-09	hypomethylated	Ubxn7	27	27
chr16	32418787	32420787	-0.52917	1.86E-09	stronglyhypometh	Tctex1d2	12	12
chr16	32643728	32645728	-0.32202	2.88E-09	hypomethylated	Tnk2	23	37
chr16	32876869	32878869	-0.24892	1.08E-08	hypomethylated	Fyttd1	42	48
chr16	33055538	33057538	-0.1683	0.003762	hypomethylated	Iqcg,Rpl35a	19	19
chr16	33055567	33057567	-0.1683	0.003762	hypomethylated	Iqcg,Rpl35a	19	19
chr16	33055599	33057599	-0.1683	0.003762	hypomethylated	Iqcg,Rpl35a	19	19
chr16	33056272	33058272	-0.26984	0.000000112	hypomethylated	Iqcg,Rpl35a	15	15
chr16	33184156	33186156	-0.15036	0.0001214	hypomethylated	Osbpl11	42	41
chr16	33250541	33252541	-0.10738	0.0083435	hypomethylated	Snx4	13	15
chr16	33967089	33969089	-0.35833	0.0026722	stronglyhypometh	Umps	4	4
chr16	35021506	35023506	-0.21097	0.0000241	hypomethylated	Ptplb	31	31
chr16	36070745	36072745	-0.25345	0.0048655	hypomethylated	Ccdc58,Fam162a	14	30
chr16	36071601	36073601	-0.17808	0.019526	hypomethylated	Ccdc58,Fam162a	13	24
chr16	36874912	36876912	-0.14429	0.00000252	hypomethylated	Eaf2	32	36
chr16	37010871	37012871	-0.24723	5.36E-09	hypomethylated	Polq	15	18
chr16	37867485	37869485	-0.080133	0.0000174	hypomethylated	Lrrc58	49	49
chr16	37915581	37917581	0.11461	0.0072045	hypermethylated	Gpr156	18	17
chr16	38346084	38348084	-0.53199	0.0067537	stronglyhypometh	Cox17	6	8
chr16	38550266	38552266	-0.41531	0.00057129	stronglyhypometh	Poglut1	4	7
chr16	38741376	38743376	-0.2077	0.033364	hypomethylated	B4galt4	33	33
chr16	44172509	44174509	-0.12048	0.043437	hypomethylated	Gm608	29	32
chr16	44393911	44395911	-0.30863	0.0066745	hypomethylated	Wdr52	3	7
chr16	44558242	44560242	-0.065723	0.0097487	hypomethylated	Boc,Mir3081	15	22
chr16	45743019	45745019	-0.8	0.0000333	stronglyhypometh	Abhd10	4	2
chr16	48993300	48995300	-0.45609	0.00106	stronglyhypometh	C330027C09Rik,Dzip3	12	15
chr16	48994225	48996225	-0.3	0.019426	hypomethylated	C330027C09Rik,Dzip3	5	6
chr16	49698406	49700406	-0.36895	0.000000258	stronglyhypometh	Ift57	7	12
chr16	55838754	55840754	-0.33477	0.00011381	stronglyhypometh	Nfkbiz	14	14
chr16	55965387	55967387	-0.070238	0.00013417	hypomethylated	Rpl24	15	16
chr16	55972916	55974916	-0.058647	0.00040349	hypomethylated	2310061J03Rik,Zbtb11	47	50
chr16	56074521	56076521	-0.27702	0.047274	hypomethylated	Senp7	8	9
chr16	57120826	57122826	-0.094684	0.0065311	hypomethylated	Tomm70a	43	44
chr16	57548354	57550354	-0.22222	0.00027275	hypomethylated	Filip1l	9	9
chr16	59639165	59641165	-0.47502	0.0020969	stronglyhypometh	Arl6	9	5
chr16	62846866	62848866	-0.42949	0.017391	stronglyhypometh	Arl13b	2	2
chr16	64770765	64772765	-0.26818	0.040906	hypomethylated	4930453N24Rik	5	5
chr16	64850910	64852910	-0.11222	0.0028912	hypomethylated	Cggbp1	23	33
chr16	70313331	70315331	-0.21076	0.0005686	hypomethylated	Gbe1	52	52
chr16	81199941	81201941	-0.19152	0.00023001	hypomethylated	Ncam2	32	38
chr16	84735426	84737426	-0.13679	0.0000292	hypomethylated	Mrpl39	31	31
chr16	87353429	87355429	-0.21958	0.0086571	hypomethylated	N6amt1	8	10
chr16	87440837	87442837	-0.26272	0.0096465	hypomethylated	Rwdd2b	6	6
chr16	87454229	87456229	0.078671	0.0019935	hypermethylated	Usp16	28	37
chr16	87698198	87700198	-0.047607	0.044548	hypomethylated	Bach1	44	53
chr16	90385641	90387641	0.034808	0.032466	hypermethylated	Hunk	45	56
chr16	90727616	90729616	-0.475	0.000000285	stronglyhypometh	Mis18a	10	10
chr16	90830103	90832103	-0.034605	0.0033514	hypomethylated	Eva1c	45	52
chr16	90935094	90937094	-0.14437	0.030234	hypomethylated	1110004E09Rik	16	20

chr16	91372027	91374027	-0.21122	0.02371	hypomethylated	Ifnar2	18	22
chr16	91405479	91407479	-0.079259	0.029212	hypomethylated	Il10rb	12	15
chr16	91597925	91599925	-0.16208	0.006814	hypomethylated	Tmem50b	14	17
chr16	91647068	91649068	-0.11356	0.00018146	hypomethylated	Gart,Son	41	46
chr16	91647217	91649217	-0.12527	0.00016861	hypomethylated	Gart,Son	36	41
chr16	92057566	92059566	-0.0070546	0.029888	hypomethylated	Mrps6,Slc5a3	45	62
chr16	92057580	92059580	-0.0070546	0.029888	hypomethylated	Mrps6,Slc5a3	45	62
chr16	92697573	92699573	-0.14185	0.0000891	hypomethylated	Runx1	33	39
chr16	93607081	93609081	-0.13773	0.024115	hypomethylated	Cbr1	8	8
chr16	93831365	93833365	0.041218	0.040741	hypermethylated	Morc3	56	57
chr16	93883145	93885145	-0.054677	0.016941	hypomethylated	Chaf1b	31	36
chr16	94591345	94593345	-0.10825	0.022807	hypomethylated	Pigp,Ttc3	28	27
chr16	94591937	94593937	-0.10825	0.022807	hypomethylated	Pigp,Ttc3	28	27
chr16	94748236	94750236	-0.77348	4.53E-12	stronglyhypometh	Dscr3	17	17
chr16	94790812	94792812	-0.031274	0.0047979	hypomethylated	Dyrk1a	101	114
chr16	94791513	94793513	-0.040277	0.0019229	hypomethylated	Dyrk1a	83	87
chr16	95923013	95925013	-0.12025	0.0024959	hypomethylated	Ets2	31	37
chr16	96304035	96306035	-0.19258	0.0023784	hypomethylated	Brwd1	5	19
chr16	96349332	96351332	-0.18021	0.016612	hypomethylated	Hmgn1	27	27
chr16	96501638	96503638	-0.27959	0.038972	hypomethylated	B3galt5	4	4
chr16	98072834	98074834	-0.16563	0.0000836	hypomethylated	Prdm15	57	57
chr17	4994073	4996073	-0.099244	0.00022461	hypomethylated	Arid1b	78	87
chr17	5491599	5493599	-0.0883	0.006836	hypomethylated	Zdhhc14	75	78
chr17	5840379	5842379	-0.12437	0.000000146	hypomethylated	Snx9	67	78
chr17	6269474	6271474	-0.43939	0.0068228	stronglyhypometh	Tmem181a	11	11
chr17	6987129	6989129	-0.021176	0.025386	hypomethylated	Ezr	29	31
chr17	7373463	7375463	-0.15901	0.0041646	hypomethylated	Rps6ka2	31	31
chr17	8475677	8477677	-0.11984	0.0067943	hypomethylated	Mpc1	47	59
chr17	8993609	8995609	-0.078164	0.0000105	hypomethylated	Pde10a	35	48
chr17	11032249	11034249	-0.04456	0.0072476	hypomethylated	Pacrg,Park2	8	12
chr17	12700570	12702570	-0.4342	0.031247	stronglyhypometh	Slc22a3	5	9
chr17	13108330	13110330	0.12349	0.0017272	hypermethylated	Mrpl18,Tcp1	11	17
chr17	13185125	13187125	-0.18832	0.020417	hypomethylated	Wtap	37	41
chr17	13185405	13187405	-0.3665	0.001329	stronglyhypometh	Wtap	19	23
chr17	14415512	14417512	-0.079181	0.00085884	hypomethylated	Smoc2	24	33
chr17	15532165	15534165	-0.65205	0.00077088	stronglyhypometh	Fam120b	5	3
chr17	15532209	15534209	-0.65205	0.00077088	stronglyhypometh	Fam120b	5	3
chr17	15635240	15637240	-0.35646	7.67E-08	stronglyhypometh	Psmb1,Tbp	31	31
chr17	15635851	15637851	-0.53918	6.56E-11	stronglyhypometh	Psmb1,Tbp	24	24
chr17	15963550	15965550	-0.25859	0.012098	hypomethylated	Rgmb	6	6
chr17	21519711	21521711	-0.30141	0.0034204	hypomethylated	Zfp677	7	7
chr17	21559190	21561190	-0.56002	0.011216	stronglyhypometh	Zfp54	4	7
chr17	22560234	22562234	-0.70101	0.0056697	stronglyhypometh	Zfp946	3	10
chr17	22954714	22956714	-0.5087	0.00061566	stronglyhypometh	Gm16386	6	6
chr17	23004101	23006101	-0.65	0.016367	stronglyhypometh	Zfp945	2	2
chr17	23782236	23784236	-0.13423	0.0050109	hypomethylated	Mmp25	10	10
chr17	23862302	23864302	-0.19482	9.96E-10	hypomethylated	Pkmyt1	42	44
chr17	23882796	23884796	-0.18351	0.011394	hypomethylated	Kremen2	9	8
chr17	23939153	23941153	-0.19306	0.0012195	hypomethylated	Srrm2	31	37
chr17	23966076	23968076	-0.22727	0.00019363	hypomethylated	Tceb2	12	11
chr17	24306374	24308374	-0.37377	9.39E-10	stronglyhypometh	Atp6v0c,Atp6v0c-ps2	13	23
chr17	24487990	24489990	-0.30079	0.000000108	hypomethylated	Abca3	27	38
chr17	24562627	24564627	-0.1864	0.0000631	hypomethylated	Eci1	17	16
chr17	24606417	24608417	-0.12978	0.000000047	hypomethylated	Pgp	58	66
chr17	24832178	24834178	-0.02509	0.0000452	hypomethylated	Gfer,Noxo1	43	49
chr17	24888947	24890947	-0.29231	0.00056537	hypomethylated	Hs3st6	29	38
chr17	25032032	25034032	-0.22176	0.0067557	hypomethylated	Eme2,Mrps34,Nme3	14	18

chr17	25032444	25034444	-0.275	0.0028412	hypomethylated	Eme2,Mrps34,Nme3	4	10
chr17	25152030	25154030	-0.086454	0.00019849	hypomethylated	Cramp1l,Ift140	27	34
chr17	25152175	25154175	-0.086454	0.00019849	hypomethylated	Cramp1l,Ift140	27	34
chr17	25269338	25271338	-0.15128	0.0000477	hypomethylated	Clcn7	22	21
chr17	25324344	25326344	-0.15516	0.00070421	hypomethylated	Unkl	48	52
chr17	25570728	25572728	-0.10443	0.00058341	hypomethylated	Cacna1h	26	27
chr17	26056659	26058659	-0.74542	0.000000338	stronglyhypometh	Rab40c	7	9
chr17	26206122	26208122	0.07078	0.0091372	hypermethylated	Rab11fip3	36	41
chr17	26227109	26229109	-0.5976	0.022395	stronglyhypometh	Decr2	3	6
chr17	26249260	26251260	-0.42726	0.00000249	stronglyhypometh	Tmem8	22	22
chr17	26274630	26276630	-0.13376	9.82E-11	hypomethylated	Axin1	31	32
chr17	26550909	26552909	0.1071	0.0000593	hypermethylated	Neurl1b	39	55
chr17	26697456	26699456	-0.2541	2.17E-08	hypomethylated	Ergic1	21	23
chr17	27053035	27055035	-0.56737	2.09E-09	stronglyhypometh	Kifc5b	8	9
chr17	27069071	27071071	-0.26584	0.032598	hypomethylated	Phf1	10	15
chr17	27193248	27195248	0.11642	0.0027428	hypermethylated	Itpr3	23	31
chr17	27692518	27694518	0.039272	0.010674	hypermethylated	Hmga1,Hmga1-rs1	66	77
chr17	27692548	27694548	0.037695	0.019344	hypermethylated	Hmga1,Hmga1-rs1	64	75
chr17	27692597	27694597	0.037695	0.019344	hypermethylated	Hmga1,Hmga1-rs1	64	75
chr17	27702672	27704672	0.17183	0.00000184	hypermethylated	AI413582	15	15
chr17	27992451	27994451	-0.058131	0.00000297	hypomethylated	Uhrf1bp1	25	28
chr17	28044669	28046669	-0.048106	0.0091219	hypomethylated	Anks1,Taf11	33	31
chr17	28045284	28047284	-0.051356	0.0018411	hypomethylated	Anks1,Taf11	36	33
chr17	28278470	28280470	-0.17449	0.000018	hypomethylated	Scube3	44	47
chr17	28313362	28315362	-0.31982	7.24E-09	hypomethylated	Zfp523	42	42
chr17	28368698	28370698	0.099918	0.046816	hypermethylated	Ppard	12	21
chr17	28464415	28466415	-0.084204	0.008307	hypomethylated	Rpl10a	32	38
chr17	28666805	28668805	0.12377	0.026811	hypermethylated	Armc12	7	7
chr17	28685431	28687431	-0.13992	0.034389	hypomethylated	Clpsl2	6	7
chr17	28711663	28713663	-0.052129	0.029009	hypomethylated	Lhfpl5	13	13
chr17	28937070	28939070	-0.20034	0.000000142	hypomethylated	Brpf3	41	48
chr17	29168604	29170604	-0.24454	9.66E-09	hypomethylated	Srsf3	33	39
chr17	29374735	29376735	-0.41717	2.08E-12	stronglyhypometh	Cpne5	30	33
chr17	29626989	29628989	-0.090978	0.00000162	hypomethylated	Pim1	73	87
chr17	29685746	29687746	-0.25745	8.22E-09	hypomethylated	Tbc1d22b,Tmem217	25	28
chr17	29686538	29688538	-0.25745	8.22E-09	hypomethylated	Tbc1d22b,Tmem217	25	28
chr17	29796545	29798545	-0.17182	0.042331	hypomethylated	Ftsjd2	22	23
chr17	30141031	30143031	-0.058649	0.0001233	hypomethylated	Zfand3	43	55
chr17	31193638	31195638	-0.138	0.0005341	hypomethylated	Abcg1	20	20
chr17	31431427	31433427	-0.24577	0.00000313	hypomethylated	Slc37a1	17	19
chr17	31522178	31524178	-0.090607	0.01156	hypomethylated	Pde9a	15	15
chr17	31700717	31702717	-0.055889	0.00040009	hypomethylated	Pknox1	64	81
chr17	31992737	31994737	-0.19139	0.00000454	hypomethylated	Sik1	21	20
chr17	32421068	32423068	-0.11536	0.00028255	hypomethylated	Brd4	55	65
chr17	32642406	32644406	-0.54167	0.0017237	stronglyhypometh	Cyp4f17	8	6
chr17	33765036	33767036	-0.13694	0.0018846	hypomethylated	Zfp414	5	5
chr17	34045389	34047389	0.15818	0.049775	hypermethylated	BC051226,Daxx	19	32
chr17	34052120	34054120	-0.56759	0.0056721	stronglyhypometh	Zbtb22	5	9
chr17	34161625	34163625	-0.11479	0.036232	hypomethylated	Mir219-1,Ring1	35	42
chr17	34162037	34164037	-0.16367	0.032633	hypomethylated	H2-Ke6,Mir219-1,Ring	16	18
chr17	34165000	34167000	-0.049603	0.037772	hypomethylated	H2-Ke6	6	7
chr17	34167796	34169796	-0.15534	1.57E-08	hypomethylated	Rxrb,Slc39a7	63	64
chr17	34168635	34170635	-0.10941	3.45E-10	hypomethylated	Rxrb,Slc39a7	47	48
chr17	34257328	34259328	-0.31535	0.000000385	hypomethylated	Brd2,H2-DMa	24	31
chr17	34740506	34742506	-0.5631	1.39E-08	stronglyhypometh	Agpat1,Rnf5	14	18
chr17	34764042	34766042	0.024441	0.016137	hypermethylated	Ppt2,Prrt1	14	30
chr17	34783124	34785124	-0.18496	0.00000702	hypomethylated	Atf6b	30	44

chr17	34806479	34808479	-0.32021	0.0080187	hypomethylated	Tnxb	13	12
chr17	34972963	34974963	-0.2492	0.0000299	hypomethylated	Dom3z,Stk19	48	55
chr17	34973848	34975848	-0.16057	0.0060566	hypomethylated	Dom3z,Stk19	28	37
chr17	34986335	34988335	-0.10535	0.00000656	hypomethylated	Nelfe,Skiv2l	35	44
chr17	34987149	34989149	-0.088683	0.00012351	hypomethylated	Nelfe,Skiv2l	36	39
chr17	35030503	35032503	-0.11993	0.0052077	hypomethylated	Zbtb12	27	37
chr17	35034443	35036443	-0.14678	0.0025006	hypomethylated	Ehmt2	39	49
chr17	35034918	35036918	-0.14422	0.00042125	hypomethylated	Ehmt2	43	53
chr17	35067197	35069197	0.5965	0.0052642	stronglyhypermeth	Neu1	2	4
chr17	35108647	35110647	-0.12969	0.035107	hypomethylated	Hspa1a,Hspa1l	9	9
chr17	35109101	35111101	-0.12969	0.035107	hypomethylated	Hspa1a,Hspa1l	9	9
chr17	35136851	35138851	-0.14925	0.0031727	hypomethylated	Vars	42	47
chr17	35225235	35227235	-0.15997	0.00015873	hypomethylated	Abhd16a	23	22
chr17	35263059	35265059	-0.16019	0.026302	hypomethylated	D17H6S53E	21	22
chr17	35271186	35273186	-0.10629	0.0036163	hypomethylated	Bag6	36	38
chr17	35301822	35303822	0.31478	0.030302	hypermethylated	Prrc2a	8	14
chr17	35377690	35379690	-0.20397	0.00029127	hypomethylated	Ddx39b	28	39
chr17	35456502	35458502	-0.11698	0.00093711	hypomethylated	H2-Q1	22	22
chr17	35478277	35480277	-0.16837	0.00033074	hypomethylated	H2-Q2	18	19
chr17	35515561	35517561	0.078437	0.00018573	hypermethylated	H2-Q4	25	35
chr17	35560821	35562821	0.12272	0.0023354	hypermethylated	H2-Q6,H2-Q8	13	14
chr17	35653060	35655060	-0.28655	0.00063485	hypomethylated	Cchcr1,Tcf19	10	14
chr17	35653769	35655769	-0.26422	0.0023904	hypomethylated	Cchcr1,Tcf19	13	17
chr17	35977442	35979442	-0.42314	0.0053264	stronglyhypometh	Mdc1	11	16
chr17	36034323	36036323	-0.2164	0.00013432	hypomethylated	2310061I04Rik	10	10
chr17	36052855	36054855	-0.11631	0.0043796	hypomethylated	Mir1894,Mrps18b,Ppp	28	43
chr17	36053140	36055140	-0.11631	0.0043796	hypomethylated	Mir1894,Mrps18b,Ppp	28	43
chr17	36053314	36055314	-0.14587	0.0014669	hypomethylated	Mir1894,Mrps18b,Ppp	26	41
chr17	36053833	36055833	-0.074207	0.022006	hypomethylated	Mir1894,Mrps18b,Ppp	6	12
chr17	36280962	36282962	-0.34267	8.72E-15	stronglyhypometh	2410017I17Rik	9	11
chr17	36408378	36410378	-0.19394	0.011383	hypomethylated	Trim39	21	26
chr17	36973085	36975085	-0.1432	0.010893	hypomethylated	Trim26	30	31
chr17	37078995	37080995	0.10882	0.020813	hypermethylated	Rnf39	29	32
chr17	37138426	37140426	-0.29863	0.00031264	hypomethylated	Zfp57	15	15
chr17	37138469	37140469	-0.29863	0.00031264	hypomethylated	Zfp57	15	15
chr17	37139610	37141610	-0.29863	0.00031264	hypomethylated	Zfp57	15	15
chr17	37406178	37408178	-0.78864	0.0012208	stronglyhypometh	H2-M3	5	7
chr17	39980080	39982080	-0.28925	0	hypomethylated	Rn45s	81	83
chr17	43704399	43706399	-0.31488	0.0000182	hypomethylated	Pla2g7	7	8
chr17	43803373	43805373	-0.18407	0.0000675	hypomethylated	Cyp39a1,Slc25a27	12	13
chr17	43803964	43805964	-0.1623	0.0014599	hypomethylated	Cyp39a1,Slc25a27	9	10
chr17	44242757	44244757	-0.27186	5.85E-13	hypomethylated	Enpp4	16	17
chr17	44913119	44915119	-0.32797	7.56E-18	hypomethylated	Supt3	23	32
chr17	45570656	45572656	-0.16953	0.0016119	hypomethylated	Cdc5l	16	17
chr17	45691664	45693664	0.04681	0.019796	hypermethylated	Nfkbie	19	19
chr17	46246952	46248952	-0.37164	0.000000323	stronglyhypometh	Mrps18a	14	14
chr17	46296980	46298980	-0.5382	0.00028909	stronglyhypometh	Gtpbp2	10	21
chr17	46338803	46340803	-0.28569	0.0015721	hypomethylated	Polh,Xpo5	20	20
chr17	46339574	46341574	-0.28569	0.0015721	hypomethylated	Polh,Xpo5	20	20
chr17	46384028	46386028	-0.13395	0.00000232	hypomethylated	Polr1c,Yipf3	17	17
chr17	46384994	46386994	-0.15428	0.000002	hypomethylated	Polr1c,Yipf3	18	18
chr17	46519714	46521714	-0.12625	0.00022076	hypomethylated	Zfp318	59	68
chr17	46847411	46849411	-0.23293	3.92E-15	hypomethylated	Pex6	42	47
chr17	46908583	46910583	-0.42143	0.0000523	stronglyhypometh	2310039H08Rik	7	10
chr17	46919605	46921605	-0.56623	0.00093121	stronglyhypometh	Rpl7l1	4	6
chr17	47026569	47028569	-0.085905	0.03203	hypomethylated	Tbcc	12	27
chr17	47748441	47750441	-0.42183	0.038013	stronglyhypometh	Bysl,Med20	5	14

chr17	47872985	47874985	-0.085371	0.00000538	hypomethylated	Tfeb	31	34
chr17	47873879	47875879	-0.18895	1.17E-08	hypomethylated	Tfeb	49	57
chr17	48498240	48500240	0.38244	0.023725	stronglyhypermeth	Treml1	3	6
chr17	52741087	52743087	-0.39709	0.000013	stronglyhypometh	Kcnh8	21	22
chr17	56147623	56149623	-0.2209	0.00015392	hypomethylated	Mpnd	17	17
chr17	56178838	56180838	-0.556	0.0000062	stronglyhypometh	Chaf1a	16	19
chr17	56218079	56220079	-0.15074	0.038938	hypomethylated	Hdgfrp2	15	27
chr17	56381410	56383410	-0.23655	0.0030887	hypomethylated	Mir7b	8	8
chr17	56442735	56444735	-0.40489	0.0000137	stronglyhypometh	Uhrf1	21	32
chr17	56464472	56466472	-0.38537	0.00000197	stronglyhypometh	Kdm4b	16	17
chr17	56723404	56725404	-0.13162	0.0000465	hypomethylated	Safb,Safb2	54	69
chr17	56724006	56726006	-0.17744	0.000000217	hypomethylated	Safb,Safb2	48	52
chr17	56751817	56753817	-0.091167	0.0055347	hypomethylated	Rpl36	21	23
chr17	56896953	56898953	-0.26163	0.0010238	hypomethylated	Nrtn	15	15
chr17	56903173	56905173	0.12936	0.030556	hypermethylated	Dus3l	15	19
chr17	57074811	57076811	0.1986	0.0068738	hypermethylated	Mllt1	26	32
chr17	57907992	57909992	-0.1737	0.042072	hypomethylated	Cntnap5c	4	4
chr17	63230666	63232666	-0.1946	0.00015429	hypomethylated	Efna5	62	61
chr17	64949988	64951988	-0.041895	0.0013546	hypomethylated	Man2a1	65	66
chr17	65962895	65964895	-0.28755	9.31E-11	hypomethylated	Vapa	26	26
chr17	66122092	66124092	-0.19265	0.0026785	hypomethylated	Rab31	8	8
chr17	66131694	66133694	-0.065464	0.0000116	hypomethylated	Ppp4r1	90	92
chr17	66459885	66461885	-0.36687	0.0000163	stronglyhypometh	Wash	15	18
chr17	66459984	66461984	-0.36687	0.0000163	stronglyhypometh	Wash	15	18
chr17	66799090	66801090	-0.23851	0.0016915	hypomethylated	Soga2	8	10
chr17	68622136	68624136	-0.28114	0.014927	hypomethylated	L3mbtl4	9	9
chr17	69732317	69734317	-0.087914	0.0007681	hypomethylated	Zbtb14	62	64
chr17	69787665	69789665	-0.2337	0.00000233	hypomethylated	A330050F15Rik	27	37
chr17	71199130	71201130	-0.28175	0.0073368	hypomethylated	Tgif1	20	19
chr17	71532317	71534317	-0.14329	0.0016027	hypomethylated	Lpin2	44	48
chr17	71824683	71826683	-0.079762	0.0000824	hypomethylated	Smchd1	25	35
chr17	71876197	71878197	-0.34263	0.022116	stronglyhypometh	Ndc80	16	27
chr17	71964554	71966554	-0.057494	0.00051251	hypomethylated	Wdr43	42	44
chr17	74153180	74155180	-0.13874	0.042741	hypomethylated	Ehd3	19	21
chr17	74694203	74696203	0.13217	0.0032938	hypermethylated	Memo1	39	39
chr17	74737326	74739326	-0.066178	0.0026005	hypomethylated	Spast	45	40
chr17	74793971	74795971	-0.33599	0.0020454	stronglyhypometh	Slc30a6	12	13
chr17	74926634	74928634	-0.03869	0.033516	hypomethylated	Birc6	50	55
chr17	75403868	75405868	-0.050656	0.010647	hypomethylated	Ltbp1	61	67
chr17	79233855	79235855	-0.34385	0.000000178	stronglyhypometh	Gpatch11,Heatr5b	25	27
chr17	79234721	79236721	-0.36457	0.0070177	stronglyhypometh	Gpatch11,Heatr5b	6	6
chr17	79336410	79338410	0.18398	0.038436	hypermethylated	Cebpz,Ndufaf7	4	4
chr17	79420156	79422156	-0.10321	0.00062084	hypomethylated	Prkd3	18	18
chr17	79754431	79756431	-0.076188	0.00028246	hypomethylated	Cdc42ep3	23	30
chr17	80295463	80297463	-0.27152	0.0059316	hypomethylated	Atl2	7	8
chr17	80879793	80881793	-0.26603	0.028662	hypomethylated	Sos1	12	9
chr17	84031235	84033235	-0.041461	0.011438	hypomethylated	Kcng3	40	40
chr17	84587287	84589287	-0.5021	0.00041024	stronglyhypometh	Zfp36l2	9	18
chr17	85489039	85491039	-0.50492	0.0014002	stronglyhypometh	Camkmt,Prepl	10	28
chr17	85489607	85491607	-0.45988	0.0058299	stronglyhypometh	Camkmt,Prepl	10	19
chr17	85489614	85491614	-0.45988	0.0058299	stronglyhypometh	Camkmt,Prepl	10	18
chr17	86019173	86021173	-0.16687	0.022073	hypomethylated	Six3	19	19
chr17	86087594	86089594	-0.073486	0.0018926	hypomethylated	Six2	31	38
chr17	86544515	86546515	0.37169	0.00026381	stronglyhypermeth	Srbd1	9	11
chr17	86566124	86568124	-0.24669	3.21E-09	hypomethylated	Prkce	64	79
chr17	87152203	87154203	-0.076933	0.038246	hypomethylated	Epas1	28	36
chr17	87361450	87363450	-0.14545	0.00045042	hypomethylated	Rhoq	56	60

chr17	87423900	87425900	-0.10193	0.0000231	hypomethylated	Cript,Pigf	29	31
chr17	87506018	87508018	-0.14451	2.9E-10	hypomethylated	Socs5	67	67
chr17	87665287	87667287	-0.35355	0.000000325	stronglyhypometh	Mcfd2	10	12
chr17	88070896	88072896	0.096755	0.036051	hypermethylated	Msh2	17	19
chr17	88373389	88375389	-0.031177	0.0044671	hypomethylated	Msh6	40	46
chr17	88928463	88930463	-0.030995	0.0024632	hypomethylated	Ppp1r21	23	31
chr17	89066999	89068999	-0.070926	0.02074	hypomethylated	Gtf2a1l	10	10
chr17	93597761	93599761	-0.325	0.0060423	hypomethylated	Adcyap1	4	4
chr18	3506954	3508954	-0.2379	3.03E-12	hypomethylated	Bambi	38	56
chr18	5590888	5592888	-0.069568	0.027813	hypomethylated	Gm10125,Zeb1	35	64
chr18	6490644	6492644	-0.11083	0.0000713	hypomethylated	Epc1,Mir1893	25	41
chr18	6490854	6492854	-0.19184	0.0000021	hypomethylated	Epc1,Mir1893	23	34
chr18	7004777	7006777	-0.21846	0.000025	hypomethylated	Mkx	21	22
chr18	7868194	7870194	-0.012266	0.022918	hypomethylated	Wac	62	69
chr18	9211853	9213853	-0.14609	5.97E-08	hypomethylated	Fzd8	49	53
chr18	9450148	9452148	-0.3382	0.0000088	stronglyhypometh	Ccny	16	16
chr18	9706645	9708645	0.046547	0.027931	hypermethylated	Colec12	23	28
chr18	9957177	9959177	-0.044008	0.0061866	hypomethylated	Thoc1	18	28
chr18	10181790	10183790	0.22207	0.0222	hypermethylated	Rock1	6	15
chr18	10324176	10326176	-0.18342	0.032945	hypomethylated	Greb1l	27	36
chr18	10616793	10618793	-0.10277	0.0000111	hypomethylated	Snrpd1	24	37
chr18	10724622	10726622	-0.084494	0.000000997	hypomethylated	Mib1	55	69
chr18	11815350	11817350	-0.39176	1.32E-19	stronglyhypometh	Rbbp8	36	45
chr18	11996782	11998782	-0.15034	0.0000553	hypomethylated	Cables1,Gm6277,Mir1	68	69
chr18	12286358	12288358	-0.23462	7.98E-09	hypomethylated	Riok3	25	30
chr18	12326238	12328238	-0.05125	0.0031867	hypomethylated	3110002H16Rik	19	33
chr18	12491532	12493532	-0.28266	0.0000133	hypomethylated	Lama3	8	12
chr18	12801041	12803041	-0.13347	0.0021818	hypomethylated	Ttc39c	20	20
chr18	14841423	14843423	-0.39011	0.00015142	stronglyhypometh	Ss18	13	13
chr18	15222091	15224091	-0.18235	0.022786	hypomethylated	Kctd1	19	19
chr18	21054579	21056579	-0.17035	0.0099926	hypomethylated	Trappc8	24	28
chr18	22502589	22504589	0.05867	0.0018134	hypermethylated	Asxl3	39	44
chr18	23200154	23202154	-0.16171	0.0072793	hypomethylated	Nol4	2	7
chr18	23961470	23963470	-0.55322	1.65E-19	stronglyhypometh	Mapre2	19	22
chr18	24112188	24114188	-0.25896	0.0000458	hypomethylated	Zfp397	12	17
chr18	24280320	24282320	0.29736	0.0000847	hypermethylated	Ino80c	10	12
chr18	24761461	24763461	-0.062345	0.0000267	hypomethylated	Elp2,Slc39a6	36	38
chr18	24866945	24868945	-0.088816	0.032441	hypomethylated	Fhod3	51	52
chr18	25326520	25328520	-0.033121	0.019466	hypomethylated	AW554918,Tpgs2	47	51
chr18	25327378	25329378	-0.078055	0.0016506	hypomethylated	AW554918,Tpgs2	25	29
chr18	25912484	25914484	-0.013793	0.043991	hypomethylated	Celf4	32	41
chr18	32226387	32228387	-0.073009	0.046064	hypomethylated	Iws1	20	24
chr18	32321742	32323742	-0.31158	2.31E-09	hypomethylated	Map3k2	34	35
chr18	32535870	32537870	-0.16929	0.00030027	hypomethylated	A830052D11Rik,Bin1	21	26
chr18	32975513	32977513	-0.20101	0.030113	hypomethylated	Tslp	2	6
chr18	34166860	34168860	0.13665	0.018065	hypermethylated	Epb4.1l4a	11	13
chr18	34917559	34919559	-0.18013	0.0000649	hypomethylated	Fam53c	18	25
chr18	34935661	34937661	-0.019547	0.001326	hypomethylated	Kdm3b	42	51
chr18	34999311	35001311	-0.1937	0.011423	hypomethylated	Reep2	9	11
chr18	35114005	35116005	-0.4501	7.41E-08	stronglyhypometh	Hspa9	25	33
chr18	35720811	35722811	-0.22687	0.030209	hypomethylated	Matr3	11	15
chr18	35757320	35759320	-0.57006	0.0081292	stronglyhypometh	Paip2	6	13
chr18	36614149	36616149	-1	0.00014006	stronglyhypometh	Pfdn1	3	3
chr18	36941933	36943933	-0.13856	0.025997	hypomethylated	Hars,Hars2	8	13
chr18	36942859	36944859	-0.15572	0.028336	hypomethylated	Hars,Hars2	7	10
chr18	37248789	37250789	0.35275	0.0074155	stronglyhypermeth	Pcdhac1	11	16
chr18	37827758	37829758	-0.47755	0.032894	stronglyhypometh	Pcdhga2	10	7

chr18	37914426	37916426	-0.37763	0.022566	stronglyhypometh	Pcdhga11	3	11
chr18	37973732	37975732	-0.99242	0.000000534	stronglyhypometh	Pcdhgc4	3	11
chr18	38095065	38097065	-0.077303	0.00029389	hypomethylated	Diap1	12	13
chr18	38369416	38371416	-0.24861	1.25E-08	hypomethylated	Pcdh1	18	24
chr18	42110848	42112848	-0.4817	0.00000111	stronglyhypometh	Prelid2	4	4
chr18	42421725	42423725	-0.47376	0.00000311	stronglyhypometh	Lars	11	11
chr18	42434006	42436006	-0.4127	0.011306	stronglyhypometh	Gm4013,Rbm27	9	25
chr18	42553250	42555250	-0.14163	0.012692	hypomethylated	Pou4f3	17	26
chr18	42670140	42672140	-0.077378	0.042642	hypomethylated	Tcerg1	37	50
chr18	44820318	44822318	-0.12135	0.00011249	hypomethylated	A930012L18Rik,Mcc	19	25
chr18	45718807	45720807	-0.12598	0.0043116	hypomethylated	Kcnn2	44	61
chr18	46756357	46758357	-0.13941	0.0000175	hypomethylated	Eif1a,Tmed7	35	45
chr18	46757189	46759189	-0.22421	0.041844	hypomethylated	Eif1a,Tmed7	12	19
chr18	46900570	46902570	-0.10086	2.66E-09	hypomethylated	Ap3s1,Atg12	41	47
chr18	46901233	46903233	-0.11929	2.65E-10	hypomethylated	Ap3s1,Atg12	37	41
chr18	47008692	47010692	-0.24593	0.0000736	hypomethylated	4833403I15Rik	18	19
chr18	47528522	47530522	-0.67857	0.0000227	stronglyhypometh	Sema6a	6	16
chr18	47696388	47698388	-0.39798	0.037351	stronglyhypometh	Gm5095	3	3
chr18	52490189	52492189	0.16667	8.35E-11	hypermethylated	Ftmt	4	6
chr18	53577661	53579661	-0.16159	0.02336	hypomethylated	Ppic	10	10
chr18	56590785	56592785	-0.11229	0.010543	hypomethylated	Gramd3	18	20
chr18	56721222	56723222	-0.25278	0.0068262	hypomethylated	Aldh7a1,Phax	19	22
chr18	56731524	56733524	-0.30445	0.00022352	hypomethylated	Aldh7a1,Phax	23	34
chr18	56732593	56734593	-0.45705	0.0000008	stronglyhypometh	Aldh7a1,Phax	19	24
chr18	58369580	58371580	-0.028506	0.015649	hypomethylated	Fbn2	32	29
chr18	58818135	58820135	-0.17384	3.92E-16	hypomethylated	Isoc1	30	38
chr18	58995417	58997417	-0.26102	0.0063196	hypomethylated	Adamts19	29	37
chr18	59333993	59335993	-0.043009	1.05E-09	hypomethylated	Chsy3	44	60
chr18	60684874	60686874	-0.2057	0.006041	hypomethylated	Dctn4	12	17
chr18	60933249	60935249	-0.098583	0.04237	hypomethylated	Rps14	27	31
chr18	61371250	61373250	-0.48587	0.0000193	stronglyhypometh	Slc26a2	16	20
chr18	61560085	61562085	-0.23341	0.0052105	hypomethylated	Ppargc1b	13	17
chr18	61714235	61716235	-0.21068	0.0000213	hypomethylated	Csnk1a1	38	44
chr18	61885985	61887985	-0.03499	0.031658	hypomethylated	1500015A07Rik,Grpel2	9	12
chr18	62482857	62484857	-0.59531	0.016999	stronglyhypometh	Htr4	5	9
chr18	62707564	62709564	-0.3677	0.018493	stronglyhypometh	Fbxo38,Spink10	17	13
chr18	63080980	63082980	-0.084664	0.026467	hypomethylated	Apcdd1	43	46
chr18	63852013	63854013	-0.54306	0.00027507	stronglyhypometh	Txnl1	5	8
chr18	64648720	64650720	-0.54467	0.00019878	stronglyhypometh	Fech	5	9
chr18	65046409	65048409	-0.08761	0.030962	hypomethylated	Nedd4l	50	61
chr18	65589650	65591650	-0.17272	0.0000269	hypomethylated	Malt1	43	46
chr18	67246989	67248989	-0.13858	0.00000186	hypomethylated	Gnal	45	56
chr18	67364012	67366012	-0.16737	0.0029879	hypomethylated	Chmp1b	15	19
chr18	67502217	67504217	-0.26867	0.045105	hypomethylated	B430212C06Rik,Cidea	7	7
chr18	67800252	67802252	-0.095896	0.0000478	hypomethylated	Cep76,Psmg2	64	73
chr18	67800990	67802990	-0.06692	0.024345	hypomethylated	Cep76,Psmg2	33	39
chr18	68459008	68461008	-0.38331	0.0000329	stronglyhypometh	Fam210a,Rnmt	19	25
chr18	70631198	70633198	0.10225	0.041647	hypermethylated	4930503L19Rik,Stard6	24	33
chr18	70689792	70691792	-0.27876	0.0041601	hypomethylated	Poli	5	4
chr18	73731358	73733358	0.039848	0.00000238	hypermethylated	Mex3c	94	113
chr18	73914133	73916133	-0.22222	0.0012354	hypomethylated	Elac1	9	19
chr18	74426941	74428941	-0.4226	0.00027144	stronglyhypometh	Mbd1	20	18
chr18	75159130	75161130	-0.10007	0.021816	hypomethylated	Rpl17,Snord58b	37	37
chr18	75159721	75161721	-0.10007	0.021816	hypomethylated	Rpl17,Snord58b	37	37
chr18	75177425	75179425	-0.086791	0.033007	hypomethylated	Dym	24	28
chr18	75978831	75980831	-0.16426	0.0000104	hypomethylated	Zbtb7c	60	69
chr18	77094143	77096143	-0.1421	0.0000468	hypomethylated	Skor2	14	14

chr18	77167765	77169765	-0.10156	0.00069702	hypomethylated	Ier3ip1	21	24
chr18	77181853	77183853	-0.083575	0.0000245	hypomethylated	Hdhd2	13	22
chr18	77182156	77184156	-0.083575	0.0000245	hypomethylated	Hdhd2	13	22
chr18	77302946	77304946	0.26487	0.0017271	hypermethylated	Pias2	25	47
chr18	77303418	77305418	0.23287	0.0026935	hypermethylated	Pias2	25	45
chr18	77951922	77953922	-0.10695	0.00000421	hypomethylated	4930465K10Rik,80304	59	86
chr18	78032288	78034288	-0.065111	0.033594	hypomethylated	Pstpip2	36	43
chr18	80242633	80244633	-0.13325	0.017388	hypomethylated	Pard6g	33	32
chr18	80909810	80911810	-0.11761	0.00018322	hypomethylated	Nfatc1	9	19
chr18	81183317	81185317	-0.033042	0.0088569	hypomethylated	Sall3	68	77
chr18	82576169	82578169	-0.32659	0.03755	hypomethylated	Galr1	8	6
chr18	82643514	82645514	-0.22807	0.0011222	hypomethylated	Mbp	17	27
chr18	84255954	84257954	-0.16847	0.0000121	hypomethylated	Tshz1,Zadh2	48	48
chr18	84256549	84258549	-0.17163	0.0000138	hypomethylated	Tshz1,Zadh2	51	51
chr18	84855025	84857025	-0.26266	0.010397	hypomethylated	Cndp2	6	8
chr18	85019805	85021805	-0.11904	0.027173	hypomethylated	Cyb5	12	13
chr18	86563343	86565343	-0.2328	0.00028942	hypomethylated	Neto1	21	26
chr18	90678545	90680545	-0.097698	0.024899	hypomethylated	Tmx3	12	15
chr19	3282046	3284046	-0.54282	0.0000172	stronglyhypometh	Ighmbp2,Mrpl21	10	12
chr19	3283010	3285010	-0.41144	0.00000895	stronglyhypometh	Ighmbp2,Mrpl21	11	13
chr19	3322300	3324300	-0.0075002	0.046686	hypomethylated	Cpt1a	16	27
chr19	3387868	3389868	-0.18803	2.15E-09	hypomethylated	Mtl5	23	23
chr19	3575749	3577749	-0.3586	0.0041736	stronglyhypometh	Ppp6r3	10	14
chr19	3766420	3768420	-0.037209	0.040528	hypomethylated	Suv420h1	54	54
chr19	3999579	4001579	-0.075218	0.0085747	hypomethylated	Nudt8	34	34
chr19	4163245	4165245	-0.62932	0.0000694	stronglyhypometh	Rps6kb2	6	7
chr19	4191047	4193047	-0.18296	0.00032791	hypomethylated	Ppp1ca,Tbc1d10c	45	48
chr19	4191173	4193173	-0.18296	0.00032791	hypomethylated	Ppp1ca,Tbc1d10c	45	48
chr19	4213391	4215391	-0.071102	0.03208	hypomethylated	Clcf1	24	28
chr19	4305955	4307955	-0.070426	0.002963	hypomethylated	Adrbk1	28	33
chr19	4509471	4511471	-0.058085	0.013145	hypomethylated	Pcx	19	21
chr19	4625617	4627617	0.083836	0.0084812	hypermethylated	Rce1	16	16
chr19	4854128	4856128	-0.6702	6.72E-11	stronglyhypometh	Ctsf	17	17
chr19	4877667	4879667	-0.33977	0.00000201	stronglyhypometh	Actn3,Zdhhc24	16	21
chr19	4877689	4879689	-0.33977	0.00000201	stronglyhypometh	Actn3,Zdhhc24	16	21
chr19	4906627	4908627	-0.14091	0.0019368	hypomethylated	Bbs1	4	5
chr19	5040403	5042403	-0.064622	0.011612	hypomethylated	Brms1	26	27
chr19	5067077	5069077	-0.26786	0.0026372	hypomethylated	Cd248	17	25
chr19	5085477	5087477	-0.61823	0.0000651	stronglyhypometh	Tmem151a	14	21
chr19	5118408	5120408	-0.34022	0.00036867	stronglyhypometh	Klc2	10	10
chr19	5273119	5275119	-0.047619	0.00086112	hypomethylated	Pacs1	12	16
chr19	5365812	5367812	-0.18332	9.11E-09	hypomethylated	Banf1,Eif1ad	43	46
chr19	5366347	5368347	-0.20423	5.83E-09	hypomethylated	Banf1,Eif1ad	43	45
chr19	5366645	5368645	-0.39575	3.4E-14	stronglyhypometh	Banf1,Eif1ad	23	25
chr19	5387335	5389335	-0.11874	0.0014091	hypomethylated	D330050I16Rik,Sart1	35	47
chr19	5424143	5426143	-0.19431	0.00000323	hypomethylated	AI837181,Drap1	49	62
chr19	5424916	5426916	-0.22542	0.000000944	hypomethylated	AI837181,Drap1	30	38
chr19	5446697	5448697	-0.17197	0.032725	hypomethylated	Fosl1	19	20
chr19	5489454	5491454	-0.1321	0.0015447	hypomethylated	Cfl1	41	46
chr19	5510489	5512489	-0.11454	0.0013001	hypomethylated	Snx32	24	25
chr19	5560575	5562575	-0.22473	0.048549	hypomethylated	Ovol1	21	26
chr19	5567073	5569073	-0.0857	0.0000114	hypomethylated	Ap5b1	48	59
chr19	5600872	5602872	-0.29113	0.0000325	hypomethylated	Rnaseh2c	16	14
chr19	5636489	5638489	-0.13744	0.00000025	hypomethylated	Rela	49	61
chr19	5845478	5847478	-0.49417	0.000000614	stronglyhypometh	Neat1	13	13
chr19	5875208	5877208	0.26964	0.0010645	hypermethylated	Frmd8	7	7
chr19	6084096	6086096	-0.64967	1.06E-08	stronglyhypometh	Cdca5,Zfpl1	18	23

chr19	6084891	6086891	-0.60225	1.73E-08	stronglyhypometh	Cdca5,Zfpl1	20	25
chr19	6141137	6143137	-0.22095	0.037989	hypomethylated	Arl2	5	5
chr19	6235840	6237840	-0.27755	0.0020754	hypomethylated	Ppp2r5b	7	7
chr19	6275895	6277895	-0.10897	0.0015029	hypomethylated	Ehd1	46	42
chr19	6305456	6307456	-0.11313	0.00098472	hypomethylated	Cdc42bpg	21	21
chr19	6333978	6335978	-0.34017	0.022352	stronglyhypometh	Men1	15	17
chr19	6334012	6336012	-0.34017	0.022352	stronglyhypometh	Men1	15	17
chr19	6334038	6336038	-0.34017	0.022352	stronglyhypometh	Men1	15	17
chr19	6399582	6401582	-0.05921	0.033599	hypomethylated	Rasgrp2	28	29
chr19	6427015	6429015	-0.026739	0.031285	hypomethylated		44	57
chr19	6983187	6985187	-0.23422	0.0076109	hypomethylated	Prdx5,Trmt112	31	33
chr19	6996298	6998298	-0.37084	0.0000495	stronglyhypometh	Esrra	17	20
chr19	6999870	7001870	-0.27653	0.0059231	hypomethylated	Tex40	15	15
chr19	7015344	7017344	-0.11139	0.00043646	hypomethylated	Bad,Gpr137	41	46
chr19	7015683	7017683	-0.11139	0.00043646	hypomethylated	Bad,Gpr137	41	46
chr19	7016940	7018940	-0.13247	0.023505	hypomethylated	Bad,Gpr137	6	6
chr19	7048537	7050537	-0.12531	0.017079	hypomethylated	Ppp1r14b	22	24
chr19	7062141	7064141	-0.077856	0.030891	hypomethylated	Vegfb	16	18
chr19	7130257	7132257	-0.12463	0.0021258	hypomethylated	Macrod1	19	21
chr19	7280774	7282774	-0.51389	0.0079861	stronglyhypometh	Otub1	3	6
chr19	7567529	7569529	-0.20091	0.00000019	hypomethylated	Atl3	29	32
chr19	7567927	7569927	-0.20091	0.00000019	hypomethylated	Atl3	29	32
chr19	8809328	8811328	0.10977	9.23E-08	hypermethylated	Wdr74	11	16
chr19	8830592	8832592	-0.18221	0.00089439	hypomethylated	Nxf1	34	34
chr19	8844485	8846485	-0.24869	0.029277	hypomethylated	Tmem223	10	10
chr19	8847974	8849974	-0.16078	0.00046057	hypomethylated	Gm2518,Tmem179b	46	47
chr19	8848957	8850957	-0.20109	0.00016435	hypomethylated	Gm2518,Tmem179b	42	44
chr19	8892890	8894890	-0.26917	0.0097928	hypomethylated	Hnrnpul2,Ttc9c	15	21
chr19	8962392	8964392	-0.18432	0.0000507	hypomethylated	1810009A15Rik,57304	12	15
chr19	8963260	8965260	-0.10565	0.00027262	hypomethylated	1810009A15Rik,57304	10	10
chr19	8966476	8968476	-0.37588	0.0018504	stronglyhypometh	Ints5	21	16
chr19	9015409	9017409	-0.10338	0.0000629	hypomethylated	Mta2	54	55
chr19	9974023	9976023	0.10045	0.041568	hypermethylated	Incenp	11	10
chr19	10115037	10117037	-0.14784	0.0000682	hypomethylated	Fads3	31	30
chr19	10462579	10464579	-0.11969	0.0052007	hypomethylated	Syt7	69	62
chr19	10598733	10600733	-0.33833	1.73E-11	stronglyhypometh	Cpsf7,Sdhaf2	42	40
chr19	10599699	10601699	-0.38761	6.3E-15	stronglyhypometh	Cpsf7,Sdhaf2	37	29
chr19	10762304	10764304	-0.079	0.036578	hypomethylated	Vps37c	25	25
chr19	10944269	10946269	-0.071429	0.000053	hypomethylated	Tmem132a	7	7
chr19	10968781	10970781	-0.057799	0.00504	hypomethylated	Prpf19	18	17
chr19	11893893	11895893	-0.49398	0.0031769	stronglyhypometh	Stx3	6	8
chr19	11985888	11987888	-0.18925	0.025545	hypomethylated	Patl1	20	31
chr19	12039333	12041333	-0.074429	0.0027874	hypomethylated	Osbp	43	47
chr19	16059479	16061479	-0.2192	0.0000626	hypomethylated	C130060C02Rik,Cep78	8	8
chr19	16206320	16208320	-0.059593	2.13E-08	hypomethylated	Gnaq	71	88
chr19	16509156	16511156	-0.16558	0.0021659	hypomethylated	Gna14	22	25
chr19	17912122	17914122	-0.20918	0.0023175	hypomethylated	Pcsk5	12	14
chr19	18705505	18707505	-0.33643	7.94E-09	stronglyhypometh	Nmrk1,Ostf1	28	25
chr19	21851831	21853831	-0.042799	0.010304	hypomethylated	Tmem2	30	42
chr19	23214715	23216715	-0.17019	4.31E-16	hypomethylated	Klf9	50	45
chr19	23760889	23762889	-0.030277	0.00023297	hypomethylated	Ptar1	41	55
chr19	23832365	23834365	-0.21296	6.32E-08	hypomethylated	Apba1	22	30
chr19	25684026	25686026	-0.047689	0.049254	hypomethylated	Dmrt3	55	62
chr19	27504310	27506310	-0.42068	0.0000175	stronglyhypometh	C030016D13Rik,D19Bv	8	10
chr19	29063983	29065983	-0.22648	0.0000809	hypomethylated	Cdc37l1	30	34
chr19	29174864	29176864	-0.083671	0.0016114	hypomethylated	Rcl1	33	44
chr19	29325317	29327317	0.079548	0.0000236	hypermethylated	Jak2	67	75

chr19	29595771	29597771	-0.07675	0.0000283	hypomethylated	A930007I19Rik,C0300	85	90
chr19	29596477	29598477	-0.091423	0.0000287	hypomethylated	A930007I19Rik,C0300	81	86
chr19	29880499	29882499	-0.15567	0.00000529	hypomethylated	9930021J03Rik	25	22
chr19	29887464	29889464	0.14422	0.021849	hypermethylated	Ranbp6	9	10
chr19	31738860	31740860	-0.54557	0.000000163	stronglyhypometh	Prkg1	5	10
chr19	32462705	32464705	-0.075468	0.031878	hypomethylated	2700046G09Rik,Sgms1	67	66
chr19	32462944	32464944	-0.13197	0.0017995	hypomethylated	2700046G09Rik,Sgms1	42	41
chr19	32831066	32833066	-0.062752	0.013697	hypomethylated	Pten	63	68
chr19	34265759	34267759	-0.33883	0.000000391	stronglyhypometh	Stambpl1	18	20
chr19	34821601	34823601	-0.13186	0.004767	hypomethylated	Slc16a12	16	16
chr19	36628128	36630128	-0.040355	0.0068295	hypomethylated	Hectd2	31	46
chr19	36907721	36909721	-0.1144	2.18E-08	hypomethylated	Tnks2	31	29
chr19	36999568	37001568	-0.062213	0.00000929	hypomethylated	Btaf1	50	52
chr19	37281034	37283034	-0.17869	0.00086224	hypomethylated	Cpeb3,March5	60	69
chr19	37281783	37283783	-0.15298	0.012856	hypomethylated	Cpeb3,March5	48	51
chr19	37449892	37451892	-0.39699	2.73E-12	stronglyhypometh	Kif11	14	13
chr19	37508330	37510330	-0.078976	0.0047553	hypomethylated	Hhex	48	54
chr19	37623907	37625907	-0.075352	0.0004832	hypomethylated	Exoc6	37	47
chr19	37771297	37773297	-0.24086	0.0000528	hypomethylated	Cyp26a1	21	22
chr19	38338271	38340271	-0.24269	0.035183	hypomethylated	Lgi1	3	3
chr19	38469469	38471469	-0.12578	0.0031307	hypomethylated	Slc35g1	25	23
chr19	40346106	40348106	-0.25078	7.07E-08	hypomethylated	Pdlim1	8	8
chr19	40968194	40970194	-0.41213	0.0000462	stronglyhypometh	Zfp518a	22	31
chr19	41922622	41924622	-0.5	0.00013703	stronglyhypometh	Frat2	8	9
chr19	42007804	42009804	-0.14913	0.020383	hypomethylated	Exosc1,Zdhhc16	14	19
chr19	42163924	42165924	-0.1885	0.00028937	hypomethylated	Pi4k2a	38	38
chr19	42220878	42222878	0.098678	0.021741	hypermethylated	Marveld1	21	33
chr19	42244056	42246056	-0.077212	0.010376	hypomethylated	Zfyve27	30	30
chr19	42329228	42331228	-0.20813	0.0067467	hypomethylated	Golga7b	20	22
chr19	42506273	42508273	-0.055095	0.0086797	hypomethylated	Crtac1	15	18
chr19	42592294	42594294	-0.25365	0.000022	hypomethylated	R3hcc1l	14	16
chr19	42687296	42689296	-0.2376	0.030318	hypomethylated	Loxl4	7	11
chr19	43599095	43601095	-0.23483	0.017346	hypomethylated	Got1	8	8
chr19	43685814	43687814	-0.49393	0.002581	stronglyhypometh	Nkx2-3	10	19
chr19	43749371	43751371	0.11763	0.0010289	hypermethylated	BC037704,Slc25a28	19	15
chr19	44144176	44146176	-0.045806	0.025978	hypomethylated	Erlin1	20	20
chr19	44144265	44146265	-0.077796	0.022417	hypomethylated	Erlin1	13	13
chr19	44144275	44146275	-0.094304	0.001783	hypomethylated	Erlin1	12	12
chr19	44482199	44484199	-0.26165	0.011532	hypomethylated	Scd1	6	6
chr19	44629905	44631905	-0.16667	0.0047014	hypomethylated	Ndufb8	6	6
chr19	45080047	45082047	-0.16128	0.044416	hypomethylated	Mrpl43,Peo1	37	36
chr19	45120262	45122262	-0.49784	0.022197	stronglyhypometh	Pdzd7,Sfxn3	3	7
chr19	45121065	45123065	-0.60606	0.0098051	stronglyhypometh	Pdzd7,Sfxn3	3	3
chr19	45224204	45226204	0.10565	0.012531	hypermethylated	Tlx1	21	30
chr19	45309726	45311726	-0.068993	0.0016997	hypomethylated	Lbx1	48	56
chr19	45734683	45736683	-0.22306	5.43E-08	hypomethylated	Fbxw4	8	20
chr19	45857781	45859781	-0.33727	0.000000239	stronglyhypometh	Mgea5	29	29
chr19	46076967	46078967	-0.11669	0.037948	hypomethylated	Hps6	25	25
chr19	46119701	46121701	-0.11695	0.016502	hypomethylated	Ldb1	25	25
chr19	46130028	46132028	-0.30961	6.42E-13	hypomethylated	Pprc1	30	34
chr19	46378226	46380226	-0.10027	0.0000215	hypomethylated	Nfkb2	35	45
chr19	46379184	46381184	-0.10214	0.00000448	hypomethylated	Nfkb2	33	40
chr19	46379419	46381419	-0.16513	0.0000538	hypomethylated	Nfkb2	27	33
chr19	46470407	46472407	0.1588	0.0061484	hypermethylated	Actr1a,Sufu	12	23
chr19	46646854	46648854	-0.48238	0.00000611	stronglyhypometh	Arl3,Sfxn2	9	15
chr19	46647575	46649575	-0.42862	0.000011	stronglyhypometh	Arl3,Sfxn2	9	16
chr19	46763395	46765395	-0.36556	0.020513	stronglyhypometh	2010012O05Rik	14	14

chr19	46835098	46837098	-0.12209	0.043759	hypomethylated	Cnnm2	35	55
chr19	47088187	47090187	-0.38514	0.00090476	stronglyhypometh	Ina,Nt5c2	26	27
chr19	47252309	47254309	-0.15867	0.00044398	hypomethylated	Neurl1a	58	58
chr19	47805245	47807245	-0.44851	0.00023778	stronglyhypometh	Sfr1	11	16
chr19	47993789	47995789	-0.31454	0.000036	hypomethylated	Itprip	19	19
chr19	48279514	48281514	-0.22021	0.026425	hypomethylated	Sorcs3	38	43
chr19	53216245	53218245	-0.45802	3.07E-11	stronglyhypometh	Add3	27	27
chr19	53217486	53219486	-0.18035	0.045035	hypomethylated	Add3	19	20
chr19	53383995	53385995	-0.12025	0.038569	hypomethylated	Mxi1	22	31
chr19	53465063	53467063	-0.21524	0.0033312	hypomethylated	Smndc1	11	15
chr19	53602807	53604807	-0.31662	1.58E-08	hypomethylated	Dusp5	46	47
chr19	53673885	53675885	-0.079641	0.00087229	hypomethylated	Smc3	32	48
chr19	53976840	53978840	-0.056881	0.0044332	hypomethylated	Pdcd4	44	45
chr19	55390522	55392522	0.052729	0.0057328	hypermethylated	Vti1a,Zdhhc6	22	25
chr19	55815299	55817299	-0.0085894	0.029555	hypomethylated	Tcf7l2	43	48
chr19	56899698	56901698	-0.02842	0.0022198	hypomethylated	Tdrd1	16	17
chr19	56899764	56901764	-0.02842	0.0022198	hypomethylated	Tdrd1	16	17
chr19	56899775	56901775	-0.02842	0.0022198	hypomethylated	Tdrd1	16	17
chr19	57526395	57528395	-0.28571	0.028582	hypomethylated	Trub1	4	4
chr19	58935474	58937474	-0.065	0.028573	hypomethylated	Hspa12a	5	8
chr19	59533125	59535125	-0.25476	0.00012805	hypomethylated	Emx2,Emx2os	26	42
chr19	60964651	60966651	-0.056105	0.00032701	hypomethylated	Grk5	31	37
chr19	61304321	61306321	0.461	0.038934	stronglyhypermeth	Csf2ra	4	7
chr2	3200559	3202559	-0.19731	0.0052921	hypomethylated	Nmt2	26	28
chr2	3429336	3431336	-0.22239	0.0037677	hypomethylated	Cdnf,Hspa14	22	29
chr2	3430086	3432086	-0.32544	0.0014411	hypomethylated	Cdnf,Hspa14	13	15
chr2	4801609	4803609	-0.15253	0.00000614	hypomethylated	Sephs1	56	70
chr2	5057821	5059821	-0.14204	0.00000608	hypomethylated	Ccdc3	28	32
chr2	5635710	5637710	0.101	0.039021	hypermethylated	Camk1d	18	27
chr2	7002348	7004348	-0.46429	0.0016735	stronglyhypometh	Celf2	2	2
chr2	10001238	10003238	-0.53268	0.00046258	stronglyhypometh	Atp5c1,Kin	7	18
chr2	10293062	10295062	-0.43326	0.0000234	stronglyhypometh	Sfmbt2	26	26
chr2	11093008	11095008	-0.069318	0.0044882	hypomethylated	Prkcq	8	25
chr2	11626162	11628162	-0.11	0.049812	hypomethylated	Il15ra	12	15
chr2	11626474	11628474	-0.11528	0.009036	hypomethylated	Il15ra	15	18
chr2	12223547	12225547	0.17267	0.0057739	hypermethylated	E030013I19Rik,Itga8	9	9
chr2	14524932	14526932	-0.078048	0.0076903	hypomethylated	Cacnb2	34	45
chr2	14975988	14977988	-0.34437	2.64E-10	stronglyhypometh	Arl5b,Nsun6	39	39
chr2	14976499	14978499	-0.34437	2.64E-10	stronglyhypometh	Arl5b,Nsun6	39	39
chr2	16276948	16278948	-0.35156	0.0000338	stronglyhypometh	Plxdc2	24	34
chr2	17652695	17654695	-0.1425	0.010564	hypomethylated	Nebl	7	8
chr2	17947875	17949875	-0.4927	0.000000506	stronglyhypometh	Gm17762	7	25
chr2	17959368	17961368	-0.36558	0.00065791	stronglyhypometh	A930004D18Rik	7	7
chr2	19292262	19294262	-0.62121	0.0048879	stronglyhypometh	Msrb2	4	4
chr2	19366289	19368289	-0.36477	0.012232	stronglyhypometh	Ptf1a	24	22
chr2	19578688	19580688	-0.085187	0.00000512	hypomethylated	Gm3230,Otud1	61	68
chr2	19579524	19581524	-0.080436	0.0000574	hypomethylated	Gm3230,Otud1	55	61
chr2	22476846	22478846	0.15947	0.015483	hypermethylated	Gad2	12	16
chr2	22750041	22752041	-0.51149	0.0012775	stronglyhypometh	Pdss1	19	22
chr2	23011065	23013065	-0.16895	0.0000133	hypomethylated	Mastl,Yme1l1	20	24
chr2	23011544	23013544	-0.12328	0.00000185	hypomethylated	Mastl,Yme1l1	14	19
chr2	24775110	24777110	0.048282	0.00065668	hypermethylated	Ehmt1	15	15
chr2	24790801	24792801	-0.31291	0.013087	hypomethylated	Arrdc1	13	17
chr2	24804321	24806321	-0.17875	4.87E-11	hypomethylated	Zmynd19	62	72
chr2	25035277	25037277	0.31416	1.08E-10	hypermethylated	Nrarp	16	31
chr2	25109958	25111958	0.066633	0.041534	hypermethylated	Ndor1,Tmem203	27	31
chr2	25110934	25112934	0.084416	0.0374	hypermethylated	Ndor1,Tmem203	17	21

chr2	25117117	25119117	0.015035	0.024012	hypermethylated	Tprn	32	38
chr2	25126985	25128985	-0.31012	3.72E-09	hypomethylated	Anapc2,Ssna1	23	31
chr2	25221146	25223146	-0.73136	0.000000525	stronglyhypometh	Uap1l1	6	8
chr2	25226840	25228840	-0.091087	0.0000158	hypomethylated	Sapcd2	20	22
chr2	25402414	25404414	-0.18043	0.00011433	hypomethylated	Traf2	7	10
chr2	25412419	25414419	-0.39924	2.9E-13	stronglyhypometh	Edf1	23	27
chr2	26062076	26064076	-0.18357	0.0061614	hypomethylated	Lhx3	12	17
chr2	26243867	26245867	-0.19153	0.000000184	hypomethylated	Pmpca,Sdccag3	25	37
chr2	26459730	26461730	-0.54154	0.0000327	stronglyhypometh	Agpat2	19	19
chr2	26766162	26768162	-0.5926	0.0066934	stronglyhypometh	Med22,Rpl7a	4	7
chr2	27740944	27742944	-0.14643	0.00032093	hypomethylated	Col5a1	39	39
chr2	28060208	28062208	-0.090052	0.00011313	hypomethylated	Olfm1	49	47
chr2	28302460	28304460	-0.14825	0.00010415	hypomethylated	Ppp1r26	32	32
chr2	28367686	28369686	-0.020864	0.028908	hypomethylated	Ralgds	46	46
chr2	28495762	28497762	0.097765	0.016104	hypermethylated	Tsc1	23	23
chr2	28554680	28556680	-0.41294	0.0000061	stronglyhypometh	1700026L06Rik,Ak8	24	24
chr2	28555171	28557171	-0.40661	0.0000102	stronglyhypometh	1700026L06Rik,Ak8	24	22
chr2	28910586	28912586	-0.10739	0.00000273	hypomethylated	1700101E01Rik	10	10
chr2	29642782	29644782	-0.31352	0.00000094	hypomethylated	Coq4,Trub2	8	12
chr2	29643191	29645191	-0.38769	0.0000242	stronglyhypometh	Coq4,Trub2	7	8
chr2	29657199	29659199	-0.22453	0.0013637	hypomethylated	Slc27a4	33	35
chr2	29701247	29703247	-0.042374	0.028017	hypomethylated	Mir219-2	29	34
chr2	29724013	29726013	-0.11497	0.0073611	hypomethylated	Cercam	13	10
chr2	29744239	29746239	-0.062226	0.0066143	hypomethylated	Odf2	48	54
chr2	29744582	29746582	-0.068805	0.0018968	hypomethylated	Odf2	47	53
chr2	29949155	29951155	-0.72028	0.0000046	stronglyhypometh	Zdhhc12	9	10
chr2	29988390	29990390	-0.10788	0.0075646	hypomethylated	Tbc1d13	14	20
chr2	30026043	30028043	-0.18687	0.0080388	hypomethylated	Endog	13	13
chr2	30141874	30143874	-0.71975	0.00020391	stronglyhypometh	Dolk,Nup188	7	8
chr2	31006835	31008835	-0.41736	0.0025844	stronglyhypometh	D330023K18Rik,Gpr10	11	16
chr2	31100442	31102442	0.053216	0.042775	hypermethylated	Ncs1	51	64
chr2	31525256	31527256	-0.4077	3E-13	stronglyhypometh	Exosc2	12	12
chr2	31543075	31545075	-0.15332	0.028893	hypomethylated	Abl1	38	40
chr2	31614464	31616464	-0.23274	0.036669	hypomethylated	Abl1	12	13
chr2	31701525	31703525	-0.21269	0.025574	hypomethylated	Fibcd1	17	25
chr2	31741800	31743800	-0.11278	0.035785	hypomethylated	Lamc3	17	17
chr2	31804822	31806822	0.087874	0.000081	hypermethylated	Aif1l	20	34
chr2	31828969	31830969	-0.46501	0.0000107	stronglyhypometh	Nup214	20	25
chr2	32142772	32144772	-0.19797	0.0022139	hypomethylated	Golga2,Swi5	8	12
chr2	32143588	32145588	-0.19797	0.0022139	hypomethylated	Golga2,Swi5	8	12
chr2	32172979	32174979	-0.63056	0.0021479	stronglyhypometh	Mir199b	6	6
chr2	32568304	32570304	-0.013296	0.02915	hypomethylated	Cdk9,Mir2861,Mir396(	27	29
chr2	32568408	32570408	-0.032384	0.013391	hypomethylated	Cdk9,Mir2861,Mir396(	26	28
chr2	32816231	32818231	-0.18688	0.0072143	hypomethylated	Lrsam1,Rpl12,Snora65	27	28
chr2	32816771	32818771	-0.22444	0.017516	hypomethylated	Lrsam1,Rpl12,Snora65	24	24
chr2	32837576	32839576	-0.17879	0.0000228	hypomethylated	Slc2a8	12	12
chr2	33496031	33498031	-0.11658	0.00000137	hypomethylated	C130021I20Rik,Lmx1b	34	52
chr2	34626609	34628609	-0.032461	0.0085778	hypomethylated	Hspa5	25	32
chr2	35136959	35138959	-0.60623	0.00059921	stronglyhypometh	Gsn	17	17
chr2	35990917	35992917	-0.45182	0.00085417	stronglyhypometh	Mrrf,Rbm18	8	10
chr2	35992224	35994224	-0.81667	0.00000199	stronglyhypometh	Mrrf,Rbm18	6	6
chr2	36084945	36086945	-0.5746	0.035241	stronglyhypometh	Ptgs1	7	9
chr2	37306773	37308773	-0.058604	0.00051933	hypomethylated	Rabgap1	13	21
chr2	38862658	38864658	-0.11495	1.89E-10	hypomethylated	Arpc5l	33	42
chr2	48804027	48806027	-0.14357	0.0024061	hypomethylated	Mbd5,Orc4	13	18
chr2	49306005	49308005	-0.06732	0.00021735	hypomethylated	Epc2	57	70
chr2	49642205	49644205	-0.20707	0.0001618	hypomethylated	Lypd6b	21	21

chr2	49920981	49922981	-0.51409	0.011961	stronglyhypometh	Lypd6	15	36
chr2	51927356	51929356	-0.056781	0.0015109	hypomethylated	Rif1	52	57
chr2	53050117	53052117	-0.36972	1.68E-16	stronglyhypometh	Arl6ip6,Prpf40a	38	55
chr2	53050221	53052221	-0.38462	2.55E-13	stronglyhypometh	Arl6ip6,Prpf40a	32	49
chr2	55288566	55290566	-0.14386	0.0000722	hypomethylated	Kcnj3	12	18
chr2	58997906	58999906	-0.1612	0.0000263	hypomethylated	Ccdc148,Pkp4	51	58
chr2	58998684	59000684	-0.1866	0.000000841	hypomethylated	Ccdc148,Pkp4	47	59
chr2	60046992	60048992	-0.10216	5.49E-08	hypomethylated	March7	57	64
chr2	61548750	61550750	-0.10084	0.00043666	hypomethylated	Psmd14	19	22
chr2	62501383	62503383	-0.12479	0.016863	hypomethylated	Gca	16	23
chr2	63936064	63938064	-0.14707	0.013187	hypomethylated	Fign	49	49
chr2	65076683	65078683	-0.13306	0.046006	hypomethylated	Cobll1	37	37
chr2	68972856	68974856	-0.33796	0.0033592	stronglyhypometh	Nostrin	2	2
chr2	69484311	69486311	-0.267	0.04265	hypomethylated	Bbs5	9	9
chr2	69560144	69562144	-0.27464	0.00000103	hypomethylated	Ppig	27	39
chr2	69659426	69661426	-0.048275	0.0081502	hypomethylated	Klhl23	48	54
chr2	69734302	69736302	-0.038385	0.00041231	hypomethylated	Ubr3	45	45
chr2	70311979	70313979	-0.44638	0.0015114	stronglyhypometh	Sp5	13	20
chr2	70399220	70401220	0.093898	0.016677	hypermethylated	1500002O10Rik,Gad1	8	15
chr2	70498565	70500565	-0.103	0.00010625	hypomethylated	Gorasp2	42	45
chr2	71048762	71050762	-0.23106	0.00035496	hypomethylated	Dync1i2	15	25
chr2	71049002	71051002	-0.23106	0.00035496	hypomethylated	Dync1i2	15	25
chr2	71205611	71207611	-0.099564	0.0031427	hypomethylated	Slc25a12	12	15
chr2	71290394	71292394	-0.20933	0.00029197	hypomethylated	Metap1d	18	19
chr2	71710328	71712328	-0.40972	0.0000194	stronglyhypometh	Pdk1	8	18
chr2	72817338	72819338	-0.26891	0.0029216	hypomethylated	1700011J10Rik,Sp3	53	63
chr2	73108982	73110982	0.19008	0.0090018	hypermethylated	Sp9	17	18
chr2	73149708	73151708	-0.71931	6.24E-17	stronglyhypometh	Cir1,Scrn3	23	23
chr2	73150649	73152649	-0.77163	4.25E-12	stronglyhypometh	Cir1,Scrn3	11	15
chr2	73730685	73732685	-0.46667	0.0050131	stronglyhypometh	Atf2	5	5
chr2	74505366	74507366	-0.15995	2.38E-10	hypomethylated	Hoxd13	39	48
chr2	74512086	74514086	-0.31836	0.00047944	hypomethylated	Hoxd12	24	25
chr2	74529004	74531004	-0.40448	0.0000488	stronglyhypometh	Hoxd10	24	29
chr2	74600036	74602036	-0.089658	0.032273	hypomethylated	Hoxd1	46	48
chr2	75496315	75498315	-0.046895	0.0020774	hypomethylated	Gm6793,Hnrnpa3	49	53
chr2	75496346	75498346	-0.046895	0.0020774	hypomethylated	Gm6793,Hnrnpa3	49	53
chr2	75669233	75671233	0.37972	0.000000357	stronglyhypermeth	Agps	23	17
chr2	76176710	76178710	0.2492	0.049923	hypermethylated	Pde11a	10	11
chr2	76207040	76209040	-0.23191	0.037324	hypomethylated	Rbm45	16	24
chr2	76243594	76245594	-0.18648	5.5E-12	hypomethylated	Osbpl6	85	90
chr2	76511155	76513155	-0.48569	0.021771	stronglyhypometh	Fkbp7,Plekha3	4	8
chr2	78708203	78710203	-0.041861	0.0092765	hypomethylated	Ube2e3	68	86
chr2	84581335	84583335	-0.54444	0.0000444	stronglyhypometh	Mir130a	9	9
chr2	84637984	84639984	-0.16161	0.018633	hypomethylated	Ube2l6	4	5
chr2	84678564	84680564	-0.0695	0.018634	hypomethylated	Slc43a1	20	28
chr2	84679479	84681479	-0.087557	0.00011152	hypomethylated	Slc43a1	23	31
chr2	84726849	84728849	-0.0536	0.00045532	hypomethylated	Rtn4rl2	20	21
chr2	84775812	84777812	0.27816	0.0079849	hypermethylated	Slc43a3	10	10
chr2	90420804	90422804	-0.091949	0.048542	hypomethylated	Ptprj	16	16
chr2	90686311	90688311	-0.092878	0.0023927	hypomethylated	Mtch2	40	42
chr2	90743942	90745942	-0.73954	0.0000094	stronglyhypometh	Kbtbd4,Ndufs3	7	9
chr2	90744878	90746878	-0.64394	0.00000148	stronglyhypometh	Kbtbd4,Ndufs3	10	12
chr2	90779614	90781614	0.083932	0.034749	hypermethylated	Celf1	47	57
chr2	90910378	90912378	0.23054	0.0041764	hypermethylated	Slc39a13	14	23
chr2	90935953	90937953	-0.43044	0.0024728	stronglyhypometh	Sfpi1	8	7
chr2	91042068	91044068	-0.034041	0.0020101	hypomethylated	Acp2	13	10
chr2	91096485	91098485	0.15244	0.0012492	hypermethylated	Pacsin3	20	22

chr2	91104271	91106271	-0.27302	7.93E-09	hypomethylated	Arfgap2	22	17
chr2	91489274	91491274	-0.15277	0.00094287	hypomethylated	Arhgap1,Zfp408	18	21
chr2	91489948	91491948	-0.056121	0.022192	hypomethylated	Arhgap1,Zfp408	14	13
chr2	91550106	91552106	-0.080701	0.026262	hypomethylated	Atg13,Harbi1	17	18
chr2	91569294	91571294	-0.23567	0.0016741	hypomethylated	Ambra1	16	17
chr2	91761345	91763345	-0.092244	0.00214	hypomethylated	Chrm4	39	47
chr2	91803720	91805720	-0.057779	0.0082124	hypomethylated	Dgkz	11	11
chr2	92299951	92301951	-0.23597	0.0050274	hypomethylated	Slc35c1	9	12
chr2	92754257	92756257	-0.27443	0.049553	hypomethylated	Syt13	3	14
chr2	93026740	93028740	-0.14138	0.00000219	hypomethylated	Trp53i11	46	48
chr2	101517596	101519596	-0.1676	0.00000401	hypomethylated	Traf6	37	41
chr2	103405466	103407466	-0.19126	0.00000322	hypomethylated	Abtb2	42	56
chr2	103637235	103639235	-0.23981	0.0012862	hypomethylated	Caprin1	23	23
chr2	103809443	103811443	-0.17692	0.0072659	hypomethylated	Lmo2	15	27
chr2	103866955	103868955	-0.19843	0.034284	hypomethylated	Fbxo3	18	22
chr2	104334646	104336646	-0.14622	0.0000123	hypomethylated	Hipk3	13	14
chr2	104429640	104431640	-0.31334	0.036686	hypomethylated	Cstf3	13	17
chr2	104552319	104554319	-0.28988	0.00067686	hypomethylated	Tcp11l1	14	18
chr2	104582958	104584958	-0.28663	0.000021	hypomethylated	Depdc7	11	16
chr2	104965685	104967685	-0.17962	0.0006164	hypomethylated	AI314831,Wt1	26	37
chr2	104966667	104968667	-0.20625	0.0000434	hypomethylated	AI314831,Wt1	26	34
chr2	106532615	106534615	-0.097265	0.031365	hypomethylated	Mpped2	35	46
chr2	109119894	109121894	-0.26715	1.05E-12	hypomethylated	Kif18a	19	17
chr2	109513856	109515856	-0.082187	0.027554	hypomethylated	Bdnf	7	9
chr2	109515053	109517053	-0.47383	0.0000255	stronglyhypometh	Bdnf	12	13
chr2	109531592	109533592	-0.25163	0.02833	hypomethylated	Bdnf	9	13
chr2	109730034	109732034	-0.28474	0.0000286	hypomethylated	Lin7c	15	22
chr2	112078997	112080997	0.069185	0.0022733	hypermethylated	Lpcat4	47	50
chr2	115890357	115892357	-0.24171	0.0053195	hypomethylated	Meis2	15	19
chr2	115890794	115892794	-0.17452	0.00413	hypomethylated	Meis2	16	20
chr2	116946185	116948185	-0.18482	0.0084479	hypomethylated	Spred1	56	57
chr2	118422946	118424946	0.23465	0.0013336	hypermethylated	Bub1b	4	9
chr2	118726350	118728350	-0.11263	4.01E-09	hypomethylated	Bahd1	82	92
chr2	118751232	118753232	-0.44846	0.00000084	stronglyhypometh	Chst14	14	18
chr2	118859525	118861525	-0.56156	0.0000428	stronglyhypometh	Rpusd2	21	24
chr2	118937552	118939552	-0.18076	0.0000392	hypomethylated	Rad51	22	26
chr2	119096962	119098962	-0.066667	0.01123	hypomethylated	Rhov	5	5
chr2	119113477	119115477	-0.38737	0.0046141	stronglyhypometh	Vps18	6	11
chr2	119175977	119177977	-0.40026	0.0099032	stronglyhypometh	Chac1	12	14
chr2	119373363	119375363	0.11487	0.044975	hypermethylated	Chp1,Exd1	16	16
chr2	119624249	119626249	-0.011265	0.000000247	hypomethylated	Tyro3	52	60
chr2	119980311	119982311	-0.30549	0.00066884	hypomethylated	Ehd4	8	8
chr2	120995941	120997941	-0.088464	0.041509	hypomethylated	Tubgcp4,Zscan29	44	52
chr2	120996885	120998885	-0.061644	0.016134	hypomethylated	Tubgcp4,Zscan29	25	29
chr2	121273963	121275963	-0.10092	0.012683	hypomethylated	Serf2	54	55
chr2	121691705	121693705	-0.075926	0.024726	hypomethylated	Casc4	18	27
chr2	122590094	122592094	-0.071445	0.02643	hypomethylated	Sqrdl	14	16
chr2	125071983	125073983	-0.28614	4.11E-09	hypomethylated	Dut	32	36
chr2	125072250	125074250	-0.28614	4.11E-09	hypomethylated	Dut	32	36
chr2	125683953	125685953	-0.3961	0.0027068	stronglyhypometh	Cops2,Galk2	17	18
chr2	125684754	125686754	-0.57083	0.014752	stronglyhypometh	Cops2,Galk2	6	10
chr2	126377759	126379759	-0.1502	0.013304	hypomethylated	Slc27a2	17	26
chr2	126501222	126503222	-0.1574	0.015559	hypomethylated	Gabpb1	10	26
chr2	126501223	126503223	-0.1574	0.015559	hypomethylated	Gabpb1	10	26
chr2	126833446	126835446	-0.28723	0.00000148	hypomethylated	Ap4e1	19	25
chr2	126959690	126961690	-0.91414	0.0023708	stronglyhypometh	Ncaph	3	3
chr2	127072710	127074710	-0.1969	0.0090167	hypomethylated	Ciao1,Tmem127	30	30

chr2	127073552	127075552	-0.23434	0.0074952	hypomethylated	Ciao1,Tmem127	29	27
chr2	127094964	127096964	-0.078381	0.00011273	hypomethylated	Stard7	35	35
chr2	127188021	127190021	-0.27714	0.00000414	hypomethylated	Adra2b	29	31
chr2	127249934	127251934	-0.13333	0.028162	hypomethylated	Gpat2	7	6
chr2	127950773	127952773	-0.26904	0.00014308	hypomethylated	Bcl2l11	31	45
chr2	129023508	129025508	-0.18161	0.00011668	hypomethylated	Slc20a1	43	46
chr2	129417574	129419574	-0.28506	0.0013933	hypomethylated	Sirpa	17	22
chr2	129417930	129419930	-0.25975	0.0020678	hypomethylated	Sirpa	18	23
chr2	129625252	129627252	-0.22476	0.0028207	hypomethylated	4932416H05Rik,Stk35	26	37
chr2	130120674	130122674	0.05048	0.015047	hypermethylated	Ebf4	64	66
chr2	130249055	130251055	-0.11769	0.00087948	hypomethylated	Pced1a,Vps16	10	17
chr2	130388492	130390492	-0.074697	0.026629	hypomethylated	4930473A02Rik,Mrps2	26	30
chr2	130455722	130457722	-0.089808	0.03996	hypomethylated	Fastkd5,Ubox5	3	3
chr2	130952147	130954147	-0.19167	0.034939	hypomethylated	Hspa12b	3	6
chr2	131011686	131013686	-0.30949	0.0020836	hypomethylated	Cdc25b	14	20
chr2	131058873	131060873	-0.25508	0.0077685	hypomethylated	Mavs	10	16
chr2	131087235	131089235	-0.15854	0.00030688	hypomethylated	Pank2	25	25
chr2	131316597	131318597	-0.19269	1.13E-12	hypomethylated	Smox	38	41
chr2	131970844	131972844	-0.63527	0.000000149	stronglyhypometh	Slc23a2	6	12
chr2	132606013	132608013	-0.41811	0.0057393	stronglyhypometh	Chgb	6	11
chr2	132672469	132674469	-0.088773	0.014861	hypomethylated	Crls1	36	38
chr2	139502913	139504913	-0.1266	0.0087965	hypomethylated	Ism1	48	55
chr2	139995381	139997381	-0.25484	0.044463	hypomethylated	Esf1,Ndufaf5	14	19
chr2	139996294	139998294	-0.2857	0.043881	hypomethylated	Esf1,Ndufaf5	11	16
chr2	143740066	143742066	-0.28672	0.000000064	hypomethylated	Dstn	25	28
chr2	144096308	144098308	-0.044234	0.000000197	hypomethylated	Mgme1,Snx5	17	17
chr2	144193718	144195718	-0.20517	0.00000224	hypomethylated	Csrp2bp,Pet117	33	33
chr2	144193767	144195767	-0.20517	0.00000224	hypomethylated	Csrp2bp,Pet117	33	33
chr2	144193770	144195770	-0.20517	0.00000224	hypomethylated	Csrp2bp,Pet117	33	33
chr2	144353134	144355134	-0.070333	0.023426	hypomethylated	Dzank1,Polr3f	12	16
chr2	144381012	144383012	-0.084871	0.00081714	hypomethylated	Sec23b	17	17
chr2	144418800	144420800	-0.92041	0.00018898	stronglyhypometh	Gm561	4	7
chr2	146046732	146048732	-0.0064258	0.037295	hypomethylated	Insm1	48	81
chr2	146337740	146339740	-0.17163	0.00000927	hypomethylated	Ralgapa2	23	24
chr2	146837795	146839795	-0.033876	0.00244	hypomethylated	Xrn2	33	44
chr2	147872705	147874705	-0.44425	0.00027184	stronglyhypometh	Foxa2	12	14
chr2	148497350	148499350	-0.28589	0.00000448	hypomethylated	Nxt1	17	20
chr2	148497376	148499376	-0.28589	0.00000448	hypomethylated	Nxt1	17	20
chr2	148505855	148507855	-0.49032	1.42E-09	stronglyhypometh	Gzf1	15	20
chr2	149655518	149657518	-0.14524	0.029355	hypomethylated	Syndig1	25	34
chr2	150493976	150495976	-0.38318	0.000019	stronglyhypometh	Acss1,E130215H24Rik	9	11
chr2	150573816	150575816	0.085326	0.029016	hypermethylated	Entpd6	18	18
chr2	150611531	150613531	-0.38953	0.00013658	stronglyhypometh	Pygb	17	16
chr2	151367234	151369234	-0.07782	0.0043195	hypomethylated	Fkbp1a	31	36
chr2	151667662	151669662	-0.10736	0.00025593	hypomethylated	Rspo4	22	25
chr2	151805955	151807955	-0.20755	0.0000334	hypomethylated	Fam110a	6	8
chr2	151906264	151908264	-0.27754	0.00000613	hypomethylated	Scrt2	38	45
chr2	151930465	151932465	-0.098156	9.54E-09	hypomethylated	Srxn1	32	34
chr2	152158161	152160161	-0.40986	0.0056141	stronglyhypometh	Rbck1	7	8
chr2	152223782	152225782	-0.34353	0.00079353	stronglyhypometh	Sox12	21	25
chr2	152736087	152738087	-0.26339	0.01101	hypomethylated	Mylk2	9	6
chr2	153270215	153272215	-0.15617	0.0000398	hypomethylated	8430427H17Rik	26	25
chr2	153458517	153460517	-0.28079	0.000000239	hypomethylated	Commd7	6	6
chr2	154233820	154235820	-0.23146	0.0021613	hypomethylated	Snta1	11	11
chr2	154438103	154440103	-0.16649	9.95E-09	hypomethylated	Zfp341	47	53
chr2	154615845	154617845	-0.046961	0.00087525	hypomethylated	Raly	46	52
chr2	155101179	155103179	-0.11517	0.0024758	hypomethylated	Map1lc3a	43	47

chr2	155889948	155891948	0.12026	0.01181	hypermethylated	Spag4	24	26
chr2	156021382	156023382	-0.073041	0.00038883	hypomethylated	Phf20	24	33
chr2	156137208	156139208	-0.14469	0.017561	hypomethylated	Cnbd2,Scand1	34	35
chr2	156218715	156220715	-0.33333	0.00031298	hypomethylated	2900097C17Rik	2	2
chr2	156372311	156374311	-0.071145	0.002552	hypomethylated	Aar2	12	12
chr2	156438440	156440440	-0.055124	0.003903	hypomethylated	Dlgap4,Gm14169	44	50
chr2	156687857	156689857	-0.12391	0.000000061	hypomethylated	1110008F13Rik,54304	52	52
chr2	156688681	156690681	-0.053145	0.010863	hypomethylated	1110008F13Rik,54304	38	37
chr2	156905001	156907001	-0.23021	0.00088109	hypomethylated	9830001H06Rik	11	17
chr2	157384845	157386845	-0.46515	1.46E-08	stronglyhypometh	Nnat	11	11
chr2	157562136	157564136	-0.21569	0.0061589	hypomethylated	Ctnnbl1	12	9
chr2	158234588	158236588	0.08725	0.00013347	hypermethylated	Ralgapb	39	46
chr2	158491468	158493468	-0.10891	0.00051455	hypomethylated	Ppp1r16b	15	16
chr2	158619555	158621555	-0.18801	8.6E-11	hypomethylated	Dhx35	13	13
chr2	160470632	160472632	-0.18078	0.000147	hypomethylated	Top1	45	46
chr2	160556045	160558045	-0.018859	0.012015	hypomethylated	Plcg1	39	49
chr2	160934792	160936792	-0.040222	0.02527	hypomethylated	Chd6	13	22
chr2	163245206	163247206	-0.35496	0.00063168	stronglyhypometh	3230401D17Rik	17	24
chr2	163263202	163265202	-0.62908	8.26E-08	stronglyhypometh	Gdap1l1	10	14
chr2	163483121	163485121	-0.039159	0.033204	hypomethylated	Pkig	34	34
chr2	163483193	163485193	-0.039159	0.033204	hypomethylated	Pkig	34	34
chr2	163819932	163821932	-0.14193	0.0082564	hypomethylated	Ywhab	27	34
chr2	163898913	163900913	-0.23173	0.00013451	hypomethylated	Stk4	24	24
chr2	164182243	164184243	-0.375	0.01129	stronglyhypometh	Slpi	2	2
chr2	164268688	164270688	-0.21873	0.00033886	hypomethylated	Sdc4	12	12
chr2	164285470	164287470	-0.092968	0.000024	hypomethylated	Sys1	51	58
chr2	164310639	164312639	0.17148	0.00059425	hypermethylated	Dbndd2	28	27
chr2	164310954	164312954	0.17037	0.00036914	hypermethylated	Dbndd2	30	28
chr2	164311376	164313376	0.19124	0.00036873	hypermethylated	Dbndd2	30	30
chr2	164610520	164612520	-0.10644	0.012192	hypomethylated	Snx21	34	48
chr2	164629613	164631613	-0.43997	0.00012216	stronglyhypometh	Acot8,Zswim3	13	17
chr2	164659096	164661096	-0.72731	4.65E-10	stronglyhypometh	Ctsa,Neurl2	10	10
chr2	164737250	164739250	-0.36312	0.029254	stronglyhypometh	Zfp335	4	8
chr2	164792487	164794487	-0.24218	0.0041757	hypomethylated	Slc12a5	28	40
chr2	165817136	165819136	-0.17974	1.46E-08	hypomethylated	Ncoa3	40	44
chr2	166630080	166632080	-0.12013	0.00025378	hypomethylated	Arfgef2	21	26
chr2	166821778	166823778	-0.5666	0.0054654	stronglyhypometh	Stau1	10	10
chr2	166887433	166889433	-0.096502	0.00000253	hypomethylated	1500012F01Rik,Znfx1	61	64
chr2	166888515	166890515	-0.1917	6.48E-08	hypomethylated	1500012F01Rik,Snord1	20	25
chr2	167246220	167248220	-0.15656	0.00051409	hypomethylated	Slc9a8	23	25
chr2	167318374	167320374	0.30478	0.00012515	hypermethylated	Rnf114,Spata2	12	17
chr2	167487044	167489044	-0.17707	0.0059171	hypomethylated	Tmem189	27	22
chr2	167513414	167515414	-0.10041	0.035934	hypomethylated	Cebpb	76	90
chr2	167756826	167758826	-0.025624	0.019755	hypomethylated	Ptpn1	38	45
chr2	167905503	167907503	-0.029468	0.0000504	hypomethylated	Pard6b	36	39
chr2	168055879	168057879	-0.047775	0.029274	hypomethylated	Dpm1,Mocs3	66	74
chr2	170322638	170324638	-0.088254	0.0056625	hypomethylated	Cyp24a1,Pfdn4	5	9
chr2	170335729	170337729	-0.074288	0.00019523	hypomethylated	Gm16796,Pfdn4	24	26
chr2	170556306	170558306	-0.48413	0.00040912	stronglyhypometh	Dok5	9	21
chr2	172195502	172197502	-0.14357	4.87E-09	hypomethylated	Aurka,Cstf1	25	27
chr2	172196006	172198006	-0.18416	2.42E-09	hypomethylated	Aurka,Cstf1	22	24
chr2	172374092	172376092	-0.20083	0.0036866	hypomethylated	Tfap2c	12	20
chr2	172375490	172377490	-0.11463	0.012293	hypomethylated	Tfap2c	34	32
chr2	172765794	172767794	-0.050601	0.0052856	hypomethylated	Bmp7	19	24
chr2	172824637	172826637	-0.13913	0.0046612	hypomethylated	Rae1	26	40
chr2	173562071	173564071	-0.076006	0.00037364	hypomethylated	Vapb	24	27
chr2	173901551	173903551	-0.099329	0.021989	hypomethylated	Stx16	13	14

chr2	173934851	173936851	-0.011777	0.043088	hypomethylated	Npepl1	33	40
chr2	174120937	174122937	-0.78551	0.0000245	stronglyhypometh	Gnas,Nespas	11	13
chr2	174240304	174242304	-0.26303	0.00079756	hypomethylated	Nelfcd	11	15
chr2	179711351	179713351	-0.030093	0.03823	hypomethylated	4921531C22Rik,Taf4a	22	22
chr2	179804297	179806297	-0.26001	9.37E-08	hypomethylated	Gtpbp5	11	16
chr2	179905292	179907292	-0.14497	2.16E-18	hypomethylated	Adrm1	50	47
chr2	180323111	180325111	-0.38015	0.007681	stronglyhypometh	Ogfr	12	18
chr2	180436805	180438805	-0.34032	0.00046435	stronglyhypometh	Dido1	16	16
chr2	180511605	180513605	-0.51006	0.0025778	stronglyhypometh	Bhlhe23	7	5
chr2	180627744	180629744	-0.16776	0.0011778	hypomethylated	Mir124a-3	65	83
chr2	181053428	181055428	-0.65629	0.0002561	stronglyhypometh	Rtel1	14	14
chr2	181194131	181196131	-0.20052	0.00034589	hypomethylated	Zbtb46	26	32
chr2	181335023	181337023	0.15572	0.0029643	hypermethylated	Prpf6	13	19
chr2	181414014	181416014	-0.22457	0.0021123	hypomethylated	Tcea2	13	30
chr2	181428629	181430629	-0.24656	0.029219	hypomethylated	Rgs19	10	14
chr3	8667038	8669038	-0.058556	0.0091447	hypomethylated	Hey1	36	45
chr3	10011605	10013605	-0.1159	0.043042	hypomethylated	Fabp5	6	8
chr3	10351301	10353301	-0.25	0.00012584	hypomethylated	Zfand1	4	8
chr3	14885425	14887425	-0.031594	0.00000687	hypomethylated	Car2	48	48
chr3	19087243	19089243	-0.0071496	0.031978	hypomethylated	Mtfr1	21	29
chr3	19211322	19213322	-0.046194	0.033661	hypomethylated	Pde7a	42	44
chr3	21974573	21976573	-0.019152	0.0042112	hypomethylated	Tbl1xr1	80	84
chr3	30498792	30500792	-0.19704	0.040022	hypomethylated	Actrt3,Mynn	3	8
chr3	30690797	30692797	0.034021	0.011927	hypermethylated	Sec62	38	47
chr3	30893692	30895692	-0.31406	0.0000353	hypomethylated	Prkci	24	26
chr3	30992982	30994982	-0.15005	0.00013276	hypomethylated	Skil	96	107
chr3	32263410	32265410	-0.19697	0.000000244	hypomethylated	4930429B21Rik,Zmat3	44	50
chr3	32408471	32410471	-0.12639	0.00018155	hypomethylated	Zfp639	28	31
chr3	32408513	32410513	-0.12639	0.00018155	hypomethylated	Zfp639	28	31
chr3	32427403	32429403	0.14789	0.0121	hypermethylated	Mfn1	10	20
chr3	32606467	32608467	-0.07132	0.0025508	hypomethylated	Actl6a	24	33
chr3	33918000	33920000	0.019513	0.020037	hypermethylated	Fxr1	57	78
chr3	35652059	35654059	-0.18302	0.0042003	hypomethylated	Atp11b	33	39
chr3	36470918	36472918	-0.24309	0.0000514	hypomethylated	Ccna2	13	14
chr3	37246574	37248574	-0.060861	0.0017368	hypomethylated	Fgf2	27	33
chr3	37318201	37320201	-0.11983	0.0054835	hypomethylated	Nudt6,Spata5	33	45
chr3	37537871	37539871	0.033657	0.018062	hypermethylated	Spry1	48	74
chr3	41367801	41369801	-0.036895	0.018996	hypomethylated	Phf17	61	75
chr3	41545539	41547539	0.094927	0.011791	hypermethylated	D3Ertd751e,Sclt1	29	30
chr3	51027368	51029368	-0.069807	0.0019726	hypomethylated	Ccrn4l	54	64
chr3	51286887	51288887	-0.31401	0.0043479	hypomethylated	Rab33b	33	38
chr3	51364745	51366745	-0.1247	0.002008	hypomethylated	Setd7	15	21
chr3	52071258	52073258	-0.09001	0.021878	hypomethylated	Foxo1	39	40
chr3	53266738	53268738	-0.1375	0.019637	hypomethylated	Nhlrc3,Proser1	15	15
chr3	53267180	53269180	-0.1375	0.019637	hypomethylated	Nhlrc3,Proser1	15	15
chr3	54539286	54541286	-0.093922	0.04247	hypomethylated	Alg5,Exosc8	25	25
chr3	54858977	54860977	-0.15992	0.0022385	hypomethylated	4931419H13Rik,Ccna1	23	28
chr3	55045447	55047447	-0.27257	0.023273	hypomethylated	Dclk1	8	8
chr3	55585431	55587431	-0.47727	0.0000223	stronglyhypometh	Mab21l1	8	11
chr3	58218610	58220610	-0.1462	0.00000848	hypomethylated	Tsc22d2	110	98
chr3	58379579	58381579	-0.14374	0.00017787	hypomethylated	2810407C02Rik	35	37
chr3	62141698	62143698	-0.28516	7.75E-13	hypomethylated	Arhgef26	35	45
chr3	63779064	63781064	-0.1884	0.00032583	hypomethylated	Gmps	30	32
chr3	65331368	65333368	-0.045918	0.0000103	hypomethylated	4931440P22Rik,Tiparp	72	78
chr3	65333336	65335336	0.20024	0.020394	hypermethylated	4931440P22Rik,Tiparp	6	6
chr3	65469149	65471149	-0.10537	0.0000293	hypomethylated	Lekr1	19	25
chr3	65469156	65471156	-0.10537	0.0000293	hypomethylated	Lekr1	19	25

chr3	65762147	65764147	-0.078323	0.011935	hypomethylated	Ccnl1	26	26
chr3	68297114	68299114	-0.25078	0.0000211	hypomethylated	Schip1	40	44
chr3	68493565	68495565	-0.11167	0.0060192	hypomethylated	Il12a	8	8
chr3	68494345	68496345	-0.11167	0.0060192	hypomethylated	Il12a	8	8
chr3	68931014	68933014	-0.32145	0.00000849	hypomethylated	Kpna4	12	14
chr3	69402783	69404783	-0.44828	0.0028944	stronglyhypometh	B3galnt1	2	2
chr3	69663818	69665818	-0.19048	0.014957	hypomethylated	Sptssb	6	8
chr3	72860865	72862865	-0.22387	0.046085	hypomethylated	Slitrk3	7	7
chr3	79394310	79396310	-0.088583	0.00030709	hypomethylated	Ppid	28	26
chr3	79432000	79434000	-0.34872	0.0045808	stronglyhypometh	4930579G24Rik,Etfdh	9	15
chr3	79432689	79434689	-0.39403	0.033265	stronglyhypometh	4930579G24Rik,Etfdh	6	6
chr3	80606713	80608713	0.08944	0.041233	hypermethylated	Gria2	9	11
chr3	80839337	80841337	-0.23199	0.0010699	hypomethylated	Pdgfc	7	16
chr3	82160993	82162993	-0.30707	0.020146	hypomethylated	Map9	27	32
chr3	83844083	83846083	-0.2873	0.00061972	hypomethylated	D930015E06Rik	16	14
chr3	84284351	84286351	0.21836	0.02501	hypermethylated	Fhdc1	11	17
chr3	84386547	84388547	-0.2505	0.00011307	hypomethylated	Arfip1	25	27
chr3	84618498	84620498	-0.15328	8.03E-09	hypomethylated	Fbxw7	40	43
chr3	85944609	85946609	-0.46261	5.84E-12	stronglyhypometh	Rnu73b,Rps3a1	23	36
chr3	86724806	86726806	0.13512	0.033078	hypermethylated	Dclk2	9	9
chr3	87328499	87330499	-0.39658	4.66E-12	stronglyhypometh	Etv3	27	36
chr3	87709242	87711242	-0.23371	0.047151	hypomethylated	Hdgf	40	42
chr3	88018092	88020092	-0.38902	0.00082256	stronglyhypometh	Mir3093,Mir9-1	11	13
chr3	88100056	88102056	-0.7716	0.000000366	stronglyhypometh	Cct3,Tsacc	9	9
chr3	88100760	88102760	-0.63636	0.00019823	stronglyhypometh	Cct3,Tsacc	9	11
chr3	88138355	88140355	-0.426	1.24E-09	stronglyhypometh	Smg5,Tmem79	5	10
chr3	88139181	88141181	-0.47614	6.67E-14	stronglyhypometh	Smg5,Tmem79	19	21
chr3	88356637	88358637	-0.28595	0.0010761	hypomethylated	Lamtor2,Ubqln4	34	40
chr3	88356849	88358849	-0.25299	0.036656	hypomethylated	Lamtor2,Ubqln4	36	37
chr3	88488715	88490715	0.11393	0.0026296	hypermethylated	2810403A07Rik	16	22
chr3	88754204	88756204	-0.092838	0.00068781	hypomethylated	Dap3	33	34
chr3	88964079	88966079	-0.2	0.019423	hypomethylated	Hcn3	2	2
chr3	88967726	88969726	-0.44887	2.35E-12	stronglyhypometh	Clk2	33	34
chr3	88980406	88982406	-0.51813	0.000000121	stronglyhypometh	Scamp3	7	7
chr3	89018108	89020108	-0.27033	0.00098812	hypomethylated	Mtx1,Thbs3	10	13
chr3	89049819	89051819	-0.10691	0.0092932	hypomethylated	Krtcap2,Trim46	18	18
chr3	89083567	89085567	-0.35998	0.0009171	stronglyhypometh	Efna1	11	17
chr3	89084873	89086873	-0.27438	0.012238	hypomethylated	Efna1	10	21
chr3	89197125	89199125	-0.29512	0.00000735	hypomethylated	Gm15417,Zbtb7b	17	19
chr3	89215785	89217785	-0.16722	0.011084	hypomethylated	Flad1	10	11
chr3	89221472	89223472	-0.20601	0.0016792	hypomethylated	Cks1b,Shc1	14	20
chr3	89222213	89224213	-0.66061	0.010112	stronglyhypometh	Cks1b,Shc1	5	5
chr3	89802608	89804608	-0.14837	0.0000588	hypomethylated	Gm19710,Hax1	7	7
chr3	89855738	89857738	-0.51732	0.00000149	stronglyhypometh	4933434E20Rik,Ubap2	19	22
chr3	89856397	89858397	-0.76009	4.86E-09	stronglyhypometh	4933434E20Rik,Ubap2	11	8
chr3	89856437	89858437	-0.82202	2.26E-09	stronglyhypometh	4933434E20Rik,Ubap2	10	7
chr3	90193849	90195849	-0.36688	3.54E-09	stronglyhypometh	Slc27a3	11	11
chr3	94113053	94115053	-0.55926	0.0046271	stronglyhypometh	Them4	4	14
chr3	94386624	94388624	-0.68485	0.047794	stronglyhypometh	Snx27	3	3
chr3	94640488	94642488	-0.085882	0.0000167	hypomethylated	Pogz	29	38
chr3	94913963	94915963	-0.4721	0.00026344	stronglyhypometh	Vps72	8	13
chr3	94937934	94939934	-0.43571	0.0063927	stronglyhypometh	Lysmd1,Scnm1	2	3
chr3	95021864	95023864	-0.30702	0.0348	hypomethylated	Gabpb2	2	10
chr3	95427901	95429901	-0.0023636	0.006082	hypomethylated	Ensa	21	25
chr3	95686151	95688151	-0.14983	0.0067434	hypomethylated	Gm129	8	12
chr3	95696918	95698918	-0.64584	2.95E-17	stronglyhypometh	Aph1a	12	12
chr3	95696977	95698977	-0.64584	2.95E-17	stronglyhypometh	Aph1a	12	12

chr3	95732179	95734179	-0.095451	0.0077719	hypomethylated	Anp32e	40	43
chr3	95907449	95909449	-0.27529	1.65E-11	hypomethylated	Otud7b	41	44
chr3	95984149	95986149	-0.33307	0.00048212	hypomethylated	Sv2a	10	10
chr3	96001509	96003509	-0.1176	0.022139	hypomethylated	Bola1	7	7
chr3	96042999	96044999	-0.31323	0.0018923	hypomethylated	Hist2h2aa1,Hist2h2aa2	8	8
chr3	96043050	96045050	-0.25058	0.00046201	hypomethylated	Hist2h2aa1,Hist2h2aa2	10	10
chr3	96048460	96050460	-0.34931	0.032273	stronglyhypometh	Hist2h2aa1,Hist2h2aa2	6	8
chr3	96048503	96050503	-0.34931	0.032273	stronglyhypometh	Hist2h2aa1,Hist2h2aa2	6	8
chr3	96399558	96401558	-0.034862	0.035307	hypomethylated	Ankrd34a	12	12
chr3	96432850	96434850	-0.61412	0.0078869	stronglyhypometh	6330549D23Rik,Rbm8	7	9
chr3	96438279	96440279	-0.14942	0.000053	hypomethylated	Pex11b	22	25
chr3	96438352	96440352	-0.14942	0.000053	hypomethylated	Pex11b	22	25
chr3	96438769	96440769	-0.21935	0.00000803	hypomethylated	Pex11b	24	27
chr3	96499297	96501297	-0.13437	0.0022877	hypomethylated	Pias3	27	35
chr3	96499998	96501998	-0.080259	0.019128	hypomethylated	Pias3	27	40
chr3	96530533	96532533	0.11024	0.047574	hypermethylated	Polr3c,Rnf115	19	26
chr3	97461134	97463134	-0.15583	0.0004748	hypomethylated	Prkab2	35	36
chr3	99688339	99690339	-0.78313	0.00000361	stronglyhypometh	Spag17	8	9
chr3	100489396	100491396	0.044423	0.037781	hypermethylated	Man1a2	37	42
chr3	101408580	101410580	0.12387	0.00015137	hypermethylated	Atp1a1	13	13
chr3	102823468	102825468	-0.12535	0.0037369	hypomethylated	Csde1	20	30
chr3	102861207	102863207	-0.36806	0.00000207	stronglyhypometh	Nras	12	16
chr3	103378203	103380203	-0.1677	0.00010302	hypomethylated	Syt6	25	27
chr3	103595198	103597198	0.15444	0.01113	hypermethylated	Hipk1	20	23
chr3	103717042	103719042	-0.062933	0.0000154	hypomethylated	Rsbn1	37	37
chr3	104441590	104443590	-0.048084	0.0083878	hypomethylated	Slc16a1	60	78
chr3	104582973	104584973	-0.34676	1.83E-09	stronglyhypometh	Ppm1j	21	21
chr3	104666998	104668998	-0.30196	0.000000449	hypomethylated	Capza1,St7l	31	34
chr3	104667423	104669423	-0.4677	1.32E-10	stronglyhypometh	Capza1,St7l	21	24
chr3	104764627	104766627	-0.20113	1.29E-08	hypomethylated	Wnt2b	30	38
chr3	104855871	104857871	-0.040223	0.03622	hypomethylated	Cttnbp2nl	15	15
chr3	105254247	105256247	-0.24376	0.00016633	hypomethylated	1700095B22Rik,Kcnd3	30	47
chr3	105604254	105606254	0.068826	0.028046	hypermethylated	Rap1a	10	10
chr3	105761415	105763415	-0.12086	0.0046469	hypomethylated	Atp5f1,Wdr77	26	27
chr3	106284348	106286348	-0.73604	0.0018595	stronglyhypometh	Dennd2d	5	7
chr3	106486904	106488904	-0.35122	0.00000842	stronglyhypometh	Lrif1	7	7
chr3	107080775	107082775	-0.10228	0.0000204	hypomethylated	Lamtor5	14	20
chr3	107136207	107138207	-0.29508	0.0000319	hypomethylated	Rbm15	11	13
chr3	107434628	107436628	-0.33856	0.023002	stronglyhypometh	Strip1	5	5
chr3	107697771	107699771	-0.54447	0.0023374	stronglyhypometh	4933431E20Rik,Gstm5	8	15
chr3	107847774	107849774	0.15633	0.049336	hypermethylated	Gstm4	7	7
chr3	107847777	107849777	0.15633	0.049336	hypermethylated	Gstm4	7	7
chr3	108003752	108005752	-0.096086	0.0000162	hypomethylated	Cyb561d1	9	9
chr3	108058843	108060843	-0.21342	0.015308	hypomethylated	Psma5	21	23
chr3	108086049	108088049	0.037783	0.0062239	hypermethylated	Sort1	52	64
chr3	108248087	108250087	-0.66667	3.17E-08	stronglyhypometh	Sars	4	6
chr3	108339440	108341440	-0.21222	0.018924	hypomethylated	1700013F07Rik,53304	10	12
chr3	108339501	108341501	-0.21222	0.018924	hypomethylated	1700013F07Rik,53304	10	12
chr3	115709366	115711366	-0.13964	0.041091	hypomethylated	Extl2,Slc30a7	18	21
chr3	116415198	116417198	-0.36111	5.83E-08	stronglyhypometh	Slc35a3	13	13
chr3	121128458	121130458	-0.061512	0.00000559	hypomethylated	Cnn3	57	62
chr3	121655243	121657243	-0.10476	0.0035105	hypomethylated	Arhgap29	37	35
chr3	121947509	121949509	-0.057332	0.015713	hypomethylated	Gclm	31	53
chr3	122121697	122123697	-0.3865	0.000000689	stronglyhypometh	Bcar3	30	33
chr3	122626314	122628314	0.10796	0.012771	hypermethylated	1810037I17Rik,49334C	18	26
chr3	122969413	122971413	-0.24139	0.0004769	hypomethylated	Sec24d	15	17
chr3	123148830	123150830	-0.15814	0.00090239	hypomethylated	Prss12	42	42

chr3	124022954	124024954	-0.00071842	0.0051849	hypomethylated	Tram1l1	30	35
chr3	126298890	126300890	-0.37113	0.000000133	stronglyhypometh	Camk2d	36	39
chr3	127255406	127257406	-0.14464	0.0051044	hypomethylated	4930422G04Rik,Larp7	8	10
chr3	127256267	127258267	-0.13393	0.0054295	hypomethylated	4930422G04Rik,Larp7	8	8
chr3	127335062	127337062	-0.2759	0.0066024	hypomethylated	Neurog2	20	31
chr3	127491830	127493830	0.18776	0.0080325	hypermethylated	Tifa	19	22
chr3	127599241	127601241	0.45	0.048399	stronglyhypermeth	5730508B09Rik	3	4
chr3	129234303	129236303	-0.44526	0.00014982	stronglyhypometh	Elovl6	5	9
chr3	129580539	129582539	-0.048889	0.020449	hypomethylated	Pla2g12a	15	15
chr3	129603342	129605342	-0.94167	0.0000349	stronglyhypometh	Casp6	2	8
chr3	129882795	129884795	-0.49213	0.008993	stronglyhypometh	Col25a1	9	17
chr3	131226731	131228731	-0.12354	3.46E-08	hypomethylated	Papss1	27	31
chr3	131747255	131749255	-0.050505	0.044937	hypomethylated	Dkk2	3	8
chr3	132753916	132755916	-0.39052	0.0051633	stronglyhypometh	Gstcd,Ints12	22	35
chr3	132754704	132756704	-0.37094	0.0091563	stronglyhypometh	Gstcd,Ints12	22	22
chr3	133902111	133904111	-0.3336	0.00010352	stronglyhypometh	Mir1895	21	25
chr3	134490970	134492970	-0.25248	0.0030882	hypomethylated	Tacr3	8	8
chr3	135100722	135102722	-0.017599	0.040271	hypomethylated	4930539J05Rik,Ube2d	50	64
chr3	135147574	135149574	-0.023089	0.021255	hypomethylated	Manba	20	23
chr3	137285635	137287635	-0.33797	0.012027	stronglyhypometh	Ddit4l	13	17
chr3	137529638	137531638	-0.062925	0.015451	hypomethylated	Dnajb14	47	47
chr3	137644513	137646513	0.31456	0.0017268	hypermethylated	Dapp1	7	7
chr3	141127527	141129527	-0.30467	0.0000114	hypomethylated	Unc5c	20	40
chr3	144232390	144234390	-0.12116	0.046209	hypomethylated	Hs2st1,Sep15	37	48
chr3	145238171	145240171	-0.37695	3.18E-15	stronglyhypometh	Znhit6	9	11
chr3	145312949	145314949	-0.46667	0.042019	stronglyhypometh	Cyr61	5	5
chr3	145420655	145422655	0.053462	0.0189	hypermethylated	Ddah1	33	44
chr3	145649833	145651833	-0.10862	0.000000034	hypomethylated	Syde2	88	105
chr3	146066650	146068650	-0.30982	6.81E-08	hypomethylated	Ssx2ip	44	47
chr3	146161799	146163799	-0.048306	0.0000592	hypomethylated	Gng5,Spata1	52	64
chr3	146162717	146164717	-0.065514	0.00000985	hypomethylated	Gng5,Spata1	43	49
chr3	151099845	151101845	-0.47299	0.00041538	stronglyhypometh	Eltd1	12	14
chr3	151500492	151502492	-0.28289	0.040574	hypomethylated	Ptgfr	5	6
chr3	151872421	151874421	0.08578	0.0000262	hypermethylated	Dnajb4,Fubp1	43	50
chr3	151873263	151875263	0.0664	0.0000409	hypermethylated	Dnajb4,Fubp1	40	48
chr3	152008444	152010444	-0.39309	0.012781	stronglyhypometh	Usp33	22	23
chr3	153388097	153390097	-0.29167	0.0000168	hypomethylated	St6galnac3	3	3
chr3	153575153	153577153	-0.33971	0.00088426	stronglyhypometh	Rabggtb,Snord45c	11	6
chr3	153575167	153577167	-0.33971	0.00088426	stronglyhypometh	Rabggtb,Snord45c	11	6
chr3	153575930	153577930	-0.33971	0.00088426	stronglyhypometh	Rabggtb,Snord45c	11	6
chr3	156223757	156225757	-0.15303	0.00000261	hypomethylated	4930570G19Rik,Negr1	41	45
chr3	156223922	156225922	-0.15303	0.00000261	hypomethylated	4930570G19Rik,Negr1	41	45
chr3	157228855	157230855	-0.096633	0.0070692	hypomethylated	Ptger3	23	28
chr3	157694718	157696718	0.033145	0.037896	hypermethylated	Srsf11	7	7
chr4	3501025	3503025	-0.095734	0.00091838	hypomethylated	Tgs1,Tmem68	27	28
chr4	3501915	3503915	-0.090701	0.022789	hypomethylated	Tgs1,Tmem68	22	23
chr4	3762747	3764747	-0.39831	0.0028595	stronglyhypometh	Rps20	10	9
chr4	6117251	6119251	0.15065	0.033494	hypermethylated	Ubxn2b	17	18
chr4	8461790	8463790	-0.19036	0.000000195	hypomethylated	Rab2a	21	33
chr4	8617067	8619067	0.0041922	0.040925	hypermethylated	Chd7	144	165
chr4	9696731	9698731	-0.072495	0.022392	hypomethylated	4930412C18Rik	5	7
chr4	9770518	9772518	0.079383	0.0096547	hypermethylated	Gdf6	30	36
chr4	10800644	10802644	-0.25922	1.08E-08	hypomethylated	2610301B20Rik	41	46
chr4	11117500	11119500	-0.12747	0.00015026	hypomethylated	Ccne2	56	71
chr4	11412104	11414104	-0.031788	0.023596	hypomethylated	1110037F02Rik	19	20
chr4	11630593	11632593	-0.08961	0.0069682	hypomethylated	Gem	11	12
chr4	12066263	12068263	-0.46939	0.00038023	stronglyhypometh	Rbm12b1	7	7

chr4	15807410	15809410	0.32531	0.047072	hypermethylated	Calb1	5	8
chr4	17779628	17781628	-0.16972	0.00000018	hypomethylated	Mmp16	15	17
chr4	21694358	21696358	-0.125	0.014166	hypomethylated	Tstd3	3	3
chr4	21773729	21775729	-0.14244	0.0000168	hypomethylated	4930528A17Rik,Sfrs18	15	26
chr4	21857472	21859472	-0.11408	0.00012987	hypomethylated	Faxc	31	36
chr4	24824229	24826229	-0.060341	0.037074	hypomethylated	Ndufaf4	17	17
chr4	26273799	26275799	-0.32071	5.92E-09	hypomethylated	Manea	7	7
chr4	32050081	32052081	-0.21495	0.00019604	hypomethylated	Map3k7	25	28
chr4	32701447	32703447	0.21653	0.010304	hypermethylated	Casp8ap2	9	14
chr4	32743093	32745093	-0.19595	0.0010808	hypomethylated	Mdn1	31	33
chr4	33068972	33070972	-0.31012	1.09E-10	hypomethylated	Rragd	45	49
chr4	33118298	33120298	0.24779	0.00068315	hypermethylated	4933421O10Rik,Ube2j	26	26
chr4	33294965	33296965	-0.1705	0.019492	hypomethylated	Srsf12	32	46
chr4	34830197	34832197	-0.27285	0.018683	hypomethylated	Zfp292	10	10
chr4	40089297	40091297	0.08985	0.019304	hypermethylated	Aco1	24	24
chr4	40801031	40803031	0.21429	0.0019205	hypermethylated	B4galt1	4	7
chr4	40894585	40896585	0.07009	0.00019578	hypermethylated	Bag1,Chmp5	39	40
chr4	40916975	40918975	-0.071924	0.00000794	hypomethylated	Nfx1	24	24
chr4	41295028	41297028	-0.39883	0.0010412	stronglyhypometh	Ubap1	18	20
chr4	41515826	41517826	-0.19315	0.0000577	hypomethylated	Dnaic1,Fam219a	37	41
chr4	41516560	41518560	-0.6781	0.0027041	stronglyhypometh	Dnaic1,Fam219a	8	9
chr4	41660549	41662549	-0.087248	0.023874	hypomethylated	Rpp25l	26	26
chr4	41678174	41680174	0.20551	0.0032888	hypermethylated	Arid3c	7	9
chr4	41720974	41722974	-0.64912	0.0000715	stronglyhypometh	Ccl27a	6	3
chr4	41721007	41723007	-0.64912	0.0000715	stronglyhypometh	Ccl27a	6	3
chr4	41721049	41723049	-0.31579	0.0082066	hypomethylated	Ccl27a	3	3
chr4	43418445	43420445	0.29167	0.024401	hypermethylated	Rusc2	4	6
chr4	43505275	43507275	-0.28229	0.00000692	hypomethylated	Ccdc107,Rmrp	23	25
chr4	43505931	43507931	-0.45455	0.00057848	stronglyhypometh	Ccdc107,Rmrp	11	19
chr4	43590606	43592606	-0.066886	0.035966	hypomethylated	Gba2,Rgp1	23	33
chr4	43666424	43668424	-0.32634	0.003538	hypomethylated	Hint2,Spag8	7	8
chr4	44312788	44314788	-0.014821	0.0012707	hypomethylated	Melk	16	21
chr4	44993282	44995282	-0.22539	0.023604	hypomethylated	Grhpr	13	16
chr4	45030496	45032496	-0.36883	0.0033869	stronglyhypometh	Polr1e	10	7
chr4	45353972	45355972	-0.099793	0.0000289	hypomethylated	Dcaf10	39	42
chr4	46150382	46152382	-0.07337	0.000000748	hypomethylated	Ncbp1,Tstd2	33	33
chr4	46151347	46153347	-0.064162	0.0016381	hypomethylated	Ncbp1,Tstd2	28	28
chr4	46209183	46211183	-0.45972	0.018783	stronglyhypometh	Xpa	4	4
chr4	46402295	46404295	0.073215	0.049183	hypermethylated	5830415F09Rik	11	11
chr4	46501200	46503200	-0.11096	0.0030873	hypomethylated	Nans	16	14
chr4	47219883	47221883	-0.12352	0.0033387	hypomethylated	Col15a1	24	24
chr4	47365176	47367176	-0.23265	1.85E-08	hypomethylated	Tgfbr1	34	41
chr4	48292461	48294461	-0.24189	0.039628	hypomethylated	Erp44,Invs	12	12
chr4	49071333	49073333	0.36636	0.012592	stronglyhypermeth	E130309F12Rik	10	18
chr4	53452284	53454284	-0.095547	0.021247	hypomethylated	Slc44a1	57	64
chr4	53643342	53645342	-0.098654	0.0029951	hypomethylated	Fsd1l	24	26
chr4	56814200	56816200	-0.28174	0.00000564	hypomethylated	BC026590,Ikbkap	21	19
chr4	58925597	58927597	-0.87403	1.65E-11	stronglyhypometh	AI314180	10	11
chr4	59047027	59049027	-0.033275	0.019016	hypomethylated	Gng10	39	41
chr4	59593434	59595434	-0.41273	0.000024	stronglyhypometh	Hsdl2	10	25
chr4	59796727	59798727	-0.082681	0.0025053	hypomethylated	Inip	6	6
chr4	59817521	59819521	-0.20486	0.00000444	hypomethylated	Snx30	25	23
chr4	61869580	61871580	-0.58681	0.00000304	stronglyhypometh	Zfp37	10	8
chr4	61946478	61948478	-0.22895	0.0000435	hypomethylated	Slc31a2	16	16
chr4	62020734	62022734	-0.41603	0.00000421	stronglyhypometh	Fkbp15,Slc31a1	13	18
chr4	62021582	62023582	-0.55707	0.0016265	stronglyhypometh	Fkbp15,Slc31a1	4	6
chr4	62068816	62070816	-0.1083	0.011446	hypomethylated	Cdc26,Prpf4	18	18

chr4	62069657	62071657	-0.11325	0.0068189	hypomethylated	Cdc26,Prpf4	19	19
chr4	62625607	62627607	-0.22217	0.0000111	hypomethylated	Zfp618	61	58
chr4	63204798	63206798	-0.91841	0.0035063	stronglyhypometh	Atp6v1g1	3	13
chr4	70195962	70197962	-0.070307	0.010774	hypomethylated	Megf9	30	30
chr4	71861277	71863277	-0.23061	0.0002124	hypomethylated	C630043F03Rik,Tle1	29	26
chr4	71861953	71863953	-0.098304	0.042722	hypomethylated	C630043F03Rik,Tle1	11	12
chr4	73896782	73898782	0.14209	0.0020207	hypermethylated	Kdm4c	26	27
chr4	83062647	83064647	-0.14627	0.010629	hypomethylated	Snapc3	17	16
chr4	83170448	83172448	-0.1922	0.00000253	hypomethylated	Ccdc171	21	30
chr4	84529230	84531230	-0.18199	0.0010088	hypomethylated	Cntln	44	53
chr4	87679311	87681311	-0.20833	0.0020812	hypomethylated	Mllt3	12	12
chr4	88368412	88370412	-0.79167	4.42E-08	stronglyhypometh	Klhl9	6	6
chr4	88782273	88784273	-0.21151	0.00041567	hypomethylated	Mtap	17	16
chr4	93002202	93004202	-0.17222	0.042723	hypomethylated	Tusc1	3	6
chr4	94269938	94271938	-0.18965	7.01E-09	hypomethylated	Plaa	17	17
chr4	94718913	94720913	-0.48861	0.0018721	stronglyhypometh	Jun	15	14
chr4	95633069	95635069	-0.14044	0.047703	hypomethylated	9530080O11Rik,Hook1	31	30
chr4	97443316	97445316	-0.32229	0.000000231	hypomethylated	Nfia	39	43
chr4	98859624	98861624	-0.42977	0.00062965	stronglyhypometh	Atg4c	8	8
chr4	98859837	98861837	-0.42977	0.00062965	stronglyhypometh	Atg4c	8	8
chr4	99601055	99603055	-0.32686	0.00000918	hypomethylated	Pgm2	16	38
chr4	100448283	100450283	-0.13304	0.000089	hypomethylated	Cachd1	27	32
chr4	100740642	100742642	-0.20132	0.000000016	hypomethylated	Raver2	47	54
chr4	101090893	101092893	0.10641	0.012975	hypermethylated	Ak4	32	45
chr4	101090916	101092916	0.10641	0.012975	hypermethylated	Ak4	32	45
chr4	101090925	101092925	0.10641	0.012975	hypermethylated	Ak4	32	45
chr4	101091307	101093307	0.10641	0.012975	hypermethylated	Ak4	32	45
chr4	101389011	101391011	-0.4982	0.0010354	stronglyhypometh	Lepr	5	7
chr4	102887489	102889489	-0.096885	0.00049028	hypomethylated	Slc35d1	18	21
chr4	106232642	106234642	-0.28281	1.02E-09	hypomethylated	Dhcr24	27	25
chr4	106583074	106585074	-0.11714	4.57E-13	hypomethylated	Ssbp3	56	63
chr4	106926138	106928138	-0.18085	0.0000351	hypomethylated	Hspb11,Lrrc42	12	12
chr4	107106323	107108323	-0.19165	0.000000189	hypomethylated	Glis1	42	54
chr4	107551417	107553417	-0.3157	0.00000651	hypomethylated	Magoh	7	9
chr4	107704695	107706695	-0.23594	0.016889	hypomethylated	Podn	11	9
chr4	108131030	108133030	-0.11132	0.0040118	hypomethylated	Zcchc11	28	28
chr4	108251058	108253058	-0.16122	0.0014675	hypomethylated	Orc1,Prpf38a	23	27
chr4	108951879	108953879	-0.055557	0.025733	hypomethylated	Eps15	45	52
chr4	109078704	109080704	0.020351	0.049369	hypermethylated	Ttc39a	21	24
chr4	109149110	109151110	-0.089183	0.0076241	hypomethylated	Rnf11	20	25
chr4	109649629	109651629	-0.23917	0.0000431	hypomethylated	Dmrta2	35	39
chr4	111086610	111088610	-0.096475	0.018392	hypomethylated	Bend5	31	33
chr4	114580893	114582893	-0.089834	0.04717	hypomethylated	9130206I24Rik,Foxd2	62	58
chr4	114581503	114583503	-0.52643	1.65E-19	stronglyhypometh	9130206I24Rik,Foxd2	29	25
chr4	114598618	114600618	-0.6325	0.0086021	stronglyhypometh	Foxe3	5	5
chr4	114731131	114733131	-0.11822	0.041054	hypomethylated	Tal1	4	6
chr4	115456589	115458589	-0.18826	0.0069575	hypomethylated	Atpaf1	22	21
chr4	115510850	115512850	-0.56354	0.000000139	stronglyhypometh	Mknk1	12	12
chr4	115690507	115692507	-0.3775	0.0000149	stronglyhypometh	Faah	5	5
chr4	115747675	115749675	-0.13032	0.016853	hypomethylated	Lrrc41,Uqcrh	37	47
chr4	115822122	115824122	-0.020124	0.022873	hypomethylated	Pomgnt1	44	38
chr4	115893518	115895518	-0.27755	0.00000376	hypomethylated	Pik3r3	26	27
chr4	116228548	116230548	-0.26316	1.87E-09	hypomethylated	Gpbp1l1,Tmem69	37	40
chr4	116229331	116231331	-0.22166	8.87E-08	hypomethylated	Gpbp1l1,Tmem69	40	43
chr4	116270235	116272235	-0.62294	0.00077138	stronglyhypometh	C530005A16Rik,Ccdc1	7	10
chr4	116494113	116496113	-0.36611	0.027609	stronglyhypometh	Hpdl	3	5
chr4	116666952	116668952	-0.16438	2.76E-08	hypomethylated	Hectd3,Urod	47	49

chr4	116666980	116668980	-0.16438	2.76E-08	hypomethylated	Hectd3,Urod	47	49
chr4	117807495	117809495	-0.072961	0.00066203	hypomethylated	St3gal3	15	24
chr4	117807519	117809519	-0.11043	0.0005265	hypomethylated	St3gal3	15	23
chr4	117964002	117966002	0.18365	0.01114	hypermethylated	Ptprf	8	9
chr4	118080941	118082941	-0.098717	0.0038468	hypomethylated	Med8,Szt2	23	23
chr4	118081868	118083868	-0.10762	0.0053533	hypomethylated	Med8,Szt2	18	18
chr4	118099697	118101697	-0.048377	0.0053672	hypomethylated	Elovl1	15	16
chr4	118109948	118111948	-0.41987	0.021884	stronglyhypometh	Cdc20	7	12
chr4	118780349	118782349	-0.11308	0.0092359	hypomethylated	Slc2a1	17	20
chr4	118904519	118906519	-0.39347	0.0000168	stronglyhypometh	AU022252,Lepre1	27	34
chr4	118905329	118907329	-0.39637	0.00000281	stronglyhypometh	AU022252,Lepre1	22	26
chr4	118905433	118907433	-0.39637	0.00000281	stronglyhypometh	AU022252,Lepre1	22	26
chr4	118967118	118969118	0.084528	0.047722	hypermethylated	Ybx1	20	24
chr4	120076885	120078885	-0.19588	0.00069729	hypomethylated	Scmh1	32	33
chr4	120242881	120244881	-0.33083	0.0000385	hypomethylated	Ctps	14	14
chr4	120338167	120340167	-0.12341	0.0054234	hypomethylated	Cited4	33	42
chr4	120770848	120772848	-0.22222	0.011373	hypomethylated	Zmpste24	9	9
chr4	122781407	122783407	-0.12772	0.013959	hypomethylated	Bmp8b	41	47
chr4	122817184	122819184	-0.21429	0.0027379	hypomethylated	Ppie	6	11
chr4	123427542	123429542	-0.13399	0.0073557	hypomethylated	Akirin1	19	20
chr4	123581255	123583255	-0.13081	0.00000592	hypomethylated	Mycbp	29	34
chr4	123593675	123595675	-0.081121	0.00074735	hypomethylated	Rragc	38	44
chr4	124376942	124378942	-0.14747	0.0032137	hypomethylated	Fhl3	26	33
chr4	124478792	124480792	-0.483	0.000000697	stronglyhypometh	Mtf1	25	27
chr4	124527974	124529974	-0.030207	0.019289	hypomethylated	1110065P20Rik,Yrdc	39	41
chr4	124558028	124560028	-0.11285	0.0026506	hypomethylated	Epha10	27	22
chr4	124662673	124664673	-0.094371	0.0004753	hypomethylated	Rspo1	24	32
chr4	124805125	124807125	-0.18307	0.0000979	hypomethylated	Zc3h12a	14	14
chr4	125167074	125169074	-0.12293	0.04285	hypomethylated	Grik3	72	90
chr4	125772896	125774896	-0.4632	0.0038412	stronglyhypometh	Lsm10	9	10
chr4	125938648	125940648	-0.15325	0.0095786	hypomethylated	Trappc3	19	24
chr4	125963037	125965037	-0.10956	0.023827	hypomethylated	Col8a2	27	23
chr4	125998947	126000947	-0.25065	0.0065894	hypomethylated	Adprhl2	9	8
chr4	126106786	126108786	-0.13929	0.032265	hypomethylated	Ago3	10	10
chr4	126145665	126147665	0.082601	0.022357	hypermethylated	Ago1	16	22
chr4	126429798	126431798	-0.17296	2.53E-08	hypomethylated	AU040320,Ncdn	41	49
chr4	126430013	126432013	-0.17296	2.53E-08	hypomethylated	AU040320,Ncdn	41	49
chr4	128560383	128562383	0.219	0.049197	hypermethylated	Trim62	32	39
chr4	128669508	128671508	-0.24417	0.012126	hypomethylated	Ak2	13	13
chr4	128669557	128671557	-0.24417	0.012126	hypomethylated	Ak2	13	13
chr4	128781859	128783859	-0.33384	0.020415	stronglyhypometh	Tmem54	20	25
chr4	128963864	128965864	-0.55664	2.82E-11	stronglyhypometh	Sync	9	11
chr4	129012269	129014269	-0.30216	0.000000643	hypomethylated	Rbbp4,Zbtb8os	24	32
chr4	129137922	129139922	-0.12289	0.032844	hypomethylated	Bsdc1	20	20
chr4	129189824	129191824	-0.36641	4.54E-14	stronglyhypometh	Marcksl1	46	56
chr4	129276950	129278950	-0.1799	0.0000515	hypomethylated	Eif3i,Tmem234	21	26
chr4	129301152	129303152	-0.5	0.011364	stronglyhypometh	Ccdc28b	2	2
chr4	129815795	129817795	-0.43657	0.0000169	stronglyhypometh	Hcrtr1	20	25
chr4	129985021	129987021	-0.034643	0.044557	hypomethylated	Fabp3	10	14
chr4	130124704	130126704	-0.12867	0.00000823	hypomethylated	Nkain1	30	28
chr4	130347451	130349451	-0.053655	0.020853	hypomethylated	Sdc3	39	44
chr4	131428553	131430553	-0.15749	0.00000508	hypomethylated	Srsf4	63	65
chr4	131768171	131770171	-0.46587	0.0009078	stronglyhypometh	Ythdf2	6	6
chr4	131909601	131911601	-0.39971	0.00030255	stronglyhypometh	Snhg3	6	7
chr4	131978361	131980361	-0.069437	0.017934	hypomethylated	Phactr4	13	14
chr4	132090473	132092473	-0.13649	0.00077058	hypomethylated	Atpif1,Dnajc8	26	26
chr4	132323274	132325274	-0.27553	0.004513	hypomethylated	Rpa2	17	15

chr4	132795732	132797732	0.11082	0.0003592	hypermethylated	Map3k6	18	23
chr4	132895230	132897230	-0.70301	0.000000146	stronglyhypometh	Wdtc1	9	20
chr4	133073877	133075877	0.33866	0.00000766	stronglyhypermeth	1810019J16Rik	12	13
chr4	133101942	133103942	-0.39128	0.00000242	stronglyhypometh	Nudc	5	5
chr4	133309526	133311526	-0.037738	0.0041525	hypomethylated	Arid1a	53	57
chr4	133523906	133525906	-0.26787	0.00046283	hypomethylated	Hmgn2	12	22
chr4	133684668	133686668	-0.096158	0.033843	hypomethylated	Sh3bgrl3	7	7
chr4	134323919	134325919	-0.068542	0.010274	hypomethylated	Ldlrap1	17	18
chr4	134409260	134411260	-0.27562	1.35E-09	hypomethylated	Tmem57	19	34
chr4	134478539	134480539	-0.095722	5.51E-08	hypomethylated	D4Wsu53e	34	42
chr4	135050419	135052419	-0.25378	0.00000104	hypomethylated	Nipal3,Stpg1	15	20
chr4	135050901	135052901	-0.17461	0.0007668	hypomethylated	Nipal3,Stpg1	14	14
chr4	135411006	135413006	-0.057625	0.00010777	hypomethylated	Srsf10	52	61
chr4	135429761	135431761	-0.8843	0.00017285	stronglyhypometh	Pnrc2	2	10
chr4	135475640	135477640	-0.23357	0.0034653	hypomethylated	Fuca1	27	30
chr4	135501367	135503367	-0.12815	0.0224	hypomethylated	Hmgcl	9	26
chr4	135543159	135545159	0.039259	0.0035822	hypermethylated	Pithd1	9	15
chr4	135577564	135579564	-0.14444	0.046477	hypomethylated	Tceb3	7	8
chr4	135840983	135842983	-0.45349	3.52E-12	stronglyhypometh	Zfp46	17	26
chr4	135865890	135867890	-0.18964	0.00014814	hypomethylated	Hnrnpr	38	44
chr4	136391850	136393850	-0.085592	0.018601	hypomethylated	Ephb2	22	40
chr4	136512731	136514731	0.088962	0.0098008	hypermethylated	Epha8	18	20
chr4	136832549	136834549	-0.10205	0.0004798	hypomethylated	Wnt4	50	60
chr4	137149103	137151103	-0.54922	0.00014535	stronglyhypometh	Usp48	17	26
chr4	137548384	137550384	-0.14399	0.00000495	hypomethylated	Eif4g3	54	63
chr4	137817165	137819165	-0.090053	0.021434	hypomethylated	Kif17	36	43
chr4	137859652	137861652	-0.1959	0.0014884	hypomethylated	Ddost	30	33
chr4	137882211	137884211	0.20404	0.020447	hypermethylated	Pink1	5	6
chr4	137923870	137925870	-0.33055	0.0037963	hypomethylated	Cda	8	11
chr4	137989586	137991586	-0.099285	0.046031	hypomethylated	Mul1	24	26
chr4	138010062	138012062	-0.079224	0.00040979	hypomethylated	Camk2n1	57	68
chr4	138630704	138632704	-0.17178	0.00012602	hypomethylated	Htr6	41	42
chr4	138686952	138688952	-0.094123	0.013841	hypomethylated	Minos1	8	11
chr4	138687028	138689028	-0.14099	0.0028836	hypomethylated	Minos1	6	6
chr4	139177808	139179808	-0.40875	0.021285	stronglyhypometh	Aldh4a1	3	4
chr4	139208452	139210452	0.29321	0.022811	hypermethylated	Tas1r2	3	3
chr4	140634260	140636260	-0.066122	0.015371	hypomethylated	Necap2	9	9
chr4	140702836	140704836	-0.1671	0.00055149	hypomethylated	Fbxo42	45	42
chr4	140856154	140858154	-0.34955	0.0000016	stronglyhypometh	Epha2	22	28
chr4	141094512	141096512	-0.24307	0.0000324	hypomethylated	Spen	28	26
chr4	141101076	141103076	-0.1374	0.0000373	hypomethylated	B330016D10Rik	52	57
chr4	141348526	141350526	-0.048889	0.0065176	hypomethylated	Casp9	15	15
chr4	141430835	141432835	-0.080402	0.022354	hypomethylated	Efhd2	9	10
chr4	142802612	142804612	-0.074074	0.042515	hypomethylated	Prdm2	9	9
chr4	147504807	147506807	-0.98437	0.00000472	stronglyhypometh	Draxin	4	15
chr4	147533704	147535704	-0.30906	0.00029813	hypomethylated	Fbxo2,Fbxo44	9	24
chr4	147533776	147535776	-0.30906	0.00029813	hypomethylated	Fbxo2,Fbxo44	9	24
chr4	147534173	147536173	-0.49359	0.0000352	stronglyhypometh	Fbxo2,Fbxo44	8	18
chr4	147821690	147823690	-0.18106	0.032483	hypomethylated	Mtor	28	29
chr4	148001105	148003105	0.15207	0.02025	hypermethylated	Tardbp	10	11
chr4	148177500	148179500	-0.18033	0.00065702	hypomethylated	Casz1	35	42
chr4	148681807	148683807	-0.046176	0.0056747	hypomethylated	Kif1b	24	28
chr4	148959746	148961746	-0.061163	0.00032592	hypomethylated	Clstn1	64	68
chr4	149329115	149331115	-0.16662	0.031401	hypomethylated	Spsb1	9	7
chr4	149610305	149612305	0.11044	0.01901	hypermethylated	Eno1,Gm5506	20	24
chr4	149610432	149612432	0.16106	0.010833	hypermethylated	Eno1,Gm5506	18	21
chr4	151370288	151372288	-0.14537	0.0042489	hypomethylated	Phf13	38	41

chr4	151647470	151649470	-0.13984	0.0014812	hypomethylated	Gpr153	32	34
chr4	151698986	151700986	0.054891	0.029152	hypermethylated	Rpl22	14	21
chr4	151851250	151853250	-0.10796	0.00013666	hypomethylated	Kcnab2,Nphp4	24	30
chr4	151851588	151853588	-0.10766	0.00036343	hypomethylated	Kcnab2,Nphp4	21	27
chr4	153515480	153517480	-0.053382	0.016054	hypomethylated	Wdr8	15	24
chr4	154010982	154012982	-0.059691	0.00000216	hypomethylated	5930403L14Rik,Prdm1	35	46
chr4	154334031	154336031	0.039498	0.045956	hypermethylated	Hes5	59	63
chr4	154440138	154442138	-0.29507	0.0086425	hypomethylated	Pex10	14	14
chr4	154782298	154784298	0.64615	0.0086207	stronglyhypermeth	2010015L04Rik	1	2
chr4	154864469	154866469	-0.097825	0.0040031	hypomethylated	Gnb1	21	39
chr4	154997977	154999977	-0.27311	0.00037362	hypomethylated	Cdk11b,Gm16023	20	22
chr4	155027493	155029493	-0.40683	0.0000532	stronglyhypometh	Mmp23	11	26
chr4	155067580	155069580	-0.27249	0.0000279	hypomethylated	B930041F14Rik	31	44
chr4	155107912	155109912	-0.15295	0.0000387	hypomethylated	Tmem240	53	57
chr4	155242675	155244675	-0.13495	0.0000341	hypomethylated	Cpsf3l,Gltpd1	16	26
chr4	155243549	155245549	-0.070462	0.001842	hypomethylated	Cpsf3l,Gltpd1	14	26
chr4	155264983	155266983	-0.21565	0.00000306	hypomethylated	Acap3,Pusl1	51	49
chr4	155265871	155267871	-0.18019	0.003516	hypomethylated	Acap3,Pusl1	39	40
chr4	155316939	155318939	-0.1002	0.00053929	hypomethylated	Ube2j2	26	39
chr4	155335426	155337426	-0.33333	0.00094987	hypomethylated	Fam132a	7	7
chr4	155366022	155368022	-0.048062	0.048891	hypomethylated	B3galt6,Sdf4	55	65
chr4	155483106	155485106	-0.54659	0.0024216	stronglyhypometh	9430015G10Rik	8	5
chr4	155576392	155578392	-0.22445	0.011201	hypomethylated	AW011738	22	19
chr4	155608966	155610966	-0.086247	0.0011359	hypomethylated	Klhl17	30	36
chr5	3542832	3544832	-0.3084	4.76E-09	hypomethylated	Fam133b	19	33
chr5	3595065	3597065	-0.12226	0.0045938	hypomethylated	Pex1,Rbm48	11	19
chr5	7959471	7961471	-0.46964	0.0049417	stronglyhypometh	Steap4	10	10
chr5	8055541	8057541	-0.30597	0.0040643	hypomethylated	Sri	9	17
chr5	8421849	8423849	-0.14726	0.00000758	hypomethylated	Dbf4,Slc25a40	48	54
chr5	8422716	8424716	-0.23993	0.00000286	hypomethylated	Dbf4,Slc25a40	29	39
chr5	8997146	8999146	-0.2892	0.0010093	hypomethylated	Crot	6	7
chr5	12382165	12384165	-0.3509	0.0013627	stronglyhypometh	Sema3d	9	9
chr5	14513917	14515917	-0.34376	0.000000407	stronglyhypometh	Pclo	29	29
chr5	15439508	15441508	-0.071091	0.0021407	hypomethylated	Cacna2d1	53	56
chr5	18731863	18733863	-0.14349	5.05E-08	hypomethylated	4921504A21Rik,Magi2	16	22
chr5	20387270	20389270	-0.084633	0.016279	hypomethylated	Phtf2,Tmem60	60	62
chr5	20457640	20459640	-0.70018	0.00053286	stronglyhypometh	A630072M18Rik,Rsbn	12	13
chr5	20561615	20563615	-0.065718	0.015166	hypomethylated	Ptpn12	35	39
chr5	20929720	20931720	-0.33782	0.0020478	stronglyhypometh	Ccdc146,Fam185a	12	13
chr5	20930495	20932495	-0.33782	0.0020478	stronglyhypometh	Ccdc146,Fam185a	12	13
chr5	21241977	21243977	-0.26483	0.000018	hypomethylated	Pmpcb	15	15
chr5	21290100	21292100	-0.21174	0.00000233	hypomethylated	Dnajc2,Psmc2	29	39
chr5	21290983	21292983	-0.40047	1.82E-12	stronglyhypometh	Dnajc2,Psmc2	27	35
chr5	21850523	21852523	-0.11654	0.0000436	hypomethylated	Reln	32	41
chr5	22056149	22058149	-0.33333	0.00011995	hypomethylated	6030443J06Rik,Orc5	3	3
chr5	23669780	23671780	-0.39576	0.00000475	stronglyhypometh	Nupl2	15	21
chr5	23857422	23859422	0.1556	0.007987	hypermethylated	Kcnh2	23	30
chr5	24032666	24034666	-0.36019	0.039142	stronglyhypometh	Gbx1	18	23
chr5	24096931	24098931	-0.12025	0.019016	hypomethylated	Mir671	15	25
chr5	25264843	25266843	-0.19916	0.0010271	hypomethylated	Actr3b	12	13
chr5	29760206	29762206	-0.30476	5.97E-08	hypomethylated	Nom1	21	21
chr5	29805010	29807010	-0.18552	0.0012934	hypomethylated	Mnx1	12	19
chr5	30062476	30064476	-0.14684	0.014896	hypomethylated	Dnajb6,Gm5129	21	24
chr5	30558157	30560157	-0.18314	0.00063537	hypomethylated	Ept1	11	11
chr5	30889542	30891542	-0.059483	0.0000926	hypomethylated	Gm9899,Kcnk3	39	46
chr5	31013267	31015267	-0.21117	0.00011509	hypomethylated	Dpysl5	14	12
chr5	31116128	31118128	-0.25055	1.65E-08	hypomethylated	Mapre3	20	22

chr5	31350012	31352012	-0.23138	0.015031	hypomethylated	Atraid,Slc5a6	22	24
chr5	31350452	31352452	-0.21879	0.020926	hypomethylated	Atraid,Slc5a6	22	25
chr5	31356183	31358183	-0.21461	0.00000295	hypomethylated	Cad	28	31
chr5	31522918	31524918	-0.94	0.0000851	stronglyhypometh	Ppm1g	5	5
chr5	31598250	31600250	-0.39489	0.00061708	stronglyhypometh	Fndc4,Gckr	7	13
chr5	31796133	31798133	-0.17947	0.0000122	hypomethylated	Gpn1	29	28
chr5	31828367	31830367	-0.29224	0.0071836	hypomethylated	Slc4a1ap,Supt7l	24	30
chr5	31915323	31917323	-0.10782	0.0000812	hypomethylated	Mrpl33	16	16
chr5	31999422	32001422	-0.15238	8.52E-09	hypomethylated	Bre,Rbks	30	30
chr5	31999983	32001983	-0.15335	1.77E-08	hypomethylated	Bre,Rbks	28	28
chr5	32760342	32762342	-0.25631	0.00000112	hypomethylated	Ppp1cb	22	33
chr5	33128275	33130275	-0.5904	0.000000174	stronglyhypometh	Pisd	11	10
chr5	33677220	33679220	-0.096753	0.00000365	hypomethylated	Maea	53	62
chr5	34063372	34065372	-0.16155	0.0017957	hypomethylated	Fgfr3	57	58
chr5	34063408	34065408	-0.14524	0.0028778	hypomethylated	Fgfr3	61	58
chr5	34063954	34065954	-0.1162	0.013847	hypomethylated	Fgfr3	67	67
chr5	34125353	34127353	-0.24264	0.0092421	hypomethylated	Letm1	7	7
chr5	34278907	34280907	-0.43335	4.09E-14	stronglyhypometh	Nelfa	15	15
chr5	34530359	34532359	-0.49653	0.00000143	stronglyhypometh	Mxd4	10	10
chr5	34630973	34632973	0.079298	0.0015221	hypermethylated	Zfyve28	24	24
chr5	34678038	34680038	-0.18751	0.00018209	hypomethylated	Rnf4	31	41
chr5	34856628	34858628	-0.18114	0.049079	hypomethylated	Tnip2	7	7
chr5	35924708	35926708	-0.214	0.0031698	hypomethylated	Acox3	17	22
chr5	36099528	36101528	-0.16647	0.00000314	hypomethylated	Ablim2	22	27
chr5	36806833	36808833	-0.4367	0.00025571	stronglyhypometh	Grpel1	15	19
chr5	36826236	36828236	-0.26964	6.19E-14	hypomethylated	Ccdc96,Tada2b	51	59
chr5	36826934	36828934	-0.26766	0.000000118	hypomethylated	Ccdc96,Tada2b	28	36
chr5	37258808	37260808	-0.13982	0.00000212	hypomethylated	Ppp2r2c	62	65
chr5	37632318	37634318	0.037223	0.044693	hypermethylated	Crmp1	31	46
chr5	38215824	38217824	-0.090152	0.00000143	hypomethylated	Msx1	16	23
chr5	38550706	38552706	-0.092353	0.015049	hypomethylated	Nsg1	20	23
chr5	38709747	38711747	-0.067716	0.0074756	hypomethylated	Drd5	17	22
chr5	38952834	38954834	-0.18074	0.0000643	hypomethylated	Wdr1	26	27
chr5	40035870	40037870	-0.70938	0.00000939	stronglyhypometh	Hs3st1	5	5
chr5	42099394	42101394	0.14592	0.01498	hypermethylated	Rab28	5	6
chr5	42155459	42157459	-0.2472	0.0070229	hypomethylated	Nkx3-2	14	15
chr5	42235554	42237554	-0.21902	0.016784	hypomethylated	Bod1l	7	7
chr5	43623701	43625701	-0.012457	0.025707	hypomethylated	Cpeb2,Gm7854	73	76
chr5	46060163	46062163	-0.17801	5.44E-09	hypomethylated	1600023N17Rik,Ncapg	11	30
chr5	46247791	46249791	-0.1153	0.0018698	hypomethylated	Lcorl	35	42
chr5	52580919	52582919	-0.13415	0.0000991	hypomethylated	9230114K14Rik,Dhx15	35	37
chr5	53131812	53133812	-0.29071	8.86E-10	hypomethylated	Pi4k2b	26	26
chr5	54388761	54390761	0.040706	0.022105	hypermethylated	Stim2	39	42
chr5	63157416	63159416	-0.074973	0.00034039	hypomethylated	Arap2	15	15
chr5	64202733	64204733	-0.17938	0.00000283	hypomethylated	0610040J01Rik	24	24
chr5	64360136	64362136	-0.29333	0.003292	hypomethylated	Rell1	5	5
chr5	65360313	65362313	-0.074473	0.0062377	hypomethylated	Fam114a1,Mir574	13	17
chr5	65360556	65362556	-0.074473	0.0062377	hypomethylated	Fam114a1,Mir574	13	17
chr5	65781735	65783735	-0.29753	1.86E-13	hypomethylated	Lias,Rpl9	21	33
chr5	65782670	65784670	-0.29177	3.36E-12	hypomethylated	Lias,Rpl9	16	16
chr5	65884074	65886074	-0.20723	0.0016316	hypomethylated	Smim14	25	26
chr5	66089095	66091095	-0.068031	0.0026209	hypomethylated	Pds5a	47	69
chr5	66542207	66544207	-0.27418	0.00095475	hypomethylated	Rbm47	7	10
chr5	66650363	66652363	0.1506	0.00069435	hypermethylated	Nsun7	29	32
chr5	67066359	67068359	-0.19927	0.003274	hypomethylated	Uchl1	9	11
chr5	67650890	67652890	-0.060666	0.0018709	hypomethylated	Tmem33	29	33
chr5	67998121	68000121	0.09466	0.00527	hypermethylated	Shisa3	24	43

chr5	72090254	72092254	-0.28333	0.03421	hypomethylated	Gabrb1	3	3
chr5	72895447	72897447	-0.12357	0.022269	hypomethylated	Corin	7	9
chr5	73038034	73040034	-0.59647	0.00043638	stronglyhypometh	Nipal1	12	13
chr5	73647857	73649857	0.1415	0.0044147	hypermethylated	Fryl	8	9
chr5	74488107	74490107	-0.18156	0.0000359	hypomethylated	Dancr,Snora26	29	33
chr5	74488554	74490554	-0.18156	0.0000359	hypomethylated	Dancr,Snora26	29	33
chr5	74590350	74592350	0.3148	0.00011706	hypermethylated	Rasl11b	15	28
chr5	74927774	74929774	1	0.004329	stronglyhypermeth	Scfd2	2	2
chr5	76568301	76570301	-0.1321	0.033065	hypomethylated	Srd5a3	13	18
chr5	77402725	77404725	-0.30073	0.000108	hypomethylated	Srp72	18	30
chr5	77524292	77526292	-0.092968	0.0000564	hypomethylated	Hopx	13	13
chr5	77693518	77695518	-0.10159	0.000000319	hypomethylated	Rest	82	81
chr5	81449617	81451617	-0.26722	0.00000829	hypomethylated	Lphn3	25	23
chr5	88982507	88984507	-0.16288	0.000000149	hypomethylated	Utp3	30	37
chr5	89104605	89106605	-0.22311	0.014272	hypomethylated	Grsf1	42	42
chr5	89193037	89195037	-0.51884	5.82E-13	stronglyhypometh	Dck	23	29
chr5	91359221	91361221	0.10667	0.00000464	hypermethylated	Mthfd2l	26	35
chr5	91945725	91947725	-0.32604	0.0085662	hypomethylated	Parm1	8	20
chr5	92392094	92394094	0.10145	0.046946	hypermethylated	Rchy1,Thap6	15	15
chr5	92565963	92567963	-0.12677	0.00065487	hypomethylated	Uso1	39	51
chr5	92777880	92779880	-0.33494	0.0090222	stronglyhypometh	Cxcl10	3	3
chr5	93237413	93239413	-0.19583	0.0013539	hypomethylated	Shroom3	6	13
chr5	93695598	93697598	0.081862	0.040696	hypermethylated	Ccng2	23	35
chr5	96638133	96640133	-0.50751	0.00000932	stronglyhypometh	Mrpl1	9	9
chr5	97425707	97427707	-0.014763	0.0000197	hypomethylated	Bmp2k	54	63
chr5	99282457	99284457	-0.14065	0.00021295	hypomethylated	Bmp3	39	45
chr5	100407957	100409957	-0.20816	0.017476	hypomethylated	Hnrnpd	16	30
chr5	100468012	100470012	-0.015615	0.023007	hypomethylated	Enoph1,Hnrpdl	41	52
chr5	100845253	100847253	-0.16763	0.00022152	hypomethylated	Sec31a	20	24
chr5	100927592	100929592	-0.21451	0.0000706	hypomethylated	Lin54	35	48
chr5	101226777	101228777	-0.10603	0.00080067	hypomethylated	Helq,Mrps18c	29	28
chr5	101274247	101276247	-0.16985	0.042812	hypomethylated	Agpat9	17	22
chr5	102498940	102500940	-0.27381	0.0000223	hypomethylated	Wdfy3	16	13
chr5	103853210	103855210	-0.017863	0.015251	hypomethylated	Ptpn13	45	51
chr5	104084743	104086743	-0.32807	0.00000522	hypomethylated	1700016H13Rik	7	7
chr5	104084751	104086751	-0.32807	0.00000522	hypomethylated	1700016H13Rik	7	7
chr5	104182591	104184591	-0.030179	0.0034059	hypomethylated	Aff1	22	34
chr5	107831531	107833531	0.060223	0.035788	hypermethylated	Ephx4	26	38
chr5	108025398	108027398	-0.1098	0.0000672	hypomethylated	Glmn,Rpap2	17	19
chr5	108025735	108027735	-0.063137	0.00015002	hypomethylated	Glmn,Rpap2	17	20
chr5	108026658	108028658	-0.074167	0.00012917	hypomethylated	Glmn,Rpap2	16	20
chr5	108026907	108028907	-0.069048	0.0097755	hypomethylated	Glmn,Rpap2	6	7
chr5	108416096	108418096	-0.0075758	0.023602	hypomethylated	Fam69a	8	11
chr5	108560932	108562932	-0.2956	0.0038905	hypomethylated	Ccdc18,Tmed5	17	17
chr5	108561610	108563610	-0.42587	0.004793	stronglyhypometh	Ccdc18,Tmed5	11	12
chr5	108696915	108698915	-0.40947	5.48E-13	stronglyhypometh	Dr1	34	35
chr5	108740943	108742943	-0.032876	0.035436	hypomethylated	Pigg	9	21
chr5	110604719	110606719	-0.1294	0.04996	hypomethylated	Gm15787,Golga3	25	22
chr5	111268035	111270035	-0.53455	0.0000675	stronglyhypometh	Chek2,Hscb	5	7
chr5	111268796	111270796	-0.49825	0.0000202	stronglyhypometh	Chek2,Hscb	7	9
chr5	111846185	111848185	-0.072761	0.00026973	hypomethylated	Mn1	85	96
chr5	112704726	112706726	-0.45623	0.024542	stronglyhypometh	Tpst2	2	19
chr5	112754388	112756388	-0.25134	0.027933	hypomethylated	Tfip11	25	32
chr5	112771114	112773114	-0.063566	0.0016948	hypomethylated	Hps4,Srrd	30	32
chr5	113918466	113920466	-0.082011	0.04074	hypomethylated	Wscd2	28	35
chr5	114184790	114186790	-0.091075	0.0020235	hypomethylated	Ficd	25	31
chr5	114221658	114223658	-0.093028	0.017284	hypomethylated	Iscu,Sart3	32	35

chr5	114221820	114223820	-0.11296	0.022421	hypomethylated	Iscu,Sart3	27	30
chr5	114443766	114445766	-0.11062	0.00067348	hypomethylated	Ssh1	24	26
chr5	114578185	114580185	-0.15497	0.0014633	hypomethylated	Alkbh2,Ung	8	9
chr5	114579443	114581443	0.084879	0.0077553	hypermethylated	Ung	23	30
chr5	114723770	114725770	-0.49932	3.85E-10	stronglyhypometh	Foxn4	22	28
chr5	114893314	114895314	-0.51666	0.0000136	stronglyhypometh	Mmab,Mvk	16	10
chr5	114894036	114896036	-0.33107	0.0083147	hypomethylated	Mmab,Mvk	17	13
chr5	115017259	115019259	-0.1887	3.87E-09	hypomethylated	Fam222a	49	59
chr5	115140944	115142944	-0.10064	0.00032513	hypomethylated	Gltp	21	21
chr5	115156787	115158787	-0.53916	8.27E-11	stronglyhypometh	Tchp	21	23
chr5	115372248	115374248	-0.60649	0.00000461	stronglyhypometh	Oasl1	11	11
chr5	115608185	115610185	0.083774	0.022513	hypermethylated	Mlec	21	29
chr5	115750999	115752999	-0.1922	0.0051591	hypomethylated	Dynll1	12	16
chr5	115776185	115778185	-0.10294	0.00018084	hypomethylated	Srsf9	17	17
chr5	115790255	115792255	-0.25334	0.00000454	hypomethylated	Gatc,Triap1	21	24
chr5	115791170	115793170	-0.023935	0.0075792	hypomethylated	Gatc,Triap1	10	16
chr5	115878693	115880693	-0.042138	0.021734	hypomethylated	Msi1	72	73
chr5	115934306	115936306	-0.34848	0.030104	stronglyhypometh	Sirt4	2	4
chr5	116080995	116082995	-0.0028935	0.024216	hypomethylated	1110006O24Rik,Rab35	62	80
chr5	116294664	116296664	-0.29338	0.034536	hypomethylated	Cit	4	4
chr5	117569137	117571137	-0.19497	0.047003	hypomethylated	Taok3	22	21
chr5	117737573	117739573	-0.25747	0.00041244	hypomethylated	Pebp1	15	19
chr5	117863008	117865008	-0.11747	0.0000026	hypomethylated	Ksr2	21	35
chr5	118476832	118478832	-0.097059	0.0078881	hypomethylated	Tesc	38	44
chr5	118618772	118620772	-0.14144	0.0000152	hypomethylated	Hrk	47	53
chr5	120119677	120121677	-0.24753	0.00051123	hypomethylated	Tbx3	34	39
chr5	120283671	120285671	-0.44356	0.00000104	stronglyhypometh	Tbx5	10	9
chr5	120565521	120567521	0.21801	0.016706	hypermethylated	Rbm19	15	21
chr5	120880894	120882894	-0.25492	0.001393	hypomethylated	Lhx5	24	22
chr5	120922772	120924772	0.14505	0.00019546	hypermethylated	Sdsl	5	4
chr5	120953632	120955632	-0.13571	0.009914	hypomethylated	Plbd2	4	4
chr5	121097830	121099830	-0.10081	0.00052791	hypomethylated	Rasal1	27	35
chr5	122043833	122045833	-0.41137	8.49E-08	stronglyhypometh	Aldh2	7	10
chr5	122109594	122111594	-0.44699	0.0000233	stronglyhypometh	Acad10,Brap	21	15
chr5	122110519	122112519	-0.41092	0.00000782	stronglyhypometh	Acad10,Brap	20	15
chr5	122286810	122288810	-0.39333	0.0021509	stronglyhypometh	Sh2b3	10	10
chr5	122298036	122300036	-0.16364	0.0094612	hypomethylated	Fam109a	26	31
chr5	122497834	122499834	-0.3313	0.0047173	hypomethylated	Cux2	19	17
chr5	122658745	122660745	-0.13586	0.011637	hypomethylated	Hvcn1	12	14
chr5	122659306	122661306	-0.2402	0.0052646	hypomethylated	Hvcn1	8	10
chr5	122733406	122735406	0.13373	0.013671	hypermethylated	Pptc7	15	42
chr5	122821516	122823516	-0.31062	2.22E-10	hypomethylated	Fam216a,Gpn3	19	24
chr5	122821972	122823972	-0.31062	2.22E-10	hypomethylated	Fam216a,Gpn3	19	24
chr5	123156565	123158565	-0.19923	0.0048266	hypomethylated	P2rx4	26	31
chr5	123350359	123352359	-0.077475	0.0021714	hypomethylated	Kdm2b	9	21
chr5	123438542	123440542	-0.23103	2.45E-09	hypomethylated	A930024E05Rik,Kdm2l	18	17
chr5	123439101	123441101	-0.22853	1.58E-10	hypomethylated	A930024E05Rik,Kdm2l	17	16
chr5	123464082	123466082	-0.19155	2.87E-12	hypomethylated	Orai1	41	40
chr5	123793456	123795456	-0.041143	0.000000303	hypomethylated	Bcl7a	69	76
chr5	123843827	123845827	-0.22817	1.11E-13	hypomethylated	Mlxip	47	51
chr5	124134300	124136300	-0.16446	0.025138	hypomethylated	Clip1	7	22
chr5	124356283	124358283	-0.38323	2.73E-15	stronglyhypometh	Denr	38	41
chr5	124565116	124567116	-0.3697	1.71E-09	stronglyhypometh	Arl6ip4	25	24
chr5	124989799	124991799	0.050896	0.047781	hypermethylated	Tmed2	30	31
chr5	125001872	125003872	-0.083813	0.026638	hypomethylated	Ddx55	28	28
chr5	125342074	125344074	-0.056594	0.0031348	hypomethylated	Ccdc92,Zfp664	94	101
chr5	125659584	125661584	-0.41307	0.013436	stronglyhypometh	Ncor2	8	9

chr5	125821444	125823444	-0.43534	0.0064549	stronglyhypometh	Scarb1	6	8
chr5	125955242	125957242	0.050785	0.020355	hypermethylated	Aacs	19	22
chr5	126011787	126013787	-0.084807	4.29E-08	hypomethylated	Tmem132b	50	61
chr5	129105980	129107980	-0.072098	0.031909	hypomethylated	5930412G12Rik,Fzd10	65	74
chr5	129106562	129108562	-0.084154	0.0055278	hypomethylated	5930412G12Rik,Fzd10	50	60
chr5	129525030	129527030	-0.22487	1.86E-11	hypomethylated	Ran	57	64
chr5	130006105	130008105	-0.15643	0.0012931	hypomethylated	Sfswap	35	35
chr5	130321865	130323865	-0.73333	0.0000362	stronglyhypometh	Sumf2	5	5
chr5	130619757	130621757	-0.4568	0.00023868	stronglyhypometh	Kctd7	14	14
chr5	130697204	130699204	0.089971	0.0024872	hypermethylated	Tmem248	41	44
chr5	130844327	130846327	-0.33063	0.0031137	hypomethylated	Caln1	5	5
chr5	130923661	130925661	-0.14212	0.0097798	hypomethylated	Caln1	25	27
chr5	133018213	133020213	-0.15244	0.0010302	hypomethylated	Auts2	39	54
chr5	134574617	134576617	-0.30696	0.0005175	hypomethylated	Gatsl2	19	27
chr5	134790616	134792616	-0.25176	0.011584	hypomethylated	Gtf2i	24	26
chr5	135028304	135030304	0.24135	0.022327	hypermethylated	Clip2	7	8
chr5	135090893	135092893	0.40104	0.017836	stronglyhypermeth	Lat2	3	4
chr5	135115198	135117198	-0.23741	1.42E-13	hypomethylated	Eif4h	14	14
chr5	135407242	135409242	-0.41509	0.011823	stronglyhypometh	Wbscr27	7	13
chr5	135554136	135556136	-0.10935	0.013793	hypomethylated	Vps37d	13	14
chr5	136254255	136256255	-0.11308	0.015409	hypomethylated	Mdh2,Styxl1	31	36
chr5	136674824	136676824	0.14497	0.043956	hypermethylated	Prkrip1	7	9
chr5	137436946	137438946	-0.30556	0.04878	hypomethylated	Fis1	2	2
chr5	137461888	137463888	-0.20967	0.00070893	hypomethylated	Plod3,Znhit1	29	34
chr5	137505164	137507164	-0.10257	0.012242	hypomethylated	Vgf	30	33
chr5	137728504	137730504	-0.18467	0.0052112	hypomethylated	Ache	28	28
chr5	137734896	137736896	-0.087706	0.0063291	hypomethylated	Ufsp1	19	23
chr5	137774810	137776810	-0.67308	0.00047385	stronglyhypometh	Slc12a9	6	3
chr5	138125845	138127845	-0.53846	3.47E-13	stronglyhypometh	Agfg2	13	12
chr5	138186223	138188223	-0.25978	0.0077025	hypomethylated	Tsc22d4	9	9
chr5	138219145	138221145	-0.15551	0.041122	hypomethylated	Ppp1r35	36	39
chr5	138557132	138559132	-0.28095	0.00088578	hypomethylated	Zscan21	4	5
chr5	138601329	138603329	-0.83333	0.0013675	stronglyhypometh	Cops6	3	3
chr5	138695709	138697709	-0.16671	0.005076	hypomethylated	Lamtor4	10	10
chr5	139230034	139232034	-0.055476	0.0000198	hypomethylated	Fam20c	64	80
chr5	139470907	139472907	-0.043438	0.0011638	hypomethylated	Pdgfa	80	85
chr5	139605706	139607706	0.11827	0.039118	hypermethylated	Prkar1b	13	15
chr5	139625176	139627176	-0.02081	0.00000439	hypomethylated	Heatr2	39	43
chr5	139675623	139677623	-0.25814	0.0026358	hypomethylated	Sun1	34	34
chr5	140797506	140799506	-0.47699	3.16E-11	stronglyhypometh	Mad1l1	11	16
chr5	140806875	140808875	-0.58482	0.0000105	stronglyhypometh	Ftsj2,Nudt1	7	12
chr5	141178332	141180332	-0.05938	0.00073246	hypomethylated	Brat1,Iqce	8	8
chr5	141179976	141181976	-0.59255	4.91E-09	stronglyhypometh	Brat1	10	11
chr5	143006030	143008030	-0.23919	0.010209	hypomethylated	Papolb	11	11
chr5	143579066	143581066	-0.21689	0.000000958	hypomethylated	Tnrc18	18	22
chr5	143721033	143723033	-0.0099072	0.039524	hypomethylated	Fscn1	43	45
chr5	144089986	144091986	-0.28497	0.0028241	hypomethylated	0610040B10Rik,Zdhhc	29	33
chr5	144164498	144166498	-0.053037	0.0017284	hypomethylated	Kdelr2	14	19
chr5	144224360	144226360	-0.053084	0.024016	hypomethylated	Daglb	28	32
chr5	144288861	144290861	-0.11536	0.00034498	hypomethylated	Rac1	20	20
chr5	144382315	144384315	-0.1844	0.0000649	hypomethylated	Cyth3	13	16
chr5	144493149	144495149	-0.12792	0.0000542	hypomethylated	Usp42	25	25
chr5	144518222	144520222	-0.25004	0.00095754	hypomethylated	D130017N08Rik	12	12
chr5	144577660	144579660	-0.29119	0.0015509	hypomethylated	Eif2ak1	8	8
chr5	144669869	144671869	-0.21852	0.00000307	hypomethylated	Aimp2,Pms2	13	26
chr5	144670708	144672708	-0.13909	0.0000324	hypomethylated	Aimp2,Pms2	8	17
chr5	144775722	144777722	-0.35539	0.04912	stronglyhypometh	Ccz1	6	6

chr5	144860304	144862304	-0.080498	0.028935	hypomethylated	Lmtk2	27	39
chr5	144984447	144986447	-0.42072	0.00056905	stronglyhypometh	Tecpr1	8	15
chr5	145015093	145017093	-0.31192	0.00000243	hypomethylated	Bri3	32	33
chr5	145305755	145307755	0.065992	0.048087	hypermethylated	Nptx2	55	63
chr5	145528660	145530660	-0.30978	0.00000198	hypomethylated	Trrap	34	41
chr5	145726699	145728699	0.1127	0.014794	hypermethylated	Smurf1	18	20
chr5	145927092	145929092	-0.078652	0.00019615	hypomethylated	Cpsf4,Ptcd1	21	33
chr5	145927973	145929973	-0.029001	0.030533	hypomethylated	Cpsf4,Ptcd1	15	24
chr5	145964427	145966427	-0.33906	0.042358	stronglyhypometh	Zkscan5	11	14
chr5	145991583	145993583	-0.094973	0.00056573	hypomethylated	Zfp655	32	35
chr5	145991627	145993627	-0.094973	0.00056573	hypomethylated	Zfp655	32	35
chr5	147033013	147035013	0.12672	0.0000254	hypermethylated	Rnf6	9	12
chr5	147643465	147645465	-0.16474	0.048694	hypomethylated	Rpl21	17	23
chr5	147759232	147761232	-0.53654	0.010286	stronglyhypometh	Gtf3a	1	10
chr5	147888116	147890116	-0.2449	0.0000111	hypomethylated	Lnx2,Polr1d	26	29
chr5	147888143	147890143	-0.2449	0.0000111	hypomethylated	Lnx2,Polr1d	26	29
chr5	147888148	147890148	-0.2449	0.0000111	hypomethylated	Lnx2,Polr1d	26	29
chr5	148080706	148082706	-0.24641	0.0021791	hypomethylated	2210019I11Rik,Pdx1	17	22
chr5	148671203	148673203	-0.2581	0.014958	hypomethylated	Pomp	23	23
chr5	148767895	148769895	-0.084676	0.00000131	hypomethylated	Mtus2	36	43
chr5	149740223	149742223	-0.1587	0.026532	hypomethylated	Katnal1	24	32
chr5	150241280	150243280	-0.6118	0.004529	stronglyhypometh	Tex26	7	4
chr5	150438890	150440890	-0.048396	0.044338	hypomethylated	Hsph1	18	17
chr5	151324197	151326197	-0.12562	0.0000132	hypomethylated	Brca2	23	23
chr5	151324204	151326204	-0.12562	0.0000132	hypomethylated	Brca2	23	23
chr5	151397100	151399100	-0.33124	2.45E-17	hypomethylated	N4bp2l1	20	20
chr5	151475401	151477401	-0.16637	0.00064837	hypomethylated	Pds5b	38	53
chr5	151754181	151756181	-0.53565	0.000000279	stronglyhypometh	Kl	12	23
chr6	3713623	3715623	-0.17782	0.0012729	hypomethylated	Calcr	9	14
chr6	4550065	4552065	-0.092804	0.0000529	hypomethylated	Casd1	39	40
chr6	4852319	4854319	-0.28964	9.19E-10	hypomethylated	Ppp1r9a	38	43
chr6	6815150	6817150	-0.60455	0.004785	stronglyhypometh	Dlx6as2	3	2
chr6	7794223	7796223	-0.042665	0.0071394	hypomethylated	C1galt1	53	54
chr6	8158226	8160226	-0.080462	0.042961	hypomethylated	Mios	33	37
chr6	8208287	8210287	-0.28338	2.82E-08	hypomethylated	Gm16039,Rpa3	17	19
chr6	8209141	8211141	-0.28338	2.82E-08	hypomethylated	Gm16039,Rpa3	17	19
chr6	11874880	11876880	-0.22654	0.0014135	hypomethylated	Phf14	15	21
chr6	12699253	12701253	-0.13549	0.017623	hypomethylated	Thsd7a	10	10
chr6	14850348	14852348	0.15004	0.011426	hypermethylated	Foxp2	18	22
chr6	17230340	17232340	-0.37945	0.0034238	stronglyhypometh	Cav2	10	8
chr6	17412956	17414956	-0.28827	9.73E-08	hypomethylated	Met	25	30
chr6	17586097	17588097	-0.13952	0.013282	hypomethylated	Capza2	36	46
chr6	17980445	17982445	-0.60269	5.83E-08	stronglyhypometh	Wnt2	23	26
chr6	22824501	22826501	-0.10455	0.0024829	hypomethylated	Ptprz1	13	23
chr6	23789300	23791300	-0.14768	0.0000002	hypomethylated	Cadps2	31	35
chr6	28371724	28373724	-0.09476	0.0085963	hypomethylated	Arf5,Gcc1	16	20
chr6	28372639	28374639	-0.15899	0.0014569	hypomethylated	Arf5,Gcc1	23	35
chr6	28429347	28431347	-0.38635	0.00000368	stronglyhypometh	Snd1	16	23
chr6	28781747	28783747	-0.075168	0.00020531	hypomethylated	Lrrc4	31	37
chr6	29162271	29164271	-0.13816	0.000000521	hypomethylated	Impdh1	18	18
chr6	29221487	29223487	-0.44829	0.0001313	stronglyhypometh	Hilpda	11	14
chr6	29297118	29299118	-0.19989	0.02413	hypomethylated	Calu	35	42
chr6	29475732	29477732	-0.13157	0.043642	hypomethylated	Irf5	45	49
chr6	29866012	29868012	-0.19391	0.000000775	hypomethylated	Strip2	27	32
chr6	29996987	29998987	0.059592	0.0034559	hypermethylated	Nrf1	40	50
chr6	30350908	30352908	-0.12823	0.025521	hypomethylated	Klhdc10	25	27
chr6	30846760	30848760	-0.053649	0.032734	hypomethylated	Copg2	7	12

chr6	31347827	31349827	-0.10271	0.00028803	hypomethylated	Gm13845,Mkln1	32	39
chr6	32538192	32540192	-0.646	0.0036581	stronglyhypometh	Plxna4	6	2
chr6	33198149	33200149	-0.47888	0.0039671	stronglyhypometh	Exoc4	13	14
chr6	34425355	34427355	-0.26843	0.00029977	hypomethylated	Bpgm	14	18
chr6	34828065	34830065	-0.16845	0.0014962	hypomethylated	3110062M04Rik	6	10
chr6	35126615	35128615	-0.071369	0.013905	hypomethylated	Nup205	41	48
chr6	35201698	35203698	-0.06038	0.044241	hypomethylated	1810058I24Rik	9	15
chr6	35201731	35203731	-0.06038	0.044241	hypomethylated	1810058I24Rik	9	15
chr6	37819810	37821810	-0.14765	1.78E-09	hypomethylated	Trim24	48	53
chr6	38204009	38206009	0.16162	0.00059435	hypermethylated	D630045J12Rik	15	15
chr6	39541581	39543581	-0.24681	0.0099479	hypomethylated	Ndufb2	24	34
chr6	39760935	39762935	0.23826	0.010487	hypermethylated	Mrps33	8	8
chr6	40274476	40276476	0.13695	0.005151	hypermethylated	Agk	14	24
chr6	40420413	40422413	-0.20528	0.0021202	hypomethylated	Ssbp1	13	13
chr6	41554480	41556480	-0.2736	0.000000696	hypomethylated	Ephb6	19	22
chr6	42274639	42276639	-0.21723	0.0013179	hypomethylated	Fam131b	19	22
chr6	42298826	42300826	-0.042598	0.0072131	hypomethylated	Zyx	47	53
chr6	43214642	43216642	-0.074664	0.0018943	hypomethylated	Arhgef5	26	24
chr6	43616174	43618174	0.12946	0.031379	hypermethylated	Tpk1	8	8
chr6	47403322	47405322	-0.00029671	0.00036187	hypomethylated	Cul1	76	86
chr6	47545029	47547029	0.17462	0.025094	hypermethylated	Ezh2	12	8
chr6	47892173	47894173	-0.24932	3.35E-08	hypomethylated	Zfp783	30	16
chr6	47902388	47904388	0.34103	0.00086188	stronglyhypermeth	Zfp956	7	9
chr6	48542881	48544881	-0.21068	0.000168	hypomethylated	Repin1	18	12
chr6	49771727	49773727	-0.2479	0.047317	hypomethylated	Npy	17	20
chr6	50546589	50548589	-0.50814	0.000029	stronglyhypometh	4921507P07Rik	4	9
chr6	51419614	51421614	-0.29519	0.000000278	hypomethylated	Cbx3,Hnrnpa2b1	48	63
chr6	51419893	51421893	-0.26702	0.00000203	hypomethylated	Cbx3,Hnrnpa2b1	46	63
chr6	51472901	51474901	-0.50455	0.0000127	stronglyhypometh	Snx10	10	11
chr6	51962548	51964548	-0.17561	0.0013917	hypomethylated	Skap2	14	14
chr6	52195766	52197766	-0.32474	0.0092909	hypomethylated	Hoxa11,Hoxa11as	15	30
chr6	52210874	52212874	-0.099773	0.030691	hypomethylated	Hoxa13	6	7
chr6	52662722	52664722	-0.21865	0.00000113	hypomethylated	Tax1bp1	35	42
chr6	53236288	53238288	-0.35131	0.000000569	stronglyhypometh	9430076C15Rik	37	56
chr6	53770819	53772819	-0.31333	0.0000078	hypomethylated	Tril	18	23
chr6	54543122	54545122	-0.48958	0.000000636	stronglyhypometh	Fkbp14,Plekha8	8	8
chr6	54544104	54546104	-0.20863	0.00052767	hypomethylated	Fkbp14,Plekha8	29	30
chr6	54630765	54632765	-0.10902	0.046605	hypomethylated	2410066E13Rik	33	40
chr6	54765909	54767909	-0.046007	0.036172	hypomethylated	Znrf2	65	74
chr6	56654693	56656693	-0.31201	0.0082062	hypomethylated	Lsm5	5	5
chr6	57642072	57644072	-0.36896	0.00042969	stronglyhypometh	Pyurf	7	7
chr6	59157863	59159863	-0.065127	0.0029018	hypomethylated	Tigd2	32	33
chr6	64678139	64680139	-0.24908	0.000031	hypomethylated	Atoh1	27	29
chr6	65727955	65729955	-0.25446	3.65E-09	hypomethylated	Prdm5	20	18
chr6	66484461	66486461	-0.12912	0.012274	hypomethylated	Mad2l1	13	20
chr6	66845390	66847390	0.10139	0.0032695	hypermethylated	Gng12	25	32
chr6	66845884	66847884	0.10139	0.0032695	hypermethylated	Gng12	25	32
chr6	67215972	67217972	-0.17485	0.0057986	hypomethylated	Serbp1	46	36
chr6	70742169	70744169	-0.59249	0.0000941	stronglyhypometh	Rpia	12	13
chr6	70793520	70795520	-0.40135	0.01139	stronglyhypometh	Eif2ak3	13	25
chr6	70905599	70907599	-0.23707	1.28E-11	hypomethylated	Foxi3	39	39
chr6	71094370	71096370	-0.58422	0.0049191	stronglyhypometh	Thnsl2	4	4
chr6	71442887	71444887	-0.10599	0.000000271	hypomethylated	Rnf103	23	32
chr6	72304476	72306476	-0.20056	0.00014109	hypomethylated	Tmem150a	25	30
chr6	72389552	72391552	-0.24196	0.0000224	hypomethylated	4930414L22Rik,Mat2a	12	18
chr6	72738950	72740950	-0.088926	0.0013043	hypomethylated	Tcf7l1	22	23
chr6	73171625	73173625	-0.46154	0.039409	stronglyhypometh	Dnahc6	2	2

chr6	77191710	77193710	-0.16222	0.01741	hypomethylated	Lrrtm1	18	23
chr6	82602859	82604859	-0.02504	0.044632	hypomethylated	Pole4	15	22
chr6	82983217	82985217	-0.091876	0.030287	hypomethylated	Dok1,Loxl3	8	11
chr6	82983465	82985465	-0.091876	0.030287	hypomethylated	Dok1,Loxl3	8	11
chr6	83003646	83005646	-0.12488	0.0014938	hypomethylated	Aup1,Htra2	47	54
chr6	83004565	83006565	-0.13933	0.0070322	hypomethylated	Aup1,Htra2	31	40
chr6	83027383	83029383	-0.15033	0.00069017	hypomethylated	Pcgf1	20	25
chr6	83050509	83052509	-0.25093	0.000000331	hypomethylated	Ccdc142	33	35
chr6	83058101	83060101	-0.16788	0.0012836	hypomethylated	Mrpl53	18	19
chr6	83071455	83073455	-0.16219	0.0078876	hypomethylated	Wbp1	22	22
chr6	83275032	83277032	-0.38622	2.56E-10	stronglyhypometh	Mob1a	21	21
chr6	83660926	83662926	-0.39697	0.020737	stronglyhypometh	Vax2,Vax2os	12	18
chr6	83863346	83865346	-0.029313	0.0048156	hypomethylated	Zfml	17	21
chr6	83957583	83959583	-0.25795	0.0089449	hypomethylated	Dysf	10	11
chr6	85452880	85454880	-0.37602	0.016148	stronglyhypometh	Fbxo41	9	9
chr6	86144244	86146244	-0.073497	0.016383	hypomethylated	Tgfa	37	37
chr6	86314676	86316676	0.15198	0.02189	hypermethylated	Fam136a	31	32
chr6	86683372	86685372	-0.18189	0.0028504	hypomethylated	Gmcl1	12	16
chr6	86798510	86800510	-0.15773	0.0037167	hypomethylated	2610306M01Rik,Aak1	49	36
chr6	86958829	86960829	-0.48571	0.0000925	stronglyhypometh	Nfu1	10	14
chr6	87679862	87681862	-0.27235	0.00000649	hypomethylated	Efcc1	16	20
chr6	87801100	87803100	-0.22363	0.0042723	hypomethylated	Cnbp	10	10
chr6	88033466	88035466	-0.15719	0.01616	hypomethylated	Rpn1	13	18
chr6	88673405	88675405	-0.19535	0.0097878	hypomethylated	Mgll	38	40
chr6	88673697	88675697	-0.19535	0.0097878	hypomethylated	Mgll	38	40
chr6	88791929	88793929	-0.027139	0.021906	hypomethylated	Abtb1,Gm15612	20	29
chr6	89312607	89314607	-0.05867	0.0040051	hypomethylated	Plxna1	13	30
chr6	91161757	91163757	-0.14082	0.021596	hypomethylated	Fbln2	15	17
chr6	91633060	91635060	-0.0578	0.01724	hypomethylated	Slc6a6	14	27
chr6	92040411	92042411	-0.078034	0.00026179	hypomethylated	Nr2c2	75	82
chr6	95066899	95068899	-0.12981	0.026065	hypomethylated	Kbtbd8	21	35
chr6	97098877	97100877	-0.60566	0.0000138	stronglyhypometh	Eogt	4	9
chr6	97129118	97131118	-0.076096	0.0081978	hypomethylated	Tmf1	14	14
chr6	97756051	97758051	-0.15164	0.00075414	hypomethylated	Mitf	19	18
chr6	100237352	100239352	-0.015949	0.000000128	hypomethylated	Rybp	33	50
chr6	105626738	105628738	-0.35138	0.00073104	stronglyhypometh	Cntn4	9	11
chr6	106718165	106720165	0.094397	0.0076403	hypermethylated	Trnt1	13	25
chr6	107478778	107480778	-0.26681	0.0086366	hypomethylated	Lrrn1	10	10
chr6	108014039	108016039	-0.88095	0.022199	stronglyhypometh	Setmar	2	2
chr6	108732052	108734052	-0.12298	0.0041976	hypomethylated	Arl8b	18	28
chr6	110594591	110596591	-0.15244	0.048466	hypomethylated	Grm7	12	16
chr6	113231300	113233300	-0.36578	0.0000935	stronglyhypometh	Cpne9	31	31
chr6	113256190	113258190	-0.095304	0.0043234	hypomethylated	Brpf1	28	30
chr6	113327106	113329106	-0.077124	0.022805	hypomethylated	Arpc4,Tada3	22	28
chr6	113391628	113393628	0.080378	0.016393	hypermethylated	Jagn1	21	21
chr6	113480675	113482675	-0.47909	0.013676	stronglyhypometh	Emc3,Fancd2	11	7
chr6	113553765	113555765	-0.1302	0.011419	hypomethylated	Brk1	18	21
chr6	113573014	113575014	-0.13698	0.02367	hypomethylated	Vhl	26	36
chr6	114231628	114233628	-0.55526	0.0014967	stronglyhypometh	Slc6a1	9	18
chr6	114346929	114348929	-0.18358	0.019232	hypomethylated	Hrh1	25	32
chr6	115550955	115552955	-0.22347	0.0000144	hypomethylated	Mkrn2	9	9
chr6	115626653	115628653	-0.07119	0.0414	hypomethylated	Raf1	7	10
chr6	115758121	115760121	-0.12185	0.00018417	hypomethylated	Rpl32,Snora7a	17	17
chr6	115802545	115804545	-0.26083	0.00000142	hypomethylated	Ift122,Mbd4	22	20
chr6	115803359	115805359	-0.16926	0.00030597	hypomethylated	Ift122,Mbd4	13	13
chr6	115945023	115947023	-0.2509	2.94E-09	hypomethylated	Plxnd1	12	12
chr6	117117552	117119552	-0.061183	0.023505	hypomethylated	Cxcl12	42	42

chr6	117855799	117857799	-0.124	0.000000799	hypomethylated	Hnrnpf	55	65
chr6	117856821	117858821	-0.078159	0.000688	hypomethylated	Hnrnpf	60	76
chr6	119428685	119430685	-0.12606	0.014342	hypomethylated	Fbxl14	81	78
chr6	119494365	119496365	-0.11706	0.012655	hypomethylated	Wnt5b	11	20
chr6	119797210	119799210	0.14029	0.002021	hypermethylated	3110021A11Rik,Erc1	33	36
chr6	120313116	120315116	-0.28208	0.0027836	hypomethylated	Ccdc77,Kdm5a	25	44
chr6	120785247	120787247	-0.010844	0.018721	hypomethylated	Bcl2l13	25	27
chr6	124691097	124693097	-0.37179	0.035109	stronglyhypometh	Grcc10	2	3
chr6	124706505	124708505	0.26058	0.014474	hypermethylated	Atn1	28	36
chr6	124757958	124759958	-0.099724	0.0030564	hypomethylated	Spsb2	15	17
chr6	124779193	124781193	-0.20481	0.0025916	hypomethylated	Cdca3,Usp5	20	26
chr6	124779465	124781465	-0.39444	0.0012936	stronglyhypometh	Cdca3,Usp5	12	12
chr6	124945737	124947737	0.12254	0.0015112	hypermethylated	Pianp	20	15
chr6	124958822	124960822	0.098907	0.000000862	hypermethylated	Zfp384	44	49
chr6	125080900	125082900	-0.46024	7.91E-13	stronglyhypometh	Nop2	18	20
chr6	125141604	125143604	-0.37756	0.0068734	stronglyhypometh	Mrpl51,Ncapd2	13	23
chr6	125298740	125300740	-0.46095	0.0045777	stronglyhypometh	Tnfrsf1a	7	9
chr6	125639436	125641436	-0.50556	0.0056893	stronglyhypometh	Ano2	3	3
chr6	127059570	127061570	-0.14175	0.024305	hypomethylated	9630033F20Rik	5	5
chr6	127402740	127404740	-0.24772	0.00048326	hypomethylated	Parp11	19	19
chr6	127836639	127838639	-0.40201	0.029369	stronglyhypometh	Tspan11	3	7
chr6	128250831	128252831	-0.37425	0.0024867	stronglyhypometh	Tead4	8	5
chr6	128312011	128314011	-0.036687	0.0024338	hypomethylated	Foxm1,Rhno1	36	40
chr6	128312840	128314840	-0.057331	0.0050702	hypomethylated	Foxm1,Rhno1	18	22
chr6	128312915	128314915	-0.057331	0.0050702	hypomethylated	Foxm1,Rhno1	18	22
chr6	128388649	128390649	-0.17278	0.0098878	hypomethylated	Fkbp4,Gm10069	14	14
chr6	133054256	133056256	-0.12322	0.047892	hypomethylated	2700089E24Rik	17	18
chr6	133984724	133986724	-0.4523	8.47E-10	stronglyhypometh	Etv6	22	30
chr6	134779216	134781216	-0.58838	0.0000227	stronglyhypometh	Crebl2	9	11
chr6	134869418	134871418	-0.32856	0.0010836	hypomethylated	Cdkn1b	17	29
chr6	136466371	136468371	-0.30909	0.0000911	hypomethylated	Atf7ip	11	10
chr6	140371619	140373619	0.075232	0.0050875	hypermethylated	Plekha5	62	69
chr6	141196789	141198789	-0.029046	0.01966	hypomethylated	Pde3a	26	30
chr6	142704205	142706205	-0.51227	1.64E-11	stronglyhypometh	Cmas	27	37
chr6	145158653	145160653	-0.31553	0.0063432	hypomethylated	Casc1,Lyrm5	13	14
chr6	145158666	145160666	-0.31553	0.0063432	hypomethylated	Casc1,Lyrm5	13	14
chr6	145198751	145200751	-0.25216	0.029074	hypomethylated	Gm15706,Kras	26	24
chr6	146450434	146452434	-0.13085	0.00012246	hypomethylated	Itpr2	23	28
chr6	146836015	146838015	-0.12289	0.001572	hypomethylated	Ppfibp1	30	40
chr6	147038596	147040596	0.087022	0.029357	hypermethylated	Klhl42	22	22
chr6	147212607	147214607	0.21489	0.00017886	hypermethylated	Pthlh	16	16
chr6	147994937	147996937	-0.64913	0.00092725	stronglyhypometh	Far2	5	6
chr6	148160896	148162896	0.40125	0.00071642	stronglyhypermeth	4732416N19Rik,Ergic2	4	4
chr6	149050202	149052202	-0.39889	0.0013816	stronglyhypometh	Dennd5b	3	5
chr7	3292028	3294028	-0.73333	0.0002469	stronglyhypometh	Myadm	4	4
chr7	3424422	3426422	-0.2669	0.0001543	hypomethylated	Cacng6	11	11
chr7	3580586	3582586	-0.16219	0.0000333	hypomethylated	Prpf31,Tfpt	9	13
chr7	3581486	3583486	-0.12116	0.016915	hypomethylated	Prpf31,Tfpt	5	9
chr7	3595870	3597870	-0.39943	6E-17	stronglyhypometh	Cnot3	36	39
chr7	3644211	3646211	-0.58561	1.03E-09	stronglyhypometh	Mboat7,Tsen34	21	29
chr7	4453282	4455282	-0.010645	0.011119	hypomethylated	Ppp1r12c	8	16
chr7	4498169	4500169	-0.41197	0.031408	stronglyhypometh	Syt5	13	12
chr7	4827654	4829654	-0.28944	0.0013439	hypomethylated	Isoc2a	3	3
chr7	4865818	4867818	0.081768	0.024836	hypermethylated	Zfp628	15	23
chr7	5012783	5014783	-0.3662	0.0000195	stronglyhypometh	U2af2	26	25
chr7	6107573	6109573	-0.40475	2.42E-10	stronglyhypometh	Zfp787	25	34
chr7	6237181	6239181	0.08102	0.032974	hypermethylated	Zfp667	8	15

chr7	6335027	6337027	-0.36305	0.0080221	stronglyhypometh	Zfp28	14	19
chr7	6649143	6651143	-0.82848	0.0000587	stronglyhypometh	Zim1	5	5
chr7	7231000	7233000	0.078471	0.00023126	hypermethylated	Vmn2r29	8	10
chr7	13419158	13421158	-0.60972	0.0000198	stronglyhypometh	Zfp110	15	11
chr7	13511764	13513764	-0.14041	1.71E-08	hypomethylated	2310014L17Rik	28	27
chr7	13620126	13622126	-0.23857	0.010766	hypomethylated	Chmp2a,Ube2m	8	12
chr7	13623327	13625327	-0.36711	0.000000441	stronglyhypometh	Ube2m	12	19
chr7	13623619	13625619	-0.58413	8.64E-09	stronglyhypometh	Ube2m	7	8
chr7	16682991	16684991	-0.32778	5.16E-18	hypomethylated	Napa	20	20
chr7	16704224	16706224	-0.34702	0.0010543	stronglyhypometh	Kptn	5	5
chr7	16985544	16987544	-0.12044	0.0071389	hypomethylated	Zc3h4	34	48
chr7	17037127	17039127	-0.25609	0.0000773	hypomethylated	Tmem160	15	25
chr7	17322792	17324792	-0.31407	0.0000169	hypomethylated	Ap2s1	17	19
chr7	17365694	17367694	-0.43939	0.00060189	stronglyhypometh	Slc1a5	7	11
chr7	17509381	17511381	-0.27833	0.0066023	hypomethylated	Calm3	6	10
chr7	17529030	17531030	-0.32136	0.022293	hypomethylated	Pnmal2	15	21
chr7	17613263	17615263	-0.41667	0.015492	stronglyhypometh	Ppp5c	3	3
chr7	19608725	19610725	-0.18961	3.85E-09	hypomethylated	Foxa3,Sympk	47	53
chr7	19608888	19610888	-0.18111	2.49E-10	hypomethylated	Foxa3,Sympk	43	52
chr7	19660548	19662548	-0.20797	0.0096604	hypomethylated	Dmwd	44	39
chr7	19671176	19673176	0.57143	0.0063387	stronglyhypermeth	Mir3100	5	9
chr7	19944832	19946832	-0.19612	0.034527	hypomethylated	Cd3eap,Ppp1r13l	11	12
chr7	19945564	19947564	-0.22005	0.0055866	hypomethylated	Cd3eap,Ppp1r13l	11	12
chr7	19966387	19968387	-0.032765	0.01368	hypomethylated	Ercc2	26	27
chr7	20043843	20045843	-0.5327	0.00053704	stronglyhypometh	Mark4	12	15
chr7	20093077	20095077	-0.2828	6.1E-17	hypomethylated	Bloc1s3,Trappc6a	23	26
chr7	20093680	20095680	-0.32337	1.04E-17	hypomethylated	Bloc1s3,Trappc6a	20	21
chr7	20161635	20163635	-0.22947	0.031561	hypomethylated	Zfp296	18	19
chr7	20214787	20216787	-0.13737	0.0055613	hypomethylated	Relb	19	19
chr7	20334922	20336922	-0.21691	0.0015248	hypomethylated	Pvrl2	5	9
chr7	24865962	24867962	-0.035451	0.0037866	hypomethylated	Zfp180	15	14
chr7	25246518	25248518	-0.39211	0.000000471	stronglyhypometh	Plaur	7	7
chr7	25266041	25268041	-0.12076	0.0002606	hypomethylated	Cadm4	31	38
chr7	25291105	25293105	0.086693	0.0048355	hypermethylated	Zfp428	23	39
chr7	25314666	25316666	-0.14243	0.00023711	hypomethylated	Irgq	11	18
chr7	25331024	25333024	-0.017484	0.037773	hypomethylated	Pinlyp,Xrcc1	30	25
chr7	25331168	25333168	-0.017484	0.037773	hypomethylated	Pinlyp,Xrcc1	30	25
chr7	25445312	25447312	-0.38838	0.044085	stronglyhypometh	Gm4598	5	6
chr7	25790914	25792914	-0.099276	0.024044	hypomethylated	Atp1a3	13	11
chr7	25857388	25859388	0.1977	0.034678	hypermethylated	Grik5	11	14
chr7	25861223	25863223	0.049582	0.031436	hypermethylated	Zfp574	18	29
chr7	25861264	25863264	0.049582	0.031436	hypermethylated	Zfp574	18	29
chr7	25917479	25919479	-0.90606	0.0014605	stronglyhypometh	Pou2f2	3	9
chr7	26035777	26037777	0.16895	0.029051	hypermethylated	Erf	9	20
chr7	26101182	26103182	-0.295	0.0058663	hypomethylated	Megf8	15	15
chr7	27980779	27982779	-0.44674	0.000000225	stronglyhypometh	BC024978,Snrpa	18	20
chr7	28118667	28120667	-0.30795	0.0015028	hypomethylated	Ltbp4	18	20
chr7	28231608	28233608	-0.12378	0.0000307	hypomethylated	Blvrb,Sptbn4	25	29
chr7	28257858	28259858	-0.013216	0.046298	hypomethylated	Sertad3	18	21
chr7	28337313	28339313	-0.19434	0.019427	hypomethylated	2310022A10Rik,Pld3	10	10
chr7	28338131	28340131	-0.19434	0.019427	hypomethylated	2310022A10Rik,Pld3	10	10
chr7	28437942	28439942	-0.27579	0.00000644	hypomethylated	Ttc9b	27	27
chr7	28515427	28517427	-0.23946	0.0013159	hypomethylated	Zfp60	5	7
chr7	28622602	28624602	-0.18341	0.028593	hypomethylated	Zfp59	13	10
chr7	28953766	28955766	-0.097228	0.0000228	hypomethylated	Fbl	23	25
chr7	28963501	28965501	-0.35048	6.44E-16	stronglyhypometh	Dyrk1b	25	38
chr7	28963512	28965512	-0.35048	6.44E-16	stronglyhypometh	Dyrk1b	25	38

chr7	29051899	29053899	-0.16966	0.00044321	hypomethylated	Eid2	34	38
chr7	29076153	29078153	-0.67388	8.36E-08	stronglyhypometh	BC089491	7	10
chr7	29157681	29159681	-0.22243	0.0047318	hypomethylated	Plekhg2	16	20
chr7	29236003	29238003	-0.21759	1.82E-11	hypomethylated	Lrfn1	41	52
chr7	29236256	29238256	-0.21605	2.96E-11	hypomethylated	Lrfn1	41	53
chr7	29383203	29385203	-0.24802	0.030999	hypomethylated	Pak4	3	3
chr7	29540192	29542192	-0.82575	0.022523	stronglyhypometh	Gm6537	2	7
chr7	29550770	29552770	-0.26245	6.97E-09	hypomethylated	Nfkbib,Sirt2	22	30
chr7	29551543	29553543	-0.27495	0.00025628	hypomethylated	Nfkbib,Sirt2	13	21
chr7	29665674	29667674	-0.72756	0.0019616	stronglyhypometh	Capn12	7	12
chr7	30552570	30554570	-0.34222	0.033942	stronglyhypometh	Zfp84	3	5
chr7	30879094	30881094	-0.31126	0.016243	hypomethylated	Zfp260	5	5
chr7	31016092	31018092	-0.38318	0.0000292	stronglyhypometh	Polr2i,Tbcb	26	24
chr7	31092771	31094771	-0.48485	0.03835	stronglyhypometh	Alkbh6	2	2
chr7	31204873	31206873	-0.21021	0.033427	hypomethylated	Hcst	7	9
chr7	31244076	31246076	0.43835	0.021409	stronglyhypermeth	Nphs1	3	5
chr7	31347358	31349358	-0.30486	0.024983	hypomethylated	Psenen,U2af1l4	16	19
chr7	31348203	31350203	-0.12089	0.038408	hypomethylated	Igflr1,Psenen,U2af1l4	21	26
chr7	31514553	31516553	-0.53833	0.00061488	stronglyhypometh	Tmem147	4	7
chr7	31741822	31743822	-0.38493	0.0000132	stronglyhypometh	Usf2	25	43
chr7	31758488	31760488	-0.41301	0.00000261	stronglyhypometh	Fam187b,Lsr	16	9
chr7	31900309	31902309	-0.27075	0.019688	hypomethylated	Hpn	12	12
chr7	35174559	35176559	0.1098	0.047707	hypermethylated	Lsm14a	27	30
chr7	35437860	35439860	-0.33759	0.00040789	stronglyhypometh	Kctd15	32	51
chr7	35903311	35905311	-0.16691	0.0000239	hypomethylated	Cebpa	60	85
chr7	36000364	36002364	-0.16572	0.00034808	hypomethylated	Lrp3	17	17
chr7	36118255	36120255	-0.31288	0.027769	hypomethylated	Rhpn2	8	17
chr7	36181111	36183111	-0.1555	0.023499	hypomethylated	C230052I12Rik,Cep89	7	7
chr7	36181786	36183786	-0.2177	0.0090887	hypomethylated	C230052I12Rik,Cep89	5	5
chr7	36370265	36372265	-0.18366	0.0014573	hypomethylated	Ankrd27,Rgs9bp	22	23
chr7	36370601	36372601	-0.19526	0.048175	hypomethylated	Ankrd27,Rgs9bp	15	16
chr7	37482136	37484136	-0.076864	4.15E-08	hypomethylated	Tshz3	60	64
chr7	38554771	38556771	0.080111	0.0068455	hypermethylated	Zfp536	49	50
chr7	38967235	38969235	-0.19318	0.0028701	hypomethylated	1600014C10Rik	10	13
chr7	38967805	38969805	-0.19847	0.0025649	hypomethylated	1600014C10Rik	10	15
chr7	46772135	46774135	0.24019	0.010116	hypermethylated	Zfp619	15	15
chr7	50568631	50570631	-0.34878	0.0037128	stronglyhypometh	Zfp715	7	8
chr7	50745393	50747393	-0.15151	0.015673	hypomethylated	Iglon5	7	18
chr7	51501091	51503091	-0.3208	0.00000594	hypomethylated	2410002F23Rik	23	29
chr7	51638495	51640495	-0.90909	0.021739	stronglyhypometh	Syt3	2	2
chr7	52002998	52004998	-0.14621	0.00002	hypomethylated	Vrk3,Zfp473	20	20
chr7	52003987	52005987	-0.18309	0.0015637	hypomethylated	Vrk3,Zfp473	9	9
chr7	52184860	52186860	-0.49662	0.00085239	stronglyhypometh	Ap2a1	11	10
chr7	52271619	52273619	-0.18001	0.011888	hypomethylated	Rras,Scaf1	20	28
chr7	52272376	52274376	-0.23336	0.00035178	hypomethylated	Rras,Scaf1	15	23
chr7	52316798	52318798	-0.30233	0.011611	hypomethylated	Nosip,Prrg2	8	8
chr7	52317022	52319022	-0.30233	0.011611	hypomethylated	Nosip,Prrg2	8	8
chr7	52382833	52384833	-0.67646	1.02E-08	stronglyhypometh	Mir5121,Rpl13a,Snord	18	14
chr7	52384105	52386105	-0.53798	0.0034529	stronglyhypometh	Rpl13a	12	8
chr7	52408767	52410767	-0.26313	0.0000182	hypomethylated	Aldh16a1,Pih1d1	12	13
chr7	52418290	52420290	0.12918	0.0017293	hypermethylated	Slc17a7	18	19
chr7	52435364	52437364	-0.48636	0.011064	stronglyhypometh	Gm581,Pth2	5	11
chr7	52621602	52623602	-0.28939	0.00000103	hypomethylated	Mtag2,Ppfia3	33	32
chr7	52622389	52624389	-0.23493	0.0090145	hypomethylated	Mtag2,Ppfia3	11	11
chr7	52651017	52653017	-0.54387	0.0090434	stronglyhypometh	Snrnp70	5	8
chr7	52660329	52662329	-0.18832	0.0000113	hypomethylated	Kcna7	33	34
chr7	52824732	52826732	-0.20062	0.00060398	hypomethylated	Bcat2	16	20

chr7	52954336	52956336	-0.26865	0.00000191	hypomethylated	Car11	18	20
chr7	52972440	52974440	-0.23274	0.0000751	hypomethylated	Rpl18,Sphk2	27	36
chr7	52973372	52975372	-0.11907	0.012996	hypomethylated	Rpl18,Sphk2	15	23
chr7	53288065	53290065	-0.1897	0.000000908	hypomethylated	Nomo1	26	30
chr7	53355607	53357607	-0.42657	0.012398	stronglyhypometh	Kcnj11	8	10
chr7	53966021	53968021	-0.37269	0.00000283	stronglyhypometh	Saal1	6	12
chr7	54050463	54052463	0.19571	0.016752	hypermethylated	Gtf2h1,Hps5	28	26
chr7	54051251	54053251	0.16434	0.024982	hypermethylated	Gtf2h1,Hps5	28	25
chr7	54100173	54102173	-0.21595	0.00041213	hypomethylated	Ldha	16	21
chr7	56043372	56045372	0.046883	0.033311	hypermethylated	Zdhhc13	19	25
chr7	56500558	56502558	-0.13888	0.0000172	hypomethylated	Nav2	33	50
chr7	56892205	56894205	-0.27787	0.0074151	hypomethylated	Dbx1	8	9
chr7	57013475	57015475	-0.051279	0.0081319	hypomethylated	Htatip2	17	19
chr7	57013881	57015881	-0.051279	0.0081319	hypomethylated	Htatip2	17	19
chr7	57229719	57231719	-0.42508	9.24E-10	stronglyhypometh	Nell1	26	38
chr7	58876199	58878199	-0.06964	0.038529	hypomethylated	Slc17a6	9	15
chr7	62089614	62091614	-0.39596	0.000000222	stronglyhypometh	Luzp2	8	6
chr7	63096440	63098440	0.020334	0.038997	hypermethylated	Cyfip1	25	25
chr7	63216900	63218900	-0.098485	0.00018354	hypomethylated	A230056P14Rik,Nipa2	27	27
chr7	63304524	63306524	-0.26762	0.0024715	hypomethylated	Herc2	19	39
chr7	64642241	64644241	-0.90506	0.0046249	stronglyhypometh	Gabrg3,Gm9962	2	19
chr7	64844903	64846903	-0.17614	1.71E-10	hypomethylated	Gabrb3	48	48
chr7	64845542	64847542	-0.12911	0.000000693	hypomethylated	Gabrb3	57	57
chr7	71083801	71085801	-0.23039	0.030944	hypomethylated	Klf13	15	26
chr7	71431555	71433555	-0.1183	0.01606	hypomethylated	Mtmr10	33	36
chr7	71536656	71538656	-0.14385	0.0060278	hypomethylated	Mcee,Mphosph10	28	26
chr7	71537122	71539122	-0.24355	0.000055	hypomethylated	Mcee,Mphosph10	17	15
chr7	72837302	72839302	-0.10626	0.009248	hypomethylated	Tm2d3	24	28
chr7	73223534	73225534	-0.17905	0.00051617	hypomethylated	Vimp	18	23
chr7	73572363	73574363	-0.12366	0.00072119	hypomethylated	Aldh1a3	19	18
chr7	74366429	74368429	-0.2206	0.0021017	hypomethylated	Lysmd4	7	11
chr7	74366501	74368501	-0.2206	0.0021017	hypomethylated	Lysmd4	7	11
chr7	74517744	74519744	-0.071885	0.0058922	hypomethylated	Mef2a	34	34
chr7	77505479	77507479	-0.10638	0.016822	hypomethylated	Nr2f2	45	65
chr7	81699666	81701666	-0.21635	0.015043	hypomethylated	Slco3a1	17	18
chr7	82992223	82994223	-0.14813	0.00046758	hypomethylated	Klhl25	37	46
chr7	85722724	85724724	-0.092655	0.012176	hypomethylated	Ntrk3	12	21
chr7	85927016	85929016	-0.1813	0.008933	hypomethylated	Mrpl46,Mrps11	10	18
chr7	86039812	86041812	-0.27751	2.56E-11	hypomethylated	Aen	15	15
chr7	86417151	86419151	-0.15275	0.047787	hypomethylated	Abhd2	13	19
chr7	86611159	86613159	-0.15349	0.000000813	hypomethylated	Polg	16	24
chr7	86954612	86956612	-0.31453	0.00080726	hypomethylated	Mesp2	15	19
chr7	87330726	87332726	-0.30094	0.000000446	hypomethylated	Sema4b	14	14
chr7	87376778	87378778	-0.17045	0.0037118	hypomethylated	Cib1,Gdpgp1	28	30
chr7	87377502	87379502	-0.22016	0.00091204	hypomethylated	Cib1,Gdpgp1	29	31
chr7	87405100	87407100	-0.433	7.92E-10	stronglyhypometh	Ngrn	20	16
chr7	87413540	87415540	-0.34562	0.000000378	stronglyhypometh	Vps33b	16	20
chr7	87487022	87489022	-0.27012	0.022008	hypomethylated	Hddc3	4	4
chr7	88092436	88094436	-0.39559	0.045334	stronglyhypometh	Sec11a	4	8
chr7	88357689	88359689	-0.064793	0.020177	hypomethylated	Pde8a	38	42
chr7	89002907	89004907	0.13918	0.04879	hypermethylated	Tm6sf1	8	11
chr7	89137185	89139185	-0.33774	0.000000306	stronglyhypometh	Bnc1	31	31
chr7	89318727	89320727	0.035487	0.00050524	hypermethylated	Sh3gl3	21	21
chr7	90014842	90016842	-0.13121	0.0013194	hypomethylated	Mex3b	68	67
chr7	95577782	95579782	-0.31862	0.030049	hypomethylated	Rab38	8	14
chr7	96551875	96553875	-0.1546	0.045332	hypomethylated	Fzd4	38	39
chr7	97277741	97279741	-0.050027	0.0074598	hypomethylated	2310010J17Rik,Picalm	47	54

chr7	97590290	97592290	-0.075241	0.040682	hypomethylated	Crebzf	55	55
chr7	97624505	97626505	-0.22234	0.012571	hypomethylated	Tmem126b	6	9
chr7	99818422	99820422	-0.53023	0.0014308	stronglyhypometh	Pcf11	7	18
chr7	99889969	99891969	-0.10714	0.0010184	hypomethylated	4632427E13Rik,Rab30	4	3
chr7	100022742	100024742	-0.1915	0.0050151	hypomethylated	4632434I11Rik,Prcp	17	18
chr7	100022762	100024762	-0.15219	0.012359	hypomethylated	4632434I11Rik,Prcp	16	17
chr7	104229260	104231260	-0.096206	0.0000869	hypomethylated	Gab2	53	56
chr7	104479832	104481832	-0.60353	3.47E-09	stronglyhypometh	Kctd21	15	17
chr7	105805079	105807079	-0.86508	0.0000562	stronglyhypometh	2210018M11Rik	6	14
chr7	105850872	105852872	-0.021593	0.028255	hypomethylated	Prkrir	44	44
chr7	105986354	105988354	-0.17066	0.010087	hypomethylated	Wnt11	14	25
chr7	106331223	106333223	-0.2285	0.0022559	hypomethylated	Dgat2	12	10
chr7	106529058	106531058	-0.27579	0.0024125	hypomethylated	Gdpd5	15	34
chr7	106631442	106633442	-0.28638	0.00000611	hypomethylated	Rps3,Snord15a	7	9
chr7	107375116	107377116	-0.19056	0.00040597	hypomethylated	Pgm2l1	26	30
chr7	107519742	107521742	-0.068916	0.016521	hypomethylated	C2cd3,Ppme1	30	28
chr7	107755099	107757099	-0.10081	0.00039997	hypomethylated	Mrpl48,Rab6a	37	44
chr7	107756510	107758510	-0.25208	0.00000731	hypomethylated	Mrpl48,Rab6a	27	32
chr7	108256288	108258288	-0.072999	0.0019481	hypomethylated	Fchsd2	31	38
chr7	108468832	108470832	-0.16054	0.000000191	hypomethylated	Stard10	47	50
chr7	108598765	108600765	-0.067514	0.0007886	hypomethylated	Pde2a	18	23
chr7	108598821	108600821	-0.067514	0.0007886	hypomethylated	Pde2a	18	23
chr7	109053350	109055350	0.045547	0.03557	hypermethylated	Lamtor1,Tomt	18	21
chr7	109082371	109084371	-0.20488	0.0032701	hypomethylated	Lrrc51	6	8
chr7	109398632	109400632	-0.52914	0.00000149	stronglyhypometh	Rhog	11	13
chr7	109589208	109591208	-0.77351	0.009235	stronglyhypometh	Rrm1	4	11
chr7	109787761	109789761	0.071429	0.048466	hypermethylated	Olfr554	3	3
chr7	111366308	111368308	-0.38143	0.0000007	stronglyhypometh	Trim6	7	10
chr7	112788053	112790053	-0.24914	0.0000372	hypomethylated	Arfip2,Timm10b	8	10
chr7	112884103	112886103	0.089786	0.022276	hypermethylated	Ilk,Rrp8	27	28
chr7	112884238	112886238	0.089786	0.022276	hypermethylated	Ilk,Rrp8	27	28
chr7	116076928	116078928	-0.12213	0.0015416	hypomethylated	Eif3f	8	9
chr7	116846250	116848250	-0.25306	8.61E-14	hypomethylated	Akip1	26	35
chr7	116925059	116927059	-0.33387	0.013355	stronglyhypometh	Nrip3	13	13
chr7	117160938	117162938	-0.32307	4.48E-10	hypomethylated	Ipo7	21	24
chr7	117204215	117206215	-0.25209	2.07E-11	hypomethylated	Zfp143	34	39
chr7	117264572	117266572	-0.082012	0.0039653	hypomethylated	Wee1	54	61
chr7	117758434	117760434	-0.32197	0.016001	hypomethylated	Sbf2	8	11
chr7	118171464	118173464	-0.1562	0.0010653	hypomethylated	Ctr9	11	11
chr7	119166019	119168019	-0.14902	0.0019026	hypomethylated	Usp47	27	31
chr7	119570219	119572219	-0.61587	0.0047005	stronglyhypometh	Parva	4	5
chr7	120349978	120351978	-0.13284	0.043069	hypomethylated	Arntl	44	48
chr7	120512853	120514853	-0.039837	0.019481	hypomethylated	Btbd10	18	24
chr7	120656375	120658375	-0.0022729	0.036093	hypomethylated	Far1	25	35
chr7	123174561	123176561	-0.092816	0.01451	hypomethylated	Sox6	27	34
chr7	123182258	123184258	-0.72443	0.00000287	stronglyhypometh	Sox6	6	13
chr7	123235893	123237893	-0.32623	4.57E-08	hypomethylated	1110004F10Rik,17000	14	14
chr7	123236663	123238663	-0.32623	4.57E-08	hypomethylated	1110004F10Rik,17000	14	14
chr7	123477704	123479704	-0.55482	0.000000297	stronglyhypometh	Rps13	19	19
chr7	125739810	125741810	-0.55685	0.0098242	stronglyhypometh	Tmc5	5	8
chr7	125855124	125857124	-0.41667	0.00022361	stronglyhypometh	Ccp110	14	14
chr7	125999512	126001512	-0.19617	0.00029556	hypomethylated	2310008H09Rik,Iqck	15	15
chr7	126327888	126329888	-0.37728	0.040101	stronglyhypometh	Gpr139	11	9
chr7	126936643	126938643	-0.1625	0.00010357	hypomethylated	2610020H08Rik,Eri2	15	16
chr7	126936700	126938700	-0.1625	0.00010357	hypomethylated	2610020H08Rik,Eri2	15	16
chr7	126937572	126939572	-0.5	0.00010339	stronglyhypometh	2610020H08Rik,Eri2	4	4
chr7	127038805	127040805	-0.18174	0.012608	hypomethylated	Dcun1d3,Lyrm1	21	21

chr7	127038972	127040972	-0.18174	0.012608	hypomethylated	Dcun1d3,Lyrm1	21	21
chr7	127039259	127041259	-0.22032	0.026779	hypomethylated	Dcun1d3,Lyrm1	12	12
chr7	127777702	127779702	-0.083361	0.015217	hypomethylated	Uqcrc2	22	25
chr7	127820133	127822133	-0.33851	0.0000514	stronglyhypometh	BC030336	31	24
chr7	127985396	127987396	-0.18441	0.040957	hypomethylated	Eef2k	17	16
chr7	128060257	128062257	-0.043012	0.013847	hypomethylated	Polr3e	54	59
chr7	128534809	128536809	-0.21433	0.00012347	hypomethylated	Hs3st2	38	47
chr7	129007624	129009624	0.21416	0.01121	hypermethylated	Scnn1b	7	8
chr7	130113077	130115077	-0.17914	2.04E-12	hypomethylated	4930413G21Rik,Rbbp6	65	68
chr7	130520495	130522495	-0.27269	0.0095488	hypomethylated	Lcmt1	7	7
chr7	132635056	132637056	-0.6	0.00079734	stronglyhypometh	Nsmce1	6	14
chr7	132851202	132853202	-0.23456	0.022948	hypomethylated	D430042O09Rik,Gtf3c	19	25
chr7	133838513	133840513	-0.36776	0.00000616	stronglyhypometh	Bola2,Slx1b	17	25
chr7	133919488	133921488	0.15305	0.049512	hypermethylated	Ypel3	19	22
chr7	133942692	133944692	-0.35076	0.012369	stronglyhypometh	Aldoa	15	16
chr7	133942768	133944768	-0.24859	0.038033	hypomethylated	Aldoa	12	13
chr7	134041792	134043792	-0.27165	0.0011023	hypomethylated	Tmem219	7	7
chr7	134071392	134073392	-0.12213	0.0011034	hypomethylated	Kctd13	33	35
chr7	134387039	134389039	-0.22584	0.0017705	hypomethylated	Zfp771	29	34
chr7	134404181	134406181	-0.048942	0.014183	hypomethylated	Dctpp1	9	9
chr7	134488828	134490828	-0.13391	0.00061624	hypomethylated	Zfp768	36	42
chr7	134715861	134717861	-0.33274	0.000000262	hypomethylated	Phkg2	4	10
chr7	134731211	134733211	-0.12876	0.0000181	hypomethylated	Gm166,Rnf40	27	30
chr7	134732109	134734109	-0.14987	0.000000391	hypomethylated	Gm166,Rnf40	25	28
chr7	134889288	134891288	-0.14656	0.013074	hypomethylated	Fbxl19	14	20
chr7	134984321	134986321	-0.16096	0.0023136	hypomethylated	Stx4a	33	31
chr7	135020214	135022214	-0.22622	0.00030534	hypomethylated	Zfp646,Zfp668	12	15
chr7	135020337	135022337	-0.14103	0.049907	hypomethylated	Zfp646,Zfp668	10	14
chr7	135039131	135041131	-0.35476	0.00022763	stronglyhypometh	Vkorc1	6	6
chr7	135109992	135111992	-0.16688	0.00030765	hypomethylated	Fus	28	33
chr7	135605027	135607027	-0.22222	0.0000749	hypomethylated	Tial1	24	30
chr7	135887383	135889383	-0.11478	0.0000152	hypomethylated	Sec23ip	17	22
chr7	136399607	136401607	-0.1806	0.00086868	hypomethylated	Ppapdc1a	22	28
chr7	137663044	137665044	-0.3248	0.040182	hypomethylated	Ate1	22	29
chr7	137663053	137665053	-0.3248	0.040182	hypomethylated	Ate1	22	29
chr7	137663475	137665475	-0.49448	0.0000563	stronglyhypometh	Ate1	11	11
chr7	138008423	138010423	-0.055736	0.00018638	hypomethylated	Plekha1	45	50
chr7	138506212	138508212	-0.0094364	0.045894	hypomethylated	2310057M21Rik	9	9
chr7	138513659	138515659	-0.45942	0.00023154	stronglyhypometh	Pstk	9	8
chr7	138553114	138555114	-0.27181	0.0011233	hypomethylated	Acadsb,Ikzf5	18	22
chr7	138685476	138687476	-0.14261	0.00000141	hypomethylated	Hmx3	41	58
chr7	139157142	139159142	-0.34613	0.00072927	stronglyhypometh	Gpr26	18	23
chr7	139506346	139508346	-0.21271	0.0025991	hypomethylated	Chst15,Gm10584	26	32
chr7	139801325	139803325	-0.9256	1.27E-10	stronglyhypometh	Lhpp	10	12
chr7	140004879	140006879	-0.10919	0.020582	hypomethylated	Fam53b	28	31
chr7	140049907	140051907	-0.27201	0.010968	hypomethylated	Fam175b	23	23
chr7	140900015	140902015	0.15503	0.000022	hypermethylated	Bccip,Uros	31	34
chr7	142601049	142603049	-0.10572	0.0049521	hypomethylated	Foxi2	17	17
chr7	144506128	144508128	-0.057417	0.024045	hypomethylated	Ebf3	13	31
chr7	144628330	144630330	-0.28705	0.0000366	hypomethylated	Glrx3,Gm12669	24	29
chr7	144628356	144630356	-0.28705	0.0000366	hypomethylated	Glrx3,Gm12669	24	29
chr7	145589413	145591413	-0.074455	2.24E-08	hypomethylated	Tcerg1l	21	27
chr7	146101189	146103189	0.041401	0.01343	hypermethylated	Bnip3	7	13
chr7	146574007	146576007	-0.030963	0.0059261	hypomethylated	Inpp5a	34	44
chr7	147128754	147130754	-0.10085	0.0000737	hypomethylated	Utf1	34	34
chr7	147340558	147342558	-0.60392	0.0017487	stronglyhypometh	Sprn	6	6
chr7	148042282	148044282	-0.14322	0.0000595	hypomethylated	Bet1l,Ric8	14	19

chr7	148042295	148044295	-0.14322	0.0000595	hypomethylated	Bet1l,Ric8	14	19
chr7	148126479	148128479	0.20917	0.0014277	hypermethylated	Athl1	5	8
chr7	148141860	148143860	-0.59375	0.0011058	stronglyhypometh	Ifitm2	4	4
chr7	148202791	148204791	-0.84041	0.0055336	stronglyhypometh	Ifitm6	3	3
chr7	148379028	148381028	-0.26139	2.87E-22	hypomethylated	Hras1,Lrrc56	69	64
chr7	148379903	148381903	-0.31802	2.64E-17	hypomethylated	Hras1,Lrrc56	27	28
chr7	148380041	148382041	-0.38809	1.98E-19	stronglyhypometh	Hras1,Lrrc56	23	24
chr7	148407392	148409392	-0.18106	0.00016914	hypomethylated	Mir210	11	12
chr7	148476904	148478904	0.20328	0.018541	hypermethylated	Drd4	5	5
chr7	148513624	148515624	-0.20284	0.016827	hypomethylated	Deaf1,Tmem80	11	16
chr7	148632548	148634548	-0.20912	0.0019747	hypomethylated	Rplp2,Snora52	23	27
chr7	148633731	148635731	-0.42992	0.022973	stronglyhypometh	Rplp2,Snora52	3	4
chr7	148640086	148642086	-0.4296	0.0090715	stronglyhypometh	Pnpla2	6	5
chr7	148652260	148654260	-0.013796	0.0020066	hypomethylated	Cd151	16	19
chr7	148660138	148662138	-0.13358	0.0023422	hypomethylated	Polr2l,Tspan4	35	44
chr7	149088311	149090311	-0.11671	0.023399	hypomethylated	Tollip	6	9
chr7	149134655	149136655	-0.027733	0.0017337	hypomethylated	Brsk2	60	70
chr7	149134990	149136990	-0.03809	0.0015545	hypomethylated	Brsk2	59	69
chr7	149246939	149248939	-0.51653	0.00032691	stronglyhypometh	Mob2	6	14
chr7	149488406	149490406	0.13333	0.020436	hypermethylated	Krtap5-4	3	3
chr7	149573775	149575775	-0.19259	0.025494	hypomethylated	Ctsd	14	14
chr7	150155169	150157169	-0.29697	0.00044473	hypomethylated	Ascl2	10	11
chr7	150688429	150690429	-0.34611	0.00000135	stronglyhypometh	Phlda2	12	16
chr7	151008071	151010071	-0.32156	0.00000237	hypomethylated	Dhcr7,Nadsyn1	8	7
chr7	151008746	151010746	-0.37101	0.00026066	stronglyhypometh	Dhcr7,Nadsyn1	3	3
chr7	151924383	151926383	-0.11114	0.0018774	hypomethylated	Ano1	6	10
chr7	152023516	152025516	-0.32224	0.00000691	hypomethylated	Fgf3	31	39
chr7	152046290	152048290	-0.10752	0.014883	hypomethylated	Fgf4	41	50
chr7	152100098	152102098	-0.26026	0.0094083	hypomethylated	Oraov1	13	13
chr8	3392007	3394007	-0.30704	0.00000993	hypomethylated	Arhgef18	15	15
chr8	3492137	3494137	-0.24432	0.0021775	hypomethylated	Zfp358	34	31
chr8	3514383	3516383	-0.15372	0.020789	hypomethylated	Pnpla6	15	17
chr8	4165566	4167566	-0.28194	0.00042279	hypomethylated	Evi5l	12	12
chr8	4225826	4227826	-0.27364	0.0000655	hypomethylated	Lrrc8e	17	18
chr8	4324209	4326209	-0.0065425	0.013593	hypomethylated	Ccl25,Elavl1	36	40
chr8	8660773	8662773	0.019559	0.02861	hypermethylated	Efnb2	33	42
chr8	9771023	9773023	-0.12355	0.00018959	hypomethylated	Fam155a	26	27
chr8	9976716	9978716	-0.10959	0.028403	hypomethylated	Abhd13,Lig4	16	16
chr8	10152922	10154922	-0.1674	0.044483	hypomethylated	Myo16	7	7
chr8	11312826	11314826	0.062833	0.0066933	hypermethylated	Col4a1,Col4a2	13	18
chr8	11757329	11759329	-0.18108	0.00012348	hypomethylated	Arhgef7	29	35
chr8	12756015	12758015	-0.1309	9.25E-11	hypomethylated	Atp11a	68	70
chr8	13059907	13061907	0.09375	0.049408	hypermethylated	Proz	6	8
chr8	13704888	13706888	-0.281	0.0030719	hypomethylated	4932443I19Rik	4	7
chr8	13756689	13758689	-0.20454	0.011535	hypomethylated	Cdc16	26	26
chr8	14094874	14096874	-0.2194	0.0089082	hypomethylated	Dlgap2	45	51
chr8	18845278	18847278	-0.18766	0.000000121	hypomethylated	Agpat5	47	56
chr8	23509093	23511093	-0.61905	0.00000301	stronglyhypometh	1810012K16Rik,Slc25a	6	7
chr8	23586171	23588171	-0.11749	0.0000193	hypomethylated	AI316807,Slc20a2	41	58
chr8	23587351	23589351	-0.1003	0.047122	hypomethylated	AI316807,Slc20a2	12	24
chr8	23916126	23918126	-0.15893	0.011957	hypomethylated	Ap3m2	20	25
chr8	23969010	23971010	-0.053135	0.040837	hypomethylated	Kat6a	77	72
chr8	24318925	24320925	-0.1876	0.0000282	hypomethylated	Agpat6	19	19
chr8	24520973	24522973	-0.2867	0.00000658	hypomethylated	Sfrp1	25	25
chr8	24779132	24781132	-0.6176	4.77E-09	stronglyhypometh	Zmat4	7	11
chr8	26126682	26128682	-0.31517	0.0000035	hypomethylated	Adam9,Tm2d2	19	33
chr8	26127394	26129394	-0.3787	0.0000383	stronglyhypometh	Adam9,Tm2d2	14	23

chr8	26212283	26214283	-0.22631	0.024027	hypomethylated	Plekha2	23	24
chr8	26311921	26313921	-0.26389	0.00043355	hypomethylated	Tacc1	12	11
chr8	26711777	26713777	-0.090953	0.00000141	hypomethylated	Whsc1l1	50	61
chr8	26829519	26831519	-0.22197	0.0000194	hypomethylated	Ppapdc1b	5	6
chr8	26864752	26866752	-0.30965	2.33E-17	hypomethylated	Ddhd2	28	29
chr8	27267640	27269640	-0.12583	1.44E-08	hypomethylated	Thap1	42	45
chr8	28086807	28088807	-0.18026	0.0019227	hypomethylated	Zfp703	33	37
chr8	28152026	28154026	-0.39614	0.0000475	stronglyhypometh	Prosc	17	24
chr8	32259787	32261787	-0.16546	0.000000835	hypomethylated	Tti2	10	16
chr8	32296803	32298803	-0.5629	2.19E-09	stronglyhypometh	Fut10	14	19
chr8	32296849	32298849	-0.5629	2.19E-09	stronglyhypometh	Fut10	14	19
chr8	34841385	34843385	-0.15225	0.0008901	hypomethylated	Gtf2e2	26	26
chr8	34841538	34843538	-0.12164	0.001701	hypomethylated	Gtf2e2	25	26
chr8	34841810	34843810	-0.12164	0.001701	hypomethylated	Gtf2e2	25	26
chr8	35040313	35042313	-0.25099	0.00012343	hypomethylated	Rbpms	21	21
chr8	35209793	35211793	-0.47578	9.73E-08	stronglyhypometh	Leprotl1	17	13
chr8	35869663	35871663	-0.21547	0.001425	hypomethylated	Dusp4	52	58
chr8	36889613	36891613	0.051113	0.037788	hypermethylated	Cldn23	11	16
chr8	41508168	41510168	-0.16365	0.0072453	hypomethylated	Zdhhc2	39	51
chr8	41596136	41598136	-0.056876	0.014322	hypomethylated	Cnot7,Vps37a	50	56
chr8	41596658	41598658	-0.059927	0.005781	hypomethylated	Cnot7,Vps37a	47	49
chr8	42219080	42221080	-0.25	0.021961	hypomethylated	Mtus1	2	6
chr8	46969838	46971838	-0.27176	0.0091984	hypomethylated	Pdlim3	3	3
chr8	47059892	47061892	-0.66667	0.000000126	stronglyhypometh	1700029J07Rik,Ufsp2	9	9
chr8	47060606	47062606	-1	4.22E-09	stronglyhypometh	1700029J07Rik,Ufsp2	6	6
chr8	47296363	47298363	-0.37418	1.87E-11	stronglyhypometh	Slc25a4	13	13
chr8	47555395	47557395	-0.053025	0.037477	hypomethylated	Acsl1	44	45
chr8	47636422	47638422	-0.12952	0.00062015	hypomethylated	Mlf1ip	22	22
chr8	47701802	47703802	-0.096074	0.030593	hypomethylated	Casp3,Ccdc111	19	21
chr8	47824098	47826098	-0.20616	6.75E-08	hypomethylated	Gm16675,Irf2	32	37
chr8	48617998	48619998	-0.21734	0.00083825	hypomethylated	Rwdd4a,Trappc11	16	30
chr8	48618824	48620824	-0.10026	0.0091883	hypomethylated	Rwdd4a,Trappc11	14	23
chr8	48760513	48762513	-0.21107	0.0000541	hypomethylated	Ing2	37	43
chr8	49194311	49196311	-0.34686	0.00000893	stronglyhypometh	Dctd	18	18
chr8	49194366	49196366	-0.34686	0.00000893	stronglyhypometh	Dctd	18	18
chr8	55161885	55163885	-0.12389	0.0006802	hypomethylated	Vegfc	32	29
chr8	59028930	59030930	-0.19277	0.046955	hypomethylated	Cep44,Fbxo8	8	14
chr8	59029363	59031363	-0.22837	0.032543	hypomethylated	Cep44,Fbxo8	8	12
chr8	59989639	59991639	-0.27237	1.04E-12	hypomethylated	Hmgb2	65	59
chr8	60131852	60133852	-0.11873	0.00000899	hypomethylated	Galnt7	9	9
chr8	61389551	61391551	-0.44275	0.0011643	stronglyhypometh	BC030500,Galntl6	11	13
chr8	61390424	61392424	-0.30209	0.0082734	hypomethylated	BC030500,Galntl6	18	18
chr8	63110656	63112656	-0.17197	0.0042679	hypomethylated	Mfap3l	29	33
chr8	63368247	63370247	-0.28724	0.0011259	hypomethylated	2700029M09Rik	24	28
chr8	63701967	63703967	-0.16042	0.0014576	hypomethylated	Sh3rf1	21	45
chr8	67212375	67214375	-0.25859	0.00044962	hypomethylated	Sc4mol	11	19
chr8	69010406	69012406	0.46805	0.0098749	stronglyhypermeth	Tma16	8	9
chr8	70799426	70801426	-0.23174	0.047922	hypomethylated	Sh2d4a	7	13
chr8	71259041	71261041	-0.28929	0.0023864	hypomethylated	Csgalnact1	7	8
chr8	72149074	72151074	-0.053042	0.0027666	hypomethylated	Zfp868	2	5
chr8	72240385	72242385	0.069662	0.0069501	hypermethylated	Zfp869	9	14
chr8	72331585	72333585	-0.28768	0.0045088	hypomethylated	Gmip	16	14
chr8	72345463	72347463	-0.077525	0.026168	hypomethylated	Lpar2	11	11
chr8	72425113	72427113	-0.25176	0.000000261	hypomethylated	Ndufa13,Tssk6	39	39
chr8	72426457	72428457	-0.25657	0.0019274	hypomethylated	Ndufa13,Tssk6	17	19
chr8	72757124	72759124	-0.31287	3.88E-11	hypomethylated	Armc6,Sugp2	18	17
chr8	72758321	72760321	-0.40959	1.96E-11	stronglyhypometh	Armc6,Sugp2	11	10

chr8	72852663	72854663	-0.12272	0.040457	hypomethylated	Gdf1	17	17
chr8	73034266	73036266	-0.14845	0.0013203	hypomethylated	Uba52	15	14
chr8	73277577	73279577	-0.66527	0.0084352	stronglyhypometh	Rab3a	6	11
chr8	73397389	73399389	-0.049679	0.02308	hypomethylated	Ccdc124	13	16
chr8	73419755	73421755	-0.049164	0.017311	hypomethylated	Rpl18a,Snora68	18	24
chr8	73428872	73430872	-0.054725	0.0074451	hypomethylated	Map1s	27	36
chr8	73795612	73797612	-0.26303	0.000000703	hypomethylated	Haus8,Myo9b	36	34
chr8	73796489	73798489	-0.088826	0.032133	hypomethylated	Haus8,Myo9b	28	32
chr8	73889746	73891746	-0.33333	0.00047847	hypomethylated	Use1	6	9
chr8	73890115	73892115	-0.33333	0.00047847	hypomethylated	Use1	6	9
chr8	73905851	73907851	0.11164	0.012514	hypermethylated	Nr2f6	34	42
chr8	73928910	73930910	-0.60655	0.00058013	stronglyhypometh	Ankle1	12	16
chr8	74133922	74135922	-0.72288	0.0012539	stronglyhypometh	Glt25d1	9	12
chr8	74195656	74197656	-0.023587	0.023995	hypomethylated	Unc13a	11	12
chr8	75016513	75018513	-0.40103	0.00000221	stronglyhypometh	Slc35e1	27	27
chr8	75072209	75074209	-0.79365	4.88E-11	stronglyhypometh	Med26	9	7
chr8	75284778	75286778	-0.18333	0.016535	hypomethylated	F2rl3	2	5
chr8	75876455	75878455	-0.068204	0.0063977	hypomethylated	Large	63	76
chr8	77516601	77518601	-0.014426	0.015588	hypomethylated	Hmgxb4	41	45
chr8	77556584	77558584	-0.15347	0.000000539	hypomethylated	Tom1	25	26
chr8	77616516	77618516	-0.31781	0.0010847	hypomethylated	Hmox1	10	12
chr8	79424072	79426072	-0.079129	0.044791	hypomethylated	Gm10649,Nr3c2	29	32
chr8	81032233	81034233	-0.069435	0.0042727	hypomethylated	Rbmxl1,Slc10a7	32	34
chr8	81551335	81553335	-0.27519	0.037813	hypomethylated	Zfp827	14	24
chr8	82162574	82164574	0.042716	0.048042	hypermethylated	Otud4	61	72
chr8	84926444	84928444	-0.57629	0.016367	stronglyhypometh	Il15	5	8
chr8	85688250	85690250	0.24035	0.024247	hypermethylated	Tbc1d9	41	49
chr8	85856385	85858385	-0.4521	6.91E-12	stronglyhypometh	Elmod2	6	6
chr8	85912789	85914789	-0.44409	0.00036098	stronglyhypometh	Clgn	11	12
chr8	86238075	86240075	-0.21371	1.34E-22	hypomethylated	Ddx39	34	38
chr8	86422996	86424996	-0.11111	0.0000112	hypomethylated	Lphn1	67	82
chr8	86589734	86591734	-0.25368	0.00048068	hypomethylated	Rfx1	37	43
chr8	86760941	86762941	-0.16574	0.0007579	hypomethylated	Zswim4	9	9
chr8	87224355	87226355	-0.22029	0.00000451	hypomethylated	Lyl1	13	17
chr8	87324239	87326239	-0.14751	0.000016	hypomethylated	Nfix	17	21
chr8	87364540	87366540	-0.17127	0.043962	hypomethylated	Rad23a	5	5
chr8	87395009	87397009	-0.24106	1.24E-08	hypomethylated	Syce2	24	31
chr8	87395156	87397156	-0.24106	1.24E-08	hypomethylated	Syce2	24	31
chr8	87424826	87426826	-0.15079	0.0035044	hypomethylated	Klf1	6	9
chr8	87492546	87494546	-0.27671	2.25E-12	hypomethylated	Prdx2	19	25
chr8	87549177	87551177	-0.24857	0.0048555	hypomethylated	2310036O22Rik,Asna1	22	24
chr8	87559813	87561813	0.02607	0.019405	hypermethylated	Tnpo2	16	19
chr8	88015515	88017515	-0.12886	0.00015333	hypomethylated	Gpt2	16	21
chr8	89146941	89148941	-0.52389	6.88E-09	stronglyhypometh	Lonp2	15	15
chr8	89268597	89270597	-0.020198	0.049348	hypomethylated	Gm10638,Siah1a	75	78
chr8	89269905	89271905	-0.029291	0.0017859	hypomethylated	Gm10638,Siah1a	23	28
chr8	89995710	89997710	-0.29952	0.0000945	hypomethylated	Cbln1,Gm2694	52	46
chr8	89996491	89998491	-0.37133	0.0000778	stronglyhypometh	Cbln1,Gm2694	31	22
chr8	90483494	90485494	-0.16663	1.06E-08	hypomethylated	Zfp423	52	56
chr8	90722111	90724111	-0.053879	0.000000864	hypomethylated	Papd5	67	77
chr8	90795301	90797301	-0.18243	0.0015181	hypomethylated	Adcy7	8	22
chr8	91044242	91046242	-0.068609	2.16E-09	hypomethylated	Nkd1	65	65
chr8	92872151	92874151	-0.1337	0.0049107	hypomethylated	Tox3	23	31
chr8	93351733	93353733	-0.22051	0.0026687	hypomethylated	Chd9	15	9
chr8	93592992	93594992	-0.62882	0.00027912	stronglyhypometh	Rbl2	4	15
chr8	94880019	94882019	-0.071002	0.0088601	hypomethylated	4933436C20Rik,Irx5	22	34
chr8	94880694	94882694	-0.047233	0.0019091	hypomethylated	4933436C20Rik,Irx5	34	47

chr8	95350226	95352226	-0.13771	0.02032	hypomethylated	Mmp2	9	13
chr8	95483945	95485945	-0.30567	0.027	hypomethylated	Slc6a2	38	41
chr8	96333737	96335737	-0.13051	0.00000062	hypomethylated	Gnao1	70	64
chr8	96337455	96339455	-0.33022	0.0011147	hypomethylated	4930488L21Rik	5	5
chr8	96695517	96697517	-0.12989	0.0059135	hypomethylated	Mt2	24	29
chr8	96701988	96703988	-0.39035	0.00053533	stronglyhypometh	Mt1	14	17
chr8	97361316	97363316	-0.12786	0.0000959	hypomethylated	Ciapin1,Coq9	30	31
chr8	97380349	97382349	-0.53605	0.000000035	stronglyhypometh	Polr2c	15	26
chr8	97604100	97606100	-0.14449	0.018232	hypomethylated	Katnb1	12	17
chr8	97875236	97877236	-0.11551	0.00040714	hypomethylated	Mmp15	46	55
chr8	97958769	97960769	-0.054212	0.042437	hypomethylated	Gtl3	9	10
chr8	98156458	98158458	-0.24946	0.00043807	hypomethylated	Gins3	21	22
chr8	98238812	98240812	-0.13595	0.0029035	hypomethylated	Setd6	17	17
chr8	98331366	98333366	-0.12993	0.00037794	hypomethylated	4930513N10Rik,Cnot1	7	10
chr8	106772458	106774458	-0.69119	0.0058265	stronglyhypometh	Cklf,Tk2	5	10
chr8	106863493	106865493	-0.24767	0.031671	hypomethylated	Cmtm3	23	27
chr8	107063706	107065706	-0.038536	0.025897	hypomethylated	Car7	19	26
chr8	107693573	107695573	-0.037116	0.00058832	hypomethylated	Cbfb	64	73
chr8	107748087	107750087	-0.039279	0.0013715	hypomethylated	D230025D16Rik	20	22
chr8	107787547	107789547	-0.046788	0.011357	hypomethylated	Fbxl8,Tradd	15	16
chr8	107788494	107790494	-0.081019	0.0034151	hypomethylated	Fbxl8,Tradd	8	8
chr8	107813428	107815428	-0.57146	0.0095811	stronglyhypometh	4931428F04Rik	9	17
chr8	107819998	107821998	-0.17773	0.029204	hypomethylated	E2f4,Exoc3l	34	34
chr8	107828500	107830500	-0.55525	0.0000904	stronglyhypometh	Elmo3	5	6
chr8	107849263	107851263	-0.10285	0.0026304	hypomethylated	Lrrc29,Tmem208	8	8
chr8	107871157	107873157	-0.28981	0.0000443	hypomethylated	Fhod1,Slc9a5	26	29
chr8	107871870	107873870	-0.075704	0.0018476	hypomethylated	Fhod1,Slc9a5	37	41
chr8	108089940	108091940	-0.31046	0.000000378	hypomethylated	Agrp,Atp6v0d1	12	14
chr8	108128128	108130128	-0.30291	0.000000101	hypomethylated	Fam65a	40	41
chr8	108159437	108161437	-0.081785	0.000000465	hypomethylated	Ctcf	80	103
chr8	108282507	108284507	-0.48044	0.00012473	stronglyhypometh	Gfod2	10	11
chr8	108350643	108352643	-0.43109	0.00010232	stronglyhypometh	Ranbp10,Tsnaxip1	13	13
chr8	108351250	108353250	-0.47917	0.0043941	stronglyhypometh	Ranbp10,Tsnaxip1	2	3
chr8	108378002	108380002	-0.098167	0.0000374	hypomethylated	Thap11	36	36
chr8	108467302	108469302	-0.15167	0.038234	hypomethylated	Lcat	6	8
chr8	108534406	108536406	-0.41271	0.01579	stronglyhypometh	Ddx28,Dus2l	19	24
chr8	108582502	108584502	0.078694	0.0098487	hypermethylated	Nfatc3	36	39
chr8	108673298	108675298	-0.35362	0.0031946	stronglyhypometh	Pla2g15	16	21
chr8	108734833	108736833	-0.18224	0.00000323	hypomethylated	Prmt7,Slc7a6os	22	28
chr8	108938238	108940238	0.13124	0.0013891	hypermethylated	Zfp90	15	17
chr8	109033790	109035790	-0.35483	0.0001037	stronglyhypometh	Cdh3	14	20
chr8	109033812	109035812	-0.3494	0.0000311	stronglyhypometh	Cdh3	15	21
chr8	109126267	109128267	-0.22018	0.00000175	hypomethylated	Cdh1	27	34
chr8	109205967	109207967	-0.21055	0.000000331	hypomethylated	Tango6	16	23
chr8	109393141	109395141	-0.15035	0.0088392	hypomethylated	Has3	19	27
chr8	109416539	109418539	-0.11627	0.000000514	hypomethylated	Chtf8,Cirh1a	38	35
chr8	109458649	109460649	-0.25865	2.2E-09	hypomethylated	Sntb2	34	44
chr8	109554225	109556225	-0.18831	0.00030627	hypomethylated	Vps4a	42	47
chr8	109579776	109581776	-0.24623	1.68E-12	hypomethylated	Cog8,Nip7	55	66
chr8	109580637	109582637	-0.31814	5.12E-11	hypomethylated	Cog8,Nip7	32	36
chr8	109673560	109675560	-0.1161	0.001941	hypomethylated	Cyb5b	22	25
chr8	109948938	109950938	-0.068903	0.0030184	hypomethylated	Nob1	13	14
chr8	109959297	109961297	-0.1151	0.0034075	hypomethylated	Wwp2	23	32
chr8	111237543	111239543	-0.10045	0.010172	hypomethylated	Zfhx3	64	66
chr8	112391502	112393502	-0.16995	0.000029	hypomethylated	Phlpp2	43	50
chr8	112602633	112604633	-0.22272	0.0026878	hypomethylated	Zfp612	6	13
chr8	112692106	112694106	-0.10102	0.021997	hypomethylated	Calb2	12	18

chr8	113551670	113553670	-0.21594	0.004286	hypomethylated	Ddx19b	5	5
chr8	113579238	113581238	-0.0351	0.01361	hypomethylated	Exosc6	49	51
chr8	113783102	113785102	-0.2341	0.00034684	hypomethylated	Glg1	12	17
chr8	114059539	114061539	-0.062928	0.002545	hypomethylated	Znrf1	59	59
chr8	114061242	114063242	-0.14032	0.0050438	hypomethylated	Znrf1	11	13
chr8	114256036	114258036	-0.13492	0.015826	hypomethylated	Bcar1	6	8
chr8	114463617	114465617	-0.15006	0.0023083	hypomethylated	Gabarapl2	21	20
chr8	114534258	114536258	-0.18463	0.00000257	hypomethylated	Kars,Terf2ip	20	30
chr8	114535205	114537205	-0.19198	0.00000257	hypomethylated	Kars,Terf2ip	19	27
chr8	115092942	115094942	-0.58323	0.00015015	stronglyhypometh	Cntnap4	4	5
chr8	116962551	116964551	-0.13257	0.00010255	hypomethylated	Wwox	11	16
chr8	119681034	119683034	-0.060463	0.012251	hypomethylated	Gan	46	53
chr8	120021190	120023190	-0.21974	0.00040151	hypomethylated	Plcg2	23	26
chr8	121969618	121971618	-0.098457	0.00020034	hypomethylated	Necab2	56	65
chr8	122625372	122627372	-0.10021	0.00000125	hypomethylated	Zdhhc7	17	18
chr8	122637051	122639051	-0.2446	1.9E-19	hypomethylated	6430548M08Rik	54	53
chr8	123011765	123013765	-0.22322	0.00017407	hypomethylated	Gse1	37	38
chr8	123058756	123060756	-0.13346	0.0000553	hypomethylated	Gse1	47	48
chr8	123112975	123114975	-0.65204	0.000000933	stronglyhypometh	Gins2	5	10
chr8	123191189	123193189	-0.096102	0.000000013	hypomethylated	Cox4i1,Emc8	42	57
chr8	123192012	123194012	-0.097351	3.35E-08	hypomethylated	Cox4i1,Emc8	25	39
chr8	123259275	123261275	0.16626	0.036187	hypermethylated	Irf8	10	9
chr8	124430732	124432732	-0.23448	0.0072537	hypomethylated	BC048644,Slc7a5	25	41
chr8	124899515	124901515	-0.10663	0.00000293	hypomethylated	Zc3h18	43	51
chr8	125202075	125204075	-0.10998	0.0063857	hypomethylated	Cbfa2t3	43	50
chr8	125565897	125567897	-0.081623	0.00000347	hypomethylated	2810013P06Rik,Ankrd	80	80
chr8	125588407	125590407	-0.044346	0.002097	hypomethylated	Spg7	30	33
chr8	125735757	125737757	-0.047745	0.028518	hypomethylated	4732415M23Rik,Chmp	26	27
chr8	125896652	125898652	-0.27736	8.48E-10	hypomethylated	Tcf25	24	27
chr8	125896723	125898723	-0.27736	8.48E-10	hypomethylated	Tcf25	24	27
chr8	125896734	125898734	-0.27736	8.48E-10	hypomethylated	Tcf25	24	27
chr8	126000761	126002761	-0.19284	0.00011899	hypomethylated	Afg3l1	11	11
chr8	126039355	126041355	-0.82143	0.014487	stronglyhypometh	Dbndd1	1	8
chr8	126176828	126178828	-0.17068	0.000000758	hypomethylated	Rhou	53	62
chr8	126328895	126330895	-0.30455	0.00000269	hypomethylated	Rab4a	20	20
chr8	126473165	126475165	-0.65114	0.0036912	stronglyhypometh	Nup133	7	20
chr8	127245038	127247038	-0.16986	1.53E-08	hypomethylated	Arv1,Ttc13	33	40
chr8	127245875	127247875	-0.13276	0.00000926	hypomethylated	Arv1,Ttc13	19	23
chr8	127275709	127277709	-0.51797	0.00000154	stronglyhypometh	Fam89a	12	12
chr8	127315918	127317918	0.16068	0.013386	hypermethylated	Trim67	33	46
chr8	127421605	127423605	0.12419	0.020957	hypermethylated	Exoc8,Sprtn	19	19
chr8	127473154	127475154	-0.025086	0.020108	hypomethylated	Egln1	40	40
chr8	127577094	127579094	-0.257	0.018617	hypomethylated	Disc1	23	30
chr8	128821478	128823478	0.26434	0.00052035	hypermethylated	Slc35f3	21	36
chr8	129117336	129119336	-0.027915	0.000000784	hypomethylated	Irf2bp2	74	75
chr8	129586955	129588955	-0.1242	0.0008094	hypomethylated	Pard3	55	58
chr8	131208553	131210553	-0.032952	0.0023798	hypomethylated	Itgb1	41	52
chr9	3199813	3201813	0.39236	0.014797	stronglyhypermeth	4930433N12Rik	2	2
chr9	4308742	4310742	-0.063876	0.00034542	hypomethylated	Aasdhppt,Kbtbd3	40	43
chr9	4308815	4310815	-0.063876	0.00034542	hypomethylated	Aasdhppt,Kbtbd3	40	43
chr9	8898832	8900832	-0.40051	0.013226	stronglyhypometh	Pgr	7	7
chr9	13552624	13554624	-0.2058	0.011437	hypomethylated	Mtmr2	22	23
chr9	13631170	13633170	0.14083	0.0052021	hypermethylated	Cep57,Fam76b	21	23
chr9	13631497	13633497	0.15231	0.0046969	hypermethylated	Cep57,Fam76b	15	17
chr9	13631551	13633551	0.15231	0.0046969	hypermethylated	Cep57,Fam76b	15	17
chr9	14419444	14421444	-0.21473	0.011149	hypomethylated	Amotl1	11	11
chr9	15110892	15112892	-0.78241	0.0042102	stronglyhypometh	4931406C07Rik,Taf1d	4	9

chr9	15162232	15164232	-0.58485	0.00076376	stronglyhypometh	5830418K08Rik	6	6
chr9	20384771	20386771	-0.35714	0.00094851	stronglyhypometh	Zfp846	7	7
chr9	20410918	20412918	-0.15769	0.00014601	hypomethylated	5730577I03Rik	9	13
chr9	20446761	20448761	-0.44855	0.0000473	stronglyhypometh	Fbxl12,Ubl5	14	14
chr9	20447250	20449250	-0.44853	0.000000343	stronglyhypometh	Fbxl12,Ubl5	14	17
chr9	20449211	20451211	-0.21075	0.0000978	hypomethylated	Fbxl12	12	12
chr9	20619478	20621478	-0.21101	0.0071818	hypomethylated	Col5a3	8	8
chr9	20757067	20759067	-0.081981	0.033675	hypomethylated	Dnmt1	13	13
chr9	20806180	20808180	-0.27787	0.000000341	hypomethylated	Mrpl4	21	22
chr9	21068742	21070742	-0.032337	0.0049912	hypomethylated	Atg4d	22	22
chr9	21350337	21352337	-0.22583	1.19E-09	hypomethylated	Carm1	55	65
chr9	21722565	21724565	-0.26192	0.00023348	hypomethylated	Rab3d	9	11
chr9	21793897	21795897	-0.22117	0.00073957	hypomethylated	Rgl3	12	17
chr9	21902696	21904696	-0.41287	0.016804	stronglyhypometh	Cnn1	9	10
chr9	24307584	24309584	-0.030234	0.000026	hypomethylated	Dpy19l1	48	51
chr9	25059168	25061168	-0.066837	0.010875	hypomethylated	Sept7	61	79
chr9	25288181	25290181	-0.10001	0.021477	hypomethylated	Eepd1	20	20
chr9	26806261	26808261	0.50103	0.027623	stronglyhypermeth	Acad8,Thyn1	6	11
chr9	26962965	26964965	-0.10656	0.0092521	hypomethylated	Jam3	10	10
chr9	30749147	30751147	-0.2597	9.61E-09	hypomethylated	Adamts8	34	42
chr9	30939384	30941384	-0.3141	0.0022549	hypomethylated	St14	3	6
chr9	31019400	31021400	-0.11905	0.028422	hypomethylated	Aplp2	5	5
chr9	32348953	32350953	-0.39924	0.0036729	stronglyhypometh	Fli1	5	8
chr9	35017787	35019787	-0.16353	0.000000753	hypomethylated	Foxred1,Srpr	45	50
chr9	35018510	35020510	-0.15229	1.36E-09	hypomethylated	Foxred1,Srpr	37	44
chr9	35366050	35368050	-0.058333	0.02647	hypomethylated	Ddx25,Pus3	16	20
chr9	35366055	35368055	-0.058333	0.02647	hypomethylated	Ddx25,Pus3	16	20
chr9	36954851	36956851	-0.45456	2.91E-08	stronglyhypometh	Pknox2	7	6
chr9	36954899	36956899	-0.43183	0.0000691	stronglyhypometh	Pknox2	5	4
chr9	37295905	37297905	-0.061583	0.0000173	hypomethylated	Msantd2	58	66
chr9	37464897	37466897	0.41979	0.017042	stronglyhypermeth	Tbrg1	2	2
chr9	40263349	40265349	-0.12334	0.00014144	hypomethylated	Gramd1b	21	20
chr9	40493046	40495046	-0.073663	0.03186	hypomethylated	Clmp	14	19
chr9	42072383	42074383	-0.066838	0.031935	hypomethylated	Sc5d	14	18
chr9	42752454	42754454	-0.19471	0.00012017	hypomethylated	Grik4	17	19
chr9	42913801	42915801	-0.067415	0.0033923	hypomethylated	Arhgef12	44	50
chr9	43013838	43015838	-0.1293	0.014558	hypomethylated	Pou2f3	16	16
chr9	43551658	43553658	-0.15551	2.73E-08	hypomethylated	Pvrl1	51	65
chr9	43874103	43876103	-0.23333	0.042412	hypomethylated	Usp2	5	6
chr9	44042129	44044129	-0.20537	0.00000653	hypomethylated	Cbl	16	22
chr9	44128365	44130365	-0.16481	0.0014796	hypomethylated	C2cd2l	9	9
chr9	44169753	44171753	-0.57498	0.02326	stronglyhypometh	Vps11	7	7
chr9	44186572	44188572	-0.15504	2.13E-10	hypomethylated	Hyou1	28	43
chr9	44214796	44216796	-0.27681	0.00000243	hypomethylated	Rps25,Trappc4	11	29
chr9	44306218	44308218	-0.18205	0.0036751	hypomethylated	Bcl9l	9	11
chr9	44575891	44577891	0.35714	0.0015755	stronglyhypermeth	Arcn1	4	8
chr9	44580091	44582091	0.099637	0.025021	hypermethylated	Ift46	7	7
chr9	44607299	44609299	-0.09824	0.0073499	hypomethylated	Tmem25	17	17
chr9	44689357	44691357	-0.0093097	0.00031826	hypomethylated	Mll1	30	41
chr9	44862270	44864270	-0.37731	0.012926	stronglyhypometh	Mpzl3	15	13
chr9	44862299	44864299	-0.37731	0.012926	stronglyhypometh	Mpzl3	15	13
chr9	44946124	44948124	0.061809	0.0059712	hypermethylated	Scn4b	17	21
chr9	45237375	45239375	-0.26914	0.042063	hypomethylated	4833428L15Rik,Dscam	26	30
chr9	45645611	45647611	-0.18795	0.0029712	hypomethylated	Bace1	16	21
chr9	48302449	48304449	-0.18417	0.037121	hypomethylated	Gm5617,Rbm7	23	33
chr9	49147766	49149766	-0.11371	0.00013046	hypomethylated	Drd2	21	26
chr9	50411005	50413005	-0.1417	0.00024895	hypomethylated	Sdhd,Timm8b	15	15

chr9	50411954	50413954	-0.2397	0.027919	hypomethylated	Sdhd,Timm8b	5	5
chr9	50582434	50584434	-0.054048	0.035363	hypomethylated	Alg9	38	40
chr9	50817178	50819178	-0.34961	0.00035405	stronglyhypometh	Sik2	26	27
chr9	50910183	50912183	-0.3676	0.0000598	stronglyhypometh	4833427G06Rik,Mir34	17	20
chr9	50911215	50913215	-0.3391	0.0045805	stronglyhypometh	Mir34b,Mir34c	17	21
chr9	51086659	51088659	-0.23589	0.034756	hypomethylated	Gm684	13	13
chr9	51976216	51978216	-0.11619	0.000036	hypomethylated	Zc3h12c	27	29
chr9	54407886	54409886	-0.49567	0.00047535	stronglyhypometh	Cib2	7	10
chr9	54546365	54548365	-0.066439	0.00072772	hypomethylated	Dnaja4	31	43
chr9	54797665	54799665	-0.30684	3.43E-11	hypomethylated	AY074887,Psma4	20	27
chr9	54798761	54800761	-0.23231	0.014147	hypomethylated	AY074887,Psma4	20	27
chr9	54996175	54998175	-0.22406	0.0000109	hypomethylated	Ube2q2	67	77
chr9	55888651	55890651	-0.30143	0.00023048	hypomethylated	Rcn2	13	11
chr9	56265652	56267652	-0.55698	0.0024855	stronglyhypometh	C230081A13Rik,Hmg2	5	15
chr9	56265857	56267857	-0.85694	0.018487	stronglyhypometh	C230081A13Rik,Hmg2	2	11
chr9	56841774	56843774	-0.20768	0.0032137	hypomethylated	Ptpn9	47	52
chr9	56923182	56925182	-0.1558	0.002071	hypomethylated	Sin3a	18	41
chr9	56977583	56979583	-0.19844	0.00012033	hypomethylated	2410133F24Rik,Man2c	12	16
chr9	57350908	57352908	-0.062747	0.014809	hypomethylated	Rpp25	33	33
chr9	57368038	57370038	-0.033838	0.014776	hypomethylated	Cox5a	42	39
chr9	57757792	57759792	-0.32019	5.18E-09	hypomethylated	Ubl7	19	23
chr9	57786941	57788941	-0.051128	0.018779	hypomethylated	Sema7a	42	44
chr9	58335409	58337409	-0.11036	0.003187	hypomethylated	6030419C18Rik	70	74
chr9	58670318	58672318	-0.11498	0.00036526	hypomethylated	Hcn4	66	84
chr9	58884248	58886248	-0.061236	0.0000511	hypomethylated	Neo1	60	60
chr9	59334181	59336181	-0.10668	0.00034362	hypomethylated	Arih1	21	41
chr9	59386473	59388473	-0.17186	2.32E-08	hypomethylated	Hexa	24	27
chr9	59464090	59466090	-0.37263	1.08E-09	stronglyhypometh	Parp6	24	29
chr9	60535341	60537341	-0.475	0.00000231	stronglyhypometh	Lrrc49	11	11
chr9	61219172	61221172	-0.1306	0.0094102	hypomethylated	Tle3	40	46
chr9	62188149	62190149	-0.34225	0.0000113	stronglyhypometh	Anp32a	27	33
chr9	62659455	62661455	-0.15781	0.028578	hypomethylated	Fem1b	27	27
chr9	62994787	62996787	-0.0089274	0.022468	hypomethylated	Skor1	30	37
chr9	62994818	62996818	-0.0017811	0.028844	hypomethylated	Skor1	29	36
chr9	63605801	63607801	-0.31046	2.39E-10	hypomethylated	Smad3	18	26
chr9	63869866	63871866	-0.30644	0.0027921	hypomethylated	Smad6	13	18
chr9	64020193	64022193	-0.18994	0.00010507	hypomethylated	Rpl4,Zwilch	14	17
chr9	64020738	64022738	-0.32759	0.0000846	hypomethylated	Rpl4,Snord16a,Zwilch	6	9
chr9	64232432	64234432	-0.15395	0.00028583	hypomethylated	Megf11	41	43
chr9	64807638	64809638	-0.10551	0.0000117	hypomethylated	Vwa9	15	17
chr9	64807743	64809743	-0.10551	0.0000117	hypomethylated	Vwa9	15	17
chr9	64879264	64881264	-0.12649	0.00061074	hypomethylated	Dpp8	28	28
chr9	65061496	65063496	-0.036168	0.021164	hypomethylated	Parp16	24	29
chr9	65141101	65143101	-0.20646	0.0010549	hypomethylated	Clpx	19	28
chr9	65307743	65309743	-0.45455	0.00019381	stronglyhypometh	Spg21	11	12
chr9	65675371	65677371	0.20894	0.002992	hypermethylated	Zfp609	13	16
chr9	65906975	65908975	-0.28348	1.19E-13	hypomethylated	Ppib	30	30
chr9	66005032	66007032	-0.12555	0.0032567	hypomethylated	Dapk2	27	28
chr9	66792924	66794924	-0.35033	0.000000922	stronglyhypometh	Rps27l	19	23
chr9	67606243	67608243	-0.11527	0.003712	hypomethylated	C2cd4b	29	32
chr9	69244804	69246804	-0.027447	0.0047305	hypomethylated	Narg2	12	15
chr9	69608747	69610747	0.4793	0.00039279	stronglyhypermeth	B230323A14Rik,Foxb1	7	10
chr9	69859356	69861356	-0.16299	0.00085142	hypomethylated	Gtf2a2	28	32
chr9	70389584	70391584	-0.25111	3.57E-17	hypomethylated	Sltm	63	71
chr9	70389676	70391676	-0.25111	3.57E-17	hypomethylated	Sltm	63	71
chr9	70525807	70527807	-0.11912	0.013341	hypomethylated	Adam10	70	63
chr9	72285335	72287335	-0.13307	0.0081396	hypomethylated	Mns1	9	9

chr9	72339763	72341763	-0.39087	0.0000859	stronglyhypometh	Tex9	5	5
chr9	72379046	72381046	-0.24726	0.0031068	hypomethylated	4930509E16Rik,Rfx7	34	35
chr9	72805591	72807591	-0.6151	1.28E-08	stronglyhypometh	Dyx1c1	9	13
chr9	72832357	72834357	-0.37941	2.77E-08	stronglyhypometh	Ccpg1,Gm5918	15	15
chr9	72833183	72835183	-0.38009	6.76E-09	stronglyhypometh	Ccpg1,Gm5918	13	13
chr9	72891671	72893671	-0.40629	0.018557	stronglyhypometh	Rab27a	8	13
chr9	74708727	74710727	-0.20225	0.00076593	hypomethylated	Onecut1	31	38
chr9	74822917	74824917	-0.44256	0.011941	stronglyhypometh	Fam214a	7	8
chr9	74884420	74886420	-0.27973	3.72E-09	hypomethylated	Arpp19	23	29
chr9	74884538	74886538	-0.27973	3.72E-09	hypomethylated	Arpp19	23	29
chr9	74918012	74920012	-0.15694	9.54E-08	hypomethylated	Myo5a	27	31
chr9	77391866	77393866	-0.28898	0.010323	hypomethylated	Lrrc1	16	19
chr9	77601616	77603616	-0.19032	0.000038	hypomethylated	Gclc	47	48
chr9	77764171	77766171	-0.23283	0.001656	hypomethylated	Elovl5	32	28
chr9	79566485	79568485	-0.29786	0.0026013	hypomethylated	Col12a1	12	15
chr9	79913709	79915709	-0.1821	0.000000537	hypomethylated	Senp6	48	68
chr9	81526159	81528159	-0.34571	0.028621	stronglyhypometh	D430036J16Rik,Htr1b	4	15
chr9	81756350	81758350	-0.27023	0.033837	hypomethylated	Mei4	5	9
chr9	82722412	82724412	0.03893	0.0045381	hypermethylated	Irak1bp1	22	34
chr9	83040214	83042214	-0.50415	0.00000112	stronglyhypometh	Hmgn3	8	9
chr9	83699849	83701849	-0.45971	0.012218	stronglyhypometh	Elovl4	3	13
chr9	83699912	83701912	-0.45481	0.015055	stronglyhypometh	Elovl4	3	11
chr9	85642941	85644941	-0.29716	0.00030631	hypomethylated	Ibtk	10	11
chr9	86464594	86466594	-0.42285	0.00029494	stronglyhypometh	Pgm3,Rwdd2a	25	23
chr9	86464659	86466659	-0.42285	0.00029494	stronglyhypometh	Pgm3,Rwdd2a	25	23
chr9	86465449	86467449	-0.40571	0.020723	stronglyhypometh	Pgm3,Rwdd2a	14	12
chr9	86775207	86777207	0.11606	0.038276	hypermethylated	Snap91	16	28
chr9	88441857	88443857	-0.92917	0.00091185	stronglyhypometh	Zfp949	4	5
chr9	89803612	89805612	-0.20541	0.00056773	hypomethylated	Rasgrf1	17	20
chr9	92144031	92146031	-0.17103	0.0078082	hypomethylated	Plscr1	4	12
chr9	92436060	92438060	-0.12231	0.007034	hypomethylated	B430319G15Rik,Plod2	40	40
chr9	94438500	94440500	-0.072812	0.0010604	hypomethylated	1190002N15Rik	29	45
chr9	95307689	95309689	-0.28726	0.0003117	hypomethylated	Chst2	15	22
chr9	95412415	95414415	-0.56493	0.0061723	stronglyhypometh	U2surp	5	10
chr9	96264718	96266718	0.023722	0.016706	hypermethylated	Atp1b3	36	35
chr9	96789841	96791841	0.11785	0.028975	hypermethylated	Acpl2	24	32
chr9	96918774	96920774	-0.02465	0.018231	hypomethylated	Spsb4	36	36
chr9	98463149	98465149	-0.088507	0.037952	hypomethylated	4930579K19Rik,Copb2	21	26
chr9	98464030	98466030	-0.17708	0.01921	hypomethylated	4930579K19Rik,Copb2	13	18
chr9	98855025	98857025	-0.062317	0.035424	hypomethylated	Foxl2,Foxl2os	63	80
chr9	99040630	99042630	-0.076672	0.026526	hypomethylated	Pik3cb	23	22
chr9	99142886	99144886	-0.30173	0.0010119	hypomethylated	Cep70	12	22
chr9	99258949	99260949	0.19591	0.046546	hypermethylated	Esyt3	4	5
chr9	100446472	100448472	-0.32703	0.0000514	hypomethylated	Nck1	13	25
chr9	102407467	102409467	0.059982	0.026292	hypermethylated	Ky	20	20
chr9	102619133	102621133	-0.47888	0.000000782	stronglyhypometh	Amotl2	23	23
chr9	102736249	102738249	-0.14604	0.0044548	hypomethylated	Ryk	50	50
chr9	103132616	103134616	-0.41183	0.022254	stronglyhypometh	Trf	4	8
chr9	103267309	103269309	-0.04203	0.017144	hypomethylated	Cdv3	28	32
chr9	103965032	103967032	-0.34667	0.00011868	stronglyhypometh	Acad11,Uba5	28	40
chr9	104954597	104956597	-0.63393	0.0072384	stronglyhypometh	Mrpl3	4	14
chr9	105296721	105298721	-0.37885	0.025865	stronglyhypometh	Aste1,Nek11	8	16
chr9	106060469	106062469	-0.29191	0.000000852	hypomethylated	Glyctk	9	10
chr9	106150285	106152285	-0.16431	0.0000173	hypomethylated	Alas1	12	10
chr9	106269962	106271962	-0.11191	0.000000657	hypomethylated	Dusp7	46	53
chr9	106340567	106342567	-0.67434	0.00067164	stronglyhypometh	Acy1	5	4
chr9	106378639	106380639	-0.45704	0.04043	stronglyhypometh	Parp3,Rrp9	8	19

chr9	106378982	106380982	-0.45704	0.04043	stronglyhypometh	Parp3,Rrp9	8	19
chr9	106723306	106725306	-0.10404	0.030006	hypomethylated	Vprbp	18	18
chr9	106789331	106791331	-0.20667	0.00080878	hypomethylated	Rbm15b	17	33
chr9	107198019	107200019	0.097436	0.042883	hypermethylated	Cish	10	10
chr9	107301210	107303210	-0.13895	1.94E-09	hypomethylated	Cacna2d2	51	55
chr9	107443539	107445539	-0.51667	0.0000158	stronglyhypometh	Cyb561d2,Nprl2	6	8
chr9	107444196	107446196	-0.53571	0.0000684	stronglyhypometh	Cyb561d2,Nprl2	5	7
chr9	107455986	107457986	-0.055159	0.0000184	hypomethylated	Rassf1	22	24
chr9	107470493	107472493	-0.13647	0.0054773	hypomethylated	Hyal2	20	20
chr9	107489048	107491048	-0.21414	0.00029099	hypomethylated	Ifrd2	18	18
chr9	107537673	107539673	0.14286	0.044509	hypermethylated	Gnai2	5	4
chr9	107673333	107675333	0.37694	0.024029	stronglyhypermeth	Rbm5	3	6
chr9	107950620	107952620	-0.32416	0.007059	hypomethylated	Gmppb	10	12
chr9	107996811	107998811	-0.44392	0.00000107	stronglyhypometh	Apeh	16	18
chr9	108207535	108209535	-0.12019	0.00047436	hypomethylated	Rhoa,Tcta	38	49
chr9	108208282	108210282	-0.13595	0.00024181	hypomethylated	Rhoa,Tcta	34	42
chr9	108409335	108411335	-0.11357	0.0033031	hypomethylated	Qars	7	8
chr9	108418417	108420417	-0.121	0.0096433	hypomethylated	Qrich1	25	35
chr9	108461831	108463831	-0.63286	0.00000921	stronglyhypometh	Impdh2	12	16
chr9	108469649	108471649	-0.22721	0.0029392	hypomethylated	Dalrd3,Mir191,Mir425	12	27
chr9	108469673	108471673	-0.22721	0.0029392	hypomethylated	Dalrd3,Mir191,Mir425	12	27
chr9	108470107	108472107	-0.50757	0.00092281	stronglyhypometh	Dalrd3,Mir191,Mir425	5	10
chr9	108481001	108483001	0.15878	0.0032339	hypermethylated	Wdr6	10	10
chr9	108551711	108553711	-0.74918	0.0083333	stronglyhypometh	Arih2	1	4
chr9	108563428	108565428	-0.24573	0.0073031	hypomethylated	Slc25a20	3	13
chr9	108593473	108595473	-0.066227	0.00010561	hypomethylated	Prkar2a	41	44
chr9	108755373	108757373	-0.35174	0.000000141	stronglyhypometh	Slc26a6	13	13
chr9	108892946	108894946	-0.42923	0.00000989	stronglyhypometh	Pfkfb4	19	11
chr9	108940140	108942140	-0.30671	5.15E-11	hypomethylated	Shisa5	17	18
chr9	108984009	108986009	-0.33693	0.0000518	stronglyhypometh	Ccdc51,Tma7	10	14
chr9	108996949	108998949	-0.12008	0.011614	hypomethylated	Plxnb1	42	51
chr9	109833277	109835277	-0.066993	0.032213	hypomethylated	Map4	21	30
chr9	110033527	110035527	-0.28924	6.19E-10	hypomethylated	Smarcc1	28	41
chr9	110206695	110208695	-0.27674	0.0096995	hypomethylated	Elp6	16	16
chr9	110378461	110380461	-0.51492	0.00000164	stronglyhypometh	Kif9,Klhl18	13	13
chr9	110378497	110380497	-0.51492	0.00000164	stronglyhypometh	Kif9,Klhl18	13	13
chr9	110434100	110436100	-0.060226	0.00000514	hypomethylated	Setd2	55	61
chr9	110558006	110560006	-0.21682	0.0045462	hypomethylated	Ccdc12	11	16
chr9	111213242	111215242	-0.15578	0.010129	hypomethylated	Trank1	46	45
chr9	112135780	112137780	-0.33352	0.009398	stronglyhypometh	2900079G21Rik,Arpp2	12	18
chr9	112135884	112137884	-0.29748	0.028702	hypomethylated	2900079G21Rik,Arpp2	13	19
chr9	113839051	113841051	-0.019899	0.012538	hypomethylated	Ubp1	54	58
chr9	114309236	114311236	-0.059743	0.0000749	hypomethylated	Glb1,Tmppe	30	36
chr9	114596948	114598948	-0.056511	0.038583	hypomethylated	Dync1li1	60	59
chr9	114639320	114641320	-0.54763	0.0037215	stronglyhypometh	Cmtm6	19	20
chr9	114690947	114692947	-0.35661	0.0019943	stronglyhypometh	Cmtm7	19	18
chr9	117948616	117950616	-0.17332	0.029127	hypomethylated	Azi2	23	36
chr9	118414435	118416435	-0.14314	0.027353	hypomethylated	Golga4	27	39
chr9	119010595	119012595	-0.46491	0.037898	stronglyhypometh	Dlec1	15	12
chr9	119249158	119251158	-0.6894	0.000000423	stronglyhypometh	Acaa1a,Myd88	15	17
chr9	119249411	119251411	-0.75802	0.000000781	stronglyhypometh	Acaa1a,Myd88	13	15
chr9	119803012	119805012	-0.0892	0.028252	hypomethylated	Wdr48	37	49
chr9	119845723	119847723	-0.062455	0.012307	hypomethylated	Gorasp1,Ttc21a	14	15
chr9	120018516	120020516	-0.15445	0.0058955	hypomethylated	Slc25a38	13	17
chr9	120035883	120037883	-0.15701	0.025838	hypomethylated	Rpsa	17	29
chr9	120479633	120481633	-0.15804	0.0055111	hypomethylated	Rpl14	25	31
chr9	121186290	121188290	-0.16216	0.032059	hypomethylated	Ulk4	15	24

chr9	121404807	121406807	-0.8	0.0000159	stronglyhypometh	Cck	3	5
chr9	121550684	121552684	-0.077859	0.012394	hypomethylated	Lyzl4,Vipr1	27	34
chr9	121550833	121552833	-0.067946	0.012568	hypomethylated	Lyzl4,Vipr1	27	32
chr9	121627298	121629298	-0.048276	0.0011185	hypomethylated	Nktr	43	41
chr9	121668150	121670150	-0.2077	0.0000478	hypomethylated	E530011L22Rik,Zfp651	57	59
chr9	121669061	121671061	-0.19652	0.00012297	hypomethylated	E530011L22Rik,Zfp651	37	33
chr9	121766649	121768649	-0.15898	0.00030056	hypomethylated	Higd1a	30	31
chr9	122025383	122027383	-0.13135	0.00033875	hypomethylated	Snrk	47	50
chr9	122259733	122261733	-0.23108	0.010878	hypomethylated	Abhd5	34	39
chr9	122480618	122482618	-0.10592	0.049328	hypomethylated	9530059O14Rik	38	42
chr9	122831195	122833195	-0.14479	0.00038771	hypomethylated	Zfp105	22	23
chr9	122859198	122861198	-0.55682	0.00000837	stronglyhypometh	1110059G10Rik,Kif15	11	9
chr9	122929443	122931443	-0.12401	0.0095775	hypomethylated	Tmem42	30	32
chr9	123386818	123388818	-0.18677	0.0013177	hypomethylated	Limd1	21	19
chr9	123437999	123439999	-0.1866	0.014933	hypomethylated	Sacm1l	40	42
chr9	123760782	123762782	-0.29733	0.00071154	hypomethylated	Fyco1	9	14
chr9	123761020	123763020	-0.094432	0.0099685	hypomethylated	Fyco1	7	8
chrX	5976262	5978262	-0.16052	0.0019102	hypomethylated	Shroom4	21	29
chrX	6998173	7000173	-0.65	0.0096765	stronglyhypometh	2010204K13Rik	2	2
chrX	7149725	7151725	0.077918	0.00011413	hypermethylated	4930524L23Rik,Ppp1r3	28	28
chrX	7151246	7153246	0.29655	0.0000183	hypermethylated	4930524L23Rik,Ppp1r3	15	15
chrX	7417956	7419956	-0.14389	0.0000065	hypomethylated	Otud5	52	64
chrX	7454431	7456431	-0.12661	0.0087245	hypomethylated	Pim2	20	20
chrX	7721552	7723552	-0.27461	0.0000225	hypomethylated	2900002K06Rik,Rbm3	6	16
chrX	7783651	7785651	-0.60507	0.014507	stronglyhypometh	Porcn	1	6
chrX	10293490	10295490	-0.22258	0.00000451	hypomethylated	Mid1ip1	24	34
chrX	11657679	11659679	-0.1148	0.034022	hypomethylated	Bcor	18	20
chrX	20193628	20195628	-0.15855	0.028968	hypomethylated	Ndufb11,Rbm10	9	14
chrX	20539104	20541104	0.54266	0.024934	stronglyhypermeth	A230072C01Rik,Uxt	3	3
chrX	34334646	34336646	-0.049658	0.0089098	hypomethylated	Slc25a5	30	31
chrX	34404241	34406241	-0.41312	0.00047927	stronglyhypometh	C330007P06Rik	7	9
chrX	34587839	34589839	-0.33459	0.00024333	stronglyhypometh	Sowahd	20	30
chrX	34665757	34667757	-0.30236	0.022586	hypomethylated	Nkap	16	17
chrX	34730337	34732337	0.1364	0.035684	hypermethylated	Ndufa1,Rnf113a1	23	33
chrX	35541969	35543969	-0.22842	0.0036502	hypomethylated	Zbtb33	24	27
chrX	38753480	38755480	-0.56469	0.00094258	stronglyhypometh	Gria3	10	10
chrX	45609110	45611110	-0.17029	0.019143	hypomethylated	Utp14a	8	10
chrX	45975756	45977756	-0.43624	0.010921	stronglyhypometh	Slc25a14	18	15
chrX	45976138	45978138	-0.43624	0.010921	stronglyhypometh	Slc25a14	18	15
chrX	49518100	49520100	-0.50535	0.0000184	stronglyhypometh	Gpc4	25	29
chrX	53598576	53600576	-0.35805	0.0021566	stronglyhypometh	Zfp449	11	15
chrX	53851096	53853096	-0.16684	0.00000455	hypomethylated	Mmgt1	26	26
chrX	53983963	53985963	-0.28047	0.00021992	hypomethylated	Fhl1	13	22
chrX	53984133	53986133	-0.28047	0.00021992	hypomethylated	Fhl1	13	22
chrX	57657156	57659156	0.096664	0.019523	hypermethylated	Atp11c	27	31
chrX	63905333	63907333	-0.61722	0.00013106	stronglyhypometh	Slitrk2	3	3
chrX	65930729	65932729	-0.17716	0.0070045	hypomethylated	Fmr1,Gm10474	41	46
chrX	65931574	65933574	-0.18537	0.0053185	hypomethylated	Fmr1,Gm10474	42	47
chrX	66612505	66614505	-0.21896	0.0018463	hypomethylated	Aff2	25	27
chrX	67618260	67620260	-0.34225	0.016193	stronglyhypometh	Ids	5	5
chrX	67638087	67640087	-0.032413	0.036503	hypomethylated	1110012L19Rik	9	9
chrX	68302430	68304430	-0.43257	1.49E-08	stronglyhypometh	Mamld1	15	20
chrX	68808167	68810167	0.021797	0.0042527	hypermethylated	Hmgb3	44	45
chrX	68915941	68917941	-0.018869	0.040691	hypomethylated	Gpr50	29	32
chrX	69207359	69209359	-0.19436	0.00000077	hypomethylated	Prrg3	26	30
chrX	70917471	70919471	-0.25958	0.00061574	hypomethylated	Slc6a8	43	42
chrX	71031366	71033366	-0.32974	0.00071231	hypomethylated	Idh3g,Ssr4	12	20

chrX	71031481	71033481	-0.32974	0.00071231	hypomethylated	Idh3g,Ssr4	12	20
chrX	71032236	71034236	-0.34034	0.00041446	stronglyhypometh	Idh3g,Ssr4	12	16
chrX	71211654	71213654	-0.37074	0.000000864	stronglyhypometh	Hcfc1	12	15
chrX	71515154	71517154	-0.52106	0.032317	stronglyhypometh	Rpl10,Snora70	8	10
chrX	72758635	72760635	-0.84615	0.036946	stronglyhypometh	Vbp1	2	2
chrX	74755565	74757565	-0.10262	0.00072899	hypomethylated	Tbl1x	37	40
chrX	91368746	91370746	-0.37171	0.027651	stronglyhypometh	Zfx	5	8
chrX	91457990	91459990	-0.57244	1.64E-08	stronglyhypometh	Eif2s3x	19	18
chrX	92222021	92224021	-0.49828	0.0015711	stronglyhypometh	Spin4	5	6
chrX	96330468	96332468	-0.14685	0.015656	hypomethylated	Efnb1	24	25
chrX	97169944	97171944	-0.23593	0.011419	hypomethylated	Eda	15	25
chrX	97821246	97823246	-0.17516	0.027168	hypomethylated	Kif4,Pdzd11	14	21
chrX	98468429	98470429	-0.19459	0.0021502	hypomethylated	Med12	22	22
chrX	98615253	98617253	-0.31734	0.019729	hypomethylated	Zmym3	11	14
chrX	98727073	98729073	-0.28801	0.012858	hypomethylated	Taf1	6	8
chrX	99383710	99385710	-0.33048	0.023037	hypomethylated	Rps4x	6	5
chrX	99447108	99449108	0.1126	0.0037147	hypermethylated	Cited1,Gm14858	15	19
chrX	101677120	101679120	-0.23215	0.0026606	hypomethylated	Uprt	11	12
chrX	102274113	102276113	-0.68013	0.000000371	stronglyhypometh	Pbdc1	16	16
chrX	103680568	103682568	-0.51886	0.010029	stronglyhypometh	Fndc3c1	6	15
chrX	104870850	104872850	-0.43269	0.0004721	stronglyhypometh	Tbx22	6	6
chrX	105010910	105012910	-0.36068	0.011731	stronglyhypometh	2610002M06Rik,Fam4	11	14
chrX	106028816	106030816	-0.46806	0.013516	stronglyhypometh	2810403D21Rik,Brwd3	18	16
chrX	126283277	126285277	-0.25584	0.00065847	hypomethylated	Diap2	19	23
chrX	130592887	130594887	-0.18495	0.00092013	hypomethylated	Cstf2	28	35
chrX	130828763	130830763	-0.55691	0.0037378	stronglyhypometh	Tmem35	8	7
chrX	131076168	131078168	-0.040314	0.048864	hypomethylated	Timm8a1	16	17
chrX	131119192	131121192	-0.30616	0.0079806	hypomethylated	Rpl36a	7	3
chrX	133572841	133574841	0.13549	0.0034068	hypermethylated	Slc25a53	6	8
chrX	133583132	133585132	-0.23807	0.0031924	hypomethylated	Fam199x	22	26
chrX	136597107	136599107	-0.25188	0.0075506	hypomethylated	E230019M04Rik,Nup6	5	5
chrX	139401271	139403271	-0.30176	0.032647	hypomethylated	Ammecr1	17	11
chrX	147954214	147956214	-0.21701	0.00000466	hypomethylated	Phf8	24	25
chrX	148435438	148437438	-0.72222	0.0094538	stronglyhypometh	Hsd17b10	2	3
chrX	148666772	148668772	-0.29673	0.0031858	hypomethylated	Kdm5c	17	17
chrX	149204004	149206004	-0.20687	0.0020267	hypomethylated	Shroom2	13	22
chrX	149671587	149673587	-0.20738	0.0057719	hypomethylated	Klf8	5	7
chrX	149931774	149933774	-0.23159	0.00000998	hypomethylated	Ubqln2	17	29
chrX	154481042	154483042	-0.19233	0.00000962	hypomethylated	Cnksr2	40	36
chrX	155851509	155853509	-0.19746	0.0019741	hypomethylated	Map7d2	32	33
chrX	155969599	155971599	-0.37895	0.026253	stronglyhypometh	A830080D01Rik	19	23
chrX	156425364	156427364	-0.089434	0.00028663	hypomethylated	Map3k15	42	46
chrX	158153967	158155967	-0.18858	0.00000738	hypomethylated	Rai2	30	35
chrX	158154484	158156484	-0.18118	0.0000183	hypomethylated	Rai2	30	38
chrX	160513981	160515981	0.035976	0.017868	hypermethylated	Siah1b	14	14
chrX	160856718	160858718	-0.34416	0.0055623	stronglyhypometh	Piga	6	8
chrX	161417523	161419523	-0.50135	0.034937	stronglyhypometh	Fancb,Mospd2	6	9
chrX	165232030	165234030	-0.32013	0.0024195	hypomethylated	Arhgap6	9	10
chrX	165758275	165760275	0.296	0.0010456	hypermethylated	Hccs	8	14
chrX	166316553	166318553	0.21402	0.031159	hypermethylated	Mid1	6	11
