# Supplementary material for: Dnmt1 links BCR-ABLp210 to epigenetic tumor stem cell priming in myeloid leukemia
Source: Leukemia. 2018 Jun 28;33(1):249–78. doi: 10.1038/s41375-018-0192-z (PMC6326950; doi:10.1038/s41375-018-0192-z)
Supplement: Supplementary file 4 — Table S3 [file 41375_2018_192_MOESM4_ESM.pdf]

Table S3: RRBS methylation changes in bone marrow of young Sca1-Dnmt1 mice compared to wild-type controls.

Samples name:		
	zr151_9	Bone Marrow young Sca1-Dnmt1
	zr322_1	Bone Marrow young WT
	zr322_3	Bone Marrow young Sca1-Dnmt1

chrom	start	end	gene_name	methDiff (zr151_9 vs zr322_1)	pValue (zr151_9 vs zr322_1)	pvalClass (zr151_9.RRBS vs zr322_1)	methDiff (zr322_1 vs zr322_3)	pValue (zr322_1 vs zr322_3)	pvalClass (zr322_1 vs zr322_3)	Number of CpGs zr151_9	Number of CpGs zr322_1	Number of CpGs zr322_3
chr1	3661579	3663579	Xkr4	-0.11289	1.39E-17	hypomethylated	0.00046535	0.000091102	inconclusive	20	89	78
chr1	4486494	4488494	Sox17	-0.20615	0.000026685	hypomethylated	0.0019038	0.29187	insignificant	7	51	48
chr1	4796973	4798973	Lypla1	-0.16029	4.21E-40	hypomethylated	-0.025368	0.0038927	hypomethylated	30	66	87
chr1	4846774	4848774	Tcea1	-0.15252	1.82E-24	hypomethylated	0.013336	0.24542	insignificant	30	109	107
chr1	4847408	4849408	Tcea1	-0.13378	1.23E-23	hypomethylated	0.0049379	0.21076	insignificant	30	103	103
chr1	5009507	5011507	Rgs20	-0.14751	4.33E-34	hypomethylated	0.023527	0.47807	insignificant	19	95	80
chr1	5072253	5074253	Atp6v1h	-0.15729	0.014246	hypomethylated	-0.031944	0.11437	insignificant	11	58	45
chr1	5577573	5579573	Oprk1	-0.041613	1	insignificant	-0.047225	0.91657	insignificant	3	16	16
chr1	5907479	5909479	Npbwr1	-0.26407	0.25528	insignificant	0.037132	0.076477	insignificant	2	4	6
chr1	6203742	6205742	Rb1cc1	-0.13191	1.3E-29	hypomethylated	-0.014695	0.55851	insignificant	50	173	154
chr1	6348411	6350411	Fam150a	-0.20099	1.19E-11	hypomethylated	-0.030807	0.96733	insignificant	15	57	58
chr1	7078000	7080000	Pcmtd1	-0.13284	1.12E-12	hypomethylated	-0.013919	0.00000949	hypomethylated	22	78	79
chr1	9289811	9291811	Sntg1	-0.085315	0.21822	insignificant	0.016823	0.36103	insignificant	7	33	34
chr1	9534488	9536488	Rrs1	-0.16218	5.78E-46	hypomethylated	-0.02392	0.0032444	hypomethylated	40	155	153
chr1	9537126	9539126	Adhfe1	-0.017189	0.08279	insignificant	0.043225	0.062807	insignificant	4	25	26
chr1	9590347	9592347	3110035E14Rik	0.082796	1	insignificant	-0.053498	0.0005959	hypomethylated	2	29	29
chr1	9690290	9692290	Mybl1	-0.12267	0.000000119	hypomethylated	0.0028675	0.46092	insignificant	13	47	65
chr1	9738463	9740463	1700034P13Rik	-0.17228	0.23398	insignificant	-0.066202	0.74064	insignificant	6	37	32
chr1	9787210	9789210	Sgk3	-0.089101	4.33E-17	hypomethylated	-0.0097098	0.045565	hypomethylated	46	151	157
chr1	9837361	9839361	Sgk3	-0.099265	7.28E-12	hypomethylated	0.017531	0.78749	insignificant	9	30	39
chr1	9897718	9899718	6030422M02Ril	-0.013063	0.0007171	hypomethylated	0.061644	0.00004632	hypermethylated	11	37	35
chr1	9932624	9934624	Snord87	-0.10449	0.000000574	hypomethylated	-0.011126	0.95464	insignificant	16	66	69
chr1	9934199	9936199	Snhg6	-0.2365	1.23E-08	hypomethylated	0.028446	1	insignificant	6	15	20
chr1	9999201	10001201	Ppp1r42		1	noCoverage	-0.044118	0.25346	insignificant	0	7	4
chr1	9999211	10001211	Ppp1r42		1	noCoverage	-0.044118	0.25346	insignificant	0	7	4
chr1	10027298	10029298	Cspp1	-0.20018	1.15E-13	hypomethylated	-0.03518	0.82087	insignificant	26	101	100
chr1	10027979	10029979	Cops5	-0.16555	3.08E-09	hypomethylated	-0.034201	0.34589	insignificant	33	108	114
chr1	10222751	10224751	Arfgef1	-0.12973	4.51E-08	hypomethylated	-0.019605	0.081947	insignificant	35	102	99
chr1	10710024	10712024	Cpa6		1	noCoverage	-0.026916	0.83675	insignificant	0	8	8
chr1	10982545	10984545	Prex2	-0.11126	1.14E-09	hypomethylated	-0.014047	0.66782	insignificant	18	86	79
chr1	11403185	11405185	A830018L16Rik	-0.1212	0.00000263	hypomethylated	0.024194	0.10639	insignificant	15	40	42
chr1	12681510	12683510	Sulf1	0.0023775	0.21182	insignificant	-0.013079	0.73901	insignificant	7	40	40
chr1	12707625	12709625	Sulf1		1	noCoverage	-0.11186	0.027858	hypomethylated	0	15	14
chr1	12981216	12983216	Slco5a1	-0.13629	3.01E-12	hypomethylated	-0.010943	0.23707	insignificant	13	100	100
chr1	13117244	13119244	Prdm14	-0.31703	0.000015937	hypomethylated	0.046953	0.080521	insignificant	5	12	10
chr1	13364164	13366164	Ncoa2	-0.14274	0.00021118	hypomethylated	-0.036795	0.61808	insignificant	10	55	50
chr1	13579945	13581945	Tram1	0.085152	1	insignificant	-0.11094	0.075215	insignificant	1	12	12
chr1	13650590	13652590	Lactb2	-0.20974	0.000096442	hypomethylated	0.040683	0.037242	inconclusive	12	40	38
chr1	13657851	13659851	Xkr9	-0.8878	0.21552	lowCoverage	-0.1228	0.44924	insignificant	1	12	11
chr1	13777010	13779010	Gm5523	0.1066	1	insignificant	0.10955	0.047471	hypermethylated	2	4	4
chr1	14746047	14748047	Msc		1	noCoverage	-0.095588	0.43321	insignificant	0	2	2
chr1	14908943	14910943	Trpa1	-0.26262	0.010486	hypomethylated	-0.019431	0.77884	insignificant	2	4	4
chr1	15301532	15303532	Kcnb2	0.13205	0.50197	insignificant	0.10723	0.3909	insignificant	8	30	31
chr1	15794738	15796738	Terf1	-0.12253	0.0027435	hypomethylated	-0.016531	0.38641	insignificant	13	70	77
chr1	15882803	15884803	Gm106	-0.21293	0.00024737	hypomethylated	-0.0053146	0.044317	hypomethylated	5	14	14
chr1	16094514	16096514	Rpl7	-0.069162	5.12E-46	hypomethylated	-0.0033244	0.43402	insignificant	73	263	280
chr1	16094962	16096962	Rdh10	-0.065374	7.3E-44	hypomethylated	-0.0013267	0.70658	insignificant	71	259	272
chr1	16509383	16511383	Stau2	-0.17146	6.65E-13	hypomethylated	-0.0075375	0.91092	insignificant	12	36	36
chr1	16609367	16611367	Ube2w	-0.089747	1.14E-10	hypomethylated	0.0086991	0.0086196	inconclusive	19	62	60
chr1	16646946	16648946	D030040B21Rik	-0.16454	0.00000171	hypomethylated	0.0058164	0.1319	insignificant	10	40	36
chr1	16654271	16656271	Tmem70	-0.22551	2.77E-26	hypomethylated	-0.034545	0.097749	insignificant	31	87	103
chr1	16677536	16679536	Ly96	-0.10713	0.79967	insignificant	-0.014572	1	insignificant	4	31	31
chr1	17087879	17089879	Jph1	-0.084329	7.28E-10	hypomethylated	0.0050614	0.13384	insignificant	11	40	40
chr1	17134453	17136453	Gdap1	-0.11257	0.55619	insignificant	-0.010956	0.26577	insignificant	2	26	26
chr1	17716497	17718497	Crispld1	-0.41747	0.000021638	stronglyHypometh	-0.0060176	0.55231	insignificant	1	12	14
chr1	19092102	19094102	Tfap2d		1	noCoverage	0.025641	0.57772	insignificant	0	8	8
chr1	19197994	19199994	Tfap2b	-0.24158	0.11757	insignificant	0.039081	0.63526	insignificant	1	7	2
chr1	19201134	19203134	Tfap2b	-0.31048	0.00003648	hypomethylated	-0.069105	0.51467	insignificant	1	8	7
chr1	20719985	20721985	Il17a	-0.061275	0.48734	insignificant	0.046204	0.08081	insignificant	2	4	5
chr1	20810294	20812294	Mcm3	-0.51714	0.00000633	stronglyHypometh	0.015885	0.019118	inconclusive	3	13	14
chr1	20879702	20881702	Paqr8	-0.11946	1.36E-14	hypomethylated	0.0045682	0.085013	insignificant	18	76	73
chr1	20940706	20942706	Efhc1	-0.10915	0.22696	insignificant	0.033119	1	insignificant	2	16	18
chr1	21069290	21071290	Tram2	-0.3133	1.95E-12	hypomethylated	-0.039323	0.000000124	hypomethylated	23	67	75
chr1	21069306	21071306	Tram2	-0.31821	1.82E-12	hypomethylated	-0.031651	0.00000052	hypomethylated	21	65	71
chr1	21207711	21209711	Tmem14a	-0.2198	0.22235	insignificant	-0.0057291	0.81981	insignificant	4	34	34
chr1	21372636	21374636	Khdc1b		1	noCoverage	-0.0042105	0.30723	insignificant	0	4	4
chr1	21952023	21954023	Kcnq5	-0.16918	0.000008397	hypomethylated	-0.017568	0.109	insignificant	5	16	25
chr1	22812563	22814563	Rims1	-0.13042	6.82E-17	hypomethylated	-0.010632	0.009948	hypomethylated	31	83	88
chr1	23109092	23111092	4933415F23Rik	-0.26564	0.26245	insignificant	-0.04415	0.096739	insignificant	1	5	4
chr1	23278107	23280107	Mir30a	-0.20222	0.66959	insignificant	-0.048519	0.69042	insignificant	3	10	9
chr1	23297539	23299539	Mir30c-2	-0.913	0.012318	stronglyHypometh	-0.055861	0.51698	insignificant	1	4	4
chr1	23390014	23392014	Ogfrl1	-0.12506	0.089079	insignificant	-0.055402	0.83683	insignificant	15	29	48
chr1	23767764	23769764	B3gat2	-0.11474	4.59E-24	hypomethylated	-0.0012038	0.1108	insignificant	33	138	141
chr1	23929128	23931128	Smap1	-0.1253	5.07E-17	hypomethylated	0.0008921	0.66411	insignificant	23	56	56
chr1	24107180	24109180	Fam135a	-0.18063	3E-11	hypomethylated	-0.0010521	0.13355	insignificant	11	31	26
chr1	24183448	24185448	Col9a1		1	noCoverage	0.0086651	0.52856	insignificant	0	12	12
chr1	24684382	24686382	Lmbrd1	-0.12165	3.32E-08	hypomethylated	0.0018138	0.42824	insignificant	17	58	58

chr1	25886552	25888552	Bai3	-0.090893	2.16E-12	hypomethylated	-0.016675	0.68143	insignificant	34	131	139
chr1	30920101	30922101	Phf3		1	noCoverage	0.01859	1	insignificant	0	8	10
chr1	31006555	31008555	Gm13363	-0.1425	2.68E-36	hypomethylated	-0.027927	0.038851	hypomethylated	31	100	105
chr1	31006600	31008600	Gm13363	-0.1457	2.58E-37	hypomethylated	-0.030519	0.018043	hypomethylated	31	90	95
chr1	31278683	31280683	4930521A18Rik	-0.0039683	0.63042	insignificant	0.013889	1	insignificant	2	6	6
chr1	32228650	32230650	Khdrbs2	-0.10389	0.000000873	hypomethylated	-0.025567	0.0057285	hypomethylated	18	86	86
chr1	33725668	33727668	1700001G17Rik	-0.10721	0.15062	insignificant	0.01079	1	insignificant	4	39	39
chr1	33726603	33728603	Prim2	-0.14263	0.14979	insignificant	-0.005358	0.86754	insignificant	4	41	40
chr1	33775740	33777740	Rab23	-0.090267	6.42E-21	hypomethylated	0.0026667	0.080106	insignificant	37	118	124
chr1	33776263	33778263	Rab23	-0.090267	6.42E-21	hypomethylated	0.0026667	0.080106	insignificant	37	118	124
chr1	33814595	33816595	Bag2	-0.035042	0.001551	hypomethylated	0.010775	0.13318	insignificant	12	44	45
chr1	33871272	33873272	Zfp451	-0.12783	0.000026842	hypomethylated	0.0057414	6.13E-08	inconclusive	8	49	63
chr1	33895759	33897759	Gm15455	0.062719	1	insignificant	-0.1536	0.20486	insignificant	3	19	23
chr1	33964466	33966466	Bend6	-0.094484	3.69E-19	hypomethylated	-0.003369	0.27431	insignificant	46	125	136
chr1	34067669	34069669	Dst	-0.18676	3.99E-16	hypomethylated	-0.01621	0.22714	insignificant	18	54	63
chr1	34495673	34497673	Imp4	-0.1911	3.78E-26	hypomethylated	-0.016182	0.63894	insignificant	14	56	65
chr1	34496517	34498517	Ccdc115	-0.19278	1.89E-09	hypomethylated	-0.0085387	0.29137	insignificant	6	36	44
chr1	34515590	34517590	Ptpn18	-0.19826	4.98E-12	hypomethylated	-0.0050776	0.019795	hypomethylated	20	74	74
chr1	34554274	34556274	Prss39	0.041667	1	lowCoverage	-0.055917	0.050927	insignificant	1	12	8
chr1	34591492	34593492	Cfc1	-0.070269	0.71582	insignificant	0.12497	1	insignificant	1	8	8
chr1	34635537	34637537	Fam123c	-0.077818	3.22E-08	hypomethylated	-0.0086942	0.00000476	hypomethylated	15	68	71
chr1	34693055	34695055	AA619741		1	noCoverage	0.1625	0.41257	insignificant	0	4	4
chr1	34857566	34859566	Arhgef4	-0.098195	2.95E-11	hypomethylated	0.000019721	0.80603	insignificant	14	52	52
chr1	34899895	34901895	Fam168b	-0.089257	4.61E-14	hypomethylated	-0.0070701	0.31452	insignificant	17	36	36
chr1	34905803	34907803	Plekhb2	-0.13266	0.00014633	hypomethylated	-0.006622	0.55169	insignificant	10	41	41
chr1	36124244	36126244	Hs6st1	-0.10193	3.68E-40	hypomethylated	0.0030482	0.95283	insignificant	62	198	199
chr1	36301147	36303147	Uggt1	-0.16524	0.00008796	hypomethylated	0.037074	0.24934	insignificant	9	31	33
chr1	36330270	36332270	Neurl3	-0.284	3.63E-09	hypomethylated	0.0034627	0.057682	insignificant	6	27	27
chr1	36363577	36365577	Arid5a	-0.078493	0.010976	hypomethylated	-0.0066316	0.28113	insignificant	20	77	71
chr1	36426026	36428026	4632411B12Rik	-0.24345	3.47E-31	hypomethylated	0.05307	0.0020545	inconclusive	13	48	41
chr1	36502084	36504084	Lman2l		1	noCoverage	-0.004987	0.73867	insignificant	0	14	14
chr1	36527441	36529441	Cnnm4	-0.088057	0.00000585	hypomethylated	0.012571	0.73538	insignificant	16	60	65
chr1	36567720	36569720	Cnnm3	-0.099225	5.78E-50	hypomethylated	-0.018258	0.00091343	hypomethylated	70	271	271
chr1	36592574	36594574	Ankrd23	-0.0021317	0.34484	insignificant	-0.15544	0.12749	insignificant	2	18	14
chr1	36604046	36606046	Ankrd39	-0.35498	0.070347	insignificant	-0.068651	0.000079763	hypomethylated	8	33	30
chr1	36615226	36617226	Sema4c	-0.21243	0.000000356	hypomethylated	0.049447	0.00097465	inconclusive	8	19	19
chr1	36740028	36742028	Fam178b	-0.06254	0.39997	insignificant	0.017053	0.28583	insignificant	8	35	40
chr1	36747331	36749331	Cox5b	-0.13831	1.78E-08	hypomethylated	-0.0082579	0.034977	hypomethylated	12	62	62
chr1	36766770	36768770	Actr1b	-0.24389	0.15763	insignificant	-0.052315	0.46063	insignificant	3	13	15
chr1	36817705	36819705	Zap70	0.10903	1	insignificant	0.017317	0.57216	insignificant	1	18	18
chr1	36996372	36998372	Tmem131	-0.1408	3.86E-13	hypomethylated	0.053641	0.056815	insignificant	28	78	85
chr1	37275105	37277105	Cnga3	-0.23746	0.000022536	hypomethylated	-0.00634	0.028717	hypomethylated	5	29	29
chr1	37355737	37357737	Inpp4a	-0.098979	2.37E-08	hypomethylated	0.0075031	0.0061027	hypermethylated	30	77	77
chr1	37486016	37488016	Unc50	-0.11876	6.14E-13	hypomethylated	0.0058339	0.79249	insignificant	22	74	74
chr1	37486948	37488948	6330578E17Rik	-0.15717	0.069197	insignificant	0.015867	0.6079	insignificant	6	48	48
chr1	37545450	37547450	Mgat4a		1	noCoverage	0.084306	0.85366	insignificant	0	23	21
chr1	37776656	37778656	2010300C02Rik	-0.081927	6.93E-08	hypomethylated	-0.00044328	0.76461	insignificant	26	67	58
chr1	37921924	37923924	Tsga10	-0.077328	9.18E-14	hypomethylated	0.007807	0.84719	insignificant	20	72	72
chr1	37928050	37930050	Lipt1	-0.27941	0.066911	insignificant	-0.0060357	0.77673	insignificant	3	8	8
chr1	37946397	37948397	Mrpl30	-0.14637	0.0000051	hypomethylated	0.016058	1	insignificant	10	56	49
chr1	37947256	37949256	Mitd1	-0.17141	0.00000118	hypomethylated	0.025076	0.68897	insignificant	10	39	34
chr1	38053854	38055854	Eif5b	-0.13824	1.37E-65	hypomethylated	-0.007171	0.020688	hypomethylated	77	192	209
chr1	38054053	38056053	Txndc9	-0.14027	8.99E-61	hypomethylated	-0.0074198	0.078629	insignificant	73	176	193
chr1	38186507	38188507	Rev1	-0.097635	3.24E-12	hypomethylated	-0.0078966	0.59286	insignificant	26	105	92
chr1	38878060	38880060	Lonrf2	-0.87664	0.05314	insignificant	-0.0572	0.56579	insignificant	1	6	8
chr1	38994993	38996993	Nms		1	noCoverage	0.087611	0.40448	insignificant	0	6	6
chr1	39043658	39045658	Pdcl3	-0.1507	6.66E-13	hypomethylated	-0.015516	0.3127	insignificant	21	82	85
chr1	39250116	39252116	Npas2	-0.13931	1.31E-22	hypomethylated	-0.0092187	0.57879	insignificant	43	135	147
chr1	39423695	39425695	Rpl31	-0.15359	1.09E-24	hypomethylated	-0.0030544	0.00038189	hypomethylated	34	118	118
chr1	39535592	39537592	Tbc1d8	-0.18462	0.00000725	hypomethylated	-0.0060935	0.61464	insignificant	11	24	24
chr1	39591646	39593646	D1Bwg0212e	-0.17352	4.25E-16	hypomethylated	-0.0089818	0.2916	insignificant	42	161	158
chr1	39605685	39607685	Snord89	0.10814	1	insignificant	-0.016707	1	insignificant	1	9	9
chr1	39634192	39636192	Rnf149		1	noCoverage	-0.026064	0.31018	insignificant	0	48	46
chr1	39777834	39779834	Rfx8	-0.46405	0.056962	insignificant	0.06732	0.87369	insignificant	3	30	31
chr1	39956757	39958757	Map4k4	-0.098076	2.4E-25	hypomethylated	-0.0010167	0.77936	insignificant	69	162	178
chr1	40322430	40324430	Il1r1	-0.35182	0.000000024	stronglyHypometh	-0.020403	0.50169	insignificant	8	22	20
chr1	40737556	40739556	Slc9a2	-0.077739	2.86E-32	hypomethylated	-0.0052944	0.010674	hypomethylated	51	188	191
chr1	40861445	40863445	Tmem182		1	noCoverage	0.0634	0.34709	insignificant	0	21	20
chr1	42751670	42753670	2610017I09Rik	-0.053857	0.09593	insignificant	0.00022305	0.44001	insignificant	23	113	114
chr1	42752990	42754990	Pou3f3	-0.072174	0.000057283	hypomethylated	-0.021551	0.051525	insignificant	48	211	211
chr1	42756180	42758180	2900092D14Rik	-0.27191	0.00047989	hypomethylated	0.01742	0.24178	insignificant	3	6	6
chr1	42907077	42909077	Mrps9	-0.2085	0.00012652	hypomethylated	-0.0045787	0.08026	insignificant	11	34	34
chr1	43008716	43010716	Gpr45	-0.1112	3.93E-10	hypomethylated	-0.0059306	0.052068	insignificant	25	88	88
chr1	43154554	43156554	AI597479	-0.10912	0.38103	insignificant	-0.012364	0.34484	insignificant	3	62	67
chr1	43155467	43157467	Tgfbrap1	-0.097455	0.37811	insignificant	-0.0075798	0.65603	insignificant	3	56	61
chr1	43220806	43222806	Fhl2	-0.072385	0.000000083	hypomethylated	0.017036	0.72749	insignificant	12	42	42
chr1	43501595	43503595	Nck2	-0.11161	2.28E-39	hypomethylated	-0.01498	0.0036185	hypomethylated	73	210	210
chr1	43786446	43788446	1500015O10Rik	-0.21123	0.31633	insignificant	0.03832	0.36611	insignificant	1	4	4
chr1	43884553	43886553	Uxs1	-0.091187	3.26E-11	hypomethylated	-0.0071745	0.030539	hypomethylated	17	61	73
chr1	43989851	43991851	Tpp2	-0.17555	7.67E-27	hypomethylated	-0.019917	0.068198	insignificant	17	55	60
chr1	44159233	44161233	1700029F09Rik	-0.10168	5.27E-14	hypomethylated	-0.0039406	0.021966	hypomethylated	17	58	58
chr1	44175618	44177618	Kdelc1	-0.22222	1.51E-11	hypomethylated	0.016929	0.47284	insignificant	4	40	35
chr1	44175812	44177812	Bivm	-0.23147	4.01E-11	hypomethylated	0.015117	0.39729	insignificant	4	34	29
chr1	44203588	44205588	Ercc5	-0.2044	2.88E-13	hypomethylated	-0.025978	0.21117	insignificant	17	64	65
chr1	44607515	44609515	Gulp1	-0.15037	1.33E-12	hypomethylated	-0.015926	0.12939	insignificant	20	80	76

chr1	45851345	45853345	Wdr75	-0.17718	4.54E-26	hypomethylated	-0.01321	0.18151	insignificant	27	87	82
chr1	45982439	45984439	Slc40a1	-0.21218	0.000035643	hypomethylated	-0.036145	0.045704	hypomethylated	7	37	37
chr1	46122582	46124582	Dnahc7b	-0.2609	0.00033309	hypomethylated	-0.028516	0.13875	insignificant	6	29	31
chr1	46910354	46912354	Slc39a10	-0.14774	1.45E-22	hypomethylated	0.014259	1	insignificant	31	77	66
chr1	50983366	50985366	Tmeff2	-0.11409	2.61E-18	hypomethylated	0.0082007	0.0036275	inconclusive	14	90	81
chr1	51535243	51537243	Obfc2a	-0.12802	3.26E-11	hypomethylated	-0.012636	0.0081155	hypomethylated	6	38	38
chr1	51972818	51974818	Myo1b	-0.1546	9.01E-13	hypomethylated	-0.0012205	0.94776	insignificant	12	24	24
chr1	52064087	52066087	Stat4	-0.24396	1	lowCoverage	0.037761	0.23773	insignificant	1	13	13
chr1	52175281	52177281	Stat1	-0.16196	4.44E-27	hypomethylated	-0.029293	0.075127	insignificant	24	97	106
chr1	52290076	52292076	Gls	-0.23803	0.055712	insignificant	-0.099724	0.000016159	hypomethylated	1	9	16
chr1	52557292	52559292	Nab1	-0.16083	0.00029097	hypomethylated	0.056324	0.45202	insignificant	11	52	39
chr1	52686548	52688548	Tmem194b	-0.095167	0.24181	insignificant	-0.0029365	0.76059	insignificant	7	42	42
chr1	52784162	52786162	Mfsd6	-0.1018	0.000010303	hypomethylated	0.0048925	0.70804	insignificant	10	28	28
chr1	52874532	52876532	Inpp1	-0.18539	0.000030731	hypomethylated	-0.010743	0.71129	insignificant	5	10	10
chr1	52900889	52902889	Hibch	-0.2031	2.28E-08	hypomethylated	-0.0046848	0.3207	insignificant	17	49	50
chr1	53009684	53011684	1700019D03Rik	-0.14427	2.66E-11	hypomethylated	0.022073	0.96423	insignificant	13	36	36
chr1	53352938	53354938	Ormdl1	-0.11542	0.00000828	hypomethylated	-0.0087926	0.054336	insignificant	9	75	84
chr1	53353840	53355840	Pms1	-0.11317	0.0000082	hypomethylated	-0.0031511	0.065957	insignificant	9	68	70
chr1	53409543	53411543	Asnsd1	-0.12824	0.000037891	hypomethylated	-0.0043936	0.83496	insignificant	9	20	20
chr1	53752288	53754288	Dnahc7a		1	noCoverage	-0.042323	0.7315	insignificant	0	37	29
chr1	53752289	53754289	Dnahc7a		1	noCoverage	-0.042323	0.7315	insignificant	0	37	29
chr1	53842059	53844059	Stk17b	-0.15928	0.0048344	hypomethylated	-0.00078606	0.74383	insignificant	14	34	44
chr1	54251878	54253878	Hecw2	-0.068445	1	insignificant	-0.0017409	0.64049	insignificant	3	28	28
chr1	54306526	54308526	Ccdc150	-0.21487	0.00000134	hypomethylated	-0.0058217	0.5489	insignificant	9	34	30
chr1	54495815	54497815	Gtf3c3	-0.50842	0.0018929	stronglyHypometh	0.025677	0.5243	insignificant	2	15	15
chr1	54614526	54616526	Pgap1	-0.1022	0.0027372	hypomethylated	-0.044154	1	insignificant	6	21	18
chr1	54983231	54985231	Ankrd44	-0.13705	5.09E-17	hypomethylated	-0.026743	0.012086	hypomethylated	13	38	51
chr1	55084322	55086322	Sf3b1	-0.22917	0.00042477	hypomethylated	0.0063316	0.12573	insignificant	7	44	46
chr1	55108613	55110613	Coq10b	-0.1346	6.78E-33	hypomethylated	-0.002453	0.40412	insignificant	47	109	109
chr1	55143991	55145991	Hspe1	-0.093251	7.63E-20	hypomethylated	0.003827	0.89464	insignificant	47	190	188
chr1	55144776	55146776	Hspd1	-0.10549	5.72E-16	hypomethylated	0.014036	0.49422	insignificant	28	106	104
chr1	55187088	55189088	Mob4	-0.1142	7.65E-24	hypomethylated	-0.003578	0.45899	insignificant	34	105	110
chr1	55293020	55295020	Mars2	-0.13088	1.87E-69	hypomethylated	-0.0036823	0.0010438	hypomethylated	52	168	167
chr1	55419551	55421551	Boll	-0.18813	0.030976	inconclusive	-0.005866	0.0080797	hypomethylated	29	167	169
chr1	55420313	55422313	Boll	-0.10907	0.000000174	hypomethylated	0.014215	0.95252	insignificant	8	50	50
chr1	55461789	55463789	Plcl1	-0.14844	5.75E-17	hypomethylated	-0.0089733	0.20629	insignificant	34	104	105
chr1	57027312	57029312	9130024F11Rik	-0.10347	8.59E-10	hypomethylated	-0.0065211	0.91436	insignificant	29	125	130
chr1	57028178	57030178	Satb2	-0.11171	5.45E-14	hypomethylated	-0.00039187	0.92576	insignificant	42	178	179
chr1	57433463	57435463	1700066M21Ril	-0.13396	8.62E-27	hypomethylated	0.0064764	0.97081	insignificant	33	101	101
chr1	57462391	57464391	9430016H08Rik	-0.095939	0.005209	inconclusive	-0.0086171	0.58937	insignificant	28	126	126
chr1	57463518	57465518	9430016H08Rik	-0.077289	0.3419	insignificant	0.0084648	0.57499	insignificant	11	38	38
chr1	57830704	57832704	Spats2l	-0.084776	3.02E-22	hypomethylated	0.013159	0.28061	insignificant	51	120	122
chr1	58026368	58028368	Kctd18	-0.14727	7.54E-21	hypomethylated	0.012545	0.46606	insignificant	39	101	103
chr1	58026928	58028928	Kctd18	-0.075538	0.00000134	hypomethylated	0.051697	0.94096	insignificant	14	34	36
chr1	58041183	58043183	Sgol2		1	noCoverage	-0.045949	0.053026	insignificant	0	14	14
chr1	58051817	58053817	Sgol2	-0.18823	8.86E-19	hypomethylated	0.0072413	0.44857	insignificant	7	64	64
chr1	58085812	58087812	Aox1		1	noCoverage	0.13447	0.27896	insignificant	0	4	8
chr1	58266240	58268240	Aox4		1	noCoverage	-0.047727	1	insignificant	0	11	9
chr1	58448979	58450979	Bzw1	-0.094283	3.22E-12	hypomethylated	-0.0003971	0.42508	insignificant	45	192	165
chr1	58480932	58482932	Clk1	0.083333	1	insignificant	-0.15	1	insignificant	1	8	10
chr1	58503476	58505476	Nif3l1	0.10091	0.32687	insignificant	-0.032854	0.0085129	hypomethylated	4	12	12
chr1	58561763	58563763	Orc2	-0.20899	0.000000099	hypomethylated	0.025966	0.45663	insignificant	10	28	29
chr1	58642442	58644442	Ndufb3	-0.25437	1.16E-09	hypomethylated	0.022195	0.078675	insignificant	7	18	31
chr1	58643177	58645177	Fam126b	-0.25437	1.16E-09	hypomethylated	-0.0011259	0.064716	insignificant	7	18	30
chr1	58851217	58853217	Casp8	-0.086503	0.00000486	hypomethylated	-0.012967	0.4142	insignificant	10	43	43
chr1	59029414	59031414	Stradb	-0.16191	4.45E-21	hypomethylated	-0.014397	0.078955	insignificant	33	105	109
chr1	59030326	59032326	Trak2	-0.14766	2.49E-15	hypomethylated	-0.0077398	0.42789	insignificant	20	77	77
chr1	59176650	59178650	Als2cr4	-0.1273	5.65E-28	hypomethylated	0.0015754	0.75392	insignificant	34	82	77
chr1	59176940	59178940	Mpp4	-0.1283	1.33E-24	hypomethylated	0.00094511	0.96398	insignificant	21	54	49
chr1	59220233	59222233	Als2	-0.15288	0.6241	insignificant	-0.034994	0.70767	insignificant	1	14	11
chr1	59293937	59295937	Als2	-0.098903	8.67E-16	hypomethylated	0.015319	0.72411	insignificant	21	58	60
chr1	59294075	59296075	Als2	-0.12571	0.056855	insignificant	0.036411	0.93775	insignificant	5	16	18
chr1	59537990	59539990	Fzd7	-0.11622	1.03E-22	hypomethylated	-0.0064996	0.41087	insignificant	51	183	180
chr1	59572107	59574107	Gm973	-0.5754	0.04605	stronglyHypometh	-0.073794	0.5523	insignificant	1	4	4
chr1	59727678	59729678	Sumo1	-0.21068	0.031126	hypomethylated	-0.0075032	0.35604	insignificant	8	24	24
chr1	59820480	59822480	Bmpr2	-0.088062	2.57E-11	hypomethylated	0.016321	0.76834	insignificant	28	112	114
chr1	59968849	59970849	Fam117b	-0.12267	6.13E-26	hypomethylated	-0.017016	0.18181	insignificant	34	130	128
chr1	60099931	60101931	Ica1l	-0.070011	2.86E-26	hypomethylated	0.013482	0.68064	insignificant	40	110	111
chr1	60154124	60156124	Carf	-0.10597	1.49E-20	hypomethylated	0.0016577	1	insignificant	29	83	94
chr1	60154751	60156751	Wdr12	-0.0951	7.59E-19	hypomethylated	-0.0070549	0.83444	insignificant	27	69	80
chr1	60154978	60156978	Wdr12	-0.0951	7.59E-19	hypomethylated	-0.0070549	0.83444	insignificant	27	69	80
chr1	60155344	60157344	Wdr12	-0.083602	5.03E-14	hypomethylated	0.014565	0.90806	insignificant	22	59	62
chr1	60236442	60238442	Nbeal1	-0.063878	7.98E-10	hypomethylated	-0.0059169	0.67629	insignificant	37	134	142
chr1	60399215	60401215	Cyp20a1	-0.19918	4.36E-30	hypomethylated	-0.031264	0.000068363	hypomethylated	19	62	60
chr1	60465462	60467462	Abi2	-0.12059	1.16E-14	hypomethylated	-0.00064475	0.75702	insignificant	24	131	126
chr1	60623609	60625609	Raph1	-0.073973	0.017647	hypomethylated	0.0058517	0.53929	insignificant	21	70	65
chr1	61684397	61686397	Pard3b	-0.13243	5.72E-22	hypomethylated	0.0089447	0.075266	insignificant	44	152	155
chr1	62748890	62750890	Nrp2	-0.16533	4.87E-19	hypomethylated	0.019865	0.27173	insignificant	23	76	71
chr1	63160841	63162841	Ino80d	-0.052998	0.01467	hypomethylated	0.0060738	0.5574	insignificant	11	22	22
chr1	63222404	63224404	Eef1b2	-0.17997	2.4E-62	hypomethylated	-0.0091191	0.0073514	hypomethylated	39	128	132
chr1	63223396	63225396	Ndufs1	-0.12501	7.17E-24	hypomethylated	-0.010211	0.035348	hypomethylated	22	68	69
chr1	63260783	63262783	Gpr1	0.40054	1	lowCoverage	0.014177	0.45073	insignificant	1	7	7
chr1	63318842	63320842	Zdbf2	-0.07876	4.46E-18	hypomethylated	0.0078478	0.6414	insignificant	60	185	188
chr1	63491477	63493477	Adam23	-0.059375	1.27E-10	hypomethylated	0.0030253	0.68916	insignificant	39	130	130
chr1	64167963	64169963	Klf7	-0.12606	2.1E-16	hypomethylated	0.016901	0.84656	insignificant	19	84	84

chr1	64578377	64580377	Creb1	-0.10011	7.74E-41	hypomethylated	-0.0014881	0.032373	hypomethylated	64	201	199
chr1	64663742	64665742	Mettl21a	-0.056923	0.038026	hypomethylated	-0.0052593	0.6274	insignificant	10	39	48
chr1	64736918	64738918	Ccnyl1	-0.1521	4.38E-59	hypomethylated	-0.014879	0.000077807	hypomethylated	61	211	203
chr1	64784324	64786324	Fzd5	-0.15289	3.87E-20	hypomethylated	0.0087188	0.11994	insignificant	20	56	54
chr1	65003398	65005398	Plekhm3	-0.036572	0.32751	insignificant	-0.073991	0.0035676	hypomethylated	3	15	16
chr1	65097737	65099737	Cryge	0.066667	1	insignificant	0.077778	0.63869	insignificant	5	12	12
chr1	65110014	65112014	Crygd	-0.10124	0.57192	insignificant	0.04808	0.34587	insignificant	2	5	6
chr1	65128841	65130841	Crygb	0.05	1	insignificant	-0.033333	1	insignificant	2	6	6
chr1	65149937	65151937	Cryga	-0.12083	0.021263	hypomethylated	-0.067262	0.19867	insignificant	6	12	12
chr1	65169767	65171767	D630023F18Rik	-0.85417	0.22857	lowCoverage	0.0625	1	insignificant	1	4	4
chr1	65225716	65227716	Idh1		1	noCoverage	-0.056458	0.68152	insignificant	0	17	18
chr1	65232258	65234258	Pikfyve	-0.15513	1.34E-14	hypomethylated	0.039537	1	insignificant	25	57	65
chr1	65233053	65235053	Idh1	-0.15071	1.38E-14	hypomethylated	0.035372	0.95877	insignificant	25	60	68
chr1	65356830	65358830	Pth2r	-0.39903	0.026896	stronglyHypometh	0.016079	0.34126	insignificant	3	10	10
chr1	65972095	65974095	Crygf	-0.28505	0.014211	hypomethylated	-0.10013	0.027771	hypomethylated	1	6	9
chr1	66220902	66222902	Mtap2	-0.1426	0.000000162	hypomethylated	-0.018888	0.12848	insignificant	17	92	92
chr1	66514020	66516020	Unc80	-0.13036	5.7E-12	hypomethylated	-0.0013812	0.017811	hypomethylated	43	127	133
chr1	66746466	66748466	Rpe	-0.17409	5.36E-08	hypomethylated	-0.0079046	0.24659	insignificant	16	40	41
chr1	66864169	66866169	1110028C15Rik	-0.11448	2.11E-15	hypomethylated	-0.0052988	0.083122	insignificant	29	98	95
chr1	66909841	66911841	Acadl	-0.13165	0.10196	insignificant	-0.029866	0.86359	insignificant	9	55	50
chr1	67085408	67087408	Lancl1		1	noCoverage	-0.044643	0.59235	insignificant	0	10	10
chr1	67085446	67087446	Lancl1		1	noCoverage	-0.044643	0.59235	insignificant	0	10	10
chr1	69154633	69156633	Erbb4	-0.10764	1	insignificant	0.018477	0.8721	insignificant	3	16	16
chr1	69732534	69734534	Ikzf2	-0.077822	4.45E-17	hypomethylated	0.011784	0.29743	insignificant	24	124	129
chr1	69872561	69874561	Spag16	-0.15108	9.74E-13	hypomethylated	0.013401	0.64508	insignificant	10	34	37
chr1	70771288	70773288	Vwc2l	-0.45272	0.00000212	stronglyHypometh	-0.016216	0.22199	insignificant	3	12	12
chr1	71149546	71151546	Bard1	-0.42462	0.00029557	stronglyHypometh	-0.041282	0.093502	insignificant	3	10	10
chr1	71602729	71604729	Atic	-0.15475	2.03E-11	hypomethylated	0.018862	0.63793	insignificant	27	75	78
chr1	71699745	71701745	Fn1	-0.10026	3.34E-24	hypomethylated	0.0060316	0.12843	insignificant	17	49	49
chr1	72258881	72260881	Mreg	-0.059669	0.0089293	hypomethylated	0.0021757	1	insignificant	6	22	22
chr1	72329946	72331946	Tmem169	-0.2789	8.33E-13	hypomethylated	-0.098176	0.0034825	hypomethylated	6	24	20
chr1	72583138	72585138	March4	-0.18744	0.0012823	hypomethylated	0.014024	1	insignificant	3	30	30
chr1	72747138	72749138	Ankar	-0.090278	0.43173	insignificant	0.15921	0.013711	hypermethylated	1	8	10
chr1	72756833	72758833	Rpl37a	-0.21147	9.02E-19	hypomethylated	-0.043432	0.097029	insignificant	8	42	50
chr1	72870076	72872076	Igfbp2	-0.11382	1.07E-08	hypomethylated	0.005697	1	insignificant	15	101	100
chr1	72921439	72923439	Igfbp5	0.53188	0.49277	insignificant	0.1673	0.86189	insignificant	1	6	4
chr1	73062473	73064473	Tnp1	-0.25721	0.10003	insignificant	0.06122	0.3069	insignificant	2	4	4
chr1	74171021	74173021	Tns1	-0.11899	0.000041784	hypomethylated	0.020492	0.20684	insignificant	15	95	100
chr1	74171114	74173114	Rufy4	-0.13003	0.006187	hypomethylated	0.019828	0.59037	insignificant	10	87	92
chr1	74199567	74201567	Cxcr2		1	noCoverage	0.12829	1	insignificant	0	4	4
chr1	74282123	74284123	Arpc2	-0.16237	2.42E-10	hypomethylated	0.00034103	0.20661	insignificant	17	102	98
chr1	74324173	74326173	Gpbar1	-0.030553	0.038287	hypomethylated	0.01008	0.1448	insignificant	6	18	18
chr1	74330607	74332607	Pnkd	-0.14041	0.000010429	hypomethylated	0.019014	0.11533	insignificant	8	76	76
chr1	74331312	74333312	Aamp	-0.13378	0.000014517	hypomethylated	0.013323	0.15098	insignificant	4	48	48
chr1	74350910	74352910	Tmbim1	-0.33696	1	insignificant	-0.068438	0.16469	insignificant	2	4	4
chr1	74378183	74380183	Pnkd	-0.13927	0.000016389	hypomethylated	0.014689	0.4159	insignificant	13	42	42
chr1	74420776	74422776	Slc11a1	0.22364	0.59478	insignificant	0.028545	0.17284	insignificant	3	12	12
chr1	74437182	74439182	Ctdsp1	-0.1145	4.7E-33	hypomethylated	0.0024794	0.32843	insignificant	48	153	162
chr1	74439883	74441883	Mir26b	-0.35398	0.000000749	stronglyHypometh	-0.0043664	0.037375	inconclusive	8	20	22
chr1	74551633	74553633	Rqcd1	-0.13239	2.97E-23	hypomethylated	-0.0082888	0.39304	insignificant	32	106	106
chr1	74590860	74592860	Plcd4	-0.18973	0.49289	insignificant	-0.072995	0.8507	insignificant	1	16	20
chr1	74633934	74635934	Bcs1l	-0.13632	4.03E-21	hypomethylated	-0.017202	0.20776	insignificant	25	84	84
chr1	74634602	74636602	Zfp142	-0.068712	0.0004902	hypomethylated	-0.010603	0.00001012	hypomethylated	18	54	54
chr1	74647028	74649028	Stk36	-0.020551	5.09E-14	hypomethylated	-0.0080473	0.84125	insignificant	8	32	32
chr1	74707327	74709327	Ttll4	-0.24055	0.003315	hypomethylated	0.010545	0.007761	inconclusive	7	56	57
chr1	74759147	74761147	Cyp27a1		1	noCoverage	-0.016856	0.87798	insignificant	0	13	14
chr1	74795529	74797529	Prkag3	-0.3917	1	lowCoverage	-0.1609	0.15836	insignificant	1	19	16
chr1	74817465	74819465	Wnt6	-0.15216	2.14E-09	hypomethylated	-0.026436	0.13961	insignificant	16	84	85
chr1	74837592	74839592	Wnt10a	-0.085784	0.0021338	hypomethylated	0.084085	0.78041	insignificant	10	28	33
chr1	74900602	74902602	Cdk5r2	-0.11059	1.08E-23	hypomethylated	-0.0040526	0.10384	insignificant	61	160	161
chr1	74931982	74933982	Fev		1	noCoverage	-0.037334	0.9313	insignificant	0	10	10
chr1	74939709	74941709	Cryba2	-0.29358	0.17818	insignificant	-0.03505	0.91583	insignificant	6	21	21
chr1	74947295	74949295	Mir375	-0.22012	9.19E-11	hypomethylated	-0.071738	0.010901	hypomethylated	9	54	52
chr1	74998225	75000225	Ihh	-0.12009	0.000073591	hypomethylated	0.039474	0.76164	insignificant	14	43	43
chr1	75138359	75140359	Fam134a	-0.080944	2.32E-25	hypomethylated	-0.011578	0.10608	insignificant	33	124	121
chr1	75138942	75140942	1810031K17Rik	-0.056603	1.82E-23	hypomethylated	-0.0078402	0.21532	insignificant	28	106	104
chr1	75164219	75166219	Zfand2b	-0.11194	0.000015686	hypomethylated	-0.016976	0.074278	insignificant	27	86	86
chr1	75164308	75166308	Zfand2b	-0.11194	0.000015686	hypomethylated	-0.016976	0.074278	insignificant	27	86	86
chr1	75176857	75178857	Atg9a		1	noCoverage	0.1	0.02761	hypermethylated	0	3	3
chr1	75187708	75189708	Ankzf1	-0.13113	6.32E-13	hypomethylated	0.0074384	0.78664	insignificant	28	92	84
chr1	75188497	75190497	Atg9a	-0.14729	0.0056486	hypomethylated	0.013918	0.69214	insignificant	9	42	34
chr1	75206403	75208403	Stk16	-0.13378	1.64E-11	hypomethylated	0.0067813	0.06789	insignificant	14	70	70
chr1	75207353	75209353	Glb1l	-0.17685	1.35E-11	hypomethylated	-0.0052923	0.0034954	hypomethylated	13	74	73
chr1	75215828	75217828	A630095N17Rik	-0.17213	6.93E-36	hypomethylated	0.030929	0.41756	insignificant	28	106	106
chr1	75231997	75233997	Dnajb2	-0.10933	2.65E-13	hypomethylated	0.011749	0.48843	insignificant	17	48	48
chr1	75232276	75234276	Dnajb2	-0.13171	9.21E-11	hypomethylated	0.010273	0.06566	insignificant	18	52	52
chr1	75260783	75262783	Ptprn	-0.27206	0.00017375	hypomethylated	0.027012	0.2191	insignificant	7	14	14
chr1	75274955	75276955	Resp18		1	noCoverage	0.025132	1	insignificant	0	8	8
chr1	75313592	75315592	Dnpep	-0.11656	3.42E-37	hypomethylated	0.00039758	0.92982	insignificant	40	145	145
chr1	75314212	75316212	Dnpep	-0.034111	0.71072	insignificant	-0.00174	0.30341	insignificant	19	78	78
chr1	75355918	75357918	Des	-0.13626	1.39E-09	hypomethylated	0.001912	0.14945	insignificant	25	138	123
chr1	75370871	75372871	Speg	-0.19608	1.19E-09	hypomethylated	-0.03718	0.000000471	hypomethylated	22	65	64
chr1	75377688	75379688	Speg	-0.11362	0.00000393	hypomethylated	-0.0018932	1	insignificant	23	62	62
chr1	75381184	75383184	Speg	-0.068658	0.15519	insignificant	0.12084	4.19E-08	hypermethylated	5	35	35
chr1	75395659	75397659	Speg		1	noCoverage	-0.020833	0.80175	insignificant	0	2	2

chr1	75446084	75448084	Accn4	-0.20477	0.00000221	hypomethylated	0.026558	0.033733	hypermethylated	17	52	52
chr1	75475106	75477106	Tmem198	-0.12393	6.4E-41	hypomethylated	0.0031284	0.10825	insignificant	66	169	168
chr1	75476046	75478046	Chpf	-0.1101	2.24E-10	hypomethylated	-0.0069508	0.71529	insignificant	23	55	55
chr1	75502651	75504651	Inha	-0.17846	0.000000215	hypomethylated	-0.016874	0.000020611	hypomethylated	23	66	65
chr1	75503027	75505027	Obsl1	-0.31311	7.74E-10	hypomethylated	-0.0178	0.00063428	hypomethylated	7	24	23
chr1	75517103	75519103	Stk11ip	-0.28412	0.000033616	hypomethylated	-0.046359	0.59495	insignificant	6	14	14
chr1	75541840	75543840	Slc4a3	-0.1992	1.87E-14	hypomethylated	-0.015425	0.43021	insignificant	18	54	60
chr1	77511663	77513663	Epha4	-0.20184	8.79E-14	hypomethylated	-0.022446	0.10859	insignificant	22	71	71
chr1	78193711	78195711	Pax3	-0.12778	0.5801	insignificant	0.010067	0.70909	insignificant	4	12	12
chr1	78305920	78307920	Sgpp2	-0.17824	3.9E-14	hypomethylated	0.0071868	0.36386	insignificant	16	50	53
chr1	78485421	78487421	Farsb	-0.5	0.1654	insignificant	-0.16176	0.8806	insignificant	1	8	6
chr1	78506634	78508634	Mogat1	-0.46677	1.09E-16	stronglyHypometh	0.090467	0.00038967	inconclusive	6	20	20
chr1	78653399	78655399	Utp14b	-0.1346	7.97E-13	hypomethylated	-0.0087466	0.96556	insignificant	31	118	106
chr1	78654014	78656014	Utp14b	-0.063442	1.53E-10	hypomethylated	0.0083916	0.85134	insignificant	31	110	98
chr1	78812523	78814523	Kcne4	-0.069951	0.63701	insignificant	-0.048236	0.29196	insignificant	6	26	31
chr1	79436665	79438665	Scg2	-0.21139	0.0016822	hypomethylated	0.065726	0.30736	insignificant	1	8	10
chr1	79668547	79670547	Ap1s3	-0.59746	0.000019429	stronglyHypometh	0.035139	0.26848	insignificant	2	13	17
chr1	79758344	79760344	Wdfy1	-0.058971	0.025956	hypomethylated	-0.0098503	0.35922	insignificant	8	38	38
chr1	79771592	79773592	Mrpl44	-0.27946	0.000000345	hypomethylated	0.10041	0.53054	insignificant	3	11	6
chr1	79855240	79857240	Serpine2	-0.15871	0.015529	hypomethylated	0.0027861	0.091	insignificant	11	43	43
chr1	80337005	80339005	Cul3	-0.15402	7.77E-20	hypomethylated	-0.010551	0.099899	insignificant	30	100	97
chr1	81072891	81074891	9430031J16Rik	-0.76266	0.0094894	stronglyHypometh	0.07448	0.57296	insignificant	2	10	10
chr1	82288014	82290014	Irs1	-0.12653	4.09E-10	hypomethylated	-0.045881	0.0037313	hypomethylated	11	42	42
chr1	82312153	82314153	Rhbdd1	-0.12103	2.92E-10	hypomethylated	-0.017285	0.15581	insignificant	21	58	58
chr1	82582495	82584495	Col4a3	-0.13895	0.000019568	hypomethylated	0.0082923	0.77169	insignificant	9	53	53
chr1	82583424	82585424	Col4a4	-0.11303	0.052575	insignificant	0.031438	0.10503	insignificant	4	23	23
chr1	82720492	82722492	Mff	-0.086594	0.082572	insignificant	0.0016509	0.19904	insignificant	22	70	70
chr1	82835057	82837057	Agfg1	-0.095421	3.73E-30	hypomethylated	-0.0077342	0.17585	insignificant	57	165	164
chr1	83035023	83037023	Slc19a3	-0.28039	0.000028532	hypomethylated	-0.015447	0.86398	insignificant	5	11	11
chr1	84281220	84283220	Pid1	-0.25421	0.64578	insignificant	0.05078	0.3696	insignificant	8	20	20
chr1	84692796	84694796	Mir5126	0.091138	1	insignificant	-0.11362	0.00025642	inconclusive	4	22	23
chr1	84835415	84837415	Fbxo36	-0.14841	2.04E-23	hypomethylated	-0.02015	0.92519	insignificant	41	177	180
chr1	84835879	84837879	Fbxo36	-0.10624	1.12E-14	hypomethylated	-0.015938	0.85899	insignificant	21	95	99
chr1	84931658	84933658	Slc16a14	-0.18417	0.0036234	hypomethylated	0.012697	0.078273	insignificant	6	18	22
chr1	87633181	87635181	A630001G21Rik	-0.125	0.74609	insignificant	0.009596	0.1547	insignificant	2	6	6
chr1	87689021	87691021	Cab39	-0.095324	2.65E-16	hypomethylated	-0.0055084	0.20986	insignificant	41	142	140
chr1	87790084	87792084	Itm2c	-0.17728	4.28E-10	hypomethylated	-0.016969	0.96729	insignificant	20	73	71
chr1	87917516	87919516	Spata3	-0.20371	0.47501	insignificant	0.079616	0.15732	insignificant	2	24	20
chr1	87941437	87943437	2810459M11Ril	-0.097819	7.36E-39	hypomethylated	-0.0057487	0.019392	hypomethylated	52	152	144
chr1	87960193	87962193	Psmd1	-0.19503	0.000035376	hypomethylated	0.0082353	0.35384	insignificant	25	80	83
chr1	88050354	88052354	Armc9	-0.12437	2.45E-14	hypomethylated	0.014555	0.54613	insignificant	24	70	70
chr1	88050389	88052389	Armc9	-0.12437	2.45E-14	hypomethylated	0.014555	0.54613	insignificant	24	70	70
chr1	88198795	88200795	B3gnt7	-0.21339	6.53E-26	hypomethylated	-0.055001	0.00000832	hypomethylated	23	87	92
chr1	88255148	88257148	C130036L24Rik	-0.085446	7.37E-16	hypomethylated	0.0015473	0.4671	insignificant	33	141	146
chr1	88256030	88258030	Ncl	-0.086914	0.051273	insignificant	-0.0075091	0.39378	insignificant	12	68	68
chr1	88284716	88286716	Nmur1	-0.29807	0.11029	insignificant	0.055579	0.49678	insignificant	4	8	8
chr1	88321903	88323903	1700019O17Rik	-0.28471	0.070494	insignificant	-0.12794	0.26354	insignificant	7	14	14
chr1	88422310	88424310	Ptma	-0.093039	7.05E-44	hypomethylated	0.000028404	0.37813	insignificant	81	279	261
chr1	88479076	88481076	Pde6d	-0.40899	0.57551	insignificant	-0.018645	0.0018125	inconclusive	2	28	29
chr1	88482674	88484674	Cops7b	-0.1572	0.719	insignificant	0.067974	0.62058	insignificant	8	35	30
chr1	88567147	88569147	Nppc	-0.16035	0.0030089	hypomethylated	0.010526	0.056276	insignificant	7	18	18
chr1	88599378	88601378	Dis3l2	-0.12273	1.28E-32	hypomethylated	0.024116	0.66704	insignificant	21	72	58
chr1	89051602	89053602	Ecel1	-0.15184	0.000036074	hypomethylated	-0.0070373	0.0995	insignificant	21	85	86
chr1	89086226	89088226	Chrnd		1	noCoverage	-0.030678	0.096886	insignificant	0	8	8
chr1	89101385	89103385	Chrng		1	noCoverage	-0.001746	0.8003	insignificant	0	10	12
chr1	89109488	89111488	Eif4e2	-0.1465	1.7E-46	hypomethylated	-0.011507	0.75327	insignificant	32	110	110
chr1	89109513	89111513	Eif4e2	-0.1465	1.7E-46	hypomethylated	-0.011507	0.75327	insignificant	32	110	110
chr1	89159938	89161938	Efhd1	-0.11895	2.59E-25	hypomethylated	0.0056597	0.052619	insignificant	32	99	96
chr1	89222572	89224572	Gigyf2	-0.18698	1.27E-24	hypomethylated	-0.030733	0.0021939	hypomethylated	33	125	131
chr1	89222603	89224603	Gigyf2	-0.18698	1.27E-24	hypomethylated	-0.030733	0.0021939	hypomethylated	33	125	131
chr1	89406937	89408937	Ngef	-0.12418	4.74E-09	hypomethylated	0.072296	0.90735	insignificant	17	38	42
chr1	89515886	89517886	Inpp5d	-0.14914	0.00000854	hypomethylated	0.0036617	0.051906	insignificant	5	20	20
chr1	89651645	89653645	Atg16l1	-0.12528	2.37E-28	hypomethylated	-0.00028172	0.035674	hypomethylated	38	157	157
chr1	89699254	89701254	Sag	0.19796	0.81373	insignificant	0.22204	0.49167	insignificant	3	8	10
chr1	89748861	89750861	Dgkd	-0.12415	1.93E-53	hypomethylated	-0.0016838	0.010984	hypomethylated	52	169	169
chr1	89990575	89992575	Ugt1a7c	-0.47467	2.52E-09	stronglyHypometh	-0.0068281	0.63154	insignificant	6	15	15
chr1	90174132	90176132	A730008H23Rik	-0.34126	4.82E-08	stronglyHypometh	-0.029227	0.00000277	hypomethylated	5	21	20
chr1	90174154	90176154	A730008H23Rik	-0.40231	0.00000297	stronglyHypometh	-0.015531	0.0070669	hypomethylated	3	17	16
chr1	90202286	90204286	Trpm8	-0.55662	0.38545	insignificant	-0.022096	0.77396	insignificant	3	18	18
chr1	90302593	90304593	Spp2	-0.28526	0.014659	hypomethylated	-0.054894	0.10919	insignificant	5	14	14
chr1	90406641	90408641	Glrp1		1	noCoverage	-0.0076172	0.79453	insignificant	0	10	14
chr1	90598766	90600766	Arl4c	-0.074972	0.0019729	hypomethylated	-0.0015839	1	insignificant	16	52	52
chr1	90966036	90968036	Sh3bp4	-0.13566	3.42E-40	hypomethylated	-0.011816	0.11734	insignificant	43	164	176
chr1	91350385	91352385	Agap1	-0.10563	1.13E-37	hypomethylated	0.0036011	0.0028257	inconclusive	83	232	250
chr1	91827751	91829751	Gbx2	0.078447	0.011191	hypermethylated	-0.030428	0.00000858	inconclusive	20	89	89
chr1	91911152	91913152	Asb18	0.026136	0.67444	insignificant	0.12234	0.12502	insignificant	2	8	10
chr1	92049976	92051976	Iqca	-0.2166	0.25011	insignificant	-0.049544	0.94858	insignificant	8	26	30
chr1	92099577	92101577	Cxcr7	-0.35648	0.036992	stronglyHypometh	-0.083434	0.54511	insignificant	4	22	22
chr1	92498999	92500999	Cops8	-0.19093	4.88E-12	hypomethylated	-0.039539	0.011808	hypomethylated	21	79	76
chr1	92810676	92812676	Mlph	-0.082527	0.00000747	hypomethylated	-0.024202	0.000067103	hypomethylated	7	25	26
chr1	92866197	92868197	Rab17	0.029675	0.21955	insignificant	-0.0076928	0.16131	insignificant	4	9	12
chr1	92894303	92896303	Lrrfip1	-0.10412	1.4E-47	hypomethylated	0.0033042	0.3281	insignificant	40	128	132
chr1	92949020	92951020	Lrrfip1	-0.12253	0.0023664	hypomethylated	-0.016974	0.96171	insignificant	8	68	78
chr1	93040679	93042679	Rbm44	0.26822	0.0015981	hypermethylated	0.17252	0.40423	insignificant	8	26	31
chr1	93075398	93077398	Ramp1	-0.18379	1.32E-15	hypomethylated	-0.0030016	0.13374	insignificant	12	57	58

chr1	93145895	93147895	Ube2f	-0.10368	4.36E-48	hypomethylated	0.0060244	0.18446	insignificant	51	148	148
chr1	93193914	93195914	Scly	-0.098694	2.06E-08	hypomethylated	0.0049404	0.29681	insignificant	24	75	68
chr1	93217651	93219651	Espnl	-0.4372	5.47E-19	stronglyHypometh	-0.016247	8.44E-08	hypomethylated	6	24	24
chr1	93246649	93248649	Klhl30	-0.675	0.35556	lowCoverage	-0.083333	0.23317	insignificant	1	4	5
chr1	93262006	93264006	Fam132b	-0.12941	0.000015307	hypomethylated	-0.011807	0.19761	insignificant	10	64	72
chr1	93295360	93297360	Ilkap	-0.19099	0.000000437	hypomethylated	0.011252	1	insignificant	6	18	18
chr1	93309799	93311799	Hes6	-0.17327	3.07E-08	hypomethylated	-0.046851	0.050747	insignificant	14	54	55
chr1	93355905	93357905	Per2	-0.1581	2.83E-11	hypomethylated	-0.013753	0.82352	insignificant	17	55	55
chr1	93390244	93392244	Traf3ip1	-0.16027	1.05E-59	hypomethylated	-0.017307	0.23147	insignificant	40	123	128
chr1	93436141	93438141	Asb1	-0.15015	1.49E-22	hypomethylated	-0.0029189	0.023966	hypomethylated	23	58	58
chr1	93436656	93438656	Asb1	-0.15015	1.49E-22	hypomethylated	-0.0029189	0.023966	hypomethylated	23	58	58
chr1	93697053	93699053	Twist2	-0.12077	8.9E-40	hypomethylated	0.0071359	0.012312	inconclusive	33	110	110
chr1	94370335	94372335	Ndufa10	-0.1161	0.0043466	hypomethylated	-0.011006	0.64126	insignificant	3	33	33
chr1	94415092	94417092	Olfr1414	-0.056973	0.056091	insignificant	-0.2165	0.00076649	hypomethylated	3	7	8
chr1	94538525	94540525	Myeov2	0.16585	1	insignificant	0.041304	0.84866	insignificant	1	6	6
chr1	94538562	94540562	Myeov2	0.16585	1	insignificant	0.041304	0.84866	insignificant	1	6	6
chr1	94727262	94729262	Gpc1	-0.10837	2.11E-46	hypomethylated	0.0037331	0.92046	insignificant	68	208	228
chr1	94802565	94804565	Dusp28	-0.21382	6.57E-08	hypomethylated	-0.0060118	0.098563	insignificant	13	56	50
chr1	94806981	94808981	Rnpepl1	-0.099527	1.48E-33	hypomethylated	0.0082607	0.5756	insignificant	47	183	185
chr1	94829984	94831984	Capn10	-0.14094	1.67E-19	hypomethylated	-0.0020516	0.00000167	hypomethylated	24	103	100
chr1	94847167	94849167	9430060I03Rik	-0.07328	0.29956	insignificant	-0.079374	0.089522	insignificant	2	6	9
chr1	94901910	94903910	Aqp12	-0.067515	0.29064	insignificant	-0.032836	0.15021	insignificant	3	6	6
chr1	94998442	95000442	Kif1a	-0.061545	0.88422	insignificant	-0.041235	0.0082553	hypomethylated	10	30	31
chr1	95030852	95032852	Agxt	0.041361	0.36444	insignificant	0.015939	0.54738	insignificant	6	16	16
chr1	95057447	95059447	2310007B03Rik	-0.17142	0.00020207	hypomethylated	-0.003353	0.057678	insignificant	7	32	33
chr1	95057525	95059525	2310007B03Rik	-0.18029	0.0099136	hypomethylated	-0.0011493	0.066683	insignificant	5	28	29
chr1	95131473	95133473	Sned1	-0.08726	4.33E-08	hypomethylated	-0.015244	0.00050478	hypomethylated	34	128	126
chr1	95202447	95204447	Mterfd2	-0.010848	0.000056593	hypomethylated	-0.1753	0.013827	hypomethylated	3	24	32
chr1	95239221	95241221	Ppp1r7	-0.10701	3.83E-15	hypomethylated	-0.0010046	0.71694	insignificant	21	124	119
chr1	95239365	95241365	Ppp1r7	-0.10776	7.3E-13	hypomethylated	-0.0024037	0.42678	insignificant	13	102	97
chr1	95269472	95271472	Ano7	0.038987	0.67538	insignificant	0.027682	0.0098066	hypermethylated	6	20	20
chr1	95374569	95376569	############	-0.15133	3.54E-33	hypomethylated	-0.018525	0.000076588	hypomethylated	35	115	114
chr1	95374637	95376637	############	-0.18147	3.45E-35	hypomethylated	-0.010847	0.000039349	hypomethylated	36	123	122
chr1	95375385	95377385	############	-0.25299	3.52E-20	hypomethylated	-0.016752	0.000013129	hypomethylated	15	66	66
chr1	95407680	95409680	Farp2	-0.11584	3E-11	hypomethylated	-0.0092213	0.015366	hypomethylated	8	72	78
chr1	95532304	95534304	Stk25	0.0062086	0.0015534	inconclusive	-0.043357	0.0065434	hypomethylated	5	12	12
chr1	95581270	95583270	Bok	-0.11485	3.17E-10	hypomethylated	-0.011364	0.085853	insignificant	31	137	144
chr1	95650609	95652609	Atg4b	-0.080179	1.03E-29	hypomethylated	0.021428	0.72826	insignificant	60	164	163
chr1	95651415	95653415	Thap4	-0.051846	4.45E-13	hypomethylated	0.035458	1	insignificant	34	93	92
chr1	95698511	95700511	Ing5	-0.073062	0.00000303	hypomethylated	0.0064561	0.18822	insignificant	20	78	78
chr1	95699541	95701541	Ing5	-0.0039951	0.16695	insignificant	0.01247	0.059365	insignificant	12	81	82
chr1	95720816	95722816	D2hgdh	-0.25754	3.55E-10	hypomethylated	-0.020411	0.70547	insignificant	3	20	20
chr1	95949130	95951130	Pdcd1	0.12495	1	insignificant	-0.047387	0.0068092	hypomethylated	2	18	15
chr1	97209204	97211204	Fam174a	-0.16305	2.94E-31	hypomethylated	0.0039721	0.18317	insignificant	15	59	60
chr1	97564171	97566171	St8sia4	-0.08184	0.15443	insignificant	-0.013023	0.78649	insignificant	4	30	31
chr1	98768748	98770748	Slco4c1	-0.22636	0.0001335	hypomethylated	-0.018154	0.19511	insignificant	7	14	14
chr1	99558595	99560595	D1Ertd622e	-0.11838	7.65E-19	hypomethylated	-0.016427	0.87254	insignificant	19	44	48
chr1	99665755	99667755	Gin1	-0.12906	4.33E-12	hypomethylated	0.0098852	0.46983	insignificant	17	93	97
chr1	99666669	99668669	Ppip5k2	-0.20033	2.19E-11	hypomethylated	0.0098855	0.18133	insignificant	8	32	40
chr1	99992209	99994209	Pam	-0.1352	0.000098848	hypomethylated	-0.029704	0.013987	hypomethylated	9	40	45
chr1	101668341	101670341	Cntnap5b		1	noCoverage	0.067738	0.46541	insignificant	0	10	10
chr1	106664395	106666395	Cdh20	-0.24698	0.28891	insignificant	0.055193	0.88251	insignificant	2	23	20
chr1	107253287	107255287	Rnf152	-0.36364	1	lowCoverage	0.01553	0.82373	insignificant	1	2	2
chr1	107559437	107561437	2310035C23Rik	-0.12811	1.75E-09	hypomethylated	-0.016387	0.47655	insignificant	17	114	111
chr1	107560253	107562253	Pign	-0.10717	0.000000711	hypomethylated	-0.01319	0.9063	insignificant	16	99	96
chr1	107676299	107678299	Tnfrsf11a	-0.10038	1.93E-37	hypomethylated	-0.010975	0.12603	insignificant	33	96	96
chr1	107885982	107887982	Zcchc2	-0.078367	1.31E-30	hypomethylated	0.00063998	0.019303	inconclusive	61	180	171
chr1	108067445	108069445	Phlpp1	-0.087607	1.2E-29	hypomethylated	-0.0071068	0.00054624	hypomethylated	64	201	202
chr1	108610867	108612867	Bcl2	-0.093624	0.00051705	hypomethylated	0.007493	1	insignificant	14	32	46
chr1	108656319	108658319	Kdsr		1	noCoverage	0.1231	0.076283	insignificant	0	10	23
chr1	108693302	108695302	Vps4b	-0.30903	0.012518	hypomethylated	0.19362	0.0058179	hypermethylated	2	6	7
chr1	108756756	108758756	Serpinb5	-0.25833	0.077078	insignificant	-0.11321	0.54757	insignificant	1	4	4
chr1	109057691	109059691	Serpinb3b	-0.1399	0.033309	hypomethylated	-0.082582	0.024131	hypomethylated	2	4	4
chr1	113761495	113763495	Dsel		1	noCoverage	-0.063622	0.80817	insignificant	0	11	12
chr1	117580713	117582713	Cntnap5a	-0.2077	0.33136	insignificant	0.029104	0.89648	insignificant	5	22	22
chr1	120207709	120209709	Tsn	-0.15315	1.9E-17	hypomethylated	0.0012477	0.70832	insignificant	23	62	71
chr1	120217419	120219419	Mki67ip	-0.18468	0.000000188	hypomethylated	0.0094811	0.0076461	hypermethylated	15	53	53
chr1	120284634	120286634	Clasp1	-0.16265	4.8E-17	hypomethylated	-0.012696	0.44602	insignificant	29	96	103
chr1	120355842	120357842	Clasp1	-0.29508	1.24E-09	hypomethylated	-0.029447	0.40455	insignificant	7	17	17
chr1	120523521	120525521	Tfcp2l1	-0.16213	4.71E-11	hypomethylated	-0.021271	0.035376	hypomethylated	15	68	68
chr1	120950196	120952196	Gli2	-0.10201	2.57E-14	hypomethylated	-0.041852	0.00001114	hypomethylated	23	62	62
chr1	121318825	121320825	Inhbb	-0.11321	7.68E-10	hypomethylated	0.030716	0.28569	insignificant	15	68	60
chr1	121401359	121403359	Ralb	-0.11223	0.098392	insignificant	-0.029244	0.83603	insignificant	2	23	24
chr1	121421743	121423743	Tmem185b	-0.12858	8.87E-17	hypomethylated	-0.0036161	0.37132	insignificant	23	94	94
chr1	121545577	121547577	Epb4.1l5	-0.20169	0.40948	insignificant	-0.012345	0.061054	insignificant	5	21	21
chr1	121733648	121735648	Ptpn4	-0.12169	0.00002279	hypomethylated	-0.0063594	1	insignificant	9	41	41
chr1	121809745	121811745	Tmem177	-0.2547	0.2032	insignificant	-0.036723	0.12875	insignificant	0	38	37
chr1	121891787	121893787	Gm101	0.10186	0.17039	insignificant	-0.021448	0.47444	insignificant	2	28	26
chr1	121902556	121904556	Sctr	-0.20248	0.00014373	hypomethylated	-0.013928	0.68992	insignificant	12	34	36
chr1	121970357	121972357	Tmem37	0.041603	0.54314	insignificant	-0.0062367	0.58047	insignificant	5	32	32
chr1	122016763	122018763	3110009E18Rik	-0.11784	0.0014329	hypomethylated	-0.018057	0.059096	insignificant	8	70	69
chr1	122017496	122019496	Dbi	-0.14951	0.0011297	hypomethylated	-0.017341	0.029144	hypomethylated	6	65	65
chr1	122017673	122019673	Dbi	-0.13556	0.083095	insignificant	-0.020322	0.0071641	hypomethylated	3	43	43
chr1	122161857	122163857	Steap3	-0.28312	0.022294	hypomethylated	-0.0073593	0.78819	insignificant	4	11	11
chr1	122236158	122238158	C1ql2	-0.13374	1.12E-16	hypomethylated	-0.011946	0.50925	insignificant	29	123	123

chr1	122498063	122500063	En1	-0.1243	3.38E-17	hypomethylated	-0.0035338	0.55398	insignificant	26	113	112
chr1	123224339	123226339	Insig2	-0.16259	2.69E-16	hypomethylated	0.03388	0.00042479	inconclusive	15	57	55
chr1	123326643	123328643	Ccdc93	-0.21181	3.66E-21	hypomethylated	0.00014169	0.67797	insignificant	19	50	50
chr1	125942136	125944136	Dpp10	-0.21873	2.36E-15	hypomethylated	0.056476	0.0097312	inconclusive	13	61	53
chr1	127332067	127334067	Actr3	-0.073947	0.000000285	hypomethylated	0.031274	0.70017	insignificant	20	68	68
chr1	127456592	127458592	Slc35f5	-0.14349	0.00000026	hypomethylated	-0.00090477	0.23441	insignificant	21	79	85
chr1	127572572	127574572	Gpr39	0.088884	0.00013907	hypermethylated	-0.036	0.51356	insignificant	4	9	8
chr1	127808791	127810791	Lypd1	-0.31602	0.013597	hypomethylated	0.0681	0.6971	insignificant	3	8	8
chr1	128727209	128729209	Nckap5	-0.24886	0.035983	hypomethylated	-0.0043324	1	insignificant	1	8	8
chr1	129100562	129102562	Mgat5	-0.24299	8.6E-21	hypomethylated	0.019429	0.16194	insignificant	20	56	66
chr1	129574598	129576598	Tmem163	-0.23625	0.39472	insignificant	0.014303	0.85443	insignificant	2	38	38
chr1	129669740	129671740	Ccnt2	-0.15367	6.05E-24	hypomethylated	-0.0089054	0.38088	insignificant	32	87	91
chr1	129764349	129766349	Rab3gap1	0.55373	0.49319	lowCoverage	-0.048605	0.10345	insignificant	1	32	34
chr1	129998882	130000882	R3hdm1	-0.095155	3.13E-11	hypomethylated	-0.0013029	0.29961	insignificant	22	83	92
chr1	129998985	130000985	Zranb3	-0.095155	3.13E-11	hypomethylated	-0.0013029	0.29961	insignificant	22	83	92
chr1	130139757	130141757	Ubxn4	-0.10923	3.64E-26	hypomethylated	0.014306	0.040558	inconclusive	27	94	95
chr1	130256233	130258233	Mcm6	-0.28579	0.000000437	hypomethylated	-0.031418	0.56623	insignificant	9	24	24
chr1	130488876	130490876	Cxcr4	-0.20572	1.72E-08	hypomethylated	-0.046776	0.024652	hypomethylated	13	36	36
chr1	131168880	131170880	Thsd7b	-0.11303	2.62E-12	hypomethylated	-0.01189	0.15717	insignificant	26	66	66
chr1	132359317	132361317	Cd55	0.052407	0.024368	hypermethylated	0.034021	0.8216	insignificant	4	14	12
chr1	132526183	132528183	Zp3r	0.13646	0.051524	insignificant	-0.024816	0.84037	insignificant	4	8	8
chr1	132611153	132613153	Pfkfb2	-0.13863	0.0015247	hypomethylated	0.012069	0.83247	insignificant	7	35	30
chr1	132612398	132614398	Pfkfb2	-0.15302	2.14E-22	hypomethylated	0.005833	0.82731	insignificant	20	70	70
chr1	132612903	132614903	Yod1	-0.17779	3.21E-27	hypomethylated	0.0046917	0.69163	insignificant	20	66	66
chr1	132627552	132629552	AA986860	-0.16548	0.000000331	hypomethylated	-0.092293	0.00000612	hypomethylated	6	20	18
chr1	132696478	132698478	Fcamr		1	noCoverage	0.039151	1	insignificant	0	6	7
chr1	132807873	132809873	Il20	-0.38485	0.023469	stronglyHypometh	-0.061772	0.16234	insignificant	1	2	2
chr1	132915421	132917421	Il10	-0.29615	0.13017	insignificant	-0.002972	0.82029	insignificant	2	4	4
chr1	132994120	132996120	Mapkapk2	-0.14884	2.61E-19	hypomethylated	-0.014987	0.32827	insignificant	23	71	81
chr1	133034811	133036811	Dyrk3	-0.35731	0.0050379	stronglyHypometh	-0.019786	0.063949	insignificant	2	12	12
chr1	133048783	133050783	Eif2d	-0.21435	0.00028601	hypomethylated	-0.0018423	1	insignificant	8	20	20
chr1	133141755	133143755	Rassf5	-0.17008	3.3E-25	hypomethylated	-0.012264	0.56963	insignificant	22	98	100
chr1	133176140	133178140	Ikbke		1	noCoverage	0.020996	0.38011	insignificant	0	8	8
chr1	133423565	133425565	Fam72a	-0.12316	4.28E-19	hypomethylated	-0.013913	0.024173	hypomethylated	23	105	105
chr1	133423938	133425938	Fam72a	-0.13267	0.00000719	hypomethylated	-0.018811	0.20272	insignificant	10	53	53
chr1	133494690	133496690	Avpr1b	-0.13077	0.57725	insignificant	0.21041	0.0066233	hypermethylated	1	2	2
chr1	133533890	133535890	Ctse		1	noCoverage	-0.20062	0.059152	insignificant	0	8	9
chr1	133639598	133641598	Slc26a9	-0.31998	0.093983	insignificant	-0.089702	0.42176	insignificant	2	25	25
chr1	133692971	133694971	Pm20d1		1	noCoverage	0.018176	0.73458	insignificant	0	10	10
chr1	133723588	133725588	Slc41a1	-0.11855	0.00000223	hypomethylated	0.023437	0.53779	insignificant	11	68	68
chr1	133762853	133764853	Rab7l1	-0.18839	0.00068833	hypomethylated	0.020762	0.19062	insignificant	10	60	62
chr1	133806034	133808034	Nucks1	-0.13128	8.16E-10	hypomethylated	0.0039803	1	insignificant	21	90	93
chr1	133858543	133860543	Slc45a3	-0.10374	1.35E-22	hypomethylated	-0.004642	0.00094367	hypomethylated	39	132	131
chr1	133866185	133868185	Slc45a3	-0.38247	0.0054224	stronglyHypometh	-0.067375	0.24276	insignificant	6	16	16
chr1	133903181	133905181	Elk4	-0.093357	2.96E-14	hypomethylated	0.0032415	0.80645	insignificant	30	164	164
chr1	133964639	133966639	Mfsd4	-0.077008	0.000015209	hypomethylated	-0.01639	0.00036225	hypomethylated	7	32	34
chr1	134036262	134038262	Cdk18	-0.41738	3.71E-11	stronglyHypometh	-0.018537	0.00061229	hypomethylated	3	12	12
chr1	134087012	134089012	Lemd1	-0.12602	7.63E-20	hypomethylated	0.0052751	0.62228	insignificant	37	128	119
chr1	134093664	134095664	Mir135b	0.0023265	1	insignificant	-0.047381	0.0044814	hypomethylated	5	14	14
chr1	134194202	134196202	Klhdc8a	-0.091018	8.28E-13	hypomethylated	-0.023989	0.00010891	hypomethylated	44	179	180
chr1	134211701	134213701	Nuak2	-0.11097	0.00002065	hypomethylated	-0.014542	0.73464	insignificant	5	55	53
chr1	134287858	134289858	Tmcc2	-0.086613	0.000000001	hypomethylated	-0.005257	0.24376	insignificant	30	84	84
chr1	134313029	134315029	Dstyk	-0.13577	5.62E-22	hypomethylated	0.00083431	0.091128	insignificant	30	106	106
chr1	134372943	134374943	Rbbp5	-0.19891	0.026734	hypomethylated	-0.0030322	0.91644	insignificant	3	62	56
chr1	134401806	134403806	Tmem81	-0.93651	0.085106	lowCoverage	-0.023629	0.69535	insignificant	1	4	4
chr1	134439517	134441517	Cntn2	-0.18832	0.14491	insignificant	-0.013024	0.26381	insignificant	5	20	22
chr1	134539435	134541435	Nfasc	0.0081349	1	insignificant	-0.0093864	0.83502	insignificant	3	6	6
chr1	134638374	134640374	Nfasc	-0.17252	0.0075402	hypomethylated	0.090155	1	insignificant	3	6	8
chr1	134775931	134777931	Lrrn2	-0.11296	1.37E-49	hypomethylated	0.0013849	0.15071	insignificant	39	151	151
chr1	134921925	134923925	Mdm4	-0.45124	1.71E-14	stronglyHypometh	0.011839	0.57593	insignificant	4	25	22
chr1	134941588	134943588	Pik3c2b	-0.11172	1.65E-20	hypomethylated	-0.011064	0.11469	insignificant	48	167	164
chr1	135026742	135028742	Ppp1r15b	-0.091036	2.78E-29	hypomethylated	-0.0044295	0.29859	insignificant	31	134	134
chr1	135259148	135261148	Etnk2	-0.17304	2.44E-18	hypomethylated	-0.013157	0.0081697	hypomethylated	32	117	114
chr1	135320789	135322789	Sox13	-0.012697	0.050906	insignificant	0.0057901	1	insignificant	6	34	31
chr1	135506857	135508857	Snrpe	-0.17145	0.00003951	hypomethylated	0.071558	0.37088	insignificant	5	24	10
chr1	135557462	135559462	Zbed6	-0.16036	0.0049997	hypomethylated	-0.0047284	0.76668	insignificant	11	34	34
chr1	135557957	135559957	Zbed6	-0.18281	1	insignificant	-0.070598	0.26345	insignificant	2	16	14
chr1	135586593	135588593	Lax1	-0.1731	1	insignificant	0.0063177	0.10377	insignificant	2	19	20
chr1	135586685	135588685	Lax1	-0.1731	1	insignificant	0.0063177	0.10377	insignificant	2	19	20
chr1	135803750	135805750	Optc	0.17027	0.67609	insignificant	0.14673	0.01497	hypermethylated	2	13	9
chr1	135804576	135806576	Optc	0.17027	0.67609	insignificant	0.14673	0.01497	hypermethylated	2	13	9
chr1	135975732	135977732	Btg2	-0.072299	0.000021385	hypomethylated	-0.0050834	0.30826	insignificant	15	46	46
chr1	136185580	136187580	Myog	-0.012521	0.46162	insignificant	0.029358	0.66058	insignificant	7	21	20
chr1	136229505	136231505	Ppfia4		1	noCoverage	-0.083936	0.82511	insignificant	0	10	9
chr1	136258576	136260576	Tmem183a	-0.11923	0.068924	insignificant	-0.014601	0.35661	insignificant	3	14	6
chr1	136301566	136303566	Cyb5r1	-0.27177	6.3E-17	hypomethylated	-0.037946	0.14087	insignificant	4	38	37
chr1	136311043	136313043	Adipor1	-0.096887	1.3E-35	hypomethylated	-0.0036208	0.011134	hypomethylated	47	148	153
chr1	136351131	136353131	Klhl12	-0.18296	4.21E-08	hypomethylated	-0.017801	0.84572	insignificant	8	31	30
chr1	136390236	136392236	Rabif	-0.11134	1.03E-08	hypomethylated	-0.0011454	0.82788	insignificant	24	86	86
chr1	136455754	136457754	Kdm5b	-0.1082	1.72E-22	hypomethylated	0.034267	0.69823	insignificant	27	64	64
chr1	136542257	136544257	Syt2	-0.093496	0.000013899	hypomethylated	0.012365	0.54936	insignificant	18	98	91
chr1	136858153	136860153	Ube2t		1	noCoverage	0.033111	0.84929	insignificant	0	12	8
chr1	137042410	137044410	Arl8a	-0.054038	0.27431	insignificant	0.020699	0.03106	inconclusive	64	166	149
chr1	137155049	137157049	Elf3	0.067521	0.49503	insignificant	0.042491	0.092032	insignificant	6	13	13
chr1	137210314	137212314	Timm17a	-0.069889	0.014359	inconclusive	0.0092929	0.00000132	hypermethylated	9	22	24

chr1	137220389	137222389	Lmod1	-0.14027	0.88313	insignificant	-0.013225	0.25211	insignificant	8	39	37
chr1	137271640	137273640	Shisa4	-0.13583	1	insignificant	0.017528	1	insignificant	3	7	7
chr1	137327068	137329068	Ipo9	-0.043155	0.4751	insignificant	-0.023074	0.26479	insignificant	8	24	24
chr1	137481932	137483932	Nav1	-0.18851	0.0064062	hypomethylated	-0.033727	1	insignificant	6	50	45
chr1	137624773	137626773	Csrp1	-0.17916	3.64E-08	hypomethylated	-0.0007351	0.19852	insignificant	15	37	37
chr1	137661661	137663661	Phlda3	-0.16537	1.71E-21	hypomethylated	0.029617	0.0056766	hypermethylated	38	150	142
chr1	137714174	137716174	Lad1	-0.15134	0.000012341	hypomethylated	-0.0065097	0.56395	insignificant	29	83	84
chr1	137736437	137738437	Tnnt2	-0.03125	0.57906	insignificant	-0.018803	0.20594	insignificant	1	6	6
chr1	137815601	137817601	Pkp1	-0.12293	0.000078917	hypomethylated	0.015174	0.6211	insignificant	12	58	58
chr1	137902919	137904919	Tmem9		1	noCoverage	0.031321	0.89325	insignificant	0	27	20
chr1	137903795	137905795	Tmem9		1	noCoverage	0.0412	0.82988	insignificant	0	32	25
chr1	137913577	137915577	Tmem9		1	noCoverage	0.046351	0.34536	insignificant	0	6	7
chr1	137913732	137915732	Tmem9		1	noCoverage	0.089602	0.10372	insignificant	0	18	19
chr1	138026977	138028977	Kif21b	-0.12487	1.56E-11	hypomethylated	0.00040881	0.14942	insignificant	23	90	92
chr1	138130841	138132841	5730559C18Rik	-0.2336	7.81E-10	hypomethylated	-0.0086176	0.028066	inconclusive	16	37	37
chr1	138157450	138159450	Gpr25		1	noCoverage	-0.077244	0.86512	insignificant	0	2	2
chr1	138242681	138244681	Camsap2	-0.125	0.027313	hypomethylated	0.1245	0.69847	insignificant	4	8	9
chr1	138310847	138312847	Ddx59	-0.086953	1.25E-17	hypomethylated	-0.0022907	0.71474	insignificant	25	70	69
chr1	138312096	138314096	Ddx59	0.016641	0.24691	insignificant	-0.009336	0.25581	insignificant	11	41	41
chr1	138363534	138365534	Kif14	0.010404	0.12886	insignificant	-0.0034356	0.83576	insignificant	7	24	24
chr1	138520477	138522477	Zfp281	-0.13182	3.49E-15	hypomethylated	-0.0099604	0.29478	insignificant	42	119	120
chr1	138850207	138852207	Nr5a2	-0.34167	0.071519	insignificant	-0.19643	0.016968	hypomethylated	3	8	8
chr1	140516273	140518273	Nek7	-0.15359	0.000061895	hypomethylated	-0.0033825	0.43243	insignificant	10	38	38
chr1	140739021	140741021	Lhx9	-0.49855	0.00030539	stronglyHypometh	0.0090374	1	insignificant	1	2	2
chr1	140744156	140746156	Lhx9	-0.21739	0.0017573	hypomethylated	-0.0052556	0.47118	insignificant	12	55	57
chr1	140859285	140861285	Dennd1b	-0.11075	6.33E-22	hypomethylated	-0.00049982	0.18968	insignificant	31	131	143
chr1	141273653	141275653	Crb1	-0.082698	0.098862	insignificant	0.02619	0.85431	insignificant	2	6	6
chr1	141317959	141319959	Zbtb41	-0.10713	1.98E-18	hypomethylated	-0.0064655	0.34459	insignificant	33	110	105
chr1	141350349	141352349	Aspm	-0.20267	0.000000908	hypomethylated	-0.014547	0.34483	insignificant	15	59	59
chr1	141456799	141458799	Cfhr1	0.041667	1	lowCoverage	-0.12112	0.19913	insignificant	1	4	2
chr1	142141833	142143833	Kcnt2	-0.072067	0.000000311	hypomethylated	0.043196	0.0010799	inconclusive	7	39	43
chr1	145549814	145551814	Cdc73	-0.052231	3.49E-10	hypomethylated	-0.0043261	0.29321	insignificant	43	135	136
chr1	145585478	145587478	Glrx2	-0.21768	2.45E-17	hypomethylated	0.0029289	0.24053	insignificant	23	93	90
chr1	145585697	145587697	Glrx2	-0.23451	1.01E-17	hypomethylated	0.014991	0.21429	insignificant	24	95	94
chr1	145585744	145587744	Glrx2	-0.23451	1.01E-17	hypomethylated	0.014991	0.21429	insignificant	24	95	94
chr1	145586576	145588576	Glrx2	-0.16664	1.81E-09	hypomethylated	-0.0053721	0.28455	insignificant	23	89	86
chr1	145623407	145625407	Uchl5	-0.116	2.07E-32	hypomethylated	0.012541	0.14076	insignificant	61	192	175
chr1	145624181	145626181	Trove2	-0.11672	9.42E-15	hypomethylated	0.0077628	0.085941	insignificant	41	128	127
chr1	145851279	145853279	Rgs2	-0.045564	0.68546	insignificant	0.010783	1	insignificant	7	34	34
chr1	148341795	148343795	Fam5c	-0.26755	0.00061174	hypomethylated	-0.018486	0.18276	insignificant	8	20	20
chr1	151808414	151810414	Pla2g4a	-0.60222	0.03363	stronglyHypometh	-0.057574	0.71623	insignificant	1	2	4
chr1	151946253	151948253	Ptgs2	-0.22385	0.0085912	hypomethylated	-0.0028265	0.31518	insignificant	5	28	28
chr1	151946896	151948896	7530420F21Rik	-0.15246	0.21114	insignificant	-0.0017947	0.84668	insignificant	3	14	14
chr1	152238967	152240967	Tpr	-0.12677	1.56E-18	hypomethylated	-0.00059078	0.074518	insignificant	36	126	128
chr1	152240185	152242185	BC003331	-0.13106	1.67E-15	hypomethylated	0.0017358	0.068197	insignificant	29	120	119
chr1	152840565	152842565	Hmcn1	-0.17961	0.0016347	hypomethylated	-0.048621	0.1552	insignificant	5	26	26
chr1	153190627	153192627	Ivns1abp	-0.12819	4.07E-34	hypomethylated	-0.01501	0.61268	insignificant	50	143	155
chr1	153274777	153276777	1190005F20Rik	-0.10926	3.37E-09	hypomethylated	0.0077476	1	insignificant	12	64	71
chr1	153275565	153277565	1200016B10Rik	-0.092669	1	insignificant	-0.0004137	0.018418	hypomethylated	4	24	31
chr1	153347953	153349953	Rnf2	-0.079601	1.9E-17	hypomethylated	0.029762	1	insignificant	18	69	69
chr1	153417502	153419502	Fam129a	-0.24631	6.46E-16	hypomethylated	0.086731	0.014383	inconclusive	17	55	36
chr1	153601503	153603503	Edem3	-0.17636	1.87E-49	hypomethylated	0.0089113	0.2253	insignificant	31	106	103
chr1	153937450	153939450	1700025G04Rik	-0.2871	0.00000186	hypomethylated	-0.030161	0.22643	insignificant	8	24	23
chr1	154233812	154235812	Tsen15	-0.39506	0.028759	stronglyHypometh	0.078112	0.032509	hypermethylated	2	18	19
chr1	154245996	154247996	Glt25d2	-0.14645	5.44E-08	hypomethylated	-0.0091956	0.32483	insignificant	13	53	49
chr1	154472241	154474241	Rgl1	-0.065968	0.00000167	hypomethylated	0.028796	0.16487	insignificant	11	22	23
chr1	154596680	154598680	Apobec4	-0.048973	1	insignificant	-0.065278	0.59914	insignificant	2	12	8
chr1	154612671	154614671	Arpc5	-0.12026	2.21E-68	hypomethylated	-0.011471	0.11654	insignificant	82	215	214
chr1	154654019	154656019	Ncf2	-0.26916	0.17883	insignificant	0.084707	0.41484	insignificant	3	12	12
chr1	154749776	154751776	Smg7	-0.17981	3.46E-30	hypomethylated	-0.0025188	0.94281	insignificant	28	92	85
chr1	154801230	154803230	Nmnat2	-0.1439	2.08E-20	hypomethylated	-0.0093916	0.9604	insignificant	17	59	74
chr1	155033577	155035577	Lamc2		1	noCoverage	-0.17778	1	insignificant	0	2	5
chr1	155179916	155181916	Lamc1	-0.18196	3.45E-23	hypomethylated	0.0095721	0.024356	inconclusive	26	80	80
chr1	155261363	155263363	E330020D12Rik	-0.35058	0.30453	insignificant	0.0097978	0.59788	insignificant	1	23	28
chr1	155271338	155273338	1700012A16Rik	-0.065966	0.00055062	hypomethylated	-0.003692	0.18881	insignificant	10	46	39
chr1	155334790	155336790	Dhx9		1	noCoverage	0.013542	0.0031209	hypermethylated	0	24	24
chr1	155396844	155398844	Npl	-0.30992	0.37785	insignificant	-0.030437	0.52942	insignificant	2	6	6
chr1	155499166	155501166	Rgs8	-0.3778	0.0003076	stronglyHypometh	-0.022968	0.58498	insignificant	7	25	25
chr1	155586482	155588482	Rgs16	-0.13229	6.01E-10	hypomethylated	0.023824	0.47183	insignificant	14	44	44
chr1	155746074	155748074	Glul	-0.15078	1.44E-28	hypomethylated	-0.012006	0.22017	insignificant	49	144	147
chr1	156573050	156575050	Cacna1e	-0.1101	0.38517	insignificant	0.015862	0.50623	insignificant	7	33	33
chr1	156946766	156948766	Ier5	-0.2	0.044462	hypomethylated	-0.011623	0.19218	insignificant	1	4	4
chr1	157004832	157006832	Stx6	-0.17394	2.44E-31	hypomethylated	-0.031808	0.088726	insignificant	45	124	121
chr1	157404249	157406249	Acbd6	-0.096097	1.75E-33	hypomethylated	0.0053348	0.36356	insignificant	48	124	126
chr1	157589157	157591157	Lhx4	-0.11791	1.64E-18	hypomethylated	0.0082148	0.68447	insignificant	30	123	125
chr1	157660029	157662029	Qsox1	-0.16657	0.0011066	hypomethylated	-0.017032	0.95468	insignificant	15	35	40
chr1	157881793	157883793	Tor1aip2	-0.11213	0.000000176	hypomethylated	-0.0088628	0.97111	insignificant	27	120	117
chr1	157881856	157883856	Tor1aip2	-0.11213	0.000000176	hypomethylated	-0.0088628	0.97111	insignificant	27	120	117
chr1	157881947	157883947	Tor1aip1	-0.11213	0.000000176	hypomethylated	-0.0088628	0.97111	insignificant	27	120	117
chr1	157882165	157884165	Tor1aip2	-0.11213	0.000000176	hypomethylated	-0.0088628	0.97111	insignificant	27	120	117
chr1	158135156	158137156	Fam163a	-0.24145	0.029131	hypomethylated	0.025137	1	insignificant	2	4	6
chr1	158233479	158235479	Tdrd5	-0.20333	2.23E-26	hypomethylated	-0.015337	0.0018377	hypomethylated	17	62	62
chr1	158239865	158241865	Nphs2	-0.2817	0.24326	insignificant	0.10349	0.028929	hypermethylated	2	14	14
chr1	158404459	158406459	Soat1	-0.038542	1	insignificant	-0.017563	1	insignificant	3	6	6
chr1	158487917	158489917	Abl2	-0.10158	0.0041708	hypomethylated	0.014376	0.081534	insignificant	10	111	133

chr1	158604470	158606470	Tor3a	-0.30613	1.62E-09	hypomethylated	0.035738	0.011418	inconclusive	8	39	41
chr1	158649041	158651041	Fam20b	-0.14861	0.005272	hypomethylated	-0.012358	0.55101	insignificant	6	24	24
chr1	158869518	158871518	Ralgps2	-0.0866	1.26E-14	hypomethylated	0.037248	0.70791	insignificant	24	60	79
chr1	158869757	158871757	Ralgps2	-0.13047	1.01E-16	hypomethylated	-0.027205	0.154	insignificant	19	34	48
chr1	159341482	159343482	2810025M15Ril	-0.059298	3.04E-27	hypomethylated	-0.004965	0.80319	insignificant	55	153	152
chr1	159342726	159344726	Rasal2	0.78398	6.88E-13	stronglyHypermeth	0.0056472	0.12318	insignificant	3	27	26
chr1	159387712	159389712	BC026585	-0.1609	0.00000029	hypomethylated	0.0013474	1	insignificant	6	39	39
chr1	159455300	159457300	Sec16b	-0.09664	1	insignificant	0.01369	0.6326	insignificant	4	14	14
chr1	160286391	160288391	Fam5b	-0.33054	0.16805	insignificant	0.0064669	0.69579	insignificant	3	7	7
chr1	160291470	160293470	Astn1	-0.20332	0.000061586	hypomethylated	0.0074545	0.46754	insignificant	8	39	39
chr1	161161456	161163456	Rfwd2	-0.10238	7.19E-11	hypomethylated	-0.0092105	0.054567	insignificant	32	115	114
chr1	161973510	161975510	4930523C07Rik	-0.11369	0.28436	insignificant	-0.028519	0.0029969	hypomethylated	11	32	36
chr1	162124390	162126390	Mrps14	-0.15534	3.88E-11	hypomethylated	-0.010534	0.066802	insignificant	8	35	35
chr1	162142908	162144908	Cacybp	-0.085024	4.35E-23	hypomethylated	-0.0047385	0.70344	insignificant	31	54	54
chr1	162723069	162725069	Rabgap1l	0.044949	1	insignificant	-0.062127	0.000067991	hypomethylated	4	24	24
chr1	162835541	162837541	Rc3h1	-0.043281	1	insignificant	-0.008051	0.071405	insignificant	28	129	130
chr1	162964296	162966296	Gas5	-0.12174	3.21E-10	hypomethylated	-0.0074316	0.47047	insignificant	36	118	114
chr1	162964390	162966390	Zbtb37	-0.12174	3.21E-10	hypomethylated	-0.0074316	0.47047	insignificant	36	118	114
chr1	163000022	163002022	Cenpl	-0.23423	3.08E-26	hypomethylated	-0.0092441	0.0042862	hypomethylated	13	48	45
chr1	163000763	163002763	Dars2	-0.22695	8.95E-26	hypomethylated	0.024271	0.0037962	inconclusive	13	45	36
chr1	163061610	163063610	Klhl20		1	noCoverage	-0.0088645	0.84304	insignificant	0	8	8
chr1	163071842	163073842	Ankrd45	-0.29069	0.0007455	hypomethylated	0.049643	0.21314	insignificant	9	26	27
chr1	163324568	163326568	Tnfsf4	-0.37738	1.09E-13	stronglyHypometh	0.075273	0.32141	insignificant	4	8	8
chr1	163423787	163425787	Tnfsf18	-0.0036526	1	insignificant	0.070931	0.50104	insignificant	1	4	4
chr1	163806792	163808792	AI848100	-0.14564	0.000000028	hypomethylated	0.00072252	0.0030189	inconclusive	8	54	54
chr1	163898318	163900318	Pigc	-0.20016	0.000080402	hypomethylated	-0.021892	0.66614	insignificant	18	48	45
chr1	164146753	164148753	Dnm3os	-0.10252	0.13935	insignificant	-0.016016	0.21452	insignificant	2	4	5
chr1	164146944	164148944	Mir199a-2	-0.10252	0.13935	insignificant	-0.016016	0.21452	insignificant	2	4	5
chr1	164152498	164154498	Mir214	-0.49359	0.085664	insignificant	-0.005698	0.51184	insignificant	1	6	6
chr1	164478476	164480476	Mettl13	-0.12353	1.09E-09	hypomethylated	0.0041091	0.4302	insignificant	11	49	45
chr1	164499958	164501958	Vamp4	-0.12519	1.94E-25	hypomethylated	-0.0039574	0.0014324	hypomethylated	29	108	109
chr1	164568280	164570280	Myoc		1	noCoverage	0.026872	1	insignificant	0	5	5
chr1	164670687	164672687	Prrc2c		1	noCoverage	-0.020785	0.37303	insignificant	0	30	30
chr1	164828843	164830843	Fmo2	-0.41547	0.00074759	stronglyHypometh	-0.046451	0.59287	insignificant	1	9	9
chr1	165243781	165245781	Prrx1	-0.14282	0.00000251	hypomethylated	-0.0083421	0.568	insignificant	6	22	22
chr1	165333772	165335772	Gorab	-0.23675	0.00000423	hypomethylated	-0.043106	0.020762	hypomethylated	5	20	20
chr1	165708809	165710809	Kifap3	-0.16779	0.0048572	hypomethylated	-0.040649	0.44234	insignificant	12	35	35
chr1	165859194	165861194	Scyl3	-0.17493	1.07E-44	hypomethylated	0.0035306	0.37199	insignificant	42	101	99
chr1	165924075	165926075	2810422O20Rik	-0.20816	7.36E-08	hypomethylated	0.014746	0.44309	insignificant	14	79	82
chr1	165924912	165926912	BC055324	-0.41778	1.11E-12	stronglyHypometh	0.017528	0.84439	insignificant	8	48	51
chr1	166178185	166180185	Slc19a2	-0.14166	4.9E-20	hypomethylated	-0.0040401	0.94573	insignificant	29	109	109
chr1	166204728	166206728	4930455F23Rik	-0.16415	0.00024237	hypomethylated	-0.00093976	0.65796	insignificant	9	28	32
chr1	166236805	166238805	Nme7	-0.19664	3.71E-09	hypomethylated	-0.0063392	0.80303	insignificant	13	72	72
chr1	166237615	166239615	Nme7	-0.36116	0.052806	insignificant	-0.055459	0.91788	insignificant	6	41	41
chr1	166388486	166390486	Atp1b1	-0.14779	0.000080407	hypomethylated	-0.012198	0.28044	insignificant	15	60	63
chr1	167090904	167092904	Tbx19	0.20249	1	lowCoverage	0.090586	0.28202	insignificant	1	16	10
chr1	167124564	167126564	Sft2d2	-0.099037	0.023329	hypomethylated	-0.0029158	0.88417	insignificant	7	22	22
chr1	167167089	167169089	Tiprl	-0.18801	0.000000789	hypomethylated	-0.0065956	0.60386	insignificant	8	28	30
chr1	167390338	167392338	Brp44	-0.12458	1.01E-29	hypomethylated	-0.0056383	0.51198	insignificant	40	155	155
chr1	167390594	167392594	Dcaf6	-0.12152	4.37E-15	hypomethylated	-0.0055546	1	insignificant	28	124	124
chr1	167564672	167566672	Mpzl1	-0.21946	1.64E-15	hypomethylated	-0.029298	0.000072287	hypomethylated	10	38	38
chr1	167638225	167640225	Rcsd1		1	noCoverage	0.014706	0.63956	insignificant	0	4	4
chr1	167692910	167694910	Creg1	-0.11281	1.04E-10	hypomethylated	-0.0131	0.161	insignificant	21	57	56
chr1	167932765	167934765	Pou2f1	-0.09265	2.54E-13	hypomethylated	-0.0094581	0.91579	insignificant	34	152	163
chr1	168058029	168060029	Gpa33	0.080192	1	insignificant	-0.043741	0.22294	insignificant	2	6	6
chr1	168059590	168061590	Gpa33	-0.18623	0.00008365	hypomethylated	0.078196	0.92504	insignificant	5	16	16
chr1	168168875	168170875	Mael	0.073519	0.000010742	hypermethylated	0.10935	0.000000042	hypermethylated	6	26	26
chr1	168183269	168185269	Ildr2	-0.09012	1.68E-14	hypomethylated	-0.013278	1	insignificant	38	103	102
chr1	168308297	168310297	Tada1	-0.13357	1.01E-14	hypomethylated	0.00068127	0.86101	insignificant	33	90	90
chr1	168339959	168341959	Pogk	-0.15845	0.000003847	hypomethylated	-0.0064505	0.16523	insignificant	7	42	42
chr1	168930547	168932547	Fam78b	-0.10989	5E-27	hypomethylated	0.01339	0.57677	insignificant	53	174	190
chr1	169215258	169217258	Uck2	-0.23079	4.87E-09	hypomethylated	0.015434	0.3941	insignificant	10	41	42
chr1	169237800	169239800	Tmco1	-0.14203	8.7E-14	hypomethylated	0.010875	0.22358	insignificant	25	101	104
chr1	169279121	169281121	Aldh9a1	-0.11295	4.45E-30	hypomethylated	-0.010539	0.00069656	hypomethylated	17	85	82
chr1	169323928	169325928	Mgst3	-0.34267	0.32325	insignificant	-0.10294	0.21792	insignificant	2	19	17
chr1	169527492	169529492	Rxrg	-0.50486	0.0031536	stronglyHypometh	-0.03737	0.12742	insignificant	4	26	25
chr1	169618688	169620688	Lmx1a	-0.1576	8.81E-34	hypomethylated	-0.0011219	0.0038085	hypomethylated	42	167	160
chr1	171461595	171463595	Nuf2	-0.12701	0.36832	insignificant	-0.01787	0.27982	insignificant	2	16	16
chr1	171677773	171679773	Rgs4		1	noCoverage	0.096667	0.84281	insignificant	0	2	3
chr1	171858069	171860069	1700084C01Rik	-0.48291	1	insignificant	-0.062769	0.81843	insignificant	1	9	10
chr1	171899336	171901336	Hsd17b7	0.035373	0.0041518	inconclusive	-0.052017	0.030511	hypomethylated	10	22	22
chr1	172105077	172107077	Uap1	-0.13124	3.41E-13	hypomethylated	0.0013321	0.53638	insignificant	17	80	80
chr1	172162001	172164001	Sh2d1b2	-0.54495	0.088102	insignificant	-0.060338	0.77335	insignificant	2	4	4
chr1	172237991	172239991	1700015E13Rik	-0.05306	0.081779	insignificant	0.041464	0.27178	insignificant	3	18	18
chr1	172519980	172521980	Nos1ap	-0.050382	1.01E-09	hypomethylated	0.004112	0.41309	insignificant	21	48	48
chr1	172573662	172575662	Olfml2b	-0.12944	1.03E-15	hypomethylated	-0.00071451	0.023378	hypomethylated	21	76	67
chr1	172797902	172799902	Atf6	-0.18743	0.00495	hypomethylated	-0.010472	0.066035	insignificant	4	16	16
chr1	172857714	172859714	Fcrla	-0.35	0.0011002	stronglyHypometh	0.0058824	1	insignificant	2	5	5
chr1	172906202	172908202	Fcgr2b	0.26734	1	lowCoverage	-0.010107	0.63689	insignificant	1	10	11
chr1	172948056	172950056	Fcgr4	-0.41684	0.25993	insignificant	0.056568	0.097983	insignificant	2	14	13
chr1	172989534	172991534	Fcgr3		1	noCoverage	-0.20032	0.13161	insignificant	0	6	6
chr1	173079843	173081843	Mpz	-0.18287	9.97E-09	hypomethylated	-0.0042246	0.24705	insignificant	13	51	56
chr1	173080734	173082734	Sdhc	-0.19166	0.000032331	hypomethylated	-0.034274	0.016816	hypomethylated	6	20	25
chr1	173143100	173145100	Nr1i3		1	noCoverage	-0.037684	1	insignificant	0	6	6
chr1	173152645	173154645	Apoa2	-0.32953	0.49545	insignificant	-0.092074	0.039542	hypomethylated	1	18	17

chr1	173154184	173156184	Apoa2	0.061339	0.45546	insignificant	0.080452	0.15122	insignificant	3	6	6
chr1	173164480	173166480	Fcer1g		1	noCoverage	-0.033211	0.07104	insignificant	0	13	13
chr1	173177243	173179243	Ndufs2	-0.11346	0.0062889	hypomethylated	-0.0047012	0.13388	insignificant	11	47	44
chr1	173179552	173181552	Adamts4	0.088153	1	insignificant	-0.08631	0.20069	insignificant	6	20	20
chr1	173199458	173201458	B4galt3	-0.29475	8.68E-10	hypomethylated	-0.02644	0.000066722	hypomethylated	10	78	79
chr1	173211317	173213317	Usp21	-0.19286	0.073504	insignificant	-0.076545	0.4996	insignificant	1	29	26
chr1	173218092	173220092	Ufc1	0.44156	1	insignificant	-0.17004	1	insignificant	1	11	15
chr1	173225113	173227113	Ufc1	-0.61905	0.00058667	stronglyHypometh	-0.070661	0.82809	insignificant	1	2	2
chr1	173258275	173260275	Dedd	-0.13728	3.28E-09	hypomethylated	0.0116	0.88979	insignificant	25	114	118
chr1	173274829	173276829	Pfdn2	-0.17339	0.06135	insignificant	-0.007023	0.13271	insignificant	10	53	53
chr1	173275776	173277776	Nit1	-0.15503	0.00000111	hypomethylated	0.00085119	0.43605	insignificant	9	35	35
chr1	173290929	173292929	Klhdc9	-0.11964	0.62837	insignificant	0.076359	0.034227	hypermethylated	2	14	14
chr1	173340811	173342811	Usf1	-0.13293	9.28E-12	hypomethylated	0.011617	0.2351	insignificant	27	108	83
chr1	173348163	173350163	Tstd1	-0.39365	0.00039666	stronglyHypometh	-0.037685	0.036191	hypomethylated	6	34	34
chr1	173366691	173368691	F11r	-0.16761	0.14844	insignificant	-0.0064416	0.32589	insignificant	10	56	55
chr1	173432608	173434608	Refbp2	-0.16407	7.72E-11	hypomethylated	-0.018111	0.95872	insignificant	14	61	52
chr1	173583168	173585168	Slamf7	-0.20889	0.051568	insignificant	-0.034286	0.14278	insignificant	1	6	6
chr1	173611185	173613185	Cd48	-0.069762	1	insignificant	-0.014345	0.65563	insignificant	1	10	10
chr1	173696262	173698262	Slamf1	-0.52551	1.02E-09	stronglyHypometh	-0.076481	0.42065	insignificant	2	4	4
chr1	173957399	173959399	Vangl2	-0.047131	3.15E-16	hypomethylated	-0.047406	0.038636	hypomethylated	9	52	49
chr1	173987727	173989727	Nhlh1	-0.72398	0.31304	lowCoverage	-0.17737	0.29398	insignificant	1	8	8
chr1	174011659	174013659	Copa	-0.10579	0.18798	insignificant	0.020395	0.29676	insignificant	11	63	64
chr1	174012880	174014880	Copa	-0.22466	0.000075349	hypomethylated	-0.031728	0.041954	hypomethylated	10	61	58
chr1	174055885	174057885	Pex19	-0.21672	0.4802	insignificant	-0.060073	0.01114	inconclusive	5	25	30
chr1	174077145	174079145	Dcaf8	-0.11598	5.72E-13	hypomethylated	-0.020606	0.35859	insignificant	21	94	90
chr1	174241537	174243537	Igsf8	-0.16703	0.0042073	hypomethylated	-0.01923	0.54581	insignificant	7	30	35
chr1	174270340	174272340	Kcnj10	-0.16563	1.42E-16	hypomethylated	-0.0023946	0.21935	insignificant	16	50	45
chr1	174305661	174307661	Pigm	-0.22031	1.99E-43	hypomethylated	-0.024895	0.012818	hypomethylated	34	112	111
chr1	174429376	174431376	Tagln2	-0.14617	0.00000678	hypomethylated	-0.008738	0.53914	insignificant	15	69	69
chr1	174450260	174452260	Ccdc19	-0.26759	0.020589	hypomethylated	0.025148	0.88462	insignificant	3	8	8
chr1	174485068	174487068	Vsig8	-0.37885	0.11995	insignificant	-0.072521	0.83564	insignificant	3	7	10
chr1	174489036	174491036	Vsig8	0.11411	1	lowCoverage	-0.016474	0.029539	hypomethylated	1	6	7
chr1	174520699	174522699	Slamf8	-0.20313	0.000036461	hypomethylated	-0.019615	0.17725	insignificant	6	18	15
chr1	174563105	174565105	Dusp23		1	noCoverage	0.09114	0.75232	insignificant	0	8	8
chr1	175263634	175265634	Darc	-0.64583	0.0016119	stronglyHypometh	0.12321	0.62922	insignificant	2	4	5
chr1	175297826	175299826	Cadm3		1	noCoverage	-0.071021	0.77134	insignificant	0	11	14
chr1	176087852	176089852	Olfr420	-0.65074	0.44366	lowCoverage	-0.12179	1	insignificant	1	4	4
chr1	176359560	176361560	Olfr414	-0.10047	0.19279	insignificant	-0.010733	1	insignificant	4	10	15
chr1	176430955	176432955	Fmn2	-0.10266	0.29448	insignificant	-0.0012253	0.0055708	hypomethylated	8	43	38
chr1	177422676	177424676	Rgs7	-0.26772	0.000011258	hypomethylated	0.048327	0.11657	insignificant	9	32	33
chr1	177555766	177557766	Fh1		1	noCoverage	-0.026443	0.179	insignificant	0	0	0
chr1	177622721	177624721	Opn3	-0.1828	8.11E-10	hypomethylated	-0.020576	0.77205	insignificant	11	22	28
chr1	177627723	177629723	Wdr64	-0.40672	0.0082408	stronglyHypometh	-0.047279	0.11493	insignificant	4	8	8
chr1	177809908	177811908	Exo1	-0.156	1.05E-15	hypomethylated	-0.036174	0.52217	insignificant	24	113	112
chr1	178144073	178146073	Pld5	-0.075	0.021142	hypomethylated	0.0625	0.082098	insignificant	2	4	4
chr1	178205443	178207443	Pld5	-0.12275	7.27E-09	hypomethylated	-0.028974	0.12912	insignificant	14	34	34
chr1	178743942	178745942	Sdccag8	-0.24535	4.27E-20	hypomethylated	-0.012688	0.0084114	hypomethylated	12	42	42
chr1	179726640	179728640	Adss		1	noCoverage	-0.04	0.51541	insignificant	0	15	15
chr1	179920565	179922565	Gm16432		1	noCoverage	0.15968	0.00064264	hypermethylated	0	10	11
chr1	180116547	180118547	Pppde1	-0.10195	0.052221	insignificant	-0.00015876	0.8594	insignificant	4	43	43
chr1	180248283	180250283	Fam36a	-0.10657	8.89E-25	hypomethylated	-0.0066499	0.68209	insignificant	30	144	146
chr1	180267915	180269915	Hnrnpu	-0.11621	4.04E-10	hypomethylated	-0.021048	0.50207	insignificant	17	54	54
chr1	180335011	180337011	Efcab2	-0.12652	4.34E-11	hypomethylated	0.0099937	0.79923	insignificant	33	138	138
chr1	180458255	180460255	Kif26b	-0.10253	1.28E-26	hypomethylated	0.0032837	0.83093	insignificant	37	171	156
chr1	181475659	181477659	Cnst	-0.1058	1.06E-34	hypomethylated	-0.003763	0.0092786	hypomethylated	61	172	171
chr1	181476398	181478398	Cnst	-0.11203	1.24E-25	hypomethylated	-0.0014319	0.091432	insignificant	44	114	112
chr1	181597361	181599361	Sccpdh	-0.1235	4.62E-27	hypomethylated	-0.0079252	0.068148	insignificant	46	139	139
chr1	181890219	181892219	Cdc42bpa	-0.098879	4.65E-10	hypomethylated	-0.0017545	0.12873	insignificant	23	82	82
chr1	182126151	182128151	Adck3	-0.18851	0.00039877	hypomethylated	0.070599	0.00010139	hypermethylated	11	30	28
chr1	182176046	182178046	Psen2	0.086905	1	insignificant	0.034405	0.63167	insignificant	4	10	10
chr1	182259606	182261606	Itpkb	-0.21175	2.13E-10	hypomethylated	-0.021535	0.26494	insignificant	16	56	56
chr1	182260680	182262680	Itpkb	-0.19731	4.4E-23	hypomethylated	-0.014368	0.063771	insignificant	43	133	135
chr1	182413635	182415635	6330403A02Rik	-0.18413	0.32548	insignificant	0.035585	0.17554	insignificant	3	16	14
chr1	182498105	182500105	Parp1	-0.12346	0.1289	insignificant	-0.010531	0.9581	insignificant	14	76	77
chr1	182570464	182572464	Lin9	-0.14001	2.9E-63	hypomethylated	-0.012688	0.00002464	hypomethylated	70	190	202
chr1	182627165	182629165	Mixl1	-0.17167	1.68E-10	hypomethylated	0.011123	0.00066107	hypermethylated	15	38	36
chr1	182655173	182657173	Acbd3	-0.12224	1.63E-30	hypomethylated	-0.0097529	0.035762	hypomethylated	39	98	98
chr1	182743734	182745734	H3f3a	-0.087918	2.16E-08	hypomethylated	-0.015892	0.93071	insignificant	26	90	100
chr1	182780281	182782281	BC031781	-0.11436	6.11E-11	hypomethylated	-0.010165	1	insignificant	17	79	80
chr1	182833404	182835404	Pycr2	-0.21688	2.08E-15	hypomethylated	-0.0187	0.0081829	hypomethylated	10	60	60
chr1	182864169	182866169	Lefty1	0.054504	0.36682	insignificant	0.012218	0.42362	insignificant	3	16	16
chr1	182871648	182873648	Tmem63a	-0.13761	1.62E-15	hypomethylated	0.0066577	0.1512	insignificant	18	63	71
chr1	182980367	182982367	9130409I23Rik	-0.16458	0.013011	hypomethylated	-0.065785	0.62689	insignificant	5	28	32
chr1	183080061	183082061	Cnih4	-0.098772	1.43E-13	hypomethylated	0.01816	0.50718	insignificant	27	88	89
chr1	183142109	183144109	Wdr26	-0.13875	1.38E-17	hypomethylated	-0.0083105	0.47885	insignificant	23	64	64
chr1	183281758	183283758	Cnih3	-0.12311	1.11E-17	hypomethylated	-0.0056568	0.028673	inconclusive	27	109	111
chr1	183441582	183443582	Ccdc121	0.2898	0.55504	insignificant	0.084695	0.0044679	hypermethylated	1	10	10
chr1	183772532	183774532	Lbr	-0.10533	3.14E-22	hypomethylated	-0.0090604	0.11291	insignificant	23	72	72
chr1	183950111	183952111	Enah	-0.060617	0.039012	hypomethylated	0.0098079	0.59571	insignificant	17	118	132
chr1	184053867	184055867	Srp9	-0.15332	1.57E-26	hypomethylated	-0.035534	0.011839	hypomethylated	23	63	62
chr1	184229964	184231964	1700047M11Ril	0.087914	1	insignificant	0.032472	0.4746	insignificant	1	6	7
chr1	184338297	184340297	Trp53bp2	-0.1304	4.12E-30	hypomethylated	-0.00011987	0.16996	insignificant	40	143	133
chr1	184447614	184449614	Capn2	-0.11049	8.45E-08	hypomethylated	-0.0062658	1	insignificant	16	64	64
chr1	184494137	184496137	Capn8	1.11E-16	0.69291	insignificant	-0.0094268	1	insignificant	2	4	4
chr1	184694036	184696036	Susd4	-0.14951	3.18E-12	hypomethylated	-0.001199	0.000000723	hypomethylated	32	122	128

chr1	184883918	184885918	Tlr5	-0.19792	0.1301	insignificant	-0.030809	0.63165	insignificant	1	8	9
chr1	185857339	185859339	Dusp10	-0.14456	1.38E-18	hypomethylated	0.016932	0.8987	insignificant	41	114	116
chr1	185857877	185859877	1700056E22Rik	-0.19225	9.42E-08	hypomethylated	0.021539	0.59696	insignificant	9	33	33
chr1	186556372	186558372	Hlx	-0.20675	1.34E-30	hypomethylated	-0.002466	0.1933	insignificant	14	61	62
chr1	186635192	186637192	Mosc1	-0.28004	0.058872	insignificant	0.027963	0.13791	insignificant	3	12	12
chr1	186669726	186671726	Mosc2		1	noCoverage	0.039394	0.53777	insignificant	0	10	10
chr1	186706915	186708915	C130074G19Rik	-0.17433	0.000000161	hypomethylated	0.031545	0.1487	insignificant	9	18	18
chr1	186823428	186825428	Mark1	-0.083944	2.16E-28	hypomethylated	-0.0023572	0.49658	insignificant	69	204	204
chr1	187027046	187029046	Rab3gap2	-0.090899	0.000020153	hypomethylated	0.0088648	0.79774	insignificant	11	42	36
chr1	187065804	187067804	Snora36b	0.095833	1	insignificant	0.073106	0.083199	insignificant	1	4	4
chr1	187065853	187067853	Mir664	0.095833	1	insignificant	0.073106	0.083199	insignificant	1	4	4
chr1	187136197	187138197	Mir194-1	0.087179	1	insignificant	-0.036305	0.26141	insignificant	3	13	14
chr1	187136459	187138459	Mir215	0.087179	1	insignificant	-0.036305	0.26141	insignificant	3	13	14
chr1	187155037	187157037	Bpnt1	-0.17311	0.00007745	hypomethylated	-0.0095202	0.59719	insignificant	8	29	29
chr1	187185973	187187973	Eprs	-0.082066	1.38E-10	hypomethylated	0.0024245	0.72412	insignificant	38	161	162
chr1	187277726	187279726	Slc30a10	-0.099509	4.41E-08	hypomethylated	-0.0013888	0.21966	insignificant	28	145	129
chr1	187320184	187322184	5033404E19Rik		1	noCoverage	-0.13976	0.64552	insignificant	0	10	13
chr1	188529871	188531871	Tgfb2	-0.21697	0.000010594	hypomethylated	0.065717	0.41507	insignificant	2	4	4
chr1	188573237	188575237	Rrp15	-0.2512	0.000000748	hypomethylated	0.0035676	0.12805	insignificant	7	35	36
chr1	188790294	188792294	D1Pas1	-0.3048	1.58E-09	hypomethylated	-0.0044617	1	insignificant	13	62	64
chr1	189038389	189040389	Gpatch2	-0.16992	1.13E-17	hypomethylated	-0.00099872	0.79334	insignificant	21	54	60
chr1	189039325	189041325	Gpatch2	-0.15293	6.88E-17	hypomethylated	0.000022843	0.78615	insignificant	20	40	46
chr1	189820737	189822737	Esrrg	-0.17845	0.0045926	hypomethylated	0.0064461	0.59939	insignificant	5	48	53
chr1	190085716	190087716	Ush2a	-0.094909	1	insignificant	-0.016302	0.076742	insignificant	5	29	30
chr1	190831719	190833719	Kctd3	-0.13546	0.0067802	hypomethylated	-0.048876	0.121	insignificant	6	25	25
chr1	191167232	191169232	Kcnk2	-0.13985	2.88E-15	hypomethylated	0.025294	0.88238	insignificant	14	107	107
chr1	191167583	191169583	Kcnk2	-0.092606	0.00000791	hypomethylated	0.070853	0.63864	insignificant	6	57	57
chr1	191511965	191513965	Cenpf	-0.13285	1.03E-08	hypomethylated	0.01071	0.53448	insignificant	12	30	30
chr1	191551146	191553146	Ptpn14	-0.14855	1.4E-46	hypomethylated	-0.0086313	0.0096185	hypomethylated	51	176	176
chr1	191746167	191748167	Smyd2	-0.00035694	0.33861	insignificant	0.010773	0.77538	insignificant	22	70	70
chr1	191994559	191996559	Prox1	-0.15775	0.000000501	hypomethylated	0.020138	0.44915	insignificant	16	61	49
chr1	192735649	192737649	Rps6kc1	-0.14539	0.014666	hypomethylated	0.0057663	0.053889	insignificant	5	24	24
chr1	192751383	192753383	Angel2	-0.12748	4.22E-27	hypomethylated	-0.0031483	0.021007	hypomethylated	33	102	108
chr1	192802877	192804877	Vash2	-0.13468	4.97E-16	hypomethylated	-0.0053787	0.4626	insignificant	30	87	87
chr1	192803175	192805175	Vash2	-0.1917	0.00000955	hypomethylated	0.0070001	0.050178	insignificant	10	27	26
chr1	192848229	192850229	A230020J21Rik	-0.11674	2.01E-42	hypomethylated	0.0023115	0.82304	insignificant	34	118	129
chr1	192850069	192852069	A230020J21Rik	-0.36554	2.19E-20	stronglyHypometh	-0.04855	0.022856	hypomethylated	4	10	10
chr1	192859700	192861700	1700022P22Rik	-0.18969	0.0068861	hypomethylated	-0.097917	0.003343	hypomethylated	1	8	8
chr1	192885899	192887899	Nsl1	-0.043545	0.024421	hypomethylated	-0.0033269	0.042459	hypomethylated	18	90	92
chr1	192886811	192888811	Nsl1	-0.012763	0.5373	insignificant	-0.0094755	0.13873	insignificant	17	62	62
chr1	192921292	192923292	Batf3	-0.071914	0.18922	insignificant	-0.0074536	0.18708	insignificant	17	92	88
chr1	192988696	192990696	Fam71a	0.18783	1	insignificant	-0.073257	0.82013	insignificant	1	6	6
chr1	193007212	193009212	Atf3	-0.19629	1.94E-32	hypomethylated	-0.021559	0.79708	insignificant	27	96	103
chr1	193148843	193150843	Tmem206	-0.15374	6.83E-15	hypomethylated	0.000049865	0.81872	insignificant	19	78	77
chr1	193220920	193222920	Ppp2r5a	-0.11475	1.44E-09	hypomethylated	-0.0012759	0.25416	insignificant	30	79	79
chr1	193398612	193400612	Ints7	-0.14633	1.47E-30	hypomethylated	-0.013016	0.067927	insignificant	20	50	50
chr1	193399413	193401413	Dtl	-0.074855	0.00000111	hypomethylated	-0.015801	0.01186	hypomethylated	8	18	18
chr1	193540902	193542902	Lpgat1	-0.10164	1.03E-24	hypomethylated	0.0022173	0.81997	insignificant	69	181	177
chr1	193541273	193543273	Lpgat1	-0.10517	9.73E-35	hypomethylated	0.0028699	0.13484	insignificant	78	211	207
chr1	193644351	193646351	Nek2	-0.09502	7.81E-15	hypomethylated	-0.013707	0.78614	insignificant	32	102	102
chr1	193729659	193731659	Slc30a1	-0.079029	3.07E-24	hypomethylated	-0.0052209	0.95143	insignificant	46	224	214
chr1	193731406	193733406	Slc30a1	-0.1341	0.10166	insignificant	0.18251	3.73E-16	hypermethylated	2	40	17
chr1	193916445	193918445	Traf5	-0.11932	0.038305	hypomethylated	0.17424	0.06265	insignificant	4	8	10
chr1	193959779	193961779	Gm10516	-0.11921	1.96E-22	hypomethylated	0.00043855	0.12827	insignificant	29	116	121
chr1	193961669	193963669	Rcor3	-0.10113	1.08E-24	hypomethylated	0.011861	0.28424	insignificant	49	117	113
chr1	194013753	194015753	Kcnh1	-0.16291	1.58E-33	hypomethylated	-0.013936	0.00057635	hypomethylated	33	111	104
chr1	194597413	194599413	Hhat	-0.38984	0.002717	stronglyHypometh	-0.094188	0.020532	hypomethylated	1	8	8
chr1	194681946	194683946	Sertad4		1	noCoverage	-0.00096604	0.8773	insignificant	0	18	18
chr1	194861892	194863892	Syt14	-0.1936	0.0026341	hypomethylated	0.020967	0.068153	insignificant	20	52	58
chr1	194956445	194958445	Diexf	0.32324	0.48806	insignificant	0.009055	0.0090643	hypermethylated	4	26	26
chr1	194978305	194980305	Irf6	-0.093024	0.000092437	hypomethylated	0.0049952	0.20626	insignificant	20	77	76
chr1	195127187	195129187	Lamb3	-0.08125	0.5299	insignificant	0.0043727	0.83653	insignificant	1	6	6
chr1	196445022	196447022	Plxna2	-0.12633	8.3E-19	hypomethylated	-0.00060706	0.79741	insignificant	38	151	149
chr1	196764014	196766014	Cd34	-0.065683	0.21952	insignificant	-0.0031771	0.36065	insignificant	11	32	37
chr1	196957764	196959764	Cr1l	-0.25	0.20388	insignificant	0.02	0.85476	insignificant	2	4	4
chr10	3133303	3135303	Cnksr3	-0.11429	1.29E-13	hypomethylated	-0.0089422	0.00025463	hypomethylated	28	141	149
chr10	3365075	3367075	Ipcef1	-0.20511	0.049773	hypomethylated	0.042524	0.82407	insignificant	2	28	27
chr10	4423140	4425140	Rgs17	-0.13814	0.013186	hypomethylated	0.015388	0.054582	insignificant	20	45	57
chr10	4521597	4523597	Mtrf1l	-0.099601	0.00043317	hypomethylated	0.011508	0.87205	insignificant	13	67	67
chr10	4540075	4542075	Fbxo5	-0.17282	7.12E-15	hypomethylated	-0.017863	0.43787	insignificant	26	97	107
chr10	4794848	4796848	Syne1	-0.39833	3.89E-21	stronglyHypometh	0.066429	0.02212	inconclusive	10	31	31
chr10	5150833	5152833	Syne1	-0.19164	1.48E-09	hypomethylated	-0.039977	0.20734	insignificant	7	26	26
chr10	5913188	5915188	Rmnd1	-0.11662	0.000027915	hypomethylated	0.0024485	0.063133	insignificant	22	100	101
chr10	5913936	5915936	1700052N19Rik	-0.11711	0.000094246	hypomethylated	0.0036609	0.072926	insignificant	17	62	62
chr10	5957432	5959432	Zbtb2	-0.12304	3.37E-36	hypomethylated	-0.018024	0.069234	insignificant	43	132	142
chr10	6080212	6082212	Akap12	0.30298	1	insignificant	-0.082127	0.29392	insignificant	1	8	8
chr10	7308597	7310597	Lrp11	-0.14003	6.56E-70	hypomethylated	0.001958	0.063173	insignificant	54	138	130
chr10	7382168	7384168	A630066F11Rik	-0.10516	8.08E-51	hypomethylated	-0.0024627	0.24623	insignificant	45	126	129
chr10	7383382	7385382	Pcmt1	-0.22172	0.000000895	hypomethylated	0.050129	0.2796	insignificant	8	20	22
chr10	7386301	7388301	Nup43	-0.52277	0.00031642	stronglyHypometh	-0.37925	1.29E-08	stronglyhypometh	2	12	22
chr10	7400006	7402006	Lats1	-0.09239	4.59E-16	hypomethylated	0.0070033	0.043275	inconclusive	55	145	153
chr10	7400956	7402956	BC020402	-0.10157	4.31E-08	hypomethylated	0.0088283	0.51721	insignificant	42	99	107
chr10	7444797	7446797	Katna1	-0.11792	0.00012271	hypomethylated	-0.073058	0.08304	insignificant	9	28	26
chr10	7500715	7502715	BC013529	-0.53921	3.26E-25	stronglyHypometh	-0.015514	0.00026818	hypomethylated	8	60	58
chr10	7511691	7513691	Ppil4	-0.145	1.51E-12	hypomethylated	-0.004476	0.36365	insignificant	20	76	76

chr10	7675921	7677921	Tab2	-0.099288	1.84E-15	hypomethylated	-0.00089522	0.46158	insignificant	35	140	142
chr10	8238623	8240623	Ust	-0.13333	1	insignificant	-0.022976	0.70405	insignificant	5	9	10
chr10	8605868	8607868	Sash1	-0.17056	3.07E-13	hypomethylated	0.013781	0.51185	insignificant	21	68	70
chr10	9395006	9397006	Samd5	-0.14697	0.00000105	hypomethylated	-0.012925	0.52706	insignificant	17	76	76
chr10	9620838	9622838	Stxbp5	-0.16393	1.78E-12	hypomethylated	-0.038039	0.61429	insignificant	18	53	53
chr10	10278005	10280005	Rab32	-0.30114	0.0019275	hypomethylated	-0.080242	0.13498	insignificant	2	4	4
chr10	10868227	10870227	Shprh	-0.11464	2.95E-13	hypomethylated	-0.00011448	0.77268	insignificant	24	82	87
chr10	11000127	11002127	Fbxo30	-0.1342	3.81E-24	hypomethylated	-0.014625	0.25388	insignificant	43	97	111
chr10	11000384	11002384	Fbxo30	-0.1342	3.81E-24	hypomethylated	-0.014625	0.25388	insignificant	43	97	111
chr10	11062242	11064242	Epm2a	-0.089382	9.48E-23	hypomethylated	-0.0015937	0.14827	insignificant	49	138	134
chr10	12684065	12686065	Stx11	-0.06751	1	insignificant	0.056584	0.06128	insignificant	9	39	40
chr10	12727255	12729255	Sf3b5	-0.12022	7.83E-08	hypomethylated	-0.03056	0.11139	insignificant	17	96	94
chr10	12809593	12811593	Plagl1	0.20338	5.26E-12	hypermethylated	0.13021	0.000000086	hypermethylated	25	77	80
chr10	12897829	12899829	Ltv1	0.21875	0.31061	insignificant	-0.01875	1	insignificant	3	8	5
chr10	12912943	12914943	Ltv1	-0.18486	0.074164	insignificant	-0.00051637	1	insignificant	4	9	9
chr10	13043966	13045966	Phactr2	-0.12996	1.74E-15	hypomethylated	-0.0088113	0.51834	insignificant	20	40	40
chr10	13194202	13196202	Phactr2	-0.12798	1	insignificant	-0.014709	0.73495	insignificant	2	14	22
chr10	13219833	13221833	Fuca2	-0.27135	3.87E-10	hypomethylated	0.010533	0.15266	insignificant	3	29	30
chr10	13271712	13273712	Adat2	-0.13922	9.15E-08	hypomethylated	-0.02569	0.48937	insignificant	11	66	66
chr10	13272948	13274948	Pex3	-0.18899	7.12E-09	hypomethylated	-0.054895	0.017697	hypomethylated	6	44	43
chr10	13588636	13590636	Aig1	-0.18959	8.98E-24	hypomethylated	-0.021976	0.005745	hypomethylated	29	82	87
chr10	13685184	13687184	Hivep2	-0.12659	1.55E-31	hypomethylated	-0.010351	0.41489	insignificant	50	161	150
chr10	14264842	14266842	Gpr126	-0.023997	7.29E-16	hypomethylated	0.0044879	6.19E-16	inconclusive	27	116	107
chr10	17442033	17444033	Cited2	-0.15956	2.85E-41	hypomethylated	-0.01295	0.86602	insignificant	38	132	144
chr10	17667873	17669873	Heca		1	noCoverage	0.0072994	0.32893	insignificant	0	14	11
chr10	17743058	17745058	3110003A17Rik	-0.7	0.2439	insignificant	-0.048611	0.14771	insignificant	2	10	10
chr10	17774745	17776745	Reps1	-0.1211	1.29E-53	hypomethylated	0.0022598	0.93394	insignificant	67	213	202
chr10	17930696	17932696	Ect2l	-0.22042	0.0093697	hypomethylated	0.0052243	0.70957	insignificant	2	14	14
chr10	17954787	17956787	Ccdc28a	-0.41561	4.58E-09	stronglyHypometh	-0.014469	0.94574	insignificant	4	34	40
chr10	18126480	18128480	Nhsl1	-0.28869	0.55599	insignificant	0.08631	1	insignificant	1	8	8
chr10	18265882	18267882	Hebp2	-0.25494	0.32844	insignificant	-0.033904	0.58814	insignificant	2	6	6
chr10	18463564	18465564	D10Bwg1379e	-0.21102	0.00058892	hypomethylated	0.0010269	0.088797	insignificant	8	34	34
chr10	18506599	18508599	Gm4922		1	noCoverage	0.031122	0.54689	insignificant	0	14	14
chr10	18563876	18565876	Perp	-0.071935	1.67E-15	hypomethylated	-0.0089452	0.0023294	hypomethylated	19	86	86
chr10	18735216	18737216	Tnfaip3	-0.0625	0.21355	insignificant	-0.025253	0.011969	hypomethylated	8	18	18
chr10	19075344	19077344	Olig3	-0.18373	0.00000233	hypomethylated	0.0062979	0.16512	insignificant	7	32	30
chr10	19310763	19312763	Ifngr1	-0.26121	0.00022743	hypomethylated	-0.0053333	0.51533	insignificant	5	11	11
chr10	19431392	19433392	Il20ra	-0.25951	7.68E-11	hypomethylated	0.015562	0.72583	insignificant	16	46	48
chr10	19571265	19573265	Slc35d3	-0.13779	0.38402	insignificant	0.0076781	0.74579	insignificant	4	18	18
chr10	19653331	19655331	Map3k5	-0.10627	1.3E-45	hypomethylated	0.0096773	0.0016954	inconclusive	78	234	245
chr10	19867725	19869725	Mtap7	-0.10271	9.07E-28	hypomethylated	-0.005822	0.23894	insignificant	74	269	270
chr10	20031274	20033274	Bclaf1	-0.12331	1.67E-24	hypomethylated	-0.0015087	0.69681	insignificant	41	136	136
chr10	20066624	20068624	Fam54a	-0.15217	2.52E-08	hypomethylated	0.054328	0.23947	insignificant	10	21	34
chr10	20444874	20446874	Pde7b	-0.20963	0.0010993	hypomethylated	0.046044	0.11288	insignificant	2	14	14
chr10	20671352	20673352	Ahi1	-0.099451	9.8E-27	hypomethylated	0.010717	0.020861	inconclusive	34	87	86
chr10	20880790	20882790	Myb	-0.18272	6.51E-27	hypomethylated	-0.015299	0.31046	insignificant	24	68	66
chr10	21014784	21016784	Hbs1l	-0.14624	2.02E-18	hypomethylated	-0.019928	0.14321	insignificant	18	91	98
chr10	21096105	21098105	Aldh8a1	0.083022	0.39221	insignificant	-0.081799	0.028742	hypomethylated	4	20	18
chr10	21311950	21313950	1700020N01Rik	-0.063528	0.67499	insignificant	-0.04391	0.195	insignificant	2	8	8
chr10	21600989	21602989	Sgk1	-0.25609	0.000000141	hypomethylated	-0.038051	0.62823	insignificant	13	48	48
chr10	21697477	21699477	Sgk1	0.053704	0.69374	insignificant	-0.099004	0.020508	hypomethylated	3	12	12
chr10	21711028	21713028	Sgk1	-0.18901	0.000016163	hypomethylated	-0.03504	0.70561	insignificant	10	44	44
chr10	21712695	21714695	Sgk1	-0.066374	1.4E-19	hypomethylated	0.00062059	0.23047	insignificant	39	138	140
chr10	21713471	21715471	Sgk1	-0.043817	1.7E-14	hypomethylated	0.0027514	0.9124	insignificant	34	116	116
chr10	21877414	21879414	Raet1a	-0.20804	1.36E-24	hypomethylated	-0.042592	0.31571	insignificant	19	62	81
chr10	21992279	21994279	H60b	-0.13403	1.86E-16	hypomethylated	-0.0082585	0.42974	insignificant	17	100	92
chr10	22025525	22027525	C920009B18Rik	-0.2364	0.093734	insignificant	-0.027313	0.65158	insignificant	1	4	2
chr10	22363816	22365816	Slc2a12	-0.13739	0.0061574	hypomethylated	-0.0146	0.013065	hypomethylated	10	54	58
chr10	22451253	22453253	Tbpl1	-0.096628	1.42E-22	hypomethylated	-0.0020608	0.094794	insignificant	24	82	82
chr10	22539934	22541934	Tcf21	-0.22917	0.092382	insignificant	0.12887	0.49554	insignificant	1	10	7
chr10	23069709	23071709	Eya4	-0.15904	1.14E-21	hypomethylated	0.0022004	0.79317	insignificant	32	124	109
chr10	23507015	23509015	Rps12	-0.25833	0.00025161	hypomethylated	-0.0081129	0.56308	insignificant	3	12	18
chr10	23515791	23517791	1110021L09Rik	-0.18535	1.06E-10	hypomethylated	0.01029	0.052732	insignificant	12	38	34
chr10	23668363	23670363	Taar3	-0.089477	0.025451	hypomethylated	-0.034739	0.023809	hypomethylated	2	14	10
chr10	23679299	23681299	Taar4	-0.17627	0.13021	insignificant	-0.064698	0.11409	insignificant	3	16	16
chr10	23718744	23720744	Taar7b		1	noCoverage	0.059158	0.77078	insignificant	0	4	4
chr10	23756419	23758419	Taar7e		1	noCoverage	0.0036553	0.0072667	inconclusive	0	6	6
chr10	23821757	23823757	Taar8c	-0.31593	0.068119	insignificant	-0.024267	0.80254	insignificant	1	4	4
chr10	23868122	23870122	Stx7	-0.16667	0.22734	insignificant	0.075758	0.35003	insignificant	3	21	22
chr10	23942322	23944322	Moxd1	-0.058151	0.00000301	hypomethylated	0.010301	0.7379	insignificant	16	54	54
chr10	24314247	24316247	Ctgf	-0.15266	0.000000131	hypomethylated	-0.0043618	0.5837	insignificant	20	61	62
chr10	24431908	24433908	Enpp1	-0.33915	0.55352	insignificant	-0.090157	0.66743	insignificant	1	15	13
chr10	24588791	24590791	Med23	-0.17508	7.65E-12	hypomethylated	0.0051555	0.61061	insignificant	17	64	69
chr10	24647276	24649276	Arg1	-0.23448	0.18949	insignificant	-0.18267	1.04E-09	hypomethylated	3	24	24
chr10	25018969	25020969	Akap7	-0.033472	0.67016	insignificant	-0.062275	0.018689	hypomethylated	2	13	13
chr10	25078603	25080603	Epb4.1l2	-0.16216	4.71E-30	hypomethylated	-0.033048	0.00066318	hypomethylated	28	92	87
chr10	25797060	25799060	Gm9767	-0.15105	1.16E-26	hypomethylated	0.013012	0.70779	insignificant	30	131	131
chr10	26094991	26096991	L3mbtl3	-0.057293	0.024054	hypomethylated	0.022686	0.16358	insignificant	13	33	42
chr10	26491317	26493317	Arhgap18	-0.28326	0.027381	hypomethylated	-0.019977	0.867	insignificant	4	25	22
chr10	27793625	27795625	Ptprk	-0.11322	6.93E-10	hypomethylated	-0.0035858	0.61931	insignificant	29	108	116
chr10	28862801	28864801	6330407J23Rik	-0.21087	1.53E-37	hypomethylated	0.031853	0.7681	insignificant	12	38	38
chr10	29031971	29033971	Echdc1	-0.17963	3.88E-11	hypomethylated	-0.030283	0.89909	insignificant	12	26	28
chr10	29032305	29034305	Echdc1	-0.17963	3.88E-11	hypomethylated	-0.030283	0.89909	insignificant	12	26	28
chr10	29255673	29257673	Rspo3	-0.18032	1	lowCoverage	-0.01633	0.68939	insignificant	1	32	32
chr10	29920346	29922346	Cenpw	-0.18507	2.12E-09	hypomethylated	-0.01985	0.020614	hypomethylated	8	24	21

chr10	30338172	30340172	Hint3	0.2869	0.51097	insignificant	-0.022196	0.6812	insignificant	1	30	30
chr10	30522913	30524913	Ncoa7	-0.11076	4.56E-18	hypomethylated	-0.0036321	0.26571	insignificant	23	60	60
chr10	30562589	30564589	Hey2	-0.2241	7.72E-13	hypomethylated	-0.019408	0.0073758	inconclusive	10	68	62
chr10	31032210	31034210	Hddc2	-0.27729	1.53E-17	hypomethylated	-0.090786	0.16362	insignificant	17	47	43
chr10	31165727	31167727	Tpd52l1	-0.098485	1	insignificant	-0.0035677	0.89492	insignificant	5	12	12
chr10	31329531	31331531	Rnf217	-0.30556	0.34784	insignificant	0.0079477	0.91354	insignificant	2	8	8
chr10	32609721	32611721	Nkain2	-0.075773	0.050956	insignificant	0.0082857	1	insignificant	5	56	70
chr10	33344406	33346406	Clvs2	-0.87619	0.42188	lowCoverage	-0.31667	0.48662	insignificant	1	6	4
chr10	33501921	33503921	Gm4794		1	noCoverage	-0.15331	0.13735	insignificant	0	11	7
chr10	33739422	33741422	Rwdd1	-0.18299	1.45E-19	hypomethylated	-0.026484	0.0021813	hypomethylated	18	68	65
chr10	33816325	33818325	Bet3l		1	noCoverage	-0.10027	0.30109	insignificant	0	6	6
chr10	33927357	33929357	Dse	-0.6453	0.00063051	stronglyHypometh	-0.064417	0.039583	hypomethylated	2	6	6
chr10	34000995	34002995	Tspyl1	-0.029814	8.22E-08	hypomethylated	0.00037574	0.30915	insignificant	11	92	92
chr10	34016226	34018226	Tspyl4	-0.30013	0.00001257	hypomethylated	0.012848	0.96176	insignificant	7	45	48
chr10	34202205	34204205	Frk	-0.21281	0.03333	hypomethylated	-0.035916	0.51434	insignificant	9	22	24
chr10	36225612	36227612	Hs3st5	-0.13698	1.02E-32	hypomethylated	0.050974	0.28012	insignificant	27	70	70
chr10	36693349	36695349	Hdac2	-0.082621	2.09E-27	hypomethylated	0.015182	0.36255	insignificant	57	169	190
chr10	36858732	36860732	Marcks	-0.19074	0.00000139	hypomethylated	-0.011027	0.19636	insignificant	6	40	56
chr10	38684320	38686320	Lama4	-0.23694	0.00000663	hypomethylated	0.0072802	0.91431	insignificant	5	10	10
chr10	38852828	38854828	Tube1	-0.21168	1.01E-10	hypomethylated	0.033444	0.19341	insignificant	11	65	70
chr10	38853701	38855701	1700025K23Rik	-0.11626	0.00000616	hypomethylated	-0.004156	0.36244	insignificant	9	53	53
chr10	39088604	39090604	Fyn	-0.099739	1.22E-26	hypomethylated	-0.0087503	0.65656	insignificant	53	144	143
chr10	39331739	39333739	Traf3ip2		1	noCoverage	-0.073329	0.68457	insignificant	0	16	21
chr10	39450965	39452965	Rev3l	-0.073346	2.17E-27	hypomethylated	0.0028111	0.72804	insignificant	69	263	243
chr10	39619044	39621044	AA474331	-0.33803	1.17E-12	stronglyHypometh	0.002861	0.55218	insignificant	6	18	18
chr10	39646729	39648729	2010001E11Rik	-0.33228	0.0051308	hypomethylated	0.04076	0.18427	insignificant	2	29	30
chr10	39679959	39681959	G630090E17Rik	-0.31949	0.88802	insignificant	0.011576	0.00011386	hypermethylated	7	35	35
chr10	39706452	39708452	BC021785		1	noCoverage	-0.067143	0.38516	insignificant	0	10	10
chr10	39745074	39747074	AI317395	-0.17036	0.086115	insignificant	-0.045216	0.70696	insignificant	3	17	20
chr10	39966785	39968785	Rpf2	-0.13729	0.00081952	hypomethylated	-0.011837	0.38607	insignificant	11	26	28
chr10	39966845	39968845	Rpf2	-0.12768	0.0090275	hypomethylated	-0.017574	0.93333	insignificant	7	18	20
chr10	39977471	39979471	Gtf3c6	-0.25101	0.000000338	hypomethylated	-0.010319	0.24413	insignificant	9	35	40
chr10	40021992	40023992	Amd2	-0.10532	0.001463	hypomethylated	-0.028498	0.53203	insignificant	12	45	48
chr10	40068113	40070113	Cdk19	-0.1604	1.52E-11	hypomethylated	0.000034687	0.76598	insignificant	11	70	70
chr10	40348816	40350816	Ddo	-0.13611	0.2651	insignificant	0.15873	0.26159	insignificant	1	8	8
chr10	40402087	40404087	9030224M15Ril	-0.17443	1.64E-29	hypomethylated	-0.017282	0.00085802	hypomethylated	29	73	76
chr10	40602339	40604339	Wasf1	-0.093981	1.03E-29	hypomethylated	0.0014477	0.84169	insignificant	73	200	200
chr10	40602949	40604949	Cdc40	-0.093981	1.03E-29	hypomethylated	0.0014477	0.84169	insignificant	73	200	200
chr10	40791390	40793390	Gpr6	0.022781	0.60567	insignificant	-0.012085	0.23099	insignificant	8	22	22
chr10	41023047	41025047	Fig4	-0.39116	0.000052071	stronglyHypometh	-0.033006	0.12102	insignificant	3	10	10
chr10	41169201	41171201	Zbtb24	-0.22245	2.78E-43	hypomethylated	-0.02036	0.00010753	hypomethylated	20	68	74
chr10	41195119	41197119	Mical1	-0.1174	2.96E-08	hypomethylated	0.018803	0.3535	insignificant	18	59	59
chr10	41209244	41211244	Ppil6	-0.14785	0.00016643	hypomethylated	0.02115	0.87116	insignificant	10	27	28
chr10	41210146	41212146	Ppil6	-0.11842	0.14159	insignificant	0.0071446	0.65901	insignificant	4	12	12
chr10	41238305	41240305	Cd164	-0.1211	1.26E-11	hypomethylated	-0.012706	0.44912	insignificant	28	119	113
chr10	41307559	41309559	Ccdc162	0.075658	1	insignificant	-0.13031	0.072859	insignificant	2	4	4
chr10	41529379	41531379	Sesn1	-0.18272	4.47E-19	hypomethylated	-0.019804	0.034911	hypomethylated	25	90	90
chr10	41529436	41531436	Sesn1	-0.18272	4.47E-19	hypomethylated	-0.019804	0.034911	hypomethylated	25	90	90
chr10	41606244	41608244	Sesn1	-0.17251	1.55E-08	hypomethylated	-0.0081342	0.52317	insignificant	25	105	111
chr10	41738188	41740188	Armc2		1	noCoverage	0.0059354	1	insignificant	0	6	6
chr10	41996548	41998548	Foxo3	-0.097549	2.87E-36	hypomethylated	0.0082569	0.26578	insignificant	60	215	214
chr10	42220859	42222859	Snx3	-0.12932	1.42E-19	hypomethylated	0.0096224	0.86705	insignificant	38	155	159
chr10	42303394	42305394	Nr2e1	-0.17769	1.56E-11	hypomethylated	0.038634	0.24068	insignificant	11	68	69
chr10	42397722	42399722	Ostm1	-0.14894	3.98E-26	hypomethylated	-0.0014552	0.0098516	hypomethylated	19	59	58
chr10	42480301	42482301	Sec63	-0.09877	2.18E-12	hypomethylated	0.011048	0.39916	insignificant	24	110	97
chr10	42579317	42581317	Scml4	-0.44936	3.48E-09	stronglyHypometh	0.054767	0.22437	insignificant	3	10	10
chr10	42893509	42895509	9030612E09Rik	-0.12058	9.66E-26	hypomethylated	-0.0012551	0.001567	hypomethylated	50	171	184
chr10	42894336	42896336	Sobp	-0.14942	9.95E-19	hypomethylated	-0.0051945	0.022019	hypomethylated	16	63	78
chr10	42940291	42942291	Pdss2	-0.15509	2.11E-21	hypomethylated	0.0016255	0.010855	inconclusive	23	93	93
chr10	43197945	43199945	Bend3	-0.10054	7.01E-20	hypomethylated	0.0035075	0.78532	insignificant	64	233	227
chr10	43260800	43262800	1700021F05Rik		1	noCoverage	0.1369	0.062363	insignificant	0	5	4
chr10	43297974	43299974	Cd24a	-0.1334	1.83E-21	hypomethylated	-0.0041972	0.080493	insignificant	38	162	150
chr10	43312232	43314232	F830002L21Rik	-0.13044	0.57788	insignificant	-0.050928	0.7275	insignificant	2	17	16
chr10	43620612	43622612	Rtn4ip1	-0.13844	6.3E-14	hypomethylated	0.060889	0.10916	insignificant	15	52	49
chr10	43621542	43623542	Qrsl1	0.0010936	0.00035761	inconclusive	0.17075	0.001106	inconclusive	4	22	19
chr10	43724652	43726652	Aim1	-0.3821	1.6E-18	stronglyHypometh	-0.13284	0.4055	insignificant	4	20	22
chr10	43889252	43891252	Speer5-ps1	-0.021549	0.71737	insignificant	-0.062915	0.014254	hypomethylated	3	9	9
chr10	43987163	43989163	Atg5	-0.17538	1.06E-36	hypomethylated	-0.013004	0.43756	insignificant	42	99	100
chr10	44178493	44180493	Prdm1	-0.17857	0.58469	insignificant	-0.018526	0.18004	insignificant	5	24	24
chr10	44786019	44788019	Prep	-0.085535	7.84E-37	hypomethylated	0.0053804	1	insignificant	58	169	168
chr10	45008110	45010110	Popdc3	-0.11843	3.26E-12	hypomethylated	-0.012623	0.19091	insignificant	22	80	81
chr10	45054567	45056567	Bves	-0.045987	0.000000358	hypomethylated	0.0043888	0.6566	insignificant	35	125	126
chr10	45190007	45192007	Lin28b	-0.79125	0.001382	stronglyHypometh	-0.092436	0.029177	hypomethylated	3	6	6
chr10	45296634	45298634	Hace1	-0.077033	0.000006338	hypomethylated	-0.015568	0.048991	hypomethylated	17	82	94
chr10	49508560	49510560	Grik2		1	noCoverage	0.20742	0.75088	insignificant	0	9	6
chr10	50311474	50313474	Ascc3	-0.084411	2.03E-14	hypomethylated	-0.0027112	0.044137	hypomethylated	30	137	137
chr10	50614456	50616456	Sim1	-0.15201	6.41E-28	hypomethylated	-0.0028904	0.093132	insignificant	36	123	127
chr10	51396565	51398565	Rfx6	-0.25808	0.082337	insignificant	0.0057714	0.23256	insignificant	3	27	28
chr10	51741491	51743491	Vgll2	-0.098449	3.55E-11	hypomethylated	0.01837	0.34441	insignificant	23	82	85
chr10	51952424	51954424	Dcbld1	-0.16672	3.15E-24	hypomethylated	-0.0096998	0.96766	insignificant	40	93	99
chr10	52101930	52103930	Gopc	-0.01104	0.003241	inconclusive	-0.014194	0.00013424	inconclusive	8	34	34
chr10	52136352	52138352	Nus1	-0.1065	2.56E-09	hypomethylated	-0.013861	0.18533	insignificant	25	119	120
chr10	52265545	52267545	Zfa		1	noCoverage	-0.0024488	0.53228	insignificant	0	16	16
chr10	52409306	52411306	Slc35f1	-0.20128	2.59E-26	hypomethylated	-0.0055856	0.3606	insignificant	27	88	93
chr10	53315766	53317766	Asf1a	-0.10485	4.27E-33	hypomethylated	-0.0065199	0.00075359	hypomethylated	61	164	188

chr10	53350245	53352245	Mcm9		1	noCoverage	-0.039067	0.60856	insignificant	0	14	16
chr10	53470715	53472715	Fam184a	-0.10282	2.38E-09	hypomethylated	0.010933	0.94386	insignificant	19	57	45
chr10	53795602	53797602	Man1a	-0.15436	1.05E-08	hypomethylated	0.0019111	0.90032	insignificant	16	46	59
chr10	55825722	55827722	Msl3l2	-0.46667	0.43396	lowCoverage	0.10507	1	insignificant	1	6	4
chr10	55948495	55950495	D630037F22Rik	-0.1583	0.030418	hypomethylated	0.0051874	0.89144	insignificant	5	15	15
chr10	56096105	56098105	Gja1		1	noCoverage	-0.056818	0.25518	insignificant	0	8	8
chr10	57205190	57207190	Hsf2	-0.11447	2.42E-26	hypomethylated	-0.0095406	0.069357	insignificant	46	143	125
chr10	57252335	57254335	Serinc1	-0.24281	0.12277	insignificant	-0.026034	0.61075	insignificant	4	8	8
chr10	57350786	57352786	Pkib	-0.27993	0.32705	insignificant	-0.026876	0.65872	insignificant	1	10	10
chr10	57513349	57515349	Smpdl3a	-0.15901	4.78E-08	hypomethylated	-0.0013349	0.69148	insignificant	15	49	49
chr10	57694761	57696761	4933403O03Rik		1	noCoverage	-0.029979	0.03613	inconclusive	0	18	12
chr10	57695423	57697423	4933403O03Rik		1	noCoverage	0.145	0.0058748	hypermethylated	0	4	4
chr10	57699408	57701408	Gm4981		1	noCoverage	-0.17244	0.047224	hypomethylated	0	4	4
chr10	57717273	57719273	Gcc2	-0.15265	0.036458	hypomethylated	0.0016741	0.2113	insignificant	10	51	54
chr10	57785213	57787213	Lims1	-0.11935	7.72E-20	hypomethylated	0.0013137	0.36015	insignificant	44	109	106
chr10	57908599	57910599	Ranbp2	-0.10672	3.64E-12	hypomethylated	-0.0099296	0.036108	hypomethylated	25	114	115
chr10	57959684	57961684	Ccdc138	-0.15613	4.53E-15	hypomethylated	0.03006	0.85987	insignificant	18	65	67
chr10	58275106	58277106	Sh3rf3	-0.099158	4.12E-21	hypomethylated	0.0059998	0.57434	insignificant	45	167	168
chr10	58683669	58685669	Ankrd57	-0.1216	4.12E-25	hypomethylated	0.00030657	0.0094816	inconclusive	39	178	178
chr10	58684595	58686595	############	-0.15478	1.51E-11	hypomethylated	-0.0018164	0.0067212	hypomethylated	13	77	77
chr10	58785043	58787043	P4ha1	-0.13334	4.15E-31	hypomethylated	-0.014354	0.39739	insignificant	48	156	154
chr10	58865432	58867432	Pla2g12b	0.26382	0.59665	insignificant	0.13174	0.038236	hypermethylated	2	14	14
chr10	59079440	59081440	Ccdc109a	-0.29967	0.00000354	hypomethylated	-0.010474	0.044207	hypermethylated	4	16	16
chr10	59164382	59166382	Cbara1	-0.15676	3.2E-09	hypomethylated	-0.01184	0.16796	insignificant	11	47	55
chr10	59341338	59343338	Dnajb12	-0.18859	0.0002362	hypomethylated	-0.0064555	0.00066346	hypomethylated	9	34	31
chr10	59414518	59416518	Ddit4	-0.16608	1.9E-17	hypomethylated	0.0050732	0.76368	insignificant	15	58	59
chr10	59464552	59466552	Ascc1	0.0085643	0.017553	inconclusive	0.1461	0.42555	insignificant	7	50	37
chr10	59465074	59467074	Ascc1	-0.089979	0.00000903	hypomethylated	0.042182	0.61196	insignificant	4	38	25
chr10	59568004	59570004	Spock2	-0.12571	5.12E-38	hypomethylated	-0.0048374	0.68829	insignificant	39	109	108
chr10	59682008	59684008	Chst3	-0.30797	2.56E-28	hypomethylated	0.10433	0.083362	insignificant	9	18	12
chr10	59739375	59741375	Psap	-0.33079	0.000026939	hypomethylated	-0.030501	0.64534	insignificant	10	66	64
chr10	59808598	59810598	4632428N05Rik	-0.2743	0.32897	insignificant	-0.07663	0.67235	insignificant	4	39	43
chr10	59861473	59863473	Gm17455	0.0048173	0.248	insignificant	-0.0022764	0.55752	insignificant	2	13	13
chr10	60159238	60161238	Cdh23	0.03619	0.017157	hypermethylated	0.082716	0.11417	insignificant	2	10	11
chr10	60215530	60217530	Slc29a3	-0.23211	6.23E-11	hypomethylated	-0.032807	0.082421	insignificant	4	14	14
chr10	60294329	60296329	Unc5b	-0.24395	0.000030846	hypomethylated	-0.036053	0.53558	insignificant	12	44	40
chr10	60610413	60612413	Sgpl1	-0.13247	4.29E-11	hypomethylated	-0.0028168	0.081577	insignificant	21	65	65
chr10	60633711	60635711	Tbata	0.14778	0.31493	insignificant	0.0058187	0.4704	insignificant	4	10	10
chr10	60637009	60639009	Tbata	0.087777	0.053265	insignificant	0.0090183	0.000097002	inconclusive	11	46	46
chr10	60736186	60738186	Adamts14	-0.034965	0.32659	insignificant	0.043224	0.54826	insignificant	9	13	27
chr10	60846271	60848271	X99384	-0.15484	0.053149	insignificant	0.091108	0.0061051	hypermethylated	7	38	25
chr10	60879719	60881719	Nodal	-0.19522	0.000046739	hypomethylated	0.038288	0.67982	insignificant	14	48	50
chr10	60915417	60917417	Eif4ebp2	-0.2607	1.09E-31	hypomethylated	0.022493	0.00027288	inconclusive	19	70	70
chr10	60937580	60939580	Lrrc20	-0.1391	0.0054149	hypomethylated	0.010803	0.95823	insignificant	6	60	43
chr10	61057231	61059231	Npffr1	-0.11824	5.58E-12	hypomethylated	-0.039008	0.47317	insignificant	17	37	34
chr10	61110368	61112368	Ppa1	-0.11666	1.7E-09	hypomethylated	-0.019691	0.85664	insignificant	23	107	129
chr10	61142068	61144068	Sar1a	-0.11483	2.34E-10	hypomethylated	0.0046171	0.24572	insignificant	21	87	95
chr10	61157261	61159261	Tysnd1	-0.11295	7.95E-39	hypomethylated	-0.012073	0.92541	insignificant	47	181	199
chr10	61177010	61179010	Aifm2	-0.18717	0.000303	hypomethylated	-0.0057701	0.079484	insignificant	11	26	26
chr10	61182396	61184396	Aifm2	-0.20181	0.0021077	hypomethylated	-0.055551	0.52474	insignificant	1	6	6
chr10	61246612	61248612	H2afy2	-0.13347	0.000000317	hypomethylated	0.0061497	0.04224	hypermethylated	8	47	50
chr10	61441856	61443856	Col13a1	-0.36972	0.57654	insignificant	-0.22289	0.058534	insignificant	0	29	22
chr10	61532769	61534769	Gm5424	0.11005	1	insignificant	-0.046644	0.082016	insignificant	2	14	14
chr10	61573761	61575761	2010107G23Rik	-0.16385	1.64E-08	hypomethylated	0.0071899	0.54978	insignificant	8	14	14
chr10	61594837	61596837	Neurog3	-0.14828	5.36E-08	hypomethylated	-0.0031655	0.52742	insignificant	23	99	99
chr10	61693966	61695966	Tspan15	-0.19239	0.00000172	hypomethylated	0.00351	0.064898	insignificant	7	23	22
chr10	61803169	61805169	Hk1	-0.2893	1	lowCoverage	-0.021442	1	insignificant	1	8	8
chr10	61885205	61887205	Hkdc1		1	noCoverage	0.025778	0.84422	insignificant	0	12	10
chr10	61910907	61912907	4930507D05Rik	-0.14611	2.41E-14	hypomethylated	-0.019781	0.0274	hypomethylated	15	73	77
chr10	61912441	61914441	Supv3l1	-0.0086197	1	insignificant	-0.0073673	0.84778	insignificant	6	47	53
chr10	61949346	61951346	Vps26a	0.032123	0.00069676	inconclusive	-0.034214	0.87996	insignificant	11	66	63
chr10	61949553	61951553	Vps26a	0.22603	0.0056751	inconclusive	-0.042056	0.25089	insignificant	5	50	47
chr10	61970503	61972503	Srgn	0.23018	0.059576	insignificant	-0.017389	0.32064	insignificant	1	20	20
chr10	62041205	62043205	2510003E04Rik	0.20041	1	insignificant	-0.032397	0.09138	insignificant	1	23	25
chr10	62065046	62067046	Ddx21	-0.44741	0.00000344	stronglyHypometh	-0.019276	0.0070543	hypomethylated	6	30	32
chr10	62113946	62115946	Ddx50	-0.15867	0.000067954	hypomethylated	0.0027768	0.015027	hypermethylated	7	15	15
chr10	62188847	62190847	Stox1	-0.54741	3.82E-22	stronglyHypometh	0.067794	0.36783	insignificant	5	27	33
chr10	62255116	62257116	Ccar1	-0.20025	0.036459	hypomethylated	-0.011927	0.25483	insignificant	11	56	56
chr10	62342762	62344762	Tet1		1	noCoverage	-0.1247	0.10019	insignificant	0	4	4
chr10	62382380	62384380	Slc25a16	-0.12848	0.000000702	hypomethylated	-0.052952	0.43387	insignificant	18	48	44
chr10	62408776	62410776	Dna2	-0.08227	0.0002985	hypomethylated	0.043376	0.0022803	hypermethylated	21	103	103
chr10	62441970	62443970	Rufy2	-0.19443	0.0000011	hypomethylated	-0.036007	0.024661	hypermethylated	13	60	67
chr10	62486259	62488259	Pbld2	-0.10418	1.44E-18	hypomethylated	0.00035893	0.89769	insignificant	27	85	85
chr10	62486597	62488597	Hnrnph3	-0.10606	0.00000104	hypomethylated	0.0044463	0.85034	insignificant	12	45	45
chr10	62561903	62563903	Atoh7	-0.062385	0.000001702	hypomethylated	-0.0012602	0.43105	insignificant	30	92	92
chr10	62705544	62707544	Herc4	-0.11456	4.8E-11	hypomethylated	0.020609	0.45861	insignificant	36	96	113
chr10	62801743	62803743	Sirt1	-0.080551	0.0047958	hypomethylated	0.0098461	0.65458	insignificant	19	71	87
chr10	62801780	62803780	Sirt1	-0.08379	0.0029061	hypomethylated	0.012691	0.90313	insignificant	19	62	75
chr10	62801783	62803783	Sirt1	-0.08379	0.0029061	hypomethylated	0.012691	0.90313	insignificant	19	61	73
chr10	62844190	62846190	Dnajc12	-0.21433	0.000003189	hypomethylated	0.048575	0.7278	insignificant	4	26	24
chr10	62848310	62850310	Dnajc12	-0.27834	0.0029294	hypomethylated	0.05618	0.89479	insignificant	6	28	28
chr10	62891845	62893845	Ctnna3	-0.12749	0.0011242	hypomethylated	0.021368	0.72044	insignificant	11	91	86
chr10	63553003	63555003	Ctnna3	-0.32428	0.04183	hypomethylated	-0.047637	0.065118	insignificant	2	22	19
chr10	66559605	66561605	Reep3	-0.11917	4.34E-11	hypomethylated	-0.0062303	0.39173	insignificant	14	37	28
chr10	66589005	66591005	Jmjd1c	-0.058472	8.5E-14	hypomethylated	-0.0027842	0.60126	insignificant	83	226	234

chr10	66748029	66750029	Nrbf2	-0.090517	0.00021445	hypomethylated	0.0074577	0.30583	insignificant	13	26	26
chr10	66999616	67001616	Egr2	-0.1409	0.000000943	hypomethylated	0.0063545	0.42453	insignificant	16	70	67
chr10	67011703	67013703	Ado	-0.20202	3.13E-10	hypomethylated	0.048004	0.074757	insignificant	11	60	60
chr10	67441345	67443345	Rtkn2	-0.048434	0.085718	insignificant	-0.00013417	0.0006202	hypomethylated	9	79	83
chr10	67741474	67743474	Arid5b	-0.17583	0.00000084	hypomethylated	0.030719	0.83488	insignificant	15	70	58
chr10	68185493	68187493	Tmem26	-0.22768	2.26E-12	hypomethylated	0.008262	0.26309	insignificant	7	34	34
chr10	68674404	68676404	Rhobtb1	-0.1211	7.59E-11	hypomethylated	0.00094577	0.47819	insignificant	28	118	127
chr10	68815660	68817660	Cdk1	-0.41738	1.42E-08	stronglyHypometh	-0.086671	0.23078	insignificant	4	12	12
chr10	68995455	68997455	Ank3	-0.59722	0.42623	lowCoverage	0.15278	0.46303	insignificant	1	4	4
chr10	69387279	69389279	Ank3	-0.3189	1.81E-13	hypomethylated	-0.043275	0.28	insignificant	9	42	38
chr10	69558868	69560868	Ccdc6	-0.1227	8.67E-28	hypomethylated	-0.0058056	0.0035812	hypomethylated	56	156	156
chr10	69707023	69709023	Slc16a9	-0.14825	3.25E-14	hypomethylated	-0.014933	0.57013	insignificant	22	68	69
chr10	69902415	69904415	Fam13c	-0.17829	1.48E-11	hypomethylated	-0.050927	0.019822	hypomethylated	11	55	53
chr10	69902657	69904657	Fam13c	-0.17829	1.48E-11	hypomethylated	-0.050927	0.019822	hypomethylated	11	55	53
chr10	70062039	70064039	Phyhipl	-0.06366	4.24E-08	hypomethylated	-0.0027668	0.19713	insignificant	42	148	148
chr10	70622382	70624382	Bicc1	-0.080331	1.94E-09	hypomethylated	-0.0013259	0.17551	insignificant	41	110	110
chr10	70700792	70702792	Tfam	-0.15952	0.000015398	hypomethylated	0.035607	0.45118	insignificant	14	30	33
chr10	70748010	70750010	Ube2d1	-0.091846	0.0093374	hypomethylated	-0.001891	0.16604	insignificant	10	28	28
chr10	70807597	70809597	Cisd1	-0.2043	2.8E-12	hypomethylated	0.074355	0.40842	insignificant	10	50	59
chr10	70809540	70811540	Ipmk	-0.12954	3.66E-48	hypomethylated	-0.0031286	0.82752	insignificant	49	146	154
chr10	71443438	71445438	1700049L16Rik	-0.84189	0.16883	lowCoverage	-0.091886	0.63824	insignificant	1	6	6
chr10	72116642	72118642	Zwint	-0.29468	0.00000589	hypomethylated	-0.12964	1	insignificant	9	24	32
chr10	74428975	74430975	Gnaz	-0.11529	2.86E-18	hypomethylated	0.026153	0.5457	insignificant	30	136	137
chr10	74495298	74497298	Rtdr1	-0.10732	0.068496	insignificant	-0.013967	0.59655	insignificant	5	34	34
chr10	74495331	74497331	Rtdr1	-0.10732	0.068496	insignificant	-0.013967	0.59655	insignificant	5	34	34
chr10	74498833	74500833	Rab36	0.011608	1	insignificant	-0.013208	0.34201	insignificant	4	22	22
chr10	74522640	74524640	Bcr	-0.10275	1.05E-31	hypomethylated	-0.00075709	0.042664	hypomethylated	41	183	183
chr10	74673817	74675817	Specc1l	-0.090668	2.29E-16	hypomethylated	0.022832	0.58868	insignificant	29	89	77
chr10	74674134	74676134	Specc1l	-0.090668	2.29E-16	hypomethylated	0.022832	0.58868	insignificant	29	89	77
chr10	74778687	74780687	Adora2a	-0.16656	1.6E-50	hypomethylated	-0.011096	0.20813	insignificant	41	124	126
chr10	74979786	74981786	Snrpd3	-0.14659	0.000000923	hypomethylated	-0.0043294	0.005881	hypomethylated	21	97	95
chr10	74980067	74982067	1110038D17Rik	-0.16645	0.25827	insignificant	0.012778	0.83055	insignificant	14	63	62
chr10	75035337	75037337	Ggt1	-0.075658	0.0034428	hypomethylated	-0.1548	0.16428	insignificant	4	16	18
chr10	75051125	75053125	Ggt5		1	noCoverage	0.0013848	0.91134	insignificant	0	18	18
chr10	75106753	75108753	Susd2	0.0273	1	insignificant	-0.075709	0.042424	hypomethylated	4	12	12
chr10	75227102	75229102	Cabin1	-0.38004	1	insignificant	-0.010989	0.65702	insignificant	2	14	14
chr10	75236119	75238119	Gstt3	-0.09697	0.59928	insignificant	0.021926	0.47517	insignificant	7	22	21
chr10	75261329	75263329	Gstt1	0.038889	1	insignificant	-0.028825	1	insignificant	3	18	18
chr10	75297626	75299626	Gstt2	-0.54583	4.05E-13	stronglyHypometh	0.10621	0.80386	insignificant	1	4	4
chr10	75322995	75324995	Mif	0.074281	0.000000144	inconclusive	0.0096415	0.0004566	inconclusive	4	30	30
chr10	75355142	75357142	Derl3	-0.18763	3.08E-12	hypomethylated	-0.0031303	0.44361	insignificant	9	84	84
chr10	75395208	75397208	Mmp11	-0.2125	0.075543	insignificant	0.077355	0.21595	insignificant	5	10	10
chr10	75397317	75399317	Chchd10	-0.13617	1.4E-32	hypomethylated	-0.0084458	0.21974	insignificant	14	60	60
chr10	75416258	75418258	Gm5134	-0.082231	0.000026392	hypomethylated	-0.028434	0.055301	insignificant	4	24	24
chr10	75494356	75496356	Zfp280b	-0.19424	0.00045248	hypomethylated	-0.022215	0.25095	insignificant	10	40	51
chr10	75573763	75575763	Slc5a4b	-0.22424	0.24684	insignificant	-0.053001	0.50512	insignificant	1	6	6
chr10	75609195	75611195	Slc5a4a	0.1911	0.00052141	hypermethylated	0.030239	0.0046573	hypermethylated	5	26	30
chr10	75670154	75672154	Prmt2	-0.091301	0.019161	hypomethylated	-0.00956	0.89801	insignificant	5	19	18
chr10	75700610	75702610	Prmt2	-0.21107	1.7E-09	hypomethylated	-0.038901	0.24853	insignificant	9	22	26
chr10	75808007	75810007	Dip2a	-0.1195	1.17E-12	hypomethylated	0.00020002	0.70055	insignificant	36	76	82
chr10	75905657	75907657	Pcnt	-0.13492	0.60932	insignificant	-0.0039138	0.89446	insignificant	5	18	18
chr10	75910825	75912825	2610028H24Rik	-0.068828	0.55416	insignificant	-0.020045	1	insignificant	3	13	12
chr10	75930715	75932715	Mcm3ap	-0.30914	0.00000219	hypomethylated	-0.029844	0.60019	insignificant	15	44	42
chr10	75931859	75933859	Ybey	-0.12802	0.25679	insignificant	0.026051	0.20526	insignificant	14	48	48
chr10	75993371	75995371	Lss	0.14608	0.82092	insignificant	-0.0035094	0.049095	hypomethylated	4	32	32
chr10	76086149	76088149	Col6a2	-0.56818	0.00027069	stronglyHypometh	-0.040404	1	insignificant	1	2	2
chr10	76424692	76426692	Pcbp3	-0.00062787	0.12958	insignificant	-0.0053114	0.79221	insignificant	8	30	37
chr10	76494483	76496483	Slc19a1	-0.21159	2.67E-10	hypomethylated	-0.014054	0.79214	insignificant	4	16	23
chr10	76494956	76496956	Slc19a1	-0.22917	3.23E-11	hypomethylated	-0.018151	0.47326	insignificant	5	21	28
chr10	76629275	76631275	Col18a1	-0.17381	0.0040631	hypomethylated	0.057008	0.0046598	hypermethylated	5	9	14
chr10	76721044	76723044	Pofut2	-0.098104	4.03E-13	hypomethylated	-0.0037434	0.96174	insignificant	22	69	70
chr10	76721968	76723968	Gm10941	-0.14358	5.8E-21	hypomethylated	-0.01752	5.42E-16	hypomethylated	27	91	92
chr10	76880120	76882120	Gm17769	-0.11528	2.33E-32	hypomethylated	-0.0094185	3.67E-10	hypomethylated	34	156	164
chr10	76881018	76883018	Adarb1	-0.14859	3.93E-14	hypomethylated	-0.020012	1.27E-14	hypomethylated	15	55	63
chr10	76978558	76980558	1810008A18Rik	-0.40691	2.52E-10	stronglyHypometh	-0.12237	0.42755	insignificant	2	8	17
chr10	76992092	76994092	Itgb2	-0.64318	0.13339	insignificant	-0.11269	0.27374	insignificant	1	4	4
chr10	77043511	77045511	Pttg1ip	-0.26608	0.0015426	hypomethylated	-0.01262	0.63536	insignificant	6	35	25
chr10	77067978	77069978	Sumo3	-0.088291	6.45E-14	hypomethylated	-0.0061436	0.81868	insignificant	40	120	140
chr10	77084065	77086065	Ube2g2	-0.17864	3.02E-15	hypomethylated	-0.020743	0.59664	insignificant	32	91	87
chr10	77173201	77175201	Gm10024	-0.27352	0.000000806	hypomethylated	0.058385	0.85242	insignificant	1	10	9
chr10	77289815	77291815	Krtap10-4	0.12694	1	insignificant	-0.092396	0.13953	insignificant	2	10	11
chr10	77297691	77299691	Krtap10-10	-0.26189	0.074692	insignificant	-0.16229	0.0058247	hypomethylated	3	12	12
chr10	77365281	77367281	Lrrc3	-0.25002	0.18475	insignificant	0.026653	0.21919	insignificant	4	30	22
chr10	77432617	77434617	Trpm2	-0.31847	0.000112	hypomethylated	-0.056004	0.66876	insignificant	3	14	14
chr10	77440394	77442394	1810043G02Rik	-0.12767	1.56E-23	hypomethylated	0.00050533	0.0049099	inconclusive	31	97	96
chr10	77472541	77474541	Pfkl	-0.13567	0.016231	hypomethylated	0.022306	0.017651	hypermethylated	8	33	32
chr10	77504031	77506031	Dnmt3l	-0.24286	0.000013364	hypomethylated	-0.13432	0.043542	hypomethylated	2	14	16
chr10	77506325	77508325	Dnmt3l		1	noCoverage	-0.057083	0.13778	insignificant	0	15	15
chr10	77531112	77533112	Icosl	-0.15088	1.49E-14	hypomethylated	-0.018451	0.01081	hypomethylated	30	92	88
chr10	77632513	77634513	D10Jhu81e		1	noCoverage	-0.1282	0.56282	insignificant	0	12	13
chr10	77647894	77649894	Pwp2		1	noCoverage	-0.017571	0.71623	insignificant	0	6	11
chr10	77707387	77709387	Trappc10	-0.12427	0.000000641	hypomethylated	-0.012901	0.097366	insignificant	12	36	49
chr10	77814445	77816445	Agpat3	-0.084997	2.57E-24	hypomethylated	0.0060191	0.44088	insignificant	41	130	134
chr10	77887414	77889414	Cstb	-0.23261	7.29E-08	hypomethylated	0.052497	0.56249	insignificant	4	33	35
chr10	77927693	77929693	Pdxk	-0.25557	0.0021632	hypomethylated	-0.0041054	0.0029304	hypomethylated	8	54	53

chr10	78054709	78056709	Syde1	-0.12459	0.78762	insignificant	-0.014782	0.22025	insignificant	10	47	47
chr10	78181038	78183038	Casp14	-0.25329	0.30287	insignificant	-0.012169	0.047526	inconclusive	3	9	6
chr10	78242240	78244240	Slc1a6	-0.23793	0.000000141	hypomethylated	-0.0013243	0.064028	insignificant	11	40	40
chr10	78996532	78998532	Ppap2c	-0.16548	0.000017283	hypomethylated	0.0092135	0.82197	insignificant	5	14	14
chr10	79017836	79019836	Mier2	-0.056856	0.15512	insignificant	-0.001147	1	insignificant	12	37	37
chr10	79100663	79102663	Shc2	-0.22929	0.000000005	hypomethylated	-0.022636	0.28677	insignificant	11	40	40
chr10	79126318	79128318	Madcam1	-0.12397	6.52E-08	hypomethylated	-0.054	0.00026083	hypomethylated	13	50	51
chr10	79131154	79133154	Gm16517	-0.16056	4.42E-25	hypomethylated	-0.023684	0.33744	insignificant	38	88	89
chr10	79143939	79145939	Cdc34	-0.15332	1.18E-40	hypomethylated	0.023955	0.11415	insignificant	42	108	90
chr10	79150764	79152764	Gzmm	-0.0078707	0.39693	insignificant	-0.010468	0.16939	insignificant	3	22	22
chr10	79166102	79168102	Bsg	-0.23113	1.31E-08	hypomethylated	0.0037376	0.11206	insignificant	8	28	33
chr10	79178378	79180378	Hcn2	-0.10088	5.32E-09	hypomethylated	-0.0048233	0.43912	insignificant	41	128	120
chr10	79209326	79211326	Polrmt	-0.31343	9.49E-10	hypomethylated	-0.0027055	0.85073	insignificant	3	13	16
chr10	79216863	79218863	Fgf22	-0.15894	1.2E-14	hypomethylated	0.054546	0.92524	insignificant	10	42	44
chr10	79229666	79231666	Rnf126	-0.18263	0.01047	hypomethylated	0.01316	0.010077	hypermethylated	15	68	72
chr10	79229686	79231686	Rnf126	-0.20046	0.0089353	hypomethylated	0.012825	0.15301	insignificant	14	60	64
chr10	79239018	79241018	Fstl3	-0.15815	1.14E-09	hypomethylated	-0.022616	0.46854	insignificant	15	61	56
chr10	79255316	79257316	Palm	-0.088757	1.78E-13	hypomethylated	0.00055727	0.8376	insignificant	20	70	70
chr10	79282765	79284765	9130017N09Rik		1	noCoverage	-0.03125	0.36	insignificant	0	2	2
chr10	79316349	79318349	Ptbp1	-0.13384	6.38E-32	hypomethylated	-0.015151	0.11577	insignificant	39	124	126
chr10	79317716	79319716	E130317F20Rik	-0.12405	3.99E-21	hypomethylated	-0.019569	0.03896	hypomethylated	30	100	101
chr10	79337379	79339379	BC005764		1	noCoverage	-0.17334	0.069645	insignificant	0	10	8
chr10	79341411	79343411	Prtn3		1	noCoverage	0.054167	0.64975	insignificant	0	6	6
chr10	79348056	79350056	Elane	0.24345	1	lowCoverage	0.021979	0.25722	insignificant	1	25	23
chr10	79352597	79354597	Cfd	0.31143	0.3331	insignificant	0.1334	0.19346	insignificant	3	9	6
chr10	79371683	79373683	Med16	-0.69367	1.47E-08	stronglyHypometh	0.071644	0.44306	insignificant	2	15	18
chr10	79378715	79380715	Kiss1r	-0.13448	0.000000157	hypomethylated	-0.00085079	0.51229	insignificant	10	46	46
chr10	79379675	79381675	Kiss1r	-0.11226	1	insignificant	0.0038667	0.68752	insignificant	3	22	22
chr10	79388816	79390816	Arid3a	-0.15436	1.52E-37	hypomethylated	-0.0060076	0.014487	hypomethylated	43	168	165
chr10	79421896	79423896	Wdr18	-0.089306	0.00000752	hypomethylated	0.043503	0.85086	insignificant	5	29	29
chr10	79432468	79434468	Grin3b	-0.12756	1.72E-20	hypomethylated	-0.011502	1.52E-11	hypomethylated	27	112	112
chr10	79447075	79449075	ORF61	-0.27005	0.00012933	hypomethylated	-0.024797	0.086734	insignificant	6	20	20
chr10	79450344	79452344	Cnn2	-0.21076	3.41E-13	hypomethylated	-0.0060282	0.11822	insignificant	34	96	94
chr10	79459359	79461359	Abca7	-0.30203	7.54E-10	hypomethylated	-0.021594	0.93014	insignificant	10	50	50
chr10	79481419	79483419	Hmha1	-0.50993	0.11837	insignificant	-0.032629	0.17178	insignificant	3	18	17
chr10	79502404	79504404	Polr2e	-0.059473	8.43E-25	hypomethylated	-0.0048527	0.000018507	hypomethylated	21	72	72
chr10	79515254	79517254	Gpx4	-0.15252	2.71E-30	hypomethylated	-0.0079637	0.083965	insignificant	39	101	108
chr10	79515781	79517781	Gpx4	-0.1333	2.47E-28	hypomethylated	-0.0059202	0.30237	insignificant	38	118	126
chr10	79565447	79567447	Sbno2	-0.20385	0.031561	hypomethylated	-0.063132	0.61202	insignificant	9	21	20
chr10	79578280	79580280	Stk11	-0.10688	2.73E-14	hypomethylated	-0.001426	0.16815	insignificant	42	148	150
chr10	79600132	79602132	Dos	-0.11065	2.89E-16	hypomethylated	0.004225	0.39598	insignificant	30	108	114
chr10	79602112	79604112	Dos	-0.03002	0.000034883	hypomethylated	0.023021	0.019692	inconclusive	7	31	38
chr10	79604059	79606059	Atp5d	-0.15958	1.3E-10	hypomethylated	-0.004379	0.029236	hypomethylated	14	81	87
chr10	79610034	79612034	Midn	-0.1301	2E-25	hypomethylated	-0.014318	0.0063964	hypomethylated	34	95	95
chr10	79629585	79631585	Cirbp	-0.098929	1.4E-20	hypomethylated	-0.0061996	0.32796	insignificant	33	74	64
chr10	79634688	79636688	1600002K03Rik	-0.26022	1.34E-43	hypomethylated	0.062162	2.86E-16	inconclusive	17	80	76
chr10	79641226	79643226	Efna2	-0.11461	3.93E-09	hypomethylated	0.01632	0.73766	insignificant	23	64	66
chr10	79688342	79690342	Mum1	-0.1291	0.00000528	hypomethylated	-0.015331	0.17012	insignificant	17	72	83
chr10	79711196	79713196	Ndufs7	-0.10835	0.0053292	hypomethylated	-0.021082	0.2141	insignificant	3	36	36
chr10	79723713	79725713	Gamt	-0.51258	0.000000215	stronglyHypometh	-0.046394	0.44172	insignificant	4	32	30
chr10	79726735	79728735	Dazap1	-0.095524	2.94E-24	hypomethylated	-0.0024856	0.00019236	hypomethylated	36	150	140
chr10	79754175	79756175	Rps15	-0.13395	2E-22	hypomethylated	0.005349	0.0026905	inconclusive	38	126	130
chr10	79763564	79765564	Apc2	-0.18326	0.063913	insignificant	0.0013477	0.70036	insignificant	5	16	16
chr10	79783293	79785293	Pcsk4	-0.52722	4.62E-09	stronglyHypometh	-0.056242	0.45719	insignificant	3	53	53
chr10	79791914	79793914	Pcsk4	-0.11894	6.69E-15	hypomethylated	-0.021666	0.78048	insignificant	20	62	78
chr10	79792218	79794218	Pcsk4	-0.15303	2.48E-14	hypomethylated	-0.030894	0.94099	insignificant	21	68	84
chr10	79811157	79813157	Adamtsl5	-0.11112	0.6794	insignificant	-0.0043278	0.41994	insignificant	25	97	90
chr10	79811191	79813191	Adamtsl5	-0.11345	0.50396	insignificant	-0.0020266	0.060304	insignificant	22	89	82
chr10	79818203	79820203	Plk5	-0.13611	3.7E-20	hypomethylated	0.0052103	0.18122	insignificant	36	100	110
chr10	79850396	79852396	Mex3d	-0.033773	0.018561	hypomethylated	0.011691	0.014799	inconclusive	12	57	48
chr10	79862224	79864224	Mbd3	-0.16302	0.0016736	hypomethylated	-0.0043981	0.68606	insignificant	7	24	25
chr10	79869566	79871566	Uqcr11	-0.21866	0.000000254	hypomethylated	-0.0097551	0.46471	insignificant	8	16	16
chr10	79896398	79898398	Tcf3	-0.29473	0.0029586	hypomethylated	-0.0062364	0.30716	insignificant	4	34	34
chr10	79956650	79958650	Onecut3	-0.081929	1.08E-13	hypomethylated	0.0029311	0.18935	insignificant	41	140	160
chr10	80024305	80026305	Rexo1	-0.21473	0.000050329	hypomethylated	0.012206	0.79741	insignificant	11	36	37
chr10	80040041	80042041	Klf16	-0.12667	5.85E-32	hypomethylated	0.0082306	0.19387	insignificant	28	160	158
chr10	80053086	80055086	Fam108a	-0.51331	1	lowCoverage	-0.084406	0.35846	insignificant	1	21	20
chr10	80064624	80066624	Adat3	-0.10543	0.000000732	hypomethylated	-0.0067498	0.28723	insignificant	20	67	68
chr10	80064626	80066626	Scamp4	-0.10543	0.000000732	hypomethylated	-0.0067498	0.28723	insignificant	20	67	68
chr10	80084524	80086524	Csnk1g2	-0.17032	2.1E-26	hypomethylated	-0.0108	0.02631	inconclusive	27	73	73
chr10	80119816	80121816	Btbd2		1	noCoverage	0.083812	1	insignificant	0	8	12
chr10	80164437	80166437	Izumo4	-0.15042	1.4E-25	hypomethylated	-0.0058385	0.03315	hypomethylated	26	113	116
chr10	80164565	80166565	Izumo4	-0.15041	7.41E-24	hypomethylated	-0.00342	0.059682	insignificant	21	97	100
chr10	80204956	80206956	Ap3d1	-0.29729	0.55897	insignificant	-0.047109	0.94655	insignificant	3	36	38
chr10	80216950	80218950	Dot1l	-0.082196	1.76E-20	hypomethylated	-0.0052618	0.28152	insignificant	49	139	139
chr10	80260479	80262479	Sf3a2	-0.076344	9.49E-23	hypomethylated	0.0076804	0.11796	insignificant	52	218	226
chr10	80261371	80263371	Plekhj1	-0.060427	0.000000162	hypomethylated	0.013704	0.79659	insignificant	23	100	108
chr10	80288400	80290400	Oaz1	-0.098289	0.000000289	hypomethylated	-0.010906	0.16797	insignificant	21	127	140
chr10	80288440	80290440	Gm9786	-0.098289	0.000000289	hypomethylated	-0.010906	0.16797	insignificant	21	127	140
chr10	80290541	80292541	Mir1982	-0.10851	0.024411	hypomethylated	-0.021649	0.094951	insignificant	4	16	16
chr10	80306784	80308784	Lingo3	-0.10018	0.011787	hypomethylated	0.0046524	0.060588	insignificant	9	38	38
chr10	80317019	80319019	3110056O03Rik	-0.074316	5.2E-41	hypomethylated	-0.014525	0.0007551	hypomethylated	34	106	105
chr10	80317954	80319954	Lsm7	-0.097287	2.33E-42	hypomethylated	-0.013949	0.00053112	hypomethylated	36	99	99
chr10	80363714	80365714	Timm13	0.049339	0.37379	insignificant	0.062029	0.41405	insignificant	4	19	24
chr10	80380990	80382990	Lmnb2	-0.1932	0.0012197	hypomethylated	-0.00002015	0.064896	insignificant	10	36	36

chr10	80391835	80393835	Gadd45b	-0.1335	7.08E-16	hypomethylated	-0.00014348	0.43467	insignificant	25	103	101
chr10	80464110	80466110	Gng7	-0.11657	0.072278	insignificant	-0.033155	0.13932	insignificant	7	34	39
chr10	80477670	80479670	Gng7		1	noCoverage	0.057267	1	insignificant	0	12	13
chr10	80488122	80490122	Diras1	-0.38684	1.4E-15	stronglyHypometh	-0.13151	0.57006	insignificant	5	35	41
chr10	80522899	80524899	Sgta		1	noCoverage	-0.20773	0.50486	insignificant	0	29	29
chr10	80531827	80533827	Thop1	-0.094032	0.011206	hypomethylated	-0.0089767	0.12684	insignificant	7	40	40
chr10	80561617	80563617	Creb3l3	-0.095758	0.00000315	hypomethylated	-0.080881	0.00021855	hypomethylated	2	14	14
chr10	80567691	80569691	Map2k2	-0.12346	4.37E-24	hypomethylated	-0.012575	0.27069	insignificant	33	110	108
chr10	80598015	80600015	Zbtb7a	-0.10833	1.35E-23	hypomethylated	0.0035693	0.16591	insignificant	57	217	212
chr10	80630465	80632465	Pias4	-0.25069	0.00000013	hypomethylated	0.0015401	0.066665	insignificant	6	50	50
chr10	80638375	80640375	Eef2	-0.071789	1.28E-11	hypomethylated	0.0015915	0.97089	insignificant	17	100	101
chr10	80640705	80642705	Snord37	0.056737	0.62032	insignificant	0.014167	0.87618	insignificant	3	30	30
chr10	80644751	80646751	Dapk3	-0.19168	1.14E-15	hypomethylated	-0.022788	0.38083	insignificant	14	61	60
chr10	80645007	80647007	Dapk3	-0.19168	1.14E-15	hypomethylated	-0.022788	0.38083	insignificant	14	61	60
chr10	80645662	80647662	Dapk3	-0.16744	1.54E-13	hypomethylated	-0.011842	0.96191	insignificant	14	50	50
chr10	80656435	80658435	2310050B05Rik	-0.090465	0.0093153	hypomethylated	0.0073043	1	insignificant	11	16	16
chr10	80694907	80696907	Zfr2	-0.14632	0.000000315	hypomethylated	-0.025292	0.40599	insignificant	15	61	48
chr10	80719289	80721289	Matk	-0.15333	5.63E-17	hypomethylated	0.0023978	0.26728	insignificant	28	80	80
chr10	80729671	80731671	Apba3	-0.27344	3.38E-21	hypomethylated	0.022326	0.84115	insignificant	16	30	31
chr10	80729916	80731916	Apba3	-0.35015	8.14E-27	stronglyHypometh	0.031245	0.35737	insignificant	12	24	25
chr10	80733341	80735341	Mir3057	0.0071581	1	insignificant	0.016414	0.21272	insignificant	5	10	10
chr10	80754012	80756012	Pip5k1c	-0.096362	0.013815	hypomethylated	0.0034769	0.015306	hypermethylated	22	82	82
chr10	80754716	80756716	Pip5k1c	-0.10769	0.000065478	hypomethylated	0.0013047	0.78839	insignificant	25	114	114
chr10	80782847	80784847	2510012J08Rik	-0.11675	1.89E-25	hypomethylated	0.014031	0.31896	insignificant	35	157	168
chr10	80790475	80792475	Tbxa2r	-0.46429	0.5131	insignificant	-0.014152	0.54066	insignificant	1	7	8
chr10	80806011	80808011	Gipc3	-0.12611	0.000000363	hypomethylated	-0.0026147	0.10958	insignificant	5	10	10
chr10	80813171	80815171	Hmg20b	-0.12068	0.00088819	hypomethylated	0.0064956	0.53284	insignificant	16	76	72
chr10	80813172	80815172	Hmg20b	-0.12068	0.00088819	hypomethylated	0.0064956	0.53284	insignificant	16	76	72
chr10	80813202	80815202	Hmg20b	-0.13299	0.002964	hypomethylated	0.0097169	0.24046	insignificant	11	66	62
chr10	80819314	80821314	F630110N24Rik	-0.081531	1.36E-39	hypomethylated	-0.0085813	0.000000351	hypomethylated	23	108	101
chr10	80828565	80830565	4930404N11Rik	0.0038162	0.71386	insignificant	0.0092252	0.37575	insignificant	8	18	18
chr10	80841115	80843115	Fzr1	-0.079569	0.00000228	hypomethylated	-0.012136	0.55216	insignificant	15	68	79
chr10	80846172	80848172	Dohh	-0.21148	0.000026064	hypomethylated	0.054008	0.35674	insignificant	12	81	78
chr10	80945454	80947454	Celf5	-0.23572	0.098917	insignificant	-0.049695	0.87192	insignificant	7	28	25
chr10	80959108	80961108	Ncln	-0.48059	0.49248	insignificant	-0.063343	0.85013	insignificant	1	4	6
chr10	80986970	80988970	Gna15		1	noCoverage	0.025455	0.80007	insignificant	0	11	13
chr10	81007791	81009791	Gna11	-0.078905	0.000000509	hypomethylated	0.012991	0.24513	insignificant	12	45	46
chr10	81021304	81023304	Aes	-0.12867	2.27E-27	hypomethylated	0.016303	0.032816	inconclusive	34	75	107
chr10	81037276	81039276	Tle2	-0.14995	2.8E-18	hypomethylated	0.010461	0.1268	insignificant	12	57	58
chr10	81063645	81065645	Tle6	-0.25505	0.00024226	hypomethylated	-0.01957	0.0062756	hypomethylated	4	49	42
chr10	81068052	81070052	BC025920	-0.22001	0.0081057	hypomethylated	-0.0036575	0.29859	insignificant	4	38	37
chr10	81090067	81092067	Sirt6	-0.30414	1.2E-14	hypomethylated	0.038475	0.74178	insignificant	7	61	72
chr10	81090284	81092284	Ankrd24	-0.3966	5.54E-12	stronglyHypometh	0.033688	1	insignificant	5	43	54
chr10	81090353	81092353	Sirt6		1	noCoverage	-0.0011103	0.12486	insignificant	0	27	38
chr10	81110813	81112813	Gm10778	-0.26085	0.00027084	hypomethylated	-0.086536	0.7769	insignificant	2	18	32
chr10	81509871	81511871	Zfp873	-0.16673	0.0087209	hypomethylated	0.0073187	0.94307	insignificant	6	26	26
chr10	81704020	81706020	Zfp938	-0.55399	1.11E-09	stronglyHypometh	-0.13519	0.071497	insignificant	2	12	14
chr10	82091582	82093582	Tdg	-0.11861	0.067965	insignificant	-0.01406	0.55919	insignificant	10	68	77
chr10	82091586	82093586	Gm9855	-0.14022	0.042886	hypomethylated	-0.019331	0.38293	insignificant	10	70	79
chr10	82160751	82162751	Hcfc2	-0.12981	1.17E-24	hypomethylated	-0.01193	0.44861	insignificant	50	140	140
chr10	82226886	82228886	Nfyb	-0.10421	0.00000117	hypomethylated	-0.01084	0.55962	insignificant	13	40	58
chr10	82320950	82322950	Txnrd1	-0.19188	2.69E-19	hypomethylated	-0.026241	1	insignificant	15	56	56
chr10	82322085	82324085	Txnrd1	-0.17426	3.51E-33	hypomethylated	-0.023873	0.25828	insignificant	29	73	77
chr10	82328370	82330370	Eid3	-0.74573	0.00017453	stronglyHypometh	-0.13601	0.8177	insignificant	1	9	4
chr10	82447241	82449241	Chst11	-0.16195	2.8E-61	hypomethylated	-0.0021293	0.0022202	hypomethylated	52	145	146
chr10	82800562	82802562	Slc41a2		1	noCoverage	-0.082275	0.8308	insignificant	0	19	17
chr10	82821965	82823965	D10Wsu102e	-0.14228	1.55E-33	hypomethylated	0.0062057	0.0891	insignificant	30	114	111
chr10	82996885	82998885	Aldh1l2	0.16667	1	insignificant	-0.033333	1	insignificant	2	10	10
chr10	83005685	83007685	A230046K03Rik	-0.078747	7.67E-14	hypomethylated	0.0082951	0.22229	insignificant	33	128	127
chr10	83111409	83113409	Appl2	0.051591	0.084145	insignificant	-0.025707	0.4985	insignificant	2	18	23
chr10	83184609	83186609	1500009L16Rik	-0.38032	0.020521	stronglyHypometh	0.04064	0.39689	insignificant	5	12	13
chr10	83903216	83905216	Nuak1	-0.14155	3.15E-42	hypomethylated	-0.0056535	8.39E-10	hypomethylated	52	176	176
chr10	83996633	83998633	Ckap4	-0.11475	3.4E-20	hypomethylated	-0.018803	0.25574	insignificant	44	105	103
chr10	84038691	84040691	Tcp11l2	-0.17379	4.94E-18	hypomethylated	-0.019935	0.54151	insignificant	12	56	52
chr10	84084181	84086181	Polr3b	-0.23604	1.1E-16	hypomethylated	0.024382	0.33747	insignificant	18	85	94
chr10	84217792	84219792	Rfx4	-0.1859	1.82E-12	hypomethylated	-0.00071252	0.46515	insignificant	21	76	76
chr10	84379360	84381360	Ric8b	-0.1941	4.65E-22	hypomethylated	0.042858	0.29202	insignificant	16	57	58
chr10	84379400	84381400	Ric8b	-0.1941	4.65E-22	hypomethylated	0.042858	0.29202	insignificant	16	57	58
chr10	84564371	84566371	AI597468	-0.11437	4.59E-22	hypomethylated	-0.008519	0.32816	insignificant	26	88	100
chr10	84565240	84567240	AI597468	-0.10263	4.82E-20	hypomethylated	-0.0078718	0.2406	insignificant	23	78	90
chr10	84590772	84592772	Mterfd3		1	noCoverage	0.017857	0.036621	hypermethylated	0	4	4
chr10	84647799	84649799	Cry1	-0.097467	1.15E-14	hypomethylated	0.00089401	0.7665	insignificant	29	73	68
chr10	84848558	84850558	Btbd11	-0.14064	1.2E-21	hypomethylated	0.002945	0.91457	insignificant	32	132	132
chr10	85060155	85062155	Btbd11	-0.23724	4.56E-14	hypomethylated	-0.03484	7.34E-12	hypomethylated	11	45	45
chr10	85333575	85335575	Pwp1	-0.17409	0.00000038	hypomethylated	0.0026145	0.82369	insignificant	10	59	59
chr10	85379690	85381690	Prdm4	-0.036342	8.89E-12	hypomethylated	-0.015499	0.017342	hypomethylated	18	64	64
chr10	85390235	85392235	Ascl4	-0.17304	6.12E-43	hypomethylated	-0.0051727	0.00090679	hypomethylated	25	70	70
chr10	85483673	85485673	Fbxo7	-0.11858	1.87E-17	hypomethylated	-0.0082836	0.57237	insignificant	33	82	97
chr10	85762156	85764156	Timp3	-0.13253	2.47E-11	hypomethylated	0.0067461	0.025011	inconclusive	14	76	65
chr10	86147271	86149271	1810014B01Rik	-0.31366	0.002642	hypomethylated	-0.041727	0.24111	insignificant	1	8	8
chr10	86167555	86169555	BC030307	-0.15337	4.07E-11	hypomethylated	-0.0069495	0.037381	inconclusive	17	83	77
chr10	86168189	86170189	BC030307	-0.13829	0.040516	hypomethylated	-0.0076396	0.72247	insignificant	4	34	28
chr10	86174509	86176509	BC030307	-0.12028	0.061108	insignificant	0.031662	0.16809	insignificant	3	15	16
chr10	86240749	86242749	Nt5dc3	-0.1337	2.32E-35	hypomethylated	-0.00577	0.0017302	hypomethylated	36	137	141
chr10	86519790	86521790	1700113H08Rik	-0.21717	0.57847	insignificant	0.078376	0.3917	insignificant	4	12	16

chr10	86956405	86958405	Ascl1	-0.16101	0.00000395	hypomethylated	0.031583	0.93946	insignificant	8	19	22
chr10	87428415	87430415	Tyms-ps	0.050947	1	insignificant	0.12744	1	insignificant	3	6	13
chr10	87608736	87610736	Nup37	-0.19675	0.00000281	hypomethylated	-0.00038634	0.96313	insignificant	12	72	72
chr10	87608815	87610815	Nup37	-0.19675	0.00000281	hypomethylated	-0.00038634	0.96313	insignificant	12	72	72
chr10	87609686	87611686	4930547N16Rik	-0.21031	0.000011932	hypomethylated	-0.0045911	0.91402	insignificant	8	54	54
chr10	87662841	87664841	Ccdc53	-0.43385	0.058589	insignificant	-0.17957	0.55785	insignificant	6	28	22
chr10	87819820	87821820	Dram1	-0.13387	0.050706	insignificant	-0.019217	0.7932	insignificant	5	10	10
chr10	87841156	87843156	Gnptab	-0.13863	8.49E-39	hypomethylated	-0.015276	0.000046096	hypomethylated	40	112	119
chr10	87921331	87923331	Sycp3	0.080123	0.8746	insignificant	0.026152	0.015596	hypermethylated	12	38	38
chr10	87966715	87968715	Chpt1	-0.21629	4.92E-11	hypomethylated	-0.05692	0.0015409	hypomethylated	10	15	24
chr10	88193158	88195158	Arl1	-0.074414	5.14E-22	hypomethylated	-0.008342	0.13144	insignificant	29	166	166
chr10	88193846	88195846	Gm4925	-0.051112	7.59E-20	hypomethylated	-0.0071	0.21978	insignificant	25	152	152
chr10	88347736	88349736	Slc5a8	-0.2824	2.01E-11	hypomethylated	0.0051926	0.38717	insignificant	6	43	47
chr10	88720535	88722535	Ano4		1	noCoverage	-0.14887	0.13587	insignificant	0	8	8
chr10	88906712	88908712	Gas2l3		1	noCoverage	0.081956	0.015109	hypermethylated	0	14	8
chr10	89083994	89085994	Slc17a8	0.047206	0.63331	insignificant	0.064174	0.6079	insignificant	4	14	14
chr10	89149030	89151030	Scyl2	-0.19817	0.00000145	hypomethylated	-0.036343	0.35616	insignificant	13	26	27
chr10	89195040	89197040	Actr6		1	noCoverage	0.045647	1	insignificant	0	9	10
chr10	89206735	89208735	Uhrf1bp1l	-0.1579	2.96E-11	hypomethylated	-0.047914	0.48728	insignificant	15	51	56
chr10	89335253	89337253	Anks1b	-0.12973	4.74E-12	hypomethylated	-0.01064	0.018941	inconclusive	18	104	104
chr10	90038435	90040435	Anks1b	-0.30765	0.088723	insignificant	0.10904	0.012098	hypermethylated	4	8	8
chr10	90544783	90546783	Ikbip	-0.13063	2.58E-30	hypomethylated	-0.011985	0.00070194	hypomethylated	41	114	115
chr10	90545488	90547488	Ikbip	-0.096352	8.01E-09	hypomethylated	-0.017721	0.050572	insignificant	18	54	55
chr10	90586708	90588708	Slc25a3	-0.10607	0.00017969	hypomethylated	0.0039247	0.74005	insignificant	6	38	38
chr10	90634327	90636327	Tmpo	-0.18374	0.000000706	hypomethylated	0.067367	0.81938	insignificant	10	80	61
chr10	91627923	91629923	Rmst	-0.90939	0.15833	lowCoverage	-0.024331	0.37188	insignificant	1	6	6
chr10	92622620	92624620	Cdk17	-0.10065	1.19E-26	hypomethylated	0.014632	0.33041	insignificant	46	198	161
chr10	92773904	92775904	Elk3		1	noCoverage	0.0027876	0.89382	insignificant	0	24	30
chr10	92915140	92917140	Lta4h	-0.11989	0.000000403	hypomethylated	0.0025778	0.62333	insignificant	10	42	46
chr10	92950512	92952512	Hal	-0.34893	0.30974	insignificant	-0.087874	0.026274	inconclusive	2	16	16
chr10	93002376	93004376	Ccdc38	-0.13452	5.77E-14	hypomethylated	0.0039729	0.47747	insignificant	11	51	52
chr10	93002778	93004778	Amdhd1	-0.10615	0.050013	insignificant	0.017773	0.41625	insignificant	3	13	14
chr10	93052403	93054403	Snrpf	-0.079932	0.47432	insignificant	-0.089049	0.012619	inconclusive	1	21	26
chr10	93102793	93104793	Ntn4	-0.17898	1.75E-43	hypomethylated	-0.027898	0.0015499	hypomethylated	41	144	156
chr10	93293299	93295299	Usp44	0.014802	0.33497	insignificant	0.25017	0.00000106	hypermethylated	3	16	14
chr10	93497745	93499745	Fgd6	-0.099879	3.4E-14	hypomethylated	0.0027884	0.1663	insignificant	29	121	121
chr10	93498493	93500493	Vezt	-0.079402	1.64E-11	hypomethylated	0.0086415	0.39874	insignificant	27	101	96
chr10	93609675	93611675	Nr2c1	-0.15068	5.39E-13	hypomethylated	-0.032915	0.045457	hypomethylated	14	30	38
chr10	93660753	93662753	Ndufa12	-0.13135	8.12E-14	hypomethylated	-0.007559	0.15452	insignificant	15	74	74
chr10	93976601	93978601	Tmcc3	-0.14772	1.82E-38	hypomethylated	-0.0071116	0.025292	hypomethylated	42	151	154
chr10	94037001	94039001	Tmcc3	-0.48318	0.013053	stronglyHypometh	-0.010744	1	insignificant	2	11	13
chr10	94038007	94040007	Tmcc3	-0.32776	0.023985	hypomethylated	0.0059803	1	insignificant	4	17	19
chr10	94150534	94152534	Ccdc41	-0.088364	3.42E-19	hypomethylated	0.0041861	1	insignificant	36	151	148
chr10	94151358	94153358	4932415G12Rik	-0.084299	1.26E-15	hypomethylated	0.010961	0.55109	insignificant	31	121	119
chr10	94407212	94409212	Plxnc1	-0.10371	4.18E-16	hypomethylated	0.0092545	0.13625	insignificant	25	99	84
chr10	94877917	94879917	Socs2	-0.10906	3E-27	hypomethylated	-0.0040986	0.41638	insignificant	44	195	195
chr10	94878118	94880118	Socs2	-0.10379	5.5E-26	hypomethylated	-0.0035026	0.51788	insignificant	43	193	193
chr10	94878846	94880846	Socs2	-0.12327	2.19E-33	hypomethylated	-0.01011	0.050528	insignificant	46	166	165
chr10	94879491	94881491	5730420D15Rik	-0.15231	3.16E-09	hypomethylated	-0.021101	0.047533	hypomethylated	15	58	58
chr10	94964561	94966561	Mrpl42	0.096669	1	insignificant	0.081095	1	insignificant	3	8	10
chr10	94976795	94978795	Ube2n	-0.11175	1.89E-44	hypomethylated	-0.0076439	0.72674	insignificant	63	178	178
chr10	95021955	95023955	Mir3058	-0.26942	0.01561	hypomethylated	-0.0079031	0.89626	insignificant	4	18	20
chr10	95026801	95028801	Nudt4	-0.21251	2.94E-37	hypomethylated	0.025786	0.000080619	inconclusive	17	72	77
chr10	95402296	95404296	Eea1	-0.11082	1.52E-15	hypomethylated	-0.0054725	0.6584	insignificant	47	149	148
chr10	96078634	96080634	Btg1	-0.10169	6.68E-16	hypomethylated	-0.013182	0.92119	insignificant	44	156	153
chr10	97154692	97156692	4921510H08Rik	0.026001	0.87067	insignificant	0.036003	0.031251	hypermethylated	11	67	66
chr10	98376785	98378785	Atp2b1	-0.10349	6.54E-37	hypomethylated	-0.0031858	0.68452	insignificant	106	279	286
chr10	98568804	98570804	Poc1b	-0.13198	3.96E-23	hypomethylated	0.0099266	0.75194	insignificant	36	104	106
chr10	98569768	98571768	Galnt4	-0.14722	6.42E-14	hypomethylated	0.015325	0.74485	insignificant	39	98	102
chr10	98724864	98726864	Dusp6	-0.12649	8.05E-17	hypomethylated	0.014957	0.20864	insignificant	23	109	117
chr10	99222292	99224292	Csl		1	noCoverage	0.025301	0.37329	insignificant	0	9	10
chr10	99477457	99479457	Kitl	-0.1855	4.19E-51	hypomethylated	-0.015618	0.008937	hypomethylated	46	99	109
chr10	99949922	99951922	Cep290	-0.12688	5.82E-12	hypomethylated	-0.0084465	0.47446	insignificant	19	67	66
chr10	99949981	99951981	Cep290	-0.15428	1.27E-10	hypomethylated	-0.01088	0.11612	insignificant	16	55	54
chr10	100051893	100053893	4930430F08Rik	-0.12196	1	insignificant	-0.0055887	1	insignificant	4	16	16
chr10	102491411	102493411	Alx1	-0.17494	0.00063766	hypomethylated	0.026159	0.47703	insignificant	10	75	76
chr10	102829476	102831476	Slc6a15	-0.19071	0.00000194	hypomethylated	0.012587	0.5831	insignificant	6	36	34
chr10	105277534	105279534	Ccdc59	-0.12951	9.79E-23	hypomethylated	0.0022225	0.24915	insignificant	35	105	110
chr10	105278436	105280436	Ccdc59	-0.12827	1.32E-10	hypomethylated	-0.010274	0.19272	insignificant	23	73	77
chr10	105906394	105908394	Ppfia2	-0.22955	0.0053825	hypomethylated	0.026724	0.65681	insignificant	6	24	24
chr10	106707886	106709886	Lin7a	-0.14736	1.14E-11	hypomethylated	0.0023187	0.81321	insignificant	7	34	27
chr10	107157083	107159083	Ptprq	-0.11452	0.075254	insignificant	-0.065337	0.80112	insignificant	3	9	10
chr10	107349190	107351190	Otogl	-0.12937	0.52338	insignificant	-0.12804	0.42409	insignificant	1	6	6
chr10	107598455	107600455	Ppp1r12a	-0.12302	4.38E-29	hypomethylated	-0.01263	0.15034	insignificant	52	207	210
chr10	107768244	107770244	Pawr	-0.10651	4.26E-43	hypomethylated	-0.011669	0.023153	hypomethylated	75	269	266
chr10	108447675	108449675	Syt1	-0.13243	0.017475	hypomethylated	-0.024506	0.11102	insignificant	3	40	42
chr10	110036705	110038705	9230102K24Rik	-0.18323	0.51021	insignificant	-0.079318	0.11611	insignificant	3	16	16
chr10	110181520	110183520	E2f7	-0.097228	1.22E-25	hypomethylated	0.0031631	0.65743	insignificant	57	185	190
chr10	110356231	110358231	Csrp2	-0.13725	1.48E-22	hypomethylated	-0.012028	0.082527	insignificant	24	75	63
chr10	110447122	110449122	Zdhhc17	-0.11549	8.91E-15	hypomethylated	-0.00049894	0.020957	hypomethylated	36	100	104
chr10	110600857	110602857	Osbpl8	-0.12597	8.52E-25	hypomethylated	-0.0010915	0.85599	insignificant	45	151	148
chr10	110734734	110736734	Bbs10		1	noCoverage	0.0064301	0.7421	insignificant	0	54	45
chr10	110909247	110911247	Nap1l1	-0.14035	5.99E-37	hypomethylated	-0.0067214	0.003695	hypomethylated	38	103	103
chr10	110936843	110938843	Gm5176		1	noCoverage	-0.033333	0.53215	insignificant	0	4	4
chr10	110942341	110944341	Phlda1	-0.094654	2.96E-24	hypomethylated	-0.00731	0.0089302	hypomethylated	49	176	170

chr10	111408750	111410750	Krr1	-0.24115	3.11E-16	hypomethylated	-0.023984	0.14349	insignificant	18	58	61
chr10	111519409	111521409	Glipr1l2	0.07109	0.16559	insignificant	0.056273	0.02187	hypermethylated	5	22	19
chr10	111707178	111709178	Kcnc2	-0.13102	0.023895	hypomethylated	0.0015202	0.93637	insignificant	8	83	83
chr10	112366082	112368082	Atxn7l3b	-0.16035	0.037377	hypomethylated	0.055806	0.79802	insignificant	7	25	25
chr10	114238426	114240426	Trhde	-0.15851	3.84E-23	hypomethylated	-0.034662	0.50843	insignificant	43	133	135
chr10	114752647	114754647	Rab21	-0.14218	0.000034372	hypomethylated	-0.008027	0.14257	insignificant	12	36	36
chr10	114799318	114801318	Tmem19	-0.095641	0.00000392	hypomethylated	-0.0042314	0.71467	insignificant	5	45	45
chr10	114821014	114823014	Zfc3h1	-0.12244	2.97E-21	hypomethylated	0.0021827	0.86868	insignificant	65	178	178
chr10	114821491	114823491	Thap2	-0.12371	5.93E-21	hypomethylated	0.0044456	0.71624	insignificant	61	156	156
chr10	115024836	115026836	Lgr5	-0.18229	0.000000477	hypomethylated	0.034039	0.85157	insignificant	10	28	34
chr10	115253339	115255339	Tspan8		1	noCoverage	0.057692	0.60283	insignificant	0	4	4
chr10	115454418	115456418	Ptprr	-0.19402	0.000000161	hypomethylated	-0.02708	0.79221	insignificant	7	30	30
chr10	115550973	115552973	4933416C03Rik	0.03996	0.20035	insignificant	-0.087702	1.15E-08	hypomethylated	11	36	36
chr10	115613313	115615313	Ptprr	-0.43035	0.16249	insignificant	-0.015665	0.6387	insignificant	1	24	24
chr10	115613406	115615406	Ptprr	-0.43035	0.16249	insignificant	-0.015665	0.6387	insignificant	1	24	24
chr10	115737429	115739429	Ptprb	-0.085977	0.00097773	hypomethylated	-0.012843	0.35539	insignificant	10	34	34
chr10	115854176	115856176	1700058G18Rik	-0.18209	0.11529	insignificant	-0.071119	0.17169	insignificant	4	33	33
chr10	115910579	115912579	Kcnmb4	-0.071894	5.12E-11	hypomethylated	-0.0085965	0.33651	insignificant	36	133	134
chr10	116018567	116020567	Cnot2	-0.1212	3.97E-28	hypomethylated	0.0017859	0.22348	insignificant	39	125	126
chr10	116387436	116389436	Rab3ip		1	noCoverage	0.021871	0.51611	insignificant	0	18	18
chr10	116422369	116424369	Best3	-0.045387	1	insignificant	-0.13666	0.0056373	hypomethylated	6	12	12
chr10	116481396	116483396	Lrrc10	-0.13741	1	insignificant	0.043941	0.0025253	hypermethylated	16	42	46
chr10	116500870	116502870	Cct2	0.30439	0.30333	insignificant	-0.13605	0.25856	insignificant	3	18	21
chr10	116585530	116587530	Frs2	-0.21805	0.0000024	hypomethylated	-0.014044	0.15817	insignificant	10	38	36
chr10	116661563	116663563	Yeats4	-0.15983	0.39196	insignificant	-0.029802	0.67971	insignificant	5	13	13
chr10	116675737	116677737	9530003J23Rik	0.039541	1	lowCoverage	-0.040816	0.035958	hypomethylated	1	7	8
chr10	116729924	116731924	Lyz1	0.14587	0.37045	insignificant	0.14065	0.010825	hypermethylated	3	7	6
chr10	116814029	116816029	Cpsf6	0.063889	1	insignificant	0.027381	0.55676	insignificant	1	12	15
chr10	117065555	117067555	Cpm	-0.15111	5.45E-09	hypomethylated	0.0090877	0.21586	insignificant	11	73	72
chr10	117147772	117149772	Mdm2	-0.059337	0.00074884	hypomethylated	-0.0074866	0.010913	hypomethylated	35	76	76
chr10	117183414	117185414	Slc35e3	0.39933	1	insignificant	0.091994	0.5019	insignificant	2	26	22
chr10	117229761	117231761	Nup107		1	noCoverage	-0.037191	0.90942	insignificant	0	25	25
chr10	117283030	117285030	Rap1b	-0.083489	0.0015186	hypomethylated	-0.015298	0.31812	insignificant	22	55	66
chr10	117577842	117579842	Mdm1	-0.10587	0.000032679	hypomethylated	-0.021106	0.35216	insignificant	28	105	106
chr10	117732094	117734094	Iltifb		1	noCoverage	-0.14286	0.39832	insignificant	0	3	6
chr10	117877102	117879102	Ifng	-0.75703	0.28947	lowCoverage	0.13501	0.012188	hypermethylated	1	8	8
chr10	118305959	118307959	Dyrk2	-0.089299	1.03E-22	hypomethylated	-0.000088999	0.68445	insignificant	42	123	124
chr10	118677111	118679111	Cand1	-0.13361	4.92E-13	hypomethylated	-0.0094426	0.15051	insignificant	22	80	81
chr10	118890369	118892369	Grip1	-0.10854	1.46E-20	hypomethylated	0.0077114	0.83543	insignificant	55	198	195
chr10	119550021	119552021	Helb	-0.30688	1	lowCoverage	-0.0085878	0.0088282	inconclusive	1	31	31
chr10	119638593	119640593	Irak3	-0.038391	0.2326	insignificant	0.012209	1	insignificant	3	40	40
chr10	119644881	119646881	Tmbim4	-0.13985	1.51E-11	hypomethylated	-0.018191	0.6459	insignificant	15	46	52
chr10	119663115	119665115	Llph		1	noCoverage	0.083748	0.20317	insignificant	0	36	25
chr10	119913991	119915991	Hmga2	-0.14444	1.17E-17	hypomethylated	-0.0063667	0.82385	insignificant	13	63	62
chr10	120336027	120338027	Msrb3	-0.063217	0.00000407	hypomethylated	0.0023325	0.86705	insignificant	11	26	27
chr10	120416386	120418386	Lemd3	-0.10803	1.76E-22	hypomethylated	-0.0075643	0.07918	insignificant	29	64	64
chr10	120470059	120472059	Wif1	-0.16602	2.32E-09	hypomethylated	-0.0051708	0.047189	hypomethylated	15	74	74
chr10	120748245	120750245	Tbc1d30	0.098718	1	insignificant	0.04967	0.67796	insignificant	3	10	8
chr10	120801145	120803145	Gns	-0.17437	5.92E-25	hypomethylated	-0.011402	0.045505	hypomethylated	26	107	106
chr10	120913306	120915306	Rassf3	-0.1807	0.000000273	hypomethylated	-0.0064253	0.3732	insignificant	10	36	36
chr10	121023850	121025850	Xpot	-0.20798	0.35473	insignificant	-0.028793	0.00021241	hypomethylated	9	29	29
chr10	121063372	121065372	Xpot	-0.079054	0.0026823	hypomethylated	-0.00041803	0.96151	insignificant	6	44	45
chr10	121077756	121079756	D930020B18Rik	-0.10137	2.11E-13	hypomethylated	-0.014475	0.0080823	hypomethylated	29	108	110
chr10	121175992	121177992	BC048403	-0.10652	0.00000115	hypomethylated	0.005312	0.39924	insignificant	23	60	60
chr10	121484249	121486249	Srgap1	-0.10739	5.1E-22	hypomethylated	0.029097	1	insignificant	44	139	142
chr10	121516935	121518935	Gm9079		1	noCoverage	0.016647	0.28104	insignificant	0	14	14
chr10	121884554	121886554	Avpr1a	-0.020773	0.055542	insignificant	0.031746	0.095368	insignificant	7	83	74
chr10	122114817	122116817	Ppm1h	-0.09312	2.99E-28	hypomethylated	-0.0047714	0.93292	insignificant	54	170	163
chr10	122422780	122424780	Mirlet7i	-0.10808	7.54E-30	hypomethylated	-0.0097816	0.11082	insignificant	69	185	185
chr10	122513561	122515561	Mon2		1	noCoverage	0.11458	1	insignificant	0	10	18
chr10	122633979	122635979	Usp15	-0.14251	0.000027638	hypomethylated	-0.060189	0.2363	insignificant	19	57	53
chr10	122700131	122702131	Fam19a2	-0.13486	5.26E-21	hypomethylated	-0.011702	0.018073	hypomethylated	23	112	112
chr10	123905129	123907129	4930503E24Rik		1	noCoverage	-0.13821	0.30671	insignificant	0	22	31
chr10	124765591	124767591	Slc16a7	-0.42121	0.000076937	stronglyHypometh	-0.11746	0.0034007	hypomethylated	1	6	6
chr10	125402274	125404274	Lrig3	-0.11274	8.33E-26	hypomethylated	-0.0057681	0.16189	insignificant	47	156	154
chr10	126414772	126416772	Ctdsp2	-0.068354	0.00033501	hypomethylated	0.0082327	0.22032	insignificant	35	124	136
chr10	126431585	126433585	Mir26a-2	-0.21013	0.012247	hypomethylated	-0.0088031	0.52335	insignificant	5	23	22
chr10	126431668	126433668	Ctdsp2	-0.21013	0.012247	hypomethylated	-0.0088031	0.52335	insignificant	5	23	22
chr10	126434495	126436495	Mir546	-0.0010791	1	insignificant	0.030657	0.26252	insignificant	2	4	4
chr10	126467870	126469870	Tsfm	-0.0975	0.0021507	hypomethylated	0.088927	0.16669	insignificant	5	29	33
chr10	126477987	126479987	Mettl1	-0.18365	7.93E-57	hypomethylated	0.0041347	0.0028516	inconclusive	26	119	110
chr10	126484301	126486301	Cyp27b1	-0.13883	0.01253	hypomethylated	0.088589	0.838	insignificant	4	21	21
chr10	126497240	126499240	March9	-0.18056	0.043916	hypomethylated	0.017885	0.11915	insignificant	8	25	26
chr10	126499658	126501658	Cdk4	-0.096165	0.00001476	hypomethylated	0.017344	0.77389	insignificant	17	89	83
chr10	126507317	126509317	Tspan31	0.26528	0.17827	insignificant	-0.00058521	0.20269	insignificant	1	16	16
chr10	126514962	126516962	Agap2	0.13999	5.47E-08	hypermethylated	0.0671	0.00000957	hypermethylated	15	37	43
chr10	126601280	126603280	B4galnt1	-0.094729	1.86E-56	hypomethylated	0.00082227	0.40101	insignificant	57	220	216
chr10	126601886	126603886	B4galnt1	-0.094138	2.28E-57	hypomethylated	0.0037646	0.31285	insignificant	58	240	240
chr10	126626879	126628879	Dtx3	-0.27244	9.11E-17	hypomethylated	0.035831	0.70415	insignificant	9	34	34
chr10	126627110	126629110	Dtx3	-0.22569	0.1092	insignificant	0.038599	0.66947	insignificant	2	10	10
chr10	126632765	126634765	Dtx3	-0.0037085	0.000099992	inconclusive	0.024508	0.37556	insignificant	5	22	20
chr10	126648678	126650678	Pip4k2c	-0.34361	9.81E-34	stronglyHypometh	0.027794	0.000016487	inconclusive	13	43	46
chr10	126700419	126702419	Dctn2	0.19444	1	insignificant	0.021429	0.81947	insignificant	2	6	9
chr10	126702317	126704317	Dctn2	-0.14076	1.7E-11	hypomethylated	-0.0023897	0.28948	insignificant	29	83	88
chr10	126725827	126727827	Ddit3	-0.12167	2.33E-13	hypomethylated	0.0061876	0.63329	insignificant	12	98	108

chr10	126726848	126728848	Ddit3	-0.19572	4.17E-16	hypomethylated	0.000013406	0.68471	insignificant	14	59	59
chr10	126748842	126750842	Mars	-0.38299	1.98E-09	stronglyHypometh	-0.16447	0.2237	insignificant	4	15	24
chr10	126788828	126790828	Inhbe	-0.083957	0.18176	insignificant	-0.025285	0.7607	insignificant	3	12	12
chr10	126856922	126858922	R3hdm2	0.0013605	0.34587	insignificant	-0.0034014	0.77074	insignificant	3	7	6
chr10	126950994	126952994	Ndufa4l2	-0.39209	0.53744	insignificant	-0.039428	0.81494	insignificant	1	15	10
chr10	126959491	126961491	Shmt2	-0.37829	0.17939	insignificant	-0.10701	0.28831	insignificant	2	14	15
chr10	126971615	126973615	Nxph4		1	noCoverage	-0.023968	0.76354	insignificant	0	35	35
chr10	127058204	127060204	Lrp1	-0.2979	8.39E-28	hypomethylated	-0.039225	1	insignificant	8	34	33
chr10	127079041	127081041	Stat6	-0.20972	5.4E-19	hypomethylated	-0.002459	0.061439	insignificant	11	34	32
chr10	127103759	127105759	Nab2	-0.16289	0.000000293	hypomethylated	0.0029644	0.16885	insignificant	9	58	62
chr10	127113120	127115120	Tmem194	-0.14187	2.73E-11	hypomethylated	-0.013445	0.094884	insignificant	17	55	73
chr10	127175593	127177593	Zbtb39	-0.16383	2.66E-30	hypomethylated	-0.022058	0.17877	insignificant	35	78	80
chr10	127188854	127190854	Gpr182	-0.10113	0.45238	insignificant	-0.00068139	1	insignificant	2	5	4
chr10	127451270	127453270	Prim1	-0.33138	6.9E-11	hypomethylated	-0.040943	0.61486	insignificant	7	39	42
chr10	127471401	127473401	Naca	-0.11782	1.04E-17	hypomethylated	0.021458	0.43634	insignificant	17	88	88
chr10	127495037	127497037	Ptges3	-0.12582	3.02E-24	hypomethylated	-0.011043	0.40686	insignificant	54	154	155
chr10	127519362	127521362	Atp5b	-0.13668	2.34E-25	hypomethylated	-0.011808	0.13754	insignificant	23	91	91
chr10	127521341	127523341	Mir677	0.26481	1	insignificant	0.096184	0.63555	insignificant	2	21	22
chr10	127528838	127530838	Baz2a	-0.15266	2.9E-15	hypomethylated	-0.011927	0.32074	insignificant	26	86	86
chr10	127630690	127632690	Gls2	-0.098468	4E-22	hypomethylated	0.035918	0.80533	insignificant	30	133	123
chr10	127648850	127650850	Spryd4	-0.35915	0.00000603	stronglyHypometh	-0.087037	0.071359	insignificant	10	31	40
chr10	127668118	127670118	Timeless	-0.12091	5.69E-08	hypomethylated	0.0042353	0.73631	insignificant	18	45	45
chr10	127674121	127676121	Timeless	-0.012323	1	insignificant	-0.13851	0.5406	insignificant	2	6	6
chr10	127690186	127692186	Apon	0.030075	1	insignificant	0.00031328	0.40868	insignificant	2	7	14
chr10	127704052	127706052	Apof		1	noCoverage	0.071171	0.44234	insignificant	0	9	7
chr10	127706631	127708631	Stat2	-0.35505	0.00000618	stronglyHypometh	0.056133	0.78294	insignificant	5	34	36
chr10	127735140	127737140	Il23a		1	noCoverage	0.056818	1	insignificant	0	6	6
chr10	127739390	127741390	Pan2		1	noCoverage	-0.012305	0.88662	insignificant	0	14	14
chr10	127758514	127760514	Cnpy2	-0.29008	0.000010945	hypomethylated	-0.023	0.00058085	inconclusive	6	38	44
chr10	127773887	127775887	Cs	-0.18477	9.33E-38	hypomethylated	-0.041113	0.000034266	hypomethylated	22	88	93
chr10	127807093	127809093	Coq10a	-0.44944	0.13982	insignificant	-0.067733	0.11209	insignificant	1	17	20
chr10	127813179	127815179	Ankrd52	-0.1044	1.03E-37	hypomethylated	-0.0034212	0.48117	insignificant	36	128	121
chr10	127846852	127848852	Rnf41	-0.15681	8.16E-16	hypomethylated	-0.0068821	0.54387	insignificant	8	38	40
chr10	127847671	127849671	Rnf41	-0.15162	6.75E-14	hypomethylated	0.0083526	0.47966	insignificant	7	36	36
chr10	127895291	127897291	Smarcc2	-0.08362	2.29E-23	hypomethylated	0.0049338	0.91042	insignificant	54	214	193
chr10	127935741	127937741	Myl6b	-0.19827	0.020865	hypomethylated	0.017061	0.32822	insignificant	13	59	59
chr10	127962915	127964915	Esyt1	-0.2693	0.0012323	hypomethylated	-0.11773	0.58577	insignificant	3	17	15
chr10	127984800	127986800	Zc3h10	-0.13614	0.00000539	hypomethylated	0.008697	0.90979	insignificant	15	50	53
chr10	127986224	127988224	Rpl41		1	noCoverage	-0.067674	0.69232	insignificant	0	6	6
chr10	128002990	128004990	Pa2g4	0.002668	5.96E-14	inconclusive	0.091313	0.34553	insignificant	8	64	48
chr10	128026557	128028557	Erbb3	-0.17567	6.03E-21	hypomethylated	-0.025767	0.005475	hypomethylated	43	125	134
chr10	128063562	128065562	Rps26	-0.2827	0.00023897	hypomethylated	0.096079	0.020557	inconclusive	9	27	32
chr10	128110974	128112974	Suox	-0.21278	0.000071178	hypomethylated	-0.048191	0.84938	insignificant	10	34	41
chr10	128133324	128135324	Rab5b		1	noCoverage	-0.023438	0.80426	insignificant	0	8	4
chr10	128142107	128144107	Cdk2	-0.11196	0.0049571	hypomethylated	0.0051077	0.94413	insignificant	14	40	34
chr10	128142313	128144313	Pmel	-0.12696	0.8261	insignificant	0.010767	0.61816	insignificant	2	8	8
chr10	128181112	128183112	Dgka	-0.38341	0.068177	insignificant	-0.012425	0.010807	inconclusive	2	25	28
chr10	128183934	128185934	Wibg	-0.2399	2.78E-22	hypomethylated	0.0081973	0.69753	insignificant	15	90	89
chr10	128184577	128186577	Wibg	-0.13671	8.1E-21	hypomethylated	-0.0032621	0.28903	insignificant	18	83	82
chr10	128241426	128243426	1110012D08Rik	-0.17147	7.26E-20	hypomethylated	-0.0023566	0.000084139	hypomethylated	29	100	106
chr10	128241731	128243731	Dnajc14	-0.18383	4.22E-22	hypomethylated	-0.0022478	0.00000206	hypomethylated	29	102	108
chr10	128257826	128259826	Sarnp	-0.18336	0.089569	insignificant	-0.042336	0.40306	insignificant	9	22	25
chr10	128328774	128330774	Gdf11	-0.1449	0.003894	hypomethylated	0.0017155	0.42244	insignificant	8	34	34
chr10	128344974	128346974	Cd63	-0.2519	0.0000195	hypomethylated	0.027644	0.055495	insignificant	6	44	42
chr10	128345921	128347921	Cd63	-0.13151	0.000026692	hypomethylated	0.027523	0.93869	insignificant	18	161	160
chr10	128369868	128371868	Itga7	-0.20228	1.48E-17	hypomethylated	-0.019584	0.021258	hypomethylated	10	64	53
chr10	128549479	128551479	Olfr769	-0.625	0.00032731	stronglyHypometh	-0.026515	0.70059	insignificant	1	11	11
chr10	128624475	128626475	Olfr773		1	noCoverage	-0.066938	0.731	insignificant	0	4	6
chr10	128908749	128910749	Olfr788	-0.42917	0.0057762	stronglyHypometh	-0.025198	1	insignificant	2	4	4
chr10	128976594	128978594	Olfr792		1	noCoverage	0.059524	0.77959	insignificant	0	4	2
chr10	129203553	129205553	Olfr808	-0.5625	0.018492	stronglyHypometh	0.070833	0.74319	insignificant	1	4	5
chr10	129617934	129619934	Olfr826		1	noCoverage	0.0074074	1	insignificant	0	3	3
chr11	3023023	3025023	Pisd-ps1	-0.12668	6.69E-09	hypomethylated	0.026598	0.23102	insignificant	5	22	26
chr11	3023029	3025029	Pisd-ps3	-0.12668	6.69E-09	hypomethylated	0.026598	0.23102	insignificant	5	22	26
chr11	3093466	3095466	Sfi1	-0.1732	3.52E-35	hypomethylated	-0.018104	0.23052	insignificant	11	42	41
chr11	3101355	3103355	Eif4enif1	-0.11685	3.67E-56	hypomethylated	-0.0040021	0.43885	insignificant	81	198	201
chr11	3101585	3103585	Eif4enif1	-0.11685	3.67E-56	hypomethylated	-0.0040021	0.43885	insignificant	81	198	201
chr11	3101997	3103997	Eif4enif1	-0.11685	3.67E-56	hypomethylated	-0.0040021	0.43885	insignificant	81	198	201
chr11	3102021	3104021	Eif4enif1	-0.11685	3.67E-56	hypomethylated	-0.0040021	0.43885	insignificant	81	198	201
chr11	3166389	3168389	Drg1		1	noCoverage	-0.046967	1	insignificant	0	14	21
chr11	3189459	3191459	Patz1	-0.11203	2.94E-44	hypomethylated	-0.0064623	0.040806	hypomethylated	36	146	145
chr11	3229733	3231733	Pik3ip1		1	noCoverage	-0.037729	0.065295	insignificant	0	5	4
chr11	3271395	3273395	Limk2	-0.21705	0.000000723	hypomethylated	-0.0013529	1	insignificant	7	11	11
chr11	3351439	3353439	8430429K09Rik	-0.16287	1.73E-27	hypomethylated	-0.0088701	0.34805	insignificant	22	100	90
chr11	3352328	3354328	Rnf185	-0.055933	0.00014405	hypomethylated	0.006998	0.028096	inconclusive	7	46	36
chr11	3387229	3389229	Pla2g3	-0.10755	0.070154	insignificant	0.014912	0.72047	insignificant	9	27	27
chr11	3404824	3406824	Inpp5j	-0.14983	0.0059026	hypomethylated	-0.038853	0.18109	insignificant	1	7	9
chr11	3413704	3415704	Selm	-0.11878	0.26424	insignificant	-0.088899	0.32814	insignificant	4	15	15
chr11	3439295	3441295	Smtn	-0.63158	0.000024827	stronglyHypometh	-0.034417	0.399	insignificant	2	21	21
chr11	3547536	3549536	Tug1	-0.12929	5.49E-25	hypomethylated	-0.00010406	0.32056	insignificant	51	132	154
chr11	3548496	3550496	Morc2a	-0.15158	1.57E-24	hypomethylated	-0.026372	0.33919	insignificant	48	128	146
chr11	3548811	3550811	Tug1	-0.18942	1.65E-24	hypomethylated	-0.039114	0.44297	insignificant	33	86	104
chr11	3794242	3796242	Dusp18	-0.034836	0.22624	insignificant	-0.028733	0.000029099	hypomethylated	5	57	56
chr11	3795129	3797129	4921536K21Rik	-0.017698	0.72107	insignificant	-0.017304	1.48E-08	hypomethylated	7	46	46
chr11	3814667	3816667	Slc35e4	-0.2	0.023737	hypomethylated	0.011682	0.45125	insignificant	3	6	6

chr11	3831964	3833964	Tcn2	-0.3464	0.30148	insignificant	0.024709	0.52851	insignificant	4	14	14
chr11	3832047	3834047	Tcn2	-0.30156	0.5562	insignificant	0.028838	0.12979	insignificant	3	12	12
chr11	3832081	3834081	Tcn2	-0.36187	0.28244	insignificant	0.011076	0.63064	insignificant	3	10	10
chr11	3862977	3864977	Pes1	-0.31192	1.27E-15	hypomethylated	-0.066159	0.0012897	hypomethylated	7	16	18
chr11	3882638	3884638	Gal3st1	-0.099492	1.1E-13	hypomethylated	0.0054776	0.019445	inconclusive	23	70	70
chr11	3888935	3890935	Gal3st1	0.17361	1	insignificant	-0.034722	0.40926	insignificant	2	4	4
chr11	3995434	3997434	Mtfp1	-0.11776	0.000040101	hypomethylated	-0.008919	0.27482	insignificant	17	61	62
chr11	4018732	4020732	Sec14l2		1	noCoverage	-0.062959	0.18203	insignificant	0	18	18
chr11	4034162	4036162	Rnf215	-0.1448	0.000022748	hypomethylated	-0.031966	0.12097	insignificant	17	55	59
chr11	4038366	4040366	Mir3060	-0.11333	0.10828	insignificant	-0.058735	0.68385	insignificant	1	8	8
chr11	4059356	4061356	Sf3a1	-0.13431	4.37E-15	hypomethylated	0.0096773	0.1868	insignificant	16	78	78
chr11	4060296	4062296	Ccdc157	-0.11936	1.69E-10	hypomethylated	0.0045838	0.79513	insignificant	14	70	70
chr11	4085835	4087835	Tbc1d10a	-0.12123	0.0014964	hypomethylated	0.00076376	0.93249	insignificant	9	91	100
chr11	4117253	4119253	Gatsl3	-0.11276	7.04E-42	hypomethylated	0.0060931	0.00020611	inconclusive	36	96	93
chr11	4156570	4158570	Lif	0.087234	0.48631	insignificant	-0.016164	0.78218	insignificant	4	22	22
chr11	4166097	4168097	Lif	-0.13526	6.75E-28	hypomethylated	-0.00029266	0.071709	insignificant	29	86	89
chr11	4341085	4343085	Hormad2	-0.10431	0.090873	insignificant	0.04809	0.44763	insignificant	6	16	16
chr11	4494818	4496818	Mtmr3	-0.075222	0.0083974	hypomethylated	0.022771	0.26591	insignificant	7	14	14
chr11	4536795	4538795	Ascc2	-0.20926	2.18E-15	hypomethylated	-0.010187	0.00055272	inconclusive	17	46	45
chr11	4603680	4605680	Zmat5		1	noCoverage	0.000092006	0.8606	insignificant	0	25	25
chr11	4604347	4606347	Uqcr10		1	noCoverage	-0.0073977	0.78194	insignificant	0	19	19
chr11	4646781	4648781	Cabp7	-0.10741	1.88E-18	hypomethylated	0.031042	0.96506	insignificant	31	104	104
chr11	4749530	4751530	Nf2	-0.12242	1	insignificant	0.01984	0.03207	hypermethylated	14	46	46
chr11	4773005	4775005	Nipsnap1	-0.21159	0.00015392	hypomethylated	-0.033379	0.44314	insignificant	5	37	39
chr11	4794345	4796345	Thoc5	-0.15388	6.6E-21	hypomethylated	-0.0037785	0.39336	insignificant	17	62	62
chr11	4848067	4850067	Ap1b1	-0.41612	1.16E-13	stronglyHypometh	-0.043413	0.058217	insignificant	12	60	56
chr11	4885907	4887907	Ap1b1	-0.15682	4.8E-17	hypomethylated	-0.020837	0.0083906	hypomethylated	26	78	78
chr11	4957130	4959130	Rasl10a	-0.1425	3.22E-32	hypomethylated	0.028712	0.0024791	inconclusive	39	130	128
chr11	4965330	4967330	Gas2l1	-0.26103	2.48E-11	hypomethylated	0.025306	0.18122	insignificant	4	19	25
chr11	4998440	5000440	Rhbdd3	-0.097851	1.82E-16	hypomethylated	0.0067346	0.93667	insignificant	35	159	164
chr11	4999080	5001080	Ewsr1	-0.098469	1.6E-11	hypomethylated	0.023508	1	insignificant	17	108	103
chr11	5052225	5054225	Emid1	-0.18634	0.00000395	hypomethylated	-0.023935	0.17162	insignificant	18	42	51
chr11	5161613	5163613	Kremen1	-0.13443	0.070693	insignificant	-0.015569	0.00012633	inconclusive	30	97	95
chr11	5344850	5346850	Znrf3	-0.1022	5.05E-08	hypomethylated	0.021987	0.45138	insignificant	7	63	63
chr11	5419969	5421969	Xbp1	-0.082175	0.000020837	hypomethylated	-0.0066807	0.29812	insignificant	30	100	100
chr11	5442220	5444220	Ccdc117	-0.1188	0.14473	insignificant	-0.019296	0.18932	insignificant	3	10	12
chr11	5607702	5609702	Mrps24		1	noCoverage	-0.1428	0.09118	insignificant	0	10	5
chr11	5641179	5643179	Urgcp	-0.071108	0.010676	hypomethylated	0.13755	0.92159	insignificant	3	17	20
chr11	5661152	5663152	2210015D19Rik	-0.12672	3.75E-08	hypomethylated	0.041696	0.77915	insignificant	8	28	40
chr11	5687485	5689485	Dbnl	-0.12349	5.48E-24	hypomethylated	0.0093859	0.1201	insignificant	27	94	100
chr11	5703799	5705799	Pgam2		1	noCoverage	-0.014094	0.39296	insignificant	0	3	5
chr11	5760868	5762868	Aebp1	-0.14901	1.59E-19	hypomethylated	-0.016656	0.033621	hypomethylated	14	63	64
chr11	5854760	5856760	Ykt6	-0.10371	2.62E-14	hypomethylated	-0.00000509	0.013014	hypomethylated	21	102	92
chr11	6073334	6075334	Gm11968		1	noCoverage	0.17143	1	insignificant	0	7	7
chr11	6167732	6169732	Ddx56	0.46756	0.17435	insignificant	0.074342	0.15139	insignificant	2	14	14
chr11	6190599	6192599	Ogdh	-0.092034	2.78E-14	hypomethylated	-0.0098379	0.34308	insignificant	34	94	97
chr11	6288366	6290366	Zmiz2	-0.12325	1.2E-18	hypomethylated	-0.014366	0.63637	insignificant	43	162	175
chr11	6314872	6316872	Ppia	-0.10755	2.64E-19	hypomethylated	-0.0050978	0.88743	insignificant	36	131	131
chr11	6344446	6346446	H2afv	0.046912	0.086946	insignificant	-0.0085551	0.0024073	hypomethylated	10	24	24
chr11	6375920	6377920	Purb	-0.10229	2.25E-20	hypomethylated	-0.0083698	1	insignificant	34	80	80
chr11	6445889	6447889	Ccm2	-0.11925	3.73E-22	hypomethylated	-0.0048054	0.26234	insignificant	33	94	94
chr11	6506057	6508057	Nacad	-0.075732	0.14356	insignificant	0.099226	1	insignificant	4	12	13
chr11	6526070	6528070	Tbrg4	-0.16042	4.37E-16	hypomethylated	0.010618	0.26379	insignificant	15	37	32
chr11	6538652	6540652	Wap	0.12912	0.77584	insignificant	-0.24112	0.29981	insignificant	2	10	10
chr11	6557535	6559535	Ramp3	-0.40648	0.00021165	stronglyHypometh	0.11087	0.54187	insignificant	1	6	4
chr11	6962491	6964491	Adcy1	-0.091209	2.32E-11	hypomethylated	-0.014121	0.020797	hypomethylated	50	145	142
chr11	7113926	7115926	Igfbp3	-0.20085	2.36E-44	hypomethylated	-0.0099489	0.000082783	hypomethylated	21	66	68
chr11	8564538	8566538	Tns3		1	noCoverage	-0.084419	0.91819	insignificant	0	31	22
chr11	8911140	8913140	Hus1	-0.16598	6.5E-09	hypomethylated	0.0047178	0.6994	insignificant	10	28	28
chr11	8947594	8949594	Gm11992	-0.15489	0.15645	insignificant	-0.031347	0.82594	insignificant	10	34	34
chr11	9017010	9019010	Upp1	0.81505	1.37E-11	stronglyHypermeth	0.0094117	0.15111	insignificant	5	18	18
chr11	9017105	9019105	Upp1	0.81505	1.37E-11	stronglyHypermeth	0.0094117	0.15111	insignificant	5	18	18
chr11	9017509	9019509	Upp1	0.81505	1.37E-11	stronglyHypermeth	-0.010025	0.13292	insignificant	5	18	20
chr11	9090944	9092944	Abca13	-0.0045958	0.70931	insignificant	0.14264	0.11574	insignificant	1	12	10
chr11	11013018	11015018	Vwc2	-0.1986	8.05E-09	hypomethylated	-0.022297	0.46918	insignificant	16	41	41
chr11	11585215	11587215	Ikzf1	-0.095795	4.31E-24	hypomethylated	0.0054559	0.53331	insignificant	62	221	228
chr11	11708965	11710965	Fignl1	-0.17324	0.38034	insignificant	0.018928	0.68322	insignificant	4	44	44
chr11	11798147	11800147	Ddc	-0.95299	0.051546	lowCoverage	-0.1788	0.039982	hypomethylated	1	6	6
chr11	11937423	11939423	Grb10	-0.15295	1.19E-17	hypomethylated	0.0057738	0.00054647	inconclusive	32	104	99
chr11	12364963	12366963	Cobl	-0.075922	2.66E-08	hypomethylated	-0.0014587	0.83914	insignificant	16	35	35
chr11	14498242	14500242	Pom121l12		1	noCoverage	-0.078431	0.35836	insignificant	0	4	4
chr11	16156726	16158726	Vstm2a		1	noCoverage	-0.050219	1	insignificant	0	14	24
chr11	16408487	16410487	Sec61g		1	noCoverage	0.035714	0.4073	insignificant	0	9	9
chr11	16651205	16653205	Egfr	-0.1443	6.22E-29	hypomethylated	-0.020246	0.12526	insignificant	33	108	106
chr11	16850412	16852412	Fbxo48	-0.30873	0.55383	insignificant	-0.023016	0.74724	insignificant	2	14	14
chr11	16851096	16853096	2810442I21Rik	-0.29028	1	insignificant	-0.040278	0.58375	insignificant	1	8	8
chr11	16950936	16952936	Cnrip1	-0.18193	0.000079697	hypomethylated	0.031036	0.35828	insignificant	10	34	39
chr11	17058300	17060300	Ppp3r1	-0.14463	9.02E-24	hypomethylated	-0.02081	0.051193	insignificant	58	151	147
chr11	17110895	17112895	Wdr92	-0.17199	4.92E-25	hypomethylated	-0.015301	0.1989	insignificant	21	70	72
chr11	17111592	17113592	Pno1	-0.32842	0.000000015	hypomethylated	-0.039182	0.19253	insignificant	3	16	16
chr11	17156620	17158620	C1d	-0.22458	3.69E-14	hypomethylated	0.032005	0.022662	inconclusive	12	52	57
chr11	18918972	18920972	Meis1	-0.14778	0.000010307	hypomethylated	-0.0057695	0.67143	insignificant	24	140	139
chr11	19823444	19825444	Spred2	-0.1356	1.47E-08	hypomethylated	-0.0053003	0.83984	insignificant	20	72	57
chr11	20012954	20014954	Actr2	-0.32303	1.92E-31	hypomethylated	0.042506	0.11211	insignificant	19	67	63
chr11	20100604	20102604	Rab1	-0.15796	3.85E-18	hypomethylated	-0.033653	0.051189	insignificant	25	89	104

chr11	20149427	20151427	Cep68		1	noCoverage	0.0037259	0.92598	insignificant	0	31	48
chr11	20232716	20234716	Slc1a4	-0.19832	0.33165	insignificant	0.026005	0.73401	insignificant	4	28	30
chr11	20442255	20444255	Sertad2	-0.1137	0.000000436	hypomethylated	-0.005239	0.36103	insignificant	37	121	120
chr11	20530979	20532979	Sertad2	-0.14682	2.5E-20	hypomethylated	-0.016201	0.010904	hypomethylated	39	129	125
chr11	20641487	20643487	Aftph	-0.17389	2.96E-12	hypomethylated	-0.0078956	0.67783	insignificant	9	34	34
chr11	20731111	20733111	1110067D22Rik	-0.10834	3.68E-10	hypomethylated	-0.0047551	0.59301	insignificant	8	55	55
chr11	20990326	20992326	Peli1	-0.11054	2.77E-12	hypomethylated	-0.022696	0.035691	hypomethylated	32	109	123
chr11	21137891	21139891	Vps54	-0.10324	3.08E-31	hypomethylated	0.00032138	0.97151	insignificant	49	131	139
chr11	21270882	21272882	Ugp2	-0.16491	0.00000156	hypomethylated	0.0018762	0.59826	insignificant	16	67	67
chr11	21471283	21473283	Wdpcp	-0.11604	0.00000332	hypomethylated	0.019826	0.52723	insignificant	14	58	61
chr11	21471937	21473937	Mdh1	-0.093779	0.00020352	hypomethylated	0.012168	0.63646	insignificant	8	36	37
chr11	21901654	21903654	Otx1	-0.044064	4.43E-15	hypomethylated	0.0080922	0.6861	insignificant	28	84	92
chr11	22185841	22187841	Ehbp1	-0.19425	0.00000227	hypomethylated	-0.0408	0.45288	insignificant	9	38	45
chr11	22411285	22413285	Tmem17	-0.18749	1.34E-32	hypomethylated	-0.018692	0.19612	insignificant	34	101	102
chr11	22759735	22761735	B3gnt2	-0.12965	3.23E-14	hypomethylated	-0.0055774	0.065496	insignificant	19	76	76
chr11	22760336	22762336	B3gnt2	-0.22949	0.00061666	hypomethylated	-0.020571	0.0029489	hypomethylated	6	28	28
chr11	22871028	22873028	Commd1	-0.049318	0.43416	insignificant	-0.047265	0.43341	insignificant	33	101	105
chr11	22882284	22884284	Commd1	-0.15687	0.4932	insignificant	-0.028836	0.00031458	hypomethylated	6	28	24
chr11	22889592	22891592	Cct4	-0.14654	5.5E-22	hypomethylated	-0.026005	0.099075	insignificant	28	62	70
chr11	23155040	23157040	Xpo1	-0.14245	0.00018717	hypomethylated	-0.018042	1	insignificant	13	121	119
chr11	23205894	23207894	Usp34	-0.12371	6.17E-22	hypomethylated	-0.0088537	0.053642	insignificant	55	147	147
chr11	23397946	23399946	Ahsa2	-0.2486	0.0038309	hypomethylated	-0.021347	0.10047	insignificant	6	29	38
chr11	23419195	23421195	1700093K21Rik		1	noCoverage	-0.15414	0.044197	hypomethylated	0	6	6
chr11	23458842	23460842	Gm12060		1	noCoverage	0.045996	0.76351	insignificant	0	7	4
chr11	23533631	23535631	0610010F05Rik		1	noCoverage	-0.19718	0.94387	insignificant	0	26	41
chr11	23564673	23566673	Pus10	-0.1423	3.78E-11	hypomethylated	0.010497	0.83418	insignificant	18	53	64
chr11	23564975	23566975	Pus10	-0.1423	3.78E-11	hypomethylated	0.010497	0.83418	insignificant	18	53	64
chr11	23565935	23567935	Pus10	-0.15377	0.00021756	hypomethylated	-0.018442	0.52685	insignificant	5	14	14
chr11	23670970	23672970	Rel	-0.059758	2.19E-08	hypomethylated	0.0069266	0.893	insignificant	30	109	101
chr11	23795270	23797270	Papolg	-0.2127	0.0688	insignificant	0.048578	1	insignificant	2	6	6
chr11	23977055	23979055	Bcl11a	-0.15689	5.89E-25	hypomethylated	-0.016623	0.47436	insignificant	27	99	99
chr11	23979694	23981694	Bcl11a	-0.12226	1.52E-30	hypomethylated	0.015362	0.43939	insignificant	19	122	114
chr11	24630722	24632722	Gm10466	0.125	1	insignificant	-0.0625	0.75125	insignificant	1	4	4
chr11	26110576	26112576	5730522E02Rik	-0.076253	0.00000264	hypomethylated	-0.010865	0.2245	insignificant	9	18	18
chr11	26286083	26288083	Fancl	-0.0931	0.064929	insignificant	0.0061905	0.60936	insignificant	6	39	38
chr11	28484296	28486296	Ccdc85a		1	noCoverage	0.15903	0.26919	insignificant	0	8	8
chr11	28752204	28754204	Efemp1	-0.14603	0.090142	insignificant	0.070649	0.30688	insignificant	3	18	25
chr11	29029750	29031750	Pnpt1	-0.13882	4.62E-14	hypomethylated	0.024763	0.19168	insignificant	15	58	55
chr11	29071906	29073906	Smek2	-0.11489	5.82E-16	hypomethylated	-0.0139	0.0059352	hypomethylated	29	156	150
chr11	29147272	29149272	Ccdc104	-0.12428	0.010313	hypomethylated	0.0040203	0.8923	insignificant	4	10	10
chr11	29272774	29274774	Cccdc88a	-0.048097	1.39E-10	hypomethylated	-0.0035649	0.94807	insignificant	38	153	153
chr11	29415033	29417033	Prorsd1	-0.41291	0.00066102	stronglyHypometh	-0.046847	0.156	insignificant	10	48	45
chr11	29425456	29427456	Mtif2	-0.29917	2.17E-32	hypomethylated	-0.010018	0.056671	insignificant	13	47	47
chr11	29446949	29448949	1700034F02Rik	-0.21792	0.57694	insignificant	-0.035414	0.50567	insignificant	4	51	52
chr11	29448040	29450040	1700034F02Rik		1	noCoverage	-0.026463	0.87873	insignificant	0	23	24
chr11	29591897	29593897	Rtn4	-0.12161	8.34E-25	hypomethylated	-0.00014146	0.096987	insignificant	44	120	140
chr11	29592773	29594773	Rtn4	-0.10792	9.35E-27	hypomethylated	-0.0086795	0.153	insignificant	46	115	134
chr11	29926033	29928033	Eml6	-0.19668	0.0014976	hypomethylated	-0.0085207	0.17802	insignificant	12	28	28
chr11	30098257	30100257	Spnb2	-0.090922	0.022795	hypomethylated	-0.0084885	0.80328	insignificant	7	45	45
chr11	30549396	30551396	Acyp2	-0.13323	0.00076871	hypomethylated	-0.0033207	0.44823	insignificant	5	22	22
chr11	30670774	30672774	Psme4	-0.13032	7.01E-18	hypomethylated	-0.016345	0.45849	insignificant	48	107	135
chr11	30790096	30792096	Gpr75		1	noCoverage	-0.0066527	1	insignificant	0	4	6
chr11	30853397	30855397	Asb3	-0.13618	1.97E-14	hypomethylated	0.048476	0.000017355	hypermethylated	17	87	92
chr11	30854131	30856131	Erlec1	-0.1314	0.00000172	hypomethylated	0.028982	0.11805	insignificant	12	47	52
chr11	30886365	30888365	Chac2		1	noCoverage	-0.030411	0.88626	insignificant	0	6	6
chr11	31270061	31272061	Stc2	-0.19794	0.000000166	hypomethylated	-0.054059	0.23382	insignificant	12	40	64
chr11	31571862	31573862	Bod1	-0.1313	0.14608	insignificant	-0.0025024	0.77431	insignificant	5	17	29
chr11	31771210	31773210	Cpeb4	-0.19524	0.031768	hypomethylated	0.0046164	0.46104	insignificant	5	30	30
chr11	31864632	31866632	4930524B15Rik	0.085737	0.32624	insignificant	0.086367	0.001817	hypermethylated	7	36	34
chr11	31899458	31901458	Nsg2	-0.16669	0.00000192	hypomethylated	-0.04644	0.93962	insignificant	9	30	31
chr11	32100279	32102279	Il9r	-0.19868	0.027844	hypomethylated	0.015759	0.18908	insignificant	3	12	12
chr11	32104414	32106414	Snrnp25	-0.41573	0.000000022	stronglyHypometh	0.017344	0.1001	insignificant	5	18	20
chr11	32122293	32124293	Rhbdf1	-0.12242	0.000017193	hypomethylated	0.00084569	1	insignificant	14	28	28
chr11	32125504	32127504	Mpg	-0.13138	7.1E-18	hypomethylated	0.018992	0.73147	insignificant	22	76	76
chr11	32167614	32169614	Nprl3	-0.075421	0.053121	insignificant	-0.013279	0.15447	insignificant	14	30	34
chr11	32175599	32177599	Hba-x	-0.18586	0.21915	insignificant	-0.26319	0.000068521	hypomethylated	2	5	4
chr11	32246810	32248810	Sh3pxd2b	-0.07606	1	insignificant	0.0052521	0.64407	insignificant	4	47	47
chr11	32354371	32356371	Ubtd2	-0.14473	1E-21	hypomethylated	-0.017158	0.02405	hypomethylated	35	122	124
chr11	32432265	32434265	Stk10	-0.10431	0.000000437	hypomethylated	0.011685	0.031617	hypermethylated	33	112	110
chr11	32541874	32543874	Fbxw11	-0.15096	1.2E-51	hypomethylated	0.0051365	0.91313	insignificant	49	126	134
chr11	32724594	32726594	1700008A04Rik		1	noCoverage	-0.12319	0.051546	insignificant	0	6	6
chr11	33047400	33049400	Fgf18	-0.11537	4.43E-35	hypomethylated	-0.0065509	0.013867	hypomethylated	54	108	117
chr11	33103588	33105588	Tlx3	-0.12933	4.79E-15	hypomethylated	-0.0078976	0.46331	insignificant	24	76	76
chr11	33413746	33415746	Ranbp17	-0.27452	2.23E-12	hypomethylated	0.096182	0.0016325	inconclusive	9	41	42
chr11	33478957	33480957	Gabrp	-0.42905	0.076704	insignificant	0.045557	1	insignificant	4	14	14
chr11	33743585	33745585	Kcnip1	-0.11743	0.012509	hypomethylated	-0.0080672	0.21493	insignificant	5	20	28
chr11	33862012	33864012	Kcnmb1	-0.13785	0.000000769	hypomethylated	-0.035545	0.0028638	hypomethylated	4	22	24
chr11	34213821	34215821	Fam196b	0.094125	1	insignificant	0.022728	0.66571	insignificant	3	20	22
chr11	34597325	34599325	Dock2		1	noCoverage	-0.057682	0.81605	insignificant	0	4	3
chr11	34647143	34649143	Ccdc99	-0.084539	0.00011305	hypomethylated	0.0017118	0.090762	insignificant	8	30	30
chr11	34933957	34935957	Slit3	-0.17569	2.23E-33	hypomethylated	-0.018544	0.50871	insignificant	25	143	143
chr11	35581996	35583996	Pank3	-0.18791	6.5E-21	hypomethylated	-0.0057331	0.018901	hypomethylated	27	100	102
chr11	35648030	35650030	Rars		1	noCoverage	0.034021	0.44788	insignificant	0	13	12
chr11	35793591	35795591	Wwc1	-0.086881	5.07E-12	hypomethylated	-0.0013031	0.029482	hypomethylated	32	113	125
chr11	40546143	40548143	Nudcd2	-0.16772	0.00000931	hypomethylated	-0.015215	0.38763	insignificant	12	40	44

chr11	40546939	40548939	Hmmr	-0.16772	0.00000931	hypomethylated	-0.015215	0.38763	insignificant	12	40	44
chr11	40568788	40570788	Ccng1	-0.12778	0.086457	insignificant	-0.0054439	0.91337	insignificant	2	18	18
chr11	41996432	41998432	Gabra1	0.03994	0.025463	inconclusive	-0.026713	0.86193	insignificant	3	14	15
chr11	43239483	43241483	Pttg1	-0.26868	6.93E-14	hypomethylated	-0.086611	4.48E-09	hypomethylated	13	41	42
chr11	43246232	43248232	Slu7	-0.16294	0.00000865	hypomethylated	-0.0014678	0.44894	insignificant	18	52	51
chr11	43286845	43288845	C1qtnf2	-0.15763	0.000000449	hypomethylated	0.0044172	1	insignificant	13	44	44
chr11	43341285	43343285	Ccnjl	-0.10627	4.94E-19	hypomethylated	-0.0021762	0.25165	insignificant	33	98	98
chr11	43494499	43496499	Pwwp2a	-0.093747	2.39E-18	hypomethylated	-0.000778	0.1286	insignificant	34	118	127
chr11	43561475	43563475	Ttc1	0.52696	0.22833	lowCoverage	-0.047252	0.50713	insignificant	1	19	18
chr11	43649834	43651834	Adra1b	-0.10072	0.000000163	hypomethylated	-0.0026397	0.42201	insignificant	24	63	63
chr11	44284050	44286050	Ublcp1	-0.30961	0.00011829	hypomethylated	-0.032209	0.05345	insignificant	6	32	32
chr11	44331465	44333465	Rnf145	-0.11186	1.69E-17	hypomethylated	0.0085246	0.89447	insignificant	45	127	115
chr11	44331878	44333878	Rnf145	-0.11186	1.69E-17	hypomethylated	0.010783	0.8945	insignificant	45	127	121
chr11	44430635	44432635	Ebf1	-0.067543	2.9E-16	hypomethylated	-0.0098791	0.96391	insignificant	19	59	59
chr11	45664465	45666465	Clint1	-0.11473	4.71E-14	hypomethylated	0.0039474	0.61997	insignificant	16	55	55
chr11	45792811	45794811	Sox30	-0.11605	1.18E-25	hypomethylated	-0.0083226	0.59767	insignificant	55	195	193
chr11	45868488	45870488	Adam19	-0.21848	2.93E-40	hypomethylated	-0.01316	0.040724	hypomethylated	15	47	42
chr11	45979861	45981861	Nipal4	-0.31217	0.20795	insignificant	-0.15434	0.018101	hypomethylated	4	27	24
chr11	46125852	46127852	Cyfip2	-0.11997	0.00014818	hypomethylated	0.014378	0.37985	insignificant	6	34	34
chr11	46249448	46251448	Med7	-0.095437	0.00037725	hypomethylated	0.0096492	0.9061	insignificant	8	63	58
chr11	46249462	46251462	Med7	-0.095437	0.00037725	hypomethylated	0.0096492	0.9061	insignificant	8	63	58
chr11	46336752	46338752	Gm12169	0.058838	1	insignificant	0.037927	0.43443	insignificant	1	8	8
chr11	46520606	46522606	Timd2	-0.14279	0.23305	insignificant	0.082116	0.64413	insignificant	2	12	13
chr11	46623300	46625300	Timd4	-0.0085784	0.3291	insignificant	0.041944	0.29439	insignificant	4	8	8
chr11	47192804	47194804	Sgcd		1	noCoverage	0.084578	0.44055	insignificant	0	8	8
chr11	48612861	48614861	Gnb2l1	-0.17602	5.19E-11	hypomethylated	-0.0034588	0.20105	insignificant	13	53	53
chr11	48630893	48632893	Trim41	-0.18832	3.06E-18	hypomethylated	-0.028328	0.32819	insignificant	12	20	20
chr11	48638639	48640639	Trim7	-0.097461	1	insignificant	0.013678	0.89705	insignificant	4	37	42
chr11	48684848	48686848	Irgm1	-0.22499	0.00018361	hypomethylated	-0.024094	0.3826	insignificant	5	34	33
chr11	48715654	48717654	Gm5431		1	noCoverage	-0.065934	0.39438	insignificant	0	3	4
chr11	48899556	48901556	Ifi47	-0.49624	0.000000136	stronglyHypometh	0.040992	0.0037541	hypermethylated	6	22	22
chr11	48960760	48962760	Olfr1395	-0.17572	0.28829	insignificant	-0.000029639	0.87577	insignificant	1	16	16
chr11	49016001	49018001	Zfp62	-0.16769	2.82E-12	hypomethylated	-0.0050353	0.11289	insignificant	12	61	58
chr11	49056692	49058692	Mgat1	-0.19605	1	lowCoverage	0.016139	0.24111	insignificant	1	28	28
chr11	49063053	49065053	Mgat1	-0.17133	5.33E-11	hypomethylated	-0.015621	0.81079	insignificant	13	34	34
chr11	49422180	49424180	Flt4	-0.13218	3.56E-30	hypomethylated	0.0071116	0.87464	insignificant	23	90	86
chr11	49526212	49528212	Cnot6	-0.11708	1	insignificant	0.016559	0.49607	insignificant	14	50	50
chr11	49606656	49608656	Gfpt2	-0.23206	9.37E-08	hypomethylated	0.03117	4.36E-12	hypermethylated	15	66	66
chr11	49659252	49661252	Mapk9	-0.13291	6.8E-23	hypomethylated	0.0066218	0.74366	insignificant	34	89	89
chr11	49714336	49716336	Rasgef1c	-0.063826	5.89E-12	hypomethylated	0.0012749	0.6475	insignificant	39	122	131
chr11	49837832	49839832	Rnf130	-0.12159	0.000012356	hypomethylated	-0.007003	0.5475	insignificant	12	40	46
chr11	49943861	49945861	Tbc1d9b	-0.030914	5.8E-10	hypomethylated	-0.0044661	0.015729	hypomethylated	27	128	127
chr11	49987352	49989352	3010026O09Rik	-0.20165	0.000000795	hypomethylated	-0.032917	0.30991	insignificant	9	34	34
chr11	50024292	50026292	Sqstm1		1	noCoverage	-0.034712	0.55374	insignificant	0	16	16
chr11	50037836	50039836	Mgat4b	-0.14103	1.66E-12	hypomethylated	-0.001991	0.40829	insignificant	20	82	88
chr11	50105838	50107838	Maml1	-0.27323	0.0099667	hypomethylated	-0.14359	0.053275	insignificant	7	24	26
chr11	50139175	50141175	Canx		1	noCoverage	-0.027778	0.6981	insignificant	0	6	6
chr11	50171381	50173381	Mir804	-0.12529	0.49677	insignificant	-0.041793	0.59851	insignificant	12	56	82
chr11	50190220	50192220	Hnrnph1	-0.079397	1.46E-12	hypomethylated	-0.0075156	0.3157	insignificant	15	98	98
chr11	50244613	50246613	Rufy1	0.4599	0.011696	stronglyHypermeth	0.039729	0.53146	insignificant	1	16	15
chr11	50414586	50416586	Adamts2	-0.13217	3.84E-56	hypomethylated	-0.006017	0.603	insignificant	67	168	181
chr11	50641233	50643233	Zfp354c	-0.97222	7.58E-15	stronglyHypometh	-0.065789	0.1479	insignificant	2	4	4
chr11	50663186	50665186	Grm6	-0.17817	3.13E-38	hypomethylated	-0.00078462	0.58653	insignificant	28	68	68
chr11	50700945	50702945	Zfp454	-0.21996	0.00030172	hypomethylated	-0.0018746	0.58044	insignificant	9	24	24
chr11	50729663	50731663	Zfp2		1	noCoverage	-0.028639	0.90336	insignificant	0	4	4
chr11	50745137	50747137	Zfp354b		1	noCoverage	0.14867	0.57567	insignificant	0	6	6
chr11	50839505	50841505	Olfr54	-0.30556	0.3201	insignificant	0.11111	0.72414	insignificant	1	6	6
chr11	50871758	50873758	Zfp354a	-0.16564	4.76E-13	hypomethylated	-0.013852	0.0013431	hypomethylated	21	88	87
chr11	51075672	51077672	Clk4	-0.20765	2.44E-22	hypomethylated	0.0057118	0.87907	insignificant	15	63	64
chr11	51076453	51078453	Clk4	-0.15042	4.51E-18	hypomethylated	0.010546	0.93249	insignificant	13	54	55
chr11	51102421	51104421	Col23a1	-0.12495	7.84E-21	hypomethylated	-0.023406	0.059968	insignificant	40	148	155
chr11	51397258	51399258	Agxt2l2	-0.13196	0.0005946	hypomethylated	0.01391	0.39158	insignificant	10	36	36
chr11	51420383	51422383	Hnrnpab	-0.209	9.81E-19	hypomethylated	0.014382	0.73083	insignificant	15	49	47
chr11	51432274	51434274	Nhp2	0.030145	0.059596	insignificant	-0.0079553	0.12851	insignificant	4	63	59
chr11	51449398	51451398	Rmnd5b	-0.34743	3.69E-17	stronglyHypometh	-0.0072744	0.54398	insignificant	15	43	43
chr11	51463455	51465455	D930048N14Rik	-0.081482	2.08E-15	hypomethylated	-0.027011	0.32194	insignificant	29	102	92
chr11	51502136	51504136	0610009B22Rik	-0.11866	1.76E-13	hypomethylated	0.021621	0.59478	insignificant	15	72	60
chr11	51570336	51572336	Sec24a	-0.36267	0.69746	insignificant	-0.045569	0.0021638	inconclusive	5	31	31
chr11	51576164	51578164	Sar1b	-0.23454	3.91E-10	hypomethylated	-0.032262	0.6796	insignificant	10	24	24
chr11	51670983	51672983	Phf15	-0.17856	2.46E-23	hypomethylated	-0.00028481	0.84262	insignificant	14	46	49
chr11	51813968	51815968	Ube2b	-0.17976	9.18E-16	hypomethylated	0.011448	0.0027472	inconclusive	26	64	64
chr11	51816722	51818722	Cdkl3	-0.23807	1.77E-20	hypomethylated	-0.0020518	0.0091285	hypomethylated	19	75	79
chr11	51911325	51913325	Ppp2ca	-0.12424	1.42E-37	hypomethylated	-0.0053494	0.80034	insignificant	53	148	165
chr11	52044496	52046496	Skp1a	-0.128	7.65E-20	hypomethylated	0.0087686	0.040838	inconclusive	28	133	136
chr11	52095752	52097752	Tcf7	-0.1671	2.29E-28	hypomethylated	0.010467	0.12194	insignificant	58	192	192
chr11	52173616	52175616	Vdac1	-0.1299	1.65E-32	hypomethylated	-0.0055198	0.16085	insignificant	31	117	122
chr11	52208929	52210929	9530068E07Rik	-0.14397	3.65E-17	hypomethylated	-0.0087587	0.054244	insignificant	17	81	81
chr11	52577207	52579207	Fstl4	-0.12901	1.86E-43	hypomethylated	0.0040275	0.1877	insignificant	47	138	139
chr11	53113981	53115981	Hspa4	-0.46487	0.000030785	stronglyHypometh	-0.071659	0.60448	insignificant	4	13	13
chr11	53137190	53139190	Zcchc10	-0.33182	0.000013325	hypomethylated	-0.045025	0.54575	insignificant	9	41	42
chr11	53163268	53165268	Aff4	-0.10102	9.56E-43	hypomethylated	-0.00052152	0.49629	insignificant	84	216	211
chr11	53244333	53246333	Uqcrq	-0.1749	0.00069672	hypomethylated	-0.022195	0.93823	insignificant	6	29	29
chr11	53269706	53271706	Shroom1	-0.18509	4.93E-27	hypomethylated	0.020388	0.000000096	inconclusive	29	86	89
chr11	53332301	53334301	###########	-0.14081	0.14147	insignificant	-0.024868	0.54128	insignificant	29	101	95
chr11	53379880	53381880	Kif3a	-0.14517	0.000034314	hypomethylated	-0.033254	0.46135	insignificant	14	33	33

chr11	53520821	53522821	Rad50	-0.13273	0.00000204	hypomethylated	-0.011163	0.39308	insignificant	12	28	28
chr11	53582515	53584515	Irf1	-0.095757	4.4E-09	hypomethylated	-0.0050066	0.93731	insignificant	26	141	152
chr11	53582974	53584974	Irf1	-0.095757	4.4E-09	hypomethylated	0.00093618	0.96868	insignificant	26	141	153
chr11	53672758	53674758	Gm12216	-0.6079	2.09E-12	stronglyHypometh	-0.062253	0.0060269	inconclusive	3	24	25
chr11	53705205	53707205	Slc22a5	-0.10201	0.00000189	hypomethylated	-0.026413	0.043441	hypomethylated	15	46	46
chr11	53793529	53795529	Slc22a21	-0.086978	0.060602	insignificant	0.041072	0.83475	insignificant	10	42	47
chr11	53841592	53843592	Slc22a4	-0.15585	1.46E-19	hypomethylated	0.0052941	0.8067	insignificant	24	60	64
chr11	53882519	53884519	Pdlim4	-0.12532	0.000000105	hypomethylated	0.18667	0.85381	insignificant	5	29	11
chr11	53913425	53915425	P4ha2	-0.18613	1.23E-30	hypomethylated	-0.00075423	0.000054716	hypomethylated	34	119	121
chr11	54116325	54118325	Acsl6	-0.14793	4.99E-16	hypomethylated	-0.0042219	0.1857	insignificant	27	85	85
chr11	54116705	54118705	Acsl6	-0.14793	4.99E-16	hypomethylated	-0.0042219	0.1857	insignificant	27	85	85
chr11	54183153	54185153	4930404A10Rik		1	noCoverage	0.066361	0.26017	insignificant	0	6	6
chr11	54250680	54252680	Fnip1	-0.16835	0.026899	hypomethylated	-0.003042	0.33816	insignificant	15	53	53
chr11	54335348	54337348	Rapgef6	-0.135	7.11E-17	hypomethylated	0.009072	0.75703	insignificant	18	74	75
chr11	54674093	54676093	Lyrm7	-0.41813	0.080813	insignificant	-0.2319	0.47977	insignificant	2	6	4
chr11	54678939	54680939	Hint1	-0.099917	5.59E-22	hypomethylated	-0.011235	0.23688	insignificant	41	131	130
chr11	54715354	54717354	Gpx3	0.58832	0.67434	insignificant	0.074779	0.036598	inconclusive	1	27	29
chr11	54715427	54717427	Gpx3	0.63881	0.23876	insignificant	0.084753	0.037479	inconclusive	1	25	27
chr11	54769624	54771624	Tnip1	-0.22938	9.81E-08	hypomethylated	0.0061279	0.14496	insignificant	10	44	44
chr11	54776442	54778442	Tnip1	-0.75863	0.000013685	stronglyHypometh	-0.067489	0.061234	insignificant	3	8	8
chr11	54891633	54893633	Ccdc69	-0.30836	1.54E-09	hypomethylated	-0.014805	0.0025801	hypomethylated	5	16	16
chr11	54910618	54912618	Gm2a	-0.34697	4.16E-14	stronglyHypometh	0.0098309	0.40408	insignificant	8	45	45
chr11	55016841	55018841	Slc36a1	-0.13506	1.29E-14	hypomethylated	0.0086304	0.69951	insignificant	9	40	40
chr11	55274640	55276640	Atox1	-0.24283	1.34E-08	hypomethylated	-0.0015192	0.35094	insignificant	9	35	36
chr11	55282253	55284253	G3bp1	-0.1233	1.31E-13	hypomethylated	-0.02124	0.047137	hypomethylated	33	97	96
chr11	55421292	55423292	Glra1	-0.047525	0.032883	hypomethylated	0.0043244	0.43872	insignificant	6	52	52
chr11	57331166	57333166	Mfap3	-0.11486	2.59E-26	hypomethylated	-0.015798	0.000083029	hypomethylated	30	116	113
chr11	57332146	57334146	Fam114a2	-0.1705	3E-10	hypomethylated	-0.010026	0.042475	hypomethylated	6	27	24
chr11	57457943	57459943	Galnt10	-0.17939	4.25E-25	hypomethylated	-0.010565	0.0008259	hypomethylated	15	85	84
chr11	57614138	57616138	Sap30l	-0.11671	4.99E-46	hypomethylated	-0.012007	0.45655	insignificant	65	220	220
chr11	57645649	57647649	Hand1	-0.26479	0.00066142	hypomethylated	-0.023017	0.1866	insignificant	10	46	46
chr11	57821565	57823565	Larp1	-0.094411	3.96E-49	hypomethylated	-0.00058906	0.13212	insignificant	79	265	269
chr11	57916654	57918654	Cnot8	-0.14433	0.60418	insignificant	0.0002343	0.7725	insignificant	5	54	54
chr11	57984155	57986155	Mrpl22	-0.24181	6.27E-13	hypomethylated	0.04823	0.88042	insignificant	11	58	58
chr11	58012057	58014057	Igtp	0.026496	0.27512	insignificant	-0.089633	0.49632	insignificant	2	13	14
chr11	58027478	58029478	Irgm2	-0.16388	0.60543	insignificant	0.050437	1	insignificant	2	20	18
chr11	58119570	58121570	Zfp692	-0.13425	6.17E-15	hypomethylated	-0.021607	0.35541	insignificant	28	58	61
chr11	58136848	58138848	Zfp672	-0.34377	1	insignificant	0.049194	0.3721	insignificant	2	18	18
chr11	58143219	58145219	Sh3bp5l	-0.27598	1.36E-15	hypomethylated	-0.0038956	0.031517	hypomethylated	20	83	88
chr11	58191533	58193533	2210415F13Rik	-0.74138	0.06331	insignificant	0.0451	0.26711	insignificant	1	4	4
chr11	58409846	58411846	Olfr324	0.17262	0.57724	insignificant	-0.029806	0.71497	insignificant	2	13	15
chr11	58452966	58454966	Trim58	-0.15664	0.000017722	hypomethylated	0.0010389	0.72577	insignificant	7	33	33
chr11	58570168	58572168	Olfr316		1	noCoverage	0.15667	0.18666	insignificant	0	5	4
chr11	58590590	58592590	Olfr315	0.057344	1	insignificant	-0.0066337	0.00016461	hypomethylated	7	20	20
chr11	58598640	58600640	Olfr314	0.074175	1	insignificant	-0.038734	0.22744	insignificant	3	14	16
chr11	58629429	58631429	Olfr313		1	noCoverage	0.0090351	1	insignificant	0	2	3
chr11	58643657	58645657	Olfr312		1	noCoverage	0.0082071	1	insignificant	0	3	6
chr11	58679741	58681741	2810021J22Rik	-0.38634	0.000000018	stronglyHypometh	-0.017376	0.80746	insignificant	11	30	30
chr11	58717727	58719727	Zfp39	-0.063334	1	insignificant	-0.0025482	0.78707	insignificant	5	28	28
chr11	58730558	58732558	Butr1	0.18678	1	insignificant	-0.22408	0.0018164	hypomethylated	2	17	17
chr11	58761412	58763412	Hist3h2ba		1	noCoverage	-0.025491	0.11716	insignificant	0	32	39
chr11	58767186	58769186	Hist3h2a	-0.092053	0.0015347	hypomethylated	0.066385	0.63216	insignificant	13	39	50
chr11	58768014	58770014	Hist3h2bb-ps	-0.08774	0.11789	insignificant	0.05702	0.5287	insignificant	8	28	38
chr11	58790617	58792617	Trim11	-0.099327	2.26E-26	hypomethylated	0.014294	0.84432	insignificant	40	142	146
chr11	58949877	58951877	Obscn	-0.063654	0.79003	insignificant	0.0057929	0.90254	insignificant	4	8	10
chr11	58977244	58979244	A230051G13Rik	-0.13198	1	insignificant	-0.0032256	1	insignificant	1	22	22
chr11	58996328	58998328	Gjc2	-0.17188	0.19032	insignificant	-0.032986	0.60183	insignificant	2	4	4
chr11	58996715	58998715	Guk1	-0.17188	0.19032	insignificant	-0.032986	0.60183	insignificant	2	4	4
chr11	59001243	59003243	Guk1	-0.20235	0.038279	hypomethylated	-0.017589	0.10616	insignificant	2	2	4
chr11	59015018	59017018	Mrpl55	-0.17347	0.00000125	hypomethylated	0.00025995	0.48026	insignificant	10	40	65
chr11	59020862	59022862	2310033P09Rik	-0.14958	0.00062345	hypomethylated	0.051986	0.031216	inconclusive	6	24	51
chr11	59041664	59043664	Arf1	-0.11291	0.00000755	hypomethylated	-0.017657	0.46494	insignificant	16	46	46
chr11	59041769	59043769	Arf1	-0.15665	8.66E-08	hypomethylated	-0.014157	0.14125	insignificant	10	28	28
chr11	59104253	59106253	Wnt3a	-0.17185	0.0018712	hypomethylated	-0.051519	0.64118	insignificant	22	38	62
chr11	59119431	59121431	Wnt9a	-0.081949	7.12E-12	hypomethylated	-0.0088299	0.58156	insignificant	47	158	157
chr11	59262589	59264589	Snap47	-0.15334	6.71E-28	hypomethylated	0.0036146	0.53812	insignificant	17	69	66
chr11	59263458	59265458	Snap47	-0.14902	5.07E-28	hypomethylated	0.0070933	0.5645	insignificant	17	63	60
chr11	59285976	59287976	Zfp867	-0.19592	0.11632	insignificant	-0.12458	0.049351	inconclusive	2	20	34
chr11	59318186	59320186	4933439C10Rik	-0.17906	3.12E-08	hypomethylated	-0.005984	0.41276	insignificant	29	86	75
chr11	59320142	59322142	4933439C10Rik	-0.53639	0.037062	stronglyHypometh	-0.088054	0.020662	hypomethylated	2	13	14
chr11	59425467	59427467	Olfr225	-0.074819	0.038576	hypomethylated	0.085049	0.0023769	hypermethylated	6	28	30
chr11	59474996	59476996	Mprip	-0.09098	1.84E-26	hypomethylated	-0.012247	0.042298	hypomethylated	43	182	182
chr11	59601159	59603159	Pld6	-0.028789	0.5232	insignificant	-0.01611	0.46575	insignificant	7	19	17
chr11	59623275	59625275	Flcn	-0.11753	0.000017304	hypomethylated	-0.0064238	0.74352	insignificant	14	64	64
chr11	59653269	59655269	Cops3	-0.038006	0.000088401	hypomethylated	0.018028	0.006244	inconclusive	10	36	36
chr11	59660574	59662574	Nt5m	-0.11679	4.23E-26	hypomethylated	-0.021296	0.15274	insignificant	37	118	128
chr11	59760715	59762715	Med9	-0.18015	0.00044137	hypomethylated	-0.0093944	0.16195	insignificant	9	57	65
chr11	59778444	59780444	Rasd1	-0.21593	0.000000734	hypomethylated	0.03558	0.33128	insignificant	11	52	52
chr11	59917514	59919514	Rai1	-0.10727	2.48E-22	hypomethylated	0.004308	0.28809	insignificant	61	184	179
chr11	59952583	59954583	Rai1	-0.082831	0.86635	insignificant	0.017759	0.26921	insignificant	17	40	40
chr11	60165881	60167881	Lrrc48	-0.026434	0.0003099	hypomethylated	0.013195	0.30461	insignificant	12	63	64
chr11	60166407	60168407	Tom1l2	-0.10144	0.052969	insignificant	0.029072	0.5012	insignificant	4	17	18
chr11	60229646	60231646	4933439F18Rik	-0.16265	7.08E-30	hypomethylated	-0.0065947	0.0076886	hypomethylated	28	77	83
chr11	60230601	60232601	Atpaf2	-0.15866	1.9E-26	hypomethylated	0.00021965	0.15268	insignificant	28	87	95
chr11	60267118	60269118	Drg2	-0.16689	3.5E-19	hypomethylated	0.004602	0.67854	insignificant	12	59	54

chr11	60350184	60352184	Alkbh5	-0.10193	1.56E-34	hypomethylated	-0.015091	0.0060107	hypomethylated	54	240	250
chr11	60512191	60514191	Llgl1	-0.13978	8.56E-35	hypomethylated	0.0065968	0.058847	insignificant	33	129	130
chr11	60540723	60542723	Flii	-0.13471	5E-24	hypomethylated	0.012482	0.84187	insignificant	30	75	79
chr11	60540899	60542899	Smcr7	-0.1552	4.01E-23	hypomethylated	0.019077	0.50066	insignificant	24	63	67
chr11	60590026	60592026	Smcr8	-0.16619	0.0013403	hypomethylated	-0.017295	0.85114	insignificant	13	62	69
chr11	60590867	60592867	Top3a	-0.24501	0.013974	hypomethylated	-0.025357	0.61857	insignificant	12	38	43
chr11	60643132	60645132	Dhrs7b	-0.14881	0.00087686	hypomethylated	0.019698	0.43096	insignificant	8	22	30
chr11	60744558	60746558	Map2k3	-0.11584	1.25E-19	hypomethylated	-0.014983	0.42367	insignificant	36	134	136
chr11	60745369	60747369	Gm16516	-0.11202	2.44E-17	hypomethylated	-0.0077397	0.52643	insignificant	31	102	105
chr11	60878305	60880305	Kcnj12	-0.4541	0.0025578	stronglyHypometh	-0.036686	0.60792	insignificant	7	19	19
chr11	60953336	60955336	Tnfrsf13b	0.0079023	1	insignificant	0.13799	0.083104	insignificant	1	4	9
chr11	60988561	60990561	Usp22	-0.14435	0.0001421	hypomethylated	-0.011959	0.75686	insignificant	9	34	34
chr11	61080629	61082629	Aldh3a2	-0.080615	0.00067712	hypomethylated	-0.036469	0.1706	insignificant	12	39	45
chr11	61267388	61269388	Rnf112	-0.52451	0.44737	lowCoverage	-0.025387	1	insignificant	1	4	4
chr11	61297945	61299945	Mfap4	-0.011019	0.31279	insignificant	-0.028185	0.081806	insignificant	8	35	29
chr11	61307705	61309705	Mapk7	-0.14753	8.66E-31	hypomethylated	-0.011871	0.00000296	hypomethylated	25	89	89
chr11	61317673	61319673	B9d1	-0.18261	3.39E-14	hypomethylated	-0.030007	0.55703	insignificant	10	30	39
chr11	61393153	61395153	Epn2	-0.022878	0.8243	insignificant	0.034378	0.24873	insignificant	9	46	47
chr11	61465822	61467822	Grap		1	noCoverage	-0.083504	0.29708	insignificant	0	4	4
chr11	61496911	61498911	Fam83g	-0.24274	1.3E-43	hypomethylated	0.024951	0.000017136	inconclusive	21	111	114
chr11	61575590	61577590	Prpsap2		1	noCoverage	0.1142	0.84218	insignificant	0	30	30
chr11	61668594	61670594	Ulk2	-0.14594	0.000042285	hypomethylated	-0.019542	0.86419	insignificant	11	34	34
chr11	61743728	61745728	Akap10	-0.17628	0.00082277	hypomethylated	0.099491	0.03732	hypermethylated	2	24	36
chr11	61889598	61891598	Specc1	-0.046791	0.3123	insignificant	-0.0033314	0.58556	insignificant	15	90	90
chr11	62061485	62063485	Adora2b	-0.16027	4.79E-31	hypomethylated	0.0057523	0.27507	insignificant	38	151	149
chr11	62093974	62095974	Ttc19	-0.083529	2.89E-52	hypomethylated	0.017173	0.26915	insignificant	60	167	152
chr11	62094897	62096897	Zswim7	-0.064997	4.46E-33	hypomethylated	0.013987	0.5129	insignificant	50	132	124
chr11	62270834	62272834	Ncor1	-0.12245	1.84E-43	hypomethylated	0.0040942	0.011553	inconclusive	48	142	146
chr11	62270961	62272961	Pigl	-0.13048	7.72E-37	hypomethylated	-0.0017443	0.0042058	hypomethylated	38	116	120
chr11	62352763	62354763	Cenpv	-0.21565	0.00000324	hypomethylated	0.078385	1	insignificant	5	10	11
chr11	62364005	62366005	Ubb	-0.1583	1.15E-08	hypomethylated	-0.0078179	0.67483	insignificant	16	149	142
chr11	62386987	62388987	Trpv2	-0.50156	0.069511	insignificant	-0.023305	0.12282	insignificant	2	14	14
chr11	62415378	62417378	2410006H16Rik	-0.16561	2.46E-12	hypomethylated	0.003394	0.0032619	hypermethylated	10	48	48
chr11	62415587	62417587	Snord49b	-0.18467	3.69E-14	hypomethylated	-0.00051046	0.020435	inconclusive	13	56	56
chr11	62415961	62417961	Snord49a	-0.18467	3.69E-14	hypomethylated	-0.00051046	0.020435	inconclusive	13	56	56
chr11	62417031	62419031	Snord65	-0.23418	1.73E-10	hypomethylated	-0.032529	0.51135	insignificant	5	14	14
chr11	62461165	62463165	Mmgt2	-0.085937	4.15E-18	hypomethylated	0.0012714	0.35266	insignificant	52	130	134
chr11	62462025	62464025	Mmgt2	-0.19511	1.17E-28	hypomethylated	0.070258	0.000000015	inconclusive	13	40	34
chr11	62691991	62693991	Fam18b	-0.17625	0.066806	insignificant	-0.004991	0.45357	insignificant	16	72	78
chr11	62874160	62876160	Tekt3	-0.40869	0.081573	insignificant	-0.0046926	0.91154	insignificant	1	6	8
chr11	62944011	62946011	Pmp22	-0.095841	0.00030015	hypomethylated	0.014573	0.5894	insignificant	7	30	31
chr11	63735786	63737786	Hs3st3b1	-0.17267	1.99E-17	hypomethylated	-0.001283	0.33056	insignificant	34	132	131
chr11	63891985	63893985	2810001G20Rik	-0.12711	7.35E-24	hypomethylated	-0.0068252	0.054794	insignificant	21	58	58
chr11	63892974	63894974	2810001G20Rik	-0.11399	1.55E-14	hypomethylated	-0.0039103	0.18291	insignificant	13	30	30
chr11	64247833	64249833	Hs3st3a1	-0.079912	0.0092889	hypomethylated	-0.0047788	0.14113	insignificant	20	71	68
chr11	64791536	64793536	Elac2	-0.17923	5.13E-22	hypomethylated	-0.015353	0.006542	hypomethylated	16	54	54
chr11	65083491	65085491	Myocd	-0.11845	0.08984	insignificant	0.0025534	0.69615	insignificant	6	34	34
chr11	65601799	65603799	Map2k4	-0.057596	0.043458	hypomethylated	0.014751	0.63931	insignificant	7	22	19
chr11	65619745	65621745	Zkscan6	-0.12016	6.73E-15	hypomethylated	-0.0050322	0.046198	hypomethylated	17	75	84
chr11	66339628	66341628	Shisa6	-0.10774	1.22E-14	hypomethylated	0.011152	0.09037	insignificant	49	142	146
chr11	66724492	66726492	Pirt	-0.20888	0.11137	insignificant	0.014975	0.019286	hypermethylated	10	30	32
chr11	66760588	66762588	Gm12298	0.045843	1	insignificant	-0.08792	0.0071324	hypomethylated	4	17	23
chr11	66837655	66839655	Tmem220		1	noCoverage	0.038134	0.86904	insignificant	0	29	18
chr11	66865171	66867171	Sco1	-0.18743	0.000000735	hypomethylated	0.006148	0.96791	insignificant	15	60	61
chr11	66866120	66868120	2310004I24Rik	-0.17395	0.000011737	hypomethylated	0.026175	1	insignificant	6	36	37
chr11	67050530	67052530	Myh4	-0.020635	1	insignificant	-0.1138	0.25407	insignificant	3	7	8
chr11	67139604	67141604	Myh13		1	noCoverage	0.10025	0.22675	insignificant	0	8	8
chr11	67507827	67509827	Rcvrn	0.1522	0.000030807	hypermethylated	0.00049537	0.63306	insignificant	5	28	28
chr11	67610772	67612772	Dhrs7c	-0.13456	0.33036	insignificant	-0.086786	0.45964	insignificant	3	13	14
chr11	67735655	67737655	Usp43	-0.13686	0.000000305	hypomethylated	-0.0073358	0.093473	insignificant	21	84	80
chr11	68200328	68202328	Ntn1	-0.15595	2.4E-16	hypomethylated	-0.02124	0.71472	insignificant	33	83	85
chr11	68244626	68246626	Pik3r5	-0.18068	5.3E-17	hypomethylated	-0.011842	0.54456	insignificant	23	75	69
chr11	68368687	68370687	Mfsd6l	-0.14797	0.31167	insignificant	0.07835	0.087633	insignificant	12	47	46
chr11	68399538	68401538	Ccdc42	0.036841	0.80584	insignificant	0.14537	0.000038524	hypermethylated	6	15	18
chr11	68504416	68506416	Myh10	-0.16991	4.38E-14	hypomethylated	-0.010642	0.21489	insignificant	15	80	78
chr11	68666633	68668633	Ndel1	-0.067935	1	insignificant	0.0090176	0.018552	hypermethylated	6	16	16
chr11	68701054	68703054	Rnf222	-0.22363	0.23073	insignificant	0.056861	0.037224	hypermethylated	4	12	13
chr11	68714067	68716067	Rpl26	-0.14403	3.43E-12	hypomethylated	-0.022328	0.0061708	hypomethylated	29	104	110
chr11	68714090	68716090	Gm15772	-0.14403	3.43E-12	hypomethylated	-0.022328	0.0061708	hypomethylated	29	104	110
chr11	68770360	68772360	Arhgef15	0.013009	0.4629	insignificant	-0.034978	0.42583	insignificant	5	18	20
chr11	68780632	68782632	Slc25a35	-0.18351	9E-18	hypomethylated	-0.021476	0.7121	insignificant	27	77	78
chr11	68787322	68789322	Rangrf	-0.36473	0.030909	stronglyHypometh	0.007034	0.43562	insignificant	2	16	15
chr11	68804180	68806180	Pfas	-0.029548	0.57869	insignificant	-0.055359	0.44591	insignificant	3	6	6
chr11	68821962	68823962	Pfas		1	noCoverage	0.0056127	0.91701	insignificant	0	10	10
chr11	68828412	68830412	1500010J02Rik	-0.13754	0.17093	insignificant	-0.0058659	0.057194	insignificant	7	48	53
chr11	68858144	68860144	Aurkb	-0.081849	0.32817	insignificant	0.033276	0.89641	insignificant	7	47	49
chr11	68872276	68874276	2310047M10Ril	-0.1496	1.39E-40	hypomethylated	-0.0040623	0.60585	insignificant	54	169	176
chr11	68883310	68885310	Tmem107	-0.13738	2.47E-18	hypomethylated	-0.01925	0.093907	insignificant	12	76	74
chr11	68901029	68903029	Vamp2	-0.12261	1.85E-15	hypomethylated	-0.0083257	0.060748	insignificant	28	115	115
chr11	68911457	68913457	Per1	-0.16295	0.00074346	hypomethylated	-0.0066008	0.25993	insignificant	3	39	42
chr11	68912659	68914659	Per1	-0.060085	0.03151	hypomethylated	-0.031905	0.085644	insignificant	10	30	30
chr11	68932954	68934954	Hes7	-0.35145	1.91E-12	stronglyHypometh	-0.068575	6.84E-09	hypomethylated	8	26	32
chr11	68969573	68971573	Alox12b	-0.23589	0.00074774	hypomethylated	-0.054765	0.23467	insignificant	7	23	23
chr11	69050524	69052524	Gucy2e		1	noCoverage	0.070208	0.74743	insignificant	0	23	24
chr11	69136487	69138487	Trappc1	-0.2481	0.0055219	hypomethylated	0.016622	0.000024223	hypermethylated	12	52	52

chr11	69137375	69139375	Cntrob	-0.36209	0.018188	stronglyHypometh	0.0050204	0.00037177	hypermethylated	6	42	42
chr11	69138759	69140759	Kcnab3	-0.25295	3.61E-30	hypomethylated	-0.026831	0.026503	hypomethylated	19	80	76
chr11	69153270	69155270	A030009H04Rik	-0.13622	0.000084856	hypomethylated	0.024679	0.39142	insignificant	8	56	46
chr11	69182928	69184928	Chd3	-0.27685	0.16526	insignificant	-0.26213	0.045037	hypomethylated	3	12	13
chr11	69208292	69210292	Lsmd1	-0.1407	7.04E-11	hypomethylated	-0.011632	0.00035774	hypomethylated	16	88	96
chr11	69208848	69210848	Cyb5d1	-0.13142	6.04E-10	hypomethylated	-0.022948	0.0011465	hypomethylated	14	65	65
chr11	69211736	69213736	Kdm6b	-0.10965	0.00000075	hypomethylated	0.03976	0.58107	insignificant	15	54	76
chr11	69373739	69375739	Efnb3	-0.09987	0.043514	hypomethylated	0.02484	0.34556	insignificant	5	29	29
chr11	69392826	69394826	Wrap53	-0.23541	3.88E-16	hypomethylated	-0.02027	0.91395	insignificant	12	39	36
chr11	69392860	69394860	Trp53	-0.25863	6.66E-20	hypomethylated	-0.01145	0.62524	insignificant	12	41	38
chr11	69434610	69436610	Sat2	-0.39862	0.00029587	stronglyHypometh	-0.018779	0.082599	insignificant	4	48	48
chr11	69445472	69447472	Fxr2	-0.12641	2.66E-20	hypomethylated	-0.0047808	0.96332	insignificant	40	111	122
chr11	69467874	69469874	Sox15	-0.656	0.022761	stronglyHypometh	0.13631	0.50856	insignificant	3	22	22
chr11	69475544	69477544	Mir1934		1	noCoverage	-0.049329	0.42541	insignificant	0	24	25
chr11	69476144	69478144	Mir1934		1	noCoverage	-0.038157	0.6993	insignificant	0	16	17
chr11	69495472	69497472	BC096441	-0.30951	0.31842	insignificant	-0.043629	0.19233	insignificant	9	34	34
chr11	69495586	69497586	BC096441	-0.30951	0.31842	insignificant	-0.043629	0.19233	insignificant	9	34	34
chr11	69499056	69501056	BC096441		1	noCoverage	-0.057788	1	insignificant	0	8	8
chr11	69509600	69511600	BC096441		1	noCoverage	-0.021212	1	insignificant	0	3	5
chr11	69571725	69573725	Polr2a	-0.12369	1.01E-11	hypomethylated	0.0042746	0.92952	insignificant	28	92	95
chr11	69578413	69580413	Zbtb4	-0.098993	9.45E-09	hypomethylated	0.013156	0.33102	insignificant	21	66	71
chr11	69609439	69611439	Chrnb1	-0.20611	8.71E-15	hypomethylated	-0.051135	0.0076342	hypomethylated	3	23	23
chr11	69615127	69617127	G630025P09Rik	-0.11918	1.33E-13	hypomethylated	0.012076	0.082961	insignificant	14	73	71
chr11	69616170	69618170	G630025P09Rik	-0.1589	1.68E-19	hypomethylated	0.013066	0.89509	insignificant	21	120	110
chr11	69631942	69633942	4933402P03Rik		1	noCoverage	0.046462	0.016813	inconclusive	0	6	8
chr11	69648351	69650351	Nlgn2	-0.27905	0.0020546	hypomethylated	0.074237	0.4464	insignificant	5	14	14
chr11	69651026	69653026	1810027O10Rik	-0.15049	9.35E-11	hypomethylated	-0.00074287	0.0069807	hypomethylated	9	57	70
chr11	69658873	69660873	Plscr3	-0.15608	7.36E-13	hypomethylated	-0.014346	0.87513	insignificant	9	34	36
chr11	69659118	69661118	Plscr3	-0.14604	8.02E-13	hypomethylated	-0.02528	0.4616	insignificant	12	46	48
chr11	69659145	69661145	Plscr3	-0.18862	4.98E-13	hypomethylated	-0.075556	0.57433	insignificant	12	50	50
chr11	69672232	69674232	Tnk1	-0.18146	0.00029615	hypomethylated	0.057355	0.042571	hypermethylated	2	17	16
chr11	69691520	69693520	Tmem95		1	noCoverage	-0.034074	0.50239	insignificant	0	15	13
chr11	69694487	69696487	Acap1	-0.13326	7.35E-19	hypomethylated	-0.027807	0.58158	insignificant	19	94	94
chr11	69714379	69716379	Neurl4	-0.14875	1.07E-44	hypomethylated	-0.018192	0.51964	insignificant	39	156	155
chr11	69714488	69716488	2810408A11Rik	-0.14601	7.76E-44	hypomethylated	-0.018483	0.40668	insignificant	37	152	151
chr11	69726693	69728693	Gps2	-0.11646	5.89E-12	hypomethylated	-0.0071695	0.95446	insignificant	20	74	76
chr11	69734132	69736132	Eif5a	-0.12572	1.55E-41	hypomethylated	-0.0025689	1	insignificant	31	78	78
chr11	69734449	69736449	Eif5a	-0.21432	4.13E-13	hypomethylated	-0.033745	0.023651	hypomethylated	6	14	16
chr11	69734587	69736587	Eif5a	-0.27388	0.0076405	hypomethylated	-0.063219	0.61677	insignificant	3	8	10
chr11	69734777	69736777	Eif5a	-0.27388	0.0076405	hypomethylated	-0.063219	0.61677	insignificant	3	8	10
chr11	69734888	69736888	Eif5a	-0.27388	0.0076405	hypomethylated	-0.063219	0.61677	insignificant	3	8	10
chr11	69748400	69750400	Ybx2	-0.11977	1.39E-44	hypomethylated	-0.0087597	0.00047081	hypomethylated	65	194	197
chr11	69761692	69763692	Slc2a4	-0.27388	9.64E-10	hypomethylated	0.010078	0.2364	insignificant	4	11	14
chr11	69777280	69779280	Cldn7	-0.25012	1.42E-11	hypomethylated	-0.070363	0.000028917	hypomethylated	10	41	42
chr11	69777922	69779922	Cldn7	-0.21694	2.05E-15	hypomethylated	-0.051658	0.00017898	hypomethylated	22	78	76
chr11	69793669	69795669	Ctdnep1	-0.12483	2.68E-12	hypomethylated	-0.002109	0.52258	insignificant	14	79	89
chr11	69793745	69795745	Rai12	-0.12483	2.68E-12	hypomethylated	-0.002109	0.52258	insignificant	14	79	89
chr11	69794737	69796737	Rai12	-0.21158	0.00000281	hypomethylated	0.031054	1	insignificant	5	25	22
chr11	69803871	69805871	Gabarap	-0.1476	1.82E-13	hypomethylated	-0.005007	0.80175	insignificant	14	50	51
chr11	69808272	69810272	Phf23	-0.18521	9.22E-30	hypomethylated	-0.011346	0.028476	hypomethylated	19	91	97
chr11	69813127	69815127	Dvl2	0.29622	0.0012571	hypermethylated	0.0053976	0.000055756	inconclusive	3	79	72
chr11	69828909	69830909	Acadvl	0.33316	0.59388	insignificant	0.074307	0.26208	insignificant	3	6	6
chr11	69831106	69833106	Dlg4	-0.44944	0.000000751	stronglyHypometh	-0.081148	0.36478	insignificant	5	10	10
chr11	69866870	69868870	Asgr1	-0.20833	0.10363	insignificant	0.012021	0.56538	insignificant	1	8	6
chr11	69942858	69944858	Mgl2		1	noCoverage	0.26	1	insignificant	0	2	2
chr11	70026411	70028411	Slc16a11	-0.14496	0.14027	insignificant	-0.0040736	0.18511	insignificant	3	46	46
chr11	70034496	70036496	Slc16a13	-0.15804	0.000504	hypomethylated	-0.108	0.037011	hypomethylated	4	13	11
chr11	70043300	70045300	Bcl6b	-0.19576	0.011841	hypomethylated	-0.022501	0.16807	insignificant	7	48	47
chr11	70051416	70053416	0610010K14Rik	-0.075595	1	lowCoverage	-0.00059524	1	insignificant	0	14	14
chr11	70053354	70055354	Rnasek		1	noCoverage	-0.013033	0.69055	insignificant	0	7	7
chr11	70223533	70225533	Pelp1	-0.085366	0.00000554	hypomethylated	0.016806	0.11528	insignificant	5	38	34
chr11	70245154	70247154	Arrb2	-0.14377	1.76E-09	hypomethylated	-0.0051635	0.0021383	hypomethylated	15	92	95
chr11	70264432	70266432	Med11	-0.12005	0.00001189	hypomethylated	0.027073	0.47737	insignificant	13	50	52
chr11	70272123	70274123	Zmynd15	-0.19628	0.00000754	hypomethylated	0.006745	0.94386	insignificant	7	32	32
chr11	70331710	70333710	Gltpd2	-0.18169	0.32708	insignificant	-0.093254	0.30742	insignificant	6	38	38
chr11	70337858	70339858	Psmb6	-0.46596	0.1234	insignificant	-0.0030376	1	insignificant	2	22	24
chr11	70352665	70354665	Pld2	-0.21194	8.18E-30	hypomethylated	-0.015521	0.64227	insignificant	26	94	94
chr11	70375382	70377382	Mink1	-0.11445	4.82E-16	hypomethylated	-0.0083738	0.18509	insignificant	29	113	110
chr11	70428395	70430395	4930544D05Rik	-0.062498	0.70089	insignificant	-0.025822	0.24977	insignificant	8	25	26
chr11	70460090	70462090	Rnf167	-0.15454	1.16E-11	hypomethylated	0.0046592	0.79585	insignificant	10	51	54
chr11	70460495	70462495	Slc25a11	-0.18061	2.18E-10	hypomethylated	0.0042952	0.44302	insignificant	7	33	36
chr11	70468152	70470152	Eno3	-0.10977	0.015034	hypomethylated	-0.0098031	0.12321	insignificant	21	126	127
chr11	70469714	70471714	Eno3	-0.10941	0.18896	insignificant	0.0089293	0.47974	insignificant	3	7	9
chr11	70482918	70484918	Camta2	-0.26226	0.000000335	hypomethylated	0.020554	0.83287	insignificant	8	30	30
chr11	70501607	70503607	Camta2		1	noCoverage	-0.0051062	0.1444	insignificant	0	2	2
chr11	70513049	70515049	Kif1c	-0.11324	1.62E-31	hypomethylated	-0.0025579	0.13312	insignificant	53	124	138
chr11	70513657	70515657	Inca1	-0.10581	1.09E-26	hypomethylated	-0.00070333	0.060267	insignificant	46	100	114
chr11	70576948	70578948	Zfp3	-0.13019	3.46E-34	hypomethylated	-0.012752	0.053163	insignificant	39	112	110
chr11	70657264	70659264	Rabep1	-0.12757	3.04E-24	hypomethylated	-0.0041009	0.0033778	hypomethylated	43	150	150
chr11	70782714	70784714	Nup88	-0.17168	1.55E-10	hypomethylated	-0.020171	0.7309	insignificant	11	54	67
chr11	70783464	70785464	Nup88	-0.13526	0.000000781	hypomethylated	-0.10714	0.47498	insignificant	5	16	21
chr11	70783475	70785475	Nup88	-0.13526	0.000000781	hypomethylated	-0.10714	0.47498	insignificant	5	16	21
chr11	70796528	70798528	C1qbp		1	noCoverage	0.04396	0.084962	insignificant	0	10	10
chr11	70817934	70819934	Dhx33		1	noCoverage	0.15873	0.098012	insignificant	0	6	6
chr11	70832112	70834112	Mis12	-0.1468	1.63E-13	hypomethylated	-0.017675	0.35163	insignificant	28	94	99

chr11	70832765	70834765	Derl2	-0.14433	1.87E-13	hypomethylated	-0.022363	0.16945	insignificant	24	73	80
chr11	71563204	71565204	Wscd1	-0.14141	0.00000254	hypomethylated	0.0090898	0.93632	insignificant	33	108	117
chr11	71855003	71857003	Fam64a	-0.15623	0.000056238	hypomethylated	-0.0060198	0.86577	insignificant	9	53	54
chr11	71949391	71951391	Pitpnm3	-0.086764	0.011571	hypomethylated	0.0024306	0.42386	insignificant	10	44	44
chr11	72016873	72018873	4933427D14Rik	-0.088613	0.035351	hypomethylated	-0.079787	0.057251	insignificant	8	21	21
chr11	72020055	72022055	Txndc17	-0.18246	1.65E-08	hypomethylated	0.00037829	0.1407	insignificant	19	87	87
chr11	72027639	72029639	4930563E22Rik	-0.15459	3.56E-11	hypomethylated	-0.023121	0.031975	hypomethylated	11	82	81
chr11	72080106	72082106	Slc13a5	-0.15371	0.0011313	hypomethylated	-0.029102	0.4353	insignificant	3	49	61
chr11	72114130	72116130	Xaf1	0.12044	1	lowCoverage	0.016366	1	insignificant	1	5	5
chr11	72126945	72128945	Fbxo39	0.087284	1	insignificant	-0.085817	0.36951	insignificant	12	31	27
chr11	72175382	72177382	Tekt1	0.22719	1	insignificant	-0.036971	0.02463	hypomethylated	1	19	32
chr11	72225215	72227215	Smtnl2	-0.30311	5.52E-10	hypomethylated	-0.069125	0.000026256	hypomethylated	14	36	35
chr11	72253879	72255879	Mybbp1a	-0.33843	2.29E-10	stronglyHypometh	0.019177	0.0002398	inconclusive	6	15	17
chr11	72303406	72305406	Spns2	-0.1538	0.00000543	hypomethylated	-0.012735	0.7043	insignificant	10	24	24
chr11	72419762	72421762	Ube2g1	-0.088839	1.38E-35	hypomethylated	-0.0029661	0.35671	insignificant	81	219	227
chr11	72502503	72504503	Ankfy1	-0.078406	0.0087271	hypomethylated	-0.020608	0.011037	hypomethylated	23	79	83
chr11	72608727	72610727	Zzef1	-0.11113	0.00000182	hypomethylated	-0.0048494	0.81979	insignificant	18	105	96
chr11	72609341	72611341	Cyb5d2	-0.10419	0.00000695	hypomethylated	-0.0019367	0.92628	insignificant	16	88	79
chr11	72773670	72775670	Atp2a3	-0.15537	1.82E-18	hypomethylated	-0.015044	0.18813	insignificant	29	106	105
chr11	72811646	72813646	P2rx1	-0.33587	0.006171	stronglyHypometh	-0.057818	0.77915	insignificant	4	8	8
chr11	72831509	72833509	Camkk1	-0.13248	5.06E-19	hypomethylated	-0.010733	0.43004	insignificant	33	140	142
chr11	72860368	72862368	1200014J11Rik	-0.12524	5.39E-12	hypomethylated	-0.0075356	0.045496	hypomethylated	19	79	80
chr11	72903096	72905096	Itgae	-0.22228	0.008454	hypomethylated	-0.14808	0.066408	insignificant	2	15	16
chr11	72951796	72953796	Gsg2	-0.19017	0.00027576	hypomethylated	0.024213	1	insignificant	7	39	36
chr11	72973031	72975031	P2rx5	-0.12187	3.56E-17	hypomethylated	0.008736	0.49671	insignificant	21	72	77
chr11	72989584	72991584	Tax1bp3	-0.12209	2.39E-17	hypomethylated	-0.0019427	0.23428	insignificant	27	145	147
chr11	72990510	72992510	Tmem93	-0.15729	3.44E-12	hypomethylated	0.0076366	0.63635	insignificant	18	101	104
chr11	72990544	72992544	Tmem93	-0.15729	3.44E-12	hypomethylated	0.0076366	0.63635	insignificant	18	101	104
chr11	73011983	73013983	Shpk	-0.12337	2.6E-12	hypomethylated	-0.027343	0.085199	insignificant	11	54	57
chr11	73012521	73014521	Ctns	-0.12337	2.6E-12	hypomethylated	-0.027343	0.085199	insignificant	11	54	57
chr11	73050760	73052760	Trpv1	-0.0053971	0.22195	insignificant	-0.0078569	0.71225	insignificant	4	10	18
chr11	73142242	73144242	Spata22	-0.12041	0.23733	insignificant	-0.029059	0.42275	insignificant	1	8	8
chr11	73415083	73417083	Olfr384	0.0020064	0.39479	insignificant	-0.048658	0.022187	hypomethylated	1	4	4
chr11	74081892	74083892	Olfr406-ps		1	noCoverage	0.030424	0.37175	insignificant	0	10	10
chr11	74403660	74405660	Rap1gap2	-0.12391	0.000011738	hypomethylated	-0.0056927	0.76595	insignificant	17	37	38
chr11	74432106	74434106	E130309D14Rik	-0.20125	3.82E-21	hypomethylated	-0.019398	0.020684	hypomethylated	21	99	103
chr11	74461996	74463996	1300001I01Rik	-0.09931	8.7E-13	hypomethylated	-0.010556	0.32544	insignificant	26	90	89
chr11	74537360	74539360	Pafah1b1	-0.10515	2.6E-53	hypomethylated	-0.0087042	0.091875	insignificant	45	129	143
chr11	74537886	74539886	Pafah1b1	-0.091181	0.000078499	hypomethylated	-0.011006	0.3205	insignificant	14	43	50
chr11	74583364	74585364	Mettl16	-0.25103	2.19E-11	hypomethylated	0.0096498	0.3771	insignificant	9	34	38
chr11	74643425	74645425	Mnt	-0.13087	6.34E-30	hypomethylated	-0.0068009	0.21897	insignificant	30	144	144
chr11	74710581	74712581	Tsr1	-0.11937	2.3E-34	hypomethylated	-0.0030954	0.7517	insignificant	25	120	122
chr11	74710582	74712582	Tsr1	-0.11937	2.3E-34	hypomethylated	-0.0030954	0.7517	insignificant	25	120	122
chr11	74717947	74719947	Snord91a	-0.95995	0.00046739	stronglyHypometh	-0.013521	0.59933	insignificant	3	11	8
chr11	74738373	74740373	Smg6	-0.086983	0.0042933	hypomethylated	-0.013782	0.46011	insignificant	10	69	86
chr11	74739300	74741300	Srr	-0.071712	0.0064191	hypomethylated	0.0022684	0.92697	insignificant	10	36	47
chr11	74879493	74881493	Smg6	-0.12276	0.0075268	hypomethylated	0.04599	1	insignificant	1	26	26
chr11	74981654	74983654	Hic1	-0.15078	6.22E-37	hypomethylated	-0.0062539	0.040781	hypomethylated	55	148	156
chr11	74983757	74985757	Hic1	-0.1534	6.81E-11	hypomethylated	0.0047177	0.022792	hypermethylated	11	65	69
chr11	74985889	74987889	Mir212	-0.13752	9.33E-29	hypomethylated	0.0029636	0.84539	insignificant	38	123	123
chr11	74986183	74988183	Mir132	-0.19129	5.12E-16	hypomethylated	-0.0051868	0.48643	insignificant	28	112	112
chr11	74992310	74994310	Ovca2	-0.25401	3.07E-09	hypomethylated	-0.027709	0.14627	insignificant	6	20	20
chr11	75003985	75005985	Dph1	-0.15865	0.022133	hypomethylated	0.0040106	0.53815	insignificant	7	33	39
chr11	75006494	75008494	Rtn4rl1	-0.13934	2.83E-49	hypomethylated	-0.0070659	0.059016	insignificant	65	196	209
chr11	75160934	75162934	Smyd4	-0.18898	2.43E-30	hypomethylated	0.0090699	0.25891	insignificant	16	91	90
chr11	75161885	75163885	Rpa1	0.40473	7.64E-09	stronglyHypermeth	0.0085042	0.00030725	hypermethylated	4	53	52
chr11	75236125	75238125	Serpinf1		1	noCoverage	-0.028505	0.15536	insignificant	0	14	18
chr11	75268219	75270219	Wdr81	-0.10161	0.2091	insignificant	0.042632	0.32034	insignificant	5	16	15
chr11	75274040	75276040	2210403K04Rik	-0.20513	0.00000832	hypomethylated	-0.058782	0.47149	insignificant	14	74	60
chr11	75276217	75278217	Mir22	-0.48584	0.00001332	stronglyHypometh	-0.093006	0.01391	hypomethylated	4	20	19
chr11	75280580	75282580	Tlcd2	-0.75505	0.28324	lowCoverage	-0.05477	0.75651	insignificant	1	6	6
chr11	75299278	75301278	Prpf8	-0.24862	5.15E-11	hypomethylated	-0.019735	0.23776	insignificant	6	46	46
chr11	75322595	75324595	Rilp	-0.24498	3.41E-28	hypomethylated	-0.023975	0.080673	insignificant	13	40	38
chr11	75326042	75328042	Scarf1	-0.27238	0.041854	hypomethylated	0.032618	0.34533	insignificant	4	11	12
chr11	75344195	75346195	Slc43a2	-0.14535	2.41E-35	hypomethylated	-0.0075086	0.0069217	hypomethylated	40	155	155
chr11	75344613	75346613	Slc43a2	-0.13856	5.01E-32	hypomethylated	-0.0045432	0.052314	insignificant	40	140	140
chr11	75400609	75402609	Pitpna	-0.11625	3.82E-27	hypomethylated	-0.0035016	0.27708	insignificant	37	101	98
chr11	75443521	75445521	Inpp5k	-0.05399	0.00042232	hypomethylated	-0.042031	0.65559	insignificant	16	63	87
chr11	75464010	75466010	Myo1c	-0.18736	1.18E-13	hypomethylated	-0.03435	0.10488	insignificant	15	55	52
chr11	75464651	75466651	Myo1c	-0.2718	1.77E-20	hypomethylated	-0.042044	0.000000733	hypomethylated	15	72	66
chr11	75468516	75470516	Myo1c	-0.11129	1.27E-13	hypomethylated	-0.00781	0.73338	insignificant	29	89	82
chr11	75491811	75493811	Crk	-0.16514	8.26E-46	hypomethylated	-0.011642	0.00045988	hypomethylated	40	135	132
chr11	75545388	75547388	Ywhae	-0.16329	1.29E-33	hypomethylated	-0.0099045	0.0017806	hypomethylated	32	140	142
chr11	75609559	75611559	Doc2b	0.07159	1	insignificant	-0.061153	0.021383	hypomethylated	2	16	16
chr11	75724528	75726528	Rph3al	-0.27273	0.38889	insignificant	-0.17273	1	insignificant	1	2	2
chr11	75812413	75814413	1700016K19Rik	-0.38233	7.85E-20	stronglyHypometh	-0.072067	0.028601	hypomethylated	8	22	22
chr11	75841284	75843284	Fam101b	-0.052197	0.00000888	hypomethylated	-0.014057	0.77397	insignificant	14	55	55
chr11	75993132	75995132	Vps53	-0.2074	1	insignificant	0.087529	0.3973	insignificant	3	20	22
chr11	76014557	76016557	Fam57a	-0.089875	4.95E-32	hypomethylated	-0.0070438	0.085943	insignificant	36	125	125
chr11	76030114	76032114	Dbil5	-0.1465	0.000003005	hypomethylated	-0.0096544	0.33077	insignificant	9	56	68
chr11	76031074	76033074	Gemin4	-0.12543	0.12956	insignificant	-0.021037	0.010099	inconclusive	2	22	22
chr11	76056237	76058237	Rnmtl1	-0.14189	3.51E-09	hypomethylated	0.0051974	0.22874	insignificant	28	87	92
chr11	76057201	76059201	Glod4	-0.15593	0.0018152	hypomethylated	0.005524	0.32139	insignificant	16	48	50
chr11	76212643	76214643	Nxn	-0.10919	3.31E-10	hypomethylated	-0.0024517	0.31002	insignificant	11	42	42
chr11	76219668	76221668	Timm22	-0.17767	0.000046781	hypomethylated	-0.016976	0.69077	insignificant	8	75	74

chr11	76322921	76324921	Abr	0.014269	0.45415	insignificant	-0.0093204	0.45824	insignificant	3	10	10
chr11	76384876	76386876	Abr	-0.2514	1.56E-13	hypomethylated	-0.11081	0.0010754	hypomethylated	14	12	32
chr11	76484971	76486971	Bhlha9	-0.020548	0.0024092	hypomethylated	0.043722	0.67111	insignificant	15	82	81
chr11	76660510	76662510	Cpd	-0.74807	1.25E-08	stronglyHypometh	0.035459	0.031212	inconclusive	1	4	5
chr11	76758157	76760157	Blmh	-0.15046	3.9E-18	hypomethylated	0.017802	0.90554	insignificant	24	73	80
chr11	76811098	76813098	Slc6a4	-0.24505	2.95E-11	hypomethylated	-0.036049	0.83333	insignificant	9	58	76
chr11	76891674	76893674	Mir423	-0.19908	3.57E-27	hypomethylated	-0.0088607	0.0098864	hypomethylated	31	88	88
chr11	76891937	76893937	Mir423	-0.20073	2.51E-19	hypomethylated	-0.013755	0.0092671	hypomethylated	22	56	56
chr11	77028926	77030926	Ssh2	-0.10755	1.38E-19	hypomethylated	-0.019153	0.19012	insignificant	25	109	109
chr11	77276414	77278414	Coro6	-0.35558	1.89E-15	stronglyHypometh	0.052628	0.26814	insignificant	9	38	40
chr11	77303180	77305180	Ankrd13b	-0.10508	1.06E-31	hypomethylated	0.013007	0.00001926	inconclusive	38	103	103
chr11	77305913	77307913	Git1	-0.13246	1.1E-29	hypomethylated	-0.016836	0.0052974	hypomethylated	51	125	136
chr11	77326775	77328775	Trp53i13	-0.11432	1.14E-20	hypomethylated	0.0054967	0.84698	insignificant	27	126	117
chr11	77327618	77329618	Abhd15	-0.11412	5.48E-39	hypomethylated	-0.0036821	0.078366	insignificant	46	167	158
chr11	77421317	77423317	Taok1	-0.28891	1.62E-10	hypomethylated	-0.024538	0.0078694	hypomethylated	5	19	19
chr11	77498640	77500640	Nufip2	-0.21816	0.0025131	hypomethylated	-0.011179	0.24181	insignificant	5	33	31
chr11	77538795	77540795	Cryba1	0.22113	0.58659	insignificant	-0.0042726	0.062196	insignificant	2	12	12
chr11	77589766	77591766	Myo18a	-0.049525	1	insignificant	0.025692	0.037971	hypermethylated	16	57	57
chr11	77707374	77709374	Pipox	-0.595	2.34E-11	stronglyHypometh	-0.036621	0.29784	insignificant	2	4	4
chr11	77742790	77744790	Sez6	-0.10218	7.59E-21	hypomethylated	-0.018222	0.14332	insignificant	40	118	120
chr11	77743444	77745444	Sez6	-0.12131	1.42E-41	hypomethylated	-0.00209	0.17772	insignificant	68	201	203
chr11	77795317	77797317	Phf12	-0.17947	7.37E-21	hypomethylated	0.003593	0.40934	insignificant	35	99	107
chr11	77844814	77846814	Dhrs13	-0.13171	1.17E-15	hypomethylated	0.0032779	0.16329	insignificant	35	128	132
chr11	77850442	77852442	Flot2	-0.30811	1.7E-37	hypomethylated	-0.048469	0.00011947	hypomethylated	32	101	95
chr11	77907174	77909174	BC017647	-0.1755	1.04E-20	hypomethylated	0.050449	0.74499	insignificant	7	14	15
chr11	77990168	77992168	Nek8	-0.15729	4.16E-17	hypomethylated	0.026883	0.40827	insignificant	19	81	76
chr11	77991267	77993267	Tlcd1	-0.090273	2.86E-10	hypomethylated	0.01521	1	insignificant	7	50	54
chr11	77995258	77997258	Snord42b	-0.19365	0.002444	hypomethylated	-0.0086152	0.72171	insignificant	2	32	34
chr11	77997086	77999086	Snord42b	0.16346	1	insignificant	-0.030782	1	insignificant	2	18	24
chr11	78000928	78002928	Rab34	-0.10836	4.28E-26	hypomethylated	0.011129	0.077944	insignificant	38	164	165
chr11	78001249	78003249	Rab34	-0.057844	1.01E-23	hypomethylated	0.016424	0.16908	insignificant	38	152	153
chr11	78058247	78060247	Sdf2	-0.1431	1.75E-13	hypomethylated	-0.014653	0.12573	insignificant	21	86	90
chr11	78059205	78061205	Supt6h	-0.22353	5.02E-08	hypomethylated	-0.0021454	0.27387	insignificant	10	63	66
chr11	78074255	78076255	2610507B11Rik	-0.14782	7.56E-33	hypomethylated	-0.020422	0.082447	insignificant	38	122	125
chr11	78114092	78116092	Spag5	-0.20507	0.079888	insignificant	-0.011461	0.55578	insignificant	7	31	32
chr11	78136699	78138699	Aldoc		1	noCoverage	-0.088943	0.62019	insignificant	0	14	11
chr11	78140923	78142923	Pigs	-0.10472	6.83E-12	hypomethylated	-0.022113	0.90626	insignificant	24	74	77
chr11	78156023	78158023	Unc119	-0.14472	1.6E-23	hypomethylated	0.058819	0.39017	insignificant	24	44	43
chr11	78235687	78237687	Slc13a2	0.051763	0.039669	inconclusive	0.10991	0.60896	insignificant	1	20	19
chr11	78278202	78280202	Slc46a1		1	noCoverage	0.12693	0.90475	insignificant	0	21	33
chr11	78311256	78313256	Sarm1	-0.39651	0.64347	insignificant	-0.0056821	0.92322	insignificant	3	12	12
chr11	78311621	78313621	Vtn	-0.12471	0.35504	insignificant	0.0042088	1	insignificant	1	6	6
chr11	78316014	78318014	Sebox	0.21142	1	insignificant	0.088666	0.68611	insignificant	1	9	8
chr11	78324797	78326797	Poldip2	-0.17504	2.38E-32	hypomethylated	-0.01921	0.44214	insignificant	19	86	87
chr11	78325670	78327670	Tmem199	-0.23121	2.92E-09	hypomethylated	0.017195	0.54934	insignificant	5	36	38
chr11	78348937	78350937	Ift20	-0.17279	4.25E-22	hypomethylated	-0.0088225	0.96362	insignificant	14	71	61
chr11	78349762	78351762	Tnfaip1	-0.10033	0.00019385	hypomethylated	-0.012721	0.022243	inconclusive	13	62	60
chr11	78364237	78366237	Tmem97	-0.22602	1.1E-12	hypomethylated	0.12663	0.09564	insignificant	7	33	22
chr11	78510927	78512927	Nlk	-0.12658	1.26E-13	hypomethylated	-0.0034426	0.77647	insignificant	16	36	38
chr11	78639096	78641096	1810012P15Rik	-0.16659	8.74E-08	hypomethylated	-0.034188	0.1352	insignificant	16	76	84
chr11	78959856	78961856	Ksr1		1	noCoverage	0.0032468	0.53315	insignificant	0	11	10
chr11	79068197	79070197	Wsb1	-0.24085	0.010344	hypomethylated	0.016841	0.32304	insignificant	4	32	35
chr11	79152393	79154393	Nf1	-0.11546	1.69E-09	hypomethylated	0.0104	0.53753	insignificant	36	147	147
chr11	79403713	79405713	Rab11fip4	-0.069657	0.000013826	hypomethylated	-0.0074403	0.00062614	hypomethylated	14	122	135
chr11	79524470	79526470	Mir193	-0.16292	3.29E-56	hypomethylated	-0.0031562	0.000000142	hypomethylated	43	178	177
chr11	79538901	79540901	Mir365-2		1	noCoverage	0.035342	0.17382	insignificant	0	4	4
chr11	79775889	79777889	Utp6	-0.17537	1.41E-08	hypomethylated	-0.0081983	0.0045725	hypomethylated	10	29	26
chr11	79805607	79807607	Suz12	-0.089745	2.23E-18	hypomethylated	-0.0031588	0.12061	insignificant	43	189	200
chr11	79894457	79896457	Crlf3	-0.09067	0.02243	hypomethylated	0.0035937	0.087992	insignificant	10	28	28
chr11	79901901	79903901	Atad5	-0.14767	5.43E-20	hypomethylated	-0.018725	0.3809	insignificant	19	48	52
chr11	79955655	79957655	1110002N22Rik	-0.25096	0.33756	insignificant	-0.034238	0.66921	insignificant	7	17	22
chr11	79966663	79968663	Adap2	-0.24861	5.59E-11	hypomethylated	0.013344	0.25177	insignificant	12	46	46
chr11	79996373	79998373	Rnf135	-0.21836	3.92E-10	hypomethylated	-0.016242	0.040697	hypomethylated	12	53	50
chr11	80021556	80023556	Rhot1	-0.117	2.37E-16	hypomethylated	0.0085293	0.076284	insignificant	34	111	113
chr11	80113413	80115413	Rhbdl3	-0.13529	3.47E-35	hypomethylated	0.0063016	0.19233	insignificant	54	204	207
chr11	80191517	80193517	5730455P16Rik	-0.2585	1	insignificant	-0.099633	0.50211	insignificant	3	7	6
chr11	80241116	80243116	Psmd11	-0.16224	3.75E-52	hypomethylated	-0.011572	0.030174	hypomethylated	53	128	128
chr11	80289547	80291547	Cdk5r1	-0.099197	5.2E-27	hypomethylated	0.031198	0.000073837	hypermethylated	61	208	193
chr11	80593527	80595527	Myo1d	-0.2	0.33927	insignificant	-0.045652	0.85782	insignificant	5	5	10
chr11	80622916	80624916	Tmem98	-0.13991	0.000090223	hypomethylated	-0.024986	0.46568	insignificant	14	37	40
chr11	80966405	80968405	Accn1	-0.1322	2.55E-23	hypomethylated	0.020655	0.0064477	inconclusive	25	99	107
chr11	81781898	81783898	Accn1	-0.19824	0.000012382	hypomethylated	-0.0068345	0.031941	hypomethylated	6	18	18
chr11	81914346	81916346	Ccl12	-0.19492	0.0099976	hypomethylated	0.011596	0.79424	insignificant	2	4	4
chr11	81993314	81995314	Ccl1	-0.09904	0.64323	insignificant	-0.11888	0.21554	insignificant	1	12	12
chr11	82201401	82203401	Tmem132e	-0.11263	6.65E-34	hypomethylated	-0.000092998	0.17153	insignificant	47	187	188
chr11	82445854	82447854	Gm11426	-0.01388	0.77098	insignificant	0.013144	0.86469	insignificant	4	10	10
chr11	82576846	82578846	Zfp830	-0.13508	2.36E-09	hypomethylated	0.0073526	0.97056	insignificant	13	83	82
chr11	82577790	82579790	Cct6b	-0.14256	2.49E-09	hypomethylated	0.017672	1	insignificant	10	63	62
chr11	82593636	82595636	Lig3	-0.12113	4.45E-33	hypomethylated	-0.01087	0.000034511	hypomethylated	46	156	156
chr11	82684246	82686246	Rffl	-0.17964	9.82E-17	hypomethylated	-0.025753	0.013821	hypomethylated	32	97	107
chr11	82684712	82686712	Rffl	-0.32144	0.00042008	hypomethylated	-0.11955	0.13909	insignificant	10	32	41
chr11	82704117	82706117	Rad51l3	-0.26922	0.0031223	hypomethylated	0.015954	0.45188	insignificant	2	14	12
chr11	82721897	82723897	Unc45b	-0.10767	0.000000039	hypomethylated	0.016906	0.32347	insignificant	11	22	22
chr11	82723754	82725754	Unc45b	0.19954	0.0039381	hypermethylated	0.064071	0.00045618	hypermethylated	3	16	18
chr11	82764603	82766603	Slfn5	-0.27156	7.27E-18	hypomethylated	-0.031336	0.9545	insignificant	5	27	27

chr11	82805332	82807332	Slfn9	-0.20479	0.20326	insignificant	0.11623	0.030763	hypermethylated	3	6	6
chr11	82834224	82836224	Slfn8		1	noCoverage	-0.0077519	1	insignificant	0	3	3
chr11	82834312	82836312	Slfn8		1	noCoverage	-0.0077519	1	insignificant	0	3	3
chr11	83036077	83038077	AI662270	-0.74473	0.36719	lowCoverage	-0.07344	0.50944	insignificant	1	8	6
chr11	83104200	83106200	AA465934	-0.15869	0.057889	insignificant	-0.021804	0.24487	insignificant	10	32	32
chr11	83112479	83114479	Pex12	-0.49288	0.023512	stronglyHypometh	-0.033182	0.000059859	inconclusive	5	30	28
chr11	83115198	83117198	Ap2b1	-0.14396	2.17E-08	hypomethylated	-0.025654	0.022955	hypomethylated	19	63	70
chr11	83222573	83224573	Rasl10b	-0.1385	1.23E-19	hypomethylated	-0.017408	0.29333	insignificant	28	84	105
chr11	83242957	83244957	Gas2l2	-0.40892	0.0032359	stronglyHypometh	0.076385	0.093264	insignificant	3	22	26
chr11	83276463	83278463	Mmp28	-0.17791	0.0060388	hypomethylated	-0.07478	0.45414	insignificant	5	11	10
chr11	83285609	83287609	Taf15	-0.11673	1.26E-15	hypomethylated	-0.0097531	0.97569	insignificant	32	143	156
chr11	83566138	83568138	Heatr6	-0.13962	2.97E-17	hypomethylated	-0.015908	0.0019773	hypomethylated	27	68	68
chr11	83663370	83665370	Hnf1b	-0.13951	0.000000152	hypomethylated	-0.036701	0.017785	hypomethylated	18	100	98
chr11	83754591	83756591	Ddx52	-0.1544	0.000000369	hypomethylated	-0.018162	0.036816	hypomethylated	20	56	55
chr11	83776929	83778929	Synrg	-0.084965	7.01E-13	hypomethylated	-0.011826	0.52293	insignificant	28	87	95
chr11	83776972	83778972	Synrg	-0.084965	7.01E-13	hypomethylated	-0.011826	0.52293	insignificant	28	87	95
chr11	83881859	83883859	Dusp14	-0.11409	4.71E-14	hypomethylated	-0.027589	0.23591	insignificant	18	59	58
chr11	83943070	83945070	Tada2a	-0.069599	0.69404	insignificant	-0.0086149	0.61743	insignificant	16	74	75
chr11	83980978	83982978	Gm11437		1	noCoverage	-0.10985	0.53451	insignificant	0	4	4
chr11	84327003	84329003	Aatf	-0.107	5.63E-09	hypomethylated	-0.0031997	0.042816	hypomethylated	20	69	61
chr11	84339036	84341036	Lhx1	-0.1593	2.84E-12	hypomethylated	-0.018758	0.00044401	hypomethylated	25	85	90
chr11	84633017	84635017	Mrm1	-0.2025	0.00000228	hypomethylated	0.00947	0.47772	insignificant	4	10	10
chr11	84642505	84644505	Dhrs11	-0.1933	1.07E-10	hypomethylated	0.03398	0.000000601	inconclusive	14	53	56
chr11	84684240	84686240	Ggnbp2	-0.12773	0.000036035	hypomethylated	-0.0054946	0.59268	insignificant	11	60	60
chr11	84692721	84694721	Myo19	-0.11625	8.51E-23	hypomethylated	-0.0053731	0.062804	insignificant	42	140	140
chr11	84693697	84695697	Pigw	-0.1337	1.53E-09	hypomethylated	0.00091615	0.0072501	inconclusive	20	66	66
chr11	84693787	84695787	Pigw	-0.13544	0.00036308	hypomethylated	0.00016984	0.084258	insignificant	12	50	50
chr11	84729858	84731858	Znhit3		1	noCoverage	-0.037879	0.75995	insignificant	0	10	10
chr11	84770255	84772255	Car4	-0.30662	0.000000664	hypomethylated	0.037212	0.88724	insignificant	8	43	40
chr11	84953457	84955457	Usp32	-0.11502	0.0001148	hypomethylated	0.0033141	0.76118	insignificant	7	50	49
chr11	84983597	84985597	1700125H20Rik	-0.26132	0.000000472	hypomethylated	-0.085311	0.19927	insignificant	10	25	22
chr11	85047667	85049667	D630032N06Rik	-0.10256	7.08E-47	hypomethylated	-0.0038804	0.98003	insignificant	69	226	226
chr11	85048622	85050622	Appbp2	-0.10939	0.00035768	hypomethylated	-0.0054555	0.0038082	inconclusive	14	58	58
chr11	85123755	85125755	Ppm1d	-0.083131	4.66E-09	hypomethylated	0.0016717	0.1095	insignificant	27	128	130
chr11	85165665	85167665	Bcas3	-0.17625	0.00029498	hypomethylated	-0.0079954	0.3299	insignificant	9	50	50
chr11	85645116	85647116	Tbx2	-0.17425	7.37E-22	hypomethylated	-0.011111	0.20433	insignificant	30	134	135
chr11	85702564	85704564	Tbx4	-0.19468	0.000000024	hypomethylated	0.025355	0.65428	insignificant	21	59	64
chr11	86014695	86016695	Brip1	-0.12794	0.000014597	hypomethylated	0.023678	0.044294	inconclusive	21	75	87
chr11	86071070	86073070	Ints2	-0.18066	1.32E-12	hypomethylated	0.19275	0.73433	insignificant	3	32	26
chr11	86171027	86173027	Med13	-0.18626	6.92E-15	hypomethylated	-0.045946	0.031347	hypomethylated	22	86	93
chr11	86297158	86299158	Rnft1	-0.096165	1.78E-17	hypomethylated	-0.0048004	0.052396	insignificant	22	100	100
chr11	86357492	86359492	Tubd1	-0.15679	1.07E-25	hypomethylated	-0.015509	0.60322	insignificant	39	124	132
chr11	86358309	86360309	Rps6kb1	-0.20992	4.9E-14	hypomethylated	-0.018421	0.44091	insignificant	11	47	48
chr11	86496484	86498484	Ptrh2	-0.14742	5.37E-15	hypomethylated	-0.011368	0.17573	insignificant	33	100	100
chr11	86496582	86498582	Ptrh2	-0.14742	5.37E-15	hypomethylated	-0.011368	0.17573	insignificant	33	100	100
chr11	86497324	86499324	Ptrh2	-0.16688	5.78E-13	hypomethylated	-0.0061191	0.23816	insignificant	16	56	56
chr11	86570994	86572994	Cltc	-0.15251	0.000000103	hypomethylated	0.0082432	0.44502	insignificant	7	54	58
chr11	86621162	86623162	Dhx40	-0.60783	0.4812	lowCoverage	-0.0061055	0.4743	insignificant	1	8	10
chr11	86807264	86809264	Ypel2	-0.48778	0.011668	stronglyHypometh	0.12055	0.22918	insignificant	5	15	14
chr11	86887639	86889639	Gdpd1		1	noCoverage	0.12722	0.6956	insignificant	0	29	32
chr11	86900279	86902279	1200011M11Ril	-0.54448	1.07E-28	stronglyHypometh	-0.071917	0.5181	insignificant	6	20	22
chr11	86921762	86923762	Fam33a	-0.13335	1	insignificant	0.019938	0.59331	insignificant	4	28	27
chr11	86922216	86924216	Prr11	-0.13335	1	insignificant	0.019938	0.59331	insignificant	4	28	27
chr11	86939578	86941578	Trim37	-0.11346	1.26E-14	hypomethylated	-0.014654	0.018682	hypomethylated	29	121	132
chr11	87172496	87174496	Ppm1e	-0.12797	1.49E-09	hypomethylated	-0.011925	0.13688	insignificant	17	54	60
chr11	87217566	87219566	Tex14	-0.18358	0.2411	insignificant	-0.01273	0.12435	insignificant	5	46	54
chr11	87217997	87219997	Rad51c	-0.16366	0.40965	insignificant	0.0071921	0.15383	insignificant	5	47	54
chr11	87240092	87242092	Tex14		1	noCoverage	0.066471	0.9202	insignificant	0	14	18
chr11	87393632	87395632	###########	-0.21875	0.031702	hypomethylated	-0.016369	1	insignificant	2	4	4
chr11	87404718	87406718	Mtmr4	-0.17603	4.44E-24	hypomethylated	-0.013555	0.94877	insignificant	32	120	129
chr11	87429665	87431665	Hsf5	0.0017231	0.30458	insignificant	-0.024281	0.00000487	hypomethylated	13	110	128
chr11	87550066	87552066	Supt4h1	-0.15843	0.00000655	hypomethylated	0.0327	0.54968	insignificant	9	43	43
chr11	87569365	87571365	Mir142	-0.28964	5.22E-16	hypomethylated	-0.023356	0.7106	insignificant	8	33	38
chr11	87573042	87575042	Bzrap1	-0.31267	1.54E-17	hypomethylated	0.035462	0.48992	insignificant	10	21	24
chr11	87665726	87667726	Mks1	-0.28179	2.19E-19	hypomethylated	0.068544	0.00018274	hypermethylated	9	22	26
chr11	87689038	87691038	Epx		1	noCoverage	-0.34189	0.041135	stronglyhypometh	0	4	4
chr11	87800087	87802087	Dynll2	-0.072483	4.28E-10	hypomethylated	0.06775	0.0033975	hypermethylated	38	112	135
chr11	87801035	87803035	Dynll2	-0.03865	0.24983	insignificant	0.20262	0.10736	insignificant	7	36	40
chr11	87860172	87862172	Srsf1	-0.1272	1.68E-10	hypomethylated	-0.007043	0.6112	insignificant	32	157	163
chr11	87880842	87882842	Vezf1	-0.10037	4.45E-15	hypomethylated	-0.0041016	0.81917	insignificant	45	145	137
chr11	87911647	87913647	Cuedc1	-0.11603	5.01E-39	hypomethylated	-0.013381	0.25019	insignificant	47	148	162
chr11	87982066	87984066	Cuedc1	-0.12173	0.090929	insignificant	-0.011756	0.54682	insignificant	15	66	73
chr11	88016919	88018919	Mrps23	-0.23664	1.31E-33	hypomethylated	-0.014265	0.01565	hypomethylated	12	60	65
chr11	88106544	88108544	1700106J16Rik	0.4148	0.14546	insignificant	0.16684	0.11788	insignificant	2	20	22
chr11	88579543	88581543	Msi2	-0.14933	0.000092251	hypomethylated	-0.0068909	0.22951	insignificant	16	90	89
chr11	88725900	88727900	Akap1	-0.28745	1	insignificant	-0.0102	0.0065981	inconclusive	9	54	52
chr11	88816756	88818756	Scpep1	-0.56732	0.24404	insignificant	-0.0363	0.57758	insignificant	1	17	12
chr11	88834248	88836248	Coil	-0.17247	3.25E-12	hypomethylated	-0.051133	0.97258	insignificant	10	70	71
chr11	88859716	88861716	Trim25	-0.22729	0.22333	insignificant	-0.0013424	0.28941	insignificant	5	33	31
chr11	88922062	88924062	Dgke	-0.49487	0.0020775	stronglyHypometh	0.030979	0.20643	insignificant	3	18	19
chr11	89163873	89165873	Nog	-0.075968	1.24E-10	hypomethylated	-0.020871	0.004648	hypomethylated	24	67	85
chr11	89864208	89866208	Pctp		1	noCoverage	-0.0026808	0.46789	insignificant	0	8	8
chr11	89890661	89892661	Tmem100	0.2611	0.31878	insignificant	-0.17088	0.028037	hypomethylated	2	27	27
chr11	90109789	90111789	Mmd	-0.12631	2.78E-11	hypomethylated	-0.025431	0.0068523	hypomethylated	29	97	105
chr11	90252231	90254231	Hlf	-0.23323	0.0031597	inconclusive	-0.089726	0.043672	inconclusive	8	18	16

chr11	90498497	90500497	Cox11	-0.18312	4.64E-15	hypomethylated	-0.070644	0.23565	insignificant	21	96	100
chr11	90499422	90501422	Stxbp4	-0.22202	1.06E-13	hypomethylated	-0.11564	0.21576	insignificant	19	67	82
chr11	90548915	90550915	Tom1l1	-0.18181	8.86E-14	hypomethylated	0.0004071	0.18066	insignificant	13	46	46
chr11	92959603	92961603	Car10	-0.16236	8.85E-19	hypomethylated	-0.011944	0.58625	insignificant	24	110	111
chr11	93746532	93748532	Mbtd1	-0.10202	5.7E-19	hypomethylated	0.0010688	0.7398	insignificant	37	166	177
chr11	93747080	93749080	Utp18	-0.12337	4.69E-10	hypomethylated	-0.0088578	0.88657	insignificant	14	70	80
chr11	93817002	93819002	Nme2	-0.094408	0.018603	hypomethylated	0.038893	0.39003	insignificant	17	55	58
chr11	93817321	93819321	Nme2	0.16623	0.21219	insignificant	0.050733	0.1662	insignificant	2	7	10
chr11	93856404	93858404	Spag9	-0.13103	2.01E-40	hypomethylated	0.0067011	0.041341	inconclusive	57	151	151
chr11	93904518	93906518	Spag9		1	noCoverage	0.12937	0.030845	hypermethylated	0	16	11
chr11	94071767	94073767	Tob1	-0.12667	1.3E-26	hypomethylated	-0.0093182	0.78473	insignificant	45	170	187
chr11	94103893	94105893	Wfikkn2	-0.14736	0.11767	insignificant	0.078694	0.094715	insignificant	9	30	30
chr11	94183225	94185225	Luc7l3	-0.1479	2.78E-14	hypomethylated	-0.0041641	0.046233	hypomethylated	24	55	57
chr11	94188314	94190314	Ankrd40	-0.15117	6.56E-13	hypomethylated	-0.015506	0.39491	insignificant	22	80	83
chr11	94254290	94256290	Abcc3	-0.30876	0.00000056	hypomethylated	-0.016785	0.1273	insignificant	4	20	22
chr11	94335512	94337512	Cacna1g	-0.17528	1.93E-45	hypomethylated	-0.023277	1.83E-09	hypomethylated	27	114	116
chr11	94346624	94348624	Spata20	-0.23149	0.017613	hypomethylated	-0.065789	0.33365	insignificant	4	25	22
chr11	94361288	94363288	Epn3	-0.057846	0.65244	insignificant	0.0029685	0.2328	insignificant	10	32	32
chr11	94382816	94384816	Mycbpap	-0.18726	0.0045489	hypomethylated	0.047204	0.024714	inconclusive	6	26	28
chr11	94410521	94412521	Rsad1	-0.29545	0.0013265	hypomethylated	-0.13192	0.25044	insignificant	4	11	8
chr11	94425387	94427387	Chad	0.12893	0.53617	insignificant	0.035161	0.52967	insignificant	7	68	78
chr11	94463100	94465100	Acsf2	-0.17312	0.0030488	hypomethylated	0.0065402	0.30197	insignificant	1	36	36
chr11	94490137	94492137	Lrrc59	-0.09695	9.07E-18	hypomethylated	0.0059103	0.51341	insignificant	27	116	109
chr11	94514104	94516104	Mrpl27	-0.21489	1.7E-11	hypomethylated	0.0044471	0.71188	insignificant	12	32	27
chr11	94515068	94517068	Eme1	-0.19257	0.00023245	hypomethylated	0.0047216	0.62089	insignificant	7	22	18
chr11	94693316	94695316	Tmem92	0.067251	1	insignificant	-0.35677	0.00013018	stronglyhypometh	1	9	12
chr11	94796583	94798583	Col1a1		1	noCoverage	0.1188	0.047835	hypermethylated	0	12	12
chr11	94827974	94829974	Hils1	-0.16746	0.033519	hypomethylated	-0.22763	0.0089242	hypomethylated	1	14	14
chr11	94851525	94853525	Ppp1r9b	-0.091852	1.34E-30	hypomethylated	-0.0054854	0.12701	insignificant	52	207	216
chr11	94870192	94872192	Samd14	-0.153	1.07E-23	hypomethylated	0.010199	0.042977	inconclusive	18	84	102
chr11	94902685	94904685	Pdk2	-0.087633	0.00082691	hypomethylated	-0.0090477	0.73374	insignificant	3	24	27
chr11	94938028	94940028	Itga3	-0.061008	0.03945	hypomethylated	-0.073839	0.061016	insignificant	10	69	75
chr11	94980430	94982430	Dlx3	-0.13944	1.34E-25	hypomethylated	-0.028553	0.015805	hypomethylated	35	116	119
chr11	95007115	95009115	A730090H04Rik	-0.040543	0.60438	insignificant	-0.0027981	0.80726	insignificant	2	46	42
chr11	95171560	95173560	Myst2	-0.19856	8.51E-10	hypomethylated	-0.038862	0.02346	hypomethylated	7	29	31
chr11	95197331	95199331	Fam117a	-0.15153	2.07E-17	hypomethylated	-0.0058978	0.57841	insignificant	29	84	74
chr11	95245235	95247235	Slc35b1	-0.20026	9.22E-09	hypomethylated	-0.014561	0.061336	insignificant	6	41	42
chr11	95274396	95276396	Spop	-0.021016	7.54E-15	hypomethylated	-0.025495	0.20601	insignificant	7	39	52
chr11	95375879	95377879	Nxph3	-0.089819	0.00010159	hypomethylated	-0.0085043	0.00000155	inconclusive	19	70	70
chr11	95449012	95451012	Ngfr	-0.22889	0.065166	insignificant	-0.0093465	0.88431	insignificant	3	15	14
chr11	95527270	95529270	Phb	0.066316	0.58714	insignificant	-0.037339	0.052301	insignificant	4	22	21
chr11	95609380	95611380	Zfp652	-0.63481	0.24905	insignificant	0.027381	0.16977	insignificant	3	26	26
chr11	95684813	95686813	Phospho1	-0.089742	7.94E-23	hypomethylated	0.010291	0.012597	inconclusive	36	131	129
chr11	95776185	95778185	B4galnt2	-0.22801	5.96E-15	hypomethylated	-0.046032	0.16231	insignificant	10	39	42
chr11	95867258	95869258	Igf2bp1	-0.1363	1.04E-24	hypomethylated	-0.001719	0.45537	insignificant	38	198	200
chr11	95895230	95897230	Snf8	-0.19331	2.82E-21	hypomethylated	-0.0070836	0.19276	insignificant	19	48	48
chr11	95926678	95928678	Ube2z	-0.55461	0.084031	insignificant	-0.0077691	0.39712	insignificant	1	38	37
chr11	95936945	95938945	Atp5g1	-0.41667	0.0057606	stronglyHypometh	0.065882	0.020862	hypermethylated	5	18	20
chr11	95937008	95939008	Atp5g1	-0.41667	0.0057606	stronglyHypometh	0.065882	0.020862	hypermethylated	5	18	20
chr11	95973276	95975276	Calcoco2	-0.075641	0.37002	insignificant	-0.051143	0.14871	insignificant	3	13	15
chr11	95994099	95996099	Ttll6		1	noCoverage	-0.096429	0.75803	insignificant	0	8	4
chr11	96054674	96056674	Hoxb13	-0.19388	1.29E-08	hypomethylated	-0.034203	0.044916	hypomethylated	12	66	65
chr11	96125477	96127477	Mir196a-1	0.082808	0.08892	insignificant	-0.050554	0.63502	insignificant	4	32	32
chr11	96131643	96133643	Hoxb9	-0.13516	2.4E-42	hypomethylated	-0.0103	0.010664	hypomethylated	34	128	117
chr11	96142218	96144218	Hoxb8	-0.17918	0.23239	insignificant	-0.0086931	0.80074	insignificant	5	20	20
chr11	96146959	96148959	Hoxb7	-0.16311	5.4E-32	hypomethylated	0.0031683	0.0094814	inconclusive	42	133	136
chr11	96159484	96161484	Hoxb6	-0.20971	6.08E-11	hypomethylated	-0.049351	0.92835	insignificant	11	46	53
chr11	96163825	96165825	Hoxb5	-0.17398	0.000000011	hypomethylated	0.0085295	1	insignificant	12	32	32
chr11	96177478	96179478	Mir10a		1	noCoverage	-0.037898	0.81953	insignificant	0	32	32
chr11	96178580	96180580	Hoxb4	-0.23153	5.7E-41	hypomethylated	-0.012471	0.00068288	hypomethylated	19	62	62
chr11	96183439	96185439	Hoxb3		1	noCoverage	0.018814	1	insignificant	0	10	10
chr11	96204082	96206082	Hoxb3	-0.15946	0.63988	insignificant	0.0070909	0.00055501	hypermethylated	8	29	29
chr11	96211945	96213945	Hoxb2	-0.12974	0.032509	hypomethylated	0.013311	0.63703	insignificant	5	42	42
chr11	96226071	96228071	Hoxb1	-0.24936	1.29E-12	hypomethylated	-0.016411	0.042832	hypomethylated	5	39	42
chr11	96324904	96326904	Skap1	-0.11442	0.004994	hypomethylated	-0.0027242	0.35282	insignificant	3	28	28
chr11	96325861	96327861	Gm11529	-0.11442	0.004994	hypomethylated	-0.0027242	0.35282	insignificant	3	28	28
chr11	96638869	96640869	Snx11	-0.097136	1.27E-17	hypomethylated	-0.0090622	0.032323	hypomethylated	17	58	58
chr11	96649449	96651449	Cbx1	-0.10935	6.3E-12	hypomethylated	-0.023943	0.0065622	hypomethylated	13	50	57
chr11	96690816	96692816	Nfe2l1	-0.1459	6.99E-10	hypomethylated	0.0044791	1	insignificant	14	92	83
chr11	96691282	96693282	Nfe2l1	-0.13756	0.0046021	hypomethylated	0.01948	0.086748	insignificant	8	60	56
chr11	96710189	96712189	Copz2	-0.14445	0.18526	insignificant	0.010644	0.79339	insignificant	13	77	79
chr11	96710706	96712706	Mir152	-0.17003	0.0036953	hypomethylated	0.0057864	1	insignificant	12	73	75
chr11	96777795	96779795	Cdk5rap3	-0.15208	0.00003464	hypomethylated	-0.037765	0.048003	hypomethylated	8	52	54
chr11	96804459	96806459	D030028A08Rik	-0.19885	0.020482	hypomethylated	-0.054147	0.33422	insignificant	4	16	20
chr11	96805333	96807333	Pnpo	-0.318	0.0019381	hypomethylated	-0.097762	0.097571	insignificant	4	20	24
chr11	96839002	96841002	Sp2	-0.16119	0.00000106	hypomethylated	0.017746	0.84036	insignificant	8	50	50
chr11	96873882	96875882	Sp6		1	noCoverage	0.01359	1	insignificant	0	2	2
chr11	96890265	96892265	Scrn2	-0.29015	4.19E-12	hypomethylated	0.030816	0.92501	insignificant	10	46	49
chr11	96901899	96903899	Mrpl10	-0.21693	0.00016815	hypomethylated	-0.005748	1	insignificant	8	38	37
chr11	96902683	96904683	Lrrc46	-0.2012	0.00016699	hypomethylated	0.020789	1	insignificant	8	36	37
chr11	96911133	96913133	Osbpl7	-0.25617	1.18E-19	hypomethylated	-0.026544	0.71731	insignificant	9	40	43
chr11	97011026	97013026	Tbkbp1	-0.084461	2.17E-31	hypomethylated	0.027092	0.70099	insignificant	66	217	232
chr11	97049206	97051206	Kpnb1	-0.11054	5.67E-39	hypomethylated	-0.0066213	0.18956	insignificant	31	136	132
chr11	97141890	97143890	Npepps	-0.021225	0.22911	insignificant	0.0149	0.053921	insignificant	2	14	13
chr11	97222864	97224864	Socs7	-0.12198	9.38E-36	hypomethylated	-0.0042184	0.92785	insignificant	64	166	166

chr11	97436440	97438440	Srcin1	-0.093674	0.01373	hypomethylated	-0.0063553	0.9304	insignificant	26	96	100
chr11	97491030	97493030	E130012A19Rik	-0.17727	0.00000314	hypomethylated	0.014926	0.04	inconclusive	6	46	43
chr11	97523725	97525725	Mllt6	-0.1578	7.65E-29	hypomethylated	0.0025357	0.13587	insignificant	49	90	121
chr11	97546265	97548265	Cisd3	-0.12612	3.63E-18	hypomethylated	-0.012515	0.47372	insignificant	16	66	66
chr11	97560698	97562698	Pcgf2	-0.15746	6.23E-27	hypomethylated	-0.010907	0.029262	hypomethylated	29	120	120
chr11	97560962	97562962	Pcgf2	-0.17384	2.99E-13	hypomethylated	-0.0097364	0.059046	insignificant	13	86	86
chr11	97561811	97563811	Pcgf2	-0.17067	6.32E-13	hypomethylated	-0.0059305	0.31811	insignificant	13	66	66
chr11	97563747	97565747	Psmb3		1	noCoverage	-0.023132	0.40529	insignificant	0	52	52
chr11	97606018	97608018	Cwc25	-0.13791	6.25E-09	hypomethylated	0.0030603	0.8179	insignificant	18	93	88
chr11	97637232	97639232	1700001P01Rik	-0.071633	0.29908	insignificant	-0.13302	0.38699	insignificant	1	12	20
chr11	97643080	97645080	Rpl23	-0.13732	1.76E-08	hypomethylated	-0.0068898	0.67547	insignificant	20	93	94
chr11	97643753	97645753	Rpl23	-0.019953	0.24351	insignificant	0.0083072	1	insignificant	3	27	27
chr11	97659985	97661985	Lasp1	-0.12062	8.96E-17	hypomethylated	-0.019498	0.71813	insignificant	24	111	133
chr11	97701093	97703093	B230217C12Rik	-0.22798	0.031085	hypomethylated	0.017611	0.67786	insignificant	6	25	31
chr11	97745468	97747468	Fbxo47	-0.20156	0.000000403	hypomethylated	-0.002781	0.87341	insignificant	3	18	18
chr11	97847760	97849760	Plxdc1	-0.16248	0.014008	hypomethylated	-0.013335	0.012932	inconclusive	8	57	54
chr11	97857487	97859487	Arl5c		1	noCoverage	-0.016331	0.38276	insignificant	0	10	6
chr11	97879686	97881686	Cacnb1	-0.75588	0.000025614	stronglyHypometh	-0.067484	0.86875	insignificant	2	6	6
chr11	97883941	97885941	Cacnb1	-0.092527	2.96E-23	hypomethylated	-0.011405	0.11212	insignificant	14	75	76
chr11	97887023	97889023	Rpl19	-0.17063	3.89E-13	hypomethylated	0.0034346	0.075562	insignificant	22	107	99
chr11	97887237	97889237	Rpl19	-0.17063	3.89E-13	hypomethylated	0.0034346	0.075562	insignificant	22	107	99
chr11	97914776	97916776	Stac2	-0.46145	1	lowCoverage	-0.056562	0.2317	insignificant	1	15	18
chr11	98010930	98012930	Fbxl20	-0.1252	0.00010405	hypomethylated	-0.031226	0.065021	insignificant	23	97	98
chr11	98054220	98056220	Med1	-0.16139	0.00000079	hypomethylated	0.0045714	0.39112	insignificant	5	54	61
chr11	98054607	98056607	Med1		1	noCoverage	0.051108	0.80877	insignificant	0	18	20
chr11	98063618	98065618	Cdk12	-0.10454	0.000013997	hypomethylated	0.0026384	0.0083974	inconclusive	19	123	123
chr11	98190959	98192959	Neurod2	-0.25476	1	insignificant	-0.060459	0.096616	insignificant	2	6	6
chr11	98209051	98211051	Ppp1r1b	-0.11459	2.17E-14	hypomethylated	0.011058	0.056021	insignificant	27	117	111
chr11	98218697	98220697	1700003D09Rik	-0.13512	2.09E-17	hypomethylated	-0.029539	0.4392	insignificant	21	68	65
chr11	98246945	98248945	Pnmt	-0.11684	0.000020372	hypomethylated	0.0051917	1	insignificant	15	43	43
chr11	98261804	98263804	Pgap3	-0.25803	0.012068	hypomethylated	0.056034	0.69045	insignificant	4	10	10
chr11	98272797	98274797	Erbb2	-0.14836	1.44E-18	hypomethylated	0.002711	0.6613	insignificant	42	131	118
chr11	98300302	98302302	1810046J19Rik	-0.22222	0.000057381	hypomethylated	-0.047586	0.69032	insignificant	5	18	13
chr11	98307147	98309147	Grb7	-0.30345	4.04E-08	hypomethylated	-0.038744	0.23305	insignificant	7	33	36
chr11	98407345	98409345	Ikzf3		1	noCoverage	0.024854	0.00012531	hypermethylated	0	22	22
chr11	98411410	98413410	Zpbp2	-0.15821	5.42E-11	hypomethylated	0.0091299	0.68431	insignificant	7	14	14
chr11	98448559	98450559	Ormdl3	-0.23095	1.36E-12	hypomethylated	-0.0047046	0.0012393	hypomethylated	14	53	52
chr11	98458604	98460604	Gm12	-0.23383	0.12625	insignificant	-0.12292	0.51047	insignificant	5	25	27
chr11	98486673	98488673	Gsdma3		1	noCoverage	-0.084098	0.42802	insignificant	0	10	7
chr11	98542867	98544867	Psmd3	-0.15387	3.53E-24	hypomethylated	-0.015689	0.0043146	hypomethylated	30	87	89
chr11	98561626	98563626	Csf3	-0.28985	1	insignificant	-0.015354	0.081481	insignificant	2	18	18
chr11	98590749	98592749	Med24	-0.55172	0.000028378	stronglyHypometh	0.16355	0.84335	insignificant	1	2	3
chr11	98602186	98604186	Thra	-0.13564	2.48E-10	hypomethylated	-0.018065	0.46461	insignificant	14	76	92
chr11	98636556	98638556	Nr1d1	-0.15389	0.0010196	hypomethylated	-0.031926	0.14496	insignificant	2	18	18
chr11	98656082	98658082	Msl1	-0.10474	4.46E-26	hypomethylated	0.0049569	0.51746	insignificant	69	217	223
chr11	98670121	98672121	Casc3	-0.11856	6.7E-14	hypomethylated	-0.011626	0.72468	insignificant	32	91	102
chr11	98671403	98673403	Casc3	-0.18987	0.042952	hypomethylated	-0.002865	1	insignificant	4	32	32
chr11	98697098	98699098	Rapgefl1	-0.14994	4.46E-40	hypomethylated	-0.021037	0.13742	insignificant	51	158	167
chr11	98723911	98725911	Wipf2	-0.05327	0.054966	insignificant	0.0029926	0.56102	insignificant	16	81	80
chr11	98768202	98770202	Cdc6	-0.15808	4.95E-16	hypomethylated	-0.0115	0.37251	insignificant	12	45	45
chr11	98768464	98770464	Cdc6	-0.15455	4.91E-16	hypomethylated	-0.0079725	0.37259	insignificant	12	46	45
chr11	98798009	98800009	Rara	-0.16741	4.18E-52	hypomethylated	-0.0052698	0.036781	hypomethylated	30	96	96
chr11	98798031	98800031	Rara	-0.16741	4.18E-52	hypomethylated	-0.0052698	0.036781	hypomethylated	30	96	96
chr11	98800024	98802024	Rara	-0.71299	0.13573	insignificant	-0.16282	0.28611	insignificant	1	13	10
chr11	98820784	98822784	Rara	-0.15658	1.5E-34	hypomethylated	0.0043573	0.37162	insignificant	38	121	122
chr11	98844330	98846330	Gjd3	-0.086166	0.005879	hypomethylated	-0.087991	0.1323	insignificant	6	18	18
chr11	98885503	98887503	Top2a		1	noCoverage	0.10399	0.0033331	hypermethylated	0	13	11
chr11	98901573	98903573	Igfbp4	-0.18902	4.16E-19	hypomethylated	0.084036	0.12466	insignificant	17	40	47
chr11	99092331	99094331	Smarce1	-0.14644	0.72913	insignificant	0.053284	0.64117	insignificant	4	31	33
chr11	99146552	99148552	Krt24	-0.21761	0.15269	insignificant	0.029575	0.58694	insignificant	4	17	16
chr11	99184255	99186255	Krt25	0.061929	1	lowCoverage	-0.065385	0.57527	insignificant	1	17	15
chr11	99199279	99201279	Krt26	0.10635	0.58336	insignificant	0.067599	0.82999	insignificant	3	30	26
chr11	99212408	99214408	Krt27	0.15753	1	lowCoverage	-0.05805	0.15516	insignificant	1	10	9
chr11	99236217	99238217	Krt28	-0.14196	0.42237	insignificant	0.089286	0.60049	insignificant	2	4	4
chr11	99354424	99356424	Krt23	0.12143	0.66318	insignificant	0.020997	0.58947	insignificant	1	6	6
chr11	99382572	99384572	Krt39	-0.047196	0.34427	insignificant	0.12537	0.62254	insignificant	5	26	27
chr11	99412177	99414177	Krtap3-3		1	noCoverage	-0.011689	0.12326	insignificant	0	4	4
chr11	99452653	99454653	Krtap1-3	-0.018039	0.61226	insignificant	0.10196	0.32013	insignificant	2	5	5
chr11	99505403	99507403	Krtap4-7	0.12608	0.59284	insignificant	0.0069844	0.58368	insignificant	2	6	6
chr11	99957538	99959538	Krt35	-0.050142	0.21404	insignificant	-0.10099	0.001664	hypomethylated	6	30	31
chr11	100068824	100070824	Krt14	0.080372	1	insignificant	-0.077494	0.128	insignificant	1	6	6
chr11	100122303	100124303	Krt17	-0.125	1	insignificant	-0.016828	0.78288	insignificant	1	4	4
chr11	100180309	100182309	Eif1	-0.15178	0.00035206	hypomethylated	-0.0074458	0.52317	insignificant	19	85	84
chr11	100217455	100219455	Hap1	-0.16423	0.000010574	hypomethylated	0.11878	0.28116	insignificant	3	19	17
chr11	100259053	100261053	Jup	-0.31422	4.33E-10	hypomethylated	0.072661	0.19262	insignificant	4	31	31
chr11	100276007	100278007	Fkbp10	-0.2984	3.18E-21	hypomethylated	-0.063436	0.058367	insignificant	15	61	64
chr11	100276133	100278133	Fkbp10	-0.32332	9.54E-15	hypomethylated	-0.068264	0.17596	insignificant	11	53	54
chr11	100302237	100304237	Klhl10	-0.0062826	0.7799	insignificant	-0.27955	0.000040588	hypomethylated	6	14	20
chr11	100302403	100304403	Klhl10	-0.0062826	0.7799	insignificant	-0.27955	0.000040588	hypomethylated	6	14	20
chr11	100389314	100391314	Acly	-0.27728	0.0019664	hypomethylated	-0.020467	0.83115	insignificant	6	27	27
chr11	100405945	100407945	Ttc25	-0.19798	1E-18	hypomethylated	-0.038656	0.043298	hypomethylated	8	28	28
chr11	100435252	100437252	Cnp	-0.099293	4.44E-11	hypomethylated	0.011877	0.54837	insignificant	16	58	58
chr11	100436204	100438204	Cnp	-0.033872	7.34E-37	hypomethylated	0.0061069	1.18E-35	inconclusive	21	88	88
chr11	100483268	100485268	Nkiras2	-0.22685	5.77E-35	hypomethylated	-0.0022467	0.5766	insignificant	12	47	47
chr11	100565585	100567585	Dhx58	-0.015394	0.066716	insignificant	-0.034444	0.0009476	hypomethylated	8	24	25

chr11	100573781	100575781	Kat2a	-0.18696	0.00013202	hypomethylated	-0.015072	0.13188	insignificant	17	55	50
chr11	100574163	100576163	Hspb9	-0.17009	0.00020338	hypomethylated	-0.016838	0.1104	insignificant	16	54	49
chr11	100599444	100601444	Rab5c	-0.09346	0.0073574	inconclusive	-0.031957	4.96E-08	inconclusive	7	64	61
chr11	100621092	100623092	Kcnh4	-0.15818	1	insignificant	-0.011018	0.48505	insignificant	6	16	16
chr11	100624245	100626245	Hcrt		1	noCoverage	0.046663	0.62616	insignificant	0	8	10
chr11	100632271	100634271	Ghdc	-0.44095	0.000012892	stronglyHypometh	0.0018359	0.59482	insignificant	3	14	15
chr11	100711899	100713899	Stat5b	-0.097118	1.79E-10	hypomethylated	-0.00083529	0.50739	insignificant	33	93	93
chr11	100719664	100721664	Stat5a	-0.25539	0.13996	insignificant	-0.038406	0.19284	insignificant	6	23	23
chr11	100720797	100722797	Stat5a	-0.1445	0.34112	insignificant	-0.0018718	0.76148	insignificant	6	69	65
chr11	100800825	100802825	Stat3	-0.090345	0.000037671	hypomethylated	0.012643	0.6563	insignificant	14	49	49
chr11	100831931	100833931	Ptrf	-0.39205	2.74E-24	stronglyHypometh	0.046927	0.032564	inconclusive	7	22	22
chr11	100869768	100871768	Atp6v0a1	-0.1038	1.49E-08	hypomethylated	-0.0022287	0.00035348	hypomethylated	20	79	76
chr11	100930407	100932407	Naglu	-0.096108	0.000000405	hypomethylated	-0.0044031	0.28308	insignificant	7	40	34
chr11	100938724	100940724	Hsd17b1		1	noCoverage	-0.1074	0.47442	insignificant	0	12	8
chr11	100942938	100944938	Coasy	-0.11988	0.21305	insignificant	0.059705	0.035001	hypermethylated	9	43	39
chr11	100947603	100949603	Mlx	-0.15397	0.010933	hypomethylated	0.070199	1	insignificant	4	28	14
chr11	100956715	100958715	Fam134c	-0.27452	1.07E-18	hypomethylated	-0.029845	0.071376	insignificant	16	69	67
chr11	100980444	100982444	Tubg1	-0.16526	8.48E-33	hypomethylated	0.0016099	0.033701	hypermethylated	39	120	120
chr11	100981138	100983138	Fam134c	-0.13921	0.000000414	hypomethylated	0.0029622	0.78498	insignificant	14	46	46
chr11	100981157	100983157	Fam134c	-0.15191	0.000095981	hypomethylated	0.0019492	0.69664	insignificant	12	42	42
chr11	101016197	101018197	Tubg2	-0.1292	0.031756	hypomethylated	-0.046308	0.39325	insignificant	7	23	36
chr11	101032616	101034616	Plekhh3	-0.14082	6.65E-09	hypomethylated	-0.021213	0.00050203	hypomethylated	18	56	62
chr11	101036430	101038430	Cntnap1	-0.18402	0.079433	insignificant	0.0044842	0.84778	insignificant	9	30	30
chr11	101036757	101038757	Ccr10	-0.17284	1	insignificant	0.016417	0.7144	insignificant	10	32	34
chr11	101087764	101089764	Ezh1	-0.29508	0.11167	insignificant	-0.12552	0.68303	insignificant	1	20	23
chr11	101106647	101108647	Ramp2	-0.1279	2.7E-18	hypomethylated	-0.0088075	0.54954	insignificant	29	90	90
chr11	101114056	101116056	Vps25	-0.15192	0.00000112	hypomethylated	-0.005813	0.29487	insignificant	9	32	32
chr11	101120880	101122880	Wnk4	-0.21942	2.95E-51	hypomethylated	0.0066831	0.83132	insignificant	40	120	120
chr11	101139516	101141516	Cntd1	0.084642	0.11431	insignificant	0.065225	0.000015278	hypermethylated	10	57	57
chr11	101140262	101142262	Ccdc56	0.21266	0.35624	insignificant	0.10379	0.000015887	hypermethylated	5	31	31
chr11	101163581	101165581	Becn1	-0.337	0.0012542	stronglyHypometh	-0.037436	0.037015	hypomethylated	3	13	14
chr11	101176564	101178564	Psme3	-0.23036	2.02E-32	hypomethylated	-0.010988	0.000057589	hypomethylated	35	66	70
chr11	101185376	101187376	Aoc2		1	noCoverage	0.02622	0.16598	insignificant	0	4	5
chr11	101190919	101192919	Aoc3	-0.10195	0.42818	insignificant	-0.082696	0.48039	insignificant	3	17	24
chr11	101228043	101230043	G6pc	-0.12566	0.24327	insignificant	0.029101	0.63303	insignificant	2	6	6
chr11	101285398	101287398	Rundc1	-0.097388	9.25E-18	hypomethylated	0.00037694	0.0055397	inconclusive	20	104	100
chr11	101286647	101288647	1700113I22Rik	-0.09066	0.00016882	hypomethylated	0.0043082	0.037891	hypermethylated	16	68	64
chr11	101302558	101304558	Rpl27	-0.24447	6.68E-24	hypomethylated	0.016138	0.00038065	inconclusive	30	100	101
chr11	101327513	101329513	Vat1	-0.289	0.020179	hypomethylated	0.066652	0.1824	insignificant	3	20	20
chr11	101328651	101330651	Rnd2	-0.18528	5.74E-44	hypomethylated	0.011051	0.63129	insignificant	23	63	64
chr11	101413171	101415171	Nbr1	-0.16715	0.081871	insignificant	-0.021566	0.2368	insignificant	2	45	49
chr11	101413269	101415269	Nbr1	-0.25859	0.011053	hypomethylated	-0.045514	0.58845	insignificant	2	27	31
chr11	101442555	101444555	Tmem106a	-0.25808	0.000080088	hypomethylated	-0.0064791	1	insignificant	8	32	34
chr11	101488262	101490262	Rdm1	-0.14079	0.00013428	hypomethylated	0.13838	0.55206	insignificant	2	23	10
chr11	101525854	101527854	Arl4d	-0.14604	5.16E-09	hypomethylated	-0.0064721	0.023446	hypomethylated	8	43	52
chr11	101593269	101595269	Dhx8	-0.25816	0.000000019	hypomethylated	-0.022353	0.10809	insignificant	5	43	36
chr11	101646624	101648624	Etv4	-0.33412	8.7E-14	stronglyHypometh	-0.02594	0.30523	insignificant	8	36	37
chr11	101846105	101848105	Dusp3	-0.13318	5.09E-10	hypomethylated	-0.036308	0.024951	hypomethylated	10	24	24
chr11	101888269	101890269	Mpp3	-0.064764	0.0019311	hypomethylated	0.016227	0.87172	insignificant	12	50	50
chr11	101949829	101951829	Mpp2	-0.39878	0.045696	stronglyHypometh	-0.085524	0.13624	insignificant	4	14	16
chr11	101962614	101964614	Ppy	-0.47157	0.000016881	stronglyHypometh	-0.09143	0.74699	insignificant	2	16	16
chr11	101969090	101971090	Pyy	-0.068806	0.10516	insignificant	-0.17367	0.000090948	hypomethylated	3	9	9
chr11	102005900	102007900	Nags	-0.12961	5.52E-17	hypomethylated	0.012594	0.37942	insignificant	21	82	82
chr11	102006679	102008679	Nags	-0.12961	5.52E-17	hypomethylated	0.012594	0.37942	insignificant	21	82	82
chr11	102017721	102019721	Tmem101	-0.36516	0.00033688	stronglyHypometh	-0.14996	0.35165	insignificant	4	9	9
chr11	102046570	102048570	Lsm12	-0.10585	0.24404	insignificant	0.03682	0.70513	insignificant	9	25	25
chr11	102050012	102052012	G6pc3	-0.10968	1	insignificant	-0.022575	0.052837	insignificant	12	43	52
chr11	102091486	102093486	Hdac5	-0.39696	0.002166	stronglyHypometh	-0.026962	0.6429	insignificant	3	6	6
chr11	102109195	102111195	BC030867	-0.14464	1E-10	hypomethylated	-0.024617	0.032566	hypomethylated	11	32	32
chr11	102145274	102147274	Tmub2	0.10212	0.00027188	hypermethylated	-0.011191	0.54664	insignificant	3	56	56
chr11	102157943	102159943	Atxn7l3	-0.20068	6.78E-26	hypomethylated	-0.011087	0.00051834	hypomethylated	21	87	85
chr11	102178601	102180601	Ubtf	-0.11319	3.99E-62	hypomethylated	-0.010503	0.28975	insignificant	95	274	301
chr11	102180410	102182410	Ubtf	-0.17449	5.7E-16	hypomethylated	-0.021694	0.016066	hypomethylated	21	126	126
chr11	102226595	102228595	Slc4a1		1	noCoverage	-0.1048	0.0092753	hypomethylated	0	15	16
chr11	102253748	102255748	Rundc3a	-0.30986	9.64E-19	hypomethylated	0.04346	0.000047964	inconclusive	9	49	49
chr11	102268831	102270831	Slc25a39	-0.1567	1.33E-20	hypomethylated	0.0083129	0.2064	insignificant	22	66	65
chr11	102290635	102292635	Grn	-0.20154	0.000000892	hypomethylated	-0.040524	0.76922	insignificant	13	24	25
chr11	102308977	102310977	Fam171a2	-0.49524	1.92E-12	stronglyHypometh	0.021514	0.927	insignificant	3	12	12
chr11	102331197	102333197	Itga2b	-0.39705	0.081563	insignificant	-0.016664	0.63681	insignificant	1	12	12
chr11	102417472	102419472	Gpatch8	0.016601	0.00044055	inconclusive	0.0061997	0.0048507	inconclusive	5	38	38
chr11	102464744	102466744	Fzd2	-0.18221	0.054756	insignificant	0.0077164	0.95967	insignificant	11	87	77
chr11	102485730	102487730	2810433D01Rik	-0.65442	0.012828	stronglyHypometh	-0.18421	0.010057	hypomethylated	3	12	12
chr11	102525901	102527901	Gm1564	-0.041449	0.41431	insignificant	-0.044579	0.042493	hypomethylated	24	154	154
chr11	102559039	102561039	Ccdc43	-0.14952	7.48E-23	hypomethylated	0.0040923	0.025338	inconclusive	23	52	52
chr11	102621752	102623752	Adam11	-0.15311	6.7E-18	hypomethylated	0.016579	0.55789	insignificant	26	90	93
chr11	102680473	102682473	Gjc1	-0.10394	4.1E-17	hypomethylated	-0.0061422	0.0021596	hypomethylated	30	93	91
chr11	102681000	102683000	Gjc1		1	noCoverage	-0.0093319	0.24577	insignificant	0	17	15
chr11	102741557	102743557	Ccdc103	-0.19292	9.58E-52	hypomethylated	-0.028621	0.0018511	hypomethylated	28	90	91
chr11	102742289	102744289	Eftud2	-0.26586	1.43E-11	hypomethylated	-0.034148	0.52742	insignificant	15	44	42
chr11	102745482	102747482	Fam187a	0.15076	0.71518	insignificant	0.11169	0.29623	insignificant	1	7	11
chr11	102758514	102760514	Gfap		1	noCoverage	-0.010349	0.42115	insignificant	0	8	8
chr11	102786438	102788438	Kif18b	-0.18975	2.48E-09	hypomethylated	0.034298	0.26227	insignificant	11	42	44
chr11	102807775	102809775	C1ql1	-0.14324	1.22E-12	hypomethylated	0.002963	0.51097	insignificant	17	42	51
chr11	102878461	102880461	Dcakd	-0.29879	9.41E-08	hypomethylated	0.027694	1	insignificant	2	10	10
chr11	102888875	102890875	Nmt1	-0.14067	0.053022	insignificant	-0.011561	0.7814	insignificant	9	30	30

chr11	102962001	102964001	Acbd4	-0.19783	1.1E-15	hypomethylated	-0.016512	0.7969	insignificant	24	85	84
chr11	102962972	102964972	Plcd3	-0.20289	5.14E-19	hypomethylated	-0.0040477	0.9172	insignificant	22	92	91
chr11	102976638	102978638	Hexim1	-0.16433	0.00036954	hypomethylated	-0.026339	0.60273	insignificant	10	52	51
chr11	102993652	102995652	Hexim2	-0.14521	1.23E-16	hypomethylated	-0.013435	0.87423	insignificant	29	69	74
chr11	102993666	102995666	Hexim2	-0.14521	1.23E-16	hypomethylated	-0.013435	0.87423	insignificant	29	69	74
chr11	102993672	102995672	Hexim2	-0.14521	1.23E-16	hypomethylated	-0.013435	0.87423	insignificant	29	69	74
chr11	103031451	103033451	Fmnl1	-0.11223	7.37E-52	hypomethylated	-0.0051451	0.60422	insignificant	53	124	132
chr11	103079746	103081746	4933400C05Rik	-0.21302	0.0031978	hypomethylated	0.12065	0.63697	insignificant	1	9	6
chr11	103128715	103130715	Map3k14	-0.14396	0.00082792	hypomethylated	-0.014456	0.96402	insignificant	13	60	60
chr11	103206016	103208016	Arhgap27	-0.49183	1.52E-09	stronglyHypometh	-0.13586	0.29853	insignificant	3	7	10
chr11	103224698	103226698	Arhgap27	-0.3352	0.000031422	stronglyHypometh	-0.12197	0.73015	insignificant	6	16	16
chr11	103273978	103275978	Plekhm1		1	noCoverage	0.0077225	0.53771	insignificant	0	8	13
chr11	103509822	103511822	Rprml	-0.13624	8.64E-11	hypomethylated	-0.030667	0.79728	insignificant	31	125	124
chr11	103559024	103561024	Gosr2	-0.11234	0.000022345	hypomethylated	0.01409	0.078695	insignificant	17	72	72
chr11	103611135	103613135	Wnt9b	-0.1445	2.73E-11	hypomethylated	-0.0028744	0.058298	insignificant	11	33	28
chr11	103634488	103636488	Wnt3	-0.12416	4.12E-14	hypomethylated	-0.0050384	0.24032	insignificant	37	110	112
chr11	103815370	103817370	Nsf	-0.12204	0.00036053	hypomethylated	-0.01245	0.23666	insignificant	13	42	42
chr11	103827184	103829184	Arf2	-0.10196	0.00000201	hypomethylated	-0.00082836	0.0066131	hypomethylated	9	88	80
chr11	103993195	103995195	Crhr1	-0.1739	9.15E-45	hypomethylated	-0.022575	0.17991	insignificant	57	182	178
chr11	104046640	104048640	4933407P14Rik	-0.26197	0.000000028	hypomethylated	-0.024073	0.11467	insignificant	6	24	24
chr11	104091749	104093749	Mapt	-0.079254	1.88E-22	hypomethylated	0.0041879	0.035251	inconclusive	18	129	133
chr11	104303605	104305605	1700081L11Rik	-0.10751	2.35E-15	hypomethylated	-0.019095	0.39498	insignificant	32	88	97
chr11	104410976	104412976	Myl4	-0.13856	2.93E-12	hypomethylated	0.00050869	0.55617	insignificant	32	104	108
chr11	104411934	104413934	Myl4	-0.20638	0.00081482	hypomethylated	0.0024821	1	insignificant	3	14	14
chr11	104468313	104470313	Itgb3	-0.12005	0.03619	hypomethylated	-0.01946	0.035804	hypomethylated	9	67	70
chr11	104986738	104988738	Mettl2	-0.12986	8.65E-15	hypomethylated	0.011857	0.85912	insignificant	23	83	86
chr11	105041843	105043843	Tlk2	-0.13024	5.04E-32	hypomethylated	-0.014042	0.038029	hypomethylated	72	272	271
chr11	105042747	105044747	1700052K11Rik	-0.1516	3.75E-20	hypomethylated	-0.021831	0.00023869	hypomethylated	40	148	146
chr11	105152959	105154959	Mrc2	-0.16166	0.00026865	hypomethylated	-0.0074527	0.10421	insignificant	10	63	69
chr11	105318049	105320049	March10	0.093681	0.00067908	hypermethylated	0.034833	0.00017878	hypermethylated	6	18	18
chr11	105450299	105452299	Tanc2	-0.091843	9.55E-16	hypomethylated	-0.0067078	0.84162	insignificant	45	203	225
chr11	105805461	105807461	Cyb561	-0.11334	2.87E-14	hypomethylated	0.026411	0.10583	insignificant	23	77	75
chr11	105828260	105830260	Ace	-0.16196	2.61E-21	hypomethylated	-0.013761	0.058469	insignificant	24	101	101
chr11	105868516	105870516	Kcnh6	-0.1012	0.0001064	hypomethylated	-0.0076482	0.38116	insignificant	7	34	34
chr11	105897185	105899185	Dcaf7	-0.14232	3.13E-15	hypomethylated	-0.0049523	0.01363	hypomethylated	34	101	102
chr11	105926420	105928420	Taco1	-0.20773	2.26E-10	hypomethylated	0.039927	0.3614	insignificant	20	86	86
chr11	105945215	105947215	Map3k3	-0.090407	7.72E-18	hypomethylated	-0.0012838	0.077592	insignificant	46	172	179
chr11	106021456	106023456	Limd2	-0.15269	4.11E-08	hypomethylated	0.039388	0.79747	insignificant	24	99	96
chr11	106054862	106056862	Strada	-0.25791	0.00020868	hypomethylated	-0.012643	0.19658	insignificant	3	34	34
chr11	106077239	106079239	Ddx42	-0.22266	0.00044793	hypomethylated	-0.022881	0.30487	insignificant	11	47	48
chr11	106077681	106079681	Ddx42	-0.21076	0.00074633	hypomethylated	0.032646	0.72166	insignificant	11	37	41
chr11	106116498	106118498	Psmc5	-0.18123	2.32E-13	hypomethylated	-0.034447	0.024703	hypomethylated	13	72	72
chr11	106117116	106119116	Ftsj3	-0.034551	0.063017	insignificant	-0.035713	0.261	insignificant	13	60	60
chr11	106134286	106136286	Smarcd2	-0.22095	0.00000014	hypomethylated	0.035838	0.00000624	inconclusive	11	75	73
chr11	106136985	106138985	Tcam1	0.51963	0.0092491	stronglyHypermeth	0.00079181	0.00015618	inconclusive	2	43	43
chr11	106163124	106165124	Gh	0.33512	1	insignificant	0.090167	1	insignificant	2	8	9
chr11	106235139	106237139	2310007L24Rik	-0.23048	0.092854	insignificant	0.036015	0.38702	insignificant	1	29	28
chr11	106349110	106351110	Ern1	-0.19561	0.15199	insignificant	-0.00688	0.58	insignificant	3	16	16
chr11	106361304	106363304	Snord104	-0.17072	4.08E-09	hypomethylated	-0.022394	0.77256	insignificant	8	54	57
chr11	106474244	106476244	Tex2	-0.59429	2.32E-08	stronglyHypometh	0.11571	0.87327	insignificant	1	7	12
chr11	106611581	106613581	Gm885		1	noCoverage	-0.050926	0.39983	insignificant	0	6	6
chr11	106640829	106642829	Polg2	-0.1335	6.23E-08	hypomethylated	-0.0041094	0.52413	insignificant	8	18	18
chr11	106640851	106642851	Polg2	-0.15019	1.87E-08	hypomethylated	-0.0046231	0.74754	insignificant	7	16	16
chr11	106644073	106646073	Mir3064	0.17012	1	insignificant	-0.043793	0.023231	hypomethylated	1	16	18
chr11	106649565	106651565	Ccdc45	-0.080641	1.85E-11	hypomethylated	-0.0048899	0.82066	insignificant	30	119	116
chr11	106649808	106651808	Ddx5	-0.093937	3.79E-13	hypomethylated	-0.0052174	0.86701	insignificant	30	93	90
chr11	106782029	106784029	Smurf2	-0.06579	0.84886	insignificant	0.017012	0.006724	hypermethylated	19	83	78
chr11	106860839	106862839	Kpna2		1	noCoverage	0.014913	0.000085907	hypermethylated	0	33	37
chr11	106888536	106890536	1810010H24Rik	-0.10421	1.76E-19	hypomethylated	0.0099912	0.60728	insignificant	48	149	144
chr11	106993236	106995236	Bptf	-0.065134	0.000000189	hypomethylated	-0.00065166	0.72034	insignificant	15	78	78
chr11	107050695	107052695	Nol11		1	noCoverage	0.050401	0.024016	inconclusive	0	16	16
chr11	107339841	107341841	Psmd12	-0.22418	4.21E-08	hypomethylated	-0.0013924	0.23105	insignificant	10	42	42
chr11	107408273	107410273	Helz	-0.13865	0.000000151	hypomethylated	-0.021188	0.28571	insignificant	29	111	125
chr11	107655778	107657778	Cacng4	-0.15168	1.16E-26	hypomethylated	-0.026429	0.000022924	hypomethylated	49	105	101
chr11	108205202	108207202	Prkca	-0.13865	1.97E-10	hypomethylated	-0.0031952	0.000013109	hypomethylated	23	66	74
chr11	108285579	108287579	Ccdc46	-0.24762	5.54E-10	hypomethylated	-0.057542	0.61252	insignificant	17	50	53
chr11	108780662	108782662	Axin2	-0.13178	4.03E-63	hypomethylated	-0.013893	0.036256	hypomethylated	85	223	223
chr11	109159495	109161495	Rgs9	-0.15789	0.14844	insignificant	0.047472	0.47263	insignificant	4	14	17
chr11	109223107	109225107	Gna13	-0.11277	1.8E-26	hypomethylated	0.00053352	0.20523	insignificant	57	204	211
chr11	109286259	109288259	Amz2	-0.19503	1.61E-11	hypomethylated	-0.044962	0.44544	insignificant	9	30	32
chr11	109333687	109335687	Arsg	-0.097483	0.000000766	hypomethylated	-0.010453	0.11209	insignificant	67	191	194
chr11	109334543	109336543	Slc16a6	-0.1108	7.39E-11	hypomethylated	-0.011164	0.032425	hypomethylated	43	141	134
chr11	109334910	109336910	Slc16a6	-0.14023	3.27E-09	hypomethylated	-0.011361	0.37944	insignificant	13	59	52
chr11	109404024	109406024	Arsg	0.08513	1	insignificant	0.022252	0.11243	insignificant	1	12	12
chr11	109472703	109474703	Wipi1	-0.59615	0.38462	lowCoverage	-0.054487	0.62823	insignificant	1	4	4
chr11	109511262	109513262	Prkar1a	-0.091768	5.63E-26	hypomethylated	0.0034827	0.0099581	inconclusive	33	174	175
chr11	109583570	109585570	Fam20a		1	noCoverage	-0.05777	0.00081959	hypomethylated	0	11	11
chr11	109957251	109959251	Abca8a		1	noCoverage	-0.0664	0.70602	insignificant	0	10	8
chr11	110199030	110201030	Abca5	-0.15788	4.79E-15	hypomethylated	-0.019188	0.00000305	hypomethylated	12	56	62
chr11	110259435	110261435	Map2k6	-0.31786	0.32241	insignificant	-0.054699	0.58729	insignificant	1	8	8
chr11	110926477	110928477	Kcnj2	-0.18694	9.82E-16	hypomethylated	-0.017204	0.92425	insignificant	10	62	60
chr11	112642523	112644523	Sox9	-0.14392	1.49E-28	hypomethylated	-0.0083744	0.169	insignificant	38	119	116
chr11	113063152	113065152	2610035D17Rik		1	noCoverage	0.026245	0.59586	insignificant	0	4	4
chr11	113484570	113486570	Sstr2	-0.040606	0.20626	insignificant	-0.049441	0.0078355	hypomethylated	10	26	26
chr11	113509842	113511842	Cog1	-0.0088551	0.000010268	hypomethylated	-0.021789	0.00013669	hypomethylated	31	132	127

chr11	113544725	113546725	D11Wsu47e	-0.12061	2.92E-13	hypomethylated	-0.01693	0.77705	insignificant	24	182	174
chr11	113545465	113547465	D11Wsu47e	-0.21003	0.36092	insignificant	-0.034458	0.96372	insignificant	7	77	66
chr11	113612474	113614474	Cdc42ep4	-0.2533	0.00069337	hypomethylated	0.043676	0.70439	insignificant	6	28	26
chr11	113613129	113615129	Cdc42ep4	-0.37322	0.23648	insignificant	0.15271	0.14549	insignificant	1	9	10
chr11	113927265	113929265	Sdk2	-0.11693	7.72E-42	hypomethylated	-0.0039364	0.027899	hypomethylated	65	209	209
chr11	114528856	114530856	Rpl38	-0.17396	7.13E-12	hypomethylated	-0.017923	0.057378	insignificant	23	123	123
chr11	114529094	114531094	Rpl38	-0.14767	2.35E-11	hypomethylated	-0.01485	0.034872	hypomethylated	24	118	118
chr11	114535781	114537781	Ttyh2	-0.26424	1.01E-11	hypomethylated	-0.0089808	0.0000088	hypomethylated	13	78	77
chr11	114587725	114589725	Dnaic2	-0.25864	0.47621	insignificant	-0.069752	0.2828	insignificant	5	27	33
chr11	114625702	114627702	Kif19a	-0.1684	1.08E-12	hypomethylated	0.004805	0.45718	insignificant	15	33	31
chr11	114657206	114659206	Btbd17	-0.39804	0.000000174	stronglyHypometh	0.022875	0.31933	insignificant	5	10	10
chr11	114711845	114713845	Gprc5c	-0.17699	6.55E-17	hypomethylated	-0.031057	0.12964	insignificant	25	96	123
chr11	114711853	114713853	Gprc5c	-0.17326	1.16E-16	hypomethylated	-0.029114	0.14096	insignificant	25	98	125
chr11	114712343	114714343	Gprc5c	-0.17311	7.83E-18	hypomethylated	-0.0074789	0.10325	insignificant	26	102	113
chr11	114750354	114752354	Cd300a	-0.24736	0.00035554	hypomethylated	0.018941	0.33129	insignificant	3	14	14
chr11	114951744	114953744	Rab37	-0.13173	1.28E-17	hypomethylated	-0.0015362	0.36876	insignificant	19	44	44
chr11	115014491	115016491	Rab37	-0.16553	5.6E-13	hypomethylated	-0.015662	0.74436	insignificant	26	64	63
chr11	115023654	115025654	Slc9a3r1	-0.14515	1.53E-31	hypomethylated	-0.0079385	0.18416	insignificant	37	143	134
chr11	115047800	115049800	Tmem104	-0.089274	7.16E-09	hypomethylated	-0.013706	0.81906	insignificant	14	52	50
chr11	115048630	115050630	Nat9	-0.089274	7.16E-09	hypomethylated	-0.013706	0.81906	insignificant	14	52	50
chr11	115128557	115130557	Grin2c	-0.28109	0.085297	insignificant	-0.054959	0.060089	insignificant	9	44	40
chr11	115138283	115140283	Fdxr	-0.25971	1	lowCoverage	-0.06485	0.25844	insignificant	1	31	31
chr11	115158860	115160860	Fads6	-0.41206	0.46472	insignificant	-0.25856	0.2446	insignificant	4	17	8
chr11	115167476	115169476	Otop2	-0.20991	0.029674	hypomethylated	-0.014201	0.1997	insignificant	2	9	9
chr11	115183232	115185232	Ush1g	-0.21635	0.023926	hypomethylated	-0.041242	0.044682	hypomethylated	12	72	72
chr11	115195047	115197047	Otop3	-0.22116	0.035938	hypomethylated	0.030297	0.016044	inconclusive	7	45	46
chr11	115229033	115231033	C630004H02Rik	-0.15163	0.000033438	hypomethylated	0.0016399	0.81096	insignificant	4	18	18
chr11	115242229	115244229	Cdr2l	-0.15065	3.95E-26	hypomethylated	-0.0048129	0.57665	insignificant	31	72	75
chr11	115264079	115266079	Ict1	-0.15111	5.59E-17	hypomethylated	-0.019658	0.70731	insignificant	24	88	88
chr11	115280439	115282439	Kctd2	-0.15436	1.71E-52	hypomethylated	-0.0078609	0.032615	hypomethylated	41	132	136
chr11	115281233	115283233	Kctd2	-0.11085	7.38E-43	hypomethylated	0.00045516	0.069692	insignificant	40	116	119
chr11	115322786	115324786	Slc16a5	-0.50202	0.057928	insignificant	0.039643	0.79063	insignificant	1	8	8
chr11	115335990	115337990	Armc7	-0.16371	5.6E-12	hypomethylated	-0.016637	0.31097	insignificant	8	43	44
chr11	115353128	115355128	Nt5c	-0.24004	0.0000012	hypomethylated	0.0073137	0.56881	insignificant	5	18	14
chr11	115375684	115377684	Hn1	-0.16581	0.00099985	hypomethylated	-0.0013201	0.40427	insignificant	2	23	23
chr11	115397544	115399544	Sumo2	-0.29344	0.00000464	hypomethylated	-0.042678	0.000071926	inconclusive	9	38	38
chr11	115424757	115426757	Nup85	-0.18224	2.13E-09	hypomethylated	0.045324	0.87821	insignificant	3	34	35
chr11	115464464	115466464	Mrps7	-0.095181	9.49E-28	hypomethylated	-0.0029988	0.90852	insignificant	54	186	190
chr11	115465220	115467220	Gga3	-0.0877	0.1732	insignificant	-0.010555	0.8494	insignificant	26	113	111
chr11	115473817	115475817	Mif4gd	-0.11895	2.4E-35	hypomethylated	0.0015387	0.56738	insignificant	52	128	127
chr11	115474267	115476267	Mif4gd	-0.15374	0.023215	hypomethylated	0.034324	0.081109	insignificant	7	28	27
chr11	115489453	115491453	Slc25a19	0.073753	0.50087	insignificant	-0.012482	0.002645	inconclusive	2	33	31
chr11	115569911	115571911	Grb2	-0.1562	3.82E-13	hypomethylated	-0.022359	0.058099	insignificant	12	71	74
chr11	115625746	115627746	2310067B10Rik	-0.15673	3.87E-11	hypomethylated	-0.015145	0.043792	hypomethylated	14	74	82
chr11	115674906	115676906	Caskin2	-0.13108	1.12E-32	hypomethylated	-0.017967	0.013644	hypomethylated	56	150	169
chr11	115675052	115677052	Tsen54	-0.12965	6.36E-33	hypomethylated	-0.010786	0.00029803	hypomethylated	51	138	147
chr11	115684371	115686371	Llgl2	-0.11942	0.00000303	hypomethylated	-0.027034	1	insignificant	35	98	108
chr11	115760452	115762452	2210020M01Ril	-0.358	0.000000431	stronglyHypometh	-0.0086937	0.0030076	inconclusive	7	26	26
chr11	115793972	115795972	Sap30bp	-0.13685	1	insignificant	-0.0094161	0.9457	insignificant	4	29	30
chr11	115794320	115796320	Recql5	-0.13685	1	insignificant	-0.0094161	0.9457	insignificant	4	29	30
chr11	115835038	115837038	Itgb4	-0.15334	1.18E-24	hypomethylated	0.035781	0.81426	insignificant	34	87	81
chr11	115874033	115876033	Galk1	-0.30157	0.014657	hypomethylated	-0.070566	1	insignificant	1	6	7
chr11	115885818	115887818	H3f3b	-0.10435	0.00016207	hypomethylated	-0.010549	0.37106	insignificant	4	26	26
chr11	115890635	115892635	Unk	-0.12578	0.000002	hypomethylated	-0.0058544	0.7615	insignificant	13	58	58
chr11	115948278	115950278	Wbp2		1	noCoverage	-0.024567	0.23237	insignificant	0	22	23
chr11	115971330	115973330	Trim47	-0.18488	4.42E-28	hypomethylated	-0.01146	0.32487	insignificant	32	77	78
chr11	115992442	115994442	Mrpl38	-0.064899	0.34112	insignificant	0.11874	0.39241	insignificant	5	36	30
chr11	116000182	116002182	Mrpl38	-0.10748	0.60179	insignificant	-0.16442	0.6188	insignificant	3	13	16
chr11	116029492	116031492	Fbf1	-0.080357	1	insignificant	0.065454	1	insignificant	4	14	15
chr11	116059168	116061168	2310004N24Rik	-0.18099	0.00064089	hypomethylated	0.0031356	0.9263	insignificant	14	88	85
chr11	116060359	116062359	Acox1	-0.39233	0.057241	insignificant	0.084783	0.11292	insignificant	6	52	55
chr11	116076315	116078315	Cdk3-ps		1	noCoverage	0.12375	0.51865	insignificant	0	12	12
chr11	116099405	116101405	Evpl	0.041319	1	insignificant	0.0035528	0.95205	insignificant	5	22	22
chr11	116141252	116143252	Galr2	-0.11489	0.00089338	hypomethylated	0.0026821	0.65945	insignificant	5	36	36
chr11	116168052	116170052	Exoc7	-0.14837	1.58E-11	hypomethylated	0.010506	0.16862	insignificant	9	28	25
chr11	116196668	116198668	Rnf157	-0.14747	1.82E-10	hypomethylated	-0.0074896	0.89169	insignificant	16	42	44
chr11	116274346	116276346	Rnf157	-0.058473	0.00000372	hypomethylated	-0.0089757	0.23555	insignificant	21	84	83
chr11	116294407	116296407	Fam100b	-0.098298	2.28E-08	hypomethylated	0.0026554	0.92639	insignificant	42	142	144
chr11	116294526	116296526	Gm7367	-0.097527	2.29E-08	hypomethylated	0.0049465	0.902	insignificant	42	143	145
chr11	116315661	116317661	Qrich2	0.060904	0.74264	insignificant	-0.052934	0.33179	insignificant	2	16	18
chr11	116351660	116353660	Prpsap1	-0.16967	0.0004243	hypomethylated	-0.0095476	0.13121	insignificant	9	58	57
chr11	116392224	116394224	Sphk1	-0.17234	2.32E-19	hypomethylated	-0.053133	0.02025	hypomethylated	27	61	80
chr11	116392757	116394757	Sphk1	-0.14051	1.59E-34	hypomethylated	-0.018072	0.0027456	hypomethylated	48	122	145
chr11	116393551	116395551	Sphk1	-0.12987	2.36E-50	hypomethylated	-0.0072103	0.0035841	hypomethylated	51	163	187
chr11	116395053	116397053	Sphk1	-0.15425	4.74E-21	hypomethylated	0.0053878	0.012496	inconclusive	19	72	78
chr11	116442761	116444761	Ube2o	-0.24637	2.7E-30	hypomethylated	-0.017095	5.49E-13	hypomethylated	22	86	88
chr11	116485566	116487566	Rhbdf2	-0.079139	0.0034202	inconclusive	-0.017813	0.022813	hypomethylated	2	27	27
chr11	116515627	116517627	Cygb	0.58451	0.27439	lowCoverage	0.059128	0.78822	insignificant	1	39	43
chr11	116517421	116519421	Gm11744	-0.19116	0.79719	insignificant	-0.12797	1	insignificant	9	25	20
chr11	116531973	116533973	1810032O08Rik	-0.17464	2.97E-09	hypomethylated	-0.021191	0.77034	insignificant	15	61	69
chr11	116532818	116534818	Snord1c	-0.2147	2.75E-08	hypomethylated	-0.010322	0.62266	insignificant	7	34	42
chr11	116534460	116536460	Snord1b	0.025884	0.68585	insignificant	-0.15365	0.42414	insignificant	3	12	14
chr11	116534910	116536910	Snord1a	0.025884	0.68585	insignificant	-0.15365	0.42414	insignificant	3	12	14
chr11	116555974	116557974	St6galnac2	-0.14948	7.68E-15	hypomethylated	0.0014436	0.051663	insignificant	16	65	65
chr11	116612480	116614480	BC018473		1	noCoverage	0.048077	1	insignificant	0	2	4

chr11	116689360	116691360	Mxra7	-0.22564	0.59752	insignificant	0.019915	1	insignificant	2	4	4
chr11	116703828	116705828	1110005A03Rik	-0.096908	8.02E-28	hypomethylated	0.0015057	0.041232	inconclusive	56	192	200
chr11	116704763	116706763	Jmjd6	-0.22433	0.0056418	hypomethylated	0.033179	0.014475	hypermethylated	7	64	70
chr11	116714328	116716328	Mfsd11	-0.15331	2.01E-09	hypomethylated	-0.0093359	0.47203	insignificant	12	66	60
chr11	116714408	116716408	Srsf2	-0.1581	1.24E-09	hypomethylated	-0.010257	0.57459	insignificant	12	64	58
chr11	116779176	116781176	Mgat5b	-0.084681	1.4E-22	hypomethylated	-0.0099034	0.72364	insignificant	69	203	196
chr11	116937096	116939096	2810008D09Rik	-0.19873	0.000000288	hypomethylated	0.0053554	0.46012	insignificant	14	27	34
chr11	116975485	116977485	Sec14l1	-0.14864	4.98E-28	hypomethylated	-0.023525	0.00052478	hypomethylated	32	124	126
chr11	117059974	117061974	############	0.066608	6.44E-17	inconclusive	0.025427	0.0023654	inconclusive	32	98	100
chr11	117126559	117128559	############	-0.12918	7.35E-09	hypomethylated	0.027526	0.58176	insignificant	19	97	84
chr11	117192028	117194028	############	-0.18219	0.00000145	hypomethylated	0.023804	0.17681	insignificant	10	44	49
chr11	117192626	117194626	############	-0.19002	0.000000296	hypomethylated	0.019861	0.039154	inconclusive	10	50	55
chr11	117514602	117516602	Tnrc6c	-0.081534	3.84E-28	hypomethylated	-0.0032342	0.20895	insignificant	64	224	221
chr11	117641935	117643935	Tmc6	-0.18781	0.1359	insignificant	0.011323	0.374	insignificant	5	42	42
chr11	117641997	117643997	Tmc6	-0.18781	0.1359	insignificant	0.011323	0.374	insignificant	5	42	42
chr11	117642610	117644610	Tmc8	-0.18047	7.58E-10	hypomethylated	-0.0089515	0.67493	insignificant	7	46	48
chr11	117642971	117644971	Tmc8	-0.19636	1.01E-09	hypomethylated	-0.011761	0.093281	insignificant	5	33	38
chr11	117669980	117671980	Syngr2	-0.12002	4.46E-12	hypomethylated	-0.001021	0.035124	hypomethylated	29	87	87
chr11	117686232	117688232	Afmid	0.02175	0.08982	insignificant	-0.020814	0.00013974	hypomethylated	11	37	39
chr11	117687328	117689328	Afmid	-0.41496	0.036552	stronglyHypometh	0.02488	0.24153	insignificant	4	18	18
chr11	117709550	117711550	Birc5	-0.12321	6.92E-08	hypomethylated	0.005196	0.018139	inconclusive	24	81	79
chr11	117734840	117736840	Tha1	-0.39534	0.014991	stronglyHypometh	0.017201	0.91789	insignificant	1	18	18
chr11	117830680	117832680	Socs3		1	noCoverage	-0.010706	0.58805	insignificant	0	38	47
chr11	117847170	117849170	Pgs1	-0.16234	1.78E-29	hypomethylated	-0.0038136	0.39198	insignificant	21	51	51
chr11	117990533	117992533	Dnahc17	0.21117	1	insignificant	0.08759	0.49738	insignificant	1	8	8
chr11	118109906	118111906	Cyth1	-0.15737	0.0009326	hypomethylated	0.0090052	0.00037878	hypermethylated	13	30	30
chr11	118203848	118205848	BC100451	-0.37132	0.0023074	stronglyHypometh	-0.075223	0.00017897	hypomethylated	4	24	24
chr11	118216725	118218725	Timp2	-0.073607	0.0147	hypomethylated	0.072884	0.26286	insignificant	6	17	22
chr11	118280366	118282366	Cant1	-0.20443	5.8E-29	hypomethylated	-0.023061	0.082618	insignificant	13	32	32
chr11	118294166	118296166	C1qtnf1	-0.72757	0.0023215	stronglyHypometh	0.038724	0.78481	insignificant	4	28	27
chr11	118337273	118339273	Engase	-0.10395	3.51E-14	hypomethylated	0.015883	0.54281	insignificant	16	55	50
chr11	118770886	118772886	Rbfox3	0.34193	0.46692	insignificant	0.47387	0.000028286	stronglyhypermeth	3	25	11
chr11	118848501	118850501	Enpp7	0.12989	0.20203	insignificant	-0.16266	0.75166	insignificant	2	13	17
chr11	118883342	118885342	Cbx2	-0.11583	1.58E-23	hypomethylated	-0.010872	0.00038836	hypomethylated	40	150	162
chr11	118902227	118904227	Cbx8	-0.11548	0.00000138	hypomethylated	0.015713	0.589	insignificant	23	135	135
chr11	118947551	118949551	Cbx4	-0.15174	3.04E-25	hypomethylated	0.009123	0.84713	insignificant	27	86	86
chr11	119088885	119090885	Ccdc40	-0.15043	5E-40	hypomethylated	0.0010881	0.10318	insignificant	39	119	112
chr11	119089813	119091813	Ccdc40	-0.16656	9.07E-36	hypomethylated	-0.01578	0.017313	hypomethylated	28	85	84
chr11	119128280	119130280	Gaa	-0.1899	6.17E-10	hypomethylated	0.034487	0.0034942	inconclusive	9	42	29
chr11	119128321	119130321	Gaa	-0.1899	6.17E-10	hypomethylated	0.034487	0.0034942	inconclusive	9	42	29
chr11	119161357	119163357	Eif4a3	-0.12818	0.40017	insignificant	0.021862	0.37121	insignificant	12	56	58
chr11	119175100	119177100	Card14	-0.30334	7.08E-15	hypomethylated	-0.021991	0.57187	insignificant	4	14	14
chr11	119215870	119217870	Slc26a11	-0.13639	0.009816	hypomethylated	0.0021407	0.04436	hypermethylated	22	59	64
chr11	119216824	119218824	Sgsh	-0.13111	0.1421	insignificant	-0.0036095	0.015391	inconclusive	20	39	45
chr11	119250785	119252785	Mir1932	-0.1159	3.9E-17	hypomethylated	-0.0056459	1	insignificant	32	133	139
chr11	119351660	119353660	A730011L01Rik	-0.13534	4.79E-11	hypomethylated	-0.024972	0.55281	insignificant	21	138	136
chr11	119351933	119353933	A730011L01Rik	-0.14581	4.8E-12	hypomethylated	-0.021645	0.54689	insignificant	21	142	140
chr11	119409134	119411134	Nptx1	-0.096423	1.78E-13	hypomethylated	0.0055464	0.64169	insignificant	38	129	125
chr11	119463308	119465308	Rptor	-0.18904	9.48E-17	hypomethylated	-0.0095079	0.00016805	hypomethylated	17	72	72
chr11	119774123	119776123	Chmp6	-0.15609	0.000043333	hypomethylated	0.012334	0.46586	insignificant	15	38	38
chr11	119803405	119805405	Baiap2	-0.10127	1.15E-43	hypomethylated	0.003147	0.52662	insignificant	62	179	179
chr11	119875080	119877080	Mir3065	-0.0024809	0.4222	insignificant	-0.012927	0.23121	insignificant	6	24	24
chr11	119876176	119878176	Aatk	-0.0033541	0.1806	insignificant	-0.0063938	0.10447	insignificant	9	30	30
chr11	119908459	119910459	Aatk	-0.27889	0.000000562	hypomethylated	-0.02436	0.077194	insignificant	3	38	33
chr11	119948141	119950141	Azi1	-0.27117	2.19E-11	hypomethylated	-0.031719	0.3645	insignificant	9	33	32
chr11	119959247	119961247	1810043H04Rik	-0.10921	0.060383	insignificant	-0.0025811	0.78902	insignificant	6	41	30
chr11	119960045	119962045	2410002I01Rik	-0.07902	1	insignificant	0.014631	0.028966	hypermethylated	2	23	12
chr11	120051170	120053170	2810410L24Rik	-0.331	0.0010269	hypomethylated	-0.011918	0.28307	insignificant	3	34	34
chr11	120093260	120095260	Bahcc1	-0.11356	1.94E-13	hypomethylated	-0.0092014	0.11787	insignificant	21	96	96
chr11	120209798	120211798	Actg1	-0.11937	2.81E-35	hypomethylated	-0.007225	0.000035476	hypomethylated	75	209	224
chr11	120221847	120223847	Fscn2	-0.045296	0.33908	insignificant	-0.067936	0.00015047	hypomethylated	9	38	38
chr11	120240060	120242060	2310003H01Rik	-0.049642	0.0049751	hypomethylated	-0.0033917	0.50113	insignificant	10	30	30
chr11	120299014	120301014	Nploc4	-0.25762	0.000038891	hypomethylated	-0.064811	0.64792	insignificant	3	32	30
chr11	120302944	120304944	Tspan10	-0.85264	0.14953	lowCoverage	-0.021404	0.83964	insignificant	1	4	4
chr11	120318442	120320442	Ccdc137	-0.18665	2.42E-27	hypomethylated	0.061567	0.94044	insignificant	22	61	68
chr11	120319377	120321377	1810049H13Rik	-0.11783	0.37387	insignificant	0.051112	0.54093	insignificant	10	38	40
chr11	120327948	120329948	Hgs	-0.15319	2.33E-24	hypomethylated	-0.013559	0.082744	insignificant	21	70	70
chr11	120328914	120330914	Arl16	-0.13845	1.94E-14	hypomethylated	-0.0057722	0.235	insignificant	15	42	42
chr11	120344982	120346982	Mrpl12	-0.16019	4.43E-09	hypomethylated	-0.018217	0.00043209	hypomethylated	19	37	37
chr11	120352150	120354150	Slc25a10	-0.14605	4.36E-12	hypomethylated	0.0019589	0.070311	insignificant	16	44	44
chr11	120391040	120393040	Gcgr	-0.13891	0.23754	insignificant	0.00086267	0.75847	insignificant	3	16	16
chr11	120411041	120413041	Fam195b	-0.27523	3.32E-18	hypomethylated	0.026321	2.89E-15	inconclusive	18	70	62
chr11	120412446	120414446	Ppp1r27	0.12426	1	insignificant	0.064844	0.15014	insignificant	1	10	10
chr11	120434250	120436250	P4hb	-0.10969	0.000071338	hypomethylated	0.00034714	0.25817	insignificant	15	82	82
chr11	120442934	120444934	Arhgdia	-0.091125	0.029703	hypomethylated	0.013108	0.42914	insignificant	7	36	36
chr11	120458734	120460734	Anapc11	-0.083253	3.26E-24	hypomethylated	0.0004858	0.049023	inconclusive	42	203	195
chr11	120458845	120460845	Anapc11	-0.083253	3.26E-24	hypomethylated	0.0004858	0.049023	inconclusive	42	203	195
chr11	120459679	120461679	Anapc11	-0.092232	0.00000977	hypomethylated	0.0042456	0.1228	insignificant	5	77	69
chr11	120468790	120470790	Npb	-0.17914	4.19E-12	hypomethylated	0.060523	0.016893	hypermethylated	7	33	34
chr11	120479204	120481204	Sirt7	-0.10676	5.6E-15	hypomethylated	0.0019177	3.36E-31	inconclusive	17	88	88
chr11	120486316	120488316	Sirt7		1	noCoverage	-0.057222	0.32821	insignificant	0	8	9
chr11	120494861	120496861	Mafg		1	noCoverage	-0.096974	0.097317	insignificant	0	34	29
chr11	120509651	120511651	Myadml2	-0.1982	0.59967	insignificant	0.070414	0.004159	inconclusive	2	6	4
chr11	120522151	120524151	Notum	-0.12091	1.04E-12	hypomethylated	-0.016692	0.45287	insignificant	26	75	73
chr11	120533286	120535286	Aspscr1	-0.15755	2.2E-43	hypomethylated	-0.016317	0.16526	insignificant	46	122	122

chr11	120574266	120576266	Lrrc45	-0.13816	0.00000637	hypomethylated	0.011257	0.66215	insignificant	4	58	58
chr11	120575081	120577081	Stra13	-0.11708	0.00252	hypomethylated	0.013351	0.57123	insignificant	3	40	40
chr11	120581781	120583781	Rac3	-0.13492	4.3E-34	hypomethylated	-0.024695	0.0054153	hypomethylated	25	104	126
chr11	120588595	120590595	Dcxr		1	noCoverage	0.10818	0.43159	insignificant	0	11	14
chr11	120593335	120595335	Cbr2	-0.18248	0.35739	insignificant	0.010923	0.8803	insignificant	3	8	8
chr11	120644585	120646585	Gps1	-0.095809	5.62E-64	hypomethylated	0.00096642	0.27563	insignificant	83	223	222
chr11	120644828	120646828	Gps1	-0.095809	5.62E-64	hypomethylated	0.00096642	0.27563	insignificant	83	223	222
chr11	120645518	120647518	Rfng	-0.12262	8.42E-55	hypomethylated	0.0029167	0.13949	insignificant	57	169	164
chr11	120657709	120659709	Dus1l	-0.18755	0.068308	insignificant	-0.025879	0.13575	insignificant	17	50	49
chr11	120685861	120687861	Fasn	-0.10845	0.000000295	hypomethylated	-0.011645	0.0075478	hypomethylated	17	98	98
chr11	120794186	120796186	Ccdc57	-0.70175	2.35E-08	stronglyHypometh	-0.050439	0.15367	insignificant	1	6	4
chr11	120808797	120810797	Slc16a3	-0.13016	1.57E-15	hypomethylated	0.018616	0.086798	insignificant	25	99	94
chr11	120809380	120811380	Slc16a3	-0.10539	4.96E-15	hypomethylated	0.021248	0.1765	insignificant	27	101	96
chr11	120852647	120854647	Csnk1d	-0.23698	1.01E-08	hypomethylated	0.021526	0.048776	inconclusive	5	16	17
chr11	121006456	121008456	Tex19.1	-0.024994	0.25905	insignificant	0.040499	0.21672	insignificant	5	24	22
chr11	121064746	121066746	Hexdc	-0.061785	0.38364	insignificant	0.010393	0.83849	insignificant	17	38	38
chr11	121065962	121067962	Hexdc	-0.043478	1	insignificant	0.010298	0.51115	insignificant	14	29	29
chr11	121067956	121069956	Hexdc	0.10239	0.19812	insignificant	0.083778	0.054895	insignificant	2	6	6
chr11	121090579	121092579	BC017643		1	noCoverage	0.32634	0.82805	insignificant	0	2	5
chr11	121097549	121099549	Narf	-0.1743	1.59E-13	hypomethylated	-0.04412	0.57816	insignificant	17	54	53
chr11	121120300	121122300	Foxk2	-0.091221	6.48E-48	hypomethylated	-0.0043199	0.0006222	hypomethylated	69	213	227
chr11	121215761	121217761	Wdr45l		1	noCoverage	-0.0053824	0.64462	insignificant	0	28	28
chr11	121249565	121251565	Rab40b	-0.33267	0.25743	insignificant	0.030529	0.10175	insignificant	2	12	12
chr11	121281686	121283686	Fn3krp	-0.1872	1.32E-18	hypomethylated	-0.0023633	0.1124	insignificant	18	43	45
chr11	121295266	121297266	Fn3k	-0.22197	0.000000572	hypomethylated	0.0035433	0.52768	insignificant	9	30	30
chr11	121312262	121314262	Tbcd	-0.19396	8.46E-08	hypomethylated	-0.06125	0.37279	insignificant	11	53	53
chr11	121380656	121382656	Zfp750	0.0010417	0.51924	insignificant	0.070048	0.44884	insignificant	4	8	8
chr11	121534465	121536465	B3gntl1	-0.12825	0.23459	insignificant	-0.025687	0.67085	insignificant	8	30	30
chr11	121562740	121564740	Metrnl	-0.1251	2.77E-17	hypomethylated	-0.0075241	0.48843	insignificant	27	80	80
chr11	121690531	121692531	Ptchd3	-0.024165	0.28341	insignificant	-0.033726	0.013993	hypomethylated	7	32	32
chr12	3234790	3236790	1700012B15Rik	-0.11316	0.44798	insignificant	0.061822	0.000015759	hypermethylated	26	49	43
chr12	3309969	3311969	Rab10	-0.15037	4.45E-11	hypomethylated	-0.0027498	0.66286	insignificant	16	58	57
chr12	3364131	3366131	Kif3c	-0.13248	0.00016985	hypomethylated	0.0023896	0.70692	insignificant	17	84	92
chr12	3425883	3427883	Asxl2	-0.12989	3.41E-10	hypomethylated	0.0040185	0.38683	insignificant	38	120	116
chr12	3426747	3428747	Asxl2	-0.12907	0.00000303	hypomethylated	0.0033806	0.043674	hypermethylated	33	102	99
chr12	3571390	3573390	Dtnb	-0.14043	6.11E-39	hypomethylated	0.0052988	0.024801	inconclusive	55	154	123
chr12	3572040	3574040	Dtnb	-0.12478	8.28E-38	hypomethylated	-0.0012815	0.01031	hypomethylated	56	144	115
chr12	3805979	3807979	Dnmt3a	-0.086057	4.92E-46	hypomethylated	-0.0011145	0.039437	hypomethylated	111	325	341
chr12	3890743	3892743	Dnmt3a	-0.20042	0.1976	insignificant	-0.034286	0.23664	insignificant	4	36	47
chr12	3953950	3955950	Pomc	-0.35185	0.12186	insignificant	-0.061227	0.4355	insignificant	3	9	8
chr12	4038915	4040915	Efr3b	-0.23859	0.011752	hypomethylated	-0.035003	0.44259	insignificant	5	15	15
chr12	4132396	4134396	Adcy3	-0.12114	6.86E-20	hypomethylated	0.025102	0.50813	insignificant	34	135	135
chr12	4133152	4135152	Adcy3	-0.049525	2.71E-20	hypomethylated	0.021353	0.0016038	inconclusive	37	135	135
chr12	4484060	4486060	Ncoa1	-0.11883	3.49E-10	hypomethylated	-0.0074043	0.27129	insignificant	15	50	60
chr12	4598813	4600813	Itsn2	-0.11731	6.56E-44	hypomethylated	-0.020923	0.00004149	hypomethylated	54	143	138
chr12	4775100	4777100	Pfn4	-0.24581	0.03242	hypomethylated	-0.028709	0.9431	insignificant	4	31	31
chr12	4776073	4778073	A830093I24Rik	-0.23748	1	insignificant	-0.030831	0.041523	hypomethylated	1	17	17
chr12	4823413	4825413	0610009D07Rik	-0.1659	2.89E-10	hypomethylated	-0.015293	0.85848	insignificant	20	61	72
chr12	4848401	4850401	BC068281	-0.22729	0.00023696	hypomethylated	-0.009787	0.94895	insignificant	9	32	43
chr12	4881165	4883165	Mfsd2b	-0.41192	1	insignificant	-0.018314	0.000034136	hypomethylated	1	24	19
chr12	4914326	4916326	Ubxn2a	-0.072786	0.00000134	hypomethylated	0.037388	0.15136	insignificant	28	76	76
chr12	4923158	4925158	Atad2b	-0.11458	2.01E-41	hypomethylated	0.0009379	0.22047	insignificant	47	133	132
chr12	5382307	5384307	2810032G03Rik	-0.12223	3.7E-12	hypomethylated	-0.0082657	0.95851	insignificant	22	61	60
chr12	5382488	5384488	Klhl29	-0.11444	0.00000282	hypomethylated	-0.011067	0.46617	insignificant	13	29	29
chr12	7983482	7985482	Apob	-0.26438	0.02672	hypomethylated	-0.093899	0.0025446	hypomethylated	4	19	23
chr12	8308760	8310760	Gdf7	-0.067743	0.65316	insignificant	0.021163	0.17456	insignificant	3	18	18
chr12	8319238	8321238	Hs1bp3	-0.20323	3.04E-20	hypomethylated	0.014187	0.0002183	inconclusive	13	36	37
chr12	8506791	8508791	Rhob	-0.10235	6.17E-08	hypomethylated	-0.0041048	0.50007	insignificant	12	57	57
chr12	8605872	8607872	Slc7a15		1	noCoverage	-0.058496	0.046003	hypomethylated	0	28	28
chr12	8679939	8681939	Pum2	-0.10554	2.57E-24	hypomethylated	0.030131	0.55546	insignificant	58	165	176
chr12	8680064	8682064	Pum2	-0.10554	2.57E-24	hypomethylated	0.010866	0.49097	insignificant	58	165	171
chr12	8680486	8682486	Pum2	-0.10554	2.57E-24	hypomethylated	0.010866	0.49097	insignificant	58	165	171
chr12	8777201	8779201	Sdc1	-0.1589	1.29E-39	hypomethylated	0.0043523	0.84897	insignificant	49	133	140
chr12	8927112	8929112	Laptm4a	-0.18855	0.00000112	hypomethylated	-0.027689	0.17702	insignificant	21	55	55
chr12	8953734	8955734	Matn3	-0.16827	1.39E-37	hypomethylated	0.0023808	0.81539	insignificant	26	77	78
chr12	8979806	8981806	Wdr35	-0.1504	0.00000168	hypomethylated	-0.044015	0.1376	insignificant	7	53	54
chr12	9035802	9037802	Ttc32	-0.21488	0.019775	hypomethylated	-0.016087	0.13363	insignificant	8	47	51
chr12	9580247	9582247	Osr1	-0.25252	1.87E-11	hypomethylated	-0.079774	0.14263	insignificant	6	23	28
chr12	10396585	10398585	Rdh14	-0.14236	7.17E-14	hypomethylated	-0.016018	0.22443	insignificant	21	72	73
chr12	11157179	11159179	Kcns3	-0.10174	0.0000065	hypomethylated	-0.0059811	0.81397	insignificant	13	49	49
chr12	11157648	11159648	Kcns3	-0.6748	0.092308	insignificant	-0.043849	1	insignificant	2	9	9
chr12	11271691	11273691	Smc6	-0.11686	6.47E-16	hypomethylated	-0.0098296	0.19771	insignificant	36	138	145
chr12	11272593	11274593	Gen1	-0.09944	2.24E-12	hypomethylated	-0.010916	0.3703	insignificant	30	118	123
chr12	11443455	11445455	Vsnl1	-0.18223	0.053585	insignificant	0.033966	0.12092	insignificant	10	36	43
chr12	11461884	11463884	Rad51ap2	-0.12348	0.59097	insignificant	0.051462	0.18459	insignificant	5	27	33
chr12	12267944	12269944	Fam49a	-0.10564	5.83E-15	hypomethylated	0.00050641	0.34963	insignificant	25	69	66
chr12	12948642	12950642	Mycn		1	noCoverage	0.0016809	0.77851	insignificant	0	4	7
chr12	13255980	13257980	Ddx1	-0.41909	4.57E-27	stronglyHypometh	0.0060605	0.030512	inconclusive	5	14	14
chr12	13274932	13276932	Nbas	-0.12155	0.000088757	hypomethylated	0.016893	0.18308	insignificant	11	39	39
chr12	14158844	14160844	Fam84a	-0.22269	0.011793	hypomethylated	-0.012128	0.2369	insignificant	7	19	18
chr12	15823591	15825591	Trib2	-0.10008	7.95E-31	hypomethylated	-0.011622	0.0013056	hypomethylated	23	81	80
chr12	16596576	16598576	Lpin1	-0.20199	0.0077881	hypomethylated	-0.13524	3.57E-11	hypomethylated	8	36	40
chr12	16659275	16661275	Ntsr2	-0.28726	8.2E-09	hypomethylated	-0.12906	0.00015011	hypomethylated	6	37	30
chr12	16807692	16809692	Greb1	-0.38249	0.014748	stronglyHypometh	-0.086873	0.3647	insignificant	5	26	25
chr12	16816770	16818770	E2f6	-0.20583	6.87E-40	hypomethylated	-0.03813	0.085461	insignificant	38	123	110

chr12	16900783	16902783	Rock2	-0.08446	1.04E-14	hypomethylated	-0.00053024	0.97017	insignificant	55	212	180
chr12	17006924	17008924	Pqlc3	-0.21098	1.13E-15	hypomethylated	-0.022246	0.55686	insignificant	6	22	22
chr12	17272400	17274400	Pdia6	-0.12075	7.2E-25	hypomethylated	-0.002033	0.32102	insignificant	44	106	90
chr12	17331536	17333536	Atp6v1c2	-0.14633	6.58E-08	hypomethylated	0.010558	0.048397	hypermethylated	19	46	46
chr12	17354298	17356298	Nol10	-0.17546	8.83E-14	hypomethylated	-0.049856	0.33021	insignificant	12	29	43
chr12	17550678	17552678	Odc1	-0.11927	6.01E-14	hypomethylated	-0.003955	0.0088282	hypomethylated	36	126	135
chr12	17696619	17698619	Hpcal1	-0.14362	7.71E-20	hypomethylated	-0.0050126	0.44773	insignificant	30	102	102
chr12	18185048	18187048			1	noCoverage		1	noCoverage	0	21	0
chr12	18520351	18522351	5730507C01Rik	-0.09495	0.00034412	hypomethylated	-0.10116	1.07E-74	hypomethylated	29	150	82
chr12	20821640	20823640	1700030C10Rik	-0.18427	0.000030221	hypomethylated	0.018602	0.63566	insignificant	5	25	16
chr12	21116616	21118616	Asap2	-0.12341	5.08E-09	hypomethylated	0.030442	2.97E-12	hypermethylated	18	164	154
chr12	21291157	21293157	Cpsf3	-0.15622	0.00000439	hypomethylated	-0.023103	1	insignificant	13	46	58
chr12	21292098	21294098	Itgb1bp1	-0.093137	0.37687	insignificant	0.029212	0.082246	insignificant	4	12	19
chr12	21321252	21323252	Iah1	-0.12015	0.15566	insignificant	-0.041574	0.000019569	hypomethylated	12	39	41
chr12	21379452	21381452	Adam17	-0.35282	6.89E-28	stronglyHypometh	-0.067145	0.0000051	hypomethylated	16	49	49
chr12	21423297	21425297	Ywhaq	-0.15342	3.59E-09	hypomethylated	0.063073	0.25661	insignificant	6	22	16
chr12	22989696	22991696	2410018L13Rik	-0.075843	0.000036042	hypomethylated	-0.024788	0.25587	insignificant	18	113	101
chr12	25182445	25184445	Taf1b	-0.056435	4.64E-10	hypomethylated	-0.028429	0.0001482	hypomethylated	9	40	27
chr12	25256151	25258151	Grhl1	-0.11827	2.23E-65	hypomethylated	0.0069653	0.0024813	inconclusive	68	179	184
chr12	25264583	25266583	Grhl1		1	noCoverage	-0.14368	0.34261	insignificant	0	6	3
chr12	25335235	25337235	Klf11	-0.10754	6.96E-27	hypomethylated	-0.0024703	0.3167	insignificant	49	134	134
chr12	25366660	25368660	Cys1	-0.21878	0.64679	insignificant	-0.28099	0.0088251	inconclusive	3	24	12
chr12	25392118	25394118	Rrm2	-0.13139	0.0011823	hypomethylated	-0.021802	0.52335	insignificant	19	91	90
chr12	25515463	25517463	Mboat2	-0.11493	1.01E-30	hypomethylated	-0.0060982	0.71517	insignificant	33	108	111
chr12	25658796	25660796	Kidins220	-0.13412	6.06E-17	hypomethylated	0.025312	0.76769	insignificant	14	44	37
chr12	25780957	25782957	Id2	-0.27639	6.38E-13	hypomethylated	-0.019036	0.80196	insignificant	11	30	30
chr12	27100121	27102121	Rnf144a		1	noCoverage	0.03125	1	insignificant	0	8	8
chr12	27153079	27155079	Cmpk2	-0.16545	2.05E-27	hypomethylated	-0.011956	0.14797	insignificant	25	78	79
chr12	28027583	28029583	Sox11	-0.11744	0.0023159	hypomethylated	0.011862	0.024943	inconclusive	10	73	91
chr12	29320818	29322818	Rps7	-0.35039	1.48E-16	stronglyHypometh	-0.009821	0.28627	insignificant	12	31	33
chr12	29333466	29335466	Rnaseh1	-0.078046	0.0047343	hypomethylated	-0.0016995	0.91722	insignificant	4	24	24
chr12	29359071	29361071	Adi1	-0.074604	0.025733	hypomethylated	-0.0069719	0.50237	insignificant	10	20	20
chr12	29435259	29437259	Tssc1	-0.18317	8.35E-08	hypomethylated	0.0080346	0.11862	insignificant	16	74	47
chr12	29435318	29437318	Tssc1	-0.19734	6.98E-08	hypomethylated	0.021582	0.56281	insignificant	16	65	40
chr12	29435692	29437692	Tssc1	-0.17013	0.00000524	hypomethylated	0.020754	0.72305	insignificant	14	56	32
chr12	30212248	30214248	Myt1l	-0.36496	1	lowCoverage	-0.12431	0.55484	insignificant	1	10	10
chr12	30218769	30220769	Myt1l	-0.21461	0.0067567	hypomethylated	-0.024801	0.14145	insignificant	4	29	29
chr12	30621900	30623900	Pxdn	-0.14952	0.000022334	hypomethylated	-0.01269	0.65048	insignificant	22	63	64
chr12	30817474	30819474	Tpo	-0.88387	0.095238	lowCoverage		1	noCoverage	1	2	0
chr12	31058240	31060240	Sntg2	-0.19308	2.4E-15	hypomethylated	-0.065196	0.00024556	hypomethylated	11	26	24
chr12	31268307	31270307	Tmem18	-0.12278	9.82E-08	hypomethylated	-0.024753	0.30371	insignificant	16	58	35
chr12	31568188	31570188	Fam150b	-0.12326	0.00000674	hypomethylated	-0.021931	0.064003	insignificant	17	42	42
chr12	31595533	31597533	Sh3yl1		1	noCoverage	0.036621	1	insignificant	0	10	10
chr12	31757832	31759832	Fam110c	-0.15117	1.23E-12	hypomethylated	-0.00022422	0.17688	insignificant	20	82	82
chr12	31949158	31951158	Lamb1	-0.17133	0.00035902	hypomethylated	-0.0061252	0.26264	insignificant	5	28	28
chr12	32036302	32038302	Dld	0.10052	1	insignificant	-0.0052426	0.097275	insignificant	2	13	10
chr12	32184405	32186405	Cbll1	-0.12733	2.93E-21	hypomethylated	-0.006438	0.96668	insignificant	23	62	62
chr12	32244834	32246834	Slc26a4	-0.23127	0.036743	hypomethylated	-0.064761	0.0011148	hypomethylated	2	14	11
chr12	32319462	32321462	Bcap29	-0.21003	1.44E-13	hypomethylated	-0.032278	0.83845	insignificant	21	64	64
chr12	32319523	32321523	Bcap29	-0.29555	1.14E-12	hypomethylated	-0.04677	0.36272	insignificant	14	44	44
chr12	32338733	32340733	Cog5	-0.17633	1.97E-25	hypomethylated	-0.033049	0.012715	hypomethylated	38	96	95
chr12	32339691	32341691	Cog5	-0.17918	5.97E-12	hypomethylated	-0.025002	0.4544	insignificant	17	52	52
chr12	32635400	32637400	Hbp1	-0.76515	0.00010746	stronglyHypometh	0.028277	0.73882	insignificant	1	8	10
chr12	32746144	32748144	Prkar2b	-0.24895	7.65E-10	hypomethylated	0.01194	0.000013955	inconclusive	8	25	25
chr12	32893203	32895203	Pik3cg		1	noCoverage	0.037076	0.52825	insignificant	0	11	9
chr12	32893335	32895335	Pik3cg		1	noCoverage	0.16667	1	insignificant	0	4	2
chr12	33062653	33064653	2010109K11Rik	-0.091149	6.72E-30	hypomethylated	-0.000097987	0.62084	insignificant	67	174	177
chr12	33504199	33506199	Nampt	-0.10076	7.84E-36	hypomethylated	-0.0016267	0.22604	insignificant	58	157	149
chr12	33637809	33639809	Sypl	-0.098583	0.000000113	hypomethylated	-0.0023497	1	insignificant	36	112	119
chr12	33638050	33640050	Sypl	-0.098583	0.000000113	hypomethylated	-0.0023497	1	insignificant	36	112	119
chr12	33831550	33833550	Atxn7l1	-0.15531	6.43E-22	hypomethylated	-0.019987	0.027201	hypomethylated	34	156	150
chr12	33832451	33834451	F730043M19Rik	-0.19187	4.39E-09	hypomethylated	0.0046793	0.38708	insignificant	15	89	86
chr12	34113488	34115488	Twistnb	-0.13942	2.14E-18	hypomethylated	-0.0091417	0.16775	insignificant	16	79	80
chr12	34612289	34614289	Ferd3l	-0.29857	0.000000156	hypomethylated	0.017311	0.23377	insignificant	6	31	24
chr12	34641535	34643535	Twist1	-0.11814	7.36E-26	hypomethylated	-0.0035479	0.0093321	hypomethylated	39	171	171
chr12	35668432	35670432	Prps1l1	0.17292	0.01372	hypermethylated	-0.053908	0.0067562	inconclusive	4	23	17
chr12	35730860	35732860	Snx13	-0.098223	7.76E-32	hypomethylated	0.0069776	0.11966	insignificant	50	100	98
chr12	36219661	36221661	Ahr	-0.096414	0.00031612	hypomethylated	0.015295	0.021558	inconclusive	7	21	29
chr12	36651207	36653207	Agr3		1	noCoverage	0.034762	0.050583	insignificant	0	7	15
chr12	36769065	36771065	Tspan13	-0.23793	0.000096958	hypomethylated	0.038614	0.90509	insignificant	6	26	24
chr12	36815965	36817965	Gm5434	0.062845	1	insignificant	-0.0078069	0.92403	insignificant	2	42	41
chr12	36882710	36884710	Ankmy2	-0.14589	3.01E-34	hypomethylated	-0.032037	0.028008	hypomethylated	23	84	82
chr12	36883412	36885412	Bzw2	-0.16351	3.89E-09	hypomethylated	-0.043591	0.91598	insignificant	8	56	54
chr12	36979985	36981985	1700108M19Rik		1	noCoverage	0.1538	0.79048	insignificant	0	16	14
chr12	37039755	37041755	Sostdc1	-0.40408	0.00047552	stronglyHypometh	-0.093029	0.79422	insignificant	2	14	14
chr12	37107105	37109105	Ispd	-0.1378	2.07E-15	hypomethylated	-0.0099874	0.35048	insignificant	22	59	64
chr12	37834132	37836132	Meox2	-0.3072	2.62E-12	hypomethylated	0.040865	0.70031	insignificant	2	12	12
chr12	38606291	38608291	Dgkb	-0.35152	0.52706	insignificant	-0.059643	0.69162	insignificant	1	15	25
chr12	39505844	39507844	Etv1	-0.24227	0.35255	insignificant	0.0043531	0.64064	insignificant	2	26	35
chr12	40763963	40765963	Arl4a	-0.074805	2.37E-25	hypomethylated	-0.00072368	0.18205	insignificant	35	151	152
chr12	40764574	40766574	Arl4a	-0.056612	0.00000138	hypomethylated	0.00065546	0.70682	insignificant	12	66	66
chr12	40860815	40862815	Scin	-0.10447	0.021682	hypomethylated	0.0013748	0.3327	insignificant	4	8	8
chr12	40949398	40951398	Ifrd1	-0.13228	5.19E-25	hypomethylated	-0.0091702	0.0022464	hypomethylated	23	58	58
chr12	40949776	40951776	Ifrd1	-0.13301	0.00000316	hypomethylated	-0.0034328	0.8503	insignificant	9	18	18
chr12	41171639	41173639	Dock4	-0.1529	2.25E-34	hypomethylated	0.023517	0.34192	insignificant	51	158	165

chr12	41172377	41174377	Zfp277	-0.14205	7.39E-28	hypomethylated	0.025901	0.17942	insignificant	47	148	154
chr12	41749676	41751676	Immp2l	-0.089146	0.00026016	hypomethylated	0.018995	0.61678	insignificant	11	84	85
chr12	45311055	45313055	Dnajb9	-0.086503	0.000000168	hypomethylated	0.0062479	0.21112	insignificant	15	44	44
chr12	45369140	45371140	Pnpla8	-0.08826	5.79E-16	hypomethylated	0.0016103	0.60658	insignificant	33	90	86
chr12	45428871	45430871	Nrcam	-0.15598	2.82E-27	hypomethylated	-0.0033217	0.00051658	hypomethylated	48	151	152
chr12	46175470	46177470	Stxbp6	-0.21614	0.20476	insignificant	-0.095229	0.00000739	hypomethylated	2	7	9
chr12	47919762	47921762	Nova1	-0.098153	0.000000614	hypomethylated	-0.00072508	0.6444	insignificant	45	160	160
chr12	50482869	50484869	Foxg1	-0.12607	0.00000326	hypomethylated	0.007229	0.50287	insignificant	16	87	90
chr12	50482993	50484993	Foxg1	-0.12995	0.00000205	hypomethylated	0.0069794	0.4847	insignificant	17	95	98
chr12	50489638	50491638	3110039M20Ril	-0.48142	0.49742	insignificant	-0.0091991	1	insignificant	1	6	6
chr12	51750210	51752210	Prkd1	-0.12209	0.000000126	hypomethylated	-0.028527	0.94996	insignificant	15	48	55
chr12	52448216	52450216	G2e3	-0.11507	8.2E-16	hypomethylated	-0.0037146	0.28475	insignificant	39	133	130
chr12	52477566	52479566	Scfd1	-0.15835	0.000075498	hypomethylated	0.019594	0.21694	insignificant	8	24	22
chr12	52693327	52695327	Coch	-0.11924	2.84E-12	hypomethylated	-0.025206	0.089211	insignificant	25	105	115
chr12	52790952	52792952	Ap4s1	-0.1815	9.56E-12	hypomethylated	-0.041109	0.33929	insignificant	20	85	80
chr12	52792901	52794901	Ap4s1	-0.10978	0.28446	insignificant	-0.018583	0.016206	inconclusive	16	67	63
chr12	52930523	52932523	Hectd1	-0.10978	1.66E-38	hypomethylated	0.019945	0.10628	insignificant	37	117	118
chr12	53072308	53074308	Heatr5a	-0.18047	0.000142	hypomethylated	-0.015976	0.89124	insignificant	10	26	26
chr12	53107488	53109488	6530401N04Rik	-0.53224	0.33187	insignificant	-0.0048565	0.01788	hypomethylated	4	22	23
chr12	53129050	53131050	Gpr33	0.21795	0.56871	insignificant	0.067949	1	insignificant	1	4	4
chr12	53197732	53199732	Nubpl	-0.18297	0.00000031	hypomethylated	-0.049494	0.93663	insignificant	9	19	18
chr12	53616063	53618063	Arhgap5		1	noCoverage	-0.016667	1	insignificant	0	2	2
chr12	53799369	53801369	Akap6	-0.045691	0.45683	insignificant	-0.064287	0.71033	insignificant	2	14	13
chr12	54348663	54350663	Npas3	-0.24771	1	lowCoverage	-0.12236	0.72612	insignificant	1	9	14
chr12	55304861	55306861	Egln3	-0.18499	0.38667	insignificant	-0.040781	1	insignificant	7	16	15
chr12	55492305	55494305	Gm7550		1	noCoverage	-0.059542	0.0086884	hypomethylated	0	31	33
chr12	55757559	55759559	1110002B05Rik	-0.13878	1.14E-16	hypomethylated	-0.0045332	0.80016	insignificant	16	36	36
chr12	55796852	55798852	Eapp	-0.29652	0.35232	insignificant	-0.0096641	0.6861	insignificant	5	22	18
chr12	55963864	55965864	Cfl2	-0.12751	0.0020233	hypomethylated	-0.012445	0.95085	insignificant	9	56	59
chr12	56180482	56182482	Srp54a		1	noCoverage	-0.0045014	0.43222	insignificant	0	74	73
chr12	56181097	56183097	2700097O09Rik		1	noCoverage	-0.005895	0.64783	insignificant	0	65	65
chr12	56402623	56404623	1110008L16Rik	-0.17049	0.0016858	hypomethylated	0.025255	1	insignificant	6	38	38
chr12	56403987	56405987	Ppp2r3c	-0.22781	0.0074974	hypomethylated	0.046523	0.91776	insignificant	3	14	14
chr12	56498811	56500811	Psma6	-0.24282	1.54E-19	hypomethylated	-0.047261	0.34417	insignificant	14	61	66
chr12	56593634	56595634	Nfkbia	-0.14713	0.00001124	hypomethylated	0.013976	0.12146	insignificant	9	45	42
chr12	56665121	56667121	Aldoart2	-0.033675	0.73	insignificant	-0.070496	0.75764	insignificant	5	10	10
chr12	56698903	56700903	Insm2	-0.1364	4.47E-48	hypomethylated	0.0069191	0.17926	insignificant	50	138	143
chr12	56936352	56938352	Brms1l	-0.14557	1.03E-60	hypomethylated	-0.023425	0.064327	insignificant	42	99	107
chr12	57636093	57638093	Nkx2-1	-0.12849	1.4E-11	hypomethylated	-0.0001515	0.041364	hypomethylated	22	117	110
chr12	57637895	57639895	Nkx2-1	-0.10496	0.79078	insignificant	-0.023108	0.047557	hypomethylated	5	42	42
chr12	57714271	57716271	Nkx2-9	-0.24269	0.00000161	hypomethylated	-0.023423	0.11674	insignificant	13	63	68
chr12	57795625	57797625	Pax9	-0.23314	4.17E-12	hypomethylated	-0.0046584	0.28583	insignificant	13	63	63
chr12	58330398	58332398	Prps1l3	-0.18097	0.000000785	hypomethylated	-0.011906	0.084609	insignificant	15	74	75
chr12	58330411	58332411	Mipol1	-0.18097	0.000000785	hypomethylated	-0.011906	0.084609	insignificant	15	74	75
chr12	58664101	58666101	4921506M07Rik		1	noCoverage	0.12858	0.16312	insignificant	0	10	10
chr12	59311790	59313790	Sstr1	-0.21988	7.74E-18	hypomethylated	-0.027648	0.054701	insignificant	14	46	46
chr12	59370245	59372245	Clec14a	-0.0045163	1	insignificant	-0.22845	0.000000107	hypomethylated	2	10	12
chr12	60113004	60115004	Sec23a	-0.1041	2.68E-21	hypomethylated	0.011605	0.72509	insignificant	15	77	67
chr12	60113379	60115379	Sip1	-0.26334	6.6E-10	hypomethylated	-0.0064346	0.024543	inconclusive	7	49	46
chr12	60162448	60164448	Trappc6b	-0.16567	0.000040737	hypomethylated	0.033382	0.78807	insignificant	5	12	12
chr12	60166905	60168905	Pnn	-0.11657	4.59E-40	hypomethylated	-0.0034908	0.70539	insignificant	35	98	98
chr12	60231439	60233439	Ctage5	-0.10638	8.78E-35	hypomethylated	-0.0014059	0.86825	insignificant	60	191	191
chr12	60320470	60322470	Fbxo33	-0.19339	2.73E-31	hypomethylated	0.008305	0.61479	insignificant	51	154	168
chr12	62624618	62626618	Lrfn5	-0.2023	4.15E-09	hypomethylated	-0.012929	0.0047901	hypomethylated	14	50	58
chr12	66017897	66019897	Gm527	-0.080539	5.1E-09	hypomethylated	-0.00723	0.000053202	hypomethylated	36	130	150
chr12	66065728	66067728	Fam179b	-0.15392	8.36E-27	hypomethylated	-0.013732	0.63987	insignificant	29	90	88
chr12	66066523	66068523	Fam179b	-0.16689	2.55E-27	hypomethylated	-0.015681	0.47226	insignificant	25	88	86
chr12	66136320	66138320	Prpf39	-0.12714	6.72E-15	hypomethylated	-0.011086	0.000010908	hypomethylated	21	100	100
chr12	66174925	66176925	Fancm	-0.090373	2.7E-16	hypomethylated	-0.0051588	0.089431	insignificant	39	133	138
chr12	66175592	66177592	Fancm	-0.11241	1.14E-17	hypomethylated	-0.0081733	0.031232	hypomethylated	30	115	115
chr12	66273567	66275567	Mis18bp1	-0.12023	4.03E-14	hypomethylated	0.0023857	0.65414	insignificant	16	48	48
chr12	66325503	66327503	Wdr20b	-0.1051	0.010415	hypomethylated	0.012085	0.16051	insignificant	3	26	26
chr12	68323536	68325536	Mdga2	-0.12336	0.035573	hypomethylated	0.013571	0.14673	insignificant	8	26	26
chr12	68770258	68772258	Gm17821	0.093874	1	insignificant	-0.085628	0.65364	insignificant	1	8	6
chr12	70268800	70270800	Lrr1	-0.039618	0.16451	insignificant	0.0056826	0.83348	insignificant	18	82	83
chr12	70284144	70286144	Mgat2	-0.10254	2.17E-28	hypomethylated	-0.011709	0.75675	insignificant	69	209	214
chr12	70285054	70287054	Rpl36al	-0.13819	1.11E-29	hypomethylated	-0.019265	0.38186	insignificant	53	138	144
chr12	70297197	70299197	9330151L19Rik	-0.11556	9.55E-54	hypomethylated	-0.0033704	0.60517	insignificant	68	200	211
chr12	70329177	70331177	Pole2	-0.35277	0.0013694	stronglyHypometh	0.046437	1	insignificant	2	10	9
chr12	70341818	70343818	Klhdc1	-0.065384	0.000018355	hypomethylated	0.022715	1	insignificant	20	66	66
chr12	70396667	70398667	Klhdc2	-0.074071	9.04E-19	hypomethylated	0.0084801	0.56303	insignificant	24	86	90
chr12	70472136	70474136	Arf6	-0.10955	5.54E-51	hypomethylated	-0.0080186	0.012927	hypomethylated	81	253	253
chr12	70782839	70784839	Sos2	-0.065429	7.82E-17	hypomethylated	0.0030727	0.30353	insignificant	39	136	136
chr12	70824948	70826948	Atp5s	-0.15779	6.74E-12	hypomethylated	-0.033042	0.026518	hypomethylated	20	71	88
chr12	70825861	70827861	L2hgdh	-0.14687	0.00001823	hypomethylated	-0.048206	0.12857	insignificant	12	41	46
chr12	70890330	70892330	4930512B01Rik	-0.26917	1.13E-17	hypomethylated	-0.042978	0.074682	insignificant	17	72	74
chr12	70891694	70893694	Cdkl1	-0.18694	1.7E-18	hypomethylated	-0.0010443	0.031597	hypomethylated	8	48	50
chr12	70993091	70995091	Atl1	-0.11525	2.25E-35	hypomethylated	0.0019335	0.000018785	inconclusive	60	181	188
chr12	70994150	70996150	Atl1	-0.14293	1.4E-13	hypomethylated	0.0042074	0.015053	inconclusive	18	50	57
chr12	71087989	71089989	Sav1	-0.10671	6.31E-11	hypomethylated	-0.024279	0.70908	insignificant	26	64	73
chr12	71203841	71205841	Nin		1	noCoverage	-0.05695	0.34233	insignificant	0	10	11
chr12	71212912	71214912	Nin	-0.16026	6.88E-08	hypomethylated	0.17268	0.22394	insignificant	8	16	24
chr12	71328670	71330670	Pygl	-0.61905	0.0000067	stronglyHypometh	-0.056364	0.092602	insignificant	1	8	8
chr12	71448601	71450601	Trim9	-0.42545	0.1199	insignificant	-0.097931	0.68072	insignificant	5	14	14
chr12	71553140	71555140	Tmx1	-0.14323	0.000014376	hypomethylated	0.088837	0.80021	insignificant	11	31	33

chr12	71925500	71927500	Frmd6	-0.12906	1.59E-28	hypomethylated	0.00027725	0.56174	insignificant	34	136	139
chr12	72037843	72039843	Actr10	-0.11573	7.6E-19	hypomethylated	0.0057874	0.96013	insignificant	24	57	64
chr12	72074609	72076609	Psma3	-0.29077	3.7E-24	hypomethylated	-0.0058844	0.080591	insignificant	16	72	64
chr12	72115953	72117953	Arid4a	-0.11528	6.32E-38	hypomethylated	-0.0050745	0.00025343	hypomethylated	90	242	234
chr12	72236834	72238834	2700049A03Rik	-0.23369	3.32E-54	hypomethylated	-0.012144	0.23423	insignificant	24	64	65
chr12	72237662	72239662	Timm9	-0.35158	0.32599	insignificant	-0.021736	0.10937	insignificant	5	38	38
chr12	72409870	72411870	Dact1	-0.072852	1.11E-12	hypomethylated	-0.011344	0.081791	insignificant	49	152	162
chr12	72931064	72933064	Daam1	-0.15596	0.0027091	hypomethylated	-0.015954	0.014806	inconclusive	14	68	69
chr12	73171978	73173978	Gpr135	-0.083747	0.75064	insignificant	-0.0017682	1	insignificant	3	28	28
chr12	73186109	73188109	Jkamp	-0.15081	1.82E-09	hypomethylated	0.0031018	0.9564	insignificant	8	36	36
chr12	73186300	73188300	Jkamp	-0.18792	0.000080219	hypomethylated	-0.033332	0.55206	insignificant	2	17	18
chr12	73337711	73339711	Rtn1	-0.064984	0.0081748	hypomethylated	0.034904	0.11677	insignificant	7	38	23
chr12	73509926	73511926	Rtn1	-0.63131	0.010575	stronglyHypometh	-0.092424	0.68569	insignificant	3	6	6
chr12	73541852	73543852	Lrrc9	-0.1786	1.07E-17	hypomethylated	-0.010491	4.98E-08	hypomethylated	14	51	52
chr12	73542235	73544235	Lrrc9	-0.14447	3.34E-17	hypomethylated	-0.0278	3.24E-09	hypomethylated	15	53	53
chr12	73636343	73638343	1810048J11Rik	-0.26691	1.25E-22	hypomethylated	0.010678	0.047502	inconclusive	14	74	70
chr12	73765815	73767815	Dhrs7	-0.27671	8.59E-13	hypomethylated	-0.054147	0.015246	hypomethylated	7	25	25
chr12	73861197	73863197	Ppm1a	-0.086576	1.77E-21	hypomethylated	0.0084029	0.10131	insignificant	52	177	192
chr12	74018752	74020752	4930447C04Rik	0.14689	1	insignificant	0.014577	0.22112	insignificant	3	20	17
chr12	74039931	74041931	Six6	-0.26015	2.32E-16	hypomethylated	-0.052749	0.35103	insignificant	11	70	90
chr12	74147699	74149699	Six1	-0.095568	0.00010295	hypomethylated	0.012456	0.49695	insignificant	14	46	38
chr12	74214232	74216232	Six4	-0.28542	2.99E-09	hypomethylated	-0.0020769	0.32498	insignificant	12	40	40
chr12	74223703	74225703	Mnat1	-0.21003	0.11316	insignificant	0.07958	0.43886	insignificant	2	28	24
chr12	74386841	74388841	Trmt5	-0.24402	1.63E-25	hypomethylated	-0.06637	0.61965	insignificant	23	63	71
chr12	74387698	74389698	Trmt5	-0.23468	4.53E-12	hypomethylated	-0.090549	0.00063034	hypomethylated	15	46	53
chr12	74647382	74649382	Tmem30b	-0.273	0.000052755	hypomethylated	-0.026118	0.000089349	hypomethylated	12	47	49
chr12	74685027	74687027	Prkch	-0.14612	9.93E-26	hypomethylated	-0.0017745	0.70691	insignificant	48	131	130
chr12	75007853	75009853	Hif1a	-0.10271	2.03E-21	hypomethylated	-0.01455	0.31875	insignificant	42	138	168
chr12	75064516	75066516	Snapc1	-0.18015	2.27E-18	hypomethylated	-0.016384	0.0086291	hypomethylated	19	73	71
chr12	75097747	75099747	Syt16	-0.13394	3.01E-15	hypomethylated	-0.019718	0.42737	insignificant	19	47	46
chr12	75384262	75386262	1700086L19Rik	-0.23465	3.38E-18	hypomethylated	-0.021094	0.000013896	hypomethylated	13	56	56
chr12	75397460	75399460	Dbpht2	0.083333	0.74019	insignificant	-0.1502	0.088753	insignificant	4	10	12
chr12	76278319	76280319	Kcnh5	-0.1839	0.00000161	hypomethylated	-0.023467	0.15338	insignificant	10	50	50
chr12	76408299	76410299	Rhoj	-0.39262	0.10586	insignificant	-0.0039551	0.40807	insignificant	6	41	41
chr12	76517768	76519768	Gphb5	0.09638	1	insignificant	0.09638	0.59181	insignificant	1	6	6
chr12	76697187	76699187	Ppp2r5e	-0.10694	4.07E-13	hypomethylated	-0.0059168	1	insignificant	28	105	101
chr12	76770524	76772524	Wdr89	-0.27679	0.067603	insignificant	-0.065696	0.40196	insignificant	6	12	12
chr12	76836716	76838716	Sgpp1	-0.12184	1.37E-11	hypomethylated	-0.00061192	0.34663	insignificant	16	40	40
chr12	76918304	76920304	Syne2	-0.13496	2.99E-21	hypomethylated	-0.00064336	0.25766	insignificant	44	142	142
chr12	77278246	77280246	Esr2	0.19651	0.010801	hypermethylated	0.05956	0.57858	insignificant	3	47	55
chr12	77347733	77349733	Tex21		1	noCoverage	-0.059275	0.049969	hypomethylated	0	6	7
chr12	77355218	77357218	Mthfd1	-0.18606	5.38E-27	hypomethylated	-0.0082645	0.00011828	hypomethylated	19	74	72
chr12	77424877	77426877	Akap5	-0.16512	5.98E-11	hypomethylated	0.0042331	0.3825	insignificant	21	93	92
chr12	77470252	77472252	Zbtb1	-0.084279	7.9E-31	hypomethylated	0.011894	0.48451	insignificant	67	232	246
chr12	77470451	77472451	Zbtb25	-0.075094	1.2E-20	hypomethylated	0.0010307	0.47181	insignificant	56	208	218
chr12	77470547	77472547	Zbtb25	-0.076319	9.69E-20	hypomethylated	0.00076767	0.95646	insignificant	49	188	198
chr12	77504162	77506162	Hspa2	-0.17089	9.44E-09	hypomethylated	0.0023072	0.02718	inconclusive	14	84	85
chr12	77504356	77506356	Hspa2	-0.17399	5.48E-10	hypomethylated	0.014479	0.23726	insignificant	14	111	117
chr12	77517585	77519585	Ppp1r36	-0.32445	8.95E-20	hypomethylated	0.059371	0.49536	insignificant	20	76	79
chr12	77544483	77546483	Gm10451	0.26481	1	lowCoverage	0.011343	1	insignificant	1	6	6
chr12	77633546	77635546	Plekhg3	-0.13125	2.55E-31	hypomethylated	-0.012215	0.00015568	hypomethylated	24	110	112
chr12	77811534	77813534	Spnb1	-0.21628	6.66E-16	hypomethylated	-0.0059358	0.010763	hypomethylated	18	40	40
chr12	77865559	77867559	Churc1	-0.19613	6.83E-20	hypomethylated	-0.017197	0.19025	insignificant	16	63	66
chr12	77896541	77898541	Gpx2	0.17374	1	insignificant	0.0078791	0.50289	insignificant	1	8	8
chr12	77923511	77925511	Rab15	-0.12106	0.040441	hypomethylated	-0.0028548	0.13223	insignificant	16	60	60
chr12	77937453	77939453	Fntb		1	noCoverage	0.017893	1	insignificant	0	14	23
chr12	78063235	78065235	Max	-0.22047	1.7E-10	hypomethylated	-0.020722	0.72266	insignificant	16	68	69
chr12	78339007	78341007	Fut8	-0.087155	4.46E-31	hypomethylated	0.0022819	0.37266	insignificant	68	202	196
chr12	79326641	79328641	Gphn	-0.14471	3.54E-28	hypomethylated	-0.016932	0.094241	insignificant	51	162	178
chr12	79848933	79850933	Mpp5	-0.10214	1.28E-18	hypomethylated	-0.0050739	0.79038	insignificant	51	198	177
chr12	79962058	79964058	Eif2s1	-0.12906	2.85E-34	hypomethylated	0.0083873	0.56616	insignificant	21	91	86
chr12	79962625	79964625	Atp6v1d	-0.11092	9.65E-09	hypomethylated	0.010542	0.82283	insignificant	9	58	58
chr12	80007925	80009925	Plek2	-0.44195	0.000000257	stronglyHypometh	-0.25142	0.26195	insignificant	3	17	12
chr12	80108264	80110264	Tmem229b	-0.10257	0.021063	hypomethylated	0.059474	0.015096	inconclusive	3	12	23
chr12	80129149	80131149	Plekhh1	-0.088027	2.06E-23	hypomethylated	-0.0062948	0.083696	insignificant	35	106	106
chr12	80230774	80232774	Arg2	-0.181	0.000000865	hypomethylated	0.0062786	0.039235	inconclusive	13	68	65
chr12	80273445	80275445	Vti1b	-0.15815	1.16E-19	hypomethylated	-0.010969	0.065911	insignificant	21	56	56
chr12	80308900	80310900	Rdh12	-0.14511	0.23473	insignificant	0.053287	0.17422	insignificant	2	6	6
chr12	80397268	80399268	Rad51l1	-0.17906	0.00000103	hypomethylated	0.048179	0.51433	insignificant	11	51	52
chr12	80397269	80399269	Rad51l1	-0.17906	0.00000103	hypomethylated	0.048179	0.51433	insignificant	11	51	52
chr12	81214000	81216000	Zfp36l1	-0.16322	6.23E-14	hypomethylated	0.0019708	0.58748	insignificant	21	92	92
chr12	81233831	81235831	2310015A10Rik	-0.1899	0.000028981	hypomethylated	-0.012091	0.82392	insignificant	3	12	12
chr12	81361358	81363358	Actn1	-0.16076	3.95E-08	hypomethylated	-0.0067705	0.84709	insignificant	4	20	20
chr12	81537588	81539588	Dcaf5	-0.081365	0.000034502	hypomethylated	0.0055364	0.87842	insignificant	15	68	69
chr12	81563081	81565081	Exd2	-0.12856	7.73E-11	hypomethylated	-0.0090007	0.11919	insignificant	10	58	66
chr12	81618976	81620976	Galntl1	-0.11101	1.42E-36	hypomethylated	-0.0032492	0.017307	hypomethylated	62	247	248
chr12	81744201	81746201	Slc39a9	-0.16798	1.33E-20	hypomethylated	0.0077708	0.60822	insignificant	20	120	120
chr12	81744848	81746848	Erh	-0.21512	3.83E-19	hypomethylated	0.013027	1	insignificant	10	66	66
chr12	81792587	81794587	3830431G21Rik	-0.14112	0.0000047	hypomethylated	-0.0016013	0.19394	insignificant	22	93	96
chr12	81861702	81863702	Gm1568	-0.22008	0.013463	hypomethylated	-0.074486	0.90416	insignificant	3	22	22
chr12	81890545	81892545	4933426M11Ril	-0.12246	1.39E-28	hypomethylated	-0.019845	0.0084365	hypomethylated	36	115	115
chr12	82025434	82027434	1700052I22Rik		1	noCoverage	-0.028194	0.022382	hypomethylated	0	26	26
chr12	82045490	82047490	Srsf5	-0.11418	2.03E-11	hypomethylated	-0.0088294	0.63586	insignificant	29	128	135
chr12	82126794	82128794	Smoc1	-0.13588	3.33E-16	hypomethylated	-0.0021353	0.0038665	hypomethylated	34	102	103
chr12	82434167	82436167	Slc8a3		1	noCoverage	0.028427	0.66528	insignificant	0	12	12

chr12	82633894	82635894	Synj2bp	-0.09246	0.3604	insignificant	-0.0080225	0.63658	insignificant	2	12	13
chr12	82695945	82697945	Med6	-0.10196	2.57E-09	hypomethylated	-0.0082582	0.71187	insignificant	12	33	33
chr12	82731355	82733355	Ttc9	-0.12418	2.45E-17	hypomethylated	-0.0054749	0.36676	insignificant	32	111	117
chr12	82882157	82884157	Map3k9	-0.11034	4.42E-15	hypomethylated	-0.015503	0.35798	insignificant	16	52	52
chr12	82960016	82962016	Pcnx	-0.098791	1.35E-09	hypomethylated	0.0081452	0.8781	insignificant	42	129	158
chr12	83270002	83272002	Sipa1l1	-0.11758	6.48E-36	hypomethylated	-0.017101	0.40265	insignificant	60	195	211
chr12	83717024	83719024	Rgs6	-0.17161	2.59E-15	hypomethylated	-0.019715	0.024711	hypomethylated	30	97	97
chr12	84828658	84830658	Dpf3	-0.20982	1	insignificant	-0.045411	0.38337	insignificant	1	16	15
chr12	84860415	84862415	Dcaf4	0.025734	0.0018067	inconclusive	0.010851	1	insignificant	5	40	41
chr12	84865998	84867998	Dcaf4		1	noCoverage	0.0059921	0.37017	insignificant	0	14	15
chr12	84938097	84940097	Zfyve1	-0.39583	5.71E-11	stronglyHypometh	-0.016286	0.92711	insignificant	3	6	6
chr12	84972183	84974183	Rbm25	-0.12659	2.61E-16	hypomethylated	0.012306	0.90703	insignificant	25	107	106
chr12	85028512	85030512	Psen1	-0.13915	1.26E-15	hypomethylated	-0.016074	0.15589	insignificant	27	66	66
chr12	85103590	85105590	Papln	-0.2691	0.00012738	hypomethylated	0.02366	1	insignificant	2	27	27
chr12	85290557	85292557	2410016O06Rik	-0.10582	2.76E-41	hypomethylated	-0.0068525	0.010209	hypomethylated	70	193	196
chr12	85327810	85329810	Acot2	-0.16932	0.03495	hypomethylated	-0.056659	0.34106	insignificant	4	58	56
chr12	85349451	85351451	Acot1	-0.14579	0.000053097	hypomethylated	-0.011392	0.6326	insignificant	10	81	77
chr12	85378328	85380328	Acot4	-0.075101	0.0010456	hypomethylated	0.09839	0.0026225	hypermethylated	4	44	33
chr12	85392100	85394100	Acot3		1	noCoverage	0.0036106	0.57125	insignificant	0	8	8
chr12	85409274	85411274	Acot5	-0.13012	0.1612	insignificant	0.066143	0.0099612	hypermethylated	5	74	76
chr12	85440603	85442603	Acot6	-0.14751	0.00000102	hypomethylated	0.012412	0.16813	insignificant	8	83	64
chr12	85454277	85456277	Dnalc1	-0.21235	0.34811	insignificant	-0.049614	0.67908	insignificant	2	18	18
chr12	85489439	85491439	C130039O16Rik	0.091199	1	insignificant	-0.17585	0.24583	insignificant	1	14	16
chr12	85534965	85536965	C130039O16Rik	-0.1509	0.065713	insignificant	-0.0043195	1	insignificant	11	22	22
chr12	85559831	85561831	C130039O16Rik	-0.079541	2.37E-14	hypomethylated	-0.0042587	0.38169	insignificant	15	48	48
chr12	85625263	85627263	Ptgr2	-0.099686	0.01291	hypomethylated	0.013567	1	insignificant	9	28	22
chr12	85657079	85659079	Zfp410	-0.14809	5.1E-10	hypomethylated	-0.0035564	0.0063409	inconclusive	26	121	119
chr12	85701917	85703917	Coq6	-0.19123	0.000011648	hypomethylated	0.009454	0.16954	insignificant	11	46	45
chr12	85702771	85704771	Fam161b	-0.19203	0.000033425	hypomethylated	0.021826	0.1905	insignificant	11	44	42
chr12	85749017	85751017	2900006K08Rik	-0.10749	0.001779	hypomethylated	-0.017253	0.068215	insignificant	14	74	111
chr12	85757149	85759149	Rnf113a2	-0.23917	9.38E-40	hypomethylated	0.024472	0.000079425	inconclusive	9	56	54
chr12	85791457	85793457	Lin52	-0.23598	1.12E-09	hypomethylated	-0.017428	0.89429	insignificant	15	64	62
chr12	85791900	85793900	Aldh6a1	-0.29176	0.002696	hypomethylated	0.02905	0.3384	insignificant	7	52	51
chr12	85909801	85911801	Vsx2	-0.16709	8.78E-15	hypomethylated	0.015694	0.74863	insignificant	23	104	101
chr12	85958416	85960416	Abcd4	-0.50397	3.05E-08	stronglyHypometh	-0.06849	0.76245	insignificant	3	15	15
chr12	85980968	85982968	Vrtn	-0.35265	0.00067722	stronglyHypometh	-0.023557	0.92182	insignificant	3	18	16
chr12	85982504	85984504	Vrtn	-0.027301	0.012534	hypomethylated	0.0098888	0.52263	insignificant	8	40	40
chr12	85982845	85984845	Vrtn	0.0066741	0.013684	inconclusive	0.040571	0.57125	insignificant	9	37	38
chr12	86039757	86041757	Tmem90a	-0.18183	0.060894	insignificant	-0.038797	1	insignificant	8	18	17
chr12	86113219	86115219	Isca2	-0.13026	0.000000407	hypomethylated	0.0066415	0.59469	insignificant	10	64	61
chr12	86114062	86116062	Npc2	-0.13931	0.060773	insignificant	-0.0063056	0.89335	insignificant	2	32	32
chr12	86215751	86217751	D030025P21Rik	-0.039006	6.84E-24	hypomethylated	-0.0091101	0.64673	insignificant	24	119	111
chr12	86217445	86219445	D030025P21Rik	-0.15473	1	insignificant	-0.0024251	0.60359	insignificant	2	27	26
chr12	86310879	86312879	Fcf1	-0.20068	7.61E-25	hypomethylated	0.01132	0.40515	insignificant	13	75	76
chr12	86311836	86313836	Fcf1	-0.28942	5.56E-34	hypomethylated	0.02084	0.0018125	inconclusive	10	44	43
chr12	86336270	86338270	Ylpm1	-0.094349	1.13E-15	hypomethylated	0.0010538	0.005304	inconclusive	35	122	122
chr12	86450782	86452782	Dlst	-0.17132	3.55E-53	hypomethylated	-0.013122	0.0094831	hypomethylated	46	115	115
chr12	86492214	86494214	Rps6kl1	-0.51735	4.62E-08	stronglyHypometh	-0.024754	0.74476	insignificant	2	10	10
chr12	86518235	86520235	Pgf	-0.17496	1.45E-11	hypomethylated	-0.0092232	0.15107	insignificant	8	33	32
chr12	86559462	86561462	Eif2b2	-0.13524	0.000000084	hypomethylated	-0.02231	0.00042505	hypomethylated	21	111	111
chr12	86621385	86623385	Acyp1	-0.12304	1	insignificant	0.038392	0.71543	insignificant	1	14	15
chr12	86628540	86630540	Fam164c	-0.12855	0.027571	hypomethylated	0.012353	0.0030954	inconclusive	6	31	30
chr12	86715667	86717667	Tmed10	-0.15245	0.16289	insignificant	0.0055689	0.63514	insignificant	2	15	12
chr12	86813850	86815850	Fos	-0.13929	3.99E-18	hypomethylated	-0.0058241	0.12188	insignificant	23	102	102
chr12	86939365	86941365	Jdp2	-0.11077	1.09E-18	hypomethylated	-0.012311	0.098603	insignificant	57	149	166
chr12	87026669	87028669	Batf	-0.14638	0.031181	hypomethylated	-0.039903	0.0038095	hypomethylated	6	24	24
chr12	87086488	87088488	Mfsd7c	-0.22574	0.000024972	hypomethylated	-0.028252	0.2729	insignificant	17	54	49
chr12	87164899	87166899	Ttll5	-0.17898	6.84E-26	hypomethylated	-0.016043	0.00011261	hypomethylated	21	70	72
chr12	87165495	87167495	0610007P14Rik	-0.16304	1.17E-13	hypomethylated	-0.018048	0.11818	insignificant	17	56	58
chr12	87419991	87421991	Tgfb3	-0.22139	0.000000288	hypomethylated	0.011749	0.00011943	inconclusive	6	24	24
chr12	87422512	87424512	1700019E19Rik	-0.31295	2.33E-11	hypomethylated	-0.021451	0.00024172	hypomethylated	17	66	65
chr12	87581827	87583827	1700020O03Rik	-0.12434	0.1486	insignificant	-0.0082049	0.69471	insignificant	6	47	47
chr12	87701066	87703066	Esrrb	-0.75266	0.007211	stronglyHypometh	-0.16526	0.025097	hypomethylated	2	14	9
chr12	87761593	87763593	Esrrb	-0.13566	3.79E-13	hypomethylated	-0.010597	0.050023	insignificant	23	87	97
chr12	88018649	88020649	Vash1	-0.13336	7.34E-21	hypomethylated	-0.0011964	0.22633	insignificant	36	93	93
chr12	88067410	88069410	Angel1		1	noCoverage	-0.0051948	0.8074	insignificant	0	12	12
chr12	88074318	88076318	Gm6772	0.1024	1	insignificant	-0.11486	0.01044	hypomethylated	4	14	14
chr12	88286992	88288992	2310044G17Rik	-0.1136	4.01E-17	hypomethylated	0.0029835	0.31677	insignificant	24	94	92
chr12	88287292	88289292	2310044G17Rik	-0.1136	4.01E-17	hypomethylated	0.0029835	0.31677	insignificant	24	94	92
chr12	88329626	88331626	Zdhhc22	-0.16983	7.58E-19	hypomethylated	0.01525	0.10688	insignificant	17	66	69
chr12	88366513	88368513	Tmem63c	-0.21543	5.58E-08	hypomethylated	0.033994	0.7373	insignificant	4	62	62
chr12	88487667	88489667	Gstz1	-0.21394	5.02E-27	hypomethylated	0.00056629	0.0021445	inconclusive	27	120	116
chr12	88488852	88490852	Gstz1	-0.2299	0.0032133	hypomethylated	0.012052	8.21E-08	inconclusive	9	51	51
chr12	88541179	88543179	Tmed8	-0.16079	4.31E-27	hypomethylated	0.012071	0.015961	inconclusive	24	60	62
chr12	88606677	88608677	Ahsa1	-0.16805	0.033184	hypomethylated	0.00929	0.8771	insignificant	2	42	43
chr12	88607236	88609236	Vipar	-0.16805	0.033184	hypomethylated	-0.010297	0.75609	insignificant	2	42	42
chr12	88729180	88731180	Sptlc2	-0.17835	2.14E-09	hypomethylated	-0.0090552	0.39026	insignificant	21	55	64
chr12	88778707	88780707	Alkbh1	-0.03696	0.29987	insignificant	-0.087546	0.0017491	hypomethylated	3	23	21
chr12	88783845	88785845	1810035L17Rik	-0.24904	0.000032231	hypomethylated	0.014767	0.72821	insignificant	8	34	32
chr12	88813249	88815249	Snw1	-0.55769	0.00024179	stronglyHypometh	0.020433	0.097003	insignificant	0	0	0
chr12	88958817	88960817	BB287469		1	noCoverage	-0.018076	0.79833	insignificant	0	4	8
chr12	88958858	88960858	Gm4027		1	noCoverage	-0.018076	0.79833	insignificant	0	4	8
chr12	89598244	89600244	Adck1	-0.13776	0.000062602	hypomethylated	-0.015027	0.15722	insignificant	12	52	53
chr12	89960319	89962319	Nrxn3	-0.346	0.165	insignificant	-0.026053	0.70954	insignificant	4	27	24
chr12	90190833	90192833	Nrxn3		1	noCoverage	0.21429	1	insignificant	0	4	4

chr12	91976878	91978878	Dio2	-0.047368	1	insignificant	-0.056796	0.87722	insignificant	1	5	4
chr12	92622849	92624849	4930534B04Rik	-0.29922	0.17775	insignificant	0.074817	0.79788	insignificant	2	8	10
chr12	92638432	92640432	Tshr	-0.15944	5.29E-08	hypomethylated	-0.0058134	0.56708	insignificant	7	18	18
chr12	92828089	92830089	Gtf2a1	-0.14749	1.96E-10	hypomethylated	-0.045694	0.031405	hypomethylated	29	105	112
chr12	92828927	92830927	Gtf2a1	-0.28639	0.20553	insignificant	0.017597	0.14763	insignificant	7	27	26
chr12	93087597	93089597	Sel1l	-0.1809	1	insignificant	0.0016351	0.79594	insignificant	3	12	12
chr12	96929435	96931435	Flrt2	-0.27041	1.39E-20	hypomethylated	-0.0094667	0.86955	insignificant	19	70	74
chr12	99497547	99499547	Galc	-0.16755	0.04357	hypomethylated	-0.077329	0.45914	insignificant	7	31	33
chr12	99816150	99818150	Kcnk10	0.00094785	0.10647	insignificant	-0.12565	0.9205	insignificant	5	20	22
chr12	99865393	99867393	Spata7	-0.29138	8.06E-18	hypomethylated	0.02369	0.33485	insignificant	5	24	24
chr12	99975615	99977615	Ptpn21	-0.07296	0.0096975	hypomethylated	-0.001301	0.14833	insignificant	22	51	52
chr12	99984177	99986177	Zc3h14	-0.090397	0.000020418	hypomethylated	-0.0011882	0.71984	insignificant	24	133	133
chr12	100139694	100141694	Eml5	-0.25	0.57419	insignificant	-0.11875	0.67113	insignificant	2	8	8
chr12	100157783	100159783	Ttc8	-0.29628	0.00000155	hypomethylated	-0.0086764	0.49983	insignificant	7	22	20
chr12	100157821	100159821	Ttc8	-0.29628	0.00000155	hypomethylated	-0.0086764	0.49983	insignificant	7	22	20
chr12	100688284	100690284	Foxn3	-0.32436	0.02081	hypomethylated	-0.049359	0.61815	insignificant	2	6	6
chr12	101121652	101123652	Tdp1	-0.10828	0.000000144	hypomethylated	0.018864	0.14149	insignificant	14	78	82
chr12	101121724	101123724	Tdp1	-0.12527	3.39E-08	hypomethylated	0.017831	0.082737	insignificant	14	80	85
chr12	101201708	101203708	Kcnk13	-0.19917	0.000059287	hypomethylated	-0.011497	0.59832	insignificant	8	57	57
chr12	101349540	101351540	Psmc1	-0.66581	0.012359	stronglyHypometh	0.0097165	0.61121	insignificant	3	14	14
chr12	101436750	101438750	Calm1	-0.11566	9.52E-31	hypomethylated	-0.014788	0.6705	insignificant	43	142	153
chr12	101759032	101761032	Ttc7b	-0.13539	0.000000519	hypomethylated	0.027408	0.76093	insignificant	14	30	34
chr12	101963238	101965238	Rps6ka5		1	noCoverage	-0.0011515	0.12418	insignificant	0	33	32
chr12	102016332	102018332	9030617O03Rik	-0.40873	0.29406	insignificant	-0.051901	0.77254	insignificant	1	6	6
chr12	102146408	102148408	Gpr68	-0.15589	0.000016931	hypomethylated	-0.054911	0.37312	insignificant	5	18	20
chr12	102260003	102262003	Ccdc88c	-0.46826	0.11542	insignificant	-0.10646	0.018779	hypomethylated	1	9	17
chr12	102267193	102269193	Ccdc88c	-0.12261	0.000053531	hypomethylated	-0.022804	0.45658	insignificant	15	47	60
chr12	102321912	102323912	D130020L05Rik	-0.26369	0.000000151	hypomethylated	-0.0098314	0.50746	insignificant	7	50	53
chr12	102949783	102951783	Tc2n	-0.38242	0.043179	stronglyHypometh	-0.056788	0.0050495	hypomethylated	2	18	24
chr12	102956712	102958712	Tc2n	-0.13459	0.00034026	hypomethylated	-0.0081736	0.75345	insignificant	6	24	24
chr12	103151381	103153381	Trip11	-0.12391	0.07236	insignificant	-0.024789	1	insignificant	3	20	20
chr12	103196453	103198453	Atxn3	-0.058907	0.00000557	hypomethylated	-0.0046454	0.45628	insignificant	10	20	20
chr12	103213183	103215183	Cpsf2	-0.10545	3.13E-12	hypomethylated	0.0073759	0.30917	insignificant	28	123	116
chr12	103366628	103368628	Slc24a4	-0.11278	1.34E-17	hypomethylated	0.0080609	0.31316	insignificant	35	130	133
chr12	103520283	103522283	Rin3	-0.11256	5.09E-15	hypomethylated	-0.0076028	0.7936	insignificant	26	106	106
chr12	103520850	103522850	Rin3	-0.1312	1.69E-16	hypomethylated	0.0017646	0.69169	insignificant	26	110	110
chr12	103677907	103679907	Lgmn	-0.16948	0.00000022	hypomethylated	-0.055717	1.34E-10	hypomethylated	6	26	26
chr12	103706343	103708343	Golga5	-0.11379	3.29E-35	hypomethylated	0.0059806	0.10545	insignificant	47	137	136
chr12	103707119	103709119	Golga5	-0.11473	3.28E-35	hypomethylated	-0.0078824	0.091157	insignificant	46	134	132
chr12	103792178	103794178	Chga	-0.11354	0.00000232	hypomethylated	-0.0064991	0.16855	insignificant	12	56	56
chr12	103923230	103925230	Itpk1	-0.024981	0.0099288	hypomethylated	0.19177	0.50467	insignificant	2	8	13
chr12	103943079	103945079	Itpk1	-0.13117	1.59E-12	hypomethylated	-0.019914	0.0036676	hypomethylated	30	86	100
chr12	103980969	103982969	D230037D09Rik	-0.14064	2.33E-22	hypomethylated	-0.010106	0.03914	hypomethylated	32	89	96
chr12	103981870	103983870	AK010878	-0.18971	4.14E-22	hypomethylated	-0.052842	0.78592	insignificant	15	32	39
chr12	103995184	103997184	Ubr7	-0.13157	1.03E-24	hypomethylated	-0.0080908	0.34484	insignificant	34	120	119
chr12	103996020	103998020	AK010878	-0.11488	1.59E-21	hypomethylated	0.0039674	0.72961	insignificant	21	80	79
chr12	104116616	104118616	Btbd7	-0.021219	1.47E-12	hypomethylated	-0.001937	0.00012522	hypomethylated	22	72	72
chr12	104136515	104138515	Cox8c	0.12175	1	insignificant	0.05111	0.19542	insignificant	1	15	14
chr12	104186068	104188068	Unc79	-0.21348	3.13E-12	hypomethylated	-0.052312	0.021301	hypomethylated	18	46	46
chr12	104480356	104482356	Prima1	-0.11838	0.0027354	hypomethylated	0.052919	0.6595	insignificant	22	86	88
chr12	104552168	104554168	Fam181a	-0.05204	0.046279	hypomethylated	-0.010592	0.000042797	hypomethylated	15	56	56
chr12	104614103	104616103	Otub2		1	noCoverage	-0.0030904	0.20552	insignificant	0	6	6
chr12	104625890	104627890	Otub2	-0.10856	2.04E-15	hypomethylated	0.012443	0.84159	insignificant	18	76	76
chr12	104625891	104627891	Otub2	-0.10856	2.04E-15	hypomethylated	0.012443	0.84159	insignificant	18	76	76
chr12	104663990	104665990	Ddx24	-0.1132	0.00036509	hypomethylated	-0.012232	0.35031	insignificant	9	30	35
chr12	104664077	104666077	Ddx24	-0.096154	1	insignificant	0.0022258	1	insignificant	6	24	24
chr12	104681890	104683890	Ifi27l2a	0.25694	1	lowCoverage	0.03588	0.67716	insignificant	1	6	6
chr12	104769774	104771774	Ppp4r4	-0.10789	4.6E-25	hypomethylated	0.010408	0.19829	insignificant	38	155	155
chr12	104976399	104978399	Serpina1b	0.1101	1	lowCoverage	-0.0095571	0.13663	insignificant	1	10	13
chr12	105101829	105103829	Serpina1a	0.16974	1	insignificant	0.012019	1	insignificant	3	10	13
chr12	105143160	105145160	Serpina1c	0.028571	1	insignificant	-0.24143	0.022546	hypomethylated	2	7	10
chr12	105195107	105197107	Serpina1e	-0.33772	0.014247	stronglyHypometh	-0.037549	0.26206	insignificant	1	11	11
chr12	105392082	105394082	Serpina3c	0.064816	1	insignificant	-0.09332	0.37254	insignificant	2	21	26
chr12	105485105	105487105	Serpina3h	-0.17278	0.12289	insignificant	-0.076331	0.35357	insignificant	2	10	10
chr12	105643917	105645917	Serpina3n	0.23438	1	lowCoverage	-0.12396	0.23918	insignificant	1	4	4
chr12	105711446	105713446	Gsc	-0.11222	0.00077941	hypomethylated	-0.0010503	0.27415	insignificant	6	43	50
chr12	105990162	105992162	Dicer1	-0.14054	5.62E-10	hypomethylated	-0.0055417	0.63899	insignificant	24	102	99
chr12	106103286	106105286	Clmn	-0.13704	0.17131	insignificant	0.0075544	1	insignificant	10	22	22
chr12	106236887	106238887	4831426I19Rik	-0.31788	0.00016615	hypomethylated	0.12656	0.79731	insignificant	3	8	6
chr12	106248019	106250019	4831426I19Rik	-0.16089	4.32E-10	hypomethylated	-0.062782	0.51714	insignificant	15	30	43
chr12	106269559	106271559	Snhg10	-0.13505	5.76E-34	hypomethylated	-0.0082052	0.01044	hypomethylated	39	168	152
chr12	106269898	106271898	Glrx5	-0.1303	2.18E-28	hypomethylated	-0.006815	0.43642	insignificant	35	152	135
chr12	106270489	106272489	Snhg10	-0.13277	6.03E-15	hypomethylated	-0.011755	0.82335	insignificant	30	124	106
chr12	106384242	106386242	Tcl1b2	-0.47645	0.000026261	stronglyHypometh	0.040217	0.33208	insignificant	1	4	4
chr12	106460947	106462947	Tcl1	0.16491	1	insignificant	-0.053718	0.52838	insignificant	2	10	10
chr12	106691185	106693185	D430019H16Rik	-0.25161	2.82E-10	hypomethylated	-0.061901	0.0000052	hypomethylated	13	48	44
chr12	106800381	106802381	Bdkrb2	-0.25036	0.00000382	hypomethylated	0.010278	0.31863	insignificant	7	22	21
chr12	106841300	106843300	Bdkrb1	-0.10422	0.34217	insignificant	-0.013211	1	insignificant	1	12	9
chr12	106922561	106924561	4933433P14Rik	-0.1592	9.71E-49	hypomethylated	-0.0070015	0.0012105	hypomethylated	56	143	143
chr12	106923451	106925451	Atg2b	-0.21962	1.85E-16	hypomethylated	-0.022627	0.0027345	hypomethylated	26	60	60
chr12	106943191	106945191	Ak7	0.041667	1	lowCoverage	0.018939	1	insignificant	1	2	4
chr12	107021911	107023911	Papola	-0.16716	9.7E-21	hypomethylated	-0.00098981	0.049094	hypomethylated	33	94	83
chr12	107247472	107249472	Vrk1	-0.20377	2.63E-15	hypomethylated	-0.05896	0.3631	insignificant	15	44	56
chr12	107685876	107687876	1700121N20Rik	-0.11954	0.41473	insignificant	0.029013	0.23922	insignificant	6	21	24
chr12	107954534	107956534	4933406K04Rik		1	noCoverage	-0.0063417	0.86688	insignificant	0	18	18

chr12	109241624	109243624	Bcl11b	-0.15263	1.91E-18	hypomethylated	0.014227	0.24997	insignificant	40	102	123
chr12	109416947	109418947	Ccnk	-0.087259	7.9E-27	hypomethylated	-0.0049171	0.12218	insignificant	60	206	212
chr12	109417494	109419494	Setd3	-0.062827	0.00019419	hypomethylated	-0.00022623	0.64511	insignificant	8	80	86
chr12	109513627	109515627	Ccdc85c	-0.16316	1.5E-13	hypomethylated	0.0081859	0.55227	insignificant	33	97	97
chr12	109543479	109545479	Hhipl1	-0.14999	1.23E-19	hypomethylated	-0.0062224	1	insignificant	28	81	81
chr12	109571590	109573590	Cyp46a1	-0.12362	1.93E-26	hypomethylated	-0.013755	0.055346	insignificant	30	122	122
chr12	109647864	109649864	Eml1	-0.20571	0.050281	insignificant	-0.034116	0.73722	insignificant	4	10	10
chr12	109660025	109662025	Eml1	-0.12941	1.79E-15	hypomethylated	0.00662	0.0019743	inconclusive	47	145	133
chr12	109791929	109793929	Evl	-0.11274	4.43E-46	hypomethylated	0.059833	0.0094322	inconclusive	69	164	185
chr12	109842975	109844975	Evl	-0.26139	0.63949	insignificant	-0.027949	1	insignificant	3	6	6
chr12	109940516	109942516	Degs2	-0.22799	0.02047	insignificant	-0.015223	0.86624	insignificant	4	33	39
chr12	110030520	110032520	Yy1	-0.068186	1.81E-29	hypomethylated	-0.0020217	0.040347	hypomethylated	88	366	376
chr12	110074086	110076086	Slc25a29	-0.020787	0.15085	insignificant	-0.025404	0.68895	insignificant	18	82	81
chr12	110074182	110076182	Mir345	0.019294	0.87678	insignificant	-0.030806	0.083836	insignificant	13	64	63
chr12	110088338	110090338	Slc25a47		1	noCoverage	0.11235	0.12586	insignificant	0	13	14
chr12	110131408	110133408	Wars	-0.15545	8.72E-12	hypomethylated	-0.0024705	0.31585	insignificant	16	38	39
chr12	110131421	110133421	Wars	-0.15545	8.72E-12	hypomethylated	-0.0024705	0.31585	insignificant	16	38	39
chr12	110131481	110133481	Wdr25	-0.15545	8.72E-12	hypomethylated	-0.0024705	0.31585	insignificant	16	38	39
chr12	110132384	110134384	Wars	-0.088474	0.000017779	hypomethylated	0.024036	0.86349	insignificant	10	26	28
chr12	110306427	110308427	Begain	-0.16156	1.19E-09	hypomethylated	-0.035764	0.48882	insignificant	10	30	36
chr12	110690032	110692032	Dlk1	-0.084207	1.82E-13	hypomethylated	0.060519	0.07705	insignificant	25	64	68
chr12	110690664	110692664	Dlk1	-0.098516	3.44E-14	hypomethylated	0.074029	0.1237	insignificant	27	68	74
chr12	110778205	110780205	Meg3	0.053747	0.54988	insignificant	-0.0051231	0.76616	insignificant	3	44	35
chr12	110781750	110783750	Mir1906-2	0.21659	1	lowCoverage	0.14803	0.45214	insignificant	1	11	14
chr12	110782607	110784607	Meg3	-0.25098	0.071863	insignificant	0.080708	0.56282	insignificant	3	19	22
chr12	110822998	110824998	Mir337	-0.28811	0.0033443	hypomethylated	0.0093541	0.72179	insignificant	3	26	27
chr12	110823289	110825289	Mir540	-0.28811	0.0033443	hypomethylated	0.0093541	0.72179	insignificant	3	26	27
chr12	110823523	110825523	Mir665	-0.28811	0.0033443	hypomethylated	0.0093541	0.72179	insignificant	3	26	27
chr12	110827656	110829656	Mir431	-0.13045	0.53754	insignificant	-0.014882	0.51855	insignificant	5	43	46
chr12	110828746	110830746	6430411K18Rik	-0.086759	1	insignificant	-0.0027394	0.51874	insignificant	8	38	38
chr12	110828924	110830924	Mir433	-0.1009	0.64485	insignificant	-0.018225	0.0086605	hypomethylated	8	38	39
chr12	110830055	110832055	Mir127	0.039389	1	insignificant	0.025188	0.76444	insignificant	7	36	37
chr12	110831715	110833715	Mir434	0.037844	0.4521	insignificant	0.034542	0.032449	hypermethylated	6	39	37
chr12	110832165	110834165	Mir432	0.056295	0.44767	insignificant	0.037563	0.021297	hypermethylated	3	33	31
chr12	110832536	110834536	Mir136	0.049272	0.44773	insignificant	0.03054	0.026547	hypermethylated	3	28	31
chr12	110919406	110921406	Mir882	-0.29096	0.000095574	hypomethylated	-0.018162	0.44159	insignificant	2	4	4
chr12	110949012	110951012	Mir380	-0.21565	0.41616	insignificant	-0.041085	0.015514	hypomethylated	9	27	32
chr12	110949526	110951526	Mir1197	-0.21565	0.41616	insignificant	-0.041085	0.015514	hypomethylated	9	27	32
chr12	110949717	110951717	Mir323	-0.22341	0.41421	insignificant	-0.048843	0.011782	hypomethylated	9	30	32
chr12	110950019	110952019	Mir758	-0.22341	0.41421	insignificant	-0.048843	0.011782	hypomethylated	9	30	32
chr12	110950690	110952690	Mir329	-0.02255	0.57627	insignificant	-0.092191	0.060655	insignificant	6	26	33
chr12	110955963	110957963	Mir495	0.31	1	insignificant	0.17333	0.15859	insignificant	2	10	10
chr12	110961522	110963522	Mir300	-0.0144	0.79822	insignificant	0.033111	0.88778	insignificant	5	20	20
chr12	110965338	110967338	Mir539	-0.27083	0.28384	insignificant	-0.052775	0.75793	insignificant	1	4	4
chr12	110971348	110973348	Mir134	-0.04881	0.33395	insignificant	-0.012453	0.69637	insignificant	3	8	8
chr12	110971941	110973941	Mir668	-0.04881	0.33395	insignificant	-0.012453	0.69637	insignificant	3	8	8
chr12	110972111	110974111	Mir485	-0.04881	0.33395	insignificant	-0.012453	0.69637	insignificant	3	8	8
chr12	110972190	110974190	Mirg	-0.04881	0.33395	insignificant	-0.012453	0.69637	insignificant	3	8	8
chr12	110972828	110974828	Mir453	-0.04881	0.33395	insignificant	-0.012453	0.69637	insignificant	3	8	8
chr12	110979618	110981618	Mir541		1	noCoverage	-0.09375	0.52677	insignificant	0	2	2
chr12	110980367	110982367	Mir409	-0.25	0.29056	insignificant	-0.057292	0.59511	insignificant	1	4	4
chr12	110980498	110982498	Mir412	-0.25	0.29056	insignificant	-0.057292	0.59511	insignificant	1	4	4
chr12	110980627	110982627	Mir369	-0.25	0.29056	insignificant	-0.057292	0.59511	insignificant	1	4	4
chr12	110980924	110982924	Mir410	-0.25	0.29056	insignificant	-0.057292	0.59511	insignificant	1	4	4
chr12	110985087	110987087	Mir3072	-0.23333	0.12488	insignificant	-0.11491	0.0025454	hypomethylated	2	4	4
chr12	111516278	111518278	Dio3os	-0.12914	1.19E-31	hypomethylated	0.01337	0.27776	insignificant	65	221	228
chr12	111516339	111518339	Dio3os	-0.12565	5.8E-30	hypomethylated	0.013696	0.53698	insignificant	65	221	228
chr12	111516439	111518439	Dio3	-0.12633	4.14E-25	hypomethylated	0.0098248	0.40998	insignificant	55	199	206
chr12	111684388	111686388	Ppp2r5c	-0.10184	2.52E-16	hypomethylated	-0.00079841	0.51633	insignificant	32	172	177
chr12	111722948	111724948	Ppp2r5c	-0.21154	0.000043516	hypomethylated	-0.021358	0.030043	inconclusive	5	33	35
chr12	111828583	111830583	B930059L03Rik	-0.13921	0.00000463	hypomethylated	-0.014679	0.42966	insignificant	18	89	89
chr12	111838604	111840604	Dync1h1	-0.13103	1.77E-27	hypomethylated	-0.013084	0.20124	insignificant	38	116	116
chr12	111920829	111922829	1700001K19Rik	-0.53171	0.0004715	stronglyHypometh	-0.080716	0.89338	insignificant	2	6	6
chr12	111934605	111936605	Hsp90aa1	-0.1249	0.0069492	hypomethylated	-0.027586	0.42411	insignificant	6	70	67
chr12	111975158	111977158	Wdr20a	-0.1368	1.19E-15	hypomethylated	-0.011202	0.94251	insignificant	26	122	139
chr12	112079149	112081149	Stk30	-0.325	0.17019	insignificant	-0.025735	0.5098	insignificant	2	4	4
chr12	112087488	112089488	Zfp839	-0.13297	7.41E-24	hypomethylated	0.0018802	0.077121	insignificant	33	92	92
chr12	112126473	112128473	Tecpr2	0.016396	0.020231	inconclusive	0.015084	0.85224	insignificant	20	80	85
chr12	112127324	112129324	Cinp	0.035906	0.050677	insignificant	0.0037834	0.80583	insignificant	17	66	67
chr12	112217231	112219231	Ankrd9	-0.13763	1.7E-24	hypomethylated	-0.013811	0.43408	insignificant	18	53	53
chr12	112277008	112279008	Rcor1	-0.081546	1.21E-25	hypomethylated	-0.0041925	0.16877	insignificant	56	227	234
chr12	112403758	112405758	Traf3	-0.12426	8.63E-34	hypomethylated	-0.021101	0.0034171	hypomethylated	45	152	162
chr12	112508321	112510321	Amn	-0.14916	0.00050583	hypomethylated	-0.0020541	0.013584	inconclusive	15	68	72
chr12	112615929	112617929	Cdc42bpb	-0.18589	3.56E-09	hypomethylated	0.023928	0.57495	insignificant	14	65	65
chr12	112679871	112681871	Tnfaip2	-0.13296	0.000022195	hypomethylated	-0.009577	0.030491	hypomethylated	11	57	57
chr12	112724034	112726034	Gm266	-0.13812	7.07E-09	hypomethylated	-0.019221	0.36177	insignificant	17	51	51
chr12	112775311	112777311	Eif5	-0.09423	7.33E-22	hypomethylated	-0.0051156	0.19349	insignificant	50	222	223
chr12	112811720	112813720	Mark3	-0.14662	9.59E-13	hypomethylated	-0.011858	0.33889	insignificant	30	100	98
chr12	112910549	112912549	Ckb	-0.37742	0.042423	stronglyHypometh	-0.033733	0.10259	insignificant	4	32	35
chr12	112915315	112917315	Trmt61a	-0.13406	0.000000726	hypomethylated	-0.0058065	0.53488	insignificant	9	51	51
chr12	112950479	112952479	2810002N01Rik	-0.089942	3.71E-25	hypomethylated	0.0018922	0.81392	insignificant	65	202	204
chr12	112951467	112953467	Bag5	-0.10775	5E-11	hypomethylated	0.0045105	0.42428	insignificant	28	72	73
chr12	112996059	112998059	Klc1	-0.069929	3.25E-19	hypomethylated	0.0071315	0.63879	insignificant	36	151	144
chr12	113051480	113053480	Zfyve21	-0.10788	2.88E-33	hypomethylated	-0.013342	0.50297	insignificant	27	112	122
chr12	113052052	113054052	Zfyve21	-0.09963	8.06E-27	hypomethylated	-0.0043706	0.42541	insignificant	19	84	84

chr12	113146266	113148266	Ppp1r13b	-0.082818	0.000088407	hypomethylated	-0.019273	0.033372	hypomethylated	15	57	57
chr12	113205188	113207188	2010107E04Rik	-0.23689	0.21198	insignificant	-0.043399	0.93685	insignificant	4	15	17
chr12	113208769	113210769	Tdrd9	-0.25631	0.1657	insignificant	-0.005939	0.47976	insignificant	6	24	22
chr12	113218962	113220962	Tdrd9		1	noCoverage	-0.020455	0.8512	insignificant	0	10	10
chr12	113368090	113370090	Mir203	-0.15012	6.55E-26	hypomethylated	0.021487	0.010679	inconclusive	34	99	86
chr12	113383418	113385418	Kif26a	-0.15577	9.79E-22	hypomethylated	-0.026972	0.25054	insignificant	39	116	120
chr12	113825994	113827994	Inf2	-0.14223	1.43E-16	hypomethylated	0.0070555	0.73437	insignificant	28	97	109
chr12	113857257	113859257	Adssl1	-0.16214	0.040402	hypomethylated	-0.021111	1	insignificant	6	68	71
chr12	113882038	113884038	Siva1	-0.10136	1.06E-08	hypomethylated	0.024117	0.31939	insignificant	14	63	56
chr12	113912487	113914487	Akt1	-0.14682	1.42E-08	hypomethylated	-0.0040404	0.029203	hypomethylated	18	58	60
chr12	113916050	113918050	Zbtb42	-0.11492	1.34E-24	hypomethylated	-0.0041776	0.66411	insignificant	51	133	135
chr12	113959384	113961384	AW555464	-0.10283	7.86E-36	hypomethylated	0.0082484	0.000058059	inconclusive	55	143	137
chr12	114046185	114048185	BC022687	-0.14569	3.09E-21	hypomethylated	-0.0040206	0.094702	insignificant	26	109	110
chr12	114067600	114069600	Cdca4	-0.086711	4.19E-09	hypomethylated	0.0012417	0.75825	insignificant	22	95	100
chr12	114099127	114101127	Gpr132	0.10113	0.33149	insignificant	-0.047727	0.57888	insignificant	3	14	14
chr12	114167706	114169706	Jag2	-0.12096	9.49E-14	hypomethylated	-0.02992	1.6E-30	hypomethylated	47	136	134
chr12	114180329	114182329	Nudt14	-0.20534	0.0018001	hypomethylated	-0.034403	0.82434	insignificant	6	29	26
chr12	114213692	114215692	Btbd6	-0.082903	0.10428	insignificant	-0.012317	0.61051	insignificant	6	71	71
chr12	114213773	114215773	Btbd6	-0.13182	0.10405	insignificant	-0.01166	0.67952	insignificant	6	75	75
chr12	114238832	114240832	Brf1	-0.075726	0.62937	insignificant	0.0024044	0.5673	insignificant	13	50	50
chr12	114251718	114253718	Pacs2	-0.10184	2.6E-18	hypomethylated	-0.0010071	0.15807	insignificant	34	130	128
chr12	114335488	114337488	Mta1	-0.12274	1.5E-15	hypomethylated	-0.011926	0.0020101	hypomethylated	49	185	199
chr12	114377446	114379446	Crip2	-0.1303	6.41E-12	hypomethylated	-0.015697	0.97569	insignificant	34	159	159
chr12	114389222	114391222	Crip1	-0.38901	1.67E-24	stronglyHypometh	-0.035518	0.40333	insignificant	11	44	43
chr12	114393631	114395631	4930427A07Rik	-0.19578	8.13E-30	hypomethylated	-0.013938	0.12626	insignificant	31	120	121
chr12	114423113	114425113	Tmem121	-0.097958	0.00018191	hypomethylated	0.040804	0.55611	insignificant	10	20	22
chr12	117284934	117286934	Zfp386	-0.059469	0.00023712	hypomethylated	-0.0041324	0.77267	insignificant	8	48	48
chr12	117315195	117317195	Vipr2	-0.15495	0.000000762	hypomethylated	-0.025534	0.0072646	hypomethylated	18	46	46
chr12	117501498	117503498	Wdr60		1	noCoverage	-0.17392	0.071488	insignificant	0	2	4
chr12	117518694	117520694	Esyt2	-0.11675	7.61E-36	hypomethylated	-0.0090786	0.5199	insignificant	26	97	97
chr12	117642874	117644874	Ncapg2	0.075608	1.07E-21	hypermethylated	-0.038614	0.35757	insignificant	19	117	118
chr12	117643634	117645634	D430020J02Rik	-0.10078	5.58E-08	hypomethylated	-0.011118	1	insignificant	13	102	106
chr12	117723192	117725192	Ptprn2	-0.11437	3.65E-17	hypomethylated	0.022967	0.0016238	inconclusive	35	145	145
chr12	118389742	118391742	Gm10421	-0.31716	0.00000328	hypomethylated	-0.054164	0.43101	insignificant	10	29	46
chr12	118488289	118490289	Mir153	0.020698	0.79535	insignificant	0.0094093	0.1609	insignificant	3	8	7
chr12	118753951	118755951	Rapgef5		1	noCoverage	-0.10666	0.55327	insignificant	0	7	6
chr12	119081333	119083333	Cdca7l	-0.10734	3.67E-12	hypomethylated	-0.0051964	0.73088	insignificant	34	103	103
chr12	119539913	119541913	Sp4	-0.096846	1.22E-29	hypomethylated	0.0035955	0.026038	inconclusive	41	174	168
chr12	120083801	120085801	Sp8	-0.14728	1.29E-16	hypomethylated	-0.00015401	0.74143	insignificant	14	32	32
chr12	120476749	120478749	Itgb8	0.047778	1	insignificant	-0.15241	0.4939	insignificant	4	18	17
chr13	3476548	3478548	2810429I04Rik	-0.2011	9.1E-23	hypomethylated	-0.07578	0.009035	hypomethylated	14	55	59
chr13	3536320	3538320	Gdi2	-0.14665	3.33E-22	hypomethylated	0.0033834	0.30495	insignificant	42	128	127
chr13	3610354	3612354	BC016423	-0.16667	1	insignificant	0	1	insignificant	2	6	6
chr13	3632321	3634321	Asb13	-0.14487	1.79E-15	hypomethylated	-0.0069783	0.00000962	hypomethylated	25	69	69
chr13	3632323	3634323	Asb13	-0.14487	1.79E-15	hypomethylated	-0.0069783	0.00000962	hypomethylated	25	69	69
chr13	3803564	3805564	Calml3	-0.33135	0.062048	insignificant	0.055556	0.53473	insignificant	1	6	6
chr13	3892824	3894824	Net1	-0.11388	0.00000997	hypomethylated	-0.0035557	1	insignificant	6	20	20
chr13	5859734	5861734	Klf6	-0.16339	1.11E-10	hypomethylated	-0.0086124	0.58986	insignificant	13	64	70
chr13	6546402	6548402	Pitrm1	-0.19015	0.0032979	hypomethylated	0.0020004	0.64087	insignificant	13	26	26
chr13	6647970	6649970	Pfkp	-0.25805	1	lowCoverage	0.066914	0.42197	insignificant	1	18	16
chr13	8870288	8872288	Wdr37	-0.17564	0.0050579	hypomethylated	-0.0021311	0.40971	insignificant	9	28	28
chr13	8995258	8997258	Gtpbp4	-0.21923	1.46E-13	hypomethylated	-0.064271	0.56319	insignificant	10	56	52
chr13	9092150	9094150	Larp4b	-0.14674	2.2E-28	hypomethylated	-0.016003	0.029155	hypomethylated	62	182	182
chr13	9274771	9276771	Dip2c	-0.1015	8.96E-08	hypomethylated	-0.0047507	0.88893	insignificant	29	129	141
chr13	12199212	12201212	Ryr2	0.069692	0.34455	insignificant	-0.000086264	0.20457	insignificant	2	21	22
chr13	12350267	12352267	Mtr	-0.11176	0.000014731	hypomethylated	0.021496	0.378	insignificant	11	49	46
chr13	12432999	12434999	Actn2	-0.111	0.15802	insignificant	-0.017379	0.52485	insignificant	4	20	22
chr13	12486641	12488641	Heatr1	-0.1411	0.000000801	hypomethylated	0.017339	0.19681	insignificant	12	54	55
chr13	12554005	12556005	Lgals8	-0.11325	0.36303	insignificant	-0.18468	0.68966	insignificant	1	3	9
chr13	12657149	12659149	Ero1lb	-0.079777	0.0057429	hypomethylated	-0.0048829	0.81311	insignificant	34	92	93
chr13	12742662	12744662	Gpr137b-ps	-0.081901	0.056091	insignificant	-0.0033199	1	insignificant	5	16	16
chr13	13485891	13487891	Gpr137b	-0.076587	0.12276	insignificant	0.010115	0.86251	insignificant	5	12	12
chr13	13528868	13530868	Nid1		1	noCoverage	0.0025024	0.0067251	inconclusive	0	20	20
chr13	13681675	13683675	Lyst	-0.16739	1.07E-15	hypomethylated	-0.036516	0.063863	insignificant	29	111	112
chr13	13875805	13877805	Gng4	-0.1339	7.45E-19	hypomethylated	-0.0058523	0.33742	insignificant	28	79	70
chr13	14045940	14047940	B3galnt2	-0.095917	0.000000578	hypomethylated	-0.0069872	0.78172	insignificant	27	158	168
chr13	14131867	14133867	Tbce	-0.21583	4.48E-08	hypomethylated	-0.01184	0.0053031	hypomethylated	10	34	34
chr13	14155058	14157058	Arid4b	-0.11817	6.46E-56	hypomethylated	-0.00017597	0.87764	insignificant	73	228	238
chr13	14155668	14157668	Arid4b	-0.11128	3.27E-37	hypomethylated	0.0074733	0.92872	insignificant	57	196	204
chr13	14704508	14706508	Psma2	-0.16566	0.022218	hypomethylated	-0.0011532	0.77618	insignificant	5	34	34
chr13	14705304	14707304	Psma2	-0.24904	1	insignificant	0.00052757	0.4432	insignificant	1	25	25
chr13	14721511	14723511	AW209491	-0.21527	1.17E-13	hypomethylated	-0.0066389	0.91512	insignificant	12	42	42
chr13	14721524	14723524	AW209491	-0.21527	1.17E-13	hypomethylated	-0.0066389	0.91512	insignificant	12	42	42
chr13	15554555	15556555	Gli3	-0.095911	4.27E-33	hypomethylated	-0.0041397	0.32589	insignificant	70	238	224
chr13	17786246	17788246	2810021B07Rik	-0.12656	0.000090807	hypomethylated	0.0059418	0.94272	insignificant	20	120	122
chr13	17786599	17788599	2810021B07Rik	-0.12117	0.00012579	hypomethylated	0.004917	0.97111	insignificant	17	112	114
chr13	17896931	17898931	Cdk13	-0.14698	4.04E-09	hypomethylated	-0.0087183	0.06583	insignificant	13	44	44
chr13	18036051	18038051	Rala	-0.14588	0.000007061	hypomethylated	0.043826	0.68511	insignificant	19	41	41
chr13	18808160	18810160	Vps41	-0.078096	0.025815	hypomethylated	-0.011763	0.27854	insignificant	15	41	40
chr13	19039239	19041239	Amph	-0.10733	6.01E-26	hypomethylated	0.012082	0.50955	insignificant	27	120	120
chr13	19487621	19489621	Stard3nl		1	noCoverage	-0.021154	1	insignificant	0	4	4
chr13	19714043	19716043	Sfrp4	-0.2301	1.9E-11	hypomethylated	0.0017013	0.22971	insignificant	5	45	42
chr13	20563961	20565961	Elmo1	-0.54167	0.054375	insignificant	-0.090212	0.79574	insignificant	2	15	9
chr13	20884987	20886987	Aoah		1	noCoverage	-0.011111	1	insignificant	0	3	6
chr13	21270827	21272827	Trim27	-0.13124	1.71E-43	hypomethylated	-0.0046276	0.10956	insignificant	66	175	169

chr13	21384555	21386555	Gpx5		1	noCoverage	-0.041667	0.75653	insignificant	0	4	4
chr13	21453688	21455688	Zscan12	-0.27736	6.62E-15	hypomethylated	-0.048421	0.0040272	hypomethylated	15	35	35
chr13	21532579	21534579	Pgbd1	-0.44986	2.09E-20	stronglyHypometh	-0.023276	0.0011986	hypomethylated	7	28	28
chr13	21532922	21534922	Pgbd1	-0.50831	1.06E-19	stronglyHypometh	0.010244	0.000089208	inconclusive	4	23	30
chr13	21536028	21538028	Mir1896		1	noCoverage	0.15347	0.048725	hypermethylated	0	8	8
chr13	21545596	21547596	Zfp187	-0.10781	0.02363	hypomethylated	0.073378	0.24187	insignificant	2	8	4
chr13	21560370	21562370	Nkapl	-0.024582	0.18422	insignificant	0.12386	0.69096	insignificant	2	22	17
chr13	21569775	21571775	Zkscan4	-0.19884	0.49074	insignificant	-0.1485	0.093733	insignificant	5	40	32
chr13	21598393	21600393	Zfp389		1	noCoverage	0.01	1	insignificant	0	10	10
chr13	21825964	21827964	Hist1h4j	-0.1444	2.3E-09	hypomethylated	0.0049164	0.10624	insignificant	5	40	36
chr13	21872423	21874423	Hist1h3i		1	noCoverage	0.058527	1	insignificant	0	12	12
chr13	21988918	21990918	Mir1983	0.27671	0.57419	insignificant	-0.14155	0.0083439	hypomethylated	1	12	12
chr13	22035962	22037962	Zfp184	-0.15277	0.000013955	hypomethylated	0.012003	0.24183	insignificant	7	43	44
chr13	22072049	22074049	Pom121l2	-0.18122	1.58E-10	hypomethylated	0.019396	0.1082	insignificant	29	102	107
chr13	22072062	22074062	Pom121l2	-0.18122	1.58E-10	hypomethylated	0.019396	0.1082	insignificant	29	102	107
chr13	22126755	22128755	Hist1h2bk		1	noCoverage	-0.022172	0.02451	hypomethylated	0	14	14
chr13	22127421	22129421	Hist1h2ah		1	noCoverage	-0.022172	0.02451	hypomethylated	0	14	14
chr13	22133221	22135221	Hist1h2ag		1	noCoverage	-0.017857	0.57803	insignificant	0	4	4
chr13	22134098	22136098	Hist1h2bj		1	noCoverage	-0.039901	0.564	insignificant	0	5	5
chr13	22134818	22136818	Hist1h2ag		1	noCoverage	-0.039901	0.564	insignificant	0	5	5
chr13	22279917	22281917	Vmn1r192		1	noCoverage	-0.051474	0.57179	insignificant	0	6	6
chr13	22614919	22616919	Vmn1r203		1	noCoverage	-0.15418	0.2793	insignificant	0	6	6
chr13	22712904	22714904	Vmn1r206	-0.25	0.00058357	hypomethylated	-0.16667	0.037407	hypomethylated	1	4	4
chr13	23253511	23255511	Vmn1r219		1	noCoverage	0.011278	1	insignificant	0	4	4
chr13	23461071	23463071	Zfp322a	-0.16357	0.0037576	hypomethylated	0.13545	0.64926	insignificant	14	34	40
chr13	23515735	23517735	Abt1	-0.20634	0.0033688	hypomethylated	0.0062733	0.02903	hypermethylated	5	36	36
chr13	23522886	23524886	C230035I16Rik	-0.3117	0.031206	hypomethylated	-0.028375	0.020836	hypomethylated	4	36	36
chr13	23621912	23623912	Hist1h4h	-0.23532	0.20431	insignificant	-0.0010289	0.77169	insignificant	4	45	28
chr13	23626286	23628286	Hist1h3g	-0.23684	1	insignificant	-0.01128	0.66706	insignificant	2	10	10
chr13	23634920	23636920	Hist1h3f	-0.2992	0.13024	insignificant	0.080165	0.33773	insignificant	1	11	11
chr13	23643512	23645512	Hist1h4f		1	noCoverage	0.022697	0.34917	insignificant	0	8	8
chr13	23645900	23647900	Hist1h1d	-0.17824	1	insignificant	-0.028986	0.14556	insignificant	4	30	29
chr13	23665339	23667339	Hist1h2ad		1	noCoverage	-0.017093	1	insignificant	0	14	13
chr13	23666059	23668059	Hist1h3d	-0.26852	0.58158	insignificant	-0.076079	0.25664	insignificant	2	12	12
chr13	23666670	23668670	Hist1h3d	-0.26852	0.58158	insignificant	-0.076079	0.25664	insignificant	2	12	12
chr13	23712963	23714963	Hist1h1e	-0.10027	5.18E-09	hypomethylated	0.037209	0.066639	insignificant	17	66	68
chr13	23712993	23714993	Hist1h1e	-0.10027	5.18E-09	hypomethylated	0.037209	0.066639	insignificant	17	66	68
chr13	23786679	23788679	Hist1h1t	0.045455	1	lowCoverage	-0.017045	1	insignificant	1	2	4
chr13	23790318	23792318	Hist1h4c	-0.091227	1	insignificant	-0.084977	0.0074686	hypomethylated	2	17	14
chr13	23802680	23804680	Hfe	-0.52764	0.01442	stronglyHypometh	0.010817	0.22076	insignificant	1	4	2
chr13	23829675	23831675	Hist1h1c	-0.15743	0.0036322	hypomethylated	-0.007346	0.39011	insignificant	14	60	60
chr13	23841956	23843956	Hist1h2ab		1	noCoverage		1	noCoverage	11	0	22
chr13	23853026	23855026	Hist1h3a	-0.12961	9.51E-18	hypomethylated	-0.0096912	0.17529	insignificant	32	78	78
chr13	23854255	23856255	Hist1h3a	-0.19455	0.3091	insignificant	0.047537	0.8113	insignificant	2	25	18
chr13	23854536	23856536	Hist1h1a	-0.19455	0.3091	insignificant	0.047537	0.8113	insignificant	2	25	18
chr13	23897895	23899895	Slc17a2		1	noCoverage	-0.10327	0.19086	insignificant	0	8	8
chr13	24025330	24027330	Hist1h2aa	0.033249	0.63785	insignificant	0.025364	0.25294	insignificant	13	31	32
chr13	24026025	24028025	Hist1h2ba	-0.052146	0.15309	insignificant	-0.0059392	0.70868	insignificant	7	14	14
chr13	24083083	24085083	Scgn	-0.035024	1	insignificant	0.032346	1	insignificant	4	8	8
chr13	24372659	24374659	Lrrc16a	-0.10465	4.47E-25	hypomethylated	0.0040157	0.93472	insignificant	29	77	77
chr13	24505991	24507991	Cmah	-0.41084	0.011933	stronglyHypometh	0.072156	0.25363	insignificant	1	6	5
chr13	24696636	24698636	Gm11346	0.056811	0.68454	insignificant	-0.034706	0.78705	insignificant	3	8	8
chr13	24729516	24731516	Fam65b	-0.13704	1.47E-57	hypomethylated	-0.0015921	0.055806	insignificant	29	119	113
chr13	24853806	24855806	Gmnn		1	noCoverage	0.031994	0.64359	insignificant	0	13	13
chr13	24892525	24894525	BC005537	-0.11889	6.94E-36	hypomethylated	0.0033619	0.71609	insignificant	44	162	164
chr13	24922527	24924527	Tdp2	-0.11951	1.11E-14	hypomethylated	0.0022161	0.1529	insignificant	32	172	164
chr13	24923358	24925358	Acot13	-0.11243	1.37E-12	hypomethylated	0.002126	0.49497	insignificant	30	146	141
chr13	24935999	24937999	D130043K22Rik	-0.1935	1	insignificant	-0.018496	0.68146	insignificant	3	39	38
chr13	25029530	25031530	Aldh5a1		1	noCoverage	-0.023407	0.078093	insignificant	0	14	14
chr13	25034020	25036020	Gpld1	0.20361	0.18379	insignificant	-0.15346	0.17424	insignificant	2	20	15
chr13	25112248	25114248	Mrs2	-0.30607	2.8E-13	hypomethylated	0.08826	0.0071512	hypermethylated	5	22	20
chr13	25146872	25148872	Dcdc2a	-0.098318	2.68E-15	hypomethylated	-0.015427	8.78E-10	hypomethylated	31	121	121
chr13	26862034	26864034	Hdgfl1	-0.24324	0.4828	insignificant	0.1839	0.0033158	hypermethylated	5	18	18
chr13	27674040	27676040	Prl8a1	-0.043651	1	insignificant	-0.15797	1	insignificant	1	2	4
chr13	29947457	29949457	Cdkal1	-0.34911	0.41616	insignificant	0.016208	0.73145	insignificant	2	8	10
chr13	30077932	30079932	E2f3	-0.32307	0.016988	hypomethylated	0.057542	0.92743	insignificant	4	36	38
chr13	30227358	30229358	Mboat1	-0.18795	0.29271	insignificant	0.0090939	0.044979	hypermethylated	17	51	54
chr13	30427224	30429224	Agtr1a	-0.21146	0.000000521	hypomethylated	-0.039238	0.85174	insignificant	9	34	33
chr13	30637185	30639185	Uqcrfs1	-0.074701	0.003323	hypomethylated	0.01323	0.73376	insignificant	10	36	36
chr13	30750964	30752964	Dusp22	-0.14063	1.26E-11	hypomethylated	0.013675	0.62763	insignificant	25	75	81
chr13	30750969	30752969	Dusp22	-0.14063	1.26E-11	hypomethylated	0.013675	0.62763	insignificant	25	75	81
chr13	30840126	30842126	Irf4	-0.21404	0.049763	hypomethylated	-0.0030796	0.16763	insignificant	11	26	26
chr13	31065916	31067916	Exoc2		1	noCoverage	0.011593	0.03256	hypermethylated	0	32	32
chr13	31649038	31651038	Foxq1	-0.15737	2.11E-15	hypomethylated	-0.017018	1	insignificant	34	120	136
chr13	31716684	31718684	Foxf2	-0.14367	1.43E-24	hypomethylated	-0.011442	0.87803	insignificant	43	176	182
chr13	31897514	31899514	Foxc1	-0.11923	1.06E-38	hypomethylated	-0.010534	0.16792	insignificant	70	235	239
chr13	32430413	32432413	Gmds	-0.1208	7.43E-26	hypomethylated	-0.0064109	0.074461	insignificant	24	93	92
chr13	32892898	32894898	Wrnip1	-0.092781	9.38E-20	hypomethylated	0.013479	0.237	insignificant	41	182	190
chr13	32943054	32945054	Serpinb1a	-0.37857	0.24891	insignificant	0.060119	0.48875	insignificant	1	6	6
chr13	33250220	33252220	Serpinb9c	0.14079	0.17048	insignificant	0.053079	0.63579	insignificant	5	10	10
chr13	33752273	33754273	Serpinb6d		1	noCoverage	0.017869	1	insignificant	0	4	4
chr13	34055527	34057527	Nqo2	-0.30218	1	insignificant	0.084374	0.75165	insignificant	1	29	21
chr13	34058021	34060021	Nqo2		1	noCoverage	0.032738	1	insignificant	0	4	4
chr13	34093742	34095742	Ripk1	-0.12987	6.75E-35	hypomethylated	0.0024149	0.53362	insignificant	37	112	113
chr13	34094618	34096618	Serpinb6a	-0.25901	0.000004604	hypomethylated	0.08711	0.78957	insignificant	3	14	16

chr13	34128509	34130509	Bphl	-0.11821	3.78E-12	hypomethylated	0.0018456	0.95335	insignificant	17	37	36
chr13	34169877	34171877	Tubb2a	-0.12682	0.000025927	hypomethylated	0.0032352	0.0016628	inconclusive	10	56	56
chr13	34222223	34224223	Tubb2b	-0.23799	0.015372	hypomethylated	-0.027697	0.49163	insignificant	10	31	31
chr13	34253845	34255845	Psmg4		1	noCoverage	-0.15837	0.14432	insignificant	0	14	11
chr13	34437051	34439051	Slc22a23	-0.13645	0.000000152	hypomethylated	-0.020302	0.20933	insignificant	17	34	55
chr13	34744550	34746550	1300014I06Rik	-0.13288	9.29E-21	hypomethylated	0.0070054	0.29101	insignificant	27	77	84
chr13	34966362	34968362	Prpf4b	-0.15793	0.00051606	hypomethylated	0.0093027	0.098629	insignificant	11	112	114
chr13	35011861	35013861	1700026J04Rik	-0.32077	0.000023808	hypomethylated	0.023467	0.69944	insignificant	2	8	8
chr13	35055678	35057678	Eci3	-0.32955	0.31887	insignificant	-0.03267	0.83467	insignificant	3	8	8
chr13	35085996	35087996	Eci2	-0.14928	0.000000228	hypomethylated	0.005583	0.25244	insignificant	7	30	30
chr13	35086013	35088013	Eci2	-0.14928	0.000000228	hypomethylated	0.005583	0.25244	insignificant	7	30	30
chr13	35832270	35834270	Cdyl	-0.13502	1.48E-60	hypomethylated	-0.029798	0.21507	insignificant	97	254	257
chr13	35998216	36000216	Rpp40		1	noCoverage	-0.28467	0.20892	insignificant	0	9	6
chr13	36058774	36060774	Ppp1r3g	-0.14834	0.000000871	hypomethylated	0.026672	0.16589	insignificant	27	85	88
chr13	36208279	36210279	Fars2	-0.096942	0.00000716	hypomethylated	-0.024568	0.82936	insignificant	15	75	68
chr13	36209226	36211226	Lyrm4	-0.18348	3.15E-16	hypomethylated	-0.0096254	0.87983	insignificant	11	72	68
chr13	36826323	36828323	Nrn1	-0.15789	2.04E-08	hypomethylated	0.015288	0.37667	insignificant	17	46	46
chr13	37916906	37918906	Rreb1	-0.10816	6.85E-30	hypomethylated	-0.009609	0.45658	insignificant	33	138	140
chr13	37917780	37919780	Rreb1	-0.10283	4.72E-38	hypomethylated	-0.0044352	0.4299	insignificant	60	248	241
chr13	37918261	37920261	Rreb1	-0.10916	5.54E-29	hypomethylated	-0.0043593	0.44589	insignificant	50	227	220
chr13	38086059	38088059	Ssr1	-0.10136	0.61812	insignificant	-0.031038	0.40049	insignificant	1	22	16
chr13	38128163	38130163	Cage1	-0.17032	2.6E-26	hypomethylated	-0.019861	0.044687	hypomethylated	23	98	100
chr13	38128806	38130806	Cage1	-0.23083	4.39E-28	hypomethylated	-0.04899	0.052863	insignificant	18	83	82
chr13	38242162	38244162	Dsp	-0.072295	2.24E-23	hypomethylated	0.012515	0.37558	insignificant	22	119	110
chr13	38295807	38297807	Snrnp48	-0.10459	0.0067568	hypomethylated	-0.023542	0.55848	insignificant	10	67	61
chr13	38436584	38438584	Bmp6	-0.11977	1.09E-11	hypomethylated	-0.0053025	1	insignificant	41	156	159
chr13	38620329	38622329	Txndc5	-0.20979	1.53E-12	hypomethylated	0.015047	0.053334	insignificant	10	33	31
chr13	38726978	38728978	Muted	-0.35696	0.0019784	stronglyHypometh	-0.1425	0.51683	insignificant	6	34	30
chr13	38750897	38752897	Eef1e1	-0.21452	0.00001488	hypomethylated	0.018908	0.64314	insignificant	6	36	37
chr13	39052406	39054406	Slc35b3	0.017233	0.11957	insignificant	0.008875	0.14567	insignificant	12	39	41
chr13	39052744	39054744	Slc35b3	0.11684	0.28894	insignificant	-0.0065751	0.77171	insignificant	3	6	6
chr13	40383380	40385380	Ofcc1		1	noCoverage	0.066667	1	insignificant	0	4	4
chr13	40825812	40827812	Tfap2a	-0.099463	6.29E-14	hypomethylated	0.010845	0.39815	insignificant	16	98	87
chr13	40829192	40831192	Tfap2a	-0.29122	0.0081175	hypomethylated	0.017058	0.10821	insignificant	6	30	30
chr13	40954500	40956500	Gcnt2		1	noCoverage	0.023752	1	insignificant	0	21	20
chr13	40981534	40983534	Gcnt2	-0.23008	0.010485	hypomethylated	-0.0073502	0.69006	insignificant	9	41	41
chr13	41012002	41014002	Gcnt2	-0.23557	0.000055162	hypomethylated	-0.038322	0.0037803	hypomethylated	8	46	46
chr13	41095378	41097378	Pak1ip1	-0.20066	1.64E-13	hypomethylated	-0.021025	0.89844	insignificant	20	77	72
chr13	41110618	41112618	Tmem14c	-0.15441	1.09E-15	hypomethylated	-0.011087	0.069337	insignificant	22	48	48
chr13	41175075	41177075	Mak		1	noCoverage	0.12017	1	insignificant	0	4	4
chr13	41315772	41317772	Elovl2	-0.080315	0.00000368	hypomethylated	0.050426	0.087873	insignificant	30	73	78
chr13	41344212	41346212	BC024659	-0.15015	4.47E-22	hypomethylated	-0.013875	0.24914	insignificant	18	88	112
chr13	41700584	41702584	Tmem170b	-0.10066	4.02E-45	hypomethylated	-0.0072132	0.035275	hypomethylated	75	233	231
chr13	41745130	41747130	Gm5082		1	noCoverage	0.058997	1	insignificant	0	10	10
chr13	41960283	41962283	Gm10790	0.0066804	0.66197	insignificant	-0.090805	0.11301	insignificant	2	20	18
chr13	42146389	42148389	Hivep1	-0.080437	5.13E-26	hypomethylated	0.0057165	0.064706	insignificant	67	203	216
chr13	42395638	42397638	Edn1	-0.15653	1	insignificant	-0.018981	0.2349	insignificant	2	14	20
chr13	42803949	42805949	Phactr1	-0.087172	0.18803	insignificant	-0.020868	0.81957	insignificant	8	40	43
chr13	43217207	43219207	Phactr1		1	noCoverage	-0.26316	0.57719	insignificant	0	8	3
chr13	43266728	43268728	Tbc1d7	-0.19042	2.24E-21	hypomethylated	-0.024504	0.086041	insignificant	18	58	58
chr13	43399541	43401541	Gfod1	-0.16802	1.04E-18	hypomethylated	-0.016811	0.50688	insignificant	18	62	61
chr13	43492744	43494744	Nol7	-0.22255	1.12E-44	hypomethylated	-0.007293	0.85025	insignificant	30	98	96
chr13	43576342	43578342	Ranbp9	-0.075788	3.27E-19	hypomethylated	-0.0033754	0.85459	insignificant	26	124	143
chr13	43655530	43657530	Ccdc90a	-0.36667	0.000000209	stronglyHypometh	-0.10489	0.92151	insignificant	3	9	14
chr13	43710165	43712165	Rnf182	-0.12025	6.88E-09	hypomethylated	0.0037322	0.7158	insignificant	22	70	73
chr13	43879475	43881475	Cd83	-0.20381	1	insignificant	-0.051239	0.71461	insignificant	7	21	44
chr13	44825271	44827271	Jarid2	-0.097313	9.16E-27	hypomethylated	-0.011732	0.16525	insignificant	45	160	148
chr13	45097465	45099465	Dtnbp1	-0.24802	0.0066702	hypomethylated	-0.01281	0.82292	insignificant	11	50	50
chr13	45484110	45486110	Mylip	-0.11966	4.63E-12	hypomethylated	-0.019247	0.060534	insignificant	34	105	125
chr13	45484325	45486325	Mylip	-0.11144	4.65E-12	hypomethylated	-0.011028	0.066313	insignificant	34	104	125
chr13	45601837	45603837	Gmpr	-0.26211	1.3E-15	hypomethylated	-0.047953	0.00000391	hypomethylated	16	66	66
chr13	46060345	46062345	Atxn1	-0.20899	7.93E-14	hypomethylated	-0.0092869	0.54076	insignificant	7	66	67
chr13	46368089	46370089	Gm1574		1	noCoverage	-0.026331	0.21233	insignificant	0	10	9
chr13	46512668	46514668	Rbm24	-0.13319	2.06E-09	hypomethylated	-0.019303	0.02223	hypomethylated	12	92	106
chr13	46596271	46598271	Cap2	-0.22295	5.88E-34	hypomethylated	0.013945	0.66641	insignificant	24	80	82
chr13	46763890	46765890	C78339	-0.13767	6.76E-28	hypomethylated	-0.014134	0.10342	insignificant	46	112	114
chr13	46823218	46825218	Nup153	0.24164	0.13527	insignificant	-0.048095	0.0000074	hypomethylated	4	54	46
chr13	47025087	47027087	Kif13a	-0.16549	0.0000003	hypomethylated	-0.015259	0.062271	insignificant	26	78	80
chr13	47137907	47139907	Kdm1b	-0.11414	9.84E-38	hypomethylated	-0.0017108	0.22708	insignificant	36	140	128
chr13	47138586	47140586	Kdm1b	-0.11017	3.4E-34	hypomethylated	-0.00014175	0.10012	insignificant	35	138	126
chr13	47201589	47203589	Dek	-0.12767	8.37E-17	hypomethylated	0.019925	0.35585	insignificant	28	106	122
chr13	47217088	47219088	Rnf144b	-0.18268	1.39E-15	hypomethylated	0.025882	0.79582	insignificant	12	64	69
chr13	47288371	47290371	Rnf144b	-0.067986	0.0004149	hypomethylated	0.033824	0.56426	insignificant	12	50	45
chr13	48355795	48357795	Id4	-0.067185	8.03E-41	hypomethylated	0.0041566	0.98267	insignificant	69	243	234
chr13	48608779	48610779	Zfp169	-0.081578	0.00011174	hypomethylated	0.022	0.0076304	inconclusive	7	33	33
chr13	48608805	48610805	Zfp169	-0.062519	0.0020031	hypomethylated	0.019387	0.0039919	inconclusive	5	29	29
chr13	48720942	48722942	Ptpdc1	-0.10657	3.6E-14	hypomethylated	-0.0066838	0.74593	insignificant	26	82	82
chr13	48757404	48759404	Barx1	-0.10319	1.44E-24	hypomethylated	0.012941	0.87847	insignificant	34	98	102
chr13	48966254	48968254	Phf2	-0.12809	5.91E-19	hypomethylated	-0.008578	0.29154	insignificant	24	70	70
chr13	49062480	49064480	C030044B11Rik	-0.092475	4.73E-48	hypomethylated	0.00015332	0.86844	insignificant	99	257	265
chr13	49063197	49065197	Fam120a	-0.079931	4.08E-31	hypomethylated	-0.0057731	0.95559	insignificant	82	206	212
chr13	49243383	49245383	Wnk2	-0.10107	0.000000201	hypomethylated	0.0035399	0.43522	insignificant	9	34	34
chr13	49281915	49283915	Ninj1	-0.11718	1.36E-38	hypomethylated	0.0055698	0.92551	insignificant	33	105	105
chr13	49311395	49313395	1110007C09Rik	0.21595	0.082744	insignificant	0.039012	0.75246	insignificant	6	38	38
chr13	49343532	49345532	Susd3	-0.61805	1.33E-14	stronglyHypometh	-0.055937	0.42274	insignificant	2	20	20

chr13	49435917	49437917	Bicd2	0.043784	1.48E-12	inconclusive	0.02633	0.040036	inconclusive	15	74	66
chr13	49515679	49517679	Ippk	-0.10631	1.06E-23	hypomethylated	-0.030641	0.23727	insignificant	40	122	118
chr13	49702439	49704439	Ogn		1	noCoverage	0.14597	0.039789	hypermethylated	0	7	7
chr13	49747718	49749718	Nol8	-0.076661	1.97E-09	hypomethylated	-0.0052307	0.0035934	hypomethylated	20	98	88
chr13	49748100	49750100	Cenpp	-0.058877	0.00000247	hypomethylated	-0.0074476	0.00051355	hypomethylated	14	83	72
chr13	49776498	49778498	Iars	-0.12135	0.67001	insignificant	0.13216	0.10004	insignificant	5	32	30
chr13	50512245	50514245	Fbxw17	-0.28084	6.12E-16	hypomethylated	-0.034063	0.065531	insignificant	13	44	44
chr13	51195266	51197266	Spin1	-0.089733	2.63E-46	hypomethylated	-0.005756	0.14754	insignificant	72	235	253
chr13	51196096	51198096	Spin1	-0.068861	2.7E-32	hypomethylated	0.0047954	0.021039	inconclusive	58	191	208
chr13	51265393	51267393	Nxnl2	-0.16728	3.47E-11	hypomethylated	-0.002708	0.0052956	hypomethylated	15	93	95
chr13	51502986	51504986	S1pr3	-0.030848	0.045414	hypomethylated	0.031095	0.059926	insignificant	19	51	48
chr13	51662453	51664453	Shc3	-0.12158	0.000000287	hypomethylated	-0.015615	0.83244	insignificant	30	89	89
chr13	51739600	51741600	Cks2	-0.13472	1.12E-11	hypomethylated	-0.0049753	0.47374	insignificant	23	119	119
chr13	51746082	51748082	Secisbp2	-0.096494	0.000001795	hypomethylated	-0.007178	0.51674	insignificant	29	87	87
chr13	51941043	51943043	Gadd45g	-0.090011	6.21E-15	hypomethylated	0.0033408	0.27813	insignificant	32	133	130
chr13	52626205	52628205	Diras2	-0.40017	0.00000296	stronglyHypometh	-0.020659	0.048487	hypomethylated	4	12	12
chr13	52677805	52679805	Syk	-0.18914	5.78E-21	hypomethylated	-0.0026779	0.060334	insignificant	27	114	104
chr13	52691250	52693250	Syk	-0.37112	1	lowCoverage	-0.018175	0.87583	insignificant	1	6	6
chr13	53025046	53027046	Auh	-0.39708	0.41724	insignificant	0.031173	0.092789	insignificant	4	8	10
chr13	53076408	53078408	Nfil3	-0.15443	6.01E-22	hypomethylated	-0.0037496	1	insignificant	39	120	110
chr13	53381478	53383478	Ror2	-0.28973	2.1E-35	hypomethylated	-0.0070344	3.74E-13	hypomethylated	21	64	66
chr13	53472730	53474730	Sptlc1	-0.17727	0.000000967	hypomethylated	0.045139	0.025226	inconclusive	4	54	54
chr13	54151027	54153027	Drd1a	-0.29091	0.035136	hypomethylated	-0.0055472	0.80935	insignificant	3	8	8
chr13	54166213	54168213	Sfxn1	-0.092697	0.00000049	hypomethylated	0.006027	0.063168	insignificant	23	106	108
chr13	54286497	54288497	Hrh2	-0.16711	0.14089	insignificant	-0.013299	0.93294	insignificant	4	14	15
chr13	54471712	54473712	Cplx2	-0.205	1.98E-11	hypomethylated	-0.030632	0.0069975	hypomethylated	13	49	49
chr13	54570201	54572201	Thoc3		1	noCoverage	0.22411	0.000056697	hypermethylated	0	50	36
chr13	54604165	54606165	4732471D19Rik	-0.13251	3.8E-54	hypomethylated	-0.0038772	0.80399	insignificant	45	141	135
chr13	54666743	54668743	4833439L19Rik	-0.20172	0.0043217	hypomethylated	0.12215	0.86822	insignificant	3	12	15
chr13	54675373	54677373	Arl10	-0.17246	1.99E-20	hypomethylated	0.016622	0.11201	insignificant	31	110	110
chr13	54690591	54692591	Higd2a	-0.27613	7.8E-28	hypomethylated	-0.048569	0.38376	insignificant	18	48	48
chr13	54691435	54693435	Nop16	-0.32464	2.88E-22	hypomethylated	-0.021198	0.00010742	hypomethylated	12	40	40
chr13	54712633	54714633	Cltb	-0.23605	3.99E-14	hypomethylated	0.019437	0.00044343	hypermethylated	11	77	77
chr13	54722144	54724144	Faf2	-0.2392	6.92E-12	hypomethylated	-0.061912	0.000017374	hypomethylated	10	45	52
chr13	54789169	54791169	Rnf44	-0.060928	0.45124	insignificant	-0.035239	0.71981	insignificant	2	36	36
chr13	54789561	54791561	Rnf44	-0.10874	0.34761	insignificant	-0.049276	0.76257	insignificant	2	28	28
chr13	54795321	54797321	Rnf44	-0.14697	2.03E-23	hypomethylated	0.0069025	1.72E-16	inconclusive	38	148	149
chr13	54851030	54853030	Gprin1	-0.15716	5.15E-30	hypomethylated	-0.03557	0.0053211	hypomethylated	28	72	68
chr13	54867801	54869801	Sncb	-0.19424	0.39125	insignificant	-0.039884	0.55435	insignificant	10	29	29
chr13	54884363	54886363	Eif4e1b	-0.1628	0.12164	insignificant	-0.019417	0.78595	insignificant	3	6	6
chr13	54884380	54886380	Eif4e1b	-0.1628	0.12164	insignificant	-0.019417	0.78595	insignificant	3	6	6
chr13	54889765	54891765	Tspan17	-0.15726	7.62E-19	hypomethylated	-0.0039107	0.1291	insignificant	22	58	58
chr13	55049792	55051792	Unc5a	-0.13475	4.44E-51	hypomethylated	-0.012039	0.33974	insignificant	66	149	158
chr13	55205669	55207669	Zfp346	-0.19801	1.11E-36	hypomethylated	-0.013696	0.34521	insignificant	28	72	72
chr13	55253178	55255178	Fgfr4	-0.20098	3.96E-12	hypomethylated	-0.038603	0.8726	insignificant	20	70	66
chr13	55310142	55312142	Nsd1	-0.22147	1.05E-10	hypomethylated	0.024352	0.00089659	inconclusive	37	159	166
chr13	55422415	55424415	Prelid1	-0.063177	1.22E-13	hypomethylated	-0.016766	0.00000658	hypomethylated	35	91	93
chr13	55423341	55425341	Rab24	-0.12403	0.0027385	hypomethylated	-0.0093186	0.055359	insignificant	16	48	52
chr13	55431091	55433091	Mxd3	-0.097179	0.00033521	hypomethylated	-0.00046321	1	insignificant	19	64	64
chr13	55464144	55466144	Lman2	-0.075637	1	insignificant	0.000057429	0.0081444	inconclusive	6	24	25
chr13	55500008	55502008	Slc34a1	-0.40025	0.043027	stronglyHypometh	-0.0014368	1	insignificant	1	4	4
chr13	55545694	55547694	Grk6	-0.12828	6.15E-35	hypomethylated	-0.015018	0.000052077	hypomethylated	36	169	177
chr13	55564627	55566627	Prr7	-0.11991	1.25E-34	hypomethylated	-0.0050764	0.1454	insignificant	36	126	127
chr13	55589437	55591437	Dbn1	-0.69146	0.000058582	stronglyHypometh	-0.084317	0.013552	hypomethylated	2	13	14
chr13	55614800	55616800	Pdlim7	-0.33495	0.000037366	stronglyHypometh	-0.11274	0.0017825	hypomethylated	3	21	16
chr13	55629899	55631899	Dok3	-0.45882	0.21934	insignificant	-0.028635	0.36958	insignificant	1	6	6
chr13	55638019	55640019	Ddx41		1	noCoverage	0.045674	0.62155	insignificant	0	19	18
chr13	55671336	55673336	Fam193b	-0.14199	1.11E-14	hypomethylated	-0.018272	0.043193	hypomethylated	26	87	98
chr13	55672481	55674481	Fam193b	-0.16455	0.59348	insignificant	-0.020413	0.53302	insignificant	3	8	8
chr13	55693495	55695495	Tmed9	-0.14164	0.00000294	hypomethylated	-0.004731	0.068807	insignificant	16	74	74
chr13	55700471	55702471	B4galt7	-0.094708	2.11E-14	hypomethylated	-0.012769	0.87085	insignificant	11	43	43
chr13	55723365	55725365	Caml	-0.21115	0.37093	insignificant	-0.064719	0.0047116	hypomethylated	6	83	77
chr13	55735387	55737387	Ddx46	-0.12518	6.22E-28	hypomethylated	0.0060082	0.18177	insignificant	37	118	118
chr13	55793484	55795484	B230219D22Rik	-0.11307	2.61E-20	hypomethylated	-0.0035476	0.46856	insignificant	33	81	80
chr13	55815010	55817010	Txndc15	-0.14146	0.069842	insignificant	0.10985	0.38009	insignificant	10	30	34
chr13	55827728	55829728	Pcbd2	-0.10067	1.05E-11	hypomethylated	-0.0053006	0.15113	insignificant	29	119	119
chr13	55884939	55886939	Catsper3	-0.14048	0.31464	insignificant	-0.023364	1	insignificant	1	4	4
chr13	55932786	55934786	Pitx1	-0.2319	4.75E-30	hypomethylated	0.022802	0.017802	inconclusive	20	54	55
chr13	56236911	56238911	H2afy	-0.12874	5.91E-14	hypomethylated	0.017114	0.0083747	hypermethylated	15	38	28
chr13	56280246	56282246	Tifab	-0.37738	1	insignificant	0.078537	0.076607	insignificant	2	8	8
chr13	56353524	56355524	Neurog1	-0.080913	0.0051743	hypomethylated	-0.052156	1	insignificant	2	24	23
chr13	56538715	56540715	Slc25a48	-0.15379	1.91E-08	hypomethylated	0.023609	0.13236	insignificant	20	101	92
chr13	56622868	56624868	Fbxl21	-0.16103	1	lowCoverage	0.0011676	0.51873	insignificant	1	34	34
chr13	56649899	56651899	Lect2	-0.97727	0.0041929	stronglyHypometh	-0.39394	0.0027874	stronglyhypometh	2	4	4
chr13	56803370	56805370	Smad5	-0.14004	3.71E-27	hypomethylated	-0.015555	0.70475	insignificant	57	179	172
chr13	56803412	56805412	Smad5	-0.14004	3.71E-27	hypomethylated	-0.015555	0.70475	insignificant	57	179	172
chr13	56804028	56806028	Smad5	-0.12319	1.73E-33	hypomethylated	-0.014713	0.84034	insignificant	51	157	152
chr13	56996949	56998949	Trpc7		1	noCoverage	-0.064782	0.036369	hypomethylated	0	6	6
chr13	58009693	58011693	Spock1	-0.21447	0.10997	insignificant	-0.021606	0.38503	insignificant	5	22	22
chr13	58229917	58231917	Hnrnpa0	-0.094688	3.26E-19	hypomethylated	0.0072161	0.088156	insignificant	48	194	191
chr13	58258009	58260009	5133401N09Rik	-0.24914	3.54E-21	hypomethylated	0.019509	0.10939	insignificant	11	31	38
chr13	58317014	58319014	Ubqln1	-0.14757	1.93E-19	hypomethylated	-0.0014206	0.008661	hypomethylated	15	60	61
chr13	58375549	58377549	Gkap1	-0.11672	1.66E-38	hypomethylated	-0.030918	0.00019731	hypomethylated	37	124	126
chr13	58456223	58458223	Kif27		1	noCoverage	-0.057692	0.59199	insignificant	0	4	2
chr13	58502608	58504608	Rmi1	-0.1005	1.47E-41	hypomethylated	-0.01559	0.49013	insignificant	79	320	331

chr13	58502957	58504957	Rmi1	-0.093939	7.39E-21	hypomethylated	-0.013729	0.029764	hypomethylated	60	264	271
chr13	58503877	58505877	Rmi1	-0.11971	0.0029002	hypomethylated	-0.023019	0.0010948	hypomethylated	18	120	113
chr13	58906956	58908956	Ntrk2	-0.1764	3.79E-18	hypomethylated	-0.013712	0.76457	insignificant	8	50	50
chr13	58908193	58910193	Ntrk2	-0.19293	1.17E-12	hypomethylated	-0.00243	0.95303	insignificant	13	51	48
chr13	59658680	59660680	Agtpbp1	-0.12378	0.00000873	hypomethylated	-0.016093	0.13803	insignificant	7	44	47
chr13	59685693	59687693	Naa35	-0.15972	1.69E-45	hypomethylated	-0.005997	0.00017067	hypomethylated	48	142	146
chr13	59777145	59779145	Golm1	-0.30684	0.000000769	hypomethylated	-0.023686	0.81929	insignificant	8	37	43
chr13	59807558	59809558	Fam75d3	0.09037	1	insignificant	-0.11899	0.27044	insignificant	4	8	10
chr13	59870326	59872326	Etohd2	-0.12141	6.03E-12	hypomethylated	-0.012352	0.42857	insignificant	20	120	120
chr13	59871150	59873150	Etohd2	-0.16952	1.2E-10	hypomethylated	-0.011397	0.95121	insignificant	14	49	49
chr13	59924508	59926508	Zcchc6	-0.16964	1.66E-11	hypomethylated	-0.016236	0.31258	insignificant	14	45	67
chr13	60278896	60280896	Gas1	-0.41423	0.0000843	stronglyHypometh	-0.0078294	0.51582	insignificant	1	10	10
chr13	60702307	60704307	Dapk1	-0.09421	3.64E-31	hypomethylated	-0.00041902	0.65022	insignificant	54	183	183
chr13	60702571	60704571	Dapk1	-0.09421	3.64E-31	hypomethylated	-0.00041902	0.65022	insignificant	54	183	183
chr13	62230249	62232249	Zfp808	-0.16576	0.35065	insignificant	-0.0079743	0.20468	insignificant	3	30	28
chr13	62397849	62399849	A130040M12Rik		1	noCoverage	-0.084453	1	insignificant	0	12	12
chr13	62484534	62486534	Gm3604		1	noCoverage	0.048676	0.29659	insignificant	0	11	11
chr13	62568172	62570172	Zfp935	-0.29515	0.000000199	hypomethylated	-0.029874	0.11768	insignificant	4	11	11
chr13	62659959	62661959	Zfp934		1	noCoverage	-0.014929	0.85873	insignificant	0	8	8
chr13	62861098	62863098	Mir713		1	noCoverage	-0.045477	0.2044	insignificant	0	12	9
chr13	63666828	63668828	Ptch1	-0.075981	8.3E-28	hypomethylated	0.0041701	0.55279	insignificant	50	135	143
chr13	63915627	63917627	0610007P08Rik	-0.0996	0.000000622	hypomethylated	0.011076	0.54732	insignificant	13	48	47
chr13	63915641	63917641	0610007P08Rik	-0.0996	0.000000622	hypomethylated	0.011076	0.54732	insignificant	13	48	47
chr13	64230638	64232638	Slc35d2	-0.49508	0.13724	insignificant	0.12451	0.14295	insignificant	2	27	26
chr13	64254507	64256507	Zfp367	-0.18404	0.00050229	hypomethylated	0.016419	0.13437	insignificant	8	78	79
chr13	64262173	64264173	Habp4	-0.13159	9.33E-33	hypomethylated	0.01666	0.067365	insignificant	44	124	108
chr13	64376296	64378296	Cdc14b	-0.11454	1	insignificant	0.002993	1	insignificant	2	20	20
chr13	64471614	64473614	Ctsl	-0.36998	2.45E-08	stronglyHypometh	0.03545	0.82499	insignificant	5	30	31
chr13	65379161	65381161	Zfp369	-0.19162	3.69E-09	hypomethylated	-0.0040739	0.7398	insignificant	10	51	47
chr13	67032765	67034765	Ptdss1	-0.087594	9.51E-23	hypomethylated	-0.01243	0.015718	hypomethylated	33	127	132
chr13	67034008	67036008	Mterfd1	-0.066027	3.66E-21	hypomethylated	-0.0083853	0.16448	insignificant	29	99	99
chr13	67198912	67200912	Zfp708		1	noCoverage	0.13529	0.10128	insignificant	0	10	7
chr13	67461508	67463508	Zfp953		1	noCoverage	0.026414	0.85905	insignificant	0	8	8
chr13	67585189	67587189	Zfp874b		1	noCoverage	-0.01872	1	insignificant	0	17	21
chr13	67601458	67603458	Zfp58	-0.21453	0.00000328	hypomethylated	-0.038731	0.0036275	hypomethylated	2	8	8
chr13	67829198	67831198	4930441O14Rik	-0.38764	0.013969	stronglyHypometh	-0.030138	0.8353	insignificant	2	8	13
chr13	67856071	67858071	4930441O14Rik	-0.36818	0.000014492	stronglyHypometh	-0.17572	0.6796	insignificant	6	16	12
chr13	67879629	67881629	Zfp493	-0.38869	0.00055148	stronglyHypometh	-0.03244	0.58054	insignificant	3	8	8
chr13	67913752	67915752	Zfp273	-0.32514	0.00000364	hypomethylated	-0.13128	0.76039	insignificant	3	22	23
chr13	67963273	67965273	BC048507	-0.35397	0.70171	insignificant	0.047616	0.0032733	hypermethylated	2	44	44
chr13	68720124	68722124	Fastkd3	-0.1195	7.71E-19	hypomethylated	0.00062539	0.040186	inconclusive	38	154	150
chr13	68720998	68722998	Fastkd3	-0.057112	2.52E-15	hypomethylated	0.0073106	0.00000195	inconclusive	30	92	92
chr13	69138419	69140419	Adcy2	-0.088884	0.39813	insignificant	0.05978	0.0000049	hypermethylated	1	31	19
chr13	69672742	69674742	Papd7	-0.083517	1.15E-44	hypomethylated	-0.008256	0.09886	insignificant	91	286	298
chr13	69749894	69751894	Nsun2	-0.074077	0.000000543	hypomethylated	-0.0046353	0.55558	insignificant	17	136	137
chr13	69750341	69752341	Srd5a1	-0.067782	0.00000112	hypomethylated	-0.0067117	0.68574	insignificant	17	122	123
chr13	69878775	69880775	Ube2ql1		1	noCoverage	-0.069013	1	insignificant	0	47	9
chr13	69947759	69949759	Med10	-0.1831	9.12E-11	hypomethylated	-0.021051	0.093306	insignificant	12	39	40
chr13	70776512	70778512	BC018507	-0.11485	1.19E-11	hypomethylated	0.014065	0.85964	insignificant	14	30	37
chr13	72101171	72103171	Irx1	-0.16753	0.14372	insignificant	0.012236	0.48263	insignificant	3	15	19
chr13	72765425	72767425	Irx2	-0.094689	5.84E-26	hypomethylated	0.0014643	0.66669	insignificant	48	205	205
chr13	72766012	72768012	D430050G20	-0.088875	1.38E-27	hypomethylated	0.0064004	0.24262	insignificant	46	180	180
chr13	73396944	73398944	Irx4	-0.094941	5.4E-22	hypomethylated	-0.0049173	0.0014468	hypomethylated	38	126	130
chr13	73465930	73467930	Ndufs6	0.44737	0.46875	lowCoverage	0.15107	0.20962	insignificant	1	6	6
chr13	73467456	73469456	Mrpl36	-0.23757	0.0020163	hypomethylated	-0.0063527	0.043946	inconclusive	2	19	19
chr13	73603830	73605830	Lpcat1	-0.11756	8.93E-09	hypomethylated	-0.018898	0.0039055	hypomethylated	19	73	86
chr13	73673194	73675194	Slc6a3	-0.30906	6.02E-15	hypomethylated	-0.0064662	0.53476	insignificant	12	41	40
chr13	73740748	73742748	Clptm1l	-0.11833	4.12E-10	hypomethylated	0.016806	0.0015725	hypermethylated	16	38	38
chr13	73763448	73765448	Tert	-0.065635	0.00000013	hypomethylated	0.011153	0.16537	insignificant	12	49	49
chr13	73838143	73840143	5430425J12Rik	-0.25455	0.048364	hypomethylated	-0.040965	0.67709	insignificant	1	2	3
chr13	73900144	73902144	Slc12a7	-0.1401	1.73E-19	hypomethylated	-0.0013788	0.079348	insignificant	28	68	68
chr13	74074285	74076285	Brd9	-0.1241	0.000022109	hypomethylated	-0.0011752	0.96089	insignificant	11	50	50
chr13	74075215	74077215	Trip13	-0.16183	0.004049	hypomethylated	0.00061891	0.82112	insignificant	4	14	14
chr13	74145866	74147866	Tppp	-0.15629	1.18E-11	hypomethylated	-0.017125	0.11319	insignificant	18	108	112
chr13	74257962	74259962	Slc9a3	-0.16421	6.45E-11	hypomethylated	-0.0055491	0.57917	insignificant	20	54	54
chr13	74346148	74348148	Exoc3		1	noCoverage	-0.08428	0.60316	insignificant	0	11	10
chr13	74429757	74431757	Ahrr	-0.18222	0.026404	hypomethylated	0.056354	0.91236	insignificant	2	16	12
chr13	74454774	74456774	Pdcd6		1	noCoverage	0.021577	0.58797	insignificant	0	16	16
chr13	74486764	74488764	Ccdc127	-0.14949	1.96E-14	hypomethylated	0.0063981	0.11424	insignificant	12	58	56
chr13	74631398	74633398	Zfp825	-0.55879	0.000000106	stronglyHypometh	-0.10161	0.081422	insignificant	4	7	7
chr13	74776319	74778319	Erap1	-0.16411	1.15E-32	hypomethylated	0.0016315	1	insignificant	19	50	50
chr13	74945369	74947369	Cast	-0.35136	2.46E-33	stronglyHypometh	-0.056384	0.0046878	hypomethylated	7	13	12
chr13	75226434	75228434	Pcsk1	-0.24948	0.00000458	hypomethylated	0.024744	0.69899	insignificant	8	50	46
chr13	75781493	75783493	Mir682	0.22893	0.59364	insignificant	0.27525	0.0001565	hypermethylated	4	12	9
chr13	75843931	75845931	Ell2	-0.10345	1.21E-34	hypomethylated	0.00035652	0.15444	insignificant	37	133	138
chr13	76081272	76083272	Rhobtb3	-0.11142	0.000035574	hypomethylated	0.032252	1	insignificant	14	31	31
chr13	76156010	76158010	Rfesd	-0.61857	0.0010815	stronglyHypometh	-0.040444	0.31877	insignificant	1	2	2
chr13	76235181	76237181	Ttc37	-0.18778	0.019778	hypomethylated	-0.013131	0.42911	insignificant	3	31	31
chr13	76236108	76238108	Arsk	-0.21202	1	insignificant	-0.020882	0.38628	insignificant	2	17	17
chr13	76521408	76523408	Mctp1	-0.10394	2.78E-15	hypomethylated	0.0032138	0.57255	insignificant	26	140	140
chr13	77273796	77275796	2210408I21Rik	-0.1154	3.45E-19	hypomethylated	-0.0028122	0.51829	insignificant	42	129	127
chr13	77274729	77276729	Ankrd32	-0.11991	0.000000048	hypomethylated	-0.0022633	0.28251	insignificant	11	42	42
chr13	77846950	77848950	Fam172a	-0.12449	2.22E-09	hypomethylated	-0.018765	0.39684	insignificant	9	37	41
chr13	78163162	78165162	Pou5f2	-0.062203	0.040952	hypomethylated	0.017655	0.90114	insignificant	3	22	23
chr13	78338243	78340243	Nr2f1	-0.11142	2.97E-25	hypomethylated	-0.0075945	0.15675	insignificant	24	90	90

chr13	81021682	81023682	Arrdc3	-0.18671	0.0020753	hypomethylated	0.011629	0.11135	insignificant	9	42	41
chr13	81102334	81104334	9330111N05Rik	-0.57838	2.48E-09	stronglyHypometh	-0.0079101	0.010298	hypomethylated	4	16	16
chr13	81772143	81774143	Gpr98	-0.23876	0.21074	insignificant	0.040034	0.11315	insignificant	2	20	19
chr13	81795804	81797804	Lysmd3	-0.113	0.000000259	hypomethylated	-0.0060162	0.61457	insignificant	19	88	88
chr13	81849415	81851415	Mblac2	-0.1577	5.44E-23	hypomethylated	-0.010859	0.0012632	hypomethylated	36	134	136
chr13	81850012	81852012	Mblac2	-0.17648	8.43E-21	hypomethylated	-0.00032263	0.012512	hypomethylated	25	96	99
chr13	81921290	81923290	Cetn3	-0.084918	4.51E-12	hypomethylated	-0.0075883	0.006405	hypomethylated	16	110	110
chr13	83866710	83868710	C130071C03Rik	-0.18348	0.0046952	hypomethylated	0.017401	0.94392	insignificant	9	22	25
chr13	83877418	83879418	Mir9-2	-0.22295	0.036038	hypomethylated	0.0060542	0.11872	insignificant	2	26	26
chr13	84360900	84362900	Tmem161b	-0.16138	1.89E-08	hypomethylated	0.0067738	0.90052	insignificant	11	30	30
chr13	85328081	85330081	Ccnh	-0.20338	0.00012834	hypomethylated	-0.080711	0.63733	insignificant	5	10	26
chr13	85429091	85431091	Rasa1	-0.15446	0.00000022	hypomethylated	-0.01546	0.18368	insignificant	7	32	32
chr13	86186400	86188400	Cox7c		1	noCoverage	0.15	0.59653	insignificant	0	4	5
chr13	89882117	89884117	Vcan		1	noCoverage	-0.029644	0.86398	insignificant	0	9	9
chr13	90228271	90230271	Tmem167	-0.12902	0.000000204	hypomethylated	-0.0024397	0.84939	insignificant	27	93	94
chr13	90229213	90231213	Xrcc4	-0.13808	0.000000148	hypomethylated	-0.00093548	0.95937	insignificant	22	76	78
chr13	91061726	91063726	Rps23	-0.093569	0.00000181	hypomethylated	0.0063307	0.37904	insignificant	11	87	72
chr13	91061780	91063780	Rps23	-0.093569	0.00000181	hypomethylated	0.0063307	0.37904	insignificant	11	87	72
chr13	91363592	91365592	Atg10	-0.11871	0.000001007	hypomethylated	-0.014168	0.36166	insignificant	9	40	35
chr13	91599701	91601701	Ssbp2	-0.094272	1.26E-41	hypomethylated	-0.0040876	0.03722	hypomethylated	87	292	300
chr13	91880125	91882125	Acot12	-0.2999	0.000000619	hypomethylated	-0.074214	0.0058024	hypomethylated	16	57	65
chr13	91881477	91883477	4833422C13Rik		1	noCoverage	0.035552	0.64785	insignificant	0	18	18
chr13	91947301	91949301	Zcchc9	-0.59109	4.44E-08	stronglyHypometh	-0.0032906	0.4833	insignificant	2	29	24
chr13	92901449	92903449	Rasgrf2	-0.15037	1.93E-24	hypomethylated	-0.0095303	0.69942	insignificant	29	82	82
chr13	93123737	93125737	Dhfr	-0.11368	3.81E-22	hypomethylated	0.00052452	0.92826	insignificant	51	154	154
chr13	93124958	93126958	Msh3	-0.20012	2.58E-14	hypomethylated	-0.0092264	0.55063	insignificant	21	50	50
chr13	93194923	93196923	Ankrd34b	-0.069619	2.87E-21	hypomethylated	-0.0072571	0.62459	insignificant	49	163	165
chr13	93253986	93255986	Fam151b	-0.54545	0.0029535	stronglyHypometh	0.010101	0.31322	insignificant	1	2	2
chr13	93300765	93302765	Zfyve16	-0.10769	6.34E-15	hypomethylated	0.011515	0.96391	insignificant	16	66	66
chr13	93380092	93382092	Serinc5	-0.17347	1.06E-14	hypomethylated	-0.0146	0.36067	insignificant	18	76	79
chr13	93564773	93566773	Thbs4	-0.071136	0.24188	insignificant	0.027654	0.71678	insignificant	6	21	21
chr13	93613741	93615741	Mtx3	-0.21829	0.36594	insignificant	-0.05625	0.030774	hypomethylated	6	26	30
chr13	93842258	93844258	Gm4814		1	noCoverage	-0.10903	0.15797	insignificant	0	6	6
chr13	93962238	93964238	Papd4	-0.26917	0.000000195	hypomethylated	0.030747	0.10357	insignificant	6	39	39
chr13	94073449	94075449	Homer1	-0.0977	9.59E-25	hypomethylated	-0.0027269	0.44097	insignificant	50	187	181
chr13	94269644	94271644	Jmy	-0.13278	0.00000594	hypomethylated	-0.023006	0.5227	insignificant	20	53	66
chr13	94443390	94445390	Dmgdh	-0.0822	0.038271	hypomethylated	0.075887	0.000044105	hypermethylated	23	54	57
chr13	94444257	94446257	Bhmt2	-0.060459	0.22717	insignificant	0.089734	0.10675	insignificant	11	38	41
chr13	94540633	94542633	Arsb	-0.15159	2.95E-14	hypomethylated	-0.013216	0.031803	hypomethylated	30	114	114
chr13	94826750	94828750	Lhfpl2	-0.16162	8.66E-46	hypomethylated	-0.011521	0.00079886	hypomethylated	39	104	104
chr13	95055236	95057236	Scamp1	-0.14001	3.66E-30	hypomethylated	-0.010355	0.034016	hypomethylated	30	77	81
chr13	95127914	95129914	Ap3b1	-0.12402	1.52E-20	hypomethylated	0.000069725	0.0014706	inconclusive	20	127	127
chr13	95557897	95559897	Tbca	-0.085238	0.000032919	hypomethylated	0.015693	0.58057	insignificant	23	87	84
chr13	95644581	95646581	Otp	-0.20661	0.00014216	hypomethylated	-0.005094	0.83103	insignificant	6	14	14
chr13	95745302	95747302	Wdr41	-0.15124	0.000000232	hypomethylated	0.00085419	0.37017	insignificant	11	30	30
chr13	95993005	95995005	Pde8b	-0.049429	0.34068	insignificant	-0.0098207	0.72078	insignificant	8	44	39
chr13	96094191	96096191	Zbed3	-0.13319	4.35E-16	hypomethylated	0.024318	0.46163	insignificant	21	73	67
chr13	96099534	96101534	Mir1940		1	noCoverage	-0.025537	0.84348	insignificant	0	11	14
chr13	96099565	96101565	Snora47		1	noCoverage	-0.025537	0.84348	insignificant	0	11	14
chr13	96145289	96147289	Aggf1	-0.29905	0.091779	insignificant	-0.15724	0.091411	insignificant	5	16	23
chr13	96214786	96216786	Crhbp	-0.036667	0.67296	insignificant	0.008655	0.71737	insignificant	5	20	12
chr13	96248610	96250610	S100z	0.067205	0.28161	insignificant	0.052125	0.74349	insignificant	6	24	25
chr13	96295195	96297195	F2rl1	-0.10024	0.00040567	hypomethylated	-0.015797	0.060069	insignificant	6	26	26
chr13	96388388	96390388	F2r	-0.12418	0.000065111	hypomethylated	-0.00037395	0.19072	insignificant	4	39	39
chr13	96661877	96663877	Iqgap2	-0.092163	2.29E-10	hypomethylated	-0.018953	0.11408	insignificant	12	48	48
chr13	96902532	96904532	Sv2c	-0.14274	0.24341	insignificant	-0.041115	0.54623	insignificant	4	59	57
chr13	97157248	97159248	Poc5	-0.16707	0.000000576	hypomethylated	0.0050545	0.32708	insignificant	7	56	48
chr13	97241115	97243115	Ankdd1b	-0.22321	0.22968	insignificant	-0.13988	0.46344	insignificant	1	4	4
chr13	97311689	97313689	Col4a3bp	-0.099578	5.81E-33	hypomethylated	-0.00061564	0.098192	insignificant	43	144	144
chr13	97312440	97314440	Polk	-0.091194	3.27E-30	hypomethylated	0.00027745	0.10631	insignificant	38	134	134
chr13	97440891	97442891	Hmgcr	-0.21136	0.10833	insignificant	-0.005208	9.62E-11	inconclusive	5	18	18
chr13	97693643	97695643	Gcnt4	-0.15874	3.36E-20	hypomethylated	-0.033419	0.43036	insignificant	30	142	136
chr13	97836241	97838241	Fam169a	0.048542	0.077804	insignificant	0.07941	0.14241	insignificant	2	8	8
chr13	97840597	97842597	Fam169a	-0.11817	2.6E-30	hypomethylated	-0.0037293	0.31722	insignificant	65	198	194
chr13	97906891	97908891	Gfm2	-0.1602	0.000027545	hypomethylated	-0.019361	0.29115	insignificant	9	50	54
chr13	97907881	97909881	Nsa2	-0.10833	0.36359	insignificant	-0.04214	0.44613	insignificant	7	33	37
chr13	97968312	97970312	Hexb	-0.45567	1.58E-17	stronglyHypometh	-0.010008	0.0019833	hypomethylated	6	22	22
chr13	98010059	98012059	Enc1	-0.11394	9.04E-37	hypomethylated	-0.0017741	0.47508	insignificant	43	160	172
chr13	98976120	98978120	Rgnef	-0.096705	1.59E-11	hypomethylated	0.015852	0.8277	insignificant	24	78	78
chr13	99032196	99034196	Ankra2	-0.12359	4.39E-32	hypomethylated	-0.0042509	0.022323	hypomethylated	35	120	123
chr13	99032947	99034947	Ankra2	-0.12437	1.91E-24	hypomethylated	0.0011355	0.0026605	inconclusive	27	90	92
chr13	99086922	99088922	Btf3	-0.23497	0.011419	hypomethylated	-0.0077886	0.62179	insignificant	15	70	66
chr13	99086961	99088961	Btf3	-0.25187	0.039953	hypomethylated	-0.0082004	0.74714	insignificant	15	62	58
chr13	99123199	99125199	Foxd1	-0.11103	9.95E-26	hypomethylated	0.011365	0.66574	insignificant	57	153	143
chr13	99407365	99409365	Tmem174	-0.076389	0.18063	insignificant	0.0125	0.51789	insignificant	2	4	4
chr13	99464786	99466786	Tmem171	-0.19031	1.25E-15	hypomethylated	-0.049605	0.0011106	hypomethylated	12	50	46
chr13	99585404	99587404	Fcho2	-0.090448	0.21021	insignificant	-0.011866	0.68044	insignificant	6	20	20
chr13	99660997	99662997	Tnpo1		1	noCoverage	0.1875	0.6332	insignificant	0	4	4
chr13	99696339	99698339	Tnpo1	-0.08167	0.042359	hypomethylated	0.0060886	0.67872	insignificant	15	58	59
chr13	99953777	99955777	Zfp366	-0.96429	0.010695	stronglyHypometh	-0.11905	0.36067	insignificant	2	4	4
chr13	100113740	100115740	Mrps27	-0.17129	0.000045625	hypomethylated	-0.021724	0.13242	insignificant	7	52	60
chr13	100286557	100288557	Mtap1b	-0.13958	0.00077375	hypomethylated	-0.015142	0.42546	insignificant	3	26	26
chr13	100670638	100672638	Cartpt	-0.3179	1.7E-11	hypomethylated	-0.077785	0.0023112	hypomethylated	11	33	31
chr13	100785594	100787594	Mccc2	0.078846	1	insignificant	-0.046154	0.44327	insignificant	1	4	4
chr13	100874025	100876025	Bdp1	-0.22221	2.98E-24	hypomethylated	0.023029	0.60433	insignificant	15	41	42

chr13	100876973	100878973	Serf1	0.01006	0.027497	inconclusive	-0.0011421	0.0013608	hypomethylated	13	111	125
chr13	100893809	100895809	Smn1	-0.18002	4.69E-21	hypomethylated	-0.017286	0.51662	insignificant	23	60	64
chr13	101322453	101324453	Ocln	-0.22879	1.66E-11	hypomethylated	-0.029701	0.12897	insignificant	9	46	46
chr13	101386899	101388899	Marveld2	-0.071952	0.23515	insignificant	-0.0067205	1	insignificant	5	12	12
chr13	101386926	101388926	Marveld2	-0.2289	0.021811	hypomethylated	-0.035189	0.59676	insignificant	2	4	4
chr13	101420297	101422297	Taf9	-0.10427	1.3E-53	hypomethylated	-0.0049368	0.0012275	hypomethylated	65	191	190
chr13	101420561	101422561	Taf9	-0.10014	2.69E-52	hypomethylated	-0.0066754	0.00095441	hypomethylated	63	187	186
chr13	101421014	101423014	Taf9	-0.094289	3.27E-38	hypomethylated	0.0012695	0.040047	inconclusive	50	155	156
chr13	101438435	101440435	Ccdc125	-0.17139	3.16E-14	hypomethylated	-0.028815	0.21104	insignificant	19	59	54
chr13	101500897	101502897	Cdk7	-0.056302	0.082844	insignificant	0.016946	0.42861	insignificant	3	18	18
chr13	101545854	101547854	Cenph		1	noCoverage	-0.034731	0.023564	inconclusive	0	22	22
chr13	101556441	101558441	Ccnb1		1	noCoverage	0.07779	0.68855	insignificant	0	32	30
chr13	101603382	101605382	Slc30a5	-0.12299	0.0037163	hypomethylated	0.022794	0.94269	insignificant	4	32	33
chr13	102462602	102464602	Pik3r1	-0.14977	0.070455	insignificant	0.0283	0.77702	insignificant	8	20	24
chr13	102538172	102540172	Pik3r1	-0.11956	3.55E-14	hypomethylated	-0.0032766	0.69132	insignificant	23	78	77
chr13	103482637	103484637	Cd180	-0.55207	0.0039812	stronglyHypometh	-0.052277	0.12028	insignificant	2	10	11
chr13	104710594	104712594	Erbb2ip	-0.093415	6.33E-45	hypomethylated	0.0044186	0.088148	insignificant	79	205	219
chr13	104898869	104900869	Sgtb	-0.13618	1.24E-08	hypomethylated	0.01291	0.0028577	inconclusive	26	113	121
chr13	104899694	104901694	Nln	-0.12335	0.00000374	hypomethylated	0.02595	0.079209	insignificant	20	89	89
chr13	104968177	104970177	Trim23	-0.15545	0.14146	insignificant	0.0050412	0.52991	insignificant	8	38	38
chr13	104968546	104970546	2410002O22Rik	-0.17063	0.35631	insignificant	-0.02714	0.55174	insignificant	5	14	14
chr13	105018034	105020034	Cenpk	-0.10775	2.45E-13	hypomethylated	-0.0099922	0.75414	insignificant	18	56	56
chr13	105018467	105020467	Cenpk	-0.13843	2.41E-13	hypomethylated	-0.024885	0.78886	insignificant	18	58	57
chr13	105018924	105020924	Cenpk	-0.13374	0.017003	hypomethylated	-0.039659	0.46285	insignificant	9	30	29
chr13	105076952	105078952	Adamts6	-0.33764	0.57416	insignificant	-0.0043084	0.45324	insignificant	2	14	15
chr13	105606318	105608318	Srek1ip1	-0.24492	1.45E-15	hypomethylated	0.0057576	0.002269	inconclusive	20	57	57
chr13	105607033	105609033	Srek1ip1	-0.24512	2.27E-14	hypomethylated	0.0032953	0.0024739	inconclusive	16	45	45
chr13	105653973	105655973	Fam159b	-0.43018	0.038506	stronglyHypometh	0.021209	0.0173	hypermethylated	1	6	6
chr13	105845010	105847010	Rgs7bp	-0.3537	0.0030553	stronglyHypometh	-0.0037037	0.51649	insignificant	1	4	4
chr13	105871201	105873201	4933425L06Rik	-0.39444	0.18073	insignificant	-0.014042	0.10698	insignificant	2	10	10
chr13	106083094	106085094	Rnf180	-0.099334	1.05E-10	hypomethylated	0.00017	0.35239	insignificant	37	109	109
chr13	106232772	106234772	Htr1a	-0.20059	1.59E-14	hypomethylated	0.029082	0.10688	insignificant	12	34	36
chr13	107726995	107728995	Ipo11	-0.34415	0.00017367	stronglyHypometh	0.12175	0.23858	insignificant	3	27	32
chr13	107736208	107738208	Dimt1	-0.091758	0.22481	insignificant	-0.052435	0.015883	hypomethylated	10	74	84
chr13	107811649	107813649	3830408C21Rik	-0.089556	1.69E-34	hypomethylated	-0.010123	0.37015	insignificant	89	257	260
chr13	107812194	107814194	3830408C21Rik	-0.072238	7.11E-18	hypomethylated	-0.0076315	0.12112	insignificant	61	173	169
chr13	108680258	108682258	Zswim6	-0.11519	2.15E-37	hypomethylated	-0.0042228	0.14261	insignificant	52	182	204
chr13	108833667	108835667	2810008M24Ril	-0.14455	4.72E-10	hypomethylated	-0.0054123	0.85064	insignificant	18	92	92
chr13	108947931	108949931	Ercc8	-0.17723	5.97E-27	hypomethylated	-0.022952	0.40066	insignificant	29	104	112
chr13	108948819	108950819	Ndufaf2	-0.13046	0.00000463	hypomethylated	-0.0028163	0.05122	insignificant	13	44	48
chr13	109003597	109005597	Elovl7	-0.16443	2.83E-36	hypomethylated	-0.015879	0.51816	insignificant	35	127	125
chr13	109105530	109107530	Depdc1b	-0.11855	1.17E-25	hypomethylated	-0.0021	0.034528	hypomethylated	45	104	118
chr13	110693016	110695016	Mir1904	-0.082773	4.5E-15	hypomethylated	-0.0092686	0.32615	insignificant	32	97	99
chr13	111070414	111072414	Rab3c	-0.23112	1	lowCoverage	0.009433	0.51657	insignificant	1	13	10
chr13	111184251	111186251	Plk2	-0.13844	7.68E-28	hypomethylated	0.0071805	0.45977	insignificant	48	168	164
chr13	112044220	112046220	Actbl2	-0.029223	0.74101	insignificant	0.029804	0.27563	insignificant	6	22	22
chr13	112280249	112282249	Gpbp1	-0.088248	5.29E-18	hypomethylated	0.004131	0.27006	insignificant	50	138	138
chr13	112475385	112477385	Mier3	-0.10939	1.37E-30	hypomethylated	-0.012656	0.97717	insignificant	79	214	229
chr13	112599191	112601191	Map3k1	-0.070253	4.61E-08	hypomethylated	0.010176	0.10185	insignificant	21	104	120
chr13	113253277	113255277	Il6st	-0.14233	1.13E-52	hypomethylated	0.00084498	0.81653	insignificant	50	124	121
chr13	113442518	113444518	Ddx4	0.052332	0.02007	hypermethylated	0.036384	0.00000479	hypermethylated	9	52	52
chr13	113449973	113451973	Slc38a9	0.75893	0.19302	lowCoverage	0.038993	0.7301	insignificant	1	14	15
chr13	113589999	113591999	Ppap2a	-0.14849	9.61E-28	hypomethylated	0.0081735	0.48964	insignificant	22	89	77
chr13	113590130	113592130	Ppap2a	-0.14849	9.61E-28	hypomethylated	0.0081735	0.48964	insignificant	22	89	77
chr13	113717000	113719000	Dhx29	-0.13002	2.44E-16	hypomethylated	0.0060261	0.8948	insignificant	24	96	96
chr13	113717588	113719588	Skiv2l2	-0.11525	2.49E-10	hypomethylated	0.0062789	0.96084	insignificant	19	64	64
chr13	113777009	113779009	Ccno	-0.13493	1.14E-22	hypomethylated	-0.032612	0.27318	insignificant	34	131	146
chr13	113783075	113785075	Gm6320	-0.17143	1.47E-41	hypomethylated	-0.011444	0.023119	hypomethylated	36	128	128
chr13	113825190	113827190	Mir449c		1	noCoverage	0.079805	1	insignificant	0	8	9
chr13	113836596	113838596	Gpx8	-0.14583	0.34603	insignificant	0.00075926	0.40357	insignificant	3	8	10
chr13	113998866	114000866	Esm1	0.096313	0.57555	insignificant	-0.052924	0.00061648	hypomethylated	6	26	26
chr13	114408772	114410772	Snx18	-0.19697	0.000065788	hypomethylated	0.010819	0.18694	insignificant	3	6	6
chr13	114583715	114585715	Arl15	-0.24312	2.94E-34	hypomethylated	-0.0004647	0.17094	insignificant	31	125	128
chr13	115178302	115180302	Ndufs4	-0.26528	0.70949	insignificant	0.035872	0.28991	insignificant	3	6	9
chr13	115248938	115250938	Fst	-0.16224	3.95E-31	hypomethylated	-0.0047467	0.11226	insignificant	38	87	93
chr13	115607444	115609444	Mocs2	-0.17152	0.00074153	hypomethylated	-0.0062822	0.2137	insignificant	20	71	81
chr13	115722249	115724249	Itga2	0.057925	1.43E-10	hypermethylated	0.061017	0.0017801	hypermethylated	8	31	31
chr13	115880366	115882366	Pelo	-0.19311	0.002221	hypomethylated	0.039593	0.84737	insignificant	4	27	29
chr13	115892172	115894172	Itga1	-0.44571	0.0014469	stronglyHypometh	-0.069042	0.169	insignificant	3	6	6
chr13	117099896	117101896	Isl1	-0.10214	0.00050837	hypomethylated	-0.0073568	0.43841	insignificant	24	82	80
chr13	117814323	117816323	Parp8	-0.12806	1.18E-25	hypomethylated	0.0069493	0.6532	insignificant	27	101	100
chr13	118008379	118010379	Emb	-0.17355	3.6E-12	hypomethylated	-0.0062453	0.00011742	hypomethylated	16	99	111
chr13	118390126	118392126	Hcn1	-0.12367	3.56E-10	hypomethylated	0.003673	0.38162	insignificant	43	120	133
chr13	119176059	119178059	Mrps30	-0.38772	0.000021664	stronglyHypometh	0.021456	0.31261	insignificant	0	35	36
chr13	119502505	119504505	Fgf10	-0.11149	0.000099987	hypomethylated	0.0042434	0.64498	insignificant	17	60	58
chr13	120197818	120199818	Nnt	-0.16981	7.3E-09	hypomethylated	-0.037966	0.51742	insignificant	8	24	37
chr13	120197945	120199945	Nnt	-0.23426	0.000006616	hypomethylated	-0.036858	0.75323	insignificant	2	12	17
chr13	120216406	120218406	Paip1	-0.09614	2.82E-29	hypomethylated	-0.0085839	0.054626	insignificant	63	178	218
chr13	120216713	120218713	Paip1	-0.09614	2.82E-29	hypomethylated	-0.0085839	0.054626	insignificant	63	178	218
chr13	120250565	120252565	4833420G17Rik	-0.16217	0.000055817	hypomethylated	0.12983	4.42E-09	hypermethylated	13	45	45
chr13	120275845	120277845	Gm7120	0.18535	0.28656	insignificant	0.081903	9.58E-136	hypermethylated	15	116	166
chr13	120276666	120278666	Gm7120	0.15664	0.8667	insignificant	0.076833	7.6E-155	hypermethylated	14	122	176
chr13	120277191	120279191	Gm7120	0.15139	0.027411	hypermethylated	0.0028586	0.0035743	hypermethylated	12	83	128
chr14	3048285	3050285	Gm2897		1	noCoverage	-0.036465	0.94854	insignificant	0	27	31
chr14	3048304	3050304	Gm2897		1	noCoverage	-0.036465	0.94854	insignificant	0	27	31

chr14	7859963	7861963	Gm10406		1	noCoverage	0.087525	0.61517	insignificant	0	15	11
chr14	7937971	7939971	Gm3696		1	noCoverage	0.014375	0.27964	insignificant	0	26	37
chr14	8164909	8166909	Gm5797		1	noCoverage	-0.12393	0.15415	insignificant	0	7	7
chr14	8649470	8651470	Flnb	-0.17431	8.75E-09	hypomethylated	-0.0032575	0.071656	insignificant	18	131	131
chr14	8834415	8836415	Abhd6	-0.20296	1	insignificant	0.022041	0.51233	insignificant	1	10	10
chr14	8911826	8913826	Rpp14	-0.34168	0.32904	insignificant	-0.057239	0.24683	insignificant	3	14	14
chr14	8929726	8931726	Pxk	-0.085818	1.45E-09	hypomethylated	0.0062382	0.68666	insignificant	31	93	94
chr14	9005506	9007506	Pdhb	-0.2117	1.23E-08	hypomethylated	0.010213	0.22321	insignificant	10	22	22
chr14	9045661	9047661	Kctd6	-0.10691	2.9E-14	hypomethylated	-0.015249	0.082865	insignificant	60	170	173
chr14	9211277	9213277	Oit1	-0.020833	1	insignificant	-0.038484	0.79489	insignificant	2	6	6
chr14	9498804	9500804	4930452B06Rik	-0.098677	0.00002553	hypomethylated	0.21443	0.56603	insignificant	3	6	7
chr14	11994546	11996546	Fhit		1	noCoverage	-0.01988	0.72383	insignificant	0	6	6
chr14	12385066	12387066	Ptprg	-0.13149	1.44E-31	hypomethylated	0.0024713	0.97849	insignificant	66	194	220
chr14	13115722	13117722	3830406C13Rik	-0.22183	3.01E-17	hypomethylated	-0.022865	0.22533	insignificant	17	43	40
chr14	13178379	13180379	Fezf2	-0.20742	0.000000935	hypomethylated	0.0091456	0.95907	insignificant	11	59	60
chr14	14116293	14118293	Synpr	-0.38431	0.000000938	stronglyHypometh	0.019609	0.47077	insignificant	6	17	20
chr14	14117152	14119152	Synpr	-0.30082	0.10355	insignificant	0.025898	1	insignificant	3	10	10
chr14	14793739	14795739	Thoc7	-0.085694	0.00016307	hypomethylated	0.05168	0.14521	insignificant	21	84	93
chr14	14844004	14846004	Atxn7	-0.1086	1.8E-13	hypomethylated	0.016057	0.37781	insignificant	41	108	114
chr14	14953418	14955418	Psmd6	-0.17568	0.22772	insignificant	-0.020936	0.88991	insignificant	2	8	8
chr14	15178008	15180008	Il3ra	-0.081151	0.84596	insignificant	0.038259	0.0006065	hypermethylated	13	76	110
chr14	15534538	15536538	Slc4a7	-0.13018	4.21E-55	hypomethylated	0.0075575	0.11802	insignificant	65	195	213
chr14	17080827	17082827	Ngly1	-0.14107	1.73E-11	hypomethylated	-0.0023947	0.86637	insignificant	10	81	80
chr14	17196719	17198719	Top2b	-0.10224	3.46E-24	hypomethylated	0.0048556	0.090647	insignificant	39	139	138
chr14	17407724	17409724	Rarb	-0.1408	0.59095	insignificant	0.019102	0.93909	insignificant	5	42	48
chr14	18492473	18494473	Thrb	-0.12904	6.04E-29	hypomethylated	-0.018402	1	insignificant	33	133	131
chr14	19071620	19073620	Nr1d2	-0.22554	0.013811	hypomethylated	0.025017	0.096637	insignificant	5	31	35
chr14	19102655	19104655	Nkiras1	-0.11785	3.25E-32	hypomethylated	0.0093203	0.42472	insignificant	38	153	158
chr14	19103500	19105500	Rpl15	-0.12604	1.39E-22	hypomethylated	0.0034664	0.32508	insignificant	24	68	76
chr14	19726141	19728141	Ube2e2	-0.039745	0.00053556	hypomethylated	-0.028146	0.51066	insignificant	15	41	60
chr14	20569478	20571478	Nid2	-0.18134	0.000022322	hypomethylated	-0.041871	0.073115	insignificant	15	59	60
chr14	20643045	20645045	2700060E02Rik	-0.14347	0.10632	insignificant	-0.0083248	0.62842	insignificant	7	47	46
chr14	20796471	20798471	Gng2	-0.27227	0.006598	hypomethylated	0.1979	0.63173	insignificant	5	16	19
chr14	20902394	20904394	1810063B07Rik	-0.14944	1.35E-13	hypomethylated	0.093037	0.7921	insignificant	10	16	18
chr14	21001004	21003004	Kcnk5	-0.17202	1	insignificant	0.006356	0.58927	insignificant	1	20	20
chr14	21088384	21090384	Kcnk16	-0.084821	0.81631	insignificant	0.19355	0.014891	hypermethylated	4	14	9
chr14	21112911	21114911	Nudt13	-0.59221	0.0095788	stronglyHypometh	-0.0712	0.92878	insignificant	5	26	29
chr14	21166383	21168383	Fam149b	-0.14885	0.60585	insignificant	0.039827	1	insignificant	7	52	39
chr14	21166443	21168443	Ecd	-0.14885	0.60585	insignificant	0.039827	1	insignificant	7	52	39
chr14	21167343	21169343	Ecd	-0.18708	1	insignificant	0.047608	0.94902	insignificant	3	40	32
chr14	21208132	21210132	Dnajc9	-0.10273	0.052671	insignificant	-0.013205	1	insignificant	2	19	19
chr14	21212777	21214777	Ttc18	-0.14359	0.00000209	hypomethylated	-0.0029972	0.10946	insignificant	19	40	41
chr14	21299355	21301355	Anxa7	0.39306	0.000102	stronglyHypermeth	-0.0069152	0.36493	insignificant	1	12	14
chr14	21364514	21366514	1810062O18Rik	-0.073874	6.65E-09	hypomethylated	-0.00052905	1	insignificant	43	110	110
chr14	21437576	21439576	Usp54	-0.59091	0.0026779	stronglyHypometh	-0.024242	1	insignificant	1	2	2
chr14	21492542	21494542	Sec24c	-0.19709	3.23E-21	hypomethylated	-0.028315	0.918	insignificant	18	38	41
chr14	21513189	21515189	Fut11	-0.10903	2.29E-30	hypomethylated	-0.0053128	0.8021	insignificant	44	136	147
chr14	21521248	21523248	Chchd1	-0.19509	3.62E-08	hypomethylated	-0.0093354	0.3756	insignificant	14	92	91
chr14	21522321	21524321	Chchd1	-0.33654	0.2102	insignificant	0.0080765	0.68903	insignificant	2	36	36
chr14	21525773	21527773	2310021P13Rik	-0.17159	1.65E-14	hypomethylated	0.010025	0.95187	insignificant	20	69	58
chr14	21553784	21555784	Ndst2		1	noCoverage		1	noCoverage	2	0	8
chr14	21613310	21615310	Camk2g	-0.12053	0.000156	hypomethylated	-0.017225	0.30691	insignificant	9	30	30
chr14	21654883	21656883	Plau	-0.18056	1	insignificant	0.054783	0.53588	insignificant	2	10	10
chr14	21747654	21749654	Vcl	-0.096813	6.67E-25	hypomethylated	0.012161	0.30661	insignificant	40	127	136
chr14	21870854	21872854	Ap3m1	-0.18025	3.5E-23	hypomethylated	-0.027785	0.19653	insignificant	18	54	54
chr14	21871664	21873664	Ap3m1	-0.2175	0.016563	hypomethylated	-0.026995	0.32717	insignificant	7	36	36
chr14	22318075	22320075	Myst4	-0.13095	1.42E-34	hypomethylated	-0.0041252	0.6113	insignificant	56	185	186
chr14	22533798	22535798	Dupd1	-0.10763	0.13925	insignificant	-0.023834	0.0062618	hypomethylated	3	15	15
chr14	22562101	22564101	Dusp13		1	noCoverage	0.024524	0.55528	insignificant	0	8	8
chr14	22567844	22569844	Samd8	-0.21417	0.0001549	hypomethylated	-0.021015	0.14252	insignificant	5	17	20
chr14	22568752	22570752	Samd8	-0.16246	1.36E-17	hypomethylated	0.0034832	0.065154	insignificant	28	69	83
chr14	22649782	22651782	Vdac2	-0.12754	2.42E-17	hypomethylated	-0.019407	0.074101	insignificant	14	63	64
chr14	22668132	22670132	Comtd1	-0.20613	1.29E-08	hypomethylated	-0.012972	9.06E-10	hypomethylated	12	26	28
chr14	22808823	22810823	Zfp503	-0.037539	0.29016	insignificant	0.025477	0.14768	insignificant	21	96	96
chr14	22837933	22839933	1700112E06Rik	-0.15078	1.29E-26	hypomethylated	-0.0049891	0.0083757	hypomethylated	18	41	42
chr14	24823427	24825427	Kcnma1	-0.096007	5.86E-22	hypomethylated	0.0059267	0.94381	insignificant	54	159	158
chr14	25065142	25067142	Dlg5	-0.055429	0.2648	insignificant	-0.072113	0.01941	hypomethylated	2	14	14
chr14	25111585	25113585	E330034G19Rik	-0.31297	0.037131	hypomethylated	0.029749	0.77489	insignificant	1	8	8
chr14	25306268	25308268	Polr3a	-0.54519	1.87E-41	stronglyHypometh	0.031879	0.32221	insignificant	5	33	33
chr14	25308902	25310902	Rps24	-0.26168	2.27E-11	hypomethylated	-0.020827	0.27297	insignificant	16	59	57
chr14	26277777	26279777	Zmiz1	-0.10498	1.18E-34	hypomethylated	-0.0030998	0.29733	insignificant	55	168	175
chr14	26352924	26354924	Mir3075	-0.57727	0.014829	stronglyHypometh	-0.0085227	1	insignificant	3	8	6
chr14	26512655	26514655	Ppif	-0.088946	1.47E-11	hypomethylated	-0.013269	0.5694	insignificant	33	130	125
chr14	26588342	26590342	Zcchc24	-0.13591	0.00000429	hypomethylated	-0.0059665	0.42806	insignificant	12	56	54
chr14	26660640	26662640	Anxa11	-0.18774	6.53E-17	hypomethylated	-0.0018888	0.13571	insignificant	25	103	102
chr14	27353226	27355226	Slmap	-0.0923	1.09E-18	hypomethylated	0.0041372	0.33841	insignificant	36	124	125
chr14	27398035	27400035	Fam116a	-0.12976	2.24E-19	hypomethylated	0.032145	0.10162	insignificant	34	101	97
chr14	27456682	27458682	Arf4	-0.094636	1.34E-23	hypomethylated	-0.0071828	0.12439	insignificant	32	125	122
chr14	27489332	27491332	Pde12	-0.080141	0.00027613	hypomethylated	0.025212	0.010258	inconclusive	14	42	45
chr14	27783737	27785737	Appl1	-0.09292	0.15513	insignificant	0.0073122	0.87178	insignificant	3	59	59
chr14	27812547	27814547	Hesx1	-0.33628	0.016466	stronglyHypometh	0.088271	0.39468	insignificant	5	12	15
chr14	27851186	27853186	Il17rd	-0.11204	2.16E-33	hypomethylated	0.0086778	0.29677	insignificant	36	137	141
chr14	28050224	28052224	Arhgef3	-0.42082	0.000088912	stronglyHypometh	-0.018114	0.76887	insignificant	3	12	12
chr14	28241032	28243032	D14Abb1e	-0.070183	0.67863	insignificant	0.033371	0.000007591	inconclusive	43	173	180
chr14	28321646	28323646	Ccdc66	-0.38189	8.11E-14	stronglyHypometh	-0.0094277	0.66234	insignificant	7	26	25

chr14	28434627	28436627	Erc2	-0.12934	5.96E-10	hypomethylated	-0.0084732	0.49243	insignificant	25	103	104
chr14	29317658	29319658	Wnt5a	-0.16432	3.48E-20	hypomethylated	-0.003784	0.2415	insignificant	33	69	69
chr14	29830393	29832393	Lrtm1	-0.23649	0.00027795	hypomethylated	-0.026313	0.0090575	hypomethylated	2	12	14
chr14	30535050	30537050	Cacna2d3	-0.12554	1.51E-31	hypomethylated	-0.0010582	0.080395	insignificant	54	149	164
chr14	30780565	30782565	Selk	-0.20918	1.32E-26	hypomethylated	-0.025751	0.025663	hypomethylated	23	76	80
chr14	30790522	30792522	Actr8	-0.14177	0.00000489	hypomethylated	-0.0074625	0.88236	insignificant	5	19	22
chr14	30821185	30823185	Chdh	-0.12907	2.07E-21	hypomethylated	0.0065716	0.031366	hypermethylated	26	82	85
chr14	30822082	30824082	Il17rb	-0.1064	5.04E-09	hypomethylated	0.0069695	0.44449	insignificant	18	60	63
chr14	30833488	30835488	Chdh	-0.36094	0.054018	insignificant	0.019487	0.59957	insignificant	3	8	8
chr14	31166672	31168672	Cacna1d	-0.056257	9.69E-08	hypomethylated	-0.0061109	0.46963	insignificant	25	84	107
chr14	31291750	31293750	Dcp1a	-0.16091	5.89E-19	hypomethylated	0.026272	0.029502	inconclusive	19	73	75
chr14	31361334	31363334	Tkt	-0.11807	1.86E-13	hypomethylated	0.091667	0.95941	insignificant	14	55	57
chr14	31466560	31468560	Rft1	-0.13205	1.61E-15	hypomethylated	-0.023403	0.75767	insignificant	13	30	30
chr14	31527034	31529034	Sfmbt1	-0.095141	2.64E-34	hypomethylated	0.0062163	0.000048108	inconclusive	47	155	143
chr14	31527352	31529352	Sfmbt1	-0.08103	2.08E-34	hypomethylated	0.0054338	0.00056695	inconclusive	63	232	212
chr14	31527800	31529800	Sfmbt1	-0.083451	1.9E-41	hypomethylated	0.0029009	0.0010685	inconclusive	83	290	266
chr14	31637779	31639779	Tmem110	-0.10605	4.18E-08	hypomethylated	0.0063877	0.069977	insignificant	22	66	66
chr14	31756475	31758475	Itih1	0.13564	0.68003	insignificant	0.040018	0.31525	insignificant	4	11	11
chr14	31763673	31765673	Nek4	-0.17223	0.000000767	hypomethylated	0.014205	0.388	insignificant	14	74	77
chr14	31813578	31815578	Glt8d1	-0.15552	8.22E-34	hypomethylated	0.0073015	0.16966	insignificant	28	144	148
chr14	31813618	31815618	Glt8d1	-0.15552	8.22E-34	hypomethylated	0.0073015	0.16966	insignificant	28	144	148
chr14	31814852	31816852	Glt8d1	-0.19134	0.0013361	hypomethylated	0.033177	0.012016	hypermethylated	7	54	60
chr14	31831323	31833323	Pbrm1	-0.13527	2.69E-09	hypomethylated	-0.0093966	0.13847	insignificant	22	85	86
chr14	31832275	31834275	Gnl3	-0.089629	0.000011018	hypomethylated	-0.0042639	0.60231	insignificant	19	59	68
chr14	31942116	31944116	2010107H07Rik	-0.18822	0.011646	hypomethylated	-0.0052887	0.61574	insignificant	4	53	52
chr14	31947038	31949038	Nt5dc2	-0.000053215	0.17102	insignificant	0.045692	0.045541	hypermethylated	5	20	20
chr14	32020497	32022497	Tnnc1	-0.21154	0.28126	insignificant	-0.26546	0.00099099	hypomethylated	1	6	6
chr14	32063674	32065674	Bap1	-0.11651	3.47E-26	hypomethylated	-0.023252	0.17224	insignificant	23	156	157
chr14	32064404	32066404	Phf7	-0.11498	1.38E-20	hypomethylated	-0.034635	0.34775	insignificant	18	124	123
chr14	32137082	32139082	Dnahc1	-0.19886	0.000000613	hypomethylated	0.014443	0.74671	insignificant	7	27	27
chr14	32148909	32150909	Capn7	-0.12591	8.76E-18	hypomethylated	0.002571	0.61162	insignificant	24	92	90
chr14	32249219	32251219	Sh3bp5	-0.15989	0.14295	insignificant	-0.0048115	0.94123	insignificant	6	14	14
chr14	32307264	32309264	Eaf1	-0.21583	8.32E-42	hypomethylated	0.0029184	0.48441	insignificant	23	59	55
chr14	32308163	32310163	Eaf1	-0.27794	1.45E-44	hypomethylated	0.023242	0.65127	insignificant	20	54	45
chr14	32390569	32392569	Colq	0.034805	0.75067	insignificant	-0.040405	0.61287	insignificant	1	5	5
chr14	32453242	32455242	Btd	-0.19576	0.000000671	hypomethylated	0.02573	0.73647	insignificant	13	44	49
chr14	32454151	32456151	Hacl1	-0.11824	0.000015051	hypomethylated	0.01658	0.81547	insignificant	12	28	28
chr14	32643601	32645601	Ankrd28	-0.08024	0.000000154	hypomethylated	0.0069962	0.2974	insignificant	26	79	80
chr14	32841288	32843288	Galntl2	-0.20184	0.074343	insignificant	0.0031216	0.79629	insignificant	2	10	10
chr14	32897913	32899913	Oxnad1	-0.17209	5.38E-15	hypomethylated	0.0085923	0.67397	insignificant	22	65	62
chr14	32898851	32900851	Dph3	-0.3079	0.0065218	hypomethylated	0.036545	0.00079346	inconclusive	10	32	34
chr14	32972077	32974077	Ncoa4	-0.17878	1.97E-12	hypomethylated	-0.0026582	0.33253	insignificant	12	44	33
chr14	32977977	32979977	Ncoa4	-0.1026	0.032218	hypomethylated	-0.010214	0.3282	insignificant	4	24	24
chr14	33014156	33016156	Parg	-0.13691	1.83E-21	hypomethylated	-0.020519	0.40627	insignificant	38	110	110
chr14	33015077	33017077	Timm23	-0.09672	9.96E-14	hypomethylated	0.00076245	0.59115	insignificant	28	72	72
chr14	33134204	33136204	Ogdhl	-0.18129	0.000013037	hypomethylated	0.010952	0.0084237	inconclusive	15	32	30
chr14	33278036	33280036	Slc18a3	-0.1548	6.96E-19	hypomethylated	0.0065133	0.063733	insignificant	22	76	72
chr14	33325706	33327706	Ercc6	-0.21041	2.11E-16	hypomethylated	-0.044154	0.78088	insignificant	21	68	79
chr14	33412112	33414112	Prrxl1	-0.21371	1.71E-13	hypomethylated	-0.0034285	0.12766	insignificant	19	60	62
chr14	33498458	33500458	3425401B19Rik	0.16248	0.36238	insignificant	-0.077189	0.40713	insignificant	4	24	24
chr14	33598148	33600148	1810011H11Rik	-0.089286	1	insignificant	0.012229	1	insignificant	2	4	4
chr14	33668941	33670941	E130203B14Rik	-0.11228	0.000000573	hypomethylated	0.0059833	0.076142	insignificant	26	71	70
chr14	34029008	34031008	Arhgap22	0.11726	0.2261	insignificant	0.10165	0.40871	insignificant	2	4	4
chr14	34260344	34262344	Mapk8	-0.086824	0.000002513	hypomethylated	-0.0079728	0.26509	insignificant	19	60	56
chr14	34401455	34403455	Ptpn20	-0.18895	0.011915	hypomethylated	-0.05703	0.0026432	hypomethylated	4	32	35
chr14	34735772	34737772	Gdf10	-0.096891	6.55E-16	hypomethylated	-0.0064554	0.75999	insignificant	41	118	126
chr14	34753224	34755224	Gdf2	-0.11244	0.42797	insignificant	-0.13998	0.16108	insignificant	1	10	10
chr14	34791950	34793950	Zfp488	-0.35223	0.020682	stronglyHypometh	-0.030258	0.35927	insignificant	5	35	34
chr14	34898175	34900175	Anxa8	-0.55669	0.042588	stronglyHypometh	-0.0072334	0.55771	insignificant	2	23	20
chr14	34965605	34967605	Ppyr1	-0.16822	0.51308	insignificant	0.031187	0.75603	insignificant	4	8	8
chr14	35014819	35016819	Gprin2	-0.14514	0.10307	insignificant	0.12932	0.015152	hypermethylated	8	24	20
chr14	35032231	35034231	Syt15	-0.17412	4.81E-30	hypomethylated	0.0082039	0.000000546	inconclusive	17	70	68
chr14	35122912	35124912	Glud1	-0.093803	2.84E-52	hypomethylated	-0.0049251	0.47239	insignificant	73	227	229
chr14	35123689	35125689	Fam35a	-0.095688	1.12E-41	hypomethylated	-0.001013	0.50904	insignificant	54	185	189
chr14	35157240	35159240	Gm3219	0.25	1	insignificant	0.12083	0.77241	insignificant	1	4	4
chr14	35168579	35170579	Fam25c	0.039877	1	insignificant	-0.097386	0.012578	hypomethylated	1	6	6
chr14	35187689	35189689	Mmrn2	-0.44766	0.00000999	stronglyHypometh	0.038167	0.13766	insignificant	5	18	19
chr14	35187855	35189855	Sncg	-0.44766	0.00000999	stronglyHypometh	0.038167	0.13766	insignificant	5	18	19
chr14	35315732	35317732	Bmpr1a	-0.10289	6.25E-13	hypomethylated	-0.0082974	0.82964	insignificant	38	117	117
chr14	35401793	35403793	Ldb3	-0.034403	0.00040321	hypomethylated	-0.0442	0.001485	hypomethylated	5	24	24
chr14	35401867	35403867	Ldb3	-0.037109	0.00035774	hypomethylated	-0.043596	0.0015457	hypomethylated	5	22	22
chr14	35486113	35488113	Wapal	-0.12262	1.42E-44	hypomethylated	-0.0071196	0.64152	insignificant	55	249	256
chr14	35632321	35634321	Grid1	-0.10989	5.41E-34	hypomethylated	-0.0016569	0.039631	hypomethylated	65	206	207
chr14	37781950	37783950	Gcap14	-0.17828	1.35E-20	hypomethylated	-0.072673	0.049136	hypomethylated	13	43	40
chr14	37911497	37913497	Cdhr1	0.26071	1	lowCoverage	-0.18849	0.3085	insignificant	1	11	10
chr14	37948325	37950325	Ghitm	-0.1944	5.19E-14	hypomethylated	0.004898	0.0046704	inconclusive	16	46	47
chr14	37948508	37950508	Ghitm	-0.37117	0.000000198	stronglyHypometh	0.016683	2.26E-08	inconclusive	4	20	21
chr14	40286376	40288376	Nrg3	-0.13467	1.27E-08	hypomethylated	0.0067308	0.041424	hypermethylated	33	92	92
chr14	41706254	41708254	Sh2d4b	-0.29593	0.72862	insignificant	-0.12245	0.20331	insignificant	9	26	31
chr14	41780096	41782096	Tspan14	-0.14228	0.000020275	hypomethylated	-0.039552	0.22141	insignificant	13	44	44
chr14	41827064	41829064	5730469M10Rik		1	noCoverage		1	noCoverage	0	8	0
chr14	41885199	41887199	Dydc1	-0.38333	0.0092087	stronglyHypometh	-0.054855	0.91248	insignificant	5	17	14
chr14	41917669	41919669	Mat1a	-0.33929	0.029315	stronglyHypometh	0.004881	1	insignificant	2	14	10
chr14	41963744	41965744	Mbl1		1	noCoverage	-0.0055286	1	insignificant	0	8	8
chr14	45606785	45608785	Ptger2	-0.16364	0.000014953	hypomethylated	0.011391	0.46176	insignificant	6	24	20

chr14	45838391	45840391	Gpr137c	-0.11666	3.53E-21	hypomethylated	-0.013384	0.0096082	hypomethylated	32	107	107
chr14	45839069	45841069	Txndc16	-0.13994	2.3E-21	hypomethylated	-0.016359	0.018378	hypomethylated	32	125	125
chr14	45938202	45940202	Ero1l	-0.098535	0.00045998	hypomethylated	0.011788	1	insignificant	7	38	38
chr14	45948498	45950498	Psmc6	-0.13819	0.00000996	hypomethylated	0.0069426	0.68488	insignificant	17	50	53
chr14	45969860	45971860	Styx	-0.18822	0.000000215	hypomethylated	0.00086336	0.12344	insignificant	14	46	46
chr14	46008471	46010471	Gnpnat1	-0.089343	0.00018219	hypomethylated	0.0058453	0.32016	insignificant	16	49	60
chr14	46149740	46151740	Fermt2	-0.14544	0.088624	insignificant	-0.028003	0.47432	insignificant	17	80	80
chr14	46277818	46279818	Ddhd1	-0.20169	0.00051826	hypomethylated	0.028716	0.18795	insignificant	10	50	52
chr14	46998141	47000141	Gm15217	0.24949	1	insignificant	0.1433	0.10579	insignificant	1	10	6
chr14	47010274	47012274	Bmp4	-0.12857	0.20673	insignificant	0.086998	0.43154	insignificant	4	10	12
chr14	47379215	47381215	Cdkn3	-0.13098	0.025796	hypomethylated	-0.021161	0.49386	insignificant	10	46	47
chr14	47450918	47452918	Cgrrf1	-0.17032	1.28E-09	hypomethylated	0.0011043	1	insignificant	15	52	53
chr14	47501639	47503639	Samd4	-0.13286	6.21E-36	hypomethylated	0.0049425	0.076251	insignificant	46	168	179
chr14	47620014	47622014	Samd4	0.14085	1	insignificant	-0.13926	0.74215	insignificant	1	14	10
chr14	47809077	47811077	Gch1	-0.20977	0.00039898	hypomethylated	0.051525	0.95737	insignificant	15	40	46
chr14	47895817	47897817	Socs4	-0.13341	6.17E-11	hypomethylated	-0.0036554	0.062348	insignificant	28	115	115
chr14	47896532	47898532	Socs4	-0.14694	8.18E-09	hypomethylated	-0.0097969	0.19146	insignificant	21	81	81
chr14	47916988	47918988	Mapk1ip1l	-0.15529	7.37E-51	hypomethylated	-0.048451	5.62E-37	hypomethylated	42	108	111
chr14	47992534	47994534	Lgals3	-0.17759	6.71E-08	hypomethylated	0.032139	7.73E-09	hypermethylated	13	44	44
chr14	48038082	48040082	Dlgap5	-0.5747	1	lowCoverage	-0.37944	0.02873	stronglyhypometh	1	21	10
chr14	48091235	48093235	Fbxo34	-0.13626	2.29E-17	hypomethylated	0.00546	0.044593	hypermethylated	31	112	97
chr14	48188109	48190109	Atg14	0.16887	0.88864	insignificant	0.0039992	0.11265	insignificant	11	36	50
chr14	48739543	48741543	Peli2	-0.089682	2.1E-15	hypomethylated	-0.0080036	0.76094	insignificant	34	166	155
chr14	48817390	48819390	Gm6498	-0.16354	0.29246	insignificant	0.38267	7.8E-21	stronglyhypermeth	6	17	16
chr14	49065026	49067026	6720456H20Rik	-0.12702	1.4E-14	hypomethylated	-0.029479	0.022796	hypomethylated	33	91	93
chr14	49287962	49289962	Otx2os1	-0.29177	8.14E-11	hypomethylated	-0.060003	0.27003	insignificant	7	50	41
chr14	49497547	49499547	4933429O19Rik	-0.75262	0.059957	insignificant	-0.070478	1	insignificant	2	28	20
chr14	49685169	49687169	Mudeng	-0.15236	2.14E-17	hypomethylated	-0.017806	0.96749	insignificant	44	100	110
chr14	49686342	49688342	Mudeng	-0.26	0.000000863	hypomethylated	0.052078	1	insignificant	2	4	4
chr14	49790901	49792901	Naa30	-0.10099	1.31E-27	hypomethylated	-0.0015966	0.16982	insignificant	59	206	211
chr14	50704354	50706354	Olfr726		1	noCoverage	-0.075	0.61812	insignificant	0	2	2
chr14	51405193	51407193	Ttc5		1	noCoverage	-0.0016514	1	insignificant	0	6	6
chr14	51415403	51417403	Ccnb1ip1	-0.06299	0.78228	insignificant	-0.11975	0.044617	hypomethylated	2	8	8
chr14	51426121	51428121	Rpph1	-0.18818	6.78E-12	hypomethylated	0.0056235	0.54433	insignificant	11	58	57
chr14	51426620	51428620	Parp2	-0.20413	2.21E-12	hypomethylated	0.014235	0.785	insignificant	11	60	57
chr14	51490229	51492229	Tep1		1	noCoverage	-0.041667	0.59013	insignificant	0	6	6
chr14	51512930	51514930	Klhl33		1	noCoverage	0.14459	0.22965	insignificant	0	6	6
chr14	51543695	51545695	Osgep	-0.11258	0.00016684	hypomethylated	0.012637	0.58957	insignificant	16	84	88
chr14	51544568	51546568	Osgep	0.041136	0.6396	insignificant	-0.0026892	0.22321	insignificant	6	42	48
chr14	51550524	51552524	Tmem55b	-0.094602	0.0002105	hypomethylated	-0.0083819	1	insignificant	8	56	56
chr14	51562977	51564977	Pnp	-0.13322	0.024151	hypomethylated	0.013672	0.43692	insignificant	10	51	45
chr14	51626425	51628425	Rnase10		1	noCoverage	0.22292	0.0099957	hypermethylated	0	8	8
chr14	51626603	51628603	Rnase10		1	noCoverage	0.22292	0.0099957	hypermethylated	0	8	8
chr14	51691117	51693117	Olfr750	-0.07037	0.054541	insignificant	-0.080952	0.046215	hypomethylated	3	6	6
chr14	51709751	51711751	Ang	-0.21861	1	lowCoverage	-0.0027742	0.42108	insignificant	1	33	32
chr14	51780934	51782934	Ear5	0.2575	0.55197	insignificant	0.17171	7.25E-11	hypermethylated	3	15	14
chr14	52503516	52505516	Mettl17	-0.19667	0.00000598	hypomethylated	-0.01509	0.79005	insignificant	13	62	66
chr14	52512284	52514284	Slc39a2	-0.19881	0.015181	hypomethylated	0.040793	0.15508	insignificant	4	11	12
chr14	52533163	52535163	Ndrg2	-0.20567	0.000000113	hypomethylated	-0.0212	0.29881	insignificant	11	30	29
chr14	52603507	52605507	Arhgef40	-0.16647	7.06E-26	hypomethylated	-0.0018969	0.91582	insignificant	21	113	117
chr14	52635539	52637539	G630016D24Rik		1	noCoverage	-0.14982	0.83957	insignificant	0	9	6
chr14	52687989	52689989	Snord58b	0.04543	1	insignificant	-0.034066	0.39198	insignificant	1	7	16
chr14	52723703	52725703	Hnrnpc	-0.37148	0.000000145	stronglyHypometh	-0.031558	0.94329	insignificant	6	18	26
chr14	52729577	52731577	Rpgrip1		1	noCoverage	0.14629	0.092567	insignificant	0	13	16
chr14	52816914	52818914	Chd8	-0.12398	1.53E-13	hypomethylated	0.004058	0.56318	insignificant	17	55	56
chr14	52829559	52831559	Snord8		1	noCoverage	-0.076625	0.0089936	hypomethylated	0	10	10
chr14	52857247	52859247	Chd8		1	noCoverage	0.14448	0.28965	insignificant	0	3	4
chr14	52897820	52899820	Tox4	-0.14014	2.69E-10	hypomethylated	-0.021502	0.84632	insignificant	18	68	93
chr14	52899070	52901070	Tox4	-0.16457	0.00000569	hypomethylated	-0.040215	0.070371	insignificant	11	31	44
chr14	52948345	52950345	Sall2		1	noCoverage	0.071429	0.031298	hypermethylated	0	7	7
chr14	52969719	52971719	Olfr1513		1	noCoverage	-0.36649	0.063003	insignificant	0	4	5
chr14	53069067	53071067	Olfr1509	-0.3637	0.03899	stronglyHypometh	-0.087045	0.0019769	hypomethylated	1	15	14
chr14	53087136	53089136	Olfr1508		1	noCoverage	0.1489	1	lowCoverage	0	4	1
chr14	54873604	54875604	Dad1	0.0047993	0.075826	insignificant	-0.057663	0.023607	hypomethylated	7	34	44
chr14	54877971	54879971	Abhd4	-0.15178	0.086456	insignificant	0.023086	0.95137	insignificant	3	38	50
chr14	54979524	54981524	Oxa1l	-0.15299	6.97E-10	hypomethylated	0.039684	1	insignificant	16	60	60
chr14	55036536	55038536	Slc7a7	-0.16793	0.48159	insignificant	-0.053234	1	insignificant	2	11	10
chr14	55044745	55046745	Mrpl52	-0.14606	0.033239	hypomethylated	0.0049485	0.82762	insignificant	15	64	63
chr14	55049440	55051440	Mmp14	-0.24614	2.52E-24	hypomethylated	0.0066669	0.083973	insignificant	23	93	92
chr14	55081983	55083983	Lrp10	-0.13076	1.84E-27	hypomethylated	-0.0012856	0.36474	insignificant	42	126	126
chr14	55093936	55095936	Rem2	-0.23131	0.027503	hypomethylated	0.1585	0.13659	insignificant	1	4	4
chr14	55173198	55175198	Haus4	-0.15401	5.63E-09	hypomethylated	0.026043	0.79776	insignificant	9	59	59
chr14	55196253	55198253	Jub	-0.1109	0.0015422	hypomethylated	0.0099286	0.000019007	hypermethylated	12	35	35
chr14	55224745	55226745	4931414P19Rik	-0.16792	1.36E-12	hypomethylated	0.043224	0.010947	hypermethylated	18	40	43
chr14	55235622	55237622	Psmb5	-0.31251	1.86E-37	hypomethylated	0.031728	0.17847	insignificant	12	68	56
chr14	55236832	55238832	Psmb5	-0.08624	0.000024278	hypomethylated	0.10051	0.000000363	hypermethylated	7	18	20
chr14	55243146	55245146	Psmb11	0.16445	0.0075055	hypermethylated	0.071268	5.71E-13	hypermethylated	2	14	14
chr14	55260201	55262201	Acin1	-0.09981	0.27046	insignificant	-0.0053519	1	insignificant	14	37	37
chr14	55304007	55306007	1700123O20Rik	-0.1463	1.53E-24	hypomethylated	0.013267	0.62667	insignificant	31	101	88
chr14	55331011	55333011	Cebpe	-0.58333	0.39437	lowCoverage	-0.010965	0.00023776	hypomethylated	1	7	4
chr14	55482995	55484995	Homez	-0.17419	0.0037657	hypomethylated	0.059398	0.86131	insignificant	9	33	35
chr14	55496375	55498375	Ppp1r3e	-0.17329	0.00034358	hypomethylated	-0.016689	0.02596	hypomethylated	3	13	13
chr14	55501261	55503261	Bcl2l2	-0.28219	1.87E-15	hypomethylated	-0.022313	0.28357	insignificant	12	34	34
chr14	55511979	55513979	Pabpn1	-0.1462	8.89E-19	hypomethylated	-0.007289	0.033291	hypomethylated	17	89	77
chr14	55531969	55533969	Slc22a17		1	noCoverage	0.1408	0.012154	hypermethylated	0	14	5

chr14	55545625	55547625	Efs	-0.1221	0.00000006	inconclusive	0.065824	0.31556	insignificant	11	40	42
chr14	55554306	55556306	Cmtm5	-0.2346	0.10673	insignificant	0.15175	0.000000829	hypermethylated	6	14	20
chr14	55567979	55569979	Myh6	-0.36408	0.24602	insignificant	-0.026151	0.095309	insignificant	1	16	19
chr14	55613386	55615386	Myh7	0.175	1	insignificant	0.069819	1	insignificant	2	9	7
chr14	55633290	55635290	Ngdn	-0.24357	0.000000235	hypomethylated	-0.028905	0.056606	insignificant	14	50	51
chr14	55690927	55692927	Zfhx2as	0.075962	1	insignificant	-0.0073679	0.91945	insignificant	2	34	35
chr14	55710885	55712885	Zfhx2	-0.013607	0.020295	hypomethylated	-0.0056152	0.91326	insignificant	11	50	50
chr14	55712620	55714620	Thtpa	-0.3132	3.7E-18	hypomethylated	0.050153	0.47304	insignificant	9	30	26
chr14	55725430	55727430	Ap1g2	-0.027409	0.30042	insignificant	0.00010809	0.25936	insignificant	4	17	14
chr14	55734274	55736274	Jph4	-0.1563	2.38E-09	hypomethylated	0.034824	0.050792	insignificant	8	42	42
chr14	55735115	55737115	Jph4	-0.43324	2.82E-13	stronglyHypometh	0.040906	0.13703	insignificant	5	18	16
chr14	56096594	56098594	Dhrs4	-0.19774	0.58884	insignificant	0.0031733	0.54618	insignificant	1	26	29
chr14	56108929	56110929	Lrrc16b	-0.13812	2.01E-14	hypomethylated	-0.024145	0.90131	insignificant	31	129	129
chr14	56128284	56130284	Cpne6	-0.35597	0.0015686	stronglyHypometh	0.0097301	0.82562	insignificant	2	4	5
chr14	56143802	56145802	Nrl		1	noCoverage	0.0092105	0.57095	insignificant	0	5	4
chr14	56158102	56160102	Pck2	-0.21753	0.00016309	hypomethylated	0.012547	0.43994	insignificant	7	33	38
chr14	56177865	56179865	Dcaf11	-0.13254	2.11E-11	hypomethylated	-0.0072937	0.033972	hypomethylated	25	114	113
chr14	56178759	56180759	Dcaf11	-0.11422	0.00000048	hypomethylated	0.0054456	0.14215	insignificant	22	102	102
chr14	56193510	56195510	Fitm1	-0.40236	0.00000121	stronglyHypometh	0.050796	0.17719	insignificant	4	8	12
chr14	56196330	56198330	Psme1	-0.19287	3.37E-13	hypomethylated	0.0069232	0.92	insignificant	12	52	52
chr14	56204091	56206091	Fam158a		1	noCoverage	0.098665	0.74994	insignificant	0	14	9
chr14	56209626	56211626	Rnf31	-0.13035	8.41E-20	hypomethylated	-0.000036871	0.40556	insignificant	29	118	126
chr14	56209858	56211858	Psme2	-0.12333	5.07E-18	hypomethylated	0.0016283	0.34714	insignificant	27	109	117
chr14	56209938	56211938	Psme2	-0.12575	2.92E-18	hypomethylated	0.0014882	0.39211	insignificant	27	107	115
chr14	56221821	56223821	Irf9	-0.4113	0.015894	stronglyHypometh	-0.034466	0.88354	insignificant	4	20	20
chr14	56222415	56224415	Irf9	-0.46215	0.0026032	stronglyHypometh	-0.052823	0.35817	insignificant	4	22	22
chr14	56236006	56238006	Rec8	-0.17262	0.35558	insignificant	0.18869	0.070554	insignificant	2	4	4
chr14	56254515	56256515	Ipo4	-0.14332	0.016375	hypomethylated	-0.030252	3.37E-14	hypomethylated	9	28	28
chr14	56262643	56264643	Tm9sf1	-0.40097	0.14715	insignificant	-0.12986	0.52461	insignificant	4	16	17
chr14	56268018	56270018	Tssk4	-0.20267	0.018786	hypomethylated	-0.12	0.00046527	hypomethylated	3	18	18
chr14	56268128	56270128	Tssk4	-0.20267	0.018786	hypomethylated	-0.12	0.00046527	hypomethylated	3	18	18
chr14	56279345	56281345	Mdp1	-0.40803	0.00094923	stronglyHypometh	-0.014841	0.4214	insignificant	3	16	20
chr14	56290071	56292071	Gmpr2	-0.021721	0.10394	insignificant	-0.057775	0.26259	insignificant	4	21	21
chr14	56290743	56292743	Gmpr2	-0.021721	0.10394	insignificant	-0.057775	0.26259	insignificant	4	21	21
chr14	56300654	56302654	Tinf2	-0.11139	0.038355	hypomethylated	0.011134	0.067605	insignificant	5	36	38
chr14	56331925	56333925	Tgm1	-0.31923	0.00000149	hypomethylated	0.049583	0.67832	insignificant	8	33	32
chr14	56332329	56334329	Tgm1	-0.48349	0.036049	stronglyHypometh	0.07894	0.53335	insignificant	2	23	22
chr14	56341013	56343013	Rabggta	-0.52437	2.39E-11	stronglyHypometh	-0.045275	0.033767	hypomethylated	4	14	14
chr14	56363529	56365529	2610027L16Rik	-0.11648	1.28E-09	hypomethylated	0.011682	0.12916	insignificant	26	66	77
chr14	56364521	56366521	Dhrs1	-0.13703	3.1E-14	hypomethylated	0.0091807	0.19995	insignificant	24	65	69
chr14	56379760	56381760	Ltb4r2	-0.12992	4.66E-15	hypomethylated	0.0059391	0.023524	hypermethylated	27	80	80
chr14	56402856	56404856	Adcy4	-0.095926	0.17973	insignificant	0.10084	0.0092438	hypermethylated	5	28	19
chr14	56442631	56444631	Nfatc4	-0.37745	2.05E-14	stronglyHypometh	-0.040772	0.01556	hypomethylated	6	52	52
chr14	56471951	56473951	Nynrin	-0.27474	1.31E-11	hypomethylated	-0.02274	0.18508	insignificant	8	26	26
chr14	56502805	56504805	Khnyn	-0.13538	1.21E-09	hypomethylated	-0.028025	0.014168	hypomethylated	13	70	50
chr14	56503093	56505093	Cbln3	-0.13538	1.21E-09	hypomethylated	-0.028025	0.014168	hypomethylated	13	70	50
chr14	56519069	56521069	Sdr39u1	-0.13269	1.55E-09	hypomethylated	0.051506	0.18155	insignificant	8	20	21
chr14	56830244	56832244	Gzmf		1	noCoverage	0.10672	0.35669	insignificant	0	10	12
chr14	56982904	56984904	Atp12a	-0.13003	1.93E-10	hypomethylated	-0.016934	0.90962	insignificant	19	64	65
chr14	57020533	57022533	Rnf17	-0.2247	0.18227	insignificant	-0.10438	0.00001513	hypomethylated	11	61	62
chr14	57190683	57192683	Cenpj	-0.30954	0.000011167	hypomethylated	-0.027277	0.87324	insignificant	2	4	4
chr14	57193455	57195455	Parp4	-0.18871	2.79E-15	hypomethylated	-0.037586	0.0029697	hypomethylated	9	43	41
chr14	57286084	57288084	Mphosph8	-0.11608	1.51E-15	hypomethylated	0.0065131	0.0012547	inconclusive	18	116	104
chr14	57397153	57399153	Pspc1	0.19048	0.57924	insignificant	0.10925	0.29528	insignificant	1	6	6
chr14	57430553	57432553	Zmym5	-0.11159	4.72E-19	hypomethylated	-0.01134	0.1524	insignificant	27	128	135
chr14	57505630	57507630	Zmym2	-0.13242	3.48E-09	hypomethylated	-0.016319	0.85143	insignificant	14	66	61
chr14	57676782	57678782	Gja3	-0.076921	0.000010255	hypomethylated	-0.026702	0.00034412	hypomethylated	22	56	67
chr14	57723539	57725539	Gjb2		1	noCoverage	-0.071933	1	insignificant	0	15	14
chr14	57752414	57754414	Gjb6	0.026998	1	insignificant	0.0094006	1	insignificant	3	9	8
chr14	58017320	58019320	Cryl1	-0.15493	0.021325	hypomethylated	0.019771	0.30148	insignificant	1	28	26
chr14	58041907	58043907	Ift88	-0.16271	1.16E-09	hypomethylated	-0.023738	0.49644	insignificant	23	85	85
chr14	58142665	58144665	Il17d	-0.078895	4.65E-09	hypomethylated	-0.014707	0.022123	hypomethylated	51	181	178
chr14	58190406	58192406	N6amt2	-0.23046	0.074492	insignificant	-0.029126	0.24884	insignificant	3	16	17
chr14	58283793	58285793	Xpo4	-0.14236	6.96E-25	hypomethylated	-0.01561	0.041836	hypomethylated	27	85	96
chr14	58364960	58366960	Lats2	-0.17518	2.37E-18	hypomethylated	0.0094761	0.01124	inconclusive	24	63	64
chr14	58416025	58418025	Sap18	-0.16163	3.16E-22	hypomethylated	-0.012433	0.22988	insignificant	12	52	52
chr14	58416054	58418054	Gm10094	-0.16163	3.16E-22	hypomethylated	-0.012433	0.22988	insignificant	12	52	52
chr14	58416260	58418260	Mir3077	-0.16163	3.16E-22	hypomethylated	0.000486	0.23058	insignificant	12	52	54
chr14	58444075	58446075	Mrp63	-0.1442	2.36E-29	hypomethylated	0.017652	0.26974	insignificant	39	157	158
chr14	58445000	58447000	F630043A04Rik	-0.11816	2.16E-23	hypomethylated	0.0030169	0.34761	insignificant	36	108	109
chr14	58509099	58511099	Zdhhc20	-0.1236	1.04E-09	hypomethylated	0.015272	1	insignificant	20	50	46
chr14	58618099	58620099	Efha1	-0.42182	0.04148	stronglyHypometh	-0.10156	0.85106	insignificant	4	16	15
chr14	58690522	58692522	Fgf9	-0.14228	9.05E-12	hypomethylated	-0.010515	0.043251	hypomethylated	18	58	50
chr14	59819064	59821064	Rcbtb1	-0.098527	8.86E-37	hypomethylated	-0.0068357	0.28777	insignificant	52	141	135
chr14	59879053	59881053	Gm6904	0.11111	1	insignificant	-0.010101	0.19614	insignificant	2	3	6
chr14	59916359	59918359	Phf11	-0.45506	0.000036534	stronglyHypometh	-0.017556	0.61761	insignificant	1	2	2
chr14	60058817	60060817	Cab39l	-0.14365	0.00057273	hypomethylated	-0.00046935	0.62488	insignificant	8	79	80
chr14	60059714	60061714	Setdb2	-0.19047	0.00051312	hypomethylated	0.0052997	0.53485	insignificant	5	67	68
chr14	60216796	60218796	Cdadc1	-0.18822	2.9E-12	hypomethylated	0.0055966	1	insignificant	10	24	24
chr14	60243117	60245117	Shisa2	-0.14226	3.38E-35	hypomethylated	-0.0089108	0.056974	insignificant	35	134	134
chr14	60870215	60872215	Nupl1	-0.087957	0.10217	insignificant	0.010095	0.070719	insignificant	9	68	68
chr14	60883064	60885064	Mtmr6	-0.17439	7.02E-17	hypomethylated	-0.012466	0.32274	insignificant	21	53	50
chr14	60996122	60998122	Fam123a	-0.14709	1.4E-32	hypomethylated	-0.020747	0.10705	insignificant	39	141	143
chr14	61252565	61254565	Spata13	-0.095545	0.255	insignificant	0.028032	0.36063	insignificant	5	57	51
chr14	61385970	61387970	C1qtnf9	-0.35338	0.01522	stronglyHypometh	-0.027047	0.71739	insignificant	2	4	4

chr14	61402402	61404402	Mipep	-0.16738	3.02E-09	hypomethylated	-0.015338	0.89197	insignificant	18	61	53
chr14	61656824	61658824	Tnfrsf19	-0.050586	0.77964	insignificant	0.092541	0.036376	hypermethylated	8	20	32
chr14	61756293	61758293	Sacs	-0.31193	0.00092116	hypomethylated	-0.052866	0.035573	hypomethylated	6	24	22
chr14	61877327	61879327	Sgcg	-0.12437	0.065664	insignificant	-0.13566	0.64961	insignificant	3	10	9
chr14	61979282	61981282	Ebpl	-0.276	0.000029787	hypomethylated	-0.020314	0.61667	insignificant	6	25	25
chr14	62058784	62060784	Kpna3	-0.091583	0.00035421	hypomethylated	0.0083765	0.94549	insignificant	7	38	38
chr14	62175723	62177723	6330409N04Rik	-0.34764	2.64E-25	stronglyHypometh	-0.0029044	0.0016828	hypomethylated	8	20	24
chr14	62216062	62218062	Trim13	-0.53932	0.030049	stronglyHypometh	-0.0023065	0.91614	insignificant	1	12	15
chr14	62217348	62219348	Trim13	-0.55762	2.66E-19	stronglyHypometh	0.022402	0.00000203	inconclusive	4	18	21
chr14	62225293	62227293	Kcnrg	-0.25556	0.0089655	hypomethylated	-0.026511	0.13698	insignificant	2	6	6
chr14	62225318	62227318	Kcnrg	-0.25556	0.0089655	hypomethylated	-0.026511	0.13698	insignificant	2	6	6
chr14	62301210	62303210	Dleu2	-0.093226	9.49E-10	hypomethylated	0.022264	0.62512	insignificant	12	68	86
chr14	62911816	62913816	Dleu7	-0.078833	1	insignificant	-0.010701	0.73898	insignificant	4	10	10
chr14	62949941	62951941	Rnaseh2b	-0.11001	2.81E-13	hypomethylated	-0.0032886	0.58473	insignificant	21	58	75
chr14	63379949	63381949	Ints6	-0.11065	0.0012875	hypomethylated	-0.00083232	0.047935	inconclusive	12	78	89
chr14	63455526	63457526	Wdfy2	-0.1174	0.000012817	hypomethylated	0.0039482	0.18782	insignificant	19	106	101
chr14	63740301	63742301	Ctsb	-0.16319	0.016885	hypomethylated	0.0033395	0.83443	insignificant	11	57	59
chr14	63796630	63798630	Fdft1	-0.1603	9.07E-16	hypomethylated	-0.036686	0.0035015	hypomethylated	20	46	46
chr14	63812362	63814362	Neil2	-0.29712	0.000061961	hypomethylated	-0.070534	0.029233	hypomethylated	9	32	32
chr14	63864097	63866097	Gata4	-0.17968	1.6E-11	hypomethylated	0.0063744	0.93873	insignificant	31	102	101
chr14	64054230	64056230	Fam167a	-0.12295	0.16827	insignificant	0.0025757	0.55381	insignificant	10	30	29
chr14	64127929	64129929	Tdh	-0.29169	0.020414	hypomethylated	-0.059494	0.12073	insignificant	3	17	14
chr14	64162790	64164790	Mtmr9	-0.29832	0.00030859	hypomethylated	0.11878	0.061447	insignificant	3	19	15
chr14	64224366	64226366	Xkr6	-0.11285	3.92E-15	hypomethylated	0.0047719	0.79201	insignificant	56	177	190
chr14	64478148	64480148	Pinx1	-0.13668	8.59E-13	hypomethylated	0.0094454	0.025513	inconclusive	13	78	80
chr14	64561542	64563542	Sox7	-0.1305	1.2E-14	hypomethylated	0.0019311	0.5459	insignificant	28	135	137
chr14	64610267	64612267	Rp1l1	-0.33338	0.046779	stronglyHypometh	-0.056751	0.48005	insignificant	4	16	16
chr14	65208493	65210493	Mir124a-1	-0.17349	0.00027022	hypomethylated	0.0087528	0.76498	insignificant	5	11	10
chr14	65270367	65272367	Kif13b	-0.18301	2.82E-23	hypomethylated	-0.0030753	0.025997	hypomethylated	21	66	69
chr14	65567881	65569881	Ints9	-0.2246	0.000001	hypomethylated	0.018725	0.91128	insignificant	4	33	29
chr14	65716943	65718943	Extl3	-0.30635	4.29E-12	hypomethylated	0.023727	0.074617	insignificant	10	28	26
chr14	65881300	65883300	Fzd3	-0.19152	7.78E-08	hypomethylated	-0.053575	0.086113	insignificant	8	40	32
chr14	65884537	65886537	Fbxo16		1	noCoverage	-0.037591	1	insignificant	0	6	8
chr14	65976512	65978512	Zfp395	-0.1123	4.16E-32	hypomethylated	-0.0035496	0.14525	insignificant	45	154	154
chr14	66044046	66046046	Pnoc	-0.1289	0.44144	insignificant	0.047959	0.76055	insignificant	3	18	18
chr14	66211847	66213847	Elp3		1	noCoverage	-0.022587	0.32899	insignificant	0	18	18
chr14	66423747	66425747	Pbk	-0.14835	1	insignificant	-0.018352	0.58346	insignificant	2	16	16
chr14	66452806	66454806	Esco2	-0.10857	0.15712	insignificant	-0.020349	0.0061571	hypomethylated	3	12	13
chr14	66455138	66457138	Ccdc25	-0.13363	5.59E-20	hypomethylated	0.013441	0.049216	inconclusive	24	57	59
chr14	66572581	66574581	Scara3	-0.13475	0.00000107	hypomethylated	0.0022115	0.37443	insignificant	13	39	39
chr14	66586319	66588319	Clu	-0.18494	6.05E-18	hypomethylated	-0.0044948	0.32384	insignificant	12	37	37
chr14	66696570	66698570	Adam2	-0.094102	0.23956	insignificant	-0.035591	4.72E-12	hypomethylated	9	36	33
chr14	66832389	66834389	Ptk2b		1	noCoverage	-0.082068	0.84532	insignificant	0	2	2
chr14	66899889	66901889	Ptk2b	-0.41886	0.000000113	stronglyHypometh	-0.048865	0.89455	insignificant	2	6	6
chr14	66914861	66916861	Trim35	-0.11446	5.61E-19	hypomethylated	-0.010346	0.4354	insignificant	34	82	82
chr14	66962211	66964211	Stmn4	-0.38889	0.0068573	stronglyHypometh	-0.14281	0.17213	insignificant	1	18	18
chr14	67253094	67255094	Adra1a	-0.27803	0.000000124	hypomethylated	-0.022374	0.12655	insignificant	8	40	44
chr14	67486686	67488686	Gm5464	-0.1185	0.00002669	hypomethylated	0.0026014	0.70117	insignificant	17	114	115
chr14	67487437	67489437	Dpysl2	-0.091491	0.0013513	hypomethylated	0.0060849	0.35544	insignificant	14	92	93
chr14	67529044	67531044	Pnma2	-0.13677	3.11E-28	hypomethylated	0.01469	0.12814	insignificant	31	150	147
chr14	67627714	67629714	Bnip3l	-0.2266	0.000000002	hypomethylated	-0.042745	0.00030461	hypomethylated	23	62	62
chr14	67691266	67693266	Ppp2r2a	-0.27686	0.018224	hypomethylated	-0.051617	0.017001	inconclusive	1	25	26
chr14	67851128	67853128	Ebf2	-0.21482	6.34E-21	hypomethylated	-0.034639	0.000020738	hypomethylated	27	97	95
chr14	67933548	67935548	Gm6878		1	noCoverage	0.16786	0.088249	insignificant	0	11	12
chr14	68333154	68335154	Kctd9	-0.12833	7.24E-36	hypomethylated	-0.0054111	0.079161	insignificant	55	161	161
chr14	68333667	68335667	Cdca2	-0.12632	1.03E-33	hypomethylated	0.00061507	0.097354	insignificant	39	114	114
chr14	68333898	68335898	Cdca2	-0.11892	1.35E-30	hypomethylated	-0.00087557	0.35143	insignificant	39	112	112
chr14	68362285	68364285	Gnrh1	0.16483	1	lowCoverage	-0.0092319	1	insignificant	1	16	20
chr14	68551629	68553629	Dock5	-0.17659	7.28E-18	hypomethylated	-0.020548	0.0048414	hypomethylated	16	51	51
chr14	68700940	68702940	Nefl	-0.11348	5.54E-20	hypomethylated	-0.00066669	0.18505	insignificant	27	90	90
chr14	68743061	68745061	Nefm	-0.36732	1.15E-08	stronglyHypometh	-0.073748	0.23217	insignificant	12	33	41
chr14	69646345	69648345	Stc1		1	noCoverage	0.12264	0.9324	insignificant	0	12	7
chr14	69789075	69791075	Nkx2-6	-0.1643	1.47E-08	hypomethylated	0.014725	0.25745	insignificant	6	40	40
chr14	69807748	69809748	Nkx3-1	-0.056665	0.00000294	hypomethylated	0.028105	0.54837	insignificant	8	57	57
chr14	69903160	69905160	Slc25a37	-0.33996	1.39E-53	stronglyHypometh	0.065107	1.85E-17	inconclusive	7	28	24
chr14	69912356	69914356	Synb	0.09	0.60851	insignificant	0.021189	0.55047	insignificant	4	10	10
chr14	69954207	69956207	Entpd4	-0.12619	3.22E-56	hypomethylated	0.01413	0.000084409	inconclusive	37	94	100
chr14	70008282	70010282	Loxl2	-0.21725	2.71E-19	hypomethylated	-0.003394	0.91702	insignificant	24	75	82
chr14	70107387	70109387	R3hcc1	-0.2963	1	insignificant	0.042773	0.13059	insignificant	3	9	13
chr14	70132377	70134377	Chmp7	-0.1356	0.00025603	hypomethylated	0.0037762	0.0034135	hypermethylated	10	61	65
chr14	70166278	70168278	Tnfrsf10b	-0.13321	0.000011113	hypomethylated	-0.0077643	0.51642	insignificant	16	66	66
chr14	70205352	70207352	Rhobtb2	-0.4719	0.16013	insignificant	-0.02099	0.56932	insignificant	1	16	13
chr14	70239226	70241226	Pebp4		1	noCoverage	0.17143	0.19966	insignificant	0	6	6
chr14	70476251	70478251	Egr3	-0.14976	4.93E-17	hypomethylated	-0.01166	0.020695	hypomethylated	22	99	106
chr14	70498951	70500951	Bin3	-0.16956	3.06E-29	hypomethylated	-0.026107	0.0024927	hypomethylated	24	62	70
chr14	70553598	70555598	9930012K11Rik		1	noCoverage	0.11423	0.054128	insignificant	0	13	11
chr14	70559309	70561309	9930012K11Rik	-0.24762	0.000033024	hypomethylated	-0.02232	0.21144	insignificant	4	18	18
chr14	70577479	70579479	Pdlim2	-0.57568	0.0053882	stronglyHypometh	0.10595	0.19168	insignificant	3	8	8
chr14	70689256	70691256	Ppp3cc	-0.09643	3.36E-08	hypomethylated	-0.0082173	0.94637	insignificant	13	40	40
chr14	70751231	70753231	Slc39a14	-0.13984	1.51E-08	hypomethylated	0.11113	0.40367	insignificant	10	24	36
chr14	70828901	70830901	Piwil2	-0.045691	0.70496	insignificant	0.027541	0.62636	insignificant	6	17	20
chr14	70842316	70844316	Mir320	-0.18042	1.02E-29	hypomethylated	-0.0031448	1	insignificant	21	105	102
chr14	70843034	70845034	Polr3d	-0.17756	2.51E-25	hypomethylated	-0.019636	0.69981	insignificant	20	84	77
chr14	70843278	70845278	Polr3d	-0.19856	2.52E-16	hypomethylated	-0.025187	0.742	insignificant	12	56	49
chr14	70856323	70858323	Phyhip	-0.2548	2.04E-30	hypomethylated	-0.055401	0.0052235	hypomethylated	11	37	46

chr14	70920067	70922067	Sftpc	-0.098828	0.0020895	hypomethylated	-0.037643	0.034328	hypomethylated	17	57	62
chr14	70929627	70931627	Lgi3	-0.11952	3.64E-12	hypomethylated	-0.011919	0.10721	insignificant	18	40	40
chr14	70944429	70946429	Reep4	-0.18243	7.1E-12	hypomethylated	-0.012001	0.33002	insignificant	2	70	59
chr14	70952862	70954862	Hr	-0.1423	9.28E-19	hypomethylated	0.017775	0.2116	insignificant	33	111	112
chr14	70976653	70978653	Nudt18	-0.12846	3.49E-31	hypomethylated	0.0035791	0.035977	inconclusive	26	98	98
chr14	70999642	71001642	Fam160b2	-0.3577	0.000020045	stronglyHypometh	-0.059558	1	insignificant	6	21	22
chr14	71042075	71044075	Fgf17		1	noCoverage	0.078211	0.10881	insignificant	0	5	7
chr14	71052891	71054891	Npm2	0.12579	0.69717	insignificant	-0.070113	0.054126	insignificant	3	15	15
chr14	71166435	71168435	Xpo7	-0.08081	0.000000304	hypomethylated	0.0019068	0.36058	insignificant	15	98	102
chr14	71288936	71290936	Gfra2	-0.13336	2.21E-15	hypomethylated	-0.0018346	0.76698	insignificant	20	92	92
chr14	73109810	73111810	Fndc3a	-0.12943	2.86E-28	hypomethylated	0.0017216	0.16718	insignificant	35	113	128
chr14	73424409	73426409	Gm9199	0.0012821	0.32476	insignificant	0.056373	0.13063	insignificant	4	12	12
chr14	73541316	73543316	Rcbtb2	-0.15643	2.97E-42	hypomethylated	0.083277	0.14018	insignificant	41	81	95
chr14	73541591	73543591	Rcbtb2	-0.15643	2.97E-42	hypomethylated	0.083277	0.14018	insignificant	41	81	95
chr14	73636697	73638697	Lpar6		1	noCoverage	0.11111	0.31147	insignificant	0	6	6
chr14	73725598	73727598	Rb1	-0.087453	6.48E-27	hypomethylated	-0.012674	0.0043413	hypomethylated	38	134	139
chr14	73785078	73787078	Itm2b	-0.2287	0.0011595	hypomethylated	0.0042156	0.48169	insignificant	7	12	18
chr14	73908855	73910855	Med4	-0.11587	3.27E-11	hypomethylated	0.075394	0.25779	insignificant	16	59	48
chr14	73951592	73953592	Sucla2	-0.12762	3.99E-18	hypomethylated	-0.0086663	0.65903	insignificant	31	81	95
chr14	75039646	75041646	Htr2a		1	noCoverage	-0.0057143	1	insignificant	0	10	10
chr14	75131151	75133151	Esd	-0.16926	6.69E-16	hypomethylated	0.024202	0.11946	insignificant	8	31	28
chr14	75347684	75349684	Lrch1	-0.25636	0.12704	insignificant	0.11506	0.71799	insignificant	2	16	16
chr14	75530690	75532690	Lrrc63	-0.16607	3.58E-14	hypomethylated	-0.012727	0.3469	insignificant	12	36	39
chr14	75535231	75537231	Lcp1		1	noCoverage	-0.022864	0.77133	insignificant	0	10	10
chr14	75641093	75643093	Cpb2		1	noCoverage	-0.0075758	1	insignificant	0	2	2
chr14	75683179	75685179	Zc3h13	-0.13665	7.24E-14	hypomethylated	-0.037174	0.28164	insignificant	17	58	84
chr14	75854788	75856788	Siah3	-0.40925	0.0022542	stronglyHypometh	-0.032272	0.054434	insignificant	2	16	19
chr14	76154300	76156300	Cog3	-0.18407	4.92E-14	hypomethylated	0.024057	0.63775	insignificant	11	26	29
chr14	76186844	76188844	Slc25a30		1	noCoverage	-0.029441	0.86258	insignificant	0	10	12
chr14	76244062	76246062	Tpt1	-0.11583	2.63E-38	hypomethylated	-0.001125	0.068733	insignificant	65	236	235
chr14	76410672	76412672	Gtf2f2	-0.075161	0.00000814	hypomethylated	-0.018421	0.24155	insignificant	8	25	30
chr14	76509697	76511697	Nufip1	-0.098466	2.49E-19	hypomethylated	0.0098357	0.16269	insignificant	35	124	120
chr14	76510622	76512622	Nufip1	-0.15897	3.75E-09	hypomethylated	0.033625	0.32083	insignificant	17	55	54
chr14	76814627	76816627	Tsc22d1	-0.16055	1.88E-54	hypomethylated	-0.0054694	0.00000052	hypomethylated	77	192	194
chr14	76887242	76889242	Tsc22d1	-0.14811	0.13301	insignificant	0.075941	0.63222	insignificant	4	23	30
chr14	76903316	76905316	Tsc22d1	-0.1623	3.82E-19	hypomethylated	-0.0079011	0.001275	hypomethylated	20	73	68
chr14	76956494	76958494	Serp2	-0.12106	1.94E-08	hypomethylated	-0.03776	0.00028099	hypomethylated	17	47	47
chr14	76956696	76958696	Serp2	-0.19342	0.00040591	hypomethylated	-0.039405	0.13761	insignificant	6	16	16
chr14	77076374	77078374	1700108F19Rik		1	noCoverage	0.023904	1	insignificant	0	6	8
chr14	77435578	77437578	Ccdc122	-0.14971	6.86E-09	hypomethylated	-0.00032258	0.037759	hypomethylated	21	84	72
chr14	77436424	77438424	9030625A04Rik	-0.18822	0.00000071	hypomethylated	0.018453	0.010733	inconclusive	15	62	51
chr14	77555622	77557622	Enox1	-0.13711	1.08E-58	hypomethylated	-0.02419	0.17008	insignificant	63	207	210
chr14	77906675	77908675	Gm6994	0.076149	1	insignificant	0.026074	0.033905	inconclusive	4	22	26
chr14	78198775	78200775	Gm1587	0.011796	0.039986	inconclusive	-0.083332	0.093996	insignificant	5	13	13
chr14	78274724	78276724	Dnajc15	-0.20085	3.74E-10	hypomethylated	0.027068	0.23194	insignificant	8	26	27
chr14	78303045	78305045	Epsti1	-0.25921	0.000044309	hypomethylated	0.04634	0.091236	insignificant	4	18	18
chr14	78936667	78938667	Akap11		1	noCoverage	-0.005491	0.0042899	hypomethylated	0	22	22
chr14	79124896	79126896	Dgkh	-0.23155	0.000095077	hypomethylated	0.0062578	0.0014806	hypermethylated	16	54	57
chr14	79247984	79249984	1300010F03Rik	-0.18422	1.46E-18	hypomethylated	-0.0052851	0.27059	insignificant	17	44	44
chr14	79647174	79649174	Zfp957		1	noCoverage	-0.043517	1	insignificant	0	6	6
chr14	79701442	79703442	1190002H23Rik	-0.14191	8.01E-10	hypomethylated	-0.027686	0.67348	insignificant	12	33	33
chr14	79790475	79792475	Naa16	-0.12564	5.42E-08	hypomethylated	0.011194	0.90513	insignificant	7	47	49
chr14	79825317	79827317	Kbtbd7	-0.11002	4.46E-15	hypomethylated	0.005441	0.35009	insignificant	19	60	60
chr14	79880000	79882000	Elf1	-0.099439	1.48E-26	hypomethylated	-0.0070627	0.098612	insignificant	45	184	184
chr14	79881075	79883075	Elf1	-0.14935	5.21E-24	hypomethylated	-0.01111	0.050786	insignificant	18	111	108
chr14	79986497	79988497	Sugt1	-0.15481	3.2E-11	hypomethylated	-0.020598	0.073482	insignificant	16	58	67
chr14	80137237	80139237	Mir759	0.24545	1	lowCoverage	-0.026604	0.41664	insignificant	1	4	4
chr14	80171119	80173119	Pcdh8	-0.20389	2.02E-12	hypomethylated	0.028733	0.80518	insignificant	12	36	36
chr14	80399108	80401108	Olfm4	-0.37289	0.00055155	stronglyHypometh	-0.05242	0.54848	insignificant	2	8	10
chr14	84842369	84844369	Pcdh17	-0.19496	0.017818	hypomethylated	-0.0076633	0.075811	insignificant	10	40	37
chr14	87540921	87542921	Diap3	-0.16085	1.57E-24	hypomethylated	0.041213	0.16994	insignificant	15	39	42
chr14	87815389	87817389	Tdrd3	-0.088868	2.24E-38	hypomethylated	0.0088863	0.9639	insignificant	85	266	280
chr14	94287951	94289951	Pcdh9	-0.24093	0.00000234	hypomethylated	-0.055807	0.80008	insignificant	7	34	40
chr14	96279484	96281484	4921530L21Rik	0.14978	1	insignificant	0.25891	0.00065047	hypermethylated	1	10	9
chr14	96918253	96920253	Klhl1	0.047727	1	insignificant	0.172	0.18996	insignificant	1	6	9
chr14	98568762	98570762	Dach1	-0.26098	0.000001259	hypomethylated	-0.066755	0.15062	insignificant	5	13	23
chr14	99444595	99446595	6720463M24Ril	-0.10411	8.49E-38	hypomethylated	0.0037381	0.049497	inconclusive	78	220	216
chr14	99445355	99447355	6720463M24Ril	-0.16177	2.35E-21	hypomethylated	0.009616	0.67545	insignificant	36	107	114
chr14	99497651	99499651	Pibf1	-0.099179	8.68E-23	hypomethylated	-0.0071048	0.00044126	inconclusive	30	113	138
chr14	99498989	99500989	Pibf1	-0.12029	2.35E-17	hypomethylated	-0.07231	0.21215	insignificant	10	37	56
chr14	99696909	99698909	Klf5	-0.093493	8.18E-24	hypomethylated	-0.00077304	0.24424	insignificant	36	140	147
chr14	101599286	101601286	1700110M21Ril	0.11505	0.0089028	hypermethylated	0.1634	3.72E-11	hypermethylated	3	9	9
chr14	102008408	102010408	Tbc1d4	-0.36729	0.000087495	stronglyHypometh	0.0221	0.026031	inconclusive	4	23	25
chr14	102039688	102041688	Commd6	-0.24167	0.000091007	hypomethylated	0.033414	1	insignificant	7	18	17
chr14	102052183	102054183	Uchl3	-0.13989	8.1E-53	hypomethylated	-0.013356	0.00041486	hypomethylated	50	134	135
chr14	102128144	102130144	Lmo7	-0.17468	1.74E-14	hypomethylated	-0.0063821	1	insignificant	28	84	81
chr14	103381854	103383854	Mir5130	-0.21452	1.17E-18	hypomethylated	-0.017114	0.61258	insignificant	17	58	40
chr14	103468432	103470432	Cln5	-0.15378	1.43E-34	hypomethylated	-0.026433	0.000000151	hypomethylated	46	121	120
chr14	103498726	103500726	Fbxl3	-0.085941	0.0094057	hypomethylated	-0.0010799	0.20116	insignificant	14	51	50
chr14	103746017	103748017	Mycbp2	-0.25866	0.34574	insignificant	0.016568	0.18662	insignificant	6	27	22
chr14	103911557	103913557	Scel	-0.34785	2.61E-14	stronglyHypometh	0.11126	0.92389	insignificant	3	6	8
chr14	104048459	104050459	Slain1	-0.1093	6.78E-22	hypomethylated	-0.0057569	0.60921	insignificant	39	102	102
chr14	104242913	104244913	Ednrb	-0.091427	0.011612	hypomethylated	-0.010076	0.44059	insignificant	4	12	12
chr14	104867216	104869216	Pou4f1	-0.11213	4.79E-09	hypomethylated	-0.0024132	0.45204	insignificant	34	79	87
chr14	104921883	104923883	Rnf219	-0.16409	7.6E-17	hypomethylated	-0.013157	0.43097	insignificant	10	32	31

chr14	105656889	105658889	Ndfip2	-0.15367	2.55E-26	hypomethylated	0.0035437	0.89381	insignificant	48	123	118
chr14	106296036	106298036	Spry2	-0.10729	5.11E-09	hypomethylated	-0.013443	0.7082	insignificant	14	117	116
chr14	106504414	106506414	Trim52		1	noCoverage	0.077807	0.60308	insignificant	0	24	26
chr14	112073336	112075336	Slitrk5	-0.11438	7.63E-21	hypomethylated	0.020279	0.3118	insignificant	34	153	149
chr14	115490436	115492436	Gpc5	-0.1815	4.94E-23	hypomethylated	0.0065552	0.11388	insignificant	30	99	94
chr14	117323536	117325536	Gpc6	-0.13282	3.18E-24	hypomethylated	-0.016829	0.62471	insignificant	20	78	96
chr14	118531987	118533987	Tgds	-0.15668	0.012183	hypomethylated	-0.0058682	0.82799	insignificant	8	22	22
chr14	118535348	118537348	Gpr180	-0.12039	1.25E-25	hypomethylated	0.0032978	0.00099797	hypermethylated	38	100	108
chr14	118636252	118638252	Sox21	-0.1445	0.000095762	hypomethylated	-0.024381	0.28322	insignificant	6	37	38
chr14	118665379	118667379	Gm9376	0.0062771	1	insignificant	-0.24975	0.014344	hypomethylated	4	16	19
chr14	119105441	119107441	Abcc4	-0.14554	3.64E-14	hypomethylated	0.0053218	0.65543	insignificant	22	71	71
chr14	119253171	119255171	Cldn10	-0.11508	3.64E-12	hypomethylated	-0.016074	0.42826	insignificant	17	62	60
chr14	119322389	119324389	Dzip1	-0.13351	0.000021124	hypomethylated	0.011826	0.0010177	inconclusive	15	52	51
chr14	119336153	119338153	Dnajc3	-0.10268	1.94E-39	hypomethylated	-0.0061351	0.22619	insignificant	51	134	134
chr14	119498656	119500656	Uggt2	-0.24983	2.31E-26	hypomethylated	0.016646	1	insignificant	16	48	48
chr14	119536486	119538486	Hs6st3	-0.11561	3.89E-29	hypomethylated	0.010027	0.073299	insignificant	74	214	219
chr14	120673890	120675890	Mbnl2	-0.2039	1	insignificant	-0.0052007	0.52506	insignificant	2	16	16
chr14	120876682	120878682	Rap2a	-0.11346	1.68E-11	hypomethylated	-0.020761	0.027028	hypomethylated	42	194	179
chr14	121309415	121311415	Ipo5	-0.1338	4.07E-38	hypomethylated	-0.040392	0.0031961	hypomethylated	63	205	201
chr14	121433795	121435795	Farp1	-0.076187	1.01E-23	hypomethylated	-0.0038502	0.00044749	hypomethylated	64	195	238
chr14	121778452	121780452	Stk24	-0.091878	3.94E-08	hypomethylated	0.0073728	0.8801	insignificant	11	59	68
chr14	121904476	121906476	Slc15a1	-0.083658	0.051238	insignificant	0.050034	0.27481	insignificant	8	30	32
chr14	122196956	122198956	Dock9	-0.12371	3.13E-36	hypomethylated	0.000098623	0.18196	insignificant	33	139	149
chr14	122276827	122278827	Ubac2	-0.12318	6.32E-24	hypomethylated	-0.0036001	0.45935	insignificant	38	135	128
chr14	122314996	122316996	Gpr18	0.036364	1	insignificant	0.21136	0.72716	insignificant	2	5	4
chr14	122432895	122434895	Timm8a2	-0.020646	0.29209	insignificant	0.010551	0.009763	hypermethylated	5	14	14
chr14	122505303	122507303	Tm9sf2	-0.12919	3.55E-41	hypomethylated	-0.010224	0.17341	insignificant	47	124	130
chr14	122506196	122508196	A330035P11Rik	-0.11314	9.93E-32	hypomethylated	-0.019232	0.2445	insignificant	39	97	104
chr14	122579915	122581915	Clybl	-0.37422	0.00000333	stronglyHypometh	-0.019132	0.10244	insignificant	3	28	32
chr14	122632860	122634860	1700108J01Rik	0.011472	1	insignificant	-0.010468	0.32923	insignificant	5	16	16
chr14	122864880	122866880	Zic5	-0.33081	0.00017322	hypomethylated	0.061047	0.39132	insignificant	2	18	18
chr14	122873605	122875605	Zic2	-0.10275	3.74E-34	hypomethylated	-0.0059602	0.88587	insignificant	65	225	237
chr14	122932549	122934549	Pcca	-0.12747	3.61E-08	hypomethylated	-0.017136	0.35705	insignificant	8	37	37
chr14	123312387	123314387	A2ld1	-0.13031	4.51E-10	hypomethylated	0.016825	0.3772	insignificant	11	34	34
chr15	3253526	3255526	Ccdc152	-0.23648	0.000052222	hypomethylated	-0.071539	0.018143	hypomethylated	7	20	18
chr15	3533230	3535230	Ghr	-0.042157	1	insignificant	0.043627	0.87425	insignificant	1	6	6
chr15	3945752	3947752	AW549877	-0.27356	3.76E-11	hypomethylated	0.015709	0.86276	insignificant	10	40	42
chr15	3975427	3977427	Oxct1	-0.010765	4.54E-22	hypomethylated	-0.0037129	0.24403	insignificant	32	120	129
chr15	3977406	3979406	BC037032	-0.12013	0.14523	insignificant	-0.0076314	0.9151	insignificant	2	18	20
chr15	4324490	4326490	Plcxd3	-0.33356	0.014691	stronglyHypometh	-0.10423	0.7093	insignificant	1	32	32
chr15	4676209	4678209	C6		1	noCoverage	0.098485	0.51073	insignificant	0	6	6
chr15	5058533	5060533	Card6	0.2271	0.47666	insignificant	0.10228	0.10726	insignificant	2	12	12
chr15	5065612	5067612	Rpl37	-0.13374	8.64E-19	hypomethylated	-0.0085103	0.35257	insignificant	28	118	118
chr15	5092860	5094860	Prkaa1	-0.06419	1.17E-15	hypomethylated	0.0047794	0.2289	insignificant	56	169	170
chr15	5134559	5136559	Ttc33	-0.17643	2.87E-08	hypomethylated	-0.0089858	0.25809	insignificant	15	61	60
chr15	5193682	5195682	Ptger4	-0.15698	6.97E-18	hypomethylated	0.01858	0.19062	insignificant	23	90	91
chr15	5193831	5195831	Ptger4	-0.15919	6.96E-14	hypomethylated	0.009561	0.11247	insignificant	20	79	81
chr15	6335747	6337747	Dab2	-0.232	0.21785	insignificant	-0.004448	0.62045	insignificant	1	57	44
chr15	6657380	6659380	Rictor	-0.075941	2.37E-15	hypomethylated	0.0047514	0.67168	insignificant	59	244	244
chr15	6824313	6826313	Osmr	-0.16833	0.022335	hypomethylated	0.0048177	0.19165	insignificant	11	44	46
chr15	7078571	7080571	Lifr	-0.10488	1.46E-32	hypomethylated	-0.0065502	0.78993	insignificant	34	139	139
chr15	7089541	7091541	Lifr	0.22135	1	lowCoverage	0.0023712	0.35579	insignificant	1	8	8
chr15	7348304	7350304	Egflam	-0.22483	0.000061128	hypomethylated	-0.037239	0.36788	insignificant	7	27	27
chr15	7760010	7762010	Gdnf	-0.10339	4.86E-22	hypomethylated	-0.014643	0.35853	insignificant	17	124	115
chr15	8049209	8051209	Wdr70	-0.090186	0.0032269	hypomethylated	-0.015504	0.11677	insignificant	7	14	14
chr15	8058312	8060312	Nup155	-0.33606	0.30446	insignificant	-0.026777	0.81375	insignificant	2	43	33
chr15	8118105	8120105	2410089E03Rik	-0.22064	0.0010519	hypomethylated	-0.00082616	0.27796	insignificant	11	51	51
chr15	8394463	8396463	Nipbl	-0.13258	2.79E-19	hypomethylated	-0.0067974	0.79332	insignificant	46	128	126
chr15	8660807	8662807	Slc1a3		1	noCoverage	0.083163	0.13726	insignificant	0	6	6
chr15	8917118	8919118	Ranbp3l	-0.44468	0.020214	stronglyHypometh	-0.12841	0.27896	insignificant	1	12	12
chr15	9000008	9002008	1110020G09Rik	-0.12189	7.08E-38	hypomethylated	-0.013924	0.011523	hypomethylated	48	157	161
chr15	9069325	9071325	Lmbrd2	-0.12145	9.83E-12	hypomethylated	-0.0071849	0.53894	insignificant	29	89	97
chr15	9070207	9072207	Skp2	-0.16052	3.64E-12	hypomethylated	0.022003	0.83105	insignificant	24	78	90
chr15	9264352	9266352	Ugt3a2		1	noCoverage	-0.047187	0.84037	insignificant	0	8	8
chr15	9678561	9680561	Spef2		1	noCoverage	-0.03	0.54802	insignificant	0	5	5
chr15	10105992	10107992	Prlr		1	noCoverage	0.014551	0.4994	insignificant	0	20	20
chr15	10400271	10402271	Dnajc21	-0.07261	0.68606	insignificant	-0.0059665	0.0067322	inconclusive	8	53	53
chr15	10414788	10416788	Rad1	-0.14046	1.55E-15	hypomethylated	-0.012855	0.50433	insignificant	19	68	68
chr15	10415692	10417692	Brix1	-0.14563	0.000048231	hypomethylated	-0.023783	0.029071	hypomethylated	7	32	32
chr15	10643295	10645295	Rai14	-0.15936	3.77E-14	hypomethylated	-0.029067	0.45597	insignificant	25	97	108
chr15	10644386	10646386	Rai14	-0.18308	8.62E-15	hypomethylated	-0.0058045	0.49428	insignificant	22	44	44
chr15	10881110	10883110	C1qtnf3	-0.11928	0.48447	insignificant	0.093342	0.029685	hypermethylated	3	11	12
chr15	10910510	10912510	Amacr	-0.091772	0.000029965	hypomethylated	-0.016497	0.10587	insignificant	13	57	69
chr15	10967723	10969723	Rxfp3	-0.38333	0.095868	insignificant	0.04375	0.86046	insignificant	3	6	6
chr15	10993544	10995544	Adamts12	-0.38947	0.000000191	stronglyHypometh	-0.034723	0.10538	insignificant	3	12	12
chr15	11329413	11331413	Tars		1	noCoverage	0.055243	0.057793	insignificant	0	6	8
chr15	11835429	11837429	Npr3	-0.13832	2.27E-08	hypomethylated	-0.014018	0.084951	insignificant	12	37	46
chr15	11925762	11927762	Sub1	-0.14825	0.0018791	hypomethylated	-0.032263	0.22009	insignificant	8	20	20
chr15	12046605	12048605	Zfr	-0.10352	2.51E-40	hypomethylated	-0.0070052	0.0034459	hypomethylated	76	221	222
chr15	12100246	12102246	Mir1898		1	noCoverage	0.069361	0.20212	insignificant	0	8	8
chr15	12133848	12135848	Mtmr12	-0.10898	3.19E-15	hypomethylated	-0.0060767	0.10794	insignificant	43	141	137
chr15	12250250	12252250	Golph3	-0.12234	3.49E-26	hypomethylated	-0.0023781	0.36061	insignificant	43	146	146
chr15	12522311	12524311	Pdzd2		1	noCoverage	0.058175	0.21333	insignificant	0	10	11
chr15	12753569	12755569	Drosha	-0.12762	4.77E-30	hypomethylated	0.015442	0.79546	insignificant	54	142	147
chr15	12754412	12756412	6030458C11Rik	-0.14134	2.86E-31	hypomethylated	0.023427	1	insignificant	36	90	95

chr15	13103394	13105394	Cdh6	-0.15229	0.0060057	hypomethylated	0.10095	0.77154	insignificant	3	48	45
chr15	16706855	16708855	Cdh9		1	noCoverage	-0.056321	1	insignificant	0	8	7
chr15	20596505	20598505	Acot10	-0.17842	0.14703	insignificant	-0.026859	1	insignificant	2	3	3
chr15	22965217	22967217	Cdh18		1	noCoverage	-0.078755	0.49152	insignificant	0	4	4
chr15	25342946	25344946	Gm5468	-0.15025	1.31E-22	hypomethylated	-0.017718	0.041844	hypomethylated	24	76	76
chr15	25343519	25345519	Basp1	-0.20651	0.016837	hypomethylated	-0.04251	0.74426	insignificant	4	30	30
chr15	25551304	25553304	Myo10	-0.10236	6.41E-09	hypomethylated	0.0050839	0.099509	insignificant	43	162	158
chr15	25772018	25774018	Fam134b	-0.095745	2.7E-22	hypomethylated	0.040297	0.03836	inconclusive	20	83	84
chr15	25913120	25915120	Zfp622	-0.093537	2.12E-17	hypomethylated	-0.0091686	0.37757	insignificant	44	158	162
chr15	26237826	26239826	March11	-0.12769	8.68E-41	hypomethylated	0.0083561	0.33601	insignificant	51	143	134
chr15	26825319	26827319	Fbxl7	-0.11772	0.00000188	hypomethylated	-0.0068393	0.67241	insignificant	17	42	42
chr15	27395431	27397431	Ank	-0.10196	3.05E-20	hypomethylated	-0.0059607	0.0032048	hypomethylated	51	213	211
chr15	27560448	27562448	Fam105b	0.27848	0.287	insignificant	0.010328	0.13455	insignificant	5	33	34
chr15	27611253	27613253	Fam105a	-0.065515	0.50547	insignificant	-0.038992	0.0011121	hypomethylated	5	28	22
chr15	27955603	27957603	Trio	-0.22847	0.000000105	hypomethylated	-0.027925	0.76327	insignificant	11	34	37
chr15	28132520	28134520	Dnahc5	0.12013	1	insignificant	-0.013077	0.55135	insignificant	2	8	8
chr15	30101347	30103347	Ctnnd2	-0.08905	1.71E-17	hypomethylated	0.0057938	0.83479	insignificant	55	189	199
chr15	31153139	31155139	Dap	-0.16771	2.55E-09	hypomethylated	-0.016385	0.59319	insignificant	11	50	54
chr15	31297514	31299514	Ankrd33b	-0.19622	0.69187	insignificant	-0.033429	0.038478	hypomethylated	5	19	21
chr15	31383444	31385444	Ropn1l		1	noCoverage	-0.0035307	1	insignificant	0	18	18
chr15	31460792	31462792	March6	-0.03539	0.025614	inconclusive	0.0019684	0.065527	insignificant	26	96	88
chr15	31497666	31499666	Cmbl	-0.23864	0.0039515	hypomethylated	-0.050825	0.033638	hypomethylated	6	16	16
chr15	31530870	31532870	Fam173b	-0.13777	6.39E-17	hypomethylated	-0.0087044	0.64152	insignificant	26	93	93
chr15	31531559	31533559	Cct5	-0.12735	0.00022558	hypomethylated	0.024373	0.027078	inconclusive	16	51	54
chr15	32106043	32108043	Tas2r119	0.11729	1	lowCoverage	0.012685	0.63843	insignificant	1	8	8
chr15	32173567	32175567	Sema5a	-0.12503	3.54E-10	hypomethylated	-0.019272	0.0051216	hypomethylated	14	60	66
chr15	32849477	32851477	Sdc2	-0.067375	0.0000372	hypomethylated	0.019158	0.71884	insignificant	9	58	58
chr15	33011883	33013883	Pgcp	-0.18163	8.69E-11	hypomethylated	0.023101	0.27823	insignificant	9	27	24
chr15	34011473	34013473	Mtdh	-0.096972	2.35E-34	hypomethylated	0.0065761	0.77197	insignificant	47	161	161
chr15	34166780	34168780	Laptm4b	-0.13308	1.11E-14	hypomethylated	-0.015223	0.80456	insignificant	32	122	129
chr15	34235435	34237435	Matn2	-0.11337	1.02E-08	hypomethylated	-0.0047122	0.61981	insignificant	14	42	42
chr15	34372980	34374980	Rpl30	-0.17786	1.23E-12	hypomethylated	-0.00033645	0.80756	insignificant	18	49	49
chr15	34372988	34374988	Rpl30	-0.17786	1.23E-12	hypomethylated	-0.00033645	0.80756	insignificant	18	49	49
chr15	34373031	34375031	Rpl30	-0.18859	2.96E-11	hypomethylated	-0.011876	0.53558	insignificant	16	47	47
chr15	34382066	34384066	BC030476	-0.44517	0.00013439	stronglyHypometh	-0.1426	0.22757	insignificant	2	10	16
chr15	34424065	34426065	Pop1	-0.20996	2.51E-11	hypomethylated	-0.013624	0.90466	insignificant	14	82	82
chr15	34425001	34427001	Hrsp12	-0.21076	2.45E-11	hypomethylated	-0.015592	0.93527	insignificant	14	70	70
chr15	34608461	34610461	Nipal2	-0.15682	0.00000103	hypomethylated	-0.0035108	0.000040306	hypomethylated	12	56	56
chr15	34766135	34768135	Kcns2	-0.11816	8.74E-18	hypomethylated	0.00094907	0.012345	inconclusive	18	98	96
chr15	35085561	35087561	Stk3	-0.086438	0.093208	insignificant	-0.021363	0.23701	insignificant	7	25	25
chr15	35224866	35226866	Osr2	-0.12093	9.21E-26	hypomethylated	-0.013348	0.61342	insignificant	34	123	123
chr15	35300300	35302300	Vps13b	-0.12097	8.42E-22	hypomethylated	-0.0010532	0.69951	insignificant	46	138	138
chr15	35868001	35870001	Cox6c	-0.45969	0.00064128	stronglyHypometh	-0.043082	0.063312	insignificant	2	4	4
chr15	36094639	36096639	Fbxo43	-0.055556	1	insignificant	0.020461	0.042173	hypermethylated	5	18	18
chr15	36102771	36104771	Polr2k	-0.28162	4.24E-08	hypomethylated	-0.018256	0.081767	insignificant	5	53	50
chr15	36108284	36110284	Spag1	-0.10533	0.0095903	hypomethylated	0.020493	0.95542	insignificant	10	40	40
chr15	36212902	36214902	Rnf19a	-0.078069	0.00000198	hypomethylated	0.060931	0.043797	hypermethylated	11	50	54
chr15	36426546	36428546	Ankrd46	-0.1558	0.046608	hypomethylated	0.060709	0.6081	insignificant	3	7	10
chr15	36538728	36540728	Pabpc1	-0.10588	1.34E-20	hypomethylated	0.008863	0.2274	insignificant	41	111	109
chr15	36937157	36939157	Zfp706	-0.1504	6.11E-10	hypomethylated	-0.002273	0.36187	insignificant	13	42	42
chr15	37161790	37163790	Grhl2	-0.14505	0.00077228	hypomethylated	-0.029388	0.56395	insignificant	13	123	118
chr15	37721755	37723755	Ncald	-0.27236	0.57314	insignificant	-0.017561	1	insignificant	1	11	11
chr15	37890810	37892810	Rrm2b	-0.14771	0.000021641	hypomethylated	-0.048825	0.0058933	hypomethylated	7	58	50
chr15	38008608	38010608	Ubr5	-0.2093	0.0000044	hypomethylated	-0.092145	0.20445	insignificant	11	40	35
chr15	38230462	38232462	Klf10	-0.15515	3.06E-32	hypomethylated	0.0087156	0.020856	inconclusive	33	135	134
chr15	38449021	38451021	Azin1	-0.20942	1.06E-08	hypomethylated	-0.011181	0.70292	insignificant	18	70	70
chr15	38590658	38592658	Atp6v1c1	-0.13421	6.51E-16	hypomethylated	-0.012821	0.11172	insignificant	23	75	72
chr15	38836825	38838825	Fzd6	-0.14611	6.91E-16	hypomethylated	-0.0097369	0.0055619	hypomethylated	38	82	80
chr15	38836878	38838878	Fzd6	-0.14611	6.91E-16	hypomethylated	-0.0097369	0.0055619	hypomethylated	38	82	80
chr15	38907477	38909477	Cthrc1	-0.33136	0.30739	insignificant	-0.017946	0.6716	insignificant	2	8	8
chr15	38943419	38945419	Dcaf13	-0.13651	1.95E-21	hypomethylated	-0.010077	0.00066627	hypomethylated	33	118	121
chr15	38944262	38946262	Slc25a32	-0.25061	1.05E-10	hypomethylated	-0.010121	0.0103	hypomethylated	13	54	50
chr15	39028877	39030877	Rims2	-0.1189	8.09E-26	hypomethylated	-0.01673	0.18338	insignificant	80	189	196
chr15	39775303	39777303	Lrp12	-0.131	5.48E-20	hypomethylated	0.0022801	0.84643	insignificant	29	89	80
chr15	40485587	40487587	Zfpm2	-0.13503	1.66E-28	hypomethylated	-0.0088472	0.26028	insignificant	47	131	141
chr15	41278027	41280027	Oxr1	-0.11194	1.82E-30	hypomethylated	-0.011383	0.074295	insignificant	58	158	161
chr15	41619579	41621579	Oxr1	-0.12234	0.00000037	hypomethylated	0.0076736	0.75752	insignificant	39	109	110
chr15	41620060	41622060	Oxr1	-0.12234	0.00000037	hypomethylated	0.0076736	0.75752	insignificant	39	109	110
chr15	41701266	41703266	Abra		1	noCoverage	-0.024366	1	insignificant	0	2	2
chr15	43002364	43004364	Rspo2		1	noCoverage	-0.043376	0.47576	insignificant	0	10	12
chr15	43307774	43309774	Ttc35	-0.14374	1.15E-30	hypomethylated	0.0040179	0.1312	insignificant	20	62	63
chr15	43701575	43703575	Tmem74	-0.16748	1	insignificant	-0.023712	0.47929	insignificant	3	13	16
chr15	44258656	44260656	Eny2	-0.14804	7.77E-15	hypomethylated	-0.027629	1	insignificant	16	81	81
chr15	44259853	44261853	Eny2	-0.084473	0.0039169	hypomethylated	0.0035532	0.70543	insignificant	8	30	30
chr15	44450186	44452186	Ebag9	-0.11409	1.61E-36	hypomethylated	-0.0064264	0.039796	hypomethylated	49	155	159
chr15	44584004	44586004	Sybu		1	noCoverage	-0.15119	0.051478	insignificant	0	8	6
chr15	44711939	44713939	A930017M01Ril	0.1061	0.12152	insignificant	-0.032074	0.33587	insignificant	4	19	19
chr15	48623535	48625535	Csmd3	-0.31022	0.03545	hypomethylated	-0.026077	1	insignificant	1	14	11
chr15	50720660	50722660	Trps1	-0.14801	0.000067881	hypomethylated	0.0016366	0.20193	insignificant	17	71	70
chr15	50721587	50723587	Trps1	-0.12142	0.0041252	hypomethylated	-0.00091502	0.20896	insignificant	14	52	51
chr15	51697007	51699007	Eif3h	-0.56092	0.021115	stronglyHypometh	0.09755	1	insignificant	3	7	9
chr15	51823306	51825306	Rad21	-0.06448	0.064741	insignificant	0.010311	0.34777	insignificant	7	32	32
chr15	51870652	51872652	Aard	-0.19425	0.00099647	hypomethylated	0.015171	0.073016	insignificant	8	60	60
chr15	52542999	52544999	Med30	-0.083967	9.82E-08	hypomethylated	-0.010006	0.18238	insignificant	18	62	62
chr15	53177738	53179738	Ext1	-0.12009	1.31E-17	hypomethylated	-0.010537	0.047852	hypomethylated	30	66	66

chr15	54401920	54403920	Mal2	-0.1958	1.02E-20	hypomethylated	0.00097059	0.69762	insignificant	28	45	54
chr15	54576482	54578482	Nov	-0.25088	1.66E-17	hypomethylated	-0.037355	0.24681	insignificant	7	26	30
chr15	54751516	54753516	Enpp2	-0.35457	2.93E-09	stronglyHypometh	-0.05019	0.54716	insignificant	3	20	22
chr15	54751701	54753701	Enpp2	-0.38488	1.27E-08	stronglyHypometh	-0.030214	0.047157	hypomethylated	3	16	16
chr15	54922033	54924033	Dscc1	-0.22731	8.94E-09	hypomethylated	0.037615	0.44832	insignificant	9	33	33
chr15	54943037	54945037	Deptor	-0.12306	7.19E-39	hypomethylated	-0.0041313	0.82475	insignificant	36	90	94
chr15	54963990	54965990	Deptor	-0.19015	0.21633	insignificant	0.056277	0.31049	insignificant	1	6	6
chr15	55138304	55140304	Col14a1	-0.27399	0.000000147	hypomethylated	0.0036751	0.88799	insignificant	10	26	25
chr15	55387962	55389962	Mtbp	-0.14125	1.1E-20	hypomethylated	-0.013718	0.027194	hypomethylated	20	66	66
chr15	55388867	55390867	Mrpl13	-0.14125	1.1E-20	hypomethylated	-0.00095942	0.030182	hypomethylated	20	66	67
chr15	55738504	55740504	Sntb1	-0.17309	3.12E-28	hypomethylated	0.0095172	0.49178	insignificant	8	30	30
chr15	56526101	56528101	Has2	-0.33703	0.00000152	stronglyHypometh	-0.082495	0.78306	insignificant	2	9	11
chr15	57525221	57527221	Zhx2	-0.14226	2.02E-18	hypomethylated	-0.02731	0.97322	insignificant	41	145	152
chr15	57723973	57725973	Derl1	-0.17958	0.36165	insignificant	0.013398	0.68992	insignificant	2	20	20
chr15	57742753	57744753	Wdr67	-0.40273	4.59E-20	stronglyHypometh	-0.017249	0.22134	insignificant	11	40	40
chr15	57816457	57818457	Fam83a	-0.32323	5.96E-25	hypomethylated	0.032773	0.22458	insignificant	6	28	38
chr15	57865849	57867849	9130401M01Ril	-0.27831	0.010339	hypomethylated	-0.023991	0.30812	insignificant	6	18	19
chr15	57908044	57910044	Zhx1	-0.15877	0.14053	insignificant	-0.019414	0.75968	insignificant	5	18	18
chr15	57966637	57968637	Atad2	-0.18033	0.000000303	hypomethylated	-0.020337	0.83594	insignificant	18	65	83
chr15	57971990	57973990	Wdyhv1	-0.11944	3.12E-12	hypomethylated	0.00071352	0.85443	insignificant	20	58	64
chr15	58046433	58048433	Fbxo32	-0.28078	0.0037937	hypomethylated	-0.048363	0.82864	insignificant	6	20	16
chr15	58246022	58248022	D15Ertd621e	-0.10978	5.06E-18	hypomethylated	-0.0070095	0.0016589	hypomethylated	26	112	112
chr15	58654982	58656982	Tmem65	-0.088534	2.26E-18	hypomethylated	0.0074279	0.16755	insignificant	22	95	100
chr15	58703203	58705203	Trmt12	-0.18548	3.01E-35	hypomethylated	-0.0046097	0.042655	hypomethylated	29	96	96
chr15	58719783	58721783	Rnf139	-0.11432	1.25E-18	hypomethylated	-0.0027222	0.33331	insignificant	30	107	107
chr15	58764364	58766364	Ndufb9	-0.18504	3.27E-21	hypomethylated	0.013305	0.5509	insignificant	21	68	68
chr15	58765285	58767285	Ndufb9	-0.16344	2.42E-13	hypomethylated	-0.0022914	0.95638	insignificant	13	36	36
chr15	58913581	58915581	Mtss1	-0.17607	0.00000273	hypomethylated	-0.00054194	0.0016027	hypomethylated	25	88	88
chr15	59145646	59147646	Sqle	-0.086243	1.82E-14	hypomethylated	0.0012629	0.089166	insignificant	28	115	130
chr15	59204752	59206752	Nsmce2	-0.1696	2.18E-19	hypomethylated	-0.038424	0.010293	hypomethylated	17	54	51
chr15	59205707	59207707	E430025E21Rik	-0.19362	0.000000667	hypomethylated	-0.059183	0.033281	hypomethylated	5	30	27
chr15	59479208	59481208	Trib1	-0.13015	3.19E-62	hypomethylated	-0.0055805	0.51268	insignificant	74	236	251
chr15	60653519	60655519	9930014A18Rik	-0.15202	5.86E-09	hypomethylated	-0.011726	0.5102	insignificant	25	120	122
chr15	61815895	61817895	Myc	-0.16168	2.87E-19	hypomethylated	0.0038052	0.13601	insignificant	23	115	120
chr15	61868541	61870541	Pvt1	-0.16467	0.14346	insignificant	0.077806	0.081391	insignificant	7	42	43
chr15	62051006	62053006	H2afy3	0.18175	0.00086573	hypermethylated	-0.10645	0.019255	hypomethylated	6	22	22
chr15	63677863	63679863	Gsdmcl1		1	noCoverage	-0.017154	0.90411	insignificant	0	13	10
chr15	63745015	63747015	Gsdmcl-ps		1	noCoverage	0.079107	0.22411	insignificant	0	10	5
chr15	63892010	63894010	Fam49b	-0.21882	1.22E-11	hypomethylated	0.0045131	0.95544	insignificant	11	36	36
chr15	64753858	64755858	Adcy8	-0.13952	0.2324	insignificant	-0.021093	0.28216	insignificant	10	50	56
chr15	65617602	65619602	Efr3a	-0.1076	1.06E-39	hypomethylated	-0.0001194	0.047693	hypomethylated	41	118	118
chr15	65808366	65810366	Hhla1	-0.072677	0.52392	insignificant	-0.13567	0.045488	hypomethylated	5	12	12
chr15	66117786	66119786	Kcnq3	-0.131	4.86E-16	hypomethylated	0.0035339	0.56896	insignificant	27	72	73
chr15	66332472	66334472	Lrrc6	-0.24444	0.0037476	hypomethylated	0.18056	0.82336	insignificant	2	6	8
chr15	66408133	66410133	Phf20l1	-0.097459	5.05E-15	hypomethylated	0.0032004	0.96946	insignificant	39	84	84
chr15	66801203	66803203	Ndrg1	-0.097239	0.0038132	hypomethylated	0.022609	0.28331	insignificant	7	58	58
chr15	67008444	67010444	St3gal1	-0.1065	4.86E-09	hypomethylated	0.0044062	0.55882	insignificant	16	64	64
chr15	68090418	68092418	Zfat	-0.16045	9.53E-13	hypomethylated	-0.0010008	0.0026163	hypomethylated	19	73	74
chr15	68757849	68759849	Khdrbs3	-0.085899	5.52E-13	hypomethylated	-0.00084644	0.20989	insignificant	41	132	136
chr15	71558268	71560268	Fam135b		1	noCoverage	0.015997	0.60259	insignificant	0	4	4
chr15	72376709	72378709	Kcnk9	-0.10993	5.46E-19	hypomethylated	-0.011778	0.18533	insignificant	51	189	189
chr15	72640754	72642754	Peg13	-0.58478	0.27801	insignificant	-0.17456	0.40518	insignificant	3	9	6
chr15	72891634	72893634	Trappc9	-0.68657	0.0031681	stronglyHypometh	-0.067596	0.028737	hypomethylated	2	4	4
chr15	72919841	72921841	Chrac1	-0.12699	6.73E-13	hypomethylated	0.029919	0.0061793	inconclusive	17	67	69
chr15	73015377	73017377	Eif2c2	-0.081744	0.15731	insignificant	0.0068271	0.91916	insignificant	11	51	38
chr15	73253621	73255621	Ptk2	-0.14741	2.66E-13	hypomethylated	-0.02262	0.084568	insignificant	17	54	54
chr15	73341989	73343989	Dennd3	-0.19247	1.39E-14	hypomethylated	-0.0050063	0.0077142	hypomethylated	11	44	48
chr15	73537935	73539935	Gpr20	-0.63011	0.00041569	stronglyHypometh	-0.28976	0.22425	insignificant	1	4	5
chr15	73552574	73554574	Ptp4a3		1	noCoverage	-0.054887	0.84875	insignificant	0	18	18
chr15	73554359	73556359	Ptp4a3	-0.15081	1.84E-30	hypomethylated	-0.00017956	0.000026068	hypomethylated	46	134	134
chr15	73577455	73579455	Ptp4a3	-0.27066	0.40741	insignificant	0.038565	1	insignificant	2	6	10
chr15	73670101	73672101	Gm628	-0.097392	0.000000836	hypomethylated	-0.015647	0.59763	insignificant	21	68	68
chr15	74345625	74347625	Bai1	-0.084927	0.000088122	hypomethylated	-0.021523	0.11008	insignificant	23	105	118
chr15	74466748	74468748	1700016M24Ril	-0.18571	0.085586	insignificant	-0.28155	0.0037518	hypomethylated	2	7	4
chr15	74475178	74477178		0.029946	0.60462	insignificant	0.12606	0.061514	insignificant	3	6	6
chr15	74503000	74505000	Arc	-0.37054	1.37E-08	stronglyHypometh	-0.13716	0.81613	insignificant	1	4	4
chr15	74538590	74540590	4933427E11Rik	-0.23488	2.89E-62	hypomethylated	-0.029003	0.000030614	hypomethylated	35	93	96
chr15	74539752	74541752	4933427E11Rik	-0.25258	3.35E-35	hypomethylated	-0.023314	0.034044	hypomethylated	22	52	55
chr15	74550663	74552663	4930572J05Rik	-0.14727	0.074305	insignificant	0.0067474	0.51495	insignificant	7	92	106
chr15	74564743	74566743	Lypd2	-0.46939	0.00017088	stronglyHypometh	-0.15947	0.000033567	hypomethylated	2	8	8
chr15	74577117	74579117	2300005B03Rik	0.083333	0.45398	insignificant	0.057239	0.012351	inconclusive	3	6	6
chr15	74583409	74585409	Lynx1	-0.3788	0.000000431	stronglyHypometh	0.038018	0.033837	inconclusive	6	23	28
chr15	74593997	74595997	Ly6d	0.22321	1	lowCoverage	0.085119	1	insignificant	1	4	4
chr15	74648505	74650505	Hemt1	-0.0027038	1	insignificant	-0.05957	0.45539	insignificant	3	10	14
chr15	74649245	74651245	Gml	-0.0027038	1	insignificant	-0.05957	0.45539	insignificant	3	10	14
chr15	74784480	74786480	Ly6e	-0.18217	1.91E-08	hypomethylated	-0.006249	1	insignificant	13	59	60
chr15	74784500	74786500	Ly6e	-0.18217	1.91E-08	hypomethylated	-0.006249	1	insignificant	13	59	60
chr15	74784535	74786535	Ly6e	-0.18217	1.91E-08	hypomethylated	-0.006249	1	insignificant	13	59	60
chr15	74813860	74815860	Ly6i	-0.14461	0.33696	insignificant	-0.055946	0.6753	insignificant	2	8	8
chr15	74942379	74944379	Ly6c2	0.17713	0.68119	insignificant	0.073592	0.85267	insignificant	1	8	12
chr15	75262259	75264259	9030619P08Rik	-0.52578	0.12966	insignificant	-0.16789	0.70449	insignificant	1	9	9
chr15	75397078	75399078	Ly6h	-0.082515	1.53E-15	hypomethylated	0.010107	0.57651	insignificant	26	118	109
chr15	75397286	75399286	Ly6h	-0.073688	7.43E-09	hypomethylated	-0.00044954	0.19634	insignificant	15	70	61
chr15	75426087	75428087	Gpihbp1		1	noCoverage	-0.096028	0.013292	hypomethylated	0	10	10
chr15	75446114	75448114	Zfp41	-0.13424	1.03E-39	hypomethylated	0.0018351	0.32211	insignificant	47	153	150

chr15	75509220	75511220	Top1mt	-0.27939	0.063396	insignificant	-0.0075861	0.48056	insignificant	3	8	8
chr15	75533717	75535717	Rhpn1	-0.28966	0.000000124	hypomethylated	-0.044786	0.03902	hypomethylated	13	36	40
chr15	75578352	75580352	Mafa	-0.15916	3.01E-18	hypomethylated	-0.00046897	0.20971	insignificant	34	94	84
chr15	75672338	75674338	Zc3h3	-0.080155	0.1501	insignificant	0.048371	0.084049	insignificant	9	52	55
chr15	75691768	75693768	Gsdmd	-0.17757	1.51E-08	hypomethylated	0.014481	0.86032	insignificant	13	54	58
chr15	75724911	75726911	Naprt1	0.050474	0.68999	insignificant	0.056328	0.095868	insignificant	4	14	14
chr15	75739164	75741164	Tigd5	-0.097326	1.7E-67	hypomethylated	0.010721	0.23375	insignificant	68	251	258
chr15	75739770	75741770	Eef1d	-0.11014	3.65E-49	hypomethylated	0.040226	0.98174	insignificant	48	184	199
chr15	75760160	75762160	Tsta3	-0.36071	1.12E-09	stronglyHypometh	0.074252	0.22345	insignificant	2	19	15
chr15	75770381	75772381	Zfp623	-0.11399	0.0027922	hypomethylated	-0.0058142	0.70104	insignificant	24	75	73
chr15	75798614	75800614	Zfp707	-0.15913	9.04E-14	hypomethylated	-0.021417	0.84788	insignificant	14	71	70
chr15	75812715	75814715	2410075B13Rik	-0.41816	0.29217	insignificant	-0.12883	0.096662	insignificant	2	13	12
chr15	75823198	75825198	Mapk15	-0.30814	0.0034523	hypomethylated	-0.022485	0.20842	insignificant	5	20	20
chr15	75839928	75841928	Fam83h	-0.19725	6.14E-19	hypomethylated	0.029993	0.00041784	inconclusive	15	72	68
chr15	75844766	75846766	Fam83h	-0.10718	0.60302	insignificant	-0.034076	0.37191	insignificant	6	14	14
chr15	75900160	75902160	Puf60	-0.14787	3.6E-19	hypomethylated	0.012107	0.84855	insignificant	13	42	42
chr15	75920443	75922443	Nrbp2	-0.14471	7.77E-17	hypomethylated	-0.0033272	0.25678	insignificant	31	86	86
chr15	75950625	75952625	BC024139		1	noCoverage	0.053922	0.73002	insignificant	0	2	2
chr15	76026140	76028140	Plec	-0.38532	1.48E-10	stronglyHypometh	0.02753	1	insignificant	2	6	6
chr15	76028639	76030639	Plec	-0.21353	7.08E-24	hypomethylated	-0.0028241	0.66858	insignificant	13	40	38
chr15	76029836	76031836	Plec	-0.18199	1.23E-12	hypomethylated	0.17008	0.27638	insignificant	7	16	14
chr15	76030265	76032265	Plec	-0.083939	0.1258	insignificant	0.15313	0.92098	insignificant	6	20	18
chr15	76031037	76033037	Plec	0.1299	1	insignificant	0.0080215	0.88365	insignificant	1	8	8
chr15	76036751	76038751	Plec	-0.29603	1.13E-10	hypomethylated	0.038959	0.95203	insignificant	9	18	18
chr15	76059927	76061927	Plec	-0.15149	0.49151	insignificant	0.032927	0.010909	hypermethylated	5	22	30
chr15	76061808	76063808	Plec	-0.2036	0.31628	insignificant	0.016205	0.68621	insignificant	4	56	56
chr15	76073870	76075870	Parp10	-0.014274	0.011218	hypomethylated	-0.044982	1	insignificant	4	9	15
chr15	76076236	76078236	Grina	-0.30093	0.17515	insignificant	0.035317	0.17047	insignificant	3	12	15
chr15	76097518	76099518	Spatc1	0.065079	1	insignificant	0.1011	0.65536	insignificant	2	12	12
chr15	76123863	76125863	Gm10345	-0.22447	2.69E-14	hypomethylated	-0.039797	0.000000268	hypomethylated	11	45	47
chr15	76156826	76158826	Exosc4	-0.12992	7.57E-26	hypomethylated	0.013382	0.92814	insignificant	26	69	72
chr15	76157963	76159963	Exosc4	-0.018192	7.87E-08	hypomethylated	-0.0067411	0.42776	insignificant	23	75	79
chr15	76160723	76162723	Gpaa1	-0.19789	5.54E-18	hypomethylated	-0.0090039	0.42186	insignificant	20	81	81
chr15	76172952	76174952	Cyc1	-0.12301	4.45E-15	hypomethylated	-0.0087652	0.9045	insignificant	27	80	86
chr15	76180723	76182723	Maf1	-0.144	2.3E-16	hypomethylated	-0.0050917	0.48259	insignificant	38	121	123
chr15	76181540	76183540	Sharpin	-0.11615	0.00000527	hypomethylated	0.008337	0.16297	insignificant	19	64	65
chr15	76198327	76200327	Fam203a	-0.17163	3.92E-15	hypomethylated	-0.01876	0.27101	insignificant	26	99	103
chr15	76209942	76211942	Heatr7a	-0.13579	2.96E-15	hypomethylated	0.00041405	0.11151	insignificant	15	84	84
chr15	76219460	76221460	Heatr7a	0.125	0.29932	insignificant	0.042717	0.59625	insignificant	3	9	14
chr15	76286867	76288867	Scx	-0.10456	0.045403	hypomethylated	0.032551	0.088156	insignificant	21	50	50
chr15	76306874	76308874	Hsf1	-0.13679	1.88E-42	hypomethylated	0.0048277	0.1596	insignificant	51	146	146
chr15	76307699	76309699	Bop1	-0.1109	5.67E-22	hypomethylated	-0.00093815	0.10476	insignificant	30	98	98
chr15	76342248	76344248	Dgat1	-0.10776	1.11E-20	hypomethylated	-0.015462	0.053265	insignificant	18	71	70
chr15	76352559	76354559	Scrt1	-0.14323	1.38E-14	hypomethylated	-0.027552	0.0049304	hypomethylated	17	143	139
chr15	76368372	76370372	Gpr172b	-0.15195	8.67E-28	hypomethylated	-0.0048791	0.26313	insignificant	31	104	110
chr15	76369176	76371176	Gpr172b	-0.21287	5.72E-12	hypomethylated	0.075717	0.91518	insignificant	10	32	40
chr15	76405788	76407788	Adck5	-0.1137	4.02E-20	hypomethylated	0.013714	0.15238	insignificant	24	69	70
chr15	76438021	76440021	Cpsf1	-0.20667	0.00086274	hypomethylated	0.093658	0.22174	insignificant	3	25	28
chr15	76447282	76449282	Slc39a4		1	noCoverage	0.14286	0.18467	insignificant	0	6	6
chr15	76456457	76458457	Vps28	-0.12857	0.00051425	hypomethylated	0.075609	0.17439	insignificant	4	8	9
chr15	76490070	76492070	Kifc2	-0.29222	1.43E-17	hypomethylated	0.0067322	1	insignificant	11	24	24
chr15	76490491	76492491	Cyhr1	-0.29222	1.43E-17	hypomethylated	0.0067322	1	insignificant	11	24	24
chr15	76490538	76492538	Cyhr1	-0.29222	1.43E-17	hypomethylated	0.0067322	1	insignificant	11	24	24
chr15	76500303	76502303	Ppp1r16a	-0.1755	1.42E-20	hypomethylated	0.01577	0.036599	inconclusive	29	75	76
chr15	76501109	76503109	Ppp1r16a	-0.15852	1.07E-22	hypomethylated	-0.019021	0.0041709	hypomethylated	32	82	84
chr15	76530971	76532971	Mfsd3	-0.21048	1.57E-27	hypomethylated	-0.0089046	0.62649	insignificant	14	32	32
chr15	76540169	76542169	Lrrc14	-0.12036	1.59E-13	hypomethylated	0.00079425	0.47108	insignificant	21	107	107
chr15	76540906	76542906	Lrrc14	-0.12012	3.48E-13	hypomethylated	0.0038353	0.62862	insignificant	21	97	97
chr15	76552603	76554603	C030006K11Rik	-0.1102	1.87E-33	hypomethylated	0.0054909	0.12869	insignificant	48	142	136
chr15	76554275	76556275	C030006K11Rik	-0.2147	3.2E-53	hypomethylated	0.040472	0.00084678	inconclusive	26	100	96
chr15	76648600	76650600	Arhgap39	-0.21528	0.041684	hypomethylated	0.00012626	0.1461	insignificant	5	12	12
chr15	76701865	76703865	Zfp251	-0.27768	0.0026015	hypomethylated	-0.034697	0.79399	insignificant	5	24	24
chr15	76708705	76710705	Zfp7	-0.37347	0.20025	insignificant	-0.034514	0.00053083	inconclusive	7	52	55
chr15	76729370	76731370	Commd5	-0.12182	0.16338	insignificant	0.011124	0.81813	insignificant	7	34	36
chr15	76733500	76735500	Rpl8	-0.18385	0.000000351	hypomethylated	0.032492	0.9283	insignificant	5	56	60
chr15	76755878	76757878	Zfp647	-0.17136	6.32E-23	hypomethylated	0.0061362	0.78708	insignificant	18	60	62
chr15	76777973	76779973	1110038F14Rik	-0.11262	1.21E-20	hypomethylated	-0.0022013	0.029691	hypomethylated	14	75	75
chr15	76853157	76855157	Mb		1	noCoverage	-0.076066	0.31113	insignificant	0	16	14
chr15	76853217	76855217	Mb		1	noCoverage	-0.076066	0.31113	insignificant	0	16	14
chr15	76874504	76876504	Apol6	-0.37599	0.00000965	stronglyHypometh	-0.11688	0.015743	hypomethylated	3	17	18
chr15	76874705	76876705	Apol6	-0.2573	0.000010066	hypomethylated	-0.092602	0.0048629	hypomethylated	5	21	22
chr15	76881098	76883098	Apol6	0.08372	1	insignificant	-0.067473	0.080183	insignificant	5	24	22
chr15	77137483	77139483	Rbfox2	-0.087565	1	insignificant	-0.0044356	0.41333	insignificant	3	32	32
chr15	77363745	77365745	Apol7c		1	noCoverage	-0.3	0.68335	insignificant	0	5	3
chr15	77448397	77450397	Gm8221	0.081686	1	lowCoverage	-0.1445	0.0016673	hypomethylated	1	6	6
chr15	77672545	77674545	Myh9	-0.23869	0.13768	insignificant	0.043275	0.5991	insignificant	6	51	52
chr15	77759424	77761424	Txn2	-0.24883	9.36E-08	hypomethylated	-0.01438	0.0022663	hypomethylated	8	34	34
chr15	77787152	77789152	Foxred2	-0.24806	3.73E-10	hypomethylated	-0.085734	0.94487	insignificant	8	18	16
chr15	77801254	77803254	Eif3d	-0.13086	0.00032608	hypomethylated	-0.033868	0.85407	insignificant	13	65	64
chr15	77949710	77951710	Cacng2	-0.059346	0.000061862	hypomethylated	-0.013711	0.012462	hypomethylated	36	134	134
chr15	78004538	78006538	Ift27	-0.1443	0.00044337	hypomethylated	0.0053274	0.32528	insignificant	3	40	40
chr15	78034586	78036586	Pvalb	-0.12028	0.0033616	hypomethylated	-0.036653	0.81062	insignificant	8	33	32
chr15	78155419	78157419	Csf2rb	-0.24728	0.16309	insignificant	0.045085	0.11562	insignificant	4	8	8
chr15	78236141	78238141	Mpst	-0.1589	0.00000154	hypomethylated	0.0067203	0.89151	insignificant	7	52	52
chr15	78236230	78238230	Mpst	-0.18099	0.00022725	hypomethylated	0.0057005	0.54166	insignificant	5	40	40

chr15	78236289	78238289	Tst	-0.1736	0.011546	hypomethylated	0.0080076	0.95588	insignificant	3	34	34
chr15	78236533	78238533	Mpst	-0.1612	0.011544	hypomethylated	0.021055	0.90631	insignificant	3	28	28
chr15	78258057	78260057	Kctd17	-0.11367	8.06E-11	hypomethylated	-0.033287	0.076676	insignificant	15	59	56
chr15	78299064	78301064	Tmprss6	-0.8468	0.14173	lowCoverage	0.048219	0.26417	insignificant	1	6	6
chr15	78325496	78327496	Il2rb		1	noCoverage	0.0019481	0.53754	insignificant	0	4	4
chr15	78360047	78362047	C1qtnf6	-0.37531	0.000028713	stronglyHypometh	-0.082508	0.39602	insignificant	5	20	20
chr15	78374775	78376775	Sstr3	-0.29092	3.76E-08	hypomethylated	0.029926	0.52526	insignificant	4	8	8
chr15	78426476	78428476	Cyth4	-0.48201	0.00053898	stronglyHypometh	-0.11257	0.31875	insignificant	3	6	6
chr15	78548543	78550543	Elfn2	-0.1194	9.39E-19	hypomethylated	0.010782	0.048421	hypermethylated	38	133	105
chr15	78603875	78605875	Mfng	-0.56559	7.35E-31	stronglyHypometh	-0.055785	0.16522	insignificant	5	4	4
chr15	78633472	78635472	Card10	-0.091683	0.00023856	hypomethylated	0.029797	0.055444	insignificant	12	39	37
chr15	78672076	78674076	Cdc42ep1	-0.11118	0.00000104	hypomethylated	-0.0019825	0.50767	insignificant	27	78	78
chr15	78685959	78687959	Lgals2	-0.10597	0.00037943	hypomethylated	0.0021429	0.64375	insignificant	4	4	4
chr15	78706619	78708619	Gga1	-0.10579	4.08E-19	hypomethylated	-0.0096642	0.27209	insignificant	39	106	105
chr15	78729215	78731215	Sh3bp1	-0.11699	0.026345	hypomethylated	-0.012436	0.57739	insignificant	13	58	64
chr15	78743348	78745348	Pdxp	-0.072618	2.79E-10	hypomethylated	-0.0065345	0.78701	insignificant	35	180	177
chr15	78756154	78758154	Lgals1	-0.4807	0.13329	insignificant	-0.011998	0.29897	insignificant	2	10	10
chr15	78764362	78766362	Nol12	-0.17163	1.67E-15	hypomethylated	-0.012053	0.000065298	hypomethylated	17	78	74
chr15	78777153	78779153	Triobp	-0.17305	0.0073817	hypomethylated	0.10834	0.54288	insignificant	2	16	16
chr15	78812485	78814485	Triobp	-0.15916	6.2E-18	hypomethylated	0.019366	0.7056	insignificant	27	86	79
chr15	78857641	78859641	H1f0	-0.12518	4.64E-34	hypomethylated	0.0048344	0.60856	insignificant	72	201	207
chr15	78860303	78862303	Gcat	-0.17825	0.23461	insignificant	-0.0024923	0.8228	insignificant	5	32	34
chr15	78871314	78873314	Galr3	-0.14158	0.43521	insignificant	-0.067799	0.73251	insignificant	18	54	53
chr15	78904652	78906652	Eif3l	-0.053637	0.000000121	hypomethylated	-0.0034717	0.13946	insignificant	9	80	88
chr15	78938412	78940412	Micall1	-0.1363	2.53E-39	hypomethylated	0.0066753	0.43568	insignificant	60	177	166
chr15	78970796	78972796	Polr2f	-0.16696	2.81E-08	hypomethylated	0.0060065	0.51318	insignificant	19	84	94
chr15	78971681	78973681	1700088E04Rik	-0.16307	0.018316	hypomethylated	0.013342	0.74465	insignificant	14	56	66
chr15	78994920	78996920	Gm10863	-0.015415	0.80579	insignificant	-0.032911	0.6501	insignificant	14	52	53
chr15	78995495	78997495	Gm10863	-0.0076078	0.55001	insignificant	-0.030349	0.89959	insignificant	14	54	55
chr15	79058603	79060603	Pick1	-0.16068	1.7E-27	hypomethylated	-0.013979	0.033975	hypomethylated	18	65	68
chr15	79058811	79060811	Pick1	-0.16068	1.7E-27	hypomethylated	-0.013979	0.033975	hypomethylated	18	65	68
chr15	79085178	79087178	Slc16a8		1	noCoverage	-0.066667	0.19643	insignificant	0	3	5
chr15	79115939	79117939	Pla2g6	0.061174	1	insignificant	-0.13013	0.070757	insignificant	3	12	16
chr15	79158801	79160801	Pla2g6	-0.43137	0.49327	insignificant	-0.086928	0.07823	insignificant	1	6	6
chr15	79177107	79179107	Maff	-0.1297	1.05E-40	hypomethylated	0.01079	0.0022092	inconclusive	39	138	137
chr15	79233733	79235733	Tmem184b	-0.09088	6.51E-10	hypomethylated	-0.02044	0.030704	hypomethylated	8	30	30
chr15	79272487	79274487	Csnk1e	-0.10021	0.000003608	hypomethylated	0.056888	0.078653	insignificant	18	30	33
chr15	79335671	79337671	Kcnj4	-0.12549	1.65E-43	hypomethylated	0.0063326	0.0068175	hypermethylated	65	148	146
chr15	79345837	79347837	Kdelr3	-0.18879	0.16006	insignificant	-0.014365	0.062947	insignificant	8	32	40
chr15	79363347	79365347	Ddx17	0.027823	1	insignificant	0.0099654	0.62252	insignificant	2	14	15
chr15	79376989	79378989	Ddx17	-0.31833	1.62E-09	hypomethylated	0.014323	0.41298	insignificant	16	58	59
chr15	79377171	79379171	Ddx17	-0.34954	1.03E-08	stronglyHypometh	0.013081	0.27841	insignificant	14	50	51
chr15	79435514	79437514	Dmc1	-0.12873	0.000015737	hypomethylated	0.031575	0.78547	insignificant	6	28	37
chr15	79488656	79490656	Cby1	-0.07236	0.00049881	hypomethylated	0.0045938	0.13899	insignificant	27	79	80
chr15	79489386	79491386	4933432B09Rik	-0.10539	0.55318	insignificant	-0.0011817	0.84134	insignificant	18	64	66
chr15	79500297	79502297	Tomm22	-0.193	5.85E-11	hypomethylated	-0.0077748	0.80405	insignificant	21	86	84
chr15	79518302	79520302	Josd1	-0.24394	0.018966	hypomethylated	-0.056119	0.00031789	hypomethylated	4	41	51
chr15	79520325	79522325	Gtpbp1	-0.1402	6.24E-22	hypomethylated	0.011129	0.40156	insignificant	32	68	69
chr15	79572960	79574960	Gm16576	-0.072361	0.0035164	hypomethylated	-0.020251	0.00063435	hypomethylated	22	73	70
chr15	79604897	79606897	Dnalc4	-0.16466	0.000001558	hypomethylated	0.034708	0.75433	insignificant	8	27	28
chr15	79635139	79637139	Nptxr	-0.14799	7.95E-08	hypomethylated	-0.0088284	0.09739	insignificant	21	56	60
chr15	79664763	79666763	Npcd	-0.16708	0.000000598	hypomethylated	-0.036202	0.268	insignificant	5	36	35
chr15	79721837	79723837	Apobec3	-0.36745	0.00000114	stronglyHypometh	0.086449	1	insignificant	2	14	10
chr15	79723568	79725568	D730005E14Rik	-0.59251	0.21022	insignificant	0.17208	0.001777	hypermethylated	1	17	12
chr15	79763076	79765076	Cbx7	-0.16044	0.63395	insignificant	0.1306	0.91403	insignificant	5	21	29
chr15	79845238	79847238	Pdgfb	-0.30341	0.0040249	hypomethylated	0.001408	0.72043	insignificant	4	16	16
chr15	79909009	79911009	Snord83b		1	noCoverage	0.012763	0.51476	insignificant	0	10	16
chr15	79913336	79915336	Snord43	-0.18798	3.76E-22	hypomethylated	0.0046244	0.016279	inconclusive	13	60	60
chr15	79913354	79915354	Snord43	-0.17944	3.79E-22	hypomethylated	0.025145	0.019108	inconclusive	13	62	61
chr15	79913836	79915836	Snord43	-0.1364	0.00011712	hypomethylated	0.064537	0.23543	insignificant	8	44	39
chr15	79920763	79922763	Syngr1	-0.13917	0.0018814	hypomethylated	-0.027721	0.6093	insignificant	21	79	78
chr15	79962583	79964583	Tab1	-0.11722	0.00000411	hypomethylated	-0.016142	0.0067395	hypomethylated	20	86	93
chr15	80003150	80005150	Mgat3	-0.076167	3.95E-08	hypomethylated	0.00019952	0.45512	insignificant	63	199	222
chr15	80063509	80065509	Smcr7l	-0.14135	5.99E-17	hypomethylated	-0.020699	0.0076924	hypomethylated	20	76	74
chr15	80084613	80086613	Atf4	-0.13982	5.44E-18	hypomethylated	0.0010185	0.19403	insignificant	22	119	115
chr15	80094736	80096736	Rps19bp1	-0.22148	0.00000132	hypomethylated	-0.025428	0.37647	insignificant	3	19	20
chr15	80116667	80118667	Cacna1i	-0.11188	0.000000425	hypomethylated	0.003596	0.05689	insignificant	50	153	149
chr15	80390900	80392900	Enthd1	-0.020777	0.48574	insignificant	0.052888	0.31835	insignificant	3	16	15
chr15	80501276	80503276	Fam83f	-0.045585	0.00023394	hypomethylated	-0.014657	0.14736	insignificant	18	90	87
chr15	80540742	80542742	Tnrc6b	-0.098734	7.71E-12	hypomethylated	0.00063659	0.92355	insignificant	14	104	104
chr15	80777948	80779948	Adsl	-0.20269	0.000036011	hypomethylated	0.010132	0.099244	insignificant	10	71	73
chr15	80807194	80809194	Sgsm3	-0.2406	0.00022389	hypomethylated	-0.029278	0.033975	hypomethylated	31	103	99
chr15	81020282	81022282	4930483J18Rik	-0.1556	8.16E-13	hypomethylated	0.037526	0.40951	insignificant	30	123	122
chr15	81021187	81023187	4930483J18Rik	-0.01208	0.0024056	hypomethylated	0.072841	0.45615	insignificant	12	72	66
chr15	81064928	81066928	Mchr1	-0.17847	5.17E-13	hypomethylated	-0.0044855	0.80662	insignificant	10	36	36
chr15	81191195	81193195	Slc25a17	-0.19168	1	insignificant	-0.0031582	0.39782	insignificant	3	6	6
chr15	81229617	81231617	Xpnpep3	-0.19259	4.1E-30	hypomethylated	0.02278	0.16701	insignificant	19	101	101
chr15	81230124	81232124	St13	-0.20145	2.57E-26	hypomethylated	0.021903	0.79432	insignificant	14	89	89
chr15	81295745	81297745	Rbx1	-0.21393	3.53E-19	hypomethylated	-0.02662	0.000000723	hypomethylated	15	93	99
chr15	81415643	81417643	Ep300	-0.12938	1.3E-58	hypomethylated	-0.0087085	0.0039642	hypomethylated	67	220	215
chr15	81493365	81495365	L3mbtl2	-0.23721	0.00032611	hypomethylated	0.11117	0.34601	insignificant	6	44	31
chr15	81527717	81529717	Chadl		1	noCoverage	0.034748	0.43207	insignificant	0	19	19
chr15	81560349	81562349	Rangap1	-0.83889	0.18113	lowCoverage	-0.22648	0.02015	hypomethylated	1	20	30
chr15	81574277	81576277	Zc3h7b	-0.11632	0.000000486	hypomethylated	0.052855	0.081763	insignificant	25	70	72
chr15	81640843	81642843	Tef	-0.093513	0.00051334	hypomethylated	-0.00044606	0.47932	insignificant	21	76	84

chr15	81688756	81690756	Tob2	-0.17638	1.47E-11	hypomethylated	0.0036417	0.86545	insignificant	27	106	106
chr15	81701892	81703892	Aco2	-0.092351	7.3E-28	hypomethylated	0.0026753	0.35343	insignificant	25	129	123
chr15	81702322	81704322	Phf5a	-0.10118	1.21E-19	hypomethylated	-0.0010268	0.22394	insignificant	19	103	97
chr15	81756643	81758643	Polr3h	-0.2826	2.02E-09	hypomethylated	0.017704	0.026207	inconclusive	8	49	49
chr15	81766188	81768188	Csdc2		1	noCoverage	-0.16533	0.83852	insignificant	0	5	6
chr15	81791297	81793297	Pmm1	-0.30009	2.07E-10	hypomethylated	-0.088313	0.016892	inconclusive	5	32	30
chr15	81809974	81811974	1700029P11Rik	-0.16292	0.32048	insignificant	-0.062564	0.33974	insignificant	2	25	25
chr15	81845798	81847798	Xrcc6	-0.15484	4.05E-17	hypomethylated	-0.010757	0.058286	insignificant	16	87	90
chr15	81846570	81848570	Pppde2		1	noCoverage	-0.040503	0.019199	hypomethylated	0	36	41
chr15	81878028	81880028	Nhp2l1	-0.20264	0.60636	insignificant	0.0099235	0.14976	insignificant	7	26	28
chr15	81899546	81901546	Mei1	-0.12729	0.0054849	hypomethylated	-0.025521	0.20315	insignificant	10	33	34
chr15	81957351	81959351	Ccdc134	-0.037505	1.12E-14	hypomethylated	0.035374	0.013895	hypermethylated	21	74	76
chr15	81976698	81978698	Srebf2	-0.091054	9.35E-14	hypomethylated	0.0037485	0.556	insignificant	34	162	168
chr15	82027551	82029551	Mir33	-0.34025	0.36861	insignificant	-0.014497	0.39873	insignificant	1	6	6
chr15	82054766	82056766	Tnfrsf13c	-0.60507	0.096625	insignificant	-0.16122	0.051505	insignificant	3	6	6
chr15	82074766	82076766	Cenpm	-0.194	4.34E-14	hypomethylated	0.0038327	0.076765	insignificant	18	78	79
chr15	82075177	82077177	Cenpm	-0.36712	0.000000145	stronglyHypometh	0.017635	0.20266	insignificant	6	26	27
chr15	82104364	82106364	############	-0.11677	2.68E-27	hypomethylated	0.0051041	0.010487	inconclusive	37	104	107
chr15	82128413	82130413	Wbp2nl		1	noCoverage	-0.0097863	0.26121	insignificant	0	6	6
chr15	82169256	82171256	Naga	-0.29359	0.09042	insignificant	-0.060791	0.51658	insignificant	2	6	6
chr15	82170608	82172608	Fam109b	-0.18621	2.58E-26	hypomethylated	0.15822	0.95795	insignificant	11	48	48
chr15	82175475	82177475	1500032L24Rik	-0.1645	3.32E-32	hypomethylated	-0.010113	0.27593	insignificant	25	65	70
chr15	82184721	82186721	Ndufa6	-0.095229	0.60867	insignificant	0.0092492	0.95344	insignificant	8	36	36
chr15	82237624	82239624	Cyp2d10		1	noCoverage	0.08153	1	insignificant	0	4	6
chr15	82728891	82730891	Gm20324	-0.11123	4.55E-30	hypomethylated	0.0059127	0.91861	insignificant	52	155	162
chr15	82742564	82744564	Tcf20	-0.51804	4.92E-13	stronglyHypometh	0.15457	0.83718	insignificant	3	8	6
chr15	82929634	82931634	Serhl	-0.22362	0.32728	insignificant	-0.041689	0.035335	hypomethylated	4	18	33
chr15	82953231	82955231	Rrp7a	-0.034866	0.00010558	hypomethylated	-0.016641	0.0066207	hypomethylated	4	30	30
chr15	82979074	82981074	Rnu12	-0.098546	1.02E-29	hypomethylated	0.0019902	0.30118	insignificant	37	129	129
chr15	82979766	82981766	Poldip3	-0.24779	0.082385	insignificant	0.0099856	0.35468	insignificant	4	14	14
chr15	83002638	83004638	Cyb5r3	-0.23236	0.22787	insignificant	0.060613	0.23445	insignificant	5	25	29
chr15	83082161	83084161	A4galt	-0.53667	0.34875	lowCoverage	-0.11896	0.34353	insignificant	1	13	16
chr15	83082204	83084204	A4galt	-0.68385	0.27347	lowCoverage	-0.066543	0.91047	insignificant	1	11	11
chr15	83180677	83182677	Arfgap3	-0.099869	0.000024251	hypomethylated	-0.0037387	0.60801	insignificant	8	37	37
chr15	83295036	83297036	Pacsin2		1	noCoverage	-0.030903	0.18898	insignificant	0	8	8
chr15	83341337	83343337	Ttll1	-0.50512	3.43E-14	stronglyHypometh	0.10186	0.37118	insignificant	3	48	47
chr15	83356291	83358291	Bik	-0.14992	4.13E-08	hypomethylated	-0.014545	0.14556	insignificant	25	102	102
chr15	83386141	83388141	Mcat	-0.0023217	0.3287	insignificant	0.04987	1	insignificant	4	12	14
chr15	83393002	83395002	Tspo	-0.15305	1	insignificant	0.14925	0.13753	insignificant	3	22	24
chr15	83555451	83557451	Scube1	-0.24403	0.00000163	hypomethylated	0.0052394	0.17467	insignificant	13	55	59
chr15	83609452	83611452	Mpped1	-0.13796	8.98E-20	hypomethylated	0.0042404	0.1509	insignificant	47	127	147
chr15	83749803	83751803	Efcab6		1	noCoverage	0.029541	0.91742	insignificant	0	16	16
chr15	83766525	83768525	Efcab6	0.040945	0.74798	insignificant	-0.099829	0.60603	insignificant	2	13	12
chr15	83895779	83897779	Efcab6		1	noCoverage	0.012963	0.65601	insignificant	0	6	6
chr15	83936184	83938184	Sult4a1	0.0032277	0.047326	hypermethylated	-0.0021395	0.018953	hypomethylated	2	18	18
chr15	83997245	83999245	Pnpla3	-0.18068	2.73E-18	hypomethylated	0.028105	0.41414	insignificant	23	83	83
chr15	84021662	84023662	Samm50	-0.198	3.48E-12	hypomethylated	0.034947	0.033047	inconclusive	11	60	62
chr15	84061472	84063472	Parvb	-0.16216	9.02E-12	hypomethylated	-0.014039	0.000014749	hypomethylated	13	87	82
chr15	84154149	84156149	Parvg	-0.66993	0.12179	insignificant	-0.0021168	0.77827	insignificant	2	18	18
chr15	84388253	84390253	Ldoc1l	-0.15311	0.0026575	hypomethylated	-0.01648	0.42058	insignificant	4	8	8
chr15	84510427	84512427	Prr5	-0.12858	2.41E-47	hypomethylated	0.011387	0.18771	insignificant	56	152	142
chr15	84549481	84551481	Arhgap8	-0.28358	6.69E-19	hypomethylated	-0.026873	0.012	hypomethylated	14	67	68
chr15	84549508	84551508	Arhgap8	-0.28358	6.69E-19	hypomethylated	-0.026873	0.012	hypomethylated	14	67	68
chr15	84686559	84688559	Phf21b	-0.12539	1.09E-15	hypomethylated	-0.0065489	0.7434	insignificant	20	58	58
chr15	84752857	84754857	Nup50	-0.11292	8.63E-45	hypomethylated	0.021506	0.023547	inconclusive	62	181	184
chr15	84782053	84784053	5031439G07Rik	0.037025	0.46706	insignificant	-0.010278	0.90719	insignificant	7	20	24
chr15	84818401	84820401	5031439G07Rik		1	noCoverage	0.010985	0.28787	insignificant	0	24	24
chr15	84846570	84848570	Upk3a		1	noCoverage	-0.0049985	0.4992	insignificant	0	12	13
chr15	84961528	84963528	Ribc2	-0.15216	5.05E-19	hypomethylated	0.0098388	6.42E-09	inconclusive	16	76	72
chr15	84962387	84964387	Smc1b	-0.22859	3.53E-17	hypomethylated	0.033962	0.033417	inconclusive	13	62	58
chr15	85035437	85037437	Fbln1	-0.14806	7.31E-21	hypomethylated	0.028683	0.093982	insignificant	21	76	78
chr15	85165810	85167810	Atxn10	-0.12268	1.3E-09	hypomethylated	-0.0043874	0.21934	insignificant	8	30	25
chr15	85408500	85410500	Wnt7b	-0.24229	0.0011871	hypomethylated	-0.0084996	0.24356	insignificant	6	20	20
chr15	85411159	85413159	Wnt7b	-0.099996	5.12E-40	hypomethylated	-0.004928	0.62372	insignificant	73	188	192
chr15	85412251	85414251	Wnt7b	-0.19495	3.13E-08	hypomethylated	-0.027428	0.119	insignificant	16	35	35
chr15	85536032	85538032	Mirlet7c-2	0.20536	1	insignificant	-0.034824	0.042849	hypomethylated	2	10	10
chr15	85564993	85566993	Ppara	-0.12117	6.49E-19	hypomethylated	0.0052648	0.6664	insignificant	45	186	189
chr15	85565205	85567205	Ppara	-0.1171	6.36E-19	hypomethylated	0.0031144	0.78676	insignificant	45	184	183
chr15	85642127	85644127	2210021J22Rik	-0.17364	1.37E-11	hypomethylated	-0.032871	0.61122	insignificant	7	26	26
chr15	85652163	85654163	Pkdrej	-0.097966	6.54E-11	hypomethylated	-0.0082684	0.55521	insignificant	15	36	37
chr15	85661733	85663733	Ttc38	-0.13924	4.77E-29	hypomethylated	-0.0079513	0.48359	insignificant	18	51	54
chr15	85689136	85691136	Gtse1	-0.29173	2.47E-09	hypomethylated	-0.010842	0.02672	hypomethylated	9	46	48
chr15	85689375	85691375	Gtse1	-0.31026	7.39E-10	hypomethylated	-0.019071	0.0047733	hypomethylated	9	48	50
chr15	85708756	85710756	Trmu	-0.16592	9.55E-09	hypomethylated	0.021127	0.55179	insignificant	13	51	40
chr15	85864207	85866207	Celsr1	-0.1742	2.08E-25	hypomethylated	-0.047311	0.044414	hypomethylated	19	67	76
chr15	85887136	85889136	Gramd4	-0.15975	0.16585	insignificant	0.036847	0.047264	inconclusive	3	39	34
chr15	86043888	86045888	Tbc1d22a	-0.10836	4.2E-11	hypomethylated	-0.021058	0.082581	insignificant	32	101	101
chr15	87454659	87456659	Fam19a5	-0.10777	9.65E-39	hypomethylated	-0.0051393	0.69362	insignificant	66	173	164
chr15	88429731	88431731	Zdhhc25	-0.079716	0.017483	hypomethylated	-0.045044	0.0035045	hypomethylated	16	47	46
chr15	88564649	88566649	Brd1	-0.059913	0.00000424	hypomethylated	0.0073149	0.90163	insignificant	15	54	54
chr15	88581140	88583140	Zbed4	-0.090724	1.87E-12	hypomethylated	-0.014259	0.053364	insignificant	55	168	166
chr15	88649075	88651075	Creld2	-0.14069	3.71E-55	hypomethylated	-0.012184	0.011058	hypomethylated	47	179	171
chr15	88649748	88651748	Alg12	-0.18403	1.28E-52	hypomethylated	-0.0067826	0.00032238	hypomethylated	37	130	122
chr15	88691623	88693623	Pim3	-0.086055	1.47E-25	hypomethylated	-0.0053085	0.23864	insignificant	56	204	204
chr15	88812423	88814423	Mov10l1	0.051555	1	insignificant	-0.041591	0.40731	insignificant	2	20	8

chr15	88889155	88891155	Panx2	-0.11075	0.00074969	hypomethylated	-0.047383	0.066187	insignificant	10	60	57
chr15	88905493	88907493	Trabd	-0.15999	3.97E-53	hypomethylated	-0.0088715	0.33827	insignificant	39	119	118
chr15	88918536	88920536	1300018J18Rik	-0.11002	4.87E-28	hypomethylated	0.000093302	0.77623	insignificant	43	104	105
chr15	88971133	88973133	Mapk12	-0.21364	0.0023754	hypomethylated	-0.043867	0.33211	insignificant	7	18	18
chr15	88980036	88982036	Mapk11	-0.29613	0.0045081	hypomethylated	-0.012297	1	insignificant	8	28	34
chr15	89004280	89006280	Plxnb2	-0.16485	0.010391	inconclusive	0.0024545	0.00011305	hypermethylated	3	28	31
chr15	89011218	89013218	Plxnb2		1	noCoverage	-0.067586	0.20088	insignificant	0	19	19
chr15	89026905	89028905	Fam116b	-0.13238	1.17E-16	hypomethylated	-0.0004491	0.52166	insignificant	13	34	34
chr15	89040989	89042989	Ppp6r2	-0.094105	6.18E-12	hypomethylated	0.0019486	0.00042092	inconclusive	34	116	115
chr15	89145742	89147742	Sbf1	-0.11835	0.093288	insignificant	0.10279	0.46913	insignificant	3	10	15
chr15	89152402	89154402	Adm2	-0.17071	1	insignificant	-0.026413	0.087415	insignificant	2	20	20
chr15	89163903	89165903	Miox		1	noCoverage	0.041288	0.13787	insignificant	0	8	6
chr15	89185152	89187152	Ncaph2	-0.18192	1.38E-25	hypomethylated	-0.0024434	0.40478	insignificant	24	90	88
chr15	89186090	89188090	Ncaph2	-0.2113	6.13E-26	hypomethylated	-0.0092418	0.26469	insignificant	17	64	63
chr15	89204249	89206249	Tymp	-0.41604	8.87E-27	stronglyHypometh	0.0018307	0.00093671	inconclusive	8	35	35
chr15	89207468	89209468	Tymp	-0.35218	1.41E-39	stronglyHypometh	0.029675	0.000000143	inconclusive	14	83	75
chr15	89209685	89211685	Odf3b	0.10475	1	insignificant	-0.020255	0.52575	insignificant	4	8	8
chr15	89216321	89218321	Klhdc7b	-0.42209	0.00013815	stronglyHypometh	0.041535	0.026185	inconclusive	5	18	20
chr15	89240694	89242694	Syce3		1	noCoverage	-0.032828	0.66492	insignificant	0	8	10
chr15	89256293	89258293	Cpt1b	0.052514	0.61417	insignificant	-0.031215	0.35721	insignificant	4	22	20
chr15	89260358	89262358	BC090627		1	noCoverage	-0.15582	0.41452	insignificant	0	12	21
chr15	89283341	89285341	Mapk8ip2	-0.14452	0.000047022	hypomethylated	0.013535	0.87627	insignificant	8	80	80
chr15	89307855	89309855	Arsa		1	noCoverage	0.021074	0.060167	insignificant	0	11	11
chr15	89329287	89331287	Shank3	-0.14458	2.82E-31	hypomethylated	-0.0009874	0.77824	insignificant	62	165	190
chr15	89422354	89424354	Rabl2	-0.13151	0.0046862	hypomethylated	-0.0020048	0.8189	insignificant	3	18	18
chr15	90053741	90055741	Alg10b	-0.17492	0.00000401	hypomethylated	-0.034914	0.42338	insignificant	8	33	44
chr15	90509819	90511819	Cpne8	-0.12989	4.12E-15	hypomethylated	-0.015732	0.4516	insignificant	19	69	68
chr15	90880379	90882379	Kif21a	-0.099446	0.0002242	hypomethylated	0.060933	0.062663	insignificant	12	41	46
chr15	91022238	91024238	Abcd2		1	noCoverage	-0.01565	0.89467	insignificant	0	16	16
chr15	91502654	91504654	Lrrk2	-0.10157	3.26E-08	hypomethylated	0.015164	0.36536	insignificant	9	30	37
chr15	91880595	91882595	Cntn1	-0.20763	0.000000133	hypomethylated	-0.0081661	0.46645	insignificant	14	50	54
chr15	91990787	91992787	Cntn1	-0.39122	0.000014639	stronglyHypometh	0.21917	0.71694	insignificant	1	15	15
chr15	92226240	92228240	Pdzrn4	-0.16763	0.000047105	hypomethylated	-0.0066155	0.88233	insignificant	24	52	52
chr15	92426539	92428539	Pdzrn4	-0.18478	0.18745	insignificant	-0.06256	0.23951	insignificant	5	10	10
chr15	93105515	93107515	Gxylt1	-0.14512	1.9E-12	hypomethylated	-0.024886	4.63E-08	hypomethylated	20	83	92
chr15	93167366	93169366	Yaf2	-0.14548	2.01E-24	hypomethylated	0.012135	0.93678	insignificant	33	89	84
chr15	93227780	93229780	Pphln1	-0.12352	8.13E-21	hypomethylated	-0.0055836	0.054943	insignificant	48	171	172
chr15	93228721	93230721	Zcrb1	-0.23506	5.54E-19	hypomethylated	-0.02427	0.491	insignificant	26	102	103
chr15	93426322	93428322	Prickle1	-0.14772	2.62E-08	hypomethylated	-0.017289	0.000010272	hypomethylated	14	48	48
chr15	94234781	94236781	Adamts20	0.014066	0.75957	insignificant	-0.0080579	0.000000133	hypomethylated	27	86	86
chr15	94373090	94375090	Irak4	-0.057342	2.92E-14	hypomethylated	0.025187	0.061758	insignificant	16	82	81
chr15	94373938	94375938	Pus7l	-0.043173	9.91E-14	hypomethylated	0.023542	0.074461	insignificant	14	76	75
chr15	94458615	94460615	Tmem117	-0.11904	7.18E-08	hypomethylated	-0.024829	0.79813	insignificant	36	82	86
chr15	95620273	95622273	Ano6	-0.10004	4.35E-26	hypomethylated	-0.020884	0.04273	hypomethylated	46	129	124
chr15	96116952	96118952	Arid2	-0.089356	2.66E-40	hypomethylated	0.0022379	0.47577	insignificant	108	337	348
chr15	96291274	96293274	Scaf11	-0.053954	0.0013051	hypomethylated	0.017283	0.015493	hypermethylated	13	35	35
chr15	96472393	96474393	Slc38a1	-0.093473	0.0031258	hypomethylated	-0.0085992	0.36139	insignificant	14	97	95
chr15	96472774	96474774	Slc38a1	-0.097458	0.026163	hypomethylated	-0.02378	0.57299	insignificant	11	57	55
chr15	96530129	96532129	Slc38a2	-0.15232	0.00000631	hypomethylated	0.012337	0.018689	hypermethylated	15	68	68
chr15	97076537	97078537	Fam113b	-0.1048	4.32E-18	hypomethylated	-0.0099795	0.0075142	hypomethylated	35	124	131
chr15	97077718	97079718	Fam113b	-0.073027	3.22E-11	hypomethylated	-0.00029403	0.64594	insignificant	32	114	116
chr15	97536253	97538253	Rpap3	0.32058	0.00042708	hypermethylated	-0.063292	0.86296	insignificant	6	35	40
chr15	97551581	97553581	Endou	-0.35218	0.035076	stronglyHypometh	-0.024124	0.92545	insignificant	1	13	12
chr15	97598097	97600097	Rapgef3	-0.24877	3.14E-20	hypomethylated	-0.037605	0.16818	insignificant	19	77	78
chr15	97613795	97615795	Slc48a1	-0.14929	3.54E-34	hypomethylated	-0.029915	0.23698	insignificant	31	62	71
chr15	97662102	97664102	Hdac7	-0.094166	6.63E-18	hypomethylated	-0.012344	0.19612	insignificant	26	60	60
chr15	97738727	97740727	Vdr	-0.11362	0.00000138	hypomethylated	-0.025826	0.78593	insignificant	18	37	41
chr15	97793709	97795709	Tmem106c	-0.063559	0.000007211	hypomethylated	0.022879	1	insignificant	22	75	94
chr15	97835155	97837155	Col2a1	-0.30239	8.71E-15	hypomethylated	-0.054362	0.00000142	hypomethylated	13	30	30
chr15	97922019	97924019	Pfkm	-0.18714	2.86E-16	hypomethylated	-0.042761	0.28989	insignificant	13	44	35
chr15	97937901	97939901	Pfkm	-0.17937	0.0000928	hypomethylated	-0.046288	0.86224	insignificant	7	18	18
chr15	97941490	97943490	Pfkm	-0.71834	0.39568	lowCoverage	-0.014677	0.90057	insignificant	1	9	7
chr15	97976133	97978133	Asb8	-0.11667	0.48814	insignificant	0.10979	0.17229	insignificant	1	6	6
chr15	97997236	97999236	AI836003	-0.16579	4.17E-08	hypomethylated	-0.016058	0.59136	insignificant	10	75	75
chr15	98087738	98089738	H1fnt		1	noCoverage	-0.09697	0.38921	insignificant	0	6	3
chr15	98126514	98128514	Zfp641	-0.12257	1.14E-08	hypomethylated	0.024894	0.90362	insignificant	19	40	40
chr15	98266900	98268900	Olfr282		1	noCoverage	0.12923	0.82063	insignificant	0	8	8
chr15	98285742	98287742	Olfr281		1	noCoverage	0.055934	0.059838	insignificant	0	8	9
chr15	98313114	98315114	Lalba		1	noCoverage	0.1662	0.53036	insignificant	0	8	8
chr15	98326904	98328904	Olfr279	-0.12608	0.073175	insignificant	-0.019467	0.2846	insignificant	4	8	8
chr15	98364652	98366652	2310037I24Rik	0.46038	0.0073168	stronglyHypermeth	0.064984	0.94397	insignificant	2	42	43
chr15	98396754	98398754	9330020H09Rik	-0.11185	2.28E-18	hypomethylated	-0.017536	0.22722	insignificant	34	128	132
chr15	98398067	98400067	9330020H09Rik	-0.11793	6.74E-15	hypomethylated	-0.012417	0.24221	insignificant	27	104	108
chr15	98438064	98440064	Adcy6	-0.14988	3.6E-16	hypomethylated	-0.017148	0.0068608	hypomethylated	33	81	81
chr15	98461650	98463650	Cacnb3	-0.23302	3.04E-18	hypomethylated	-0.03359	0.017344	hypomethylated	10	49	47
chr15	98464194	98466194	Cacnb3	-0.17388	8.81E-46	hypomethylated	-0.02126	0.076488	insignificant	39	133	134
chr15	98493320	98495320	Ddx23	-0.30518	0.0027346	hypomethylated	-0.011153	0.0070823	inconclusive	9	35	36
chr15	98537657	98539657	Ccdc65		1	noCoverage	0.044331	0.64261	insignificant	0	17	14
chr15	98558629	98560629	Fkbp11	-0.34771	0.31112	insignificant	-0.1148	0.1048	insignificant	2	6	6
chr15	98593549	98595549	Arf3	-0.12282	2.36E-18	hypomethylated	-0.0090436	0.060852	insignificant	13	51	51
chr15	98608581	98610581	Wnt10b	-0.019776	0.000000922	hypomethylated	-0.0059166	1	insignificant	4	12	12
chr15	98619287	98621287	Wnt1	-0.19709	5.34E-17	hypomethylated	-0.0049108	0.58282	insignificant	15	57	57
chr15	98661939	98663939	Mll2	-0.17106	0.0001004	hypomethylated	0.040314	0.55774	insignificant	4	16	18
chr15	98701614	98703614	Mll2	-0.15943	4.32E-34	hypomethylated	-0.0079962	0.3692	insignificant	55	132	143
chr15	98711845	98713845	Rhebl1	-0.41038	0.060392	insignificant	-0.0016234	1	insignificant	5	15	15

chr15	98728971	98730971	Dhh	-0.51943	0.000000153	stronglyHypometh	-0.11636	0.93377	insignificant	7	18	22
chr15	98748529	98750529	Lmbr1l		1	noCoverage	0.049469	0.016913	hypermethylated	0	42	46
chr15	98764821	98766821	Tuba1b	-0.13821	1.73E-10	hypomethylated	-0.0078826	0.00086649	hypomethylated	21	60	60
chr15	98783932	98785932	Tuba1a	0.21683	0.00014421	hypermethylated	0.20933	0.00000156	hypermethylated	4	8	8
chr15	98859321	98861321	Tuba1c	-0.166	2.82E-24	hypomethylated	-0.0036221	0.23662	insignificant	21	96	91
chr15	98884604	98886604	Prph	-0.14853	2.13E-43	hypomethylated	0.010146	0.13038	insignificant	31	120	120
chr15	98904403	98906403	Troap	-0.24117	3.51E-10	hypomethylated	0.0049862	0.31342	insignificant	15	70	70
chr15	98918159	98920159	C1ql4		1	noCoverage	-0.19353	2.75E-14	hypomethylated	0	16	24
chr15	98928914	98930914	Dnajc22	-0.0095606	1	insignificant	-0.0024397	0.75099	insignificant	5	32	32
chr15	98956275	98958275	Spats2	-0.1045	1.41E-12	hypomethylated	-0.0088922	0.29127	insignificant	35	189	193
chr15	99054406	99056406	Kcnh3	-0.15205	5.72E-20	hypomethylated	-0.016431	0.00074889	hypomethylated	41	140	140
chr15	99082392	99084392	Mcrs1	-0.28615	0.000000119	hypomethylated	-0.015178	0.10196	insignificant	9	22	22
chr15	99124839	99126839	Prpf40b	-0.15518	1.12E-18	hypomethylated	0.053263	0.36276	insignificant	25	99	105
chr15	99200897	99202897	Fmnl3	-0.58988	0.000029579	stronglyHypometh	-0.13996	0.10812	insignificant	2	16	15
chr15	99222377	99224377	Tmbim6	-0.23224	7.44E-24	hypomethylated	-0.01811	0.001277	hypomethylated	17	74	78
chr15	99222649	99224649	Tmbim6	-0.23224	7.44E-24	hypomethylated	-0.01811	0.001277	hypomethylated	17	74	78
chr15	99305161	99307161	Bcdin3d	-0.041531	0.023169	hypomethylated	-0.067151	0.014809	hypomethylated	5	9	9
chr15	99358448	99360448	Faim2	-0.54352	0.01296	stronglyHypometh	-0.0066565	0.41059	insignificant	1	12	14
chr15	99408486	99410486	Aqp2	0.16879	0.0013741	hypermethylated	0.093417	0.28034	insignificant	4	19	19
chr15	99420458	99422458	Aqp5	-0.13252	1.03E-21	hypomethylated	-0.0048591	0.00000925	hypomethylated	51	176	185
chr15	99430830	99432830	Aqp6		1	noCoverage	0.02582	0.34393	insignificant	0	6	6
chr15	99482052	99484052	Racgap1	-0.24635	0.0001	hypomethylated	-0.093789	0.10614	insignificant	4	17	14
chr15	99500148	99502148	Accn2	-0.12178	2.22E-32	hypomethylated	0.00030156	0.0082934	inconclusive	51	143	148
chr15	99531717	99533717	Smarcd1	-0.20191	3.76E-31	hypomethylated	-0.0062918	0.0010372	hypomethylated	36	107	113
chr15	99547023	99549023	Gpd1	-0.0081773	1	insignificant	-0.031599	0.00082181	inconclusive	4	23	23
chr15	99555048	99557048	2310016M24Ril	-0.11437	0.000000125	hypomethylated	-0.0097843	0.96738	insignificant	12	51	72
chr15	99602946	99604946	Lass5	-0.097039	0.064522	insignificant	0.053194	0.57164	insignificant	4	22	23
chr15	99705887	99707887	Lima1	-0.20836	0.087147	insignificant	0.032626	0.22585	insignificant	10	34	36
chr15	99762089	99764089	1700030F18Rik		1	noCoverage	-0.048359	0.61238	insignificant	0	12	12
chr15	99799504	99801504	Larp4	-0.048523	0.63632	insignificant	-0.066927	0.55222	insignificant	1	19	21
chr15	99802210	99804210	Larp4	-0.16519	6.61E-23	hypomethylated	-0.011023	0.047962	hypomethylated	28	93	93
chr15	99803718	99805718	Larp4	-0.2723	0.0000018	hypomethylated	-0.059227	0.40813	insignificant	6	22	32
chr15	99868094	99870094	Dip2b	-0.10694	4.39E-34	hypomethylated	0.0034425	0.073776	insignificant	40	153	152
chr15	100057289	100059289	Atf1	-0.087257	2.4E-21	hypomethylated	-0.0098182	0.082035	insignificant	50	189	179
chr15	100110267	100112267	Tmprss12	-0.067134	0.11766	insignificant	-0.0096623	0.77695	insignificant	4	28	28
chr15	100134247	100136247	Mettl7a1	-0.33985	0.0062391	stronglyHypometh	-0.047588	0.13932	insignificant	6	35	34
chr15	100164359	100166359	Mettl7a3		1	noCoverage	0.023797	0.55233	insignificant	0	23	28
chr15	100182630	100184630	AB099516	0.004567	0.0056797	hypermethylated	0.057606	0.0099231	inconclusive	8	34	31
chr15	100253486	100255486	Slc11a2	-0.18456	8.84E-28	hypomethylated	-0.0008245	0.096755	insignificant	22	84	84
chr15	100298464	100300464	Letmd1	-0.077182	4.21E-21	hypomethylated	0.010365	0.0046105	inconclusive	35	132	139
chr15	100325670	100327670	Csrnp2	-0.32259	0.000000347	hypomethylated	-0.091608	0.0017206	hypomethylated	7	31	40
chr15	100382378	100384378	Tfcp2	-0.10418	3.55E-13	hypomethylated	0.00035241	0.44807	insignificant	15	44	44
chr15	100445092	100447092	Dazap2	-0.14209	0.004518	hypomethylated	-0.0032165	0.88763	insignificant	7	40	40
chr15	100467286	100469286	Smagp	-0.18322	1.44E-10	hypomethylated	-0.066677	0.099909	insignificant	17	47	47
chr15	100467296	100469296	Smagp	-0.19137	1.45E-10	hypomethylated	-0.065203	0.21836	insignificant	17	44	41
chr15	100518351	100520351	Cela1	-0.19821	0.068599	insignificant	0.00037879	0.20681	insignificant	2	4	8
chr15	100559807	100561807	Galnt6	-0.14242	0.089067	insignificant	0.036772	4.62E-11	inconclusive	9	37	39
chr15	100591177	100593177	Slc4a8	-0.1348	3.14E-24	hypomethylated	-0.00062121	0.0048107	hypomethylated	29	87	89
chr15	100700113	100702113	Scn8a	-0.13355	3.85E-25	hypomethylated	-0.020104	0.84351	insignificant	55	161	178
chr15	100765701	100767701	Scn8a	-0.22378	0.000000121	hypomethylated	0.015592	0.10565	insignificant	7	30	33
chr15	100957967	100959967	Acvrl1	-0.32621	0.00046043	hypomethylated	-0.0096592	0.82693	insignificant	6	33	32
chr15	101003555	101005555	Acvr1b	-0.11309	1.93E-43	hypomethylated	0.0025723	0.014605	inconclusive	49	161	168
chr15	101053637	101055637	Grasp	-0.17548	3.36E-34	hypomethylated	-0.02308	0.03797	hypomethylated	31	62	68
chr15	101096276	101098276	Nr4a1	-0.19194	4.5E-23	hypomethylated	-0.017823	0.050351	insignificant	12	70	69
chr15	101113731	101115731	9430023L20Rik	-0.11571	5.69E-21	hypomethylated	-0.011869	0.42369	insignificant	21	80	83
chr15	101122642	101124642	6030408B16Rik		1	noCoverage	0.18336	0.19045	insignificant	0	21	21
chr15	101198782	101200782	Mir1941	-0.10921	0.027818	hypomethylated	-0.081574	0.022544	hypomethylated	4	14	14
chr15	101200556	101202556	Krt80	0.072153	1	insignificant	-0.1017	0.23784	insignificant	2	10	10
chr15	101241833	101243833	Krt7	0.085342	0.0020132	hypermethylated	0.040135	0.021978	hypermethylated	16	46	51
chr15	101269235	101271235	Krt83	-0.17125	0.10622	insignificant	-0.067835	0.058738	insignificant	3	22	22
chr15	101277175	101279175	1700011A15Rik		1	noCoverage	-0.091265	0.22196	insignificant	0	18	18
chr15	101294196	101296196	Krt81	-0.85	0.12088	lowCoverage	0.091176	0.035811	hypermethylated	1	2	2
chr15	101302908	101304908	Krt86		1	noCoverage	-0.016807	0.76783	insignificant	0	4	4
chr15	101333246	101335246	Gm6042	-0.021687	0.15847	insignificant	-0.10003	0.54913	insignificant	2	12	12
chr15	101363251	101365251	Krt84	-0.13696	0.15978	insignificant	-0.20362	0.71129	insignificant	1	6	6
chr15	101381090	101383090	Krt82		1	noCoverage	0.010714	0.44129	insignificant	0	4	4
chr15	101393381	101395381	Krt85	0.15195	1	insignificant	-0.054071	0.62441	insignificant	1	6	6
chr15	101419003	101421003	Krt85	-0.11922	0.35469	insignificant	-0.059045	0.20308	insignificant	1	17	17
chr15	101524736	101526736	Krt6a	-0.53535	0.030766	stronglyHypometh	-0.095705	0.014716	hypomethylated	1	11	10
chr15	101543322	101545322	Krt5	0.04788	0.35849	insignificant	0.045043	0.34202	insignificant	3	10	10
chr15	101593935	101595935	Krt74	-0.061667	0.62627	insignificant	-0.10491	0.022618	hypomethylated	4	10	10
chr15	101681217	101683217	Krt1	-0.26396	0.0056031	hypomethylated	-0.013965	0.74927	insignificant	3	20	21
chr15	101700049	101702049	Krt77	-0.8588	0.14953	lowCoverage	-0.086477	0.70759	insignificant	1	6	5
chr15	101755166	101757166	Krt4		1	noCoverage	-0.05172	0.48866	insignificant	0	4	5
chr15	101834773	101836773	Krt8		1	noCoverage	-0.1056	0.080281	insignificant	0	9	9
chr15	101857646	101859646	Krt18	-0.34238	0.00000014	stronglyHypometh	-0.057949	0.32041	insignificant	13	81	79
chr15	101903203	101905203	Eif4b	-0.078886	0.00069252	hypomethylated	-0.00010536	0.33453	insignificant	6	49	38
chr15	101966646	101968646	Spryd3	-0.14818	5.36E-10	hypomethylated	-0.024588	0.10019	insignificant	15	48	48
chr15	101973616	101975616	Igfbp6	-0.0048304	0.00000885	hypomethylated	0.10743	0.00000728	hypermethylated	13	47	50
chr15	102062366	102064366	Itgb7		1	noCoverage	-0.0016922	0.13237	insignificant	0	14	15
chr15	102076783	102078783	Rarg	-0.15931	0.00071806	hypomethylated	-0.0050298	0.5619	insignificant	8	50	51
chr15	102087914	102089914	Rarg	-0.17531	0.39439	insignificant	-0.079656	0.25593	insignificant	4	25	23
chr15	102108886	102110886	Mfsd5	-0.13806	1.42E-16	hypomethylated	-0.0086089	0.71655	insignificant	47	121	121
chr15	102125723	102127723	Espl1	-0.066695	0.22463	insignificant	0.013027	0.7482	insignificant	18	49	51
chr15	102155546	102157546	Pfdn5	-0.21513	8.3E-14	hypomethylated	-0.061105	0.92284	insignificant	14	40	36

chr15	102161139	102163139	Myg1	-0.38241	0.00000241	stronglyHypometh	0.0070844	0.023853	hypermethylated	10	37	37
chr15	102181190	102183190	Aaas	-0.11541	0.21944	insignificant	0.011126	0.8378	insignificant	5	10	10
chr15	102196702	102198702	Sp7		1	noCoverage	0.15745	0.086742	insignificant	0	5	5
chr15	102235746	102237746	Sp1	-0.11547	1.5E-11	hypomethylated	0.019065	0.55552	insignificant	33	139	151
chr15	102288600	102290600	Prr13	-0.43961	7.73E-18	stronglyHypometh	0.00077992	0.88336	insignificant	6	18	19
chr15	102300062	102302062	Pcbp2	-0.12555	2.17E-26	hypomethylated	0.0049554	0.73223	insignificant	30	146	143
chr15	102347435	102349435	Map3k12	-0.19485	4.56E-25	hypomethylated	-0.032277	0.023423	hypomethylated	24	50	52
chr15	102347676	102349676	Tarbp2	-0.15111	2.94E-14	hypomethylated	-0.010676	0.11975	insignificant	15	42	44
chr15	102355052	102357052	Npff	-0.41518	0.00024509	stronglyHypometh	0.018577	0.86303	insignificant	2	8	8
chr15	102355373	102357373	Npff	-0.29762	0.0057537	hypomethylated	0.031111	0.70429	insignificant	2	4	4
chr15	102455852	102457852	Atf7	-0.17373	3.23E-11	hypomethylated	-0.012126	1	insignificant	7	26	26
chr15	102501478	102503478	Atp5g2	-0.14936	0.001263	hypomethylated	-0.00090088	0.7789	insignificant	7	57	57
chr15	102502297	102504297	Atp5g2	-0.2247	0.0051241	hypomethylated	-0.017818	0.92377	insignificant	5	28	28
chr15	102552609	102554609	Calcoco1	0.0018315	0.61485	insignificant	0.10044	0.3519	insignificant	6	14	16
chr15	102750561	102752561	Hoxc13	-0.14074	2.22E-12	hypomethylated	-0.035585	0.10447	insignificant	52	148	167
chr15	102766283	102768283	Hoxc12	-0.20631	0.21485	insignificant	-0.19051	0.11333	insignificant	9	32	47
chr15	102783956	102785956	Hoxc11	-0.15994	4.92E-21	hypomethylated	0.0038713	1	insignificant	30	100	100
chr15	102796295	102798295	Hoxc10	-0.22772	4.63E-10	hypomethylated	-0.047004	0.31711	insignificant	10	64	76
chr15	102802780	102804780	Mir196a-2	-0.20645	9.66E-11	hypomethylated	0.00066908	0.95669	insignificant	13	38	38
chr15	102806462	102808462	Hoxc9	-0.038211	0.0057138	hypomethylated	0.021053	0.27834	insignificant	11	70	69
chr15	102819969	102821969	Hoxc8	-0.16066	1.84E-09	hypomethylated	-0.004826	0.17782	insignificant	27	58	59
chr15	102838992	102840992	Hoxc6	-0.26707	2.61E-09	hypomethylated	-0.033216	0.18935	insignificant	9	20	22
chr15	102843438	102845438	Hoxc5	-0.16274	0.000000486	hypomethylated	-0.016798	0.65519	insignificant	9	28	28
chr15	102844340	102846340	Mir615	-0.15273	1.36E-12	hypomethylated	-0.02578	0.64083	insignificant	20	92	88
chr15	102863825	102865825	Hoxc4	-0.18421	2.5E-13	hypomethylated	0.0040025	0.35992	insignificant	18	66	65
chr15	102993715	102995715	Smug1	-0.31711	0.0011742	hypomethylated	-0.079648	0.083603	insignificant	8	20	20
chr15	103085847	103087847	Nfe2	-0.24569	1.78E-10	hypomethylated	0.00014433	0.048043	inconclusive	7	14	14
chr15	103102348	103104348	Copz1	-0.315	0.0000017	hypomethylated	-0.054302	0.00072234	hypomethylated	3	19	19
chr15	103170517	103172517	Zfp385a	-0.24076	0.000022176	hypomethylated	0.023144	0.29049	insignificant	6	42	36
chr15	103283255	103285255	Nckap1l	-0.27639	0.0013868	hypomethylated	0.040575	0.54562	insignificant	4	8	8
chr15	103332728	103334728	Pde1b	-0.17083	0.00000667	hypomethylated	-0.02086	0.66935	insignificant	5	35	30
chr15	103368423	103370423	Ppp1r1a	-0.13531	0.0018132	hypomethylated	0.019098	0.62633	insignificant	7	26	26
chr16	3743098	3745098	Zfp263	-0.17494	2.61E-25	hypomethylated	0.0089194	0.031839	inconclusive	20	84	84
chr16	3846222	3848222	Zfp174	-0.23716	0.00060012	hypomethylated	-0.043857	0.35558	insignificant	7	45	53
chr16	3872374	3874374	Zfp597	-0.18448	0.00039943	hypomethylated	0.026001	0.14644	insignificant	10	42	47
chr16	3883618	3885618	Nat15	-0.15893	1.68E-24	hypomethylated	-0.017789	0.070403	insignificant	35	105	105
chr16	3908008	3910008	Cluap1	-0.15773	3.41E-11	hypomethylated	-0.020967	0.18272	insignificant	22	66	66
chr16	3908689	3910689	1700037C18Rik	-0.1975	1.16E-09	hypomethylated	-0.020393	0.26221	insignificant	14	48	48
chr16	4001680	4003680	Slx4	-0.11026	0.60174	insignificant	0.0040226	0.59655	insignificant	2	10	10
chr16	4077810	4079810	Trap1	-0.59991	1.44E-36	stronglyHypometh	-0.047901	0.59168	insignificant	9	24	26
chr16	4213404	4215404	Crebbp	-0.095553	1.45E-16	hypomethylated	0.015592	0.85163	insignificant	50	160	153
chr16	4419587	4421587	Adcy9	-0.093006	1.44E-30	hypomethylated	-0.011622	0.022462	hypomethylated	71	216	221
chr16	4559720	4561720	Tfap4		1	noCoverage	0.056473	0.000039586	hypermethylated	0	60	62
chr16	4593712	4595712	Glis2	-0.15452	1.17E-36	hypomethylated	-0.005877	0.000053871	hypomethylated	34	151	154
chr16	4638944	4640944	Vasn		1	noCoverage	0.06378	0.22716	insignificant	0	8	8
chr16	4679720	4681720	Coro7		1	noCoverage	-0.10497	0.29894	insignificant	0	6	5
chr16	4683069	4685069	Dnaja3	-0.28968	5.76E-15	hypomethylated	-0.048176	0.1748	insignificant	21	54	54
chr16	4725360	4727360	Hmox2	-0.1833	3.3E-22	hypomethylated	-0.014523	0.3498	insignificant	19	60	62
chr16	4789935	4791935	5730403B10Rik	-0.086872	2.76E-12	hypomethylated	0.010627	0.95259	insignificant	15	54	54
chr16	4834415	4836415	4930562C15Rik		1	noCoverage	0.0050118	0.11809	insignificant	0	12	12
chr16	4879851	4881851	Fam100a	-0.11526	3.55E-10	hypomethylated	0.008176	0.3753	insignificant	24	75	71
chr16	4885251	4887251	Mgrn1	-0.12247	2.04E-23	hypomethylated	0.014787	0.36839	insignificant	28	124	125
chr16	4938110	4940110	Nudt16l1	-0.15448	6.06E-14	hypomethylated	0.029477	0.18866	insignificant	8	28	28
chr16	4964330	4966330	Anks3	-0.19578	2.91E-16	hypomethylated	0.0074486	0.000084522	inconclusive	7	14	14
chr16	5013646	5015646	Rogdi	-0.14341	0.000000409	hypomethylated	0.088224	0.32281	insignificant	7	27	31
chr16	5049160	5051160	Ubn1	-0.10546	1.19E-50	hypomethylated	-0.0092875	0.12724	insignificant	76	225	231
chr16	5050003	5052003	Ubn1	-0.1097	7.84E-41	hypomethylated	-0.0056414	0.53807	insignificant	57	153	161
chr16	5132574	5134574	Ppl	-0.090952	0.000076715	hypomethylated	0.01954	0.14948	insignificant	15	35	34
chr16	5146201	5148201	Sec14l5	-0.092413	0.12495	insignificant	-0.010858	0.96754	insignificant	9	57	56
chr16	5204105	5206105	Nagpa	-0.30362	0.087732	insignificant	0.091115	0.5648	insignificant	1	8	10
chr16	5256049	5258049	Fam86	-0.20182	9.81E-16	hypomethylated	-0.022155	0.098969	insignificant	7	21	21
chr16	5883885	5885885	Rbfox1	-0.14231	6.71E-15	hypomethylated	0.016483	0.85811	insignificant	15	85	85
chr16	7068927	7070927	Rbfox1	0.21314	1	lowCoverage	0.016393	0.59148	insignificant	1	6	6
chr16	8469905	8471905	Mettl22	-0.096552	0.0011414	hypomethylated	-0.038281	0.0013044	hypomethylated	16	57	60
chr16	8512521	8514521	Abat	-0.12493	0.001353	hypomethylated	-0.0050817	0.26047	insignificant	14	60	62
chr16	8636799	8638799	Pmm2	-0.1329	6.07E-44	hypomethylated	-0.0031536	0.27557	insignificant	28	87	87
chr16	8637794	8639794	Tmem186	-0.30674	9.37E-24	hypomethylated	0.032388	0.00000005	inconclusive	11	39	40
chr16	8672246	8674246	Carhsp1	-0.13166	6.73E-12	hypomethylated	-0.0054201	0.47155	insignificant	18	58	58
chr16	8738435	8740435	Usp7	-0.051322	4.87E-34	hypomethylated	-0.009976	0.53989	insignificant	41	218	225
chr16	8829192	8831192	1810013L24Rik	-0.11863	2.96E-33	hypomethylated	-0.0076065	0.007489	hypomethylated	68	212	204
chr16	9992626	9994626	Grin2a	-0.18516	0.16145	insignificant	0.0051583	0.94039	insignificant	9	60	55
chr16	10169320	10171320	Rpl39l	-0.028374	0.004084	hypomethylated	0.043235	0.7551	insignificant	8	42	42
chr16	10191998	10193998	Atf7ip2	-0.13704	0.000012193	hypomethylated	-0.0085749	0.00075117	hypomethylated	20	78	82
chr16	10314061	10316061	Emp2	-0.11193	0.19996	insignificant	-0.01834	0.62028	insignificant	5	16	16
chr16	10395541	10397541	Tekt5		1	noCoverage	-0.10365	0.86736	insignificant	0	16	17
chr16	10411030	10413030	Nubp1	-0.10304	0.000026574	hypomethylated	-0.010034	0.28365	insignificant	17	60	52
chr16	10447443	10449443	Fam18a	0.13136	0.66839	insignificant	0.13893	0.2061	insignificant	1	10	16
chr16	10487371	10489371	Ciita	-0.27872	0.61322	insignificant	-0.12481	0.6628	insignificant	2	14	14
chr16	10543147	10545147	Dexi	-0.33658	7.49E-08	stronglyHypometh	0.006204	0.41032	insignificant	5	20	19
chr16	10544479	10546479	Clec16a	-0.097828	4.73E-13	hypomethylated	0.012708	0.76344	insignificant	22	117	115
chr16	10785629	10787629	Socs1	-0.14935	5.32E-09	hypomethylated	-0.0030668	0.74735	insignificant	16	66	66
chr16	10788748	10790748	Prm3		1	noCoverage	-0.081136	0.6287	insignificant	0	0	0
chr16	10796916	10798916	Prm1		1	noCoverage	-0.035577	0.73051	insignificant	0	2	4
chr16	10834151	10836151	A630055G03Rik	-0.11602	2.68E-19	hypomethylated	-0.007445	0.12525	insignificant	26	83	83
chr16	11065390	11067390	Snn	-0.11382	2.22E-14	hypomethylated	0.0043469	0.07123	insignificant	34	126	126

chr16	11088023	11090023	Txndc11	-0.29807	0.000013183	hypomethylated	0.0082354	0.34187	insignificant	1	8	8
chr16	11134625	11136625	Txndc11	-0.16789	1.23E-32	hypomethylated	-0.0564	0.23294	insignificant	27	77	68
chr16	11176486	11178486	Zc3h7a	-0.0059188	0.036748	inconclusive	-0.013342	0.53419	insignificant	7	24	24
chr16	11203385	11205385	Rsl1d1	-0.3016	0.000000716	hypomethylated	0.0038078	0.4408	insignificant	7	33	30
chr16	11254418	11256418	Gspt1	-0.1269	0.013325	hypomethylated	0.014972	0.74739	insignificant	17	63	63
chr16	11254538	11256538	Gspt1	-0.16875	0.041302	hypomethylated	0.01573	0.43008	insignificant	11	44	44
chr16	11321984	11323984	Snx29	-0.24092	7.45E-53	hypomethylated	-0.043228	0.00000731	hypomethylated	39	126	120
chr16	11404740	11406740	Snx29	-0.10087	0.094946	insignificant	-0.026366	0.73769	insignificant	3	10	10
chr16	11909516	11911516	Cpped1	-0.18392	0.00016062	hypomethylated	0.017216	0.56018	insignificant	3	12	12
chr16	11983205	11985205	Shisa9	-0.092382	8.66E-26	hypomethylated	-0.00025883	0.35643	insignificant	31	172	168
chr16	13108828	13110828	Ercc4	-0.10912	2.18E-25	hypomethylated	-0.0077913	0.90339	insignificant	20	75	74
chr16	13255573	13257573	Mkl2	-0.13689	1.21E-23	hypomethylated	0.002639	0.052469	insignificant	60	144	158
chr16	13448615	13450615	Mir193b	-0.2335	4.35E-12	hypomethylated	-0.0057466	0.0645	insignificant	15	45	44
chr16	13452932	13454932	Mir365-1	0.088542	1	insignificant	0.013542	0.70202	insignificant	1	4	4
chr16	13668263	13670263	Parn	-0.43856	1.6E-13	stronglyHypometh	0.027196	4.27E-08	inconclusive	5	20	20
chr16	13670950	13672950	Bfar	-0.21947	4.18E-16	hypomethylated	0.018185	0.17818	insignificant	22	81	78
chr16	13671112	13673112	3110001I22Rik	-0.17006	1.24E-12	hypomethylated	0.009939	0.45077	insignificant	22	75	72
chr16	13779791	13781791	Rrn3	-0.1369	1.18E-23	hypomethylated	-0.0070404	0.074092	insignificant	22	63	63
chr16	13818369	13820369	Ntan1	-0.15505	0.35987	insignificant	-0.039364	0.034103	hypomethylated	5	45	47
chr16	13903228	13905228	Pdxdc1	-0.18831	0.000000032	hypomethylated	-0.016402	0.15426	insignificant	6	46	46
chr16	13939787	13941787	Mpv17l	-0.10137	1.66E-10	hypomethylated	0.030086	0.81401	insignificant	18	58	58
chr16	13984714	13986714	2900011O08Rik		1	noCoverage	-0.029782	0.53319	insignificant	0	14	12
chr16	13985729	13987729	2900011O08Rik		1	noCoverage	0.030573	0.052625	insignificant	0	26	26
chr16	14158718	14160718	Mir484	-0.17466	7.33E-15	hypomethylated	-0.0052599	0.59558	insignificant	12	30	30
chr16	14162367	14164367	Nde1	-0.10323	1.71E-12	hypomethylated	0.017288	0.42046	insignificant	17	97	93
chr16	14291501	14293501	Myh11	-0.29028	1	lowCoverage	0.015412	0.8908	insignificant	1	8	8
chr16	14317425	14319425	0610037P05Rik	-0.36927	0.089552	insignificant	-0.11538	0.056108	insignificant	4	21	19
chr16	14360652	14362652	Abcc1	-0.074636	9.02E-27	hypomethylated	0.0087952	0.96844	insignificant	27	78	79
chr16	14561409	14563409	A630010A05Rik		1	noCoverage	0.055505	0.57704	insignificant	0	6	3
chr16	14704951	14706951	Snai2	-0.42017	0.27027	insignificant	-0.0074697	0.86088	insignificant	3	6	6
chr16	15594611	15596611	Ube2v2	-0.5867	0.00000662	stronglyHypometh	0.15876	0.51286	insignificant	1	18	20
chr16	15636958	15638958	Prkdc	-0.088265	0.43219	insignificant	0.0011928	0.29988	insignificant	45	116	106
chr16	15637493	15639493	Mcm4	-0.11161	0.00013749	hypomethylated	-0.0042477	0.56078	insignificant	30	70	70
chr16	15863415	15865415	Mzt2	-0.26289	0.000000199	hypomethylated	-0.027979	0.15562	insignificant	9	26	28
chr16	15886378	15888378	Cebpd	-0.12151	3.32E-67	hypomethylated	0.00032554	0.036076	hypermethylated	73	213	210
chr16	16146926	16148926	2310008H04Rik	-0.0625	0.26761	insignificant	-0.0030048	1	insignificant	5	14	14
chr16	16212437	16214437	Pkp2	-0.17579	1.41E-31	hypomethylated	-0.00098768	0.60756	insignificant	24	57	57
chr16	16302074	16304074	Yars2	-0.099798	0.000000583	hypomethylated	-0.0053333	0.45904	insignificant	23	112	112
chr16	16359123	16361123	Dnm1l	-0.5694	5.62E-25	stronglyHypometh	-0.062645	0.73835	insignificant	6	21	23
chr16	16829456	16831456	Spag6	-0.125	0.21483	insignificant	-0.0065476	0.48502	insignificant	2	8	8
chr16	16864078	16866078	Igll1	-0.26875	0.57371	insignificant	-0.10208	0.24948	insignificant	1	2	2
chr16	16869348	16871348	Top3b	-0.13505	3.01E-22	hypomethylated	-0.0089385	0.96345	insignificant	16	49	49
chr16	16869983	16871983	Top3b	-0.16593	2.08E-32	hypomethylated	-0.015687	0.51179	insignificant	19	67	67
chr16	16895561	16897561	Ppm1f	-0.14929	0.00011351	hypomethylated	0.011867	0.72713	insignificant	7	30	30
chr16	16982474	16984474	Mapk1	-0.10656	2.04E-23	hypomethylated	-0.0080949	0.045218	hypomethylated	44	136	136
chr16	17069403	17071403	Ypel1	-0.13616	0.00000762	hypomethylated	-0.033202	0.18868	insignificant	15	36	57
chr16	17111306	17113306	Ppil2		1	noCoverage	-0.022589	0.25261	insignificant	0	16	18
chr16	17124235	17126235	Mir130b		1	noCoverage	-0.15199	0.88513	insignificant	0	10	8
chr16	17124589	17126589	Mir130b		1	noCoverage	-0.20975	0.48976	insignificant	0	8	6
chr16	17132476	17134476	Sdf2l1	-0.32292	0.00085286	hypomethylated	0.1206	0.68424	insignificant	4	8	10
chr16	17144302	17146302	Ccdc116	-0.47061	0.000000464	stronglyHypometh	0.026673	0.90328	insignificant	2	6	6
chr16	17146071	17148071	Ydjc	-0.10395	1.91E-09	hypomethylated	0.010081	0.038523	hypermethylated	25	78	90
chr16	17201585	17203585	Ube2l3	-0.18073	1	lowCoverage	-0.013774	0.75727	insignificant	1	13	13
chr16	17207227	17209227	Rimbp3	-0.11843	9.54E-17	hypomethylated	0.03276	0.79388	insignificant	25	98	98
chr16	17232679	17234679	Hic2	-0.14237	5.73E-15	hypomethylated	-0.0052322	0.42509	insignificant	28	100	100
chr16	17275392	17277392	Tmem191c	-0.17251	0.00000867	hypomethylated	0.0027787	0.076799	insignificant	11	59	76
chr16	17405092	17407092	Snap29	-0.14074	7.43E-18	hypomethylated	0.0099638	0.4259	insignificant	32	112	124
chr16	17406407	17408407	Snap29	0.43033	0.24818	insignificant	-0.026257	0.010436	hypomethylated	1	16	16
chr16	17451079	17453079	Crkl	-0.15016	0.000000351	hypomethylated	-0.000037566	0.71879	insignificant	15	129	131
chr16	17488783	17490783	Aifm3	-0.29037	1.25E-24	hypomethylated	-0.024782	0.022901	hypomethylated	18	44	44
chr16	17508063	17510063	Lztr1	-0.18194	4.94E-24	hypomethylated	-0.023316	0.012951	hypomethylated	23	86	86
chr16	17531145	17533145	Thap7	-0.06298	0.85659	insignificant	0.072903	0.90728	insignificant	7	33	33
chr16	17560977	17562977	P2rx6	-0.21761	1	lowCoverage	0.042504	0.00067526	hypermethylated	1	27	34
chr16	17561340	17563340	P2rx6		1	noCoverage	0.080529	0.00040742	hypermethylated	0	17	24
chr16	17576764	17578764	Slc7a4		1	noCoverage	-0.085621	0.51095	insignificant	0	4	4
chr16	17618446	17620446	Smpd4	-0.18045	3.25E-18	hypomethylated	-0.022113	0.0029864	hypomethylated	17	78	82
chr16	17645562	17647562	Ccdc74a	-0.17784	3.29E-22	hypomethylated	0.013137	0.23361	insignificant	32	124	123
chr16	17758713	17760713	Klhl22	-0.20506	2.61E-43	hypomethylated	-0.036944	2.76E-11	hypomethylated	32	117	118
chr16	17796374	17798374	Scarf2	-0.15349	6.44E-49	hypomethylated	-0.012945	0.031056	hypomethylated	56	169	177
chr16	17832211	17834211	B830017H08Rik	-0.092553	9.04E-14	hypomethylated	0.018252	0.81374	insignificant	39	170	174
chr16	17893295	17895295	Tssk1	-0.04114	0.11332	insignificant	-0.0022964	0.80857	insignificant	9	45	45
chr16	17897729	17899729	Tssk2	-0.093502	0.00000156	hypomethylated	-0.047254	0.085546	insignificant	3	10	9
chr16	17911441	17913441	Dgcr14	-0.52757	0.0012904	stronglyHypometh	-0.027569	0.66441	insignificant	1	4	2
chr16	17915152	17917152	Gsc2	-0.13868	0.000030127	hypomethylated	-0.032435	0.017738	hypomethylated	3	20	20
chr16	17928312	17930312	Slc25a1	-0.13603	2.36E-16	hypomethylated	0.010546	0.74779	insignificant	22	52	56
chr16	17979657	17981657	Vpreb2	0.030973	0.51287	insignificant	0.02135	1	insignificant	5	11	10
chr16	18051952	18053952	Dgcr6	-0.31975	0.00031371	hypomethylated	-0.06306	0.37326	insignificant	3	8	8
chr16	18089283	18091283	Prodh	-0.36164	0.00029877	stronglyHypometh	-0.11765	0.67886	insignificant	5	20	19
chr16	18126798	18128798	Rtn4r	-0.12399	1.38E-21	hypomethylated	-0.0041835	0.43961	insignificant	48	171	167
chr16	18213626	18215626	4933432I09Rik	0.20288	0.016086	hypermethylated	0.036912	0.51533	insignificant	4	13	12
chr16	18213956	18215956	4933432I09Rik	0.21705	0.015804	hypermethylated	0.092879	0.30777	insignificant	4	11	10
chr16	18235229	18237229	Zdhhc8	-0.25969	0.0041193	hypomethylated	0.063806	0.15547	insignificant	6	50	45
chr16	18247975	18249975	Trmt2a	-0.11217	1.48E-25	hypomethylated	-0.0054884	0.25136	insignificant	36	127	121
chr16	18248787	18250787	Ranbp1	-0.14145	4.15E-12	hypomethylated	-0.023526	0.10423	insignificant	17	42	36
chr16	18289261	18291261	Dgcr8	-0.1054	5.33E-12	hypomethylated	0.0033024	0.3188	insignificant	21	76	78

chr16	18344025	18346025	D16H22S680E	-0.11065	8.38E-09	hypomethylated	0.003774	0.36028	insignificant	7	30	30
chr16	18347366	18349366	Arvcf	-0.15362	2.78E-12	hypomethylated	-0.010764	0.056888	insignificant	28	109	108
chr16	18425509	18427509	Txnrd2	-0.081456	0.00000417	hypomethylated	-0.0020538	0.48759	insignificant	11	50	50
chr16	18497860	18499860	Gnb1l	-0.093172	3.23E-16	hypomethylated	0.0043988	0.85741	insignificant	22	107	107
chr16	18497963	18499963	Gnb1l	-0.093172	3.23E-16	hypomethylated	0.0043988	0.85741	insignificant	22	107	107
chr16	18587062	18589062	Tbx1	-0.16616	9.18E-26	hypomethylated	0.0063437	0.000040441	inconclusive	37	104	109
chr16	18622496	18624496	Gp1bb	-0.45973	0.00057821	stronglyHypometh	0.021564	0.0087225	hypermethylated	3	43	50
chr16	18630031	18632031	############	-0.16941	3.04E-11	hypomethylated	-0.034986	0.036939	hypomethylated	23	57	58
chr16	18775939	18777939	Cldn5	0.011499	0.19885	insignificant	0.00098422	0.94122	insignificant	24	97	97
chr16	18811386	18813386	Ufd1l	-0.16647	7.45E-15	hypomethylated	-0.015147	1	insignificant	12	98	94
chr16	18811732	18813732	Cdc45	-0.18679	1.43E-15	hypomethylated	-0.016704	1	insignificant	12	82	74
chr16	18812065	18814065	Cdc45	-0.19244	3.47E-15	hypomethylated	-0.021213	1	insignificant	10	74	66
chr16	18835672	18837672	2510002D24Rik	-0.14125	0.0016924	hypomethylated	-0.0076852	0.016392	hypomethylated	10	30	30
chr16	18875842	18877842	Hira	-0.11866	2.91E-32	hypomethylated	-0.0094219	0.020821	hypomethylated	53	171	160
chr16	18876730	18878730	Mrpl40	-0.1117	1.45E-31	hypomethylated	-0.010571	0.008408	hypomethylated	48	149	149
chr16	19759326	19761326	B3gnt5	-0.21345	2.5E-12	hypomethylated	-0.013273	0.21911	insignificant	15	42	50
chr16	20096626	20098626	Klhl24	-0.13784	6.23E-15	hypomethylated	-0.011334	0.37572	insignificant	37	109	106
chr16	20140135	20142135	Yeats2	-0.074785	0.00052571	hypomethylated	-0.010773	0.92968	insignificant	20	90	90
chr16	20241431	20243431	Map6d1		1	noCoverage	0.057517	1	insignificant	0	3	6
chr16	20302435	20304435	Parl	-0.23421	0.00000138	hypomethylated	-0.01391	0.4821	insignificant	5	16	16
chr16	20426467	20428467	Abcc5	-0.3449	1E-14	stronglyHypometh	-0.031131	0.014499	hypomethylated	13	50	48
chr16	20497889	20499889	Eif2b5	-0.2701	2.64E-17	hypomethylated	-0.023699	0.35979	insignificant	11	63	67
chr16	20516136	20518136	Dvl3	-0.16958	0.00000531	hypomethylated	-0.0078086	0.79033	insignificant	24	74	71
chr16	20534576	20536576	Ap2m1	-0.23556	8.52E-15	hypomethylated	-0.0096737	0.016141	hypomethylated	17	60	60
chr16	20547675	20549675	Abcf3	-0.28333	0.020844	hypomethylated	-0.034997	0.012335	hypomethylated	6	28	33
chr16	20548629	20550629	Gm15760	-0.29988	0.19871	insignificant	0.011974	0.75438	insignificant	3	24	28
chr16	20588654	20590654	Vwa5b2	-0.1439	0.0006465	hypomethylated	-0.031397	0.24025	insignificant	6	24	24
chr16	20603524	20605524	Mir1224	-0.13527	0.19926	insignificant	-0.068385	0.34524	insignificant	3	9	9
chr16	20610673	20612673	Ece2	-0.18857	1.03E-09	hypomethylated	-0.022916	0.92377	insignificant	13	64	64
chr16	20610822	20612822	Ece2	-0.18997	0.00000021	hypomethylated	-0.027086	0.40968	insignificant	7	50	50
chr16	20621351	20623351	Camk2n2	-0.13068	5.11E-22	hypomethylated	-0.017743	0.0059732	hypomethylated	27	80	82
chr16	20628923	20630923	Ece2	-0.05871	4.3E-12	hypomethylated	0.0020473	0.20167	insignificant	19	70	70
chr16	20650724	20652724	Psmd2	-0.15628	4.53E-20	hypomethylated	0.0091433	0.14032	insignificant	15	69	69
chr16	20671821	20673821	Eif4g1	-0.10862	1.56E-23	hypomethylated	-0.0078171	0.48216	insignificant	40	104	105
chr16	20694129	20696129	Fam131a	-0.45433	0.014255	stronglyHypometh	-0.026187	0.62448	insignificant	1	16	23
chr16	20716709	20718709	Polr2h	-0.18056	4.31E-21	hypomethylated	-0.02982	0.043216	hypomethylated	9	61	73
chr16	20716898	20718898	Polr2h	-0.13224	1.68E-20	hypomethylated	-0.030358	0.23419	insignificant	10	63	75
chr16	20730671	20732671	Thpo	-0.29248	1	insignificant	0.02716	0.22042	insignificant	2	8	8
chr16	20732199	20734199	Chrd	-0.16909	1.19E-20	hypomethylated	-0.0049763	0.81712	insignificant	38	104	114
chr16	20734584	20736584	Thpo	-0.030518	0.60324	insignificant	0.00082184	0.15371	insignificant	3	16	16
chr16	21203867	21205867	Ephb3	-0.14099	0.000035709	hypomethylated	-0.0026009	0.961	insignificant	44	121	124
chr16	21422190	21424190	Vps8	-0.12567	4.92E-15	hypomethylated	-0.0073723	0.0072391	hypomethylated	20	85	85
chr16	21694738	21696738	2510009E07Rik	-0.1487	6.57E-20	hypomethylated	-0.0055247	0.7872	insignificant	17	49	49
chr16	21947617	21949617	Tmem41a	-0.82337	0.17021	lowCoverage	0.097571	1	insignificant	1	6	6
chr16	21995523	21997523	Liph	0.12054	1	lowCoverage	-0.15511	0.27778	insignificant	1	4	4
chr16	21995615	21997615	Liph	0.12054	1	lowCoverage	-0.15511	0.27778	insignificant	1	4	4
chr16	22008556	22010556	Senp2	-0.11862	1.33E-28	hypomethylated	-0.0051123	0.020983	hypomethylated	26	104	105
chr16	22163372	22165372	Igf2bp2	-0.15423	0.0096662	hypomethylated	-0.02832	0.20926	insignificant	8	30	30
chr16	22266002	22268002	Tra2b	-0.30226	1.98E-10	hypomethylated	-0.016833	0.9155	insignificant	8	42	41
chr16	22439643	22441643	Etv5	-0.17807	0.00018522	hypomethylated	0.001593	0.90152	insignificant	13	72	62
chr16	22657304	22659304	Dgkg	-0.18052	0.20461	insignificant	-0.044483	0.85974	insignificant	3	6	6
chr16	22856917	22858917	Dnajb11	-0.11231	4.07E-14	hypomethylated	-0.011343	0.9637	insignificant	24	72	73
chr16	22857642	22859642	Dnajb11	-0.11231	4.07E-14	hypomethylated	-0.015361	0.96368	insignificant	24	72	67
chr16	23057372	23059372	Kng1		1	noCoverage	-0.045652	0.5203	insignificant	0	8	8
chr16	23106551	23108551	Eif4a2	-0.10905	1.73E-24	hypomethylated	-0.0017445	0.16769	insignificant	42	171	172
chr16	23108025	23110025	Snord2	-0.12197	0.000020554	hypomethylated	-0.013032	0.0082502	hypomethylated	11	84	83
chr16	23127803	23129803	Rfc4	-0.091738	0.0012721	hypomethylated	0.0041143	0.66607	insignificant	12	48	48
chr16	23145608	23147608	Adipoq	0.075	1	insignificant	-0.023118	0.74499	insignificant	2	4	4
chr16	23223834	23225834	St6gal1	-0.17987	2.4E-10	hypomethylated	0.00039058	0.84609	insignificant	12	44	48
chr16	23609004	23611004	Rtp4	-0.3801	0.000024247	stronglyHypometh	0.031946	0.61256	insignificant	3	8	8
chr16	23930880	23932880	Rtp2	0.0046627	0.82139	insignificant	-0.029089	0.1549	insignificant	8	35	32
chr16	23988698	23990698	Bcl6	-0.09412	2.44E-08	hypomethylated	0.00074076	0.78028	insignificant	26	82	83
chr16	24392435	24394435	Lpp	-0.13737	0.14975	insignificant	-0.008771	0.052079	insignificant	42	149	145
chr16	26105870	26107870	Leprel1	-0.11086	0.000019478	hypomethylated	-0.060754	0.21929	insignificant	5	12	24
chr16	26371925	26373925	Cldn1	-0.37885	0.000038912	stronglyHypometh	0.023781	0.000031036	inconclusive	3	10	8
chr16	26462220	26464220	Cldn16	-0.1391	0.081071	insignificant	0.026444	0.34831	insignificant	2	15	14
chr16	26526857	26528857	Tmem207		1	noCoverage	-0.071785	0.29825	insignificant	0	10	12
chr16	26580790	26582790	Il1rap	-0.047161	0.00065684	inconclusive	0.0028421	0.0011289	inconclusive	16	67	66
chr16	27388062	27390062	Ccdc50	-0.097102	0.00000702	hypomethylated	-0.0064108	0.64398	insignificant	11	47	48
chr16	28445313	28447313	Fgf12	-0.12686	1.25E-34	hypomethylated	-0.0028415	0.50817	insignificant	45	149	148
chr16	28753288	28755288	Fgf12	-0.24459	0.55318	insignificant	0.0065401	0.46464	insignificant	2	14	14
chr16	28929784	28931784	1600021P15Rik	-0.16489	1.02E-09	hypomethylated	-0.0094865	0.80042	insignificant	19	92	92
chr16	29208780	29210780	Hrasls	-0.43538	0.12644	insignificant	0.0065434	0.33406	insignificant	3	10	10
chr16	29541569	29543569	Atp13a4	-0.37919	0.1868	insignificant	-0.024051	0.40582	insignificant	2	10	10
chr16	29578419	29580419	Opa1	-0.079167	1	insignificant	0.015165	0.73156	insignificant	2	12	15
chr16	30007752	30009752	4632428C04Rik	-0.14321	4.44E-08	hypomethylated	0.020249	0.027779	inconclusive	21	68	71
chr16	30064442	30066442	Hes1	-0.15098	3.53E-09	hypomethylated	0.010977	0.042286	hypermethylated	10	36	36
chr16	30283340	30285340	Lrrc15	-0.26959	0.0079953	hypomethylated	-0.031515	0.66582	insignificant	3	22	23
chr16	30388616	30390616	Atp13a3	-0.12474	1.99E-23	hypomethylated	-0.0094129	0.87141	insignificant	29	80	93
chr16	30550664	30552664	Tmem44	-0.15281	0.035931	hypomethylated	0.06613	0.80634	insignificant	4	30	32
chr16	30587675	30589675	Lsg1	-0.26227	0.000058339	hypomethylated	0.012738	0.011156	hypermethylated	5	35	33
chr16	30598808	30600808	Fam43a	-0.14556	1.47E-45	hypomethylated	-0.0068542	6.46E-11	hypomethylated	50	164	163
chr16	31314682	31316682	Apod	0.1497	1	lowCoverage	-0.16704	0.57844	insignificant	1	7	9
chr16	31421382	31423382	Bdh1	-0.025	1	insignificant	0.0625	0.056173	insignificant	2	5	4
chr16	31427838	31429838	Bdh1	-0.14366	4.69E-25	hypomethylated	-0.0064887	0.010774	hypomethylated	30	108	110

chr16	31663124	31665124	Dlg1	-0.12242	1.24E-24	hypomethylated	-0.0038135	0.016978	hypomethylated	46	172	177
chr16	31877895	31879895	Mfi2	-0.12997	0.82149	insignificant	-0.073108	0.0010068	hypomethylated	5	30	32
chr16	31932936	31934936	Pigz	-0.23209	0.051447	insignificant	-0.050043	0.34343	insignificant	9	67	61
chr16	31947631	31949631	Ncbp2	-0.10198	4.62E-34	hypomethylated	-0.013795	0.043194	hypomethylated	50	177	174
chr16	31948607	31950607	0610012G03Rik	-0.21206	9.96E-10	hypomethylated	-0.027562	0.32605	insignificant	18	71	68
chr16	32003373	32005373	Senp5	-0.35003	0.038145	stronglyHypometh	0.040289	0.13713	insignificant	3	13	13
chr16	32079359	32081359	Pak2		1	noCoverage	0.064924	0.020682	hypermethylated	0	18	16
chr16	32098887	32100887	1500031L02Rik	-0.17603	6.24E-15	hypomethylated	-0.0050734	0.18454	insignificant	20	91	91
chr16	32099813	32101813	Pigx	-0.20252	8.51E-14	hypomethylated	-0.0098445	0.025563	hypomethylated	16	51	51
chr16	32246312	32248312	Wdr53	-0.09448	9.9E-14	hypomethylated	0.0012072	0.97079	insignificant	33	121	121
chr16	32246338	32248338	Wdr53	-0.086824	3.72E-13	hypomethylated	0.0036943	0.94018	insignificant	32	119	119
chr16	32247111	32249111	Wdr53	-0.092829	5.24E-10	hypomethylated	0.013917	0.29795	insignificant	20	48	48
chr16	32270694	32272694	2310010M20Ril	0.125	1	insignificant	0.017857	0.13215	insignificant	1	4	4
chr16	32276546	32278546	Rnf168	-0.10011	1.58E-21	hypomethylated	0.0070779	0.93133	insignificant	32	100	100
chr16	32331337	32333337	Ubxn7	-0.14103	7.09E-28	hypomethylated	-0.0019875	0.085652	insignificant	26	103	103
chr16	32399591	32401591	Tm4sf19	-0.24957	0.0001916	hypomethylated	-0.10743	0.096159	insignificant	5	18	18
chr16	32418787	32420787	Tctex1d2	-0.16567	0.000010692	hypomethylated	0.01465	0.41962	insignificant	12	54	54
chr16	32430006	32432006	Pcyt1a	-0.1674	1.16E-35	hypomethylated	-0.014559	0.002771	hypomethylated	42	135	140
chr16	32430105	32432105	Pcyt1a	-0.17413	1.44E-33	hypomethylated	-0.016205	0.086944	insignificant	41	131	136
chr16	32430315	32432315	Pcyt1a	-0.14383	5.17E-27	hypomethylated	-0.014554	0.58449	insignificant	41	124	129
chr16	32607981	32609981	Tfrc	-0.10102	1	insignificant	0.038102	0.75447	insignificant	4	34	34
chr16	32643728	32645728	Tnk2	-0.10065	4.11E-11	hypomethylated	-0.017563	0.15392	insignificant	24	133	157
chr16	32668283	32670283	Gm10818	-0.45	0.24299	insignificant	-0.10605	0.025856	hypomethylated	2	10	10
chr16	32797521	32799521	Muc20	-0.27835	0.060396	insignificant	-0.01859	1	insignificant	2	8	8
chr16	32868425	32870425	1700021K19Rik	-0.34118	0.008193	stronglyHypometh	-0.040588	0.85804	insignificant	3	14	14
chr16	32876869	32878869	Fyttd1	-0.11675	8.37E-18	hypomethylated	-0.0005329	0.51761	insignificant	41	116	129
chr16	32913185	32915185	Lrch3	-0.1888	5.67E-27	hypomethylated	-0.0029281	0.17168	insignificant	40	95	101
chr16	33055538	33057538	Rpl35a	-0.10557	0.15987	insignificant	0.008824	0.0079851	hypermethylated	19	60	55
chr16	33055567	33057567	Rpl35a	-0.10557	0.15987	insignificant	0.008824	0.0079851	hypermethylated	19	60	55
chr16	33055599	33057599	Rpl35a	-0.10557	0.15987	insignificant	0.008824	0.0079851	hypermethylated	19	60	55
chr16	33056272	33058272	Rpl35a	-0.075766	0.78761	insignificant	-0.0020439	0.10028	insignificant	15	52	47
chr16	33061606	33063606	Lmln	-0.1739	4.04E-16	hypomethylated	-0.0061669	0.72178	insignificant	37	112	114
chr16	33104446	33106446	Mir1947	-0.088068	0.26943	insignificant	0.033956	0.33674	insignificant	4	8	8
chr16	33184156	33186156	Osbpl11	-0.1641	6.36E-26	hypomethylated	-0.011637	0.019917	hypomethylated	41	111	112
chr16	33250541	33252541	Snx4	-0.081684	7.14E-16	hypomethylated	0.022796	0.11387	insignificant	14	70	62
chr16	33379860	33381860	Zfp148	-0.10511	7.14E-25	hypomethylated	0.0045931	0.0022113	inconclusive	39	152	155
chr16	33517414	33519414	Slc12a8	-0.22528	0.00044754	hypomethylated	-0.014542	1	insignificant	9	22	22
chr16	33683551	33685551	Heg1	-0.10214	4.79E-16	hypomethylated	-0.0092909	0.28151	insignificant	41	108	110
chr16	33828750	33830750	Itgb5	-0.17011	2.81E-12	hypomethylated	-0.024698	0.1961	insignificant	29	106	106
chr16	33967089	33969089	Umps	-0.21131	0.0004084	hypomethylated	0.031858	0.10074	insignificant	4	14	14
chr16	34514113	34516113	Kalrn		1	noCoverage	0.07029	0.040789	hypermethylated	0	6	6
chr16	34689701	34691701	Ccdc14	-0.23849	0.21828	insignificant	-0.016331	0.24505	insignificant	3	35	42
chr16	35021506	35023506	Ptplb	-0.13083	2.22E-11	hypomethylated	-0.017773	0.59616	insignificant	35	115	112
chr16	35154721	35156721	Adcy5	-0.12695	1.07E-44	hypomethylated	-0.016662	0.0021034	hypomethylated	73	259	269
chr16	35364004	35366004	Sec22a	-0.11293	0.00000168	hypomethylated	-0.021098	0.21424	insignificant	12	26	26
chr16	35490959	35492959	Pdia5	-0.53399	4.38E-09	stronglyHypometh	-0.10323	1.86E-08	hypomethylated	3	19	17
chr16	35540447	35542447	Sema5b	-0.18718	1.91E-44	hypomethylated	-0.031573	0.014822	hypomethylated	51	136	138
chr16	35769442	35771442	Hspbap1	-0.10868	0.0010026	hypomethylated	0.0042579	0.52966	insignificant	13	42	42
chr16	35769471	35771471	Hspbap1	-0.10378	0.35288	insignificant	0.01111	0.22193	insignificant	7	30	30
chr16	35871468	35873468	Parp14	-0.51254	0.06209	insignificant	0.0023587	0.091432	insignificant	3	36	36
chr16	35937555	35939555	Parp9	-0.14236	0.000000459	hypomethylated	0.012101	0.79027	insignificant	12	77	77
chr16	35939113	35941113	Parp9	-0.11677	0.011361	hypomethylated	0.0019096	0.39092	insignificant	4	24	24
chr16	35982448	35984448	Kpna1	-0.11675	2.79E-36	hypomethylated	-0.022541	0.0050996	hypomethylated	60	186	200
chr16	36040275	36042275	Wdr5b	-0.2625	0.00000914	hypomethylated	0.028416	0.52509	insignificant	4	8	8
chr16	36070745	36072745	Ccdc58	-0.11428	0.00000192	hypomethylated	-0.002278	0.0082233	hypomethylated	13	112	112
chr16	36071601	36073601	Fam162a	-0.11071	0.00000475	hypomethylated	-0.0034253	0.027828	hypomethylated	13	95	96
chr16	36080360	36082360	Ccdc58	-0.24106	0.0016605	hypomethylated	-0.041458	0.48136	insignificant	1	24	23
chr16	36693123	36695123	Ildr1	-0.26275	4.01E-18	hypomethylated	0.047139	0.8463	insignificant	13	33	33
chr16	36785048	36787048	Slc15a2	0.30417	0.096527	insignificant	0.13142	0.0029115	hypermethylated	3	6	9
chr16	36827485	36829485	Iqcb1	-0.17136	2.14E-22	hypomethylated	0.016263	0.47848	insignificant	16	47	55
chr16	36828345	36830345	Eaf2	-0.19849	9.46E-15	hypomethylated	0.023953	0.34205	insignificant	7	17	25
chr16	36874912	36876912	Eaf2	-0.13114	4.65E-35	hypomethylated	-0.012279	0.0073953	hypomethylated	36	114	122
chr16	36884096	36886096	Golgb1	0.125	1	lowCoverage	0.077381	1	insignificant	1	6	6
chr16	37010871	37012871	Polq	-0.21766	4.48E-13	hypomethylated	-0.00076414	0.18288	insignificant	15	73	69
chr16	37385044	37387044	Stxbp5l	-0.16982	0.016819	hypomethylated	0.011868	0.053322	insignificant	6	18	18
chr16	37538979	37540979	Rabl3	-0.17033	4.57E-26	hypomethylated	0.0074137	0.044721	inconclusive	21	52	52
chr16	37539855	37541855	Gtf2e1	-0.47826	1	lowCoverage	0.19498	0.38681	insignificant	1	4	4
chr16	37654454	37656454	Ndufb4	-0.16771	0.021985	hypomethylated	-0.026835	0.61657	insignificant	4	16	16
chr16	37776140	37778140	Fstl1	-0.11979	0.00025095	hypomethylated	-0.000053209	0.3669	insignificant	10	67	64
chr16	37867485	37869485	Lrrc58	-0.10862	8.44E-39	hypomethylated	-0.0090024	0.015817	hypomethylated	48	126	126
chr16	37915581	37917581	Gpr156	-0.14	8.1E-16	hypomethylated	-0.0022318	0.0086171	hypomethylated	19	66	65
chr16	38088086	38090086	Gsk3b	-0.095301	7.09E-23	hypomethylated	-0.0053968	0.18419	insignificant	29	171	157
chr16	38089346	38091346	Gsk3b	-0.11514	1.24E-16	hypomethylated	-0.0023688	0.78228	insignificant	19	111	97
chr16	38346084	38348084	Cox17	-0.23689	0.0073558	hypomethylated	-0.019094	0.00000259	hypomethylated	7	42	42
chr16	38361258	38363258	Popdc2	-0.12164	0.45824	insignificant	0.079822	0.0064404	hypermethylated	7	40	38
chr16	38361295	38363295	Popdc2	-0.12164	0.45824	insignificant	0.079822	0.0064404	hypermethylated	7	40	38
chr16	38433225	38435225	Pla1a	-0.034432	0.73107	insignificant	0.04584	0.030429	hypermethylated	4	14	14
chr16	38452769	38454769	Adprh	-0.091027	0.0075254	hypomethylated	0.012645	0.63544	insignificant	6	28	31
chr16	38458012	38460012	Cd80		1	noCoverage	-0.057312	0.72097	insignificant	0	7	10
chr16	38522747	38524747	4930455C21Rik	-0.053117	0.00026247	hypomethylated	-0.01196	0.011209	hypomethylated	6	41	41
chr16	38550266	38552266	Poglut1	-0.2427	0.026064	hypomethylated	-0.03957	0.0084869	hypomethylated	7	37	37
chr16	38561971	38563971	Tmem39a	-0.15388	1.26E-23	hypomethylated	-0.013196	0.036028	hypomethylated	18	59	59
chr16	38713148	38715148	Arhgap31	0.02157	0.0056466	inconclusive	-0.017596	0.81638	insignificant	6	56	55
chr16	38741376	38743376	B4galt4	-0.13748	5.91E-13	hypomethylated	0.00056615	0.45668	insignificant	33	85	85
chr16	38800316	38802316	Upk1b	-0.38882	0.000021023	stronglyHypometh	0.014613	0.050194	insignificant	2	4	5

chr16	38901457	38903457	Igsf11	-0.11402	2.38E-18	hypomethylated	-0.014585	0.49877	insignificant	37	86	94
chr16	42954692	42956692	BC002163	0.053314	1	insignificant	0.018993	0.53898	insignificant	1	20	21
chr16	43246396	43248396	Zbtb20		1	noCoverage	-0.04126	0.6462	insignificant	0	13	12
chr16	43761354	43763354	Drd3	0.033467	1	insignificant	-0.17761	0.11474	insignificant	1	12	12
chr16	43889014	43891014	2610015P09Rik	-0.1392	8.14E-12	hypomethylated	-0.024625	0.39651	insignificant	16	40	43
chr16	43889789	43891789	2610015P09Rik	-0.15873	3.66E-09	hypomethylated	-0.021131	0.083454	insignificant	11	28	28
chr16	43979163	43981163	Zdhhc23	-0.014931	0.014895	hypomethylated	-0.020512	0.59309	insignificant	39	118	114
chr16	44016549	44018549	Gramd1c		1	noCoverage	-0.073291	0.68726	insignificant	0	6	6
chr16	44138921	44140921	Naa50	-0.024518	0.034958	inconclusive	0.0020674	0.90958	insignificant	34	157	155
chr16	44139132	44141132	Atp6v1a	-0.024518	0.034958	inconclusive	0.0020674	0.90958	insignificant	34	157	155
chr16	44172509	44174509	Gm608	-0.16525	3.55E-24	hypomethylated	-0.0064338	0.37001	insignificant	30	96	96
chr16	44332951	44334951	Sidt1	-0.26471	0.00000105	hypomethylated	0.083378	0.80046	insignificant	5	19	22
chr16	44346513	44348513	Spice1	-0.10501	0.000000703	hypomethylated	-0.0082878	1	insignificant	12	76	76
chr16	44393911	44395911	Wdr52	-0.066729	0.39968	insignificant	-0.11462	0.38666	insignificant	6	26	26
chr16	44558242	44560242	Boc	-0.16273	1.02E-18	hypomethylated	0.0033111	0.31197	insignificant	22	86	85
chr16	44558983	44560983	Boc		1	noCoverage	-0.19348	0.74184	insignificant	0	5	4
chr16	44723411	44725411	BC027231	-0.10433	6.45E-10	hypomethylated	-0.0037422	0.54987	insignificant	34	101	104
chr16	44942790	44944790	Cd200r3	-0.20821	0.026197	hypomethylated	0.09023	1	insignificant	2	11	8
chr16	45157941	45159941	Atg3	-0.080151	0.00002126	hypomethylated	0.0084023	0.54659	insignificant	18	80	91
chr16	45158786	45160786	Slc35a5	-0.13426	0.00000237	hypomethylated	0.0094685	0.19925	insignificant	18	58	69
chr16	45493082	45495082	Gm609		1	noCoverage	-0.055556	0.35294	insignificant	0	6	3
chr16	45654231	45656231	BC016579	-0.17949	0.30341	insignificant	-0.098859	0.17692	insignificant	1	10	13
chr16	45693771	45695771	Tmprss7	-0.12178	0.1336	insignificant	-0.035738	0.68293	insignificant	2	4	4
chr16	45724644	45726644	Tagln3		1	noCoverage	-0.3742	0.24383	insignificant	0	2	7
chr16	45743019	45745019	Abhd10	-0.15065	0.000000652	hypomethylated	0.013627	0.85434	insignificant	7	16	16
chr16	45844491	45846491	Phldb2	-0.12839	0.10482	insignificant	-0.015958	0.81449	insignificant	7	29	26
chr16	46010526	46012526	Plcxd2	-0.15419	0.36284	insignificant	-0.01871	0.14803	insignificant	4	22	18
chr16	46497080	46499080	Pvrl3	-0.14276	0.000000261	hypomethylated	0.0076626	0.49806	insignificant	26	105	100
chr16	48282847	48284847	Dppa4	-0.02079	1	insignificant	-0.071117	0.57277	insignificant	1	16	12
chr16	48309386	48311386	Dppa2	-0.26541	0.017716	hypomethylated	0.014487	0.78725	insignificant	5	18	15
chr16	48412529	48414529	Gm5485		1	noCoverage	-0.06875	0.22887	insignificant	0	4	4
chr16	48993300	48995300	C330027C09Rik	-0.085483	0.0079593	hypomethylated	0.08256	0.0028148	hypermethylated	5	74	28
chr16	48994225	48996225	C330027C09Rik	-0.11606	0.092363	insignificant	0.00055719	0.88502	insignificant	2	22	22
chr16	49698406	49700406	Ift57	-0.099142	0.0027598	hypomethylated	0.06452	0.021412	hypermethylated	7	51	29
chr16	49854766	49856766	Cd47	-0.14362	5.25E-36	hypomethylated	-0.025185	0.0029505	hypomethylated	41	164	164
chr16	52030661	52032661	Cblb	-0.15338	8.51E-17	hypomethylated	-0.0165	0.19086	insignificant	17	76	79
chr16	52453110	52455110	Alcam	-0.17733	1	insignificant	-0.0074092	0.95277	insignificant	2	42	42
chr16	55822251	55824251	Nfkbiz	-0.16315	0.00058738	hypomethylated	-0.012336	0.20175	insignificant	10	20	20
chr16	55838754	55840754	Nfkbiz	-0.17855	2.71E-11	hypomethylated	-0.015308	0.79984	insignificant	14	34	42
chr16	55895392	55897392	Fam55c	-0.15711	2.99E-21	hypomethylated	0.022296	0.8069	insignificant	20	60	66
chr16	55895398	55897398	Fam55c	-0.16245	6.25E-20	hypomethylated	0.022194	0.59521	insignificant	20	60	66
chr16	55934961	55936961	Cep97	-0.4072	0.18201	insignificant	-0.024764	0.23527	insignificant	1	8	8
chr16	55965387	55967387	Rpl24	-0.22314	1.05E-15	hypomethylated	-0.025656	0.66818	insignificant	15	56	56
chr16	55972916	55974916	Zbtb11	-0.10879	6.44E-44	hypomethylated	-0.012154	0.18561	insignificant	56	162	162
chr16	56029830	56031830	Pcnp		1	noCoverage	-0.049439	0.70614	insignificant	0	31	29
chr16	56037887	56039887	Rg9mtd1	-0.12034	2.71E-19	hypomethylated	0.036331	0.085638	insignificant	15	98	96
chr16	56074521	56076521	Senp7	-0.10592	0.000000108	hypomethylated	-0.0015941	0.38383	insignificant	7	89	90
chr16	56717467	56719467	Tfg	-0.16411	0.15144	insignificant	-0.013442	0.6716	insignificant	4	16	16
chr16	57071459	57073459	2310005G13Rik		1	noCoverage	0.027661	0.63398	insignificant	0	6	7
chr16	57120826	57122826	Tomm70a	-0.10488	1.45E-39	hypomethylated	0.0014906	0.66258	insignificant	47	128	122
chr16	57167445	57169445	Nit2	0.80312	0.00048438	stronglyHypermeth	-0.01451	1	insignificant	1	16	16
chr16	57231579	57233579	Tbc1d23	-0.13446	0.084007	insignificant	-0.00007497	0.82614	insignificant	5	28	28
chr16	57548354	57550354	Filip1l	-0.13109	0.00000751	hypomethylated	-0.055716	0.0028384	hypomethylated	4	28	47
chr16	57754850	57756850	Col8a1		1	noCoverage	0.036846	0.40829	insignificant	0	12	14
chr16	58407647	58409647	Dcbld2	-0.099708	1.94E-25	hypomethylated	-0.0086641	0.22938	insignificant	47	161	174
chr16	58523425	58525425	St3gal6	-0.12679	0.000062963	hypomethylated	0.032016	0.50295	insignificant	12	45	46
chr16	58669320	58671320	Cpox	-0.13076	0.0020946	hypomethylated	0.025947	0.06355	insignificant	12	85	82
chr16	58727156	58729156	Cldnd1	-0.13349	4.85E-13	hypomethylated	-0.018957	0.14991	insignificant	24	71	66
chr16	59098816	59100816	Olfr192		1	noCoverage	0.035354	1	insignificant	0	3	3
chr16	59406207	59408207	Gabrr3	0.12852	0.5822	insignificant	0.016	1	insignificant	2	9	9
chr16	59456409	59458409	Gabrr3	0.013552	1	insignificant	0.0016369	1	insignificant	5	25	35
chr16	59470600	59472600	Mina	-0.15467	0.000037632	hypomethylated	-0.0097252	0.2001	insignificant	13	66	69
chr16	59639165	59641165	4930547E14Rik	-0.11748	0.00000116	hypomethylated	-0.010772	0.051662	insignificant	5	26	26
chr16	60605357	60607357	Epha6	-0.26513	0.000074369	hypomethylated	-0.030694	0.39713	insignificant	4	10	14
chr16	62786542	62788542	Nsun3	-0.18954	7.22E-15	hypomethylated	0.038311	0.025256	inconclusive	12	54	57
chr16	62846866	62848866	Arl13b	-0.038258	1	insignificant	-0.027146	0.40867	insignificant	2	8	8
chr16	62853159	62855159	Pros1	-0.12217	9.55E-10	hypomethylated	-0.0099401	0.30775	insignificant	11	36	36
chr16	64478960	64480960	Csnka2ip		1	noCoverage	-0.14539	0.087499	insignificant	0	9	8
chr16	64770765	64772765	4930453N24Rik	-0.21016	0.0042978	hypomethylated	0.057677	0.93026	insignificant	5	18	23
chr16	64850910	64852910	Cggbp1	-0.13558	1.58E-08	hypomethylated	-0.0053636	0.53435	insignificant	20	122	129
chr16	65562942	65564942	Chmp2b	-0.19899	0.00021401	inconclusive	0.029945	0.038373	hypermethylated	7	23	24
chr16	65814877	65816877	Vgll3	-0.1352	2.18E-09	hypomethylated	-0.0015359	0.86383	insignificant	22	109	109
chr16	69863989	69865989	Speer2	0.056304	1	insignificant	-0.10248	0.011461	hypomethylated	3	21	27
chr16	70313331	70315331	Gbe1	-0.10127	1.1E-23	hypomethylated	-0.0027318	0.1551	insignificant	51	121	120
chr16	72662393	72664393	Robo1	-0.11779	0.00018299	hypomethylated	0.0067677	0.71316	insignificant	18	80	75
chr16	74411157	74413157	Robo2	-0.14679	1.8E-10	hypomethylated	0.0339	0.20429	insignificant	8	54	45
chr16	75592135	75594135	Rbm11	-0.3921	3.83E-15	stronglyHypometh	-0.0035005	0.24905	insignificant	14	47	52
chr16	75767063	75769063	Hspa13	-0.23983	9.67E-11	hypomethylated	-0.022109	0.6466	insignificant	10	28	26
chr16	76373294	76375294	Nrip1	-0.14552	7.81E-13	hypomethylated	-0.024321	0.04031	inconclusive	23	63	63
chr16	77013313	77015313	Usp25	-0.10154	8.54E-27	hypomethylated	0.0042319	0.84154	insignificant	32	108	102
chr16	77328572	77330572	2810055G20Rik	-0.10989	0.80678	insignificant	0.0013942	0.010487	inconclusive	6	19	18
chr16	77598180	77600180	Mir99a	-0.66667	0.002551	stronglyHypometh	-0.18214	0.0026125	hypomethylated	1	4	4
chr16	77598901	77600901	Mirlet7c-1	-0.66667	0.002551	stronglyHypometh	-0.18214	0.0026125	hypomethylated	1	4	4
chr16	78300935	78302935	Cxadr	-0.13728	5.99E-23	hypomethylated	-0.010257	0.30385	insignificant	41	115	111
chr16	78377002	78379002	Gm7334	-0.18601	0.00040776	hypomethylated	0.033037	0.56361	insignificant	14	77	76

chr16	78377030	78379030	Gm7334	-0.21888	0.00000085	hypomethylated	0.041942	0.72317	insignificant	14	67	66
chr16	78576913	78578913	D16Ertd472e	-0.07946	0.02206	hypomethylated	0.038216	0.00053471	hypermethylated	6	24	25
chr16	78930192	78932192	Chodl	-0.6374	0.0000022	stronglyHypometh	-0.079842	0.46631	insignificant	2	4	4
chr16	81199941	81201941	Ncam2	-0.11081	4.64E-12	hypomethylated	0.0061411	0.69637	insignificant	34	117	116
chr16	84735426	84737426	Mrpl39	-0.070419	0.00060392	hypomethylated	-0.01073	0.12896	insignificant	31	98	98
chr16	84773367	84775367	Jam2	-0.16321	1.27E-10	hypomethylated	0.027176	0.85131	insignificant	12	53	58
chr16	84834368	84836368	Gabpa	-0.11123	4.63E-45	hypomethylated	0.0042481	0.28673	insignificant	55	178	166
chr16	84835819	84837819	Atp5j	-0.041211	0.00000004	hypomethylated	0.001759	0.63109	insignificant	26	86	86
chr16	85173952	85175952	App	-0.16273	1.26E-12	hypomethylated	-0.004423	0.000011889	hypomethylated	6	14	14
chr16	85803360	85805360	Adamts1	-0.21401	6.08E-12	hypomethylated	0.057731	0.0085895	inconclusive	7	28	28
chr16	85901370	85903370	Adamts5	-0.22743	5.31E-22	hypomethylated	-0.0031167	0.00061284	hypomethylated	12	43	46
chr16	87353429	87355429	N6amt1	-0.17544	0.014453	hypomethylated	-0.01846	0.14431	insignificant	11	44	45
chr16	87440837	87442837	Rwdd2b	-0.26114	0.00062872	hypomethylated	-0.060778	0.76141	insignificant	6	16	14
chr16	87454229	87456229	Usp16	-0.19122	1.7E-39	hypomethylated	0.00092585	0.30288	insignificant	30	78	79
chr16	87496114	87498114	Cct8	-0.22507	0.01369	hypomethylated	-0.012171	0.51081	insignificant	7	22	22
chr16	87552574	87554574	ORF63	-0.17095	0.09171	insignificant	-0.030414	0.20472	insignificant	4	12	12
chr16	87698198	87700198	Bach1	-0.10546	6.74E-21	hypomethylated	-0.0076678	0.18514	insignificant	52	134	134
chr16	88708207	88710207	2310061N02Rik		1	noCoverage	0.0028385	0.82671	insignificant	0	10	10
chr16	88751873	88753873	Krtap13	0.083333	1	insignificant	-0.072619	0.15165	insignificant	2	4	4
chr16	88828253	88830253	Krtap15	0.03146	0.075869	insignificant	-0.080183	0.0061745	hypomethylated	4	6	6
chr16	89048144	89050144	Krtap16-8		1	noCoverage	0.065909	0.65105	insignificant	0	4	4
chr16	89571428	89573428	Krtap11-1	-0.043713	0.59472	insignificant	-0.014956	0.18464	insignificant	4	10	8
chr16	89818597	89820597	Tiam1	0.18297	1	insignificant	0.022539	0.34307	insignificant	2	14	14
chr16	90141481	90143481	Gm10789	-0.18998	2.15E-11	hypomethylated	-0.053065	0.62288	insignificant	16	88	76
chr16	90219986	90221986	Sod1	-0.1523	1.36E-23	hypomethylated	0.000052396	0.89529	insignificant	28	89	83
chr16	90284670	90286670	Scaf4	-0.13424	1E-13	hypomethylated	0.030364	0.000047832	inconclusive	23	79	80
chr16	90385641	90387641	Hunk	-0.11361	8.34E-31	hypomethylated	-0.0023957	0.48205	insignificant	50	181	181
chr16	90727616	90729616	2610039C10Rik	-0.10859	0.00000603	hypomethylated	-0.0072492	0.18041	insignificant	8	43	43
chr16	90737568	90739568	Mrap		1	noCoverage	-0.044566	0.074074	insignificant	0	24	24
chr16	90810658	90812658	Urb1	-0.48333	0.023245	stronglyHypometh	-0.0071364	0.61774	insignificant	3	10	11
chr16	90830103	90832103	4931408A02Rik	-0.12305	5.12E-32	hypomethylated	-0.0012357	0.000034471	hypomethylated	56	164	161
chr16	90935094	90937094	1110004E09Rik	-0.10984	1.07E-08	hypomethylated	-0.002701	0.19012	insignificant	16	62	62
chr16	91010493	91012493	4930404I05Rik	-0.099478	4.49E-24	hypomethylated	0.01874	0.53079	insignificant	45	147	136
chr16	91011340	91013340	Synj1	-0.10133	5.73E-08	hypomethylated	0.027713	0.10252	insignificant	16	85	74
chr16	91011553	91013553	Synj1	-0.10583	0.000000123	hypomethylated	0.014997	0.014642	inconclusive	15	58	56
chr16	91044624	91046624	Gcfc1	-0.16161	0.23931	insignificant	-0.019189	0.000042896	inconclusive	10	67	72
chr16	91069390	91071390	4932438H23Rik	0.33302	0.55823	insignificant	0.066412	0.30947	insignificant	3	28	28
chr16	91224794	91226794	Olig2	-0.18875	3.85E-10	hypomethylated	-0.03397	0.81106	insignificant	15	54	60
chr16	91269013	91271013	Olig1	-0.098281	9.65E-10	hypomethylated	0.011271	0.12804	insignificant	27	112	99
chr16	91372027	91374027	Ifnar2	-0.11535	2.35E-20	hypomethylated	0.0014548	0.83231	insignificant	18	72	72
chr16	91405479	91407479	Il10rb	-0.16333	9.49E-12	hypomethylated	-0.0041325	0.79075	insignificant	17	36	36
chr16	91484459	91486459	Ifnar1	-0.22694	7.31E-25	hypomethylated	0.0083181	0.56477	insignificant	25	112	113
chr16	91546338	91548338	Ifngr2	-0.12137	0.0005145	hypomethylated	-0.01851	0.26078	insignificant	16	69	67
chr16	91597925	91599925	Tmem50b	-0.19943	1.59E-19	hypomethylated	-0.045529	0.39579	insignificant	17	71	64
chr16	91619244	91621244	Dnajc28	-0.28333	0.12221	insignificant	0.058716	1	insignificant	3	8	13
chr16	91647068	91649068	Son	-0.11533	6.18E-17	hypomethylated	0.0038357	0.97177	insignificant	42	154	162
chr16	91647217	91649217	Son	-0.1073	9.39E-13	hypomethylated	0.0050631	0.82032	insignificant	36	142	150
chr16	91688973	91690973	Donson	-0.13487	1.91E-29	hypomethylated	-0.012744	0.0096619	hypomethylated	29	81	77
chr16	91728615	91730615	Itsn1	-0.1027	4.85E-14	hypomethylated	-0.010205	0.28932	insignificant	51	144	144
chr16	91728844	91730844	Cryzl1	-0.1027	4.85E-14	hypomethylated	-0.010205	0.28932	insignificant	51	144	144
chr16	91931875	91933875	Atp5o	-0.15552	0.00002199	hypomethylated	0.046524	0.3529	insignificant	9	26	24
chr16	92057566	92059566	Slc5a3	-0.10971	2.21E-39	hypomethylated	-0.01478	0.0013127	hypomethylated	43	201	202
chr16	92057580	92059580	Mrps6	-0.10971	2.21E-39	hypomethylated	-0.01478	0.0013127	hypomethylated	43	201	202
chr16	92300547	92302547	Fam165b	-0.53263	0.000049494	stronglyHypometh	-0.19388	0.0002607	hypomethylated	5	14	14
chr16	92359713	92361713	Kcne1	0.14679	0.34058	insignificant	-0.040234	0.71186	insignificant	2	18	18
chr16	92466391	92468391	Rcan1	0.0054348	1	insignificant	-0.010148	1	insignificant	9	46	38
chr16	92497391	92499391	Clic6		1	noCoverage	0.0081243	0.060081	insignificant	0	66	66
chr16	92697573	92699573	Runx1	-0.16322	6.11E-23	hypomethylated	-0.0052325	0.28173	insignificant	36	141	134
chr16	93368964	93370964	Mir802	-0.10952	0.18058	insignificant	-0.27817	0.00011851	hypomethylated	2	6	6
chr16	93604060	93606060	Setd4	-0.1015	0.000036867	hypomethylated	-0.007486	0.46681	insignificant	6	22	22
chr16	93607081	93609081	Cbr1	-0.092228	0.000000219	hypomethylated	0.0007069	0.20423	insignificant	8	50	50
chr16	93682463	93684463	Cbr3	-0.11388	2.81E-12	hypomethylated	0.00064698	0.51162	insignificant	40	151	146
chr16	93711151	93713151	Dopey2	-0.13388	2.22E-25	hypomethylated	-0.008848	0.070921	insignificant	31	98	102
chr16	93831365	93833365	Morc3	-0.095123	8.28E-37	hypomethylated	-0.005259	0.59573	insignificant	56	173	162
chr16	93883145	93885145	Chaf1b	-0.16699	1.63E-41	hypomethylated	-0.0013634	0.19993	insignificant	36	78	74
chr16	93929812	93931812	Cldn14	-0.11615	0.2965	insignificant	0.20608	0.28019	insignificant	1	8	6
chr16	93930062	93932062	Cldn14	-0.21597	0.21218	insignificant	0.10625	0.64673	insignificant	1	6	6
chr16	94009082	94011082	Cldn14	-0.12143	0.018555	hypomethylated	-0.0085891	0.43999	insignificant	4	18	19
chr16	94084504	94086504	Sim2	-0.086706	3.19E-29	hypomethylated	-0.00045942	0.54667	insignificant	48	166	195
chr16	94549028	94551028	Ripply3	-0.29888	1.21E-21	hypomethylated	0.011487	0.000012521	inconclusive	7	32	32
chr16	94591345	94593345	Ttc3	-0.10406	3.42E-22	hypomethylated	-0.011575	0.070059	insignificant	22	85	85
chr16	94591937	94593937	Pigp	-0.10406	3.42E-22	hypomethylated	-0.011575	0.070059	insignificant	22	85	85
chr16	94592157	94594157	Pigp	-0.08538	2.3E-18	hypomethylated	-0.0039313	0.20157	insignificant	18	77	77
chr16	94592317	94594317	Pigp	-0.08538	2.3E-18	hypomethylated	-0.0039313	0.20157	insignificant	18	77	77
chr16	94592622	94594622	Pigp	-0.12057	0.36897	insignificant	-0.0061024	0.48655	insignificant	2	29	29
chr16	94748236	94750236	Dscr3	-0.094187	0.0023941	hypomethylated	-0.017178	1	insignificant	13	50	63
chr16	94790812	94792812	Dyrk1a	-0.075902	7.36E-52	hypomethylated	-0.0075982	0.047941	hypomethylated	121	319	327
chr16	94791513	94793513	Dyrk1a	-0.10418	2.83E-51	hypomethylated	-0.0088845	0.010776	hypomethylated	94	206	206
chr16	95478257	95480257	Kcnj15	-0.07967	0.35393	insignificant	0.086386	1	insignificant	2	4	4
chr16	95923013	95925013	Ets2	-0.15968	1.88E-45	hypomethylated	-0.023757	0.028253	hypomethylated	38	92	108
chr16	96212510	96214510	Psmg1	-0.10152	9.15E-13	hypomethylated	-0.0010267	0.19655	insignificant	13	47	48
chr16	96304035	96306035	Brwd1	-0.09225	0.00000819	hypomethylated	-0.021645	0.22879	insignificant	22	72	87
chr16	96349332	96351332	Hmgn1	-0.086649	1.2E-10	hypomethylated	-0.0027702	0.23814	insignificant	27	84	80
chr16	96366025	96368025	Wrb	-0.10707	0.00032754	hypomethylated	-0.015469	0.18857	insignificant	17	49	49
chr16	96413864	96415864	Lca5l	-0.1037	1	insignificant	0.07498	1	insignificant	4	10	11

chr16	96421076	96423076	Sh3bgr	-0.31034	0.00029433	hypomethylated	0.093809	0.58874	insignificant	4	25	24
chr16	96456407	96458407	B3galt5		1	noCoverage	-0.1625	0.49246	insignificant	0	8	4
chr16	96501638	96503638	B3galt5	-0.15703	0.000038309	hypomethylated	-0.056642	0.92483	insignificant	4	12	16
chr16	96582363	96584363	Igsf5	0.10655	0.4914	insignificant	-0.042334	0.4293	insignificant	7	16	16
chr16	96582367	96584367	Igsf5	0.10655	0.4914	insignificant	-0.042334	0.4293	insignificant	7	16	16
chr16	96582370	96584370	Igsf5	0.10655	0.4914	insignificant	-0.042334	0.4293	insignificant	7	16	16
chr16	97392342	97394342	Dscam		1	noCoverage	-0.0037518	1	insignificant	0	22	22
chr16	97577334	97579334	Bace2	-0.145	1.56E-14	hypomethylated	0.0028737	0.57794	insignificant	19	90	90
chr16	97832802	97834802	Tmprss2		1	noCoverage	0.31722	0.25445	insignificant	0	12	12
chr16	97985362	97987362	Ripk4	-0.17771	0.34654	insignificant	0.022072	1	insignificant	6	22	22
chr16	98072834	98074834	Prdm15	-0.13476	2.73E-21	hypomethylated	-0.0035293	0.01684	hypomethylated	57	122	121
chr16	98183786	98185786	Zfp295	-0.065494	0.0023303	hypomethylated	0.034833	0.000012783	inconclusive	8	49	43
chr16	98303604	98305604	A630089N07Rik	-0.36386	0.083945	insignificant	0.020626	0.80932	insignificant	2	10	10
chr17	3084183	3086183	Pisd-ps2	-0.17658	1	insignificant	-0.016751	0.17968	insignificant	2	47	48
chr17	3113971	3115971	Scaf8	-0.10109	8.13E-39	hypomethylated	-0.0018978	0.23487	insignificant	55	200	203
chr17	3325572	3327572	Tiam2	-0.21371	0.0082826	hypomethylated	-0.10009	0.000000992	hypomethylated	6	96	78
chr17	3396206	3398206	Tiam2		1	noCoverage	-0.045035	0.25622	insignificant	0	17	16
chr17	3531553	3533553	Cldn20	-0.46318	0.13027	insignificant	-0.0061059	0.70659	insignificant	2	7	6
chr17	3557713	3559713	Tfb1m	-0.80556	0.015504	stronglyHypometh	-0.055556	0.73887	insignificant	3	4	4
chr17	4994073	4996073	Arid1b	-0.089959	7.67E-26	hypomethylated	0.0041397	0.23359	insignificant	77	296	280
chr17	5440260	5442260	5730437N04Rik	-0.23867	0.32847	insignificant	-0.036604	0.7866	insignificant	3	22	24
chr17	5491599	5493599	Zdhhc14	-0.13584	5.34E-72	hypomethylated	-0.0082842	0.33365	insignificant	76	233	221
chr17	5840379	5842379	Snx9	-0.10703	7.46E-16	hypomethylated	0.0096629	0.047593	inconclusive	72	186	188
chr17	5940279	5942279	Synj2	-0.018666	0.00026423	hypomethylated	0.0093017	0.12281	insignificant	26	126	126
chr17	5974585	5976585	Synj2	-0.28198	0.0056338	hypomethylated	0.04138	0.57344	insignificant	1	8	8
chr17	6078827	6080827	Gtf2h5	-0.10135	1	insignificant	-0.0062078	0.74266	insignificant	8	26	26
chr17	6079739	6081739	Serac1	-0.062632	1	insignificant	0.0031579	1	insignificant	8	20	20
chr17	6105829	6107829	Tulp4	-0.16541	4.62E-26	hypomethylated	-0.025424	0.78343	insignificant	30	109	96
chr17	6269474	6271474	Tmem181a		1	noCoverage	0.059792	0.67519	insignificant	0	95	20
chr17	6317474	6319474	Dynlt1a		1	noCoverage	0.038912	0.66527	insignificant	0	8	8
chr17	6428259	6430259	Dynlt1b		1	noCoverage	-0.041869	0.00025811	hypomethylated	0	21	33
chr17	6987129	6989129	Ezr	-0.12017	2.13E-17	hypomethylated	0.02301	0.81497	insignificant	35	128	115
chr17	7152705	7154705	Rsph3b	-0.16879	0.06828	insignificant	0.042079	9.98E-11	hypermethylated	4	30	32
chr17	7165505	7167505	Tagap1		1	noCoverage	-0.069152	0.00010471	hypomethylated	0	32	16
chr17	7182208	7184208	Rnaset2b		1	noCoverage	-0.24667	0.58661	insignificant	0	5	3
chr17	7373463	7375463	Rps6ka2	-0.13759	3.16E-25	hypomethylated	0.0031991	0.63995	insignificant	28	78	79
chr17	7589654	7591654	Gm9992	-0.23566	0.015603	hypomethylated	-0.037841	0.46928	insignificant	4	12	12
chr17	8117864	8119864	Tagap	-0.43701	0.12333	insignificant	-0.017167	0.74189	insignificant	2	22	22
chr17	8137478	8139478	Rsph3a	-0.22406	0.005068	hypomethylated	0.0096783	0.20964	insignificant	2	58	64
chr17	8357382	8359382	Fgfr1op	-0.11919	1	lowCoverage	-0.0070969	0.17667	insignificant	1	60	60
chr17	8475677	8477677	Brp44l	-0.14214	2.75E-24	hypomethylated	-0.0026561	0.78212	insignificant	51	186	186
chr17	8502967	8504967	Sft2d1	-0.17248	0.000020188	hypomethylated	-0.071236	0.00079548	hypomethylated	16	59	56
chr17	8532270	8534270	Prr18	-0.11129	1.89E-35	hypomethylated	-0.008771	0.155	insignificant	39	129	144
chr17	8532603	8534603	Prr18	-0.089363	1.63E-34	hypomethylated	-0.0029477	0.23187	insignificant	43	141	156
chr17	8626287	8628287	T	-0.13145	2.61E-10	hypomethylated	-0.034623	0.14351	insignificant	23	114	115
chr17	8993609	8995609	Pde10a	-0.14281	4.64E-33	hypomethylated	-0.00036164	0.59574	insignificant	39	116	103
chr17	9180197	9182197	1700010I14Rik	-0.47984	1	lowCoverage	0.018546	0.52301	insignificant	1	11	11
chr17	9360887	9362887	6530411M01Ril	-0.28453	0.023605	hypomethylated	0.12349	0.4386	insignificant	4	14	17
chr17	9862569	9864569	Pabpc6	-0.19588	0.016541	hypomethylated	0.0039546	1	insignificant	3	7	6
chr17	10511783	10513783	B930003M22Ril	-0.062728	3.8E-14	hypomethylated	-0.013994	0.44073	insignificant	53	187	236
chr17	10512226	10514226	B930003M22Ril	-0.08258	8.92E-11	hypomethylated	-0.018536	0.17165	insignificant	32	130	163
chr17	11032249	11034249	Park2	-0.18254	1.95E-09	hypomethylated	-0.052163	0.75115	insignificant	12	47	53
chr17	11033057	11035057	Park2	-0.16807	0.00000387	hypomethylated	-0.051449	0.92358	insignificant	10	43	49
chr17	12311149	12313149	Agpat4	-0.2079	0.00000352	hypomethylated	0.01102	0.12608	insignificant	5	22	21
chr17	12511526	12513526	4732491K20Rik	-0.1105	1.41E-09	hypomethylated	0.044569	0.000000636	inconclusive	8	41	40
chr17	12700570	12702570	Slc22a3	-0.16619	0.55922	insignificant	-0.059107	0.13612	insignificant	5	24	24
chr17	12776054	12778054	Slc22a2	0.014929	0.33624	insignificant	-0.040056	0.0013259	hypomethylated	7	19	19
chr17	12933176	12935176	Airn	-0.38357	0.064112	insignificant	-0.085849	0.49017	insignificant	5	19	20
chr17	12962572	12964572	Igf2r	0.05774	0.044647	hypermethylated	0.053811	0.83966	insignificant	11	34	37
chr17	13102866	13104866	Pnldc1	0.061208	0.41242	insignificant	-0.0092736	0.61075	insignificant	12	32	30
chr17	13108330	13110330	Tcp1	-0.10405	6.67E-11	hypomethylated	0.0034479	0.29503	insignificant	12	54	55
chr17	13108957	13110957	Tcp1	-0.19526	0.00017308	hypomethylated	0.023168	0.4371	insignificant	5	52	53
chr17	13133262	13135262	Acat3	-0.49627	2.28E-26	stronglyHypometh	-0.15061	1.82E-33	hypomethylated	7	24	16
chr17	13185125	13187125	Wtap	-0.083644	2.64E-11	hypomethylated	-0.0015662	0.032485	hypomethylated	33	119	125
chr17	13185405	13187405	Wtap	-0.099302	0.000000015	hypomethylated	-0.0083055	0.002074	hypomethylated	22	67	73
chr17	13199704	13201704	Sod2	-0.10755	3.92E-12	hypomethylated	0.00040424	0.41254	insignificant	26	114	110
chr17	13546437	13548437	Gm9880		1	noCoverage		1	noCoverage	0	6	0
chr17	13896547	13898547	Mllt4	-0.10549	4.05E-31	hypomethylated	-0.015288	0.006926	hypomethylated	106	292	303
chr17	13898401	13900401	Tcte2	-0.1624	4.14E-23	hypomethylated	0.01254	0.00014606	inconclusive	30	77	77
chr17	14084379	14086379	Gm7168	0.1484	1	insignificant	-0.08734	0.18329	insignificant	2	10	10
chr17	14340838	14342838	Dact2		1	noCoverage	-0.085003	0.74624	insignificant	0	26	21
chr17	14415512	14417512	Smoc2	-0.0876	2.27E-10	hypomethylated	-0.0032501	0.090888	insignificant	29	99	99
chr17	14535949	14537949	Smoc2		1	noCoverage	-0.13635	0.27388	insignificant	0	7	6
chr17	14831269	14833269	Thbs2	-0.10682	0.39168	insignificant	0.08367	1	insignificant	2	10	10
chr17	15079188	15081188	1600012H06Rik	-0.10652	1.12E-15	hypomethylated	0.016116	0.026285	hypermethylated	26	107	107
chr17	15080129	15082129	Wdr27	-0.10493	4.63E-11	hypomethylated	0.02549	0.0029768	hypermethylated	17	71	71
chr17	15512787	15514787	Dll1	-0.15531	2.17E-35	hypomethylated	0.0036173	0.55858	insignificant	35	108	100
chr17	15532165	15534165	Fam120b	-0.51297	4.28E-08	stronglyHypometh	-0.07639	1	insignificant	3	10	10
chr17	15532209	15534209	Fam120b	-0.51297	4.28E-08	stronglyHypometh	-0.07639	1	insignificant	3	10	10
chr17	15635240	15637240	Tbp	-0.092213	4.44E-17	hypomethylated	-0.020663	0.03724	hypomethylated	30	98	95
chr17	15635851	15637851	Tbp	-0.15352	2.12E-15	hypomethylated	-0.020915	0.36025	insignificant	27	92	90
chr17	15664265	15666265	Pdcd2	-0.30606	3.96E-12	hypomethylated	-0.010937	0.57949	insignificant	4	21	16
chr17	15700287	15702287	Prdm9	-0.029809	0.014802	inconclusive	-0.0061838	0.000000177	inconclusive	13	53	57
chr17	15840930	15842930	Chd1	-0.12068	2.13E-54	hypomethylated	-0.012157	0.093778	insignificant	66	241	257
chr17	15963550	15965550	Rgmb	-0.16027	0.0010784	hypomethylated	-0.081607	0.42142	insignificant	6	26	28

chr17	17200066	17202066	Zfp960	-0.24706	0.0019708	hypomethylated	0.095116	0.62358	insignificant	2	5	4
chr17	17510295	17512295	Riok2	-0.23057	0.00000161	hypomethylated	0.0058842	0.29771	insignificant	12	35	34
chr17	17538649	17540649	Lix1	-0.67071	0.000000729	stronglyHypometh	-0.0028115	1	insignificant	1	4	4
chr17	17761453	17763453	Lnpep	-0.15326	4.11E-42	hypomethylated	-0.0086122	0.023267	hypomethylated	41	141	141
chr17	17966151	17968151	Mir99b	0.005161	0.29662	insignificant	0.038307	0.42617	insignificant	9	36	38
chr17	17966315	17968315	Mirlet7e	0.005161	0.29662	insignificant	0.038307	0.42617	insignificant	9	36	38
chr17	17966775	17968775	Mir125a	0.005161	0.29662	insignificant	0.038307	0.42617	insignificant	9	36	38
chr17	17966938	17968938	Ncrna00085	0.005161	0.29662	insignificant	0.038307	0.42617	insignificant	9	36	38
chr17	18287893	18289893	Vmn2r92		1	noCoverage	-0.019917	0.56175	insignificant	0	8	8
chr17	20185169	20187169	Vmn2r104		1	noCoverage	0.0095779	0.43021	insignificant	0	6	7
chr17	21081417	21083417	Ppp2r1a	-0.16099	0.000046351	hypomethylated	-0.0063927	0.79194	insignificant	11	95	87
chr17	21144904	21146904	Zfp160	-0.5439	0.0032029	stronglyHypometh	-0.038186	0.80363	insignificant	1	12	9
chr17	21519711	21521711	Zfp677	-0.23349	0.000000128	hypomethylated	0.073334	0.68352	insignificant	6	32	33
chr17	21559190	21561190	Zfp54	-0.21881	0.000058476	hypomethylated	-0.015614	0.86067	insignificant	5	32	29
chr17	21586315	21588315	Zfp51	-0.21861	0.34658	insignificant	-0.018294	0.82725	insignificant	3	28	28
chr17	21624951	21626951	Zfp53	-0.20502	9.34E-09	hypomethylated	-0.010733	0.5597	insignificant	7	37	42
chr17	21671502	21673502	Zfp52	-0.18246	0.029344	hypomethylated	-0.019919	0.24645	insignificant	8	32	32
chr17	21703009	21705009	Zfp948		1	noCoverage	0.017895	0.5804	insignificant	0	12	10
chr17	21786577	21788577	3110052M02Ril	-0.2191	0.00000242	hypomethylated	-0.019856	0.7325	insignificant	1	10	10
chr17	21843707	21845707	Zfp760	-0.23813	0.015343	hypomethylated	-0.025438	0.33462	insignificant	5	35	34
chr17	21869690	21871690	Zfp229	-0.15794	0.00017313	hypomethylated	-0.001409	0.9332	insignificant	7	14	14
chr17	22098525	22100525	Zfp943	-0.12326	1.61E-13	hypomethylated	0.010257	0.86704	insignificant	9	47	45
chr17	22099417	22101417	Zfp942	-0.12852	0.00023226	hypomethylated	0.001534	0.94529	insignificant	3	27	27
chr17	22099431	22101431	Zfp942	-0.12852	0.00023226	hypomethylated	0.001534	0.94529	insignificant	3	27	27
chr17	22497419	22499419	Zfp758	-0.17151	1	insignificant	-0.034712	0.076111	insignificant	3	21	23
chr17	22498367	22500367	Zfp944		1	noCoverage	-0.20355	1	insignificant	0	9	7
chr17	22560234	22562234	Zfp946	-0.17255	0.000069781	hypomethylated	0.043727	0.48539	insignificant	7	26	15
chr17	22954714	22956714	Gm16386	-0.31683	0.00034891	hypomethylated	-0.049574	0.4726	insignificant	4	12	12
chr17	23004101	23006101	Zfp945	-0.33293	0.069546	insignificant	-0.075983	0.00028683	hypomethylated	2	4	4
chr17	23330195	23332195	Zfp40	-0.23429	0.000035033	hypomethylated	0.022547	0.71715	insignificant	5	10	10
chr17	23701193	23703193	Zfp213	-0.45222	0.017407	stronglyHypometh	0.044182	0.77973	insignificant	1	10	11
chr17	23736822	23738822	Zscan10	-0.51535	0.014304	stronglyHypometh	0.085703	1	insignificant	2	4	6
chr17	23782236	23784236	Mmp25	-0.41631	1.39E-17	stronglyHypometh	-0.020809	0.0074067	inconclusive	10	35	35
chr17	23796489	23798489	Ccdc64b	-0.15789	8.33E-12	hypomethylated	0.073567	0.053873	insignificant	11	50	46
chr17	23809574	23811574	Hcfc1r1	-0.1554	5.98E-17	hypomethylated	-0.013009	0.16047	insignificant	14	64	64
chr17	23810737	23812737	Hcfc1r1	-0.11837	1.28E-09	hypomethylated	-0.021888	0.48369	insignificant	9	26	26
chr17	23814416	23816416	Cldn6	-0.17183	7.04E-26	hypomethylated	-0.021957	0.0001066	hypomethylated	9	35	33
chr17	23815331	23817331	Cldn6	-0.18524	0.000035249	hypomethylated	-0.060467	0.33079	insignificant	7	35	33
chr17	23862302	23864302	Pkmyt1	-0.13146	1.79E-31	hypomethylated	0.012466	0.37041	insignificant	34	143	117
chr17	23877297	23879297	Kremen2	-0.12556	0.42727	insignificant	0.074522	0.012054	hypermethylated	3	50	50
chr17	23882796	23884796	Kremen2	-0.25428	4.28E-10	hypomethylated	-0.021976	0.37542	insignificant	9	29	34
chr17	23908558	23910558	Flywch1		1	noCoverage	-0.025811	0.104	insignificant	0	17	9
chr17	23923044	23925044	Flywch2	-0.25084	0.02764	hypomethylated	-0.084092	4.47E-20	hypomethylated	2	37	35
chr17	23939153	23941153	Srrm2	-0.092584	1.71E-11	hypomethylated	-0.0023813	0.00015071	hypomethylated	33	166	148
chr17	23966076	23968076	Tceb2	-0.29187	0.015459	hypomethylated	0.026422	0.0061773	inconclusive	6	21	19
chr17	23972734	23974734	Prss33	-0.056278	0.67827	insignificant	-0.033526	0.78823	insignificant	1	4	4
chr17	24004038	24006038	Prss21	0.19444	1	insignificant	-0.064784	0.38542	insignificant	2	18	11
chr17	24016842	24018842	Dcpp1	-0.1359	0.21412	insignificant	-0.32515	9.71E-14	hypomethylated	1	6	6
chr17	24034688	24036688	Dcpp2		1	noCoverage	-0.0097222	0.32628	insignificant	0	4	3
chr17	24053424	24055424	Dcpp3		1	noCoverage		1	noCoverage	0	6	0
chr17	24092240	24094240	Sbpl		1	noCoverage		1	noCoverage	0	4	0
chr17	24174209	24176209	Prss27		1	noCoverage		1	noCoverage	0	6	0
chr17	24210452	24212452	Kctd5	-0.16909	1	insignificant	-0.046089	0.81486	insignificant	3	20	24
chr17	24278556	24280556	Pdpk1	-0.18423	0.00000405	hypomethylated	-0.03177	0.12423	insignificant	5	20	20
chr17	24278561	24280561	Pdpk1	-0.18423	0.00000405	hypomethylated	-0.03177	0.12423	insignificant	5	20	20
chr17	24306374	24308374	Atp6v0c-ps2	-0.18424	0.000000154	hypomethylated	0.0067211	0.34308	insignificant	18	74	74
chr17	24336902	24338902	Tbc1d24	-0.035455	0.64933	insignificant	-0.017226	0.19885	insignificant	1	4	4
chr17	24342507	24344507	Ntn3	-0.15806	4.42E-20	hypomethylated	-0.012175	0.62362	insignificant	22	108	106
chr17	24346332	24348332	Ntn3	-0.1959	0.11706	insignificant	0.051875	0.51766	insignificant	3	16	16
chr17	24357714	24359714	1600002H07Rik	-0.62167	0.000014006	stronglyHypometh	-0.11354	0.022424	hypomethylated	1	10	10
chr17	24388354	24390354	Ccnf	-0.1721	0.000071991	hypomethylated	0.004908	0.44604	insignificant	9	53	51
chr17	24487990	24489990	Abca3	-0.10089	7.02E-14	hypomethylated	0.012549	0.94927	insignificant	31	153	155
chr17	24550619	24552619	Rnps1	-0.16839	9.16E-13	hypomethylated	0.00082848	0.38131	insignificant	11	75	70
chr17	24551458	24553458	D330041H03Rik	-0.16321	1.56E-12	hypomethylated	0.001103	0.37664	insignificant	11	68	66
chr17	24562627	24564627	Eci1	-0.13796	3.31E-10	hypomethylated	0.064807	0.035145	hypermethylated	13	72	47
chr17	24580046	24582046	E4f1	-0.033289	0.06793	insignificant	-0.037608	0.00047273	hypomethylated	9	28	28
chr17	24592256	24594256	E4f1	-0.98246	0.0011881	stronglyHypometh	-0.31579	0.086494	insignificant	1	6	3
chr17	24606417	24608417	Pgp	-0.11534	1.4E-50	hypomethylated	-0.0020872	0.093112	insignificant	71	210	205
chr17	24616023	24618023	Mlst8		1	noCoverage	0.029263	0.72358	insignificant	0	6	4
chr17	24624727	24626727	Caskin1	-0.094455	2.54E-10	hypomethylated	0.0085238	0.65866	insignificant	38	153	153
chr17	24664883	24666883	Traf7	-0.37856	5.45E-08	stronglyHypometh	-0.035084	0.074992	insignificant	10	23	22
chr17	24685894	24687894	Pkd1	-0.093148	7.19E-33	hypomethylated	0.0035031	0.21863	insignificant	28	146	133
chr17	24768626	24770626	Nthl1	-0.22357	0.039199	inconclusive	0.068949	0.42887	insignificant	5	8	8
chr17	24781902	24783902	Slc9a3r2	-0.89176	0.11475	lowCoverage	0.0025696	0.23263	insignificant	1	8	8
chr17	24787250	24789250	Slc9a3r2		1	noCoverage	0	1	insignificant	0	3	3
chr17	24795370	24797370	Npw		1	noCoverage	0.11418	0.66416	insignificant	0	27	28
chr17	24805696	24807696	Zfp598	-0.17759	1.29E-18	hypomethylated	-0.0048322	0.3611	insignificant	31	133	131
chr17	24826894	24828894	Syngr3	-0.10497	0.0062617	hypomethylated	0.045934	0.22512	insignificant	4	28	28
chr17	24832178	24834178	Noxo1	-0.17139	3.73E-29	hypomethylated	-0.018463	0.002566	hypomethylated	44	131	147
chr17	24833101	24835101	Gfer	-0.12265	9.02E-08	hypomethylated	0.012969	0.62289	insignificant	15	34	37
chr17	24844598	24846598	Tbl3	-0.34855	1.84E-12	stronglyHypometh	0.040123	0.041657	inconclusive	9	53	34
chr17	24855002	24857002	Snora78	-0.20486	0.000004533	hypomethylated	0.05676	0.061174	insignificant	3	68	57
chr17	24856007	24858007	Rps2	-0.16324	7.41E-09	hypomethylated	0.2121	1.28E-14	hypermethylated	11	121	107
chr17	24856733	24858733	Snora64	-0.11114	0.003137	hypomethylated	0.27062	5.94E-21	hypermethylated	8	95	74
chr17	24856777	24858777	Snora78	-0.11603	0.0019336	hypomethylated	0.27634	1.14E-18	hypermethylated	8	91	72

chr17	24856910	24858910	Snora78	-0.13406	0.0011386	hypomethylated	0.28825	7.5E-20	hypermethylated	8	67	62
chr17	24861333	24863333	Ndufb10	-0.064971	0.6126	insignificant	0.028861	0.22147	insignificant	4	19	18
chr17	24863773	24865773	Rpl3l	0.12167	0.07886	insignificant	0.11071	0.0037987	hypermethylated	8	38	32
chr17	24872586	24874586	Sepx1	-0.071012	0.017774	hypomethylated	0.013228	0.77825	insignificant	12	44	41
chr17	24888947	24890947	Hs3st6	-0.12949	1.19E-19	hypomethylated	0.016102	0.14929	insignificant	41	120	120
chr17	24940326	24942326	4930528F23Rik	0.69673	0.26703	lowCoverage	0.099765	4.14E-10	hypermethylated	1	45	11
chr17	24986434	24988434	Hagh	-0.16017	1.61E-28	hypomethylated	-0.0018838	0.044294	hypomethylated	37	103	117
chr17	24986611	24988611	Hagh	-0.15402	3.04E-27	hypomethylated	-0.0011938	0.050341	insignificant	36	101	115
chr17	24987247	24989247	Fahd1	-0.14878	2.44E-19	hypomethylated	-0.00041431	0.074596	insignificant	29	63	68
chr17	25014714	25016714	Igfals	-0.055061	0.097888	insignificant	0.179	0.10751	insignificant	1	5	9
chr17	25022618	25024618	Spsb3	-0.40512	2.37E-09	stronglyHypometh	0.094881	1	insignificant	4	17	10
chr17	25023295	25025295	Spsb3	-0.25359	9.87E-11	hypomethylated	0.076118	1.17E-08	hypermethylated	9	37	29
chr17	25023425	25025425	Spsb3	-0.25359	9.87E-11	hypomethylated	0.076118	1.17E-08	hypermethylated	9	37	29
chr17	25031064	25033064	Mrps34	-0.17415	1.94E-11	hypomethylated	0.0018982	0.061627	insignificant	16	80	79
chr17	25032032	25034032	Eme2	-0.15705	1.39E-10	hypomethylated	-0.0016234	0.4013	insignificant	16	96	95
chr17	25032444	25034444	Nme3	-0.334	0.040692	stronglyHypometh	-0.030149	0.073201	insignificant	6	70	70
chr17	25073922	25075922	Mapk8ip3	-0.13642	0.000000286	hypomethylated	-0.02556	0.45257	insignificant	6	18	18
chr17	25152030	25154030	Ift140	-0.14398	1.06E-28	hypomethylated	-0.0035778	0.4213	insignificant	29	102	115
chr17	25152175	25154175	Cramp1l	-0.13828	2.9E-25	hypomethylated	-0.0014265	0.8522	insignificant	29	96	110
chr17	25252850	25254850	Telo2		1	noCoverage		1	noCoverage	0	13	0
chr17	25252912	25254912	Telo2		1	noCoverage		1	noCoverage	0	5	0
chr17	25269338	25271338	Clcn7	-0.26982	3.31E-24	hypomethylated	-0.059597	0.000022418	hypomethylated	20	48	50
chr17	25298405	25300405	Ccdc154	0.17936	1	insignificant	0.013246	0.88163	insignificant	1	10	10
chr17	25320505	25322505	BC003965	-0.1049	2.04E-30	hypomethylated	0.001615	0.51283	insignificant	37	117	127
chr17	25324344	25326344	Unkl	-0.13478	5.72E-25	hypomethylated	-0.02472	0.0053046	hypomethylated	41	157	160
chr17	25358526	25360526	Unkl	-0.11496	0.00094966	hypomethylated	0.029888	0.000000478	hypermethylated	4	41	32
chr17	25376114	25378114	0610007P22Rik	-0.18779	2.89E-08	hypomethylated	-0.088763	1.37E-26	hypomethylated	26	66	99
chr17	25377061	25379061	Gnptg	-0.1595	0.000000021	hypomethylated	-0.022015	4.09E-12	hypomethylated	20	28	74
chr17	25410068	25412068	Gm17801	-0.11701	3.35E-19	hypomethylated	-0.017138	0.04158	hypomethylated	24	94	108
chr17	25410376	25412376	Ube2i	-0.12678	5.83E-21	hypomethylated	-0.021412	0.022092	hypomethylated	24	76	81
chr17	25410859	25412859	Ube2i		1	noCoverage	-0.024493	0.67212	insignificant	0	4	4
chr17	25444590	25446590	Prss28		1	noCoverage	0.06498	0.36591	insignificant	0	8	8
chr17	25570728	25572728	Cacna1h	-0.085996	2.02E-09	hypomethylated	0.00066464	0.60992	insignificant	27	117	116
chr17	25607534	25609534	Tekt4	-0.05685	1	insignificant	0.035276	0.60821	insignificant	2	15	12
chr17	25634233	25636233	Sstr5	-0.3895	1	lowCoverage	-0.13074	0.013228	hypomethylated	1	12	14
chr17	25707631	25709631	Sox8	-0.16595	0.000034595	hypomethylated	-0.033059	0.37288	insignificant	13	58	58
chr17	25715118	25717118	Lmf1	-0.40641	0.00017988	stronglyHypometh	-0.043336	0.59039	insignificant	3	6	6
chr17	25853116	25855116	Gng13	-0.07758	0.056704	insignificant	0.0015005	0.54547	insignificant	7	48	46
chr17	25863695	25865695	Rpusd1		1	noCoverage	-0.025253	0.75255	insignificant	0	16	16
chr17	25864360	25866360	Chtf18	0.082738	0.07711	insignificant	-0.0125	1	insignificant	2	14	14
chr17	25871984	25873984	Mslnl	-0.091423	0.75762	insignificant	0.025685	0.37765	insignificant	2	16	16
chr17	25909720	25911720	Narfl	-0.14149	0.14773	insignificant	0.012812	0.73212	insignificant	9	65	62
chr17	25922524	25924524	Ccdc78	-0.047356	0.6057	insignificant	-0.095936	0.02588	hypomethylated	2	6	6
chr17	25929229	25931229	Fam173a	-0.16837	4.75E-10	hypomethylated	-0.010504	0.92479	insignificant	11	50	50
chr17	25933990	25935990	Metrn	-0.24415	0.051153	insignificant	-0.073057	0.49314	insignificant	6	26	24
chr17	25945029	25947029	Fbxl16	-0.1124	1.73E-24	hypomethylated	0.0085024	0.0088911	inconclusive	46	170	168
chr17	25959571	25961571	Wdr24	-0.47087	0.17979	insignificant	0.0073523	0.67464	insignificant	2	23	22
chr17	25964987	25966987	Jmjd8	0.20431	0.49069	insignificant	0.0011036	0.064791	insignificant	2	15	15
chr17	25970306	25972306	Stub1	-0.14447	0.0054712	hypomethylated	-0.0043098	0.11237	insignificant	2	28	28
chr17	25981796	25983796	Rhot2	0.016957	0.7629	insignificant	-0.039361	0.18413	insignificant	2	34	29
chr17	25998460	26000460	Wdr90	-0.367	0.000003054	stronglyHypometh	-0.067896	0.20458	insignificant	3	22	22
chr17	26005683	26007683	Fam195a	-0.045885	0.15095	insignificant	0.015847	0.34448	insignificant	1	6	6
chr17	26011444	26013444	0610011F06Rik	-0.13248	0.0065999	hypomethylated	0.011584	0.59754	insignificant	11	64	68
chr17	26056659	26058659	Rab40c	-0.23388	0.00019254	hypomethylated	-0.028989	0.63698	insignificant	6	48	47
chr17	26078907	26080907	Nhlrc4	-0.23681	0.00000223	hypomethylated	-0.014956	0.16458	insignificant	8	24	24
chr17	26081876	26083876	Nhlrc4		1	noCoverage	-0.012054	1	insignificant	0	4	4
chr17	26206122	26208122	Rab11fip3	-0.10274	3.67E-29	hypomethylated	-0.011833	0.020864	hypomethylated	41	121	120
chr17	26227109	26229109	Decr2	-0.27005	0.00000159	hypomethylated	-0.032828	0.016433	hypomethylated	3	33	34
chr17	26249260	26251260	Tmem8	-0.091978	0.00000137	hypomethylated	-0.0017381	0.12081	insignificant	24	78	78
chr17	26259447	26261447	Mrpl28	-0.082369	0.40735	insignificant	0.0028927	1	insignificant	5	28	28
chr17	26274630	26276630	Axin1	-0.10834	1.67E-17	hypomethylated	-0.0085021	0.026736	hypomethylated	32	90	85
chr17	26336032	26338032	Pdia2	-0.086807	0.00000744	hypomethylated	-0.0067267	0.0182	hypomethylated	14	48	48
chr17	26338295	26340295	Rgs11	-0.185	0.000000365	hypomethylated	-0.0072265	0.37621	insignificant	7	14	18
chr17	26381086	26383086	Itfg3	-0.53057	0.27907	lowCoverage	-0.0033079	0.31913	insignificant	1	19	29
chr17	26388854	26390854	Luc7l	-0.11885	1.04E-21	hypomethylated	-0.015965	0.16103	insignificant	27	82	82
chr17	26550909	26552909	Neurl1b	-0.0872	2.79E-15	hypomethylated	0.0051815	0.94468	insignificant	41	151	158
chr17	26645417	26647417	Dusp1	-0.079583	2.29E-09	hypomethylated	-0.0098769	0.74867	insignificant	19	81	86
chr17	26697456	26699456	Ergic1	-0.11451	1.34E-12	hypomethylated	-0.0014279	0.13495	insignificant	17	83	83
chr17	26812340	26814340	Atp6v0e	-0.034031	0.47903	insignificant	-0.00091278	0.63398	insignificant	21	86	84
chr17	26851594	26853594	A930001N09Rik	-0.10018	5.93E-10	hypomethylated	-0.019628	0.15183	insignificant	41	137	137
chr17	26917023	26919023	Bnip1	-0.42801	0.000000217	stronglyHypometh	-0.055841	0.68483	insignificant	5	15	12
chr17	27053035	27055035	Kifc5b	-0.21659	0.00062291	hypomethylated	0.00031925	0.64531	insignificant	7	42	61
chr17	27069071	27071071	Phf1	-0.21549	2.84E-13	hypomethylated	0.00084395	0.74346	insignificant	15	65	65
chr17	27076413	27078413	Cuta	-0.10781	5.13E-13	hypomethylated	-0.013799	0.15285	insignificant	15	71	71
chr17	27076423	27078423	Cuta	-0.1128	3.35E-14	hypomethylated	-0.014264	0.10392	insignificant	15	69	69
chr17	27085064	27087064	Mir3083	-0.1045	2.03E-12	hypomethylated	0.001547	0.26595	insignificant	29	111	117
chr17	27109123	27111123	Zbtb9	-0.043053	3.28E-12	hypomethylated	0.0093762	0.10674	insignificant	32	96	116
chr17	27165203	27167203	Ggnbp1	-0.54876	7.31E-18	stronglyHypometh	-0.098783	0.074475	insignificant	6	28	28
chr17	27165571	27167571	Ggnbp1	-0.54496	1.65E-17	stronglyHypometh	-0.10451	0.033738	hypomethylated	5	26	26
chr17	27193248	27195248	Itpr3	-0.13506	1.26E-28	hypomethylated	-0.015552	0.048843	hypomethylated	31	83	83
chr17	27304709	27306709	Ip6k3	0.066667	1	insignificant	0.068514	1	insignificant	3	6	6
chr17	27341383	27343383	Lemd2	-0.2606	0.41998	insignificant	-0.033073	0.55831	insignificant	3	14	18
chr17	27640249	27642249	Grm4		1	noCoverage	-0.033131	0.5018	insignificant	0	8	8
chr17	27692518	27694518	Hmga1	-0.09764	2.97E-45	hypomethylated	0.016279	0.047745	inconclusive	73	250	271
chr17	27692548	27694548	Hmga1	-0.097675	6.87E-44	hypomethylated	0.016361	0.039447	inconclusive	71	242	263

chr17	27692597	27694597	Hmga1-rs1	-0.10254	7.65E-45	hypomethylated	0.015559	0.041298	inconclusive	71	230	251
chr17	27702672	27704672	AI413582	-0.091408	0.000000149	hypomethylated	-0.0010004	0.40981	insignificant	13	40	40
chr17	27760397	27762397	Nudt3	-0.11634	5.04E-17	hypomethylated	0.0056138	0.038094	inconclusive	22	58	74
chr17	27772187	27774187	Rps10	-0.071847	0.74181	insignificant	-0.03733	0.0002081	hypomethylated	15	53	58
chr17	27791626	27793626	Pacsin1	-0.065174	0.0012072	hypomethylated	0.012709	1.87E-08	inconclusive	13	68	73
chr17	27821198	27823198	Pacsin1	-0.077904	0.0034327	hypomethylated	0.016669	0.000017212	hypermethylated	27	86	86
chr17	27957487	27959487	D17Wsu92e	0.085978	0.27596	insignificant	-0.033159	0.15042	insignificant	6	46	52
chr17	27976031	27978031	Snrpc	-0.3183	0.0037512	hypomethylated	0.14641	0.91586	insignificant	3	12	14
chr17	27992451	27994451	Uhrf1bp1	-0.12367	9.58E-40	hypomethylated	-0.0085953	0.12277	insignificant	28	112	113
chr17	28044669	28046669	Anks1	-0.089372	4.55E-30	hypomethylated	0.021008	0.081038	insignificant	33	74	90
chr17	28045284	28047284	Anks1	-0.1072	2.15E-33	hypomethylated	0.010568	0.33053	insignificant	35	83	101
chr17	28217529	28219529	Tcp11	-0.20032	0.000000535	hypomethylated	0.053614	0.52881	insignificant	8	44	46
chr17	28217584	28219584	Tcp11	-0.21413	0.00000561	hypomethylated	0.069029	0.49439	insignificant	3	32	34
chr17	28278470	28280470	Scube3	-0.08721	7.38E-27	hypomethylated	0.010052	0.39639	insignificant	40	190	182
chr17	28313362	28315362	Zfp523	-0.12435	6.8E-23	hypomethylated	-0.022925	0.0046463	hypomethylated	42	98	99
chr17	28343722	28345722	Def6	-0.1637	0.21686	insignificant	-0.0044074	0.14241	insignificant	7	33	33
chr17	28368698	28370698	Ppard	-0.12522	0.0000035	hypomethylated	0.0033929	0.37697	insignificant	17	57	53
chr17	28449474	28451474	Fance	-0.1291	0.00000172	hypomethylated	0.049552	0.80469	insignificant	15	103	95
chr17	28464415	28466415	Rpl10a	-0.061306	1.64E-16	hypomethylated	0.0075836	0.19421	insignificant	39	143	143
chr17	28487545	28489545	Tead3	-0.15931	1.39E-12	hypomethylated	0.033956	0.65239	insignificant	27	105	103
chr17	28487750	28489750	Tead3	-0.14578	0.00000041	hypomethylated	0.042269	0.15424	insignificant	22	89	82
chr17	28502088	28504088	Tulp1	-0.57222	0.04605	stronglyHypometh	-0.11984	0.16619	insignificant	1	6	6
chr17	28623057	28625057	Fkbp5	0.070126	0.0019484	hypermethylated	-0.00064458	0.061967	insignificant	5	45	44
chr17	28666805	28668805	4930511I11Rik	0.040832	0.87795	insignificant	-0.013171	0.4102	insignificant	6	22	23
chr17	28685431	28687431	Gm749	-0.070041	0.016563	hypomethylated	0.11959	0.0088828	hypermethylated	4	26	26
chr17	28697659	28699659	Clps	0.034169	0.075576	insignificant	0.0069773	0.86569	insignificant	3	6	7
chr17	28711663	28713663	Lhfpl5	-0.081533	1.86E-16	hypomethylated	0.012496	0.64997	insignificant	13	55	48
chr17	28759399	28761399	Srpk1	-0.12407	0.000014575	hypomethylated	0.035374	0.5734	insignificant	14	48	49
chr17	28827286	28829286	Mapk14	-0.10083	4.1E-41	hypomethylated	-0.0021438	0.8015	insignificant	52	194	188
chr17	28828512	28830512	Mapk14	-0.12221	1.93E-29	hypomethylated	0.0069969	0.51374	insignificant	31	100	100
chr17	28905261	28907261	Mapk13	-0.13162	0.000000151	hypomethylated	0.010987	0.67475	insignificant	29	101	101
chr17	28937070	28939070	Brpf3	-0.11352	6.33E-25	hypomethylated	0.024301	0.33445	insignificant	46	120	122
chr17	28994355	28996355	Pnpla1	-0.19612	0.0082847	hypomethylated	-0.046683	0.21145	insignificant	2	11	12
chr17	29089160	29091160	Kctd20	-0.11047	9.07E-19	hypomethylated	-0.0023038	0.018328	hypomethylated	28	80	81
chr17	29144882	29146882	Stk38	-0.30654	2.36E-37	hypomethylated	-0.013086	1.35E-17	hypomethylated	9	48	50
chr17	29168604	29170604	Srsf3	-0.12295	3.64E-12	hypomethylated	-0.0043052	0.36753	insignificant	39	95	96
chr17	29215906	29217906	Gm16197	-0.46376	6.93E-11	stronglyHypometh	-0.053591	0.093176	insignificant	5	36	31
chr17	29229716	29231716	Cdkn1a	-0.14802	1.81E-19	hypomethylated	-0.0022825	0.37106	insignificant	18	75	75
chr17	29271000	29273000	Rab44	-0.16437	0.022223	hypomethylated	-0.16664	0.00091689	hypomethylated	3	14	14
chr17	29374735	29376735	Cpne5	-0.091602	7.23E-13	hypomethylated	-0.00055054	0.36119	insignificant	24	101	98
chr17	29400916	29402916	Ppil1	-0.30928	8.19E-20	hypomethylated	0.0083972	0.00049754	inconclusive	12	42	42
chr17	29404732	29406732	BC004004	-0.079238	0.0005168	hypomethylated	-0.0033445	0.000000297	hypomethylated	26	79	82
chr17	29484849	29486849	Mtch1	-0.25714	1.93E-09	hypomethylated	-0.045723	0.25199	insignificant	8	20	21
chr17	29496858	29498858	Fgd2	-0.30167	0.037314	hypomethylated	-0.023393	1	insignificant	3	8	8
chr17	29626989	29628989	Pim1	-0.095708	4.2E-33	hypomethylated	-0.0045646	0.027766	hypomethylated	87	278	292
chr17	29685746	29687746	Tbc1d22b	-0.19005	4.83E-25	hypomethylated	-0.019687	0.012928	hypomethylated	28	66	71
chr17	29686538	29688538	Tmem217	-0.19005	4.83E-25	hypomethylated	-0.019687	0.012928	hypomethylated	28	66	71
chr17	29750775	29752775	Rnf8	-0.13166	3.29E-15	hypomethylated	-0.018248	0.26557	insignificant	22	99	111
chr17	29796545	29798545	Ftsjd2	-0.15722	7.74E-09	hypomethylated	0.048343	0.63882	insignificant	16	77	80
chr17	30024827	30026827	Mdga1	-0.11769	3.71E-30	hypomethylated	0.0089735	0.60091	insignificant	36	139	137
chr17	30141031	30143031	Zfand3	-0.11388	5.85E-27	hypomethylated	-0.0092055	0.37506	insignificant	43	198	196
chr17	30713232	30715232	Btbd9	-0.22222	0.000081232	hypomethylated	-0.05523	0.023955	hypomethylated	6	30	74
chr17	30749604	30751604	Glo1	-0.44325	0.017074	stronglyHypometh	-0.020234	0.56053	insignificant	2	9	10
chr17	30762880	30764880	Dnahc8	-0.64416	0.0034986	stronglyHypometh	0.10205	0.24098	insignificant	1	2	4
chr17	31037811	31039811	Glp1r	-0.11186	3.63E-13	hypomethylated	0.0023686	0.11576	insignificant	15	56	57
chr17	31090627	31092627	Umodl1	0.11178	0.025522	hypermethylated	-0.025792	0.23189	insignificant	6	19	24
chr17	31193638	31195638	Abcg1	-0.10002	8.71E-15	hypomethylated	0.0073018	0.25239	insignificant	20	51	53
chr17	31334694	31336694	Tmprss3	0.092991	1	insignificant	-0.11137	0.16785	insignificant	2	10	9
chr17	31335919	31337919	Tmprss3	0.13061	1	lowCoverage	-0.14301	0.000011845	hypomethylated	1	14	11
chr17	31344010	31346010	Ubash3a	-0.50673	0.030568	stronglyHypometh	-0.020648	0.57612	insignificant	2	15	12
chr17	31431427	31433427	Slc37a1	-0.15696	3.82E-11	hypomethylated	-0.0068782	0.93913	insignificant	16	75	75
chr17	31522178	31524178	Pde9a	-0.12677	2.69E-12	hypomethylated	-0.021922	0.066674	insignificant	15	42	42
chr17	31656059	31658059	Ndufv3	-0.23264	1.65E-08	hypomethylated	0.0032728	0.57736	insignificant	5	32	30
chr17	31700717	31702717	Pknox1	-0.084404	4.96E-32	hypomethylated	-0.0092121	0.11413	insignificant	65	265	269
chr17	31774076	31776076	Cbs	0.080986	0.55823	insignificant	0.060198	0.00050282	hypermethylated	8	80	72
chr17	31774086	31776086	Cbs	0.080986	0.55823	insignificant	0.060198	0.00050282	hypermethylated	8	80	72
chr17	31795699	31797699	U2af1	-0.062479	0.0028058	hypomethylated	-0.0059892	0.33589	insignificant	6	42	41
chr17	31813889	31815889	Cryaa	-0.06545	0.019896	hypomethylated	0.011478	0.28335	insignificant	7	38	38
chr17	31992737	31994737	Sik1	-0.20101	4.62E-09	hypomethylated	-0.051919	0.60696	insignificant	17	77	81
chr17	32171453	32173453	Rrp1b	-0.33932	8.92E-58	stronglyHypometh	0.092679	0.000080365	inconclusive	9	36	29
chr17	32172106	32174106	Rrp1b	-0.1464	1.63E-18	hypomethylated	0.12314	0.61407	insignificant	9	43	22
chr17	32184229	32186229	Rrp1b		1	noCoverage	-0.049496	0.5967	insignificant	0	10	10
chr17	32228368	32230368	Pdxk-ps		1	noCoverage	0.059956	0.25194	insignificant	0	3	3
chr17	32303797	32305797	Notch3	-0.096008	0.023433	hypomethylated	-0.0029033	1	insignificant	11	24	24
chr17	32421068	32423068	Brd4	-0.097731	1.21E-36	hypomethylated	-0.0037935	0.386	insignificant	55	146	156
chr17	32458098	32460098	Akap8	-0.16822	0.6182	insignificant	0.0018946	0.71048	insignificant	3	34	34
chr17	32524762	32526762	Wiz	-0.20016	3.7E-22	hypomethylated	0.028919	0.58392	insignificant	15	35	37
chr17	32525895	32527895	Wiz	-0.20016	3.7E-22	hypomethylated	-0.014953	0.53674	insignificant	15	35	35
chr17	32538958	32540958	A530088E08Rik	0.029209	0.00011839	inconclusive	0.059754	0.0029641	hypermethylated	2	13	13
chr17	32540526	32542526	A530088E08Rik	0.19109	0.35307	insignificant	0.061823	0.00013467	hypermethylated	3	11	11
chr17	32588667	32590667	Cyp4f39	-0.29651	9.32E-14	hypomethylated	-0.060591	0.039841	hypomethylated	7	28	28
chr17	32642406	32644406	Cyp4f17	-0.33775	0.012214	stronglyHypometh	-0.039272	0.76034	insignificant	6	28	26
chr17	32795427	32797427	Cyp4f40	-0.58958	0.21863	insignificant	0.076854	0.11222	insignificant	2	8	8
chr17	32925232	32927232	Zfp871		1	noCoverage	-0.10545	0.73121	insignificant	0	4	7
chr17	33084306	33086306	Cyp4f13	-0.33279	0.0023898	hypomethylated	0.30782	0.064492	insignificant	1	5	3

chr17	33101775	33103775	Zfp472	-0.18989	5.73E-08	hypomethylated	-0.021162	0.23739	insignificant	13	56	56
chr17	33102661	33104661	Cyp4f41-ps	-0.21159	0.00000575	hypomethylated	-0.025974	0.30315	insignificant	7	34	34
chr17	33129083	33131083	Zfp952	-0.19841	0.080144	insignificant	-0.024017	0.61207	insignificant	5	18	26
chr17	33225311	33227311	Zfp563	-0.30617	5.43E-08	hypomethylated	-0.0094222	1	insignificant	10	37	37
chr17	33276628	33278628	Morc2b	-0.6875	0.41176	lowCoverage	-0.53194	0.68265	insignificant	1	2	10
chr17	33495823	33497823	Zfp81	-0.30502	0.0023439	hypomethylated	-0.049247	0.19194	insignificant	2	7	10
chr17	33568843	33570843	Actl9		1	noCoverage	0.0014815	1	insignificant	0	4	4
chr17	33660168	33662168	Adamts10	-0.10456	1.34E-10	hypomethylated	0.014926	0.13673	insignificant	9	41	41
chr17	33765036	33767036	Zfp414	-0.19913	0.11682	insignificant	-0.0090581	0.85805	insignificant	5	10	10
chr17	33822403	33824403	Hnrnpm	-0.12235	6.53E-08	hypomethylated	-0.013179	0.088795	insignificant	6	22	22
chr17	33918520	33920520	Angptl4		1	noCoverage	-0.081044	0.48796	insignificant	0	9	11
chr17	33946467	33948467	Kank3	-0.20062	0.00004592	hypomethylated	0.085397	0.60836	insignificant	9	29	28
chr17	33960558	33962558	Ndufa7	-0.33602	0.000061006	stronglyHypometh	0.01882	0.43027	insignificant	5	20	20
chr17	33961443	33963443	Rps28	-0.25695	0.015947	hypomethylated	0.04938	0.24139	insignificant	4	16	16
chr17	33979035	33981035	Cd320	-0.41667	1	lowCoverage	-0.076944	0.85993	insignificant	1	6	6
chr17	34027578	34029578	Kifc1	-0.2523	6.37E-10	hypomethylated	0.027465	0.64659	insignificant	6	13	18
chr17	34045389	34047389	Daxx	-0.16081	6.23E-20	hypomethylated	-0.025511	0.000004886	hypomethylated	26	120	131
chr17	34045545	34047545	Daxx	-0.15702	1.53E-19	hypomethylated	-0.02229	0.00000957	hypomethylated	26	113	121
chr17	34052120	34054120	Zbtb22	-0.2421	1.09E-08	hypomethylated	0.011821	0.0046467	inconclusive	4	34	22
chr17	34055422	34057422	Tapbp	-0.23882	4.4E-22	hypomethylated	-0.014285	2.27E-08	hypomethylated	27	84	83
chr17	34065838	34067838	Rgl2	-0.13185	1.43E-09	hypomethylated	-0.0059265	0.30868	insignificant	36	70	70
chr17	34076667	34078667	Wdr46	-0.17085	5.27E-21	hypomethylated	-0.0050424	0.60709	insignificant	34	109	111
chr17	34077275	34079275	H2-Ke2	-0.17085	5.27E-21	hypomethylated	-0.011174	0.63272	insignificant	34	109	109
chr17	34077288	34079288	H2-Ke2	-0.17085	5.27E-21	hypomethylated	-0.011174	0.63272	insignificant	34	109	109
chr17	34088433	34090433	B3galt4	-0.27945	0.00000312	hypomethylated	-0.026447	0.71753	insignificant	7	28	28
chr17	34091826	34093826	Vps52	-0.14145	2.38E-32	hypomethylated	0.0026619	0.25871	insignificant	40	139	129
chr17	34092586	34094586	Rps18	-0.2466	7.46E-35	hypomethylated	0.0037709	0.0009547	inconclusive	29	93	83
chr17	34115736	34117736	AA388235	-0.56944	0.23724	insignificant	0.04594	0.79862	insignificant	1	2	2
chr17	34117436	34119436	AA388235	-0.26923	0.10979	insignificant	0.064103	1	insignificant	3	8	9
chr17	34137278	34139278	H2-K1	-0.13971	0.22381	insignificant	-0.0063725	0.27138	insignificant	3	8	10
chr17	34161625	34163625	Ring1	-0.17508	1.84E-24	hypomethylated	0.012843	0.013632	inconclusive	37	115	118
chr17	34162037	34164037	H2-Ke6	-0.19478	1.19E-10	hypomethylated	0.040259	0.44696	insignificant	10	53	56
chr17	34165000	34167000	H2-Ke6	-0.55555	9.72E-17	stronglyHypometh	-0.085902	8.26E-10	hypomethylated	4	18	17
chr17	34167796	34169796	Rxrb	-0.13252	9.13E-51	hypomethylated	-0.0032973	0.15133	insignificant	57	181	171
chr17	34168635	34170635	Rxrb	-0.11075	3.67E-32	hypomethylated	-0.008019	0.032562	hypomethylated	41	135	125
chr17	34175381	34177381	Col11a2	-0.23605	0.80639	insignificant	0.037638	3.48E-08	hypermethylated	12	43	40
chr17	34228323	34230323	H2-Oa	-0.37121	0.080444	insignificant	-0.018271	0.72785	insignificant	1	6	6
chr17	34257328	34259328	Brd2	-0.15554	2.37E-18	hypomethylated	0.0073718	0.66262	insignificant	19	91	92
chr17	34258692	34260692	Brd2	-0.16418	0.000095681	hypomethylated	-0.02345	0.48155	insignificant	7	48	57
chr17	34271615	34273615	H2-DMa	-0.20987	4.54E-09	hypomethylated	-0.029801	0.040976	hypomethylated	9	47	46
chr17	34289135	34291135	H2-DMb1	-0.18939	0.17599	insignificant	-0.20727	0.37223	insignificant	3	12	13
chr17	34323500	34325500	Tap1	-0.17307	0.00000168	hypomethylated	-0.017323	0.041811	hypomethylated	16	56	56
chr17	34324275	34326275	Tap1	-0.17307	0.00000168	hypomethylated	-0.017323	0.041811	hypomethylated	16	56	56
chr17	34334139	34336139	Psmb8	-0.17687	4.57E-10	hypomethylated	-0.02891	0.56339	insignificant	11	65	59
chr17	34340423	34342423	Tap2	-0.19137	2.31E-08	hypomethylated	-0.062516	0.020355	hypomethylated	6	30	32
chr17	34399171	34401171	H2-Ab1	-0.1911	0.37087	insignificant	-0.024007	0.58774	insignificant	3	18	18
chr17	34424716	34426716	H2-Aa		1	noCoverage	0.081701	0.49807	insignificant	0	4	6
chr17	34654297	34656297	Btnl6	0.12008	0.38357	insignificant	0.2078	0.096914	insignificant	2	8	8
chr17	34700239	34702239	Notch4	-0.19444	0.68806	insignificant	0.061926	0.23324	insignificant	2	14	18
chr17	34725750	34727750	Gpsm3	-0.19123	0.37243	insignificant	-0.044845	0.30171	insignificant	2	20	21
chr17	34728415	34730415	Pbx2	-0.1832	1.39E-18	hypomethylated	-0.028743	0.62235	insignificant	43	105	96
chr17	34733806	34735806	Ager		1	noCoverage	-0.13531	0.67988	insignificant	0	4	4
chr17	34740506	34742506	Rnf5	-0.13053	0.0015919	hypomethylated	-0.0064212	1	insignificant	10	58	60
chr17	34741805	34743805	Agpat1	-0.11973	3.45E-09	hypomethylated	0.01029	0.07063	insignificant	16	49	60
chr17	34744958	34746958	Agpat1	0.27426	1	lowCoverage	0.014723	0.89967	insignificant	1	8	10
chr17	34752916	34754916	Egfl8	-0.67469	0.000029503	stronglyHypometh	0.010195	0.018236	inconclusive	2	16	16
chr17	34764042	34766042	Ppt2	-0.10768	2.64E-18	hypomethylated	0.0050554	0.45494	insignificant	14	94	81
chr17	34765630	34767630	Prrt1		1	noCoverage	-0.061959	0.52864	insignificant	0	6	6
chr17	34780827	34782827	Fkbpl	-0.22346	0.11327	insignificant	-0.026464	0.92496	insignificant	6	42	42
chr17	34783124	34785124	Atf6b	-0.094206	0.82293	insignificant	0.019089	0.00090426	hypermethylated	35	113	114
chr17	34806479	34808479	Tnxb	0.058981	0.27019	insignificant	0.041509	0.11659	insignificant	12	34	34
chr17	34941371	34943371	Cyp21a1	0.06369	1	lowCoverage	-0.025798	0.39725	insignificant	1	8	8
chr17	34960399	34962399	Stk19		1	noCoverage	-0.15931	0.019673	hypomethylated	0	12	14
chr17	34972963	34974963	Dom3z	-0.13809	9.72E-54	hypomethylated	-0.01696	0.067408	insignificant	44	189	197
chr17	34973848	34975848	Stk19	-0.13135	1.25E-08	hypomethylated	0.0038235	0.049904	hypermethylated	29	141	146
chr17	34986335	34988335	Rdbp	-0.12998	2.91E-28	hypomethylated	-0.010199	0.75357	insignificant	38	113	118
chr17	34987149	34989149	Skiv2l	-0.13012	4.77E-23	hypomethylated	-0.0060383	0.52117	insignificant	39	109	109
chr17	34999459	35001459	Cfb	0.055876	0.28744	insignificant	-0.019434	0.74198	insignificant	4	16	16
chr17	35030503	35032503	Zbtb12	-0.11074	1.05E-12	hypomethylated	-0.017352	0.092109	insignificant	31	134	153
chr17	35034443	35036443	Ehmt2	-0.12233	1.26E-26	hypomethylated	-0.0046759	0.67637	insignificant	44	137	142
chr17	35034918	35036918	Ehmt2	-0.13157	3.99E-33	hypomethylated	-0.0065441	0.68117	insignificant	48	147	152
chr17	35050410	35052410	Slc44a4	-0.14679	0.84421	insignificant	0.070802	0.13071	insignificant	6	19	33
chr17	35067197	35069197	Neu1	-0.094566	0.73856	insignificant	0.0019135	0.81082	insignificant	2	37	42
chr17	35087953	35089953	1110038B12Rik	-0.16212	1.09E-14	hypomethylated	-0.0060535	0.6365	insignificant	16	65	62
chr17	35089413	35091413	1110038B12Rik	-0.27388	0.34754	insignificant	-0.0030057	0.018782	inconclusive	5	23	19
chr17	35089416	35091416	1110038B12Rik	-0.27388	0.34754	insignificant	-0.0030057	0.018782	inconclusive	5	23	19
chr17	35108647	35110647	Hspa1l	0.16081	0.048866	hypermethylated	-0.049377	0.068432	insignificant	5	51	51
chr17	35109101	35111101	Hspa1a	0.10796	0.097288	insignificant	0.01851	0.0048906	hypermethylated	5	32	32
chr17	35117804	35119804	Lsm2	-0.078402	3.16E-09	hypomethylated	-0.011319	0.16504	insignificant	17	97	96
chr17	35118074	35120074	Lsm2	-0.091899	2.14E-10	hypomethylated	-0.010347	0.068229	insignificant	17	99	98
chr17	35136851	35138851	Vars	-0.13376	1.58E-47	hypomethylated	-0.018563	0.007836	hypomethylated	39	187	194
chr17	35152523	35154523	D17H6S56E-3	-0.58662	0.00000251	stronglyHypometh	0.0020223	0.56548	insignificant	4	17	14
chr17	35183551	35185551	Msh5		1	noCoverage	-0.03169	0.71587	insignificant	0	12	12
chr17	35183668	35185668	Msh5		1	noCoverage	-0.03169	0.71587	insignificant	0	12	12
chr17	35186187	35188187	Clic1	-0.23511	0.000000329	hypomethylated	-0.061252	0.052209	insignificant	10	36	46

chr17	35194979	35196979	Ddah2	-0.32963	0.33665	insignificant	-0.034816	0.019109	hypomethylated	2	32	32
chr17	35203129	35205129	AU023871	-0.2374	0.00000204	hypomethylated	0.0069013	0.06508	insignificant	6	16	18
chr17	35203269	35205269	Ly6g6c	-0.10537	0.13175	insignificant	-0.0025043	1	insignificant	5	22	22
chr17	35212886	35214886	Ly6g6e		1	noCoverage	0.19499	0.10981	insignificant	0	4	5
chr17	35222540	35224540	Ly6g6f	0.30914	0.17666	insignificant	0.038793	0.016824	hypermethylated	2	13	12
chr17	35225235	35227235	Abhd16a	-0.11555	2.41E-10	hypomethylated	0.0043126	0.24675	insignificant	22	96	99
chr17	35244244	35246244	Ly6g5c		1	noCoverage	-0.1921	0.016187	hypomethylated	0	14	17
chr17	35257440	35259440	Gpank1	-0.14896	1.51E-11	hypomethylated	0.0081486	0.62946	insignificant	32	123	125
chr17	35258392	35260392	Csnk2b	-0.3492	5.5E-09	stronglyHypometh	0.030574	0.50753	insignificant	13	63	65
chr17	35258896	35260896	Gpank1	-0.64306	0.065719	insignificant	0.02766	0.0021373	inconclusive	2	27	29
chr17	35263059	35265059	D17H6S53E	-0.21093	9.58E-19	hypomethylated	0.0019637	0.00068029	inconclusive	22	60	60
chr17	35268697	35270697	Apom	0.1757	0.24555	insignificant	-0.077277	0.42059	insignificant	2	36	38
chr17	35271186	35273186	Bag6	-0.13623	2.14E-12	hypomethylated	0.0025138	0.18393	insignificant	33	132	131
chr17	35301822	35303822	Prrc2a	-0.12343	4.51E-26	hypomethylated	0.000067797	0.73919	insignificant	12	67	74
chr17	35325385	35327385	Lst1	-0.59	0.00011108	stronglyHypometh	0.10551	1	insignificant	1	11	11
chr17	35330451	35332451	Ltb	0.22232	0.010755	inconclusive	0.029093	0.925	insignificant	4	10	12
chr17	35338941	35340941	Tnf	0.0065365	0.26977	insignificant	0.10576	0.32625	insignificant	2	9	7
chr17	35342296	35344296	Lta	0.18528	0.73733	insignificant	-0.0096288	0.21859	insignificant	6	21	22
chr17	35372540	35374540	Atp6v1g2	-0.1659	6.77E-14	hypomethylated	-0.0029689	0.91616	insignificant	15	62	63
chr17	35372760	35374760	Nfkbil1	-0.16035	6.99E-08	hypomethylated	0.0015594	0.80154	insignificant	12	56	57
chr17	35377690	35379690	Ddx39b	-0.12182	5.07E-17	hypomethylated	-0.0090567	0.080127	insignificant	24	120	120
chr17	35399038	35401038	H2-D1	-0.14475	2.97E-21	hypomethylated	-0.031591	0.0021607	hypomethylated	34	97	112
chr17	35456502	35458502	H2-Q1	-0.17674	7.88E-31	hypomethylated	-0.016511	0.000011017	hypomethylated	23	96	102
chr17	35478277	35480277	H2-Q2	-0.12817	0.00000209	hypomethylated	-0.0063398	0.41987	insignificant	19	78	80
chr17	35515561	35517561	H2-Q4	-0.14805	1.93E-30	hypomethylated	-0.014997	0.44231	insignificant	30	115	130
chr17	35530043	35532043	H2-Q8	0.022213	0.072395	insignificant	-0.023649	0.48327	insignificant	6	71	75
chr17	35560821	35562821	H2-Q6	-0.072343	0.16174	insignificant	-0.057108	0.00000165	hypomethylated	14	84	95
chr17	35575099	35577099	H2-Q7		1	noCoverage	0.022749	0.015392	inconclusive	0	66	69
chr17	35606033	35608033	H2-Q10	-0.20294	8.59E-17	hypomethylated	-0.058086	0.00001723	hypomethylated	27	87	94
chr17	35641983	35643983	Pou5f1	-0.28321	0.0067537	hypomethylated	-0.013571	0.41774	insignificant	5	14	14
chr17	35653060	35655060	Cchcr1	-0.31842	3.19E-12	hypomethylated	-0.034532	0.098532	insignificant	12	41	41
chr17	35653769	35655769	Tcf19	-0.19479	0.0075816	hypomethylated	-0.03199	0.0015263	hypomethylated	15	47	47
chr17	35669145	35671145	Psors1c2	-0.57641	0.000029088	stronglyHypometh	-0.064061	0.078323	insignificant	1	8	15
chr17	35688072	35690072	Cdsn	-0.09873	0.03165	hypomethylated	-0.073877	0.15248	insignificant	11	26	26
chr17	35804537	35806537	Vars2	-0.14542	4.83E-13	hypomethylated	0.0047323	0.00094477	inconclusive	14	68	68
chr17	35810627	35812627	Gtf2h4	-0.66055	0.40318	lowCoverage	0.000097468	0.4216	insignificant	1	43	37
chr17	35837535	35839535	Ddr1	-0.38638	4.37E-26	stronglyHypometh	-0.060706	0.5117	insignificant	7	38	37
chr17	35838989	35840989	Ddr1	-0.46105	0.000033631	stronglyHypometh	-0.024036	0.0018438	inconclusive	2	32	32
chr17	35957657	35959657	Ier3	-0.16849	2.05E-14	hypomethylated	0.0091901	0.086619	insignificant	20	90	90
chr17	35959301	35961301	Flot1	-0.20266	2.2E-13	hypomethylated	-0.0036683	0.00001615	hypomethylated	18	73	75
chr17	35975246	35977246	Tubb5	0.04	1	insignificant	-0.02625	0.7558	insignificant	1	10	10
chr17	35977442	35979442	Mdc1	-0.16608	3.87E-15	hypomethylated	0.0020801	0.023753	inconclusive	19	87	92
chr17	35997262	35999262	Nrm	-0.099038	5.05E-08	hypomethylated	-0.0075658	0.0026922	hypomethylated	16	64	57
chr17	36001539	36003539	Ppp1r18	-0.14262	0.027288	hypomethylated	0.00094377	0.38837	insignificant	14	63	63
chr17	36002072	36004072	Ppp1r18	-0.18315	9.56E-28	hypomethylated	-0.0064618	0.11324	insignificant	18	80	81
chr17	36002559	36004559	Ppp1r18	-0.16601	2.95E-28	hypomethylated	0.0035543	0.12528	insignificant	20	103	99
chr17	36015722	36017722	Dhx16	-0.237	4.7E-40	hypomethylated	-0.056806	0.00013845	hypomethylated	20	91	108
chr17	36034323	36036323	Atat1	-0.068194	0.0066192	hypomethylated	0.061004	0.70491	insignificant	10	26	29
chr17	36047013	36049013	Atat1	-0.72003	6.35E-32	stronglyHypometh	-0.12284	0.00040182	hypomethylated	3	26	23
chr17	36052855	36054855	Ppp1r10	-0.097639	1.62E-13	hypomethylated	0.02098	0.33074	insignificant	29	139	142
chr17	36053140	36055140	Ppp1r10	-0.092019	2.53E-13	hypomethylated	0.023296	0.3301	insignificant	29	137	138
chr17	36053314	36055314	Mrps18b	-0.09192	1.39E-13	hypomethylated	0.0256	0.32014	insignificant	29	125	126
chr17	36053833	36055833	Mir1894	-0.056236	0.0064327	hypomethylated	-0.0046426	0.16412	insignificant	6	48	48
chr17	36097759	36099759	Abcf1	-0.1974	0.20471	insignificant	-0.0054619	1	insignificant	2	20	23
chr17	36106695	36108695	Abcf1	-0.41398	0.30636	insignificant	0.010726	0.69284	insignificant	1	8	8
chr17	36115899	36117899	Gnl1	-0.11102	1.16E-42	hypomethylated	0.0066678	0.35651	insignificant	61	174	174
chr17	36115968	36117968	Prr3	-0.11102	1.16E-42	hypomethylated	0.0066678	0.35651	insignificant	61	174	174
chr17	36116412	36118412	Prr3	-0.13926	8.16E-54	hypomethylated	0.0027074	0.28619	insignificant	59	162	162
chr17	36116770	36118770	Prr3	-0.13496	1.95E-70	hypomethylated	0.0060843	0.000010906	inconclusive	48	132	132
chr17	36130448	36132448	A930015D03Rik	-0.18132	0.000000371	hypomethylated	-0.079691	0.17976	insignificant	3	30	35
chr17	36157505	36159505	H2-T24	-0.095581	0.14235	insignificant	0.044706	0.32577	insignificant	2	12	12
chr17	36169646	36171646	A930015D03Rik		1	noCoverage	-0.16654	0.42689	insignificant	0	4	4
chr17	36178905	36180905	Gm6034	-0.27914	0.062985	insignificant	-0.017322	0.53512	insignificant	4	8	8
chr17	36280962	36282962	2410017I17Rik	-0.11108	1.75E-10	hypomethylated	0.01319	0.71428	insignificant	8	76	83
chr17	36314721	36316721	Gm6623		1	noCoverage	0.0046156	1	insignificant	0	20	20
chr17	36408378	36410378	Trim39	-0.08283	0.0062847	hypomethylated	-0.006044	0.31001	insignificant	21	90	90
chr17	36779589	36781589	H2-M9		1	noCoverage	-0.1316	0.22252	insignificant	0	4	4
chr17	36948119	36950119	H2-M10.6	-0.092895	0.62393	insignificant	-0.036271	0.76869	insignificant	3	8	8
chr17	36973085	36975085	Trim26	-0.14636	1.02E-22	hypomethylated	-0.003172	0.00049888	hypomethylated	22	88	87
chr17	37005518	37007518	Trim10	-0.26007	2.39E-10	hypomethylated	-0.020644	0.86481	insignificant	6	28	28
chr17	37078995	37080995	Rnf39	-0.158	9.03E-19	hypomethylated	-0.011439	0.41418	insignificant	32	90	89
chr17	37094536	37096536	Znrd1as	-0.14935	0.038244	hypomethylated	-0.024046	0.54935	insignificant	11	48	48
chr17	37095373	37097373	Znrd1as	-0.11361	0.098808	insignificant	0.0042754	0.42246	insignificant	9	42	42
chr17	37138426	37140426	Zfp57	-0.16139	0.000058334	hypomethylated	-0.023318	0.68357	insignificant	13	50	49
chr17	37138469	37140469	Zfp57	-0.16139	0.000058334	hypomethylated	-0.023318	0.68357	insignificant	13	50	49
chr17	37139610	37141610	Zfp57	-0.12135	0.00014209	hypomethylated	-0.005747	0.57569	insignificant	13	45	46
chr17	37160343	37162343	Mog		1	noCoverage	-0.21451	0.13268	insignificant	0	6	6
chr17	37181910	37183910	Gabbr1	-0.079587	0.000043787	hypomethylated	-0.011734	0.91824	insignificant	18	64	84
chr17	37369313	37371313	Olfr97	-0.53068	0.091345	insignificant	-0.2372	0.074146	insignificant	1	4	4
chr17	37406178	37408178	H2-M3	-0.22887	0.00000037	hypomethylated	-0.037186	0.93622	insignificant	5	16	22
chr17	37931390	37933390	Olfr123	-0.061043	0.0635	insignificant	-0.048529	0.52261	insignificant	3	6	6
chr17	38690136	38692136	Gm20410		1	noCoverage	-0.038043	0.56941	insignificant	0	4	4
chr17	39980080	39982080		-0.07093	0	hypomethylated	-0.095673	0	hypomethylated	92	366	357
chr17	41070633	41072633	Mut	-0.14925	9.24E-18	hypomethylated	-0.0066835	0.25593	insignificant	25	76	76
chr17	41071500	41073500	Cenpq	-0.10653	0.00000053	hypomethylated	-0.0035897	1	insignificant	13	36	36

chr17	42451895	42453895	3110082D06Rik	-0.0099947	0.00027389	hypomethylated	-0.0079094	0.71929	insignificant	17	95	104
chr17	42829233	42831233	Gpr115		1	noCoverage	-0.045163	0.77332	insignificant	0	2	2
chr17	43013373	43015373	Cd2ap	-0.14978	6.77E-08	hypomethylated	-0.0024761	0.89304	insignificant	12	103	100
chr17	43152503	43154503	Tnfrsf21	-0.1445	1.1E-19	hypomethylated	-0.0070724	0.89292	insignificant	27	90	98
chr17	43639722	43641722	Mep1a		1	noCoverage	0.13479	0.23136	insignificant	0	6	6
chr17	43704399	43706399	Pla2g7	-0.24043	0.000095876	hypomethylated	-0.0096199	0.85678	insignificant	7	34	32
chr17	43767248	43769248	Tdrd6	0.1434	0.0031862	hypermethylated	-0.14517	0.1679	insignificant	5	44	46
chr17	43803373	43805373	Cyp39a1	-0.13013	6.41E-09	hypomethylated	-0.0013804	0.45863	insignificant	16	44	44
chr17	43803964	43805964	Cyp39a1	-0.12999	8.42E-08	hypomethylated	0.0044795	0.26051	insignificant	13	34	34
chr17	43937799	43939799	Rcan2	-0.12249	1.14E-29	hypomethylated	-0.0043522	0.079856	insignificant	26	112	112
chr17	44089139	44091139	Rcan2	-0.030961	0.58151	insignificant	-0.0298	0.76408	insignificant	7	23	26
chr17	44214794	44216794	Enpp5	-0.15501	0.000083751	hypomethylated	0.0088166	0.54177	insignificant	2	19	19
chr17	44214796	44216796	Enpp5	-0.15501	0.000083751	hypomethylated	0.0088166	0.54177	insignificant	2	19	19
chr17	44242757	44244757	Enpp4	-0.1174	0.000015874	hypomethylated	-0.029607	0.13833	insignificant	10	44	39
chr17	44324520	44326520	Clic5	-0.18498	7.67E-10	hypomethylated	0.0001439	0.84004	insignificant	8	25	24
chr17	44873597	44875597	Runx2	-0.3	0.10841	insignificant	0.0065476	1	insignificant	2	8	8
chr17	44913119	44915119	Supt3h	-0.10468	1.91E-16	hypomethylated	-0.026422	0.0057394	hypomethylated	24	98	102
chr17	45570656	45572656	Cdc5l	-0.10359	0.00023693	hypomethylated	-0.017341	0.40649	insignificant	16	77	76
chr17	45642789	45644789	Aars2	-0.17818	7.27E-22	hypomethylated	-0.0045144	0.65593	insignificant	10	32	32
chr17	45659382	45661382	Tcte1	-0.22022	0.0035423	inconclusive	-0.11175	0.93931	insignificant	6	18	18
chr17	45686626	45688626	Tmem151b	-0.26731	0.15696	insignificant	0.10412	0.2796	insignificant	1	4	4
chr17	45691664	45693664	Nfkbie	-0.15325	2.59E-17	hypomethylated	0.0079051	0.87993	insignificant	19	87	81
chr17	45700100	45702100	Slc35b2	-0.13876	7.78E-11	hypomethylated	0.035443	0.461	insignificant	13	61	53
chr17	45710210	45712210	Hsp90ab1	-0.076039	0.60521	insignificant	-0.0014076	0.41618	insignificant	4	31	27
chr17	45730589	45732589	Slc29a1	-0.12569	3.33E-11	hypomethylated	-0.0059819	0.44538	insignificant	12	42	42
chr17	45732521	45734521	Slc29a1	-0.22059	1.46E-14	hypomethylated	0.0023669	0.84531	insignificant	10	22	22
chr17	45736552	45738552	Gm7325	-0.053942	0.015817	inconclusive	0.027615	0.000077292	hypermethylated	20	54	56
chr17	45822320	45824320	Mrpl14	-0.14922	2.19E-18	hypomethylated	-0.021395	0.000044731	hypomethylated	39	144	166
chr17	45823167	45825167	Mrpl14	-0.12565	0.0014342	hypomethylated	-0.037424	1.69E-08	hypomethylated	11	46	68
chr17	46169326	46171326	Vegfa	-0.27288	0.018567	hypomethylated	-0.052575	0.51366	insignificant	4	6	10
chr17	46246952	46248952	Mrps18a	-0.095797	9.66E-08	hypomethylated	0.0074064	0.84837	insignificant	15	60	61
chr17	46290500	46292500	Mad2l1bp	-0.55217	0.00061452	stronglyHypometh	-0.096408	0.62974	insignificant	2	19	16
chr17	46296980	46298980	Gtpbp2	-0.18832	0.000000205	hypomethylated	0.015114	0.20075	insignificant	15	77	73
chr17	46338803	46340803	Xpo5	-0.16129	1.68E-17	hypomethylated	-0.026726	0.026632	hypomethylated	17	62	62
chr17	46339574	46341574	Polh	-0.16129	1.68E-17	hypomethylated	-0.026726	0.026632	hypomethylated	17	62	62
chr17	46384028	46386028	Yipf3	-0.21198	2.51E-26	hypomethylated	-0.016815	0.49302	insignificant	19	54	52
chr17	46384994	46386994	Polr1c	-0.17227	3.27E-26	hypomethylated	-0.0034663	0.8152	insignificant	20	56	54
chr17	46390113	46392113	Gm88	-0.10981	8.5E-41	hypomethylated	0.0079872	0.00097142	inconclusive	24	111	111
chr17	46419962	46421962	Tjap1	0.025866	0.77198	insignificant	0.0569	1	insignificant	2	16	15
chr17	46433369	46435369	Dlk2	-0.14868	1.27E-09	hypomethylated	-0.012979	0.63369	insignificant	18	49	55
chr17	46464972	46466972	Abcc10		1	noCoverage	-0.082377	0.14029	insignificant	0	10	20
chr17	46519714	46521714	Zfp318	-0.077239	3.5E-27	hypomethylated	0.0011268	0.49148	insignificant	71	208	218
chr17	46564890	46566890	Crip3		1	noCoverage	0.16087	0.20298	insignificant	0	9	9
chr17	46624624	46626624	Ttbk1		1	noCoverage	-0.0039148	0.0031517	hypomethylated	0	16	16
chr17	46632737	46634737	BC048355	-0.11003	1.34E-10	hypomethylated	-0.006678	0.80964	insignificant	22	67	69
chr17	46683337	46685337	Srf	-0.22039	1.71E-11	hypomethylated	0.020181	0.1237	insignificant	4	11	17
chr17	46693111	46695111	Srf	-0.21279	0.19676	insignificant	0.041787	0.092115	insignificant	6	34	27
chr17	46766453	46768453	Ptk7		1	noCoverage	-0.029672	0.88389	insignificant	0	21	22
chr17	46782093	46784093	Mrpl2	-0.20854	3.49E-17	hypomethylated	-0.040546	0.75109	insignificant	22	100	100
chr17	46782196	46784196	Mrpl2	-0.19685	1.67E-15	hypomethylated	-0.040371	0.74039	insignificant	21	98	98
chr17	46786286	46788286	Cul7	-0.10622	1.7E-12	hypomethylated	0.014609	0.5606	insignificant	20	107	103
chr17	46811204	46813204	Rrp36	-0.12406	4.74E-12	hypomethylated	0.071004	0.56118	insignificant	8	18	21
chr17	46817085	46819085	Mea1	-0.11874	0.00036137	hypomethylated	0.0048501	0.32375	insignificant	18	100	96
chr17	46817879	46819879	Mea1	-0.30634	0.0070012	hypomethylated	0.000065123	0.064962	insignificant	9	85	89
chr17	46841951	46843951	Ppp2r5d	-0.47637	0.0085828	stronglyHypometh	-0.30259	0.50627	insignificant	2	17	18
chr17	46847411	46849411	Pex6	-0.12615	4.9E-21	hypomethylated	-0.018963	0.0036837	hypomethylated	48	158	156
chr17	46866114	46868114	Gnmt	0.45841	1	lowCoverage	0.08002	0.60265	insignificant	1	18	18
chr17	46889161	46891161	Cnpy3	-0.1195	0.34382	insignificant	0.062435	0.0087763	hypermethylated	12	34	39
chr17	46908583	46910583	2310039H08Rik	-0.3804	0.000000005	stronglyHypometh	-0.018337	0.93333	insignificant	5	62	62
chr17	46919605	46921605	Rpl7l1	-0.1995	1	insignificant	-0.021949	0.00055316	hypomethylated	7	47	47
chr17	46968362	46970362	BC032203	-0.27559	0.21585	insignificant	0.07409	0.52662	insignificant	2	12	13
chr17	47026569	47028569	Tbcc	-0.16439	4.75E-10	hypomethylated	0.00010818	0.000039587	inconclusive	16	104	104
chr17	47046433	47048433	Prph2	-0.10386	0.49877	insignificant	0.045198	0.2788	insignificant	12	49	50
chr17	47276890	47278890	Trerf1	-0.19673	0.011441	hypomethylated	-0.018954	0.36186	insignificant	9	91	92
chr17	47366178	47368178	Trerf1	0.23397	0.22962	insignificant	0.096276	1	insignificant	2	18	22
chr17	47504835	47506835	Mrps10	-0.11295	1.95E-09	hypomethylated	0.078564	0.89926	insignificant	17	56	66
chr17	47505145	47507145	Mrps10	-0.1061	3.13E-09	hypomethylated	0.063805	0.83688	insignificant	18	61	72
chr17	47521341	47523341	Guca1b	0.17184	0.35988	insignificant	0.071481	0.002822	hypermethylated	2	14	16
chr17	47537533	47539533	Guca1a	-0.32517	0.045313	hypomethylated	-0.043566	0.7184	insignificant	2	7	12
chr17	47572587	47574587	AI661453	-0.16946	2E-50	hypomethylated	0.041863	0.000000269	inconclusive	16	75	71
chr17	47574324	47576324	AI661453	-0.59	0.00000227	stronglyHypometh	0.021413	0.4218	insignificant	2	4	4
chr17	47640999	47642999	Ccnd3	0.10466	0.44864	insignificant	-0.003983	1	insignificant	8	34	34
chr17	47729415	47731415	Ccnd3	-0.21742	0.034178	hypomethylated	-0.060844	0.36394	insignificant	6	47	45
chr17	47747544	47749544	Med20	-0.096819	5.62E-15	hypomethylated	-0.0082734	0.62581	insignificant	25	130	132
chr17	47748441	47750441	Med20	-0.14062	5.15E-09	hypomethylated	-0.018926	0.045187	hypomethylated	12	47	47
chr17	47766638	47768638	Usp49	-0.13664	1.44E-08	hypomethylated	0.0024668	0.0015536	inconclusive	23	75	76
chr17	47831155	47833155	Frs3	-0.1054	6.36E-19	hypomethylated	-0.014708	0.062199	insignificant	41	124	133
chr17	47862790	47864790	Pgc	-0.91958	0.020768	stronglyHypometh	-0.13271	0.16272	insignificant	2	11	6
chr17	47872985	47874985	Tfeb	-0.1511	4.04E-23	hypomethylated	-0.017102	0.77099	insignificant	39	109	109
chr17	47873879	47875879	Tfeb	-0.12693	4.65E-27	hypomethylated	-0.0027639	0.70849	insignificant	51	197	196
chr17	47921692	47923692	Tfeb	-0.1221	0.35046	insignificant	0.039162	0.81071	insignificant	3	30	30
chr17	47970207	47972207	Mdfi	-0.14173	1.08E-24	hypomethylated	0.0039039	0.66433	insignificant	14	75	69
chr17	48061581	48063581	Foxp4	-0.17614	2.31E-46	hypomethylated	0.015476	0.010336	inconclusive	20	61	52
chr17	48225581	48227581	1700067P10Rik		1	noCoverage	0.011868	0.75772	insignificant	0	14	14
chr17	48304207	48306207	A530064D06Rik	0.08407	1	insignificant	0.044855	0.049524	hypermethylated	1	13	17

chr17	48454486	48456486	B430306N03Rik		1	noCoverage	-0.062956	0.50585	insignificant	0	10	11
chr17	48498240	48500240	Treml1	0.025383	0.45125	insignificant	0.022332	0.38379	insignificant	6	14	14
chr17	48548404	48550404	AI314976	-0.12345	1.09E-42	hypomethylated	-0.0096816	0.0020196	hypomethylated	50	131	154
chr17	48549145	48551145	AI314976	-0.090909	0.16418	insignificant	-0.022064	0.45105	insignificant	8	11	34
chr17	48572053	48574053	Apobec2	0.087963	1	lowCoverage	-0.15438	0.020358	hypomethylated	1	6	11
chr17	49070906	49072906	Lrfn2	-0.067804	0.00000537	hypomethylated	-0.0025833	0.046101	inconclusive	9	30	32
chr17	49566688	49568688	Mocs1	-0.2214	0.000014072	hypomethylated	0.050658	0.56708	insignificant	9	24	26
chr17	49703662	49705662	Daam2	0.73113	0.20608	insignificant	-0.017509	0.049492	hypomethylated	2	22	22
chr17	49753496	49755496	Kif6	-0.13508	0.00000179	hypomethylated	0.0062986	0.75957	insignificant	8	28	28
chr17	50329822	50331822	Rftn1	-0.14881	0.29066	insignificant	0.072619	1	insignificant	1	4	6
chr17	50432924	50434924	Dazl	0.03393	1	insignificant	-0.01792	0.0055123	hypomethylated	6	46	54
chr17	50647871	50649871	Plcl2	-0.078692	1.18E-14	hypomethylated	0.0023088	0.052534	insignificant	72	201	185
chr17	51318674	51320674	Tbc1d5	-0.15375	0.000001057	hypomethylated	-0.056018	0.13126	insignificant	10	41	50
chr17	51951379	51953379	Satb1	-0.11805	3.53E-42	hypomethylated	-0.0027124	0.80088	insignificant	52	161	161
chr17	51971972	51973972	Satb1	-0.081519	3.86E-10	hypomethylated	-0.00076653	0.50702	insignificant	24	84	84
chr17	52741087	52743087	Kcnh8	-0.17693	4.58E-22	hypomethylated	-0.012077	0.46954	insignificant	21	78	76
chr17	53617558	53619558	Rab5a	-0.13601	1.28E-43	hypomethylated	-0.015051	0.01736	hypomethylated	73	184	184
chr17	53705295	53707295	Kat2b	-0.10049	5.1E-20	hypomethylated	0.0024443	0.015808	inconclusive	21	122	108
chr17	53722450	53724450	Kat2b	0.092997	0.59474	insignificant	-0.030114	0.63057	insignificant	2	14	16
chr17	53828640	53830640	Sgol1	-0.08873	0.0010011	hypomethylated	0.00037919	1	insignificant	14	67	47
chr17	53985283	53987283	Sult1c2	-0.31818	0.14782	insignificant	0.016043	0.82496	insignificant	1	2	2
chr17	55584014	55586014	St6gal2	-0.038334	0.0027794	hypomethylated	-0.019629	0.013637	hypomethylated	18	91	105
chr17	55763970	55765970	Vmn2r118	0.11273	0.76978	insignificant	0.15093	0.013658	hypermethylated	1	4	4
chr17	56018195	56020195	Zfp119a		1	noCoverage	-0.018751	0.68866	insignificant	0	4	4
chr17	56030515	56032515	Zfp959	-0.24714	0.00016944	hypomethylated	-0.0355	0.12514	insignificant	3	29	27
chr17	56084682	56086682	Zfp119b		1	noCoverage	0.12145	0.077576	insignificant	0	14	9
chr17	56097609	56099609	Ccdc94	-0.32124	8.98E-12	hypomethylated	0.018229	0.86703	insignificant	4	30	30
chr17	56108904	56110904	Shd	-0.12449	0.000001617	hypomethylated	-0.0059763	0.32216	insignificant	18	74	74
chr17	56124933	56126933	Fsd1	-0.63095	0.14342	insignificant	0.0072829	0.86522	insignificant	1	4	4
chr17	56144991	56146991	Stap2	-0.29864	0.37355	insignificant	0.02113	0.25696	insignificant	5	11	13
chr17	56147623	56149623	Mpnd	-0.12257	5.78E-14	hypomethylated	0.0068112	0.46907	insignificant	20	71	74
chr17	56175999	56177999	Sh3gl1	-0.17028	1.72E-09	hypomethylated	-0.024767	0.46804	insignificant	15	36	36
chr17	56178838	56180838	Chaf1a	-0.11247	0.0003676	hypomethylated	0.036796	0.071081	insignificant	18	85	72
chr17	56214412	56216412	Ubxn6	-0.25148	0.000037042	hypomethylated	-0.028138	0.88218	insignificant	11	43	44
chr17	56218079	56220079	Hdgfrp2	-0.16038	0.000048139	hypomethylated	-0.019871	0.058524	insignificant	8	65	63
chr17	56256721	56258721	Plin5	-0.28459	1.55E-09	hypomethylated	-0.053557	0.77388	insignificant	11	48	47
chr17	56256971	56258971	Plin5	-0.38853	8.57E-13	stronglyHypometh	-0.038039	0.40259	insignificant	7	46	45
chr17	56261369	56263369	Lrg1	0.0017846	0.6519	insignificant	-0.044013	0.3066	insignificant	8	32	32
chr17	56273275	56275275	Sema6b		1	noCoverage	-0.016689	0.60347	insignificant	0	12	12
chr17	56279766	56281766	Sema6b	-0.17163	0.000010855	hypomethylated	-0.021334	0.0015485	hypomethylated	12	86	86
chr17	56300913	56302913	Tnfaip8l1	-0.11153	1	insignificant	0.017569	0.51114	insignificant	7	24	25
chr17	56323343	56325343	D17Wsu104e	0.1273	9.76E-13	hypermethylated	0.033724	0.4646	insignificant	3	46	48
chr17	56358312	56360312	Dpp9	0.10959	0.030611	hypermethylated	-0.025518	0.057875	insignificant	3	38	36
chr17	56381410	56383410	Mir7b	-0.22701	2.69E-08	hypomethylated	0.011433	0.2048	insignificant	9	32	32
chr17	56395215	56397215	Fem1a	-0.12029	0.000000173	hypomethylated	0.0054511	0.042148	hypermethylated	24	182	183
chr17	56416190	56418190	Ticam1	0.095353	0.29072	insignificant	-0.026108	0.8533	insignificant	4	8	8
chr17	56429934	56431934	Plin3	-0.80507	3.45E-08	stronglyHypometh	-0.16324	0.0019379	hypomethylated	1	4	6
chr17	56439709	56441709	Arrdc5	-0.17141	1	insignificant	0.028951	0.85734	insignificant	3	11	10
chr17	56441759	56443759	Uhrf1	-0.080346	7.22E-11	inconclusive	-0.0018635	0.000019674	inconclusive	26	81	81
chr17	56442735	56444735	Uhrf1	-0.11982	2.15E-08	hypomethylated	-0.010924	0.96364	insignificant	24	86	87
chr17	56464472	56466472	Kdm4b	-0.10431	4.85E-15	hypomethylated	0.0021371	0.62017	insignificant	16	122	110
chr17	56615903	56617903	Ptprs	0.00087309	0.80442	insignificant	-0.0012062	0.034216	inconclusive	6	63	58
chr17	56651906	56653906	Znrf4	-0.052941	0.58599	insignificant	0.023682	0.29729	insignificant	1	8	8
chr17	56723404	56725404	Safb	-0.10078	8.36E-24	hypomethylated	-0.0041039	0.016563	hypomethylated	55	262	262
chr17	56724006	56726006	Safb2	-0.09914	6.41E-22	hypomethylated	-0.0024975	0.069475	insignificant	38	200	200
chr17	56749194	56751194	2410015M20Ril	-0.28458	5.3E-14	hypomethylated	-0.013456	0.94079	insignificant	4	12	12
chr17	56751817	56753817	Rpl36	-0.13012	1.91E-15	hypomethylated	0.017147	0.37691	insignificant	30	106	110
chr17	56811679	56813679	Ranbp3	-0.18664	3.18E-17	hypomethylated	-0.014748	0.39683	insignificant	32	121	127
chr17	56856184	56858184	Ndufa11	-0.1099	2.8E-22	hypomethylated	0.0035717	0.85241	insignificant	23	90	86
chr17	56856344	56858344	Vmac	-0.11707	5.29E-16	hypomethylated	0.0084771	0.46744	insignificant	16	71	67
chr17	56857122	56859122	Vmac	-0.078569	0.16291	insignificant	0.050722	0.1341	insignificant	8	42	39
chr17	56872650	56874650	Fut4-ps1	-0.094304	0.097204	insignificant	0.010718	0.45112	insignificant	3	11	10
chr17	56896953	56898953	Nrtn	-0.17389	8.92E-25	hypomethylated	0.023016	0.61551	insignificant	12	34	34
chr17	56903173	56905173	Dus3l	-0.16383	4.24E-10	hypomethylated	0.0072514	0.41396	insignificant	19	100	88
chr17	56908698	56910698	Prr22	-0.032191	0.66567	insignificant	-0.0010306	0.83094	insignificant	4	28	28
chr17	56970431	56972431	Rfx2	-0.088817	0.014087	hypomethylated	0.011413	0.71857	insignificant	18	74	74
chr17	57014055	57016055	1700061G19Rik		1	noCoverage	0.061508	0.36677	insignificant	0	6	6
chr17	57074811	57076811	Mllt1	-0.10705	9.12E-23	hypomethylated	0.0061866	0.013293	hypermethylated	26	65	65
chr17	57128686	57130686	Clpp	-0.17429	4.09E-18	hypomethylated	-0.013717	0.46596	insignificant	15	38	46
chr17	57135761	57137761	Alkbh7	-0.053053	0.0093035	hypomethylated	-0.034167	0.033798	hypomethylated	7	15	24
chr17	57150711	57152711	Gtf2f1	0.71564	0.00000075	stronglyHypermeth	0.0039494	0.057777	insignificant	2	45	45
chr17	57170930	57172930	Khsrp	-0.13381	7.5E-10	hypomethylated	0.055194	0.016971	inconclusive	10	36	34
chr17	57181077	57183077	Slc25a41	0.032966	0.69403	insignificant	-0.078145	0.37087	insignificant	2	6	6
chr17	57199286	57201286	Slc25a23		1	noCoverage	0.15328	0.70072	insignificant	0	32	44
chr17	57200699	57202699	Crb3	-0.19864	2.42E-11	hypomethylated	-0.045338	0.51939	insignificant	9	46	56
chr17	57227205	57229205	Tubb4a	-0.46775	0.12488	insignificant	-0.058663	0.097903	insignificant	1	6	6
chr17	57243807	57245807	Tnfsf9	-0.11829	0.065412	insignificant	-0.0013897	0.94686	insignificant	8	20	20
chr17	57333609	57335609	Tnfsf14		1	noCoverage	0.14246	0.39934	insignificant	0	12	10
chr17	57367559	57369559	C3	-0.54841	4.73E-14	stronglyHypometh	0.025293	0.7773	insignificant	2	4	4
chr17	57387064	57389064	Trip10	-0.13257	1	insignificant	0.018433	0.23248	insignificant	4	36	40
chr17	57387873	57389873	Trip10	-0.18109	0.089294	insignificant	-0.0091772	0.87035	insignificant	5	55	55
chr17	57417522	57419522	Vav1	-0.13571	0.13491	insignificant	0.086936	0.35396	insignificant	2	15	19
chr17	57497108	57499108	Emr1	0.23889	1	lowCoverage	0.016667	0.41217	insignificant	1	6	6
chr17	57907992	57909992	Cntnap5c	-0.17491	0.000049019	hypomethylated	-0.028637	0.64345	insignificant	4	19	19
chr17	59152745	59154745	Nudt12	-0.2399	0.3245	insignificant	-0.079643	0.43279	insignificant	1	6	6

chr17	63230666	63232666	Efna5	-0.15393	1.49E-55	hypomethylated	-0.015296	0.1005	insignificant	53	152	176
chr17	63849929	63851929	Fbxl17		1	noCoverage	0.072821	0.18684	insignificant	0	6	6
chr17	64286320	64288320	Fert2	0.21765	1	insignificant	0.20727	0.86206	insignificant	4	20	20
chr17	64949988	64951988	Man2a1	-0.10301	7.53E-49	hypomethylated	0.0051931	0.29526	insignificant	65	222	213
chr17	65962895	65964895	Vapa	-0.11957	1.79E-14	hypomethylated	-0.016391	0.40843	insignificant	27	93	93
chr17	65989096	65991096	Txndc2	0.056818	1	lowCoverage	-0.034848	0.066885	insignificant	1	8	8
chr17	66122092	66124092	Rab31	-0.17928	0.000000328	hypomethylated	0.00086907	0.56098	insignificant	7	26	26
chr17	66131694	66133694	Ppp4r1	-0.080786	6.81E-42	hypomethylated	-0.0038767	0.093514	insignificant	91	253	266
chr17	66234263	66236263	Ralbp1		1	noCoverage	0.017228	1	insignificant	0	19	19
chr17	66235095	66237095	Ralbp1		1	noCoverage	-0.018544	0.77911	insignificant	0	6	6
chr17	66426386	66428386	Ankrd12	-0.091877	2.28E-24	hypomethylated	-0.012943	0.063882	insignificant	29	90	90
chr17	66450831	66452831	Ndufv2	-0.23759	8.23E-20	hypomethylated	-0.0025629	0.63858	insignificant	19	68	69
chr17	66459885	66461885	ORF19	-0.17572	2.29E-29	hypomethylated	-0.016836	0.056494	insignificant	18	48	50
chr17	66459984	66461984	ORF19	-0.17572	2.29E-29	hypomethylated	-0.016836	0.056494	insignificant	18	48	50
chr17	66471859	66473859	Ddx11	-0.20687	0.00000191	hypomethylated	0.0073328	0.36508	insignificant	10	48	48
chr17	66799090	66801090	1110012J17Rik	-0.13861	0.012204	hypomethylated	-0.011696	0.90152	insignificant	9	40	40
chr17	66869010	66871010	Rab12	-0.16667	0.049734	hypomethylated	0.027327	0.67129	insignificant	3	6	6
chr17	67703799	67705799	Ptprm	-0.077165	1.21E-17	hypomethylated	0.010957	0.70189	insignificant	33	108	108
chr17	68045604	68047604	Lama1	-0.14188	0.00045866	hypomethylated	-0.010332	0.59895	insignificant	9	22	22
chr17	68353448	68355448	Arhgap28		1	noCoverage	-0.018588	0.75694	insignificant	0	14	14
chr17	68622136	68624136	L3mbtl4	-0.18675	5.86E-11	hypomethylated	-0.035899	0.014147	hypomethylated	13	49	44
chr17	69505149	69507149	Epb4.1l3	-0.098343	2.4E-27	hypomethylated	-0.0068717	0.84909	insignificant	33	138	138
chr17	69732317	69734317	Zfp161	-0.096678	1.81E-29	hypomethylated	-0.0081915	0.0030243	hypomethylated	55	201	219
chr17	69764786	69766786	C030034I22Rik	-0.16154	4.2E-09	hypomethylated	-0.0053101	0.25258	insignificant	9	42	42
chr17	69787665	69789665	A330050F15Rik	-0.15723	1.69E-34	hypomethylated	-0.0019042	0.8518	insignificant	37	117	118
chr17	70870449	70872449	Dlgap1	-0.51251	0.022601	stronglyHypometh	0.014932	0.64611	insignificant	2	16	14
chr17	71199130	71201130	Tgif1	-0.30159	6.41E-24	hypomethylated	-0.086326	0.17686	insignificant	16	78	82
chr17	71200550	71202550	Tgif1	-0.17398	2.35E-20	hypomethylated	-0.012574	0.3143	insignificant	22	110	110
chr17	71201074	71203074	Tgif1	-0.17223	7.7E-20	hypomethylated	-0.02753	0.65683	insignificant	22	141	144
chr17	71202872	71204872	Tgif1	-0.59609	0.00095538	stronglyHypometh	-0.0136	0.000034847	inconclusive	1	32	29
chr17	71339856	71341856	Myl12b	-0.11171	0.000003492	hypomethylated	-0.0051372	0.090687	insignificant	15	68	67
chr17	71351873	71353873	Myl12a	-0.14086	0.000019594	hypomethylated	-0.011161	0.28566	insignificant	6	42	40
chr17	71532317	71534317	Lpin2	-0.1015	3.07E-17	hypomethylated	0.0013774	1	insignificant	46	153	157
chr17	71660305	71662305	Emilin2	-0.14421	0.00000777	hypomethylated	-0.037291	0.34355	insignificant	9	28	24
chr17	71691144	71693144	Gm4566	0.1715	1	insignificant	-0.053073	0.89122	insignificant	3	23	20
chr17	71824683	71826683	Smchd1	-0.09945	8.45E-10	hypomethylated	-0.013076	0.14513	insignificant	25	96	96
chr17	71876197	71878197	Ndc80	-0.073199	0.00085992	hypomethylated	0.013945	0.34309	insignificant	16	74	72
chr17	71900400	71902400	Spdya	-0.11117	4.18E-38	hypomethylated	-0.0051772	0.22827	insignificant	41	134	134
chr17	71947621	71949621	Trmt61b	-0.18312	8.42E-37	hypomethylated	-0.021756	0.091024	insignificant	28	106	105
chr17	71948101	71950101	Trmt61b	-0.23413	1.1E-15	hypomethylated	-0.031169	0.050504	insignificant	11	56	55
chr17	71964554	71966554	Wdr43	-0.15398	1.07E-29	hypomethylated	-0.00028964	0.15088	insignificant	41	159	161
chr17	72130286	72132286	Clip4	-0.15385	4.25E-16	hypomethylated	0.014908	3.86E-08	inconclusive	22	67	61
chr17	72953647	72955647	Alk	-0.13531	0.39325	insignificant	0.029883	0.70203	insignificant	2	11	19
chr17	73185043	73187043	Ypel5	-0.11784	9.64E-34	hypomethylated	-0.004103	0.196	insignificant	61	240	233
chr17	73266644	73268644	Lbh	-0.15111	1.17E-31	hypomethylated	-0.012256	0.49185	insignificant	23	139	136
chr17	73456324	73458324	Lclat1	-0.065048	2.67E-12	hypomethylated	0.0043222	0.37647	insignificant	30	108	108
chr17	74059791	74061791	Galnt14	-0.063515	1.54E-19	hypomethylated	-0.0047734	0.79679	insignificant	11	58	58
chr17	74153180	74155180	Ehd3	-0.31187	2.36E-14	hypomethylated	-0.0087017	0.36963	insignificant	16	58	54
chr17	74694203	74696203	Memo1	-0.098942	6.65E-14	hypomethylated	0.0041545	0.0013769	inconclusive	28	129	135
chr17	74715551	74717551	Dpy30		1	noCoverage	0.0042857	1	insignificant	0	10	10
chr17	74715734	74717734	Dpy30		1	noCoverage	0.0042857	1	insignificant	0	10	10
chr17	74715773	74717773	Dpy30		1	noCoverage	0.0042857	1	insignificant	0	10	10
chr17	74737326	74739326	Spast	-0.13559	2.98E-34	hypomethylated	-0.021852	0.17379	insignificant	47	150	171
chr17	74793971	74795971	Slc30a6	-0.16819	0.00015978	hypomethylated	0.0015371	0.57521	insignificant	13	40	41
chr17	74887853	74889853	Yipf4	-0.1678	1.32E-17	hypomethylated	0.012247	0.16806	insignificant	21	70	66
chr17	74926634	74928634	Birc6	-0.11583	9.76E-49	hypomethylated	-0.013719	0.0066515	hypomethylated	62	202	204
chr17	75116089	75118089	Ttc27	-0.19662	0.000017982	hypomethylated	-0.025833	0.90142	insignificant	8	43	40
chr17	75403868	75405868	Ltbp1	-0.14551	7.86E-51	hypomethylated	-0.0090138	0.055046	insignificant	62	175	175
chr17	75577221	75579221	Ltbp1	0.16637	0.26403	insignificant	0.12158	0.00055974	hypermethylated	2	12	12
chr17	75951286	75953286	Fam98a	-0.23744	0.061961	insignificant	0.0069337	0.60472	insignificant	2	4	4
chr17	78598587	78600587	Crim1	-0.11195	7.16E-21	hypomethylated	0.0043004	0.39472	insignificant	54	182	192
chr17	78906402	78908402	Vit		1	noCoverage	0.010443	1	insignificant	0	5	5
chr17	79135900	79137900	Strn	-0.20711	6.09E-11	hypomethylated	-0.034036	0.03804	hypomethylated	16	40	38
chr17	79233855	79235855	Ccdc75	-0.11804	1.44E-16	hypomethylated	0.0082576	0.57041	insignificant	27	82	82
chr17	79234721	79236721	Heatr5b	-0.091783	0.063226	insignificant	0.0054061	0.92954	insignificant	6	26	26
chr17	79281912	79283912	Eif2ak2	-0.33785	0.0000018	stronglyHypometh	-0.10776	0.10268	insignificant	6	24	21
chr17	79314838	79316838	1110001A16Rik	-0.28701	4.3E-09	hypomethylated	-0.011713	0.8709	insignificant	5	14	14
chr17	79335474	79337474	2410091C18Rik	-0.10118	8.37E-09	hypomethylated	0.014771	0.48543	insignificant	12	42	37
chr17	79336410	79338410	2410091C18Rik	-0.16481	0.032298	hypomethylated	0.068027	0.48852	insignificant	4	16	18
chr17	79420156	79422156	Prkd3	-0.10169	8.26E-08	hypomethylated	0.0058756	0.49568	insignificant	18	80	80
chr17	79754431	79756431	Cdc42ep3	-0.11666	1.3E-20	hypomethylated	-0.0068698	0.00025953	hypomethylated	24	129	136
chr17	80013239	80015239	Fam82a1	-0.16318	0.000009767	hypomethylated	0.032064	0.30878	insignificant	33	87	93
chr17	80295368	80297368	Atl2	-0.082995	0.00000889	hypomethylated	-0.026933	0.13275	insignificant	13	50	50
chr17	80295463	80297463	Atl2	-0.030265	0.0083474	hypomethylated	-0.012105	0.25355	insignificant	7	28	28
chr17	80461674	80463674	Hnrpll	-0.083448	0.00053576	hypomethylated	-0.0085029	0.47318	insignificant	8	52	52
chr17	80606645	80608645	Srsf7	-0.12118	0.59883	insignificant	0.018083	0.51291	insignificant	6	42	42
chr17	80622828	80624828	Gemin6	-0.46656	0.27542	insignificant	-0.014147	0.6344	insignificant	3	20	20
chr17	80688551	80690551	Morn2	-0.18702	3.16E-12	hypomethylated	-0.0071351	0.60061	insignificant	17	55	55
chr17	80689816	80691816	Morn2	-0.46804	2.04E-09	stronglyHypometh	0.1521	0.5077	insignificant	2	11	15
chr17	80705746	80707746	Arhgef33		1	noCoverage	0.10753	0.20039	insignificant	0	8	8
chr17	80772882	80774882	Gm10190	0.10107	0.049101	hypermethylated	0.036513	2.34E-08	hypermethylated	12	88	88
chr17	80879793	80881793	Sos1	-0.13753	1.74E-10	hypomethylated	-0.00033006	0.50895	insignificant	9	26	26
chr17	80963174	80965174	Cdkl4		1	noCoverage	0.061385	0.036492	hypermethylated	0	22	27
chr17	81127433	81129433	Map4k3	-0.15348	0.00044307	hypomethylated	0.012322	0.2636	insignificant	15	39	39
chr17	81342971	81344971	Tmem178	-0.10396	2.3E-19	hypomethylated	-0.00848	0.0063156	hypomethylated	46	152	152

chr17	81464425	81466425	Thumpd2	-0.16955	0.13244	insignificant	-0.069948	3.72E-18	hypomethylated	12	45	43
chr17	82137717	82139717	Slc8a1	-0.35974	0.014869	stronglyHypometh	-0.028318	0.73291	insignificant	3	16	16
chr17	83613622	83615622	Pkdcc	-0.12019	3.78E-42	hypomethylated	0.013393	0.00089567	inconclusive	56	165	171
chr17	83749270	83751270	Eml4	-0.088059	5.67E-13	hypomethylated	0.018875	0.35558	insignificant	23	76	82
chr17	83913673	83915673	Cox7a2l		1	noCoverage	0.059006	1	insignificant	0	2	2
chr17	84031235	84033235	Kcng3	-0.17672	9.79E-63	hypomethylated	-0.0073552	0.000051656	hypomethylated	37	94	95
chr17	84104502	84106502	Mta3	-0.10242	0.001203	hypomethylated	0.000069294	0.77169	insignificant	12	74	95
chr17	84587287	84589287	Zfp36l2	-0.13749	0.0050654	hypomethylated	0.015187	0.67059	insignificant	10	57	56
chr17	84910234	84912234	Plekhh2	-0.15478	2.31E-25	hypomethylated	0.008318	0.73491	insignificant	30	91	93
chr17	85081470	85083470	Abcg8	-0.77596	0.22963	lowCoverage	-0.12079	0.032806	hypomethylated	1	6	6
chr17	85082263	85084263	Abcg5	-0.54483	0.061572	insignificant	-0.047048	0.057348	insignificant	2	10	10
chr17	85190126	85192126	Lrpprc	-0.11021	1	insignificant	0.0029389	1	insignificant	3	10	10
chr17	85355080	85357080	Ppm1b	-0.34135	0.01032	stronglyHypometh	0.0069613	0.2316	insignificant	2	28	29
chr17	85356340	85358340	Ppm1b	-0.086949	4.65E-13	hypomethylated	-0.0013441	0.14277	insignificant	33	159	160
chr17	85356763	85358763	1110020A21Rik	-0.071678	5.95E-11	hypomethylated	-0.001282	0.45123	insignificant	31	145	145
chr17	85357050	85359050	1110020A21Rik	-0.071678	5.95E-11	hypomethylated	-0.001282	0.45123	insignificant	31	145	145
chr17	85426686	85428686	Slc3a1	-0.43843	0.039784	stronglyHypometh	-0.050923	0.077772	insignificant	2	14	14
chr17	85489039	85491039	1700106N22Rik	-0.11102	2.11E-08	hypomethylated	-0.0053375	0.94729	insignificant	26	100	103
chr17	85489607	85491607	1700106N22Rik	-0.11821	3.04E-09	hypomethylated	-0.0098283	0.19906	insignificant	17	70	75
chr17	85489614	85491614	1700106N22Rik	-0.11821	3.04E-09	hypomethylated	-0.0098283	0.19906	insignificant	16	68	73
chr17	86017615	86019615	Six3	-0.26431	1.36E-10	hypomethylated	-0.031673	0.3421	insignificant	11	45	50
chr17	86017705	86019705	Six3	-0.28939	0.0005536	hypomethylated	-0.045847	0.36135	insignificant	8	37	42
chr17	86018751	86020751	Six3	-0.19303	0.018307	hypomethylated	0.056382	0.21715	insignificant	2	22	18
chr17	86019173	86021173	Six3	-0.16617	1.82E-13	hypomethylated	0.00017474	0.7052	insignificant	19	64	60
chr17	86087594	86089594	Six2	-0.14732	6.97E-36	hypomethylated	-0.011001	0.032899	hypomethylated	34	136	132
chr17	86544515	86546515	Srbd1	-0.28401	0.010434	hypomethylated	0.033873	0.0079683	inconclusive	10	24	17
chr17	86566124	86568124	Prkce	-0.11424	3.25E-37	hypomethylated	-0.0069254	0.67635	insignificant	80	235	249
chr17	87152203	87154203	Epas1	-0.096587	2.07E-09	hypomethylated	-0.011647	0.97531	insignificant	35	126	131
chr17	87347227	87349227	Atp6v1e2	-0.85333	0.00011576	stronglyHypometh	0.036606	0.4853	insignificant	2	10	9
chr17	87361450	87363450	Rhoq	-0.11153	4.23E-22	hypomethylated	0.0033402	0.42459	insignificant	59	148	163
chr17	87423900	87425900	Cript	-0.18281	1.35E-15	hypomethylated	-0.023905	0.016305	inconclusive	31	66	102
chr17	87424741	87426741	Pigf	-0.21229	0.000000736	hypomethylated	0.0086811	0.80693	insignificant	5	30	40
chr17	87506018	87508018	Socs5	-0.13456	1.01E-59	hypomethylated	-0.0040108	0.00098382	hypomethylated	65	175	175
chr17	87665287	87667287	Mcfd2	-0.27224	2.17E-31	hypomethylated	-0.05145	0.95667	insignificant	10	29	36
chr17	87681225	87683225	Ttc7	-0.15057	1.51E-39	hypomethylated	0.0045711	1	insignificant	53	172	178
chr17	87682154	87684154	4833418N02Rik	-0.14505	2.43E-18	hypomethylated	0.010638	0.85403	insignificant	24	96	98
chr17	87846275	87848275	Calm2	-0.12268	0.0002673	hypomethylated	-0.0092902	0.58644	insignificant	11	48	52
chr17	88034318	88036318	Epcam	-0.12724	1	insignificant	0.09108	0.27779	insignificant	8	53	40
chr17	88070896	88072896	Msh2	-0.11433	4.06E-10	hypomethylated	-0.0095637	0.6688	insignificant	25	102	107
chr17	88197334	88199334	Kcnk12	-0.14286	2.81E-11	hypomethylated	0.0031977	0.24931	insignificant	21	87	83
chr17	88373389	88375389	Msh6	-0.12645	5.5E-35	hypomethylated	-0.016038	4.82E-17	hypomethylated	41	143	159
chr17	88464625	88466625	Fbxo11	-0.10476	2.42E-20	hypomethylated	-0.0015192	0.10055	insignificant	39	119	128
chr17	88839051	88841051	Foxn2	-0.12848	2.23E-51	hypomethylated	0.0033288	0.0044083	hypermethylated	67	132	149
chr17	88928463	88930463	Klraq1	-0.10038	1.05E-11	hypomethylated	-0.013125	0.36098	insignificant	28	87	87
chr17	89024894	89026894	Ston1		1	noCoverage	-0.25331	0.043424	hypomethylated	0	8	8
chr17	89066999	89068999	Gtf2a1l	0.080225	0.61264	insignificant	0.12804	0.070324	insignificant	9	35	28
chr17	89191316	89193316	Lhcgr	-0.23759	0.017879	hypomethylated	0.14727	0.26156	insignificant	9	28	26
chr17	91492142	91494142	Nrxn1	-0.17587	0.0002161	hypomethylated	0.030446	0.54003	insignificant	5	14	18
chr17	93597761	93599761	Adcyap1	-0.62404	0.000000554	stronglyHypometh	-0.056124	0.79412	insignificant	4	10	10
chr17	95148439	95150439	2700099C18Rik	-0.12514	1.73E-09	hypomethylated	-0.025587	0.0075073	hypomethylated	11	60	66
chr17	95149232	95151232	Mettl4	-0.098494	0.000019073	hypomethylated	-0.035002	0.042877	hypomethylated	9	22	28
chr17	95233760	95235760	2610044O15Rik	-0.24544	0.000000343	hypomethylated	-0.040817	0.91156	insignificant	6	16	18
chr18	3335413	3337413	Gm6225	-0.10614	4.23E-14	hypomethylated	-0.011709	0.93752	insignificant	28	96	97
chr18	3337587	3339587	Gm6225	-0.11839	0.60946	insignificant	0.02202	0.8509	insignificant	3	42	47
chr18	3382222	3384222	Cul2	-0.14746	9.05E-26	hypomethylated	0.010801	0.021403	inconclusive	24	75	66
chr18	3506954	3508954	Bambi	-0.093625	4.53E-23	hypomethylated	-0.0032291	0.62664	insignificant	55	159	158
chr18	4352951	4354951	Map3k8	-0.19196	9.54E-16	hypomethylated	-0.033748	0.044416	hypomethylated	18	66	76
chr18	4374589	4376589	Mtpap	-0.11748	1	insignificant	-0.0087296	0.42407	insignificant	3	23	22
chr18	4633926	4635926	9430020K01Rik	-0.13652	1.53E-51	hypomethylated	-0.0015415	0.77713	insignificant	55	122	127
chr18	5045586	5047586	Svil		1	noCoverage	0.055556	0.7226	insignificant	0	4	6
chr18	5334437	5336437	Zfp438	-0.12713	1.1E-11	hypomethylated	0.019159	0.83163	insignificant	17	57	49
chr18	5590888	5592888	Gm10125	-0.099116	8.99E-33	hypomethylated	-0.0074381	0.55301	insignificant	51	139	159
chr18	5592435	5594435	Gm10125	-0.096481	1.21E-13	hypomethylated	-0.005077	0.043949	hypomethylated	32	104	104
chr18	6136096	6138096	Arhgap12	-0.31167	3.85E-10	hypomethylated	-0.0086982	1	insignificant	4	8	8
chr18	6241522	6243522	Kif5b	-0.10951	0.036395	hypomethylated	0.018011	0.00021233	hypermethylated	4	22	25
chr18	6490644	6492644	Epc1	-0.18859	2.74E-10	hypomethylated	0.0011426	0.22632	insignificant	35	106	107
chr18	6490854	6492854	Epc1	-0.20347	6.31E-09	hypomethylated	-0.0010795	0.10488	insignificant	33	90	91
chr18	6516085	6518085	Epc1	-0.12183	0.0016103	hypomethylated	0.0050521	0.67247	insignificant	10	51	52
chr18	7004777	7006777	Mkx	-0.23585	3.14E-26	hypomethylated	-0.0069879	0.055403	insignificant	19	50	51
chr18	7867855	7869855	Wac	-0.10424	4.31E-43	hypomethylated	-0.006995	0.90805	insignificant	34	198	190
chr18	7868194	7870194	Wac	-0.10583	1.77E-51	hypomethylated	-0.011753	0.082565	insignificant	50	240	235
chr18	9211853	9213853	Fzd8	-0.11343	1.78E-36	hypomethylated	-0.0065167	0.040846	hypomethylated	54	180	180
chr18	9450148	9452148	Ccny	-0.094048	0.00016367	hypomethylated	0.030379	0.55215	insignificant	10	43	42
chr18	9706645	9708645	Colec12	-0.11249	0.000073125	hypomethylated	-0.026037	0.13374	insignificant	23	107	99
chr18	9957177	9959177	Thoc1	-0.22318	4.15E-11	hypomethylated	-0.013058	0.46735	insignificant	20	81	81
chr18	10181790	10183790	Rock1	-0.064822	0.054435	insignificant	0.0087445	0.095247	insignificant	12	98	111
chr18	10324176	10326176	Greb1l	-0.10049	7.13E-20	hypomethylated	-0.018461	0.033947	hypomethylated	31	88	92
chr18	10610350	10612350	Esco1	-0.41003	1	insignificant	-0.045491	0.26705	insignificant	4	30	25
chr18	10616793	10618793	Snrpd1	-0.14185	1.02E-15	hypomethylated	-0.0056951	0.34772	insignificant	30	94	97
chr18	10724622	10726622	Mib1	-0.1043	3E-38	hypomethylated	-0.0096967	0.38874	insignificant	62	154	182
chr18	11051507	11053507	Gata6	-0.15628	0.000036375	hypomethylated	0.031245	0.30779	insignificant	3	48	50
chr18	11815350	11817350	Rbbp8	-0.1416	2.13E-21	hypomethylated	-0.013577	0.069585	insignificant	34	129	133
chr18	11996782	11998782	Cables1	-0.098035	5.92E-39	hypomethylated	-0.010009	0.51601	insignificant	63	206	224
chr18	11998947	12000947	Cables1	-0.21555	0.000018597	hypomethylated	0.007294	0.90566	insignificant	12	32	32
chr18	12279962	12281962	6030446N20Rik	-0.18478	1.38E-10	hypomethylated	0.00029498	0.0023419	inconclusive	6	18	18

chr18	12286358	12288358	Riok3	-0.16236	6.13E-15	hypomethylated	-0.011457	0.000038326	hypomethylated	26	85	94
chr18	12326238	12328238	3110002H16Rik	-0.19794	2.38E-34	hypomethylated	-0.014477	5.72E-08	hypomethylated	28	106	107
chr18	12394895	12396895	Npc1	-0.17014	0.00000116	hypomethylated	-0.011431	0.23501	insignificant	16	46	46
chr18	12464229	12466229	Ankrd29	-0.10592	0.000000637	hypomethylated	0.029295	0.42419	insignificant	5	10	10
chr18	12491532	12493532	Lama3	-0.17539	6.34E-10	hypomethylated	-0.027948	0.011908	hypomethylated	11	44	44
chr18	12801041	12803041	Ttc39c	-0.18198	6.46E-26	hypomethylated	-0.024801	1	insignificant	20	35	47
chr18	12872319	12874319	Mir1948	-0.2635	0.069643	insignificant	-0.040761	1	insignificant	2	6	6
chr18	12898863	12900863	Cabyr	-0.16619	4.34E-09	hypomethylated	0.0032665	0.6286	insignificant	12	53	54
chr18	13100229	13102229	Osbpl1a	-0.081805	0.00000001	hypomethylated	0.0082124	1	insignificant	10	42	42
chr18	13129760	13131760	Impact	-0.16376	0.68034	insignificant	-0.13841	0.064282	insignificant	3	22	17
chr18	14131242	14133242	Zfp521	-0.011183	0.17506	insignificant	-0.064887	0.85721	insignificant	2	12	12
chr18	14841423	14843423	Ss18	-0.13006	7.53E-11	hypomethylated	0.0084021	0.42324	insignificant	13	42	41
chr18	14863659	14865659	Psma8		1	noCoverage	0.067823	0.60518	insignificant	0	19	16
chr18	14940753	14942753	Taf4b	-0.16231	2.43E-20	hypomethylated	-0.01297	0.14956	insignificant	26	104	118
chr18	15222091	15224091	Kctd1	-0.20921	0.000000138	hypomethylated	-0.008309	0.4235	insignificant	16	54	54
chr18	15562193	15564193	Aqp4	0.29365	1	insignificant	0.061508	1	insignificant	2	6	7
chr18	16967558	16969558	Cdh2	-0.11817	3.19E-18	hypomethylated	0.0028057	1	insignificant	27	79	80
chr18	20404840	20406840	Dsg1c		1	noCoverage	0.029365	0.51519	insignificant	0	6	8
chr18	20715616	20717616	Dsg2	-0.21779	0.0040849	hypomethylated	-0.0090722	0.23491	insignificant	4	44	44
chr18	20904905	20906905	B4galt6	-0.028293	0.66882	insignificant	0.011288	0.40212	insignificant	8	20	20
chr18	21054579	21056579	Trappc8	-0.13173	2.16E-11	hypomethylated	0.035731	0.44913	insignificant	26	87	86
chr18	21102125	21104125	Rnf125	0.27995	0.00045305	hypermethylated	0.085083	0.0093292	hypermethylated	5	27	31
chr18	21158841	21160841	Rnf138	-0.10902	3.71E-28	hypomethylated	0.0026069	0.36105	insignificant	39	123	124
chr18	21229844	21231844	Mep1b	-0.325	0.02159	hypomethylated	-0.076923	0.75857	insignificant	2	5	4
chr18	21458640	21460640	Fam59a	-0.13629	8.53E-12	hypomethylated	0.00087803	0.020042	inconclusive	22	56	56
chr18	21810869	21812869	Klhl14	-0.15908	0.00013017	hypomethylated	0.0009682	0.57419	insignificant	12	83	86
chr18	22502589	22504589	Asxl3	-0.11184	5.04E-25	hypomethylated	-0.003974	0.9723	insignificant	34	127	109
chr18	23197164	23199164	Nol4	-0.13783	4.23E-08	hypomethylated	0.01714	0.82302	insignificant	14	66	62
chr18	23200154	23202154	Nol4	-0.14498	0.44341	insignificant	-0.019278	0.88274	insignificant	2	21	18
chr18	23909833	23911833	Mapre2	-0.1658	2.59E-17	hypomethylated	-0.024285	0.25045	insignificant	16	69	70
chr18	23911225	23913225	Mapre2	-0.2609	1	insignificant	-0.0022054	1	insignificant	2	19	19
chr18	23961470	23963470	Mapre2	-0.11445	2.71E-12	hypomethylated	-0.015295	0.31935	insignificant	22	78	85
chr18	24112188	24114188	Zfp397	-0.1367	0.0096223	hypomethylated	-0.042246	0.044396	hypomethylated	14	87	88
chr18	24147134	24149134	Zfp35		1	noCoverage	-0.012859	0.85703	insignificant	0	18	18
chr18	24179272	24181272	Zfp191	-0.10476	0.083567	insignificant	0.031648	0.17599	insignificant	14	40	40
chr18	24280320	24282320	Ino80c	-0.12888	1.21E-08	hypomethylated	0.044567	0.19976	insignificant	12	39	39
chr18	24362844	24364844	Galnt1	-0.086792	3.96E-50	hypomethylated	-0.0041244	0.18372	insignificant	75	220	219
chr18	24363494	24365494	Galnt1	-0.090185	2.64E-52	hypomethylated	-0.0082914	0.058684	insignificant	75	224	222
chr18	24628371	24630371	2700062C07Rik	-0.25288	1.43E-20	hypomethylated	-0.021597	0.32095	insignificant	16	41	47
chr18	24688705	24690705	Rprd1a	-0.43631	0.11544	insignificant	0.023988	0.27379	insignificant	3	20	28
chr18	24761461	24763461	Elp2	-0.11035	5.61E-25	hypomethylated	-0.0033901	0.59693	insignificant	46	161	165
chr18	24762318	24764318	Elp2	-0.1547	0.21173	insignificant	0.00000363	0.60778	insignificant	7	37	38
chr18	24811191	24813191	Mocos	-0.17495	5.62E-17	hypomethylated	0.011584	0.10438	insignificant	11	49	47
chr18	24866945	24868945	Fhod3	-0.15129	8.83E-34	hypomethylated	-0.012647	0.16944	insignificant	49	151	154
chr18	25326520	25328520	AW554918	-0.13035	1.07E-24	hypomethylated	-0.024636	0.036134	hypomethylated	51	112	126
chr18	25327378	25329378	5730494M16Ril	-0.13417	9.73E-15	hypomethylated	-0.013197	0.33504	insignificant	29	73	72
chr18	25912484	25914484	Celf4	-0.095121	2.79E-10	hypomethylated	-0.014506	0.91324	insignificant	32	117	117
chr18	31793036	31795036	Sap130	-0.13604	6.2E-30	hypomethylated	-0.0047501	0.34437	insignificant	29	129	129
chr18	31918533	31920533	Ammecr1l	-0.18059	1.85E-15	hypomethylated	-0.0072781	0.006946	hypomethylated	17	88	88
chr18	31947812	31949812	Polr2d	-0.10869	0.00021159	hypomethylated	0.011744	0.68967	insignificant	8	44	42
chr18	31947829	31949829	Polr2d	-0.10869	0.00021159	hypomethylated	0.011744	0.68967	insignificant	8	44	42
chr18	31962710	31964710	Wdr33	-0.12342	0.000000116	hypomethylated	-0.028505	0.045551	hypomethylated	15	93	98
chr18	32071557	32073557	Sft2d3	-0.87861	0.00060411	stronglyHypometh	-0.089515	0.75225	insignificant	4	12	11
chr18	32090160	32092160	Lims2	-0.072018	4.23E-11	hypomethylated	-0.0056389	0.26438	insignificant	9	48	48
chr18	32196585	32198585	Myo7b		1	noCoverage	-0.26032	0.00005122	hypomethylated	0	6	3
chr18	32226387	32228387	Iws1	-0.17002	2.18E-20	hypomethylated	-0.0010716	0.082371	insignificant	20	73	73
chr18	32321742	32323742	Map3k2	-0.11607	1.71E-27	hypomethylated	0.0074268	0.037337	inconclusive	34	131	119
chr18	32398984	32400984	Ercc3	-0.21064	1.91E-15	hypomethylated	0.014233	0.90957	insignificant	11	44	36
chr18	32535870	32537870	Bin1	-0.15689	1.88E-15	hypomethylated	0.0020309	0.25747	insignificant	18	84	78
chr18	32975513	32977513	Tslp	-0.25621	5.38E-17	hypomethylated	0.0014051	0.47785	insignificant	7	26	24
chr18	32995878	32997878	Wdr36	-0.12176	6.07E-20	hypomethylated	-0.010371	0.13578	insignificant	20	60	60
chr18	33097694	33099694	Camk4	-0.11147	5.9E-13	hypomethylated	0.0062462	0.29856	insignificant	23	99	94
chr18	33623075	33625075	D0H4S114	-0.15762	2.86E-08	hypomethylated	-0.026226	0.35183	insignificant	22	35	34
chr18	33623288	33625288	D0H4S114	-0.15762	2.86E-08	hypomethylated	-0.026226	0.35183	insignificant	22	35	34
chr18	33623401	33625401	D0H4S114	-0.15762	2.86E-08	hypomethylated	-0.026226	0.35183	insignificant	22	35	34
chr18	33623683	33625683	D0H4S114	-0.17134	0.000006222	hypomethylated	-0.033835	0.41239	insignificant	7	23	22
chr18	34166860	34168860	Epb4.1l4a	-0.36739	4E-31	stronglyHypometh	0.029665	0.000075987	inconclusive	12	34	35
chr18	34379637	34381637	Apc	-0.14937	3.82E-38	hypomethylated	0.0075712	0.068652	insignificant	43	116	114
chr18	34489798	34491798	Srp19	-0.18653	8.28E-35	hypomethylated	-0.028879	0.029863	hypomethylated	41	113	108
chr18	34533069	34535069	Reep5	-0.45663	0.000000048	stronglyHypometh	-0.00055827	0.00088932	hypomethylated	4	35	38
chr18	34568076	34570076	Pkd2l2	-0.049821	0.17822	insignificant	-0.089736	0.67049	insignificant	3	16	16
chr18	34666477	34668477	Fam13b	-0.15066	0.22984	insignificant	0.19811	0.21784	insignificant	8	36	36
chr18	34700981	34702981	Wnt8a	-0.28735	0.01169	hypomethylated	-0.21083	4.14E-10	hypomethylated	2	32	22
chr18	34757122	34759122	4933408B17Rik		1	noCoverage	0.13698	0.50211	insignificant	0	7	7
chr18	34783277	34785277	Kif20a	-0.21493	1.06E-16	hypomethylated	-0.016356	0.52536	insignificant	9	66	68
chr18	34783589	34785589	Kif20a	-0.19821	1.04E-16	hypomethylated	-0.015622	0.49571	insignificant	9	64	63
chr18	34783686	34785686	Kif20a	-0.19821	1.04E-16	hypomethylated	-0.015622	0.49571	insignificant	9	64	63
chr18	34784464	34786464	Kif20a	0.022846	0.73982	insignificant	-0.020087	0.00000608	inconclusive	4	37	37
chr18	34811390	34813390	Cdc23	0.32088	1	lowCoverage	0.1591	0.36929	insignificant	1	25	24
chr18	34880041	34882041	Gfra3	-0.21242	0.042706	hypomethylated	-0.040431	0.021452	hypomethylated	4	12	12
chr18	34911187	34913187	Cdc25c		1	noCoverage	0.088719	0.85154	insignificant	0	10	7
chr18	34917559	34919559	Fam53c	-0.20278	4.47E-20	hypomethylated	0.01111	0.60181	insignificant	28	91	92
chr18	34935661	34937661	Kdm3b	-0.13477	3.09E-28	hypomethylated	0.0043903	0.75799	insignificant	43	137	137
chr18	34999311	35001311	Reep2	-0.16863	0.0026003	hypomethylated	-0.048685	0.63956	insignificant	12	34	34
chr18	35019860	35021860	Egr1	-0.15043	4.02E-29	hypomethylated	0.0036505	0.49907	insignificant	31	92	92

chr18	35091657	35093657	Etf1	-0.092432	1	insignificant	0.023553	0.88162	insignificant	4	66	66
chr18	35114005	35116005	Hspa9	-0.10898	2E-19	hypomethylated	-0.010855	0.00000013	hypomethylated	36	127	128
chr18	35277565	35279565	Ctnna1	-0.17922	1.78E-16	hypomethylated	0.0098799	0.498	insignificant	29	146	131
chr18	35713220	35715220	Mir1949	-0.1758	0.038528	hypomethylated	-0.036047	0.099257	insignificant	7	28	26
chr18	35720811	35722811	Matr3	-0.099533	0.00059479	hypomethylated	-0.017563	0.95575	insignificant	11	65	93
chr18	35757320	35759320	Paip2	-0.12309	0.0012069	hypomethylated	0.0025214	0.5776	insignificant	9	62	58
chr18	35786881	35788881	Slc23a1	-0.2627	0.016807	hypomethylated	-0.092648	0.3607	insignificant	1	12	12
chr18	35809021	35811021	2010001M09Ril	0.117	1	insignificant	-0.17073	0.17087	insignificant	3	6	9
chr18	35821840	35823840	Spata24	-0.5967	0.000000159	stronglyHypometh	0.063452	0.68587	insignificant	1	4	4
chr18	35862798	35864798	Dnajc18	-0.15671	0.054507	insignificant	-0.045631	0.25946	insignificant	7	22	27
chr18	35881145	35883145	Ecscr	-0.18629	0.27821	insignificant	-0.13691	0.41157	insignificant	2	10	13
chr18	35900208	35902208	Tmem173	0.24812	0.16646	insignificant	0.090394	0.00046506	hypermethylated	2	13	13
chr18	35930212	35932212	Ube2d2	-0.13007	2.96E-32	hypomethylated	0.0035437	0.57002	insignificant	40	158	163
chr18	35988471	35990471	Cxxc5	-0.080935	4.22E-21	hypomethylated	-0.00063308	0.39934	insignificant	55	180	180
chr18	36123488	36125488	Psd2	-0.2799	0.000000004	hypomethylated	-0.010199	0.21702	insignificant	10	58	58
chr18	36356814	36358814	Nrg2	-0.18586	2.33E-12	hypomethylated	-0.017273	0.4621	insignificant	10	32	32
chr18	36439815	36441815	Pura	-0.11325	3.88E-16	hypomethylated	0.0018804	0.5591	insignificant	36	105	105
chr18	36507277	36509277	0610010O12Rik	-0.071175	1.66E-12	hypomethylated	0.013388	0.97497	insignificant	20	105	113
chr18	36614149	36616149	Pfdn1	-0.18849	0.11434	insignificant	0.055882	1	insignificant	5	14	18
chr18	36675459	36677459	Hbegf	-0.21106	0.000000972	hypomethylated	-0.00068428	0.92294	insignificant	2	10	10
chr18	36686805	36688805	Slc4a9	-0.12308	0.25733	insignificant	0.00039329	1	insignificant	1	5	7
chr18	36719256	36721256	Ankhd1	-0.11986	2.43E-50	hypomethylated	-0.0027052	0.33482	insignificant	44	174	165
chr18	36822713	36824713	Eif4ebp3	-0.035524	1	insignificant	-0.015091	0.81511	insignificant	9	22	22
chr18	36829965	36831965	Sra1	-0.27469	0.00086078	hypomethylated	0.090232	1	insignificant	2	6	6
chr18	36837868	36839868	Slc35a4	-0.14792	1.68E-14	hypomethylated	-0.024299	0.51259	insignificant	8	54	60
chr18	36839020	36841020	Slc35a4		1	noCoverage	-0.16667	0.76812	insignificant	0	3	6
chr18	36893723	36895723	Tmco6	-0.064734	0.63523	insignificant	0.033495	0.000034702	hypermethylated	13	72	73
chr18	36903309	36905309	Ik	-0.1621	0.58101	insignificant	0.028098	0.80625	insignificant	3	59	65
chr18	36904202	36906202	Ik	-0.17009	0.57828	insignificant	0.04536	0.59332	insignificant	3	43	47
chr18	36918892	36920892	Wdr55	-0.29775	0.0000094	hypomethylated	-0.011071	0.10247	insignificant	4	32	39
chr18	36941933	36943933	Hars2	-0.14344	2.11E-14	hypomethylated	0.0052899	0.218	insignificant	11	74	71
chr18	36942859	36944859	Hars	-0.15062	2.13E-09	hypomethylated	0.015252	1	insignificant	8	40	39
chr18	36952576	36954576	Zmat2	-0.22086	0.34515	insignificant	0.030993	0.24082	insignificant	3	22	22
chr18	36960416	36962416	Vaultrc5	-0.20471	2.06E-17	hypomethylated	0.0064602	0.3784	insignificant	8	28	28
chr18	37111342	37113342	Pcdha4	0.040387	0.01995	inconclusive	-0.025999	0.31286	insignificant	4	8	8
chr18	37111394	37113394	Pcdha4-g	0.040387	0.01995	inconclusive	-0.025999	0.31286	insignificant	4	8	8
chr18	37119093	37121093	Pcdha5		1	noCoverage	0.051654	0.78219	insignificant	0	16	27
chr18	37151120	37153120	Pcdha8	0.23697	0.68303	insignificant	-0.07784	0.13797	insignificant	2	29	27
chr18	37156533	37158533	Pcdha9		1	noCoverage	-0.084928	0.037457	hypomethylated	0	24	20
chr18	37163973	37165973	Pcdha10		1	noCoverage	0.088881	0.52048	insignificant	0	18	20
chr18	37169511	37171511	Pcdha11	-0.035641	0.85013	insignificant	-0.10645	0.0022639	hypomethylated	4	10	10
chr18	37178883	37180883	Pcdha12	-0.6451	0.099684	insignificant	-0.053966	0.91517	insignificant	3	34	36
chr18	37248789	37250789	Pcdhac1	-0.059006	0.0024461	hypomethylated	0.0058965	0.5943	insignificant	16	80	82
chr18	37302622	37304622	Pcdhac2	-0.10383	0.00000241	hypomethylated	-0.0087969	0.0041338	hypomethylated	22	108	109
chr18	37423651	37425651	Pcdhb1	-0.18308	0.00000133	hypomethylated	0.096124	0.2412	insignificant	9	43	35
chr18	37453493	37455493	Pcdhb2	-0.49811	0.000020437	stronglyHypometh	0.00085546	0.014083	inconclusive	4	22	20
chr18	37479034	37481034	Pcdhb5	-0.27772	0.14381	insignificant	-0.011575	1	insignificant	5	26	26
chr18	37492681	37494681	Pcdhb6	-0.090217	0.64137	insignificant	0.17057	0.0098164	hypermethylated	2	23	25
chr18	37500355	37502355	Pcdhb7	0.31253	1	lowCoverage	0.008587	0.71292	insignificant	1	15	12
chr18	37559508	37561508	Pcdhb9		1	noCoverage	-0.14566	1	insignificant	0	7	7
chr18	37594274	37596274	Pcdhb12	-0.17857	0.40545	insignificant	0.080952	0.37734	insignificant	4	4	4
chr18	37601170	37603170	Pcdhb13	-0.02865	0.21272	insignificant	-0.20942	0.000016217	hypomethylated	6	29	25
chr18	37643674	37645674	Pcdhb17	-0.043322	0.26093	insignificant	-0.049917	0.0037273	hypomethylated	5	23	24
chr18	37648118	37650118	Pcdhb18	-0.37136	1	insignificant	-0.12741	0.5513	insignificant	2	8	8
chr18	37677006	37679006	Pcdhb22	-0.039252	0.39603	insignificant	-0.10434	0.0014654	hypomethylated	5	20	20
chr18	37798377	37800377	Slc25a2		1	noCoverage	0.025722	0.73587	insignificant	0	16	16
chr18	37803858	37805858	Pcdha4-g	-0.33333	0.075398	insignificant	-0.18519	0.11514	insignificant	1	2	6
chr18	37820598	37822598	Pcdhga1	0.13173	0.24557	insignificant	-0.029136	0.60753	insignificant	3	40	41
chr18	37827758	37829758	Pcdhga2	-0.54819	3.18E-14	stronglyHypometh	-0.018942	1	insignificant	4	42	42
chr18	37832988	37834988	Pcdhga3	-0.16021	0.12451	insignificant	0.10702	0.029951	hypermethylated	2	47	36
chr18	37839111	37841111	Pcdhgb1	-0.38289	0.0059307	stronglyHypometh	-0.017426	0.15996	insignificant	4	21	20
chr18	37844053	37846053	Pcdhga4	0.26314	0.02205	hypermethylated	-0.040487	0.047214	hypomethylated	6	52	52
chr18	37848512	37850512	Pcdhgb2	-0.23055	0.1863	insignificant	-0.064608	0.0014544	hypomethylated	4	29	27
chr18	37853154	37855154	Pcdhga5	-0.16719	1	insignificant	-0.049375	0.76719	insignificant	6	44	43
chr18	37873487	37875487	Pcdhga7		1	noCoverage	0.15476	0.71827	insignificant	0	6	2
chr18	37879207	37881207	Pcdhgb4	-0.071473	0.43711	insignificant	-0.010853	0.028274	hypomethylated	4	28	29
chr18	37884359	37886359	Pcdhga8	-0.019957	0.00019474	hypomethylated	0.030753	0.0080438	inconclusive	11	48	39
chr18	37889807	37891807	Pcdhgb5	0.28338	0.030314	hypermethylated	-0.044521	0.029633	hypomethylated	4	32	33
chr18	37895589	37897589	Pcdhga9	-0.2446	0.000000966	hypomethylated	-0.018367	0.48186	insignificant	6	37	39
chr18	37900747	37902747	Pcdhgb6		1	noCoverage	0.077432	0.65399	insignificant	0	13	3
chr18	37905841	37907841	Pcdhga10	-0.16187	0.034771	hypomethylated	-0.039279	0.33922	insignificant	14	78	73
chr18	37910432	37912432	Pcdhgb7	-0.15214	0.054614	insignificant	0.052388	0.27967	insignificant	3	16	15
chr18	37914426	37916426	Pcdhga11	-0.29268	0.031531	hypomethylated	0.011693	0.072061	insignificant	7	61	61
chr18	37965063	37967063	Pcdhgc3	-0.1108	0.000012939	hypomethylated	0.01489	0.2453	insignificant	14	134	136
chr18	37973732	37975732	Pcdhgc4	-0.016023	7.33E-08	hypomethylated	0.022364	0.90535	insignificant	12	52	53
chr18	37978199	37980199	Pcdhgc5		1	noCoverage	-0.072456	0.22311	insignificant	0	12	14
chr18	38095065	38097065	Diap1	-0.083189	0.000037176	hypomethylated	-0.0080781	0.94645	insignificant	13	46	46
chr18	38114212	38116212	Rell2	-0.16961	2.97E-24	hypomethylated	-0.0098768	0.14708	insignificant	18	83	84
chr18	38114642	38116642	Hdac3	-0.15929	1.82E-16	hypomethylated	-0.0076139	0.23193	insignificant	14	75	76
chr18	38129385	38131385	Fchsd1	-0.16182	0.000000546	hypomethylated	0.005461	0.60986	insignificant	11	52	52
chr18	38157228	38159228	Arap3	-0.25735	0.000000641	hypomethylated	0.040842	1	insignificant	7	24	32
chr18	38369416	38371416	Pcdh1	-0.16489	2.24E-12	hypomethylated	-0.027956	0.65473	insignificant	20	72	72
chr18	38408902	38410902	0610009O20Rik	-0.16327	1	insignificant	-0.0324	0.57997	insignificant	3	43	39
chr18	38409852	38411852	0610009O20Rik	-0.11133	1	insignificant	-0.01769	1	insignificant	3	31	31
chr18	38455288	38457288	Rnf14	-0.16786	1.04E-30	hypomethylated	-0.0023956	0.21988	insignificant	32	79	84

chr18	38455458	38457458	Rnf14	-0.16786	1.04E-30	hypomethylated	-0.0023956	0.21988	insignificant	32	79	84
chr18	38498647	38500647	Gnpda1	-0.38421	0.00010098	stronglyHypometh	-0.073579	0.45577	insignificant	2	24	24
chr18	38577628	38579628	Ndfip1	-0.10789	9.41E-16	hypomethylated	-0.0057406	0.2085	insignificant	46	148	148
chr18	38760922	38762922	Spry4	0.20679	0.080507	insignificant	0.010045	0.086523	insignificant	5	36	36
chr18	39151798	39153798	Arhgap26	-0.11103	1.42E-38	hypomethylated	0.0066552	0.13654	insignificant	57	142	148
chr18	39646899	39648899	Nr3c1	-0.11862	0.15277	insignificant	0.013558	0.91026	insignificant	4	48	50
chr18	39932150	39934150	Pabpc2	0.077062	0.065194	insignificant	0.027062	0.063658	insignificant	6	46	39
chr18	40379053	40381053	Yipf5	-0.29383	0.00000108	hypomethylated	-0.063145	0.12901	insignificant	14	37	36
chr18	40417014	40419014	Kctd16	0.26638	0.066844	insignificant	0.01573	0.05663	insignificant	4	27	27
chr18	42110848	42112848	Prelid2	-0.58332	5.35E-13	stronglyHypometh	0.013556	0.40411	insignificant	3	19	13
chr18	42212363	42214363	Sh3rf2	-0.08099	0.054047	insignificant	-0.024281	0.14476	insignificant	7	22	22
chr18	42421725	42423725	Lars	-0.15952	0.00000573	hypomethylated	0.0027556	0.4475	insignificant	12	37	36
chr18	42434006	42436006	Rbm27	-0.19387	2.26E-09	hypomethylated	-0.0072615	0.8442	insignificant	15	67	66
chr18	42434851	42436851	Gm4013	-0.24558	0.00013649	hypomethylated	-0.0033447	0.93063	insignificant	9	60	59
chr18	42553250	42555250	Pou4f3	-0.12995	0.0001123	hypomethylated	-0.011473	0.78879	insignificant	25	72	72
chr18	42670140	42672140	Tcerg1	-0.11737	7.55E-28	hypomethylated	0.0074897	0.81968	insignificant	52	149	158
chr18	42739306	42741306	Gpr151		1	noCoverage	-0.13889	0.19208	insignificant	0	6	6
chr18	43058643	43060643	Ppp2r2b	-0.073017	0.36205	insignificant	-0.079818	0.035619	inconclusive	8	38	34
chr18	43219125	43221125	Ppp2r2b	-0.75797	0.000025102	stronglyHypometh	-0.059511	1	insignificant	1	10	10
chr18	43366350	43368350	Stk32a	-0.16531	2.48E-11	hypomethylated	-0.022181	0.40423	insignificant	12	57	58
chr18	43552985	43554985	Dpysl3	-0.10991	0.50572	insignificant	-0.10638	3.83E-08	hypomethylated	1	9	9
chr18	43847427	43849427	Jakmip2	-0.28358	1	lowCoverage	0.098538	0.61928	insignificant	1	12	16
chr18	44318957	44320957	Gm10267	-0.4875	0.17217	insignificant	0.097917	0.21646	insignificant	2	4	4
chr18	44355831	44357831	Spink11		1	noCoverage	-0.097619	0.43046	insignificant	0	4	2
chr18	44539153	44541153	Dcp2	-0.16997	5.1E-43	hypomethylated	-0.020073	0.19131	insignificant	31	96	100
chr18	44820318	44822318	A930012L18Rik	-0.17104	2.17E-15	hypomethylated	-0.0090301	0.48201	insignificant	24	81	83
chr18	44821920	44823920	A930012L18Rik		1	noCoverage	-0.015873	0.83157	insignificant	0	6	6
chr18	44971836	44973836	Mcc	-0.090249	0.14521	insignificant	0.0030711	0.69686	insignificant	6	28	28
chr18	44987318	44989318	Ythdc2	-0.1448	4.79E-22	hypomethylated	-0.052371	0.1224	insignificant	33	112	104
chr18	45718807	45720807	Kcnn2	-0.15114	5.04E-38	hypomethylated	-0.0079317	0.12525	insignificant	53	165	160
chr18	46357674	46359674	1700018A14Rik	-0.14366	3.16E-32	hypomethylated	0.0019668	0.25182	insignificant	58	157	157
chr18	46358472	46360472	1700018A14Rik	-0.1027	0.16281	insignificant	0.028625	0.000094121	hypermethylated	22	77	75
chr18	46372261	46374261	Trim36	-0.11536	0.0007399	hypomethylated	0.064437	0.43265	insignificant	6	24	27
chr18	46440504	46442504	Pggt1b	-0.17314	0.003073	hypomethylated	0.040232	0.56924	insignificant	2	11	11
chr18	46471582	46473582	Ccdc112	-0.12449	1	insignificant	0.0083399	0.75781	insignificant	2	16	16
chr18	46625470	46627470	Mospd4	-0.15476	0.18555	insignificant	0.025073	0.87406	insignificant	1	2	2
chr18	46756357	46758357	Eif1a	-0.17481	8.59E-40	hypomethylated	0.0031095	0.030731	inconclusive	36	108	109
chr18	46757189	46759189	Eif1a	-0.2312	3.38E-12	hypomethylated	0.012836	0.14198	insignificant	10	50	51
chr18	46887996	46889996	Cdo1	-0.18059	0.014089	hypomethylated	0.075208	0.94419	insignificant	5	22	30
chr18	46900570	46902570	Ap3s1	-0.13514	4.22E-41	hypomethylated	-0.0053304	0.0089751	hypomethylated	46	136	133
chr18	46901233	46903233	Ap3s1	-0.12729	9.29E-32	hypomethylated	-0.016708	0.00019936	hypomethylated	41	116	116
chr18	47008692	47010692	4833403I15Rik	0.018068	0.40738	insignificant	0.00014609	0.000000149	hypermethylated	15	56	56
chr18	47080468	47082468	Gm949	-0.3655	0.24736	insignificant	-0.033117	0.81256	insignificant	2	26	26
chr18	47117529	47119529	Commd10	-0.11811	0.000046078	hypomethylated	0.0022927	0.91987	insignificant	14	30	30
chr18	47528522	47530522	Sema6a	-0.15361	0.23464	insignificant	-0.014813	0.29263	insignificant	3	53	67
chr18	47696388	47698388	Gm5095	-0.43069	0.010227	stronglyHypometh	-0.045684	0.67671	insignificant	3	12	12
chr18	49915255	49917255	Dtwd2	-0.027766	0.70487	insignificant	0.03718	0.90778	insignificant	5	33	32
chr18	49991666	49993666	Dmxl1	-0.080404	3.06E-09	hypomethylated	0.00015688	0.87454	insignificant	34	95	96
chr18	50138080	50140080	Tnfaip8	-0.15487	0.75195	insignificant	0.01458	0.078664	insignificant	2	37	35
chr18	50211935	50213935	Tnfaip8	-0.1928	4.41E-22	hypomethylated	0.00042713	0.95513	insignificant	14	36	36
chr18	50286992	50288992	Hsd17b4	-0.13824	1.09E-09	hypomethylated	-0.014933	0.72469	insignificant	9	42	42
chr18	51276551	51278551	Prr16	-0.13628	0.00000118	hypomethylated	-0.0070493	0.83058	insignificant	15	111	114
chr18	52490189	52492189	Ftmt	-0.069236	0.42971	insignificant	0.11944	0.0024937	hypermethylated	2	18	18
chr18	52624346	52626346	Srfbp1	-0.030901	0.000027614	inconclusive	0.039684	0.000035782	hypermethylated	21	85	82
chr18	52926464	52928464	Sncaip	-0.10249	6.54E-23	hypomethylated	-0.0020078	0.76698	insignificant	39	115	115
chr18	52926746	52928746	Sncaip	-0.11277	1.12E-27	hypomethylated	0.0015638	0.73771	insignificant	44	135	135
chr18	52926797	52928797	Sncaip	-0.11505	1.32E-27	hypomethylated	0.0057932	0.76235	insignificant	44	139	139
chr18	53335018	53337018	Snx2	-0.11943	3.88E-11	hypomethylated	0.00021859	0.12945	insignificant	23	94	106
chr18	53404315	53406315	Snx24	-0.099036	7.02E-31	hypomethylated	-0.020894	0.22687	insignificant	45	131	133
chr18	53577661	53579661	Ppic	-0.16105	1.98E-10	hypomethylated	0.0081922	0.17446	insignificant	8	28	28
chr18	53623199	53625199	Prdm6	-0.11843	2.83E-60	hypomethylated	0.0020733	0.70742	insignificant	73	211	210
chr18	53904201	53906201	Cep120	-0.15295	0.000000193	hypomethylated	0.013884	0.51921	insignificant	5	44	49
chr18	54020766	54022766	Csnk1g3	-0.12151	3.17E-42	hypomethylated	-0.018109	0.074016	insignificant	53	156	150
chr18	55149834	55151834	Zfp608	-0.1692	5.48E-10	hypomethylated	-0.012088	0.64618	insignificant	5	39	39
chr18	56590785	56592785	Gramd3	-0.21087	1.24E-21	hypomethylated	-0.010654	0.84121	insignificant	18	47	48
chr18	56721222	56723222	Phax	-0.14631	0.00043268	hypomethylated	0.028052	0.00072665	hypermethylated	15	66	58
chr18	56721952	56723952	Aldh7a1	0.20436	1	insignificant	0.10225	0.42867	insignificant	2	26	18
chr18	56731524	56733524	Phax	-0.098096	1.25E-14	hypomethylated	-0.0075204	0.060131	insignificant	29	125	104
chr18	56732593	56734593	Phax	-0.085684	3.41E-12	hypomethylated	-0.017128	0.015206	hypomethylated	21	99	78
chr18	56747001	56749001	1700065I17Rik	-0.11181	0.12834	insignificant	-0.080661	0.12184	insignificant	3	6	6
chr18	56866466	56868466	Lmnb1	-0.094852	1.1E-32	hypomethylated	-0.0066096	0.0071714	hypomethylated	50	223	210
chr18	57085202	57087202	March3	-0.24847	4.21E-10	hypomethylated	-0.05023	0.62297	insignificant	3	16	16
chr18	57135033	57137033	C330018D20Rik	-0.1868	0.011623	hypomethylated	-0.11652	0.77766	insignificant	5	11	15
chr18	57291743	57293743	Megf10	-0.21918	2.62E-08	hypomethylated	0.042315	0.78217	insignificant	8	34	34
chr18	57513386	57515386	Prrc1	-0.10256	1.18E-10	hypomethylated	0.0020915	0.21206	insignificant	26	82	84
chr18	57711521	57713521	4930511M06Ril	-0.22765	0.070672	insignificant	-0.0076954	0.19736	insignificant	2	4	6
chr18	57712049	57714049	4930511M06Ril	-0.22765	0.070672	insignificant	-0.0076954	0.19736	insignificant	2	4	6
chr18	57712051	57714051	4930511M06Ril	-0.22765	0.070672	insignificant	-0.0076954	0.19736	insignificant	2	4	6
chr18	58037331	58039331	Slc12a2	-0.090752	7.35E-29	hypomethylated	-0.001682	0.00078313	hypomethylated	82	257	261
chr18	58369580	58371580	Fbn2	-0.046186	0.00000025	hypomethylated	0.0025358	0.65841	insignificant	19	98	104
chr18	58818135	58820135	Isoc1	-0.11014	8.04E-31	hypomethylated	-0.0088488	0.2795	insignificant	42	134	133
chr18	58995417	58997417	Adamts19	-0.12011	8.1E-22	hypomethylated	0.0074701	0.80041	insignificant	40	130	130
chr18	59221034	59223034	A730017C20Rik		1	noCoverage	-0.00016288	1	insignificant	0	21	20
chr18	59333993	59335993	Chsy3	-0.11553	2.38E-39	hypomethylated	0.0019505	0.34965	insignificant	57	211	202
chr18	60661637	60663637	2010002N04Rik	-0.33333	0.025927	stronglyHypometh	-0.023459	0.34803	insignificant	2	4	4

chr18	60684874	60686874	Dctn4	-0.11495	1.17E-15	hypomethylated	0.0048113	0.44634	insignificant	15	50	49
chr18	60719439	60721439	Rbm22	-0.15022	5.64E-17	hypomethylated	-0.0039624	0.31554	insignificant	18	58	68
chr18	60751370	60753370	Myoz3	0.16182	0.35351	insignificant	-0.19858	0.12006	insignificant	1	12	12
chr18	60769758	60771758	Synpo	-0.24397	0.6631	insignificant	0.0054215	0.0090471	hypermethylated	1	14	12
chr18	60783959	60785959	Synpo	-0.14943	5.47E-08	hypomethylated	-0.032058	0.15838	insignificant	8	33	32
chr18	60933249	60935249	Rps14	-0.10576	9.5E-12	hypomethylated	-0.0066681	0.094147	insignificant	24	108	113
chr18	60962502	60964502	Cd74	-0.54545	0.21923	insignificant	0.010101	1	insignificant	2	4	4
chr18	61008618	61010618	Tcof1	-0.19482	0.00011231	hypomethylated	0.15539	0.22519	insignificant	4	15	18
chr18	61070893	61072893	Arsi	-0.19276	1.3E-11	hypomethylated	0.044327	0.015536	hypermethylated	19	94	92
chr18	61084285	61086285	Camk2a	0.01117	0.64393	insignificant	-0.11267	0.2682	insignificant	7	16	15
chr18	61122207	61124207	Camk2a	0.29639	0.72597	insignificant	0.19183	0.11605	insignificant	2	7	4
chr18	61173853	61175853	Slc6a7	-0.037698	0.39553	insignificant	-0.013835	0.43444	insignificant	1	12	11
chr18	61195853	61197853	Cdx1	-0.033873	0.35698	insignificant	-0.03902	0.17812	insignificant	11	36	36
chr18	61203803	61205803	Pdgfrb	-0.35897	0.30215	insignificant	0.007195	0.32984	insignificant	4	14	14
chr18	61336703	61338703	Hmgxb3	-0.089567	0.00054842	hypomethylated	-0.0072499	0.632	insignificant	10	24	24
chr18	61336704	61338704	Hmgxb3	-0.089567	0.00054842	hypomethylated	-0.0072499	0.632	insignificant	10	24	24
chr18	61371250	61373250	Slc26a2	-0.18417	5.8E-33	hypomethylated	-0.0026909	0.00001353	hypomethylated	13	46	46
chr18	61560085	61562085	Ppargc1b	-0.14167	1.51E-17	hypomethylated	0.0070293	0.63285	insignificant	20	51	49
chr18	61696190	61698190	Arhgef37	-0.21603	0.14126	insignificant	-0.031426	0.016166	hypomethylated	8	30	30
chr18	61714235	61716235	Csnk1a1	-0.096637	1.33E-18	hypomethylated	0.0082008	0.61006	insignificant	33	160	154
chr18	61807548	61809548	E330013P06	0.40783	0.57653	insignificant	0.099027	0.25381	insignificant	2	18	16
chr18	61808912	61810912	E330013P06	0.09576	1	insignificant	0.057881	0.50308	insignificant	2	4	4
chr18	61867289	61869289	Pcyox1l	-0.11774	0.042745	hypomethylated	-0.17204	0.056324	insignificant	2	10	14
chr18	61885043	61887043	1500015A07Rik	-0.13206	2.16E-35	hypomethylated	-0.02434	0.37779	insignificant	22	103	100
chr18	61885985	61887985	Grpel2	-0.20569	1.04E-12	hypomethylated	-0.042486	0.0015105	hypomethylated	10	55	52
chr18	61946316	61948316	Afap1l1	-0.31469	2.12E-37	hypomethylated	-0.028918	4.18E-17	hypomethylated	11	30	37
chr18	62071478	62073478	Ablim3		1	noCoverage	0.015203	0.64791	insignificant	0	26	26
chr18	62071506	62073506	Ablim3		1	noCoverage	0.030785	0.60136	insignificant	0	14	14
chr18	62111728	62113728	Sh3tc2	0.372	0.22093	insignificant	0.085154	1	insignificant	4	34	34
chr18	62339613	62341613	Adrb2	-0.18182	0.000031022	hypomethylated	0.023338	0.89294	insignificant	7	42	44
chr18	62482857	62484857	Htr4	-0.088408	0.00025148	hypomethylated	0.042301	0.065484	insignificant	9	47	47
chr18	62707564	62709564	Spink10	-0.12151	3.87E-16	hypomethylated	-0.0069594	0.96183	insignificant	13	71	71
chr18	62708397	62710397	Fbxo38	-0.12129	0.0011579	hypomethylated	-0.012834	0.71233	insignificant	5	32	32
chr18	63080980	63082980	Apcdd1	-0.16156	1.76E-39	hypomethylated	-0.032526	6.14E-11	hypomethylated	45	144	143
chr18	63136569	63138569	Napg	-0.094046	8.81E-09	hypomethylated	0.01255	0.63191	insignificant	14	55	58
chr18	63546837	63548837	Fam38b	-0.059286	9.82E-10	hypomethylated	-0.038875	0.000000518	hypomethylated	9	30	30
chr18	63852013	63854013	Txnl1	-0.30617	4.44E-11	hypomethylated	0.019339	0.076723	insignificant	5	41	41
chr18	63867348	63869348	Wdr7	-0.12903	0.000079718	hypomethylated	0.026866	1	insignificant	8	18	21
chr18	64413012	64415012	St8sia3	-0.11869	0.54038	insignificant	0.1352	0.23547	insignificant	2	4	4
chr18	64499017	64501017	Onecut2	-0.11162	2.5E-15	hypomethylated	-0.011729	0.13789	insignificant	47	177	172
chr18	64648720	64650720	Fech	-0.13536	0.00068282	hypomethylated	-0.0043649	0.081759	insignificant	5	22	22
chr18	64676211	64678211	Nars	-0.21652	5.86E-09	hypomethylated	-0.042867	0.16818	insignificant	5	12	13
chr18	64820654	64822654	Atp8b1	-0.10141	5.81E-21	hypomethylated	0.0016366	0.023604	inconclusive	14	76	69
chr18	65046409	65048409	Nedd4l	-0.14014	9.15E-42	hypomethylated	-0.0035497	0.000098685	hypomethylated	58	168	171
chr18	65553542	65555542	Alpk2	-0.25056	0.16631	insignificant	0.072354	0.6498	insignificant	1	4	4
chr18	65589650	65591650	Malt1	-0.10104	4.05E-22	hypomethylated	-0.0045714	0.018387	hypomethylated	47	158	164
chr18	65738883	65740883	Zfp532	-0.083815	4.5E-21	hypomethylated	-0.0042585	0.168	insignificant	36	100	100
chr18	65959231	65961231	Sec11c	-0.1851	0.000064028	hypomethylated	-0.013102	0.95096	insignificant	6	42	42
chr18	66032147	66034147	Grp	-0.080425	0.11893	insignificant	-0.006119	0.37459	insignificant	10	50	50
chr18	66129832	66131832	Cplx4	0.069444	1	insignificant	0.028968	0.72612	insignificant	1	6	6
chr18	66162289	66164289	Lman1	-0.00043877	0.18065	insignificant	-0.049114	0.00011017	hypomethylated	2	19	17
chr18	66451492	66453492	Ccbe1	-0.22345	0.00076705	hypomethylated	-0.13688	0.039671	hypomethylated	7	29	24
chr18	66617257	66619257	Pmaip1	-0.18136	9.04E-08	hypomethylated	-0.0091226	0.58906	insignificant	29	115	117
chr18	67246989	67248989	Gnal	-0.13144	8.16E-22	hypomethylated	0.01671	0.27634	insignificant	52	137	130
chr18	67292479	67294479	Gnal	-0.14309	5.74E-17	hypomethylated	-0.0024419	0.12718	insignificant	22	79	81
chr18	67364012	67366012	Chmp1b	-0.16961	0.00000553	hypomethylated	0.0086436	0.1745	insignificant	17	67	87
chr18	67405484	67407484	Mppe1	-0.31493	0.0073509	hypomethylated	0.019694	1	insignificant	3	16	24
chr18	67447876	67449876	Impa2	-0.1412	1.07E-13	hypomethylated	-0.011447	0.10298	insignificant	13	54	54
chr18	67502217	67504217	Cidea	-0.16509	0.000000935	hypomethylated	-0.012909	0.4034	insignificant	7	41	42
chr18	67503757	67505757	Cidea	-0.20057	0.10433	insignificant	0.088364	0.070505	insignificant	3	8	8
chr18	67549384	67551384	Tubb6	-0.18631	5.62E-39	hypomethylated	0.0078585	0.15921	insignificant	45	109	109
chr18	67608790	67610790	Afg3l2	-0.12597	0.89423	insignificant	0.0094279	0.46676	insignificant	8	37	38
chr18	67623502	67625502	Slmo1	-0.1033	0.00021958	hypomethylated	-0.025226	0.0021628	hypomethylated	6	86	83
chr18	67708827	67710827	Spire1	0.16934	1	insignificant	-0.13482	1	insignificant	2	15	12
chr18	67712375	67714375	Spire1	0.21579	1	lowCoverage	0.13662	0.74722	insignificant	1	10	10
chr18	67800252	67802252	Psmg2	-0.11887	7.01E-53	hypomethylated	-0.004374	0.00073575	hypomethylated	70	210	211
chr18	67800990	67802990	Psmg2	-0.10724	1.18E-22	hypomethylated	-0.0058386	0.0011487	hypomethylated	39	112	113
chr18	67884275	67886275	Ptpn2	-0.19514	0.34559	insignificant	0.086827	0.70351	insignificant	3	30	30
chr18	67933529	67935529	Seh1l	-0.097597	5.11E-38	hypomethylated	-0.0033752	0.63543	insignificant	47	94	94
chr18	67958760	67960760	Cep192	-0.26725	5.71E-12	hypomethylated	-0.022665	0.082546	insignificant	14	102	103
chr18	68091910	68093910	D18Ertd653e	-0.099325	3E-39	hypomethylated	-0.010978	0.088957	insignificant	60	156	168
chr18	68459008	68461008	Rnmt	-0.14106	7.59E-11	hypomethylated	-0.010351	0.18835	insignificant	21	88	82
chr18	68459987	68461987	4933403F05Rik	-0.27068	0.0047018	hypomethylated	0.02078	0.54973	insignificant	5	26	20
chr18	69503145	69505145	Tcf4	-0.15047	3.18E-13	hypomethylated	-0.011369	0.15896	insignificant	17	64	66
chr18	69504374	69506374	Tcf4	-0.11966	4.06E-17	hypomethylated	-0.004426	0.57738	insignificant	34	110	117
chr18	70213023	70215023	Rab27b	-0.28235	0.32805	insignificant	0.0048391	0.82026	insignificant	1	10	10
chr18	70631198	70633198	Stard6	-0.14709	7.72E-20	hypomethylated	0.00028849	0.000042048	inconclusive	30	108	103
chr18	70632134	70634134	4930503L19Rik	-0.2163	1.59E-08	hypomethylated	0.016614	0.90393	insignificant	12	28	28
chr18	70689792	70691792	Poli	-0.25229	1.52E-27	hypomethylated	0.12412	0.078061	insignificant	4	18	18
chr18	70689975	70691975	Poli	0.052615	0.44182	insignificant	0.15516	0.000010832	hypermethylated	2	14	14
chr18	70726945	70728945	Mbd2	-0.12625	3.23E-21	hypomethylated	-0.011096	0.10206	insignificant	61	168	174
chr18	73731358	73733358	Mex3c	-0.10443	2.67E-77	hypomethylated	-0.0052143	0.0025423	hypomethylated	113	306	306
chr18	73863395	73865395	Smad4	-0.10498	0.000046115	hypomethylated	-0.0025459	0.9189	insignificant	19	70	70
chr18	73914133	73916133	Elac1	-0.22777	2.86E-13	hypomethylated	0.043725	0.10975	insignificant	17	55	47
chr18	73975046	73977046	Me2	-0.12826	8.43E-13	hypomethylated	-0.016005	0.020003	hypomethylated	15	56	60

chr18	74224603	74226603	Mapk4	-0.19048	0.000028506	hypomethylated	-0.043893	0.16186	insignificant	10	39	50
chr18	74367472	74369472	Ska1		1	noCoverage	0.11526	1	insignificant	0	4	2
chr18	74374865	74376865	Cxxc1	-0.079935	6.5E-15	hypomethylated	0.016251	0.59554	insignificant	32	144	146
chr18	74426941	74428941	Mbd1	-0.12752	1.09E-13	hypomethylated	0.029707	0.72656	insignificant	21	82	80
chr18	74441753	74443753	Ccdc11	-0.69808	0.10476	insignificant	0.13764	0.5613	insignificant	1	13	10
chr18	74601272	74603272	Myo5b	-0.11138	3.46E-19	hypomethylated	-0.0032249	0.22024	insignificant	47	166	161
chr18	74937865	74939865	Acaa2	-0.1336	0.017148	hypomethylated	0.013981	0.53679	insignificant	3	26	26
chr18	74938109	74940109	Acaa2	-0.17126	0.00093035	hypomethylated	0.013236	1	insignificant	6	36	36
chr18	75120917	75122917	Lipg	-0.12392	5.01E-08	hypomethylated	0.0024567	0.91972	insignificant	8	48	48
chr18	75159130	75161130	Rpl17	-0.16916	9.62E-43	hypomethylated	-0.00071257	0.29027	insignificant	35	106	111
chr18	75159721	75161721	Snord58b	-0.13146	1.23E-38	hypomethylated	-0.0091276	0.35134	insignificant	35	99	99
chr18	75164553	75166553	BC031181	-0.31563	9.05E-15	hypomethylated	0.0034753	0.010982	hypermethylated	17	64	62
chr18	75177425	75179425	Dym	-0.1401	5.75E-26	hypomethylated	0.0040028	0.08636	insignificant	24	86	92
chr18	75526018	75528018	Smad7	-0.1279	1.13E-61	hypomethylated	0.0038371	0.63825	insignificant	51	199	201
chr18	75857350	75859350	Ctif	-0.31677	0.00039263	hypomethylated	-0.1306	0.0015884	hypomethylated	4	14	14
chr18	75978831	75980831	Zbtb7c	-0.10659	9.71E-25	hypomethylated	-0.00094501	0.24081	insignificant	57	218	219
chr18	76400578	76402578	Smad2	-0.11418	7.37E-46	hypomethylated	-0.016179	0.00025705	hypomethylated	90	290	279
chr18	77094143	77096143	Skor2	-0.24382	1.76E-16	hypomethylated	-0.015446	0.0097851	hypomethylated	14	67	67
chr18	77167765	77169765	Ier3ip1	-0.1202	3.84E-12	hypomethylated	-0.0033788	0.66972	insignificant	24	57	57
chr18	77181853	77183853	Hdhd2	-0.12243	2.91E-11	hypomethylated	-0.0044903	0.54294	insignificant	22	56	56
chr18	77182156	77184156	Hdhd2	-0.12243	2.91E-11	hypomethylated	-0.0044903	0.54294	insignificant	22	56	56
chr18	77302946	77304946	Pias2	-0.099981	2.42E-18	hypomethylated	0.01086	0.36114	insignificant	27	143	161
chr18	77303418	77305418	Pias2	-0.086176	2.1E-16	hypomethylated	0.0084461	0.62169	insignificant	27	142	158
chr18	77423585	77425585	St8sia5	-0.11045	1.22E-39	hypomethylated	0.0046327	0.026056	inconclusive	48	160	162
chr18	77519696	77521696	Loxhd1	-0.01137	0.33529	insignificant	0.052215	0.077182	insignificant	5	22	22
chr18	77803875	77805875	Rnf165	-0.1393	4.48E-19	hypomethylated	-0.030924	0.00041098	hypomethylated	29	96	123
chr18	77951922	77953922	4930465K10Rik	-0.088337	2.81E-36	hypomethylated	-0.0048869	0.031226	hypomethylated	75	251	251
chr18	77952749	77954749	8030462N17Rik	-0.083612	0.00000013	hypomethylated	-0.0013568	0.8838	insignificant	32	134	133
chr18	78006519	78008519	Haus1	-0.11197	0.0033405	hypomethylated	-0.02115	0.37867	insignificant	6	12	12
chr18	78011506	78013506	Atp5a1	-0.15513	1.7E-30	hypomethylated	-0.0084516	0.085549	insignificant	20	91	91
chr18	78032288	78034288	Pstpip2	-0.14016	5.65E-40	hypomethylated	0.0048093	0.00061856	inconclusive	37	128	128
chr18	78134205	78136205	5430411K18Rik	-0.15343	0.000013795	hypomethylated	0.00181	0.15223	insignificant	12	50	50
chr18	78793689	78795689	Slc14a2		1	noCoverage	0.055218	0.43611	insignificant	0	8	10
chr18	79306130	79308130	Setbp1	-0.11276	7.26E-16	hypomethylated	-0.016448	0.11737	insignificant	42	183	176
chr18	80242633	80244633	Pard6g	-0.14306	1.11E-24	hypomethylated	0.0039927	0.0040594	inconclusive	27	108	110
chr18	80348221	80350221	Adnp2	-0.26906	0.079369	insignificant	-0.022654	0.74841	insignificant	2	18	18
chr18	80397358	80399358	Rbfa	-0.37183	8.94E-11	stronglyHypometh	0.0031401	0.16238	insignificant	9	41	42
chr18	80402536	80404536	Txnl4a	-0.26374	2E-23	hypomethylated	-0.019448	0.45498	insignificant	8	70	80
chr18	80402584	80404584	Txnl4a	-0.27088	3.45E-26	hypomethylated	-0.019438	0.056528	insignificant	8	74	84
chr18	80402605	80404605	Txnl4a	-0.27088	3.45E-26	hypomethylated	-0.019438	0.056528	insignificant	8	74	84
chr18	80443841	80445841	Hsbp1l1	0.023413	0.25188	insignificant	0.1294	1	insignificant	1	9	7
chr18	80451964	80453964	Pqlc1	-0.1293	0.000063023	hypomethylated	-0.015323	0.63368	insignificant	10	26	31
chr18	80452044	80454044	Pqlc1	-0.1293	0.000063023	hypomethylated	-0.011524	0.6335	insignificant	10	26	30
chr18	80666406	80668406	Ctdp1	-0.087755	0.00018589	hypomethylated	-0.010277	0.79725	insignificant	17	80	80
chr18	80904912	80906912	Nfatc1	-0.17575	1.68E-09	hypomethylated	-0.032444	0.69984	insignificant	20	70	74
chr18	80909810	80911810	Nfatc1	-0.13385	7.22E-14	hypomethylated	-0.001262	0.076847	insignificant	20	73	73
chr18	81130797	81132797	Atp9b	-0.53517	0.060017	insignificant	-0.024879	0.00064299	hypomethylated	4	16	16
chr18	81183317	81185317	Sall3	-0.093837	7.71E-39	hypomethylated	0.0055438	0.56431	insignificant	72	257	274
chr18	82576169	82578169	Galr1	-0.14255	0.46704	insignificant	-0.037448	0.0011134	inconclusive	8	20	20
chr18	82643514	82645514	Mbp	-0.18829	8.78E-18	hypomethylated	-0.0075897	0.00016749	hypomethylated	17	74	79
chr18	82722854	82724854	Mbp	0.013889	1	insignificant	-0.073611	0.0072363	hypomethylated	2	8	8
chr18	82722899	82724899	Mbp	0.013889	1	insignificant	-0.073611	0.0072363	hypomethylated	2	8	8
chr18	82862126	82864126	Zfp236	-0.16366	3.37E-16	hypomethylated	0.047566	0.28563	insignificant	21	93	91
chr18	83079270	83081270	Zfp516	-0.11671	2.43E-10	hypomethylated	-0.013512	0.70458	insignificant	7	84	85
chr18	83083023	83085023	Zfp516	0.042943	0.016363	inconclusive	0.014192	0.31564	insignificant	13	99	110
chr18	83089952	83091952	Zfp516	-0.45947	0.026986	stronglyHypometh	-0.066433	0.049954	hypomethylated	2	10	12
chr18	84255954	84257954	Zadh2	-0.1067	8.17E-11	hypomethylated	-0.0082373	0.088239	insignificant	43	130	130
chr18	84256549	84258549	Zadh2	-0.1067	8.17E-11	hypomethylated	-0.0082373	0.088239	insignificant	43	130	130
chr18	84758896	84760896	Zfp407	-0.14623	0.00002453	hypomethylated	0.049609	0.41135	insignificant	14	40	33
chr18	84855025	84857025	Cndp2	-0.20172	5.94E-20	hypomethylated	-0.035682	0.0024458	hypomethylated	10	43	43
chr18	84888633	84890633	Fam69c	-0.10407	1.01E-47	hypomethylated	0.00038012	0.0022008	inconclusive	35	133	144
chr18	85019805	85021805	Cyb5	-0.22464	1.67E-09	hypomethylated	-0.017388	0.22595	insignificant	9	53	55
chr18	85120916	85122916	Fbxo15	-0.068615	7.33E-09	hypomethylated	0.0029991	0.66234	insignificant	12	58	58
chr18	86563343	86565343	Neto1	-0.16581	3.58E-17	hypomethylated	-0.048229	0.3031	insignificant	26	69	72
chr18	86881439	86883439	Cbln2	-0.2027	0.000067947	hypomethylated	0.023106	0.70099	insignificant	8	28	39
chr18	89140181	89142181	Rttn	-0.19943	3.74E-09	hypomethylated	-0.01026	0.066122	insignificant	10	30	31
chr18	89365818	89367818	Cd226		1	noCoverage	-0.074074	0.8125	insignificant	0	3	6
chr18	89938528	89940528	Dok6	-0.11161	1.63E-16	hypomethylated	-0.022328	0.054963	insignificant	27	64	64
chr18	90678545	90680545	Tmx3	-0.022602	0.000078296	hypomethylated	-0.0076532	0.19555	insignificant	13	68	68
chr19	3282046	3284046	Mrpl21	-0.19385	4.62E-13	hypomethylated	-0.03242	0.56559	insignificant	16	34	46
chr19	3283010	3285010	Ighmbp2	-0.17834	3.87E-15	hypomethylated	-0.017311	0.000077199	hypomethylated	13	28	34
chr19	3322300	3324300	Cpt1a	-0.15638	3.48E-13	hypomethylated	-0.011227	0.96004	insignificant	21	66	66
chr19	3387868	3389868	Mtl5	-0.087419	5.41E-18	hypomethylated	0.0077821	0.93149	insignificant	23	75	79
chr19	3414457	3416457	Gal	-0.18167	3.54E-08	hypomethylated	-0.02758	0.00004234	hypomethylated	12	34	34
chr19	3575749	3577749	Ppp6r3	-0.20095	1.17E-12	hypomethylated	-0.0089907	0.042915	hypomethylated	11	66	67
chr19	3686564	3688564	Lrp5	-0.29666	3.14E-09	hypomethylated	-0.056856	0.002068	hypomethylated	10	44	44
chr19	3707332	3709332	1810055G02Rik	-0.10829	0.000000127	hypomethylated	-0.0011552	0.516	insignificant	11	68	69
chr19	3766420	3768420	Suv420h1	-0.10147	9.78E-42	hypomethylated	0.0040626	0.62674	insignificant	56	162	183
chr19	3850772	3852772	Chka	-0.10305	5.12E-10	hypomethylated	0.0094539	0.82229	insignificant	29	123	123
chr19	3905230	3907230	Tcirg1	-0.18982	1.62E-15	hypomethylated	-0.04133	0.4707	insignificant	9	43	36
chr19	3906983	3908983	Tcirg1	-0.036745	0.494	insignificant	0.052037	0.51886	insignificant	5	19	12
chr19	3907133	3909133	Tcirg1	0.0239	0.0076975	inconclusive	0.19505	1	insignificant	5	19	14
chr19	3912717	3914717	Ndufs8	-0.2	0.21454	insignificant	-0.03165	0.42241	insignificant	3	6	6
chr19	3929716	3931716	Aldh3b1		1	noCoverage	0.059722	0.83677	insignificant	0	8	8
chr19	3934185	3936185	Unc93b1	-0.49994	3.33E-24	stronglyHypometh	-0.051003	0.21804	insignificant	5	33	29

chr19	3971327	3973327	Aldh3b2	-0.095034	0.14744	insignificant	0.084774	0.024098	hypermethylated	3	10	10
chr19	3985660	3987660	Acy3	-0.012813	0.50363	insignificant	-0.0086546	0.1161	insignificant	6	15	14
chr19	3991751	3993751	Tbx10		1	noCoverage	-0.057727	0.074848	insignificant	0	6	6
chr19	3999579	4001579	Nudt8	-0.17546	8.9E-17	hypomethylated	-0.022667	0.000002473	hypomethylated	34	97	95
chr19	4002384	4004384	Doc2g	0.045801	1	insignificant	-0.10355	0.18474	insignificant	1	19	20
chr19	4012725	4014725	Ndufv1		1	noCoverage	-0.035307	0.17895	insignificant	0	14	14
chr19	4037912	4039912	Gstp1	0.597	0.073979	insignificant	-0.027288	0.48035	insignificant	2	16	17
chr19	4042221	4044221	Gstp2	-0.94898	9.43E-18	stronglyHypometh	-0.0060448	0.53795	insignificant	3	7	6
chr19	4081487	4083487	Cabp2		1	noCoverage	-0.078112	0.014723	hypomethylated	0	6	6
chr19	4082518	4084518	Cabp2	-0.01278	0.70062	insignificant	0.0030457	0.71735	insignificant	6	38	37
chr19	4096350	4098350	Cdk2ap2	-0.21572	5.8E-44	hypomethylated	0.00019356	0.0083576	inconclusive	38	97	92
chr19	4099116	4101116	Pitpnm1	-0.18948	1.58E-18	hypomethylated	-0.019559	0.00041957	hypomethylated	26	83	94
chr19	4099621	4101621	Pitpnm1	-0.1621	2.06E-14	hypomethylated	-0.019069	0.0076529	hypomethylated	23	75	86
chr19	4124959	4126959	Tmem134	-0.24588	4.93E-11	hypomethylated	-0.031355	0.00000461	hypomethylated	11	54	55
chr19	4125827	4127827	Aip	-0.32727	1.03E-13	hypomethylated	-0.037919	0.00000397	hypomethylated	11	62	61
chr19	4147662	4149662	Coro1b	-0.12113	0.000031319	hypomethylated	0.083388	0.8386	insignificant	7	38	42
chr19	4153645	4155645	Ptprcap	-0.39053	0.000049903	stronglyHypometh	0.0042111	0.90885	insignificant	3	13	12
chr19	4163245	4165245	Rps6kb2	-0.17901	0.000036797	hypomethylated	-0.0058755	0.33566	insignificant	6	21	20
chr19	4191047	4193047	Tbc1d10c	-0.12115	1.63E-22	hypomethylated	0.0065962	0.093438	insignificant	47	146	148
chr19	4191173	4193173	Ppp1ca	-0.12115	1.63E-22	hypomethylated	0.0065962	0.093438	insignificant	47	146	148
chr19	4201603	4203603	Rad9	-0.20257	8.07E-11	hypomethylated	-0.027277	0.42608	insignificant	14	36	36
chr19	4213391	4215391	Clcf1	-0.19092	1.21E-22	hypomethylated	-0.005784	0.3761	insignificant	30	105	96
chr19	4305955	4307955	Adrbk1	-0.10189	8.94E-15	hypomethylated	0.00080478	0.76926	insignificant	40	64	64
chr19	4397077	4399077	Kdm2a	-0.093457	6.5E-33	hypomethylated	0.009136	0.0021583	hypermethylated	60	184	206
chr19	4439424	4441424	Rhod	-0.22026	0.027815	hypomethylated	-0.048596	0.013539	hypomethylated	15	50	50
chr19	4477143	4479143	Syt12	-0.35326	0.16498	insignificant	-0.13508	0.21063	insignificant	5	10	11
chr19	4509471	4511471	Pcx	-0.12772	1.03E-16	hypomethylated	-0.0061748	0.12848	insignificant	19	108	111
chr19	4615667	4617667	Pcx	-0.069454	5.31E-19	hypomethylated	-0.058305	0.00049814	hypomethylated	7	38	33
chr19	4625617	4627617	Gm960	-0.081265	1.04E-09	hypomethylated	0.0099966	0.90461	insignificant	19	94	94
chr19	4710222	4712222	Spnb3	-0.081776	5.49E-10	hypomethylated	-0.0029377	0.85431	insignificant	39	116	116
chr19	4755524	4757524	Rbm4b	-0.15287	5.89E-18	hypomethylated	-0.0075748	0.88158	insignificant	17	85	86
chr19	4793877	4795877	Rbm4	-0.33339	2.48E-09	stronglyHypometh	-0.029768	0.096831	insignificant	6	28	26
chr19	4811634	4813634	Rbm14	-0.16228	0.000000294	hypomethylated	0.025778	0.00000912	inconclusive	11	36	36
chr19	4838365	4840365	Ccdc87	-0.18452	2.81E-08	hypomethylated	0.012314	0.2269	insignificant	14	56	56
chr19	4839322	4841322	Ccdc87	-0.30379	5.98E-08	hypomethylated	-0.096719	0.52042	insignificant	14	59	55
chr19	4854128	4856128	Ctsf	-0.15832	0.00095341	hypomethylated	0.022772	0.22269	insignificant	6	62	64
chr19	4877667	4879667	Zdhhc24	-0.2127	2.47E-11	hypomethylated	-0.01217	0.28894	insignificant	21	70	70
chr19	4877689	4879689	Zdhhc24	-0.2127	2.47E-11	hypomethylated	-0.01217	0.28894	insignificant	21	70	70
chr19	4877884	4879884	Zdhhc24	-0.18917	4.46E-09	hypomethylated	-0.0079584	0.052842	insignificant	14	52	52
chr19	4906627	4908627	Dpp3	-0.49101	0.000026542	stronglyHypometh	-0.087554	0.41385	insignificant	4	23	20
chr19	4943092	4945092	Peli3	-0.23956	0.000000431	hypomethylated	0.062689	0.71863	insignificant	3	20	28
chr19	4961305	4963305	Mrpl11	-0.20028	1.16E-09	hypomethylated	-0.011553	0.62843	insignificant	6	12	12
chr19	4989971	4991971	Npas4		1	noCoverage	-0.04437	0.50254	insignificant	0	36	34
chr19	5023005	5025005	Slc29a2	0.036284	0.000073801	inconclusive	-0.033462	0.13506	insignificant	3	39	41
chr19	5037825	5039825	B3gnt1	-0.14302	0.00010407	hypomethylated	-0.000029648	0.88846	insignificant	18	127	126
chr19	5040403	5042403	Brms1	-0.19223	6.44E-35	hypomethylated	-0.012805	0.0002143	hypomethylated	27	75	75
chr19	5049807	5051807	Rin1	-0.24393	0.00012632	hypomethylated	-0.081972	0.2263	insignificant	7	18	16
chr19	5067077	5069077	Cd248	-0.18966	1.52E-11	hypomethylated	-0.0063277	0.7572	insignificant	21	94	96
chr19	5085477	5087477	Tmem151a	-0.19553	0.35968	insignificant	-0.054375	0.55866	insignificant	20	50	55
chr19	5087552	5089552	Yif1a	0.10136	1	insignificant	-0.06133	0.15493	insignificant	1	10	10
chr19	5098418	5100418	Cnih2	-0.10809	6.36E-14	hypomethylated	-0.0054434	0.14422	insignificant	24	50	50
chr19	5106996	5108996	Klc2	0.25486	0.0073603	hypermethylated	-0.052735	0.030094	inconclusive	7	47	47
chr19	5118408	5120408	Klc2	-0.15597	0.000016558	hypomethylated	0.048011	0.016879	hypermethylated	10	31	31
chr19	5273119	5275119	Sf3b2	-0.097325	1.06E-17	hypomethylated	-0.0052379	0.067457	insignificant	22	66	66
chr19	5295455	5297455	Gal3st3	-0.088994	1.13E-11	hypomethylated	0.0024028	0.000048246	inconclusive	20	70	70
chr19	5297330	5299330	Gal3st3	-0.43695	0.0045811	stronglyHypometh	-0.019723	0.58029	insignificant	10	33	33
chr19	5334740	5336740	Catsper1	-0.17745	1	insignificant	-0.037748	0.33329	insignificant	3	8	8
chr19	5349574	5351574	Cst6	0.04322	0.29021	insignificant	-0.050514	0.011608	hypomethylated	5	20	18
chr19	5365812	5367812	Eif1ad	-0.13353	1.06E-24	hypomethylated	-0.014102	0.29362	insignificant	36	143	160
chr19	5366347	5368347	Banf1	-0.11984	4.01E-24	hypomethylated	-0.0088068	0.33383	insignificant	36	126	142
chr19	5366645	5368645	Banf1	-0.1471	4.94E-18	hypomethylated	0.0059216	1	insignificant	23	70	76
chr19	5387335	5389335	D330050I16Rik	-0.15229	3.25E-27	hypomethylated	-0.0032394	0.00018328	hypomethylated	49	135	132
chr19	5388703	5390703	D330050I16Rik	-0.2419	0.05009	insignificant	0.078246	0.13644	insignificant	3	41	41
chr19	5424143	5426143	AI837181	-0.14429	2.38E-36	hypomethylated	-0.0038892	0.16567	insignificant	41	141	139
chr19	5424916	5426916	Drap1	-0.071215	0.10503	insignificant	0.00077228	0.046968	inconclusive	23	71	69
chr19	5446697	5448697	Fosl1	-0.14714	3.76E-09	hypomethylated	-0.0030754	0.9323	insignificant	17	64	58
chr19	5457549	5459549	Ccdc85b	-0.13587	0.034557	hypomethylated	0.0081581	0.6895	insignificant	7	28	28
chr19	5459693	5461693	Fibp	-0.16687	0.0074814	hypomethylated	0.021048	0.7371	insignificant	5	36	37
chr19	5468498	5470498	Ctsw	-0.076795	0.12193	insignificant	0.047849	0.38859	insignificant	2	10	10
chr19	5473689	5475689	Efemp2	-0.11593	0.16223	insignificant	-0.0086114	0.091846	insignificant	42	123	122
chr19	5473734	5475734	Efemp2	-0.11593	0.16223	insignificant	-0.0086114	0.091846	insignificant	42	123	122
chr19	5488336	5490336	Mus81	-0.25748	0.0058799	hypomethylated	-0.029653	0.045429	inconclusive	9	65	47
chr19	5489454	5491454	Cfl1	-0.10575	8.32E-44	hypomethylated	-0.018678	0.47139	insignificant	45	146	135
chr19	5510489	5512489	Snx32	-0.17213	1.12E-13	hypomethylated	-0.0094881	0.16345	insignificant	22	72	72
chr19	5560575	5562575	Ovol1	-0.025891	0.000000025	hypomethylated	0.027093	0.71259	insignificant	21	85	87
chr19	5567073	5569073	Gm962	-0.144	1.95E-27	hypomethylated	-0.016491	0.044848	hypomethylated	50	165	190
chr19	5600872	5602872	Rnaseh2c	-0.14408	2.74E-28	hypomethylated	0.039582	0.0042499	inconclusive	12	66	44
chr19	5609707	5611707	Kat5	-0.13085	1.38E-19	hypomethylated	0.003971	0.471	insignificant	17	80	77
chr19	5610094	5612094	Kat5	-0.14933	0.22893	insignificant	0.023106	0.060706	insignificant	1	24	21
chr19	5636489	5638489	Rela	-0.11888	3.95E-21	hypomethylated	-0.013717	0.61928	insignificant	53	184	184
chr19	5663707	5665707	Sipa1	-0.21599	0.14237	insignificant	-0.0051233	0.11735	insignificant	11	28	28
chr19	5688130	5690130	Map3k11	-0.15365	4.83E-52	hypomethylated	-0.0012204	0.23811	insignificant	57	181	178
chr19	5688908	5690908	Pcnxl3	-0.1962	1.11E-36	hypomethylated	-0.0034668	0.72921	insignificant	43	120	120
chr19	5703475	5705475	Kcnk7	-0.42911	0.00083023	stronglyHypometh	0.23713	1	insignificant	1	14	14
chr19	5726317	5728317	Ehbp1l1	-0.12848	0.000022125	hypomethylated	-0.0085061	0.097634	insignificant	11	36	36

chr19	5729666	5731666	Fam89b	-0.021032	0.000000679	inconclusive	0.023062	0.16751	insignificant	15	67	67
chr19	5731721	5733721	Sssca1	-0.74024	0.075251	insignificant	-0.036425	0.26237	insignificant	0	12	14
chr19	5739903	5741903	Ltbp3	-0.27355	7.57E-12	hypomethylated	0.0021214	0.70594	insignificant	16	48	49
chr19	5771401	5773401	Scyl1	-0.10185	0.2226	insignificant	0.081481	0.0093407	hypermethylated	5	12	12
chr19	5802671	5804671	Malat1	-0.15675	0.000015392	hypomethylated	0.0030304	0.16777	insignificant	14	41	41
chr19	5845478	5847478	Neat1	-0.28856	0.000000101	hypomethylated	-0.044599	0.0013273	inconclusive	10	32	30
chr19	5875208	5877208	Frmd8	-0.10553	0.000070739	hypomethylated	-0.023207	0.3556	insignificant	8	28	29
chr19	5877465	5879465	Slc25a45	-0.53305	0.1193	insignificant	-0.02078	0.78196	insignificant	1	10	7
chr19	5894107	5896107	Tigd3	-0.34304	0.00011543	stronglyHypometh	0.020342	0.015123	hypermethylated	8	28	28
chr19	5912866	5914866	Dpf2	-0.0068263	0.20498	insignificant	0.027941	0.45484	insignificant	2	20	21
chr19	5964206	5966206	Pola2	-0.065645	0.11511	insignificant	0.016291	0.46514	insignificant	6	30	30
chr19	6015247	6017247	Capn1	-0.3163	3.85E-11	hypomethylated	-0.017354	0.069312	insignificant	10	70	66
chr19	6015825	6017825	Capn1	-0.40646	0.065325	insignificant	-0.0055807	0.05525	insignificant	4	40	36
chr19	6045575	6047575	Syvn1	-0.09623	4.07E-19	hypomethylated	-0.0019131	0.87358	insignificant	34	118	121
chr19	6046144	6048144	Syvn1	-0.098015	2E-14	hypomethylated	-0.0077706	0.33637	insignificant	35	122	123
chr19	6056887	6058887	Fau	-0.18283	5.37E-09	hypomethylated	-0.028007	0.764	insignificant	9	60	64
chr19	6056966	6058966	Fau	-0.18283	5.37E-09	hypomethylated	-0.028007	0.764	insignificant	9	60	64
chr19	6057751	6059751	Mrpl49	-0.14502	0.000042883	hypomethylated	-0.036158	0.54566	insignificant	6	28	26
chr19	6060206	6062206	Znhit2-ps	-0.15968	4.2E-27	hypomethylated	-0.0099653	0.094171	insignificant	34	162	152
chr19	6067850	6069850	1110014N23Rik	0.24278	0.000027088	hypermethylated	-0.047254	0.0054592	hypomethylated	5	42	47
chr19	6077187	6079187	1110014N23Rik	-0.0061224	0.084906	insignificant	0.28928	0.035341	inconclusive	1	14	10
chr19	6084096	6086096	Cdca5	-0.12321	2.64E-13	hypomethylated	-0.01277	0.73495	insignificant	19	97	97
chr19	6084891	6086891	Zfpl1	-0.08378	2.23E-09	hypomethylated	0.0077289	0.092993	insignificant	21	104	109
chr19	6104797	6106797	Naaladl1	-0.21958	0.34457	insignificant	-0.0034084	0.57735	insignificant	3	10	10
chr19	6118586	6120586	Snx15	-0.25216	1	insignificant	-0.0034228	0.83849	insignificant	1	10	10
chr19	6141137	6143137	Arl2	-0.3042	3.24E-10	hypomethylated	0.0076027	0.035638	hypermethylated	5	47	47
chr19	6183409	6185409	1700123I01Rik	0.049323	0.20833	insignificant	0.017475	0.46319	insignificant	7	29	32
chr19	6225400	6227400	Gpha2	-0.034152	0.33226	insignificant	-0.043864	0.29573	insignificant	2	14	16
chr19	6235840	6237840	Ppp2r5b	-0.32947	0.00000297	hypomethylated	0.0097958	0.83476	insignificant	6	18	17
chr19	6240667	6242667	Atg2a		1	noCoverage	-0.0066811	0.81992	insignificant	0	45	36
chr19	6275895	6277895	Ehd1	-0.11853	8E-28	hypomethylated	-0.00084267	1	insignificant	50	176	176
chr19	6305456	6307456	Cdc42bpg	-0.12893	2.51E-24	hypomethylated	0.02922	0.72919	insignificant	21	83	77
chr19	6333978	6335978	Men1	-0.1417	3.62E-10	hypomethylated	0.0040294	0.60518	insignificant	20	72	70
chr19	6334012	6336012	Men1	-0.1417	3.62E-10	hypomethylated	0.0040294	0.60518	insignificant	20	72	70
chr19	6334038	6336038	Men1	-0.1417	3.62E-10	hypomethylated	0.0040294	0.60518	insignificant	20	72	70
chr19	6340249	6342249	Map4k2	-0.095818	5.83E-15	hypomethylated	-0.0040133	0.64372	insignificant	29	90	90
chr19	6362689	6364689	Sf1	-0.10137	6.04E-37	hypomethylated	-0.015056	0.011248	hypomethylated	77	229	229
chr19	6383428	6385428	Pygm	-0.022876	0.10751	insignificant	-0.12156	0.28955	insignificant	6	26	26
chr19	6399582	6401582	Rasgrp2	-0.12851	1.26E-14	hypomethylated	-0.028642	0.57188	insignificant	23	47	69
chr19	6427015	6429015	Nrxn2	-0.11409	5.05E-28	hypomethylated	-0.0083694	0.83191	insignificant	51	155	154
chr19	6915091	6917091	Rps6ka4	-0.20405	5.58E-10	hypomethylated	-0.040279	0.14675	insignificant	13	33	33
chr19	6932701	6934701	Ccdc88b	-0.31667	0.011768	hypomethylated	0.0084824	0.4142	insignificant	3	6	6
chr19	6983187	6985187	Trmt112	-0.098886	5.05E-15	hypomethylated	0.0024523	0.97211	insignificant	34	153	153
chr19	6984135	6986135	Prdx5	-0.061742	0.70809	insignificant	-0.023475	1	insignificant	27	142	138
chr19	6996298	6998298	Esrra	-0.19738	0.000001042	hypomethylated	0.0071434	0.52088	insignificant	14	60	60
chr19	6999870	7001870	Kcnk4	-0.29649	9.68E-08	hypomethylated	0.069398	0.11154	insignificant	13	85	85
chr19	7009005	7011005	Kcnk4		1	noCoverage	-0.091667	0.20821	insignificant	0	2	2
chr19	7015344	7017344	Bad	-0.13846	1.17E-24	hypomethylated	-0.0094267	0.79415	insignificant	38	133	125
chr19	7015683	7017683	Gpr137	-0.13846	1.17E-24	hypomethylated	-0.0094267	0.79415	insignificant	38	133	125
chr19	7015997	7017997	Gpr137	-0.12468	6.87E-14	hypomethylated	-0.0043634	0.62668	insignificant	26	104	97
chr19	7016940	7018940	Gpr137	-0.23248	0.00012005	hypomethylated	-0.0034987	0.87031	insignificant	6	14	14
chr19	7044242	7046242	Plcb3	-0.20952	0.002352	hypomethylated	0.042822	0.73709	insignificant	4	39	34
chr19	7048537	7050537	Ppp1r14b	-0.12806	4.05E-39	hypomethylated	-0.0084243	0.00000249	hypomethylated	27	117	121
chr19	7054930	7056930	Fkbp2	-0.13523	0.0059304	hypomethylated	0.079325	0.34937	insignificant	9	32	36
chr19	7054951	7056951	Fkbp2	-0.16613	0.00025086	hypomethylated	0.092982	0.64916	insignificant	9	26	30
chr19	7062141	7064141	Vegfb	-0.17877	3.78E-11	hypomethylated	-0.063758	1.75E-09	hypomethylated	18	49	52
chr19	7066762	7068762	Dnajc4	-0.27294	0.29938	insignificant	-0.022284	0.36887	insignificant	2	10	10
chr19	7069620	7071620	Trpt1	-0.26261	4.57E-17	hypomethylated	0.01482	1.34E-11	inconclusive	15	59	66
chr19	7070527	7072527	Nudt22	-0.26418	2.54E-11	hypomethylated	-0.000015864	0.000018227	hypomethylated	9	37	38
chr19	7093959	7095959	Fermt3	-0.41005	0.00067717	stronglyHypometh	-0.027685	1	insignificant	2	6	6
chr19	7114516	7116516	Stip1	-0.24598	4.34E-12	hypomethylated	-0.0034696	0.036078	hypomethylated	15	43	42
chr19	7130257	7132257	Macrod1	-0.18248	3.45E-14	hypomethylated	-0.037393	0.098393	insignificant	19	82	91
chr19	7280774	7282774	Otub1	0.17866	0.2819	insignificant	-0.0030225	1	insignificant	4	46	44
chr19	7292106	7294106	Cox8a	-0.50267	2.3E-09	stronglyHypometh	-0.0045419	0.000049516	hypomethylated	3	39	40
chr19	7315712	7317712	Naa40	-0.16131	4.14E-10	hypomethylated	-0.041769	9.78E-13	hypomethylated	14	38	38
chr19	7343253	7345253	Rcor2	-0.23494	2.51E-21	hypomethylated	-0.0033961	0.35748	insignificant	16	71	71
chr19	7416334	7418334	Mark2	-0.23503	1.72E-13	hypomethylated	-0.051643	0.010271	hypomethylated	11	24	29
chr19	7457516	7459516	AI846148	-0.17452	3.94E-13	hypomethylated	-0.036546	0.27315	insignificant	14	35	37
chr19	7491114	7493114	2700081O15Rik	-0.11401	1.69E-62	hypomethylated	-0.0065411	0.010712	hypomethylated	78	213	217
chr19	7567529	7569529	Atl3	-0.18815	1.02E-17	hypomethylated	0.034894	0.020583	hypermethylated	29	89	92
chr19	7567927	7569927	Atl3	-0.18815	1.02E-17	hypomethylated	0.034894	0.020583	hypermethylated	29	89	92
chr19	7686058	7688058	Hrasls5	-0.29675	0.065672	insignificant	0.021115	0.18194	insignificant	3	20	20
chr19	8206472	8208472	Slc22a28		1	noCoverage	0.18391	1	insignificant	0	5	4
chr19	8479595	8481595	Slc22a30		1	noCoverage	0.011706	1	insignificant	0	8	5
chr19	8666671	8668671	Slc22a8		1	noCoverage	0.013276	0.73184	insignificant	0	4	4
chr19	8737494	8739494	Chrm1	-0.44444	0.50046	insignificant	0.0030193	0.70115	insignificant	2	3	7
chr19	8788387	8790387	Slc3a2	-0.022243	0.32453	insignificant	0.026947	0.21052	insignificant	5	43	42
chr19	8796976	8798976	Snhg1		1	noCoverage	0.054832	0.80245	insignificant	0	34	26
chr19	8797859	8799859	Snord22		1	noCoverage	0.12708	0.4028	insignificant	0	14	10
chr19	8809328	8811328	Wdr74	-0.13867	7.53E-20	hypomethylated	-0.014288	0.16243	insignificant	10	44	40
chr19	8814207	8816207	1700092M07Ril	-0.16564	0.052042	insignificant	-0.044522	0.064748	insignificant	7	32	32
chr19	8814913	8816913	Stx5a	-0.16291	0.023433	hypomethylated	-0.0097465	0.65588	insignificant	7	42	45
chr19	8815292	8817292	Stx5a	-0.1555	0.023416	hypomethylated	-0.0023416	0.61121	insignificant	7	44	45
chr19	8830592	8832592	Nxf1	-0.1092	1.17E-10	hypomethylated	-0.0087967	0.1917	insignificant	34	104	104
chr19	8844485	8846485	Tmem223	-0.10591	0.19553	insignificant	0.23392	1.26E-39	hypermethylated	10	40	42

chr19	8847974	8849974	Gm2518	-0.11463	7.01E-24	hypomethylated	-0.01566	0.041498	hypomethylated	46	148	150
chr19	8848957	8850957	Tmem179b	-0.10224	1.69E-19	hypomethylated	-0.0074139	0.6643	insignificant	42	124	127
chr19	8873047	8875047	Polr2g	-0.11581	1	insignificant	-0.001285	0.43261	insignificant	1	10	10
chr19	8875985	8877985	Zbtb3	-0.31032	3.35E-11	hypomethylated	-0.018117	0.62402	insignificant	4	14	14
chr19	8876019	8878019	Zbtb3	-0.31032	3.35E-11	hypomethylated	-0.018117	0.62402	insignificant	4	14	14
chr19	8892890	8894890	Hnrnpul2	-0.10718	3.77E-15	hypomethylated	0.018536	0.63658	insignificant	28	140	141
chr19	8893784	8895784	Hnrnpul2	-0.10631	4.78E-48	hypomethylated	-0.0004571	0.046397	hypomethylated	43	178	172
chr19	8910956	8912956	Bscl2	0.057257	1	insignificant	0.019679	0.54673	insignificant	5	46	43
chr19	8913736	8915736	Gng3		1	noCoverage	0.010629	0.089467	insignificant	0	16	16
chr19	8945048	8947048	Ubxn1	-0.13314	8.11E-38	hypomethylated	-0.010761	0.000022213	hypomethylated	25	81	81
chr19	8962392	8964392	1810009A15Rik	-0.1958	1.03E-11	hypomethylated	-0.018982	0.47803	insignificant	13	65	65
chr19	8963260	8965260	5730408K05Rik	-0.26425	9.33E-10	hypomethylated	-0.048429	0.26216	insignificant	6	27	27
chr19	8966476	8968476	Ints5	-0.17376	4.55E-27	hypomethylated	0.020435	0.024203	inconclusive	16	69	72
chr19	8971600	8973600	Ganab	-0.23693	1.43E-14	hypomethylated	-0.0036466	0.28734	insignificant	18	70	74
chr19	8993882	8995882	B3gat3	-0.12998	0.000000664	hypomethylated	0.0047225	0.55123	insignificant	14	48	51
chr19	9003183	9005183	Eml3	-0.1625	8.45E-23	hypomethylated	-0.0064243	0.47897	insignificant	28	124	130
chr19	9003846	9005846	Rom1	-0.13215	9.72E-22	hypomethylated	-0.0066927	0.17527	insignificant	28	110	116
chr19	9015409	9017409	Mta2	-0.13893	4.49E-49	hypomethylated	0.0032005	0.000070134	inconclusive	54	153	151
chr19	9027339	9029339	Tut1	-0.12132	1	insignificant	0.037407	1	insignificant	4	18	14
chr19	9040530	9042530	Eef1g	-0.23094	0.0056213	hypomethylated	-0.056801	0.46494	insignificant	3	27	28
chr19	9062773	9064773	Ahnak	-0.14175	5.05E-29	hypomethylated	-0.013425	0.074985	insignificant	28	91	105
chr19	9162446	9164446	Scgb1a1	0.26228	1	insignificant	0.065228	0.20061	insignificant	1	8	8
chr19	9974023	9976023	Incenp	-0.18352	1.27E-08	hypomethylated	-0.031363	0.09838	insignificant	7	34	35
chr19	10056192	10058192	Fth1		1	noCoverage	0.0050756	0.53552	insignificant	0	22	18
chr19	10091717	10093717	Rab3il1	-0.098018	1.54E-09	hypomethylated	-0.0034448	0.80923	insignificant	11	52	52
chr19	10115037	10117037	Fads3	-0.13157	1.85E-20	hypomethylated	-0.018035	0.15648	insignificant	32	111	112
chr19	10175993	10177993	Fads2	-0.13883	9.09E-11	hypomethylated	-0.00023381	0.95897	insignificant	22	61	61
chr19	10256377	10258377	Fads1	-0.1352	6.86E-39	hypomethylated	-0.0058727	0.14998	insignificant	41	125	125
chr19	10277691	10279691	1810006K21Rik	-0.17071	5.48E-12	hypomethylated	-0.018402	0.004063	hypomethylated	14	42	41
chr19	10278433	10280433	1810006K21Rik	-0.15648	4.66E-13	hypomethylated	-0.027314	0.42049	insignificant	10	26	24
chr19	10315238	10317238	Gm98	-0.13008	0.000033435	hypomethylated	0.020061	0.91907	insignificant	7	38	38
chr19	10379367	10381367	Dagla	-0.12047	7.91E-08	hypomethylated	0.029354	0.018201	inconclusive	7	18	18
chr19	10462579	10464579	Syt7	-0.079736	2.46E-42	hypomethylated	0.005457	0.0052657	inconclusive	67	185	185
chr19	10531937	10533937	Lrrc10b	-0.065146	0.098338	insignificant	0.03237	0.73281	insignificant	13	36	46
chr19	10557387	10559387	Ppp1r32	-0.10476	0.33935	insignificant	-0.035498	0.042091	hypomethylated	2	5	7
chr19	10598733	10600733	Cpsf7	-0.12079	3.09E-19	hypomethylated	-0.012945	0.00016702	hypomethylated	37	164	160
chr19	10599699	10601699	Sdhaf2	-0.129	1.99E-12	hypomethylated	-0.017266	0.010212	hypomethylated	30	128	124
chr19	10651212	10653212	Cybasc3	-0.21955	5.41E-11	hypomethylated	-0.015099	0.64694	insignificant	12	55	56
chr19	10651587	10653587	Cybasc3	-0.25484	1.35E-11	hypomethylated	-0.0081326	0.85564	insignificant	11	48	48
chr19	10678748	10680748	Dak	-0.23677	0.00038076	hypomethylated	0.043572	0.32315	insignificant	5	18	23
chr19	10679114	10681114	Ddb1	-0.23677	0.00038076	hypomethylated	0.043572	0.32315	insignificant	5	18	23
chr19	10707722	10709722	Vwce	-0.31128	1.09E-24	hypomethylated	-0.037771	0.17136	insignificant	12	52	52
chr19	10762304	10764304	Vps37c	-0.11578	1.99E-16	hypomethylated	-0.014246	1	insignificant	25	44	51
chr19	10813464	10815464	Cd5	-0.22396	0.44231	insignificant	-0.21563	0.026334	hypomethylated	1	4	4
chr19	10814436	10816436	A430093F15Rik	-0.22396	0.44231	insignificant	-0.21563	0.026334	hypomethylated	1	4	4
chr19	10904548	10906548	Cd6	-0.34511	0.0019459	stronglyHypometh	0.0097184	0.91664	insignificant	3	6	6
chr19	10916033	10918033	Slc15a3	-0.4707	0.000000224	stronglyHypometh	-0.070179	0.8503	insignificant	2	16	15
chr19	10944269	10946269	Tmem109	0.29645	0.018159	hypermethylated	0.0039692	0.097224	insignificant	3	15	15
chr19	10956233	10958233	Tmem109	-0.15592	0.000066118	hypomethylated	-0.030893	0.31334	insignificant	5	34	38
chr19	10968781	10970781	Prpf19	-0.12841	5.04E-22	hypomethylated	-0.00009199	0.023271	hypomethylated	21	73	74
chr19	11010649	11012649	Gpr44	0.020833	1	lowCoverage	0.020833	1	insignificant	1	4	4
chr19	11023756	11025756	Ccdc86	-0.23697	0.00000173	hypomethylated	-0.041962	0.047808	hypomethylated	9	31	30
chr19	11239810	11241810	1700025F22Rik		1	noCoverage	-0.30931	0.14984	insignificant	0	8	7
chr19	11271213	11273213	4930526L06Rik	0.27028	1	insignificant	0.12405	0.53557	insignificant	1	20	20
chr19	11358303	11360303	Ms4a5	-0.91637	0.0089629	stronglyHypometh	-0.072877	0.15847	insignificant	2	19	19
chr19	11821048	11823048	Gif	-0.043056	0.22969	insignificant	-0.15739	0.000000987	hypomethylated	1	8	8
chr19	11843904	11845904	Mrpl16	-0.41295	5.03E-12	stronglyHypometh	-0.015864	0.92962	insignificant	7	16	16
chr19	11893383	11895383	Stx3	-0.22907	1.3E-11	hypomethylated	-0.025792	0.62007	insignificant	18	54	67
chr19	11893893	11895893	Stx3	-0.22878	0.000046626	hypomethylated	-0.024438	0.32399	insignificant	6	34	34
chr19	11945704	11947704	Olfr1419	0.15444	0.35473	insignificant	0.082937	0.1415	insignificant	3	6	6
chr19	11985888	11987888	Patl1	-0.075691	1.17E-10	hypomethylated	0.0026195	0.057633	insignificant	18	94	101
chr19	12039333	12041333	Osbp	-0.11874	1.59E-22	hypomethylated	-0.011749	0.70106	insignificant	51	146	156
chr19	12184034	12186034	Olfr1428	-0.091164	0.6185	insignificant	0.047397	0.0060076	hypermethylated	1	21	21
chr19	12978720	12980720	Gm5512		1	noCoverage	-0.10296	0.33919	insignificant	0	10	16
chr19	14672473	14674473	Tle4	-0.044031	0.00000302	hypomethylated	0.024444	0.16186	insignificant	26	139	140
chr19	15999515	16001515	Psat1	-0.40554	0.02484	stronglyHypometh	0.020109	0.30722	insignificant	1	12	16
chr19	16059479	16061479	Cep78	-0.013604	0.019026	hypomethylated	0.0039796	0.10991	insignificant	8	44	44
chr19	16206320	16208320	Gnaq	-0.094581	8.77E-51	hypomethylated	0.0040957	0.55317	insignificant	83	265	264
chr19	16237587	16239587	E030024N20Rik	0.20317	0.12223	insignificant	0.035008	0.57838	insignificant	1	5	5
chr19	16509156	16511156	Gna14	-0.19575	9.99E-37	hypomethylated	-0.038197	0.16438	insignificant	23	88	84
chr19	16855417	16857417	Vps13a	-0.091275	0.0001255	hypomethylated	-0.0081687	0.77977	insignificant	19	50	50
chr19	16948320	16950320	Foxb2	-0.1323	0.10517	insignificant	0.0023543	0.013135	hypermethylated	6	33	31
chr19	17029607	17031607	Prune2	-0.20215	0.60075	insignificant	-0.097984	0.35979	insignificant	4	31	20
chr19	17431157	17433157	Gcnt1		1	noCoverage	0.16202	0.66332	insignificant	0	3	2
chr19	17467532	17469532	Rfk	-0.083233	1.57E-15	hypomethylated	-0.0082083	0.79866	insignificant	19	84	84
chr19	17912122	17914122	Pcsk5	-0.19932	0.0000027	hypomethylated	-0.037039	0.00057148	hypomethylated	14	35	33
chr19	18705505	18707505	BC016495	-0.11469	1.7E-27	hypomethylated	0.0045604	0.31736	insignificant	28	100	106
chr19	18706303	18708303	BC016495	-0.20333	0.080638	insignificant	-0.028684	0.46712	insignificant	4	18	24
chr19	18744269	18746269	2410127L17Rik	-0.11196	1.4E-10	hypomethylated	-0.0006662	0.79007	insignificant	32	117	117
chr19	18786725	18788725	D030056L22Rik	-0.07885	0.00000249	hypomethylated	0.0061178	0.65816	insignificant	24	57	56
chr19	18823472	18825472	Trpm6	-0.29819	9.22E-13	hypomethylated	0.046433	0.63988	insignificant	13	58	61
chr19	19185686	19187686	Rorb		1	noCoverage	0.31936	0.45294	insignificant	0	8	8
chr19	20675471	20677471	Aldh1a1		1	noCoverage	0.049653	0.75524	insignificant	0	5	8
chr19	21345767	21347767	Zfand5	-0.10587	1.57E-44	hypomethylated	-0.009025	0.096723	insignificant	69	237	227
chr19	21726798	21728798	Fam108b	-0.089092	5.67E-22	hypomethylated	-0.0034296	0.3242	insignificant	64	197	193

chr19	21727281	21729281	1110059E24Rik	-0.085115	4.33E-21	hypomethylated	-0.0069758	0.50274	insignificant	60	187	182
chr19	21851831	21853831	Tmem2	-0.14249	3.96E-28	hypomethylated	0.01297	0.48328	insignificant	38	110	113
chr19	22212606	22214606	Trpm3	-0.23553	0.042842	hypomethylated	-0.02349	0.14236	insignificant	6	18	18
chr19	22521697	22523697	Trpm3	-0.25267	0.55265	insignificant	0.0938	0.50926	insignificant	2	12	12
chr19	23214715	23216715	Klf9	-0.10763	1.72E-40	hypomethylated	-0.0049678	0.005918	hypomethylated	49	200	206
chr19	23348367	23350367	Smc5	-0.15561	0.0078759	hypomethylated	-0.061028	0.093364	insignificant	10	28	28
chr19	23760889	23762889	Ptar1	-0.10964	4.41E-34	hypomethylated	0.00014597	0.23377	insignificant	44	144	145
chr19	23832365	23834365	Apba1	-0.11403	1.99E-11	hypomethylated	-0.015662	0.028832	hypomethylated	24	103	109
chr19	24105509	24107509	Fam189a2	0.012959	0.000000751	inconclusive	0.001205	0.0001254	inconclusive	17	59	60
chr19	24248630	24250630	Tjp2	-0.097966	1.24E-15	hypomethylated	0.0024211	0.91168	insignificant	30	110	110
chr19	24299516	24301516	Tjp2	-0.20178	0.000020573	hypomethylated	0.04688	0.076135	insignificant	7	36	34
chr19	24355076	24357076	Fxn	-0.49251	0.43023	lowCoverage	-0.03936	1	insignificant	1	6	8
chr19	24551964	24553964	Fam122a	-0.18555	0.0007586	hypomethylated	0.036085	0.17615	insignificant	1	25	28
chr19	24630317	24632317	Pip5k1b	-0.11168	8.09E-35	hypomethylated	-0.0051467	0.004581	hypomethylated	44	150	149
chr19	24747497	24749497	E030010A14Rik	0.17659	1	insignificant	-0.090079	0.55693	insignificant	2	6	8
chr19	24936332	24938332	Pgm5	-0.35461	8.42E-10	stronglyHypometh	0.20639	0.1404	insignificant	1	3	2
chr19	24975799	24977799	Foxd4		1	noCoverage	0.065607	1	insignificant	0	6	6
chr19	25036106	25038106	Cbwd1	-0.16733	0.019587	hypomethylated	-0.005075	0.92485	insignificant	7	22	22
chr19	25073018	25075018	Dock8	-0.11278	1.83E-45	hypomethylated	-0.0010729	0.95764	insignificant	63	163	157
chr19	25310691	25312691	Kank1	-0.12281	6.23E-12	hypomethylated	-0.0059075	0.53938	insignificant	42	138	140
chr19	25579195	25581195	Dmrt1	-0.16146	1.19E-13	hypomethylated	-0.031418	0.0084249	hypomethylated	22	72	72
chr19	25684026	25686026	Dmrt3	-0.13042	2.35E-57	hypomethylated	-0.012298	0.39131	insignificant	61	216	218
chr19	25745900	25747900	Dmrt2	-0.16574	1.84E-19	hypomethylated	-0.01926	0.82428	insignificant	15	74	69
chr19	26678649	26680649	Smarca2	-0.13336	9.23E-34	hypomethylated	-0.003404	0.002243	hypomethylated	42	189	196
chr19	26821892	26823892	Smarca2	-0.24324	0.57564	insignificant	0.037716	0.72328	insignificant	1	14	14
chr19	27290509	27292509	Vldlr	-0.094523	1.8E-23	hypomethylated	-0.0077755	0.32838	insignificant	51	162	161
chr19	27396108	27398108	Kcnv2	0.036663	0.30392	insignificant	0.045932	0.0010222	hypermethylated	3	18	18
chr19	27503526	27505526	C030016D13Rik	-0.1335	1.91E-23	hypomethylated	-0.023634	0.24373	insignificant	27	96	101
chr19	27504310	27506310	D19Bwg1357e	-0.075395	0.0010684	hypomethylated	-0.017769	0.53101	insignificant	8	48	53
chr19	28085656	28087656	Rfx3	-0.18759	5.44E-19	hypomethylated	-0.03525	0.027848	hypomethylated	27	92	86
chr19	28754567	28756567	Glis3	-0.11565	0.0001177	hypomethylated	0.036628	0.5766	insignificant	5	21	21
chr19	28908655	28910655	Slc1a1	-0.18528	2.76E-15	hypomethylated	-0.01237	0.10718	insignificant	21	71	73
chr19	29037409	29039409	Ppapdc2	-0.14305	1.12E-48	hypomethylated	-0.0038113	0.036967	hypomethylated	28	96	96
chr19	29038644	29040644	4430402I18Rik	-0.14281	4.5E-29	hypomethylated	-0.012103	0.00048778	hypomethylated	21	52	52
chr19	29063983	29065983	Cdc37l1	-0.17546	2.22E-18	hypomethylated	-0.015586	0.96661	insignificant	34	84	84
chr19	29120972	29122972	1700018L02Rik	-0.17718	0.000011478	hypomethylated	-0.01439	0.13234	insignificant	20	75	70
chr19	29122392	29124392	1700018L02Rik	-0.16635	0.001754	hypomethylated	0.014853	0.37522	insignificant	9	22	18
chr19	29174864	29176864	Rcl1	-0.12989	1.09E-33	hypomethylated	0.0050628	0.84655	insignificant	46	120	118
chr19	29208768	29210768	Mir101b	-0.017521	0.33873	insignificant	-0.026099	0.17469	insignificant	5	10	10
chr19	29325317	29327317	Jak2	-0.084224	5.2E-34	hypomethylated	-0.0060876	0.026018	hypomethylated	72	215	204
chr19	29399808	29401808	Insl6	0.23972	0.0056459	hypermethylated	0.079158	0.000012749	hypermethylated	3	21	23
chr19	29409160	29411160	Rln1	-0.24451	0.0025807	hypomethylated	0.35549	0.018625	stronglyhypermeth	0	2	0
chr19	29436361	29438361	5033414D02Rik	-0.28121	0.0098758	hypomethylated	0.062262	0.08062	insignificant	4	18	19
chr19	29440927	29442927	Cd274	-0.32142	0.00305	hypomethylated	-0.097573	0.25715	insignificant	3	19	14
chr19	29595771	29597771	C030046E11Rik	-0.11815	7.61E-76	hypomethylated	-0.0032321	0.04384	hypomethylated	93	253	261
chr19	29596477	29598477	C030046E11Rik	-0.11808	6.25E-71	hypomethylated	-0.012803	0.0096153	hypomethylated	88	238	245
chr19	29771430	29773430	Mlana	0.023148	0.74225	insignificant	-0.061314	0.03016	hypomethylated	3	6	6
chr19	29880499	29882499	9930021J03Rik	-0.12682	0.000000337	hypomethylated	0.0039423	0.77794	insignificant	21	62	62
chr19	29887464	29889464	Ranbp6	-0.13955	7.44E-11	hypomethylated	0.025144	0.0015758	inconclusive	9	38	38
chr19	30077279	30079279	Trpd52l3	0.049306	1	insignificant	0.020961	0.92266	insignificant	3	24	23
chr19	30104002	30106002	Uhrf2	-0.15113	3.82E-36	hypomethylated	-0.0057078	0.0000061	hypomethylated	52	157	153
chr19	30614169	30616169	Ppp1r2-ps3		1	noCoverage	0.0061504	0.90118	insignificant	0	34	34
chr19	31156330	31158330	Cstf2t	-0.14193	0.000000764	hypomethylated	0.014626	0.019483	inconclusive	26	82	82
chr19	31738860	31740860	Prkg1	-0.12638	1.4E-15	hypomethylated	0.014039	0.18862	insignificant	13	51	51
chr19	31942250	31944250	A1cf	0.13718	1	insignificant	-0.023606	0.36975	insignificant	2	22	21
chr19	32462705	32464705	2700046G09Rik	-0.12836	7.46E-54	hypomethylated	-0.015411	0.69349	insignificant	65	139	167
chr19	32462944	32464944	Sgms1	-0.14173	7.02E-34	hypomethylated	-0.019096	0.57677	insignificant	40	99	123
chr19	32559258	32561258	Minpp1	-0.14473	6.57E-37	hypomethylated	-0.020302	0.097214	insignificant	45	105	111
chr19	32786788	32788788	Atad1	-0.18767	1.29E-11	hypomethylated	0.011245	0.94666	insignificant	9	18	18
chr19	32831066	32833066	Pten	-0.14024	2.34E-47	hypomethylated	-0.030618	0.00000519	hypomethylated	63	213	223
chr19	34265759	34267759	Stambpl1	-0.14646	8.5E-13	hypomethylated	-0.036837	0.003045	hypomethylated	19	65	65
chr19	34364148	34366148	Fas	-0.22068	0.080845	insignificant	0.018909	0.053959	insignificant	8	35	36
chr19	34549625	34551625	Ch25h	-0.37335	0.077289	insignificant	0.026881	0.90297	insignificant	1	8	8
chr19	34601964	34603964	Lipa	-0.65957	0.014119	stronglyHypometh	-0.27361	0.31463	insignificant	1	2	3
chr19	34624183	34626183	Ifit2		1	noCoverage	-0.037881	0.33335	insignificant	0	6	6
chr19	34673917	34675917	Gm14446		1	noCoverage	-0.083333	1	insignificant	0	4	3
chr19	34676458	34678458	Gm14446		1	noCoverage	-0.030896	0.85217	insignificant	0	4	4
chr19	34821601	34823601	Slc16a12	-0.26743	6.29E-21	hypomethylated	-0.065564	0.014936	hypomethylated	16	36	34
chr19	34895263	34897263	Pank1	-0.39226	0.019811	stronglyHypometh	-0.011473	0.62621	insignificant	1	15	15
chr19	34952407	34954407	Pank1	-0.10056	9.83E-22	hypomethylated	-0.0027802	0.034773	hypomethylated	53	163	166
chr19	34953945	34955945	Pank1	-0.3525	3.74E-13	stronglyHypometh	0.017159	0.68766	insignificant	5	12	13
chr19	34995847	34997847	Kif20b	-0.23577	3.23E-21	hypomethylated	0.025203	0.89501	insignificant	17	55	68
chr19	36131850	36133850	Htr7	-0.14756	0.000004093	hypomethylated	-0.02325	0.234	insignificant	34	97	95
chr19	36157205	36159205	Rpp30	-0.21019	0.000049475	hypomethylated	0.02796	0.3461	insignificant	8	26	26
chr19	36628128	36630128	Hectd2	-0.10837	1.96E-23	hypomethylated	-0.0068091	0.96428	insignificant	37	106	122
chr19	36811094	36813094	Ppp1r3c	-0.25606	0.43928	insignificant	-0.10517	0.30929	insignificant	12	47	42
chr19	36907721	36909721	Tnks2	-0.12439	1.32E-37	hypomethylated	0.00019098	0.55725	insignificant	31	114	116
chr19	36999568	37001568	Btaf1	-0.099181	8.65E-44	hypomethylated	0.0016426	0.0070422	inconclusive	52	154	159
chr19	37281034	37283034	March5	-0.091589	9.72E-11	hypomethylated	-0.013255	0.0034832	hypomethylated	68	178	174
chr19	37281783	37283783	March5	-0.092847	0.00094158	hypomethylated	-0.015907	0.17029	insignificant	52	127	123
chr19	37449892	37451892	Kif11	-0.14696	0.00000296	hypomethylated	-0.004858	0.17185	insignificant	9	67	58
chr19	37508330	37510330	Hhex	-0.097301	8.35E-31	hypomethylated	-0.0019026	0.7883	insignificant	48	244	249
chr19	37623907	37625907	Exoc6	-0.10496	2.46E-17	hypomethylated	-0.012851	0.83941	insignificant	41	100	107
chr19	37759169	37761169	Cyp26c1	0.016763	0.33446	insignificant	0.027621	0.02863	hypermethylated	6	21	25
chr19	37771297	37773297	Cyp26a1	-0.15201	4.73E-16	hypomethylated	-0.0085064	1	insignificant	22	109	109

chr19	38118067	38120067	Myof	0.087342	0.038183	hypermethylated	0.014215	0.000043746	hypermethylated	3	18	18
chr19	38128514	38130514	Cep55	-0.10078	0.10108	insignificant	-0.0051564	0.8634	insignificant	14	56	69
chr19	38128531	38130531	Cep55	-0.10078	0.10108	insignificant	-0.0051564	0.8634	insignificant	14	56	69
chr19	38170568	38172568	O3far1	-0.12557	0.00000163	hypomethylated	-0.012829	0.72078	insignificant	22	76	82
chr19	38199301	38201301	Rbp4	-0.083125	0.000076864	hypomethylated	-0.0092297	0.3859	insignificant	13	34	34
chr19	38206270	38208270	Pde6c	-0.021295	0.056012	insignificant	-0.018636	0.52098	insignificant	4	12	12
chr19	38298622	38300622	5730455O13Rik	-0.16444	2.55E-10	hypomethylated	0.0087342	0.59445	insignificant	9	22	22
chr19	38338271	38340271	Lgi1	-0.37007	0.000000645	stronglyHypometh	0.12477	0.8103	insignificant	3	6	9
chr19	38469469	38471469	Tmem20	-0.12079	1.61E-20	hypomethylated	0.0061868	0.5246	insignificant	25	70	72
chr19	38597686	38599686	Plce1	-0.07265	0.020369	hypomethylated	0.0071497	0.053561	insignificant	5	18	14
chr19	38893727	38895727	Noc3l	-0.12351	0.000071713	hypomethylated	0.014068	0.94456	insignificant	15	30	48
chr19	38910068	38912068	Tbc1d12	-0.10501	8.46E-28	hypomethylated	-0.02187	0.47924	insignificant	42	161	180
chr19	39004479	39006479	Hells	-0.13971	1.17E-09	hypomethylated	-0.0095196	0.54025	insignificant	27	94	90
chr19	39080508	39082508	Cyp2c55	-0.15164	0.00016518	hypomethylated	-0.090466	0.049631	hypomethylated	3	12	14
chr19	40346106	40348106	Pdlim1	-0.16321	3.77E-14	hypomethylated	-0.013203	0.07919	insignificant	8	30	30
chr19	40588226	40590226	Sorbs1	-0.098204	0.000077941	hypomethylated	0.029121	0.114	insignificant	6	18	18
chr19	40588302	40590302	Sorbs1	-0.27273	0.000000738	hypomethylated	0.00048701	0.017047	inconclusive	2	4	4
chr19	40662953	40664953	Aldh18a1		1	noCoverage	0.015063	0.34024	insignificant	0	10	10
chr19	40686705	40688705	Tctn3		1	noCoverage	-0.06086	1	insignificant	0	13	10
chr19	40733283	40735283	Entpd1	-0.3097	0.00018235	hypomethylated	-0.021244	0.78883	insignificant	4	16	18
chr19	40904768	40906768	Ccnj	-0.12578	1.33E-41	hypomethylated	-0.00081912	0.16805	insignificant	64	172	172
chr19	40968194	40970194	Zfp518a	-0.081492	5.21E-12	hypomethylated	-0.0031889	0.42505	insignificant	28	91	97
chr19	41069025	41071025	Blnk	-0.41952	0.00047115	stronglyHypometh	0.026114	0.62388	insignificant	3	34	32
chr19	41102764	41104764	Dntt	0.15275	1	insignificant	0.033427	0.84472	insignificant	1	5	4
chr19	41151603	41153603	Opalin	-0.39769	0.27671	insignificant	-0.13266	1	insignificant	2	6	6
chr19	41281264	41283264	Tll2	-0.16291	0.00063435	hypomethylated	0.0046862	0.75927	insignificant	4	32	32
chr19	41338494	41340494	Tm9sf3	-0.14206	2.65E-10	hypomethylated	0.058408	0.87929	insignificant	13	50	40
chr19	41459560	41461560	Pik3ap1	-0.15122	0.0017192	hypomethylated	-0.04977	0.40763	insignificant	6	35	33
chr19	41818346	41820346	Slit1	-0.2082	0.012344	hypomethylated	0.0071679	0.75449	insignificant	5	22	20
chr19	41903459	41905459	Frat1	-0.11047	6.57E-44	hypomethylated	-0.0020754	0.00056747	hypomethylated	49	178	191
chr19	41922622	41924622	Frat2	-0.26324	0.00054958	hypomethylated	-0.045867	0.94032	insignificant	9	72	70
chr19	41970643	41972643	Rrp12	-0.35081	0.015036	stronglyHypometh	-0.08321	0.0063141	hypomethylated	5	23	28
chr19	41985360	41987360	Pgam1	-0.07188	2.29E-11	hypomethylated	0.000061544	0.36237	insignificant	27	121	116
chr19	42006961	42008961	Zdhhc16	-0.17304	1.39E-36	hypomethylated	-0.006931	0.090695	insignificant	43	130	131
chr19	42007804	42009804	Exosc1	-0.19394	1.3E-23	hypomethylated	-0.017585	0.0031135	hypomethylated	16	64	64
chr19	42055252	42057252	Ubtd1	-0.11883	4.02E-61	hypomethylated	-0.0053577	0.091274	insignificant	70	201	203
chr19	42055626	42057626	Ubtd1	-0.12576	5.36E-57	hypomethylated	-0.0017821	0.14504	insignificant	60	175	170
chr19	42119340	42121340	Hoga1	-0.28744	0.0099545	hypomethylated	-0.054415	0.05978	insignificant	3	20	20
chr19	42163924	42165924	Pi4k2a	-0.13407	6.55E-19	hypomethylated	-0.0082751	0.40994	insignificant	31	107	108
chr19	42203483	42205483	Avpi1	-0.13837	0.053808	insignificant	-0.012734	0.13217	insignificant	9	45	45
chr19	42220878	42222878	Marveld1	-0.16683	2.2E-14	hypomethylated	-0.01167	0.19692	insignificant	26	118	121
chr19	42244056	42246056	Zfyve27	-0.12255	4.07E-31	hypomethylated	0.00078175	0.17301	insignificant	30	92	92
chr19	42276742	42278742	Sfrp5	-0.43333	0.000076879	stronglyHypometh	-0.023061	0.6818	insignificant	3	10	10
chr19	42329228	42331228	Golga7b	-0.13731	2.86E-09	hypomethylated	-0.013944	0.67386	insignificant	22	79	79
chr19	42354660	42356660	Mir3085		1	noCoverage	-0.010363	1	insignificant	0	8	8
chr19	42506273	42508273	Crtac1	-0.08348	0.046443	hypomethylated	0.0028743	0.78425	insignificant	20	95	100
chr19	42592294	42594294	D19Ertd386e	0.0027028	0.10124	insignificant	0.008992	0.72756	insignificant	12	117	118
chr19	42687296	42689296	Loxl4	-0.36053	8.05E-09	stronglyHypometh	-0.12521	0.0026547	hypomethylated	6	17	26
chr19	42827265	42829265	Pyroxd2	-0.41492	0.0021931	stronglyHypometh	0.064105	0.74284	insignificant	2	11	15
chr19	42854466	42856466	Hps1	-0.25	1	lowCoverage	0.020833	0.87787	insignificant	1	8	8
chr19	43513925	43515925	Cnnm1	-0.09869	1.49E-62	hypomethylated	-0.0026697	0.52975	insignificant	55	191	192
chr19	43599095	43601095	Got1	-0.26181	0.015475	hypomethylated	-0.028066	0.58577	insignificant	3	50	47
chr19	43685814	43687814	Nkx2-3	-0.22328	0.000000129	hypomethylated	-0.034539	0.35075	insignificant	17	72	72
chr19	43749371	43751371	Slc25a28	-0.12374	2.96E-09	hypomethylated	-0.011225	0.28102	insignificant	15	75	75
chr19	43763178	43765178	Entpd7	-0.20307	2.24E-16	hypomethylated	-0.021328	0.84294	insignificant	19	42	43
chr19	43826512	43828512	Cutc	-0.14594	1.62E-20	hypomethylated	0.0033245	0.01549	inconclusive	18	107	107
chr19	43827490	43829490	Cutc	-0.036952	0.0030387	hypomethylated	0.00075412	0.57502	insignificant	5	53	53
chr19	44061010	44063010	Cpn1		1	noCoverage	-0.023148	0.061659	insignificant	0	6	6
chr19	44144176	44146176	Erlin1	-0.1087	4.62E-09	hypomethylated	-0.026991	0.26701	insignificant	20	44	44
chr19	44144265	44146265	Erlin1	-0.08973	0.00002013	hypomethylated	-0.019322	0.83072	insignificant	13	42	42
chr19	44144275	44146275	Erlin1	-0.078264	0.000011418	hypomethylated	-0.017974	0.72821	insignificant	12	40	40
chr19	44181967	44183967	Chuk	0.10063	0.34314	insignificant	0.052875	0.62003	insignificant	5	24	26
chr19	44220936	44222936	Bloc1s2	-0.23889	0.036713	hypomethylated	-0.016667	1	insignificant	2	6	6
chr19	44367165	44369165	Scd2	-0.30909	6.9E-11	hypomethylated	0.0055332	0.32771	insignificant	8	22	22
chr19	44406815	44408815	Scd4	0.072619	0.31063	insignificant	0.25349	0.61257	insignificant	2	7	7
chr19	44482199	44484199	Scd1	-0.27092	3.1E-09	hypomethylated	0.065841	0.65678	insignificant	6	20	20
chr19	44620338	44622338	Sec31b	0.064633	0.75056	insignificant	0.13232	0.0036965	inconclusive	4	19	10
chr19	44629905	44631905	Ndufb8	-0.091497	0.0061763	hypomethylated	0.0013605	0.65816	insignificant	6	14	14
chr19	44636343	44638343	Hif1an	-0.19577	8.8E-22	hypomethylated	-0.0068884	0.011911	hypomethylated	18	58	57
chr19	44830883	44832883	Pax2	-0.18753	7.03E-25	hypomethylated	-0.046942	0.30253	insignificant	46	123	123
chr19	45004608	45006608	Fam178a	-0.122	8.39E-20	hypomethylated	-0.024356	0.038313	hypomethylated	19	70	79
chr19	45062833	45064833	Sema4g	-0.12045	1.02E-22	hypomethylated	0.0056787	5.72E-09	inconclusive	34	106	104
chr19	45080047	45082047	Peo1	-0.12807	5.69E-23	hypomethylated	0.024896	0.22877	insignificant	36	165	156
chr19	45080932	45082932	Mrpl43	-0.14674	0.0063874	hypomethylated	0.061522	2.05E-08	hypermethylated	21	79	72
chr19	45088665	45090665	Lzts2	-0.16136	7.37E-14	hypomethylated	0.052041	0.10145	insignificant	16	62	49
chr19	45088699	45090699	Lzts2	-0.16136	7.37E-14	hypomethylated	0.052041	0.10145	insignificant	16	62	49
chr19	45091624	45093624	Lzts2	-0.1448	7.62E-27	hypomethylated	-0.0023024	0.015249	hypomethylated	22	69	69
chr19	45120262	45122262	Sfxn3	-0.32202	0.002904	hypomethylated	-0.08368	0.16435	insignificant	4	14	18
chr19	45121065	45123065	Sfxn3	-0.39834	0.0073657	stronglyHypometh	-0.14336	0.0031385	hypomethylated	3	18	18
chr19	45149628	45151628	Kazald1	-0.10076	0.000015732	hypomethylated	0.02129	0.73765	insignificant	22	76	69
chr19	45224204	45226204	Tlx1	-0.13988	0.0007762	hypomethylated	0.013362	0.82736	insignificant	28	62	70
chr19	45309726	45311726	Lbx1	-0.11691	1.88E-28	hypomethylated	-0.014197	0.038248	hypomethylated	44	113	114
chr19	45437223	45439223	Btrc	-0.24361	0.000046226	hypomethylated	0.027455	0.88487	insignificant	10	40	40
chr19	45634104	45636104	Dpcd	-0.2136	1.37E-12	hypomethylated	-0.0020757	0.056817	insignificant	7	44	44
chr19	45635033	45637033	Poll	-0.19434	1.67E-12	hypomethylated	0.002891	0.055036	insignificant	6	34	34

chr19	45734683	45736683	Fbxw4	-0.17365	5.84E-28	hypomethylated	0.0018474	0.00074959	inconclusive	20	55	59
chr19	45817374	45819374	Fgf8	0.27598	0.015145	hypermethylated	-0.023633	0.58389	insignificant	3	32	32
chr19	45824053	45826053	Npm3	-0.071672	0.00018851	hypomethylated	-0.0081846	0.0055914	hypomethylated	8	26	26
chr19	45857781	45859781	Mgea5	-0.13823	6.43E-11	hypomethylated	-0.0024818	0.86891	insignificant	24	80	76
chr19	45890293	45892293	Kcnip2	-0.27974	4.54E-13	hypomethylated	-0.016796	0.000000333	hypomethylated	18	60	60
chr19	46072978	46074978	9130011E15Rik	-0.31568	2.11E-10	hypomethylated	-0.022129	0.18195	insignificant	10	29	29
chr19	46076967	46078967	Hps6	-0.178	9.48E-17	hypomethylated	-0.014134	0.0010856	hypomethylated	24	84	86
chr19	46114111	46116111	Ldb1	-0.090143	0.61384	insignificant	0.02405	0.55019	insignificant	3	24	27
chr19	46119701	46121701	Ldb1	-0.1074	1.19E-14	hypomethylated	-0.0060344	0.032109	hypomethylated	22	83	88
chr19	46130028	46132028	Pprc1	-0.12646	4.12E-23	hypomethylated	-0.013706	0.20919	insignificant	32	86	103
chr19	46149352	46151352	Nolc1	-0.22578	0.000011722	hypomethylated	0.0030479	0.000012074	hypermethylated	35	110	105
chr19	46205388	46207388	Elovl3	-0.23258	0.017985	hypomethylated	-0.032403	0.90346	insignificant	7	40	37
chr19	46222815	46224815	Pitx3	-0.16073	0.00012337	hypomethylated	-0.042638	0.83295	insignificant	7	49	48
chr19	46226047	46228047	Gbf1	-0.33144	0.000002288	hypomethylated	-0.089288	0.12595	insignificant	6	24	18
chr19	46378226	46380226	Nfkb2	-0.14044	4.59E-27	hypomethylated	0.0029867	0.70208	insignificant	37	133	134
chr19	46379184	46381184	Nfkb2	-0.14616	1.13E-21	hypomethylated	0.0052769	0.77458	insignificant	36	110	118
chr19	46379419	46381419	Nfkb2	-0.13252	1.53E-08	hypomethylated	-0.00018461	0.59937	insignificant	29	96	104
chr19	46401646	46403646	Psd	-0.11969	3.36E-24	hypomethylated	-0.015536	0.52807	insignificant	27	133	147
chr19	46401673	46403673	Fbxl15	-0.11885	6.79E-24	hypomethylated	-0.015071	0.3158	insignificant	28	135	149
chr19	46413150	46415150	Cuedc2		1	noCoverage	0.10941	0.19287	insignificant	0	13	14
chr19	46430369	46432369	Tmem180	0.0050237	1	insignificant	-0.27008	0.37407	insignificant	2	12	13
chr19	46470225	46472225	Sufu	-0.14529	6.6E-32	hypomethylated	-0.029771	0.00016506	hypomethylated	30	103	96
chr19	46470407	46472407	Sufu	-0.15912	2.07E-13	hypomethylated	-0.035669	0.000058171	hypomethylated	24	79	72
chr19	46575137	46577137	Trim8	-0.11883	6.35E-33	hypomethylated	-0.0016846	0.17256	insignificant	68	210	216
chr19	46646854	46648854	Sfxn2	-0.18676	9.15E-10	hypomethylated	-0.0095166	0.24818	insignificant	7	62	67
chr19	46647575	46649575	Arl3	-0.054652	0.000000155	hypomethylated	-0.0091553	0.18843	insignificant	8	54	54
chr19	46696890	46698890	D19Wsu162e	0.16187	0.27453	insignificant	0.044637	0.53267	insignificant	7	20	20
chr19	46763395	46765395	2010012O05Rik	-0.13062	4.81E-08	hypomethylated	0.017076	0.60631	insignificant	11	27	27
chr19	46780932	46782932	As3mt		1	noCoverage	0.096984	0.0035412	hypermethylated	0	10	10
chr19	46835098	46837098	Cnnm2	-0.13594	5.19E-26	hypomethylated	-0.0034554	0.21531	insignificant	36	148	149
chr19	47036841	47038841	Nt5c2	-0.58571	0.000070481	stronglyHypometh	0.0091133	0.48235	insignificant	1	4	2
chr19	47044057	47046057	Nt5c2	-0.27083	0.29666	insignificant	0.34305	0.062323	insignificant	2	13	12
chr19	47088187	47090187	Ina	-0.099116	1.13E-08	hypomethylated	0.012316	1	insignificant	25	114	118
chr19	47089679	47091679	Nt5c2	-0.12448	0.059378	insignificant	0.0088187	0.9297	insignificant	6	16	16
chr19	47125335	47127335	Pcgf6	-0.092602	0.36818	insignificant	0.077664	0.72743	insignificant	8	28	32
chr19	47141237	47143237	Taf5	-0.23247	9.78E-23	hypomethylated	0.0079993	0.082581	insignificant	28	90	88
chr19	47164255	47166255	Pdcd11	-0.10135	5.6E-10	hypomethylated	0.0050046	0.52649	insignificant	13	77	72
chr19	47165115	47167115	Pdcd11	-0.039707	0.00001321	hypomethylated	0.0084945	0.0098827	hypermethylated	7	51	54
chr19	47212784	47214784	Calhm2	-0.025974	1	insignificant	0.013542	0.22549	insignificant	1	7	7
chr19	47252309	47254309	Neurl1a	-0.14923	6.58E-50	hypomethylated	-0.015095	0.031575	hypomethylated	51	183	198
chr19	47302332	47304332	Neurl1a	-0.30592	3.69E-13	hypomethylated	0.10063	0.21659	insignificant	9	25	28
chr19	47538901	47540901	Sh3pxd2a	-0.13213	5.27E-10	hypomethylated	-0.0090051	0.000054995	hypomethylated	22	74	74
chr19	47611510	47613510	Obfc1	-0.10737	1.24E-09	hypomethylated	0.043953	0.12379	insignificant	9	36	36
chr19	47653508	47655508	Slk	-0.10107	1.73E-35	hypomethylated	0.0096062	0.14599	insignificant	45	116	100
chr19	47805245	47807245	Sfr1	-0.10854	0.00017721	hypomethylated	-0.011812	0.35263	insignificant	14	80	80
chr19	47911851	47913851	Wdr96	-0.024897	1	insignificant	-0.036335	0.57626	insignificant	6	29	29
chr19	47928478	47930478	Gsto1	-0.1647	0.000000162	hypomethylated	-0.031237	0.66072	insignificant	13	50	49
chr19	47939034	47941034	Gsto2	-0.17656	0.29264	insignificant	-0.079307	0.059898	insignificant	13	48	45
chr19	47939283	47941283	Gsto2	-0.22358	0.022455	hypomethylated	-0.074898	0.065137	insignificant	12	46	43
chr19	47993789	47995789	Itprip	-0.19034	0.0008186	hypomethylated	0.011561	0.69959	insignificant	17	41	42
chr19	48011201	48013201	Ccdc147	-0.22087	1.83E-22	hypomethylated	0.041614	7.18E-19	inconclusive	9	23	26
chr19	48279514	48281514	Sorcs3	-0.15188	1.22E-24	hypomethylated	0.0080014	0.54895	insignificant	36	139	141
chr19	50753102	50755102	Sorcs1	-0.061783	0.5697	insignificant	-0.0073313	0.32651	insignificant	20	50	51
chr19	52337812	52339812	Ins1	0.095353	1	insignificant	-0.025759	0.7035	insignificant	2	6	6
chr19	52996670	52998670	AA387883	-0.17603	0.027723	hypomethylated	-0.029951	0.64794	insignificant	2	17	16
chr19	53113032	53115032	Xpnpep1	-0.17139	5.81E-18	hypomethylated	0.018283	0.025558	hypermethylated	18	54	54
chr19	53216245	53218245	Add3	-0.10213	0.000072723	hypomethylated	0.0063426	1	insignificant	22	88	88
chr19	53217486	53219486	Add3	-0.11968	0.0051895	hypomethylated	0.011486	0.11818	insignificant	15	46	46
chr19	53329695	53331695	1700001K23Rik	-0.97917	0.033898	lowCoverage	-0.14028	0.0611	insignificant	1	6	9
chr19	53383995	53385995	Mxi1	-0.11233	0.00022376	hypomethylated	0.015078	0.50223	insignificant	20	87	85
chr19	53402902	53404902	Mxi1	-0.044348	0.000023487	hypomethylated	-0.0048638	0.11592	insignificant	22	173	178
chr19	53403935	53405935	Mxi1	-0.040473	0.000030166	hypomethylated	-0.0057159	0.09375	insignificant	22	160	165
chr19	53465063	53467063	Smndc1	-0.0851	0.0061275	hypomethylated	-0.00031234	0.16054	insignificant	17	68	71
chr19	53602807	53604807	Dusp5	-0.11066	1.29E-20	hypomethylated	-0.006703	0.0064024	hypomethylated	45	202	201
chr19	53673885	53675885	Smc3	-0.17766	1E-23	hypomethylated	0.0014976	0.77288	insignificant	45	112	127
chr19	53750795	53752795	Rbm20	-0.13751	1.61E-29	hypomethylated	-0.012496	0.19884	insignificant	32	111	111
chr19	53976840	53978840	Pdcd4	-0.11875	2.3E-42	hypomethylated	-0.00087304	0.25521	insignificant	45	118	124
chr19	54017795	54019795	Shoc2	-0.14674	4.75E-37	hypomethylated	-0.0072821	0.37121	insignificant	54	140	135
chr19	54018365	54020365	Shoc2	-0.14077	8.75E-35	hypomethylated	-0.0075731	0.51956	insignificant	53	138	133
chr19	54019117	54021117	Shoc2	-0.1692	7.18E-13	hypomethylated	0.01142	0.78605	insignificant	30	70	71
chr19	54118671	54120671	Adra2a	-0.1408	4.32E-27	hypomethylated	0.0018197	0.80089	insignificant	44	141	141
chr19	55173937	55175937	Gpam	-0.06721	3.91E-09	hypomethylated	-0.011347	0.23805	insignificant	14	39	39
chr19	55254374	55256374	Tectb		1	noCoverage	-0.077266	0.055887	insignificant	0	19	19
chr19	55326858	55328858	Acsl5	-0.28278	9.88E-10	hypomethylated	-0.060698	0.16705	insignificant	14	48	51
chr19	55389840	55391840	Vti1a	-0.15287	9.89E-34	hypomethylated	-0.010779	0.1246	insignificant	32	142	135
chr19	55390522	55392522	Vti1a	-0.1523	1.55E-27	hypomethylated	-0.0046588	0.055314	insignificant	21	73	72
chr19	55815299	55817299	Tcf7l2	-0.13706	1.25E-30	hypomethylated	-0.031465	0.019875	hypomethylated	41	116	123
chr19	55968810	55970810	Tcf7l2	-0.071628	0.0018008	hypomethylated	-0.012969	0.83639	insignificant	8	45	42
chr19	56470618	56472618	Casp7	-0.048928	0.000022362	hypomethylated	-0.073085	0.000000802	hypomethylated	15	70	92
chr19	56535126	56537126	9930023K05Rik	-0.70038	0.096134	insignificant	0.10829	0.16628	insignificant	2	7	9
chr19	56621750	56623750	Nhlrc2	-0.12484	2.17E-23	hypomethylated	0.0034863	0.000014487	inconclusive	27	112	106
chr19	56622516	56624516	Dclre1a	-0.1504	1.36E-25	hypomethylated	0.0018097	0.000000127	inconclusive	29	113	107
chr19	56795861	56797861	Adrb1	-0.087759	7.04E-09	hypomethylated	-0.014744	1	insignificant	26	82	97
chr19	56899698	56901698	Tdrd1	-0.11648	0.00021837	hypomethylated	0.038724	0.062257	insignificant	16	45	49
chr19	56899764	56901764	Tdrd1	-0.11648	0.00021837	hypomethylated	0.038724	0.062257	insignificant	16	45	49

chr19	56899775	56901775	Tdrd1	-0.11648	0.00021837	hypomethylated	0.038724	0.062257	insignificant	16	45	49
chr19	56947905	56949905	Vwa2	-0.12573	3.42E-23	hypomethylated	0.02405	0.74163	insignificant	24	80	94
chr19	57083065	57085065	Afap1l2	-0.61842	1.39E-12	stronglyHypometh	-0.026089	1	insignificant	2	35	36
chr19	57271982	57273982	Ablim1	-0.2212	1.58E-18	hypomethylated	0.031078	0.000020722	inconclusive	8	23	23
chr19	57290522	57292522	Ablim1	0.076923	1	insignificant	-0.011423	1	insignificant	2	8	8
chr19	57434498	57436498	Fam160b1	-0.11233	1.05E-19	hypomethylated	-0.0027261	0.93015	insignificant	38	147	150
chr19	57526395	57528395	Trub1	-0.14596	0.0082178	hypomethylated	-0.00055239	0.83091	insignificant	4	14	15
chr19	57684523	57686523	Atrnl1	-0.10682	6.85E-39	hypomethylated	0.0011916	0.0082068	inconclusive	54	190	194
chr19	58528956	58530956	Gfra1	-0.18593	3.65E-15	hypomethylated	0.011798	0.18417	insignificant	28	67	66
chr19	58802376	58804376	Pnliprp1	-0.65	0.0067439	stronglyHypometh	-0.10145	0.79476	insignificant	2	4	7
chr19	58833212	58835212	Pnliprp2	-0.25747	0.0015763	hypomethylated	-0.13733	0.022747	hypomethylated	6	14	14
chr19	58868904	58870904	1700019N19Rik	-0.17374	0.000021278	hypomethylated	0.069575	0.018138	hypermethylated	10	23	22
chr19	58935474	58937474	Hspa12a	-0.30184	0.000000175	hypomethylated	0.0062942	0.42711	insignificant	5	25	25
chr19	59016914	59018914	Eno4		1	noCoverage	-0.093789	0.5552	insignificant	0	7	7
chr19	59150559	59152559	4930506M07Rik		1	noCoverage	-0.1697	0.00017957	hypomethylated	0	14	11
chr19	59244519	59246519	Vax1	-0.17836	0.005646	hypomethylated	-0.04979	0.0031552	hypomethylated	10	60	60
chr19	59293137	59295137	Kcnk18	-0.1526	0.85862	insignificant	-0.0070724	0.85271	insignificant	8	32	34
chr19	59334367	59336367	Slc18a2	-0.14972	2.27E-08	hypomethylated	-0.017434	0.3995	insignificant	17	45	45
chr19	59420270	59422270	Pdzd8	-0.12082	1.3E-16	hypomethylated	-0.0097123	0.0037703	hypomethylated	38	126	121
chr19	59532179	59534179	Emx2	-0.13365	1.87E-25	hypomethylated	-0.0095574	0.13958	insignificant	43	124	132
chr19	59533125	59535125	Emx2	-0.14184	1.28E-28	hypomethylated	-0.010483	0.0000511	hypomethylated	42	140	140
chr19	60019267	60021267	Rab11fip2	-0.085065	0.0016767	hypomethylated	0.007652	0.94605	insignificant	12	60	60
chr19	60019557	60021557	Rab11fip2	-0.070087	0.0057821	hypomethylated	0.0088817	0.46686	insignificant	4	24	24
chr19	60302600	60304600	D19Ertd737e	0.016498	1	insignificant	-0.029592	1	insignificant	2	10	7
chr19	60830889	60832889	Nanos1	-0.08798	1.3E-09	hypomethylated	-0.010921	0.064026	insignificant	59	183	180
chr19	60866596	60868596	Eif3a	-0.11873	0.0025524	hypomethylated	-0.00094634	0.1848	insignificant	3	61	74
chr19	60886472	60888472	Fam45a	-0.10739	2.02E-09	hypomethylated	-0.033024	0.7084	insignificant	14	44	38
chr19	60964651	60966651	Grk5	-0.18145	5.86E-24	hypomethylated	0.021565	0.23821	insignificant	35	108	104
chr19	61304321	61306321	Csf2ra	-0.21286	4.43E-08	hypomethylated	0.038088	0.46287	insignificant	7	16	17
chr2	3034659	3036659	Fam171a1	-0.099127	9.06E-18	hypomethylated	-0.0063596	0.57381	insignificant	29	83	105
chr2	3200559	3202559	Nmt2	-0.11209	0.000000296	hypomethylated	-0.01059	0.083579	insignificant	29	144	137
chr2	3249900	3251900	Rpp38		1	noCoverage	0.03061	0.011247	inconclusive	0	14	12
chr2	3339920	3341920	Meig1	-0.24589	1.18E-10	hypomethylated	-0.027164	0.13019	insignificant	4	20	20
chr2	3340402	3342402	Dclre1c	-0.267	5.76E-13	hypomethylated	-0.04773	0.097443	insignificant	6	34	34
chr2	3392258	3394258	Suv39h2	-0.32004	0.000000421	hypomethylated	0.06398	0.12642	insignificant	3	7	8
chr2	3429336	3431336	Cdnf	-0.17921	1.69E-24	hypomethylated	-0.014304	0.8684	insignificant	24	97	98
chr2	3430086	3432086	Hspa14	-0.16463	1.32E-15	hypomethylated	-0.0093129	0.85816	insignificant	14	51	52
chr2	3629729	3631729	Fam107b	-0.16242	5.77E-18	hypomethylated	-0.005769	0.03126	hypomethylated	28	68	68
chr2	4072908	4074908	Frmd4a	-0.27522	1	lowCoverage	0.015964	0.20934	insignificant	1	15	12
chr2	4321021	4323021	Frmd4a	-0.067969	0.019163	hypomethylated	-0.010322	0.11111	insignificant	9	32	47
chr2	4479799	4481799	Frmd4a	-0.24687	0.000044109	hypomethylated	-0.03313	0.2358	insignificant	7	22	24
chr2	4573132	4575132	Prpf18	-0.27116	1	insignificant	-0.03019	0.86924	insignificant	2	17	16
chr2	4637876	4639876	Bend7	-0.088293	8.43E-33	hypomethylated	-0.0082817	0.087607	insignificant	46	156	186
chr2	4801609	4803609	Sephs1	-0.11484	1.28E-40	hypomethylated	-0.012209	0.049872	hypomethylated	60	206	194
chr2	4839041	4841041	Phyh	-0.10136	0.68879	insignificant	0.00071188	0.18282	insignificant	11	57	58
chr2	4896167	4898167	Ucma	-0.86044	0.13187	lowCoverage	-0.15837	0.025055	hypomethylated	1	15	16
chr2	4933837	4935837	Mcm10	-0.55101	0.0026855	stronglyHypometh	-0.10348	0.23875	insignificant	2	18	16
chr2	4984984	4986984	Optn	-0.24144	0.032727	hypomethylated	-0.13522	0.0071224	hypomethylated	6	16	13
chr2	5057821	5059821	Ccdc3	-0.094503	2E-22	hypomethylated	0.0071179	0.43297	insignificant	31	113	111
chr2	5635710	5637710	Camk1d	-0.13694	6.15E-16	hypomethylated	-0.0059562	0.47153	insignificant	28	80	82
chr2	5765079	5767079	Nudt5	-0.20564	0.0054349	hypomethylated	0.018922	0.41936	insignificant	13	67	68
chr2	5766006	5768006	Cdc123	-0.22068	0.084401	insignificant	-0.0084224	0.52578	insignificant	10	30	30
chr2	5816399	5818399	Sec61a2		1	noCoverage	0.13282	0.76042	insignificant	0	17	14
chr2	5871514	5873514	Upf2	-0.16054	1.29E-23	hypomethylated	-0.015186	0.12987	insignificant	28	135	138
chr2	6051231	6053231	Gm10857	-0.1637	7.55E-10	hypomethylated	0.019962	0.14242	insignificant	9	29	28
chr2	6052914	6054914	Gm10857	0.13469	1	insignificant	0.062456	0.40063	insignificant	3	25	22
chr2	6052928	6054928	Gm10857	0.13469	1	insignificant	0.062456	0.40063	insignificant	3	25	22
chr2	6134040	6136040	A230108P19Rik	-0.23165	0.021106	hypomethylated	-0.082014	0.68101	insignificant	6	33	41
chr2	6242802	6244802	Usp6nl	-0.12376	4.55E-35	hypomethylated	-0.0076135	0.15559	insignificant	73	185	199
chr2	6272781	6274781	Usp6nl	-0.20833	0.2975	insignificant	-0.0091991	0.52443	insignificant	2	6	6
chr2	6849768	6851768	Celf2	0.045455	1	insignificant	-0.010101	0.59756	insignificant	1	2	4
chr2	7002348	7004348	Celf2	-0.16791	0.291	insignificant	-0.0078829	0.34353	insignificant	2	4	4
chr2	9800227	9802227	Gata3	-0.17659	1.42E-16	hypomethylated	-0.00126	0.23048	insignificant	18	78	73
chr2	9801872	9803872	4930412O13Rik	-0.41379	3.09E-37	stronglyHypometh	-0.052394	0.082993	insignificant	15	54	54
chr2	9970236	9972236	Taf3	-0.15007	0.00000388	hypomethylated	-0.003416	0.091226	insignificant	9	55	55
chr2	10001238	10003238	Kin	-0.24005	2.18E-09	hypomethylated	-0.011344	0.93852	insignificant	7	28	29
chr2	10291077	10293077	Sfmbt2	-0.40251	1	lowCoverage	0.02749	0.8893	insignificant	1	12	12
chr2	10293062	10295062	Sfmbt2	-0.15911	2.13E-14	hypomethylated	-0.0030974	0.012295	hypomethylated	18	113	112
chr2	10404304	10406304	Mir467a-6	-0.8	0.2381	lowCoverage	-0.05	1	insignificant	1	2	4
chr2	11093008	11095008	Prkcq	-0.058129	0.0013338	hypomethylated	-0.0025524	0.49076	insignificant	21	70	66
chr2	11423717	11425717	Pfkfb3	-0.34533	1.19E-19	stronglyHypometh	-0.093613	0.0069436	hypomethylated	19	58	56
chr2	11475556	11477556	Pfkfb3	-0.24306	0.012171	hypomethylated	0.010961	0.89942	insignificant	8	32	34
chr2	11524826	11526826	Rbm17	-0.29625	1.52E-13	hypomethylated	0.030206	0.18047	insignificant	11	40	44
chr2	11626162	11628162	Il15ra	-0.18419	1.05E-15	hypomethylated	0.0080038	0.71816	insignificant	21	50	50
chr2	11626474	11628474	Il15ra	-0.26908	6.57E-21	hypomethylated	-0.039683	0.18378	insignificant	24	60	60
chr2	11698379	11700379	Ankrd16	-0.14999	5.54E-14	hypomethylated	0.0051992	0.88924	insignificant	19	57	62
chr2	11699154	11701154	Fbxo18	-0.15902	3.15E-17	hypomethylated	-0.0067399	0.26617	insignificant	15	56	62
chr2	12223547	12225547	E030013I19Rik	-0.18487	4.05E-14	hypomethylated	-0.035975	0.39088	insignificant	9	34	33
chr2	12341087	12343087	Fam188a	-0.13524	0.46459	insignificant	-0.063365	0.36349	insignificant	5	10	11
chr2	12844667	12846667	Pter	-0.34174	0.0010266	stronglyHypometh	0.0081009	1	insignificant	6	14	14
chr2	12932491	12934491	C1ql3	-0.18357	0.00021263	hypomethylated	-0.02493	0.21105	insignificant	10	52	44
chr2	13466291	13468291	Trdmt1	-0.20815	0.000000161	hypomethylated	0.05801	0.53524	insignificant	2	11	6
chr2	13494937	13496937	Vim	-0.14862	1.36E-20	hypomethylated	0.0072572	0.75419	insignificant	22	80	80
chr2	13715147	13717147	St8sia6	-0.063596	0.030434	hypomethylated	-0.0090576	0.65779	insignificant	8	49	49
chr2	13977662	13979662	Ptpla	0.3183	0.62937	insignificant	0.004443	0.26567	insignificant	3	26	26

chr2	13994738	13996738	Stam	-0.13425	0.000060595	hypomethylated	-0.012588	0.54098	insignificant	19	88	88
chr2	14150040	14152040	Mrc1	-0.42349	0.036115	stronglyHypometh	0.086379	0.062378	insignificant	4	12	12
chr2	14524932	14526932	Cacnb2	-0.13753	3.27E-33	hypomethylated	-0.018879	0.47069	insignificant	33	128	129
chr2	14970939	14972939	Nsun6	-0.41319	0.0015157	stronglyHypometh	-0.061343	0.49952	insignificant	3	6	6
chr2	14975988	14977988	Arl5b	-0.12774	2.5E-21	hypomethylated	-0.031883	0.19862	insignificant	41	122	116
chr2	14976499	14978499	Nsun6	-0.11963	1.67E-20	hypomethylated	-0.023778	0.38852	insignificant	41	118	116
chr2	16276948	16278948	Plxdc2	-0.19073	2.04E-22	hypomethylated	-0.019562	0.13619	insignificant	27	90	92
chr2	17652695	17654695	Nebl	-0.1141	1.44E-11	hypomethylated	-0.00015484	0.49491	insignificant	7	35	34
chr2	17918573	17920573	H2afb1		1	noCoverage	-0.044204	0.044268	hypomethylated	0	8	8
chr2	17947875	17949875	Gm17762	-0.14701	0.00013536	hypomethylated	-0.00586	0.30576	insignificant	16	103	102
chr2	17959368	17961368	A930004D18Rik	0.062236	0.3849	insignificant	0.012873	0.0026986	hypermethylated	5	14	14
chr2	17970076	17972076	2810030E01Rik	-0.15958	1	insignificant	0.026312	0.89621	insignificant	2	32	28
chr2	17975897	17977897	Mllt10	-0.28843	0.31787	insignificant	0.044187	0.41737	insignificant	3	18	14
chr2	18314457	18316457	Dnajc1	-0.29467	7.84E-10	hypomethylated	0.19251	0.11458	insignificant	4	9	11
chr2	18593088	18595088	Commd3	-0.096676	3.4E-11	hypomethylated	0.0094184	0.78088	insignificant	17	86	80
chr2	18597644	18599644	Bmi1	-0.084621	7.03E-35	hypomethylated	-0.010083	0.010387	hypomethylated	76	239	233
chr2	18619648	18621648	BC061194	-0.21504	8.2E-09	hypomethylated	-0.033326	0.84552	insignificant	15	52	52
chr2	18918945	18920945	4930426L09Rik	-0.1024	2.87E-29	hypomethylated	-0.0033375	0.49741	insignificant	61	188	188
chr2	18919748	18921748	Pip4k2a	-0.094126	6.72E-14	hypomethylated	-0.010376	0.0082975	hypomethylated	24	85	87
chr2	19119744	19121744	Armc3	-0.51465	0.00000432	stronglyHypometh	0.05694	0.21816	insignificant	2	11	7
chr2	19292262	19294262	Msrb2	-0.27083	0.037858	hypomethylated	0.0056391	0.76545	insignificant	3	8	4
chr2	19366289	19368289	Ptf1a	-0.11718	0.00053984	hypomethylated	-0.0050209	0.67856	insignificant	18	107	107
chr2	19475537	19477537	4921504E06Rik	-0.22269	0.030714	hypomethylated	-0.013291	0.66317	insignificant	6	22	22
chr2	19578688	19580688	Otud1	-0.097267	5.25E-51	hypomethylated	-0.0010975	0.079537	insignificant	68	241	236
chr2	19579524	19581524	Gm3230	-0.10087	3.43E-45	hypomethylated	-0.0016564	0.042281	hypomethylated	61	197	195
chr2	20430673	20432673	Etl4	0.029035	1	insignificant	-0.024632	0.22017	insignificant	1	15	20
chr2	20440139	20442139	Etl4	-0.23879	7.48E-18	hypomethylated	0.018715	0.80548	insignificant	21	58	58
chr2	20889348	20891348	Gm13375	-0.10412	5.56E-20	hypomethylated	-0.0012471	0.53927	insignificant	84	248	237
chr2	20889500	20891500	Gm13375	-0.10138	1.19E-19	hypomethylated	-0.0020548	0.49898	insignificant	82	244	233
chr2	21126350	21128350	Thnsl1	-0.14533	3.26E-13	hypomethylated	-0.049274	0.32627	insignificant	8	54	53
chr2	21126992	21128992	Thnsl1	-0.14533	3.26E-13	hypomethylated	-0.049274	0.32627	insignificant	8	54	53
chr2	21288193	21290193	Gpr158		1	noCoverage	-0.0080873	0.11083	insignificant	0	25	26
chr2	22148129	22150129	Myo3a	-0.21494	0.00013427	hypomethylated	0.017218	1	insignificant	5	15	15
chr2	22476846	22478846	Gad2	-0.18239	6.05E-11	hypomethylated	-0.0014832	0.82646	insignificant	16	50	50
chr2	22628846	22630846	Apbb1ip	-0.24015	1	insignificant	-0.0065088	0.69579	insignificant	1	19	19
chr2	22750041	22752041	Pdss1	-0.11503	1.19E-12	hypomethylated	0.0023925	0.046696	inconclusive	22	75	77
chr2	22895760	22897760	Abi1		1	noCoverage	0.10342	0.51538	insignificant	0	6	6
chr2	22922720	22924720	Acbd5	-0.22986	4.82E-08	hypomethylated	-0.040227	0.6603	insignificant	19	77	71
chr2	22923591	22925591	Acbd5	-0.20169	1.55E-09	hypomethylated	-0.027198	0.93783	insignificant	22	92	86
chr2	23011065	23013065	Mastl	-0.13753	3.82E-11	hypomethylated	0.025882	0.050382	insignificant	23	60	70
chr2	23011544	23013544	Mastl	-0.26214	2.59E-14	hypomethylated	-0.0075284	0.000000365	hypomethylated	18	58	62
chr2	23427624	23429624	Spopl	-0.19672	1	insignificant	-0.081091	0.5577	insignificant	2	11	11
chr2	24007691	24009691	Il1f8	0.16802	1	insignificant	-0.040807	0.80613	insignificant	1	4	4
chr2	24191379	24193379	Il1rn	-0.11111	0.51252	insignificant	-0.002584	0.41794	insignificant	1	4	2
chr2	24239916	24241916	Psd4	-0.56532	1	lowCoverage	-0.0012734	0.5396	insignificant	1	14	14
chr2	24618567	24620567	Cacna1b	-0.10459	0.00019623	hypomethylated	0.025886	0.18323	insignificant	6	46	46
chr2	24618672	24620672	Cacna1b	-0.086989	1	insignificant	0.040493	0.94021	insignificant	1	30	30
chr2	24775110	24777110	Ehmt1	-0.098484	1.22E-23	hypomethylated	0.0013451	4.53E-13	inconclusive	16	63	55
chr2	24790801	24792801	Arrdc1	-0.21559	0.12787	insignificant	0.063175	0.000026787	hypermethylated	14	49	54
chr2	24804321	24806321	Zmynd19	-0.13091	4.62E-44	hypomethylated	-0.028241	0.01623	hypomethylated	72	180	219
chr2	24816941	24818941	Wdr85	-0.24601	0.064077	insignificant	-0.05445	0.26366	insignificant	4	14	27
chr2	24830552	24832552	Pnpla7	-0.14794	0.026728	hypomethylated	-0.0038163	0.72409	insignificant	10	73	75
chr2	24830618	24832618	Mrpl41	-0.15532	0.016209	hypomethylated	-0.0033361	0.8437	insignificant	10	69	71
chr2	24908898	24910898	Nelf	-0.20661	2.11E-19	hypomethylated	-0.014568	0.46041	insignificant	22	68	68
chr2	24950725	24952725	Noxa1	-0.16081	0.0051169	hypomethylated	-0.12515	0.15128	insignificant	3	10	8
chr2	25035277	25037277	Nrarp	-0.10682	0.0000051	hypomethylated	-0.0050545	0.02718	hypomethylated	23	112	114
chr2	25052333	25054333	A830007P12Rik		1	noCoverage	0.068209	0.44856	insignificant	0	4	4
chr2	25067009	25069009	Cobra1	-0.4305	3.78E-31	stronglyHypometh	-0.011289	4.16E-31	hypomethylated	11	42	42
chr2	25080222	25082222	Tubb4b	-0.20164	0.081276	insignificant	0.062499	0.41992	insignificant	9	30	32
chr2	25092307	25094307	Gm757	-0.1512	0.025627	hypomethylated	-0.099248	0.00035758	hypomethylated	4	16	18
chr2	25095417	25097417	2310002J15Rik	-0.10055	0.03482	hypomethylated	-0.011858	0.36085	insignificant	9	36	36
chr2	25097448	25099448	Rnf208	-0.17909	2.89E-44	hypomethylated	0.042616	0.89455	insignificant	11	37	39
chr2	25109958	25111958	Tmem203	-0.13919	9.31E-17	hypomethylated	0.0040391	0.1207	insignificant	31	126	127
chr2	25110934	25112934	Ndor1	-0.14898	4.77E-09	hypomethylated	0.0095265	0.1806	insignificant	21	78	79
chr2	25117117	25119117	Tprn	-0.1631	6.07E-24	hypomethylated	0.0026021	0.24364	insignificant	32	138	142
chr2	25126985	25128985	Anapc2	-0.078675	0.17734	insignificant	0.0049098	0.88315	insignificant	27	94	91
chr2	25127938	25129938	Anapc2	0.43822	7.55E-11	stronglyHypermeth	-0.0020217	0.13229	insignificant	5	34	35
chr2	25144430	25146430	Lrrc26	-0.12993	4.04E-16	hypomethylated	-0.0072548	0.77919	insignificant	24	90	90
chr2	25174683	25176683	Grin1	0.11251	5.58E-12	hypermethylated	-0.021922	0.033421	inconclusive	9	48	49
chr2	25187262	25189262	Man1b1	-0.25794	5.06E-14	hypomethylated	0.01868	0.37839	insignificant	14	79	80
chr2	25188091	25190091	AA543186	-0.4496	1.85E-15	stronglyHypometh	0.0090349	0.058314	insignificant	7	22	22
chr2	25211852	25213852	Dpp7	-0.27763	0.06679	insignificant	-0.031475	0.29487	insignificant	3	10	10
chr2	25221146	25223146	Uap1l1	-0.56372	4.81E-09	stronglyHypometh	-0.026119	0.081079	insignificant	8	36	43
chr2	25226840	25228840	2010317E24Rik	-0.15862	6.56E-25	hypomethylated	0.030121	0.058432	insignificant	26	80	83
chr2	25250393	25252393	Entpd2	-0.20173	1.09E-22	hypomethylated	-0.0022249	0.42795	insignificant	21	87	87
chr2	25257602	25259602	Npdc1	-0.1088	5.52E-14	hypomethylated	0.0011368	0.96507	insignificant	18	50	50
chr2	25283193	25285193	Abca2	-0.097295	3.94E-48	hypomethylated	0.025923	0.096328	insignificant	56	136	127
chr2	25297298	25299298	Mir3087	-0.19844	0.0025261	hypomethylated	-0.039868	0.015676	hypomethylated	8	35	35
chr2	25311362	25313362	Clic3		1	noCoverage	0.065385	0.61358	insignificant	0	2	2
chr2	25316614	25318614	BC029214	0.72017	1.04E-16	stronglyHypermeth	0.083862	0.14761	insignificant	3	7	8
chr2	25355297	25357297	Fbxw5	-0.17256	2.96E-12	hypomethylated	-0.011845	0.17446	insignificant	14	51	42
chr2	25356026	25358026	C8g	-0.25698	2.63E-15	hypomethylated	0.030996	0.27425	insignificant	15	57	50
chr2	25402414	25404414	Traf2	-0.12331	0.000039842	hypomethylated	0.0020065	0.20599	insignificant	10	48	48
chr2	25412419	25414419	Edf1	-0.11353	2.71E-19	hypomethylated	0.011533	0.015462	inconclusive	27	94	95
chr2	25430391	25432391	Phpt1	-0.23072	2.71E-31	hypomethylated	-0.015544	0.019712	inconclusive	12	48	48

chr2	25463966	25465966	B230208H17Rik	0.23096	0.001054	hypermethylated	0.064441	0.36766	insignificant	12	56	57
chr2	25473198	25475198	4921530D09Rik	-0.20337	0.74966	insignificant	-0.1137	0.8044	insignificant	2	8	6
chr2	25477522	25479522	Tmem141	-0.13978	0.23959	insignificant	-0.012839	0.36162	insignificant	4	18	18
chr2	25477525	25479525	Tmem141	-0.13978	0.23959	insignificant	-0.012839	0.36162	insignificant	4	18	18
chr2	25531305	25533305	Lcn6		1	noCoverage	0.039284	0.86281	insignificant	0	4	4
chr2	25537245	25539245	Lcn10	-0.41154	0.01039	stronglyHypometh	-0.10851	0.80135	insignificant	3	17	17
chr2	25561398	25563398	Bmyc	-0.17481	0.0044739	hypomethylated	-0.041133	0.54644	insignificant	13	82	78
chr2	25677672	25679672	Lcn9	-0.24465	0.0000078	hypomethylated	-0.037669	0.0060761	hypomethylated	6	12	12
chr2	25702768	25704768	Sohlh1		1	noCoverage	-0.0041771	0.77494	insignificant	0	4	4
chr2	25718373	25720373	Kcnt1	-0.090686	0.23655	insignificant	0.004902	0.75583	insignificant	12	24	24
chr2	25732380	25734380	Kcnt1	-0.16369	3.02E-10	hypomethylated	-0.0037602	0.19454	insignificant	19	66	65
chr2	25838802	25840802	Camsap1	-0.24373	7.73E-18	hypomethylated	0.047901	0.042697	inconclusive	8	37	33
chr2	25877280	25879280	Ubac1	-0.21885	0.00000101	hypomethylated	0.034833	0.061308	insignificant	5	22	17
chr2	25978331	25980331	Nacc2	-0.099141	0.22338	insignificant	-0.000049635	0.78604	insignificant	9	39	39
chr2	26062076	26064076	Lhx3	-0.15804	0.000010349	hypomethylated	-0.012264	0.31188	insignificant	12	47	47
chr2	26150077	26152077	4932418E24Rik	-0.15965	0.59993	insignificant	-0.013813	0.84316	insignificant	2	7	6
chr2	26170052	26172052	Gpsm1	-0.14679	3.48E-23	hypomethylated	0.024655	0.73127	insignificant	22	78	79
chr2	26174289	26176289	Gpsm1		1	noCoverage	0.098958	0.52768	insignificant	0	4	4
chr2	26207630	26209630	Dnlz	-0.15646	0.00005445	hypomethylated	0.12588	0.92649	insignificant	4	14	8
chr2	26236173	26238173	Snapc4	0.21457	1	insignificant	0.086445	0.12343	insignificant	2	9	19
chr2	26243867	26245867	Pmpca	-0.10867	2.82E-18	hypomethylated	-0.002413	0.87656	insignificant	37	144	139
chr2	26244836	26246836	Sdccag3	-0.12185	0.00025136	hypomethylated	0.0144	0.9596	insignificant	5	29	28
chr2	26264708	26266708	Inpp5e	-0.29808	0.030619	hypomethylated	0.12182	0.61907	insignificant	2	4	5
chr2	26300736	26302736	Sec16a	-0.080361	0.00025079	hypomethylated	-0.010976	0.76	insignificant	14	77	78
chr2	26359342	26361342	Notch1	-0.1177	0.00091839	hypomethylated	0.022099	0.69788	insignificant	8	32	30
chr2	26435575	26437575	Egfl7	-0.10284	0.43935	insignificant	-0.083739	0.36999	insignificant	2	4	4
chr2	26438383	26440383	Egfl7	-0.085957	1	insignificant	-0.039769	0.61054	insignificant	2	12	12
chr2	26441149	26443149	Egfl7	-0.19548	1.62E-22	hypomethylated	-0.017373	0.076018	insignificant	24	74	86
chr2	26459730	26461730	Agpat2	-0.20374	0.096213	insignificant	0.012152	0.52043	insignificant	9	63	65
chr2	26482976	26484976	Fam69b	-0.12189	0.003688	hypomethylated	0.0096747	0.69543	insignificant	13	34	34
chr2	26493505	26495505	Snora43		1	noCoverage	-0.0447	0.057739	insignificant	0	24	24
chr2	26494841	26496841	Snora17	-0.246	0.00000116	hypomethylated	-0.039221	0.011328	inconclusive	7	78	82
chr2	26495764	26497764	Snora17	0.63192	0.28028	insignificant	-0.091132	0.024762	hypomethylated	2	25	29
chr2	26758333	26760333	Surf6	-0.16762	3.53E-17	hypomethylated	-0.0041502	0.43381	insignificant	12	40	40
chr2	26765326	26767326	Rpl7a	-0.18919	5.42E-08	hypomethylated	-0.015988	0.41297	insignificant	10	66	66
chr2	26766162	26768162	Med22	0.12046	0.000085699	hypermethylated	-0.046218	0.4521	insignificant	2	49	48
chr2	26770940	26772940	Surf2	-0.23342	2.91E-12	hypomethylated	-0.023932	0.020343	hypomethylated	17	74	77
chr2	26772050	26774050	Surf2	-0.064549	0.000003746	hypomethylated	0.0054202	0.028756	inconclusive	5	32	32
chr2	26788588	26790588	Gm711	-0.12565	0.036479	hypomethylated	-0.0045101	0.045481	inconclusive	8	75	64
chr2	26789031	26791031	Surf4	-0.18412	0.030338	hypomethylated	0.027406	0.45644	insignificant	3	32	34
chr2	26819906	26821906	Rexo4	-0.19078	0.64282	insignificant	0.1337	0.17062	insignificant	1	19	23
chr2	26864485	26866485	5930434B04Rik	-0.1785	8.86E-16	hypomethylated	-0.0028019	0.22758	insignificant	17	66	70
chr2	26883518	26885518	Slc2a6		1	noCoverage	0.037786	0.12284	insignificant	0	12	12
chr2	27020026	27022026	Dbh		1	noCoverage	0.052381	0.76425	insignificant	0	2	2
chr2	27282345	27284345	Vav2	-0.10643	2.44E-13	hypomethylated	-0.015826	0.11529	insignificant	22	52	52
chr2	27330918	27332918	Brd3	-0.11002	6.51E-35	hypomethylated	-0.0065438	0.0083561	hypomethylated	39	119	114
chr2	27331193	27333193	Brd3	-0.098383	1.43E-25	hypomethylated	-0.0018906	0.031559	hypomethylated	22	72	72
chr2	27369666	27371666	Wdr5	-0.1536	7.64E-25	hypomethylated	-0.023136	0.000010182	hypomethylated	16	61	82
chr2	27531720	27533720	Rxra	-0.092144	6.57E-14	hypomethylated	0.0074988	0.80728	insignificant	72	218	236
chr2	27740944	27742944	Col5a1	-0.12261	2.59E-18	hypomethylated	-0.004917	0.97355	insignificant	39	120	120
chr2	28047612	28049612	Olfm1	-0.14107	5.68E-31	hypomethylated	-0.011113	0.54609	insignificant	37	113	110
chr2	28060208	28062208	Olfm1	-0.14943	1.17E-47	hypomethylated	-0.010714	0.026747	hypomethylated	52	138	150
chr2	28302460	28304460	Ppp1r26	-0.12326	1.2E-20	hypomethylated	-0.0009504	0.51632	insignificant	32	75	75
chr2	28321844	28323844	1700007K13Rik	-0.13738	1.24E-16	hypomethylated	0.0076135	0.29079	insignificant	28	80	80
chr2	28322585	28324585	Mrps2	-0.18077	1.38E-26	hypomethylated	0.0036635	0.28411	insignificant	36	109	108
chr2	28367686	28369686	Ralgds	-0.11017	2.07E-21	hypomethylated	-0.0030755	0.57789	insignificant	46	144	150
chr2	28387983	28389983	Ralgds	-0.16028	5.37E-17	hypomethylated	-0.033286	0.062123	insignificant	18	56	67
chr2	28418882	28420882	Cel	-0.20121	0.025154	hypomethylated	0.098768	1	insignificant	2	4	7
chr2	28477502	28479502	Gfi1b	-0.04411	0.69311	insignificant	-0.060941	0.56195	insignificant	1	13	7
chr2	28495762	28497762	Tsc1	-0.14068	3.75E-24	hypomethylated	-0.015343	0.12818	insignificant	23	99	90
chr2	28554680	28556680	Ak8	-0.22855	3.56E-16	hypomethylated	-0.022837	0.54027	insignificant	16	64	72
chr2	28555171	28557171	1700026L06Rik	-0.22373	1E-14	hypomethylated	-0.029582	0.27482	insignificant	14	59	67
chr2	28694925	28696925	Ddx31	-0.12811	9.24E-14	hypomethylated	-0.0013723	0.43998	insignificant	26	110	102
chr2	28695880	28697880	Ddx31	-0.13564	1.16E-13	hypomethylated	-0.0098162	0.53987	insignificant	17	60	60
chr2	28771941	28773941	Barhl1	-0.15768	6.68E-21	hypomethylated	-0.017671	0.09339	insignificant	29	123	120
chr2	28771960	28773960	Barhl1	-0.15649	6.8E-21	hypomethylated	-0.014676	0.10117	insignificant	29	125	121
chr2	28910586	28912586	1700101E01Rik	-0.15703	1.1E-16	hypomethylated	-0.018099	0.036556	hypomethylated	9	35	35
chr2	28914782	28916782	Ttf1	-0.15164	0.00000011	hypomethylated	-0.0070191	0.59769	insignificant	19	49	49
chr2	28979511	28981511	Setx	-0.17842	7.6E-24	hypomethylated	-0.0013006	0.93919	insignificant	19	104	107
chr2	29108513	29110513	6530402F18Rik	-0.25493	4.45E-09	hypomethylated	-0.059887	0.57392	insignificant	8	19	18
chr2	29201355	29203355	Med27	-0.20068	1.18E-21	hypomethylated	0.0044668	0.50798	insignificant	11	64	66
chr2	29474239	29476239	Rapgef1	-0.11759	1.02E-47	hypomethylated	-0.015691	0.80857	insignificant	42	117	131
chr2	29642782	29644782	Coq4	-0.16695	0.0067695	hypomethylated	-0.03412	0.90968	insignificant	11	54	51
chr2	29643191	29645191	Trub2	0.0050657	0.052638	insignificant	-0.046637	0.0063821	hypomethylated	7	47	44
chr2	29657199	29659199	Slc27a4	-0.12293	2.44E-25	hypomethylated	-0.0033996	0.48767	insignificant	35	92	92
chr2	29681908	29683908	Urm1	-0.22496	1.52E-18	hypomethylated	-0.0013728	0.048272	inconclusive	13	45	45
chr2	29701247	29703247	Mir219-2	-0.066887	0.000087058	hypomethylated	0.012052	0.010798	inconclusive	29	99	100
chr2	29724013	29726013	Cercam	-0.074032	0.000046036	hypomethylated	0.00057482	0.00033712	inconclusive	13	58	58
chr2	29744239	29746239	Odf2	-0.12701	1.42E-25	hypomethylated	-0.013588	0.17761	insignificant	47	159	160
chr2	29744582	29746582	Odf2	-0.12083	2.02E-24	hypomethylated	-0.012803	0.21159	insignificant	46	157	158
chr2	29789928	29791928	Gle1	-0.20474	0.35425	insignificant	0.0098784	0.26786	insignificant	3	39	39
chr2	29820079	29822079	Spna2	-0.087402	7.83E-15	hypomethylated	0.0032263	0.56584	insignificant	22	127	134
chr2	29904399	29906399	Wdr34	-0.16638	1.04E-21	hypomethylated	0.0059523	4.61E-13	inconclusive	15	52	52
chr2	29916562	29918562	Set	-0.22044	4.64E-19	hypomethylated	-0.020499	0.0065595	hypomethylated	22	73	70
chr2	29933285	29935285	Pkn3	-0.040666	0.035856	inconclusive	-0.0087351	0.00014543	inconclusive	13	42	42

chr2	29949155	29951155	Zdhhc12	-0.13784	8.44E-12	hypomethylated	-0.00077405	0.41416	insignificant	11	45	44
chr2	29979974	29981974	Zer1	-0.137	3.14E-12	hypomethylated	0.013148	0.00000134	inconclusive	13	32	32
chr2	29988390	29990390	Tbc1d13	-0.25061	8.24E-19	hypomethylated	-0.010428	0.10046	insignificant	15	36	36
chr2	30026043	30028043	Endog	-0.1948	8.53E-09	hypomethylated	0.0011314	0.76483	insignificant	13	32	32
chr2	30033979	30035979	D2Wsu81e	-0.29373	0.00002727	hypomethylated	0.0041931	0.35891	insignificant	2	4	4
chr2	30061219	30063219	Ccbl1	0.030254	0.50911	insignificant	-0.0057753	0.0045156	hypomethylated	10	38	38
chr2	30092288	30094288	Lrrc8a	-0.12948	2.99E-47	hypomethylated	-0.010355	0.16945	insignificant	31	124	128
chr2	30093151	30095151	1700084E18Rik	-0.12185	1.58E-45	hypomethylated	-0.018308	0.25609	insignificant	26	102	102
chr2	30140952	30142952	Nup188	-0.14767	0.00000182	hypomethylated	-0.0067992	0.31742	insignificant	11	60	66
chr2	30141874	30143874	Dolk	-0.16834	0.00074936	hypomethylated	-0.025673	0.42726	insignificant	6	36	32
chr2	30214751	30216751	Sh3glb2	-0.1637	0.000000258	hypomethylated	-0.064673	0.00041687	hypomethylated	14	48	48
chr2	30218854	30220854	Fam73b	-0.17071	3.19E-11	hypomethylated	-0.013618	0.017156	hypomethylated	11	58	56
chr2	30246935	30248935	Dolpp1	-0.10179	2.15E-16	hypomethylated	0.0024291	0.75556	insignificant	31	98	98
chr2	30270569	30272569	Ppp2r4	-0.12736	1.5E-53	hypomethylated	0.0057336	0.90927	insignificant	37	132	132
chr2	30271268	30273268	Crat	-0.1846	1.77E-33	hypomethylated	0.0044461	0.068345	insignificant	19	65	64
chr2	30329719	30331719	Ier5l	-0.15071	0.000000354	hypomethylated	-0.0020598	0.63142	insignificant	20	62	62
chr2	30449563	30451563	Cstad	-0.21122	4.35E-11	hypomethylated	0.0066893	0.59722	insignificant	11	39	40
chr2	30662496	30664496	Mettl11a		1	noCoverage	-0.033974	0.63789	insignificant	0	25	26
chr2	30683820	30685820	Asb6	-0.24888	0.000000638	hypomethylated	-0.055183	0.076477	insignificant	5	10	10
chr2	30699886	30701886	Prrx2	-0.12318	1.5E-16	hypomethylated	-0.012831	0.80907	insignificant	45	125	122
chr2	30807520	30809520	Tor1b	-0.10179	1.67E-09	hypomethylated	-0.010753	0.058604	insignificant	20	89	89
chr2	30823438	30825438	Tor1a	-0.34818	2.72E-08	stronglyHypometh	-0.053234	0.24905	insignificant	2	4	4
chr2	30836798	30838798	Usp20	0.026445	0.000000678	inconclusive	0.0028669	0.26843	insignificant	19	88	89
chr2	30837461	30839461	BC005624	-0.13486	0.00013924	hypomethylated	0.0051167	0.0073762	hypermethylated	3	42	43
chr2	30997528	30999528	Fnbp1	-0.079788	0.00000568	hypomethylated	0.015613	0.83417	insignificant	9	39	38
chr2	31006835	31008835	Gpr107	-0.14013	0.000000441	hypomethylated	0.0022351	0.11299	insignificant	11	52	53
chr2	31100442	31102442	Ncs1	-0.085868	1.47E-19	hypomethylated	-0.010561	0.0012508	hypomethylated	64	196	182
chr2	31324789	31326789	Ass1	-0.12599	8.68E-08	hypomethylated	-0.01649	0.15765	insignificant	14	90	90
chr2	31427244	31429244	Fubp3	-0.11519	2.09E-20	hypomethylated	0.011774	0.20548	insignificant	15	69	76
chr2	31494556	31496556	Prdm12	-0.15978	2.84E-16	hypomethylated	0.016777	0.12645	insignificant	10	84	84
chr2	31525256	31527256	Exosc2	-0.15322	2.33E-21	hypomethylated	0.0079119	0.50789	insignificant	12	32	32
chr2	31543075	31545075	Abl1	-0.19631	1.02E-26	hypomethylated	-0.044244	0.13429	insignificant	29	128	125
chr2	31614464	31616464	Abl1	-0.20128	0.00072106	hypomethylated	0.0056759	0.21316	insignificant	6	64	64
chr2	31666038	31668038	Qrfp	0.39234	0.15519	insignificant	-0.019527	0.06117	insignificant	4	14	14
chr2	31701525	31703525	Fibcd1	-0.095376	8.51E-09	hypomethylated	0.018018	0.17045	insignificant	24	99	110
chr2	31741800	31743800	Lamc3	-0.067775	2.7E-10	hypomethylated	-0.034139	1.31E-11	hypomethylated	19	76	76
chr2	31804822	31806822	Aif1l	-0.094549	7.66E-19	hypomethylated	0.0076818	0.12249	insignificant	31	110	103
chr2	31828969	31830969	Nup214	-0.17662	3.96E-10	hypomethylated	-0.0075428	0.92889	insignificant	28	70	70
chr2	31939225	31941225	Fam78a	-0.10988	2.05E-22	hypomethylated	0.0042749	0.20098	insignificant	37	118	135
chr2	31950170	31952170	Ppapdc3	-0.82167	0.034583	stronglyHypometh	-0.030544	0.58453	insignificant	2	20	19
chr2	32005667	32007667	Prrc2b	-0.088034	0.000050693	hypomethylated	-0.00060201	0.39025	insignificant	17	46	49
chr2	32091202	32093202	Pomt1	-0.17324	5.47E-09	hypomethylated	-0.010794	0.50978	insignificant	13	50	50
chr2	32126324	32128324		-0.057624	0.19214	insignificant	-0.086227	7.35E-08	hypomethylated	5	30	30
chr2	32142772	32144772	Golga2	-0.22217	0.00010235	hypomethylated	0.016973	0.26038	insignificant	12	32	32
chr2	32143588	32145588	Swi5	-0.22217	0.00010235	hypomethylated	0.016973	0.26038	insignificant	12	32	32
chr2	32172979	32174979	Mir199b	-0.29565	0.000065047	hypomethylated	0.020097	0.086533	insignificant	6	12	12
chr2	32217753	32219753	Ciz1	-0.22149	7.89E-08	hypomethylated	0.052706	0.085207	insignificant	15	51	54
chr2	32237435	32239435	1110008P14Rik	-0.10256	1	insignificant	0.041967	0.51347	insignificant	4	15	16
chr2	32250409	32252409	Ptges2	-0.11852	2.64E-23	hypomethylated	-0.0037271	0.20905	insignificant	20	105	104
chr2	32286671	32288671	Slc25a25	-0.60354	0.075081	insignificant	0.081247	0.23234	insignificant	2	6	6
chr2	32304976	32306976	Naif1	-0.1951	3.26E-39	hypomethylated	-0.03462	0.042447	hypomethylated	37	103	125
chr2	32306990	32308990	Naif1	-0.12543	0.00000172	hypomethylated	-0.039364	0.0059101	hypomethylated	9	28	24
chr2	32389878	32391878	Fam102a	-0.15744	8.59E-40	hypomethylated	-0.021227	0.0015977	hypomethylated	46	126	128
chr2	32425377	32427377	Dpm2	-0.15966	0.0020143	hypomethylated	-0.029757	0.040854	hypomethylated	8	30	38
chr2	32442009	32444009	St6galnac4	-0.2	4.67E-23	hypomethylated	-0.0022112	0.34841	insignificant	24	69	69
chr2	32461480	32463480	St6galnac6	-0.11802	3.63E-15	hypomethylated	0.0094117	0.000000637	inconclusive	12	85	84
chr2	32501114	32503114	Eng	-0.12878	7.91E-14	hypomethylated	0.085226	0.15543	insignificant	10	34	30
chr2	32549695	32551695	Fpgs	-0.26423	0.000045794	hypomethylated	0.015427	0.64054	insignificant	6	39	39
chr2	32568304	32570304	Cdk9	-0.1285	1.23E-27	hypomethylated	0.0034888	0.096922	insignificant	29	77	75
chr2	32568408	32570408	Cdk9	-0.096305	2.66E-24	hypomethylated	0.01932	0.015933	hypermethylated	28	74	73
chr2	32575591	32577591	Sh2d3c	-0.52821	9.12E-13	stronglyHypometh	-0.015756	0.63934	insignificant	1	4	7
chr2	32611790	32613790	Tor2a	-0.15807	9.63E-26	hypomethylated	0.0040117	0.65446	insignificant	23	89	87
chr2	32630340	32632340	Ptrh1	-0.08589	0.00000103	hypomethylated	-0.014352	0.027536	hypomethylated	16	57	57
chr2	32630903	32632903	Ttc16	-0.12407	0.000076597	hypomethylated	-0.016567	0.25545	insignificant	17	65	65
chr2	32702752	32704752	Stxbp1		1	noCoverage	-0.063908	0.031181	hypomethylated	0	18	9
chr2	32702757	32704757	Stxbp1		1	noCoverage	-0.063908	0.031181	hypomethylated	0	18	9
chr2	32730653	32732653	Fam129b	-0.15107	0.00000124	hypomethylated	-0.014592	0.030838	inconclusive	22	91	82
chr2	32816231	32818231	Rpl12	-0.12538	0.0012645	hypomethylated	0.0074602	0.010984	hypermethylated	28	98	98
chr2	32816771	32818771	Rpl12	-0.13884	0.00000173	hypomethylated	0.0015047	0.57994	insignificant	24	102	102
chr2	32817820	32819820	Snora65	-0.40598	0.2389	insignificant	-0.014754	0.22729	insignificant	1	16	16
chr2	32837576	32839576	Slc2a8	-0.22462	0.000000105	hypomethylated	0.02284	0.27437	insignificant	12	28	35
chr2	33070480	33072480	Angptl2	-0.13376	0.16515	insignificant	-0.045982	0.42885	insignificant	2	8	8
chr2	33226998	33228998	Ralgps1	-0.18244	5.61E-17	hypomethylated	0.055014	0.88569	insignificant	11	28	30
chr2	33286844	33288844	Zbtb34	-0.41146	1.99E-21	stronglyHypometh	-0.083369	0.29451	insignificant	16	57	61
chr2	33324052	33326052	Zbtb43	-0.17924	0.00037866	hypomethylated	0.058053	0.087076	insignificant	11	39	40
chr2	33496031	33498031	Lmx1b	-0.11499	5.95E-24	hypomethylated	-0.0062031	0.01049	hypomethylated	44	154	164
chr2	34227556	34229556	Pbx3	-0.13595	1.63E-40	hypomethylated	0.021856	0.40694	insignificant	59	212	199
chr2	34286544	34288544	Mapkap1	-0.12258	0.23697	insignificant	0.026502	0.044758	inconclusive	4	15	16
chr2	34626609	34628609	Hspa5	-0.14981	9.45E-14	hypomethylated	-0.0022711	0.90509	insignificant	24	107	106
chr2	34655311	34657311	Rabepk	-0.19767	0.60045	insignificant	0.006871	1	insignificant	3	6	6
chr2	34681755	34683755	Fbxw2	-0.2672	0.76194	insignificant	-0.017052	0.79337	insignificant	3	23	31
chr2	34726482	34728482	Psmd5	-0.12948	2.28E-09	hypomethylated	-0.01072	0.16849	insignificant	11	26	26
chr2	34728955	34730955	D730039F16Rik	-0.54162	3.75E-10	stronglyHypometh	-0.0025247	0.50878	insignificant	7	11	12
chr2	34769496	34771496	Phf19	-0.072261	2.01E-13	hypomethylated	0.00015964	0.67713	insignificant	26	85	87
chr2	34964011	34966011	Cep110	-0.15969	6.61E-17	hypomethylated	-0.0023273	0.55919	insignificant	19	92	92

chr2	35056640	35058640	Rab14	-0.10144	0.00062218	hypomethylated	-0.0010584	0.91563	insignificant	9	41	41
chr2	35136959	35138959	Gsn	-0.15633	1.15E-10	hypomethylated	0.010208	0.0056863	hypermethylated	9	57	57
chr2	35192529	35194529	Stom	-0.27232	0.00000113	hypomethylated	-0.015796	0.0096584	hypomethylated	8	20	20
chr2	35316945	35318945	Ggta1	-0.17871	1.47E-18	hypomethylated	-0.03018	0.020042	hypomethylated	25	57	64
chr2	35412977	35414977	Dab2ip	-0.0082825	0.71007	insignificant	-0.11771	0.027385	hypomethylated	1	8	9
chr2	35476500	35478500	Dab2ip	-0.15816	0.042739	hypomethylated	-0.006469	0.36338	insignificant	5	28	28
chr2	35835144	35837144	Ttll11	-0.066482	0.000019175	hypomethylated	-0.0096648	0.76381	insignificant	17	50	60
chr2	35903992	35905992	Morn5	-0.13968	1.84E-08	hypomethylated	0.0099098	0.58336	insignificant	7	61	62
chr2	35904812	35906812	Ndufa8	-0.16597	0.141	insignificant	-0.045562	0.23262	insignificant	1	17	16
chr2	35959580	35961580	Lhx6	-0.16074	1.36E-13	hypomethylated	-0.034545	0.0058162	hypomethylated	25	92	90
chr2	35960928	35962928	Lhx6	-0.23431	0.000000133	hypomethylated	0.07537	0.593	insignificant	2	7	4
chr2	35990917	35992917	Mrrf	-0.19338	0.000000093	hypomethylated	-0.0016256	0.49643	insignificant	15	51	51
chr2	35992224	35994224	Mrrf	-0.18687	0.0022103	hypomethylated	0.0010861	0.54018	insignificant	9	35	35
chr2	36084945	36086945	Ptgs1	-0.13438	0.00014118	hypomethylated	-0.0099076	0.84209	insignificant	5	28	28
chr2	36244430	36246430	Mir684-1		1	noCoverage	-0.037821	1	insignificant	0	5	10
chr2	37186268	37188268	Olfr368	-0.46314	0.12661	insignificant	0.094426	1	insignificant	2	6	7
chr2	37214852	37216852	Pdcl		1	noCoverage	-0.041304	0.3372	insignificant	0	8	10
chr2	37286439	37288439	Zbtb6	-0.026998	0.00000604	hypomethylated	-0.081642	0.26912	insignificant	7	20	24
chr2	37297804	37299804	Rabgap1	-0.24524	6.11E-08	hypomethylated	-0.046576	0.63212	insignificant	7	17	14
chr2	37298641	37300641	Zbtb26	-0.70522	0.031731	stronglyHypometh	-0.038778	0.53439	insignificant	2	14	17
chr2	37306773	37308773	Rabgap1	-0.24194	6.61E-29	hypomethylated	-0.00010783	0.32915	insignificant	19	77	81
chr2	37630768	37632768	Crb2	0.069197	0.59838	insignificant	0.008941	0.12154	insignificant	9	54	54
chr2	38142904	38144904	Dennd1a	-0.0625	1	insignificant	0.020979	0.69745	insignificant	5	16	16
chr2	38205827	38207827	Lhx2	-0.16124	3.54E-10	hypomethylated	-0.0055805	0.20024	insignificant	16	85	81
chr2	38366216	38368216	Nek6	-0.058872	0.000000534	hypomethylated	0.00044593	0.82837	insignificant	16	122	116
chr2	38366395	38368395	Nek6	-0.058872	0.000000534	hypomethylated	0.00044593	0.82837	insignificant	16	122	116
chr2	38499426	38501426	Psmb7	-0.22398	0.0016806	hypomethylated	0.044228	0.083104	insignificant	1	12	7
chr2	38781981	38783981	Nr6a1	-0.26919	0.01592	hypomethylated	0.044848	0.50488	insignificant	4	26	28
chr2	38786499	38788499	Olfml2a	-0.18267	0.00054866	hypomethylated	0.085524	1	insignificant	3	16	20
chr2	38852828	38854828	Wdr38	-0.095078	4.81E-09	hypomethylated	-0.025317	8.93E-20	hypomethylated	7	41	44
chr2	38860651	38862651	Rpl35	-0.1728	7.81E-22	hypomethylated	-0.077818	7.56E-11	hypomethylated	21	52	54
chr2	38862658	38864658	Arpc5l	-0.13806	1.59E-18	hypomethylated	-0.025394	0.057412	insignificant	41	94	92
chr2	38920926	38922926	Golga1	-0.1181	4.78E-15	hypomethylated	0.0030561	0.53021	insignificant	20	64	64
chr2	39017755	39019755	Scai	-0.2619	0.015524	hypomethylated	-0.064536	0.19594	insignificant	1	4	4
chr2	39046250	39048250	Scai	-0.30698	0.00076179	hypomethylated	-0.038954	0.17747	insignificant	5	41	38
chr2	39081858	39083858	Ppp6c	-0.17778	0.038107	hypomethylated	-0.071111	0.42075	insignificant	4	18	18
chr2	43409848	43411848	Kynu	0.071875	1	insignificant	-0.061648	0.10986	insignificant	1	8	8
chr2	44965657	44967657	Gm13476	-0.15678	3.37E-18	hypomethylated	0.0073904	0.035667	inconclusive	17	67	68
chr2	44966779	44968779	Gm13476	-0.45566	0.031312	stronglyHypometh	-0.1196	0.036052	hypomethylated	1	12	16
chr2	48668628	48670628	Acvr2a	-0.1054	2.86E-22	hypomethylated	-0.0086493	0.50371	insignificant	54	155	161
chr2	48804027	48806027	Mbd5	-0.18123	1.92E-11	hypomethylated	-0.017185	0.0089228	hypomethylated	15	62	62
chr2	48804787	48806787	Orc4	-0.21984	0.30172	insignificant	-0.014545	0.041934	hypomethylated	2	24	24
chr2	49306005	49308005	Epc2	-0.10456	6.55E-35	hypomethylated	-0.011244	0.10852	insignificant	68	218	210
chr2	49473833	49475833	Kif5c	-0.12873	1.71E-22	hypomethylated	-0.0044042	0.44093	insignificant	35	110	110
chr2	49642205	49644205	Lypd6b	-0.12526	9.66E-10	hypomethylated	0.0022823	0.95986	insignificant	21	62	62
chr2	49920981	49922981	Lypd6	-0.1116	0.000082411	hypomethylated	-0.0044371	0.027511	hypomethylated	18	99	99
chr2	50152197	50154197	Mmadhc		1	noCoverage	-0.0047377	0.34761	insignificant	0	14	14
chr2	51482030	51484030	Tas2r134	0.1489	1	lowCoverage	0.023897	0.35072	insignificant	1	2	4
chr2	51790164	51792164	Rbm43	-0.14395	0.010176	hypomethylated	-0.0047159	0.41599	insignificant	9	69	67
chr2	51790529	51792529	Rbm43	-0.25057	0.048999	hypomethylated	0.0085127	0.18967	insignificant	6	26	24
chr2	51892632	51894632	Tnfaip6	-0.19427	0.084129	insignificant	0.091216	0.026141	hypermethylated	6	14	14
chr2	51927356	51929356	Rif1	-0.11257	4.1E-11	hypomethylated	-0.0075549	0.85445	insignificant	45	142	141
chr2	52194318	52196318	Neb	0.064274	1	insignificant	0.020003	1	insignificant	2	8	8
chr2	52280394	52282394	Arl5a	-0.21799	0.000000019	hypomethylated	-0.03327	0.34269	insignificant	10	24	24
chr2	52532101	52534101	Cacnb4	-0.19886	1	insignificant	-0.0091315	0.63762	insignificant	1	8	8
chr2	52601183	52603183	Stam2	-0.19252	0.0010907	hypomethylated	-0.0037971	0.53292	insignificant	10	42	41
chr2	52715901	52717901	Fmnl2	-0.11854	2.06E-29	hypomethylated	0.010366	0.40406	insignificant	68	194	211
chr2	53050117	53052117	Arl6ip6	-0.093047	1.94E-14	hypomethylated	-0.00077111	0.82633	insignificant	39	196	200
chr2	53050221	53052221	Prpf40a	-0.087083	1.32E-09	hypomethylated	-0.00045504	0.8363	insignificant	33	184	188
chr2	53937963	53939963	Rprm	-0.2629	0.0025827	hypomethylated	0.085675	0.52097	insignificant	2	12	12
chr2	54287797	54289797	Galnt13	-0.13981	2.67E-18	hypomethylated	0.0077128	0.15251	insignificant	56	169	170
chr2	55288566	55290566	Kcnj3	-0.15677	0.000000996	hypomethylated	-0.019578	0.44769	insignificant	15	64	56
chr2	56967449	56969449	Nr4a2	-0.2154	9.18E-12	hypomethylated	-0.016335	0.27165	insignificant	13	54	59
chr2	56976414	56978414	Nr4a2	-0.10653	4.8E-10	hypomethylated	-0.019519	0.6121	insignificant	28	70	70
chr2	57089088	57091088	Gpd2	-0.16691	6.61E-23	hypomethylated	0.015965	0.15114	insignificant	34	78	87
chr2	57089792	57091792	Gpd2	-0.16691	6.61E-23	hypomethylated	0.0065126	0.15077	insignificant	34	78	86
chr2	58012533	58014533	Cytip	-0.41667	0.50351	insignificant	-0.018056	0.66389	insignificant	2	8	12
chr2	58210169	58212169	Acvr1c		1	noCoverage	-0.0043151	0.91031	insignificant	0	27	27
chr2	58419239	58421239	Acvr1	-0.10448	9.2E-24	hypomethylated	-0.0076022	0.37493	insignificant	66	156	150
chr2	58606595	58608595	Upp2	-0.24205	0.026341	hypomethylated	0.0077146	0.40299	insignificant	2	8	8
chr2	58997906	58999906	Pkp4	-0.11192	4.67E-23	hypomethylated	-0.004772	0.031639	hypomethylated	56	214	217
chr2	58998684	59000684	Pkp4	-0.11402	2.78E-22	hypomethylated	-0.0033037	0.17019	insignificant	60	221	223
chr2	59321709	59323709	Dapl1	-0.19646	0.083898	insignificant	0.065852	0.19518	insignificant	2	6	8
chr2	59449100	59451100	Tanc1	-0.13884	6.78E-45	hypomethylated	-0.0075914	0.54919	insignificant	48	100	100
chr2	59720663	59722663	Wdsub1	-0.0837	1	insignificant	-0.010193	0.21361	insignificant	6	16	16
chr2	60046992	60048992	March7	-0.085697	1.69E-18	hypomethylated	-0.0023781	0.7637	insignificant	57	175	174
chr2	60221288	60223288	Ly75	-0.071825	0.02694	hypomethylated	0.018043	0.36374	insignificant	7	26	28
chr2	60391318	60393318	Pla2r1	-0.056341	1	insignificant	0.059044	0.18015	insignificant	1	12	8
chr2	60719495	60721495	Rbms1	-0.097459	0.027723	hypomethylated	-0.021833	0.039693	hypomethylated	13	38	48
chr2	60801261	60803261	Rbms1	-0.023255	1	insignificant	-0.036213	0.0006429	hypomethylated	2	22	22
chr2	61430153	61432153	Tank	-0.14937	4.4E-21	hypomethylated	0.0034454	0.10546	insignificant	20	66	65
chr2	61548750	61550750	Psmd14	-0.27147	8.39E-15	hypomethylated	-0.022665	0.049178	hypomethylated	20	64	64
chr2	61641509	61643509	Tbr1	-0.1492	8.23E-24	hypomethylated	0.021392	0.58604	insignificant	21	64	65
chr2	61883596	61885596	Slc4a10	-0.33987	0.05126	insignificant	-0.045377	0.046599	hypomethylated	1	8	8
chr2	62250288	62252288	Dpp4	-0.53935	1	lowCoverage	-0.19607	1	insignificant	1	12	8

chr2	62412078	62414078	Fap	-0.29023	0.033048	hypomethylated	0.012235	0.28336	insignificant	4	16	16
chr2	62484312	62486312	Ifih1	0.41667	1	insignificant	0.29444	1	insignificant	2	8	6
chr2	62501383	62503383	Gca	-0.17646	6.08E-11	hypomethylated	0.006375	0.20925	insignificant	23	63	63
chr2	63022344	63024344	Kcnh7	-0.3039	0.042694	hypomethylated	-0.021698	0.8844	insignificant	3	14	13
chr2	63936064	63938064	Fign	-0.16925	4.66E-52	hypomethylated	0.0032492	0.3226	insignificant	55	116	100
chr2	64860823	64862823	Grb14	-0.17144	5.99E-08	hypomethylated	-0.022884	1	insignificant	8	28	27
chr2	65076683	65078683	Cobll1	-0.10346	1.68E-24	hypomethylated	-0.016949	0.29632	insignificant	32	122	122
chr2	65405549	65407549	Scn3a	0.021428	0.060536	insignificant	-0.040051	0.023295	hypomethylated	3	14	15
chr2	65962850	65964850	Galnt3	-0.15718	0.0039405	hypomethylated	0.037156	0.13799	insignificant	10	46	50
chr2	66094674	66096674	Ttc21b	-0.082875	1	lowCoverage	-0.0089838	0.86823	insignificant	1	14	14
chr2	66277936	66279936	Gm13629	-0.51667	0.00000405	stronglyHypometh	0.048019	0.50075	insignificant	2	4	4
chr2	67954769	67956769	B3galt1	0.28819	1	insignificant	0.23819	0.001962	hypermethylated	2	6	5
chr2	68310038	68312038	Stk39	-0.13134	0.061954	insignificant	-0.0017026	0.41113	insignificant	5	71	64
chr2	68419469	68421469	4933409G03Rik		1	noCoverage	-0.25326	0.11313	insignificant	0	2	4
chr2	68698613	68700613	Lass6	-0.11486	1.58E-18	hypomethylated	0.025911	0.24471	insignificant	34	118	123
chr2	68972856	68974856	Nostrin	-0.32847	0.024512	hypomethylated	-0.045849	0.92134	insignificant	2	6	6
chr2	69424124	69426124	Lrp2	-0.15901	0.00000676	hypomethylated	-0.028502	0.93058	insignificant	8	59	60
chr2	69484311	69486311	Bbs5	-0.08545	0.001974	hypomethylated	0.015304	0.55085	insignificant	9	42	44
chr2	69507176	69509176	Kbtbd10	0.04104	0.79761	insignificant	-0.013296	0.099793	insignificant	13	48	48
chr2	69550663	69552663	Fastkd1	-0.19802	0.00028672	hypomethylated	-0.0068913	0.63687	insignificant	7	26	26
chr2	69560144	69562144	Ppig	-0.13238	3.32E-17	hypomethylated	0.022544	0.19407	insignificant	33	133	126
chr2	69626793	69628793	Phospho2	-0.15446	3.37E-09	hypomethylated	-0.026059	0.27414	insignificant	10	56	63
chr2	69627543	69629543	4930578N16Rik	-0.12144	0.021174	hypomethylated	-0.024981	0.17179	insignificant	7	42	47
chr2	69659426	69661426	Klhl23	-0.11039	2.53E-18	hypomethylated	0.0057479	0.0019504	inconclusive	50	139	148
chr2	69698618	69700618	Ssb	-0.11761	5.44E-08	hypomethylated	0.0040407	0.37979	insignificant	8	68	60
chr2	69723661	69725661	Mettl5	-0.21149	0.007219	hypomethylated	-0.037735	0.69121	insignificant	9	34	40
chr2	69734302	69736302	Ubr3	-0.079253	1.03E-08	hypomethylated	-0.0041466	0.16996	insignificant	48	170	169
chr2	69876182	69878182	Myo3b	-0.32779	0.000000112	hypomethylated	-0.16309	0.043096	hypomethylated	3	8	9
chr2	70311979	70313979	Sp5	-0.1714	0.00000494	hypomethylated	-0.011476	0.4112	insignificant	17	66	70
chr2	70345875	70347875	4933404M02Ril	-0.10515	0.000000108	hypomethylated	0.015767	0.74931	insignificant	18	121	118
chr2	70399220	70401220	Gad1	-0.18867	0.000019996	hypomethylated	0.0099876	0.42147	insignificant	9	44	46
chr2	70498565	70500565	Gorasp2	-0.11773	3.33E-33	hypomethylated	-0.010095	0.39059	insignificant	38	131	133
chr2	70663537	70665537	Tlk1	-0.097859	1.19E-10	hypomethylated	-0.012051	0.38851	insignificant	45	136	136
chr2	70892800	70894800	Dcaf17	-0.15711	3.27E-10	hypomethylated	-0.013918	0.25987	insignificant	10	48	48
chr2	70893663	70895663	Mettl8	-0.27778	2.97E-19	hypomethylated	0.010016	0.054718	insignificant	10	46	46
chr2	70955110	70957110	Cybrd1	-0.13495	1.81E-09	hypomethylated	-0.0032207	0.87382	insignificant	17	61	67
chr2	71048762	71050762	Dync1i2	-0.17482	0.00000182	hypomethylated	-0.014247	0.60133	insignificant	17	66	66
chr2	71049002	71051002	Dync1i2	-0.17482	0.00000182	hypomethylated	-0.014247	0.60133	insignificant	17	66	66
chr2	71205611	71207611	Slc25a12	-0.088805	0.00000158	hypomethylated	-0.0072505	0.61608	insignificant	15	55	59
chr2	71226316	71228316	Hat1	-0.12046	0.000085459	hypomethylated	-0.033593	0.07131	insignificant	9	31	30
chr2	71290394	71292394	Metap1d	-0.19982	7.73E-08	hypomethylated	-0.0024639	0.36457	insignificant	20	51	51
chr2	71366501	71368501	Dlx1	-0.10208	5.65E-31	hypomethylated	0.010643	1	insignificant	33	128	133
chr2	71375948	71377948	Dlx1as	-0.26059	0.0036083	hypomethylated	0.072865	0.79307	insignificant	2	42	41
chr2	71384811	71386811	Dlx2	-0.16801	3.83E-12	hypomethylated	0.021476	0.24454	insignificant	10	39	44
chr2	71556473	71558473	Gm1631	0.14672	1	insignificant	0.069823	0.55139	insignificant	3	9	10
chr2	71624139	71626139	Itga6	-0.11977	1.38E-13	hypomethylated	-0.010763	0.064218	insignificant	57	184	183
chr2	71710328	71712328	Pdk1	-0.19601	0.00065363	hypomethylated	-0.027146	0.73041	insignificant	12	68	65
chr2	71818343	71820343	Rapgef4	0.74404	0.029726	stronglyHypermeth	-0.026976	0.37462	insignificant	1	45	57
chr2	72122693	72124693	B230120H23Rik	-0.11956	5.81E-09	hypomethylated	-0.026262	0.022421	hypomethylated	33	109	119
chr2	72122757	72124757	B230120H23Rik	-0.11956	5.81E-09	hypomethylated	-0.026262	0.022421	hypomethylated	33	109	119
chr2	72313275	72315275	Cdca7	-0.10493	0.00000119	hypomethylated	0.0092896	0.34149	insignificant	19	78	81
chr2	72817338	72819338	Sp3	-0.094363	5.97E-24	hypomethylated	-0.020134	0.43882	insignificant	54	196	205
chr2	72818503	72820503	1700011J10Rik	-0.15458	3.01E-19	hypomethylated	-0.016799	0.50267	insignificant	24	91	88
chr2	73052504	73054504	Ola1	-0.35615	2.48E-09	stronglyHypometh	0.067583	0.22083	insignificant	8	33	26
chr2	73108982	73110982	Sp9	-0.10941	0.00000625	hypomethylated	0.0058034	0.65938	insignificant	17	80	94
chr2	73149708	73151708	Scrn3	-0.11541	0.000000131	hypomethylated	0.0073428	0.058262	insignificant	23	88	91
chr2	73150649	73152649	Cir1	-0.078796	0.0011008	hypomethylated	-0.0021816	0.28142	insignificant	15	53	53
chr2	73224455	73226455	Gpr155	-0.15768	4.64E-21	hypomethylated	0.0074083	0.94568	insignificant	8	30	30
chr2	73367467	73369467	Wipf1	-0.15708	0.00012027	hypomethylated	0.023255	0.24995	insignificant	12	49	46
chr2	73418363	73420363	Chrna1	-0.079545	0.14473	insignificant	-0.033961	0.27577	insignificant	3	6	6
chr2	73613403	73615403	Chn1	-0.18883	0.00009894	hypomethylated	-0.0076689	0.73901	insignificant	9	36	36
chr2	73730685	73732685	Atf2	-0.37909	0.000000118	stronglyHypometh	0.0051308	0.63437	insignificant	8	40	41
chr2	73749351	73751351	Atp5g3	-0.077589	0.011626	hypomethylated	0.0026827	0.75161	insignificant	9	29	26
chr2	74417005	74419005	Lnp	-0.096999	0.014125	hypomethylated	0.057867	0.1369	insignificant	4	22	22
chr2	74497476	74499476	Evx2	-0.33865	0.0022935	stronglyHypometh	-0.061721	0.66022	insignificant	4	28	28
chr2	74505366	74507366	Hoxd13	-0.11907	2.62E-20	hypomethylated	0.011553	0.95681	insignificant	39	151	158
chr2	74512086	74514086	Hoxd12	-0.15563	1.54E-14	hypomethylated	-0.022222	0.052647	insignificant	25	104	104
chr2	74519449	74521449	Hoxd11	-0.12886	3.86E-30	hypomethylated	-0.0010543	0.8731	insignificant	82	265	269
chr2	74529004	74531004	Hoxd10	-0.24066	5.42E-20	hypomethylated	-0.032822	0.014943	hypomethylated	26	114	121
chr2	74534819	74536819	Hoxd9	-0.13754	9.79E-37	hypomethylated	0.001572	0.15446	insignificant	61	185	189
chr2	74542545	74544545	Hoxd8	-0.10253	4.44E-18	hypomethylated	-0.0056128	0.41416	insignificant	63	174	184
chr2	74549049	74551049	Hoxd3	-0.11179	1.84E-13	hypomethylated	-0.002174	0.00000001	hypomethylated	21	54	59
chr2	74559034	74561034	Hoxd4	-0.18612	0.65696	insignificant	-0.019759	0.19858	insignificant	3	24	24
chr2	74563126	74565126	Mir10b	0.049044	0.0053996	hypermethylated	-0.017045	0.73295	insignificant	11	38	38
chr2	74600036	74602036	Hoxd1	-0.13864	3.38E-21	hypomethylated	-0.0097736	0.040539	hypomethylated	43	155	143
chr2	74662868	74664868	Mtx2	-0.096718	0.00028522	hypomethylated	0.0084845	0.09399	insignificant	13	88	77
chr2	75496315	75498315	Hnrnpa3	-0.11923	2.82E-26	hypomethylated	-0.0078172	0.16238	insignificant	53	183	179
chr2	75496346	75498346	Gm6793	-0.11923	2.82E-26	hypomethylated	-0.0078172	0.16238	insignificant	53	183	179
chr2	75542698	75544698	Nfe2l2	-0.19462	2.74E-08	hypomethylated	-0.027492	0.18249	insignificant	9	44	44
chr2	75669233	75671233	Agps	-0.10481	5.75E-13	hypomethylated	-0.0048467	0.38792	insignificant	24	116	116
chr2	75776519	75778519	Ttc30b	-0.21928	1	insignificant	0.065365	0.41791	insignificant	2	28	34
chr2	75820024	75822024	Ttc30a1		1	noCoverage	-0.010101	0.25498	insignificant	0	4	6
chr2	76176710	76178710	Pde11a	-0.0041257	0.0068857	hypomethylated	0.011375	0.12336	insignificant	4	33	33
chr2	76207040	76209040	Rbm45	-0.19651	0.00000928	hypomethylated	0.0087678	0.0014061	inconclusive	14	81	78
chr2	76243594	76245594	Osbpl6	-0.10968	2.18E-47	hypomethylated	-0.0038069	0.80083	insignificant	84	198	188

chr2	76486051	76488051	Prkra	-0.15526	9.24E-11	hypomethylated	0.010632	0.80287	insignificant	11	57	54
chr2	76511155	76513155	Plekha3	-0.0295	0.038954	hypomethylated	-0.030957	0.014087	hypomethylated	6	24	24
chr2	76512371	76514371	Plekha3	-0.13101	3.85E-20	hypomethylated	-0.010689	0.068182	insignificant	50	162	164
chr2	76820604	76822604	Ttn	0.086796	0.076235	insignificant	0.015992	0.78962	insignificant	2	11	8
chr2	77008692	77010692	Ccdc141	0.27264	0.21285	insignificant	0.049182	0.45023	insignificant	5	25	26
chr2	77118682	77120682	Sestd1	-0.070999	7.75E-17	hypomethylated	0.016413	0.42113	insignificant	29	56	56
chr2	77654873	77656873	Zfp385b	-0.16391	1	lowCoverage	0.0048406	0.88204	insignificant	1	50	49
chr2	78708203	78710203	Ube2e3	-0.047294	0.0005641	hypomethylated	-0.0024628	0.22157	insignificant	59	250	244
chr2	79094582	79096582	Itga4	-0.11845	1.9E-09	hypomethylated	-0.014062	0.95259	insignificant	13	31	30
chr2	79269145	79271145	Cerkl	-0.12422	1	insignificant	-0.0071256	0.26099	insignificant	1	8	11
chr2	79296793	79298793	Neurod1	-0.21219	6.91E-09	hypomethylated	-0.030503	1	insignificant	6	20	28
chr2	79474581	79476581	Ssfa2	-0.10273	1.16E-16	hypomethylated	-0.00081278	0.5246	insignificant	46	159	147
chr2	79546936	79548936	Ppp1r1c	-0.33935	0.31603	insignificant	0.036421	0.36005	insignificant	2	20	22
chr2	80131628	80133628	Prdx6b	0.05	1	insignificant	-0.033333	0.64358	insignificant	1	4	4
chr2	80154622	80156622	Dnajc10	-0.14136	2.11E-12	hypomethylated	-0.019457	0.0030053	hypomethylated	24	88	88
chr2	80287553	80289553	Frzb	-0.24694	0.34491	insignificant	-0.0095879	0.37458	insignificant	2	19	19
chr2	80421122	80423122	Nckap1	-0.13108	0.0010123	hypomethylated	0.024214	1.57E-17	inconclusive	28	104	108
chr2	80456370	80458370	Dusp19	-0.41299	0.020763	stronglyHypometh	-0.058289	0.54733	insignificant	3	6	6
chr2	80477968	80479968	Nup35	-0.16708	0.000000289	hypomethylated	0.02509	0.081579	insignificant	8	52	56
chr2	80478420	80480420	Nup35	-0.16708	0.000000289	hypomethylated	0.02509	0.081579	insignificant	8	52	56
chr2	81892814	81894814	Zfp804a	-0.10569	1.94E-11	hypomethylated	0.0046988	0.62314	insignificant	34	91	89
chr2	83483734	83485734	Zc3h15	-0.1301	1.11E-26	hypomethylated	-0.001087	0.693	insignificant	28	88	88
chr2	83563553	83565553	Itgav	-0.12195	9.97E-27	hypomethylated	0.0016963	0.84308	insignificant	50	142	142
chr2	83651884	83653884	Fam171b	-0.14354	4.88E-13	hypomethylated	-0.031847	0.54588	insignificant	22	57	52
chr2	84498235	84500235	Gm13718	0.023805	0.47899	insignificant	-0.07065	0.08717	insignificant	5	26	32
chr2	84510865	84512865	Tmx2		1	noCoverage	0.041976	0.9036	insignificant	0	21	16
chr2	84517558	84519558	Med19	-0.16059	0.013243	hypomethylated	0.029905	0.80257	insignificant	8	44	47
chr2	84518204	84520204	Tmx2	-0.16218	0.02926	hypomethylated	-0.005074	1	insignificant	7	26	26
chr2	84555321	84557321	Zdhhc5	-0.21416	0.0074439	hypomethylated	0.00079763	0.14678	insignificant	5	23	23
chr2	84567425	84569425	Clp1	-0.36486	1	insignificant	-0.059076	0.65298	insignificant	1	47	47
chr2	84573360	84575360	Ypel4	-0.41342	1.16E-09	stronglyHypometh	-0.18633	0.56256	insignificant	3	6	10
chr2	84581335	84583335	Mir130a	-0.1784	0.10377	insignificant	0.017149	0.79139	insignificant	6	20	22
chr2	84637984	84639984	Ube2l6	-0.3601	6.83E-08	stronglyHypometh	-0.02452	0.029463	hypomethylated	4	21	21
chr2	84666177	84668177	Timm10	-0.18891	0.0018196	hypomethylated	-0.030108	0.26461	insignificant	9	64	53
chr2	84678564	84680564	Slc43a1	-0.16071	8.98E-09	hypomethylated	0.018433	1	insignificant	12	85	88
chr2	84679479	84681479	Slc43a1	-0.15292	0.000000189	hypomethylated	-0.0084369	0.50196	insignificant	15	95	95
chr2	84679782	84681782	Slc43a1	-0.14109	0.00000474	hypomethylated	-0.0030158	0.69823	insignificant	10	60	60
chr2	84726849	84728849	Rtn4rl2	-0.11357	4.29E-14	hypomethylated	-0.0069268	0.83995	insignificant	17	80	85
chr2	84775812	84777812	Slc43a3	-0.27502	6.43E-13	hypomethylated	-0.0090658	0.1981	insignificant	10	35	28
chr2	84875991	84877991	Ssrp1	-0.12721	2.33E-12	hypomethylated	-0.014965	0.85339	insignificant	10	48	48
chr2	84876357	84878357	Ssrp1	-0.12721	2.33E-12	hypomethylated	-0.014965	0.85339	insignificant	10	48	48
chr2	84876607	84878607	Ssrp1	-0.14502	2.51E-12	hypomethylated	-0.032777	0.80566	insignificant	10	49	48
chr2	84889616	84891616	Tnks1bp1	-0.19693	0.14081	insignificant	0.030083	0.072088	insignificant	3	18	19
chr2	84975516	84977516	Aplnr	-0.059958	0.30201	insignificant	-0.00050477	0.17474	insignificant	7	16	14
chr2	85036856	85038856	Lrrc55	-0.013187	1	insignificant	-0.07769	0.84672	insignificant	2	10	7
chr2	85504612	85506612	Olfr154		1	noCoverage	0.048841	0.33839	insignificant	0	4	4
chr2	85624630	85626630	Olfr1015		1	noCoverage	-0.24419	0.21773	insignificant	0	9	6
chr2	85859796	85861796	Olfr1033	-0.36296	1	lowCoverage	0.11474	0.45109	insignificant	1	15	10
chr2	86076990	86078990	Olfr1048	0.03125	1	lowCoverage	-0.005787	1	insignificant	1	8	3
chr2	86667832	86669832	Olfr1094	0.21111	1	lowCoverage	-0.045299	0.80203	insignificant	1	6	3
chr2	87498614	87500614	Olfr1134		1	noCoverage	0.13333	0.63306	insignificant	0	4	5
chr2	89892479	89894479	Olfr140	0.083333	1	lowCoverage	-0.0059524	1	insignificant	1	4	4
chr2	90420804	90422804	Ptprj	-0.095652	0.00000309	hypomethylated	-0.011595	0.71792	insignificant	16	52	52
chr2	90584526	90586526	Fnbp4	-0.12681	1.9E-25	hypomethylated	0.015151	0.93518	insignificant	36	96	96
chr2	90621900	90623900	Agbl2	-0.23556	5.36E-10	hypomethylated	0.027368	0.046761	inconclusive	17	55	56
chr2	90686311	90688311	Mtch2	-0.10382	1.4E-22	hypomethylated	-0.0033717	0.00000229	hypomethylated	38	118	118
chr2	90724942	90726942	C1qtnf4	-0.15681	1.97E-12	hypomethylated	-0.021481	0.84317	insignificant	12	36	40
chr2	90743942	90745942	Kbtbd4	-0.047751	0.042204	hypomethylated	-0.050601	0.36151	insignificant	9	36	33
chr2	90744878	90746878	Ndufs3	-0.039624	0.053023	insignificant	-0.05728	0.26398	insignificant	12	46	45
chr2	90758207	90760207	Ptpmt1		1	noCoverage	0.26732	0.30381	insignificant	0	4	2
chr2	90779614	90781614	Celf1	-0.10842	9.51E-28	hypomethylated	-0.0034884	0.19772	insignificant	39	186	189
chr2	90874783	90876783	Rapsn	-0.0027868	1	insignificant	-0.0024119	0.8968	insignificant	4	8	8
chr2	90893172	90895172	Psmc3	-0.20144	0.0011741	hypomethylated	0.010188	0.75942	insignificant	4	36	24
chr2	90910378	90912378	Slc39a13	-0.097556	1.6E-11	hypomethylated	-0.0070287	0.012879	hypomethylated	18	58	68
chr2	90935953	90937953	Sfpi1	-0.27027	1.44E-10	hypomethylated	0.0078711	0.20454	insignificant	6	27	27
chr2	90957300	90959300	Mybpc3	-0.44375	0.0048507	stronglyHypometh	-0.073104	0.507	insignificant	2	8	8
chr2	91019478	91021478	Madd	-0.23206	0.065928	insignificant	-0.11032	0.022548	hypomethylated	2	10	10
chr2	91023204	91025204	Madd	-0.31878	0.0044556	hypomethylated	-0.047095	1	insignificant	2	9	6
chr2	91035192	91037192	Nr1h3	-0.083469	0.016491	hypomethylated	0.20423	0.76022	insignificant	3	6	6
chr2	91035273	91037273	Nr1h3	-0.083469	0.016491	hypomethylated	0.20423	0.76022	insignificant	3	6	6
chr2	91042068	91044068	Acp2	-0.1689	0.00000679	hypomethylated	-0.0056103	0.21589	insignificant	9	34	34
chr2	91076302	91078302	A330069E16Rik	-0.29929	0.00000415	hypomethylated	-0.10463	0.31938	insignificant	7	40	34
chr2	91077223	91079223	Ddb2	-0.44384	0.55507	insignificant	-0.17914	0.48363	insignificant	1	21	17
chr2	91095969	91097969	Pacsin3	-0.13692	2.04E-19	hypomethylated	0.0020785	0.43169	insignificant	28	96	96
chr2	91096485	91098485	Pacsin3	-0.05356	3.93E-08	hypomethylated	0.015722	0.87414	insignificant	19	70	70
chr2	91104271	91106271	Arfgap2	-0.21131	2.1E-14	hypomethylated	-0.012803	0.016754	hypomethylated	23	91	95
chr2	91284799	91286799	1110051M20Ril	-0.38007	0.0017757	stronglyHypometh	-0.02621	0.0039821	hypomethylated	5	20	20
chr2	91296687	91298687	Lrp4	-0.14966	3.5E-33	hypomethylated	-0.03372	0.000000947	hypomethylated	29	111	121
chr2	91489274	91491274	Arhgap1	-0.11908	0.000000012	hypomethylated	0.033664	0.0039677	hypermethylated	11	75	74
chr2	91489948	91491948	Zfp408	-0.28652	0.000000138	hypomethylated	0.060498	0.17566	insignificant	7	63	62
chr2	91550106	91552106	Harbi1	-0.1824	1.23E-20	hypomethylated	0.035712	0.32768	insignificant	18	46	45
chr2	91550733	91552733	Harbi1	-0.18293	1.45E-13	hypomethylated	0.13641	1	insignificant	6	15	14
chr2	91569294	91571294	Ambra1	-0.1576	0.000000163	hypomethylated	-0.028139	0.065972	insignificant	14	69	61
chr2	91761345	91763345	Chrm4	-0.17214	1.48E-34	hypomethylated	-0.0016216	0.00012054	hypomethylated	47	134	134
chr2	91771856	91773856	Mdk	-0.24121	0.00000164	hypomethylated	-0.022773	0.39971	insignificant	4	47	52

chr2	91771950	91773950	Mdk		1	noCoverage	-0.026232	0.5741	insignificant	0	38	43
chr2	91790505	91792505	Dgkz	-0.65985	0.0051647	stronglyHypometh	-0.15189	0.71877	insignificant	2	8	11
chr2	91803720	91805720	Dgkz	-0.075254	6.11E-09	hypomethylated	0.0011107	0.79134	insignificant	11	55	55
chr2	91864327	91866327	Creb3l1	-0.22406	0.000000756	hypomethylated	-0.0092718	0.74732	insignificant	9	29	29
chr2	92023338	92025338	Phf21a	-0.095541	7.64E-24	hypomethylated	0.044207	0.47405	insignificant	43	138	142
chr2	92024639	92026639	Phf21a	-0.2219	1.27E-15	hypomethylated	-0.015387	0.3347	insignificant	22	57	57
chr2	92031133	92033133	Mir1955	-0.28958	0.00093758	hypomethylated	0.013225	1	insignificant	2	4	4
chr2	92211193	92213193	Gyltl1b	-0.17682	0.37539	insignificant	0.0949	0.058996	insignificant	5	26	26
chr2	92213832	92215832	Pex16	-0.36137	1.43E-28	stronglyHypometh	-0.030587	0.010669	hypomethylated	10	64	66
chr2	92214395	92216395	Pex16	-0.20564	8.91E-13	hypomethylated	-0.029945	0.067304	insignificant	10	46	48
chr2	92222074	92224074	1700029I15Rik		1	noCoverage	0.029365	0.6197	insignificant	0	6	6
chr2	92241420	92243420	Mapk8ip1	-0.36366	4.52E-13	stronglyHypometh	-0.064531	0.000001	hypomethylated	12	50	48
chr2	92274226	92276226	Cry2	0.056426	9.34E-16	inconclusive	-0.010686	0.0010203	hypomethylated	11	52	52
chr2	92299951	92301951	Slc35c1	-0.24793	3.09E-14	hypomethylated	0.014258	0.1079	insignificant	12	39	41
chr2	92438863	92440863	Chst1	-0.13441	2.66E-30	hypomethylated	-0.016146	0.20021	insignificant	32	125	124
chr2	92754257	92756257	Syt13	-0.16791	0.00000716	hypomethylated	0.010857	0.00066403	hypermethylated	14	28	28
chr2	92886301	92888301	Prdm11	-0.097288	1	insignificant	-0.0069678	0.59072	insignificant	13	65	60
chr2	93026740	93028740	Trp53i11	-0.12262	8.7E-35	hypomethylated	0.000053912	0.37302	insignificant	46	155	157
chr2	93174644	93176644	Tspan18	-0.12409	0.001753	hypomethylated	0.0083138	0.3268	insignificant	11	28	28
chr2	93302653	93304653	Gm10804	-0.022628	0.71103	insignificant	0.044485	0.48603	insignificant	6	16	16
chr2	93303103	93305103	Gm10804	0.027731	0.00017359	inconclusive	0.02041	0.038461	inconclusive	8	20	20
chr2	93481590	93483590	Alx4	-0.13914	1.25E-42	hypomethylated	-0.017413	0.61255	insignificant	50	167	166
chr2	93662725	93664725	Ext2	-0.73761	1.95E-09	stronglyHypometh	-0.098026	0.0048587	hypomethylated	1	28	28
chr2	93689933	93691933	Accs	-0.23432	0.00000165	hypomethylated	-0.02716	0.78192	insignificant	9	20	20
chr2	93709260	93711260	Accsl	-0.21597	0.05472	insignificant	0.033235	0.77189	insignificant	2	4	4
chr2	93797257	93799257	Gm13889	-0.060849	0.79259	insignificant	0.045945	0.34778	insignificant	6	22	22
chr2	93850887	93852887	Alkbh3	-0.21139	1.07E-13	hypomethylated	0.11291	0.17133	insignificant	8	26	30
chr2	94081610	94083610	Mir129-2	-0.090894	1	insignificant	0.10897	0.83195	insignificant	4	36	26
chr2	94245863	94247863	2810002D19Rik	-0.14129	1.48E-13	hypomethylated	-0.015496	0.11979	insignificant	34	112	114
chr2	94246846	94248846	Ttc17	-0.13793	0.000096903	hypomethylated	-0.0166	0.28896	insignificant	9	30	30
chr2	94278304	94280304	Api5	-0.075617	0.0004161	hypomethylated	-0.010564	0.90191	insignificant	7	18	18
chr2	97306830	97308830	Lrrc4c	-0.35987	4.46E-08	stronglyHypometh	0.025588	0.11062	insignificant	2	20	24
chr2	101517596	101519596	Traf6	-0.1486	2.82E-39	hypomethylated	-0.01202	0.051258	insignificant	42	116	114
chr2	101637864	101639864	Prr5l	-0.29244	0.12399	insignificant	-0.07106	0.41751	insignificant	1	17	21
chr2	101725418	101727418	Commd9	-0.20219	0.0092264	hypomethylated	-0.025453	0.18513	insignificant	3	18	19
chr2	102026534	102028534	Ldlrad3	-0.20849	0.00017403	hypomethylated	0.0037971	0.13804	insignificant	12	32	32
chr2	102241057	102243057	Trim44	-0.11179	1.18E-14	hypomethylated	-0.00093279	0.00081866	hypomethylated	22	52	60
chr2	102291949	102293949	Fjx1	-0.19927	2.64E-09	hypomethylated	-0.022481	0.21467	insignificant	9	39	39
chr2	102389177	102391177	Pamr1	-0.23246	0.0014579	hypomethylated	0.0069586	0.19016	insignificant	3	16	16
chr2	102497839	102499839	Slc1a2	-0.14728	6.27E-32	hypomethylated	-0.0078074	0.1433	insignificant	37	131	131
chr2	102912831	102914831	Apip	-0.10026	0.0010403	hypomethylated	-0.0056909	0.55543	insignificant	13	67	65
chr2	102913670	102915670	Pdhx	-0.07084	0.043882	hypomethylated	-0.0098169	0.23481	insignificant	8	47	45
chr2	103143353	103145353	Ehf	-0.18287	0.0039556	hypomethylated	0.031155	0.15058	insignificant	1	8	6
chr2	103325310	103327310	Cat		1	noCoverage	0.0027056	0.38033	insignificant	0	6	6
chr2	103405466	103407466	Abtb2	-0.13412	2.52E-26	hypomethylated	-0.017566	0.16526	insignificant	52	165	174
chr2	103601407	103603407	Nat10	-0.15618	2.38E-10	hypomethylated	-0.0024561	0.35744	insignificant	13	48	48
chr2	103637235	103639235	Caprin1	-0.10861	6.55E-13	hypomethylated	-0.014861	0.030824	hypomethylated	22	89	93
chr2	103637797	103639797	Caprin1	-0.36086	0.0065714	stronglyHypometh	0.035178	0.1143	insignificant	3	14	14
chr2	103797151	103799151	Lmo2	-0.22226	0.078831	insignificant	-0.0079259	0.75858	insignificant	4	20	20
chr2	103808713	103810713	Lmo2	-0.20138	1.04E-10	hypomethylated	-0.096868	0.44845	insignificant	7	42	62
chr2	103809443	103811443	Lmo2	-0.21708	1.08E-30	hypomethylated	-0.041784	0.58599	insignificant	19	90	110
chr2	103866955	103868955	Fbxo3	-0.11053	0.00000398	hypomethylated	-0.0083436	0.059961	insignificant	11	67	66
chr2	103910166	103912166	Cd59b	0.054167	1	insignificant	-0.072693	0.014136	hypomethylated	2	12	12
chr2	103934957	103936957	Cd59a	0.24887	0.0013965	hypermethylated	0.082263	0.00084273	hypermethylated	6	18	18
chr2	103961925	103963925	A930018P22Rik	-0.13002	6.75E-10	hypomethylated	-0.0171	0.92176	insignificant	30	82	82
chr2	104250491	104252491	D430041D05Rik	-0.082865	0.018515	hypomethylated	-0.013952	0.67421	insignificant	13	72	74
chr2	104334646	104336646	Hipk3	-0.20794	1.84E-10	hypomethylated	-0.00089204	0.0000014	hypomethylated	12	60	60
chr2	104429640	104431640	Cstf3	-0.13124	2.42E-09	hypomethylated	-0.010858	0.41304	insignificant	11	57	52
chr2	104552319	104554319	Tcp11l1	-0.0773	0.68278	insignificant	0.01192	0.00000364	inconclusive	16	70	74
chr2	104582958	104584958	Depdc7	-0.083119	0.000000395	hypomethylated	-0.001387	0.50958	insignificant	14	34	34
chr2	104656853	104658853	Qser1	-0.10724	7.41E-12	hypomethylated	0.0010358	0.26988	insignificant	28	69	81
chr2	104690007	104692007	Prrg4		1	noCoverage	-0.08046	0.71315	insignificant	0	0	0
chr2	104725480	104727480	Ccdc73	-0.11572	0.41102	insignificant	-0.036903	0.59463	insignificant	1	14	13
chr2	104857184	104859184	Eif3m	-0.11489	1.62E-11	hypomethylated	-0.0027406	0.9131	insignificant	8	17	17
chr2	104965685	104967685	Wt1	-0.13259	1.02E-19	hypomethylated	-0.012761	0.00094035	hypomethylated	27	114	119
chr2	104966667	104968667	Wt1	-0.13888	2.77E-18	hypomethylated	-0.0075881	0.003274	hypomethylated	27	122	133
chr2	105063498	105065498	0610012H03Rik	-0.15638	4.72E-19	hypomethylated	-0.0011328	0.25582	insignificant	8	30	30
chr2	105239476	105241476	Rcn1	-0.24152	8.86E-16	hypomethylated	-0.0063657	0.00092825	hypomethylated	21	58	58
chr2	105510487	105512487	Pax6os1		1	noCoverage	-0.21429	0.29767	insignificant	0	6	6
chr2	105515601	105517601	Pax6	-0.1748	1.32E-23	hypomethylated	0.0041625	0.0018172	inconclusive	16	78	80
chr2	105743794	105745794	Immp1l	-0.19314	3.13E-11	hypomethylated	-0.026236	0.041603	hypomethylated	9	38	41
chr2	105744657	105746657	Elp4		1	noCoverage	-0.073673	1	insignificant	0	8	11
chr2	106532615	106534615	Mpped2	-0.1329	1.8E-22	hypomethylated	-0.012973	0.063497	insignificant	42	135	144
chr2	106534757	106536757	Mpped2	-0.045868	1	insignificant	-0.052362	0.66637	insignificant	2	19	11
chr2	106810548	106812548	2700007P21Rik	-0.069255	0.45999	insignificant	-0.14291	0.021366	hypomethylated	5	18	19
chr2	106814554	106816554	2700007P21Rik		1	noCoverage	0.02276	0.73289	insignificant	0	10	10
chr2	107129745	107131745	Kcna4	-0.17804	0.16425	insignificant	-0.096395	0.80323	insignificant	4	34	30
chr2	109119894	109121894	Kif18a	-0.13936	3.91E-27	hypomethylated	-0.0047527	4.63E-09	hypomethylated	12	50	50
chr2	109513856	109515856	Bdnf	-0.20925	0.000000114	hypomethylated	0.012985	1	insignificant	6	30	30
chr2	109515053	109517053	Bdnf	-0.21166	0.000011984	hypomethylated	0.00061693	0.014535	hypermethylated	9	38	39
chr2	109531592	109533592	Bdnf	-0.054337	0.30699	insignificant	-0.020858	0.13624	insignificant	9	57	57
chr2	109532719	109534719	Bdnf	-0.12214	3.75E-33	hypomethylated	-0.013145	0.039295	hypomethylated	33	143	143
chr2	109730034	109732034	Lin7c	-0.10499	9.8E-14	hypomethylated	0.0078793	0.84277	insignificant	32	106	106
chr2	109753476	109755476	Gm13939	-0.35333	0.057283	insignificant	0.039524	1	insignificant	3	10	10
chr2	109756803	109758803	Lgr4	-0.11334	4.69E-53	hypomethylated	-0.00087141	0.01588	hypomethylated	60	166	170

chr2	110790260	110792260	Ano3		1	noCoverage	0.051674	0.68052	insignificant	0	6	6
chr2	110790401	110792401	Ano3		1	noCoverage	0.051674	0.68052	insignificant	0	6	6
chr2	111714010	111716010	Olfr1305		1	noCoverage	-0.16667	0.38513	insignificant	0	4	4
chr2	112078997	112080997	Lpcat4	-0.16476	9.51E-36	hypomethylated	-0.053218	0.67471	insignificant	48	143	141
chr2	112101125	112103125	Nop10	0.071845	0.71122	insignificant	0.020839	0.36081	insignificant	6	30	25
chr2	112105470	112107470	Slc12a6	-0.14216	3.27E-28	hypomethylated	-0.0098085	0.0093447	hypomethylated	42	114	114
chr2	112123844	112125844	Slc12a6	0.14744	1	insignificant	-0.060897	1	insignificant	2	6	6
chr2	112218367	112220367	2410042D21Rik	-0.19336	1.6E-20	hypomethylated	-0.019485	0.65074	insignificant	38	84	92
chr2	112294181	112296181	2900064A13Rik	-0.18557	2.12E-10	hypomethylated	-0.0026474	0.76537	insignificant	16	50	51
chr2	112332120	112334120	Aven	-0.088096	4.66E-38	hypomethylated	0.0034982	0.88416	insignificant	53	133	133
chr2	113166892	113168892	Fmn1	-0.11331	0.0014044	hypomethylated	-0.026393	0.13772	insignificant	4	50	45
chr2	113598805	113600805	Grem1	-0.15599	2.22E-08	hypomethylated	-0.051985	0.81759	insignificant	15	39	44
chr2	113838300	113840300	A530058N18Rik	-0.096896	6.27E-09	hypomethylated	-0.015675	0.95513	insignificant	13	52	79
chr2	113878547	113880547	Actc1	-0.16367	0.22807	insignificant	0.0076373	0.083789	insignificant	2	17	21
chr2	113879131	113881131	C130080G10Rik	-0.17946	0.22648	insignificant	0.072375	0.05596	insignificant	2	19	16
chr2	114001075	114003075	Aqr	-0.6161	0.38889	lowCoverage	-0.10648	0.33716	insignificant	1	2	4
chr2	114027168	114029168	Zfp770	-0.12347	0.013698	hypomethylated	0.025509	0.47977	insignificant	6	17	18
chr2	115337937	115339937	3110099E03Rik	-0.40123	0.34729	insignificant	-0.15123	0.46263	insignificant	1	6	6
chr2	115890357	115892357	Meis2	-0.10813	3.98E-10	hypomethylated	-0.018566	0.27584	insignificant	23	59	56
chr2	115890794	115892794	Meis2	-0.1005	3.07E-12	hypomethylated	-0.0022539	0.50264	insignificant	24	65	65
chr2	115901832	115903832	2810405F15Rik	-0.3263	0.076493	insignificant	-0.040027	0.78015	insignificant	3	11	9
chr2	116946185	116948185	Spred1	-0.092902	9.26E-19	hypomethylated	-0.012037	0.084982	insignificant	66	283	289
chr2	117074474	117076474	Fam98b	-0.15494	1	insignificant	-0.032851	1	insignificant	3	34	26
chr2	117168613	117170613	Rasgrp1	-0.16805	1	insignificant	-0.035112	1	insignificant	3	16	11
chr2	117936657	117938657	Thbs1	-0.30598	8.47E-10	hypomethylated	-0.020535	0.81006	insignificant	5	19	16
chr2	118082702	118084702	Fsip1		1	noCoverage	0.023553	0.31406	insignificant	0	22	22
chr2	118199155	118201155	Gpr176	-0.28356	0.018592	hypomethylated	-0.037564	0.21217	insignificant	12	35	32
chr2	118213352	118215352	Eif2ak4	-0.19511	1E-12	hypomethylated	-0.06441	0.62658	insignificant	15	79	84
chr2	118213847	118215847	Eif2ak4	-0.19511	1E-12	hypomethylated	-0.06441	0.62658	insignificant	15	79	84
chr2	118375414	118377414	Bmf	-0.23813	1	lowCoverage	-0.0068211	0.82277	insignificant	1	18	18
chr2	118422946	118424946	Bub1b	-0.19428	0.00044424	hypomethylated	0.052748	0.93397	insignificant	8	24	25
chr2	118488312	118490312	Pak6	-0.14433	0.2515	insignificant	0.10276	0.55947	insignificant	10	32	36
chr2	118501981	118503981	Pak6	-0.12126	3.12E-14	hypomethylated	-0.024374	0.000021442	hypomethylated	15	96	106
chr2	118529699	118531699	Gm1337	-0.16655	0.000014411	hypomethylated	-0.0081526	0.70014	insignificant	6	20	21
chr2	118554174	118556174	Plcb2	-0.15139	1	insignificant	0.064484	0.6401	insignificant	3	12	10
chr2	118570497	118572497	5430417L22Rik	-0.12559	4.09E-64	hypomethylated	-0.0064951	4.37E-08	hypomethylated	77	230	240
chr2	118588397	118590397	A430105I19Rik	-0.31654	0.0024575	hypomethylated	0.001061	0.12779	insignificant	6	27	27
chr2	118597504	118599504	Phgr1	-0.09375	0.58487	insignificant	0.072411	0.35166	insignificant	2	8	8
chr2	118604454	118606454	Disp2	-0.22326	0.071774	insignificant	0.02685	0.51039	insignificant	5	38	38
chr2	118638738	118640738	D2Ertd750e	-0.11911	6.56E-12	hypomethylated	0.0080719	0.79956	insignificant	18	78	79
chr2	118686735	118688735	Ivd	-0.18339	0.000088714	hypomethylated	-0.030898	0.083074	insignificant	5	32	34
chr2	118726350	118728350	Bahd1	-0.1076	1.58E-36	hypomethylated	-0.007392	0.11938	insignificant	72	267	280
chr2	118751232	118753232	Chst14	-0.14648	0.000000364	hypomethylated	-0.020558	0.61785	insignificant	17	72	74
chr2	118855129	118857129	Ccdc32		1	noCoverage	0.050102	0.40854	insignificant	0	11	7
chr2	118859525	118861525	Rpusd2	-0.12582	2E-12	hypomethylated	-0.0025062	0.31193	insignificant	20	65	65
chr2	118871854	118873854	Casc5	-0.13617	2.28E-09	hypomethylated	0.0095517	0.66789	insignificant	12	63	48
chr2	118937552	118939552	Rad51	-0.18012	1.47E-21	hypomethylated	-0.0013023	0.0057098	inconclusive	21	81	84
chr2	118982770	118984770	Fam82a2	-0.29835	7.16E-09	hypomethylated	-0.0044499	0.00054713	hypomethylated	7	47	48
chr2	118999244	119001244	Gm14137	-0.038564	1	insignificant	0.027653	0.51144	insignificant	4	20	20
chr2	119033455	119035455	Zfyve19	-0.30396	0.00014245	hypomethylated	-0.048312	0.004632	inconclusive	6	50	50
chr2	119034531	119036531	Dnajc17	-0.1599	0.0059202	hypomethylated	0.075054	0.022736	hypermethylated	3	20	26
chr2	119062095	119064095	Spint1	-0.15317	9.73E-16	hypomethylated	0.0074781	0.038218	inconclusive	33	117	120
chr2	119096962	119098962	Rhov	-0.13787	0.0033827	hypomethylated	0.21152	1	insignificant	5	16	21
chr2	119113477	119115477	Vps18	-0.18377	0.00014874	hypomethylated	0.0066743	0.20863	insignificant	13	60	58
chr2	119150519	119152519	Dll4	-0.16006	3.72E-25	hypomethylated	-0.018285	0.37411	insignificant	35	119	121
chr2	119151933	119153933	Dll4	-0.16225	1.31E-18	hypomethylated	-0.013931	0.047412	hypomethylated	23	90	92
chr2	119175977	119177977	Chac1	-0.17542	0.0015081	hypomethylated	-0.0054862	0.34453	insignificant	12	40	43
chr2	119303365	119305365	Ino80	-0.33198	4.74E-21	hypomethylated	-0.074412	0.0041307	hypomethylated	14	73	68
chr2	119372442	119374442	1500003O03Rik	-0.13661	2.36E-29	hypomethylated	-0.0072557	0.064353	insignificant	38	130	133
chr2	119373363	119375363	Exd1	-0.11905	5.11E-11	hypomethylated	0.0079001	0.37765	insignificant	16	72	72
chr2	119419031	119421031	1700020I14Rik	-0.11489	3.68E-11	hypomethylated	0.0043159	0.4455	insignificant	13	76	76
chr2	119488534	119490534	Ndufaf1	-0.18778	0.00029665	hypomethylated	0.013534	0.75083	insignificant	9	18	20
chr2	119499803	119501803	Rtf1	-0.12037	3.32E-29	hypomethylated	0.0010895	0.0048482	inconclusive	43	153	162
chr2	119567072	119569072	Itpka	-0.1344	3.19E-64	hypomethylated	-0.004363	0.33816	insignificant	59	191	189
chr2	119584261	119586261	Ltk	-0.15106	4.17E-13	hypomethylated	0.0023972	0.785	insignificant	20	85	85
chr2	119586167	119588167	Ltk	-0.8278	1.15E-11	stronglyHypometh	-0.093837	0.78862	insignificant	3	8	8
chr2	119624249	119626249	Tyro3	-0.15036	2.33E-52	hypomethylated	-0.021494	0.00000805	hypomethylated	48	138	151
chr2	119721963	119723963	Mga	-0.10187	0.001445	hypomethylated	-0.00964	0.03091	inconclusive	10	90	90
chr2	119797434	119799434	Mapkbp1	-0.12469	0.010841	hypomethylated	-0.0017334	0.82632	insignificant	5	34	34
chr2	119852218	119854218	Jmjd7	0.55194	1	lowCoverage	-0.16607	2.97E-10	hypomethylated	1	30	47
chr2	119980311	119982311	Ehd4	-0.25164	1.91E-08	hypomethylated	0.002638	0.43384	insignificant	8	12	18
chr2	120228631	120230631	Ganc	-0.1845	0.35489	insignificant	-0.0040507	0.88331	insignificant	5	38	31
chr2	120229852	120231852	Ganc		1	noCoverage	-0.015468	0.92329	insignificant	0	10	10
chr2	120288328	120290328	Capn3	-0.022905	0.47686	insignificant	0.11487	0.27396	insignificant	4	12	12
chr2	120392406	120394406	Snap23	-0.079509	0.30044	insignificant	0.02562	0.96375	insignificant	8	49	50
chr2	120434171	120436171	Haus2	-0.29937	1.19E-36	hypomethylated	0.04473	0.34587	insignificant	12	24	25
chr2	120435051	120437051	Lrrc57	-0.45569	5.96E-44	stronglyHypometh	0.065741	0.77792	insignificant	7	6	6
chr2	120557253	120559253	Cdan1	-0.20395	0.053795	insignificant	-0.0091542	0.11288	insignificant	4	36	36
chr2	120676154	120678154	AV039307	-0.25841	0.000000913	hypomethylated	-0.025543	0.72985	insignificant	10	59	59
chr2	120676320	120678320	AV039307	-0.31388	0.000000313	hypomethylated	-0.04493	0.96525	insignificant	10	51	51
chr2	120796451	120798451	Ubr1	-0.15413	0.000000295	hypomethylated	-0.0066341	0.14606	insignificant	9	27	27
chr2	120801797	120803797	Tmem62	-0.07928	0.000000102	hypomethylated	-0.0053999	0.071292	insignificant	20	73	73
chr2	120833143	120835143	Ccndbp1	-0.15864	6.91E-12	hypomethylated	0.01082	0.49963	insignificant	15	57	57
chr2	120995941	120997941	Tubgcp4	-0.12889	2.9E-23	hypomethylated	-0.0070068	0.32821	insignificant	33	148	168
chr2	120996885	120998885	Zscan29	-0.11467	1.02E-15	hypomethylated	0.0026925	0.82923	insignificant	20	72	86

chr2	121114337	121116337	Mtap1a	-0.19763	3.28E-30	hypomethylated	0.0051996	0.016125	hypermethylated	14	61	53
chr2	121183376	121185376	Ckmt1	-0.1947	5.4E-12	hypomethylated	0.023042	0.024285	inconclusive	8	43	41
chr2	121206676	121208676	Strc		1	noCoverage	-0.039819	1	insignificant	0	4	4
chr2	121238637	121240637	Pdia3	-0.10396	1.75E-31	hypomethylated	-0.0026444	0.78306	insignificant	40	172	166
chr2	121239528	121241528	Catsper2	-0.11262	1.39E-19	hypomethylated	-0.0096834	0.64416	insignificant	29	121	124
chr2	121268337	121270337	Ell3	-0.42977	6.47E-08	stronglyHypometh	-0.068091	0.27449	insignificant	2	18	16
chr2	121273963	121275963	Serf2	-0.092135	2.02E-22	hypomethylated	-0.0013214	0.37599	insignificant	50	178	178
chr2	121281823	121283823	2310003F16Rik	-0.18997	0.0019755	hypomethylated	0.0093063	0.90103	insignificant	4	51	54
chr2	121282500	121284500	2310003F16Rik	-0.19144	0.0019646	hypomethylated	-0.0083689	0.8999	insignificant	4	49	50
chr2	121299759	121301759	Mfap1b	-0.23754	0.12407	insignificant	0.084241	0.78209	insignificant	2	12	10
chr2	121331458	121333458	Wdr76	0.18139	0.73491	insignificant	-0.10842	0.028657	hypomethylated	4	39	43
chr2	121332392	121334392	Mfap1a	-0.27794	0.32506	insignificant	-0.092331	0.52645	insignificant	1	16	16
chr2	121632793	121634793	Frmd5	-0.24491	0.00091357	hypomethylated	-0.036952	0.16902	insignificant	3	16	16
chr2	121691705	121693705	Casc4	-0.15853	0.000000129	hypomethylated	-0.032509	0.63325	insignificant	20	101	116
chr2	121781188	121783188	Ctdspl2	-0.10492	3.99E-19	hypomethylated	-0.0020851	0.41235	insignificant	29	106	101
chr2	121781828	121783828	Mageb3	-0.095276	0.000010395	hypomethylated	0.0079999	0.90815	insignificant	11	52	47
chr2	121853358	121855358	Eif3j	-0.12579	2.17E-17	hypomethylated	0.015083	0.00080646	inconclusive	26	143	142
chr2	121944122	121946122	Spg11	-0.22555	2.61E-08	hypomethylated	-0.11119	0.12491	insignificant	10	26	26
chr2	121972422	121974422	B2m	-0.21356	0.0047963	hypomethylated	0.003869	0.36578	insignificant	5	34	34
chr2	122011007	122013007	4933406J08Rik		1	noCoverage	-0.14143	0.24881	insignificant	0	8	9
chr2	122059574	122061574	Sord	-0.19283	1.83E-11	hypomethylated	-0.006472	0.69356	insignificant	11	66	66
chr2	122123635	122125635	Duoxa2		1	noCoverage	-0.02817	0.89793	insignificant	0	17	13
chr2	122123901	122125901	Duox2		1	noCoverage	-0.017767	0.89925	insignificant	0	20	16
chr2	122139466	122141466	Duoxa1	-0.50505	0.50875	insignificant	-0.12754	0.75731	insignificant	2	15	18
chr2	122194654	122196654	Shf	-0.11008	0.023241	hypomethylated	0.010344	0.67098	insignificant	24	42	41
chr2	122293533	122295533	Bambi-ps1		1	noCoverage	-0.0056086	0.90361	insignificant	0	30	30
chr2	122437013	122439013	Gatm	0.15786	1	insignificant	-0.068514	0.64848	insignificant	3	26	26
chr2	122462622	122464622	AA467197	0.15691	1	insignificant	-0.055483	0.0038872	hypomethylated	4	43	50
chr2	122528399	122530399	Slc30a4	-0.086513	0.000000355	hypomethylated	-0.004697	0.64303	insignificant	16	55	56
chr2	122563240	122565240	Pldn	-0.11248	0.38814	insignificant	-0.010916	0.053867	insignificant	3	43	43
chr2	122590094	122592094	Sqrdl	-0.10891	0.0017178	hypomethylated	0.0015496	0.13656	insignificant	6	47	50
chr2	124435031	124437031	Sema6d	-0.11642	6.61E-26	hypomethylated	-0.0077238	0.00014104	hypomethylated	33	142	142
chr2	124949396	124951396	Myef2	-0.14861	0.10447	insignificant	0.0037679	0.34774	insignificant	7	25	26
chr2	125071983	125073983	Dut	-0.049846	1.28E-15	hypomethylated	-0.0066049	0.14555	insignificant	20	153	152
chr2	125072250	125074250	Dut	-0.049846	1.28E-15	hypomethylated	-0.0066049	0.14555	insignificant	20	153	152
chr2	125332174	125334174	Fbn1	-0.094415	0.00068575	hypomethylated	0.066816	0.87066	insignificant	9	24	21
chr2	125450849	125452849	Cep152	-0.19664	1	lowCoverage	0.025889	0.048453	inconclusive	1	38	36
chr2	125497835	125499835	Eid1	-0.16899	0.090234	insignificant	0.027223	0.32185	insignificant	7	69	74
chr2	125549884	125551884	Shc4		1	noCoverage	-0.050023	1	insignificant	0	23	21
chr2	125608606	125610606	Secisbp2l		1	noCoverage	-0.049759	0.62202	insignificant	0	4	6
chr2	125683953	125685953	Galk2	-0.17075	1.59E-08	hypomethylated	-0.0068077	0.62698	insignificant	12	70	69
chr2	125684754	125686754	Cops2	-0.14967	0.0017458	hypomethylated	0.0016183	0.80267	insignificant	6	32	32
chr2	125976876	125978876	Dtwd1	-0.11726	0.000047171	hypomethylated	-0.0021612	0.33146	insignificant	8	66	60
chr2	126377759	126379759	Slc27a2	-0.21297	1.04E-08	hypomethylated	-0.019688	0.32838	insignificant	17	95	96
chr2	126444401	126446401	Hdc	-0.2387	0.22478	insignificant	0.010094	0.17653	insignificant	2	26	28
chr2	126501222	126503222	Gabpb1	-0.17643	4.39E-10	hypomethylated	0.0047794	0.17377	insignificant	16	66	69
chr2	126501223	126503223	Gabpb1	-0.17643	4.39E-10	hypomethylated	0.0047794	0.17377	insignificant	16	66	69
chr2	126532098	126534098	Usp8	-0.14303	2.29E-09	hypomethylated	0.009699	0.075878	insignificant	18	112	112
chr2	126701997	126703997	Trpm7	-0.1816	0.033773	hypomethylated	-0.053543	0.000033961	hypomethylated	10	42	39
chr2	126758971	126760971	2010106G01Rik		1	noCoverage	0	1	insignificant	0	10	10
chr2	126833446	126835446	Ap4e1	-0.18608	6.47E-14	hypomethylated	0.033592	0.89603	insignificant	11	64	64
chr2	126895392	126897392	Blvra	-0.063563	4.42E-09	hypomethylated	-0.021572	0.51191	insignificant	13	63	60
chr2	126959690	126961690	Ncaph		1	noCoverage	-0.075314	0.40083	insignificant	0	23	23
chr2	126968326	126970326	Itpripl1	-0.1444	0.000000714	hypomethylated	-0.0065856	0.35713	insignificant	12	52	52
chr2	126969193	126971193	Itpripl1		1	noCoverage	0.0048872	0.55621	insignificant	0	6	6
chr2	127033139	127035139	Snrnp200	-0.24292	6.07E-16	hypomethylated	-0.055367	0.2209	insignificant	15	46	47
chr2	127034016	127036016	1810024B03Rik	-0.26981	1.69E-13	hypomethylated	-0.10305	0.087066	insignificant	14	48	43
chr2	127072710	127074710	Tmem127	-0.14976	4.32E-25	hypomethylated	-0.0033333	0.0021279	hypomethylated	27	91	100
chr2	127073552	127075552	Ciao1	-0.15867	4.18E-25	hypomethylated	-0.014527	0.0016427	hypomethylated	27	86	88
chr2	127094964	127096964	Stard7	-0.12706	4.09E-17	hypomethylated	-0.004825	0.78128	insignificant	30	96	96
chr2	127160894	127162894	Dusp2	-0.12571	5.31E-32	hypomethylated	-0.012904	0.32089	insignificant	35	126	132
chr2	127163374	127165374	Astl	-0.52703	3.81E-09	stronglyHypometh	0.046144	0.88159	insignificant	1	2	2
chr2	127188021	127190021	Adra2b	-0.12568	1.69E-19	hypomethylated	-0.0058315	0.83282	insignificant	22	100	102
chr2	127249934	127251934	Gpat2	-0.0092778	0.2001	insignificant	-0.01305	1	insignificant	6	32	32
chr2	127270301	127272301	Fahd2a		1	noCoverage	-0.065676	0.59189	insignificant	0	11	12
chr2	127308235	127310235	Kcnip3	0.11187	0.013437	inconclusive	-0.0072079	0.47518	insignificant	7	70	74
chr2	127347106	127349106	Kcnip3	-0.24914	0.5024	insignificant	-0.0027961	0.47696	insignificant	6	30	30
chr2	127367153	127369153	Prom2	-0.50554	3.6E-17	stronglyHypometh	-0.10136	0.000000861	hypomethylated	5	20	21
chr2	127410413	127412413	Zfp661	-0.16447	0.0069891	hypomethylated	0.024908	0.64015	insignificant	5	14	14
chr2	127412161	127414161	Mrps5	-0.12303	1.56E-16	hypomethylated	-0.0033156	0.24257	insignificant	17	97	98
chr2	127614590	127616590	Nphp1	-0.35162	0.00088872	stronglyHypometh	-0.032107	0.0013729	hypomethylated	4	43	41
chr2	127618224	127620224	1500011K16Rik	-0.010736	1	insignificant	-0.03594	0.42179	insignificant	3	55	72
chr2	127657595	127659595	Bub1		1	noCoverage	-0.017789	0.21594	insignificant	0	4	6
chr2	127679363	127681363	Acoxl	-0.39377	0.10828	insignificant	-0.050965	0.48318	insignificant	1	9	8
chr2	127950773	127952773	Bcl2l11	-0.12413	0.00000273	hypomethylated	0.0022881	0.314	insignificant	21	159	160
chr2	128523732	128525732	Mertk	-0.13987	3.44E-24	hypomethylated	0.0031592	0.81682	insignificant	20	62	62
chr2	128643038	128645038	Tmem87b	-0.22042	1.39E-17	hypomethylated	-0.015822	0.31516	insignificant	17	45	53
chr2	128688667	128690667	Fbln7	-0.22857	0.22781	insignificant	-0.054411	0.65368	insignificant	2	21	21
chr2	128769756	128771756	Zc3h8	0.43552	1	lowCoverage	0.25205	0.087106	insignificant	1	12	8
chr2	128792137	128794137	Zc3h6	-0.10665	9.32E-33	hypomethylated	0.014577	0.0054998	inconclusive	54	167	172
chr2	128890682	128892682	Ttl	-0.07962	6.87E-11	hypomethylated	0.039112	0.57807	insignificant	20	72	75
chr2	128925731	128927731	Polr1b	-0.283	8.72E-15	hypomethylated	-0.0064968	0.052395	insignificant	8	41	40
chr2	128954435	128956435	Chchd5	-0.22423	1.12E-26	hypomethylated	0.009194	0.28155	insignificant	10	54	56
chr2	129023508	129025508	Slc20a1	-0.11096	1.43E-33	hypomethylated	-0.0099929	0.041726	hypomethylated	50	145	145
chr2	129122948	129124948	Gm14023	-0.31227	0.0015939	hypomethylated	-0.10942	0.1271	insignificant	3	12	16

chr2	129417574	129419574	Sirpa	-0.12149	4.36E-08	hypomethylated	-0.006103	0.076551	insignificant	15	67	66
chr2	129417930	129419930	Sirpa	-0.15385	6.5E-09	hypomethylated	-0.000786	0.10996	insignificant	16	73	72
chr2	129625252	129627252	Stk35	-0.1197	3.75E-21	hypomethylated	-0.0047399	0.41498	insignificant	33	162	153
chr2	129627195	129629195	Stk35	-0.15497	0.021194	hypomethylated	-0.031397	0.1079	insignificant	9	36	36
chr2	129925129	129927129	AU015228	-0.0010857	0.67756	insignificant	-0.0065599	0.81249	insignificant	6	17	18
chr2	130005100	130007100	Snrpb	-0.30408	0.032996	hypomethylated	-0.027373	0.70132	insignificant	3	12	12
chr2	130099147	130101147	Nop56	-0.11328	0.00000284	hypomethylated	-0.026128	0.2056	insignificant	23	101	104
chr2	130100250	130102250	Snord110	-0.12245	0.025913	hypomethylated	-0.017209	0.083562	insignificant	5	48	49
chr2	130110187	130112187	Idh3b	-0.76223	0.035338	stronglyHypometh	0.069979	0.73253	insignificant	0	2	4
chr2	130120674	130122674	Ebf4	-0.099341	6.93E-40	hypomethylated	-0.010594	0.48968	insignificant	64	193	195
chr2	130223365	130225365	Cpxm1	-0.13726	0.27876	insignificant	-0.043063	0.022128	hypomethylated	10	35	36
chr2	130229994	130231994	1700020A23Rik	0.08547	0.43551	insignificant	0.064373	0.88575	insignificant	2	12	10
chr2	130230031	130232031	1700020A23Rik	0.08547	0.43551	insignificant	0.064373	0.88575	insignificant	2	12	10
chr2	130231257	130233257	4933425O20Rik	-0.21795	0.38628	insignificant	-0.11966	0.42637	insignificant	1	6	6
chr2	130249055	130251055	Vps16	-0.10716	2.14E-18	hypomethylated	0.00063897	0.96845	insignificant	12	68	63
chr2	130250024	130252024	Fam113a	-0.19075	0.00017934	hypomethylated	0.042977	0.85813	insignificant	7	50	53
chr2	130250377	130252377	Fam113a	-0.22818	0.000054065	hypomethylated	0.054472	0.23508	insignificant	7	36	39
chr2	130275013	130277013	Ptpra	-0.10067	1.02E-19	hypomethylated	0.02577	0.096096	insignificant	32	129	140
chr2	130388492	130390492	Mrps26	-0.096406	9.7E-10	hypomethylated	-0.0088422	0.97173	insignificant	26	99	99
chr2	130400908	130402908	Oxt	-0.1322	0.013837	hypomethylated	-0.025914	0.30867	insignificant	11	36	36
chr2	130408277	130410277	Avp		1	noCoverage	-0.085317	0.28706	insignificant	0	7	6
chr2	130455722	130457722	Ubox5	-0.36837	5.09E-18	stronglyHypometh	-0.076381	0.010905	hypomethylated	5	12	12
chr2	130455763	130457763	Ubox5	-0.3992	0.000000264	stronglyHypometh	-0.10881	0.036715	hypomethylated	3	8	8
chr2	130468539	130470539	Prosapip1	-0.23533	4.89E-20	hypomethylated	0.0097403	0.67125	insignificant	13	41	40
chr2	130492576	130494576	Itpa	-0.30214	9.54E-15	hypomethylated	-0.053518	0.015449	hypomethylated	12	39	46
chr2	130523255	130525255	Slc4a11	-0.23109	0.064148	insignificant	0.032526	0.23243	insignificant	4	14	14
chr2	130665846	130667846	4930402H24Rik	-0.093262	0.00000131	hypomethylated	-0.019241	0.19463	insignificant	19	60	60
chr2	130731231	130733231	Atrn	-0.083205	0.000000146	hypomethylated	0.0043996	0.56971	insignificant	29	88	101
chr2	130732132	130734132	A730017L22Rik	-0.083887	0.000000762	hypomethylated	-0.007091	0.50759	insignificant	25	81	86
chr2	130868418	130870418	Gfra4		1	noCoverage	0.089655	0.33272	insignificant	0	4	4
chr2	130868824	130870824	Gfra4	-0.37824	0.098854	insignificant	-0.004416	0.6954	insignificant	1	10	10
chr2	130889550	130891550	Adam33		1	noCoverage	0.044282	0.30151	insignificant	0	17	16
chr2	130952147	130954147	Hspa12b	-0.19503	0.00042619	hypomethylated	-0.023621	0.16935	insignificant	6	26	26
chr2	130985756	130987756	1700037H04Rik	-0.10143	0.40182	insignificant	-0.0384	0.099667	insignificant	1	12	12
chr2	131005748	131007748	Cenpb	-0.10334	0.0059056	hypomethylated	-0.0018051	0.79894	insignificant	14	36	36
chr2	131011686	131013686	Cdc25b	-0.20342	1.25E-08	hypomethylated	-0.022776	0.020623	hypomethylated	13	54	54
chr2	131035175	131037175	2310035K24Rik	-0.20449	0.094609	insignificant	0.030859	0.069315	insignificant	10	26	26
chr2	131058873	131060873	Mavs	-0.028252	7.93E-12	hypomethylated	-0.016571	0.00000735	hypomethylated	11	56	56
chr2	131087235	131089235	Pank2	-0.094437	6.95E-10	hypomethylated	-0.022555	0.33833	insignificant	25	90	108
chr2	131178628	131180628	Rnf24	-0.074201	0.49269	insignificant	0.053617	0.0011692	inconclusive	7	37	39
chr2	131316597	131318597	Smox	-0.12773	1.36E-14	hypomethylated	-0.0015694	0.50657	insignificant	24	96	70
chr2	131388021	131390021	Adra1d	-0.14696	1.24E-14	hypomethylated	0.011533	0.79818	insignificant	18	77	77
chr2	131775596	131777596	Prnd	0.43417	0.081636	insignificant	-0.06785	0.22426	insignificant	2	21	20
chr2	131855724	131857724	Rassf2	-0.11487	2.12E-09	hypomethylated	-0.076821	0.00091498	hypomethylated	3	14	14
chr2	131970844	131972844	Slc23a2	-0.35216	4.52E-10	stronglyHypometh	-0.043996	0.68603	insignificant	8	36	36
chr2	132073524	132075524	5730494N06Rik	-0.38178	3.43E-08	stronglyHypometh	0.0058174	0.035174	hypermethylated	10	30	30
chr2	132078916	132080916	Pcna	0.70347	0.070782	insignificant	-0.016618	0.35698	insignificant	2	24	23
chr2	132087992	132089992	Cds2	-0.071637	0.000045038	hypomethylated	0.012214	0.82045	insignificant	10	113	94
chr2	132211183	132213183	Prokr2	-0.035541	1	insignificant	-0.11931	0.28499	insignificant	7	20	16
chr2	132403984	132405984	Gpcpd1	-0.19429	0.10984	insignificant	-0.16027	0.72206	insignificant	4	12	8
chr2	132422931	132424931	AU019990	-0.28929	0.33121	insignificant	-0.076786	0.32326	insignificant	3	10	10
chr2	132606013	132608013	Chgb	-0.14308	0.000071943	hypomethylated	-0.014797	0.045135	hypomethylated	11	40	36
chr2	132641070	132643070	Mcm8	-0.15237	3.17E-12	hypomethylated	-0.011908	0.16118	insignificant	16	66	66
chr2	132641790	132643790	Trmt6	-0.10776	0.000000199	hypomethylated	-0.0086089	0.70012	insignificant	12	52	52
chr2	132671401	132673401	Crls1	-0.070663	0.75023	insignificant	0.028262	0.44455	insignificant	35	118	126
chr2	132672469	132674469	Crls1	-0.088551	0.028411	hypomethylated	-0.0088345	0.039838	hypomethylated	38	118	118
chr2	132771772	132773772	Fermt1	-0.066094	0.59428	insignificant	0.010848	0.86762	insignificant	2	12	10
chr2	133377934	133379934	Bmp2	-0.12043	5.81E-50	hypomethylated	-0.0090406	0.000014601	hypomethylated	59	188	188
chr2	134469857	134471857	Tmx4		1	noCoverage	-0.029118	0.36142	insignificant	0	11	11
chr2	134610899	134612899	Plcb1	-0.12748	9.13E-19	hypomethylated	-0.025994	0.17186	insignificant	38	116	116
chr2	135566565	135568565	Plcb4	0.082353	1	insignificant	-0.057495	0.67892	insignificant	1	14	14
chr2	135882662	135884662	6330527O06Rik	-0.30637	0.39735	insignificant	-0.10478	0.58948	insignificant	2	6	6
chr2	136538185	136540185	Snap25	-0.19597	0.00000322	hypomethylated	0.040937	0.90305	insignificant	8	38	44
chr2	136715953	136717953	2210009G21Rik	-0.1576	5.13E-09	hypomethylated	0.028519	0.66479	insignificant	17	66	71
chr2	136716998	136718998	2210009G21Rik	-0.14286	0.000000102	hypomethylated	-0.0020673	0.039496	inconclusive	14	58	58
chr2	136717142	136719142	2210009G21Rik	-0.1452	0.00034881	hypomethylated	-0.0048269	0.85827	insignificant	8	40	40
chr2	136942067	136944067	Jag1	-0.1338	5.08E-36	hypomethylated	-0.0076902	0.33692	insignificant	46	146	142
chr2	138081319	138083319	Btbd3	-0.086983	3.87E-14	hypomethylated	0.0032337	0.0088014	hypermethylated	31	110	110
chr2	138103228	138105228	Btbd3	0.11982	0.38035	insignificant	-0.14954	0.039629	hypomethylated	4	28	27
chr2	139502913	139504913	Ism1	-0.13562	8.13E-27	hypomethylated	-0.00027617	0.34666	insignificant	50	145	148
chr2	139892496	139894496	Tasp1		1	noCoverage	0.11187	0.56544	insignificant	0	8	8
chr2	139892521	139894521	Tasp1		1	noCoverage	0.078054	0.1146	insignificant	0	12	12
chr2	139995381	139997381	2310003L22Rik	-0.27298	3.45E-25	hypomethylated	-0.060044	0.000015553	hypomethylated	15	66	75
chr2	139996294	139998294	Esf1	-0.28922	2.69E-11	hypomethylated	-0.032661	0.00000386	hypomethylated	11	56	61
chr2	140220165	140222165	Macrod2	-0.11114	1.44E-50	hypomethylated	0.00067807	0.61847	insignificant	47	140	131
chr2	140220170	140222170	Macrod2	-0.11114	1.44E-50	hypomethylated	0.00067807	0.61847	insignificant	47	140	131
chr2	142727200	142729200	Kif16b	-0.26626	4.57E-15	hypomethylated	-0.021946	0.00051457	hypomethylated	12	37	34
chr2	142887804	142889804	Snrpb2	-0.20011	4.08E-10	hypomethylated	0.059717	0.00091794	inconclusive	17	68	74
chr2	143370868	143372868	Pcsk2	-0.11737	1.29E-18	hypomethylated	-0.025386	0.26598	insignificant	36	103	103
chr2	143740066	143742066	Dstn	-0.15317	7.14E-12	hypomethylated	-0.02088	0.048352	hypomethylated	23	113	112
chr2	143836999	143838999	Rrbp1	0.64277	0.000014659	stronglyHypermeth	-0.0022348	0.73032	insignificant	2	46	46
chr2	144091938	144093938	Snord17	-0.7193	0.35294	lowCoverage	-0.11432	0.69604	insignificant	1	4	4
chr2	144095770	144097770	8430406I07Rik	-0.10783	5.56E-24	hypomethylated	-0.00071998	0.095701	insignificant	28	98	97
chr2	144096308	144098308	Snx5	-0.0082279	0.013167	hypomethylated	-0.0051186	0.080926	insignificant	17	50	53
chr2	144096638	144098638	Snx5	-0.061663	0.30271	insignificant	-0.092805	0.72585	insignificant	5	14	17

chr2	144157098	144159098	Ovol2	-0.17531	2.02E-26	hypomethylated	-0.015004	0.0079125	hypomethylated	20	77	79
chr2	144157816	144159816	Ovol2	-0.11987	0.00000035	hypomethylated	0.018381	0.53501	insignificant	6	49	49
chr2	144193718	144195718	Gm20571	-0.2052	1.96E-43	hypomethylated	-0.038391	0.24469	insignificant	35	96	115
chr2	144193767	144195767	Csrp2bp	-0.2052	1.96E-43	hypomethylated	-0.038391	0.24469	insignificant	35	96	115
chr2	144193770	144195770	Csrp2bp	-0.2052	1.96E-43	hypomethylated	-0.038391	0.24469	insignificant	35	96	115
chr2	144284015	144286015	LOC668917	0.27938	0.00000558	inconclusive	-0.018205	0.44028	insignificant	2	15	16
chr2	144352480	144354480	Polr3f	-0.18822	0.000000758	hypomethylated	-0.0052059	0.82746	insignificant	17	53	62
chr2	144353134	144355134	6330439K17Rik	-0.16107	0.00000356	hypomethylated	-0.0072584	0.48735	insignificant	16	47	56
chr2	144381012	144383012	Sec23b	-0.24097	1.7E-26	hypomethylated	0.0079322	0.26789	insignificant	16	20	16
chr2	144418800	144420800	Gm561	-0.18018	0.03125	hypomethylated	-0.033387	0.50217	insignificant	9	51	52
chr2	144424688	144426688	Dtd1	-0.033192	0.21752	insignificant	-0.00049094	0.65052	insignificant	12	42	48
chr2	145067346	145069346	Slc24a3		1	noCoverage	-0.082616	0.56731	insignificant	0	34	32
chr2	145610851	145612851	Rin2		1	noCoverage	0.0354	0.5024	insignificant	0	4	4
chr2	145727976	145729976	Naa20	-0.12055	9.77E-17	hypomethylated	-0.023098	0.045363	hypomethylated	32	96	94
chr2	145759519	145761519	4930529M08Ril	-0.33483	4.72E-09	stronglyHypometh	-0.043542	0.35198	insignificant	12	62	52
chr2	145760436	145762436	Crnkl1	-0.39165	0.55029	insignificant	-0.10128	0.20777	insignificant	9	46	46
chr2	146046732	146048732	Insm1	-0.086062	8.84E-17	hypomethylated	0.0028729	0.93428	insignificant	59	243	259
chr2	146337740	146339740	Ralgapa2	-0.1222	2.36E-17	hypomethylated	-0.0047683	0.32334	insignificant	25	64	63
chr2	146680624	146682624	Plk1s1	-0.23214	0.13886	insignificant	-0.024587	0.00057833	hypomethylated	2	21	21
chr2	146837795	146839795	Xrn2	-0.10857	6.39E-20	hypomethylated	-0.00081465	0.10544	insignificant	43	133	127
chr2	146911081	146913081	Nkx2-4	-0.10864	7.88E-08	hypomethylated	0.064301	1.95E-09	hypermethylated	15	44	25
chr2	147008818	147010818	Nkx2-2as	-0.1261	0.000000249	hypomethylated	-0.010073	0.25896	insignificant	24	82	94
chr2	147012138	147014138	Nkx2-2as	-0.18028	0.000014688	hypomethylated	0.040743	0.036303	hypermethylated	13	51	41
chr2	147189729	147191729	Pax1	-0.20624	0.000063395	hypomethylated	-0.025039	0.70111	insignificant	9	83	83
chr2	147872705	147874705	Foxa2	-0.15685	0.06921	insignificant	-0.011249	0.38146	insignificant	10	41	48
chr2	148220112	148222112	Sstr4	-0.12724	2.52E-21	hypomethylated	-0.0016363	0.66938	insignificant	28	90	91
chr2	148233924	148235924	Thbd	-0.1768	0.046316	hypomethylated	-0.0054554	1	insignificant	7	21	21
chr2	148269271	148271271	Cd93	-0.29717	1	insignificant	-0.02166	1	insignificant	1	4	4
chr2	148497350	148499350	Nxt1	-0.16452	5.36E-19	hypomethylated	-0.0054513	0.058599	insignificant	12	86	83
chr2	148497376	148499376	Nxt1	-0.16452	5.36E-19	hypomethylated	-0.0054513	0.058599	insignificant	12	86	83
chr2	148505855	148507855	Gzf1	-0.24548	8.47E-22	hypomethylated	-0.0096383	0.11458	insignificant	19	68	68
chr2	148558156	148560156	Napb	-0.011436	0.30526	insignificant	0.031082	0.55225	insignificant	2	22	19
chr2	148575096	148577096	Cstl1	-0.068394	0.44065	insignificant	-0.13863	0.068879	insignificant	3	14	6
chr2	148606744	148608744	8030411F24Rik		1	noCoverage		1	noCoverage	0	6	0
chr2	148614096	148616096	Cst12	-0.32351	0.08259	insignificant	0.030024	0.1325	insignificant	5	20	20
chr2	149655518	149657518	Tmem90b	-0.12126	2.69E-21	hypomethylated	-0.0068532	0.22053	insignificant	33	133	136
chr2	149962414	149964414	Zfp120	-0.51768	0.051445	insignificant	0.056728	0.67527	insignificant	2	4	4
chr2	150006490	150008490	Gm14139	0.18945	1	insignificant	0.080811	0.57376	insignificant	2	10	10
chr2	150082252	150084252	Gm14124		1	noCoverage	0.091746	0.70115	insignificant	0	4	6
chr2	150310799	150312799	Zfp345		1	noCoverage	0.026399	0.032813	hypermethylated	0	4	4
chr2	150434259	150436259	2310001A20Rik	-0.15028	0.24236	insignificant	-0.040587	0.011923	hypomethylated	5	10	10
chr2	150493976	150495976	E130215H24Rik	-0.23632	0.000011315	hypomethylated	-0.027667	0.31363	insignificant	11	26	26
chr2	150573816	150575816	Entpd6	-0.09681	4.94E-14	hypomethylated	-0.0012253	0.34025	insignificant	18	64	64
chr2	150611531	150613531	Pygb	-0.26223	1.85E-17	hypomethylated	-0.01276	0.0088439	hypomethylated	16	54	49
chr2	150730467	150732467	Abhd12	-0.28583	0.000026149	hypomethylated	-0.031583	0.8324	insignificant	6	16	16
chr2	150734329	150736329	Gins1	-0.19391	7.42E-09	hypomethylated	0.010878	0.41173	insignificant	12	28	28
chr2	150865115	150867115	Nanp	-0.17539	0.34809	insignificant	-0.0033461	0.81691	insignificant	2	6	6
chr2	151301887	151303887	4921509C19Rik	-0.27283	0.09231	insignificant	0.0059065	0.88907	insignificant	2	6	6
chr2	151319043	151321043	Nsfl1c	-0.11779	0.015172	hypomethylated	0.01537	0.67531	insignificant	5	38	34
chr2	151367234	151369234	Fkbp1a	-0.056365	0.00016572	hypomethylated	0.0071893	0.62651	insignificant	36	120	119
chr2	151526743	151528743	5430405G05Rik	-0.12719	1.32E-41	hypomethylated	-0.0098875	0.053615	insignificant	53	172	172
chr2	151567029	151569029	Psmf1	-0.18193	1	insignificant	0.021483	1	insignificant	2	20	20
chr2	151667662	151669662	Rspo4	-0.072761	1.04E-20	hypomethylated	0.0073474	0.16939	insignificant	23	70	74
chr2	151736067	151738067	Angpt4	0.017749	0.7922	insignificant	0.091278	0.52983	insignificant	5	16	27
chr2	151793353	151795353	Mir1953	-0.57407	0.31162	insignificant	0.11267	0.31134	insignificant	1	2	2
chr2	151799710	151801710	Fam110a	-0.31386	0.00000673	hypomethylated	-0.055655	0.00039372	hypomethylated	7	18	18
chr2	151805955	151807955	Fam110a	-0.22054	1.02E-14	hypomethylated	0.031123	0.48314	insignificant	8	16	16
chr2	151821246	151823246	2310046K01Rik		1	noCoverage	-0.010369	0.0029903	hypomethylated	0	35	34
chr2	151824601	151826601	2310046K01Rik	-0.13294	0.38259	insignificant	-0.10627	0.097254	insignificant	2	14	15
chr2	151906264	151908264	Scrt2	-0.10861	0.000000002	hypomethylated	-0.010699	0.18567	insignificant	37	123	123
chr2	151930465	151932465	Srxn1	-0.088655	1.45E-22	hypomethylated	0.022331	0.42758	insignificant	32	105	117
chr2	151968344	151970344	Tcf15	-0.094312	3.61E-14	hypomethylated	0.0027003	0.89132	insignificant	35	144	138
chr2	152051575	152053575	Csnk2a1	-0.23704	5.52E-09	hypomethylated	-0.02403	0.021745	hypomethylated	10	23	25
chr2	152118607	152120607	Tbc1d20	-0.082715	6.02E-10	hypomethylated	0.012931	0.58605	insignificant	25	76	76
chr2	152158161	152160161	Rbck1	-0.20248	0.0011406	hypomethylated	0.0042279	0.64194	insignificant	6	47	41
chr2	152158375	152160375	Rbck1	-0.19831	0.013613	hypomethylated	0.020054	0.58158	insignificant	4	35	29
chr2	152169796	152171796	Trib3	-0.4723	0.00026303	stronglyHypometh	-0.072303	0.028986	hypomethylated	3	26	22
chr2	152202302	152204302	Nrsn2	-0.23198	0.000000404	hypomethylated	0.036847	0.23772	insignificant	5	15	18
chr2	152223782	152225782	Sox12	-0.08706	4.73E-19	hypomethylated	-0.00050342	0.40124	insignificant	21	88	86
chr2	152240322	152242322	6820408C15Rik	-0.10652	0.62638	insignificant	-0.019044	0.91961	insignificant	6	33	33
chr2	152397479	152399479	Defb21		1	noCoverage	-0.35721	1.42E-08	stronglyhypometh	0	6	6
chr2	152406048	152408048	Defb19	-0.095238	0.11714	insignificant	0.13769	0.82449	insignificant	1	7	9
chr2	152429062	152431062	Defb36	-0.10423	0.17137	insignificant	-0.016229	1	insignificant	3	30	30
chr2	152451743	152453743	Rem1	-0.23449	2.01E-33	hypomethylated	-0.04987	0.000000735	hypomethylated	29	97	96
chr2	152494196	152496196	H13	-0.20963	0.00011672	hypomethylated	-0.029493	0.3255	insignificant	3	46	46
chr2	152511883	152513883	Mcts2	-0.017334	0.7966	insignificant	-0.069364	0.046091	hypomethylated	16	62	62
chr2	152561009	152563009	Id1	-0.30556	0.0022539	hypomethylated	-0.030337	0.38323	insignificant	3	8	8
chr2	152578908	152580908	Cox4i2	-0.23832	0.28347	insignificant	0.080037	0.30062	insignificant	5	17	18
chr2	152657418	152659418	Bcl2l1	-0.06724	8.74E-31	hypomethylated	-0.0048907	0.00039361	hypomethylated	10	34	35
chr2	152672699	152674699	Tpx2	-0.20701	6.71E-11	hypomethylated	0.0050685	0.099665	insignificant	8	21	22
chr2	152736087	152738087	Mylk2	-0.14989	0.0000003	hypomethylated	-0.099907	0.002186	hypomethylated	6	22	24
chr2	152758944	152760944	Foxs1	-0.1625	0.29404	insignificant		1	noCoverage	1	4	0
chr2	152777141	152779141	Dusp15	-0.16235	1.89E-14	hypomethylated	-0.016583	0.77587	insignificant	17	60	61
chr2	152777318	152779318	Dusp15	-0.17588	0.0011874	hypomethylated	-0.018536	0.84741	insignificant	9	38	39
chr2	152787220	152789220	Ttll9		1	noCoverage	0.079654	0.60663	insignificant	0	18	14

chr2	152841119	152843119	Pdrg1	-0.35196	0.0015876	stronglyHypometh	-0.021798	0.73775	insignificant	2	11	11
chr2	152856587	152858587	Xkr7	-0.1507	1.03E-15	hypomethylated	-0.026561	0.0015485	hypomethylated	27	111	120
chr2	152933203	152935203	Hck	-0.21205	3.19E-11	hypomethylated	0.010897	0.16936	insignificant	8	45	38
chr2	152986036	152988036	Tm9sf4		1	noCoverage	-0.16519	0.62957	insignificant	0	6	8
chr2	153066267	153068267	Pofut1	-0.10935	4.68E-13	hypomethylated	0.012049	0.28366	insignificant	33	127	129
chr2	153067094	153069094	Plagl2	-0.11672	7.67E-12	hypomethylated	0.013821	0.84678	insignificant	19	79	80
chr2	153116151	153118151	Kif3b	-0.099503	0.000013812	hypomethylated	-0.020277	0.73648	insignificant	43	105	105
chr2	153170874	153172874	Asxl1	-0.12079	2.72E-17	hypomethylated	-0.0217	0.18422	insignificant	49	135	167
chr2	153270215	153272215	8430427H17Rik	-0.16214	9.24E-15	hypomethylated	0.053216	0.14851	insignificant	23	67	74
chr2	153355707	153357707	8430427H17Rik	-0.063989	7.38E-14	hypomethylated	0.031405	0.012716	inconclusive	27	98	101
chr2	153458517	153460517	Commd7	-0.14189	4.15E-10	hypomethylated	-0.023996	0.041118	hypomethylated	6	13	16
chr2	153474189	153476189	Dnmt3b	-0.093236	1.61E-15	hypomethylated	-0.0022079	0.51288	insignificant	38	125	117
chr2	153566022	153568022	Mapre1	-0.10298	1.61E-12	hypomethylated	-0.0077798	0.079076	insignificant	23	72	72
chr2	153699780	153701780	Bpifb2	0.07381	1	insignificant	-0.0003663	1	insignificant	2	4	4
chr2	153955082	153957082	Bpifa3	-0.9	0.085106	lowCoverage	-0.057258	1	insignificant	1	6	4
chr2	154015553	154017553	Bpifb1	-0.39286	0.01587	stronglyHypometh	-0.23935	0.015618	hypomethylated	2	6	4
chr2	154233820	154235820	Snta1	-0.42396	4.87E-15	stronglyHypometh	-0.010786	0.4316	insignificant	9	40	37
chr2	154261219	154263219	Cbfa2t2	-0.091803	0.00000127	hypomethylated	0.0061489	0.91409	insignificant	24	139	135
chr2	154373639	154375639	1700003F12Rik	-0.17892	1	insignificant	-0.024061	0.62595	insignificant	4	50	54
chr2	154376511	154378511	1700007I08Rik	0.30754	0.014927	hypermethylated	0.054466	0.00045718	hypermethylated	5	45	45
chr2	154384589	154386589	E2f1	0.23143	0.7721	insignificant	0.015409	0.015608	inconclusive	1	14	14
chr2	154395588	154397588	E2f1	-0.075097	0.00000942	hypomethylated	-0.033211	0.0013832	hypomethylated	20	76	75
chr2	154429409	154431409	Pxmp4	-0.33447	0.30141	insignificant	0.0072786	0.80332	insignificant	1	10	10
chr2	154438103	154440103	Zfp341	-0.13986	1.26E-22	hypomethylated	-0.014404	0.0036972	hypomethylated	41	158	161
chr2	154481761	154483761	Chmp4b	-0.18064	2.81E-13	hypomethylated	0.0031599	0.02649	inconclusive	23	64	65
chr2	154615845	154617845	Raly	-0.13042	6.6E-37	hypomethylated	0.010651	0.17861	insignificant	48	142	146
chr2	154718642	154720642	Eif2s2	-0.16297	0.00000999	hypomethylated	0.026724	0.73443	insignificant	8	38	38
chr2	154900233	154902233	Ahcy	-0.11717	0.0030909	hypomethylated	-0.028523	0.0023035	hypomethylated	7	30	28
chr2	154958216	154960216	Itch	-0.12463	1.86E-44	hypomethylated	0.0053128	0.023851	inconclusive	35	104	104
chr2	155061268	155063268	Dynlrb1	-0.24621	0.000000722	hypomethylated	-0.019338	0.13116	insignificant	15	37	31
chr2	155101179	155103179	Map1lc3a	-0.1552	1.24E-36	hypomethylated	-0.010543	0.20713	insignificant	46	150	146
chr2	155206591	155208591	Trp53inp2	-0.17993	5.25E-23	hypomethylated	-0.023758	0.462	insignificant	27	66	64
chr2	155340582	155342582	Ggt7	-0.1622	1	insignificant	0.0013939	0.55183	insignificant	3	8	8
chr2	155342778	155344778	Acss2	0.080621	7.03E-12	hypermethylated	0.26276	4.07E-63	hypermethylated	22	88	66
chr2	155418442	155420442	Gss	-0.09054	0.11465	insignificant	-0.054799	0.71734	insignificant	5	28	28
chr2	155447615	155449615	Mir499		1	noCoverage	0.046369	0.88082	insignificant	0	12	12
chr2	155555211	155557211	Edem2		1	noCoverage	0.0057766	0.85047	insignificant	0	13	12
chr2	155575952	155577952	Procr	-0.29711	0.0086568	hypomethylated	-0.048678	0.051002	insignificant	8	32	32
chr2	155600079	155602079	Mmp24	-0.12539	3.83E-45	hypomethylated	-0.0082632	0.030237	hypomethylated	60	163	151
chr2	155652661	155654661	Eif6		1	noCoverage	0.37466	0.27466	insignificant	0	10	4
chr2	155781293	155783293	Cep250	-0.18021	1.71E-10	hypomethylated	0.052457	0.097881	insignificant	21	94	63
chr2	155832860	155834860	Ergic3	-0.31204	3.73E-33	hypomethylated	-0.024018	0.00000257	hypomethylated	13	42	47
chr2	155833713	155835713	6430550D23Rik	-0.30054	5.21E-26	hypomethylated	-0.020144	0.00000029	hypomethylated	9	34	40
chr2	155878683	155880683	Fer1l4	0.13667	1	insignificant	-0.11333	0.30637	insignificant	2	8	5
chr2	155889948	155891948	Spag4	-0.21725	5.24E-28	hypomethylated	-0.031103	0.0011343	hypomethylated	24	73	70
chr2	155937701	155939701	Rbm12	-0.15688	5.49E-08	hypomethylated	0.042224	0.5681	insignificant	7	18	22
chr2	155968888	155970888	Romo1	-0.090994	0.000000079	hypomethylated	0.022309	0.088775	insignificant	23	104	84
chr2	155968970	155970970	Romo1	-0.090994	0.000000079	hypomethylated	0.022309	0.088775	insignificant	23	104	84
chr2	155969079	155971079	Romo1	-0.090994	0.000000079	hypomethylated	0.022309	0.088775	insignificant	23	104	84
chr2	155969922	155971922	Nfs1	-0.11312	1.04E-08	hypomethylated	0.01866	0.27616	insignificant	19	72	50
chr2	156005976	156007976	Rbm39	-0.16377	1.17E-09	hypomethylated	-0.0055868	0.51807	insignificant	7	26	14
chr2	156021382	156023382	Phf20	-0.10034	1.5E-09	hypomethylated	-0.0020443	0.16087	insignificant	28	89	86
chr2	156137208	156139208	4921517L17Rik	-0.16616	9.76E-30	hypomethylated	-0.0084888	0.63778	insignificant	34	88	98
chr2	156245787	156247787	Epb4.1l1	-0.085934	0.000038336	hypomethylated	0.026452	0.000023224	hypermethylated	25	133	133
chr2	156300572	156302572	Epb4.1l1	-0.092285	4.45E-12	hypomethylated	-0.0076031	0.37959	insignificant	21	88	103
chr2	156372311	156374311	0610011L14Rik	-0.23575	0.000000326	hypomethylated	-0.045143	0.72767	insignificant	8	32	27
chr2	156438440	156440440	Dlgap4	-0.10394	5.17E-22	hypomethylated	0.0065721	0.26909	insignificant	40	159	169
chr2	156545720	156547720	Dlgap4	-0.10005	3.06E-40	hypomethylated	0.0096541	0.7613	insignificant	39	102	102
chr2	156546014	156548014	Dlgap4	-0.12686	1.23E-45	hypomethylated	0.0051041	0.69696	insignificant	42	110	110
chr2	156600199	156602199	Myl9	-0.26499	2.98E-13	hypomethylated	0.028321	0.0021747	inconclusive	14	49	48
chr2	156664812	156666812	Tgif2	-0.14185	1.06E-13	hypomethylated	-0.022741	0.83467	insignificant	22	130	129
chr2	156687857	156689857	1110008F13Rik	-0.087533	2.13E-20	hypomethylated	-0.0088549	0.80275	insignificant	54	189	186
chr2	156688681	156690681	5430405H02Rik	-0.085448	5.54E-27	hypomethylated	0.0084261	0.13262	insignificant	44	149	151
chr2	156817847	156819847	Ndrg3	-0.20442	0.0091669	hypomethylated	0.00115	0.42071	insignificant	3	20	20
chr2	156832811	156834811	Dsn1	-0.30203	1.04E-09	hypomethylated	0.014847	0.39685	insignificant	6	22	22
chr2	156905001	156907001	9830001H06Rik	-0.25137	0.00000457	hypomethylated	-0.0916	0.048445	hypomethylated	17	49	63
chr2	156960958	156962958	Samhd1	-0.83057	1.47E-10	stronglyHypometh	0.015551	0.00011408	inconclusive	0	11	10
chr2	157030270	157032270	Rbl1	-0.089595	0.0058317	hypomethylated	-0.014855	0.53459	insignificant	18	58	58
chr2	157103833	157105833	Rpn2	-0.12049	1.31E-37	hypomethylated	-0.010198	0.95122	insignificant	47	143	151
chr2	157105285	157107285	Rpn2	-0.17742	0.020503	hypomethylated	0.017745	0.95	insignificant	8	33	34
chr2	157172391	157174391	Ghrh	-0.24483	1.25E-13	hypomethylated	-0.034202	0.00089469	hypomethylated	8	28	32
chr2	157192329	157194329	Manbal	-0.20897	1.76E-16	hypomethylated	0.065872	0.57258	insignificant	17	56	65
chr2	157249028	157251028	Src	-0.09115	1.47E-16	hypomethylated	0.000076944	1	insignificant	47	122	115
chr2	157384845	157386845	Nnat	-0.015601	0.48537	insignificant	0.045338	0.68887	insignificant	8	83	83
chr2	157392097	157394097	Blcap	-0.27785	1	insignificant	-0.025495	0.69552	insignificant	2	20	22
chr2	157562136	157564136	Ctnnbl1	-0.21982	8.79E-10	hypomethylated	0.036175	0.48969	insignificant	9	39	42
chr2	157739388	157741388	Vstm2l	-0.11681	3.12E-12	hypomethylated	0.029146	0.55387	insignificant	25	144	151
chr2	157853529	157855529	Rprd1b	-0.14686	0.000086176	hypomethylated	-0.031499	0.013549	hypomethylated	4	64	64
chr2	158016743	158018743	1700060C20Rik	-0.55978	0.035756	stronglyHypometh	0.030502	1	insignificant	2	10	10
chr2	158082976	158084976	Bpi	-0.3321	0.022907	hypomethylated	-0.082333	0.031107	hypomethylated	3	26	26
chr2	158200373	158202373	Snhg11	-0.39567	0.51489	insignificant	-0.00000875	0.1465	insignificant	2	9	11
chr2	158234588	158236588	Ralgapb	-0.12518	2.6E-27	hypomethylated	-0.0037799	0.021762	hypomethylated	43	120	115
chr2	158327347	158329347	Adig	0.03843	1	insignificant	0.026344	0.47804	insignificant	2	12	12
chr2	158435493	158437493	Slc32a1	-0.25143	1.65E-22	hypomethylated	-0.033996	0.17386	insignificant	13	32	32
chr2	158449648	158451648	Actr5	-0.11264	6.98E-33	hypomethylated	0.018929	0.12664	insignificant	33	84	74

chr2	158463318	158465318	Mir3474	-0.041101	0.41896	insignificant	-0.0076699	0.2351	insignificant	2	33	32
chr2	158491468	158493468	Ppp1r16b	-0.15711	7.09E-12	hypomethylated	-0.018636	0.0041115	hypomethylated	18	70	70
chr2	158491870	158493870	Ppp1r16b	-0.12509	2.27E-20	hypomethylated	-0.0012667	0.35203	insignificant	35	124	124
chr2	158592834	158594834	Fam83d	-0.10757	1.35E-16	hypomethylated	0.0013174	0.57112	insignificant	28	98	108
chr2	158619555	158621555	Dhx35	-0.15185	4.47E-13	hypomethylated	-0.025409	0.093773	insignificant	12	63	59
chr2	160192801	160194801	Mafb	-0.12746	1.33E-10	hypomethylated	-0.0050971	0.21748	insignificant	23	123	125
chr2	160470632	160472632	Top1	-0.14904	2.9E-39	hypomethylated	0.004815	0.95739	insignificant	41	146	151
chr2	160556045	160558045	Plcg1	-0.076646	0.00000179	hypomethylated	0.0026939	0.55503	insignificant	40	190	201
chr2	160698726	160700726	Zhx3	-0.12363	0.01839	hypomethylated	-0.052508	0.12899	insignificant	7	20	24
chr2	160705405	160707405	Lpin3	-0.84688	0.00000797	stronglyHypometh	0.029072	0.0047202	inconclusive	1	8	6
chr2	160712903	160714903	Lpin3	-0.46296	2.1E-32	stronglyHypometh	-0.062963	0.5061	insignificant	3	6	6
chr2	160738064	160740064	Emilin3	-0.080902	0.017182	hypomethylated	-0.017884	0.25085	insignificant	11	44	44
chr2	160934792	160936792	Chd6	-0.17987	3.03E-15	hypomethylated	0.01304	0.035095	hypermethylated	14	53	53
chr2	162485898	162487898	9430021M05Rik	-0.11168	1.09E-33	hypomethylated	0.0027705	0.030576	inconclusive	56	159	150
chr2	162486883	162488883	Ptprt	-0.11416	1.42E-13	hypomethylated	-0.013483	0.000022113	hypomethylated	13	50	50
chr2	162756243	162758243	Srsf6	-0.077613	2.47E-19	hypomethylated	-0.0028739	0.71486	insignificant	57	221	220
chr2	162768200	162770200	L3mbtl1	-0.37665	1.11E-33	stronglyHypometh	-0.11566	0.00013451	hypomethylated	12	40	39
chr2	162812216	162814216	Sgk2		1	noCoverage	-0.37193	0.05387	insignificant	0	6	6
chr2	162842207	162844207	Ift52	-0.10139	0.0016728	hypomethylated	-0.022683	0.87148	insignificant	15	43	42
chr2	162879370	162881370	Mybl2	-0.10985	8.64E-18	hypomethylated	-0.01017	0.26515	insignificant	28	109	110
chr2	163050189	163052189	Tox2	-0.069395	0.01235	hypomethylated	-0.050316	0.34745	insignificant	2	16	16
chr2	163223686	163225686	Jph2		1	noCoverage	-0.076147	0.25582	insignificant	0	14	14
chr2	163245206	163247206	3230401D17Rik	-0.18033	3.07E-10	hypomethylated	0.014707	0.88681	insignificant	21	93	92
chr2	163263202	163265202	Gdap1l1	-0.12327	0.10384	insignificant	-0.0084953	0.53269	insignificant	8	65	62
chr2	163297280	163299280	2310001K24Rik	-0.053081	0.078176	insignificant	0.0076062	0.87386	insignificant	11	46	46
chr2	163317053	163319053	R3hdml	-0.25375	0.0066386	hypomethylated	-0.18717	0.0012701	hypomethylated	5	16	16
chr2	163367457	163369457	0610008F07Rik	-0.024621	0.57947	insignificant	-0.1077	0.11336	insignificant	1	4	4
chr2	163371923	163373923	Hnf4a	0.023411	0.63152	insignificant	0.012081	0.029553	inconclusive	6	16	16
chr2	163427049	163429049	Ttpal	-0.19847	7.76E-27	hypomethylated	-0.012293	0.10734	insignificant	25	80	79
chr2	163469585	163471585	0610039K10Rik	-0.10451	2.95E-20	hypomethylated	-0.016636	0.027234	hypomethylated	30	115	120
chr2	163470879	163472879	0610039K10Rik	-0.41149	0.33312	insignificant	-0.049888	0.053815	insignificant	5	26	29
chr2	163483121	163485121	Pkig	-0.10294	6.97E-45	hypomethylated	0.0016128	0.58444	insignificant	40	88	88
chr2	163483193	163485193	Pkig	-0.10294	6.97E-45	hypomethylated	0.0016128	0.58444	insignificant	40	88	88
chr2	163518772	163520772	Pkig	0.030449	1	insignificant	-0.076276	0.40111	insignificant	1	4	4
chr2	163575913	163577913	Ada	-0.16914	2.67E-21	hypomethylated	-0.03352	0.0019783	hypomethylated	15	53	53
chr2	163678485	163680485	Kcnk15	-0.079856	4.97E-12	hypomethylated	-0.0035912	0.034099	hypomethylated	25	104	99
chr2	163744419	163746419	Rims4	-0.08972	0.16203	insignificant	-0.0093514	0.82598	insignificant	14	65	65
chr2	163819932	163821932	Ywhab	-0.083322	7.43E-15	hypomethylated	0.00064371	0.44541	insignificant	35	122	122
chr2	163896838	163898838	Tomm34	-0.32126	1.01E-19	hypomethylated	-0.045388	5.32E-08	hypomethylated	12	35	37
chr2	163898913	163900913	Stk4	-0.20731	5.66E-26	hypomethylated	-0.010789	0.12902	insignificant	21	58	59
chr2	163996849	163998849	Kcns1	0.23548	1	insignificant	-0.10618	0.027667	hypomethylated	2	10	16
chr2	164008478	164010478	Wfdc5	0.13021	1	insignificant	-0.015327	1	insignificant	1	8	8
chr2	164025853	164027853	Wfdc15a		1	noCoverage	0.19434	0.15606	insignificant	0	8	13
chr2	164157500	164159500	Svs5	0.010606	1	insignificant	-0.029704	0.74323	insignificant	4	10	10
chr2	164182243	164184243	Slpi	-0.19156	0.29878	insignificant	-0.035199	0.89559	insignificant	2	9	8
chr2	164227693	164229693	Rbpjl	-0.22644	8.8E-09	hypomethylated	0.0071984	0.85444	insignificant	5	26	26
chr2	164230114	164232114	Matn4	-0.10417	1	insignificant	0.042659	0.58773	insignificant	1	4	4
chr2	164268688	164270688	Sdc4	-0.31295	5.91E-25	hypomethylated	0.023528	0.0012182	inconclusive	12	42	41
chr2	164285470	164287470	Sys1	-0.17452	2.35E-45	hypomethylated	-0.0058478	0.37064	insignificant	57	142	142
chr2	164310639	164312639	Dbndd2	-0.11539	0.00000339	hypomethylated	0.001284	0.64999	insignificant	28	105	142
chr2	164310954	164312954	Dbndd2	-0.12926	0.000000518	hypomethylated	-0.0065127	0.42482	insignificant	30	113	149
chr2	164311376	164313376	Dbndd2	-0.13704	0.000000117	hypomethylated	-0.006549	0.24592	insignificant	30	117	153
chr2	164312610	164314610	Dbndd2	-0.32643	1	insignificant	-0.017642	0.91803	insignificant	2	12	11
chr2	164322024	164324024	Pigt	-0.10145	0.24	insignificant	-0.0047919	0.47579	insignificant	10	36	36
chr2	164387215	164389215	Wfdc2	-0.41442	0.0089107	stronglyHypometh	-0.11751	0.00030796	hypomethylated	6	36	27
chr2	164509606	164511606	Wfdc13	-0.23442	0.063388	insignificant	0.00064351	0.82108	insignificant	2	12	9
chr2	164568767	164570767	Dnttip1	-0.84028	0.24806	lowCoverage	-0.069444	0.7654	insignificant	1	6	6
chr2	164570514	164572514	Dnttip1	-0.1349	6.99E-13	hypomethylated	0.00016856	0.026776	inconclusive	15	40	44
chr2	164594428	164596428	Ube2c	-0.16454	0.067247	insignificant	0.0055212	0.85838	insignificant	11	106	107
chr2	164605234	164607234	Tnnc2	0.076389	1	insignificant	-0.10456	0.3799	insignificant	3	8	14
chr2	164610520	164612520	Snx21	-0.10879	4.17E-14	hypomethylated	-0.01862	0.092488	insignificant	39	140	147
chr2	164629613	164631613	Zswim3	-0.17305	0.00000179	hypomethylated	0.042645	0.26871	insignificant	13	42	44
chr2	164630381	164632381	Acot8	-0.33213	0.013542	hypomethylated	0.091129	0.20445	insignificant	2	19	22
chr2	164647185	164649185	Zswim1	-0.15713	0.00022215	hypomethylated	0.031828	0.69547	insignificant	10	57	59
chr2	164654034	164656034	1700020C07Rik	0.081657	1	lowCoverage	0.023254	0.82929	insignificant	1	8	9
chr2	164657372	164659372	Ctsa	-0.29785	2.84E-08	hypomethylated	-0.027199	0.095283	insignificant	8	41	41
chr2	164659096	164661096	Ctsa	-0.30693	0.000000311	hypomethylated	-0.048126	0.085574	insignificant	4	39	34
chr2	164703867	164705867	Pcif1	-0.14558	6.77E-25	hypomethylated	-0.015506	0.044697	hypomethylated	64	163	163
chr2	164737250	164739250	Zfp335	-0.19523	0.027281	hypomethylated	-0.017432	0.13318	insignificant	9	32	35
chr2	164772750	164774750	Mmp9	-0.47916	0.0019011	stronglyHypometh	-0.022258	0.10546	insignificant	3	32	34
chr2	164792487	164794487	Slc12a5	-0.10487	0.00000557	hypomethylated	0.0026048	0.66595	insignificant	35	124	128
chr2	164860279	164862279	Ncoa5	-0.36741	0.00000507	stronglyHypometh	0.00050652	0.30745	insignificant	7	43	52
chr2	164880135	164882135	Cd40		1	noCoverage	-0.02159	0.66891	insignificant	0	6	6
chr2	164916250	164918250	1700025C18Rik		1	noCoverage	0.021816	0.30961	insignificant	0	8	7
chr2	165060237	165062237	Cdh22	-0.22832	1	insignificant	0.20533	0.90113	insignificant	1	29	35
chr2	165113327	165115327	Elmo2	-0.067513	0.7472	insignificant	0.065119	0.046307	hypermethylated	6	12	12
chr2	165142393	165144393	Elmo2	-0.68056	0.00037902	stronglyHypometh	-0.069192	0.30688	insignificant	2	4	4
chr2	165151979	165153979	Elmo2	-0.13271	0.000094637	hypomethylated	-0.020732	0.40422	insignificant	17	67	70
chr2	165187619	165189619	Zfp663	-0.2068	0.27563	insignificant	0.038803	0.92752	insignificant	2	13	13
chr2	165213759	165215759	Zfp334	0.4472	1	lowCoverage	0.29095	0.085794	insignificant	1	7	6
chr2	165298697	165300697	Slc13a3	0.71249	1.14E-29	stronglyHypermeth	0.016845	0.37603	insignificant	4	14	14
chr2	165318814	165320814	2810408M09Rik	-0.047319	0.058054	insignificant	0.1044	0.61801	insignificant	12	41	35
chr2	165328477	165330477	Slc2a10	-0.18672	0.00000454	hypomethylated	0.039997	0.80233	insignificant	9	34	32
chr2	165479797	165481797	Eya2	-0.38832	1.81E-15	stronglyHypometh	-0.035151	0.61287	insignificant	4	12	12
chr2	165710188	165712188	Zmynd8	-0.10221	0.26891	insignificant	0.047713	0.39904	insignificant	1	24	22

chr2	165817136	165819136	Ncoa3	-0.11477	2.95E-16	hypomethylated	-0.0076594	0.32895	insignificant	33	166	164
chr2	165981156	165983156	Sulf2	-0.0015132	0.56186	insignificant	0.015228	0.0016216	hypermethylated	2	32	31
chr2	166098407	166100407	Gm11468	-0.00091756	1	insignificant	-0.017102	1	insignificant	1	4	4
chr2	166539332	166541332	Prex1	-0.10996	1	lowCoverage	0.082062	0.76021	insignificant	1	30	33
chr2	166618266	166620266	Trp53rk	-0.14587	0.0004347	hypomethylated	-0.013994	0.35535	insignificant	13	77	77
chr2	166630080	166632080	Arfgef2	-0.19625	9.79E-18	hypomethylated	-0.0012642	0.93041	insignificant	25	67	64
chr2	166730595	166732595	Cse1l	-0.091507	1.43E-15	hypomethylated	0.0062263	0.20933	insignificant	27	74	74
chr2	166821778	166823778	Stau1	-0.12442	0.022746	hypomethylated	0.01457	0.71097	insignificant	10	28	28
chr2	166839812	166841812	Ddx27	-0.10916	6.46E-08	hypomethylated	0.029435	0.91204	insignificant	7	51	45
chr2	166887433	166889433	1500012F01Rik	-0.14809	1.35E-66	hypomethylated	-0.014121	0.034285	hypomethylated	56	201	200
chr2	166888515	166890515	Snord12	-0.17197	1.09E-31	hypomethylated	-0.011933	0.56769	insignificant	20	92	92
chr2	166889792	166891792	Snord12	-0.068033	1	insignificant	0.037462	0.15146	insignificant	3	16	16
chr2	167014299	167016299	Kcnb1	-0.12766	4.38E-16	hypomethylated	0.011062	0.019247	hypermethylated	60	164	170
chr2	167066037	167068037	Ptgis	-0.26086	0.000074892	hypomethylated	-0.037106	0.77682	insignificant	2	13	13
chr2	167174678	167176678	B4galt5	-0.16082	2.84E-08	hypomethylated	0.0059785	0.81931	insignificant	10	86	81
chr2	167246220	167248220	Slc9a8	-0.15048	7.62E-21	hypomethylated	0.0060533	0.19448	insignificant	23	75	74
chr2	167317144	167319144	Rnf114	-0.16673	3.74E-25	hypomethylated	0.0062706	0.55803	insignificant	34	103	99
chr2	167318374	167320374	Rnf114	-0.22141	1.05E-16	hypomethylated	-0.016919	0.17248	insignificant	15	63	57
chr2	167362726	167364726	Snai1	-0.12145	1.19E-12	hypomethylated	0.0079082	0.77635	insignificant	30	119	116
chr2	167457505	167459505	Ube2v1	-0.13725	3.07E-27	hypomethylated	-0.030756	0.0018822	hypomethylated	15	66	70
chr2	167487044	167489044	Tmem189	-0.13884	5.36E-12	hypomethylated	0.0020354	0.33322	insignificant	22	72	76
chr2	167513414	167515414	Cebpb	-0.067314	1.88E-37	hypomethylated	0.0049349	0.052306	insignificant	97	305	305
chr2	167515707	167517707	A530013C23Rik	0.34648	1	lowCoverage	-0.23579	0.0014583	hypomethylated	1	11	14
chr2	167756826	167758826	Ptpn1	-0.14274	2.5E-16	hypomethylated	0.014331	0.083218	insignificant	32	145	141
chr2	167836093	167838093	Fam65c		1	noCoverage	0.19676	0.82585	insignificant	0	6	4
chr2	167905503	167907503	Pard6b	-0.126	6.73E-40	hypomethylated	-0.012033	0.35549	insignificant	42	117	112
chr2	168032562	168034562	Adnp		1	noCoverage	-0.029295	0.47952	insignificant	0	9	9
chr2	168055121	168057121	Mocs3	-0.10434	3.32E-20	hypomethylated	0.0099909	0.24097	insignificant	91	332	344
chr2	168055879	168057879	Dpm1	-0.08897	1.47E-14	hypomethylated	0.0064544	0.000027335	inconclusive	79	283	296
chr2	168094831	168096831	Kcng1	-0.099814	0.035093	hypomethylated	0.02293	1	insignificant	5	16	13
chr2	168415691	168417691	Nfatc2	-0.12473	0.000000142	hypomethylated	-0.018979	0.25306	insignificant	18	50	60
chr2	168415783	168417783	Nfatc2	-0.17536	3.78E-09	hypomethylated	-0.036781	0.67561	insignificant	14	38	48
chr2	168415848	168417848	Nfatc2	-0.21923	8.52E-11	hypomethylated	-0.04496	0.78893	insignificant	9	24	30
chr2	168567300	168569300	Atp9a	-0.086699	5.79E-11	hypomethylated	0.0047055	0.26287	insignificant	41	85	112
chr2	168592701	168594701	Sall4	-0.4473	0.0082609	stronglyHypometh	-0.068466	0.066116	insignificant	4	14	17
chr2	168781087	168783087	Zfp64	0.052141	1	insignificant	-0.087738	0.20285	insignificant	3	11	10
chr2	169458145	169460145	Tshz2	-0.19786	1	insignificant	-0.080952	0.74742	insignificant	4	11	18
chr2	169896171	169898171	A630075F10Rik		1	noCoverage	-0.38485	0.00017369	stronglyhypometh	0	10	10
chr2	169896278	169898278	A630075F10Rik		1	noCoverage	-0.38485	0.00017369	stronglyhypometh	0	10	10
chr2	169956720	169958720	Zfp217	-0.13849	0.14158	insignificant	-0.025477	0.0014319	hypomethylated	6	36	59
chr2	169968175	169970175	Zfp217	0.025815	0.45418	insignificant	0.079584	0.066013	insignificant	3	21	22
chr2	170253345	170255345	Bcas1	0.037146	0.68814	insignificant	-0.0078954	0.9049	insignificant	3	15	14
chr2	170320927	170322927	Pfdn4	-0.18309	6.6E-11	hypomethylated	-0.0052385	0.54549	insignificant	10	60	62
chr2	170322638	170324638	Cyp24a1	-0.12847	0.0000328	hypomethylated	0.018108	0.88612	insignificant	6	43	43
chr2	170335729	170337729	Pfdn4	-0.12967	7.04E-14	hypomethylated	-0.024217	0.10981	insignificant	17	85	79
chr2	170556306	170558306	Dok5	-0.20096	0.00027646	hypomethylated	-0.030473	0.94501	insignificant	7	76	78
chr2	172072991	172074991	Mc3r	-0.76993	1.99E-21	stronglyHypometh	0.046841	0.28931	insignificant	5	6	8
chr2	172170076	172172076	2010011I20Rik	-0.14827	6.17E-25	hypomethylated	0.0098743	0.70536	insignificant	41	111	117
chr2	172195502	172197502	Cstf1	-0.10945	3.59E-16	hypomethylated	-0.0002874	0.0020197	hypomethylated	25	120	122
chr2	172196006	172198006	Aurka	-0.099337	8E-13	hypomethylated	0.00023157	0.046191	inconclusive	20	106	108
chr2	172265077	172267077	2410001C21Rik	-0.12364	2.67E-10	hypomethylated	0.011273	0.85173	insignificant	16	56	60
chr2	172297053	172299053	1700029J11Rik	-0.019992	0.0027731	hypomethylated	-0.033138	1	insignificant	6	16	15
chr2	172374092	172376092	Tfap2c	-0.10409	2.23E-13	hypomethylated	0.0148	0.92975	insignificant	17	79	86
chr2	172375490	172377490	Tfap2c	-0.14437	1.46E-14	hypomethylated	-0.012217	0.20959	insignificant	27	139	141
chr2	172765794	172767794	Bmp7	-0.23108	9.24E-14	hypomethylated	-0.0093357	0.05348	insignificant	19	59	59
chr2	172804342	172806342	Spo11	0.030899	0.31813	insignificant	-0.0086567	0.35927	insignificant	6	40	38
chr2	172824637	172826637	Rae1	-0.073349	1.26E-14	hypomethylated	-0.0033228	0.1027	insignificant	40	138	136
chr2	172846402	172848402	Rbm38	-0.093231	3.2E-13	hypomethylated	-0.016949	0.093468	insignificant	33	128	115
chr2	172977573	172979573	Pck1	-0.026879	0.27967	insignificant	0.0051128	0.077193	insignificant	4	16	16
chr2	173044423	173046423	Zbp1		1	noCoverage	-0.086036	1	insignificant	0	13	12
chr2	173102034	173104034	Pmepa1	-0.060424	0.000027069	hypomethylated	0.0075795	0.71452	insignificant	19	56	62
chr2	173347092	173349092	1700021F07Rik	-0.25624	0.0016691	hypomethylated	0.079814	0.91497	insignificant	4	25	24
chr2	173484345	173486345	Rab22a	-0.10961	1.08E-16	hypomethylated	0.0023348	0.89255	insignificant	25	76	76
chr2	173485040	173487040	Ppp4r1l-ps	-0.20663	6.33E-33	hypomethylated	-0.0029648	0.00041174	hypomethylated	25	68	68
chr2	173562071	173564071	Vapb	-0.11039	1.06E-19	hypomethylated	-0.0024367	0.058492	insignificant	29	82	81
chr2	173901551	173903551	Stx16	-0.14949	1.79E-09	hypomethylated	-0.01342	0.66664	insignificant	17	49	54
chr2	173934851	173936851	Npepl1	-0.11352	3.49E-14	hypomethylated	0.0061751	0.13757	insignificant	45	129	118
chr2	174092626	174094626	Mir296		1	noCoverage	-0.019964	0.51353	insignificant	0	12	12
chr2	174108820	174110820	Gnas	-0.20264	5.28E-22	hypomethylated	0.04117	0.00022013	inconclusive	29	75	86
chr2	174120937	174122937	Gnas	0.28787	0.03862	hypermethylated	0.054847	0.35201	insignificant	8	33	33
chr2	174122359	174124359	Gnas	0.10963	0.5272	insignificant	-0.009135	0.00078076	hypomethylated	21	74	74
chr2	174152414	174154414	Gnas	0.054704	0.57502	insignificant	-0.037685	0.023948	hypomethylated	31	95	90
chr2	174154589	174156589	Gnas	-0.098767	1.86E-29	hypomethylated	-0.0082022	0.64275	insignificant	55	241	249
chr2	174154592	174156592	Gnas	-0.098767	1.86E-29	hypomethylated	-0.0082022	0.64275	insignificant	55	241	249
chr2	174240304	174242304	Th1l	-0.26476	8.04E-26	hypomethylated	0.092436	0.80599	insignificant	15	60	63
chr2	174264493	174266493	Ctsz	-0.37025	0.00030027	stronglyHypometh	-0.18908	0.31412	insignificant	2	11	11
chr2	174289602	174291602	Atp5e	-0.17666	0.00000985	hypomethylated	0.0038136	0.60315	insignificant	5	20	20
chr2	174298442	174300442	Slmo2	-0.13667	0.003004	hypomethylated	-0.014414	0.57651	insignificant	8	20	20
chr2	174468034	174470034	Zfp831		1	noCoverage	-0.1	0.17419	insignificant	0	6	6
chr2	174585273	174587273	Edn3	-0.16035	0.00000021	hypomethylated	-0.01547	0.6102	insignificant	12	46	46
chr2	174893264	174895264	Gm14393	0.1593	1	insignificant	0.08675	0.051751	insignificant	1	7	6
chr2	177248446	177250446	Gm14420		1	noCoverage	0.10355	0.32237	insignificant	0	25	28
chr2	177281930	177283930	Gm14403	-0.18649	0.0089164	hypomethylated	0.1783	0.00064808	hypermethylated	2	19	19
chr2	177756988	177758988	Etohi1	-0.22753	0.00022071	hypomethylated	-0.053188	0.6753	insignificant	9	32	29
chr2	177852679	177854679	Phactr3	-0.2116	3.18E-21	hypomethylated	-0.0054301	0.000022997	hypomethylated	19	60	67

chr2	177875637	177877637	Phactr3	-0.20357	0.000000244	hypomethylated	-0.021557	0.22269	insignificant	9	23	22
chr2	178148238	178150238	9030418K01Rik	-0.10798	1.12E-12	hypomethylated	-0.011105	0.0003488	hypomethylated	46	136	136
chr2	178149177	178151177	9030418K01Rik	-0.14031	2.07E-11	hypomethylated	0.021866	0.0038762	inconclusive	14	38	40
chr2	179176182	179178182	Cdh4	-0.097721	5.44E-24	hypomethylated	-0.0052182	0.51834	insignificant	65	196	197
chr2	179710557	179712557	4921531C22Rik	-0.098169	3.77E-30	hypomethylated	0.0048753	0.61465	insignificant	78	202	244
chr2	179711351	179713351	Taf4a	-0.1609	2.2E-19	hypomethylated	-0.016892	0.14597	insignificant	22	50	50
chr2	179758691	179760691	Lsm14b	-0.085314	2.39E-15	hypomethylated	-0.0037231	0.0057235	hypomethylated	42	175	186
chr2	179776187	179778187	Ss18l1	-0.090402	8.6E-33	hypomethylated	0.0039287	0.37118	insignificant	73	271	266
chr2	179777107	179779107	Psma7	-0.077711	4.38E-16	hypomethylated	0.0026289	0.96778	insignificant	39	145	145
chr2	179804297	179806297	Gtpbp5	-0.13941	0.0042079	hypomethylated	-0.00018942	0.24137	insignificant	9	64	66
chr2	179838927	179840927	Hrh3	-0.20773	0.000022402	hypomethylated	0.0084291	0.73995	insignificant	3	45	42
chr2	179853070	179855070	Osbpl2	-0.077588	5.27E-09	hypomethylated	0.000087656	0.90676	insignificant	38	123	122
chr2	179905292	179907292	Adrm1	-0.10259	3.95E-24	hypomethylated	-0.0012273	0.25244	insignificant	50	150	150
chr2	179960564	179962564	Lama5	-0.19187	0.00010715	hypomethylated	-0.0038764	0.038317	inconclusive	16	63	63
chr2	179991083	179993083	Rps21	-0.17113	3.86E-08	hypomethylated	-0.002411	0.73126	insignificant	22	75	71
chr2	179991240	179993240	Mir3091	-0.17113	3.86E-08	hypomethylated	-0.002411	0.73126	insignificant	22	75	71
chr2	180008170	180010170	Cables2	-0.36006	0.0000042	stronglyHypometh	0.014767	0.089973	insignificant	2	28	28
chr2	180024584	180026584	BC066135	0.047383	0.67985	insignificant	-0.13435	0.00018291	hypomethylated	4	14	14
chr2	180069384	180071384	Gata5	-0.12801	4.04E-13	hypomethylated	-0.024806	0.18112	insignificant	14	42	43
chr2	180122752	180124752	Mir1a-1		1	noCoverage	-0.2	0.31624	insignificant	0	5	3
chr2	180194682	180196682	Slco4a1		1	noCoverage	-0.06053	0.29677	insignificant	0	10	10
chr2	180233680	180235680	Ntsr1	-0.20234	0.000018654	hypomethylated	0.017863	0.83555	insignificant	14	58	54
chr2	180323111	180325111	Ogfr	-0.1372	1.72E-11	hypomethylated	0.010656	0.25505	insignificant	10	87	73
chr2	180331926	180333926	Col9a3	-0.14105	0.45167	insignificant	-0.0082955	0.04246	inconclusive	14	50	48
chr2	180377396	180379396	Tcfl5	0	1	insignificant	-0.092105	0.040748	hypomethylated	0	1	2
chr2	180436805	180438805	Dido1	-0.19127	1.75E-08	hypomethylated	0.010008	0.0018327	inconclusive	14	34	34
chr2	180444704	180446704	2310003C23Rik	-0.1646	1.42E-16	hypomethylated	-0.0051522	0.23068	insignificant	29	115	100
chr2	180446250	180448250	2310003C23Rik		1	noCoverage	-0.012049	0.31292	insignificant	0	13	12
chr2	180511605	180513605	Bhlhe23	-0.26391	0.13317	insignificant	-0.090818	0.28798	insignificant	5	16	16
chr2	180627744	180629744	Mir124a-3	-0.11883	1.41E-40	hypomethylated	-0.0031567	0.19066	insignificant	78	168	188
chr2	180655641	180657641	Ythdf1	-0.08044	0.023487	hypomethylated	-0.0095776	0.72264	insignificant	17	46	40
chr2	180689404	180691404	Nkain4	-0.83542	0.17188	lowCoverage	-0.014583	0.81914	insignificant	1	4	4
chr2	180700929	180702929	Arfgap1	-0.16647	3.6E-37	hypomethylated	-0.041712	0.97431	insignificant	28	102	119
chr2	180701004	180703004	Arfgap1	-0.16647	3.6E-37	hypomethylated	-0.041712	0.97431	insignificant	28	102	119
chr2	180773882	180775882	Chrna4	-0.1641	1.67E-10	hypomethylated	-0.01158	0.089209	insignificant	9	42	42
chr2	180869930	180871930	Kcnq2	-0.1501	0.032033	hypomethylated	0.098119	0.0022343	hypermethylated	7	22	28
chr2	180921047	180923047	Ppdpf	-0.18284	0.020122	hypomethylated	-0.014641	0.46471	insignificant	11	38	39
chr2	180937494	180939494	Ptk6	0.19233	0.074965	insignificant	0.052822	0.10586	insignificant	4	20	21
chr2	180953720	180955720	BC051628	-0.20579	0.017801	hypomethylated	-0.05271	0.71385	insignificant	3	19	22
chr2	180976732	180978732	BC006779		1	noCoverage	0.26856	0.36608	insignificant	0	6	4
chr2	181022671	181024671	Gmeb2	0.48944	0.0017443	stronglyHypermeth	-0.021271	9.51E-09	inconclusive	5	18	27
chr2	181049205	181051205	Stmn3	-0.14773	0.42471	insignificant	-0.025552	0.64378	insignificant	1	4	4
chr2	181053428	181055428	Rtel1	-0.14563	5.42E-10	hypomethylated	0.033208	0.50864	insignificant	14	53	56
chr2	181054510	181056510	Rtel1	-0.15675	0.077016	insignificant	0.013733	0.49767	insignificant	4	22	22
chr2	181098635	181100635	Zgpat	-0.11468	3.09E-10	hypomethylated	-0.010105	0.79556	insignificant	17	80	75
chr2	181099127	181101127	Zgpat	-0.12802	6.15E-13	hypomethylated	-0.009696	0.7619	insignificant	21	97	95
chr2	181100109	181102109	Arfrp1	-0.18276	0.000000101	hypomethylated	-0.032405	0.11059	insignificant	13	59	56
chr2	181114939	181116939	Lime1		1	noCoverage	-0.063988	0.45098	insignificant	0	6	6
chr2	181194131	181196131	Zbtb46	-0.14131	1.27E-09	hypomethylated	0.020725	0.93999	insignificant	30	98	105
chr2	181226910	181228910	Abhd16b	-0.1238	0.026265	hypomethylated	-0.058784	0.21445	insignificant	5	19	20
chr2	181230957	181232957	Tpd52l2		1	noCoverage	0.021041	0.92372	insignificant	0	16	18
chr2	181254209	181256209	Dnajc5	-0.1126	1.34E-22	hypomethylated	-0.010341	0.90324	insignificant	24	97	98
chr2	181316678	181318678	Uckl1		1	noCoverage	-0.025461	0.41879	insignificant	0	14	14
chr2	181327166	181329166	Znf512b	-0.047532	0.00000583	hypomethylated	-0.01307	0.02089	hypomethylated	30	100	113
chr2	181333852	181335852	Samd10	-0.14868	1.17E-16	hypomethylated	-0.00022022	0.48333	insignificant	14	32	32
chr2	181335023	181337023	Prpf6	-0.10456	1.43E-20	hypomethylated	-0.001495	0.55976	insignificant	21	76	76
chr2	181406345	181408345	Sox18	-0.20348	0.00000133	hypomethylated	0.020172	0.35277	insignificant	3	16	16
chr2	181414014	181416014	Tcea2	-0.12228	1.06E-18	hypomethylated	-0.0081474	0.38795	insignificant	24	98	97
chr2	181428629	181430629	Rgs19	-0.12964	0.00000143	hypomethylated	0.046128	0.78715	insignificant	15	45	44
chr2	181571607	181573607	Pcmtd2	-0.37719	1	lowCoverage	-0.04386	0.29643	insignificant	1	6	6
chr2	181598064	181600064	Polr3k	-0.13125	0.000000596	hypomethylated	0.023001	0.83993	insignificant	3	26	26
chr3	3507029	3509029	Hnf4g	-0.29352	0.000279	hypomethylated	-0.037547	0.057307	insignificant	3	20	20
chr3	5217553	5219553	Zfhx4	-0.15634	0.00014684	hypomethylated	-0.01451	0.15839	insignificant	10	43	43
chr3	5576150	5578150	Pxmp3	-0.15657	0.00000391	hypomethylated	-0.011789	0.81974	insignificant	13	44	44
chr3	5576151	5578151	Pxmp3	-0.15657	0.00000391	hypomethylated	-0.011789	0.81974	insignificant	13	44	44
chr3	5576239	5578239	Pxmp3	-0.09525	0.00057803	hypomethylated	-0.017185	0.53473	insignificant	10	36	36
chr3	7365603	7367603	Pkia	-0.13571	0.000011479	hypomethylated	-0.022036	0.030596	hypomethylated	12	65	65
chr3	7502425	7504425	Fam164a	-0.10199	0.0033966	hypomethylated	0.00024959	0.80291	insignificant	17	71	71
chr3	7613427	7615427	Il7	-0.59259	0.00036926	stronglyHypometh	-0.36122	0.4503	insignificant	0	0	3
chr3	8463098	8465098	Gm6194		1	noCoverage	-0.063889	0.55781	insignificant	0	4	2
chr3	8508526	8510526	Stmn2	-0.28546	0.55323	insignificant	-0.017839	0.90781	insignificant	2	15	15
chr3	8667038	8669038	Hey1	-0.089971	1.03E-18	hypomethylated	-0.0060601	0.36357	insignificant	39	108	105
chr3	8923857	8925857	Mrps28	-0.090764	0.16989	insignificant	0.020445	0.3216	insignificant	6	24	26
chr3	8964054	8966054	Tpd52	-0.34571	0.0003922	stronglyHypometh	-0.04488	0.11338	insignificant	5	10	10
chr3	9004515	9006515	Tpd52		1	noCoverage	-0.25904	0.091396	insignificant	0	6	10
chr3	9249566	9251566	Zbtb10	-0.086381	7.01E-17	hypomethylated	0.0052004	0.87416	insignificant	58	257	255
chr3	9610085	9612085	Zfp704	-0.21674	2.66E-15	hypomethylated	-0.011582	0.38664	insignificant	6	16	16
chr3	10011605	10013605	Fabp5	-0.11714	7.43E-11	hypomethylated	0.0014694	0.014573	hypermethylated	7	71	70
chr3	10331439	10333439	Impa1	-0.13333	0.58949	insignificant	-0.014912	0.38806	insignificant	3	6	6
chr3	10351301	10353301	Zfand1	-0.12075	0.0049471	hypomethylated	-0.052821	0.67317	insignificant	4	29	41
chr3	10365972	10367972	Chmp4c	-0.3375	1	lowCoverage	0.00625	1	insignificant	1	8	8
chr3	10440124	10442124	Snx16	-0.21529	0.34749	insignificant	0.018454	0.85971	insignificant	3	12	12
chr3	13470654	13472654	Ralyl	-0.090097	0.022357	hypomethylated	0.0081067	0.586	insignificant	13	30	35
chr3	14532787	14534787	Lrrcc1	-0.21598	2.27E-15	hypomethylated	-0.027464	0.00044892	hypomethylated	11	38	38
chr3	14577670	14579670	E2f5	-0.11063	7.47E-09	hypomethylated	-0.011168	0.33932	insignificant	13	90	97

chr3	14611256	14613256	1810022K09Rik	-0.14717	7.07E-23	hypomethylated	-0.0076948	0.53821	insignificant	16	64	64
chr3	14640726	14642726	Car13	0.41336	0.00000986	stronglyHypermeth	0.0034069	0.83116	insignificant	1	40	40
chr3	14862537	14864537	Car3	-0.42043	1.9E-09	stronglyHypometh	-0.04246	0.95587	insignificant	7	39	44
chr3	14885425	14887425	Car2	-0.11431	4.12E-30	hypomethylated	0.010268	0.65308	insignificant	43	124	128
chr3	16082182	16084182	Ythdf3	-0.091377	1.04E-24	hypomethylated	-0.0019794	0.79416	insignificant	31	105	114
chr3	17694661	17696661	Mir124a-2	-0.15066	0.017872	hypomethylated	-0.018697	0.89342	insignificant	3	30	31
chr3	17847443	17849443	Cypt12	-0.12145	0.54929	insignificant	-0.055568	0.12697	insignificant	2	15	15
chr3	17953324	17955324	Bhlhe22	-0.099203	5.04E-35	hypomethylated	-0.0030962	0.94992	insignificant	48	128	128
chr3	19087243	19089243	Mtfr1	-0.15811	6.25E-19	hypomethylated	0.014136	0.91949	insignificant	23	66	79
chr3	19211322	19213322	Pde7a	-0.092206	9.29E-19	hypomethylated	0.0023026	0.7025	insignificant	33	108	108
chr3	19407594	19409594	Dnajc5b		1	noCoverage	0.025183	1	insignificant	0	4	4
chr3	19430246	19432246	Dnajc5b	-0.017857	0.67251	insignificant	-0.22522	1	insignificant	2	4	14
chr3	19595396	19597396	Crh	-0.15685	0.28523	insignificant	-0.18128	0.0040304	hypomethylated	2	8	14
chr3	19793870	19795870	4632415L05Rik	-0.1531	0.000000119	hypomethylated	-0.016238	0.4196	insignificant	7	67	63
chr3	19935310	19937310	Hps3	-0.3149	0.0689	insignificant	-0.062162	0.09263	insignificant	5	20	19
chr3	19956810	19958810	Hltf	-0.12739	1.14E-14	hypomethylated	0.010028	0.74776	insignificant	16	64	64
chr3	20054995	20056995	Gyg	-0.13929	0.000024527	hypomethylated	-0.025418	0.39519	insignificant	4	19	19
chr3	21974573	21976573	Tbl1xr1	-0.11326	5.22E-30	hypomethylated	-0.022377	0.56632	insignificant	73	225	241
chr3	22148862	22150862	Rprl2	0.044893	1	lowCoverage	-0.010648	1	insignificant	1	9	8
chr3	26052229	26054229	Nlgn1	-0.18393	0.0018983	hypomethylated	0.027972	0.42704	insignificant	5	12	12
chr3	26230831	26232831	Nlgn1		1	noCoverage	0.12497	0.1349	insignificant	0	15	18
chr3	27052776	27054776	Ect2	-0.18023	1	insignificant	-0.079945	1	insignificant	4	12	12
chr3	27052800	27054800	Ect2	-0.18023	1	insignificant	-0.079945	1	insignificant	4	12	12
chr3	27080925	27082925	Nceh1	-0.20375	1.91E-22	hypomethylated	-0.002912	0.73513	insignificant	12	65	64
chr3	27269272	27271272	Ghsr	-0.18754	0.014082	hypomethylated	0.0094199	0.63239	insignificant	8	57	58
chr3	27609361	27611361	Fndc3b	-0.11183	1.5E-28	hypomethylated	-0.0039975	0.49851	insignificant	49	143	135
chr3	27836601	27838601	Pld1	-0.15257	8.66E-16	hypomethylated	-0.0085175	0.27098	insignificant	17	78	78
chr3	28161135	28163135	Tnik	-0.15876	3.91E-18	hypomethylated	-0.0040385	0.039212	hypomethylated	12	104	96
chr3	28679232	28681232	Eif5a2	-0.18001	2.16E-39	hypomethylated	0.0065347	0.092265	insignificant	45	114	117
chr3	28703432	28705432	Rpl22l1	-0.08081	9.59E-16	hypomethylated	-0.0036845	0.18661	insignificant	22	84	85
chr3	28980498	28982498	Egfem1	-0.19284	1.1E-38	hypomethylated	-0.00074708	0.0015588	hypomethylated	42	122	133
chr3	29314744	29316744	Mir551b	0.10595	1	insignificant	-0.10548	0.083668	insignificant	3	10	10
chr3	30408409	30410409	Mecom	-0.21117	0.000035017	hypomethylated	-0.014917	0.71326	insignificant	5	14	14
chr3	30498792	30500792	Mynn	-0.27591	0.0051362	hypomethylated	0.00018182	0.81101	insignificant	7	42	48
chr3	30500008	30502008	Mynn	-0.19616	8.56E-27	hypomethylated	-0.0094523	0.86812	insignificant	29	110	119
chr3	30546740	30548740	Lrrc34	-0.29025	0.027036	hypomethylated	-0.12198	0.52998	insignificant	2	14	14
chr3	30690797	30692797	Sec62	-0.10115	8.45E-15	hypomethylated	-0.010146	0.050099	insignificant	44	134	148
chr3	30753871	30755871	Gpr160	-0.10032	6.71E-09	hypomethylated	-0.012033	0.75384	insignificant	23	72	80
chr3	30893692	30895692	Prkci	-0.11422	3.76E-08	hypomethylated	-0.0026241	0.26024	insignificant	26	126	128
chr3	30992982	30994982	Skil	-0.055031	2.11E-45	hypomethylated	-0.0056509	0.22174	insignificant	101	325	336
chr3	31047841	31049841	Cldn11	-0.27227	1.36E-13	hypomethylated	-0.067738	0.13944	insignificant	12	48	62
chr3	31209241	31211241	Slc7a14	-0.3127	0.0061662	hypomethylated	0.064616	0.67479	insignificant	1	9	8
chr3	31800624	31802624	Kcnmb2	0.14357	0.62732	insignificant	-0.11139	0.17938	insignificant	4	20	22
chr3	32263410	32265410	4930429B21Rik	-0.20655	8.56E-29	hypomethylated	-0.010626	0.000001776	hypomethylated	39	159	162
chr3	32264587	32266587	4930429B21Rik	-0.30141	3.19E-13	hypomethylated	-0.038884	0.0071257	hypomethylated	11	66	62
chr3	32390891	32392891	Kcnmb3	-0.24148	0.047248	hypomethylated	-0.044792	1	insignificant	1	4	4
chr3	32408471	32410471	Zfp639	-0.16271	1.28E-30	hypomethylated	0.0077392	0.0030832	inconclusive	29	79	79
chr3	32408513	32410513	Zfp639	-0.16068	1.27E-30	hypomethylated	0.0097731	0.0030922	inconclusive	29	80	79
chr3	32427403	32429403	Mfn1	-0.14846	1.55E-18	hypomethylated	-0.0013654	0.12344	insignificant	16	48	49
chr3	32515457	32517457	Gnb4	-0.23789	1.12E-12	hypomethylated	-0.051318	0.000033047	hypomethylated	15	38	38
chr3	32606467	32608467	Actl6a	-0.12208	2.53E-25	hypomethylated	-0.0048628	0.39827	insignificant	30	98	88
chr3	32715547	32717547	Usp13	-0.13945	1.7E-29	hypomethylated	-0.015543	0.18565	insignificant	32	107	114
chr3	33042004	33044004	Pex5l	-0.13486	1.16E-38	hypomethylated	-0.011204	0.0039993	hypomethylated	24	70	70
chr3	33698116	33700116	Ttc14	-0.13175	4.2E-32	hypomethylated	-0.020772	6.56E-09	hypomethylated	42	114	114
chr3	33918000	33920000	Fxr1	-0.096512	8.09E-44	hypomethylated	-0.000080963	0.0038673	hypomethylated	65	187	172
chr3	33980248	33982248	Dnajc19	-0.23864	0.00081421	hypomethylated	-0.0023606	0.83658	insignificant	3	10	10
chr3	34536385	34538385	Mir1897		1	noCoverage	-0.097552	0.38248	insignificant	0	10	10
chr3	34547926	34549926	Sox2	-0.11038	1.96E-27	hypomethylated	0.0033586	0.32576	insignificant	46	151	158
chr3	35652059	35654059	Atp11b	-0.075234	3.25E-15	hypomethylated	0.0045035	0.69115	insignificant	33	101	100
chr3	35828996	35830996	Dcun1d1		1	noCoverage	0.011982	1	insignificant	0	14	11
chr3	35897834	35899834	A330050B17Rik	-0.20383	0.057653	insignificant	-0.0033809	0.061792	insignificant	1	18	18
chr3	35899600	35901600	Mccc1	-0.65779	0.0092759	stronglyHypometh	-0.011604	0.81765	insignificant	2	6	6
chr3	35963921	35965921	Acad9	-0.013326	0.058637	insignificant	0.01053	1	insignificant	17	54	48
chr3	36049001	36051001	D3Ertd254e	-0.2328	8.23E-53	hypomethylated	-0.029948	0.079167	insignificant	26	59	61
chr3	36450527	36452527	Exosc9	-0.10958	0.0000028	hypomethylated	0.014619	0.27717	insignificant	10	26	26
chr3	36470918	36472918	Ccna2	-0.19104	0.069834	insignificant	-0.05711	0.56712	insignificant	13	42	40
chr3	36512311	36514311	Bbs7	-0.32657	2.19E-12	hypomethylated	-0.0090434	0.00018946	hypomethylated	9	29	28
chr3	36589089	36591089	Trpc3		1	noCoverage	0.018045	0.8041	insignificant	0	8	8
chr3	36761027	36763027	4932438A13Rik	-0.11126	8.67E-10	hypomethylated	-0.0065731	0.58259	insignificant	23	65	94
chr3	36961577	36963577	Adad1	-0.076615	0.45188	insignificant	-0.065347	0.0094902	hypomethylated	18	77	89
chr3	37210475	37212475	Bbs12	-0.10746	0.00010286	hypomethylated	-0.01136	0.0062957	hypomethylated	11	22	22
chr3	37211368	37213368	Cetn4	-0.10746	0.00010286	hypomethylated	-0.01136	0.0062957	hypomethylated	11	22	22
chr3	37246574	37248574	Fgf2	-0.12777	1.43E-21	hypomethylated	-0.007481	0.73816	insignificant	33	81	88
chr3	37318201	37320201	Spata5	-0.11581	7.97E-13	hypomethylated	0.034711	0.36	insignificant	45	158	168
chr3	37318512	37320512	Spata5	-0.11445	0.000000838	hypomethylated	0.01022	0.083801	insignificant	33	121	127
chr3	37537871	37539871	Spry1	-0.10825	8.5E-29	hypomethylated	-0.0045508	0.4972	insignificant	69	175	175
chr3	38383738	38385738	Ankrd50	-0.13266	0.037807	hypomethylated	0.013063	1	insignificant	4	8	8
chr3	38784861	38786861	Fat4	-0.14741	4.92E-34	hypomethylated	-0.010767	0.087391	insignificant	57	193	193
chr3	40438688	40440688	Intu	-0.091954	0.000000249	hypomethylated	0.0018009	0.95231	insignificant	11	54	54
chr3	40511792	40513792	Slc25a31	0.044907	0.58105	insignificant	-0.015428	0.64157	insignificant	13	56	54
chr3	40548534	40550534	Hspa4l	-0.085635	2.67E-08	hypomethylated	-0.005428	0.076841	insignificant	19	134	123
chr3	40602872	40604872	Plk4	-0.078941	3.27E-16	hypomethylated	-0.012094	0.16002	insignificant	33	114	114
chr3	40650776	40652776	Gm2011		1	noCoverage	0.17649	0.76625	insignificant	0	4	4
chr3	40697198	40699198	3110057O12Rik	-0.18084	3.21E-24	hypomethylated	-0.0043022	0.7011	insignificant	21	69	70
chr3	40753552	40755552	Larp1b	-0.075513	1.71E-35	hypomethylated	0.0098183	0.43527	insignificant	72	257	286

chr3	40886968	40888968	Pgrmc2	-0.12907	3.92E-11	hypomethylated	-0.014099	0.1142	insignificant	13	68	76
chr3	41358655	41360655	Phf17		1	noCoverage	-0.010331	0.72657	insignificant	0	11	9
chr3	41366304	41368304	Phf17	-0.21286	0.00000144	hypomethylated	-0.071679	0.0050832	hypomethylated	8	24	39
chr3	41366672	41368672	Phf17	-0.19837	0.00000207	hypomethylated	-0.052858	0.47398	insignificant	12	32	47
chr3	41367801	41369801	Phf17	-0.095134	1.71E-30	hypomethylated	-0.0043646	0.01469	hypomethylated	63	205	216
chr3	41545539	41547539	D3Ertd751e	-0.078148	2.06E-09	hypomethylated	0.043207	0.76083	insignificant	31	75	77
chr3	45181319	45183319	Pcdh10	-0.21268	0.00081405	hypomethylated	0.016514	0.5832	insignificant	3	48	48
chr3	46251863	46253863	Pabpc4l	-0.17813	9.42E-09	hypomethylated	0.02208	0.24909	insignificant	9	34	33
chr3	48413022	48415022	1700018B24Rik	0.1	1	insignificant	0.040972	0.028698	hypermethylated	2	4	4
chr3	49561238	49563238	Pcdh18	-0.098673	0.089692	insignificant	-0.012283	0.062106	insignificant	11	41	37
chr3	51027368	51029368	Ccrn4l	-0.10366	2.75E-28	hypomethylated	0.0036602	0.13261	insignificant	64	167	178
chr3	51200469	51202469	4930583H14Rik	-0.22222	0.066774	insignificant	-0.054989	0.012005	hypomethylated	3	6	14
chr3	51212877	51214877	Ndufc1	-0.10278	0.000000155	hypomethylated	-0.015433	1	insignificant	6	22	23
chr3	51218937	51220937	Naa15	-0.11968	1.72E-24	hypomethylated	-0.0064557	0.0093817	hypomethylated	56	177	178
chr3	51286887	51288887	Rab33b	-0.084237	1.06E-13	hypomethylated	0.0033703	0.092127	insignificant	40	131	134
chr3	51362678	51364678	5031434O11Rik	-0.11641	6.73E-13	hypomethylated	-0.016312	0.81964	insignificant	27	102	108
chr3	51364745	51366745	Setd7	-0.17791	3.82E-14	hypomethylated	-0.012819	0.48443	insignificant	16	63	65
chr3	51908928	51910928	Maml3		1	noCoverage	-0.085833	0.018899	hypomethylated	0	10	10
chr3	52071258	52073258	Foxo1	-0.083891	4.77E-24	hypomethylated	0.0099988	0.29124	insignificant	33	211	204
chr3	52821145	52823145	Cog6	-0.22185	0.000000557	hypomethylated	-0.020796	0.0048984	hypomethylated	6	36	36
chr3	52844468	52846468	Lhfp	-0.19789	6.64E-12	hypomethylated	0.007145	0.77492	insignificant	16	64	56
chr3	53266738	53268738	2810046L04Rik	-0.14112	1.79E-12	hypomethylated	0.036647	0.45408	insignificant	10	25	30
chr3	53267180	53269180	Nhlrc3	-0.30669	6.15E-14	hypomethylated	-0.082124	0.215	insignificant	10	31	37
chr3	53291714	53293714	Stoml3	-0.062637	0.18374	insignificant	-0.14743	0.01424	hypomethylated	3	6	7
chr3	53461277	53463277	Frem2	-0.31135	0.0018416	hypomethylated	-0.073669	0.094271	insignificant	2	28	27
chr3	53667729	53669729	Ufm1	-0.13424	2.09E-11	hypomethylated	0.17403	0.31532	insignificant	7	27	18
chr3	53959025	53961025	Trpc4	-0.24083	0.027644	hypomethylated	-0.0087434	0.43465	insignificant	2	16	16
chr3	54496026	54498026	Fam48a	-0.20541	0.0024596	hypomethylated	-0.030422	0.0087594	hypomethylated	7	53	60
chr3	54538460	54540460	Alg5	-0.091043	5.41E-16	hypomethylated	0.002271	0.77436	insignificant	52	179	186
chr3	54539286	54541286	Exosc8	-0.18974	0.000080528	hypomethylated	0.011018	0.0031766	hypermethylated	24	86	91
chr3	54558503	54560503	Smad9	-0.14361	0.43246	insignificant	0.012215	0.56244	insignificant	14	64	56
chr3	54611713	54613713	Rfxap	-0.18627	0.37902	insignificant	0.089322	0.78169	insignificant	3	28	32
chr3	54719809	54721809	6030405A18Rik		1	noCoverage	0.041667	0.48336	insignificant	0	0	0
chr3	54858977	54860977	Ccna1	-0.1021	8.13E-13	hypomethylated	0.0031869	0.64295	insignificant	26	112	113
chr3	54915029	54917029	Spg20	-0.20142	1.25E-20	hypomethylated	0.031875	0.0011917	inconclusive	20	71	79
chr3	54915087	54917087	Spg20	-0.20142	1.25E-20	hypomethylated	0.031875	0.0011917	inconclusive	20	71	79
chr3	54984965	54986965	Sohlh2	-0.10083	0.17137	insignificant	0.004704	0.42405	insignificant	21	84	84
chr3	55045447	55047447	Dclk1	-0.17623	0.00000197	hypomethylated	0.010176	0.94572	insignificant	8	28	30
chr3	55585431	55587431	Mab21l1	-0.20982	0.0000087	hypomethylated	-0.019545	0.19856	insignificant	12	44	44
chr3	55987623	55989623	Nbea	-0.067914	0.000001	hypomethylated	0.0052571	0.059966	insignificant	17	68	70
chr3	57379798	57381798	Wwtr1	-0.17533	0.00012779	hypomethylated	-0.01298	0.00055741	hypomethylated	9	36	36
chr3	57379832	57381832	Wwtr1	-0.11946	0.0030272	hypomethylated	-0.0080515	0.000094829	hypomethylated	8	32	32
chr3	57455606	57457606	Commd2	-0.30172	0.000015978	hypomethylated	-0.016461	0.24718	insignificant	4	8	8
chr3	57538987	57540987	Rnf13	-0.15064	7.19E-08	hypomethylated	-0.0013142	0.61537	insignificant	12	32	32
chr3	57651679	57653679	Pfn2	-0.11292	3.92E-11	hypomethylated	-0.026007	0.057244	insignificant	26	52	52
chr3	58218610	58220610	Tsc22d2	-0.094502	1.01E-64	hypomethylated	0.0044246	0.89755	insignificant	104	325	330
chr3	58328742	58330742	Eif2a	-0.14131	9.73E-28	hypomethylated	0.010771	0.035144	inconclusive	45	128	132
chr3	58329806	58331806	Eif2a	-0.11844	2.1E-10	hypomethylated	0.041503	0.36102	insignificant	16	43	47
chr3	58379579	58381579	2810407C02Rik	-0.12965	6.57E-18	hypomethylated	0.022723	0.32268	insignificant	37	106	107
chr3	58496310	58498310	Siah2	-0.27292	1.63E-09	hypomethylated	-0.029733	0.3423	insignificant	14	40	40
chr3	58809899	58811899	Med12l	-0.09456	5.23E-55	hypomethylated	0.0090962	0.28546	insignificant	60	189	196
chr3	58934546	58936546	Med12l	-0.43981	0.082215	insignificant	-0.10053	0.54746	insignificant	1	4	2
chr3	59066753	59068753	P2ry12	-0.083974	0.57049	insignificant	0.051404	0.19298	insignificant	2	8	8
chr3	60304173	60306173	Mbnl1	-0.10664	2.04E-29	hypomethylated	0.006615	0.52057	insignificant	46	133	141
chr3	60805716	60807716	P2ry1	-0.13343	3.46E-26	hypomethylated	0.0087942	0.31977	insignificant	18	80	80
chr3	61167428	61169428	Rap2b	-0.13073	1.71E-31	hypomethylated	-0.015985	0.25962	insignificant	27	96	97
chr3	62141698	62143698	Arhgef26	-0.12924	2.83E-14	hypomethylated	-0.0039827	0.17316	insignificant	33	121	127
chr3	62310910	62312910	Dhx36	-0.15239	7.5E-12	hypomethylated	-0.015988	0.46989	insignificant	8	34	34
chr3	63098793	63100793	Mme	-0.14232	3.35E-08	hypomethylated	0.061143	0.83982	insignificant	10	41	36
chr3	63733307	63735307	E130311K13Rik	-0.24418	5.13E-15	hypomethylated	0.010434	0.00000117	inconclusive	7	18	18
chr3	63768655	63770655	Slc33a1	-0.061111	0.046843	inconclusive	-0.0031469	0.01199	hypomethylated	8	22	22
chr3	63779064	63781064	Gmps	-0.22221	2E-35	hypomethylated	0.0088022	0.20067	insignificant	36	93	88
chr3	64311607	64313607	Vmn2r5		1	noCoverage	-0.059325	0.64717	insignificant	0	2	4
chr3	64912564	64914564	Kcnab1	-0.23529	0.3138	insignificant	-0.16221	0.5103	insignificant	4	26	23
chr3	65196475	65198475	Ssr3		1	noCoverage	-0.0040206	0.49954	insignificant	0	31	31
chr3	65331368	65333368	Tiparp	-0.087606	1.13E-25	hypomethylated	0.006266	0.40766	insignificant	74	251	271
chr3	65333336	65335336	Tiparp	-0.080156	0.014465	hypomethylated	0.015272	0.26796	insignificant	6	12	12
chr3	65469149	65471149	Lekr1	-0.099551	7.46E-15	hypomethylated	-0.0076885	0.65394	insignificant	24	87	88
chr3	65469156	65471156	Lekr1	-0.099551	7.46E-15	hypomethylated	-0.0076885	0.65394	insignificant	24	87	88
chr3	65762147	65764147	Ccnl1	-0.19089	1.12E-27	hypomethylated	-0.016127	0.046313	hypomethylated	26	83	83
chr3	66100741	66102741	Veph1	-0.59559	0.053374	insignificant	-0.049077	0.73336	insignificant	1	2	2
chr3	66785693	66787693	Shox2	-0.23094	4.54E-08	hypomethylated	-0.014131	1	insignificant	6	41	37
chr3	66788593	66790593	Rsrc1	-0.14768	0.097766	insignificant	-0.018564	1	insignificant	2	70	68
chr3	67177018	67179018	Mlf1	-0.10519	1.48E-09	hypomethylated	-0.034935	0.48605	insignificant	22	54	58
chr3	67233036	67235036	Gfm1	-0.18095	9.54E-11	hypomethylated	0.0012931	0.347	insignificant	6	37	44
chr3	67267829	67269829	Gfm1	-0.24286	0.00000108	hypomethylated	0.044258	1	insignificant	5	10	14
chr3	67319445	67321445	Rarres1	-0.15015	0.000000156	hypomethylated	-0.016577	0.82925	insignificant	4	14	14
chr3	67385689	67387689	Mfsd1	-0.20168	3.53E-10	hypomethylated	0.015465	0.042745	hypermethylated	9	27	27
chr3	67695141	67697141	Iqcj	-0.02757	0.48458	insignificant	-0.053715	0.14394	insignificant	11	24	24
chr3	67867723	67869723	Schip1		1	noCoverage	0.047619	0.13605	insignificant	0	16	16
chr3	68297114	68299114	Schip1	-0.15674	4.58E-42	hypomethylated	-0.010186	0.030075	hypomethylated	44	108	108
chr3	68493565	68495565	Il12a	-0.070023	0.47402	insignificant	-0.013773	0.94482	insignificant	8	31	33
chr3	68494345	68496345	Il12a	-0.054158	0.47378	insignificant	-0.026541	0.88906	insignificant	8	29	31
chr3	68672507	68674507	1110032F04Rik	-0.10124	4.98E-29	hypomethylated	-0.007657	0.099426	insignificant	48	145	148
chr3	68807893	68809893	Smc4	-0.13468	9.77E-14	hypomethylated	0.037088	0.25392	insignificant	20	83	76

chr3	68808492	68810492	Smc4	-0.12009	5.65E-18	hypomethylated	0.017286	0.22554	insignificant	25	95	88
chr3	68848664	68850664	Trim59	-0.12753	0.0090506	hypomethylated	0.00000626	0.055191	insignificant	6	71	72
chr3	68931014	68933014	Kpna4	-0.19883	0.00039	hypomethylated	-0.025292	0.31165	insignificant	9	56	56
chr3	69025340	69027340	Arl14	-0.083598	0.028124	hypomethylated	0.16273	0.0029782	hypermethylated	4	8	8
chr3	69119839	69121839	Ppm1l	-0.11278	2.71E-29	hypomethylated	-0.0015183	0.90184	insignificant	71	224	235
chr3	69402783	69404783	B3galnt1	-0.23125	0.0011685	hypomethylated	0.022444	0.94942	insignificant	2	22	22
chr3	69524976	69526976	Nmd3	-0.14426	2.28E-31	hypomethylated	-0.0059802	0.27401	insignificant	42	124	124
chr3	69663818	69665818	1110032A04Rik	-0.20763	0.00000147	hypomethylated	-0.035742	0.28288	insignificant	7	43	43
chr3	72860865	72862865	Slitrk3	-0.096689	0.023755	hypomethylated	-0.0066392	0.023747	inconclusive	7	42	43
chr3	73512337	73514337	Bche		1	noCoverage	-0.051932	0.057952	insignificant	0	3	6
chr3	75360454	75362454	Serpini1	-0.20488	1.1E-12	hypomethylated	0.015592	0.16964	insignificant	16	52	52
chr3	75360721	75362721	Pdcd10	-0.36601	1.05E-13	stronglyHypometh	0.069962	0.030516	inconclusive	6	18	18
chr3	75760753	75762753	Golim4	-0.090972	0.22849	insignificant	-0.035848	0.90483	insignificant	4	18	26
chr3	75877519	75879519	Fstl5	-0.10627	0.36142	insignificant	0.044241	0.66539	insignificant	2	37	33
chr3	79148297	79150297	Gm17359	-0.17678	0.000058135	hypomethylated	-0.074346	0.0020366	hypomethylated	4	12	12
chr3	79371601	79373601	Fnip2	-0.18895	1.42E-18	hypomethylated	0.057952	0.00000534	inconclusive	30	79	93
chr3	79394310	79396310	Ppid	-0.11143	1.76E-22	hypomethylated	-0.0078382	0.34409	insignificant	28	82	84
chr3	79432000	79434000	4930579G24Rik	-0.21678	0.000002112	hypomethylated	-0.021407	0.63593	insignificant	7	38	40
chr3	79432689	79434689	Etfdh	-0.37899	0.00071211	stronglyHypometh	-0.003665	0.28607	insignificant	5	14	14
chr3	79646584	79648584	Tmem144	-0.018797	0.0045822	hypomethylated	0.040417	0.058694	insignificant	2	22	18
chr3	79688851	79690851	Fam198b	-0.37045	0.0035736	stronglyHypometh	-0.018059	0.91008	insignificant	3	6	6
chr3	80606713	80608713	Gria2	-0.13124	0.0061822	hypomethylated	-0.065675	0.013309	hypomethylated	11	45	40
chr3	80839337	80841337	Pdgfc	-0.15714	9.45E-09	hypomethylated	-0.037628	0.32089	insignificant	10	60	65
chr3	81735537	81737537	Ctso	-0.2401	1.41E-09	hypomethylated	0.0025301	0.00024136	inconclusive	10	28	28
chr3	82160993	82162993	Mtap9	-0.14099	1.94E-18	hypomethylated	0.00031915	0.90485	insignificant	32	75	76
chr3	82680405	82682405	Rbm46	-0.29731	1	insignificant	-0.047175	0.017786	hypomethylated	2	8	8
chr3	82707896	82709896	4930564K09Rik	-0.43418	3.27E-11	stronglyHypometh	0.091406	0.0019331	inconclusive	3	28	32
chr3	82853712	82855712	Fgb	0.18626	1	insignificant	0.073252	0.73546	insignificant	1	11	10
chr3	82858459	82860459	Plrg1	-0.2246	0.00000138	hypomethylated	0.024079	0.00032425	inconclusive	9	66	69
chr3	83569242	83571242	Sfrp2	-0.14005	3.58E-31	hypomethylated	-0.00026327	0.68247	insignificant	38	144	144
chr3	83645530	83647530	Tlr2	-0.28947	0.5491	insignificant	0.010526	0.44635	insignificant	2	4	4
chr3	83844083	83846083	D930015E06Rik	-0.10341	9.32E-14	hypomethylated	-0.012857	0.27163	insignificant	12	58	58
chr3	84024799	84026799	Trim2	-0.03142	0.64186	insignificant	-0.0015254	0.0015359	inconclusive	1	15	17
chr3	84284351	84286351	Fhdc1	-0.11198	0.000027524	hypomethylated	0.013841	0.022802	inconclusive	13	45	38
chr3	84386547	84388547	Arfip1	-0.14968	6.41E-12	hypomethylated	0.001337	0.001439	inconclusive	28	72	75
chr3	84469113	84471113	Tmem154	-0.20271	0.26032	insignificant	0.00079265	0.13272	insignificant	10	20	20
chr3	84618498	84620498	Fbxw7	-0.129	1.95E-33	hypomethylated	-0.010595	0.17103	insignificant	39	143	154
chr3	84755132	84757132	Fbxw7	-0.16422	0.36699	insignificant	0.014356	1	insignificant	4	8	8
chr3	85377050	85379050	Pet112l	-0.16483	0.00000461	hypomethylated	-0.012505	0.57139	insignificant	6	14	14
chr3	85550131	85552131	Fam160a1	-0.087829	0.39704	insignificant	-0.064752	1	insignificant	2	4	5
chr3	85691440	85693440	Glt28d2	-0.11785	1	insignificant	0.024376	0.6986	insignificant	3	21	16
chr3	85806413	85808413	Prss48	0.11261	0.80176	insignificant	0.071147	0.85369	insignificant	10	41	42
chr3	85887355	85889355	Sh3d19	-0.052535	0.00040416	hypomethylated	-0.036684	0.51243	insignificant	8	37	37
chr3	85942780	85944780	Rnu73b	-0.19459	0.22027	insignificant	-0.18853	0.078281	insignificant	1	11	10
chr3	85944609	85946609	Rnu73b	-0.097528	3.32E-14	hypomethylated	-0.0097633	0.010091	hypomethylated	31	120	124
chr3	85946590	85948590	Rps3a	-0.66159	1.05E-10	stronglyHypometh	-0.10896	0.021609	hypomethylated	1	2	2
chr3	86027611	86029611	Lrba	-0.081995	1.41E-30	hypomethylated	0.0026991	0.14548	insignificant	54	203	198
chr3	86724806	86726806	Dclk2	-0.22846	0.75634	insignificant	0.031711	0.00015349	hypermethylated	10	37	38
chr3	86789507	86791507	Cd1d2	0.218	1	insignificant	-0.16264	1	insignificant	1	7	12
chr3	86803262	86805262	Cd1d1	-0.13554	0.0082738	hypomethylated	0.059461	0.052156	insignificant	2	20	20
chr3	86978669	86980669	Kirrel	-0.40603	0.26372	insignificant	0.061058	0.55182	insignificant	1	18	19
chr3	87328499	87330499	Etv3	-0.12323	0.000000884	hypomethylated	-0.0069828	0.056812	insignificant	16	113	114
chr3	87421672	87423672	Arhgef11		1	noCoverage	0.051749	0.61678	insignificant	0	6	7
chr3	87572875	87574875	Pear1	-0.31096	7.53E-09	hypomethylated	0.088945	9.36E-15	inconclusive	7	26	22
chr3	87599084	87601084	Insrr	-0.1957	2.16E-10	hypomethylated	0.014109	0.88491	insignificant	10	53	53
chr3	87599872	87601872	Insrr	-0.25306	0.1126	insignificant	0.015757	0.70486	insignificant	4	27	27
chr3	87689484	87691484	Prcc	-0.45961	1	lowCoverage	0.037832	0.24519	insignificant	1	16	15
chr3	87709242	87711242	Hdgf	-0.12379	5.19E-26	hypomethylated	-0.019587	0.37627	insignificant	42	126	139
chr3	87722465	87724465	Mrpl24		1	noCoverage	0.37949	0.061341	insignificant	0	13	2
chr3	87733235	87735235	Isg20l2	-0.16734	0.14941	insignificant	-0.051266	0.37616	insignificant	7	62	79
chr3	87734117	87736117	Rrnad1	-0.11363	0.000009053	inconclusive	-0.10284	0.0065089	inconclusive	9	47	65
chr3	87774014	87776014	Nes	-0.12767	5.71E-23	hypomethylated	-0.013714	0.65343	insignificant	36	120	124
chr3	87804278	87806278	Bcan	-0.26669	0.000014266	hypomethylated	0.0017518	0.0098384	inconclusive	3	31	32
chr3	87846029	87848029	Gpatch4	-0.20049	0.030945	hypomethylated	-0.021094	0.12383	insignificant	11	64	66
chr3	87862417	87864417	Apoa1bp	-0.25544	0.042597	hypomethylated	-0.02496	0.21063	insignificant	4	42	46
chr3	87884972	87886972	Iqgap3	-0.13486	0.15536	insignificant	-0.0049726	0.883	insignificant	4	32	32
chr3	87945316	87947316	Mef2d	-0.095496	1.24E-20	hypomethylated	-0.0030399	0.22457	insignificant	47	182	182
chr3	88018092	88020092	Mir3093	-0.1428	0.00000178	hypomethylated	0.01446	0.82125	insignificant	13	94	87
chr3	88018519	88020519	Mir9-1	-0.14169	0.00357	hypomethylated	0.0080126	0.14984	insignificant	10	82	75
chr3	88058606	88060606	Rhbg	-0.29706	5.22E-17	hypomethylated	-0.0017524	0.90269	insignificant	8	16	16
chr3	88100056	88102056	Cct3	-0.17325	0.0070104	hypomethylated	-0.037191	0.91431	insignificant	3	21	18
chr3	88100760	88102760	Cct3	0.007504	0.00014024	inconclusive	0.013558	0.024821	inconclusive	4	25	22
chr3	88138355	88140355	Smg5	-0.10385	0.00000156	hypomethylated	-0.0049028	0.19474	insignificant	10	38	38
chr3	88139181	88141181	Smg5	-0.22293	2.31E-11	hypomethylated	0.0019831	0.80036	insignificant	18	86	86
chr3	88167510	88169510	Paqr6	0.035576	0.10639	insignificant	0.047679	0.000026981	hypermethylated	7	32	32
chr3	88173642	88175642	Bglap-rs1	-0.1444	0.088499	insignificant	0.019239	0.5199	insignificant	7	16	19
chr3	88214238	88216238	Pmf1	-0.13157	0.000063585	hypomethylated	0.070743	0.48694	insignificant	9	24	26
chr3	88229061	88231061	Slc25a44		1	noCoverage	-0.083341	0.029293	hypomethylated	0	10	10
chr3	88259683	88261683	Sema4a	0.45226	1	lowCoverage	0.077257	0.41171	insignificant	1	4	4
chr3	88262806	88264806	Sema4a	-0.033018	0.32343	insignificant	0.12532	1	insignificant	3	9	8
chr3	88263023	88265023	Sema4a	-0.033018	0.32343	insignificant	0.14476	0.86421	insignificant	3	9	9
chr3	88265104	88267104	Sema4a	-0.23514	0.035045	hypomethylated	-0.13247	0.045564	hypomethylated	3	10	10
chr3	88297221	88299221	Lmna	-0.47559	0.000000406	stronglyHypometh	0.0036428	0.4005	insignificant	2	10	9
chr3	88307254	88309254	Lmna		1	noCoverage	-0.021778	1	insignificant	0	9	13
chr3	88335316	88337316	Mex3a	-0.16954	0.00000011	hypomethylated	-0.04169	0.86007	insignificant	18	93	105

chr3	88340304	88342304	Mir1905	0.23701	0.018195	hypermethylated	0.059388	0.47543	insignificant	6	45	41
chr3	88352201	88354201	Rab25	-0.045273	0.081484	insignificant	0.030917	0.73315	insignificant	4	6	7
chr3	88356637	88358637	Ubqln4	-0.15879	3.73E-26	hypomethylated	-0.017241	0.8034	insignificant	30	108	111
chr3	88356849	88358849	Ubqln4	-0.15565	4.89E-23	hypomethylated	-0.0038528	0.83459	insignificant	31	107	105
chr3	88382592	88384592	Ssr2	-0.11488	0.000095992	hypomethylated	0.0072936	0.89257	insignificant	6	32	32
chr3	88419128	88421128	Arhgef2	0.1004	1	insignificant	-0.035331	0.59966	insignificant	7	17	21
chr3	88424027	88426027	Arhgef2	-0.12518	4.05E-24	hypomethylated	-0.014467	0.19178	insignificant	47	140	142
chr3	88424315	88426315	Arhgef2	-0.12518	4.05E-24	hypomethylated	-0.01525	0.19176	insignificant	47	140	143
chr3	88488715	88490715	2810403A07Rik	-0.14393	1.79E-10	hypomethylated	0.0013825	0.51585	insignificant	16	87	73
chr3	88519775	88521775	Rit1	-0.22235	3.18E-15	hypomethylated	-0.040395	0.23389	insignificant	14	39	37
chr3	88638149	88640149	Gon4l	-0.18031	0.23053	insignificant	0.018368	0.0022887	hypermethylated	2	52	58
chr3	88717872	88719872	Msto1		1	noCoverage	0.13398	0.032208	hypermethylated	0	14	14
chr3	88754204	88756204	Dap3	-0.1395	2.45E-55	hypomethylated	0.005368	0.027608	inconclusive	33	111	107
chr3	88768733	88770733	Ash1l		1	noCoverage	-0.066919	0.60643	insignificant	0	6	6
chr3	88893953	88895953	Rusc1	-0.1742	5.41E-38	hypomethylated	-0.0079416	0.03263	hypomethylated	30	114	108
chr3	88897285	88899285	Fdps	0.17786	1	lowCoverage	0.041414	0.50175	insignificant	1	16	13
chr3	88905867	88907867	Fdps	-0.38838	0.010108	stronglyHypometh	-0.014149	0.7918	insignificant	4	9	9
chr3	88964079	88966079	Hcn3	-0.28993	1.19E-08	hypomethylated	-0.13924	0.077542	insignificant	3	9	6
chr3	88967726	88969726	Clk2	-0.12077	8.83E-16	hypomethylated	-0.010177	0.93508	insignificant	34	90	94
chr3	88980406	88982406	Scamp3	-0.28362	6.14E-14	hypomethylated	0.0042006	0.13849	insignificant	7	23	28
chr3	88986146	88988146	Fam189b	-0.44548	0.0022834	stronglyHypometh	-0.087767	0.00064804	hypomethylated	2	12	12
chr3	89005849	89007849	Gba		1	noCoverage	0.26587	0.66575	insignificant	0	4	2
chr3	89005861	89007861	Gba		1	noCoverage	0.26587	0.66575	insignificant	0	4	2
chr3	89018108	89020108	Thbs3	-0.14601	0.094482	insignificant	-0.017441	0.77575	insignificant	8	45	45
chr3	89018257	89020257	Thbs3	-0.17132	0.38077	insignificant	-0.017315	0.59932	insignificant	6	30	30
chr3	89031120	89033120	Mir92b	-0.20071	0.0016733	hypomethylated	-0.041936	0.72666	insignificant	3	31	42
chr3	89031973	89033973	Muc1	-0.7134	0.09778	insignificant	-0.22319	1	insignificant	1	14	28
chr3	89049121	89051121	Trim46	-0.26271	2.83E-31	hypomethylated	-0.042442	0.10505	insignificant	19	55	62
chr3	89049359	89051359	Krtcap2	-0.26271	2.83E-31	hypomethylated	-0.013382	0.16529	insignificant	19	55	55
chr3	89049819	89051819	Trim46	-0.25709	6.03E-31	hypomethylated	-0.0099112	0.18381	insignificant	18	51	51
chr3	89069473	89071473	Dpm3	-0.27108	0.00008571	hypomethylated	0.016912	0.76908	insignificant	4	14	15
chr3	89074492	89076492	Slc50a1	-0.17516	0.22881	insignificant	0.0098321	0.26942	insignificant	4	14	14
chr3	89083567	89085567	Efna1	-0.14231	4.77E-08	hypomethylated	0.010883	0.83453	insignificant	11	74	73
chr3	89084873	89086873	Efna1	-0.16686	0.00015508	hypomethylated	0.0032247	0.30963	insignificant	11	59	58
chr3	89153932	89155932	Adam15	-0.27165	1.88E-16	hypomethylated	-0.033724	0.11779	insignificant	25	67	70
chr3	89169161	89171161	Dcst1	-0.34722	0.000000114	stronglyHypometh	-0.058234	0.27022	insignificant	2	16	16
chr3	89197125	89199125	Zbtb7b	-0.16317	8.18E-09	hypomethylated	-0.0093649	0.16366	insignificant	19	66	74
chr3	89215785	89217785	Flad1	-0.33374	7E-21	stronglyHypometh	-0.070767	0.00017374	hypomethylated	10	29	31
chr3	89221472	89223472	Shc1	-0.18257	3.94E-16	hypomethylated	-0.064405	0.61591	insignificant	14	70	76
chr3	89222213	89224213	Cks1b	-0.18817	0.000034887	hypomethylated	-0.032696	0.53473	insignificant	5	38	38
chr3	89224542	89226542	Shc1	-0.048568	0.047943	hypomethylated	0.053268	0.18613	insignificant	5	14	14
chr3	89233758	89235758	Pygo2	-0.097498	4.39E-11	hypomethylated	-0.006101	0.068802	insignificant	24	78	78
chr3	89239625	89241625	Pbxip1	-0.27919	0.056953	insignificant	-0.040533	0.90535	insignificant	4	12	12
chr3	89262039	89264039	Pmvk		1	noCoverage	-0.00026385	0.23813	insignificant	0	32	31
chr3	89262492	89264492	Pmvk		1	noCoverage	0.0083383	1	insignificant	0	22	21
chr3	89323085	89325085	Kcnn3	-0.22075	0.12158	insignificant	-0.035543	0.65533	insignificant	10	37	36
chr3	89517943	89519943	Adar	-0.2	0.000044352	hypomethylated	-0.0054431	0.45882	insignificant	10	50	45
chr3	89533639	89535639	Adar	-0.25666	0.18919	insignificant	-0.10126	0.65039	insignificant	4	14	18
chr3	89568554	89570554	Chrnb2	-0.4	0.31391	insignificant	0.13671	0.46272	insignificant	1	3	4
chr3	89576530	89578530	Ube2q1	-0.11809	6.24E-57	hypomethylated	-0.004585	0.66475	insignificant	46	142	154
chr3	89634291	89636291	She	-0.14532	7.85E-47	hypomethylated	0.0029999	0.050963	insignificant	79	217	226
chr3	89717084	89719084	Il6ra	-0.21704	7.22E-29	hypomethylated	0.015655	0.0011495	inconclusive	19	65	65
chr3	89767430	89769430	Atp8b2		1	noCoverage	0.12233	1	insignificant	0	16	10
chr3	89802608	89804608	Hax1	-0.31325	1.41E-14	hypomethylated	0.069665	0.22417	insignificant	7	26	22
chr3	89855738	89857738	4933434E20Rik	-0.13674	0.00000198	hypomethylated	0.00032417	0.055322	insignificant	17	101	99
chr3	89856397	89858397	Ubap2l	-0.13561	0.085347	insignificant	-0.0050308	0.011479	hypomethylated	9	38	38
chr3	89856437	89858437	Ubap2l	-0.13101	0.054175	insignificant	-0.0053103	0.11906	insignificant	8	36	36
chr3	89872941	89874941	Mir190b	-0.082262	0.32614	insignificant	-0.11044	0.081545	insignificant	5	14	14
chr3	89907053	89909053	Nup210l	0.021606	0.70298	insignificant	-0.062265	0.44554	insignificant	5	19	22
chr3	90017569	90019569	Gm9846		1	noCoverage	-0.019464	0.32444	insignificant	0	18	18
chr3	90017570	90019570	Gm9846		1	noCoverage	-0.019464	0.32444	insignificant	0	18	18
chr3	90023738	90025738	Rab13	-0.088964	0.46665	insignificant	-0.023329	0.15491	insignificant	9	38	38
chr3	90034518	90036518	Jtb	-0.11005	7.86E-16	hypomethylated	-0.0021234	0.0047453	hypomethylated	23	76	82
chr3	90047434	90049434	Creb3l4	-0.21308	0.070326	insignificant	0.074896	0.12359	insignificant	2	28	28
chr3	90051113	90053113	Slc39a1	-0.19008	0.000000536	hypomethylated	-0.021588	0.88999	insignificant	6	31	44
chr3	90057202	90059202	Crtc2	-0.1188	7.89E-13	hypomethylated	-0.01569	0.55664	insignificant	26	100	97
chr3	90069455	90071455	Dennd4b	-0.025344	0.20833	insignificant	0.032847	0.00026461	hypermethylated	14	65	66
chr3	90193849	90195849	Slc27a3	-0.1903	0.00032009	hypomethylated	0.0073578	0.91224	insignificant	10	18	18
chr3	90237558	90239558	Ints3	-0.35762	0.5235	insignificant	-0.048712	0.026335	hypomethylated	4	31	34
chr3	90269788	90271788	Npr1	-0.34607	0.00076288	stronglyHypometh	-0.049637	0.78132	insignificant	3	23	23
chr3	90279122	90281122	Ilf2	-0.31649	0.000000962	hypomethylated	-0.0098779	0.13473	insignificant	6	42	41
chr3	90294935	90296935	Snapin	-0.25451	0.0027877	hypomethylated	0.031564	0.39106	insignificant	2	20	22
chr3	90317681	90319681	S100a13	-0.23338	0.00000284	hypomethylated	-0.021597	0.043029	inconclusive	8	32	32
chr3	90318252	90320252	S100a13	-0.29448	8.98E-08	hypomethylated	-0.10615	0.92162	insignificant	4	14	17
chr3	90471992	90473992	S100a8	-0.21364	0.35977	insignificant	0.070094	0.50311	insignificant	1	4	4
chr3	92053568	92055568	Sprr2a1		1	noCoverage	0.014805	0.29905	insignificant	0	6	4
chr3	92119626	92121626	Sprr2b		1	noCoverage	-0.016667	1	insignificant	0	4	4
chr3	92819608	92821608	Crct1	-0.86364	0.056452	insignificant	-0.37197	0.3971	insignificant	1	4	4
chr3	92876442	92878442	Gm4858		1	noCoverage	-0.12083	0.45643	insignificant	0	4	4
chr3	93000194	93002194	Flg2	0.066667	1	lowCoverage	-0.047148	0.42588	insignificant	1	6	7
chr3	93196620	93198620	Rptn		1	noCoverage	0.0017045	1	insignificant	0	6	6
chr3	93323417	93325417	S100a11	-0.2018	2.66E-09	hypomethylated	-0.030251	0.71631	insignificant	8	24	29
chr3	93599319	93601319	Tdpoz4		1	noCoverage	-0.25556	0.00052757	hypomethylated	0	4	6
chr3	94113053	94115053	Them4	-0.077028	0.57372	insignificant	-0.074012	0.078917	insignificant	10	51	48
chr3	94165365	94167365	C2cd4d	-0.02617	0.00000338	hypomethylated	-0.10908	0.12171	insignificant	5	16	16

chr3	94202140	94204140	Lingo4	-0.17182	0.00019722	hypomethylated	0.0022675	0.56265	insignificant	2	32	32
chr3	94216239	94218239	Tdrkh	-0.26791	0.000001639	hypomethylated	0.0049599	0.9503	insignificant	7	14	14
chr3	94246257	94248257	Mrpl9	-0.16288	2.71E-08	hypomethylated	-0.013457	0.8581	insignificant	13	39	39
chr3	94281752	94283752	Celf3	-0.22206	0.0021722	hypomethylated	0.044885	0.54621	insignificant	6	24	28
chr3	94386624	94388624	Snx27		1	noCoverage	0.1575	0.86747	insignificant	0	6	7
chr3	94590437	94592437	Cgn	-0.18414	0.000000503	hypomethylated	-0.020673	0.62717	insignificant	7	24	25
chr3	94640488	94642488	Pogz	-0.10401	4.95E-19	hypomethylated	-0.0010918	0.84342	insignificant	33	98	104
chr3	94690880	94692880	Psmb4	-0.50121	0.000027161	stronglyHypometh	0.011366	0.29453	insignificant	1	13	14
chr3	94757936	94759936	Rfx5	-0.18846	0.36233	insignificant	0.11367	0.17769	insignificant	5	22	25
chr3	94777652	94779652	Pi4kb	-0.1175	2.11E-25	hypomethylated	-0.0030219	0.001107	hypomethylated	26	85	80
chr3	94819160	94821160	Zfp687	-0.09426	3.41E-12	hypomethylated	0.00096369	0.57725	insignificant	9	96	96
chr3	94846467	94848467	Psmd4	-0.35805	0.0082877	stronglyHypometh	0.017843	0.65573	insignificant	5	18	17
chr3	94910780	94912780	Pip5k1a	-0.1911	2.52E-09	hypomethylated	-0.046026	0.00095966	hypomethylated	5	16	16
chr3	94913963	94915963	Vps72	-0.26982	0.0012136	hypomethylated	-0.0052143	0.0030545	hypomethylated	7	67	74
chr3	94937009	94939009	Lysmd1	0.041757	1	insignificant	-0.037962	0.15571	insignificant	2	19	11
chr3	94937934	94939934	Scnm1	-0.4177	0.00012711	stronglyHypometh	-0.043242	0.36907	insignificant	3	14	14
chr3	94946282	94948282	Tnfaip8l2	-0.14643	0.58849	insignificant	-0.034013	0.0073563	hypomethylated	3	15	15
chr3	95021864	95023864	Gabpb2	-0.12584	0.00084414	hypomethylated	-0.02123	0.31677	insignificant	6	32	32
chr3	95031701	95033701	Cdc42se1	-0.19197	2.07E-18	hypomethylated	-0.010634	0.80295	insignificant	31	80	79
chr3	95031873	95033873	Cdc42se1	-0.19197	2.07E-18	hypomethylated	-0.010634	0.80295	insignificant	31	80	79
chr3	95032599	95034599	Cdc42se1	-0.20302	2.6E-18	hypomethylated	-0.010351	0.57448	insignificant	25	64	63
chr3	95045031	95047031	Gm128	0.038866	1	insignificant	-0.049872	0.56049	insignificant	2	18	11
chr3	95085856	95087856	Fam63a	-0.13041	0.000000444	hypomethylated	0.018338	0.64127	insignificant	23	70	71
chr3	95085998	95087998	Fam63a	-0.13041	0.000000444	hypomethylated	0.018338	0.64127	insignificant	23	70	71
chr3	95111098	95113098	Anxa9	0.10513	1	insignificant	-0.11089	0.13464	insignificant	3	10	11
chr3	95118173	95120173	Lass2	-0.17342	9.23E-29	hypomethylated	-0.032561	0.20134	insignificant	31	93	99
chr3	95161124	95163124	Setdb1	-0.52069	0.0093236	stronglyHypometh	-0.019375	0.034083	hypomethylated	4	21	21
chr3	95230270	95232270	Gm4349	0.081452	0.21195	insignificant	0.038185	0.00000076	hypermethylated	3	26	21
chr3	95237311	95239311	Arnt	-0.079546	0.055074	insignificant	0.027644	0.95866	insignificant	4	32	34
chr3	95362598	95364598	Hormad1	-0.15848	0.071232	insignificant	0.017329	0.22656	insignificant	18	50	50
chr3	95427901	95429901	Ensa	-0.22951	0.00028207	hypomethylated	-0.013752	0.039524	inconclusive	21	78	78
chr3	95461642	95463642	Mcl1	-0.084242	4.93E-36	hypomethylated	-0.0012753	0.2465	insignificant	79	235	226
chr3	95491781	95493781	Adamtsl4	-0.15999	6.86E-13	hypomethylated	-0.0042834	0.24308	insignificant	16	50	50
chr3	95558900	95560900	Tars2	0.31111	0.63453	insignificant	-0.0094017	0.64848	insignificant	4	6	6
chr3	95622876	95624876	Rprd2	-0.18216	0.14663	insignificant	0.0055574	0.90683	insignificant	8	48	48
chr3	95659676	95661676	Prpf3	-0.10359	3.34E-09	hypomethylated	-0.0089853	0.63174	insignificant	18	64	67
chr3	95674542	95676542	Mrps21	-0.31157	0.000056784	hypomethylated	0.0097593	0.84617	insignificant	2	24	24
chr3	95686151	95688151	C920021L13Rik	-0.17793	0.000003906	hypomethylated	-0.020195	0.11814	insignificant	19	47	44
chr3	95696918	95698918	Aph1a	-0.19336	9.32E-18	hypomethylated	-0.019661	0.00043645	hypomethylated	11	38	39
chr3	95696977	95698977	Aph1a	-0.19336	9.32E-18	hypomethylated	-0.019661	0.00043645	hypomethylated	11	38	39
chr3	95708562	95710562	Car14	-0.30883	4.76E-09	hypomethylated	-0.0072692	0.75659	insignificant	2	14	14
chr3	95732179	95734179	Anp32e	-0.093764	2.1E-13	hypomethylated	-0.0018164	0.6501	insignificant	46	146	142
chr3	95799762	95801762	Plekho1	-0.10473	5.74E-23	hypomethylated	-0.0019658	0.3587	insignificant	45	111	112
chr3	95862378	95864378	Vps45	-0.41563	0.00000902	stronglyHypometh	-0.10382	0.73223	insignificant	2	8	7
chr3	95907449	95909449	Otud7b	-0.12562	1.37E-23	hypomethylated	-0.0059227	0.096153	insignificant	40	92	100
chr3	95964892	95966892	Mtmr11	-0.1697	0.40441	insignificant	-0.043088	0.64765	insignificant	1	10	9
chr3	95975472	95977472	Sf3b4	-0.14486	1.77E-08	hypomethylated	0.015211	0.75944	insignificant	15	53	54
chr3	95984149	95986149	Sv2a	-0.24504	2.12E-17	hypomethylated	-0.023545	0.57266	insignificant	10	26	26
chr3	96001509	96003509	Bola1	-0.14874	5.7E-11	hypomethylated	-0.011452	0.063815	insignificant	7	28	28
chr3	96022838	96024838	Hist2h2ab	-0.23238	0.0049122	hypomethylated	-0.037848	0.87422	insignificant	2	18	20
chr3	96024043	96026043	Hist2h2be	-0.2267	0.0029047	hypomethylated	-0.054581	0.23337	insignificant	6	26	30
chr3	96024767	96026767	Hist2h2ac	-0.26459	0.00034173	hypomethylated	0.0055768	0.21308	insignificant	8	34	38
chr3	96042999	96044999	Hist2h3c2-ps	0.14903	0.17408	insignificant	-0.047267	0.0012011	hypomethylated	3	43	43
chr3	96043050	96045050	Hist2h3c2-ps	0.2693	0.00000234	hypermethylated	-0.048276	0.011213	hypomethylated	5	41	41
chr3	96044173	96046173	Hist2h2aa2	0.15924	0.00000517	hypermethylated	-0.075157	0.30415	insignificant	5	39	39
chr3	96044216	96046216	Hist2h2aa2	0.15924	0.00000517	hypermethylated	-0.075157	0.30415	insignificant	5	39	39
chr3	96048460	96050460	Hist2h2aa1	-0.21373	0.00000199	hypomethylated	-0.018147	0.42839	insignificant	9	31	31
chr3	96048503	96050503	Hist2h2aa2	-0.21373	0.00000199	hypomethylated	-0.018147	0.42839	insignificant	9	31	31
chr3	96049673	96051673	Hist2h2aa1		1	noCoverage	0.013889	0.80673	insignificant	0	12	12
chr3	96067240	96069240	Hist2h4	-0.21664	0.000224	hypomethylated	-0.0031518	0.57025	insignificant	6	36	36
chr3	96071616	96073616	Hist2h3b	0.26851	0.088023	insignificant	0.025042	0.86647	insignificant	2	31	43
chr3	96072622	96074622	Hist2h2bb	-0.1827	0.60972	insignificant	-0.011925	0.038701	hypomethylated	7	52	61
chr3	96218756	96220756	Terc		1	noCoverage	-0.030051	0.88394	insignificant	0	6	6
chr3	96328107	96330107	Hfe2	-0.19309	0.043848	hypomethylated	-0.026573	0.008893	hypomethylated	3	22	23
chr3	96360879	96362879	Txnip	-0.14158	0.00092232	hypomethylated	0.024329	0.96408	insignificant	16	77	75
chr3	96398081	96400081	Polr3gl		1	noCoverage	-0.03859	0.72221	insignificant	0	15	21
chr3	96399558	96401558	Ankrd34a	-0.21155	1.77E-09	hypomethylated	-0.042214	0.72092	insignificant	12	37	37
chr3	96404055	96406055	Lix1l	-0.13811	1.1E-29	hypomethylated	-0.020677	0.00000703	hypomethylated	19	72	66
chr3	96432850	96434850	Rbm8a	0.15886	0.40589	insignificant	-0.0039672	0.79571	insignificant	5	39	38
chr3	96433742	96435742	6330549D23Rik		1	noCoverage	0.0079365	0.68979	insignificant	0	14	14
chr3	96438279	96440279	Pex11b	-0.11539	1.76E-10	hypomethylated	-0.0071468	0.21433	insignificant	21	80	89
chr3	96438352	96440352	Pex11b	-0.11539	1.76E-10	hypomethylated	-0.0071468	0.21433	insignificant	21	80	89
chr3	96438769	96440769	Pex11b	-0.16967	6.29E-14	hypomethylated	-0.011286	0.0014791	hypomethylated	21	88	97
chr3	96448506	96450506	Itga10	0.14145	0.18251	insignificant	0.016447	1	insignificant	2	4	4
chr3	96473053	96475053	Ankrd35	0.39028	0.11084	insignificant	-0.14167	0.0018614	hypomethylated	2	16	21
chr3	96499297	96501297	Pias3	-0.16054	9.15E-27	hypomethylated	-0.00036992	0.71026	insignificant	29	93	98
chr3	96499998	96501998	Pias3	-0.12996	2.25E-26	hypomethylated	-0.017685	0.58962	insignificant	32	114	120
chr3	96512483	96514483	Nudt17	-0.13985	0.00026617	hypomethylated	0.0014452	0.38838	insignificant	5	10	10
chr3	96530533	96532533	Rnf115	-0.11666	2.16E-11	hypomethylated	0.0091308	0.03261	inconclusive	22	84	99
chr3	96531362	96533362	Rnf115	-0.086294	1.85E-08	hypomethylated	0.021674	0.53234	insignificant	21	72	87
chr3	96835338	96837338	Gja5	-0.15474	0.015004	hypomethylated	0.16339	0.29579	insignificant	4	13	13
chr3	96961699	96963699	Acp6	-0.14895	1.14E-13	hypomethylated	0.038316	0.85688	insignificant	7	61	44
chr3	97414113	97416113	Chd1l	0.13816	0.21376	insignificant	-0.0070802	1	insignificant	2	8	8
chr3	97461134	97463134	Prkab2	-0.14489	4E-37	hypomethylated	-0.014338	0.12379	insignificant	31	97	104
chr3	97571937	97573937	Pde4dip	-0.068879	0.38391	insignificant	-0.02794	0.41386	insignificant	7	18	18

chr3	97672235	97674235	Pde4dip	-0.36362	0.45294	insignificant	0.031304	0.44794	insignificant	4	12	16
chr3	97692630	97694630	Pde4dip		1	noCoverage	-0.027506	0.91083	insignificant	0	4	4
chr3	97704149	97706149	Sec22b	-0.16828	4.78E-16	hypomethylated	0.017577	0.96346	insignificant	12	52	52
chr3	97816460	97818460	Notch2	-0.089783	5.83E-18	hypomethylated	0.0058462	0.5293	insignificant	29	120	120
chr3	98143892	98145892	Phgdh	-0.28643	0.000066102	hypomethylated	-0.09091	0.11132	insignificant	3	14	14
chr3	98185403	98187403	Zfp697	-0.24706	0.0083837	hypomethylated	-0.028011	0.54011	insignificant	3	10	10
chr3	98944012	98946012	Wars2	-0.13361	2.19E-10	hypomethylated	-0.022096	0.79626	insignificant	13	61	71
chr3	99056682	99058682	Tbx15	-0.088011	3.3E-11	hypomethylated	-0.0037934	0.31257	insignificant	20	115	119
chr3	99688339	99690339	Spag17	-0.31358	0.0053019	hypomethylated	0.00074284	0.54294	insignificant	9	30	30
chr3	99965440	99967440	Gdap2	-0.16909	1.54E-17	hypomethylated	-0.0050026	0.4498	insignificant	35	112	121
chr3	99966326	99968326	Wdr3	-0.21437	7.59E-15	hypomethylated	-0.014226	0.07468	insignificant	22	72	81
chr3	100293115	100295115	Fam46c	-0.17057	2.85E-15	hypomethylated	-0.037598	0.0022754	hypomethylated	30	56	64
chr3	100489396	100491396	Man1a2	-0.1081	2.12E-25	hypomethylated	-0.0046303	0.063348	insignificant	40	116	129
chr3	100725415	100727415	Trim45	-0.16697	1.6E-21	hypomethylated	-0.021997	0.16158	insignificant	14	55	57
chr3	100773586	100775586	Ttf2	-0.22778	0.00000128	hypomethylated	-0.068729	0.072456	insignificant	5	21	21
chr3	100914089	100916089	Ptgfrn	-0.15521	1.91E-24	hypomethylated	-0.026503	0.0016694	hypomethylated	30	89	87
chr3	101091862	101093862	Cd2	0.027941	1	insignificant	0.04189	0.018742	hypermethylated	2	10	10
chr3	101180047	101182047	Igsf3	-0.13814	1.18E-20	hypomethylated	-0.0098648	0.078075	insignificant	43	115	119
chr3	101408580	101410580	Atp1a1	-0.18582	8.53E-23	hypomethylated	-0.0064748	0.42409	insignificant	15	41	41
chr3	101728376	101730376	Slc22a15	-0.11098	0.59439	insignificant	0.0031153	0.83605	insignificant	2	40	40
chr3	101889432	101891432	Casq2	-0.22889	0.00000125	hypomethylated	-0.12826	0.038596	hypomethylated	4	11	12
chr3	102007886	102009886	Vangl1	-0.13336	5.12E-13	hypomethylated	-0.009051	0.17149	insignificant	42	116	116
chr3	102008616	102010616	Vangl1	-0.14216	0.72629	insignificant	-0.019096	0.084058	insignificant	9	18	18
chr3	102272850	102274850	Ngf	-0.16446	0.0076488	hypomethylated	0.0064791	0.33993	insignificant	8	30	30
chr3	102537692	102539692	Tspan2	-0.1782	2.46E-12	hypomethylated	-0.027555	0.39842	insignificant	19	80	77
chr3	102740023	102742023	Sycp1	-0.12484	0.083256	insignificant	-0.0012083	0.62795	insignificant	10	25	24
chr3	102798662	102800662	Sike1	-0.059327	0.18138	insignificant	0.022172	0.59716	insignificant	14	66	67
chr3	102823468	102825468	Csde1	-0.086518	5.25E-11	hypomethylated	-0.00029603	0.68673	insignificant	21	104	101
chr3	102861207	102863207	Nras	-0.098069	0.0039432	hypomethylated	0.010566	0.67392	insignificant	16	70	66
chr3	102876936	102878936	Ampd1	-0.32143	0.0021538	hypomethylated	0.14894	0.44235	insignificant	1	4	4
chr3	102974633	102976633	Bcas2	-0.11778	0.016733	hypomethylated	-0.016721	0.71196	insignificant	14	68	64
chr3	103082215	103084215	Trim33	-0.073229	3.36E-10	hypomethylated	0.004315	0.91705	insignificant	30	135	138
chr3	103378203	103380203	Syt6	-0.19599	2.67E-27	hypomethylated	-0.0091542	0.034281	hypomethylated	26	67	70
chr3	103541924	103543924	Olfml3	-0.39286	0.00002315	stronglyHypometh	-0.1076	0.034101	hypomethylated	1	6	6
chr3	103595198	103597198	Hipk1	-0.29018	5.92E-38	hypomethylated	-0.027998	8.88E-13	hypomethylated	20	79	79
chr3	103612439	103614439	Ap4b1	-0.14431	8.79E-17	hypomethylated	-0.0061307	0.17392	insignificant	12	85	85
chr3	103613310	103615310	Dclre1b	-0.16393	5.16E-14	hypomethylated	-0.021236	0.14334	insignificant	8	56	56
chr3	103717042	103719042	Rsbn1	-0.10555	8.8E-33	hypomethylated	0.011819	0.70676	insignificant	33	128	127
chr3	103771032	103773032	Phtf1	-0.1472	2.85E-25	hypomethylated	-0.018088	0.02837	hypomethylated	28	78	78
chr3	103771180	103773180	Phtf1	-0.1472	2.85E-25	hypomethylated	-0.018088	0.02837	hypomethylated	28	78	78
chr3	103772239	103774239	Phtf1	-0.20916	4.52E-21	hypomethylated	-0.032155	0.01169	hypomethylated	19	58	55
chr3	104024329	104026329	Magi3	-0.38409	0.036612	stronglyHypometh	-0.010202	1	insignificant	4	22	20
chr3	104315779	104317779	Lrig2	-0.21211	1.1E-09	hypomethylated	-0.017933	0.56761	insignificant	8	31	31
chr3	104441590	104443590	Slc16a1	-0.10555	1.36E-56	hypomethylated	-0.011627	0.055489	insignificant	72	213	230
chr3	104582973	104584973	Ppm1j	-0.17435	9.79E-33	hypomethylated	-0.0094877	0.019626	hypomethylated	20	102	103
chr3	104590951	104592951	Rhoc	-0.11337	3.54E-14	hypomethylated	0.035091	0.017694	inconclusive	25	74	73
chr3	104621223	104623223	Mov10	-0.15909	1	insignificant	0.044996	0.17644	insignificant	1	11	11
chr3	104666998	104668998	Capza1	-0.15784	6.14E-35	hypomethylated	0.00091037	0.49508	insignificant	33	124	123
chr3	104667423	104669423	Capza1	-0.14176	7.59E-14	hypomethylated	0.0054695	0.34888	insignificant	24	95	93
chr3	104764627	104766627	Wnt2b	-0.11678	4.29E-31	hypomethylated	-0.0383	0.00034884	hypomethylated	34	95	108
chr3	104855871	104857871	Cttnbp2nl	-0.24047	6.94E-21	hypomethylated	-0.014467	0.12848	insignificant	13	44	42
chr3	104856064	104858064	Cttnbp2nl	-0.33581	8.08E-08	stronglyHypometh	0.0088371	0.074526	insignificant	6	24	22
chr3	105254247	105256247	Kcnd3	-0.12375	7.61E-32	hypomethylated	0.0018087	0.76089	insignificant	37	142	160
chr3	105260733	105262733	Kcnd3	-0.11671	0.15911	insignificant	-0.045562	1	insignificant	2	31	32
chr3	105490489	105492489	Ddx20	-0.13998	9.2E-13	hypomethylated	-0.0059301	0.04905	hypomethylated	7	24	22
chr3	105506516	105508516	6530418L21Rik	-0.16731	3.33E-17	hypomethylated	-0.008577	0.20335	insignificant	14	36	36
chr3	105507375	105509375	6530418L21Rik	-0.16731	3.33E-17	hypomethylated	-0.008577	0.20335	insignificant	14	36	36
chr3	105604254	105606254	Rap1a	-0.15855	0.000033448	hypomethylated	-0.011343	0.92832	insignificant	10	25	35
chr3	105706338	105708338	Adora3		1	noCoverage	0.12006	0.80702	insignificant	0	4	4
chr3	105761415	105763415	Wdr77	-0.14652	6.35E-40	hypomethylated	-0.011253	0.057351	insignificant	27	76	76
chr3	106284348	106286348	Dennd2d	-0.20713	0.19963	insignificant	-0.0091315	0.02434	hypomethylated	3	34	35
chr3	106349744	106351744	Dram2	-0.10525	9.5E-20	hypomethylated	-0.010017	0.19197	insignificant	24	108	113
chr3	106350679	106352679	Dram2	-0.095318	0.38271	insignificant	-0.015605	0.40881	insignificant	4	42	43
chr3	106486904	106488904	4933421E11Rik	-0.21916	0.00000123	hypomethylated	0.037116	0.42097	insignificant	7	18	19
chr3	106523599	106525599	4933421E11Rik	-0.20176	0.035823	hypomethylated	-0.014703	0.88949	insignificant	3	46	40
chr3	106838079	106840079	Kcna3	-0.10162	3.15E-10	hypomethylated	0.0037956	0.3223	insignificant	24	160	167
chr3	106903484	106905484	Kcna2	-0.14431	1.02E-24	hypomethylated	-0.0080014	0.2989	insignificant	34	116	122
chr3	107080775	107082775	Hbxip	-0.14641	0.000045873	hypomethylated	0.028302	0.93194	insignificant	14	68	71
chr3	107136207	107138207	Rbm15	-0.20245	1.26E-10	hypomethylated	-0.03205	0.18757	insignificant	11	24	24
chr3	107261816	107263816	Kcnc4	-0.11734	1.16E-16	hypomethylated	-0.0063965	0.61745	insignificant	36	132	132
chr3	107320936	107322936	Slc6a17	-0.09192	0.0075522	hypomethylated	-0.016462	0.71684	insignificant	7	15	16
chr3	107396948	107398948	Alx3	-0.14374	8.16E-29	hypomethylated	-0.0049048	0.31276	insignificant	33	122	130
chr3	107434628	107436628	Fam40a	-0.23792	0.013101	hypomethylated	0.09351	0.35985	insignificant	2	33	34
chr3	107499466	107501466	Ahcyl1		1	noCoverage	-0.019737	0.79094	insignificant	0	10	10
chr3	107697771	107699771	Gstm5	-0.18318	2.99E-11	hypomethylated	-0.031452	0.2516	insignificant	17	55	55
chr3	107699131	107701131	Gstm5	-0.19483	0.22072	insignificant	-0.052524	0.30177	insignificant	5	11	11
chr3	107734663	107736663	Gstm7	-0.22727	0.049933	hypomethylated	-0.12013	0.40462	insignificant	2	4	4
chr3	107746667	107748667	Gstm6	0.15341	0.36375	insignificant	-0.1421	0.092317	insignificant	1	10	11
chr3	107789354	107791354	Gstm2	0.057423	0.62354	insignificant	0.12164	0.69213	insignificant	1	7	7
chr3	107820891	107822891	Gstm1		1	noCoverage	0.17979	0.5079	insignificant	0	12	16
chr3	107847774	107849774	Gstm4	-0.14806	0.000000078	hypomethylated	-0.011664	0.38148	insignificant	7	24	24
chr3	107847777	107849777	Gstm4	-0.14806	0.000000078	hypomethylated	-0.011664	0.38148	insignificant	7	24	24
chr3	107889545	107891545	Ampd2	-0.12895	3.63E-08	hypomethylated	0.000518	0.29352	insignificant	4	21	21
chr3	107894985	107896985	Gnat2	0.23875	0.35518	insignificant	-0.049668	0.42356	insignificant	1	16	20
chr3	107949032	107951032	Gnai3	-0.060312	0.0035923	hypomethylated	0.0624	0.61815	insignificant	8	20	22

chr3	107988252	107990252	Amigo1	-0.11446	1.02E-31	hypomethylated	0.0050756	0.00015465	inconclusive	39	142	136
chr3	108003752	108005752	Cyb561d1	-0.046682	0.00005622	hypomethylated	0.0023187	0.82891	insignificant	9	26	26
chr3	108013852	108015852	Atxn7l2		1	noCoverage	0.0039788	0.34247	insignificant	0	8	8
chr3	108029517	108031517	Sypl2	-0.28952	0.0029263	hypomethylated	-0.033276	0.052169	insignificant	6	18	18
chr3	108058843	108060843	Psma5	-0.15448	9.47E-31	hypomethylated	-0.011803	0.9649	insignificant	21	72	77
chr3	108086049	108088049	Sort1	-0.12172	3.81E-46	hypomethylated	-0.0072515	0.70728	insignificant	62	183	180
chr3	108185721	108187721	Psrc1	-0.12518	0.000023142	hypomethylated	-0.0040027	0.82548	insignificant	7	48	43
chr3	108185755	108187755	Psrc1	-0.12518	0.000023142	hypomethylated	-0.0040027	0.82548	insignificant	7	48	43
chr3	108218412	108220412	Celsr2	-0.15699	8.1E-23	hypomethylated	-0.0050235	0.075896	insignificant	36	98	98
chr3	108248087	108250087	Sars	-0.33996	1	insignificant	0.015199	0.30233	insignificant	4	14	14
chr3	108339440	108341440	5330417C22Rik	-0.21573	0.093307	insignificant	0.0053973	0.56065	insignificant	3	31	36
chr3	108339501	108341501	1700013F07Rik	-0.21573	0.093307	insignificant	0.0053973	0.56065	insignificant	3	31	36
chr3	108357320	108359320	Scarna2	-0.29145	0.000000423	hypomethylated	-0.013634	0.0005614	hypomethylated	14	36	36
chr3	108365384	108367384	Tmem167b	-0.27454	0.19522	insignificant	-0.036553	0.23276	insignificant	2	23	23
chr3	108373616	108375616	Taf13	-0.078899	0.70694	insignificant	0.0054419	0.27286	insignificant	12	52	54
chr3	108393195	108395195	Wdr47	-0.13816	9.58E-13	hypomethylated	0.0069376	0.69483	insignificant	23	68	63
chr3	108455830	108457830	Clcc1	-0.11104	0.000000311	hypomethylated	0.0017038	0.44276	insignificant	13	66	66
chr3	108455857	108457857	Clcc1	-0.11104	0.000000311	hypomethylated	0.0017038	0.44276	insignificant	13	66	66
chr3	108455907	108457907	Clcc1	-0.11104	0.000000311	hypomethylated	0.0017038	0.44276	insignificant	13	66	66
chr3	108525217	108527217	Gpsm2	-0.12331	0.50917	insignificant	0.0056139	0.28476	insignificant	8	26	26
chr3	108692926	108694926	Fndc7	0.072222	1	insignificant	-0.51111	0.29934	insignificant	1	6	6
chr3	108714622	108716622	Prpf38b	-0.14288	6.91E-15	hypomethylated	-0.0024824	0.32716	insignificant	19	54	54
chr3	108742001	108744001	4921515J06Rik	0.0014723	0.63481	insignificant	-0.27502	6.23E-21	hypomethylated	11	37	37
chr3	108742474	108744474	4921515J06Rik	0.0014723	0.63481	insignificant	-0.27502	6.23E-21	hypomethylated	11	37	37
chr3	108830525	108832525	Fam102b	-0.1141	2.76E-17	hypomethylated	0.00077761	0.79502	insignificant	22	46	46
chr3	108882416	108884416	4930443G12Rik		1	noCoverage	0.019316	1	insignificant	0	5	6
chr3	108925066	108927066	Slc25a24	-0.37719	2E-12	stronglyHypometh	-0.083135	0.00048685	hypomethylated	9	39	46
chr3	109142600	109144600	Vav3	-0.10936	2.09E-52	hypomethylated	-0.014292	0.23614	insignificant	66	149	166
chr3	109946390	109948390	Ntng1		1	noCoverage	0.036789	0.54894	insignificant	0	24	34
chr3	110053916	110055916	Prmt6	-0.24352	0.00096019	hypomethylated	0.030538	0.0024834	inconclusive	6	24	24
chr3	113277680	113279680	Amy1	-0.25455	1	insignificant	-0.038199	0.82637	insignificant	1	5	4
chr3	113333067	113335067	Rnpc3	-0.12198	0.37182	insignificant	-0.010565	0.81779	insignificant	5	22	22
chr3	113732457	113734457	Col11a1	-0.3675	1E-12	stronglyHypometh	-0.051352	0.50361	insignificant	4	22	24
chr3	115417973	115419973	S1pr1	-0.15916	3.46E-08	hypomethylated	-0.022894	0.27046	insignificant	6	22	22
chr3	115590100	115592100	Dph5	-0.16573	4.85E-11	hypomethylated	0.0076916	0.079382	insignificant	10	40	40
chr3	115590977	115592977	A930005H10Rik		1	noCoverage	0.016785	0.019083	hypermethylated	0	17	17
chr3	115591038	115593038	A930005H10Rik		1	noCoverage	0.019784	0.022139	hypermethylated	0	13	13
chr3	115591048	115593048	A930005H10Rik		1	noCoverage	0.019784	0.022139	hypermethylated	0	13	13
chr3	115642454	115644454	Slc30a7	0.12313	1	insignificant	0.10797	0.042498	hypermethylated	1	8	6
chr3	115709366	115711366	Extl2	-0.18271	5.43E-21	hypomethylated	-0.010148	0.00033758	hypomethylated	20	72	79
chr3	115710214	115712214	Extl2	-0.16946	6.56E-14	hypomethylated	-0.013166	0.23164	insignificant	15	48	55
chr3	115710324	115712324	Slc30a7	-0.23561	5.08E-08	hypomethylated	-0.025083	0.32074	insignificant	6	24	31
chr3	115956402	115958402	Gpr88		1	noCoverage	0.033384	1	insignificant	0	12	12
chr3	116126950	116128950	Cdc14a	-0.20265	0.00000175	hypomethylated	0.00063311	0.59411	insignificant	3	22	26
chr3	116211093	116213093	Rtcd1		1	noCoverage	0.090969	0.31319	insignificant	0	5	5
chr3	116214996	116216996	Dbt	-0.17869	1.57E-24	hypomethylated	-0.0023605	0.40046	insignificant	13	51	50
chr3	116296925	116298925	Sass6	-0.1505	1.12E-13	hypomethylated	-0.0073698	0.87094	insignificant	29	94	94
chr3	116415198	116417198	Slc35a3	-0.097581	0.000072756	hypomethylated	0.0074216	1	insignificant	13	34	34
chr3	116511084	116513084	Agl	-0.16239	0.00074952	hypomethylated	-0.062688	0.76682	insignificant	6	13	12
chr3	116561484	116563484	Frrs1		1	noCoverage	-0.044426	0.63674	insignificant	0	8	8
chr3	116563597	116565597	Frrs1	-0.1897	6.39E-26	hypomethylated	0.0028029	0.95708	insignificant	9	30	30
chr3	116671870	116673870	4930455H04Rik		1	noCoverage	-0.00042088	0.75988	insignificant	0	6	6
chr3	117277378	117279378	4833424O15Rik	-0.099181	1.02E-23	hypomethylated	-0.0063556	0.4422	insignificant	36	122	127
chr3	117571854	117573854	Snx7	-0.096591	0.0025419	hypomethylated	-0.035625	0.34874	insignificant	14	37	36
chr3	118135774	118137774	Mir137	-0.18246	0.14056	insignificant	0.031928	0.47885	insignificant	3	40	36
chr3	118264095	118266095	Dpyd	-0.17383	0.061721	insignificant	0.000027481	0.79764	insignificant	5	30	30
chr3	119486306	119488306	Ptbp2	-0.3492	0.60328	insignificant	-0.13708	0.46938	insignificant	1	11	18
chr3	120874613	120876613	Rwdd3	-0.35924	0.42937	insignificant	-0.061979	0.026046	hypomethylated	4	17	16
chr3	120966234	120968234	Tmem56	-0.13024	0.69601	insignificant	-0.041711	0.2688	insignificant	5	26	26
chr3	120993734	120995734	Alg14	-0.25484	0.085059	insignificant	-0.058213	0.63454	insignificant	4	26	26
chr3	121128458	121130458	Cnn3	-0.11596	9.49E-21	hypomethylated	-0.023645	0.42023	insignificant	62	168	189
chr3	121235262	121237262	Slc44a3		1	noCoverage	-0.002307	0.59784	insignificant	0	6	6
chr3	121425454	121427454	F3	-0.21791	3.86E-08	hypomethylated	-0.028057	0.18411	insignificant	9	60	80
chr3	121518133	121520133	Abcd3	-0.10932	3.19E-09	hypomethylated	-0.013912	0.16811	insignificant	15	54	54
chr3	121655243	121657243	Arhgap29	-0.16066	9.25E-18	hypomethylated	-0.0027315	0.12998	insignificant	30	83	89
chr3	121746377	121748377	Abca4	-0.18696	0.014846	hypomethylated	-0.038484	1	insignificant	5	21	14
chr3	121947509	121949509	Gclm	-0.084377	1.51E-20	hypomethylated	-0.0067329	0.23275	insignificant	40	152	153
chr3	121976331	121978331	Dnttip2	-0.10911	0.0002032	hypomethylated	-0.0086322	0.76073	insignificant	23	82	83
chr3	121996621	121998621	Mir760	-0.15392	0.00000422	hypomethylated	0.017072	0.15969	insignificant	4	64	52
chr3	122121697	122123697	Bcar3	-0.18045	6.03E-14	hypomethylated	0.027906	0.0012189	inconclusive	32	67	71
chr3	122322585	122324585	Fnbp1l	-0.14423	3.15E-10	hypomethylated	-0.015702	0.028899	hypomethylated	24	45	63
chr3	122431075	122433075	Pde5a	-0.13062	2.18E-28	hypomethylated	-0.012426	0.14273	insignificant	44	119	114
chr3	122597309	122599309	Fabp2	0.16435	1	insignificant	-0.010648	1	insignificant	1	12	8
chr3	122626314	122628314	1810037I17Rik	-0.14095	3.59E-10	hypomethylated	-0.010632	0.74491	insignificant	23	58	58
chr3	122687365	122689365	Usp53	-0.16001	5.86E-16	hypomethylated	0.012196	0.89721	insignificant	14	35	44
chr3	122969413	122971413	Sec24d	-0.11885	0.00013956	hypomethylated	0.0010741	0.62151	insignificant	14	90	100
chr3	123088908	123090908	Mettl14		1	noCoverage	0.028788	0.68178	insignificant	0	4	5
chr3	123148830	123150830	Prss12	-0.12284	3.94E-35	hypomethylated	-0.0097919	0.35221	insignificant	42	130	130
chr3	123210984	123212984	Snora24	-0.13222	0.0020345	hypomethylated	-0.021426	0.076056	insignificant	16	54	55
chr3	123211254	123213254	Snora24	-0.13222	0.0020345	hypomethylated	-0.021426	0.076056	insignificant	16	54	55
chr3	124022954	124024954	Tram1l1	-0.16475	4.92E-27	hypomethylated	0.020479	4.11E-10	inconclusive	32	99	96
chr3	125106008	125108008	Ndst4	-0.2047	0.020887	hypomethylated	-0.11386	0.086439	insignificant	4	14	14
chr3	125641468	125643468	Ugt8a	-0.06162	0.000053649	hypomethylated	-0.026039	0.00029835	hypomethylated	21	89	89
chr3	126065769	126067769	Arsj	-0.45446	0.0039535	stronglyHypometh	-0.065402	1	insignificant	5	38	37
chr3	126298890	126300890	Camk2d	-0.091356	9.02E-16	hypomethylated	0.0038788	0.215	insignificant	38	150	152

chr3	126646304	126648304	Ank2	-0.19268	0.043606	hypomethylated	-0.043375	0.071497	insignificant	3	20	20
chr3	127247145	127249145	Mir302b	-0.22083	0.022486	hypomethylated	0.012121	0.40159	insignificant	2	4	4
chr3	127247280	127249280	Mir302c	-0.22083	0.022486	hypomethylated	0.012121	0.40159	insignificant	2	4	4
chr3	127247413	127249413	Mir302a	-0.22083	0.022486	hypomethylated	0.012121	0.40159	insignificant	2	4	4
chr3	127247541	127249541	Mir302d	-0.22083	0.022486	hypomethylated	0.012121	0.40159	insignificant	2	4	4
chr3	127247650	127249650	Mir367	-0.22083	0.022486	hypomethylated	0.012121	0.40159	insignificant	2	4	4
chr3	127255406	127257406	4930422G04Rik	-0.14221	0.000069145	hypomethylated	-0.0078892	0.3369	insignificant	8	40	44
chr3	127256267	127258267	Larp7	-0.16744	0.000067113	hypomethylated	-0.022625	0.32743	insignificant	8	28	27
chr3	127335062	127337062	Neurog2	-0.11099	1.69E-08	hypomethylated	-0.014682	0.32262	insignificant	35	114	128
chr3	127483445	127485445	Alpk1		1	noCoverage	-0.1193	0.035103	hypomethylated	0	10	10
chr3	127491830	127493830	Tifa	-0.13821	8E-14	hypomethylated	-0.018511	0.57235	insignificant	21	76	76
chr3	127540410	127542410	Ap1ar	-0.10935	0.0015835	hypomethylated	-0.036847	0.0054596	hypomethylated	3	25	35
chr3	127599241	127601241	5730508B09Rik	-0.3287	0.2962	insignificant	0.019408	0.13575	insignificant	3	10	10
chr3	128901835	128903835	Pitx2	-0.21712	6.45E-15	hypomethylated	0.03068	0.70461	insignificant	11	70	65
chr3	128901841	128903841	Pitx2	-0.21712	6.45E-15	hypomethylated	0.03068	0.70461	insignificant	11	70	65
chr3	128915854	128917854	Pitx2	-0.20648	4.38E-12	hypomethylated	0.02084	0.36222	insignificant	13	64	58
chr3	129035667	129037667	Enpep		1	noCoverage	-0.16392	1	insignificant	0	6	7
chr3	129135590	129137590	Gm5712	0.10896	0.1474	insignificant	0.035645	0.59502	insignificant	5	18	18
chr3	129234303	129236303	Elovl6	-0.15686	0.00000434	hypomethylated	-0.00090078	0.17622	insignificant	9	31	30
chr3	129534314	129536314	Gar1	-0.070521	0.23834	insignificant	0.004168	0.73472	insignificant	10	44	44
chr3	129538656	129540656	Cfi	0.55357	0.47273	lowCoverage	-0.039021	0.6981	insignificant	1	6	6
chr3	129580539	129582539	Pla2g12a	-0.096174	9.11E-11	hypomethylated	-0.0075427	0.45551	insignificant	15	54	54
chr3	129603342	129605342	Casp6	-0.10138	0.0096245	hypomethylated	-0.0017813	0.025807	hypomethylated	9	48	48
chr3	129673124	129675124	Ccdc109b	-0.41343	0.11269	insignificant	-0.019348	0.66829	insignificant	1	24	24
chr3	129763825	129765825	Sec24b	-0.094233	0.023974	hypomethylated	0.024141	0.065148	insignificant	9	35	36
chr3	129882795	129884795	Col25a1	-0.099621	0.50238	insignificant	0.0020852	0.78552	insignificant	5	37	47
chr3	130319365	130321365	Agxt2l1	-0.50914	0.0012055	stronglyHypometh	-0.032648	0.47234	insignificant	3	20	20
chr3	130319492	130321492	Agxt2l1	-0.50291	0.0015437	stronglyHypometh	-0.034279	0.67154	insignificant	3	18	18
chr3	130432222	130434222	Rpl34	-0.13076	3.5E-26	hypomethylated	-0.025246	0.0039915	hypomethylated	25	117	114
chr3	130433226	130435226	Rpl34	-0.15755	0.75258	insignificant	-0.06248	0.27819	insignificant	12	66	63
chr3	130433247	130435247	Rpl34	-0.15253	0.7512	insignificant	-0.064358	0.26085	insignificant	11	64	61
chr3	130812388	130814388	Lef1	-0.15286	2.58E-32	hypomethylated	-0.0052715	0.76958	insignificant	37	102	111
chr3	130974955	130976955	Hadh	-0.26528	0.011599	hypomethylated	0.0077417	0.25618	insignificant	6	26	28
chr3	131006145	131008145	Cyp2u1		1	noCoverage	-0.036111	1	insignificant	0	6	3
chr3	131226731	131228731	Papss1	-0.092054	5.63E-11	hypomethylated	-0.00030074	0.25722	insignificant	27	103	107
chr3	131747255	131749255	Dkk2	-0.27639	0.022029	hypomethylated	-0.028161	0.39696	insignificant	3	17	16
chr3	132346107	132348107	Tbck	-0.17307	0.00000304	hypomethylated	0.013439	1	insignificant	12	56	56
chr3	132346843	132348843	Tbck	-0.1562	0.00008526	hypomethylated	0.0099228	0.81945	insignificant	10	41	42
chr3	132613255	132615255	Npnt	-0.21319	0.000005874	hypomethylated	-0.06042	0.048638	hypomethylated	13	47	43
chr3	132753916	132755916	Ints12	-0.14709	2.05E-11	hypomethylated	-0.013607	0.74932	insignificant	26	112	102
chr3	132754704	132756704	Gstcd	-0.17351	3.55E-11	hypomethylated	-0.018804	0.5358	insignificant	16	80	74
chr3	132972079	132974079	Ppa2	-0.19229	3.84E-31	hypomethylated	-0.0090648	0.0042091	hypomethylated	19	104	112
chr3	133207354	133209354	Tet2	-0.10199	1.44E-20	hypomethylated	-0.0026065	0.48305	insignificant	19	142	132
chr3	133902111	133904111	Cxxc4	-0.10063	9.59E-09	hypomethylated	-0.015549	0.34548	insignificant	17	67	91
chr3	134490970	134492970	Tacr3	-0.34778	2.49E-13	stronglyHypometh	-0.0090007	0.7848	insignificant	8	48	46
chr3	134874526	134876526	Cenpe	-0.10053	0.15396	insignificant	0.00869	0.4169	insignificant	8	45	47
chr3	134969663	134971663	Nhedc2	-0.28467	0.29016	insignificant	0.080327	0.77484	insignificant	3	9	10
chr3	135086397	135088397	Cisd2	-0.078205	5.19E-13	hypomethylated	-0.012954	0.49486	insignificant	16	38	41
chr3	135100722	135102722	Ube2d3	-0.14499	8.9E-30	hypomethylated	-0.019496	0.48379	insignificant	53	212	213
chr3	135101629	135103629	4930539J05Rik	-0.15671	2.01E-23	hypomethylated	-0.022969	0.37314	insignificant	39	142	146
chr3	135147574	135149574	Manba	-0.18135	5.1E-12	hypomethylated	-0.031119	0.69708	insignificant	26	91	93
chr3	135354511	135356511	Nfkb1	-0.099359	0.074399	insignificant	0.0040212	0.28964	insignificant	11	48	49
chr3	135487454	135489454	Slc39a8	-0.26545	9.53E-12	hypomethylated	-0.067569	0.70517	insignificant	8	30	32
chr3	135487620	135489620	Slc39a8	-0.17712	5.44E-14	hypomethylated	-0.028951	0.84217	insignificant	16	58	60
chr3	135487753	135489753	Slc39a8	-0.17133	5.85E-14	hypomethylated	-0.028747	0.80467	insignificant	16	62	64
chr3	136332733	136334733	Ppp3ca	-0.092217	4.8E-41	hypomethylated	-0.0027785	0.42558	insignificant	99	280	280
chr3	137003041	137005041	Emcn		1	noCoverage	0.014693	0.81099	insignificant	0	6	6
chr3	137215586	137217586	Gm4861		1	noCoverage	0.23248	0.48257	insignificant	0	4	4
chr3	137285635	137287635	Ddit4l	-0.15742	0.0015279	hypomethylated	-0.0075121	0.025301	hypomethylated	16	58	58
chr3	137526562	137528562	H2afz	-0.09277	1.41E-30	hypomethylated	-0.001522	0.18225	insignificant	42	224	221
chr3	137529638	137531638	Dnajb14	-0.093273	4.7E-26	hypomethylated	0.0029247	0.82088	insignificant	43	143	144
chr3	137580518	137582518	Lamtor3	0.19429	0.000000835	hypermethylated	-0.030182	0.37115	insignificant	2	34	38
chr3	137644513	137646513	Dapp1	-0.36637	0.00013327	stronglyHypometh	0.046564	0.21444	insignificant	6	16	16
chr3	137730352	137732352	Gm5105	-0.04758	0.66718	insignificant	0.0059029	1	insignificant	4	32	28
chr3	137805501	137807501	Rg9mtd2	-0.10066	2.53E-10	hypomethylated	0.0057354	0.19395	insignificant	34	121	126
chr3	137806352	137808352	Mttp	-0.096384	4.14E-08	hypomethylated	0.0037438	0.19296	insignificant	32	97	102
chr3	137879736	137881736	Adh7		1	noCoverage	-0.2625	0.1966	insignificant	0	2	4
chr3	137939608	137941608	Adh1		1	noCoverage	0.125	1	insignificant	0	3	2
chr3	138105127	138107127	Adh5	-0.12264	3.6E-18	hypomethylated	-0.00518	0.39037	insignificant	31	96	95
chr3	138152346	138154346	Metap1	-0.16913	1.52E-32	hypomethylated	-0.014242	0.0000049	hypomethylated	21	74	94
chr3	138188154	138190154	Eif4e	-0.084139	0.02353	hypomethylated	-0.0042007	0.55228	insignificant	6	28	39
chr3	138188384	138190384	Mir1956	-0.10457	2.82E-12	hypomethylated	0.013075	0.52297	insignificant	17	70	77
chr3	138404158	138406158	Tspan5	-0.11161	8.64E-29	hypomethylated	-0.0033695	0.83491	insignificant	50	133	143
chr3	138738163	138740163	Rap1gds1	-0.081262	0.000010658	hypomethylated	-0.0026413	0.66084	insignificant	17	98	98
chr3	138867856	138869856	B930007M17Ril	0.84492	0.0003215	stronglyHypermeth	-0.024105	1	insignificant	4	46	49
chr3	141127527	141129527	Unc5c	-0.14759	2.74E-14	hypomethylated	-0.0020364	0.49758	insignificant	34	156	150
chr3	142058660	142060660	Pdlim5	-0.14777	3.74E-11	hypomethylated	-0.010243	0.55497	insignificant	10	38	40
chr3	142192301	142194301	Gbp7	-0.29156	0.013521	hypomethylated	-0.00074435	0.17795	insignificant	3	29	27
chr3	142192305	142194305	Gbp7	-0.29156	0.013521	hypomethylated	-0.00074435	0.17795	insignificant	3	29	27
chr3	142222015	142224015	Gbp3		1	noCoverage	-0.088986	0.21394	insignificant	0	14	15
chr3	142363043	142365043	Ccbl2	-0.17868	1.69E-11	hypomethylated	-0.0071146	0.12703	insignificant	13	57	54
chr3	142427210	142429210	Gtf2b	-0.10247	0.00092954	hypomethylated	-0.0036224	0.96663	insignificant	16	92	92
chr3	142544968	142546968	Pkn2	-0.092154	1.15E-12	hypomethylated	-0.0010762	0.25101	insignificant	21	87	85
chr3	143865296	143867296	Lmo4		1	noCoverage	-0.041835	0.74001	insignificant	0	20	21
chr3	143865360	143867360	Lmo4		1	noCoverage	-0.041835	0.74001	insignificant	0	20	21

chr3	143868219	143870219	Lmo4	-0.15109	0.00000177	hypomethylated	-0.0058348	0.53919	insignificant	15	58	58
chr3	144232390	144234390	############	-0.094004	2.08E-33	hypomethylated	0.039339	0.09674	insignificant	34	171	170
chr3	144233180	144235180	############	-0.11129	2.87E-20	hypomethylated	-0.0064549	0.052798	insignificant	18	88	88
chr3	144383287	144385287	Sh3glb1	-0.31818	0.13456	insignificant	0.0021593	1	insignificant	4	12	12
chr3	144762005	144764005	Clca5	-0.09375	0.52237	insignificant	-0.076593	0.43968	insignificant	1	4	4
chr3	144780552	144782552	Odf2l	-0.18817	2.3E-09	hypomethylated	-0.036026	1	insignificant	24	80	90
chr3	144780563	144782563	Odf2l	-0.18817	2.3E-09	hypomethylated	-0.036026	1	insignificant	24	80	90
chr3	145238171	145240171	Znhit6	-0.12411	0.00010466	hypomethylated	-0.033409	0.22941	insignificant	11	40	40
chr3	145312949	145314949	Cyr61	-0.30972	0.00083196	hypomethylated	-0.019653	0.31102	insignificant	4	26	27
chr3	145420655	145422655	Ddah1	-0.12551	3.88E-23	hypomethylated	-0.0029426	0.73423	insignificant	34	111	121
chr3	145586341	145588341	Bcl10	-0.1398	5.29E-11	hypomethylated	-0.02548	0.13459	insignificant	28	76	77
chr3	145599995	145601995	2410004B18Rik	-0.21101	1.65E-49	hypomethylated	-0.015079	0.010744	hypomethylated	38	76	88
chr3	145649833	145651833	Syde2	-0.098695	4.43E-48	hypomethylated	-0.0033926	0.039485	hypomethylated	102	318	316
chr3	145771000	145773000	Wdr63	0.14583	1	insignificant	0.046591	0.092292	insignificant	2	12	15
chr3	145811796	145813796	Mcoln2	-0.1932	3.1E-14	hypomethylated	-0.011881	0.069501	insignificant	30	94	90
chr3	145812137	145814137	Mcoln2	-0.18723	6.84E-14	hypomethylated	-0.012308	0.1375	insignificant	32	96	92
chr3	145882924	145884924	Lpar3	-0.1128	1.13E-41	hypomethylated	-0.0045906	3.03E-17	hypomethylated	46	114	112
chr3	146041908	146043908	Gm10636		1	noCoverage	0.2139	1	insignificant	0	2	4
chr3	146066650	146068650	Ssx2ip	-0.17147	9.83E-44	hypomethylated	0.014975	0.00000919	inconclusive	46	146	146
chr3	146112456	146114456	Ctbs	-0.0060895	2.46E-11	hypomethylated	-0.016403	0.025595	hypomethylated	6	44	44
chr3	146161799	146163799	Gng5	-0.10152	1.49E-25	hypomethylated	-0.0010476	0.29242	insignificant	58	157	164
chr3	146162717	146164717	Spata1	-0.09483	2.38E-18	hypomethylated	0.00032025	0.62431	insignificant	46	114	121
chr3	146184387	146186387	Rpf1	-0.56785	5.46E-08	stronglyHypometh	-0.041024	0.0016476	inconclusive	3	28	28
chr3	146259112	146261112	Uox		1	noCoverage	0.039359	0.80541	insignificant	0	10	8
chr3	146475910	146477910	Prkacb	-0.11624	0.00000214	hypomethylated	0.019005	0.12375	insignificant	11	44	45
chr3	146514330	146516330	Ttll7	-0.19456	2.23E-13	hypomethylated	-0.006799	0.087506	insignificant	13	32	32
chr3	151099845	151101845	Eltd1	-0.046206	0.87779	insignificant	0.0012483	0.30408	insignificant	13	42	42
chr3	151500492	151502492	Ptgfr	-0.48474	8.9E-21	stronglyHypometh	-0.0037057	0.00078678	hypomethylated	4	19	18
chr3	151828864	151830864	Gipc2	-0.20263	0.0177	hypomethylated	-0.075986	0.00000614	hypomethylated	7	35	32
chr3	151872421	151874421	Fubp1	-0.094268	2.52E-23	hypomethylated	-0.0024059	0.21006	insignificant	47	201	197
chr3	151873263	151875263	Dnajb4	-0.12028	2.3E-23	hypomethylated	-0.0075288	0.12746	insignificant	40	140	139
chr3	151929284	151931284	Nexn	-0.57234	0.00022072	stronglyHypometh	0.025931	0.22083	insignificant	3	27	31
chr3	152003371	152005371	Fam73a	-0.37491	0.000075403	stronglyHypometh	-0.16113	0.12873	insignificant	3	11	11
chr3	152008444	152010444	Usp33	-0.10729	0.00000671	hypomethylated	-0.020585	0.025317	hypomethylated	14	90	97
chr3	152057973	152059973	Zzz3	-0.097318	2.73E-34	hypomethylated	-0.014668	0.079886	insignificant	77	224	236
chr3	152058657	152060657	Zzz3	-0.099983	4.82E-37	hypomethylated	-0.013799	0.040455	hypomethylated	80	240	252
chr3	152331104	152333104	Ak5	-0.23127	0.00040227	hypomethylated	0.007078	0.049136	inconclusive	8	33	33
chr3	152376063	152378063	Pigk	-0.11855	0.0011326	hypomethylated	0.0083358	0.56391	insignificant	12	71	71
chr3	153388097	153390097	St6galnac3	-0.15189	0.000000233	hypomethylated	-0.0058649	0.011509	hypomethylated	3	22	22
chr3	153506210	153508210	Asb17	0.022894	1	insignificant	-0.25908	0.2073	insignificant	2	13	12
chr3	153575153	153577153	Rabggtb	-0.13243	0.000000397	hypomethylated	-0.0062727	0.016145	hypomethylated	11	44	44
chr3	153575167	153577167	Rabggtb	-0.13243	0.000000397	hypomethylated	-0.0062727	0.016145	hypomethylated	11	44	44
chr3	153575930	153577930	Rabggtb	-0.13243	0.000000397	hypomethylated	-0.0062727	0.016145	hypomethylated	11	44	44
chr3	153607396	153609396	Acadm	-0.24837	0.000619	hypomethylated	-0.033371	0.19066	insignificant	6	23	23
chr3	153635399	153637399	Slc44a5	-0.16534	0.59877	insignificant	-0.031743	0.21795	insignificant	7	42	42
chr3	153993524	153995524	AI606473	-0.19358	0.068712	insignificant	-0.02322	0.61893	insignificant	3	16	24
chr3	154258975	154260975	Cryz	-0.11169	2.3E-11	hypomethylated	-0.025452	0.73079	insignificant	23	92	92
chr3	154260062	154262062	Cryz	-0.22915	1.13E-13	hypomethylated	-0.027369	0.3896	insignificant	11	32	30
chr3	156223757	156225757	Negr1	-0.13211	1.25E-34	hypomethylated	-0.0068487	0.27362	insignificant	34	125	113
chr3	156223922	156225922	Negr1	-0.13548	7.23E-35	hypomethylated	-0.010213	0.25849	insignificant	34	122	113
chr3	157196360	157198360	Zranb2	-0.1168	6.92E-35	hypomethylated	-0.013012	0.23226	insignificant	56	171	173
chr3	157228855	157230855	Ptger3	-0.080095	3.08E-24	hypomethylated	-0.0025552	0.12889	insignificant	31	107	106
chr3	157588027	157590027	Cth		1	noCoverage	-0.063095	0.27421	insignificant	0	15	15
chr3	157609429	157611429	Ankrd13c	-0.13335	4.63E-48	hypomethylated	-0.015482	0.086284	insignificant	65	161	179
chr3	157694718	157696718	Srsf11	-0.61235	2.19E-41	stronglyHypometh	-0.10594	0.86822	insignificant	7	14	17
chr3	157698666	157700666	Lrrc40	-0.089295	0.00252	hypomethylated	-0.0041658	0.41789	insignificant	17	71	71
chr3	157699603	157701603	Srsf11	-0.11667	0.21992	insignificant	-0.011695	0.77742	insignificant	4	12	12
chr3	158225185	158227185	Lrrc7	-0.46143	0.055734	insignificant	0.087021	0.33468	insignificant	4	12	12
chr3	159157396	159159396	Depdc1a		1	noCoverage	-0.053205	0.85269	insignificant	0	7	4
chr4	3501025	3503025	Tgs1	-0.16454	1.91E-13	hypomethylated	-0.042636	0.1223	insignificant	26	78	74
chr4	3501915	3503915	Tmem68	-0.18655	2.18E-16	hypomethylated	0.019593	0.021184	inconclusive	22	64	66
chr4	3604267	3606267	Lyn	-0.14397	1.26E-19	hypomethylated	-0.018319	0.30929	insignificant	27	81	77
chr4	3762747	3764747	Rps20	-0.058554	0.00000333	hypomethylated	0.033608	0.86818	insignificant	12	34	36
chr4	3865034	3867034	Chchd7	-0.089694	0.000012716	hypomethylated	0.01276	0.32079	insignificant	22	115	117
chr4	3865060	3867060	Chchd7	-0.089694	0.000012716	hypomethylated	0.01276	0.32079	insignificant	22	115	117
chr4	3865552	3867552	Plag1	-0.093771	0.000012614	hypomethylated	0.0043283	0.29395	insignificant	22	110	112
chr4	4065592	4067592	Penk	-0.070569	0.0002414	hypomethylated	-0.0044647	0.90052	insignificant	16	71	71
chr4	4720453	4722453	Impad1	-0.3439	0.000000183	stronglyHypometh	0.0085995	0.73492	insignificant	4	8	8
chr4	5570325	5572325	Fam110b	-0.1381	1.52E-10	hypomethylated	0.00084665	0.96036	insignificant	26	76	76
chr4	6117251	6119251	Ubxn2b	-0.16194	1.08E-15	hypomethylated	0.007213	0.31479	insignificant	18	50	50
chr4	6291826	6293826	Sdcbp	-0.14492	9.16E-10	hypomethylated	0.0024755	0.070799	insignificant	7	42	43
chr4	6381418	6383418	Nsmaf	0.081051	0.084167	insignificant	-0.06613	0.0016402	hypomethylated	5	23	25
chr4	6917870	6919870	Tox	-0.13177	0.0015863	hypomethylated	-0.022649	0.93263	insignificant	14	39	47
chr4	8166188	8168188	Car8	-0.11718	0.0032952	hypomethylated	-0.028715	0.7149	insignificant	8	14	19
chr4	8461790	8463790	Rab2a	-0.095458	4.39E-09	hypomethylated	-0.002175	0.31804	insignificant	31	91	95
chr4	8617067	8619067	Chd7	-0.06819	3.85E-51	hypomethylated	-0.00077196	0.92717	insignificant	153	431	435
chr4	9195463	9197463	Clvs1	-0.25006	0.20797	insignificant	0.023332	0.5768	insignificant	3	10	10
chr4	9596309	9598309	Asph	-0.36136	0.52114	insignificant	-0.0075965	0.50435	insignificant	1	22	22
chr4	9696731	9698731	4930412C18Rik	-0.10638	0.000044174	hypomethylated	0.017378	0.35634	insignificant	5	27	30
chr4	9770518	9772518	Gdf6	-0.15233	4.05E-23	hypomethylated	-0.036614	0.53939	insignificant	30	128	133
chr4	10800644	10802644	2610301B20Rik	-0.097703	8.72E-24	hypomethylated	-0.0099281	0.01161	hypomethylated	46	167	165
chr4	10934766	10936766	Plekhf2	-0.01858	0.00025113	hypomethylated	-0.011544	0.0046491	hypomethylated	13	66	67
chr4	11003351	11005351	2310030N02Rik	-0.62304	0.073466	insignificant	-0.018663	0.17063	insignificant	2	20	20
chr4	11060243	11062243	Mir684-2		1	noCoverage	-0.18208	0.83202	insignificant	0	9	10
chr4	11082587	11084587	Trp53inp1	-0.19215	1.42E-11	hypomethylated	0.00078085	0.92694	insignificant	17	77	85

chr4	11117500	11119500	Ccne2	-0.12647	4.33E-34	hypomethylated	-0.0073336	0.13948	insignificant	65	188	193
chr4	11117855	11119855	Ccne2	-0.12401	5.13E-31	hypomethylated	-0.01069	0.029064	hypomethylated	51	149	154
chr4	11181406	11183406	Ints8	0.60548	0.00044556	stronglyHypermeth	-0.018056	0.10484	insignificant	1	23	21
chr4	11249278	11251278	Dpy19l4	-0.12619	0.12977	insignificant	-0.0011411	0.7669	insignificant	4	15	22
chr4	11313930	11315930	Esrp1	-0.14921	1.52E-43	hypomethylated	-0.0032424	0.10725	insignificant	35	120	128
chr4	11412104	11414104	1110037F02Rik	-0.12892	0.00000105	hypomethylated	0.019224	0.47769	insignificant	23	71	71
chr4	11485118	11487118	Rad54b	-0.13386	0.00000152	hypomethylated	-0.021324	0.84842	insignificant	14	50	54
chr4	11630593	11632593	Gem	-0.12149	0.00012623	hypomethylated	-0.0095819	0.27355	insignificant	11	65	63
chr4	11892860	11894860	Pdp1	-0.099085	7.75E-35	hypomethylated	-0.0031357	0.55046	insignificant	46	120	120
chr4	11893597	11895597	Pdp1	-0.084045	0.5481	insignificant	-0.052487	0.49763	insignificant	4	15	14
chr4	12015104	12017104	Tmem67	-0.23367	0.032719	hypomethylated	0.14527	0.75128	insignificant	2	12	19
chr4	12015516	12017516	C430048L16Rik	-0.20029	0.17264	insignificant	0.087708	0.72796	insignificant	2	14	25
chr4	12099162	12101162	Fam92a	-0.66667	0.46875	lowCoverage	-0.16667	0.014128	inconclusive	1	2	2
chr4	12832983	12834983	Gm11818	-0.18198	0.06208	insignificant	-0.036616	0.62997	insignificant	7	24	24
chr4	13669448	13671448	Runx1t1	-0.064726	5.24E-10	hypomethylated	-0.016838	0.0014843	hypomethylated	38	149	149
chr4	13677443	13679443	Runx1t1	-0.20351	0.0047618	hypomethylated	0.020641	0.0028983	inconclusive	11	43	36
chr4	13710928	13712928	Runx1t1	-0.21667	1	insignificant	-0.011253	0.65455	insignificant	2	12	12
chr4	14753734	14755734	Otud6b	-0.64583	3.06E-13	stronglyHypometh	-0.48408	0.00013646	stronglyhypometh	1	2	13
chr4	14790365	14792365	Tmem55a	-0.17927	0.000011058	hypomethylated	-0.025759	0.17114	insignificant	8	41	40
chr4	15076278	15078278	Necab1	-0.275	0.56597	insignificant	-0.036574	0.57473	insignificant	2	12	12
chr4	15191966	15193966	Tmem64	-0.095295	1.57E-26	hypomethylated	-0.0020522	0.87934	insignificant	50	166	167
chr4	15807410	15809410	Calb1	-0.097922	0.000022314	hypomethylated	-0.031708	1	insignificant	7	43	41
chr4	15872654	15874654	Decr1		1	noCoverage	-0.030825	0.83435	insignificant	0	4	4
chr4	15884113	15886113	Nbn	-0.11894	3.93E-36	hypomethylated	0.0016331	0.48617	insignificant	39	94	94
chr4	15941024	15943024	Osgin2	-0.0751	9.54E-19	hypomethylated	-0.0017357	0.83107	insignificant	23	90	90
chr4	16090645	16092645	Ripk2	-0.21088	1.6E-40	hypomethylated	0.033952	0.84649	insignificant	24	63	67
chr4	17779628	17781628	Mmp16	-0.11426	0.035039	hypomethylated	0.007496	1	insignificant	11	63	58
chr4	19497251	19499251	Cpne3		1	noCoverage	-0.0033333	1	insignificant	0	8	8
chr4	19501213	19503213	Fam82b	-0.24785	0.00000215	hypomethylated	0.013726	0.0063562	hypermethylated	10	33	26
chr4	19636140	19638140	Wwp1	-0.12888	1.5E-16	hypomethylated	0.02855	0.82325	insignificant	20	40	43
chr4	19934574	19936574	Ttpa	-0.1522	2.65E-18	hypomethylated	0.012673	0.69255	insignificant	32	89	90
chr4	19968198	19970198	Ggh	-0.13421	5.99E-26	hypomethylated	0.012298	0.15316	insignificant	25	78	76
chr4	21613110	21615110	Prdm13	-0.35318	0.0053843	stronglyHypometh	-0.069661	0.44895	insignificant	7	35	39
chr4	21653849	21655849	Ccnc	-0.16959	6.68E-30	hypomethylated	-0.016978	0.65384	insignificant	19	68	68
chr4	21694358	21696358	2610029I01Rik	-0.42303	0.000000027	stronglyHypometh	0.11827	0.00031531	inconclusive	3	12	11
chr4	21702416	21704416	Usp45	-0.1908	3.75E-11	hypomethylated	-0.0012638	0.35702	insignificant	12	51	51
chr4	21773729	21775729	Sfrs18	-0.15795	6.78E-15	hypomethylated	-0.017273	0.58608	insignificant	26	83	83
chr4	21775595	21777595	Sfrs18	-0.12884	0.05199	insignificant	-0.031897	0.46382	insignificant	7	14	14
chr4	21805821	21807821	Coq3	-0.19613	8.73E-09	hypomethylated	0.015257	0.60164	insignificant	12	72	72
chr4	21857472	21859472	6230409E13Rik	-0.12867	7.75E-24	hypomethylated	-0.015439	0.32827	insignificant	41	136	135
chr4	22283711	22285711	Fbxl4	-0.10737	7.16E-29	hypomethylated	-0.0052327	0.85051	insignificant	35	90	96
chr4	22415278	22417278	Pou3f2	-0.21196	1.5E-19	hypomethylated	-0.024881	0.014823	hypomethylated	17	73	72
chr4	24422608	24424608	Mms22l	-0.13272	1.08E-26	hypomethylated	-0.0276	0.0027487	hypomethylated	40	120	107
chr4	24778233	24780233	Klhl32	-0.091105	1.04E-15	hypomethylated	0.0025854	0.81915	insignificant	13	26	26
chr4	24824229	24826229	Ndufaf4	-0.20595	1.06E-21	hypomethylated	0.00000326	0.53933	insignificant	16	65	69
chr4	24899565	24901565	Gpr63		1	noCoverage	-0.048764	1	insignificant	0	4	4
chr4	25208968	25210968	1810074P20Rik	-0.232	0.023456	hypomethylated	0.36477	0.50636	insignificant	0	3	0
chr4	25727150	25729150	Fut9	-0.39698	0.019249	stronglyHypometh	-0.20837	0.10349	insignificant	3	7	6
chr4	26273799	26275799	Manea	-0.12427	0.000052213	hypomethylated	-0.016803	0.55583	insignificant	7	21	21
chr4	28739294	28741294	Epha7	-0.10177	4.11E-22	hypomethylated	-0.0043563	0.0026019	hypomethylated	35	168	168
chr4	32050081	32052081	Map3k7	-0.097094	0.00000318	hypomethylated	-0.00000505	0.93291	insignificant	36	92	92
chr4	32503409	32505409	Bach2		1	noCoverage	-0.022807	0.15665	insignificant	0	4	4
chr4	32701447	32703447	Casp8ap2	-0.27902	0.00000044	hypomethylated	-0.024993	0.45828	insignificant	11	55	55
chr4	32743093	32745093	Mdn1	-0.12672	3.88E-23	hypomethylated	-0.0080141	0.014388	hypomethylated	34	77	77
chr4	33010480	33012480	Ankrd6	0.123	1	insignificant	0.062859	0.72636	insignificant	4	15	15
chr4	33037801	33039801	Ankrd6	-0.15774	5.76E-17	hypomethylated	-0.060156	0.024568	hypomethylated	16	54	56
chr4	33068972	33070972	Rragd	-0.11883	1.98E-28	hypomethylated	-0.0021271	0.53104	insignificant	38	108	119
chr4	33117399	33119399	Ube2j1	-0.099447	6.49E-25	hypomethylated	-0.015066	0.051563	insignificant	45	150	149
chr4	33118298	33120298	4933421O10Rik	-0.088823	3.1E-11	hypomethylated	-0.012154	0.04599	hypomethylated	26	79	78
chr4	33218530	33220530	Gabrr1	0.0098894	0.40376	insignificant	-0.033664	0.63017	insignificant	3	6	9
chr4	33276712	33278712	Pm20d2		1	noCoverage	-0.039931	0.65964	insignificant	0	8	8
chr4	33294965	33296965	Srsf12	-0.18559	2.99E-21	hypomethylated	0.0036816	0.25906	insignificant	33	126	128
chr4	33335762	33337762	Pnrc1	0.29067	1	lowCoverage	-0.049922	0.48741	insignificant	1	14	11
chr4	33396285	33398285	Rngtt	-0.24352	1.2E-23	hypomethylated	-0.037296	0.00053004	hypomethylated	30	88	82
chr4	34010606	34012606	Cnr1	-0.10815	1.72E-30	hypomethylated	-0.012704	0.20309	insignificant	45	176	187
chr4	34137042	34139042	Spaca1	0.21956	0.35358	insignificant	0.000024208	0.46031	insignificant	5	26	26
chr4	34496863	34498863	Akirin2	-0.10945	1.55E-42	hypomethylated	-0.0040069	0.00048603	hypomethylated	58	170	183
chr4	34561206	34563206	Rars2	-0.13304	4.27E-19	hypomethylated	0.0016996	0.0012254	inconclusive	21	67	57
chr4	34562191	34564191	Orc3	-0.1699	0.000064956	hypomethylated	0.0076202	0.00018942	inconclusive	24	55	54
chr4	34634687	34636687	Slc35a1	-0.2788	1	lowCoverage	-0.10952	0.1099	insignificant	0	18	16
chr4	34830197	34832197	Zfp292	-0.2558	0.000000909	hypomethylated	0.052534	0.02087	inconclusive	10	38	39
chr4	35104715	35106715	Mob3b	-0.77175	0.076056	insignificant	-0.026605	0.91249	insignificant	1	20	14
chr4	35173129	35175129	3110043O21Rik	-0.092308	0.069766	insignificant	-0.0057966	0.74984	insignificant	3	8	16
chr4	36898777	36900777	Lingo2	-0.1634	0.000041642	hypomethylated	-0.015024	0.048998	hypomethylated	10	58	57
chr4	40089297	40091297	Aco1	-0.17108	9.68E-18	hypomethylated	-0.021619	0.73041	insignificant	20	64	66
chr4	40186858	40188858	Ddx58	-0.31254	0.054913	insignificant	0.0053044	0.21955	insignificant	4	21	20
chr4	40216874	40218874	2010003O02Rik	-0.10465	0.77749	insignificant	-0.086615	0.0016789	hypomethylated	6	25	23
chr4	40226401	40228401	Ndufb6		1	noCoverage	0	1	insignificant	0	12	12
chr4	40418212	40420212	Tmem215	-0.36846	0.00000275	stronglyHypometh	-0.096607	0.032982	hypomethylated	3	28	28
chr4	40419162	40421162	Tmem215	-0.22589	0.0099064	hypomethylated	-0.045824	0.89008	insignificant	8	42	42
chr4	40649949	40651949	Aptx	-0.1741	6.44E-13	hypomethylated	-0.0030986	0.35132	insignificant	14	47	46
chr4	40650220	40652220	Aptx	-0.24867	0.000013062	hypomethylated	-0.043167	1	insignificant	4	17	17
chr4	40668500	40670500	Dnaja1	-0.15291	8.65E-27	hypomethylated	-0.00097965	0.070165	insignificant	47	140	140
chr4	40668949	40670949	Mir207	-0.15291	8.65E-27	hypomethylated	-0.00097965	0.070165	insignificant	47	140	140
chr4	40668954	40670954	Dnaja1	-0.15291	8.65E-27	hypomethylated	-0.00097965	0.070165	insignificant	47	140	140

chr4	40668962	40670962	Dnaja1	-0.15291	8.65E-27	hypomethylated	-0.00097965	0.070165	insignificant	47	140	140
chr4	40801031	40803031	B4galt1	-0.24248	0.0026436	hypomethylated	-0.013054	0.76001	insignificant	7	38	38
chr4	40894585	40896585	Chmp5	-0.10341	7.19E-34	hypomethylated	-0.0041052	0.86194	insignificant	36	104	104
chr4	40895327	40897327	Bag1	-0.12832	0.014141	hypomethylated	-0.011531	0.27909	insignificant	5	30	30
chr4	40916975	40918975	Nfx1	-0.13927	3.17E-17	hypomethylated	-0.019498	0.68449	insignificant	24	82	94
chr4	40939727	40941727	Mir3094		1	noCoverage	-0.12602	0.48322	insignificant	0	15	14
chr4	40995169	40997169	Aqp7	-0.26855	0.00000139	hypomethylated	-0.10538	0.036313	hypomethylated	2	10	10
chr4	41045216	41047216	Aqp3	-0.46747	0.0040406	stronglyHypometh	0.020369	0.042853	hypermethylated	1	9	9
chr4	41071372	41073372	Nol6	-0.19851	0.0013529	hypomethylated	-0.020762	0.32442	insignificant	7	21	22
chr4	41082053	41084053	Ube2r2	-0.11846	4.32E-12	hypomethylated	-0.0011449	0.033886	hypomethylated	27	122	133
chr4	41222168	41224168	Ubap2	-0.11753	0.60425	insignificant	0.0132	0.35097	insignificant	3	30	30
chr4	41295028	41297028	Ubap1	-0.12511	3.91E-09	hypomethylated	0.012095	0.040538	inconclusive	17	76	66
chr4	41411180	41413180	Nudt2	-0.19487	3.38E-18	hypomethylated	0.019651	0.050704	insignificant	18	95	96
chr4	41411881	41413881	Kif24	-0.28155	6.28E-15	hypomethylated	0.058007	0.31634	insignificant	2	37	41
chr4	41450109	41452109	AI464131	-0.50252	4.57E-08	stronglyHypometh	-0.06741	0.00179	hypomethylated	4	21	22
chr4	41454797	41456797	1110017D15Rik	0.11625	0.10063	insignificant	-0.0012695	0.015907	inconclusive	7	22	22
chr4	41515826	41517826	Dnaic1	-0.11341	1.52E-32	hypomethylated	-0.0073944	0.0063929	hypomethylated	43	118	120
chr4	41516560	41518560	2310028H24Rik	-0.12877	1	insignificant	0.031559	0.57461	insignificant	9	28	30
chr4	41587335	41589335	Enho		1	noCoverage	0.034784	0.42848	insignificant	0	8	9
chr4	41642477	41644477	Cntfr		1	noCoverage	0.022939	0.70846	insignificant	0	16	21
chr4	41644123	41646123	Cntfr	-0.2094	6.79E-23	hypomethylated	-0.0071789	0.0092601	hypomethylated	11	43	38
chr4	41660549	41662549	2810432D09Rik	-0.15027	0.002502	hypomethylated	-0.012953	0.032158	hypomethylated	22	71	72
chr4	41670195	41672195	Arid3c		1	noCoverage	-0.10833	0.1005	insignificant	0	10	13
chr4	41678174	41680174	Arid3c	-0.12499	0.0010122	hypomethylated	0.021579	0.28973	insignificant	9	48	56
chr4	41688186	41690186	Sigmar1		1	noCoverage	-0.064762	0.60696	insignificant	0	10	10
chr4	41701125	41703125	Galt	-0.31183	0.0039619	hypomethylated	0.019822	0.011345	hypermethylated	5	26	26
chr4	41706315	41708315	Il11ra1	-0.20384	5.94E-10	hypomethylated	0.014535	0.33576	insignificant	4	16	16
chr4	41708182	41710182	Il11ra1		1	noCoverage	-0.014349	0.14315	insignificant	0	6	6
chr4	41720974	41722974	Ccl27a	-0.20139	0.00024417	hypomethylated	-0.035629	0.91513	insignificant	6	12	15
chr4	41721007	41723007	Ccl27a	-0.20139	0.00024417	hypomethylated	-0.023789	0.91501	insignificant	6	12	14
chr4	41721049	41723049	Ccl27a	-0.19444	0.038678	hypomethylated	0.037415	0.49855	insignificant	3	6	6
chr4	42929122	42931122	N28178	-0.082682	3.18E-31	hypomethylated	0.01371	0.96877	insignificant	50	120	131
chr4	42964965	42966965	Dnajb5	0.029533	1	insignificant	-0.027826	1	insignificant	1	4	4
chr4	43013379	43015379	Vcp		1	noCoverage	0.035562	0.72568	insignificant	0	22	22
chr4	43023173	43025173	Fancg	-0.15368	1	insignificant	-0.015892	0.35045	insignificant	4	8	8
chr4	43038628	43040628	Pigo	-0.13974	0.00021159	hypomethylated	0.094954	0.74973	insignificant	7	18	21
chr4	43044256	43046256	Stoml2	-0.12818	0.0018951	hypomethylated	-0.010948	0.69708	insignificant	4	20	20
chr4	43059075	43061075	B230312A22Rik	-0.16579	0.075802	insignificant	0.065672	0.93451	insignificant	2	13	19
chr4	43070855	43072855	Unc13b	-0.12755	2.14E-18	hypomethylated	-0.00141	0.51484	insignificant	28	89	89
chr4	43070944	43072944	Unc13b	-0.12755	2.14E-18	hypomethylated	-0.00141	0.51484	insignificant	28	89	89
chr4	43393853	43395853	Rusc2	-0.062881	0.00084292	hypomethylated	0.0037163	0.8608	insignificant	30	111	110
chr4	43418445	43420445	Rusc2	0.41207	0.0044543	stronglyHypermeth	0.17505	0.93993	insignificant	7	28	17
chr4	43442006	43444006	Fam166b	-0.15132	0.41475	insignificant	-0.11243	0.16444	insignificant	3	9	12
chr4	43454148	43456148	Tesk1	-0.16571	7.11E-32	hypomethylated	-0.013392	0.27924	insignificant	34	63	61
chr4	43496581	43498581	Sit1		1	noCoverage	-0.081731	0.43154	insignificant	0	4	3
chr4	43505275	43507275	Rmrp	-0.16997	1.99E-09	hypomethylated	-0.0096372	0.3418	insignificant	12	101	103
chr4	43505931	43507931	Rmrp	-0.1132	0.000002	hypomethylated	0.011439	0.62822	insignificant	6	55	62
chr4	43512532	43514532	E130306D19Rik	-0.052422	0.04154	hypomethylated	0.009582	0.75263	insignificant	5	28	24
chr4	43518897	43520897	Car9	-0.20437	0.0039936	hypomethylated	-0.042026	0.62475	insignificant	2	6	6
chr4	43536260	43538260	Tpm2	-0.1594	3.54E-08	hypomethylated	-0.0094412	0.24284	insignificant	13	96	93
chr4	43574505	43576505	Creb3	-0.1651	1.63E-27	hypomethylated	-0.011489	0.21563	insignificant	22	80	86
chr4	43575455	43577455	Tln1	-0.22563	1.83E-08	hypomethylated	0.027545	0.61801	insignificant	6	22	23
chr4	43590606	43592606	Rgp1	-0.14018	4.11E-29	hypomethylated	-0.0051312	0.0014531	hypomethylated	28	118	118
chr4	43591736	43593736	Rgp1	-0.15031	0.000000757	hypomethylated	0.017478	0.01728	inconclusive	12	52	54
chr4	43597366	43599366	Rgp1	-0.14971	0.63201	insignificant	-0.019873	0.66868	insignificant	2	6	6
chr4	43643806	43645806	Npr2	-0.14141	1.02E-31	hypomethylated	-0.027118	3.4E-18	hypomethylated	52	156	149
chr4	43666424	43668424	Spag8	-0.11945	0.21065	insignificant	-0.089796	0.0000112	hypomethylated	6	27	28
chr4	43680842	43682842	Tmem8b	-0.11177	1.52E-14	hypomethylated	0.0036269	0.078453	insignificant	19	68	66
chr4	43681731	43683731	4930412F15Rik	-0.079663	8.82E-16	hypomethylated	0.0035056	0.3909	insignificant	21	72	72
chr4	43739069	43741069	Hrct1	-0.23461	0.0020147	hypomethylated	-0.12213	0.0038335	hypomethylated	4	12	15
chr4	43741905	43743905	5430416O09Rik	-0.47031	0.00019906	stronglyHypometh	0.0041022	1	insignificant	5	12	12
chr4	43849389	43851389	Olfr157	-0.050388	0.15398	insignificant	-0.032134	0.042358	hypomethylated	2	6	7
chr4	43866163	43868163	Olfr155	-0.14904	0.45785	insignificant	-0.024038	0.25342	insignificant	4	8	8
chr4	43887401	43889401	Reck	-0.1525	1.56E-35	hypomethylated	-0.021647	0.075377	insignificant	47	171	168
chr4	43969573	43971573	Glipr2	-0.0017133	0.00020024	hypomethylated	0.014416	0.9315	insignificant	8	70	70
chr4	43995375	43997375	Ccin	0.15701	8.9E-11	hypermethylated	-0.044507	0.030855	inconclusive	14	74	61
chr4	44024514	44026514	Clta	-0.15527	8.62E-15	hypomethylated	-0.0091578	0.0086567	hypomethylated	24	64	64
chr4	44180410	44182410	Rnf38	-0.10063	2.22E-26	hypomethylated	-0.0096389	0.089366	insignificant	61	172	178
chr4	44181155	44183155	Rnf38	-0.10618	0.000007038	hypomethylated	-0.032481	0.012298	hypomethylated	17	54	54
chr4	44312788	44314788	Melk	-0.17939	1.79E-14	hypomethylated	0.031454	1	insignificant	18	62	63
chr4	44723312	44725312	Pax5	-0.29501	4.33E-22	hypomethylated	-0.068548	0.69961	insignificant	14	44	37
chr4	44768430	44770430	Zcchc7	-0.16801	3.37E-27	hypomethylated	-0.015194	0.00069648	hypomethylated	18	94	91
chr4	44993282	44995282	Grhpr	-0.14371	2.94E-18	hypomethylated	-0.017134	0.12706	insignificant	16	55	54
chr4	45025284	45027284	Zbtb5	-0.11667	1	insignificant	-0.002381	0.83016	insignificant	1	10	10
chr4	45030496	45032496	Polr1e	-0.18836	2.56E-15	hypomethylated	0.00023859	0.42226	insignificant	7	40	40
chr4	45097476	45099476	Fbxo10	-0.19382	0.070693	insignificant	-0.036425	0.95329	insignificant	0	25	25
chr4	45120985	45122985	Tomm5	0.26689	0.57338	insignificant	-0.040235	0.57883	insignificant	1	20	20
chr4	45196777	45198777	Frmpd1	-0.13738	3.93E-27	hypomethylated	-0.0031852	0.72222	insignificant	42	112	114
chr4	45333475	45335475	Exosc3	-0.32982	7.29E-31	hypomethylated	0.0090081	0.018579	inconclusive	6	20	21
chr4	45353972	45355972	Dcaf10	-0.13361	6.83E-43	hypomethylated	0.00087107	0.10194	insignificant	46	128	128
chr4	45421638	45423638	Mcart1	-0.34103	9.71E-11	stronglyHypometh	-0.16232	0.000000828	hypomethylated	14	29	42
chr4	45543700	45545700	Shb	-0.12972	0.014524	hypomethylated	-0.0155	0.42172	insignificant	7	51	51
chr4	45695460	45697460	Gm829	0.0088541	0.39932	insignificant	-0.022305	0.44705	insignificant	4	13	13
chr4	45810893	45812893	Aldh1b1	-0.13328	0.10564	insignificant	-0.0070142	0.083856	insignificant	10	54	54
chr4	45839699	45841699	Igfbpl1	-0.081124	0.15668	insignificant	-0.0067276	0.82388	insignificant	7	18	18

chr4	46051093	46053093	Tmod1	-0.096918	4.82E-27	hypomethylated	0.0044419	0.58464	insignificant	40	126	126
chr4	46150382	46152382	Ncbp1	-0.16005	1.24E-40	hypomethylated	-0.019487	0.0000064	hypomethylated	33	93	78
chr4	46151347	46153347	Tstd2	-0.16018	1.99E-36	hypomethylated	-0.031093	7.71E-08	hypomethylated	28	88	72
chr4	46209183	46211183	Xpa	-0.0034911	0.21991	insignificant	-0.074782	0.0030776	hypomethylated	3	18	18
chr4	46356065	46358065	Foxe1	-0.12562	3.32E-57	hypomethylated	-0.023317	0.000033284	hypomethylated	85	259	265
chr4	46402295	46404295	5830415F09Rik	-0.12829	0.00048519	hypomethylated	-0.019159	0.73278	insignificant	11	40	40
chr4	46417055	46419055	Hemgn	-0.092109	0.26555	insignificant	-0.17896	0.096423	insignificant	2	7	7
chr4	46462988	46464988	Anp32b	-0.11148	1.76E-45	hypomethylated	-0.0016806	0.17883	insignificant	79	217	235
chr4	46501200	46503200	Nans	-0.23852	4.95E-18	hypomethylated	-0.0043937	0.0089035	hypomethylated	13	64	63
chr4	46549016	46551016	Trim14		1	noCoverage	0.0053471	0.77142	insignificant	0	12	12
chr4	46579312	46581312	Coro2a	0.053214	0.25834	insignificant	0.047824	0.7454	insignificant	2	10	10
chr4	46614801	46616801	Coro2a	-0.13508	2.35E-23	hypomethylated	0.02031	0.42222	insignificant	12	46	35
chr4	46663071	46665071	Tbc1d2	-0.17326	2.07E-08	hypomethylated	0.0089875	0.83632	insignificant	6	18	18
chr4	47004586	47006586	Gabbr2	-0.13401	0.000000929	hypomethylated	-0.0039746	0.93191	insignificant	19	86	86
chr4	47070178	47072178	Anks6	-0.16457	2.1E-15	hypomethylated	-0.014484	0.091728	insignificant	18	77	76
chr4	47103824	47105824	Galnt12	-0.1288	1.64E-08	hypomethylated	-0.013214	0.89725	insignificant	12	64	69
chr4	47219883	47221883	Col15a1	-0.09706	1.04E-11	hypomethylated	0.011043	0.46801	insignificant	26	105	104
chr4	47365176	47367176	Tgfbr1	-0.14612	1.42E-14	hypomethylated	0.016304	0.85045	insignificant	39	107	107
chr4	47486532	47488532	Sec61b	-0.14204	0.00044716	hypomethylated	-0.014651	0.25274	insignificant	7	46	46
chr4	47487239	47489239	Alg2	-0.15449	0.36575	insignificant	-0.015068	0.71418	insignificant	3	16	16
chr4	48063119	48065119	Nr4a3	-0.16964	0.000000749	hypomethylated	-0.044634	0.38987	insignificant	8	53	50
chr4	48136790	48138790	Stx17	-0.1417	1.24E-28	hypomethylated	-0.010784	0.034419	hypomethylated	27	72	72
chr4	48291673	48293673	Invs	-0.16472	1.12E-08	hypomethylated	-0.0079043	0.58246	insignificant	10	68	68
chr4	48292461	48294461	Erp44	-0.13552	0.000013627	hypomethylated	-0.010264	0.96185	insignificant	9	46	46
chr4	48486294	48488294	Tex10	-0.088862	9.94E-10	hypomethylated	0.018505	0.53683	insignificant	14	36	37
chr4	48551817	48553817	5730528L13Rik	-0.091844	1.24E-28	hypomethylated	-0.0070551	0.00017988	hypomethylated	63	147	171
chr4	48551952	48553952	5730528L13Rik	-0.091844	1.24E-28	hypomethylated	-0.0070551	0.00017988	hypomethylated	63	147	171
chr4	48552370	48554370	5730528L13Rik	-0.091844	1.24E-28	hypomethylated	-0.0070551	0.00017988	hypomethylated	63	147	171
chr4	48597064	48599064	Tmeff1	-0.13718	1.44E-24	hypomethylated	-0.049446	0.12449	insignificant	56	181	183
chr4	48675385	48677385	Murc	0.13191	1	insignificant	0.088411	0.039672	hypermethylated	1	18	19
chr4	49071333	49073333	E130309F12Rik	-0.18205	0.00020338	hypomethylated	-0.025348	0.42724	insignificant	13	66	66
chr4	49533088	49535088	Zfp189	-0.13959	3.72E-08	hypomethylated	-0.013873	0.95253	insignificant	11	43	53
chr4	49533955	49535955	Mrpl50	-0.20029	0.046195	inconclusive	-0.065374	0.19402	insignificant	12	37	47
chr4	49643931	49645931	Rnf20	-0.17348	0.000025425	hypomethylated	0.041469	0.26601	insignificant	10	32	38
chr4	49694855	49696855	Grin3a		1	noCoverage	0.045401	0.60748	insignificant	0	6	6
chr4	49857953	49859953	Grin3a	-0.16545	3.73E-19	hypomethylated	-0.039604	0.58556	insignificant	24	88	86
chr4	52451120	52453120	Smc2	-0.16844	0.000029777	hypomethylated	-0.0027023	0.40123	insignificant	14	52	58
chr4	52924664	52926664	Olfr272	0.016148	1	insignificant	-0.12945	0.00000372	hypomethylated	4	14	14
chr4	53001155	53003155	Nipsnap3a		1	noCoverage	0.11112	0.0059231	hypermethylated	0	12	12
chr4	53023795	53025795	Nipsnap3b	-0.091641	0.0017779	hypomethylated	0.041866	0.2705	insignificant	12	35	53
chr4	53172767	53174767	Abca1	-0.14296	0.0045265	hypomethylated	-0.0001684	0.016389	hypomethylated	10	40	40
chr4	53283104	53285104	AI427809	-0.053198	0.11729	insignificant	0.024872	0.92543	insignificant	11	29	29
chr4	53452284	53454284	Slc44a1	-0.091599	1.59E-43	hypomethylated	0.00466	0.89901	insignificant	63	198	195
chr4	53643342	53645342	Fsd1l	-0.092714	0.0012705	hypomethylated	0.0057255	0.56962	insignificant	19	88	99
chr4	53726053	53728053	Fktn	-0.1209	0.00000615	hypomethylated	0.01442	0.81075	insignificant	11	74	73
chr4	53791576	53793576	Tal2	-0.22668	0.0088791	hypomethylated	-0.027364	1	insignificant	5	18	18
chr4	53837916	53839916	Tmem38b	-0.13741	0.011019	hypomethylated	-0.019747	0.6805	insignificant	13	65	60
chr4	54959816	54961816	Zfp462	-0.38005	0.000058643	stronglyHypometh	-0.054916	0.27132	insignificant	3	14	14
chr4	55361913	55363913	Rad23b	-0.098563	7.35E-43	hypomethylated	0.0078246	0.13432	insignificant	72	217	226
chr4	55545347	55547347	Klf4	-0.13325	2E-19	hypomethylated	-0.015001	0.00016576	hypomethylated	19	145	163
chr4	56814200	56816200	BC026590	-0.15924	4.97E-09	hypomethylated	-0.01644	0.48578	insignificant	26	96	103
chr4	56815203	56817203	BC026590	-0.34382	1.3E-19	stronglyHypometh	-0.095243	0.015854	hypomethylated	8	28	35
chr4	56878083	56880083	Ctnnal1		1	noCoverage	0.00071316	0.72574	insignificant	0	13	13
chr4	56908170	56910170	D730040F13Rik	0.0083333	1	insignificant	-0.0047664	1	insignificant	3	9	8
chr4	57003263	57005263	6430704M03Ril	-0.065784	1	insignificant	0.13349	0.11113	insignificant	1	7	6
chr4	57156028	57158028	Epb4.1l4b	-0.10569	2.55E-43	hypomethylated	-0.0084158	0.54646	insignificant	67	146	158
chr4	57314709	57316709	Ptpn3	-0.13379	0.060719	insignificant	0.026797	0.76941	insignificant	4	20	20
chr4	57580119	57582119	Palm2	-0.1351	5.31E-10	hypomethylated	0.0014638	0.5729	insignificant	14	36	46
chr4	57857119	57859119	Akap2	-0.14786	0.00053355	hypomethylated	0.058022	1	insignificant	4	10	8
chr4	57866433	57868433	Akap2	-0.1338	0.000081336	hypomethylated	-0.070373	0.034347	hypomethylated	4	11	8
chr4	57969283	57971283	Txn1		1	noCoverage	-0.017252	0.84568	insignificant	0	10	10
chr4	58297833	58299833	Musk	0.077778	0.77991	insignificant	0.07381	0.0036624	hypermethylated	4	8	8
chr4	58566165	58568165	Lpar1	-0.17678	3.09E-12	hypomethylated	-0.056733	1	insignificant	14	46	42
chr4	58566363	58568363	Lpar1	-0.53176	7.21E-08	stronglyHypometh	-0.13738	0.84291	insignificant	3	10	6
chr4	58925597	58927597	AI314180	-0.071813	0.14921	insignificant	0.058304	0.92147	insignificant	11	37	29
chr4	58955499	58957499	Zkscan16		1	noCoverage	0.094147	0.18344	insignificant	0	6	6
chr4	59015064	59017064	Dnajc25	-0.11653	2.37E-14	hypomethylated	0.012225	0.026048	inconclusive	31	113	112
chr4	59047027	59049027	Gng10	-0.077553	6E-19	hypomethylated	0.0098685	0.40403	insignificant	42	98	110
chr4	59201421	59203421	Ugcg	-0.092382	5.15E-27	hypomethylated	-0.0011003	0.000047954	hypomethylated	34	142	139
chr4	59451505	59453505	Susd1	-0.26129	0.00000453	hypomethylated	-0.044099	0.48497	insignificant	4	19	18
chr4	59562236	59564236	Rod1	-0.1181	0.16122	insignificant	-0.018549	0.0026246	hypomethylated	1	54	43
chr4	59593434	59595434	Hsdl2	-0.12874	0.045086	hypomethylated	-0.010084	0.77334	insignificant	15	70	74
chr4	59638092	59640092	E130308A19Rik	-0.097792	2.35E-38	hypomethylated	0.0047963	0.52292	insignificant	43	160	173
chr4	59638198	59640198	E130308A19Rik	-0.097792	2.35E-38	hypomethylated	0.0047963	0.52292	insignificant	43	160	173
chr4	59796727	59798727	1110054O05Rik	-0.10811	0.0010651	hypomethylated	-0.019168	0.60832	insignificant	6	24	28
chr4	59817521	59819521	Snx30	-0.10697	3.89E-18	hypomethylated	-0.016104	0.0014117	hypomethylated	24	108	111
chr4	61811875	61813875	Mup21	0.02674	1	insignificant	-0.079381	0.25752	insignificant	2	14	14
chr4	61869580	61871580	Zfp37	-0.13545	0.018686	hypomethylated	-0.012623	0.014537	hypomethylated	10	24	24
chr4	61946478	61948478	Slc31a2	-0.15132	1.08E-09	hypomethylated	0.0060207	0.87341	insignificant	13	43	48
chr4	62020734	62022734	Slc31a1	-0.13664	0.0023402	hypomethylated	0.0048196	0.74288	insignificant	13	74	72
chr4	62021582	62023582	Slc31a1	-0.13972	0.072342	insignificant	0.0025217	0.48967	insignificant	4	40	40
chr4	62068816	62070816	Prpf4	-0.19526	9.78E-23	hypomethylated	-0.024058	0.37814	insignificant	17	71	68
chr4	62069657	62071657	Cdc26	-0.21835	1.54E-24	hypomethylated	-0.030171	0.34089	insignificant	18	65	63
chr4	62131906	62133906	Wdr31	-0.083031	0.023433	hypomethylated	0.010779	0.078011	insignificant	5	14	14
chr4	62140100	62142100	Bspry	-0.16013	3.67E-09	hypomethylated	-0.0062738	0.6255	insignificant	8	37	50

chr4	62163234	62165234	Hdhd3	-0.30464	0.00000023	hypomethylated	-0.0021692	0.030866	inconclusive	2	10	10
chr4	62181097	62183097	Alad		1	noCoverage	0.14011	0.00042961	hypermethylated	0	13	9
chr4	62185402	62187402	4933430I17Rik	-0.14823	0.000097874	hypomethylated	-0.022076	0.27628	insignificant	4	42	42
chr4	62186048	62188048	Pole3	-0.12796	0.0020808	hypomethylated	-0.020838	0.5479	insignificant	2	28	28
chr4	62219880	62221880	Rgs3	-0.78571	6.11E-09	stronglyHypometh	-0.017477	0.22967	insignificant	2	7	7
chr4	62279705	62281705	Rgs3	-0.39718	0.50082	insignificant	-0.1098	0.5397	insignificant	1	14	14
chr4	62625607	62627607	Zfp618	-0.11518	6.84E-18	hypomethylated	-0.029835	0.34033	insignificant	51	140	159
chr4	62815176	62817176	Ambp	-0.054545	0.30317	insignificant	-0.0024621	0.50102	insignificant	1	4	4
chr4	62875445	62877445	Col27a1	-0.12458	2.75E-14	hypomethylated	-0.0018753	0.10481	insignificant	36	126	127
chr4	62916884	62918884	Mir455	-0.041374	0.57081	insignificant	0.010427	0.64794	insignificant	2	30	37
chr4	63022482	63024482	Orm2	-0.068824	0.62288	insignificant	0.16984	0.073871	insignificant	2	8	11
chr4	63064479	63066479	Akna	-0.21429	0.0027246	hypomethylated	0.29716	0.49021	insignificant	2	6	12
chr4	63156985	63158985	Whrn	-0.15134	3.52E-10	hypomethylated	-0.014498	0.060923	insignificant	10	33	33
chr4	63204798	63206798	Atp6v1g1	-0.17128	2.85E-14	hypomethylated	0.018802	0.70568	insignificant	12	59	70
chr4	63261798	63263798	Gm11213	-0.26376	0.065538	insignificant	0.024934	0.77363	insignificant	1	9	9
chr4	63406147	63408147	Tnfsf15		1	noCoverage	-0.18788	0.86101	insignificant	0	5	5
chr4	63522318	63524318	Tnfsf8		1	noCoverage	-0.018279	0.8431	insignificant	0	8	8
chr4	64784207	64786207	Pappa	-0.11446	4.21E-13	hypomethylated	0.017066	0.083104	insignificant	39	86	95
chr4	65265019	65267019	Trim32	-0.51948	0.49306	lowCoverage	0.046144	0.5641	insignificant	1	7	8
chr4	66065517	66067517	Astn2	-0.074724	0.00003712	hypomethylated	-0.025504	0.0074562	hypomethylated	5	28	28
chr4	68615431	68617431	Dbc1	-0.52267	9.96E-13	stronglyHypometh	0.042311	0.76586	insignificant	2	8	8
chr4	70071401	70073401	Cdk5rap2	-0.010175	2.36E-09	hypomethylated	-0.0068273	0.002026	hypomethylated	11	51	50
chr4	70195962	70197962	Megf9	-0.050527	2.34E-11	hypomethylated	-0.0048928	0.21565	insignificant	35	96	96
chr4	71861277	71863277	C630043F03Rik	-0.12078	7.51E-10	hypomethylated	-0.0004461	0.87805	insignificant	29	123	123
chr4	71861953	71863953	Tle1	-0.18828	0.0018808	hypomethylated	-0.007537	1	insignificant	8	30	30
chr4	73436506	73438506	Rasef	-0.099174	1.7E-13	hypomethylated	-0.0041739	1	insignificant	29	95	93
chr4	73658506	73660506	Frmd3	-0.19086	0.00000046	hypomethylated	0.021151	0.63493	insignificant	4	22	29
chr4	73887400	73889400	Kdm4c		1	noCoverage	-0.1332	1	insignificant	0	7	7
chr4	73896782	73898782	Kdm4c	-0.1135	6.36E-14	hypomethylated	0.000037159	0.61171	insignificant	27	95	95
chr4	74924190	74926190	3110001D03Rik	-0.17002	0.011394	hypomethylated	0.012545	0.2753	insignificant	4	22	22
chr4	80479133	80481133	Tyrp1		1	noCoverage	0.040694	1	insignificant	0	10	7
chr4	80555589	80557589	D4Bwg0951e	-0.24321	0.0028812	hypomethylated	-0.074841	0.62302	insignificant	9	41	47
chr4	81088709	81090709	Mpdz	-0.14892	1.17E-22	hypomethylated	0.00011668	0.0056062	inconclusive	14	54	56
chr4	82151212	82153212	Nfib	-0.10721	4.55E-21	hypomethylated	-0.0099734	0.048797	hypomethylated	65	202	197
chr4	82505565	82507565	Zdhhc21	-0.14493	0.000000315	hypomethylated	-0.0019562	0.48818	insignificant	8	26	26
chr4	82970093	82972093	Ttc39b	-0.12515	0.000006536	hypomethylated	-0.012404	0.21291	insignificant	9	26	26
chr4	83062647	83064647	Snapc3	-0.14674	1.34E-15	hypomethylated	-0.0076533	0.0062187	hypomethylated	16	75	74
chr4	83132294	83134294	Psip1	-0.12719	0.00492	hypomethylated	-0.017822	0.49294	insignificant	4	24	24
chr4	83170448	83172448	4930473A06Rik	-0.11205	1.95E-12	hypomethylated	-0.010196	0.063093	insignificant	33	130	130
chr4	84320990	84322990	Bnc2	-0.087689	0.00000137	hypomethylated	-0.017425	0.042433	hypomethylated	33	115	110
chr4	84529230	84531230	Cntln	-0.080762	4.25E-13	hypomethylated	-0.0066857	0.18979	insignificant	36	136	136
chr4	84850359	84852359	Sh3gl2	-0.11767	0.039555	hypomethylated	0.028364	0.96335	insignificant	5	43	42
chr4	86204207	86206207	Fam154a	-0.45926	0.57667	insignificant	-0.13056	0.76223	insignificant	2	6	6
chr4	86220576	86222576	Rraga	-0.13145	1.06E-18	hypomethylated	-0.0021341	0.078734	insignificant	16	97	96
chr4	86257926	86259926	Haus6	-0.20354	0.0000031	hypomethylated	-0.02749	0.000019872	hypomethylated	14	48	48
chr4	86393458	86395458	Dennd4c	-0.1405	2.13E-13	hypomethylated	0.056959	0.67952	insignificant	24	72	80
chr4	86503271	86505271	Rps6	-0.18695	8.29E-20	hypomethylated	-0.039495	0.057046	insignificant	16	41	42
chr4	86519317	86521317	Acer2	-0.22069	8.01E-36	hypomethylated	0.055891	0.0010061	inconclusive	20	37	41
chr4	86873867	86875867	Slc24a2	-0.52195	0.000062198	stronglyHypometh	-0.10914	0.090426	insignificant	2	8	8
chr4	86876444	86878444	Slc24a2		1	noCoverage	-0.1741	0.57933	insignificant	0	15	12
chr4	87679311	87681311	Mllt3	-0.14345	0.0057651	hypomethylated	0.0069286	0.12451	insignificant	6	42	42
chr4	87739533	87741533	BC057079	-0.16904	1.02E-12	hypomethylated	0.0046009	0.33765	insignificant	13	42	42
chr4	88399771	88401771	Gm13271	0.11875	1	insignificant	0.062754	0.086739	insignificant	3	8	9
chr4	88418897	88420897	Gm13285		1	noCoverage	0.02252	0.14865	insignificant	0	33	33
chr4	88424753	88426753	Gm13285		1	noCoverage	0.0072412	4.5E-10	inconclusive	0	39	47
chr4	88427034	88429034	Ifnz		1	noCoverage	0.000079008	0.39275	insignificant	0	18	37
chr4	88435798	88437798	Gm13278		1	noCoverage	-0.012076	0.62227	insignificant	0	22	20
chr4	88436453	88438453	Gm13285		1	noCoverage	0.00077289	0.74742	insignificant	0	26	24
chr4	88438645	88440645	Gm13275		1	noCoverage	0.018838	0.80062	insignificant	0	19	27
chr4	88441645	88443645	Gm13278		1	noCoverage	-0.0075188	1	insignificant	0	9	9
chr4	88442300	88444300	Gm13285		1	noCoverage	0.34449	0.42122	insignificant	0	9	17
chr4	88444478	88446478	Gm13275		1	noCoverage	-0.0121	0.90546	insignificant	0	18	18
chr4	88445223	88447223	Gm13285		1	noCoverage	0.11272	1	insignificant	0	18	21
chr4	88448157	88450157	Gm13285		1	noCoverage	0.21103	0.00000971	inconclusive	0	16	19
chr4	88451089	88453089	Gm13285	-0.48595	5.26E-33	stronglyHypometh	-0.044406	9.35E-59	hypomethylated	8	25	25
chr4	88472319	88474319	Ifna6	-0.75	0.27273	lowCoverage	0.029762	1	insignificant	1	4	4
chr4	88782273	88784273	Mtap	-0.13641	0.00000318	hypomethylated	-0.00097856	0.89264	insignificant	17	55	50
chr4	88928096	88930096	Cdkn2a		1	noCoverage	-0.13935	0.501	insignificant	0	9	10
chr4	88940523	88942523	Cdkn2a	-0.10304	4.71E-13	hypomethylated	-0.0049581	0.15917	insignificant	51	173	164
chr4	88956941	88958941	Cdkn2b		1	noCoverage	0	0.69318	insignificant	0	8	8
chr4	89353888	89355888	Dmrta1	-0.095817	0.000000708	hypomethylated	-0.0026251	0.93755	insignificant	25	64	64
chr4	91038746	91040746	Elavl2	-0.18414	0.0015926	hypomethylated	-0.032226	0.10236	insignificant	9	46	46
chr4	91043187	91045187	Elavl2	-0.095004	1	insignificant	0.066322	0.6238	insignificant	3	24	20
chr4	91066675	91068675	Elavl2	-0.11978	0.000025822	hypomethylated	-0.012095	0.35481	insignificant	11	46	46
chr4	93002202	93004202	Tusc1	-0.12645	0.0013436	hypomethylated	0.0040619	0.89564	insignificant	6	22	22
chr4	94269938	94271938	Plaa	-0.1426	9.81E-14	hypomethylated	-0.021254	0.0027811	hypomethylated	17	50	50
chr4	94280210	94282210	Ift74		1	noCoverage	-0.033708	1	insignificant	0	13	12
chr4	94330847	94332847	Mir872	-0.16157	0.14597	insignificant	0.047694	1	insignificant	2	4	4
chr4	94645791	94647791	Mysm1	-0.21613	0.073199	insignificant	0.02351	0.078804	insignificant	5	12	14
chr4	94718913	94720913	Jun	-0.2089	1.27E-08	hypomethylated	-0.053117	0.06819	insignificant	10	58	73
chr4	95223197	95225197	Fggy	-0.5239	0.020221	stronglyHypometh	0.032929	0.53071	insignificant	4	18	22
chr4	95633069	95635069	Hook1	-0.091075	5.11E-11	hypomethylated	-0.001446	0.88288	insignificant	26	94	94
chr4	97247633	97249633	Nfia		1	noCoverage	-0.012589	0.38013	insignificant	0	8	9
chr4	97251282	97253282	Nfia	-0.06099	0.015215	hypomethylated	-0.042334	0.013728	hypomethylated	4	8	8
chr4	97443316	97445316	Nfia	-0.074826	0.00044824	hypomethylated	0.018061	0.87688	insignificant	43	105	113

chr4	98061516	98063516	Inadl	-0.080682	0.0003009	hypomethylated	0.0017159	0.78413	insignificant	20	80	80
chr4	98392444	98394444	L1td1	-0.35222	1.04E-21	stronglyHypometh	0.0029799	0.00019268	inconclusive	7	30	30
chr4	98484228	98486228	Kank4	-0.22568	3.19E-12	hypomethylated	0.10435	0.82033	insignificant	11	26	32
chr4	98589500	98591500	Usp1	-0.1216	4.18E-36	hypomethylated	-0.0041059	0.68735	insignificant	61	161	161
chr4	98787606	98789606	Dock7	-0.23123	0.024321	hypomethylated	-0.078864	0.81223	insignificant	7	25	22
chr4	98859624	98861624	Atg4c	-0.13665	9.85E-11	hypomethylated	0.016561	0.1797	insignificant	7	44	47
chr4	98859837	98861837	Atg4c	-0.13665	9.85E-11	hypomethylated	0.016561	0.1797	insignificant	7	44	47
chr4	99321989	99323989	Foxd3	-0.16686	8.32E-49	hypomethylated	-0.0013324	0.66672	insignificant	47	182	206
chr4	99381320	99383320	Alg6	-0.25454	0.00032651	hypomethylated	0.035683	0.68948	insignificant	5	18	19
chr4	99495190	99497190	Efcab7	-0.14093	0.000000497	hypomethylated	-0.024441	0.3549	insignificant	8	34	41
chr4	99495809	99497809	Itgb3bp	-0.14093	0.000000497	hypomethylated	-0.024441	0.3549	insignificant	8	34	41
chr4	99601055	99603055	Pgm2	-0.16224	0.0019895	hypomethylated	-0.013587	0.56296	insignificant	26	113	126
chr4	99767395	99769395	Ror1	-0.11486	7.7E-19	hypomethylated	0.0010356	0.25811	insignificant	22	90	95
chr4	100150471	100152471	Ube2u	-0.69167	0.00088122	stronglyHypometh	-0.16087	0.012979	hypomethylated	2	10	18
chr4	100448283	100450283	Cachd1	-0.13698	1.26E-24	hypomethylated	-0.022069	0.21519	insignificant	28	67	65
chr4	100740642	100742642	Raver2	-0.1008	1.53E-40	hypomethylated	-0.0092322	0.00001404	hypomethylated	49	173	176
chr4	101019632	101021632	Mir101a		1	noCoverage	0.059653	0.04523	hypermethylated	0	8	8
chr4	101090893	101092893	Ak4	-0.16235	1.08E-26	hypomethylated	-0.021183	0.16735	insignificant	37	113	123
chr4	101090916	101092916	Ak4	-0.16235	1.08E-26	hypomethylated	-0.021183	0.16735	insignificant	37	113	123
chr4	101090925	101092925	Ak4	-0.16235	1.08E-26	hypomethylated	-0.021183	0.16735	insignificant	37	113	123
chr4	101091307	101093307	Ak4	-0.14749	1.07E-25	hypomethylated	-0.020305	0.30626	insignificant	37	111	121
chr4	101168252	101170252	Dnajc6	-0.12702	7.15E-30	hypomethylated	-0.013772	0.028915	hypomethylated	48	125	128
chr4	101179580	101181580	Dnajc6	-0.20321	0.010401	hypomethylated	0.062145	0.48797	insignificant	4	30	26
chr4	101222198	101224198	Dnajc6	-0.15087	9.95E-13	hypomethylated	0.0061811	0.29241	insignificant	21	73	74
chr4	101389011	101391011	Lepr	-0.16977	0.00072779	hypomethylated	0.002603	0.94605	insignificant	4	28	32
chr4	101658444	101660444	Gm12789		1	noCoverage	0.0090278	1	insignificant	0	8	8
chr4	102241664	102243664	Pde4b	-0.20098	0.13754	insignificant	-0.022895	0.7351	insignificant	4	24	24
chr4	102261447	102263447	Pde4b		1	noCoverage	0.16046	0.011308	hypermethylated	0	10	17
chr4	102431967	102433967	Sgip1	-0.18375	0.0028894	hypomethylated	0.031906	0.063886	insignificant	7	18	18
chr4	102786069	102788069	Mier1	-0.088442	8.31E-60	hypomethylated	0.005145	0.076693	insignificant	46	159	156
chr4	102786904	102788904	Wdr78	-0.075079	1.94E-44	hypomethylated	0.0047291	0.020145	inconclusive	34	108	105
chr4	102790994	102792994	Mier1	-0.096789	0.000000321	hypomethylated	-0.010481	0.82888	insignificant	25	101	100
chr4	102887489	102889489	Slc35d1	-0.062502	0.39843	insignificant	0.0038197	0.19015	insignificant	21	85	83
chr4	102985450	102987450	Oma1	-0.08686	0.00017617	hypomethylated	0.0075427	0.038185	hypermethylated	23	74	77
chr4	104039164	104041164	Dab1	-0.13606	1.3E-25	hypomethylated	0.017686	0.82283	insignificant	45	137	142
chr4	104039175	104041175	Dab1	-0.13606	1.3E-25	hypomethylated	0.017686	0.82283	insignificant	45	139	144
chr4	104437921	104439921	C8b	-0.6527	0.000013017	stronglyHypometh	-0.15308	0.0055922	hypomethylated	2	8	8
chr4	104585060	104587060	1700024P16Rik	-0.14649	0.00004214	hypomethylated	0.013853	0.51468	insignificant	5	22	22
chr4	104782503	104784503	Prkaa2	0.031402	1	insignificant	0.019127	0.12473	insignificant	2	8	8
chr4	104828951	104830951	Ppap2b	-0.16942	5.37E-27	hypomethylated	0.0055314	0.000048942	inconclusive	41	132	136
chr4	105987817	105989817	Usp24	-0.10316	3.66E-38	hypomethylated	-0.000010179	0.74981	insignificant	75	212	227
chr4	106136930	106138930	Pcsk9	-0.44	1	insignificant	-0.16059	0.53963	insignificant	2	5	4
chr4	106164848	106166848	Bsnd		1	noCoverage	0.033333	0.57375	insignificant	0	2	2
chr4	106232642	106234642	Dhcr24	-0.13516	1.82E-19	hypomethylated	0.0060831	0.02578	hypermethylated	32	124	122
chr4	106289843	106291843	BC055111	-0.17785	1.52E-09	hypomethylated	-0.0046806	0.82354	insignificant	6	14	14
chr4	106294053	106296053	Ttc22	-0.035238	0.22196	insignificant	-0.016605	0.039581	hypomethylated	16	63	72
chr4	106322673	106324673	Pars2	-0.24392	0.000042472	hypomethylated	-0.023168	0.079937	insignificant	10	20	20
chr4	106351291	106353291	Ttc4	0.019482	0.78254	insignificant	0.0082995	0.1927	insignificant	5	28	28
chr4	106351549	106353549	Ttc4	0.052632	1	insignificant	0.052632	0.29916	insignificant	1	4	4
chr4	106472436	106474436	Acot11	-0.37948	3.75E-16	stronglyHypometh	0.020712	0.60867	insignificant	7	22	24
chr4	106583074	106585074	Ssbp3	-0.074001	2.42E-17	hypomethylated	-0.0037952	0.41388	insignificant	65	262	262
chr4	106739471	106741471	Mrpl37	-0.10891	1.63E-13	hypomethylated	-0.0016439	0.16788	insignificant	13	54	54
chr4	106741772	106743772	Cyb5rl		1	noCoverage	0.037402	0.6875	insignificant	0	8	9
chr4	106850234	106852234	Tmem59	-0.20214	4E-41	hypomethylated	-0.001625	0.094764	insignificant	28	92	95
chr4	106850971	106852971	2210012G02Rik	-0.11918	1.7E-25	hypomethylated	0.013979	0.83867	insignificant	25	66	69
chr4	106925538	106927538	Hspb11	-0.11377	4.35E-30	hypomethylated	0.0051999	0.18887	insignificant	27	90	90
chr4	106926138	106928138	Lrrc42	-0.12073	0.00015205	hypomethylated	0.013953	0.35923	insignificant	12	28	28
chr4	106986035	106988035	Yipf1	-0.24179	0.012108	hypomethylated	0.097683	0.31118	insignificant	3	23	32
chr4	107039388	107041388	Tmem48	-0.13672	6.1E-18	hypomethylated	0.0074694	0.41666	insignificant	17	56	56
chr4	107106323	107108323	Glis1	-0.12261	1.57E-29	hypomethylated	-0.010016	0.66323	insignificant	42	180	192
chr4	107356767	107358767	Dmrtb1	-0.20495	0.0020325	hypomethylated	-0.070442	0.094696	insignificant	3	21	24
chr4	107473863	107475863	Lrp8	-0.13137	3.9E-35	hypomethylated	0.0063402	0.91125	insignificant	54	154	163
chr4	107551417	107553417	Magoh	-0.20995	7.07E-11	hypomethylated	0.046327	0.033442	inconclusive	8	50	44
chr4	107561503	107563503	0610037L13Rik	-0.13938	0.00043784	hypomethylated	0.024531	0.0053431	inconclusive	15	61	56
chr4	107596194	107598194	Cpt2	-0.12646	0.000000218	hypomethylated	0.027451	0.15358	insignificant	5	18	18
chr4	107639938	107641938	Slc1a7	-0.085847	0.33288	insignificant	0.18671	0.00052745	hypermethylated	2	10	14
chr4	107704695	107706695	Podn	-0.28876	0.00000252	hypomethylated	-0.0085153	0.000046942	hypomethylated	5	28	28
chr4	107791104	107793104	Scp2	-0.10913	0.13445	insignificant	-0.0046398	0.025828	inconclusive	6	20	24
chr4	107837070	107839070	Echdc2	-0.10901	1.31E-12	hypomethylated	-0.014936	0.25536	insignificant	11	46	47
chr4	107890527	107892527	Zyg11a	-0.11667	0.0019207	hypomethylated	0.10056	0.92461	insignificant	7	14	16
chr4	107999756	108001756	2010305A19Rik	-0.1657	0.22461	insignificant	0.023139	0.15045	insignificant	6	34	34
chr4	108055954	108057954	Fam159a	-0.12039	9.52E-11	hypomethylated	-0.039	0.79588	insignificant	15	42	54
chr4	108079318	108081318	Gpx7	0.12155	1	insignificant	-0.039799	0.030894	hypomethylated	1	20	22
chr4	108131030	108133030	Zcchc11	-0.13824	1.5E-19	hypomethylated	-0.010215	0.00080156	hypomethylated	21	113	128
chr4	108251058	108253058	Orc1	-0.20001	1.64E-18	hypomethylated	-0.0069654	0.0059002	hypomethylated	31	94	91
chr4	108251941	108253941	Prpf38a	-0.15253	6.52E-09	hypomethylated	0.001015	0.56583	insignificant	19	53	53
chr4	108291560	108293560	Cc2d1b	-0.11212	4.82E-39	hypomethylated	-0.0028585	0.037354	hypomethylated	40	124	128
chr4	108506189	108508189	Btf3l4	-0.12642	0.0087296	hypomethylated	-0.016062	0.96755	insignificant	14	84	84
chr4	108506282	108508282	Txndc12	-0.16558	0.00024215	hypomethylated	-0.019184	0.3083	insignificant	14	57	57
chr4	108519461	108521461	Kti12	-0.15723	6.53E-21	hypomethylated	-0.015263	0.062519	insignificant	33	103	103
chr4	108550674	108552674	Rab3b	-0.21093	1.32E-17	hypomethylated	-0.022271	0.16269	insignificant	10	45	46
chr4	108672409	108674409	Nrd1	-0.18199	1.04E-13	hypomethylated	-0.015968	0.97356	insignificant	16	76	77
chr4	108951879	108953879	Eps15	-0.12924	2.01E-29	hypomethylated	0.0008652	0.016928	inconclusive	47	103	115
chr4	109078704	109080704	Ttc39a	-0.14639	1.81E-15	hypomethylated	-0.035124	1	insignificant	20	75	90
chr4	109087251	109089251	Ttc39a	0.098485	1	insignificant	-0.036932	0.46079	insignificant	2	6	6

chr4	109149110	109151110	Rnf11	-0.15593	4.69E-15	hypomethylated	0.0059341	0.54429	insignificant	20	70	70
chr4	109337977	109339977	Cdkn2c	-0.10501	3.56E-38	hypomethylated	0.0018656	0.3948	insignificant	49	204	199
chr4	109348231	109350231	Faf1	-0.1015	7.43E-22	hypomethylated	-0.0080087	0.77108	insignificant	50	127	134
chr4	109649629	109651629	Dmrta2	-0.12765	7.06E-08	hypomethylated	-0.0039949	0.32711	insignificant	29	126	136
chr4	110069395	110071395	Agbl4	-0.10969	5.94E-08	hypomethylated	0.011353	0.52949	insignificant	10	20	20
chr4	111086610	111088610	Bend5	-0.091857	0.00092989	hypomethylated	0.018071	0.57241	insignificant	28	90	99
chr4	111391614	111393614	Spata6	-0.089667	2.94E-08	hypomethylated	0.0067693	0.65351	insignificant	24	66	69
chr4	114078328	114080328	Gm12824	-0.088601	1.83E-14	hypomethylated	-0.012809	0.75744	insignificant	41	139	139
chr4	114493324	114495324	Gm12830	-0.044764	0.0017123	hypomethylated	0.029458	0.70495	insignificant	13	46	45
chr4	114580893	114582893	9130206I24Rik	-0.12485	8.51E-38	hypomethylated	-0.0097643	0.30405	insignificant	40	170	190
chr4	114581503	114583503	9130206I24Rik	-0.16477	8.14E-15	hypomethylated	-0.0093377	0.38725	insignificant	19	78	82
chr4	114598618	114600618	Foxe3	-0.19642	0.012635	hypomethylated	-0.012802	0.25017	insignificant	5	18	18
chr4	114659833	114661833	Cmpk1	-0.14348	1.79E-10	hypomethylated	-0.022312	0.08181	insignificant	13	28	28
chr4	114671722	114673722	Stil	-0.074588	0.00016386	hypomethylated	-0.0044594	0.11344	insignificant	3	45	49
chr4	114731131	114733131	Tal1	-0.2485	0.00032644	hypomethylated	0.016511	0.56205	insignificant	7	26	26
chr4	114760312	114762312	Pdzk1ip1		1	noCoverage	0.13309	0.1615	insignificant	0	7	4
chr4	115255732	115257732	Gm12839		1	noCoverage	0.010894	0.19648	insignificant	0	32	28
chr4	115409677	115411677	4732418C07Rik	-0.050561	0.00095134	hypomethylated	-0.0036863	0.40294	insignificant	7	44	44
chr4	115456589	115458589	Atpaf1	-0.074206	0.0027935	hypomethylated	-0.0024182	0.49883	insignificant	17	71	80
chr4	115499696	115501696	Mob3c	-0.11289	2.05E-12	hypomethylated	0.0025433	0.41142	insignificant	22	75	75
chr4	115510850	115512850	Mknk1	-0.17234	0.0013861	hypomethylated	0.0086953	0.58321	insignificant	9	40	41
chr4	115556004	115558004	Kncn	-0.16604	0.0014318	hypomethylated	0.068147	0.29403	insignificant	4	14	13
chr4	115612531	115614531	Dmbx1	-0.11418	1.38E-12	hypomethylated	0.0061541	0.61521	insignificant	15	32	32
chr4	115690507	115692507	Faah	-0.12842	0.00052081	hypomethylated	-0.033888	0.32635	insignificant	5	16	12
chr4	115747069	115749069	Lrrc41	-0.14225	8.65E-34	hypomethylated	-0.0045512	0.87281	insignificant	27	147	143
chr4	115747675	115749675	Uqcrh	-0.12206	7.19E-35	hypomethylated	-0.0063451	0.6791	insignificant	35	151	147
chr4	115796294	115798294	Rad54l	-0.29857	2.29E-11	hypomethylated	-0.022245	0.40839	insignificant	3	11	11
chr4	115796295	115798295	Rad54l	-0.29857	2.29E-11	hypomethylated	-0.022245	0.40839	insignificant	3	11	11
chr4	115817221	115819221	1520402A15Rik		1	noCoverage	-0.19228	0.15401	insignificant	0	18	11
chr4	115822122	115824122	Pomgnt1	-0.17066	7.12E-19	hypomethylated	-0.052361	0.0091646	hypomethylated	44	91	127
chr4	115840203	115842203	Tspan1	-0.29697	0.17202	insignificant	-0.2004	0.00014363	hypomethylated	1	10	10
chr4	115844976	115846976	1700042G07Rik	-0.14441	0.40841	insignificant	-0.041545	0.90169	insignificant	7	16	16
chr4	115893518	115895518	Pik3r3	-0.19695	2.86E-15	hypomethylated	0.0065305	0.66768	insignificant	23	74	80
chr4	116136788	116138788	Mast2	-0.089937	0.00098185	hypomethylated	-0.01553	0.91875	insignificant	15	54	55
chr4	116179153	116181153	Ipp	-0.13791	7.03E-15	hypomethylated	-0.015018	0.90818	insignificant	26	81	80
chr4	116228548	116230548	Tmem69	-0.16483	4.42E-27	hypomethylated	-0.012326	0.025332	hypomethylated	36	96	94
chr4	116229331	116231331	Gpbp1l1	-0.13822	1.14E-26	hypomethylated	0.0047223	0.24084	insignificant	39	122	124
chr4	116268334	116270334	Ccdc17	-0.12477	0.00000013	hypomethylated	-0.011713	0.82902	insignificant	12	40	40
chr4	116270235	116272235	C530005A16Rik	-0.20777	0.12992	insignificant	-0.015169	0.13596	insignificant	7	29	29
chr4	116300102	116302102	Nasp	-0.29464	0.58748	insignificant	0.14821	0.085124	insignificant	2	7	5
chr4	116324256	116326256	Akr1a1	-0.20714	0.000024504	hypomethylated	-0.0059934	0.63924	insignificant	3	6	6
chr4	116357203	116359203	Prdx1	-0.1322	2.33E-08	hypomethylated	-0.0070933	0.39105	insignificant	21	88	100
chr4	116380534	116382534	Ccdc163	-0.19176	1	insignificant	0.026155	0.47653	insignificant	5	20	22
chr4	116380990	116382990	Mmachc	-0.37279	1	insignificant	0.03181	0.73257	insignificant	2	10	12
chr4	116392559	116394559	Tesk2	-0.18933	2.25E-20	hypomethylated	0.020976	0.84784	insignificant	17	94	89
chr4	116479338	116481338	Mutyh	-0.51166	1.07E-12	stronglyHypometh	-0.035106	0.14233	insignificant	6	32	32
chr4	116494113	116496113	Hpdl	-0.44589	0.0014792	stronglyHypometh	-0.016989	0.926	insignificant	5	15	15
chr4	116549006	116551006	Zswim5	-0.11515	3.8E-69	hypomethylated	0.0013448	0.030796	inconclusive	84	224	222
chr4	116666952	116668952	Hectd3	-0.11392	2.43E-22	hypomethylated	0.010812	0.062803	insignificant	49	134	135
chr4	116666980	116668980	Hectd3	-0.11392	2.43E-22	hypomethylated	0.010812	0.062803	insignificant	49	134	135
chr4	116691012	116693012	Eif2b3	-0.087895	4.29E-12	hypomethylated	-0.034683	0.00015752	hypomethylated	24	93	96
chr4	116767960	116769960	Ptch2	-0.23108	0.000015046	hypomethylated	0.028744	0.53285	insignificant	11	32	32
chr4	116798330	116800330	Btbd19		1	noCoverage	-0.20264	0.55962	insignificant	0	7	8
chr4	116798417	116800417	Tctex1d4		1	noCoverage	-0.20264	0.55962	insignificant	0	7	8
chr4	116806557	116808557	Plk3	-0.043806	0.54283	insignificant	0.002737	0.74568	insignificant	6	31	35
chr4	116827179	116829179	Snord38a	-0.15237	0.000000503	hypomethylated	-0.023923	0.041128	hypomethylated	23	95	88
chr4	116828453	116830453	Snord55	-0.084023	0.032671	hypomethylated	0.01736	0.9656	insignificant	15	66	68
chr4	116828737	116830737	Snord55	0.41644	0.2801	insignificant	0.021452	0.085824	insignificant	3	27	28
chr4	116855229	116857229	Kif2c	-0.52664	0.080939	insignificant	-0.007598	0.54112	insignificant	3	31	33
chr4	116923593	116925593	Tmem53	-0.12603	1.51E-09	hypomethylated	0.017604	0.28279	insignificant	15	43	43
chr4	116924522	116926522	Gm1661	-0.14088	0.0001305	hypomethylated	0.051087	0.29841	insignificant	9	31	32
chr4	117169520	117171520	Rnf220		1	noCoverage	0.19154	0.62969	insignificant	0	14	7
chr4	117354830	117356830	Dmap1	-0.37063	0.00000107	stronglyHypometh	-0.16177	0.50672	insignificant	6	13	17
chr4	117506862	117508862	Slc6a9	-0.12088	1.1E-24	hypomethylated	-0.0008757	0.49594	insignificant	29	70	70
chr4	117545075	117547075	B4galt2	-0.085133	0.59455	insignificant	0.18697	0.00000159	hypermethylated	6	24	24
chr4	117556074	117558074	Atp6v0b	-0.060475	0.090261	insignificant	-0.002823	0.069125	insignificant	1	29	26
chr4	117559934	117561934	Atp6v0b	-0.23048	2.13E-09	hypomethylated	0.02739	0.9079	insignificant	8	38	34
chr4	117564608	117566608	Dph2	-0.68254	0.015946	stronglyHypometh	-0.015873	1	insignificant	2	9	9
chr4	117587604	117589604	Ipo13	-0.16716	0.000000687	hypomethylated	0.0095366	0.49703	insignificant	16	57	58
chr4	117602368	117604368	Artn	-0.13064	0.1535	insignificant	0.027836	0.070132	insignificant	3	10	12
chr4	117807495	117809495	St3gal3	-0.13869	1.57E-08	hypomethylated	-0.011184	0.10502	insignificant	16	54	54
chr4	117807519	117809519	St3gal3	-0.15569	4.05E-10	hypomethylated	-0.012005	0.031726	hypomethylated	16	50	50
chr4	117852648	117854648	Kdm4a	-0.26583	0.0021508	hypomethylated	0.068785	0.64575	insignificant	5	15	17
chr4	117964002	117966002	Ptprf	-0.18743	3.19E-08	hypomethylated	-0.016425	0.36249	insignificant	10	33	32
chr4	118031618	118033618	Hyi	-0.092651	0.00060709	hypomethylated	-0.01061	0.27651	insignificant	5	41	46
chr4	118080941	118082941	Med8	-0.17774	3.1E-47	hypomethylated	-0.024905	0.30933	insignificant	24	77	78
chr4	118081868	118083868	Szt2	-0.17947	3.52E-46	hypomethylated	-0.014324	0.34564	insignificant	19	65	66
chr4	118099697	118101697	Elovl1	-0.20812	6.73E-16	hypomethylated	-0.038764	0.0070125	hypomethylated	15	42	40
chr4	118101305	118103305	Elovl1	0.11253	1	insignificant	0.0013247	1	insignificant	1	12	12
chr4	118109948	118111948	Cdc20	-0.35373	0.002863	stronglyHypometh	0.084119	0.23895	insignificant	11	56	57
chr4	118130100	118132100	Mpl	-0.2041	0.000011279	hypomethylated	-0.081101	0.033698	hypomethylated	4	22	23
chr4	118162454	118164454	Tie1		1	noCoverage	0.044002	0.92621	insignificant	0	6	6
chr4	118198879	118200879	2610528J11Rik		1	noCoverage	-0.048181	0.020565	inconclusive	0	12	6
chr4	118216331	118218331	Tmem125	-0.62121	4.16E-10	stronglyHypometh	-0.21143	0.00061495	hypomethylated	4	23	24
chr4	118292403	118294403	Ebna1bp2	-0.040779	0.0011007	hypomethylated	0.065102	0.91296	insignificant	4	35	37

chr4	118293010	118295010	Wdr65	-0.017102	0.00065546	hypomethylated	0.070343	0.68888	insignificant	4	30	32
chr4	118298008	118300008	D4Ertd617e	0.056127	0.24581	insignificant	0.047396	0.4677	insignificant	2	4	4
chr4	118406135	118408135	Olfr1339		1	noCoverage	0.20895	0.76606	insignificant	0	8	6
chr4	118427141	118429141	Olfr1338	0.030631	0.26662	insignificant	-0.13723	0.15105	insignificant	2	4	4
chr4	118780349	118782349	Slc2a1	-0.13337	0.00034493	hypomethylated	0.035872	0.74188	insignificant	12	87	78
chr4	118862616	118864616	Ermap	-0.21617	0.2061	insignificant	0.01617	0.45699	insignificant	1	10	12
chr4	118866914	118868914	Ccdc23	-0.14013	9.93E-17	hypomethylated	-0.001861	0.13228	insignificant	25	106	106
chr4	118867129	118869129	Ccdc23	-0.16179	6.17E-21	hypomethylated	0.0050812	0.0098105	inconclusive	25	110	110
chr4	118904519	118906519	Lepre1	-0.15054	4.69E-16	hypomethylated	0.035657	0.074533	insignificant	24	103	109
chr4	118905329	118907329	AU022252	-0.13662	7.67E-12	hypomethylated	-0.017397	0.037261	hypomethylated	16	77	78
chr4	118905433	118907433	Lepre1	-0.13662	7.67E-12	hypomethylated	-0.017397	0.037261	hypomethylated	16	77	78
chr4	118927045	118929045	Cldn19	0.075647	0.051539	insignificant	0.00042763	0.45089	insignificant	7	17	16
chr4	118927073	118929073	Cldn19	0.075647	0.051539	insignificant	0.00042763	0.45089	insignificant	7	17	16
chr4	118967118	118969118	Ybx1	-0.13993	3.01E-20	hypomethylated	0.041232	0.21269	insignificant	25	76	83
chr4	118993128	118995128	Ppih	-0.30682	1.59E-15	hypomethylated	-0.085299	0.84429	insignificant	9	11	20
chr4	119088126	119090126	Ccdc30	-0.16611	0.00002725	hypomethylated	0.0042851	0.72847	insignificant	8	26	26
chr4	119094288	119096288	Zmynd12	-0.11147	9.42E-21	hypomethylated	0.000093074	0.84457	insignificant	22	138	138
chr4	119095025	119097025	Zmynd12	-0.11258	1.34E-14	hypomethylated	0.0061239	0.098778	insignificant	16	80	80
chr4	119165203	119167203	Rimkla	-0.070844	0.000015425	hypomethylated	0.01408	0.77374	insignificant	11	30	35
chr4	119211292	119213292	Foxj3	-0.10185	6.56E-51	hypomethylated	-0.0029838	0.29034	insignificant	99	285	282
chr4	119211374	119213374	AA415398	-0.10185	6.56E-51	hypomethylated	-0.0029838	0.29034	insignificant	99	285	282
chr4	119309336	119311336	Guca2a	-0.042945	0.75847	insignificant	0.019616	0.76146	insignificant	5	16	18
chr4	119486282	119488282	Hivep3		1	noCoverage	0.022522	1	insignificant	0	30	19
chr4	119833028	119835028	Edn2	-0.27275	0.023904	hypomethylated	-0.002935	0.95758	insignificant	3	30	30
chr4	119959866	119961866	Foxo6	-0.097624	6.01E-36	hypomethylated	-0.0046045	0.040248	hypomethylated	47	124	124
chr4	120076885	120078885	Scmh1	-0.12754	6.81E-19	hypomethylated	-0.013063	0.10738	insignificant	33	103	101
chr4	120242881	120244881	Ctps	-0.12689	1.1E-19	hypomethylated	-0.011486	0.26289	insignificant	14	37	40
chr4	120260349	120262349	Gm8439	-0.7	0.31148	lowCoverage	0.16458	0.60635	insignificant	1	4	8
chr4	120338167	120340167	Cited4	-0.16007	4.86E-45	hypomethylated	-0.001736	0.26161	insignificant	41	115	113
chr4	120419781	120421781	Kcnq4	-0.14419	1.26E-48	hypomethylated	-0.0062938	0.0029015	hypomethylated	60	155	155
chr4	120445302	120447302	Nfyc	-0.13175	0.039229	hypomethylated	-0.048413	0.21348	insignificant	2	6	6
chr4	120498320	120500320	Nfyc		1	noCoverage	-0.0019134	5.47E-11	inconclusive	0	14	12
chr4	120597610	120599610	Dem1	-0.1151	0.00010701	hypomethylated	0.0047583	0.94469	insignificant	7	28	28
chr4	120624306	120626306	Zfp69	-0.24454	8.14E-23	hypomethylated	-0.054842	0.0002848	hypomethylated	13	34	37
chr4	120689852	120691852	Smap2	-0.1473	0.0019029	hypomethylated	0.030265	0.70502	insignificant	6	22	24
chr4	120711170	120713170	Col9a2	-0.3084	5.81E-24	hypomethylated	-0.081347	0.61378	insignificant	15	46	58
chr4	120770848	120772848	Zmpste24	-0.28654	0.000070319	hypomethylated	-0.0048	0.86213	insignificant	6	22	20
chr4	120781831	120783831	Tmco2		1	noCoverage	0.021186	0.4046	insignificant	0	31	32
chr4	122512469	122514469	Ppt1	0.21326	0.50783	insignificant	0.018676	0.83602	insignificant	1	9	6
chr4	122563124	122565124	Cap1	-0.21178	5.94E-17	hypomethylated	-0.0095269	0.9601	insignificant	14	41	41
chr4	122638431	122640431	Mfsd2a	-0.097403	0.071758	insignificant	0.017164	0.00000833	hypermethylated	7	26	26
chr4	122672341	122674341	Mycl1	-0.12741	2.52E-21	hypomethylated	0.0030988	0.77649	insignificant	40	157	163
chr4	122692839	122694839	Trit1	-0.22688	1.17E-16	hypomethylated	0.068747	0.83083	insignificant	10	26	31
chr4	122781407	122783407	Bmp8b	-0.16541	3.94E-10	hypomethylated	-0.0060539	0.4263	insignificant	45	150	148
chr4	122792490	122794490	Oxct2b		1	noCoverage	0.045497	0.0020605	hypermethylated	0	27	28
chr4	122817184	122819184	Ppie	-0.034514	0.0002029	hypomethylated	-0.018439	0.30157	insignificant	8	25	28
chr4	122859746	122861746	Hpcal4	-0.18355	3.87E-11	hypomethylated	-0.016413	0.81646	insignificant	16	71	71
chr4	122877795	122879795	Nt5c1a	-0.23719	7.68E-08	hypomethylated	-0.018808	0.3338	insignificant	12	34	34
chr4	122909798	122911798	Heyl	-0.14449	8.55E-08	hypomethylated	-0.004756	0.10846	insignificant	25	68	68
chr4	122959153	122961153	Pabpc4	-0.15235	1.91E-14	hypomethylated	-0.024942	0.91447	insignificant	18	63	64
chr4	122959527	122961527	Pabpc4	-0.15235	1.91E-14	hypomethylated	-0.024942	0.91447	insignificant	18	63	64
chr4	123000877	123002877	Bmp8a		1	noCoverage	-0.091418	0.034031	hypomethylated	0	3	3
chr4	123020046	123022046	Bmp8a	-0.26898	6.99E-13	hypomethylated	-0.0071096	0.60019	insignificant	7	22	22
chr4	123089154	123091154	D830031N03Rik	-0.14511	9.07E-15	hypomethylated	-0.0015692	1	insignificant	28	69	67
chr4	123395404	123397404	BC002163	-0.082374	0.0014031	hypomethylated	0.0001137	0.34798	insignificant	8	48	48
chr4	123395429	123397429	BC002163	-0.1	0.00016464	hypomethylated	0.0048662	0.54767	insignificant	8	40	40
chr4	123427542	123429542	Akirin1	-0.13632	1.16E-17	hypomethylated	-0.027434	0.000009232	hypomethylated	19	63	76
chr4	123581255	123583255	Mycbp	-0.092553	4.73E-16	hypomethylated	-0.0001063	0.50991	insignificant	34	92	86
chr4	123593675	123595675	Rragc	-0.099766	1.04E-32	hypomethylated	-0.0054615	0.88847	insignificant	38	167	171
chr4	124333888	124335888	Pou3f1	-0.10182	2.09E-21	hypomethylated	0.0030123	0.69554	insignificant	87	263	268
chr4	124370798	124372798	Utp11l	-0.23242	0.000039561	hypomethylated	-0.037645	0.88471	insignificant	6	18	17
chr4	124376942	124378942	Fhl3	-0.1358	2.02E-23	hypomethylated	0.0082759	0.76861	insignificant	40	135	142
chr4	124391104	124393104	Sf3a3	-0.12652	7.51E-12	hypomethylated	0.0038055	0.14275	insignificant	8	36	34
chr4	124420024	124422024	Mir698	-0.11667	1	insignificant	0.035913	0.60595	insignificant	6	20	20
chr4	124478792	124480792	Mtf1	-0.19283	9.13E-30	hypomethylated	-0.0037777	0.0025597	hypomethylated	19	80	75
chr4	124527002	124529002	Yrdc	-0.10539	1.78E-58	hypomethylated	0.0031469	0.0085367	inconclusive	56	203	203
chr4	124527974	124529974	1110065P20Rik	-0.06236	1.07E-18	hypomethylated	0.0020517	0.26287	insignificant	36	128	128
chr4	124539415	124541415	Maneal	-0.18893	8.16E-40	hypomethylated	0.021872	0.00000637	inconclusive	24	74	76
chr4	124558028	124560028	Epha10	-0.16615	2.23E-11	hypomethylated	0.026354	0.018503	inconclusive	23	95	90
chr4	124613291	124615291	9930104L06Rik	-0.18769	3.82E-17	hypomethylated	0.024993	0.32108	insignificant	14	63	56
chr4	124614161	124616161	Cdca8	0.38866	0.67243	insignificant	0.29128	1	insignificant	1	17	10
chr4	124662673	124664673	Rspo1	-0.12299	1.19E-27	hypomethylated	0.0021487	0.13466	insignificant	31	106	106
chr4	124706257	124708257	Gnl2	-0.10377	4.92E-13	hypomethylated	-0.01259	0.34107	insignificant	19	74	74
chr4	124742901	124744901	Dnali1	-0.1034	7.43E-25	hypomethylated	0.005304	0.053885	insignificant	35	122	122
chr4	124742936	124744936	Snip1	-0.1034	7.43E-25	hypomethylated	0.005304	0.053885	insignificant	35	122	122
chr4	124761390	124763390	Meaf6	-0.12968	3.08E-24	hypomethylated	-0.0081724	0.85151	insignificant	43	142	142
chr4	124805125	124807125	Zc3h12a	-0.17788	1.32E-29	hypomethylated	-0.029674	2.34E-12	hypomethylated	14	48	47
chr4	125167074	125169074	Grik3	-0.11106	1.54E-33	hypomethylated	0.0024127	0.56328	insignificant	90	229	239
chr4	125180288	125182288	Grik3		1	noCoverage	-0.28636	0.018112	hypomethylated	0	4	2
chr4	125700902	125702902	Csf3r		1	noCoverage	0.011458	1	insignificant	0	8	8
chr4	125723171	125725171	Mrps15	-0.12515	8.54E-17	hypomethylated	0.00034072	0.57085	insignificant	9	64	64
chr4	125734808	125736808	Oscp1	-0.14724	4.76E-25	hypomethylated	-0.02122	0.00016581	hypomethylated	38	115	113
chr4	125772896	125774896	Lsm10	-0.13107	2.04E-09	hypomethylated	0.04596	0.42269	insignificant	10	47	47
chr4	125780200	125782200	Stk40	-0.16398	4.43E-25	hypomethylated	0.0099391	0.26323	insignificant	30	108	105
chr4	125824246	125826246	Fam176b	-0.27316	3.28E-41	hypomethylated	0.10536	0.49457	insignificant	17	40	40

chr4	125839909	125841909	1700029G01Rik	-0.093253	4.13E-17	hypomethylated	0.029488	0.089023	insignificant	16	52	55
chr4	125879954	125881954	Thrap3	-0.26709	5.4E-10	hypomethylated	0.029806	0.92387	insignificant	8	62	52
chr4	125933563	125935563	Mtap7d1	-0.22175	0.00074598	hypomethylated	-0.0092652	0.92868	insignificant	9	52	52
chr4	125938648	125940648	Trappc3	-0.21142	2.49E-38	hypomethylated	-0.020453	4.25E-11	hypomethylated	23	71	70
chr4	125963037	125965037	Col8a2	-0.19977	4.12E-12	hypomethylated	0.00039085	0.48315	insignificant	24	80	80
chr4	125998947	126000947	Tekt2	-0.13022	2.05E-24	hypomethylated	0.018198	3.09E-12	inconclusive	8	33	34
chr4	126106786	126108786	Eif2c3	-0.15001	1.29E-14	hypomethylated	-0.017114	0.055929	insignificant	10	39	39
chr4	126145665	126147665	Eif2c1	-0.081943	0.00043004	hypomethylated	-0.0017038	0.64667	insignificant	16	71	71
chr4	126210702	126212702	Eif2c4	-0.094287	8.65E-12	hypomethylated	-0.0082991	0.013872	hypomethylated	29	85	85
chr4	126233223	126235223	Clspn	-0.13556	2.64E-16	hypomethylated	-0.0056776	0.32781	insignificant	26	94	88
chr4	126286097	126288097	5730409E04Rik	-0.10039	3.2E-23	hypomethylated	-0.0029812	0.27051	insignificant	20	48	48
chr4	126353886	126355886	Psmb2	-0.25692	1.04E-10	hypomethylated	-0.047075	0.21308	insignificant	14	46	43
chr4	126413513	126415513	Tfap2e		1	noCoverage	-0.09462	0.77929	insignificant	0	14	16
chr4	126429798	126431798	AU040320	-0.14184	1.22E-37	hypomethylated	-0.015082	0.013005	hypomethylated	51	171	177
chr4	126430013	126432013	AU040320	-0.14184	1.22E-37	hypomethylated	-0.015082	0.013005	hypomethylated	51	171	177
chr4	126430673	126432673	Ncdn	-0.17956	2.94E-34	hypomethylated	-0.016406	0.00012118	hypomethylated	35	126	127
chr4	126645167	126647167	Zmym4	-0.1851	0.010983	hypomethylated	-0.033127	0.64218	insignificant	13	48	46
chr4	126697544	126699544	Sfpq	-0.1552	3.68E-34	hypomethylated	-0.028555	0.057616	insignificant	37	109	113
chr4	126738376	126740376	Zmym1	0.34195	0.095177	insignificant	-0.098252	0.68542	insignificant	2	21	32
chr4	126753626	126755626	Zmym6	-0.10928	1	insignificant	0.0037936	0.72275	insignificant	4	58	61
chr4	126802289	126804289	Gm12942	-0.16784	4.69E-09	hypomethylated	-0.046561	0.59124	insignificant	12	32	30
chr4	126845514	126847514	Dlgap3	-0.11796	7.04E-39	hypomethylated	0.028734	0.54002	insignificant	48	133	125
chr4	126991222	126993222	Gja4	-0.26293	1	insignificant	0.054172	0.026284	hypermethylated	3	10	12
chr4	127006369	127008369	Gjb3	-0.20847	0.0079895	hypomethylated	-0.026161	0.08458	insignificant	7	55	56
chr4	127031325	127033325	Gjb4	-0.043263	1	insignificant	-0.10721	0.58366	insignificant	5	14	14
chr4	127648085	127650085	CK137956	-0.19788	0.015274	hypomethylated	0.023312	0.030383	hypermethylated	7	38	40
chr4	128287342	128289342	Zscan20	-0.15101	2.05E-08	hypomethylated	-0.0096434	0.57951	insignificant	6	18	18
chr4	128295863	128297863	Tlr12		1	noCoverage	-0.14186	0.90277	insignificant	0	13	13
chr4	128330945	128332945	Phc2	-0.12333	3.86E-30	hypomethylated	-0.009817	0.0066765	hypomethylated	48	139	146
chr4	128364981	128366981	Phc2	-0.073162	0.3676	insignificant	0.062424	0.87633	insignificant	3	8	8
chr4	128403814	128405814	Phc2	-0.080503	3.08E-12	hypomethylated	0.019168	0.47294	insignificant	32	134	99
chr4	128483356	128485356	Zfp362	-0.098737	5.5E-19	hypomethylated	0.007358	0.27017	insignificant	45	138	147
chr4	128560383	128562383	Trim62	-0.15467	1.9E-30	hypomethylated	-0.0098677	0.74228	insignificant	35	124	119
chr4	128639661	128641661	Adc	-0.33996	1.68E-36	stronglyHypometh	-0.042772	0.0064799	hypomethylated	12	78	78
chr4	128669508	128671508	Ak2	-0.15101	9.13E-09	hypomethylated	-0.0059467	0.81011	insignificant	13	32	32
chr4	128669557	128671557	Ak2	-0.15101	9.13E-09	hypomethylated	-0.0059467	0.81011	insignificant	13	32	32
chr4	128734514	128736514	Rnf19b	-0.08664	2.95E-28	hypomethylated	0.0031994	0.79684	insignificant	69	207	203
chr4	128781859	128783859	Tmem54	-0.215	5.8E-10	hypomethylated	0.010585	0.271	insignificant	18	71	70
chr4	128798535	128800535	Hpca	-0.27151	0.0031614	hypomethylated	-0.091683	0.046497	hypomethylated	14	46	48
chr4	128798984	128800984	Hpca	-0.33279	0.00011948	hypomethylated	-0.29251	0.020652	hypomethylated	2	7	9
chr4	128813303	128815303	Fndc5	-0.11201	0.14738	insignificant	-0.0075228	0.5033	insignificant	10	47	47
chr4	128866038	128868038	Yars	-0.16584	2.84E-15	hypomethylated	-0.020403	0.67381	insignificant	20	56	62
chr4	128866726	128868726	S100pbp	-0.20703	9.72E-13	hypomethylated	0.0020656	0.81435	insignificant	11	32	32
chr4	128925687	128927687	C77080	-0.21337	1.98E-12	hypomethylated	0.003872	0.00000601	inconclusive	25	62	64
chr4	128963864	128965864	Sync	-0.17086	0.00000177	hypomethylated	-0.0092524	0.95158	insignificant	9	37	38
chr4	129012269	129014269	Zbtb8os	-0.15715	5.04E-10	hypomethylated	-0.025249	0.1324	insignificant	26	139	152
chr4	129012614	129014614	Rbbp4	-0.23162	0.00058254	hypomethylated	-0.056194	0.00042943	hypomethylated	7	81	90
chr4	129055272	129057272	Zbtb8a	-0.020551	0.437	insignificant	-0.044806	0.099469	insignificant	2	10	12
chr4	129118062	129120062	Zbtb8b	-0.23341	0.70861	insignificant	0.040887	0.026393	hypermethylated	2	39	38
chr4	129137922	129139922	Bsdc1	-0.25048	1.2E-27	hypomethylated	-0.0027666	0.93179	insignificant	22	52	53
chr4	129167433	129169433	1700125D06Rik	-0.028559	0.49941	insignificant	0.053496	2.33E-10	hypermethylated	29	96	100
chr4	129168014	129170014	Tssk3	0.013648	0.52144	insignificant	0.054671	5.94E-12	hypermethylated	29	88	92
chr4	129189824	129191824	Marcksl1	-0.1122	4.38E-24	hypomethylated	0.0011118	0.81275	insignificant	38	158	163
chr4	129219890	129221890	Hdac1	-0.17228	0.0018703	hypomethylated	0.0015451	3.48E-08	hypermethylated	6	52	54
chr4	129250885	129252885	Lck	0.2549	1	insignificant	-0.01463	0.000000245	hypomethylated	2	14	14
chr4	129255824	129257824	Fam167b	-0.41946	0.30863	insignificant	0.038876	1	insignificant	3	6	8
chr4	129276950	129278950	2510006D16Rik	-0.22066	3.97E-14	hypomethylated	-0.041846	0.17006	insignificant	25	74	81
chr4	129277892	129279892	Eif3i	-0.27243	1.66E-09	hypomethylated	-0.052091	0.031578	inconclusive	14	39	47
chr4	129296337	129298337	Iqcc	-0.19152	1	insignificant	-0.08194	4.29E-09	hypomethylated	6	18	16
chr4	129301152	129303152	Ccdc28b	-0.17424	0.11456	insignificant	0.10433	0.26051	insignificant	2	12	10
chr4	129350011	129352011	Kpna6	0.11277	1	insignificant	0.031351	0.68449	insignificant	1	12	9
chr4	129374082	129376082	Tmem39b	-0.13542	1	insignificant	-0.021805	0.057985	insignificant	5	8	15
chr4	129419526	129421526	Khdrbs1	-0.17206	7.61E-13	hypomethylated	-0.020844	0.75531	insignificant	23	72	74
chr4	129496722	129498722	Ptp4a2	-0.088364	5.68E-25	hypomethylated	-0.0010477	0.0031294	hypomethylated	60	212	229
chr4	129496951	129498951	Ptp4a2	-0.08557	3.02E-25	hypomethylated	0.00033482	0.0068414	inconclusive	59	210	227
chr4	129661321	129663321	Bai2	-0.13001	1.1E-49	hypomethylated	-0.01322	0.86426	insignificant	53	145	166
chr4	129724083	129726083	Col16a1	-0.14973	1.92E-08	hypomethylated	-0.031071	0.26044	insignificant	17	46	62
chr4	129783799	129785799	Pef1	-0.16852	3.84E-09	hypomethylated	-0.014312	0.70204	insignificant	9	46	46
chr4	129815795	129817795	Hcrtr1	-0.14702	4.04E-10	hypomethylated	0.028518	0.11084	insignificant	22	84	78
chr4	129816406	129818406	Hcrtr1	-0.053764	0.000000617	hypomethylated	0.075971	0.28349	insignificant	8	37	32
chr4	129852046	129854046	Tinagl1	-0.21965	3.77E-08	hypomethylated	-0.00025268	0.00040034	hypomethylated	7	16	16
chr4	129852366	129854366	Tinagl1	-0.37232	0.29879	insignificant	0.0093074	1	insignificant	2	6	6
chr4	129985021	129987021	Fabp3	-0.14216	0.0033311	hypomethylated	-0.0011654	0.60123	insignificant	7	59	56
chr4	130036378	130038378	Snrnp40	-0.16743	2.99E-14	hypomethylated	0.00418	0.097751	insignificant	17	64	64
chr4	130037190	130039190	Zcchc17	-0.13761	0.000015415	hypomethylated	0.013347	0.72441	insignificant	12	42	42
chr4	130124704	130126704	Nkain1	-0.15054	1.25E-12	hypomethylated	0.0094787	0.72695	insignificant	26	94	94
chr4	130218273	130220273	Pum1	-0.10626	5.27E-26	hypomethylated	-0.011073	0.37033	insignificant	39	122	119
chr4	130347451	130349451	Sdc3	-0.093337	3.94E-15	hypomethylated	-0.0065124	0.18429	insignificant	41	148	149
chr4	130499299	130501299	Matn1	0.37577	0.30614	insignificant	0.19442	0.80574	insignificant	1	7	12
chr4	131394193	131396193	Ptpru	-0.28818	0.19932	insignificant	0.1026	0.0013462	inconclusive	7	24	31
chr4	131398385	131400385	Mecr	-0.21304	5.78E-15	hypomethylated	-0.016095	0.48587	insignificant	19	79	78
chr4	131428553	131430553	Srsf4	-0.10852	9.57E-32	hypomethylated	-0.01015	0.029033	hypomethylated	63	214	218
chr4	131604993	131606993	Epb4.1	-0.14872	0.104	insignificant	-0.052311	0.44896	insignificant	15	25	37
chr4	131628012	131630012	Epb4.1		1	noCoverage	-0.10079	0.45145	insignificant	0	3	2
chr4	131631228	131633228	Epb4.1	-0.20208	0.003366	hypomethylated	0.026907	0.10993	insignificant	7	22	22

chr4	131700401	131702401	Oprd1	-0.14417	2.02E-14	hypomethylated	-0.0075183	0.27652	insignificant	14	38	38
chr4	131768171	131770171	Ythdf2	-0.17778	0.49931	insignificant	-0.027244	0.89051	insignificant	6	38	37
chr4	131817464	131819464	Gmeb1	-0.65417	0.0071423	stronglyHypometh	-0.02492	0.72079	insignificant	3	8	10
chr4	131826101	131828101	Rnu11	-0.43861	0.002605	stronglyHypometh	-0.082787	0.039751	hypomethylated	2	32	32
chr4	131829289	131831289	Taf12	-0.23179	0.042965	hypomethylated	-0.007784	0.86386	insignificant	7	46	46
chr4	131859271	131861271	Rab42-ps	-0.11194	0.023174	hypomethylated	-0.027744	0.024195	hypomethylated	4	14	15
chr4	131863592	131865592	Snhg12	-0.35163	0.057958	insignificant	-0.093057	0.59393	insignificant	3	34	40
chr4	131864379	131866379	Snora16a	-0.2682	0.12861	insignificant	-0.041007	0.22943	insignificant	2	16	31
chr4	131885453	131887453	Trnau1ap	-0.28449	5.82E-13	hypomethylated	0.017456	0.22304	insignificant	6	20	20
chr4	131909601	131911601	Snhg3	-0.44535	2.05E-13	stronglyHypometh	-0.027213	0.15266	insignificant	7	22	24
chr4	131978361	131980361	Phactr4	-0.14736	3.11E-10	hypomethylated	0.013145	0.84472	insignificant	14	51	52
chr4	132019836	132021836	Med18	0.0056627	7.68E-09	hypermethylated	-0.031128	0.0012867	inconclusive	6	37	35
chr4	132066371	132068371	Sesn2	-0.22442	6.11E-10	hypomethylated	-0.0041459	0.75105	insignificant	8	18	18
chr4	132089574	132091574	Atpif1	-0.12908	0.00000134	hypomethylated	-0.019301	0.90032	insignificant	8	32	39
chr4	132090473	132092473	Dnajc8	-0.16116	2.19E-32	hypomethylated	0.0012425	0.055436	insignificant	26	68	77
chr4	132193960	132195960	Eya3	-0.19079	8.4E-09	hypomethylated	-0.038972	0.15346	insignificant	19	61	78
chr4	132193970	132195970	Eya3	-0.19079	8.4E-09	hypomethylated	-0.038972	0.15346	insignificant	19	61	78
chr4	132288461	132290461	Xkr8	-0.4048	4.39E-41	stronglyHypometh	-0.06065	0.10867	insignificant	12	60	59
chr4	132313086	132315086	Smpdl3b	-0.45074	0.052132	insignificant	-0.043545	0.55278	insignificant	2	6	6
chr4	132323274	132325274	Rpa2	0.045786	0.0061481	inconclusive	0.014274	0.82535	insignificant	10	111	113
chr4	132352279	132354279	BC013712	-0.44129	0.0024966	stronglyHypometh	0.10219	0.34362	insignificant	2	8	10
chr4	132440373	132442373	Stx12	-0.15693	4.6E-28	hypomethylated	0.030735	0.58069	insignificant	17	61	66
chr4	132478466	132480466	Fam76a	-0.1021	9.46E-12	hypomethylated	-0.0031254	0.37102	insignificant	16	63	71
chr4	132529009	132531009	Fgr	-0.13166	0.00007634	hypomethylated	-0.0046139	0.152	insignificant	7	44	38
chr4	132566420	132568420	Ahdc1	-0.2049	5.23E-40	hypomethylated	-0.02051	0.077339	insignificant	39	73	74
chr4	132685547	132687547	Wasf2	-0.16439	1.95E-24	hypomethylated	-0.010919	0.10981	insignificant	24	72	68
chr4	132768451	132770451	Gpr3	-0.075767	0.00030677	hypomethylated	0.042084	0.00012939	hypermethylated	11	56	52
chr4	132775723	132777723	Cd164l2	-0.093762	0.09588	insignificant	0.052906	0.76156	insignificant	5	34	34
chr4	132795732	132797732	Map3k6	-0.10997	2.53E-14	hypomethylated	0.027643	0.3329	insignificant	23	72	62
chr4	132833705	132835705	Tmem222	-0.37202	0.5831	insignificant	0.14999	0.00045	hypermethylated	3	14	18
chr4	132895230	132897230	Wdtc1	-0.11392	6.64E-12	hypomethylated	-0.0055608	0.70001	insignificant	18	76	72
chr4	132924686	132926686	Slc9a1	-0.19803	2.71E-10	hypomethylated	-0.031735	0.00038801	hypomethylated	9	30	30
chr4	133035047	133037047	Fam46b	-0.36944	2.1E-30	stronglyHypometh	-0.053074	0.000013251	hypomethylated	17	52	52
chr4	133054465	133056465	Trnp1	-0.34307	0.000011173	stronglyHypometh	-0.046737	0.5307	insignificant	4	6	6
chr4	133073877	133075877	1810019J16Rik	-0.049759	0.60848	insignificant	0.081596	0.00095253	hypermethylated	13	93	77
chr4	133101942	133103942	Nudc	-0.154	2.72E-09	hypomethylated	0.17758	0.63741	insignificant	5	13	12
chr4	133108304	133110304	Nr0b2	-0.40472	0.00000753	stronglyHypometh	-0.066178	0.81691	insignificant	3	6	6
chr4	133129659	133131659	Gpatch3	-0.19903	6.92E-21	hypomethylated	0.033673	0.0061174	hypermethylated	22	80	82
chr4	133139287	133141287	Gpn2	-0.2381	1.96E-08	hypomethylated	-0.059164	0.00025807	hypomethylated	17	47	66
chr4	133158083	133160083	Sfn	0.14667	0.59429	insignificant	-0.019167	0.60451	insignificant	5	10	13
chr4	133189344	133191344	Zdhhc18	-0.15385	0.012467	hypomethylated	-0.012698	0.61722	insignificant	14	39	38
chr4	133228562	133230562	Pigv	-0.11625	2.52E-12	hypomethylated	-0.0077359	0.60196	insignificant	20	49	46
chr4	133309526	133311526	Arid1a	-0.13021	4.05E-34	hypomethylated	-0.011122	0.064248	insignificant	55	127	128
chr4	133443714	133445714	Rps6ka1	-0.12391	4.13E-09	hypomethylated	-0.016683	0.61621	insignificant	10	34	34
chr4	133523906	133525906	Hmgn2	-0.10643	4.18E-09	hypomethylated	0.0020333	0.95944	insignificant	19	70	71
chr4	133574731	133576731	Lin28a	-0.39064	0.029075	stronglyHypometh	-0.054994	0.020249	hypomethylated	2	17	17
chr4	133650988	133652988	Cd52		1	noCoverage	0.14444	1	lowCoverage	0	6	1
chr4	133657513	133659513	Ubxn11	-0.11791	0.024293	hypomethylated	0.0024552	0.33071	insignificant	2	43	44
chr4	133684668	133686668	Sh3bgrl3	-0.29631	1.52E-24	hypomethylated	-0.02186	0.52648	insignificant	8	30	30
chr4	133743000	133745000	Ccdc21	-0.20572	1.64E-10	hypomethylated	0.028649	0.25491	insignificant	9	26	28
chr4	133794314	133796314	Cnksr1	0.28027	0.34195	insignificant	0.054701	0.053999	insignificant	2	22	23
chr4	133801506	133803506	Zfp593	-0.28415	1.56E-09	hypomethylated	-0.18927	0.000011016	hypomethylated	9	14	30
chr4	133843168	133845168	Pdik1l	-0.11712	4.11E-34	hypomethylated	0.0072656	0.49909	insignificant	38	123	122
chr4	133843761	133845761	Pdik1l	-0.23867	1E-11	hypomethylated	-0.023768	0.22768	insignificant	9	22	28
chr4	133870034	133872034	Trim63	-0.052302	0.74916	insignificant	0.089122	1	insignificant	3	13	10
chr4	133897984	133899984	Slc30a2	-0.24211	4.17E-19	hypomethylated	-0.010532	0.0023964	hypomethylated	17	77	80
chr4	133928462	133930462	Extl1	-0.24857	0.000000591	hypomethylated	-0.014448	0.32433	insignificant	2	8	8
chr4	134023234	134025234	Stmn1	-0.062305	0.38372	insignificant	-0.0095931	0.19178	insignificant	21	135	149
chr4	134051892	134053892	Paqr7	-0.18209	8.41E-13	hypomethylated	-0.032206	0.02723	inconclusive	18	49	49
chr4	134065913	134067913	2610002D18Rik	-0.12391	2.53E-12	hypomethylated	0.0020281	0.95971	insignificant	13	53	36
chr4	134088667	134090667	Fam54b	-0.22069	0.0020659	hypomethylated	-0.054139	0.27765	insignificant	7	45	44
chr4	134260205	134262205	Man1c1	-0.079253	0.0048007	hypomethylated	-0.0061033	0.0005668	hypomethylated	10	45	45
chr4	134323919	134325919	Ldlrap1	-0.16945	0.000016332	hypomethylated	-0.0079134	0.012178	hypomethylated	16	47	47
chr4	134409260	134411260	Tmem57	-0.1751	2.35E-26	hypomethylated	-0.03105	0.0037998	hypomethylated	33	119	138
chr4	134419450	134421450	Rhd	-0.29985	0.11158	insignificant	-0.021702	0.017847	hypomethylated	7	27	27
chr4	134470831	134472831	Tmem50a	-0.2038	0.13553	insignificant	0.02418	0.00000664	inconclusive	9	31	34
chr4	134478539	134480539	D4Wsu53e	-0.094077	2.01E-45	hypomethylated	0.015931	0.20495	insignificant	38	193	202
chr4	134485894	134487894	Syf2	-0.17616	1.32E-26	hypomethylated	0.014397	0.067344	insignificant	19	86	96
chr4	134675559	134677559	Runx3		1	noCoverage	-0.12485	0.33993	insignificant	0	8	12
chr4	134828675	134830675	Clic4	-0.12191	1.21E-09	hypomethylated	-0.020394	0.51825	insignificant	13	61	74
chr4	134909129	134911129	Srrm1	-0.11778	3.85E-10	hypomethylated	0.035889	0.78075	insignificant	10	28	29
chr4	134941587	134943587	A330049M08Rik		1	noCoverage	-0.068904	0.082904	insignificant	0	6	12
chr4	134954142	134956142	A330049M08Ril	-0.47356	0.000000274	stronglyHypometh	0.019285	0.84192	insignificant	4	24	24
chr4	134989720	134991720	Rcan3	-0.1701	2.13E-23	hypomethylated	-0.015658	0.00000122	hypomethylated	12	30	30
chr4	135050419	135052419	Nipal3	-0.1387	7.28E-13	hypomethylated	-0.072859	1.75E-11	hypomethylated	20	60	72
chr4	135050901	135052901	4930555I21Rik	-0.11223	0.098406	insignificant	0.0039679	0.66039	insignificant	14	42	42
chr4	135129535	135131535	Grhl3	-0.42281	0.00092712	stronglyHypometh	-0.10554	0.55372	insignificant	3	24	24
chr4	135181569	135183569	1700029M20Ril	-0.072478	0.20467	insignificant	-0.032218	0.21563	insignificant	3	28	24
chr4	135241371	135243371	Il28ra	-0.10412	0.00000214	hypomethylated	-0.003753	0.62331	insignificant	19	69	70
chr4	135283134	135285134	Il22ra1	0.031658	0.36113	insignificant	-0.0068356	0.020129	inconclusive	2	21	23
chr4	135314629	135316629	Myom3	0.034722	1	insignificant	-0.091797	1	insignificant	1	6	7
chr4	135411006	135413006	Srsf10	-0.097101	6.57E-15	hypomethylated	-0.0077605	0.020962	hypomethylated	55	195	196
chr4	135429761	135431761	Pnrc2	0.06729	0.0072107	inconclusive	-0.0070137	0.20287	insignificant	5	38	38
chr4	135450318	135452318	Cnr2	-0.33737	0.021817	stronglyHypometh	-0.061056	0.3915	insignificant	2	4	4
chr4	135475640	135477640	Fuca1	-0.14983	3.58E-21	hypomethylated	0.010018	0.17895	insignificant	27	77	70

chr4	135501367	135503367	Hmgcl	-0.24458	1.64E-19	hypomethylated	-0.060412	0.0056065	hypomethylated	17	77	83
chr4	135528509	135530509	Lypla2	-0.21126	0.049725	hypomethylated	-0.019858	0.19304	insignificant	3	6	6
chr4	135543159	135545159	1110049F12Rik	-0.18298	0.33919	insignificant	0.04207	0.43541	insignificant	14	49	53
chr4	135577564	135579564	Tceb3	-0.19764	0.00000204	hypomethylated	-0.02363	0.0071155	hypomethylated	8	43	43
chr4	135609286	135611286	Rpl11	-0.38926	1	lowCoverage	-0.020483	0.94049	insignificant	1	26	25
chr4	135698736	135700736	Id3	-0.097357	0.000000388	hypomethylated	0.011786	0.05785	insignificant	8	101	105
chr4	135727308	135729308	E2f2	-0.097404	2.52E-18	hypomethylated	0.01792	0.60509	insignificant	35	121	107
chr4	135761279	135763279	Asap3	-0.11188	7.12E-12	hypomethylated	0.001493	0.79813	insignificant	16	67	58
chr4	135802871	135804871	Tcea3	-0.18674	0.00000211	hypomethylated	0.020413	0.88651	insignificant	15	85	79
chr4	135840983	135842983	Zfp46	-0.18203	2.89E-17	hypomethylated	-0.016744	0.80148	insignificant	18	60	66
chr4	135865890	135867890	Hnrnpr	-0.13197	5.29E-25	hypomethylated	-0.0067247	0.04751	hypomethylated	47	150	154
chr4	135978438	135980438	Htr1d	-0.23316	0.00026841	hypomethylated	-0.054798	0.77269	insignificant	8	35	38
chr4	136024675	136026675	Luzp1	-0.1159	1.14E-33	hypomethylated	0.00052542	0.75783	insignificant	42	113	132
chr4	136158638	136160638	Kdm1a		1	noCoverage	0.043479	0.02942	hypermethylated	0	24	10
chr4	136391850	136393850	Ephb2	-0.15742	7.59E-12	hypomethylated	0.0025346	0.37013	insignificant	35	103	106
chr4	136454759	136456759	C1qa	-0.15812	0.59999	insignificant	-0.18798	0.33891	insignificant	1	9	11
chr4	136512731	136514731	Epha8	-0.14902	0.0095957	hypomethylated	-0.011189	0.005544	hypomethylated	23	60	60
chr4	136832549	136834549	Wnt4	-0.12055	1.49E-37	hypomethylated	0.0042204	0.049818	inconclusive	62	170	205
chr4	136913652	136915652	Cdc42	-0.292	0.82933	insignificant	0.14046	0.0050565	hypermethylated	5	14	16
chr4	136986435	136988435	Cela3b		1	noCoverage	-0.0076754	0.74145	insignificant	0	4	4
chr4	137023717	137025717	Hspg2	-0.16387	2.92E-16	hypomethylated	-0.030038	0.28928	insignificant	26	88	90
chr4	137149103	137151103	Usp48	-0.1127	8.94E-16	hypomethylated	-0.021576	0.70208	insignificant	23	92	103
chr4	137236617	137238617	Rap1gap	-0.16817	4.71E-20	hypomethylated	-0.01599	0.061863	insignificant	17	86	73
chr4	137352292	137354292	Alpl	-0.25038	0.000000162	hypomethylated	-0.029252	0.11033	insignificant	8	26	18
chr4	137417151	137419151	Ece1	-0.19763	8.53E-18	hypomethylated	-0.0087949	0.75776	insignificant	17	84	84
chr4	137548384	137550384	Eif4g3	-0.077081	3.17E-22	hypomethylated	0.0018519	0.04043	hypermethylated	66	220	210
chr4	137771541	137773541	Hp1bp3	-0.085361	1.03E-35	hypomethylated	-0.022372	0.00077515	hypomethylated	68	217	223
chr4	137772045	137774045	Hp1bp3	-0.098066	4.16E-37	hypomethylated	-0.018789	0.036851	hypomethylated	70	224	231
chr4	137805325	137807325	Sh2d5	-0.12842	2.37E-16	hypomethylated	0.059265	0.00006335	hypermethylated	12	54	36
chr4	137817165	137819165	Kif17	-0.1258	4.21E-37	hypomethylated	-0.019923	0.024224	hypomethylated	47	132	132
chr4	137859652	137861652	Ddost	-0.13858	1.83E-20	hypomethylated	-0.0020781	0.052844	insignificant	35	80	103
chr4	137882211	137884211	Pink1	-0.30358	0.0612	insignificant	-0.16504	0.035208	inconclusive	6	35	37
chr4	137923870	137925870	Cda	-0.22832	7.66E-13	hypomethylated	-0.019211	0.006369	hypomethylated	10	39	36
chr4	137989586	137991586	Mul1	-0.25656	1.07E-29	hypomethylated	-0.065299	0.67063	insignificant	27	89	85
chr4	138010062	138012062	Camk2n1	-0.095217	1.5E-21	hypomethylated	-0.0038092	0.48216	insignificant	55	199	188
chr4	138280239	138282239	Pla2g2c	-0.32077	5.8E-16	hypomethylated	-0.02057	0.050653	insignificant	10	23	28
chr4	138281166	138283166	Ubxn10	-0.66667	0.000013036	stronglyHypometh	-0.44744	0.024859	stronglyhypometh	3	6	6
chr4	138313513	138315513	Pla2g2f	-0.15527	0.0033414	hypomethylated	0.21258	1	insignificant	1	8	12
chr4	138375172	138377172	Pla2g5	-0.091667	1	insignificant	0.087821	0.13124	insignificant	1	6	6
chr4	138469862	138471862	Otud3	-0.26315	3.87E-14	hypomethylated	0.089852	3.39E-09	inconclusive	9	33	40
chr4	138522026	138524026	Rnf186	-0.019283	0.0047567	hypomethylated	-0.011888	0.54807	insignificant	13	48	62
chr4	138527819	138529819	Tmco4	-0.18291	0.000041343	hypomethylated	-0.026365	0.29429	insignificant	12	67	68
chr4	138630704	138632704	Htr6	-0.132	2.11E-14	hypomethylated	-0.010857	0.060989	insignificant	43	138	143
chr4	138648885	138650885	Nbl1	0.12825	0.0060924	inconclusive	0.060295	0.00000151	hypermethylated	10	54	56
chr4	138686952	138688952	Minos1	-0.26961	4.33E-16	hypomethylated	-0.1268	0.33544	insignificant	11	28	24
chr4	138687028	138689028	Minos1	-0.35186	3.67E-11	stronglyHypometh	-0.1969	0.037727	hypomethylated	6	18	14
chr4	138747845	138749845	Capzb	-0.15763	0.000000163	hypomethylated	0.0016891	0.59603	insignificant	25	106	100
chr4	138747893	138749893	Capzb	-0.15763	0.000000163	hypomethylated	0.0016891	0.59603	insignificant	25	106	100
chr4	138865658	138867658	Akr7a5	-0.11272	6.75E-54	hypomethylated	-0.015176	0.46494	insignificant	35	120	139
chr4	138866615	138868615	Pqlc2	-0.14765	8.33E-25	hypomethylated	0.016437	0.45477	insignificant	16	76	86
chr4	138907507	138909507	C230096C10Rik	-0.16401	1.14E-13	hypomethylated	0.0031554	0.28706	insignificant	24	76	71
chr4	138908213	138910213	Mrto4	-0.20678	5.68E-13	hypomethylated	0.012727	0.20371	insignificant	19	52	53
chr4	138935573	138937573	Ubr4	-0.16501	2.53E-27	hypomethylated	-0.0013822	0.095026	insignificant	29	68	81
chr4	139130050	139132050	Iffo2	-0.11327	1.71E-36	hypomethylated	-0.0036071	0.048282	hypomethylated	66	198	207
chr4	139177808	139179808	Aldh4a1	-0.35007	0.010156	stronglyHypometh	-0.088544	0.92977	insignificant	4	13	28
chr4	139208452	139210452	Tas1r2	0.10397	0.067826	insignificant	0.27111	4.67E-08	hypermethylated	3	6	6
chr4	139388883	139390883	Pax7	-0.17014	3.07E-18	hypomethylated	0.043901	0.14426	insignificant	15	58	45
chr4	139802726	139804726	Igsf21	-0.18563	0.06393	insignificant	-0.060608	0.39055	insignificant	5	24	25
chr4	140204671	140206671	Arhgef10l	-0.099062	0.089858	insignificant	-0.007186	0.73078	insignificant	1	16	31
chr4	140221820	140223820	Arhgef10l	-0.40135	0.3975	insignificant	0.089767	0.00033906	hypermethylated	3	14	14
chr4	140256387	140258387	Rcc2	-0.07876	3.59E-12	hypomethylated	-0.0017411	0.53828	insignificant	39	145	154
chr4	140366563	140368563	Padi3	0.12796	1	insignificant	0.024765	1	insignificant	2	6	6
chr4	140401693	140403693	Padi1		1	noCoverage	0.0074074	1	insignificant	0	2	2
chr4	140461274	140463274	Padi2	-0.50171	1.52E-29	stronglyHypometh	-0.05009	0.58424	insignificant	4	18	26
chr4	140516185	140518185	Sdhb	-0.19256	2.02E-27	hypomethylated	-0.022651	0.0010044	hypomethylated	29	83	80
chr4	140541787	140543787	Atp13a2	-0.13762	1.35E-25	hypomethylated	-0.015655	0.86913	insignificant	29	67	66
chr4	140565338	140567338	Mfap2	-0.11314	0.0004237	hypomethylated	0.0026959	0.17077	insignificant	9	31	34
chr4	140565551	140567551	Mfap2	-0.089666	0.00033036	hypomethylated	-0.0058292	0.20579	insignificant	10	33	36
chr4	140609576	140611576	Crocc	-0.32209	0.017494	hypomethylated	-0.019253	0.017584	hypomethylated	7	33	34
chr4	140616460	140618460	Crocc	-0.19381	0.0047532	hypomethylated	0.023973	0.46848	insignificant	0	33	33
chr4	140634260	140636260	Necap2	-0.58556	1.03E-39	stronglyHypometh	-0.11869	0.00408	hypomethylated	9	41	35
chr4	140643259	140645259	Spata21	-0.038126	0.54229	insignificant	-0.030926	0.46312	insignificant	5	16	12
chr4	140702836	140704836	Fbxo42	-0.10716	3.62E-51	hypomethylated	-0.0085101	0.040545	hypomethylated	31	160	178
chr4	140768870	140770870	6330545A04Rik	0.065666	0.0026569	hypermethylated	-0.024801	0.42469	insignificant	4	38	38
chr4	140797798	140799798	Arhgef19	-0.33333	0.82974	insignificant	-0.14848	0.31526	insignificant	6	10	14
chr4	140856154	140858154	Epha2	-0.11821	0.00037923	hypomethylated	-0.021487	0.12595	insignificant	21	89	86
chr4	140923134	140925134	Fam131c	0.0047254	7.4E-14	inconclusive	-0.038302	0.000000674	hypomethylated	16	78	104
chr4	140954014	140956014	Clcnka	-0.12084	1	insignificant	-0.0063323	0.13151	insignificant	5	10	10
chr4	140954621	140956621	Clcnka	-0.11603	0.32461	insignificant	-0.0023667	0.013137	hypomethylated	7	25	28
chr4	140975693	140977693	Hspb7	0.19439	0.2003	insignificant	-0.051278	0.24104	insignificant	2	13	15
chr4	140992020	140994020	Gm694	0.092781	1	insignificant	-0.015644	0.15547	insignificant	2	16	16
chr4	140999587	141001587	Zbtb17	-0.1293	9.16E-41	hypomethylated	-0.010794	0.021018	hypomethylated	46	112	116
chr4	141094512	141096512	Spen	-0.095469	1.35E-12	hypomethylated	0.004662	0.56481	insignificant	28	80	80
chr4	141101076	141103076	B330016D10Rik	-0.1187	1.08E-24	hypomethylated	-0.014351	0.066101	insignificant	59	140	150
chr4	141155839	141157839	Fblim1	0.051751	0.82592	insignificant	-0.097803	0.30878	insignificant	7	30	43

chr4	141169284	141171284	AI507597	0.0060802	0.10035	insignificant	0.12908	0.3629	insignificant	4	23	27
chr4	141179749	141181749	Slc25a34	0.026801	0.13051	insignificant	-0.038086	0.038184	inconclusive	5	18	20
chr4	141220030	141222030	Plekhm2	-0.20991	1.95E-30	hypomethylated	-0.0010332	0.0014723	hypomethylated	22	69	71
chr4	141279334	141281334	Ddi2	-0.13104	2.26E-09	hypomethylated	-0.024733	0.32823	insignificant	15	30	62
chr4	141301589	141303589	Agmat	-0.63203	0.0017729	stronglyHypometh	-0.25561	0.00000364	hypomethylated	3	21	11
chr4	141346559	141348559	Dnajc16	-0.25031	2.67E-10	hypomethylated	-0.028422	0.35064	insignificant	10	47	46
chr4	141348526	141350526	Casp9	-0.15562	2.27E-11	hypomethylated	-0.022012	0.24448	insignificant	15	32	32
chr4	141430835	141432835	Efhd2	-0.016934	1.04E-12	inconclusive	0.0021914	0.0074335	hypermethylated	10	38	26
chr4	141639212	141641212	Gm10565	-0.15483	1.43E-22	hypomethylated	-0.00067078	0.097721	insignificant	42	137	128
chr4	141640219	141642219	Gm10565	-0.15059	0.0056317	hypomethylated	0.04048	0.00059974	hypermethylated	12	64	75
chr4	141766123	141768123	Kazn	-0.57759	0.0010088	stronglyHypometh	0.042414	0.021024	hypermethylated	1	2	2
chr4	142802612	142804612	Prdm2	-0.14054	0.00025966	hypomethylated	-0.0032922	0.22966	insignificant	9	43	55
chr4	142889410	142891410	Pdpn	-0.43695	0.00010844	stronglyHypometh	0.01755	0.61024	insignificant	3	6	6
chr4	142938652	142940652	Lrrc38	-0.14223	6.36E-34	hypomethylated	-0.0080061	0.020644	hypomethylated	36	123	119
chr4	142983343	142985343	Pramel1	0.051913	0.73609	insignificant	-0.069323	0.92753	insignificant	9	44	41
chr4	143001328	143003328	Pramef8	-0.25687	0.051717	insignificant	-0.11211	0.009362	hypomethylated	8	36	51
chr4	143998367	144000367	Pramef12		1	noCoverage	-0.23143	0.13014	insignificant	0	11	12
chr4	144481729	144483729	Dhrs3	-0.17612	6.94E-18	hypomethylated	-0.011866	0.000070245	hypomethylated	19	56	56
chr4	144482039	144484039	Dhrs3	-0.17612	6.94E-18	hypomethylated	-0.011866	0.000070245	hypomethylated	19	56	56
chr4	144905050	144907050	Tnfrsf8		1	noCoverage	-0.13223	0.88193	insignificant	0	14	10
chr4	145053112	145055112	Smarca5-ps	-0.13882	0.00011745	hypomethylated	0.014359	0.043594	hypermethylated	10	88	85
chr4	145259599	145261599	Gm13242		1	noCoverage	0.0048334	0.071226	insignificant	0	9	8
chr4	145426121	145428121	Gm13238		1	noCoverage	0.15551	0.0072528	hypermethylated	0	54	61
chr4	146033924	146035924	Gm13034	-0.31037	1.04E-13	hypomethylated	-0.038463	9.33E-10	hypomethylated	6	147	141
chr4	147222484	147224484	Zfp933	-0.021672	0.0016399	inconclusive	0.056403	0.0011818	hypermethylated	6	17	22
chr4	147242087	147244087	Fv1	-0.27527	0.000028233	hypomethylated	-0.12276	0.0013384	hypomethylated	14	66	66
chr4	147242828	147244828	Fv1	-0.013422	0.000018199	inconclusive	-0.21625	0.00037262	hypomethylated	7	32	32
chr4	147278928	147280928	Mfn2		1	noCoverage	-0.0125	1	insignificant	0	8	8
chr4	147310885	147312885	Plod1	-0.2393	0.00000543	hypomethylated	-0.0092669	0.32248	insignificant	12	26	28
chr4	147314003	147316003	2510039O18Rik	-0.10647	4.88E-27	hypomethylated	-0.0036471	0.5803	insignificant	38	139	145
chr4	147358897	147360897	Nppb	-0.2011	0.49543	insignificant	-0.0090465	0.13193	insignificant	3	10	10
chr4	147412185	147414185	Mthfr	-0.16652	4.75E-14	hypomethylated	-0.025977	0.12659	insignificant	22	72	80
chr4	147412876	147414876	Mthfr	-0.11595	6.52E-14	hypomethylated	0.010758	0.0024298	inconclusive	16	62	71
chr4	147414297	147416297	Mthfr	-0.095548	0.023269	hypomethylated	0.02361	0.16889	insignificant	7	32	32
chr4	147462173	147464173	Agtrap	-0.34301	9.59E-08	stronglyHypometh	0.021081	0.033028	inconclusive	6	25	23
chr4	147504807	147506807	2610109H07Rik	-0.010869	0.10746	insignificant	0.052759	7.37E-37	hypermethylated	12	66	59
chr4	147513598	147515598	Mad2l2	-0.19328	3.72E-14	hypomethylated	-0.0034292	0.17975	insignificant	16	60	60
chr4	147526034	147528034	Fbxo6	0.15182	0.047313	hypermethylated	0.056098	0.75768	insignificant	7	21	23
chr4	147526138	147528138	Fbxo6	0.046576	0.67901	insignificant	0.028988	0.6505	insignificant	7	18	20
chr4	147526244	147528244	Fbxo6	0.14692	0.29516	insignificant	0.019991	0.13806	insignificant	6	18	20
chr4	147533704	147535704	Fbxo44	-0.098158	2.08E-17	hypomethylated	0.011041	0.0000085	inconclusive	20	82	88
chr4	147533776	147535776	Fbxo2	-0.098158	2.08E-17	hypomethylated	0.011041	0.0000085	inconclusive	20	82	88
chr4	147534173	147536173	Fbxo44	-0.10616	2.35E-14	hypomethylated	0.0070215	0.00000143	inconclusive	18	60	69
chr4	147662074	147664074	Ptchd2	-0.12473	0.036915	hypomethylated	0.030134	0.11529	insignificant	9	23	21
chr4	147818860	147820860	Ubiad1		1	noCoverage	0.1692	0.65272	insignificant	0	10	5
chr4	147821690	147823690	Mtor	-0.14128	1.02E-08	hypomethylated	0.015805	0.30416	insignificant	19	132	122
chr4	147931535	147933535	Exosc10	-0.15652	3.46E-15	hypomethylated	-0.037988	0.51501	insignificant	26	53	58
chr4	147964621	147966621	Srm	-0.14161	4.15E-29	hypomethylated	-0.012918	0.5884	insignificant	33	91	95
chr4	148001105	148003105	Tardbp	-0.73611	4E-49	stronglyHypometh	-0.078159	0.0070424	hypomethylated	10	8	9
chr4	148177500	148179500	Casz1	-0.12661	1.17E-19	hypomethylated	-0.0022468	0.22892	insignificant	32	109	120
chr4	148473921	148475921	Pex14	-0.52062	1	lowCoverage	-0.06758	0.0034063	hypomethylated	1	31	30
chr4	148477261	148479261	Dffa	-0.16458	2.81E-10	hypomethylated	-0.049566	0.0011338	hypomethylated	8	44	38
chr4	148500850	148502850	Cort	-0.64247	0.000068666	stronglyHypometh	-0.0041121	0.13682	insignificant	2	27	30
chr4	148540816	148542816	Pgd	-0.25626	0.082575	insignificant	-0.12887	0.31397	insignificant	3	25	18
chr4	148681807	148683807	Kif1b	-0.12261	1.77E-33	hypomethylated	-0.0089114	0.022968	hypomethylated	27	96	96
chr4	148800740	148802740	Ube4b	-0.525	0.56602	insignificant	-0.275	0.22908	insignificant	1	4	2
chr4	148829077	148831077	Rbp7		1	noCoverage	-0.072821	0.65899	insignificant	0	2	2
chr4	148858441	148860441	Lzic	-0.12771	1.49E-14	hypomethylated	-0.011038	0.041614	hypomethylated	27	81	83
chr4	148859251	148861251	Nmnat1	-0.15584	1.16E-17	hypomethylated	-0.025685	0.0075852	hypomethylated	21	69	69
chr4	148891349	148893349	Ctnnbip1	-0.12093	8.08E-10	hypomethylated	0.0096991	0.41515	insignificant	17	40	40
chr4	148918225	148920225	Ctnnbip1	-0.015526	0.49997	insignificant	-0.033448	0.27848	insignificant	8	24	24
chr4	148959746	148961746	Clstn1	-0.10271	9.8E-34	hypomethylated	-0.0084913	0.056743	insignificant	72	208	209
chr4	149050198	149052198	Pik3cd	-0.20832	0.12161	insignificant	-0.036518	0.00465	hypomethylated	9	31	31
chr4	149072817	149074817	Pik3cd	-0.45455	1	lowCoverage	-0.040699	1	insignificant	1	10	10
chr4	149075738	149077738	Pik3cd	-0.4873	0.03051	stronglyHypometh	-0.18239	0.76773	insignificant	3	16	15
chr4	149112143	149114143	Tmem201	-0.19248	0.043214	hypomethylated	0.0011079	1	insignificant	3	6	6
chr4	149112145	149114145	Tmem201	-0.19248	0.043214	hypomethylated	0.0011079	1	insignificant	2	4	4
chr4	149148376	149150376	Slc25a33	-0.16011	5.73E-09	hypomethylated	0.10273	0.7171	insignificant	11	6	14
chr4	149329115	149331115	Spsb1	0.053673	2.66E-15	inconclusive	0.13159	0.0027603	inconclusive	10	27	22
chr4	149441562	149443562	Mir34a		1	noCoverage	-0.024731	0.14304	insignificant	0	6	8
chr4	149492452	149494452	Slc2a5	-0.015507	0.056329	insignificant	0.028496	0.5623	insignificant	5	16	12
chr4	149610305	149612305	Eno1	-0.15719	1E-22	hypomethylated	-0.016296	0.0097916	hypomethylated	24	82	97
chr4	149610432	149612432	Gm5506	-0.15647	7.64E-22	hypomethylated	-0.012419	0.52972	insignificant	21	74	89
chr4	149655024	149657024	Rere	-0.093918	0.000065209	hypomethylated	0.0019216	0.72022	insignificant	22	88	84
chr4	150026283	150028283	Slc45a1	-0.37293	0.000022827	stronglyHypometh	0.0371	0.03352	inconclusive	3	16	16
chr4	150228199	150230199	Errfi1	-0.10218	5.01E-17	hypomethylated	-0.0064004	0.26946	insignificant	43	147	160
chr4	150284030	150286030	Park7	-0.47969	0.0034449	stronglyHypometh	-0.16314	0.60546	insignificant	6	32	28
chr4	150301107	150303107	Tnfrsf9		1	noCoverage	0.34927	0.62119	insignificant	0	3	3
chr4	150418731	150420731	Per3	-0.11484	2.11E-09	hypomethylated	-0.0056861	0.018169	hypomethylated	14	42	54
chr4	150432062	150434062	Vamp3	-0.13379	0.021488	hypomethylated	0.011944	0.082794	insignificant	5	28	29
chr4	151235877	151237877	Camta1	-0.12383	8.17E-29	hypomethylated	-0.012743	0.30529	insignificant	37	144	159
chr4	151306828	151308828	Dnajc11	-0.094573	6.12E-20	hypomethylated	-0.0072486	0.40725	insignificant	48	145	143
chr4	151370288	151372288	Phf13	-0.09058	2.42E-26	hypomethylated	0.003181	0.84586	insignificant	44	171	165
chr4	151381999	151383999	Klhl21	-0.11962	2.94E-38	hypomethylated	-0.017385	0.0055911	hypomethylated	30	96	93
chr4	151401780	151403780	Zbtb48	-0.40183	1	insignificant	-0.088073	0.56436	insignificant	2	46	40

chr4	151412435	151414435	Nol9	-0.11987	2.39E-22	hypomethylated	-0.0090846	0.78421	insignificant	54	154	154
chr4	151412599	151414599	Nol9	-0.11987	2.39E-22	hypomethylated	-0.0090846	0.78421	insignificant	54	154	154
chr4	151469827	151471827	Plekhg5	-0.64301	0.00000852	stronglyHypometh	-0.12412	0.027645	hypomethylated	2	9	10
chr4	151489451	151491451	Tnfrsf25	-0.15848	3.46E-15	hypomethylated	-0.01672	0.88414	insignificant	17	42	42
chr4	151503034	151505034	Espn	-0.2563	0.00051618	hypomethylated	0.025988	0.25691	insignificant	7	48	48
chr4	151510434	151512434	Espn	0.10009	1	insignificant	0.032727	0.49217	insignificant	1	8	8
chr4	151526316	151528316	Espn	-0.13592	1.02E-09	hypomethylated	-0.013448	0.22492	insignificant	23	56	56
chr4	151531975	151533975	Hes2	-0.041601	0.31248	insignificant	0.035891	0.34515	insignificant	5	34	34
chr4	151551208	151553208	Acot7	-0.12002	2.49E-50	hypomethylated	-0.0054745	0.047418	inconclusive	56	144	149
chr4	151559242	151561242	Acot7	-0.33764	0.000040708	stronglyHypometh	0.077971	0.74479	insignificant	5	10	10
chr4	151647470	151649470	Gpr153	-0.087794	1.3E-15	hypomethylated	-0.0056814	0.37981	insignificant	35	125	127
chr4	151665771	151667771	Hes3	-0.039171	1	insignificant	0.047904	0.34805	insignificant	5	16	14
chr4	151670459	151672459	Icmt	-0.13458	7.25E-62	hypomethylated	-0.0091831	0.21269	insignificant	58	153	153
chr4	151692734	151694734	Rnf207	-0.38766	0.00000697	stronglyHypometh	-0.048869	0.10192	insignificant	8	45	45
chr4	151698986	151700986	Rpl22	-0.17427	1.61E-34	hypomethylated	-0.016003	7.29E-12	hypomethylated	21	84	89
chr4	151711759	151713759	Chd5	-0.11061	1.08E-44	hypomethylated	-0.010336	0.063604	insignificant	68	186	186
chr4	151851250	151853250	Nphp4	-0.16649	7.19E-33	hypomethylated	-0.019078	0.022933	hypomethylated	33	108	104
chr4	151851588	151853588	Kcnab2	-0.16555	1.26E-30	hypomethylated	-0.022721	0.0016128	hypomethylated	28	92	88
chr4	152070648	152072648	Gm833	0.050825	0.10189	insignificant	-0.12595	0.81828	insignificant	5	24	30
chr4	152856939	152858939	Ajap1	-0.080033	0.010576	hypomethylated	0.0067779	0.39858	insignificant	18	92	92
chr4	153330345	153332345	A430005L14Rik	-0.1085	0.000000056	hypomethylated	-0.0082556	0.095219	insignificant	21	112	112
chr4	153348669	153350669	BC046331	-0.13822	4.89E-28	hypomethylated	-0.0030324	0.77209	insignificant	39	110	123
chr4	153349190	153351190	Dffb	-0.19147	1.12E-10	hypomethylated	-0.012315	0.62289	insignificant	17	50	63
chr4	153384911	153386911	Lrrc47	-0.13594	2.77E-40	hypomethylated	-0.015037	0.023274	hypomethylated	49	140	139
chr4	153416786	153418786	Ccdc27	-0.02176	0.81123	insignificant	-0.034417	0.92902	insignificant	6	28	30
chr4	153471282	153473282	Trp73	0.08513	0.21079	insignificant	-0.030859	0.49719	insignificant	2	21	20
chr4	153514317	153516317	Trp73	-0.2066	0.000000676	hypomethylated	-0.029528	0.16655	insignificant	17	98	106
chr4	153515480	153517480	Wdr8	-0.17104	1.97E-14	hypomethylated	-0.017424	0.000013497	hypomethylated	20	88	77
chr4	153534793	153536793	Tprgl	-0.21715	0.00000102	hypomethylated	-0.058979	0.00001038	hypomethylated	14	66	57
chr4	153543821	153545821	Megf6	-0.11256	8.59E-13	hypomethylated	-0.014765	0.56048	insignificant	19	105	106
chr4	153674004	153676004	Arhgef16	-0.13566	5.55E-20	hypomethylated	0.010965	0.19791	insignificant	24	62	62
chr4	154010982	154012982	Prdm16	-0.11775	1.19E-37	hypomethylated	-0.014243	0.1048	insignificant	36	132	132
chr4	154229308	154231308	Ttc34	-0.093504	1.1E-11	hypomethylated	-0.0060964	0.79746	insignificant	24	124	130
chr4	154242693	154244693	Mmel1	-0.16633	0.00000148	hypomethylated	-0.054835	0.018001	hypomethylated	4	12	8
chr4	154334031	154336031	Hes5	-0.097046	4.05E-54	hypomethylated	-0.00021063	0.0063002	hypomethylated	66	155	150
chr4	154337241	154339241	Pank4	-0.12734	2.26E-10	hypomethylated	-0.027947	0.31744	insignificant	25	106	117
chr4	154385093	154387093	Plch2	-0.0671	0.15537	insignificant	0.016897	0.60418	insignificant	2	5	6
chr4	154440138	154442138	Pex10	-0.12483	4.24E-11	hypomethylated	0.0017731	0.0019003	inconclusive	19	87	87
chr4	154459685	154461685	Morn1	-0.10511	4.73E-20	hypomethylated	-0.0040778	0.25726	insignificant	46	108	135
chr4	154460406	154462406	Rer1	-0.13781	3.14E-08	hypomethylated	-0.002941	0.78115	insignificant	23	31	54
chr4	154596644	154598644	Ski	-0.077235	6.1E-36	hypomethylated	0.013233	0.58092	insignificant	81	171	169
chr4	154623074	154625074	2610002J02Rik	-0.053868	0.000000188	hypomethylated	0.0082636	0.77824	insignificant	9	50	58
chr4	154719881	154721881	Prkcz	-0.17872	0.000065646	hypomethylated	0.049716	0.59663	insignificant	5	14	14
chr4	154735500	154737500	Prkcz	-0.023772	0.0078664	hypomethylated	0.0038721	0.84821	insignificant	9	65	66
chr4	154772178	154774178	Gabrd	-0.27228	0.12857	insignificant	0.040688	0.018183	hypermethylated	9	22	25
chr4	154842223	154844223	Tmem52	-0.22208	0.060784	insignificant	0.0019208	1	insignificant	4	10	10
chr4	154864469	154866469	Gnb1	-0.15505	1.68E-36	hypomethylated	-0.0065981	0.86662	insignificant	35	99	108
chr4	154936923	154938923	Nadk	-0.16554	6.49E-08	hypomethylated	0.0084316	0.30852	insignificant	20	73	69
chr4	154974524	154976524	Slc35e2	-0.21887	0.000019362	hypomethylated	-0.063093	0.079751	insignificant	10	87	87
chr4	154997977	154999977	Cdk11b	-0.11493	1.31E-08	hypomethylated	-0.021795	0.54131	insignificant	17	95	90
chr4	155027493	155029493	Mmp23	-0.10244	3.41E-13	hypomethylated	-0.021606	0.10292	insignificant	24	72	89
chr4	155043336	155045336	Mib2	0.041667	1	insignificant	-0.46169	0.0019831	stronglyhypometh	3	8	17
chr4	155067580	155069580	B930041F14Rik	-0.067961	1.4E-19	hypomethylated	-0.0033301	0.59423	insignificant	39	180	177
chr4	155077923	155079923	Ssu72	-0.1474	4.59E-19	hypomethylated	-0.0086731	0.037086	hypomethylated	31	149	151
chr4	155107912	155109912	Gm5151	-0.13977	1.06E-31	hypomethylated	-0.0049916	0.40009	insignificant	52	172	177
chr4	155134300	155136300	2610204G22Rik	-0.096948	0.00000443	hypomethylated	-0.0033678	0.85062	insignificant	18	72	72
chr4	155135207	155137207	Atad3a	-0.13501	0.0046771	hypomethylated	-0.0063166	0.85083	insignificant	7	30	30
chr4	155148670	155150670	Vwa1	-0.34796	0.0045617	stronglyHypometh	0.048505	0.6124	insignificant	4	17	16
chr4	155176726	155178726	Mrpl20	-0.26882	0.013478	hypomethylated	-0.031848	0.008363	inconclusive	9	32	32
chr4	155185597	155187597	Ccnl2	-0.1077	1.5E-22	hypomethylated	-0.0077583	0.4361	insignificant	29	137	138
chr4	155204754	155206754	Aurkaip1	-0.24111	1.21E-22	hypomethylated	-0.041126	0.06614	insignificant	15	62	58
chr4	155212788	155214788	Mxra8	-0.06297	1	insignificant	-0.0025852	0.035435	inconclusive	4	16	17
chr4	155220520	155222520	Dvl1	-0.11287	3.48E-28	hypomethylated	0.011094	0.63913	insignificant	54	155	160
chr4	155242675	155244675	Cpsf3l	-0.15538	1.6E-16	hypomethylated	-0.0094225	0.0050895	inconclusive	26	113	129
chr4	155243549	155245549	Gltpd1	-0.10431	1.55E-09	hypomethylated	0.019882	0.0002667	hypermethylated	24	95	115
chr4	155264983	155266983	Acap3	-0.11116	4.09E-32	hypomethylated	0.0086784	0.51647	insignificant	59	195	196
chr4	155265871	155267871	Pusl1	-0.085004	3.79E-26	hypomethylated	-0.0050616	0.36054	insignificant	41	158	154
chr4	155316939	155318939	Ube2j2	-0.11997	1.8E-17	hypomethylated	-0.0024699	0.73411	insignificant	39	117	138
chr4	155335426	155337426	Fam132a	-0.28693	0.0059301	hypomethylated	-0.12523	0.049904	hypomethylated	7	24	41
chr4	155366022	155368022	Sdf4	-0.095343	2.65E-31	hypomethylated	0.0047197	0.56254	insignificant	59	293	288
chr4	155366787	155368787	B3galt6	-0.15235	2.14E-28	hypomethylated	0.0094977	0.24015	insignificant	32	163	162
chr4	155399450	155401450	Tnfrsf18	-0.56872	0.0064542	stronglyHypometh	0.20785	0.040223	hypermethylated	1	16	18
chr4	155483106	155485106	9430015G10Rik	-0.12381	0.36236	insignificant	-0.026511	0.6682	insignificant	9	44	52
chr4	155576392	155578392	AW011738	-0.19079	0.000000648	hypomethylated	-0.029363	0.52821	insignificant	16	66	72
chr4	155589035	155591035	2310042D19Rik	0.0625	0.56115	insignificant	-0.072557	1	insignificant	2	4	4
chr4	155608966	155610966	Klhl17	-0.080732	0.00000124	hypomethylated	0.0042791	0.017463	inconclusive	30	126	131
chr5	3343311	3345311	Cdk6	-0.15222	1.34E-18	hypomethylated	-0.013098	0.037615	hypomethylated	22	101	115
chr5	3542832	3544832	Fam133b	-0.078648	4.73E-25	hypomethylated	-0.0026707	0.57723	insignificant	32	120	120
chr5	3595065	3597065	Pex1	-0.016043	0.000079968	hypomethylated	0.0026234	0.5798	insignificant	11	70	78
chr5	3596547	3598547	C030048B08Rik	0.031603	0.75989	insignificant	0.066354	0.0027829	hypermethylated	3	17	17
chr5	3647936	3649936	Gatad1	-0.029412	0.47033	insignificant	-0.0092481	0.88562	insignificant	6	6	12
chr5	3802164	3804164	Krit1	-0.14392	7.65E-51	hypomethylated	-0.0078485	0.49168	insignificant	50	154	158
chr5	3802180	3804180	Krit1	-0.14392	7.65E-51	hypomethylated	-0.0078485	0.49168	insignificant	50	154	158
chr5	3803109	3805109	Ankib1	-0.16712	4.77E-21	hypomethylated	0.014977	0.8341	insignificant	19	63	63
chr5	3844172	3846172	4932412H11Rik	0	1	insignificant	-0.054113	0.10315	insignificant	1	6	6

chr5	3927185	3929185	Akap9	-0.15128	9.28E-18	hypomethylated	-0.014737	0.0034133	hypomethylated	29	98	106
chr5	4104697	4106697	Cyp51	-0.1327	0.59895	insignificant	-0.018678	0.68459	insignificant	4	10	10
chr5	4758216	4760216	Fzd1	-0.31002	0.000007025	hypomethylated	-0.09731	0.10765	insignificant	5	24	21
chr5	5380251	5382251	Cdk14	-0.09958	5.44E-30	hypomethylated	0.0096882	0.9734	insignificant	54	127	125
chr5	5514789	5516789	Cldn12	-0.093574	8.69E-10	hypomethylated	0.026319	0.029711	inconclusive	14	39	42
chr5	5514849	5516849	Cldn12	-0.17707	4.84E-11	hypomethylated	0.019901	0.0060701	inconclusive	11	25	28
chr5	5514976	5516976	Cldn12	-0.44806	0.00000014	stronglyHypometh	-0.074235	0.0024027	hypomethylated	3	5	8
chr5	5559501	5561501	Gtpbp10		1	noCoverage	0.0002682	0.84692	insignificant	0	38	38
chr5	5572798	5574798	Gm8773	-0.38594	2.29E-12	stronglyHypometh	-0.13645	8.22E-10	hypomethylated	8	50	47
chr5	5694077	5696077	Steap2	-0.13461	5.02E-12	hypomethylated	-0.0082723	1	insignificant	16	75	78
chr5	5694568	5696568	Steap2	-0.39435	1	lowCoverage	-0.11268	0.66003	insignificant	1	8	10
chr5	5749317	5751317	Steap1	-0.16207	4.54E-28	hypomethylated	-0.01335	2.02E-09	hypomethylated	19	52	52
chr5	7344378	7346378	Zfp804b	-0.18211	0.58931	insignificant	-0.021768	0.73125	insignificant	2	37	42
chr5	7959471	7961471	Steap4	-0.20099	0.018287	hypomethylated	-0.0030935	1	insignificant	2	26	26
chr5	8055541	8057541	Sri	-0.19852	5.66E-21	hypomethylated	0.018905	0.068797	insignificant	11	63	64
chr5	8368081	8370081	Adam22	-0.20358	7.53E-12	hypomethylated	-0.0057006	0.26808	insignificant	9	18	18
chr5	8421849	8423849	Slc25a40	-0.13427	1.39E-17	hypomethylated	-0.0086818	0.004584	inconclusive	45	138	140
chr5	8422716	8424716	Dbf4	-0.12519	6.63E-09	hypomethylated	-0.0074693	0.44929	insignificant	35	88	90
chr5	8622952	8624952	Rundc3b	-0.032609	1	insignificant	-0.020261	0.35719	insignificant	4	4	4
chr5	8659091	8661091	Abcb1a	-0.18383	0.0011793	hypomethylated	0.046816	0.65115	insignificant	4	31	31
chr5	8797146	8799146	Abcb1b	-0.21285	0.00000392	hypomethylated	-0.024984	0.27276	insignificant	9	44	44
chr5	8892720	8894720	Abcb4	-0.1665	2.45E-18	hypomethylated	-0.0067271	0.79388	insignificant	19	97	96
chr5	8997146	8999146	Crot	-0.10692	0.000045919	hypomethylated	0.01047	0.44103	insignificant	6	36	36
chr5	9099736	9101736	4930420K17Rik	-0.090588	2.97E-14	hypomethylated	-0.015175	0.024708	hypomethylated	42	156	156
chr5	9161776	9163776	Dmtf1	-0.2	1	insignificant	0.0083333	0.88603	insignificant	3	5	5
chr5	9265192	9267192	9330182L06Rik	-0.11809	4.47E-11	hypomethylated	-0.024	0.79245	insignificant	30	87	107
chr5	9725352	9727352	Grm3	-0.275	0.53846	insignificant	0.075	1	insignificant	1	8	6
chr5	12382165	12384165	Sema3d	-0.16694	0.00024582	hypomethylated	0.025466	0.26999	insignificant	11	34	34
chr5	13398308	13400308	Sema3a	-0.29275	1	lowCoverage	-0.092753	0.079376	insignificant	1	18	5
chr5	13790618	13792618	Speer3		1	noCoverage	0.012656	1	insignificant	0	4	4
chr5	14024275	14026275	Sema3e	-0.11421	0.000065317	hypomethylated	0.076594	0.12799	insignificant	11	62	50
chr5	14513917	14515917	Pclo	-0.13429	4.24E-13	hypomethylated	-0.015612	0.5189	insignificant	23	76	90
chr5	15439508	15441508	Cacna2d1	-0.13896	1.04E-17	hypomethylated	-0.024293	0.0014995	hypomethylated	61	186	197
chr5	17079633	17081633	Sema3c	-0.20541	8.73E-26	hypomethylated	-0.0038529	0.18321	insignificant	28	56	60
chr5	18731863	18733863	Magi2	-0.14889	8.59E-09	hypomethylated	-0.028286	0.43184	insignificant	16	75	75
chr5	19412335	19414335	Magi2		1	noCoverage	-0.11602	0.77429	insignificant	0	7	6
chr5	20387270	20389270	Tmem60	-0.079757	2.74E-26	hypomethylated	-0.0094168	0.078115	insignificant	52	256	267
chr5	20387942	20389942	Phtf2	-0.09592	2.45E-09	hypomethylated	-0.0078175	0.7281	insignificant	11	82	82
chr5	20455806	20457806	A630072M18Ril	-0.053334	7.83E-14	hypomethylated	0.019646	0.53875	insignificant	36	171	171
chr5	20457640	20459640	A630072M18Ril	-0.12206	0.053393	insignificant	-0.0033667	0.69124	insignificant	13	57	57
chr5	20561615	20563615	Ptpn12	-0.13985	8.69E-18	hypomethylated	0.017603	0.32752	insignificant	37	104	107
chr5	20691084	20693084	Pion	-0.13423	7.29E-08	hypomethylated	-0.0086033	0.76578	insignificant	10	46	46
chr5	20929720	20931720	Fam185a	-0.14173	0.000046322	hypomethylated	0.0071744	0.9613	insignificant	9	67	72
chr5	20930495	20932495	Fam185a	-0.14453	0.000010438	hypomethylated	-0.00046655	0.32493	insignificant	9	66	70
chr5	21150801	21152801	Armc10	-0.1502	2.56E-22	hypomethylated	-0.017354	0.2159	insignificant	20	89	94
chr5	21151423	21153423	Armc10	-0.15115	1.83E-17	hypomethylated	-0.035202	0.0061381	hypomethylated	20	74	79
chr5	21207163	21209163	Napepld	-0.15498	2.43E-16	hypomethylated	-0.0045277	0.10433	insignificant	21	68	72
chr5	21241977	21243977	Pmpcb	-0.12804	0.000000296	hypomethylated	0.008527	0.95325	insignificant	15	38	40
chr5	21290100	21292100	Psmc2	-0.11947	6.48E-26	hypomethylated	0.0067881	0.58303	insignificant	35	119	120
chr5	21290983	21292983	Psmc2	-0.14257	1.6E-19	hypomethylated	-0.010497	0.009158	hypomethylated	26	91	99
chr5	21371422	21373422	Slc26a5		1	noCoverage	-0.02517	0.044552	hypomethylated	0	4	4
chr5	21850523	21852523	Reln	-0.12673	3.35E-26	hypomethylated	-0.0053941	0.030531	hypomethylated	31	122	122
chr5	22056149	22058149	Orc5	-0.23469	0.000055093	hypomethylated	0.15281	0.52863	insignificant	3	7	8
chr5	22251010	22253010	Lhfpl3	-0.10233	2.12E-09	hypomethylated	0.016411	0.16263	insignificant	17	79	74
chr5	22252702	22254702	Lhfpl3	0.048704	1	insignificant	-0.01744	0.14547	insignificant	2	19	19
chr5	22939246	22941246	Mll5	-0.14738	0.00000123	hypomethylated	-0.010636	0.51178	insignificant	9	102	96
chr5	23216894	23218894	Mir3096		1	noCoverage	0.045776	0.51774	insignificant	0	26	26
chr5	23218484	23220484	AI506816		1	noCoverage	0.088077	0.7457	insignificant	0	14	14
chr5	23289479	23291479	Pus7	-0.23337	0.12808	insignificant	0.18885	0.57747	insignificant	2	15	5
chr5	23292561	23294561	Rint1	-0.24539	2.75E-15	hypomethylated	0.016212	0.13581	insignificant	24	58	62
chr5	23349963	23351963	Tomm7	-0.09677	1	insignificant	0.045356	0.83775	insignificant	3	24	32
chr5	23536501	23538501	Fam126a		1	noCoverage	0.0006912	0.55613	insignificant	0	20	20
chr5	23605407	23607407	Klhl7	-0.19483	0.000098706	hypomethylated	0.040911	0.47582	insignificant	5	29	19
chr5	23669780	23671780	Nupl2	-0.15622	0.00000238	hypomethylated	-0.030568	0.95868	insignificant	17	47	50
chr5	23857422	23859422	Kcnh2	-0.0662	3.29E-09	hypomethylated	-0.0067902	0.000025403	hypomethylated	25	92	92
chr5	23869636	23871636	Nos3	0.1548	0.0071376	hypermethylated	0.16258	0.21849	insignificant	8	28	28
chr5	23898973	23900973	Abcb8	-0.1245	0.00063929	hypomethylated	-0.010899	0.67984	insignificant	9	20	21
chr5	23918268	23920268	Accn3	-0.099725	0.36604	insignificant	-0.0068507	0.41641	insignificant	3	28	23
chr5	23929348	23931348	Cdk5	-0.13139	1.51E-18	hypomethylated	-0.0054161	0.43248	insignificant	37	110	111
chr5	23930049	23932049	Slc4a2	-0.13121	5.81E-14	hypomethylated	-0.014559	0.13584	insignificant	36	94	95
chr5	23951053	23953053	Tmub1	0.038503	0.00011462	hypermethylated	0.056494	0.69873	insignificant	11	38	40
chr5	23953664	23955664	Tmub1	-0.15582	0.000000163	hypomethylated	0.0066884	0.85955	insignificant	6	14	14
chr5	23956994	23958994	Agap3	-0.11994	2.83E-51	hypomethylated	-0.00057802	0.11397	insignificant	57	162	183
chr5	24032666	24034666	Gbx1	-0.11256	3.1E-14	hypomethylated	0.0048931	0.041166	inconclusive	23	73	76
chr5	24083285	24085285	Abcf2	-0.12809	0.00015647	hypomethylated	0.018636	0.52419	insignificant	7	32	32
chr5	24091567	24093567	Chpf2	0.02598	0.00063839	hypermethylated	-0.015865	0.6893	insignificant	3	62	58
chr5	24096931	24098931	Mir671	-0.31129	2.33E-16	hypomethylated	0.040205	0.0072714	hypermethylated	20	71	70
chr5	24107820	24109820	Smarcd3	-0.13004	0.000000393	hypomethylated	-0.017096	0.46597	insignificant	13	30	36
chr5	24150270	24152270	1700022A21Rik		1	noCoverage	-0.18568	0.0081975	hypomethylated	0	6	6
chr5	24190632	24192632	Nub1	-0.1471	1.41E-17	hypomethylated	0.013997	0.13964	insignificant	12	73	76
chr5	24236498	24238498	Wdr86	-0.39411	0.000094669	stronglyHypometh	-0.0044629	0.48652	insignificant	4	35	44
chr5	24263661	24265661	Crygn	-0.036577	0.57391	insignificant	-0.0052142	0.45676	insignificant	5	16	17
chr5	24348179	24350179	Rheb	-0.094025	6.44E-12	hypomethylated	-0.009807	0.29499	insignificant	21	93	100
chr5	24414327	24416327	Prkag2	-0.11978	0.0094079	hypomethylated	-0.009052	0.54213	insignificant	1	43	43
chr5	24605792	24607792	2900005J15Rik	-0.16922	3.3E-20	hypomethylated	-0.017416	0.67464	insignificant	25	102	102

chr5	24606460	24608460	Prkag2	-0.17087	9.28E-15	hypomethylated	0.032618	0.18115	insignificant	19	55	51
chr5	24727710	24729710	Galnt11	-0.090801	2.65E-10	hypomethylated	-0.019391	0.47962	insignificant	31	79	91
chr5	25004601	25006601	Mll3	-0.092524	8.47E-10	hypomethylated	-0.00039222	0.7619	insignificant	52	207	184
chr5	25004614	25006614	4831440E17Rik	-0.095163	4.43E-09	hypomethylated	-0.00057569	0.76104	insignificant	50	203	180
chr5	25020884	25022884	Cct8l1	0.011899	0.71882	insignificant	0.043356	0.19748	insignificant	4	17	17
chr5	25034835	25036835	1700096K18Rik	-0.13196	3.35E-25	hypomethylated	0.0021049	0.087342	insignificant	31	88	88
chr5	25211615	25213615	Xrcc2		1	noCoverage	-0.05424	0.54974	insignificant	0	11	11
chr5	25264843	25266843	Actr3b	-0.2646	0.000000025	hypomethylated	-0.054525	0.0039877	hypomethylated	11	40	41
chr5	27142896	27144896	Dpp6	-0.13494	0.000048381	hypomethylated	0.01909	0.071593	insignificant	17	85	84
chr5	27374738	27376738	Dpp6	-0.16095	0.030361	hypomethylated	-0.005282	0.53507	insignificant	8	63	62
chr5	27587474	27589474	Dpp6	-0.28216	0.044646	hypomethylated	-0.13114	0.000068231	hypomethylated	7	35	31
chr5	28117879	28119879	Paxip1	-0.12768	6.88E-24	hypomethylated	-0.015059	0.39798	insignificant	16	65	75
chr5	28167486	28169486	Htr5a		1	noCoverage	-0.031507	0.15402	insignificant	0	23	21
chr5	28396951	28398951	Insig1	-0.12367	1.03E-27	hypomethylated	-0.0243	0.25976	insignificant	34	112	110
chr5	28491235	28493235	En2	-0.018788	1.29E-11	hypomethylated	-0.001076	0.80302	insignificant	90	241	244
chr5	28642728	28644728	Rbm33	-0.09772	2.53E-43	hypomethylated	-0.0091401	0.26237	insignificant	60	199	211
chr5	28792523	28794523	9530036O11Rik	-0.33446	0.00071599	stronglyHypometh	-0.02698	0.0020656	hypomethylated	3	19	15
chr5	29704930	29706930	Lmbr1	-0.38276	2.29E-10	stronglyHypometh	0.14621	0.70816	insignificant	4	17	16
chr5	29760206	29762206	Nom1	-0.15678	0.000000744	hypomethylated	-0.056749	0.1345	insignificant	19	62	62
chr5	29805010	29807010	Mnx1	-0.20065	2.9E-11	hypomethylated	0.0012578	0.059251	insignificant	16	66	70
chr5	29894781	29896781	Ube3c	-0.10843	9.17E-12	hypomethylated	0.0023389	0.027961	hypermethylated	38	146	146
chr5	30061437	30063437	Dnajb6	-0.12767	6.57E-30	hypomethylated	-0.002065	0.041389	hypomethylated	31	122	121
chr5	30062476	30064476	Dnajb6	-0.1379	7.35E-20	hypomethylated	-0.0048788	0.18518	insignificant	24	90	89
chr5	30338700	30340700	Il6	-0.31429	0.000018367	hypomethylated	0.098006	0.69722	insignificant	2	7	8
chr5	30430735	30432735	Fam59b	-0.14472	2.56E-20	hypomethylated	0.008103	0.19161	insignificant	34	126	124
chr5	30480861	30482861	Hadhb	-0.20044	0.019474	hypomethylated	-0.030977	0.85269	insignificant	7	30	33
chr5	30481520	30483520	Hadha	-0.33673	0.31054	insignificant	-0.13173	0.30263	insignificant	2	7	10
chr5	30532262	30534262	Gpr113	-0.12969	0.27593	insignificant	-0.12629	0.04785	hypomethylated	1	8	13
chr5	30558157	30560157	Ept1	-0.17825	4.77E-08	hypomethylated	-0.10199	3.14E-12	hypomethylated	12	40	44
chr5	30606913	30608913	Ccdc164	-0.085506	0.62901	insignificant	-0.021389	0.82349	insignificant	9	36	36
chr5	30764305	30766305	Otof		1	noCoverage	0.021134	0.21968	insignificant	0	7	11
chr5	30767449	30769449	1700001C02Rik	-0.23835	5.43E-20	hypomethylated	0.035969	0.037388	inconclusive	17	49	52
chr5	30848209	30850209	Cib4		1	noCoverage	-0.0067626	0.035398	hypomethylated	0	7	4
chr5	30889542	30891542	Kcnk3	-0.090977	2.77E-18	hypomethylated	-0.0020824	0.89273	insignificant	36	133	134
chr5	30949311	30951311	4930471M23Ril	-0.1885	1.02E-15	hypomethylated	-0.015088	0.96116	insignificant	27	66	62
chr5	30968274	30970274	Cenpa	-0.16396	0.000035798	hypomethylated	-0.0099986	0.84086	insignificant	9	54	51
chr5	31013267	31015267	Dpysl5	-0.20268	1.76E-14	hypomethylated	0.020051	0.078171	insignificant	9	67	73
chr5	31116128	31118128	Mapre3	-0.15336	2.35E-21	hypomethylated	0.0026486	0.90223	insignificant	23	62	60
chr5	31171019	31173019	Tmem214	-0.14744	9.17E-11	hypomethylated	0.0021092	0.45209	insignificant	18	60	60
chr5	31190224	31192224	Agbl5	-0.15601	1.87E-16	hypomethylated	-0.0068202	0.32841	insignificant	21	116	106
chr5	31190378	31192378	Agbl5	-0.16446	9.47E-15	hypomethylated	-0.00068134	0.18775	insignificant	18	102	92
chr5	31210095	31212095	Ost4	-0.092215	0.000000022	hypomethylated	0.000055253	0.80244	insignificant	10	44	44
chr5	31210161	31212161	Ost4	-0.092215	0.000000022	hypomethylated	0.000055253	0.80244	insignificant	10	44	44
chr5	31215158	31217158	Emilin1	-0.24725	1	lowCoverage	0.14591	0.30001	insignificant	1	14	12
chr5	31223267	31225267	Khk	-0.1255	0.00079853	hypomethylated	-0.021425	0.45905	insignificant	10	36	36
chr5	31247800	31249800	Cgref1	0.4892	0.50731	insignificant	0.22694	0.00000364	hypermethylated	1	17	12
chr5	31247853	31249853	Cgref1	0.40896	0.52105	insignificant	0.21376	0.00002451	hypermethylated	1	12	11
chr5	31251438	31253438	Abhd1		1	noCoverage	0.010922	1	insignificant	0	22	22
chr5	31251478	31253478	Abhd1		1	noCoverage	0.010922	1	insignificant	0	22	22
chr5	31262734	31264734	Preb	-0.16146	0.13564	insignificant	-0.000044732	0.77037	insignificant	10	34	34
chr5	31350012	31352012	0610007C21Rik	-0.19437	4.86E-18	hypomethylated	0.010335	0.35602	insignificant	24	67	61
chr5	31350452	31352452	Slc5a6	-0.20678	9.8E-19	hypomethylated	0.0072207	0.43216	insignificant	24	69	63
chr5	31350935	31352935	Slc5a6	-0.31529	5.92E-16	hypomethylated	0.031821	0.42418	insignificant	4	18	14
chr5	31351297	31353297	Slc5a6	-0.41591	0.000000109	stronglyHypometh	-0.065666	1	insignificant	1	8	8
chr5	31356183	31358183	Cad	-0.14327	1.42E-29	hypomethylated	-0.011961	0.30364	insignificant	24	96	92
chr5	31395900	31397900	Slc30a3	-0.16819	2.68E-13	hypomethylated	-0.0096145	0.54973	insignificant	17	66	68
chr5	31409691	31411691	Dnajc5g	-0.0094831	0.55312	insignificant	0.051588	0.85893	insignificant	5	17	16
chr5	31441268	31443268	Ucn	-0.14792	0.08315	insignificant	0.013792	0.15568	insignificant	4	22	22
chr5	31482517	31484517	Gtf3c2		1	noCoverage	-0.02641	1	insignificant	0	23	20
chr5	31494676	31496676	Snx17	-0.16608	9.56E-51	hypomethylated	-0.011898	0.081671	insignificant	33	116	118
chr5	31495512	31497512	Eif2b4	-0.19366	4.56E-32	hypomethylated	-0.013909	0.0056089	hypomethylated	17	56	58
chr5	31504415	31506415	Ppm1g	-0.16546	0.00000326	inconclusive	0.0057837	0.46027	insignificant	10	46	48
chr5	31504676	31506676	Ppm1g	-0.25523	3.53E-15	inconclusive	0.011908	0.0073286	hypermethylated	4	12	14
chr5	31522918	31524918	Ppm1g	-0.1863	0.0012109	hypomethylated	-0.007453	0.44939	insignificant	3	14	13
chr5	31542290	31544290	Nrbp1	-0.12286	6.66E-29	hypomethylated	-0.0062896	0.038874	hypomethylated	49	157	161
chr5	31553078	31555078	Krtcap3	0.051152	1	insignificant	-0.047456	0.56361	insignificant	2	14	14
chr5	31593487	31595487	Ift172	-0.26382	5.42E-09	hypomethylated	0.030387	0.28716	insignificant	2	8	8
chr5	31598250	31600250	Fndc4	-0.3544	0.000000198	stronglyHypometh	-0.015905	0.0049642	hypomethylated	9	33	31
chr5	31598953	31600953	Gckr		1	noCoverage	0.022944	1	insignificant	0	14	12
chr5	31753808	31755808	Zfp512	-0.28603	0.0012214	hypomethylated	-0.00089159	0.78803	insignificant	3	18	18
chr5	31796133	31798133	Gpn1	-0.1999	8.03E-17	hypomethylated	-0.00052351	0.26934	insignificant	28	79	83
chr5	31828367	31830367	Slc4a1ap	-0.26692	3.22E-49	hypomethylated	-0.051061	0.000012012	hypomethylated	25	90	94
chr5	31829135	31831135	Supt7l	-0.30643	6.41E-41	hypomethylated	-0.060725	0.00002754	hypomethylated	16	68	72
chr5	31915323	31917323	Mrpl33	-0.069062	0.14323	insignificant	-0.047766	0.1008	insignificant	17	43	41
chr5	31999422	32001422	Bre	-0.093681	3.51E-26	hypomethylated	0.015231	0.18816	insignificant	30	82	86
chr5	31999983	32001983	Rbks	-0.087767	2.25E-14	hypomethylated	0.010124	0.77133	insignificant	28	64	68
chr5	32437844	32439844	Fosl2	-0.11001	1.92E-24	hypomethylated	0.0077848	0.3258	insignificant	38	159	159
chr5	32534080	32536080	Plb1	0.19841	1	lowCoverage	0.019841	1	insignificant	1	7	4
chr5	32760342	32762342	Ppp1cb	-0.12522	0.000000116	hypomethylated	0.010991	0.12097	insignificant	23	108	114
chr5	32912605	32914605	Yes1	-0.11546	6.56E-55	hypomethylated	-0.0051731	0.02882	hypomethylated	56	123	141
chr5	33128275	33130275	Pisd	-0.2499	0.000000214	hypomethylated	-0.064172	0.8862	insignificant	6	36	39
chr5	33196879	33198879	C330019G07Rik		1	noCoverage	0.13887	0.12486	insignificant	0	2	2
chr5	33205369	33207369	Depdc5	-0.13282	0.00018107	hypomethylated	0.017499	0.76472	insignificant	8	70	72
chr5	33360464	33362464	Ywhah	-0.11794	2.56E-33	hypomethylated	-0.0038586	0.22296	insignificant	37	125	128
chr5	33445867	33447867	Slc5a1	-0.049721	0.0022209	hypomethylated	-0.063625	0.35699	insignificant	3	20	34

chr5	33560887	33562887	Spon2	-0.74118	0.00012764	stronglyHypometh	-0.043077	0.19143	insignificant	1	11	11
chr5	33617653	33619653	Ctbp1	-0.10913	0.000000156	hypomethylated	0.00069261	0.2769	insignificant	14	42	42
chr5	33677220	33679220	Maea	-0.14142	3.66E-49	hypomethylated	0.01102	0.2154	insignificant	55	149	162
chr5	33720344	33722344	4933407H18Rik	-0.17415	0.00000034	hypomethylated	-0.0066868	0.0082529	hypomethylated	11	74	79
chr5	33972284	33974284	Fam53a	-0.091384	1.07E-09	hypomethylated	-0.0065794	0.28576	insignificant	19	74	74
chr5	33999795	34001795	Tacc3	-0.11643	0.00033541	hypomethylated	0.0043601	0.049551	hypermethylated	16	83	82
chr5	34000481	34002481	Tmem129	-0.11711	0.0028341	hypomethylated	0.0032745	0.098588	insignificant	12	63	62
chr5	34063372	34065372	Fgfr3	-0.12531	1.91E-54	hypomethylated	0.0036091	0.54428	insignificant	62	155	146
chr5	34063408	34065408	Fgfr3	-0.12447	5.16E-56	hypomethylated	0.005945	0.59448	insignificant	62	167	158
chr5	34063954	34065954	Fgfr3	-0.13015	1.26E-59	hypomethylated	0.0081089	0.90436	insignificant	71	203	194
chr5	34125353	34127353	Letm1	-0.16562	0.000064354	hypomethylated	-0.064207	0.30981	insignificant	7	16	22
chr5	34278907	34280907	Whsc2	-0.16658	3.46E-13	hypomethylated	0.014432	0.058035	insignificant	15	54	53
chr5	34325122	34327122	Gm1673	-0.077439	0.00008741	hypomethylated	-0.011572	0.53472	insignificant	8	115	130
chr5	34337632	34339632	Nat8l	-0.087801	5.17E-12	hypomethylated	0.00061392	0.00058313	inconclusive	49	184	179
chr5	34512073	34514073	Haus3	-0.11553	0.0069967	hypomethylated	-0.0042787	0.44991	insignificant	11	41	41
chr5	34512097	34514097	Haus3	-0.11553	0.0069967	hypomethylated	-0.0042787	0.44991	insignificant	11	41	41
chr5	34530359	34532359	Mxd4	-0.1336	5.26E-08	hypomethylated	-0.0043163	0.9406	insignificant	10	26	26
chr5	34630973	34632973	Zfyve28	-0.065665	4.11E-10	hypomethylated	0.013149	0.76634	insignificant	24	70	74
chr5	34678038	34680038	Rnf4	-0.12629	5.98E-17	hypomethylated	0.0048813	0.78449	insignificant	37	107	104
chr5	34856628	34858628	Tnip2	-0.10722	1.18E-10	hypomethylated	0.014031	0.88224	insignificant	7	22	22
chr5	34867432	34869432	Sh3bp2	-0.34211	1	insignificant	-0.0047847	0.73417	insignificant	2	19	19
chr5	34885013	34887013	Sh3bp2	-0.15043	0.022238	hypomethylated	-0.05873	0.07949	insignificant	3	6	6
chr5	34891224	34893224	Sh3bp2	-0.11771	1.51E-09	hypomethylated	0.0094736	0.22971	insignificant	17	72	70
chr5	34891336	34893336	Sh3bp2	-0.096307	2.27E-08	hypomethylated	0.0086536	0.30872	insignificant	17	70	68
chr5	34915362	34917362	Add1	-0.16389	1.63E-26	hypomethylated	0.0031485	0.85459	insignificant	32	85	87
chr5	34979763	34981763	Mfsd10	-0.19697	1	insignificant	-0.0085639	0.82733	insignificant	1	6	6
chr5	35002027	35004027	Grk4	-0.082851	1.05E-14	hypomethylated	0.0057265	0.56408	insignificant	30	137	137
chr5	35002797	35004797	Nop14	-0.063183	3.44E-10	hypomethylated	0.0037245	0.0049449	inconclusive	29	131	131
chr5	35103388	35105388	Htt	-0.20464	0.000000134	hypomethylated	-0.013511	0.67964	insignificant	16	60	67
chr5	35291096	35293096	Rgs12	-0.13717	8.2E-09	hypomethylated	-0.0090376	0.0054702	hypomethylated	28	85	85
chr5	35398732	35400732	Dok7	-0.13557	0.000000391	hypomethylated	-0.0024777	0.59444	insignificant	22	58	58
chr5	35448346	35450346	Lrpap1	-0.10088	1.7E-17	hypomethylated	-0.02349	0.00000225	hypomethylated	10	22	24
chr5	35620214	35622214	Adra2c	-0.071125	4.66E-12	hypomethylated	-0.0038965	0.5145	insignificant	38	159	160
chr5	35730765	35732765	Hmx1	-0.097356	9.23E-29	hypomethylated	0.010532	0.031244	inconclusive	62	186	191
chr5	35924708	35926708	Acox3	-0.17638	4.74E-09	hypomethylated	0.0012693	0.5693	insignificant	17	66	70
chr5	36099528	36101528	Ablim2	-0.13625	0.000000279	hypomethylated	0.0060517	0.85294	insignificant	17	104	100
chr5	36234967	36236967	Afap1	-0.18396	8.28E-23	hypomethylated	-0.026924	0.029413	hypomethylated	29	114	127
chr5	36545669	36547669	Psapl1	0.025	1	insignificant	-0.07619	0.41469	insignificant	1	4	4
chr5	36740788	36742788	Sorcs2	-0.18841	0.0026907	hypomethylated	-0.071195	0.024045	hypomethylated	10	29	33
chr5	36806833	36808833	Grpel1	-0.16466	3.25E-13	hypomethylated	-0.021355	0.018861	hypomethylated	14	96	91
chr5	36826236	36828236	Ccdc96	-0.087123	2.51E-30	hypomethylated	0.00035039	0.044579	inconclusive	59	189	192
chr5	36826934	36828934	Tada2b	-0.13439	6.64E-17	hypomethylated	0.005271	0.0018737	inconclusive	38	118	123
chr5	36925046	36927046	Tbc1d14	-0.20942	1	insignificant	-0.086193	0.70477	insignificant	1	22	20
chr5	37087208	37089208	D5Ertd579e	-0.20643	0.000024943	hypomethylated	-0.0074334	0.88101	insignificant	7	33	33
chr5	37258808	37260808	Ppp2r2c	-0.092649	1.3E-25	hypomethylated	-0.0077991	0.13513	insignificant	60	159	176
chr5	37380221	37382221	Wfs1	-0.097204	0.00028309	hypomethylated	-0.0034171	0.26493	insignificant	15	46	46
chr5	37632318	37634318	Crmp1	-0.11984	3.64E-23	hypomethylated	-0.0099235	0.000028808	hypomethylated	41	174	168
chr5	37636030	37638030	Crmp1	-0.13926	7.91E-08	hypomethylated	-0.026007	0.38331	insignificant	34	109	109
chr5	37728120	37730120	Evc	-0.2628	0.00000285	hypomethylated	-0.018577	0.96316	insignificant	16	48	48
chr5	37728716	37730716	Evc2	-0.42222	0.56239	insignificant	-0.062963	0.76398	insignificant	3	18	18
chr5	38108392	38110392	Stk32b	-0.26856	0.000000488	hypomethylated	-0.019856	0.62225	insignificant	3	37	38
chr5	38125757	38127757	Cytl1		1	noCoverage	-0.14994	0.031223	hypomethylated	0	8	8
chr5	38210802	38212802	Msx1as	-0.66962	0.36934	lowCoverage	-0.048308	0.70809	insignificant	1	13	12
chr5	38215824	38217824	Msx1	-0.17691	0.000000394	hypomethylated	-0.018443	0.16289	insignificant	22	74	75
chr5	38429473	38431473	Stx18	-0.13118	1.1E-12	hypomethylated	-0.012933	0.37465	insignificant	20	56	56
chr5	38550706	38552706	Nsg1	-0.11419	4.01E-28	hypomethylated	0.0029942	1.15E-17	inconclusive	20	85	85
chr5	38610720	38612720	Lyar	-0.15058	2.62E-15	hypomethylated	-0.012198	0.93736	insignificant	30	101	107
chr5	38611667	38613667	Zbtb49	-0.18047	6.04E-12	hypomethylated	-0.017835	0.58734	insignificant	21	55	55
chr5	38650613	38652613	Tmem128	-0.18457	0.60128	insignificant	0.0036101	0.5435	insignificant	3	39	44
chr5	38667642	38669642	Otop1	-0.25515	5.38E-13	hypomethylated	-0.0050551	0.047085	hypomethylated	16	82	83
chr5	38709747	38711747	Drd5	-0.16123	1.13E-11	hypomethylated	-0.018497	0.0011164	hypomethylated	23	120	122
chr5	38874624	38876624	Slc2a9	-0.53203	0.0010544	stronglyHypometh	-0.10693	0.071607	insignificant	1	4	4
chr5	38952834	38954834	Wdr1	-0.10399	1.75E-11	hypomethylated	-0.024306	0.080611	insignificant	31	82	84
chr5	40035870	40037870	Hs3st1	-0.20253	2.29E-10	hypomethylated	-0.018407	0.16967	insignificant	5	16	16
chr5	42099394	42101394	Rab28	-0.14359	0.12838	insignificant	-0.049135	0.019459	hypomethylated	5	20	25
chr5	42155459	42157459	Nkx3-2	-0.17198	6.33E-22	hypomethylated	0.000039807	0.067989	insignificant	16	56	56
chr5	42235554	42237554	Bod1l	-0.1883	0.022437	hypomethylated	-0.048813	0.063228	insignificant	5	24	24
chr5	43623701	43625701	Cpeb2	-0.10269	2.37E-56	hypomethylated	-0.00024333	0.017185	hypomethylated	75	278	294
chr5	43626593	43628593	Gm7854	-0.086352	1	insignificant	0.040664	0.63424	insignificant	4	44	45
chr5	43992160	43994160	C1qtnf7	0.13158	1	lowCoverage	0.095865	0.44153	insignificant	1	2	4
chr5	44052684	44054684	Cc2d2a	-0.38987	0.000000513	stronglyHypometh	-0.05574	0.52482	insignificant	3	10	10
chr5	44173388	44175388	Fbxl5	-0.076429	0.000000554	hypomethylated	-0.0038377	0.010593	hypomethylated	13	54	54
chr5	44259065	44261065	Cd38	-0.425	0.01034	stronglyHypometh	-0.12734	0.10513	insignificant	1	8	8
chr5	44492975	44494975	Prom1	-0.36343	0.0014518	stronglyHypometh	0.085624	0.40846	insignificant	4	12	12
chr5	44617845	44619845	Tapt1	-0.09912	2.63E-16	hypomethylated	0.020199	0.029299	inconclusive	34	86	95
chr5	45883612	45885612	Lap3	-0.082014	0.1841	insignificant	0.0025392	0.46923	insignificant	24	114	104
chr5	45910467	45912467	Med28	-0.11744	9.17E-22	hypomethylated	0.0041539	0.03356	hypermethylated	23	72	72
chr5	46060163	46062163	Ncapg	-0.067358	1.66E-08	hypomethylated	-0.012293	0.40291	insignificant	24	146	146
chr5	46247791	46249791	Lcorl	-0.098471	3.31E-13	hypomethylated	-0.00014888	0.46624	insignificant	37	157	160
chr5	46248779	46250779	Lcorl	-0.11334	0.067515	insignificant	-0.032088	0.28012	insignificant	7	22	14
chr5	48373393	48375393	Slit2	-0.10095	9.37E-10	hypomethylated	-0.0097196	0.30677	insignificant	17	104	104
chr5	48762630	48764630	Pacrgl	-0.1131	1	insignificant	-0.023254	0.38169	insignificant	2	32	32
chr5	48990921	48992921	Kcnip4	-0.38642	0.00032222	stronglyHypometh	-0.0092722	0.84979	insignificant	2	10	10
chr5	49280796	49282796	Kcnip4	-0.48758	8.59E-10	stronglyHypometh	-0.12849	0.0019786	hypomethylated	4	10	10
chr5	50450235	50452235	Gpr125	0.19073	0.15731	insignificant	-0.016918	0.061	insignificant	3	28	28

chr5	52580919	52582919	9230114K14Rik	-0.10124	9.53E-12	hypomethylated	0.0005748	1	insignificant	36	110	110
chr5	52581758	52583758	Dhx15	-0.077195	0.39972	insignificant	-0.0082992	0.61292	insignificant	8	16	16
chr5	52754042	52756042	Sod3	-0.17936	0.0024828	hypomethylated	-0.035231	0.10557	insignificant	3	8	8
chr5	52957519	52959519	Lgi2	-0.077925	0.000014023	hypomethylated	0.0019367	0.59981	insignificant	5	32	39
chr5	53060940	53062940	Sepsecs	0.17407	0.000000154	inconclusive	-0.013367	0.13965	insignificant	7	40	40
chr5	53131812	53133812	Pi4k2b	-0.20863	7.12E-17	hypomethylated	0.00033546	0.00093421	inconclusive	31	83	80
chr5	53173305	53175305	Zcchc4	-0.12016	0.000021974	hypomethylated	-0.015689	1	insignificant	7	33	30
chr5	53224373	53226373	Anapc4	-0.15545	1.44E-10	hypomethylated	-0.021325	0.29241	insignificant	14	52	53
chr5	53439591	53441591	Slc34a2	-0.13909	2.34E-11	hypomethylated	0.098002	0.96464	insignificant	20	48	61
chr5	53604691	53606691	Sel1l3	-0.071225	0.0064628	hypomethylated	0.009128	0.85826	insignificant	14	38	38
chr5	53657344	53659344	1810013D10Rik	-0.18537	2.67E-15	hypomethylated	0.0074113	0.52382	insignificant	19	67	66
chr5	53946017	53948017	Rbpj	-0.085794	0.23673	insignificant	0.080248	0.55405	insignificant	2	10	10
chr5	53980453	53982453	Rbpj	-0.14193	1E-53	hypomethylated	-0.028763	0.079378	insignificant	76	217	220
chr5	53980812	53982812	Rbpj	-0.15389	1.14E-45	hypomethylated	-0.031275	0.019158	hypomethylated	58	159	154
chr5	54199865	54201865	Tbc1d19	-0.072027	0.0056413	hypomethylated	0.017444	0.55625	insignificant	11	42	42
chr5	54388761	54390761	Stim2	-0.16917	5.61E-16	hypomethylated	-0.013864	0.94266	insignificant	41	118	114
chr5	58108259	58110259	Pcdh7	-0.15192	2.95E-16	hypomethylated	-0.0080567	0.070111	insignificant	27	109	111
chr5	58109158	58111158	4932441J04Rik	-0.15628	6.7E-23	hypomethylated	-0.014166	0.0009936	hypomethylated	43	176	175
chr5	62199081	62201081	G6pd2		1	noCoverage	0.038889	0.41099	insignificant	0	4	4
chr5	63157416	63159416	Arap2	-0.07737	8.38E-09	hypomethylated	0.019389	1	insignificant	15	47	42
chr5	64039341	64041341	3110047P20Rik	-0.1089	0.013676	hypomethylated	-0.003742	0.67471	insignificant	6	31	38
chr5	64202733	64204733	0610040J01Rik	-0.10535	5.09E-12	hypomethylated	0.0086651	0.080826	insignificant	23	89	87
chr5	64360136	64362136	Rell1	-0.145	0.20929	insignificant	-0.011667	0.6367	insignificant	5	10	10
chr5	64483188	64485188	Pgm1	-0.18115	2.04E-37	hypomethylated	0.029402	0.22772	insignificant	35	92	94
chr5	64550449	64552449	Tbc1d1	-0.13695	2.94E-12	hypomethylated	-0.0085518	0.016603	hypomethylated	30	121	130
chr5	65193761	65195761	Klf3	-0.10996	2.65E-42	hypomethylated	0.0063827	0.047113	inconclusive	65	188	193
chr5	65351273	65353273	Tlr6	-0.36254	0.24219	insignificant	0.03137	1	insignificant	3	10	12
chr5	65360313	65362313	Fam114a1	-0.11326	2.9E-11	hypomethylated	-0.0040422	0.89318	insignificant	10	49	63
chr5	65360556	65362556	Mir574	-0.11326	2.9E-11	hypomethylated	-0.0040422	0.89318	insignificant	10	49	63
chr5	65589934	65591934	Wdr19	-0.21159	5.07E-14	hypomethylated	-0.024849	0.054693	insignificant	7	30	30
chr5	65726878	65728878	Rfc1	-0.14341	1.04E-08	hypomethylated	-0.0043108	0.12912	insignificant	9	30	30
chr5	65738649	65740649	Klb	-0.025956	0.072023	insignificant	0.03902	0.60504	insignificant	14	41	40
chr5	65781735	65783735	Lias	-0.14594	6.24E-22	hypomethylated	0.00013463	0.12572	insignificant	30	95	107
chr5	65782670	65784670	Rpl9	-0.11347	6.02E-12	hypomethylated	0.0045948	0.87207	insignificant	16	49	49
chr5	65827081	65829081	Ugdh	-0.28466	4.85E-09	hypomethylated	0.00052508	0.95937	insignificant	10	36	36
chr5	65884074	65886074	1110003E01Rik	-0.11538	2.39E-24	hypomethylated	0.00074489	0.499	insignificant	25	82	82
chr5	65927499	65929499	Ube2k	-0.15097	7.5E-35	hypomethylated	-0.020507	0.0015709	hypomethylated	42	124	123
chr5	66089095	66091095	Pds5a	-0.14977	2.9E-33	hypomethylated	-0.020057	0.000062361	hypomethylated	35	224	236
chr5	66153759	66155759	N4bp2	-0.075799	2.4E-10	hypomethylated	0.0022247	0.73841	insignificant	42	191	187
chr5	66253807	66255807	Rhoh	-0.3941	0.52948	insignificant	-0.023395	0.71666	insignificant	1	12	12
chr5	66357362	66359362	Chrna9	0.17292	1	insignificant	0.077273	0.74032	insignificant	1	8	8
chr5	66542207	66544207	Rbm47	-0.20502	0.0022525	hypomethylated	-0.015549	0.95726	insignificant	6	36	40
chr5	66543163	66545163	Rbm47	0.25247	1	lowCoverage	-0.065827	0.79152	insignificant	1	17	21
chr5	66650363	66652363	Nsun7	-0.19604	7.76E-28	hypomethylated	-0.039093	0.37676	insignificant	28	88	86
chr5	67009910	67011910	Apbb2	-0.12054	4.1E-12	hypomethylated	-0.013111	0.28576	insignificant	22	94	94
chr5	67066359	67068359	Uchl1	-0.21494	7.99E-16	hypomethylated	0.017586	0.00056649	inconclusive	9	45	40
chr5	67136078	67138078	Limch1	-0.19565	1.02E-28	hypomethylated	0.0061616	0.000012255	inconclusive	32	101	101
chr5	67490365	67492365	Phox2b	-0.18442	0.048534	hypomethylated	0.0036783	0.33708	insignificant	5	22	14
chr5	67650890	67652890	Tmem33	-0.1335	1.04E-30	hypomethylated	0.0051691	0.5174	insignificant	32	104	105
chr5	67697195	67699195	Slc30a9	-0.13939	4.02E-41	hypomethylated	-0.015418	0.5241	insignificant	37	120	115
chr5	67819038	67821038	Bend4	-0.045477	0.000026675	hypomethylated	-0.0091016	0.93369	insignificant	23	75	76
chr5	67854136	67856136	C330024D21Rik	-0.0083573	0.44581	insignificant	-0.037452	0.79441	insignificant	8	24	24
chr5	67998121	68000121	Shisa3	-0.12829	1.81E-36	hypomethylated	0.0030475	0.4778	insignificant	35	163	156
chr5	68238670	68240670	Atp8a1	-0.035649	0.00037907	hypomethylated	-0.048419	0.28046	insignificant	9	46	47
chr5	69947180	69949180	Guf1	-0.10817	1.54E-21	hypomethylated	-0.0096416	0.19502	insignificant	32	123	123
chr5	69983524	69985524	Gnpda2		1	noCoverage	0.087119	0.84696	insignificant	0	22	22
chr5	71233856	71235856	Gabrg1		1	noCoverage	-0.051515	0.68861	insignificant	0	8	8
chr5	71487088	71489088	Gabra2		1	noCoverage	0.022574	0.94564	insignificant	0	26	26
chr5	72049547	72051547	Gabra4	-0.24766	0.57348	insignificant	-0.08495	0.74125	insignificant	1	10	10
chr5	72090254	72092254	Gabrb1	-0.17035	0.0071682	hypomethylated	-0.0025584	0.57323	insignificant	3	18	18
chr5	72559422	72561422	Commd8	-0.076923	0.61023	insignificant	0.010005	0.52788	insignificant	4	17	17
chr5	72593567	72595567	Atp10d	-0.17244	1	lowCoverage	-0.025141	1	insignificant	1	36	36
chr5	72593581	72595581	Atp10d	-0.17244	1	lowCoverage	-0.025141	1	insignificant	1	36	36
chr5	72895447	72897447	Corin	-0.20038	0.00012344	hypomethylated	0.043434	0.25502	insignificant	7	14	15
chr5	72950884	72952884	Nfxl1		1	noCoverage	0.0073859	0.75093	insignificant	0	36	36
chr5	72978789	72980789	Gm5868	-0.18947	0.43635	insignificant	0.15755	0.80173	insignificant	2	4	5
chr5	73033991	73035991	Cnga1	-0.55128	0.065782	insignificant	0.016178	1	insignificant	2	6	6
chr5	73038034	73040034	Nipal1	-0.22829	8.44E-08	hypomethylated	-0.00095464	0.8701	insignificant	11	34	35
chr5	73127744	73129744	Txk		1	noCoverage	-0.047619	0.61582	insignificant	0	4	4
chr5	73259687	73261687	Tec	-0.037541	1.29E-08	hypomethylated	0.00019037	0.26396	insignificant	20	64	64
chr5	73304555	73306555	Slain2	-0.10212	7.06E-31	hypomethylated	-0.005006	0.020995	hypomethylated	53	201	206
chr5	73397142	73399142	Slc10a4	-0.095648	0.00095951	hypomethylated	-0.0077142	0.00000533	hypomethylated	16	77	77
chr5	73647857	73649857	Fryl	-0.15357	0.0051071	hypomethylated	-0.013871	0.10014	insignificant	7	36	33
chr5	73683032	73685032	Ociad1	-0.12305	0.000000533	hypomethylated	-0.001538	0.95069	insignificant	18	67	67
chr5	73796316	73798316	Cwh43	-0.2201	1.31E-11	hypomethylated	0.0073719	0.76928	insignificant	9	35	38
chr5	73871293	73873293	Dcun1d4	-0.057197	0.3865	insignificant	0.028589	0.31256	insignificant	2	4	8
chr5	73881264	73883264	Dcun1d4	-0.081801	6.5E-11	hypomethylated	0.010928	0.45818	insignificant	29	136	138
chr5	74038970	74040970	Sgcb	-0.20947	0.32643	insignificant	0.018709	0.33917	insignificant	2	8	8
chr5	74041618	74043618	Spata18	-0.12468	8.59E-11	hypomethylated	-0.0098483	0.91604	insignificant	9	78	85
chr5	74464436	74466436	Usp46	-0.1275	1.32E-22	hypomethylated	-0.012529	0.045612	hypomethylated	29	77	78
chr5	74488107	74490107	2700023E23Rik	-0.12064	4.98E-27	hypomethylated	-0.0063771	0.41421	insignificant	31	92	97
chr5	74488554	74490554	Snora26	-0.12064	4.98E-27	hypomethylated	-0.0063771	0.41421	insignificant	31	92	97
chr5	74590350	74592350	Rasl11b	-0.088091	9.18E-09	hypomethylated	-0.0073664	0.12422	insignificant	23	91	103
chr5	74927774	74929774	Scfd2	-0.12964	0.0016165	inconclusive	-0.047824	0.000042401	inconclusive	4	14	14
chr5	74930506	74932506	Fip1l1	-0.16376	1.33E-26	hypomethylated	-0.0094632	0.16287	insignificant	21	64	71

chr5	75098928	75100928	Lnx1		1	noCoverage	-0.15015	0.50087	insignificant	0	9	10
chr5	75440651	75442651	Chic2	-0.2366	6.88E-11	hypomethylated	-0.016054	0.063721	insignificant	13	45	41
chr5	75470625	75472625	Gsx2	-0.14465	8.94E-12	hypomethylated	-0.0019272	0.27192	insignificant	26	120	105
chr5	75547315	75549315	Pdgfra	-0.18866	0.055467	insignificant	-0.02579	0.075531	insignificant	7	28	28
chr5	75551190	75553190	Pdgfra	-0.16311	0.000000647	hypomethylated	0.0098531	0.95761	insignificant	15	48	51
chr5	75970011	75972011	Kit	-0.19313	6.86E-12	hypomethylated	-0.0089402	0.068701	insignificant	9	48	60
chr5	75970015	75972015	Kit	-0.19313	6.86E-12	hypomethylated	-0.0089402	0.068701	insignificant	9	48	60
chr5	76374453	76376453	Kdr	-0.3503	0.00014638	stronglyHypometh	0.33746	0.047818	stronglyhypermeth	3	10	23
chr5	76568301	76570301	Srd5a3	-0.22903	4.56E-20	hypomethylated	-0.00071049	0.000090627	hypomethylated	14	55	52
chr5	76611904	76613904	Tmem165	-0.14764	1.17E-41	hypomethylated	-0.0054625	0.046225	hypomethylated	41	116	116
chr5	76733573	76735573	Clock	-0.12005	3.61E-42	hypomethylated	-0.0021997	0.055434	insignificant	51	98	98
chr5	76792802	76794802	Nmu	-0.22001	0.013566	hypomethylated	-0.026939	0.39831	insignificant	8	21	20
chr5	76912101	76914101	Gm7271	-0.20172	0.0024637	hypomethylated	-0.040259	0.40883	insignificant	6	22	24
chr5	76957347	76959347	Exoc1	-0.31234	3.91E-10	hypomethylated	-0.018712	0.76565	insignificant	10	22	25
chr5	77379435	77381435	Paics	-0.13038	9.43E-11	hypomethylated	0.0035836	0.0032629	inconclusive	13	64	67
chr5	77380603	77382603	Paics	-0.14501	0.00062662	hypomethylated	0.010101	0.43814	insignificant	7	35	35
chr5	77402725	77404725	Srp72	-0.12822	1.98E-14	hypomethylated	-0.010603	0.45088	insignificant	13	89	90
chr5	77432079	77434079	Arl9	0.1455	0.19974	insignificant	0.15251	0.000041883	hypermethylated	3	17	16
chr5	77444047	77446047	1700023E05Rik	-0.21376	0.054356	insignificant	-0.018697	0.3973	insignificant	21	58	58
chr5	77524292	77526292	Hopx	-0.091847	0.10374	insignificant	-0.0063435	0.00000532	hypomethylated	13	30	30
chr5	77544148	77546148	Hopx		1	noCoverage	0.21111	0.0062127	hypermethylated	0	8	8
chr5	77640478	77642478	Spink2		1	noCoverage	0.18682	0.75835	insignificant	0	10	9
chr5	77693518	77695518	Rest	-0.11937	8.54E-33	hypomethylated	-0.004591	0.04916	hypomethylated	74	230	245
chr5	77738508	77740508	Polr2b	-0.14314	5.78E-32	hypomethylated	-0.0076553	0.78313	insignificant	40	110	110
chr5	77739111	77741111	Noa1	-0.17346	0.2272	insignificant	-0.029487	0.67919	insignificant	6	32	32
chr5	77837070	77839070	Igfbp7	-0.32144	0.00000113	hypomethylated	0.082844	0.08385	insignificant	14	45	55
chr5	81449617	81451617	Lphn3	-0.096627	0.00000987	hypomethylated	-0.0051492	0.4848	insignificant	12	71	75
chr5	84846407	84848407	Epha5		1	noCoverage	0.23237	0.1784	insignificant	0	7	6
chr5	86494608	86496608	Cenpc1	-0.21562	0.00044958	hypomethylated	0.031795	1	insignificant	3	51	51
chr5	86601768	86603768	Uba6	-0.17876	0.088663	insignificant	0.025609	0.33127	insignificant	5	25	27
chr5	87232514	87234514	Ythdc1	-0.13874	1.91E-35	hypomethylated	-0.00934	0.017395	hypomethylated	34	122	132
chr5	87766220	87768220	Ugt2a3	0.15513	1	insignificant	-0.061709	0.7299	insignificant	1	12	16
chr5	87967220	87969220	Sult1b1		1	noCoverage	-0.0024038	1	insignificant	0	4	4
chr5	88353657	88355657	Csn3	-0.47591	0.0083943	stronglyHypometh	-0.086267	0.25993	insignificant	3	8	8
chr5	88407475	88409475	4931407G18Rik		1	noCoverage	-0.082493	0.028386	hypomethylated	0	14	14
chr5	88884035	88886035	Ambn		1	noCoverage	0.064611	0.84468	insignificant	0	4	4
chr5	88982507	88984507	Utp3	-0.087577	2.92E-12	hypomethylated	0.019434	0.37008	insignificant	25	122	125
chr5	89011598	89013598	Rufy3	-0.25078	0.080881	insignificant	0.049362	0.79694	insignificant	3	15	12
chr5	89104605	89106605	Grsf1	-0.06665	7.49E-15	hypomethylated	0.00091337	0.24951	insignificant	42	181	181
chr5	89105196	89107196	Grsf1	-0.13262	0.00025961	hypomethylated	-0.0084322	0.57466	insignificant	14	36	36
chr5	89148895	89150895	Mob1b	-0.18382	2.31E-34	hypomethylated	-0.010987	1	insignificant	35	88	76
chr5	89193037	89195037	Dck	-0.14008	1.62E-14	hypomethylated	0.02247	0.017198	hypermethylated	29	91	87
chr5	89315284	89317284	Slc4a4	-0.12479	1.42E-10	hypomethylated	0.0056098	0.72624	insignificant	31	118	130
chr5	89955453	89957453	Npffr2		1	noCoverage	-0.20363	0.76019	insignificant	0	10	8
chr5	90044460	90046460	Gm7056	-0.3125	0.15766	insignificant	-0.079167	0.072005	insignificant	1	3	4
chr5	90312359	90314359	Adamts3	-0.0021992	0.046069	hypomethylated	0.072778	0.95019	insignificant	6	60	47
chr5	90653021	90655021	Cox18		1	noCoverage	0.02167	0.75137	insignificant	0	14	16
chr5	90795211	90797211	Ankrd17	-0.098991	0.00014298	hypomethylated	-0.0032007	0.65379	insignificant	11	73	74
chr5	90946974	90948974	Afm	-0.20296	0.24363	insignificant	0.0046113	0.78608	insignificant	2	2	4
chr5	91187324	91189324	Cxcl5		1	noCoverage	0.026737	0.8045	insignificant	0	23	22
chr5	91200460	91202460	Pf4	-0.27422	0.079973	insignificant	0.08391	0.11256	insignificant	5	15	21
chr5	91214128	91216128	Cxcl3	-0.38293	0.3616	insignificant	-0.0039257	0.0027972	inconclusive	7	36	32
chr5	91222559	91224559	Cxcl15		1	noCoverage	0.037446	1	insignificant	0	4	4
chr5	91331924	91333924	Cxcl2	-0.23346	0.049571	hypomethylated	0.04143	0.29202	insignificant	2	10	10
chr5	91359221	91361221	Mthfd2l	-0.10073	6.84E-35	hypomethylated	0.0044969	0.021515	inconclusive	35	74	74
chr5	91502642	91504642	Ereg	-0.11533	0.0014383	hypomethylated	0.076339	0.79454	insignificant	10	48	30
chr5	91567640	91569640	Areg	-0.24994	9.95E-09	hypomethylated	-0.026114	0.034295	hypomethylated	9	46	46
chr5	91945725	91947725	Parm1	-0.17021	0.000018158	hypomethylated	-0.0059144	0.54073	insignificant	13	88	91
chr5	92390907	92392907	Thap6	-0.11993	1.4E-11	hypomethylated	0.010912	0.04491	inconclusive	25	74	80
chr5	92392094	92394094	Thap6	-0.10977	0.00000427	hypomethylated	0.028103	0.45072	insignificant	15	36	42
chr5	92472032	92474032	Cdkl2	-0.21699	4.53E-31	hypomethylated	-0.019135	0.0034746	hypomethylated	13	32	32
chr5	92472044	92474044	Cdkl2	-0.24212	7.89E-36	hypomethylated	-0.020998	0.00022208	hypomethylated	10	26	26
chr5	92511943	92513943	G3bp2	-0.093455	3.1E-20	hypomethylated	-0.0021392	0.07289	insignificant	37	95	95
chr5	92512583	92514583	G3bp2	-0.23386	0.69705	insignificant	-0.010847	0.71001	insignificant	11	14	14
chr5	92512761	92514761	G3bp2	-0.76235	0.000000033	stronglyHypometh	-0.046443	0.19506	insignificant	11	4	4
chr5	92565963	92567963	Uso1	-0.12678	1.64E-20	hypomethylated	0.011438	1	insignificant	49	106	118
chr5	92707207	92709207	Naaa	-0.35098	0.090504	insignificant	-0.052679	0.00021828	hypomethylated	2	30	24
chr5	92739050	92741050	Sdad1		1	noCoverage	0.0067278	0.45209	insignificant	0	14	14
chr5	92777880	92779880	Art3	-0.046898	0.79672	insignificant	0.10831	0.75362	insignificant	3	6	7
chr5	92864225	92866225	Nup54		1	noCoverage	0.058842	0.11428	insignificant	0	24	29
chr5	92934634	92936634	Scarb2	-0.12857	0.37712	insignificant	0.0098822	0.86782	insignificant	6	28	28
chr5	92983113	92985113		-0.33397	0.23395	insignificant	-0.050337	0.68322	insignificant	4	10	10
chr5	92999562	93001562	Fam47e	0.073087	0.077603	insignificant	0.036168	0.32595	insignificant	3	12	12
chr5	93031076	93033076	Stbd1	-0.21965	2.5E-15	hypomethylated	0.013521	0.68409	insignificant	14	34	38
chr5	93104153	93106153	Ccdc158	-0.145	0.000000221	hypomethylated	-0.0083484	0.53551	insignificant	14	68	65
chr5	93111460	93113460	Shroom3	-0.11027	0.11572	insignificant	-0.069723	0.61801	insignificant	6	26	26
chr5	93237413	93239413	Shroom3	0.039174	1	insignificant	-0.037166	0.0000035	hypomethylated	6	44	44
chr5	93326023	93328023	Shroom3	-0.14135	2.31E-08	hypomethylated	-0.0084169	0.066406	insignificant	25	94	91
chr5	93474048	93476048	Ankrd56	-0.15331	0.0011699	hypomethylated	-0.0097533	0.074624	insignificant	13	80	81
chr5	93521482	93523482	############	-0.12821	6.44E-18	hypomethylated	0.004722	0.23681	insignificant	26	143	147
chr5	93635521	93637521	Ccni	-0.061413	0.004231	hypomethylated	0.0041766	0.39022	insignificant	6	41	41
chr5	93695598	93697598	Ccng2	-0.15385	2.69E-36	hypomethylated	0.0058752	0.13175	insignificant	37	138	145
chr5	96590774	96592774	Cnot6l	-0.13777	1.65E-25	hypomethylated	-0.028592	0.87386	insignificant	28	132	147
chr5	96591009	96593009	Cnot6l	-0.14504	7.35E-21	hypomethylated	-0.019562	0.25473	insignificant	22	90	97
chr5	96638133	96640133	Mrpl1	-0.22079	0.000011391	hypomethylated	-0.012809	0.029756	inconclusive	9	20	20

chr5	96801973	96803973	Fras1	-0.19265	3.36E-09	hypomethylated	-0.029208	0.20927	insignificant	18	60	60
chr5	97221403	97223403	Anxa3	-0.40744	0.53841	insignificant	-0.044749	1	insignificant	2	14	12
chr5	97425707	97427707	Bmp2k	-0.086797	7.76E-31	hypomethylated	-0.0021258	0.061391	insignificant	67	186	190
chr5	97540615	97542615	Paqr3	-0.086068	0.0012417	hypomethylated	-0.017931	0.083625	insignificant	3	26	33
chr5	97821349	97823349	Naa11	0.012127	1	insignificant	0.026107	0.49597	insignificant	7	16	16
chr5	98459981	98461981	Antxr2	-0.1128	0.38152	insignificant	0.019613	0.36067	insignificant	13	37	43
chr5	98608887	98610887	Prdm8	-0.14477	0.0025766	hypomethylated	-0.017584	0.2741	insignificant	9	24	24
chr5	98682202	98684202	Fgf5	-0.14259	1.49E-27	hypomethylated	0.0052978	0.70882	insignificant	39	119	123
chr5	98757322	98759322	1700007G11Rik	-0.12724	1.8E-09	hypomethylated	-0.0056746	0.21465	insignificant	22	68	68
chr5	99282457	99284457	Bmp3	-0.1321	2.03E-16	hypomethylated	-0.0067545	0.14674	insignificant	44	123	129
chr5	99466098	99468098	Prkg2	-0.1318	0.00001026	hypomethylated	-0.019312	0.36371	insignificant	23	58	58
chr5	99681946	99683946	Rasgef1b	-0.15909	0.03039	hypomethylated	-0.012492	0.83526	insignificant	11	20	30
chr5	100158079	100160079	A930011G23Rik		1	noCoverage	-0.17841	0.13668	insignificant	0	29	20
chr5	100407957	100409957	Hnrnpd	-0.098936	1.99E-16	hypomethylated	-0.0024794	0.40751	insignificant	16	100	100
chr5	100468012	100470012	Enoph1	-0.093563	5.96E-43	hypomethylated	-0.011093	0.067302	insignificant	59	207	212
chr5	100468241	100470241	Hnrpdl	-0.091188	3.64E-09	hypomethylated	-0.016096	0.57087	insignificant	40	152	156
chr5	100845253	100847253	Sec31a	-0.23635	5.93E-24	hypomethylated	-0.011033	0.30245	insignificant	22	65	72
chr5	100858554	100860554	5430416N02Rik	-0.16762	2.45E-15	hypomethylated	0.0076104	0.089697	insignificant	14	48	48
chr5	100927592	100929592	Lin54	-0.15099	8.93E-39	hypomethylated	-0.0072623	0.05343	insignificant	41	90	90
chr5	100946327	100948327	Cops4	-0.17881	0.00000166	hypomethylated	-0.02389	0.31782	insignificant	10	51	51
chr5	101103275	101105275	Coq2	-0.13962	0.00011105	hypomethylated	0.097759	0.71828	insignificant	6	32	32
chr5	101148702	101150702	Hpse	-0.14595	2.45E-10	hypomethylated	0.025063	0.60058	insignificant	5	27	28
chr5	101226777	101228777	Mrps18c	-0.15489	5.96E-44	hypomethylated	-0.012297	0.1838	insignificant	29	91	98
chr5	101227619	101229619	Helq	-0.15937	1	insignificant	-0.025447	0.36109	insignificant	2	19	26
chr5	101249954	101251954	Fam175a	-0.36697	1	lowCoverage	0.049955	0.0012569	hypermethylated	1	19	19
chr5	101274247	101276247	Agpat9	-0.15007	1.21E-18	hypomethylated	0.0050447	0.66397	insignificant	14	62	62
chr5	102093730	102095730	Nkx6-1	-0.15311	0.00000372	hypomethylated	0.025284	0.25513	insignificant	11	49	39
chr5	102193148	102195148	Cds1	-0.082326	1.96E-17	hypomethylated	0.0044818	0.14295	insignificant	51	182	188
chr5	102498940	102500940	Wdfy3	-0.10223	2.57E-09	hypomethylated	0.0034784	0.37573	insignificant	17	59	59
chr5	102909409	102911409	Arhgap24	-0.23801	0.000000506	hypomethylated	-0.012951	0.88772	insignificant	10	36	39
chr5	103853210	103855210	Ptpn13	-0.08281	1.8E-13	hypomethylated	0.0065838	0.26294	insignificant	48	211	201
chr5	104058422	104060422	Slc10a6		1	noCoverage	-0.07265	0.79156	insignificant	0	12	14
chr5	104084743	104086743	1700016H13Rik	-0.18567	0.000000846	hypomethylated	-0.0070074	0.33101	insignificant	7	30	31
chr5	104084751	104086751	1700016H13Rik	-0.18567	0.000000846	hypomethylated	-0.0070074	0.33101	insignificant	7	30	31
chr5	104182180	104184180	Aff1	-0.17263	5.06E-44	hypomethylated	-0.0089523	0.10855	insignificant	28	148	146
chr5	104182591	104184591	Aff1	-0.14601	6.26E-40	hypomethylated	-0.00069857	0.31429	insignificant	23	122	123
chr5	104450815	104452815	Hsd17b11	-0.19268	0.00071274	hypomethylated	0.011891	0.70376	insignificant	5	14	14
chr5	104475029	104477029	Nudt9	-0.15252	5.31E-29	hypomethylated	-0.018631	0.70162	insignificant	23	79	77
chr5	104543107	104545107	Sparcl1	-0.035736	0.25997	insignificant	0.12102	0.68831	insignificant	7	20	16
chr5	104630635	104632635	Dmp1	0.18717	0.054741	insignificant	-0.083139	0.4278	insignificant	3	6	6
chr5	104863136	104865136	Spp1		1	noCoverage	-0.1	0.58525	insignificant	0	4	4
chr5	104887475	104889475	Pkd2	-0.10314	1.07E-23	hypomethylated	-0.011752	0.13149	insignificant	56	152	140
chr5	104936370	104938370	BC005561	-0.091881	1.24E-13	hypomethylated	-0.0015421	0.39434	insignificant	29	106	104
chr5	105289087	105291087	Zfp951		1	noCoverage	-0.033314	0.17137	insignificant	0	9	10
chr5	105411736	105413736	Abcg3	-0.22519	0.01082	hypomethylated	-0.020483	0.44895	insignificant	3	36	40
chr5	105843793	105845793	Lrrc8b	-0.12526	1.54E-14	hypomethylated	-0.011931	0.42409	insignificant	36	107	113
chr5	105947489	105949489	Lrrc8c	-0.087278	9.47E-21	hypomethylated	-0.01354	0.37334	insignificant	38	140	148
chr5	106127987	106129987	Lrrc8d	-0.11402	3.77E-52	hypomethylated	0.0037219	0.19206	insignificant	83	227	217
chr5	106128828	106130828	Lrrc8d	-0.11545	4.67E-51	hypomethylated	0.015112	0.54629	insignificant	80	236	223
chr5	106304586	106306586	Zfp326	-0.10584	3.07E-11	hypomethylated	0.0073709	0.22105	insignificant	25	126	127
chr5	106887185	106889185	Barhl2	-0.14189	9.17E-20	hypomethylated	-0.025215	0.63376	insignificant	45	150	166
chr5	107125849	107127849	Zfp644	-0.28846	0.0012155	hypomethylated	0.22582	0.55669	insignificant	2	4	6
chr5	107354909	107356909	Hfm1	-0.064057	0.28015	insignificant	0.0041776	0.58344	insignificant	14	72	65
chr5	107392340	107394340	Cdc7	-0.13382	2.63E-16	hypomethylated	-0.014208	0.000041716	hypomethylated	27	101	101
chr5	107718614	107720614	Tgfbr3		1	noCoverage	-0.014881	0.71693	insignificant	0	12	12
chr5	107759212	107761212	Brdt	-0.14731	0.033573	hypomethylated	0.0075207	0.7241	insignificant	4	20	20
chr5	107831531	107833531	Ephx4	-0.09091	7.01E-10	hypomethylated	0.018075	0.051784	insignificant	31	111	80
chr5	107859567	107861567	Lpcat2b	-0.084162	0.61028	insignificant	-0.074074	0.34153	insignificant	3	6	6
chr5	107962729	107964729	1700028K03Rik	-0.14175	0.0033489	hypomethylated	0.0161	0.64302	insignificant	9	34	34
chr5	108025398	108027398	Rpap2	-0.13531	9.24E-11	hypomethylated	0.065218	0.38973	insignificant	11	50	36
chr5	108025735	108027735	Rpap2	-0.14877	3.39E-11	hypomethylated	0.072008	0.2813	insignificant	11	52	38
chr5	108026658	108028658	Rpap2	-0.1944	3.91E-12	hypomethylated	0.057929	0.26445	insignificant	11	55	41
chr5	108026907	108028907	Rpap2	-0.28424	9.66E-11	hypomethylated	0.039324	0.091819	insignificant	6	24	23
chr5	108153363	108155363	Gfi1	-0.12968	0.000000516	hypomethylated	0.0041032	0.70977	insignificant	12	98	94
chr5	108258271	108260271	1700013N18Rik	0.064941	0.61322	insignificant	-0.019109	0.0023049	hypomethylated	5	23	29
chr5	108328608	108330608	Rpl5	-0.23106	6.4E-45	hypomethylated	0.012279	0.025222	inconclusive	34	124	124
chr5	108416096	108418096	Fam69a	-0.22049	0.00018746	hypomethylated	-0.003642	0.9545	insignificant	6	55	60
chr5	108493760	108495760	Mtf2	-0.097317	8.32E-09	hypomethylated	0.014737	0.17266	insignificant	14	69	72
chr5	108560932	108562932	Ccdc18	-0.14236	1.43E-08	hypomethylated	0.012803	0.61493	insignificant	13	88	92
chr5	108561610	108563610	Tmed5	-0.11324	0.018846	hypomethylated	-0.0026301	0.32317	insignificant	5	46	50
chr5	108696915	108698915	Dr1	-0.11467	8.68E-18	hypomethylated	-0.0048677	0.01891	hypomethylated	32	99	104
chr5	108740943	108742943	Pigg	-0.13864	1.09E-14	hypomethylated	0.027064	0.31202	insignificant	19	60	52
chr5	108816391	108818391	Pde6b	0.12756	1	insignificant	-0.0099359	0.12172	insignificant	2	6	8
chr5	108863397	108865397	Atp5k	-0.43123	0.519	insignificant	-0.12323	0.018045	hypomethylated	2	18	17
chr5	108889350	108891350	Pcgf3	-0.16755	4.15E-25	hypomethylated	0.032954	0.00068554	hypermethylated	28	82	70
chr5	108979046	108981046	Cplx1	-0.13562	0.00094437	hypomethylated	0.018584	0.04999	hypermethylated	8	48	48
chr5	109057828	109059828	Tmem175	-0.14102	1	insignificant	0.00048528	0.66446	insignificant	2	16	15
chr5	109089788	109091788	Dgkq	-0.38889	0.047392	stronglyHypometh	-0.040373	0.074045	insignificant	2	6	6
chr5	109122247	109124247	Fgfrl1	-0.10753	1.6E-19	hypomethylated	-0.0097399	0.20843	insignificant	54	153	160
chr5	109237773	109239773	Vmn2r8	-0.50595	0.087815	insignificant	-0.13095	0.5275	insignificant	1	4	4
chr5	109758399	109760399	Vmn2r16	-0.029954	0.66774	insignificant	-0.23432	0.1703	insignificant	1	2	4
chr5	109988012	109990012	Crlf2	-0.31605	0.00015021	hypomethylated	-0.16701	0.0000011	hypomethylated	11	24	30
chr5	110263161	110265161	Gm10416	-0.098638	0.55157	insignificant	0.053501	0.48836	insignificant	2	12	11
chr5	110424545	110426545	Zfp932	-0.33474	1	insignificant	0.026613	0.75109	insignificant	1	37	34
chr5	110527987	110529987	Plcxd1	-0.1875	1	insignificant	-0.29861	0.60421	insignificant	2	6	6

chr5	110528577	110530577	Plcxd1	-0.083442	0.00000433	hypomethylated	-0.069649	0.0023886	hypomethylated	11	45	45
chr5	110537216	110539216	Gtpbp6	-0.23092	0.025811	hypomethylated	0.0025279	0.8226	insignificant	1	16	16
chr5	110538110	110540110	Zfp605	-0.32083	0.00094655	hypomethylated	-0.058864	0.82437	insignificant	1	14	16
chr5	110563858	110565858	Chfr	-0.20272	0.0001685	hypomethylated	0.015625	0.40001	insignificant	16	47	46
chr5	110604719	110606719	Golga3	-0.18139	2.54E-32	hypomethylated	-0.0079012	0.043304	hypomethylated	18	75	78
chr5	110659059	110661059	Ankle2	-0.11438	5.55E-11	hypomethylated	-0.043803	0.019452	hypomethylated	27	87	84
chr5	110698918	110700918	Pgam5	-0.21455	0.34505	insignificant	0.092334	0.22431	insignificant	4	21	26
chr5	110714337	110716337	Pole	-0.11135	1.61E-15	hypomethylated	-0.011972	0.032711	hypomethylated	33	88	92
chr5	110715187	110717187	Pxmp2	-0.10017	0.0017999	hypomethylated	-0.011866	0.04209	hypomethylated	15	42	46
chr5	110782114	110784114	Gm1679	-0.45776	0.000032641	stronglyHypometh	-0.072212	0.069375	insignificant	7	30	30
chr5	110816146	110818146	Fbrsl1	-0.21093	0.00025742	hypomethylated	-0.03528	0.057456	insignificant	7	18	18
chr5	110877522	110879522	Fbrsl1	-0.17994	1.39E-09	hypomethylated	0.0023589	0.16611	insignificant	11	40	40
chr5	110972363	110974363	Galnt9	-0.075563	3.36E-10	hypomethylated	0.00082341	0.5453	insignificant	25	120	121
chr5	111081469	111083469	Ddx51	-0.080878	0.20868	insignificant	-0.00043654	0.53176	insignificant	19	133	133
chr5	111082401	111084401	Noc4l	-0.027543	0.000000467	hypomethylated	-0.004789	5.59E-10	hypomethylated	11	83	83
chr5	111199736	111201736	Ep400	-0.12726	0.13488	insignificant	-0.0055634	0.34658	insignificant	4	23	18
chr5	111239100	111241100	Ulk1	-0.13149	0.00088864	hypomethylated	0.0022032	0.40517	insignificant	9	35	36
chr5	111268035	111270035	Chek2	-0.15811	0.0024942	hypomethylated	-0.040784	0.10406	insignificant	2	30	26
chr5	111268796	111270796	Hscb	0.054338	0.71052	insignificant	0.017695	0.00000221	hypermethylated	7	50	46
chr5	111307821	111309821	Ttc28	-0.095772	0.00000358	hypomethylated	0.0095987	0.011574	hypermethylated	15	125	113
chr5	111758782	111760782	Pitpnb	-0.17131	1.92E-31	hypomethylated	-0.02928	0.90947	insignificant	25	78	86
chr5	111846185	111848185	Mn1	-0.12366	7.65E-42	hypomethylated	-0.017315	1.3E-19	hypomethylated	93	280	293
chr5	112009580	112011580	C130026L21Rik	-0.23918	0.00000534	hypomethylated	-0.08324	0.82132	insignificant	12	42	32
chr5	112657968	112659968	Miat	-0.18151	0.000000894	hypomethylated	-0.052889	0.70487	insignificant	6	14	12
chr5	112683840	112685840	Crybb1	-0.3511	0.024922	stronglyHypometh	-0.023716	1	insignificant	3	14	14
chr5	112704726	112706726	Tpst2	-0.10938	0.013293	hypomethylated	0.032986	0.00028671	inconclusive	4	80	73
chr5	112754388	112756388	Tfip11	-0.14283	6.1E-30	hypomethylated	0.028752	0.0018562	inconclusive	25	96	101
chr5	112771114	112773114	Hps4	-0.11143	1.11E-23	hypomethylated	0.0031934	0.038651	inconclusive	36	88	90
chr5	112772060	112774060	Srrd	-0.12428	0.000000002	hypomethylated	0.021129	1	insignificant	12	28	30
chr5	112785053	112787053	Hps4	-0.13659	0.36998	insignificant	0.010899	0.084447	insignificant	2	14	14
chr5	112821233	112823233	Asphd2	-0.23992	0.022923	hypomethylated	-0.14945	0.30712	insignificant	4	16	13
chr5	112877445	112879445	Gm6588	0.02324	0.82177	insignificant	-0.075761	0.040032	hypomethylated	9	46	45
chr5	113006205	113008205	Sez6l	-0.0085365	0.0014088	inconclusive	-0.0451	0.40826	insignificant	13	47	43
chr5	113444534	113446534	Adrbk2	-0.15047	0.25182	insignificant	-0.066748	0.058504	insignificant	6	20	27
chr5	113510604	113512604	Crybb3		1	noCoverage	-0.10165	0.084608	insignificant	0	6	7
chr5	113592333	113594333	2900026A02Rik	-0.21083	0.67062	insignificant	0.036487	0.48504	insignificant	9	43	36
chr5	113654928	113656928	Tmem211	-0.21522	0.4705	insignificant	0.04148	0.73355	insignificant	4	13	16
chr5	113739806	113741806	Sgsm1		1	noCoverage	0.044444	1	insignificant	0	9	9
chr5	113771105	113773105		-0.071088	0.48314	insignificant	-0.015797	0.34393	insignificant	1	9	10
chr5	113785313	113787313	Aym1	-0.11258	0.00019901	hypomethylated	-0.02207	0.19208	insignificant	3	14	14
chr5	113918466	113920466	Wscd2	-0.18682	6.86E-22	hypomethylated	0.02501	0.0021327	inconclusive	33	91	92
chr5	114174781	114176781	1700069L16Rik	-0.040849	0.82094	insignificant	-0.063252	0.74029	insignificant	4	25	18
chr5	114184790	114186790	Ficd	-0.11615	8.55E-17	hypomethylated	-0.0092941	0.027792	hypomethylated	35	126	113
chr5	114221658	114223658	Sart3	-0.12843	8.97E-25	hypomethylated	-0.0080088	0.072868	insignificant	28	168	171
chr5	114221820	114223820	Iscu	-0.14056	6.91E-21	hypomethylated	-0.0087126	0.05928	insignificant	23	146	149
chr5	114250361	114252361	Tmem119	-0.34314	1	insignificant	-0.15425	0.49072	insignificant	2	6	6
chr5	114280510	114282510	Selplg	-0.057221	0.7229	insignificant	-0.014943	0.37969	insignificant	3	6	9
chr5	114358715	114360715	Coro1c	-0.09097	0.000024076	hypomethylated	-0.0077975	0.74624	insignificant	23	70	62
chr5	114443766	114445766	Ssh1	-0.15557	6.7E-19	hypomethylated	-0.019119	0.040887	hypomethylated	25	77	72
chr5	114452834	114454834	Dao	0.20909	1	insignificant	0.12914	0.083523	insignificant	1	13	13
chr5	114549341	114551341	Usp30	-0.10329	3.02E-33	hypomethylated	-0.0051154	0.52382	insignificant	39	126	126
chr5	114578185	114580185	Alkbh2	-0.41167	2.89E-22	stronglyHypometh	-0.003173	0.000044963	hypomethylated	8	31	31
chr5	114579443	114581443	Ung	-0.13415	3.98E-09	hypomethylated	0.0022622	0.023956	inconclusive	30	102	102
chr5	114580164	114582164	Ung	-0.10112	1.29E-10	hypomethylated	-0.019762	0.0030183	hypomethylated	36	138	134
chr5	114614526	114616526	Acacb	0.092033	1	insignificant	-0.12912	0.13492	insignificant	2	4	4
chr5	114723770	114725770	Foxn4	-0.13499	2.69E-17	hypomethylated	-0.02278	0.33208	insignificant	27	106	104
chr5	114763949	114765949	Myo1h	0.015873	1	insignificant	-0.052808	0.39653	insignificant	2	12	13
chr5	114829615	114831615	Ube3b	-0.11694	4.33E-21	hypomethylated	-0.0016702	0.34028	insignificant	29	84	86
chr5	114830514	114832514	Kctd10	-0.21217	1.34E-20	hypomethylated	-0.027658	0.95486	insignificant	13	28	30
chr5	114893314	114895314	Mvk	-0.21364	1.85E-14	hypomethylated	-0.026281	0.34406	insignificant	10	42	36
chr5	114894036	114896036	Mmab	-0.33333	6.2E-21	hypomethylated	-0.015639	0.0030586	hypomethylated	10	51	44
chr5	115017259	115019259	BC057022	-0.10251	1.42E-39	hypomethylated	-0.01604	0.022213	hypomethylated	69	175	187
chr5	115108430	115110430	Trpv4	0.15223	0.51857	insignificant	0.019437	0.000003589	inconclusive	10	65	65
chr5	115140944	115142944	Gltp	-0.18332	3.28E-26	hypomethylated	0.0097536	0.0014556	inconclusive	23	88	93
chr5	115156787	115158787	Tchp	-0.18439	5.88E-20	hypomethylated	0.039092	0.20356	insignificant	20	55	57
chr5	115223500	115225500	Git2	-0.089217	2.69E-26	hypomethylated	0.007847	0.14006	insignificant	52	168	175
chr5	115223501	115225501	Git2	-0.089217	2.69E-26	hypomethylated	0.007847	0.14006	insignificant	52	168	175
chr5	115224148	115226148	Ankrd13a	-0.07998	2.16E-23	hypomethylated	0.0087791	0.048249	inconclusive	48	148	148
chr5	115224992	115226992	4930515G01Rik	-0.059378	7.66E-11	hypomethylated	0.016686	0.22683	insignificant	26	82	82
chr5	115263985	115265985	1500011B03Rik	0.26415	1	insignificant	0.023784	0.29892	insignificant	1	14	14
chr5	115273477	115275477	2610524H06Rik		1	noCoverage	0.0041126	1	insignificant	0	6	14
chr5	115300446	115302446		-0.5	0.078947	insignificant	-0.20504	0.007506	hypomethylated	2	6	8
chr5	115345942	115347942	Oasl2		1	noCoverage	-0.014202	0.64863	insignificant	0	26	23
chr5	115372248	115374248	Oasl1	-0.041941	0.000000728	hypomethylated	-0.032201	0.044927	hypomethylated	8	40	40
chr5	115391210	115393210	2210016L21Rik	-0.039685	0.46768	insignificant	0.088171	0.70996	insignificant	3	8	9
chr5	115421071	115423071	Hnf1a	-0.081642	0.036392	hypomethylated	0.070755	1	insignificant	3	30	32
chr5	115460532	115462532	Sppl3	-0.10574	1.52E-36	hypomethylated	-0.0062581	0.37059	insignificant	66	256	256
chr5	115569322	115571322	Acads	-0.25428	3.81E-10	hypomethylated	0.14786	0.43238	insignificant	8	12	15
chr5	115584984	115586984	Unc119b	-0.15625	0.59523	insignificant	-0.010417	0.60874	insignificant	3	8	8
chr5	115608185	115610185	Mlec	-0.1892	8.67E-27	hypomethylated	-0.019835	0.049818	hypomethylated	29	86	77
chr5	115636130	115638130	Cabp1		1	noCoverage	-0.096847	0.0023423	hypomethylated	0	18	16
chr5	115684859	115686859	Pop5	-0.14964	1.3E-15	hypomethylated	0.066109	0.18568	insignificant	29	102	95
chr5	115722904	115724904	Rnf10	-0.38646	0.004271	stronglyHypometh	0.10289	0.42978	insignificant	4	16	18
chr5	115728710	115730710	Coq5	-0.10584	4.27E-14	hypomethylated	0.011298	0.37659	insignificant	13	57	53
chr5	115750999	115752999	Dynll1	-0.056683	0.044401	hypomethylated	-0.01187	0.92159	insignificant	17	61	70

chr5	115776185	115778185	Srsf9	-0.0048449	0.029854	hypomethylated	-0.000087351	0.062306	insignificant	15	125	125
chr5	115790255	115792255	Triap1	-0.13748	3.14E-34	hypomethylated	-0.0066854	0.11757	insignificant	21	89	89
chr5	115791170	115793170	Gatc	-0.14069	1E-18	hypomethylated	-0.01591	0.65326	insignificant	13	52	52
chr5	115798964	115800964	Cox6a1	-0.21872	0.095499	insignificant	0.6474	2.86E-69	stronglyhypermeth	1	40	8
chr5	115878693	115880693	Msi1	-0.11707	6.32E-46	hypomethylated	-0.001276	0.41237	insignificant	66	181	177
chr5	115915274	115917274	Pla2g1b	-0.14428	0.33618	insignificant	0.12373	0.48818	insignificant	1	12	14
chr5	115934306	115936306	Sirt4	-0.69764	0.021823	stronglyHypometh	-0.052889	0.70205	insignificant	2	27	27
chr5	115955710	115957710	Pxn	-0.17202	1.12E-21	hypomethylated	0.016092	0.054295	insignificant	23	70	72
chr5	116008475	116010475	Rplp0	-0.19386	5.14E-29	hypomethylated	-0.020138	0.16904	insignificant	23	101	101
chr5	116014271	116016271	Gcn1l1	-0.13036	3.34E-47	hypomethylated	-0.016425	1.07E-09	hypomethylated	50	122	127
chr5	116080995	116082995	Rab35	-0.098154	3.26E-40	hypomethylated	0.0066507	0.049394	inconclusive	73	267	248
chr5	116081825	116083825	1110006O24Rik	-0.094916	5.07E-16	hypomethylated	0.012818	0.093824	insignificant	49	185	166
chr5	116181568	116183568	Ccdc64	-0.06206	5.5E-24	hypomethylated	0.000051464	0.048669	inconclusive	75	269	275
chr5	116294664	116296664	Cit	-0.24864	0.033191	hypomethylated	0.018291	0.7896	insignificant	4	18	18
chr5	116474437	116476437	Prkab1	-0.18772	0.00000101	hypomethylated	0.051752	0.014777	hypermethylated	5	83	82
chr5	116533255	116535255	Tmem233	0.10198	0.39841	insignificant	-0.046208	0.62401	insignificant	3	20	20
chr5	116738994	116740994	Ccdc60	-0.34661	7.12E-20	stronglyHypometh	-0.038886	0.0072029	hypomethylated	9	21	29
chr5	117041826	117043826	Srrm4	-0.33036	1.13E-25	hypomethylated	-0.073409	0.0054178	hypomethylated	6	27	24
chr5	117566002	117568002	Suds3	-0.20703	0.000040769	hypomethylated	-0.023188	0.21286	insignificant	12	36	34
chr5	117569137	117571137	Taok3	-0.1121	3.05E-18	hypomethylated	0.0022146	0.036768	inconclusive	18	116	116
chr5	117737573	117739573	Pebp1	-0.21614	7.94E-14	hypomethylated	-0.015217	0.0034781	hypomethylated	19	68	69
chr5	117768274	117770274	Vsig10	-0.14671	1.68E-45	hypomethylated	-0.015235	0.0099362	hypomethylated	41	133	133
chr5	117806313	117808313	Wsb2	-0.087601	1.91E-34	hypomethylated	-0.028092	0.013329	hypomethylated	32	138	151
chr5	117839032	117841032	Rfc5	-0.24792	0.000084059	hypomethylated	0.0096219	0.048861	inconclusive	14	37	40
chr5	117863008	117865008	Ksr2	-0.1192	2.69E-20	hypomethylated	0.022263	0.51557	insignificant	43	91	96
chr5	117863837	117865837	Ksr2	-0.11288	2.47E-19	hypomethylated	0.01302	0.38387	insignificant	34	72	72
chr5	118316127	118318127	Nos1	-0.051908	0.091103	insignificant	-0.043786	0.003499	hypomethylated	9	64	58
chr5	118425778	118427778	Fbxo21	-0.20229	1.51E-26	hypomethylated	0.021862	0.081123	insignificant	27	90	92
chr5	118476832	118478832	Tesc	-0.13728	4.07E-36	hypomethylated	-0.0080631	0.054296	insignificant	38	149	144
chr5	118618772	118620772	Hrk	-0.11968	1.76E-36	hypomethylated	-0.0045548	0.89122	insignificant	53	154	154
chr5	118694235	118696235	2410131K14Rik	-0.11224	0.00005004	hypomethylated	0.0011862	0.9341	insignificant	25	86	86
chr5	118694870	118696870	Rnft2	-0.11224	0.00005004	hypomethylated	0.0011862	0.9341	insignificant	25	86	86
chr5	118695034	118697034	Rnft2	-0.11374	0.00002729	hypomethylated	0.0062946	0.74825	insignificant	25	72	72
chr5	119009727	119011727	Med13l	-0.085833	2.33E-33	hypomethylated	0.013897	0.97846	insignificant	77	311	289
chr5	120119677	120121677	Tbx3	-0.11743	0.000000114	hypomethylated	-0.026081	0.022141	hypomethylated	17	134	135
chr5	120283671	120285671	Tbx5	-0.10735	0.0065119	hypomethylated	0.019775	0.6293	insignificant	12	39	40
chr5	120565521	120567521	Rbm19	-0.10213	6.95E-15	hypomethylated	0.044613	0.11677	insignificant	17	64	66
chr5	120880894	120882894	Lhx5	-0.17775	4.64E-18	hypomethylated	-0.011205	0.6881	insignificant	27	90	91
chr5	120922772	120924772	Sdsl	-0.068816	0.061142	insignificant	-0.063598	0.0018655	hypomethylated	4	12	10
chr5	120925555	120927555	Sds	-0.31616	6.45E-09	hypomethylated	-0.023464	0.63027	insignificant	10	23	27
chr5	120953632	120955632	Plbd2	-0.26024	0.00013595	hypomethylated	0.032258	0.39333	insignificant	6	20	22
chr5	120960200	120962200	Slc24a6	0.0044643	1	insignificant	0.019561	1	insignificant	4	20	23
chr5	121038031	121040031	Iqcd	-0.18273	1.13E-16	hypomethylated	0.013279	0.059575	insignificant	15	55	48
chr5	121062138	121064138	Ddx54	-0.12625	5.64E-12	hypomethylated	-0.0063447	0.97477	insignificant	29	126	127
chr5	121062598	121064598	1110008J03Rik	-0.20963	1.01E-08	hypomethylated	-0.020728	0.46048	insignificant	21	110	109
chr5	121084244	121086244	Ccdc42b	0.3285	0.34057	insignificant	-0.0049622	0.76586	insignificant	1	6	7
chr5	121097830	121099830	Rasal1	-0.15679	1.62E-28	hypomethylated	0.0057671	0.2013	insignificant	26	96	95
chr5	121161678	121163678	Dtx1	-0.20007	0.018937	inconclusive	-0.0063727	0.16778	insignificant	6	20	20
chr5	121245539	121247539	Oas1e		1	noCoverage	0.0063492	1	insignificant	0	6	6
chr5	121261643	121263643	Oas1b	-0.43303	0.000061812	stronglyHypometh	-0.034561	0.20547	insignificant	3	20	18
chr5	121261646	121263646	Oas1b	-0.43303	0.000061812	stronglyHypometh	-0.034561	0.20547	insignificant	3	20	18
chr5	121262523	121264523	Oas1c	-0.37964	0.00081731	stronglyHypometh	-0.040032	0.086784	insignificant	2	16	14
chr5	121296375	121298375	Oas1f	-0.29167	0.27462	insignificant	-0.034524	0.81826	insignificant	1	4	4
chr5	121459527	121461527	Rph3a	-0.32076	0.13039	insignificant	-0.21767	0.57843	insignificant	4	13	10
chr5	121641406	121643406	Ptpn11	-0.3058	7.15E-20	hypomethylated	-0.026971	4.36E-09	hypomethylated	14	61	57
chr5	121653509	121655509	Rpl6	-0.13181	0.0041343	hypomethylated	0.022853	0.49967	insignificant	13	59	66
chr5	121846990	121848990	Naa25	-0.10307	9.92E-29	hypomethylated	-0.0024403	0.3286	insignificant	41	116	116
chr5	121901717	121903717	Tmem116	-0.1095	0.00000339	hypomethylated	-0.0090531	0.01746	hypomethylated	20	125	132
chr5	121902483	121904483	Erp29	-0.073341	0.62886	insignificant	-0.0019614	0.28427	insignificant	5	48	48
chr5	121995901	121997901	Mapkapk5	-0.24077	0.59688	insignificant	-0.0081004	0.77773	insignificant	3	37	42
chr5	122043833	122045833	Aldh2	-0.20448	8.86E-08	hypomethylated	-0.003698	0.67039	insignificant	11	23	22
chr5	122068947	122070947	Acad12	-0.085169	2.7E-19	hypomethylated	-0.01395	0.0001746	hypomethylated	9	54	52
chr5	122109594	122111594	Brap	-0.12946	0.000000991	hypomethylated	-0.010125	0.18319	insignificant	16	94	89
chr5	122110519	122112519	Acad10	-0.11596	0.00000661	hypomethylated	-0.014192	0.19159	insignificant	16	77	72
chr5	122160617	122162617	Atxn2	-0.091318	5.63E-36	hypomethylated	-0.0080698	0.077134	insignificant	85	255	259
chr5	122286810	122288810	Sh2b3	-0.16152	0.092511	insignificant	-0.018057	0.57632	insignificant	9	53	60
chr5	122298036	122300036	Fam109a	-0.18733	1.23E-41	hypomethylated	0.0036324	0.000012076	inconclusive	26	123	125
chr5	122497834	122499834	Cux2	-0.20199	1.12E-22	hypomethylated	0.0074871	0.23346	insignificant	17	76	71
chr5	122588071	122590071	Ccdc63	0.087951	1	insignificant	-0.046011	0.069238	insignificant	1	13	13
chr5	122607287	122609287	Ppp1cc	-0.090442	0.000000538	hypomethylated	-0.0009014	0.061369	insignificant	33	164	160
chr5	122658745	122660745	Hvcn1	-0.18868	1.66E-16	hypomethylated	-0.0044276	0.0036704	hypomethylated	10	40	40
chr5	122659306	122661306	Hvcn1	-0.19271	6.83E-11	hypomethylated	0.011779	0.76365	insignificant	8	30	30
chr5	122714469	122716469	Tctn1		1	noCoverage	0.08486	0.33665	insignificant	0	16	15
chr5	122733406	122735406	Pptc7	-0.13534	1.48E-26	hypomethylated	-0.00013042	0.090662	insignificant	30	94	102
chr5	122803421	122805421	Vps29	-0.15441	2.72E-42	hypomethylated	-0.022989	0.00018191	hypomethylated	42	138	145
chr5	122804204	122806204	Rad9b	-0.083051	4.06E-15	hypomethylated	-0.020623	0.87522	insignificant	43	124	127
chr5	122821516	122823516	Gpn3	-0.12785	5.91E-21	hypomethylated	-0.019019	0.11561	insignificant	16	83	83
chr5	122821972	122823972	1500011H22Rik	-0.12028	9.67E-21	hypomethylated	-0.010105	0.1624	insignificant	16	82	82
chr5	122840936	122842936	Arpc3	-0.20841	9.36E-13	hypomethylated	-0.033836	0.033494	hypomethylated	22	77	75
chr5	122871452	122873452	Anapc7	-0.15544	4.6E-22	hypomethylated	0.012356	0.91923	insignificant	28	99	99
chr5	122952234	122954234	Atp2a2		1	noCoverage	-0.012349	0.000053333	hypomethylated	0	88	84
chr5	123064527	123066527	Ift81		1	noCoverage	-0.18252	0.25773	insignificant	0	14	18
chr5	123092919	123094919	P2rx7	-0.5388	0.0000039	stronglyHypometh	-0.013585	0.28064	insignificant	4	26	26
chr5	123156565	123158565	P2rx4	-0.06516	8.9E-10	hypomethylated	0.014097	0.50131	insignificant	28	75	76
chr5	123229419	123231419	Camkk2	-0.49304	3.99E-53	stronglyHypometh	0.02099	0.00000158	inconclusive	20	28	28

chr5	123271348	123273348	Anapc5	-0.12603	0.69857	insignificant	0.017689	0.80458	insignificant	1	11	11
chr5	123299196	123301196	Rnf34	-0.22036	4.46E-15	hypomethylated	-0.026073	0.13318	insignificant	16	52	52
chr5	123350359	123352359	Kdm2b	-0.20627	0.0046247	hypomethylated	-0.033321	0.15485	insignificant	8	44	50
chr5	123438542	123440542	Kdm2b	-0.1468	1.68E-12	hypomethylated	-0.012841	0.47723	insignificant	15	75	76
chr5	123439101	123441101	Kdm2b	-0.12428	4.83E-11	hypomethylated	-0.014657	0.74873	insignificant	14	71	72
chr5	123464082	123466082	Orai1	-0.15948	3.26E-57	hypomethylated	0.0011428	0.01131	inconclusive	39	130	128
chr5	123525283	123527283	Tmem120b	-0.15188	1.06E-25	hypomethylated	-0.026744	0.1914	insignificant	27	84	82
chr5	123582638	123584638	Rhof	-0.33382	2.78E-17	stronglyHypometh	-0.020959	0.13976	insignificant	7	36	37
chr5	123677198	123679198	Psmd9	-0.12448	2.06E-18	hypomethylated	0.020614	0.43065	insignificant	13	48	49
chr5	123793456	123795456	Bcl7a	-0.11481	1.24E-47	hypomethylated	0.00013559	0.29873	insignificant	79	213	218
chr5	123843827	123845827	Mlxip	-0.16216	5.17E-42	hypomethylated	-0.017736	0.0073798	hypomethylated	41	157	155
chr5	123938333	123940333	Lrrc43		1	noCoverage	0.11538	0.19538	insignificant	0	5	5
chr5	123959468	123961468	B3gnt4	-0.025787	0.10618	insignificant	-0.043148	0.72679	insignificant	3	31	28
chr5	123974173	123976173	Diablo	-0.10168	3.26E-11	hypomethylated	-0.0026414	0.26925	insignificant	32	101	93
chr5	124023024	124025024	Vps33a		1	noCoverage	0.11398	0.012945	hypermethylated	0	14	14
chr5	124134300	124136300	Clip1	-0.12446	0.000047973	hypomethylated	-0.0063799	0.03832	hypomethylated	21	76	76
chr5	124198734	124200734	Kntc1	-0.15461	1.68E-11	hypomethylated	0.016842	0.72572	insignificant	16	83	93
chr5	124199421	124201421	Rsrc2	-0.14955	0.00000575	hypomethylated	0.033556	0.41202	insignificant	11	69	74
chr5	124330029	124332029	Gpr81	-0.22987	0.0045279	hypomethylated	-0.00012057	1	insignificant	4	8	8
chr5	124356283	124358283	Denr	-0.18589	6.7E-30	hypomethylated	0.022313	2.42E-09	inconclusive	44	129	125
chr5	124379697	124381697	Ccdc62	-0.11223	1	insignificant	-0.02539	0.20214	insignificant	22	88	95
chr5	124422636	124424636	Hip1r	-0.10876	7.28E-11	hypomethylated	-0.0087995	0.10453	insignificant	21	93	93
chr5	124545807	124547807	Abcb9	-0.17305	9.72E-12	hypomethylated	-0.025545	0.20523	insignificant	11	36	36
chr5	124561346	124563346	Ogfod2	0.32893	1	insignificant	-0.029496	0.33035	insignificant	4	44	52
chr5	124565116	124567116	Arl6ip4	-0.063235	0.020281	inconclusive	-0.0008052	1	insignificant	27	86	86
chr5	124666427	124668427	Pitpnm2	0.12374	1	insignificant	0.084848	0.26811	insignificant	1	4	6
chr5	124777097	124779097	2810006K23Rik	-0.11751	4.38E-16	hypomethylated	-0.016333	0.17748	insignificant	31	114	108
chr5	124804637	124806637	Cdk2ap1	-0.075962	0.07366	insignificant	-0.012872	1	insignificant	8	40	40
chr5	124875923	124877923	Sbno1	-0.22779	5.93E-14	hypomethylated	0.066746	0.16866	insignificant	7	40	47
chr5	124888938	124890938	Setd8	-0.18816	2.52E-15	hypomethylated	-0.028737	0.028042	hypomethylated	12	72	74
chr5	124932163	124934163	Snrnp35	-0.23768	5.96E-23	hypomethylated	-0.018498	0.060622	insignificant	17	85	83
chr5	124981400	124983400	Rilpl1	-0.069606	0.000072253	hypomethylated	0.045669	0.092613	insignificant	4	36	36
chr5	124989799	124991799	Tmed2	-0.085037	1.84E-12	hypomethylated	-0.007167	0.39468	insignificant	37	124	124
chr5	125001872	125003872	Ddx55	-0.17972	3.68E-25	hypomethylated	-0.0055538	0.020221	inconclusive	33	101	100
chr5	125028156	125030156	Gtf2h3	-0.10972	8.03E-20	hypomethylated	-0.0043828	0.64468	insignificant	15	70	70
chr5	125029066	125031066	Eif2b1	-0.099212	2.6E-10	hypomethylated	-0.0037675	0.11967	insignificant	7	38	38
chr5	125047757	125049757	Tctn2	-0.16582	4.73E-13	hypomethylated	0.0536	0.31922	insignificant	14	48	48
chr5	125078647	125080647	Atp6v0a2	-0.44477	1	lowCoverage	-0.12777	0.14033	insignificant	1	15	20
chr5	125204454	125206454	Dnahc10	-0.0049076	1	insignificant	0.09297	0.016687	hypermethylated	1	33	27
chr5	125342074	125344074	Zfp664	-0.093606	3.23E-55	hypomethylated	-0.0067735	0.28993	insignificant	101	373	362
chr5	125342591	125344591	Ccdc92	-0.11045	5.7E-23	hypomethylated	-0.011315	0.062637	insignificant	64	292	282
chr5	125482844	125484844	Fam101a	-0.16593	0.0020713	hypomethylated	0.012157	0.75552	insignificant	7	26	26
chr5	125659584	125661584	Ncor2	-0.13118	2.29E-14	inconclusive	0.00012869	0.0014164	hypermethylated	12	42	42
chr5	125821444	125823444	Scarb1	-0.1513	0.035439	hypomethylated	-0.012333	0.34168	insignificant	8	40	40
chr5	125870387	125872387	Ubc	-0.22421	0.13223	insignificant	-0.035737	0.090613	insignificant	3	34	34
chr5	125914418	125916418	Dhx37	-0.11185	8.06E-08	hypomethylated	0.10047	0.22016	insignificant	11	25	28
chr5	125920937	125922937	Bri3bp	-0.11302	5.23E-28	hypomethylated	0.01033	0.7075	insignificant	44	133	133
chr5	125955242	125957242	Aacs	-0.058338	2.51E-09	hypomethylated	0.028691	0.35819	insignificant	24	80	84
chr5	126011787	126013787	Tmem132b	-0.14758	1.59E-37	hypomethylated	-0.023408	0.0007439	hypomethylated	50	140	133
chr5	127721195	127723195	Tmem132c	-0.15182	1.37E-35	hypomethylated	0.01949	0.0065443	hypermethylated	39	148	139
chr5	128097762	128099762	Slc15a4	-0.20929	4.14E-09	hypomethylated	0.042031	0.14813	insignificant	8	46	50
chr5	128111631	128113631	Glt1d1	-0.095116	0.22128	insignificant	0.057046	0.74213	insignificant	11	29	36
chr5	128938937	128940937	Tmem132d		1	noCoverage	-0.087333	0.23994	insignificant	0	22	27
chr5	129105980	129107980	Fzd10	-0.14653	3.37E-37	hypomethylated	-0.020172	0.0044075	hypomethylated	75	232	232
chr5	129106562	129108562	5930412G12Rik	-0.1354	1.68E-27	hypomethylated	-0.017651	0.010506	hypomethylated	61	205	206
chr5	129525030	129527030	Ran	-0.089113	3.29E-37	hypomethylated	0.00091099	0.00030901	inconclusive	55	242	231
chr5	130006105	130008105	Sfswap	-0.12612	1.4E-15	hypomethylated	0.0053455	0.71996	insignificant	36	110	108
chr5	130089088	130091088	Mmp17	-0.1467	8.22E-38	hypomethylated	0.003283	0.70165	insignificant	48	155	155
chr5	130175963	130177963	Zfp11	-0.44744	9.63E-09	stronglyHypometh	-0.052234	0.22948	insignificant	3	6	6
chr5	130214386	130216386	############	-0.047918	0.049654	hypomethylated	-0.11144	0.17673	insignificant	2	10	12
chr5	130220402	130222402	Mrps17	-0.17426	1.75E-09	hypomethylated	0.015273	0.124	insignificant	17	46	44
chr5	130229949	130231949	Gbas	-0.11091	3.35E-15	hypomethylated	-0.010144	0.82135	insignificant	28	88	86
chr5	130292260	130294260	Cct6a	-0.17768	2.46E-16	hypomethylated	-0.006402	0.62982	insignificant	11	56	61
chr5	130293129	130295129	Cct6a	-0.19322	2.78E-10	hypomethylated	0.039237	0.51961	insignificant	7	42	44
chr5	130321865	130323865	Sumf2	-0.2357	0.0015841	hypomethylated	0.057141	0.23692	insignificant	5	10	10
chr5	130363340	130365340	Chchd2	-0.63313	0.00045207	stronglyHypometh	0.010739	0.56407	insignificant	3	19	19
chr5	130370592	130372592	2410018M08Ril	-0.25259	0.81638	insignificant	-0.06909	0.66626	insignificant	9	39	44
chr5	130383409	130385409	4930579G22Rik	-0.18166	3.08E-08	hypomethylated	0.061684	0.59902	insignificant	14	50	54
chr5	130416982	130418982	Vkorc1l1	-0.11662	0.00000159	hypomethylated	0.003782	0.90414	insignificant	21	64	65
chr5	130478698	130480698	Gusb	-0.24561	0.030728	hypomethylated	0.15543	0.43552	insignificant	1	6	8
chr5	130500201	130502201	Asl	0.20486	1	insignificant	0.064956	0.3511	insignificant	4	23	23
chr5	130504175	130506175	Crcp	-0.20334	4.05E-13	hypomethylated	-0.019568	0.1497	insignificant	22	57	58
chr5	130554355	130556355	Tpst1	-0.1552	6.06E-40	hypomethylated	-0.0099733	0.057295	insignificant	41	106	112
chr5	130619757	130621757	Kctd7	-0.12724	2.09E-17	hypomethylated	-0.00020693	0.9689	insignificant	17	89	82
chr5	130646688	130648688	Rabgef1	-0.093907	3.98E-22	hypomethylated	-0.01081	0.063423	insignificant	37	57	82
chr5	130662049	130664049	Rabgef1	-0.25358	0.016812	hypomethylated	0.060295	0.3815	insignificant	1	6	6
chr5	130694613	130696613	0610007L01Rik	0.074789	1	insignificant	0.12457	0.16262	insignificant	3	10	10
chr5	130697204	130699204	0610007L01Rik	-0.095453	4.96E-23	hypomethylated	-0.0042747	0.20726	insignificant	44	118	118
chr5	130730488	130732488	Tyw1	-0.13641	0.075661	insignificant	0.070217	0.1015	insignificant	12	83	93
chr5	130731332	130733332	Tyw1	-0.0061224	1	insignificant	0.20029	0.4911	insignificant	1	7	17
chr5	130731906	130733906	Tyw1	0.044954	1	insignificant	0.036681	0.23013	insignificant	2	15	25
chr5	130844327	130846327	Caln1	-0.31532	0.000040314	hypomethylated	0.073872	0.13212	insignificant	5	20	20
chr5	130923661	130925661	Caln1	-0.14359	0.00000709	hypomethylated	-0.02982	0.10155	insignificant	25	101	101
chr5	131783392	131785392	Wbscr17	-0.21079	0.00013109	hypomethylated	-0.0037505	0.51857	insignificant	9	21	21
chr5	133018213	133020213	Auts2	-0.047675	0.000000203	hypomethylated	0.011844	0.88463	insignificant	37	191	165

chr5	134574617	134576617	Gatsl2	-0.18103	6.84E-13	hypomethylated	-0.0063436	0.17527	insignificant	20	83	81
chr5	134652637	134654637	Wbscr16		1	noCoverage	0.16667	0.0083376	hypermethylated	0	8	8
chr5	134658907	134660907	Gtf2ird2	-0.099753	2.28E-23	hypomethylated	-0.0011134	0.50204	insignificant	25	111	108
chr5	134790616	134792616	Gtf2i	-0.20898	7.97E-28	hypomethylated	-0.0091351	0.9358	insignificant	25	69	72
chr5	134932132	134934132	Gtf2ird1	-0.068482	2.18E-20	hypomethylated	0.0091825	0.70371	insignificant	22	71	71
chr5	134932581	134934581	Gtf2ird1	-0.071197	9.46E-15	hypomethylated	0.036642	0.0054691	inconclusive	14	48	48
chr5	135028304	135030304	Clip2	-0.50866	4.88E-09	stronglyHypometh	-0.15456	0.02014	hypomethylated	7	20	25
chr5	135035963	135037963	Syna	-0.11022	0.41824	insignificant	-0.005239	0.60302	insignificant	4	19	17
chr5	135057559	135059559	Rfc2	-0.26933	5.81E-11	hypomethylated	-0.017261	0.044405	hypomethylated	12	62	60
chr5	135090893	135092893	Lat2	0.28309	0.3235	insignificant	-0.10485	0.26597	insignificant	2	21	24
chr5	135115198	135117198	Eif4h	-0.17306	2.88E-17	hypomethylated	0.016821	0.063187	insignificant	14	44	45
chr5	135164460	135166460	Limk1	-0.14325	2.46E-12	hypomethylated	0.0061533	0.025033	inconclusive	23	84	84
chr5	135407242	135409242	Wbscr27	-0.11669	0.00003119	hypomethylated	0.029629	0.60466	insignificant	4	60	59
chr5	135422804	135424804	Cldn4	-0.27394	0.013329	hypomethylated	0.0071812	0.90728	insignificant	3	20	18
chr5	135461083	135463083	Cldn3	-0.16297	0.00000436	hypomethylated	-0.015091	0.63521	insignificant	16	98	98
chr5	135477220	135479220	Wbscr25	-0.21938	0.00090028	hypomethylated	0.02293	0.70252	insignificant	2	21	20
chr5	135484021	135486021	Abhd11	-0.15912	5.69E-09	hypomethylated	0.0022966	0.24869	insignificant	9	53	50
chr5	135484168	135486168	Abhd11	-0.15912	5.69E-09	hypomethylated	0.0022966	0.24869	insignificant	9	53	50
chr5	135484325	135486325	Abhd11	-0.13693	0.000000133	hypomethylated	0.010124	0.49025	insignificant	9	51	48
chr5	135498441	135500441	Stx1a	-0.1829	2.51E-23	hypomethylated	-0.017457	0.31809	insignificant	23	76	88
chr5	135539075	135541075	Dnajc30	-0.10266	1.42E-12	hypomethylated	-0.0042282	0.53739	insignificant	27	122	127
chr5	135539980	135541980	Wbscr22	-0.08711	5.73E-10	hypomethylated	0.0024557	0.78329	insignificant	24	101	101
chr5	135554136	135556136	Vps37d	-0.3455	0.00000439	stronglyHypometh	-0.048479	0.3637	insignificant	12	44	42
chr5	135581782	135583782	Mlxipl	-0.14749	4.78E-17	hypomethylated	0.056998	0.17797	insignificant	6	41	44
chr5	135624580	135626580	Tbl2	-0.1568	2.76E-09	hypomethylated	0.011421	0.0077834	inconclusive	14	80	73
chr5	135643241	135645241	Bcl7b	-0.1336	9.21E-13	hypomethylated	-0.0050417	0.62975	insignificant	20	81	81
chr5	135662192	135664192	Baz1b	-0.10457	1.72E-34	hypomethylated	0.001658	0.27121	insignificant	58	217	213
chr5	135726917	135728917	Fzd9	-0.11698	1.13E-13	hypomethylated	0.011174	0.0050865	inconclusive	23	93	89
chr5	135825861	135827861	Fkbp6	0.060695	0.000000469	hypermethylated	-0.066659	0.49624	insignificant	13	38	38
chr5	135828165	135830165	Trim50		1	noCoverage	-0.035256	0.42191	insignificant	0	6	6
chr5	135844822	135846822	Nsun5	-0.25867	5.79E-17	hypomethylated	-0.0027012	0.00049331	hypomethylated	12	39	39
chr5	135870416	135872416	Pom121	-0.015156	0.56509	insignificant	0.022447	0.61194	insignificant	4	39	39
chr5	136020992	136022992	Hip1	-0.19151	1.93E-09	hypomethylated	-0.020089	0.13926	insignificant	7	36	38
chr5	136048913	136050913	Ccl24		1	noCoverage	-0.082164	0.80072	insignificant	0	6	6
chr5	136107523	136109523	Rhbdd2	-0.12593	0.01376	hypomethylated	0.022451	0.41435	insignificant	2	54	55
chr5	136164083	136166083	Por	-0.10558	0.000000188	hypomethylated	0.0080396	0.71044	insignificant	19	80	80
chr5	136220042	136222042	Tmem120a	-0.10119	0.00000128	hypomethylated	-0.013776	0.063991	insignificant	11	34	34
chr5	136253518	136255518	Mdh2	-0.18719	8.62E-46	hypomethylated	-0.026151	0.000011874	hypomethylated	44	134	146
chr5	136254255	136256255	Styxl1	-0.14712	6.63E-24	hypomethylated	-0.01298	0.00031918	hypomethylated	33	108	119
chr5	136362788	136364788	Hspb1	-0.23234	0.18111	insignificant	0.010273	0.13425	insignificant	4	51	52
chr5	136410511	136412511	Ywhag	-0.17184	0.038697	hypomethylated	0.041688	0.91574	insignificant	8	20	23
chr5	136450346	136452346	Srcrb4d	-0.29298	0.0088094	hypomethylated	0.17693	0.0055835	hypermethylated	2	4	4
chr5	136454974	136456974	Zp3	-0.33881	0.03446	stronglyHypometh	-0.0026178	0.11789	insignificant	1	10	18
chr5	136469746	136471746	Dtx2		1	noCoverage	-0.038657	0.82915	insignificant	0	16	16
chr5	136558785	136560785	Rasa4	-0.27917	0.18606	insignificant	-0.037584	1	insignificant	4	8	9
chr5	136591560	136593560	Polr2j	-0.067392	3.06E-12	hypomethylated	-0.047043	0.2505	insignificant	7	45	60
chr5	136611944	136613944	Lrwd1	-0.14873	0.035816	hypomethylated	-0.037214	0.31986	insignificant	6	27	32
chr5	136613650	136615650	Alkbh4		1	noCoverage	-0.31501	0.24686	insignificant	0	5	10
chr5	136674824	136676824	Prkrip1	-0.18697	0.00055479	hypomethylated	-0.00080795	0.0010341	hypomethylated	9	40	41
chr5	136720773	136722773	Sh2b2	-0.15671	0.088389	insignificant	0.016949	0.8039	insignificant	4	24	24
chr5	137043275	137045275	Cux1	-0.10541	0.000000211	hypomethylated	-0.019656	0.67385	insignificant	34	118	143
chr5	137043301	137045301	Cux1	-0.10541	0.000000211	hypomethylated	-0.019656	0.67385	insignificant	34	118	143
chr5	137358977	137360977	Emid2	-0.11433	0.0071372	hypomethylated	0.066263	7.44E-10	hypermethylated	8	31	33
chr5	137383019	137385019	Rabl5	0.020554	0.01138	inconclusive	0.22194	0.015224	hypermethylated	4	14	16
chr5	137394015	137396015	4933404O12Rik	-0.18637	1.34E-17	hypomethylated	-0.031073	0.66311	insignificant	15	51	54
chr5	137428144	137430144	Fis1	0.14517	8.84E-10	hypermethylated	-0.009278	0.035037	hypomethylated	5	56	66
chr5	137436946	137438946	Fis1	-0.16224	0.0041657	hypomethylated	0.060602	0.91992	insignificant	5	22	23
chr5	137461888	137463888	Plod3	-0.13787	7.01E-26	hypomethylated	-0.0042255	0.26389	insignificant	35	133	138
chr5	137466302	137468302	Mir702	0.04036	0.38492	insignificant	0.054265	0.082595	insignificant	7	41	42
chr5	137505164	137507164	Vgf	-0.15589	2.26E-64	hypomethylated	0.0030561	0.0092341	inconclusive	36	123	120
chr5	137521930	137523930	Ap1s1	-0.16986	2.05E-17	hypomethylated	0.043423	0.0001561	inconclusive	12	44	44
chr5	137548142	137550142	Serpine1	-0.40216	0.00079115	stronglyHypometh	-0.015511	0.00025647	hypomethylated	4	21	21
chr5	137592077	137594077	Trim56	-0.53947	0.0001118	stronglyHypometh	-0.009063	0.0012602	inconclusive	1	19	30
chr5	137728504	137730504	Ache	-0.16807	9.66E-17	hypomethylated	0.0074602	0.78965	insignificant	23	69	78
chr5	137734896	137736896	Ufsp1	-0.19171	3.09E-41	hypomethylated	-0.0011798	0.017553	hypomethylated	19	59	55
chr5	137748902	137750902	Srrt	0.3538	1	lowCoverage	-0.028143	1	insignificant	1	12	12
chr5	137755469	137757469	Slc12a9	-0.010692	0.049813	inconclusive	0.025858	0.000036773	hypermethylated	17	52	51
chr5	137774810	137776810	Slc12a9	-0.28073	0.087473	insignificant	-0.054289	0.73539	insignificant	3	29	25
chr5	137790336	137792336	Ephb4	-0.20068	0.00000021	hypomethylated	-0.014711	0.38307	insignificant	5	34	34
chr5	137918292	137920292	Zan	-0.22992	0.15349	insignificant	-0.034832	0.018106	hypomethylated	2	26	28
chr5	137927044	137929044	Epo		1	noCoverage	0.15934	0.0082146	hypermethylated	0	6	6
chr5	137943657	137945657	Pop7	-0.15695	4.95E-18	hypomethylated	0.0080649	0.126	insignificant	22	83	85
chr5	137959107	137961107	Gigyf1		1	noCoverage	0.022139	1	insignificant	0	15	16
chr5	137974457	137976457	Gnb2	-0.2317	0.0039126	hypomethylated	0.036656	1	insignificant	4	10	12
chr5	137993782	137995782	Actl6b	-0.12078	0.00083185	hypomethylated	-0.0022829	0.64596	insignificant	23	56	56
chr5	138042268	138044268	Mospd3	-0.037666	0.0048166	hypomethylated	0.0037178	0.040995	inconclusive	5	47	48
chr5	138052632	138054632	Pcolce	0.51096	0.078718	insignificant	0.19444	0.31382	insignificant	2	11	12
chr5	138066306	138068306	Fbxo24		1	noCoverage	0.24643	0.0040857	hypermethylated	0	10	10
chr5	138069350	138071350	Lrch4-sap25	-0.14999	3.05E-08	hypomethylated	-0.00055059	0.18887	insignificant	18	57	57
chr5	138086942	138088942	Irs3		1	noCoverage	-0.13413	0.28977	insignificant	0	14	14
chr5	138125845	138127845	Agfg2	-0.094668	0.00000039	hypomethylated	-0.011047	0.084904	insignificant	6	42	42
chr5	138125921	138127921	Agfg2		1	noCoverage	0.0029315	0.92185	insignificant	0	26	26
chr5	138181226	138183226	6430598A04Rik	-0.33294	1.43E-11	hypomethylated	-0.066728	0.000046286	hypomethylated	7	37	41
chr5	138186223	138188223	Tsc22d4	-0.202	5.41E-10	hypomethylated	0.0031538	0.28844	insignificant	8	23	22
chr5	138219145	138221145	Ppp1r35	-0.21132	9.54E-33	hypomethylated	-0.040486	0.0000079	hypomethylated	38	98	120

chr5	138227929	138229929	Mepce	-0.48013	0.0012583	stronglyHypometh	-0.044243	0.29287	insignificant	9	48	48
chr5	138228029	138230029	Zcwpw1	-0.48013	0.0012583	stronglyHypometh	-0.044243	0.29287	insignificant	9	48	48
chr5	138277506	138279506	Pilra	-0.087439	0.0033839	hypomethylated	-0.024463	0.00087987	hypomethylated	5	25	26
chr5	138299277	138301277	Pilrb1		1	noCoverage	0.03125	1	insignificant	0	4	4
chr5	138421748	138423748	Azgp1	-0.19519	0.065007	insignificant	0.23487	0.035956	hypermethylated	1	3	4
chr5	138525311	138527311	Zkscan1	-0.1535	3.91E-15	hypomethylated	-0.0011777	0.1672	insignificant	16	60	60
chr5	138557132	138559132	Zscan21	-0.18584	8.01E-08	hypomethylated	-0.0098135	0.046111	hypomethylated	8	60	57
chr5	138596972	138598972	Zfp113	-0.39841	0.00010913	stronglyHypometh	-0.043112	0.51444	insignificant	3	11	10
chr5	138601329	138603329	Cops6	-0.36364	0.16918	insignificant	-0.064317	1	insignificant	2	6	7
chr5	138606632	138608632	Mcm7	-0.18033	0.00000153	hypomethylated	-0.11105	0.014353	hypomethylated	2	2	2
chr5	138612248	138614248	Ap4m1	-0.10513	4.42E-08	hypomethylated	0.027906	0.93102	insignificant	20	96	95
chr5	138613090	138615090	Mcm7	-0.12922	0.23306	insignificant	0.05107	0.46696	insignificant	4	41	41
chr5	138627762	138629762	Cnpy4	-0.36003	9.91E-09	stronglyHypometh	-0.21721	0.082114	insignificant	6	20	20
chr5	138628414	138630414	Taf6	-0.61111	0.000014666	stronglyHypometh	-0.086034	0.19699	insignificant	1	2	4
chr5	138634541	138636541	Mblac1	-0.099045	1.52E-14	hypomethylated	-0.0029416	0.43861	insignificant	11	39	51
chr5	138695709	138697709	0910001L09Rik	-0.15869	8.89E-09	hypomethylated	-0.0067224	0.72277	insignificant	7	34	36
chr5	138705280	138707280	BC037034	0.57544	0.046557	stronglyHypermeth	-0.034583	0.20316	insignificant	1	10	12
chr5	138713982	138715982	Gpc2	-0.33025	1.69E-10	hypomethylated	0.078031	0.80342	insignificant	5	14	14
chr5	138720736	138722736	Stag3	-0.24785	0.0037691	hypomethylated	0.038023	0.27781	insignificant	12	82	82
chr5	138721165	138723165	Gpc2	-0.32105	0.011858	hypomethylated	0.044122	0.032282	inconclusive	9	52	57
chr5	138881703	138883703	Zfp157	-0.18906	0.084389	insignificant	-0.036254	0.12611	insignificant	7	22	22
chr5	139005922	139007922	1700123K08Rik	-0.42308	0.1871	insignificant	-0.17308	0.060696	insignificant	1	2	2
chr5	139060971	139062971	Zfp68	-0.21246	0.00016912	hypomethylated	-0.0040167	0.39321	insignificant	3	10	10
chr5	139063086	139065086	A430033K04Rik		1	noCoverage	0.031718	0.44025	insignificant	0	12	13
chr5	139230034	139232034	Fam20c	-0.10243	1.53E-61	hypomethylated	-0.0039023	0.31206	insignificant	70	234	240
chr5	139295033	139297033	Gm5294	-0.094182	0.19605	insignificant	-0.015695	0.00059331	inconclusive	6	18	15
chr5	139470907	139472907	Pdgfa	-0.1158	2.38E-31	hypomethylated	-0.0017693	0.010606	hypomethylated	71	192	205
chr5	139605706	139607706	Prkar1b	-0.1286	0.000000709	hypomethylated	0.01853	0.095877	insignificant	14	54	54
chr5	139625176	139627176	Heatr2	-0.1004	1.96E-36	hypomethylated	-0.012639	0.067777	insignificant	42	148	136
chr5	139675623	139677623	Sun1	-0.10112	3.13E-18	hypomethylated	0.0020306	0.68392	insignificant	29	123	128
chr5	139727277	139729277	Get4	-0.058961	9.49E-11	hypomethylated	0.0012089	0.4394	insignificant	12	89	89
chr5	139728450	139730450	Get4		1	noCoverage	-0.0032572	0.72592	insignificant	0	21	21
chr5	139801418	139803418	Adap1	-0.11149	1.27E-18	hypomethylated	-0.0062339	0.001348	hypomethylated	23	72	72
chr5	139821120	139823120	Cox19	0.16755	0.57224	insignificant	0.0032114	0.092236	insignificant	4	34	28
chr5	139827570	139829570	Cyp2w1	0.03026	0.20738	insignificant	0.011688	0.47009	insignificant	3	14	14
chr5	139845699	139847699	Mir339	0.042328	1	insignificant	-0.051202	0.27887	insignificant	3	6	6
chr5	139852650	139854650	D830046C22Rik	0.079609	1	insignificant	0.075836	0.8389	insignificant	1	6	6
chr5	139855620	139857620	Gpr146	-0.18475	1.89E-09	hypomethylated	0.032937	0.90154	insignificant	22	82	94
chr5	139881340	139883340	C130050O18Rik	-0.55714	0.29547	insignificant	0.0042857	0.83349	insignificant	3	7	10
chr5	139936488	139938488	3110082I17Rik		1	noCoverage	-0.0064133	1	insignificant	0	10	10
chr5	139960445	139962445	Zfand2a		1	noCoverage	0.096893	0.88395	insignificant	0	43	41
chr5	140018851	140020851	Uncx	-0.16364	1.35E-18	hypomethylated	-0.016123	0.1118	insignificant	44	159	160
chr5	140212287	140214287	Micall2	0.039594	0.00029185	hypermethylated	-0.033535	0.31666	insignificant	8	32	32
chr5	140251632	140253632	Ints1	-0.1745	1.82E-12	hypomethylated	-0.017897	0.30297	insignificant	10	34	34
chr5	140266489	140268489	Mafk	-0.12111	1.56E-12	hypomethylated	0.013417	0.55831	insignificant	23	110	96
chr5	140289197	140291197	Tmem184a	-0.63574	0.0013238	stronglyHypometh	-0.21748	0.23331	insignificant	1	14	14
chr5	140302797	140304797	Psmg3	-0.59989	0.34286	lowCoverage	-0.10758	0.87976	insignificant	1	2	2
chr5	140382896	140384896	Elfn1	-0.099517	0.0079361	hypomethylated	-0.0054748	0.0092809	hypomethylated	19	100	100
chr5	140797506	140799506	Mad1l1	-0.21998	6.35E-12	hypomethylated	-0.029633	0.26746	insignificant	15	36	36
chr5	140806875	140808875	Nudt1	-0.051612	0.000015484	hypomethylated	0.11599	0.083096	insignificant	12	35	38
chr5	140807852	140809852	Ftsj2	0.45284	0.60066	insignificant	-0.010998	0.000053688	hypomethylated	4	15	14
chr5	140865201	140867201	Snx8	-0.14687	7.03E-11	hypomethylated	-0.011009	0.82176	insignificant	18	53	58
chr5	140894258	140896258	Eif3b	-0.10247	2.42E-29	hypomethylated	-0.011929	0.49807	insignificant	36	141	133
chr5	140980562	140982562	Chst12	-0.18187	0.019106	hypomethylated	0.015705	0.82757	insignificant	5	30	26
chr5	141041021	141043021	Grifin	-0.079453	0.62319	insignificant	-0.0045278	1	insignificant	1	11	12
chr5	141082294	141084294	Lfng	-0.12975	6.05E-13	hypomethylated	-0.021955	1	insignificant	25	133	140
chr5	141124985	141126985	Ttyh3	-0.17822	1.08E-10	hypomethylated	-0.031662	0.08555	insignificant	9	26	26
chr5	141178332	141180332	Baat1	-0.24863	2.78E-22	hypomethylated	0.053397	0.36196	insignificant	7	23	25
chr5	141179976	141181976	Baat1	-0.23813	1.28E-19	hypomethylated	0.042522	0.54063	insignificant	10	46	43
chr5	141306385	141308385	Gna12	-0.21667	0.00000707	hypomethylated	-0.02549	0.86112	insignificant	4	20	17
chr5	141716487	141718487	Sdk1	-0.095143	3.3E-49	hypomethylated	0.0019645	0.0567	insignificant	102	258	258
chr5	142876450	142878450	Foxk1	-0.10613	5.38E-26	hypomethylated	0.0068636	0.012979	inconclusive	43	120	122
chr5	142938884	142940884	C330006K01Rik	-0.17677	0.00000118	hypomethylated	-0.023375	0.29905	insignificant	24	76	81
chr5	143006030	143008030	Radil	-0.099652	0.02911	hypomethylated	0.15262	0.000039378	hypermethylated	11	36	41
chr5	143027031	143029031	Radil	-0.12092	0.39169	insignificant	-0.011305	0.27137	insignificant	3	31	30
chr5	143084706	143086706	Mmd2	-0.38924	3.2E-12	stronglyHypometh	-0.066815	0.072574	insignificant	11	23	23
chr5	143104537	143106537	Wipi2	-0.095494	0.001259	hypomethylated	0.002273	0.51565	insignificant	31	161	154
chr5	143177054	143179054	Slc29a4	-0.12724	9.03E-24	hypomethylated	0.0055391	0.2813	insignificant	30	96	91
chr5	143579066	143581066	Tnrc18	-0.19466	3.73E-11	hypomethylated	-0.028384	0.11853	insignificant	17	70	71
chr5	143656917	143658917	Fbxl18	-0.54021	5.21E-18	stronglyHypometh	-0.11825	0.19118	insignificant	5	36	34
chr5	143668403	143670403	Actb	-0.26249	3.09E-10	hypomethylated	-0.026203	0.055654	insignificant	9	36	38
chr5	143721033	143723033	Fscn1	-0.13546	9.76E-57	hypomethylated	-0.023261	0.000012605	hypomethylated	47	124	145
chr5	143874699	143876699	Rnf216		1	noCoverage	0.092869	0.66425	insignificant	0	10	11
chr5	143941695	143943695	4933411G11Rik	-0.078738	0.057128	insignificant	0.0060046	0.51321	insignificant	23	61	61
chr5	143942422	143944422	Rbak	0.082655	1.84E-10	hypermethylated	0.010867	0.53925	insignificant	10	33	33
chr5	143995886	143997886	Zfp12	-0.20488	0.040395	hypomethylated	0.055971	0.00053444	hypermethylated	6	40	45
chr5	144077039	144079039	E130309D02Rik	-0.12203	0.00000277	hypomethylated	-0.0030148	0.00037269	inconclusive	12	32	32
chr5	144089986	144091986	0610040B10Rik	-0.22309	1.12E-31	hypomethylated	-0.0095001	0.000015283	hypomethylated	30	90	90
chr5	144090905	144092905	Zdhhc4	-0.21532	3.86E-12	hypomethylated	-0.00043101	0.032037	hypomethylated	12	28	28
chr5	144118016	144120016	Grid2ip	-0.14384	0.000000244	hypomethylated	-0.0014663	0.030557	hypomethylated	9	38	32
chr5	144124586	144126586	Grid2ip	-0.21509	0.000036485	hypomethylated	-0.091332	0.24759	insignificant	11	48	55
chr5	144164498	144166498	Kdelr2	-0.098314	6.34E-21	hypomethylated	0.0082403	0.85744	insignificant	19	60	67
chr5	144224360	144226360	Daglb	-0.1754	1.3E-11	hypomethylated	-0.017088	0.60658	insignificant	27	92	91
chr5	144288861	144290861	Rac1	-0.095669	2.94E-09	hypomethylated	-0.013866	0.79259	insignificant	26	63	60
chr5	144308573	144310573	2810453I06Rik	-0.096982	6.1E-18	hypomethylated	0.03799	0.50388	insignificant	20	57	74

chr5	144382315	144384315	Cyth3	-0.10565	1.85E-10	hypomethylated	-0.00247	1	insignificant	16	95	95
chr5	144493149	144495149	Usp42	-0.08596	2.38E-09	hypomethylated	0.0018161	0.67512	insignificant	25	78	72
chr5	144518222	144520222	D130017N08Rik	-0.3925	3.45E-17	stronglyHypometh	-0.072047	0.000000508	hypomethylated	11	46	48
chr5	144577660	144579660	Eif2ak1	-0.14916	8.55E-08	hypomethylated	-0.0095175	0.80845	insignificant	8	68	69
chr5	144655936	144657936	Ankrd61	0.038105	1	insignificant	0.0035249	0.8294	insignificant	3	12	14
chr5	144669869	144671869	Pms2	-0.21165	9.7E-39	hypomethylated	-0.025967	1	insignificant	18	54	54
chr5	144670708	144672708	Pms2	-0.37323	9.81E-58	stronglyHypometh	0.033863	0.26128	insignificant	12	23	26
chr5	144775722	144777722	Ccz1	-0.42792	0.01695	stronglyHypometh	-0.057436	0.01114	hypomethylated	5	18	16
chr5	144860304	144862304	Lmtk2	-0.16123	2.66E-46	hypomethylated	-0.026259	0.000051045	hypomethylated	28	95	101
chr5	144950154	144952154	Bhlha15	0.011275	0.011742	hypermethylated	-0.20173	0.80152	insignificant	11	26	34
chr5	144984447	144986447	Tecpr1	-0.22108	3.3E-09	hypomethylated	-0.019007	0.081478	insignificant	14	50	45
chr5	145015093	145017093	Bri3	-0.15444	4.03E-17	hypomethylated	-0.0072604	0.75145	insignificant	27	100	111
chr5	145118981	145120981	Baiap2l1	-0.0017563	2.02E-10	hypomethylated	0.027742	0.041477	inconclusive	4	50	40
chr5	145305755	145307755	Nptx2	-0.10458	3.04E-14	hypomethylated	0.004686	0.0012794	hypermethylated	74	258	275
chr5	145522447	145524447	Tmem130	-0.36243	0.0011136	stronglyHypometh	-0.20664	0.00007367	hypomethylated	3	8	8
chr5	145528660	145530660	Trrap	-0.10832	2.71E-22	hypomethylated	0.0015277	0.031709	inconclusive	35	144	147
chr5	145726699	145728699	Smurf1	-0.1342	3.06E-09	hypomethylated	0.0039134	0.011361	inconclusive	18	58	58
chr5	145843737	145845737	Arpc1a	-0.30453	1.03E-15	hypomethylated	-0.10828	0.30878	insignificant	9	26	50
chr5	145874124	145876124	Arpc1b	-0.14803	5.62E-32	hypomethylated	-0.0039161	0.28361	insignificant	26	139	139
chr5	145900265	145902265	Bud31	-0.11831	4.77E-27	hypomethylated	-0.0020888	0.47628	insignificant	31	125	126
chr5	145900958	145902958	Pdap1	-0.12119	5.11E-09	hypomethylated	-0.0032392	0.12284	insignificant	15	71	72
chr5	145927092	145929092	Cpsf4	-0.14877	1.71E-21	hypomethylated	-0.012003	0.0077402	hypomethylated	35	127	123
chr5	145927973	145929973	Ptcd1	-0.1688	5.53E-20	hypomethylated	0.011197	0.019865	inconclusive	27	96	95
chr5	145952461	145954461	Atp5j2		1	noCoverage	-0.0625	0.39995	insignificant	0	2	2
chr5	145962751	145964751	Zkscan5	0.33157	0.016953	hypermethylated	0.21728	0.29001	insignificant	4	17	12
chr5	145964427	145966427	Zkscan5	-0.27637	2.5E-09	hypomethylated	-0.025697	0.48782	insignificant	14	49	45
chr5	145991583	145993583	Zfp655	-0.1164	3.35E-30	hypomethylated	-0.0044499	0.51028	insignificant	38	128	124
chr5	145991627	145993627	Zfp655	-0.1164	3.35E-30	hypomethylated	-0.0044499	0.51028	insignificant	38	128	124
chr5	146043211	146045211	Zfp498	-0.12648	0.82352	insignificant	-0.030598	0.35549	insignificant	9	29	30
chr5	147033013	147035013	Rnf6	-0.14461	2.39E-22	hypomethylated	-0.028864	0.000063927	hypomethylated	12	36	36
chr5	147042250	147044250	Cdk8	-0.12302	3.09E-14	hypomethylated	-0.0032135	0.020391	hypomethylated	27	141	137
chr5	147195581	147197581	Wasf3	-0.21835	1.98E-12	hypomethylated	-0.0090363	0.14974	insignificant	28	80	89
chr5	147395980	147397980	Gpr12	-0.13194	0.000000155	hypomethylated	-0.001007	0.069771	insignificant	23	80	93
chr5	147606532	147608532	Usp12	-0.2554	1.23E-13	hypomethylated	-0.0133	7.48E-12	hypomethylated	12	41	42
chr5	147643465	147645465	Rpl21	-0.094895	2.52E-08	hypomethylated	0.093162	0.0018541	hypermethylated	19	97	86
chr5	147655646	147657646	Rasl11a	-0.17604	1.41E-13	hypomethylated	-0.044632	0.0030279	hypomethylated	24	65	86
chr5	147775350	147777350	Mtif3	-0.66667	1.98E-12	stronglyHypometh	-0.0055337	0.000000199	hypomethylated	1	8	8
chr5	147888116	147890116	Polr1d	-0.12416	2.56E-13	hypomethylated	0.019974	0.14964	insignificant	22	82	90
chr5	147888143	147890143	Polr1d	-0.12726	8.43E-14	hypomethylated	0.020146	0.26797	insignificant	22	80	88
chr5	147888148	147890148	Lnx2	-0.12726	8.43E-14	hypomethylated	0.020146	0.26797	insignificant	22	80	88
chr5	147999271	148001271	Gsx1	-0.13289	4.14E-48	hypomethylated	0.018818	0.40894	insignificant	52	152	157
chr5	148080706	148082706	Pdx1	-0.18684	3.95E-26	hypomethylated	-0.0059956	1	insignificant	17	64	64
chr5	148118825	148120825	Cdx2		1	noCoverage	-0.046429	0.14417	insignificant	0	4	4
chr5	148134016	148136016	Prhoxnb	-0.65694	7.11E-08	stronglyHypometh	-0.11387	0.2136	insignificant	2	12	13
chr5	148212065	148214065	Flt3	-0.36639	1.43E-26	stronglyHypometh	-0.017167	0.00088464	hypomethylated	6	26	27
chr5	148241155	148243155	Pan3	-0.13065	6.26E-43	hypomethylated	-0.044485	0.000082389	hypomethylated	61	239	241
chr5	148537564	148539564	Flt1	-0.15239	4.55E-49	hypomethylated	-0.0066096	0.000000736	hypomethylated	29	108	109
chr5	148671203	148673203	Pomp	-0.24828	2.84E-17	hypomethylated	-0.0032933	0.00082977	hypomethylated	17	74	78
chr5	148706378	148708378	Slc46a3	-0.18485	2.14E-10	hypomethylated	-0.052634	0.067189	insignificant	9	52	48
chr5	148767895	148769895	Mtus2	-0.15888	5.25E-48	hypomethylated	-0.0068783	0.10119	insignificant	42	112	124
chr5	149211480	149213480	Slc7a1	-0.26529	0.000000323	hypomethylated	-0.038807	0.051772	insignificant	5	25	24
chr5	149552415	149554415	2210417A02Rik	0.12391	1	insignificant	-0.10757	0.72585	insignificant	3	16	12
chr5	149740223	149742223	Katnal1	-0.16466	1.63E-23	hypomethylated	-0.0092991	0.28728	insignificant	29	107	109
chr5	149864613	149866613	Hmgb1	-0.11753	0.79228	insignificant	0.017401	0.13329	insignificant	14	38	32
chr5	149995135	149997135	Uspl1	-0.21117	0.0000007	hypomethylated	0.035647	0.3562	insignificant	7	24	24
chr5	149995639	149997639	Uspl1	-0.25978	5.11E-09	hypomethylated	0.010688	0.080595	insignificant	7	33	29
chr5	150213380	150215380	6330406I15Rik	-0.31194	0.0000057	hypomethylated	0.048942	0.14799	insignificant	3	16	16
chr5	150241280	150243280	4930588N13Rik	-0.253	0.0021555	hypomethylated	-0.024031	0.22245	insignificant	4	24	26
chr5	150330253	150332253	Wdr95		1	noCoverage	-0.013965	0.73642	insignificant	0	8	8
chr5	150438890	150440890	Hsph1	-0.27962	2.7E-28	hypomethylated	-0.013742	0.12074	insignificant	17	54	53
chr5	150479831	150481831	B3galtl	-0.14414	2.98E-25	hypomethylated	-0.021344	0.013035	hypomethylated	31	83	83
chr5	150820249	150822249	Rxfp2	0.019444	0.05892	insignificant	0.013889	0.39737	insignificant	2	8	3
chr5	151061504	151063504	Fry	-0.016081	0.45259	insignificant	-0.028486	0.85873	insignificant	8	35	42
chr5	151324197	151326197	Brca2	-0.070621	8.73E-10	hypomethylated	0.006799	0.80122	insignificant	23	40	48
chr5	151324204	151326204	Brca2	-0.070621	8.73E-10	hypomethylated	0.006799	0.80122	insignificant	23	40	48
chr5	151397100	151399100	N4bp2l1	-0.24674	7.35E-19	hypomethylated	0.023632	0.11572	insignificant	17	49	48
chr5	151468187	151470187	N4bp2l2	-0.25817	0.042327	hypomethylated	0.081657	0.32549	insignificant	4	8	11
chr5	151475401	151477401	Pds5b	-0.083296	3.56E-12	hypomethylated	0.00053753	0.58667	insignificant	43	159	163
chr5	151754181	151756181	Kl	-0.16748	0.00000256	hypomethylated	-0.035762	0.21296	insignificant	19	63	72
chr5	151992768	151994768	Stard13		1	noCoverage	-0.012343	0.86744	insignificant	0	18	18
chr5	152453783	152455783	Rfc3	-0.18981	6.57E-19	hypomethylated	0.013178	0.41817	insignificant	11	22	22
chr6	3237518	3239518	Gm8579		1	noCoverage	-0.17118	0.00000867	hypomethylated	0	15	6
chr6	3447392	3449392	Ccdc132	-0.17362	0.00048482	hypomethylated	-0.050442	0.46043	insignificant	8	36	34
chr6	3713623	3715623	Calcr	-0.25245	3.44E-12	hypomethylated	-0.002944	0.028471	hypomethylated	9	51	52
chr6	3714713	3716713	Calcr		1	noCoverage	-0.081944	0.69829	insignificant	0	4	4
chr6	3952986	3954986	Gng11	-0.19681	0.65022	insignificant	-0.025082	0.0011038	hypomethylated	7	35	36
chr6	4036927	4038927	Bet1	-0.18364	0.58214	insignificant	-0.036176	0.61563	insignificant	1	24	25
chr6	4454696	4456696	Col1a2	-0.23101	0.00027605	hypomethylated	0.00296	0.72217	insignificant	7	22	22
chr6	4550065	4552065	Casd1	-0.010023	3.65E-13	hypomethylated	-0.0090131	0.016024	hypomethylated	41	92	92
chr6	4696305	4698305	Peg10	-0.033186	1	insignificant	-0.24432	1.01E-14	hypomethylated	5	30	30
chr6	4697204	4699204	Sgce	-0.036235	0.73607	insignificant	-0.27808	5.97E-15	hypomethylated	5	18	18
chr6	4852319	4854319	Ppp1r9a	-0.05818	1.46E-19	hypomethylated	-0.015427	0.72316	insignificant	39	148	150
chr6	5248373	5250373	Pon2	-0.24127	0.000000365	hypomethylated	-0.032278	0.0010437	hypomethylated	7	19	17
chr6	5332385	5334385	Asb4	-0.23101	0.000084089	hypomethylated	0.098187	0.020507	hypermethylated	7	49	48
chr6	5674638	5676638	Dync1i1	0.843	0.18914	lowCoverage	-0.0011488	1	insignificant	1	24	23

chr6	6167173	6169173	Slc25a13	-0.24185	0.000000375	hypomethylated	-0.041934	0.52929	insignificant	9	19	18
chr6	6812333	6814333	Dlx6	-0.10943	1.46E-21	hypomethylated	0.00077286	0.14043	insignificant	23	108	105
chr6	6815150	6817150	Dlx6as2	-0.34239	0.012071	stronglyHypometh	-0.16297	0.050144	insignificant	2	5	8
chr6	6832068	6834068	Dlx5		1	noCoverage	0.013599	0.019299	hypermethylated	0	8	9
chr6	6905017	6907017	Acn9	-0.080303	0.000000297	hypomethylated	-0.031877	0.00000169	hypomethylated	17	68	69
chr6	7504070	7506070	Tac1	-0.25308	0.00017786	hypomethylated	-0.045643	0.0014242	hypomethylated	4	35	32
chr6	7643182	7645182	Asns	-0.70054	0.33871	lowCoverage	-0.011504	0.88582	insignificant	1	6	6
chr6	7794223	7796223	C1galt1	-0.084065	3.82E-17	hypomethylated	-0.0051641	1	insignificant	54	116	116
chr6	8158226	8160226	Mios	-0.11571	1.81E-28	hypomethylated	-0.0041002	0.24616	insignificant	40	134	134
chr6	8208287	8210287	Gm16039	-0.15796	1.69E-08	hypomethylated	-0.020999	0.5182	insignificant	9	76	70
chr6	8209141	8211141	Rpa3	-0.13799	3.43E-08	hypomethylated	-0.022141	0.65446	insignificant	9	62	56
chr6	8458595	8460595	Glcci1	-0.084133	3.62E-41	hypomethylated	0.0038579	0.87333	insignificant	70	243	234
chr6	8899018	8901018	Nxph1	-0.21422	3.32E-13	hypomethylated	-0.0069609	0.34204	insignificant	13	40	35
chr6	11857446	11859446	Ndufa4	-0.11841	4.66E-20	hypomethylated	-0.0057586	0.073293	insignificant	23	79	79
chr6	11874880	11876880	Phf14	-0.12762	8.39E-15	hypomethylated	0.0070581	0.027494	inconclusive	15	75	75
chr6	12699253	12701253	Thsd7a	-0.16921	2.26E-09	hypomethylated	0.012364	0.51951	insignificant	8	24	24
chr6	13018758	13020758	Tmem106b	-0.14695	4.36E-17	hypomethylated	0.022423	0.44543	insignificant	25	75	75
chr6	13558063	13560063	Tmem168	-0.14663	0.055684	insignificant	-0.0014709	1	insignificant	6	16	16
chr6	13627966	13629966	B630005N14Rik	-0.11508	0.0007585	hypomethylated	-0.0097887	0.20232	insignificant	5	24	24
chr6	13789848	13791848	Gpr85	-0.36872	0.0061117	stronglyHypometh	-0.20596	0.071822	insignificant	3	15	19
chr6	14850348	14852348	Foxp2	-0.15204	0.000000446	hypomethylated	-0.003321	0.0019857	hypomethylated	16	87	87
chr6	15669660	15671660	Mdfic	-0.18651	6.61E-18	hypomethylated	-0.00093287	0.082652	insignificant	19	58	59
chr6	17014148	17016148	Tes	-0.12425	1.85E-10	hypomethylated	-0.0035924	0.050973	insignificant	17	41	48
chr6	17230340	17232340	Cav2	-0.15558	0.000046396	hypomethylated	0.024931	0.88984	insignificant	9	43	35
chr6	17255369	17257369	Cav1	-0.26336	0.000000259	hypomethylated	0.014414	0.84886	insignificant	8	26	26
chr6	17412956	17414956	Met	-0.18195	2.36E-20	hypomethylated	-0.058972	0.0087839	hypomethylated	22	87	84
chr6	17586097	17588097	Capza2	-0.1137	3.76E-28	hypomethylated	0.0015625	0.96693	insignificant	45	115	90
chr6	17698215	17700215	St7	-0.26563	1	lowCoverage	0.013851	0.31124	insignificant	1	19	19
chr6	17980445	17982445	Wnt2	-0.038469	0.77658	insignificant	-0.0019084	0.63175	insignificant	19	80	80
chr6	18059061	18061061	Asz1	-0.049204	0.036239	hypomethylated	0.06835	0.00084699	hypermethylated	6	16	17
chr6	18119686	18121686	Cftr	-0.15168	0.000013073	hypomethylated	-0.00036127	0.26771	insignificant	7	16	19
chr6	18464825	18466825	Cttnbp2	-0.14297	0.017398	hypomethylated	0.021973	0.65278	insignificant	4	44	44
chr6	18797634	18799634	Naa38	-0.14324	8.87E-16	hypomethylated	-0.0084732	0.081897	insignificant	34	92	100
chr6	18815317	18817317	Ankrd7	0.11083	1	lowCoverage	-0.018816	1	insignificant	1	15	15
chr6	21165108	21167108	Kcnd2	-0.2594	0.000000417	hypomethylated	0.037191	0.09903	insignificant	3	28	30
chr6	21802515	21804515	Tspan12	-0.4141	7.49E-08	stronglyHypometh	-0.065915	0.0018269	hypomethylated	3	8	8
chr6	21898614	21900614	Ing3	-0.12264	0.0003352	hypomethylated	-0.028704	0.0070828	hypomethylated	15	65	60
chr6	21934909	21936909	A430107O13Rik	-0.17794	0.000000963	hypomethylated	0.035963	0.7432	insignificant	3	37	26
chr6	22306081	22308081	Fam3c	-0.089244	0.00000459	hypomethylated	-0.000278	0.70032	insignificant	22	72	72
chr6	22824501	22826501	Ptprz1	-0.12226	6.11E-08	hypomethylated	-0.0022862	0.78789	insignificant	19	67	68
chr6	23198264	23200264	Fezf1	-0.14188	1.08E-15	hypomethylated	-0.014464	0.16074	insignificant	21	70	72
chr6	23789300	23791300	Cadps2	-0.1182	2.08E-18	hypomethylated	-0.0037376	0.25799	insignificant	33	151	157
chr6	24477143	24479143	Asb15	-0.23347	0.00000316	hypomethylated	-0.014484	0.72859	insignificant	5	10	14
chr6	24614995	24616995	Wasl	-0.10804	0.00060778	hypomethylated	0.015852	0.57196	insignificant	22	69	69
chr6	24682244	24684244	Hyal6	-0.095291	0.05778	insignificant	-0.059557	0.15801	insignificant	4	10	10
chr6	24906125	24908125	Tmem229a	-0.26297	1.48E-08	hypomethylated	-0.0038756	0.57448	insignificant	11	26	29
chr6	25639980	25641980	Gpr37	-0.18187	0.1213	insignificant	-0.064212	0.71439	insignificant	7	27	28
chr6	25759226	25761226	Pot1a	-0.32407	1	insignificant	-0.035384	0.64396	insignificant	1	18	16
chr6	27886750	27888750	Grm8		1	noCoverage	-0.2312	0.22312	insignificant	0	6	9
chr6	28084369	28086369	Grm8	-0.13087	1.54E-15	hypomethylated	-0.0022476	0.000010787	hypomethylated	22	66	66
chr6	28211601	28213601	Zfp800	-0.063924	6.55E-15	hypomethylated	-0.0043499	0.34972	insignificant	34	78	78
chr6	28371724	28373724	Arf5	-0.20093	1.19E-13	hypomethylated	0.03417	0.80784	insignificant	21	54	45
chr6	28372639	28374639	Arf5	-0.18994	2.95E-17	hypomethylated	0.0019752	0.81785	insignificant	34	90	79
chr6	28376900	28378900	Fscn3	-0.025463	0.58094	insignificant	-0.051455	0.67882	insignificant	2	6	4
chr6	28399340	28401340	Pax4	-0.57023	0.078695	insignificant	-0.037655	0.48023	insignificant	1	4	4
chr6	28429347	28431347	Snd1	-0.14845	7.86E-13	hypomethylated	-0.011157	0.018017	hypomethylated	22	86	92
chr6	28781747	28783747	Snd1	-0.064659	1.33E-25	hypomethylated	0.0086634	0.72105	insignificant	37	76	76
chr6	29009220	29011220	Lep	-0.034757	1	insignificant	0.016564	0.84812	insignificant	5	25	25
chr6	29114724	29116724	Rbm28	-0.58995	0.55413	insignificant	-0.083598	0.21609	insignificant	3	21	18
chr6	29162271	29164271	Impdh1	-0.16528	0.000000233	hypomethylated	0.032079	0.079983	insignificant	18	51	54
chr6	29221487	29223487	Hilpda	-0.16576	0.000000105	hypomethylated	-0.047281	0.13536	insignificant	17	38	38
chr6	29221625	29223625	Hilpda	-0.19534	0.00000308	hypomethylated	-0.066615	0.6172	insignificant	15	28	30
chr6	29268139	29270139	Fam71f1	-0.63811	0.02589	stronglyHypometh	-0.040006	0.08513	insignificant	1	11	11
chr6	29297118	29299118	Calu	-0.16927	6.35E-30	hypomethylated	-0.025069	0.00063066	hypomethylated	37	140	140
chr6	29345635	29347635		-0.10938	4.51E-09	hypomethylated	-0.0093829	0.0039568	hypomethylated	32	104	106
chr6	29382152	29384152	Flnc	-0.10046	8.31E-12	hypomethylated	0.0015447	0.59031	insignificant	25	95	96
chr6	29416782	29418782	Atp6v1f	-0.18009	0.00002433	hypomethylated	-0.01357	0.30044	insignificant	7	48	49
chr6	29457934	29459934	Kcp		1	noCoverage	-0.073697	0.77016	insignificant	0	3	0
chr6	29475732	29477732	Irf5	-0.1321	3.77E-47	hypomethylated	0.0029689	0.42453	insignificant	50	161	163
chr6	29559607	29561607	Tnpo3	-0.41714	1.59E-12	stronglyHypometh	-0.051246	0.0071704	inconclusive	17	61	61
chr6	29643255	29645255	Tspan33	-0.14812	0.00026075	hypomethylated	-0.014091	0.07268	insignificant	22	63	57
chr6	29684496	29686496	Smo	-0.10454	3.03E-31	hypomethylated	-0.0080586	0.37144	insignificant	50	147	141
chr6	29717442	29719442	Ahcyl2	-0.13812	3.81E-26	hypomethylated	0.0012777	0.056929	insignificant	16	100	102
chr6	29866012	29868012	Fam40b	-0.14664	4.15E-26	hypomethylated	-0.00090275	0.0040427	hypomethylated	27	124	126
chr6	29996987	29998987	Nrf1	-0.11776	1.65E-21	hypomethylated	-0.0041514	0.72422	insignificant	51	150	143
chr6	30115992	30117992	Mir182	-0.56203	0.000026512	stronglyHypometh	-0.02848	0.8641	insignificant	2	4	4
chr6	30254517	30256517	Gm2058	-0.087985	0.0096876	inconclusive	0.13664	8.86E-09	hypermethylated	6	32	20
chr6	30254539	30256539	Gm2058	-0.10673	0.025278	inconclusive	0.16534	7.7E-09	hypermethylated	6	30	18
chr6	30350908	30352908	Klhdc10	-0.19744	4.1E-40	hypomethylated	-0.0058836	0.002899	hypomethylated	28	96	96
chr6	30459706	30461706	1700025E21Rik	0.0086353	0.44544	insignificant	0.046213	0.12444	insignificant	2	16	18
chr6	30461285	30463285	1700025E21Rik	0.090608	0.37261	insignificant	0.035124	0.12172	insignificant	3	18	18
chr6	30490641	30492641	Cpa2	0.054348	1	lowCoverage	-0.070652	0.089848	insignificant	1	4	4
chr6	30517375	30519375	Cpa4	-0.059722	0.12831	insignificant	-0.076669	0.0036027	hypomethylated	6	19	16
chr6	30643682	30645682	Tsga14	-0.080482	1	insignificant	-0.0033586	0.48938	insignificant	3	20	21
chr6	30846760	30848760	Tsga13	-0.11382	0.0024473	hypomethylated	0.0066219	1	insignificant	7	31	29

chr6	30908990	30910990	Klf14	-0.17859	0.02008	hypomethylated	-0.047296	0.89826	insignificant	4	10	10
chr6	31347827	31349827	Mkln1	-0.12519	4.15E-20	hypomethylated	-0.01174	0.14974	insignificant	39	135	134
chr6	31513937	31515937	Podxl	-0.27346	0.34715	insignificant	0.050563	0.016502	hypermethylated	2	40	36
chr6	32538192	32540192	Plxna4	-0.17394	0.00067324	hypomethylated	-0.047813	0.47967	insignificant	2	16	22
chr6	33010152	33012152	Chchd3	-0.12515	0.000085033	hypomethylated	0.018526	0.097554	insignificant	7	37	34
chr6	33198149	33200149	Exoc4	-0.15408	0.00000363	hypomethylated	-0.031155	0.77597	insignificant	9	42	51
chr6	34267489	34269489	Akr1b3	-0.17214	0.00019545	hypomethylated	-0.027156	0.14165	insignificant	2	30	30
chr6	34303163	34305163	Akr1b8	0.11664	0.14113	insignificant	-0.016991	0.072708	insignificant	4	17	15
chr6	34333246	34335246	Akr1b10	-0.42782	0.00046245	stronglyHypometh	-0.00023624	0.90167	insignificant	5	11	10
chr6	34425355	34427355	Bpgm	-0.21478	4.19E-16	hypomethylated	-0.037385	0.00091532	hypomethylated	18	52	50
chr6	34729431	34731431	Agbl3	-0.11367	2E-15	hypomethylated	-0.0086772	0.74802	insignificant	25	108	108
chr6	34828065	34830065	3110062M04Ril	-0.11836	1.66E-10	hypomethylated	-0.0016637	0.047757	hypomethylated	13	32	32
chr6	34860831	34862831	Wdr91	-0.32134	1.04E-16	hypomethylated	-0.12331	0.041069	hypomethylated	5	22	24
chr6	34869959	34871959	Stra8		1	noCoverage	0.028589	0.00032163	hypermethylated	0	27	27
chr6	35083737	35085737	Cnot4	-0.17898	0.00012811	hypomethylated	-0.0060002	0.9538	insignificant	17	36	44
chr6	35126615	35128615	Nup205	-0.099103	1.1E-16	hypomethylated	0.020066	0.71082	insignificant	48	161	170
chr6	35201698	35203698	1810058I24Rik	-0.19778	0.000017559	hypomethylated	0.045875	0.65836	insignificant	15	56	47
chr6	35201731	35203731	1810058I24Rik	-0.19778	0.000017559	hypomethylated	0.045875	0.65836	insignificant	15	56	47
chr6	35489888	35491888	Mtpn	-0.21345	0.0018177	hypomethylated	0.14571	0.74266	insignificant	3	6	7
chr6	36338234	36340234	Chrm2	-0.23887	3.81E-15	hypomethylated	-0.009501	0.44807	insignificant	7	14	14
chr6	36370741	36372741	Mir490	0.06369	1	insignificant	0.018455	1	insignificant	3	16	16
chr6	36472169	36474169	Chrm2	-0.13735	0.15832	insignificant	0.016695	0.43779	insignificant	3	6	6
chr6	37249976	37251976	Dgki	-0.17787	8.8E-10	hypomethylated	0.00019914	0.054585	insignificant	8	30	26
chr6	37392148	37394148	Creb3l2	-0.34159	0.59486	insignificant	-0.020681	0.26635	insignificant	3	32	32
chr6	37819810	37821810	Trim24	-0.10366	7.26E-24	hypomethylated	0.025932	0.36021	insignificant	51	165	184
chr6	38204009	38206009	D630045J12Rik	-0.094807	0.000047925	hypomethylated	0.012104	0.888	insignificant	15	45	32
chr6	38249259	38251259	Zc3hav1l	-0.55583	6.13E-15	stronglyHypometh	0.019227	0.79659	insignificant	2	4	4
chr6	38304603	38306603	Zc3hav1		1	noCoverage	0.004738	0.75219	insignificant	0	14	13
chr6	38382924	38384924	Ubn2	-0.076872	2.03E-20	hypomethylated	-0.0080742	0.090252	insignificant	65	249	245
chr6	38483860	38485860	1110001J03Rik	-0.16521	3.54E-14	hypomethylated	0.019106	0.45162	insignificant	8	49	37
chr6	38500443	38502443	Luc7l2	-0.13181	0.00001347	hypomethylated	0.0058784	0.74696	insignificant	11	43	43
chr6	38587239	38589239	Klrg2	-0.33706	0.21393	insignificant	0.046104	0.91375	insignificant	6	19	20
chr6	38612068	38614068	Clec2l	-0.1302	2.42E-29	hypomethylated	0.0023266	0.014162	inconclusive	43	82	82
chr6	38787223	38789223	Hipk2	0.092131	1	lowCoverage	0.077037	0.12469	insignificant	1	7	10
chr6	38826189	38828189	Hipk2	-0.11795	0.000000409	hypomethylated	-0.017628	0.34744	insignificant	31	101	122
chr6	39068348	39070348	Parp12	0.060434	0.062592	insignificant	0.033382	0.00017699	inconclusive	16	83	83
chr6	39156772	39158772	Jhdm1d	-0.04917	0.00017935	hypomethylated	0.0041835	0.71683	insignificant	17	78	79
chr6	39330426	39332426	Rab19	-0.36138	0.5519	insignificant	-0.12313	0.17933	insignificant	3	16	13
chr6	39370368	39372368	Mkrn1	0.60329	0.10723	insignificant	0.06924	0.026969	hypermethylated	2	18	18
chr6	39507833	39509833	Dennd2a	-0.2015	0.023385	hypomethylated	0.018354	0.51243	insignificant	9	47	45
chr6	39522874	39524874	Adck2	-0.16409	7.13E-19	hypomethylated	-0.031695	0.43299	insignificant	21	60	59
chr6	39541581	39543581	Ndufb2	-0.0015553	1.47E-26	inconclusive	-0.012804	0.48525	insignificant	30	94	94
chr6	39675462	39677462	Braf		1	noCoverage	0.11319	0.63864	insignificant	0	20	21
chr6	39760935	39762935	Mrps33	-0.10193	0.000064549	hypomethylated	0.0014839	0.18457	insignificant	8	34	36
chr6	40059251	40061251	Gm5567	-0.36391	2.07E-08	stronglyHypometh	0.044042	0.83321	insignificant	3	10	9
chr6	40274476	40276476	Agk	-0.16227	6.19E-22	hypomethylated	-0.010263	0.60712	insignificant	20	47	54
chr6	40386132	40388132	E330009J07Rik	-0.15151	6.75E-11	hypomethylated	-0.047785	0.17015	insignificant	15	30	34
chr6	40420413	40422413	Ssbp1	-0.2175	3.13E-13	hypomethylated	-0.025059	0.23174	insignificant	10	48	48
chr6	40520386	40522386	Olfr460		1	noCoverage	-0.19695	0.20777	insignificant	0	12	12
chr6	41554480	41556480	Ephb6	-0.12216	8.16E-12	hypomethylated	-0.012853	0.4836	insignificant	15	98	98
chr6	42164326	42166326	Tas2r144	0.083333	1	insignificant	-0.012821	1	insignificant	1	4	4
chr6	42194933	42196933	Gstk1	-0.12613	1.05E-11	hypomethylated	-0.0018949	0.72839	insignificant	12	45	45
chr6	42210968	42212968	Tmem139		1	noCoverage	-0.039232	0.23805	insignificant	0	6	9
chr6	42214037	42216037	Casp2	-0.15228	0.00000429	hypomethylated	-0.039147	0.87898	insignificant	6	50	54
chr6	42274639	42276639	Fam131b	-0.12681	1.39E-13	hypomethylated	-0.0060198	0.88679	insignificant	23	83	84
chr6	42298826	42300826	Zyx	-0.10974	1.84E-22	hypomethylated	0.0068066	0.78834	insignificant	48	178	181
chr6	42323267	42325267	2010310C07Rik	-0.22382	0.00000254	hypomethylated	0.0068568	0.93092	insignificant	4	14	16
chr6	42354527	42356527	Tas2r135	-0.085818	0.21432	insignificant	-0.08183	0.053181	insignificant	1	6	5
chr6	42595040	42597040	Fam115c	-0.27664	5.36E-08	hypomethylated	0.052175	0.17279	insignificant	5	25	24
chr6	42643058	42645058	Fam115a	-0.14476	0.17944	insignificant	-0.062826	0.8825	insignificant	2	9	10
chr6	43122986	43124986	Olfr13	-0.79061	0.26042	lowCoverage	0.052522	0.47569	insignificant	1	6	7
chr6	43165913	43167913	Olfr434	0.041477	1	insignificant	-0.019334	0.26946	insignificant	1	4	4
chr6	43184608	43186608	Olfr47	-0.37059	0.026343	stronglyHypometh	-0.063922	0.0031168	hypomethylated	1	6	6
chr6	43214642	43216642	Arhgef5	-0.2725	1.4E-20	hypomethylated	-0.016078	0.48792	insignificant	24	59	58
chr6	43259553	43261553	Nobox	0.047409	0.76592	insignificant	0.060636	0.49698	insignificant	2	12	9
chr6	43616174	43618174	Tpk1	-0.080525	0.0032882	hypomethylated	-0.029372	0.40811	insignificant	8	39	38
chr6	47403322	47405322	Cul1	-0.091992	4.08E-37	hypomethylated	0.0023464	0.27089	insignificant	86	253	243
chr6	47545029	47547029	Ezh2	-0.1583	5.77E-08	hypomethylated	-0.005036	0.63624	insignificant	8	40	40
chr6	47618090	47620090	Rn4.5s		1	noCoverage	-0.0078046	6.31E-09	inconclusive	0	39	39
chr6	47622398	47624398	Rn4.5s		1	noCoverage	0.00055556	0.10251	insignificant	0	49	38
chr6	47635375	47637375	Rn4.5s		1	noCoverage	0.038365	0.88809	insignificant	0	3	12
chr6	47702924	47704924	Rn4.5s		1	noCoverage	-0.26945	0.30849	insignificant	0	37	20
chr6	47780504	47782504	Zfp786	-0.10225	0.24782	insignificant	-0.014252	0.40659	insignificant	5	10	10
chr6	47784659	47786659	Zfp398	-0.085671	4.03E-23	hypomethylated	-0.0012274	0.84819	insignificant	33	116	116
chr6	47826553	47828553	Zfp282	-0.11971	2.97E-17	hypomethylated	-0.0021874	0.010544	hypomethylated	39	124	124
chr6	47869566	47871566	Zfp212	-0.11433	4.23E-08	hypomethylated	-0.0062175	0.12365	insignificant	22	68	68
chr6	47892173	47894173	Zfp783	-0.084185	0.00000828	hypomethylated	0.15396	9.86E-10	hypermethylated	21	101	99
chr6	47902388	47904388	Zfp956	-0.17967	0.00000008	hypomethylated	0.022148	0.52927	insignificant	7	34	34
chr6	47998113	48000113	Zfp777	-0.08314	1.76E-24	hypomethylated	-0.0036143	0.019364	hypomethylated	67	196	192
chr6	48036592	48038592	Zfp746	-0.11054	1	insignificant	0.074777	0.81026	insignificant	4	33	34
chr6	48344584	48346584	Krba1	-0.17459	5.53E-12	hypomethylated	-0.022043	0.16378	insignificant	11	70	68
chr6	48395089	48397089	Zfp467	-0.13255	0.000043148	hypomethylated	0.0059793	0.064328	insignificant	12	58	59
chr6	48395824	48397824	Zfp467	-0.36557	0.00012811	stronglyHypometh	-0.12317	0.0041309	hypomethylated	4	8	8
chr6	48397227	48399227	Sspo	-0.34662	0.0027113	stronglyHypometh	-0.1549	0.000465	hypomethylated	2	9	9
chr6	48453337	48455337	Zfp862	-0.17267	1.57E-10	hypomethylated	0.035505	0.4635	insignificant	6	27	22

chr6	48486567	48488567	Atp6v0e2	-0.6122	0.000034003	stronglyHypometh	-0.27887	0.17895	insignificant	4	30	3
chr6	48503797	48505797	Lrrc61	-0.18435	0.086197	insignificant	-0.013988	0.5302	insignificant	4	14	14
chr6	48538443	48540443	Gm5111	-0.37407	1	lowCoverage	0.057506	1	insignificant	1	6	5
chr6	48542881	48544881	Repin1	-0.14835	5.37E-12	hypomethylated	-0.012714	0.00033962	hypomethylated	24	86	80
chr6	48577165	48579165	AI854703	-0.15233	2.85E-10	hypomethylated	-0.029698	0.05399	insignificant	14	28	28
chr6	48688045	48690045	Gimap1	-0.083489	0.68962	insignificant	0.036526	0.25464	insignificant	3	16	16
chr6	48695195	48697195	Gimap5	-0.50058	5.49E-10	stronglyHypometh	-0.061955	0.24722	insignificant	2	6	6
chr6	49022793	49024793	2410003K15Rik	-0.12357	5.09E-10	hypomethylated	0.0033337	0.93624	insignificant	13	79	79
chr6	49164953	49166953	Igf2bp3	-0.15172	9.54E-08	hypomethylated	0.03197	0.35903	insignificant	20	86	79
chr6	49214051	49216051	Tra2a	-0.23803	1.99E-12	hypomethylated	-0.015469	0.0040973	hypomethylated	14	46	46
chr6	49268351	49270351	Ccdc126	-0.1284	0.59791	insignificant	0.024336	0.84516	insignificant	5	44	44
chr6	49316737	49318737	D330028D13Rik	-0.14583	1.55E-10	hypomethylated	-0.0054662	0.44918	insignificant	5	61	43
chr6	49344602	49346602	Stk31		1	noCoverage	-0.023634	0.28486	insignificant	0	22	22
chr6	49771727	49773727	Npy	-0.14913	9.98E-12	hypomethylated	-0.006872	0.58406	insignificant	17	93	93
chr6	50059239	50061239	Mpp6	-0.10803	9.02E-36	hypomethylated	-0.019695	0.00013087	hypomethylated	67	161	182
chr6	50211768	50213768	Dfna5	-0.1469	0.72018	insignificant	-0.013777	0.62007	insignificant	14	30	30
chr6	50406169	50408169	Osbpl3	-0.15345	0.17343	insignificant	-0.012998	1	insignificant	7	24	20
chr6	50515641	50517641	5430402O13Rik	-0.29055	5.78E-13	hypomethylated	-0.055842	0.083285	insignificant	12	46	46
chr6	50516473	50518473	Cycs	-0.37143	0.000062334	stronglyHypometh	-0.065476	1	insignificant	5	14	14
chr6	50546589	50548589	4921507P07Rik	-0.15139	0.22246	insignificant	0.013198	1	insignificant	6	37	33
chr6	51219909	51221909	Mir148a	-0.13375	1.84E-37	hypomethylated	-0.0014135	0.090253	insignificant	35	134	135
chr6	51381668	51383668	Nfe2l3	-0.12265	0.000055921	hypomethylated	-0.010348	0.81363	insignificant	18	47	47
chr6	51419614	51421614	Cbx3	-0.08842	5.22E-16	hypomethylated	0.0010261	0.59126	insignificant	47	203	183
chr6	51419893	51421893	Cbx3	-0.088634	8.34E-16	hypomethylated	-0.00081663	0.58972	insignificant	47	193	173
chr6	51962548	51964548	Skap2	-0.1683	5.1E-22	hypomethylated	-0.010633	0.14586	insignificant	14	42	40
chr6	52108316	52110316	Hoxa1	-0.15102	0.020081	hypomethylated	0.0052867	0.17956	insignificant	18	59	52
chr6	52114830	52116830	Hoxa2	0.048203	0.045842	hypermethylated	0.060251	0.088997	insignificant	8	20	20
chr6	52141702	52143702	Hoxa3	-0.16261	0.00050986	hypomethylated	-0.0070107	0.64078	insignificant	7	51	51
chr6	52150122	52152122	2700086A05Rik	-0.042989	0.091271	insignificant	0.022151	0.88294	insignificant	6	28	32
chr6	52154586	52156586	Hoxa5	0.079254	0.22175	insignificant	0.046225	0.00000578	hypermethylated	14	52	55
chr6	52158623	52160623	Hoxa3	-0.128	1.11E-11	hypomethylated	-0.016317	0.015809	hypomethylated	21	56	56
chr6	52168572	52170572	Hoxa7	0.016054	0.32653	insignificant	0.17985	0.19662	insignificant	1	7	4
chr6	52177369	52179369	Hoxa9	-0.22134	0.34793	insignificant	-0.0029018	0.7036	insignificant	8	38	38
chr6	52180164	52182164	Mir196b	-0.15342	0.000000696	hypomethylated	0.084409	0.3531	insignificant	11	53	53
chr6	52194241	52196241	Hoxa11as	-0.12668	1.94E-20	hypomethylated	-0.005044	0.71988	insignificant	34	150	148
chr6	52195766	52197766	Hoxa11	-0.15572	1.25E-13	hypomethylated	0.014122	0.4503	insignificant	23	76	80
chr6	52210874	52212874	Hoxa13	-0.16099	0.0044659	hypomethylated	-0.024611	0.16588	insignificant	6	34	35
chr6	52262491	52264491	Evx1	-0.23046	4.05E-11	hypomethylated	-0.0068364	0.91451	insignificant	16	66	66
chr6	52590294	52592294	Hibadh	-0.10765	4.52E-09	hypomethylated	-0.018186	0.28175	insignificant	11	38	38
chr6	52662722	52664722	Tax1bp1	-0.11757	2.19E-23	hypomethylated	-0.01011	0.17382	insignificant	34	118	118
chr6	53018618	53020618	Jazf1	-0.3969	3.97E-23	stronglyHypometh	-0.033675	0.000014546	hypomethylated	9	28	29
chr6	53236288	53238288	9430076C15Rik	-0.14913	8.22E-26	hypomethylated	0.012698	0.26073	insignificant	46	156	157
chr6	53522367	53524367	Creb5	-0.28396	1	lowCoverage	-0.052011	0.33595	insignificant	1	9	8
chr6	53770819	53772819	Tril	-0.28161	2.55E-30	hypomethylated	-0.015952	0.00062857	hypomethylated	22	68	68
chr6	53988925	53990925	Chn2	-0.12168	1.18E-17	hypomethylated	-0.0075278	0.25924	insignificant	39	134	138
chr6	54221815	54223815	Chn2	-0.7021	0.16	lowCoverage	0.13124	0.53581	insignificant	1	5	4
chr6	54276005	54278005	Prr15	-0.36315	0.00014588	stronglyHypometh	-0.14884	0.011802	hypomethylated	4	36	10
chr6	54401876	54403876	Wipf3	-0.41778	0.51188	insignificant	0.048693	0.032421	hypermethylated	4	10	10
chr6	54516376	54518376	Scrn1	-0.23239	7.77E-10	hypomethylated	-0.02588	0.25949	insignificant	6	16	17
chr6	54543122	54545122	Fkbp14	-0.11449	3.57E-08	hypomethylated	-0.016552	1	insignificant	10	26	22
chr6	54544104	54546104	Plekha8	-0.10616	7.13E-24	hypomethylated	-0.0072146	0.83002	insignificant	32	100	101
chr6	54630765	54632765	2410066E13Rik	-0.15288	5.76E-18	hypomethylated	-0.0092114	0.80381	insignificant	37	100	92
chr6	54765909	54767909	Znrf2	-0.086922	9.35E-34	hypomethylated	-0.0024894	0.36648	insignificant	75	194	207
chr6	54921655	54923655	Nod1	-0.11094	0.000000802	hypomethylated	-0.0014428	0.79809	insignificant	14	57	57
chr6	54922606	54924606	Nod1		1	noCoverage	-0.13121	0.90346	insignificant	0	6	5
chr6	54942861	54944861	Ggct	-0.10364	1	insignificant	0.066339	0.46046	insignificant	4	26	28
chr6	54986994	54988994	Gars	-0.069225	0.0023644	hypomethylated	-0.0056828	0.11734	insignificant	14	84	90
chr6	55285292	55287292	Aqp1		1	noCoverage	0.16828	0.00000968	hypermethylated	0	14	17
chr6	55400973	55402973	Adcyap1r1	-0.21671	2.62E-10	hypomethylated	0.031179	0.9508	insignificant	10	39	44
chr6	55966508	55968508	Ppp1r17		1	noCoverage	-0.052482	0.1997	insignificant	0	4	4
chr6	56319628	56321628	Pde1c		1	noCoverage	0.056692	1	insignificant	0	6	4
chr6	56654693	56656693	Lsm5	-0.11352	0.032761	hypomethylated	0.011644	0.61046	insignificant	5	21	18
chr6	56663898	56665898	Avl9	-0.14744	1.68E-31	hypomethylated	0.029476	0.050612	insignificant	33	130	129
chr6	56747807	56749807	Kbtbd2	-0.32533	0.00034081	hypomethylated	-0.024435	0.36625	insignificant	3	12	12
chr6	56781052	56783052	Fkbp9	-0.14136	8.2E-21	hypomethylated	-0.0080277	0.090271	insignificant	22	32	38
chr6	56873926	56875926	Nt5c3	-0.15614	0.00058342	hypomethylated	0.018837	0.35612	insignificant	14	36	36
chr6	57485420	57487420	Ppm1k	0.035786	0.07602	insignificant	-0.014233	0.42176	insignificant	6	28	26
chr6	57642072	57644072	Pigy	-0.3969	1.68E-17	stronglyHypometh	-0.078013	0.00000019	hypomethylated	6	22	20
chr6	57651448	57653448	Lancl2	-0.11104	1.01E-11	hypomethylated	0.0067272	0.9586	insignificant	13	57	57
chr6	59157863	59159863	Tigd2	-0.11087	3.27E-27	hypomethylated	-0.0053486	0.13871	insignificant	33	95	101
chr6	60778990	60780990	Snca	-0.4522	1	lowCoverage	-0.051925	0.10796	insignificant	1	12	10
chr6	61129318	61131318	Fam190a	-0.0925	1.1E-21	hypomethylated	-0.011083	0.15565	insignificant	45	156	166
chr6	63205850	63207850	Grid2	-0.13448	1.47E-13	hypomethylated	0.0024591	0.24583	insignificant	34	153	153
chr6	64678139	64680139	Atoh1	-0.12358	7.59E-25	hypomethylated	0.01244	0.054607	insignificant	26	100	92
chr6	64991660	64993660	Smarcad1	-0.10123	4.19E-10	hypomethylated	-0.019301	0.34115	insignificant	34	103	119
chr6	65620604	65622604	A930038C07Rik	-0.17039	4.55E-18	hypomethylated	0.005158	0.42337	insignificant	23	111	109
chr6	65727955	65729955	Prdm5	-0.15714	7.9E-25	hypomethylated	0.02189	0.93073	insignificant	20	69	61
chr6	65901564	65903564	4930544G11Rik	0.058707	0.86016	insignificant	0.054366	0.389	insignificant	6	30	30
chr6	66484461	66486461	Mad2l1	-0.17147	3.02E-08	hypomethylated	-0.028707	0.0088866	hypomethylated	20	75	74
chr6	66666883	66668883	Vmn1r36		1	noCoverage	-0.090368	1	insignificant	0	4	4
chr6	66845390	66847390	Gng12	-0.084489	3.43E-12	hypomethylated	-0.0063439	0.039992	hypomethylated	34	126	126
chr6	66845884	66847884	Gng12	-0.099612	2.33E-12	hypomethylated	-0.0061487	0.017033	hypomethylated	34	130	130
chr6	66987401	66989401	E230016M11Ril	-0.41238	0.010172	stronglyHypometh	0.087479	0.000016352	hypermethylated	2	21	28
chr6	67215972	67217972	Serbp1	-0.13347	1.69E-25	hypomethylated	-0.0035134	0.92408	insignificant	36	132	131
chr6	67485816	67487816	Tacstd2	-0.15278	0.30619	insignificant	-0.055033	0.87409	insignificant	4	8	8

chr6	69277511	69279511	Rprl1		1	noCoverage	0.13186	0.72908	insignificant	0	14	17
chr6	70742169	70744169	Rpia	-0.30537	1.67E-16	hypomethylated	-0.026189	0.2642	insignificant	11	38	38
chr6	70793520	70795520	Eif2ak3	-0.14324	0.00097936	hypomethylated	-0.0022594	0.40128	insignificant	25	107	113
chr6	70905599	70907599	Foxi3	-0.18376	2.27E-39	hypomethylated	-0.014092	0.00017369	hypomethylated	30	119	115
chr6	71094370	71096370	Thnsl2	0.021896	0.49182	insignificant	-0.076212	0.92981	insignificant	2	13	12
chr6	71148881	71150881	Fabp1		1	noCoverage	-0.049849	0.86948	insignificant	0	6	6
chr6	71221012	71223012	Krcc1	-0.11	0.00032484	hypomethylated	0.016531	0.27113	insignificant	14	82	66
chr6	71271805	71273805	Cd8b1	-0.35768	0.023106	stronglyHypometh	0.052469	0.19892	insignificant	3	11	14
chr6	71442887	71444887	Rnf103	-0.16716	1.12E-37	hypomethylated	-0.023535	0.000023749	hypomethylated	31	146	140
chr6	71492847	71494847	Chmp3	-0.16805	2.05E-21	hypomethylated	-0.039853	0.02512	hypomethylated	17	62	57
chr6	71582680	71584680	Kdm3a	-0.077266	0.61249	insignificant	0.014488	0.78193	insignificant	3	59	59
chr6	71582899	71584899	Kdm3a	-0.087667	0.61347	insignificant	-0.0027927	0.88827	insignificant	3	52	52
chr6	71656853	71658853	Reep1	-0.16822	3.31E-28	hypomethylated	0.022505	0.97214	insignificant	26	73	78
chr6	71780324	71782324	Immt	-0.12097	1.04E-21	hypomethylated	0.035099	0.74229	insignificant	22	117	95
chr6	71858046	71860046	Polr1a	-0.31332	5.72E-08	hypomethylated	0.021859	0.00000347	inconclusive	5	40	37
chr6	71858756	71860756	Ptcd3	-0.32036	2.88E-08	hypomethylated	0.025148	0.00000306	inconclusive	5	35	32
chr6	72046606	72048606	St3gal5	-0.11734	1.84E-12	hypomethylated	-0.019741	0.082247	insignificant	16	70	67
chr6	72185571	72187571	Atoh8		1	noCoverage	-0.064627	0.25871	insignificant	0	8	7
chr6	72295169	72297169	Usp39		1	noCoverage	0.004462	0.30345	insignificant	0	32	29
chr6	72296310	72298310	0610030E20Rik	-0.14845	2.93E-17	hypomethylated	0.019242	0.16262	insignificant	18	76	81
chr6	72304476	72306476	Tmem150a	-0.11447	0.00000232	hypomethylated	-0.0033355	0.023445	inconclusive	32	110	104
chr6	72312375	72314375	Rnf181	-0.020651	0.0033897	inconclusive	0.028355	0.037481	inconclusive	3	18	20
chr6	72330462	72332462	Vamp5	-0.32127	1.31E-09	hypomethylated	-0.23956	2.05E-19	hypomethylated	8	36	26
chr6	72340661	72342661	Vamp8		1	noCoverage	-0.1502	0.033582	hypomethylated	0	10	11
chr6	72363326	72365326	Ggcx	-0.18517	0.000020819	hypomethylated	-0.048388	0.0091854	hypomethylated	3	26	22
chr6	72389552	72391552	Mat2a	-0.11408	0.0000062	hypomethylated	-0.010639	1	insignificant	13	122	122
chr6	72493432	72495432	Capg		1	noCoverage	-0.083696	0.043552	hypomethylated	0	20	20
chr6	72498442	72500442	Capg	-0.041547	0.0017457	inconclusive	0.071217	0.000054722	hypermethylated	2	16	16
chr6	72566742	72568742	Tgoln1	-0.062986	7.68E-08	hypomethylated	-0.034367	0.10842	insignificant	11	39	34
chr6	72566994	72568994	Tgoln1	-0.062158	0.02028	hypomethylated	-0.13481	0.00073915	hypomethylated	9	18	14
chr6	72738950	72740950	Tcf7l1	-0.15868	6.53E-17	hypomethylated	-0.017133	0.027257	hypomethylated	25	76	78
chr6	72849973	72851973	Kcmf1	-0.087267	1	insignificant	-0.053378	0.83041	insignificant	24	100	97
chr6	72908226	72910226	Tmsb10	-0.10604	0.0212	hypomethylated	0.019362	0.59277	insignificant	4	18	18
chr6	72908477	72910477	Tmsb10	-0.34583	0.40889	insignificant	0.070445	0.39159	insignificant	2	6	6
chr6	72908742	72910742	Tmsb10	-0.34583	0.40889	insignificant	0.070445	0.39159	insignificant	2	6	6
chr6	73171625	73173625	Dnahc6	-0.1835	0.20761	insignificant	0.011635	0.49479	insignificant	6	14	12
chr6	73197498	73199498	Suclg1	-0.21328	2.79E-14	hypomethylated	-0.0078046	0.049511	hypomethylated	22	78	76
chr6	77191710	77193710	Lrrtm1	-0.18096	8.76E-24	hypomethylated	-0.019526	0.0070521	hypomethylated	23	70	68
chr6	77929661	77931661	Ctnna2	-0.17943	0.016448	hypomethylated	-0.0047625	0.56794	insignificant	7	41	39
chr6	79968636	79970636	Lrrtm4		1	noCoverage	-0.02963	0.82325	insignificant	0	9	9
chr6	81872662	81874662	AW146020	-0.12523	3.97E-23	hypomethylated	0.002489	0.39642	insignificant	30	82	82
chr6	81915943	81917943	Mrpl19	-0.26316	0.09376	insignificant	0.033717	1	insignificant	2	4	4
chr6	81990621	81992621	Fam176a	0.049234	0.69065	insignificant	-0.011045	0.53582	insignificant	5	51	55
chr6	82351468	82353468	Tacr1	-0.21922	2.04E-10	hypomethylated	-0.0031801	0.41835	insignificant	21	58	58
chr6	82602859	82604859	Pole4	-0.15022	1.15E-14	hypomethylated	0.035882	0.33256	insignificant	20	54	59
chr6	82724448	82726448	Hk2	-0.56261	0.125	insignificant	-0.33448	1	insignificant	2	10	19
chr6	82889744	82891744	Sema4f	-0.28194	1	lowCoverage	-0.0056811	0.53965	insignificant	1	4	4
chr6	82983217	82985217	Loxl3	-0.10015	1.43E-08	hypomethylated	0.0065683	0.46837	insignificant	13	55	55
chr6	82983465	82985465	Dok1	-0.18682	0.000000028	hypomethylated	0.03049	0.67476	insignificant	7	33	32
chr6	83003646	83005646	Aup1	-0.13499	4.29E-67	hypomethylated	-0.010166	0.47716	insignificant	57	179	170
chr6	83004565	83006565	Aup1	-0.13692	6.41E-42	hypomethylated	-0.002454	0.43611	insignificant	40	127	122
chr6	83020219	83022219	Tlx2	-0.10709	3.69E-20	hypomethylated	0.010881	0.5198	insignificant	25	84	89
chr6	83027383	83029383	Pcgf1	-0.12489	2.52E-14	hypomethylated	-0.0088716	0.65897	insignificant	20	82	83
chr6	83035358	83037358	Lbx2	-0.24705	8.61E-08	hypomethylated	-0.038573	0.16033	insignificant	6	16	16
chr6	83050509	83052509	Ccdc142	-0.13165	7.37E-43	hypomethylated	-0.032528	0.0018091	hypomethylated	33	110	128
chr6	83058101	83060101	Mrpl53	-0.12161	4.72E-13	hypomethylated	-0.0071151	0.68649	insignificant	19	48	56
chr6	83064499	83066499	Mogs	-0.11438	1.05E-25	hypomethylated	-0.011496	0.0054946	hypomethylated	38	130	140
chr6	83071455	83073455	Wbp1	-0.13339	2.04E-25	hypomethylated	0.031834	0.34358	insignificant	22	75	61
chr6	83075023	83077023	Ino80b	-0.17987	0.00017474	hypomethylated	-0.019091	0.83507	insignificant	9	28	28
chr6	83084801	83086801	Rtkn	-0.21401	2.86E-10	hypomethylated	0.0065704	0.74091	insignificant	16	53	54
chr6	83086077	83088077	Rtkn	-0.25595	0.58451	insignificant	0.092066	0.78482	insignificant	1	12	13
chr6	83105397	83107397	1700003E16Rik		1	noCoverage	0.099996	0.27827	insignificant	0	4	6
chr6	83106373	83108373	Wdr54		1	noCoverage	-0.03965	0.53336	insignificant	0	8	6
chr6	83114917	83116917	Dctn1		1	noCoverage	-0.0040105	0.25155	insignificant	0	49	49
chr6	83128582	83130582	Dctn1	0.073034	0.77512	insignificant	-0.020372	0.59088	insignificant	2	6	6
chr6	83186368	83188368	Slc4a5		1	noCoverage	-0.078205	0.83238	insignificant	0	6	6
chr6	83267598	83269598	Mthfd2	-0.1295	0.00034285	hypomethylated	0.001218	0.28728	insignificant	5	10	10
chr6	83275032	83277032	Mob1a	-0.090734	1.01E-16	hypomethylated	0.0053462	0.55782	insignificant	21	74	73
chr6	83298477	83300477	Bola3	-0.17597	1.24E-27	hypomethylated	-0.0060681	0.038091	hypomethylated	23	64	59
chr6	83390748	83392748	B230319C09Rik	-0.17512	6.74E-24	hypomethylated	-0.02426	0.004555	hypomethylated	30	82	82
chr6	83391672	83393672	Tet3	-0.23776	0.00016905	hypomethylated	-0.092837	0.0002937	hypomethylated	11	36	34
chr6	83660257	83662257	Vax2	-0.18201	0.000000297	hypomethylated	-0.018137	0.34227	insignificant	14	50	44
chr6	83660926	83662926	Vax2os2	-0.2004	0.00000208	hypomethylated	-0.027945	0.060738	insignificant	8	32	24
chr6	83662195	83664195	Vax2os2	-0.39982	0.016891	stronglyHypometh	-0.13173	0.039259	hypomethylated	2	18	11
chr6	83711639	83713639	Ankrd53	-0.23313	0.075805	insignificant	-0.040014	0.74674	insignificant	4	10	11
chr6	83725806	83727806	Tex261	-0.07169	0.2437	insignificant	-0.0064602	0.73733	insignificant	7	19	19
chr6	83744050	83746050	Nagk	-0.10956	0.000000334	hypomethylated	0.023985	0.72276	insignificant	16	102	87
chr6	83744151	83746151	Nagk	-0.1614	0.000000131	hypomethylated	0.0168	0.73093	insignificant	16	112	95
chr6	83781735	83783735	Paip2b	-0.25605	0.000000159	hypomethylated	-0.0010226	0.0063758	hypomethylated	5	42	42
chr6	83863346	83865346	Zfml	-0.079664	3.39E-20	hypomethylated	-0.0021275	0.4058	insignificant	22	99	97
chr6	83957583	83959583	Dysf	-0.14583	0.000014871	hypomethylated	0.010547	4.14E-08	inconclusive	10	55	47
chr6	85019507	85021507	Exoc6b	-0.36948	0.3769	insignificant	0.020316	0.17981	insignificant	3	8	8
chr6	85025134	85027134	Npm3-ps1	-0.38472	0.24051	insignificant	0.0013945	0.47676	insignificant	2	37	34
chr6	85076088	85078088	Gm5878	-0.14145	0.00080364	hypomethylated	0.016979	0.00015979	inconclusive	13	45	45
chr6	85087758	85089758	Spr		1	noCoverage	0.030769	1	insignificant	0	4	4

chr6	85136924	85138924	Emx1	-0.032504	0.0033379	hypomethylated	-0.0054514	0.23002	insignificant	20	92	90
chr6	85324628	85326628	Rab11fip5	-0.1213	0.000012473	hypomethylated	0.0029363	0.10813	insignificant	9	50	50
chr6	85372879	85374879	Noto	-0.28476	0.000027259	hypomethylated	-0.026106	0.054737	insignificant	7	30	30
chr6	85380969	85382969	Smyd5	-0.1128	1.09E-10	hypomethylated	-0.023482	0.865	insignificant	21	38	54
chr6	85400498	85402498	Cct7	-0.10524	1.97E-14	hypomethylated	-0.0026035	0.016905	hypomethylated	20	163	158
chr6	85401296	85403296	1700040I03Rik	-0.093974	4.3E-09	hypomethylated	-0.011902	0.058346	insignificant	16	111	118
chr6	85401964	85403964	1700040I03Rik		1	noCoverage	-0.008795	0.82426	insignificant	0	29	36
chr6	85452880	85454880	Fbxo41	-0.22222	1.2E-09	hypomethylated	-0.12196	0.13263	insignificant	9	27	24
chr6	85463536	85465536	Egr4	-0.13768	9.74E-10	hypomethylated	-0.018978	0.77711	insignificant	19	68	68
chr6	85536524	85538524	Alms1	-0.11692	0.0011969	hypomethylated	-0.0045418	0.47931	insignificant	2	30	28
chr6	85819131	85821131	Cml2	-0.90455	0.19444	lowCoverage	-0.23093	0.38664	insignificant	1	6	6
chr6	85864712	85866712	Tprkb	-0.12289	1.53E-16	hypomethylated	0.027334	0.12707	insignificant	21	101	81
chr6	85865671	85867671	Cml1	-0.10724	2.54E-15	hypomethylated	0.033704	0.20997	insignificant	20	82	62
chr6	85911661	85913661	Dusp11	-0.30973	0.0011935	hypomethylated	-0.050022	2.02E-09	hypomethylated	6	35	38
chr6	85966184	85968184	Figla	-0.40461	0.27304	insignificant	-0.15975	0.40949	insignificant	2	24	24
chr6	86144244	86146244	Tgfa	-0.15124	1.44E-18	hypomethylated	-0.02022	0.000000894	hypomethylated	44	132	143
chr6	86314676	86316676	Fam136a	-0.079226	0.83488	insignificant	-0.011751	0.13939	insignificant	32	104	107
chr6	86320533	86322533	Snrpg	-0.14987	1.98E-13	hypomethylated	-0.0073656	0.63219	insignificant	8	51	57
chr6	86353212	86355212	Tia1	-0.25478	2.04E-17	hypomethylated	-0.031262	0.0083265	hypomethylated	19	72	64
chr6	86387391	86389391	C87436	-0.21339	2.47E-21	hypomethylated	-0.026849	0.87636	insignificant	12	77	80
chr6	86387639	86389639	C87436	-0.21708	8.67E-22	hypomethylated	-0.022979	1	insignificant	12	81	82
chr6	86476159	86478159	Pcbp1	-0.09797	1.04E-26	hypomethylated	-0.0062002	0.029469	hypomethylated	56	200	202
chr6	86577167	86579167	Asprv1	0.094556	0.26969	insignificant	-0.0052111	0.54407	insignificant	2	21	21
chr6	86619153	86621153	Mxd1	-0.13124	5.18E-19	hypomethylated	-0.0093815	0.12226	insignificant	35	107	107
chr6	86683372	86685372	Gmcl1	-0.19763	0.00000119	hypomethylated	-0.013708	0.12594	insignificant	15	56	57
chr6	86743578	86745578	Anxa4	-0.47111	0.033598	stronglyHypometh	-0.099683	0.52125	insignificant	1	4	5
chr6	86798510	86800510	Aak1	-0.12434	6.12E-24	hypomethylated	-0.0043366	0.010304	hypomethylated	39	121	103
chr6	86799434	86801434	2610306M01Ril	-0.128	8.66E-22	hypomethylated	-0.0096365	0.0113	hypomethylated	36	89	87
chr6	86958829	86960829	Nfu1	-0.11504	0.00000188	hypomethylated	-0.0047241	0.53944	insignificant	13	40	41
chr6	86991839	86993839	Gfpt1	-0.12993	1.72E-12	hypomethylated	-0.025145	0.39225	insignificant	19	97	104
chr6	87300909	87302909	Gkn1	0.017008	1	insignificant	-0.0068012	0.8117	insignificant	4	8	8
chr6	87322358	87324358	Gkn2	0.2013	0.31033	insignificant	-0.012392	0.10297	insignificant	2	14	14
chr6	87377995	87379995	Bmp10	0.015924	1	insignificant	-0.0083124	0.28918	insignificant	6	32	31
chr6	87446294	87448294	Arhgap25		1	noCoverage	-0.014853	0.82932	insignificant	0	5	6
chr6	87540695	87542695	Prokr1	-0.075556	0.40983	insignificant	0.0526	1	insignificant	2	25	24
chr6	87622162	87624162	Aplf	-0.17157	1	insignificant	0.22107	1	insignificant	2	6	8
chr6	87679862	87681862	Ccdc48	-0.073666	9.27E-10	hypomethylated	0.004988	0.025682	hypermethylated	20	83	80
chr6	87759748	87761748	Rab43	-0.12399	4.27E-37	hypomethylated	-0.012137	0.54324	insignificant	42	137	137
chr6	87761773	87763773	Rab43	-0.19977	0.000041939	hypomethylated	0.17428	0.61343	insignificant	6	17	24
chr6	87788753	87790753	Isy1	-0.66903	0.017785	stronglyHypometh	-0.028621	0.086498	insignificant	1	15	15
chr6	87801100	87803100	Cnbp	-0.0984	0.000067602	hypomethylated	-0.0036722	0.77013	insignificant	10	24	24
chr6	87836933	87838933	Copg		1	noCoverage	-0.006265	0.46182	insignificant	0	20	20
chr6	87836965	87838965	Copg		1	noCoverage	-0.006265	0.46182	insignificant	0	20	20
chr6	87862969	87864969	8430410A17Rik	-0.11211	4.17E-11	hypomethylated	-0.0074167	0.6434	insignificant	11	73	72
chr6	87930676	87932676	Gm5577	-0.057792	1.79E-11	hypomethylated	-0.0063512	0.04681	hypomethylated	48	196	204
chr6	87931476	87933476	H1fx	0.010231	0.33342	insignificant	0.0047533	0.14517	insignificant	16	71	71
chr6	87963684	87965684	Rab7	0.17847	0.28123	insignificant	-0.00066435	0.87938	insignificant	3	16	23
chr6	88033466	88035466	Rpn1	-0.11549	1.84E-20	hypomethylated	-0.00072489	0.84779	insignificant	16	82	80
chr6	88147657	88149657	Gata2	-0.16093	1.68E-25	hypomethylated	-0.026331	0.067205	insignificant	25	96	109
chr6	88171261	88173261	Dnajb8	0.095102	0.71632	insignificant	-0.012213	0.72667	insignificant	9	41	41
chr6	88396533	88398533	Eefsec	-0.10313	1	insignificant	-0.043641	0.45577	insignificant	10	36	30
chr6	88468794	88470794	Sec61a1	-0.046829	0.078699	insignificant	-0.0060858	0.57292	insignificant	16	38	43
chr6	88577439	88579439	Kbtbd12	-0.18259	0.023843	hypomethylated	0.05291	0.81332	insignificant	1	19	20
chr6	88673405	88675405	Mgll	-0.090546	1.89E-24	hypomethylated	0.00055238	0.013177	inconclusive	40	134	126
chr6	88673697	88675697	Mgll	-0.090546	1.89E-24	hypomethylated	0.00055238	0.013177	inconclusive	40	134	126
chr6	88791929	88793929	Podxl2	-0.11373	1.29E-09	hypomethylated	-0.006299	0.00000535	hypomethylated	28	105	105
chr6	88824038	88826038	Podxl2	-0.12843	4.15E-40	hypomethylated	-0.0089517	1.2E-15	hypomethylated	51	163	179
chr6	88848774	88850774	Mcm2		1	noCoverage	0.020481	0.62082	insignificant	0	12	13
chr6	88851244	88853244	Tpra1	-0.022339	0.000011066	hypomethylated	0.010254	0.054412	insignificant	12	67	69
chr6	89045108	89047108	4933427D06Rik		1	noCoverage	0.069429	0.44138	insignificant	0	6	7
chr6	89312607	89314607	Plxna1	-0.014601	0.00007069	hypomethylated	0.014884	0.088896	insignificant	22	65	70
chr6	89592981	89594981	Txnrd3	-0.11781	1.36E-43	hypomethylated	-0.015794	0.022521	hypomethylated	42	124	124
chr6	89695475	89697475	Vmn1r41	-0.825	8.33E-10	stronglyHypometh	-0.15207	0.53711	insignificant	2	4	4
chr6	89795531	89797531	Vmn1r42	-0.19318	1	insignificant	0.0091991	1	insignificant	1	4	4
chr6	90275179	90277179	Chst13	0.080994	0.10843	insignificant	-0.066274	0.011778	hypomethylated	3	4	4
chr6	90318487	90320487	Zxdc	-0.10611	5.67E-29	hypomethylated	-0.012855	0.059581	insignificant	51	154	164
chr6	90378474	90380474	Ccdc37	-0.21946	0.0039415	hypomethylated	0.068144	0.000050032	inconclusive	13	34	37
chr6	90411619	90413619	Klf15	-0.10088	0.000003038	hypomethylated	-0.0013423	0.59249	insignificant	33	127	131
chr6	90499841	90501841	Aldh1l1	-0.19133	0.000047767	hypomethylated	-0.0068247	0.15528	insignificant	10	35	35
chr6	90553873	90555873	Slc41a3	0.24359	0.23823	insignificant	-0.053477	0.92592	insignificant	3	22	22
chr6	90568209	90570209	Slc41a3	0.0086611	0.8263	insignificant	-0.084011	0.36715	insignificant	7	30	30
chr6	90666523	90668523	Iqsec1	-0.25	0.010861	hypomethylated	-0.089183	0.47386	insignificant	6	6	13
chr6	90760117	90762117	Iqsec1	-0.31071	1	lowCoverage	0.24594	0.012693	hypermethylated	1	4	4
chr6	91066820	91068820	Nup210	-0.11103	1.38E-30	hypomethylated	-0.040239	0.000000472	hypomethylated	20	54	61
chr6	91105808	91107808	Hdac11	-0.21538	0.20005	insignificant	0.063836	1	insignificant	1	10	11
chr6	91161757	91163757	Fbln2	-0.14385	1.63E-08	hypomethylated	0.0019125	0.29443	insignificant	19	52	52
chr6	91422744	91424744	Tmem43	-0.13139	4.15E-11	hypomethylated	0.0042534	0.10078	insignificant	17	83	83
chr6	91423417	91425417	Chchd4	-0.10793	7.25E-09	hypomethylated	0.0025896	0.032428	inconclusive	8	55	55
chr6	91465028	91467028	Lsm3	-0.1159	6.35E-12	hypomethylated	-0.012161	0.058521	insignificant	23	102	104
chr6	91465882	91467882	Lsm3	-0.27157	0.030337	hypomethylated	-0.030022	0.000000168	hypomethylated	4	52	52
chr6	91633060	91635060	Slc6a6	-0.096519	1.67E-12	hypomethylated	0.010582	0.57047	insignificant	26	73	82
chr6	91827046	91829046	C130022K22Rik	-0.15183	2.17E-11	hypomethylated	-0.011352	0.23636	insignificant	9	58	56
chr6	91936103	91938103	Fgd5	0.080879	0.78541	insignificant	0.02581	0.39187	insignificant	4	16	16
chr6	92040411	92042411	Nr2c2	-0.1106	2.98E-54	hypomethylated	0.014904	0.67908	insignificant	69	235	247
chr6	92134017	92136017	Mrps25	-0.14218	0.000000371	hypomethylated	-0.0027007	1	insignificant	8	16	16

chr6	92164805	92166805	Zfyve20	-0.15063	0.23547	insignificant	-0.0055067	0.50438	insignificant	3	24	24
chr6	92194642	92196642	Trh	-0.090078	0.020116	hypomethylated	-0.0058528	0.00013157	hypomethylated	15	46	43
chr6	92431386	92433386	Prickle2		1	noCoverage	-0.19475	0.052259	insignificant	0	4	4
chr6	92484855	92486855	Prickle2		1	noCoverage	-0.075312	0.82694	insignificant	0	4	4
chr6	92656178	92658178	Prickle2	-0.17262	0.000014937	hypomethylated	0.067643	0.81877	insignificant	4	8	9
chr6	92889575	92891575	9530026P05Rik	-0.36126	0.055071	insignificant	0.0015392	1	insignificant	4	9	9
chr6	94233898	94235898	Magi1	-0.17364	0.00063149	hypomethylated	0.023369	0.32663	insignificant	10	36	27
chr6	94449307	94451307	Slc25a26	-0.16756	0.00000295	hypomethylated	-0.026846	0.33013	insignificant	14	41	41
chr6	94650139	94652139	Lrig1	-0.073064	0.0047748	hypomethylated	0.011101	0.88346	insignificant	8	27	27
chr6	95066899	95068899	Kbtbd8	-0.085668	2.36E-16	hypomethylated	-0.012763	0.0045723	hypomethylated	25	99	100
chr6	96062147	96064147	Fam19a1	0.16667	1	insignificant	-0.024784	0.31066	insignificant	2	10	11
chr6	97098877	97100877	A130022J15Rik	-0.3416	0.00000189	stronglyHypometh	-0.0022032	0.51272	insignificant	8	20	20
chr6	97129118	97131118	Tmf1	-0.10398	4.58E-13	hypomethylated	-0.0019543	0.56194	insignificant	14	48	48
chr6	97159785	97161785	Arl6ip5	-0.24416	0.002854	hypomethylated	-0.0013224	1	insignificant	2	14	14
chr6	97202774	97204774	Lmod3	0.089865	0.38492	insignificant	-0.061369	0.12368	insignificant	7	15	15
chr6	97567651	97569651	Frmd4b	-0.37048	0.0027891	stronglyHypometh	0.04027	0.0051509	hypermethylated	0	14	20
chr6	97756051	97758051	Mitf	-0.13589	1.02E-08	hypomethylated	0.0021401	0.92718	insignificant	19	57	56
chr6	97878810	97880810	Mitf	0.51365	1	lowCoverage	0.31074	0.052658	insignificant	1	9	10
chr6	98978292	98980292	Foxp1	-0.068214	0.02272	hypomethylated	0.0026377	0.1955	insignificant	20	76	76
chr6	99385339	99387339	Foxp1		1	noCoverage	-0.018182	0.48761	insignificant	0	2	3
chr6	99616765	99618765	Eif4e3	-0.10769	0.000053902	hypomethylated	0.095911	0.65302	insignificant	10	13	15
chr6	99641672	99643672	Gpr27	-0.069086	3.61E-59	hypomethylated	0.0063914	0.051986	insignificant	90	324	323
chr6	99676386	99678386	Prok2		1	noCoverage	0.081029	0.87291	insignificant	0	10	11
chr6	100237352	100239352	Rybp	-0.13597	2.66E-19	hypomethylated	-0.026582	0.23758	insignificant	40	146	138
chr6	100621151	100623151	Shq1	-0.28111	0.079512	insignificant	-0.088803	0.66198	insignificant	4	15	13
chr6	100653727	100655727	Gxylt2	-0.12499	1.3E-14	hypomethylated	-0.031567	0.077646	insignificant	17	61	73
chr6	100782631	100784631	Ppp4r2	-0.090752	5.64E-12	hypomethylated	-0.0092752	0.091679	insignificant	30	125	135
chr6	101327891	101329891	Pdzrn3	-0.24079	0.000000445	hypomethylated	-0.043533	0.9476	insignificant	8	28	28
chr6	103459869	103461869	Chl1	-0.17612	1	insignificant	0.00039683	0.83589	insignificant	3	22	22
chr6	105626738	105628738	Cntn4	-0.19089	0.075186	insignificant	0.030804	0.38151	insignificant	5	40	25
chr6	106067758	106069758	Cntn4		1	noCoverage	0.14286	0.1588	insignificant	0	2	2
chr6	106718165	106720165	Trnt1	-0.13454	2.17E-21	hypomethylated	0.025438	0.45435	insignificant	19	54	46
chr6	106750068	106752068	Crbn	-0.25146	0.000017521	hypomethylated	0.0048716	0.88178	insignificant	8	4	4
chr6	107478778	107480778	Lrrn1	-0.21093	0.00024591	hypomethylated	-0.13511	0.2336	insignificant	11	48	57
chr6	108014039	108016039	Setmar	-0.23649	0.59351	insignificant	-0.089055	0.73227	insignificant	2	18	17
chr6	108162089	108164089	Itpr1	-0.19153	4.35E-22	hypomethylated	0.008426	0.063855	insignificant	21	70	61
chr6	108609622	108611622	Bhlhe40	-0.16184	5.98E-12	hypomethylated	0.0028003	0.8265	insignificant	11	50	50
chr6	108732052	108734052	Arl8b	-0.079975	0.000000066	hypomethylated	0.0059239	0.63069	insignificant	31	92	90
chr6	108777634	108779634	Edem1	-0.12602	3.8E-22	hypomethylated	-0.020169	0.0047439	hypomethylated	45	135	141
chr6	110594591	110596591	Grm7	-0.1917	0.000000026	hypomethylated	-0.019159	0.84704	insignificant	19	94	95
chr6	112222751	112224751	Lmcd1		1	noCoverage	0.024021	0.25194	insignificant	0	23	22
chr6	112408498	112410498	Cav3		1	noCoverage	0.021136	0.080811	insignificant	0	11	16
chr6	112439802	112441802	Oxtr	-0.13935	7.69E-10	hypomethylated	0.0057028	0.32719	insignificant	6	28	28
chr6	112897260	112899260	Srgap3	-0.1788	1.05E-11	hypomethylated	-0.03303	0.000000157	hypomethylated	4	10	10
chr6	112995320	112997320	Thumpd3	-0.16748	2.98E-19	hypomethylated	-0.0024162	0.63621	insignificant	18	46	46
chr6	113026632	113028632	Setd5	-0.11863	1.22E-39	hypomethylated	-0.0058202	0.1204	insignificant	46	165	159
chr6	113027238	113029238	Gt(ROSA)26Sor	-0.12908	5.07E-11	hypomethylated	0.0001032	0.025093	inconclusive	27	93	94
chr6	113145378	113147378	Lhfpl4		1	noCoverage	-0.0811	0.91322	insignificant	0	18	16
chr6	113186836	113188836	Mtmr14	-0.18038	1.6E-26	hypomethylated	-0.021454	0.1706	insignificant	28	87	96
chr6	113231300	113233300	Cpne9	-0.28021	1.95E-36	hypomethylated	-0.011874	0.000011466	hypomethylated	29	85	90
chr6	113256190	113258190	Brpf1	-0.144	1.62E-25	hypomethylated	0.00024298	0.021049	inconclusive	28	125	119
chr6	113275969	113277969	Ogg1	-0.24305	3.01E-30	hypomethylated	-0.025008	0.97041	insignificant	20	81	89
chr6	113327106	113329106	Arpc4	-0.19647	1.76E-21	hypomethylated	-0.023836	0.89549	insignificant	25	91	88
chr6	113327514	113329514	Tada3	-0.19891	1.71E-22	hypomethylated	-0.019675	0.89845	insignificant	28	95	96
chr6	113338253	113340253	Ttll3	-0.02761	1	insignificant	0.066991	0.24615	insignificant	7	29	26
chr6	113341490	113343490	Ttll3	-0.18947	1.1E-33	hypomethylated	-0.005054	0.040763	hypomethylated	21	84	85
chr6	113369334	113371334	Rpusd3	-0.20793	0.00068561	hypomethylated	-0.056906	0.12744	insignificant	5	34	35
chr6	113385749	113387749	Cidec	0.21915	1	insignificant	-0.035147	0.24146	insignificant	4	24	24
chr6	113391628	113393628	Jagn1	-0.14293	1.25E-18	hypomethylated	0.00078252	0.20292	insignificant	21	56	56
chr6	113407477	113409477	Il17re		1	noCoverage	-0.21561	0.87197	insignificant	0	9	6
chr6	113407888	113409888	Il17re		1	noCoverage	-0.21561	0.87197	insignificant	0	9	6
chr6	113420448	113422448	Il17rc	0.042251	1	insignificant	-0.028124	0.2714	insignificant	2	25	24
chr6	113432562	113434562	Creld1	-0.21053	2.23E-26	hypomethylated	0.0034873	0.0067008	inconclusive	27	119	112
chr6	113451812	113453812	Prrt3	-0.21074	9.66E-09	hypomethylated	0.13135	1.44E-10	hypermethylated	8	16	16
chr6	113480675	113482675	Fancd2	-0.097718	0.00020249	hypomethylated	0.010589	0.30923	insignificant	6	46	52
chr6	113481632	113483632	Tmem111	-0.15198	0.099743	insignificant	-0.067255	0.71582	insignificant	1	28	26
chr6	113550709	113552709	4931417G12Rik	-0.76561	0.11765	lowCoverage	-0.04112	0.03939	hypomethylated	1	15	21
chr6	113553765	113555765	6720456B07Rik	-0.1829	9.22E-12	hypomethylated	0.0033455	0.89771	insignificant	23	119	120
chr6	113573014	113575014	Vhl	-0.15886	1.53E-29	hypomethylated	-0.011901	0.000010867	hypomethylated	29	112	112
chr6	113587460	113589460	Irak2	-0.55497	0.25169	insignificant	0.063285	0.237	insignificant	3	39	40
chr6	113646492	113648492	Tatdn2	-0.079031	3.31E-20	hypomethylated	-0.0033423	0.10787	insignificant	46	189	188
chr6	113690675	113692675	Sec13		1	noCoverage	0.097619	0.60401	insignificant	0	12	12
chr6	113841370	113843370	Atp2b2	-0.11903	0.055249	insignificant	0.014036	0.00016995	inconclusive	5	36	36
chr6	114080234	114082234	Slc6a11	-0.13934	2.89E-08	hypomethylated	0.028071	0.26513	insignificant	8	16	16
chr6	114231628	114233628	Slc6a1	-0.13885	0.00000599	hypomethylated	0.01513	0.64538	insignificant	16	49	49
chr6	114346929	114348929	Hrh1	-0.14786	2.66E-11	hypomethylated	0.012406	0.0020143	inconclusive	26	92	87
chr6	114592142	114594142	Atg7	-0.2449	5.31E-16	hypomethylated	-0.021537	0.0085465	hypomethylated	16	39	39
chr6	114987892	114989892	1500001M20Ril	-0.075203	3.95E-09	hypomethylated	0.053544	0.41529	insignificant	11	27	21
chr6	115083919	115085919	Syn2	-0.10106	2.04E-24	hypomethylated	0.0057519	0.66317	insignificant	31	109	107
chr6	115201867	115203867	Timp4	0.13684	1	insignificant	-0.17003	0.37091	insignificant	2	6	6
chr6	115310238	115312238	Pparg	-0.071085	4.28E-18	hypomethylated	-0.0076425	0.0032642	hypomethylated	31	130	116
chr6	115493721	115495721	Tsen2	-0.15097	0.16296	insignificant	-0.058099	0.61492	insignificant	8	27	27
chr6	115550955	115552955	Mkrn2	-0.15476	7.71E-08	hypomethylated	-0.0097542	0.71573	insignificant	9	21	21
chr6	115626653	115628653	Raf1	-0.23581	1.28E-08	hypomethylated	-0.013122	0.11731	insignificant	10	34	34
chr6	115709017	115711017	Tmem40		1	noCoverage	-0.1719	0.32769	insignificant	0	7	7

chr6	115723574	115725574	Cand2	-0.23886	0.00050321	hypomethylated	-0.067986	0.28953	insignificant	6	31	26
chr6	115758121	115760121	Rpl32	-0.1964	1.03E-32	hypomethylated	0.0071543	0.65708	insignificant	17	93	94
chr6	115758761	115760761	Rpl32		1	noCoverage	-0.0026303	0.019417	inconclusive	0	20	21
chr6	115802545	115804545	Ift122	-0.13744	4.68E-29	hypomethylated	0.0042357	0.0016754	inconclusive	16	78	76
chr6	115803359	115805359	Mbd4	-0.097723	6.71E-16	hypomethylated	0.02404	0.63056	insignificant	9	52	52
chr6	115880944	115882944	Rho	-0.59216	0.0030032	stronglyHypometh	0.021903	0.78764	insignificant	3	38	28
chr6	115945023	115947023	Plxnd1	-0.19076	2.9E-09	hypomethylated	-0.0050457	0.018866	hypomethylated	12	32	30
chr6	116143392	116145392	Tmcc1	-0.091837	0.00021854	hypomethylated	0.012925	0.38089	insignificant	13	28	28
chr6	116157050	116159050	D6Wsu116e	-0.329	5.03E-14	hypomethylated	-0.027311	0.39766	insignificant	12	42	36
chr6	116213252	116215252	Anubl1	-0.12443	3.16E-19	hypomethylated	0.022303	0.17353	insignificant	46	116	115
chr6	116287140	116289140	March8	-0.10005	2.46E-16	hypomethylated	-0.0060225	0.5045	insignificant	44	106	105
chr6	116411196	116413196	Alox5	-0.29054	1	insignificant	0.20946	0.48835	insignificant	2	4	2
chr6	116455533	116457533	Olfr212		1	noCoverage	0.02185	0.016153	hypermethylated	0	12	13
chr6	116532962	116534962	Olfr215	0.088312	1	insignificant	-0.054545	0.70489	insignificant	1	2	2
chr6	116599701	116601701	8430408G22Rik	-0.16515	1	insignificant	0.34938	0.0070549	stronglyhypermeth	3	12	8
chr6	116623854	116625854	Rassf4	-0.12387	0.71078	insignificant	0.030698	0.29205	insignificant	1	20	20
chr6	116666776	116668776	Tmem72	-0.080013	0.1042	insignificant	-0.075671	0.30104	insignificant	6	15	15
chr6	117117552	117119552	Cxcl12	-0.10842	1.35E-13	hypomethylated	0.0087443	0.22621	insignificant	42	128	128
chr6	117790259	117792259	Zfp637	-0.21715	2.07E-19	hypomethylated	-0.010515	0.0074762	hypomethylated	16	64	62
chr6	117812094	117814094	Zfp239	-0.21725	0.0007194	hypomethylated	0.24218	0.39498	insignificant	4	10	14
chr6	117849357	117851357	Hnrnpf	-0.37985	0.2636	insignificant	0.062155	0.03796	inconclusive	1	7	6
chr6	117855799	117857799	Hnrnpf	-0.11108	6.03E-53	hypomethylated	0.0062378	0.32034	insignificant	66	194	194
chr6	117856821	117858821	Hnrnpf	-0.11083	1.66E-49	hypomethylated	0.00166	0.79804	insignificant	71	214	214
chr6	117866311	117868311	Hnrnpf	-0.20273	5.54E-12	hypomethylated	-0.029688	0.20806	insignificant	16	79	92
chr6	117887353	117889353	Fxyd4		1	noCoverage	-0.011111	1	insignificant	0	8	8
chr6	118015402	118017402	Rasgef1a	-0.25259	0.34058	insignificant	-0.016434	1	insignificant	4	16	16
chr6	118089158	118091158	Csgalnact2		1	noCoverage	-0.10936	0.78853	insignificant	0	8	7
chr6	118147762	118149762	Ret	0.15455	0.35266	insignificant	0.092641	1	insignificant	2	10	10
chr6	118369435	118371435	Bms1	-0.13211	0.006268	hypomethylated	0.0028647	0.037739	hypermethylated	1	18	18
chr6	118429291	118431291	Zfp9	-0.07763	0.0053384	inconclusive	-0.12313	0.78741	insignificant	3	12	10
chr6	118512274	118514274	Ankrd26	-0.17668	0.014193	hypomethylated	-0.024855	0.94259	insignificant	7	20	22
chr6	119058207	119060207	Cacna1c	-0.069189	8.36E-22	hypomethylated	0.010687	0.79087	insignificant	31	159	154
chr6	119124270	119126270	Dcp1b	-0.21986	0.000000323	hypomethylated	0.0045927	0.74576	insignificant	9	52	52
chr6	119146427	119148427	Dcp1b		1	noCoverage	-0.12784	0.73997	insignificant	0	3	6
chr6	119279222	119281222	Cacna2d4	-0.10151	0.00000291	hypomethylated	0.021939	0.3526	insignificant	9	54	53
chr6	119280784	119282784	Cacna2d4	0.31329	0.34116	insignificant	0.061196	0.45068	insignificant	2	24	24
chr6	119428685	119430685	Fbxl14	-0.072051	8.86E-11	hypomethylated	-0.0082681	0.01719	hypomethylated	73	238	235
chr6	119494365	119496365	Wnt5b	-0.17307	0.000045591	hypomethylated	0.0014866	0.040344	inconclusive	19	49	49
chr6	119797210	119799210	3110021A11Rik	-0.10075	5.44E-29	hypomethylated	-0.019486	0.23043	insignificant	47	136	142
chr6	119798168	119800168	Erc1	-0.084816	0.00022927	hypomethylated	-0.020293	0.94855	insignificant	17	62	65
chr6	119851715	119853715	Rad52	-0.19057	8.07E-22	hypomethylated	-0.0054963	0.25203	insignificant	24	62	60
chr6	119984329	119986329	Mir706	-0.20268	0.45777	insignificant	0.02203	0.16182	insignificant	3	17	17
chr6	119988673	119990673	Wnk1	-0.14251	0.000018774	hypomethylated	-0.023217	0.000083158	hypomethylated	12	72	80
chr6	120244577	120246577	B4galnt3	-0.14275	4.32E-14	hypomethylated	0.035175	0.036859	inconclusive	15	50	51
chr6	120307378	120309378	Ccdc77	0.4407	0.031365	stronglyHypermeth	0.047952	0.32542	insignificant	1	27	27
chr6	120313116	120315116	Kdm5a	-0.14302	5.9E-47	hypomethylated	-0.0031688	0.0058157	hypomethylated	35	151	142
chr6	120412214	120414214	Il17ra	-0.099656	4.82E-16	hypomethylated	0.023106	0.57428	insignificant	27	94	102
chr6	120443825	120445825	Cecr6	-0.18507	1.58E-30	hypomethylated	0.0028248	0.0083475	inconclusive	21	46	52
chr6	120481317	120483317	Cecr5	-0.11656	0.00000472	hypomethylated	-0.010962	0.020847	hypomethylated	5	49	52
chr6	120615438	120617438	Cecr2	-0.088843	6.28E-52	hypomethylated	0.000031203	0.16493	insignificant	110	335	313
chr6	120772703	120774703	Atp6v1e1	-0.42727	0.31605	insignificant	0.086566	0.16506	insignificant	2	11	13
chr6	120785247	120787247	Bcl2l13	-0.094021	0.000000253	hypomethylated	-0.00081132	0.08403	insignificant	21	87	84
chr6	120866838	120868838	Bid	-0.33948	0.000018336	stronglyHypometh	-0.079099	0.77666	insignificant	5	20	19
chr6	121132684	121134684	Pex26	-0.30263	0.035141	hypomethylated	-0.057421	0.063445	insignificant	2	22	22
chr6	121159787	121161787	Tuba8	-0.32268	0.209	insignificant	-0.0093187	0.47993	insignificant	8	16	16
chr6	121194923	121196923	Usp18	-0.14345	0.000069571	hypomethylated	-0.011442	0.096946	insignificant	13	38	38
chr6	121249313	121251313	Slc6a13	-0.060749	0.46595	insignificant	-0.10268	0.064259	insignificant	5	22	22
chr6	121295714	121297714	Slc6a12	-0.41854	0.17835	insignificant	-0.075301	0.042162	hypomethylated	4	12	12
chr6	121423696	121425696	Iqsec3	-0.19746	0.57616	insignificant	-0.064816	0.23765	insignificant	4	20	20
chr6	121585190	121587190	A2m		1	noCoverage	-0.049874	0.12185	insignificant	0	10	6
chr6	122258027	122260027	M6pr	-0.1475	1.56E-14	hypomethylated	0.017147	0.3605	insignificant	6	28	28
chr6	122436323	122438323	Rimklb	-0.1003	0.000056685	hypomethylated	0.0048624	0.00024673	inconclusive	8	88	59
chr6	122502826	122504826	Aicda	-0.57024	0.00046037	stronglyHypometh	0.019048	0.79569	insignificant	2	4	4
chr6	122560089	122562089	Gdf3	-0.67333	0.24138	lowCoverage	0.055833	0.42778	insignificant	1	5	4
chr6	122575441	122577441	Dppa3	-0.27978	0.037536	hypomethylated	0.024086	0.71028	insignificant	5	37	34
chr6	122692763	122694763	Slc2a3	-0.041148	0.48513	insignificant	0.062477	0.6635	insignificant	4	24	24
chr6	122769201	122771201	Foxj2	-0.1254	3.59E-34	hypomethylated	0.032931	0.0024589	inconclusive	33	126	131
chr6	122823574	122825574	Necap1	-0.10825	0.056641	insignificant	-0.010574	0.35888	insignificant	7	38	38
chr6	124365085	124367085	Pex5	-0.17791	0.13676	insignificant	-0.018963	0.90997	insignificant	4	14	14
chr6	124414802	124416802	Clstn3	0.32938	1	lowCoverage	0.062195	0.70721	insignificant	1	8	9
chr6	124442130	124444130	C1rl	-0.37619	0.19184	insignificant	-0.042976	0.25109	insignificant	1	10	10
chr6	124461638	124463638	C1ra	-0.40952	0.032237	stronglyHypometh	0.13214	0.77662	insignificant	2	7	6
chr6	124519447	124521447	C1rb		1	noCoverage	-0.052273	0.84612	insignificant	0	6	6
chr6	124612121	124614121	Lpcat3	-0.13098	7.08E-16	hypomethylated	0.000024114	0.096204	insignificant	33	152	159
chr6	124661306	124663306	Phb2	-0.17737	1.23E-09	hypomethylated	-0.0023486	0.72286	insignificant	13	56	55
chr6	124662196	124664196	Emg1	-0.17291	0.0006944	hypomethylated	0.0073113	0.82681	insignificant	7	35	34
chr6	124668003	124670003	Mir141	-0.13739	0.4181	insignificant	0.023401	0.38713	insignificant	10	28	28
chr6	124668408	124670408	Mir141	-0.12881	0.5232	insignificant	-0.0088484	0.34351	insignificant	10	26	27
chr6	124688727	124690727	Ptpn6	-0.63704	0.000068553	stronglyHypometh	-0.13704	0.10106	insignificant	3	6	6
chr6	124691097	124693097	Grcc10	-0.24672	0.0043729	hypomethylated	-0.0011482	0.80828	insignificant	3	15	15
chr6	124693852	124695852	Atn1	-0.13082	0.00071574	inconclusive	0.055659	0.00000383	hypermethylated	10	46	46
chr6	124706505	124708505	Atn1	-0.064499	9.68E-08	hypomethylated	-0.032161	0.049161	hypomethylated	31	110	124
chr6	124719527	124721527	Lrrc23	-0.29452	0.00046283	inconclusive	-0.03916	0.18326	insignificant	12	56	56
chr6	124729736	124731736	Lrrc23	-0.28099	0.0952	insignificant	-0.026667	0.90445	insignificant	1	12	9
chr6	124757958	124759958	Spsb2	-0.14686	5.93E-18	hypomethylated	-0.0055241	0.71389	insignificant	20	69	69

chr6	124764314	124766314	Tpi1	-0.31784	0.54087	insignificant	-0.030293	0.14581	insignificant	4	16	16
chr6	124779193	124781193	Cdca3	-0.18813	2.62E-22	hypomethylated	-0.018001	0.84512	insignificant	15	72	74
chr6	124779465	124781465	Usp5	-0.22101	0.00000465	hypomethylated	-0.02199	0.68485	insignificant	7	44	46
chr6	124807705	124809705	Leprel2	0.043019	0.37273	insignificant	-0.025246	0.75924	insignificant	14	41	40
chr6	124861723	124863723	Lag3		1	noCoverage	-0.14709	0.25146	insignificant	0	11	13
chr6	124867964	124869964	Ptms	-0.1169	0.000000949	hypomethylated	0.16437	0.042542	hypermethylated	2	15	26
chr6	124880405	124882405	Mlf2	-0.10668	8.9E-23	hypomethylated	0.027991	0.32922	insignificant	26	104	107
chr6	124915415	124917415	Cops7a	-0.12221	3.96E-29	hypomethylated	-0.013705	0.27554	insignificant	24	76	71
chr6	124915547	124917547	Cops7a	-0.14457	5.29E-08	hypomethylated	-0.015566	0.35667	insignificant	13	48	43
chr6	124945737	124947737	C530028O21Rik	-0.12563	5.59E-09	hypomethylated	0.025916	0.000017408	hypermethylated	22	47	37
chr6	124947454	124949454	C530028O21Rik	-0.2265	0.10833	insignificant	0.055794	1	insignificant	3	14	16
chr6	124947627	124949627	C530028O21Rik	-0.2265	0.10833	insignificant	0.055794	1	insignificant	3	14	16
chr6	124958822	124960822	Zfp384	-0.12521	8.58E-13	hypomethylated	0.013772	0.78496	insignificant	42	126	121
chr6	124988865	124990865	Ing4	-0.19046	0.20852	insignificant	0.00075651	0.53584	insignificant	2	28	28
chr6	124998944	125000944	Acrbp	0.034071	0.82662	insignificant	0.066896	0.024998	hypermethylated	6	12	12
chr6	125016937	125018937	Lpar5	0.20708	0.24522	insignificant	0.1962	0.18961	insignificant	2	12	17
chr6	125045180	125047180	Chd4	-0.15172	5.15E-21	hypomethylated	-0.00909	0.012497	hypomethylated	41	121	132
chr6	125080900	125082900	Nop2	-0.16666	1.5E-10	hypomethylated	-0.0076302	0.01387	hypomethylated	19	70	70
chr6	125094258	125096258	Iffo1	-0.15764	1.94E-15	hypomethylated	-0.019686	0.45064	insignificant	21	83	83
chr6	125094268	125096268	Iffo1	-0.15764	1.94E-15	hypomethylated	-0.019686	0.45064	insignificant	21	83	83
chr6	125115601	125117601	Gapdh	-0.43871	0.12656	insignificant	-0.28738	0.051668	insignificant	2	8	20
chr6	125136449	125138449	Ncapd2	0.020008	1	insignificant	-0.018295	0.8122	insignificant	2	25	21
chr6	125141217	125143217	Mrpl51	-0.076533	1.46E-15	hypomethylated	-0.0085469	0.19134	insignificant	21	108	107
chr6	125141604	125143604	Ncapd2	-0.049343	5.37E-08	hypomethylated	0.010131	0.42251	insignificant	16	90	86
chr6	125164598	125166598	Vamp1	-0.10072	0.00006222	hypomethylated	0.012064	0.48616	insignificant	6	32	32
chr6	125180293	125182293	E130112N10Rik	-0.53846	0.000000571	stronglyHypometh	-0.06892	0.32987	insignificant	1	4	12
chr6	125181878	125183878	E130112N10Rik	0.00076927	0.0074683	inconclusive	-0.12285	0.71543	insignificant	3	18	20
chr6	125187045	125189045	E130112N10Rik	-0.4881	0.12192	insignificant	-0.020924	1	insignificant	3	6	6
chr6	125214542	125216542	4930417O13Rik	-0.91204	1.15E-10	stronglyHypometh	-0.010926	0.040891	hypomethylated	1	6	6
chr6	125236060	125238060	Tuba3a	-0.07834	0.17679	insignificant	-0.031961	0.64457	insignificant	4	12	12
chr6	125263888	125265888	Ltbr	-0.17107	0.043903	hypomethylated	0.056037	1	insignificant	5	14	9
chr6	125270357	125272357	Scnn1a	0.21161	0.080394	insignificant	0.071724	0.011374	hypermethylated	5	16	20
chr6	125298740	125300740	Tnfrsf1a	-0.20904	0.00000123	hypomethylated	-0.0041699	0.42286	insignificant	13	52	52
chr6	125330522	125332522	Plekhg6	-0.16956	0.00008595	hypomethylated	0.032117	0.87343	insignificant	8	26	26
chr6	125444773	125446773	Cd9	-0.34589	0.00087847	stronglyHypometh	0.081555	0.7002	insignificant	8	25	22
chr6	125639436	125641436	Ano2	-0.44313	0.00066717	stronglyHypometh	0.052562	0.46382	insignificant	3	8	8
chr6	126114978	126116978	Ntf3	-0.20307	0.00061499	hypomethylated	0.054099	0.76138	insignificant	7	42	46
chr6	126116762	126118762	Ntf3	-0.16989	0.0011706	hypomethylated	-0.011181	0.42067	insignificant	8	20	20
chr6	126690692	126692692	Kcna6		1	noCoverage	0.36012	0.1588	insignificant	0	5	4
chr6	126799162	126801162	Ndufa9		1	noCoverage	0.072823	0.91898	insignificant	0	5	12
chr6	126964559	126966559	Fgf6	-0.21238	0.000096502	hypomethylated	-0.030291	0.069127	insignificant	4	32	34
chr6	127059570	127061570	9630033F20Rik	-0.1972	3.42E-12	hypomethylated	-0.0053731	0.018733	inconclusive	7	16	12
chr6	127101066	127103066	Ccnd2	-0.18542	1	insignificant	-0.015917	0.34663	insignificant	2	24	31
chr6	127402740	127404740	Parp11	-0.20658	4.18E-29	hypomethylated	0.060437	0.0040192	inconclusive	15	92	72
chr6	127718747	127720747	Prmt8	-0.10463	7.68E-08	hypomethylated	0.004819	0.41695	insignificant	30	132	146
chr6	127836639	127838639	Tspan11	-0.22562	0.00076076	hypomethylated	-0.0086715	0.94054	insignificant	6	22	19
chr6	128093596	128095596	Tspan9	-0.17878	0.0087566	hypomethylated	-0.02301	0.26912	insignificant	2	28	28
chr6	128250831	128252831	Tead4	-0.12501	0.099997	insignificant	-0.0084602	0.0026969	hypomethylated	5	24	24
chr6	128305869	128307869	Tulp3	0.20549	0.18207	insignificant	-0.21779	0.000042898	hypomethylated	5	22	14
chr6	128312011	128314011	Foxm1	-0.13802	1.1E-34	hypomethylated	-0.0035858	0.0065328	hypomethylated	40	122	137
chr6	128312840	128314840	5930416I19Rik	-0.13685	6.09E-25	hypomethylated	0.012386	0.17653	insignificant	22	42	74
chr6	128312915	128314915	5930416I19Rik	-0.13685	6.09E-25	hypomethylated	-0.02297	0.14702	insignificant	22	42	62
chr6	128348783	128350783	Nrip2	-0.07526	0.16042	insignificant	-0.0068586	0.59074	insignificant	5	20	17
chr6	128387774	128389774	Gm10069	-0.10096	4.31E-27	hypomethylated	0.027171	0.0027224	inconclusive	50	166	170
chr6	128388649	128390649	Fkbp4	-0.13566	1.06E-16	hypomethylated	0.11712	4.88E-08	inconclusive	12	28	30
chr6	129225387	129227387	Cd69		1	noCoverage	-0.32222	0.72811	insignificant	0	5	3
chr6	129346314	129348314	Clec1b	-0.24921	0.26314	insignificant	-0.06031	0.68972	insignificant	1	8	8
chr6	129402018	129404018	Clec1a		1	noCoverage	-0.041667	0.79653	insignificant	0	3	3
chr6	129482209	129484209	Gabarapl1	-0.41984	8.84E-12	stronglyHypometh	-0.074004	0.37702	insignificant	4	23	26
chr6	130079853	130081853	Klra15	0.15714	1	lowCoverage	0.15714	1	insignificant	1	12	2
chr6	130079897	130081897	Klra15	0.15714	1	lowCoverage	0.15714	1	insignificant	1	12	3
chr6	130079916	130081916	Klra15	0.15714	1	lowCoverage	0.15714	1	insignificant	1	12	3
chr6	131243262	131245262	Magohb	-0.19872	0.000093764	hypomethylated	-0.013989	0.67068	insignificant	9	39	40
chr6	131338468	131340468	Csda	-0.25556	1	insignificant	-0.042222	0.021759	inconclusive	2	27	25
chr6	132264761	132266761	Prpmp5		1	noCoverage	0.019048	0.75651	insignificant	0	5	4
chr6	133054256	133056256	2700089E24Rik	-0.12099	1.52E-08	hypomethylated	0.0035171	0.21717	insignificant	18	59	59
chr6	133984724	133986724	Etv6	-0.086684	0.00028517	hypomethylated	-0.0068274	0.33499	insignificant	27	115	109
chr6	134345346	134347346	Bcl2l14	-0.78571	0.047441	stronglyHypometh	-0.05103	0.31366	insignificant	2	8	8
chr6	134516931	134518931	Lrp6	-0.10528	0.026367	hypomethylated	-0.01749	0.45582	insignificant	15	42	55
chr6	134589957	134591957	Loh12cr1	-0.10304	0.000000204	hypomethylated	-0.013532	0.29859	insignificant	23	93	71
chr6	134742646	134744646	Dusp16	-0.19538	1.13E-26	hypomethylated	0.089359	2.44E-10	hypermethylated	23	74	43
chr6	134779216	134781216	Crebl2	-0.13846	0.094207	insignificant	-0.030529	0.38345	insignificant	7	49	47
chr6	134846978	134848978	2810454H06Rik	-0.07345	0.80416	insignificant	-0.011643	0.020456	inconclusive	19	90	68
chr6	134847833	134849833	Gpr19	-0.11372	0.000000906	hypomethylated	-0.01632	0.35846	insignificant	16	84	62
chr6	134847943	134849943	Gpr19	-0.12752	0.000001795	hypomethylated	-0.021729	0.59199	insignificant	11	64	46
chr6	134869418	134871418	Cdkn1b	-0.13774	7.73E-14	hypomethylated	-0.014902	0.000026249	hypomethylated	27	124	128
chr6	134931018	134933018	Apold1	0.065762	1	insignificant	-0.058772	0.49134	insignificant	1	12	12
chr6	134960629	134962629	Ddx47		1	noCoverage	-0.0094573	0.21896	insignificant	0	28	28
chr6	135014679	135016679	Gprc5a	0.72075	0.15964	lowCoverage	0.092138	3.84E-13	hypermethylated	1	45	50
chr6	135068301	135070301	Gprc5d		1	noCoverage	-0.00096219	0.62023	insignificant	0	8	8
chr6	135118233	135120233	Hebp1	-0.20238	0.35042	insignificant	-0.0056898	0.26468	insignificant	2	4	4
chr6	135147004	135149004	8430419L09Rik	-0.13653	6.33E-24	hypomethylated	-0.049859	0.12946	insignificant	24	90	76
chr6	136466371	136468371	Atf7ip	-0.15665	0.000066379	hypomethylated	-0.0088385	0.000016286	hypomethylated	10	74	79
chr6	136610414	136612414	Plbd1	-0.50405	0.59382	insignificant	-0.054322	7.43E-12	inconclusive	2	10	8
chr6	136730263	136732263	Gucy2c		1	noCoverage	0.038823	1	insignificant	0	8	8

chr6	136752900	136754900	Hist4h4	0.1012	0.2935	insignificant	-0.097356	1	insignificant	2	7	8
chr6	136755768	136757768	H2afj	-0.11221	2.52E-09	hypomethylated	-0.0021229	0.59808	insignificant	13	51	48
chr6	136776363	136778363	BC049715	-0.14008	1.83E-13	hypomethylated	0.055345	0.56116	insignificant	19	46	43
chr6	136824326	136826326	Mgp	0.027958	0.63273	insignificant	-0.066	0.00084907	hypomethylated	4	22	18
chr6	136870701	136872701	Erp27	0.14115	0.60584	insignificant	0.044839	0.27289	insignificant	2	19	22
chr6	137118239	137120239	Rerg	-0.074988	5.22E-10	hypomethylated	-0.05153	0.0000154	hypomethylated	4	32	33
chr6	137119017	137121017	Rerg	-0.18009	0.32411	insignificant	0.25741	0.0097439	hypermethylated	2	18	8
chr6	137199819	137201819	Ptpro	-0.22023	1.54E-11	hypomethylated	-0.097228	0.039208	hypomethylated	11	48	51
chr6	137597641	137599641	Eps8	-0.13671	0.00059015	hypomethylated	-0.086509	4.7E-11	hypomethylated	13	46	36
chr6	137682602	137684602	Strap	-0.19403	0.00019903	hypomethylated	-0.021548	0.076283	insignificant	6	28	16
chr6	137702097	137704097	Dera	-0.1596	0.0081544	hypomethylated	-0.020335	0.18622	insignificant	5	28	24
chr6	138606965	138608965	Igbp1b	-0.54326	0.0096273	stronglyHypometh	-0.073813	0.04731	hypomethylated	2	9	8
chr6	139450430	139452430	Rergl	0.22115	1	lowCoverage	0.031438	0.50218	insignificant	1	4	4
chr6	139788965	139790965	Pik3c2g	0.16667	0.34747	insignificant	0.029167	0.5219	insignificant	4	8	8
chr6	140371619	140373619	Plekha5	-0.11148	2.54E-33	hypomethylated	-0.00021952	7.78E-08	hypomethylated	69	197	200
chr6	140570183	140572183	Aebp2	-0.21575	1.61E-10	hypomethylated	0.035387	3.86E-08	hypermethylated	12	87	85
chr6	140571209	140573209	Aebp2	-0.17299	2.76E-12	hypomethylated	0.019069	0.00022634	hypermethylated	28	160	158
chr6	140571818	140573818	Aebp2	-0.10354	8.36E-16	hypomethylated	0.00046956	0.93846	insignificant	38	159	159
chr6	141196789	141198789	Pde3a	-0.11855	4.46E-13	hypomethylated	-0.0047773	0.2421	insignificant	21	93	94
chr6	142293216	142295216	Pyroxd1	-0.24198	0.0014423	hypomethylated	-0.016057	0.099072	insignificant	13	80	79
chr6	142334762	142336762	Golt1b	-0.11894	4.5E-46	hypomethylated	-0.0011138	0.86902	insignificant	49	144	147
chr6	142335607	142337607	Golt1b	-0.10992	8.55E-38	hypomethylated	0.0050094	0.44807	insignificant	33	101	101
chr6	142519876	142521876	Kcnj8	-0.11552	0.000099693	hypomethylated	-0.018443	0.71316	insignificant	7	14	18
chr6	142650794	142652794	Abcc9	-0.35445	3.62E-10	stronglyHypometh	-0.1957	0.0035279	hypomethylated	5	12	14
chr6	142704205	142706205	Cmas	-0.10991	1.02E-11	hypomethylated	0.0062924	0.096783	insignificant	21	104	107
chr6	142752986	142754986	Gm766	0.2375	1	lowCoverage	0.060833	1	insignificant	1	4	5
chr6	142912972	142914972	St8sia1		1	noCoverage	0.10327	0.0087465	hypermethylated	0	23	29
chr6	143048627	143050627	5730419I09Rik	-0.16633	0.0046308	hypomethylated	-0.0080837	0.47985	insignificant	5	35	43
chr6	143114749	143116749	Etnk1	-0.11694	2.58E-41	hypomethylated	-0.0032563	0.00099618	hypomethylated	60	190	190
chr6	143193407	143195407	D6Ertd474e	0.15634	1	insignificant	-0.052656	1	insignificant	2	6	6
chr6	145069258	145071258	Lrmp	-0.42033	0.0068404	stronglyHypometh	-0.094044	1	insignificant	2	11	10
chr6	145158653	145160653	Lyrm5	-0.042831	0.33207	insignificant	-0.013073	0.06772	insignificant	11	78	76
chr6	145158666	145160666	Lyrm5	-0.042831	0.33207	insignificant	-0.013073	0.06772	insignificant	11	78	76
chr6	145159490	145161490	Casc1	0.018337	0.61333	insignificant	-0.0078682	0.15518	insignificant	8	54	51
chr6	145198751	145200751	Kras	-0.12699	9.81E-12	hypomethylated	0.014355	0.049663	inconclusive	32	104	100
chr6	145335144	145337144	1700073E17Rik	-0.3538	0.24592	insignificant	0.015487	0.90948	insignificant	1	12	8
chr6	145563482	145565482	Tuba3b	-0.45396	0.000000298	stronglyHypometh	-0.058593	0.25108	insignificant	8	44	44
chr6	145813860	145815860	Bhlhe41	-0.12423	1.63E-41	hypomethylated	0.019754	0.55333	insignificant	42	130	120
chr6	146450434	146452434	Itpr2	-0.10221	0.00000182	hypomethylated	-0.00038404	0.32335	insignificant	19	106	64
chr6	146525432	146527432	Fgfr1op2	-0.11324	0.000010595	hypomethylated	0.0074234	0.17213	insignificant	29	138	138
chr6	146526357	146528357	4933424B01Rik	-0.11316	0.0092294	hypomethylated	0.013092	0.85779	insignificant	15	74	75
chr6	146590100	146592100	Med21	-0.25643	4.88E-11	hypomethylated	-0.034152	0.40353	insignificant	9	64	68
chr6	146672451	146674451	Stk38l	-0.072452	0.0056811	hypomethylated	-0.00089513	0.76027	insignificant	19	62	64
chr6	146753051	146755051	Arntl2		1	noCoverage	0.010446	0.52992	insignificant	0	6	6
chr6	146836015	146838015	Ppfibp1	-0.11186	5.77E-29	hypomethylated	-0.010105	0.07936	insignificant	34	133	128
chr6	146990291	146992291	Mrps35	-0.14894	1.22E-19	hypomethylated	-0.041118	0.000004207	hypomethylated	21	97	94
chr6	147035554	147037554	Gm5887	0.39184	0.051583	insignificant	-0.015564	0.0022489	hypomethylated	2	18	6
chr6	147038596	147040596	Klhdc5	-0.14187	1.28E-09	hypomethylated	-0.028358	1	insignificant	22	52	40
chr6	147212607	147214607	Pthlh	-0.17345	4.65E-12	hypomethylated	-0.00040052	0.22974	insignificant	16	41	41
chr6	147423392	147425392	Ccdc91	-0.10002	1.45E-18	hypomethylated	-0.0070527	0.02653	hypomethylated	35	94	90
chr6	147994937	147996937	Far2	-0.12304	0.082217	insignificant	0.040835	1	insignificant	6	20	15
chr6	148159809	148161809	4732416N19Rik	-0.11364	3.65E-19	hypomethylated	-0.0097101	0.089442	insignificant	44	132	132
chr6	148160896	148162896	Ergic2	-0.46338	3.46E-18	stronglyHypometh	0.013296	0.77087	insignificant	4	16	20
chr6	148302177	148304177	Rps4y2	0.035243	0.020814	hypermethylated	0.073563	1.25E-09	hypermethylated	23	83	91
chr6	148392874	148394874	Tmtc1	-0.15364	1.4E-32	hypomethylated	-0.017791	0.0033235	hypomethylated	15	65	65
chr6	148779989	148781989	Ipo8	-0.14687	0.13229	insignificant	0.0008421	0.76618	insignificant	5	20	20
chr6	148844648	148846648	Caprin2	-0.2509	2.83E-42	hypomethylated	-0.004033	0.00049298	hypomethylated	15	50	56
chr6	148894954	148896954	Fam60a	-0.093415	0.61295	insignificant	0.040045	0.72353	insignificant	8	36	40
chr6	149050202	149052202	Dennd5b	-0.42817	8.02E-08	stronglyHypometh	-0.081997	0.020478	hypomethylated	5	10	10
chr6	149089118	149091118	4833442J19Rik	-0.14157	0.59571	insignificant	0.011572	1	insignificant	6	24	24
chr6	149137234	149139234	Amn1	-0.041908	0.67426	insignificant	-0.01161	0.66124	insignificant	3	13	12
chr6	149256935	149258935	2810474O19Rik	-0.12385	1.83E-14	hypomethylated	-0.00070282	0.86416	insignificant	26	71	64
chr6	149356407	149358407	Bicd1	-0.32371	1.15E-14	hypomethylated	-0.033886	0.012082	hypomethylated	5	14	14
chr7	3169204	3171204	AU018091	0.14653	1	insignificant	-0.10047	0.7106	insignificant	3	8	16
chr7	3217626	3219626	Mir290	-0.18377	2.77E-12	hypomethylated	-0.02735	0.18919	insignificant	12	32	48
chr7	3217784	3219784	D7Ertd143e	-0.10263	1.02E-11	hypomethylated	-0.016955	0.30967	insignificant	14	34	50
chr7	3217919	3219919	Mir291a	-0.13119	4.08E-13	hypomethylated	-0.022404	0.12012	insignificant	12	28	44
chr7	3218189	3220189	Mir292	-0.13119	4.08E-13	hypomethylated	-0.01465	0.12025	insignificant	12	28	45
chr7	3218482	3220482	Mir291b	-0.13119	4.08E-13	hypomethylated	-0.01465	0.12025	insignificant	12	28	45
chr7	3219343	3221343	Mir293	-0.013487	0.30019	insignificant	-0.016035	0.75107	insignificant	6	20	33
chr7	3219641	3221641	Mir294	0.4614	0.52715	insignificant	0.18398	0.065295	insignificant	2	4	5
chr7	3249741	3251741	Nlrp12	-0.16709	0.59192	insignificant	0.035098	0.7834	insignificant	1	17	18
chr7	3288038	3290038	Myadm	-0.17246	0.0001142	hypomethylated	-0.021455	0.9621	insignificant	8	40	40
chr7	3289555	3291555	Myadm	-0.25342	0.065758	insignificant	-0.023197	0.28894	insignificant	4	15	16
chr7	3289556	3291556	Myadm	-0.25342	0.065758	insignificant	-0.023197	0.28894	insignificant	4	15	16
chr7	3292028	3294028	Myadm	-0.55758	6.46E-09	stronglyHypometh	-0.067522	0.2018	insignificant	4	17	19
chr7	3424422	3426422	Cacng6	-0.37353	3.36E-10	stronglyHypometh	-0.021012	0.000047096	hypomethylated	11	34	34
chr7	3567735	3569735	Oscar	-0.2142	3.17E-09	hypomethylated	-0.023343	0.075218	insignificant	7	30	34
chr7	3567974	3569974	Ndufa3	-0.2142	3.17E-09	hypomethylated	-0.023343	0.075218	insignificant	7	30	34
chr7	3580586	3582586	Prpf31	-0.19433	2.3E-10	hypomethylated	-0.025137	0.00085001	hypomethylated	9	58	55
chr7	3581486	3583486	Tfpt	-0.20538	0.000021809	hypomethylated	-0.027744	0.00032861	hypomethylated	5	44	41
chr7	3595870	3597870	Cnot3	-0.089458	3.91E-18	hypomethylated	-0.0038579	0.79204	insignificant	36	127	123
chr7	3617442	3619442	Tmc4	-0.17764	0.0000048	inconclusive	-0.070449	0.00016791	inconclusive	5	29	33
chr7	3629155	3631155	Tmc4	0.16057	0.37263	insignificant	0.0032867	0.30762	insignificant	5	23	23
chr7	3644211	3646211	Tsen34	-0.095332	1.79E-14	hypomethylated	-0.0039696	0.17871	insignificant	34	178	178

chr7	3645109	3647109	Tsen34	-0.14462	3.44E-19	hypomethylated	0.030955	0.0012976	inconclusive	21	102	112
chr7	3645127	3647127	Tsen34	-0.14462	3.44E-19	hypomethylated	0.030955	0.0012976	inconclusive	21	102	112
chr7	3654642	3656642	Rps9	-0.2043	1	insignificant	-0.015501	0.43239	insignificant	2	53	54
chr7	4014806	4016806	Lair1		1	noCoverage	0.088636	0.31604	insignificant	0	4	8
chr7	4070134	4072134	Ttyh1	-0.17254	1.76E-20	hypomethylated	0.010759	0.15037	insignificant	22	51	50
chr7	4072330	4074330	9430041J12Rik	-0.18981	0.12725	insignificant	-0.085709	0.22679	insignificant	1	10	10
chr7	4087657	4089657	Leng8	-0.1293	4.15E-11	hypomethylated	-0.018857	0.24986	insignificant	17	88	79
chr7	4088528	4090528	D030047H15Rik	-0.12005	6.07E-09	hypomethylated	-0.017578	0.31049	insignificant	15	84	75
chr7	4101474	4103474	Leng9	-0.13798	0.00042132	hypomethylated	0.044603	0.000097722	hypermethylated	5	26	26
chr7	4188355	4190355	Lilra5	-0.31106	0.2525	insignificant	0.0011441	0.68478	insignificant	3	18	18
chr7	4415343	4417343	Eps8l1	0.078947	1	lowCoverage	-0.12105	0.14197	insignificant	1	2	2
chr7	4422486	4424486	Eps8l1	-0.032577	0.4232	insignificant	0.095118	0.0028422	hypermethylated	3	22	16
chr7	4453282	4455282	Ppp1r12c	-0.1535	1.58E-09	hypomethylated	0.049071	0.82453	insignificant	10	66	54
chr7	4474045	4476045	Tnni3	-0.14793	0.027983	hypomethylated	0.037043	0.60309	insignificant	6	38	40
chr7	4498169	4500169	Syt5	-0.14058	0.000057583	hypomethylated	-0.027414	0.9094	insignificant	7	60	62
chr7	4610552	4612552	Ppp6r1	-0.13868	0.00030457	hypomethylated	-0.0060025	0.08946	insignificant	6	57	58
chr7	4636565	4638565	Hspbp1	-0.026262	0.00086922	hypomethylated	-0.00008713	0.013443	hypomethylated	2	26	26
chr7	4641529	4643529	Brsk1	-0.10877	0.47357	insignificant	0.019696	0.93536	insignificant	3	33	33
chr7	4676684	4678684	Tmem150b	-0.39107	0.082159	insignificant	-0.065932	0.38093	insignificant	2	10	10
chr7	4676853	4678853	Tmem150b	-0.28051	0.17746	insignificant	-0.024082	0.30277	insignificant	2	8	8
chr7	4690728	4692728	Suv420h2	-0.1557	0.00086971	hypomethylated	-0.0226	0.71114	insignificant	9	34	34
chr7	4704504	4706504	Cox6b2	-0.49992	0.00000455	stronglyHypometh	-0.0069731	1	insignificant	2	10	7
chr7	4704696	4706696	Cox6b2	-0.49992	0.00000455	stronglyHypometh	-0.0069731	1	insignificant	2	10	7
chr7	4722872	4724872	Fam71e2	0.079971	1	insignificant	0.045822	0.57967	insignificant	1	14	14
chr7	4729743	4731743	Il11	-0.13907	0.11313	insignificant	-0.069485	0.000523	hypomethylated	2	16	17
chr7	4733541	4735541	Tmem190	-0.175	0.33404	insignificant	0.29274	0.21032	insignificant	1	4	5
chr7	4741162	4743162	2210411K11Rik	-0.53554	7.11E-42	stronglyHypometh	-0.063848	0.77882	insignificant	4	31	32
chr7	4743566	4745566	Rpl28	-0.16165	0.000000822	hypomethylated	-0.017917	0.034348	hypomethylated	8	98	104
chr7	4763942	4765942	Ube2s	-0.13972	0.19179	insignificant	0.072831	0.34036	insignificant	17	100	99
chr7	4796298	4798298	Isoc2b	-0.24314	0.00094283	hypomethylated	-0.01827	0.05599	insignificant	7	27	25
chr7	4817781	4819781	Isoc2b	-0.26606	0.00000159	hypomethylated	-0.058835	0.56731	insignificant	6	45	46
chr7	4827654	4829654	Isoc2a	-0.36756	1.12E-08	stronglyHypometh	-0.0045966	0.92185	insignificant	3	6	6
chr7	4865818	4867818	Zfp628	-0.14553	4.19E-08	hypomethylated	0.035132	0.76031	insignificant	18	61	64
chr7	4872852	4874852	Nat14	-0.12573	0.000000827	hypomethylated	0.040255	0.18251	insignificant	14	39	39
chr7	4915950	4917950	Sbk2	-0.28634	0.0004826	hypomethylated	0.075388	0.67002	insignificant	2	14	10
chr7	4947703	4949703	Zfp579	-0.3179	4.22E-21	hypomethylated	0.051437	0.56397	insignificant	8	22	22
chr7	4964844	4966844	Fiz1	-0.15272	0.20182	insignificant	0.11886	0.58641	insignificant	5	26	26
chr7	4965855	4967855	Fiz1	-0.09064	1.03E-11	hypomethylated	0.01813	0.72715	insignificant	27	109	104
chr7	4966109	4968109	Zfp524	-0.085479	3.28E-10	hypomethylated	0.014945	0.83966	insignificant	27	103	102
chr7	4966299	4968299	Fiz1	-0.078012	3.21E-09	hypomethylated	0.010986	0.75277	insignificant	24	87	86
chr7	4966330	4968330	Fiz1	-0.078012	3.21E-09	hypomethylated	0.010986	0.75277	insignificant	24	87	86
chr7	4970977	4972977	Zfp865	-0.21557	1.59E-14	hypomethylated	-0.012427	0.22343	insignificant	20	83	96
chr7	4990048	4992048	Zfp784	-0.14188	0.70401	insignificant	0.044582	0.18348	insignificant	5	26	21
chr7	5002133	5004133	Zfp580	-0.12299	1	insignificant	0.086787	0.0013199	hypermethylated	3	34	25
chr7	5007327	5009327	Ccdc106	-0.1363	0.18696	insignificant	0.0058676	0.55656	insignificant	8	34	34
chr7	5012783	5014783	U2af2	-0.088399	5.89E-13	hypomethylated	0.000045846	0.53041	insignificant	21	137	137
chr7	5030906	5032906	Epn1	-0.14361	1.51E-40	hypomethylated	0.0064465	0.25768	insignificant	33	85	97
chr7	5068513	5070513	Rfpl4	-0.8964	0.08871	lowCoverage	-0.08321	0.011416	hypomethylated	1	14	14
chr7	5077552	5079552	Rasl2-9-ps	-0.3881	0.000000172	stronglyHypometh	0.040203	0.86395	insignificant	8	28	34
chr7	5301142	5303142	Gm5065	0.1437	1	insignificant	0.03909	1	insignificant	1	6	6
chr7	5302637	5304637	Nlrp2	0.1437	1	insignificant	0.03909	1	insignificant	1	6	6
chr7	5562915	5564915	Vmn1r61	-0.021429	0.42668	insignificant	-0.11829	0.09556	insignificant	2	4	4
chr7	5995762	5997762	Nlrp4c	-0.36236	0.22743	insignificant	-0.0056741	0.027301	hypomethylated	2	12	12
chr7	6107573	6109573	Zfp787	-0.16125	4.5E-17	hypomethylated	-0.0053948	0.92641	insignificant	17	74	74
chr7	6123114	6125114	Zfp444	-0.11171	7.88E-16	hypomethylated	0.0023293	0.15894	insignificant	21	58	64
chr7	6147691	6149691	Galp	-0.03729	1	insignificant	0.036199	0.6788	insignificant	1	4	4
chr7	6172879	6174879	Zscan5b	-0.05555	0.13085	insignificant	0.052712	0.63501	insignificant	5	25	25
chr7	6203311	6205311	Gm6792		1	noCoverage	0.01654	0.52415	insignificant	0	19	22
chr7	6206523	6208523	Gm6792		1	noCoverage	-0.048563	0.19531	insignificant	0	2	2
chr7	6237181	6239181	Zfp667	-0.12555	3.06E-14	hypomethylated	-0.0060014	0.53056	insignificant	17	35	35
chr7	6282036	6284036	Zfp583	-0.17214	1	insignificant	-0.025607	0.64021	insignificant	5	26	26
chr7	6335027	6337027	Zfp28	-0.19991	0.000012539	hypomethylated	-0.0057251	0.66243	insignificant	12	55	59
chr7	6366885	6368885	Gm16532	-0.22721	0.34611	insignificant	0.010885	0.76309	insignificant	2	14	14
chr7	6391612	6393612	Olfr1344		1	noCoverage	-0.096099	0.0033972	hypomethylated	0	12	12
chr7	6454935	6456935	Olfr1348		1	noCoverage	-0.0019069	0.5253	insignificant	0	5	5
chr7	6649143	6651143	Zim1	-0.18316	0.088564	insignificant	-0.057517	0.74416	insignificant	5	14	17
chr7	6658470	6660470	Peg3as		1	noCoverage	-0.060519	0.26759	insignificant	0	10	8
chr7	6682451	6684451	Usp29	-0.024275	0.43533	insignificant	-0.1539	0.00000319	hypomethylated	5	12	12
chr7	6755297	6757297	Mir3099	0.26792	1	lowCoverage	-0.10639	0.21237	insignificant	1	10	10
chr7	6929373	6931373	Zim3	0.1584	0.0039391	hypermethylated	0.048752	0.0090568	hypermethylated	15	40	44
chr7	6947095	6949095	Aurkc		1	noCoverage	-0.039992	0.59543	insignificant	0	18	20
chr7	7074187	7076187	Zfp954		1	noCoverage	0.081077	0.84852	insignificant	0	6	6
chr7	7123063	7125063	Zfp418	0.7419	0.25086	lowCoverage	-0.0011191	0.30201	insignificant	1	20	20
chr7	7231000	7233000	Vmn2r29	0.012399	0.66923	insignificant	-0.20254	1.28E-104	hypomethylated	15	38	36
chr7	7252222	7254222	Clcn4-2	-0.066347	0.64112	insignificant	-0.0030184	0.000014024	inconclusive	12	33	37
chr7	11080730	11082730	Zik1	-0.33985	0.024499	stronglyHypometh	-0.1227	0.072013	insignificant	4	10	10
chr7	12907477	12909477	Vmn1r83	0.33333	1	lowCoverage	0.3141	0.038266	hypermethylated	1	4	4
chr7	13062653	13064653	Zfp606	-0.1221	7.55E-08	hypomethylated	0.010202	0.9655	insignificant	18	80	80
chr7	13419158	13421158	Zfp110	-0.13137	0.00000141	hypomethylated	0.00033397	0.066415	insignificant	15	63	72
chr7	13465526	13467526	Zfp128	-0.43864	0.51683	insignificant	0.03789	0.0184	hypermethylated	2	14	14
chr7	13482163	13484163	Zscan22	-0.20604	2.51E-08	hypomethylated	-0.0038797	0.23657	insignificant	8	49	50
chr7	13506659	13508659	Rps5	-0.15871	0.00000312	hypomethylated	-0.0041184	0.67763	insignificant	11	60	60
chr7	13511764	13513764	2310014L17Rik	-0.082799	2.01E-15	hypomethylated	0.030827	0.067405	insignificant	28	70	75
chr7	13550212	13552212	Zfp324	-0.12967	0.000038046	hypomethylated	-0.019444	0.7106	insignificant	8	46	53
chr7	13562196	13564196	Zfp446	-0.12992	0.0010874	hypomethylated	0.034696	0.49115	insignificant	19	49	46

chr7	13595149	13597149	Zbtb45	-0.24499	2.08E-09	hypomethylated	-0.026836	0.14998	insignificant	7	24	24
chr7	13608500	13610500	Trim28	-0.13014	6.69E-09	hypomethylated	-0.033167	0.37115	insignificant	19	50	60
chr7	13620126	13622126	Ube2m	-0.10181	1.05E-09	inconclusive	-0.018716	2.09E-12	inconclusive	13	32	33
chr7	13623327	13625327	Ube2m	-0.15622	1.89E-09	hypomethylated	-0.0045009	1	insignificant	19	56	56
chr7	13623619	13625619	Ube2m	-0.25139	3.43E-09	hypomethylated	-0.012023	0.12374	insignificant	7	24	24
chr7	13640113	13642113	Mzf1		1	noCoverage	0.013508	0.00719	inconclusive	0	30	30
chr7	13762067	13764067	Vmn1r88		1	noCoverage	-0.049567	0.47528	insignificant	0	11	11
chr7	13861661	13863661	Lig1	-0.21212	0.051509	insignificant	-0.030818	0.17353	insignificant	3	6	6
chr7	13863174	13865174	Lig1	-0.17907	2.82E-23	hypomethylated	-0.045395	0.44929	insignificant	27	61	73
chr7	13863613	13865613	Lig1	-0.17907	2.82E-23	hypomethylated	-0.045395	0.44929	insignificant	27	61	73
chr7	13864070	13866070	Lig1	-0.151	0.00032235	hypomethylated	-0.081404	0.038044	hypomethylated	14	17	29
chr7	14035189	14037189	Bsph1		1	noCoverage	0.018849	1	insignificant	0	4	4
chr7	14574937	14576937	Sult2a4	0.083196	0.13461	insignificant	0.04689	0.78976	insignificant	4	58	52
chr7	15208415	15210415	Gm18756	-0.076578	3.91E-10	hypomethylated	-0.03202	0.0060822	hypomethylated	18	76	81
chr7	16334718	16336718	Obox5		1	noCoverage	-0.016667	0.53325	insignificant	0	6	6
chr7	16457734	16459734	Crx		1	noCoverage	0.35901	0.061881	insignificant	0	4	13
chr7	16507720	16509720	Sepw1	0.014048	0.69907	insignificant	0.1034	0.000033178	hypermethylated	3	27	35
chr7	16524209	16526209	Gltscr2	0.0030715	1	insignificant	-0.0068158	0.30065	insignificant	7	22	29
chr7	16531457	16533457	Gltscr2	-0.43564	1	lowCoverage	-0.029775	0.080785	insignificant	1	32	32
chr7	16552884	16554884	Ehd2	-0.26341	0.027754	hypomethylated	0.024683	0.34036	insignificant	1	6	10
chr7	16682991	16684991	Napa	-0.13752	1.21E-10	hypomethylated	-0.036784	0.42422	insignificant	21	67	62
chr7	16704224	16706224	Kptn	-0.39342	0.000000206	stronglyHypometh	-0.1522	0.098017	insignificant	5	16	16
chr7	16714648	16716648	Slc8a2	-0.24749	7.48E-20	hypomethylated	-0.043188	0.36335	insignificant	12	30	29
chr7	16759728	16761728	Meis3	-0.073501	0.000046742	hypomethylated	-0.0079927	0.28924	insignificant	14	42	42
chr7	16827680	16829680	Gpr77	-0.02675	0.39905	insignificant	0.093808	0.00024255	hypermethylated	2	12	8
chr7	16844687	16846687	C5ar1	-0.0019653	0.83963	insignificant	0.02554	0.10032	insignificant	4	8	8
chr7	16844889	16846889	C5ar1	-0.0019653	0.83963	insignificant	0.02554	0.10032	insignificant	4	8	8
chr7	16859041	16861041	Ccdc9	-0.14516	0.00070047	hypomethylated	0.029425	0.50519	insignificant	7	26	42
chr7	16870008	16872008	Ccdc9	-0.19048	0.000017117	hypomethylated	0.0082372	0.42662	insignificant	2	41	45
chr7	16893931	16895931	Bbc3	-0.095604	1.21E-32	hypomethylated	-0.0092136	0.046951	hypomethylated	50	165	174
chr7	16973134	16975134	Sae1	-0.079943	0.000010561	hypomethylated	-0.0251	0.080862	insignificant	7	34	36
chr7	16985544	16987544	Zc3h4	-0.11744	5.76E-23	hypomethylated	-0.0037683	0.61918	insignificant	46	169	176
chr7	17037127	17039127	Tmem160	-0.12104	1.91E-16	hypomethylated	-0.013149	0.14699	insignificant	19	89	81
chr7	17062129	17064129	Npas1	-0.049734	0.83015	insignificant	-0.0077716	0.1124	insignificant	5	19	19
chr7	17200342	17202342	Grlf1	-0.2319	0.64191	insignificant	0.013215	0.049038	hypermethylated	3	24	35
chr7	17322792	17324792	Ap2s1	-0.10457	2.17E-10	hypomethylated	0.045176	0.80541	insignificant	17	58	59
chr7	17365694	17367694	Slc1a5	-0.074501	0.016996	hypomethylated	-0.016723	0.96445	insignificant	14	63	81
chr7	17400237	17402237	Strn4	-0.097508	8.8E-09	hypomethylated	-0.0090169	0.30403	insignificant	44	136	110
chr7	17402081	17404081	Strn4	-0.19372	4.57E-17	hypomethylated	-0.032411	0.0000001	hypomethylated	15	33	32
chr7	17427413	17429413	Prkd2	-0.13648	8.47E-12	hypomethylated	0.037715	0.14657	insignificant	11	53	44
chr7	17459665	17461665	Dact3	-0.10508	1.96E-11	hypomethylated	0.06874	0.40309	insignificant	13	40	47
chr7	17476134	17478134	Gng8	-0.29844	0.10955	insignificant	0.0095393	0.6462	insignificant	3	19	14
chr7	17490838	17492838	Ptgir		1	noCoverage		1	noCoverage	0	9	0
chr7	17509381	17511381	Calm3	-0.11299	0.0043842	hypomethylated	-0.03359	0.0095603	hypomethylated	4	40	49
chr7	17529030	17531030	Pnmal2	-0.15623	0.000000149	hypomethylated	-0.0027788	0.37615	insignificant	21	58	58
chr7	17578936	17580936	Ccdc8	-0.21534	0.022713	hypomethylated	-0.066999	0.038402	hypomethylated	6	42	40
chr7	17613263	17615263	Ppp5c	-0.36423	0.000000227	stronglyHypometh	-0.041131	0.24922	insignificant	3	15	19
chr7	17642028	17644028	Hif3a	-0.33116	0.00023995	hypomethylated	-0.016082	0.53754	insignificant	5	27	27
chr7	19271341	19273341	Psg20	-0.38043	0.052228	insignificant	-0.14631	0.55308	insignificant	1	4	4
chr7	19406940	19408940	Psg17	0.11993	1	insignificant	0.028733	0.4776	insignificant	3	8	8
chr7	19424314	19426314	Mill2	-0.2966	0.012574	hypomethylated	-0.016834	0.00088031	inconclusive	4	19	8
chr7	19469038	19471038	Pglyrp1	-0.37935	0.0013664	stronglyHypometh	0.020652	0.46581	insignificant	3	8	8
chr7	19495753	19497753	Ccdc61	-0.38191	3.07E-08	stronglyHypometh	-0.11545	0.0020875	hypomethylated	1	2	2
chr7	19510236	19512236	Nova2	-0.19874	0.21167	insignificant	0.069097	0.00031787	hypermethylated	5	71	64
chr7	19575593	19577593	Mypop	-0.099811	1.15E-15	hypomethylated	0.0011497	0.061968	insignificant	21	74	69
chr7	19588413	19590413	Irf2bp1	-0.17638	4.51E-63	hypomethylated	0.0077274	0.29333	insignificant	44	134	140
chr7	19608725	19610725	Sympk	-0.1387	8.95E-46	hypomethylated	-0.023495	0.00072392	hypomethylated	56	153	162
chr7	19608888	19610888	Foxa3	-0.12996	3.12E-38	hypomethylated	-0.020439	0.0025159	hypomethylated	52	143	148
chr7	19639035	19641035	Rsph6a	-0.1179	0.84032	insignificant	0.085528	0.00016207	hypermethylated	4	28	28
chr7	19660548	19662548	Dmwd	-0.1331	2.09E-40	hypomethylated	0.0010417	0.17133	insignificant	39	97	85
chr7	19668197	19670197	Dmpk	-0.0479	0.75645	insignificant	-0.012399	0.53536	insignificant	1	6	7
chr7	19671176	19673176	Mir3100	-0.17776	0.00000123	hypomethylated	-0.048373	0.00012795	hypomethylated	7	29	30
chr7	19678892	19680892	Six5	-0.12319	1.3E-14	hypomethylated	-0.031632	0.081642	insignificant	45	142	141
chr7	19704207	19706207	Fbxo46	-0.26836	6.8E-10	hypomethylated	0.028842	0.0047465	hypermethylated	4	39	41
chr7	19734186	19736186	Snrpd2	-0.16581	9.02E-11	hypomethylated	0.0081991	0.51708	insignificant	9	38	47
chr7	19734545	19736545	Qpctl	-0.15969	1.13E-10	hypomethylated	0.051044	0.81192	insignificant	9	26	37
chr7	19765518	19767518	Eml2	-0.15901	0.00000589	hypomethylated	0.0046668	0.3389	insignificant	11	42	48
chr7	19765813	19767813	Mir330	-0.058435	3.44E-09	inconclusive	-0.01935	0.0085029	inconclusive	16	52	58
chr7	19796886	19798886	Gpr4	-0.19764	9E-14	hypomethylated	0.050872	0.74034	insignificant	9	40	42
chr7	19812737	19814737	Opa3	-0.15667	8.18E-12	hypomethylated	-0.032184	0.0072471	hypomethylated	12	80	81
chr7	19857203	19859203	Vasp	-0.22391	2.03E-10	hypomethylated	-0.0079421	0.039405	hypomethylated	12	55	54
chr7	19867015	19869015	Rtn2	-0.16702	5.92E-12	hypomethylated	-0.0086029	0.21365	insignificant	25	70	70
chr7	19875447	19877447	Rtn2	-0.19161	0.089094	insignificant	0.029227	0.30704	insignificant	4	14	14
chr7	19895394	19897394	Fosb	-0.22954	0.4896	insignificant	-0.030645	1.62E-08	inconclusive	11	38	38
chr7	19929419	19931419	Ercc1	-0.20309	0.00011314	hypomethylated	-0.0044483	0.89879	insignificant	11	49	53
chr7	19944832	19946832	Cd3eap	-0.18334	4.82E-18	hypomethylated	0.0082513	0.057431	insignificant	11	60	54
chr7	19945564	19947564	Ppp1r13l	-0.20234	6.04E-16	hypomethylated	-0.0061913	0.62478	insignificant	10	63	54
chr7	19966387	19968387	Ercc2	-0.14712	0.0011911	hypomethylated	-0.00075019	0.22077	insignificant	25	68	92
chr7	19970991	19972991	Mir343	0.04375	1	insignificant	-0.10157	0.0020578	hypomethylated	1	8	8
chr7	19995442	19997442	Ckm	-0.11715	1	insignificant	0.052604	1	insignificant	6	27	27
chr7	20043843	20045843	Mark4	-0.16929	0.00000368	hypomethylated	-0.017193	0.12587	insignificant	12	33	37
chr7	20093077	20095077	Trappc6a	-0.18862	2.7E-18	hypomethylated	-0.0026117	0.37576	insignificant	27	58	73
chr7	20093680	20095680	Bloc1s3	-0.2318	2.19E-16	hypomethylated	0.0038417	0.12057	insignificant	19	55	59
chr7	20103079	20105079	Nkpd1		1	noCoverage	-0.040547	1	insignificant	0	17	17
chr7	20147747	20149747	Ppp1r37	-0.10774	1.67E-08	hypomethylated	0.23505	1	insignificant	5	9	18

chr7	20158692	20160692	Gemin7	-0.21085	1.97E-10	hypomethylated	-0.018812	0.0039162	inconclusive	12	61	70
chr7	20161635	20163635	Zfp296	-0.13743	0.00000413	hypomethylated	-0.0092044	0.3218	insignificant	20	80	76
chr7	20189817	20191817	Clasrp	0.4826	3.82E-10	stronglyHypermeth	0.083716	0.27688	insignificant	2	30	33
chr7	20214787	20216787	Relb	-0.14602	1.35E-10	hypomethylated	-0.015908	0.30008	insignificant	19	34	34
chr7	20250379	20252379	Clptm1	-0.20826	0.000020366	hypomethylated	0.0034375	0.0096907	hypermethylated	14	56	66
chr7	20262213	20264213	Apoc2	-0.020079	0.20769	insignificant	-0.013214	0.74505	insignificant	6	15	15
chr7	20300778	20302778	Tomm40	-0.12331	0.014734	hypomethylated	-0.0052035	0.36806	insignificant	8	55	56
chr7	20334922	20336922	Pvrl2	-0.34833	0.00000318	stronglyHypometh	-0.0122	0.050647	insignificant	5	21	20
chr7	20355881	20357881	Bcam	-0.20018	0.093183	insignificant	-0.015017	0.0001077	hypomethylated	2	25	25
chr7	20382158	20384158	Cblc	-0.40775	0.0011028	stronglyHypometh	-0.030183	0.81133	insignificant	2	23	16
chr7	20408104	20410104	Bcl3	-0.17808	0.000000403	hypomethylated	0.025847	0.49001	insignificant	13	46	52
chr7	20446648	20448648	Ceacam16	0.125	1	insignificant	0.029648	0.5811	insignificant	1	2	3
chr7	20506492	20508492	Pvr	-0.25388	0.030502	hypomethylated	0.14078	0.000084102	hypermethylated	3	28	32
chr7	20536092	20538092	2210010C17Rik		1	noCoverage	0.16117	0.00000361	hypermethylated	0	14	13
chr7	24169915	24171915	Nlrp5	-0.42226	0.01488	stronglyHypometh	-0.12044	0.013609	hypomethylated	3	9	13
chr7	24537929	24539929	Vmn1r174		1	noCoverage	0.010714	0.24473	insignificant	0	8	10
chr7	24865962	24867962	Zfp180	-0.22063	0.68046	insignificant	-0.085214	1.08E-09	hypomethylated	11	33	54
chr7	24918181	24920181	Zfp235	-0.24314	0.22395	insignificant	-0.041934	0.020911	inconclusive	3	18	25
chr7	25022617	25024617	Zfp109	-0.052843	0.068782	insignificant	0.093103	0.010681	hypermethylated	4	20	22
chr7	25038812	25040812	Zfp108	-0.39726	0.015996	stronglyHypometh	0.10548	0.32651	insignificant	5	12	12
chr7	25054436	25056436	Zfp93	-0.1199	0.54722	insignificant	0.05814	0.00025707	hypermethylated	4	7	22
chr7	25084568	25086568	Zfp61	-0.24397	4.53E-08	hypomethylated	0.054731	6.16E-12	inconclusive	6	23	30
chr7	25101685	25103685	Zfp94	-0.10161	0.00066001	hypomethylated	0.14461	1	insignificant	8	28	33
chr7	25154281	25156281	Kcnn4	-0.33964	0.55431	insignificant	0.05758	0.78034	insignificant	3	7	6
chr7	25183646	25185646	1500002O20Rik	-0.13946	0.00099613	hypomethylated	0.068842	0.2087	insignificant	4	14	11
chr7	25246518	25248518	Plaur	-0.24463	0.000010604	hypomethylated	-0.040903	1	insignificant	8	30	26
chr7	25266041	25268041	Cadm4	-0.092201	6.26E-14	hypomethylated	-0.0019299	0.26767	insignificant	35	150	165
chr7	25291105	25293105	Zfp428	-0.11438	6.29E-09	hypomethylated	-0.017484	0.79212	insignificant	30	132	136
chr7	25314666	25316666	Irgq	-0.16337	2.36E-11	hypomethylated	0.020201	0.000011531	hypermethylated	18	76	67
chr7	25331024	25333024	Xrcc1	-0.14474	1.5E-27	hypomethylated	-0.01227	0.070129	insignificant	28	88	113
chr7	25331168	25333168	Xrcc1	-0.16237	2.1E-28	hypomethylated	-0.021107	0.034593	hypomethylated	28	90	115
chr7	25371561	25373561	Ethe1	-0.42057	0.074288	insignificant	0.14738	0.072571	insignificant	4	35	26
chr7	25372660	25374660	Ethe1	-0.67029	0.17804	insignificant	-0.047256	0.15364	insignificant	0	21	18
chr7	25395346	25397346	Phldb3	-0.20317	1.19E-10	hypomethylated	-0.015726	0.048866	hypomethylated	21	78	80
chr7	25420588	25422588	Lypd3	-0.16091	0.21669	insignificant	0.10442	0.34995	insignificant	13	53	52
chr7	25445312	25447312	Gm4598	-0.069989	0.3022	insignificant	0.10592	0.000081815	hypermethylated	8	31	32
chr7	25457069	25459069	Tex101	-0.068667	0.030811	hypomethylated	-0.075584	0.000046294	hypomethylated	7	24	24
chr7	25493277	25495277	BC049730	-0.094351	0.28515	insignificant	-0.042968	0.094664	insignificant	3	14	14
chr7	25654075	25656075	Dmrtc2	0.17697	0.67389	insignificant	0.087016	0.0091862	hypermethylated	6	54	54
chr7	25654710	25656710	Lypd4	0.19875	0.67311	insignificant	0.098263	0.0088394	hypermethylated	6	50	50
chr7	25668732	25670732	Rps19	-0.12402	7.61E-26	hypomethylated	-0.016081	0.0018388	hypomethylated	35	110	108
chr7	25681529	25683529	Cd79a		1	noCoverage	-0.071205	0.00031672	hypomethylated	0	12	12
chr7	25687004	25689004	Arhgef1	-0.1615	1.3E-22	hypomethylated	-0.0062108	0.00047075	hypomethylated	23	103	112
chr7	25688364	25690364	Arhgef1	-0.19289	3.85E-09	hypomethylated	0.0083019	0.58275	insignificant	13	44	44
chr7	25757747	25759747	Rabac1	-0.20436	1	insignificant	-0.0054388	0.86404	insignificant	1	12	12
chr7	25790914	25792914	Atp1a3	-0.15304	0.000072129	hypomethylated	-0.02986	1	insignificant	11	30	27
chr7	25857388	25859388	Grik5	-0.19106	6.79E-15	hypomethylated	0.0048304	0.60565	insignificant	10	31	27
chr7	25861223	25863223	Zfp574	-0.13569	5.71E-25	hypomethylated	0.012507	0.12271	insignificant	20	116	112
chr7	25861264	25863264	Zfp574	-0.13569	5.71E-25	hypomethylated	0.012507	0.12271	insignificant	20	116	112
chr7	25917479	25919479	Pou2f2	-0.15273	0.0038371	hypomethylated	0.016857	0.72965	insignificant	7	21	25
chr7	26022870	26024870	Gsk3a	-0.17693	7.32E-08	hypomethylated	0.00082903	0.068551	insignificant	6	35	35
chr7	26035777	26037777	Erf	-0.17079	4.82E-11	hypomethylated	0.024434	0.90217	insignificant	16	43	44
chr7	26066197	26068197	Cic	-0.1105	0.000000356	hypomethylated	0.016949	0.89361	insignificant	28	117	124
chr7	26085377	26087377	Prr19	-0.18993	1	insignificant	0.0049158	0.70693	insignificant	3	16	20
chr7	26090126	26092126	Tmem145	-0.12473	4.9E-10	hypomethylated	0.00068589	0.50185	insignificant	23	64	66
chr7	26101182	26103182	Megf8	-0.28753	0.021094	hypomethylated	-0.0026427	0.83889	insignificant	17	59	59
chr7	26179432	26181432	4732471J01Rik	-0.29167	3.56E-21	hypomethylated	-0.016011	0.55408	insignificant	6	16	16
chr7	26181006	26183006	4732471J01Rik	-0.082506	0.0000044	hypomethylated	-0.024915	0.59732	insignificant	10	28	28
chr7	26262644	26264644	4732471J01Rik		1	noCoverage	0.045093	0.096829	insignificant	0	10	15
chr7	26325023	26327023	Ceacam2	-0.44706	0.01636	stronglyHypometh	-0.059126	0.89767	insignificant	2	16	20
chr7	26400911	26402911	Gm7092	-0.13709	0.074492	insignificant	-0.069702	0.082303	insignificant	3	9	9
chr7	26403511	26405511	Atp5sl	-0.14768	0.000089927	hypomethylated	0.02551	0.94849	insignificant	3	23	20
chr7	26411642	26413642	B3gnt8	-0.13998	0.00011983	hypomethylated	-0.017783	1	insignificant	17	68	68
chr7	26443171	26445171	Exosc5	-0.15705	0.000036335	hypomethylated	0.0025835	0.61151	insignificant	6	56	56
chr7	26443780	26445780	Bckdha	-0.17572	0.000021431	hypomethylated	0.0051313	0.5862	insignificant	6	58	58
chr7	26460185	26462185	Tmem91	-0.15918	0.49367	insignificant	-0.0039599	0.42791	insignificant	7	20	20
chr7	26465176	26467176	B9d2		1	noCoverage	-0.031538	0.2986	insignificant	0	6	6
chr7	26471020	26473020	Tgfb1	-0.12564	3.06E-08	hypomethylated	-0.012292	0.126	insignificant	17	53	58
chr7	26504072	26506072	Ccdc97	-0.036166	1.67E-15	hypomethylated	-0.026631	0.46229	insignificant	6	22	22
chr7	26539720	26541720	Hnrnpul1	-0.17859	0.000021508	hypomethylated	-0.0062014	0.31717	insignificant	8	44	44
chr7	26539739	26541739	Hnrnpul1	-0.17859	0.000021508	hypomethylated	-0.0062014	0.31717	insignificant	8	44	44
chr7	26601549	26603549	Cyp2s1	-0.29196	0.00069578	hypomethylated	0.01209	0.41294	insignificant	2	4	4
chr7	27219131	27221131	Nlrp4a	-0.41945	0.32976	insignificant	-0.076805	0.3821	insignificant	2	4	4
chr7	27937732	27939732	Cyp2t4		1	noCoverage	-0.014245	0.81046	insignificant	0	6	6
chr7	27951821	27953821	Egln2	-0.16037	0.13972	insignificant	0.033477	0.21873	insignificant	5	26	28
chr7	27963902	27965902	Rab4b	-0.15606	1	insignificant	0.0056574	0.55953	insignificant	3	8	13
chr7	27980779	27982779	BC024978	-0.11451	5.37E-19	hypomethylated	0.010584	0.081834	insignificant	22	80	80
chr7	28013616	28015616	Itpkc	-0.28041	0.000067131	hypomethylated	-0.025823	0.009056	hypomethylated	5	30	30
chr7	28017031	28019031	Adck4		1	noCoverage	0.11742	0.51405	insignificant	0	6	6
chr7	28042779	28044779	Numbl	-0.092679	4.81E-23	hypomethylated	0.013256	0.18797	insignificant	34	95	85
chr7	28118667	28120667	Ltbp4	-0.15076	7.34E-08	hypomethylated	0.017954	0.37537	insignificant	11	67	87
chr7	28122631	28124631	Ltbp4	-0.24148	0.68114	insignificant	-0.021557	1	insignificant	7	22	30
chr7	28141027	28143027	Spnb4	-0.23037	1.12E-08	hypomethylated	0.0050878	0.023384	inconclusive	5	32	32
chr7	28179525	28181525	Spnb4	0.0035147	0.5911	insignificant	-0.020424	0.046431	hypomethylated	2	27	29
chr7	28181217	28183217	Spnb4	-0.057804	0.88972	insignificant	-0.011373	0.0082068	hypomethylated	7	77	75

chr7	28181572	28183572	Spnb4	-0.088864	0.23868	insignificant	-0.011832	0.14391	insignificant	7	52	52
chr7	28231608	28233608	Spnb4	-0.096888	2.07E-22	hypomethylated	0.0062505	0.55677	insignificant	31	90	90
chr7	28231996	28233996	Blvrb	-0.16626	6.38E-12	hypomethylated	0.013732	0.0057665	inconclusive	16	58	58
chr7	28257858	28259858	Sertad3	-0.19495	0.000000788	hypomethylated	-0.025035	0.39486	insignificant	18	59	77
chr7	28270971	28272971	Sertad1	-0.22091	6.49E-42	hypomethylated	-0.014974	0.00058256	hypomethylated	27	52	52
chr7	28283342	28285342	Prx	0.14858	1	insignificant	0.041213	1	insignificant	1	4	4
chr7	28307279	28309279	Hipk4	0.063751	0.38131	insignificant	0.0096408	0.76973	insignificant	6	24	24
chr7	28337313	28339313	2310022A10Rik	-0.19732	0.00000168	hypomethylated	-0.087334	0.48825	insignificant	7	33	32
chr7	28338131	28340131	Pld3	-0.14578	0.00000281	hypomethylated	-0.035804	0.60232	insignificant	7	31	32
chr7	28344494	28346494	2310022A10Rik		1	noCoverage	0.011869	0.017752	hypermethylated	0	9	10
chr7	28375578	28377578	Akt2	-0.11502	0.10765	insignificant	0.012281	0.37243	insignificant	6	18	26
chr7	28437942	28439942	Ttc9b	-0.087607	0.010324	hypomethylated	-0.023304	0.019944	hypomethylated	23	101	108
chr7	28458679	28460679	C030039L03Rik	-0.14069	1.15E-20	hypomethylated	-0.0056819	0.019953	hypomethylated	30	93	87
chr7	28459617	28461617	C030039L03Rik		1	noCoverage	0.021368	1	insignificant	0	4	4
chr7	28473358	28475358	C030039L03Rik	-0.63095	0.000000723	stronglyHypometh	-0.09573	0.063007	insignificant	1	12	12
chr7	28515427	28517427	Zfp60	-0.1818	2.98E-09	hypomethylated	0.016486	0.080697	insignificant	7	37	38
chr7	28549691	28551691	Gm10046		1	noCoverage	0.078149	0.45432	insignificant	0	32	30
chr7	28622602	28624602	Zfp59	-0.25833	3.13E-10	hypomethylated	-0.021483	0.66902	insignificant	10	28	28
chr7	28644603	28646603	Zfp607	0.24867	0.4742	insignificant	-0.043298	0.32169	insignificant	2	24	26
chr7	28714449	28716449	1700049G17Rik		1	noCoverage	0.012454	0.80734	insignificant	0	15	15
chr7	28835111	28837111	Psmc4	-0.22187	0.0011274	hypomethylated	-0.052611	0.9094	insignificant	5	16	16
chr7	28855254	28857254	Fcgbp	-0.090455	0.38318	insignificant	0.0013291	0.91985	insignificant	3	29	29
chr7	28913484	28915484	9530053A07Rik	-0.24679	0.028043	hypomethylated	-0.05967	0.33819	insignificant	1	7	7
chr7	28953766	28955766	Fbl	-0.1376	1.91E-32	hypomethylated	-0.0078468	0.00014661	hypomethylated	21	86	85
chr7	28963501	28965501	Dyrk1b	-0.11562	6.36E-11	hypomethylated	-0.010805	0.79097	insignificant	27	135	126
chr7	28963512	28965512	Dyrk1b	-0.11562	6.36E-11	hypomethylated	-0.010805	0.79097	insignificant	27	135	126
chr7	29051899	29053899	Eid2	-0.12606	1.87E-18	hypomethylated	0.0080267	0.68936	insignificant	30	115	115
chr7	29061724	29063724	Eid2b	-0.21931	1.4E-37	hypomethylated	-0.046943	2.17E-08	hypomethylated	27	99	110
chr7	29076153	29078153	BC089491	-0.063118	0.00012485	hypomethylated	-0.0087349	0.16339	insignificant	8	28	27
chr7	29086804	29088804	Dll3	-0.098783	0.0016462	hypomethylated	0.039251	0.45764	insignificant	6	33	32
chr7	29123738	29125738	Supt5h	0.090795	2.05E-27	hypermethylated	-0.0412	0.15194	insignificant	7	42	42
chr7	29134707	29136707	Rps16	-0.13455	0.00000333	hypomethylated	-0.0060233	0.36631	insignificant	8	69	69
chr7	29135558	29137558	LOC100302567	-0.12955	0.00000197	hypomethylated	-0.0055311	0.22573	insignificant	8	63	63
chr7	29157681	29159681	Plekhg2	-0.015704	0.23544	insignificant	0.010974	0.43361	insignificant	16	80	80
chr7	29164247	29166247	Zfp36	-0.26323	0.015477	hypomethylated	0.029506	0.029824	hypermethylated	5	36	39
chr7	29177014	29179014	Paf1	-0.18585	1.16E-17	hypomethylated	0.0079099	0.0010581	inconclusive	15	56	56
chr7	29177709	29179709	Med29	-0.35997	0.00003767	stronglyHypometh	-0.093107	0.80534	insignificant	2	12	8
chr7	29221210	29223210	Samd4b	-0.12627	2.79E-19	hypomethylated	-0.0075154	0.17236	insignificant	20	80	85
chr7	29221465	29223465	Gmfg	-0.15943	1.89E-19	hypomethylated	-0.020355	0.12703	insignificant	16	59	64
chr7	29236003	29238003	Lrfn1	-0.10446	3.07E-15	hypomethylated	0.010123	0.35299	insignificant	42	118	101
chr7	29236256	29238256	Lrfn1	-0.11324	4.74E-17	hypomethylated	0.0093332	0.28812	insignificant	43	120	103
chr7	29280959	29282959	1700028B04Rik	-0.11683	0.10193	insignificant	-0.10879	0.056338	insignificant	3	28	25
chr7	29324903	29326903	Sycn	-0.57169	0.035233	stronglyHypometh	-0.010892	0.29807	insignificant	1	20	20
chr7	29332273	29334273	Nccrp1	-0.081337	0.45976	insignificant	-0.073079	0.41786	insignificant	4	8	8
chr7	29383203	29385203	Pak4	-0.41913	0.000005	stronglyHypometh	-0.033738	0.10586	insignificant	3	12	16
chr7	29416034	29418034	C330005M16Ril	-0.15839	0.00000562	hypomethylated	-0.016702	0.076324	insignificant	8	17	24
chr7	29476867	29478867	Fbxo27	-0.29043	1.44E-13	hypomethylated	0.07378	0.32332	insignificant	5	49	46
chr7	29477162	29479162	Fbxo27	-0.24435	1.11E-12	hypomethylated	0.081889	0.60739	insignificant	5	41	38
chr7	29500808	29502808	Fbxo17	-0.18364	9.03E-10	hypomethylated	-0.027984	0.15782	insignificant	14	20	20
chr7	29526009	29528009	Mrps12	-0.15357	1.56E-12	hypomethylated	-0.0043748	0.35683	insignificant	20	73	73
chr7	29526800	29528800	Mrps12	-0.1847	7.58E-15	hypomethylated	-0.012519	0.92178	insignificant	17	63	63
chr7	29550770	29552770	Sirt2	-0.16024	9E-16	hypomethylated	-0.0011378	0.000020038	hypomethylated	24	76	76
chr7	29551543	29553543	Sirt2	-0.21354	0.000000358	hypomethylated	0.018399	0.056451	insignificant	15	58	58
chr7	29572987	29574987	Rinl	0.47283	0.55175	insignificant	0.059196	1	insignificant	3	15	12
chr7	29594908	29596908	Hnrnpl	-0.13902	3.76E-14	hypomethylated	0.017774	0.5149	insignificant	22	56	56
chr7	29609356	29611356	Ech1	-0.2678	1.15E-08	hypomethylated	0.075653	0.83625	insignificant	5	24	24
chr7	29665674	29667674	Capn12	-0.15723	0.0071771	inconclusive	0.11668	0.000001657	hypermethylated	8	28	28
chr7	29747299	29749299	Actn4	-0.22637	4.85E-10	hypomethylated	0.051364	0.89966	insignificant	16	63	67
chr7	29766833	29768833	Eif3k	-0.2312	0.00000135	hypomethylated	-0.028731	0.011579	hypomethylated	8	29	34
chr7	29766872	29768872	Map4k1	-0.26774	0.000044285	hypomethylated	-0.020837	0.05424	insignificant	6	28	32
chr7	29910170	29912170	Ryr1	-0.039583	0.64836	insignificant	-0.052083	0.71883	insignificant	1	2	4
chr7	29918951	29920951	Rasgrp4	-0.27611	0.0048909	hypomethylated	-0.011838	0.14297	insignificant	7	24	19
chr7	29941229	29943229	Fam98c	-0.81786	0.11667	lowCoverage	0.069015	1	insignificant	1	14	14
chr7	29953666	29955666	Spred3	-0.21076	0.000084626	hypomethylated	-0.0061924	0.16839	insignificant	7	46	46
chr7	29954228	29956228	Ggn	-0.19326	0.000047023	hypomethylated	-0.0059945	0.33181	insignificant	10	72	70
chr7	29954408	29956408	Ggn	-0.19326	0.000047023	hypomethylated	-0.0059945	0.33181	insignificant	10	72	70
chr7	29999052	30001052	Catsperg1	-0.1571	0.0011505	hypomethylated	-0.079599	0.20391	insignificant	4	16	16
chr7	30017541	30019541	Kcnk6	-0.21034	9.63E-15	hypomethylated	0.011471	0.82439	insignificant	7	26	18
chr7	30022341	30024341	Yif1b	-0.1496	3.23E-14	hypomethylated	-0.023073	0.46292	insignificant	16	69	70
chr7	30033486	30035486	2200002D01Rik	-0.24752	7.31E-11	hypomethylated	-0.11748	0.041408	hypomethylated	6	12	12
chr7	30066996	30068996	Spint2	-0.22488	0.000053721	hypomethylated	-0.088517	0.3508	insignificant	4	19	16
chr7	30073338	30075338	Ppp1r14a	-0.069221	0.00000002	hypomethylated	0.0026422	0.01848	hypermethylated	27	83	84
chr7	30088023	30090023	Dpf1	-0.23553	7.31E-32	hypomethylated	-0.0233	0.34393	insignificant	25	117	118
chr7	30552570	30554570	Zfp84	-0.33019	0.00000439	hypomethylated	-0.056731	0.039769	hypomethylated	8	22	23
chr7	30557312	30559312	Mir1964	-0.056548	1	insignificant	-0.039541	1	insignificant	2	4	4
chr7	30568808	30570808	Zfp30	0.046933	0.000000181	inconclusive	0.033862	3.49E-10	inconclusive	13	49	48
chr7	30600090	30602090	Zfp790	-0.26	0.00057513	hypomethylated	0.00088639	0.44546	insignificant	8	40	36
chr7	30643997	30645997	Zfp420	-0.26423	0.00095247	hypomethylated	-0.065451	0.46623	insignificant	6	16	15
chr7	30691123	30693123	Zfp27	0.018522	0.62319	insignificant	0.0082899	0.039294	hypermethylated	4	16	21
chr7	30736912	30738912	Zfp74	-0.18458	0.23115	insignificant	-0.044048	0.63184	insignificant	2	8	8
chr7	30767973	30769973	Zfp568	-0.11809	2.29E-24	hypomethylated	-0.013653	0.21842	insignificant	18	66	68
chr7	30836415	30838415	Zfp14		1	noCoverage	0.06323	0.0013235	inconclusive	0	7	14
chr7	30857842	30859842	Zfp82		1	noCoverage	-0.036469	0.7487	insignificant	0	16	14
chr7	30879094	30881094	Zfp260	-0.30504	0.0010133	hypomethylated	-0.076614	0.12815	insignificant	4	20	20
chr7	30905966	30907966	Zfp382	-0.16718	5.24E-25	hypomethylated	0.03055	0.19836	insignificant	16	52	50

chr7	30953940	30955940	Gm5113	-0.39948	0.00013388	stronglyHypometh	-0.14425	0.64556	insignificant	3	14	24
chr7	30954746	30956746	Zfp146	-0.39573	1	insignificant	-0.15993	0.92735	insignificant	1	8	16
chr7	30980067	30982067	Capns1	-0.41172	1.74E-12	stronglyHypometh	-0.010298	0.000016269	hypomethylated	3	31	32
chr7	31016092	31018092	Polr2i	-0.1054	0.00031468	hypomethylated	0.012782	0.0012224	hypermethylated	20	126	111
chr7	31017048	31019048	Tbcb	0.033385	1.57E-09	hypermethylated	0.056999	0.040853	hypermethylated	18	80	65
chr7	31065440	31067440	Wdr62	-0.39459	0.15911	insignificant	-0.043775	0.34697	insignificant	2	12	12
chr7	31092771	31094771	Alkbh6		1	noCoverage	0.01265	0.19803	insignificant	0	12	12
chr7	31098834	31100834	AI428936	0.0048961	0.37081	insignificant	-0.0067147	0.38044	insignificant	12	29	29
chr7	31107394	31109394	Sdhaf1	-0.12041	2.36E-09	hypomethylated	0.009271	0.81576	insignificant	6	24	24
chr7	31147791	31149791	Lrfn3	-0.14878	0.000002413	hypomethylated	0.018887	0.27711	insignificant	13	43	43
chr7	31197806	31199806	Tyrobp	-0.53136	0.000000112	stronglyHypometh	0.16978	0.42952	insignificant	1	4	6
chr7	31204873	31206873	Hcst	-0.12422	0.00012269	hypomethylated	0.0078871	0.81749	insignificant	8	42	42
chr7	31207322	31209322	Nfkbid	-0.1901	0.023816	hypomethylated	-0.0074443	0.16539	insignificant	3	52	52
chr7	31230580	31232580	Aplp1	-0.49542	4.68E-30	stronglyHypometh	-0.036748	0.000000528	hypomethylated	9	35	34
chr7	31242534	31244534	Nphs1	-0.16719	0.00002067	hypomethylated	0.0093414	0.78595	insignificant	6	18	18
chr7	31244076	31246076	Nphs1	-0.017837	0.0039866	hypomethylated	-0.0011934	0.27503	insignificant	5	21	20
chr7	31250610	31252610	Nphs1	-0.18096	0.082065	insignificant	0.076798	0.58079	insignificant	5	34	31
chr7	31277676	31279676	Prodh2	-0.086957	1	insignificant	-0.35951	0.015238	stronglyhypometh	2	8	8
chr7	31299140	31301140	Gm1082	-0.28033	0.029481	hypomethylated	-0.033502	0.41369	insignificant	3	12	13
chr7	31320028	31322028	Arhgap33	-0.16131	0.00000154	hypomethylated	-0.015638	0.58189	insignificant	9	26	27
chr7	31337291	31339291	BC053749	0.049766	0.001362	inconclusive	-0.041484	0.0013266	hypomethylated	8	41	40
chr7	31337320	31339320	Hspb6	0.049766	0.001362	inconclusive	-0.041484	0.0013266	hypomethylated	8	41	40
chr7	31344665	31346665	Lin37		1	noCoverage	-0.098482	0.071864	insignificant	0	8	13
chr7	31347358	31349358	U2af1l4	-0.20008	9.84E-22	hypomethylated	-0.0082309	0.24437	insignificant	17	70	73
chr7	31348203	31350203	Psenen	-0.13612	1.68E-16	hypomethylated	-0.013059	0.0086917	inconclusive	22	96	97
chr7	31349445	31351445	Tmem149	-0.057865	0.71751	insignificant	-0.026443	1	insignificant	8	38	39
chr7	31373745	31375745	Zbtb32	-0.13595	1.77E-10	hypomethylated	-0.036207	0.000000624	inconclusive	20	141	148
chr7	31411170	31413170	Cox6b1	-0.36734	0.000000419	stronglyHypometh	-0.027438	0.006628	hypomethylated	6	33	32
chr7	31435247	31437247	Rbm42	-0.33575	0.56038	insignificant	-0.049882	0.38107	insignificant	3	35	34
chr7	31450013	31452013	Haus5	-0.11471	1	insignificant	0.0034759	0.80453	insignificant	4	10	10
chr7	31482944	31484944	2200002J24Rik	0.40947	1	lowCoverage	0.14119	0.099381	insignificant	1	12	10
chr7	31514553	31516553	Gapdhs	-0.13407	0.000019996	hypomethylated	0.019528	0.38461	insignificant	4	28	28
chr7	31547774	31549774	Dmkn	-0.042561	1	insignificant	-0.07161	0.088431	insignificant	6	16	20
chr7	31571923	31573923	Krtdap	-0.12197	0.0022481	hypomethylated	0.015273	0.87783	insignificant	8	29	34
chr7	31664047	31666047	Cd22	-0.23154	0.13644	insignificant	-0.032826	0.32212	insignificant	5	12	12
chr7	31665361	31667361	Cd22	-0.11118	0.00024369	hypomethylated	-0.11513	0.059433	insignificant	2	4	4
chr7	31699851	31701851	Mag	0.0026702	0.84723	insignificant	-0.0012868	1	insignificant	3	8	8
chr7	31729036	31731036	Hamp		1	noCoverage	-0.0609	0.33255	insignificant	0	6	6
chr7	31741822	31743822	Usf2	-0.1085	1.46E-11	hypomethylated	0.0097213	0.0077906	inconclusive	33	138	141
chr7	31758488	31760488	Lsr	-0.16199	6.48E-13	hypomethylated	-0.024027	0.67167	insignificant	10	44	48
chr7	31826837	31828837	Fxyd5	-0.19326	0.0771	insignificant	-0.035462	0.33614	insignificant	2	31	28
chr7	31827341	31829341	Fxyd5	0.010919	0.64964	insignificant	0.0044195	0.023111	inconclusive	2	8	6
chr7	31836473	31838473	Fxyd7	-0.12655	0.0028733	hypomethylated	-0.019638	0.50472	insignificant	5	43	49
chr7	31861716	31863716	Fxyd3	-0.1308	0.13194	insignificant	0.085985	0.066525	insignificant	2	4	4
chr7	31900309	31902309	Hpn	-0.20023	0.00000041	hypomethylated	0.049665	0.86417	insignificant	10	35	35
chr7	31911964	31913964	Scn1b	-0.13275	3.29E-13	hypomethylated	-0.015703	0.21386	insignificant	15	47	50
chr7	31936069	31938069	Gramd1a	-0.197	1.23E-08	hypomethylated	-0.078526	0.000034108	hypomethylated	10	39	34
chr7	34918287	34920287	Wtip	-0.15017	1	lowCoverage	0.011807	0.030717	inconclusive	1	46	46
chr7	34953548	34955548	Uba2	-0.19947	0.019606	hypomethylated	-0.044524	0.000016235	hypomethylated	12	40	40
chr7	34981666	34983666	Pdcd2l		1	noCoverage	-0.036856	0.018368	hypomethylated	0	18	16
chr7	35015324	35017324	Gpi1	-0.096702	0.000000176	hypomethylated	0.0043983	0.76189	insignificant	5	26	26
chr7	35174559	35176559	Lsm14a	-0.020453	0.094784	insignificant	-0.0068068	0.000010758	hypomethylated	22	121	123
chr7	35437860	35439860	Kctd15	-0.12348	9.14E-12	hypomethylated	0.0096835	0.55782	insignificant	19	145	147
chr7	35597730	35599730	Chst8	0.47389	0.00024947	stronglyHypermeth	0.116	0.040589	hypermethylated	3	17	12
chr7	35841585	35843585	Cebpg	-0.10782	0.00017173	hypomethylated	0.015782	0.32953	insignificant	12	40	43
chr7	35903311	35905311	Cebpa	-0.10891	1.03E-43	hypomethylated	-0.0033285	0.27998	insignificant	68	256	253
chr7	35970403	35972403	Slc7a10	-0.13357	1.82E-32	hypomethylated	0.019641	0.10042	insignificant	25	97	94
chr7	36000364	36002364	Lrp3	-0.0721	3.48E-09	hypomethylated	-0.001557	0.22626	insignificant	17	62	62
chr7	36103459	36105459	Gpatch1	0.55838	0.0083186	stronglyHypermeth	0.019397	0.012805	hypermethylated	1	16	16
chr7	36118255	36120255	Rhpn2	-0.16186	0.000000635	hypomethylated	0.000161	0.1978	insignificant	11	72	74
chr7	36181111	36183111	Ccdc123	-0.066992	0.000013781	hypomethylated	0.12075	0.56332	insignificant	5	37	33
chr7	36181786	36183786	C230052I12Rik	-0.092762	0.00000036	hypomethylated	0.1829	0.20384	insignificant	5	27	23
chr7	36233110	36235110	Slc7a9	-0.16905	1	insignificant	0.069049	0.21976	insignificant	1	8	8
chr7	36233116	36235116	Slc7a9	-0.16905	1	insignificant	0.069049	0.21976	insignificant	1	8	8
chr7	36233197	36235197	Slc7a9	-0.16905	1	insignificant	0.069049	0.21976	insignificant	1	8	8
chr7	36322763	36324763	Tdrd12	0.12313	1	insignificant	0.02971	0.57571	insignificant	2	10	10
chr7	36340947	36342947	Nudt19	-0.15921	0.00095717	hypomethylated	-0.014779	0.5696	insignificant	3	8	8
chr7	36370265	36372265	Ankrd27	-0.16903	1.43E-14	hypomethylated	-0.0059308	0.04401	inconclusive	19	97	94
chr7	36370601	36372601	Rgs9bp	-0.1881	2.06E-10	hypomethylated	0.0038209	0.024033	hypermethylated	12	62	58
chr7	36432501	36434501	Pdcd5		1	noCoverage	0.0054233	0.9123	insignificant	0	12	12
chr7	36539473	36541473	Dpy19l3	-0.143	0.00024983	hypomethylated	-0.028296	0.92411	insignificant	12	32	32
chr7	36586610	36588610	E130304I02Rik	-0.11871	2.16E-11	hypomethylated	-0.019758	0.40857	insignificant	25	64	70
chr7	36588008	36590008	E130304I02Rik	-0.11878	0.019312	hypomethylated	-0.007841	0.08037	insignificant	4	8	8
chr7	37482136	37484136	Tshz3	-0.11023	1.43E-30	hypomethylated	0.017546	0.35262	insignificant	53	198	192
chr7	38554771	38556771	Zfp536	-0.15504	1.88E-28	hypomethylated	-0.0017717	0.57586	insignificant	48	145	150
chr7	38804571	38806571	C80913	-0.12866	0.000001094	hypomethylated	-0.018818	0.33465	insignificant	9	20	20
chr7	38892509	38894509	Ccne1	-0.12303	1.28E-38	hypomethylated	-0.0045272	0.60007	insignificant	39	126	126
chr7	38967235	38969235	1600014C10Rik	-0.16478	0.000070326	hypomethylated	0.0064612	0.66466	insignificant	13	40	40
chr7	38967805	38969805	1600014C10Rik	-0.19261	0.000058527	hypomethylated	0.015021	0.71099	insignificant	13	33	33
chr7	39013013	39015013	Plekhf1		1	noCoverage	0.01768	0.86046	insignificant	0	28	20
chr7	39056367	39058367	Pop4		1	noCoverage	-0.016667	0.76721	insignificant	0	12	12
chr7	46519904	46521904	Gm16387		1	noCoverage	-0.0030556	1	insignificant	0	5	4
chr7	46772135	46774135	Zfp619	-0.16516	0.00000344	hypomethylated	0.0038067	0.43284	insignificant	8	56	56
chr7	46842887	46844887	Gm2058	-0.10047	0.011188	hypomethylated	-0.013868	0.57762	insignificant	5	61	53
chr7	48153647	48155647	Vstm2b	-0.14788	3.39E-32	hypomethylated	0.0097107	0.89439	insignificant	40	112	111

chr7	48648630	48650630	AI987944		1	noCoverage	-0.010398	0.27896	insignificant	0	13	13
chr7	48648749	48650749	AI987944		1	noCoverage	-0.010398	0.27896	insignificant	0	13	13
chr7	48755260	48757260	AW146154	0.51804	1	insignificant	0.0039405	0.01634	inconclusive	1	14	16
chr7	48887900	48889900	Zfp788		1	noCoverage	0.028572	0.92087	insignificant	0	17	17
chr7	48922383	48924383	Vmn2r-ps54	-0.010743	0.59284	insignificant	-0.028799	0.11893	insignificant	1	15	15
chr7	49703450	49705450	4933421I07Rik	0	1	insignificant	-0.073696	0.055366	insignificant	1	4	7
chr7	49948088	49950088	Gm5595	-0.38655	0.00000045	stronglyHypometh	-0.0091002	0.8897	insignificant	3	6	6
chr7	50189159	50191159	Vmn2r63	0.12412	1	insignificant	-0.0025057	1	insignificant	1	4	4
chr7	50431957	50433957	Zfp936	-0.294	0.019891	hypomethylated	-0.12525	0.28173	insignificant	3	10	10
chr7	50568631	50570631	Zfp715	-0.20161	3.47E-09	hypomethylated	-0.020167	0.72855	insignificant	8	16	19
chr7	50605774	50607774	Siglec5	-0.51958	0.00015108	stronglyHypometh	0.013987	1	insignificant	3	12	12
chr7	50684470	50686470	Lim2	-0.28214	0.109	insignificant	-0.032837	0.67306	insignificant	2	8	8
chr7	50698512	50700512	Etfb	-0.18889	7.62E-12	hypomethylated	-0.01753	0.50929	insignificant	16	38	39
chr7	50745393	50747393	Iglon5	-0.19898	0.0081246	hypomethylated	-0.013488	0.10122	insignificant	9	41	41
chr7	50788541	50790541	Cd33		1	noCoverage	0.16477	0.63207	insignificant	0	4	4
chr7	50816739	50818739	Zfp658	0.38542	0.30826	insignificant	-0.12623	0.38996	insignificant	3	16	14
chr7	50833955	50835955	Zfp719	-0.20861	1	lowCoverage	0.0033525	0.18886	insignificant	1	16	16
chr7	50861538	50863538	Zfp819	-0.36408	1.48E-19	stronglyHypometh	0.012358	0.2829	insignificant	10	40	40
chr7	50926400	50928400	Ctu1	0.1354	0.19658	insignificant	0.014074	0.049451	inconclusive	6	18	17
chr7	50966936	50968936	Klk13	-0.35954	0.048075	stronglyHypometh	0.0092648	1	insignificant	2	8	8
chr7	51023469	51025469	Klk12	0.03125	1	insignificant	-0.060096	0.5076	insignificant	1	8	8
chr7	51046260	51048260	Klk9	-0.19716	0.041144	hypomethylated	-0.11486	0.0011499	hypomethylated	5	20	20
chr7	51051946	51053946	Klk8	-0.10624	0.0178	hypomethylated	-0.012921	0.66587	insignificant	9	36	36
chr7	51078913	51080913	Klk6	0.13456	1	insignificant	0.026604	0.6119	insignificant	2	8	6
chr7	51079819	51081819	Klk6	0.17941	1	insignificant	0.071457	0.60938	insignificant	2	6	6
chr7	51096638	51098638	Klk5	-0.63424	0.10249	insignificant	0.0066834	0.88482	insignificant	1	6	6
chr7	51491492	51493492	1700028J19Rik	-0.027687	0.864	insignificant	-0.042114	0.91397	insignificant	5	18	18
chr7	51501091	51503091	2410002F23Rik	-0.11164	7.58E-25	hypomethylated	-0.0058869	0.32025	insignificant	24	90	91
chr7	51564633	51566633	Shank1	-0.15291	0.00000021	hypomethylated	-0.006408	1	insignificant	18	95	95
chr7	51630400	51632400	1700008O03Rik	-0.39731	0.17901	insignificant	-0.10993	0.0022566	hypomethylated	1	8	8
chr7	51638495	51640495	Syt3	-0.19489	0.084409	insignificant	0.031687	0.69189	insignificant	2	24	20
chr7	51696856	51698856	Lrrc4b	-0.30839	4.92E-09	hypomethylated	-0.037969	0.32401	insignificant	4	18	18
chr7	51722425	51724425	Josd2	-0.15626	5.55E-16	hypomethylated	-0.02295	0.00000281	hypomethylated	23	51	52
chr7	51750957	51752957	Fam71e1	-0.12555	5.54E-15	hypomethylated	0.032681	0.27904	insignificant	28	107	99
chr7	51751883	51753883	2310044H10Rik	-0.064317	0.000000208	hypomethylated	0.058756	0.53271	insignificant	16	71	68
chr7	51780039	51782039	Mybpc2	-0.22448	0.025187	hypomethylated	-0.056649	0.0017562	hypomethylated	8	19	23
chr7	51787441	51789441	Spib	0.029791	0.2936	insignificant	0.014591	0.43456	insignificant	4	13	13
chr7	51804185	51806185	Nr1h2	-0.16593	0.070254	insignificant	-0.020512	0.46368	insignificant	2	6	6
chr7	51809293	51811293	Nr1h2	-0.49798	3.39E-10	stronglyHypometh	-0.024611	0.58591	insignificant	3	26	26
chr7	51845255	51847255	Kcnc3	-0.13756	2.47E-42	hypomethylated	-0.0024913	0.89968	insignificant	57	151	165
chr7	51963112	51965112	Izumo2		1	noCoverage	-0.021529	0.70136	insignificant	0	26	26
chr7	52002998	52004998	Vrk3	-0.22507	6.71E-25	hypomethylated	-0.013685	0.18579	insignificant	18	56	56
chr7	52003987	52005987	Zfp473	-0.20842	2.3E-11	hypomethylated	-0.018527	0.53398	insignificant	9	30	30
chr7	52070739	52072739	Nup62-il4i1	-0.12689	9.65E-17	hypomethylated	-0.010178	0.65253	insignificant	20	63	63
chr7	52070789	52072789	Nup62	-0.12689	9.65E-17	hypomethylated	-0.010178	0.65253	insignificant	20	63	63
chr7	52071028	52073028	Atf5	-0.12689	9.65E-17	hypomethylated	-0.015546	0.6524	insignificant	20	63	66
chr7	52072028	52074028	Nup62-il4i1	-0.067363	0.000000497	hypomethylated	-0.012265	0.41393	insignificant	9	30	33
chr7	52103596	52105596	Akt1s1	-0.18925	0.0045735	hypomethylated	-0.017277	0.90361	insignificant	5	47	52
chr7	52104068	52106068	Mir707	-0.17283	0.13352	insignificant	-0.0055976	0.60259	insignificant	2	37	38
chr7	52104449	52106449	Akt1s1		1	noCoverage	-0.021531	0.31626	insignificant	0	24	25
chr7	52111550	52113550	Pnkp	-0.20233	9.98E-19	hypomethylated	-0.024304	0.10344	insignificant	11	40	40
chr7	52125158	52127158	Ptov1	-0.16388	1	insignificant	0.083708	0.10371	insignificant	2	19	25
chr7	52147736	52149736	Med25	-0.18271	9.01E-23	hypomethylated	0.00057903	0.73916	insignificant	22	58	58
chr7	52150448	52152448	Fuz	-0.18982	0.000033808	hypomethylated	-0.026125	0.94231	insignificant	9	36	36
chr7	52184860	52186860	Ap2a1	-0.13503	0.00000037	hypomethylated	-0.021716	0.88366	insignificant	12	68	68
chr7	52197609	52199609	Tsks	0.15732	0.70229	insignificant	-0.00085322	0.23427	insignificant	10	36	37
chr7	52241702	52243702	Prmt1	-0.61449	4.63E-08	stronglyHypometh	0.046958	0.90793	insignificant	1	6	6
chr7	52252029	52254029	Irf3	-0.15021	1	insignificant	-0.0028276	0.23054	insignificant	3	41	41
chr7	52252949	52254949	Irf3	-0.11174	1	insignificant	-0.0079663	0.69387	insignificant	3	20	26
chr7	52271619	52273619	Rras	-0.16526	4.38E-14	hypomethylated	-0.0072994	0.22368	insignificant	28	71	71
chr7	52272376	52274376	Rras	-0.17598	7.3E-13	hypomethylated	-0.005327	0.365	insignificant	23	61	61
chr7	52308251	52310251	Prr12	-0.14569	7.02E-10	hypomethylated	0.006944	0.36176	insignificant	7	47	51
chr7	52316798	52318798	Nosip	-0.30528	0.00024176	hypomethylated	-0.01856	0.63699	insignificant	5	28	30
chr7	52317022	52319022	Prrg2	-0.30528	0.00024176	hypomethylated	-0.01856	0.63699	insignificant	5	28	30
chr7	52347583	52349583	Rcn3	-0.051693	1	insignificant	-0.036051	1	insignificant	2	6	6
chr7	52359192	52361192	Fcgrt	0.009342	1	insignificant	-0.076878	0.018906	hypomethylated	4	25	27
chr7	52378481	52380481	Rps11	-0.12971	5.46E-14	hypomethylated	0.014832	0.32931	insignificant	22	98	98
chr7	52379759	52381759	Rps11	-0.14052	0.21954	insignificant	0.012279	0.85856	insignificant	3	8	8
chr7	52381805	52383805	Snord35a	0.28984	0.57721	insignificant	-0.058477	0.45842	insignificant	3	15	15
chr7	52382038	52384038	Snord35a	0.28984	0.57721	insignificant	-0.058477	0.45842	insignificant	3	15	15
chr7	52382315	52384315	Snord35a	-0.00022782	0.52782	insignificant	-0.026337	0.27129	insignificant	13	45	46
chr7	52382833	52384833	Snord34	-0.097726	0.00000882	hypomethylated	-0.015168	0.027624	hypomethylated	18	50	51
chr7	52384105	52386105	Rpl13a	-0.14305	0.000000249	hypomethylated	0.011286	0.043423	inconclusive	12	36	38
chr7	52391802	52393802	Flt3l	-0.175	0.0048	hypomethylated	0.0071429	0.80993	insignificant	5	20	20
chr7	52408767	52410767	Pih1d1	-0.22911	0.000084735	hypomethylated	-0.01238	0.85964	insignificant	10	38	38
chr7	52409908	52411908	Pih1d1		1	noCoverage	0.0028756	0.45579	insignificant	0	27	28
chr7	52418290	52420290	Slc17a7	-0.2823	6.85E-12	hypomethylated	-0.0088253	0.39303	insignificant	18	57	57
chr7	52434967	52436967	Gm581		1	noCoverage	-0.084666	0.59457	insignificant	0	20	16
chr7	52435364	52437364	Pth2	-0.41625	0.000000263	stronglyHypometh	-0.022411	0.01367	inconclusive	2	32	28
chr7	52460262	52462262	Ccdc155	0.23726	1	lowCoverage	0.050353	0.28915	insignificant	1	19	21
chr7	52467253	52469253	Dkkl1	-0.13428	0.000029063	hypomethylated	-0.11184	0.0038515	hypomethylated	6	22	24
chr7	52470122	52472122	Tead2	-0.15353	2.91E-09	hypomethylated	0.024071	0.010042	hypermethylated	21	60	70
chr7	52494209	52496209	Cd37	0.037049	0.83774	insignificant	-0.0038732	0.0016409	inconclusive	8	34	34
chr7	52537242	52539242	1700039E15Rik	-0.054411	0.37541	insignificant	-0.050515	0.082713	insignificant	8	26	26
chr7	52589150	52591150	Trpm4	-0.019367	1	insignificant	0.0097309	0.4429	insignificant	2	6	6

chr7	52589638	52591638	Hrc	-0.018305	1	insignificant	0.041019	1	insignificant	2	8	8
chr7	52621602	52623602	Mtag2	-0.13086	6.67E-21	hypomethylated	0.0015621	0.066144	insignificant	29	129	132
chr7	52622389	52624389	Lin7b	-0.11926	0.000017768	hypomethylated	0.0011471	0.68715	insignificant	14	69	81
chr7	52625934	52627934	Lin7b	0.25427	0.67577	insignificant	-0.16075	0.0018329	hypomethylated	1	14	18
chr7	52651017	52653017	Snrnp70	-0.10198	0.00019756	hypomethylated	-0.0026596	0.96317	insignificant	14	62	62
chr7	52660329	52662329	Kcna7	-0.12447	2.34E-23	hypomethylated	-0.011049	0.029747	hypomethylated	34	144	141
chr7	52668064	52670064	Ntf5	-0.20731	5.94E-31	hypomethylated	-0.021786	0.00000833	hypomethylated	18	45	46
chr7	52675315	52677315	Lhb	-0.047797	0.000055653	hypomethylated	0.0091113	0.29427	insignificant	21	56	57
chr7	52689208	52691208	Gys1	-0.20302	4.14E-09	hypomethylated	0.026994	0.69047	insignificant	9	30	34
chr7	52689834	52691834	Ruvbl2	-0.21226	0.00000183	hypomethylated	0.073208	0.45172	insignificant	3	16	20
chr7	52715256	52717256	Ftl1	-0.17298	0.013268	hypomethylated	0.061654	0.3168	insignificant	4	31	32
chr7	52722268	52724268	Bax		1	noCoverage	0.0018082	0.00000263	hypermethylated	0	38	40
chr7	52744166	52746166	Dhdh		1	noCoverage	-0.13538	0.03694	hypomethylated	0	18	18
chr7	52780699	52782699	Plekha4	-0.13298	0.0022212	hypomethylated	0.043433	0.16643	insignificant	7	74	80
chr7	52781638	52783638	Ppp1r15a	0.014196	0.75681	insignificant	0.10661	0.000093654	hypermethylated	1	22	28
chr7	52809297	52811297	Hsd17b14		1	noCoverage	0.026295	0.57199	insignificant	0	12	16
chr7	52824732	52826732	0610005C13Rik	-0.18361	0.0017165	hypomethylated	-0.0051387	0.0072322	inconclusive	18	66	70
chr7	52870860	52872860	Fut1	-0.30013	0.4905	insignificant	-0.00011421	0.63523	insignificant	2	12	14
chr7	52871975	52873975	Fut1	-0.22954	0.000010601	hypomethylated	-0.034934	0.00012097	hypomethylated	9	30	32
chr7	52876180	52878180	Izumo1		1	noCoverage	-0.056406	0.53448	insignificant	0	8	8
chr7	52881906	52883906	Rasip1	-0.225	0.17234	insignificant	0.015432	1	insignificant	2	6	9
chr7	52894346	52896346	Mamstr	0.080066	0.73211	insignificant	0.10551	0.013352	hypermethylated	6	40	36
chr7	52934928	52936928	Sec1	-0.10558	0.39187	insignificant	-0.12798	0.018041	hypomethylated	3	23	17
chr7	52938783	52940783	Ntn5	0.098386	1	insignificant	-0.033074	0.037675	hypomethylated	1	11	10
chr7	52954336	52956336	Car11	-0.17615	0.00000148	hypomethylated	-0.014516	0.3305	insignificant	13	52	52
chr7	52959616	52961616	Dbp	-0.24532	1.21E-25	hypomethylated	-0.0095015	0.068032	insignificant	14	42	42
chr7	52970577	52972577	Sphk2	-0.14181	0.36601	insignificant	0.0076013	0.86491	insignificant	2	24	24
chr7	52972440	52974440	Rpl18	-0.14255	4.2E-20	hypomethylated	-0.0065704	0.21665	insignificant	36	117	117
chr7	52973372	52975372	Sphk2	-0.21215	1.02E-14	hypomethylated	0.0023954	0.043413	inconclusive	19	69	71
chr7	52975589	52977589	Fam83e	-0.054047	0.094487	insignificant	-0.029837	0.75126	insignificant	6	17	17
chr7	52981186	52983186	Spaca4	0.12383	1	insignificant	0.18067	0.75035	insignificant	3	13	18
chr7	53014925	53016925	Sult2b1	-0.38015	0.000000367	stronglyHypometh	0.0055259	0.5835	insignificant	7	25	28
chr7	53038316	53040316	Lmtk3	-0.11026	0.15653	insignificant	0.032758	0.71491	insignificant	7	36	45
chr7	53069686	53071686	Cyth2	-0.78479	0.30337	lowCoverage	-0.22415	0.44113	insignificant	1	6	9
chr7	53122051	53124051	Grin2d		1	noCoverage	0.24449	1	insignificant	0	4	2
chr7	53127209	53129209	Kdelr1	-0.22214	1.63E-24	hypomethylated	0.029138	0.88822	insignificant	12	54	52
chr7	53151438	53153438	Tmem143	-0.11984	1	insignificant	0.093042	0.93229	insignificant	7	20	25
chr7	53152081	53154081	Syngr4	-0.066486	1	insignificant	0.13932	0.42303	insignificant	7	16	19
chr7	53176219	53178219	Emp3	-0.076418	0.77778	insignificant	0.0061126	0.35381	insignificant	3	10	8
chr7	53176796	53178796	Emp3	0.16503	0.44818	insignificant	0.019541	0.60172	insignificant	2	8	6
chr7	53182767	53184767	Ccdc114	-0.056397	0.29234	insignificant	-0.014262	0.55761	insignificant	7	22	18
chr7	53285656	53287656	Abcc6		1	noCoverage	-0.21818	0.18347	insignificant	0	2	2
chr7	53288065	53290065	Nomo1	-0.13122	6.72E-23	hypomethylated	-0.010301	0.00083665	hypomethylated	30	91	91
chr7	53355607	53357607	Kcnj11	-0.28411	2.24E-16	hypomethylated	-0.042856	1	insignificant	5	24	27
chr7	53435403	53437403	Abcc8	-0.13995	0.00060842	hypomethylated	-0.015398	0.46784	insignificant	4	23	23
chr7	53493860	53495860	Ush1c	-0.22934	0.092595	insignificant	-0.058773	0.73789	insignificant	2	10	10
chr7	53495356	53497356	Otog	-0.0014805	0.62519	insignificant	-0.077815	0.038952	hypomethylated	4	15	16
chr7	53630843	53632843	Myod1	-0.095016	0.070231	insignificant	-0.00043769	0.27025	insignificant	11	73	73
chr7	53650837	53652837	Kcnc1	-0.10015	8.33E-46	hypomethylated	-0.0014937	0.061402	insignificant	43	169	160
chr7	53650866	53652866	Kcnc1	-0.10015	8.33E-46	hypomethylated	-0.0014937	0.061402	insignificant	43	169	160
chr7	53895177	53897177	Sergef	-0.15446	7.12E-12	hypomethylated	-0.011649	0.017628	hypomethylated	13	38	36
chr7	53922718	53924718	Tph1	-0.12869	0.3248	insignificant	0.081894	0.89029	insignificant	7	16	18
chr7	53927907	53929907	Tph1		1	noCoverage	0.0287	0.8462	insignificant	0	6	6
chr7	53966021	53968021	Saal1	-0.14209	0.0013822	hypomethylated	-0.0012076	0.1161	insignificant	12	30	30
chr7	53998350	54000350	Saa1	-0.79116	0.027132	stronglyHypometh	0.039027	0.58841	insignificant	2	19	20
chr7	54006202	54008202	Saa2		1	noCoverage	0.1113	1	insignificant	0	5	5
chr7	54050463	54052463	Gtf2h1	-0.15496	3.79E-16	hypomethylated	-0.013684	0.65699	insignificant	26	109	124
chr7	54051251	54053251	Hps5	-0.16042	3.65E-16	hypomethylated	-0.010352	0.72417	insignificant	26	95	107
chr7	54100173	54102173	Ldha	-0.16659	0.000016663	hypomethylated	-0.0039519	0.38233	insignificant	16	75	70
chr7	54101451	54103451	Ldha	0.44315	1.07E-09	stronglyHypermeth	0.098874	8E-11	hypermethylated	4	28	23
chr7	54175300	54177300	Tsg101	-0.12268	9.99E-27	hypomethylated	-0.011479	0.4782	insignificant	11	44	45
chr7	54213888	54215888	Uevld	-0.12882	1	insignificant	0.017808	0.46702	insignificant	2	30	30
chr7	54263784	54265784	Spty2d1	-0.33789	2.56E-08	stronglyHypometh	-0.0066964	0.89492	insignificant	4	8	8
chr7	54305009	54307009	Tmem86a	-0.13123	7.85E-29	hypomethylated	0.0028684	0.14602	insignificant	27	80	82
chr7	54387968	54389968	Ptpn5	-0.15393	0.59508	insignificant	0.020324	0.865	insignificant	5	30	40
chr7	54389054	54391054	Ptpn5		1	noCoverage	0.071589	1	insignificant	0	10	7
chr7	56043372	56045372	Zdhhc13	-0.074571	6.87E-11	hypomethylated	0.010622	0.96365	insignificant	25	66	66
chr7	56103421	56105421	Csrp3	-0.067857	0.40378	insignificant	-0.17686	0.71449	insignificant	2	6	6
chr7	56136411	56138411	E2f8	-0.12505	4.5E-42	hypomethylated	0.0023274	0.0072425	inconclusive	43	173	163
chr7	56213442	56215442	Nav2	-0.27494	6.39E-18	hypomethylated	-0.0062343	0.65712	insignificant	16	49	49
chr7	56500558	56502558	Nav2	-0.10933	0.000000587	hypomethylated	-0.0059558	0.60572	insignificant	36	172	172
chr7	56892205	56894205	Dbx1	-0.2543	1.13E-08	hypomethylated	-0.034665	0.3826	insignificant	9	58	55
chr7	57013475	57015475	Htatip2	-0.13904	6.17E-14	hypomethylated	0.0083861	0.24369	insignificant	20	60	66
chr7	57013881	57015881	Htatip2	-0.13904	6.17E-14	hypomethylated	0.0083861	0.24369	insignificant	20	60	66
chr7	57032727	57034727	Prmt3	-0.09833	4.05E-15	hypomethylated	-0.023527	0.046192	hypomethylated	37	102	114
chr7	57164668	57166668	Slc6a5	-0.2643	0.00000521	hypomethylated	-0.023312	0.35055	insignificant	7	24	24
chr7	57166069	57168069	Slc6a5	-0.14412	1.61E-22	hypomethylated	0.0051991	0.14143	insignificant	27	65	70
chr7	57229719	57231719	Nell1	-0.12124	1.05E-12	hypomethylated	0.0042604	0.078532	insignificant	28	122	129
chr7	58765398	58767398	Ano5	-0.26522	0.011067	hypomethylated	0.064687	0.0040409	hypermethylated	8	21	22
chr7	58876199	58878199	Slc17a6	-0.11845	0.0019529	hypomethylated	-0.0026579	0.95846	insignificant	14	44	44
chr7	59261084	59263084	Svip	-0.1323	9.05E-11	hypomethylated	0.036286	0.87051	insignificant	9	18	19
chr7	62089614	62091614	Luzp2	-0.26712	0.00069056	hypomethylated	-0.013411	0.93933	insignificant	4	18	18
chr7	63048517	63050517	Tubgcp5	-0.13946	0.000000068	hypomethylated	-0.021093	0.000000486	hypomethylated	15	66	71
chr7	63096440	63098440	Cyfip1	-0.16145	2.54E-16	hypomethylated	-0.013311	0.73827	insignificant	25	68	73
chr7	63216900	63218900	A230056P14Rik	-0.089909	4.04E-22	hypomethylated	-0.0080789	0.76088	insignificant	27	112	100

chr7	63217820	63219820	Nipa2	-0.083393	0.000069886	hypomethylated	-0.0014074	0.050194	insignificant	6	34	26
chr7	63274943	63276943	Nipa1	-0.091542	0.06416	insignificant	-0.056522	0.001471	hypomethylated	7	26	26
chr7	63304524	63306524	Herc2	-0.12463	8.69E-14	hypomethylated	-0.0021915	0.32186	insignificant	26	115	112
chr7	63494140	63496140	Oca2		1	noCoverage	-0.09603	0.10768	insignificant	0	10	10
chr7	64641641	64643641	Gm9962	-0.12042	0.0000002	hypomethylated	0.032949	0.69165	insignificant	27	99	111
chr7	64642241	64644241	Gabrg3	-0.072413	0.023674	hypomethylated	0.047938	0.091298	insignificant	17	55	73
chr7	64765379	64767379	Gabra5	-0.16055	0.0069286	hypomethylated	-0.027139	0.52138	insignificant	3	10	10
chr7	64844903	64846903	Gabrb3	-0.15522	1.86E-28	hypomethylated	-0.031588	0.73261	insignificant	50	104	112
chr7	64845542	64847542	Gabrb3	-0.15831	4.41E-35	hypomethylated	-0.02258	0.46726	insignificant	59	134	142
chr7	65912571	65914571	Atp10a	-0.083654	1.68E-13	hypomethylated	0.02438	0.36761	insignificant	21	81	71
chr7	66483119	66485119	Ube3a	-0.087432	0.00000606	hypomethylated	-0.008679	0.20189	insignificant	23	103	104
chr7	66483121	66485121	Ube3a	-0.087432	0.00000606	hypomethylated	-0.008679	0.20189	insignificant	23	103	104
chr7	66931449	66933449	Snord116		1	noCoverage	-0.078041	0.383	insignificant	0	10	12
chr7	67004191	67006191	Snord116		1	noCoverage	0.50952	0.22477	insignificant	0	5	3
chr7	67006708	67008708	Snord116l2		1	noCoverage	-0.072989	0.012688	hypomethylated	0	13	12
chr7	67011765	67013765	Snord116l2		1	noCoverage	-0.071572	0.087893	insignificant	0	8	9
chr7	67150042	67152042	Snrpn	0.14895	0.1765	insignificant	0.10253	0.032168	hypermethylated	2	6	6
chr7	69520864	69522864	Magel2	-0.078964	0.15999	insignificant	-0.01242	0.39945	insignificant	16	46	48
chr7	69565025	69567025	Mkrn3	-0.17691	0.0017632	hypomethylated	-0.06748	0.19228	insignificant	9	18	18
chr7	70357412	70359412	Chrna7	-0.083174	0.51296	insignificant	-0.042631	0.29657	insignificant	9	27	26
chr7	70588657	70590657	Otud7a	-0.096177	4.04E-15	hypomethylated	-0.025169	0.015343	hypomethylated	35	109	109
chr7	71083801	71085801	Klf13	-0.12989	2.45E-10	hypomethylated	-0.024916	0.87159	insignificant	25	104	105
chr7	71349691	71351691	Mir211		1	noCoverage	-0.13566	0.066756	insignificant	0	8	9
chr7	71431555	71433555	Mtmr10	-0.11385	1.07E-18	hypomethylated	-0.0050117	0.13236	insignificant	28	113	112
chr7	71518981	71520981	Fan1		1	noCoverage	0.0093193	1	insignificant	0	10	10
chr7	71536656	71538656	Mcee	-0.14247	1.43E-25	hypomethylated	-0.013735	0.21149	insignificant	24	98	101
chr7	71537122	71539122	Mphosph10	-0.17342	5.93E-16	hypomethylated	-0.021526	0.073041	insignificant	12	65	68
chr7	71645591	71647591	Apba2	-0.13731	2.35E-21	hypomethylated	-0.006839	0.84259	insignificant	47	159	160
chr7	72017926	72019926	Ndnl2	-0.033578	1	insignificant	-0.19248	0.5562	insignificant	2	13	21
chr7	72301414	72303414	Fam189a1	-0.084882	3.29E-18	hypomethylated	-0.01167	0.00011189	hypomethylated	35	94	98
chr7	72516130	72518130	Tjp1	-0.23065	0.000007883	hypomethylated	0.071886	0.079069	insignificant	10	30	34
chr7	72837302	72839302	Tm2d3	-0.13376	2.61E-22	hypomethylated	-0.0087746	0.18326	insignificant	21	78	78
chr7	73006021	73008021	Pcsk6	-0.085836	2.82E-35	hypomethylated	0.020796	0.82122	insignificant	49	117	119
chr7	73204221	73206221	Snrpa1	-0.1093	3.64E-12	hypomethylated	0.0098761	1	insignificant	32	106	102
chr7	73223534	73225534	H47	-0.12709	5.32E-13	hypomethylated	-0.0087425	0.090139	insignificant	21	94	92
chr7	73253400	73255400	Chsy1	-0.076348	3.59E-14	hypomethylated	-0.0072374	0.076787	insignificant	73	227	247
chr7	73533227	73535227	Lrrk1	0.42308	1	insignificant	-0.24123	0.17649	insignificant	1	8	19
chr7	73572363	73574363	Aldh1a3	-0.16825	6.22E-14	hypomethylated	0.010754	0.56522	insignificant	18	42	42
chr7	73833774	73835774	Lins	-0.20953	5.95E-14	hypomethylated	-0.0072282	0.58751	insignificant	13	48	48
chr7	73834447	73836447	Lins	-0.17879	8.67E-11	hypomethylated	-0.0014487	0.6926	insignificant	10	36	36
chr7	73983620	73985620	Adamts17	-0.14061	2.77E-28	hypomethylated	-0.011993	0.0046832	hypomethylated	44	122	122
chr7	74366429	74368429	Lysmd4	-0.16952	4.98E-08	hypomethylated	0.021801	0.51142	insignificant	8	58	58
chr7	74366501	74368501	Lysmd4	-0.16952	4.98E-08	hypomethylated	0.021801	0.51142	insignificant	8	58	58
chr7	74517744	74519744	Mef2a	-0.11608	7.79E-27	hypomethylated	0.011162	0.11065	insignificant	36	110	110
chr7	74790122	74792122	Lrrc28	-0.24197	0.000000117	hypomethylated	-0.011415	0.40474	insignificant	5	33	37
chr7	74904628	74906628	Synm	-0.074549	5.33E-08	hypomethylated	0.0095242	0.40148	insignificant	10	42	36
chr7	75096142	75098142	Igf1r	-0.095032	1.4E-37	hypomethylated	-0.0095385	0.011528	hypomethylated	57	247	257
chr7	75409119	75411119	Pgpep1l	-0.23028	0.57789	insignificant	-0.16508	0.000029603	hypomethylated	3	16	16
chr7	75894124	75896124	Arrdc4	-0.16865	0.61541	insignificant	-0.099973	0.64267	insignificant	3	7	7
chr7	77505479	77507479	Nr2f2	-0.08507	3.56E-33	hypomethylated	0.0087568	0.0014893	inconclusive	60	211	202
chr7	77511632	77513632	Nr2f2	-0.5081	0.000046024	stronglyHypometh	-0.13425	0.21663	insignificant	2	8	9
chr7	80519405	80521405	Rgma	-0.13866	3.36E-62	hypomethylated	-0.017957	0.00021917	hypomethylated	54	137	155
chr7	80703281	80705281	1810026B05Rik	-0.077825	0.0073799	hypomethylated	-0.0085202	0.66704	insignificant	5	56	50
chr7	80884192	80886192	Fam174b	-0.10722	2.37E-14	hypomethylated	-0.0040933	0.60977	insignificant	15	62	63
chr7	81158568	81160568	St8sia2	-0.10663	0.00024249	hypomethylated	0.0098012	1	insignificant	8	31	31
chr7	81699666	81701666	Slco3a1	-0.12855	1.46E-23	hypomethylated	-0.0041374	0.62137	insignificant	16	58	58
chr7	82453272	82455272	Sv2b	-0.12166	1	insignificant	-0.024218	0.67728	insignificant	6	46	37
chr7	82599419	82601419	Akap13	-0.11834	0.88938	insignificant	-0.0012769	0.1514	insignificant	25	113	113
chr7	82992223	82994223	Klhl25	-0.14986	4.5E-29	hypomethylated	0.005492	0.094088	insignificant	46	135	125
chr7	85722724	85724724	Ntrk3	-0.13436	0.00041456	hypomethylated	-0.00092222	0.63217	insignificant	9	58	63
chr7	85927016	85929016	Mrps11	-0.15317	0.00000138	hypomethylated	0.0011022	0.55021	insignificant	11	72	69
chr7	85927975	85929975	Mrpl46	-0.18522	0.000010926	hypomethylated	0.018465	0.21589	insignificant	5	34	31
chr7	85992097	85994097	Det1	-0.15451	6.98E-15	hypomethylated	0.0091509	0.85503	insignificant	7	14	14
chr7	86039812	86041812	Aen	-0.145	3.64E-11	hypomethylated	-0.010484	0.10362	insignificant	17	42	50
chr7	86058120	86060120	Isg20		1	noCoverage	-0.32523	0.54707	insignificant	0	2	3
chr7	86197368	86199368	Acan	-0.15249	2.51E-09	hypomethylated	0.013125	0.39281	insignificant	22	89	91
chr7	86275904	86277904	Hapln3	-0.32095	0.000077897	hypomethylated	-0.0073413	0.0027929	inconclusive	7	14	14
chr7	86293946	86295946	Mfge8	-0.16349	4.7E-25	hypomethylated	0.0096386	0.043699	inconclusive	10	26	26
chr7	86417151	86419151	Abhd2	-0.20635	6.7E-32	hypomethylated	-0.011729	0.64903	insignificant	17	72	71
chr7	86536223	86538223	Fanci	-0.2749	3.44E-13	hypomethylated	-0.030361	0.016248	hypomethylated	9	30	30
chr7	86611159	86613159	Polg	-0.15713	7.78E-22	hypomethylated	-0.00027584	0.41698	insignificant	22	80	80
chr7	86649149	86651149	Mir9-3	-0.15681	5.1E-33	hypomethylated	-0.023681	0.031326	hypomethylated	27	98	98
chr7	86762543	86764543	Rhcg	-0.14799	0.052982	insignificant	0.013773	0.43387	insignificant	1	22	22
chr7	86804081	86806081	5730590G19Rik	-0.11169	6.08E-11	hypomethylated	0.0087532	0.089191	insignificant	24	86	86
chr7	86859072	86861072	Kif7	-0.10466	5.98E-12	hypomethylated	-0.015561	0.13599	insignificant	24	81	78
chr7	86887048	86889048	Wdr93	-0.13657	0.00022624	hypomethylated	-0.010023	0.28758	insignificant	17	67	76
chr7	86887911	86889911	Pex11a	-0.20431	0.084284	insignificant	-0.011568	0.6925	insignificant	6	16	16
chr7	86954612	86956612	Mesp2	-0.17328	8.26E-10	hypomethylated	-0.0061057	1.88E-08	inconclusive	19	58	57
chr7	86987238	86989238	Anpep	-0.078761	0.31196	insignificant	-0.0011242	0.11023	insignificant	4	13	12
chr7	87065526	87067526	Ap3s2	-0.17867	0.00043196	hypomethylated	-0.039222	0.020409	hypomethylated	2	16	16
chr7	87080150	87082150	2610034B18Rik	-0.033776	0.00082316	hypomethylated	0.011415	0.077471	insignificant	10	31	30
chr7	87168699	87170699	Zfp710	-0.15846	0.000000118	hypomethylated	0.0030389	0.80358	insignificant	13	39	39
chr7	87170113	87172113	Zfp710	-0.14652	4.54E-28	hypomethylated	-0.004867	0.5289	insignificant	45	121	132
chr7	87260236	87262236	Idh2	-0.18283	0.00048235	hypomethylated	-0.0078738	0.60178	insignificant	3	24	24
chr7	87297901	87299901	Mir1965	-0.12857	0.10175	insignificant	-0.04796	0.37422	insignificant	1	4	4

chr7	87330726	87332726	Sema4b	-0.13909	1.49E-08	hypomethylated	-0.010187	0.086526	insignificant	15	65	65
chr7	87376778	87378778	D330012F22Rik	-0.15885	5.8E-21	hypomethylated	0.0087693	0.51684	insignificant	28	80	83
chr7	87377502	87379502	Cib1	-0.16654	1.67E-21	hypomethylated	0.020552	0.00051822	inconclusive	29	84	87
chr7	87390261	87392261	Ttll13	-0.34734	0.000030714	stronglyHypometh	0.019088	0.036542	hypermethylated	5	31	30
chr7	87405100	87407100	Ngrn	-0.33898	1.28E-13	stronglyHypometh	0.0048505	0.025745	inconclusive	16	56	56
chr7	87413540	87415540	Vps33b	-0.18417	1.86E-17	hypomethylated	-0.019398	0.45595	insignificant	18	56	56
chr7	87438350	87440350	Prc1	-0.22198	3.82E-21	hypomethylated	0.020069	0.0016235	inconclusive	15	94	101
chr7	87469340	87471340	Unc45a	0.010678	0.43325	insignificant	-0.0074463	0.79301	insignificant	6	36	36
chr7	87485105	87487105	Unc45a	-0.3748	0.1563	insignificant	0.011328	1	insignificant	2	6	6
chr7	87487022	87489022	Hddc3	-0.40705	0.00059464	stronglyHypometh	-0.055256	0.76927	insignificant	4	14	14
chr7	87516012	87518012	Man2a2	-0.12618	4.4E-19	hypomethylated	0.013261	0.71195	insignificant	32	98	94
chr7	87532832	87534832	Fes	-0.25788	1	lowCoverage	-0.059542	0.57575	insignificant	1	15	18
chr7	87547648	87549648	Furin	-0.10583	0.000001894	hypomethylated	0.0070593	0.67368	insignificant	26	72	90
chr7	87679884	87681884	Blm	-0.17515	0.024279	hypomethylated	-0.032328	0.64744	insignificant	10	55	54
chr7	87680005	87682005	Blm	-0.30968	0.004184	hypomethylated	-0.059958	0.11337	insignificant	5	27	26
chr7	87833763	87835763	Crtc3	-0.094722	2.5E-09	hypomethylated	-0.010149	0.58803	insignificant	15	30	30
chr7	87948217	87950217	Iqgap1	-0.097444	1	insignificant	-0.0034898	0.49139	insignificant	4	29	30
chr7	88005993	88007993	Zscan2	-0.096641	0.01258	hypomethylated	-0.0085722	0.40357	insignificant	3	29	34
chr7	88046155	88048155	Nmb	-0.34821	1	insignificant	0.015055	0.35487	insignificant	1	12	12
chr7	88049957	88051957	Nmb	-0.31494	0.000000153	hypomethylated	0.011381	0.033126	inconclusive	3	10	10
chr7	88092436	88094436	Sec11a	-0.25555	0.00000108	hypomethylated	-0.12672	0.092816	insignificant	8	24	36
chr7	88137569	88139569	Zfp592	-0.1153	1.82E-57	hypomethylated	-0.00011718	0.034651	hypomethylated	82	189	195
chr7	88201485	88203485	Alpk3	-0.077532	0.36576	insignificant	-0.092636	0.10551	insignificant	11	26	34
chr7	88357689	88359689	Pde8a	-0.10657	3.57E-19	hypomethylated	0.0094745	0.93704	insignificant	51	190	190
chr7	88490120	88492120	Rps17	-0.22821	6.59E-16	hypomethylated	0.15162	0.28696	insignificant	8	20	24
chr7	88599562	88601562	Cpeb1	-0.10291	2.95E-20	hypomethylated	0.016634	0.93907	insignificant	28	104	104
chr7	88643216	88645216	BC048679	0.14583	1	insignificant	-0.020833	1	insignificant	1	8	10
chr7	88711867	88713867	Fsd2	0.15	1	lowCoverage	0.025	1	insignificant	1	4	8
chr7	88715177	88717177	Whamm	-0.093977	9.39E-17	hypomethylated	0.0034582	0.69679	insignificant	43	135	127
chr7	88851811	88853811	Homer2	-0.094025	0.0016964	hypomethylated	0.0012868	0.76521	insignificant	14	37	36
chr7	88906838	88908838	Fam103a1	-0.184	1.18E-30	hypomethylated	-0.029893	0.0068751	hypomethylated	24	128	129
chr7	88934339	88936339	3110040N11Rik		1	noCoverage	-0.065827	0.81795	insignificant	0	17	16
chr7	88974317	88976317	Btbd1	-0.13288	1	insignificant	0.0053273	1	insignificant	3	44	44
chr7	89002907	89004907	Tm6sf1	-0.22137	0.00000766	hypomethylated	-0.0041836	0.012629	hypomethylated	9	48	49
chr7	89079345	89081345	Hdgfrp3	-0.34814	1.07E-15	stronglyHypometh	-0.06679	3.5E-12	hypomethylated	18	60	71
chr7	89137185	89139185	Bnc1	-0.19309	4.16E-32	hypomethylated	-0.010336	0.0000022	hypomethylated	30	86	86
chr7	89318727	89320727	Sh3gl3	-0.047768	0.014748	inconclusive	0.010609	0.00012423	hypermethylated	21	64	64
chr7	89483203	89485203	Adamtsl3	-0.10976	8.83E-11	hypomethylated	0.0036552	0.00012003	inconclusive	24	96	100
chr7	89796123	89798123	Eftud1	-0.14368	1E-29	hypomethylated	0.0046463	0.39666	insignificant	28	84	88
chr7	89797038	89799038	Fam154b	-0.17728	4.37E-26	hypomethylated	-0.0048514	0.10668	insignificant	19	54	54
chr7	90014842	90016842	Mex3b	-0.10979	2.1E-32	hypomethylated	-0.0058717	0.017654	hypomethylated	63	187	192
chr7	91032851	91034851	Mesdc1	-0.13622	5.69E-09	hypomethylated	-0.025665	0.00001258	hypomethylated	23	64	64
chr7	91039509	91041509	Mesdc2	-0.17997	0.019726	hypomethylated	0.023436	0.86932	insignificant	5	54	54
chr7	91235015	91237015	9930013L23Rik	-0.13446	4.54E-08	hypomethylated	-0.018024	0.39436	insignificant	14	30	30
chr7	91300403	91302403	Fam108c	-0.10238	2.64E-12	hypomethylated	-0.024224	0.61154	insignificant	28	109	106
chr7	91558469	91560469	Arnt2	-0.11746	0.56723	insignificant	-0.016776	0.14972	insignificant	8	57	52
chr7	91754452	91756452	Fah	-0.21914	7.98E-20	hypomethylated	-0.0098317	0.41721	insignificant	13	45	45
chr7	92564186	92566186	Vmn2r69		1	noCoverage	-0.016376	0.84076	insignificant	0	18	20
chr7	93535168	93537168	Olfr304		1	noCoverage	-0.16468	0.25624	insignificant	0	6	7
chr7	93765153	93767153	Olfr294		1	noCoverage	0.055484	1	insignificant	0	37	36
chr7	94394304	94396304	Nox4	-0.15889	4.2E-12	hypomethylated	-0.013735	0.068095	insignificant	18	60	60
chr7	95425602	95427602	Ctsc	-0.26974	0.000033286	hypomethylated	0.0048677	0.82384	insignificant	2	8	8
chr7	95577782	95579782	Rab38	-0.18534	0.00000126	hypomethylated	0.0067431	0.32945	insignificant	10	45	47
chr7	96487297	96489297	Tmem135	-0.09155	0.0098292	hypomethylated	-0.0022066	0.54467	insignificant	10	26	26
chr7	96551875	96553875	Fzd4	-0.18792	3.57E-26	hypomethylated	-0.027764	0.21254	insignificant	35	117	117
chr7	96666096	96668096	Prss23	-0.11758	0.000001026	hypomethylated	0.075515	0.048534	hypermethylated	10	32	36
chr7	96780327	96782327	Me3	-0.12411	0.59808	insignificant	-0.010974	0.87294	insignificant	1	28	28
chr7	97052139	97054139	Ccdc81		1	noCoverage	-0.20065	0.092925	insignificant	0	10	10
chr7	97089695	97091695	l7Rn6	-0.33695	0.00000896	stronglyHypometh	0.091392	0.038654	hypermethylated	5	49	44
chr7	97129486	97131486	Eed	-0.18239	0.58955	insignificant	-0.01521	0.73498	insignificant	1	34	34
chr7	97176668	97178668	E230029C05Rik		1	noCoverage	0.012155	0.85959	insignificant	0	14	13
chr7	97277741	97279741	Picalm	-0.1321	1.18E-40	hypomethylated	-0.014322	0.085838	insignificant	50	153	167
chr7	97413942	97415942	Ccdc83	-0.49492	0.0002527	stronglyHypometh	-0.0059651	0.30325	insignificant	1	16	23
chr7	97534490	97536490	Sytl2	-0.28602	0.00000659	hypomethylated	-0.064237	0.01486	hypomethylated	6	13	13
chr7	97590290	97592290	Crebzf	-0.10743	5.19E-27	hypomethylated	-0.0055678	0.084905	insignificant	54	190	197
chr7	97605718	97607718	Tmem126a	-0.15461	5.73E-09	hypomethylated	-0.099973	0.000000198	hypomethylated	8	22	22
chr7	97624505	97626505	Tmem126b	-0.14115	3.71E-08	hypomethylated	0.030486	0.49741	insignificant	6	38	40
chr7	98238295	98240295	Dlg2	-0.3546	0.00050129	stronglyHypometh	0.011752	0.72708	insignificant	5	20	20
chr7	99785652	99787652	Ankrd42	0.074299	0.41612	insignificant	-0.0099923	0.81324	insignificant	3	14	14
chr7	99818422	99820422	Pcf11	-0.11348	5.08E-08	hypomethylated	0.022024	0.010415	hypermethylated	11	46	46
chr7	99889223	99891223	Rab30	-0.17138	5.01E-24	hypomethylated	0.0072438	0.049867	inconclusive	12	40	40
chr7	99889969	99891969	4632427E13Rik	-0.24123	0.000000332	hypomethylated	-0.075233	0.61825	insignificant	3	10	8
chr7	100022742	100024742	4632434I11Rik	-0.15638	1.2E-16	hypomethylated	-0.0028755	0.039127	hypomethylated	20	72	67
chr7	100022762	100024762	Prcp	-0.14351	2.04E-16	hypomethylated	-0.0027422	0.02319	hypomethylated	19	70	65
chr7	100227388	100229388	Fam181b	-0.15625	6.79E-29	hypomethylated	-0.01287	0.1544	insignificant	27	151	140
chr7	103358146	103360146	Odz4	-0.11597	2.08E-25	hypomethylated	0.0074055	0.93182	insignificant	53	156	158
chr7	104099036	104101036	Nars2	-0.11699	2.77E-08	hypomethylated	0.0043383	0.11064	insignificant	6	40	40
chr7	104229260	104231260	Gab2	-0.10994	7.23E-24	hypomethylated	-0.001918	0.91896	insignificant	61	145	156
chr7	104479832	104481832	Kctd21	-0.16243	0.020794	hypomethylated	-0.011487	0.43977	insignificant	11	39	43
chr7	104547512	104549512	Ndufc2	-0.12509	0.00000173	hypomethylated	0.083056	1.01E-28	hypermethylated	13	58	58
chr7	104566020	104568020	Thrsp	-0.16461	0.055936	insignificant	-0.040148	0.55765	insignificant	2	5	4
chr7	104600713	104602713	Kctd14	-0.27946	0.19944	insignificant	-0.0084766	0.61272	insignificant	4	39	40
chr7	104628465	104630465	Ints4	-0.1725	0.002145	hypomethylated	-0.023027	0.20367	insignificant	4	34	34
chr7	104727405	104729405	Rsf1	-0.11668	3.93E-26	hypomethylated	-0.018482	0.44677	insignificant	43	130	151
chr7	104728007	104730007	1810020D17Rik	-0.11668	3.93E-26	hypomethylated	-0.017789	0.44682	insignificant	43	130	149

chr7	104844200	104846200	Clns1a	-0.10944	2.37E-09	hypomethylated	0.0050301	0.97257	insignificant	37	114	114
chr7	104886757	104888757	Aqp11	0.29477	0.85445	insignificant	-0.040656	2.35E-10	hypomethylated	8	49	48
chr7	104990448	104992448	Pak1	-0.351	1	lowCoverage	0.023606	0.45118	insignificant	1	18	18
chr7	105268003	105270003	Myo7a		1	noCoverage	-0.013091	0.70579	insignificant	0	4	4
chr7	105326703	105328703	Capn5	0.024829	5.22E-09	inconclusive	-0.041454	0.0074857	hypomethylated	7	48	54
chr7	105458037	105460037	Acer3	-0.098614	0.059154	insignificant	-0.0073451	0.9488	insignificant	4	22	23
chr7	105509798	105511798	Tsku	-0.14758	0.000050629	hypomethylated	0.010919	0.9178	insignificant	14	49	48
chr7	105509838	105511838	Tsku	-0.12102	0.0043645	hypomethylated	0.023298	0.9517	insignificant	8	37	36
chr7	105641731	105643731	Lrrc32		1	noCoverage	-0.10379	0.84026	insignificant	0	6	4
chr7	105805079	105807079	2210018M11Ril	-0.14438	1	insignificant	0.0013641	0.0057529	hypermethylated	2	35	37
chr7	105850872	105852872	Prkrir	-0.10148	4.48E-29	hypomethylated	0.001433	0.33185	insignificant	39	162	162
chr7	105986354	105988354	Wnt11	-0.13256	3.16E-16	hypomethylated	-0.029127	0.018799	hypomethylated	17	79	100
chr7	106289654	106291654	Uvrag	-0.11583	0.0037373	hypomethylated	-0.0078383	0.35778	insignificant	5	23	23
chr7	106331223	106333223	Dgat2	-0.11564	0.0091781	hypomethylated	0.021961	0.29381	insignificant	8	39	39
chr7	106414956	106416956	Mtap6	-0.2349	3.29E-19	hypomethylated	-0.023844	0.013829	hypomethylated	17	58	59
chr7	106415855	106417855	Mtap6	-0.11391	6.74E-21	hypomethylated	-0.020926	0.0023572	hypomethylated	40	129	135
chr7	106529058	106531058	Gdpd5	-0.14425	3.19E-15	hypomethylated	0.0028506	0.015662	hypermethylated	27	111	106
chr7	106613513	106615513	Klhl35		1	noCoverage	-0.043599	0.049876	hypomethylated	0	4	7
chr7	106631442	106633442	Snord15a	-0.16938	0.00030703	hypomethylated	0.0018022	0.83267	insignificant	9	40	40
chr7	106632219	106634219	Snord15a	-0.57151	0.083007	insignificant	-0.13632	1	insignificant	2	9	9
chr7	106682995	106684995	Arrb1	-0.129	4.42E-12	hypomethylated	-0.028284	0.014534	hypomethylated	19	84	92
chr7	106775613	106777613	Gm4980	-0.19091	0.52381	insignificant	-0.024242	0.77994	insignificant	1	4	4
chr7	106859850	106861850	Slco2b1	0.069264	1	insignificant	-0.058941	0.15342	insignificant	1	6	6
chr7	106976927	106978927	Neu3		1	noCoverage	-0.13571	0.025833	hypomethylated	0	7	6
chr7	107153913	107155913	Chrdl2	-0.10834	0.016548	hypomethylated	-0.013125	0.78116	insignificant	3	20	23
chr7	107270010	107272010	Pold3	-0.11462	0.14693	insignificant	-0.0043571	0.56263	insignificant	4	11	10
chr7	107306786	107308786	Lipt2	-0.10642	0.0048355	hypomethylated	0.020226	0.44716	insignificant	10	67	63
chr7	107326192	107328192	Kcne3		1	noCoverage	0.038411	0.0058179	inconclusive	0	40	34
chr7	107375116	107377116	Pgm2l1	-0.13446	1.43E-12	hypomethylated	-0.010929	0.11459	insignificant	19	85	77
chr7	107412053	107414053	Gpx2-ps1	0.12867	0.59826	insignificant	-0.050154	0.86801	insignificant	2	8	8
chr7	107433029	107435029	P4ha3	-0.37987	0.21297	insignificant	-0.051482	0.3187	insignificant	2	14	12
chr7	107519742	107521742	C2cd3	-0.1298	1.29E-30	hypomethylated	-0.0089615	0.014854	hypomethylated	23	100	89
chr7	107520406	107522406	Ppme1	-0.1065	2.12E-21	hypomethylated	-0.0047666	0.0001205	hypomethylated	15	76	65
chr7	107620499	107622499	Ucp3	0.055971	1	insignificant	0.0098954	0.70422	insignificant	6	20	16
chr7	107640853	107642853	Ucp2	-0.18681	1.75E-12	hypomethylated	0.0025225	0.58315	insignificant	15	77	74
chr7	107684613	107686613	Chchd8		1	noCoverage	0.011127	0.6272	insignificant	0	16	18
chr7	107731644	107733644	Mrpl48	-0.30753	0.023199	hypomethylated	0.053839	0.16249	insignificant	2	5	6
chr7	107755099	107757099	Rab6a	-0.11478	7.3E-27	hypomethylated	-0.018525	0.0028929	hypomethylated	41	129	129
chr7	107756510	107758510	Mrpl48	-0.14767	3.37E-17	hypomethylated	-0.020393	0.025341	hypomethylated	29	91	91
chr7	107805479	107807479	Plekhb1	0.077018	0.12825	insignificant	0.0058738	0.47341	insignificant	5	22	24
chr7	107806970	107808970	Plekhb1		1	noCoverage	-0.066505	0.2233	insignificant	0	13	18
chr7	107810908	107812908	Plekhb1	-0.49764	2.04E-26	stronglyHypometh	0.096975	0.000057053	inconclusive	3	38	40
chr7	107854215	107856215	Fam168a	-0.2285	0.000008176	hypomethylated	-0.040704	0.078721	insignificant	7	17	24
chr7	108030923	108032923	Mir3102	0.0046134	1	insignificant	0.0091265	0.91531	insignificant	1	27	27
chr7	108080675	108082675	Arhgef17	-0.30604	2.48E-14	hypomethylated	-0.072656	0.49123	insignificant	3	14	14
chr7	108160505	108162505	P2ry2	-0.13245	7.28E-08	hypomethylated	0.000094201	0.18605	insignificant	11	21	21
chr7	108256288	108258288	Fchsd2	-0.109	3.36E-17	hypomethylated	0.0021155	0.51543	insignificant	34	133	135
chr7	108450602	108452602	Atg16l2	-0.24343	0.000073838	hypomethylated	-0.017325	0.37266	insignificant	6	18	18
chr7	108468832	108470832	Stard10	-0.10761	1.75E-25	hypomethylated	0.0030079	0.021744	inconclusive	51	155	152
chr7	108495582	108497582	Arap1	-0.14931	1.22E-31	hypomethylated	0.0066295	0.084256	insignificant	16	64	64
chr7	108533039	108535039	Arap1	-0.05506	1	insignificant	-0.036029	0.66126	insignificant	1	6	6
chr7	108541901	108543901	Arap1	-0.11299	0.056904	insignificant	-0.038433	0.589	insignificant	5	10	10
chr7	108598765	108600765	Pde2a	-0.10909	9.54E-12	hypomethylated	-0.003041	0.56752	insignificant	18	70	80
chr7	108598821	108600821	Pde2a	-0.10909	9.54E-12	hypomethylated	-0.003041	0.56752	insignificant	18	70	80
chr7	108622889	108624889	Mir139	-0.50211	0.0020259	stronglyHypometh	-0.19251	0.00044997	hypomethylated	2	6	6
chr7	108704316	108706316	Art2a-ps		1	noCoverage	0	0.6599	insignificant	0	6	6
chr7	108709379	108711379	Art2a-ps	0.15885	1	insignificant	-0.060193	0.10919	insignificant	2	8	8
chr7	108811281	108813281	Clpb	-0.051131	6.37E-09	hypomethylated	-0.018969	0.85564	insignificant	18	70	70
chr7	108965826	108967826	Phox2a		1	noCoverage	0.028475	0.79594	insignificant	0	28	32
chr7	108986338	108988338	Inppl1	-0.03287	0.012199	hypomethylated	0.036436	0.92513	insignificant	16	80	80
chr7	108986740	108988740	Inppl1	-0.31346	0.15968	insignificant	0.38966	0.15706	insignificant	2	8	8
chr7	109019197	109021197	Folr1		1	noCoverage	0.041229	0.20478	insignificant	0	14	14
chr7	109043928	109045928	3200002M19Ril	-0.055427	0.0022117	hypomethylated	0.026341	0.049992	inconclusive	15	63	62
chr7	109053350	109055350	2400001E08Rik	-0.15907	5.71E-21	hypomethylated	-0.0055831	0.28978	insignificant	21	69	69
chr7	109054873	109056873	2400001E08Rik	-0.41932	3.25E-17	stronglyHypometh	0.048947	0.71299	insignificant	5	26	31
chr7	109082371	109084371	Lrrc51	-0.24113	0.00014109	hypomethylated	-0.029431	0.93005	insignificant	10	70	70
chr7	109117356	109119356	Numa1	-0.11125	0.0018739	hypomethylated	-0.025094	0.20469	insignificant	4	49	48
chr7	109166669	109168669	Il18bp		1	noCoverage	-0.002948	0.74583	insignificant	0	4	4
chr7	109213226	109215226	Trpc2	-0.13424	2.89E-27	hypomethylated	0.07328	0.014388	inconclusive	17	49	53
chr7	109213646	109215646	Rnf121	-0.21028	1.49E-13	hypomethylated	0.016814	0.26336	insignificant	16	53	55
chr7	109248743	109250743	Art5		1	noCoverage	-0.036027	1	insignificant	0	7	8
chr7	109265227	109267227	Chrna10	0.071252	0.48906	insignificant	-0.023718	0.51296	insignificant	2	28	28
chr7	109358634	109360634	Nup98	-0.14771	0.059223	insignificant	-0.0018052	0.49343	insignificant	7	51	53
chr7	109370587	109372587	Pgap2	-0.18326	8.15E-12	hypomethylated	-0.0081025	0.80547	insignificant	18	58	60
chr7	109398632	109400632	Rhog	-0.18419	0.0026634	hypomethylated	-0.036211	0.32507	insignificant	5	36	30
chr7	109415337	109417337	Stim1	-0.13281	1.13E-10	hypomethylated	0.022813	0.059478	insignificant	11	70	83
chr7	109589208	109591208	Rrm1	-0.11523	0.0038008	hypomethylated	-0.004745	0.68026	insignificant	7	44	44
chr7	109701799	109703799	Olfr549	-0.577	0.016726	stronglyHypometh	-0.022035	0.32138	insignificant	3	12	12
chr7	109713983	109715983	Trim21		1	noCoverage	-0.14408	0.88349	insignificant	0	18	21
chr7	109725982	109727982	Olfr550	0.0052297	0.65032	insignificant	-0.029528	0.072467	insignificant	8	23	24
chr7	109751869	109753869	Olfr552		1	noCoverage	-0.071423	0.20272	insignificant	0	8	9
chr7	109787761	109789761	Olfr554	-0.082793	0.85173	insignificant	0.030123	0.11283	insignificant	2	6	6
chr7	109806336	109808336	Olfr555	-0.96429	0.00077914	stronglyHypometh	-0.064286	0.013917	hypomethylated	1	7	6
chr7	109835840	109837840	Trim68	-0.18675	0.051345	insignificant	-0.034739	0.40881	insignificant	5	14	15
chr7	109922039	109924039	Olfr561	0.064167	0.48534	insignificant	-0.074804	0.15872	insignificant	3	12	12

chr7	110024635	110026635	Olfr568	0.031746	0.024197	inconclusive	-0.040675	0.026635	hypomethylated	3	7	6
chr7	110304259	110306259	Olfr589		1	noCoverage	-0.091667	0.25759	insignificant	0	2	2
chr7	111084895	111086895	Olfr632	-0.38194	0.043053	stronglyHypometh	-0.041008	0.01545	hypomethylated	3	8	11
chr7	111094081	111096081	Olfr633	-0.082009	0.36974	insignificant	0.038164	0.42702	insignificant	1	8	11
chr7	111187311	111189311	Olfr641	-0.095238	0.509	insignificant	-0.050977	0.45009	insignificant	1	6	6
chr7	111198866	111200866	Olfr642	-0.17352	0.26747	insignificant	0.047249	0.075262	insignificant	1	18	19
chr7	111217543	111219543	Olfr644		1	noCoverage	-0.18611	0.23708	insignificant	0	4	5
chr7	111253794	111255794	Olfr646		1	noCoverage	0.13049	0.6778	insignificant	0	10	10
chr7	111338719	111340719	Olfr649	-0.32115	0.58252	insignificant	0.19231	0.060975	insignificant	2	4	4
chr7	111366308	111368308	Trim6	-0.26531	0.000000959	hypomethylated	-0.014874	0.94183	insignificant	7	26	32
chr7	111765170	111767170	Olfr656	-0.25561	0.046835	hypomethylated	0.015639	0.21471	insignificant	1	4	4
chr7	111795819	111797819	Olfr658	-0.24168	0.21257	insignificant	-0.082809	0.35198	insignificant	1	11	11
chr7	112028222	112030222	Olfr665		1	noCoverage	-0.010417	1	insignificant	0	4	4
chr7	112216982	112218982	Olfr678	-0.10768	0.31101	insignificant	-0.17391	0.076617	insignificant	3	7	7
chr7	112292823	112294823	Olfr683		1	noCoverage	0.14986	0.25508	insignificant	0	10	9
chr7	112548525	112550525	Fam160a2	-0.10639	0.025635	hypomethylated	0.0050354	0.88951	insignificant	7	43	43
chr7	112573333	112575333	Cckbr	-0.35026	0.070776	insignificant	-0.047455	0.59708	insignificant	3	27	22
chr7	112630711	112632711	Prkcdbp	-0.093547	0.0053666	hypomethylated	0.0070899	0.28983	insignificant	3	20	20
chr7	112701873	112703873	Smpd1	-0.15989	0.051089	insignificant	-0.018346	0.23957	insignificant	2	37	35
chr7	112730049	112732049	Apbb1	-0.16608	0.02324	hypomethylated	-0.015552	0.35358	insignificant	5	12	12
chr7	112748630	112750630	Hpx	-0.18627	1	insignificant	-0.077579	0.67728	insignificant	1	6	4
chr7	112782013	112784013	Arfip2	-0.12688	0.000000171	hypomethylated	-0.025212	0.000018493	hypomethylated	22	53	54
chr7	112788053	112790053	Fxc1	-0.13689	0.0014781	hypomethylated	-0.010549	0.89401	insignificant	8	45	50
chr7	112788930	112790930	Arfip2		1	noCoverage	-0.07704	1	insignificant	0	19	24
chr7	112884103	112886103	Ilk	-0.19549	2.56E-36	hypomethylated	-0.015385	1	insignificant	37	125	116
chr7	112884238	112886238	Ilk	-0.18246	4.35E-36	hypomethylated	-0.01493	0.90148	insignificant	37	123	114
chr7	112885014	112887014	Rrp8	-0.20312	1.56E-10	hypomethylated	-0.020604	0.099659	insignificant	19	65	55
chr7	112900721	112902721	Dchs1	0.094314	0.20614	insignificant	0.22912	0.65816	insignificant	1	9	6
chr7	112936064	112938064	Dchs1	0.026289	0.48918	insignificant	0.1937	0.19895	insignificant	4	29	34
chr7	112959601	112961601	Mrpl17	-0.15694	0.49445	insignificant	-0.012639	1	insignificant	3	12	12
chr7	113833243	113835243	Olfr694		1	noCoverage	-0.20893	0.49767	insignificant	0	4	5
chr7	113896900	113898900	Olfr698		1	noCoverage	-0.02	0.78286	insignificant	0	4	5
chr7	113987126	113989126	Olfr703	0.122	1	insignificant	-0.018276	0.24613	insignificant	1	6	6
chr7	114035621	114037621	Olfr707		1	noCoverage	-0.094551	0.36618	insignificant	0	8	12
chr7	114351958	114353958	Rbmxl2	-0.13514	0.00020449	hypomethylated	-0.0062316	0.18457	insignificant	73	252	250
chr7	114513303	114515303	Syt9	-0.1026	2.98E-21	hypomethylated	0.0071049	0.5315	insignificant	47	175	175
chr7	114709946	114711946	Olfml1	-0.13188	0.034828	hypomethylated	0.040989	0.74684	insignificant	4	17	17
chr7	114737564	114739564	Ppfibp2	-0.20178	1.01E-15	hypomethylated	-0.0047582	0.014996	hypomethylated	12	54	54
chr7	114901510	114903510	Cyb5r2	-0.51902	4.31E-09	stronglyHypometh	-0.0024977	0.69778	insignificant	4	27	28
chr7	115303385	115305385	Olfr485		1	noCoverage	0.083333	0.45679	insignificant	0	6	6
chr7	115754822	115756822	Olfr506	-0.275	0.085701	insignificant	-0.12752	0.024812	hypomethylated	1	6	6
chr7	115855868	115857868	Olfr512	-0.076291	0.097969	insignificant	-0.035815	0.023324	hypomethylated	1	4	4
chr7	116076928	116078928	Eif3f	-0.26166	7.11E-22	hypomethylated	-0.021921	0.61809	insignificant	8	20	20
chr7	116153393	116155393	Tub	-0.10119	1.34E-39	hypomethylated	-0.012338	0.0069712	hypomethylated	37	112	112
chr7	116226838	116228838	Ric3		1	noCoverage		1	noCoverage	0	24	0
chr7	116313822	116315822	Lmo1		1	noCoverage	-0.050439	0.62157	insignificant	0	12	12
chr7	116582567	116584567	Stk33	-0.13556	7.79E-09	hypomethylated	0.010897	0.15934	insignificant	18	61	61
chr7	116637252	116639252	Trim66	-0.041584	0.30584	insignificant	-0.011975	0.81494	insignificant	2	10	10
chr7	116651648	116653648	Trim66	0.060517	0.25265	insignificant	-0.11215	0.2386	insignificant	3	9	15
chr7	116661708	116663708	Rpl27a	-0.31663	9.04E-12	hypomethylated	-0.085759	0.10404	insignificant	6	17	19
chr7	116662645	116664645	Snora3	-0.39595	1.08E-09	stronglyHypometh	-0.036358	0.26822	insignificant	3	7	6
chr7	116760082	116762082	St5	-0.1145	0.000010331	hypomethylated	0.02203	0.47613	insignificant	8	18	18
chr7	116846250	116848250	D930014E17Rik	-0.16047	3.64E-19	hypomethylated	-0.024126	0.19204	insignificant	28	94	93
chr7	116867285	116869285	BC051019		1	noCoverage	-0.025641	1	insignificant	0	4	4
chr7	116895777	116897777	Tmem9b	-0.22091	8.54E-19	hypomethylated	-0.02164	0.013656	hypomethylated	24	51	51
chr7	116925059	116927059	Nrip3	-0.009878	0.00000298	hypomethylated	-0.0048659	0.27635	insignificant	9	60	60
chr7	117009193	117011193	Scube2	-0.11902	0.075712	insignificant	-0.002093	0.58828	insignificant	12	33	33
chr7	117103936	117105936	Dennd5a	-0.1556	1	insignificant	-0.040698	0.59044	insignificant	1	17	22
chr7	117129744	117131744	Tmem41b	0.13549	0.31236	insignificant	-0.027182	0.058492	insignificant	8	49	50
chr7	117160938	117162938	Ipo7	-0.21321	2.8E-20	hypomethylated	-0.025996	0.23836	insignificant	25	64	68
chr7	117204215	117206215	Zfp143	-0.15715	3.02E-25	hypomethylated	0.010597	0.94645	insignificant	36	121	114
chr7	117264572	117266572	Wee1	-0.094088	8.43E-36	hypomethylated	-0.0035734	0.8688	insignificant	64	265	266
chr7	117364216	117366216	Swap70	-0.090627	6.62E-14	hypomethylated	0.007896	0.72084	insignificant	22	80	80
chr7	117758434	117760434	Sbf2	-0.15295	0.00011794	hypomethylated	-0.020107	0.3188	insignificant	9	56	54
chr7	117770182	117772182	Adm	-0.20716	0.00014044	hypomethylated	0.0089438	0.31275	insignificant	7	22	22
chr7	118171464	118173464	Ctr9	-0.13028	4.96E-09	hypomethylated	0.0015344	0.50455	insignificant	12	56	56
chr7	118226544	118228544	Eif4g2	-0.15248	0.00000056	hypomethylated	-0.014661	0.59428	insignificant	11	63	65
chr7	118266189	118268189	1700012D14Rik	-0.21703	0.000036524	hypomethylated	-0.041196	0.48565	insignificant	7	17	17
chr7	118923491	118925491	Galntl4	-0.18792	0.0099323	hypomethylated	0.027562	0.00034543	hypermethylated	6	59	62
chr7	119166019	119168019	Usp47	-0.13649	7.05E-24	hypomethylated	-0.034694	0.86579	insignificant	27	100	142
chr7	119302571	119304571	Dkk3	-0.39952	0.0010296	stronglyHypometh	0.012751	0.92143	insignificant	4	8	8
chr7	119368349	119370349	Mical2	-0.35664	5.87E-19	stronglyHypometh	-0.062621	0.0092024	hypomethylated	13	72	65
chr7	119570219	119572219	Parva	-0.19972	0.02995	hypomethylated	-0.0048285	0.069988	insignificant	5	22	22
chr7	119821833	119823833	Tead1	-0.10828	1.77E-14	hypomethylated	0.0010661	0.0049041	inconclusive	46	134	137
chr7	120096475	120098475	Rassf10	-0.11202	1.27E-30	hypomethylated	-0.0042496	0.031609	hypomethylated	43	180	176
chr7	120349978	120351978	Arntl	-0.074853	1.23E-17	hypomethylated	0.010206	0.46747	insignificant	52	156	178
chr7	120512853	120514853	Btbd10	-0.058164	0.00000532	hypomethylated	-0.0050602	0.19294	insignificant	25	105	103
chr7	120656375	120658375	Far1	-0.080687	2.29E-09	hypomethylated	0.0021892	0.08629	insignificant	31	153	153
chr7	120908511	120910511	Spon1	-0.11969	0.00000295	hypomethylated	-0.0096547	0.31645	insignificant	20	77	77
chr7	121261295	121263295	Rras2	-0.22051	0.19771	insignificant	0.014561	0.62596	insignificant	2	10	15
chr7	121419630	121421630	Psma1		1	noCoverage	-0.025	0.70965	insignificant	0	8	8
chr7	121557767	121559767	Pde3b	-0.086311	2.34E-41	hypomethylated	0.00014022	0.23986	insignificant	74	229	231
chr7	121558675	121560675	4933406I18Rik	-0.087188	4.21E-23	hypomethylated	-0.0070061	0.15249	insignificant	64	243	252
chr7	121706486	121708486	Cyp2r1	-0.20453	1.1E-12	hypomethylated	-0.05686	0.46016	insignificant	9	30	31
chr7	121861156	121863156	Calcb	-0.43007	0.50884	insignificant	-0.056289	0.45987	insignificant	1	6	6

chr7	121888279	121890279	Insc	-0.16278	0.042022	hypomethylated	-0.047148	0.93659	insignificant	7	18	18
chr7	123174561	123176561	Sox6	-0.20203	1.68E-31	hypomethylated	-0.027261	0.058767	insignificant	29	93	89
chr7	123182258	123184258	Sox6	-0.23375	0.00000477	hypomethylated	0.0064335	0.61563	insignificant	13	40	40
chr7	123235893	123237893	1110004F10Rik	0.087531	0.36045	insignificant	0.0039165	0.42007	insignificant	14	53	54
chr7	123236663	123238663	1700003G18Rik	0.087531	0.36045	insignificant	0.0039165	0.42007	insignificant	14	53	54
chr7	123333355	123335355	Plekha7		1	noCoverage	0.10642	0.097556	insignificant	0	9	8
chr7	123477704	123479704	Rps13	-0.15567	9.26E-08	hypomethylated	-0.032443	0.050854	insignificant	12	77	75
chr7	123586972	123588972	Pik3c2a	-0.1209	0.0252	hypomethylated	-0.0074703	0.74923	insignificant	17	46	46
chr7	123646887	123648887	Nucb2	-0.13286	5.34E-11	hypomethylated	0.011493	0.14474	insignificant	18	73	73
chr7	124523492	124525492	Xylt1	-0.10444	1.02E-25	hypomethylated	-0.0053979	0.088464	insignificant	31	133	133
chr7	125259661	125261661	Rps15a		1	noCoverage	0.099876	0.20978	insignificant	0	12	12
chr7	125273139	125275139	Arl6ip1	-0.28601	0.00079397	hypomethylated	-0.020266	0.255	insignificant	2	18	20
chr7	125386420	125388420	4930583K01Rik	-0.097586	1.64E-23	hypomethylated	0.016771	0.060337	insignificant	50	180	181
chr7	125387151	125389151	Smg1	-0.16546	2.38E-13	hypomethylated	0.025335	0.004015	inconclusive	19	76	75
chr7	125635489	125637489	Itpripl2	-0.64286	0.0040588	stronglyHypometh	-0.12857	0.46224	insignificant	0	0	0
chr7	125676827	125678827	Coq7		1	noCoverage	0.014951	0.71002	insignificant	0	14	14
chr7	125728200	125730200	Tmc7	-0.085478	0.01191	hypomethylated	0.038907	0.56491	insignificant	6	24	25
chr7	125739810	125741810	Tmc5	-0.15316	0.22651	insignificant	-0.0067768	0.0073705	hypomethylated	8	50	52
chr7	125776249	125778249	Tmc5		1	noCoverage	0.076767	0.073289	insignificant	0	5	6
chr7	125849252	125851252	Gde1	-0.14539	0.0011112	hypomethylated	-0.017369	4.72E-11	hypomethylated	4	26	26
chr7	125855124	125857124	6330503K22Rik	-0.19405	9.4E-12	hypomethylated	-0.011239	0.82093	insignificant	14	64	64
chr7	125882784	125884784	9030624J02Rik		1	noCoverage	0.061274	0.59575	insignificant	0	33	20
chr7	125998288	126000288	Iqck	-0.13352	2.24E-20	hypomethylated	-0.016872	0.016317	hypomethylated	28	98	121
chr7	125999127	126001127	2310008H09Rik	-0.12184	2.8E-15	hypomethylated	-0.0091094	0.59497	insignificant	28	84	101
chr7	125999512	126001512	2310008H09Rik	-0.145	6.85E-13	hypomethylated	-0.0086434	0.64939	insignificant	15	38	41
chr7	126138725	126140725	Gprc5b	-0.16225	0.053274	insignificant	0.0031214	0.25945	insignificant	5	26	26
chr7	126327888	126329888	Gpr139	-0.21846	0.000059826	hypomethylated	-0.057406	0.32921	insignificant	7	38	62
chr7	126602786	126604786	Gp2		1	noCoverage	0.088304	0.80854	insignificant	0	6	6
chr7	126666996	126668996	Pdilt	-0.52941	0.00054103	stronglyHypometh	0.040196	0.53155	insignificant	2	4	4
chr7	126668778	126670778	Acsm5	-0.036564	0.59998	insignificant	-0.032728	0.12179	insignificant	2	11	14
chr7	126704221	126706221	Acsm2		1	noCoverage	0.12424	0.7458	insignificant	0	6	7
chr7	126864279	126866279	Thumpd1	-0.088088	0.17014	insignificant	0.003751	0.76325	insignificant	4	14	14
chr7	126903436	126905436	Acsm3	-0.65154	0.27885	lowCoverage	0.23846	0.0011818	hypermethylated	1	16	10
chr7	126936643	126938643	2610020H08Rik	-0.097299	5.75E-12	hypomethylated	-0.0045245	0.67064	insignificant	15	52	52
chr7	126936700	126938700	2610020H08Rik	-0.097299	5.75E-12	hypomethylated	-0.0045245	0.67064	insignificant	15	52	52
chr7	126937572	126939572	Eri2	-0.1699	0.60197	insignificant	0.054297	0.73788	insignificant	4	11	12
chr7	127038805	127040805	Lyrm1	-0.14388	1.53E-29	hypomethylated	-0.010913	0.24797	insignificant	21	89	89
chr7	127038972	127040972	Dcun1d3	-0.14047	2.58E-28	hypomethylated	-0.0097029	0.34222	insignificant	21	83	83
chr7	127039259	127041259	Dcun1d3	-0.19642	7.68E-23	hypomethylated	-0.014659	0.48112	insignificant	12	41	41
chr7	127244939	127246939	Tmem159	-0.20642	7.02E-08	hypomethylated	0.017905	0.21905	insignificant	5	50	47
chr7	127345502	127347502	Crym	-0.20274	0.77101	insignificant	0.1276	0.00082712	hypermethylated	6	18	21
chr7	127346477	127348477	Abca14	-0.20349	0.29241	insignificant	0.052732	0.00077733	hypermethylated	8	22	22
chr7	127740138	127742138	E130201H02Rik	-0.17817	4.29E-11	hypomethylated	-0.014755	0.75667	insignificant	14	58	61
chr7	127777702	127779702	Uqcrc2	-0.16393	1.6E-22	hypomethylated	-0.0019527	0.27724	insignificant	26	63	63
chr7	127813764	127815764	Pdzd9		1	noCoverage	0.0025095	0.73137	insignificant	0	8	4
chr7	127813857	127815857	Pdzd9		1	noCoverage	0.0025095	0.73137	insignificant	0	8	4
chr7	127820133	127822133	BC030336	-0.10214	4.07E-15	hypomethylated	0.014246	0.50555	insignificant	20	74	72
chr7	127882070	127884070	Vwa3a		1	noCoverage	0.0035848	0.64905	insignificant	0	6	5
chr7	127985396	127987396	Eef2k	-0.13937	1.41E-10	hypomethylated	0.013493	0.52075	insignificant	17	42	42
chr7	128060257	128062257	Polr3e	-0.11332	1.58E-46	hypomethylated	0.0035942	0.34667	insignificant	60	186	180
chr7	128125826	128127826	Cdr2	-0.23468	1	lowCoverage	0.018345	0.72469	insignificant	1	21	13
chr7	128176958	128178958	Mettl9	-0.11807	1.2E-34	hypomethylated	-0.0067151	0.6372	insignificant	53	176	174
chr7	128534809	128536809	Hs3st2	-0.13272	5.52E-39	hypomethylated	0.011815	0.0038777	inconclusive	42	171	171
chr7	128850767	128852767	Usp31	-0.05702	0.000000026	hypomethylated	0.035576	0.069804	insignificant	15	38	39
chr7	128877020	128879020	Scnn1g	-0.198	4.3E-13	hypomethylated	0.0010801	0.18814	insignificant	10	38	38
chr7	129007624	129009624	Scnn1b	-0.22336	0.00000462	hypomethylated	-0.086465	0.59071	insignificant	8	25	20
chr7	129125207	129127207	Cog7	-0.2832	6.22E-25	hypomethylated	0.022636	0.63741	insignificant	10	31	40
chr7	129164712	129166712	Gga2	-0.37002	0.35206	insignificant	-0.05573	0.1271	insignificant	3	10	10
chr7	129209711	129211711	Ubfd1	-0.1613	9.57E-22	hypomethylated	-0.011591	0.053769	insignificant	29	97	96
chr7	129210577	129212577	Ubfd1	-0.15299	3.56E-17	hypomethylated	-0.011056	0.030175	hypomethylated	23	78	78
chr7	129245362	129247362	Ndufab1	-0.10329	0.0074453	hypomethylated	0.051705	0.62143	insignificant	8	20	23
chr7	129275554	129277554	Dctn5	-0.17112	0.01758	hypomethylated	-0.0529	0.34679	insignificant	7	28	49
chr7	129276460	129278460	Palb2	-0.17112	0.01758	hypomethylated	-0.0513	0.37597	insignificant	7	28	40
chr7	129301950	129303950	Plk1	-0.21472	2.08E-21	hypomethylated	0.013569	0.0089709	inconclusive	19	79	72
chr7	129362009	129364009	2010110P09Rik	-0.36499	0.5899	insignificant	0.039337	0.44305	insignificant	2	28	26
chr7	129431638	129433638	Prkcb	-0.066157	2.17E-20	hypomethylated	0.0022548	0.036804	inconclusive	28	78	82
chr7	129814257	129816257	Cacng3	-0.16186	0.000000255	hypomethylated	0.048868	0.16727	insignificant	7	26	28
chr7	130113077	130115077	Rbbp6	-0.11373	3.84E-37	hypomethylated	-0.013573	0.7825	insignificant	68	189	186
chr7	130266398	130268398	Tnrc6a	-0.12359	7.48E-09	hypomethylated	0.0030628	0.15659	insignificant	17	80	71
chr7	130513429	130515429	Arhgap17	-0.13277	0.000000134	hypomethylated	-0.011439	0.45883	insignificant	6	45	45
chr7	130520495	130522495	Lcmt1	-0.17263	0.000014759	hypomethylated	-0.044234	0.50417	insignificant	7	28	28
chr7	130643963	130645963	Zkscan2		1	noCoverage	-0.045075	1	insignificant	0	24	24
chr7	132511374	132513374	4930571K23Rik	-0.49485	0.000053514	stronglyHypometh	-0.098499	0.024975	hypomethylated	4	8	8
chr7	132587189	132589189	Jmjd5	-0.13066	5.79E-08	hypomethylated	-0.02347	0.026204	hypomethylated	26	100	97
chr7	132635056	132637056	Nsmce1	0.11651	9.45E-09	hypermethylated	-0.011007	0.00083516	inconclusive	13	45	44
chr7	132694795	132696795	Il4ra	-0.19031	0.14007	insignificant	-0.037544	0.27158	insignificant	7	41	40
chr7	132850389	132852389	D430042O09Rik	-0.11472	1.98E-17	hypomethylated	-0.010967	0.21979	insignificant	29	104	105
chr7	132851202	132853202	Gtf3c1	-0.074226	0.048069	hypomethylated	-0.018971	0.057303	insignificant	22	70	71
chr7	133225925	133227925	Gsg1l	-0.16772	0.11046	insignificant	-0.019436	0.4295	insignificant	6	21	21
chr7	133343922	133345922	Xpo6	-0.12622	0.0079591	hypomethylated	-0.02196	0.060453	insignificant	11	38	34
chr7	133415132	133417132	Sbk1	-0.10441	3.57E-26	hypomethylated	-0.0052366	0.09048	insignificant	49	152	152
chr7	133513048	133515048	Spns1	-0.19309	0.71332	insignificant	-0.026178	0.83987	insignificant	12	28	28
chr7	133521448	133523448	Spns1		1	noCoverage	-0.031228	0.63742	insignificant	0	12	12
chr7	133540251	133542251	Nfatc2ip	-0.22055	1.07E-11	hypomethylated	0.0019139	0.15783	insignificant	6	22	22
chr7	133571280	133573280	Rabep2	-0.11027	7.25E-31	hypomethylated	0.017265	0.87033	insignificant	24	131	138

chr7	133629868	133631868	Tufm	-0.17329	1.95E-17	hypomethylated	-0.0093262	0.30774	insignificant	15	40	40
chr7	133646816	133648816	Atxn2l	-0.10069	1.31E-15	hypomethylated	-0.0056596	0.632	insignificant	31	105	104
chr7	133709880	133711880	Eif3c	-0.22111	4.45E-09	hypomethylated	0.0032988	0.00037608	hypermethylated	8	20	22
chr7	133726750	133728750	Cln3	-0.30045	0.00000642	hypomethylated	-0.04346	0.83322	insignificant	4	16	16
chr7	133727521	133729521	Apobr	-0.51781	0.000036454	stronglyHypometh	0.031942	0.29079	insignificant	2	6	6
chr7	133727794	133729794	Cln3	-0.28371	1	insignificant	0.048999	0.008986	hypermethylated	1	6	6
chr7	133738424	133740424	Il27	-0.65278	0.048326	stronglyHypometh	-0.05018	0.58053	insignificant	1	4	4
chr7	133791822	133793822	Ccdc101	-0.12895	9.2E-10	hypomethylated	0.0030171	0.39929	insignificant	16	80	80
chr7	133838513	133840513	Bola2	-0.12139	1.74E-17	hypomethylated	-0.018758	0.40542	insignificant	15	126	126
chr7	133839297	133841297	Slx1b	-0.14176	0.000000442	hypomethylated	-0.025298	0.38267	insignificant	7	78	79
chr7	133848268	133850268	Coro1a	0.30375	0.37116	insignificant	-0.016405	0.54742	insignificant	2	32	32
chr7	133902139	133904139	Mapk3	-0.13691	1.66E-13	hypomethylated	-0.018177	0.57303	insignificant	21	68	68
chr7	133908927	133910927	Gdpd3	-0.093485	0.19154	insignificant	-0.046366	0.053964	insignificant	5	14	23
chr7	133919488	133921488	Ypel3	-0.14834	7.59E-15	hypomethylated	-0.015751	0.62758	insignificant	26	76	78
chr7	133923996	133925996	Tbx6	-0.2761	4.17E-08	hypomethylated	0.13104	0.0033488	inconclusive	7	18	18
chr7	133935985	133937985	Ppp4c	-0.1932	0.000000282	hypomethylated	-0.0005876	1	insignificant	11	23	20
chr7	133942692	133944692	Aldoa	-0.2074	2.23E-09	hypomethylated	0.018471	0.0010647	hypermethylated	14	73	74
chr7	133942768	133944768	Aldoa	-0.22387	3.69E-09	hypomethylated	0.024284	0.0018474	hypermethylated	11	61	62
chr7	133943961	133945961	Aldoa		1	noCoverage	-0.023752	0.67595	insignificant	0	4	4
chr7	133959398	133961398	Fam57b	0.12477	0.60597	insignificant	-0.048884	0.14606	insignificant	2	10	10
chr7	133965816	133967816	Fam57b	-0.48457	0.000015142	stronglyHypometh	-0.062623	0.89768	insignificant	2	9	10
chr7	133967166	133969166	Fam57b	-0.56944	0.00000021	stronglyHypometh	-0.064581	0.3714	insignificant	2	4	4
chr7	133975010	133977010	4930451I11Rik	-0.21912	0.000086776	hypomethylated	0.034714	0.34772	insignificant	2	13	13
chr7	133990066	133992066	Doc2a	-0.11175	5.58E-23	hypomethylated	0.016784	0.003975	inconclusive	36	126	129
chr7	134004485	134006485	Hirip3	-0.12758	0.055926	insignificant	-0.013084	0.069112	insignificant	12	58	58
chr7	134004977	134006977	Hirip3	-0.17918	0.053505	insignificant	-0.010002	0.87167	insignificant	11	40	40
chr7	134041792	134043792	Tmem219	-0.11486	0.023391	hypomethylated	-0.017704	0.079299	insignificant	6	26	26
chr7	134071392	134073392	Kctd13	-0.095144	3.92E-16	hypomethylated	0.014311	0.64112	insignificant	33	93	95
chr7	134093095	134095095	Asphd1	-0.19008	0.0022189	hypomethylated	-0.00077807	0.74151	insignificant	7	35	35
chr7	134093242	134095242	Asphd1	-0.19008	0.0022189	hypomethylated	-0.00077807	0.74151	insignificant	7	35	35
chr7	134118862	134120862	Cdipt	-0.18504	1.11E-25	hypomethylated	-0.034593	0.78511	insignificant	18	77	78
chr7	134118901	134120901	Cdipt	-0.18504	1.11E-25	hypomethylated	-0.034593	0.78511	insignificant	18	77	78
chr7	134158108	134160108	2900092E17Rik	0.087965	0.60669	insignificant	-0.015171	0.059145	insignificant	9	32	34
chr7	134164725	134166725	Maz	-0.15434	1.38E-15	hypomethylated	0.00045043	0.4966	insignificant	11	30	30
chr7	134169993	134171993	Maz	-0.10211	0.000014042	hypomethylated	-0.0074853	0.71202	insignificant	25	79	107
chr7	134185934	134187934	Kif22	-0.16149	0.47865	insignificant	-0.011722	0.27689	insignificant	6	18	19
chr7	134233949	134235949	AI467606	-0.27421	0.11076	insignificant	0.073538	0.74335	insignificant	2	8	9
chr7	134265543	134267543	Qprt	-0.67205	0.0032721	stronglyHypometh	-0.04798	0.8076	insignificant	1	6	6
chr7	134281331	134283331	Spn	-0.38773	0.080854	insignificant	-0.068117	0.67194	insignificant	2	4	4
chr7	134339514	134341514	Cd2bp2	-0.25627	0.070342	insignificant	-0.19359	0.14895	insignificant	1	16	20
chr7	134351982	134353982	Tbc1d10b	-0.10151	0.000098011	hypomethylated	-0.0086522	0.085658	insignificant	15	58	58
chr7	134375956	134377956	Zfp553	-0.090313	2.49E-17	hypomethylated	-0.0059765	0.18006	insignificant	32	89	108
chr7	134376644	134378644	Gm4532	-0.1003	9E-11	hypomethylated	-0.0082319	0.031451	hypomethylated	19	65	75
chr7	134387039	134389039	Zfp771	-0.086843	5.61E-11	hypomethylated	0.0093515	0.7776	insignificant	29	82	88
chr7	134404181	134406181	Dctpp1	-0.21976	0.0000262	hypomethylated	0.0444	0.90279	insignificant	9	27	21
chr7	134417573	134419573	Sephs2	-0.092687	1	insignificant	0.0012169	0.51419	insignificant	2	14	14
chr7	134438861	134440861	Itgal	-0.36019	0.0085383	stronglyHypometh	0.021788	0.81654	insignificant	3	19	19
chr7	134488828	134490828	Zfp768	-0.15082	1.01E-29	hypomethylated	-0.031416	0.57013	insignificant	41	112	140
chr7	134565548	134567548	Zfp688	-0.30263	0.00050165	hypomethylated	-0.018735	0.87295	insignificant	2	4	4
chr7	134592672	134594672	Zfp689	0.018534	0.12652	insignificant	0.014658	0.33347	insignificant	2	12	12
chr7	134614127	134616127	Prr14	-0.22321	3.81E-19	hypomethylated	-0.012701	0.72778	insignificant	29	88	96
chr7	134627734	134629734	Fbrs	-0.00011103	0.29246	insignificant	0.019594	0.5191	insignificant	8	24	24
chr7	134656383	134658383	1700008J07Rik	-0.21391	0.000000231	hypomethylated	-0.026913	0.64693	insignificant	8	16	16
chr7	134670410	134672410	Snora30		1	noCoverage	0.1814	0.00076731	hypermethylated	0	14	16
chr7	134715861	134717861	Phkg2	-0.212	7.54E-10	hypomethylated	0.012339	1	insignificant	8	44	44
chr7	134731211	134733211	Rnf40	-0.20314	3.14E-29	hypomethylated	-0.024164	0.0047449	hypomethylated	29	93	91
chr7	134732109	134734109	Gm166	-0.23413	9.21E-11	hypomethylated	0.013267	0.042963	inconclusive	25	94	95
chr7	134757947	134759947	Zfp629	-0.1577	9.46E-18	hypomethylated	-0.0044457	0.058796	insignificant	29	90	84
chr7	134851000	134853000	Mir762	-0.11666	6.77E-19	hypomethylated	-0.0096558	0.77115	insignificant	34	89	100
chr7	134852280	134854280	Mir762	-0.081792	0.000055108	hypomethylated	-0.0087159	0.8539	insignificant	16	34	34
chr7	134855249	134857249	Ctf1	-0.36551	0.52656	insignificant	-0.018793	0.70274	insignificant	1	36	37
chr7	134869130	134871130	Ctf2	-0.43056	0.096328	insignificant	-0.019268	0.072373	insignificant	2	12	12
chr7	134889288	134891288	Fbxl19	-0.1382	0.014195	hypomethylated	0.027991	0.92981	insignificant	13	48	44
chr7	134912328	134914328	Orai3	-0.26509	4.12E-16	hypomethylated	-0.05503	0.003358	hypomethylated	12	39	51
chr7	134919902	134921902	Setd1a	-0.0672	0.000000151	hypomethylated	-0.010367	0.0095673	hypomethylated	44	167	167
chr7	134943122	134945122	Hsd3b7		1	noCoverage	0.02472	0.72695	insignificant	0	6	6
chr7	134984321	134986321	Stx4a	-0.16415	4.28E-31	hypomethylated	-0.0092358	0.0034033	hypomethylated	32	100	100
chr7	135020214	135022214	Zfp646	-0.21236	0.000000156	hypomethylated	-0.015291	0.43314	insignificant	12	66	66
chr7	135020337	135022337	Zfp646	-0.12995	0.20721	insignificant	-0.026871	0.39096	insignificant	11	66	66
chr7	135039131	135041131	Vkorc1	-0.32861	0.00010078	hypomethylated	0.014954	0.096055	insignificant	6	39	36
chr7	135046586	135048586	Bckdk	-0.54864	0.000086581	stronglyHypometh	-0.042028	0.11844	insignificant	5	38	36
chr7	135055030	135057030	Myst1	-0.35231	6.31E-24	stronglyHypometh	-0.034915	0.013646	hypomethylated	16	43	44
chr7	135073627	135075627	Prss8	-0.080795	0.16542	insignificant	-0.069561	0.01888	hypomethylated	3	11	10
chr7	135109992	135111992	Fus	-0.14753	1.14E-19	hypomethylated	-0.0010569	0.026811	hypomethylated	25	92	96
chr7	135146891	135148891	Trim72	-0.10285	6.14E-10	hypomethylated	-0.0029964	3.07E-11	hypomethylated	10	32	30
chr7	135205153	135207153	Itgam	-0.241	0.38085	insignificant	-0.013013	0.93332	insignificant	3	16	16
chr7	135379870	135381870	Armc5	-0.12632	3.65E-27	hypomethylated	-0.028908	3.42E-08	hypomethylated	45	167	155
chr7	135381545	135383545	9130023H24Rik	-0.15961	1.43E-08	hypomethylated	-0.058392	0.73933	insignificant	15	66	50
chr7	135389384	135391384	Tgfb1i1	-0.17381	0.041996	hypomethylated	0.029431	0.53995	insignificant	6	12	12
chr7	135561686	135563686	Rgs10	-0.1692	9.96E-26	hypomethylated	0.041554	0.00028165	inconclusive	26	109	108
chr7	135605027	135607027	Tial1	-0.23409	1.18E-14	hypomethylated	-0.022323	0.016257	inconclusive	30	98	98
chr7	135666096	135668096	Bag3	-0.14473	1.06E-37	hypomethylated	-0.0059963	0.064861	insignificant	34	130	128
chr7	135753878	135755878	Inpp5f	-0.15229	1.72E-26	hypomethylated	0.0041456	0.60874	insignificant	35	94	94
chr7	135883943	135885943	Mcmbp	-0.2999	0.000000431	hypomethylated	0.0011355	0.42056	insignificant	7	36	36
chr7	135887383	135889383	Sec23ip	-0.16367	8.68E-15	hypomethylated	-0.016679	0.010934	hypomethylated	23	68	69

chr7	136399607	136401607	Ppapdc1a	-0.12274	3.37E-11	hypomethylated	0.0037802	0.00024747	inconclusive	18	68	72
chr7	136734376	136736376	Wdr11	-0.12917	1.25E-26	hypomethylated	0.0026475	0.53784	insignificant	23	57	57
chr7	137663044	137665044	Ate1	-0.11682	3.68E-13	hypomethylated	-0.024516	0.0065771	hypomethylated	28	103	116
chr7	137663053	137665053	Ate1	-0.11682	3.68E-13	hypomethylated	-0.024516	0.0065771	hypomethylated	28	103	116
chr7	137663475	137665475	Ate1	-0.073352	0.136	insignificant	-0.036778	0.18762	insignificant	10	36	40
chr7	137835069	137837069	Tacc2	-0.082538	0.00072532	hypomethylated	-0.016712	0.82166	insignificant	14	53	53
chr7	138008423	138010423	Plekha1	-0.08774	5.02E-46	hypomethylated	-0.010303	0.0057851	hypomethylated	45	173	172
chr7	138078716	138080716	Htra1	-0.11122	2.36E-15	hypomethylated	-0.017415	0.086528	insignificant	28	175	186
chr7	138316915	138318915	5430419D17Rik	0.24747	1	lowCoverage	0.078903	0.85099	insignificant	1	6	6
chr7	138477135	138479135	Fam24a		1	noCoverage	-0.021593	0.20634	insignificant	0	8	8
chr7	138506212	138508212	2310057M21Ril	0.078241	0.15004	insignificant	0.11823	0.51657	insignificant	6	30	22
chr7	138513659	138515659	Pstk	-0.12259	0.000021938	hypomethylated	0.00021284	0.21739	insignificant	10	52	55
chr7	138553114	138555114	Acadsb	-0.20409	3.99E-27	hypomethylated	-0.0011848	0.015815	hypomethylated	20	88	88
chr7	138553992	138555992	Ikzf5	-0.31925	0.32954	insignificant	0.035694	0.068908	insignificant	3	24	24
chr7	138685476	138687476	Hmx3	-0.1018	0.011379	hypomethylated	-0.016902	0.48866	insignificant	41	157	176
chr7	138696575	138698575	Hmx2	-0.16977	6.85E-39	hypomethylated	-0.0091362	0.33823	insignificant	30	105	105
chr7	138702904	138704904	Bub3	-0.1842	9.95E-12	hypomethylated	-0.025724	0.30614	insignificant	17	48	56
chr7	139157142	139159142	Gpr26	-0.092747	0.00018056	hypomethylated	-0.0069397	0.65477	insignificant	31	114	124
chr7	139346424	139348424	Cpxm2	-0.525	0.018094	stronglyHypometh	0.090385	0.009026	hypermethylated	1	2	2
chr7	139506346	139508346	Gm10584	-0.096523	1.17E-17	hypomethylated	0.0023965	0.10542	insignificant	34	108	107
chr7	139508838	139510838	Chst15	0.056533	0.18329	insignificant	-0.01591	0.1208	insignificant	5	22	22
chr7	139768081	139770081	Oat	-0.36797	0.066219	insignificant	-0.052612	0.50228	insignificant	3	13	11
chr7	139791320	139793320	Nkx1-2	-0.15034	0.81696	insignificant	-0.044428	1	insignificant	8	18	16
chr7	139801325	139803325	Lhpp	-0.15499	0.060155	insignificant	-0.0049695	0.54334	insignificant	11	44	44
chr7	139978618	139980618	Fam53b	-0.18182	0.18	insignificant	-0.08658	0.48071	insignificant	2	4	4
chr7	140004879	140006879	Fam53b	-0.13749	1.66E-28	hypomethylated	-0.005679	0.14587	insignificant	37	100	99
chr7	140044330	140046330	Mettl10	-0.55358	0.031711	stronglyHypometh	-0.038255	0.77238	insignificant	3	22	22
chr7	140049907	140051907	Fam175b	-0.16784	3.75E-30	hypomethylated	-0.013744	0.41629	insignificant	24	81	83
chr7	140315166	140317166	Ctbp2	-0.10265	8.98E-39	hypomethylated	0.0018646	0.83167	insignificant	30	155	156
chr7	140828357	140830357	2700050L05Rik	-0.15871	3.42E-08	hypomethylated	0.0036105	0.4882	insignificant	19	46	51
chr7	140900015	140902015	Bccip	-0.16218	3.57E-39	hypomethylated	-0.0069565	0.0074031	hypomethylated	31	95	98
chr7	140900978	140902978	Uros	-0.3466	8.11E-21	stronglyHypometh	-0.017757	0.1818	insignificant	9	22	25
chr7	140967573	140969573	Fank1	-0.13193	5.61E-39	hypomethylated	0.008849	0.69856	insignificant	45	147	147
chr7	140968486	140970486	Dhx32	-0.13924	1.44E-22	hypomethylated	-0.0013636	0.059899	insignificant	18	48	48
chr7	141416780	141418780	Adam12	-0.1524	0.000048679	hypomethylated	0.0033263	0.00059307	hypermethylated	15	48	52
chr7	141861369	141863369	Dock1	-0.10054	4.57E-21	hypomethylated	0.023051	0.32309	insignificant	29	85	68
chr7	142601049	142603049	Foxi2	-0.15192	2.25E-10	hypomethylated	-0.052129	0.014254	hypomethylated	17	38	53
chr7	142728506	142730506	Ptpre	-0.12174	3.23E-43	hypomethylated	0.0066018	0.41408	insignificant	30	96	99
chr7	142908062	142910062	Mki67	-0.16307	9.14E-11	hypomethylated	0.070508	0.92515	insignificant	5	13	10
chr7	144085293	144087293	Mgmt		1	noCoverage	-0.015594	0.31319	insignificant	0	12	12
chr7	144506128	144508128	Ebf3	-0.12085	6.76E-11	hypomethylated	-0.010644	0.89324	insignificant	27	134	135
chr7	144602439	144604439	9430038I01Rik	-0.17825	0.0026724	hypomethylated	0.025321	0.11562	insignificant	2	22	24
chr7	144628330	144630330	Glrx3	-0.12536	1.4E-15	hypomethylated	-0.010189	0.28229	insignificant	29	109	110
chr7	144628356	144630356	Gm12669	-0.12536	1.4E-15	hypomethylated	-0.010189	0.28229	insignificant	29	109	110
chr7	145589413	145591413	Tcerg1l	-0.19126	5.86E-12	hypomethylated	-0.0133	0.40608	insignificant	24	85	81
chr7	146037068	146039068	Ppp2r2d	-0.10839	2.63E-15	hypomethylated	0.0035871	0.75524	insignificant	54	176	194
chr7	146037950	146039950	Mapk1ip1	-0.11385	1.45E-09	hypomethylated	0.010725	0.40315	insignificant	39	134	150
chr7	146101189	146103189	Bnip3	-0.27146	4.43E-19	hypomethylated	0.059125	0.87441	insignificant	10	46	49
chr7	146131432	146133432	Jakmip3	-0.11254	1.97E-08	hypomethylated	-0.0076967	0.79774	insignificant	28	122	122
chr7	146270899	146272899	Dpysl4	-0.088427	6.52E-11	hypomethylated	-0.005023	0.91438	insignificant	32	123	129
chr7	146374393	146376393	Stk32c	-0.12291	0.000014706	hypomethylated	0.0026915	0.4636	insignificant	10	56	56
chr7	146397886	146399886	Lrrc27	-0.13208	0.000054848	hypomethylated	0.0024249	1	insignificant	7	64	55
chr7	146398173	146400173	Lrrc27	-0.13208	0.000054848	hypomethylated	0.0024249	1	insignificant	7	64	55
chr7	146399206	146401206	Lrrc27	0.66854	0.026685	stronglyHypermeth	0.045446	0.58787	insignificant	1	30	24
chr7	146433380	146435380	Pwwp2b	-0.17202	7.54E-33	hypomethylated	-0.020301	0.072906	insignificant	76	175	174
chr7	146574007	146576007	Inpp5a	-0.1439	5.71E-37	hypomethylated	0.0024222	0.43615	insignificant	41	115	117
chr7	146768696	146770696	Nkx6-2	-0.1359	0.000066824	hypomethylated	0.032519	0.37975	insignificant	6	37	28
chr7	147019072	147021072	Gpr123	-0.21195	0.000070037	hypomethylated	-0.0035838	0.33692	insignificant	11	36	36
chr7	147079594	147081594	Kndc1	-0.19504	5.06E-21	hypomethylated	-0.024182	0.36902	insignificant	25	56	56
chr7	147128754	147130754	Utf1	-0.14262	8.95E-42	hypomethylated	-0.003861	0.023906	hypomethylated	36	100	100
chr7	147164654	147166654	Adam8		1	noCoverage	-0.12223	0.3807	insignificant	0	12	10
chr7	147221289	147223289	Zfp511	-0.20304	1.22E-14	hypomethylated	-0.0082488	0.32988	insignificant	17	93	86
chr7	147222249	147224249	Tubgcp2	-0.18375	0.00000131	hypomethylated	0.0067249	0.83116	insignificant	13	75	71
chr7	147234987	147236987	Msx3		1	noCoverage	-0.0035757	0.48148	insignificant	0	16	16
chr7	147268200	147270200	Caly		1	noCoverage	-0.21591	0.32337	insignificant	0	2	6
chr7	147268447	147270447	Caly		1	noCoverage	-0.17235	0.56885	insignificant	0	4	8
chr7	147310583	147312583	Paox	-0.14078	0.0021634	hypomethylated	-0.0094575	1	insignificant	5	16	16
chr7	147322462	147324462	Mtg1	-0.17846	9.02E-20	hypomethylated	-0.009314	0.10176	insignificant	16	58	66
chr7	147340558	147342558	Sprn	-0.27191	0.056753	insignificant	-0.01261	1	insignificant	6	22	19
chr7	147348847	147350847	Olfr522		1	noCoverage	-0.030159	0.71171	insignificant	0	11	9
chr7	147361002	147363002	Olfr523	-0.21818	0.40196	insignificant	-0.14596	0.13436	insignificant	3	10	10
chr7	147403165	147405165	Cd163l1	0.2125	1	insignificant	-0.097467	0.69237	insignificant	2	8	8
chr7	147531886	147533886	Olfr60	0.37054	0.54117	insignificant	0.17054	0.37903	insignificant	2	4	4
chr7	147559507	147561507	Olfr530		1	noCoverage	-0.072304	0.78122	insignificant	0	4	4
chr7	148020394	148022394	1190003J15Rik	-0.13134	0.12089	insignificant	-0.070086	0.080545	insignificant	1	8	8
chr7	148030463	148032463	Scgb1c1	-0.071734	0.045269	inconclusive	-0.035486	0.53569	insignificant	7	18	18
chr7	148032814	148034814	Odf3		1	noCoverage	0.034495	0.74875	insignificant	0	15	20
chr7	148042282	148044282	Ric8	-0.19484	1.52E-19	hypomethylated	0.019011	0.16952	insignificant	20	70	71
chr7	148042295	148044295	Ric8	-0.19484	1.52E-19	hypomethylated	0.019011	0.16952	insignificant	20	70	71
chr7	148067292	148069292	Psmd13	-0.15555	3.02E-12	hypomethylated	-0.014458	0.3252	insignificant	24	94	101
chr7	148067768	148069768	Psmd13	-0.15845	2.41E-09	hypomethylated	-0.012171	0.43105	insignificant	20	69	76
chr7	148068208	148070208	Psmd13	-0.16559	0.000000672	hypomethylated	-0.013769	0.66691	insignificant	13	31	38
chr7	148090962	148092962	BC024386		1	noCoverage	0.077221	0.52568	insignificant	0	8	9
chr7	148105800	148107800	Nlrp6	-0.074651	0.32845	insignificant	0.0009699	0.098298	insignificant	2	22	27
chr7	148126479	148128479	Athl1	-0.13918	4.71E-09	hypomethylated	0.0052427	0.1643	insignificant	5	27	33

chr7	148141860	148143860	Ifitm2	-0.4581	0.28708	insignificant	0.071702	0.016912	hypermethylated	4	16	16
chr7	148196643	148198643	Ifitm3		1	noCoverage	-0.035586	1	insignificant	0	10	7
chr7	148202791	148204791	Ifitm6	-0.80952	0.0021127	stronglyHypometh	-0.051997	0.53485	insignificant	2	14	14
chr7	148246172	148248172	B4galnt4	-0.11642	1.07E-34	hypomethylated	-0.0055517	0.033234	hypomethylated	53	135	143
chr7	148263127	148265127	Pkp3	0.038766	1	insignificant	-0.017685	0.0273	hypomethylated	5	18	18
chr7	148264667	148266667	Pkp3	-0.089855	0.091486	insignificant	0.020506	0.25778	insignificant	7	22	23
chr7	148303705	148305705	Ano9	-0.30375	1	insignificant	-0.096685	0.33129	insignificant	3	14	14
chr7	148316184	148318184	Ptdss2	-0.24561	1.69E-48	hypomethylated	-0.021663	1.91E-10	hypomethylated	18	64	64
chr7	148358750	148360750	Rnh1	-0.19198	0.32552	insignificant	-0.00095529	0.07218	insignificant	2	18	18
chr7	148379028	148381028	Lrrc56	-0.13417	7.02E-48	hypomethylated	-0.009084	0.00011134	hypomethylated	64	177	174
chr7	148379903	148381903	Lrrc56	-0.13948	9.91E-11	hypomethylated	-0.0098088	0.01163	hypomethylated	28	95	100
chr7	148380041	148382041	Lrrc56	-0.14852	1.1E-11	hypomethylated	-0.0074521	0.54354	insignificant	24	69	74
chr7	148399979	148401979	1600016N20Rik	0.092822	0.00082278	hypermethylated	-0.0054212	0.0025198	hypomethylated	9	56	56
chr7	148400758	148402758	Rassf7	-0.011015	0.027827	inconclusive	-0.0052995	0.18633	insignificant	20	94	94
chr7	148407392	148409392	Mir210	-0.21133	3.42E-09	hypomethylated	-0.011353	0.67751	insignificant	10	61	61
chr7	148413686	148415686	Phrf1	-0.20267	1.12E-30	hypomethylated	0.0088491	0.0055423	inconclusive	18	74	75
chr7	148462685	148464685	Sct	-0.2901	0.070376	insignificant	0.011255	0.31287	insignificant	2	4	4
chr7	148465030	148467030	Sct	-0.23184	0.000000226	hypomethylated	-0.0289	0.9419	insignificant	5	18	18
chr7	148476904	148478904	Drd4	-0.31408	9.23E-11	hypomethylated	-0.086943	0.93503	insignificant	5	13	10
chr7	148513028	148515028	Tmem80	-0.095525	0.000023357	hypomethylated	-0.0058637	0.033204	inconclusive	21	115	117
chr7	148513624	148515624	Deaf1	-0.14364	0.000000538	hypomethylated	-0.01474	0.87144	insignificant	12	55	57
chr7	148523900	148525900	Eps8l2	-0.19376	2.18E-28	hypomethylated	-0.031022	3.68E-08	hypomethylated	17	46	46
chr7	148524682	148526682	Deaf1	-0.11469	1.78E-24	hypomethylated	-0.028386	0.00000321	hypomethylated	17	44	44
chr7	148551009	148553009	B230206H07Rik		1	noCoverage	-0.027083	1	insignificant	0	4	4
chr7	148577058	148579058	Taldo1	-0.27986	8.3E-25	hypomethylated	-0.050533	0.089385	insignificant	12	46	40
chr7	148591433	148593433		0.065143	1	insignificant	0.010116	0.2781	insignificant	1	16	18
chr7	148600024	148602024	Pddc1	-0.2513	2.36E-08	hypomethylated	0.073889	0.69821	insignificant	6	41	36
chr7	148615319	148617319	Slc25a22	-0.22541	0.0012643	hypomethylated	0.0057733	0.45393	insignificant	4	14	14
chr7	148623773	148625773	Lrdd	-0.07753	0.00079158	hypomethylated	-0.0047341	0.83457	insignificant	4	19	19
chr7	148629254	148631254	Lrdd	-0.56328	4.44E-16	stronglyHypometh	-0.033155	0.0088255	hypomethylated	5	15	14
chr7	148632548	148634548	Rplp2	-0.091579	7.42E-16	hypomethylated	0.0090309	0.34366	insignificant	20	86	86
chr7	148633731	148635731	Snora52	-0.15223	0.0074572	hypomethylated	0.011093	0.47279	insignificant	3	20	20
chr7	148640086	148642086	Pnpla2	-0.38587	2.83E-09	stronglyHypometh	-0.061357	0.031626	hypomethylated	8	28	36
chr7	148645992	148647992	Efcab4a	-0.17938	1.6E-15	hypomethylated	-0.012592	0.8499	insignificant	20	90	90
chr7	148652260	148654260	Cd151	-0.20192	0.000000569	hypomethylated	-0.047354	0.00000357	hypomethylated	18	56	58
chr7	148653687	148655687	Cd151	-0.33242	0.030561	hypomethylated	-0.03727	1	insignificant	2	12	15
chr7	148660138	148662138	Polr2l	-0.12639	2.97E-19	hypomethylated	-0.0030687	0.94144	insignificant	41	141	151
chr7	148661052	148663052	Polr2l	-0.098826	2.49E-12	hypomethylated	-0.0043261	0.88183	insignificant	32	112	122
chr7	148725756	148727756	Chid1	-0.10585	0.24444	insignificant	-0.030427	0.3637	insignificant	5	23	25
chr7	148747078	148749078	Ap2a2	-0.099748	0.00000481	hypomethylated	-0.0028682	0.79027	insignificant	35	108	108
chr7	149023976	149025976	Muc5b	0.25	1	lowCoverage	0.031746	0.34267	insignificant	1	6	6
chr7	149088311	149090311	Tollip	-0.062576	0.00048448	hypomethylated	0.011803	0.32689	insignificant	6	34	34
chr7	149134655	149136655	Brsk2	-0.1248	4.96E-39	hypomethylated	0.0032451	0.43365	insignificant	60	191	183
chr7	149134990	149136990	Brsk2	-0.12062	1.05E-37	hypomethylated	0.0027341	0.6039	insignificant	60	189	181
chr7	149246939	149248939	Mob2	-0.14806	7.04E-08	hypomethylated	0.019347	0.051382	insignificant	15	40	41
chr7	149488406	149490406	Krtap5-4	-0.20031	0.012034	hypomethylated	-0.038616	0.36139	insignificant	3	6	6
chr7	149558164	149560164	6330512M04Ril	-0.072991	0.05574	insignificant	-0.013077	0.47306	insignificant	15	46	46
chr7	149573775	149575775	Ctsd	-0.072884	0.000000334	hypomethylated	0.000093242	0.27308	insignificant	12	43	43
chr7	149619881	149621881	Syt8	0.068502	1	insignificant	-0.3393	0.0033908	stronglyhypometh	2	4	6
chr7	149627372	149629372	Tnni2	-0.41093	0.001087	stronglyHypometh	-0.035677	0.01065	hypomethylated	3	16	16
chr7	149645774	149647774	Lsp1	-0.79342	0.00000317	stronglyHypometh	0.036152	0.080294	insignificant	1	8	8
chr7	149656747	149658747	Lsp1	-0.32496	0.76227	insignificant	0.0057282	0.48183	insignificant	2	8	8
chr7	149683740	149685740	Tnnt3	-0.57926	0.000005912	stronglyHypometh	-0.028125	0.19203	insignificant	2	4	6
chr7	149718021	149720021	Mrpl23	-0.12933	0.000000114	hypomethylated	-0.035009	0.59007	insignificant	20	68	66
chr7	149744503	149746503	Nctc1	-0.39916	0.052738	insignificant	0.0069009	1	insignificant	1	6	6
chr7	149763052	149765052	Mir675	-0.1122	0.16289	insignificant	0.073819	0.00019836	hypermethylated	7	32	30
chr7	149764051	149766051	Mir675	-0.29887	0.027991	hypomethylated	0.069923	0.00000739	hypermethylated	2	22	20
chr7	149840901	149842901	Mir483		1	noCoverage	-0.074156	0.016809	hypomethylated	0	18	18
chr7	149843386	149845386	Igf2	-0.18752	1.18E-21	hypomethylated	0.0055762	0.51474	insignificant	31	95	92
chr7	149844597	149846597	Igf2as	-0.14793	7.42E-28	hypomethylated	-0.0077067	0.11131	insignificant	57	173	173
chr7	149844709	149846709	Igf2	-0.14793	7.42E-28	hypomethylated	-0.0077067	0.11131	insignificant	57	173	173
chr7	149846940	149848940	Igf2as		1	noCoverage	-0.30997	0.16671	insignificant	0	5	2
chr7	150085871	150087871	Th		1	noCoverage	-0.0018166	0.62299	insignificant	0	4	4
chr7	150155169	150157169	Ascl2	-0.1701	2.93E-08	hypomethylated	-0.025111	0.10245	insignificant	6	42	40
chr7	150189950	150191950	Tspan32	-0.4752	0.053432	insignificant	-0.03423	0.57032	insignificant	1	6	7
chr7	150190563	150192563	Tspan32	-0.4752	0.053432	insignificant	-0.03423	0.57032	insignificant	1	6	7
chr7	150237654	150239654	Cd81	-0.17595	3.06E-20	hypomethylated	-0.0074662	0.12628	insignificant	22	94	94
chr7	150239273	150241273	Cd81	-0.21754	0.0063974	hypomethylated	0.040849	0.59713	insignificant	7	50	50
chr7	150254272	150256272	Tssc4	-0.11803	0.0021333	hypomethylated	-0.0046618	0.49339	insignificant	13	95	95
chr7	150280547	150282547	Trpm5		1	noCoverage	0.011905	1	insignificant	0	4	4
chr7	150292158	150294158	Kcnq1	-0.10896	0.00017392	hypomethylated	-0.0035106	0.53825	insignificant	16	90	90
chr7	150646955	150648955	Cdkn1c	-0.13575	0.019071	hypomethylated	0.044065	0.18287	insignificant	16	43	61
chr7	150660020	150662020	Slc22a18	-0.2235	0.000003146	hypomethylated	0.10239	0.0011701	hypermethylated	7	22	22
chr7	150688429	150690429	Phlda2	-0.0094894	0.34466	insignificant	-0.036543	0.55859	insignificant	12	50	56
chr7	150734995	150736995	Nap1l4	-0.10449	0.000057876	hypomethylated	0.032437	0.86707	insignificant	11	51	51
chr7	150785947	150787947	Cars	-0.083984	0.0031922	hypomethylated	0.0076214	0.19681	insignificant	7	20	20
chr7	150926265	150928265	Osbpl5	-0.19587	0.0035449	hypomethylated	-0.060776	0.20434	insignificant	2	22	20
chr7	150952898	150954898	Mrgprg	-0.29205	1	insignificant	0.087762	0.35946	insignificant	2	4	4
chr7	151008071	151010071	Dhcr7	-0.3035	2.51E-15	hypomethylated	-0.084753	0.0053977	hypomethylated	10	30	27
chr7	151008746	151010746	Nadsyn1	-0.33726	2.06E-11	stronglyHypometh	-0.13053	0.0024864	hypomethylated	3	16	13
chr7	151360424	151362424	Shank2	-0.30485	9.21E-08	hypomethylated	-0.066291	0.54141	insignificant	16	56	57
chr7	151469289	151471289	Shank2	-0.16129	9.6E-15	hypomethylated	0.010248	0.41851	insignificant	12	56	56
chr7	151656646	151658646	Cttn	-0.22194	0.77917	insignificant	-0.0034587	1	insignificant	1	2	2
chr7	151739634	151741634	Ppfia1	-0.14214	2.26E-11	hypomethylated	-0.008507	0.11884	insignificant	19	47	47
chr7	151768341	151770341	Fadd	-0.11449	0.0030297	hypomethylated	-0.023779	0.14769	insignificant	5	20	20

chr7	151924383	151926383	Ano1	-0.20335	1.67E-10	hypomethylated	-0.038059	0.016701	hypomethylated	10	20	20
chr7	152023516	152025516	Fgf3	-0.10938	4.44E-10	hypomethylated	0.0042261	0.23444	insignificant	31	162	159
chr7	152046290	152048290	Fgf4	-0.14966	5.59E-16	hypomethylated	-0.012516	0.057722	insignificant	46	153	160
chr7	152081436	152083436	Fgf15	-0.14587	1.23E-16	hypomethylated	0.016647	0.68275	insignificant	25	86	93
chr7	152100098	152102098	Oraov1	-0.12279	0.000078716	hypomethylated	-0.0075795	0.55143	insignificant	13	46	50
chr7	152125830	152127830	Ccnd1	-0.12693	1.82E-09	hypomethylated	-0.013823	0.73446	insignificant	14	66	73
chr7	152469832	152471832	Tpcn2	-0.56897	0.000044066	stronglyHypometh	-0.0037481	0.418	insignificant	1	2	2
chr8	3279617	3281617	Insr	-0.14338	0.31737	insignificant	-0.046816	0.68752	insignificant	4	17	14
chr8	3352414	3354414	A430078G23Rik		1	noCoverage	-0.017267	0.86376	insignificant	0	23	25
chr8	3392007	3394007	Arhgef18	0.13695	0.00082841	hypermethylated	0.075204	0.084857	insignificant	15	34	35
chr8	3492137	3494137	Zfp358	-0.1632	2.95E-24	hypomethylated	0.0073196	0.21208	insignificant	28	67	52
chr8	3499518	3501518	Mcoln1	-0.13383	0.00039004	hypomethylated	-0.024202	0.29444	insignificant	6	60	59
chr8	3514383	3516383	Pnpla6	-0.19217	5.24E-25	hypomethylated	0.034239	0.091072	insignificant	20	56	56
chr8	3586449	3588449	Camsap3	-0.10961	7.05E-09	hypomethylated	-0.0040683	0.94358	insignificant	27	90	90
chr8	3620550	3622550	2900053A13Rik	-0.16783	1.95E-12	hypomethylated	-0.0008517	0.0052741	inconclusive	11	76	76
chr8	3621296	3623296	Xab2	-0.11738	0.60236	insignificant	0.0037273	0.2359	insignificant	6	44	44
chr8	3630159	3632159	Stxbp2	-0.11379	0.00000038	hypomethylated	0.0082844	0.9207	insignificant	10	62	64
chr8	3664761	3666761	1810033B17Rik	-0.48954	0.0015327	stronglyHypometh	0.16502	0.58659	insignificant	1	7	6
chr8	3675476	3677476	Trappc5	-0.28683	0.00000469	hypomethylated	-0.0089852	0.21911	insignificant	10	28	31
chr8	3803689	3805689	Rprl3		1	noCoverage	0.16214	0.15991	insignificant	0	16	13
chr8	4165566	4167566	Evi5l	-0.11282	0.0013711	hypomethylated	0.0077338	0.032807	hypermethylated	12	53	53
chr8	4225826	4227826	Lrrc8e	-0.22392	1.11E-10	hypomethylated	0.012925	0.73595	insignificant	16	47	52
chr8	4237739	4239739	Map2k7	-0.041893	1	insignificant	-0.0011065	0.26928	insignificant	7	62	64
chr8	4252101	4254101	Snapc2	-0.1389	0.48656	insignificant	0.0093885	0.11274	insignificant	3	50	49
chr8	4259274	4261274	Timm44	-0.067922	0.45203	insignificant	0.0013923	0.10533	insignificant	6	33	31
chr8	4275905	4277905	Timm44		1	noCoverage	-0.13308	0.59737	insignificant	0	10	10
chr8	4324209	4326209	Ccl25	-0.1045	1.7E-15	hypomethylated	0.015804	0.91879	insignificant	36	139	137
chr8	4325100	4327100	Elavl1		1	noCoverage	0.04332	0.17548	insignificant	0	41	39
chr8	4348587	4350587	Ccl25	0	0.80828	insignificant	0.0074013	0.26033	insignificant	2	4	4
chr8	4492404	4494404	Lass4	-0.16858	0.00068256	hypomethylated	0.029442	0.13858	insignificant	2	34	31
chr8	4612169	4614169	Zfp958		1	noCoverage	-0.18207	0.67976	insignificant	0	21	18
chr8	4779534	4781534	Shcbp1	-0.18703	0.0033442	hypomethylated	-0.043686	0.38456	insignificant	5	16	16
chr8	8660773	8662773	Efnb2	-0.1045	2.61E-37	hypomethylated	0.0076796	0.27329	insignificant	41	146	138
chr8	9771023	9773023	Fam155a	-0.11457	1.03E-16	hypomethylated	0.018025	0.83245	insignificant	25	92	101
chr8	9976323	9978323	Lig4	-0.085516	2.3E-19	hypomethylated	-0.0067621	0.025236	hypomethylated	26	88	88
chr8	9976716	9978716	Abhd13	-0.059462	1.63E-11	hypomethylated	-0.010227	0.11038	insignificant	16	68	73
chr8	10005632	10007632	Tnfsf13b	-0.20431	0.46094	insignificant	0.016066	0.044059	inconclusive	5	24	24
chr8	10152922	10154922	Myo16	-0.23034	0.00000137	hypomethylated	0.076467	0.43887	insignificant	7	18	22
chr8	10928457	10930457	3930402G23Rik	-0.03208	0.088653	insignificant	-0.011837	0.33819	insignificant	5	15	14
chr8	11008430	11010430	Irs2	-0.093364	9.88E-29	hypomethylated	-0.007391	0.021154	hypomethylated	52	229	234
chr8	11311828	11313828	Col4a2	-0.13533	8.72E-25	hypomethylated	-0.013278	0.86861	insignificant	25	108	112
chr8	11312826	11314826	Col4a1	-0.12218	9.5E-17	hypomethylated	0.00022086	0.62654	insignificant	13	56	56
chr8	11478499	11480499	Rab20	-0.16041	0.036999	hypomethylated	-0.002684	0.10796	insignificant	4	34	34
chr8	11496505	11498505	Carkd	-0.14968	2.15E-32	hypomethylated	-0.040913	0.000077079	hypomethylated	28	80	99
chr8	11496567	11498567	Carkd	-0.16391	9.58E-36	hypomethylated	-0.04918	0.000000896	hypomethylated	28	84	103
chr8	11550771	11552771	Cars2	-0.44522	3.75E-16	stronglyHypometh	-0.054956	0.0090942	hypomethylated	8	36	37
chr8	11555065	11557065	Ing1	-0.080433	6.73E-29	hypomethylated	0.0078858	0.62167	insignificant	61	210	213
chr8	11635036	11637036	Ankrd10	-0.11628	1.86E-10	hypomethylated	0.0039877	0.0071822	inconclusive	23	106	128
chr8	11635754	11637754	Ankrd10	-0.27207	0.0004781	hypomethylated	-0.03519	0.29396	insignificant	11	55	76
chr8	11694514	11696514	Gm6524	0.32451	1	insignificant	-0.043293	0.25468	insignificant	1	12	12
chr8	11727104	11729104	Arhgef7	-0.10272	2.74E-28	hypomethylated	-0.0038848	0.13643	insignificant	72	223	239
chr8	11757329	11759329	Arhgef7	-0.12093	1.06E-17	hypomethylated	-0.014412	0.0039291	hypomethylated	36	111	111
chr8	12384770	12386770	Gm5607	-0.11814	5.94E-22	hypomethylated	0.013437	0.45779	insignificant	33	147	154
chr8	12394518	12396518	Sox1	-0.16191	1.19E-19	hypomethylated	-0.019923	0.067262	insignificant	11	47	47
chr8	12672100	12674100	Tubgcp3	-0.087795	0.00021196	hypomethylated	0.033507	1	insignificant	12	47	42
chr8	12756015	12758015	Atp11a	-0.11281	3.52E-55	hypomethylated	-0.0024433	0.010377	hypomethylated	75	194	194
chr8	12914892	12916892	Mcf2l	-0.26612	0.5724	insignificant	-0.026121	0.68025	insignificant	1	10	10
chr8	12948417	12950417	Mcf2l	-0.48189	0.00009033	stronglyHypometh	-0.031187	0.0000024	hypomethylated	1	12	12
chr8	13025033	13027033	F7	-0.10412	0.000005617	hypomethylated	-0.043616	0.16251	insignificant	5	10	10
chr8	13036307	13038307	F10	-0.030649	1	insignificant	0.024907	0.63055	insignificant	4	8	8
chr8	13059907	13061907	Proz	-0.17033	0.0056296	hypomethylated	0.053753	0.76234	insignificant	7	26	26
chr8	13104720	13106720	Cul4a	-0.11103	7.99E-31	hypomethylated	-0.013264	0.42159	insignificant	48	201	214
chr8	13105343	13107343	Pcid2	-0.15221	2.31E-16	hypomethylated	-0.027042	1	insignificant	30	123	136
chr8	13158134	13160134	Lamp1	-0.2828	0.00000177	hypomethylated	-0.056053	0.00018253	hypomethylated	8	65	55
chr8	13189098	13191098	Grtp1		1	noCoverage	-0.04044	0.60513	insignificant	0	10	10
chr8	13287012	13289012	Tmco3	-0.10159	2.27E-19	hypomethylated	-0.0060774	0.76293	insignificant	28	118	118
chr8	13288126	13290126	Tmco3	-0.045779	0.4976	insignificant	0.00089116	0.35613	insignificant	3	40	40
chr8	13338673	13340673	Tfdp1	-0.099241	2.84E-08	hypomethylated	-0.0066904	0.12307	insignificant	44	116	121
chr8	13396778	13398778	Atp4b	-0.053488	1	insignificant	-0.055869	0.38122	insignificant	1	5	7
chr8	13404080	13406080	Grk1	-0.069793	0.01529	hypomethylated	-0.098148	0.00063922	hypomethylated	5	10	10
chr8	13434458	13436458	Fam70b	-0.3032	0.1116	insignificant	0.020634	0.86198	insignificant	4	10	10
chr8	13494535	13496535	Gas6		1	noCoverage	-0.090909	0.54209	insignificant	0	3	3
chr8	13562448	13564448	1700029H14Rik	-0.297	0.0063662	hypomethylated	-0.037289	0.12795	insignificant	6	16	15
chr8	13677587	13679587	Rasa3	-0.53333	0.00000112	stronglyHypometh	-0.032609	0.15949	insignificant	0	0	0
chr8	13704888	13706888	4932443I19Rik	-0.032052	0.77923	insignificant	-0.0074288	0.46736	insignificant	5	23	24
chr8	13756689	13758689	Cdc16	-0.11518	2.35E-14	hypomethylated	0.0029705	0.19758	insignificant	26	81	84
chr8	13784614	13786614	Upf3a	-0.15815	7.35E-36	hypomethylated	0.0059712	0.16659	insignificant	42	144	149
chr8	13868640	13870640	Zfp828	-0.13233	7.63E-12	hypomethylated	-0.023993	0.080842	insignificant	23	86	86
chr8	13890271	13892271	2410022L05Rik	-0.10152	2.78E-08	hypomethylated	-0.0011296	0.0061902	hypomethylated	13	50	50
chr8	13906805	13908805	Fbxo25	-0.087718	5.41E-09	hypomethylated	0.0029623	0.51373	insignificant	22	85	85
chr8	13974777	13976777	2610019F03Rik	-0.48369	0.21291	insignificant	-0.02099	0.21635	insignificant	2	20	24
chr8	14090327	14092327	Erich1	-0.15588	0.000000165	hypomethylated	0.037567	0.0052374	inconclusive	8	30	32
chr8	14094874	14096874	Dlgap2	-0.095072	7.22E-12	hypomethylated	-0.022109	0.00063813	hypomethylated	37	180	176
chr8	14887535	14889535	Cln8	-0.19158	0.000048067	hypomethylated	-0.0007035	0.91722	insignificant	5	12	12
chr8	14910716	14912716	Arhgef10	-0.13642	1	insignificant	-0.025893	0.73345	insignificant	1	27	38

chr8	15010024	15012024	Kbtbd11	-0.11158	2.7E-22	hypomethylated	-0.0037856	0.57606	insignificant	34	125	125
chr8	15046069	15048069	BB014433		1	noCoverage	-0.11619	0.16677	insignificant	0	4	4
chr8	17535385	17537385	Csmd1	-0.16147	0.019691	hypomethylated	0.012785	0.66032	insignificant	4	15	15
chr8	18594172	18596172	Mcph1	-0.10447	2.52E-14	hypomethylated	0.0010402	0.52861	insignificant	16	119	119
chr8	18845278	18847278	Agpat5	-0.10414	8.95E-27	hypomethylated	0.00044103	0.016963	inconclusive	49	174	173
chr8	18978116	18980116	Defb40		1	noCoverage	0.16114	0.2008	insignificant	0	11	10
chr8	19193327	19195327	Defb14	0.13782	1	insignificant	0.029873	1	insignificant	2	4	4
chr8	19893010	19895010	6820431F20Rik		1	noCoverage	0.1293	0.69084	insignificant	0	24	17
chr8	20020278	20022278	6820431F20Rik		1	noCoverage	0.032066	0.13919	insignificant	0	20	20
chr8	20020392	20022392	6820431F20Rik		1	noCoverage	-0.013564	0.91779	insignificant	0	11	11
chr8	22041664	22043664	Gm15056	0.18502	1	insignificant	-0.13244	0.87632	insignificant	2	14	14
chr8	22084427	22086427	AY761185		1	noCoverage	-0.0412	0.05064	insignificant	0	12	12
chr8	22436072	22438072	Defa5		1	noCoverage	0.022222	1	insignificant	0	3	3
chr8	22775268	22777268	Gm15315		1	noCoverage	-0.09	0.18914	insignificant	0	3	3
chr8	23056233	23058233	Defb13	0.16429	1	insignificant	0.0025474	0.4192	insignificant	1	8	8
chr8	23170191	23172191	Alg11	-0.16301	0.23468	insignificant	-0.021541	0.20073	insignificant	5	59	64
chr8	23170546	23172546	Alg11	-0.18386	1	insignificant	-0.013672	0.84072	insignificant	4	57	62
chr8	23235525	23237525	Nek5	-0.22422	0.062655	insignificant	-0.059001	0.1799	insignificant	3	10	10
chr8	23276907	23278907	Nek3	-0.669	0.4902	lowCoverage	-0.064831	0.66193	insignificant	1	6	4
chr8	23296291	23298291	Ckap2	-0.11538	1	insignificant	0.040505	0.26867	insignificant	3	18	19
chr8	23302331	23304331	Vps36	-0.16503	0.13712	insignificant	-0.0057745	0.20479	insignificant	4	40	40
chr8	23392912	23394912	Tpte	-0.27896	0.0036708	hypomethylated	-0.044242	1	insignificant	4	11	12
chr8	23509093	23511093	Slc25a15	-0.14444	0.58764	insignificant	0.0025463	1	insignificant	7	9	18
chr8	23520811	23522811	Mrps31	-0.24043	4.25E-18	hypomethylated	-0.027824	0.60831	insignificant	12	45	45
chr8	23586171	23588171	Slc20a2	-0.089404	4.67E-17	hypomethylated	0.010585	0.89374	insignificant	39	144	142
chr8	23587351	23589351	Slc20a2	-0.099373	2.45E-09	hypomethylated	-0.0017929	0.78738	insignificant	14	48	48
chr8	23704285	23706285	Vdac3	-0.082701	0.00013281	hypomethylated	-0.0035355	0.53743	insignificant	11	40	40
chr8	23747281	23749281	A930013F10Rik		1	noCoverage	0.043339	0.013405	inconclusive	0	7	6
chr8	23867215	23869215	Plat	-0.30721	0.000000157	hypomethylated	-0.027289	1	insignificant	4	29	29
chr8	23916126	23918126	Ap3m2	-0.17661	2.85E-24	hypomethylated	-0.0037968	0.70694	insignificant	21	68	61
chr8	23969010	23971010	Myst3	-0.10688	1.99E-27	hypomethylated	-0.010049	0.042305	hypomethylated	70	183	207
chr8	24084353	24086353	Ank1	-0.13171	1.83E-75	hypomethylated	-0.010055	0.011257	hypomethylated	54	164	166
chr8	24167746	24169746	Ank1	-0.15714	7.2E-29	hypomethylated	-0.00025103	0.89619	insignificant	40	117	112
chr8	24252026	24254026	Mir486	-0.29316	0.0091883	hypomethylated	-0.075739	0.029444	hypomethylated	3	12	12
chr8	24253134	24255134	Ank1	-0.29316	0.0091883	hypomethylated	-0.075739	0.029444	hypomethylated	3	12	12
chr8	24262742	24264742	Nkx6-3	-0.079755	0.18913	insignificant	-0.073947	0.89555	insignificant	6	31	29
chr8	24318925	24320925	Agpat6	-0.15728	6.69E-17	hypomethylated	0.034773	0.19611	insignificant	19	40	41
chr8	24348140	24350140	Gins4	-0.077993	0.52922	insignificant	-0.06507	0.0078064	hypomethylated	5	21	20
chr8	24367552	24369552	Golga7	-0.13079	0.095199	insignificant	0.0015864	0.94447	insignificant	5	32	32
chr8	24520973	24522973	Sfrp1	-0.10248	9.97E-23	hypomethylated	-0.0050801	0.00085933	hypomethylated	36	120	121
chr8	24779132	24781132	Zmat4	-0.073174	0.18136	insignificant	0.029757	0.29557	insignificant	10	37	36
chr8	25707481	25709481	Ido1		1	noCoverage	-0.052701	0.4038	insignificant	0	6	4
chr8	26059276	26061276	Adam9	-0.45229	0.18911	insignificant	-0.19187	0.2414	insignificant	1	11	8
chr8	26126682	26128682	Tm2d2	-0.13365	8.73E-23	hypomethylated	-0.006823	0.82522	insignificant	30	127	126
chr8	26127394	26129394	Adam9	-0.16685	3.08E-15	hypomethylated	-0.017305	0.0085278	hypomethylated	19	94	93
chr8	26212283	26214283	Plekha2	-0.15725	4.62E-26	hypomethylated	0.0068935	0.64519	insignificant	19	92	94
chr8	26311921	26313921	Tacc1	0.29815	9.52E-20	inconclusive	0.022533	1.17E-16	inconclusive	5	54	57
chr8	26628243	26630243	Fgfr1	-0.093064	5.37E-29	hypomethylated	-0.00086862	0.87354	insignificant	49	160	169
chr8	26707959	26709959	Letm2		1	noCoverage	-0.020833	1	insignificant	0	12	8
chr8	26711777	26713777	Whsc1l1	-0.12807	1.44E-68	hypomethylated	0.00070332	0.72693	insignificant	62	152	147
chr8	26829519	26831519	Ppapdc1b	-0.46368	7.05E-09	stronglyHypometh	-0.041733	0.0092735	hypomethylated	5	24	23
chr8	26864752	26866752	Ddhd2	-0.092878	0.000000829	hypomethylated	-0.021781	0.04195	hypomethylated	29	70	70
chr8	26895062	26897062	Lsm1	-0.10791	1.55E-29	hypomethylated	-0.0084069	0.8059	insignificant	31	120	114
chr8	26895681	26897681	Bag4	-0.10359	1.49E-23	hypomethylated	-0.012006	0.72574	insignificant	21	92	86
chr8	26917984	26919984	Star	-0.07132	0.018971	hypomethylated	0.029882	0.84244	insignificant	2	4	4
chr8	26951251	26953251	Ash2l	-0.14213	0.013387	hypomethylated	0.021696	0.69704	insignificant	4	18	14
chr8	26958142	26960142	Kcnu1	0.25333	1	insignificant	0.38162	0.0030304	stronglyhypermeth	1	5	10
chr8	26959094	26961094	Kcnu1	0.3369	0.16018	insignificant	0.25977	0.00010121	hypermethylated	4	8	8
chr8	27126073	27128073	Fnta		1	noCoverage	-0.14828	0.0025183	hypomethylated	0	22	24
chr8	27228851	27230851	Rnf170	-0.11289	3.83E-26	hypomethylated	-0.0053385	0.32728	insignificant	49	158	172
chr8	27229696	27231696	Rnf170	-0.13054	1.5E-16	hypomethylated	0.0059193	0.40966	insignificant	34	111	132
chr8	27267640	27269640	Thap1	-0.099093	8.37E-25	hypomethylated	0.0048368	0.88408	insignificant	49	163	159
chr8	28086807	28088807	Zfp703	-0.18912	1.13E-25	hypomethylated	0.01039	0.25769	insignificant	30	91	82
chr8	28133330	28135330	Erlin2	-0.10098	0.00000184	hypomethylated	0.010375	0.61735	insignificant	18	88	88
chr8	28152026	28154026	Prosc	-0.18987	1.61E-08	hypomethylated	-0.027753	0.24857	insignificant	20	73	72
chr8	28195312	28197312	Gpr124	-0.15278	4.38E-43	hypomethylated	-0.0017595	0.33333	insignificant	52	128	128
chr8	28239104	28241104	Brf2	-0.35187	0.0079754	stronglyHypometh	-0.18697	0.48528	insignificant	3	7	6
chr8	28285118	28287118	Rab11fip1	-0.35	0.54499	insignificant	-0.083333	0.50151	insignificant	1	2	2
chr8	28369798	28371798	Eif4ebp1	-0.10808	1.82E-23	hypomethylated	-0.011117	0.045408	hypomethylated	23	119	116
chr8	28405516	28407516	Gm9731	-0.078863	0.080753	insignificant	-0.061988	0.47861	insignificant	8	43	42
chr8	28453865	28455865	Tex24		1	noCoverage	-0.083333	0.49475	insignificant	0	2	2
chr8	28478182	28480182	Chrnb3	-0.30738	0.051258	insignificant	0.12621	0.32085	insignificant	2	8	8
chr8	28557141	28559141	Poteg	0.093764	1	lowCoverage	-0.10468	0.042432	hypomethylated	1	10	8
chr8	29195325	29197325	Gm8096	-0.048727	0.64371	insignificant	-0.032217	0.16358	insignificant	1	8	8
chr8	30330108	30332108	Unc5d	-0.15696	1.86E-14	hypomethylated	-0.013966	0.84103	insignificant	19	50	61
chr8	32199133	32201133	Dusp26	-0.11539	0.0016819	hypomethylated	-0.0048235	0.88548	insignificant	12	41	41
chr8	32221317	32223317	Rnf122	-0.16691	1.08E-27	hypomethylated	0.0010594	0.20895	insignificant	25	98	103
chr8	32259787	32261787	BC019943	-0.21664	1.9E-13	hypomethylated	-0.016743	0.58554	insignificant	12	68	68
chr8	32279196	32281196	Mak16	-0.17263	7.86E-12	hypomethylated	-0.013493	0.54712	insignificant	16	41	35
chr8	32296803	32298803	Fut10	-0.17549	4.37E-08	hypomethylated	0.013248	0.69046	insignificant	14	54	61
chr8	32296849	32298849	Fut10	-0.17549	4.37E-08	hypomethylated	0.013248	0.69046	insignificant	14	54	61
chr8	33028675	33030675	Nrg1	-0.30402	0.062979	insignificant	0.019086	0.28283	insignificant	2	11	12
chr8	34495796	34497796	Purg	-0.0762	2.31E-14	hypomethylated	-0.0028434	0.10399	insignificant	37	117	109
chr8	34495999	34497999	Purg	-0.0762	2.31E-14	hypomethylated	-0.0028434	0.10399	insignificant	37	117	109
chr8	34593782	34595782	Hmgb1-rs17	-0.096054	0.10348	insignificant	-0.073396	0.00025128	hypomethylated	3	17	17

chr8	34709092	34711092	Ppp2cb	-0.088745	1.11E-16	hypomethylated	-0.0042982	0.19183	insignificant	26	150	133
chr8	34752448	34754448	Ubxn8	-0.16017	0.00013256	hypomethylated	0.0013532	0.50788	insignificant	5	28	28
chr8	34762709	34764709	Gsr	-0.15506	4.07E-43	hypomethylated	0.021072	0.00027911	inconclusive	29	86	87
chr8	34841385	34843385	Gtf2e2	-0.15578	4.82E-12	hypomethylated	-0.010877	0.094905	insignificant	26	86	101
chr8	34841538	34843538	Gtf2e2	-0.14073	3.47E-11	hypomethylated	-0.011721	0.17543	insignificant	25	84	99
chr8	34841810	34843810	Gtf2e2	-0.14073	3.47E-11	hypomethylated	-0.011721	0.17543	insignificant	25	84	99
chr8	35040313	35042313	Rbpms	-0.15481	0.000042591	hypomethylated	-0.0073102	0.29433	insignificant	18	76	78
chr8	35171565	35173565	Dctn6	-0.52415	0.48696	lowCoverage	0.18208	0.56794	insignificant	1	6	10
chr8	35177083	35179083	Mboat4		1	noCoverage	0.043532	0.27711	insignificant	0	10	8
chr8	35209793	35211793	Leprotl1	-0.10976	3.93E-14	hypomethylated	-0.0039324	0.54937	insignificant	13	34	37
chr8	35216616	35218616	Tmem66	-0.256	1.49E-15	hypomethylated	-0.039386	0.0072378	hypomethylated	9	20	20
chr8	35869663	35871663	Dusp4	-0.13157	1.08E-31	hypomethylated	-0.0043068	0.48823	insignificant	48	165	170
chr8	36028744	36030744	Tnks		1	noCoverage	0.0032862	0.75295	insignificant	0	14	12
chr8	36437794	36439794	Ppp1r3b	-0.14126	6.21E-12	hypomethylated	-0.0069289	0.68164	insignificant	31	96	98
chr8	36558587	36560587	Eri1	-0.073255	0.0011309	hypomethylated	0.022039	0.81737	insignificant	13	44	45
chr8	36649851	36651851	Mfhas1	-0.12312	5.56E-42	hypomethylated	-0.0029271	0.0022593	hypomethylated	77	213	198
chr8	36889613	36891613	Cldn23	-0.14946	5.29E-11	hypomethylated	-0.017309	0.20857	insignificant	13	46	47
chr8	37156881	37158881	D8Ertd82e	-0.15115	2.76E-29	hypomethylated	-0.0093495	0.029101	hypomethylated	35	125	131
chr8	37312570	37314570	Lonrf1	-0.13538	0.59731	insignificant	-0.0037424	0.017068	inconclusive	3	18	18
chr8	37796108	37798108	Dlc1	0.26532	1	insignificant	0.15282	0.022493	hypermethylated	2	9	10
chr8	38015490	38017490	A730069N07Rik		1	noCoverage	-0.16629	0.53624	insignificant	0	7	8
chr8	38055628	38057628	AI429214	-0.28479	0.00000169	hypomethylated	-0.025549	1	insignificant	7	20	25
chr8	40067920	40069920	Tusc3	-0.10648	0.00000142	hypomethylated	-0.0074937	0.25816	insignificant	15	80	80
chr8	41372307	41374307	Fgf20	-0.27679	0.000044666	hypomethylated	-0.017319	0.28049	insignificant	5	10	10
chr8	41392404	41394404	Efha2	-0.080322	0.000000194	hypomethylated	-0.0021755	0.63952	insignificant	26	112	112
chr8	41508168	41510168	Zdhhc2	-0.094309	3.88E-23	hypomethylated	0.0021672	0.2039	insignificant	48	177	180
chr8	41596136	41598136	Vps37a	-0.11608	5.64E-32	hypomethylated	0.0066061	0.83265	insignificant	50	171	171
chr8	41596658	41598658	Vps37a	-0.12309	3.96E-30	hypomethylated	0.010953	0.69886	insignificant	47	139	141
chr8	41720146	41722146	Mtmr7	-0.19101	0.66955	insignificant	0.070937	0.74033	insignificant	8	26	26
chr8	41946720	41948720	Slc7a2	-0.16735	0.000018314	hypomethylated	0.0046377	0.36241	insignificant	8	22	20
chr8	41946746	41948746	Slc7a2	-0.16735	0.000018314	hypomethylated	0.0046377	0.36241	insignificant	8	22	20
chr8	42010586	42012586	Pdgfrl	-0.17472	0.000023101	hypomethylated	0.017957	1	insignificant	4	20	20
chr8	42101821	42103821	B430010I23Rik	-0.19411	0.00000603	hypomethylated	-0.042163	0.22041	insignificant	4	20	18
chr8	42140148	42142148	Mtus1		1	noCoverage	0.17186	0.058426	insignificant	0	4	4
chr8	42219080	42221080	Mtus1	-0.085714	0.027028	hypomethylated	0.014286	0.68741	insignificant	2	20	20
chr8	42300510	42302510	Fgl1	-0.034524	0.14303	insignificant	0.037649	0.64124	insignificant	3	7	8
chr8	42324112	42326112	Pcm1	-0.11592	0.00021884	hypomethylated	-0.01166	0.2316	insignificant	19	96	96
chr8	42460051	42462051	Asah1	-0.11038	3.17E-09	hypomethylated	0.035623	0.89974	insignificant	14	43	48
chr8	42502472	42504472	Frg1	-0.099283	0.014378	hypomethylated	-0.018775	0.00039725	hypomethylated	5	28	28
chr8	44267475	44269475	Triml2	0.069617	0.60439	insignificant	-0.082563	0.037341	hypomethylated	2	14	14
chr8	44392363	44394363	Zfp42	0.1531	1	lowCoverage	-0.034807	0.82284	insignificant	1	27	28
chr8	46034561	46036561	Fat1		1	noCoverage	-0.038672	0.26411	insignificant	0	9	10
chr8	46153563	46155563	Mtnr1a	-0.22028	0.000027498	hypomethylated	-0.05845	0.55504	insignificant	4	24	24
chr8	46347385	46349385	F11		1	noCoverage	-0.089196	0.38922	insignificant	0	4	4
chr8	46380189	46382189	Klkb1	-0.018239	0.59143	insignificant	0.032547	0.25323	insignificant	1	12	12
chr8	46418550	46420550	Cyp4v3	-0.18381	0.01797	hypomethylated	-0.016741	0.58683	insignificant	9	39	41
chr8	46495893	46497893	Tlr3		1	noCoverage	0.010101	1	insignificant	0	2	2
chr8	46969838	46971838	Pdlim3	-0.23534	3.61E-08	hypomethylated	0.015096	0.44503	insignificant	3	16	16
chr8	47019002	47021002	Ccdc110	-0.27652	0.055809	insignificant	-0.072054	0.19826	insignificant	4	19	19
chr8	47059892	47061892	Ufsp2	-0.12758	0.000031886	hypomethylated	0.060495	0.36408	insignificant	9	70	76
chr8	47060606	47062606	1700029J07Rik	-0.13072	0.0016499	hypomethylated	0.046082	0.63509	insignificant	6	58	62
chr8	47085204	47087204	Ankrd37	0.49665	0.035015	stronglyHypermeth	-0.0023985	0.021565	inconclusive	1	22	21
chr8	47094955	47096955	Lrp2bp	-0.60662	0.42	lowCoverage	0.023591	1	insignificant	1	4	4
chr8	47209500	47211500	Snx25		1	noCoverage	-0.12917	0.18157	insignificant	0	4	4
chr8	47296363	47298363	Slc25a4	-0.13774	0.00000523	hypomethylated	-0.0071852	0.36361	insignificant	13	36	36
chr8	47380025	47382025	Helt	-0.15064	0.000010502	hypomethylated	0.070373	0.96327	insignificant	9	55	65
chr8	47555395	47557395	Acsl1	-0.103	2.69E-28	hypomethylated	-0.015373	0.010008	hypomethylated	50	152	153
chr8	47636422	47638422	Mlf1ip	-0.13062	2.71E-24	hypomethylated	-0.015413	0.0023111	hypomethylated	22	68	68
chr8	47701802	47703802	Casp3	-0.17295	4.35E-10	hypomethylated	-0.0014816	0.20978	insignificant	21	83	79
chr8	47702554	47704554	Ccdc111	-0.13075	0.000008371	hypomethylated	0.0027179	0.88616	insignificant	18	59	55
chr8	47824098	47826098	Irf2	-0.12836	3.47E-15	hypomethylated	-0.0064383	0.2731	insignificant	32	121	118
chr8	48071278	48073278	Enpp6	-0.98485	0.037975	lowCoverage	-0.43939	0.095665	insignificant	1	6	11
chr8	48374756	48376756	Stox2	-0.063492	0.088824	insignificant	0.031651	0.038037	hypermethylated	6	18	18
chr8	48437702	48439702	Stox2	0.11782	0.30986	insignificant	-0.2929	0.86925	insignificant	2	6	4
chr8	48617998	48619998	Rwdd4a	-0.1527	1.25E-09	hypomethylated	-0.016474	0.00021453	hypomethylated	17	102	100
chr8	48618824	48620824	D030016E14Rik	-0.1142	0.000000432	hypomethylated	-0.01025	0.093229	insignificant	16	80	78
chr8	48760513	48762513	Ing2	-0.11169	2.63E-25	hypomethylated	0.0048924	0.17886	insignificant	43	143	138
chr8	48799285	48801285	Cdkn2aip	-0.085396	7.71E-41	hypomethylated	-0.0063784	1.96E-15	hypomethylated	38	143	143
chr8	48906496	48908496	Cldn24	-0.091667	0.16771	insignificant	-0.075	0.18451	insignificant	3	6	6
chr8	49075905	49077905	Wwc2	-0.1421	2.8E-17	hypomethylated	0.024673	0.1948	insignificant	22	102	90
chr8	49183445	49185445	Dctd	0.14583	1	lowCoverage	-0.079167	0.48959	insignificant	1	4	4
chr8	49194311	49196311	Dctd	-0.10017	3.13E-09	hypomethylated	-0.0037804	0.022665	hypomethylated	19	89	89
chr8	49194366	49196366	Dctd	-0.10017	3.13E-09	hypomethylated	-0.0037804	0.022665	hypomethylated	19	89	89
chr8	49760044	49762044	Odz3		1	noCoverage	0.072951	1	insignificant	0	33	26
chr8	54596079	54598079	Aga	-0.22268	3.66E-10	hypomethylated	-0.021798	0.0048197	hypomethylated	9	22	22
chr8	54724419	54726419	Neil3	-0.72702	0.080325	insignificant	0.013989	0.00035037	inconclusive	2	15	15
chr8	55161885	55163885	Vegfc	-0.11408	4.04E-10	hypomethylated	-0.010285	0.79495	insignificant	29	82	85
chr8	55615351	55617351	Spcs3	-0.08924	2.55E-13	hypomethylated	0.011693	0.39357	insignificant	10	30	31
chr8	55634683	55636683	Asb5	0.13203	1	insignificant	0.04625	0.14057	insignificant	2	12	9
chr8	55685133	55687133	Spata4	-0.60499	0.00000655	stronglyHypometh	-0.20205	0.77407	insignificant	3	8	7
chr8	55809721	55811721	Wdr17		1	noCoverage	-0.016265	0.47296	insignificant	0	12	12
chr8	56039080	56041080	Gpm6a		1	noCoverage	-0.089744	0.58592	insignificant	0	4	4
chr8	58772348	58774348	Hpgd	-0.12441	0.000079577	hypomethylated	-0.041154	0.28375	insignificant	8	18	19
chr8	59028930	59030930	Fbxo8	-0.14186	0.000028946	hypomethylated	-0.024954	0.39068	insignificant	13	100	100
chr8	59029363	59031363	Fbxo8	-0.12812	0.000045951	hypomethylated	-0.024274	0.70002	insignificant	13	94	94

chr8	59798779	59800779	Hand2	-0.13991	1.6E-12	hypomethylated	0.0038565	0.00047645	inconclusive	19	70	56
chr8	59966657	59968657	Sap30	-0.11851	1	insignificant	-0.0085743	1	insignificant	1	23	23
chr8	59989639	59991639	Hmgb2	-0.11314	5.89E-26	hypomethylated	-0.0028903	0.01252	hypomethylated	48	205	206
chr8	60131852	60133852	Galnt7	-0.18268	5.52E-13	hypomethylated	0.014814	0.0034851	inconclusive	9	36	39
chr8	61179593	61181593	Galntl6	0	1	lowCoverage	-0.083333	0.50011	insignificant	1	2	2
chr8	61389551	61391551	BC030500	-0.14488	0.00016642	hypomethylated	0.021509	0.68149	insignificant	10	32	30
chr8	61390424	61392424	Galntl6	-0.16375	4.55E-08	hypomethylated	0.0023494	1	insignificant	15	46	44
chr8	62983920	62985920	Aadat	-0.22224	3.35E-13	hypomethylated	0.11546	0.75344	insignificant	4	17	15
chr8	63110656	63112656	Mfap3l	-0.12977	2.11E-29	hypomethylated	0.016003	0.14788	insignificant	37	150	148
chr8	63368247	63370247	2700029M09Ril	-0.12838	1.98E-17	hypomethylated	-0.025335	0.11748	insignificant	24	84	88
chr8	63433596	63435596	Clcn3	-0.80769	0.01217	stronglyHypometh	-0.22575	0.81792	insignificant	1	4	6
chr8	63462108	63464108	Clcn3	-0.14635	4.91E-10	hypomethylated	0.040698	0.2274	insignificant	16	49	54
chr8	63471016	63473016	Nek1	-0.13818	1.89E-19	hypomethylated	0.045567	0.20933	insignificant	28	119	119
chr8	63701967	63703967	Sh3rf1	-0.11098	9.78E-26	hypomethylated	0.011928	0.0002853	inconclusive	49	153	134
chr8	63965530	63967530	Cbr4	-0.16049	8.36E-10	hypomethylated	-0.032133	0.33051	insignificant	8	42	42
chr8	64405885	64407885	Ddx60	-0.12984	0.034654	hypomethylated	0.043036	0.69041	insignificant	3	20	20
chr8	65429242	65431242	Spock3	-0.17393	0.000014836	hypomethylated	0.002023	0.038395	hypermethylated	11	84	79
chr8	66430967	66432967	Gm4975	0.16163	0.19864	insignificant	-0.15643	0.0028212	hypomethylated	2	17	18
chr8	66684790	66686790	Tll1	-0.18809	1	insignificant	-0.015121	1	insignificant	3	27	25
chr8	67171837	67173837	Cpe	-0.065375	0.10728	insignificant	-0.018266	0.080976	insignificant	7	18	18
chr8	67212375	67214375	Sc4mol	-0.1096	0.000025503	hypomethylated	-0.0045511	0.80563	insignificant	11	63	64
chr8	67373716	67375716	Klhl2	-0.12132	6.88E-12	hypomethylated	-0.053801	0.9646	insignificant	34	110	94
chr8	67470083	67472083	Tmem192	-0.11422	2.81E-23	hypomethylated	0.012423	0.25132	insignificant	40	132	134
chr8	67511543	67513543	Trim75	-0.42564	0.014265	stronglyHypometh	0.052137	0.80259	insignificant	3	7	6
chr8	68140938	68142938	March1	-0.31588	0.000000652	hypomethylated	-0.055612	0.38776	insignificant	4	8	8
chr8	68141423	68143423	March1	-0.31588	0.000000652	hypomethylated	-0.055612	0.38776	insignificant	4	8	8
chr8	69010406	69012406	1810029B16Rik	-0.13737	5.65E-11	hypomethylated	-0.01014	0.36852	insignificant	4	24	21
chr8	69034638	69036638	Tktl2	-0.15899	0.032615	hypomethylated	-0.022909	0.12263	insignificant	4	18	20
chr8	69211993	69213993	Npy5r	-0.3018	0.00000111	hypomethylated	-0.0013041	0.36344	insignificant	6	16	19
chr8	69220320	69222320	Npy1r	-0.45826	1.61E-08	stronglyHypometh	-0.072214	0.0040255	hypomethylated	5	10	10
chr8	69383115	69385115	Naf1	-0.10106	3.73E-46	hypomethylated	0.0055277	0.044242	inconclusive	54	209	205
chr8	70017773	70019773	Nat2	-0.29408	8.71E-08	hypomethylated	-0.042473	0.033958	hypomethylated	11	22	22
chr8	70017846	70019846	Nat2	-0.29408	8.71E-08	hypomethylated	-0.042473	0.033958	hypomethylated	11	22	22
chr8	70046752	70048752	Nat3	0.23207	0.10086	insignificant	-0.080161	0.37258	insignificant	6	13	13
chr8	70498473	70500473	Psd3	0.021474	1	insignificant	-0.18288	0.00040933	hypomethylated	4	13	8
chr8	70799426	70801426	Sh2d4a	-0.40166	1.18E-10	stronglyHypometh	-0.029985	0.013335	hypomethylated	10	46	42
chr8	71259041	71261041	Csgalnact1	-0.35804	1E-11	stronglyHypometh	-0.066862	0.026531	hypomethylated	10	29	26
chr8	71316852	71318852	Ints10	-0.044882	2.55E-08	hypomethylated	0.012379	0.87792	insignificant	15	96	95
chr8	71403453	71405453	Lpl	-0.16803	0.0047036	hypomethylated	0.029472	0.51002	insignificant	10	53	51
chr8	71611634	71613634	Atp6v1b2	-0.17969	6.9E-09	hypomethylated	-0.010655	0.37032	insignificant	14	50	50
chr8	71613121	71615121	Slc18a1	-0.24215	0.01611	hypomethylated	-0.057308	0.15537	insignificant	4	18	16
chr8	71793978	71795978	D130040H23Rik	-0.26894	1	lowCoverage	0.024086	0.90695	insignificant	1	8	8
chr8	72149074	72151074	Zfp868	-0.29802	0.00000213	hypomethylated	-0.040807	0.0012092	hypomethylated	3	31	31
chr8	72149447	72151447	Zfp868		1	noCoverage	-0.13579	0.34258	insignificant	0	8	8
chr8	72177454	72179454	Zfp964	-0.18028	0.00012978	hypomethylated	-0.016253	0.16257	insignificant	7	47	47
chr8	72240385	72242385	Zfp869	-0.12942	0.00000258	hypomethylated	0.01714	0.17388	insignificant	16	44	42
chr8	72298810	72300810	Zfp866	-0.78333	0.00000177	stronglyHypometh	-0.083547	0.66938	insignificant	1	4	4
chr8	72314061	72316061	Atp13a1	-0.12025	7.42E-31	hypomethylated	0.004597	0.062794	insignificant	27	68	62
chr8	72331585	72333585	Gmip	-0.13544	2.26E-09	hypomethylated	-0.00027735	0.073837	insignificant	9	68	67
chr8	72345463	72347463	Lpar2	-0.19505	0.00000116	hypomethylated	-0.038455	0.20122	insignificant	11	36	34
chr8	72355602	72357602	Pbx4	-0.12059	0.14656	insignificant	-0.039264	0.24135	insignificant	8	31	22
chr8	72411291	72413291	Cilp2	-0.20586	0.084932	insignificant	0.003854	0.82657	insignificant	5	51	50
chr8	72425113	72427113	Tssk6	-0.14348	9.37E-18	hypomethylated	0.040647	0.70392	insignificant	39	136	134
chr8	72426457	72428457	Tssk6	-0.225	5.72E-13	hypomethylated	0.00042884	0.9142	insignificant	13	56	50
chr8	72498282	72500282	Gatad2a	-0.042825	0.002368	hypomethylated	0.011598	0.69406	insignificant	13	45	45
chr8	72520278	72522278	Gatad2a	-0.11974	0.0072681	hypomethylated	0.00010797	0.39645	insignificant	12	52	52
chr8	72565711	72567711	Sugp1	-0.14468	5.51E-18	hypomethylated	0.015722	0.15888	insignificant	17	80	80
chr8	72566633	72568633	Sugp1	-0.23267	3.34E-11	hypomethylated	0.0011356	0.94792	insignificant	12	32	32
chr8	72595830	72597830	Tm6sf2	-0.46042	0.069306	insignificant	-0.065215	0.33451	insignificant	3	6	6
chr8	72606427	72608427	Hapln4	-0.2302	4.34E-27	hypomethylated	-0.04591	0.34869	insignificant	11	24	24
chr8	72644743	72646743	Ncan	-0.25309	0.14592	insignificant	-0.056008	0.92617	insignificant	3	12	12
chr8	72654231	72656231	Nr2c2ap	-0.20647	9.3E-16	hypomethylated	-0.017642	0.35577	insignificant	22	60	59
chr8	72662611	72664611	2310045N01Rik	-0.32883	0.35098	insignificant	-0.13355	0.71846	insignificant	1	16	23
chr8	72663096	72665096	Rfxank	-0.32883	0.35098	insignificant	-0.13355	0.71846	insignificant	1	16	23
chr8	72695309	72697309	Tmem161a		1	noCoverage	0.020419	0.0047591	inconclusive	0	10	8
chr8	72736180	72738180	Slc25a42	-0.13036	0.14452	insignificant	0.015971	0.78254	insignificant	5	44	37
chr8	72757124	72759124	Sugp2	-0.099252	1.84E-17	hypomethylated	0.0016259	0.23955	insignificant	25	118	118
chr8	72758321	72760321	Armc6	-0.10305	5.78E-09	hypomethylated	-0.0075745	0.59193	insignificant	18	76	76
chr8	72805897	72807897	Homer3	-0.11065	4.78E-20	hypomethylated	-0.0085461	0.00000197	hypomethylated	31	100	102
chr8	72825683	72827683	Cope	-0.1836	2.22E-13	hypomethylated	-0.029579	0.33903	insignificant	14	49	56
chr8	72826351	72828351	Cope	-0.19548	0.00016196	hypomethylated	-0.047332	1	insignificant	7	35	42
chr8	72838673	72840673	Lass1	-0.29139	5.96E-14	hypomethylated	-0.085776	0.28766	insignificant	10	34	42
chr8	72852663	72854663	Gdf1	-0.092417	0.0049408	hypomethylated	0.045345	0.11957	insignificant	14	73	69
chr8	72877172	72879172	Upf1	-0.12721	0.0077292	hypomethylated	0.0069044	0.40051	insignificant	17	60	60
chr8	72896446	72898446	Comp	-0.19674	0.000000559	hypomethylated	-0.026764	0.25158	insignificant	17	50	50
chr8	72963472	72965472	Crtc1	-0.10804	2.13E-16	hypomethylated	0.013728	0.50692	insignificant	13	44	49
chr8	73000842	73002842	Klhl26	-0.21586	0.0010373	hypomethylated	-0.06399	0.34139	insignificant	5	15	14
chr8	73011257	73013257	Tmem59l	-0.17777	1.83E-12	hypomethylated	-0.035955	0.2227	insignificant	18	80	85
chr8	73016054	73018054	Crlf1	-0.11836	3.61E-24	hypomethylated	-0.0061317	0.34505	insignificant	50	162	166
chr8	73030638	73032638	2810428I15Rik	0.7076	0.02204	stronglyHypermeth	0.11204	0.58331	insignificant	1	17	26
chr8	73034266	73036266	Uba52	-0.31257	1.23E-36	hypomethylated	-0.017534	0.75006	insignificant	13	85	83
chr8	73047079	73049079	2810422J05Rik	-0.28458	1	lowCoverage	0.039869	0.013133	hypermethylated	1	14	14
chr8	73050641	73052641	Fkbp8	-0.099787	1.06E-08	hypomethylated	-0.028498	0.005575	hypomethylated	7	61	68
chr8	73050744	73052744	Fkbp8	-0.080722	1.75E-08	hypomethylated	-0.0094332	0.0097065	hypomethylated	7	59	68
chr8	73062573	73064573	Ell	-0.10764	0.0039973	hypomethylated	-0.010529	0.068476	insignificant	12	57	57

chr8	73117379	73119379	Isyna1	-0.11376	0.00000016	hypomethylated	0.020739	0.63729	insignificant	23	137	137
chr8	73132213	73134213	Ssbp4	-0.11123	1.02E-23	hypomethylated	-0.0020842	0.26322	insignificant	24	119	117
chr8	73139742	73141742	Lrrc25		1	noCoverage	-0.066976	0.73735	insignificant	0	2	2
chr8	73183580	73185580	Pgpep1	-0.20384	2.38E-16	hypomethylated	-0.016432	0.55634	insignificant	20	56	56
chr8	73196129	73198129	Lsm4	-0.26276	0.000000555	hypomethylated	-0.13619	0.21963	insignificant	14	52	74
chr8	73220637	73222637	Jund	0.23908	1	insignificant	-0.062506	0.44389	insignificant	2	24	19
chr8	73241441	73243441	Gm3336	-0.034249	0.044771	inconclusive	0.044533	0.00013071	hypermethylated	4	27	31
chr8	73277577	73279577	Rab3a	-0.14411	1.34E-12	hypomethylated	-0.019343	0.070842	insignificant	12	98	106
chr8	73284820	73286820	Mpv17l2	-0.35703	0.55461	insignificant	-0.042581	0.066862	insignificant	1	16	16
chr8	73290562	73292562	Ifi30	-0.15313	0.0034157	hypomethylated	-0.10392	0.000000323	hypomethylated	8	12	14
chr8	73299760	73301760	2010320M18Rik	-0.1079	2.04E-21	hypomethylated	-0.0073795	0.90391	insignificant	36	126	120
chr8	73300611	73302611	Pik3r2	-0.088959	0.000016324	hypomethylated	-0.002762	0.57577	insignificant	11	48	48
chr8	73316332	73318332	Mast3	-0.20656	0.59588	insignificant	0.0053006	0.065135	insignificant	0	10	10
chr8	73331347	73333347	Il12rb1	-0.15963	0.16788	insignificant	-0.0020991	0.90011	insignificant	4	14	14
chr8	73363619	73365619	Arrdc2	-0.20849	0.000000468	hypomethylated	0.01953	0.066473	insignificant	9	25	24
chr8	73380907	73382907	Kcnn1		1	noCoverage	-0.06466	0.18571	insignificant	0	6	6
chr8	73397389	73399389	Ccdc124	-0.16207	1.16E-17	hypomethylated	-0.0024254	9.47E-11	hypomethylated	19	60	60
chr8	73416656	73418656	Slc5a5	-0.2519	0.2835	insignificant	-0.0698	0.23083	insignificant	4	18	20
chr8	73419755	73421755	Rpl18a	-0.13678	0.0048049	hypomethylated	-0.018893	0.0003584	inconclusive	32	89	101
chr8	73421311	73423311	Rpl18a	-0.11272	0.000000771	hypomethylated	0.0035804	0.48458	insignificant	10	21	21
chr8	73421342	73423342	Rpl18a	-0.11272	0.000000771	hypomethylated	0.0035804	0.48458	insignificant	10	21	21
chr8	73428872	73430872	Mtap1s	-0.14394	6.87E-23	hypomethylated	0.0023102	0.94994	insignificant	30	115	116
chr8	73795612	73797612	Myo9b	-0.11885	4.64E-20	hypomethylated	0.0050767	0.090524	insignificant	38	100	107
chr8	73796489	73798489	Haus8	-0.12194	3.44E-15	hypomethylated	0.0020627	0.82143	insignificant	30	78	85
chr8	73889746	73891746	Use1	-0.20433	0.037118	hypomethylated	-0.03204	0.86777	insignificant	7	49	56
chr8	73890115	73892115	Use1	-0.20433	0.037118	hypomethylated	-0.03204	0.86777	insignificant	7	49	56
chr8	73894196	73896196	Ocel1	-0.1456	6.05E-15	hypomethylated	-0.0084738	0.82696	insignificant	25	99	103
chr8	73905851	73907851	Nr2f6	-0.1445	8.67E-08	hypomethylated	-0.025356	0.078269	insignificant	40	104	113
chr8	73919700	73921700	Ushbp1	-0.042671	1	insignificant	0.026746	0.29528	insignificant	16	77	79
chr8	73919753	73921753	5430437P03Rik	-0.055115	1	insignificant	0.02394	0.28299	insignificant	16	77	79
chr8	73928910	73930910	Ankle1	-0.11406	0.00087998	hypomethylated	-0.0038598	0.0077073	hypomethylated	7	71	68
chr8	73987556	73989556	Mrpl34	-0.12786	0.000000582	hypomethylated	0.011935	0.59701	insignificant	14	69	69
chr8	73987824	73989824	Mrpl34	-0.12958	0.000000572	hypomethylated	0.010222	0.7327	insignificant	14	61	61
chr8	73992092	73994092	Dda1	-0.11351	1.63E-12	hypomethylated	0.0013282	0.02128	inconclusive	41	112	110
chr8	74009966	74011966	Gtpbp3	-0.34848	2.94E-09	stronglyHypometh	-0.059244	0.000000115	hypomethylated	10	41	49
chr8	74011001	74013001	Gtpbp3	-0.29013	2.2E-12	hypomethylated	0.0020534	0.000000215	inconclusive	10	38	44
chr8	74035668	74037668	Plvap	-0.40058	2.09E-08	stronglyHypometh	-0.0068288	0.95186	insignificant	7	31	38
chr8	74061336	74063336	Bst2	-0.23601	1.55E-13	hypomethylated	0.042263	0.15551	insignificant	4	27	24
chr8	74065828	74067828	Fam125a	-0.15584	1.1E-09	hypomethylated	0.032492	0.065801	insignificant	11	91	90
chr8	74082770	74084770	Tmem221	-0.26633	0.0085914	hypomethylated	-0.015598	0.82291	insignificant	2	21	23
chr8	74090548	74092548	Nxnl1	-0.028464	0.60995	insignificant	-0.1069	0.038847	hypomethylated	3	6	6
chr8	74091825	74093825	Slc27a1	-0.19543	9.73E-14	hypomethylated	0.008282	0.89632	insignificant	10	31	31
chr8	74115082	74117082	Pgls	-0.12601	1.95E-08	hypomethylated	-0.010981	0.11619	insignificant	23	95	95
chr8	74120544	74122544	Fam129c		1	noCoverage	-0.14569	0.15279	insignificant	0	6	6
chr8	74133922	74135922	Glt25d1	-0.21543	0.0087949	hypomethylated	-0.059858	0.31129	insignificant	6	60	59
chr8	74195656	74197656	Unc13a	-0.12719	0.000023071	hypomethylated	0.00038863	0.84404	insignificant	12	30	31
chr8	74199281	74201281	Jak3	-0.41167	0.3098	insignificant	-0.02517	0.81286	insignificant	3	11	10
chr8	74212150	74214150	Insl3	-0.39571	0.010309	stronglyHypometh	0.054008	0.0075618	hypermethylated	4	17	16
chr8	74404966	74406966	Zfp709	-0.12787	5.13E-09	hypomethylated	0.063123	0.90805	insignificant	11	52	54
chr8	74431504	74433504	Zfp882	-0.15732	0.013668	hypomethylated	0.11201	0.0031376	hypermethylated	9	34	34
chr8	74445723	74447723	Zfp617	-0.21744	3.9E-22	hypomethylated	-0.00045498	0.035985	hypomethylated	28	107	109
chr8	74473964	74475964	Zfp961	-0.5505	0.00028084	stronglyHypometh	-0.068681	0.045248	hypomethylated	5	32	32
chr8	74580558	74582558	Olfr372	0.092593	1	insignificant	-0.016103	0.45658	insignificant	2	6	6
chr8	74622660	74624660	Olfr373	0.19792	1	insignificant	-0.0066288	0.39329	insignificant	1	4	4
chr8	74658190	74660190	Tpm4	-0.11499	0.00000615	hypomethylated	-0.022111	0.3615	insignificant	20	99	107
chr8	74684098	74686098	Rab8a	-0.17211	2.93E-24	hypomethylated	-0.0099717	0.043227	hypomethylated	29	98	100
chr8	74712566	74714566	Hsh2d	-0.54722	0.00000146	stronglyHypometh	-0.14199	0.54997	insignificant	3	8	6
chr8	74742628	74744628	Fam32a	-0.19185	5.11E-10	hypomethylated	0.11506	0.70566	insignificant	12	48	52
chr8	74763030	74765030	Ap1m1	-0.28548	3.74E-23	hypomethylated	-0.04076	0.023749	hypomethylated	14	70	68
chr8	74841960	74843960	Klf2	-0.12081	1.3E-34	hypomethylated	-0.0052844	0.020246	hypomethylated	43	113	149
chr8	74945373	74947373	Eps15l1	-0.12081	0.36911	insignificant	-0.011837	0.84227	insignificant	3	22	23
chr8	74966778	74968778	1700030K09Rik	-0.43151	3.12E-13	stronglyHypometh	-0.11578	0.0015021	hypomethylated	6	22	31
chr8	74967677	74969677	Calr3	-0.10913	0.0028141	hypomethylated	0.092622	0.74243	insignificant	5	10	11
chr8	74999132	75001132	Cherp	-0.37953	4.71E-14	stronglyHypometh	-0.055414	1.42E-10	hypomethylated	13	38	43
chr8	75016513	75018513	Slc35e1	-0.1302	1.54E-16	hypomethylated	-0.0041963	0.89112	insignificant	28	78	81
chr8	75072209	75074209	Med26	-0.068014	0.27219	insignificant	-0.017419	0.57955	insignificant	7	20	20
chr8	75094947	75096947	9130011J15Rik	-0.14012	0.00000458	hypomethylated	-0.019105	0.12219	insignificant	11	71	81
chr8	75095001	75097001	Tmem38a	-0.14912	0.00000133	hypomethylated	-0.015065	0.0041965	inconclusive	10	61	71
chr8	75169609	75171609	Nwd1	-0.40406	0.00027742	stronglyHypometh	-0.027216	0.89971	insignificant	3	6	9
chr8	75246186	75248186	Sin3b	-0.079485	0.0011529	hypomethylated	0.026297	0.90487	insignificant	8	54	58
chr8	75284778	75286778	F2rl3	-0.37566	0.088114	insignificant	-0.058878	0.8737	insignificant	2	6	10
chr8	75876455	75878455	Large	-0.12851	3.72E-36	hypomethylated	-0.00087234	0.24475	insignificant	65	204	203
chr8	77516601	77518601	Hmgxb4	-0.15442	6.16E-39	hypomethylated	-0.0022951	0.00013368	hypomethylated	44	126	137
chr8	77556584	77558584	Tom1	-0.13052	9.36E-28	hypomethylated	-0.034657	0.000017523	hypomethylated	19	83	86
chr8	77616516	77618516	Hmox1	-0.15518	1.56E-12	hypomethylated	-0.016891	0.4656	insignificant	10	30	33
chr8	77632426	77634426	Mcm5	-0.20581	1.35E-17	hypomethylated	0.0055042	0.8667	insignificant	13	65	60
chr8	77736842	77738842	Rasd2	-0.088816	1.73E-17	hypomethylated	-0.013479	0.068151	insignificant	50	186	183
chr8	79424072	79426072	Gm10649	-0.12676	7.28E-21	hypomethylated	-0.017582	0.77074	insignificant	30	122	122
chr8	79425406	79427406	Nr3c2	-0.15004	0.0000015	hypomethylated	-0.0024411	0.39059	insignificant	15	73	69
chr8	80039954	80041954	0610038B21Rik	-0.16575	3.1E-09	hypomethylated	-0.0002772	0.46562	insignificant	21	72	72
chr8	80041806	80043806	Arhgap10	-0.088738	0.000000402	hypomethylated	-0.002583	0.68509	insignificant	15	57	57
chr8	80072295	80074295	Prmt10	-0.15877	0.023173	hypomethylated	0.0074567	0.62697	insignificant	4	14	14
chr8	80134552	80136552	Tmem184c		1	noCoverage	-0.11053	0.050859	insignificant	0	5	4
chr8	80960551	80962551	Pou4f2	-0.18117	1.6E-27	hypomethylated	0.0081821	0.1071	insignificant	18	58	58
chr8	81032233	81034233	Slc10a7	-0.079931	4.82E-14	hypomethylated	-0.0020781	0.38691	insignificant	32	180	180

chr8	81032827	81034827	Slc10a7	-0.10677	3.53E-12	hypomethylated	-0.0048173	0.84401	insignificant	19	81	81
chr8	81345051	81347051	Lsm6	-0.036298	4.79E-15	hypomethylated	0.0089321	1.21E-08	inconclusive	6	12	12
chr8	81551335	81553335	Zfp827	-0.13176	1E-14	hypomethylated	0.019811	0.040304	hypermethylated	25	77	83
chr8	81818855	81820855	Mmaa	-0.083335	9.78E-08	hypomethylated	0.00008561	0.026282	inconclusive	12	59	59
chr8	81923367	81925367	Smad1	-0.16334	9.92E-10	hypomethylated	-0.019169	0.0033146	hypomethylated	10	52	52
chr8	82162574	82164574	Otud4	-0.086559	3.58E-42	hypomethylated	-0.0027943	0.14706	insignificant	76	235	243
chr8	82234718	82236718	Anapc10	-0.098971	0.00068113	hypomethylated	-0.002301	0.28753	insignificant	12	75	74
chr8	82235639	82237639	Abce1	0.093079	0.000000199	hypermethylated	0.0049116	0.029626	hypermethylated	5	35	34
chr8	82581907	82583907	Hhip	-0.13568	0.60652	insignificant	0.017955	1	insignificant	6	26	26
chr8	83016943	83018943	Gypa	-0.03122	1	insignificant	-0.040076	0.18063	insignificant	1	18	18
chr8	83133937	83135937	Frem3	-0.18228	2.29E-08	hypomethylated	-0.015812	0.15219	insignificant	6	28	28
chr8	83263358	83265358	Smarca5		1	noCoverage	-0.17937	1.4E-27	hypomethylated	0	38	34
chr8	83404378	83406378	Gab1	-0.14925	0.00072381	hypomethylated	0.019737	0.85069	insignificant	10	34	32
chr8	83538805	83540805	Usp38	-0.21467	7.67E-15	hypomethylated	-0.03479	0.014783	hypomethylated	17	63	63
chr8	84926444	84928444	Il15	-0.32176	7.17E-08	hypomethylated	-0.01737	0.024345	inconclusive	3	23	24
chr8	85298025	85300025	Zfp330	-0.1529	0.35942	insignificant	-0.024907	0.80517	insignificant	6	16	18
chr8	85386254	85388254	Rnf150	-0.080995	1.73E-09	hypomethylated	-0.0034102	0.16621	insignificant	23	115	120
chr8	85688250	85690250	Tbc1d9	-0.12264	3.14E-22	hypomethylated	0.0099409	0.0072017	inconclusive	43	157	166
chr8	85813246	85815246	Ucp1	-0.38846	0.0016253	stronglyHypometh	-0.061719	0.48658	insignificant	3	9	14
chr8	85856385	85858385	Elmod2	-0.18211	0.00000268	hypomethylated	-0.0056558	0.044016	hypomethylated	6	49	51
chr8	85912789	85914789	Clgn	-0.16806	2.14E-11	hypomethylated	-0.040625	0.00015044	hypomethylated	16	51	61
chr8	85982290	85984290	Scoc		1	noCoverage	-0.001419	0.51576	insignificant	0	5	7
chr8	86089656	86091656	Ndufb7	-0.30433	1.78E-13	hypomethylated	0.0047081	0.15561	insignificant	14	66	62
chr8	86118390	86120390	Tecr	-0.1601	1	insignificant	-0.0034047	0.88829	insignificant	1	32	32
chr8	86131073	86133073	Dnajb1	-0.10425	3.83E-31	hypomethylated	-0.0032373	0.031389	hypomethylated	45	160	163
chr8	86175576	86177576	Gipc1	-0.13034	1.17E-08	hypomethylated	-0.0058945	0.00013204	hypomethylated	15	85	83
chr8	86189538	86191538	Ptger1		1	noCoverage	-0.032213	1	insignificant	0	4	6
chr8	86223078	86225078	Pkn1	-0.14649	0.00027152	hypomethylated	0.01034	0.076048	insignificant	7	31	31
chr8	86238075	86240075	Ddx39	-0.12488	6E-27	hypomethylated	0.0054334	0.093097	insignificant	43	148	137
chr8	86265210	86267210	Cd97	-0.035378	0.00012515	hypomethylated	0.034383	0.18963	insignificant	4	25	25
chr8	86422996	86424996	Lphn1	-0.093493	1.86E-12	hypomethylated	0.0020786	0.21392	insignificant	84	244	254
chr8	86478592	86480592	Asf1b	-0.1669	4.5E-31	hypomethylated	-0.034286	0.02752	hypomethylated	31	151	148
chr8	86495876	86497876	Prkaca	-0.08745	4.15E-36	hypomethylated	-0.0063458	0.44124	insignificant	37	129	129
chr8	86520570	86522570	Samd1	-0.090015	2.25E-22	hypomethylated	0.0013074	0.081314	insignificant	69	260	247
chr8	86524604	86526604	1700067K01Rik	-0.065543	0.014801	hypomethylated	-0.045852	0.0022357	hypomethylated	11	30	30
chr8	86535532	86537532	Mir1199	-0.19406	3.84E-20	hypomethylated	0.017596	0.044129	hypermethylated	33	121	118
chr8	86544372	86546372	Palm3	0.065313	0.004919	inconclusive	0.021664	0.061106	insignificant	13	40	35
chr8	86566474	86568474	Il27ra		1	noCoverage	-0.055165	0.44885	insignificant	0	12	12
chr8	86568878	86570878	Rln3	0.30598	1	insignificant	0.10068	0.76589	insignificant	1	15	15
chr8	86589734	86591734	Rfx1	-0.11898	6.44E-09	hypomethylated	-0.014712	0.23649	insignificant	39	135	141
chr8	86628661	86630661	Dcaf15	-0.34659	0.000014976	stronglyHypometh	0.055522	0.6754	insignificant	2	18	18
chr8	86670936	86672936	4930432K21Rik	-0.17617	0.000013781	hypomethylated	0.0087081	1	insignificant	6	32	32
chr8	86671652	86673652	Cc2d1a	-0.15458	0.000097564	hypomethylated	0.0072053	0.75049	insignificant	5	30	30
chr8	86700451	86702451	Nanos3		1	noCoverage	0.011508	0.28608	insignificant	0	12	12
chr8	86702686	86704686	Mir181d	-0.23835	0.26419	insignificant	0.08879	0.14784	insignificant	3	14	14
chr8	86702860	86704860	Mir181d	-0.19485	0.18932	insignificant	0.0079248	0.85634	insignificant	1	4	4
chr8	86732806	86734806	Mir3074-2	-0.14226	0.75549	insignificant	-0.016015	0.93659	insignificant	5	12	12
chr8	86760941	86762941	Zswim4	-0.15747	0.0053652	hypomethylated	-0.021171	0.063155	insignificant	11	57	56
chr8	86781223	86783223	Ccdc130		1	noCoverage	-0.31884	0.14541	insignificant	0	5	3
chr8	86794259	86796259	Ccdc130	-0.36868	1.64E-26	stronglyHypometh	-0.015814	0.075867	insignificant	15	53	53
chr8	86938262	86940262	Cacna1a	-0.13008	9.46E-31	hypomethylated	-0.012828	0.013915	hypomethylated	41	118	114
chr8	87186751	87188751	Ier2	-0.18975	1.15E-33	hypomethylated	-0.014768	0.91928	insignificant	30	82	82
chr8	87211761	87213761	Nacc1	-0.17386	3.72E-09	hypomethylated	-0.024171	0.56602	insignificant	19	82	80
chr8	87212145	87214145	Trmt1	-0.19674	6.22E-18	hypomethylated	-0.026721	0.21306	insignificant	23	84	82
chr8	87224355	87226355	Lyl1	-0.19328	5.16E-19	hypomethylated	-0.011725	1	insignificant	11	42	41
chr8	87324239	87326239	Nfix	-0.074344	4.4E-09	hypomethylated	0.0013238	0.91395	insignificant	18	56	70
chr8	87346722	87348722	Dand5	-0.31667	0.10392	insignificant	-0.083333	1	insignificant	1	3	6
chr8	87355180	87357180	Gadd45gip1	-0.19965	4.01E-09	hypomethylated	0.011635	0.030376	hypermethylated	29	79	78
chr8	87356164	87358164	Dand5	-0.18607	6.28E-08	hypomethylated	0.024135	0.12853	insignificant	17	53	50
chr8	87364540	87366540	Rad23a	-0.051619	0.22948	insignificant	-0.027594	1	insignificant	5	10	16
chr8	87379884	87381884	Farsa	-0.18503	0.59575	insignificant	0.0097293	0.80745	insignificant	1	43	43
chr8	87395009	87397009	Syce2	-0.12107	7.67E-11	hypomethylated	0.018219	0.078117	insignificant	32	128	119
chr8	87395156	87397156	Syce2	-0.12107	7.67E-11	hypomethylated	0.018219	0.078117	insignificant	32	128	119
chr8	87424826	87426826	Klf1	-0.1791	0.000000115	hypomethylated	-0.0020981	0.82778	insignificant	7	18	18
chr8	87431522	87433522	Dnase2a	-0.18197	5.49E-12	hypomethylated	0.018172	0.73993	insignificant	21	86	89
chr8	87469889	87471889	Rtbdn	-0.19426	4.43E-23	hypomethylated	0.044302	0.78616	insignificant	10	50	51
chr8	87489910	87491910	Rnaseh2a	0.028568	0.77151	insignificant	-0.028572	0.042441	hypomethylated	3	32	29
chr8	87492546	87494546	Prdx2	-0.054347	0.01154	inconclusive	0.011697	0.00036355	hypermethylated	25	75	68
chr8	87502617	87504617	Junb	-0.20647	1.11E-18	hypomethylated	-0.0072879	0.11977	insignificant	7	57	57
chr8	87513493	87515493	Hook2	-0.17157	0.089709	insignificant	-0.0032344	0.91438	insignificant	4	26	26
chr8	87549177	87551177	2310036O22Rik	-0.25387	1.73E-10	hypomethylated	-0.047292	0.051602	insignificant	24	78	77
chr8	87549731	87551731	2310036O22Rik	-0.26708	3.79E-15	hypomethylated	-0.048011	0.061358	insignificant	26	92	91
chr8	87559813	87561813	Tnpo2	-0.16987	5.59E-13	hypomethylated	0.0015083	0.77808	insignificant	16	40	40
chr8	87560066	87562066	Tnpo2	-0.16758	4.14E-15	hypomethylated	-0.011965	0.43221	insignificant	19	58	60
chr8	87583017	87585017	Fbxw9	-0.32188	0.0017257	hypomethylated	-0.0049764	0.43608	insignificant	6	77	80
chr8	87594655	87596655	Dhps	-0.020621	0.0096327	hypomethylated	-0.0055574	0.0051138	hypomethylated	7	54	54
chr8	87603861	87605861	BC056474	-0.18946	0.00000226	hypomethylated	0.00072922	0.36054	insignificant	18	68	62
chr8	87604645	87606645	BC056474	-0.30882	4.46E-09	hypomethylated	0.068997	0.50117	insignificant	4	22	16
chr8	87822530	87824530	Orc6	-0.20795	8.92E-12	hypomethylated	-0.027532	0.15869	insignificant	19	109	111
chr8	87823396	87825396	Vps35	-0.24834	5.01E-08	hypomethylated	-0.028337	0.1103	insignificant	9	70	69
chr8	88015515	88017515	Gpt2	-0.15279	1.69E-17	hypomethylated	-0.017911	0.37309	insignificant	24	71	74
chr8	88079170	88081170	Dnaja2	-0.13238	0.00000495	hypomethylated	-0.031339	0.012678	hypomethylated	15	64	71
chr8	88214908	88216908	Neto2		1	noCoverage	-0.044643	0.1222	insignificant	0	3	6
chr8	88363900	88365900	Phkb	-0.12676	3.36E-08	hypomethylated	-0.011324	0.82594	insignificant	29	91	89
chr8	89090489	89092489	Abcc12	-0.45372	1	insignificant	-0.22468	0.079925	insignificant	2	31	34

chr8	89146941	89148941	Lonp2	-0.17943	3.62E-13	hypomethylated	-0.016678	0.31363	insignificant	16	46	52
chr8	89268597	89270597	Gm10638	-0.074694	5.94E-41	hypomethylated	0.010134	0.60435	insignificant	84	219	230
chr8	89269905	89271905	Gm10638	-0.10484	5.07E-20	hypomethylated	0.0047173	0.10916	insignificant	31	103	102
chr8	89995710	89997710	Gm2694	-0.13397	2.76E-36	hypomethylated	-0.0093209	0.084007	insignificant	41	192	209
chr8	89996491	89998491	Cbln1	-0.1275	1.06E-21	hypomethylated	-0.0052839	0.56112	insignificant	22	100	114
chr8	90483494	90485494	Zfp423	-0.15342	1.51E-30	hypomethylated	0.0032004	0.20925	insignificant	55	158	161
chr8	90641657	90643657	Tmem188	-0.28071	4.96E-14	hypomethylated	0.0022114	0.55378	insignificant	11	63	62
chr8	90660783	90662783	Heatr3	-0.11278	9.92E-17	hypomethylated	0.01423	0.47268	insignificant	22	100	101
chr8	90722111	90724111	Papd5	-0.079828	2.14E-22	hypomethylated	-0.00033593	0.88915	insignificant	68	232	240
chr8	90795301	90797301	Adcy7	-0.17069	4.14E-13	hypomethylated	0.0023417	0.29323	insignificant	21	78	82
chr8	90811964	90813964	Adcy7		1	noCoverage	0.040579	0.42286	insignificant	0	15	16
chr8	90886090	90888090	Brd7	-0.11834	0.00011632	hypomethylated	0.0061884	0.62412	insignificant	11	34	30
chr8	91044242	91046242	Nkd1	-0.088132	2.18E-29	hypomethylated	0.0087169	0.50807	insignificant	65	199	214
chr8	91170245	91172245	Nod2	-0.33595	0.0010717	stronglyHypometh	-0.0017687	0.22576	insignificant	3	14	14
chr8	91219926	91221926	Cyld	-0.11618	0.0036748	hypomethylated	-0.013838	0.2097	insignificant	18	65	66
chr8	91219942	91221942	Cyld	-0.11618	0.0036748	hypomethylated	-0.013838	0.2097	insignificant	18	65	66
chr8	91568061	91570061	Sall1	-0.25544	0.000000675	hypomethylated	-0.012714	0.080745	insignificant	6	36	43
chr8	92872151	92874151	Tox3	-0.1791	4.76E-56	hypomethylated	0.0010506	1.08E-11	inconclusive	32	75	78
chr8	93351733	93353733	Chd9	-0.081102	0.71452	insignificant	-0.014196	0.44148	insignificant	7	33	33
chr8	93592992	93594992	Rbl2	-0.15138	0.034998	hypomethylated	-0.017402	0.049183	hypomethylated	6	67	71
chr8	93836423	93838423	Fto		1	noCoverage	0.037001	0.59386	insignificant	0	39	36
chr8	93837121	93839121	Fto		1	noCoverage	0.11546	0.79925	insignificant	0	27	26
chr8	94325273	94327273	Irx3	-0.23329	1.69E-41	hypomethylated	-0.039147	0.00055301	hypomethylated	16	87	80
chr8	94880019	94882019	4933436C20Rik	-0.12876	2.83E-12	hypomethylated	-0.014848	0.38815	insignificant	25	132	130
chr8	94880694	94882694	Irx5	-0.11838	3.66E-19	hypomethylated	-0.012849	0.45475	insignificant	34	190	188
chr8	95197300	95199300	Irx6	-0.15203	1.49E-09	hypomethylated	-0.0012644	0.0031621	hypomethylated	21	94	94
chr8	95350226	95352226	Mmp2	-0.14427	9.15E-14	hypomethylated	-0.010436	0.25475	insignificant	13	47	53
chr8	95378248	95380248	Lpcat2	-0.14157	2.96E-14	hypomethylated	0.069868	0.0017385	inconclusive	16	61	62
chr8	95483945	95485945	Slc6a2	-0.12362	1.25E-16	hypomethylated	0.010456	0.14051	insignificant	38	94	105
chr8	96333737	96335737	Gnao1	-0.12263	1.26E-35	hypomethylated	-0.014394	0.34591	insignificant	75	228	242
chr8	96337455	96339455	Gnao1	-0.19248	0.000000937	hypomethylated	0.012325	0.15914	insignificant	5	12	16
chr8	96536540	96538540	Amfr	-0.10997	0.000000055	hypomethylated	-0.0094809	1	insignificant	14	52	50
chr8	96560097	96562097	Ogfod1	-0.15825	2.8E-14	hypomethylated	-0.0032146	0.0011017	hypomethylated	20	72	76
chr8	96560939	96562939	Nudt21	-0.18961	1.75E-09	hypomethylated	0.0035073	0.2218	insignificant	11	38	36
chr8	96660103	96662103	Mt4	-0.46061	0.0004173	stronglyHypometh	-0.086813	0.60929	insignificant	2	4	4
chr8	96675506	96677506	Mt3	-0.27855	6.26E-10	hypomethylated	-0.010677	0.0082227	hypomethylated	2	16	14
chr8	96695517	96697517	Mt2	-0.1566	8.85E-56	hypomethylated	-0.0031122	0.02032	hypomethylated	27	101	93
chr8	96701988	96703988	Mt1	-0.19195	1.82E-09	hypomethylated	-0.01072	0.17779	insignificant	13	59	59
chr8	96737500	96739500	Nup93	-0.067061	0.000010309	hypomethylated	-0.0053876	0.012947	hypomethylated	20	76	76
chr8	96847210	96849210	Mir138-2	-0.13791	0.005541	hypomethylated	0.03505	0.403	insignificant	7	16	16
chr8	96852107	96854107	Slc12a3	-0.35471	0.0029826	stronglyHypometh	-0.022191	0.71885	insignificant	1	8	5
chr8	96909399	96911399	Herpud1	-0.10615	1.03E-08	hypomethylated	-0.017499	0.015096	hypomethylated	12	58	58
chr8	96959003	96961003	9330175E14Rik	-0.4313	0.0015958	stronglyHypometh	-0.084021	0.0011231	hypomethylated	5	20	20
chr8	96995662	96997662	Nlrc5	0.06	1	insignificant	-0.20149	0.079442	insignificant	5	10	12
chr8	97055927	97057927	Cpne2	-0.11307	4.12E-35	hypomethylated	-0.010566	0.27608	insignificant	43	117	122
chr8	97124840	97126840	Rspry1	-0.11184	6.68E-33	hypomethylated	-0.012912	1	insignificant	41	141	157
chr8	97125626	97127626	Fam192a	-0.099859	1.58E-24	hypomethylated	-0.000088684	0.85559	insignificant	20	91	91
chr8	97189654	97191654	Arl2bp	-0.10463	1.48E-14	hypomethylated	0.029819	0.0026049	inconclusive	26	107	109
chr8	97190005	97192005	Arl2bp	-0.10463	1.48E-14	hypomethylated	0.029819	0.0026049	inconclusive	26	107	109
chr8	97268583	97270583	Ccl22		1	noCoverage	-0.10298	0.10889	insignificant	0	14	14
chr8	97295079	97297079	Cx3cl1	-0.21665	0.00038484	hypomethylated	-0.037422	0.41863	insignificant	9	28	29
chr8	97333352	97335352	Ccl17	-0.19167	0.0035899	hypomethylated	-0.025368	0.48233	insignificant	2	4	4
chr8	97361316	97363316	Coq9	-0.12397	3.28E-10	hypomethylated	0.033479	0.47363	insignificant	23	120	129
chr8	97362240	97364240	Ciapin1	-0.10738	0.000027688	hypomethylated	-0.000076772	0.64503	insignificant	12	62	62
chr8	97380349	97382349	Polr2c	-0.15576	0.000000216	hypomethylated	-0.0027889	0.66023	insignificant	19	82	82
chr8	97400212	97402212	Dok4		1	noCoverage	-0.014247	0.69863	insignificant	0	17	18
chr8	97441998	97443998	Ccdc102a		1	noCoverage	-0.0059524	0.019533	hypomethylated	0	14	14
chr8	97446593	97448593	Gpr114	-0.51364	0.055881	insignificant	-0.076311	0.27063	insignificant	2	10	10
chr8	97500008	97502008	Gpr56	-0.25075	0.06817	insignificant	0.041164	0.93213	insignificant	3	14	14
chr8	97507298	97509298	Gpr56	-0.36693	0.41778	lowCoverage	0.17809	0.4873	insignificant	1	9	12
chr8	97578002	97580002	Ccdc135	-0.82576	0.2	lowCoverage	-0.00075758	1	insignificant	1	4	4
chr8	97604100	97606100	Katnb1	-0.17805	2.04E-14	hypomethylated	0.024741	0.62965	insignificant	12	53	48
chr8	97666440	97668440	Kifc3	-0.46666	0.050485	insignificant	-0.017218	0.0017556	hypomethylated	2	16	16
chr8	97855183	97857183	AA960436	-0.12536	7.48E-42	hypomethylated	0.0056425	0.063262	insignificant	57	148	157
chr8	97855850	97857850	Zfp319	-0.16763	1.07E-14	hypomethylated	-0.00039581	0.076073	insignificant	23	76	84
chr8	97875236	97877236	Mmp15	-0.1472	2.58E-61	hypomethylated	-0.028001	0.037885	hypomethylated	47	149	172
chr8	97958769	97960769	Gtl3	-0.22987	1.54E-12	hypomethylated	-0.023918	0.15254	insignificant	10	46	46
chr8	98012720	98014720	Csnk2a2	-0.30075	5.3E-38	hypomethylated	-0.014265	3.31E-08	hypomethylated	12	30	26
chr8	98056999	98058999	Ccdc113	-0.080828	0.46972	insignificant	-0.053683	0.23166	insignificant	5	23	24
chr8	98099097	98101097	Prss54	0.090476	1	lowCoverage	-0.12747	0.17206	insignificant	1	5	4
chr8	98156458	98158458	Gins3	-0.15744	2.25E-13	hypomethylated	0.0059338	0.51058	insignificant	20	90	92
chr8	98225936	98227936	Ndrg4	-0.24994	0.0031519	hypomethylated	-0.01209	0.20555	insignificant	4	39	40
chr8	98238812	98240812	Setd6	-0.08878	0.079331	insignificant	0.0018023	0.0072172	hypermethylated	17	48	48
chr8	98329729	98331729	4930513N10Rik	-0.099054	5.07E-20	hypomethylated	0.0044014	0.38515	insignificant	40	150	154
chr8	98331366	98333366	4930513N10Rik	-0.14379	0.058141	insignificant	0.057489	0.055946	insignificant	10	28	28
chr8	98377391	98379391	Slc38a7	0.33347	0.025041	stronglyHypermeth	-0.11321	0.051807	insignificant	3	17	25
chr8	98412265	98414265	Got2		1	noCoverage	-0.090909	0.048259	hypomethylated	0	5	4
chr8	101940357	101942357	Cdh8	-0.21424	0.05397	insignificant	-0.039382	0.00000723	inconclusive	6	32	19
chr8	105309011	105311011	Cdh11	-0.27274	1.41E-20	hypomethylated	-0.0098266	0.001362	hypomethylated	12	52	51
chr8	106693412	106695412	Bean1	-0.10377	4.43E-20	hypomethylated	-0.00082709	0.28053	insignificant	24	84	84
chr8	106772458	106774458	Cklf	-0.19549	0.000024983	hypomethylated	0.080087	0.50782	insignificant	4	26	25
chr8	106773802	106775802	Cklf	-0.24028	1.99E-17	hypomethylated	0.024973	0.34998	insignificant	11	39	39
chr8	106773831	106775831	Cklf	-0.24028	1.99E-17	hypomethylated	0.024973	0.34998	insignificant	11	39	39
chr8	106817081	106819081	Cmtm1	0.1177	1	lowCoverage	-0.056488	0.46256	insignificant	1	7	9
chr8	106845136	106847136	Cmtm2b	-0.34747	0.00012511	stronglyHypometh	-0.039093	0.0017972	hypomethylated	7	28	27

chr8	106863493	106865493	Cmtm3	-0.16838	1.26E-35	hypomethylated	0.011546	0.40325	insignificant	26	121	127
chr8	106919596	106921596	Cmtm4	-0.20905	3.72E-09	hypomethylated	0.032171	0.38478	insignificant	3	54	40
chr8	107033787	107035787	Ccdc79	0.019642	0.658	insignificant	0.011875	0.26162	insignificant	3	14	14
chr8	107058537	107060537	Nae1	-0.11178	0.37633	insignificant	0.0054119	0.35439	insignificant	6	18	18
chr8	107063706	107065706	Car7	-0.15349	6.64E-15	hypomethylated	-0.024754	0.034234	hypomethylated	23	71	76
chr8	107114367	107116367	Pdp2	-0.029588	0.0089986	hypomethylated	0.044513	0.62038	insignificant	3	18	18
chr8	107165628	107167628	Fam96b	-0.11866	1	insignificant	-0.061018	0.075358	insignificant	4	19	19
chr8	107354543	107356543	Ces2b	-0.28836	0.43898	insignificant	-0.0051638	1	insignificant	1	7	8
chr8	107470255	107472255	Ces2f	-0.9222	0.13077	lowCoverage	-0.042588	0.14077	insignificant	1	12	12
chr8	107693573	107695573	Cbfb	-0.080218	4.33E-27	hypomethylated	0.0022388	0.8407	insignificant	70	176	191
chr8	107748087	107750087	D230025D16Rik	-0.18188	1.12E-28	hypomethylated	0.012348	0.12629	insignificant	21	76	64
chr8	107779051	107781051	B3gnt9-ps	-0.78113	0.051095	lowCoverage	-0.071629	0.096401	insignificant	1	5	6
chr8	107787547	107789547	Fbxl8	-0.12679	4.03E-09	hypomethylated	-0.021225	0.039596	hypomethylated	18	64	69
chr8	107788494	107790494	Fbxl8	-0.14435	0.00000002	hypomethylated	0.00075075	0.19041	insignificant	8	22	22
chr8	107792773	107794773	Hsf4	-0.17694	7.66E-26	hypomethylated	-0.013893	0.004723	hypomethylated	31	114	114
chr8	107799346	107801346	Nol3	-0.27163	0.0081435	hypomethylated	-0.0036206	0.83821	insignificant	2	24	24
chr8	107813428	107815428	4931428F04Rik	-0.091377	0.43409	insignificant	0.00052998	4.13E-10	hypermethylated	9	75	79
chr8	107819998	107821998	E2f4	-0.18316	4.44E-13	hypomethylated	-0.014863	1	insignificant	33	86	87
chr8	107820562	107822562	E2f4	-0.17869	7.4E-11	hypomethylated	0.0013255	0.34088	insignificant	31	86	90
chr8	107828500	107830500	Elmo3	-0.33315	0.0058179	hypomethylated	-0.017619	0.86498	insignificant	5	14	14
chr8	107849263	107851263	Tmem208	-0.22183	0.00000341	hypomethylated	0.010458	0.7568	insignificant	7	32	32
chr8	107850176	107852176	Tmem208	-0.39564	0.0016463	stronglyHypometh	0.0039667	0.65593	insignificant	3	32	32
chr8	107871157	107873157	Slc9a5	-0.14426	2.05E-14	hypomethylated	0.007152	0.91101	insignificant	24	95	94
chr8	107871870	107873870	Fhod1	-0.13606	4.01E-22	hypomethylated	-0.0016107	0.017004	hypomethylated	32	96	96
chr8	107898280	107900280	Plekhg4	-0.13763	0.057258	insignificant	0.045926	0.68339	insignificant	23	90	92
chr8	107936521	107938521	Lrrc36	0.092043	0.000000256	inconclusive	-0.074586	0.13369	insignificant	4	29	29
chr8	107937387	107939387	Lrrc36	0.072678	0.000000152	inconclusive	-0.09395	0.1483	insignificant	4	30	29
chr8	107995322	107997322	Tppp3	0.046965	0.51463	insignificant	-0.015405	0.37615	insignificant	6	26	26
chr8	108020770	108022770	Zdhhc1	-0.16636	0.000046747	hypomethylated	-0.0028037	0.94082	insignificant	9	21	21
chr8	108041645	108043645	Hsd11b2	-0.15315	6.63E-22	hypomethylated	-0.0073973	0.069425	insignificant	22	75	82
chr8	108089940	108091940	Atp6v0d1	-0.21745	7.47E-12	hypomethylated	-0.01947	0.11449	insignificant	14	50	50
chr8	108092198	108094198	Agrp	-0.85676	0.14213	lowCoverage	-0.015691	0.42631	insignificant	1	16	16
chr8	108128128	108130128	Fam65a	-0.11596	3.31E-44	hypomethylated	0.0014245	0.62196	insignificant	47	117	117
chr8	108138365	108140365	Mir1966	0.065704	1	insignificant	-0.015372	0.23494	insignificant	2	26	26
chr8	108159437	108161437	Ctcf	-0.109	3.02E-55	hypomethylated	-0.0027345	0.0010324	hypomethylated	96	294	307
chr8	108213805	108215805	Rltpr	0.2325	0.010254	hypermethylated	0.18365	0.00000794	hypermethylated	3	14	14
chr8	108224053	108226053	Pard6a	-0.018346	1.1E-32	hypomethylated	-0.012036	0.20057	insignificant	19	111	112
chr8	108224548	108226548	Pard6a	-0.17732	4.24E-33	hypomethylated	-0.014157	1	insignificant	16	89	88
chr8	108224995	108226995	Pard6a	-0.13462	2.61E-28	hypomethylated	-0.030927	1.31E-11	hypomethylated	8	69	66
chr8	108231202	108233202	4933405L10Rik	-0.26341	0.09732	insignificant	-0.033567	0.76184	insignificant	2	42	42
chr8	108232068	108234068	E130303B06Rik	-0.67532	0.00000458	stronglyHypometh	-0.059203	0.091366	insignificant	3	29	29
chr8	108282507	108284507	Gfod2	-0.1403	0.18824	insignificant	0.021549	0.20317	insignificant	7	45	41
chr8	108350643	108352643	Tsnaxip1	-0.17302	1.31E-17	hypomethylated	-0.037931	0.0655	insignificant	10	46	60
chr8	108351250	108353250	Tsnaxip1	-0.23139	0.00021114	hypomethylated	-0.056802	0.92588	insignificant	3	13	19
chr8	108375908	108377908	Cenpt		1	noCoverage	-0.12025	0.13981	insignificant	0	16	16
chr8	108378002	108380002	Thap11	-0.15884	3.97E-33	hypomethylated	0.0079662	0.10357	insignificant	33	88	105
chr8	108383533	108385533	Nutf2	-0.18925	4.27E-16	hypomethylated	-0.014845	0.74896	insignificant	22	63	66
chr8	108383559	108385559	Nutf2-ps1	-0.18925	4.27E-16	hypomethylated	-0.014845	0.74896	insignificant	22	63	66
chr8	108403850	108405850	Edc4	-0.17358	2.73E-11	hypomethylated	-0.037519	0.21033	insignificant	10	51	50
chr8	108416469	108418469	Nrn1l	-0.053601	0.11339	insignificant	0.0056469	1	insignificant	4	12	12
chr8	108423373	108425373	Pskh1	-0.11529	0.017075	hypomethylated	0.058831	0.53383	insignificant	8	34	47
chr8	108457762	108459762	Ctrl	-0.12092	0.0001729	hypomethylated	-0.048096	0.064551	insignificant	2	12	12
chr8	108467302	108469302	Lcat	-0.24299	0.041438	hypomethylated	-0.04987	0.52844	insignificant	6	15	18
chr8	108489939	108491939	Slc12a4	-0.1124	1.79E-09	hypomethylated	-0.02379	0.94189	insignificant	22	52	50
chr8	108520323	108522323	Dpep2	-0.29545	1	insignificant	-0.045455	0.23072	insignificant	2	4	4
chr8	108534406	108536406	Dus2l	-0.11496	1.35E-14	hypomethylated	-0.012652	1	insignificant	22	117	129
chr8	108535386	108537386	Ddx28	-0.32889	0.0020375	hypomethylated	-0.049271	0.75079	insignificant	7	35	31
chr8	108582502	108584502	Nfatc3	-0.16129	4.25E-25	hypomethylated	-0.004298	0.16071	insignificant	41	125	135
chr8	108660874	108662874	Esrp2	-0.18887	8.78E-08	hypomethylated	0.019144	0.8512	insignificant	15	61	58
chr8	108673298	108675298	Pla2g15	-0.24696	3.02E-24	hypomethylated	-0.022656	0.50116	insignificant	16	50	52
chr8	108691774	108693774	Slc7a6	-0.14422	2.84E-20	hypomethylated	-0.03183	0.27876	insignificant	26	91	90
chr8	108733953	108735953	Prmt7	-0.098316	2.85E-29	hypomethylated	-0.0039995	0.36332	insignificant	41	150	149
chr8	108734833	108736833	Slc7a6os	-0.124	0.00000152	hypomethylated	-0.019031	0.26145	insignificant	26	74	73
chr8	108861888	108863888	Smpd3	-0.18314	0.82416	insignificant	-0.020755	0.43051	insignificant	4	10	10
chr8	108938238	108940238	Zfp90	-0.29581	1.49E-18	hypomethylated	-0.024395	0.00021419	hypomethylated	15	46	46
chr8	109033790	109035790	Cdh3	-0.17516	2.83E-17	hypomethylated	-0.00040966	0.47368	insignificant	15	86	85
chr8	109033812	109035812	Cdh3	-0.17088	6.17E-18	hypomethylated	-0.0002618	0.78684	insignificant	16	88	87
chr8	109126267	109128267	Cdh1	-0.16627	2.88E-24	hypomethylated	-0.0096019	0.00021816	hypomethylated	38	120	123
chr8	109205967	109207967	Tmco7	-0.14557	0.00000643	hypomethylated	-0.017672	0.78019	insignificant	19	58	64
chr8	109393141	109395141	Has3	-0.12901	0.00000334	hypomethylated	-0.0017981	0.2674	insignificant	23	81	78
chr8	109416539	109418539	Cirh1a	-0.11484	7.84E-28	hypomethylated	0.014341	0.47494	insignificant	31	137	144
chr8	109417493	109419493	Chtf8	-0.4126	0.0027897	stronglyHypometh	0.031633	0.39539	insignificant	1	18	23
chr8	109458649	109460649	Sntb2	-0.085413	0.000000126	hypomethylated	0.0031205	0.66601	insignificant	27	152	153
chr8	109554225	109556225	Vps4a	-0.12167	1.87E-31	hypomethylated	0.0044371	0.90179	insignificant	44	112	120
chr8	109572514	109574514	Cog8	-0.11506	0.00081941	hypomethylated	-0.017401	0.37507	insignificant	5	10	10
chr8	109579776	109581776	Nip7	-0.13629	3.16E-34	hypomethylated	-0.0066412	0.94007	insignificant	60	200	200
chr8	109580637	109582637	Cog8	-0.18921	1.58E-18	hypomethylated	-0.016503	0.97474	insignificant	33	117	116
chr8	109673560	109675560	Cyb5b	-0.16462	2.62E-17	hypomethylated	0.033808	0.9044	insignificant	25	72	79
chr8	109816369	109818369	Nfat5	-0.14153	2.01E-48	hypomethylated	-0.0015645	0.71145	insignificant	68	197	213
chr8	109927105	109929105	Nqo1	-0.60375	0.44531	lowCoverage	0.014738	0.56192	insignificant	1	18	18
chr8	109948938	109950938	Nob1	-0.22361	6.94E-42	hypomethylated	0.045011	4.67E-12	inconclusive	13	60	65
chr8	109959297	109961297	Wwp2	-0.11671	2.29E-17	hypomethylated	0.0016041	0.5797	insignificant	22	68	70
chr8	111237543	111239543	Zfhx3	-0.15708	2.8E-56	hypomethylated	0.00097433	2.43E-14	inconclusive	65	172	166
chr8	111459759	111461759	Mir3108	0.019756	0.60464	insignificant	-0.039717	0.38547	insignificant	2	8	8
chr8	111864808	111866808	Gm1943	-0.4875	0.11818	insignificant	0.17083	0.10589	insignificant	2	8	8

chr8	112088885	112090885	Txnl4b	-0.12468	0.00012662	hypomethylated	-0.0088033	0.079259	insignificant	15	92	92
chr8	112089501	112091501	Dhx38	-0.12307	0.0025252	hypomethylated	-0.01039	0.48655	insignificant	9	50	50
chr8	112132573	112134573	Dhodh	-0.15045	9.07E-08	hypomethylated	0.010049	0.74217	insignificant	8	22	22
chr8	112137416	112139416	Pkd1l3	-0.048962	0.73549	insignificant	0.00043047	0.75692	insignificant	4	30	31
chr8	112217194	112219194	2400003C14Rik	-0.12673	0.000029567	hypomethylated	-0.002831	0.84026	insignificant	10	27	26
chr8	112228448	112230448	Zfp821	-0.11007	6E-25	hypomethylated	-0.014637	0.028617	hypomethylated	32	131	130
chr8	112228987	112230987	Zfp821	-0.10407	5.83E-25	hypomethylated	-0.0086327	0.030887	hypomethylated	32	129	130
chr8	112301539	112303539	Ap1g1	-0.11371	6.83E-28	hypomethylated	-0.009361	0.27934	insignificant	44	140	140
chr8	112391502	112393502	Phlpp2	-0.12808	2.67E-30	hypomethylated	-0.012854	0.12785	insignificant	53	157	157
chr8	112486105	112488105	Marveld3	-0.47642	2.13E-14	stronglyHypometh	-0.072403	0.000041405	hypomethylated	5	31	33
chr8	112602633	112604633	Zfp612	-0.17296	5.93E-09	hypomethylated	0.026602	0.91436	insignificant	9	57	61
chr8	112692106	112694106	Calb2	-0.28851	1.07E-16	hypomethylated	0.0099791	0.14925	insignificant	13	55	57
chr8	112740859	112742859	Ftsjd1	-0.19085	7.61E-12	hypomethylated	-0.042565	0.39395	insignificant	8	26	31
chr8	113141537	113143537	Vac14	-0.22074	3.96E-15	hypomethylated	-0.02255	0.080961	insignificant	18	67	67
chr8	113244383	113246383	Mtss1l	-0.13475	1.02E-25	hypomethylated	-0.0049473	0.078901	insignificant	41	102	115
chr8	113329803	113331803	Il34		1	noCoverage	0.053347	1	insignificant	0	2	2
chr8	113369923	113371923	Cog4	-0.21152	9.26E-26	hypomethylated	-0.011683	0.25464	insignificant	15	70	66
chr8	113370703	113372703	Sf3b3	-0.22592	1.44E-14	hypomethylated	-0.047123	0.065196	insignificant	6	22	18
chr8	113442764	113444764	St3gal2	-0.17798	0.0052515	hypomethylated	-0.015949	0.38406	insignificant	7	56	66
chr8	113551670	113553670	Ddx19b	-0.20238	0.0018558	hypomethylated	-0.061535	0.15858	insignificant	5	26	21
chr8	113555651	113557651	Ddx19b	0.035223	1	insignificant	0.012474	0.24437	insignificant	2	16	19
chr8	113556866	113558866	Aars	-0.22046	0.00000158	hypomethylated	0.01368	0.014402	hypermethylated	15	52	52
chr8	113579238	113581238	Exosc6	-0.082154	3.06E-30	hypomethylated	-0.001051	0.070869	insignificant	52	150	149
chr8	113617644	113619644	Pdpr	-0.11971	2.95E-10	hypomethylated	-0.0077115	0.10524	insignificant	25	148	154
chr8	113783102	113785102	Glg1	-0.25657	4.34E-08	hypomethylated	-0.058429	0.53016	insignificant	12	74	74
chr8	113824122	113826122	Rfwd3	-0.3105	1	lowCoverage	0.092495	0.0053214	hypermethylated	1	26	29
chr8	113861803	113863803	Mlkl	-0.14599	1.15E-23	hypomethylated	0.0028616	0.34702	insignificant	15	44	44
chr8	113917721	113919721	Fa2h	-0.1429	9.91E-14	hypomethylated	0.0030803	0.95451	insignificant	11	30	30
chr8	114046007	114048007	Wdr59		1	noCoverage	-0.10446	0.70959	insignificant	0	6	6
chr8	114059539	114061539	Znrf1	-0.12005	4.2E-39	hypomethylated	-0.018665	0.039648	hypomethylated	59	166	164
chr8	114061242	114063242	Znrf1	-0.12235	4.92E-15	hypomethylated	-0.0058255	0.19546	insignificant	11	47	47
chr8	114154222	114156222	Ldhd	0.012829	0.23054	insignificant	-0.020925	0.39352	insignificant	9	22	20
chr8	114166342	114168342	Zfp1	-0.12486	0.11692	insignificant	-0.0009464	0.8772	insignificant	5	42	42
chr8	114256036	114258036	Bcar1	-0.26995	0.024115	hypomethylated	-0.087262	0.000000635	hypomethylated	12	26	24
chr8	114267749	114269749	Bcar1	-0.13343	2.78E-09	hypomethylated	0.026806	0.84054	insignificant	17	56	56
chr8	114378210	114380210	Cfdp1		1	noCoverage	-0.0028345	0.071429	insignificant	0	29	29
chr8	114400575	114402575	Tmem170	-0.18562	0.011258	hypomethylated	-0.0060211	0.067124	insignificant	5	40	42
chr8	114434099	114436099	Chst5	-0.34289	0.00042746	stronglyHypometh	-0.073667	0.67982	insignificant	8	31	32
chr8	114457691	114459691	Tmem231		1	noCoverage	0.048186	0.10484	insignificant	0	18	20
chr8	114463617	114465617	Gabarapl2	-0.10941	6.63E-10	hypomethylated	-0.00063254	0.087358	insignificant	25	116	114
chr8	114516202	114518202	Adat1	-0.59881	0.00078045	stronglyHypometh	-0.16064	0.87924	insignificant	2	11	14
chr8	114534258	114536258	Terf2ip	-0.10308	0.000018332	hypomethylated	0.004741	0.25588	insignificant	22	112	110
chr8	114535205	114537205	Kars	-0.090964	0.001493	hypomethylated	0.0057698	1	insignificant	21	102	100
chr8	115092942	115094942	Cntnap4	-0.07281	1	insignificant	-0.023696	0.21125	insignificant	5	14	18
chr8	116158485	116160485	Mon1b	-0.24991	4.48E-22	hypomethylated	-0.023028	0.07304	insignificant	21	62	67
chr8	116158698	116160698	Mon1b	-0.25759	1.41E-22	hypomethylated	-0.0194	0.089835	insignificant	21	64	68
chr8	116372739	116374739	Adamts18	-0.32191	0.12927	insignificant	-0.10987	0.082391	insignificant	7	17	17
chr8	116656478	116658478	Nudt7	-0.35279	4.56E-17	stronglyHypometh	-0.0032584	0.42317	insignificant	2	19	19
chr8	116728539	116730539	Vat1l	-0.18109	0.0014496	hypomethylated	-0.012231	0.71335	insignificant	10	48	48
chr8	116962551	116964551	Wwox	-0.13996	1.96E-09	hypomethylated	-0.025987	0.048945	hypomethylated	11	48	48
chr8	118230794	118232794	Maf	-0.17883	3.55E-30	hypomethylated	-0.017296	0.035569	hypomethylated	31	110	110
chr8	119027914	119029914	Dynlrb2	-0.34906	0.53931	insignificant	-0.036711	0.89353	insignificant	1	6	6
chr8	119256891	119258891	Cdyl2	-0.13849	8.1E-09	hypomethylated	-0.050881	0.017926	hypomethylated	16	57	62
chr8	119444639	119446639	Cenpn	-0.10955	3.73E-08	hypomethylated	-0.018612	0.25309	insignificant	22	84	87
chr8	119445336	119447336	Cenpn	-0.21253	1.14E-12	hypomethylated	0.0046889	0.40299	insignificant	13	40	46
chr8	119466292	119468292	Atmin	-0.089458	3.6E-12	hypomethylated	-0.00001159	0.0080941	hypomethylated	28	108	111
chr8	119502843	119504843	1700030J22Rik	-0.085953	3.89E-09	hypomethylated	0.0063429	1	insignificant	9	31	31
chr8	119517349	119519349	Gcsh	-0.16112	0.00072474	hypomethylated	-0.021812	0.060984	insignificant	14	31	38
chr8	119606349	119608349	Pkd1l2	-0.30848	0.063715	insignificant	-0.076309	0.45908	insignificant	5	16	16
chr8	119618764	119620764	Bcmo1	0.12564	1	insignificant	0.017682	0.33906	insignificant	1	10	10
chr8	119681034	119683034	Gan	-0.096816	3.47E-22	hypomethylated	0.0031716	0.8465	insignificant	39	163	166
chr8	119779918	119781918	4933407C03Rik	-0.095603	2.29E-35	hypomethylated	0.00079671	0.0099814	inconclusive	72	259	248
chr8	120021190	120023190	Plcg2	-0.17147	6.15E-27	hypomethylated	-0.0061706	0.24852	insignificant	26	83	79
chr8	120195415	120197415	Sdr42e1		1	noCoverage	0.032407	0.59845	insignificant	0	2	2
chr8	120325829	120327829	Mphosph6	-0.14402	0.000055977	hypomethylated	-0.018685	0.26531	insignificant	11	43	43
chr8	120806654	120808654	Cdh13	-0.20172	6.07E-15	hypomethylated	-0.011588	0.72296	insignificant	22	62	66
chr8	121867437	121869437	Hsbp1	-0.18503	2.38E-14	hypomethylated	-0.027394	0.56528	insignificant	27	88	91
chr8	121917791	121919791	Mlycd	-0.082717	6.74E-16	hypomethylated	0.0041038	1	insignificant	56	156	158
chr8	121960061	121962061	Osgin1	-0.42281	0.0000071	stronglyHypometh	0.029347	1	insignificant	3	10	10
chr8	121969618	121971618	Necab2	-0.11144	1.12E-22	hypomethylated	-0.0064079	0.48844	insignificant	57	154	162
chr8	122025598	122027598	Slc38a8	-0.022761	0.052016	insignificant	-0.019573	0.75249	insignificant	3	12	12
chr8	122082661	122084661	Mbtps1	-0.21512	0.00000047	hypomethylated	-0.034543	6.25E-10	hypomethylated	16	46	44
chr8	122098134	122100134	Lrrc50	-0.094134	1.11E-16	hypomethylated	-0.014037	0.048464	hypomethylated	29	132	140
chr8	122099100	122101100	Hsdl1	-0.17893	4.1E-11	hypomethylated	-0.0064601	0.011622	hypomethylated	10	54	56
chr8	122135646	122137646	Adad2	0.039517	1	insignificant	0.10501	0.1076	insignificant	4	36	34
chr8	122189264	122191264	Wfdc1		1	noCoverage	-0.024504	0.47989	insignificant	0	10	11
chr8	122222908	122224908	Atp2c2	-0.24763	4.05E-12	hypomethylated	-0.015176	0.017043	hypomethylated	16	52	43
chr8	122302316	122304316	4632415K11Rik	-0.30699	0.0026229	hypomethylated	0.070005	0.55146	insignificant	4	24	27
chr8	122364479	122366479	Cotl1		1	noCoverage	0.15027	0.72089	insignificant	0	12	16
chr8	122385204	122387204	Klhl36	-0.234	2.35E-48	hypomethylated	-0.00045674	3.12E-13	hypomethylated	20	80	82
chr8	122433751	122435751	Usp10	-0.092494	2.09E-08	hypomethylated	-0.0045545	0.48561	insignificant	42	125	131
chr8	122515369	122517369	Crispld2	-0.28698	1.93E-13	hypomethylated	-0.011997	0.77497	insignificant	10	51	51
chr8	122625372	122627372	Zdhhc7	-0.21562	1.81E-28	hypomethylated	-0.0037164	0.0027918	hypomethylated	19	82	82
chr8	122637051	122639051	6430548M08Ril	-0.099634	4.69E-31	hypomethylated	-0.0059831	0.14224	insignificant	59	191	191
chr8	122667828	122669828	6430548M08Ril	-0.12976	0.035182	hypomethylated	-0.038333	0.41969	insignificant	2	4	5

chr8	122701369	122703369	Fam92b		1	noCoverage	0.068943	0.030277	hypermethylated	0	10	10
chr8	123011765	123013765	Gse1	-0.12092	3.03E-23	hypomethylated	-0.0050757	0.50535	insignificant	30	132	131
chr8	123058756	123060756	Gse1	-0.095817	1.65E-31	hypomethylated	-0.0072725	0.065797	insignificant	46	180	181
chr8	123060308	123062308	Gse1	-0.1004	2.78E-23	hypomethylated	-0.0030111	0.096076	insignificant	59	202	206
chr8	123112975	123114975	Gins2	-0.16956	0.00091355	hypomethylated	-0.0066295	0.33342	insignificant	9	42	42
chr8	123158282	123160282	1190005I06Rik	-0.1667	6.11E-32	hypomethylated	0.013714	0.17457	insignificant	17	49	57
chr8	123191189	123193189	Cox4i1	-0.11815	1.7E-39	hypomethylated	-0.005308	0.060843	insignificant	47	145	145
chr8	123192012	123194012	Cox4nb	-0.20998	7.88E-29	hypomethylated	-0.032453	0.000040088	hypomethylated	30	91	89
chr8	123259275	123261275	Irf8	-0.25745	0.000001028	hypomethylated	-0.048544	0.92874	insignificant	4	47	81
chr8	123607373	123609373	Foxf1a	-0.11749	1.31E-20	hypomethylated	-0.006251	0.055664	insignificant	66	222	222
chr8	123639070	123641070	Foxc2	-0.14618	4.28E-24	hypomethylated	0.011874	0.000084629	inconclusive	37	131	140
chr8	123650584	123652584	Foxl1	-0.13427	2.64E-10	hypomethylated	-0.017549	0.42925	insignificant	17	74	67
chr8	124065854	124067854	1700018B08Rik	-0.18535	0.20943	insignificant	0.043436	0.00020497	hypermethylated	3	49	51
chr8	124102706	124104706	Fbxo31	-0.28676	0.17843	insignificant	0.050648	0.75468	insignificant	2	8	8
chr8	124113367	124115367	Map1lc3b	-0.11511	7.51E-10	hypomethylated	0.010245	0.4087	insignificant	14	60	63
chr8	124175833	124177833	Zcchc14	-0.080098	1.6E-23	hypomethylated	0.0097565	0.37873	insignificant	76	283	284
chr8	124253462	124255462	Jph3	-0.12262	1.11E-44	hypomethylated	-0.0068439	1.8E-09	hypomethylated	56	175	168
chr8	124353469	124355469	Klhdc4	-0.21404	0.00012482	hypomethylated	0.078298	0.13973	insignificant	5	12	12
chr8	124430732	124432732	BC048644	-0.13913	2.26E-33	hypomethylated	-0.022663	0.021432	hypomethylated	24	116	121
chr8	124431586	124433586	Slc7a5		1	noCoverage	-0.12599	0.44015	insignificant	0	22	28
chr8	124468812	124470812	Car5a	0.094231	1	insignificant	-0.035491	0.84535	insignificant	3	12	11
chr8	124473432	124475432	Banp	-0.11817	1.82E-38	hypomethylated	-0.0040242	0.00030563	hypomethylated	46	155	154
chr8	124805040	124807040	Zfpm1	-0.075661	5.83E-30	hypomethylated	-0.0057632	0.82549	insignificant	56	215	205
chr8	124899515	124901515	Zc3h18	-0.10342	4.22E-38	hypomethylated	-0.0039208	0.0062175	hypomethylated	48	196	197
chr8	124944984	124946984	Il17c	-0.079666	0.44626	insignificant	0.09892	0.26495	insignificant	3	16	16
chr8	124956840	124958840	Cyba	0.076657	0.3397	insignificant	-0.071069	0.083905	insignificant	4	28	28
chr8	124967322	124969322	Mvd	-0.22238	1.03E-24	hypomethylated	-0.019522	0.49258	insignificant	11	44	44
chr8	124984592	124986592	Snai3		1	noCoverage	0.0064876	0.75939	insignificant	0	24	24
chr8	124999042	125001042	Ctu2	-0.11225	1.56E-44	hypomethylated	-0.0021494	0.95545	insignificant	68	167	167
chr8	124999964	125001964	Rnf166	-0.12519	5.11E-37	hypomethylated	-0.0057544	0.89313	insignificant	44	98	98
chr8	125075229	125077229	Fam38a	-0.016205	0.50663	insignificant	0.0041764	0.51777	insignificant	14	52	53
chr8	125090914	125092914	Cdt1	-0.16022	2.58E-27	hypomethylated	-0.014604	0.72612	insignificant	23	83	85
chr8	125134525	125136525	Trappc2l	-0.17277	1.02E-10	hypomethylated	-0.0060039	0.018905	hypomethylated	11	45	50
chr8	125135387	125137387	Galns	-0.20265	4.33E-14	hypomethylated	-0.0076847	0.0055826	hypomethylated	11	47	52
chr8	125202075	125204075	Cbfa2t3	-0.14452	4.36E-28	hypomethylated	-0.0053646	0.000010665	hypomethylated	43	133	138
chr8	125298404	125300404	Acsf3	-0.17498	3.03E-13	hypomethylated	-0.01862	0.098075	insignificant	10	52	48
chr8	125565897	125567897	Ankrd11	-0.097636	5.09E-51	hypomethylated	-0.0015831	0.069518	insignificant	83	221	220
chr8	125588407	125590407	Spg7	-0.13009	1.23E-28	hypomethylated	-0.0052374	0.66263	insignificant	36	105	106
chr8	125625249	125627249	Rpl13	-0.12239	4.72E-20	hypomethylated	0.0064022	0.57748	insignificant	37	127	122
chr8	125625957	125627957	Snord68	-0.16079	7.7E-23	hypomethylated	-0.0061015	0.13244	insignificant	37	130	128
chr8	125640273	125642273	Cpne7	-0.20666	6.87E-52	hypomethylated	-0.015426	2.3E-19	hypomethylated	34	129	133
chr8	125682180	125684180	Sult5a1	0.043579	1	insignificant	-0.12086	0.15774	insignificant	2	12	8
chr8	125709134	125711134	Dpep1	-0.22558	0.000032831	hypomethylated	-0.045708	0.017915	hypomethylated	7	36	36
chr8	125735757	125737757	4732415M23Rik	-0.13696	6.41E-15	hypomethylated	-0.019862	0.29462	insignificant	32	100	101
chr8	125736688	125738688	Chmp1a	-0.27962	1.89E-17	hypomethylated	-0.047304	0.059455	insignificant	15	40	42
chr8	125747740	125749740	Cdk10	-0.20197	0.11024	insignificant	-0.037902	0.03164	hypomethylated	9	67	71
chr8	125760109	125762109	Spata2L	-0.69411	5.74E-18	stronglyHypometh	-0.012394	0.054251	insignificant	2	12	12
chr8	125777094	125779094	Zfp276	-0.087673	4.04E-50	hypomethylated	-0.00081502	0.58715	insignificant	56	209	225
chr8	125778075	125780075	Zfp276	-0.114	4.27E-33	hypomethylated	-0.025872	0.9495	insignificant	39	138	143
chr8	125842476	125844476	Fanca	0.031126	0.4386	insignificant	0.013817	0.52156	insignificant	5	20	21
chr8	125855612	125857612	Spire2	-0.15156	2.67E-21	hypomethylated	-0.036548	0.58962	insignificant	26	116	124
chr8	125896652	125898652	Tcf25	-0.11047	2.59E-29	hypomethylated	0.0066547	0.11402	insignificant	22	101	101
chr8	125896723	125898723	Tcf25	-0.11047	2.59E-29	hypomethylated	0.01531	0.11441	insignificant	22	101	102
chr8	125896734	125898734	Tcf25	-0.11047	2.59E-29	hypomethylated	0.01531	0.11441	insignificant	22	101	102
chr8	125934463	125936463	Tubb3	0.038002	0.35608	insignificant	0.0042923	0.71963	insignificant	11	51	50
chr8	125965885	125967885	Def8	-0.27076	7.95E-15	hypomethylated	-0.03994	0.47738	insignificant	13	53	50
chr8	126000761	126002761	Afg3l1	-0.092822	5.71E-13	hypomethylated	-0.027055	0.030414	hypomethylated	11	69	71
chr8	126039355	126041355	Dbndd1	-0.48024	8.98E-12	stronglyHypometh	0.026542	0.000000132	inconclusive	7	28	29
chr8	126041734	126043734	Gas8	-0.2413	0.000012218	hypomethylated	-0.023792	0.0021727	hypomethylated	13	38	40
chr8	126176828	126178828	Rhou	-0.091607	4.27E-26	hypomethylated	0.0010955	0.92186	insignificant	62	168	168
chr8	126328895	126330895	Rab4a	-0.19785	4.36E-12	hypomethylated	-0.0011793	0.30078	insignificant	19	64	64
chr8	126418636	126420636	Acta1	-0.37773	0.0025624	stronglyHypometh	0.079476	0.22465	insignificant	7	23	23
chr8	126473165	126475165	Nup133	-0.19085	0.000081874	hypomethylated	0.011611	0.42047	insignificant	17	61	63
chr8	126507022	126509022	Abcb10	-0.10636	0.10108	insignificant	0.034571	0.037292	hypermethylated	13	38	34
chr8	126545209	126547209	Taf5l	-0.060523	3.29E-09	hypomethylated	-0.0080569	0.05018	insignificant	16	94	94
chr8	126545540	126547540	Mir1967		1	noCoverage	-0.00044911	0.58657	insignificant	0	14	14
chr8	126754293	126756293	Galnt2	-0.059009	3.37E-10	hypomethylated	-0.0073982	0.32679	insignificant	43	205	202
chr8	126957836	126959836	Pgbd5	-0.093514	4.69E-29	hypomethylated	-0.01593	0.25675	insignificant	64	182	183
chr8	127043666	127045666	Cog2		1	noCoverage	0.00059589	1	insignificant	0	30	30
chr8	127099010	127101010	Capn9	0.083176	0.22756	insignificant	-0.046932	0.55082	insignificant	4	14	14
chr8	127245038	127247038	Arv1	-0.13291	8.63E-39	hypomethylated	-0.022414	0.00000588	hypomethylated	41	123	125
chr8	127245875	127247875	Ttc13	-0.17508	6.01E-24	hypomethylated	-0.018354	0.00014942	hypomethylated	22	74	78
chr8	127275709	127277709	Fam89a	-0.089683	0.0035764	hypomethylated	-0.0096669	0.76953	insignificant	12	45	45
chr8	127315918	127317918	Trim67	-0.12107	7.13E-35	hypomethylated	-0.0048132	0.38381	insignificant	40	138	134
chr8	127385932	127387932	Gnpat	-0.16729	0.00098378	hypomethylated	0.014056	0.87998	insignificant	5	30	28
chr8	127386929	127388929	2810004N23Rik	-0.10495	0.0065401	hypomethylated	0.078514	0.25971	insignificant	5	22	24
chr8	127420785	127422785	Gm505	-0.094744	5.89E-32	hypomethylated	0.00040985	0.576	insignificant	39	190	190
chr8	127421605	127423605	Exoc8	-0.16955	1.84E-15	hypomethylated	-0.0051155	0.39265	insignificant	15	85	87
chr8	127473154	127475154	Egln1	-0.16919	3.7E-37	hypomethylated	-0.021066	0.5325	insignificant	40	122	124
chr8	127535896	127537896	Tsnax	-0.11091	2.95E-32	hypomethylated	0.019874	0.55658	insignificant	50	136	144
chr8	127577094	127579094	Disc1	-0.15237	4.33E-18	hypomethylated	-0.023589	0.04155	hypomethylated	28	94	103
chr8	128016610	128018610	Sipa1l2	-0.50045	0.000060171	stronglyHypometh	-0.16711	0.0061906	hypomethylated	4	8	8
chr8	128192717	128194717	4933403G14Rik	-0.21671	9.03E-34	hypomethylated	0.0093201	0.44029	insignificant	31	95	116
chr8	128257102	128259102	Ntpcr	-0.32219	4.49E-26	hypomethylated	-0.01152	0.37775	insignificant	16	38	36
chr8	128422193	128424193	Pcnxl2	-0.525	0.22247	insignificant	-0.15852	0.078622	insignificant	2	4	4

chr8	128433349	128435349	BC021891	-0.13574	1.12E-32	hypomethylated	0.0024193	0.21326	insignificant	53	193	193
chr8	128518001	128520001	Kcnk1	-0.11653	3E-10	hypomethylated	-0.018101	0.14226	insignificant	25	59	59
chr8	128821478	128823478	Slc35f3	-0.15009	1.32E-28	hypomethylated	-0.0028469	0.1623	insignificant	28	93	93
chr8	128945400	128947400	1810063B05Rik	-0.1948	3.99E-15	hypomethylated	0.026909	0.82613	insignificant	27	82	84
chr8	128998965	129000965	Gm17296	-0.12699	0.013007	hypomethylated	0.013462	5.34E-08	inconclusive	14	49	48
chr8	129117336	129119336	Irf2bp2	-0.091742	1.19E-37	hypomethylated	-0.010309	0.013671	hypomethylated	74	259	276
chr8	129469730	129471730	Tomm20	-0.15729	0.000001527	hypomethylated	-0.051138	8.89E-12	hypomethylated	8	38	38
chr8	129494979	129496979	Rbm34	-0.14048	0.030827	hypomethylated	0.02086	0.033626	hypermethylated	3	6	6
chr8	129586955	129588955	Pard3	-0.10205	6.44E-29	hypomethylated	0.00037189	0.43012	insignificant	57	201	204
chr8	129822511	129824511	Pard3	0.067688	1	insignificant	0.042383	0.45901	insignificant	4	16	15
chr8	129822551	129824551	Pard3	0.067688	1	insignificant	0.042383	0.45901	insignificant	4	16	15
chr8	130881972	130883972	Nrp1	-0.15201	0.0000012	hypomethylated	0.030839	0.46077	insignificant	15	68	61
chr8	130882140	130884140	Mir1903	-0.14488	0.000001973	hypomethylated	0.027443	0.51412	insignificant	15	64	59
chr8	131208553	131210553	Itgb1	-0.083717	2.73E-20	hypomethylated	-0.010185	0.083523	insignificant	33	156	162
chr9	3199813	3201813	4930433N12Rik	-0.053992	0.00000268	hypomethylated	0.1401	0.15538	insignificant	4	21	30
chr9	3403084	3405084	Cwf19l2	-0.24467	0.0016421	hypomethylated	-0.037725	0.15503	insignificant	5	29	26
chr9	3531353	3533353	Gucy1a2	-0.36884	0.0033161	stronglyHypometh	-0.0010968	0.13439	insignificant	2	8	8
chr9	4308742	4310742	Kbtbd3	-0.13386	1.34E-35	hypomethylated	0.011647	0.70353	insignificant	33	141	146
chr9	4308815	4310815	Kbtbd3	-0.13386	1.34E-35	hypomethylated	0.011647	0.70353	insignificant	33	141	146
chr9	4309494	4311494	Aasdhppt	-0.15675	9.13E-17	hypomethylated	0.0039864	0.62222	insignificant	16	79	82
chr9	6167611	6169611	Pdgfd	-0.21853	0.57651	insignificant	-0.025647	0.0020234	hypomethylated	3	24	24
chr9	7177046	7179046	Dync2h1	-0.26576	2.37E-10	hypomethylated	-0.024178	1	insignificant	2	16	16
chr9	7183565	7185565	Dcun1d5	-0.18146	3.14E-15	hypomethylated	-0.004072	0.13758	insignificant	18	56	56
chr9	7444821	7446821	Mmp3	-0.45426	0.1156	insignificant	-0.056828	0.54075	insignificant	1	4	4
chr9	7570457	7572457	Mmp27		1	noCoverage	0.028488	0.87411	insignificant	0	6	6
chr9	7763076	7765076	Tmem123	-0.12921	0.00099024	hypomethylated	0.0072964	0.67628	insignificant	6	28	27
chr9	7835255	7837255	Birc2	-0.19974	4.06E-09	hypomethylated	-0.0124	0.24346	insignificant	15	38	38
chr9	8004596	8006596	Yap1	-0.12212	1.78E-13	hypomethylated	-0.0018097	0.21707	insignificant	11	51	51
chr9	8042823	8044823	9230110C19Rik	-0.26027	0.60079	insignificant	-0.00072595	0.29076	insignificant	2	13	17
chr9	8134294	8136294	AK129341	-0.041505	0.029217	hypomethylated	0.0018184	0.60789	insignificant	8	46	50
chr9	8220888	8222888	1700128F08Rik	-0.40537	0.17569	insignificant	-0.0085667	0.398	insignificant	2	20	15
chr9	8543141	8545141	Trpc6	-0.16066	0.0012411	hypomethylated	-0.002532	0.674	insignificant	10	34	34
chr9	8898832	8900832	Pgr	-0.16681	0.00007734	hypomethylated	-0.00037236	0.41002	insignificant	6	30	30
chr9	9239013	9241013	Arhgap42	-0.25612	0.000000618	hypomethylated	-0.037792	0.56549	insignificant	14	45	44
chr9	13552624	13554624	Mtmr2	-0.10124	1.38E-20	hypomethylated	0.026621	0.32169	insignificant	17	77	79
chr9	13631170	13633170	Fam76b	-0.11954	1.19E-13	hypomethylated	-0.0059353	0.51967	insignificant	21	98	95
chr9	13631497	13633497	Cep57	-0.14085	4.4E-14	hypomethylated	0.0047214	0.48641	insignificant	15	62	59
chr9	13631551	13633551	Cep57	-0.14085	4.4E-14	hypomethylated	0.0047214	0.48641	insignificant	15	62	59
chr9	14079744	14081744	Sesn3	-0.1894	1.08E-25	hypomethylated	0.0040185	0.088026	insignificant	27	84	84
chr9	14185686	14187686	Endod1	-0.57854	0.000000408	stronglyHypometh	-0.032338	0.11679	insignificant	2	18	18
chr9	14304062	14306062	Cwc15	-0.17796	5.02E-32	hypomethylated	-0.012036	0.8411	insignificant	29	85	86
chr9	14304926	14306926	Kdm4d	-0.19854	2.99E-30	hypomethylated	-0.012733	0.55275	insignificant	24	60	60
chr9	14419444	14421444	Amotl1	-0.23256	7.79E-10	hypomethylated	0.034856	0.039109	inconclusive	11	24	25
chr9	14556566	14558566	Fut4	-0.18063	0.1273	insignificant	-0.00043458	0.33542	insignificant	3	14	14
chr9	14575474	14577474	1700012B09Rik	-0.36265	0.00035403	stronglyHypometh	0.1778	0.020562	hypermethylated	2	7	7
chr9	14587408	14589408	Ankrd49	-0.10775	0.00015736	hypomethylated	0.013837	0.31711	insignificant	14	42	38
chr9	14588150	14590150	Mre11a	-0.10775	0.00015736	hypomethylated	0.013837	0.31711	insignificant	14	42	38
chr9	14663697	14665697	Gpr83	-0.13046	0.0016806	hypomethylated	0.011661	0.0062171	inconclusive	15	83	76
chr9	14708395	14710395	Folr4	-0.56977	0.14336	insignificant	0.07917	0.71617	insignificant	1	8	7
chr9	14849922	14851922	Panx1	-0.1958	0.000004967	hypomethylated	0.11777	0.77549	insignificant	13	31	37
chr9	15084311	15086311	Med17	-0.15288	0.000000238	hypomethylated	-0.020813	0.2502	insignificant	10	35	40
chr9	15109657	15111657	Taf1d	-0.17849	6.94E-08	hypomethylated	0.016637	0.0010142	hypermethylated	21	129	129
chr9	15110611	15112611	4931406C07Rik	-0.11062	0.00057637	hypomethylated	0.017198	0.40762	insignificant	17	107	107
chr9	15110892	15112892	4931406C07Rik	-0.22697	0.14272	insignificant	0.028732	0.011416	hypermethylated	8	60	60
chr9	15162232	15164232	5830418K08Rik	-0.12201	0.000088127	hypomethylated	-0.012938	0.41367	insignificant	6	24	24
chr9	15513212	15515212	Slc36a4	-0.10095	9.56E-13	hypomethylated	-0.0008563	0.61826	insignificant	35	90	90
chr9	18095710	18097710	Chordc1	-0.15284	9.41E-20	hypomethylated	-0.0095163	0.58235	insignificant	21	80	80
chr9	18278003	18280003	Zfp558	-0.14295	1.2E-09	hypomethylated	0.0090844	0.56904	insignificant	8	28	28
chr9	18791433	18793433	Olfr834	-0.021825	0.15398	insignificant	0.17298	0.16301	insignificant	1	4	4
chr9	19425534	19427534	Zfp317	-0.12496	0.024249	hypomethylated	0.0048926	0.58819	insignificant	3	31	30
chr9	20189602	20191602	Zfp560	-0.16853	0.0025104	hypomethylated	0.0097277	0.038532	inconclusive	10	57	54
chr9	20297190	20299190	Zfp426	-0.19049	6.31E-11	hypomethylated	-0.031071	0.00000383	hypomethylated	6	31	28
chr9	20325863	20327863	Zfp266	-0.21083	0.0017091	hypomethylated	0.0074447	0.27096	insignificant	4	44	48
chr9	20384771	20386771	Zfp846	-0.17395	0.1249	insignificant	-0.0025382	0.74	insignificant	7	14	14
chr9	20410918	20412918	5730577I03Rik	-0.11623	1.63E-10	hypomethylated	-0.0252	0.084747	insignificant	17	38	38
chr9	20446761	20448761	Ubl5	-0.17775	3.31E-08	hypomethylated	-0.029026	0.048526	hypomethylated	13	50	56
chr9	20447250	20449250	Fbxl12	-0.22119	0.00000159	hypomethylated	-0.024193	1	insignificant	10	40	46
chr9	20449211	20451211	Ubl5	-0.42747	2.19E-15	stronglyHypometh	-0.06844	0.15092	insignificant	10	26	21
chr9	20455573	20457573	Pin1	-0.13154	0.000000208	hypomethylated	-0.029968	1	insignificant	19	56	52
chr9	20619478	20621478	Col5a3	-0.23041	0.010934	hypomethylated	0.018464	0.012446	inconclusive	7	33	32
chr9	20672085	20674085	A230050P20Rik	-0.17201	3.45E-19	hypomethylated	-0.015071	0.60941	insignificant	20	79	81
chr9	20691618	20693618	Ppan	0.13375	0.064059	insignificant	0.0043805	0.33028	insignificant	22	87	86
chr9	20757067	20759067	Dnmt1	-0.046453	0.037893	inconclusive	0.20956	1.32E-23	hypermethylated	15	36	41
chr9	20781237	20783237	S1pr2	-0.1931	0.000000701	hypomethylated	0.055127	0.16936	insignificant	3	11	11
chr9	20806180	20808180	Mrpl4	-0.18471	2.43E-14	hypomethylated	-0.011433	0.41731	insignificant	22	82	80
chr9	20819403	20821403	Icam1	-0.32461	1.26E-11	hypomethylated	-0.043408	0.10331	insignificant	11	35	35
chr9	20832816	20834816	Icam4	-0.25609	9.7E-28	hypomethylated	0.027395	0.13149	insignificant	14	54	51
chr9	20835481	20837481	Icam5	-0.18671	7.99E-13	hypomethylated	-0.012424	0.15987	insignificant	13	36	40
chr9	20836772	20838772	Icam5	-0.19105	2.16E-12	hypomethylated	-0.0066538	0.11642	insignificant	14	57	61
chr9	20871537	20873537	Fdx1l	0.016019	0.76149	insignificant	-0.010153	0.32167	insignificant	2	32	37
chr9	20877958	20879958	Fdx1l	-0.19771	0.000032947	hypomethylated	-0.042319	0.48992	insignificant	5	18	22
chr9	20896432	20898432	Raver1	-0.20667	0.0037603	hypomethylated	0.11694	0.46948	insignificant	6	25	28
chr9	20954350	20956350	Cdc37	-0.29812	0.021867	hypomethylated	0.027924	0.070825	insignificant	5	32	33
chr9	20969157	20971157	Pde4a	-0.11785	1.45E-18	hypomethylated	0.0051328	0.11619	insignificant	34	112	97
chr9	21043776	21045776	Keap1	-0.63998	0.0099436	stronglyHypometh	-0.28503	0.033911	hypomethylated	2	9	7

chr9	21052887	21054887	S1pr5	-0.27746	2.31E-08	hypomethylated	0.03658	0.26628	insignificant	9	48	49
chr9	21068742	21070742	Atg4d	-0.1367	1.06E-32	hypomethylated	0.015007	0.081932	insignificant	22	42	36
chr9	21116777	21118777	Ap1m2	-0.1649	0.67184	insignificant	0.030795	0.68492	insignificant	6	38	35
chr9	21124162	21126162	Slc44a2	-0.066511	0.031273	hypomethylated	0.094917	0.16872	insignificant	10	32	34
chr9	21141271	21143271	Slc44a2	0.050798	0.70142	insignificant	-0.054425	0.85994	insignificant	3	18	15
chr9	21171446	21173446	Ilf3	-0.17545	2.58E-11	hypomethylated	-0.018404	0.027349	hypomethylated	21	98	97
chr9	21215280	21217280	Qtrt1	-0.25153	9.16E-13	hypomethylated	0.01813	0.97125	insignificant	16	74	74
chr9	21228388	21230388	Dnm2	-0.21765	0.000029348	hypomethylated	-0.021659	0.018392	hypomethylated	13	62	71
chr9	21314630	21316630	Tmed1	-0.46015	0.00007098	stronglyHypometh	0.095599	0.0015161	hypermethylated	4	24	24
chr9	21329620	21331620	AB124611	0.19735	1	lowCoverage	-0.088	0.63519	insignificant	1	10	10
chr9	21350337	21352337	Carm1	-0.10614	2.9E-26	hypomethylated	-0.00021942	0.0087967	hypomethylated	51	190	195
chr9	21396165	21398165	1810026J23Rik	-0.12337	1.04E-47	hypomethylated	-0.0096519	0.12741	insignificant	36	153	154
chr9	21397190	21399190	1810026J23Rik	-0.13161	9.44E-58	hypomethylated	-0.0039093	0.0091148	hypomethylated	39	165	166
chr9	21412773	21414773	Gm7904	-0.023166	0.82828	insignificant	-0.01042	0.098888	insignificant	3	15	16
chr9	21419612	21421612	Smarca4	-0.24014	0.00022087	hypomethylated	0.0084912	0.78185	insignificant	12	68	56
chr9	21527037	21529037	Ldlr	-0.14543	0.00000292	hypomethylated	-0.0033714	0.57351	insignificant	14	66	66
chr9	21602990	21604990	Kank2	-0.087686	4.67E-09	hypomethylated	-0.0029982	0.73218	insignificant	13	53	53
chr9	21638953	21640953	Gm6484	-0.25505	0.0021876	hypomethylated	-0.0043051	1	insignificant	4	8	8
chr9	21657079	21659079	Dock6	-0.15056	9.66E-09	hypomethylated	0.042289	0.87985	insignificant	14	47	48
chr9	21722565	21724565	Rab3d	-0.32125	6.04E-11	hypomethylated	-0.062103	0.00017595	hypomethylated	6	28	28
chr9	21730914	21732914	Ccdc159	-0.35557	8.38E-08	stronglyHypometh	-0.028576	0.34016	insignificant	8	35	35
chr9	21731906	21733906	Tmem205	-0.63369	0.55744	insignificant	-0.039668	0.01912	hypomethylated	3	22	22
chr9	21759196	21761196	2310047B19Rik	-0.15022	0.00059842	hypomethylated	-0.017862	0.065283	insignificant	9	55	57
chr9	21793897	21795897	Rgl3	-0.30603	1.18E-17	hypomethylated	-0.028335	0.70639	insignificant	16	74	64
chr9	21806478	21808478	Prkcsh	-0.13828	9.2E-19	hypomethylated	0.04176	0.090821	insignificant	17	55	55
chr9	21807078	21809078	Prkcsh	-0.24409	6.53E-14	hypomethylated	0.053158	0.010224	inconclusive	14	36	36
chr9	21856467	21858467	Elavl3	-0.14218	0.000054722	hypomethylated	0.024684	0.20695	insignificant	8	31	28
chr9	21875789	21877789	Ecsit	-0.26152	0.00000232	hypomethylated	-0.016623	0.058995	insignificant	8	25	25
chr9	21889826	21891826	Ecsit	-0.29618	4.91E-17	hypomethylated	0.0019618	0.42985	insignificant	14	52	57
chr9	21902696	21904696	Cnn1	-0.039939	0.41341	insignificant	0.05731	0.26314	insignificant	9	24	25
chr9	21935042	21937042	Acp5	-0.1146	2.47E-09	hypomethylated	-0.02805	0.03923	inconclusive	13	60	65
chr9	21936309	21938309	Acp5	-0.30963	0.000035152	hypomethylated	0.0085692	0.67751	insignificant	9	44	44
chr9	21940190	21942190	Acp5	-0.12292	0.2299	insignificant	-0.024944	0.40587	insignificant	2	4	4
chr9	21960289	21962289	Pigyl	-0.13787	4.3E-24	hypomethylated	-0.031131	0.000006435	hypomethylated	33	135	134
chr9	21991609	21993609	Zfp872	-0.015892	1.27E-09	hypomethylated	0.045774	0.022524	inconclusive	9	34	33
chr9	22029146	22031146	Zfp809	-0.23564	1.25E-15	hypomethylated	-0.022099	0.3277	insignificant	7	36	36
chr9	22029155	22031155	Zfp809	-0.23564	1.25E-15	hypomethylated	-0.022099	0.3277	insignificant	7	36	36
chr9	22112082	22114082	Zfp810	-0.40114	1.56E-25	stronglyHypometh	-0.048374	0.051959	insignificant	7	37	35
chr9	22193650	22195650	Anln	-0.16154	3.22E-08	hypomethylated	-0.055708	0.63323	insignificant	10	31	34
chr9	22272800	22274800	Rp9	-0.50852	0.25562	insignificant	-0.020103	0.11898	insignificant	3	10	10
chr9	22279158	22281158	Bbs9		1	noCoverage	-0.21901	0.58238	insignificant	0	16	12
chr9	23026519	23028519	Bmper	-0.12792	1.2E-36	hypomethylated	-0.0075285	0.1029	insignificant	60	160	160
chr9	24307584	24309584	Dpy19l1	-0.090316	6.29E-38	hypomethylated	-0.0058921	0.0024087	hypomethylated	50	130	130
chr9	24956280	24958280	Herpud2	0.14087	0.23946	insignificant	-0.0081561	0.76961	insignificant	4	14	14
chr9	25059168	25061168	############	-0.1125	2.18E-43	hypomethylated	-0.011491	0.048127	hypomethylated	68	212	219
chr9	25288181	25290181	Eepd1	-0.16986	1.47E-16	hypomethylated	-0.0023464	0.14967	insignificant	20	56	57
chr9	26614002	26616002	Glb1l2	-0.12997	0.000000641	hypomethylated	-0.0063584	0.17421	insignificant	10	26	26
chr9	26806261	26808261	Thyn1	-0.14301	0.049478	hypomethylated	-0.0042548	0.42195	insignificant	5	28	37
chr9	26807134	26809134	Thyn1		1	noCoverage	0.032105	0.06953	insignificant	0	12	12
chr9	26836759	26838759	Ncapd3	-0.11992	2.78E-29	hypomethylated	-0.019273	0.023854	hypomethylated	27	135	129
chr9	26837679	26839679	Vps26b	-0.11828	2.08E-12	hypomethylated	0.0072704	0.87518	insignificant	13	62	54
chr9	26962965	26964965	Jam3	-0.31239	3.57E-10	hypomethylated	-0.028066	0.0419	hypomethylated	11	42	42
chr9	27105812	27107812	Igsf9b	-0.13333	9.59E-24	hypomethylated	0.01434	0.081787	insignificant	29	99	90
chr9	27159269	27161269	AI414108	-0.0080128	0.24744	insignificant	-0.057692	0.74628	insignificant	2	4	4
chr9	27597853	27599853	Opcml	-0.34067	1	lowCoverage	0.0044867	0.71089	insignificant	1	10	10
chr9	30233913	30235913	Snx19	-0.14527	2.59E-11	hypomethylated	-0.020476	0.12118	insignificant	14	70	70
chr9	30749147	30751147	Adamts8	-0.076116	1.62E-21	hypomethylated	0.012414	0.63129	insignificant	41	136	125
chr9	30837228	30839228	Zbtb44	-0.081513	1.69E-13	hypomethylated	0.0026648	0.87978	insignificant	26	145	150
chr9	30939384	30941384	St14	-0.27498	0.01268	hypomethylated	-0.0070101	0.069501	insignificant	8	25	24
chr9	31019400	31021400	Mir3471-1	-0.105	0.0039649	hypomethylated	0.015412	1	insignificant	5	10	10
chr9	31081482	31083482	Gm7244	0.1107	1	lowCoverage	0.047487	0.4717	insignificant	1	25	24
chr9	31192776	31194776	Nfrkb	-0.11076	3.85E-21	hypomethylated	-0.0014821	0.0016046	hypomethylated	30	95	92
chr9	31271823	31273823	Tmem45b	-0.15518	0.022703	hypomethylated	0.080691	0.21228	insignificant	4	9	9
chr9	31720870	31722870	Barx2	-0.1601	1.06E-08	hypomethylated	0.026664	0.82933	insignificant	18	89	96
chr9	32151822	32153822	Kcnj5		1	noCoverage	0.0049383	0.85054	insignificant	0	9	6
chr9	32179002	32181002	Kcnj1		1	noCoverage	-0.059375	0.69543	insignificant	0	4	4
chr9	32348953	32350953	Fli1	-0.15331	0.00096828	hypomethylated	-0.012349	0.52264	insignificant	8	56	56
chr9	32502626	32504626	Ets1	-0.10079	0.00068034	hypomethylated	0.0033411	0.95035	insignificant	12	62	75
chr9	34295315	34297315	Kirrel3	-0.12471	7.28E-11	hypomethylated	0.0011329	0.28774	insignificant	21	88	88
chr9	34923312	34925312	4930581F22Rik	-0.10591	0.017059	hypomethylated	0.040627	0.92659	insignificant	7	86	89
chr9	34924395	34926395	4930581F22Rik	-0.11523	1	insignificant	0.11859	0.61208	insignificant	3	41	49
chr9	34983572	34985572	Dcps	-0.29257	0.021631	hypomethylated	-0.082432	0.0366	hypomethylated	2	14	16
chr9	35007490	35009490	Tirap	-0.15676	0.0086862	hypomethylated	0.028093	1	insignificant	7	14	14
chr9	35007550	35009550	Tirap	-0.26923	0.062993	insignificant	-0.0025641	1	insignificant	2	4	4
chr9	35017787	35019787	Srpr	-0.1271	2.36E-27	hypomethylated	-0.0063272	0.49565	insignificant	48	150	148
chr9	35018510	35020510	Foxred1	-0.16181	1.58E-16	hypomethylated	-0.0039983	0.055682	insignificant	40	127	127
chr9	35074465	35076465	Rpusd4	-0.045215	0.000011445	hypomethylated	-0.00041872	0.02449	hypomethylated	13	108	104
chr9	35075352	35077352	Fam118b	-0.25238	0.0033283	hypomethylated	0.0041792	0.00042598	inconclusive	3	48	48
chr9	35075365	35077365	Fam118b	-0.27532	0.003408	hypomethylated	0.0045591	0.00032043	inconclusive	3	44	44
chr9	35366050	35368050	Pus3	-0.10418	0.0002924	hypomethylated	-0.0045889	0.096198	insignificant	20	58	54
chr9	35366055	35368055	Ddx25	-0.10418	0.0002924	hypomethylated	-0.0045889	0.096198	insignificant	20	58	54
chr9	35377654	35379654	Hyls1	-0.17376	0.000000193	hypomethylated	0.0033178	0.73568	insignificant	14	66	66
chr9	36575163	36577163	Stt3a	-0.16776	0.00014631	hypomethylated	-0.019725	0.61401	insignificant	8	80	81
chr9	36604653	36606653	Ei24	-0.2601	2.12E-20	hypomethylated	0.015684	0.00050714	hypermethylated	15	63	63
chr9	36604978	36606978	Ei24	-0.60514	3.84E-11	stronglyHypometh	-0.05161	0.20947	insignificant	4	18	18

chr9	36954851	36956851	Pknox2	-0.19928	1.15E-10	hypomethylated	0.0042549	0.01292	inconclusive	10	35	32
chr9	36954899	36956899	Pknox2	-0.21217	8.29E-08	hypomethylated	0.005573	0.023048	inconclusive	8	27	24
chr9	37014917	37016917	Tmem218	0.052018	0.13154	insignificant	-0.087232	1	insignificant	5	15	20
chr9	37063323	37065323	Slc37a2		1	noCoverage	-0.063288	0.27102	insignificant	0	6	6
chr9	37155977	37157977	Ccdc15	-0.17473	0.000042517	hypomethylated	-0.025929	0.40732	insignificant	6	14	14
chr9	37174190	37176190	Hepacam	0.14524	1	insignificant	-0.11552	0.017647	hypomethylated	1	10	6
chr9	37208630	37210630	Robo4	0.086902	0.29781	insignificant	-0.014832	0.14741	insignificant	4	16	18
chr9	37240760	37242760	Robo3	-0.43613	0.000055717	stronglyHypometh	-0.14409	0.47847	insignificant	3	12	15
chr9	37295905	37297905	BC024479	-0.12575	1.39E-23	hypomethylated	0.0012425	0.23757	insignificant	60	162	163
chr9	37334673	37336673	Esam	-0.1615	8.41E-10	hypomethylated	0.0097621	0.50167	insignificant	10	34	34
chr9	37345839	37347839	Vsig2	-0.13452	0.21196	insignificant	-0.0085238	0.35894	insignificant	2	7	10
chr9	37360330	37362330	Nrgn	-0.20624	0.000000409	hypomethylated	0.01477	0.56676	insignificant	1	8	8
chr9	37420431	37422431	Siae	-0.1718	0.58529	insignificant	-0.003466	0.09902	insignificant	1	44	44
chr9	37421199	37423199	Spa17	0.3825	0.0003103	stronglyHypermeth	-0.0078579	0.42924	insignificant	4	40	40
chr9	37464897	37466897	Tbrg1	-0.2118	0.000000419	hypomethylated	-0.042105	0.81616	insignificant	2	6	6
chr9	39127197	39129197	Olfr948	0.08428	1	lowCoverage	-0.17188	0.48272	insignificant	1	8	8
chr9	39411237	39413237	AW551984	-0.37358	0.00000133	stronglyHypometh	-0.028492	0.56577	insignificant	5	16	16
chr9	39411709	39413709	AW551984	-0.37358	0.00000133	stronglyHypometh	-0.028492	0.56577	insignificant	5	16	16
chr9	39800396	39802396	Olfr978		1	noCoverage	-0.082789	0.54907	insignificant	0	6	6
chr9	39857142	39859142	1700001J11Rik	0.051596	0.58329	insignificant	0.028381	0.033965	hypermethylated	7	13	14
chr9	39998900	40000900	Zfp202	-0.20862	3.24E-27	hypomethylated	-0.026002	0.0017487	hypomethylated	32	88	88
chr9	40075800	40077800	Scn3b	-0.28466	0.000016331	hypomethylated	-0.0078898	0.94766	insignificant	4	31	31
chr9	40263349	40265349	Gramd1b	-0.19519	5.74E-28	hypomethylated	-0.011424	0.17352	insignificant	19	70	72
chr9	40493046	40495046	9030425E11Rik	-0.13413	2.59E-11	hypomethylated	0.009253	0.93437	insignificant	23	73	80
chr9	40608355	40610355	Hspa8	-0.13359	5.45E-19	hypomethylated	0.0041233	0.10399	insignificant	37	130	134
chr9	40681209	40683209	Bsx	-0.32199	5.8E-16	hypomethylated	-0.0078209	0.56235	insignificant	7	26	26
chr9	40772195	40774195	4931429I11Rik	-0.0625	0.39087	insignificant	0.012962	0.61846	insignificant	11	28	28
chr9	40965577	40967577	Ubash3b	-0.20872	2.01E-20	hypomethylated	-0.022453	0.00016438	hypomethylated	19	50	54
chr9	41388421	41390421	2610203C20Rik	-0.28535	0.54918	insignificant	0.17414	0.62197	insignificant	1	12	12
chr9	41389008	41391008	Mir125b-1	-0.28535	0.54918	insignificant	0.17414	0.62197	insignificant	1	12	12
chr9	41932372	41934372	Sorl1	0.78058	0.44444	lowCoverage	0.086701	0.000094106	inconclusive	1	31	35
chr9	42072383	42074383	Sc5d	-0.12724	0.000035309	hypomethylated	-0.011127	0.0025821	hypomethylated	17	59	56
chr9	42280309	42282309	Tbcel	-0.12	0.034649	hypomethylated	0.024118	0.39911	insignificant	7	20	20
chr9	42752454	42754454	Grik4	-0.14117	2.94E-18	hypomethylated	-0.011387	0.0023258	hypomethylated	19	48	48
chr9	42913801	42915801	Arhgef12	-0.11625	1.61E-19	hypomethylated	-0.0060421	0.0014901	hypomethylated	51	128	128
chr9	42924653	42926653	Tmem136	0.25618	0.3634	insignificant	-0.044288	0.013371	hypomethylated	1	17	20
chr9	43013838	43015838	Pou2f3	-0.12764	0.14996	insignificant	0.019455	0.33884	insignificant	16	41	41
chr9	43047899	43049899	Oaf	-0.18983	0.012584	hypomethylated	0.094491	0.9188	insignificant	2	23	18
chr9	43117845	43119845	Trim29	0.22995	0.010192	hypermethylated	0.056513	0.11538	insignificant	3	30	30
chr9	43551658	43553658	Pvrl1	-0.11717	2.5E-37	hypomethylated	-0.00094619	0.58494	insignificant	47	187	187
chr9	43850466	43852466	Thy1	-0.22222	0.056168	insignificant	0.023466	0.61121	insignificant	4	14	14
chr9	43874103	43876103	Usp2	-0.18533	0.00016466	hypomethylated	0.011742	0.46683	insignificant	10	37	46
chr9	43892002	43894002	Usp2	-0.11787	5.19E-18	hypomethylated	0.0057347	0.39162	insignificant	24	86	92
chr9	43908852	43910852	Mfrp	-0.23128	0.16763	insignificant	-0.10122	0.31055	insignificant	4	8	11
chr9	43914327	43916327	C1qtnf5	-0.066357	0.40465	insignificant	0.033085	0.42647	insignificant	10	24	26
chr9	43914353	43916353	C1qtnf5	-0.066357	0.40465	insignificant	0.033085	0.42647	insignificant	10	24	26
chr9	43914391	43916391	C1qtnf5	-0.066357	0.40465	insignificant	0.033085	0.42647	insignificant	10	24	26
chr9	43921134	43923134	Rnf26	-0.18977	0.056196	insignificant	0.025874	0.27772	insignificant	12	85	91
chr9	43941740	43943740	Mcam	-0.13972	4.19E-14	hypomethylated	0.0084652	0.52336	insignificant	17	106	105
chr9	44042129	44044129	Cbl	-0.20093	2.18E-13	hypomethylated	-0.015399	0.046242	inconclusive	16	68	80
chr9	44059547	44061547	Pdzd3	0.028873	0.36416	insignificant	-0.013231	1	insignificant	8	22	22
chr9	44076270	44078270	Nlrx1	-0.43697	0.009843	stronglyHypometh	0.029197	0.13912	insignificant	4	29	29
chr9	44076574	44078574	Nlrx1		1	noCoverage	0.0087302	1	insignificant	0	12	12
chr9	44076682	44078682	Nlrx1		1	noCoverage	0.0087302	1	insignificant	0	12	12
chr9	44096327	44098327	Abcg4	-0.14614	0.58282	insignificant	0.026632	0.88656	insignificant	5	18	18
chr9	44113754	44115754	Hinfp	-0.26455	0.00038339	hypomethylated	0.018929	0.41307	insignificant	12	80	82
chr9	44128365	44130365	C2cd2l	-0.20232	1.5E-10	hypomethylated	-0.019284	0.91694	insignificant	7	12	12
chr9	44133927	44135927	Dpagt1	-0.20941	1.77E-20	hypomethylated	-0.0059324	0.088032	insignificant	19	62	62
chr9	44141797	44143797	H2afx	-0.12923	1.36E-63	hypomethylated	-0.00099094	0.15231	insignificant	51	148	160
chr9	44150464	44152464	Hmbs	-0.16705	6.2E-17	hypomethylated	0.008764	0.12645	insignificant	16	40	40
chr9	44169753	44171753	Vps11	-0.40546	0.000065917	stronglyHypometh	-0.059481	0.094013	insignificant	4	32	32
chr9	44186572	44188572	Hyou1	-0.12282	4.54E-31	hypomethylated	0.00074899	0.0007777	inconclusive	41	154	155
chr9	44205258	44207258	Slc37a4	-0.2326	0.00013213	hypomethylated	-0.11584	0.53602	insignificant	3	24	20
chr9	44214796	44216796	Rps25	-0.12723	0.000000112	hypomethylated	-0.0097887	1	insignificant	26	106	106
chr9	44215631	44217631	Trappc4	-0.1655	0.0028696	hypomethylated	-0.047642	0.3619	insignificant	6	49	49
chr9	44226090	44228090	Ccdc84	-0.36172	3.09E-17	stronglyHypometh	-0.03799	0.083001	insignificant	14	54	53
chr9	44248951	44250951	Foxr1	0.18481	1	insignificant	0.043093	0.1406	insignificant	2	22	24
chr9	44262850	44264850	Upk2		1	noCoverage	0.081752	0.37182	insignificant	0	3	7
chr9	44306218	44308218	Bcl9l	-0.18886	6.64E-11	hypomethylated	-0.0084639	0.53762	insignificant	13	46	46
chr9	44334504	44336504	Cxcr5		1	noCoverage	-0.12014	0.40389	insignificant	0	8	8
chr9	44411974	44413974	Ddx6	-0.11534	2.89E-23	hypomethylated	-0.01013	0.30887	insignificant	36	171	174
chr9	44543277	44545277	Phldb1		1	noCoverage	0.073314	0.28779	insignificant	0	15	16
chr9	44575891	44577891	Arcn1	-0.083462	7.88E-20	hypomethylated	0.017259	0.01244	inconclusive	7	22	22
chr9	44580091	44582091	Ift46	-0.1672	8.55E-21	hypomethylated	-0.036585	0.37622	insignificant	7	31	31
chr9	44607299	44609299	Tmem25	-0.10248	1.69E-13	hypomethylated	0.000055091	0.17037	insignificant	17	72	72
chr9	44689357	44691357	Mll1	-0.14741	1.71E-36	hypomethylated	-0.010699	0.025528	hypomethylated	35	112	121
chr9	44773683	44775683	Ube4a	-0.10758	0.00000215	hypomethylated	-0.0098426	0.46425	insignificant	6	25	24
chr9	44849426	44851426	Mpzl2	-0.21956	0.079227	insignificant	0.045573	0.3611	insignificant	3	25	28
chr9	44862270	44864270	Mpzl3	-0.25641	2.24E-12	hypomethylated	-0.000054025	0.16192	insignificant	12	40	40
chr9	44862299	44864299	Mpzl3	-0.25641	2.24E-12	hypomethylated	-0.000054025	0.16192	insignificant	12	40	40
chr9	44924958	44926958	Scn2b	0.23611	1	insignificant	-0.15675	0.12849	insignificant	1	6	6
chr9	44943689	44945689	Gm10684	0.010169	1	insignificant	-0.0068336	0.84857	insignificant	1	5	5
chr9	44946124	44948124	Scn4b	-0.14935	5.64E-15	hypomethylated	-0.011515	0.11213	insignificant	18	52	52
chr9	45077229	45079229	Il10ra		1	noCoverage	-0.02623	0.4393	insignificant	0	15	15
chr9	45126182	45128182	Tmprss13	-0.4183	0.0013006	stronglyHypometh	-0.064973	0.24757	insignificant	2	18	14

chr9	45177267	45179267	Fxyd6	-0.22371	3.97E-11	hypomethylated	-0.013235	0.03216	inconclusive	20	70	70
chr9	45213159	45215159	Fxyd2	-0.14177	0.54746	insignificant	0.066162	1	insignificant	2	9	10
chr9	45237375	45239375	Dscaml1	-0.14956	1.87E-12	hypomethylated	0.0033586	0.36093	insignificant	20	114	112
chr9	45636721	45638721	Cep164		1	noCoverage	-0.055861	0.012667	inconclusive	0	14	14
chr9	45645611	45647611	Bace1	-0.22387	0.000000321	hypomethylated	0.014621	0.7738	insignificant	14	89	89
chr9	45713651	45715651	Pcsk7	-0.10808	8.93E-44	hypomethylated	-0.0014788	0.66299	insignificant	56	188	190
chr9	45714960	45716960	Pcsk7	-0.12931	5.27E-12	hypomethylated	-0.0095504	0.11385	insignificant	14	40	40
chr9	45744141	45746141	Tagln	-0.24813	0.00019116	hypomethylated	0.053488	0.11121	insignificant	9	20	19
chr9	45792954	45794954	Pafah1b2	-0.15437	1	insignificant	0.017332	0.92289	insignificant	4	21	24
chr9	45819902	45821902	Sik3	-0.10201	4.95E-22	hypomethylated	0.0031667	0.94183	insignificant	50	105	110
chr9	46047926	46049926	Apoa4	-0.030357	0.073865	insignificant	-0.056696	0.012075	hypomethylated	4	8	8
chr9	46075690	46077690	Apoa5		1	noCoverage	-0.022523	0.81843	insignificant	0	2	2
chr9	46080146	46082146	Zfp259	-0.13414	4.32E-14	hypomethylated	-0.0067245	0.60206	insignificant	20	48	46
chr9	46090094	46092094	Bud13	-0.10472	1.78E-20	hypomethylated	-0.0081881	0.4118	insignificant	15	40	42
chr9	47337434	47339434	Cadm1	-0.10865	7.81E-29	hypomethylated	0.011571	0.26276	insignificant	58	243	223
chr9	48288716	48290716	Rexo2	-0.024283	0.36201	insignificant	0.082568	0.9478	insignificant	7	19	21
chr9	48302449	48304449	Gm5617	-0.1518	0.000000867	hypomethylated	-0.007811	0.00049966	hypomethylated	26	101	101
chr9	48303435	48305435	Rbm7	-0.14472	0.000023725	hypomethylated	-0.0054159	0.0005404	hypomethylated	23	91	91
chr9	48644050	48646050	Zbtb16	-0.1482	2.12E-14	hypomethylated	-0.0035025	0.30207	insignificant	21	74	82
chr9	48719204	48721204	Htr3a		1	noCoverage	0.067949	1	insignificant	0	6	6
chr9	48792489	48794489	Usp28	-0.10349	1.46E-23	hypomethylated	-0.0068991	0.16206	insignificant	40	157	158
chr9	48862736	48864736	Zw10	-0.16637	0.000020308	hypomethylated	0.014564	0.93685	insignificant	11	57	59
chr9	48909883	48911883	Tmprss5	-0.086508	0.4814	insignificant	-0.025611	0.79304	insignificant	1	6	6
chr9	49147766	49149766	Drd2	-0.13774	7.54E-13	hypomethylated	0.043353	0.59571	insignificant	19	84	83
chr9	49294330	49296330	Ttc12		1	noCoverage	-0.20989	0.7391	insignificant	0	4	8
chr9	49607174	49609174	Ncam1	-0.21411	0.00027873	hypomethylated	-0.035319	0.883	insignificant	4	20	20
chr9	50336746	50338746	Pts	-0.15341	0.00000205	hypomethylated	-0.018381	0.40061	insignificant	10	38	32
chr9	50369373	50371373	Tex12	0.075188	1	lowCoverage	-0.12377	0.72102	insignificant	1	4	4
chr9	50411005	50413005	Timm8b	-0.17017	1.18E-22	hypomethylated	0.090951	0.71576	insignificant	19	85	86
chr9	50411954	50413954	Sdhd	-0.10313	1.7E-12	hypomethylated	0.24115	0.037309	hypermethylated	10	35	36
chr9	50424425	50426425	Pih1d2	-0.12198	1.15E-12	hypomethylated	-0.0019151	0.0040328	hypomethylated	25	97	109
chr9	50467885	50469885	Dlat	-0.19214	0.032643	hypomethylated	-0.0058969	0.16101	insignificant	3	32	32
chr9	50536089	50538089	Dixdc1	-0.11364	0.0026113	hypomethylated	-0.04846	0.066023	insignificant	10	28	36
chr9	50554626	50556626	2310030G06Rik	-0.16431	0.055966	insignificant	0.077848	0.22097	insignificant	2	18	17
chr9	50559862	50561862	Cryab	-0.081782	0.00000147	hypomethylated	-0.0013516	0.50839	insignificant	7	28	23
chr9	50560459	50562459	Hspb2	-0.13645	9.41E-13	hypomethylated	-0.017993	0.2784	insignificant	8	30	25
chr9	50575342	50577342	Fdxacb1	-0.15012	5.03E-12	hypomethylated	-0.010306	0.18062	insignificant	16	82	84
chr9	50576257	50578257	1110032A03Rik	-0.13105	7.75E-10	hypomethylated	0.004957	0.25794	insignificant	14	76	78
chr9	50582434	50584434	Alg9	-0.13367	3.15E-21	hypomethylated	0.0011379	0.42327	insignificant	38	104	108
chr9	50664039	50666039	Ppp2r1b	-0.13398	3.18E-24	hypomethylated	-0.0065472	0.021214	hypomethylated	26	115	104
chr9	50817178	50819178	Sik2	-0.14613	0.00028718	hypomethylated	-0.0073182	0.80574	insignificant	20	45	45
chr9	50885199	50887199	Layn	-0.625	0.024323	stronglyHypometh	-0.043497	0.31299	insignificant	2	14	14
chr9	50910183	50912183	4833427G06Rik	-0.14641	4.3E-09	hypomethylated	-0.030725	0.11569	insignificant	18	75	89
chr9	50911215	50913215	Mir34c	-0.17491	2.61E-10	hypomethylated	-0.033374	0.13328	insignificant	18	79	90
chr9	50911750	50913750	Mir34c	-0.20086	1.59E-08	hypomethylated	-0.060805	0.03167	hypomethylated	9	50	64
chr9	51086659	51088659	Gm684	0.082419	0.00018746	inconclusive	0.019955	0.00028807	hypermethylated	17	54	48
chr9	51572456	51574456	Arhgap20	-0.088549	1.63E-16	hypomethylated	-0.012552	0.95857	insignificant	56	220	220
chr9	51771638	51773638	Fdx1	-0.29734	0.000000194	hypomethylated	-0.038949	0.90643	insignificant	1	2	2
chr9	51854254	51856254	Rdx	-0.1276	0.0037942	hypomethylated	-0.0053572	1	insignificant	5	20	20
chr9	51855045	51857045	Rdx	-0.076058	4.76E-30	hypomethylated	-0.0062669	0.35467	insignificant	60	217	217
chr9	51856810	51858810	Rdx	-0.082881	1.17E-08	hypomethylated	-0.00085216	0.18549	insignificant	18	55	52
chr9	51976216	51978216	Zc3h12c	-0.11312	4.63E-14	hypomethylated	-0.0082052	0.00095349	hypomethylated	34	126	128
chr9	53056217	53058217	Ddx10	-0.34501	0.57772	insignificant	0.018695	0.58351	insignificant	2	21	20
chr9	53108774	53110774	Exph5		1	noCoverage	0.06002	0.43048	insignificant	0	12	12
chr9	53191127	53193127	Kdelc2	-0.13205	1.5E-15	hypomethylated	-0.010863	0.057348	insignificant	22	84	84
chr9	53212390	53214390	4930550C14Rik	0.020738	0.61663	insignificant	0.034543	0.056327	insignificant	2	8	8
chr9	53344151	53346151	Npat	-0.11951	0.00000263	hypomethylated	-0.015108	0.032187	hypomethylated	20	121	113
chr9	53344776	53346776	Npat	-0.10935	0.000022196	hypomethylated	-0.019492	0.13666	insignificant	18	103	102
chr9	53475612	53477612	Cul5	-0.086704	0.39639	insignificant	0.0034951	1	insignificant	4	15	14
chr9	53618341	53620341	Slc35f2	-0.083862	0.022199	hypomethylated	-0.018216	0.93205	insignificant	14	52	60
chr9	53697057	53699057	Sln		1	noCoverage	-0.20204	0.05132	insignificant	0	9	7
chr9	53823108	53825108	Elmod1	-0.11349	1	insignificant	0.031847	0.94257	insignificant	4	28	28
chr9	53916218	53918218	Tnfaip8l3	-0.0875	0.23056	insignificant	-0.0032107	0.91365	insignificant	5	14	16
chr9	54133292	54135292	Gldn	-0.11627	0.61802	insignificant	-0.045035	0.032175	hypomethylated	7	51	56
chr9	54349433	54351433	Dmxl2	-0.14301	4.27E-19	hypomethylated	-0.025155	1.59E-08	hypomethylated	38	130	119
chr9	54385790	54387790	Sh2d7	-0.091453	1	insignificant	-0.10316	0.36925	insignificant	3	13	8
chr9	54407886	54409886	Cib2	-0.16276	0.027726	hypomethylated	-0.026685	0.15394	insignificant	10	38	38
chr9	54433317	54435317	Idh3a	-0.070786	1.1E-16	hypomethylated	-0.0049081	0.17271	insignificant	22	91	101
chr9	54546365	54548365	Dnaja4	-0.13135	7.84E-31	hypomethylated	-0.012629	0.01521	hypomethylated	39	115	120
chr9	54582301	54584301	Wdr61	-0.17424	0.029219	hypomethylated	-0.043617	0.39639	insignificant	5	24	21
chr9	54582317	54584317	Wdr61	-0.17424	0.029219	hypomethylated	-0.043617	0.39639	insignificant	5	24	21
chr9	54582335	54584335	Wdr61	-0.17424	0.029219	hypomethylated	-0.043617	0.39639	insignificant	5	24	21
chr9	54611614	54613614	Crabp1	-0.1559	0.0034805	hypomethylated	0.033949	0.18292	insignificant	7	40	34
chr9	54710561	54712561	Ireb2	-0.12222	9.62E-26	hypomethylated	-0.026237	0.000060964	hypomethylated	30	88	88
chr9	54764096	54766096	Agphd1	-0.14478	0.0000058	hypomethylated	-0.0064364	0.1831	insignificant	6	28	28
chr9	54797665	54799665	Psma4	-0.17962	2.51E-17	hypomethylated	0.030477	0.12263	insignificant	26	82	84
chr9	54798761	54800761	AY074887	-0.081007	0.52895	insignificant	0.021429	0.3136	insignificant	27	65	66
chr9	54827686	54829686	Chrna5	-0.17611	0.0021911	hypomethylated	0.004398	0.33406	insignificant	14	92	93
chr9	54874366	54876366	Chrna3		1	noCoverage	-0.1062	0.21209	insignificant	0	18	16
chr9	54896351	54898351	Chrnb4	-0.11062	1.39E-10	hypomethylated	-0.054877	0.017502	hypomethylated	3	21	21
chr9	54996175	54998175	Ube2q2	-0.087963	1.23E-36	hypomethylated	-0.011066	0.18851	insignificant	62	241	245
chr9	55055741	55057741	Fbxo22	-0.16052	4.66E-17	hypomethylated	0.062712	0.0035356	hypermethylated	20	44	59
chr9	55173755	55175755	AI118078	-0.13037	8.37E-31	hypomethylated	-0.013508	0.011463	hypomethylated	21	68	69
chr9	55387955	55389955	Isl2	-0.16709	9.23E-17	hypomethylated	-0.0040128	0.17375	insignificant	29	101	107
chr9	55874442	55876442	Gm7444	-0.1457	0.1289	insignificant	-0.10572	0.0017343	hypomethylated	4	10	7

chr9	55888651	55890651	Rcn2	-0.1077	0.00000279	hypomethylated	0.0065131	0.40307	insignificant	13	62	62
chr9	55936782	55938782	Pstpip1	-0.27658	1.55E-08	hypomethylated	0.0086786	0.097146	insignificant	12	42	42
chr9	56265652	56267652	Hmg20a	-0.12778	0.0065912	hypomethylated	-0.0033867	0.91441	insignificant	12	50	48
chr9	56265857	56267857	C230081A13Rik	-0.13849	0.039746	hypomethylated	-0.0080389	0.084252	insignificant	8	34	32
chr9	56699770	56701770	Odf3l1		1	noCoverage	-0.14242	0.28958	insignificant	0	6	6
chr9	56711910	56713910	Cspg4	-0.3297	0.000000912	hypomethylated	-0.024269	0.63755	insignificant	7	34	34
chr9	56776178	56778178	Snx33	-0.077646	0.24874	insignificant	-0.0050315	0.47944	insignificant	5	28	32
chr9	56784306	56786306	Imp3	-0.088751	0.00031865	hypomethylated	-0.0099518	0.76611	insignificant	15	63	66
chr9	56797730	56799730	Snupn	-0.1393	3.53E-14	hypomethylated	0.028686	0.74866	insignificant	12	59	60
chr9	56841774	56843774	Ptpn9	-0.12518	1.33E-14	hypomethylated	-0.0081108	0.02027	hypomethylated	51	149	148
chr9	56918846	56920846	Sin3a	-0.15665	7.33E-16	hypomethylated	-0.011616	0.057309	insignificant	12	96	80
chr9	56923182	56925182	Sin3a	-0.11466	1.97E-12	hypomethylated	-0.011384	0.016008	hypomethylated	25	172	186
chr9	56977583	56979583	Man2c1	-0.14543	7.72E-10	hypomethylated	0.015565	0.77269	insignificant	16	56	55
chr9	56994841	56996841	Neil1	-0.23084	8.25E-17	hypomethylated	-0.032735	0.22387	insignificant	6	34	28
chr9	57006106	57008106	Commd4	-0.2582	1	insignificant	0.016581	0.31519	insignificant	5	18	18
chr9	57110406	57112406	1700017B05Rik	-0.2282	0.0083807	hypomethylated	0.02567	0.41204	insignificant	7	28	28
chr9	57315831	57317831	Scamp5	-0.121	0.000215	hypomethylated	0.0086823	0.18411	insignificant	14	46	48
chr9	57350908	57352908	Rpp25	-0.14372	1.05E-28	hypomethylated	-0.0055103	1	insignificant	28	112	115
chr9	57368038	57370038	Cox5a	-0.097294	1.98E-35	hypomethylated	-0.014014	0.26836	insignificant	42	130	140
chr9	57384334	57386334	2310046O06Rik	-0.061624	0.00000002	hypomethylated	0.009014	0.020732	inconclusive	21	86	80
chr9	57407750	57409750	Scamp2	-0.19249	0.36367	insignificant	-0.043636	0.67705	insignificant	4	48	53
chr9	57436258	57438258	Ulk3	-0.1697	0.00058654	hypomethylated	-0.0012856	0.5493	insignificant	12	48	47
chr9	57454088	57456088	Lman1l		1	noCoverage	-0.045833	1	insignificant	0	4	4
chr9	57468581	57470581	Lman1l	-0.19198	0.47404	insignificant	0.0037037	0.2823	insignificant	2	18	18
chr9	57492987	57494987	Csk	-0.18426	2.25E-33	hypomethylated	-0.006213	0.024654	hypomethylated	42	78	78
chr9	57544419	57546419	Cyp1a1	-0.48932	1.47E-08	stronglyHypometh	-0.0072505	0.23177	insignificant	2	10	10
chr9	57555375	57557375	Edc3	-0.33599	0.000000154	stronglyHypometh	-0.019453	0.26248	insignificant	9	47	52
chr9	57613667	57615667	Clk3		1	noCoverage	-0.045812	0.50724	insignificant	0	14	12
chr9	57682041	57684041	Arid3b	-0.24857	2.24E-12	hypomethylated	-0.18928	0.00000073	hypomethylated	8	17	16
chr9	57757792	57759792	Ubl7	-0.2177	4.38E-16	hypomethylated	-0.043801	0.14423	insignificant	22	50	47
chr9	57786941	57788941	Sema7a	-0.15549	8.12E-57	hypomethylated	-0.0047311	0.000031947	hypomethylated	52	144	154
chr9	57950032	57952032	Ccdc33	-0.19299	0.0064232	hypomethylated	0.041859	0.098162	insignificant	4	8	8
chr9	57975894	57977894	Stra6	-0.030941	0.00077214	hypomethylated	0.071006	0.37192	insignificant	5	34	37
chr9	57976155	57978155	Stra6	0.031293	0.00331	inconclusive	0.083033	0.29065	insignificant	5	22	25
chr9	57981471	57983471	Stra6	-0.47618	3.66E-10	stronglyHypometh	0.017659	0.91295	insignificant	3	6	6
chr9	58050092	58052092	Islr2	-0.15	0.02788	hypomethylated	0.11398	0.76196	insignificant	3	6	7
chr9	58050151	58052151	Islr2	-0.125	0.029218	hypomethylated	0.10369	0.56632	insignificant	5	10	11
chr9	58050341	58052341	Islr2	-0.11838	0.022251	hypomethylated	0.061197	0.41402	insignificant	5	20	21
chr9	58052126	58054126	1600029O15Rik	-0.14318	0.23013	insignificant	0.020894	0.76727	insignificant	3	24	24
chr9	58097593	58099593	Pml	-0.26786	0.56437	insignificant	-0.017857	0.83948	insignificant	3	8	8
chr9	58099970	58101970	Stoml1	-0.32086	1.13E-11	hypomethylated	-0.094885	0.91428	insignificant	8	28	28
chr9	58161019	58163019	Loxl1		1	noCoverage	-0.14688	0.5881	insignificant	0	20	19
chr9	58218176	58220176	Tbc1d21	0.24986	0.33968	insignificant	0.026501	0.52831	insignificant	2	9	9
chr9	58335409	58337409	6030419C18Rik	-0.13232	6.37E-53	hypomethylated	-0.012113	0.5968	insignificant	71	181	181
chr9	58388747	58390747	Cd276	-0.32222	0.016929	hypomethylated	0.028393	1	insignificant	5	10	10
chr9	58429046	58431046	Nptn	-0.12533	2.02E-21	hypomethylated	-0.014938	0.43093	insignificant	26	76	75
chr9	58670318	58672318	Hcn4	-0.1058	1.15E-45	hypomethylated	-0.0059441	0.90382	insignificant	84	247	234
chr9	58884248	58886248	Neo1	-0.093379	3.26E-20	hypomethylated	0.018029	0.034412	hypermethylated	45	178	154
chr9	59138378	59140378	Adpgk	-0.14857	4.05E-27	hypomethylated	-0.0078205	0.37553	insignificant	40	89	80
chr9	59201315	59203315	Bbs4	-0.65	0.094286	insignificant	0.018014	0.90651	insignificant	1	12	14
chr9	59334181	59336181	Arih1	-0.095269	0.20091	insignificant	-0.0077896	0.15402	insignificant	24	100	105
chr9	59386473	59388473	Hexa	-0.16055	5.34E-25	hypomethylated	-0.0047539	0.021493	hypomethylated	26	59	64
chr9	59425143	59427143	Celf6	-0.097465	0.015751	hypomethylated	0.012355	0.12914	insignificant	19	88	95
chr9	59464090	59466090	Parp6	-0.053159	0.038638	hypomethylated	0.0082671	0.085651	insignificant	21	86	84
chr9	59503414	59505414	Pkm2	-0.15087	2.5E-15	hypomethylated	-0.013014	0.22508	insignificant	13	79	79
chr9	59554570	59556570	Gramd2	-0.35714	0.00000757	stronglyHypometh	-0.19583	0.00070144	hypomethylated	4	13	15
chr9	59597980	59599980	Myo9a	-0.11978	0.000000356	hypomethylated	-0.0083299	0.83322	insignificant	22	118	125
chr9	59598456	59600456	Senp8	-0.11205	0.00000141	hypomethylated	-0.0078414	0.80231	insignificant	21	116	123
chr9	59797886	59799886	Nr2e3	0.13654	0.21959	insignificant	0.10321	0.0027954	hypermethylated	4	10	10
chr9	59798100	59800100	Gm7616	0.13654	0.21959	insignificant	0.10321	0.0027954	hypermethylated	4	10	10
chr9	60358842	60360842	Thsd4	-0.11185	0.48623	insignificant	-0.093447	0.004209	hypomethylated	1	14	12
chr9	60535341	60537341	Lrrc49	-0.21727	0.0029025	hypomethylated	0.002791	0.94156	insignificant	11	29	33
chr9	60535941	60537941	Lrrc49	-0.28185	0.58652	insignificant	0.073901	0.73822	insignificant	2	7	10
chr9	60559927	60561927	Larp6	-0.09377	0.000037722	hypomethylated	-0.0029347	0.0071399	hypomethylated	22	98	98
chr9	60641354	60643354	Uaca	-0.202	1.08E-23	hypomethylated	0.0018826	0.86561	insignificant	15	61	54
chr9	61219172	61221172	Tle3	-0.1174	1E-19	hypomethylated	0.0033624	0.71026	insignificant	44	137	142
chr9	61762317	61764317	Rplp1	-0.15952	0.0044015	hypomethylated	-0.010223	0.49513	insignificant	9	52	53
chr9	61794606	61796606	Kif23	-0.17194	0.0000725	hypomethylated	-0.024267	0.10728	insignificant	12	33	34
chr9	61874597	61876597	Paqr5	0.2751	0.36818	insignificant	0.006655	0.17871	insignificant	1	18	18
chr9	62129986	62131986	Spesp1	-0.37794	9.95E-11	stronglyHypometh	-0.098859	0.019845	hypomethylated	3	14	14
chr9	62188149	62190149	Anp32a	-0.06976	9.66E-11	hypomethylated	-0.004985	0.56405	insignificant	21	136	150
chr9	62384851	62386851	Coro2b	-0.5229	0.0043322	stronglyHypometh	-0.26113	0.0045962	hypomethylated	1	8	8
chr9	62524661	62526661	Itga11	-0.36429	0.16335	insignificant	-0.075626	0.91971	insignificant	3	14	18
chr9	62659455	62661455	Fem1b	-0.12261	1.08E-18	hypomethylated	-0.0035497	0.020304	hypomethylated	31	86	86
chr9	62685593	62687593	Cln6	-0.13153	0.0022719	hypomethylated	-0.019227	0.015761	hypomethylated	14	64	70
chr9	62704910	62706910	Calml4	-0.39712	4.29E-09	stronglyHypometh	-0.0013439	0.018735	inconclusive	8	20	20
chr9	62828686	62830686	Pias1	-0.12805	0.00000691	hypomethylated	-0.0019559	0.21494	insignificant	8	27	32
chr9	62994787	62996787	Skor1	-0.13906	4.23E-16	hypomethylated	-0.014207	0.0014877	hypomethylated	32	112	112
chr9	62994818	62996818	Skor1	-0.12875	2.87E-13	hypomethylated	-0.016336	0.00052104	hypomethylated	31	114	114
chr9	62996768	62998768	Skor1	-0.0887	1.07E-17	hypomethylated	0.0022437	0.85097	insignificant	23	118	122
chr9	63225659	63227659	Map2k5	-0.11458	0.23784	insignificant	-0.017949	0.50067	insignificant	4	12	12
chr9	63247051	63249051	2300009A05Rik	-0.45595	0.25086	insignificant	-0.27933	0.019217	hypomethylated	1	6	18
chr9	63449461	63451461	Aagab	-0.16213	1.51E-41	hypomethylated	-0.0041755	0.15746	insignificant	36	104	104
chr9	63450255	63452255	Iqch	-0.092263	2.44E-31	hypomethylated	-0.0041876	0.7052	insignificant	34	88	88
chr9	63605801	63607801	Smad3	-0.18906	3.59E-29	hypomethylated	0.031548	0.022226	inconclusive	24	81	72

chr9	63869866	63871866	Smad6	-0.17096	0.014332	hypomethylated	-0.019683	0.000027434	hypomethylated	20	62	62
chr9	64020193	64022193	Rpl4	-0.15908	0.000000206	hypomethylated	-0.0079763	0.91386	insignificant	14	80	80
chr9	64020738	64022738	Zwilch	-0.15437	0.000049157	hypomethylated	0.046655	0.034725	inconclusive	6	38	41
chr9	64026103	64028103	Snapc5	-0.076067	0.0029646	hypomethylated	-0.00057263	0.585	insignificant	10	52	52
chr9	64128413	64130413	Tipin	-0.17704	0.0004555	hypomethylated	0.026555	0.40809	insignificant	4	85	85
chr9	64188264	64190264	Dis3l	-0.16453	2.32E-35	hypomethylated	0.0037591	0.21051	insignificant	34	120	99
chr9	64188580	64190580	Dis3l	-0.1782	8.74E-29	hypomethylated	0.0050448	0.29339	insignificant	28	98	77
chr9	64189064	64191064	Dis3l	-0.26597	1.38E-12	hypomethylated	-0.0007905	0.80507	insignificant	7	34	27
chr9	64232432	64234432	Megf11	-0.13704	9.88E-20	hypomethylated	-0.0080612	0.54537	insignificant	41	139	138
chr9	64585563	64587563	Rab11a	-0.34482	3.87E-08	stronglyHypometh	-0.081672	0.1494	insignificant	6	14	14
chr9	64657817	64659817	Dennd4a	-0.12454	4.41E-17	hypomethylated	-0.0069212	0.66173	insignificant	41	114	114
chr9	64799414	64801414	Slc24a1	-0.15542	0.44541	insignificant	0.052182	0.84914	insignificant	9	18	18
chr9	64807638	64809638	2010321M09Ril	-0.15574	1.06E-26	hypomethylated	-0.01512	0.020035	hypomethylated	17	46	48
chr9	64807743	64809743	2010321M09Ril	-0.15574	1.06E-26	hypomethylated	-0.01512	0.020035	hypomethylated	17	46	48
chr9	64879264	64881264	Dpp8	-0.14603	1.59E-10	hypomethylated	-0.0069596	0.63521	insignificant	32	90	90
chr9	64948301	64950301	Igdcc4	-0.26154	1.05E-14	hypomethylated	-0.075381	0.022394	hypomethylated	20	50	57
chr9	64987995	64989995	Igdcc3	-0.097684	5.38E-17	hypomethylated	0.0057677	0.56892	insignificant	42	161	162
chr9	65061496	65063496	Parp16	-0.10455	1.58E-16	hypomethylated	0.028563	0.23881	insignificant	31	104	107
chr9	65141101	65143101	Clpx	-0.14617	0.00000159	hypomethylated	-0.050334	0.00000284	hypomethylated	26	83	81
chr9	65192874	65194874	Pdcd7	-0.097004	1.68E-17	hypomethylated	0.019587	0.1292	insignificant	39	118	125
chr9	65207866	65209866	Gm514	0.37143	1	insignificant	0.27341	0.34283	insignificant	2	7	7
chr9	65245294	65247294	Rasl12	-0.15636	1	insignificant	0.017447	0.6637	insignificant	5	14	14
chr9	65282588	65284588	Mtfmt	-0.17559	2.09E-19	hypomethylated	0.018388	0.10297	insignificant	15	55	50
chr9	65307743	65309743	Spg21	-0.11628	0.065057	insignificant	-0.013391	0.85159	insignificant	8	48	42
chr9	65427841	65429841	Plekho2	-0.20831	6.75E-12	hypomethylated	0.037687	0.29111	insignificant	16	49	53
chr9	65434011	65436011	Pif1	-0.17707	1.56E-11	hypomethylated	-0.013215	0.70899	insignificant	23	78	78
chr9	65477388	65479388	Rbpms2	-0.12073	2.02E-23	hypomethylated	0.003708	0.43792	insignificant	48	198	180
chr9	65523354	65525354	Oaz2-ps	-0.18836	0.01268	hypomethylated	-0.0056892	0.59857	insignificant	7	71	62
chr9	65675371	65677371	Zfp609	-0.10098	9.1E-13	hypomethylated	-0.011873	0.77892	insignificant	13	66	66
chr9	65737129	65739129	2810417H13Rik	-0.086311	0.00021437	hypomethylated	0.027481	0.76134	insignificant	4	24	21
chr9	65755816	65757816	Csnk1g1	-0.18956	1.46E-19	hypomethylated	0.0082002	0.0060045	inconclusive	18	82	90
chr9	65756601	65758601	Trip4	-0.15124	2.22E-16	hypomethylated	0.010021	0.13072	insignificant	18	77	82
chr9	65906975	65908975	Ppib	-0.096086	1.53E-14	hypomethylated	0.0045123	0.92962	insignificant	30	90	90
chr9	65917538	65919538	Snx22	-0.27601	0.00056747	hypomethylated	-0.029646	0.050713	insignificant	4	12	12
chr9	65972693	65974693	Snx1	-0.0025345	0.62345	insignificant	0.040044	0.88242	insignificant	3	44	38
chr9	65973417	65975417	Fam96a	-0.16475	0.58222	insignificant	0.057981	0.26515	insignificant	2	34	28
chr9	66005032	66007032	Dapk2	-0.119	1.62E-31	hypomethylated	0.0020357	1.08E-09	inconclusive	30	78	74
chr9	66197256	66199256	Herc1	-0.11385	1.54E-22	hypomethylated	-0.0043518	0.26737	insignificant	28	96	107
chr9	66440787	66442787	Usp3	-0.15756	1.06E-18	hypomethylated	-0.0099646	0.22862	insignificant	17	48	46
chr9	66560492	66562492	Car12	-0.40277	0.52416	insignificant	-0.11981	0.016326	hypomethylated	2	10	10
chr9	66643230	66645230	Aph1b	-0.10678	0.036621	hypomethylated	-0.0045049	0.15336	insignificant	10	62	59
chr9	66682513	66684513	Aph1c	-0.16354	9.07E-13	hypomethylated	0.01567	0.57229	insignificant	6	42	42
chr9	66792924	66794924	Rps27l	-0.12618	1.17E-12	hypomethylated	-0.011869	0.86228	insignificant	17	77	84
chr9	66891678	66893678	Tpm1	-0.12648	3.33E-17	hypomethylated	-0.024656	2.8E-14	hypomethylated	11	46	46
chr9	66897020	66899020	Tpm1	-0.11988	0.017602	hypomethylated	-0.013194	0.92172	insignificant	20	58	58
chr9	67084533	67086533	Mir190		1	noCoverage		1	noCoverage	0	6	0
chr9	67606243	67608243	C2cd4b	-0.1562	2.49E-41	hypomethylated	-0.017603	0.12092	insignificant	37	144	145
chr9	67680137	67682137	C2cd4a	-0.5	0.024656	stronglyHypometh	0.045455	0.093027	insignificant	1	2	4
chr9	67687202	67689202	Vps13c	-0.19196	0.030283	hypomethylated	-0.0091866	0.57993	insignificant	5	42	42
chr9	68500608	68502608	Rora	-0.14279	1.82E-20	hypomethylated	0.0036413	0.065637	insignificant	16	53	69
chr9	69244804	69246804	Narg2	-0.049211	5.49E-17	hypomethylated	-0.013066	0.16507	insignificant	15	40	40
chr9	69300489	69302489	Anxa2	-0.14496	0.000000685	hypomethylated	0.030713	0.94001	insignificant	12	37	33
chr9	69608747	69610747	Foxb1	-0.21498	0.00000186	hypomethylated	-0.032823	1	insignificant	7	26	26
chr9	69836307	69838307	Bnip2	-0.15391	5.32E-23	hypomethylated	-0.008768	0.13563	insignificant	35	99	94
[truncated: 119,662 more chars]
