# Supplementary material for: Dnmt1 links BCR-ABLp210 to epigenetic tumor stem cell priming in myeloid leukemia
Source: Leukemia. 2018 Jun 28;33(1):249–78. doi: 10.1038/s41375-018-0192-z (PMC6326950; doi:10.1038/s41375-018-0192-z)
Supplement: Supplementary file 5 — Table S4 [file 41375_2018_192_MOESM5_ESM.pdf]

Table S4: RRBS methylation changes in bone marrow of old Sca1-Dnmt1 mice compared to wild-typ

Samples name:		
	zr151_8	Bone Marrow old Sca1-Dnmt1
	zr322_2	Bone Marrow old WT
	zr322_4	Bone Marrow old Sca1-Dnmt1

chrom	start	end	gene_name	methDiff (zr151_8.RRBS vs zr322_2)	pValue (zr151_8 vs zr322_2)	pvalClass (zr151_8 vs zr322_2)	methDiff (zr322_2 vs zr322_4)	pValue (zr322_2 vs zr322_4)	pvalClass (zr322_2 vs zr322_4)	Number of CpGs zr151_8	Number of CpGs zr322_2	Number of CpGs zr322_4
chr1	3661579	3663579	Xkr4	-0.08382368	2.41E-14	hypomethylated	-0.012333	0.10666	insignificant	20	89	89
chr1	4486494	4488494	Sox17	-0.20199147	0.0016132	hypomethylated	0.0023461	0.77926	insignificant	7	54	46
chr1	4796973	4798973	Lypla1	-0.23558779	4.4E-31	hypomethylated	-0.098633	0.23425	insignificant	30	85	66
chr1	4846774	4848774	Tcea1	-0.12733839	1.08E-20	hypomethylated	0.031134	0.20408	insignificant	29	120	103
chr1	4847408	4849408	Tcea1	-0.10881817	4.42E-20	hypomethylated	0.021275	0.40171	insignificant	29	116	99
chr1	5009507	5011507	Rgs20	-0.13626318	7.84E-17	hypomethylated	0.016335	0.68906	insignificant	21	93	87
chr1	5072253	5074253	Atp6v1h	-0.15463345	0.016382	hypomethylated	0.0094433	0.63467	insignificant	14	45	53
chr1	5577573	5579573	Oprk1	0.06620408	0.02032	hypermethylated	0.0069347	0.84507	insignificant	5	16	19
chr1	5907479	5909479	Npbwr1	0.27676768	0.13998	insignificant	0.17248	0.28967	insignificant	2	9	4
chr1	6203742	6205742	Rb1cc1	-0.11099187	5.56E-39	hypomethylated	-0.00043402	0.50225	insignificant	37	176	145
chr1	6348411	6350411	Fam150a	-0.25087242	3.11E-17	hypomethylated	-0.030425	0.18196	insignificant	15	57	55
chr1	7078000	7080000	Pcmtd1	-0.08450915	7.68E-18	hypomethylated	-0.0044739	0.39513	insignificant	23	78	78
chr1	9289811	9291811	Sntg1	-0.11112364	0.072341	insignificant	0.061209	0.0008709	hypermethylated	7	33	34
chr1	9534488	9536488	Rrs1	-0.19085598	1.28E-45	hypomethylated	0.0097822	0.85832	insignificant	39	158	154
chr1	9537126	9539126	Adhfe1	-0.26005172	0.0046139	hypomethylated	-0.068378	0.53419	insignificant	3	30	26
chr1	9590347	9592347	3110035E14Ri	-0.19087847	0.23618	insignificant	0.049744	0.0022735	hypermethylated	2	30	29
chr1	9690290	9692290	Mybl1	-0.12419443	0.00000004	hypomethylated	-0.037341	0.90064	insignificant	9	47	74
chr1	9738463	9740463	1700034P13Ri	-0.09974639	1	insignificant	-0.014693	0.94537	insignificant	1	34	30
chr1	9787210	9789210	Sgk3	-0.09278795	1.59E-15	hypomethylated	0.0015435	0.22705	insignificant	48	161	151
chr1	9837361	9839361	Sgk3	-0.11984979	1.05E-11	hypomethylated	-0.02823	0.31504	insignificant	12	39	30
chr1	9897718	9899718	6030422M02F	0.2167575	0.0072827	hypermethylated	-0.0052607	0.49163	insignificant	6	34	34
chr1	9932624	9934624	Snord87	-0.09745417	1.44E-11	hypomethylated	0.011596	0.56226	insignificant	20	66	66
chr1	9934199	9936199	Snhg6	-0.26069818	5.31E-12	hypomethylated	0.040345	0.82802	insignificant	6	17	17
chr1	9999201	10001201	Ppp1r42	0.18939394	1	lowCoverage	0.12689	0.12748	insignificant	1	11	4
chr1	9999211	10001211	Ppp1r42	0.18939394	1	lowCoverage	0.12689	0.12748	insignificant	1	11	4
chr1	10027298	10029298	Cspp1	-0.17362098	1.15E-13	hypomethylated	0.0030768	0.25665	insignificant	25	100	99
chr1	10027979	10029979	Cops5	-0.13823582	2.54E-09	hypomethylated	0.0031373	1	insignificant	37	113	116
chr1	10222751	10224751	Arfgef1	-0.1390992	3.68E-12	hypomethylated	0.02814	0.44805	insignificant	29	104	108
chr1	10710024	10712024	Cpa6	-0.88801129	0.12179	lowCoverage	-0.11386	0.00084324	hypomethylated	1	8	8
chr1	10982545	10984545	Prex2	-0.06145121	3.42E-08	hypomethylated	0.0071879	0.43805	insignificant	21	84	78
chr1	11403185	11405185	A830018L16Ri	-0.24624161	1.72E-13	hypomethylated	0.013043	0.51984	insignificant	12	46	40
chr1	12681510	12683510	Sulf1	-0.1661469	0.014733	hypomethylated	0.01267	0.16275	insignificant	7	40	40
chr1	12707625	12709625	Sulf1		1	noCoverage	-0.047341	0.89983	insignificant	0	14	14
chr1	12981216	12983216	Slco5a1	-0.12116137	1.4E-10	hypomethylated	-0.010985	0.77106	insignificant	11	100	107
chr1	13117244	13119244	Prdm14	-0.34169811	0.00064968	stronglyHypometh	-0.03722	0.85755	insignificant	5	12	12
chr1	13364164	13366164	Ncoa2	-0.14153827	0.000000011	hypomethylated	-0.0040501	0.95713	insignificant	10	51	54
chr1	13579945	13581945	Tram1	0.04390097	0.46661	insignificant	0.30548	0.13657	insignificant	1	24	12
chr1	13650590	13652590	Lactb2	-0.19238897	0.000012312	hypomethylated	0.028859	0.86328	insignificant	9	44	38
chr1	13657851	13659851	Xkr9		1	noCoverage	0.00042952	0.27736	insignificant	0	12	11
chr1	13777010	13779010	Gm5523	-0.03188259	0.71591	insignificant	-0.00059497	0.45123	insignificant	2	4	5
chr1	14746047	14748047	Msc		1	noCoverage	0.049667	0.21979	insignificant	0	2	2
chr1	14908943	14910943	Trpa1	-0.20208333	0.092959	insignificant	0.10009	0.43895	insignificant	2	4	8
chr1	15301532	15303532	Kcnb2	0.01791697	0.4595	insignificant	-0.021752	0.84888	insignificant	9	30	30
chr1	15794738	15796738	Terf1	-0.075458	0.41438	insignificant	-0.020932	0.90324	insignificant	10	72	70
chr1	15882803	15884803	Gm106	-0.21166274	0.000000319	hypomethylated	0.059277	0.74606	insignificant	5	14	14
chr1	16094514	16096514	Rpl7	-0.09695874	2.07E-65	hypomethylated	0.0080383	0.77732	insignificant	75	282	276
chr1	16094962	16096962	Rdh10	-0.09584435	6.16E-64	hypomethylated	0.0076353	0.74328	insignificant	73	272	268
chr1	16509383	16511383	Stau2	-0.16450545	1.38E-11	hypomethylated	-0.013879	0.53018	insignificant	12	36	36
chr1	16609367	16611367	Ube2w	-0.19767173	0.000000355	hypomethylated	-0.016894	0.44394	insignificant	17	60	60
chr1	16646946	16648946	D030040B21R	-0.17173942	0.00000493	hypomethylated	0.043752	0.591	insignificant	10	36	42
chr1	16654271	16656271	Tmem70	-0.22132122	1.15E-25	hypomethylated	0.018711	0.31801	insignificant	33	109	109
chr1	16677536	16679536	Ly96	-0.22148938	0.43106	insignificant	-0.049587	1	insignificant	4	32	33
chr1	17087879	17089879	Jph1	-0.08287304	1.03E-08	hypomethylated	0.017141	0.19897	insignificant	11	51	41
chr1	17134453	17136453	Gdap1	-0.21339714	0.0021419	hypomethylated	-0.037528	0.28178	insignificant	5	26	26
chr1	17716497	17718497	Crispld1	-0.36550744	0.00018822	stronglyHypometh	0.063026	0.66156	insignificant	1	14	14
chr1	19092102	19094102	Tfap2d		1	noCoverage	-0.072917	0.82766	insignificant	0	8	8
chr1	19197994	19199994	Tfap2b	0.13247863	1	insignificant	-0.059946	1	insignificant	1	5	2
chr1	19201134	19203134	Tfap2b	-0.01005857	0.50989	insignificant	0.39235	0.043244	stronglyhypermeth	1	7	8
chr1	20719985	20721985	Il17a	-0.09752155	0.056444	insignificant	0.015625	1	insignificant	2	4	4
chr1	20810294	20812294	Mcm3	-0.58637771	0.0003835	stronglyHypometh	-0.002048	0.73739	insignificant	3	19	14
chr1	20879702	20881702	Paqr8	-0.14595965	1.99E-11	hypomethylated	-0.011701	0.63381	insignificant	11	80	75
chr1	20940706	20942706	Efhc1	-0.23073064	0.088942	insignificant	-0.096892	0.2034	insignificant	2	20	18
chr1	21069290	21071290	Tram2	-0.02430263	6.18E-13	hypomethylated	0.020959	0.30546	insignificant	28	74	75
chr1	21069306	21071306	Tram2	-0.04337843	3.85E-13	hypomethylated	0.021871	0.40288	insignificant	28	70	71
chr1	21207711	21209711	Tmem14a	-0.21634502	0.019571	hypomethylated	0.019551	0.5066	insignificant	3	34	34
chr1	21372636	21374636	Khdc1b		1	noCoverage	-0.13095	0.24212	insignificant	0	4	4
chr1	21952023	21954023	Kcnq5	-0.13110623	0.0002371	hypomethylated	0.054893	0.63835	insignificant	5	25	24
chr1	22812563	22814563	Rims1	-0.09839723	0.000023124	hypomethylated	-0.0036675	0.73942	insignificant	31	88	88
chr1	23109092	23111092	4933415F23Ri	-0.24608628	0.27101	insignificant	0.025182	0.37415	insignificant	1	4	4
chr1	23278107	23280107	Mir30a	-0.60237252	0.0099116	stronglyHypometh	0.091735	0.056659	insignificant	3	9	9
chr1	23297539	23299539	Mir30c-2		1	noCoverage	-0.046338	1	insignificant	0	6	5
chr1	23390014	23392014	Ogfrl1	-0.18587117	0.0021187	hypomethylated	-0.059322	0.79036	insignificant	9	33	44
chr1	23767764	23769764	B3gat2	-0.11875843	2.54E-24	hypomethylated	-0.003966	0.50479	insignificant	22	148	137
chr1	23929128	23931128	Smap1	-0.09179904	7.5E-09	hypomethylated	0.018626	0.57097	insignificant	24	67	56
chr1	24107180	24109180	Fam135a	-0.13832996	0.00012824	hypomethylated	-0.011229	0.79326	insignificant	11	31	35
chr1	24183448	24185448	Col9a1		1	noCoverage	0.089381	0.091646	insignificant	0	12	12
chr1	24684382	24686382	Lmbrd1	-0.15546082	1.3E-09	hypomethylated	-0.0072732	0.39249	insignificant	10	60	58
chr1	25886552	25888552	Bai3	-0.07780276	0.000015688	hypomethylated	0.020843	0.058947	insignificant	34	137	132

chr1	30920101	30922101	Phf3		1	noCoverage	0.033487	0.74689	insignificant	0	10	8
chr1	31006555	31008555	Gm13363	-0.1186661	8E-33	hypomethylated	0.006778	0.94617	insignificant	29	105	100
chr1	31006600	31008600	Gm13363	-0.11949359	3.23E-33	hypomethylated	0.0070964	0.80597	insignificant	29	95	90
chr1	31278683	31280683	4930521A18R	0.05533274	0.73101	insignificant	0.032816	1	insignificant	2	6	6
chr1	32228650	32230650	Khdrbs2	-0.08661831	0.000011356	hypomethylated	-0.0034102	0.40615	insignificant	18	86	86
chr1	33725668	33727668	1700001G17R	-0.09187935	0.16253	insignificant	0.0056305	0.55601	insignificant	6	39	39
chr1	33726603	33728603	Prim2	-0.10156153	0.16244	insignificant	0.01799	0.55675	insignificant	6	40	40
chr1	33775740	33777740	Rab23	-0.12160335	3.02E-22	hypomethylated	-0.01735	0.77633	insignificant	37	142	125
chr1	33776263	33778263	Rab23	-0.12160335	3.02E-22	hypomethylated	-0.01735	0.77633	insignificant	37	142	125
chr1	33814595	33816595	Bag2	-0.09521442	0.000000662	hypomethylated	0.0069049	0.79057	insignificant	12	48	44
chr1	33871272	33873272	Zfp451	-0.16930121	4.7E-09	hypomethylated	0.027078	0.000001415	hypermethylated	13	67	67
chr1	33895759	33897759	Gm15455	0.06520437	0.24918	insignificant	0.058576	0.0039179	hypermethylated	3	21	23
chr1	33964466	33966466	Bend6	-0.08378309	4.59E-18	hypomethylated	-0.00045299	0.61981	insignificant	50	125	125
chr1	34067669	34069669	Dst	-0.20795692	1.04E-11	hypomethylated	-0.030375	0.87425	insignificant	21	48	54
chr1	34495673	34497673	Imp4	-0.18883844	8.27E-19	hypomethylated	0.010972	1	insignificant	13	56	56
chr1	34496517	34498517	Ccdc115	-0.19462001	0.0013355	hypomethylated	0.01987	0.69163	insignificant	5	36	36
chr1	34515590	34517590	Ptpn18	-0.17469675	0.000000139	hypomethylated	0.0022731	0.56505	insignificant	19	74	74
chr1	34554274	34556274	Prss39		1	noCoverage	-0.00017087	0.06273	insignificant	0	12	10
chr1	34591492	34593492	Cfc1	0.1196176	0.081217	insignificant	0.098543	0.0048425	hypermethylated	1	10	6
chr1	34617788	34619788	Prss40		1	noCoverage	0.028329	1	insignificant	0	6	9
chr1	34635537	34637537	Fam123c	-0.07063221	0.00000136	hypomethylated	0.0073233	0.68634	insignificant	14	69	68
chr1	34857566	34859566	Arhgef4	-0.08528395	1.66E-08	hypomethylated	0.0056377	0.27367	insignificant	14	52	52
chr1	34899895	34901895	Fam168b	-0.10813779	1.02E-21	hypomethylated	-0.015128	0.81051	insignificant	17	37	36
chr1	34905803	34907803	Plekhb2	-0.06556615	0.000032815	hypomethylated	-0.01337	0.95285	insignificant	11	38	38
chr1	36124244	36126244	Hs6st1	-0.10901343	1.12E-17	hypomethylated	0.0014795	0.12928	insignificant	60	200	198
chr1	36301147	36303147	Uggt1	-0.02236758	0.016635	hypomethylated	0.0082503	0.20184	insignificant	9	36	36
chr1	36330270	36332270	Neurl3	-0.31113529	0.046133	hypomethylated	0.054239	0.040084	hypermethylated	6	19	20
chr1	36363577	36365577	Arid5a	-0.11925431	3.31E-15	hypomethylated	0.00025016	0.22012	insignificant	20	74	71
chr1	36426026	36428026	4632411B12R	-0.20181476	2.9E-09	hypomethylated	0.045429	0.18045	insignificant	13	48	42
chr1	36502084	36504084	Lman2l		1	noCoverage	0.013408	1	insignificant	0	14	10
chr1	36527441	36529441	Cnnm4	-0.09386373	2.62E-09	hypomethylated	-0.0040296	0.3288	insignificant	16	66	60
chr1	36567720	36569720	Cnnm3	-0.06956696	3.87E-34	hypomethylated	0.0037946	0.55901	insignificant	66	286	260
chr1	36592574	36594574	Ankrd23	0.01281805	0.00024026	inconclusive	0.13051	0.023209	hypermethylated	2	16	14
chr1	36604046	36606046	Ankrd39	-0.08190644	0.010398	hypomethylated	-0.018791	1	insignificant	3	33	30
chr1	36615226	36617226	Sema4c	-0.15247061	0.016498	hypomethylated	0.056991	1	insignificant	7	19	19
chr1	36740028	36742028	Fam178b		1	noCoverage	0.033141	0.022464	hypermethylated	0	35	35
chr1	36747331	36749331	Cox5b	-0.17020902	4.05E-09	hypomethylated	-0.054885	0.93051	insignificant	4	65	68
chr1	36766770	36768770	Actr1b	-0.34816106	1	insignificant	-0.12221	0.87113	insignificant	3	15	13
chr1	36817705	36819705	Zap70		1	noCoverage	0.048836	1	insignificant	0	19	18
chr1	36996372	36998372	Tmem131	-0.12494713	8.34E-14	hypomethylated	-0.00089458	0.50186	insignificant	27	82	82
chr1	37275105	37277105	Cnga3	-0.28655659	0.00002109	hypomethylated	0.036957	0.67484	insignificant	4	34	34
chr1	37355737	37357737	Inpp4a	-0.11309684	0.000000412	hypomethylated	0.0082416	0.22772	insignificant	34	80	77
chr1	37486016	37488016	Unc50	-0.10876468	7.79E-11	hypomethylated	0.00087509	0.79308	insignificant	22	74	74
chr1	37486948	37488948	6330578E17Ri	0.03330791	0.70064	insignificant	0.026034	0.44628	insignificant	8	48	48
chr1	37545450	37547450	Mgat4a	0.43638246	0.14475	insignificant	-0.011193	0.15248	insignificant	4	23	23
chr1	37776656	37778656	2010300C02Ri	-0.06215515	1.9E-10	hypomethylated	0.0049889	0.27298	insignificant	26	76	76
chr1	37921924	37923924	Tsga10	-0.07659543	1.06E-18	hypomethylated	0.018415	0.67255	insignificant	20	72	68
chr1	37928050	37930050	Lipt1	-0.22540595	0.0090745	hypomethylated	0.080662	0.14265	insignificant	3	9	8
chr1	37946397	37948397	Mrpl30	-0.13669689	0.000016348	hypomethylated	0.067566	0.36256	insignificant	10	57	49
chr1	37947256	37949256	Mitd1	-0.17735186	0.00000163	hypomethylated	0.035376	0.89849	insignificant	10	39	34
chr1	38053854	38055854	Eif5b	-0.130759	1.36E-52	hypomethylated	0.0069517	0.23642	insignificant	70	191	193
chr1	38054053	38056053	Txndc9	-0.13198648	2.85E-48	hypomethylated	0.0075185	0.18396	insignificant	68	175	177
chr1	38186507	38188507	Rev1	-0.09558362	0.000084454	hypomethylated	-0.0029649	0.67049	insignificant	30	94	105
chr1	38878060	38880060	Lonrf2		1	noCoverage	-0.089087	0.39097	insignificant	0	6	6
chr1	38994993	38996993	Nms		1	noCoverage	0.10454	0.0030935	hypermethylated	0	6	6
chr1	39043658	39045658	Pdcl3	-0.16082834	9.99E-15	hypomethylated	-0.0041012	0.19999	insignificant	22	77	77
chr1	39250116	39252116	Npas2	-0.12123112	2.19E-25	hypomethylated	0.0078328	0.52388	insignificant	49	146	141
chr1	39423695	39425695	Rpl31	-0.05881424	0.00054723	hypomethylated	0.0045968	0.86534	insignificant	34	139	118
chr1	39535592	39537592	Tbc1d8	-0.19510732	0.000000588	hypomethylated	-0.000031566	0.91773	insignificant	7	24	24
chr1	39591646	39593646	D1Bwg0212e	-0.13652124	2.66E-17	hypomethylated	-0.014576	0.17489	insignificant	38	167	166
chr1	39605685	39607685	Snord89	0.20222476	1	insignificant	0.10172	0.0043871	hypermethylated	1	9	9
chr1	39634192	39636192	Rnf149	0.87608098	0.13634	lowCoverage	0.023919	0.9017	insignificant	1	48	43
chr1	39708027	39710027	Creg2		1	noCoverage	-0.012412	0.76899	insignificant	0	14	11
chr1	39777834	39779834	Rfx8	-0.13948342	1	insignificant	0.2009	0.000013235	hypermethylated	1	33	35
chr1	39956757	39958757	Map4k4	-0.09456835	2.39E-18	hypomethylated	0.0043051	0.33053	insignificant	69	162	177
chr1	40322430	40324430	Il1r1	-0.20711018	1.11E-12	hypomethylated	0.046389	0.65007	insignificant	9	20	18
chr1	40636071	40638071	Slc9a4	-0.67694805	0.040703	stronglyHypometh	-0.02996	0.44088	insignificant	1	12	12
chr1	40737556	40739556	Slc9a2	-0.04469038	5.94E-14	hypomethylated	0.01343	0.096612	insignificant	42	198	191
chr1	40861445	40863445	Tmem182		1	noCoverage	0.06107	0.32239	insignificant	0	20	20
chr1	42751670	42753670	2610017I09Ril	-0.12121374	0.000087256	hypomethylated	0.0097871	0.82014	insignificant	25	114	113
chr1	42752990	42754990	Pou3f3	-0.09959075	3.4E-11	hypomethylated	0.0050135	0.80749	insignificant	43	196	211
chr1	42756180	42758180	2900092D14R	-0.35513508	0.000023513	stronglyHypometh	-0.019725	0.34255	insignificant	3	6	6
chr1	42907077	42909077	Mrps9	-0.19459086	0.00027317	hypomethylated	0.025014	0.95911	insignificant	4	34	38
chr1	43008716	43010716	Gpr45	-0.10275203	5.89E-08	hypomethylated	-0.010097	0.89123	insignificant	21	88	88
chr1	43154554	43156554	AI597479	-0.09085291	0.61614	insignificant	0.010634	0.86416	insignificant	8	62	62
chr1	43155467	43157467	Tgfbrap1	-0.07822062	0.61151	insignificant	0.016799	0.5869	insignificant	8	56	56
chr1	43220806	43222806	Fhl2	-0.16706619	7.23E-10	hypomethylated	-0.005787	0.363	insignificant	10	42	42
chr1	43501595	43503595	Nck2	-0.09686496	2.54E-36	hypomethylated	0.0082619	0.97925	insignificant	63	215	210
chr1	43786446	43788446	1500015O10R	0.08017677	1	lowCoverage	0.048125	0.76696	insignificant	1	4	4
chr1	43884553	43886553	Uxs1	-0.10105158	7.86E-14	hypomethylated	0.010771	0.41814	insignificant	16	76	73
chr1	43989851	43991851	Tpp2	-0.14739975	8.85E-24	hypomethylated	0.017227	0.96803	insignificant	15	60	55
chr1	44159233	44161233	1700029F09Ri	-0.09704093	2.8E-12	hypomethylated	0.0073026	0.043499	inconclusive	15	64	58
chr1	44175618	44177618	Kdelc1	-0.13558042	0.00024991	hypomethylated	-0.098951	0.57704	insignificant	6	39	35
chr1	44175812	44177812	Bivm	-0.15240266	0.00057426	hypomethylated	-0.10584	0.57517	insignificant	6	33	29
chr1	44203588	44205588	Ercc5	-0.13591044	0.000001735	hypomethylated	0.038729	0.091959	insignificant	18	86	80

chr1	44607515	44609515	Gulp1	-0.14441812	0.0044645	hypomethylated	-0.012973	0.9081	insignificant	22	90	89
chr1	45851345	45853345	Wdr75	-0.15264333	4.65E-23	hypomethylated	0.0044914	0.08703	insignificant	25	82	82
chr1	45982439	45984439	Slc40a1	-0.18243733	0.03058	hypomethylated	-0.024408	0.36906	insignificant	6	48	55
chr1	46122582	46124582	Dnahc7b	-0.23064165	0.00040809	hypomethylated	0.017206	0.40242	insignificant	3	29	29
chr1	46910354	46912354	Slc39a10	-0.14231907	6.27E-29	hypomethylated	0.015883	0.21925	insignificant	31	78	66
chr1	50983366	50985366	Tmeff2	-0.07633324	1.32E-14	hypomethylated	0.034418	0.14216	insignificant	11	90	90
chr1	51535243	51537243	Obfc2a	-0.11176957	8.36E-10	hypomethylated	0.006079	0.5844	insignificant	11	38	38
chr1	51972818	51974818	Myo1b	-0.13620291	1.99E-09	hypomethylated	-0.015813	0.30107	insignificant	12	24	24
chr1	52064087	52066087	Stat4		1	noCoverage	-0.0012725	0.47214	insignificant	0	13	13
chr1	52175281	52177281	Stat1	-0.13542574	1.31E-22	hypomethylated	-0.0016815	0.81251	insignificant	26	97	97
chr1	52290076	52292076	Gls	-0.13606412	0.24615	insignificant	0.0041911	0.013599	hypermethylated	1	16	16
chr1	52557292	52559292	Nab1	-0.16191067	0.0092025	hypomethylated	0.03329	0.87582	insignificant	8	37	36
chr1	52686548	52688548	Tmem194b	-0.09406482	0.15808	insignificant	0.005938	0.52563	insignificant	7	51	42
chr1	52784162	52786162	Mfsd6	-0.09647159	0.0000015	hypomethylated	0.0062083	0.74737	insignificant	10	28	28
chr1	52874532	52876532	Inpp1	-0.19716305	0.0001945	hypomethylated	0.076573	0.56401	insignificant	5	10	11
chr1	52900889	52902889	Hibch	-0.18473841	0.000000609	hypomethylated	0.013538	0.55446	insignificant	15	50	50
chr1	53009684	53011684	1700019D03R	-0.17356553	7.44E-12	hypomethylated	-0.0004915	0.66659	insignificant	10	36	36
chr1	53352938	53354938	Ormdl1	-0.11017069	0.006876	hypomethylated	-0.0042429	0.29763	insignificant	4	84	84
chr1	53353840	53355840	Pms1	-0.14242136	0.0039757	hypomethylated	-0.031022	0.23166	insignificant	4	72	70
chr1	53409543	53411543	Asnsd1	-0.11734147	0.000011018	hypomethylated	-0.0082145	0.62331	insignificant	9	21	20
chr1	53752288	53754288	Dnahc7a		1	noCoverage	-0.0040252	0.43943	insignificant	0	40	31
chr1	53752289	53754289	Dnahc7a		1	noCoverage	-0.0040252	0.43943	insignificant	0	40	31
chr1	53842059	53844059	Stk17b	-0.18839522	5.22E-10	hypomethylated	-0.019947	0.087569	insignificant	13	45	44
chr1	54251878	54253878	Hecw2	-0.0888945	0.23026	insignificant	0.036963	0.26409	insignificant	3	28	28
chr1	54306526	54308526	Ccdc150	-0.1549422	0.0058585	hypomethylated	0.033386	0.12701	insignificant	5	30	30
chr1	54495815	54497815	Gtf3c3	-0.62413788	0.000063233	stronglyHypometh	-0.07676	0.43338	insignificant	2	23	19
chr1	54614526	54616526	Pgap1	-0.08142947	0.002254	hypomethylated	-0.0012207	0.90834	insignificant	6	26	26
chr1	54983231	54985231	Ankrd44	-0.23029766	2.14E-22	hypomethylated	-0.050613	0.53268	insignificant	10	50	46
chr1	55084322	55086322	Sf3b1	-0.15294894	0.06245	insignificant	-0.013928	0.04541	hypomethylated	7	40	40
chr1	55108613	55110613	Coq10b	-0.12681997	1.76E-36	hypomethylated	0.013728	0.15251	insignificant	42	135	109
chr1	55143991	55145991	Hspe1	-0.08168303	1.6E-15	hypomethylated	0.0019977	0.025589	inconclusive	34	189	185
chr1	55144776	55146776	Hspd1	-0.087366	1.35E-13	hypomethylated	0.0077922	0.031308	inconclusive	18	105	100
chr1	55187088	55189088	Mob4	-0.10831465	3.28E-16	hypomethylated	-0.0063009	0.54176	insignificant	35	115	105
chr1	55293020	55295020	Mars2	-0.13123106	7.36E-62	hypomethylated	-0.0056647	0.89712	insignificant	50	169	162
chr1	55419551	55421551	Boll	-0.04005756	0.0000082	inconclusive	-0.019267	0.08712	insignificant	33	169	171
chr1	55420313	55422313	Boll	-0.12088072	0.00028903	hypomethylated	-0.0070797	0.28042	insignificant	8	50	50
chr1	55461789	55463789	Plcl1	-0.10087341	0.00016516	hypomethylated	0.028689	0.54804	insignificant	34	121	108
chr1	57027312	57029312	9130024F11Ri	-0.09645337	0.000000692	hypomethylated	0.0142	0.15274	insignificant	27	129	127
chr1	57028178	57030178	Satb2	-0.12105457	5.28E-12	hypomethylated	-0.0032002	0.18818	insignificant	36	184	175
chr1	57433463	57435463	1700066M21F	-0.11935554	4.96E-30	hypomethylated	-0.0051099	0.51593	insignificant	33	97	93
chr1	57462391	57464391	9430016H08R	-0.13637414	0.000022114	inconclusive	-0.016609	0.16109	insignificant	28	125	125
chr1	57463518	57465518	9430016H08R	-0.1818135	0.0010485	hypomethylated	-0.01157	0.50237	insignificant	15	38	38
chr1	57830704	57832704	Spats2l	-0.09465191	1.52E-21	hypomethylated	0.019497	0.62874	insignificant	43	138	107
chr1	58026368	58028368	Kctd18	-0.13101265	6.45E-20	hypomethylated	0.019551	0.24818	insignificant	39	106	106
chr1	58026928	58028928	Kctd18	-0.07475975	0.0000866	hypomethylated	0.027787	0.27023	insignificant	14	34	34
chr1	58041183	58043183	Sgol2	0.28905841	1	lowCoverage	0.10229	0.02758	hypermethylated	1	14	14
chr1	58051817	58053817	Sgol2	-0.17941363	2.26E-13	hypomethylated	0.0050423	0.47909	insignificant	7	64	64
chr1	58085812	58087812	Aox1		1	noCoverage	-0.05	1	insignificant	0	8	8
chr1	58266240	58268240	Aox4		1	noCoverage	-0.017237	0.89815	insignificant	0	9	10
chr1	58448979	58450979	Bzw1	-0.10511044	1.41E-12	hypomethylated	-0.0085698	0.42345	insignificant	37	180	177
chr1	58480932	58482932	Clk1	0.17728758	1	insignificant	0.050914	0.55121	insignificant	1	6	13
chr1	58503476	58505476	Nif3l1	0.13191741	0.085623	insignificant	0.068598	0.053946	insignificant	4	12	12
chr1	58561763	58563763	Orc2	-0.18757191	0.000008378	hypomethylated	0.0084307	0.83973	insignificant	10	29	32
chr1	58642442	58644442	Ndufb3	-0.19251624	0.00043106	hypomethylated	0.0049998	1	insignificant	7	30	26
chr1	58643177	58645177	Fam126b	-0.19251624	0.00043106	hypomethylated	0.0049998	1	insignificant	7	30	26
chr1	58851217	58853217	Casp8	-0.13683713	1.2E-11	hypomethylated	0.011365	0.38443	insignificant	10	43	43
chr1	59029414	59031414	Stradb	-0.14733809	5.03E-19	hypomethylated	0.001577	0.81162	insignificant	27	118	108
chr1	59030326	59032326	Trak2	-0.13549863	8.6E-15	hypomethylated	0.0070479	0.75729	insignificant	17	86	76
chr1	59176650	59178650	Als2cr4	-0.11726001	1.96E-30	hypomethylated	-0.0020718	0.90434	insignificant	32	93	77
chr1	59176940	59178940	Mpp4	-0.13044516	3.63E-28	hypomethylated	-0.0069953	1	insignificant	21	54	49
chr1	59220233	59222233	Als2	0.18219083	0.35732	insignificant	-0.010342	1	insignificant	1	14	11
chr1	59293937	59295937	Als2	-0.0618936	2.22E-08	hypomethylated	0.00041223	0.25103	insignificant	21	58	58
chr1	59294075	59296075	Als2	-0.09074126	0.10368	insignificant	-0.013732	0.86207	insignificant	5	16	16
chr1	59537990	59539990	Fzd7	-0.12950544	3.16E-25	hypomethylated	-0.017769	0.97199	insignificant	46	193	175
chr1	59572107	59574107	Gm973	-0.60961538	0.020528	stronglyHypometh	-0.056044	1	insignificant	2	4	4
chr1	59727678	59729678	Sumo1	-0.16283149	0.12942	insignificant	0.03298	0.27424	insignificant	4	27	24
chr1	59820480	59822480	Bmpr2	-0.09630644	3.85E-13	hypomethylated	-0.00080401	0.61678	insignificant	28	112	112
chr1	59968849	59970849	Fam117b	-0.10548378	3.92E-19	hypomethylated	0.00086975	0.73271	insignificant	31	130	128
chr1	60099931	60101931	Ica1l	-0.067599	1.16E-16	hypomethylated	-0.00075478	0.56454	insignificant	40	110	110
chr1	60154124	60156124	Carf	-0.15988355	6.61E-26	hypomethylated	-0.0098294	0.8556	insignificant	24	84	77
chr1	60154751	60156751	Wdr12	-0.15133632	1.38E-24	hypomethylated	-0.013563	0.73197	insignificant	24	74	67
chr1	60154978	60156978	Wdr12	-0.15133632	1.38E-24	hypomethylated	-0.013563	0.73197	insignificant	24	74	67
chr1	60155344	60157344	Wdr12	-0.14498212	4.26E-18	hypomethylated	-0.0042222	0.87046	insignificant	22	62	57
chr1	60236442	60238442	Nbeal1	-0.10140101	1.37E-14	hypomethylated	-0.019588	0.11456	insignificant	40	134	134
chr1	60399215	60401215	Cyp20a1	-0.15601493	6E-16	hypomethylated	0.045438	0.18033	insignificant	19	63	62
chr1	60465462	60467462	Abi2	-0.09173579	3.06E-11	hypomethylated	-0.00024122	0.92449	insignificant	24	144	138
chr1	60623609	60625609	Raph1	-0.07665116	0.00024247	hypomethylated	0.050077	1	insignificant	15	65	69
chr1	61684397	61686397	Pard3b	-0.1380934	1.43E-29	hypomethylated	0.0022401	0.51808	insignificant	46	159	152
chr1	62748890	62750890	Nrp2	-0.11838892	9.76E-10	hypomethylated	0.013339	0.55292	insignificant	23	87	73
chr1	63160841	63162841	Ino80d	-0.03408753	0.18518	insignificant	0.0069263	0.32095	insignificant	11	22	22
chr1	63222404	63224404	Eef1b2	-0.13671326	2.33E-62	hypomethylated	0.028451	0.35189	insignificant	37	157	128
chr1	63223396	63225396	Ndufs1	-0.09576433	1.19E-36	hypomethylated	0.026415	0.53874	insignificant	22	92	68
chr1	63260783	63262783	Gpr1		1	noCoverage	0.12127	0.32339	insignificant	0	8	8
chr1	63318842	63320842	Zdbf2	-0.09342787	8.19E-14	hypomethylated	-0.0022472	0.52834	insignificant	39	195	186
chr1	63491477	63493477	Adam23	0.00255041	0.00012942	inconclusive	0.029125	1	insignificant	39	131	133

chr1	64167963	64169963	Klf7	-0.07725839	3.43E-18	hypomethylated	0.0020162	0.77513	insignificant	20	84	84
chr1	64578377	64580377	Creb1	-0.09874719	2.61E-40	hypomethylated	0.00644	0.46824	insignificant	70	215	198
chr1	64663742	64665742	Mettl21a	-0.09300927	0.0045944	hypomethylated	0.00088563	1	insignificant	10	38	38
chr1	64736918	64738918	Ccnyl1	-0.14657944	5.34E-49	hypomethylated	-0.035271	0.30344	insignificant	58	229	209
chr1	64784324	64786324	Fzd5	-0.15819067	6.02E-21	hypomethylated	0.012479	0.52562	insignificant	20	54	54
chr1	65003398	65005398	Plekhm3	0.17230099	1	insignificant	-0.0033923	0.60892	insignificant	3	16	16
chr1	65079322	65081322	Akr1cl	-0.1969697	0.56617	insignificant	0.0075758	1	insignificant	2	4	4
chr1	65097737	65099737	Cryge	0.16917293	0.59235	insignificant	0.038079	0.2102	insignificant	5	12	12
chr1	65110014	65112014	Crygd	-0.34605031	0.000019324	stronglyHypometh	0.14568	0.028076	hypermethylated	2	6	3
chr1	65149937	65151937	Cryga	-0.10876068	0.12436	insignificant	-0.071581	0.36545	insignificant	6	12	12
chr1	65225716	65227716	Idh1		1	noCoverage	-0.027003	0.69991	insignificant	0	18	18
chr1	65232258	65234258	Pikfyve	-0.15969839	2.72E-19	hypomethylated	-0.0037806	0.95865	insignificant	20	57	57
chr1	65233053	65235053	Idh1	-0.15588013	2.91E-19	hypomethylated	-0.0077582	0.91755	insignificant	20	60	60
chr1	65356830	65358830	Pth2r	-0.35108466	0.097205	insignificant	0.063601	0.33236	insignificant	2	10	10
chr1	65972095	65974095	Crygf	-0.24289596	0.086515	insignificant	-0.015217	0.42804	insignificant	2	7	7
chr1	66220902	66222902	Mtap2	-0.15012501	0.00000988	hypomethylated	0.019348	0.60747	insignificant	18	92	92
chr1	66514020	66516020	Unc80	-0.12029207	2.22E-16	hypomethylated	0.0018716	0.72848	insignificant	34	118	131
chr1	66746466	66748466	Rpe	-0.15453356	6.09E-09	hypomethylated	0.051843	0.39209	insignificant	12	58	40
chr1	66864169	66866169	1110028C15Ri	-0.11716176	1.69E-25	hypomethylated	0.005668	0.20356	insignificant	28	97	95
chr1	66909841	66911841	Acadl	-0.12076458	0.10156	insignificant	-0.0056964	0.88447	insignificant	7	55	54
chr1	66981940	66983940	Myl1	-0.18888889	0.57467	insignificant	0.18611	0.10968	insignificant	2	6	6
chr1	69154633	69156633	Erbb4		1	noCoverage	-0.016303	0.51389	insignificant	0	16	16
chr1	69732534	69734534	Ikzf2	-0.08284153	2.75E-10	hypomethylated	-0.012223	0.56653	insignificant	27	109	125
chr1	69872561	69874561	Spag16	-0.10951274	0.000011956	hypomethylated	-0.011082	0.83596	insignificant	10	34	34
chr1	70771288	70773288	Vwc2l	-0.50002387	0.000013015	stronglyHypometh	-0.14291	0.14105	insignificant	3	14	12
chr1	71149546	71151546	Bard1	-0.44173993	0.00000949	stronglyHypometh	0.14585	0.16026	insignificant	3	10	17
chr1	71602729	71604729	Atic	-0.12237753	0.0034954	hypomethylated	0.039892	0.96857	insignificant	20	81	79
chr1	71699745	71701745	Fn1	-0.1007981	5.07E-18	hypomethylated	0.0022489	0.058568	insignificant	17	48	49
chr1	72258881	72260881	Mreg	-0.05941529	0.16704	insignificant	-0.0063586	0.67712	insignificant	6	22	22
chr1	72329946	72331946	Tmem169	-0.36291715	7.55E-11	stronglyHypometh	-0.017977	0.19443	insignificant	6	24	24
chr1	72583138	72585138	March4	-0.21092434	0.0017462	hypomethylated	0.038206	0.61564	insignificant	3	30	31
chr1	72747138	72749138	Ankar	-0.06042526	0.43203	insignificant	0.057075	0.37194	insignificant	1	11	10
chr1	72756833	72758833	Rpl37a	-0.16909845	8.91E-08	hypomethylated	0.023131	0.13975	insignificant	8	42	41
chr1	72870076	72872076	Igfbp2	-0.10485102	0.00000827	hypomethylated	-0.00050597	0.37948	insignificant	15	100	96
chr1	72921439	72923439	Igfbp5		1	noCoverage	0.034524	0.18101	insignificant	0	6	6
chr1	73062473	73064473	Tnp1	-0.02467532	1	insignificant	0.12333	0.20669	insignificant	2	4	4
chr1	74171021	74173021	Tns1	-0.132978	0.0030425	hypomethylated	0.039943	0.28865	insignificant	16	100	100
chr1	74171114	74173114	Rufy4	-0.1417091	0.0055684	hypomethylated	0.041881	0.27974	insignificant	11	92	92
chr1	74199567	74201567	Cxcr2		1	noCoverage	-0.091667	0.12202	insignificant	0	4	4
chr1	74282123	74284123	Arpc2	-0.18189704	6.78E-08	hypomethylated	-0.030163	0.37266	insignificant	17	109	102
chr1	74324173	74326173	Gpbar1	0.0164203	0.55269	insignificant	0.062136	0.002972	hypermethylated	6	18	18
chr1	74330607	74332607	Pnkd	-0.14165455	0.000097406	hypomethylated	0.0020004	0.80868	insignificant	7	76	76
chr1	74331312	74333312	Aamp	-0.12929629	0.000223	hypomethylated	0.00047306	0.95839	insignificant	6	48	48
chr1	74350910	74352910	Tmbim1	-0.271875	0.56668	insignificant	0.21164	0.10247	insignificant	2	4	7
chr1	74378183	74380183	Pnkd	-0.15916318	2.66E-10	hypomethylated	0.075246	0.86351	insignificant	12	50	50
chr1	74420776	74422776	Slc11a1	0.2058651	1	insignificant	0.054726	0.55868	insignificant	2	12	12
chr1	74437182	74439182	Ctdsp1	-0.10799486	1.26E-16	hypomethylated	-0.0029623	0.40054	insignificant	48	160	153
chr1	74439883	74441883	Mir26b	-0.24080431	0.00085392	hypomethylated	0.086562	0.49547	insignificant	7	25	20
chr1	74551633	74553633	Rqcd1	-0.10556052	1.11E-18	hypomethylated	0.00059881	0.37892	insignificant	32	110	106
chr1	74590860	74592860	Plcd4		1	noCoverage	-0.11268	0.34631	insignificant	0	19	18
chr1	74633934	74635934	Bcs1l	-0.1477371	3.73E-12	hypomethylated	-0.013882	0.65818	insignificant	17	81	84
chr1	74634602	74636602	Zfp142	-0.18476172	0.00041356	hypomethylated	-0.00059362	0.32983	insignificant	9	56	54
chr1	74647028	74649028	Stk36	-0.13892298	4.93E-08	hypomethylated	-0.010495	0.1434	insignificant	7	32	32
chr1	74707327	74709327	Ttll4	-0.25337939	0.0012521	hypomethylated	-0.012464	0.061442	insignificant	9	57	56
chr1	74759147	74761147	Cyp27a1		1	noCoverage	0.21123	0.19954	insignificant	0	16	14
chr1	74795529	74797529	Prkag3		1	noCoverage	-0.024667	0.7963	insignificant	0	16	16
chr1	74817465	74819465	Wnt6	-0.13965615	0.000000345	hypomethylated	0.013229	0.46995	insignificant	14	96	87
chr1	74837592	74839592	Wnt10a	-0.11585707	0.000066793	hypomethylated	0.04412	0.51863	insignificant	5	31	31
chr1	74900602	74902602	Cdk5r2	-0.10145895	2.3E-26	hypomethylated	0.0062787	0.97778	insignificant	52	158	160
chr1	74931982	74933982	Fev	-0.33290653	0.54808	insignificant	-0.10466	0.93389	insignificant	2	10	9
chr1	74939709	74941709	Cryba2	-0.33023059	0.31227	insignificant	0.056968	0.076194	insignificant	3	21	21
chr1	74947295	74949295	Mir375	-0.16010162	2.17E-09	hypomethylated	0.028065	0.66165	insignificant	9	52	58
chr1	74998225	75000225	Ihh	-0.13690251	0.010771	hypomethylated	0.021471	0.83971	insignificant	6	43	44
chr1	75138359	75140359	Fam134a	-0.10400743	4.61E-17	hypomethylated	-0.010495	0.53937	insignificant	33	124	115
chr1	75138942	75140942	1810031K17Ri	-0.09495073	9.67E-15	hypomethylated	-0.012758	0.31866	insignificant	28	106	98
chr1	75164219	75166219	Zfand2b	-0.05871147	1.21E-15	hypomethylated	0.0046176	0.5945	insignificant	25	88	86
chr1	75164308	75166308	Zfand2b	-0.05871147	1.21E-15	hypomethylated	0.0046176	0.5945	insignificant	25	88	86
chr1	75176857	75178857	Atg9a	0	1	insignificant	0	1	insignificant	2	6	3
chr1	75187708	75189708	Ankzf1	-0.16320504	1.25E-14	hypomethylated	-0.028321	0.22541	insignificant	24	95	84
chr1	75188497	75190497	Atg9a	-0.13327821	0.015075	hypomethylated	0.023554	0.5657	insignificant	5	41	34
chr1	75206403	75208403	Stk16	-0.1415823	8.21E-11	hypomethylated	-0.0045293	0.7384	insignificant	13	70	70
chr1	75207353	75209353	Glb1l	-0.15166062	5.99E-10	hypomethylated	-0.011558	0.52008	insignificant	12	71	71
chr1	75215828	75217828	A630095N17R	-0.14753463	8.56E-24	hypomethylated	-0.0053303	0.063694	insignificant	28	109	103
chr1	75231997	75233997	Dnajb2	-0.10995143	1.33E-09	hypomethylated	0.0020599	0.37155	insignificant	16	48	48
chr1	75232276	75234276	Dnajb2	-0.14119711	3.55E-09	hypomethylated	0.013985	0.2245	insignificant	17	52	52
chr1	75260783	75262783	Ptprn	-0.18993757	0.062251	insignificant	-0.0029157	0.53737	insignificant	7	15	14
chr1	75274955	75276955	Resp18		1	noCoverage	0.059075	0.11268	insignificant	0	8	8
chr1	75313592	75315592	Dnpep	-0.10921606	2.42E-35	hypomethylated	0.0062672	0.17436	insignificant	37	149	142
chr1	75314212	75316212	Dnpep	0.02966711	0.51821	insignificant	0.0082022	0.43826	insignificant	19	78	78
chr1	75355918	75357918	Des	-0.1094199	1.15E-08	hypomethylated	-0.0020991	0.18002	insignificant	25	129	124
chr1	75370871	75372871	Speg	-0.01467535	0.00067858	hypomethylated	0.03356	0.39	insignificant	21	71	65
chr1	75377688	75379688	Speg	-0.19970017	2.67E-16	hypomethylated	-0.006932	0.31806	insignificant	23	65	61
chr1	75381184	75383184	Speg	-0.27602625	0.000000685	hypomethylated	0.044487	0.0019859	hypermethylated	2	35	40
chr1	75395659	75397659	Speg		1	noCoverage	0.044286	0.4264	insignificant	0	2	2
chr1	75446084	75448084	Accn4	-0.23288824	1.96E-09	hypomethylated	0.039772	0.0060624	hypermethylated	18	53	52

chr1	75475106	75477106	Tmem198	-0.1408322	1.92E-56	hypomethylated	0.00041392	0.0013873	hypermethylated	61	168	168
chr1	75476046	75478046	Chpf	-0.18776181	2.36E-27	hypomethylated	-0.0055587	0.0022581	inconclusive	23	55	55
chr1	75502651	75504651	Inha	-0.11938482	0.000000867	hypomethylated	0.041681	0.13051	insignificant	23	72	66
chr1	75503027	75505027	Obsl1	-0.16744938	5.01E-12	hypomethylated	0.061013	0.14613	insignificant	8	24	24
chr1	75517103	75519103	Stk11ip	-0.26192273	1.01E-10	hypomethylated	0.12869	0.47914	insignificant	6	14	16
chr1	75541840	75543840	Slc4a3	-0.16416117	2.8E-12	hypomethylated	0.012347	0.055078	insignificant	19	56	56
chr1	77511663	77513663	Epha4	-0.10360411	0.0059352	hypomethylated	0.01419	0.66463	insignificant	13	77	78
chr1	78193711	78195711	Pax3	-0.37373737	0.039567	stronglyHypometh	-0.18012	0.15952	insignificant	4	11	8
chr1	78305920	78307920	Sgpp2	-0.14518145	4.37E-10	hypomethylated	0.014896	0.26676	insignificant	18	50	50
chr1	78485421	78487421	Farsb	-0.50595238	0.53422	insignificant	-0.14574	1	insignificant	1	8	6
chr1	78506634	78508634	Mogat1	-0.35222465	7.3E-12	stronglyHypometh	-0.0051386	0.11456	insignificant	7	20	20
chr1	78653399	78655399	Utp14b	-0.13404959	4.16E-12	hypomethylated	-0.0079838	0.052243	insignificant	20	111	106
chr1	78654014	78656014	Utp14b	-0.07020265	1.31E-10	hypomethylated	-0.0032023	0.14137	insignificant	20	107	102
chr1	78812523	78814523	Kcne4	0.06120832	0.67556	insignificant	-0.012971	1	insignificant	6	28	27
chr1	79436665	79438665	Scg2	-0.33531446	1.91E-10	stronglyHypometh	-0.043223	0.57372	insignificant	1	9	5
chr1	79668547	79670547	Ap1s3	-0.62248536	0.00000026	stronglyHypometh	-0.084593	0.41965	insignificant	2	17	13
chr1	79758344	79760344	Wdfy1	-0.069301	0.16962	insignificant	0.000068186	0.00080779	inconclusive	10	38	40
chr1	79771592	79773592	Mrpl44	-0.34908425	0.000050292	stronglyHypometh	0.024653	1	insignificant	3	6	6
chr1	79855240	79857240	Serpine2	-0.14250668	0.0011688	hypomethylated	0.0098947	0.94516	insignificant	10	47	43
chr1	80337005	80339005	Cul3	-0.16062662	1.4E-24	hypomethylated	0.0037928	0.73356	insignificant	30	110	110
chr1	81072891	81074891	9430031J16Rik		1	noCoverage	-0.12271	0.85146	insignificant	0	10	10
chr1	82288014	82290014	Irs1	-0.13925797	2.85E-08	hypomethylated	-0.017261	0.8809	insignificant	17	43	54
chr1	82312153	82314153	Rhbdd1	-0.13577093	0.000000197	hypomethylated	0.0052801	0.86405	insignificant	16	52	52
chr1	82582495	82584495	Col4a3	-0.17161388	1.53E-08	hypomethylated	0.016902	0.24738	insignificant	7	53	53
chr1	82583424	82585424	Col4a4	-0.0840584	0.026015	hypomethylated	0.032376	0.15068	insignificant	3	23	23
chr1	82720492	82722492	Mff	-0.06975161	0.8404	insignificant	-0.017751	0.12516	insignificant	23	76	76
chr1	82835057	82837057	Agfg1	-0.09550983	2.63E-32	hypomethylated	0.0045266	0.42887	insignificant	52	165	164
chr1	83035023	83037023	Slc19a3	-0.29612579	0.00019683	hypomethylated	0.032745	0.00018704	hypermethylated	5	11	11
chr1	84281220	84283220	Pid1	-0.26675108	0.15576	insignificant	-0.039855	0.13484	insignificant	7	22	20
chr1	84692796	84694796	Mir5126	0.11219915	1	insignificant	0.075127	0.13905	insignificant	4	22	22
chr1	84835415	84837415	Fbxo36	-0.14602448	4.69E-22	hypomethylated	-0.0089247	0.95384	insignificant	48	177	177
chr1	84835879	84837879	Fbxo36	-0.13628924	2.25E-08	hypomethylated	0.013377	0.92217	insignificant	22	96	96
chr1	84931658	84933658	Slc16a14	-0.21079966	0.0095694	hypomethylated	-0.020678	0.28268	insignificant	5	18	19
chr1	87689021	87691021	Cab39	-0.09652328	1.37E-16	hypomethylated	-0.0096747	0.79735	insignificant	40	166	139
chr1	87790084	87792084	Itm2c	-0.13888577	2.05E-08	hypomethylated	-0.0062931	0.077698	insignificant	18	71	71
chr1	87917516	87919516	Spata3	-0.32384734	0.64995	insignificant	-0.057919	0.68224	insignificant	2	23	23
chr1	87941437	87943437	2810459M11R	-0.09071042	2.85E-34	hypomethylated	0.0012866	0.80696	insignificant	52	144	144
chr1	87960193	87962193	Psmd1	-0.21308703	0.015149	hypomethylated	0.0059046	0.13073	insignificant	25	80	75
chr1	88050354	88052354	Armc9	-0.12937899	2.09E-16	hypomethylated	-0.00058572	0.71392	insignificant	19	70	70
chr1	88050389	88052389	Armc9	-0.12937899	2.09E-16	hypomethylated	-0.00058572	0.71392	insignificant	19	70	70
chr1	88198795	88200795	B3gnt7	-0.21984129	5.94E-37	hypomethylated	0.0070289	0.1793	insignificant	18	97	87
chr1	88255148	88257148	C130036L24Ri	-0.10049535	7.21E-12	hypomethylated	0.0014038	0.26872	insignificant	25	154	146
chr1	88256030	88258030	Ncl	-0.09648285	0.35144	insignificant	0.0061349	0.95868	insignificant	12	69	68
chr1	88284716	88286716	Nmur1	-0.37562724	0.00055749	stronglyHypometh	-0.10832	0.10564	insignificant	4	10	8
chr1	88321903	88323903	1700019O17R	0.00626688	1	insignificant	-0.00082221	0.62186	insignificant	4	14	14
chr1	88422310	88424310	Ptma	-0.05675175	1.03E-19	hypomethylated	0.007213	0.20336	insignificant	68	280	268
chr1	88479076	88481076	Pde6d		1	noCoverage	0.042916	0.000087498	inconclusive	0	28	28
chr1	88482674	88484674	Cops7b	0.00542563	0.30603	insignificant	-0.015202	0.001712	hypomethylated	5	30	30
chr1	88567147	88569147	Nppc	-0.18880764	0.000020737	hypomethylated	-0.000074258	0.073453	insignificant	7	23	18
chr1	88599378	88601378	Dis3l2	-0.13313784	2.09E-27	hypomethylated	0.0049119	0.73073	insignificant	21	58	58
chr1	89051602	89053602	Ecel1	-0.17045611	0.000041506	hypomethylated	-0.012141	0.22241	insignificant	16	86	86
chr1	89086226	89088226	Chrnd		1	noCoverage	-0.092411	0.11202	insignificant	0	8	8
chr1	89101385	89103385	Chrng	0.43168898	1	lowCoverage	0.035856	0.43658	insignificant	1	11	14
chr1	89109488	89111488	Eif4e2	-0.15017504	3.44E-35	hypomethylated	0.0026531	0.49865	insignificant	31	110	110
chr1	89109513	89111513	Eif4e2	-0.15017504	3.44E-35	hypomethylated	0.0026531	0.49865	insignificant	31	110	110
chr1	89159938	89161938	Efhd1	-0.14802372	7.86E-17	hypomethylated	0.0045275	0.9155	insignificant	30	104	96
chr1	89222572	89224572	Gigyf2	-0.18793653	6.18E-17	hypomethylated	0.016744	0.021767	hypermethylated	31	131	125
chr1	89222603	89224603	Gigyf2	-0.18793653	6.18E-17	hypomethylated	0.016744	0.021767	hypermethylated	31	131	125
chr1	89406937	89408937	Ngef	-0.12624544	2.26E-11	hypomethylated	-0.0015795	0.76342	insignificant	17	38	38
chr1	89479596	89481596	Neu2		1	noCoverage	0.075	0.11196	insignificant	0	4	4
chr1	89515886	89517886	Inpp5d	-0.15650584	0.00000471	hypomethylated	0.061908	0.090195	insignificant	5	20	21
chr1	89651645	89653645	Atg16l1	-0.12157089	3.43E-30	hypomethylated	0.0088732	0.0032029	hypermethylated	44	164	157
chr1	89699254	89701254	Sag	-0.65516256	0.0041418	stronglyHypometh	-0.1609	0.38819	insignificant	1	8	8
chr1	89748861	89750861	Dgkd	-0.11902461	4.61E-51	hypomethylated	0.0088791	0.62305	insignificant	52	174	172
chr1	89990575	89992575	Ugt1a7c	-0.38702883	0.00000433	stronglyHypometh	-0.024833	0.91962	insignificant	3	14	14
chr1	90174132	90176132	A730008H23R	-0.48586194	7.4E-12	stronglyHypometh	-0.12019	0.51852	insignificant	5	16	13
chr1	90174154	90176154	A730008H23R	-0.61385892	4.24E-09	stronglyHypometh	-0.12279	0.35531	insignificant	3	12	9
chr1	90202286	90204286	Trpm8	-0.51040564	0.044546	stronglyHypometh	0.081105	0.49325	insignificant	3	21	18
chr1	90302593	90304593	Spp2	0.09239527	0.86701	insignificant	0.058076	0.15139	insignificant	6	14	14
chr1	90406641	90408641	Glrp1		1	noCoverage	0.071714	0.31571	insignificant	0	10	10
chr1	90598766	90600766	Arl4c	-0.09457817	5.91E-08	hypomethylated	-0.0053341	0.48012	insignificant	16	52	52
chr1	90966036	90968036	Sh3bp4	-0.12574166	1.46E-41	hypomethylated	-0.0016465	0.97724	insignificant	36	185	164
chr1	91350385	91352385	Agap1	-0.06923711	3.59E-27	hypomethylated	0.0010438	0.0039523	hypermethylated	95	246	235
chr1	91827751	91829751	Gbx2	0.09714705	0.56848	insignificant	0.018512	0.013862	hypermethylated	21	89	88
chr1	91911152	91913152	Asb18	-0.28579545	0.13599	insignificant	-0.085241	0.7453	insignificant	1	10	10
chr1	92049976	92051976	Iqca	-0.09286582	0.17824	insignificant	0.018823	0.5742	insignificant	8	26	26
chr1	92099577	92101577	Cxcr7	-0.37895511	0.00000362	stronglyHypometh	-0.0172	0.03031	hypomethylated	4	20	20
chr1	92498999	92500999	Cops8	-0.13825456	2.92E-11	hypomethylated	0.013229	0.74298	insignificant	18	80	89
chr1	92810676	92812676	Mlph	-0.23263645	2.56E-10	hypomethylated	0.018756	0.49502	insignificant	5	26	26
chr1	92866197	92868197	Rab17	-0.11111111	0.37162	insignificant	-0.027006	0.12924	insignificant	3	10	8
chr1	92894303	92896303	Lrrfip1	-0.08950509	1.24E-22	hypomethylated	-0.0022399	0.17242	insignificant	36	135	127
chr1	92949020	92951020	Lrrfip1	-0.1173799	0.0016503	hypomethylated	0.0040927	0.63643	insignificant	5	60	60
chr1	93040679	93042679	Rbm44	0.13174471	0.51653	insignificant	-0.022844	0.028879	hypomethylated	8	28	31
chr1	93075398	93077398	Ramp1	-0.1700108	1.05E-12	hypomethylated	0.012134	0.056206	insignificant	12	58	58
chr1	93145895	93147895	Ube2f	-0.07940927	4.82E-20	hypomethylated	0.11166	0.13664	insignificant	42	136	137

chr1	93193914	93195914	Scly	-0.11329435	1.62E-12	hypomethylated	0.0052205	0.43671	insignificant	20	68	70
chr1	93217651	93219651	Espnl	-0.57884359	6.13E-19	stronglyHypometh	-0.0064952	0.34582	insignificant	5	24	24
chr1	93262006	93264006	Fam132b	0.00519065	0.051864	insignificant	0.0015547	0.67632	insignificant	6	64	64
chr1	93295360	93297360	Ilkap	-0.17898428	0.000000316	hypomethylated	0.027368	0.52173	insignificant	6	18	18
chr1	93309799	93311799	Hes6	-0.1374422	0.000019804	hypomethylated	0.00046401	0.59821	insignificant	14	56	52
chr1	93355905	93357905	Per2	-0.10800911	7.69E-15	hypomethylated	0.0092977	0.49352	insignificant	17	55	55
chr1	93390244	93392244	Traf3ip1	-0.13029778	4.56E-42	hypomethylated	-0.017412	0.13781	insignificant	41	123	123
chr1	93436141	93438141	Asb1	-0.14099265	1.56E-16	hypomethylated	0.00056077	0.93419	insignificant	17	58	58
chr1	93436656	93438656	Asb1	-0.14099265	1.56E-16	hypomethylated	0.00056077	0.93419	insignificant	17	58	58
chr1	93697053	93699053	Twist2	-0.09974513	1.2E-22	hypomethylated	0.016566	0.025876	hypermethylated	36	110	110
chr1	94370335	94372335	Ndufa10	-0.11777738	0.000048547	hypomethylated	0.0013199	0.18824	insignificant	3	33	33
chr1	94415092	94417092	Olfr1414	-0.25002706	0.028742	hypomethylated	0.25202	0.0052909	hypermethylated	3	8	8
chr1	94538525	94540525	Myeov2		1	noCoverage	0.25838	0.83922	insignificant	0	9	6
chr1	94538562	94540562	Myeov2		1	noCoverage	0.25838	0.83922	insignificant	0	9	6
chr1	94727262	94729262	Gpc1	-0.11586319	1E-43	hypomethylated	-0.0087112	0.25114	insignificant	67	229	228
chr1	94802565	94804565	Dusp28	-0.18530802	0.0014347	hypomethylated	0.015239	0.55807	insignificant	13	54	54
chr1	94806981	94808981	Rnpepl1	-0.0966804	2.4E-32	hypomethylated	0.0080202	0.0085817	hypermethylated	64	198	188
chr1	94829984	94831984	Capn10	-0.13121968	1E-23	hypomethylated	0.0081339	0.9553	insignificant	21	100	100
chr1	94847167	94849167	9430060I03Ril	-0.01197917	0.45067	insignificant	-0.099858	0.85084	insignificant	2	8	6
chr1	94901910	94903910	Aqp12	-0.05740741	0.51559	insignificant	-0.009958	0.89424	insignificant	3	6	6
chr1	94998442	95000442	Kif1a	-0.13969249	0.0033256	hypomethylated	-0.027718	0.019895	hypomethylated	10	31	30
chr1	95030852	95032852	Agxt	-0.00572891	0.60174	insignificant	0.056169	0.39589	insignificant	6	16	20
chr1	95057447	95059447	2310007B03R	0.08207973	0.23361	insignificant	0.0060176	0.39355	insignificant	5	32	29
chr1	95057525	95059525	2310007B03R	0.18346319	0.36565	insignificant	0.0026818	0.48355	insignificant	3	28	25
chr1	95131473	95133473	Sned1	-0.09142771	2.27E-16	hypomethylated	0.0025048	0.89022	insignificant	29	128	128
chr1	95202447	95204447	Mterfd2	-0.53546789	0.00010205	stronglyHypometh	-0.0083102	0.14849	insignificant	1	27	32
chr1	95239221	95241221	Ppp1r7	-0.09818556	2.02E-09	hypomethylated	-0.0097129	0.54929	insignificant	23	120	118
chr1	95239365	95241365	Ppp1r7	-0.10212757	3.01E-08	hypomethylated	-0.011741	0.48852	insignificant	15	98	96
chr1	95269472	95271472	Ano7	0.06506181	1	insignificant	0.09931	0.13621	insignificant	6	20	20
chr1	95374569	95376569	###########	-0.12277224	1.07E-32	hypomethylated	-0.00077338	0.46778	insignificant	33	118	114
chr1	95374637	95376637	###########	-0.14622013	9.82E-34	hypomethylated	0.019957	0.63451	insignificant	33	126	122
chr1	95375385	95377385	###########	-0.2110552	6.24E-17	hypomethylated	0.044052	0.82263	insignificant	16	66	66
chr1	95407680	95409680	Farp2	-0.09717281	2.83E-11	hypomethylated	0.061616	0.71265	insignificant	12	78	52
chr1	95532304	95534304	Stk25	-0.08638194	0.10984	insignificant	0.0014815	0.43391	insignificant	5	12	12
chr1	95581270	95583270	Bok	-0.09906217	0.000000103	hypomethylated	-0.00033329	0.72527	insignificant	26	139	145
chr1	95650609	95652609	Atg4b	0.02232488	8.71E-08	inconclusive	0.0037627	0.8694	insignificant	53	173	169
chr1	95651415	95653415	Thap4	0.11147584	0.3353	insignificant	0.0053705	0.51047	insignificant	30	101	101
chr1	95698511	95700511	Ing5	-0.12221504	0.00010523	hypomethylated	-0.0066942	0.33678	insignificant	14	78	77
chr1	95699541	95701541	Ing5	-0.0827454	0.01647	hypomethylated	0.0029307	0.45169	insignificant	19	81	81
chr1	95720816	95722816	D2hgdh	-0.20819506	0.000000607	hypomethylated	-0.028046	0.92355	insignificant	2	21	19
chr1	95949130	95951130	Pdcd1	-0.40457201	0.44989	insignificant	0.036315	0.53416	insignificant	2	18	18
chr1	97209204	97211204	Fam174a	-0.15823277	2.59E-27	hypomethylated	0.027182	0.58665	insignificant	16	75	62
chr1	97564171	97566171	St8sia4	-0.12403696	0.025208	hypomethylated	0.013716	0.084613	insignificant	4	30	34
chr1	98768748	98770748	Slco4c1	-0.23478386	0.00000012	hypomethylated	0.054943	1	insignificant	4	14	14
chr1	99558595	99560595	D1Ertd622e	-0.12457833	6.82E-16	hypomethylated	0.000034344	0.72419	insignificant	19	41	41
chr1	99665755	99667755	Gin1	-0.12525244	5.74E-10	hypomethylated	0.0047763	0.69806	insignificant	17	85	83
chr1	99666669	99668669	Ppip5k2	-0.27614199	4.09E-09	hypomethylated	-0.10563	0.93228	insignificant	8	36	32
chr1	99992209	99994209	Pam	-0.01443759	0.64777	insignificant	-0.0019862	0.96464	insignificant	8	40	40
chr1	101668341	101670341	Cntnap5b		1	noCoverage	0.025221	0.81627	insignificant	0	10	10
chr1	106664395	106666395	Cdh20	-0.27029321	0.0074484	hypomethylated	0.10908	0.52524	insignificant	2	24	21
chr1	107253287	107255287	Rnf152	-0.35714286	1	lowCoverage	-0.14286	0.47293	insignificant	1	2	2
chr1	107559437	107561437	2310035C23Ri	-0.14558959	0.00000188	hypomethylated	-0.0072418	0.81816	insignificant	23	112	112
chr1	107560253	107562253	Pign	-0.11571886	0.00013797	hypomethylated	0.0056226	0.55004	insignificant	22	95	94
chr1	107676299	107678299	Tnfrsf11a	-0.08176163	1.73E-28	hypomethylated	0.0029393	0.18612	insignificant	30	97	96
chr1	107885982	107887982	Zcchc2	-0.12093864	8.39E-44	hypomethylated	-0.032184	0.6049	insignificant	64	202	164
chr1	108067445	108069445	Phlpp1	-0.08949013	4.12E-23	hypomethylated	-0.01053	0.78221	insignificant	58	216	199
chr1	108610867	108612867	Bcl2	-0.09583255	1.08E-09	hypomethylated	0.0044359	1	insignificant	14	46	46
chr1	108656319	108658319	Kdsr	-0.28258094	0.61316	insignificant	-0.16043	0.74766	insignificant	5	15	10
chr1	108693302	108695302	Vps4b	-0.12612613	0.68664	insignificant	-0.015015	0.8909	insignificant	2	6	6
chr1	108756756	108758756	Serpinb5	0.07407407	1	insignificant	-0.0064815	0.64098	insignificant	1	4	4
chr1	109057691	109059691	Serpinb3b	-0.05122818	1	insignificant	0.0030053	0.13472	insignificant	1	4	4
chr1	113761495	113763495	Dsel		1	noCoverage	0.0059332	0.92458	insignificant	0	15	11
chr1	117580713	117582713	Cntnap5a	-0.25799413	1	lowCoverage	0.0035394	0.9021	insignificant	1	22	22
chr1	120207709	120209709	Tsn	-0.1606897	1.47E-15	hypomethylated	0.0057076	0.3056	insignificant	23	63	63
chr1	120217419	120219419	Mki67ip	-0.20743114	0.000000075	hypomethylated	0.14667	5.11E-21	hypermethylated	8	49	50
chr1	120284634	120286634	Clasp1	-0.16370553	2.26E-21	hypomethylated	0.016089	0.77622	insignificant	21	101	95
chr1	120355842	120357842	Clasp1	-0.27336198	1.04E-08	hypomethylated	-0.010453	0.88062	insignificant	8	17	17
chr1	120523521	120525521	Tfcp2l1	-0.15027584	0.000000125	hypomethylated	0.00076461	0.88049	insignificant	18	65	65
chr1	120950196	120952196	Gli2	-0.13165359	0.00014263	hypomethylated	0.0093396	0.12013	insignificant	22	62	69
chr1	121318825	121320825	Inhbb	-0.08131411	1.17E-10	hypomethylated	0.015113	1	insignificant	15	69	70
chr1	121401359	121403359	Ralb	-0.03644845	0.34893	insignificant	-0.0054737	0.2161	insignificant	2	24	24
chr1	121421743	121423743	Tmem185b	-0.11823468	8.88E-21	hypomethylated	0.0018828	0.84716	insignificant	23	95	101
chr1	121545577	121547577	Epb4.1l5	0.04308577	1	insignificant	0.013912	0.38338	insignificant	6	18	18
chr1	121733648	121735648	Ptpn4	-0.11265364	0.0001554	hypomethylated	0.0029683	1	insignificant	10	41	41
chr1	121809745	121811745	Tmem177	-0.21452343	0.12827	insignificant	-0.058056	0.95615	insignificant	0	36	32
chr1	121891787	121893787	Gm101	0.00959157	1	insignificant	0.06447	0.67213	insignificant	6	25	22
chr1	121902556	121904556	Sctr	-0.12636427	0.00029936	hypomethylated	-0.0072405	0.74656	insignificant	12	39	36
chr1	121970357	121972357	Tmem37		1	noCoverage	0.017207	0.0012077	inconclusive	0	32	32
chr1	122016763	122018763	3110009E18Ri	-0.1511551	0.00065933	hypomethylated	0.013856	0.33722	insignificant	13	67	62
chr1	122017496	122019496	Dbi	-0.20037127	0.0030053	hypomethylated	-0.00015761	0.11444	insignificant	9	58	58
chr1	122017673	122019673	Dbi	-0.25053734	0.0054078	hypomethylated	-0.0060406	0.0039596	hypomethylated	3	36	36
chr1	122161857	122163857	Steap3	-0.30725108	0.00000352	hypomethylated	-0.12715	0.016349	hypomethylated	4	11	9
chr1	122236158	122238158	C1ql2	-0.10550171	4.77E-11	hypomethylated	-0.012471	0.30666	insignificant	24	123	121
chr1	122498063	122500063	En1	-0.11242658	3.14E-14	hypomethylated	0.0044322	0.68267	insignificant	26	112	110
chr1	123224339	123226339	Insig2	-0.16838227	1.82E-13	hypomethylated	0.0054339	0.12215	insignificant	15	55	53

chr1	123326643	123328643	Ccdc93	-0.18619666	1.66E-19	hypomethylated	0.036929	0.18995	insignificant	17	53	52
chr1	125942136	125944136	Dpp10	-0.19810018	4.39E-19	hypomethylated	-0.02356	0.82158	insignificant	13	61	55
chr1	127332067	127334067	Actr3	-0.13811259	7.5E-16	hypomethylated	0.018045	0.37097	insignificant	24	69	68
chr1	127456592	127458592	Slc35f5	-0.14157336	6.09E-09	hypomethylated	-0.001527	0.031651	inconclusive	28	79	79
chr1	127572572	127574572	Gpr39	-0.32843323	0.10962	insignificant	-0.0081331	1	insignificant	4	9	9
chr1	127808791	127810791	Lypd1	-0.15494003	0.00000102	hypomethylated	-0.022029	0.56089	insignificant	3	8	8
chr1	128727209	128729209	Nckap5	-0.25167112	0.0084729	hypomethylated	0.29148	0.60694	insignificant	1	8	4
chr1	129100562	129102562	Mgat5	-0.23748125	1.13E-31	hypomethylated	-0.0023901	0.23337	insignificant	15	56	56
chr1	129574598	129576598	Tmem163		1	noCoverage	0.0050316	0.015768	hypermethylated	0	38	38
chr1	129669740	129671740	Ccnt2	-0.14211753	1E-29	hypomethylated	0.021347	0.21905	insignificant	25	88	80
chr1	129764349	129766349	Rab3gap1	0.51376958	0.22758	insignificant	-0.045665	0.732	insignificant	1	33	32
chr1	129998882	130000882	R3hdm1	-0.07669083	0.000000314	hypomethylated	0.030263	0.63934	insignificant	19	84	82
chr1	129998985	130000985	Zranb3	-0.07669083	0.000000314	hypomethylated	0.030263	0.63934	insignificant	19	84	82
chr1	130139757	130141757	Ubxn4	-0.1152583	1.54E-20	hypomethylated	0.013552	0.29479	insignificant	29	94	93
chr1	130256233	130258233	Mcm6	-0.38986803	7.75E-26	stronglyHypometh	0.010308	0.048281	inconclusive	7	24	24
chr1	130488876	130490876	Cxcr4	-0.17127525	0.0000006	hypomethylated	-0.015167	0.20205	insignificant	9	36	41
chr1	131168880	131170880	Thsd7b	-0.09679996	0.00000116	hypomethylated	0.013858	0.42274	insignificant	17	66	66
chr1	132359317	132361317	Cd55	-0.21563105	0.71226	insignificant	0.030574	0.013267	hypermethylated	3	14	14
chr1	132526183	132528183	Zp3r	-0.0577381	0.79837	insignificant	-0.010119	0.34512	insignificant	2	8	8
chr1	132611153	132613153	Pfkfb2	-0.15367755	0.091603	insignificant	0.0058155	0.48499	insignificant	3	30	30
chr1	132612398	132614398	Pfkfb2	-0.12754792	1.82E-27	hypomethylated	0.013882	0.92924	insignificant	17	72	70
chr1	132612903	132614903	Yod1	-0.16198191	7.98E-36	hypomethylated	0.021301	0.35133	insignificant	20	68	66
chr1	132627552	132629552	AA986860	-0.18114956	4.91E-08	hypomethylated	0.023458	0.24899	insignificant	7	19	20
chr1	132696478	132698478	Fcamr		1	noCoverage	0.11173	0.029667	hypermethylated	0	6	4
chr1	132807873	132809873	Il20	0.025	1	lowCoverage	0.0080508	0.24851	insignificant	1	5	2
chr1	132915421	132917421	Il10	-0.02777778	1	insignificant	0.044833	0.55248	insignificant	2	4	4
chr1	132994120	132996120	Mapkapk2	-0.11150723	2.31E-19	hypomethylated	0.0044748	0.76126	insignificant	23	72	69
chr1	133034811	133036811	Dyrk3	-0.47082257	0.000000667	stronglyHypometh	-0.084811	0.56524	insignificant	2	13	13
chr1	133048783	133050783	Eif2d	-0.12194077	0.041934	hypomethylated	0.049002	0.84228	insignificant	6	25	20
chr1	133141755	133143755	Rassf5	-0.14628573	1.21E-15	hypomethylated	0.0037708	0.57223	insignificant	21	95	100
chr1	133176140	133178140	Ikbke		1	noCoverage	0.082814	0.073075	insignificant	0	8	8
chr1	133423565	133425565	Fam72a	-0.10414709	4.42E-10	hypomethylated	-0.0016939	0.080415	insignificant	17	109	112
chr1	133423938	133425938	Fam72a	-0.10235135	0.0062844	hypomethylated	-0.0095848	1	insignificant	9	57	60
chr1	133533890	133535890	Ctse		1	noCoverage	-0.001611	1	insignificant	0	8	8
chr1	133639598	133641598	Slc26a9	0.41763106	0.10299	insignificant	-0.24279	0.09192	insignificant	1	27	14
chr1	133692971	133694971	Pm20d1		1	noCoverage	-0.028594	1	insignificant	0	10	10
chr1	133723588	133725588	Slc41a1	0.00790218	0.32975	insignificant	-0.057219	0.40074	insignificant	7	76	71
chr1	133762853	133764853	Rab7l1	-0.19488496	0.00025063	hypomethylated	-0.0068855	1	insignificant	4	67	66
chr1	133806034	133808034	Nucks1	-0.12891108	1.36E-14	hypomethylated	-0.0036391	0.64733	insignificant	21	96	82
chr1	133858543	133860543	Slc45a3	-0.11996716	2.56E-20	hypomethylated	-0.0073774	0.065067	insignificant	38	135	135
chr1	133866185	133868185	Slc45a3	-0.24349302	0.20389	insignificant	-0.021713	0.57456	insignificant	4	17	16
chr1	133903181	133905181	Elk4	-0.08398312	9.41E-20	hypomethylated	-0.00014016	0.76228	insignificant	24	163	164
chr1	133964639	133966639	Mfsd4	-0.15271582	1.73E-11	hypomethylated	0.030375	0.80129	insignificant	7	32	34
chr1	134036262	134038262	Cdk18	-0.46170163	4.41E-18	stronglyHypometh	0.00060651	0.60053	insignificant	4	12	13
chr1	134087012	134089012	Lemd1	-0.11967726	1.94E-15	hypomethylated	-0.0031984	0.0070244	inconclusive	29	128	130
chr1	134093664	134095664	Mir135b	-0.15077919	0.60029	insignificant	0.076594	0.00000154	hypermethylated	5	14	14
chr1	134194202	134196202	Klhdc8a	-0.06903031	1.45E-12	hypomethylated	-0.00031327	0.38961	insignificant	39	183	188
chr1	134211701	134213701	Nuak2	-0.07356706	0.00011313	hypomethylated	0.024259	0.91525	insignificant	5	58	54
chr1	134287858	134289858	Tmcc2	-0.08575692	2.06E-09	hypomethylated	0.0059088	0.37324	insignificant	20	101	101
chr1	134313029	134315029	Dstyk	-0.12070865	4.41E-15	hypomethylated	-0.000027752	0.1254	insignificant	28	106	96
chr1	134372943	134374943	Rbbp5	-0.26018095	0.00043858	hypomethylated	-0.0092519	0.2297	insignificant	7	63	58
chr1	134401806	134403806	Tmem81		1	noCoverage	0.044238	0.35729	insignificant	0	4	4
chr1	134439517	134441517	Cntn2	-0.07995345	0.67848	insignificant	0.017278	0.83645	insignificant	5	18	22
chr1	134539435	134541435	Nfasc	0.06465124	0.6821	insignificant	-0.16755	0.079452	insignificant	3	6	6
chr1	134638374	134640374	Nfasc	-0.18539772	0.002942	hypomethylated	0.019281	0.88385	insignificant	3	6	6
chr1	134775931	134777931	Lrrn2	-0.11017978	3.34E-45	hypomethylated	0.0040159	0.14174	insignificant	39	157	150
chr1	134921925	134923925	Mdm4	-0.4106169	3.72E-10	stronglyHypometh	0.084486	0.36139	insignificant	4	20	22
chr1	134941588	134943588	Pik3c2b	-0.11449916	1.39E-28	hypomethylated	-0.0017337	0.80652	insignificant	38	167	167
chr1	135026742	135028742	Ppp1r15b	-0.08046708	8.83E-19	hypomethylated	-0.0045269	0.87427	insignificant	29	136	133
chr1	135259148	135261148	Etnk2	-0.14466736	2.28E-19	hypomethylated	-0.0081866	0.72383	insignificant	25	116	114
chr1	135320789	135322789	Sox13	0.03575803	0.10924	insignificant	-0.015277	0.094713	insignificant	6	34	31
chr1	135506857	135508857	Snrpe	0.07226854	0.865	insignificant	0.058319	0.028551	hypermethylated	5	24	17
chr1	135557462	135559462	Zbed6	-0.18260388	0.000077507	hypomethylated	-0.003909	0.65507	insignificant	12	36	36
chr1	135557957	135559957	Zbed6	-0.20382749	1	insignificant	-0.013996	0.3948	insignificant	4	16	16
chr1	135586593	135588593	Lax1		1	noCoverage	0.05206	0.013452	hypermethylated	0	21	20
chr1	135586685	135588685	Lax1		1	noCoverage	0.05206	0.013452	hypermethylated	0	21	20
chr1	135803750	135805750	Optc	0.30641555	0.17448	insignificant	-0.0030644	0.72681	insignificant	2	9	18
chr1	135804576	135806576	Optc	0.30641555	0.17448	insignificant	-0.091487	0.79259	insignificant	2	9	14
chr1	135975732	135977732	Btg2	-0.07900669	0.00000191	hypomethylated	0.0021581	0.43371	insignificant	12	46	46
chr1	136185580	136187580	Myog	-0.15976253	0.053385	insignificant	-0.1316	0.1551	insignificant	4	24	22
chr1	136229505	136231505	Ppfia4		1	noCoverage	0.038374	1	insignificant	0	9	9
chr1	136258576	136260576	Tmem183a	-0.13793103	0.0015621	hypomethylated	-0.012931	0.8367	insignificant	3	6	6
chr1	136301566	136303566	Cyb5r1	-0.23553208	2.89E-14	hypomethylated	-0.016991	0.37953	insignificant	5	45	45
chr1	136311043	136313043	Adipor1	-0.09034759	1.36E-31	hypomethylated	0.0049756	0.51024	insignificant	50	149	148
chr1	136351131	136353131	Klhl12	-0.17527157	1.06E-09	hypomethylated	-0.029218	0.73497	insignificant	8	32	32
chr1	136390236	136392236	Rabif	-0.10675672	1.09E-09	hypomethylated	0.018402	0.82716	insignificant	25	86	86
chr1	136455754	136457754	Kdm5b	-0.17071024	1.78E-19	hypomethylated	-0.056047	0.39075	insignificant	26	52	73
chr1	136542257	136544257	Syt2	-0.1203973	0.00000146	hypomethylated	0.0056218	0.61132	insignificant	18	106	97
chr1	136858153	136860153	Ube2t		1	noCoverage	0.087269	0.74436	insignificant	0	12	8
chr1	137042410	137044410	Arl8a	-0.08831493	1.21E-24	hypomethylated	0.0081723	0.23737	insignificant	63	172	155
chr1	137155049	137157049	Elf3	0.02663477	0.50213	insignificant	0.068386	0.9036	insignificant	6	11	13
chr1	137210314	137212314	Timm17a	-0.1644639	0.18624	insignificant	0.052623	0.065556	insignificant	9	22	24
chr1	137220389	137222389	Lmod1	-0.1505104	0.78192	insignificant	0.074803	0.038261	hypermethylated	6	38	38
chr1	137271640	137273640	Shisa4	0.10640925	0.14563	insignificant	-0.026527	0.018529	hypomethylated	3	7	7
chr1	137327068	137329068	Ipo9	-0.11415176	0.69589	insignificant	-0.0017479	0.89621	insignificant	6	24	24

chr1	137481932	137483932	Nav1	-0.18337062	8.38E-09	hypomethylated	-0.002077	0.30334	insignificant	5	49	41
chr1	137624773	137626773	Csrp1	-0.16111789	0.00000483	hypomethylated	0.056348	1	insignificant	14	44	37
chr1	137661661	137663661	Phlda3	-0.11572063	2.26E-10	hypomethylated	0.009747	0.90098	insignificant	33	149	137
chr1	137714174	137716174	Lad1	-0.12386112	0.000018258	hypomethylated	0.032018	0.00000518	hypermethylated	14	91	83
chr1	137736437	137738437	Tnnt2	-0.04814815	0.62301	insignificant	-0.014259	0.19014	insignificant	1	6	6
chr1	137815601	137817601	Pkp1	-0.07025057	0.00000166	hypomethylated	0.0070693	0.67409	insignificant	8	62	58
chr1	137902919	137904919	Tmem9		1	noCoverage	0.087212	1	insignificant	0	20	24
chr1	137903795	137905795	Tmem9		1	noCoverage	0.068817	0.91323	insignificant	0	25	28
chr1	137913577	137915577	Tmem9	-0.3780209	0.50579	insignificant	0.099635	1	insignificant	2	6	7
chr1	137913732	137915732	Tmem9	-0.29262954	1	insignificant	0.029842	0.72536	insignificant	2	18	19
chr1	138026977	138028977	Kif21b	-0.12847127	3.37E-15	hypomethylated	0.004325	0.90107	insignificant	18	93	90
chr1	138130841	138132841	5730559C18Ri	-0.23951211	1.48E-13	hypomethylated	-0.041003	0.038093	hypomethylated	16	39	36
chr1	138157450	138159450	Gpr25	-0.62692308	0.22018	lowCoverage	0.24429	0.64475	insignificant	1	2	6
chr1	138242681	138244681	Camsap2	-0.15115632	0.004131	hypomethylated	0.11008	0.33485	insignificant	4	8	9
chr1	138310847	138312847	Ddx59	-0.08640619	4.64E-20	hypomethylated	0.014449	0.7175	insignificant	20	69	70
chr1	138312096	138314096	Ddx59	0.12141594	0.30786	insignificant	0.018328	0.013401	hypermethylated	6	41	42
chr1	138363534	138365534	Kif14	-0.24721727	0.000056844	hypomethylated	-0.011649	0.60287	insignificant	3	28	24
chr1	138520477	138522477	Zfp281	-0.11002686	1.43E-13	hypomethylated	-0.01367	0.77929	insignificant	41	138	132
chr1	138850207	138852207	Nr5a2	0.3021978	0.57851	insignificant	0.052198	0.6402	insignificant	2	7	6
chr1	140516273	140518273	Nek7	-0.15387187	0.00000122	hypomethylated	-0.0076731	0.45496	insignificant	11	38	38
chr1	140739021	140741021	Lhx9	-0.47645768	0.00000367	stronglyHypometh	0.11398	0.41527	insignificant	1	2	2
chr1	140744156	140746156	Lhx9	-0.19718985	0.000000954	hypomethylated	0.005511	1	insignificant	15	64	62
chr1	140859285	140861285	Dennd1b	-0.11664673	3.76E-19	hypomethylated	-0.010302	0.2713	insignificant	27	144	129
chr1	141273653	141275653	Crb1	-0.09318182	0.24298	insignificant	-0.035248	0.75558	insignificant	2	6	7
chr1	141317959	141319959	Zbtb41	-0.03288202	0.029883	hypomethylated	-0.01114	0.35189	insignificant	29	109	105
chr1	141350349	141352349	Aspm	-0.23549336	5.96E-08	hypomethylated	0.013437	0.88431	insignificant	17	59	65
chr1	141456799	141458799	Cfhr1		1	noCoverage	-0.22543	0.80907	insignificant	0	4	4
chr1	142141833	142143833	Kcnt2	-0.25619687	2.39E-08	hypomethylated	0.018563	0.82114	insignificant	8	46	36
chr1	145549814	145551814	Cdc73	-0.07348748	9.65E-09	hypomethylated	-0.0046051	0.38581	insignificant	43	135	135
chr1	145585478	145587478	Glrx2	-0.21947734	6.02E-22	hypomethylated	0.010453	0.19499	insignificant	17	93	93
chr1	145585697	145587697	Glrx2	-0.24049546	1.28E-22	hypomethylated	0.0062076	0.13094	insignificant	17	97	96
chr1	145585744	145587744	Glrx2	-0.24049546	1.28E-22	hypomethylated	0.0062076	0.13094	insignificant	17	97	96
chr1	145586576	145588576	Glrx2	-0.15385917	3.72E-15	hypomethylated	0.0060968	0.76309	insignificant	16	88	87
chr1	145623407	145625407	Uchl5	-0.10402982	1.57E-09	hypomethylated	0.012562	0.22189	insignificant	57	193	187
chr1	145624181	145626181	Trove2	-0.11931375	0.0090145	hypomethylated	0.0053805	0.39814	insignificant	44	134	126
chr1	145851279	145853279	Rgs2	-0.04642833	1	insignificant	0.017717	0.49792	insignificant	7	34	35
chr1	148341795	148343795	Fam5c	-0.25155565	0.000011218	hypomethylated	0.0064082	0.70127	insignificant	10	20	20
chr1	151808414	151810414	Pla2g4a	-0.59642857	0.43956	lowCoverage	-0.070567	0.75041	insignificant	1	4	4
chr1	151946253	151948253	Ptgs2	0.12686378	0.45768	insignificant	-0.0066295	0.45592	insignificant	7	28	28
chr1	151946896	151948896	7530420F21Ri	0.19070725	0.11448	insignificant	-0.027578	0.40803	insignificant	4	14	14
chr1	152238967	152240967	Tpr	-0.12016741	7.49E-13	hypomethylated	0.0014355	0.42241	insignificant	37	126	128
chr1	152240185	152242185	BC003331	-0.12246965	3.65E-12	hypomethylated	0.010985	0.90124	insignificant	30	118	118
chr1	152840565	152842565	Hmcn1	-0.37950137	1.73E-13	stronglyHypometh	-0.044276	0.14068	insignificant	4	26	26
chr1	153190627	153192627	Ivns1abp	-0.12400449	6.95E-40	hypomethylated	-0.0032158	0.8754	insignificant	50	148	137
chr1	153274777	153276777	1190005F20Ri	-0.13071385	1.23E-11	hypomethylated	-0.0029044	0.22614	insignificant	11	73	64
chr1	153275565	153277565	1200016B10R	-0.12303383	0.59567	insignificant	-0.03373	0.84307	insignificant	3	24	24
chr1	153347953	153349953	Rnf2	-0.1187504	2.29E-10	hypomethylated	0.016865	0.38871	insignificant	17	69	69
chr1	153417502	153419502	Fam129a	-0.18070595	8.3E-30	hypomethylated	0.051783	0.0074763	hypermethylated	13	51	45
chr1	153601503	153603503	Edem3	-0.1600847	1.83E-48	hypomethylated	0.017234	0.17356	insignificant	29	106	104
chr1	153937450	153939450	1700025G04R	-0.19222687	1.52E-09	hypomethylated	0.051471	0.21526	insignificant	8	27	23
chr1	154233812	154235812	Tsen15	-0.53405345	0.50577	insignificant	-0.11375	0.095849	insignificant	2	10	14
chr1	154245996	154247996	Glt25d2	-0.13512878	0.0000167	hypomethylated	-0.0019067	0.96284	insignificant	12	64	52
chr1	154472241	154474241	Rgl1	-0.06007956	0.000000241	hypomethylated	0.03552	0.52779	insignificant	11	22	23
chr1	154596680	154598680	Apobec4	-0.11131193	0.089388	insignificant	0.046136	0.57131	insignificant	2	12	12
chr1	154612671	154614671	Arpc5	-0.11353511	1.05E-58	hypomethylated	0.0034445	0.64427	insignificant	80	226	223
chr1	154654019	154656019	Ncf2	-0.30326434	0.1792	insignificant	0.01495	0.94157	insignificant	3	12	12
chr1	154749776	154751776	Smg7	-0.1664395	1.14E-26	hypomethylated	0.020951	0.42367	insignificant	24	95	91
chr1	154801230	154803230	Nmnat2	-0.13632097	2.1E-09	hypomethylated	0.01154	0.87696	insignificant	11	69	71
chr1	155033577	155035577	Lamc2		1	noCoverage	-0.15833	0.081017	insignificant	0	2	2
chr1	155179916	155181916	Lamc1	-0.18121254	8.63E-29	hypomethylated	0.016675	0.67392	insignificant	26	84	80
chr1	155261363	155263363	E330020D12R	-0.62468412	0.023589	stronglyHypometh	-0.026066	1	insignificant	1	23	23
chr1	155271338	155273338	1700012A16R	-0.10750638	0.0036262	hypomethylated	0.022633	0.72006	insignificant	9	39	39
chr1	155334790	155336790	Dhx9	-0.32051282	0.34745	insignificant	-0.0037199	0.2495	insignificant	7	24	24
chr1	155396844	155398844	Npl	-0.42300195	0.1465	insignificant	-0.054208	0.70958	insignificant	3	6	6
chr1	155499166	155501166	Rgs8	-0.32548012	0.55532	insignificant	-0.049753	0.70114	insignificant	3	25	25
chr1	155586482	155588482	Rgs16	-0.0989523	0.012572	hypomethylated	-0.047127	0.80194	insignificant	9	48	44
chr1	155746074	155748074	Glul	-0.08928169	1.1E-29	hypomethylated	0.014416	0.6369	insignificant	51	147	144
chr1	156573050	156575050	Cacna1e	-0.12480706	1	insignificant	-0.0056455	0.5954	insignificant	6	33	33
chr1	156946766	156948766	Ier5	-0.19305556	0.23678	insignificant	-0.011237	0.37114	insignificant	1	4	4
chr1	157004832	157006832	Stx6	-0.16033985	8.04E-40	hypomethylated	-0.021274	0.94576	insignificant	46	121	116
chr1	157404249	157406249	Acbd6	-0.12697935	2.82E-30	hypomethylated	-0.01465	0.53385	insignificant	46	127	126
chr1	157589157	157591157	Lhx4	-0.097726	8.44E-17	hypomethylated	0.0029231	0.95436	insignificant	28	128	130
chr1	157660029	157662029	Qsox1	-0.14454965	0.00000382	hypomethylated	0.0059592	0.3738	insignificant	8	40	37
chr1	157881793	157883793	Tor1aip2	-0.10024839	0.00000037	hypomethylated	-0.0057398	0.4271	insignificant	25	119	117
chr1	157881856	157883856	Tor1aip2	-0.10024839	0.00000037	hypomethylated	-0.0057398	0.4271	insignificant	25	119	117
chr1	157881947	157883947	Tor1aip1	-0.10024839	0.00000037	hypomethylated	-0.0057398	0.4271	insignificant	25	119	117
chr1	157882165	157884165	Tor1aip2	-0.10024839	0.00000037	hypomethylated	-0.0057398	0.4271	insignificant	25	119	117
chr1	158135156	158137156	Fam163a	-0.19892473	0.01647	hypomethylated	0.021075	0.63499	insignificant	2	4	4
chr1	158233479	158235479	Tdrd5	-0.1746909	0.0012526	hypomethylated	0.0054112	0.5629	insignificant	17	62	62
chr1	158239865	158241865	Nphs2	-0.18333333	1	insignificant	0.13683	0.074763	insignificant	2	20	14
chr1	158404459	158406459	Soat1		1	noCoverage	0.0045956	1	insignificant	0	6	6
chr1	158487917	158489917	Abl2	-0.03209994	0.01555	hypomethylated	0.007554	1	insignificant	16	137	111
chr1	158604470	158606470	Tor3a	-0.3327878	2.4E-12	hypomethylated	-0.028506	0.21577	insignificant	9	39	36
chr1	158649041	158651041	Fam20b	-0.1364417	0.00000118	hypomethylated	0.044514	0.28595	insignificant	11	33	25
chr1	158869518	158871518	Ralgps2	-0.08862827	3.47E-19	hypomethylated	-0.0201	0.38295	insignificant	24	77	67

chr1	158869757	158871757	Ralgps2	-0.11119515	1.9E-21	hypomethylated	-0.016913	0.73111	insignificant	19	51	41
chr1	159038776	159040776	1700057K13Ri	0.0952381	1	insignificant	-0.031746	0.20981	insignificant	1	6	6
chr1	159341482	159343482	2810025M15F	-0.0427569	1.08E-30	hypomethylated	0.0053439	0.89908	insignificant	55	161	166
chr1	159342726	159344726	Rasal2	0.67889657	0.00011417	stronglyHypermeth	-0.0044284	0.61161	insignificant	3	26	26
chr1	159387712	159389712	BC026585	-0.20659369	0.000073383	hypomethylated	0.013605	0.46704	insignificant	10	39	39
chr1	159455300	159457300	Sec16b	0.10731088	0.48674	insignificant	0.000029032	0.14598	insignificant	2	16	14
chr1	160286391	160288391	Fam5b	-0.34548633	0.53768	insignificant	-0.035246	0.49893	insignificant	2	7	7
chr1	160291470	160293470	Astn1	-0.27179068	0.0066219	hypomethylated	-0.061976	0.85744	insignificant	5	43	38
chr1	161161456	161163456	Rfwd2	-0.11547606	1.07E-18	hypomethylated	-0.0087386	0.34729	insignificant	34	116	113
chr1	161973510	161975510	4930523C07Ri	-0.17178979	0.0026831	hypomethylated	0.0028679	0.27444	insignificant	11	40	40
chr1	162124390	162126390	Mrps14	-0.13912102	2.89E-11	hypomethylated	-0.018874	0.97063	insignificant	8	35	43
chr1	162142908	162144908	Cacybp	-0.08580695	1.22E-21	hypomethylated	0.010393	0.58342	insignificant	31	54	58
chr1	162723069	162725069	Rabgap1l	-0.23782051	0.34278	insignificant	-0.027404	0.44301	insignificant	4	24	24
chr1	162835541	162837541	Rc3h1	-0.09480424	3.32E-34	hypomethylated	0.0061333	0.49649	insignificant	23	136	130
chr1	162964296	162966296	Gas5	-0.11050242	1.59E-11	hypomethylated	0.007004	0.51777	insignificant	23	129	114
chr1	162964390	162966390	Zbtb37	-0.11050242	1.59E-11	hypomethylated	0.007004	0.51777	insignificant	23	129	114
chr1	163000022	163002022	Cenpl	-0.27822419	1.53E-20	hypomethylated	0.0017951	0.86946	insignificant	13	42	39
chr1	163000763	163002763	Dars2	-0.27330042	8.35E-19	hypomethylated	-0.00024927	0.6491	insignificant	13	38	36
chr1	163061610	163063610	Klhl20		1	noCoverage	-0.014359	0.75159	insignificant	0	8	8
chr1	163071842	163073842	Ankrd45	-0.02826632	0.051677	insignificant	0.0009494	1	insignificant	9	26	26
chr1	163089445	163091445	Ankrd45	0.32234848	0.043409	hypermethylated	-0.073646	0.66355	insignificant	2	8	8
chr1	163324568	163326568	Tnfsf4	-0.35040713	4.34E-17	stronglyHypometh	0.043539	0.86037	insignificant	4	9	8
chr1	163423787	163425787	Tnfsf18	0.10256008	0.77353	insignificant	0.16894	0.76903	insignificant	1	5	4
chr1	163806792	163808792	AI848100	0.07380525	0.68333	insignificant	0.0052572	0.38783	insignificant	8	54	54
chr1	163898318	163900318	Pigc	-0.18268751	1.77E-16	hypomethylated	0.010625	0.79199	insignificant	18	52	44
chr1	164146753	164148753	Dnm3os	-0.36805556	0.16149	insignificant	-0.010227	1	insignificant	2	4	4
chr1	164146944	164148944	Mir199a-2	-0.36805556	0.16149	insignificant	-0.010227	1	insignificant	2	4	4
chr1	164152498	164154498	Mir214	0.0719697	1	insignificant	0.031843	0.63186	insignificant	1	6	6
chr1	164478476	164480476	Mettl13	-0.11969166	0.000031493	hypomethylated	0.0038976	0.49228	insignificant	16	45	45
chr1	164499958	164501958	Vamp4	-0.14298682	4.1E-18	hypomethylated	-0.018982	0.58593	insignificant	22	116	108
chr1	164568280	164570280	Myoc		1	noCoverage	-0.035951	0.21261	insignificant	0	5	5
chr1	164670687	164672687	Prrc2c		1	noCoverage	0.0067235	0.94721	insignificant	0	30	30
chr1	164828843	164830843	Fmo2	-0.61716301	0.00000326	stronglyHypometh	-0.067698	0.23678	insignificant	1	8	10
chr1	165243781	165245781	Prrx1	-0.14522328	0.0018251	hypomethylated	0.013983	0.058805	insignificant	6	22	22
chr1	165333772	165335772	Gorab	-0.21791255	0.00055038	hypomethylated	-0.013163	0.21365	insignificant	5	20	20
chr1	165708809	165710809	Kifap3	-0.15809524	0.0029488	hypomethylated	-0.018996	0.92623	insignificant	12	35	35
chr1	165859194	165861194	Scyl3	-0.13941915	9.52E-41	hypomethylated	0.021121	0.20698	insignificant	40	111	99
chr1	165924075	165926075	2810422O20R	-0.22904851	0.000000418	hypomethylated	-0.040761	0.16581	insignificant	14	81	76
chr1	165924912	165926912	BC055324	-0.45713266	6.37E-09	stronglyHypometh	-0.05615	0.1804	insignificant	8	51	46
chr1	166178185	166180185	Slc19a2	-0.11602574	1.77E-13	hypomethylated	0.027259	0.042753	hypermethylated	28	111	111
chr1	166204728	166206728	4930455F23Ri	-0.15677232	0.00000156	hypomethylated	-0.012666	0.87861	insignificant	9	31	35
chr1	166236805	166238805	Nme7	-0.18154466	6.13E-13	hypomethylated	0.0087223	0.94501	insignificant	16	72	74
chr1	166237615	166239615	Nme7	-0.21125091	0.00036281	hypomethylated	0.041529	0.57329	insignificant	8	43	41
chr1	166388486	166390486	Atp1b1	-0.1300312	0.021849	hypomethylated	0.0054791	0.093726	insignificant	9	57	58
chr1	167090904	167092904	Tbx19	-0.01786245	0.49015	insignificant	0.069956	0.85983	insignificant	1	11	15
chr1	167124564	167126564	Sft2d2	-0.10343182	0.000004986	hypomethylated	-0.013307	0.19138	insignificant	7	22	22
chr1	167167089	167169089	Tiprl	-0.06708569	0.000021132	hypomethylated	0.034716	0.40298	insignificant	9	28	28
chr1	167390338	167392338	Brp44	-0.0938518	2.5E-14	hypomethylated	-0.010811	0.40091	insignificant	33	161	155
chr1	167390594	167392594	Dcaf6	-0.08072283	0.00018315	hypomethylated	-0.013201	0.78301	insignificant	21	130	124
chr1	167564672	167566672	Mpzl1	-0.27714859	5.76E-16	hypomethylated	0.054743	0.40922	insignificant	9	40	38
chr1	167638225	167640225	Rcsd1		1	noCoverage	-0.029412	1	insignificant	0	2	2
chr1	167692910	167694910	Creg1	-0.02288907	0.00000635	hypomethylated	-0.012123	0.46464	insignificant	18	56	56
chr1	167932765	167934765	Pou2f1	-0.07761951	5.36E-11	hypomethylated	0.0076471	0.76252	insignificant	33	163	153
chr1	168058029	168060029	Gpa33	0.17618867	1	lowCoverage	0.053824	0.12792	insignificant	1	6	6
chr1	168059590	168061590	Gpa33	-0.05594826	9.61E-08	hypomethylated	0.13174	0.016038	hypermethylated	2	16	16
chr1	168168875	168170875	Mael	0.08998015	0.077719	insignificant	-0.0010406	0.3544	insignificant	6	26	26
chr1	168183269	168185269	Ildr2	-0.10511765	2.53E-10	hypomethylated	-0.0065302	0.010236	inconclusive	38	105	102
chr1	168308297	168310297	Tada1	-0.19338967	2.59E-20	hypomethylated	-0.05018	0.52347	insignificant	29	96	90
chr1	168339959	168341959	Pogk	-0.13352044	0.0057079	hypomethylated	0.028938	0.099561	insignificant	7	42	42
chr1	168427719	168429719	Gm4846	-0.21190476	0.1251	insignificant	-0.034286	1	insignificant	2	10	10
chr1	168930547	168932547	Fam78b	-0.10928732	8.53E-33	hypomethylated	0.024498	0.40247	insignificant	45	210	178
chr1	169215258	169217258	Uck2	-0.1937119	2.18E-11	hypomethylated	0.025549	0.38912	insignificant	10	42	42
chr1	169237800	169239800	Tmco1	-0.1943188	1.5E-27	hypomethylated	0.038096	0.42199	insignificant	21	106	104
chr1	169279121	169281121	Aldh9a1	-0.1091005	9.97E-24	hypomethylated	0.0049686	0.07627	insignificant	16	82	83
chr1	169323928	169325928	Mgst3	-0.39818239	0.32027	insignificant	-0.052304	0.87536	insignificant	4	22	19
chr1	169527492	169529492	Rxrg	-0.43742116	1.11E-08	stronglyHypometh	0.068527	0.63475	insignificant	4	33	25
chr1	169618688	169620688	Lmx1a	-0.15086096	1.17E-13	hypomethylated	-0.0077224	0.43246	insignificant	38	167	160
chr1	171461595	171463595	Nuf2	-0.1890068	0.22972	insignificant	-0.055527	1	insignificant	2	22	16
chr1	171677773	171679773	Rgs4		1	noCoverage	-0.0034562	0.19169	insignificant	0	2	2
chr1	171858069	171860069	1700084C01Rik		1	noCoverage	-0.10268	0.51883	insignificant	0	12	10
chr1	171899336	171901336	Hsd17b7	-0.14493066	4.12E-10	hypomethylated	0.024575	0.01958	hypermethylated	10	22	22
chr1	172105077	172107077	Uap1	-0.08400509	0.2393	insignificant	-0.0012015	0.25468	insignificant	21	80	80
chr1	172237991	172239991	1700015E13Ri	0.0207235	0.40803	insignificant	0.006703	0.0076059	inconclusive	4	18	18
chr1	172519980	172521980	Nos1ap	-0.05943417	8.28E-10	hypomethylated	0.0072466	0.61045	insignificant	21	48	48
chr1	172573662	172575662	Olfml2b	-0.08890241	3.2E-25	hypomethylated	0.0046079	0.4636	insignificant	21	73	74
chr1	172797902	172799902	Atf6	-0.26468254	0.37549	insignificant	-0.048826	0.7965	insignificant	4	20	16
chr1	172857714	172859714	Fcrla	-0.0625	0.20546	insignificant	0.016667	1	insignificant	2	4	4
chr1	172906202	172908202	Fcgr2b	-0.64571429	0.32911	lowCoverage	0.099048	0.72806	insignificant	1	10	10
chr1	172948056	172950056	Fcgr4	0.01639656	0.71221	insignificant	0.18371	0.18073	insignificant	1	10	12
chr1	172989534	172991534	Fcgr3		1	noCoverage	-0.11814	0.066406	insignificant	0	6	6
chr1	173079843	173081843	Mpz	-0.14906274	0.000065115	hypomethylated	0.0039752	0.74354	insignificant	7	51	51
chr1	173080734	173082734	Sdhc	-0.10042285	0.0079982	hypomethylated	0.025616	0.59404	insignificant	4	20	20
chr1	173126399	173128399	Pcp4l1		1	noCoverage	-0.014815	0.63904	insignificant	0	6	6
chr1	173143100	173145100	Nr1i3		1	noCoverage	0.057312	0.0019044	hypermethylated	0	6	6
chr1	173152645	173154645	Apoa2	-0.30656775	0.51113	insignificant	0.023155	0.20198	insignificant	2	18	18

chr1	173154184	173156184	Apoa2	0.03968254	0.22175	insignificant	0.12097	0.042551	hypermethylated	3	6	6
chr1	173164480	173166480	Fcer1g	-0.6694791	0.0046072	stronglyHypometh	-0.024157	0.26655	insignificant	1	15	18
chr1	173177243	173179243	Ndufs2	-0.11058782	0.040232	hypomethylated	0.037041	0.2711	insignificant	9	44	44
chr1	173179552	173181552	Adamts4	-0.49733186	0.021563	stronglyHypometh	-0.082334	0.048799	inconclusive	5	21	23
chr1	173199458	173201458	B4galt3	-0.14095759	3.04E-08	hypomethylated	0.045492	0.45364	insignificant	14	78	78
chr1	173211317	173213317	Usp21	-0.11107643	0.026812	hypomethylated	0.015989	0.8313	insignificant	1	23	26
chr1	173218092	173220092	Ufc1		1	noCoverage	-0.42753	0.38218	insignificant	0	6	11
chr1	173225113	173227113	Ufc1	-0.08802309	1	insignificant	0.01592	0.5274	insignificant	1	2	2
chr1	173258275	173260275	Dedd	-0.13452208	3.74E-12	hypomethylated	0.0002641	0.48896	insignificant	36	122	122
chr1	173274829	173276829	Pfdn2	-0.20103587	0.18063	insignificant	0.010493	0.80827	insignificant	12	53	53
chr1	173275776	173277776	Nit1	-0.1664104	0.000063593	hypomethylated	-0.0034457	0.62892	insignificant	10	35	35
chr1	173290929	173292929	Klhdc9	-0.26536748	0.011669	hypomethylated	0.017966	1	insignificant	2	13	12
chr1	173340811	173342811	Usf1	-0.11860659	2.92E-11	hypomethylated	-0.002463	0.54112	insignificant	25	101	102
chr1	173348163	173350163	Tstd1	-0.03278172	0.22734	insignificant	-0.040183	0.30511	insignificant	4	40	34
chr1	173366691	173368691	F11r	-0.16806885	0.0087071	hypomethylated	-0.0036624	0.33105	insignificant	11	55	55
chr1	173432608	173434608	Refbp2	-0.12511497	0.0002448	hypomethylated	0.023422	0.95938	insignificant	14	61	61
chr1	173465424	173467424	Itln1		1	noCoverage	0.10278	1	insignificant	0	4	4
chr1	173583168	173585168	Slamf7	-0.525	0.25296	insignificant	-0.0069444	0.49845	insignificant	2	6	6
chr1	173611185	173613185	Cd48	-0.29256198	0.67404	insignificant	-0.0062982	0.89451	insignificant	2	11	10
chr1	173696262	173698262	Slamf1	-0.54882155	1.39E-15	stronglyHypometh	-0.028385	0.60506	insignificant	2	4	4
chr1	173957399	173959399	Vangl2	-0.10350714	1.76E-26	hypomethylated	0.013513	0.040478	hypermethylated	7	52	52
chr1	173987727	173989727	Nhlh1	0.30755583	1	lowCoverage	0.030593	0.63053	insignificant	1	9	8
chr1	174011659	174013659	Copa	-0.08583374	0.006602	hypomethylated	0.015732	0.8452	insignificant	5	67	63
chr1	174012880	174014880	Copa	-0.21045644	0.00023525	hypomethylated	0.024188	0.6647	insignificant	4	61	61
chr1	174055885	174057885	Pex19	-0.07597228	0.78881	insignificant	0.08633	0.77929	insignificant	7	25	20
chr1	174077145	174079145	Dcaf8	-0.12398054	9.77E-13	hypomethylated	-0.0012741	0.53747	insignificant	21	93	90
chr1	174228195	174230195	Atp1a2	-0.93333333	0.033333	stronglyHypometh	-0.07619	0.12106	insignificant	2	5	5
chr1	174241537	174243537	Igsf8	-0.15025553	0.00056892	hypomethylated	0.0050506	0.58327	insignificant	7	30	35
chr1	174270340	174272340	Kcnj10	-0.13291851	0.0018342	hypomethylated	-0.013011	0.043786	inconclusive	14	53	45
chr1	174305661	174307661	Pigm	-0.18491965	1.74E-29	hypomethylated	0.016839	0.02296	hypermethylated	35	111	111
chr1	174429376	174431376	Tagln2	-0.14697827	1.09E-08	hypomethylated	0.014517	1	insignificant	16	69	69
chr1	174485068	174487068	Vsig8	-0.24372558	0.61725	insignificant	0.0023719	0.20031	insignificant	2	10	13
chr1	174489036	174491036	Vsig8		1	noCoverage	-0.029054	0.88439	insignificant	0	7	6
chr1	174520699	174522699	Slamf8	-0.04048996	0.000000288	hypomethylated	0.21948	0.15186	insignificant	6	18	17
chr1	174563105	174565105	Dusp23		1	noCoverage	-0.0049741	1	insignificant	0	8	6
chr1	175263634	175265634	Darc	-0.08427602	1	insignificant	0.071078	0.60437	insignificant	2	4	4
chr1	175297826	175299826	Cadm3		1	noCoverage	0.025662	0.23824	insignificant	0	11	11
chr1	176087852	176089852	Olfr420	-0.52747253	0.20309	insignificant	-0.0042767	0.91819	insignificant	1	4	4
chr1	176359560	176361560	Olfr414	-0.05546537	0.2683	insignificant	0.002904	1	insignificant	4	11	11
chr1	176430955	176432955	Fmn2	-0.2448843	0.00035671	hypomethylated	0.0014299	0.45555	insignificant	8	38	38
chr1	177422676	177424676	Rgs7	-0.254457	7.37E-11	hypomethylated	-0.054206	0.56401	insignificant	8	32	28
chr1	177555766	177557766	Fh1	-0.52063983	0.0041958	stronglyHypometh	-0.00088409	0.33469	insignificant	0	0	0
chr1	177618484	177620484	Opn3	0.13888889	1	lowCoverage	-0.24206	0.68731	insignificant	1	2	3
chr1	177622721	177624721	Opn3	-0.13211145	0.00034989	hypomethylated	0.014674	0.48089	insignificant	11	28	28
chr1	177627723	177629723	Wdr64	0.13258787	0.37196	insignificant	0.056659	0.18891	insignificant	4	8	8
chr1	177809908	177811908	Exo1	-0.15141307	2.18E-11	hypomethylated	0.002263	0.94341	insignificant	23	106	106
chr1	178205443	178207443	Pld5	-0.12499249	6.7E-09	hypomethylated	0.0071488	0.27132	insignificant	14	36	36
chr1	178743942	178745942	Sdccag8	-0.28242094	9.3E-13	hypomethylated	-0.047791	0.58148	insignificant	11	43	42
chr1	179726640	179728640	Adss		1	noCoverage	0.067414	0.86325	insignificant	0	19	18
chr1	180116547	180118547	Pppde1	-0.12533201	0.00000216	hypomethylated	-0.024688	0.36949	insignificant	4	43	51
chr1	180248283	180250283	Fam36a	-0.11312597	8.54E-28	hypomethylated	-0.0055472	0.57592	insignificant	28	136	143
chr1	180267915	180269915	Hnrnpu	-0.11477858	7.33E-08	hypomethylated	-0.016953	0.96555	insignificant	15	54	53
chr1	180335011	180337011	Efcab2	-0.13428138	4.68E-11	hypomethylated	0.0080553	0.56859	insignificant	29	142	139
chr1	180458255	180460255	Kif26b	-0.07858902	3.19E-09	hypomethylated	0.0050574	0.43446	insignificant	37	180	162
chr1	181475659	181477659	Cnst	-0.09629769	1.44E-30	hypomethylated	-0.010322	0.081163	insignificant	57	170	171
chr1	181476398	181478398	Cnst	-0.11323554	7.87E-27	hypomethylated	-0.0082332	0.25816	insignificant	40	112	113
chr1	181597361	181599361	Sccpdh	-0.0847266	6.24E-10	hypomethylated	0.00043613	0.21544	insignificant	46	138	131
chr1	181890219	181892219	Cdc42bpa	-0.05396123	6.24E-10	hypomethylated	0.01596	0.027003	hypermethylated	23	94	89
chr1	182126151	182128151	Adck3	-0.26217866	6.34E-11	hypomethylated	0.0035538	0.81643	insignificant	8	31	30
chr1	182176046	182178046	Psen2	0.07261905	1	insignificant	0.019229	0.55656	insignificant	3	10	10
chr1	182186431	182188431	Psen2	0.56017316	0.27473	insignificant	0.36295	0.00060713	stronglyhypermeth	1	11	6
chr1	182259606	182261606	Itpkb	-0.19732555	3.97E-12	hypomethylated	0.0024963	1	insignificant	17	51	56
chr1	182260680	182262680	Itpkb	-0.08439151	4.64E-11	hypomethylated	0.027382	0.15344	insignificant	41	127	127
chr1	182413635	182415635	6330403A02R	-0.12529453	0.15249	insignificant	0.078978	0.55234	insignificant	3	13	15
chr1	182498105	182500105	Parp1	-0.07776109	0.31274	insignificant	-0.0057373	0.64543	insignificant	12	77	76
chr1	182570464	182572464	Lin9	-0.13243455	3.11E-64	hypomethylated	0.0094681	0.34441	insignificant	77	192	190
chr1	182627165	182629165	Mixl1	-0.08932059	4.01E-18	hypomethylated	0.033612	0.25081	insignificant	21	39	36
chr1	182655173	182657173	Acbd3	-0.11965963	1.18E-27	hypomethylated	0.0067715	0.61244	insignificant	33	103	100
chr1	182743734	182745734	H3f3a	-0.09115861	4.71E-21	hypomethylated	0.0020964	0.47715	insignificant	32	101	101
chr1	182780281	182782281	BC031781	-0.10864936	3.17E-10	hypomethylated	0.027763	0.44476	insignificant	17	90	92
chr1	182833404	182835404	Pycr2	-0.23384737	3.16E-16	hypomethylated	-0.0011497	0.34212	insignificant	12	60	60
chr1	182864169	182866169	Lefty1	0.09023217	0.15967	insignificant	-0.0007105	0.60032	insignificant	3	16	16
chr1	182871648	182873648	Tmem63a	-0.12113383	9.22E-09	hypomethylated	0.026843	0.56863	insignificant	21	71	63
chr1	182980367	182982367	9130409I23Ril	-0.29142936	0.24347	insignificant	0.0099008	0.37277	insignificant	4	29	28
chr1	183080061	183082061	Cnih4	-0.09117894	0.000000866	hypomethylated	0.0038145	0.96621	insignificant	27	88	88
chr1	183142109	183144109	Wdr26	-0.1077389	1.6E-16	hypomethylated	0.013502	0.44153	insignificant	23	67	64
chr1	183281758	183283758	Cnih3	-0.14810577	1.73E-15	hypomethylated	0.00070576	0.88148	insignificant	33	112	107
chr1	183441582	183443582	Ccdc121	-0.17538314	0.54804	insignificant	0.058045	0.10025	insignificant	2	10	10
chr1	183772532	183774532	Lbr	-0.09332468	9.72E-16	hypomethylated	-0.0048081	0.96764	insignificant	23	72	72
chr1	183950111	183952111	Enah	-0.07517288	0.00098359	hypomethylated	0.0036482	0.96018	insignificant	21	137	118
chr1	184053867	184055867	Srp9	-0.16115921	7.87E-32	hypomethylated	0.018002	0.10182	insignificant	22	65	63
chr1	184229964	184231964	1700047M11F	-0.04086022	1	insignificant	0.02049	0.26086	insignificant	1	6	6
chr1	184338297	184340297	Trp53bp2	-0.12676914	3.3E-18	hypomethylated	-0.0021348	0.53405	insignificant	40	129	142
chr1	184447614	184449614	Capn2	-0.13657539	0.000077304	hypomethylated	-0.013557	0.31905	insignificant	16	68	65
chr1	184494137	184496137	Capn8	-0.45238095	0.36233	insignificant	0.036905	1	insignificant	2	4	4

chr1	184694036	184696036	Susd4	-0.15823033	5.3E-17	hypomethylated	0.012192	0.20153	insignificant	24	123	122
chr1	184883918	184885918	Tlr5	0.47862079	0.23867	insignificant	0.036618	0.58244	insignificant	1	9	9
chr1	185857339	185859339	Dusp10	-0.15780963	6.04E-27	hypomethylated	0.014647	0.85907	insignificant	31	122	118
chr1	185857877	185859877	1700056E22Ri	-0.17752753	0.00067131	hypomethylated	0.019738	0.73677	insignificant	9	34	33
chr1	186556372	186558372	Hlx	-0.21651958	4.74E-46	hypomethylated	0.0027218	0.070897	insignificant	16	61	61
chr1	186635192	186637192	Mosc1	0.1444035	0.22468	insignificant	0.0091706	0.11242	insignificant	2	12	12
chr1	186669726	186671726	Mosc2		1	noCoverage	0.010656	0.89015	insignificant	0	10	10
chr1	186706915	186708915	C130074G19R	-0.24799376	0.000000198	hypomethylated	-0.056801	0.37085	insignificant	9	23	18
chr1	186823428	186825428	Mark1	-0.07496076	4.8E-20	hypomethylated	0.011794	0.030463	hypermethylated	69	207	200
chr1	187027046	187029046	Rab3gap2	-0.00281378	1	insignificant	-0.016619	0.70155	insignificant	8	36	44
chr1	187065804	187067804	Snora36b	0.08695652	0.58301	insignificant	0.048495	0.17919	insignificant	2	4	4
chr1	187065853	187067853	Mir664	0.08695652	0.58301	insignificant	0.048495	0.17919	insignificant	2	4	4
chr1	187136197	187138197	Mir194-1	-0.13928571	0.10948	insignificant	-0.055655	0.021151	hypomethylated	3	12	14
chr1	187136459	187138459	Mir215	-0.13928571	0.10948	insignificant	-0.055655	0.021151	hypomethylated	3	12	14
chr1	187155037	187157037	Bpnt1	-0.13545953	0.0096073	hypomethylated	0.042321	0.055414	insignificant	8	32	29
chr1	187185973	187187973	Eprs	-0.05141173	0.52314	insignificant	0.00098322	0.45985	insignificant	32	162	161
chr1	187277726	187279726	Slc30a10	-0.10675743	3.05E-09	hypomethylated	0.023987	0.53175	insignificant	35	140	130
chr1	187320184	187322184	5033404E19Rik		1	noCoverage	-0.077431	0.33694	insignificant	0	13	15
chr1	188529871	188531871	Tgfb2	-0.25297	0.00032073	hypomethylated	0.028971	0.23329	insignificant	2	4	4
chr1	188573237	188575237	Rrp15	-0.21502739	0.00000987	hypomethylated	0.051046	0.44511	insignificant	5	37	37
chr1	188790294	188792294	D1Pas1	-0.11792351	0.00060321	hypomethylated	0.011779	0.41629	insignificant	11	62	64
chr1	189038389	189040389	Gpatch2	-0.17737506	5.2E-18	hypomethylated	-0.0044416	0.18658	insignificant	20	48	54
chr1	189039325	189041325	Gpatch2	-0.15265532	1.35E-16	hypomethylated	-0.00035564	0.46212	insignificant	20	40	40
chr1	189820737	189822737	Esrrg	-0.15265444	0.000011043	hypomethylated	0.015173	0.082586	insignificant	6	50	45
chr1	190085716	190087716	Ush2a	-0.02579419	0.39194	insignificant	0.035772	0.0096567	hypermethylated	5	32	30
chr1	190831719	190833719	Kctd3	-0.1592029	0.000045434	hypomethylated	-0.060724	1	insignificant	6	20	24
chr1	191167232	191169232	Kcnk2	-0.11967072	4.88E-18	hypomethylated	-0.021685	0.25883	insignificant	14	110	107
chr1	191167583	191169583	Kcnk2	-0.09893582	0.0000088	hypomethylated	-0.024492	1	insignificant	6	60	57
chr1	191511965	191513965	Cenpf	-0.12937923	3.38E-08	hypomethylated	-0.0049885	0.22882	insignificant	12	30	30
chr1	191551146	191553146	Ptpn14	-0.06901282	5.4E-41	hypomethylated	0.00086371	0.032912	hypermethylated	45	178	176
chr1	191746167	191748167	Smyd2	0.08127561	9.75E-15	hypermethylated	0.020772	0.12225	insignificant	17	70	70
chr1	191994559	191996559	Prox1	-0.11925078	2.2E-09	hypomethylated	0.077479	0.58889	insignificant	13	67	46
chr1	192735649	192737649	Rps6kc1	-0.14459546	0.03729	hypomethylated	0.012459	1	insignificant	5	26	24
chr1	192751383	192753383	Angel2	-0.10034632	6.03E-17	hypomethylated	0.0067501	0.82278	insignificant	28	114	102
chr1	192802877	192804877	Vash2	-0.14427889	1.27E-19	hypomethylated	-0.010525	0.31401	insignificant	27	86	86
chr1	192803175	192805175	Vash2	-0.22809827	0.000031234	hypomethylated	-0.004609	0.27696	insignificant	7	26	26
chr1	192848229	192850229	A230020J21Ri	-0.11140911	1.51E-39	hypomethylated	0.049106	0.08269	insignificant	38	130	124
chr1	192850069	192852069	A230020J21Ri	-0.32383764	1.42E-12	hypomethylated	0.017883	0.70847	insignificant	4	10	10
chr1	192859700	192861700	1700022P22Ri	-0.04565508	0.53158	insignificant	0.035827	0.87099	insignificant	1	8	8
chr1	192885899	192887899	Nsl1	0.02503968	1	insignificant	0.0025646	0.052026	insignificant	16	90	90
chr1	192886811	192888811	Nsl1	0.05749944	0.33242	insignificant	-0.00062564	0.0035456	inconclusive	15	62	62
chr1	192921292	192923292	Batf3	0.00252992	0.21839	insignificant	0.011038	0.39993	insignificant	15	84	84
chr1	192988696	192990696	Fam71a	0.22420635	1	insignificant	0.13624	0.2414	insignificant	1	6	6
chr1	193007212	193009212	Atf3	-0.19959311	4.85E-23	hypomethylated	-0.0021395	0.97407	insignificant	29	92	96
chr1	193148843	193150843	Tmem206	-0.14695101	4.56E-08	hypomethylated	0.0040076	0.13952	insignificant	19	77	77
chr1	193220920	193222920	Ppp2r5a	-0.09585808	0.000016112	hypomethylated	0.010761	0.30921	insignificant	25	86	78
chr1	193398612	193400612	Ints7	-0.13519967	4.7E-23	hypomethylated	-0.013082	0.80738	insignificant	20	50	63
chr1	193399413	193401413	Dtl	-0.06527351	0.00045861	hypomethylated	0.0049104	0.904	insignificant	8	18	19
chr1	193540902	193542902	Lpgat1	-0.09502256	2.75E-32	hypomethylated	-0.0048158	0.69583	insignificant	56	187	180
chr1	193541273	193543273	Lpgat1	-0.10018209	3.72E-55	hypomethylated	-0.00014267	0.48681	insignificant	65	217	210
chr1	193644351	193646351	Nek2	-0.10089832	3.83E-13	hypomethylated	-0.0084866	0.97004	insignificant	29	103	102
chr1	193729659	193731659	Slc30a1	-0.06888248	7.72E-21	hypomethylated	-0.003811	0.38073	insignificant	41	224	236
chr1	193731406	193733406	Slc30a1	-0.15088003	0.13527	insignificant	-0.038901	0.18374	insignificant	2	39	39
chr1	193916445	193918445	Traf5	-0.09640523	0.29055	insignificant	0.20556	0.0054472	hypermethylated	4	8	10
chr1	193959779	193961779	Gm10516	-0.08792061	0.000000116	hypomethylated	0.0041674	0.54714	insignificant	18	129	129
chr1	193961669	193963669	Rcor3	-0.13702872	2.47E-31	hypomethylated	-0.0041909	0.43986	insignificant	42	117	113
chr1	194013753	194015753	Kcnh1	-0.0947863	4.2E-23	hypomethylated	-0.01505	0.94268	insignificant	39	110	116
chr1	194597413	194599413	Hhat	-0.26582877	0.10587	insignificant	0.10527	0.15829	insignificant	1	8	8
chr1	194681946	194683946	Sertad4		1	noCoverage	0.0027831	0.73653	insignificant	0	19	20
chr1	194861892	194863892	Syt14	-0.11693216	0.10265	insignificant	-0.00086267	0.91736	insignificant	16	58	56
chr1	194956445	194958445	Diexf	0.07347652	0.12246	insignificant	0.0084751	0.47494	insignificant	4	26	26
chr1	194978305	194980305	Irf6	-0.09151089	0.019235	hypomethylated	-0.0055198	0.51162	insignificant	22	77	76
chr1	195127187	195129187	Lamb3		1	noCoverage	-0.055825	0.41971	insignificant	0	6	6
chr1	196445022	196447022	Plxna2	-0.10652145	0.000000032	hypomethylated	0.0090142	0.065703	insignificant	32	148	146
chr1	196764014	196766014	Cd34	-0.12218173	0.05283	insignificant	0.023107	0.31505	insignificant	11	33	32
chr1	196957764	196959764	Cr1l	-0.16402116	0.069644	insignificant	0.12169	0.013751	hypermethylated	2	6	4
chr10	3133303	3135303	Cnksr3	-0.09530096	3.11E-13	hypomethylated	0.0080328	0.089997	insignificant	30	148	148
chr10	3365075	3367075	Ipcef1	-0.27368587	0.0050969	hypomethylated	0.062269	0.88394	insignificant	3	26	25
chr10	4423140	4425140	Rgs17	-0.16045583	0.000029065	hypomethylated	-0.0028169	0.1348	insignificant	12	51	51
chr10	4521597	4523597	Mtrf1l	-0.11475813	0.00079136	hypomethylated	0.013793	0.20131	insignificant	13	69	69
chr10	4540075	4542075	Fbxo5	-0.14617465	2.21E-13	hypomethylated	0.020383	0.91742	insignificant	25	109	94
chr10	4794848	4796848	Syne1	-0.37679963	4.38E-15	stronglyHypometh	0.016227	0.0014197	inconclusive	10	31	31
chr10	5150833	5152833	Syne1	-0.19033947	1.75E-12	hypomethylated	-0.032989	0.000082484	hypomethylated	11	26	26
chr10	5913188	5915188	Rmnd1	-0.02897376	1	insignificant	-0.010794	0.5133	insignificant	25	100	104
chr10	5913936	5915936	1700052N19R	0.02407227	0.10311	insignificant	0.0049937	0.66814	insignificant	17	62	62
chr10	5957432	5959432	Zbtb2	-0.10109099	2.69E-34	hypomethylated	-0.0033199	0.57091	insignificant	52	149	164
chr10	6080212	6082212	Akap12	-0.71691698	0.026992	stronglyHypometh	0.15316	0.80922	insignificant	3	8	16
chr10	7308597	7310597	Lrp11	-0.13745607	7.27E-65	hypomethylated	0.015344	0.099295	insignificant	54	151	138
chr10	7382168	7384168	A630066F11Ri	-0.11638958	1.62E-46	hypomethylated	-0.017069	0.13728	insignificant	45	131	132
chr10	7383382	7385382	Pcmt1	-0.24799156	0.000028665	hypomethylated	-0.017382	0.0006586	hypomethylated	8	22	22
chr10	7386301	7388301	Nup43	-0.37305195	0.00016548	stronglyHypometh	0.0069683	0.80224	insignificant	2	22	22
chr10	7400006	7402006	Lats1	-0.12612603	2.87E-18	hypomethylated	-0.014564	0.040509	inconclusive	45	170	158
chr10	7400956	7402956	BC020402	-0.13924255	8.48E-12	hypomethylated	-0.011768	0.6589	insignificant	34	119	112
chr10	7444797	7446797	Katna1	-0.17015815	0.00036388	hypomethylated	-0.00827	0.71883	insignificant	9	26	26
chr10	7500715	7502715	BC013529	-0.58159377	3.64E-22	stronglyHypometh	-0.025552	0.011363	hypomethylated	7	61	60

chr10	7511691	7513691	Ppil4	-0.14832212	0.000000126	hypomethylated	0.0028302	0.52764	insignificant	19	77	76
chr10	7551267	7553267	Zc3h12d	-0.26041667	0.19305	insignificant	0.030948	0.72921	insignificant	1	8	8
chr10	7675921	7677921	Tab2	-0.10111103	9.87E-23	hypomethylated	-0.0022693	0.72769	insignificant	37	142	137
chr10	8238623	8240623	Ust	-0.11196283	1	insignificant	-0.084762	0.79453	insignificant	5	10	9
chr10	8605868	8607868	Sash1	-0.18948494	4.23E-08	hypomethylated	-0.025441	0.96378	insignificant	15	70	68
chr10	9395006	9397006	Samd5	-0.14254734	4.63E-12	hypomethylated	-0.016231	0.9658	insignificant	17	78	72
chr10	9620838	9622838	Stxbp5	-0.09460246	0.00012278	hypomethylated	-0.012423	0.6159	insignificant	18	53	50
chr10	10278005	10280005	Rab32	-0.2546875	0.00027616	hypomethylated	0.065706	0.75652	insignificant	2	4	4
chr10	10868227	10870227	Shprh	-0.07239564	0.061606	insignificant	0.014746	0.70239	insignificant	22	82	82
chr10	11000127	11002127	Fbxo30	-0.14311667	1.23E-37	hypomethylated	-0.0088865	0.086858	insignificant	39	104	132
chr10	11000384	11002384	Fbxo30	-0.14311667	1.23E-37	hypomethylated	-0.0088865	0.086858	insignificant	39	104	132
chr10	11062242	11064242	Epm2a	-0.11710475	4.79E-31	hypomethylated	-0.026169	0.63004	insignificant	49	132	124
chr10	12684065	12686065	Stx11	-0.04583202	0.000000232	inconclusive	0.023566	0.09767	insignificant	9	42	40
chr10	12727255	12729255	Sf3b5	-0.09330338	0.000017389	hypomethylated	-0.0062349	1	insignificant	15	94	95
chr10	12809593	12811593	Plagl1	-0.12857227	0.79141	insignificant	-0.082982	1	insignificant	25	93	75
chr10	12897829	12899829	Ltv1	0.01388889	0.68223	insignificant	0.097222	0.00040662	hypermethylated	2	6	6
chr10	12912943	12914943	Ltv1	-0.26839827	0.000035752	hypomethylated	0.074739	0.14377	insignificant	4	9	12
chr10	13043966	13045966	Phactr2	-0.11072887	0.000000209	hypomethylated	0.0093483	0.67761	insignificant	20	40	41
chr10	13194202	13196202	Phactr2		1	noCoverage	0.014122	1	insignificant	0	23	13
chr10	13219833	13221833	Fuca2	-0.27505555	0.00015065	hypomethylated	0.019182	0.63435	insignificant	3	29	29
chr10	13271712	13273712	Adat2	0.02113329	0.01945	inconclusive	0.012695	0.37476	insignificant	16	66	66
chr10	13272948	13274948	Pex3	0.0378779	0.00039301	inconclusive	0.024974	0.59379	insignificant	11	44	44
chr10	13588636	13590636	Aig1	-0.15455839	1.65E-23	hypomethylated	0.019108	0.011973	hypermethylated	31	87	82
chr10	13685184	13687184	Hivep2	-0.10796155	1.01E-32	hypomethylated	0.0050961	0.19519	insignificant	38	157	151
chr10	14264842	14266842	Gpr126	-0.08280477	4E-23	hypomethylated	0.032255	0.00000021	hypermethylated	26	125	101
chr10	17442033	17444033	Cited2	-0.13619252	1.36E-29	hypomethylated	0.030618	0.57638	insignificant	41	147	132
chr10	17667873	17669873	Heca		1	noCoverage	0.0074514	0.56333	insignificant	0	11	11
chr10	17743058	17745058	3110003A17Rik		1	noCoverage	0.071821	0.58397	insignificant	0	10	15
chr10	17774745	17776745	Reps1	-0.10881414	3E-38	hypomethylated	-0.00066149	0.42741	insignificant	62	217	193
chr10	17930696	17932696	Ect2l	-0.17832106	1	insignificant	0.068919	0.1224	insignificant	2	14	14
chr10	17954787	17956787	Ccdc28a	-0.48141757	8.2E-10	stronglyHypometh	0.012173	0.71406	insignificant	5	30	34
chr10	18126480	18128480	Nhsl1	-0.27580972	0.32506	insignificant	-0.009143	1	insignificant	3	8	8
chr10	18265882	18267882	Hebp2	0.27271166	0.21087	insignificant	0.047886	0.36513	insignificant	2	6	6
chr10	18463564	18465564	D10Bwg1379e	-0.19172385	0.00024851	hypomethylated	0.0093527	0.37023	insignificant	11	32	34
chr10	18506599	18508599	Gm4922		1	noCoverage	0.16822	0.24585	insignificant	0	18	15
chr10	18563876	18565876	Perp	-0.0591379	1.76E-24	hypomethylated	0.014446	0.57078	insignificant	19	86	86
chr10	18735216	18737216	Tnfaip3	-0.04947883	0.61311	insignificant	0.18628	0.60478	insignificant	8	23	22
chr10	19075344	19077344	Olig3	-0.25591575	0.00051275	hypomethylated	0.00068478	0.93521	insignificant	6	30	30
chr10	19310763	19312763	Ifngr1	-0.26131444	0.0001604	hypomethylated	-0.030969	0.31649	insignificant	5	11	10
chr10	19431392	19433392	Il20ra	-0.29034402	0.0001972	hypomethylated	-0.067814	0.56141	insignificant	16	51	44
chr10	19571265	19573265	Slc35d3	-0.12497078	1	insignificant	-0.00056834	0.82525	insignificant	3	18	18
chr10	19653331	19655331	Map3k5	-0.08590857	2.2E-19	hypomethylated	-0.0020574	0.2417	insignificant	73	258	235
chr10	19867725	19869725	Mtap7	-0.10906612	3.29E-30	hypomethylated	0.0061721	0.80148	insignificant	80	269	270
chr10	20031274	20033274	Bclaf1	-0.0866913	4.97E-13	hypomethylated	0.060319	0.88434	insignificant	41	140	145
chr10	20066624	20068624	Fam54a	-0.20274959	3.05E-15	hypomethylated	0.0088261	0.71424	insignificant	10	24	22
chr10	20444874	20446874	Pde7b	-0.2319637	0.00045069	hypomethylated	0.083022	0.016227	hypermethylated	2	14	14
chr10	20671352	20673352	Ahi1	-0.12460182	2.7E-20	hypomethylated	0.014721	0.55268	insignificant	31	86	86
chr10	20880790	20882790	Myb	-0.18536798	1.57E-38	hypomethylated	0.00046774	1	insignificant	24	66	66
chr10	21014784	21016784	Hbs1l	-0.10263345	1.64E-10	hypomethylated	0.024018	0.89603	insignificant	19	98	91
chr10	21096105	21098105	Aldh8a1	-0.07519728	0.2351	insignificant	0.051343	0.57302	insignificant	4	20	20
chr10	21311950	21313950	1700020N01R	-0.2919289	0.011665	hypomethylated	0.11387	0.0015912	hypermethylated	2	8	8
chr10	21600989	21602989	Sgk1	-0.2518265	0.00000587	hypomethylated	0.008046	0.84212	insignificant	11	43	48
chr10	21697477	21699477	Sgk1	-0.02944386	1	insignificant	0.042786	0.12753	insignificant	5	12	12
chr10	21711028	21713028	Sgk1	-0.23080972	0.00085918	hypomethylated	-0.0041823	0.048203	hypomethylated	10	45	44
chr10	21712695	21714695	Sgk1	-0.15992571	9.3E-38	hypomethylated	-0.033422	0.00048877	hypomethylated	42	144	140
chr10	21713471	21715471	Sgk1	-0.15354509	2.91E-30	hypomethylated	-0.038553	0.0025771	hypomethylated	37	118	119
chr10	21877414	21879414	Raet1a	-0.20136985	4.79E-30	hypomethylated	-0.023642	0.46538	insignificant	17	64	78
chr10	21992279	21994279	H60b	-0.11721711	0.00000541	hypomethylated	0.018321	0.80829	insignificant	17	98	92
chr10	22025525	22027525	C920009B18R	0.10648148	1	insignificant	0.030093	1	insignificant	1	4	2
chr10	22363816	22365816	Slc2a12	-0.2267416	0.000000952	hypomethylated	-0.015059	0.90314	insignificant	10	54	60
chr10	22451253	22453253	Tbpl1	-0.10945509	4.89E-15	hypomethylated	-0.009747	0.77224	insignificant	24	82	82
chr10	22539934	22541934	Tcf21	0.09612079	0.45631	insignificant	0.12666	0.039026	hypermethylated	1	9	8
chr10	23069709	23071709	Eya4	-0.1466246	3.11E-20	hypomethylated	0.0029499	0.70796	insignificant	35	123	124
chr10	23507015	23509015	Rps12	-0.21963716	0.00000252	hypomethylated	0.029488	0.76928	insignificant	3	22	22
chr10	23515791	23517791	1110021L09Ri	-0.19028224	9.78E-15	hypomethylated	0.017665	0.20732	insignificant	12	38	34
chr10	23668363	23670363	Taar3	0.13894174	0.13489	insignificant	0.050369	0.13327	insignificant	2	10	10
chr10	23679299	23681299	Taar4	-0.4935852	0.10562	insignificant	-0.06061	0.023661	inconclusive	3	14	14
chr10	23718744	23720744	Taar7b		1	noCoverage	-0.11042	1	insignificant	0	4	4
chr10	23756419	23758419	Taar7e		1	noCoverage	0.12278	0.049289	hypermethylated	0	6	6
chr10	23821757	23823757	Taar8c	-0.4613831	0.026467	stronglyHypometh	-0.1471	0.12108	insignificant	1	6	4
chr10	23868122	23870122	Stx7	-0.17241379	0.047023	hypomethylated	0.057961	0.78421	insignificant	3	29	23
chr10	23942322	23944322	Moxd1	-0.16520522	0.00019457	hypomethylated	0.023372	0.92768	insignificant	14	62	62
chr10	24314247	24316247	Ctgf	-0.13255306	0.00070142	hypomethylated	0.019108	0.41056	insignificant	10	61	61
chr10	24431908	24433908	Enpp1	0.21911303	0.28233	insignificant	0.0064699	0.40097	insignificant	1	14	14
chr10	24588791	24590791	Med23	-0.17111719	3.79E-08	hypomethylated	-0.0040398	0.81611	insignificant	15	67	60
chr10	24647276	24649276	Arg1	-0.05406683	1	insignificant	-0.036223	0.063612	insignificant	3	24	24
chr10	25018969	25020969	Akap7	0.1959576	0.34247	insignificant	0.048517	0.13483	insignificant	6	13	13
chr10	25078603	25080603	Epb4.1l2	-0.14027037	1.22E-23	hypomethylated	0.0038075	0.96409	insignificant	21	86	86
chr10	25797060	25799060	Gm9767	-0.04757252	0.65894	insignificant	0.026033	0.65579	insignificant	33	137	131
chr10	26094991	26096991	L3mbtl3	-0.04589161	0.00095513	hypomethylated	0.025112	1	insignificant	13	37	42
chr10	26491317	26493317	Arhgap18	-0.31065031	0.19643	insignificant	-0.028283	0.532	insignificant	3	25	22
chr10	27793625	27795625	Ptprk	-0.10910484	1.49E-16	hypomethylated	0.003536	0.52404	insignificant	34	116	116
chr10	28862801	28864801	6330407J23Ri	-0.17655475	1.67E-22	hypomethylated	0.010797	0.90025	insignificant	12	43	40
chr10	29031971	29033971	Echdc1	-0.16173041	1.92E-09	hypomethylated	0.00052629	1	insignificant	11	26	26
chr10	29032305	29034305	Echdc1	-0.16173041	1.92E-09	hypomethylated	0.00052629	1	insignificant	11	26	26

chr10	29255673	29257673	Rspo3	-0.16447874	0.58076	insignificant	0.035834	0.33375	insignificant	4	31	31
chr10	29920346	29922346	Cenpw	-0.14013177	7.2E-10	hypomethylated	0.038159	0.81695	insignificant	7	21	24
chr10	30338172	30340172	Hint3	0.3000416	1	insignificant	0.041296	0.37464	insignificant	2	30	30
chr10	30522913	30524913	Ncoa7	-0.10327083	2.53E-16	hypomethylated	-0.008343	1	insignificant	21	60	54
chr10	30562589	30564589	Hey2	-0.1690987	0.00000285	hypomethylated	-0.02004	0.4571	insignificant	8	65	62
chr10	31032210	31034210	Hddc2	-0.26223252	1.01E-14	hypomethylated	-0.01873	0.78229	insignificant	17	53	46
chr10	31165727	31167727	Tpd52l1	-0.06011438	1	insignificant	0.013435	0.10076	insignificant	5	12	12
chr10	31329531	31331531	Rnf217	-0.31216398	0.049482	hypomethylated	-0.1321	0.72986	insignificant	2	8	12
chr10	32609721	32611721	Nkain2	-0.08021176	0.0013193	hypomethylated	-0.0072308	0.36973	insignificant	5	56	70
chr10	33344406	33346406	Clvs2	-0.38541667	0.14007	insignificant	0.36878	0.054234	insignificant	1	7	4
chr10	33739422	33741422	Rwdd1	-0.13735839	1.31E-11	hypomethylated	0.013894	0.5191	insignificant	16	68	65
chr10	33816325	33818325	Bet3l		1	noCoverage	0.045509	0.58497	insignificant	0	6	6
chr10	33927357	33929357	Dse	-0.19116162	0.52546	insignificant	-0.053199	0.70037	insignificant	2	6	6
chr10	34000995	34002995	Tspyl1	-0.18580136	0.00012821	hypomethylated	-0.071294	0.11229	insignificant	14	100	92
chr10	34016226	34018226	Tspyl4	-0.13409638	0.10387	insignificant	0.023308	0.030126	hypermethylated	6	51	47
chr10	34202205	34204205	Frk	-0.0679355	0.11027	insignificant	0.073729	0.039498	hypermethylated	9	24	24
chr10	36225612	36227612	Hs3st5	-0.16781537	1.54E-12	hypomethylated	-0.0075805	0.0088484	inconclusive	28	77	70
chr10	36693349	36695349	Hdac2	-0.11942538	7.2E-51	hypomethylated	0.0036274	0.59428	insignificant	59	174	169
chr10	36858732	36860732	Marcks	-0.19301449	0.0012627	hypomethylated	-0.026306	0.11845	insignificant	3	57	55
chr10	38684320	38686320	Lama4	-0.21521164	0.0024429	hypomethylated	0.10524	0.45474	insignificant	5	10	11
chr10	38852828	38854828	Tube1	-0.1060823	0.00000246	hypomethylated	0.070413	0.61707	insignificant	13	65	69
chr10	38853701	38855701	1700025K23Ri	-0.11103619	0.0049381	hypomethylated	0.0057799	0.21009	insignificant	10	54	53
chr10	39088604	39090604	Fyn	-0.09364793	4.07E-28	hypomethylated	0.0044955	0.45059	insignificant	48	144	147
chr10	39331739	39333739	Traf3ip2	-0.36937408	0.52704	insignificant	0.020599	0.66172	insignificant	1	16	18
chr10	39450965	39452965	Rev3l	-0.07855409	1.3E-36	hypomethylated	0.012017	0.10774	insignificant	59	282	241
chr10	39619044	39621044	AA474331	-0.18819198	4.58E-10	hypomethylated	0.001951	0.3964	insignificant	9	18	20
chr10	39646729	39648729	2010001E11Ri	-0.26261865	0.032224	hypomethylated	0.095428	0.15338	insignificant	2	40	29
chr10	39679959	39681959	G630090E17R	-0.27265139	0.22106	insignificant	-0.023973	0.7496	insignificant	4	35	35
chr10	39745074	39747074	AI317395	-0.07476796	0.10847	insignificant	-0.013941	0.17309	insignificant	3	21	20
chr10	39966785	39968785	Rpf2	-0.11632841	0.0071317	hypomethylated	0.035378	0.0021025	hypermethylated	11	26	26
chr10	39966845	39968845	Rpf2	-0.10621658	0.049449	hypomethylated	0.041457	0.023926	hypermethylated	7	18	18
chr10	39977471	39979471	Gtf3c6	-0.20316267	0.0014596	hypomethylated	0.025973	0.38698	insignificant	4	40	37
chr10	40021992	40023992	Amd2	-0.1089657	0.00022181	hypomethylated	-0.0082746	0.01224	hypomethylated	9	45	45
chr10	40068113	40070113	Cdk19	-0.15545737	0.000000142	hypomethylated	0.015031	0.84241	insignificant	24	82	70
chr10	40348816	40350816	Ddo	0.10558608	1	insignificant	-0.02382	0.313	insignificant	3	7	7
chr10	40402087	40404087	9030224M15F	-0.14828	7.85E-24	hypomethylated	0.0020808	0.029303	hypermethylated	27	74	73
chr10	40602339	40604339	Wasf1	-0.10493799	5.35E-27	hypomethylated	0.0034537	0.11508	insignificant	63	200	206
chr10	40602949	40604949	Cdc40	-0.10493799	5.35E-27	hypomethylated	0.0034537	0.11508	insignificant	63	200	206
chr10	40791390	40793390	Gpr6		1	noCoverage	0.04601	0.049832	hypermethylated	0	22	22
chr10	41023047	41025047	Fig4	-0.3547338	0.0098362	stronglyHypometh	0.033406	0.71305	insignificant	2	10	10
chr10	41169201	41171201	Zbtb24	-0.14833967	0.000087412	hypomethylated	-0.03673	0.15986	insignificant	19	72	68
chr10	41195119	41197119	Mical1	-0.11894968	0.00000244	hypomethylated	0.01057	0.74885	insignificant	18	59	59
chr10	41209244	41211244	Ppil6	-0.11851314	0.0093552	hypomethylated	0.026226	0.16725	insignificant	10	27	27
chr10	41210146	41212146	Ppil6	-0.10885565	1	insignificant	0.039359	0.094873	insignificant	4	12	12
chr10	41238305	41240305	Cd164	-0.11294517	6.49E-12	hypomethylated	0.0096201	0.89595	insignificant	24	126	122
chr10	41307559	41309559	Ccdc162	-0.03611111	0.68587	insignificant	0.020833	1	insignificant	2	4	4
chr10	41529379	41531379	Sesn1	-0.15806862	1.22E-14	hypomethylated	0.022579	0.21219	insignificant	29	103	90
chr10	41529436	41531436	Sesn1	-0.15806862	1.22E-14	hypomethylated	0.022579	0.21219	insignificant	29	103	90
chr10	41606244	41608244	Sesn1	-0.16991741	0.000020464	hypomethylated	0.055956	0.60164	insignificant	23	107	114
chr10	41738188	41740188	Armc2		1	noCoverage	0.04162	0.024108	hypermethylated	0	21	6
chr10	41996548	41998548	Foxo3	-0.06322327	8.7E-15	hypomethylated	-0.00016374	0.6209	insignificant	39	209	218
chr10	42198371	42200371	Lace1	-0.55	0.16923	insignificant	-0.016667	0.59839	insignificant	0	0	0
chr10	42220859	42222859	Snx3	-0.1276341	1.41E-16	hypomethylated	-0.0099716	0.2657	insignificant	40	162	155
chr10	42303394	42305394	Nr2e1	-0.2038411	4.19E-14	hypomethylated	-0.014153	0.091207	insignificant	8	68	68
chr10	42397722	42399722	Ostm1	-0.1507496	1.58E-32	hypomethylated	-0.003494	0.10006	insignificant	18	58	58
chr10	42480301	42482301	Sec63	-0.091294	0.0073946	hypomethylated	0.009676	0.12263	insignificant	15	110	97
chr10	42579317	42581317	Scml4	-0.55581699	1.37E-11	stronglyHypometh	-0.073651	0.77953	insignificant	3	10	10
chr10	42893509	42895509	9030612E09Ri	-0.04153459	0.0069397	hypomethylated	0.0098597	0.16289	insignificant	46	194	169
chr10	42894336	42896336	Sobp	-0.07377708	0.00026242	hypomethylated	0.018052	0.40546	insignificant	16	83	63
chr10	42940291	42942291	Pdss2	-0.09416739	1.39E-20	hypomethylated	0.0019164	0.58938	insignificant	19	90	90
chr10	43197945	43199945	Bend3	-0.08150315	1.96E-18	hypomethylated	0.004548	0.72435	insignificant	71	242	227
chr10	43297974	43299974	Cd24a	-0.12258624	7.09E-22	hypomethylated	-0.0016009	0.45209	insignificant	36	170	162
chr10	43312232	43314232	F830002L21Ri	-0.6317617	0.0004088	stronglyHypometh	0.073503	0.21329	insignificant	2	16	16
chr10	43620612	43622612	Rtn4ip1	-0.20968853	4.46E-13	hypomethylated	-0.00035264	0.27777	insignificant	11	52	47
chr10	43621542	43623542	Qrsl1	-0.2863345	1.86E-08	hypomethylated	0.010432	0.92146	insignificant	3	22	17
chr10	43724652	43726652	Aim1	-0.2855846	2.59E-17	hypomethylated	0.0026073	0.2136	insignificant	3	24	20
chr10	43889252	43891252	Speer5-ps1	-0.10072464	1	insignificant	0.053269	0.18861	insignificant	3	9	9
chr10	43987163	43989163	Atg5	-0.14772181	9.61E-32	hypomethylated	0.006173	1	insignificant	38	96	95
chr10	44178493	44180493	Prdm1	-0.16205578	0.58985	insignificant	-0.0004772	0.36522	insignificant	5	26	24
chr10	44786019	44788019	Prep	-0.08968937	5.67E-33	hypomethylated	-0.0026031	0.70519	insignificant	61	170	169
chr10	45008110	45010110	Popdc3	-0.10373193	1.69E-17	hypomethylated	0.014513	0.87273	insignificant	22	80	80
chr10	45054567	45056567	Bves	-0.06807378	3.15E-09	hypomethylated	-0.0068863	0.39496	insignificant	35	125	125
chr10	45296634	45298634	Hace1	-0.08571504	0.00069293	hypomethylated	-0.004665	0.34108	insignificant	17	82	82
chr10	49508560	49510560	Grik2		1	noCoverage	-0.1939	0.86707	insignificant	0	4	6
chr10	50311474	50313474	Ascc3	-0.08597091	3.94E-10	hypomethylated	0.013604	0.08217	insignificant	38	129	137
chr10	50614456	50616456	Sim1	-0.17460337	2.19E-22	hypomethylated	0.011046	0.24247	insignificant	37	131	123
chr10	51396565	51398565	Rfx6	-0.26904068	0.12644	insignificant	-0.000042971	0.30728	insignificant	1	27	27
chr10	51741491	51743491	Vgll2	-0.12720329	0.000036518	hypomethylated	-0.0070291	0.72368	insignificant	19	75	68
chr10	51952424	51954424	Dcbld1	-0.148028	1.23E-26	hypomethylated	0.019751	0.81651	insignificant	28	91	98
chr10	52101930	52103930	Gopc	-0.08955467	0.00000135	hypomethylated	0.033287	0.57512	insignificant	8	34	35
chr10	52136352	52138352	Nus1	-0.09469845	1.05E-13	hypomethylated	0.0025404	0.56068	insignificant	27	112	120
chr10	52265545	52267545	Zfa		1	noCoverage	-0.030228	0.80188	insignificant	0	16	16
chr10	52409306	52411306	Slc35f1	-0.20264091	1.24E-30	hypomethylated	0.0064258	0.38596	insignificant	32	88	88
chr10	53315766	53317766	Asf1a	-0.10973647	2.94E-33	hypomethylated	-0.0011003	0.88045	insignificant	61	191	164
chr10	53350245	53352245	Mcm9		1	noCoverage	-0.077661	0.44044	insignificant	0	14	14

chr10	53470715	53472715	Fam184a	-0.07044002	0.000084988	hypomethylated	0.028437	0.55497	insignificant	19	61	45
chr10	53795602	53797602	Man1a	-0.15469914	0.000068893	hypomethylated	-0.0036465	0.56861	insignificant	16	58	58
chr10	55825722	55827722	Msl3l2	-0.41904762	0.23529	insignificant	0.080952	0.84149	insignificant	1	5	4
chr10	55948495	55950495	D630037F22R	-0.14680135	0.22534	insignificant	0.0026239	0.71084	insignificant	5	15	15
chr10	57205190	57207190	Hsf2	-0.11400869	5.6E-13	hypomethylated	-0.013296	0.47558	insignificant	52	144	143
chr10	57252335	57254335	Serinc1	-0.11819155	0.11589	insignificant	-0.0041368	0.8332	insignificant	4	8	8
chr10	57350786	57352786	Pkib	-0.17287582	0.20367	insignificant	0.10004	0.44891	insignificant	2	20	10
chr10	57513349	57515349	Smpdl3a	-0.15489845	1.4E-09	hypomethylated	0.0055448	0.63718	insignificant	14	49	49
chr10	57694761	57696761	4933403O03Rik		1	noCoverage	0.012799	0.4664	insignificant	0	12	12
chr10	57695423	57697423	4933403O03Rik		1	noCoverage	0.00553	0.84527	insignificant	0	3	3
chr10	57699408	57701408	Gm4981		1	noCoverage	0.099451	1	insignificant	0	6	4
chr10	57717273	57719273	Gcc2	-0.13119958	0.037098	hypomethylated	0.019358	0.79829	insignificant	8	52	51
chr10	57785213	57787213	Lims1	-0.13024986	8.43E-23	hypomethylated	-0.0099667	0.27283	insignificant	44	113	108
chr10	57908599	57910599	Ranbp2	-0.09036	4.89E-12	hypomethylated	-0.0027065	0.80881	insignificant	17	114	114
chr10	57959684	57961684	Ccdc138	-0.18840051	1.77E-13	hypomethylated	0.0055313	0.41816	insignificant	16	67	67
chr10	58275106	58277106	Sh3rf3	-0.0878783	2.32E-12	hypomethylated	0.0088327	0.39339	insignificant	40	179	167
chr10	58683669	58685669	Ankrd57	-0.09295576	1.4E-16	hypomethylated	-0.0011704	0.83232	insignificant	32	180	179
chr10	58684595	58686595	###########	-0.15336798	1.03E-10	hypomethylated	-0.0013483	1	insignificant	10	77	78
chr10	58785043	58787043	P4ha1	-0.09636968	1.65E-14	hypomethylated	0.010092	0.15232	insignificant	42	155	154
chr10	58865432	58867432	Pla2g12b	0.10032468	0.59488	insignificant	0.01449	0.20349	insignificant	2	14	14
chr10	59079440	59081440	Ccdc109a	-0.23098265	0.000030485	hypomethylated	0.1127	0.027507	hypermethylated	2	18	16
chr10	59164382	59166382	Cbara1	-0.17201721	8.44E-08	hypomethylated	-0.028356	0.67555	insignificant	6	52	58
chr10	59341338	59343338	Dnajb12	-0.26465749	0.0093706	hypomethylated	0.042685	0.44871	insignificant	9	36	28
chr10	59414518	59416518	Ddit4	-0.16065601	2.25E-12	hypomethylated	0.016119	0.38215	insignificant	14	63	60
chr10	59464552	59466552	Ascc1	0.05123899	0.00016997	inconclusive	0.047152	0.24024	insignificant	7	29	37
chr10	59465074	59467074	Ascc1	-0.13079664	0.000000877	hypomethylated	-0.033644	0.44251	insignificant	4	20	25
chr10	59568004	59570004	Spock2	-0.10170067	5.12E-21	hypomethylated	0.016629	0.041303	hypermethylated	39	108	108
chr10	59682008	59684008	Chst3	-0.39882402	1.07E-31	stronglyHypometh	0.0070417	0.6278	insignificant	9	12	10
chr10	59739375	59741375	Psap	-0.31240438	0.015308	hypomethylated	0.045591	0.072653	insignificant	11	72	64
chr10	59808598	59810598	4632428N05R	0.72748495	0.023466	stronglyHypermeth	-0.0078068	0.12927	insignificant	2	42	39
chr10	59861473	59863473	Gm17455	-0.09979489	0.0021195	hypomethylated	0.0093307	0.27573	insignificant	2	13	13
chr10	60159238	60161238	Cdh23	-0.57502498	0.000064528	stronglyHypometh	-0.077155	0.78292	insignificant	2	11	10
chr10	60215530	60217530	Slc29a3	-0.16201893	0.00000377	hypomethylated	0.0040543	1	insignificant	4	14	14
chr10	60294329	60296329	Unc5b	-0.11392816	0.000011253	hypomethylated	-0.014767	0.0034393	inconclusive	10	44	40
chr10	60610413	60612413	Sgpl1	-0.12775696	6.07E-12	hypomethylated	0.0020141	0.61248	insignificant	21	65	66
chr10	60633711	60635711	Tbata	0.23242128	1	insignificant	0.09505	0.24573	insignificant	4	11	10
chr10	60637009	60639009	Tbata	0.07032446	0.0005492	hypermethylated	0.036898	0.00079348	hypermethylated	10	46	46
chr10	60736186	60738186	Adamts14	-0.04766551	0.030205	hypomethylated	-0.028397	1	insignificant	9	25	13
chr10	60846271	60848271	X99384	0.83035529	0.012995	stronglyHypermeth	0.041862	0.1036	insignificant	1	39	38
chr10	60879719	60881719	Nodal	-0.05616474	0.6865	insignificant	0.019387	0.1089	insignificant	8	48	50
chr10	60915417	60917417	Eif4ebp2	-0.29524951	4.06E-29	hypomethylated	-0.011393	0.79551	insignificant	19	70	70
chr10	60937580	60939580	Lrrc20	-0.20454015	0.0001242	hypomethylated	-0.034137	0.59535	insignificant	6	44	43
chr10	61057231	61059231	Npffr1	-0.07441163	1.47E-08	hypomethylated	0.0026087	0.21002	insignificant	17	41	28
chr10	61110368	61112368	Ppa1	-0.14095087	1.7E-21	hypomethylated	-0.0027614	0.053753	insignificant	30	106	106
chr10	61142068	61144068	Sar1a	-0.06350338	0.046228	hypomethylated	-0.021572	0.13545	insignificant	21	87	87
chr10	61157261	61159261	Tysnd1	-0.11435712	6.51E-39	hypomethylated	0.00097463	0.52983	insignificant	56	192	181
chr10	61177010	61179010	Aifm2	-0.18293888	0.097301	insignificant	0.0078782	0.88946	insignificant	8	26	26
chr10	61182396	61184396	Aifm2	-0.08751994	0.50162	insignificant	-0.016043	0.84866	insignificant	1	6	6
chr10	61246612	61248612	H2afy2	-0.13221881	0.00000256	hypomethylated	-0.0095537	0.38154	insignificant	8	47	47
chr10	61441856	61443856	Col13a1	-0.12226147	1	insignificant	0.02506	0.084397	insignificant	0	29	22
chr10	61532769	61534769	Gm5424	0.19137271	1	lowCoverage	0.064161	0.063607	insignificant	1	14	14
chr10	61573761	61575761	2010107G23R	-0.26574853	6.43E-09	hypomethylated	0.091752	0.15664	insignificant	8	16	18
chr10	61594837	61596837	Neurog3	-0.16282889	2.07E-09	hypomethylated	0.0033423	0.19936	insignificant	23	99	99
chr10	61693966	61695966	Tspan15	-0.19894439	0.000027501	hypomethylated	-0.055255	0.33727	insignificant	7	24	22
chr10	61803169	61805169	Hk1		1	noCoverage	-0.089213	0.90465	insignificant	0	9	8
chr10	61885205	61887205	Hkdc1		1	noCoverage	0.10208	0.095666	insignificant	0	12	9
chr10	61910907	61912907	4930507D05R	-0.14019019	5.16E-10	hypomethylated	-0.0084799	0.42015	insignificant	14	76	75
chr10	61912441	61914441	Supv3l1	-0.17444665	0.23124	insignificant	-0.010099	0.38834	insignificant	8	50	49
chr10	61949346	61951346	Vps26a	-0.13328134	0.00010847	hypomethylated	0.00088526	0.25365	insignificant	9	64	70
chr10	61949553	61951553	Vps26a	-0.15356618	0.00031877	hypomethylated	0.0042189	0.86446	insignificant	3	48	52
chr10	61970503	61972503	Srgn	0.09610364	0.000000576	hypermethylated	0.010097	0.14326	insignificant	3	20	17
chr10	62041205	62043205	2510003E04Ri	-0.08859884	0.42034	insignificant	-0.066857	0.83996	insignificant	1	25	22
chr10	62065046	62067046	Ddx21	-0.41455061	0.000003835	stronglyHypometh	-0.0059615	0.32652	insignificant	4	30	28
chr10	62113946	62115946	Ddx50	-0.1037275	0.041624	hypomethylated	0.061811	0.92369	insignificant	7	15	16
chr10	62188847	62190847	Stox1	-0.5784955	4.67E-20	stronglyHypometh	0.022129	0.24659	insignificant	5	38	29
chr10	62255116	62257116	Ccar1	-0.16626934	0.0013414	hypomethylated	0.039509	0.62816	insignificant	10	56	56
chr10	62342762	62344762	Tet1		1	noCoverage	0.29238	0.0063257	hypermethylated	0	5	4
chr10	62382380	62384380	Slc25a16	-0.07359244	0.39774	insignificant	-0.018325	0.45117	insignificant	14	46	44
chr10	62408776	62410776	Dna2	-0.13570413	0.00000219	hypomethylated	0.017879	0.15195	insignificant	21	102	101
chr10	62441970	62443970	Rufy2	-0.16070057	0.000057807	hypomethylated	-0.0038982	0.50418	insignificant	10	62	67
chr10	62486259	62488259	Pbld2	-0.11166015	7.24E-21	hypomethylated	-0.0032876	0.90581	insignificant	27	85	85
chr10	62486597	62488597	Hnrnph3	-0.12657974	0.000000121	hypomethylated	-0.010086	0.47945	insignificant	12	45	45
chr10	62523364	62525364	Pbld1	-0.13727669	0.44464	insignificant	0.075608	0.10014	insignificant	2	12	8
chr10	62561903	62563903	Atoh7	-0.08681649	1.43E-14	hypomethylated	-0.0078883	0.092738	insignificant	26	95	92
chr10	62705544	62707544	Herc4	-0.10624057	2.45E-08	hypomethylated	0.013148	0.35662	insignificant	31	115	113
chr10	62801743	62803743	Sirt1	-0.08385599	0.00036283	hypomethylated	0.00057986	0.88497	insignificant	20	57	72
chr10	62801780	62803780	Sirt1	-0.09123245	0.00022057	hypomethylated	-0.0052231	0.54493	insignificant	20	48	63
chr10	62801783	62803783	Sirt1	-0.09123245	0.00022057	hypomethylated	-0.0052231	0.54493	insignificant	20	47	62
chr10	62844190	62846190	Dnajc12	-0.18598431	0.00000028	hypomethylated	-0.013191	1	insignificant	3	23	27
chr10	62848310	62850310	Dnajc12	-0.21908329	0.026799	hypomethylated	0.021019	0.076213	insignificant	4	28	28
chr10	62891845	62893845	Ctnna3	-0.15999724	0.15661	insignificant	-0.016744	0.83983	insignificant	9	73	83
chr10	63553003	63555003	Ctnna3	-0.85299423	0.25	lowCoverage	-0.13097	0.48734	insignificant	1	22	18
chr10	66559605	66561605	Reep3	-0.10380276	1.66E-09	hypomethylated	0.015739	0.59275	insignificant	14	38	37
chr10	66589005	66591005	Jmjd1c	-0.02497818	8.97E-17	hypomethylated	0.0014769	0.93249	insignificant	83	232	223
chr10	66748029	66750029	Nrbf2	-0.08643644	0.0001021	hypomethylated	0.025411	0.4684	insignificant	8	37	38

chr10	66999616	67001616	Egr2	-0.15709454	0.00016146	hypomethylated	-0.036062	0.13841	insignificant	16	75	73
chr10	67011703	67013703	Ado	-0.26493197	1.22E-08	hypomethylated	-0.038314	0.0084121	inconclusive	11	60	54
chr10	67441345	67443345	Rtkn2	0.32471935	0.52407	insignificant	0.049901	0.46726	insignificant	9	76	84
chr10	67741474	67743474	Arid5b	-0.16904369	2.47E-08	hypomethylated	0.006647	0.6998	insignificant	12	70	70
chr10	68185493	68187493	Tmem26	-0.10206081	0.000000457	hypomethylated	0.014843	0.53429	insignificant	7	34	34
chr10	68674404	68676404	Rhobtb1	-0.11737258	2.28E-09	hypomethylated	0.0087492	0.38129	insignificant	25	123	118
chr10	68815660	68817660	Cdk1	-0.27484668	0.0041695	hypomethylated	0.095366	0.22588	insignificant	4	12	12
chr10	68995455	68997455	Ank3		1	noCoverage	0.044845	1	insignificant	0	4	4
chr10	69387279	69389279	Ank3	-0.29580678	1.25E-15	hypomethylated	-0.0011957	0.3249	insignificant	11	43	41
chr10	69558868	69560868	Ccdc6	-0.10527035	3.82E-39	hypomethylated	0.030696	0.017102	hypermethylated	59	174	160
chr10	69707023	69709023	Slc16a9	-0.12141169	7.47E-12	hypomethylated	0.017837	0.95961	insignificant	19	74	74
chr10	69902415	69904415	Fam13c	-0.15087652	0.000000482	hypomethylated	0.00096929	0.49418	insignificant	9	53	52
chr10	69902657	69904657	Fam13c	-0.15087652	0.000000482	hypomethylated	0.00096929	0.49418	insignificant	9	53	52
chr10	70062039	70064039	Phyhipl	-0.08474327	2.62E-08	hypomethylated	0.0025481	0.32377	insignificant	32	148	157
chr10	70622382	70624382	Bicc1	-0.08172845	5.55E-13	hypomethylated	0.013647	1	insignificant	41	118	110
chr10	70700792	70702792	Tfam	-0.17795011	0.000000807	hypomethylated	-0.041428	0.38744	insignificant	14	30	28
chr10	70748010	70750010	Ube2d1	-0.09702535	0.0010924	hypomethylated	-0.01544	1	insignificant	10	28	28
chr10	70807597	70809597	Cisd1	-0.24973723	4.26E-16	hypomethylated	0.016559	0.19495	insignificant	16	60	60
chr10	70809540	70811540	Ipmk	0.02304243	4.08E-10	inconclusive	0.011773	0.95251	insignificant	51	160	158
chr10	71443438	71445438	1700049L16Ri	0.19074074	1	insignificant	-0.031481	0.78418	insignificant	1	6	3
chr10	72116642	72118642	Zwint	-0.24191318	0.13344	insignificant	0.032676	0.29485	insignificant	2	24	23
chr10	74428975	74430975	Gnaz	-0.12727509	7.82E-26	hypomethylated	0.018656	1	insignificant	32	134	129
chr10	74495298	74497298	Rtdr1	-0.35644997	0.000000297	stronglyHypometh	0.010403	0.86376	insignificant	6	36	34
chr10	74495331	74497331	Rtdr1	-0.35644997	0.000000297	stronglyHypometh	0.010403	0.86376	insignificant	6	36	34
chr10	74498833	74500833	Rab36	-0.12190652	0.60617	insignificant	0.0044664	0.946	insignificant	4	22	22
chr10	74522640	74524640	Bcr	-0.1144854	2.91E-28	hypomethylated	0.010421	0.041029	hypermethylated	46	205	183
chr10	74673817	74675817	Specc1l	-0.07567635	2.18E-14	hypomethylated	0.029856	0.11488	insignificant	20	96	77
chr10	74674134	74676134	Specc1l	-0.07567635	2.18E-14	hypomethylated	0.029856	0.11488	insignificant	20	96	77
chr10	74778687	74780687	Adora2a	-0.13834421	1.3E-40	hypomethylated	0.0012704	0.40132	insignificant	35	126	131
chr10	74979786	74981786	Snrpd3	-0.15683428	1.03E-08	hypomethylated	-0.0090765	0.77909	insignificant	20	103	95
chr10	74980067	74982067	1110038D17R	-0.16348946	0.029444	hypomethylated	-0.023887	0.098785	insignificant	13	63	61
chr10	75023075	75025075	AI646023	0.01025641	0.42512	insignificant	0.04092	0.49989	insignificant	1	12	17
chr10	75035337	75037337	Ggt1	0.09864589	1	insignificant	0.10746	0.000040659	hypermethylated	5	16	17
chr10	75051125	75053125	Ggt5		1	noCoverage	-0.010861	0.62192	insignificant	0	16	16
chr10	75106753	75108753	Susd2	0.03410681	1	insignificant	-0.0020904	0.11749	insignificant	1	12	12
chr10	75227102	75229102	Cabin1	-0.42424242	1	insignificant	0.012456	0.73099	insignificant	2	12	12
chr10	75236119	75238119	Gstt3	-0.0964591	0.60302	insignificant	0.0066217	0.58096	insignificant	4	21	22
chr10	75261329	75263329	Gstt1	-0.0031746	1	insignificant	-0.034816	0.19681	insignificant	4	18	19
chr10	75297626	75299626	Gstt2	-0.52536825	5.86E-08	stronglyHypometh	-0.048524	0.26047	insignificant	1	4	4
chr10	75322995	75324995	Mif	-0.42039527	0.000000308	stronglyHypometh	0.019734	0.056354	insignificant	2	30	30
chr10	75355142	75357142	Derl3	-0.14052627	0.00000141	hypomethylated	-0.01451	0.2479	insignificant	8	84	84
chr10	75395208	75397208	Mmp11	-0.2827381	0.00074317	hypomethylated	-0.0625	0.62834	insignificant	5	14	10
chr10	75397317	75399317	Chchd10	-0.14025148	1.22E-21	hypomethylated	-0.011023	0.75814	insignificant	14	60	60
chr10	75416258	75418258	Gm5134	-0.09484099	0.0014104	hypomethylated	0.028967	0.3087	insignificant	2	24	24
chr10	75494356	75496356	Zfp280b	-0.14784187	0.0078365	hypomethylated	0.02825	0.45653	insignificant	13	51	40
chr10	75573763	75575763	Slc5a4b	-0.4375	0.15619	insignificant	0.049131	0.33709	insignificant	1	6	6
chr10	75609195	75611195	Slc5a4a	-0.03042192	0.060249	insignificant	0.033675	0.02391	hypermethylated	5	30	27
chr10	75670154	75672154	Prmt2	0.07069277	0.022948	hypermethylated	0.040479	0.091778	insignificant	5	18	19
chr10	75700610	75702610	Prmt2	-0.22421594	1.8E-27	hypomethylated	-0.01832	0.46696	insignificant	11	26	26
chr10	75715580	75717580	S100b	0.06897452	1	insignificant	-0.056025	0.67872	insignificant	1	4	4
chr10	75808007	75810007	Dip2a	-0.11091137	1.06E-13	hypomethylated	0.021317	0.059414	insignificant	31	81	81
chr10	75905657	75907657	Pcnt	-0.13690476	0.60001	insignificant	-0.010939	0.82613	insignificant	5	18	18
chr10	75910825	75912825	2610028H24R	0.08112689	0.54693	insignificant	-0.039148	0.90314	insignificant	3	12	12
chr10	75930715	75932715	Mcm3ap	-0.32650543	3.37E-12	hypomethylated	-0.021533	0.24497	insignificant	10	43	42
chr10	75931859	75933859	Ybey	-0.09140621	0.000000767	hypomethylated	-0.021768	0.0010078	inconclusive	8	48	48
chr10	75993371	75995371	Lss	-0.13890833	0.0000022	hypomethylated	-0.0073923	0.016947	hypomethylated	8	34	32
chr10	76086149	76088149	Col6a2	-0.51875	4.82E-08	stronglyHypometh	-0.015517	0.062752	insignificant	1	2	2
chr10	76424692	76426692	Pcbp3	-0.23656503	0.0084657	hypomethylated	0.03291	0.23944	insignificant	2	30	31
chr10	76494483	76496483	Slc19a1	-0.26227599	8.84E-11	hypomethylated	-0.034557	0.63333	insignificant	4	24	16
chr10	76494956	76496956	Slc19a1	-0.2540018	9.92E-12	hypomethylated	-0.032883	0.6136	insignificant	4	29	21
chr10	76629275	76631275	Col18a1	-0.12945016	0.012408	hypomethylated	0.17933	0.040296	hypermethylated	5	12	14
chr10	76721044	76723044	Pofut2	-0.08815317	1.62E-08	hypomethylated	-0.013856	0.10302	insignificant	22	70	69
chr10	76721968	76723968	Gm10941	-0.15457001	9.95E-19	hypomethylated	-0.0005966	0.11855	insignificant	27	92	91
chr10	76880120	76882120	Gm17769	-0.14185341	1.91E-50	hypomethylated	-0.0023507	0.000062959	hypomethylated	33	157	156
chr10	76881018	76883018	Adarb1	-0.22333788	8.19E-30	hypomethylated	-0.014689	5.52E-10	hypomethylated	14	56	55
chr10	76978558	76980558	1810008A18R	0.1956456	0.000014248	inconclusive	-0.05242	0.74176	insignificant	4	17	17
chr10	76992092	76994092	Itgb2	-0.59495614	0.033446	stronglyHypometh	-0.070204	0.47248	insignificant	1	4	4
chr10	77043511	77045511	Pttg1ip	-0.20650163	0.00017153	hypomethylated	-0.00016359	0.57493	insignificant	6	23	22
chr10	77067978	77069978	Sumo3	-0.0911623	1.17E-14	hypomethylated	-0.00139	0.51416	insignificant	28	119	133
chr10	77084065	77086065	Ube2g2	-0.10057653	0.16797	insignificant	0.0095368	0.08326	insignificant	30	86	86
chr10	77173201	77175201	Gm10024	-0.09220396	0.011815	hypomethylated	0.056497	0.86501	insignificant	1	8	10
chr10	77289815	77291815	Krtap10-4	-0.09469744	0.66923	insignificant	-0.035997	0.90125	insignificant	2	10	10
chr10	77297691	77299691	Krtap10-10	-0.40370759	0.0021296	stronglyHypometh	-0.02298	0.70921	insignificant	3	12	12
chr10	77365281	77367281	Lrrc3	-0.27796202	0.044012	hypomethylated	-0.0349	0.69376	insignificant	5	32	30
chr10	77432617	77434617	Trpm2	-0.38192979	0.00000103	stronglyHypometh	-0.013772	0.91218	insignificant	3	12	15
chr10	77440394	77442394	1810043G02R	-0.11371805	2.15E-21	hypomethylated	-0.01274	0.86918	insignificant	33	104	94
chr10	77472541	77474541	Pfkl	-0.14319105	0.38117	insignificant	-0.0038475	0.8075	insignificant	5	32	32
chr10	77504031	77506031	Dnmt3l	-0.09060267	0.10352	insignificant	-0.033944	0.16895	insignificant	2	16	16
chr10	77506325	77508325	Dnmt3l		1	noCoverage	-0.028168	0.2357	insignificant	0	15	14
chr10	77531112	77533112	Icosl	-0.16032416	2.63E-19	hypomethylated	0.0077744	0.67573	insignificant	26	89	93
chr10	77632513	77634513	D10Jhu81e		1	noCoverage	0.032029	0.45982	insignificant	0	12	12
chr10	77647894	77649894	Pwp2		1	noCoverage	0.10953	0.60927	insignificant	0	6	15
chr10	77707387	77709387	Trappc10	-0.12366047	2.92E-08	hypomethylated	0.0056422	0.80027	insignificant	12	36	36
chr10	77814445	77816445	Agpat3	-0.08657765	3.22E-27	hypomethylated	-0.00032251	0.74909	insignificant	44	141	130
chr10	77887414	77889414	Cstb	-0.2420093	3.61E-09	hypomethylated	0.035123	0.082907	insignificant	4	43	33

chr10	77927693	77929693	Pdxk	-0.2473309	0.0014414	hypomethylated	-0.00099093	0.68915	insignificant	8	53	53
chr10	78054709	78056709	Syde1	0.01714102	0.78473	insignificant	0.086504	0.62626	insignificant	8	47	47
chr10	78080819	78082819	Olfr1357		1	noCoverage	-0.051938	0.38882	insignificant	0	9	9
chr10	78181038	78183038	Casp14	-0.02006443	0.80274	insignificant	0.014848	0.87608	insignificant	3	6	6
chr10	78242240	78244240	Slc1a6	-0.15450562	0.048464	hypomethylated	-0.024099	0.1064	insignificant	10	40	40
chr10	78996532	78998532	Ppap2c	-0.1587426	2.74E-10	hypomethylated	0.0029257	0.76505	insignificant	5	14	14
chr10	79017836	79019836	Mier2	-0.04400569	0.0044016	hypomethylated	0.040345	0.73497	insignificant	9	37	37
chr10	79100663	79102663	Shc2	-0.29589886	2.37E-12	hypomethylated	-0.000863	0.82919	insignificant	11	40	40
chr10	79126318	79128318	Madcam1	-0.36520655	2.99E-12	stronglyHypometh	0.07692	0.069303	insignificant	11	48	48
chr10	79131154	79133154	Gm16517	-0.13543331	3.92E-27	hypomethylated	0.0036312	0.68028	insignificant	38	95	88
chr10	79143939	79145939	Cdc34	-0.1528788	3.35E-37	hypomethylated	0.0026242	0.21991	insignificant	42	97	104
chr10	79150764	79152764	Gzmm	0.03484848	0.66901	insignificant	0.038202	0.69722	insignificant	1	22	22
chr10	79166102	79168102	Bsg	-0.24470776	0.000056253	hypomethylated	0.004429	1	insignificant	8	28	33
chr10	79178378	79180378	Hcn2	-0.08180685	7.18E-10	hypomethylated	0.0086033	0.79114	insignificant	47	145	125
chr10	79209326	79211326	Polrmt	-0.30504433	0.0013786	hypomethylated	-0.016387	0.24486	insignificant	3	13	16
chr10	79216863	79218863	Fgf22	0.00536165	0.47063	insignificant	0.014142	0.79225	insignificant	10	44	42
chr10	79229666	79231666	Rnf126	-0.17510336	0.44048	insignificant	-0.0035758	0.08619	insignificant	15	68	68
chr10	79229686	79231686	Rnf126	-0.1921506	0.18236	insignificant	-0.0043225	0.2511	insignificant	14	60	60
chr10	79239018	79241018	Fstl3	-0.12793139	3.75E-13	hypomethylated	-0.0016835	0.09698	insignificant	15	61	54
chr10	79255316	79257316	Palm	-0.08401767	4.05E-08	hypomethylated	0.00013309	0.25316	insignificant	20	75	70
chr10	79316349	79318349	Ptbp1	-0.12199219	1.42E-25	hypomethylated	-0.00024738	1	insignificant	39	128	125
chr10	79317716	79319716	E130317F20Ri	-0.10549579	1.08E-13	hypomethylated	-0.001392	0.6348	insignificant	30	104	101
chr10	79337379	79339379	BC005764		1	noCoverage	-0.18997	0.5058	insignificant	0	8	8
chr10	79348056	79350056	Elane		1	noCoverage	0.045658	0.00045371	hypermethylated	0	23	24
chr10	79352597	79354597	Cfd	-0.18939394	0.727	insignificant	0.095328	0.12708	insignificant	3	9	6
chr10	79371683	79373683	Med16	-0.66688543	2.21E-09	stronglyHypometh	0.073639	0.24494	insignificant	2	18	18
chr10	79378715	79380715	Kiss1r	-0.151331	1.3E-09	hypomethylated	-0.0080757	0.95957	insignificant	16	49	46
chr10	79379675	79381675	Kiss1r	-0.13589778	0.001384	hypomethylated	-0.035813	0.66473	insignificant	8	25	22
chr10	79388816	79390816	Arid3a	-0.15606118	3.43E-32	hypomethylated	-0.0090661	0.53067	insignificant	42	166	174
chr10	79421896	79423896	Wdr18	-0.16719795	0.000000012	hypomethylated	-0.022481	0.69144	insignificant	9	28	30
chr10	79432468	79434468	Grin3b	-0.17927979	4.09E-24	hypomethylated	0.008814	0.34943	insignificant	29	112	112
chr10	79447075	79449075	ORF61	-0.13855592	0.00000393	hypomethylated	-0.011637	0.70983	insignificant	3	24	20
chr10	79450344	79452344	Cnn2	-0.21040327	6.51E-15	hypomethylated	0.015343	0.76997	insignificant	22	110	94
chr10	79459359	79461359	Abca7	-0.24522847	0.00000023	hypomethylated	0.031261	0.45685	insignificant	10	54	49
chr10	79481419	79483419	Hmha1	0.43403893	0.47076	lowCoverage	0.010978	0.82022	insignificant	1	20	20
chr10	79502404	79504404	Polr2e	-0.08436357	5.89E-18	hypomethylated	0.0026219	0.24575	insignificant	23	72	72
chr10	79515254	79517254	Gpx4	-0.14458557	1.89E-28	hypomethylated	0.010519	0.59461	insignificant	37	94	96
chr10	79515781	79517781	Gpx4	-0.12377895	4.84E-26	hypomethylated	0.00034086	0.97208	insignificant	37	111	114
chr10	79565447	79567447	Sbno2	-0.2475	0.097902	insignificant	-0.12548	0.097159	insignificant	10	25	20
chr10	79578280	79580280	Stk11	-0.14414861	6.24E-14	hypomethylated	-0.036809	0.65458	insignificant	42	171	148
chr10	79600132	79602132	Dos	-0.09738546	1.11E-13	hypomethylated	0.006333	0.1297	insignificant	37	108	109
chr10	79602112	79604112	Dos	0.06432723	0.0022154	inconclusive	-0.053042	0.2694	insignificant	6	35	32
chr10	79604059	79606059	Atp5d	-0.15289837	8.48E-10	hypomethylated	-0.01561	0.59194	insignificant	14	81	81
chr10	79610034	79612034	Midn	-0.1248997	7.12E-22	hypomethylated	-0.0029672	0.73065	insignificant	34	94	92
chr10	79629585	79631585	Cirbp	-0.07044718	0.064449	insignificant	-0.0065796	0.65874	insignificant	33	64	64
chr10	79634688	79636688	1600002K03Ri	-0.13251641	3.86E-21	hypomethylated	0.029139	0.184	insignificant	20	79	77
chr10	79641226	79643226	Efna2	-0.13855224	0.00000208	hypomethylated	-0.030781	0.47654	insignificant	18	67	64
chr10	79688342	79690342	Mum1	-0.12335892	0.0031105	hypomethylated	0.00053193	0.88626	insignificant	14	74	72
chr10	79711196	79713196	Ndufs7	-0.20897174	2.24E-08	hypomethylated	-0.023224	0.86408	insignificant	3	35	36
chr10	79723713	79725713	Gamt	-0.43699996	0.000090715	stronglyHypometh	-0.054005	0.77392	insignificant	5	33	26
chr10	79726735	79728735	Dazap1	-0.09287192	9.07E-32	hypomethylated	0.0084174	0.83082	insignificant	46	149	141
chr10	79754175	79756175	Rps15	-0.15809423	3.42E-26	hypomethylated	-0.0014344	0.89192	insignificant	37	133	125
chr10	79763564	79765564	Apc2	0.00290731	0.44713	insignificant	-0.013276	0.030002	inconclusive	4	16	16
chr10	79783293	79785293	Pcsk4	-0.48623475	2.17E-09	stronglyHypometh	0.023443	0.44989	insignificant	3	52	52
chr10	79791914	79793914	Pcsk4	-0.12940894	1.34E-16	hypomethylated	0.001674	0.29886	insignificant	23	72	62
chr10	79792218	79794218	Pcsk4	-0.15293655	6.81E-14	hypomethylated	0.017219	0.44259	insignificant	24	78	68
chr10	79811157	79813157	Adamtsl5	-0.07759783	0.2372	insignificant	-0.01023	0.11978	insignificant	21	87	94
chr10	79811191	79813191	Adamtsl5	-0.07371899	0.003189	inconclusive	-0.013802	0.4011	insignificant	18	79	86
chr10	79818203	79820203	Plk5	-0.13789385	7.4E-23	hypomethylated	0.010778	0.27529	insignificant	29	100	106
chr10	79850396	79852396	Mex3d	-0.12112188	5.27E-09	hypomethylated	0.0013262	0.92692	insignificant	11	61	66
chr10	79862224	79864224	Mbd3	-0.17041234	0.00045198	hypomethylated	-0.037768	0.32552	insignificant	3	24	30
chr10	79869566	79871566	Uqcr11	-0.19663985	0.0003755	hypomethylated	-0.012661	1	insignificant	8	18	16
chr10	79896398	79898398	Tcf3	-0.24886752	0.019545	hypomethylated	0.008328	0.19191	insignificant	8	27	33
chr10	79956650	79958650	Onecut3	-0.0906571	1.03E-13	hypomethylated	0.0052819	0.10035	insignificant	34	158	159
chr10	80024305	80026305	Rexo1	-0.18523203	0.038001	hypomethylated	0.061319	0.19969	insignificant	3	35	37
chr10	80040041	80042041	Klf16	-0.12242546	1.49E-32	hypomethylated	0.0054056	0.15079	insignificant	23	160	151
chr10	80053086	80055086	Fam108a	-0.46227885	1	lowCoverage	-0.010408	0.022458	hypomethylated	1	20	19
chr10	80064624	80066624	Adat3	-0.1224128	0.00000441	hypomethylated	-0.018521	0.96833	insignificant	14	67	67
chr10	80064626	80066626	Scamp4	-0.1224128	0.00000441	hypomethylated	-0.018521	0.96833	insignificant	14	67	67
chr10	80084524	80086524	Csnk1g2	-0.1265681	1.76E-14	hypomethylated	0.0053522	0.074006	insignificant	27	76	72
chr10	80119816	80121816	Btbd2		1	noCoverage	0.021087	1	insignificant	0	4	8
chr10	80164437	80166437	Izumo4	-0.15342596	6.81E-17	hypomethylated	0.0015557	0.53728	insignificant	27	114	114
chr10	80164565	80166565	Izumo4	-0.15493309	3.02E-14	hypomethylated	0.00041487	0.83305	insignificant	22	98	98
chr10	80204956	80206956	Ap3d1		1	noCoverage	0.011533	0.55968	insignificant	0	37	37
chr10	80216950	80218950	Dot1l	-0.0777413	9.54E-26	hypomethylated	0.00050746	0.79816	insignificant	49	139	146
chr10	80260479	80262479	Sf3a2	-0.07355736	5.92E-29	hypomethylated	-0.0038287	0.62208	insignificant	60	218	219
chr10	80261371	80263371	Plekhj1	-0.04655712	0.00000103	hypomethylated	-0.0004283	0.90971	insignificant	23	100	100
chr10	80288400	80290400	Oaz1	-0.11076745	0.000022469	hypomethylated	-0.0062095	0.22575	insignificant	29	139	139
chr10	80288440	80290440	Gm9786	-0.11076745	0.000022469	hypomethylated	-0.0062095	0.22575	insignificant	29	139	139
chr10	80290541	80292541	Mir1982	-0.13743684	0.0009378	hypomethylated	0.03395	0.45027	insignificant	4	16	16
chr10	80306784	80308784	Lingo3	-0.04924411	0.82865	insignificant	0.014194	0.53278	insignificant	9	38	38
chr10	80317019	80319019	3110056O03R	-0.14387319	1.83E-37	hypomethylated	0.030396	0.012789	hypermethylated	29	110	105
chr10	80317954	80319954	Lsm7	-0.16059488	2.8E-38	hypomethylated	0.030716	0.0085396	hypermethylated	31	104	99
chr10	80363714	80365714	Timm13	-0.04531926	0.11033	insignificant	-0.062751	0.025311	hypomethylated	4	22	18
chr10	80380990	80382990	Lmnb2	-0.20374268	0.00062298	hypomethylated	0.018502	0.91632	insignificant	6	37	36

chr10	80391835	80393835	Gadd45b	-0.13059869	2.16E-10	hypomethylated	-0.0034008	0.88583	insignificant	25	101	103
chr10	80464110	80466110	Gng7	0.10696841	0.7162	insignificant	0.050645	0.74924	insignificant	6	34	37
chr10	80477670	80479670	Gng7		1	noCoverage	-0.00048733	0.89059	insignificant	0	12	12
chr10	80488122	80490122	Diras1	-0.39375099	9.31E-12	stronglyHypometh	-0.062841	0.68411	insignificant	5	35	41
chr10	80522899	80524899	Sgta		1	noCoverage	-0.086402	0.084343	insignificant	0	38	33
chr10	80531827	80533827	Thop1	-0.09618994	0.042472	hypomethylated	-0.0091385	0.53686	insignificant	7	40	41
chr10	80561617	80563617	Creb3l3	-0.382762	0.082585	insignificant	0.06522	0.74453	insignificant	2	14	16
chr10	80567691	80569691	Map2k2	-0.07763259	1.78E-26	hypomethylated	0.0089465	0.1009	insignificant	28	110	110
chr10	80598015	80600015	Zbtb7a	-0.11771774	2.51E-28	hypomethylated	-0.0039801	0.80923	insignificant	66	222	217
chr10	80630465	80632465	Pias4	-0.26348232	0.00038743	hypomethylated	-0.032605	0.79451	insignificant	4	48	52
chr10	80638375	80640375	Eef2	-0.10934856	5.62E-09	hypomethylated	0.0025564	0.9728	insignificant	12	100	100
chr10	80640705	80642705	Snord37	0.14205446	1	insignificant	0.047749	0.48182	insignificant	2	27	27
chr10	80644751	80646751	Dapk3	-0.19006234	8.44E-15	hypomethylated	0.005501	0.11796	insignificant	14	65	68
chr10	80645007	80647007	Dapk3	-0.19006234	8.44E-15	hypomethylated	0.005501	0.11796	insignificant	14	65	68
chr10	80645662	80647662	Dapk3	-0.16673884	6.47E-13	hypomethylated	-0.016388	0.30931	insignificant	14	54	54
chr10	80656435	80658435	2310050B05R	-0.12791005	0.0063641	hypomethylated	-0.046772	0.53962	insignificant	11	16	20
chr10	80694907	80696907	Zfr2	-0.1261545	0.00029749	hypomethylated	-0.0092122	0.28297	insignificant	10	48	62
chr10	80719289	80721289	Matk	-0.16440875	1.6E-26	hypomethylated	0.0010989	0.1259	insignificant	24	87	80
chr10	80729671	80731671	Apba3	-0.17728505	2.76E-10	hypomethylated	0.068503	0.011201	hypermethylated	13	30	31
chr10	80729916	80731916	Apba3	-0.26597784	1.95E-16	hypomethylated	0.074392	0.033824	hypermethylated	12	24	25
chr10	80733341	80735341	Mir3057	0.04961874	0.27135	insignificant	0.021898	0.055818	insignificant	5	12	10
chr10	80754012	80756012	Pip5k1c	-0.10038066	0.00042538	hypomethylated	0.011674	0.47178	insignificant	19	81	82
chr10	80754716	80756716	Pip5k1c	-0.08624597	0.000016475	hypomethylated	0.029011	0.68003	insignificant	29	113	114
chr10	80782847	80784847	2510012J08Ri	-0.10861003	2.89E-21	hypomethylated	0.029268	1	insignificant	35	164	161
chr10	80790475	80792475	Tbxa2r		1	noCoverage	-0.10317	0.17112	insignificant	0	9	8
chr10	80806011	80808011	Gipc3	-0.13129085	0.0037679	hypomethylated	-0.0049688	0.66255	insignificant	5	17	10
chr10	80813171	80815171	Hmg20b	-0.11444627	0.012824	hypomethylated	0.018638	0.048493	hypermethylated	12	72	72
chr10	80813172	80815172	Hmg20b	-0.11444627	0.012824	hypomethylated	0.018638	0.048493	hypermethylated	12	72	72
chr10	80813202	80815202	Hmg20b	-0.12694855	0.027434	hypomethylated	0.019232	0.039219	hypermethylated	7	62	62
chr10	80819314	80821314	F630110N24R	-0.0889076	1.79E-22	hypomethylated	0.0037899	0.73399	insignificant	23	103	101
chr10	80828565	80830565	4930404N11R	0.00680457	0.47515	insignificant	0.0070308	0.89409	insignificant	8	18	18
chr10	80841115	80843115	Fzr1	-0.07415401	0.00093837	hypomethylated	0.0044223	0.80117	insignificant	13	68	75
chr10	80846172	80848172	Dohh	-0.30099675	6.34E-11	hypomethylated	-0.036528	0.33115	insignificant	13	83	79
chr10	80945454	80947454	Celf5	-0.16379631	0.63557	insignificant	0.11225	0.54053	insignificant	7	29	25
chr10	80959108	80961108	Ncln	-0.27025768	1	lowCoverage	0.28857	0.48053	insignificant	1	8	4
chr10	80986970	80988970	Gna15		1	noCoverage	0.0040856	0.56145	insignificant	0	12	11
chr10	81007791	81009791	Gna11	-0.07975423	0.00000721	hypomethylated	0.0046158	0.44743	insignificant	7	45	45
chr10	81021304	81023304	Aes	-0.13190663	5.42E-23	hypomethylated	-0.031284	0.96622	insignificant	35	94	102
chr10	81037276	81039276	Tle2	-0.15698885	9.73E-18	hypomethylated	-0.013155	0.75264	insignificant	15	57	56
chr10	81063645	81065645	Tle6	-0.22948106	0.020809	hypomethylated	0.017702	1	insignificant	8	48	45
chr10	81068052	81070052	BC025920	-0.1949928	0.0023865	hypomethylated	-0.0078234	0.10946	insignificant	3	38	37
chr10	81090067	81092067	Sirt6	-0.28181959	7.53E-18	hypomethylated	0.052376	0.00012024	hypermethylated	9	69	72
chr10	81090284	81092284	Ankrd24	-0.3550808	3.68E-15	stronglyHypometh	0.061547	0.00000013	hypermethylated	7	48	54
chr10	81090353	81092353	Sirt6	-0.46091412	0.50094	insignificant	0.073809	0.012566	hypermethylated	2	32	38
chr10	81110813	81112813	Gm10778	-0.30222223	0.0019052	hypomethylated	0.027019	0.62578	insignificant	2	35	32
chr10	81273930	81275930	Gm10778		1	noCoverage	0.2334	0.087921	insignificant	0	25	16
chr10	81509871	81511871	Zfp873	-0.21619632	0.022924	hypomethylated	-0.039343	0.0038469	hypomethylated	6	26	26
chr10	81704020	81706020	Zfp938	-0.58316435	0.000092537	stronglyHypometh	-0.0096966	0.43331	insignificant	2	13	14
chr10	82091582	82093582	Tdg	-0.11621498	1	insignificant	-0.023728	0.65888	insignificant	8	77	74
chr10	82091586	82093586	Gm9855	-0.12593106	1	insignificant	-0.021344	0.63219	insignificant	8	79	75
chr10	82160751	82162751	Hcfc2	-0.11384437	2.8E-17	hypomethylated	0.0099395	0.043047	hypermethylated	43	139	140
chr10	82226886	82228886	Nfyb	-0.08886408	0.00000015	hypomethylated	-0.0059437	0.22473	insignificant	13	58	49
chr10	82320950	82322950	Txnrd1	-0.17071188	2.56E-08	hypomethylated	0.03152	0.11564	insignificant	10	56	48
chr10	82322085	82324085	Txnrd1	-0.15009094	2.14E-24	hypomethylated	0.0052527	1	insignificant	28	73	73
chr10	82328370	82330370	Eid3	-0.32497915	0.61392	insignificant	-0.26412	0.52142	insignificant	1	4	4
chr10	82447241	82449241	Chst11	-0.14819672	1.37E-43	hypomethylated	0.026619	0.031874	hypermethylated	42	152	147
chr10	82800562	82802562	Slc41a2		1	noCoverage	0.10347	0.18601	insignificant	0	17	21
chr10	82821965	82823965	D10Wsu102e	-0.1438764	9.99E-29	hypomethylated	0.00075551	0.29964	insignificant	34	112	111
chr10	82996885	82998885	Aldh1l2	-0.21997245	0.61826	insignificant	-0.10053	0.84381	insignificant	2	11	10
chr10	83005685	83007685	A230046K03R	-0.08582055	3.33E-12	hypomethylated	0.0088202	1	insignificant	32	128	127
chr10	83111409	83113409	Appl2	0.14190884	0.00031808	hypermethylated	0.006076	0.27862	insignificant	2	18	18
chr10	83184609	83186609	1500009L16Ri	-0.33719907	0.062382	insignificant	0.019257	0.8906	insignificant	2	16	16
chr10	83903216	83905216	Nuak1	-0.1186404	4.06E-28	hypomethylated	0.0081754	0.025627	hypermethylated	50	175	179
chr10	83996633	83998633	Ckap4	-0.10288103	2.12E-21	hypomethylated	-0.0032642	0.17384	insignificant	40	106	105
chr10	84038691	84040691	Tcp11l2	-0.14999287	4.36E-17	hypomethylated	0.032052	0.089565	insignificant	12	65	52
chr10	84084181	84086181	Polr3b	-0.21878728	4.68E-12	hypomethylated	0.025378	0.57214	insignificant	14	83	88
chr10	84217792	84219792	Rfx4	-0.15109462	2.19E-09	hypomethylated	-0.019963	0.42543	insignificant	23	79	84
chr10	84379360	84381360	Ric8b	-0.04965735	0.00010017	hypomethylated	-0.0095371	0.64661	insignificant	16	59	54
chr10	84379400	84381400	Ric8b	-0.04965735	0.00010017	hypomethylated	-0.0095371	0.64661	insignificant	16	59	54
chr10	84564371	84566371	AI597468	-0.1680542	1.22E-18	hypomethylated	-0.052682	0.5185	insignificant	26	113	88
chr10	84565240	84567240	AI597468	-0.16683018	1.52E-16	hypomethylated	-0.070272	0.6472	insignificant	23	101	78
chr10	84590772	84592772	Mterfd3		1	noCoverage	-0.0013655	0.61428	insignificant	0	4	4
chr10	84647799	84649799	Cry1	-0.08878593	5.34E-21	hypomethylated	0.0048	0.61093	insignificant	28	73	68
chr10	84848558	84850558	Btbd11	-0.15935616	4.16E-13	hypomethylated	-0.023197	0.14225	insignificant	28	128	121
chr10	85060155	85062155	Btbd11	0.08012492	0.00044747	inconclusive	0.05699	0.0002627	hypermethylated	7	45	45
chr10	85333575	85335575	Pwp1	-0.14818957	0.000000977	hypomethylated	0.023279	0.96705	insignificant	8	57	57
chr10	85379690	85381690	Prdm4	-0.11009522	1.65E-19	hypomethylated	0.0037759	0.065301	insignificant	18	64	64
chr10	85390235	85392235	Ascl4	-0.13355028	9.14E-30	hypomethylated	0.0050066	0.33315	insignificant	27	70	70
chr10	85483673	85485673	Fbxo7	-0.11385087	2.03E-20	hypomethylated	-0.0065059	0.47851	insignificant	33	82	82
chr10	85762156	85764156	Timp3	-0.14104458	3.11E-14	hypomethylated	0.044592	0.16147	insignificant	10	74	74
chr10	86147271	86149271	1810014B01R	-0.2040308	0.075669	insignificant	0.04737	0.9075	insignificant	1	8	8
chr10	86167555	86169555	BC030307	-0.14763158	0.000011129	hypomethylated	0.015032	0.22186	insignificant	17	77	74
chr10	86168189	86170189	BC030307	-0.15954969	0.012028	hypomethylated	0.030753	1	insignificant	4	28	25
chr10	86174509	86176509	BC030307	0.06797068	1	insignificant	0.014774	0.62395	insignificant	1	15	16
chr10	86240749	86242749	Nt5dc3	-0.13349496	1.8E-38	hypomethylated	0.025634	0.015015	hypermethylated	33	147	141

chr10	86519790	86521790	1700113H08R	-0.31908794	0.00086572	hypomethylated	-0.1302	0.089316	insignificant	4	15	12
chr10	86956405	86958405	Ascl1	-0.16246371	0.00019099	hypomethylated	0.036057	0.65561	insignificant	8	19	20
chr10	87428415	87430415	Tyms-ps	0.08132184	0.59814	insignificant	-0.081688	0.65716	insignificant	3	6	6
chr10	87608736	87610736	Nup37	-0.20759991	0.0000001	hypomethylated	-0.059092	0.0010731	hypomethylated	12	72	68
chr10	87608815	87610815	Nup37	-0.20759991	0.0000001	hypomethylated	-0.059092	0.0010731	hypomethylated	12	72	68
chr10	87609686	87611686	4930547N16R	-0.23335871	0.000000681	hypomethylated	-0.090202	0.00000115	hypomethylated	8	54	50
chr10	87662841	87664841	Ccdc53	-0.27423176	0.0014651	hypomethylated	-0.015475	0.54839	insignificant	8	22	22
chr10	87819820	87821820	Dram1	-0.14900794	0.00072648	hypomethylated	0.021254	0.4904	insignificant	5	10	10
chr10	87841156	87843156	Gnptab	-0.11334541	8.87E-31	hypomethylated	0.0083378	0.73686	insignificant	47	118	112
chr10	87921331	87923331	Sycp3	-0.01967545	0.00033314	hypomethylated	0.0057479	0.17842	insignificant	12	38	38
chr10	87966715	87968715	Chpt1	-0.21595419	0.000016468	hypomethylated	-0.034042	1	insignificant	9	24	24
chr10	88193158	88195158	Arl1	-0.10945702	1.64E-12	hypomethylated	0.0023796	0.67655	insignificant	34	164	164
chr10	88193846	88195846	Gm4925	-0.10745016	2.3E-11	hypomethylated	-0.0047742	0.82706	insignificant	30	153	153
chr10	88347736	88349736	Slc5a8	-0.29545138	0.00000146	hypomethylated	-0.043314	0.81442	insignificant	3	44	42
chr10	88720535	88722535	Ano4	-0.60793651	0.4	lowCoverage	0.075018	0.7916	insignificant	1	8	8
chr10	88906712	88908712	Gas2l3	-0.24172851	1	insignificant	-0.0030385	0.83752	insignificant	1	14	14
chr10	89083994	89085994	Slc17a8	-0.22286822	0.053889	insignificant	0.079961	0.26556	insignificant	2	14	12
chr10	89149030	89151030	Scyl2	-0.19015761	4.51E-17	hypomethylated	-0.0033124	0.71596	insignificant	13	26	26
chr10	89195040	89197040	Actr6		1	noCoverage	-0.036426	0.47586	insignificant	0	9	6
chr10	89206735	89208735	Uhrf1bp1l	-0.106927	9.93E-09	hypomethylated	0.01929	0.77237	insignificant	20	65	51
chr10	89335253	89337253	Anks1b	-0.12793283	4.21E-13	hypomethylated	-0.016479	0.61344	insignificant	17	105	94
chr10	90038435	90040435	Anks1b	0.12602675	0.27161	insignificant	0.00067006	0.40377	insignificant	4	12	9
chr10	90544783	90546783	Ikbip	-0.11156998	0.00000208	hypomethylated	0.0091158	0.0027338	hypermethylated	21	114	107
chr10	90545488	90547488	Ikbip	-0.08846417	1	insignificant	-0.006965	0.17275	insignificant	2	54	49
chr10	90586708	90588708	Slc25a3	-0.10562635	0.0078344	hypomethylated	-0.00033916	0.69498	insignificant	6	38	38
chr10	90634327	90636327	Tmpo	-0.19517611	1.02E-12	hypomethylated	0.015848	0.47709	insignificant	10	64	72
chr10	91627923	91629923	Rmst		1	noCoverage	0.04734	0.29026	insignificant	0	6	6
chr10	92622620	92624620	Cdk17	-0.0785836	5.3E-24	hypomethylated	-0.00867	0.88383	insignificant	55	198	198
chr10	92773904	92775904	Elk3	-0.11413043	0.60763	insignificant	-0.01289	0.88969	insignificant	3	24	29
chr10	92915140	92917140	Lta4h	-0.11818998	0.000000267	hypomethylated	-0.01305	0.58961	insignificant	10	42	42
chr10	92950512	92952512	Hal	-0.67691	0.12838	insignificant	-0.036834	0.65888	insignificant	2	16	16
chr10	93002376	93004376	Ccdc38	-0.14205712	1.82E-12	hypomethylated	0.00005805	0.59485	insignificant	12	52	52
chr10	93002778	93004778	Amdhd1	-0.13185597	0.094235	insignificant	0.011284	0.046183	hypermethylated	3	14	14
chr10	93052403	93054403	Snrpf	0.12268188	0.10873	insignificant	0.0014593	0.26424	insignificant	1	26	16
chr10	93102793	93104793	Ntn4	-0.15087795	2.27E-36	hypomethylated	0.0069737	0.17369	insignificant	40	150	146
chr10	93293299	93295299	Usp44	-0.73014361	0.030785	stronglyHypometh	-0.12049	0.14243	insignificant	3	16	12
chr10	93497745	93499745	Fgd6	-0.12370827	2.08E-17	hypomethylated	0.00059853	0.086029	insignificant	22	118	122
chr10	93498493	93500493	Vezt	-0.10736112	5.61E-15	hypomethylated	0.0025713	0.17137	insignificant	22	96	100
chr10	93609675	93611675	Nr2c1	-0.36931341	4.67E-16	stronglyHypometh	-0.22966	0.23049	insignificant	14	39	30
chr10	93660753	93662753	Ndufa12	-0.13267256	0.02301	hypomethylated	-0.024464	0.55615	insignificant	12	74	78
chr10	93976601	93978601	Tmcc3	-0.14566329	3.33E-29	hypomethylated	-0.007178	0.19651	insignificant	47	163	150
chr10	94037001	94039001	Tmcc3	0.2537221	0.31502	insignificant	-0.027562	0.91637	insignificant	1	13	13
chr10	94038007	94040007	Tmcc3	0.21745898	0.32671	insignificant	-0.067104	0.70796	insignificant	2	19	19
chr10	94150534	94152534	Ccdc41	-0.06434315	0.000000206	hypomethylated	0.005032	0.32624	insignificant	34	152	148
chr10	94151358	94153358	4932415G12R	-0.0550206	0.000039499	hypomethylated	0.00015165	0.41774	insignificant	30	121	118
chr10	94407212	94409212	Plxnc1	-0.18497859	1.14E-12	hypomethylated	-0.054637	0.34143	insignificant	22	91	84
chr10	94877917	94879917	Socs2	-0.09622975	3.25E-24	hypomethylated	0.0086103	0.18959	insignificant	42	192	196
chr10	94878118	94880118	Socs2	-0.09585596	1.66E-24	hypomethylated	0.0086794	0.19543	insignificant	41	190	194
chr10	94878846	94880846	Socs2	-0.11632403	7E-27	hypomethylated	0.0056029	0.094626	insignificant	45	165	166
chr10	94879491	94881491	5730420D15R	-0.15020469	5.5E-11	hypomethylated	-0.0017953	0.57989	insignificant	14	61	58
chr10	94964561	94966561	Mrpl42	0.12203704	0.73965	insignificant	0.14777	0.73504	insignificant	4	10	13
chr10	94976795	94978795	Ube2n	-0.09403325	3.39E-43	hypomethylated	0.0077826	0.3188	insignificant	62	199	178
chr10	95021955	95023955	Mir3058	-0.28922776	0.00063	hypomethylated	-0.0056288	0.53528	insignificant	1	18	23
chr10	95026801	95028801	Nudt4	-0.22198088	1.89E-39	hypomethylated	-0.0013561	0.16285	insignificant	17	77	77
chr10	95402296	95404296	Eea1	-0.08178714	2.31E-13	hypomethylated	0.0081852	0.0097523	hypermethylated	47	148	148
chr10	96078634	96080634	Btg1	-0.12176507	2.73E-16	hypomethylated	-0.016213	0.72471	insignificant	40	145	156
chr10	97154692	97156692	4921510H08R	-0.0515909	0.88896	insignificant	0.035665	0.00076737	hypermethylated	11	66	66
chr10	98376785	98378785	Atp2b1	-0.09386378	1.02E-42	hypomethylated	0.010434	0.3737	insignificant	109	284	279
chr10	98568804	98570804	Poc1b	-0.14051519	4.27E-19	hypomethylated	0.023621	0.94571	insignificant	24	106	103
chr10	98569768	98571768	Galnt4	-0.15290054	2.41E-18	hypomethylated	0.026052	0.39003	insignificant	28	100	101
chr10	98724864	98726864	Dusp6	-0.15138697	1.7E-12	hypomethylated	-0.014781	0.41109	insignificant	18	104	104
chr10	99222292	99224292	Csl		1	noCoverage	-0.0071709	0.83532	insignificant	0	10	9
chr10	99477457	99479457	Kitl	-0.16318167	3.82E-38	hypomethylated	0.0032732	0.64135	insignificant	34	110	107
chr10	99949922	99951922	Cep290	-0.1145771	0.00000534	hypomethylated	0.015891	0.019184	inconclusive	17	72	66
chr10	99949981	99951981	Cep290	-0.13045548	0.00000992	hypomethylated	0.022172	0.052991	insignificant	14	60	54
chr10	100051893	100053893	4930430F08Rik		1	noCoverage	-0.03799	0.61623	insignificant	0	17	16
chr10	102491411	102493411	Alx1	-0.21163256	0.00001238	hypomethylated	0.015523	0.49767	insignificant	16	75	75
chr10	102698956	102700956	Lrriq1	-0.39166667	1	lowCoverage	-0.19167	0.69256	insignificant	1	6	5
chr10	102829476	102831476	Slc6a15	-0.20550325	0.000003267	hypomethylated	-0.020012	1	insignificant	6	35	38
chr10	105277534	105279534	Ccdc59	-0.1281165	2.81E-25	hypomethylated	-0.0046053	0.62177	insignificant	29	106	109
chr10	105278436	105280436	Ccdc59	-0.11974853	7.64E-10	hypomethylated	-0.005024	0.64972	insignificant	17	74	77
chr10	105906394	105908394	Ppfia2	-0.23823719	0.34612	insignificant	0.036619	0.6758	insignificant	4	25	22
chr10	106707886	106709886	Lin7a	-0.18035996	7.97E-17	hypomethylated	-0.016688	0.95446	insignificant	6	34	34
chr10	107157083	107159083	Ptprq	0.17261905	1	insignificant	0.15088	0.38985	insignificant	2	10	8
chr10	107349190	107351190	Otogl	-0.39215686	0.031288	stronglyHypometh	-0.19014	0.039537	hypomethylated	1	6	6
chr10	107598455	107600455	Ppp1r12a	-0.11374591	7.26E-37	hypomethylated	-0.0036174	0.86533	insignificant	52	215	212
chr10	107768244	107770244	Pawr	-0.08530472	6.34E-27	hypomethylated	0.0021886	0.47083	insignificant	68	269	265
chr10	108447675	108449675	Syt1	-0.21432158	0.000000926	hypomethylated	-0.019253	0.6355	insignificant	3	45	42
chr10	110036705	110038705	9230102K24Ri	-0.41496128	0.0040221	stronglyHypometh	0.11184	0.54545	insignificant	6	16	16
chr10	110181520	110183520	E2f7	-0.06869881	1.27E-12	hypomethylated	-0.0090959	0.85532	insignificant	64	189	185
chr10	110356231	110358231	Csrp2	-0.13974024	1.01E-14	hypomethylated	0.012938	0.63591	insignificant	24	79	71
chr10	110447122	110449122	Zdhhc17	-0.1070759	2.04E-20	hypomethylated	-0.003867	0.086835	insignificant	24	104	101
chr10	110600857	110602857	Osbpl8	-0.1151287	1.2E-31	hypomethylated	0.0084465	0.75762	insignificant	45	157	149
chr10	110734734	110736734	Bbs10		1	noCoverage	-0.010815	0.75185	insignificant	0	60	54
chr10	110909247	110911247	Nap1l1	-0.12549911	7.23E-30	hypomethylated	-0.0056553	0.49588	insignificant	41	103	104

chr10	110936843	110938843	Gm5176		1	noCoverage	0.14258	0.30817	insignificant	0	4	4
chr10	110942341	110944341	Phlda1	-0.06913888	5.33E-15	hypomethylated	-0.0089981	0.30653	insignificant	45	165	173
chr10	111408750	111410750	Krr1	-0.18697922	5.43E-10	hypomethylated	0.03094	0.02227	hypermethylated	18	63	58
chr10	111519409	111521409	Glipr1l2	0.01468404	0.48204	insignificant	-0.04818	0.033628	hypomethylated	5	22	22
chr10	111707178	111709178	Kcnc2	-0.14332841	0.22914	insignificant	-0.0059403	0.39132	insignificant	6	83	83
chr10	112366082	112368082	Atxn7l3b	-0.17945716	0.073353	insignificant	-0.0053144	0.87962	insignificant	3	25	25
chr10	114238426	114240426	Trhde	-0.08695954	0.000000188	hypomethylated	0.025004	0.28157	insignificant	37	133	137
chr10	114752647	114754647	Rab21	-0.20665183	0.000059944	hypomethylated	-0.048349	0.0035046	inconclusive	12	39	36
chr10	114799318	114801318	Tmem19	-0.0994802	0.000027445	hypomethylated	-0.0028636	0.66968	insignificant	5	45	45
chr10	114821014	114823014	Zfc3h1	-0.13394864	5.66E-27	hypomethylated	-0.0028843	0.32517	insignificant	65	182	178
chr10	114821491	114823491	Thap2	-0.13676926	8.25E-27	hypomethylated	-0.0040024	0.33651	insignificant	61	160	156
chr10	115024836	115026836	Lgr5	-0.16892596	0.0010246	hypomethylated	0.014152	0.0043185	inconclusive	10	28	31
chr10	115454418	115456418	Ptprr	-0.17458378	0.19851	insignificant	0.0070515	0.68966	insignificant	7	30	30
chr10	115550973	115552973	4933416C03Ri	-0.01556901	0.07899	insignificant	-0.041934	0.14127	insignificant	10	36	36
chr10	115613313	115615313	Ptprr	-0.36792313	0.0083925	stronglyHypometh	0.14921	0.78501	insignificant	1	24	24
chr10	115613406	115615406	Ptprr	-0.36792313	0.0083925	stronglyHypometh	0.14921	0.78501	insignificant	1	24	24
chr10	115737429	115739429	Ptprb	-0.04900795	0.61968	insignificant	-0.0055709	0.50388	insignificant	11	34	34
chr10	115854176	115856176	1700058G18R	-0.66073307	0.0062224	stronglyHypometh	0.032975	0.01843	hypermethylated	2	30	33
chr10	115910579	115912579	Kcnmb4	-0.06451944	0.00000133	hypomethylated	0.0054708	0.0063064	hypermethylated	37	133	133
chr10	116018567	116020567	Cnot2	-0.11155428	1.68E-28	hypomethylated	0.0012373	0.087751	insignificant	37	130	132
chr10	116387436	116389436	Rab3ip		1	noCoverage	-0.026628	0.43115	insignificant	0	18	18
chr10	116422369	116424369	Best3	-0.15079365	0.10086	insignificant	-0.1119	0.039764	hypomethylated	6	12	12
chr10	116481396	116483396	Lrrc10	0.03562999	0.54676	insignificant	0.06828	0.00025353	hypermethylated	15	44	48
chr10	116500870	116502870	Cct2	-0.64648822	0.00000749	stronglyHypometh	0.043056	0.027774	hypermethylated	2	18	18
chr10	116585530	116587530	Frs2	-0.2188694	7.59E-12	hypomethylated	0.013645	0.5673	insignificant	11	37	36
chr10	116661563	116663563	Yeats4	-0.15857095	1	insignificant	0.0010774	0.19351	insignificant	5	13	13
chr10	116675737	116677737	9530003J23Rik		1	noCoverage	-0.094777	1	insignificant	0	8	7
chr10	116729924	116731924	Lyz1	-0.07198635	0.1134	insignificant	-0.19613	0.40894	insignificant	2	8	8
chr10	116814029	116816029	Cpsf6	0.27443294	1	insignificant	0.46802	0.00000485	stronglyhypermeth	1	25	12
chr10	117065555	117067555	Cpm	-0.15355957	9.23E-12	hypomethylated	-0.0053471	0.33674	insignificant	12	72	64
chr10	117147772	117149772	Mdm2	-0.05247381	0.62452	insignificant	-0.019724	0.48634	insignificant	35	76	72
chr10	117183414	117185414	Slc35e3	0.34718967	0.30122	insignificant	0.019696	0.12473	insignificant	2	26	24
chr10	117229761	117231761	Nup107		1	noCoverage	-0.010116	0.60562	insignificant	0	25	25
chr10	117283030	117285030	Rap1b	-0.09440635	0.020132	hypomethylated	0.018267	0.10548	insignificant	18	67	67
chr10	117577842	117579842	Mdm1	-0.1306981	0.000000336	hypomethylated	0.020785	0.79107	insignificant	25	108	107
chr10	117732094	117734094	Iltifb		1	noCoverage	0.081818	0.078286	insignificant	0	6	3
chr10	117877102	117879102	Ifng		1	noCoverage	0.017361	0.23835	insignificant	0	8	8
chr10	118305959	118307959	Dyrk2	-0.10027121	2.11E-16	hypomethylated	0.0087429	0.030435	hypermethylated	40	128	124
chr10	118677111	118679111	Cand1	-0.14905272	1.39E-08	hypomethylated	0.030981	0.02722	inconclusive	17	80	73
chr10	118890369	118892369	Grip1	-0.11840025	6.03E-23	hypomethylated	-0.0063708	0.9723	insignificant	50	196	194
chr10	119550021	119552021	Helb	-0.27942924	0.53072	insignificant	0.08223	0.88161	insignificant	1	30	34
chr10	119638593	119640593	Irak3	-0.09284722	0.00079496	hypomethylated	-0.012329	0.56695	insignificant	3	40	40
chr10	119644881	119646881	Tmbim4	-0.14043805	2.08E-13	hypomethylated	0.0086162	0.23673	insignificant	14	46	46
chr10	119663115	119665115	Llph		1	noCoverage	-0.059944	0.39755	insignificant	0	23	20
chr10	119913991	119915991	Hmga2	-0.03623273	2.46E-15	hypomethylated	0.080399	0.9209	insignificant	11	71	64
chr10	120336027	120338027	Msrb3	-0.10768935	0.045814	hypomethylated	-0.050623	0.44566	insignificant	4	31	26
chr10	120416386	120418386	Lemd3	-0.08427342	3.01E-15	hypomethylated	0.0076661	0.4446	insignificant	29	79	64
chr10	120470059	120472059	Wif1	-0.15673629	2.73E-10	hypomethylated	0.017939	0.44621	insignificant	17	74	77
chr10	120748245	120750245	Tbc1d30	0.13461538	0.21213	insignificant	0.028352	0.27313	insignificant	3	10	8
chr10	120801145	120803145	Gns	-0.15695088	8.9E-22	hypomethylated	-0.00030701	0.18882	insignificant	16	105	107
chr10	120913306	120915306	Rassf3	-0.11557915	0.24639	insignificant	0.0054567	0.50741	insignificant	10	35	34
chr10	121023850	121025850	Xpot	-0.24807023	1	insignificant	-0.013794	0.0074578	hypomethylated	9	29	29
chr10	121063372	121065372	Xpot	-0.11301973	0.016544	hypomethylated	-0.00048019	0.8965	insignificant	6	44	44
chr10	121077756	121079756	D930020B18R	-0.07230886	0.27717	insignificant	-0.0022815	0.94695	insignificant	32	112	108
chr10	121175992	121177992	BC048403	-0.09742594	7.46E-09	hypomethylated	0.013216	0.33537	insignificant	25	60	60
chr10	121484249	121486249	Srgap1	-0.11155975	6.7E-12	hypomethylated	0.0037803	0.86153	insignificant	44	157	150
chr10	121516935	121518935	Gm9079	-0.30037742	0.29667	insignificant	0.069615	0.21628	insignificant	1	14	14
chr10	121884554	121886554	Avpr1a	-0.13427218	0.00000048	hypomethylated	0.014208	0.90333	insignificant	7	75	75
chr10	122114817	122116817	Ppm1h	-0.0919831	9.63E-39	hypomethylated	0.00035362	0.71698	insignificant	44	169	164
chr10	122422780	122424780	Mirlet7i	-0.09670997	3.03E-34	hypomethylated	0.0081321	0.27087	insignificant	70	170	183
chr10	122513561	122515561	Mon2		1	noCoverage	-0.001607	0.27822	insignificant	0	22	16
chr10	122633979	122635979	Usp15	-0.09158058	0.000041439	hypomethylated	0.033362	0.24273	insignificant	12	53	59
chr10	122700131	122702131	Fam19a2	-0.09765002	9.91E-29	hypomethylated	0.016433	0.32098	insignificant	25	112	103
chr10	123905129	123907129	4930503E24Ri	0.1285822	1	lowCoverage	0.085313	0.18519	insignificant	1	26	10
chr10	124765591	124767591	Slc16a7	0.01619353	1	insignificant	0.0066347	0.87985	insignificant	1	7	6
chr10	125402274	125404274	Lrig3	-0.11177347	4.38E-31	hypomethylated	0.0021844	0.050587	insignificant	47	166	153
chr10	126414772	126416772	Ctdsp2	-0.06896173	8.1E-09	hypomethylated	0.0048519	0.40258	insignificant	21	150	145
chr10	126431585	126433585	Mir26a-2	0.06075367	1	insignificant	0.017385	0.22322	insignificant	5	22	26
chr10	126431668	126433668	Ctdsp2	0.06075367	1	insignificant	0.017385	0.22322	insignificant	5	22	26
chr10	126434495	126436495	Mir546	0.00992063	0.77617	insignificant	-0.07159	1	insignificant	2	4	8
chr10	126467870	126469870	Tsfm	-0.15004002	1	insignificant	0.0067782	0.50291	insignificant	1	21	22
chr10	126477987	126479987	Mettl1	-0.18379567	8.48E-45	hypomethylated	-0.006959	0.52107	insignificant	28	109	116
chr10	126484301	126486301	Cyp27b1	-0.0091951	0.5074	insignificant	0.01732	0.21197	insignificant	4	27	24
chr10	126497240	126499240	March9	-0.11167637	0.000054573	hypomethylated	0.085271	0.018561	inconclusive	5	38	25
chr10	126499658	126501658	Cdk4	-0.15001356	6.48E-14	hypomethylated	0.0003587	0.60546	insignificant	17	79	84
chr10	126507317	126509317	Tspan31	0.17612046	0.12488	insignificant	-0.11302	0.0025803	hypomethylated	1	14	14
chr10	126514962	126516962	Agap2	0.10738333	0.000000619	hypermethylated	0.042556	0.0050028	hypermethylated	11	37	37
chr10	126601280	126603280	B4galnt1	-0.03588331	1E-14	hypomethylated	-0.0036652	0.31714	insignificant	52	233	229
chr10	126601886	126603886	B4galnt1	-0.06531362	3.37E-15	hypomethylated	-0.0086374	0.22262	insignificant	53	249	245
chr10	126617701	126619701	Slc26a10		1	noCoverage	0.51587	1	lowCoverage	0	3	1
chr10	126626879	126628879	Dtx3	-0.38498914	2.21E-14	stronglyHypometh	-0.033761	0.0046993	hypomethylated	5	34	34
chr10	126627110	126629110	Dtx3	-0.83846154	0.13776	lowCoverage	-0.04388	0.88146	insignificant	1	10	10
chr10	126632765	126634765	Dtx3	0.06265159	0.0078486	hypermethylated	0.052678	0.34884	insignificant	3	20	21
chr10	126648678	126650678	Pip4k2c	-0.3406023	3.58E-15	stronglyHypometh	-0.0047727	0.44541	insignificant	13	45	45
chr10	126700419	126702419	Dctn2		1	noCoverage	-0.087031	0.82595	insignificant	0	6	5

chr10	126702317	126704317	Dctn2	-0.14944988	3.43E-15	hypomethylated	-0.00076887	0.50222	insignificant	22	88	85
chr10	126725827	126727827	Ddit3	-0.11752515	1.02E-08	hypomethylated	-0.00095158	0.55508	insignificant	10	102	96
chr10	126726848	126728848	Ddit3	-0.17744224	6.35E-16	hypomethylated	0.0040658	0.80288	insignificant	12	59	59
chr10	126748842	126750842	Mars	-0.33793531	0.0015276	stronglyHypometh	-0.0075449	0.32967	insignificant	4	15	13
chr10	126788828	126790828	Inhbe	-0.22132532	0.00000466	hypomethylated	0.038798	0.84655	insignificant	3	12	12
chr10	126856922	126858922	R3hdm2	0.03703704	0.66331	insignificant	0.10131	0.0059552	hypermethylated	3	6	6
chr10	126950994	126952994	Ndufa4l2	-0.41117925	0.003682	stronglyHypometh	-0.022602	0.49253	insignificant	1	13	17
chr10	126959491	126961491	Shmt2		1	noCoverage	0.047843	0.19857	insignificant	0	14	13
chr10	126971615	126973615	Nxph4		1	noCoverage	0.041615	0.19382	insignificant	0	39	35
chr10	127058204	127060204	Lrp1	-0.27150299	6.67E-31	hypomethylated	-0.012145	0.48907	insignificant	11	34	33
chr10	127079041	127081041	Stat6	-0.20116649	5.07E-16	hypomethylated	-0.024547	0.61717	insignificant	10	32	34
chr10	127103759	127105759	Nab2	-0.18261373	2.9E-09	hypomethylated	0.025362	0.17968	insignificant	8	42	50
chr10	127113120	127115120	Tmem194	-0.16245904	7.17E-14	hypomethylated	-0.014477	0.50026	insignificant	20	56	56
chr10	127141311	127143311	Myo1a	0.31884398	0.58163	insignificant	0.12533	0.79684	insignificant	2	8	6
chr10	127175593	127177593	Zbtb39	-0.16898926	7.88E-35	hypomethylated	-0.0081623	0.47117	insignificant	33	93	91
chr10	127188854	127190854	Gpr182	-0.43611111	0.031591	stronglyHypometh	0.0061966	0.66906	insignificant	1	4	4
chr10	127451270	127453270	Prim1	-0.15033758	5.01E-11	hypomethylated	0.010719	0.6751	insignificant	8	43	39
chr10	127471401	127473401	Naca	-0.11501145	5.59E-10	hypomethylated	-0.00031706	0.93234	insignificant	11	88	88
chr10	127495037	127497037	Ptges3	-0.10804368	1.76E-24	hypomethylated	-0.003273	0.73776	insignificant	39	158	147
chr10	127519362	127521362	Atp5b	-0.11543406	8.25E-17	hypomethylated	0.030158	0.11743	insignificant	21	91	92
chr10	127521341	127523341	Mir677		1	noCoverage	-0.065981	0.8881	insignificant	0	21	23
chr10	127528838	127530838	Baz2a	-0.14150874	4.01E-15	hypomethylated	0.0063709	0.77651	insignificant	22	85	91
chr10	127630690	127632690	Gls2	-0.13477287	3.16E-37	hypomethylated	0.014447	0.11423	insignificant	28	128	124
chr10	127648850	127650850	Spryd4	-0.35674356	0.0050894	stronglyHypometh	-0.027466	0.00000222	hypomethylated	4	33	25
chr10	127668118	127670118	Timeless	-0.12771757	0.0042701	hypomethylated	0.010032	0.055472	insignificant	11	45	45
chr10	127674121	127676121	Timeless	-0.12323956	0.0015966	hypomethylated	0.067001	0.82065	insignificant	2	6	6
chr10	127690186	127692186	Apon		1	noCoverage	0.030141	0.84114	insignificant	0	14	7
chr10	127704052	127706052	Apof		1	noCoverage	0.15014	1	insignificant	0	7	9
chr10	127706631	127708631	Stat2	-0.32425855	6.26E-11	hypomethylated	-0.12482	0.0012463	hypomethylated	6	37	34
chr10	127735140	127737140	Il23a		1	noCoverage	0.072391	0.26641	insignificant	0	6	6
chr10	127739390	127741390	Pan2	-0.17283951	1	insignificant	-0.063282	0.21507	insignificant	2	18	13
chr10	127758514	127760514	Cnpy2	-0.33995547	0.0021991	stronglyHypometh	-0.011707	0.12298	insignificant	5	41	41
chr10	127773887	127775887	Cs	-0.14987361	4E-21	hypomethylated	-0.013606	0.45483	insignificant	27	88	85
chr10	127807093	127809093	Coq10a	0.07125687	1	insignificant	0.074688	0.38542	insignificant	2	20	17
chr10	127813179	127815179	Ankrd52	-0.09981668	7.36E-26	hypomethylated	0.0072114	0.43152	insignificant	39	133	135
chr10	127846852	127848852	Rnf41	-0.15173075	7.11E-15	hypomethylated	0.015744	0.19439	insignificant	7	38	37
chr10	127847671	127849671	Rnf41	-0.15632136	6.67E-15	hypomethylated	0.033075	0.25216	insignificant	6	36	32
chr10	127895291	127897291	Smarcc2	-0.09016621	6.65E-25	hypomethylated	0.0017065	0.8051	insignificant	53	224	205
chr10	127935741	127937741	Myl6b	-0.09267825	0.74923	insignificant	0.040201	0.43961	insignificant	4	65	60
chr10	127962915	127964915	Esyt1	-0.33950193	0.000011696	stronglyHypometh	-0.0028218	0.48682	insignificant	3	12	14
chr10	127984800	127986800	Zc3h10	-0.12640374	0.00007989	hypomethylated	0.021813	0.46866	insignificant	15	53	56
chr10	127986224	127988224	Rpl41		1	noCoverage	0.071859	0.099722	insignificant	0	6	6
chr10	128002990	128004990	Pa2g4	-0.02583895	1.59E-10	hypomethylated	0.015353	0.10179	insignificant	8	66	64
chr10	128026557	128028557	Erbb3	-0.13080954	1.35E-20	hypomethylated	-0.001546	0.3328	insignificant	38	138	137
chr10	128063562	128065562	Rps26	-0.224555	0.0064378	hypomethylated	0.077649	0.64727	insignificant	6	27	29
chr10	128110974	128112974	Suox	-0.17305103	7.07E-09	hypomethylated	-0.017787	0.90777	insignificant	10	36	33
chr10	128133324	128135324	Rab5b		1	noCoverage	-0.37487	0.26855	insignificant	0	12	7
chr10	128142107	128144107	Cdk2	-0.11272764	0.000023716	hypomethylated	0.015555	0.23364	insignificant	12	40	40
chr10	128142313	128144313	Pmel	-0.25930492	0.024212	hypomethylated	0.045445	0.70904	insignificant	4	8	8
chr10	128181112	128183112	Dgka	-0.39096419	0.070206	insignificant	0.04099	0.52323	insignificant	2	30	27
chr10	128183934	128185934	Wibg	-0.25497542	1.86E-17	hypomethylated	0.049272	0.83463	insignificant	17	86	86
chr10	128184577	128186577	Wibg	-0.20010255	9.21E-16	hypomethylated	0.051408	0.89119	insignificant	18	80	82
chr10	128241426	128243426	1110012D08R	-0.15081678	8.39E-18	hypomethylated	-0.0060816	0.67236	insignificant	21	113	114
chr10	128241731	128243731	Dnajc14	-0.15916599	1.71E-19	hypomethylated	-0.0047712	0.8129	insignificant	21	115	116
chr10	128257826	128259826	Sarnp	-0.06014493	0.19195	insignificant	-0.014799	0.80233	insignificant	4	23	22
chr10	128258687	128260687	Sarnp	-0.66	0.19192	insignificant	-0.0475	1	insignificant	1	5	4
chr10	128328774	128330774	Gdf11	-0.14036538	0.003895	hypomethylated	0.068072	0.62336	insignificant	6	34	37
chr10	128344974	128346974	Cd63	-0.26266183	0.000000298	hypomethylated	-0.0038607	0.27304	insignificant	6	49	42
chr10	128345921	128347921	Cd63	-0.13606434	8.59E-10	hypomethylated	-0.0028918	0.84819	insignificant	14	171	160
chr10	128369868	128371868	Itga7	-0.20644385	3.16E-18	hypomethylated	0.00099772	0.41829	insignificant	16	53	59
chr10	128549479	128551479	Olfr769		1	noCoverage	-0.025162	0.47125	insignificant	0	11	8
chr10	128624475	128626475	Olfr773		1	noCoverage	0.17319	0.68797	insignificant	0	4	4
chr10	128908749	128910749	Olfr788	-0.175	0.43008	insignificant	0.048485	0.54933	insignificant	2	4	4
chr10	128976594	128978594	Olfr792		1	noCoverage	-0.13971	1	insignificant	0	4	2
chr10	129203553	129205553	Olfr808	-0.85909091	0.18182	lowCoverage	-0.075758	0.53564	insignificant	1	4	4
chr10	129617934	129619934	Olfr826		1	noCoverage	-0.0016667	0.71229	insignificant	0	6	3
chr11	3023023	3025023	Pisd-ps1	-0.14164332	0.0012102	hypomethylated	0.018895	0.93138	insignificant	5	26	26
chr11	3023029	3025029	Pisd-ps3	-0.14164332	0.0012102	hypomethylated	0.018895	0.93138	insignificant	5	26	26
chr11	3093466	3095466	Sfi1	-0.1458545	3.18E-139	hypomethylated	0.066372	2.46E-23	hypermethylated	9	42	29
chr11	3101355	3103355	Eif4enif1	-0.09792981	4.68E-41	hypomethylated	-0.003705	0.65242	insignificant	68	201	204
chr11	3101585	3103585	Eif4enif1	-0.09792981	4.68E-41	hypomethylated	-0.003705	0.65242	insignificant	68	201	204
chr11	3101997	3103997	Eif4enif1	-0.09473018	4.73E-41	hypomethylated	-0.0019664	0.67256	insignificant	68	198	198
chr11	3102021	3104021	Eif4enif1	-0.09473018	4.73E-41	hypomethylated	-0.0019664	0.67256	insignificant	68	198	198
chr11	3166389	3168389	Drg1		1	noCoverage	0.16268	1	insignificant	0	14	8
chr11	3189459	3191459	Patz1	-0.10361504	4.85E-28	hypomethylated	-0.0056653	0.15364	insignificant	32	145	145
chr11	3229733	3231733	Pik3ip1		1	noCoverage	0.041471	0.77664	insignificant	0	4	4
chr11	3271395	3273395	Limk2	-0.19390426	0.00093484	hypomethylated	0.19968	0.4213	insignificant	8	11	18
chr11	3309238	3311238	Limk2	0.19791667	1	insignificant	0.054693	0.62259	insignificant	1	14	14
chr11	3351439	3353439	8430429K09Ri	-0.14798585	5.34E-34	hypomethylated	0.0064913	0.07691	insignificant	22	102	96
chr11	3352328	3354328	Rnf185	-0.05759556	1.84E-20	hypomethylated	-0.012337	1	insignificant	7	44	42
chr11	3387229	3389229	Pla2g3	0.04015403	0.11884	insignificant	-0.030346	0.5642	insignificant	8	26	26
chr11	3404824	3406824	Inpp5j	0.13936266	0.72648	insignificant	0.13935	0.85319	insignificant	1	9	8
chr11	3413704	3415704	Selm	-0.32960317	0.17705	insignificant	0.032521	0.63244	insignificant	4	15	15
chr11	3439295	3441295	Smtn	-0.62604618	0.0026111	stronglyHypometh	0.077988	1	insignificant	2	21	18
chr11	3547536	3549536	Tug1	-0.11625871	4.77E-28	hypomethylated	0.00098958	0.24426	insignificant	46	138	136

chr11	3548496	3550496	Morc2a	-0.1511157	7.24E-30	hypomethylated	-0.0038222	0.39561	insignificant	46	136	133
chr11	3548811	3550811	Tug1	-0.18958197	1.03E-30	hypomethylated	-0.014203	0.71406	insignificant	33	88	91
chr11	3794242	3796242	Dusp18	-0.03239709	0.046492	inconclusive	0.0040369	0.082275	insignificant	7	53	53
chr11	3795129	3797129	4921536K21Ri	-0.06791321	0.23377	insignificant	0.013353	0.0094009	hypermethylated	7	46	46
chr11	3814667	3816667	Slc35e4	-0.15302579	0.012796	hypomethylated	0.022095	0.36434	insignificant	3	6	6
chr11	3831964	3833964	Tcn2	-0.33552953	0.18296	insignificant	-0.055343	0.38321	insignificant	3	14	14
chr11	3832047	3834047	Tcn2	-0.30357233	0.32724	insignificant	-0.070365	0.24198	insignificant	3	12	12
chr11	3832081	3834081	Tcn2	-0.36428679	0.16083	insignificant	-0.084438	0.40326	insignificant	3	10	10
chr11	3862977	3864977	Pes1	-0.27014708	7.27E-13	hypomethylated	0.0063375	0.17205	insignificant	7	16	16
chr11	3882638	3884638	Gal3st1	-0.05933327	0.000013799	hypomethylated	-0.016789	0.098407	insignificant	20	69	69
chr11	3888935	3890935	Gal3st1	0.21924603	0.58206	insignificant	0.093092	0.049799	hypermethylated	2	4	4
chr11	3995434	3997434	Mtfp1	-0.10318297	0.00023972	hypomethylated	0.010355	0.30089	insignificant	17	63	63
chr11	4018732	4020732	Sec14l2		1	noCoverage	0.0081875	1	insignificant	0	18	18
chr11	4034162	4036162	Rnf215	-0.14556722	8.24E-08	hypomethylated	0.03433	0.048253	hypermethylated	16	59	51
chr11	4038366	4040366	Mir3060	-0.14916667	0.020355	hypomethylated	0.04143	0.58263	insignificant	1	8	8
chr11	4059356	4061356	Sf3a1	-0.13695114	9.51E-10	hypomethylated	-0.016515	0.16047	insignificant	11	74	74
chr11	4060296	4062296	Ccdc157	-0.11756777	0.000018465	hypomethylated	-0.017649	0.5606	insignificant	9	66	66
chr11	4085835	4087835	Tbc1d10a	-0.1210058	0.026871	hypomethylated	0.017201	0.0019352	hypermethylated	6	84	84
chr11	4117253	4119253	Gatsl3	-0.08236269	1.06E-54	hypomethylated	0.0019689	0.000064328	hypermethylated	36	100	96
chr11	4156570	4158570	Lif	-0.22429373	0.53805	insignificant	0.0072736	0.24184	insignificant	3	22	22
chr11	4166097	4168097	Lif	-0.10938669	2.55E-25	hypomethylated	0.019843	0.84759	insignificant	29	96	88
chr11	4341085	4343085	Hormad2	-0.06395716	0.52314	insignificant	0.090945	0.10077	insignificant	6	16	16
chr11	4494818	4496818	Mtmr3	-0.07936243	0.025505	hypomethylated	0.0046436	1	insignificant	7	14	14
chr11	4536795	4538795	Ascc2	-0.25579739	7.19E-17	hypomethylated	-0.026181	0.083793	insignificant	12	44	42
chr11	4603680	4605680	Zmat5		1	noCoverage	0.01888	1	insignificant	0	25	25
chr11	4604347	4606347	Uqcr10		1	noCoverage	0.024842	1	insignificant	0	19	19
chr11	4646781	4648781	Cabp7	-0.09839693	1.93E-15	hypomethylated	0.0099883	0.93676	insignificant	29	105	104
chr11	4749530	4751530	Nf2	-0.07614189	0.27384	insignificant	-0.011364	0.39543	insignificant	11	45	45
chr11	4773005	4775005	Nipsnap1	-0.187968	0.025027	hypomethylated	0.012314	0.59148	insignificant	5	39	39
chr11	4794345	4796345	Thoc5	-0.15401108	7.4E-23	hypomethylated	0.0051173	0.19242	insignificant	17	62	62
chr11	4848067	4850067	Ap1b1	-0.03505609	1.35E-12	hypomethylated	0.013471	0.17722	insignificant	16	56	56
chr11	4885907	4887907	Ap1b1	-0.18728721	1.46E-22	hypomethylated	0.021856	0.10024	insignificant	23	79	78
chr11	4957130	4959130	Rasl10a	-0.16043211	6.88E-47	hypomethylated	0.029064	0.000031163	hypermethylated	37	134	131
chr11	4965330	4967330	Gas2l1	-0.36096706	2.42E-12	stronglyHypometh	-0.04335	0.14704	insignificant	4	22	20
chr11	4998440	5000440	Rhbdd3	-0.06678127	5.6E-12	hypomethylated	0.0023531	0.74985	insignificant	29	154	144
chr11	4999080	5001080	Ewsr1	-0.09596328	6.46E-10	hypomethylated	0.021129	0.34401	insignificant	16	104	93
chr11	5052225	5054225	Emid1	-0.16280674	0.00011164	hypomethylated	-0.010845	0.10956	insignificant	18	42	52
chr11	5161613	5163613	Kremen1	-0.13496872	0.059066	insignificant	-0.001336	0.27906	insignificant	30	97	95
chr11	5344850	5346850	Znrf3	-0.10622981	0.00000122	hypomethylated	-0.022187	0.92036	insignificant	7	63	60
chr11	5419969	5421969	Xbp1	-0.11139865	1.87E-13	hypomethylated	-0.0023137	0.59299	insignificant	21	100	100
chr11	5442220	5444220	Ccdc117	-0.20583888	0.14581	insignificant	-0.069703	0.90774	insignificant	3	11	10
chr11	5607702	5609702	Mrps24		1	noCoverage	-0.032392	0.4477	insignificant	0	10	10
chr11	5641179	5643179	Urgcp	-0.08089191	0.026691	hypomethylated	-0.001875	0.83707	insignificant	3	17	18
chr11	5661152	5663152	2210015D19R	-0.43547964	1.63E-12	stronglyHypometh	-0.3392	0.63311	insignificant	8	42	36
chr11	5662379	5664379	2210015D19R	0.34460784	0.52728	insignificant	-0.092562	0.088698	insignificant	2	17	24
chr11	5687485	5689485	Dbnl	-0.11134356	1.05E-20	hypomethylated	0.012826	0.39781	insignificant	27	94	100
chr11	5703799	5705799	Pgam2		1	noCoverage	-0.099677	0.50484	insignificant	0	7	3
chr11	5760868	5762868	Aebp1	-0.13840549	2.63E-16	hypomethylated	0.034086	0.83032	insignificant	15	63	64
chr11	5778259	5780259	Pold2		1	noCoverage	-0.045256	0.0027826	hypomethylated	0	22	22
chr11	5854760	5856760	Ykt6	-0.10248667	1.18E-13	hypomethylated	0.012918	0.54738	insignificant	25	101	100
chr11	6073334	6075334	Gm11968		1	noCoverage	0.082638	0.48567	insignificant	0	9	9
chr11	6167732	6169732	Ddx56	0.01142399	0.14459	insignificant	0.33629	0.0019313	stronglyhypermeth	2	14	8
chr11	6174840	6176840	Tmed4	-0.07380952	0.74376	insignificant	-0.019697	0.23055	insignificant	8	28	28
chr11	6190599	6192599	Ogdh	-0.08873812	2.29E-19	hypomethylated	0.003498	0.66385	insignificant	34	98	93
chr11	6288366	6290366	Zmiz2	-0.09390793	3.18E-18	hypomethylated	0.0032971	0.017874	inconclusive	35	171	159
chr11	6314872	6316872	Ppia	-0.10085073	1.35E-22	hypomethylated	0.0015732	0.25894	insignificant	31	133	131
chr11	6344446	6346446	H2afv	0.03209972	1	insignificant	0.012826	0.46128	insignificant	10	24	24
chr11	6375920	6377920	Purb	-0.08397592	8.93E-23	hypomethylated	0.019139	0.74255	insignificant	34	93	80
chr11	6445889	6447889	Ccm2	-0.1271167	5.84E-27	hypomethylated	0.041568	0.7593	insignificant	22	103	100
chr11	6506057	6508057	Nacad	0.01933805	0.82355	insignificant	0.032978	0.7561	insignificant	4	12	12
chr11	6526070	6528070	Tbrg4	-0.14435035	2.51E-16	hypomethylated	0.010083	1	insignificant	15	32	32
chr11	6538652	6540652	Wap	-0.21896334	0.0058752	hypomethylated	-0.11919	0.39377	insignificant	2	10	10
chr11	6557535	6559535	Ramp3	-0.27079689	0.001561	hypomethylated	0.017266	0.17927	insignificant	1	7	6
chr11	6962491	6964491	Adcy1	-0.07506682	1.81E-09	hypomethylated	0.0042322	0.53199	insignificant	46	139	142
chr11	7113926	7115926	Igfbp3	-0.15965759	1.51E-21	hypomethylated	0.023253	0.081882	insignificant	18	66	66
chr11	8564538	8566538	Tns3		1	noCoverage	-0.075174	0.60799	insignificant	0	31	26
chr11	8911140	8913140	Hus1	-0.14284147	0.000000802	hypomethylated	-0.015567	0.88435	insignificant	10	28	28
chr11	8947594	8949594	Gm11992	-0.16884153	0.0059226	hypomethylated	0.0075645	0.92717	insignificant	7	30	34
chr11	9017010	9019010	Upp1	-0.15330296	0.00031539	hypomethylated	0.034432	0.0026507	hypermethylated	5	22	22
chr11	9017105	9019105	Upp1	-0.15330296	0.00031539	hypomethylated	0.034432	0.0026507	hypermethylated	5	22	22
chr11	9017509	9019509	Upp1	-0.15330296	0.00031539	hypomethylated	0.060454	0.0022208	hypermethylated	5	22	24
chr11	9090944	9092944	Abca13	0.04733336	1	insignificant	-0.0052679	0.77238	insignificant	1	12	10
chr11	11013018	11015018	Vwc2	-0.19114271	6.53E-11	hypomethylated	0.0034405	0.83266	insignificant	16	41	41
chr11	11585215	11587215	Ikzf1	-0.10266604	1.12E-19	hypomethylated	0.010835	0.66458	insignificant	54	228	221
chr11	11708965	11710965	Fignl1	-0.19044747	1	insignificant	-0.0079787	0.895	insignificant	1	46	47
chr11	11798147	11800147	Ddc	0.17337398	1	insignificant	0.091292	0.0031423	hypermethylated	2	6	6
chr11	11937423	11939423	Grb10	-0.18019884	8.88E-13	hypomethylated	0.0015192	0.062627	insignificant	26	109	100
chr11	12364963	12366963	Cobl	-0.08296861	0.000000016	hypomethylated	0.04785	1	insignificant	16	35	37
chr11	14498242	14500242	Pom121l12		1	noCoverage	0.040205	1	insignificant	0	4	4
chr11	16156726	16158726	Vstm2a	-0.40985798	1	insignificant	-0.23547	0.23943	insignificant	2	19	14
chr11	16408487	16410487	Sec61g		1	noCoverage	-0.075556	0.8344	insignificant	0	10	9
chr11	16651205	16653205	Egfr	-0.12151972	1.09E-21	hypomethylated	0.01182	0.7048	insignificant	29	116	114
chr11	16850412	16852412	Fbxo48	-0.26465618	0.19335	insignificant	0.12495	0.56778	insignificant	4	16	14
chr11	16851096	16853096	2810442I21Ril	-0.26969697	0.57073	insignificant	0.037121	0.51067	insignificant	1	8	8
chr11	16950936	16952936	Cnrip1	-0.1878185	0.00000753	hypomethylated	-0.02719	0.43188	insignificant	8	41	35

chr11	17058300	17060300	Ppp3r1	-0.13885128	1.37E-29	hypomethylated	-0.0011106	0.27755	insignificant	46	175	150
chr11	17110895	17112895	Wdr92	-0.16306703	4.38E-22	hypomethylated	0.0081001	0.94119	insignificant	21	70	70
chr11	17111592	17113592	Pno1	-0.30789773	0.00000218	hypomethylated	0.012059	0.24697	insignificant	3	16	16
chr11	17156620	17158620	C1d	-0.26551679	1.03E-09	hypomethylated	-0.015907	0.86508	insignificant	12	54	54
chr11	18918972	18920972	Meis1	-0.14327358	5.23E-08	hypomethylated	0.014842	0.034046	hypermethylated	28	144	141
chr11	19823444	19825444	Spred2	-0.16509621	0.0000025	hypomethylated	-0.040034	0.68826	insignificant	19	63	62
chr11	20012954	20014954	Actr2	-0.37336899	3.2E-41	stronglyHypometh	-0.0733	0.00029083	hypomethylated	15	70	60
chr11	20100604	20102604	Rab1	-0.14492103	2.22E-16	hypomethylated	0.025785	0.49829	insignificant	18	106	101
chr11	20149427	20151427	Cep68	-0.11689248	1	insignificant	-0.015331	0.74837	insignificant	3	48	27
chr11	20232716	20234716	Slc1a4	-0.24195909	0.56916	insignificant	-0.01817	0.75752	insignificant	1	32	30
chr11	20345399	20347399			1	noCoverage	-0.068783	0.57971	insignificant	0	18	7
chr11	20442255	20444255	Sertad2	-0.11780364	5.47E-11	hypomethylated	-0.01296	0.20799	insignificant	31	118	120
chr11	20530979	20532979	Sertad2	-0.15617076	1.05E-20	hypomethylated	0.012408	0.048296	hypermethylated	37	129	136
chr11	20641487	20643487	Aftph	-0.17956376	1.5E-09	hypomethylated	-0.02027	0.50435	insignificant	9	35	34
chr11	20731111	20733111	1110067D22R	-0.11739291	7.44E-08	hypomethylated	-0.033365	0.0022429	hypomethylated	9	55	55
chr11	20990326	20992326	Peli1	-0.07790378	5.03E-11	hypomethylated	0.013242	0.70392	insignificant	29	121	98
chr11	21137891	21139891	Vps54	-0.10092518	1.34E-33	hypomethylated	-0.00094732	0.9433	insignificant	49	139	131
chr11	21270882	21272882	Ugp2	-0.18001343	0.000058158	hypomethylated	-0.0079434	0.22816	insignificant	12	67	67
chr11	21471283	21473283	Wdpcp	-0.1113137	3.88E-09	hypomethylated	0.022484	0.31285	insignificant	12	58	58
chr11	21471937	21473937	Mdh1	-0.07994103	0.000089736	hypomethylated	0.02051	0.11897	insignificant	8	36	36
chr11	21901654	21903654	Otx1	-0.1276487	2.19E-16	hypomethylated	-0.017316	0.023968	hypomethylated	20	93	84
chr11	22185841	22187841	Ehbp1	-0.17968618	9.49E-08	hypomethylated	-0.0083609	0.90056	insignificant	10	47	45
chr11	22411285	22413285	Tmem17	-0.15278498	3.3E-20	hypomethylated	-0.010104	0.78121	insignificant	34	101	100
chr11	22759735	22761735	B3gnt2	-0.14241337	1.24E-11	hypomethylated	-0.017033	0.26103	insignificant	24	78	76
chr11	22760336	22762336	B3gnt2	-0.24607245	0.00086032	hypomethylated	-0.032666	0.81333	insignificant	11	30	28
chr11	22871028	22873028	Commd1	-0.09377319	0.025661	hypomethylated	-0.059528	0.16712	insignificant	28	107	98
chr11	22882284	22884284	Commd1	-0.09684811	0.062078	insignificant	-0.019865	0.21455	insignificant	5	24	28
chr11	22889592	22891592	Cct4	-0.12642113	1.83E-16	hypomethylated	-0.0069505	0.69821	insignificant	27	68	63
chr11	23155040	23157040	Xpo1	-0.14208584	0.00000118	hypomethylated	0.009909	0.2634	insignificant	13	127	108
chr11	23205894	23207894	Usp34	-0.11251199	3.07E-25	hypomethylated	0.0090284	0.20094	insignificant	50	152	147
chr11	23397946	23399946	Ahsa2	-0.17028802	0.00003036	hypomethylated	0.055466	0.86381	insignificant	12	35	29
chr11	23419195	23421195	1700093K21Rik		1	noCoverage	0.012488	0.79975	insignificant	0	7	6
chr11	23458842	23460842	Gm12060		1	noCoverage	-0.082522	0.52759	insignificant	0	8	7
chr11	23533631	23535631	0610010F05Rik		1	noCoverage	0.0083246	0.94295	insignificant	0	45	31
chr11	23564673	23566673	Pus10	-0.15037419	3.67E-14	hypomethylated	0.0039022	0.40725	insignificant	23	62	53
chr11	23564975	23566975	Pus10	-0.15037419	3.67E-14	hypomethylated	0.0039022	0.40725	insignificant	23	62	53
chr11	23565935	23567935	Pus10	-0.21568627	0.000000748	hypomethylated	-0.065624	0.56725	insignificant	5	15	14
chr11	23670970	23672970	Rel	-0.06750275	0.000000555	hypomethylated	-0.0012557	0.96478	insignificant	28	108	108
chr11	23795270	23797270	Papolg	-0.14412118	0.61662	insignificant	0.039015	0.11587	insignificant	2	8	6
chr11	23977055	23979055	Bcl11a	-0.15501557	9.78E-14	hypomethylated	0.0051015	0.56442	insignificant	31	99	99
chr11	23979694	23981694	Bcl11a	-0.14014643	9.38E-35	hypomethylated	0.0016704	0.65205	insignificant	22	128	117
chr11	24630722	24632722	Gm10466	0.35714286	0.31776	insignificant	0.16964	0.2583	insignificant	1	4	4
chr11	26110576	26112576	5730522E02Ri	-0.06959655	0.00022696	hypomethylated	-0.0031307	0.70699	insignificant	9	18	18
chr11	26286083	26288083	Fancl	-0.09073153	0.026382	hypomethylated	0.0057252	0.74851	insignificant	9	39	38
chr11	28484296	28486296	Ccdc85a		1	noCoverage	-0.08809	0.4375	insignificant	0	6	6
chr11	28752204	28754204	Efemp1	-0.15784263	0.61837	insignificant	0.045028	0.85865	insignificant	3	22	25
chr11	29029750	29031750	Pnpt1	-0.14610171	2.65E-19	hypomethylated	-0.0059229	0.24116	insignificant	14	58	58
chr11	29071906	29073906	Smek2	-0.09575367	3.14E-15	hypomethylated	0.033001	0.24564	insignificant	25	158	159
chr11	29147272	29149272	Ccdc104	-0.11454257	0.089012	insignificant	-0.013576	1	insignificant	4	10	10
chr11	29272774	29274774	Ccdc88a	-0.08092339	1.07E-18	hypomethylated	0.014276	0.77447	insignificant	45	162	152
chr11	29415033	29417033	Prorsd1	-0.26335912	0.10472	insignificant	-0.0031093	0.18398	insignificant	11	46	47
chr11	29425456	29427456	Mtif2	-0.2335269	8.82E-33	hypomethylated	0.019564	0.96674	insignificant	13	56	55
chr11	29446949	29448949	1700034F02Ri	-0.20854841	0.35991	insignificant	-0.038323	0.62537	insignificant	7	46	54
chr11	29448040	29450040	1700034F02Ri	-0.20653463	0.5688	insignificant	0.013203	0.78039	insignificant	3	25	24
chr11	29591897	29593897	Rtn4	-0.11924486	2.62E-27	hypomethylated	-0.00086511	0.94916	insignificant	44	144	145
chr11	29592773	29594773	Rtn4	-0.10023256	3.88E-28	hypomethylated	-0.0048928	0.84879	insignificant	46	139	140
chr11	29926033	29928033	Eml6	-0.19935094	0.0052019	hypomethylated	-0.018568	0.28658	insignificant	6	28	28
chr11	30098257	30100257	Spnb2	-0.11504888	0.14836	insignificant	0.00079171	0.45562	insignificant	4	45	45
chr11	30549396	30551396	Acyp2	-0.11433127	0.0082016	hypomethylated	0.0063909	0.90839	insignificant	5	22	22
chr11	30670774	30672774	Psme4	-0.1103691	2.34E-19	hypomethylated	0.0055927	0.11898	insignificant	32	121	135
chr11	30790096	30792096	Gpr75		1	noCoverage	0.015634	0.80143	insignificant	0	4	6
chr11	30853397	30855397	Asb3	-0.12784219	1.6E-10	hypomethylated	0.0028177	0.24342	insignificant	9	95	91
chr11	30854131	30856131	Erlec1	-0.12119783	0.00087233	hypomethylated	0.0027564	0.87335	insignificant	5	55	51
chr11	30886365	30888365	Chac2		1	noCoverage	-0.042185	0.66763	insignificant	0	6	6
chr11	31270061	31272061	Stc2	-0.13012612	0.00030946	hypomethylated	0.0013492	0.51072	insignificant	7	54	54
chr11	31571862	31573862	Bod1	-0.14728655	0.38922	insignificant	-0.026365	0.14891	insignificant	3	30	29
chr11	31771210	31773210	Cpeb4	-0.19942681	0.58926	insignificant	0.0073779	0.85034	insignificant	4	30	29
chr11	31864632	31866632	4930524B15R	-0.30142449	0.00066118	hypomethylated	-0.011392	0.70508	insignificant	7	41	36
chr11	31899458	31901458	Nsg2	-0.09093488	0.000003412	hypomethylated	0.017984	0.69697	insignificant	9	31	30
chr11	32100279	32102279	Il9r	-0.25833734	0.012322	hypomethylated	-0.048359	0.74689	insignificant	3	9	9
chr11	32104414	32106414	Snrnp25	-0.20507134	6.28E-11	hypomethylated	0.063454	0.069016	insignificant	6	19	18
chr11	32122293	32124293	Rhbdf1	-0.18884581	1.45E-11	hypomethylated	-0.065998	0.20582	insignificant	14	36	28
chr11	32125504	32127504	Mpg	-0.14939953	1.02E-20	hypomethylated	-0.010866	0.66451	insignificant	27	78	76
chr11	32167614	32169614	Nprl3	-0.19104981	2.34E-09	hypomethylated	0.0014748	0.11674	insignificant	14	30	30
chr11	32175599	32177599	Hba-x	-0.30666667	0.10942	insignificant	-0.13167	0.25698	insignificant	2	4	4
chr11	32246810	32248810	Sh3pxd2b		1	noCoverage	-0.002627	0.60233	insignificant	0	47	47
chr11	32354371	32356371	Ubtd2	-0.13623051	3.14E-21	hypomethylated	-0.011525	0.10431	insignificant	31	122	122
chr11	32432265	32434265	Stk10	-0.09896925	0.00000108	hypomethylated	0.01653	0.30044	insignificant	32	123	116
chr11	32541874	32543874	Fbxw11	-0.14151496	3.54E-52	hypomethylated	0.027585	1	insignificant	49	137	138
chr11	32724594	32726594	1700008A04Rik		1	noCoverage	0.012351	0.56897	insignificant	0	6	6
chr11	33047400	33049400	Fgf18	-0.03702121	0.75665	insignificant	-0.0060553	0.30888	insignificant	48	103	108
chr11	33103588	33105588	Tlx3	-0.11183252	0.00000617	hypomethylated	-0.012302	0.12627	insignificant	24	76	76
chr11	33413746	33415746	Ranbp17	-0.22050457	0.0000069	hypomethylated	-0.007991	0.45771	insignificant	10	42	41
chr11	33478957	33480957	Gabrp		1	noCoverage	-0.061741	0.17147	insignificant	0	15	14
chr11	33743585	33745585	Kcnip1	-0.09856743	0.0013876	hypomethylated	-0.025785	0.058495	insignificant	5	20	28

chr11	33862012	33864012	Kcnmb1	0.03899232	0.013427	inconclusive	0.082908	0.019158	hypermethylated	4	23	22
chr11	34213821	34215821	Fam196b	-0.22719701	0.38723	insignificant	0.012933	0.283	insignificant	3	21	19
chr11	34647143	34649143	Ccdc99	-0.08210203	0.027242	hypomethylated	-0.0058685	0.85642	insignificant	4	30	30
chr11	34933957	34935957	Slit3	-0.13383269	1.72E-23	hypomethylated	-0.01485	0.60363	insignificant	20	137	154
chr11	35581996	35583996	Pank3	-0.16953361	1.62E-17	hypomethylated	0.019649	0.66292	insignificant	27	100	100
chr11	35648030	35650030	Rars		1	noCoverage	0.11142	0.24754	insignificant	0	12	8
chr11	35793591	35795591	Wwc1	-0.0848886	0.00000043	hypomethylated	0.0060691	0.55425	insignificant	31	125	126
chr11	40508705	40510705	Mat2b		1	noCoverage	0.0075231	0.38291	insignificant	0	16	18
chr11	40546143	40548143	Nudcd2	-0.15308013	0.0098379	hypomethylated	-0.0004582	0.85025	insignificant	11	40	40
chr11	40546939	40548939	Hmmr	-0.15308013	0.0098379	hypomethylated	-0.0004582	0.85025	insignificant	11	40	40
chr11	40568788	40570788	Ccng1	-0.08976934	0.15387	insignificant	-0.015681	0.1651	insignificant	2	18	18
chr11	41996432	41998432	Gabra1	-0.61807359	0.00016558	stronglyHypometh	-0.1779	0.27359	insignificant	3	14	17
chr11	43239483	43241483	Pttg1	-0.24235971	5.07E-09	hypomethylated	-0.049964	0.10699	insignificant	7	42	42
chr11	43239750	43241750	Pttg1		1	noCoverage	-0.13411	0.65523	insignificant	0	10	10
chr11	43246232	43248232	Slu7	-0.16661543	1.96E-09	hypomethylated	-0.0080127	0.43742	insignificant	11	52	52
chr11	43286845	43288845	C1qtnf2	-0.17500606	1.69E-08	hypomethylated	0.019557	0.70548	insignificant	13	44	45
chr11	43341285	43343285	Ccnjl	-0.11371671	1.88E-19	hypomethylated	-0.0063477	0.79497	insignificant	41	98	98
chr11	43494499	43496499	Pwwp2a	-0.08512407	7.86E-20	hypomethylated	-0.0074666	0.22912	insignificant	30	123	124
chr11	43561475	43563475	Ttc1	-0.33556481	1	lowCoverage	0.14559	0.8278	insignificant	1	16	20
chr11	43649834	43651834	Adra1b	-0.09913943	5.83E-11	hypomethylated	0.0016502	0.25109	insignificant	24	63	63
chr11	44284050	44286050	Ublcp1	-0.30585439	1.07E-08	hypomethylated	-0.041993	0.10316	insignificant	6	35	36
chr11	44331465	44333465	Rnf145	-0.10443537	7.27E-18	hypomethylated	0.0036921	0.67689	insignificant	40	122	100
chr11	44331878	44333878	Rnf145	-0.10443537	7.27E-18	hypomethylated	0.0036921	0.67689	insignificant	40	122	100
chr11	44430635	44432635	Ebf1	-0.08897886	0.000000368	hypomethylated	-0.0034617	0.044468	hypomethylated	18	67	59
chr11	45664465	45666465	Clint1	-0.09500447	1.33E-08	hypomethylated	0.010071	0.88871	insignificant	10	83	75
chr11	45792811	45794811	Sox30	-0.11994869	6.19E-29	hypomethylated	-0.013532	0.00036979	inconclusive	51	211	192
chr11	45865112	45867112			1	noCoverage	0.0175	0.071503	insignificant	0	10	10
chr11	45868488	45870488	Adam19	-0.27306639	1.95E-52	hypomethylated	-0.067991	0.77458	insignificant	15	52	47
chr11	45979861	45981861	Nipal4	-0.18510582	1	lowCoverage	-0.0064564	0.37708	insignificant	1	24	24
chr11	46125852	46127852	Cyfip2	-0.13341707	4.92E-09	hypomethylated	0.018588	0.52618	insignificant	11	34	34
chr11	46249448	46251448	Med7	-0.08999052	0.11861	insignificant	0.0013457	0.90505	insignificant	9	60	57
chr11	46249462	46251462	Med7	-0.08999052	0.11861	insignificant	0.0013457	0.90505	insignificant	9	60	57
chr11	46336752	46338752	Gm12169	-0.0184932	1	insignificant	0.04373	0.23202	insignificant	1	8	8
chr11	46520606	46522606	Timd2	-0.01212121	1	insignificant	0.073853	0.24852	insignificant	2	11	11
chr11	46623300	46625300	Timd4	-0.36065976	2.01E-09	stronglyHypometh	-0.12582	0.015317	hypomethylated	4	8	9
chr11	47192804	47194804	Sgcd		1	noCoverage	-0.0060568	0.89374	insignificant	0	8	8
chr11	48612861	48614861	Gnb2l1	-0.18544719	0.00011838	hypomethylated	-0.030659	0.52659	insignificant	11	54	55
chr11	48630893	48632893	Trim41	-0.17904679	1.93E-17	hypomethylated	-0.0045683	0.6509	insignificant	11	32	20
chr11	48638639	48640639	Trim7	-0.05646505	0.70923	insignificant	0.011667	0.41095	insignificant	4	41	39
chr11	48684848	48686848	Irgm1	-0.18823954	0.000000357	hypomethylated	0.043237	0.71432	insignificant	7	33	29
chr11	48899556	48901556	Ifi47	-0.52923822	0.000027405	stronglyHypometh	-0.011379	0.51917	insignificant	4	22	22
chr11	48960760	48962760	Olfr1395	0.06413396	1	insignificant	0.021163	0.55174	insignificant	1	16	16
chr11	49016001	49018001	Zfp62	-0.16699396	1.67E-12	hypomethylated	0.011727	0.19027	insignificant	12	56	56
chr11	49056692	49058692	Mgat1	-0.20248151	1	insignificant	0.054042	0.45479	insignificant	1	28	28
chr11	49063053	49065053	Mgat1	-0.16871816	0.00000631	hypomethylated	-0.02247	0.82397	insignificant	5	34	36
chr11	49422180	49424180	Flt4	-0.15160702	9.84E-28	hypomethylated	-0.010387	0.45866	insignificant	27	86	86
chr11	49526212	49528212	Cnot6	-0.21342051	0.01192	hypomethylated	0.0022171	0.00007285	inconclusive	14	50	50
chr11	49606656	49608656	Gfpt2	-0.28925202	0.000000144	hypomethylated	-0.0081362	0.77599	insignificant	16	66	66
chr11	49659252	49661252	Mapk9	-0.13080632	9.87E-30	hypomethylated	-0.0057286	0.18804	insignificant	33	96	88
chr11	49714336	49716336	Rasgef1c	-0.08380704	8.18E-08	hypomethylated	0.000010134	0.73095	insignificant	32	133	122
chr11	49837832	49839832	Rnf130	-0.12318863	9.38E-08	hypomethylated	-0.012022	0.24913	insignificant	12	66	40
chr11	49943861	49945861	Tbc1d9b	-0.09348704	7.59E-08	hypomethylated	-0.0040196	0.84708	insignificant	25	126	126
chr11	49987352	49989352	3010026O09R	-0.15574925	0.0016347	hypomethylated	0.015208	0.84986	insignificant	7	37	34
chr11	50024292	50026292	Sqstm1		1	noCoverage	0.055546	0.022867	hypermethylated	0	16	16
chr11	50037836	50039836	Mgat4b	-0.14122899	5.42E-17	hypomethylated	0.0036017	0.78159	insignificant	22	83	82
chr11	50105838	50107838	Maml1	-0.19479832	0.01197	hypomethylated	-0.020709	0.84202	insignificant	7	21	21
chr11	50139175	50141175	Canx		1	noCoverage	0.013889	1	insignificant	0	6	6
chr11	50171381	50173381	Mir804	-0.26307704	0.30068	insignificant	-0.16947	0.71023	insignificant	17	70	72
chr11	50190220	50192220	Hnrnph1	-0.10792165	1.83E-19	hypomethylated	0.0072741	0.83187	insignificant	15	98	98
chr11	50244613	50246613	Rufy1	-0.06918056	1	insignificant	-0.014793	0.51727	insignificant	4	15	15
chr11	50414586	50416586	Adamts2	-0.14282163	7.14E-57	hypomethylated	-0.017415	0.93405	insignificant	63	170	177
chr11	50641233	50643233	Zfp354c	-0.44279101	1.26E-16	stronglyHypometh	0.044276	1	insignificant	2	4	5
chr11	50663186	50665186	Grm6	-0.14981984	6.53E-41	hypomethylated	0.047219	0.00193	hypermethylated	28	83	69
chr11	50700945	50702945	Zfp454	-0.23408075	0.000000072	hypomethylated	0.00742	0.41374	insignificant	7	24	24
chr11	50729663	50731663	Zfp2		1	noCoverage	0.013803	0.90967	insignificant	0	4	4
chr11	50745137	50747137	Zfp354b		1	noCoverage	0.0065079	0.53222	insignificant	0	5	5
chr11	50797204	50799204	Olfr1377	0.3452381	1	lowCoverage	0.09881	0.6557	insignificant	1	4	4
chr11	50839505	50841505	Olfr54		1	noCoverage	0.13349	0.48432	insignificant	0	6	6
chr11	50871758	50873758	Zfp354a	-0.19760561	0.00078046	hypomethylated	-0.0082847	0.43694	insignificant	20	91	90
chr11	51075672	51077672	Clk4	-0.19652192	7.94E-20	hypomethylated	0.0054439	0.085827	insignificant	15	63	63
chr11	51076453	51078453	Clk4	-0.15263908	5.98E-16	hypomethylated	-0.0072379	0.30635	insignificant	13	56	54
chr11	51102421	51104421	Col23a1	-0.11716936	9.88E-30	hypomethylated	0.014422	0.69374	insignificant	27	159	148
chr11	51397258	51399258	Agxt2l2	-0.11483196	0.81862	insignificant	0.0049255	0.63146	insignificant	9	36	36
chr11	51420383	51422383	Hnrnpab	-0.16478235	2.05E-10	hypomethylated	0.0081519	0.30272	insignificant	15	44	47
chr11	51432274	51434274	Nhp2	-0.24073598	0.00062141	hypomethylated	-0.019975	0.52038	insignificant	6	64	54
chr11	51449398	51451398	Rmnd5b	-0.39006716	3.81E-26	stronglyHypometh	0.0030168	0.49223	insignificant	15	53	47
chr11	51463455	51465455	D930048N14R	-0.15988635	1.12E-19	hypomethylated	-0.015283	0.195	insignificant	31	99	88
chr11	51464575	51466575	D930048N14R	-0.26231061	0.58685	insignificant	0.025884	0.86157	insignificant	4	8	8
chr11	51502136	51504136	0610009B22R	-0.13045353	3.77E-13	hypomethylated	-0.013939	0.43638	insignificant	14	68	68
chr11	51570336	51572336	Sec24a		1	noCoverage	0.022054	0.20358	insignificant	0	31	31
chr11	51576164	51578164	Sar1b	-0.16197124	0.000027273	hypomethylated	0.019783	0.51681	insignificant	10	24	24
chr11	51670983	51672983	Phf15	-0.16094838	1.33E-20	hypomethylated	0.014793	0.60331	insignificant	14	49	46
chr11	51813968	51815968	Ube2b	-0.0918685	7.65E-17	hypomethylated	0.010544	0.20081	insignificant	18	66	65
chr11	51816722	51818722	Cdkl3	-0.15425186	2.55E-17	hypomethylated	-0.0026838	0.58512	insignificant	18	75	75
chr11	51911325	51913325	Ppp2ca	-0.08309644	3.53E-27	hypomethylated	0.0055789	0.8729	insignificant	47	149	133

chr11	52044496	52046496	Skp1a	-0.11665199	1.12E-14	hypomethylated	0.010659	0.65021	insignificant	26	137	133
chr11	52095752	52097752	Tcf7	-0.12030121	1.16E-22	hypomethylated	0.013551	0.38329	insignificant	52	197	192
chr11	52173616	52175616	Vdac1	-0.0819191	5.74E-11	hypomethylated	0.0015953	0.97353	insignificant	28	117	122
chr11	52208929	52210929	9530068E07Ri	-0.11330042	4.54E-19	hypomethylated	0.022892	0.37457	insignificant	17	79	79
chr11	52577207	52579207	Fstl4	-0.12426201	4.75E-27	hypomethylated	-0.0048991	0.5914	insignificant	50	139	138
chr11	53113981	53115981	Hspa4	-0.41369584	0.00053077	stronglyHypometh	-0.057267	0.26038	insignificant	4	13	10
chr11	53137190	53139190	Zcchc10	-0.31969356	0.000000316	hypomethylated	-0.00016833	0.13829	insignificant	5	42	42
chr11	53163268	53165268	Aff4	-0.08818279	8.07E-39	hypomethylated	0.0081058	1	insignificant	73	223	211
chr11	53244333	53246333	Uqcrq	-0.18293405	0.0037911	hypomethylated	-0.031783	0.78438	insignificant	6	24	29
chr11	53269706	53271706	Shroom1	-0.13580012	0.0010066	hypomethylated	-0.0070493	0.31841	insignificant	29	88	85
chr11	53332301	53334301	###########	-0.10162104	0.67365	insignificant	0.0037654	0.94282	insignificant	29	106	96
chr11	53379880	53381880	Kif3a	-0.10520999	0.00039077	hypomethylated	0.012143	0.42615	insignificant	14	39	33
chr11	53520821	53522821	Rad50	-0.11706393	0.000078847	hypomethylated	0.0067796	0.43918	insignificant	12	28	28
chr11	53582515	53584515	Irf1	-0.09799167	1.68E-09	hypomethylated	-0.010088	0.79976	insignificant	19	144	141
chr11	53582974	53584974	Irf1	-0.09799167	1.68E-09	hypomethylated	-0.010088	0.79976	insignificant	19	144	141
chr11	53672758	53674758	Gm12216	-0.45899251	1.77E-11	stronglyHypometh	0.13077	0.049424	hypermethylated	3	25	23
chr11	53705205	53707205	Slc22a5	-0.0859542	6.97E-08	hypomethylated	-0.023714	0.5769	insignificant	15	48	46
chr11	53793529	53795529	Slc22a21	-0.166257	0.059161	insignificant	-0.054693	0.37522	insignificant	9	45	43
chr11	53841592	53843592	Slc22a4	-0.09438528	0.000038252	hypomethylated	0.010425	0.89314	insignificant	23	60	60
chr11	53882519	53884519	Pdlim4	-0.4771021	1.75E-08	stronglyHypometh	-0.24861	0.2131	insignificant	3	13	18
chr11	53913425	53915425	P4ha2	-0.17761314	1.42E-21	hypomethylated	0.022102	0.0197	hypermethylated	26	119	119
chr11	54080781	54082781	Il3	-0.62717042	0.0021498	stronglyHypometh	0.10384	0.043817	hypermethylated	2	12	12
chr11	54116325	54118325	Acsl6	-0.13520904	8.11E-13	hypomethylated	0.029746	0.0025332	hypermethylated	24	95	85
chr11	54116705	54118705	Acsl6	-0.13520904	8.11E-13	hypomethylated	0.029746	0.0025332	hypermethylated	24	95	85
chr11	54183153	54185153	4930404A10Rik		1	noCoverage	-0.0077839	0.81685	insignificant	0	7	8
chr11	54250680	54252680	Fnip1	-0.15805927	0.38656	insignificant	-0.0049871	0.22598	insignificant	8	53	53
chr11	54335348	54337348	Rapgef6	-0.13080527	2.47E-13	hypomethylated	-0.0012045	0.93763	insignificant	18	76	76
chr11	54674093	54676093	Lyrm7	-0.1865055	0.083926	insignificant	0.013675	0.63428	insignificant	2	4	4
chr11	54678939	54680939	Hint1	-0.09388249	8.24E-15	hypomethylated	0.000897	0.53911	insignificant	41	130	130
chr11	54715354	54717354	Gpx3	0.73157045	1	lowCoverage	0.050175	0.024943	hypermethylated	1	25	15
chr11	54715427	54717427	Gpx3	0.78447205	0.38021	lowCoverage	0.012842	0.092381	insignificant	1	23	13
chr11	54769624	54771624	Tnip1	-0.2022092	8.59E-11	hypomethylated	0.0079539	0.58953	insignificant	10	46	46
chr11	54776442	54778442	Tnip1		1	noCoverage	0.052455	0.45128	insignificant	0	8	8
chr11	54846973	54848973	Anxa6		1	noCoverage	-0.010417	0.37752	insignificant	0	4	4
chr11	54891633	54893633	Ccdc69	-0.30103608	7.78E-14	hypomethylated	0.076634	0.045263	hypermethylated	6	20	16
chr11	54910618	54912618	Gm2a	-0.33660267	1.11E-12	stronglyHypometh	0.020369	0.00016545	inconclusive	9	45	45
chr11	54998579	55000579	Slc36a2	0.2767094	0.56912	insignificant	0.18307	0.16753	insignificant	2	12	10
chr11	55016841	55018841	Slc36a1	-0.14056953	0.00000845	hypomethylated	0.0069222	0.45121	insignificant	6	40	40
chr11	55274640	55276640	Atox1	-0.33711962	4.94E-10	stronglyHypometh	0.022446	0.069671	insignificant	10	37	43
chr11	55282253	55284253	G3bp1	-0.11652944	2.42E-09	hypomethylated	-0.013107	0.80012	insignificant	17	102	103
chr11	55295207	55297207	Gm12238		1	noCoverage	0.0062112	0.36872	insignificant	0	2	2
chr11	55421292	55423292	Glra1	-0.17882888	0.021514	hypomethylated	-0.02601	0.26615	insignificant	6	52	52
chr11	56824192	56826192	Gria1		1	noCoverage	-0.024437	0.38527	insignificant	0	9	9
chr11	57331166	57333166	Mfap3	-0.11773473	5.23E-18	hypomethylated	-0.001314	0.95465	insignificant	28	119	115
chr11	57332146	57334146	Fam114a2	-0.17663282	0.00000954	hypomethylated	0.045543	0.4715	insignificant	5	30	26
chr11	57457943	57459943	Galnt10	-0.13879476	3.37E-17	hypomethylated	0.035248	0.70953	insignificant	12	101	86
chr11	57614138	57616138	Sap30l	-0.10494927	1.82E-45	hypomethylated	0.0072954	0.20358	insignificant	62	222	222
chr11	57645649	57647649	Hand1	-0.07457478	0.29935	insignificant	0.026109	0.96412	insignificant	10	54	46
chr11	57821565	57823565	Larp1	-0.0853275	6.16E-34	hypomethylated	0.015315	0.077826	insignificant	75	298	268
chr11	57916654	57918654	Cnot8	-0.15520801	0.39263	insignificant	0.050945	0.000060579	hypermethylated	2	54	54
chr11	57984155	57986155	Mrpl22	-0.23476463	0.011956	hypomethylated	0.0086102	0.52538	insignificant	9	58	58
chr11	58012057	58014057	Igtp		1	noCoverage	0.16513	1	insignificant	0	14	11
chr11	58027478	58029478	Irgm2	-0.44804348	0.51855	insignificant	0.042884	0.25305	insignificant	2	20	20
chr11	58119570	58121570	Zfp692	-0.11770417	0.000000871	hypomethylated	0.0031372	0.89563	insignificant	27	61	59
chr11	58136848	58138848	Zfp672	-0.39928699	1	insignificant	0.024011	0.72663	insignificant	3	18	18
chr11	58143219	58145219	Sh3bp5l	-0.22659522	7.07E-11	hypomethylated	0.019963	0.082491	insignificant	12	84	84
chr11	58191533	58193533	2210415F13Ri	-0.09455391	0.71676	insignificant	0.11504	0.029284	hypermethylated	1	4	4
chr11	58269482	58271482	Olfr30		1	noCoverage	0.12354	0.16254	insignificant	0	6	6
chr11	58409846	58411846	Olfr324	-0.13247932	0.49094	insignificant	0.058782	0.10832	insignificant	1	13	13
chr11	58452966	58454966	Trim58	-0.18399617	0.0011814	hypomethylated	-0.0087033	0.47159	insignificant	7	36	36
chr11	58478062	58480062	Olfr179		1	noCoverage	0.12413	0.058557	insignificant	0	9	6
chr11	58590590	58592590	Olfr315	0.05564044	1	insignificant	-0.0055869	0.36507	insignificant	5	20	21
chr11	58598640	58600640	Olfr314	-0.19182572	0.71063	insignificant	0.0078647	0.098111	insignificant	2	13	15
chr11	58629429	58631429	Olfr313		1	noCoverage	-0.073653	0.28698	insignificant	0	2	2
chr11	58643657	58645657	Olfr312		1	noCoverage	-0.053686	0.55943	insignificant	0	6	3
chr11	58679741	58681741	2810021J22Ri	-0.39928181	0.000000122	stronglyHypometh	-0.013411	0.00795	hypomethylated	5	30	30
chr11	58717727	58719727	Zfp39	-0.03038829	0.6911	insignificant	0.026508	0.24916	insignificant	5	28	28
chr11	58730558	58732558	Butr1	0.23730937	1	insignificant	-0.085185	1	insignificant	2	17	13
chr11	58752408	58754408	Rnf187	-0.57317073	0.00000064	stronglyHypometh	-0.02439	0.21463	insignificant	0	0	0
chr11	58761412	58763412	Hist3h2ba		1	noCoverage	-0.0084259	0.39608	insignificant	0	39	32
chr11	58767186	58769186	Hist3h2a	-0.09357129	0.57207	insignificant	0.000026353	1	insignificant	3	48	50
chr11	58768014	58770014	Hist3h2bb-ps	-0.08940818	0.41138	insignificant	-0.013562	0.65612	insignificant	3	36	38
chr11	58790617	58792617	Trim11	-0.12772498	6.15E-27	hypomethylated	0.008108	0.20988	insignificant	30	146	143
chr11	58949877	58951877	Obscn	-0.04028119	0.30633	insignificant	0.02576	0.081337	insignificant	3	10	10
chr11	58977244	58979244	A230051G13Rik		1	noCoverage	-0.0029349	0.12936	insignificant	0	24	22
chr11	58996328	58998328	Gjc2	-0.39	0.016853	stronglyHypometh	0.044211	0.18783	insignificant	1	4	4
chr11	58996715	58998715	Guk1	-0.39	0.016853	stronglyHypometh	0.044211	0.18783	insignificant	1	4	4
chr11	59001243	59003243	Guk1	-0.59851695	0.0043289	stronglyHypometh	-0.029337	0.37092	insignificant	2	4	4
chr11	59015018	59017018	Mrpl55	-0.18417355	0.096976	insignificant	0.024504	0.016657	hypermethylated	9	65	65
chr11	59020862	59022862	2310033P09Ri	-0.04427358	0.08731	insignificant	0.000037264	0.31481	insignificant	2	35	42
chr11	59041664	59043664	Arf1	-0.08928667	0.000023388	hypomethylated	0.017722	0.34261	insignificant	16	46	46
chr11	59041769	59043769	Arf1	-0.12837728	0.000000431	hypomethylated	0.031146	0.072506	insignificant	10	28	28
chr11	59104253	59106253	Wnt3a	-0.15229849	1.38E-08	hypomethylated	-0.001758	0.96101	insignificant	23	55	55
chr11	59119431	59121431	Wnt9a	-0.09113906	4.68E-10	hypomethylated	-0.0030825	0.072866	insignificant	35	167	157
chr11	59262589	59264589	Snap47	-0.16447526	1.65E-17	hypomethylated	0.0036673	0.14619	insignificant	17	70	66

chr11	59263458	59265458	Snap47	-0.16994684	1.57E-17	hypomethylated	0.012523	0.093275	insignificant	17	65	61
chr11	59285976	59287976	Zfp867	-0.11013666	0.57692	insignificant	0.11571	0.11272	insignificant	2	32	33
chr11	59318186	59320186	4933439C10Ri	-0.21272613	1.54E-11	hypomethylated	-0.0092227	0.45621	insignificant	28	89	75
chr11	59320142	59322142	4933439C10Ri	-0.06703519	1	insignificant	0.050209	0.07629	insignificant	1	14	14
chr11	59425467	59427467	Olfr225	-0.11018843	0.0066034	hypomethylated	0.027843	0.0001808	hypermethylated	6	28	28
chr11	59474996	59476996	Mprip	-0.0798219	5.96E-19	hypomethylated	0.019185	0.091304	insignificant	50	188	183
chr11	59601159	59603159	Pld6	-0.25377468	0.076861	insignificant	0.072023	0.81931	insignificant	4	20	20
chr11	59623275	59625275	Flcn	-0.1402204	0.001385	hypomethylated	-0.03564	0.0083707	hypomethylated	8	70	67
chr11	59653269	59655269	Cops3	-0.1145736	0.014957	hypomethylated	-0.0047837	0.13323	insignificant	9	37	36
chr11	59660574	59662574	Nt5m	-0.10460695	2.3E-23	hypomethylated	0.0013574	0.22236	insignificant	21	128	128
chr11	59760715	59762715	Med9	-0.13610002	0.098503	insignificant	0.00045851	0.82681	insignificant	9	66	66
chr11	59778444	59780444	Rasd1	-0.16277321	0.045735	hypomethylated	0.029137	0.86	insignificant	7	59	54
chr11	59917514	59919514	Rai1	-0.10823715	1.05E-24	hypomethylated	-0.0069053	0.47054	insignificant	39	178	178
chr11	59952583	59954583	Rai1	-0.13590762	2.93E-11	hypomethylated	0.037042	0.097083	insignificant	17	45	44
chr11	60165881	60167881	Lrrc48	-0.1155433	9.94E-10	hypomethylated	0.0075176	0.20787	insignificant	6	64	64
chr11	60166407	60168407	Tom1l2	-0.11983626	0.020446	hypomethylated	0.026865	1	insignificant	1	18	18
chr11	60229646	60231646	4933439F18Ri	-0.1168151	1.49E-28	hypomethylated	0.0056173	0.34039	insignificant	28	83	78
chr11	60230601	60232601	Atpaf2	-0.1193452	2.03E-27	hypomethylated	0.0084012	0.25511	insignificant	28	95	92
chr11	60267118	60269118	Drg2	-0.1589997	1.18E-16	hypomethylated	0.019317	0.05382	insignificant	14	54	61
chr11	60350184	60352184	Alkbh5	-0.09831628	4.7E-32	hypomethylated	0.0077008	0.75966	insignificant	51	250	253
chr11	60512191	60514191	Llgl1	-0.13755706	1.79E-23	hypomethylated	0.0040996	0.46708	insignificant	33	130	131
chr11	60540723	60542723	Flii	-0.12467792	3.19E-11	hypomethylated	-0.0096264	0.29992	insignificant	25	87	74
chr11	60540899	60542899	Smcr7	-0.14674594	2.24E-08	hypomethylated	0.0029449	0.82171	insignificant	19	68	62
chr11	60590026	60592026	Smcr8	-0.18253056	0.0033108	hypomethylated	0.00015503	0.74069	insignificant	6	65	55
chr11	60590867	60592867	Top3a	-0.02464189	0.46839	insignificant	-0.011195	0.000008	inconclusive	5	44	38
chr11	60643132	60645132	Dhrs7b	-0.02477796	0.06866	insignificant	-0.0017761	0.82637	insignificant	7	30	30
chr11	60744558	60746558	Map2k3	-0.1333775	5.69E-29	hypomethylated	0.006456	0.081181	insignificant	30	139	134
chr11	60745369	60747369	Gm16516	-0.12593669	6.44E-25	hypomethylated	0.014841	0.20201	insignificant	27	104	103
chr11	60878305	60880305	Kcnj12	0.04092254	0.81133	insignificant	0.028853	0.31121	insignificant	3	19	19
chr11	60953336	60955336	Tnfrsf13b	0.00897973	1	insignificant	0.005465	0.85179	insignificant	1	4	4
chr11	60988561	60990561	Usp22	-0.12739932	0.0042005	hypomethylated	0.0015329	0.67482	insignificant	9	34	35
chr11	61080629	61082629	Aldh3a2	-0.17393497	0.000034884	hypomethylated	0.036252	0.89373	insignificant	10	31	39
chr11	61267388	61269388	Rnf112	-0.48533654	0.4087	lowCoverage	0.039474	0.69549	insignificant	1	4	4
chr11	61297945	61299945	Mfap4	0.04348315	1	insignificant	0.032758	0.02412	hypermethylated	8	35	34
chr11	61307705	61309705	Mapk7	-0.14847208	2.42E-21	hypomethylated	0.013662	0.56819	insignificant	21	84	84
chr11	61317673	61319673	B9d1	-0.26957215	6.74E-16	hypomethylated	-0.02252	0.40865	insignificant	11	31	35
chr11	61393153	61395153	Epn2	-0.12959	0.000035513	hypomethylated	0.011951	0.95585	insignificant	9	46	46
chr11	61465822	61467822	Grap		1	noCoverage	0.057268	0.28599	insignificant	0	4	4
chr11	61496911	61498911	Fam83g	-0.26428897	1.77E-24	hypomethylated	0.015973	0.023151	hypermethylated	23	118	111
chr11	61575590	61577590	Prpsap2		1	noCoverage	0.061528	0.29233	insignificant	0	27	21
chr11	61668594	61670594	Ulk2	-0.13022487	0.000000844	hypomethylated	-0.0067503	0.85023	insignificant	9	34	35
chr11	61743728	61745728	Akap10	-0.1682536	0.000068389	hypomethylated	-0.010526	0.70902	insignificant	2	36	24
chr11	61889598	61891598	Specc1	-0.07774331	0.068509	insignificant	0.0071366	0.53385	insignificant	15	90	90
chr11	62061485	62063485	Adora2b	-0.20305817	8.58E-51	hypomethylated	-0.01306	0.33348	insignificant	43	151	151
chr11	62093974	62095974	Ttc19	-0.09728315	2.22E-60	hypomethylated	-0.0041047	0.70222	insignificant	60	151	150
chr11	62094897	62096897	Zswim7	-0.07222341	9.52E-42	hypomethylated	-0.0044038	0.9479	insignificant	50	122	122
chr11	62270834	62272834	Ncor1	-0.13648082	9.23E-29	hypomethylated	-0.015398	0.13978	insignificant	42	150	142
chr11	62270961	62272961	Pigl	-0.13959665	6.29E-19	hypomethylated	-0.022356	0.91235	insignificant	32	124	116
chr11	62352763	62354763	Cenpv	-0.18905797	0.003343	hypomethylated	-0.018673	0.68435	insignificant	5	10	10
chr11	62364005	62366005	Ubb	-0.12095203	4.03E-08	hypomethylated	0.023859	0.18415	insignificant	15	154	138
chr11	62386987	62388987	Trpv2	-0.46818008	0.00059754	stronglyHypometh	-0.022827	0.8661	insignificant	4	15	15
chr11	62415378	62417378	2410006H16R	-0.14645088	9.12E-11	hypomethylated	-0.0062346	0.0097032	hypomethylated	10	50	48
chr11	62415587	62417587	Snord49b	-0.16699846	3.93E-13	hypomethylated	-0.0066815	0.011602	hypomethylated	14	58	56
chr11	62415961	62417961	Snord49a	-0.17015085	3.91E-13	hypomethylated	-0.0098339	0.011582	hypomethylated	14	57	56
chr11	62417031	62419031	Snord65	-0.41679588	2.42E-09	stronglyHypometh	-0.0082445	0.44553	insignificant	5	14	14
chr11	62461165	62463165	Mmgt2	-0.0327668	4.65E-24	hypomethylated	0.014258	0.2303	insignificant	52	136	129
chr11	62462025	62464025	Mmgt2	-0.26838763	9.05E-23	hypomethylated	0.05837	0.042913	hypermethylated	12	41	34
chr11	62691991	62693991	Fam18b	-0.15383502	0.83925	insignificant	-0.028205	0.22622	insignificant	9	81	78
chr11	62874160	62876160	Tekt3		1	noCoverage	-0.04025	0.92445	insignificant	0	6	6
chr11	62944011	62946011	Pmp22	-0.12232427	0.00054054	hypomethylated	-0.0024926	0.71426	insignificant	7	37	37
chr11	63735786	63737786	Hs3st3b1	-0.16350627	2.1E-11	hypomethylated	-0.0010389	0.56272	insignificant	28	131	131
chr11	63891985	63893985	2810001G20R	-0.12630589	2.98E-20	hypomethylated	-0.0022869	0.59817	insignificant	21	58	58
chr11	63892974	63894974	2810001G20R	-0.11255338	7.98E-14	hypomethylated	-0.0069893	0.94516	insignificant	13	30	30
chr11	64247833	64249833	Hs3st3a1	-0.14346153	0.00013443	hypomethylated	0.0001579	0.20155	insignificant	12	76	77
chr11	64791536	64793536	Elac2	-0.20957668	3.24E-19	hypomethylated	0.0016354	0.40687	insignificant	15	56	54
chr11	65083491	65085491	Myocd	-0.15213075	1	lowCoverage	-0.027874	0.474	insignificant	1	34	34
chr11	65601799	65603799	Map2k4		1	noCoverage	-0.039982	0.11505	insignificant	0	22	18
chr11	65619745	65621745	Zkscan6	-0.1274138	1.85E-13	hypomethylated	-0.026545	0.015508	hypomethylated	17	69	74
chr11	65982053	65984053	Dnahc9	-0.59027778	0.067881	insignificant	-0.11806	0.32332	insignificant	2	12	12
chr11	66339628	66341628	Shisa6	-0.10456334	1.09E-18	hypomethylated	0.0069518	0.16713	insignificant	48	154	142
chr11	66724492	66726492	Pirt	-0.0979159	0.0035275	inconclusive	-0.032068	0.62733	insignificant	11	30	30
chr11	66760588	66762588	Gm12298		1	noCoverage	0.1725	0.27252	insignificant	0	17	16
chr11	66837655	66839655	Tmem220		1	noCoverage	-0.098264	0.56731	insignificant	0	16	19
chr11	66865171	66867171	Sco1	-0.1660982	0.00010563	hypomethylated	0.028207	0.16844	insignificant	12	65	63
chr11	66866120	66868120	2310004I24Ril	-0.16852217	0.000020121	hypomethylated	0.031375	0.81017	insignificant	6	41	37
chr11	67012615	67014615	Myh1		1	noCoverage	-0.11905	0.38272	insignificant	0	7	6
chr11	67139604	67141604	Myh13		1	noCoverage	-0.03786	0.57746	insignificant	0	8	4
chr11	67507827	67509827	Rcvrn	0.08924793	0.017297	hypermethylated	0.066663	0.0020873	hypermethylated	5	28	28
chr11	67610772	67612772	Dhrs7c	0.03690476	1	insignificant	-0.028114	0.79386	insignificant	3	10	13
chr11	67735655	67737655	Usp43	-0.07254235	0.0007781	hypomethylated	-0.0015604	0.12743	insignificant	20	80	84
chr11	67778984	67780984	Stx8		1	noCoverage	0.05	0.77248	insignificant	0	4	4
chr11	67779144	67781144	Wdr16		1	noCoverage	0.05	0.77248	insignificant	0	4	4
chr11	68200328	68202328	Ntn1	-0.16127063	1.17E-28	hypomethylated	0.0089585	0.93397	insignificant	25	86	83
chr11	68244626	68246626	Pik3r5	-0.14559513	2.5E-11	hypomethylated	0.027437	0.23127	insignificant	21	74	72
chr11	68368687	68370687	Mfsd6l	-0.14749698	0.12787	insignificant	0.052535	0.23877	insignificant	7	52	45

chr11	68399538	68401538	Ccdc42	-0.0588519	0.5529	insignificant	0.0087093	0.70935	insignificant	1	18	15
chr11	68504416	68506416	Myh10	-0.14611314	1.46E-12	hypomethylated	0.0015026	0.87592	insignificant	20	83	78
chr11	68666633	68668633	Ndel1		1	noCoverage	0.018126	0.4391	insignificant	0	16	16
chr11	68701054	68703054	Rnf222	0.03877168	0.78995	insignificant	-0.030755	0.088351	insignificant	3	12	12
chr11	68714067	68716067	Rpl26	-0.11931509	0.038299	hypomethylated	0.014335	0.62191	insignificant	19	117	111
chr11	68714090	68716090	Gm15772	-0.11931509	0.038299	hypomethylated	0.014335	0.62191	insignificant	19	117	111
chr11	68770360	68772360	Arhgef15	-0.13126218	0.43632	insignificant	-0.048788	0.086741	insignificant	3	18	19
chr11	68780632	68782632	Slc25a35	-0.18069279	3.05E-25	hypomethylated	0.0075028	0.6782	insignificant	26	79	77
chr11	68787322	68789322	Rangrf	-0.33406928	1	insignificant	0.0031957	0.062421	insignificant	2	15	15
chr11	68804180	68806180	Pfas	0.04489189	0.51614	insignificant	0.0622	0.029317	hypermethylated	3	6	6
chr11	68821962	68823962	Pfas		1	noCoverage	0.0069798	0.82947	insignificant	0	10	10
chr11	68828412	68830412	1500010J02Ri	-0.19314565	0.85886	insignificant	0.017888	0.42749	insignificant	11	44	41
chr11	68858144	68860144	Aurkb	-0.08999613	1	insignificant	0.0023585	0.85316	insignificant	4	47	47
chr11	68872276	68874276	2310047M10F	-0.13573436	1.06E-36	hypomethylated	0.00093224	0.87861	insignificant	51	173	168
chr11	68883310	68885310	Tmem107	-0.13230006	1.51E-12	hypomethylated	0.0080496	0.90702	insignificant	12	73	73
chr11	68901029	68903029	Vamp2	-0.12792613	1.04E-19	hypomethylated	0.0079599	0.84561	insignificant	32	123	115
chr11	68911457	68913457	Per1	-0.17185394	0.0000019	hypomethylated	-0.024057	0.35788	insignificant	3	35	39
chr11	68912659	68914659	Per1	0.00104035	0.68808	insignificant	-0.030386	0.95916	insignificant	8	28	30
chr11	68932954	68934954	Hes7	-0.37872616	1.05E-17	stronglyHypometh	0.069615	0.034298	inconclusive	7	32	26
chr11	68938878	68940878	Aloxe3		1	noCoverage	0.072261	0.65865	insignificant	0	12	12
chr11	68969573	68971573	Alox12b	-0.19134872	1.88E-10	hypomethylated	-0.057235	0.14438	insignificant	7	21	20
chr11	69050524	69052524	Gucy2e		1	noCoverage	0.030017	0.80859	insignificant	0	22	23
chr11	69136487	69138487	Trappc1	-0.23282172	0.59702	insignificant	-0.016598	0.50865	insignificant	3	52	52
chr11	69137375	69139375	Cntrob	-0.36471199	0.59115	insignificant	-0.024781	0.90564	insignificant	3	42	42
chr11	69138759	69140759	Kcnab3	-0.22464489	7.19E-24	hypomethylated	0.0047916	0.08647	insignificant	20	81	76
chr11	69153270	69155270	A030009H04R	-0.14320948	0.00070323	hypomethylated	0.022975	0.92182	insignificant	4	46	49
chr11	69182928	69184928	Chd3	-0.30922832	0.39344	insignificant	-0.14477	1	insignificant	1	12	17
chr11	69208292	69210292	Lsmd1	-0.10916908	9.53E-10	hypomethylated	-0.00032047	0.69866	insignificant	16	95	91
chr11	69208848	69210848	Cyb5d1	-0.0964758	7.11E-09	hypomethylated	0.0010373	0.42193	insignificant	16	65	61
chr11	69211736	69213736	Kdm6b	-0.4151121	4.24E-17	stronglyHypometh	-0.014352	0.75035	insignificant	19	63	65
chr11	69373739	69375739	Efnb3	-0.11612195	0.000083194	hypomethylated	0.017993	0.45977	insignificant	5	29	28
chr11	69392826	69394826	Wrap53	-0.14277598	4.77E-11	hypomethylated	0.024822	0.31509	insignificant	12	39	36
chr11	69392860	69394860	Trp53	-0.17722465	3.71E-15	hypomethylated	0.021476	0.46138	insignificant	13	41	38
chr11	69434610	69436610	Sat2	-0.24364172	0.22258	insignificant	-0.068845	0.000068568	hypomethylated	2	51	46
chr11	69445472	69447472	Fxr2	-0.11702923	4.19E-18	hypomethylated	-0.00038721	0.63722	insignificant	34	114	114
chr11	69467874	69469874	Sox15	-0.74571971	0.00059685	stronglyHypometh	-0.086891	0.68465	insignificant	2	18	22
chr11	69475544	69477544	Mir1934		1	noCoverage	0.0035639	0.36385	insignificant	0	24	24
chr11	69476144	69478144	Mir1934		1	noCoverage	0.0056818	1	insignificant	0	16	16
chr11	69495472	69497472	BC096441	-0.22216369	0.18017	insignificant	0.0092102	0.27325	insignificant	6	34	34
chr11	69495586	69497586	BC096441	-0.22216369	0.18017	insignificant	0.0092102	0.27325	insignificant	6	34	34
chr11	69499056	69501056	BC096441	-0.4022179	1	lowCoverage	0.018744	0.91028	insignificant	1	8	8
chr11	69509600	69511600	BC096441		1	noCoverage	-0.057018	0.66504	insignificant	0	4	4
chr11	69571725	69573725	Polr2a	-0.12271665	5.94E-11	hypomethylated	-0.0016557	0.14685	insignificant	22	93	92
chr11	69578413	69580413	Zbtb4	-0.09277468	3.91E-09	hypomethylated	0.024912	0.65668	insignificant	19	70	70
chr11	69609439	69611439	Chrnb1	-0.37667313	0.00002974	stronglyHypometh	-0.13038	0.18738	insignificant	2	23	30
chr11	69615127	69617127	G630025P09R	-0.15379848	2.39E-12	hypomethylated	-0.0012333	0.15356	insignificant	15	75	65
chr11	69616170	69618170	G630025P09R	-0.15014263	3.63E-22	hypomethylated	-0.010796	0.70245	insignificant	22	122	104
chr11	69631942	69633942	4933402P03Ri	-0.68578216	0.36937	lowCoverage	-0.078263	0.41917	insignificant	1	9	6
chr11	69648351	69650351	Nlgn2	-0.25126596	0.003668	hypomethylated	0.0025488	0.65104	insignificant	4	14	14
chr11	69651026	69653026	1810027O10R	-0.12378791	5.03E-12	hypomethylated	0.024893	0.69972	insignificant	15	73	57
chr11	69658873	69660873	Plscr3	-0.16758383	5.81E-18	hypomethylated	0.010313	0.0818	insignificant	6	34	37
chr11	69659118	69661118	Plscr3	-0.17895411	4.38E-17	hypomethylated	0.0092776	0.19436	insignificant	9	46	49
chr11	69659145	69661145	Plscr3	-0.16947079	4.46E-17	hypomethylated	0.018761	0.19416	insignificant	9	48	49
chr11	69672232	69674232	Tnk1	-0.42978315	0.063154	insignificant	-0.0071731	0.95291	insignificant	2	17	17
chr11	69691520	69693520	Tmem95	0.12660538	1	insignificant	0.023879	0.28775	insignificant	2	12	12
chr11	69694487	69696487	Acap1	-0.23732786	4.14E-30	hypomethylated	0.013363	0.3676	insignificant	16	91	94
chr11	69714379	69716379	Neurl4	-0.12863179	5.17E-49	hypomethylated	0.0046737	0.10626	insignificant	42	155	161
chr11	69714488	69716488	2810408A11R	-0.12686144	8.96E-50	hypomethylated	0.0058422	0.12997	insignificant	40	151	157
chr11	69726693	69728693	Gps2	-0.15507083	3.6E-09	hypomethylated	-0.037422	0.7264	insignificant	10	78	76
chr11	69734132	69736132	Eif5a	-0.10173624	8.59E-12	hypomethylated	0.0094239	0.21262	insignificant	31	78	78
chr11	69734449	69736449	Eif5a	-0.17820436	0.000018086	hypomethylated	0.058319	0.42801	insignificant	6	15	14
chr11	69734587	69736587	Eif5a	-0.22596678	0.00060264	hypomethylated	0.12777	0.66689	insignificant	3	9	8
chr11	69734777	69736777	Eif5a	-0.22596678	0.00060264	hypomethylated	0.12777	0.66689	insignificant	3	9	8
chr11	69734888	69736888	Eif5a	-0.22596678	0.00060264	hypomethylated	0.12777	0.66689	insignificant	3	9	8
chr11	69748400	69750400	Ybx2	-0.11003759	1.5E-43	hypomethylated	0.011777	0.66483	insignificant	64	196	193
chr11	69761692	69763692	Slc2a4	-0.21981166	1.33E-09	hypomethylated	0.032422	0.41409	insignificant	4	15	14
chr11	69777280	69779280	Cldn7	0.00748378	0.13612	insignificant	-0.042901	0.096638	insignificant	13	42	41
chr11	69777922	69779922	Cldn7	-0.04403079	0.33162	insignificant	-0.0059786	0.087985	insignificant	25	76	75
chr11	69793669	69795669	Ctdnep1	-0.14000222	4.54E-09	hypomethylated	0.01356	0.27424	insignificant	19	75	89
chr11	69793745	69795745	Rai12	-0.14000222	4.54E-09	hypomethylated	0.01356	0.27424	insignificant	19	75	89
chr11	69794737	69796737	Rai12	-0.23973645	0.0024925	hypomethylated	-0.089952	1	insignificant	4	21	17
chr11	69803871	69805871	Gabarap	-0.1312312	3.67E-15	hypomethylated	0.042778	0.30767	insignificant	12	56	51
chr11	69808272	69810272	Phf23	-0.16392469	3.74E-33	hypomethylated	0.006806	0.5349	insignificant	21	104	93
chr11	69813127	69815127	Dvl2	0.22061848	0.70649	insignificant	0.014026	0.51566	insignificant	5	72	72
chr11	69828909	69830909	Acadvl	-0.12228881	0.48096	insignificant	0.027441	0.68875	insignificant	1	6	7
chr11	69831106	69833106	Dlg4	-0.34146282	0.000014743	stronglyHypometh	0.038877	0.22812	insignificant	5	10	10
chr11	69866870	69868870	Asgr1	-0.09084967	1	insignificant	-0.035294	0.49437	insignificant	1	6	4
chr11	69942858	69944858	Mgl2		1	noCoverage	-0.21591	1	insignificant	0	2	2
chr11	70026411	70028411	Slc16a11	-0.12626511	1	insignificant	0.043188	0.53958	insignificant	2	46	55
chr11	70034496	70036496	Slc16a13	-0.18046434	0.021314	hypomethylated	0.093484	0.71535	insignificant	4	14	12
chr11	70043300	70045300	Bcl6b	-0.16845801	0.000000976	hypomethylated	0.010083	0.36272	insignificant	5	47	48
chr11	70051416	70053416	0610010K14Ri	-0.07473958	1	insignificant	-0.01224	0.70969	insignificant	3	14	14
chr11	70223533	70225533	Pelp1	-0.10263074	0.001981	hypomethylated	-0.035585	0.021258	hypomethylated	5	34	34
chr11	70245154	70247154	Arrb2	-0.12656936	5.64E-08	hypomethylated	0.037569	0.095114	insignificant	14	94	92
chr11	70264432	70266432	Med11	-0.14617222	1.42E-08	hypomethylated	0.018755	0.92056	insignificant	13	52	52

chr11	70272123	70274123	Zmynd15	-0.21724424	0.000000157	hypomethylated	-0.011603	0.73308	insignificant	11	32	32
chr11	70328118	70330118	Vmo1		1	noCoverage	0.28609	0.00000759	hypermethylated	0	8	8
chr11	70331710	70333710	Gltpd2	0.03981978	0.088199	insignificant	0.031063	0.040745	hypermethylated	5	40	38
chr11	70337858	70339858	Psmb6	0.24778295	1	insignificant	0.038471	0.15066	insignificant	1	23	23
chr11	70352665	70354665	Pld2	-0.20122907	3.17E-27	hypomethylated	-0.024821	0.40342	insignificant	32	101	94
chr11	70375382	70377382	Mink1	-0.10623487	8.94E-19	hypomethylated	-0.011302	0.35296	insignificant	32	111	111
chr11	70428395	70430395	4930544D05R	0.01490898	0.0061725	hypermethylated	-0.028703	7.89E-08	inconclusive	5	25	25
chr11	70460090	70462090	Rnf167	-0.17179959	1.04E-11	hypomethylated	-0.0052253	0.27613	insignificant	7	55	54
chr11	70460495	70462495	Slc25a11	-0.19729327	3.31E-10	hypomethylated	0.0012876	0.096592	insignificant	7	37	36
chr11	70468152	70470152	Eno3	-0.0782238	1	insignificant	-0.0076025	0.45859	insignificant	13	126	126
chr11	70469714	70471714	Eno3	0.15725735	0.26138	insignificant	-0.018194	0.20296	insignificant	3	6	9
chr11	70482918	70484918	Camta2	-0.25618583	0.000000047	hypomethylated	-0.0091407	0.0014766	inconclusive	8	30	30
chr11	70501607	70503607	Camta2		1	noCoverage	0.13493	0.14429	insignificant	0	5	2
chr11	70513049	70515049	Kif1c	-0.11957266	1.28E-26	hypomethylated	-0.0097532	0.64804	insignificant	50	140	145
chr11	70513657	70515657	Inca1	-0.10994773	5.48E-20	hypomethylated	-0.011768	0.56152	insignificant	44	114	121
chr11	70576948	70578948	Zfp3	-0.11364658	3.13E-29	hypomethylated	0.0028794	0.62753	insignificant	50	110	110
chr11	70626063	70628063	A430084P05Rik		1	noCoverage	0.076872	0.78253	insignificant	0	4	4
chr11	70657264	70659264	Rabep1	-0.11590833	4.7E-21	hypomethylated	0.003838	0.45572	insignificant	46	150	142
chr11	70782714	70784714	Nup88	-0.05770973	0.000079367	hypomethylated	-0.018851	0.83824	insignificant	11	54	63
chr11	70783464	70785464	Nup88	-0.2753318	1.04E-12	hypomethylated	-0.070275	0.34978	insignificant	4	16	19
chr11	70783475	70785475	Nup88	-0.2753318	1.04E-12	hypomethylated	-0.070275	0.34978	insignificant	4	16	19
chr11	70796528	70798528	C1qbp		1	noCoverage	-0.017849	1	insignificant	0	10	10
chr11	70817934	70819934	Dhx33		1	noCoverage	0.051852	1	insignificant	0	6	6
chr11	70832112	70834112	Mis12	-0.12723051	9.55E-15	hypomethylated	0.0052983	1	insignificant	27	101	97
chr11	70832765	70834765	Derl2	-0.12274895	1.77E-14	hypomethylated	0.0041749	1	insignificant	27	80	78
chr11	70847119	70849119	6330403K07Rik		1	noCoverage	-0.05904	0.65494	insignificant	0	15	15
chr11	71563204	71565204	Wscd1	-0.13923873	9.66E-08	hypomethylated	0.0072756	0.61654	insignificant	24	117	117
chr11	71855003	71857003	Fam64a	-0.18628769	0.0004212	hypomethylated	-0.022834	0.28134	insignificant	11	57	53
chr11	71949391	71951391	Pitpnm3	-0.09007118	0.00023941	hypomethylated	-0.016602	0.13951	insignificant	10	44	44
chr11	72016873	72018873	4933427D14R	-0.00652154	0.054831	insignificant	0.080171	0.90641	insignificant	8	23	21
chr11	72020055	72022055	Txndc17	-0.17868527	1.63E-16	hypomethylated	0.055704	0.44261	insignificant	23	86	86
chr11	72027639	72029639	4930563E22Ri	-0.13675622	0.00000013	hypomethylated	0.000091804	0.35082	insignificant	11	77	80
chr11	72080106	72082106	Slc13a5	-0.06126496	0.54099	insignificant	0.047636	0.52025	insignificant	3	45	49
chr11	72114130	72116130	Xaf1		1	noCoverage	-0.060734	0.093264	insignificant	0	5	5
chr11	72126945	72128945	Fbxo39		1	noCoverage	-0.058442	0.045678	hypomethylated	0	30	30
chr11	72175382	72177382	Tekt1	0.21006454	1	insignificant	0.0063533	0.54111	insignificant	2	21	19
chr11	72225215	72227215	Smtnl2	-0.51344254	0.000014132	stronglyHypometh	-0.049833	0.90229	insignificant	7	34	37
chr11	72253879	72255879	Mybbp1a	-0.52129273	0.00000874	stronglyHypometh	0.028549	0.074999	insignificant	4	17	15
chr11	72303406	72305406	Spns2	-0.13661578	0.000014427	hypomethylated	-0.0080955	0.81616	insignificant	10	24	24
chr11	72419762	72421762	Ube2g1	-0.07916632	2.41E-29	hypomethylated	0.0074906	0.80738	insignificant	73	232	232
chr11	72502503	72504503	Ankfy1	-0.18781233	1.8E-10	hypomethylated	-0.0072272	0.13701	insignificant	29	82	74
chr11	72608727	72610727	Zzef1	-0.10050528	0.000000169	hypomethylated	-0.0089432	0.88957	insignificant	16	105	111
chr11	72609341	72611341	Cyb5d2	-0.09719381	0.000000282	hypomethylated	-0.012579	0.81404	insignificant	16	88	95
chr11	72773670	72775670	Atp2a3	-0.13051683	1.91E-14	hypomethylated	0.0031436	0.3316	insignificant	22	106	104
chr11	72811646	72813646	P2rx1	-0.296875	0.011719	hypomethylated	0.034226	0.71785	insignificant	2	8	8
chr11	72831509	72833509	Camkk1	-0.144814	2.29E-19	hypomethylated	-0.0097069	0.067034	insignificant	39	134	133
chr11	72860368	72862368	1200014J11Ri	-0.10963715	2.59E-09	hypomethylated	0.032652	0.087088	insignificant	22	83	79
chr11	72903096	72905096	Itgae	-0.18488951	0.083236	insignificant	0.030614	1	insignificant	2	15	15
chr11	72951796	72953796	Gsg2	-0.22641882	0.0080147	hypomethylated	0.0047877	0.19712	insignificant	7	39	39
chr11	72973031	72975031	P2rx5	-0.13104702	3.62E-25	hypomethylated	-0.011215	0.6838	insignificant	18	72	75
chr11	72989584	72991584	Tax1bp3	-0.11658961	3.59E-16	hypomethylated	0.0020549	0.72581	insignificant	25	145	145
chr11	72990510	72992510	Tmem93	-0.1275238	0.000000964	hypomethylated	0.0027809	1	insignificant	16	100	101
chr11	72990544	72992544	Tmem93	-0.1275238	0.000000964	hypomethylated	0.0027809	1	insignificant	16	100	101
chr11	73011983	73013983	Shpk	-0.19566799	1.84E-18	hypomethylated	-0.018715	0.75751	insignificant	8	54	58
chr11	73012521	73014521	Ctns	-0.19566799	1.84E-18	hypomethylated	-0.018715	0.75751	insignificant	8	54	58
chr11	73050760	73052760	Trpv1	0.08738381	0.10337	insignificant	-0.0098663	0.90012	insignificant	2	10	18
chr11	73138136	73140136	Aspa		1	noCoverage	-0.16917	0.75857	insignificant	0	4	5
chr11	73142242	73144242	Spata22	-0.23391657	0.0099843	hypomethylated	0.0093645	0.28423	insignificant	1	8	8
chr11	73415083	73417083	Olfr384	0.1201484	1	insignificant	0.024337	0.0010112	hypermethylated	1	4	4
chr11	74081892	74083892	Olfr406-ps		1	noCoverage	0.15997	0.039822	hypermethylated	0	10	12
chr11	74403660	74405660	Rap1gap2	-0.15658686	0.000019481	hypomethylated	0.054204	0.43035	insignificant	16	43	41
chr11	74432106	74434106	E130309D14R	-0.16512868	1.74E-17	hypomethylated	0.01037	0.15639	insignificant	21	103	99
chr11	74461996	74463996	1300001I01Ril	-0.09597025	3E-11	hypomethylated	0.0031238	0.31927	insignificant	26	89	90
chr11	74537360	74539360	Pafah1b1	-0.02551298	5.96E-17	hypomethylated	0.0045202	0.73235	insignificant	45	140	138
chr11	74537886	74539886	Pafah1b1	0.06007662	0.078999	insignificant	0.028299	0.32599	insignificant	17	47	52
chr11	74583364	74585364	Mettl16	-0.20197155	1.75E-09	hypomethylated	0.11749	0.40328	insignificant	10	31	34
chr11	74643425	74645425	Mnt	-0.13961095	9.19E-31	hypomethylated	-0.008903	0.8623	insignificant	30	149	144
chr11	74710581	74712581	Tsr1	-0.17224639	5.02E-37	hypomethylated	0.013169	0.61229	insignificant	33	134	118
chr11	74710582	74712582	Tsr1	-0.17224639	5.02E-37	hypomethylated	0.013169	0.61229	insignificant	33	134	118
chr11	74717947	74719947	Snord91a	0.13937166	1	insignificant	0.080042	0.50686	insignificant	2	11	11
chr11	74738373	74740373	Smg6	-0.07639598	0.0076439	hypomethylated	-0.003788	1	insignificant	12	73	60
chr11	74739300	74741300	Srr	-0.06315259	0.0075708	hypomethylated	0.0098143	0.94584	insignificant	12	54	46
chr11	74879493	74881493	Smg6	-0.14822606	0.015311	hypomethylated	0.079624	0.0018868	hypermethylated	1	20	26
chr11	74981654	74983654	Hic1	-0.14567418	4.14E-35	hypomethylated	0.0010221	1	insignificant	54	145	156
chr11	74983757	74985757	Hic1	-0.15057415	0.000000226	hypomethylated	0.011618	0.20804	insignificant	12	71	70
chr11	74985889	74987889	Mir212	-0.10312894	1.87E-09	hypomethylated	-0.0064057	0.83446	insignificant	29	124	123
chr11	74986183	74988183	Mir132	-0.13756251	0.0013583	hypomethylated	-0.0016115	0.25453	insignificant	19	117	116
chr11	74992310	74994310	Ovca2	-0.22558532	0.00000236	hypomethylated	0.03759	0.53345	insignificant	5	20	20
chr11	75003985	75005985	Dph1	-0.14557878	0.14487	insignificant	0.080252	0.031766	hypermethylated	1	36	38
chr11	75006494	75008494	Rtn4rl1	-0.13818229	2.14E-28	hypomethylated	-0.0028589	0.027459	inconclusive	64	192	202
chr11	75160934	75162934	Smyd4	-0.19913346	4.39E-23	hypomethylated	0.026094	0.90841	insignificant	14	90	82
chr11	75161885	75163885	Rpa1	0.54409256	0.033792	stronglyHypermeth	0.060209	0.14032	insignificant	3	52	44
chr11	75236125	75238125	Serpinf1		1	noCoverage	-0.024892	0.038049	hypomethylated	0	14	14
chr11	75268219	75270219	Wdr81	-0.31564872	0.022869	hypomethylated	-0.14431	1	insignificant	5	21	19
chr11	75274040	75276040	2210403K04Ri	-0.1643166	0.00030359	hypomethylated	-0.00096719	0.96498	insignificant	12	65	69

chr11	75276217	75278217	Mir22	-0.41957909	0.00000362	stronglyHypometh	0.087787	0.41934	insignificant	4	19	20
chr11	75280580	75282580	Tlcd2	0.06153846	1	insignificant	-0.16361	0.80142	insignificant	2	6	10
chr11	75299278	75301278	Prpf8	-0.21084104	3.31E-08	hypomethylated	-0.046771	0.27143	insignificant	14	51	52
chr11	75322595	75324595	Rilp	-0.12636899	4.91E-16	hypomethylated	-0.0048208	0.29068	insignificant	14	36	36
chr11	75326042	75328042	Scarf1	-0.50757662	0.0054679	stronglyHypometh	0.10333	0.25671	insignificant	2	11	11
chr11	75344195	75346195	Slc43a2	-0.14275622	7.48E-30	hypomethylated	-0.0021639	0.51492	insignificant	43	155	155
chr11	75344613	75346613	Slc43a2	-0.1422661	5.48E-28	hypomethylated	-0.0042831	0.42032	insignificant	38	140	140
chr11	75400609	75402609	Pitpna	-0.12206012	5.47E-15	hypomethylated	-0.00059914	0.04226	inconclusive	33	103	101
chr11	75443521	75445521	Inpp5k	-0.0320021	0.88411	insignificant	-0.043178	0.71724	insignificant	11	68	87
chr11	75464010	75466010	Myo1c	0.02875999	0.014935	hypermethylated	0.0056495	0.59079	insignificant	15	52	58
chr11	75464651	75466651	Myo1c	-0.15705069	0.4628	insignificant	-0.043961	0.10751	insignificant	19	71	79
chr11	75468516	75470516	Myo1c	-0.0815383	0.000000142	hypomethylated	0.017851	0.55561	insignificant	26	89	90
chr11	75491811	75493811	Crk	-0.14763139	7.07E-30	hypomethylated	-0.0078312	0.38096	insignificant	37	132	132
chr11	75545388	75547388	Ywhae	-0.15299292	2.33E-23	hypomethylated	0.012821	0.22244	insignificant	32	140	142
chr11	75609559	75611559	Doc2b	0.3115437	1	lowCoverage	0.21027	0.29131	insignificant	1	24	16
chr11	75724528	75726528	Rph3al	0.07017544	1	insignificant	0.070175	0.28234	insignificant	1	3	2
chr11	75812413	75814413	1700016K19Ri	-0.44141829	4.43E-21	stronglyHypometh	-0.10913	0.50107	insignificant	4	14	22
chr11	75841284	75843284	Fam101b	-0.06078997	2.43E-10	hypomethylated	0.001412	0.21919	insignificant	14	55	55
chr11	75993132	75995132	Vps53		1	noCoverage	-0.00049518	0.67253	insignificant	0	21	21
chr11	76014557	76016557	Fam57a	-0.09088944	1.47E-31	hypomethylated	-0.010363	0.859	insignificant	36	127	125
chr11	76030114	76032114	Dbil5	-0.14237839	2.95E-11	hypomethylated	0.028414	0.51709	insignificant	11	56	62
chr11	76031074	76033074	Gemin4	-0.13948764	0.0000093	hypomethylated	0.037981	0.18798	insignificant	2	22	22
chr11	76056237	76058237	Rnmtl1	-0.12159877	0.000001849	hypomethylated	0.017941	0.12723	insignificant	22	96	92
chr11	76057201	76059201	Glod4	-0.15376251	0.000088908	hypomethylated	0.0087987	0.060855	insignificant	12	49	50
chr11	76212643	76214643	Nxn	-0.12012929	1.61E-08	hypomethylated	0.012758	0.8778	insignificant	11	42	42
chr11	76219668	76221668	Timm22	-0.15426788	0.00020792	hypomethylated	0.0015888	0.75006	insignificant	19	74	76
chr11	76322921	76324921	Abr		1	noCoverage	-0.021579	0.52711	insignificant	0	10	10
chr11	76384876	76386876	Abr	-0.20735255	1.76E-20	hypomethylated	-0.072051	0.06482	insignificant	14	12	32
chr11	76484971	76486971	Bhlha9	-0.10754857	0.019255	hypomethylated	-0.0059473	0.64157	insignificant	9	81	81
chr11	76660510	76662510	Cpd	-0.54032258	1.97E-09	stronglyHypometh	0.021216	0.18203	insignificant	1	2	2
chr11	76758157	76760157	Blmh	-0.10131539	8.88E-14	hypomethylated	0.031481	0.55837	insignificant	25	73	82
chr11	76811098	76813098	Slc6a4	-0.09960235	0.000025956	hypomethylated	0.01916	0.36206	insignificant	9	79	69
chr11	76891674	76893674	Mir423	-0.18692654	4.33E-29	hypomethylated	0.002264	0.96952	insignificant	28	87	88
chr11	76891937	76893937	Mir423	-0.17950802	8.6E-17	hypomethylated	0.0094924	0.7677	insignificant	19	55	55
chr11	77028926	77030926	Ssh2	-0.09551414	2.31E-17	hypomethylated	0.00071058	0.47744	insignificant	23	110	106
chr11	77276414	77278414	Coro6	-0.47420391	1.06E-29	stronglyHypometh	-0.071724	0.96188	insignificant	8	37	39
chr11	77303180	77305180	Ankrd13b	-0.12620633	1.62E-27	hypomethylated	-0.015786	0.39004	insignificant	34	104	112
chr11	77305913	77307913	Git1	-0.12013873	1.51E-35	hypomethylated	0.0028065	0.76068	insignificant	39	135	133
chr11	77326775	77328775	Trp53i13	-0.1093982	1.64E-13	hypomethylated	-0.0036012	0.59427	insignificant	33	119	119
chr11	77327618	77329618	Abhd15	-0.11157906	4.98E-32	hypomethylated	-0.0034669	0.92897	insignificant	44	162	164
chr11	77421317	77423317	Taok1	-0.26210336	1.38E-09	hypomethylated	0.0042876	0.66872	insignificant	5	19	19
chr11	77498640	77500640	Nufip2	-0.17582844	0.00027791	hypomethylated	0.044545	0.76754	insignificant	5	38	26
chr11	77538795	77540795	Cryba1	-0.1625	0.27707	insignificant	0.047024	0.15412	insignificant	2	10	10
chr11	77589766	77591766	Myo18a	-0.04046828	0.70396	insignificant	0.0069823	0.055411	insignificant	14	57	57
chr11	77707374	77709374	Pipox	-0.53640351	0.000010181	stronglyHypometh	0.1136	0.54097	insignificant	2	4	4
chr11	77742790	77744790	Sez6	-0.10481809	3.45E-14	hypomethylated	-0.0020046	0.6126	insignificant	36	118	119
chr11	77743444	77745444	Sez6	-0.12005853	2.14E-28	hypomethylated	0.0088637	0.34209	insignificant	62	209	208
chr11	77795317	77797317	Phf12	-0.17011848	9.57E-28	hypomethylated	0.034435	0.011888	hypermethylated	35	128	94
chr11	77844814	77846814	Dhrs13	-0.11915328	2.9E-14	hypomethylated	0.019524	0.24125	insignificant	34	143	124
chr11	77850442	77852442	Flot2	-0.22244113	9.99E-29	hypomethylated	0.0093609	0.065123	insignificant	32	101	95
chr11	77907174	77909174	BC017647	-0.13409511	7.38E-17	hypomethylated	0.038546	0.57375	insignificant	7	14	14
chr11	77990168	77992168	Nek8	-0.18015646	7.88E-17	hypomethylated	-0.00085339	0.13046	insignificant	20	72	77
chr11	77991267	77993267	Tlcd1	-0.20354949	5.86E-10	hypomethylated	-0.063268	0.054493	insignificant	12	44	50
chr11	77995258	77997258	Snord42b	-0.20230314	0.0063813	hypomethylated	-0.042844	0.35185	insignificant	4	34	38
chr11	77997086	77999086	Snord42b	-0.66666667	0.10546	insignificant	-0.00030303	0.31157	insignificant	2	20	20
chr11	78000928	78002928	Rab34	-0.09174302	0.0014675	hypomethylated	0.0087589	0.17991	insignificant	43	170	155
chr11	78001249	78003249	Rab34	-0.04744526	0.0075127	hypomethylated	0.0096425	0.072592	insignificant	43	158	143
chr11	78005893	78007893	Proca1	0.15166667	1	insignificant	0.061374	0.81915	insignificant	2	10	10
chr11	78058247	78060247	Sdf2	-0.16468905	2.35E-13	hypomethylated	-0.0058142	0.89762	insignificant	21	91	90
chr11	78059205	78061205	Supt6h	-0.22633263	0.000000243	hypomethylated	0.0020435	0.16827	insignificant	8	68	68
chr11	78074255	78076255	2610507B11R	-0.11520404	3.26E-23	hypomethylated	0.018801	0.87268	insignificant	39	130	124
chr11	78114092	78116092	Spag5	-0.25229744	1	insignificant	-0.047668	1	insignificant	3	29	31
chr11	78136699	78138699	Aldoc		1	noCoverage	0.036419	0.078971	insignificant	0	14	11
chr11	78140923	78142923	Pigs	-0.07572514	0.0005472	hypomethylated	0.0005814	0.93457	insignificant	24	74	74
chr11	78156023	78158023	Unc119	-0.11750965	2.48E-19	hypomethylated	-0.0030075	0.61517	insignificant	24	36	36
chr11	78235687	78237687	Slc13a2	0.04803891	0.026083	inconclusive	-0.010071	0.024246	inconclusive	3	20	19
chr11	78278202	78280202	Slc46a1		1	noCoverage	0.24416	1	insignificant	0	29	10
chr11	78311256	78313256	Sarm1	-0.26822555	0.51037	insignificant	0.028331	0.4785	insignificant	3	12	12
chr11	78311621	78313621	Vtn	0.17218137	0.34919	insignificant	0.065039	0.53243	insignificant	1	6	6
chr11	78316014	78318014	Sebox	0.19293831	1	lowCoverage	-0.0078404	0.77398	insignificant	1	8	8
chr11	78324797	78326797	Poldip2	-0.08753735	0.000000236	hypomethylated	0.071527	0.17963	insignificant	19	103	74
chr11	78325670	78327670	Tmem199	-0.12772015	0.00000109	hypomethylated	0.15136	0.70823	insignificant	5	50	22
chr11	78348937	78350937	Ift20	-0.16281036	3.17E-13	hypomethylated	0.010594	0.19056	insignificant	16	57	57
chr11	78349762	78351762	Tnfaip1	-0.14637879	0.0030017	inconclusive	-0.0010158	0.45577	insignificant	15	57	56
chr11	78364237	78366237	Tmem97	-0.16818288	1.87E-12	hypomethylated	0.18131	0.29508	insignificant	7	40	22
chr11	78510927	78512927	Nlk	-0.12828418	9.73E-14	hypomethylated	0.013225	0.95493	insignificant	16	36	36
chr11	78639096	78641096	1810012P15Ri	-0.11677462	0.000000534	hypomethylated	0.0075678	0.41771	insignificant	19	78	84
chr11	78959856	78961856	Ksr1		1	noCoverage	-0.02	0.73324	insignificant	0	10	10
chr11	79068197	79070197	Wsb1	-0.23432554	0.0076169	hypomethylated	-0.053582	1	insignificant	4	32	29
chr11	79152393	79154393	Nf1	-0.11245785	0.000003	hypomethylated	-0.0031642	0.84053	insignificant	23	136	151
chr11	79403713	79405713	Rab11fip4	0.01659009	0.097948	insignificant	-0.0088801	0.64185	insignificant	14	120	120
chr11	79524470	79526470	Mir193	-0.16904049	5.71E-55	hypomethylated	0.0015668	0.8513	insignificant	49	193	178
chr11	79538901	79540901	Mir365-2		1	noCoverage	-0.013154	0.66689	insignificant	0	4	4
chr11	79775889	79777889	Utp6	-0.17759946	4.81E-09	hypomethylated	0.0097283	0.0041734	inconclusive	8	26	25
chr11	79805607	79807607	Suz12	-0.1047547	3.54E-11	hypomethylated	-0.017789	0.81297	insignificant	34	193	197

chr11	79894457	79896457	Crlf3	-0.09058981	0.0037789	hypomethylated	-0.0019242	0.32843	insignificant	10	28	28
chr11	79901901	79903901	Atad5	-0.171757	1.47E-16	hypomethylated	0.0090199	0.40176	insignificant	19	51	50
chr11	79955655	79957655	1110002N22R	-0.29192925	0.73572	insignificant	0.033156	0.30728	insignificant	7	20	18
chr11	79966663	79968663	Adap2	-0.2263125	0.000015499	hypomethylated	0.035483	0.003813	hypermethylated	12	46	46
chr11	79996373	79998373	Rnf135	-0.21351761	0.000000221	hypomethylated	-0.008737	0.11817	insignificant	12	54	48
chr11	80021556	80023556	Rhot1	-0.11380518	1.45E-12	hypomethylated	-0.018615	0.7554	insignificant	24	114	106
chr11	80113413	80115413	Rhbdl3	-0.11146222	2E-29	hypomethylated	0.01671	0.18354	insignificant	63	214	194
chr11	80191517	80193517	5730455P16Ri	-0.19402985	0.15277	insignificant	-0.012677	0.23473	insignificant	3	6	6
chr11	80241116	80243116	Psmd11	-0.14228924	2.21E-44	hypomethylated	0.008385	0.039745	hypermethylated	51	134	128
chr11	80289547	80291547	Cdk5r1	-0.12458253	1.13E-41	hypomethylated	0.0074363	0.080928	insignificant	52	199	195
chr11	80593527	80595527	Myo1d	-0.13787879	0.58778	insignificant	-0.037879	0.88145	insignificant	5	10	10
chr11	80622916	80624916	Tmem98	-0.08448902	0.0087461	hypomethylated	0.0088293	0.85679	insignificant	14	37	37
chr11	80966405	80968405	Accn1	-0.12097806	4.36E-18	hypomethylated	0.0022992	0.86696	insignificant	23	99	101
chr11	81781898	81783898	Accn1	0.12260817	0.0037569	inconclusive	-0.047065	0.69646	insignificant	3	14	18
chr11	81914346	81916346	Ccl12	-0.04675386	0.76848	insignificant	0.012343	0.17963	insignificant	2	4	4
chr11	81993314	81995314	Ccl1	-0.30080972	0.18199	insignificant	0.00034563	0.60027	insignificant	1	13	12
chr11	82201401	82203401	Tmem132e	-0.12927068	2.98E-31	hypomethylated	0.0065678	0.8365	insignificant	50	193	187
chr11	82445854	82447854	Gm11426	0.13729261	0.16976	insignificant	0.089043	0.022946	hypermethylated	4	10	10
chr11	82576846	82578846	Zfp830	-0.10450572	2.37E-09	hypomethylated	0.0063145	0.83311	insignificant	11	82	76
chr11	82577790	82579790	Cct6b	-0.10426558	8.36E-09	hypomethylated	0.011867	0.84821	insignificant	8	62	56
chr11	82593636	82595636	Lig3	-0.12041867	5.04E-40	hypomethylated	0.0064445	0.54625	insignificant	46	153	156
chr11	82684246	82686246	Rffl	-0.13275391	2.63E-08	hypomethylated	0.030526	0.36758	insignificant	28	103	108
chr11	82684712	82686712	Rffl	-0.19188444	0.0025157	hypomethylated	0.03698	0.44801	insignificant	10	39	41
chr11	82704117	82706117	Rad51l3	-0.27805361	0.000051731	hypomethylated	-0.0025524	0.82189	insignificant	2	12	12
chr11	82721897	82723897	Unc45b	-0.1055787	0.0000008	hypomethylated	0.12921	0.56381	insignificant	9	22	25
chr11	82723754	82725754	Unc45b	0.08360131	1	insignificant	0.054196	0.018277	hypermethylated	3	19	20
chr11	82764603	82766603	Slfn5	-0.25488491	6.34E-23	hypomethylated	-0.0066871	0.39259	insignificant	7	27	27
chr11	82805332	82807332	Slfn9	-0.20197044	0.3354	insignificant	0.0036706	1	insignificant	3	6	6
chr11	82834224	82836224	Slfn8		1	noCoverage	-0.0098039	1	insignificant	0	3	3
chr11	82834312	82836312	Slfn8		1	noCoverage	-0.0098039	1	insignificant	0	3	3
chr11	83036077	83038077	AI662270	-0.71574862	0.37374	lowCoverage	-0.19394	0.088928	insignificant	1	8	8
chr11	83104200	83106200	AA465934	-0.15548702	0.2283	insignificant	0.0035922	0.18307	insignificant	6	32	32
chr11	83112479	83114479	Pex12	-0.47651144	1	insignificant	-0.052449	0.5939	insignificant	2	27	31
chr11	83115198	83117198	Ap2b1	-0.14005199	0.00000352	hypomethylated	-0.014451	0.89381	insignificant	19	63	70
chr11	83222573	83224573	Rasl10b	-0.08493363	3.81E-11	hypomethylated	-0.014542	0.4367	insignificant	31	86	106
chr11	83242957	83244957	Gas2l2	-0.41038946	0.064884	insignificant	0.035993	0.637	insignificant	1	18	25
chr11	83276463	83278463	Mmp28	-0.1049435	0.00000714	hypomethylated	0.030952	1	insignificant	5	10	10
chr11	83285609	83287609	Taf15	-0.10979488	5.04E-08	hypomethylated	0.0020308	0.41435	insignificant	29	158	151
chr11	83566138	83568138	Heatr6	-0.12093327	1.48E-21	hypomethylated	0.0026165	0.079139	insignificant	27	70	68
chr11	83663370	83665370	Hnf1b	-0.10070291	0.0013927	hypomethylated	-0.0094571	0.36464	insignificant	22	100	100
chr11	83754591	83756591	Ddx52	-0.12270142	0.0030868	hypomethylated	0.0095305	0.88594	insignificant	20	55	55
chr11	83776929	83778929	Synrg	-0.09237175	6.02E-12	hypomethylated	-0.00059528	0.055567	insignificant	28	87	95
chr11	83776972	83778972	Synrg	-0.09237175	6.02E-12	hypomethylated	-0.00059528	0.055567	insignificant	28	87	95
chr11	83881859	83883859	Dusp14	-0.10566101	1.77E-24	hypomethylated	0.0014374	0.022886	hypermethylated	20	58	58
chr11	83943070	83945070	Tada2a	-0.11772214	0.000010061	hypomethylated	-0.002458	0.051192	insignificant	14	72	72
chr11	83980978	83982978	Gm11437		1	noCoverage	-0.23889	1	insignificant	0	6	4
chr11	84327003	84329003	Aatf	-0.10304024	1.75E-09	hypomethylated	0.0010814	0.41258	insignificant	20	69	69
chr11	84339036	84341036	Lhx1	-0.08653248	0.001815	hypomethylated	0.015152	0.24515	insignificant	14	85	85
chr11	84633017	84635017	Mrm1	-0.1880661	0.00021324	hypomethylated	0.022287	0.55637	insignificant	3	10	10
chr11	84642505	84644505	Dhrs11	-0.2437657	9.35E-24	hypomethylated	0.019928	0.094981	insignificant	13	56	56
chr11	84684240	84686240	Ggnbp2	-0.13264438	0.000000532	hypomethylated	-0.001785	0.36512	insignificant	11	60	60
chr11	84692721	84694721	Myo19	-0.11055281	7.02E-19	hypomethylated	0.0033324	0.52085	insignificant	39	136	137
chr11	84693697	84695697	Pigw	-0.1527307	1.86E-12	hypomethylated	-0.0045478	0.57666	insignificant	17	66	66
chr11	84693787	84695787	Pigw	-0.14881554	0.00000325	hypomethylated	-0.0059879	0.48111	insignificant	9	50	50
chr11	84729858	84731858	Znhit3		1	noCoverage	-0.0091667	1	insignificant	0	10	10
chr11	84770255	84772255	Car4	-0.23977998	0.0012031	hypomethylated	-0.0052018	0.30309	insignificant	4	38	38
chr11	84953457	84955457	Usp32	-0.12843436	6.69E-08	hypomethylated	0.00023164	0.37549	insignificant	11	49	49
chr11	84983597	84985597	1700125H20R	-0.15877232	5.17E-10	hypomethylated	0.0166	0.50732	insignificant	10	22	22
chr11	85047667	85049667	D630032N06R	-0.08732918	1.05E-23	hypomethylated	-0.010253	0.70024	insignificant	70	224	226
chr11	85048622	85050622	Appbp2	-0.1999538	0.16752	insignificant	-0.028145	0.11181	insignificant	15	56	58
chr11	85123755	85125755	Ppm1d	-0.09342052	4.02E-26	hypomethylated	0.01956	0.57378	insignificant	33	154	146
chr11	85165665	85167665	Bcas3	-0.17337379	0.00080951	hypomethylated	-0.0081674	0.93879	insignificant	12	55	50
chr11	85645116	85647116	Tbx2	-0.14419714	1.63E-28	hypomethylated	-0.0099051	0.0084061	hypomethylated	30	141	141
chr11	85702564	85704564	Tbx4	-0.08435866	7.83E-13	hypomethylated	0.0010487	0.69322	insignificant	17	65	59
chr11	86014695	86016695	Brip1	-0.11326678	0.000016584	hypomethylated	0.055768	0.31173	insignificant	16	81	87
chr11	86071070	86073070	Ints2	-0.17633289	6.11E-10	hypomethylated	-0.015426	0.48278	insignificant	4	28	26
chr11	86171027	86173027	Med13	-0.23374392	5.07E-25	hypomethylated	0.033811	0.0065319	inconclusive	22	74	82
chr11	86297158	86299158	Rnft1	-0.11423463	5.37E-12	hypomethylated	-0.0069334	0.2838	insignificant	22	88	100
chr11	86357492	86359492	Tubd1	-0.12938981	5E-22	hypomethylated	0.020135	0.9306	insignificant	36	130	123
chr11	86358309	86360309	Rps6kb1	-0.15602473	5.32E-10	hypomethylated	0.03441	0.13133	insignificant	11	49	46
chr11	86496484	86498484	Ptrh2	-0.10290983	7.19E-19	hypomethylated	0.0057847	0.57213	insignificant	31	104	103
chr11	86496582	86498582	Ptrh2	-0.10290983	7.19E-19	hypomethylated	0.0057847	0.57213	insignificant	31	104	103
chr11	86497324	86499324	Ptrh2	-0.14543802	7.89E-12	hypomethylated	0.0031439	0.27745	insignificant	15	56	59
chr11	86570994	86572994	Cltc	-0.16246172	0.0046789	hypomethylated	-0.017715	0.61203	insignificant	5	50	62
chr11	86621162	86623162	Dhx40	0.15389151	0.62496	insignificant	-0.012122	0.25793	insignificant	1	8	12
chr11	86807264	86809264	Ypel2	-0.50793651	0.25684	insignificant	-0.039187	0.71419	insignificant	1	14	16
chr11	86887639	86889639	Gdpd1		1	noCoverage	-0.0057745	0.85348	insignificant	0	20	30
chr11	86900279	86902279	1200011M11F	-0.50947838	8.46E-28	stronglyHypometh	-0.0278	0.70009	insignificant	6	22	20
chr11	86921762	86923762	Fam33a		1	noCoverage	0.064288	0.81716	insignificant	0	35	23
chr11	86922216	86924216	Prr11		1	noCoverage	0.048214	0.81781	insignificant	0	30	23
chr11	86939578	86941578	Trim37	-0.10424012	4.27E-12	hypomethylated	0.0030099	0.48288	insignificant	30	122	121
chr11	87172496	87174496	Ppm1e	-0.10229462	0.0047999	hypomethylated	0.010018	0.82531	insignificant	17	58	55
chr11	87217566	87219566	Tex14	-0.06081694	0.54951	insignificant	0.005516	0.29881	insignificant	3	45	43
chr11	87217997	87219997	Rad51c	-0.06081694	0.54951	insignificant	0.005516	0.29881	insignificant	3	45	43
chr11	87240092	87242092	Tex14	-0.35391156	1	lowCoverage	-0.072556	0.049742	hypomethylated	1	14	14

chr11	87393632	87395632	###########	-0.03309524	0.77532	insignificant	0.08	0.42189	insignificant	2	4	4
chr11	87404718	87406718	Mtmr4	-0.12282664	5.8E-09	hypomethylated	0.0099049	0.797	insignificant	32	120	129
chr11	87429665	87431665	Hsf5	0.01833981	0.81705	insignificant	0.00057108	0.19576	insignificant	11	130	114
chr11	87550066	87552066	Supt4h1	-0.15419117	5.79E-08	hypomethylated	0.058957	0.38148	insignificant	15	50	43
chr11	87569365	87571365	Mir142	-0.19386819	0.30269	insignificant	0.013533	0.20981	insignificant	8	40	38
chr11	87573042	87575042	Bzrap1	-0.25528006	9.5E-10	hypomethylated	0.047574	0.61803	insignificant	6	24	24
chr11	87665726	87667726	Mks1	-0.22198107	2.17E-10	hypomethylated	0.10337	0.0054796	hypermethylated	9	22	26
chr11	87689038	87691038	Epx		1	noCoverage	0.10379	1	insignificant	0	4	3
chr11	87800087	87802087	Dynll2	-0.08793085	0.00012941	hypomethylated	0.056325	0.51858	insignificant	15	96	122
chr11	87801035	87803035	Dynll2	-0.05215139	1	insignificant	0.13601	0.94081	insignificant	5	29	46
chr11	87860172	87862172	Srsf1	-0.15566498	7.02E-12	hypomethylated	-0.036838	0.56162	insignificant	30	173	155
chr11	87880842	87882842	Vezf1	-0.08804584	1.09E-26	hypomethylated	0.011901	0.8991	insignificant	42	156	137
chr11	87911647	87913647	Cuedc1	-0.11268407	2.37E-43	hypomethylated	0.0071474	0.81255	insignificant	55	158	148
chr11	87982066	87984066	Cuedc1	-0.02795808	0.064888	insignificant	-0.0019406	0.40694	insignificant	12	66	72
chr11	88016919	88018919	Mrps23	-0.23421894	3.21E-25	hypomethylated	-0.0055138	0.54398	insignificant	14	66	66
chr11	88106544	88108544	1700106J16Ri	0.38055556	0.15521	insignificant	0.027428	0.034348	inconclusive	1	16	22
chr11	88579543	88581543	Msi2	-0.11068863	0.032166	hypomethylated	-0.0039313	0.53323	insignificant	14	84	90
chr11	88725900	88727900	Akap1	0.06178642	0.022101	hypermethylated	0.040562	0.8006	insignificant	8	54	52
chr11	88816756	88818756	Scpep1		1	noCoverage	0.092012	0.059412	insignificant	0	15	18
chr11	88828231	88830231	Gm15698		1	noCoverage	0.21767	0.01256	hypermethylated	0	10	12
chr11	88834248	88836248	Coil	-0.17442218	2.95E-24	hypomethylated	-0.00024738	0.21597	insignificant	13	75	75
chr11	88859716	88861716	Trim25		1	noCoverage	-0.018185	0.13594	insignificant	0	31	33
chr11	88922062	88924062	Dgke	-0.35254907	0.01442	stronglyHypometh	-0.017685	0.45611	insignificant	4	19	19
chr11	89163873	89165873	Nog	-0.12106577	5.96E-12	hypomethylated	0.0081407	0.88758	insignificant	20	75	71
chr11	89864208	89866208	Pctp	-0.12890625	0.57249	insignificant	0.00029113	1	insignificant	2	8	8
chr11	89890661	89892661	Tmem100	-0.58674939	0.52145	insignificant	-0.29116	0.20764	insignificant	1	20	26
chr11	90109789	90111789	Mmd	-0.07260696	0.000047089	hypomethylated	0.01797	0.17293	insignificant	27	105	98
chr11	90252231	90254231	Hlf	0.61791235	0.000000571	stronglyHypermeth	0.011282	0.40045	insignificant	2	16	16
chr11	90498497	90500497	Cox11	-0.13314613	4.87E-13	hypomethylated	-0.0033236	0.30209	insignificant	20	101	105
chr11	90499422	90501422	Stxbp4	-0.12901954	3.1E-12	hypomethylated	-0.007682	0.41181	insignificant	19	76	82
chr11	90548915	90550915	Tom1l1	-0.05403419	0.0031998	hypomethylated	0.011819	0.82503	insignificant	13	46	46
chr11	92959603	92961603	Car10	-0.18568902	1.69E-19	hypomethylated	-0.012096	0.97116	insignificant	32	114	114
chr11	93746532	93748532	Mbtd1	-0.04427081	7.11E-12	hypomethylated	0.015849	0.83574	insignificant	47	177	180
chr11	93747080	93749080	Utp18	0.01858822	0.000000621	inconclusive	0.036098	0.77023	insignificant	19	76	84
chr11	93817002	93819002	Nme2	-0.15130853	0.15323	insignificant	0.012844	0.63096	insignificant	16	58	58
chr11	93817321	93819321	Nme2	-0.09429825	1	insignificant	0.027924	0.020726	hypermethylated	2	10	10
chr11	93856404	93858404	Spag9	-0.11071544	4.03E-37	hypomethylated	0.017869	0.0053432	hypermethylated	52	163	151
chr11	93904518	93906518	Spag9		1	noCoverage	0.024045	0.76898	insignificant	0	14	15
chr11	94071767	94073767	Tob1	-0.10897662	5.14E-19	hypomethylated	0.012418	0.76792	insignificant	40	180	179
chr11	94103893	94105893	Wfikkn2	-0.07060264	0.15887	insignificant	0.061825	0.080744	insignificant	7	31	30
chr11	94183225	94185225	Luc7l3	-0.17078479	8.64E-16	hypomethylated	-0.014224	0.33723	insignificant	24	55	58
chr11	94188314	94190314	Ankrd40	-0.14848851	1.32E-23	hypomethylated	-0.0013766	0.58062	insignificant	20	79	79
chr11	94254290	94256290	Abcc3	-0.23596702	0.00064819	hypomethylated	0.05757	0.69762	insignificant	3	20	20
chr11	94335512	94337512	Cacna1g	-0.11526722	1.02E-27	hypomethylated	0.014128	0.48833	insignificant	31	115	114
chr11	94346624	94348624	Spata20	-0.15974978	0.095413	insignificant	0.0066348	0.020346	hypermethylated	3	22	22
chr11	94361288	94363288	Epn3	-0.02515958	0.27521	insignificant	0.056961	0.32673	insignificant	6	32	35
chr11	94382816	94384816	Mycbpap	-0.31396366	0.065931	insignificant	0.025751	0.0062689	hypermethylated	9	26	28
chr11	94410521	94412521	Rsad1	-0.19634002	0.0052549	hypomethylated	-0.029673	0.54989	insignificant	4	11	8
chr11	94425387	94427387	Chad	-0.06749235	0.15469	insignificant	0.012	0.82533	insignificant	9	69	70
chr11	94463100	94465100	Acsf2	-0.18836526	0.0054695	hypomethylated	0.026935	0.00071324	hypermethylated	4	34	36
chr11	94490137	94492137	Lrrc59	-0.10023326	1.12E-13	hypomethylated	0.0008258	1	insignificant	26	109	115
chr11	94514104	94516104	Mrpl27	-0.24373212	0.00000215	hypomethylated	-0.051308	0.15425	insignificant	12	32	32
chr11	94515068	94517068	Eme1	-0.24182453	0.00048926	hypomethylated	-0.054386	0.27366	insignificant	7	23	22
chr11	94796583	94798583	Col1a1		1	noCoverage	0.14449	0.92396	insignificant	0	12	12
chr11	94827974	94829974	Hils1	0.10342556	1	insignificant	0.14111	0.15714	insignificant	1	11	14
chr11	94851525	94853525	Ppp1r9b	-0.08924618	1.02E-22	hypomethylated	-0.0083532	0.72508	insignificant	52	197	214
chr11	94870192	94872192	Samd14	-0.12314048	4.56E-23	hypomethylated	0.037426	0.0061856	hypermethylated	23	100	93
chr11	94902685	94904685	Pdk2	-0.082293	0.01723	hypomethylated	-0.031867	0.079318	insignificant	3	24	24
chr11	94938028	94940028	Itga3	0.13527993	0.00042549	hypermethylated	-0.051902	0.046438	inconclusive	7	66	79
chr11	94980430	94982430	Dlx3	-0.14735855	3.02E-19	hypomethylated	-0.010603	0.6951	insignificant	34	120	118
chr11	95007115	95009115	A730090H04R	0.44080894	2.15E-11	stronglyHypermeth	0.0060062	0.55962	insignificant	2	46	46
chr11	95171560	95173560	Myst2	-0.17910325	1.16E-11	hypomethylated	0.016931	0.13391	insignificant	7	31	32
chr11	95197331	95199331	Fam117a	-0.17483229	4.7E-28	hypomethylated	0.0044286	0.3619	insignificant	27	78	84
chr11	95245235	95247235	Slc35b1	-0.31237221	9.11E-10	hypomethylated	-0.017086	0.69907	insignificant	6	42	42
chr11	95274396	95276396	Spop	-0.01346653	3.56E-08	hypomethylated	0.11007	0.34668	insignificant	8	47	56
chr11	95375879	95377879	Nxph3	-0.07704105	0.00000281	hypomethylated	-0.0038696	0.58638	insignificant	18	70	72
chr11	95449012	95451012	Ngfr	-0.17830882	0.0012978	hypomethylated	0.088867	0.65342	insignificant	3	16	14
chr11	95527270	95529270	Phb	-0.03376921	0.37339	insignificant	-0.13476	0.54203	insignificant	4	16	21
chr11	95609380	95611380	Zfp652	-0.30760363	0.0086884	hypomethylated	-0.10389	0.76536	insignificant	3	25	25
chr11	95684813	95686813	Phospho1	-0.13527199	1.41E-37	hypomethylated	-0.0023769	0.46823	insignificant	30	131	124
chr11	95776185	95778185	B4galnt2	-0.19742854	9.28E-10	hypomethylated	0.020315	0.0027679	hypermethylated	10	34	39
chr11	95867258	95869258	Igf2bp1	-0.1515087	1.92E-15	hypomethylated	-0.0067679	0.93594	insignificant	30	189	196
chr11	95895230	95897230	Snf8	-0.17161099	1.29E-18	hypomethylated	-0.013448	0.072113	insignificant	21	48	48
chr11	95926678	95928678	Ube2z	-0.5853533	0.039846	stronglyHypometh	-0.11611	0.13778	insignificant	1	40	44
chr11	95936945	95938945	Atp5g1	-0.4682197	0.011157	stronglyHypometh	-0.039344	1	insignificant	5	20	18
chr11	95937008	95939008	Atp5g1	-0.4682197	0.011157	stronglyHypometh	-0.039344	1	insignificant	5	20	18
chr11	95994099	95996099	Ttll6		1	noCoverage	-0.2017	1	insignificant	0	8	4
chr11	96054674	96056674	Hoxb13	-0.13692715	0.000041997	hypomethylated	0.0072849	0.00053522	hypermethylated	11	65	65
chr11	96125477	96127477	Mir196a-1	-0.1460552	0.75207	insignificant	-0.059329	0.43372	insignificant	6	32	32
chr11	96131643	96133643	Hoxb9	-0.12343509	4.08E-27	hypomethylated	0.010773	0.076934	insignificant	30	117	120
chr11	96142218	96144218	Hoxb8	-0.18502695	0.43965	insignificant	-0.1122	0.47293	insignificant	3	19	26
chr11	96146959	96148959	Hoxb7	-0.18743742	1.26E-51	hypomethylated	-0.0031805	0.035861	inconclusive	39	136	133
chr11	96159484	96161484	Hoxb6	-0.18052567	8.11E-10	hypomethylated	0.0091582	0.64809	insignificant	11	46	46
chr11	96163825	96165825	Hoxb5	-0.14875318	0.0019903	hypomethylated	0.013767	0.2595	insignificant	13	33	32
chr11	96177478	96179478	Mir10a		1	noCoverage	-0.03523	0.0052974	inconclusive	0	32	33

chr11	96178580	96180580	Hoxb4	-0.18744688	7.52E-51	hypomethylated	0.0053335	0.010037	inconclusive	20	69	62
chr11	96183439	96185439	Hoxb3	0.17777778	1	lowCoverage	-0.12016	0.32585	insignificant	1	10	10
chr11	96204082	96206082	Hoxb3	-0.01589218	0.090204	insignificant	-0.072429	0.029077	hypomethylated	6	39	29
chr11	96211945	96213945	Hoxb2	-0.06202435	0.042793	hypomethylated	-0.0039699	0.5477	insignificant	5	42	42
chr11	96226071	96228071	Hoxb1	-0.22149737	1.43E-14	hypomethylated	0.053088	0.21942	insignificant	3	39	39
chr11	96324904	96326904	Skap1	-0.1000773	0.000025723	hypomethylated	0.041284	0.0082777	inconclusive	3	14	30
chr11	96325861	96327861	Gm11529	-0.1000773	0.000025723	hypomethylated	-0.015846	0.0058062	hypomethylated	3	14	28
chr11	96638869	96640869	Snx11	-0.08229355	7.57E-16	hypomethylated	-0.013924	0.3554	insignificant	17	58	58
chr11	96649449	96651449	Cbx1	-0.15708252	3.93E-13	hypomethylated	-0.014048	0.15539	insignificant	11	49	43
chr11	96685327	96687327	Nfe2l1	-0.09615385	0.75518	insignificant	-0.38901	0.22875	insignificant	1	2	5
chr11	96685441	96687441	Nfe2l1	-0.51121795	0.24751	insignificant	-0.24336	0.80886	insignificant	2	6	9
chr11	96690816	96692816	Nfe2l1	-0.14185614	3.33E-08	hypomethylated	0.00071662	0.38104	insignificant	12	90	89
chr11	96691282	96693282	Nfe2l1	-0.12614196	0.016647	hypomethylated	0.015396	0.30744	insignificant	6	58	57
chr11	96710189	96712189	Copz2	-0.14245742	0.037609	hypomethylated	0.032431	0.019134	hypermethylated	11	73	81
chr11	96710706	96712706	Mir152	-0.16828428	0.00046095	hypomethylated	0.042086	0.020523	hypermethylated	10	68	77
chr11	96777795	96779795	Cdk5rap3	-0.05370731	0.0013886	hypomethylated	0.0020219	0.037563	hypermethylated	5	52	55
chr11	96804459	96806459	D030028A08R	-0.21806426	0.0012361	hypomethylated	-0.016486	1	insignificant	4	16	16
chr11	96805333	96807333	Pnpo	-0.24939046	0.0012147	hypomethylated	0.06261	0.011159	hypermethylated	4	18	20
chr11	96839002	96841002	Sp2	-0.15975479	0.000096775	hypomethylated	-0.020836	0.57099	insignificant	4	50	43
chr11	96873882	96875882	Sp6		1	noCoverage	-0.021212	1	insignificant	0	2	5
chr11	96890265	96892265	Scrn2	-0.20819231	3.09E-12	hypomethylated	-0.00020728	0.055259	insignificant	13	48	47
chr11	96901899	96903899	Mrpl10	0.13926904	0.035287	hypermethylated	-0.011389	0.72864	insignificant	4	38	38
chr11	96902683	96904683	Lrrc46	0.15940076	0.034293	hypermethylated	-0.033272	0.67522	insignificant	4	37	36
chr11	96911133	96913133	Osbpl7	-0.21783534	2.2E-21	hypomethylated	0.027134	1	insignificant	9	40	43
chr11	97011026	97013026	Tbkbp1	-0.06958438	8.64E-26	hypomethylated	0.0066835	0.13685	insignificant	67	215	237
chr11	97049206	97051206	Kpnb1	-0.11664325	7.38E-37	hypomethylated	0.00012177	0.055294	insignificant	29	138	127
chr11	97141890	97143890	Npepps		1	noCoverage	0.14286	0.36424	insignificant	0	9	6
chr11	97222864	97224864	Socs7	-0.11858525	9.68E-42	hypomethylated	0.0079588	0.06586	insignificant	62	168	168
chr11	97310473	97312473	Arhgap23	-0.09097744	0.20227	insignificant	-0.10909	0.0016616	hypomethylated	1	2	2
chr11	97436440	97438440	Srcin1	-0.09952506	9.71E-09	hypomethylated	-0.0097999	0.32	insignificant	32	88	96
chr11	97491030	97493030	E130012A19R	-0.21217262	0.00804	hypomethylated	-0.053052	0.3578	insignificant	8	43	44
chr11	97523725	97525725	Mllt6	-0.14361434	3.4E-27	hypomethylated	-0.0049825	0.00000179	inconclusive	31	91	106
chr11	97546265	97548265	Cisd3	-0.13779976	1.17E-18	hypomethylated	-0.022565	0.58381	insignificant	17	66	66
chr11	97560698	97562698	Pcgf2	-0.15874349	5.68E-28	hypomethylated	0.0074885	0.45937	insignificant	34	126	115
chr11	97560962	97562962	Pcgf2	-0.17551373	1.5E-15	hypomethylated	0.016066	0.24369	insignificant	18	92	81
chr11	97561811	97563811	Pcgf2	-0.16906582	5.81E-16	hypomethylated	0.026936	0.78314	insignificant	14	68	61
chr11	97563747	97565747	Psmb3		1	noCoverage	0.02732	0.41685	insignificant	0	58	52
chr11	97606018	97608018	Cwc25	-0.16818978	4.32E-10	hypomethylated	-0.023791	0.65105	insignificant	16	92	86
chr11	97637232	97639232	1700001P01Ri	-0.03870032	0.35971	insignificant	-0.14268	1	insignificant	1	14	15
chr11	97643080	97645080	Rpl23	-0.14996302	0.000000184	hypomethylated	0.0033808	0.52908	insignificant	19	96	100
chr11	97643753	97645753	Rpl23	0.3055538	0.29172	insignificant	0.007385	0.33954	insignificant	2	27	27
chr11	97659985	97661985	Lasp1	-0.16697301	1.4E-20	hypomethylated	-0.030575	0.42955	insignificant	29	93	130
chr11	97701093	97703093	B230217C12R	-0.32112364	0.55543	insignificant	-0.087304	0.56433	insignificant	3	25	31
chr11	97745468	97747468	Fbxo47	-0.19184728	0.00000755	hypomethylated	-0.0061115	0.92464	insignificant	3	18	18
chr11	97847760	97849760	Plxdc1	-0.1101895	0.027119	hypomethylated	-0.0041238	0.051866	insignificant	9	57	53
chr11	97857487	97859487	Arl5c	0.13079844	1	lowCoverage	0.016574	0.68826	insignificant	1	8	8
chr11	97879686	97881686	Cacnb1	-0.72627551	0.0014581	stronglyHypometh	-0.028855	1	insignificant	2	7	10
chr11	97883941	97885941	Cacnb1	-0.30694836	3.64E-21	hypomethylated	0.0083058	0.12728	insignificant	11	65	65
chr11	97887023	97889023	Rpl19	-0.16467511	4.04E-14	hypomethylated	0.0069895	0.94783	insignificant	22	105	98
chr11	97887237	97889237	Rpl19	-0.16467511	4.04E-14	hypomethylated	0.0069895	0.94783	insignificant	22	105	98
chr11	97914776	97916776	Stac2		1	noCoverage	0.042659	0.53277	insignificant	0	13	14
chr11	98010930	98012930	Fbxl20	-0.10655637	0.00000706	hypomethylated	0.0080358	0.22438	insignificant	23	97	104
chr11	98054220	98056220	Med1	-0.16992498	0.00031242	hypomethylated	0.002807	0.1844	insignificant	9	56	55
chr11	98054607	98056607	Med1		1	noCoverage	-0.013024	0.39525	insignificant	0	20	19
chr11	98063618	98065618	Cdk12	-0.08665086	0.000000382	hypomethylated	-0.0027166	0.348	insignificant	23	118	123
chr11	98190959	98192959	Neurod2	-0.35018751	1	insignificant	-0.057858	0.20022	insignificant	2	7	6
chr11	98209051	98211051	Ppp1r1b	-0.05119187	3.87E-10	hypomethylated	0.0077624	0.46096	insignificant	30	117	117
chr11	98218697	98220697	1700003D09R	-0.12025694	8.34E-19	hypomethylated	-0.0095792	0.37527	insignificant	21	67	65
chr11	98246945	98248945	Pnmt	-0.16368247	0.00000077	hypomethylated	-0.042782	0.07133	insignificant	15	44	43
chr11	98261804	98263804	Pgap3	-0.31122004	0.0045643	hypomethylated	-0.0040626	0.39986	insignificant	4	10	10
chr11	98272797	98274797	Erbb2	-0.14708146	3.24E-21	hypomethylated	-0.0015598	0.39872	insignificant	39	115	118
chr11	98300302	98302302	1810046J19Ri	-0.18711002	0.0022872	hypomethylated	0.042482	1	insignificant	5	16	14
chr11	98307147	98309147	Grb7	-0.15390169	0.038546	hypomethylated	0.051768	0.72714	insignificant	5	36	33
chr11	98407345	98409345	Ikzf3	-0.42468891	0.34419	insignificant	0.038135	0.6577	insignificant	5	22	22
chr11	98411410	98413410	Zpbp2	-0.16586335	5.85E-09	hypomethylated	0.010636	0.6148	insignificant	7	14	14
chr11	98448559	98450559	Ormdl3	-0.13648376	0.049363	hypomethylated	-0.0038826	0.96139	insignificant	16	52	52
chr11	98458604	98460604	Gm12	-0.14107332	0.65514	insignificant	0.074815	0.54185	insignificant	5	24	24
chr11	98486673	98488673	Gsdma3		1	noCoverage	0.0026761	0.11654	insignificant	0	7	10
chr11	98542867	98544867	Psmd3	-0.14287911	2.69E-22	hypomethylated	0.00083217	0.04714	hypermethylated	24	87	89
chr11	98561626	98563626	Csf3	0.25368401	1	insignificant	0.002647	0.88395	insignificant	1	18	18
chr11	98590749	98592749	Med24	-0.52631579	0.0081358	stronglyHypometh	0.030607	0.25151	insignificant	1	2	2
chr11	98602186	98604186	Thra	-0.122673	3.65E-13	hypomethylated	-0.0003793	0.03017	inconclusive	16	76	86
chr11	98636556	98638556	Nr1d1	-0.15042315	0.0046929	hypomethylated	-0.012617	0.42976	insignificant	2	18	18
chr11	98656082	98658082	Msl1	-0.12812719	3.11E-36	hypomethylated	0.00034698	0.96082	insignificant	74	229	235
chr11	98670121	98672121	Casc3	-0.1250327	4.54E-13	hypomethylated	-0.0035765	0.86112	insignificant	20	91	97
chr11	98671403	98673403	Casc3	-0.0489441	0.5512	insignificant	-0.0040634	0.70605	insignificant	5	32	32
chr11	98697098	98699098	Rapgefl1	-0.10303591	8.38E-24	hypomethylated	0.011617	0.36958	insignificant	48	166	167
chr11	98723911	98725911	Wipf2	-0.02902795	0.003856	hypomethylated	0.0058243	0.7414	insignificant	11	83	90
chr11	98768202	98770202	Cdc6	-0.16877833	9.57E-11	hypomethylated	0.016503	0.67406	insignificant	12	45	45
chr11	98768464	98770464	Cdc6	-0.16510924	9.55E-11	hypomethylated	0.033692	0.64679	insignificant	12	46	52
chr11	98798009	98800009	Rara	-0.17722224	5.5E-46	hypomethylated	0.0022378	0.72347	insignificant	34	98	96
chr11	98798031	98800031	Rara	-0.17722224	5.5E-46	hypomethylated	0.0022378	0.72347	insignificant	34	98	96
chr11	98800024	98802024	Rara	-0.60588998	0.016022	stronglyHypometh	0.04878	0.65034	insignificant	1	13	10
chr11	98820784	98822784	Rara	-0.10384802	2.86E-23	hypomethylated	0.017851	0.54591	insignificant	42	126	121
chr11	98844330	98846330	Gjd3	-0.10870351	0.028277	hypomethylated	-0.097596	0.38649	insignificant	3	18	18

chr11	98885503	98887503	Top2a		1	noCoverage	-0.014809	1	insignificant	0	14	14
chr11	98901573	98903573	Igfbp4	-0.17013509	1.48E-14	hypomethylated	0.071223	0.13288	insignificant	17	49	37
chr11	99092331	99094331	Smarce1	-0.23108859	0.68474	insignificant	-0.078172	0.94884	insignificant	3	31	30
chr11	99146552	99148552	Krt24	-0.18605878	0.60119	insignificant	0.069089	0.5561	insignificant	4	17	16
chr11	99184255	99186255	Krt25	0.21740443	0.37702	insignificant	0.074212	1	insignificant	4	15	15
chr11	99199279	99201279	Krt26	0.07595671	0.33885	insignificant	-0.0037269	0.52056	insignificant	3	30	22
chr11	99212408	99214408	Krt27	0.08842082	1	lowCoverage	-0.034305	0.13478	insignificant	1	10	7
chr11	99236217	99238217	Krt28	0.2122807	0.40597	insignificant	0.13616	0.066155	insignificant	2	4	4
chr11	99354424	99356424	Krt23	0.10560224	1	insignificant	0.16493	0.8641	insignificant	1	6	8
chr11	99382572	99384572	Krt39	0.08297027	0.4578	insignificant	-0.11303	0.20032	insignificant	6	26	26
chr11	99412177	99414177	Krtap3-3		1	noCoverage	-0.13889	0.19686	insignificant	0	4	2
chr11	99418167	99420167	Krtap3-2		1	noCoverage	0.09375	0.66441	insignificant	0	4	4
chr11	99452653	99454653	Krtap1-3	0.13560606	0.66686	insignificant	0.064574	0.48385	insignificant	1	5	5
chr11	99505403	99507403	Krtap4-7	-0.14442873	0.34317	insignificant	-0.0097289	0.65327	insignificant	1	8	7
chr11	99957538	99959538	Krt35	-0.05830578	0.012743	hypomethylated	0.03578	0.36172	insignificant	7	30	30
chr11	100007233	100009233	Krt19	-0.15	0.66815	insignificant	0.16548	0.092366	insignificant	2	6	6
chr11	100068824	100070824	Krt14	-0.07620566	0.19908	insignificant	0.0597	0.71247	insignificant	2	6	6
chr11	100122303	100124303	Krt17	-0.58041958	0.41333	lowCoverage	0.036768	0.87445	insignificant	1	4	4
chr11	100180309	100182309	Eif1	-0.10247749	0.015437	hypomethylated	0.013005	0.90391	insignificant	12	86	82
chr11	100217455	100219455	Hap1	-0.16264619	0.006819	hypomethylated	0.11957	0.54339	insignificant	2	18	19
chr11	100259053	100261053	Jup	-0.27376264	9.04E-12	hypomethylated	0.005746	0.31125	insignificant	5	32	31
chr11	100276007	100278007	Fkbp10	-0.20070606	1.33E-13	hypomethylated	-0.0089685	0.43561	insignificant	16	61	61
chr11	100276133	100278133	Fkbp10	-0.20945354	1.22E-08	hypomethylated	-0.0085556	1	insignificant	12	53	53
chr11	100302237	100304237	Klhl10	-0.42350746	2.18E-09	stronglyHypometh	-0.36132	0.0019325	stronglyhypometh	8	15	26
chr11	100302403	100304403	Klhl10	-0.26285795	1.41E-08	hypomethylated	-0.21347	0.27088	insignificant	6	15	19
chr11	100334096	100336096	Klhl11		1	noCoverage	-0.0059777	0.46641	insignificant	0	26	24
chr11	100389314	100391314	Acly	-0.25050051	0.00000694	hypomethylated	0.027676	0.56996	insignificant	6	25	26
chr11	100405945	100407945	Ttc25	-0.14942979	8.71E-11	hypomethylated	-0.0082002	0.76669	insignificant	8	28	28
chr11	100435252	100437252	Cnp	-0.09422484	5.71E-10	hypomethylated	0.018234	0.79619	insignificant	19	63	57
chr11	100436204	100438204	Cnp	-0.22154931	2.97E-27	hypomethylated	0.058283	0.019604	hypermethylated	19	95	91
chr11	100483268	100485268	Nkiras2	-0.18427653	5.43E-37	hypomethylated	0.037148	1	insignificant	14	47	47
chr11	100565585	100567585	Dhx58	0.06958184	0.024014	hypermethylated	0.040635	0.000045875	hypermethylated	8	24	25
chr11	100573781	100575781	Kat2a	-0.1886132	1.42E-09	hypomethylated	0.055408	0.14215	insignificant	13	53	52
chr11	100574163	100576163	Hspb9	-0.19784374	1.41E-09	hypomethylated	0.031354	0.26292	insignificant	13	48	51
chr11	100599444	100601444	Rab5c	-0.20537303	0.00011323	inconclusive	-0.013579	0.3472	insignificant	11	64	60
chr11	100621092	100623092	Kcnh4	-0.20308521	1.2E-09	hypomethylated	-0.032429	0.00010342	hypomethylated	6	16	18
chr11	100624245	100626245	Hcrt		1	noCoverage	-0.052821	0.27678	insignificant	0	10	6
chr11	100632271	100634271	Ghdc	-0.45890569	0.0000011	stronglyHypometh	0.042369	0.42488	insignificant	2	15	16
chr11	100711899	100713899	Stat5b	-0.11382322	2.38E-13	hypomethylated	-0.0088946	0.010032	hypomethylated	23	93	93
chr11	100719664	100721664	Stat5a	-0.26363009	0.0021185	hypomethylated	0.0083836	1	insignificant	6	23	23
chr11	100720797	100722797	Stat5a	-0.16311953	0.016012	hypomethylated	0.0035458	0.79658	insignificant	9	67	65
chr11	100800825	100802825	Stat3	-0.10020036	0.0009519	hypomethylated	0.0091953	0.75518	insignificant	10	49	50
chr11	100831931	100833931	Ptrf	-0.26465099	5.96E-09	hypomethylated	0.03598	0.85678	insignificant	8	22	26
chr11	100869768	100871768	Atp6v0a1	-0.10446285	5.71E-11	hypomethylated	0.018356	0.15976	insignificant	16	76	79
chr11	100930407	100932407	Naglu	-0.099057	0.00000158	hypomethylated	0.00077352	0.077412	insignificant	7	35	40
chr11	100938724	100940724	Hsd17b1		1	noCoverage	0.066088	0.85721	insignificant	0	12	12
chr11	100942938	100944938	Coasy	-0.11528528	0.71611	insignificant	-0.039892	0.91294	insignificant	9	40	38
chr11	100947603	100949603	Mlx	0.20470529	0.50703	insignificant	0.066759	0.92136	insignificant	4	25	8
chr11	100956715	100958715	Fam134c	-0.21218684	7.63E-11	hypomethylated	-0.013182	0.32341	insignificant	15	67	67
chr11	100980444	100982444	Tubg1	-0.15434641	3.21E-38	hypomethylated	0.0041552	0.16359	insignificant	37	120	120
chr11	100981138	100983138	Fam134c	-0.13327372	4.59E-09	hypomethylated	-0.005066	0.24107	insignificant	14	46	46
chr11	100981157	100983157	Fam134c	-0.14537932	0.000075757	hypomethylated	-0.0050993	0.49243	insignificant	12	42	42
chr11	101016197	101018197	Tubg2	-0.06324988	0.8416	insignificant	0.049002	0.13292	insignificant	7	26	27
chr11	101032616	101034616	Plekhh3	-0.11262749	0.000013682	hypomethylated	-0.0037669	0.18725	insignificant	13	58	56
chr11	101036430	101038430	Cntnap1	-0.12426161	0.6947	insignificant	-0.041311	0.49071	insignificant	3	30	30
chr11	101036757	101038757	Ccr10	-0.11834327	0.53721	insignificant	-0.032114	0.74347	insignificant	4	38	37
chr11	101087764	101089764	Ezh1	0.30602782	0.5981	insignificant	0.078252	0.3272	insignificant	1	24	18
chr11	101106647	101108647	Ramp2	-0.15661669	9.82E-23	hypomethylated	0.010355	0.57541	insignificant	23	90	90
chr11	101114056	101116056	Vps25	-0.13147791	6.99E-10	hypomethylated	0.013049	0.67051	insignificant	9	34	32
chr11	101120880	101122880	Wnk4	-0.17194494	1.79E-39	hypomethylated	0.010578	0.83526	insignificant	31	124	124
chr11	101139516	101141516	Cntd1	-0.08023561	0.021944	hypomethylated	0.014672	0.001662	hypermethylated	13	61	57
chr11	101140262	101142262	Ccdc56	0.02092426	0.0043463	inconclusive	0.023447	0.035539	hypermethylated	5	34	31
chr11	101163581	101165581	Becn1	-0.38833333	0.029879	stronglyHypometh	-0.052669	0.90336	insignificant	3	16	13
chr11	101176564	101178564	Psme3	-0.18877011	3.71E-40	hypomethylated	0.0065297	0.11431	insignificant	30	71	74
chr11	101185376	101187376	Aoc2	0.075	1	insignificant	0.0041872	0.55544	insignificant	1	4	4
chr11	101190919	101192919	Aoc3	0.07730952	1	insignificant	0.076521	0.22873	insignificant	2	25	25
chr11	101228043	101230043	G6pc	-0.01703704	0.2479	insignificant	0.13841	0.46458	insignificant	2	8	7
chr11	101285398	101287398	Rundc1	-0.10376132	1.6E-13	hypomethylated	0.037442	0.038962	hypermethylated	20	96	101
chr11	101286647	101288647	1700113I22Ril	-0.12680622	0.000085041	hypomethylated	0.055814	0.001801	hypermethylated	16	60	65
chr11	101302558	101304558	Rpl27	-0.27585623	1.32E-46	hypomethylated	0.0077368	0.56661	insignificant	27	107	97
chr11	101308725	101310725	Ifi35	0.51232247	0.46667	lowCoverage	0.060082	0.19861	insignificant	1	10	10
chr11	101327513	101329513	Vat1	0.25481717	0.14058	insignificant	-0.013954	0.16275	insignificant	2	23	23
chr11	101328651	101330651	Rnd2	-0.18747536	6.06E-41	hypomethylated	-0.032065	0.90306	insignificant	21	63	63
chr11	101413171	101415171	Nbr1	-0.13638444	0.051132	insignificant	0.0085339	1	insignificant	2	45	45
chr11	101413269	101415269	Nbr1	-0.20124873	0.0040273	hypomethylated	0.0032447	0.57419	insignificant	2	27	27
chr11	101442555	101444555	Tmem106a	-0.28645136	8.52E-08	hypomethylated	0.010393	0.10786	insignificant	8	34	34
chr11	101488262	101490262	Rdm1	-0.13541667	0.000011167	hypomethylated	0.093155	0.68364	insignificant	6	24	10
chr11	101525854	101527854	Arl4d	-0.12813831	0.00000159	hypomethylated	0.01628	0.46054	insignificant	6	44	43
chr11	101593269	101595269	Dhx8	-0.24271924	0.000026073	hypomethylated	0.070286	0.65001	insignificant	5	31	36
chr11	101646624	101648624	Etv4	-0.03950076	2.34E-18	hypomethylated	-0.008962	0.020514	hypomethylated	6	36	36
chr11	101846105	101848105	Dusp3	-0.08317558	0.000098136	hypomethylated	-0.006227	1	insignificant	10	24	24
chr11	101888269	101890269	Mpp3	-0.08181326	0.000000823	hypomethylated	-0.017252	0.26371	insignificant	12	50	49
chr11	101949829	101951829	Mpp2	-0.34453223	0.1346	insignificant	0.072632	0.61466	insignificant	2	14	16
chr11	101962614	101964614	Ppy	-0.43479799	0.00071229	stronglyHypometh	0.013707	0.69757	insignificant	2	16	11
chr11	101969090	101971090	Pyy	-0.26619029	0.08002	insignificant	-0.068656	0.5596	insignificant	4	8	9

chr11	102005900	102007900	Nags	-0.15317402	2.1E-16	hypomethylated	-0.0092689	0.40573	insignificant	22	82	82
chr11	102006679	102008679	Nags	-0.15317402	2.1E-16	hypomethylated	-0.0092689	0.40573	insignificant	22	82	82
chr11	102017721	102019721	Tmem101	-0.25367647	0.0015225	hypomethylated	0.06299	0.1514	insignificant	4	8	8
chr11	102046570	102048570	Lsm12	-0.13347045	0.24381	insignificant	-0.026783	1	insignificant	9	26	25
chr11	102050012	102052012	G6pc3	-0.10821874	0.0027019	inconclusive	0.016631	0.88814	insignificant	14	43	52
chr11	102091486	102093486	Hdac5	-0.34615385	0.0027219	stronglyHypometh	0.0088882	1	insignificant	3	6	6
chr11	102109195	102111195	BC030867	-0.01389281	0.026145	inconclusive	0.027794	0.22867	insignificant	14	35	32
chr11	102145274	102147274	Tmub2	0.09076723	0.00000124	hypermethylated	0.058086	0.078158	insignificant	3	53	55
chr11	102157943	102159943	Atxn7l3	-0.16829386	5.06E-31	hypomethylated	0.00036967	0.72423	insignificant	24	105	79
chr11	102178601	102180601	Ubtf	-0.08818686	1.01E-44	hypomethylated	-0.0091524	0.66797	insignificant	99	268	282
chr11	102180410	102182410	Ubtf	-0.13805928	1.85E-14	hypomethylated	-0.014799	0.80656	insignificant	24	130	125
chr11	102226595	102228595	Slc4a1	0.26486045	1	lowCoverage	0.11067	0.25468	insignificant	1	14	18
chr11	102253748	102255748	Rundc3a	-0.1151777	6.74E-08	hypomethylated	0.047161	0.029372	hypermethylated	11	49	38
chr11	102268831	102270831	Slc25a39	-0.04839084	0.00000991	hypomethylated	0.022968	0.79672	insignificant	17	66	71
chr11	102290635	102292635	Grn	-0.15128476	0.2768	insignificant	0.019112	0.09934	insignificant	13	24	24
chr11	102308977	102310977	Fam171a2	-0.18733342	1	insignificant	0.017511	0.57859	insignificant	3	12	12
chr11	102331197	102333197	Itga2b	-0.33192439	1	lowCoverage	0.028414	0.14481	insignificant	1	12	12
chr11	102417472	102419472	Gpatch8	-0.10073934	6.36E-12	hypomethylated	0.053191	0.0037649	inconclusive	5	38	32
chr11	102464744	102466744	Fzd2	-0.18565927	0.086878	insignificant	-0.01193	0.29248	insignificant	12	87	87
chr11	102485730	102487730	2810433D01R	-0.0833925	0.34385	insignificant	-0.010532	0.27951	insignificant	1	12	12
chr11	102525901	102527901	Gm1564	0.0115488	0.10904	insignificant	0.023478	0.18955	insignificant	24	156	154
chr11	102559039	102561039	Ccdc43	-0.13442822	1.22E-20	hypomethylated	0.033475	0.10282	insignificant	23	54	52
chr11	102621752	102623752	Adam11	-0.15649757	6.53E-23	hypomethylated	0.015423	0.12663	insignificant	26	104	104
chr11	102680473	102682473	Gjc1	-0.09633955	2E-17	hypomethylated	0.015522	0.47744	insignificant	28	94	91
chr11	102681000	102683000	Gjc1		1	noCoverage	0.080113	0.33573	insignificant	0	15	15
chr11	102741557	102743557	Ccdc103	-0.17078876	2.64E-36	hypomethylated	0.0080196	0.40906	insignificant	27	93	90
chr11	102742289	102744289	Eftud2	-0.2288107	3.23E-13	hypomethylated	0.017959	0.68153	insignificant	15	44	44
chr11	102745482	102747482	Fam187a	0.01914701	0.7696	insignificant	0.14942	0.20705	insignificant	1	2	2
chr11	102758514	102760514	Gfap		1	noCoverage	0.020938	0.55621	insignificant	0	8	9
chr11	102786438	102788438	Kif18b	-0.02750974	0.15401	insignificant	0.057592	0.062692	insignificant	11	53	45
chr11	102807775	102809775	C1ql1	-0.13478256	8.53E-20	hypomethylated	0.0097258	0.54733	insignificant	17	42	51
chr11	102878461	102880461	Dcakd	-0.25806452	0.000043077	hypomethylated	-0.015913	0.9179	insignificant	4	10	7
chr11	102888875	102890875	Nmt1	-0.13188726	0.000745	hypomethylated	-0.0047926	0.50287	insignificant	5	30	30
chr11	102962001	102964001	Acbd4	-0.1671847	2.06E-09	hypomethylated	-0.010308	0.73532	insignificant	21	86	84
chr11	102962972	102964972	Plcd3	-0.18356731	1.43E-12	hypomethylated	0.0030117	0.85872	insignificant	21	89	91
chr11	102976638	102978638	Hexim1	-0.13388481	0.036009	hypomethylated	-0.0086133	0.68908	insignificant	9	52	48
chr11	102993652	102995652	Hexim2	-0.14898151	6.47E-21	hypomethylated	-0.0072123	0.32341	insignificant	29	75	75
chr11	102993666	102995666	Hexim2	-0.14898151	6.47E-21	hypomethylated	-0.0072123	0.32341	insignificant	29	75	75
chr11	102993672	102995672	Hexim2	-0.14898151	6.47E-21	hypomethylated	-0.0072123	0.32341	insignificant	29	75	75
chr11	103031451	103033451	Fmnl1	-0.07843076	8.63E-43	hypomethylated	-0.0018185	0.4201	insignificant	52	132	132
chr11	103079746	103081746	4933400C05Ri	-0.22070461	0.074952	insignificant	-0.031899	0.34155	insignificant	1	9	9
chr11	103128715	103130715	Map3k14	-0.17167448	1.84E-09	hypomethylated	0.011414	0.21249	insignificant	14	60	60
chr11	103206016	103208016	Arhgap27	-0.18714332	0.000013589	hypomethylated	0.059805	0.25737	insignificant	2	10	7
chr11	103224698	103226698	Arhgap27	-0.16964206	0.0013007	hypomethylated	0.018211	1	insignificant	6	24	16
chr11	103273978	103275978	Plekhm1		1	noCoverage	0.013889	0.8114	insignificant	0	9	8
chr11	103509822	103511822	Rprml	-0.06751648	0.002584	hypomethylated	0.0072506	0.62197	insignificant	27	121	122
chr11	103559024	103561024	Gosr2	-0.08795081	0.0020011	hypomethylated	-0.0027195	0.91965	insignificant	20	73	67
chr11	103611135	103613135	Wnt9b	-0.14007183	1.07E-08	hypomethylated	0.0062542	0.76834	insignificant	6	28	28
chr11	103634488	103636488	Wnt3	-0.14264299	3.13E-11	hypomethylated	0.0060354	0.12149	insignificant	29	114	110
chr11	103815370	103817370	Nsf	-0.24630091	0.0000013	hypomethylated	-0.079629	0.000069937	hypomethylated	11	43	52
chr11	103827184	103829184	Arf2	-0.09420309	0.0000041	hypomethylated	0.001005	0.83555	insignificant	9	80	81
chr11	103993195	103995195	Crhr1	-0.17134744	5.21E-43	hypomethylated	0.01434	0.73308	insignificant	62	186	181
chr11	104046640	104048640	4933407P14Ri	0.10925145	0.14589	insignificant	-0.020306	0.63198	insignificant	8	24	25
chr11	104091749	104093749	Mapt	-0.09460293	1.24E-15	hypomethylated	-0.0018579	0.074024	insignificant	19	131	133
chr11	104303605	104305605	1700081L11Ri	-0.09663198	7.27E-10	hypomethylated	0.0071833	0.55499	insignificant	34	91	88
chr11	104410976	104412976	Myl4	-0.12948847	0.001173	hypomethylated	-0.0037705	0.79422	insignificant	29	108	108
chr11	104411934	104413934	Myl4	0.2082093	0.64344	insignificant	0.10541	0.59231	insignificant	5	14	16
chr11	104468313	104470313	Itgb3	-0.11411039	0.061631	insignificant	0.035365	0.19745	insignificant	8	63	68
chr11	104986738	104988738	Mettl2	-0.10274646	1.5E-09	hypomethylated	0.012696	0.46421	insignificant	25	83	75
chr11	105041843	105043843	Tlk2	-0.11847904	3.43E-30	hypomethylated	0.014514	0.14452	insignificant	63	268	275
chr11	105042747	105044747	1700052K11Ri	-0.14284721	2.02E-19	hypomethylated	0.011872	0.78739	insignificant	37	144	149
chr11	105152959	105154959	Mrc2	-0.14296709	0.0064522	hypomethylated	0.020101	0.49409	insignificant	12	62	66
chr11	105318049	105320049	March10	0.36958601	5.43E-09	stronglyHypermeth	-0.017284	0.47113	insignificant	3	18	18
chr11	105450299	105452299	Tanc2	-0.09355236	1.5E-15	hypomethylated	0.0068464	1	insignificant	46	214	213
chr11	105805461	105807461	Cyb561	-0.12226488	3.01E-12	hypomethylated	0.0066373	0.34147	insignificant	23	77	75
chr11	105828260	105830260	Ace	-0.15671351	2.7E-10	hypomethylated	-0.0044822	0.053314	insignificant	17	101	101
chr11	105868516	105870516	Kcnh6	-0.10453004	0.0038679	hypomethylated	0.019306	0.22173	insignificant	7	34	34
chr11	105897185	105899185	Dcaf7	-0.13406456	3.13E-24	hypomethylated	0.01949	0.1113	insignificant	33	103	101
chr11	105926420	105928420	Taco1	-0.23314552	3.56E-13	hypomethylated	-0.0035656	0.44239	insignificant	20	86	84
chr11	105945215	105947215	Map3k3	-0.10773324	9.29E-19	hypomethylated	0.0078972	0.8206	insignificant	36	174	164
chr11	106021456	106023456	Limd2	-0.10963963	8.59E-08	hypomethylated	-0.00062575	0.21552	insignificant	22	107	92
chr11	106054862	106056862	Strada	0.07880425	0.089284	insignificant	0.040352	0.56594	insignificant	5	34	30
chr11	106077239	106079239	Ddx42	-0.19324721	0.0037248	hypomethylated	0.023771	0.21125	insignificant	7	47	46
chr11	106077681	106079681	Ddx42	-0.20748367	0.0061225	hypomethylated	0.045638	0.11195	insignificant	7	40	39
chr11	106116498	106118498	Psmc5	-0.13204594	3.89E-09	hypomethylated	0.013199	0.061731	insignificant	13	76	72
chr11	106117116	106119116	Ftsj3	-0.06159054	0.091454	insignificant	-0.011204	0.8198	insignificant	13	60	60
chr11	106134286	106136286	Smarcd2	-0.18443976	0.00043268	hypomethylated	-0.014689	0.4221	insignificant	11	75	73
chr11	106136985	106138985	Tcam1	0.09937812	0.0083323	hypermethylated	0.018001	0.018236	hypermethylated	4	38	43
chr11	106163124	106165124	Gh		1	noCoverage	0.074501	1	insignificant	0	3	6
chr11	106235139	106237139	2310007L24Ri	0.10993086	0.17034	insignificant	-0.0053742	0.9548	insignificant	3	28	29
chr11	106349110	106351110	Ern1	-0.20558756	0.034487	hypomethylated	0.026981	0.39951	insignificant	3	16	16
chr11	106361304	106363304	Snord104	-0.15307897	9.35E-11	hypomethylated	0.013105	0.19808	insignificant	10	59	54
chr11	106474244	106476244	Tex2	-0.63426573	0.000000267	stronglyHypometh	-0.17905	0.0087829	hypomethylated	1	8	8
chr11	106611581	106613581	Gm885		1	noCoverage	0.12899	0.84103	insignificant	0	6	8
chr11	106640829	106642829	Polg2	-0.11742738	0.000099534	hypomethylated	-0.011497	0.60269	insignificant	8	18	18

chr11	106640851	106642851	Polg2	-0.13186449	0.000026193	hypomethylated	-0.017653	0.42996	insignificant	7	16	16
chr11	106644073	106646073	Mir3064	-0.27320632	0.52859	insignificant	0.0029271	0.0026696	hypermethylated	1	18	18
chr11	106649565	106651565	Ccdc45	-0.12026898	3.29E-27	hypomethylated	0.025316	0.84086	insignificant	23	116	122
chr11	106649808	106651808	Ddx5	-0.12683915	2.03E-30	hypomethylated	0.033019	0.73976	insignificant	23	90	96
chr11	106782029	106784029	Smurf2	-0.06241145	0.71375	insignificant	0.0034083	0.272	insignificant	19	78	78
chr11	106860839	106862839	Kpna2		1	noCoverage	-0.16297	0.0081844	hypomethylated	0	23	31
chr11	106888536	106890536	1810010H24R	-0.11100665	1.67E-23	hypomethylated	0.032577	0.97464	insignificant	48	158	150
chr11	106993236	106995236	Bptf	-0.06395794	0.00019959	hypomethylated	-0.011468	0.045051	hypomethylated	9	78	78
chr11	107050695	107052695	Nol11		1	noCoverage	-0.051509	0.32785	insignificant	0	16	16
chr11	107339841	107341841	Psmd12	-0.22485444	9.6E-09	hypomethylated	0.013178	0.75295	insignificant	10	43	42
chr11	107408273	107410273	Helz	-0.12595607	2.25E-09	hypomethylated	-0.0045863	0.38506	insignificant	33	117	120
chr11	107577790	107579790	Cacng1	-0.23809524	0.16461	insignificant	-0.038587	0.64504	insignificant	1	7	10
chr11	107655778	107657778	Cacng4	-0.1334397	2.58E-36	hypomethylated	0.017337	0.11085	insignificant	44	108	108
chr11	108205202	108207202	Prkca	-0.08200222	2.85E-12	hypomethylated	0.021055	0.11702	insignificant	23	84	65
chr11	108285579	108287579	Ccdc46	-0.19656507	4.37E-08	hypomethylated	0.0302	0.88708	insignificant	16	57	50
chr11	108542922	108544922	Ccdc46	-0.31458333	0.34798	insignificant	0.029167	0.83821	insignificant	1	8	8
chr11	108542931	108544931	Ccdc46	-0.31458333	0.34798	insignificant	0.029167	0.83821	insignificant	1	8	8
chr11	108780662	108782662	Axin2	-0.11645791	2.92E-49	hypomethylated	0.00032617	0.70359	insignificant	87	226	224
chr11	109159495	109161495	Rgs9	-0.09326013	0.1017	insignificant	-0.0097375	0.7289	insignificant	4	13	14
chr11	109223107	109225107	Gna13	-0.10756055	1.87E-36	hypomethylated	0.0054673	0.87414	insignificant	57	216	216
chr11	109286259	109288259	Amz2	-0.15229519	8.98E-13	hypomethylated	0.026993	0.39209	insignificant	10	30	30
chr11	109333687	109335687	Arsg	-0.12381752	3.83E-29	hypomethylated	-0.010646	1	insignificant	60	192	205
chr11	109334543	109336543	Slc16a6	-0.11186187	7.42E-19	hypomethylated	-0.006477	0.86753	insignificant	36	138	146
chr11	109334910	109336910	Slc16a6	-0.13108464	0.00000313	hypomethylated	-0.029827	0.84789	insignificant	13	56	64
chr11	109404024	109406024	Arsg	-0.9256377	0.076412	lowCoverage	-0.0014831	0.64244	insignificant	1	13	12
chr11	109472703	109474703	Wipi1	-0.75	0.35088	lowCoverage	-0.12647	0.84241	insignificant	1	4	4
chr11	109511262	109513262	Prkar1a	-0.09306503	3.82E-24	hypomethylated	0.0057732	0.040461	hypermethylated	33	162	175
chr11	109957251	109959251	Abca8a		1	noCoverage	0.083438	0.79368	insignificant	0	10	10
chr11	110199030	110201030	Abca5	-0.14948751	0.000011772	hypomethylated	-0.0087386	0.31758	insignificant	12	54	59
chr11	110259435	110261435	Map2k6	-0.24802867	0.013492	hypomethylated	0.047805	0.90244	insignificant	4	9	8
chr11	110926477	110928477	Kcnj2	-0.15957194	3.67E-12	hypomethylated	0.0011479	0.76838	insignificant	8	60	60
chr11	112642523	112644523	Sox9	-0.15704353	2.45E-19	hypomethylated	0.019164	0.97035	insignificant	38	125	116
chr11	113063152	113065152	2610035D17Rik		1	noCoverage	-0.034783	0.31833	insignificant	0	4	4
chr11	113484570	113486570	Sstr2	-0.21860596	0.00013408	hypomethylated	0.065518	0.0037188	hypermethylated	10	26	26
chr11	113509842	113511842	Cog1	0.09401835	0.00029125	inconclusive	0.0067767	0.013116	hypermethylated	22	136	127
chr11	113544725	113546725	D11Wsu47e	-0.06611866	7.59E-13	hypomethylated	-0.00093898	0.46814	insignificant	31	179	168
chr11	113545465	113547465	D11Wsu47e	-0.16142756	0.0048344	hypomethylated	-0.014191	0.75837	insignificant	12	74	63
chr11	113570840	113572840	Cpsf4l		1	noCoverage	0.08977	0.073477	insignificant	0	12	9
chr11	113571331	113573331	Cpsf4l		1	noCoverage	0.10632	0.0056106	hypermethylated	0	17	14
chr11	113612474	113614474	Cdc42ep4	-0.28959216	0.0025533	hypomethylated	-0.0041889	0.92851	insignificant	6	37	26
chr11	113613129	113615129	Cdc42ep4	-0.62528875	0.34043	lowCoverage	0.12888	0.0037582	hypermethylated	1	10	10
chr11	113927265	113929265	Sdk2	-0.12391648	1.96E-47	hypomethylated	0.0033698	0.58638	insignificant	61	208	208
chr11	114528856	114530856	Rpl38	-0.19644141	2.05E-14	hypomethylated	0.05819	0.003698	hypermethylated	26	125	91
chr11	114529094	114531094	Rpl38	-0.14942346	5.45E-14	hypomethylated	0.075546	0.0077508	hypermethylated	27	119	86
chr11	114535781	114537781	Ttyh2	-0.18707414	0.016635	hypomethylated	0.042278	0.65943	insignificant	14	81	79
chr11	114587725	114589725	Dnaic2	-0.23311549	0.74387	insignificant	0.042785	0.047216	hypermethylated	6	30	27
chr11	114625702	114627702	Kif19a	-0.15071268	3.32E-08	hypomethylated	0.01407	0.68836	insignificant	15	31	37
chr11	114657206	114659206	Btbd17	0.05577031	0.0039803	inconclusive	0.071075	0.12277	insignificant	4	10	10
chr11	114711845	114713845	Gprc5c	-0.16093868	2.19E-11	hypomethylated	0.0040351	0.17919	insignificant	20	101	110
chr11	114711853	114713853	Gprc5c	-0.15770578	2.14E-11	hypomethylated	0.0055039	0.15767	insignificant	20	103	112
chr11	114712343	114714343	Gprc5c	-0.15734785	5.66E-12	hypomethylated	0.013347	0.052252	insignificant	22	107	108
chr11	114750354	114752354	Cd300a	-0.32514116	0.00020283	hypomethylated	0.033554	0.90839	insignificant	3	16	10
chr11	114951744	114953744	Rab37	-0.17720031	0.000000613	hypomethylated	0.0002972	0.44987	insignificant	17	45	45
chr11	115014491	115016491	Rab37	-0.15959053	0.00000105	hypomethylated	-0.032799	0.22136	insignificant	13	63	62
chr11	115023654	115025654	Slc9a3r1	-0.12536686	3.75E-32	hypomethylated	-0.0031931	0.35294	insignificant	37	142	134
chr11	115047800	115049800	Tmem104	-0.01606792	0.15465	insignificant	0.00020723	0.14535	insignificant	14	52	52
chr11	115048630	115050630	Nat9	-0.01606792	0.15465	insignificant	0.00020723	0.14535	insignificant	14	52	52
chr11	115128557	115130557	Grin2c	-0.10202061	0.36695	insignificant	-0.024226	0.50891	insignificant	7	40	44
chr11	115138283	115140283	Fdxr	-0.20569013	0.57007	insignificant	0.047424	0.65858	insignificant	3	31	31
chr11	115158860	115160860	Fads6	-0.0425641	0.01499	hypomethylated	0.00016515	0.73238	insignificant	4	10	8
chr11	115167476	115169476	Otop2	-0.51537425	0.00038881	stronglyHypometh	-0.016936	0.87107	insignificant	2	9	8
chr11	115183232	115185232	Ush1g	-0.05311041	1	insignificant	-0.12466	0.0076217	hypomethylated	9	78	72
chr11	115195047	115197047	Otop3	-0.22782062	0.077367	insignificant	0.015631	1	insignificant	4	46	45
chr11	115229033	115231033	C630004H02R	-0.1378796	0.0014981	hypomethylated	0.0048764	0.93837	insignificant	4	18	18
chr11	115242229	115244229	Cdr2l	-0.17924337	3.34E-31	hypomethylated	0.0070589	0.83148	insignificant	29	72	76
chr11	115264079	115266079	Ict1	-0.13230716	1.19E-16	hypomethylated	0.010401	0.96843	insignificant	26	89	90
chr11	115280439	115282439	Kctd2	-0.14989992	8.07E-48	hypomethylated	0.0088275	0.039584	hypermethylated	45	136	126
chr11	115281233	115283233	Kctd2	-0.13894138	1.77E-43	hypomethylated	-0.0018855	0.10546	insignificant	44	120	109
chr11	115322786	115324786	Slc16a5	0.12017013	1	lowCoverage	-0.0084345	1	insignificant	1	8	8
chr11	115335990	115337990	Armc7	-0.14414163	1.12E-11	hypomethylated	0.024896	0.29018	insignificant	11	44	44
chr11	115353128	115355128	Nt5c	-0.27682496	5.19E-08	hypomethylated	0.021691	0.29352	insignificant	5	14	24
chr11	115375684	115377684	Hn1	-0.15800151	0.0045947	hypomethylated	0.011158	1	insignificant	2	23	23
chr11	115397544	115399544	Sumo2	-0.40029168	0.00032204	stronglyHypometh	-0.026724	0.85293	insignificant	7	38	36
chr11	115424757	115426757	Nup85	-0.15178155	0.00013908	hypomethylated	0.0043333	0.54624	insignificant	3	39	28
chr11	115464464	115466464	Mrps7	-0.06573696	1.74E-36	hypomethylated	-0.0091644	0.43128	insignificant	37	190	186
chr11	115465220	115467220	Gga3	0.09645921	1	insignificant	0.000083469	0.42443	insignificant	11	114	113
chr11	115473817	115475817	Mif4gd	-0.08274414	6.25E-09	hypomethylated	0.0065397	0.7403	insignificant	52	136	125
chr11	115474267	115476267	Mif4gd	-0.14540005	0.023063	hypomethylated	0.025532	0.26232	insignificant	7	29	27
chr11	115489453	115491453	Slc25a19	-0.46296746	1	lowCoverage	-0.018636	0.78358	insignificant	1	35	42
chr11	115569911	115571911	Grb2	-0.12713754	3.38E-10	hypomethylated	0.0087114	0.044015	inconclusive	14	74	74
chr11	115625746	115627746	2310067B10R	-0.07245439	0.24638	insignificant	0.017129	0.86356	insignificant	14	82	80
chr11	115674906	115676906	Caskin2	-0.14327964	1.92E-26	hypomethylated	-0.03816	0.55785	insignificant	51	174	164
chr11	115675052	115677052	Tsen54	-0.15785852	8.81E-26	hypomethylated	-0.043984	0.54261	insignificant	46	154	146
chr11	115684371	115686371	Llgl2	-0.06827254	1.24E-08	hypomethylated	0.00712	0.2432	insignificant	35	123	108
chr11	115760452	115762452	2210020M01F	-0.49580229	1.93E-09	stronglyHypometh	-0.040821	0.072532	insignificant	5	27	26

chr11	115793972	115795972	Sap30bp		1	noCoverage	-0.020613	0.49524	insignificant	0	27	29
chr11	115794320	115796320	Recql5		1	noCoverage	-0.020613	0.49524	insignificant	0	27	29
chr11	115835038	115837038	Itgb4	-0.16571958	1.91E-15	hypomethylated	-0.0070916	0.62791	insignificant	27	81	81
chr11	115874033	115876033	Galk1	-0.03258471	0.78571	insignificant	0.14427	0.6913	insignificant	1	9	9
chr11	115885818	115887818	H3f3b	-0.13995817	0.01547	hypomethylated	-0.072677	0.54199	insignificant	4	28	26
chr11	115890635	115892635	Unk	-0.0853501	0.49383	insignificant	0.0029047	0.4754	insignificant	8	58	58
chr11	115948278	115950278	Wbp2		1	noCoverage	0.015111	0.93867	insignificant	0	26	20
chr11	115971330	115973330	Trim47	-0.15392723	8.73E-36	hypomethylated	-0.0020766	1	insignificant	32	81	80
chr11	115992442	115994442	Mrpl38	0.00997398	0.57864	insignificant	-0.036688	0.62834	insignificant	3	32	32
chr11	116000182	116002182	Mrpl38	-0.15858674	0.58752	insignificant	0.023953	0.00323	hypermethylated	2	12	12
chr11	116029492	116031492	Fbf1	-0.0780112	1	insignificant	0.01654	0.11967	insignificant	4	14	14
chr11	116059168	116061168	2310004N24R	-0.1932944	0.000000136	hypomethylated	0.0020429	1	insignificant	14	86	82
chr11	116060359	116062359	Acox1	-0.40217316	0.0094744	stronglyHypometh	0.057524	0.65106	insignificant	6	57	50
chr11	116076315	116078315	Cdk3-ps	0.31809641	1	lowCoverage	-0.014951	1	insignificant	1	12	12
chr11	116099405	116101405	Evpl	-0.27506185	0.25933	insignificant	-0.071159	0.099923	insignificant	3	22	22
chr11	116135531	116137531	Srp68	0.02727273	1	insignificant	0.028893	0.79579	insignificant	2	6	6
chr11	116141252	116143252	Galr2	-0.14851427	1.75E-09	hypomethylated	-0.010663	0.96193	insignificant	5	36	36
chr11	116168052	116170052	Exoc7	-0.12024537	0.000000746	hypomethylated	0.0031384	0.49069	insignificant	9	28	28
chr11	116196668	116198668	Rnf157	-0.19970772	3.2E-24	hypomethylated	-0.0021631	0.73941	insignificant	20	43	44
chr11	116274346	116276346	Rnf157	-0.08160845	0.000019589	hypomethylated	0.00071316	0.9653	insignificant	21	84	84
chr11	116294407	116296407	Fam100b	-0.10399394	3.27E-09	hypomethylated	-0.002494	0.26315	insignificant	30	142	141
chr11	116294526	116296526	Gm7367	-0.10326671	3.26E-09	hypomethylated	-0.0017668	0.26321	insignificant	30	143	141
chr11	116315661	116317661	Qrich2	0.14459081	0.089797	insignificant	-0.00081526	0.84011	insignificant	3	16	16
chr11	116351660	116353660	Prpsap1	-0.16124205	0.00000186	hypomethylated	-0.082219	0.00018887	hypomethylated	11	61	53
chr11	116392224	116394224	Sphk1	-0.1762835	1.86E-22	hypomethylated	-0.02401	0.53478	insignificant	28	61	74
chr11	116392757	116394757	Sphk1	-0.14304289	7.59E-42	hypomethylated	-0.0013235	0.60349	insignificant	49	124	131
chr11	116393551	116395551	Sphk1	-0.12900736	3.68E-54	hypomethylated	0.0025676	0.41306	insignificant	52	165	168
chr11	116395053	116397053	Sphk1	-0.21653333	8.06E-37	hypomethylated	-0.016457	0.66155	insignificant	17	77	78
chr11	116442761	116444761	Ube2o	-0.231601	8.94E-21	hypomethylated	-0.00065619	0.0094934	hypomethylated	16	86	85
chr11	116485566	116487566	Rhbdf2	0.54946166	7.7E-09	stronglyHypermeth	0.10827	0.58942	insignificant	1	26	28
chr11	116515627	116517627	Cygb		1	noCoverage	-0.075479	0.015552	hypomethylated	0	44	38
chr11	116517421	116519421	Gm11744	-0.09081585	1	insignificant	0.083571	0.56909	insignificant	2	20	26
chr11	116531973	116533973	1810032O08R	-0.17038747	3.26E-17	hypomethylated	0.023775	0.37378	insignificant	17	76	61
chr11	116532818	116534818	Snord1c	-0.18813725	9.26E-12	hypomethylated	0.051058	0.55053	insignificant	9	45	37
chr11	116534460	116536460	Snord1b	0.07909732	0.16584	insignificant	0.04452	0.45978	insignificant	4	12	15
chr11	116534910	116536910	Snord1a	0.07909732	0.16584	insignificant	0.098375	0.33289	insignificant	4	12	12
chr11	116555974	116557974	St6galnac2	-0.18502816	5.04E-08	hypomethylated	0.034406	0.010894	hypermethylated	11	65	61
chr11	116689360	116691360	Mxra7	0.01102941	0.49016	insignificant	0.082458	0.07754	insignificant	2	4	4
chr11	116703828	116705828	1110005A03R	-0.10475717	4.79E-42	hypomethylated	0.0079132	0.48793	insignificant	56	197	177
chr11	116704763	116706763	Jmjd6	-0.24932172	0.00088248	hypomethylated	0.0242	0.083408	insignificant	7	69	53
chr11	116714328	116716328	Mfsd11	-0.14226982	6.57E-08	hypomethylated	0.0055083	0.69999	insignificant	11	74	60
chr11	116714408	116716408	Srsf2	-0.14627032	4.05E-08	hypomethylated	0.0066639	0.86375	insignificant	11	72	58
chr11	116779176	116781176	Mgat5b	-0.07977494	1.46E-19	hypomethylated	-0.00053932	0.76718	insignificant	60	205	196
chr11	116937096	116939096	2810008D09R	-0.13195138	0.07879	insignificant	0.020675	0.094679	insignificant	13	33	36
chr11	116975485	116977485	Sec14l1	-0.13568445	1.86E-22	hypomethylated	-0.0043579	0.63338	insignificant	35	127	124
chr11	117059974	117061974	###########	0.04202994	1.1E-19	inconclusive	0.02216	0.38015	insignificant	23	104	100
chr11	117126559	117128559	###########	-0.13678566	7.06E-09	hypomethylated	0.013333	0.91288	insignificant	16	97	88
chr11	117192028	117194028	###########	-0.15404717	0.00026443	hypomethylated	0.029019	0.35338	insignificant	9	45	44
chr11	117192626	117194626	###########	-0.15979008	0.00017428	hypomethylated	0.031852	0.10615	insignificant	9	51	50
chr11	117514602	117516602	Tnrc6c	-0.06144494	0.00000384	hypomethylated	0.0043111	0.89844	insignificant	49	214	212
chr11	117641935	117643935	Tmc6	-0.19666445	0.58909	insignificant	-0.0021489	0.60886	insignificant	5	42	42
chr11	117641997	117643997	Tmc6	-0.19666445	0.58909	insignificant	-0.0021489	0.60886	insignificant	5	42	42
chr11	117642610	117644610	Tmc8	-0.1889064	3.23E-10	hypomethylated	-0.025819	0.70424	insignificant	4	48	48
chr11	117642971	117644971	Tmc8	-0.21958301	8.63E-11	hypomethylated	-0.025881	0.61675	insignificant	5	38	38
chr11	117669980	117671980	Syngr2	-0.11778284	0.00004342	hypomethylated	-0.0055243	0.50595	insignificant	20	89	87
chr11	117686232	117688232	Afmid	-0.17029307	0.00099372	hypomethylated	0.0096457	0.1592	insignificant	13	37	37
chr11	117687328	117689328	Afmid	-0.33234923	0.00087776	hypomethylated	-0.027004	0.87026	insignificant	5	18	18
chr11	117709550	117711550	Birc5	-0.09869446	3.47E-10	hypomethylated	-0.0084709	0.35699	insignificant	20	80	80
chr11	117734840	117736840	Tha1	-0.47930219	0.050765	insignificant	-0.038167	0.63561	insignificant	2	18	18
chr11	117830680	117832680	Socs3		1	noCoverage	0.0016524	0.36398	insignificant	0	52	48
chr11	117847170	117849170	Pgs1	-0.16933579	1.38E-38	hypomethylated	0.02275	0.96535	insignificant	21	53	46
chr11	117990533	117992533	Dnahc17	-0.61163245	0.030909	stronglyHypometh	0.26892	1	insignificant	1	5	6
chr11	118109906	118111906	Cyth1	-0.17483799	0.029618	hypomethylated	-0.034021	0.47826	insignificant	13	31	30
chr11	118203848	118205848	BC100451	-0.13573581	0.015224	hypomethylated	0.0052981	1	insignificant	5	24	24
chr11	118216725	118218725	Timp2	-0.09739259	0.0016017	hypomethylated	0.029986	0.018406	hypermethylated	6	18	18
chr11	118280366	118282366	Cant1	-0.17876985	4.19E-30	hypomethylated	0.015346	0.43084	insignificant	13	32	32
chr11	118294166	118296166	C1qtnf1	0.30080693	1	lowCoverage	0.089294	0.00008065	hypermethylated	1	27	29
chr11	118337273	118339273	Engase	-0.10523086	4.35E-12	hypomethylated	0.0067261	0.67077	insignificant	16	50	50
chr11	118770886	118772886	Rbfox3	0.00261752	1	insignificant	-0.064446	0.10477	insignificant	3	8	8
chr11	118848501	118850501	Enpp7	0.07987708	0.82387	insignificant	-0.09463	0.6982	insignificant	4	13	13
chr11	118883342	118885342	Cbx2	-0.11828141	3.3E-20	hypomethylated	0.0076228	0.50685	insignificant	34	162	160
chr11	118902227	118904227	Cbx8	-0.11688105	1.66E-09	hypomethylated	-0.0072782	0.15181	insignificant	31	135	137
chr11	118947551	118949551	Cbx4	-0.17153838	6.97E-29	hypomethylated	-0.014453	0.6028	insignificant	25	90	86
chr11	119088885	119090885	Ccdc40	-0.14796115	2.36E-34	hypomethylated	0.0096749	0.40843	insignificant	33	115	112
chr11	119089813	119091813	Ccdc40	-0.14734366	5.5E-35	hypomethylated	0.010184	0.52105	insignificant	26	87	84
chr11	119128280	119130280	Gaa	-0.21266297	1.6E-10	hypomethylated	-0.050429	0.020014	hypomethylated	8	32	42
chr11	119128321	119130321	Gaa	-0.21266297	1.6E-10	hypomethylated	-0.050429	0.020014	hypomethylated	8	32	42
chr11	119161357	119163357	Eif4a3		1	noCoverage	-0.0054885	0.68377	insignificant	0	58	58
chr11	119175100	119177100	Card14	-0.32021443	1.65E-10	hypomethylated	-0.032316	0.88745	insignificant	5	14	14
chr11	119215870	119217870	Slc26a11	-0.13447025	0.31908	insignificant	-0.021318	0.94363	insignificant	14	63	51
chr11	119216824	119218824	Sgsh	-0.08143137	1	insignificant	-0.011266	0.66427	insignificant	9	44	33
chr11	119250785	119252785	Mir1932	-0.12732317	2.25E-18	hypomethylated	0.0031548	0.091278	insignificant	34	139	140
chr11	119351660	119353660	A730011L01Ri	-0.05539282	7.86E-12	hypomethylated	0.00215	0.43704	insignificant	12	137	137
chr11	119351933	119353933	A730011L01Ri	-0.06456057	4.66E-13	hypomethylated	0.0092952	0.54989	insignificant	12	141	141
chr11	119409134	119411134	Nptx1	-0.1518214	1.15E-10	hypomethylated	-0.038562	0.91942	insignificant	31	140	125

chr11	119463308	119465308	Rptor	-0.16790106	3.07E-11	hypomethylated	-0.017756	0.23993	insignificant	19	78	72
chr11	119774123	119776123	Chmp6	-0.19723338	9.92E-09	hypomethylated	-0.008736	0.16685	insignificant	13	40	45
chr11	119803405	119805405	Baiap2	-0.09721584	4.37E-41	hypomethylated	0.0095555	0.14399	insignificant	62	194	179
chr11	119875080	119877080	Mir3065	0.06525031	0.49938	insignificant	0.059892	0.66427	insignificant	6	24	24
chr11	119876176	119878176	Aatk	-0.0487329	0.88305	insignificant	-0.025951	1	insignificant	9	30	30
chr11	119908459	119910459	Aatk	-0.26610009	0.00048221	hypomethylated	0.050219	0.3592	insignificant	5	38	33
chr11	119948141	119950141	Azi1	-0.14417239	0.000035248	hypomethylated	0.028469	0.092393	insignificant	9	38	30
chr11	119959247	119961247	1810043H04R	-0.1051613	0.00035633	hypomethylated	-0.0053119	0.93826	insignificant	2	30	30
chr11	119960045	119962045	2410002I01Rik		1	noCoverage	0.0021854	0.078764	insignificant	0	12	12
chr11	120051170	120053170	2810410L24Ri	-0.3112605	0.00063275	hypomethylated	0.023355	0.60478	insignificant	3	34	28
chr11	120093260	120095260	Bahcc1	-0.12769267	4.14E-14	hypomethylated	0.0074584	0.11062	insignificant	23	95	94
chr11	120209798	120211798	Actg1	-0.1222169	7.09E-37	hypomethylated	-0.0048543	0.61	insignificant	71	228	224
chr11	120221847	120223847	Fscn2	-0.04813834	0.18249	insignificant	0.015157	0.84308	insignificant	9	37	38
chr11	120240060	120242060	2310003H01R	-0.0512749	0.0026484	hypomethylated	-0.011396	0.29118	insignificant	10	30	30
chr11	120299014	120301014	Nploc4	-0.21466935	0.00033291	hypomethylated	0.078981	0.66973	insignificant	3	32	34
chr11	120302944	120304944	Tspan10		1	noCoverage	0.058805	0.40889	insignificant	0	4	4
chr11	120318442	120320442	Ccdc137	-0.18604827	6.56E-21	hypomethylated	0.036879	0.86863	insignificant	16	63	66
chr11	120319377	120321377	1810049H13R	0.06800291	0.051062	insignificant	0.02185	1	insignificant	4	39	38
chr11	120327948	120329948	Hgs	-0.13095853	1.68E-19	hypomethylated	-0.00019071	0.70108	insignificant	21	70	70
chr11	120328914	120330914	Arl16	-0.12008929	3.23E-11	hypomethylated	-0.00025734	0.6966	insignificant	15	42	42
chr11	120344982	120346982	Mrpl12	-0.10150561	3.91E-14	hypomethylated	0.0050079	0.67346	insignificant	19	37	37
chr11	120352150	120354150	Slc25a10	-0.15780363	7.75E-20	hypomethylated	-0.010516	0.25443	insignificant	8	44	46
chr11	120391040	120393040	Gcgr	-0.14441434	1	lowCoverage	-0.0026733	0.85793	insignificant	1	16	16
chr11	120411041	120413041	Fam195b	-0.22236228	6.1E-41	hypomethylated	-0.0096213	0.031735	hypomethylated	20	63	61
chr11	120412446	120414446	Ppp1r27	-0.01377841	0.3465	insignificant	0.015947	0.75828	insignificant	3	12	10
chr11	120434250	120436250	P4hb	-0.10934337	0.028506	hypomethylated	0.011352	0.29932	insignificant	10	82	82
chr11	120442934	120444934	Arhgdia	-0.03275076	0.10583	insignificant	0.024113	0.12787	insignificant	7	35	38
chr11	120458734	120460734	Anapc11	-0.01398952	1.77E-09	hypomethylated	0.0078018	0.62914	insignificant	42	193	189
chr11	120458845	120460845	Anapc11	-0.01816328	1.76E-09	hypomethylated	0.0083604	0.60694	insignificant	42	195	190
chr11	120459679	120461679	Anapc11	-0.11412171	0.00070784	hypomethylated	0.016253	0.33412	insignificant	5	66	65
chr11	120468790	120470790	Npb	-0.17788045	6.22E-20	hypomethylated	-0.049677	0.00033125	hypomethylated	10	33	33
chr11	120479204	120481204	Sirt7	0.02220042	1.42E-08	inconclusive	0.021713	0.37643	insignificant	20	90	88
chr11	120486316	120488316	Sirt7		1	noCoverage	-0.043056	1	insignificant	0	10	8
chr11	120509651	120511651	Myadml2	-0.42363124	1	insignificant	-0.22434	0.46867	insignificant	2	6	4
chr11	120522151	120524151	Notum	-0.03302565	0.00013543	hypomethylated	0.0046854	0.78123	insignificant	26	73	74
chr11	120533286	120535286	Aspscr1	-0.13731124	5.63E-35	hypomethylated	-0.0045232	0.97327	insignificant	40	124	108
chr11	120574266	120576266	Lrrc45	-0.11906951	0.00015502	hypomethylated	0.040735	0.057545	insignificant	4	60	64
chr11	120575081	120577081	Stra13	-0.09575847	0.033225	hypomethylated	0.026613	0.18298	insignificant	3	41	40
chr11	120581781	120583781	Rac3	-0.13741787	1.12E-40	hypomethylated	0.009187	0.058929	insignificant	18	109	115
chr11	120588595	120590595	Dcxr	0.10984848	0.22213	insignificant	0.071786	0.18015	insignificant	3	11	11
chr11	120593335	120595335	Cbr2	-0.5565047	0.0017213	stronglyHypometh	-0.028823	0.2833	insignificant	1	8	8
chr11	120644585	120646585	Gps1	-0.09732598	2.18E-47	hypomethylated	-0.0073635	0.82907	insignificant	74	223	218
chr11	120644828	120646828	Gps1	-0.09732598	2.18E-47	hypomethylated	-0.0073635	0.82907	insignificant	74	223	218
chr11	120645518	120647518	Rfng	-0.1206676	5.38E-43	hypomethylated	-0.0079585	0.58224	insignificant	54	169	164
chr11	120657709	120659709	Dus1l	-0.15228277	0.081901	insignificant	-0.034262	0.0013738	hypomethylated	17	48	47
chr11	120685861	120687861	Fasn	-0.09009564	0.000011649	hypomethylated	0.0070023	0.69393	insignificant	17	98	98
chr11	120794186	120796186	Ccdc57	-0.33877996	0.000074866	stronglyHypometh	0.21966	0.85577	insignificant	1	9	10
chr11	120808797	120810797	Slc16a3	-0.15843518	3.15E-19	hypomethylated	0.019273	0.80907	insignificant	25	99	92
chr11	120809380	120811380	Slc16a3	-0.16256108	1.9E-20	hypomethylated	0.017115	0.94953	insignificant	27	101	94
chr11	120852647	120854647	Csnk1d	-0.22970238	0.0077879	hypomethylated	0.045916	0.77128	insignificant	5	16	18
chr11	121006456	121008456	Tex19.1	0.06025683	0.14437	insignificant	0.0016321	0.87605	insignificant	5	24	22
chr11	121064746	121066746	Hexdc		1	noCoverage	-0.0031807	1	insignificant	0	49	38
chr11	121065962	121067962	Hexdc		1	noCoverage	-0.008921	0.84986	insignificant	0	40	29
chr11	121067956	121069956	Hexdc	-0.22619048	0.018742	hypomethylated	0.0081936	1	insignificant	2	6	7
chr11	121090579	121092579	BC017643	-0.5862069	0.26042	lowCoverage	0.0082986	0.08233	insignificant	1	2	2
chr11	121097549	121099549	Narf	-0.15628869	6.25E-22	hypomethylated	-0.0046455	0.50651	insignificant	17	52	50
chr11	121120300	121122300	Foxk2	-0.08494252	8.66E-65	hypomethylated	-0.0013105	0.18398	insignificant	62	217	217
chr11	121215761	121217761	Wdr45l		1	noCoverage	0.033875	0.43643	insignificant	0	28	29
chr11	121249565	121251565	Rab40b	0.1788604	1	insignificant	0.053047	0.23228	insignificant	1	12	12
chr11	121281686	121283686	Fn3krp	-0.15756199	8.72E-14	hypomethylated	0.0085594	0.73201	insignificant	15	44	51
chr11	121295266	121297266	Fn3k	-0.21061834	0.000000209	hypomethylated	0.036815	0.81465	insignificant	9	33	30
chr11	121312262	121314262	Tbcd	-0.11625803	3.99E-08	hypomethylated	0.032635	0.59333	insignificant	5	53	55
chr11	121380656	121382656	Zfp750	-0.1696533	0.13733	insignificant	0.025795	0.57833	insignificant	4	8	8
chr11	121534465	121536465	B3gntl1	-0.17698566	0.60988	insignificant	0.017906	0.11851	insignificant	8	33	33
chr11	121562740	121564740	Metrnl	-0.13215995	8.17E-17	hypomethylated	-0.0050813	0.64142	insignificant	25	92	81
chr11	121690531	121692531	Ptchd3	-0.00607067	0.86102	insignificant	0.045197	0.080186	insignificant	7	32	32
chr12	3234790	3236790	1700012B15R	-0.12389019	4.25E-08	hypomethylated	0.010405	0.00000394	inconclusive	21	55	55
chr12	3309969	3311969	Rab10	-0.15840879	1.36E-12	hypomethylated	-0.018187	0.82835	insignificant	11	58	58
chr12	3364131	3366131	Kif3c	-0.07228319	0.00021622	hypomethylated	-0.015627	0.20728	insignificant	21	86	91
chr12	3425883	3427883	Asxl2	-0.12842528	2.46E-14	hypomethylated	-0.00033488	0.65772	insignificant	32	126	120
chr12	3426747	3428747	Asxl2	-0.15590309	2.38E-10	hypomethylated	0.0042916	0.0081329	inconclusive	26	108	102
chr12	3571390	3573390	Dtnb	-0.12533866	1.84E-27	hypomethylated	-0.0061369	0.46998	insignificant	40	165	158
chr12	3572040	3574040	Dtnb	-0.12899842	1.5E-28	hypomethylated	-0.0056228	0.78417	insignificant	40	152	146
chr12	3805979	3807979	Dnmt3a	-0.06298956	4.55E-22	hypomethylated	0.012362	0.056038	insignificant	102	334	325
chr12	3890743	3892743	Dnmt3a	-0.14125523	1	insignificant	0.026727	0.60067	insignificant	2	38	47
chr12	3953950	3955950	Pomc	-0.07380661	0.75868	insignificant	-0.079993	0.33241	insignificant	3	8	8
chr12	4038915	4040915	Efr3b	-0.26530817	0.013377	hypomethylated	-0.047294	0.51251	insignificant	5	16	15
chr12	4081573	4083573	Dnajc27		1	noCoverage	-0.014797	0.57765	insignificant	0	26	26
chr12	4132396	4134396	Adcy3	-0.15329107	8.1E-22	hypomethylated	-0.0058144	0.38711	insignificant	31	137	134
chr12	4133152	4135152	Adcy3	-0.20029985	1.66E-29	hypomethylated	-0.0037809	0.16773	insignificant	31	137	134
chr12	4234294	4236294	Cenpo		1	noCoverage	0.046305	0.72832	insignificant	0	14	16
chr12	4484060	4486060	Ncoa1	-0.11408575	2.38E-13	hypomethylated	0.0068981	0.84337	insignificant	16	50	60
chr12	4598813	4600813	Itsn2	-0.10439219	4.78E-47	hypomethylated	0.00016128	0.29673	insignificant	55	143	138
chr12	4775100	4777100	Pfn4	-0.24656292	0.011849	hypomethylated	-0.023481	0.95527	insignificant	4	31	32
chr12	4776073	4778073	A830093I24Ri	-0.23688194	1	lowCoverage	-0.024649	0.86359	insignificant	1	17	18

chr12	4823413	4825413	0610009D07R	-0.16296606	1.3E-11	hypomethylated	-0.012503	0.89173	insignificant	20	65	70
chr12	4848401	4850401	BC068281	-0.21656598	2.57E-08	hypomethylated	-0.0051443	0.16459	insignificant	9	39	32
chr12	4881165	4883165	Mfsd2b		1	noCoverage	0.070941	0.028081	inconclusive	0	24	23
chr12	4914326	4916326	Ubxn2a	-0.11467942	2.09E-11	hypomethylated	-0.023471	0.56293	insignificant	28	87	84
chr12	4923158	4925158	Atad2b	-0.11653957	1.85E-47	hypomethylated	0.0075921	0.62764	insignificant	41	146	132
chr12	5382307	5384307	2810032G03R	-0.12345494	9.4E-10	hypomethylated	-0.0024137	0.71274	insignificant	17	60	61
chr12	5382488	5384488	Klhl29	-0.12456957	0.000010865	hypomethylated	0.0046403	0.87277	insignificant	8	29	29
chr12	7983482	7985482	Apob	0.1383895	0.55254	insignificant	0.081864	1	insignificant	2	21	19
chr12	8308760	8310760	Gdf7	-0.41250022	0.21185	insignificant	0.021527	0.68488	insignificant	2	18	18
chr12	8319238	8321238	Hs1bp3	-0.20653488	0.00027243	hypomethylated	-0.040116	0.066221	insignificant	13	37	36
chr12	8506791	8508791	Rhob	-0.11080545	0.000000562	hypomethylated	0.033654	0.34142	insignificant	14	57	57
chr12	8605872	8607872	Slc7a15		1	noCoverage	-0.022464	0.20886	insignificant	0	31	23
chr12	8679939	8681939	Pum2	-0.08856405	3.77E-18	hypomethylated	0.016621	0.22345	insignificant	62	173	158
chr12	8680064	8682064	Pum2	-0.08856405	3.77E-18	hypomethylated	0.016621	0.22345	insignificant	62	173	158
chr12	8680486	8682486	Pum2	-0.08856405	3.77E-18	hypomethylated	0.016621	0.22345	insignificant	62	173	158
chr12	8777201	8779201	Sdc1	-0.15512167	1.12E-37	hypomethylated	0.0057513	0.5615	insignificant	38	139	134
chr12	8927112	8929112	Laptm4a	-0.15580153	0.015971	hypomethylated	-0.017005	0.33388	insignificant	15	59	52
chr12	8953734	8955734	Matn3	-0.14283762	2.48E-29	hypomethylated	0.0094764	0.16967	insignificant	27	78	78
chr12	8979806	8981806	Wdr35	-0.16894995	0.000000127	hypomethylated	-0.032591	0.69814	insignificant	8	53	52
chr12	9035802	9037802	Ttc32	-0.1965469	0.023698	hypomethylated	0.0068692	0.70453	insignificant	8	47	47
chr12	9580247	9582247	Osr1	-0.28383304	2.53E-08	hypomethylated	-0.070361	0.68526	insignificant	6	23	28
chr12	10396585	10398585	Rdh14	-0.11940662	1.66E-10	hypomethylated	0.0027492	0.68885	insignificant	21	73	72
chr12	11157179	11159179	Kcns3	-0.10927163	0.00000212	hypomethylated	0.021924	0.14048	insignificant	11	50	49
chr12	11157648	11159648	Kcns3		1	noCoverage	0.089672	0.49532	insignificant	0	8	8
chr12	11271691	11273691	Smc6	-0.02446568	8.09E-12	hypomethylated	0.0065474	0.47731	insignificant	36	138	144
chr12	11272593	11274593	Gen1	-0.06112056	2.25E-14	hypomethylated	0.0054869	0.4732	insignificant	30	118	118
chr12	11443455	11445455	Vsnl1	-0.0872799	0.60424	insignificant	0.037755	1	insignificant	9	36	37
chr12	11461884	11463884	Rad51ap2	-0.14980967	0.59028	insignificant	-0.014706	0.59491	insignificant	5	27	27
chr12	12267944	12269944	Fam49a	-0.12539477	7.61E-16	hypomethylated	0.0165	0.33964	insignificant	25	68	74
chr12	12948642	12950642	Mycn	-0.18939394	1	lowCoverage	0.091029	0.77007	insignificant	1	11	7
chr12	13255980	13257980	Ddx1	-0.41620184	6.28E-10	stronglyHypometh	0.010978	0.14961	insignificant	3	14	14
chr12	13274932	13276932	Nbas	-0.14102693	0.00024222	hypomethylated	0.017195	0.64222	insignificant	6	39	39
chr12	14158844	14160844	Fam84a	-0.20763561	0.14405	insignificant	-0.00064146	0.27335	insignificant	4	23	18
chr12	15823591	15825591	Trib2	-0.1260727	4.22E-17	hypomethylated	0.0059171	0.76876	insignificant	21	81	81
chr12	16596576	16598576	Lpin1	-0.4089426	0.00010586	stronglyHypometh	-0.050448	0.072785	insignificant	8	37	41
chr12	16659275	16661275	Ntsr2	-0.25869403	1.96E-12	hypomethylated	0.019378	0.15543	insignificant	7	36	36
chr12	16807692	16809692	Greb1	-0.17024803	0.0003931	hypomethylated	0.013144	0.43771	insignificant	6	31	27
chr12	16816770	16818770	E2f6	-0.19773493	4.16E-53	hypomethylated	0.019126	0.00049164	hypermethylated	38	127	127
chr12	16900783	16902783	Rock2	-0.10469743	2.3E-20	hypomethylated	-0.012523	0.029613	hypomethylated	45	200	200
chr12	17006924	17008924	Pqlc3	-0.17573242	1.02E-10	hypomethylated	0.014618	0.90308	insignificant	6	22	22
chr12	17272400	17274400	Pdia6	-0.11434621	3.77E-15	hypomethylated	0.021782	0.16441	insignificant	39	109	99
chr12	17331536	17333536	Atp6v1c2	-0.09842324	0.061932	insignificant	0.011087	0.64572	insignificant	13	46	46
chr12	17354298	17356298	Nol10	-0.1136005	1.4E-13	hypomethylated	0.04654	0.065904	insignificant	12	36	45
chr12	17550678	17552678	Odc1	-0.09580651	0.00000175	hypomethylated	-0.001821	0.42531	insignificant	33	126	141
chr12	17696619	17698619	Hpcal1	-0.13404276	5.67E-20	hypomethylated	0.020934	0.088267	insignificant	35	104	102
chr12	18185048	18187048			1	noCoverage	0.010377	0.70067	insignificant	0	21	21
chr12	18520351	18522351	5730507C01R	-0.05111071	1.02E-08	hypomethylated	-0.045375	0.000010327	hypomethylated	24	155	146
chr12	20821640	20823640	1700030C10R	0.00740959	0.67224	insignificant	-0.032277	0.44025	insignificant	7	25	25
chr12	21116616	21118616	Asap2	-0.03040213	0.15153	insignificant	0.027862	0.0076156	hypermethylated	22	153	152
chr12	21291157	21293157	Cpsf3	-0.15947368	4.61E-10	hypomethylated	0.0038447	0.79887	insignificant	12	47	48
chr12	21292098	21294098	Itgb1bp1	-0.12795019	0.00019288	hypomethylated	-0.024162	0.062217	insignificant	4	13	12
chr12	21321252	21323252	Iah1	-0.13677498	0.000030308	hypomethylated	-0.029239	0.12611	insignificant	10	39	60
chr12	21379452	21381452	Adam17	-0.38035955	2.82E-35	stronglyHypometh	-0.04705	0.0026017	hypomethylated	16	38	47
chr12	21423297	21425297	Ywhaq	-0.20188596	4.71E-09	hypomethylated	-0.011049	0.44846	insignificant	8	16	16
chr12	22989696	22991696	2410018L13Ri	-0.19327138	0.000000185	hypomethylated	-0.034915	0.88593	insignificant	18	126	119
chr12	25182445	25184445	Taf1b	-0.0628456	0.000000168	hypomethylated	0.024465	0.3023	insignificant	12	40	45
chr12	25256151	25258151	Grhl1	-0.02359818	2.49E-50	hypomethylated	-0.0014478	0.058692	insignificant	66	179	184
chr12	25335235	25337235	Klf11	-0.1004877	1.56E-32	hypomethylated	0.0013113	0.32928	insignificant	49	134	134
chr12	25366660	25368660	Cys1	-0.16948512	0.41498	insignificant	0.031108	0.27871	insignificant	3	28	27
chr12	25392118	25394118	Rrm2	-0.20123771	2.97E-08	hypomethylated	-0.062671	0.81256	insignificant	14	93	84
chr12	25515463	25517463	Mboat2	-0.10797112	2.33E-27	hypomethylated	-0.003437	0.53261	insignificant	30	127	112
chr12	25658796	25660796	Kidins220	-0.12618787	1.03E-12	hypomethylated	0.0069383	0.85912	insignificant	14	44	44
chr12	25780957	25782957	Id2	-0.19585183	0.057794	insignificant	0.01366	0.26829	insignificant	8	31	30
chr12	27100121	27102121	Rnf144a		1	noCoverage	0.015865	0.16035	insignificant	0	8	8
chr12	27153079	27155079	Cmpk2	-0.11758163	1.04E-15	hypomethylated	-0.0088358	0.55302	insignificant	23	67	79
chr12	28027583	28029583	Sox11	-0.13916045	0.017888	hypomethylated	-0.039243	0.85145	insignificant	11	82	79
chr12	29320818	29322818	Rps7	-0.35172211	5.28E-22	stronglyHypometh	0.025349	0.21586	insignificant	12	32	33
chr12	29333466	29335466	Rnaseh1	-0.09897035	0.028367	hypomethylated	0.00028483	0.88215	insignificant	7	24	24
chr12	29359071	29361071	Adi1	-0.11730413	1.14E-11	hypomethylated	-0.013819	0.61218	insignificant	10	20	20
chr12	29435259	29437259	Tssc1	-0.13045809	0.0019093	hypomethylated	-0.0045045	0.71905	insignificant	10	74	75
chr12	29435318	29437318	Tssc1	-0.1361769	0.0018487	hypomethylated	0.0031585	0.23649	insignificant	10	65	64
chr12	29435692	29437692	Tssc1	-0.12507225	0.18914	insignificant	-0.011576	0.22639	insignificant	8	59	56
chr12	30212248	30214248	Myt1l		1	noCoverage	0.074791	0.48132	insignificant	0	10	11
chr12	30218769	30220769	Myt1l	-0.24575585	0.58505	insignificant	-0.041203	0.59719	insignificant	4	32	30
chr12	30621900	30623900	Pxdn	-0.12664885	0.000000203	hypomethylated	0.0064994	0.43197	insignificant	20	63	63
chr12	30817474	30819474	Tpo		1	noCoverage	-0.078947	0.67989	insignificant	0	4	2
chr12	31058240	31060240	Sntg2	-0.00186307	5.05E-20	hypomethylated	-0.0089405	0.0046833	hypomethylated	8	26	28
chr12	31268307	31270307	Tmem18	-0.11984741	0.00060843	hypomethylated	0.030451	0.95496	insignificant	8	58	50
chr12	31568188	31570188	Fam150b	-0.10848504	0.000012851	hypomethylated	-0.0058896	0.73085	insignificant	17	42	42
chr12	31595533	31597533	Sh3yl1		1	noCoverage	0.013853	0.38337	insignificant	0	17	16
chr12	31757832	31759832	Fam110c	-0.06376243	9.16E-10	hypomethylated	0.010423	0.91201	insignificant	21	82	77
chr12	31949158	31951158	Lamb1	-0.09037525	0.064273	insignificant	-0.0069795	0.86008	insignificant	5	42	38
chr12	32036302	32038302	Dld	-0.05025138	0.38664	insignificant	0.12066	0.1264	insignificant	2	12	10
chr12	32184405	32186405	Cbll1	-0.1595259	8.87E-12	hypomethylated	-0.006146	0.53016	insignificant	19	60	62
chr12	32244834	32246834	Slc26a4	0.36184595	0.11171	insignificant	0.03809	0.12843	insignificant	2	14	13

chr12	32319462	32321462	Bcap29	-0.18641669	2.91E-13	hypomethylated	0.016111	0.090198	insignificant	21	64	64
chr12	32319523	32321523	Bcap29	-0.25840945	1.76E-16	hypomethylated	0.024068	1	insignificant	14	44	44
chr12	32338733	32340733	Cog5	-0.16527759	1.46E-20	hypomethylated	-0.015162	0.30297	insignificant	27	97	96
chr12	32339691	32341691	Cog5	-0.16760745	1.35E-10	hypomethylated	-0.028895	0.0436	hypomethylated	9	53	52
chr12	32635400	32637400	Hbp1	-0.67700893	0.035543	stronglyHypometh	0.17334	0.010887	hypermethylated	1	8	10
chr12	32746144	32748144	Prkar2b	-0.12725575	5.1E-13	hypomethylated	0.064361	0.38877	insignificant	9	32	24
chr12	32893203	32895203	Pik3cg	-0.15221414	0.57102	insignificant	0.11582	0.58213	insignificant	3	11	12
chr12	32893335	32895335	Pik3cg		1	noCoverage	0.23947	0.85288	insignificant	0	4	5
chr12	33062653	33064653	2010109K11Ri	-0.09193995	2.79E-38	hypomethylated	0.0033217	0.65471	insignificant	60	191	176
chr12	33504199	33506199	Nampt	-0.11156494	8.66E-34	hypomethylated	-0.012534	0.23415	insignificant	61	166	163
chr12	33637809	33639809	Sypl	-0.0901413	4.24E-12	hypomethylated	0.030794	0.039864	hypermethylated	25	116	118
chr12	33638050	33640050	Sypl	-0.0901413	4.24E-12	hypomethylated	0.031843	0.039854	hypermethylated	25	116	117
chr12	33831550	33833550	Atxn7l1	-0.14447694	7.57E-12	hypomethylated	0.0075141	0.066931	insignificant	34	157	155
chr12	33832451	33834451	F730043M19F	-0.15820654	0.000068872	hypomethylated	0.0175	0.47859	insignificant	15	86	90
chr12	34113488	34115488	Twistnb	-0.05237182	1.45E-08	hypomethylated	-0.0050338	0.96816	insignificant	16	79	79
chr12	34612289	34614289	Ferd3l	-0.31624685	0.000015263	hypomethylated	-0.037659	0.8384	insignificant	5	24	31
chr12	34641535	34643535	Twist1	-0.12760178	1.61E-30	hypomethylated	0.0085995	0.53849	insignificant	41	171	171
chr12	35668432	35670432	Prps1l1	-0.02401329	1	insignificant	-0.023073	0.17344	insignificant	4	21	20
chr12	35730860	35732860	Snx13	-0.09914953	7.1E-42	hypomethylated	0.011862	0.91801	insignificant	54	114	98
chr12	36219661	36221661	Ahr	-0.09341438	0.25414	insignificant	0.050493	1	insignificant	7	21	29
chr12	36651207	36653207	Agr3		1	noCoverage	-0.030346	0.030007	inconclusive	0	4	14
chr12	36769065	36771065	Tspan13	-0.23754181	6.71E-10	hypomethylated	0.070233	0.78527	insignificant	6	26	25
chr12	36815965	36817965	Gm5434	0.01619289	0.65757	insignificant	-0.0092189	0.2607	insignificant	4	46	44
chr12	36882710	36884710	Ankmy2	-0.18223058	8.35E-45	hypomethylated	-0.01301	0.56666	insignificant	22	82	67
chr12	36883412	36885412	Bzw2	-0.20730954	5.26E-08	hypomethylated	0.017525	0.71906	insignificant	7	53	39
chr12	36979985	36981985	1700108M19Rik		1	noCoverage	-0.085053	0.018638	hypomethylated	0	15	12
chr12	37039755	37041755	Sostdc1	-0.0846022	0.1201	insignificant	0.11389	0.44765	insignificant	2	14	13
chr12	37107105	37109105	Ispd	-0.12963306	1.11E-12	hypomethylated	0.0070539	0.58767	insignificant	17	64	59
chr12	37834132	37836132	Meox2	-0.42342836	7.59E-13	stronglyHypometh	-0.17577	0.61819	insignificant	2	12	19
chr12	38606291	38608291	Dgkb	-0.2588767	0.53755	insignificant	-0.0010629	0.13123	insignificant	1	21	25
chr12	39505844	39507844	Etv1	-0.23659671	0.3648	insignificant	0.012316	0.86457	insignificant	2	43	30
chr12	40763963	40765963	Arl4a	-0.07404084	2.22E-22	hypomethylated	-0.0011184	0.88951	insignificant	43	151	152
chr12	40764574	40766574	Arl4a	-0.0583173	0.00010084	hypomethylated	-0.0042783	0.46002	insignificant	17	66	66
chr12	40860815	40862815	Scin	-0.15634484	0.0000034	hypomethylated	-0.0059825	0.37207	insignificant	4	8	8
chr12	40949398	40951398	Ifrd1	-0.11998418	1.85E-24	hypomethylated	-0.0026867	0.17178	insignificant	23	58	58
chr12	40949776	40951776	Ifrd1	-0.12447067	0.000000145	hypomethylated	0.0041208	1	insignificant	9	18	18
chr12	41171639	41173639	Dock4	-0.15547817	1.44E-28	hypomethylated	-0.0089865	0.46922	insignificant	48	160	158
chr12	41172377	41174377	Zfp277	-0.1469631	4.6E-23	hypomethylated	-0.013163	0.36066	insignificant	46	150	148
chr12	41749676	41751676	Immp2l	-0.11541095	2.76E-10	hypomethylated	0.013823	0.33131	insignificant	13	81	81
chr12	45311055	45313055	Dnajb9	-0.07519633	0.000011348	hypomethylated	0.0012961	0.36634	insignificant	11	47	44
chr12	45369140	45371140	Pnpla8	-0.10062935	4.97E-18	hypomethylated	-0.0020161	0.86564	insignificant	33	90	86
chr12	45428871	45430871	Nrcam	-0.12944491	1.4E-12	hypomethylated	-0.001882	0.1488	insignificant	43	156	153
chr12	46175470	46177470	Stxbp6	-0.1609186	0.03337	hypomethylated	-0.019617	0.52212	insignificant	2	7	9
chr12	47919762	47921762	Nova1	-0.06356067	2.63E-08	hypomethylated	0.0046861	0.27565	insignificant	30	160	155
chr12	50482869	50484869	Foxg1	-0.09955886	0.00016595	hypomethylated	0.0040383	0.96538	insignificant	16	91	87
chr12	50482993	50484993	Foxg1	-0.10470563	0.00011206	hypomethylated	0.0041132	0.80056	insignificant	19	99	95
chr12	50489638	50491638	3110039M20Rik		1	noCoverage	0.049306	0.68458	insignificant	0	6	6
chr12	51750210	51752210	Prkd1	-0.11867963	2.9E-14	hypomethylated	-0.0025718	0.24618	insignificant	15	48	48
chr12	52448216	52450216	G2e3	-0.11519885	6.85E-17	hypomethylated	-0.0029722	0.049304	hypomethylated	40	134	130
chr12	52477566	52479566	Scfd1	-0.18044153	2.03E-09	hypomethylated	0.049227	0.15193	insignificant	8	24	21
chr12	52693327	52695327	Coch	-0.0942539	0.00044141	hypomethylated	0.014794	0.32736	insignificant	12	110	105
chr12	52790952	52792952	Ap4s1	-0.15901498	2.15E-17	hypomethylated	0.0056265	0.55787	insignificant	20	81	70
chr12	52792901	52794901	Ap4s1	-0.11763489	0.3277	insignificant	0.035853	0.36898	insignificant	16	65	64
chr12	52930523	52932523	Hectd1	-0.0942465	3.55E-22	hypomethylated	0.052213	0.87635	insignificant	37	128	114
chr12	53072308	53074308	Heatr5a	-0.16606055	0.023134	hypomethylated	0.00045436	0.64259	insignificant	10	27	26
chr12	53107488	53109488	6530401N04Rik		1	noCoverage	0.064843	0.043383	inconclusive	0	22	22
chr12	53197732	53199732	Nubpl	-0.17984802	0.00000295	hypomethylated	-0.0058183	0.74414	insignificant	9	19	19
chr12	53799369	53801369	Akap6	-0.28908312	0.016074	hypomethylated	0.042576	0.011117	hypermethylated	2	13	13
chr12	54348663	54350663	Npas3		1	noCoverage	0.39748	0.13112	insignificant	0	5	10
chr12	55304861	55306861	Egln3	-0.22596154	1	insignificant	-0.014396	0.32943	insignificant	5	16	16
chr12	55492305	55494305	Gm7550		1	noCoverage	-0.0026677	0.79507	insignificant	0	37	42
chr12	55757559	55759559	1110002B05R	-0.12602961	1.26E-14	hypomethylated	0.010937	0.69706	insignificant	16	36	36
chr12	55796852	55798852	Eapp	-0.29511867	1	insignificant	0.091491	0.43658	insignificant	2	22	18
chr12	55896649	55898649	Snx6	0.31742424	1	insignificant	0.084824	0.000018249	inconclusive	1	18	12
chr12	55963864	55965864	Cfl2	-0.15535102	0.01198	hypomethylated	0.018856	0.68285	insignificant	12	60	60
chr12	56180482	56182482	Srp54a		1	noCoverage	0.091437	0.48167	insignificant	0	74	82
chr12	56181097	56183097	2700097O09Rik		1	noCoverage	0.1049	1	insignificant	0	66	74
chr12	56330464	56332464	Srp54c		1	noCoverage	0.023317	0.85986	insignificant	0	13	8
chr12	56402623	56404623	1110008L16Ri	-0.17813541	0.0018006	hypomethylated	0.013449	0.54728	insignificant	8	38	38
chr12	56403987	56405987	Ppp2r3c	-0.28884712	0.0085673	hypomethylated	-0.042932	1	insignificant	5	15	14
chr12	56498811	56500811	Psma6	-0.15476243	1.88E-14	hypomethylated	0.0031038	0.55132	insignificant	15	61	62
chr12	56593634	56595634	Nfkbia	-0.26736477	0.00000012	hypomethylated	-0.00035265	0.16167	insignificant	6	44	44
chr12	56665121	56667121	Aldoart2	-0.12691447	0.17847	insignificant	-0.055408	1	insignificant	5	10	10
chr12	56698903	56700903	Insm2	-0.11103584	5.78E-20	hypomethylated	0.0025562	0.25385	insignificant	51	145	138
chr12	56936352	56938352	Brms1l	-0.13642667	1.42E-58	hypomethylated	0.013152	0.29548	insignificant	42	96	92
chr12	57636093	57638093	Nkx2-1	-0.14245755	6.27E-22	hypomethylated	0.0093431	0.50098	insignificant	24	121	121
chr12	57637895	57639895	Nkx2-1	-0.17554229	0.061051	insignificant	-0.026589	0.80521	insignificant	4	40	40
chr12	57714271	57716271	Nkx2-9	-0.32112079	4.79E-10	hypomethylated	-0.049642	0.25093	insignificant	13	62	68
chr12	57795625	57797625	Pax9	-0.20776098	1.11E-08	hypomethylated	0.020725	0.8917	insignificant	16	65	63
chr12	58330398	58332398	Prps1l3	-0.17073037	0.000010872	hypomethylated	0.016041	0.36024	insignificant	8	75	75
chr12	58330411	58332411	Mipol1	-0.17073037	0.000010872	hypomethylated	0.016041	0.36024	insignificant	8	75	75
chr12	58664101	58666101	4921506M07Rik		1	noCoverage	0.026964	0.25339	insignificant	0	10	10
chr12	59311790	59313790	Sstr1	-0.17345079	9.04E-09	hypomethylated	0.0046202	0.2769	insignificant	11	46	47
chr12	59370245	59372245	Clec14a	-0.19331681	0.58707	insignificant	-0.014187	0.00055796	hypomethylated	2	10	12
chr12	60113004	60115004	Sec23a	0.01241571	7.29E-19	inconclusive	0.0016352	0.34049	insignificant	12	74	74

chr12	60113379	60115379	Sip1	-0.24870507	0.00034477	hypomethylated	0.05698	0.1543	insignificant	4	46	46
chr12	60162448	60164448	Trappc6b	-0.05160277	1	insignificant	0.095524	0.028929	hypermethylated	5	18	12
chr12	60166905	60168905	Pnn	-0.11400806	2.28E-38	hypomethylated	0.021096	0.19048	insignificant	35	98	98
chr12	60231439	60233439	Ctage5	-0.06797701	5.62E-24	hypomethylated	-0.0036498	0.79918	insignificant	57	196	191
chr12	60320470	60322470	Fbxo33	-0.07917975	1.17E-15	hypomethylated	-0.019427	0.41223	insignificant	47	154	168
chr12	62624618	62626618	Lrfn5	-0.17900499	7.53E-10	hypomethylated	0.031855	0.78534	insignificant	13	60	54
chr12	66017897	66019897	Gm527	-0.10450864	1.94E-23	hypomethylated	-0.0018405	0.24619	insignificant	23	144	150
chr12	66065728	66067728	Fam179b	-0.14395049	9.08E-21	hypomethylated	0.010646	0.18146	insignificant	34	90	90
chr12	66066523	66068523	Fam179b	-0.15830234	5.09E-21	hypomethylated	0.0054558	0.2339	insignificant	30	88	88
chr12	66136320	66138320	Prpf39	-0.10621336	7.37E-12	hypomethylated	0.025003	0.86172	insignificant	19	98	100
chr12	66174925	66176925	Fancm	-0.08659537	1.58E-10	hypomethylated	0.0041175	0.75256	insignificant	39	133	133
chr12	66175592	66177592	Fancm	-0.09365544	6.71E-10	hypomethylated	0.0052176	0.87447	insignificant	27	111	111
chr12	66273567	66275567	Mis18bp1	-0.1214033	9.33E-13	hypomethylated	-0.0092519	0.87595	insignificant	16	48	48
chr12	66325503	66327503	Wdr20b	0.0567405	0.61104	insignificant	0.011111	0.12984	insignificant	5	26	26
chr12	67385388	67387388	Rpl10l		1	noCoverage	0.14129	0.068046	insignificant	0	13	10
chr12	68323536	68325536	Mdga2	-0.12067737	0.000020019	hypomethylated	0.0036933	0.51147	insignificant	8	26	26
chr12	68770258	68772258	Gm17821	0.26170772	0.32319	insignificant	0.068749	0.11676	insignificant	1	6	6
chr12	70268800	70270800	Lrr1	-0.18355822	0.00018204	hypomethylated	-0.0058617	1	insignificant	20	75	82
chr12	70284144	70286144	Mgat2	-0.10513383	8.16E-33	hypomethylated	0.0033828	0.4228	insignificant	55	218	222
chr12	70285054	70287054	Rpl36al	-0.15359012	4.74E-31	hypomethylated	0.012609	0.0097083	hypermethylated	43	147	149
chr12	70297197	70299197	9330151L19Ri	-0.11565098	3.69E-37	hypomethylated	0.010337	0.37222	insignificant	61	207	199
chr12	70329177	70331177	Pole2	-0.54153546	0.00000355	stronglyHypometh	-0.042835	0.91436	insignificant	2	11	11
chr12	70341818	70343818	Klhdc1	-0.06197841	0.005219	hypomethylated	0.012095	0.26639	insignificant	14	65	65
chr12	70396667	70398667	Klhdc2	-0.06014951	0.000000415	hypomethylated	-0.00028143	0.24659	insignificant	24	107	86
chr12	70472136	70474136	Arf6	-0.10825597	6.98E-42	hypomethylated	0.0027172	0.17262	insignificant	75	254	253
chr12	70782839	70784839	Sos2	-0.06932434	3.79E-18	hypomethylated	-0.00010278	0.74157	insignificant	37	140	136
chr12	70824948	70826948	Atp5s	-0.15307383	6.16E-13	hypomethylated	-0.0070595	0.21526	insignificant	20	87	71
chr12	70825861	70827861	L2hgdh	-0.1173971	0.000021616	hypomethylated	0.014106	0.3367	insignificant	12	50	41
chr12	70890330	70892330	4930512B01R	-0.16660076	8.54E-12	hypomethylated	0.067366	0.11454	insignificant	13	67	68
chr12	70891694	70893694	Cdkl1	-0.09891071	1.1E-11	hypomethylated	0.046843	0.91217	insignificant	11	49	49
chr12	70993091	70995091	Atl1	-0.10263712	3.92E-35	hypomethylated	0.0091652	0.6003	insignificant	55	185	182
chr12	70994150	70996150	Atl1	-0.1426116	1.25E-19	hypomethylated	0.022946	1	insignificant	18	52	51
chr12	71087989	71089989	Sav1	-0.04774803	0.0040566	hypomethylated	-0.011028	0.027102	hypomethylated	26	62	62
chr12	71203841	71205841	Nin	0.18071429	1	lowCoverage	-0.14941	0.41194	insignificant	1	10	13
chr12	71212912	71214912	Nin	-0.12961487	5.74E-10	hypomethylated	0.11338	0.17636	insignificant	8	16	29
chr12	71254157	71256157	Abhd12b		1	noCoverage	0.12615	0.32829	insignificant	0	7	7
chr12	71328670	71330670	Pygl	-0.55964912	2.06E-09	stronglyHypometh	0.046441	0.11897	insignificant	1	8	8
chr12	71448601	71450601	Trim9	-0.55278617	0.011826	stronglyHypometh	0.08539	0.86835	insignificant	3	14	14
chr12	71553140	71555140	Tmx1	-0.19926042	0.00013276	hypomethylated	-0.024567	0.30205	insignificant	11	33	30
chr12	71925500	71927500	Frmd6	-0.08462021	6.53E-13	hypomethylated	0.024076	0.068489	insignificant	29	160	148
chr12	72037843	72039843	Actr10	-0.10665381	3.71E-14	hypomethylated	0.0058339	0.39495	insignificant	24	53	63
chr12	72074609	72076609	Psma3	-0.20489107	6.49E-26	hypomethylated	0.1307	0.10403	insignificant	16	95	58
chr12	72115953	72117953	Arid4a	-0.10594152	1.97E-41	hypomethylated	-0.0031394	0.78743	insignificant	92	248	242
chr12	72236834	72238834	2700049A03R	-0.1639906	5.85E-71	hypomethylated	0.0068459	0.77652	insignificant	22	67	65
chr12	72237662	72239662	Timm9	-0.31244138	0.036887	hypomethylated	0.018213	0.49443	insignificant	3	40	39
chr12	72409870	72411870	Dact1	-0.09567267	2.52E-12	hypomethylated	0.0046382	0.9777	insignificant	51	168	163
chr12	72931064	72933064	Daam1	-0.13121209	0.0065041	hypomethylated	0.0060709	0.69885	insignificant	14	64	62
chr12	73171978	73173978	Gpr135	-0.26348517	1	insignificant	-0.10904	0.27657	insignificant	1	15	27
chr12	73186109	73188109	Jkamp	-0.19388024	0.00007452	hypomethylated	-0.022724	0.53973	insignificant	10	49	28
chr12	73186300	73188300	Jkamp	-0.18744381	0.048673	hypomethylated	0.061648	0.95509	insignificant	4	29	10
chr12	73286016	73288016	4930403N07Rik		1	noCoverage	-0.05	1	insignificant	0	4	4
chr12	73337711	73339711	Rtn1	-0.04813112	0.25795	insignificant	-0.002893	0.48019	insignificant	7	22	22
chr12	73509926	73511926	Rtn1	0.09325397	1	insignificant	0.45833	0.00075244	stronglyhypermeth	3	6	6
chr12	73541852	73543852	Lrrc9	-0.14951684	0.000000193	hypomethylated	-0.039644	0.055336	insignificant	12	51	52
chr12	73542235	73544235	Lrrc9	-0.1064433	0.000000206	hypomethylated	-0.03917	0.085858	insignificant	13	53	54
chr12	73636343	73638343	1810048J11Ri	-0.25375574	2.91E-12	hypomethylated	0.026449	0.064932	insignificant	11	69	69
chr12	73765815	73767815	Dhrs7	-0.21929332	2.11E-16	hypomethylated	-0.026483	0.89886	insignificant	7	25	25
chr12	73861197	73863197	Ppm1a	-0.08607175	7.43E-22	hypomethylated	0.0062015	0.31516	insignificant	34	176	177
chr12	74018752	74020752	4930447C04Ri	0.20544128	0.22825	insignificant	0.039083	0.76171	insignificant	3	15	17
chr12	74039931	74041931	Six6	-0.23722529	8.61E-18	hypomethylated	-0.012771	0.10588	insignificant	11	72	75
chr12	74147699	74149699	Six1	-0.11183	0.0024261	hypomethylated	0.0070957	0.87206	insignificant	8	46	46
chr12	74214232	74216232	Six4	-0.30134974	0.12746	insignificant	0.026033	0.25406	insignificant	9	42	41
chr12	74223703	74225703	Mnat1		1	noCoverage	-0.017503	0.43444	insignificant	0	30	29
chr12	74386841	74388841	Trmt5	-0.20705665	1.01E-30	hypomethylated	-0.0163	0.75208	insignificant	23	64	68
chr12	74387698	74389698	Trmt5	-0.30614299	8.01E-10	hypomethylated	-0.016781	0.13131	insignificant	13	46	50
chr12	74647382	74649382	Tmem30b	-0.21380582	0.47542	insignificant	0.026443	0.6758	insignificant	18	47	47
chr12	74685027	74687027	Prkch	-0.16949032	2.65E-30	hypomethylated	-0.03633	0.13553	insignificant	47	141	124
chr12	75007853	75009853	Hif1a	-0.08763005	2.49E-23	hypomethylated	0.013339	0.027712	hypermethylated	42	148	162
chr12	75064516	75066516	Snapc1	-0.17853932	1.46E-17	hypomethylated	-0.018375	0.19169	insignificant	18	71	76
chr12	75097747	75099747	Syt16	-0.09087254	0.012464	hypomethylated	0.013664	1	insignificant	19	47	47
chr12	75384262	75386262	1700086L19Ri	-0.17074323	5.72E-09	hypomethylated	0.0015818	0.094053	insignificant	13	52	52
chr12	75397460	75399460	Dbpht2	-0.08963953	0.00012479	hypomethylated	-0.014184	0.19317	insignificant	4	10	12
chr12	76278319	76280319	Kcnh5	-0.18063316	0.026677	hypomethylated	-0.018303	0.46842	insignificant	10	51	50
chr12	76408299	76410299	Rhoj	-0.34951599	0.037945	stronglyHypometh	0.05895	0.54823	insignificant	4	40	39
chr12	76517768	76519768	Gphb5	-0.43333333	0.156	insignificant	-0.0074074	1	insignificant	2	6	6
chr12	76697187	76699187	Ppp2r5e	-0.10826388	8.16E-10	hypomethylated	-0.0010216	0.35448	insignificant	23	101	101
chr12	76770524	76772524	Wdr89	-0.15333333	0.00027352	hypomethylated	0.016012	0.83824	insignificant	6	12	12
chr12	76836716	76838716	Sgpp1	-0.12292661	1.57E-11	hypomethylated	0.0028111	0.75536	insignificant	16	40	40
chr12	76918304	76920304	Syne2	-0.13421111	7.5E-17	hypomethylated	0.0036774	0.068788	insignificant	31	143	141
chr12	77278246	77280246	Esr2	-0.3323001	0.56248	insignificant	0.069429	0.3995	insignificant	3	56	50
chr12	77347733	77349733	Tex21		1	noCoverage	0.074412	1	insignificant	0	6	7
chr12	77355218	77357218	Mthfd1	-0.14445682	2.13E-23	hypomethylated	0.023489	0.01299	hypermethylated	19	73	73
chr12	77424877	77426877	Akap5	-0.11965281	2.04E-08	hypomethylated	0.017466	0.58942	insignificant	20	94	93
chr12	77470252	77472252	Zbtb1	-0.07181099	2.44E-23	hypomethylated	0.010079	0.3621	insignificant	71	254	231
chr12	77470451	77472451	Zbtb25	-0.0658353	4.42E-15	hypomethylated	0.011194	0.63227	insignificant	60	230	207

chr12	77470547	77472547	Zbtb25	-0.07435536	2.67E-17	hypomethylated	0.014171	0.71607	insignificant	53	210	187
chr12	77504162	77506162	Hspa2	-0.10101093	0.0022398	hypomethylated	-0.0075136	0.23083	insignificant	16	84	88
chr12	77504356	77506356	Hspa2	-0.08512974	0.058392	insignificant	-0.013759	0.22024	insignificant	20	116	115
chr12	77517585	77519585	Ppp1r36	-0.21521638	0.00004387	hypomethylated	-0.031377	0.5029	insignificant	17	79	78
chr12	77544483	77546483	Gm10451		1	noCoverage	0.15057	0.25232	insignificant	0	6	6
chr12	77633546	77635546	Plekhg3	-0.12013538	3.41E-32	hypomethylated	0.001745	0.71038	insignificant	23	110	110
chr12	77811534	77813534	Spnb1	-0.21170274	0.000000363	hypomethylated	-0.029853	0.95885	insignificant	17	40	40
chr12	77865559	77867559	Churc1	-0.17035048	1.5E-15	hypomethylated	0.032906	0.27651	insignificant	16	66	66
chr12	77896541	77898541	Gpx2	0.18383686	1	lowCoverage	0.0038382	0.75614	insignificant	1	8	8
chr12	77923511	77925511	Rab15	-0.10862861	0.0039362	hypomethylated	-0.012022	0.45882	insignificant	16	60	60
chr12	77937453	77939453	Fntb		1	noCoverage	0.0043092	0.69812	insignificant	0	14	21
chr12	78063235	78065235	Max	-0.21993352	5.93E-14	hypomethylated	-0.012453	0.10532	insignificant	15	72	72
chr12	78339007	78341007	Fut8	-0.06397521	9.09E-09	hypomethylated	0.0082544	0.97941	insignificant	64	211	196
chr12	79326641	79328641	Gphn	-0.13803598	9.41E-43	hypomethylated	-0.0031546	0.27439	insignificant	48	180	180
chr12	79848933	79850933	Mpp5	-0.10655315	2.28E-18	hypomethylated	-0.017126	0.78958	insignificant	49	196	198
chr12	79962058	79964058	Eif2s1	-0.04569063	3.14E-24	hypomethylated	0.011032	0.97295	insignificant	23	90	86
chr12	79962625	79964625	Atp6v1d	0.06479118	0.00017357	inconclusive	-0.00071448	0.72911	insignificant	11	57	58
chr12	80007925	80009925	Plek2	-0.24398605	0.0000024	hypomethylated	-0.04677	0.57744	insignificant	3	13	12
chr12	80108264	80110264	Tmem229b	-0.16977113	0.0097346	hypomethylated	-0.063089	0.91629	insignificant	3	11	10
chr12	80129149	80131149	Plekhh1	-0.08946066	1.45E-14	hypomethylated	-0.0010986	0.51095	insignificant	35	106	106
chr12	80230774	80232774	Arg2	-0.15897503	0.0021799	hypomethylated	-0.002096	0.57274	insignificant	15	67	69
chr12	80273445	80275445	Vti1b	0.10461545	0.19582	insignificant	-0.010096	0.1695	insignificant	15	56	56
chr12	80308900	80310900	Rdh12	0.12303587	0.42184	insignificant	0.022181	1	insignificant	3	6	6
chr12	80397268	80399268	Rad51l1	-0.2053286	7.32E-08	hypomethylated	-0.010777	0.38418	insignificant	9	56	44
chr12	80397269	80399269	Rad51l1	-0.2053286	7.32E-08	hypomethylated	-0.010777	0.38418	insignificant	9	56	44
chr12	81214000	81216000	Zfp36l1	-0.13512186	1.04E-17	hypomethylated	0.019491	0.099367	insignificant	20	92	92
chr12	81233831	81235831	2310015A10R	-0.19526515	0.00001178	hypomethylated	0.025061	0.39022	insignificant	3	12	12
chr12	81361358	81363358	Actn1	-0.24155039	0.0000011	hypomethylated	-0.070341	0.89522	insignificant	4	22	20
chr12	81537588	81539588	Dcaf5	-0.0610849	0.000000997	hypomethylated	0.0024404	0.87911	insignificant	23	68	68
chr12	81563081	81565081	Exd2	-0.1260226	2.2E-10	hypomethylated	0.0053596	0.81738	insignificant	10	68	57
chr12	81618976	81620976	Galntl1	-0.09387145	6.9E-32	hypomethylated	0.0030879	0.060779	insignificant	65	247	253
chr12	81744201	81746201	Slc39a9	-0.07354238	2.82E-12	hypomethylated	0.036028	0.00722	hypermethylated	23	120	120
chr12	81744848	81746848	Erh	-0.09096832	8.08E-13	hypomethylated	0.046396	0.019609	hypermethylated	16	66	66
chr12	81792587	81794587	3830431G21R	-0.15537603	1.11E-09	hypomethylated	0.0050743	0.69173	insignificant	21	93	93
chr12	81861702	81863702	Gm1568	0.18312839	0.038784	hypermethylated	0.06476	0.093904	insignificant	3	22	22
chr12	81890545	81892545	4933426M11F	-0.1158922	1.88E-25	hypomethylated	0.013764	0.13929	insignificant	36	129	116
chr12	82025434	82027434	1700052I22Rik		1	noCoverage	0.054195	0.46594	insignificant	0	25	26
chr12	82045490	82047490	Srsf5	-0.14730132	9.18E-10	hypomethylated	-0.0028305	0.19532	insignificant	29	140	131
chr12	82126794	82128794	Smoc1	-0.09792728	0.00014472	hypomethylated	0.0024633	0.10498	insignificant	31	103	103
chr12	82434167	82436167	Slc8a3	-0.3394286	0.54069	insignificant	-0.0052041	0.62058	insignificant	2	12	12
chr12	82633894	82635894	Synj2bp	-0.08012821	1	insignificant	0.10021	0.12459	insignificant	2	12	10
chr12	82695945	82697945	Med6	-0.09706135	9.09E-19	hypomethylated	0.0011474	0.70271	insignificant	11	33	33
chr12	82731355	82733355	Ttc9	-0.1162184	1.64E-31	hypomethylated	-0.014556	0.97237	insignificant	24	127	130
chr12	82882157	82884157	Map3k9	-0.10535906	4.11E-15	hypomethylated	-0.0057852	0.48444	insignificant	16	52	52
chr12	82960016	82962016	Pcnx	-0.10737172	8.08E-19	hypomethylated	0.012679	0.47222	insignificant	33	161	155
chr12	83270002	83272002	Sipa1l1	-0.09481242	9.77E-21	hypomethylated	-0.0002518	0.33175	insignificant	67	211	210
chr12	83717024	83719024	Rgs6	-0.1645458	3.27E-16	hypomethylated	0.000053052	0.35325	insignificant	28	102	90
chr12	84828658	84830658	Dpf3		1	noCoverage	-0.017164	0.909	insignificant	0	16	16
chr12	84860415	84862415	Dcaf4	-0.07212291	0.060515	insignificant	0.0048549	1	insignificant	2	40	40
chr12	84865998	84867998	Dcaf4		1	noCoverage	-0.1143	0.053865	insignificant	0	16	17
chr12	84938097	84940097	Zfyve1	-0.38967803	1.75E-11	stronglyHypometh	-0.01944	0.82594	insignificant	3	6	6
chr12	84972183	84974183	Rbm25	-0.09382985	0.00000174	hypomethylated	-0.0027471	0.23202	insignificant	13	110	94
chr12	85028512	85030512	Psen1	-0.14443932	5.47E-24	hypomethylated	0.022437	0.18428	insignificant	20	66	66
chr12	85103590	85105590	Papln	-0.24967128	0.1137	insignificant	-0.010132	0.28026	insignificant	2	27	30
chr12	85290557	85292557	2410016O06R	-0.09895175	3.91E-44	hypomethylated	-0.0027212	0.58162	insignificant	60	184	181
chr12	85327810	85329810	Acot2		1	noCoverage	0.010928	0.86731	insignificant	0	41	41
chr12	85349451	85351451	Acot1	-0.00331396	0.038465	hypomethylated	0.0071357	0.13633	insignificant	8	83	80
chr12	85378328	85380328	Acot4	-0.14124329	0.0023381	hypomethylated	0.023604	0.49412	insignificant	4	32	32
chr12	85392100	85394100	Acot3	0.13392857	1	lowCoverage	0.043135	0.46948	insignificant	1	8	8
chr12	85409274	85411274	Acot5	-0.1080244	0.71581	insignificant	0.0061811	0.40774	insignificant	5	70	70
chr12	85440603	85442603	Acot6	-0.15564959	2.39E-08	hypomethylated	0.035401	0.21279	insignificant	8	85	64
chr12	85454277	85456277	Dnalc1	-0.21788397	0.35075	insignificant	-0.057769	0.73616	insignificant	2	18	19
chr12	85489439	85491439	C130039O16R	0.42725796	1	insignificant	0.053717	0.73807	insignificant	1	27	22
chr12	85534965	85536965	C130039O16R	-0.13645549	0.1699	insignificant	-0.015067	0.0018987	hypomethylated	8	22	22
chr12	85559831	85561831	C130039O16R	-0.06657997	1.51E-10	hypomethylated	-0.0018635	0.95891	insignificant	15	48	48
chr12	85625263	85627263	Ptgr2	-0.14159323	0.0095837	hypomethylated	-0.0044179	0.78086	insignificant	6	22	22
chr12	85657079	85659079	Zfp410	-0.12590751	0.00000684	hypomethylated	0.027317	0.49842	insignificant	30	122	120
chr12	85701917	85703917	Coq6	-0.21573692	0.000000907	hypomethylated	-0.019477	0.71236	insignificant	6	46	47
chr12	85702771	85704771	Fam161b	-0.22970981	0.00000266	hypomethylated	-0.034603	0.3769	insignificant	7	44	45
chr12	85749017	85751017	2900006K08Ri	-0.10924814	0.000005056	hypomethylated	-0.017882	0.55517	insignificant	20	78	77
chr12	85757149	85759149	Rnf113a2	-0.29613236	2.41E-23	hypomethylated	0.11889	0.95494	insignificant	7	53	54
chr12	85791457	85793457	Lin52	-0.25229319	3.6E-19	hypomethylated	-0.012407	0.73346	insignificant	15	61	61
chr12	85791900	85793900	Aldh6a1	-0.37361702	3.55E-08	stronglyHypometh	-0.027096	0.89761	insignificant	8	50	50
chr12	85909801	85911801	Vsx2	-0.1571417	1.08E-08	hypomethylated	0.0087663	0.29296	insignificant	23	102	97
chr12	85958416	85960416	Abcd4	-0.52479723	3.93E-11	stronglyHypometh	-0.078297	0.50642	insignificant	3	14	15
chr12	85980968	85982968	Vrtn	-0.38166441	0.0045358	stronglyHypometh	0.01477	0.9249	insignificant	3	16	16
chr12	85982504	85984504	Vrtn	-0.2417968	0.00077019	hypomethylated	-0.0088828	1	insignificant	8	43	42
chr12	85982845	85984845	Vrtn	-0.18493263	0.17891	insignificant	0.0025281	0.47107	insignificant	9	40	39
chr12	86039757	86041757	Tmem90a	-0.09392306	0.0067249	hypomethylated	0.09705	0.43012	insignificant	9	25	18
chr12	86113219	86115219	Isca2	-0.14817832	0.00094407	hypomethylated	0.061983	0.90281	insignificant	8	61	61
chr12	86114062	86116062	Npc2	-0.14661862	0.056646	insignificant	0.057123	0.37374	insignificant	2	32	32
chr12	86215751	86217751	D030025P21R	-0.17460012	3.02E-23	hypomethylated	-0.0020695	0.2046	insignificant	15	117	114
chr12	86217445	86219445	D030025P21R	-0.16000829	1	insignificant	0.034469	0.88654	insignificant	3	26	28
chr12	86310879	86312879	Fcf1	-0.28763327	2.01E-37	hypomethylated	-0.0071053	0.6718	insignificant	12	77	73
chr12	86311836	86313836	Fcf1	-0.39220805	9.57E-46	stronglyHypometh	-0.0069236	0.54873	insignificant	9	44	42

chr12	86336270	86338270	Ylpm1	-0.04974834	1.03E-14	hypomethylated	0.012891	0.30692	insignificant	30	127	123
chr12	86450782	86452782	Dlst	-0.16263565	1.23E-58	hypomethylated	-0.0062751	0.88069	insignificant	46	119	120
chr12	86492214	86494214	Rps6kl1	-0.48964052	0.000000965	stronglyHypometh	-0.026686	0.0020379	hypomethylated	2	10	14
chr12	86518235	86520235	Pgf	-0.16929081	1.76E-16	hypomethylated	-0.018089	0.14185	insignificant	8	32	32
chr12	86559462	86561462	Eif2b2	-0.19817794	1.02E-09	hypomethylated	0.022267	0.50657	insignificant	19	106	100
chr12	86621385	86623385	Acyp1	-0.10889765	1	insignificant	0.010428	0.28564	insignificant	1	14	14
chr12	86628540	86630540	Fam164c	-0.26036949	0.0011432	hypomethylated	0.046936	0.24976	insignificant	4	31	31
chr12	86680312	86682312	Nek9	-0.49315789	0.0032853	stronglyHypometh	0.054461	0.49293	insignificant	1	2	2
chr12	86715667	86717667	Tmed10	0.31044959	1	insignificant	0.020972	0.2411	insignificant	2	15	13
chr12	86813850	86815850	Fos	-0.17331199	1.67E-31	hypomethylated	-0.010152	0.40679	insignificant	26	104	102
chr12	86939365	86941365	Jdp2	-0.11270384	1.04E-10	hypomethylated	0.0018588	0.57053	insignificant	57	176	157
chr12	87026669	87028669	Batf	-0.35264893	0.022541	stronglyHypometh	0.044808	0.12764	insignificant	3	24	24
chr12	87086488	87088488	Mfsd7c	-0.16934674	2.09E-10	hypomethylated	-0.010941	0.24557	insignificant	13	57	53
chr12	87164899	87166899	Ttll5	-0.17622238	7.11E-19	hypomethylated	0.0060819	0.083944	insignificant	15	70	71
chr12	87165495	87167495	0610007P14Ri	-0.15860924	1.29E-10	hypomethylated	0.0090168	0.95572	insignificant	13	56	57
chr12	87419991	87421991	Tgfb3	-0.35636981	5.18E-10	stronglyHypometh	0.0059996	0.55753	insignificant	5	24	24
chr12	87422512	87424512	1700019E19Ri	-0.25639725	5.09E-21	hypomethylated	0.046632	0.74942	insignificant	16	65	65
chr12	87581827	87583827	1700020O03R	-0.15952638	0.0099228	hypomethylated	-0.026054	0.44924	insignificant	10	50	49
chr12	87701066	87703066	Esrrb	-0.74146702	0.0018006	stronglyHypometh	0.10512	0.8962	insignificant	1	14	12
chr12	87761593	87763593	Esrrb	-0.0635866	0.29277	insignificant	0.020041	0.12476	insignificant	21	97	86
chr12	88018649	88020649	Vash1	-0.13168051	2.81E-13	hypomethylated	-0.00008072	0.25094	insignificant	25	93	92
chr12	88074318	88076318	Gm6772	0.13640069	0.60549	insignificant	-0.02098	0.34439	insignificant	1	14	14
chr12	88286992	88288992	2310044G17R	-0.14644731	9.55E-15	hypomethylated	0.0022779	0.3236	insignificant	18	94	93
chr12	88287292	88289292	2310044G17R	-0.14644731	9.55E-15	hypomethylated	0.0022779	0.3236	insignificant	18	94	93
chr12	88329626	88331626	Zdhhc22	-0.15511164	6.7E-13	hypomethylated	0.0055612	0.71235	insignificant	21	72	77
chr12	88366513	88368513	Tmem63c	-0.33576311	0.0035022	stronglyHypometh	0.02307	0.029481	inconclusive	7	63	62
chr12	88487667	88489667	Gstz1	-0.17642504	1.07E-29	hypomethylated	-0.010129	0.23758	insignificant	26	116	115
chr12	88488852	88490852	Gstz1	-0.23174613	0.0030255	hypomethylated	0.02301	0.11774	insignificant	9	49	45
chr12	88541179	88543179	Tmed8	-0.11230829	4.47E-23	hypomethylated	0.011661	0.51847	insignificant	21	62	62
chr12	88606677	88608677	Ahsa1	-0.16721726	0.37537	insignificant	-0.0094152	0.81915	insignificant	2	47	45
chr12	88607236	88609236	Vipar	-0.16721726	0.37537	insignificant	-0.0094152	0.81915	insignificant	2	47	45
chr12	88729180	88731180	Sptlc2	-0.13709199	6.03E-11	hypomethylated	0.020285	0.054668	insignificant	20	66	63
chr12	88778707	88780707	Alkbh1	-0.05624319	1	insignificant	-0.070303	0.27161	insignificant	2	24	23
chr12	88783845	88785845	1810035L17Ri	-0.28973652	4.35E-08	hypomethylated	-0.04535	0.40158	insignificant	9	32	30
chr12	88813249	88815249	Snw1	-0.52727273	0.0085868	stronglyHypometh	0.075668	0.17585	insignificant	0	0	0
chr12	88958817	88960817	BB287469		1	noCoverage	-0.025	0.63918	insignificant	0	8	4
chr12	88958858	88960858	Gm4027		1	noCoverage	-0.025	0.63918	insignificant	0	8	4
chr12	89598244	89600244	Adck1	-0.1774062	0.000000735	hypomethylated	-0.025666	0.46126	insignificant	18	54	54
chr12	89960319	89962319	Nrxn3	-0.33985314	0.5552	insignificant	0.029092	0.57712	insignificant	1	24	24
chr12	91976878	91978878	Dio2	-0.43188406	0.0066068	stronglyHypometh	-0.028679	0.53415	insignificant	1	5	5
chr12	92622849	92624849	4930534B04R	-0.29125616	1	insignificant	0.031624	0.2485	insignificant	2	8	8
chr12	92638432	92640432	Tshr	-0.18484805	0.000000232	hypomethylated	-0.0061997	0.64456	insignificant	7	18	18
chr12	92828089	92830089	Gtf2a1	-0.14321424	1.69E-11	hypomethylated	-0.029751	0.80442	insignificant	30	118	112
chr12	92828927	92830927	Gtf2a1	-0.30837643	0.011722	inconclusive	0.026087	0.45981	insignificant	7	27	26
chr12	93087597	93089597	Sel1l		1	noCoverage	0.017414	0.62079	insignificant	0	12	12
chr12	96929435	96931435	Flrt2	-0.26654381	1.08E-14	hypomethylated	-0.003708	0.76957	insignificant	16	74	71
chr12	99497547	99499547	Galc	-0.15774116	0.091725	insignificant	0.01216	0.24363	insignificant	7	30	30
chr12	99816150	99818150	Kcnk10	-0.14428534	0.34963	insignificant	-0.10191	0.92497	insignificant	6	20	20
chr12	99865393	99867393	Spata7	-0.29925347	2.74E-09	hypomethylated	-0.005173	0.16044	insignificant	8	25	24
chr12	99975615	99977615	Ptpn21	-0.0588219	0.000017424	hypomethylated	0.011497	0.93873	insignificant	22	61	55
chr12	99984177	99986177	Zc3h14	-0.08248544	4.09E-09	hypomethylated	0.0049065	0.1959	insignificant	23	132	132
chr12	100139694	100141694	Eml5	-0.125	1	insignificant	0.0073529	0.8527	insignificant	2	8	8
chr12	100157783	100159783	Ttc8	-0.1535551	0.012684	hypomethylated	0.043411	0.22425	insignificant	7	25	20
chr12	100157821	100159821	Ttc8	-0.1535551	0.012684	hypomethylated	0.043411	0.22425	insignificant	7	25	20
chr12	100688284	100690284	Foxn3	0.03518519	1	insignificant	-0.0090067	1	insignificant	2	6	6
chr12	101121652	101123652	Tdp1	-0.11226821	0.000013047	hypomethylated	-0.014597	0.17878	insignificant	19	85	75
chr12	101121724	101123724	Tdp1	-0.13053201	0.00000524	hypomethylated	-0.01742	0.16365	insignificant	19	88	77
chr12	101201708	101203708	Kcnk13	-0.18045811	0.000000192	hypomethylated	-0.00212	0.57886	insignificant	6	59	57
chr12	101349540	101351540	Psmc1	0.04644174	1	insignificant	0.01149	0.61347	insignificant	3	14	14
chr12	101436750	101438750	Calm1	-0.20054248	6.74E-35	hypomethylated	-0.07903	0.82944	insignificant	49	160	152
chr12	101759032	101761032	Ttc7b	-0.1312601	5.09E-11	hypomethylated	-0.0038938	0.93141	insignificant	16	26	36
chr12	101963238	101965238	Rps6ka5	-0.25078017	1	lowCoverage	-0.001118	0.438	insignificant	1	32	28
chr12	102016332	102018332	9030617O03Rik		1	noCoverage	0.061328	0.06296	insignificant	0	6	6
chr12	102146408	102148408	Gpr68	-0.45963251	0.017759	stronglyHypometh	-0.10236	0.59883	insignificant	4	20	17
chr12	102260003	102262003	Ccdc88c	0.04621849	1	lowCoverage	-0.1016	0.082521	insignificant	1	10	16
chr12	102267193	102269193	Ccdc88c	-0.20884683	0.15068	insignificant	0.00059692	0.50244	insignificant	10	47	64
chr12	102321912	102323912	D130020L05Ri	-0.23297551	0.000000841	hypomethylated	0.015383	0.036944	inconclusive	7	50	50
chr12	102949783	102951783	Tc2n	-0.89846154	0.11377	lowCoverage	-0.0042514	1	insignificant	1	20	20
chr12	102956712	102958712	Tc2n	-0.14747303	0.000058604	hypomethylated	-0.023927	0.29088	insignificant	6	24	24
chr12	103151381	103153381	Trip11	-0.10580076	0.12763	insignificant	0.025098	0.6063	insignificant	3	20	20
chr12	103196453	103198453	Atxn3	-0.05515128	0.00044029	hypomethylated	0.00062832	0.38734	insignificant	10	20	20
chr12	103213183	103215183	Cpsf2	-0.11700772	1.58E-14	hypomethylated	0.0057943	0.03228	hypermethylated	26	118	116
chr12	103366628	103368628	Slc24a4	-0.06929287	8.51E-09	hypomethylated	-0.00017856	0.45098	insignificant	32	130	130
chr12	103520283	103522283	Rin3	-0.09809573	2.35E-10	hypomethylated	0.0025229	0.54089	insignificant	22	109	106
chr12	103520850	103522850	Rin3	-0.11684377	1.61E-11	hypomethylated	-0.0069735	0.45111	insignificant	22	113	109
chr12	103677907	103679907	Lgmn	-0.00752109	0.046976	hypomethylated	0.039256	0.02225	hypermethylated	7	26	26
chr12	103706343	103708343	Golga5	-0.12133505	6.92E-31	hypomethylated	0.0087849	0.31063	insignificant	47	141	136
chr12	103707119	103709119	Golga5	-0.12842725	6.82E-31	hypomethylated	-0.0056111	0.32674	insignificant	46	139	132
chr12	103792178	103794178	Chga	-0.14509955	2.17E-20	hypomethylated	-0.0088416	0.62473	insignificant	12	56	56
chr12	103923230	103925230	Itpk1	-0.19277438	0.091732	insignificant	0.25057	0.00000798	hypermethylated	4	8	8
chr12	103943079	103945079	Itpk1	-0.12497775	1.67E-15	hypomethylated	-0.017115	0.50239	insignificant	34	99	111
chr12	103980969	103982969	D230037D09R	-0.12585595	0.0029787	hypomethylated	-0.026337	0.65157	insignificant	30	102	89
chr12	103981870	103983870	AK010878	-0.08564173	0.081357	insignificant	0.041465	0.87423	insignificant	15	34	33
chr12	103995184	103997184	Ubr7	-0.12853945	1.79E-29	hypomethylated	0.0003209	0.094673	insignificant	27	126	122
chr12	103996020	103998020	AK010878	-0.1341377	1.07E-22	hypomethylated	0.0064751	0.63224	insignificant	19	86	79

chr12	104116616	104118616	Btbd7	-0.10183347	9.09E-13	hypomethylated	0.010037	0.26419	insignificant	19	72	74
chr12	104136515	104138515	Cox8c	0.10084256	1	insignificant	0.049861	0.31177	insignificant	1	17	15
chr12	104186068	104188068	Unc79	-0.17487897	4.25E-14	hypomethylated	0.02713	0.50464	insignificant	13	52	52
chr12	104480356	104482356	Prima1	-0.14917789	0.68299	insignificant	0.0040617	0.774	insignificant	21	88	87
chr12	104552168	104554168	Fam181a	0.00540917	0.17271	insignificant	-0.0056735	0.77553	insignificant	14	56	56
chr12	104614103	104616103	Otub2		1	noCoverage	-0.035335	0.11089	insignificant	0	6	9
chr12	104625890	104627890	Otub2	-0.10183332	1.54E-17	hypomethylated	0.0068932	0.68068	insignificant	18	76	76
chr12	104625891	104627891	Otub2	-0.10183332	1.54E-17	hypomethylated	0.0068932	0.68068	insignificant	18	76	76
chr12	104663990	104665990	Ddx24	-0.13373016	0.00000316	hypomethylated	-0.022625	1	insignificant	3	30	30
chr12	104664077	104666077	Ddx24		1	noCoverage	-0.034425	0.53195	insignificant	0	24	24
chr12	104681890	104683890	Ifi27l2a	-0.10960591	0.39006	insignificant	-0.018832	0.29043	insignificant	1	6	6
chr12	104769774	104771774	Ppp4r4	-0.1016604	2E-26	hypomethylated	-0.0017442	0.52992	insignificant	34	163	154
chr12	104976399	104978399	Serpina1b		1	noCoverage	0.10988	0.31788	insignificant	0	13	18
chr12	105101829	105103829	Serpina1a	-0.41830983	0.28948	insignificant	-0.26275	0.017871	hypomethylated	2	11	12
chr12	105143160	105145160	Serpina1c	0.16666667	0.46119	insignificant	0.15889	0.017496	hypermethylated	2	10	10
chr12	105195107	105197107	Serpina1e	-0.29664328	0.030027	hypomethylated	-0.11159	0.64756	insignificant	1	12	14
chr12	105228153	105230153	Serpina11		1	noCoverage	0.0125	1	insignificant	0	4	4
chr12	105228167	105230167	Serpina11		1	noCoverage	0.0125	1	insignificant	0	4	4
chr12	105349933	105351933	Serpina3a		1	noCoverage	-0.0031526	1	insignificant	0	18	18
chr12	105392082	105394082	Serpina3c	-0.11344048	0.084122	insignificant	-0.13295	0.03705	hypomethylated	3	22	26
chr12	105485105	105487105	Serpina3h	-0.23876984	0.10789	insignificant	-0.035066	0.19162	insignificant	2	10	10
chr12	105643917	105645917	Serpina3n	0.29464286	1	insignificant	-0.026906	1	insignificant	1	4	4
chr12	105711446	105713446	Gsc	-0.20815386	1.23E-08	hypomethylated	-0.018533	0.079246	insignificant	11	45	45
chr12	105990162	105992162	Dicer1	-0.14102986	0.000000119	hypomethylated	-0.00000605	0.42392	insignificant	19	110	95
chr12	106103286	106105286	Clmn	-0.09472449	0.00013267	hypomethylated	0.022817	0.19038	insignificant	10	22	22
chr12	106236887	106238887	4831426I19Ril	-0.20214599	0.020658	hypomethylated	0.10619	0.80672	insignificant	3	8	6
chr12	106248019	106250019	4831426I19Ril	-0.13790105	9.9E-10	hypomethylated	0.024525	0.77069	insignificant	15	30	33
chr12	106269559	106271559	Snhg10	-0.09851429	3.23E-31	hypomethylated	-0.001185	0.85718	insignificant	30	172	165
chr12	106269898	106271898	Glrx5	-0.12254532	5.46E-30	hypomethylated	-0.0018517	0.54379	insignificant	28	154	147
chr12	106270489	106272489	Snhg10	-0.09341081	2.3E-14	hypomethylated	0.023556	0.13797	insignificant	25	120	121
chr12	106384242	106386242	Tcl1b2	0.01815789	0.16102	insignificant	0.094286	0.58232	insignificant	2	4	4
chr12	106396913	106398913	Tcl1b1		1	noCoverage	0.020833	0.70531	insignificant	0	6	6
chr12	106460947	106462947	Tcl1	-0.9072807	0.018234	stronglyHypometh	-0.091582	0.19323	insignificant	2	10	10
chr12	106691185	106693185	D430019H16R	-0.14015459	1.22E-14	hypomethylated	-0.015631	0.23425	insignificant	11	52	48
chr12	106800381	106802381	Bdkrb2	-0.22468623	0.00000026	hypomethylated	0.010112	0.57537	insignificant	8	22	20
chr12	106841300	106843300	Bdkrb1	-0.01842287	1	insignificant	0.06954	0.0047117	hypermethylated	1	11	9
chr12	106922561	106924561	4933433P14Ri	-0.14865207	2.66E-34	hypomethylated	-0.0017972	0.35883	insignificant	42	143	143
chr12	106923451	106925451	Atg2b	-0.16207503	2.27E-09	hypomethylated	-0.0026381	0.54228	insignificant	17	60	60
chr12	106943191	106945191	Ak7	0.33522727	1	lowCoverage	0.27273	0.088781	insignificant	1	4	4
chr12	107021911	107023911	Papola	-0.18269028	1.05E-27	hypomethylated	-0.01231	0.63718	insignificant	34	78	87
chr12	107247472	107249472	Vrk1	-0.16874465	1.31E-25	hypomethylated	-0.012967	0.81014	insignificant	12	45	45
chr12	107685876	107687876	1700121N20R	-0.05909499	1	insignificant	0.010315	0.75347	insignificant	6	22	21
chr12	107954534	107956534	4933406K04Rik		1	noCoverage	-0.0056518	0.7391	insignificant	0	18	18
chr12	109241624	109243624	Bcl11b	-0.14248643	1.76E-17	hypomethylated	0.023147	0.23408	insignificant	40	108	119
chr12	109416947	109418947	Ccnk	-0.08187255	5.44E-15	hypomethylated	-0.0090418	0.69046	insignificant	54	212	206
chr12	109417494	109419494	Setd3	-0.11532306	0.00012077	hypomethylated	-0.011191	0.91588	insignificant	9	86	80
chr12	109513627	109515627	Ccdc85c	-0.17509964	2.72E-14	hypomethylated	-0.00072431	0.026118	hypomethylated	29	96	97
chr12	109543479	109545479	Hhipl1	-0.10835566	1.05E-11	hypomethylated	0.00074279	0.6608	insignificant	26	81	81
chr12	109571590	109573590	Cyp46a1	-0.12072473	2.83E-13	hypomethylated	0.01236	0.099496	insignificant	22	122	122
chr12	109647864	109649864	Eml1	-0.10092221	0.3399	insignificant	0.052507	0.76803	insignificant	4	10	10
chr12	109660025	109662025	Eml1	-0.11499611	3.22E-23	hypomethylated	-0.0027555	0.5716	insignificant	45	145	131
chr12	109791929	109793929	Evl	-0.12488161	6.04E-40	hypomethylated	-0.0064573	0.47106	insignificant	57	170	181
chr12	109842975	109844975	Evl	-0.28062678	0.2782	insignificant	0.17873	0.15539	insignificant	3	6	6
chr12	109940516	109942516	Degs2	-0.21681263	0.033667	hypomethylated	0.013697	0.61561	insignificant	4	39	39
chr12	110030520	110032520	Yy1	-0.0727623	4.94E-36	hypomethylated	-0.004722	0.58414	insignificant	84	369	377
chr12	110074086	110076086	Slc25a29	-0.09256431	0.00000651	hypomethylated	0.044741	0.75553	insignificant	19	81	84
chr12	110074182	110076182	Mir345	-0.09209113	0.0057009	hypomethylated	0.052965	0.42143	insignificant	14	63	66
chr12	110088338	110090338	Slc25a47		1	noCoverage	-0.0092831	0.34758	insignificant	0	14	13
chr12	110131408	110133408	Wars	-0.13753099	1.99E-10	hypomethylated	0.027377	0.81627	insignificant	12	47	33
chr12	110131421	110133421	Wars	-0.13753099	1.99E-10	hypomethylated	0.027377	0.81627	insignificant	12	47	33
chr12	110131481	110133481	Wdr25	-0.13753099	1.99E-10	hypomethylated	0.027377	0.81627	insignificant	12	47	33
chr12	110132384	110134384	Wars	-0.07264084	0.0072759	hypomethylated	0.020315	0.86765	insignificant	6	26	21
chr12	110306427	110308427	Begain	-0.1448293	0.000000009	hypomethylated	0.0074244	0.69006	insignificant	14	46	35
chr12	110690032	110692032	Dlk1	-0.07716193	8E-12	hypomethylated	0.02623	0.10137	insignificant	17	73	64
chr12	110690664	110692664	Dlk1	-0.09821581	8.72E-10	hypomethylated	0.021883	0.0055297	hypermethylated	19	79	68
chr12	110778205	110780205	Meg3	0.16202727	0.73001	insignificant	0.21629	0.16885	insignificant	3	41	40
chr12	110781750	110783750	Mir1906-2	0.11845325	1	lowCoverage	-0.014072	0.322	insignificant	1	11	11
chr12	110782607	110784607	Meg3	-0.07401708	0.14635	insignificant	-0.0099308	0.035844	hypomethylated	3	23	19
chr12	110822998	110824998	Mir337	-0.24407425	0.00061494	hypomethylated	0.003313	0.012325	hypermethylated	8	26	26
chr12	110823289	110825289	Mir540	-0.24407425	0.00061494	hypomethylated	0.003313	0.012325	hypermethylated	8	26	26
chr12	110823523	110825523	Mir665	-0.24407425	0.00061494	hypomethylated	0.003313	0.012325	hypermethylated	8	26	26
chr12	110827656	110829656	Mir431	0.15977059	0.61323	insignificant	0.023307	0.00013709	hypermethylated	5	46	49
chr12	110828746	110830746	6430411K18Ri	-0.04578143	0.64207	insignificant	0.033095	0.0018356	hypermethylated	8	38	38
chr12	110828924	110830924	Mir433	-0.08471416	0.36158	insignificant	0.016016	0.0082983	hypermethylated	8	41	38
chr12	110830055	110832055	Mir127	-0.01487171	0.29552	insignificant	0.023328	0.043006	hypermethylated	9	39	38
chr12	110831715	110833715	Mir434	-0.14654924	0.046763	hypomethylated	0.0054848	0.83385	insignificant	8	37	34
chr12	110832165	110834165	Mir432	-0.15488673	0.28306	insignificant	-0.014981	0.36077	insignificant	5	31	28
chr12	110832536	110834536	Mir136	-0.13280124	0.2873	insignificant	0.0071049	0.3653	insignificant	5	33	28
chr12	110919406	110921406	Mir882	0.07565789	1	insignificant	0.0031579	1	insignificant	2	4	4
chr12	110949012	110951012	Mir380	0.03825983	0.031631	hypermethylated	0.01889	0.30286	insignificant	9	32	27
chr12	110949526	110951526	Mir1197	0.03825983	0.031631	hypermethylated	0.01889	0.30286	insignificant	9	32	27
chr12	110949717	110951717	Mir323	0.03604634	0.031891	hypermethylated	0.016677	0.30391	insignificant	9	33	27
chr12	110950019	110952019	Mir758	0.03604634	0.031891	hypermethylated	0.016677	0.30391	insignificant	9	33	27
chr12	110950690	110952690	Mir329	0.08167614	0.018938	hypermethylated	0.0078556	0.82773	insignificant	6	32	25
chr12	110955963	110957963	Mir495		1	noCoverage	-0.38722	0.70024	insignificant	0	10	10

chr12	110960667	110962667	Mir376b	-0.09722222	0.46906	insignificant	-0.063756	0.098397	insignificant	6	12	14
chr12	110960990	110962990	Mir376a	-0.09722222	0.46906	insignificant	-0.063756	0.098397	insignificant	6	12	14
chr12	110961522	110963522	Mir300	-0.06354776	1	insignificant	-0.040951	0.13979	insignificant	8	20	22
chr12	110965338	110967338	Mir539	0.04398148	0.58123	insignificant	-0.0064387	0.72029	insignificant	1	9	4
chr12	110971348	110973348	Mir134	-0.13375687	0.14623	insignificant	0.055804	0.2839	insignificant	2	8	8
chr12	110971941	110973941	Mir668	-0.13375687	0.14623	insignificant	0.055804	0.2839	insignificant	2	8	8
chr12	110972111	110974111	Mir485	-0.13375687	0.14623	insignificant	0.055804	0.2839	insignificant	2	8	8
chr12	110972190	110974190	Mirg	-0.13375687	0.14623	insignificant	0.055804	0.2839	insignificant	2	8	8
chr12	110972828	110974828	Mir453	-0.13375687	0.14623	insignificant	0.055804	0.2839	insignificant	2	8	8
chr12	110979618	110981618	Mir541		1	noCoverage	0.034722	0.35739	insignificant	0	2	2
chr12	110980367	110982367	Mir409	0.09166667	1	insignificant	0.02601	0.60866	insignificant	1	4	4
chr12	110980498	110982498	Mir412	0.09166667	1	insignificant	0.02601	0.60866	insignificant	1	4	4
chr12	110980627	110982627	Mir369	0.09166667	1	insignificant	0.02601	0.60866	insignificant	1	4	4
chr12	110980924	110982924	Mir410	0.09166667	1	insignificant	0.02601	0.60866	insignificant	1	4	4
chr12	110985087	110987087	Mir3072	-0.83653846	0.026923	stronglyHypometh	0.16346	0.30645	insignificant	1	4	4
chr12	111516278	111518278	Dio3os	-0.13627687	1.19E-41	hypomethylated	0.011717	0.026221	hypermethylated	58	223	223
chr12	111516339	111518339	Dio3os	-0.13279899	3.28E-40	hypomethylated	0.01136	0.013	hypermethylated	58	223	223
chr12	111516439	111518439	Dio3	-0.13493896	4.5E-30	hypomethylated	0.0091816	0.013315	hypermethylated	50	201	201
chr12	111684388	111686388	Ppp2r5c	-0.10074791	3.19E-13	hypomethylated	0.0075508	0.45718	insignificant	32	186	171
chr12	111722948	111724948	Ppp2r5c	-0.1453358	0.000010885	hypomethylated	-0.031911	0.66259	insignificant	5	33	29
chr12	111828583	111830583	B930059L03Ri	-0.11755315	0.000049978	hypomethylated	0.048763	0.0014721	hypermethylated	20	90	89
chr12	111838604	111840604	Dync1h1	-0.10695111	5.67E-24	hypomethylated	0.017139	0.89212	insignificant	34	132	116
chr12	111920829	111922829	1700001K19Ri	-0.42181533	0.00055125	stronglyHypometh	0.047406	0.1034	insignificant	2	6	6
chr12	111934605	111936605	Hsp90aa1	-0.1086289	0.064151	insignificant	-0.021168	0.46262	insignificant	6	67	70
chr12	111975158	111977158	Wdr20a	-0.1380491	4.59E-15	hypomethylated	-0.014863	0.52272	insignificant	21	131	122
chr12	112079149	112081149	Stk30	-0.4038961	0.001512	stronglyHypometh	-0.11312	0.84641	insignificant	2	5	4
chr12	112087488	112089488	Zfp839	-0.12960917	6.01E-35	hypomethylated	0.019547	0.88444	insignificant	33	98	92
chr12	112126473	112128473	Tecpr2	-0.12102468	1.24E-21	hypomethylated	0.00032211	0.72081	insignificant	20	80	80
chr12	112127324	112129324	Cinp	-0.11249123	4.89E-19	hypomethylated	-0.00022501	0.94013	insignificant	17	66	66
chr12	112217231	112219231	Ankrd9	-0.12850029	5.14E-17	hypomethylated	0.0053588	0.6812	insignificant	18	53	53
chr12	112277008	112279008	Rcor1	-0.07123541	5.06E-18	hypomethylated	0.0028807	1	insignificant	44	234	227
chr12	112403758	112405758	Traf3	-0.09841187	7.4E-24	hypomethylated	0.012123	0.54907	insignificant	46	153	144
chr12	112508321	112510321	Amn	0.03185139	0.0013729	hypermethylated	-0.021514	0.54858	insignificant	10	71	65
chr12	112615929	112617929	Cdc42bpb	-0.25488394	1.97E-13	hypomethylated	-0.051428	0.3132	insignificant	14	65	75
chr12	112679871	112681871	Tnfaip2	-0.12573216	0.000000845	hypomethylated	0.0044516	0.16741	insignificant	7	57	57
chr12	112724034	112726034	Gm266	-0.10436557	0.000069457	hypomethylated	0.010272	0.813	insignificant	17	51	51
chr12	112775311	112777311	Eif5	-0.09055504	1.32E-17	hypomethylated	0.0013307	0.00000109	hypermethylated	72	226	223
chr12	112811720	112813720	Mark3	-0.12930605	7.81E-16	hypomethylated	0.016757	0.032233	hypermethylated	28	101	100
chr12	112910549	112912549	Ckb	-0.36064334	0.065165	insignificant	0.0059959	1	insignificant	2	32	32
chr12	112915315	112917315	Trmt61a	-0.150629	8.4E-11	hypomethylated	0.0039566	0.76862	insignificant	8	51	51
chr12	112950479	112952479	2810002N01R	-0.09258243	4.06E-32	hypomethylated	-0.0031998	0.45074	insignificant	61	205	202
chr12	112951467	112953467	Bag5	-0.10569356	7.85E-12	hypomethylated	-0.0037753	0.33893	insignificant	28	72	72
chr12	112996059	112998059	Klc1	-0.0856006	1.8E-43	hypomethylated	0.0054421	0.40414	insignificant	37	146	144
chr12	113051480	113053480	Zfyve21	-0.097169	5.32E-45	hypomethylated	-0.010578	0.92058	insignificant	21	112	119
chr12	113052052	113054052	Zfyve21	-0.08842863	4.54E-32	hypomethylated	-0.01179	0.78683	insignificant	17	84	91
chr12	113146266	113148266	Ppp1r13b	-0.03747646	2.72E-11	hypomethylated	0.011828	0.96354	insignificant	15	58	57
chr12	113205188	113207188	2010107E04Rik		1	noCoverage	-0.10623	0.53678	insignificant	0	17	15
chr12	113208769	113210769	Tdrd9	-0.19218849	0.036305	hypomethylated	0.011902	0.33869	insignificant	5	24	22
chr12	113218962	113220962	Tdrd9		1	noCoverage	-0.014848	0.84355	insignificant	0	11	10
chr12	113346448	113348448	Mir3073		1	noCoverage	0.09358	0.71409	insignificant	0	9	7
chr12	113368090	113370090	Mir203	-0.12484444	1.45E-29	hypomethylated	0.021434	0.00000379	hypermethylated	38	100	100
chr12	113383418	113385418	Kif26a	-0.14749258	1.49E-16	hypomethylated	0.0075125	0.87949	insignificant	39	123	120
chr12	113825994	113827994	Inf2	-0.15115039	2.48E-17	hypomethylated	0.0011134	0.30937	insignificant	31	109	101
chr12	113857257	113859257	Adssl1	-0.15752202	0.022752	hypomethylated	-0.006107	0.064061	insignificant	9	68	68
chr12	113882038	113884038	Siva1	-0.12226844	1.52E-11	hypomethylated	-0.035955	1	insignificant	15	70	76
chr12	113912487	113914487	Akt1	-0.14284751	5.31E-08	hypomethylated	-0.011272	0.42163	insignificant	17	59	57
chr12	113916050	113918050	Zbtb42	-0.14356732	2.82E-23	hypomethylated	0.0063351	0.36442	insignificant	48	146	146
chr12	113959384	113961384	AW555464	-0.14148145	2.41E-35	hypomethylated	-0.013418	0.0089008	inconclusive	56	149	139
chr12	114046185	114048185	BC022687	-0.1283613	6.33E-13	hypomethylated	0.047875	0.34043	insignificant	27	107	105
chr12	114067600	114069600	Cdca4	-0.09691704	5.29E-09	hypomethylated	-0.013904	0.46468	insignificant	14	84	80
chr12	114099127	114101127	Gpr132	-0.26611803	0.080565	insignificant	-0.027383	0.55835	insignificant	5	14	18
chr12	114167706	114169706	Jag2	-0.06442554	4.28E-11	hypomethylated	0.016334	1.89E-11	hypermethylated	47	130	131
chr12	114180329	114182329	Nudt14	-0.34459958	0.071127	insignificant	-0.11261	0.44643	insignificant	5	34	28
chr12	114213692	114215692	Btbd6	-0.07856232	0.10676	insignificant	-0.0041062	0.70871	insignificant	18	72	71
chr12	114213773	114215773	Btbd6	-0.11541983	0.10714	insignificant	-0.015606	0.65011	insignificant	18	75	73
chr12	114238832	114240832	Brf1	-0.06185916	0.37302	insignificant	0.0061579	0.30208	insignificant	11	50	50
chr12	114251718	114253718	Pacs2	-0.10433094	1.28E-21	hypomethylated	0.00029746	0.15758	insignificant	44	128	128
chr12	114335488	114337488	Mta1	-0.10687857	5.55E-29	hypomethylated	-0.0099005	0.021248	inconclusive	61	200	217
chr12	114377446	114379446	Crip2	-0.10327577	4.82E-19	hypomethylated	0.015591	0.0009118	hypermethylated	28	163	159
chr12	114389222	114391222	Crip1	-0.25167419	1.09E-09	hypomethylated	0.018169	0.55212	insignificant	16	43	46
chr12	114393631	114395631	4930427A07R	-0.192567	3.19E-19	hypomethylated	-0.004182	0.83446	insignificant	29	120	120
chr12	114423113	114425113	Tmem121	-0.10983825	0.00000595	hypomethylated	0.0096562	0.46114	insignificant	10	20	20
chr12	114781118	114783118	Adam6a	-0.24488636	0.59257	insignificant	0.019861	0.22492	insignificant	4	10	10
chr12	117284934	117286934	Zfp386	-0.07737928	0.000025967	hypomethylated	0.012713	0.18403	insignificant	8	48	48
chr12	117315195	117317195	Vipr2	-0.16783306	0.000000648	hypomethylated	0.0074406	0.23957	insignificant	19	52	46
chr12	117501498	117503498	Wdr60		1	noCoverage	0.02	1	insignificant	0	2	2
chr12	117518694	117520694	Esyt2	-0.09920858	1.05E-22	hypomethylated	-0.0091214	0.80695	insignificant	33	99	97
chr12	117642874	117644874	Ncapg2	0.00572256	0.37157	insignificant	0.025801	0.93272	insignificant	25	114	117
chr12	117643634	117645634	D430020J02Ri	-0.10271061	4.27E-12	hypomethylated	0.0025862	0.66287	insignificant	19	102	102
chr12	117723192	117725192	Ptprn2	-0.13029656	1.75E-18	hypomethylated	-0.011625	0.0002351	inconclusive	33	148	145
chr12	118389742	118391742	Gm10421	-0.29107885	0.000060354	hypomethylated	0.075817	0.6264	insignificant	3	29	30
chr12	118753951	118755951	Rapgef5		1	noCoverage	0.024183	0.50497	insignificant	0	6	6
chr12	119081333	119083333	Cdca7l	-0.09652496	3.79E-10	hypomethylated	0.009881	0.94496	insignificant	23	114	104
chr12	119539913	119541913	Sp4	-0.09172774	9.62E-26	hypomethylated	0.00014322	0.25174	insignificant	38	169	160
chr12	120083801	120085801	Sp8	-0.14999042	1.57E-19	hypomethylated	-0.015076	1	insignificant	14	36	31

chr12	120476749	120478749	Itgb8	0.58111625	0.32009	lowCoverage	-0.013316	0.0042596	inconclusive	1	18	17
chr13	3476548	3478548	2810429I04Ril	-0.22164524	4.83E-64	hypomethylated	0.046466	0.28027	insignificant	15	53	53
chr13	3536320	3538320	Gdi2	-0.10973263	6.29E-17	hypomethylated	0.014891	1	insignificant	38	132	116
chr13	3632321	3634321	Asb13	-0.1138818	1.64E-23	hypomethylated	0.014039	0.0014003	hypermethylated	23	69	70
chr13	3632323	3634323	Asb13	-0.1138818	1.64E-23	hypomethylated	0.014039	0.0014003	hypermethylated	23	69	70
chr13	3803564	3805564	Calml3	-0.26875	0.038688	hypomethylated	-0.074811	0.04225	hypomethylated	1	8	6
chr13	3892824	3894824	Net1	-0.14961533	0.010079	hypomethylated	-0.035884	0.30619	insignificant	6	22	20
chr13	5859734	5861734	Klf6	-0.15790855	3.97E-08	hypomethylated	-0.012537	0.96887	insignificant	12	70	71
chr13	6546402	6548402	Pitrm1	-0.19974639	0.21887	insignificant	-0.01065	0.2289	insignificant	2	26	26
chr13	6647970	6649970	Pfkp	-0.31374073	0.55346	insignificant	0.053977	1	insignificant	3	16	17
chr13	8870288	8872288	Wdr37	-0.20938685	0.00000526	hypomethylated	-0.030472	0.65951	insignificant	9	30	28
chr13	8995258	8997258	Gtpbp4	-0.18398378	1.51E-13	hypomethylated	-0.052868	0.20168	insignificant	8	57	51
chr13	9092150	9094150	Larp4b	-0.10438256	3.26E-23	hypomethylated	0.00859	0.72751	insignificant	58	186	181
chr13	9274771	9276771	Dip2c	-0.06330208	8.79E-08	hypomethylated	0.0038847	0.46368	insignificant	33	148	132
chr13	12199212	12201212	Ryr2	-0.05109953	0.47077	insignificant	0.010576	0.70386	insignificant	2	21	21
chr13	12350267	12352267	Mtr	-0.10704657	0.00021994	hypomethylated	0.0052544	0.78117	insignificant	11	49	48
chr13	12432999	12434999	Actn2	-0.08742609	0.056419	insignificant	0.018867	0.16794	insignificant	4	20	20
chr13	12486641	12488641	Heatr1	-0.14050859	0.000014981	hypomethylated	-0.0069862	0.6947	insignificant	9	60	54
chr13	12554005	12556005	Lgals8	0.19529915	1	insignificant	0.22183	0.40851	insignificant	1	6	6
chr13	12657149	12659149	Ero1lb	-0.08818101	0.0015574	hypomethylated	0.0081673	0.7871	insignificant	28	92	92
chr13	12742662	12744662	Gpr137b-ps	-0.07710627	0.012258	hypomethylated	-0.0032426	1	insignificant	5	16	16
chr13	13485891	13487891	Gpr137b	-0.0800377	0.041179	hypomethylated	0.024764	0.87785	insignificant	5	12	12
chr13	13528868	13530868	Nid1		1	noCoverage	0.01253	0.59034	insignificant	0	20	20
chr13	13681675	13683675	Lyst	-0.1512671	1.57E-13	hypomethylated	-0.0084987	0.073554	insignificant	28	110	105
chr13	13875805	13877805	Gng4	-0.06856677	1.5E-21	hypomethylated	0.0077606	0.093955	insignificant	22	79	74
chr13	14045940	14047940	B3galnt2	-0.08303423	0.00053395	hypomethylated	0.0068075	0.15095	insignificant	23	161	161
chr13	14131867	14133867	Tbce	-0.14221763	0.018952	hypomethylated	0.041858	0.94382	insignificant	10	34	34
chr13	14155058	14157058	Arid4b	-0.1038271	1.79E-51	hypomethylated	0.029133	0.011976	hypermethylated	62	233	238
chr13	14155668	14157668	Arid4b	-0.12694069	1.48E-37	hypomethylated	0.01391	0.0046745	hypermethylated	49	199	200
chr13	14704508	14706508	Psma2	-0.15307663	0.14971	insignificant	0.021428	0.28189	insignificant	3	34	34
chr13	14705304	14707304	Psma2	-0.26862424	1	insignificant	0.015006	0.41508	insignificant	1	25	25
chr13	14721511	14723511	AW209491	-0.12897162	0.000016064	hypomethylated	-0.019438	0.28566	insignificant	12	42	42
chr13	14721524	14723524	AW209491	-0.12897162	0.000016064	hypomethylated	-0.019438	0.28566	insignificant	12	42	42
chr13	15554555	15556555	Gli3	-0.103326	5.92E-36	hypomethylated	-0.0071044	0.62396	insignificant	70	235	226
chr13	17786246	17788246	2810021B07R	-0.12080951	1.4E-10	hypomethylated	0.028284	0.24398	insignificant	17	125	123
chr13	17786599	17788599	2810021B07R	-0.11772396	1.33E-10	hypomethylated	0.027828	0.33269	insignificant	17	117	115
chr13	17896931	17898931	Cdk13	0.17265072	0.000019772	hypermethylated	0.03949	0.077797	insignificant	10	52	44
chr13	18036051	18038051	Rala	-0.14129919	0.000017034	hypomethylated	0.019692	0.50814	insignificant	17	39	39
chr13	18808160	18810160	Vps41	-0.14046143	0.000000039	hypomethylated	-0.012409	0.33595	insignificant	13	41	40
chr13	19039239	19041239	Amph	-0.11143839	4.53E-22	hypomethylated	0.019979	0.0454	inconclusive	33	120	120
chr13	19487621	19489621	Stard3nl		1	noCoverage	-0.045089	0.71412	insignificant	0	4	4
chr13	19714043	19716043	Sfrp4	-0.25539666	2.38E-15	hypomethylated	0.023007	0.46804	insignificant	5	42	42
chr13	19789629	19791629	Txndc3		1	noCoverage	0.085714	0.047827	hypermethylated	0	2	2
chr13	20563961	20565961	Elmo1	0.07936508	1	lowCoverage	-0.040053	0.17342	insignificant	1	9	17
chr13	21270827	21272827	Trim27	-0.12269184	1.55E-24	hypomethylated	0.0023053	0.54148	insignificant	50	177	173
chr13	21384555	21386555	Gpx5		1	noCoverage	0.13438	0.69353	insignificant	0	4	4
chr13	21453688	21455688	Zscan12	-0.22450815	2.09E-14	hypomethylated	0.041635	0.037282	hypermethylated	13	37	36
chr13	21532579	21534579	Pgbd1	-0.45127734	1.22E-13	stronglyHypometh	-0.067098	0.11423	insignificant	6	28	28
chr13	21532922	21534922	Pgbd1	-0.54568174	6.26E-11	stronglyHypometh	-0.029559	0.58605	insignificant	3	27	27
chr13	21545596	21547596	Zfp187	-0.19883041	0.085324	insignificant	0.012079	0.87974	insignificant	2	4	8
chr13	21560370	21562370	Nkapl	0.10957896	0.009141	inconclusive	0.0097607	0.33402	insignificant	2	22	22
chr13	21569775	21571775	Zkscan4	-0.25014118	0.017819	hypomethylated	0.031409	0.66921	insignificant	5	35	37
chr13	21825964	21827964	Hist1h4j	-0.1601259	0.00000457	hypomethylated	0.0088381	0.27335	insignificant	5	40	36
chr13	21872423	21874423	Hist1h3i		1	noCoverage	-0.016667	1	insignificant	0	12	12
chr13	21988918	21990918	Mir1983	0.28388578	1	insignificant	-0.098969	0.0050287	hypomethylated	1	12	12
chr13	22035962	22037962	Zfp184	0.21250814	0.72988	insignificant	0.0011906	0.57188	insignificant	7	46	43
chr13	22072049	22074049	Pom121l2	-0.12488307	0.000041876	hypomethylated	0.029404	0.30991	insignificant	19	107	102
chr13	22072062	22074062	Pom121l2	-0.12488307	0.000041876	hypomethylated	0.029404	0.30991	insignificant	19	107	102
chr13	22126755	22128755	Hist1h2bk		1	noCoverage	-0.20909	0.37939	insignificant	0	24	14
chr13	22127421	22129421	Hist1h2ah		1	noCoverage	-0.20909	0.37939	insignificant	0	24	14
chr13	22133221	22135221	Hist1h2ag		1	noCoverage	-0.0026709	0.69831	insignificant	0	4	4
chr13	22134098	22136098	Hist1h2bj		1	noCoverage	0.03	0.73696	insignificant	0	5	5
chr13	22134818	22136818	Hist1h2ag		1	noCoverage	0.03	0.73696	insignificant	0	5	5
chr13	22178746	22180746	Vmn1r188		1	noCoverage	0.11919	0.22804	insignificant	0	4	4
chr13	22194534	22196534	Vmn1r189		1	noCoverage	0.10227	0.29105	insignificant	0	2	2
chr13	22279917	22281917	Vmn1r192		1	noCoverage	0.015648	0.13403	insignificant	0	6	6
chr13	22614919	22616919	Vmn1r203		1	noCoverage	0.016353	0.029724	hypermethylated	0	6	6
chr13	22712904	22714904	Vmn1r206	0.12708333	0.3155	insignificant	-0.015774	0.72275	insignificant	1	4	2
chr13	23253511	23255511	Vmn1r219		1	noCoverage	-0.054167	0.35544	insignificant	0	4	4
chr13	23461071	23463071	Zfp322a	-0.15834946	0.00013456	hypomethylated	0.014662	0.31113	insignificant	12	36	34
chr13	23515735	23517735	Abt1	-0.09637726	0.55304	insignificant	0.033643	0.91317	insignificant	5	36	36
chr13	23522886	23524886	C230035I16Ril	-0.28092905	0.063929	insignificant	0.0079877	0.65714	insignificant	4	36	32
chr13	23621912	23623912	Hist1h4h	-0.25627496	0.047862	hypomethylated	-0.0073704	0.84677	insignificant	4	36	28
chr13	23626286	23628286	Hist1h3g	-0.21052632	1	insignificant	0.040454	0.6404	insignificant	2	10	10
chr13	23634920	23636920	Hist1h3f	-0.31818182	0.60372	insignificant	0.02668	0.79929	insignificant	1	11	11
chr13	23643512	23645512	Hist1h4f		1	noCoverage	-0.077438	0.89947	insignificant	0	8	10
chr13	23645900	23647900	Hist1h1d	-0.11075405	0.51423	insignificant	-0.024199	0.56458	insignificant	2	29	29
chr13	23665339	23667339	Hist1h2ad		1	noCoverage	-0.060741	0.37925	insignificant	0	4	4
chr13	23666059	23668059	Hist1h3d	-0.20603159	0.58853	insignificant	-0.025862	0.36821	insignificant	2	12	12
chr13	23666670	23668670	Hist1h3d	-0.20603159	0.58853	insignificant	-0.025862	0.36821	insignificant	2	12	12
chr13	23712963	23714963	Hist1h1e	-0.13407593	0.0000084	hypomethylated	0.0029129	0.91361	insignificant	14	59	59
chr13	23712993	23714993	Hist1h1e	-0.13407593	0.0000084	hypomethylated	0.0029129	0.91361	insignificant	14	59	59
chr13	23786679	23788679	Hist1h1t		1	noCoverage	-0.05	0.18857	insignificant	0	5	4
chr13	23790318	23792318	Hist1h4c	-0.07339015	0.48445	insignificant	-0.024184	0.58674	insignificant	2	16	15
chr13	23829675	23831675	Hist1h1c	-0.16256909	0.24113	insignificant	0.013299	0.14476	insignificant	11	60	60

chr13	23853026	23855026	Hist1h3a	-0.10486147	1.13E-21	hypomethylated	0.020843	1	insignificant	32	85	78
chr13	23854255	23856255	Hist1h3a	0.15614145	1	insignificant	0.010328	0.8197	insignificant	2	20	20
chr13	23854536	23856536	Hist1h1a	0.15614145	1	insignificant	0.010328	0.8197	insignificant	2	20	20
chr13	23897895	23899895	Slc17a2		1	noCoverage	0.0024123	0.26122	insignificant	0	8	8
chr13	24025330	24027330	Hist1h2aa	-0.06319579	0.12151	insignificant	-0.010001	0.42837	insignificant	13	33	37
chr13	24026025	24028025	Hist1h2ba	-0.01355509	1	insignificant	-0.013753	0.15567	insignificant	7	15	19
chr13	24083083	24085083	Scgn	-0.18963921	0.021007	hypomethylated	-0.020578	0.80331	insignificant	4	8	8
chr13	24372659	24374659	Lrrc16a	-0.09823515	1.1E-16	hypomethylated	-0.001561	0.91096	insignificant	25	88	85
chr13	24505991	24507991	Cmah	-0.7890923	0.034559	stronglyHypometh	-0.10457	0.41371	insignificant	1	6	5
chr13	24696636	24698636	Gm11346	-0.33281598	0.63545	insignificant	0.054748	0.74231	insignificant	3	8	8
chr13	24729516	24731516	Fam65b	-0.14640203	9.11E-40	hypomethylated	-0.011314	0.29536	insignificant	23	111	117
chr13	24853806	24855806	Gmnn		1	noCoverage	0.12236	0.8744	insignificant	0	13	15
chr13	24892525	24894525	BC005537	-0.10132847	1.52E-20	hypomethylated	-0.0031342	0.4797	insignificant	43	170	162
chr13	24922527	24924527	Tdp2	-0.09075743	2.27E-08	hypomethylated	0.0030546	0.78389	insignificant	33	163	169
chr13	24923358	24925358	Acot13	-0.09387043	6.94E-08	hypomethylated	-0.0026414	0.58186	insignificant	31	140	143
chr13	24935999	24937999	D130043K22Rik		1	noCoverage	0.0083403	0.61944	insignificant	0	41	38
chr13	25029530	25031530	Aldh5a1	-0.32724823	0.42697	lowCoverage	-0.034152	0.052322	insignificant	1	18	14
chr13	25034020	25036020	Gpld1		1	noCoverage	0.23023	1	insignificant	0	17	17
chr13	25112248	25114248	Mrs2	-0.34520752	6.34E-20	stronglyHypometh	0.053782	0.37905	insignificant	5	22	20
chr13	25146872	25148872	Dcdc2a	-0.12080104	3.13E-12	hypomethylated	-0.0026861	0.07557	insignificant	27	121	121
chr13	26862034	26864034	Hdgfl1	0.18066417	0.064375	insignificant	0.11439	0.16356	insignificant	5	18	18
chr13	27674040	27676040	Prl8a1	-0.27350427	0.043413	hypomethylated	-0.027778	0.2883	insignificant	1	2	3
chr13	29045551	29047551	Sox4	0.12700783	1	insignificant	0.051435	0.14839	insignificant	2	19	20
chr13	29947457	29949457	Cdkal1	-0.35064935	0.47236	insignificant	-0.092316	0.85262	insignificant	2	11	12
chr13	30077932	30079932	E2f3	-0.30778929	0.000027925	hypomethylated	0.080853	1	insignificant	7	36	36
chr13	30227358	30229358	Mboat1	-0.14039582	0.034073	inconclusive	-0.026725	0.24603	insignificant	18	54	57
chr13	30427224	30429224	Agtr1a	-0.19199959	3.01E-10	hypomethylated	0.0014868	0.35934	insignificant	9	37	34
chr13	30637185	30639185	Uqcrfs1	-0.05384662	0.082058	insignificant	-0.00072188	0.94344	insignificant	10	36	36
chr13	30750964	30752964	Dusp22	-0.12639761	2.99E-13	hypomethylated	0.017206	0.033809	hypermethylated	25	90	76
chr13	30750969	30752969	Dusp22	-0.12639761	2.99E-13	hypomethylated	0.017206	0.033809	hypermethylated	25	90	76
chr13	30840126	30842126	Irf4	-0.05989871	0.0063732	inconclusive	0.016764	1	insignificant	10	26	26
chr13	31065916	31067916	Exoc2		1	noCoverage	-0.0069699	0.39905	insignificant	0	32	32
chr13	31649038	31651038	Foxq1	-0.11186406	3.85E-10	hypomethylated	0.0089201	0.94685	insignificant	24	123	120
chr13	31716684	31718684	Foxf2	-0.15167317	1.63E-21	hypomethylated	-0.016858	0.66868	insignificant	41	176	193
chr13	31897514	31899514	Foxc1	-0.11143258	1.77E-50	hypomethylated	0.0092691	0.021371	hypermethylated	53	247	239
chr13	32430413	32432413	Gmds	-0.12972918	2.15E-16	hypomethylated	-0.0021285	0.94492	insignificant	25	93	93
chr13	32892898	32894898	Wrnip1	-0.0797138	4.67E-09	hypomethylated	0.0018671	0.52398	insignificant	38	178	174
chr13	32943054	32945054	Serpinb1a	0.10863698	1	insignificant	0.019748	0.77597	insignificant	1	6	6
chr13	33250220	33252220	Serpinb9c	-0.47030454	0.000019959	stronglyHypometh	0.040298	0.76413	insignificant	5	10	10
chr13	33752273	33754273	Serpinb6d		1	noCoverage	0.036387	0.66534	insignificant	0	4	4
chr13	34055527	34057527	Nqo2	-0.28712131	1	lowCoverage	0.11595	0.17357	insignificant	1	26	28
chr13	34058021	34060021	Nqo2		1	noCoverage	-0.12378	0.38545	insignificant	0	4	4
chr13	34093742	34095742	Ripk1	-0.11039794	6.99E-26	hypomethylated	0.015854	0.14796	insignificant	32	119	112
chr13	34094618	34096618	Serpinb6a	-0.15607769	0.00000811	hypomethylated	0.10169	0.78767	insignificant	3	21	14
chr13	34128509	34130509	Bphl	-0.1236256	7.68E-20	hypomethylated	0.003295	0.46707	insignificant	17	40	36
chr13	34169877	34171877	Tubb2a	-0.14184577	0.0038174	hypomethylated	0.01496	0.96385	insignificant	3	56	56
chr13	34222223	34224223	Tubb2b	-0.20838339	0.5784	insignificant	0.015207	0.26492	insignificant	4	31	31
chr13	34253845	34255845	Psmg4	-0.40675439	0.13239	insignificant	0.12781	0.17188	insignificant	1	12	14
chr13	34437051	34439051	Slc22a23	-0.10665678	0.000000012	hypomethylated	0.018731	0.88124	insignificant	18	54	46
chr13	34744550	34746550	1300014I06Ril	-0.1256607	6.57E-24	hypomethylated	-0.0012741	0.29217	insignificant	27	83	83
chr13	34966362	34968362	Prpf4b	-0.09263357	0.62291	insignificant	0.0085556	0.0071476	hypermethylated	11	114	118
chr13	35011861	35013861	1700026J04Ri	-0.10178019	0.23328	insignificant	-0.037993	0.82371	insignificant	2	8	8
chr13	35055678	35057678	Eci3	-0.26599327	0.06809	insignificant	0.049446	0.51895	insignificant	2	8	14
chr13	35085996	35087996	Eci2	-0.1726998	0.000000182	hypomethylated	-0.021135	0.73554	insignificant	9	30	30
chr13	35086013	35088013	Eci2	-0.1726998	0.000000182	hypomethylated	-0.021135	0.73554	insignificant	9	30	30
chr13	35832270	35834270	Cdyl	-0.10662715	2.13E-56	hypomethylated	0.01068	0.52971	insignificant	92	257	248
chr13	35998216	36000216	Rpp40		1	noCoverage	-0.02787	0.88357	insignificant	0	9	9
chr13	36058774	36060774	Ppp1r3g	-0.1592425	0.00000537	hypomethylated	-0.017081	0.97353	insignificant	17	90	84
chr13	36208279	36210279	Fars2	-0.17296669	1.95E-15	hypomethylated	-0.0096893	0.24915	insignificant	13	69	72
chr13	36209226	36211226	Lyrm4	-0.21452351	1.13E-08	hypomethylated	-0.027808	0.4815	insignificant	9	70	73
chr13	36826323	36828323	Nrn1	-0.06350382	0.57357	insignificant	0.035368	0.0019157	hypermethylated	12	55	44
chr13	37916906	37918906	Rreb1	-0.10422787	4.54E-27	hypomethylated	0.0066691	0.5671	insignificant	29	147	124
chr13	37917780	37919780	Rreb1	-0.09304686	1.85E-33	hypomethylated	0.0013991	0.78072	insignificant	56	248	225
chr13	37918261	37920261	Rreb1	-0.104241	2.74E-33	hypomethylated	0.0024665	0.79837	insignificant	54	219	204
chr13	38086059	38088059	Ssr1	-0.06855365	1	insignificant	0.0064637	1	insignificant	1	16	16
chr13	38128163	38130163	Cage1	-0.16217312	2.51E-34	hypomethylated	0.011782	0.20057	insignificant	20	109	100
chr13	38128806	38130806	Cage1	-0.19374302	1.89E-37	hypomethylated	0.0093207	0.12689	insignificant	16	93	82
chr13	38242162	38244162	Dsp	-0.08471406	3.52E-11	hypomethylated	-0.0091379	0.262	insignificant	15	119	118
chr13	38295807	38297807	Snrnp48	-0.13961258	0.15105	insignificant	0.0075856	1	insignificant	7	68	67
chr13	38436584	38438584	Bmp6	-0.1126635	1.55E-14	hypomethylated	-0.01467	0.20062	insignificant	31	160	156
chr13	38620329	38622329	Txndc5	-0.10955695	1.61E-08	hypomethylated	0.013547	0.19763	insignificant	10	28	28
chr13	38726978	38728978	Muted	-0.26661989	0.045068	hypomethylated	0.071491	0.0032065	hypermethylated	6	30	34
chr13	38750897	38752897	Eef1e1	-0.34920589	0.0095043	stronglyHypometh	-0.087901	0.58913	insignificant	6	42	37
chr13	39052406	39054406	Slc35b3	-0.09081399	0.0039149	hypomethylated	0.01062	0.32098	insignificant	12	41	41
chr13	39052744	39054744	Slc35b3	-0.27460317	1	insignificant	-0.052381	0.63987	insignificant	3	6	6
chr13	40383380	40385380	Ofcc1		1	noCoverage	0.1239	1	insignificant	0	5	3
chr13	40825812	40827812	Tfap2a	-0.09854507	6.56E-12	hypomethylated	0.0040162	1	insignificant	16	97	97
chr13	40829192	40831192	Tfap2a	-0.34223302	0.00015501	stronglyHypometh	-0.078975	0.56449	insignificant	7	30	33
chr13	40954500	40956500	Gcnt2	0.7047479	0.1296	insignificant	0.032863	0.032451	hypermethylated	1	20	20
chr13	40981534	40983534	Gcnt2	-0.2603415	0.00000231	hypomethylated	-0.013848	0.27265	insignificant	6	40	40
chr13	41012002	41014002	Gcnt2	-0.23288715	0.000087686	hypomethylated	-0.021646	0.60155	insignificant	8	46	50
chr13	41095378	41097378	Pak1ip1	-0.20148973	5.14E-13	hypomethylated	0.0043126	0.11264	insignificant	19	80	81
chr13	41110618	41112618	Tmem14c	-0.14640728	2.25E-13	hypomethylated	-0.021494	0.21074	insignificant	18	52	48
chr13	41175075	41177075	Mak		1	noCoverage	-0.075287	0.58649	insignificant	0	4	4
chr13	41205357	41207357	Gcm2	0.02352941	1	insignificant	-0.044828	1	insignificant	1	4	4

chr13	41315772	41317772	Elovl2	-0.03563334	0.00042724	hypomethylated	0.0021775	0.17692	insignificant	20	74	74
chr13	41344212	41346212	BC024659	-0.11860005	2.13E-11	hypomethylated	-0.0061228	0.45204	insignificant	18	104	105
chr13	41700584	41702584	Tmem170b	-0.09160435	6.57E-47	hypomethylated	-0.0015552	0.55414	insignificant	66	248	231
chr13	41745130	41747130	Gm5082		1	noCoverage	0.16806	0.10134	insignificant	0	13	10
chr13	41960283	41962283	Gm10790	0.11597088	0.74411	insignificant	0.098038	0.10141	insignificant	3	18	20
chr13	42146389	42148389	Hivep1	-0.09441773	1.27E-22	hypomethylated	-0.0013008	0.26454	insignificant	68	221	205
chr13	42395638	42397638	Edn1		1	noCoverage	-0.032715	1	insignificant	0	15	23
chr13	42803949	42805949	Phactr1	-0.14745169	0.0013518	hypomethylated	-0.066108	0.046949	hypomethylated	6	41	44
chr13	43217207	43219207	Phactr1		1	noCoverage	0.1732	0.72609	insignificant	0	3	6
chr13	43266728	43268728	Tbc1d7	-0.1467849	3.29E-10	hypomethylated	-0.0081277	0.77659	insignificant	18	61	60
chr13	43399541	43401541	Gfod1	-0.05606512	0.00024501	hypomethylated	-0.049796	0.50785	insignificant	18	69	63
chr13	43492744	43494744	Nol7	-0.23714834	9E-27	hypomethylated	-0.010488	0.34083	insignificant	25	103	100
chr13	43576342	43578342	Ranbp9	-0.02527414	0.0086618	hypomethylated	-0.0071	0.62581	insignificant	28	124	120
chr13	43655530	43657530	Ccdc90a	-0.24498928	0.00000167	hypomethylated	0.0083254	0.75638	insignificant	2	14	14
chr13	43710165	43712165	Rnf182	-0.14140142	6.84E-14	hypomethylated	0.012599	0.96205	insignificant	23	70	71
chr13	43879475	43881475	Cd83		1	noCoverage	-0.0016743	0.84547	insignificant	0	30	30
chr13	44825271	44827271	Jarid2	-0.08103741	7.96E-27	hypomethylated	0.0065099	0.34461	insignificant	38	168	158
chr13	45097465	45099465	Dtnbp1	-0.24439886	0.00000423	hypomethylated	-0.0061593	0.0016002	hypomethylated	11	57	50
chr13	45484110	45486110	Mylip	-0.12746636	2.72E-14	hypomethylated	0.0052858	0.32451	insignificant	25	122	121
chr13	45484325	45486325	Mylip	-0.12746636	2.72E-14	hypomethylated	0.0052858	0.32451	insignificant	25	122	121
chr13	45601837	45603837	Gmpr	-0.23068152	8.55E-14	hypomethylated	-0.016029	0.052719	insignificant	14	81	66
chr13	46060345	46062345	Atxn1	-0.19294891	2.88E-13	hypomethylated	0.005719	0.96865	insignificant	11	66	66
chr13	46368089	46370089	Gm1574		1	noCoverage	0.040672	1	insignificant	0	10	9
chr13	46512668	46514668	Rbm24	-0.12075411	1.52E-08	hypomethylated	-0.020678	1	insignificant	26	107	105
chr13	46596271	46598271	Cap2	-0.18985271	1.88E-27	hypomethylated	-0.019891	0.58707	insignificant	23	85	87
chr13	46763890	46765890	C78339	-0.10158236	4.74E-17	hypomethylated	0.011378	0.050436	insignificant	42	112	112
chr13	46823218	46825218	Nup153	0.27506005	0.060822	insignificant	0.0020583	0.00024162	inconclusive	4	56	56
chr13	47025087	47027087	Kif13a	-0.12449369	0.00025607	hypomethylated	-0.0050311	0.47271	insignificant	26	84	76
chr13	47137907	47139907	Kdm1b	-0.0766992	1.18E-14	hypomethylated	0.019812	0.0097776	hypermethylated	35	140	127
chr13	47138586	47140586	Kdm1b	-0.07877381	3.5E-15	hypomethylated	0.021469	0.03741	hypermethylated	34	138	125
chr13	47201589	47203589	Dek	-0.12864582	1.17E-12	hypomethylated	-0.0036636	0.78752	insignificant	25	106	106
chr13	47217088	47219088	Rnf144b	-0.19153548	3.52E-15	hypomethylated	-0.0015507	0.3866	insignificant	8	67	69
chr13	47288371	47290371	Rnf144b	-0.15561255	3.68E-08	hypomethylated	0.018644	0.89675	insignificant	10	50	50
chr13	48355795	48357795	Id4	-0.09136134	3.83E-21	hypomethylated	-0.0041462	0.65014	insignificant	64	240	232
chr13	48608779	48610779	Zfp169	-0.30289092	0.000000194	hypomethylated	0.012365	0.022175	inconclusive	5	35	33
chr13	48608805	48610805	Zfp169	-0.32124795	0.00000544	hypomethylated	0.012895	0.02612	inconclusive	3	31	29
chr13	48720942	48722942	Ptpdc1	-0.12032362	6.7E-20	hypomethylated	-0.018924	0.50928	insignificant	23	85	82
chr13	48757404	48759404	Barx1	-0.09415873	3.46E-17	hypomethylated	0.01325	0.0053263	hypermethylated	37	110	104
chr13	48966254	48968254	Phf2	-0.12719381	1.22E-23	hypomethylated	0.0012573	0.85484	insignificant	23	77	70
chr13	49062480	49064480	C030044B11R	-0.08314039	4.19E-41	hypomethylated	0.00000307	0.82808	insignificant	96	274	261
chr13	49063197	49065197	Fam120a	-0.07180096	2.17E-27	hypomethylated	-0.0015892	1	insignificant	81	220	210
chr13	49243383	49245383	Wnk2	-0.03239144	0.03391	hypomethylated	0.0093385	0.28403	insignificant	9	34	34
chr13	49281915	49283915	Ninj1	-0.12636764	1.57E-45	hypomethylated	-0.0034499	0.75317	insignificant	33	109	105
chr13	49311395	49313395	1110007C09Ri	0.05172098	0.1738	insignificant	0.063272	0.29335	insignificant	6	39	29
chr13	49343532	49345532	Susd3	-0.64195613	4.38E-12	stronglyHypometh	-0.050336	0.51826	insignificant	2	26	22
chr13	49435917	49437917	Bicd2	-0.11158035	2.05E-15	hypomethylated	-0.0085411	0.04621	inconclusive	13	67	72
chr13	49515679	49517679	Ippk	-0.13217903	2.98E-34	hypomethylated	0.027772	0.027987	hypermethylated	45	125	123
chr13	49702439	49704439	Ogn		1	noCoverage	0.14606	0.11719	insignificant	0	9	7
chr13	49747718	49749718	Nol8	-0.12214453	2.6E-14	hypomethylated	-0.0090129	0.029166	inconclusive	17	91	91
chr13	49748100	49750100	Cenpp	-0.11970269	6.17E-10	hypomethylated	-0.013548	0.31461	insignificant	14	76	76
chr13	49776498	49778498	Iars	-0.34225589	1	lowCoverage	-0.050947	0.18945	insignificant	1	30	28
chr13	50512245	50514245	Fbxw17	-0.24891202	0.00000298	hypomethylated	-0.014216	0.65043	insignificant	9	44	44
chr13	51195266	51197266	Spin1	-0.1289223	2.5E-56	hypomethylated	-0.029856	0.67695	insignificant	72	255	237
chr13	51196096	51198096	Spin1	-0.1092902	5.95E-33	hypomethylated	-0.034892	0.58514	insignificant	61	213	195
chr13	51265393	51267393	Nxnl2	-0.19111205	3.65E-14	hypomethylated	-0.0017093	0.72084	insignificant	11	95	93
chr13	51502986	51504986	S1pr3	-0.14403016	0.000000726	hypomethylated	0.023191	0.92874	insignificant	16	48	52
chr13	51662453	51664453	Shc3	-0.12662424	1.48E-15	hypomethylated	0.0037897	0.10548	insignificant	30	93	89
chr13	51739600	51741600	Cks2	-0.14048332	0.0097496	hypomethylated	-0.0038191	0.36763	insignificant	30	123	119
chr13	51746082	51748082	Secisbp2	-0.10294593	0.000036146	hypomethylated	0.01005	0.16128	insignificant	22	92	87
chr13	51941043	51943043	Gadd45g	-0.12540542	3.72E-15	hypomethylated	-0.000829	0.91483	insignificant	28	130	130
chr13	52626205	52628205	Diras2	-0.28618326	0.038004	hypomethylated	-0.025448	0.38905	insignificant	4	12	12
chr13	52677805	52679805	Syk	-0.16822836	3.19E-14	hypomethylated	0.0033914	0.52619	insignificant	27	118	116
chr13	52691250	52693250	Syk		1	noCoverage	-0.30917	0.6819	insignificant	0	10	7
chr13	53025046	53027046	Auh	0.25037824	1	lowCoverage	-0.021381	0.71151	insignificant	1	8	8
chr13	53076408	53078408	Nfil3	-0.13369107	2.88E-12	hypomethylated	0.0036538	0.94726	insignificant	37	119	130
chr13	53381478	53383478	Ror2	-0.21972977	1.43E-18	hypomethylated	0.031497	0.21506	insignificant	18	68	64
chr13	53472730	53474730	Sptlc1	-0.19496431	0.000011114	hypomethylated	-0.046653	0.74039	insignificant	3	53	54
chr13	53568149	53570149	Msx2		1	noCoverage	0.22302	0.57388	insignificant	0	2	3
chr13	54151027	54153027	Drd1a	-0.26136364	0.065806	insignificant	0.035354	0.50653	insignificant	3	8	8
chr13	54166213	54168213	Sfxn1	-0.09537048	1.42E-13	hypomethylated	-0.0054497	0.4171	insignificant	23	106	104
chr13	54286497	54288497	Hrh2	-0.11750111	0.59716	insignificant	0.044343	0.88905	insignificant	2	19	14
chr13	54471712	54473712	Cplx2	-0.09324014	9.93E-09	hypomethylated	-0.015509	0.72879	insignificant	16	49	53
chr13	54570201	54572201	Thoc3		1	noCoverage	-0.017848	0.74349	insignificant	0	42	44
chr13	54604165	54606165	4732471D19R	-0.13280279	1.22E-54	hypomethylated	-0.0091237	0.46477	insignificant	45	132	138
chr13	54666743	54668743	4833439L19Ri	-0.09357265	0.49217	insignificant	-0.0028891	0.64145	insignificant	3	12	12
chr13	54675373	54677373	Arl10	-0.15576156	5.09E-09	hypomethylated	0.012691	0.87286	insignificant	21	111	111
chr13	54690591	54692591	Higd2a	-0.25292305	1.24E-27	hypomethylated	0.004045	0.36052	insignificant	17	48	48
chr13	54691435	54693435	Nop16	-0.32900905	2.31E-21	hypomethylated	-0.0043774	0.044872	inconclusive	13	40	40
chr13	54712633	54714633	Cltb	-0.29637813	1.4E-13	hypomethylated	0.013039	0.0016866	hypermethylated	10	81	77
chr13	54722144	54724144	Faf2	-0.0043764	6.91E-11	hypomethylated	0.010436	0.91683	insignificant	11	45	45
chr13	54789169	54791169	Rnf44	0.41679208	0.3471	insignificant	0.0052311	0.84948	insignificant	2	37	36
chr13	54789561	54791561	Rnf44	0.39192246	0.35735	insignificant	0.011582	0.89471	insignificant	2	29	28
chr13	54795321	54797321	Rnf44	-0.07016005	0.00025562	hypomethylated	-0.0021901	0.00066269	inconclusive	29	150	149
chr13	54851030	54853030	Gprin1	-0.13111873	1.46E-24	hypomethylated	-0.01011	0.34551	insignificant	21	77	68
chr13	54867801	54869801	Sncb		1	noCoverage	-0.015686	0.24747	insignificant	0	29	29

chr13	54884363	54886363	Eif4e1b	-0.55952381	0.0015357	stronglyHypometh	0.034392	0.44926	insignificant	3	7	6
chr13	54884380	54886380	Eif4e1b	-0.55952381	0.0015357	stronglyHypometh	0.034392	0.44926	insignificant	3	7	6
chr13	54889765	54891765	Tspan17	-0.15285402	2.02E-19	hypomethylated	0.0010334	0.20594	insignificant	22	63	58
chr13	55049792	55051792	Unc5a	-0.121007	1.21E-57	hypomethylated	0.003607	0.88936	insignificant	61	156	148
chr13	55205669	55207669	Zfp346	-0.17554743	2.46E-31	hypomethylated	0.040659	0.33494	insignificant	28	74	72
chr13	55253178	55255178	Fgfr4	-0.19945063	8.19E-17	hypomethylated	0.0010065	0.46267	insignificant	16	81	70
chr13	55310142	55312142	Nsd1	-0.27655137	3.38E-17	hypomethylated	0.053355	0.0014532	hypermethylated	35	159	161
chr13	55422415	55424415	Prelid1	-0.14655771	4.44E-13	hypomethylated	-0.0024071	0.6815	insignificant	30	95	100
chr13	55423341	55425341	Rab24	-0.20586759	0.078845	insignificant	-0.028827	0.6836	insignificant	15	53	59
chr13	55431091	55433091	Mxd3	-0.10450501	0.000016971	hypomethylated	0.0068498	0.095314	insignificant	22	73	64
chr13	55464144	55466144	Lman2	-0.1777347	0.31737	insignificant	-0.011758	0.008142	hypomethylated	8	24	24
chr13	55500008	55502008	Slc34a1	-0.67708333	0.011766	stronglyHypometh	-0.27608	0.90111	insignificant	1	6	4
chr13	55516593	55518593	Pfn3		1	noCoverage	0.01732	1	insignificant	0	17	12
chr13	55545694	55547694	Grk6	-0.11004287	6.57E-29	hypomethylated	-0.0010811	0.10715	insignificant	36	172	167
chr13	55564627	55566627	Prr7	-0.12565986	7.91E-28	hypomethylated	0.006453	0.21736	insignificant	35	128	126
chr13	55589437	55591437	Dbn1	-0.56242533	0.054966	insignificant	0.01771	0.0050348	inconclusive	2	18	14
chr13	55614800	55616800	Pdlim7	-0.3751287	0.23359	insignificant	-0.080991	0.70746	insignificant	3	16	16
chr13	55629899	55631899	Dok3	-0.33826779	0.21919	insignificant	-0.051282	1	insignificant	2	6	6
chr13	55638019	55640019	Ddx41	-0.13369506	1	insignificant	0.10632	0.43677	insignificant	4	19	20
chr13	55671336	55673336	Fam193b	-0.13114384	2.34E-17	hypomethylated	0.008284	0.78414	insignificant	26	106	100
chr13	55672481	55674481	Fam193b	-0.21847291	0.22932	insignificant	0.098966	0.28265	insignificant	3	15	10
chr13	55693495	55695495	Tmed9	-0.1406439	2.09E-09	hypomethylated	0.016662	0.63013	insignificant	16	74	74
chr13	55700471	55702471	B4galt7	-0.09027635	3.54E-16	hypomethylated	0.02477	0.6147	insignificant	11	40	40
chr13	55723365	55725365	Caml	-0.20477815	1	insignificant	0.020246	1	insignificant	3	84	80
chr13	55735387	55737387	Ddx46	-0.10414409	6.28E-18	hypomethylated	0.0009942	0.62262	insignificant	34	118	118
chr13	55793484	55795484	B230219D22R	-0.10885879	2.28E-09	hypomethylated	0.0061893	0.53904	insignificant	33	88	85
chr13	55815010	55817010	Txndc15	-0.17699491	0.23221	insignificant	-0.021293	0.63227	insignificant	6	31	30
chr13	55827728	55829728	Pcbd2	-0.09557083	2.88E-11	hypomethylated	0.0060291	0.84773	insignificant	29	116	119
chr13	55884939	55886939	Catsper3	-0.69949495	0.019285	stronglyHypometh	-0.032828	1	insignificant	2	4	4
chr13	55932786	55934786	Pitx1	-0.23463243	7.6E-18	hypomethylated	0.046629	0.029747	hypermethylated	17	54	50
chr13	56236911	56238911	H2afy	-0.12056853	0.00000163	hypomethylated	0.0017082	0.045174	hypermethylated	16	47	38
chr13	56280246	56282246	Tifab		1	noCoverage	-0.0061008	0.88911	insignificant	0	8	7
chr13	56353524	56355524	Neurog1	-0.12926744	0.00000704	hypomethylated	-0.0021628	0.93882	insignificant	2	23	23
chr13	56538715	56540715	Slc25a48	-0.13085155	3.47E-13	hypomethylated	0.019977	0.57831	insignificant	13	100	92
chr13	56622868	56624868	Fbxl21	-0.16456204	0.37174	insignificant	-0.0030551	1	insignificant	6	34	34
chr13	56649899	56651899	Lect2		1	noCoverage	0.059315	0.78485	insignificant	0	4	4
chr13	56803370	56805370	Smad5	-0.11334623	6.71E-34	hypomethylated	0.036758	0.10946	insignificant	53	188	177
chr13	56803412	56805412	Smad5	-0.11334623	6.71E-34	hypomethylated	0.036758	0.10946	insignificant	53	188	177
chr13	56804028	56806028	Smad5	-0.11513604	2.81E-40	hypomethylated	0.03631	0.067726	insignificant	47	167	158
chr13	56996949	56998949	Trpc7		1	noCoverage	0.042621	0.065641	insignificant	0	6	6
chr13	58009693	58011693	Spock1	-0.24114618	0.10079	insignificant	-0.035942	0.088567	insignificant	5	22	22
chr13	58229917	58231917	Hnrnpa0	-0.09221023	3.08E-26	hypomethylated	0.0095058	0.10309	insignificant	44	188	192
chr13	58258009	58260009	5133401N09R	-0.20414738	3.29E-22	hypomethylated	0.014078	0.05129	insignificant	9	31	31
chr13	58317014	58319014	Ubqln1	-0.13209334	6.44E-20	hypomethylated	0.0082572	0.42514	insignificant	13	64	60
chr13	58375549	58377549	Gkap1	-0.08592444	3.64E-22	hypomethylated	0.001418	0.000013659	hypermethylated	34	124	130
chr13	58456223	58458223	Kif27		1	noCoverage	-0.015491	1	insignificant	0	4	4
chr13	58502608	58504608	Rmi1	-0.10166648	1.09E-63	hypomethylated	0.01157	0.000039472	hypermethylated	77	308	310
chr13	58502957	58504957	Rmi1	-0.10617115	7.37E-41	hypomethylated	0.015127	0.0081756	hypermethylated	59	256	250
chr13	58503877	58505877	Rmi1	-0.15369273	2.42E-22	hypomethylated	0.054324	0.0045067	hypermethylated	19	112	99
chr13	58906956	58908956	Ntrk2	-0.12824696	3.23E-10	hypomethylated	0.026353	0.76952	insignificant	7	46	52
chr13	58908193	58910193	Ntrk2	-0.13170982	1.42E-11	hypomethylated	0.045406	0.79235	insignificant	10	61	56
chr13	59658680	59660680	Agtpbp1	-0.10541459	0.00003634	hypomethylated	0.022446	0.83008	insignificant	7	53	54
chr13	59685693	59687693	Naa35	-0.06446627	2.85E-18	hypomethylated	-0.011271	0.079269	insignificant	43	124	146
chr13	59777145	59779145	Golm1	-0.21745995	0.009018	hypomethylated	0.083878	0.75737	insignificant	3	47	38
chr13	59807558	59809558	Fam75d3	0.04285714	1	insignificant	-0.26271	0.0046634	hypomethylated	2	9	10
chr13	59870326	59872326	Etohd2	-0.1042143	6.53E-09	hypomethylated	0.0075845	0.5169	insignificant	21	127	119
chr13	59871150	59873150	Etohd2	-0.15949117	0.000027529	hypomethylated	-0.015818	0.92145	insignificant	14	50	49
chr13	59924508	59926508	Zcchc6	-0.14611943	2.91E-17	hypomethylated	0.014867	0.61208	insignificant	18	45	45
chr13	60278896	60280896	Gas1	-0.21525424	0.021234	hypomethylated	0.037048	1	insignificant	1	10	10
chr13	60305831	60307831	Gm5084		1	noCoverage	0.12124	0.033593	hypermethylated	0	8	8
chr13	60702307	60704307	Dapk1	-0.07339496	4.4E-21	hypomethylated	0.0062759	0.46132	insignificant	59	177	183
chr13	60702571	60704571	Dapk1	-0.07339496	4.4E-21	hypomethylated	0.0062759	0.46132	insignificant	59	177	183
chr13	62230249	62232249	Zfp808	-0.1265517	1	insignificant	0.036432	0.42695	insignificant	2	30	30
chr13	62397849	62399849	A130040M12Rik		1	noCoverage	0.000019882	0.033381	inconclusive	0	12	12
chr13	62484534	62486534	Gm3604		1	noCoverage	-0.056943	0.428	insignificant	0	11	11
chr13	62568172	62570172	Zfp935	-0.24557994	0.000000403	hypomethylated	-0.026725	0.90733	insignificant	4	11	11
chr13	62659959	62661959	Zfp934		1	noCoverage	-0.059453	0.87461	insignificant	0	8	8
chr13	62861098	62863098	Mir713		1	noCoverage	-0.042712	0.1477	insignificant	0	9	9
chr13	63400791	63402791	Mir23b	-0.12948718	0.32976	insignificant	0.073291	0.38051	insignificant	2	4	4
chr13	63401019	63403019	Mir27b	-0.12948718	0.32976	insignificant	0.073291	0.38051	insignificant	2	4	4
chr13	63401515	63403515	Mir24-1	-0.12948718	0.32976	insignificant	0.073291	0.38051	insignificant	2	4	4
chr13	63666828	63668828	Ptch1	-0.08256581	5.27E-38	hypomethylated	0.024861	0.19362	insignificant	51	151	143
chr13	63915627	63917627	0610007P08Ri	-0.19224501	4.42E-09	hypomethylated	-0.095882	0.26461	insignificant	16	57	47
chr13	63915641	63917641	0610007P08Ri	-0.19224501	4.42E-09	hypomethylated	-0.095882	0.26461	insignificant	16	57	47
chr13	64230638	64232638	Slc35d2	-0.49606625	0.051265	insignificant	-0.086079	0.20637	insignificant	5	23	22
chr13	64254507	64256507	Zfp367	-0.18669881	0.000031683	hypomethylated	-0.0026966	0.45281	insignificant	13	80	78
chr13	64262173	64264173	Habp4	-0.1382969	6.55E-27	hypomethylated	0.00852	0.0010535	hypermethylated	38	123	108
chr13	64376296	64378296	Cdc14b	-0.11877311	1	insignificant	0.01456	0.72392	insignificant	2	20	20
chr13	64414018	64416018	1110018J18Rik		1	noCoverage	-0.14758	1	insignificant	0	5	4
chr13	64471614	64473614	Ctsl	-0.19002805	0.00000414	hypomethylated	0.11982	0.091312	insignificant	5	36	30
chr13	65379161	65381161	Zfp369	-0.27963417	2.65E-08	hypomethylated	-0.10087	0.78888	insignificant	5	59	47
chr13	67032765	67034765	Ptdss1	-0.11362545	8.2E-27	hypomethylated	-0.0087638	0.12612	insignificant	37	130	127
chr13	67034008	67036008	Mterfd1	-0.10018687	1.31E-20	hypomethylated	-0.0056242	0.25505	insignificant	25	99	99
chr13	67198912	67200912	Zfp708		1	noCoverage	-0.060448	0.020659	hypomethylated	0	6	6
chr13	67228163	67230163	Zfp759		1	noCoverage	-0.15747	0.052807	insignificant	0	24	14

chr13	67273117	67275117	Rsl1		1	noCoverage	0.030556	0.73351	insignificant	0	9	6
chr13	67461508	67463508	Zfp953		1	noCoverage	-0.081767	0.30511	insignificant	0	8	8
chr13	67552560	67554560	Zfp874a	0.11785714	1	insignificant	0.017857	0.078133	insignificant	2	12	12
chr13	67585189	67587189	Zfp874b	-0.81545455	0.060606	insignificant	-0.022702	0.49503	insignificant	2	20	17
chr13	67601458	67603458	Zfp58	-0.22117821	0.00000171	hypomethylated	0.029187	0.78518	insignificant	2	8	8
chr13	67654088	67656088	Zfp748		1	noCoverage	0.05	1	insignificant	0	6	6
chr13	67829198	67831198	4930441O14R	-0.45302752	0.24077	insignificant	0.013158	0.24008	insignificant	1	9	9
chr13	67856071	67858071	4930441O14R	-0.41106151	0.00014956	stronglyHypometh	-0.034736	0.63214	insignificant	6	16	16
chr13	67879629	67881629	Zfp493	-0.36282468	0.29825	insignificant	-0.096139	0.082668	insignificant	3	8	8
chr13	67913752	67915752	Zfp273	-0.29269285	0.000000459	hypomethylated	-0.0036806	0.49993	insignificant	3	22	19
chr13	67963273	67965273	BC048507	0.01670417	0.37388	insignificant	0.07855	0.33533	insignificant	3	49	44
chr13	68720124	68722124	Fastkd3	-0.1027285	6E-17	hypomethylated	0.011303	0.12319	insignificant	30	162	150
chr13	68720998	68722998	Fastkd3	-0.12825651	9.27E-17	hypomethylated	0.0091505	0.0054321	inconclusive	18	100	92
chr13	69138419	69140419	Adcy2	-0.17954913	0.0017049	hypomethylated	0.02941	0.57935	insignificant	1	32	14
chr13	69672742	69674742	Papd7	-0.0797519	2.6E-33	hypomethylated	-0.0038167	0.38472	insignificant	81	315	277
chr13	69749894	69751894	Nsun2	-0.09575308	8.49E-13	hypomethylated	0.008674	0.30788	insignificant	20	136	136
chr13	69750341	69752341	Srd5a1	-0.08888822	3.06E-12	hypomethylated	0.0073156	0.76366	insignificant	20	122	122
chr13	69947759	69949759	Med10	-0.20694843	3.35E-12	hypomethylated	-0.04727	0.43257	insignificant	12	43	50
chr13	70776512	70778512	BC018507	-0.1199344	6.99E-09	hypomethylated	0.0081852	0.32547	insignificant	14	36	30
chr13	72101171	72103171	Irx1	-0.23324939	0.13806	insignificant	-0.1071	0.42643	insignificant	3	19	26
chr13	72765425	72767425	Irx2	-0.10211246	4.07E-35	hypomethylated	0.0057506	0.63625	insignificant	55	208	216
chr13	72766012	72768012	D430050G20	-0.10300081	2.8E-38	hypomethylated	0.0048424	0.13484	insignificant	52	183	184
chr13	73396944	73398944	Irx4	-0.05053513	0.0082405	inconclusive	-0.01857	0.030008	hypomethylated	38	130	122
chr13	73465930	73467930	Ndufs6		1	noCoverage	-0.061111	0.48166	insignificant	0	6	6
chr13	73467456	73469456	Mrpl36	-0.2040518	0.014181	hypomethylated	0.012857	0.57683	insignificant	3	19	19
chr13	73603830	73605830	Lpcat1	-0.09305225	0.14728	insignificant	0.013634	0.35981	insignificant	5	86	84
chr13	73673194	73675194	Slc6a3	-0.20452491	1.41E-12	hypomethylated	-0.0022569	0.70031	insignificant	9	40	40
chr13	73740748	73742748	Clptm1l	-0.08923007	7.68E-15	hypomethylated	0.040785	0.90415	insignificant	16	54	38
chr13	73763448	73765448	Tert	0.06671131	6.52E-12	inconclusive	0.007314	0.00038324	hypermethylated	12	52	49
chr13	73838143	73840143	5430425J12Ri	-0.27597403	0.047662	hypomethylated	0.071429	1	insignificant	1	2	2
chr13	73900144	73902144	Slc12a7	-0.12866078	3.65E-15	hypomethylated	-0.0075593	0.60802	insignificant	28	68	74
chr13	74074285	74076285	Brd9	-0.1350442	0.00000258	hypomethylated	-0.0051452	0.7569	insignificant	11	50	50
chr13	74075215	74077215	Trip13	-0.17344577	0.00067144	hypomethylated	-0.00085691	1	insignificant	4	14	14
chr13	74145866	74147866	Tppp	-0.13598622	4.46E-08	hypomethylated	0.01068	0.90554	insignificant	18	108	108
chr13	74257962	74259962	Slc9a3	-0.1517283	6.86E-10	hypomethylated	-0.0094117	0.75408	insignificant	15	59	54
chr13	74346148	74348148	Exoc3		1	noCoverage	-0.065714	1	insignificant	0	11	10
chr13	74429757	74431757	Ahrr	-0.25502699	0.20594	insignificant	-0.038721	0.57657	insignificant	2	12	14
chr13	74454774	74456774	Pdcd6		1	noCoverage	0.027048	0.52515	insignificant	0	16	22
chr13	74486764	74488764	Ccdc127	-0.11732763	1.67E-10	hypomethylated	0.008675	0.79598	insignificant	11	57	56
chr13	74631398	74633398	Zfp825	-0.46326531	2.39E-08	stronglyHypometh	0.0024697	0.88549	insignificant	4	7	7
chr13	74776319	74778319	Erap1	-0.16258487	2.91E-24	hypomethylated	-0.010951	0.50462	insignificant	19	50	50
chr13	74945369	74947369	Cast	-0.30302976	4.58E-25	hypomethylated	-0.0037873	0.23641	insignificant	7	13	7
chr13	75226434	75228434	Pcsk1	-0.1476423	0.000000368	hypomethylated	0.063175	0.91973	insignificant	8	46	46
chr13	75781493	75783493	Mir682	0.00281804	0.68511	insignificant	0.046032	0.0022939	hypermethylated	4	9	9
chr13	75843931	75845931	Ell2	-0.0958917	2.57E-20	hypomethylated	-0.0088798	0.087716	insignificant	33	138	133
chr13	76081272	76083272	Rhobtb3	-0.16289025	0.00019171	hypomethylated	-0.01655	1	insignificant	14	32	31
chr13	76156010	76158010	Rfesd	-0.54385965	0.053833	insignificant	-0.00017726	0.18302	insignificant	1	2	2
chr13	76235181	76237181	Ttc37	-0.16560016	0.23415	insignificant	0.044728	0.71493	insignificant	1	31	32
chr13	76236108	76238108	Arsk		1	noCoverage	0.0080972	1	insignificant	0	17	17
chr13	76521408	76523408	Mctp1	-0.09135375	9.58E-28	hypomethylated	0.0074008	0.097349	insignificant	35	140	140
chr13	77273796	77275796	2210408I21Ril	-0.11360431	2.76E-15	hypomethylated	0.004438	0.3285	insignificant	48	126	126
chr13	77274729	77276729	Ankrd32	-0.12738214	0.000013562	hypomethylated	0.0025176	0.31985	insignificant	11	42	42
chr13	77846950	77848950	Fam172a	-0.12531628	0.00078706	hypomethylated	-0.0090499	0.93119	insignificant	9	38	38
chr13	78163162	78165162	Pou5f2	-0.23443969	7.83E-09	hypomethylated	0.060375	0.3385	insignificant	4	23	23
chr13	78338243	78340243	Nr2f1	-0.07906228	1.5E-24	hypomethylated	0.0027176	0.21268	insignificant	24	94	90
chr13	81021682	81023682	Arrdc3	-0.18223455	0.002558	hypomethylated	0.027662	0.34483	insignificant	9	49	41
chr13	81102334	81104334	9330111N05R	-0.49809773	0.000000197	stronglyHypometh	-0.0035587	0.023735	inconclusive	4	17	16
chr13	81772143	81774143	Gpr98	-0.24132371	0.027218	hypomethylated	-0.0040596	0.1268	insignificant	2	19	19
chr13	81795804	81797804	Lysmd3	-0.10653074	0.000015587	hypomethylated	0.0013169	0.85552	insignificant	19	88	88
chr13	81849415	81851415	Mblac2	-0.15465365	1.18E-18	hypomethylated	-0.00036015	0.72371	insignificant	37	132	132
chr13	81850012	81852012	Mblac2	-0.15817977	1.82E-14	hypomethylated	0.0073505	0.77076	insignificant	26	96	96
chr13	81921290	81923290	Cetn3	-0.07739596	0.000000263	hypomethylated	-0.0071686	0.44813	insignificant	16	110	110
chr13	83866710	83868710	C130071C03R	-0.16359891	0.012163	hypomethylated	0.033175	0.024787	hypermethylated	9	24	22
chr13	83877418	83879418	Mir9-2	-0.12828634	0.47452	insignificant	0.018483	0.20197	insignificant	4	25	26
chr13	84360900	84362900	Tmem161b	-0.13140194	0.00004188	hypomethylated	0.006671	0.42555	insignificant	11	30	30
chr13	85328081	85330081	Ccnh	-0.22771382	0.000000011	hypomethylated	-0.013714	0.85561	insignificant	5	10	10
chr13	85429091	85431091	Rasa1	-0.14745763	0.000000122	hypomethylated	-0.028354	0.55953	insignificant	7	32	24
chr13	86186400	86188400	Cox7c		1	noCoverage	0.05	0.43545	insignificant	0	4	4
chr13	89882117	89884117	Vcan		1	noCoverage	0.090242	0.38258	insignificant	0	9	9
chr13	90228271	90230271	Tmem167	-0.14692683	2.34E-12	hypomethylated	-0.021421	0.70579	insignificant	25	95	92
chr13	90229213	90231213	Xrcc4	-0.14872274	2.59E-12	hypomethylated	-0.017757	0.39974	insignificant	20	77	76
chr13	91061726	91063726	Rps23	-0.09113544	0.00012492	hypomethylated	0.0058011	0.77016	insignificant	7	87	80
chr13	91061780	91063780	Rps23	-0.10146345	0.00012642	hypomethylated	-0.0045269	0.80239	insignificant	7	88	80
chr13	91363592	91365592	Atg10	-0.10856168	0.010615	hypomethylated	-0.0054165	0.74115	insignificant	9	35	35
chr13	91599701	91601701	Ssbp2	-0.08688647	4.65E-43	hypomethylated	0.0040696	0.18643	insignificant	89	304	283
chr13	91880125	91882125	Acot12	-0.23522931	0.00036536	hypomethylated	0.0018254	0.22369	insignificant	12	57	58
chr13	91881477	91883477	4833422C13Ri	0.21983573	0.36332	insignificant	0.092852	0.063298	insignificant	4	18	18
chr13	91947301	91949301	Zcchc9	-0.49926635	3.33E-08	stronglyHypometh	0.2083	0.0037708	inconclusive	2	39	27
chr13	92901449	92903449	Rasgrf2	-0.1094151	2.35E-08	hypomethylated	-0.0025967	0.51676	insignificant	27	82	82
chr13	93123737	93125737	Dhfr	-0.09964448	5.76E-19	hypomethylated	-0.00055865	0.60875	insignificant	53	154	154
chr13	93124958	93126958	Msh3	-0.17609386	2.56E-09	hypomethylated	0.0040856	0.11745	insignificant	23	50	50
chr13	93194923	93196923	Ankrd34b	-0.07504997	3.22E-09	hypomethylated	0.0046744	0.19021	insignificant	47	163	163
chr13	93253986	93255986	Fam151b	-0.54	0.01652	stronglyHypometh	0.098889	0.85384	insignificant	1	2	2
chr13	93300765	93302765	Zfyve16	-0.12821084	4.1E-16	hypomethylated	-0.001609	0.265	insignificant	16	66	66
chr13	93380092	93382092	Serinc5	-0.16022696	2.32E-09	hypomethylated	-0.010879	0.30321	insignificant	18	76	78

chr13	93564773	93566773	Thbs4	-0.14097304	1	insignificant	-0.015448	0.68971	insignificant	6	21	21
chr13	93613741	93615741	Mtx3	-0.31201796	0.05692	insignificant	-0.096656	0.72092	insignificant	6	30	26
chr13	93842258	93844258	Gm4814		1	noCoverage	0.010338	0.52925	insignificant	0	6	6
chr13	93962238	93964238	Papd4	-0.32938535	0.000010919	hypomethylated	-0.029395	1	insignificant	6	33	33
chr13	94073449	94075449	Homer1	-0.10632659	2.81E-25	hypomethylated	-0.0064863	0.14764	insignificant	46	200	168
chr13	94269644	94271644	Jmy	-0.13457787	5.16E-08	hypomethylated	-0.023864	0.36611	insignificant	20	67	67
chr13	94443390	94445390	Dmgdh	-0.14592764	0.000000729	hypomethylated	-0.0031886	0.44948	insignificant	23	54	56
chr13	94444257	94446257	Bhmt2	-0.20960065	0.000000954	hypomethylated	-0.034663	0.032142	hypomethylated	11	38	41
chr13	94540633	94542633	Arsb	-0.14928287	1.58E-18	hypomethylated	0.014052	0.79194	insignificant	31	114	118
chr13	94826750	94828750	Lhfpl2	-0.12966126	3.12E-33	hypomethylated	0.017189	0.0025599	hypermethylated	39	109	104
chr13	95055236	95057236	Scamp1	-0.08896217	2.03E-13	hypomethylated	0.033368	0.10607	insignificant	30	104	81
chr13	95127914	95129914	Ap3b1	-0.12467555	7.02E-12	hypomethylated	0.020035	0.57784	insignificant	22	123	117
chr13	95557897	95559897	Tbca	-0.05848902	0.0016668	hypomethylated	0.014359	0.5323	insignificant	25	89	83
chr13	95644581	95646581	Otp	-0.22653412	0.041271	hypomethylated	-0.045713	0.72607	insignificant	6	20	14
chr13	95745302	95747302	Wdr41	-0.18324556	4.49E-11	hypomethylated	-0.032811	0.77415	insignificant	11	31	30
chr13	95993005	95995005	Pde8b	0.00582463	0.30724	insignificant	0.02087	0.0054311	hypermethylated	8	44	44
chr13	96094191	96096191	Zbed3	-0.15279908	1.6E-12	hypomethylated	0.0029251	0.90052	insignificant	17	72	72
chr13	96145289	96147289	Aggf1	0.02683341	0.0027831	inconclusive	0.089786	0.00000258	hypermethylated	2	26	16
chr13	96214786	96216786	Crhbp	0.44106061	0.076857	insignificant	0.29786	0.0056933	hypermethylated	2	25	12
chr13	96248610	96250610	S100z	-0.0964041	0.081894	insignificant	-0.10778	0.059862	insignificant	8	26	24
chr13	96295195	96297195	F2rl1	-0.08766128	0.0020002	hypomethylated	-0.0062062	0.75118	insignificant	6	26	28
chr13	96388388	96390388	F2r	-0.13019781	0.00000509	hypomethylated	0.014835	0.51483	insignificant	4	40	41
chr13	96661877	96663877	Iqgap2	-0.05651958	0.00000169	hypomethylated	0.0093153	0.64965	insignificant	12	57	48
chr13	96902532	96904532	Sv2c	-0.15077652	0.60627	insignificant	-0.014174	0.67467	insignificant	4	53	53
chr13	97157248	97159248	Poc5	-0.14941144	7.32E-10	hypomethylated	0.0011621	0.23265	insignificant	9	56	46
chr13	97241115	97243115	Ankdd1b	-0.0013369	1	insignificant	0.044118	0.25	insignificant	2	4	4
chr13	97311689	97313689	Col4a3bp	-0.08065353	1.71E-26	hypomethylated	0.0058173	0.85323	insignificant	36	141	144
chr13	97312440	97314440	Polk	-0.0836955	2.8E-27	hypomethylated	0.0078172	0.97441	insignificant	31	131	134
chr13	97440891	97442891	Hmgcr	-0.19750083	0.31072	insignificant	0.012436	0.0026208	inconclusive	5	18	18
chr13	97693643	97695643	Gcnt4	-0.14350619	1.26E-13	hypomethylated	-0.020454	0.33331	insignificant	29	139	136
chr13	97804317	97806317	1700029F12Ri	0.18500928	0.68418	insignificant	0.075918	0.60079	insignificant	1	7	7
chr13	97836241	97838241	Fam169a	-0.20216728	0.0026891	hypomethylated	0.016311	1	insignificant	2	8	8
chr13	97840597	97842597	Fam169a	-0.09040927	1.72E-33	hypomethylated	-0.00024099	0.95882	insignificant	54	199	195
chr13	97906891	97908891	Gfm2	-0.14690644	0.00047133	hypomethylated	0.04888	0.065099	insignificant	9	54	54
chr13	97907881	97909881	Nsa2	-0.13561676	0.099583	insignificant	0.03883	0.11528	insignificant	7	37	37
chr13	97968312	97970312	Hexb	-0.4280425	1.79E-27	stronglyHypometh	-0.013643	0.017854	inconclusive	7	22	25
chr13	98010059	98012059	Enc1	-0.11066689	4.32E-27	hypomethylated	-0.0073184	0.43397	insignificant	41	164	163
chr13	98976120	98978120	Rgnef	-0.14132253	4.31E-18	hypomethylated	-0.0047012	0.49989	insignificant	25	85	83
chr13	99032196	99034196	Ankra2	-0.11273491	1.71E-18	hypomethylated	-0.010298	0.12422	insignificant	30	120	118
chr13	99032947	99034947	Ankra2	-0.10854169	1E-14	hypomethylated	-0.0079584	0.4499	insignificant	22	90	88
chr13	99086922	99088922	Btf3	-0.20975436	0.00023353	hypomethylated	0.031226	0.66226	insignificant	17	66	63
chr13	99086961	99088961	Btf3	-0.22088714	0.0030034	hypomethylated	0.037998	0.75599	insignificant	16	58	55
chr13	99123199	99125199	Foxd1	-0.11439146	4.67E-20	hypomethylated	0.0068584	0.76425	insignificant	43	145	143
chr13	99407365	99409365	Tmem174	-0.6577381	0.034091	stronglyHypometh	0.092262	0.29084	insignificant	2	4	4
chr13	99464786	99466786	Tmem171	-0.07635033	0.011751	hypomethylated	-0.025603	0.2365	insignificant	14	46	50
chr13	99585404	99587404	Fcho2	0.10166981	0.47347	insignificant	0.017679	0.25416	insignificant	6	20	20
chr13	99696339	99698339	Tnpo1	-0.09835773	0.6228	insignificant	0.00069805	0.3058	insignificant	3	69	58
chr13	100113740	100115740	Mrps27	-0.14784546	0.00023516	hypomethylated	0.00075482	0.44422	insignificant	7	52	55
chr13	100286557	100288557	Mtap1b	-0.13973114	0.0034004	hypomethylated	-0.0057094	0.7125	insignificant	2	26	26
chr13	100670638	100672638	Cartpt	-0.25249984	1.56E-24	hypomethylated	0.076298	0.012369	hypermethylated	12	31	35
chr13	100785594	100787594	Mccc2	0.08333333	1	insignificant	0.055556	0.36245	insignificant	1	4	4
chr13	100874025	100876025	Bdp1	-0.25149161	2.22E-35	hypomethylated	0.030552	0.42172	insignificant	14	43	42
chr13	100876973	100878973	Serf1	-0.02715969	0.08123	insignificant	-0.0087061	0.97271	insignificant	17	124	123
chr13	100893809	100895809	Smn1	-0.15560559	1.9E-18	hypomethylated	-0.0021899	0.49269	insignificant	19	60	60
chr13	101262564	101264564	Gtf2h2	-0.18232323	0.07462	insignificant	0.01438	0.70078	insignificant	5	14	14
chr13	101322453	101324453	Ocln	-0.18149002	0.00004606	hypomethylated	-0.0041811	0.38734	insignificant	13	47	46
chr13	101386899	101388899	Marveld2	-0.08670644	0.00014949	hypomethylated	0.033935	0.74377	insignificant	5	24	18
chr13	101386926	101388926	Marveld2	-0.12172226	0.00000979	hypomethylated	0.083909	0.70895	insignificant	2	16	10
chr13	101420297	101422297	Taf9	-0.08772225	2.12E-22	hypomethylated	0.015688	0.34788	insignificant	67	199	191
chr13	101420561	101422561	Taf9	-0.08362064	2.27E-21	hypomethylated	0.015171	0.34251	insignificant	65	195	187
chr13	101421014	101423014	Taf9	-0.09695088	5.02E-14	hypomethylated	0.0011375	0.57251	insignificant	50	160	157
chr13	101438435	101440435	Ccdc125	-0.14733517	4.66E-09	hypomethylated	0.0035316	0.17063	insignificant	19	54	59
chr13	101500897	101502897	Cdk7	-0.20462147	0.16939	insignificant	-0.14172	0.9114	insignificant	3	22	20
chr13	101545854	101547854	Cenph	-0.2933572	1	insignificant	-0.0064033	0.0037177	hypomethylated	2	22	22
chr13	101556441	101558441	Ccnb1		1	noCoverage	0.024218	0.83277	insignificant	0	30	30
chr13	101603382	101605382	Slc30a5	-0.11351	0.6081	insignificant	0.0048848	0.66979	insignificant	4	33	32
chr13	102462602	102464602	Pik3r1	-0.16588983	0.00000227	hypomethylated	0.010036	0.36599	insignificant	8	20	24
chr13	102538172	102540172	Pik3r1	-0.1483454	2.05E-10	hypomethylated	-0.012414	0.63307	insignificant	23	79	78
chr13	103482637	103484637	Cd180	-0.39933055	0.51726	insignificant	0.10102	0.83092	insignificant	2	9	9
chr13	104710594	104712594	Erbb2ip	-0.07045233	3.47E-41	hypomethylated	-0.0012767	0.93476	insignificant	74	226	217
chr13	104898869	104900869	Sgtb	-0.11733631	0.00000284	hypomethylated	0.0382	0.051291	insignificant	17	122	114
chr13	104899694	104901694	Nln	-0.09840837	0.0044164	hypomethylated	0.049067	0.37073	insignificant	11	90	90
chr13	104968177	104970177	Trim23	-0.1528238	0.1483	insignificant	-0.0051383	0.27355	insignificant	2	38	29
chr13	104968546	104970546	2410002O22R	-0.19313258	0.085561	insignificant	-0.027323	0.33398	insignificant	2	14	14
chr13	105018034	105020034	Cenpk	-0.09866394	0.000000103	hypomethylated	0.035857	0.41962	insignificant	12	56	56
chr13	105018467	105020467	Cenpk	-0.09866394	0.000000103	hypomethylated	0.035857	0.41962	insignificant	12	56	56
chr13	105018924	105020924	Cenpk	-0.05939664	1	insignificant	0.049064	1	insignificant	3	28	28
chr13	105076952	105078952	Adamts6		1	noCoverage	0.01575	0.72008	insignificant	0	14	14
chr13	105606318	105608318	Srek1ip1	-0.20602727	0.000000278	hypomethylated	0.017015	0.32442	insignificant	19	59	57
chr13	105607033	105609033	Srek1ip1	-0.19734819	0.00000326	hypomethylated	0.018829	0.36725	insignificant	14	47	45
chr13	105653973	105655973	Fam159b	-0.50555556	0.0034579	stronglyHypometh	-0.020455	0.15748	insignificant	1	6	6
chr13	105845010	105847010	Rgs7bp	-0.32291667	0.2923	insignificant	0.036458	0.75229	insignificant	1	4	4
chr13	105871201	105873201	4933425L06Rik		1	noCoverage	0.03126	0.12755	insignificant	0	10	10
chr13	106083094	106085094	Rnf180	-0.07767299	0.000000137	hypomethylated	0.018338	0.094345	insignificant	27	111	106
chr13	106232772	106234772	Htr1a	-0.24315965	6.46E-13	hypomethylated	-0.04415	0.10548	insignificant	9	36	35

chr13	107726995	107728995	Ipo11	-0.47091095	0.010421	stronglyHypometh	-0.018391	0.1373	insignificant	3	34	37
chr13	107736208	107738208	Dimt1	-0.12806528	0.32286	insignificant	-0.011688	0.6512	insignificant	9	78	76
chr13	107811649	107813649	3830408C21Ri	-0.07975662	4.2E-36	hypomethylated	-0.0031051	0.44587	insignificant	87	256	255
chr13	107812194	107814194	3830408C21Ri	-0.06171296	7.96E-15	hypomethylated	0.0017247	0.47602	insignificant	61	169	169
chr13	108680258	108682258	Zswim6	-0.10145029	1.54E-34	hypomethylated	0.0063455	0.40695	insignificant	49	173	184
chr13	108833667	108835667	2810008M24R	-0.12829832	3.25E-08	hypomethylated	0.021957	0.88055	insignificant	18	101	92
chr13	108947931	108949931	Ercc8	-0.13487286	7.28E-12	hypomethylated	0.044112	0.15599	insignificant	32	116	109
chr13	108948819	108950819	Ndufaf2	-0.11785715	0.00083929	hypomethylated	-0.0028551	0.062982	insignificant	11	49	46
chr13	109003597	109005597	Elovl7	-0.17472542	1.26E-36	hypomethylated	-0.017355	0.51832	insignificant	35	132	131
chr13	109105530	109107530	Depdc1b	-0.10852142	7.42E-24	hypomethylated	-0.0041465	0.36312	insignificant	40	123	117
chr13	110693016	110695016	Mir1904	-0.0714888	1.08E-18	hypomethylated	0.01406	0.18594	insignificant	23	102	99
chr13	111070414	111072414	Rab3c		1	noCoverage	-0.017537	1	insignificant	0	9	10
chr13	111184251	111186251	Plk2	-0.12375311	5.03E-37	hypomethylated	0.01233	0.45088	insignificant	53	166	165
chr13	112044220	112046220	Actbl2	0.04362568	0.047355	hypermethylated	0.050227	0.12235	insignificant	6	23	22
chr13	112280249	112282249	Gpbp1	-0.10675351	1.91E-26	hypomethylated	-0.019952	0.21527	insignificant	39	138	137
chr13	112475385	112477385	Mier3	-0.09920668	1.35E-26	hypomethylated	0.011235	0.055136	insignificant	70	220	227
chr13	112599191	112601191	Map3k1	-0.08449622	0.000000923	hypomethylated	-0.0021348	0.57039	insignificant	28	121	116
chr13	113253277	113255277	Il6st	-0.15905089	2.3E-60	hypomethylated	-0.015289	0.95021	insignificant	52	128	124
chr13	113442518	113444518	Ddx4	0.03050933	0.36409	insignificant	0.02732	0.017983	hypermethylated	9	52	52
chr13	113449973	113451973	Slc38a9	0.7851153	0.1849	lowCoverage	0.064618	0.2227	insignificant	1	20	13
chr13	113589999	113591999	Ppap2a	-0.13253327	1.67E-14	hypomethylated	0.001744	0.93927	insignificant	22	91	80
chr13	113590130	113592130	Ppap2a	-0.13253327	1.67E-14	hypomethylated	0.001744	0.93927	insignificant	22	91	80
chr13	113717000	113719000	Dhx29	-0.1303826	5.72E-18	hypomethylated	0.036837	0.13997	insignificant	25	96	96
chr13	113717588	113719588	Skiv2l2	-0.12262883	4.09E-14	hypomethylated	0.030664	0.269	insignificant	17	64	64
chr13	113777009	113779009	Ccno	-0.1264079	6.2E-14	hypomethylated	-0.0032812	0.61914	insignificant	28	126	137
chr13	113783075	113785075	Gm6320	-0.16734092	4.64E-35	hypomethylated	-0.0061434	0.43863	insignificant	36	132	130
chr13	113825190	113827190	Mir449c		1	noCoverage	0.060485	0.28249	insignificant	0	8	8
chr13	113836596	113838596	Gpx8	-0.1953086	0.34632	insignificant	-0.012108	0.78897	insignificant	3	8	8
chr13	113998866	114000866	Esm1	-0.03775013	0.39342	insignificant	0.047447	0.040674	hypermethylated	7	26	26
chr13	114408772	114410772	Snx18	-0.17076023	0.032336	hypomethylated	0.015351	0.83237	insignificant	3	6	6
chr13	114583715	114585715	Arl15	-0.21491072	9.53E-29	hypomethylated	0.013431	0.0039937	inconclusive	29	131	119
chr13	115178302	115180302	Ndufs4	0.37878788	0.0050028	stronglyHypermeth	-0.17538	0.37693	insignificant	1	8	6
chr13	115248938	115250938	Fst	-0.13186982	2.06E-09	hypomethylated	-0.0025933	0.075989	insignificant	40	90	96
chr13	115607444	115609444	Mocs2	-0.15152024	0.00000132	hypomethylated	0.025612	0.83894	insignificant	20	80	81
chr13	115722249	115724249	Itga2	-0.06042199	0.00086061	inconclusive	-0.037673	0.28592	insignificant	8	32	31
chr13	115880366	115882366	Pelo	-0.18836427	0.096501	insignificant	0.0020906	0.53214	insignificant	4	27	27
chr13	115892172	115894172	Itga1	-0.16884543	0.70476	insignificant	-0.0030945	0.9357	insignificant	3	6	6
chr13	117099896	117101896	Isl1	0.00583375	0.004075	inconclusive	0.012315	0.22689	insignificant	23	80	81
chr13	117814323	117816323	Parp8	-0.11825211	1.03E-23	hypomethylated	0.0091746	0.43658	insignificant	27	104	101
chr13	118008379	118010379	Emb	-0.07683153	2.82E-14	hypomethylated	0.003652	0.82227	insignificant	15	88	105
chr13	118390126	118392126	Hcn1	-0.10784726	0.000001795	hypomethylated	-0.0057032	0.13986	insignificant	32	119	125
chr13	119176059	119178059	Mrps30	-0.36512249	3.34E-10	stronglyHypometh	0.0776	0.63182	insignificant	0	36	35
chr13	119502505	119504505	Fgf10	-0.03412549	0.000000428	hypomethylated	-0.011538	0.36749	insignificant	20	60	61
chr13	120197818	120199818	Nnt	-0.17491527	3.09E-12	hypomethylated	0.016962	0.52187	insignificant	11	23	24
chr13	120197945	120199945	Nnt	-0.25201429	0.000000895	hypomethylated	0.02822	0.54977	insignificant	5	11	12
chr13	120216406	120218406	Paip1	-0.08285232	5.23E-42	hypomethylated	0.029532	0.9359	insignificant	90	191	192
chr13	120216713	120218713	Paip1	-0.08285232	5.23E-42	hypomethylated	0.029532	0.9359	insignificant	90	191	192
chr13	120250565	120252565	4833420G17R	-0.12952492	0.3248	insignificant	0.018014	0.16494	insignificant	10	47	45
chr13	120275845	120277845	Gm7120	-0.17244706	2.55E-11	inconclusive	-0.17696	1.4E-16	hypomethylated	42	60	55
chr13	120276666	120278666	Gm7120	-0.15670681	1.93E-15	inconclusive	-0.2309	2.45E-16	hypomethylated	44	61	51
chr13	120277191	120279191	Gm7120	-0.26282474	0.0010395	inconclusive	-0.32651	0.71981	insignificant	33	15	9
chr14	3048285	3050285	Gm2897		1	noCoverage	-0.04542	0.95288	insignificant	0	28	38
chr14	3048304	3050304	Gm2897		1	noCoverage	-0.04542	0.95288	insignificant	0	28	38
chr14	7859963	7861963	Gm10406		1	noCoverage	0.081283	0.34457	insignificant	0	14	16
chr14	7937971	7939971	Gm3696		1	noCoverage	0.016253	0.31867	insignificant	0	35	27
chr14	8164909	8166909	Gm5797		1	noCoverage	0.041574	0.5455	insignificant	0	8	7
chr14	8649470	8651470	Flnb	-0.17324766	0.000016974	hypomethylated	0.010363	1	insignificant	12	138	132
chr14	8834415	8836415	Abhd6	-0.13493333	0.58663	insignificant	0.087567	0.56949	insignificant	4	25	10
chr14	8911826	8913826	Rpp14		1	noCoverage	-0.021392	0.77341	insignificant	0	14	16
chr14	8929726	8931726	Pxk	-0.0935852	1.3E-14	hypomethylated	-0.0089953	0.25277	insignificant	30	92	102
chr14	9005506	9007506	Pdhb	-0.20724117	0.000029793	hypomethylated	-0.026572	0.10453	insignificant	10	22	22
chr14	9045661	9047661	Kctd6	-0.0939109	3.3E-15	hypomethylated	0.0026974	0.29636	insignificant	50	174	170
chr14	9211277	9213277	Oit1	-0.16296296	0.50495	insignificant	0.088173	0.19475	insignificant	2	6	6
chr14	9498804	9500804	4930452B06R	-0.28986275	7.11E-12	hypomethylated	-0.079108	0.21023	insignificant	4	10	9
chr14	12385066	12387066	Ptprg	-0.06428011	2.36E-26	hypomethylated	-0.0030484	0.18953	insignificant	59	227	222
chr14	13115722	13117722	3830406C13R	-0.25013418	1.92E-09	hypomethylated	-0.001357	0.89717	insignificant	16	44	44
chr14	13178379	13180379	Fezf2	-0.19335816	0.00000019	hypomethylated	-0.010022	0.68378	insignificant	14	59	62
chr14	13655593	13657593	Cadps		1	noCoverage	-0.14771	0.65583	insignificant	0	2	9
chr14	14116293	14118293	Synpr	-0.34354342	0.18178	insignificant	0.08865	0.5455	insignificant	3	17	20
chr14	14117152	14119152	Synpr	-0.28835979	0.33497	insignificant	0.084241	0.57917	insignificant	3	9	10
chr14	14793739	14795739	Thoc7	-0.12636896	0.00042876	hypomethylated	0.023288	0.48268	insignificant	19	91	91
chr14	14844004	14846004	Atxn7	-0.06205103	0.93318	insignificant	0.0026807	0.39523	insignificant	28	121	121
chr14	14953418	14955418	Psmd6	-0.15677083	1	insignificant	0.029697	0.57179	insignificant	2	8	8
chr14	15178008	15180008	Il3ra	-0.29293567	0.043824	hypomethylated	-0.049331	0.00035609	hypomethylated	7	107	110
chr14	15534538	15536538	Slc4a7	-0.13266768	2.85E-50	hypomethylated	0.0017941	0.30414	insignificant	46	215	213
chr14	17080827	17082827	Ngly1	-0.13589657	2.08E-17	hypomethylated	0.00000994	0.3175	insignificant	16	80	81
chr14	17082322	17084322	Ngly1		1	noCoverage	-0.014448	0.010819	hypomethylated	0	2	2
chr14	17196719	17198719	Top2b	-0.11182848	2.46E-08	hypomethylated	-0.0040948	0.43609	insignificant	47	137	133
chr14	17407724	17409724	Rarb	-0.12751612	0.59441	insignificant	-0.018653	1	insignificant	5	45	45
chr14	18492473	18494473	Thrb	-0.10717684	6.91E-27	hypomethylated	0.0058544	0.86535	insignificant	28	135	130
chr14	19071620	19073620	Nr1d2	-0.33107962	6.29E-24	hypomethylated	-0.02904	0.0050498	hypomethylated	4	31	37
chr14	19102655	19104655	Nkiras1	-0.10798864	2.29E-15	hypomethylated	-0.0041711	0.80745	insignificant	34	159	157
chr14	19103500	19105500	Rpl15	-0.09928948	0.0031461	hypomethylated	0.0026315	0.26048	insignificant	20	75	75
chr14	19164358	19166358	Ube2e1	-0.26425926	0.00000673	hypomethylated	0.072583	0.0066323	inconclusive	7	28	18
chr14	19726141	19728141	Ube2e2	-0.09145112	6.56E-08	hypomethylated	-0.011468	0.73435	insignificant	6	67	60

chr14	20569478	20571478	Nid2	-0.07456465	0.17665	insignificant	-0.043355	0.86654	insignificant	10	62	59
chr14	20643045	20645045	2700060E02Ri	-0.10591876	0.039419	hypomethylated	0.025512	0.54137	insignificant	6	48	48
chr14	20796471	20798471	Gng2	-0.5647658	0.0006073	stronglyHypometh	-0.085549	0.15631	insignificant	6	16	19
chr14	20902394	20904394	1810063B07R	-0.16075717	5.67E-19	hypomethylated	-0.03449	0.14893	insignificant	10	16	16
chr14	21001004	21003004	Kcnk5	-0.14505051	1	insignificant	0.0091851	0.90198	insignificant	1	20	20
chr14	21088384	21090384	Kcnk16	0.10592622	0.13541	insignificant	-0.065612	0.64461	insignificant	4	14	10
chr14	21112911	21114911	Nudt13	-0.59667444	0.10984	insignificant	0.10124	0.63203	insignificant	1	26	28
chr14	21166383	21168383	Fam149b	-0.13263404	0.37822	insignificant	0.024395	0.71128	insignificant	6	43	39
chr14	21166443	21168443	Ecd	-0.13263404	0.37822	insignificant	0.024395	0.71128	insignificant	6	43	39
chr14	21167343	21169343	Ecd	-0.18778688	0.38646	insignificant	0.0088001	0.84969	insignificant	6	32	32
chr14	21208132	21210132	Dnajc9	-0.0948698	0.0056856	hypomethylated	-0.0022384	0.72322	insignificant	3	19	19
chr14	21212777	21214777	Ttc18	-0.13170934	2.07E-10	hypomethylated	0.011511	0.63465	insignificant	16	51	40
chr14	21299355	21301355	Anxa7	0.43562937	0.00035053	stronglyHypermeth	0.066677	0.014218	hypermethylated	1	12	13
chr14	21364514	21366514	1810062O18R	-0.08180497	1.84E-15	hypomethylated	-0.0059958	0.31633	insignificant	41	113	110
chr14	21437576	21439576	Usp54	-0.63888889	0.00000246	stronglyHypometh	-0.021242	1	insignificant	1	2	2
chr14	21492542	21494542	Sec24c	-0.24763724	1.38E-23	hypomethylated	-0.034944	0.26286	insignificant	16	43	38
chr14	21513189	21515189	Fut11	-0.08484534	1.13E-25	hypomethylated	-0.0095615	0.16641	insignificant	44	136	136
chr14	21521248	21523248	Chchd1	-0.22108834	2.27E-13	hypomethylated	0.0071816	0.40432	insignificant	20	97	91
chr14	21522321	21524321	Chchd1	-0.37182272	0.01904	stronglyHypometh	-0.012519	0.52276	insignificant	5	36	39
chr14	21525773	21527773	2310021P13Ri	-0.12049987	4.29E-11	hypomethylated	0.015442	0.5153	insignificant	20	62	49
chr14	21553784	21555784	Ndst2		1	noCoverage	-0.077381	0.12652	insignificant	0	8	8
chr14	21613310	21615310	Camk2g	-0.08212933	0.014683	hypomethylated	0.065032	0.2889	insignificant	9	40	33
chr14	21654883	21656883	Plau	-0.20595238	1	insignificant	0.038492	0.48966	insignificant	2	10	10
chr14	21747654	21749654	Vcl	-0.10624248	3E-16	hypomethylated	0.012428	0.10095	insignificant	34	150	135
chr14	21870854	21872854	Ap3m1	-0.15900324	4.04E-14	hypomethylated	-0.0067536	0.81078	insignificant	14	54	54
chr14	21871664	21873664	Ap3m1	-0.21223645	0.032955	hypomethylated	0.016184	0.43715	insignificant	3	36	36
chr14	22318075	22320075	Myst4	-0.13386864	5.77E-49	hypomethylated	-0.0031779	0.10674	insignificant	47	187	185
chr14	22533798	22535798	Dupd1	0.17626243	1	insignificant	0.0091117	1	insignificant	2	14	15
chr14	22562101	22564101	Dusp13		1	noCoverage	0.10678	0.73528	insignificant	0	8	8
chr14	22567844	22569844	Samd8	-0.16505548	0.000000406	hypomethylated	-0.029897	0.044833	hypomethylated	5	17	14
chr14	22568752	22570752	Samd8	-0.14830856	6.59E-22	hypomethylated	-0.0044391	0.7787	insignificant	27	80	72
chr14	22649782	22651782	Vdac2	-0.10172287	1.16E-16	hypomethylated	0.021188	0.85255	insignificant	14	69	64
chr14	22668132	22670132	Comtd1	-0.12934981	0.35717	insignificant	-0.019886	0.54397	insignificant	11	27	26
chr14	22808823	22810823	Zfp503	0.0824906	0.000000683	hypermethylated	0.093103	0.0029994	hypermethylated	20	101	95
chr14	22837933	22839933	1700112E06Ri	-0.081325	3.2E-12	hypomethylated	0.038683	0.049691	hypermethylated	17	45	45
chr14	23913793	23915793	Gm10248		1	noCoverage	0.016667	0.78439	insignificant	0	6	6
chr14	24823427	24825427	Kcnma1	-0.09157502	8E-16	hypomethylated	0.0045509	0.5294	insignificant	52	159	158
chr14	25065142	25067142	Dlg5	-0.41981915	0.000000185	stronglyHypometh	-0.070874	0.65595	insignificant	4	16	14
chr14	25111585	25113585	E330034G19R	-0.03095238	0.69479	insignificant	0.10159	0.40097	insignificant	1	8	9
chr14	25306268	25308268	Polr3a	-0.55603077	1.65E-56	stronglyHypometh	0.043053	0.89142	insignificant	5	37	37
chr14	25308902	25310902	Rps24	-0.23244599	6.02E-10	hypomethylated	0.10667	0.025003	hypermethylated	13	56	65
chr14	26277777	26279777	Zmiz1	-0.115987	9.97E-46	hypomethylated	-0.000745	0.60136	insignificant	55	168	170
chr14	26352924	26354924	Mir3075	-0.66117216	0.00010025	stronglyHypometh	-0.082839	0.17547	insignificant	3	6	6
chr14	26512655	26514655	Ppif	-0.05481537	3.75E-12	hypomethylated	0.013282	0.37365	insignificant	32	126	125
chr14	26588342	26590342	Zcchc24	-0.11959909	0.00081993	hypomethylated	-0.0074214	0.062871	insignificant	4	56	56
chr14	26660640	26662640	Anxa11	-0.17738988	3.24E-09	hypomethylated	-0.0048984	0.68593	insignificant	23	100	99
chr14	27353226	27355226	Slmap	-0.12252449	4.28E-31	hypomethylated	-0.0049181	0.60555	insignificant	36	124	125
chr14	27398035	27400035	Fam116a	-0.12019602	5.53E-20	hypomethylated	0.038577	0.90517	insignificant	32	102	97
chr14	27456682	27458682	Arf4	-0.10818528	9.48E-25	hypomethylated	-0.0091104	0.64857	insignificant	32	123	124
chr14	27489332	27491332	Pde12	-0.18091358	0.000000657	hypomethylated	0.039821	0.23763	insignificant	14	41	38
chr14	27783737	27785737	Appl1	-0.0927345	0.10254	insignificant	0.0072491	0.59409	insignificant	3	59	59
chr14	27812547	27814547	Hesx1	-0.09514024	0.0042834	inconclusive	0.056367	0.013833	hypermethylated	5	14	14
chr14	27851186	27853186	Il17rd	-0.10746744	7.4E-20	hypomethylated	0.0036955	0.11898	insignificant	36	137	137
chr14	28050224	28052224	Arhgef3	-0.37632184	0.028295	stronglyHypometh	0.058881	1	insignificant	3	12	12
chr14	28241032	28243032	D14Abb1e	-0.07149008	0.24673	insignificant	0.029072	0.00011708	hypermethylated	40	172	177
chr14	28321646	28323646	Ccdc66	-0.41694492	1.28E-13	stronglyHypometh	-0.019476	0.32906	insignificant	7	26	25
chr14	28434627	28436627	Erc2	-0.09612157	8.95E-13	hypomethylated	0.012432	0.43914	insignificant	18	117	97
chr14	29317658	29319658	Wnt5a	-0.15626151	1.59E-26	hypomethylated	-0.0066653	0.34406	insignificant	26	69	73
chr14	29830393	29832393	Lrtm1	-0.34657947	0.21248	insignificant	-0.015619	1	insignificant	1	12	12
chr14	30535050	30537050	Cacna2d3	-0.12037594	1.64E-27	hypomethylated	0.00051353	0.5193	insignificant	46	160	153
chr14	30780565	30782565	Selk	-0.19043897	2.28E-32	hypomethylated	0.014952	0.88713	insignificant	23	79	76
chr14	30790522	30792522	Actr8	-0.1381601	0.000069721	hypomethylated	0.12824	0.63675	insignificant	5	22	22
chr14	30821185	30823185	Chdh	-0.11096555	7.34E-14	hypomethylated	-0.0051035	0.78589	insignificant	26	84	84
chr14	30822082	30824082	Il17rb	-0.10207633	0.000000233	hypomethylated	-0.0078073	0.66686	insignificant	18	61	61
chr14	30833488	30835488	Chdh	-0.04942689	0.45973	insignificant	-0.17993	0.037641	hypomethylated	4	8	11
chr14	31166672	31168672	Cacna1d	-0.07648177	0.00000328	hypomethylated	-0.013103	0.31251	insignificant	33	99	99
chr14	31291750	31293750	Dcp1a	-0.13932647	5.88E-17	hypomethylated	0.0032344	0.51142	insignificant	17	74	73
chr14	31361334	31363334	Tkt	-0.1111025	3.4E-12	hypomethylated	0.012939	0.73744	insignificant	14	55	63
chr14	31466560	31468560	Rft1	-0.11084207	2.81E-12	hypomethylated	0.00011511	0.65893	insignificant	13	30	30
chr14	31527034	31529034	Sfmbt1	-0.11103735	3.15E-42	hypomethylated	0.0031548	0.11391	insignificant	38	158	149
chr14	31527352	31529352	Sfmbt1	-0.0881294	8.81E-41	hypomethylated	0.010084	0.087486	insignificant	46	240	226
chr14	31527800	31529800	Sfmbt1	-0.09340268	2.19E-44	hypomethylated	0.0072555	0.084595	insignificant	63	292	278
chr14	31637779	31639779	Tmem110	-0.10818112	5.34E-10	hypomethylated	0.0028197	0.57873	insignificant	22	72	66
chr14	31756475	31758475	Itih1	-0.37645776	0.20808	insignificant	0.08396	0.005139	hypermethylated	1	11	11
chr14	31763673	31765673	Nek4	-0.24933424	3.35E-12	hypomethylated	0.0011919	0.62239	insignificant	12	68	68
chr14	31813578	31815578	Glt8d1	-0.17244749	2.64E-31	hypomethylated	-0.0086631	0.36867	insignificant	32	154	149
chr14	31813618	31815618	Glt8d1	-0.17244749	2.64E-31	hypomethylated	-0.0086631	0.36867	insignificant	32	154	149
chr14	31814852	31816852	Glt8d1	-0.25855713	0.10758	insignificant	-0.037792	0.0015766	hypomethylated	11	62	62
chr14	31831323	31833323	Pbrm1	-0.1294746	4.25E-10	hypomethylated	0.0032391	0.43278	insignificant	23	94	81
chr14	31832275	31834275	Gnl3	-0.095186	0.00000213	hypomethylated	-0.01294	0.12149	insignificant	19	62	59
chr14	31942116	31944116	2010107H07R	-0.16575529	0.050009	insignificant	0.015644	0.9598	insignificant	4	52	50
chr14	31947038	31949038	Nt5dc2	-0.00574412	0.15117	insignificant	0.032949	0.01353	hypermethylated	4	24	20
chr14	32020497	32022497	Tnnc1	-0.81980519	0.21667	lowCoverage	0.033667	0.33892	insignificant	1	7	6
chr14	32063674	32065674	Bap1	-0.00207597	0.00000744	hypomethylated	-0.011095	0.31861	insignificant	26	156	149
chr14	32064404	32066404	Phf7	0.04140084	0.00072882	inconclusive	-0.013494	0.1032	insignificant	18	124	117

chr14	32137082	32139082	Dnahc1	-0.22628926	0.00000763	hypomethylated	0.011775	0.097671	insignificant	7	29	27
chr14	32148909	32150909	Capn7	-0.12732967	1.52E-10	hypomethylated	-0.0070001	0.40928	insignificant	24	90	90
chr14	32249219	32251219	Sh3bp5	-0.20361182	0.056829	insignificant	-0.031739	0.69459	insignificant	6	21	14
chr14	32307264	32309264	Eaf1	-0.17484856	3.86E-40	hypomethylated	0.03618	0.53358	insignificant	20	82	59
chr14	32308163	32310163	Eaf1	-0.21351205	5.26E-35	hypomethylated	0.052162	0.83148	insignificant	18	82	54
chr14	32390569	32392569	Colq	-0.35572344	0.72628	insignificant	-0.027242	0.65691	insignificant	2	5	5
chr14	32453242	32455242	Btd	-0.18148387	0.000034112	hypomethylated	0.031717	0.16249	insignificant	12	44	44
chr14	32454151	32456151	Hacl1	-0.11427057	0.00015701	hypomethylated	0.016373	0.72081	insignificant	12	28	28
chr14	32643601	32645601	Ankrd28	-0.11616539	1.17E-18	hypomethylated	-0.0023715	0.93592	insignificant	26	77	78
chr14	32841288	32843288	Galntl2	-0.50016835	0.0067104	stronglyHypometh	-0.093025	0.6146	insignificant	2	9	10
chr14	32897913	32899913	Oxnad1	-0.17879848	5.74E-23	hypomethylated	-0.019048	0.28858	insignificant	20	58	62
chr14	32898851	32900851	Dph3	-0.34535313	0.000000583	stronglyHypometh	0.0061945	0.16778	insignificant	10	32	32
chr14	32972077	32974077	Ncoa4	-0.18236854	1.67E-25	hypomethylated	0.059478	0.67885	insignificant	13	42	33
chr14	32977977	32979977	Ncoa4	-0.10416667	0.0042893	hypomethylated	-0.007471	0.46826	insignificant	4	24	24
chr14	33014156	33016156	Parg	-0.10117578	1.87E-13	hypomethylated	-0.010379	0.40614	insignificant	38	112	110
chr14	33015077	33017077	Timm23	-0.05114657	0.00013202	hypomethylated	0.0028394	0.92407	insignificant	27	74	72
chr14	33134204	33136204	Ogdhl	-0.12758741	0.0023674	hypomethylated	0.00437	0.073052	insignificant	9	33	32
chr14	33278036	33280036	Slc18a3	-0.16842227	1.01E-11	hypomethylated	0.0055537	0.22899	insignificant	22	79	80
chr14	33325706	33327706	Ercc6	-0.21388759	0.000000299	hypomethylated	-0.014743	0.20565	insignificant	21	68	70
chr14	33412112	33414112	Prrxl1	-0.16502184	6.16E-11	hypomethylated	-0.022367	0.74783	insignificant	19	65	63
chr14	33498458	33500458	3425401B19Rik		1	noCoverage	0.01142	0.89383	insignificant	0	24	24
chr14	33598148	33600148	1810011H11R	-0.44791667	0.078642	insignificant	0.085417	0.21817	insignificant	1	4	4
chr14	33668941	33670941	E130203B14R	-0.14024407	0.000000289	hypomethylated	0.006367	0.37087	insignificant	26	70	71
chr14	34029008	34031008	Arhgap22	-0.00660173	0.80754	insignificant	-0.041439	0.81487	insignificant	2	4	4
chr14	34260344	34262344	Mapk8	-0.06653612	0.00044048	hypomethylated	-0.0025987	0.56085	insignificant	13	56	56
chr14	34401455	34403455	Ptpn20	-0.14406803	0.27523	insignificant	0.073164	0.000069437	hypermethylated	2	34	34
chr14	34735772	34737772	Gdf10	-0.11821317	9.96E-12	hypomethylated	-0.0059777	0.6219	insignificant	37	112	143
chr14	34753224	34755224	Gdf2	-0.11708683	1	insignificant	0.002343	0.14036	insignificant	1	14	10
chr14	34791950	34793950	Zfp488	0.03126149	0.82393	insignificant	-0.095768	0.65813	insignificant	3	35	30
chr14	34898175	34900175	Anxa8	-0.20458664	0.40924	insignificant	-0.025813	0.24061	insignificant	1	22	21
chr14	34965605	34967605	Ppyr1	-0.07508454	0.077179	insignificant	0.10047	0.7904	insignificant	4	8	10
chr14	35014819	35016819	Gprin2	0.0202652	0.000002985	hypermethylated	0.079219	0.060861	insignificant	8	24	24
chr14	35032231	35034231	Syt15	-0.23274715	1.73E-21	hypomethylated	0.0090674	0.97158	insignificant	17	70	68
chr14	35122912	35124912	Glud1	-0.08973736	6.34E-37	hypomethylated	0.0016526	0.97867	insignificant	78	227	227
chr14	35123689	35125689	Fam35a	-0.08964218	2.28E-25	hypomethylated	0.0024886	0.81457	insignificant	59	185	185
chr14	35157240	35159240	Gm3219	-0.10683761	1	insignificant	0.00080128	0.58666	insignificant	1	4	4
chr14	35168579	35170579	Fam25c	-0.15459937	0.33153	insignificant	-0.016817	0.22681	insignificant	2	6	6
chr14	35187689	35189689	Mmrn2	-0.09962786	0.0054569	hypomethylated	0.1734	0.58948	insignificant	5	18	18
chr14	35187855	35189855	Sncg	-0.09962786	0.0054569	hypomethylated	0.1734	0.58948	insignificant	5	18	18
chr14	35315732	35317732	Bmpr1a	-0.12921095	4.03E-22	hypomethylated	-0.011785	1	insignificant	38	113	117
chr14	35401793	35403793	Ldb3	0.01521061	0.72398	insignificant	0.0060777	0.74906	insignificant	4	24	24
chr14	35401867	35403867	Ldb3	0.01380819	0.72651	insignificant	0.0050503	0.50697	insignificant	4	22	22
chr14	35486113	35488113	Wapal	-0.11852397	4.39E-33	hypomethylated	0.01493	0.96541	insignificant	51	251	250
chr14	35632321	35634321	Grid1	-0.09476029	5.52E-39	hypomethylated	0.0089786	0.011164	hypermethylated	62	211	206
chr14	37781950	37783950	Gcap14	-0.1765477	2.22E-18	hypomethylated	-0.061955	0.068847	insignificant	13	43	40
chr14	37911497	37913497	Cdhr1	0.48717949	1	lowCoverage	0.039377	0.3625	insignificant	1	13	7
chr14	37948325	37950325	Ghitm	-0.18613412	2.73E-09	hypomethylated	0.00030384	0.61072	insignificant	16	46	46
chr14	37948508	37950508	Ghitm	-0.39082072	0.0043593	stronglyHypometh	-0.0089187	0.0020152	inconclusive	4	20	20
chr14	40286376	40288376	Nrg3	-0.11961688	0.0005552	hypomethylated	0.0016256	0.077763	insignificant	34	116	92
chr14	41706254	41708254	Sh2d4b	-0.27234883	0.11016	insignificant	0.021151	0.95166	insignificant	8	26	26
chr14	41780096	41782096	Tspan14	-0.1340521	0.000000376	hypomethylated	0.00283	0.40932	insignificant	12	44	44
chr14	41827064	41829064	5730469M10Rik		1	noCoverage	-0.11905	1	insignificant	0	9	8
chr14	41885199	41887199	Dydc1	-0.420625	0.0012389	stronglyHypometh	0.067157	0.38986	insignificant	2	16	12
chr14	41917669	41919669	Mat1a	0.02241715	0.73168	insignificant	-0.16299	0.0026523	hypomethylated	3	6	14
chr14	41963744	41965744	Mbl1		1	noCoverage	0.069219	0.7453	insignificant	0	8	8
chr14	45606785	45608785	Ptger2	-0.1778474	0.00032439	hypomethylated	-0.045904	0.54964	insignificant	6	21	24
chr14	45838391	45840391	Gpr137c	-0.08919722	0.00000854	hypomethylated	0.0034188	0.32561	insignificant	28	110	107
chr14	45839069	45841069	Txndc16	-0.11062675	0.0000047	hypomethylated	0.0081555	0.028982	hypermethylated	28	128	125
chr14	45938202	45940202	Ero1l	-0.08556165	0.000098905	hypomethylated	0.005747	0.080426	insignificant	7	44	38
chr14	45948498	45950498	Psmc6	-0.11374747	0.035046	hypomethylated	-0.0028254	0.93207	insignificant	17	55	53
chr14	45969860	45971860	Styx	-0.16758258	0.00000542	hypomethylated	-0.010865	0.41544	insignificant	13	56	58
chr14	46008471	46010471	Gnpnat1	-0.04616543	0.0005749	hypomethylated	0.019565	0.18591	insignificant	16	55	61
chr14	46149740	46151740	Fermt2	-0.09948826	0.37244	insignificant	-0.017703	0.26908	insignificant	15	88	83
chr14	46277818	46279818	Ddhd1	-0.23385102	0.000000093	hypomethylated	0.0044652	0.68083	insignificant	10	52	52
chr14	46998141	47000141	Gm15217	-0.66882353	0.20946	lowCoverage	0.11465	0.68189	insignificant	1	10	8
chr14	47010274	47012274	Bmp4	-0.20430672	0.066844	insignificant	0.30363	1	insignificant	4	14	18
chr14	47379215	47381215	Cdkn3	-0.14008284	0.062663	insignificant	-0.017913	0.091863	insignificant	10	47	46
chr14	47450918	47452918	Cgrrf1	-0.15497324	6.43E-08	hypomethylated	0.027966	0.27511	insignificant	18	68	55
chr14	47501639	47503639	Samd4	-0.13148795	2.29E-29	hypomethylated	0.006134	0.069822	insignificant	45	171	174
chr14	47620014	47622014	Samd4	0.20045668	1	lowCoverage	-0.040245	0.61251	insignificant	1	9	10
chr14	47809077	47811077	Gch1	-0.21680459	1.31E-17	hypomethylated	0.025404	0.010776	hypermethylated	12	45	45
chr14	47895817	47897817	Socs4	-0.11530615	0.000017751	hypomethylated	0.010511	0.80658	insignificant	16	116	115
chr14	47896532	47898532	Socs4	-0.12483279	0.000070072	hypomethylated	0.016226	0.75921	insignificant	16	82	81
chr14	47916988	47918988	Mapk1ip1l	-0.13438407	9.3E-46	hypomethylated	-0.0062964	0.00064357	hypomethylated	42	105	111
chr14	47992534	47994534	Lgals3	-0.10805952	0.00051794	hypomethylated	0.026912	0.81952	insignificant	5	44	44
chr14	48038082	48040082	Dlgap5		1	noCoverage	-0.11167	0.78302	insignificant	0	15	10
chr14	48091235	48093235	Fbxo34	-0.1336274	5.02E-12	hypomethylated	0.028896	0.061122	insignificant	38	110	103
chr14	48188109	48190109	Atg14	0.30750027	0.00000351	hypermethylated	-0.037844	0.37982	insignificant	9	36	36
chr14	48739543	48741543	Peli2	-0.09729065	1.77E-23	hypomethylated	-0.011623	0.48652	insignificant	32	157	152
chr14	48817390	48819390	Gm6498	0.09227571	0.0063714	hypermethylated	0.046836	0.00080576	hypermethylated	5	17	16
chr14	49065026	49067026	6720456H20R	-0.11240464	1.55E-12	hypomethylated	-0.00016168	1	insignificant	35	88	87
chr14	49287962	49289962	Otx2os1	-0.22044083	0.00000629	hypomethylated	-0.037513	0.76959	insignificant	5	40	37
chr14	49497547	49499547	4933429O19R	0.13619439	1	insignificant	0.049736	0.86219	insignificant	3	28	20
chr14	49685169	49687169	Mudeng	-0.09344909	3.95E-12	hypomethylated	-0.0085339	0.21597	insignificant	40	128	110
chr14	49686342	49688342	Mudeng	-0.31071429	1.63E-11	hypomethylated	-0.05265	0.89381	insignificant	2	7	4

chr14	49790901	49792901	Naa30	-0.1064507	2.01E-14	hypomethylated	0.0041861	0.41889	insignificant	51	216	205
chr14	50858558	50860558	Olfr731		1	noCoverage	-0.098485	0.68754	insignificant	0	2	2
chr14	51405193	51407193	Ttc5		1	noCoverage	-0.13507	1	insignificant	0	6	3
chr14	51415403	51417403	Ccnb1ip1	-0.41391391	0.048117	stronglyHypometh	-0.016904	0.79894	insignificant	3	8	8
chr14	51426121	51428121	Rpph1	-0.21657686	3.05E-09	hypomethylated	-0.0023932	0.68881	insignificant	16	59	63
chr14	51426620	51428620	Parp2	-0.20810228	3.07E-09	hypomethylated	-0.010315	0.92072	insignificant	16	59	61
chr14	51512930	51514930	Klhl33	-0.76805556	0.065217	insignificant	-0.10139	0.87261	insignificant	1	6	7
chr14	51543695	51545695	Osgep	-0.12390879	0.00000118	hypomethylated	0.0056441	0.084101	insignificant	12	92	90
chr14	51544568	51546568	Osgep	0.49255267	0.15332	insignificant	0.032827	0.32391	insignificant	2	48	48
chr14	51550524	51552524	Tmem55b	-0.07699563	0.000043393	hypomethylated	-0.0026112	0.94561	insignificant	8	56	56
chr14	51562977	51564977	Pnp	-0.18254537	0.026745	hypomethylated	0.020327	0.69692	insignificant	11	54	50
chr14	51574815	51576815	Pnp2	-0.28582721	1	lowCoverage	-0.054965	0.1302	insignificant	1	40	38
chr14	51626425	51628425	Rnase10		1	noCoverage	0.20528	0.17423	insignificant	0	9	8
chr14	51626603	51628603	Rnase10		1	noCoverage	0.20528	0.17423	insignificant	0	9	8
chr14	51691117	51693117	Olfr750	-0.01605815	0.63193	insignificant	-0.011029	0.29732	insignificant	1	6	6
chr14	51709751	51711751	Ang	-0.20400864	1	lowCoverage	0.023393	0.46479	insignificant	1	27	38
chr14	51780934	51782934	Ear5		1	noCoverage	0.13234	0.000000298	hypermethylated	0	14	16
chr14	51875787	51877787	Ear11		1	noCoverage	-0.0043446	1	insignificant	0	4	4
chr14	52503516	52505516	Mettl17	-0.15417309	0.14005	insignificant	-0.017459	0.43382	insignificant	8	60	68
chr14	52512284	52514284	Slc39a2	-0.16291209	0.005679	hypomethylated	-0.17234	0.050353	insignificant	4	12	16
chr14	52533163	52535163	Ndrg2	-0.20036433	0.000000468	hypomethylated	0.0055942	1	insignificant	11	30	30
chr14	52603507	52605507	Arhgef40	-0.11464644	7.2E-18	hypomethylated	0.015912	0.0032382	hypermethylated	22	126	119
chr14	52635539	52637539	G630016D24Rik		1	noCoverage	-0.096212	0.34517	insignificant	0	8	9
chr14	52640408	52642408	Zfp219		1	noCoverage	0.1	0.82223	insignificant	0	5	4
chr14	52687989	52689989	Snord58b	0.10071225	1	insignificant	0.07301	0.0059671	hypermethylated	1	10	10
chr14	52723703	52725703	Hnrnpc	-0.18793013	0.000042344	hypomethylated	0.039916	0.82393	insignificant	6	28	26
chr14	52729577	52731577	Rpgrip1		1	noCoverage	-0.064041	0.74507	insignificant	0	16	15
chr14	52816914	52818914	Chd8	-0.12552878	2.36E-15	hypomethylated	0.013223	0.96402	insignificant	12	54	54
chr14	52829559	52831559	Snord8		1	noCoverage	0.0052799	0.65122	insignificant	0	10	10
chr14	52857247	52859247	Chd8		1	noCoverage	0.17639	0.29515	insignificant	0	4	4
chr14	52897820	52899820	Tox4	-0.07303835	1.23E-14	hypomethylated	0.0079306	0.71707	insignificant	19	95	86
chr14	52899070	52901070	Tox4	-0.0465594	0.000035765	hypomethylated	0.015506	0.35163	insignificant	12	47	44
chr14	52948345	52950345	Sall2		1	noCoverage	0.053258	0.11449	insignificant	0	8	7
chr14	52969719	52971719	Olfr1513		1	noCoverage	-0.052619	0.089845	insignificant	0	5	5
chr14	53069067	53071067	Olfr1509	0.04044497	1	insignificant	-0.032403	0.021946	inconclusive	1	14	16
chr14	53087136	53089136	Olfr1508		1	noCoverage	0.25992	0.17405	insignificant	0	4	4
chr14	54873604	54875604	Dad1	-0.11293379	0.3695	insignificant	0.011121	0.91994	insignificant	12	42	42
chr14	54877971	54879971	Abhd4	0.08474708	1	insignificant	-0.00063501	0.91558	insignificant	2	51	47
chr14	54979524	54981524	Oxa1l	-0.14987536	0.001097	hypomethylated	0.028096	0.025036	inconclusive	5	66	62
chr14	55036536	55038536	Slc7a7	-0.36446879	0.04184	stronglyHypometh	-0.011177	0.26846	insignificant	4	10	10
chr14	55044745	55046745	Mrpl52	-0.15157381	0.00000872	hypomethylated	0.01027	0.93449	insignificant	16	64	65
chr14	55049440	55051440	Mmp14	-0.24100968	8.88E-23	hypomethylated	0.010499	0.000052961	hypermethylated	23	93	92
chr14	55081983	55083983	Lrp10	-0.11229463	1.22E-22	hypomethylated	0.000092413	0.59209	insignificant	35	126	126
chr14	55093936	55095936	Rem2	-0.57944139	0.00011799	stronglyHypometh	-0.15728	0.064399	insignificant	2	4	4
chr14	55136307	55138307	Prmt5	-0.20666667	1	insignificant	0.035926	0.18759	insignificant	2	10	12
chr14	55173198	55175198	Haus4	-0.18171721	8.38E-11	hypomethylated	0.0079662	0.80155	insignificant	9	58	58
chr14	55196253	55198253	Jub	-0.08850877	0.10317	insignificant	0.01509	0.16445	insignificant	12	38	35
chr14	55224745	55226745	4931414P19Ri	-0.1903699	0.000000279	hypomethylated	-0.0058146	3.27E-17	inconclusive	18	43	44
chr14	55235622	55237622	Psmb5	-0.32201563	1.91E-26	hypomethylated	0.035016	0.31725	insignificant	12	73	64
chr14	55236832	55238832	Psmb5	-0.29602035	0.000021476	hypomethylated	0.0057937	0.32486	insignificant	6	21	20
chr14	55243146	55245146	Psmb11	-0.00384943	0.40091	insignificant	0.095227	1.99E-08	hypermethylated	2	14	12
chr14	55260201	55262201	Acin1	-0.2162937	0.0040034	hypomethylated	0.081972	0.015978	hypermethylated	9	37	42
chr14	55304007	55306007	1700123O20R	-0.14779153	1.45E-28	hypomethylated	0.0095591	0.50239	insignificant	25	94	97
chr14	55331011	55333011	Cebpe	-0.5819398	0.4375	lowCoverage	-0.34648	0.30099	insignificant	1	4	6
chr14	55482995	55484995	Homez	-0.19735694	0.0020132	hypomethylated	0.007338	0.43345	insignificant	8	37	33
chr14	55496375	55498375	Ppp1r3e	-0.15921429	0.0014173	hypomethylated	-0.061537	0.014159	hypomethylated	3	15	12
chr14	55501261	55503261	Bcl2l2	-0.26922903	6.08E-13	hypomethylated	0.065456	0.78135	insignificant	12	34	34
chr14	55511979	55513979	Pabpn1	-0.1499524	6.45E-15	hypomethylated	0.023665	0.44491	insignificant	20	80	78
chr14	55531969	55533969	Slc22a17		1	noCoverage	-0.056067	8.78E-10	inconclusive	0	13	8
chr14	55545625	55547625	Efs	0.2320567	3.32E-27	hypermethylated	-0.019069	0.68687	insignificant	4	40	36
chr14	55554306	55556306	Cmtm5	0.18579251	0.094946	insignificant	-0.034842	0.49481	insignificant	6	24	24
chr14	55567979	55569979	Myh6	-0.40710117	0.26298	insignificant	-0.019793	0.32578	insignificant	1	19	19
chr14	55586623	55588623	D830015G02R	0.13588449	1	insignificant	0.16962	0.56133	insignificant	1	9	9
chr14	55594613	55596613	Mir208b	-0.11247086	0.40097	insignificant	-0.057601	0.42984	insignificant	1	4	4
chr14	55613386	55615386	Myh7		1	noCoverage	-0.20089	0.01178	hypomethylated	0	13	8
chr14	55633290	55635290	Ngdn	-0.21052126	8.33E-10	hypomethylated	0.011428	0.49415	insignificant	13	52	50
chr14	55690927	55692927	Zfhx2as	0.05958089	1	insignificant	-0.043838	0.060733	insignificant	4	34	34
chr14	55710885	55712885	Zfhx2	-0.1039206	0.23588	insignificant	0.0064629	0.91823	insignificant	7	51	52
chr14	55712620	55714620	Thtpa	-0.30376479	4.63E-28	hypomethylated	-0.040725	0.045787	hypomethylated	9	26	22
chr14	55725430	55727430	Ap1g2	-0.09975631	0.21804	insignificant	-0.061472	0.72301	insignificant	3	13	13
chr14	55734274	55736274	Jph4	-0.11078448	0.11191	insignificant	0.029109	0.2154	insignificant	9	44	40
chr14	55735115	55737115	Jph4	-0.29947127	6.24E-08	hypomethylated	0.065626	0.14386	insignificant	5	18	16
chr14	56096594	56098594	Dhrs4	-0.19183113	0.35464	insignificant	0.0024584	0.48791	insignificant	2	26	26
chr14	56108929	56110929	Lrrc16b	-0.10043154	1.48E-13	hypomethylated	0.0016041	0.57215	insignificant	20	135	121
chr14	56128284	56130284	Cpne6	-0.06359649	1	insignificant	-0.0059524	0.81667	insignificant	2	4	4
chr14	56143802	56145802	Nrl		1	noCoverage	-0.0094551	0.48346	insignificant	0	4	5
chr14	56158102	56160102	Pck2	-0.29603654	0.000000181	hypomethylated	-0.065372	0.58622	insignificant	7	37	38
chr14	56177865	56179865	Dcaf11	-0.11556565	9.57E-16	hypomethylated	0.012758	0.36281	insignificant	25	113	114
chr14	56178759	56180759	Dcaf11	-0.10690867	9.52E-09	hypomethylated	0.0083972	0.15637	insignificant	22	102	102
chr14	56193510	56195510	Fitm1	-0.03492679	0.62154	insignificant	-0.011832	1	insignificant	4	8	8
chr14	56196330	56198330	Psme1	-0.17550897	1.26E-14	hypomethylated	0.043139	0.041385	hypermethylated	9	52	47
chr14	56204091	56206091	Fam158a	0.42280702	0.32308	lowCoverage	-0.11985	0.87032	insignificant	1	10	13
chr14	56209626	56211626	Rnf31	-0.12621607	1.59E-18	hypomethylated	0.01476	0.048302	hypermethylated	17	121	125
chr14	56209858	56211858	Psme2	-0.11637026	6.02E-16	hypomethylated	0.01681	0.059432	insignificant	15	113	117
chr14	56209938	56211938	Psme2	-0.11794541	3.6E-16	hypomethylated	0.017551	0.040334	hypermethylated	15	111	115

chr14	56221821	56223821	Irf9	-0.12323593	0.26275	insignificant	0.0054106	0.37597	insignificant	2	20	20
chr14	56222415	56224415	Irf9	-0.0813968	0.19649	insignificant	0.002854	1	insignificant	4	23	22
chr14	56236006	56238006	Rec8	0.196875	0.18372	insignificant	0.21139	0.48774	insignificant	2	4	4
chr14	56254515	56256515	Ipo4	-0.83155923	9.14E-14	stronglyHypometh	0.018859	1	insignificant	1	28	29
chr14	56262643	56264643	Tm9sf1	-0.4005848	0.38763	insignificant	-0.022183	0.020384	inconclusive	4	16	20
chr14	56268018	56270018	Tssk4	-0.09937551	0.26332	insignificant	-0.011199	0.57775	insignificant	3	19	18
chr14	56268128	56270128	Tssk4	-0.09937551	0.26332	insignificant	-0.011199	0.57775	insignificant	3	19	18
chr14	56279345	56281345	Mdp1	-0.48918705	1.22E-13	stronglyHypometh	-0.15641	0.93729	insignificant	3	16	20
chr14	56290071	56292071	Gmpr2	-0.02942577	0.52507	insignificant	0.050442	0.48545	insignificant	4	21	21
chr14	56290743	56292743	Gmpr2	-0.02942577	0.52507	insignificant	0.050442	0.48545	insignificant	4	21	21
chr14	56300654	56302654	Tinf2	-0.12068894	0.015474	hypomethylated	-0.019767	0.47346	insignificant	5	38	36
chr14	56331925	56333925	Tgm1	-0.29670546	9.85E-10	hypomethylated	0.061919	0.016619	hypermethylated	7	29	32
chr14	56332329	56334329	Tgm1	-0.4776745	0.45487	lowCoverage	0.054159	0.37861	insignificant	1	19	22
chr14	56341013	56343013	Rabggta	-0.51149634	1.04E-16	stronglyHypometh	0.05792	0.076205	insignificant	4	15	14
chr14	56363529	56365529	2610027L16Ri	-0.08648529	0.028421	hypomethylated	-0.0095655	0.92236	insignificant	14	78	66
chr14	56364521	56366521	Dhrs1	-0.14107083	0.00010423	hypomethylated	0.0010288	0.29236	insignificant	14	70	65
chr14	56379760	56381760	Ltb4r2	-0.06997834	2.99E-13	hypomethylated	0.0068856	0.20031	insignificant	26	80	80
chr14	56402856	56404856	Adcy4	-0.30611389	0.00005765	hypomethylated	-0.25712	0.028016	hypomethylated	5	29	27
chr14	56442631	56444631	Nfatc4	-0.29493087	1.11E-13	hypomethylated	0.063748	0.17086	insignificant	6	56	52
chr14	56471951	56473951	Nynrin	-0.2625778	0.000000304	hypomethylated	-0.0017935	0.74509	insignificant	5	26	26
chr14	56502805	56504805	Khnyn	-0.09520606	0.000000008	hypomethylated	0.0186	1	insignificant	13	60	50
chr14	56503093	56505093	Cbln3	-0.09520606	0.000000008	hypomethylated	0.0186	1	insignificant	13	60	50
chr14	56519069	56521069	Sdr39u1	-0.13025346	0.000022547	hypomethylated	0.049926	0.93116	insignificant	8	20	19
chr14	56830244	56832244	Gzmf		1	noCoverage	0.013775	0.57641	insignificant	0	11	12
chr14	56982904	56984904	Atp12a	-0.20578021	1.23E-10	hypomethylated	-0.057357	0.41789	insignificant	19	70	62
chr14	57020533	57022533	Rnf17	0.00996636	1	insignificant	0.094656	0.097192	insignificant	10	58	58
chr14	57190683	57192683	Cenpj	-0.5023595	0.000030186	stronglyHypometh	-0.20337	0.30361	insignificant	2	6	4
chr14	57193455	57195455	Parp4	-0.12230207	0.00073947	hypomethylated	-0.020244	0.34505	insignificant	8	41	40
chr14	57286084	57288084	Mphosph8	-0.10211756	5.01E-13	hypomethylated	0.017211	0.44142	insignificant	27	120	118
chr14	57397153	57399153	Pspc1	0.14712302	1	insignificant	-0.097321	0.39749	insignificant	1	6	6
chr14	57430553	57432553	Zmym5	-0.09710434	2.31E-13	hypomethylated	0.0017691	0.50622	insignificant	28	135	135
chr14	57505630	57507630	Zmym2	-0.11755508	1.81E-08	hypomethylated	-0.010864	0.51216	insignificant	14	65	61
chr14	57676782	57678782	Gja3	0.00773342	0.0028053	inconclusive	0.034945	0.57388	insignificant	22	64	64
chr14	57723539	57725539	Gjb2		1	noCoverage	0.07033	0.62385	insignificant	0	14	17
chr14	57752414	57754414	Gjb6	-0.23771044	0.018986	hypomethylated	0.028292	0.39356	insignificant	3	9	9
chr14	58017320	58019320	Cryl1	-0.14403305	0.6047	insignificant	0.056893	0.8066	insignificant	1	30	26
chr14	58041907	58043907	Ift88	-0.14400703	8.92E-21	hypomethylated	-0.0030061	0.6358	insignificant	18	85	85
chr14	58142665	58144665	Il17d	-0.07979363	4.55E-37	hypomethylated	0.011127	0.54629	insignificant	49	178	158
chr14	58190406	58192406	N6amt2		1	noCoverage	0.0036612	0.91738	insignificant	0	16	16
chr14	58283793	58285793	Xpo4	-0.11097869	7.81E-13	hypomethylated	0.012237	0.070748	insignificant	28	90	85
chr14	58364960	58366960	Lats2	-0.1957868	8.12E-16	hypomethylated	0.002122	0.41037	insignificant	24	62	62
chr14	58416025	58418025	Sap18	-0.12258746	1.31E-18	hypomethylated	0.080874	0.17632	insignificant	12	76	59
chr14	58416054	58418054	Gm10094	-0.12258746	1.31E-18	hypomethylated	0.080874	0.17632	insignificant	12	76	59
chr14	58416260	58418260	Mir3077	-0.12258746	1.31E-18	hypomethylated	0.080874	0.17632	insignificant	12	76	59
chr14	58444075	58446075	Mrp63	-0.13845087	1.19E-31	hypomethylated	0.021639	0.91723	insignificant	30	157	156
chr14	58445000	58447000	F630043A04Ri	-0.12562112	6.33E-26	hypomethylated	-0.0045054	0.41329	insignificant	27	108	108
chr14	58509099	58511099	Zdhhc20	-0.1247963	2.13E-11	hypomethylated	0.022534	0.43957	insignificant	20	50	46
chr14	58618099	58620099	Efha1	0.10185162	0.40719	insignificant	0.017553	0.69664	insignificant	2	15	18
chr14	58690522	58692522	Fgf9	-0.14379216	4.03E-08	hypomethylated	-0.0078921	0.62163	insignificant	17	50	56
chr14	59819064	59821064	Rcbtb1	-0.09609291	6.81E-31	hypomethylated	-0.001172	0.92719	insignificant	49	145	141
chr14	59879053	59881053	Gm6904		1	noCoverage	-0.12821	0.10995	insignificant	0	3	3
chr14	59916359	59918359	Phf11	0.06179775	1	insignificant	-0.019933	0.55598	insignificant	1	2	2
chr14	60058817	60060817	Cab39l	-0.13283447	0.00065111	hypomethylated	0.016465	0.46537	insignificant	10	85	79
chr14	60059714	60061714	Setdb2	-0.17499018	0.00038117	hypomethylated	0.015978	0.1936	insignificant	10	73	68
chr14	60216796	60218796	Cdadc1	-0.19602068	0.000000647	hypomethylated	0.014363	0.80365	insignificant	6	24	24
chr14	60243117	60245117	Shisa2	-0.15814744	1.12E-39	hypomethylated	-0.047076	0.95738	insignificant	35	141	132
chr14	60870215	60872215	Nupl1	-0.14831601	0.002953	hypomethylated	0.006003	0.42614	insignificant	13	68	75
chr14	60883064	60885064	Mtmr6	-0.15314836	4.24E-14	hypomethylated	0.027435	0.066702	insignificant	21	53	50
chr14	60996122	60998122	Fam123a	-0.10413388	3.35E-16	hypomethylated	0.0069921	0.039691	hypermethylated	42	154	141
chr14	61252565	61254565	Spata13	0.06517786	0.00021065	inconclusive	0.023088	0.45122	insignificant	5	52	50
chr14	61385970	61387970	C1qtnf9	0.0010101	0.4927	insignificant	0.034343	0.55626	insignificant	2	4	4
chr14	61402402	61404402	Mipep	-0.03168139	0.57262	insignificant	0.0076087	0.62176	insignificant	17	53	58
chr14	61656824	61658824	Tnfrsf19	-0.3551195	0.00000412	stronglyHypometh	0.023842	0.00514	hypermethylated	8	31	31
chr14	61756293	61758293	Sacs	-0.01802993	0.83159	insignificant	-0.018492	0.20882	insignificant	6	24	28
chr14	61877327	61879327	Sgcg	-0.09259259	0.015396	hypomethylated	-0.0025852	0.90592	insignificant	2	9	9
chr14	61979282	61981282	Ebpl	-0.31976604	0.12335	insignificant	-0.036495	0.25027	insignificant	8	27	25
chr14	62058784	62060784	Kpna3	-0.08855378	0.000015144	hypomethylated	0.020107	0.24751	insignificant	7	38	38
chr14	62175723	62177723	6330409N04R	-0.38904897	9.44E-24	stronglyHypometh	0.028805	0.5091	insignificant	5	24	24
chr14	62216062	62218062	Trim13	-0.75855943	0.059164	insignificant	-0.082653	0.50109	insignificant	1	12	16
chr14	62217348	62219348	Trim13	-0.42127582	3.51E-27	stronglyHypometh	0.18026	0.4819	insignificant	4	29	20
chr14	62225293	62227293	Kcnrg		1	noCoverage	-0.059589	0.86947	insignificant	0	6	6
chr14	62225318	62227318	Kcnrg		1	noCoverage	-0.059589	0.86947	insignificant	0	6	6
chr14	62301210	62303210	Dleu2	-0.08142412	0.00027893	hypomethylated	-0.016751	0.12491	insignificant	12	78	82
chr14	62911816	62913816	Dleu7		1	noCoverage	0.0014953	1	insignificant	0	10	10
chr14	62949941	62951941	Rnaseh2b	-0.10457201	2.46E-10	hypomethylated	0.0071171	0.18192	insignificant	16	68	58
chr14	63379949	63381949	Ints6	-0.13528561	0.00000485	hypomethylated	-0.018427	0.65077	insignificant	9	80	76
chr14	63455526	63457526	Wdfy2	-0.11541519	0.000033048	hypomethylated	0.0064614	0.58171	insignificant	17	108	106
chr14	63740301	63742301	Ctsb	-0.06150296	0.22297	insignificant	0.011568	0.37143	insignificant	12	54	55
chr14	63796630	63798630	Fdft1	-0.21171994	6.58E-12	hypomethylated	-0.066723	0.3999	insignificant	16	48	42
chr14	63812362	63814362	Neil2	-0.27991395	0.00023695	hypomethylated	0.045157	0.86145	insignificant	7	32	34
chr14	63864097	63866097	Gata4	-0.23669391	0.000000424	hypomethylated	-0.0064174	0.80472	insignificant	34	108	103
chr14	64054230	64056230	Fam167a	-0.11398682	1	insignificant	0.043471	0.14523	insignificant	3	29	29
chr14	64127929	64129929	Tdh	-0.0003872	0.56998	insignificant	0.018952	0.11518	insignificant	3	16	15
chr14	64162790	64164790	Mtmr9	-0.14347158	0.21137	insignificant	0.15406	0.38082	insignificant	3	6	9
chr14	64224366	64226366	Xkr6	-0.1326251	6.08E-19	hypomethylated	0.0085478	0.34805	insignificant	42	178	185

chr14	64478148	64480148	Pinx1	-0.18968531	1.04E-11	hypomethylated	0.028884	0.18395	insignificant	14	80	73
chr14	64561542	64563542	Sox7	-0.03321528	6.95E-26	hypomethylated	0.0062589	0.91868	insignificant	22	136	129
chr14	64610267	64612267	Rp1l1	0.0364899	1	insignificant	0.013301	0.0047491	hypermethylated	4	16	16
chr14	65208493	65210493	Mir124a-1	-0.22766831	0.0011308	hypomethylated	-0.023478	0.64926	insignificant	5	10	10
chr14	65270367	65272367	Kif13b	-0.18021523	2.51E-30	hypomethylated	-0.007799	0.38821	insignificant	23	66	66
chr14	65567881	65569881	Ints9	-0.21513088	0.00000406	hypomethylated	0.074509	0.73753	insignificant	5	30	26
chr14	65716943	65718943	Extl3	-0.31823561	1.98E-11	hypomethylated	-0.039255	0.37906	insignificant	9	28	29
chr14	65881300	65883300	Fzd3	-0.10648279	0.00004355	hypomethylated	0.045837	0.30893	insignificant	8	40	39
chr14	65884537	65886537	Fbxo16		1	noCoverage	-0.11603	0.41325	insignificant	0	7	8
chr14	65976512	65978512	Zfp395	-0.10789515	3.01E-34	hypomethylated	0.0092927	0.069564	insignificant	45	154	156
chr14	66044046	66046046	Pnoc	0.24081386	0.042698	hypermethylated	0.12824	0.22783	insignificant	3	18	18
chr14	66211847	66213847	Elp3		1	noCoverage	0.1718	1	insignificant	0	18	12
chr14	66423747	66425747	Pbk	-0.15654025	1	insignificant	-0.028564	0.30302	insignificant	2	16	16
chr14	66452806	66454806	Esco2	-0.11365744	0.10448	insignificant	-0.0098571	0.057969	insignificant	3	12	12
chr14	66455138	66457138	Ccdc25	-0.12370932	3.93E-08	hypomethylated	-0.0027222	0.54545	insignificant	25	59	57
chr14	66572581	66574581	Scara3	-0.14426843	0.0024088	hypomethylated	-0.0016977	0.34579	insignificant	13	39	39
chr14	66586319	66588319	Clu	-0.18241922	6.42E-19	hypomethylated	-0.0017923	0.34668	insignificant	12	37	37
chr14	66696570	66698570	Adam2	-0.5219141	0.0022466	stronglyHypometh	0.030732	0.30927	insignificant	7	34	34
chr14	66832389	66834389	Ptk2b		1	noCoverage	-0.012669	0.37223	insignificant	0	2	2
chr14	66899889	66901889	Ptk2b	-0.56090226	0.000000149	stronglyHypometh	-0.034586	0.67054	insignificant	2	4	4
chr14	66914861	66916861	Trim35	-0.12983427	1.4E-30	hypomethylated	0.011617	1	insignificant	34	87	82
chr14	66962211	66964211	Stmn4	0.10370626	0.71353	insignificant	0.058239	0.37457	insignificant	1	21	20
chr14	67253094	67255094	Adra1a	-0.2468002	0.000013815	hypomethylated	-0.015622	0.73767	insignificant	8	42	44
chr14	67486686	67488686	Gm5464	-0.1065254	0.00000356	hypomethylated	0.012681	0.97082	insignificant	18	119	114
chr14	67487437	67489437	Dpysl2	-0.08910043	0.00017452	hypomethylated	0.0088982	0.82625	insignificant	14	92	92
chr14	67529044	67531044	Pnma2	-0.14646312	1.13E-25	hypomethylated	0.0027174	0.88002	insignificant	32	147	144
chr14	67627714	67629714	Bnip3l	-0.17361437	0.0035828	hypomethylated	-0.015738	0.39872	insignificant	21	62	62
chr14	67691266	67693266	Ppp2r2a	0.12172941	0.086618	insignificant	0.13029	0.80058	insignificant	2	26	19
chr14	67851128	67853128	Ebf2	-0.17168644	1.46E-22	hypomethylated	0.0033016	0.73364	insignificant	27	93	95
chr14	67933548	67935548	Gm6878		1	noCoverage	0.13347	0.13979	insignificant	0	10	9
chr14	68333154	68335154	Kctd9	-0.10850268	2.02E-24	hypomethylated	0.0081003	0.55124	insignificant	52	165	161
chr14	68333667	68335667	Cdca2	-0.10788968	6.84E-20	hypomethylated	0.003252	0.62939	insignificant	36	114	114
chr14	68333898	68335898	Cdca2	-0.10026909	2.67E-17	hypomethylated	0.003078	0.51163	insignificant	36	112	112
chr14	68362285	68364285	Gnrh1	0.26919366	1	lowCoverage	-0.018439	0.50819	insignificant	1	18	20
chr14	68551629	68553629	Dock5	-0.17146309	8.31E-30	hypomethylated	-0.0067219	0.76245	insignificant	19	53	51
chr14	68700940	68702940	Nefl	-0.10621706	1.35E-15	hypomethylated	-0.0040471	0.88698	insignificant	18	90	90
chr14	68743061	68745061	Nefm	-0.00578901	0.10205	insignificant	0.012478	0.89883	insignificant	8	39	41
chr14	69646345	69648345	Stc1	-0.25553996	1	lowCoverage	0.31286	0.49648	insignificant	1	11	11
chr14	69789075	69791075	Nkx2-6	-0.15788733	0.0017315	hypomethylated	0.0063441	0.37465	insignificant	6	42	40
chr14	69807748	69809748	Nkx3-1	-0.201271	0.000476	hypomethylated	0.0023194	0.43841	insignificant	8	60	58
chr14	69903160	69905160	Slc25a37	-0.38377392	1.13E-13	stronglyHypometh	0.025233	0.22291	insignificant	6	25	26
chr14	69912356	69914356	Synb	0.07498299	0.61084	insignificant	-0.0050563	0.62465	insignificant	4	10	10
chr14	69954207	69956207	Entpd4	-0.13033533	2.14E-46	hypomethylated	-0.0032773	0.00026597	inconclusive	36	104	94
chr14	70008282	70010282	Loxl2	-0.17520927	3.69E-20	hypomethylated	-0.013386	0.5683	insignificant	24	76	80
chr14	70107387	70109387	R3hcc1		1	noCoverage	-0.14907	1	insignificant	0	9	9
chr14	70132377	70134377	Chmp7	-0.12831755	0.000093125	hypomethylated	0.0046094	0.56231	insignificant	10	67	63
chr14	70166278	70168278	Tnfrsf10b	-0.11840729	0.00000194	hypomethylated	0.0018354	0.83939	insignificant	16	66	66
chr14	70205352	70207352	Rhobtb2	-0.54813596	0.46814	lowCoverage	-0.044598	0.0023137	hypomethylated	1	16	16
chr14	70239226	70241226	Pebp4		1	noCoverage	0.092546	0.2053	insignificant	0	6	6
chr14	70476251	70478251	Egr3	-0.05726527	0.00047735	hypomethylated	0.031714	0.035086	hypermethylated	18	100	99
chr14	70498951	70500951	Bin3	-0.10790228	5.04E-12	hypomethylated	0.019317	0.42096	insignificant	20	63	65
chr14	70553598	70555598	9930012K11Rik		1	noCoverage	0.06378	0.25933	insignificant	0	14	14
chr14	70559309	70561309	9930012K11Ri	-0.34347647	0.000000444	stronglyHypometh	-0.019445	0.35035	insignificant	4	19	16
chr14	70577479	70579479	Pdlim2	-0.38119013	0.008667	stronglyHypometh	-0.060403	0.91393	insignificant	3	8	8
chr14	70689256	70691256	Ppp3cc	-0.02982053	0.061004	insignificant	0.0018604	0.05978	insignificant	13	47	40
chr14	70751231	70753231	Slc39a14	-0.09556261	0.00000342	hypomethylated	0.0038614	0.64419	insignificant	11	23	34
chr14	70828901	70830901	Piwil2	-0.5713165	0.02926	stronglyHypometh	-0.03899	0.92306	insignificant	3	20	29
chr14	70842316	70844316	Mir320	-0.15721796	8.37E-21	hypomethylated	0.025581	0.41295	insignificant	16	94	103
chr14	70843034	70845034	Polr3d	-0.14089819	3.55E-17	hypomethylated	0.022923	0.12303	insignificant	16	77	80
chr14	70843278	70845278	Polr3d	-0.15121138	5.28E-14	hypomethylated	0.027069	0.077773	insignificant	11	49	52
chr14	70856323	70858323	Phyhip	-0.06823641	7.97E-10	hypomethylated	-0.039354	1	insignificant	11	48	46
chr14	70920067	70922067	Sftpc	0.02082111	0.0001972	inconclusive	0.061531	0.048561	hypermethylated	17	67	62
chr14	70929627	70931627	Lgi3	-0.10540555	2.12E-08	hypomethylated	0.01754	1	insignificant	18	40	40
chr14	70944429	70946429	Reep4	-0.15845149	0.000005622	hypomethylated	0.028875	0.53537	insignificant	2	70	70
chr14	70952862	70954862	Hr	-0.14221038	1.04E-24	hypomethylated	-0.0041448	0.5231	insignificant	32	115	108
chr14	70976653	70978653	Nudt18	-0.15054017	1.03E-24	hypomethylated	-0.025772	0.72325	insignificant	20	101	98
chr14	70999642	71001642	Fam160b2	-0.25263797	0.000024336	hypomethylated	0.024588	0.017449	hypermethylated	6	21	20
chr14	71042075	71044075	Fgf17		1	noCoverage	-0.086269	0.49981	insignificant	0	5	6
chr14	71052891	71054891	Npm2	-0.04486643	0.14034	insignificant	0.018198	0.79389	insignificant	3	15	15
chr14	71166435	71168435	Xpo7	-0.09564563	0.58114	insignificant	0.0054168	0.46073	insignificant	15	98	99
chr14	71288936	71290936	Gfra2	-0.13117455	6.06E-17	hypomethylated	0.056379	0.022477	hypermethylated	22	92	103
chr14	73109810	73111810	Fndc3a	-0.11375809	6.08E-23	hypomethylated	0.022738	0.2793	insignificant	38	118	117
chr14	73424409	73426409	Gm9199	0.02401361	0.19631	insignificant	0.0212	0.025924	hypermethylated	4	14	12
chr14	73541316	73543316	Rcbtb2	-0.13710118	3.93E-40	hypomethylated	0.010487	0.080692	insignificant	35	96	95
chr14	73541591	73543591	Rcbtb2	-0.13710118	3.93E-40	hypomethylated	0.010487	0.080692	insignificant	35	96	95
chr14	73636697	73638697	Lpar6		1	noCoverage	0.18015	1	insignificant	0	6	8
chr14	73725598	73727598	Rb1	-0.11783968	1.23E-25	hypomethylated	-0.0096513	0.28267	insignificant	37	146	141
chr14	73785078	73787078	Itm2b	-0.15652357	0.00054325	hypomethylated	0.021232	0.63718	insignificant	3	23	18
chr14	73908855	73910855	Med4	-0.12500035	2.1E-14	hypomethylated	0.040739	1	insignificant	7	62	61
chr14	73951592	73953592	Sucla2	-0.05332031	0.00029574	hypomethylated	0.0050046	1	insignificant	31	94	86
chr14	75039646	75041646	Htr2a		1	noCoverage	-0.097733	0.57349	insignificant	0	12	11
chr14	75131151	75133151	Esd	-0.20644537	5.17E-12	hypomethylated	0.027489	0.23663	insignificant	10	20	38
chr14	75347684	75349684	Lrch1	-0.24534493	1	insignificant	0.020352	0.73898	insignificant	2	13	16
chr14	75530690	75532690	Lrrc63	-0.1661718	5.05E-12	hypomethylated	0.0064677	0.64584	insignificant	8	36	36
chr14	75535231	75537231	Lcp1		1	noCoverage	0.11219	0.37315	insignificant	0	10	11

chr14	75683179	75685179	Zc3h13	-0.11373963	1.59E-17	hypomethylated	0.0062574	0.62678	insignificant	17	69	58
chr14	75854788	75856788	Siah3	-0.39899311	0.045962	stronglyHypometh	0.049861	0.22157	insignificant	1	16	16
chr14	76154300	76156300	Cog3	-0.16521701	1.26E-16	hypomethylated	0.077744	0.12578	insignificant	13	26	29
chr14	76186844	76188844	Slc25a30		1	noCoverage	-0.0088319	0.61305	insignificant	0	15	10
chr14	76244062	76246062	Tpt1	-0.10276546	1.21E-48	hypomethylated	0.0056241	0.90639	insignificant	44	240	235
chr14	76410672	76412672	Gtf2f2	-0.0889058	0.0015508	hypomethylated	-0.0042767	0.50551	insignificant	8	30	28
chr14	76509697	76511697	Nufip1	-0.13528798	1.29E-21	hypomethylated	0.021564	0.011812	hypermethylated	35	122	120
chr14	76510622	76512622	Nufip1	-0.17689362	1.07E-08	hypomethylated	0.051079	0.47525	insignificant	17	52	54
chr14	76814627	76816627	Tsc22d1	-0.15985399	5.95E-45	hypomethylated	0.0070369	0.12892	insignificant	71	189	188
chr14	76887242	76889242	Tsc22d1	-0.27910322	0.0028656	hypomethylated	0.13309	0.88158	insignificant	3	28	30
chr14	76903316	76905316	Tsc22d1	-0.17233954	8.81E-13	hypomethylated	0.0089638	0.054408	insignificant	14	68	69
chr14	76956494	76958494	Serp2	-0.11641212	0.00001232	hypomethylated	-0.0032278	0.95902	insignificant	17	47	47
chr14	76956696	76958696	Serp2	-0.16432187	0.014086	hypomethylated	0.0045339	0.78889	insignificant	6	16	16
chr14	77435578	77437578	Ccdc122	-0.14584179	0.00000116	hypomethylated	-0.024371	0.31198	insignificant	21	85	70
chr14	77436424	77438424	9030625A04R	-0.20494679	2.29E-09	hypomethylated	-0.010378	0.11512	insignificant	15	63	48
chr14	77555622	77557622	Enox1	-0.12428211	2.88E-55	hypomethylated	-0.018559	0.78251	insignificant	55	201	205
chr14	77906675	77908675	Gm6994	0.15602536	1	insignificant	0.046036	0.26901	insignificant	4	22	22
chr14	78198775	78200775	Gm1587	0.04485002	0.66859	insignificant	-0.031782	0.57269	insignificant	5	13	16
chr14	78274724	78276724	Dnajc15	-0.08274087	0.1123	insignificant	0.062014	0.28858	insignificant	8	26	26
chr14	78303045	78305045	Epsti1	-0.26854756	0.0011317	hypomethylated	0.01531	0.94639	insignificant	3	18	18
chr14	78936667	78938667	Akap11	0.08694084	0.54089	insignificant	0.0020924	0.26408	insignificant	1	22	22
chr14	79124896	79126896	Dgkh	-0.23404133	1	insignificant	0.041641	0.010837	hypermethylated	17	54	54
chr14	79247984	79249984	1300010F03Ri	-0.17927961	3.09E-24	hypomethylated	0.019697	0.35567	insignificant	14	44	44
chr14	79647174	79649174	Zfp957		1	noCoverage	0.041358	0.3251	insignificant	0	6	6
chr14	79701442	79703442	1190002H23R	-0.15015148	1.54E-14	hypomethylated	-0.034161	0.38139	insignificant	13	34	34
chr14	79790475	79792475	Naa16	-0.13904254	0.000000124	hypomethylated	0.013057	0.18207	insignificant	10	48	48
chr14	79825317	79827317	Kbtbd7	-0.11647265	1.46E-19	hypomethylated	-0.015319	0.59989	insignificant	20	62	69
chr14	79880000	79882000	Elf1	-0.1053629	4.22E-21	hypomethylated	-0.0056193	0.084838	insignificant	43	195	185
chr14	79881075	79883075	Elf1	-0.13367007	1.33E-13	hypomethylated	0.0093074	0.57267	insignificant	16	126	113
chr14	79986497	79988497	Sugt1	-0.11276267	6.77E-14	hypomethylated	-0.02011	0.54987	insignificant	16	60	58
chr14	80171119	80173119	Pcdh8	-0.15847125	0.0038525	hypomethylated	0.012273	1	insignificant	12	33	36
chr14	80399108	80401108	Olfm4	-0.09739411	0.00000432	hypomethylated	0.040097	0.22803	insignificant	3	10	10
chr14	84842369	84844369	Pcdh17	-0.18991576	0.001848	hypomethylated	0.062778	0.57834	insignificant	8	42	37
chr14	87540921	87542921	Diap3	-0.17022516	1.17E-22	hypomethylated	-0.0093581	0.61431	insignificant	15	40	40
chr14	87815389	87817389	Tdrd3	-0.09860182	9.91E-47	hypomethylated	-0.0017136	0.24978	insignificant	84	287	284
chr14	90295030	90297030	Gm5088		1	noCoverage	0.17405	0.33791	insignificant	0	7	7
chr14	94287951	94289951	Pcdh9	-0.08035921	0.000060354	hypomethylated	0.010237	0.32971	insignificant	7	40	34
chr14	96279484	96281484	4921530L21Ri	-0.13939394	0.13425	insignificant	0.10707	0.105	insignificant	1	9	9
chr14	96918253	96920253	Klhl1	0.06169134	0.71559	insignificant	0.022925	0.86863	insignificant	1	7	7
chr14	98568762	98570762	Dach1	-0.21566266	1.25E-09	hypomethylated	0.021104	0.44802	insignificant	5	14	19
chr14	99444595	99446595	6720463M24F	-0.11638259	9.44E-50	hypomethylated	-0.010268	0.0018139	hypomethylated	72	231	232
chr14	99445355	99447355	6720463M24F	-0.18628643	4.17E-39	hypomethylated	-0.0026765	0.2313	insignificant	36	111	114
chr14	99497651	99499651	Pibf1	-0.11162703	8.31E-16	hypomethylated	0.010317	0.42971	insignificant	31	126	127
chr14	99498989	99500989	Pibf1	-0.15069263	1.24E-10	hypomethylated	0.046309	0.3904	insignificant	12	52	52
chr14	99696909	99698909	Klf5	-0.09660908	2.96E-17	hypomethylated	-0.0058559	0.79699	insignificant	39	140	140
chr14	101599286	101601286	1700110M21F	-0.03494455	0.50074	insignificant	0.089066	0.000056377	hypermethylated	3	9	8
chr14	102008408	102010408	Tbc1d4	-0.36744934	0.00042638	stronglyHypometh	0.051932	0.60348	insignificant	4	23	25
chr14	102039688	102041688	Commd6	-0.2404591	0.69373	insignificant	0.017451	0.00086158	hypermethylated	7	22	17
chr14	102052183	102054183	Uchl3	-0.12174275	9.34E-49	hypomethylated	0.018995	0.001677	hypermethylated	52	134	134
chr14	102128144	102130144	Lmo7	-0.15030921	3.36E-11	hypomethylated	0.035403	0.026037	hypermethylated	14	84	82
chr14	103381854	103383854	Mir5130	-0.18936178	1.43E-09	hypomethylated	-0.054452	0.11309	insignificant	20	58	72
chr14	103468432	103470432	Cln5	-0.0814497	0.000039754	hypomethylated	-0.000086577	0.59216	insignificant	48	120	120
chr14	103498726	103500726	Fbxl3	-0.08118287	0.11627	insignificant	-0.01452	0.90235	insignificant	15	61	48
chr14	103746017	103748017	Mycbp2	-0.27157164	1	insignificant	0.010655	0.95634	insignificant	3	22	22
chr14	103911557	103913557	Scel	-0.2860167	0.000000229	hypomethylated	-0.076535	0.047187	hypomethylated	3	6	6
chr14	104048459	104050459	Slain1	-0.10982458	4.39E-19	hypomethylated	-0.0012285	0.36304	insignificant	39	108	102
chr14	104242913	104244913	Ednrb	-0.16366041	0.47009	insignificant	-0.024441	0.56286	insignificant	4	12	12
chr14	104867216	104869216	Pou4f1	-0.10261636	0.000001728	hypomethylated	-0.00068531	0.26192	insignificant	34	87	80
chr14	104921883	104923883	Rnf219	-0.13551853	2.35E-15	hypomethylated	0.017534	0.2055	insignificant	14	35	30
chr14	105656889	105658889	Ndfip2	-0.13695136	3.48E-16	hypomethylated	-0.0070079	0.37264	insignificant	40	116	124
chr14	106296036	106298036	Spry2	-0.08888554	0.00025641	hypomethylated	0.0072722	0.90352	insignificant	16	117	117
chr14	106504414	106506414	Trim52		1	noCoverage	-0.024362	0.79447	insignificant	0	32	20
chr14	112073336	112075336	Slitrk5	-0.1168336	2.03E-10	hypomethylated	-0.010247	0.85777	insignificant	41	150	157
chr14	115490436	115492436	Gpc5	-0.19063803	5.34E-31	hypomethylated	0.020516	0.22439	insignificant	29	94	94
chr14	117323536	117325536	Gpc6	-0.12459051	7.38E-17	hypomethylated	0.0016072	0.68167	insignificant	25	90	78
chr14	118531987	118533987	Tgds	-0.1812694	0.055821	insignificant	-0.0057537	0.71285	insignificant	4	22	22
chr14	118535348	118537348	Gpr180	-0.10767285	1.33E-23	hypomethylated	0.0092002	0.37098	insignificant	39	112	108
chr14	118636252	118638252	Sox21	-0.16020986	9.03E-08	hypomethylated	0.0073831	0.95839	insignificant	2	34	38
chr14	118665379	118667379	Gm9376	0.03206993	1	insignificant	-0.0087789	0.28634	insignificant	2	16	16
chr14	119105441	119107441	Abcc4	-0.14078064	1.19E-09	hypomethylated	0.0099698	0.11096	insignificant	17	72	71
chr14	119253171	119255171	Cldn10	-0.10003383	2.43E-10	hypomethylated	0.0072211	0.56804	insignificant	12	62	54
chr14	119322389	119324389	Dzip1	-0.19379632	0.019849	hypomethylated	-0.01614	0.38712	insignificant	18	51	51
chr14	119336153	119338153	Dnajc3	-0.08183884	1.57E-15	hypomethylated	0.00065179	0.95941	insignificant	48	144	134
chr14	119498656	119500656	Uggt2	-0.24343658	1.89E-22	hypomethylated	-0.03187	0.11815	insignificant	16	48	48
chr14	119536486	119538486	Hs6st3	-0.11286024	6.61E-24	hypomethylated	0.013258	0.00097492	hypermethylated	74	229	219
chr14	120673890	120675890	Mbnl2		1	noCoverage	-0.032672	0.4633	insignificant	0	16	16
chr14	120876682	120878682	Rap2a	-0.09795969	1.25E-10	hypomethylated	0.0034584	0.87765	insignificant	24	195	194
chr14	121309415	121311415	Ipo5	-0.10351882	8.54E-33	hypomethylated	0.0037657	0.35232	insignificant	56	216	204
chr14	121433795	121435795	Farp1	-0.06879683	0.000019609	hypomethylated	-0.0012308	0.60459	insignificant	57	188	233
chr14	121778452	121780452	Stk24	-0.0392727	0.000000782	hypomethylated	0.062129	0.50792	insignificant	18	61	62
chr14	121904476	121906476	Slc15a1	0.28360625	0.00032779	hypermethylated	0.13399	0.00024766	hypermethylated	8	32	30
chr14	122097639	122099639	Dock9		1	noCoverage	0.0084034	1	insignificant	0	4	4
chr14	122196956	122198956	Dock9	-0.13561205	2.09E-36	hypomethylated	-0.010925	0.25364	insignificant	37	151	151
chr14	122276827	122278827	Ubac2	-0.12502133	8.07E-17	hypomethylated	0.003525	0.81114	insignificant	29	127	135
chr14	122314996	122316996	Gpr18	0.08333333	1	insignificant	0.21354	0.41047	insignificant	2	5	4

chr14	122432895	122434895	Timm8a2	0.01610382	0.58554	insignificant	0.029355	0.26846	insignificant	5	14	14
chr14	122505303	122507303	Tm9sf2	-0.10414164	3E-47	hypomethylated	0.031239	0.52157	insignificant	47	148	120
chr14	122506196	122508196	A330035P11R	-0.09413373	7.05E-36	hypomethylated	0.020651	0.48237	insignificant	39	119	97
chr14	122579915	122581915	Clybl	-0.35849296	0.0000048	stronglyHypometh	-0.063588	1	insignificant	3	32	32
chr14	122632860	122634860	1700108J01Ri	-0.09113937	0.00011088	hypomethylated	0.02932	0.40682	insignificant	5	16	16
chr14	122850337	122852337	Gm5089	0.19211538	1	lowCoverage	-0.030107	1	insignificant	1	10	8
chr14	122850368	122852368	Gm5089	0.19211538	1	lowCoverage	-0.030107	1	insignificant	1	10	8
chr14	122864880	122866880	Zic5	-0.17325708	0.002632	hypomethylated	-0.062046	0.51756	insignificant	6	18	18
chr14	122873605	122875605	Zic2	-0.0956526	1.61E-29	hypomethylated	0.00063334	0.87108	insignificant	61	224	225
chr14	122932549	122934549	Pcca	-0.13564743	3.73E-08	hypomethylated	-0.012025	0.57655	insignificant	10	46	37
chr14	123312387	123314387	A2ld1	-0.13501923	6.72E-13	hypomethylated	-0.021089	0.1251	insignificant	11	34	34
chr15	3253526	3255526	Ccdc152	0.01412115	1	insignificant	0.050391	0.030145	hypermethylated	7	18	18
chr15	3533230	3535230	Ghr	0.22244624	1	lowCoverage	-0.010741	0.8871	insignificant	1	6	6
chr15	3929573	3931573	Fbxo4	-0.51578947	0.00016405	stronglyHypometh	0.002729	0.62116	insignificant	1	2	2
chr15	3945752	3947752	AW549877	-0.30786285	6.88E-11	hypomethylated	-0.061466	0.37253	insignificant	10	40	40
chr15	3975427	3977427	Oxct1	-0.06346418	8.94E-26	hypomethylated	-0.0038428	0.68068	insignificant	33	126	129
chr15	3977406	3979406	BC037032	-0.10852058	1	insignificant	0.030216	1	insignificant	4	21	20
chr15	4324490	4326490	Plcxd3	-0.26889804	1	insignificant	-0.00049374	0.30692	insignificant	2	31	31
chr15	4676209	4678209	C6		1	noCoverage	0.12096	0.37281	insignificant	0	6	6
chr15	5058533	5060533	Card6	-0.24083694	0.20126	insignificant	-0.032307	1	insignificant	3	11	9
chr15	5065612	5067612	Rpl37	-0.12897519	1.68E-17	hypomethylated	0.0061626	0.39423	insignificant	24	118	118
chr15	5092860	5094860	Prkaa1	-0.08358904	1.75E-17	hypomethylated	0.0075134	0.4273	insignificant	42	176	156
chr15	5134559	5136559	Ttc33	-0.14500099	0.000007539	hypomethylated	0.011477	0.8291	insignificant	10	64	60
chr15	5193682	5195682	Ptger4	-0.17266408	9.12E-11	hypomethylated	-0.010534	0.57476	insignificant	17	91	89
chr15	5193831	5195831	Ptger4	-0.16796274	0.00000109	hypomethylated	-0.013382	0.52658	insignificant	14	81	79
chr15	6335747	6337747	Dab2	-0.20643232	0.081672	insignificant	0.033997	0.74445	insignificant	2	57	46
chr15	6528870	6530870	Fyb	0.015625	1	insignificant	-0.034375	1	insignificant	2	4	2
chr15	6657380	6659380	Rictor	-0.07739891	3.42E-22	hypomethylated	0.0013774	0.49166	insignificant	67	250	245
chr15	6824313	6826313	Osmr	-0.18478859	0.61085	insignificant	0.015454	0.88858	insignificant	7	44	43
chr15	7078571	7080571	Lifr	-0.08203603	4.69E-13	hypomethylated	-0.0026125	0.51464	insignificant	41	145	139
chr15	7089541	7091541	Lifr	0.31611131	1	lowCoverage	0.050982	0.56379	insignificant	1	8	8
chr15	7348304	7350304	Egflam	-0.18009407	0.000002311	hypomethylated	0.032203	0.068322	insignificant	8	27	27
chr15	7760010	7762010	Gdnf	-0.10457905	1.94E-18	hypomethylated	-0.0012481	0.67562	insignificant	14	127	124
chr15	8049209	8051209	Wdr70	-0.09094154	0.011622	hypomethylated	-0.011823	1	insignificant	7	14	14
chr15	8058312	8060312	Nup155	0.76119339	0.092259	insignificant	-0.001514	0.076755	insignificant	1	44	44
chr15	8118105	8120105	2410089E03Ri	-0.15693162	0.0014474	hypomethylated	0.033555	0.95908	insignificant	7	47	51
chr15	8394463	8396463	Nipbl	-0.12916034	7.93E-11	hypomethylated	0.01469	0.75812	insignificant	44	128	128
chr15	8660807	8662807	Slc1a3		1	noCoverage	0.14388	0.017615	hypermethylated	0	6	6
chr15	8917118	8919118	Ranbp3l	-0.27416667	0.13366	insignificant	0.10857	0.44633	insignificant	1	12	12
chr15	9000008	9002008	1110020G09R	-0.11443839	1.21E-32	hypomethylated	-0.0018098	0.014587	inconclusive	48	167	157
chr15	9069325	9071325	Lmbrd2	-0.13654825	2.23E-13	hypomethylated	-0.0023523	0.28278	insignificant	26	93	89
chr15	9070207	9072207	Skp2	-0.1759457	3.75E-12	hypomethylated	-0.02388	0.069095	insignificant	22	83	81
chr15	9264352	9266352	Ugt3a2		1	noCoverage	0.25994	0.42092	insignificant	0	8	4
chr15	9678561	9680561	Spef2		1	noCoverage	0.019928	0.74492	insignificant	0	4	4
chr15	10105992	10107992	Prlr		1	noCoverage	-0.0098604	0.40609	insignificant	0	20	20
chr15	10400271	10402271	Dnajc21	-0.17405007	0.0028931	hypomethylated	-0.037285	0.5528	insignificant	8	54	52
chr15	10414788	10416788	Rad1	-0.12880845	1.12E-18	hypomethylated	0.001452	0.64173	insignificant	18	73	68
chr15	10415692	10417692	Brix1	-0.13351421	0.0040981	hypomethylated	0.01528	0.11359	insignificant	7	37	32
chr15	10643295	10645295	Rai14	-0.1304112	3.27E-15	hypomethylated	-0.0046096	0.55014	insignificant	27	90	82
chr15	10644386	10646386	Rai14	-0.16476255	2.12E-15	hypomethylated	0.0082661	0.37504	insignificant	22	44	44
chr15	10881110	10883110	C1qtnf3	-0.02420937	0.69826	insignificant	0.10933	0.089399	insignificant	3	11	11
chr15	10910510	10912510	Amacr	-0.07741312	0.0078277	hypomethylated	-0.0007034	1	insignificant	9	62	62
chr15	10967723	10969723	Rxfp3	-0.38675214	0.017741	stronglyHypometh	0.0095198	0.8679	insignificant	3	6	6
chr15	10993544	10995544	Adamts12	-0.45321802	0.000023563	stronglyHypometh	-0.084151	0.67649	insignificant	3	14	12
chr15	11329413	11331413	Tars		1	noCoverage	0.057396	0.11237	insignificant	0	6	8
chr15	11835429	11837429	Npr3	-0.13103824	4.81E-15	hypomethylated	0.068729	0.022289	hypermethylated	16	41	43
chr15	11925762	11927762	Sub1	-0.11956897	0.14955	insignificant	0.030431	0.27146	insignificant	3	20	20
chr15	12046605	12048605	Zfr	-0.11047805	3.25E-46	hypomethylated	-0.0065255	0.60837	insignificant	65	220	218
chr15	12100246	12102246	Mir1898		1	noCoverage	0.10117	0.15521	insignificant	0	8	11
chr15	12133848	12135848	Mtmr12	-0.10197685	1.37E-18	hypomethylated	0.002705	0.33151	insignificant	41	136	135
chr15	12250250	12252250	Golph3	-0.106952	3.07E-19	hypomethylated	0.0077997	0.36659	insignificant	39	165	146
chr15	12522311	12524311	Pdzd2		1	noCoverage	0.055456	0.069591	insignificant	0	10	10
chr15	12753569	12755569	Drosha	-0.11498575	3.22E-36	hypomethylated	0.0090574	0.015232	hypermethylated	54	148	152
chr15	12754412	12756412	6030458C11Ri	-0.12706151	2.02E-26	hypomethylated	0.010363	0.096899	insignificant	36	90	100
chr15	13103394	13105394	Cdh6	-0.21028861	0.027453	hypomethylated	-0.0050199	0.046439	hypomethylated	3	51	36
chr15	16706855	16708855	Cdh9		1	noCoverage	0.0634	0.38481	insignificant	0	8	8
chr15	18749083	18751083	Cdh10	-0.4514411	0.0006696	stronglyHypometh	0.10015	0.36693	insignificant	1	2	2
chr15	20596505	20598505	Acot10	-0.06224693	1	insignificant	0.22107	0.000065947	hypermethylated	2	3	3
chr15	22965217	22967217	Cdh18		1	noCoverage	-0.021199	0.20906	insignificant	0	4	4
chr15	25342946	25344946	Gm5468	-0.13177149	1.12E-11	hypomethylated	-0.0021545	0.70259	insignificant	24	77	76
chr15	25343519	25345519	Basp1	-0.17843303	0.080865	insignificant	-0.0047182	0.57942	insignificant	4	31	30
chr15	25551304	25553304	Myo10	-0.1289952	4.45E-19	hypomethylated	0.063505	0.17697	insignificant	52	165	159
chr15	25772018	25774018	Fam134b	-0.13933721	1.05E-24	hypomethylated	0.041609	0.92583	insignificant	18	83	75
chr15	25913120	25915120	Zfp622	-0.05098322	0.00000192	hypomethylated	0.00046309	0.51958	insignificant	42	157	157
chr15	26237826	26239826	March11	-0.10371755	4.37E-26	hypomethylated	0.026236	0.000013692	hypermethylated	51	130	134
chr15	26825319	26827319	Fbxl7	-0.158409	6.49E-16	hypomethylated	-0.016493	0.03869	hypomethylated	17	42	42
chr15	27395431	27397431	Ank	-0.09584967	6.53E-21	hypomethylated	-0.016682	0.040463	hypomethylated	58	214	208
chr15	27560448	27562448	Fam105b	0.10106981	0.70278	insignificant	-0.051987	0.30417	insignificant	5	33	34
chr15	27611253	27613253	Fam105a	-0.02461938	0.52828	insignificant	0.0021529	0.63559	insignificant	5	22	22
chr15	27955603	27957603	Trio	-0.17056963	0.00000218	hypomethylated	-0.038655	0.88813	insignificant	8	34	28
chr15	28132520	28134520	Dnahc5	0.13717532	1	insignificant	-0.03612	0.87143	insignificant	2	8	8
chr15	30101347	30103347	Ctnnd2	-0.08639834	8.15E-11	hypomethylated	-0.00091037	0.17567	insignificant	53	196	199
chr15	31153139	31155139	Dap	-0.16302704	8.25E-12	hypomethylated	0.011881	0.52667	insignificant	18	57	47
chr15	31297514	31299514	Ankrd33b	-0.29857401	0.016516	hypomethylated	0.064006	0.00062802	hypermethylated	5	19	21
chr15	31383444	31385444	Ropn1l		1	noCoverage	0.093717	0.82958	insignificant	0	18	20

chr15	31460792	31462792	March6	-0.09673094	0.00007598	hypomethylated	-0.00028367	0.7121	insignificant	24	97	88
chr15	31497666	31499666	Cmbl	-0.11106454	0.65021	insignificant	-0.15261	0.0084911	hypomethylated	6	16	16
chr15	31530870	31532870	Fam173b	-0.13601257	6.47E-17	hypomethylated	0.0043101	0.9063	insignificant	24	93	88
chr15	31531559	31533559	Cct5	-0.13352713	0.000037974	hypomethylated	-0.010104	0.76917	insignificant	16	52	46
chr15	32106043	32108043	Tas2r119	-0.27414773	0.083115	insignificant	-0.018953	1	insignificant	1	8	8
chr15	32173567	32175567	Sema5a	-0.17872933	0.00046432	hypomethylated	0.0030794	0.96882	insignificant	14	66	66
chr15	32849477	32851477	Sdc2	-0.08155295	0.18269	insignificant	-0.014463	0.017796	hypomethylated	9	59	58
chr15	33011883	33013883	Pgcp	-0.17807125	9.73E-13	hypomethylated	0.008318	0.17012	insignificant	9	24	24
chr15	34011473	34013473	Mtdh	-0.09860374	2.23E-38	hypomethylated	-0.0050008	0.83631	insignificant	49	169	159
chr15	34166780	34168780	Laptm4b	-0.14053634	1.32E-19	hypomethylated	0.0054368	0.27536	insignificant	32	122	122
chr15	34235435	34237435	Matn2	-0.1169443	3.09E-09	hypomethylated	0.0035573	0.55834	insignificant	14	42	42
chr15	34372980	34374980	Rpl30	-0.17314202	1.17E-12	hypomethylated	0.0046568	0.88151	insignificant	18	51	51
chr15	34372988	34374988	Rpl30	-0.17314202	1.17E-12	hypomethylated	0.0046568	0.88151	insignificant	18	51	51
chr15	34373031	34375031	Rpl30	-0.19909368	3.83E-13	hypomethylated	0.0067763	0.50432	insignificant	16	49	49
chr15	34382066	34384066	BC030476	-0.15482035	0.068742	insignificant	0.06985	0.25311	insignificant	1	19	9
chr15	34424065	34426065	Pop1	-0.16979826	0.000032184	hypomethylated	0.013253	0.54743	insignificant	12	78	80
chr15	34425001	34427001	Hrsp12	-0.17005922	0.000032088	hypomethylated	0.017127	0.54436	insignificant	12	70	70
chr15	34608461	34610461	Nipal2	-0.32292511	0.00067389	hypomethylated	-0.011096	0.90808	insignificant	6	56	56
chr15	34766135	34768135	Kcns2	-0.09084712	0.000056107	hypomethylated	0.0065344	0.5928	insignificant	21	99	96
chr15	35085561	35087561	Stk3	-0.0898513	0.25278	insignificant	-0.013204	0.39151	insignificant	4	25	26
chr15	35224866	35226866	Osr2	-0.11489968	2.64E-14	hypomethylated	-0.01211	0.88122	insignificant	31	123	123
chr15	35300300	35302300	Vps13b	-0.12972023	4.56E-25	hypomethylated	-0.0036335	0.37263	insignificant	44	138	139
chr15	35868001	35870001	Cox6c	-0.42805693	0.000000522	stronglyHypometh	0.051532	0.24401	insignificant	1	4	4
chr15	36094639	36096639	Fbxo43	-0.06296296	0.61625	insignificant	0.0616	0.10992	insignificant	5	18	18
chr15	36102771	36104771	Polr2k	-0.3098885	0.00000132	hypomethylated	-0.021718	0.32658	insignificant	4	57	52
chr15	36108284	36110284	Spag1	-0.1138173	0.14457	insignificant	0.0054191	0.77638	insignificant	6	40	40
chr15	36212902	36214902	Rnf19a	-0.06613523	5.86E-10	hypomethylated	-0.01641	0.12136	insignificant	11	59	51
chr15	36426546	36428546	Ankrd46	0.00281167	1	insignificant	0.038322	0.00015374	hypermethylated	2	10	10
chr15	36538728	36540728	Pabpc1	-0.12307337	9.18E-18	hypomethylated	-0.0030374	0.40379	insignificant	42	115	111
chr15	36937157	36939157	Zfp706	-0.1476746	0.00071504	hypomethylated	-0.040672	0.40354	insignificant	13	42	42
chr15	37161790	37163790	Grhl2	-0.0342524	0.12935	insignificant	-0.0046876	1	insignificant	15	99	108
chr15	37721755	37723755	Ncald	-0.2069562	0.58327	insignificant	0.0027106	0.9396	insignificant	1	11	11
chr15	37890810	37892810	Rrm2b	-0.11550267	0.00000252	hypomethylated	-0.0084532	0.82165	insignificant	10	53	50
chr15	38008608	38010608	Ubr5	-0.10298459	0.012919	hypomethylated	0.0028778	0.017941	inconclusive	18	47	35
chr15	38230462	38232462	Klf10	-0.1599639	1.06E-37	hypomethylated	-0.0080618	0.67204	insignificant	35	134	134
chr15	38449021	38451021	Azin1	-0.20166777	0.00060176	hypomethylated	-0.009089	0.0087874	inconclusive	15	70	70
chr15	38590658	38592658	Atp6v1c1	-0.11932879	3.49E-10	hypomethylated	0.010129	0.71906	insignificant	23	72	72
chr15	38836825	38838825	Fzd6	-0.07675312	0.00039882	hypomethylated	-0.0048055	0.63271	insignificant	34	84	80
chr15	38836878	38838878	Fzd6	-0.06646954	0.0003998	hypomethylated	0.0054781	0.70162	insignificant	34	83	80
chr15	38907477	38909477	Cthrc1	-0.33028455	0.10825	insignificant	0.0019327	0.75074	insignificant	4	8	8
chr15	38943419	38945419	Dcaf13	-0.12903787	5.25E-10	hypomethylated	0.026037	0.1914	insignificant	23	113	113
chr15	38944262	38946262	Slc25a32	-0.09544986	0.0050257	hypomethylated	0.10977	0.044258	hypermethylated	10	45	49
chr15	39028877	39030877	Rims2	-0.10480154	3.53E-44	hypomethylated	0.056182	0.48118	insignificant	85	227	197
chr15	39775303	39777303	Lrp12	-0.13974071	2.74E-23	hypomethylated	0.00036507	0.81146	insignificant	29	84	80
chr15	40485587	40487587	Zfpm2	-0.10366134	7.35E-24	hypomethylated	0.024895	0.019102	hypermethylated	42	134	131
chr15	41278027	41280027	Oxr1	-0.10845259	2.87E-26	hypomethylated	-0.002791	0.88523	insignificant	54	162	158
chr15	41619579	41621579	Oxr1	-0.11324728	0.0014844	hypomethylated	-0.00033651	0.0032506	inconclusive	39	114	109
chr15	41620060	41622060	Oxr1	-0.11324728	0.0014844	hypomethylated	-0.00033651	0.0032506	inconclusive	39	114	109
chr15	41701266	41703266	Abra		1	noCoverage	0.022727	0.25591	insignificant	0	3	2
chr15	43002364	43004364	Rspo2		1	noCoverage	-0.063818	0.68382	insignificant	0	12	12
chr15	43307774	43309774	Ttc35	-0.14391746	2.18E-18	hypomethylated	-0.01149	0.3571	insignificant	21	62	62
chr15	43701575	43703575	Tmem74		1	noCoverage	-0.037819	0.34159	insignificant	0	16	16
chr15	44258656	44260656	Eny2	-0.14458396	2.36E-19	hypomethylated	0.0079172	0.41433	insignificant	16	74	74
chr15	44259853	44261853	Eny2	-0.09918173	0.00040959	hypomethylated	0.012942	0.67414	insignificant	8	30	30
chr15	44450186	44452186	Ebag9	-0.11647885	1.1E-30	hypomethylated	-0.0092444	0.76461	insignificant	47	152	155
chr15	44584004	44586004	Sybu		1	noCoverage	0.0030983	0.68651	insignificant	0	7	3
chr15	44711939	44713939	A930017M01F	-0.09237614	0.053697	insignificant	0.0080705	0.060652	insignificant	4	19	19
chr15	44946480	44948480	Kcnv1	-0.61805556	0.39535	lowCoverage	-0.082341	0.8243	insignificant	1	4	4
chr15	48623535	48625535	Csmd3	-0.35089542	0.34399	insignificant	-0.028087	0.44222	insignificant	1	14	14
chr15	50720660	50722660	Trps1	-0.13009615	0.00000347	hypomethylated	-0.0016161	0.6913	insignificant	12	60	72
chr15	50721587	50723587	Trps1	-0.08336039	0.053843	insignificant	0.0037465	0.6206	insignificant	9	43	53
chr15	51697007	51699007	Eif3h	-0.67354497	0.079614	insignificant	-0.02427	0.88283	insignificant	3	9	9
chr15	51707986	51709986	Utp23	0.01785714	0.6438	insignificant	0.14286	0.53215	insignificant	3	8	8
chr15	51823306	51825306	Rad21	0.05840437	0.30355	insignificant	-0.00092861	0.60796	insignificant	7	32	32
chr15	51870652	51872652	Aard	-0.20995564	0.000000171	hypomethylated	0.0021436	0.76293	insignificant	8	62	60
chr15	52542999	52544999	Med30	-0.06754134	0.0045173	hypomethylated	0.0013629	0.66923	insignificant	18	62	62
chr15	53177738	53179738	Ext1	-0.08693487	0.000010462	hypomethylated	-0.036955	0.94863	insignificant	30	66	63
chr15	54241328	54243328	Colec10	-0.33398693	0.18359	insignificant	-0.083987	1	insignificant	2	6	4
chr15	54401920	54403920	Mal2	-0.17365737	9.66E-23	hypomethylated	0.0031767	0.69911	insignificant	28	52	45
chr15	54576482	54578482	Nov	-0.24043504	1.67E-19	hypomethylated	-0.014705	0.41776	insignificant	7	26	29
chr15	54751516	54753516	Enpp2	-0.41394139	1.81E-14	stronglyHypometh	-0.013882	0.75312	insignificant	3	20	19
chr15	54751701	54753701	Enpp2	-0.45076931	8.76E-12	stronglyHypometh	0.036837	0.53821	insignificant	3	16	13
chr15	54922033	54924033	Dscc1	-0.09647095	0.0052127	hypomethylated	-0.023159	0.20912	insignificant	10	34	31
chr15	54943037	54945037	Deptor	-0.11938876	2.89E-23	hypomethylated	-0.0035331	0.91586	insignificant	36	94	90
chr15	54963990	54965990	Deptor	-0.19598291	0.19205	insignificant	0.060347	0.20465	insignificant	1	6	6
chr15	55138304	55140304	Col14a1	-0.12198505	3.25E-09	hypomethylated	0.06458	0.21334	insignificant	10	25	25
chr15	55387962	55389962	Mtbp	-0.14100935	2.06E-26	hypomethylated	0.0063184	0.6673	insignificant	16	74	66
chr15	55388867	55390867	Mrpl13	-0.14100935	2.06E-26	hypomethylated	0.0063184	0.6673	insignificant	16	74	66
chr15	55738504	55740504	Sntb1	-0.19194966	2.39E-24	hypomethylated	0.00095656	0.015815	hypermethylated	10	30	30
chr15	56526101	56528101	Has2	-0.4419335	1.39E-11	stronglyHypometh	-0.1765	1	insignificant	2	8	6
chr15	57525221	57527221	Zhx2	-0.13567209	3.3E-25	hypomethylated	0.0078189	0.35936	insignificant	34	167	145
chr15	57723973	57725973	Derl1	-0.18327683	0.13625	insignificant	-0.0099207	0.79385	insignificant	3	20	20
chr15	57742753	57744753	Wdr67	-0.39558281	1.31E-24	stronglyHypometh	0.018822	0.31417	insignificant	11	43	40
chr15	57816457	57818457	Fam83a	-0.33492574	2.34E-27	stronglyHypometh	-0.024716	0.27379	insignificant	6	28	29
chr15	57865849	57867849	9130401M01F	-0.25818146	0.1982	insignificant	0.0047443	0.43298	insignificant	4	18	18

chr15	57908044	57910044	Zhx1		1	noCoverage	-0.019756	0.5532	insignificant	0	18	20
chr15	57966637	57968637	Atad2	-0.1353005	4.16E-13	hypomethylated	0.039145	0.27042	insignificant	18	67	68
chr15	57971990	57973990	Wdyhv1	-0.08693177	2.25E-09	hypomethylated	0.006161	0.83257	insignificant	16	69	62
chr15	58046433	58048433	Fbxo32	-0.21020439	0.077586	insignificant	0.037662	0.47644	insignificant	6	16	16
chr15	58246022	58248022	D15Ertd621e	-0.09495955	4.34E-13	hypomethylated	0.012198	0.16838	insignificant	24	105	109
chr15	58654982	58656982	Tmem65	-0.0849211	7.11E-19	hypomethylated	0.0094247	0.080952	insignificant	22	100	100
chr15	58703203	58705203	Trmt12	-0.1456992	2.56E-30	hypomethylated	0.00041834	0.88559	insignificant	24	96	96
chr15	58719783	58721783	Rnf139	-0.11856248	1.19E-25	hypomethylated	-0.005505	0.84156	insignificant	30	109	107
chr15	58764364	58766364	Ndufb9	-0.18674624	5.91E-12	hypomethylated	-0.028948	0.63946	insignificant	17	72	68
chr15	58765285	58767285	Ndufb9	-0.17127342	0.00000954	hypomethylated	-0.043036	1	insignificant	9	40	36
chr15	58913581	58915581	Mtss1	-0.16304974	0.00000993	hypomethylated	0.0042385	0.11978	insignificant	23	88	88
chr15	59145646	59147646	Sqle	-0.09322284	1.08E-26	hypomethylated	-0.00026288	0.39832	insignificant	26	131	131
chr15	59204752	59206752	Nsmce2	-0.20693458	4.61E-11	hypomethylated	-0.078392	0.60367	insignificant	22	56	61
chr15	59205707	59207707	E430025E21Ri	-0.1215725	0.00070263	hypomethylated	0.0056826	0.37568	insignificant	10	27	37
chr15	59479208	59481208	Trib1	-0.11519302	3.55E-60	hypomethylated	-0.0024577	0.69252	insignificant	75	251	250
chr15	60653519	60655519	9930014A18R	-0.1227802	0.000053178	hypomethylated	-0.016	0.34821	insignificant	26	110	119
chr15	61815895	61817895	Myc	-0.15587828	3.3E-16	hypomethylated	-0.0012828	0.4732	insignificant	23	115	115
chr15	61868541	61870541	Pvt1		1	noCoverage	0.00088318	0.39845	insignificant	0	38	38
chr15	62051006	62053006	H2afy3	0.10612648	0.13314	insignificant	0.023886	0.00010271	hypermethylated	4	22	22
chr15	63677863	63679863	Gsdmcl1		1	noCoverage	0.064798	0.016327	hypermethylated	0	12	16
chr15	63745015	63747015	Gsdmcl-ps		1	noCoverage	-0.0014886	1	insignificant	0	4	4
chr15	63892010	63894010	Fam49b	-0.17481505	3.09E-10	hypomethylated	0.042629	0.27651	insignificant	11	36	36
chr15	64214481	64216481	Asap1	-0.21111111	0.34273	insignificant	-0.016667	1	insignificant	3	6	6
chr15	64753858	64755858	Adcy8	-0.12507568	0.0004647	hypomethylated	-0.0073422	0.0093897	hypomethylated	10	50	56
chr15	65617602	65619602	Efr3a	-0.06916744	2.25E-20	hypomethylated	0.0025552	0.00023271	hypermethylated	35	120	118
chr15	65808366	65810366	Hhla1	-0.5219719	0.028873	stronglyHypometh	0.028896	0.9393	insignificant	2	12	12
chr15	66117786	66119786	Kcnq3	-0.12980411	3.9E-19	hypomethylated	-0.0084407	0.31007	insignificant	29	77	79
chr15	66332472	66334472	Lrrc6	-0.23330075	0.59392	insignificant	-0.03092	0.43429	insignificant	2	6	6
chr15	66408133	66410133	Phf20l1	-0.10767973	8.68E-31	hypomethylated	-0.0027686	0.53106	insignificant	35	84	84
chr15	66801203	66803203	Ndrg1	-0.09055649	0.0000014	hypomethylated	0.03786	0.95609	insignificant	8	56	66
chr15	67008444	67010444	St3gal1	-0.09699272	0.000000729	hypomethylated	-0.028243	0.57007	insignificant	15	64	64
chr15	68090418	68092418	Zfat	-0.15073757	3E-10	hypomethylated	0.0040503	1	insignificant	19	78	74
chr15	68757849	68759849	Khdrbs3	-0.0390011	0.000066342	hypomethylated	0.0099574	0.33503	insignificant	34	144	132
chr15	71558268	71560268	Fam135b		1	noCoverage	0.0093317	0.66078	insignificant	0	4	4
chr15	72376709	72378709	Kcnk9	-0.07516101	2.31E-10	hypomethylated	-0.000024871	0.74773	insignificant	54	193	191
chr15	72640754	72642754	Peg13	0.3264411	1	lowCoverage	-0.03403	0.40481	insignificant	1	6	6
chr15	72891634	72893634	Trappc9		1	noCoverage	0.06829	0.18673	insignificant	0	4	4
chr15	72919841	72921841	Chrac1	-0.17011134	2.34E-16	hypomethylated	-0.021161	0.0019443	hypomethylated	16	69	68
chr15	73015377	73017377	Eif2c2	0.00023246	1	insignificant	-0.013266	0.28451	insignificant	5	52	52
chr15	73253621	73255621	Ptk2	-0.12465574	9.06E-11	hypomethylated	0.030109	0.0048157	hypermethylated	23	54	57
chr15	73341989	73343989	Dennd3	-0.20489898	4.19E-15	hypomethylated	0.023565	0.64631	insignificant	11	46	47
chr15	73537935	73539935	Gpr20	0.07038462	0.052798	insignificant	0.23182	1	insignificant	2	5	5
chr15	73552574	73554574	Ptp4a3		1	noCoverage	0.10278	0.10219	insignificant	0	18	18
chr15	73554359	73556359	Ptp4a3	-0.14060079	1.06E-18	hypomethylated	0.0024677	0.35041	insignificant	41	132	129
chr15	73577455	73579455	Ptp4a3	-0.19459037	0.64847	insignificant	0.050239	0.90052	insignificant	2	8	6
chr15	73670101	73672101	Gm628	-0.09546621	0.000000279	hypomethylated	-0.0030669	0.34697	insignificant	14	68	68
chr15	74345625	74347625	Bai1	-0.10557761	0.000000163	hypomethylated	-0.021663	0.51038	insignificant	23	115	107
chr15	74466748	74468748	1700016M24F	-0.41944444	0.5749	insignificant	0.018867	0.60567	insignificant	2	4	4
chr15	74475178	74477178		-0.49981397	0.00074741	stronglyHypometh	0.019729	1	insignificant	1	6	6
chr15	74503000	74505000	Arc	-0.2184265	0.00006694	hypomethylated	0.31788	0.15916	insignificant	1	4	2
chr15	74538590	74540590	4933427E11Ri	-0.18434834	4.04E-55	hypomethylated	0.018923	0.0036187	hypermethylated	35	97	94
chr15	74539752	74541752	4933427E11Ri	-0.17676827	9.17E-21	hypomethylated	0.02849	0.098644	insignificant	22	56	52
chr15	74550663	74552663	4930572J05Ri	-0.13966427	0.066571	insignificant	0.025483	0.91582	insignificant	6	113	91
chr15	74558456	74560456	Slurp1	0.09259259	1	insignificant	-0.018519	0.41204	insignificant	2	6	6
chr15	74564743	74566743	Lypd2	-0.16885175	0.16735	insignificant	-0.045114	0.75375	insignificant	2	8	8
chr15	74577117	74579117	2300005B03R	-0.13275058	0.12165	insignificant	-0.050824	0.56249	insignificant	3	6	6
chr15	74583409	74585409	Lynx1	-0.40508615	0.00069602	stronglyHypometh	-0.10448	0.81472	insignificant	2	31	30
chr15	74593997	74595997	Ly6d		1	noCoverage	0.011602	1	insignificant	0	4	4
chr15	74648505	74650505	Hemt1	-0.12012431	0.23503	insignificant	0.054253	0.17225	insignificant	6	14	14
chr15	74649245	74651245	Gml	-0.12012431	0.23503	insignificant	0.054253	0.17225	insignificant	6	14	14
chr15	74784480	74786480	Ly6e	-0.14218363	0.000011716	hypomethylated	-0.0057084	0.90216	insignificant	7	59	61
chr15	74784500	74786500	Ly6e	-0.14218363	0.000011716	hypomethylated	-0.0057084	0.90216	insignificant	7	59	61
chr15	74784535	74786535	Ly6e	-0.14218363	0.000011716	hypomethylated	-0.0057084	0.90216	insignificant	7	59	61
chr15	74813860	74815860	Ly6i	-0.15744143	0.23626	insignificant	0.030009	0.18952	insignificant	1	8	8
chr15	75262259	75264259	9030619P08Ri	-0.25147116	0.66304	insignificant	0.10265	0.19535	insignificant	2	7	8
chr15	75397078	75399078	Ly6h	-0.08252032	2.78E-08	hypomethylated	0.016836	0.62102	insignificant	26	113	112
chr15	75397286	75399286	Ly6h	-0.06476299	0.0019039	hypomethylated	0.017404	0.75696	insignificant	15	65	64
chr15	75426087	75428087	Gpihbp1	-0.918	0.090909	lowCoverage	-0.0805	0.13727	insignificant	1	10	10
chr15	75446114	75448114	Zfp41	-0.0776032	2.94E-11	hypomethylated	0.0068106	0.61609	insignificant	47	153	151
chr15	75509220	75511220	Top1mt	-0.23373869	0.32592	insignificant	0.041761	1	insignificant	2	8	8
chr15	75533717	75535717	Rhpn1	-0.20086328	3.28E-12	hypomethylated	0.003206	0.95699	insignificant	11	36	36
chr15	75578352	75580352	Mafa	-0.1432058	3.55E-10	hypomethylated	0.036357	0.35584	insignificant	23	95	88
chr15	75672338	75674338	Zc3h3	-0.26719032	0.000074484	hypomethylated	-0.022424	0.15892	insignificant	4	57	55
chr15	75691768	75693768	Gsdmd	-0.2032511	1.14E-09	hypomethylated	0.001117	1	insignificant	12	56	48
chr15	75724911	75726911	Naprt1	0.05406853	0.85326	insignificant	0.066442	0.05986	insignificant	4	14	14
chr15	75739164	75741164	Tigd5	-0.10800582	9.28E-67	hypomethylated	0.0066085	0.013461	hypermethylated	68	257	253
chr15	75739770	75741770	Eef1d	-0.15051483	3E-41	hypomethylated	0.01429	0.0036196	hypermethylated	49	195	192
chr15	75760160	75762160	Tsta3	-0.59417351	1.75E-09	stronglyHypometh	0.022879	0.49848	insignificant	2	16	16
chr15	75770381	75772381	Zfp623	-0.15153151	0.0015819	hypomethylated	-0.069395	0.32953	insignificant	26	83	72
chr15	75798614	75800614	Zfp707	-0.1538386	2.68E-16	hypomethylated	-0.0023565	0.17448	insignificant	14	79	72
chr15	75812715	75814715	2410075B13R	0.15744746	1	insignificant	0.084074	0.8556	insignificant	3	12	15
chr15	75823198	75825198	Mapk15	-0.32545516	0.0001029	hypomethylated	-0.098403	0.10113	insignificant	6	24	20
chr15	75839928	75841928	Fam83h	-0.20368507	2.54E-20	hypomethylated	-0.0031381	0.93388	insignificant	18	68	72
chr15	75844766	75846766	Fam83h	-0.16926792	1	insignificant	0.000084692	0.76385	insignificant	5	14	14
chr15	75900160	75902160	Puf60	-0.15291254	1.6E-18	hypomethylated	0.047363	0.66128	insignificant	12	44	43

chr15	75911300	75913300	Puf60	0.14379085	1	insignificant	-0.13313	0.82614	insignificant	2	6	13
chr15	75911376	75913376	Puf60	0.14379085	1	insignificant	-0.13313	0.82614	insignificant	2	6	13
chr15	75920443	75922443	Nrbp2	-0.14318074	3.9E-19	hypomethylated	0.017313	0.32183	insignificant	25	83	85
chr15	76026140	76028140	Plec	-0.54960317	1.11E-09	stronglyHypometh	0.0039683	0.64467	insignificant	2	6	6
chr15	76028639	76030639	Plec	-0.20823099	8.21E-25	hypomethylated	-0.0096709	0.096736	insignificant	13	38	38
chr15	76029836	76031836	Plec	-0.15701719	5.55E-10	hypomethylated	0.0084528	0.54951	insignificant	7	14	14
chr15	76030265	76032265	Plec	-0.00562483	0.16512	insignificant	-0.0029636	0.19988	insignificant	6	18	18
chr15	76031037	76033037	Plec	0.19135552	1	insignificant	-0.010632	0.78887	insignificant	1	8	8
chr15	76036751	76038751	Plec	-0.18938174	6.2E-11	hypomethylated	0.02716	0.14789	insignificant	7	18	18
chr15	76059927	76061927	Plec	-0.14708643	0.32021	insignificant	0.014902	0.65791	insignificant	6	22	22
chr15	76061808	76063808	Plec	-0.22638258	0.057477	insignificant	-0.016254	0.89105	insignificant	4	50	56
chr15	76073870	76075870	Parp10	-0.03352562	0.57805	insignificant	-0.03878	1	insignificant	1	12	14
chr15	76076236	76078236	Grina	-0.26425311	1	lowCoverage	0.12737	0.21963	insignificant	1	12	13
chr15	76097518	76099518	Spatc1	0.01041667	1	insignificant	-0.092758	0.68501	insignificant	2	12	12
chr15	76123863	76125863	Gm10345	-0.24618583	1.78E-11	hypomethylated	-0.0049956	0.027242	hypomethylated	11	45	46
chr15	76156826	76158826	Exosc4	-0.12932517	1.64E-25	hypomethylated	-0.0026176	0.85946	insignificant	26	69	73
chr15	76157963	76159963	Exosc4	0.01003359	1	insignificant	0.028291	0.17596	insignificant	23	75	76
chr15	76160723	76162723	Gpaa1	-0.14575885	3.75E-09	hypomethylated	0.011275	0.7007	insignificant	23	81	81
chr15	76172952	76174952	Cyc1	-0.10642631	7.44E-27	hypomethylated	-0.010664	0.44559	insignificant	27	80	86
chr15	76180723	76182723	Maf1	-0.15274795	9.12E-39	hypomethylated	-0.0078242	0.73175	insignificant	30	124	124
chr15	76181540	76183540	Sharpin	-0.11304135	1.5E-16	hypomethylated	-0.0067038	0.55536	insignificant	11	64	64
chr15	76198327	76200327	Fam203a	-0.14558668	8.02E-19	hypomethylated	0.012321	0.97558	insignificant	26	99	105
chr15	76209942	76211942	Heatr7a	-0.07005955	0.0029368	hypomethylated	-0.0070593	0.71381	insignificant	13	79	79
chr15	76219460	76221460	Heatr7a	0.20185376	0.0017952	hypermethylated	0.090501	0.034305	hypermethylated	4	13	12
chr15	76286867	76288867	Scx	-0.29800532	1.36E-08	hypomethylated	-0.066538	0.00056731	hypomethylated	21	61	50
chr15	76306874	76308874	Hsf1	-0.13604411	1.49E-37	hypomethylated	0.0045263	0.79162	insignificant	49	154	146
chr15	76307699	76309699	Bop1	-0.10611655	7.94E-19	hypomethylated	0.0069735	0.82595	insignificant	30	104	98
chr15	76342248	76344248	Dgat1	-0.10246736	1.86E-18	hypomethylated	-0.0018567	0.47933	insignificant	16	70	73
chr15	76352559	76354559	Scrt1	-0.14366582	9.93E-11	hypomethylated	-0.012557	0.86301	insignificant	27	126	142
chr15	76368372	76370372	Gpr172b	-0.14184013	5.96E-22	hypomethylated	0.030909	0.0076387	hypermethylated	26	104	98
chr15	76369176	76371176	Gpr172b	-0.18870636	7.13E-15	hypomethylated	0.12197	0.01119	hypermethylated	9	34	35
chr15	76405788	76407788	Adck5	-0.11590043	7.18E-15	hypomethylated	0.017036	0.13785	insignificant	24	68	68
chr15	76447282	76449282	Slc39a4		1	noCoverage	-0.032126	0.33449	insignificant	0	6	6
chr15	76456457	76458457	Vps28	-0.14744854	0.00063719	hypomethylated	0.025696	0.33378	insignificant	4	9	9
chr15	76490070	76492070	Kifc2	-0.20910759	3.79E-09	hypomethylated	0.059019	0.75012	insignificant	8	31	24
chr15	76490491	76492491	Cyhr1	-0.20910759	3.79E-09	hypomethylated	0.059019	0.75012	insignificant	8	31	24
chr15	76490538	76492538	Cyhr1	-0.20910759	3.79E-09	hypomethylated	0.059019	0.75012	insignificant	8	31	24
chr15	76500303	76502303	Ppp1r16a	-0.16374834	1.69E-17	hypomethylated	-0.016642	0.20224	insignificant	29	77	71
chr15	76501109	76503109	Ppp1r16a	-0.1293922	3.54E-19	hypomethylated	-0.0054302	0.1523	insignificant	32	84	82
chr15	76530971	76532971	Mfsd3	-0.18305688	2.67E-24	hypomethylated	0.0078748	0.92021	insignificant	14	35	32
chr15	76540169	76542169	Lrrc14	-0.11677587	5.42E-11	hypomethylated	0.014822	0.97263	insignificant	22	110	108
chr15	76540906	76542906	Lrrc14	-0.11707414	8.32E-11	hypomethylated	0.0056925	0.9153	insignificant	22	100	97
chr15	76552603	76554603	C030006K11R	-0.13928429	3.23E-29	hypomethylated	0.0010319	0.34231	insignificant	38	126	131
chr15	76554275	76556275	C030006K11R	-0.26539035	4.72E-58	hypomethylated	0.016302	0.000000137	hypermethylated	26	104	104
chr15	76648600	76650600	Arhgap39	-0.15168508	1	insignificant	-0.006474	0.88445	insignificant	3	12	12
chr15	76701865	76703865	Zfp251	-0.20963854	0.0028984	hypomethylated	0.047772	0.27498	insignificant	5	23	23
chr15	76708705	76710705	Zfp7	0.14375875	0.4466	insignificant	-0.011017	0.37334	insignificant	1	56	53
chr15	76729370	76731370	Commd5	-0.05204215	0.11336	insignificant	-0.00805	0.15025	insignificant	3	41	38
chr15	76733500	76735500	Rpl8	-0.24126721	1.03E-09	hypomethylated	-0.0090148	0.079717	insignificant	7	61	62
chr15	76755878	76757878	Zfp647	-0.17224753	1.69E-17	hypomethylated	-0.00089581	1	insignificant	18	60	60
chr15	76777973	76779973	1110038F14Ri	-0.1115521	8.14E-15	hypomethylated	-0.0053755	0.83407	insignificant	12	81	75
chr15	76853157	76855157	Mb	0.27196115	1	lowCoverage	0.026223	1	insignificant	1	14	16
chr15	76853217	76855217	Mb	0.27196115	1	lowCoverage	0.026223	1	insignificant	1	14	16
chr15	76874504	76876504	Apol6	-0.12699769	0.042532	hypomethylated	-0.0047869	0.69142	insignificant	3	18	18
chr15	76874705	76876705	Apol6	-0.00627761	0.021768	hypomethylated	-0.010166	0.14374	insignificant	5	22	22
chr15	76881098	76883098	Apol6		1	noCoverage	-0.037411	0.94211	insignificant	0	22	22
chr15	77137483	77139483	Rbfox2		1	noCoverage	0.026491	0.85017	insignificant	0	32	34
chr15	77448397	77450397	Gm8221		1	noCoverage	0.011752	0.86184	insignificant	0	6	6
chr15	77672545	77674545	Myh9	-0.26396864	0.35334	insignificant	0.12849	0.65191	insignificant	2	46	40
chr15	77759424	77761424	Txn2	-0.24043522	2.15E-10	hypomethylated	0.01681	0.57061	insignificant	8	34	30
chr15	77787152	77789152	Foxred2	-0.22585948	0.00000036	hypomethylated	-0.092097	0.9435	insignificant	8	18	16
chr15	77801254	77803254	Eif3d	-0.10761174	0.00000445	hypomethylated	0.01368	0.56666	insignificant	15	63	63
chr15	77949710	77951710	Cacng2	-0.14630836	1.53E-13	hypomethylated	-0.0056619	0.10966	insignificant	27	132	141
chr15	78004538	78006538	Ift27	-0.12914354	0.00031922	hypomethylated	0.029184	0.010094	hypermethylated	3	40	40
chr15	78034586	78036586	Pvalb	-0.12453875	0.00004415	hypomethylated	-0.036006	0.15793	insignificant	8	33	32
chr15	78074240	78076240	Ncf4		1	noCoverage	-0.048969	0.70759	insignificant	0	4	4
chr15	78155419	78157419	Csf2rb	-0.25514902	0.53411	insignificant	-0.0036595	0.15941	insignificant	4	8	8
chr15	78236141	78238141	Mpst	-0.05012349	0.53623	insignificant	0.0065059	0.96424	insignificant	7	52	55
chr15	78236230	78238230	Mpst	-0.03248873	0.86113	insignificant	0.0077454	0.69394	insignificant	5	40	43
chr15	78236289	78238289	Tst	-0.04799811	0.28885	insignificant	0.012479	0.8315	insignificant	3	34	37
chr15	78236533	78238533	Mpst	-0.03678044	0.18425	insignificant	0.0013309	0.9544	insignificant	3	28	32
chr15	78258057	78260057	Kctd17	-0.13357697	2.6E-09	hypomethylated	-0.010288	0.6266	insignificant	19	57	54
chr15	78299064	78301064	Tmprss6	0.26666667	1	lowCoverage	0.16366	0.20981	insignificant	1	6	7
chr15	78325496	78327496	Il2rb		1	noCoverage	-0.071255	0.14085	insignificant	0	4	4
chr15	78360047	78362047	C1qtnf6	-0.15943514	0.51036	insignificant	-0.0023818	0.89228	insignificant	5	20	21
chr15	78374775	78376775	Sstr3	-0.13436058	0.00046186	hypomethylated	-0.013581	0.80341	insignificant	4	8	8
chr15	78426476	78428476	Cyth4	-0.36486486	0.014618	stronglyHypometh	0.036007	0.90707	insignificant	3	6	6
chr15	78548543	78550543	Elfn2	-0.17288306	9.39E-29	hypomethylated	-0.0086924	0.046774	hypomethylated	34	129	133
chr15	78603875	78605875	Mfng	-0.61507937	5.69E-19	stronglyHypometh	0.025576	0.63737	insignificant	3	4	4
chr15	78633472	78635472	Card10	-0.19975097	1.97E-13	hypomethylated	0.014607	1	insignificant	12	40	39
chr15	78672076	78674076	Cdc42ep1	-0.07763639	0.000011081	hypomethylated	0.024365	0.15108	insignificant	27	84	78
chr15	78685959	78687959	Lgals2	-0.19240196	0.00000145	hypomethylated	0.069906	0.85686	insignificant	4	4	4
chr15	78706619	78708619	Gga1	-0.09485331	3.99E-23	hypomethylated	0.021798	0.10917	insignificant	34	112	100
chr15	78729215	78731215	Sh3bp1	-0.16071771	0.000000958	hypomethylated	-0.014896	0.36931	insignificant	12	60	57
chr15	78743348	78745348	Pdxp	-0.0913908	4.84E-13	hypomethylated	0.012919	0.033041	hypermethylated	41	201	172

chr15	78756154	78758154	Lgals1	-0.53202899	0.52502	insignificant	-0.10996	0.74377	insignificant	1	10	10
chr15	78764362	78766362	Nol12	-0.16383688	9.36E-13	hypomethylated	0.013731	0.42849	insignificant	20	74	75
chr15	78777153	78779153	Triobp	-0.1952381	0.0137	hypomethylated	-0.023921	0.39239	insignificant	2	15	16
chr15	78812485	78814485	Triobp	-0.17789779	2.92E-22	hypomethylated	0.0067649	0.5099	insignificant	30	79	69
chr15	78857641	78859641	H1f0	-0.08811524	3.05E-28	hypomethylated	0.0037163	0.37251	insignificant	74	212	201
chr15	78860303	78862303	Gcat	-0.19604535	1	insignificant	-0.061544	0.8918	insignificant	1	33	26
chr15	78871314	78873314	Galr3	-0.04638226	0.00016495	hypomethylated	-0.01279	0.000020513	hypomethylated	18	49	49
chr15	78904652	78906652	Eif3l	-0.22245624	0.0011285	hypomethylated	0.01472	0.001355	hypermethylated	12	84	84
chr15	78938412	78940412	Micall1	-0.12927151	1.46E-31	hypomethylated	-0.0038962	0.22232	insignificant	59	164	165
chr15	78970796	78972796	Polr2f	-0.16377529	0.000000121	hypomethylated	-0.031719	0.016367	inconclusive	19	98	92
chr15	78971681	78973681	1700088E04Ri	-0.16634003	0.0007626	hypomethylated	-0.049618	0.035166	inconclusive	14	65	64
chr15	78994920	78996920	Gm10863	-0.13455807	3.51E-18	hypomethylated	0.034308	0.61042	insignificant	14	47	52
chr15	78995495	78997495	Gm10863	-0.12850674	5.9E-16	hypomethylated	0.033675	0.42194	insignificant	14	49	54
chr15	79058603	79060603	Pick1	-0.08507534	9.11E-16	hypomethylated	-0.00061563	0.14212	insignificant	18	65	65
chr15	79058811	79060811	Pick1	-0.08507534	9.11E-16	hypomethylated	-0.00061563	0.14212	insignificant	18	65	65
chr15	79115939	79117939	Pla2g6	-0.04959517	0.78362	insignificant	-0.047628	0.79278	insignificant	3	10	8
chr15	79158801	79160801	Pla2g6		1	noCoverage	0.026786	0.8184	insignificant	0	6	6
chr15	79177107	79179107	Maff	-0.12476429	8.53E-31	hypomethylated	0.01013	0.00010521	hypermethylated	39	132	132
chr15	79233733	79235733	Tmem184b	-0.08543907	0.00027417	hypomethylated	-0.0079996	0.36805	insignificant	5	30	30
chr15	79272487	79274487	Csnk1e	-0.09660367	8.26E-13	hypomethylated	-0.0074343	0.94296	insignificant	18	45	30
chr15	79335671	79337671	Kcnj4	-0.16768786	1.75E-49	hypomethylated	-0.039569	0.062937	insignificant	64	159	146
chr15	79345837	79347837	Kdelr3	0.07670081	0.53835	insignificant	0.019638	0.14773	insignificant	2	32	32
chr15	79363347	79365347	Ddx17	0.06741071	1	insignificant	-0.0036571	0.00024339	inconclusive	2	14	18
chr15	79376989	79378989	Ddx17	-0.30431598	0.0018701	hypomethylated	0.013008	0.24495	insignificant	16	58	58
chr15	79377171	79379171	Ddx17	-0.33348424	0.0069429	stronglyHypometh	0.0096631	0.34997	insignificant	14	50	50
chr15	79435514	79437514	Dmc1	-0.18150096	0.000014251	hypomethylated	0.012895	0.0087817	hypermethylated	6	32	32
chr15	79488656	79490656	Cby1	-0.07066028	0.00023439	hypomethylated	0.0066783	0.48489	insignificant	21	79	79
chr15	79489386	79491386	4933432B09R	-0.0602872	0.000014694	inconclusive	0.012347	0.23115	insignificant	18	63	65
chr15	79500297	79502297	Tomm22	-0.20395933	7.07E-08	hypomethylated	0.0086198	0.38307	insignificant	16	89	81
chr15	79518302	79520302	Josd1	-0.23338993	0.34619	insignificant	0.010321	0.25027	insignificant	4	41	41
chr15	79520325	79522325	Gtpbp1	-0.09892088	5.82E-14	hypomethylated	0.044655	0.31763	insignificant	27	84	72
chr15	79572960	79574960	Gm16576	-0.25014724	2.67E-12	hypomethylated	-0.061352	0.01137	hypomethylated	19	76	70
chr15	79604897	79606897	Dnalc4	-0.15644536	0.0019598	hypomethylated	0.022208	0.10643	insignificant	8	28	28
chr15	79635139	79637139	Nptxr	-0.11242268	0.038185	hypomethylated	0.073331	0.070626	insignificant	14	55	59
chr15	79664763	79666763	Npcd	-0.1565949	5.16E-09	hypomethylated	-0.029061	0.86284	insignificant	5	28	36
chr15	79721837	79723837	Apobec3	-0.41621718	7.6E-10	stronglyHypometh	0.011561	1	insignificant	2	16	10
chr15	79723568	79725568	D730005E14R	-0.73361345	0.30252	lowCoverage	-0.20339	0.010109	hypomethylated	1	17	13
chr15	79763076	79765076	Cbx7	-0.17225813	0.61764	insignificant	-0.00515	0.30421	insignificant	4	19	21
chr15	79845238	79847238	Pdgfb	-0.0318776	0.00035427	hypomethylated	0.017789	0.93054	insignificant	4	16	16
chr15	79909009	79911009	Snord83b		1	noCoverage	0.074576	0.14855	insignificant	0	16	14
chr15	79913336	79915336	Snord43	-0.25545431	3.43E-11	hypomethylated	0.011646	0.010091	hypermethylated	11	55	60
chr15	79913354	79915354	Snord43	-0.25545431	3.43E-11	hypomethylated	0.023561	0.0086845	hypermethylated	11	55	61
chr15	79913836	79915836	Snord43	-0.29664259	0.004562	hypomethylated	0.02427	0.026889	hypermethylated	6	37	39
chr15	79920763	79922763	Syngr1	-0.16990116	2.55E-11	hypomethylated	0.032123	0.000082608	inconclusive	22	82	78
chr15	79962583	79964583	Tab1	-0.08315011	0.00012393	hypomethylated	0.016186	0.1798	insignificant	20	96	99
chr15	80003150	80005150	Mgat3	-0.12889687	1.11E-24	hypomethylated	-0.029776	0.95887	insignificant	62	201	202
chr15	80063509	80065509	Smcr7l	-0.15088161	1.22E-16	hypomethylated	0.0035502	0.86628	insignificant	20	80	76
chr15	80084613	80086613	Atf4	-0.1450673	3.06E-22	hypomethylated	0.00013031	0.31956	insignificant	22	113	106
chr15	80094736	80096736	Rps19bp1	-0.29025611	0.00053067	hypomethylated	-0.058398	0.72452	insignificant	3	19	19
chr15	80116667	80118667	Cacna1i	-0.11189158	0.00000566	hypomethylated	0.030123	0.000017926	hypermethylated	37	138	138
chr15	80390900	80392900	Enthd1	-0.10242424	0.34529	insignificant	0.0011435	0.87673	insignificant	3	15	12
chr15	80501276	80503276	Fam83f	-0.05788518	0.44077	insignificant	-0.0070454	0.82575	insignificant	18	87	87
chr15	80540742	80542742	Tnrc6b	-0.09150929	0.00021503	hypomethylated	-0.0040081	0.74868	insignificant	18	104	105
chr15	80777948	80779948	Adsl	-0.20622379	0.0097015	hypomethylated	0.040165	0.68054	insignificant	7	76	76
chr15	80807194	80809194	Sgsm3	-0.1570414	0.26534	insignificant	-0.045298	0.099988	insignificant	17	118	112
chr15	81020282	81022282	4930483J18Ri	-0.14510942	1.03E-09	hypomethylated	0.0051973	0.19545	insignificant	25	125	124
chr15	81021187	81023187	4930483J18Ri	-0.03689821	0.17602	insignificant	-0.033325	0.015221	hypomethylated	12	66	73
chr15	81064928	81066928	Mchr1	-0.15603616	0.000000247	hypomethylated	-0.0028566	0.41866	insignificant	10	38	36
chr15	81191195	81193195	Slc25a17		1	noCoverage	-0.068614	0.81117	insignificant	0	8	6
chr15	81229617	81231617	Xpnpep3	-0.18440279	2.24E-24	hypomethylated	0.020828	0.016611	hypermethylated	16	105	97
chr15	81230124	81232124	St13	-0.19114818	1.55E-21	hypomethylated	0.022991	0.0071809	hypermethylated	11	93	85
chr15	81295745	81297745	Rbx1	-0.13399483	2.88E-08	hypomethylated	0.0014762	0.76459	insignificant	13	87	94
chr15	81415643	81417643	Ep300	-0.11282207	2.18E-47	hypomethylated	0.0065772	0.80943	insignificant	70	220	220
chr15	81493365	81495365	L3mbtl2	-0.31185356	5.89E-08	hypomethylated	0.063455	0.72444	insignificant	6	31	24
chr15	81527717	81529717	Chadl	0.19392231	1	lowCoverage	-0.18363	0.026698	hypomethylated	1	19	21
chr15	81560349	81562349	Rangap1	0.16705441	1	lowCoverage	-0.069578	0.57326	insignificant	1	18	18
chr15	81574277	81576277	Zc3h7b	-0.09931777	4.19E-08	hypomethylated	0.0079399	0.96173	insignificant	21	79	72
chr15	81640843	81642843	Tef	-0.11689459	1.35E-08	hypomethylated	-0.0077271	0.35197	insignificant	17	80	80
chr15	81688756	81690756	Tob2	-0.13557713	6.7E-10	hypomethylated	0.0010057	0.97308	insignificant	27	102	104
chr15	81701892	81703892	Aco2	-0.12585999	2.7E-15	hypomethylated	0.0078281	0.57302	insignificant	19	116	125
chr15	81702322	81704322	Phf5a	-0.10585274	9.53E-10	hypomethylated	0.0078065	0.70369	insignificant	15	91	97
chr15	81756643	81758643	Polr3h	-0.29552182	0.000000234	hypomethylated	0.016018	0.8836	insignificant	8	49	50
chr15	81766188	81768188	Csdc2	-0.67750871	0.0073835	stronglyHypometh	0.099991	0.69191	insignificant	2	9	5
chr15	81791297	81793297	Pmm1	-0.24332489	0.000088374	hypomethylated	-0.034238	0.45837	insignificant	5	32	30
chr15	81809974	81811974	1700029P11Ri	0.11111111	1	insignificant	0.067058	7.23E-08	hypermethylated	2	25	25
chr15	81845798	81847798	Xrcc6	-0.14875614	3.93E-18	hypomethylated	-0.0065739	0.68017	insignificant	14	93	83
chr15	81846570	81848570	Pppde2	-0.10289656	1	lowCoverage	0.041761	0.53471	insignificant	1	40	37
chr15	81878028	81880028	Nhp2l1	-0.24551797	0.60398	insignificant	-0.0034849	0.61152	insignificant	7	28	29
chr15	81899546	81901546	Mei1	-0.12327252	0.0020302	hypomethylated	0.031834	0.9624	insignificant	10	34	34
chr15	81957351	81959351	Ccdc134	-0.03990571	0.00015629	hypomethylated	0.012711	0.76748	insignificant	21	85	76
chr15	81976698	81978698	Srebf2	-0.10894066	3.87E-19	hypomethylated	-0.0037738	0.38331	insignificant	42	162	160
chr15	82027551	82029551	Mir33	-0.00754008	1	insignificant	-0.0015877	0.66259	insignificant	1	7	6
chr15	82054766	82056766	Tnfrsf13c		1	noCoverage	0.1038	0.65958	insignificant	0	6	6
chr15	82074766	82076766	Cenpm	-0.19655236	9.59E-15	hypomethylated	0.0088315	0.66127	insignificant	18	79	78
chr15	82075177	82077177	Cenpm	-0.3623639	7.93E-17	stronglyHypometh	0.011683	0.61958	insignificant	6	26	26

chr15	82104364	82106364	###########	-0.11692033	6.47E-27	hypomethylated	0.0069966	0.16306	insignificant	37	112	104
chr15	82128413	82130413	Wbp2nl	-0.35515873	0.35373	insignificant	-0.095425	0.5564	insignificant	1	6	3
chr15	82169256	82171256	Naga	-0.31223418	0.020705	hypomethylated	0.0096121	0.90634	insignificant	2	6	6
chr15	82170608	82172608	Fam109b	-0.18436737	7.15E-14	hypomethylated	-0.022653	0.96134	insignificant	11	48	38
chr15	82175475	82177475	1500032L24Ri	-0.10719936	0.00000314	hypomethylated	-0.011339	0.21582	insignificant	25	70	66
chr15	82184721	82186721	Ndufa6	-0.11719538	0.60887	insignificant	0.012714	0.33914	insignificant	5	38	39
chr15	82237624	82239624	Cyp2d10		1	noCoverage	0.039596	0.2935	insignificant	0	4	7
chr15	82728891	82730891	Gm20324	-0.10540829	2.23E-30	hypomethylated	0.014774	0.94242	insignificant	46	171	135
chr15	82742564	82744564	Tcf20	-0.74241453	8.59E-23	stronglyHypometh	-0.076389	0.28125	insignificant	3	6	6
chr15	82853568	82855568	Nfam1		1	noCoverage	0.10495	0.4052	insignificant	0	14	15
chr15	82929634	82931634	Serhl	-0.16210723	1	lowCoverage	-0.0026899	0.42252	insignificant	1	28	28
chr15	82953231	82955231	Rrp7a	-0.27708772	0.0011626	hypomethylated	0.099692	0.00023354	hypermethylated	3	31	32
chr15	82979074	82981074	Rnu12	-0.11966789	2.11E-25	hypomethylated	0.0011335	0.49251	insignificant	40	129	129
chr15	82979766	82981766	Poldip3	-0.24535534	0.21194	insignificant	0.031848	0.39067	insignificant	4	14	14
chr15	83002638	83004638	Cyb5r3	-0.16211822	0.00050986	inconclusive	-0.026683	0.090396	insignificant	4	28	28
chr15	83082161	83084161	A4galt	-0.47447172	0.35079	lowCoverage	0.025752	0.66596	insignificant	1	14	14
chr15	83082204	83084204	A4galt	-0.60554562	0.27164	lowCoverage	0.023224	0.8679	insignificant	1	12	12
chr15	83180677	83182677	Arfgap3	-0.10196883	0.02811	hypomethylated	-0.0051136	0.62895	insignificant	8	37	37
chr15	83295036	83297036	Pacsin2	0.56259921	0.46626	lowCoverage	0.10666	0.20425	insignificant	1	18	8
chr15	83341337	83343337	Ttll1	-0.31684381	8.44E-08	hypomethylated	0.014971	0.058853	insignificant	7	42	44
chr15	83356291	83358291	Bik	-0.1164737	5.53E-09	hypomethylated	-0.00055762	0.0039561	hypomethylated	28	102	104
chr15	83386141	83388141	Mcat	0.0636166	1	insignificant	0.095486	0.0030152	hypermethylated	6	12	12
chr15	83393002	83395002	Tspo		1	noCoverage	-0.018395	0.35028	insignificant	0	24	19
chr15	83555451	83557451	Scube1	-0.20430242	0.010834	hypomethylated	0.014421	0.63595	insignificant	15	65	58
chr15	83609452	83611452	Mpped1	-0.1134045	1.78E-31	hypomethylated	0.0043596	0.47705	insignificant	43	155	138
chr15	83749803	83751803	Efcab6		1	noCoverage	-0.037803	0.52434	insignificant	0	18	16
chr15	83766525	83768525	Efcab6	0.0943718	0.70928	insignificant	0.02066	0.61064	insignificant	3	12	12
chr15	83895779	83897779	Efcab6		1	noCoverage	-0.11218	0.031376	hypomethylated	0	6	6
chr15	83936184	83938184	Sult4a1	0.61891885	0.000000635	stronglyHypermeth	0.0015661	0.16881	insignificant	1	18	18
chr15	83997245	83999245	Pnpla3	-0.15785635	1.94E-15	hypomethylated	0.029994	0.36878	insignificant	15	76	78
chr15	84021662	84023662	Samm50	-0.18525261	4.03E-15	hypomethylated	-0.0029503	0.91568	insignificant	11	64	65
chr15	84061472	84063472	Parvb	-0.13789555	3.41E-13	hypomethylated	-0.006023	0.026107	inconclusive	14	85	80
chr15	84154149	84156149	Parvg	-0.64392768	0.031722	stronglyHypometh	-0.0080691	0.65384	insignificant	2	18	18
chr15	84277527	84279527	1810041L15Ri	-0.78709273	0.20988	lowCoverage	-0.02816	1	insignificant	1	6	6
chr15	84388253	84390253	Ldoc1l	-0.14842803	0.013612	hypomethylated	-0.0029744	0.42233	insignificant	4	8	8
chr15	84510427	84512427	Prr5	-0.14719638	1.54E-79	hypomethylated	0.018641	0.60962	insignificant	54	157	141
chr15	84549481	84551481	Arhgap8	-0.20714089	5.32E-17	hypomethylated	0.10124	0.70006	insignificant	11	61	69
chr15	84549508	84551508	Arhgap8	-0.20714089	5.32E-17	hypomethylated	0.10124	0.70006	insignificant	11	61	69
chr15	84686559	84688559	Phf21b	-0.13297584	1.01E-13	hypomethylated	0.020444	0.38768	insignificant	13	63	54
chr15	84752857	84754857	Nup50	-0.11536669	8.88E-40	hypomethylated	0.015985	0.20321	insignificant	61	186	186
chr15	84782053	84784053	5031439G07R	-0.44458216	0.50545	insignificant	-0.034645	0.053794	insignificant	6	20	20
chr15	84818401	84820401	5031439G07Rik		1	noCoverage	0.010542	0.0000281	hypermethylated	0	24	24
chr15	84846570	84848570	Upk3a	-0.30059524	1	insignificant	-0.0035119	0.91203	insignificant	2	12	12
chr15	84961528	84963528	Ribc2	-0.1763421	6.31E-08	hypomethylated	-0.034354	0.81042	insignificant	14	77	72
chr15	84962387	84964387	Smc1b	-0.27662714	0.000000761	hypomethylated	-0.0065957	0.042938	inconclusive	7	60	58
chr15	85035437	85037437	Fbln1	-0.12190726	3.16E-11	hypomethylated	0.0026795	0.0025627	hypermethylated	23	76	76
chr15	85165810	85167810	Atxn10	-0.1174119	0.00062976	hypomethylated	-0.021812	0.59292	insignificant	8	25	30
chr15	85408500	85410500	Wnt7b	-0.10895331	0.10824	insignificant	0.0030859	0.94114	insignificant	5	20	20
chr15	85411159	85413159	Wnt7b	-0.10457906	1.36E-57	hypomethylated	0.018432	0.84823	insignificant	75	205	188
chr15	85412251	85414251	Wnt7b	-0.16866195	1.76E-18	hypomethylated	-0.0026058	0.53755	insignificant	15	35	35
chr15	85536032	85538032	Mirlet7c-2	0.1956446	1	insignificant	-0.012678	0.27354	insignificant	2	10	10
chr15	85564993	85566993	Ppara	-0.12740154	5.83E-16	hypomethylated	0.0015726	0.68992	insignificant	46	190	190
chr15	85565205	85567205	Ppara	-0.12712278	1.34E-15	hypomethylated	-0.0060568	0.54352	insignificant	46	186	184
chr15	85642127	85644127	2210021J22Ri	-0.16534245	4.03E-15	hypomethylated	0.0029114	0.081678	insignificant	7	26	26
chr15	85652163	85654163	Pkdrej	-0.10029239	2.35E-23	hypomethylated	-0.0020753	0.3982	insignificant	15	37	36
chr15	85661733	85663733	Ttc38	-0.19585602	8.33E-12	hypomethylated	-0.005928	0.51742	insignificant	17	56	54
chr15	85689136	85691136	Gtse1	-0.29002089	6.33E-08	hypomethylated	-0.011798	0.47089	insignificant	9	48	48
chr15	85689375	85691375	Gtse1	-0.3031305	2.29E-08	hypomethylated	-0.008296	0.25672	insignificant	10	50	50
chr15	85708756	85710756	Trmu	-0.17665463	5.09E-11	hypomethylated	-0.014287	1	insignificant	13	45	43
chr15	85864207	85866207	Celsr1	-0.13631166	1.05E-12	hypomethylated	0.012837	0.79577	insignificant	20	73	67
chr15	85887136	85889136	Gramd4	0.08864378	0.76565	insignificant	0.015434	0.02781	inconclusive	4	34	34
chr15	86043888	86045888	Tbc1d22a	-0.11338937	2.13E-10	hypomethylated	-0.020584	0.32454	insignificant	20	102	98
chr15	87454659	87456659	Fam19a5	-0.08992112	8.56E-27	hypomethylated	0.0091979	0.52679	insignificant	66	177	175
chr15	88429731	88431731	Zdhhc25	0.05454732	0.79852	insignificant	0.015868	0.20602	insignificant	14	46	47
chr15	88564649	88566649	Brd1	-0.06935066	3.01E-08	hypomethylated	-0.0042939	0.80881	insignificant	16	54	54
chr15	88581140	88583140	Zbed4	-0.09147157	5.42E-22	hypomethylated	-0.0056645	0.70712	insignificant	52	164	167
chr15	88649075	88651075	Creld2	-0.13206	9.26E-56	hypomethylated	0.004362	0.86036	insignificant	47	175	176
chr15	88649748	88651748	Alg12	-0.20427037	1.05E-49	hypomethylated	-0.02286	0.732	insignificant	37	131	127
chr15	88691623	88693623	Pim3	-0.0866431	5.68E-34	hypomethylated	-0.0080736	0.57111	insignificant	56	204	204
chr15	88812423	88814423	Mov10l1		1	noCoverage	-0.03772	0.03506	inconclusive	0	28	24
chr15	88889155	88891155	Panx2	-0.10446624	1.24E-10	hypomethylated	-0.017174	0.86859	insignificant	10	84	57
chr15	88905493	88907493	Trabd	-0.15749414	9.55E-68	hypomethylated	0.0031448	1	insignificant	39	115	114
chr15	88918536	88920536	1300018J18Ri	-0.12526345	9.79E-41	hypomethylated	-0.0090857	0.50992	insignificant	43	124	104
chr15	88971133	88973133	Mapk12	-0.22773967	0.00000034	hypomethylated	-0.037069	0.25278	insignificant	7	19	18
chr15	88980036	88982036	Mapk11	-0.11748004	1	insignificant	0.043389	0.024415	hypermethylated	3	28	28
chr15	89004280	89006280	Plxnb2	-0.2275222	0.00098121	inconclusive	-0.058208	0.29504	insignificant	7	31	31
chr15	89011218	89013218	Plxnb2	-0.46213417	1	lowCoverage	0.034164	1	insignificant	1	20	19
chr15	89026905	89028905	Fam116b	-0.12666034	9.85E-15	hypomethylated	0.010187	0.30789	insignificant	13	34	34
chr15	89040989	89042989	Ppp6r2	-0.0935755	1.42E-12	hypomethylated	0.0040407	0.47046	insignificant	25	117	119
chr15	89145742	89147742	Sbf1	-0.12759217	0.38432	insignificant	-0.019425	0.018038	hypomethylated	3	18	10
chr15	89152402	89154402	Adm2	0.10150053	1	insignificant	0.017056	0.86823	insignificant	2	20	20
chr15	89163903	89165903	Miox		1	noCoverage	-0.029336	0.72719	insignificant	0	6	6
chr15	89185152	89187152	Ncaph2	-0.13711517	2.81E-38	hypomethylated	0.021039	0.63712	insignificant	23	102	91
chr15	89186090	89188090	Ncaph2	-0.18558654	2.03E-38	hypomethylated	0.019455	0.67784	insignificant	17	68	64
chr15	89204249	89206249	Tymp	-0.37972779	1.15E-20	stronglyHypometh	-0.1168	0.18419	insignificant	7	35	45

chr15	89207468	89209468	Tymp	-0.28179844	4.9E-41	hypomethylated	0.072722	0.00046687	hypermethylated	21	83	83
chr15	89209685	89211685	Odf3b	-0.155	1	insignificant	-0.019583	1	insignificant	4	8	8
chr15	89216321	89218321	Klhdc7b	-0.28522991	4.72E-09	hypomethylated	0.052083	0.00065002	hypermethylated	6	18	20
chr15	89240694	89242694	Syce3		1	noCoverage	-0.16168	0.22211	insignificant	0	8	10
chr15	89256293	89258293	Cpt1b	0.04748653	1	insignificant	-0.0144	0.40454	insignificant	3	21	20
chr15	89260358	89262358	BC090627		1	noCoverage	-0.12291	0.89562	insignificant	0	16	12
chr15	89283341	89285341	Mapk8ip2	-0.17216376	0.000028983	hypomethylated	-0.002284	0.94102	insignificant	8	80	87
chr15	89329287	89331287	Shank3	-0.13436228	1.26E-40	hypomethylated	0.0031404	0.55493	insignificant	63	213	190
chr15	89422354	89424354	Rabl2	-0.12358392	0.034549	hypomethylated	0.0035229	0.62293	insignificant	3	18	18
chr15	90053741	90055741	Alg10b	-0.03057635	0.00031742	hypomethylated	0.080925	0.21584	insignificant	10	34	37
chr15	90509819	90511819	Cpne8	-0.12238829	1.84E-15	hypomethylated	0.0033076	0.70416	insignificant	19	68	68
chr15	90880379	90882379	Kif21a	-0.12629261	0.20917	insignificant	0.0028912	0.22476	insignificant	6	40	40
chr15	91022238	91024238	Abcd2	-0.21922359	1	insignificant	-0.025446	0.36869	insignificant	2	17	16
chr15	91502654	91504654	Lrrk2	-0.13407463	0.053053	insignificant	-0.042204	1	insignificant	9	36	30
chr15	91880595	91882595	Cntn1	-0.20629742	4.29E-11	hypomethylated	-0.049708	0.41242	insignificant	16	50	54
chr15	91990787	91992787	Cntn1	-0.52853522	0.066068	insignificant	-0.085489	0.28657	insignificant	2	20	10
chr15	92226240	92228240	Pdzrn4	-0.06490814	0.089902	insignificant	0.013534	0.97301	insignificant	17	63	63
chr15	92426539	92428539	Pdzrn4	-0.22452381	0.00065241	hypomethylated	-0.013367	1	insignificant	5	10	11
chr15	93105515	93107515	Gxylt1	-0.08597317	1.28E-20	hypomethylated	-0.011092	0.65803	insignificant	20	95	96
chr15	93167366	93169366	Yaf2	-0.13544711	1.34E-20	hypomethylated	0.0076174	0.75196	insignificant	33	83	83
chr15	93227780	93229780	Pphln1	-0.10757383	1.37E-21	hypomethylated	0.0093661	0.18533	insignificant	48	172	172
chr15	93228721	93230721	Zcrb1	-0.18801963	8.84E-22	hypomethylated	0.019773	0.00062418	hypermethylated	24	103	103
chr15	93426322	93428322	Prickle1	-0.17606192	4.53E-10	hypomethylated	-0.022561	0.88659	insignificant	14	51	48
chr15	94234781	94236781	Adamts20	-0.00974784	0.87974	insignificant	0.00069307	0.0013312	inconclusive	27	87	86
chr15	94373090	94375090	Irak4	-0.13995206	2.91E-08	hypomethylated	-0.0070744	0.67561	insignificant	15	83	81
chr15	94373938	94375938	Pus7l	-0.12989198	2.71E-08	hypomethylated	0.0011247	0.3836	insignificant	15	75	75
chr15	94420255	94422255	Twf1	0.25480769	0.01298	hypermethylated	-0.010144	1	insignificant	1	20	19
chr15	94458615	94460615	Tmem117	-0.11119548	7.55E-09	hypomethylated	-0.021677	0.47635	insignificant	36	98	82
chr15	95620273	95622273	Ano6	-0.09871073	3.91E-21	hypomethylated	-0.016531	0.92381	insignificant	41	129	124
chr15	96116952	96118952	Arid2	-0.09626688	1.76E-38	hypomethylated	-0.013514	0.12181	insignificant	104	370	342
chr15	96291274	96293274	Scaf11	-0.06425352	0.0020039	hypomethylated	-0.0068936	0.87284	insignificant	12	35	35
chr15	96472393	96474393	Slc38a1	-0.07830871	0.16562	insignificant	0.026342	0.97003	insignificant	10	89	89
chr15	96472774	96474774	Slc38a1	-0.05482061	0.71278	insignificant	0.017618	0.85829	insignificant	7	49	55
chr15	96530129	96532129	Slc38a2	-0.15385208	0.000019173	hypomethylated	-0.010677	0.0081295	hypomethylated	14	70	68
chr15	96886387	96888387	Slc38a4		1	noCoverage	0.048491	0.87714	insignificant	0	9	10
chr15	97076537	97078537	Fam113b	-0.08951505	2.99E-08	hypomethylated	0.0042777	0.55863	insignificant	49	136	126
chr15	97077718	97079718	Fam113b	-0.09940164	3.33E-08	hypomethylated	-0.004043	0.79201	insignificant	45	116	120
chr15	97536253	97538253	Rpap3	0.53879981	0.00021998	stronglyHypermeth	-0.013281	0.85729	insignificant	2	35	35
chr15	97551581	97553581	Endou	-0.41164622	0.000202	stronglyHypometh	-0.045449	0.62847	insignificant	3	12	12
chr15	97598097	97600097	Rapgef3	-0.2175059	1.18E-18	hypomethylated	0.0052611	0.82018	insignificant	19	72	78
chr15	97613795	97615795	Slc48a1	-0.14512884	2.11E-29	hypomethylated	-0.019495	0.18881	insignificant	31	67	62
chr15	97662102	97664102	Hdac7	-0.07542089	5.54E-14	hypomethylated	0.0058585	0.44009	insignificant	24	60	60
chr15	97738727	97740727	Vdr	-0.07690907	2.98E-11	hypomethylated	0.012525	0.83097	insignificant	18	40	36
chr15	97793709	97795709	Tmem106c	-0.0836207	0.00000301	hypomethylated	0.013626	0.27248	insignificant	19	89	92
chr15	97835155	97837155	Col2a1	-0.25739208	4.68E-14	hypomethylated	0.0062342	0.041486	inconclusive	11	30	30
chr15	97922019	97924019	Pfkm	-0.17671651	2.71E-11	hypomethylated	-0.020432	0.74757	insignificant	12	52	46
chr15	97937901	97939901	Pfkm	-0.15360605	0.0026449	hypomethylated	0.09157	0.033906	hypermethylated	5	18	18
chr15	97941490	97943490	Pfkm	-0.22119309	0.56191	insignificant	0.10922	1	insignificant	1	7	8
chr15	97976133	97978133	Asb8	-0.13752053	0.2229	insignificant	-0.0021039	0.69349	insignificant	1	6	6
chr15	97997236	97999236	AI836003	-0.15138185	7.02E-11	hypomethylated	0.002925	0.4229	insignificant	8	75	75
chr15	98064881	98066881	Olfr286		1	noCoverage	-0.075	0.20767	insignificant	0	4	4
chr15	98087738	98089738	H1fnt		1	noCoverage	-0.037267	1	insignificant	0	7	6
chr15	98126514	98128514	Zfp641	-0.14015246	7.12E-14	hypomethylated	0.0095138	0.79664	insignificant	19	41	40
chr15	98266900	98268900	Olfr282		1	noCoverage	0.12687	1	insignificant	0	8	13
chr15	98285742	98287742	Olfr281	0.09822485	1	insignificant	-0.11383	0.57546	insignificant	2	13	8
chr15	98313114	98315114	Lalba		1	noCoverage	0.13189	0.55594	insignificant	0	8	8
chr15	98326904	98328904	Olfr279	0.08905434	0.77746	insignificant	0.05562	0.00025522	hypermethylated	3	11	8
chr15	98364652	98366652	2310037I24Rik	0.54700651	0.00000125	stronglyHypermeth	-0.037536	0.53646	insignificant	2	43	42
chr15	98396754	98398754	9330020H09Rik	-0.10369301	4.04E-15	hypomethylated	-0.0017577	0.17445	insignificant	24	128	128
chr15	98398067	98400067	9330020H09Rik	-0.15201837	3.7E-15	hypomethylated	-0.00042247	0.51858	insignificant	20	106	104
chr15	98438064	98440064	Adcy6	-0.13869729	3.55E-17	hypomethylated	-0.011768	0.88335	insignificant	23	81	81
chr15	98461650	98463650	Cacnb3	-0.23927025	5.27E-08	hypomethylated	0.0062265	0.85672	insignificant	6	46	45
chr15	98464194	98466194	Cacnb3	-0.15707489	1.14E-64	hypomethylated	0.014516	0.076755	insignificant	50	136	147
chr15	98493320	98495320	Ddx23	-0.27008721	0.00079261	hypomethylated	-0.069721	0.00000914	hypomethylated	9	35	32
chr15	98537657	98539657	Ccdc65		1	noCoverage	0.073446	0.087265	insignificant	0	14	14
chr15	98558629	98560629	Fkbp11	0.19655797	1	insignificant	-0.0012477	0.34746	insignificant	3	6	6
chr15	98593549	98595549	Arf3	-0.10881613	3.08E-15	hypomethylated	0.017733	0.26548	insignificant	13	51	51
chr15	98608581	98610581	Wnt10b	-0.21857017	1.84E-08	hypomethylated	-0.014999	0.92275	insignificant	2	16	12
chr15	98619287	98621287	Wnt1	-0.1652523	0.000000431	hypomethylated	-0.0062118	0.80312	insignificant	18	57	59
chr15	98638356	98640356	Ddn	-0.54	0.000095954	stronglyHypometh	0.033529	0.84107	insignificant	1	2	2
chr15	98661939	98663939	Mll2	-0.17147274	0.093208	insignificant	0.051667	1	insignificant	4	18	18
chr15	98701614	98703614	Mll2	-0.10073092	6.55E-09	hypomethylated	0.0018656	0.23944	insignificant	42	133	145
chr15	98711845	98713845	Rhebl1		1	noCoverage	-0.087155	0.073254	insignificant	0	12	18
chr15	98728971	98730971	Dhh	-0.24941481	7.67E-08	hypomethylated	0.13386	0.12583	insignificant	5	13	26
chr15	98748529	98750529	Lmbr1l	-0.20219576	0.37022	insignificant	0.010799	0.64928	insignificant	7	45	44
chr15	98764821	98766821	Tuba1b	-0.18748261	2.23E-20	hypomethylated	-0.073037	0.33092	insignificant	21	66	69
chr15	98783932	98785932	Tuba1a	0.21668343	0.0039216	hypermethylated	-0.087907	0.0011464	hypomethylated	4	8	8
chr15	98859321	98861321	Tuba1c	-0.15860478	1.11E-18	hypomethylated	0.0099617	0.25316	insignificant	19	94	98
chr15	98884604	98886604	Prph	-0.13966277	1.77E-38	hypomethylated	0.021546	0.17516	insignificant	31	120	120
chr15	98904403	98906403	Troap	-0.2074112	0.00000418	hypomethylated	0.034956	0.7154	insignificant	10	70	70
chr15	98918159	98920159	C1ql4		1	noCoverage	-0.054535	0.54139	insignificant	0	24	24
chr15	98928914	98930914	Dnajc22		1	noCoverage	-0.023683	0.94334	insignificant	0	37	32
chr15	98956275	98958275	Spats2	-0.09457042	1.09E-08	hypomethylated	0.0036583	0.17753	insignificant	36	199	193
chr15	99054406	99056406	Kcnh3	-0.13241552	1.25E-18	hypomethylated	0.0075817	0.078549	insignificant	33	146	144
chr15	99082392	99084392	Mcrs1	-0.25018565	0.00000478	hypomethylated	0.08652	0.94239	insignificant	9	22	25

chr15	99124839	99126839	Prpf40b	-0.17935533	3.46E-20	hypomethylated	-0.011877	0.405	insignificant	21	100	92
chr15	99200897	99202897	Fmnl3	-0.29831554	0.00021532	hypomethylated	0.0030815	0.64637	insignificant	2	15	17
chr15	99222377	99224377	Tmbim6	-0.20555698	8.28E-28	hypomethylated	0.0072565	0.59388	insignificant	25	81	76
chr15	99222649	99224649	Tmbim6	-0.20555698	8.28E-28	hypomethylated	0.0072565	0.59388	insignificant	25	81	76
chr15	99288179	99290179	Nckap5l	-0.20849802	0.0051864	hypomethylated	0.010252	0.55029	insignificant	2	23	16
chr15	99305161	99307161	Bcdin3d	-0.13002512	0.072286	insignificant	-0.033699	0.21056	insignificant	5	9	9
chr15	99358448	99360448	Faim2	-0.2760989	0.12574	insignificant	0.082958	1	insignificant	1	10	12
chr15	99408486	99410486	Aqp2	-0.03511558	0.85649	insignificant	-0.0061406	0.39795	insignificant	4	25	27
chr15	99420458	99422458	Aqp5	-0.11942969	1.58E-15	hypomethylated	0.020834	0.000000124	hypermethylated	51	188	175
chr15	99430830	99432830	Aqp6		1	noCoverage	0.085109	0.05686	insignificant	0	6	6
chr15	99482052	99484052	Racgap1	-0.17683983	0.0012433	hypomethylated	0.057244	0.10417	insignificant	4	14	14
chr15	99500148	99502148	Accn2	-0.0786701	1.31E-19	hypomethylated	0.0076903	0.60439	insignificant	48	164	153
chr15	99531717	99533717	Smarcd1	-0.17556785	4.72E-34	hypomethylated	0.011463	0.68969	insignificant	38	112	111
chr15	99547023	99549023	Gpd1	-0.36273113	0.0082185	stronglyHypometh	0.070291	0.11123	insignificant	2	23	24
chr15	99555048	99557048	2310016M24F	-0.11061199	3.39E-08	hypomethylated	0.0030222	0.57113	insignificant	12	58	64
chr15	99602946	99604946	Lass5	-0.16458042	0.00083971	hypomethylated	-0.039896	0.048252	hypomethylated	4	24	22
chr15	99705887	99707887	Lima1	-0.10187404	0.088013	insignificant	0.10781	0.12979	insignificant	10	31	35
chr15	99762089	99764089	1700030F18Rik		1	noCoverage	0.077073	0.38545	insignificant	0	12	12
chr15	99799504	99801504	Larp4	0.23178672	0.1271	insignificant	-0.018472	0.20135	insignificant	1	22	21
chr15	99802210	99804210	Larp4	-0.13559963	6.39E-29	hypomethylated	0.0096227	1	insignificant	28	92	90
chr15	99803718	99805718	Larp4	-0.23010155	0.00000521	hypomethylated	-0.0074927	0.10644	insignificant	4	28	28
chr15	99868094	99870094	Dip2b	-0.04420701	0.0022141	hypomethylated	0.0086093	0.31784	insignificant	31	154	149
chr15	100057289	100059289	Atf1	-0.06301005	8.08E-19	hypomethylated	0.0093382	0.60649	insignificant	50	182	182
chr15	100110267	100112267	Tmprss12	-0.00092053	0.44108	insignificant	-0.0087978	0.60468	insignificant	4	26	26
chr15	100134247	100136247	Mettl7a1	-0.33618114	0.0096264	stronglyHypometh	0.056759	0.2174	insignificant	4	34	34
chr15	100164359	100166359	Mettl7a3		1	noCoverage	0.029515	0.93997	insignificant	0	28	28
chr15	100182630	100184630	AB099516	0.17054137	2.89E-10	hypermethylated	-0.010591	0.1333	insignificant	9	34	34
chr15	100253486	100255486	Slc11a2	-0.17188111	3.21E-31	hypomethylated	0.0012115	0.55909	insignificant	22	84	84
chr15	100298464	100300464	Letmd1	-0.12139556	3.67E-27	hypomethylated	0.0060126	0.45826	insignificant	35	139	139
chr15	100325670	100327670	Csrnp2	-0.29727368	0.000000626	hypomethylated	-0.036901	0.38391	insignificant	8	33	31
chr15	100382378	100384378	Tfcp2	-0.09449359	0.00000033	hypomethylated	-0.00097964	1	insignificant	15	44	44
chr15	100445092	100447092	Dazap2	-0.15209238	0.37837	insignificant	-0.015842	0.65258	insignificant	11	43	59
chr15	100467286	100469286	Smagp	-0.10701356	0.000006415	hypomethylated	0.058233	0.90083	insignificant	17	47	50
chr15	100467296	100469296	Smagp	-0.11696831	0.00000396	hypomethylated	0.062648	0.90087	insignificant	17	41	44
chr15	100518351	100520351	Cela1	0.04885658	0.73655	insignificant	0.034084	0.7643	insignificant	2	4	4
chr15	100559807	100561807	Galnt6	-0.3110439	0.00033538	hypomethylated	-0.013882	0.035321	hypomethylated	6	37	36
chr15	100591177	100593177	Slc4a8	-0.10329475	1.69E-16	hypomethylated	0.014959	0.11499	insignificant	30	89	90
chr15	100700113	100702113	Scn8a	-0.11444604	2.92E-14	hypomethylated	-0.0013976	0.69253	insignificant	62	163	178
chr15	100765701	100767701	Scn8a	0.0129192	0.17099	insignificant	0.020006	0.40283	insignificant	7	30	30
chr15	100957967	100959967	Acvrl1	-0.23654645	0.00000631	hypomethylated	-0.01248	0.45776	insignificant	12	32	33
chr15	101003555	101005555	Acvr1b	-0.08269653	2.35E-38	hypomethylated	0.012755	0.36639	insignificant	55	181	172
chr15	101053637	101055637	Grasp	-0.16490291	1.1E-31	hypomethylated	0.0040411	0.9638	insignificant	31	66	62
chr15	101096276	101098276	Nr4a1	-0.20990735	1.48E-26	hypomethylated	-0.029816	0.032961	hypomethylated	17	60	69
chr15	101113731	101115731	9430023L20Ri	-0.10520135	9.73E-14	hypomethylated	0.01721	0.047647	hypermethylated	21	80	71
chr15	101122642	101124642	6030408B16Rik		1	noCoverage	0.14214	0.05723	insignificant	0	21	22
chr15	101198782	101200782	Mir1941	-0.10611008	0.21065	insignificant	0.090562	0.25673	insignificant	3	14	14
chr15	101200556	101202556	Krt80	0.05839827	1	insignificant	0.191	0.034736	hypermethylated	2	10	10
chr15	101241833	101243833	Krt7	-0.01598014	0.41745	insignificant	0.0012205	0.19397	insignificant	15	46	47
chr15	101269235	101271235	Krt83	-0.06357278	0.35471	insignificant	-0.011491	0.67403	insignificant	3	22	22
chr15	101277175	101279175	1700011A15Rik		1	noCoverage	0.10129	0.45385	insignificant	0	18	18
chr15	101294196	101296196	Krt81		1	noCoverage	0.049052	0.59448	insignificant	0	2	2
chr15	101302908	101304908	Krt86		1	noCoverage	-0.0125	0.85232	insignificant	0	4	4
chr15	101333246	101335246	Gm6042	0.03494125	1	insignificant	-0.023882	0.61009	insignificant	2	14	12
chr15	101363251	101365251	Krt84	-0.38970814	0.000024444	stronglyHypometh	-0.014484	0.28017	insignificant	1	6	6
chr15	101381090	101383090	Krt82		1	noCoverage	-0.0041667	1	insignificant	0	4	4
chr15	101393381	101395381	Krt85		1	noCoverage	-0.082464	0.35956	insignificant	0	6	6
chr15	101419003	101421003	Krt85	0.10277778	1	insignificant	-0.044692	0.52788	insignificant	1	12	24
chr15	101524736	101526736	Krt6a	-0.11167735	0.53638	insignificant	0.012313	0.012389	hypermethylated	1	10	10
chr15	101543322	101545322	Krt5	-0.19962574	0.72279	insignificant	0.01775	0.33339	insignificant	4	10	10
chr15	101593935	101595935	Krt74		1	noCoverage	0.097368	0.89795	insignificant	0	7	7
chr15	101681217	101683217	Krt1	-0.22119151	0.43208	insignificant	0.061917	0.082038	insignificant	3	21	21
chr15	101700049	101702049	Krt77	-0.82413547	0.19231	lowCoverage	0.051282	0.18635	insignificant	1	6	6
chr15	101755166	101757166	Krt4	-0.27272727	0.40911	insignificant	-0.0093704	0.68884	insignificant	1	4	4
chr15	101834773	101836773	Krt8	-0.38988095	0.30818	insignificant	-0.28508	0.63571	insignificant	2	6	8
chr15	101857646	101859646	Krt18	-0.287509	5.14E-15	hypomethylated	0.0061141	0.61653	insignificant	17	78	81
chr15	101903203	101905203	Eif4b	-0.10113095	0.000010939	hypomethylated	-0.027215	0.60092	insignificant	6	32	47
chr15	101932418	101934418	Tenc1		1	noCoverage	0.12928	0.06248	insignificant	0	10	8
chr15	101966646	101968646	Spryd3	-0.14354425	3.09E-08	hypomethylated	-0.019734	0.65572	insignificant	12	50	48
chr15	101973616	101975616	Igfbp6	-0.1000006	0.00000213	hypomethylated	0.031802	0.000000729	hypermethylated	9	54	55
chr15	102037697	102039697	Zfp740	-0.3916532	0.003088	stronglyHypometh	0.035998	0.42975	insignificant	2	10	10
chr15	102062366	102064366	Itgb7		1	noCoverage	-0.19971	0.35471	insignificant	0	17	14
chr15	102076783	102078783	Rarg	-0.12582952	0.0072509	hypomethylated	0.029829	0.9059	insignificant	3	60	52
chr15	102087914	102089914	Rarg	-0.13953655	0.018097	inconclusive	-0.028769	0.13296	insignificant	4	17	22
chr15	102108886	102110886	Mfsd5	-0.08417605	0.00000126	hypomethylated	-0.0092385	0.16046	insignificant	39	122	122
chr15	102125723	102127723	Espl1	-0.18808917	0.070763	insignificant	0.0079228	0.8005	insignificant	15	50	49
chr15	102155546	102157546	Pfdn5	-0.13915012	4.36E-09	hypomethylated	0.022754	0.36657	insignificant	14	36	40
chr15	102161139	102163139	Myg1	-0.29703307	0.0021684	hypomethylated	-0.01508	0.83272	insignificant	10	32	32
chr15	102181190	102183190	Aaas	-0.1325	0.59185	insignificant	0.055	0.57342	insignificant	5	10	10
chr15	102196702	102198702	Sp7		1	noCoverage	0.10607	0.31373	insignificant	0	8	8
chr15	102235746	102237746	Sp1	-0.11070497	1.64E-13	hypomethylated	0.0053559	0.85906	insignificant	27	152	139
chr15	102288600	102290600	Prr13	-0.33486399	5.31E-08	stronglyHypometh	0.025548	0.79541	insignificant	6	18	18
chr15	102300062	102302062	Pcbp2	-0.11923692	2.25E-31	hypomethylated	0.0011718	0.7086	insignificant	28	143	138
chr15	102347435	102349435	Map3k12	-0.16607855	2.67E-27	hypomethylated	0.0076067	0.33723	insignificant	17	50	52
chr15	102347676	102349676	Tarbp2	-0.14287685	4.01E-16	hypomethylated	0.01743	0.91484	insignificant	8	42	44
chr15	102355052	102357052	Npff	-0.34966284	0.0034718	stronglyHypometh	0.0026442	0.27355	insignificant	4	8	8

chr15	102355373	102357373	Npff	-0.19932567	0.0075721	hypomethylated	0.075	0.42763	insignificant	2	4	4
chr15	102455852	102457852	Atf7	-0.13850209	1.14E-08	hypomethylated	0.011259	0.77633	insignificant	7	26	26
chr15	102501478	102503478	Atp5g2	-0.14622467	0.000020071	hypomethylated	-0.0039053	0.38777	insignificant	4	57	57
chr15	102502297	102504297	Atp5g2	-0.23608059	0.04477	hypomethylated	0.0024151	0.66781	insignificant	3	28	29
chr15	102552609	102554609	Calcoco1	-0.13449939	0.00074046	inconclusive	0.020103	0.080014	insignificant	6	15	16
chr15	102750561	102752561	Hoxc13	-0.14080733	2.45E-24	hypomethylated	-0.0046102	0.4117	insignificant	42	148	144
chr15	102766283	102768283	Hoxc12		1	noCoverage	-0.06732	0.44274	insignificant	0	55	32
chr15	102783956	102785956	Hoxc11	-0.15616025	4.04E-21	hypomethylated	0.0057471	0.86017	insignificant	34	93	95
chr15	102796295	102798295	Hoxc10	-0.2243052	0.00000283	hypomethylated	0.012731	0.58733	insignificant	18	69	64
chr15	102802780	102804780	Mir196a-2	-0.23409436	2.8E-11	hypomethylated	0.025013	0.26095	insignificant	13	38	38
chr15	102806462	102808462	Hoxc9	-0.14048379	0.000019286	hypomethylated	0.0038078	0.92867	insignificant	6	67	64
chr15	102819969	102821969	Hoxc8	-0.16845799	3.72E-18	hypomethylated	0.022973	0.40326	insignificant	23	58	58
chr15	102838992	102840992	Hoxc6	-0.19293398	0.31688	insignificant	0.0076506	0.26846	insignificant	9	20	20
chr15	102843438	102845438	Hoxc5	-0.09938509	0.00000044	hypomethylated	0.15002	0.10379	insignificant	9	30	31
chr15	102844340	102846340	Mir615	-0.09035113	0.000035904	hypomethylated	0.024793	0.1049	insignificant	19	84	84
chr15	102863825	102865825	Hoxc4	-0.18549262	1.2E-12	hypomethylated	0.022599	0.22795	insignificant	14	65	65
chr15	102993715	102995715	Smug1	-0.24234458	0.0031949	hypomethylated	0.022005	0.61289	insignificant	8	20	20
chr15	103085847	103087847	Nfe2	-0.25741356	1.84E-20	hypomethylated	-0.023025	0.64434	insignificant	7	14	14
chr15	103102348	103104348	Copz1	-0.2920853	0.000074398	hypomethylated	0.049223	0.67492	insignificant	6	19	19
chr15	103170517	103172517	Zfp385a	-0.29610989	2.73E-08	hypomethylated	-0.051809	0.02645	inconclusive	6	25	44
chr15	103283255	103285255	Nckap1l	-0.24464286	0.015332	hypomethylated	-0.040861	0.5931	insignificant	4	8	8
chr15	103332728	103334728	Pde1b	-0.13212945	0.000076596	hypomethylated	0.023493	0.4945	insignificant	5	29	36
chr15	103368423	103370423	Ppp1r1a	-0.03629164	0.45691	insignificant	0.011017	0.57122	insignificant	7	26	26
chr16	3743098	3745098	Zfp263	-0.17697865	5.62E-30	hypomethylated	-0.001485	0.51726	insignificant	17	84	84
chr16	3846222	3848222	Zfp174	-0.0430746	0.28152	insignificant	0.0042853	0.96315	insignificant	7	53	48
chr16	3872374	3874374	Zfp597	-0.16824492	0.015761	hypomethylated	-0.02907	0.55048	insignificant	7	39	42
chr16	3883618	3885618	Nat15	-0.14718546	4.61E-29	hypomethylated	0.01501	0.3439	insignificant	39	105	105
chr16	3908008	3910008	Cluap1	-0.1744918	2.74E-10	hypomethylated	-0.0033869	0.32154	insignificant	20	66	68
chr16	3908689	3910689	1700037C18Ri	-0.18485017	3.26E-09	hypomethylated	0.0010187	0.78853	insignificant	14	48	50
chr16	4001680	4003680	Slx4		1	noCoverage	0.0045455	0.87344	insignificant	0	10	10
chr16	4077810	4079810	Trap1	-0.58952958	1.02E-50	stronglyHypometh	-0.069281	0.47881	insignificant	9	25	24
chr16	4213404	4215404	Crebbp	-0.07953761	1.43E-09	hypomethylated	0.0041307	0.10533	insignificant	48	154	164
chr16	4419587	4421587	Adcy9	-0.06737456	1.12E-24	hypomethylated	-0.0018355	0.068477	insignificant	64	218	218
chr16	4523053	4525053	Srl	-0.92424242	0.090909	lowCoverage	0.004329	0.72922	insignificant	1	9	6
chr16	4559720	4561720	Tfap4	-0.27434247	0.11324	insignificant	0.016663	0.86376	insignificant	7	60	59
chr16	4593712	4595712	Glis2	-0.16196785	1.47E-37	hypomethylated	0.0040829	1	insignificant	32	151	156
chr16	4624946	4626946	Pam16	-0.26875	1	lowCoverage	0.36458	0.013957	stronglyhypermeth	0	14	0
chr16	4638944	4640944	Vasn		1	noCoverage	0.035856	0.3945	insignificant	0	8	8
chr16	4679720	4681720	Coro7		1	noCoverage	0.056255	0.2083	insignificant	0	5	5
chr16	4683069	4685069	Dnaja3	-0.1982951	4.93E-11	hypomethylated	0.010043	0.51717	insignificant	13	54	58
chr16	4725360	4727360	Hmox2	-0.18363703	2.62E-27	hypomethylated	0.0071942	0.8286	insignificant	19	64	64
chr16	4789935	4791935	5730403B10R	-0.09529835	3.36E-09	hypomethylated	-0.0018826	0.81634	insignificant	15	48	48
chr16	4834415	4836415	4930562C15Rik		1	noCoverage	0.0051824	0.59796	insignificant	0	13	12
chr16	4879851	4881851	Fam100a	-0.14189256	7.51E-20	hypomethylated	0.024319	0.90485	insignificant	24	81	77
chr16	4885251	4887251	Mgrn1	-0.15509833	8.27E-25	hypomethylated	-0.0036211	0.43523	insignificant	31	132	124
chr16	4938110	4940110	Nudt16l1	-0.16569401	2.49E-21	hypomethylated	-0.030184	0.73802	insignificant	8	28	35
chr16	4964330	4966330	Anks3	0.02873749	0.000000178	inconclusive	0.14898	0.49158	insignificant	7	22	14
chr16	5013646	5015646	Rogdi	-0.03997761	0.30527	insignificant	0.033878	0.81058	insignificant	5	27	29
chr16	5049160	5051160	Ubn1	-0.09541532	3.13E-43	hypomethylated	0.00078357	0.48498	insignificant	71	236	225
chr16	5050003	5052003	Ubn1	-0.09653174	1.98E-33	hypomethylated	0.011658	0.15522	insignificant	56	164	154
chr16	5132574	5134574	Ppl	-0.14029251	0.023244	hypomethylated	0.014887	0.31735	insignificant	9	34	34
chr16	5146201	5148201	Sec14l5	-0.08981118	0.0056354	hypomethylated	0.022243	0.022984	inconclusive	9	56	57
chr16	5204105	5206105	Nagpa	-0.37578311	0.012329	stronglyHypometh	0.040025	0.90841	insignificant	1	10	10
chr16	5256049	5258049	Fam86	-0.18424717	2.94E-15	hypomethylated	-0.0065399	0.66394	insignificant	7	25	25
chr16	5883885	5885885	Rbfox1	-0.14947631	1.4E-11	hypomethylated	-0.011929	0.55468	insignificant	14	85	85
chr16	7068927	7070927	Rbfox1		1	noCoverage	0.087553	0.53381	insignificant	0	6	6
chr16	8469905	8471905	Mettl22	-0.14184733	0.65166	insignificant	-0.0061289	0.14884	insignificant	16	58	59
chr16	8512521	8514521	Abat	-0.18506664	0.00046336	hypomethylated	-0.0025562	0.91839	insignificant	13	65	60
chr16	8636799	8638799	Pmm2	-0.13192188	6.26E-49	hypomethylated	0.010275	0.3061	insignificant	30	87	87
chr16	8637794	8639794	Tmem186	-0.35231614	1.45E-21	stronglyHypometh	-0.0099899	1	insignificant	11	38	40
chr16	8672246	8674246	Carhsp1	-0.11078557	0.000001718	hypomethylated	0.010142	0.82668	insignificant	19	58	58
chr16	8738435	8740435	Usp7	-0.06642626	3.4E-24	hypomethylated	-0.013282	0.08095	insignificant	37	220	202
chr16	8829192	8831192	1810013L24Ri	-0.11876295	4.3E-23	hypomethylated	0.0036278	0.20675	insignificant	49	212	213
chr16	9992626	9994626	Grin2a	-0.30277318	0.0066985	hypomethylated	-0.02128	0.31218	insignificant	5	57	55
chr16	10169320	10171320	Rpl39l	-0.16243129	0.00000021	hypomethylated	-0.11153	0.03558	hypomethylated	8	42	42
chr16	10191998	10193998	Atf7ip2	-0.10029013	0.019602	hypomethylated	0.010577	0.65706	insignificant	20	78	78
chr16	10314061	10316061	Emp2	-0.11830357	0.13184	insignificant	0.041388	0.3194	insignificant	5	16	16
chr16	10395541	10397541	Tekt5		1	noCoverage	-0.01472	1	insignificant	0	14	14
chr16	10411030	10413030	Nubp1	-0.20378656	4.34E-26	hypomethylated	-0.0076273	0.81503	insignificant	17	54	56
chr16	10447443	10449443	Fam18a	-0.55347744	1	lowCoverage	0.031038	0.15925	insignificant	1	13	12
chr16	10487371	10489371	Ciita	0.32952977	0.11654	insignificant	0.040192	0.048472	hypermethylated	2	14	14
chr16	10543147	10545147	Dexi	-0.3632977	0.0004524	stronglyHypometh	-0.041538	0.75893	insignificant	6	19	18
chr16	10544479	10546479	Clec16a	-0.09227383	1.66E-12	hypomethylated	-0.00026096	0.31009	insignificant	21	115	115
chr16	10785629	10787629	Socs1	-0.17338152	5.62E-10	hypomethylated	-0.0049403	0.72724	insignificant	13	66	66
chr16	10788748	10790748	Prm3		1	noCoverage	0.041667	0.14224	insignificant	0	0	0
chr16	10796916	10798916	Prm1		1	noCoverage	0.017628	0.19997	insignificant	0	4	4
chr16	10834151	10836151	A630055G03R	-0.12350501	1.1E-17	hypomethylated	-0.0022479	0.013746	inconclusive	22	85	84
chr16	11065390	11067390	Snn	-0.11681014	3.59E-22	hypomethylated	0.0005624	0.95078	insignificant	29	130	126
chr16	11088023	11090023	Txndc11	-0.0254903	0.50858	insignificant	-0.084139	0.059818	insignificant	1	8	8
chr16	11134625	11136625	Txndc11	-0.10296762	1.47E-20	hypomethylated	0.00079316	0.35756	insignificant	25	72	68
chr16	11176486	11178486	Zc3h7a	-0.2780103	0.095722	insignificant	-0.0097104	0.52569	insignificant	5	24	24
chr16	11203385	11205385	Rsl1d1	-0.19557723	0.000000334	hypomethylated	0.029198	1	insignificant	9	32	32
chr16	11254418	11256418	Gspt1	-0.08004597	0.3394	insignificant	0.0023011	0.42283	insignificant	17	63	63
chr16	11254538	11256538	Gspt1	-0.10426844	0.041737	hypomethylated	0.0057834	0.6607	insignificant	11	44	44
chr16	11312901	11314901	Tnfrsf17		1	noCoverage	-0.050559	1	insignificant	0	4	5

chr16	11321984	11323984	Snx29	-0.18377886	4.4E-30	hypomethylated	0.0035197	0.05299	insignificant	37	130	114
chr16	11404740	11406740	Snx29	-0.04691173	0.81572	insignificant	0.051433	0.014392	hypermethylated	3	10	8
chr16	11909516	11911516	Cpped1	-0.18211118	0.0019188	hypomethylated	0.0041455	0.57917	insignificant	3	12	12
chr16	11983205	11985205	Shisa9	-0.11322558	4.02E-20	hypomethylated	-0.0089631	0.94716	insignificant	23	168	168
chr16	13108828	13110828	Ercc4	-0.09962132	1.1E-23	hypomethylated	0.008176	0.12892	insignificant	20	75	75
chr16	13255573	13257573	Mkl2	-0.11304616	5.15E-16	hypomethylated	0.012029	0.15039	insignificant	54	162	157
chr16	13448615	13450615	Mir193b	-0.20810894	7.17E-16	hypomethylated	-0.011366	0.019339	hypomethylated	17	45	44
chr16	13452932	13454932	Mir365-1	0.01190476	0.32077	insignificant	-0.11555	0.38098	insignificant	2	4	7
chr16	13668263	13670263	Parn	-0.46779072	3.57E-12	stronglyHypometh	-0.011494	0.57818	insignificant	4	20	20
chr16	13670950	13672950	Bfar	-0.19829359	1.26E-09	hypomethylated	0.03509	0.81906	insignificant	22	81	78
chr16	13671112	13673112	3110001I22Ril	-0.1512467	0.000000419	hypomethylated	0.022783	0.93407	insignificant	22	75	72
chr16	13779791	13781791	Rrn3	-0.13486867	1.04E-14	hypomethylated	-0.0054656	0.36524	insignificant	22	63	63
chr16	13818369	13820369	Ntan1	-0.12947825	0.6021	insignificant	-0.013176	0.21568	insignificant	7	47	46
chr16	13903228	13905228	Pdxdc1	-0.16737955	0.000000328	hypomethylated	0.066533	0.0023975	hypermethylated	7	46	40
chr16	13939787	13941787	Mpv17l	-0.21477135	7.77E-08	hypomethylated	-0.027996	0.9396	insignificant	11	58	67
chr16	13984714	13986714	2900011O08Rik		1	noCoverage	-0.028185	0.23508	insignificant	0	13	12
chr16	13985729	13987729	2900011O08Rik		1	noCoverage	-0.012333	0.86691	insignificant	0	25	26
chr16	14158718	14160718	Mir484	-0.1602665	2.87E-14	hypomethylated	-0.025562	0.89918	insignificant	12	30	34
chr16	14159367	14161367	4921513D23Rik		1	noCoverage	0.10787	0.11439	insignificant	0	12	12
chr16	14162367	14164367	Nde1	-0.02876295	5.96E-09	hypomethylated	0.025612	0.35993	insignificant	12	111	94
chr16	14291501	14293501	Myh11	-0.20213613	0.21082	insignificant	0.15087	0.011943	hypermethylated	2	18	8
chr16	14317425	14319425	0610037P05Ri	-0.33923293	0.61061	insignificant	0.04354	0.63965	insignificant	4	22	20
chr16	14360652	14362652	Abcc1	-0.08629209	3.4E-20	hypomethylated	-0.007258	0.30286	insignificant	23	79	79
chr16	14561409	14563409	A630010A05Rik		1	noCoverage	0.036607	0.20619	insignificant	0	6	6
chr16	14704951	14706951	Snai2		1	noCoverage	-0.090909	0.35925	insignificant	0	6	6
chr16	15594611	15596611	Ube2v2	-0.58904933	1.75E-08	stronglyHypometh	0.059952	0.62653	insignificant	1	20	17
chr16	15636958	15638958	Prkdc	-0.10420309	0.38534	insignificant	-0.0064741	0.73071	insignificant	32	121	117
chr16	15637493	15639493	Mcm4	-0.1567137	3.97E-18	hypomethylated	-0.020068	0.90289	insignificant	28	72	70
chr16	15863415	15865415	Mzt2	-0.22667842	0.000013639	hypomethylated	0.017154	0.39407	insignificant	5	26	25
chr16	15886378	15888378	Cebpd	-0.11276446	3.33E-45	hypomethylated	0.00066748	0.55006	insignificant	68	217	214
chr16	16212437	16214437	Pkp2	-0.15647359	2.61E-16	hypomethylated	0.017134	0.4687	insignificant	24	58	57
chr16	16302074	16304074	Yars2	-0.08565723	0.00000198	hypomethylated	0.00032543	0.071608	insignificant	18	113	119
chr16	16359123	16361123	Dnm1l	-0.51060067	2.44E-28	stronglyHypometh	-0.04755	0.7512	insignificant	6	21	23
chr16	16829456	16831456	Spag6	0.26833333	0.00241	hypermethylated	0.0064799	0.72384	insignificant	2	8	8
chr16	16864078	16866078	Igll1	-0.7037037	0.4359	lowCoverage	-0.096561	0.097059	insignificant	1	2	2
chr16	16869348	16871348	Top3b	-0.16544556	1.04E-27	hypomethylated	-0.0021523	0.41465	insignificant	16	50	49
chr16	16869983	16871983	Top3b	-0.17520717	7.58E-34	hypomethylated	0.0052215	0.032729	hypermethylated	19	70	67
chr16	16895561	16897561	Ppm1f	-0.24855315	0.000000859	hypomethylated	-0.060135	0.87327	insignificant	7	30	30
chr16	16982474	16984474	Mapk1	-0.09809905	2.9E-22	hypomethylated	0.0026156	0.17739	insignificant	42	136	136
chr16	17069403	17071403	Ypel1	-0.0918792	0.0018574	hypomethylated	0.0048116	0.6949	insignificant	13	73	66
chr16	17111306	17113306	Ppil2	-0.6022409	0.34066	lowCoverage	0.059383	0.0020946	hypermethylated	1	16	15
chr16	17124235	17126235	Mir130b		1	noCoverage	-0.040899	0.61653	insignificant	0	8	8
chr16	17124589	17126589	Mir130b		1	noCoverage	-0.021199	0.8002	insignificant	0	6	6
chr16	17132476	17134476	Sdf2l1	-0.29861111	0.0037501	hypomethylated	-0.029762	0.68703	insignificant	4	8	8
chr16	17144302	17146302	Ccdc116	-0.51592846	0.000000323	stronglyHypometh	-0.00048601	0.55223	insignificant	2	6	6
chr16	17146071	17148071	Ydjc	-0.12895919	3.01E-10	hypomethylated	0.023016	0.015017	hypermethylated	24	80	78
chr16	17201585	17203585	Ube2l3		1	noCoverage	0.0015563	0.37817	insignificant	0	13	14
chr16	17207227	17209227	Rimbp3	-0.12371364	4.42E-23	hypomethylated	-0.0052286	0.56582	insignificant	29	103	99
chr16	17232679	17234679	Hic2	-0.11956554	5.53E-16	hypomethylated	-0.02278	0.17194	insignificant	26	100	100
chr16	17275392	17277392	Tmem191c	-0.17615594	0.000023749	hypomethylated	0.00142	0.39877	insignificant	11	71	68
chr16	17405092	17407092	Snap29	-0.12226911	1.05E-19	hypomethylated	-0.019001	0.27108	insignificant	29	121	114
chr16	17406407	17408407	Snap29	-0.02936054	0.66871	insignificant	-0.17894	0.60773	insignificant	3	17	12
chr16	17451079	17453079	Crkl	-0.1570213	4.77E-08	hypomethylated	-0.0070037	0.46213	insignificant	13	133	127
chr16	17488783	17490783	Aifm3	-0.28400522	5.92E-25	hypomethylated	0.01006	0.46533	insignificant	17	44	44
chr16	17508063	17510063	Lztr1	-0.15348872	4.75E-14	hypomethylated	0.0026841	0.3362	insignificant	24	87	86
chr16	17531145	17533145	Thap7	-0.23521463	0.34488	insignificant	-0.013449	0.014203	inconclusive	7	33	36
chr16	17560977	17562977	P2rx6	-0.21178143	0.35633	insignificant	-0.014137	0.86587	insignificant	7	28	28
chr16	17561340	17563340	P2rx6	-0.13351782	0.60132	insignificant	-0.015934	0.66636	insignificant	7	20	20
chr16	17576764	17578764	Slc7a4	-0.57916667	0.42222	lowCoverage	0.062545	0.68146	insignificant	1	4	4
chr16	17618446	17620446	Smpd4	-0.13516346	1.23E-19	hypomethylated	0.0068971	0.76702	insignificant	17	82	82
chr16	17645562	17647562	Ccdc74a	-0.15602153	5.56E-15	hypomethylated	0.024706	0.0050486	hypermethylated	31	132	123
chr16	17758713	17760713	Klhl22	-0.14345326	2.44E-28	hypomethylated	0.012361	0.62154	insignificant	29	119	117
chr16	17796374	17798374	Scarf2	-0.1457631	1.01E-31	hypomethylated	0.0073603	0.20777	insignificant	56	171	169
chr16	17832211	17834211	B830017H08R	-0.08165053	0.000000725	hypomethylated	0.0087603	0.25077	insignificant	39	170	176
chr16	17893295	17895295	Tssk1	-0.00342532	0.00012198	hypomethylated	0.036326	0.00011954	hypermethylated	9	47	47
chr16	17897729	17899729	Tssk2	-0.06808194	0.0079319	hypomethylated	-0.1203	0.091046	insignificant	3	13	13
chr16	17911441	17913441	Dgcr14	-0.62433862	0.0011526	stronglyHypometh	0.061936	0.052871	insignificant	1	2	3
chr16	17915152	17917152	Gsc2	-0.1887645	0.42946	insignificant	-0.027214	0.031969	inconclusive	4	23	23
chr16	17928312	17930312	Slc25a1	-0.13867404	2.65E-20	hypomethylated	0.0235	0.89105	insignificant	19	54	56
chr16	17979657	17981657	Vpreb2	0.04075611	1	insignificant	0.030908	0.1112	insignificant	5	10	10
chr16	18051952	18053952	Dgcr6	-0.40408117	0.0086058	stronglyHypometh	-0.02767	0.91568	insignificant	3	8	8
chr16	18089283	18091283	Prodh	-0.30268664	0.000010392	hypomethylated	0.011414	0.76854	insignificant	5	24	24
chr16	18126798	18128798	Rtn4r	-0.11706296	1.27E-25	hypomethylated	0.0036264	0.85454	insignificant	50	171	167
chr16	18213626	18215626	4933432I09Ril	0.11601439	0.36409	insignificant	0.053554	0.27321	insignificant	4	13	12
chr16	18213956	18215956	4933432I09Ril	0.10074428	0.35629	insignificant	0.035792	0.47861	insignificant	4	11	10
chr16	18235229	18237229	Zdhhc8	-0.29702222	0.0067394	hypomethylated	0.060762	0.040569	hypermethylated	6	45	37
chr16	18247975	18249975	Trmt2a	-0.11235517	9.32E-37	hypomethylated	0.011385	0.077814	insignificant	33	127	127
chr16	18248787	18250787	Ranbp1	-0.12746195	6.78E-23	hypomethylated	0.01763	0.40093	insignificant	14	42	42
chr16	18289261	18291261	Dgcr8	-0.11084205	0.0026858	hypomethylated	-0.011168	0.91073	insignificant	15	78	78
chr16	18344025	18346025	D16H22S680E	-0.10455607	8.54E-13	hypomethylated	-0.0073702	0.34482	insignificant	7	30	30
chr16	18347366	18349366	Arvcf	-0.09034843	0.00039898	hypomethylated	0.0082304	0.41524	insignificant	27	108	108
chr16	18425509	18427509	Txnrd2	-0.06715894	0.000037171	hypomethylated	0.0077356	0.68064	insignificant	11	50	50
chr16	18497860	18499860	Gnb1l	-0.08783831	0.00000262	hypomethylated	-0.0032729	1	insignificant	18	107	107
chr16	18497963	18499963	Gnb1l	-0.08783831	0.00000262	hypomethylated	-0.0032729	1	insignificant	18	107	107
chr16	18587062	18589062	Tbx1	-0.06024888	0.00000708	hypomethylated	0.068578	0.54579	insignificant	29	105	104

chr16	18622496	18624496	Gp1bb	-0.18312156	0.0015802	hypomethylated	-0.009073	0.24724	insignificant	3	49	50
chr16	18630031	18632031	###########	-0.09976578	0.000001059	hypomethylated	0.036108	0.087125	insignificant	22	56	60
chr16	18775939	18777939	Cldn5	-0.1298833	0.47837	insignificant	0.0018565	0.92273	insignificant	20	99	97
chr16	18811386	18813386	Ufd1l	-0.00272184	0.11369	insignificant	0.0039951	0.87922	insignificant	12	91	94
chr16	18811732	18813732	Cdc45	-0.03901065	0.050898	insignificant	-0.0039161	0.71391	insignificant	12	74	78
chr16	18812065	18814065	Cdc45	-0.02580506	0.061355	insignificant	-0.010098	0.93323	insignificant	10	67	70
chr16	18835672	18837672	2510002D24R	-0.19192967	0.000015019	hypomethylated	0.003296	0.74142	insignificant	10	32	32
chr16	18875842	18877842	Hira	-0.11622768	3.65E-36	hypomethylated	0.00027655	0.32537	insignificant	51	160	168
chr16	18876730	18878730	Mrpl40	-0.10426185	2.56E-32	hypomethylated	-0.002857	0.36684	insignificant	46	149	157
chr16	19759326	19761326	B3gnt5	-0.21538956	1.8E-14	hypomethylated	0.0056307	0.73226	insignificant	13	46	50
chr16	20096626	20098626	Klhl24	-0.14153226	1.19E-23	hypomethylated	-0.0063104	0.060491	insignificant	34	109	106
chr16	20140135	20142135	Yeats2	-0.11902171	0.000000184	hypomethylated	0.0002107	0.73266	insignificant	24	90	104
chr16	20241431	20243431	Map6d1		1	noCoverage	-0.030246	1	insignificant	0	6	3
chr16	20302435	20304435	Parl	-0.21379114	0.00000495	hypomethylated	-0.0085	0.52382	insignificant	6	16	16
chr16	20426467	20428467	Abcc5	-0.26537219	5.39E-17	hypomethylated	0.013435	0.60676	insignificant	14	51	49
chr16	20497889	20499889	Eif2b5	-0.25410709	2.08E-11	hypomethylated	-0.0057857	0.069516	insignificant	11	63	67
chr16	20516136	20518136	Dvl3	-0.16775917	3.41E-12	hypomethylated	0.0079084	0.17668	insignificant	21	68	67
chr16	20534576	20536576	Ap2m1	-0.20690946	5.92E-10	hypomethylated	0.034204	0.0073415	hypermethylated	15	60	60
chr16	20547675	20549675	Abcf3	0.03665147	0.21315	insignificant	-0.061298	0.02138	hypomethylated	5	28	34
chr16	20548629	20550629	Gm15760	-0.41345316	0.11152	insignificant	-0.14274	0.090951	insignificant	3	27	32
chr16	20588654	20590654	Vwa5b2	-0.11913685	0.000079818	hypomethylated	0.0054194	0.72179	insignificant	9	24	24
chr16	20603524	20605524	Mir1224	-0.18059147	0.071316	insignificant	-0.093902	0.41244	insignificant	3	8	6
chr16	20610673	20612673	Ece2	-0.19607387	0.000000211	hypomethylated	-0.019985	0.21258	insignificant	11	67	64
chr16	20610822	20612822	Ece2	-0.20316065	0.000000706	hypomethylated	-0.02736	0.14199	insignificant	7	53	50
chr16	20621351	20623351	Camk2n2	-0.11648956	2.43E-20	hypomethylated	-0.012932	0.31244	insignificant	20	81	86
chr16	20628923	20630923	Ece2	-0.16463455	2.51E-08	hypomethylated	-0.0076219	0.50168	insignificant	19	70	70
chr16	20650724	20652724	Psmd2	-0.17087346	5.15E-16	hypomethylated	0.01013	0.311	insignificant	14	70	69
chr16	20671821	20673821	Eif4g1	-0.13199705	2.03E-34	hypomethylated	-0.0093121	0.84299	insignificant	33	128	104
chr16	20694129	20696129	Fam131a	-0.28833333	0.09237	insignificant	0.049069	0.65395	insignificant	1	20	29
chr16	20716709	20718709	Polr2h	-0.10179096	0.00034154	hypomethylated	-0.0073786	0.95557	insignificant	9	74	73
chr16	20716898	20718898	Polr2h	-0.07720861	0.0030706	hypomethylated	-0.0073234	0.79506	insignificant	10	76	75
chr16	20730671	20732671	Thpo	-0.02357827	0.49449	insignificant	-0.10081	0.73878	insignificant	2	8	8
chr16	20732199	20734199	Chrd	-0.13859473	8.81E-32	hypomethylated	-0.016717	0.74823	insignificant	42	111	110
chr16	20734584	20736584	Thpo	-0.1819766	0.00013482	hypomethylated	-0.020404	0.78801	insignificant	7	16	16
chr16	21203867	21205867	Ephb3	-0.16143217	1.05E-20	hypomethylated	-0.0136	0.72244	insignificant	42	123	124
chr16	21422190	21424190	Vps8	-0.1259761	8.31E-16	hypomethylated	0.0070599	0.74572	insignificant	17	87	86
chr16	21694738	21696738	2510009E07Ri	-0.11212559	3.43E-14	hypomethylated	0.012123	0.81541	insignificant	17	49	49
chr16	21891041	21893041	Map3k13		1	noCoverage	-0.40455	0.0016737	stronglyhypometh	0	6	6
chr16	21947617	21949617	Tmem41a		1	noCoverage	0.33561	0.18697	insignificant	0	9	6
chr16	21995523	21997523	Liph		1	noCoverage	-0.14672	0.15595	insignificant	0	4	4
chr16	21995615	21997615	Liph		1	noCoverage	-0.14672	0.15595	insignificant	0	4	4
chr16	22008556	22010556	Senp2	-0.15494223	3.1E-26	hypomethylated	-0.04186	0.81271	insignificant	30	108	104
chr16	22163372	22165372	Igf2bp2	-0.15070271	0.041906	hypomethylated	-0.022854	0.24659	insignificant	8	30	30
chr16	22266002	22268002	Tra2b	-0.37835625	3.85E-17	stronglyHypometh	-0.082619	0.87027	insignificant	8	44	43
chr16	22439643	22441643	Etv5	-0.22328267	0.0083033	hypomethylated	0.0049491	0.50967	insignificant	13	65	60
chr16	22657304	22659304	Dgkg	-0.19035604	0.033019	hypomethylated	0.0028083	0.56399	insignificant	3	6	6
chr16	22856917	22858917	Dnajb11	-0.10068108	1.43E-10	hypomethylated	0.014062	0.11397	insignificant	24	77	67
chr16	22857642	22859642	Dnajb11	-0.10212987	1.43E-10	hypomethylated	0.012613	0.11402	insignificant	24	76	67
chr16	23057372	23059372	Kng1		1	noCoverage	0.13319	0.063138	insignificant	0	8	8
chr16	23106551	23108551	Eif4a2	-0.09991164	6.58E-22	hypomethylated	0.0020156	0.72374	insignificant	42	168	171
chr16	23108025	23110025	Snord2	-0.10319037	0.00000185	hypomethylated	0.00045879	0.97048	insignificant	9	85	84
chr16	23127803	23129803	Rfc4	-0.07313667	0.71778	insignificant	0.013961	0.0010789	hypermethylated	9	48	48
chr16	23223834	23225834	St6gal1	-0.16369485	0.000000048	hypomethylated	0.030254	0.4645	insignificant	13	60	44
chr16	23609004	23611004	Rtp4	-0.43095238	0.00002384	stronglyHypometh	0.10693	0.79998	insignificant	3	8	10
chr16	23930880	23932880	Rtp2	0.0201178	0.63359	insignificant	-0.0013872	0.3936	insignificant	8	34	32
chr16	23988698	23990698	Bcl6	-0.07329957	7.81E-08	hypomethylated	0.0073614	0.27385	insignificant	23	82	82
chr16	24392435	24394435	Lpp	-0.12449732	0.00014813	hypomethylated	-0.0022515	0.029911	inconclusive	31	160	143
chr16	26105870	26107870	Leprel1	-0.10004789	0.096754	insignificant	0.022737	0.00042523	hypermethylated	5	12	12
chr16	26371925	26373925	Cldn1	-0.39986264	0.077492	insignificant	0.003873	0.2193	insignificant	3	10	8
chr16	26462220	26464220	Cldn16	0.01447254	1	insignificant	-0.088667	0.33538	insignificant	2	14	14
chr16	26526857	26528857	Tmem207		1	noCoverage	0.074369	0.45932	insignificant	0	12	12
chr16	26580790	26582790	Il1rap	0.01561821	0.043864	hypermethylated	0.024578	0.84895	insignificant	16	69	74
chr16	27388062	27390062	Ccdc50	-0.08321604	0.018012	hypomethylated	0.0053321	1	insignificant	11	48	47
chr16	28445313	28447313	Fgf12	-0.06728069	2.88E-09	hypomethylated	0.0016275	0.85602	insignificant	44	151	149
chr16	28753288	28755288	Fgf12	-0.27762691	0.30301	insignificant	-0.0222	0.52748	insignificant	2	14	14
chr16	28929784	28931784	1600021P15Ri	-0.11613897	6.18E-09	hypomethylated	0.0053364	0.94002	insignificant	28	92	95
chr16	29208780	29210780	Hrasls	0.08971339	1	insignificant	0.056693	0.86112	insignificant	2	10	10
chr16	29541569	29543569	Atp13a4	0.17345129	0.59401	insignificant	0.12187	0.00021045	hypermethylated	2	11	10
chr16	29578419	29580419	Opa1	-0.17508013	0.60305	insignificant	0.027503	0.58231	insignificant	2	12	12
chr16	30007752	30009752	4632428C04Ri	-0.11557361	0.000000775	hypomethylated	-0.0013795	0.67055	insignificant	18	69	69
chr16	30064442	30066442	Hes1	-0.16405837	0.00000267	hypomethylated	-0.010581	1	insignificant	8	36	36
chr16	30283340	30285340	Lrrc15	-0.29914909	1	insignificant	-0.045804	0.95822	insignificant	2	26	22
chr16	30388616	30390616	Atp13a3	-0.06781679	1.4E-19	hypomethylated	0.0032859	0.47071	insignificant	29	80	80
chr16	30550664	30552664	Tmem44	-0.14703342	0.37948	insignificant	0.017573	0.18163	insignificant	4	30	30
chr16	30587675	30589675	Lsg1	0.22884842	0.19567	insignificant	0.0061324	0.1515	insignificant	2	35	33
chr16	30598808	30600808	Fam43a	-0.14439095	5.19E-33	hypomethylated	-0.0077888	0.097184	insignificant	47	168	163
chr16	31314682	31316682	Apod	-0.36351068	0.046117	stronglyHypometh	-0.0083286	0.84197	insignificant	2	7	7
chr16	31421382	31423382	Bdh1	0.02232143	1	insignificant	0.0084135	0.54024	insignificant	2	4	4
chr16	31427838	31429838	Bdh1	-0.11593599	2.93E-18	hypomethylated	0.0077038	0.57688	insignificant	26	108	108
chr16	31663124	31665124	Dlg1	-0.11572776	1.08E-24	hypomethylated	0.00091425	0.94697	insignificant	43	178	169
chr16	31877895	31879895	Mfi2	-0.02419105	1	insignificant	-0.0058584	0.029138	hypomethylated	2	30	30
chr16	31932936	31934936	Pigz	-0.20246212	0.034615	hypomethylated	0.0087173	0.29849	insignificant	8	66	66
chr16	31947631	31949631	Ncbp2	-0.0967251	2.82E-33	hypomethylated	-0.00044516	0.59698	insignificant	49	177	163
chr16	31948607	31950607	0610012G03R	-0.20865695	7.61E-12	hypomethylated	-0.0040326	0.31772	insignificant	17	71	67
chr16	32003373	32005373	Senp5	-0.29310967	0.19382	insignificant	-0.055179	0.33716	insignificant	3	12	10

chr16	32079359	32081359	Pak2		1	noCoverage	0.010104	0.45367	insignificant	0	28	21
chr16	32098887	32100887	1500031L02Ri	-0.19874842	6.89E-14	hypomethylated	-0.013843	0.47537	insignificant	18	95	93
chr16	32099813	32101813	Pigx	-0.24558599	3.56E-14	hypomethylated	-0.040236	0.077011	insignificant	14	54	53
chr16	32246312	32248312	Wdr53	-0.08873282	2.27E-08	hypomethylated	-0.0063824	0.22881	insignificant	41	121	125
chr16	32246338	32248338	Wdr53	-0.08164477	7.93E-08	hypomethylated	-0.0046129	0.2055	insignificant	40	119	123
chr16	32247111	32249111	Wdr53	-0.08409137	0.000022595	hypomethylated	-0.010965	0.77362	insignificant	20	48	52
chr16	32276546	32278546	Rnf168	-0.09192072	1.82E-15	hypomethylated	-0.0016005	0.3574	insignificant	29	100	96
chr16	32331337	32333337	Ubxn7	-0.14389361	7.55E-31	hypomethylated	0.020057	0.13515	insignificant	27	107	103
chr16	32399591	32401591	Tm4sf19	0.03912347	0.8213	insignificant	-0.065392	0.31177	insignificant	2	22	21
chr16	32418787	32420787	Tctex1d2	-0.1610743	0.00000384	hypomethylated	0.0020904	0.80279	insignificant	12	54	57
chr16	32430006	32432006	Pcyt1a	-0.15617412	3.91E-25	hypomethylated	0.0079802	0.69605	insignificant	43	135	135
chr16	32430105	32432105	Pcyt1a	-0.14172239	5.14E-22	hypomethylated	0.0036226	0.31269	insignificant	43	131	131
chr16	32430315	32432315	Pcyt1a	-0.11358366	1.31E-16	hypomethylated	-0.000081986	0.52817	insignificant	43	124	124
chr16	32607981	32609981	Tfrc	-0.09005475	0.34875	insignificant	-0.016702	0.60233	insignificant	4	34	34
chr16	32643728	32645728	Tnk2	-0.09305044	1E-15	hypomethylated	0.052343	0.43403	insignificant	30	163	140
chr16	32668283	32670283	Gm10818	0.08710526	0.58845	insignificant	0.028001	0.12836	insignificant	1	10	13
chr16	32797521	32799521	Muc20	-0.14827536	0.73491	insignificant	0.050728	0.17622	insignificant	2	8	8
chr16	32868425	32870425	1700021K19Ri	-0.30633286	0.094265	insignificant	0.011421	0.54144	insignificant	3	14	14
chr16	32876869	32878869	Fyttd1	-0.16829202	5.7E-18	hypomethylated	-0.044119	0.82632	insignificant	36	126	117
chr16	32913185	32915185	Lrch3	-0.18378728	4.12E-33	hypomethylated	0.031871	0.83203	insignificant	27	109	95
chr16	33055538	33057538	Rpl35a	-0.1698741	7.56E-14	hypomethylated	-0.016132	0.071014	insignificant	20	55	55
chr16	33055567	33057567	Rpl35a	-0.1698741	7.56E-14	hypomethylated	-0.016132	0.071014	insignificant	20	55	55
chr16	33055599	33057599	Rpl35a	-0.1698741	7.56E-14	hypomethylated	-0.016132	0.071014	insignificant	20	55	55
chr16	33056272	33058272	Rpl35a	-0.15839708	9.77E-11	hypomethylated	-0.023737	0.069559	insignificant	16	47	47
chr16	33061606	33063606	Lmln	-0.1024453	2.77E-16	hypomethylated	0.015744	0.52427	insignificant	35	118	110
chr16	33104446	33106446	Mir1947	0.01375836	1	insignificant	-0.022242	0.26535	insignificant	4	8	8
chr16	33184156	33186156	Osbpl11	-0.16981394	7.72E-42	hypomethylated	-0.014549	0.85835	insignificant	39	115	112
chr16	33250541	33252541	Snx4	-0.13946497	1.48E-15	hypomethylated	0.036335	0.028231	hypermethylated	14	70	62
chr16	33379860	33381860	Zfp148	-0.11026436	1.67E-16	hypomethylated	0.00086211	0.1491	insignificant	38	153	153
chr16	33517414	33519414	Slc12a8	-0.23138268	0.58667	insignificant	0.00098064	0.17762	insignificant	5	24	24
chr16	33683551	33685551	Heg1	-0.08664383	5.53E-13	hypomethylated	0.0090691	0.15801	insignificant	41	108	108
chr16	33828750	33830750	Itgb5	-0.16454429	7.59E-11	hypomethylated	0.0015046	0.70603	insignificant	24	111	104
chr16	33967089	33969089	Umps	-0.24345238	0.039392	hypomethylated	-0.0015793	0.22259	insignificant	4	14	19
chr16	34514113	34516113	Kalrn		1	noCoverage	0.01717	0.26127	insignificant	0	9	6
chr16	34689701	34691701	Ccdc14	-0.26963734	1	insignificant	-0.019024	0.66118	insignificant	2	44	35
chr16	35021506	35023506	Ptplb	-0.10058468	0.0017653	hypomethylated	0.018205	0.32313	insignificant	33	116	120
chr16	35154721	35156721	Adcy5	-0.115915	5.21E-57	hypomethylated	0.0051938	0.86887	insignificant	67	269	265
chr16	35364004	35366004	Sec22a	-0.1050785	0.00000966	hypomethylated	-0.0099244	0.084406	insignificant	12	26	26
chr16	35490959	35492959	Pdia5	-0.40789474	4.05E-08	stronglyHypometh	0.088048	0.17468	insignificant	4	19	17
chr16	35540447	35542447	Sema5b	-0.15773837	4.04E-47	hypomethylated	0.00738	0.013015	hypermethylated	51	133	140
chr16	35769442	35771442	Hspbap1	-0.10389476	0.00041757	hypomethylated	-0.003181	0.85845	insignificant	15	58	42
chr16	35769471	35771471	Hspbap1	-0.10731632	0.15556	insignificant	0.0040203	0.55606	insignificant	9	46	30
chr16	35871468	35873468	Parp14	0.35134588	0.19195	insignificant	-0.0065154	0.4947	insignificant	3	35	36
chr16	35937555	35939555	Parp9	-0.15992067	2.21E-11	hypomethylated	-0.0090461	0.6718	insignificant	14	77	77
chr16	35939113	35941113	Parp9	-0.14683144	0.00028625	hypomethylated	-0.012386	0.61714	insignificant	4	24	24
chr16	35982448	35984448	Kpna1	-0.09595218	2.02E-29	hypomethylated	-0.0020258	0.77423	insignificant	54	194	184
chr16	36040275	36042275	Wdr5b	-0.28814935	1.01E-09	hypomethylated	-0.039578	1	insignificant	4	8	10
chr16	36070745	36072745	Ccdc58	-0.10127227	0.00089658	hypomethylated	-0.000061048	0.46183	insignificant	9	110	109
chr16	36071601	36073601	Fam162a	-0.09187277	0.00088762	hypomethylated	0.010819	0.38096	insignificant	9	95	95
chr16	36080360	36082360	Ccdc58	-0.02136752	0.66217	insignificant	0.071443	0.2373	insignificant	1	26	24
chr16	36693123	36695123	Ildr1	-0.19028298	0.000000698	hypomethylated	-0.0061285	0.072923	insignificant	8	30	30
chr16	36827485	36829485	Iqcb1	-0.13389305	4.05E-12	hypomethylated	-0.016956	0.44416	insignificant	16	47	49
chr16	36828345	36830345	Eaf2	-0.15693322	1.21E-08	hypomethylated	-0.019775	0.60234	insignificant	7	17	17
chr16	36874912	36876912	Eaf2	-0.12666337	2.23E-23	hypomethylated	0.0001236	0.607	insignificant	33	114	114
chr16	36884096	36886096	Golgb1	0.18589744	1	insignificant	0.08837	0.27085	insignificant	1	6	7
chr16	37010871	37012871	Polq	-0.23483266	8.23E-13	hypomethylated	-0.017945	0.86762	insignificant	16	70	65
chr16	37385044	37387044	Stxbp5l	-0.17777778	0.13049	insignificant	0.037003	0.026345	inconclusive	6	18	18
chr16	37538979	37540979	Rabl3	-0.19261416	9.82E-35	hypomethylated	0.0028146	0.082456	insignificant	21	52	51
chr16	37539855	37541855	Gtf2e1	-0.5962963	0.41538	lowCoverage	0.013844	1	insignificant	1	4	4
chr16	37654454	37656454	Ndufb4	-0.11388889	0.08012	insignificant	0.033433	0.74613	insignificant	4	21	16
chr16	37776140	37778140	Fstl1	-0.10664743	1.48E-12	hypomethylated	0.040787	0.019793	hypermethylated	9	63	67
chr16	37867485	37869485	Lrrc58	-0.12449912	1.07E-46	hypomethylated	0.015719	0.04534	hypermethylated	44	144	127
chr16	37915581	37917581	Gpr156	-0.14991819	2.95E-12	hypomethylated	0.015762	0.67858	insignificant	17	66	66
chr16	38088086	38090086	Gsk3b	-0.10928502	2.37E-19	hypomethylated	-0.0036885	0.24642	insignificant	30	157	171
chr16	38089346	38091346	Gsk3b	-0.12591381	7.08E-10	hypomethylated	-0.0068047	0.79523	insignificant	19	97	111
chr16	38346084	38348084	Cox17	-0.13308809	0.36394	insignificant	0.041114	0.0084301	hypermethylated	6	42	42
chr16	38361258	38363258	Popdc2	-0.10392635	0.276	insignificant	-0.0011733	0.54782	insignificant	10	38	40
chr16	38361295	38363295	Popdc2	-0.10392635	0.276	insignificant	-0.0011733	0.54782	insignificant	10	38	40
chr16	38433225	38435225	Pla1a	0.12971939	0.31811	insignificant	0.047007	0.11573	insignificant	4	14	14
chr16	38452769	38454769	Adprh	-0.08921389	0.24729	insignificant	-0.025827	0.77019	insignificant	6	30	28
chr16	38458012	38460012	Cd80	-0.49518398	0.24928	insignificant	-0.026884	0.11732	insignificant	2	10	10
chr16	38522747	38524747	4930455C21Ri	-0.13239028	0.015573	hypomethylated	0.009165	1	insignificant	9	43	30
chr16	38550266	38552266	Poglut1	-0.07380992	0.17863	insignificant	0.03265	0.034456	inconclusive	4	36	38
chr16	38561971	38563971	Tmem39a	-0.19000187	1.98E-28	hypomethylated	0.0061517	0.22836	insignificant	18	56	56
chr16	38713148	38715148	Arhgap31	-0.13214152	0.091766	insignificant	-0.0034857	0.2778	insignificant	8	55	56
chr16	38741376	38743376	B4galt4	-0.16487424	8.6E-37	hypomethylated	-0.016457	0.54651	insignificant	33	85	82
chr16	38800316	38802316	Upk1b	-0.29820682	0.0067965	hypomethylated	-0.022782	0.80825	insignificant	2	4	4
chr16	38901457	38903457	Igsf11	-0.1336219	2.31E-18	hypomethylated	-0.011231	1	insignificant	37	90	90
chr16	42954692	42956692	BC002163	-0.42054224	0.048464	stronglyHypometh	-0.11196	0.015949	inconclusive	1	19	17
chr16	43246396	43248396	Zbtb20		1	noCoverage	0.017556	0.69776	insignificant	0	14	12
chr16	43761354	43763354	Drd3	-0.11351812	0.35935	insignificant	-0.13617	0.84483	insignificant	1	12	11
chr16	43889014	43891014	2610015P09Ri	-0.23132691	8.17E-16	hypomethylated	-0.097424	0.47188	insignificant	14	46	40
chr16	43889789	43891789	2610015P09Ri	-0.26877016	5.73E-14	hypomethylated	-0.12084	0.4554	insignificant	11	32	28
chr16	43979163	43981163	Zdhhc23	-0.09669855	0.000964	hypomethylated	0.030876	0.65396	insignificant	33	133	102
chr16	44016549	44018549	Gramd1c		1	noCoverage	-0.10129	0.12044	insignificant	0	6	6

chr16	44138921	44140921	Naa50	-0.11102242	5.82E-21	hypomethylated	0.0058204	0.97828	insignificant	35	156	157
chr16	44139132	44141132	Atp6v1a	-0.11102242	5.82E-21	hypomethylated	0.0058204	0.97828	insignificant	35	156	157
chr16	44172509	44174509	Gm608	-0.0852146	6.84E-13	hypomethylated	0.018117	0.11437	insignificant	28	108	96
chr16	44332951	44334951	Sidt1	-0.29913887	0.000054123	hypomethylated	-0.018995	0.79093	insignificant	3	19	22
chr16	44346513	44348513	Spice1	-0.09582722	0.000041573	hypomethylated	-0.0074794	1	insignificant	16	76	76
chr16	44393911	44395911	Wdr52	0.06247342	1	insignificant	0.0065768	0.17032	insignificant	5	26	26
chr16	44558242	44560242	Boc	-0.15753625	4.42E-19	hypomethylated	0.0062022	0.73484	insignificant	26	85	89
chr16	44558983	44560983	Boc		1	noCoverage	0.017444	0.22356	insignificant	0	4	9
chr16	44723411	44725411	BC027231	-0.16300086	4.83E-19	hypomethylated	0.0019714	0.18995	insignificant	35	110	103
chr16	44866209	44868209	Cd200r2	0.13675214	0.59057	insignificant	0.069788	0.073303	insignificant	2	9	8
chr16	44942790	44944790	Cd200r3	-0.40781441	0.00000428	stronglyHypometh	0.015708	0.5873	insignificant	3	8	8
chr16	45157941	45159941	Atg3	-0.14130877	1.3E-10	hypomethylated	-0.0055344	0.87569	insignificant	19	86	79
chr16	45158786	45160786	Slc35a5	-0.2048803	4.61E-11	hypomethylated	-0.01148	0.81945	insignificant	18	64	57
chr16	45654231	45656231	BC016579	-0.1000457	0.10634	insignificant	0.00074905	0.54047	insignificant	1	13	13
chr16	45693771	45695771	Tmprss7	-0.16057692	0.70292	insignificant	0.078846	0.15026	insignificant	2	4	4
chr16	45743019	45745019	Abhd10	-0.13185855	0.00031748	hypomethylated	0.005907	0.8375	insignificant	7	16	16
chr16	45844491	45846491	Phldb2	-0.04398199	0.56191	insignificant	-0.00037862	0.37909	insignificant	3	26	26
chr16	46010526	46012526	Plcxd2	-0.15192042	1	insignificant	0.071844	0.21035	insignificant	4	22	23
chr16	46497080	46499080	Pvrl3	-0.1463639	0.00028917	hypomethylated	0.017764	1	insignificant	14	111	99
chr16	48282847	48284847	Dppa4	-0.17086551	1	insignificant	-0.011624	0.39793	insignificant	4	14	12
chr16	48309386	48311386	Dppa2	0.07000754	0.77257	insignificant	0.063657	0.39132	insignificant	5	15	14
chr16	48815968	48817968	Retnlb		1	noCoverage	0.26413	0.21948	insignificant	0	4	4
chr16	48993300	48995300	C330027C09R	-0.12420148	0.0010559	hypomethylated	-0.00066944	0.28772	insignificant	8	68	64
chr16	48994225	48996225	C330027C09R	-0.16916512	0.057284	insignificant	0.020718	0.7182	insignificant	5	22	22
chr16	49698406	49700406	Ift57	-0.11897036	0.0017483	hypomethylated	-0.029926	0.0011353	hypomethylated	7	51	54
chr16	49854766	49856766	Cd47	-0.10644031	9.55E-25	hypomethylated	0.012459	0.8042	insignificant	31	172	164
chr16	52030661	52032661	Cblb	-0.14197783	1.34E-14	hypomethylated	0.0027445	0.84984	insignificant	15	81	76
chr16	52453110	52455110	Alcam	-0.17318138	0.59123	insignificant	-0.0029924	1	insignificant	3	42	47
chr16	55822251	55824251	Nfkbiz	-0.14919304	1.67E-10	hypomethylated	-0.0066004	0.75339	insignificant	10	27	20
chr16	55838754	55840754	Nfkbiz	-0.09120032	0.049416	hypomethylated	0.003375	0.94907	insignificant	14	36	34
chr16	55895392	55897392	Fam55c	-0.14512005	0.00000136	hypomethylated	-0.024326	0.11598	insignificant	21	62	61
chr16	55895398	55897398	Fam55c	-0.14851141	0.000027536	hypomethylated	-0.025532	0.14286	insignificant	21	62	61
chr16	55934961	55936961	Cep97	-0.38751877	0.058102	insignificant	-0.036136	0.62065	insignificant	1	8	8
chr16	55965387	55967387	Rpl24	-0.18027089	2.11E-08	hypomethylated	0.011152	0.2159	insignificant	13	56	56
chr16	55972916	55974916	Zbtb11	-0.08435782	4.28E-38	hypomethylated	0.0085514	0.79274	insignificant	53	173	162
chr16	56029830	56031830	Pcnp		1	noCoverage	-0.08703	0.64393	insignificant	0	33	30
chr16	56037887	56039887	Rg9mtd1	-0.18013264	1.15E-12	hypomethylated	-0.013178	0.15492	insignificant	17	93	98
chr16	56074521	56076521	Senp7	-0.14637918	0.000000235	hypomethylated	0.004096	0.49586	insignificant	7	90	92
chr16	56717467	56719467	Tfg	-0.15553977	0.38084	insignificant	0.047299	0.064263	insignificant	3	16	16
chr16	57071459	57073459	2310005G13Rik		1	noCoverage	-0.00087146	1	insignificant	0	9	9
chr16	57120826	57122826	Tomm70a	-0.13061525	6.13E-42	hypomethylated	-0.0036493	0.55218	insignificant	41	127	124
chr16	57167445	57169445	Nit2	0.80399786	0.00034252	stronglyHypermeth	-0.014896	0.82784	insignificant	1	16	16
chr16	57231579	57233579	Tbc1d23	-0.05792117	0.51701	insignificant	0.028968	0.47152	insignificant	5	28	28
chr16	57548354	57550354	Filip1l	-0.08033852	0.000031959	hypomethylated	0.059276	0.80807	insignificant	9	43	32
chr16	57754850	57756850	Col8a1		1	noCoverage	-0.034194	1	insignificant	0	6	15
chr16	58407647	58409647	Dcbld2	-0.10607074	2.31E-23	hypomethylated	-0.0063783	0.22014	insignificant	42	169	161
chr16	58523425	58525425	St3gal6	-0.14362459	0.00000121	hypomethylated	0.012133	1	insignificant	15	46	46
chr16	58638516	58640516	E330017A01Rik		1	noCoverage	-0.10433	0.42881	insignificant	0	14	14
chr16	58669320	58671320	Cpox	-0.11626228	0.042176	hypomethylated	0.014399	0.26746	insignificant	8	83	85
chr16	58727156	58729156	Cldnd1	-0.09043347	8.62E-20	hypomethylated	-0.0090283	1	insignificant	24	71	76
chr16	59098816	59100816	Olfr192		1	noCoverage	0.11111	0.56608	insignificant	0	3	3
chr16	59406207	59408207	Gabrr3	-0.0844494	0.59483	insignificant	0.0041236	0.78185	insignificant	2	8	8
chr16	59456409	59458409	Gabrr3	-0.11391813	0.013294	hypomethylated	-0.014644	0.10341	insignificant	5	15	25
chr16	59470600	59472600	Mina	-0.22660646	0.04807	hypomethylated	-0.020655	0.22434	insignificant	13	67	67
chr16	59639165	59641165	4930547E14Ri	-0.02565827	0.72118	insignificant	0.0012459	0.82165	insignificant	5	26	26
chr16	60605357	60607357	Epha6	-0.04801932	0.50771	insignificant	0.0043949	0.83924	insignificant	4	10	10
chr16	62786542	62788542	Nsun3	-0.24213063	4.1E-13	hypomethylated	-0.052958	0.76214	insignificant	13	58	54
chr16	62846866	62848866	Arl13b	-0.12120497	0.14075	insignificant	-0.013205	0.27792	insignificant	2	8	8
chr16	62853159	62855159	Pros1	-0.11846804	5.8E-10	hypomethylated	0.0067246	0.9018	insignificant	11	36	36
chr16	64478960	64480960	Csnka2ip		1	noCoverage	0.02299	0.6453	insignificant	0	8	8
chr16	64770765	64772765	4930453N24R	-0.20845005	0.0017916	hypomethylated	0.0085815	0.7259	insignificant	5	18	18
chr16	64850910	64852910	Cggbp1	-0.14414801	3.75E-17	hypomethylated	0.017292	0.61934	insignificant	28	115	108
chr16	65562942	65564942	Chmp2b	0.25837227	0.054456	insignificant	-0.059983	0.42174	insignificant	2	23	22
chr16	65814877	65816877	Vgll3	-0.12715855	0.000021468	hypomethylated	-0.0060126	0.84386	insignificant	25	109	110
chr16	69863989	69865989	Speer2	0.0449509	1	insignificant	-0.19088	0.024521	hypomethylated	3	21	32
chr16	70313331	70315331	Gbe1	-0.097162	1.99E-29	hypomethylated	0.021431	0.88613	insignificant	48	126	120
chr16	72662393	72664393	Robo1	-0.14771778	3.67E-10	hypomethylated	0.02714	0.25344	insignificant	20	78	77
chr16	74411157	74413157	Robo2	-0.15658947	3.35E-10	hypomethylated	0.010426	0.44121	insignificant	9	60	59
chr16	75592135	75594135	Rbm11	-0.39260336	1.42E-28	stronglyHypometh	0.013624	0.9276	insignificant	10	50	50
chr16	75767063	75769063	Hspa13	-0.22109704	2.48E-13	hypomethylated	0.033708	0.34041	insignificant	8	28	28
chr16	76373294	76375294	Nrip1	-0.13366525	9.41E-17	hypomethylated	-0.0033092	0.20181	insignificant	21	66	66
chr16	77013313	77015313	Usp25	-0.10000572	5.11E-29	hypomethylated	0.0037735	0.84053	insignificant	28	127	99
chr16	77328572	77330572	2810055G20R	-0.15599789	0.069681	insignificant	0.0045024	0.78241	insignificant	6	18	18
chr16	77598180	77600180	Mir99a	0.0625	1	insignificant	-0.0375	0.67732	insignificant	1	4	4
chr16	77598901	77600901	Mirlet7c-1	0.0625	1	insignificant	-0.0375	0.67732	insignificant	1	4	4
chr16	78300935	78302935	Cxadr	-0.14327671	1.49E-28	hypomethylated	-0.0071971	1	insignificant	37	117	116
chr16	78377002	78379002	Gm7334	-0.20110149	0.0036128	hypomethylated	-0.027571	0.59147	insignificant	12	76	75
chr16	78377030	78379030	Gm7334	-0.23775987	0.00000742	hypomethylated	-0.030653	0.59953	insignificant	12	66	65
chr16	78576913	78578913	D16Ertd472e	-0.0579683	0.46692	insignificant	0.014646	0.11971	insignificant	6	38	40
chr16	78930192	78932192	Chodl	-0.50681818	0.0028222	stronglyHypometh	-0.0012626	1	insignificant	2	4	4
chr16	81199941	81201941	Ncam2	-0.09795651	1.56E-11	hypomethylated	0.00099865	0.21282	insignificant	36	118	120
chr16	84735426	84737426	Mrpl39	-0.09870001	1.06E-22	hypomethylated	-0.0015102	0.7065	insignificant	29	98	98
chr16	84773367	84775367	Jam2	-0.15575355	1.67E-15	hypomethylated	0.0029038	0.92517	insignificant	15	53	60
chr16	84834368	84836368	Gabpa	-0.099709	3.81E-33	hypomethylated	0.012935	0.072544	insignificant	45	178	178
chr16	84835819	84837819	Atp5j	-0.03606416	0.26819	insignificant	0.030575	0.16102	insignificant	26	86	88

chr16	85173952	85175952	App	-0.1235259	0.00000106	hypomethylated	0.035114	0.65639	insignificant	6	14	14
chr16	85803360	85805360	Adamts1	-0.27462533	8.12E-12	hypomethylated	0.04723	0.13578	insignificant	8	28	27
chr16	85901370	85903370	Adamts5	-0.23982149	3.7E-23	hypomethylated	0.0049094	0.22446	insignificant	12	44	44
chr16	87353429	87355429	N6amt1	-0.27434465	0.00000148	hypomethylated	-0.0061246	0.81012	insignificant	10	44	44
chr16	87440837	87442837	Rwdd2b	-0.26187046	0.0001733	hypomethylated	0.09902	0.34336	insignificant	6	14	18
chr16	87454229	87456229	Usp16	-0.19275059	1.04E-33	hypomethylated	-0.032449	0.96782	insignificant	30	78	78
chr16	87496114	87498114	Cct8	-0.26259894	0.37656	insignificant	-0.028006	0.61767	insignificant	7	22	22
chr16	87552574	87554574	ORF63	-0.28687918	0.11183	insignificant	-0.078819	0.86169	insignificant	4	8	12
chr16	87698198	87700198	Bach1	-0.10437197	3.76E-22	hypomethylated	0.0019593	0.70646	insignificant	44	134	130
chr16	88708207	88710207	2310061N02R	0.08127706	1	insignificant	-0.088156	0.63117	insignificant	2	10	10
chr16	88828253	88830253	Krtap15	0.01472431	0.16994	insignificant	0.066558	0.52749	insignificant	2	6	6
chr16	89404019	89406019	Krtap16-7		1	noCoverage	0.11683	1	insignificant	0	4	4
chr16	89571428	89573428	Krtap11-1	0.07823452	0.36712	insignificant	0.14384	1	insignificant	4	10	10
chr16	89818597	89820597	Tiam1		1	noCoverage	-0.0037349	0.55737	insignificant	0	14	14
chr16	90141481	90143481	Gm10789	-0.15449194	4.76E-13	hypomethylated	0.080398	0.74603	insignificant	25	85	84
chr16	90219986	90221986	Sod1	-0.14760406	3.03E-31	hypomethylated	-0.0037243	0.90547	insignificant	20	95	90
chr16	90284670	90286670	Scaf4	-0.14646398	5.19E-19	hypomethylated	0.010378	0.063729	insignificant	23	80	78
chr16	90385641	90387641	Hunk	-0.11484338	2.15E-43	hypomethylated	0.0038304	0.11127	insignificant	46	182	189
chr16	90727616	90729616	2610039C10R	-0.1158838	0.0026611	hypomethylated	0.0045289	0.46938	insignificant	10	43	43
chr16	90737568	90739568	Mrap		1	noCoverage	-0.0036041	0.73718	insignificant	0	24	24
chr16	90810658	90812658	Urb1	-0.45163934	0.00000533	stronglyHypometh	0.0051291	0.7407	insignificant	3	10	10
chr16	90830103	90832103	4931408A02R	-0.08185743	1.14E-44	hypomethylated	0.0045642	0.025687	hypermethylated	53	161	161
chr16	90935094	90937094	1110004E09Ri	-0.11067849	2.39E-13	hypomethylated	0.0027936	0.93878	insignificant	16	62	62
chr16	91010493	91012493	4930404I05Ril	-0.09340842	4.77E-24	hypomethylated	0.0028315	0.21013	insignificant	41	153	153
chr16	91011340	91013340	Synj1	-0.10649566	3.88E-14	hypomethylated	-0.0014628	0.40784	insignificant	16	86	85
chr16	91011553	91013553	Synj1	-0.10741068	2.15E-12	hypomethylated	0.002768	0.077711	insignificant	15	59	58
chr16	91044624	91046624	Gcfc1	-0.09591168	0.0037899	inconclusive	0.034332	0.036878	inconclusive	10	74	67
chr16	91069390	91071390	4932438H23R	-0.67907884	0.35797	lowCoverage	-0.013649	0.15374	insignificant	1	29	28
chr16	91224794	91226794	Olig2	-0.12346298	0.000000519	hypomethylated	0.022858	0.41721	insignificant	18	57	50
chr16	91269013	91271013	Olig1	-0.09107551	7.32E-11	hypomethylated	-0.00004935	0.50819	insignificant	27	114	112
chr16	91372027	91374027	Ifnar2	-0.16903946	1.07E-09	hypomethylated	-0.0080499	0.62254	insignificant	14	80	78
chr16	91405479	91407479	Il10rb	-0.17809575	1.01E-09	hypomethylated	-0.023402	0.092011	insignificant	15	40	40
chr16	91484459	91486459	Ifnar1	-0.26072487	1.37E-32	hypomethylated	-0.024476	1	insignificant	25	113	112
chr16	91546338	91548338	Ifngr2	-0.10813504	0.0077447	hypomethylated	0.0038261	0.96122	insignificant	15	67	76
chr16	91597925	91599925	Tmem50b	-0.1793808	0.00000013	hypomethylated	0.034082	0.47624	insignificant	16	71	71
chr16	91619244	91621244	Dnajc28	-0.52513369	0.000034071	stronglyHypometh	-0.22045	1	insignificant	4	11	8
chr16	91647068	91649068	Son	-0.13771543	1.58E-14	hypomethylated	-0.022284	0.0065727	hypomethylated	37	162	153
chr16	91647217	91649217	Son	-0.13648074	1.94E-12	hypomethylated	-0.023382	0.00051895	hypomethylated	33	150	141
chr16	91688973	91690973	Donson	-0.12734668	1.24E-18	hypomethylated	-0.0036926	0.49109	insignificant	33	77	77
chr16	91728615	91730615	Itsn1	-0.09661834	2.82E-12	hypomethylated	-0.00015807	0.97025	insignificant	54	144	144
chr16	91728844	91730844	Cryzl1	-0.09661834	2.82E-12	hypomethylated	-0.00015807	0.97025	insignificant	54	144	144
chr16	91931875	91933875	Atp5o	-0.0503663	0.47835	insignificant	0.021896	0.79013	insignificant	8	24	24
chr16	92057566	92059566	Slc5a3	-0.10418222	1.54E-37	hypomethylated	0.0084569	0.022067	hypermethylated	46	200	197
chr16	92057580	92059580	Mrps6	-0.10418222	1.54E-37	hypomethylated	0.0084569	0.022067	hypermethylated	46	200	197
chr16	92300547	92302547	Fam165b	-0.4111176	0.10257	insignificant	0.065622	0.25804	insignificant	6	16	14
chr16	92359713	92361713	Kcne1	-0.04921232	0.6628	insignificant	0.041118	0.16025	insignificant	2	14	14
chr16	92466391	92468391	Rcan1	-0.42307692	3.41E-08	stronglyHypometh	-0.2235	0.000000496	hypomethylated	8	13	33
chr16	92497391	92499391	Clic6	-0.16409848	1	insignificant	0.015568	0.94659	insignificant	4	66	66
chr16	92697573	92699573	Runx1	-0.06617003	0.00000186	hypomethylated	0.013449	0.40312	insignificant	38	135	134
chr16	93368964	93370964	Mir802	-0.03101852	1	insignificant	-0.021606	1	insignificant	3	6	6
chr16	93604060	93606060	Setd4	-0.09947738	1.02E-08	hypomethylated	-0.00046824	0.60535	insignificant	10	22	22
chr16	93607081	93609081	Cbr1	-0.06817719	0.0011285	hypomethylated	0.0036263	0.21089	insignificant	8	50	50
chr16	93682463	93684463	Cbr3	-0.11913812	1.03E-18	hypomethylated	0.0092606	0.89868	insignificant	32	144	147
chr16	93711151	93713151	Dopey2	-0.12292204	3.26E-21	hypomethylated	-0.016981	0.77124	insignificant	30	105	98
chr16	93831365	93833365	Morc3	-0.10556244	1.99E-28	hypomethylated	-0.010924	0.11192	insignificant	56	170	172
chr16	93883145	93885145	Chaf1b	-0.13901951	2.06E-28	hypomethylated	-0.020076	0.78084	insignificant	35	74	69
chr16	93929812	93931812	Cldn14	-0.15598739	0.067051	insignificant	0.063894	0.25553	insignificant	2	7	10
chr16	93930062	93932062	Cldn14	-0.15598739	0.067051	insignificant	0.020005	0.50057	insignificant	2	7	6
chr16	94009082	94011082	Cldn14	0.05927466	0.43329	insignificant	0.078733	0.0043159	hypermethylated	5	18	18
chr16	94084504	94086504	Sim2	-0.10820328	5.24E-32	hypomethylated	0.0050404	0.053908	insignificant	40	156	156
chr16	94549028	94551028	Ripply3	-0.39510836	5.25E-18	stronglyHypometh	-0.040403	0.053388	insignificant	7	27	35
chr16	94591345	94593345	Ttc3	-0.11446369	1.06E-32	hypomethylated	-0.0060499	0.42075	insignificant	20	85	85
chr16	94591937	94593937	Pigp	-0.11446369	1.06E-32	hypomethylated	-0.0060499	0.42075	insignificant	20	85	85
chr16	94592157	94594157	Pigp	-0.09720903	4.77E-29	hypomethylated	-0.0077186	0.32735	insignificant	16	77	77
chr16	94592317	94594317	Pigp	-0.09720903	4.77E-29	hypomethylated	-0.0077186	0.32735	insignificant	16	77	77
chr16	94592622	94594622	Pigp		1	noCoverage	-0.0096895	0.85102	insignificant	0	29	29
chr16	94748236	94750236	Dscr3	-0.10618925	0.0012103	hypomethylated	-0.032852	0.38624	insignificant	16	60	63
chr16	94790812	94792812	Dyrk1a	-0.08473189	3.93E-60	hypomethylated	0.0070756	0.43872	insignificant	103	326	326
chr16	94791513	94793513	Dyrk1a	-0.11359465	3.52E-61	hypomethylated	0.01102	0.4088	insignificant	86	213	201
chr16	95478257	95480257	Kcnj15	-0.12037037	0.2521	insignificant	0.022487	0.013789	hypermethylated	2	4	4
chr16	95923013	95925013	Ets2	-0.14803686	7.43E-28	hypomethylated	0.0063715	0.197	insignificant	32	92	92
chr16	96212510	96214510	Psmg1	-0.1059279	0.000000013	hypomethylated	0.0049546	0.91346	insignificant	13	47	48
chr16	96304035	96306035	Brwd1	-0.08793962	0.0000572	hypomethylated	-0.0017041	0.88327	insignificant	21	79	64
chr16	96349332	96351332	Hmgn1	-0.09814853	7.58E-26	hypomethylated	-0.018749	0.59886	insignificant	24	87	87
chr16	96366025	96368025	Wrb	-0.0274267	1	insignificant	0.011523	0.43321	insignificant	3	49	49
chr16	96413864	96415864	Lca5l		1	noCoverage	-0.08189	0.89723	insignificant	0	12	11
chr16	96421076	96423076	Sh3bgr	-0.33765692	0.0083685	stronglyHypometh	0.010115	0.55386	insignificant	5	24	22
chr16	96501638	96503638	B3galt5	-0.27241162	0.00000157	hypomethylated	0.0014356	0.8407	insignificant	4	8	8
chr16	96582363	96584363	Igsf5	0.01369048	0.20309	insignificant	0.048736	0.38299	insignificant	7	16	16
chr16	96582367	96584367	Igsf5	0.01369048	0.20309	insignificant	0.048736	0.38299	insignificant	7	16	16
chr16	96582370	96584370	Igsf5	0.01369048	0.20309	insignificant	0.048736	0.38299	insignificant	7	16	16
chr16	97392342	97394342	Dscam		1	noCoverage	0.021761	0.5147	insignificant	0	22	22
chr16	97577334	97579334	Bace2	-0.14166819	2.06E-09	hypomethylated	0.0096953	0.89679	insignificant	16	98	90
chr16	97832802	97834802	Tmprss2		1	noCoverage	0.12776	0.52732	insignificant	0	9	12
chr16	97985362	97987362	Ripk4		1	noCoverage	0.037807	0.30391	insignificant	0	22	22

chr16	98072834	98074834	Prdm15	-0.12027965	0.0024308	hypomethylated	0.0052224	0.12173	insignificant	45	130	120
chr16	98183786	98185786	Zfp295	-0.13693584	0.000000418	hypomethylated	0.016797	0.95255	insignificant	8	42	44
chr16	98303604	98305604	A630089N07Rik		1	noCoverage	0.043166	0.15773	insignificant	0	10	10
chr17	3084183	3086183	Pisd-ps2		1	noCoverage	-0.076937	0.20487	insignificant	0	45	52
chr17	3113971	3115971	Scaf8	-0.09784506	3.3E-39	hypomethylated	0.0049848	0.70474	insignificant	53	208	202
chr17	3325572	3327572	Tiam2	-0.12738685	0.0038569	hypomethylated	0.012593	0.51378	insignificant	16	88	88
chr17	3396206	3398206	Tiam2		1	noCoverage	0.077073	0.54165	insignificant	0	16	17
chr17	3531553	3533553	Cldn20		1	noCoverage	-0.0052381	0.72293	insignificant	0	6	6
chr17	3557713	3559713	Tfb1m	-0.65714286	0.042683	stronglyHypometh	0.14643	0.38909	insignificant	1	4	4
chr17	4994073	4996073	Arid1b	-0.09621785	5.4E-27	hypomethylated	0.033632	0.90224	insignificant	71	291	288
chr17	5440260	5442260	5730437N04Rik		1	noCoverage	0.0043964	0.69935	insignificant	0	22	22
chr17	5491599	5493599	Zdhhc14	-0.12573629	2.24E-77	hypomethylated	-0.003574	0.7323	insignificant	76	256	224
chr17	5840379	5842379	Snx9	-0.11023578	1.17E-30	hypomethylated	0.012579	0.44152	insignificant	61	203	184
chr17	5940279	5942279	Synj2	-0.13711226	9.13E-08	hypomethylated	0.011863	0.50464	insignificant	32	126	119
chr17	5974585	5976585	Synj2	-0.20095694	0.026205	hypomethylated	0.076543	0.59321	insignificant	3	11	8
chr17	6078827	6080827	Gtf2h5	-0.14787133	0.60699	insignificant	-0.066636	0.027614	hypomethylated	8	29	31
chr17	6079739	6081739	Serac1	-0.1433263	0.60389	insignificant	-0.091825	0.0049774	hypomethylated	8	21	25
chr17	6105829	6107829	Tulp4	-0.13244794	3.19E-12	hypomethylated	-0.00040468	0.22852	insignificant	42	117	117
chr17	6269474	6271474	Tmem181a	-0.08324504	0.016084	hypomethylated	0.0031121	0.60819	insignificant	15	96	95
chr17	6317474	6319474	Dynlt1a	-0.24906061	1	lowCoverage	-0.035472	0.42401	insignificant	0	8	8
chr17	6428259	6430259	Dynlt1b		1	noCoverage	0.015388	0.045447	inconclusive	0	33	35
chr17	6634807	6636807	Tmem181d-ps		1	noCoverage	-0.029557	0.024056	inconclusive	0	11	10
chr17	6987129	6989129	Ezr	-0.10967713	3.17E-10	hypomethylated	0.013272	0.8532	insignificant	32	130	132
chr17	7152705	7154705	Rsph3b	0.14473898	0.87366	insignificant	0.012679	0.57023	insignificant	4	39	32
chr17	7165505	7167505	Tagap1		1	noCoverage	0.0035862	0.080458	insignificant	0	32	32
chr17	7182208	7184208	Rnaset2b		1	noCoverage	0.20714	0.42542	insignificant	0	8	5
chr17	7373463	7375463	Rps6ka2	-0.12105727	2.65E-20	hypomethylated	-0.0051211	0.89002	insignificant	28	78	78
chr17	7589654	7591654	Gm9992	-0.71343954	0.092245	insignificant	-0.047279	0.012871	hypomethylated	2	12	12
chr17	8117864	8119864	Tagap	-0.43837274	0.06284	insignificant	-0.033948	1	insignificant	2	22	22
chr17	8137478	8139478	Rsph3a	-0.21040894	0.0034452	hypomethylated	0.0014216	0.60837	insignificant	1	58	58
chr17	8357382	8359382	Fgfr1op		1	noCoverage	-0.0015945	0.41898	insignificant	0	68	60
chr17	8475677	8477677	Brp44l	-0.14646116	1.23E-29	hypomethylated	-0.000092007	0.10138	insignificant	55	193	187
chr17	8502967	8504967	Sft2d1	-0.16805406	0.000024101	hypomethylated	-0.032117	1	insignificant	14	60	59
chr17	8532270	8534270	Prr18	-0.10475643	5.24E-25	hypomethylated	0.013231	0.44293	insignificant	36	137	125
chr17	8532603	8534603	Prr18	-0.11893975	5.86E-25	hypomethylated	-0.002616	0.42978	insignificant	36	149	131
chr17	8626287	8628287	T	-0.09793812	0.21682	insignificant	0.023594	0.012808	hypermethylated	18	113	106
chr17	8993609	8995609	Pde10a	-0.13978465	1.18E-34	hypomethylated	0.012801	0.44396	insignificant	36	128	103
chr17	9180197	9182197	1700010I14Rik		1	noCoverage	0.13493	1	insignificant	0	11	14
chr17	9360887	9362887	6530411M01F	-0.30020792	0.556	insignificant	-0.023759	0.72315	insignificant	1	14	14
chr17	9862569	9864569	Pabpc6	-0.16613046	0.56241	insignificant	-0.073869	0.6754	insignificant	3	20	6
chr17	10511783	10513783	B930003M22F	-0.07914025	4.53E-20	hypomethylated	-0.0095916	0.25781	insignificant	40	197	185
chr17	10512226	10514226	B930003M22F	-0.08272786	1.95E-10	hypomethylated	-0.011311	0.064984	insignificant	26	143	131
chr17	11032249	11034249	Park2	-0.07678292	0.0010712	hypomethylated	0.017961	0.29038	insignificant	8	43	43
chr17	11033057	11035057	Park2	-0.0906181	0.00051555	hypomethylated	0.018766	0.85511	insignificant	6	39	39
chr17	12311149	12313149	Agpat4	-0.22400199	0.000000965	hypomethylated	-0.038114	0.51023	insignificant	5	22	22
chr17	12511526	12513526	4732491K20Ri	-0.07974225	0.00009128	hypomethylated	0.024108	0.000039004	hypermethylated	9	40	40
chr17	12700570	12702570	Slc22a3	-0.22070969	0.41848	insignificant	-0.065838	0.012336	hypomethylated	5	24	24
chr17	12776054	12778054	Slc22a2	-0.0091107	0.34176	insignificant	0.045491	0.00035106	hypermethylated	5	19	19
chr17	12933176	12935176	Airn	-0.02474694	1	insignificant	-0.050025	0.0012291	hypomethylated	2	19	19
chr17	12962572	12964572	Igf2r	-0.13994576	0.010348	hypomethylated	-0.11283	0.00000898	hypomethylated	11	34	34
chr17	13102866	13104866	Pnldc1	-0.05601273	0.074675	insignificant	-0.051661	0.91081	insignificant	8	34	34
chr17	13108330	13110330	Tcp1	-0.14908169	1.13E-19	hypomethylated	-0.0032249	0.21315	insignificant	12	58	60
chr17	13108957	13110957	Tcp1	-0.25153975	4.3E-09	hypomethylated	0.0085678	0.69469	insignificant	5	56	54
chr17	13114722	13116722	Snora20	0.26302083	1	insignificant	0.10917	0.4603	insignificant	2	8	8
chr17	13133262	13135262	Acat3	-0.31978273	6.55E-14	hypomethylated	0.089568	0.0013619	hypermethylated	8	34	29
chr17	13185125	13187125	Wtap	-0.07488846	0.00000195	hypomethylated	-0.010653	0.086682	insignificant	37	136	128
chr17	13185405	13187405	Wtap	-0.06646153	0.060513	insignificant	-0.0050244	0.3454	insignificant	19	76	76
chr17	13199704	13201704	Sod2	-0.06994398	9.53E-08	hypomethylated	-0.011336	0.046373	hypomethylated	27	110	110
chr17	13546437	13548437	Gm9880	0.1392364	1	insignificant	-0.030406	1	insignificant	1	6	5
chr17	13896547	13898547	Mllt4	-0.10561181	1.67E-61	hypomethylated	0.0086268	0.23371	insignificant	100	309	286
chr17	13898401	13900401	Tcte2	-0.15595935	1.26E-20	hypomethylated	-0.018102	0.5045	insignificant	30	77	74
chr17	14084379	14086379	Gm7168	-0.21581541	0.072401	insignificant	0.054795	0.36437	insignificant	3	10	10
chr17	14340838	14342838	Dact2		1	noCoverage	0.015081	0.70121	insignificant	0	26	26
chr17	14415512	14417512	Smoc2	-0.09622516	1.24E-18	hypomethylated	0.013865	0.16857	insignificant	31	107	102
chr17	14535949	14537949	Smoc2		1	noCoverage	-0.010512	0.67691	insignificant	0	7	6
chr17	14831269	14833269	Thbs2	-0.26191681	1	insignificant	0.011156	0.039366	inconclusive	2	10	10
chr17	15079188	15081188	1600012H06R	-0.14391816	1.91E-14	hypomethylated	-0.052713	0.33299	insignificant	27	127	114
chr17	15080129	15082129	Wdr27	-0.17135046	0.00000018	hypomethylated	-0.074949	0.26019	insignificant	17	91	78
chr17	15177575	15179575	2210404J11Rik		1	noCoverage	-0.00046296	1	insignificant	0	36	35
chr17	15512787	15514787	Dll1	-0.16649479	2.34E-28	hypomethylated	-0.007313	0.5009	insignificant	26	109	100
chr17	15532165	15534165	Fam120b	-0.44033164	0.00000449	stronglyHypometh	0.11659	0.90352	insignificant	3	10	10
chr17	15532209	15534209	Fam120b	-0.44033164	0.00000449	stronglyHypometh	0.11659	0.90352	insignificant	3	10	10
chr17	15635240	15637240	Tbp	-0.12383559	9.78E-08	hypomethylated	-0.0067984	0.53279	insignificant	27	98	95
chr17	15635851	15637851	Tbp	-0.14396061	0.00001017	hypomethylated	0.0023245	0.89127	insignificant	24	92	89
chr17	15664265	15666265	Pdcd2	-0.30318962	0.000000147	hypomethylated	-0.0025292	0.94557	insignificant	4	20	20
chr17	15700287	15702287	Prdm9	-0.1319672	0.060351	insignificant	0.010497	0.05029	insignificant	14	54	53
chr17	15840930	15842930	Chd1	-0.09781721	3.64E-24	hypomethylated	0.011723	0.073989	insignificant	84	282	269
chr17	15963550	15965550	Rgmb	-0.11168534	0.00000108	hypomethylated	0.036392	0.41345	insignificant	6	28	27
chr17	17200066	17202066	Zfp960	-0.4916388	0.000000381	stronglyHypometh	0.066677	0.34763	insignificant	2	4	4
chr17	17510295	17512295	Riok2	-0.24364054	2.91E-11	hypomethylated	-0.0027033	0.60249	insignificant	11	36	34
chr17	17538649	17540649	Lix1	-0.44150327	0.0012053	stronglyHypometh	0.019281	0.34486	insignificant	1	4	4
chr17	17761453	17763453	Lnpep	-0.14628401	6.8E-52	hypomethylated	-0.012304	0.36916	insignificant	43	146	141
chr17	17966151	17968151	Mir99b	-0.06049606	0.0021732	hypomethylated	-0.055897	0.56199	insignificant	11	37	38
chr17	17966315	17968315	Mirlet7e	-0.06049606	0.0021732	hypomethylated	-0.055897	0.56199	insignificant	11	37	38
chr17	17966775	17968775	Mir125a	-0.06049606	0.0021732	hypomethylated	-0.055897	0.56199	insignificant	11	37	38

chr17	17966938	17968938	Ncrna00085	-0.06049606	0.0021732	hypomethylated	-0.055897	0.56199	insignificant	11	37	38
chr17	18287893	18289893	Vmn2r92		1	noCoverage	0.012182	0.61177	insignificant	0	9	8
chr17	19909326	19911326	Vmn2r103		1	noCoverage	0.029457	0.59625	insignificant	0	6	6
chr17	20185169	20187169	Vmn2r104		1	noCoverage	-0.012369	0.26745	insignificant	0	7	7
chr17	21081417	21083417	Ppp2r1a	-0.16287703	0.006757	hypomethylated	-0.010868	0.47619	insignificant	18	95	94
chr17	21144904	21146904	Zfp160	-0.67777778	0.0046661	stronglyHypometh	-0.013095	1	insignificant	4	13	12
chr17	21519711	21521711	Zfp677	-0.25665256	9.11E-10	hypomethylated	-0.011101	0.5302	insignificant	6	35	32
chr17	21559190	21561190	Zfp54	-0.20370782	0.00028374	hypomethylated	-0.0071243	0.7898	insignificant	6	29	30
chr17	21586315	21588315	Zfp51	-0.20613368	0.011558	hypomethylated	0.025563	0.79392	insignificant	3	28	29
chr17	21624951	21626951	Zfp53	-0.16239552	3.57E-16	hypomethylated	0.067282	0.96348	insignificant	10	42	41
chr17	21671502	21673502	Zfp52	-0.15697319	0.00048729	hypomethylated	0.039023	0.12432	insignificant	4	30	37
chr17	21703009	21705009	Zfp948	-0.34131535	1	insignificant	-0.12709	0.36257	insignificant	3	13	10
chr17	21786577	21788577	3110052M02R	-0.21075036	0.00020951	hypomethylated	0.031043	0.28928	insignificant	1	5	5
chr17	21843707	21845707	Zfp760	-0.25070422	0.11639	insignificant	0.078794	0.21864	insignificant	3	35	38
chr17	21869690	21871690	Zfp229	-0.12232787	0.0023136	hypomethylated	0.070648	0.21705	insignificant	7	19	14
chr17	22098525	22100525	Zfp943	-0.12718994	0.000000149	hypomethylated	0.010791	1	insignificant	9	45	41
chr17	22099417	22101417	Zfp942	-0.12441363	0.0062176	hypomethylated	0.029699	0.66143	insignificant	3	27	23
chr17	22099431	22101431	Zfp942	-0.12441363	0.0062176	hypomethylated	0.029699	0.66143	insignificant	3	27	23
chr17	22497419	22499419	Zfp758		1	noCoverage	-0.041589	0.39467	insignificant	0	23	25
chr17	22498367	22500367	Zfp944		1	noCoverage	0.0025752	1	insignificant	0	7	7
chr17	22560234	22562234	Zfp946	-0.21903153	0.085785	insignificant	0.071162	0.36251	insignificant	8	22	22
chr17	22954714	22956714	Gm16386	-0.30050781	0.18108	insignificant	0.048288	0.23226	insignificant	2	12	12
chr17	23004101	23006101	Zfp945	-0.26492537	1	insignificant	0.021834	0.00032278	hypermethylated	2	4	5
chr17	23330195	23332195	Zfp40	-0.23121184	0.0099865	hypomethylated	0.0041111	0.83852	insignificant	5	10	10
chr17	23701193	23703193	Zfp213	-0.45893021	0.061718	insignificant	0.030547	0.92867	insignificant	1	10	10
chr17	23736822	23738822	Zscan10	-0.66538462	0.0011331	stronglyHypometh	-0.10217	0.32765	insignificant	2	6	4
chr17	23782236	23784236	Mmp25	-0.29278314	3.99E-15	hypomethylated	0.01992	0.30425	insignificant	10	40	36
chr17	23796489	23798489	Ccdc64b	-0.19248109	9.67E-12	hypomethylated	-0.03507	0.32291	insignificant	11	48	44
chr17	23809574	23811574	Hcfc1r1	-0.13065446	2.11E-16	hypomethylated	0.02666	0.92362	insignificant	14	64	64
chr17	23810737	23812737	Hcfc1r1	-0.10355505	2.74E-09	hypomethylated	0.0078926	0.55114	insignificant	9	26	26
chr17	23814416	23816416	Cldn6	-0.24823906	1.17E-29	hypomethylated	0.025114	0.056878	insignificant	8	36	34
chr17	23815331	23817331	Cldn6	-0.19247945	4.45E-08	hypomethylated	0.065246	0.91265	insignificant	7	36	34
chr17	23862302	23864302	Pkmyt1	-0.11632797	1.96E-26	hypomethylated	0.0039235	0.55573	insignificant	34	143	135
chr17	23877297	23879297	Kremen2	0.0927941	0.61999	insignificant	-0.017843	0.0011679	inconclusive	4	42	46
chr17	23882796	23884796	Kremen2	-0.16623538	9.83E-09	hypomethylated	0.030906	0.39045	insignificant	8	36	36
chr17	23908558	23910558	Flywch1	-0.12784287	0.21524	insignificant	0.019625	0.47683	insignificant	3	20	17
chr17	23923044	23925044	Flywch2	-0.23712167	0.00000102	hypomethylated	-0.087334	0.047961	hypomethylated	3	37	32
chr17	23939153	23941153	Srrm2	-0.02557354	0.55352	insignificant	-0.0041948	0.059812	insignificant	34	184	184
chr17	23966076	23968076	Tceb2	-0.29238823	0.000094944	hypomethylated	-0.0062928	0.12878	insignificant	11	19	21
chr17	23972734	23974734	Prss33	0.04712302	0.66214	insignificant	-0.040911	0.76976	insignificant	1	4	4
chr17	24004038	24006038	Prss21		1	noCoverage	0.03335	0.22789	insignificant	0	18	18
chr17	24016842	24018842	Dcpp1	0.1986715	0.17724	insignificant	-0.23585	0.076839	insignificant	1	6	6
chr17	24034688	24036688	Dcpp2	-0.19940476	0.34914	insignificant	0.0024351	1	insignificant	1	4	4
chr17	24053424	24055424	Dcpp3	0.16666667	0.20222	insignificant	0.11728	0.046711	hypermethylated	1	6	9
chr17	24092240	24094240	Sbpl		1	noCoverage	-0.022625	0.2529	insignificant	0	4	4
chr17	24174209	24176209	Prss27	-0.04355988	1	insignificant	0.13365	0.087935	insignificant	2	6	7
chr17	24210452	24212452	Kctd5	-0.20199653	1	insignificant	-0.054837	0.84992	insignificant	3	24	20
chr17	24278556	24280556	Pdpk1	-0.17228355	0.000018128	hypomethylated	-0.063552	0.43631	insignificant	7	20	18
chr17	24278561	24280561	Pdpk1	-0.17228355	0.000018128	hypomethylated	-0.063552	0.43631	insignificant	7	20	18
chr17	24306374	24308374	Atp6v0c-ps2	-0.14901146	0.00093258	hypomethylated	0.0031172	0.02358	hypermethylated	17	74	74
chr17	24336902	24338902	Tbc1d24	0.15071203	0.59908	insignificant	-0.033741	1	insignificant	1	4	4
chr17	24342507	24344507	Ntn3	-0.14885415	1.14E-15	hypomethylated	-0.0052069	1	insignificant	28	116	111
chr17	24346332	24348332	Ntn3	-0.22634712	0.36354	insignificant	0.010301	0.10733	insignificant	4	19	16
chr17	24357714	24359714	1600002H07R	-0.57162229	0.00013428	stronglyHypometh	-0.022019	0.045162	hypomethylated	1	13	12
chr17	24388354	24390354	Ccnf	-0.21281623	3.79E-16	hypomethylated	-0.024082	0.10771	insignificant	15	56	57
chr17	24487990	24489990	Abca3	-0.09587071	9.57E-16	hypomethylated	-0.0056092	0.95156	insignificant	37	155	149
chr17	24550619	24552619	Rnps1	-0.05427784	1.09E-14	hypomethylated	0.01354	0.50636	insignificant	11	79	71
chr17	24551458	24553458	D330041H03R	-0.06671344	1.07E-14	hypomethylated	-0.0017001	0.46042	insignificant	11	73	64
chr17	24562627	24564627	Eci1	-0.19590067	0.000000188	hypomethylated	-0.043718	0.82443	insignificant	16	78	75
chr17	24580046	24582046	E4f1	0.04728809	0.56961	insignificant	0.013663	0.30333	insignificant	8	28	28
chr17	24592256	24594256	E4f1	0.14309764	0.19018	insignificant	0.10228	0.19666	insignificant	2	6	7
chr17	24606417	24608417	Pgp	-0.11276559	6.69E-43	hypomethylated	0.0011355	0.15048	insignificant	66	218	205
chr17	24616023	24618023	Mlst8		1	noCoverage	0.062266	0.70439	insignificant	0	6	6
chr17	24624727	24626727	Caskin1	-0.08934181	9.83E-12	hypomethylated	0.0081762	0.34569	insignificant	31	159	153
chr17	24664883	24666883	Traf7	-0.28790811	2E-10	hypomethylated	0.047845	0.7795	insignificant	10	23	22
chr17	24685894	24687894	Pkd1	-0.1114582	1.38E-32	hypomethylated	-0.00089827	0.11549	insignificant	31	154	142
chr17	24768626	24770626	Nthl1	-0.04897013	0.00086384	inconclusive	0.066171	1	insignificant	3	8	9
chr17	24781902	24783902	Slc9a3r2	-0.29902136	0.24986	insignificant	0.043261	0.2208	insignificant	1	10	10
chr17	24787250	24789250	Slc9a3r2		1	noCoverage	-0.040741	0.8307	insignificant	0	3	3
chr17	24795370	24797370	Npw	-0.2741942	1	insignificant	-0.02629	0.79317	insignificant	1	28	28
chr17	24805696	24807696	Zfp598	-0.15457443	2.76E-15	hypomethylated	-0.0077102	0.057963	insignificant	31	131	132
chr17	24826894	24828894	Syngr3	-0.16939668	0.0045695	hypomethylated	-0.034027	0.89341	insignificant	4	28	27
chr17	24832178	24834178	Noxo1	-0.16554867	1.29E-36	hypomethylated	0.018165	0.3947	insignificant	45	140	132
chr17	24833101	24835101	Gfer	-0.08578258	0.00000854	hypomethylated	0.01009	0.48135	insignificant	15	34	34
chr17	24844598	24846598	Tbl3	-0.34467596	3.95E-09	stronglyHypometh	0.028569	0.38092	insignificant	9	54	56
chr17	24855002	24857002	Snora78	-0.02595535	0.65261	insignificant	0.0019198	0.89307	insignificant	3	71	68
chr17	24856007	24858007	Rps2	-0.12356366	0.022765	hypomethylated	0.067706	0.000000428	hypermethylated	15	126	121
chr17	24856733	24858733	Snora64	-0.10195528	0.0002301	hypomethylated	0.085017	6.64E-08	hypermethylated	12	95	93
chr17	24856777	24858777	Snora78	-0.10579042	0.00013877	hypomethylated	0.089585	9.36E-08	hypermethylated	12	91	89
chr17	24856910	24858910	Snora78	-0.12445304	0.000050188	hypomethylated	0.12197	5.75E-09	hypermethylated	12	67	65
chr17	24861333	24863333	Ndufb10		1	noCoverage	0.00467	0.83593	insignificant	0	19	19
chr17	24863773	24865773	Rpl3l	0.13853668	0.000020472	hypermethylated	0.085555	0.074638	insignificant	9	38	38
chr17	24868629	24870629	Rpl3l		1	noCoverage	-0.31167	0.0007777	hypomethylated	0	10	10
chr17	24872586	24874586	Sepx1	-0.10316541	0.017129	hypomethylated	0.027211	0.033642	hypermethylated	12	44	41
chr17	24888947	24890947	Hs3st6	-0.14903326	1.93E-26	hypomethylated	0.014051	0.68454	insignificant	31	139	119

chr17	24940326	24942326	4930528F23Ri	-0.2511698	0.34817	insignificant	0.039788	0.52387	insignificant	2	41	36
chr17	24986434	24988434	Hagh	-0.14969856	2.75E-36	hypomethylated	-0.00054671	0.41533	insignificant	34	117	117
chr17	24986611	24988611	Hagh	-0.14340587	2.41E-32	hypomethylated	-0.0008718	0.40069	insignificant	33	115	115
chr17	24987247	24989247	Fahd1	-0.1452487	6.04E-26	hypomethylated	0.0015811	0.38107	insignificant	26	68	68
chr17	25014714	25016714	Igfals	-0.10338346	0.0046682	hypomethylated	0.0089286	0.13301	insignificant	2	4	4
chr17	25022618	25024618	Spsb3	-0.34084318	1.91E-14	stronglyHypometh	0.080856	0.74394	insignificant	5	19	16
chr17	25023295	25025295	Spsb3	-0.21027852	6.61E-32	hypomethylated	0.050343	0.14844	insignificant	10	39	36
chr17	25023425	25025425	Spsb3	-0.21027852	6.61E-32	hypomethylated	0.050343	0.14844	insignificant	10	39	36
chr17	25031064	25033064	Mrps34	-0.11438761	0.00000246	hypomethylated	0.02143	0.060883	insignificant	17	80	83
chr17	25032032	25034032	Eme2	-0.13427407	0.000000209	hypomethylated	0.015752	0.014673	hypermethylated	16	96	100
chr17	25032444	25034444	Nme3	-0.29481935	0.0026689	hypomethylated	-0.015476	0.62911	insignificant	6	70	74
chr17	25073922	25075922	Mapk8ip3	-0.12940051	0.000000638	hypomethylated	-0.0019309	0.43037	insignificant	6	18	18
chr17	25152030	25154030	Ift140	-0.13082918	2.11E-17	hypomethylated	0.015966	0.9738	insignificant	30	113	108
chr17	25152175	25154175	Cramp1l	-0.12374588	6.53E-15	hypomethylated	0.015363	0.69474	insignificant	30	107	102
chr17	25252850	25254850	Telo2		1	noCoverage	0.050794	0.88023	insignificant	0	6	6
chr17	25252912	25254912	Telo2		1	noCoverage	0.050794	0.88023	insignificant	0	6	6
chr17	25269338	25271338	Clcn7	-0.22850833	1.91E-23	hypomethylated	0.014042	0.46959	insignificant	20	54	46
chr17	25298405	25300405	Ccdc154		1	noCoverage	-0.016288	0.6854	insignificant	0	12	12
chr17	25320505	25322505	BC003965	-0.10547761	3.18E-25	hypomethylated	0.01283	0.095585	insignificant	43	127	122
chr17	25324344	25326344	Unkl	-0.09845614	6.12E-17	hypomethylated	-0.009148	0.50355	insignificant	50	161	154
chr17	25358526	25360526	Unkl	0.0153504	0.007832	inconclusive	-0.0005604	0.51336	insignificant	10	44	41
chr17	25376114	25378114	0610007P22Ri	-0.10782997	0.00000405	hypomethylated	0.014984	0.017415	inconclusive	24	111	118
chr17	25377061	25379061	Gnptg	-0.14057242	3.75E-09	hypomethylated	0.010493	0.10556	insignificant	19	78	84
chr17	25410068	25412068	Gm17801	-0.09518114	1.21E-13	hypomethylated	0.0016512	0.46977	insignificant	32	104	105
chr17	25410376	25412376	Ube2i	-0.10066908	2.51E-14	hypomethylated	0.00087373	0.41475	insignificant	25	86	87
chr17	25410859	25412859	Ube2i		1	noCoverage	0.0049584	0.79235	insignificant	0	4	4
chr17	25444590	25446590	Prss28		1	noCoverage	0.078063	0.17714	insignificant	0	8	8
chr17	25454598	25456598	Prss29	-0.11098398	0.47295	insignificant	0.091681	0.41837	insignificant	1	2	2
chr17	25570728	25572728	Cacna1h	-0.08786897	0.00000343	hypomethylated	0.006686	0.86187	insignificant	14	117	117
chr17	25607534	25609534	Tekt4		1	noCoverage	0.022963	0.0041206	hypermethylated	0	12	15
chr17	25634233	25636233	Sstr5		1	noCoverage	0.011555	0.7367	insignificant	0	14	14
chr17	25707631	25709631	Sox8	-0.12829784	0.0000013	hypomethylated	0.011025	0.40218	insignificant	11	61	58
chr17	25715118	25717118	Lmf1	-0.41919192	0.000176	stronglyHypometh	-0.023914	1	insignificant	3	6	6
chr17	25853116	25855116	Gng13	-0.15930543	0.00049482	hypomethylated	-0.049765	0.64353	insignificant	7	49	46
chr17	25863695	25865695	Rpusd1		1	noCoverage	0.0016693	0.77727	insignificant	0	18	19
chr17	25864360	25866360	Chtf18	0.08684807	0.14088	insignificant	-0.13151	0.6719	insignificant	2	14	17
chr17	25871984	25873984	Mslnl	-0.07854868	0.38565	insignificant	0.058141	0.010258	hypermethylated	2	16	16
chr17	25909720	25911720	Narfl	-0.16056565	0.094618	insignificant	-0.018105	1	insignificant	3	62	62
chr17	25922524	25924524	Ccdc78	-0.26411632	0.0041766	hypomethylated	0.021732	0.65776	insignificant	2	6	6
chr17	25929229	25931229	Fam173a	-0.1592267	6.74E-10	hypomethylated	0.011484	0.85671	insignificant	10	50	50
chr17	25933990	25935990	Metrn	-0.18772246	0.1372	insignificant	-0.036939	0.085487	insignificant	0	24	24
chr17	25945029	25947029	Fbxl16	-0.09038806	3.94E-23	hypomethylated	0.0043168	0.39724	insignificant	44	170	169
chr17	25959571	25961571	Wdr24		1	noCoverage	0.045294	0.18772	insignificant	0	22	21
chr17	25964987	25966987	Jmjd8		1	noCoverage	0.003733	0.47644	insignificant	0	17	17
chr17	25970306	25972306	Stub1	-0.13963948	0.014523	hypomethylated	0.014053	0.16889	insignificant	2	28	28
chr17	25974072	25976072	Rhbdl1		1	noCoverage	0.12744	0.0060796	hypermethylated	0	18	18
chr17	25981796	25983796	Rhot2	0.05830683	0.13346	insignificant	0.0081988	0.47045	insignificant	3	34	34
chr17	25998460	26000460	Wdr90	-0.3865519	0.0037102	stronglyHypometh	-0.014893	0.71905	insignificant	3	16	18
chr17	26005683	26007683	Fam195a	0.17608187	1	insignificant	0.13443	0.11951	insignificant	1	15	11
chr17	26011444	26013444	0610011F06Ri	-0.02178396	0.016413	inconclusive	0.01269	0.40001	insignificant	5	70	68
chr17	26056659	26058659	Rab40c	0.0337995	0.20887	insignificant	0.020831	0.54574	insignificant	4	47	47
chr17	26078907	26080907	Nhlrc4	-0.14905808	0.018857	hypomethylated	0.010321	0.00023568	hypermethylated	8	24	24
chr17	26081876	26083876	Nhlrc4		1	noCoverage	-0.091457	1	insignificant	0	3	6
chr17	26206122	26208122	Rab11fip3	-0.12231051	1.18E-22	hypomethylated	0.0067853	0.64537	insignificant	28	112	124
chr17	26227109	26229109	Decr2	-0.26730166	6.89E-11	hypomethylated	0.0048951	0.0011897	inconclusive	5	34	33
chr17	26249260	26251260	Tmem8	-0.12198048	1.53E-08	hypomethylated	0.0046738	0.62542	insignificant	23	82	79
chr17	26259447	26261447	Mrpl28	-0.08482158	1	insignificant	0.0058492	0.7028	insignificant	5	28	28
chr17	26274630	26276630	Axin1	-0.09408743	2.62E-14	hypomethylated	-0.008593	0.086498	insignificant	31	85	85
chr17	26336032	26338032	Pdia2	-0.10790556	1.59E-14	hypomethylated	-0.011301	0.21897	insignificant	14	52	48
chr17	26338295	26340295	Rgs11	-0.15806323	0.00018646	hypomethylated	0.0083102	1	insignificant	7	14	22
chr17	26381086	26383086	Itfg3	0.48886957	1	insignificant	0.23058	0.017047	hypermethylated	1	19	15
chr17	26388854	26390854	Luc7l	-0.09909746	5.39E-14	hypomethylated	0.0048705	0.37518	insignificant	27	82	82
chr17	26550909	26552909	Neurl1b	-0.10314014	3.26E-26	hypomethylated	0.0074238	0.4761	insignificant	43	151	151
chr17	26645417	26647417	Dusp1	-0.07637189	0.000005015	hypomethylated	0.003263	0.74782	insignificant	16	86	81
chr17	26697456	26699456	Ergic1	-0.11034427	2.41E-08	hypomethylated	-0.0083003	0.20838	insignificant	12	83	88
chr17	26812340	26814340	Atp6v0e	-0.07420699	0.21946	insignificant	-0.012991	0.62204	insignificant	21	85	84
chr17	26851594	26853594	A930001N09R	-0.09384432	2.67E-14	hypomethylated	0.087015	0.26467	insignificant	42	143	140
chr17	26917023	26919023	Bnip1	-0.39608455	0.00010315	stronglyHypometh	0.022194	0.82535	insignificant	6	12	12
chr17	27053035	27055035	Kifc5b	-0.17962697	0.0038352	hypomethylated	0.040021	0.20995	insignificant	7	51	43
chr17	27069071	27071071	Phf1	-0.20219311	1.35E-09	hypomethylated	-0.006965	0.50122	insignificant	9	72	65
chr17	27076413	27078413	Cuta	-0.10219908	1.28E-09	hypomethylated	-0.010074	0.72611	insignificant	17	72	71
chr17	27076423	27078423	Cuta	-0.10837146	2.07E-10	hypomethylated	-0.01195	0.69063	insignificant	17	70	69
chr17	27085064	27087064	Mir3083	-0.11006018	2.68E-17	hypomethylated	0.018363	0.089878	insignificant	24	120	121
chr17	27109123	27111123	Zbtb9	-0.04921992	0.00000283	hypomethylated	-0.013948	0.26332	insignificant	35	105	110
chr17	27165203	27167203	Ggnbp1	-0.58788954	6.32E-15	stronglyHypometh	-0.0078818	0.00033795	inconclusive	6	27	28
chr17	27165571	27167571	Ggnbp1	-0.58732831	8.68E-15	stronglyHypometh	-0.0070004	0.0011861	inconclusive	5	25	26
chr17	27193248	27195248	Itpr3	-0.09407952	1.96E-15	hypomethylated	-0.0082896	0.72518	insignificant	28	83	93
chr17	27304709	27306709	Ip6k3	0.07967033	0.60484	insignificant	-0.12221	0.84086	insignificant	2	6	6
chr17	27341383	27343383	Lemd2	-0.20737499	0.00787	hypomethylated	0.00095379	0.67794	insignificant	3	14	14
chr17	27640249	27642249	Grm4	0.11074651	1	lowCoverage	-0.059708	0.11433	insignificant	1	8	8
chr17	27692518	27694518	Hmga1	-0.12689373	8.64E-44	hypomethylated	-0.034405	0.32004	insignificant	72	258	250
chr17	27692548	27694548	Hmga1	-0.12650719	2.82E-43	hypomethylated	-0.034153	0.33827	insignificant	70	250	242
chr17	27692597	27694597	Hmga1-rs1	-0.12663155	1.01E-43	hypomethylated	-0.029235	0.27227	insignificant	70	238	230
chr17	27702672	27704672	AI413582	-0.08672455	2.58E-08	hypomethylated	0.0054871	0.60003	insignificant	15	47	40
chr17	27760397	27762397	Nudt3	-0.11843594	9.64E-17	hypomethylated	0.00097703	0.92963	insignificant	27	66	66

chr17	27772187	27774187	Rps10	-0.00606463	0.8762	insignificant	0.0027281	0.28663	insignificant	17	53	53
chr17	27791626	27793626	Pacsin1	-0.15825964	0.000000368	hypomethylated	0.0011116	0.27707	insignificant	15	69	70
chr17	27821198	27823198	Pacsin1	-0.08739743	0.78324	insignificant	0.03619	0.0034462	hypermethylated	26	86	80
chr17	27957487	27959487	D17Wsu92e	-0.14606015	0.14901	insignificant	-0.00095061	0.41629	insignificant	4	52	52
chr17	27976031	27978031	Snrpc		1	noCoverage	0.1187	1	insignificant	0	12	12
chr17	27992451	27994451	Uhrf1bp1	-0.0939902	5.15E-19	hypomethylated	0.0013483	1	insignificant	25	112	112
chr17	28044669	28046669	Anks1	-0.1117825	4.39E-33	hypomethylated	-0.022155	0.11435	insignificant	31	82	66
chr17	28045284	28047284	Anks1	-0.12951122	3.54E-39	hypomethylated	-0.0099865	0.12223	insignificant	33	98	80
chr17	28217529	28219529	Tcp11	-0.22124358	2.9E-15	hypomethylated	0.064475	0.95241	insignificant	8	42	48
chr17	28217584	28219584	Tcp11	-0.27015767	4.47E-09	hypomethylated	0.071757	0.84406	insignificant	3	30	36
chr17	28278470	28280470	Scube3	-0.02100814	7.5E-14	hypomethylated	0.0083805	0.67342	insignificant	39	185	182
chr17	28313362	28315362	Zfp523	-0.16332325	3.37E-23	hypomethylated	-0.01354	0.1322	insignificant	41	102	98
chr17	28343722	28345722	Def6	-0.16796924	0.58644	insignificant	0.047379	0.49744	insignificant	4	33	28
chr17	28368698	28370698	Ppard	-0.13918309	0.000000352	hypomethylated	-0.015837	0.5224	insignificant	10	53	53
chr17	28449474	28451474	Fance	-0.04960773	1.57E-14	hypomethylated	0.031972	0.2775	insignificant	15	93	95
chr17	28464415	28466415	Rpl10a	-0.10862327	1.74E-13	hypomethylated	0.010276	0.43273	insignificant	35	146	155
chr17	28487545	28489545	Tead3	-0.16576037	5.82E-09	hypomethylated	0.010203	0.00000167	hypermethylated	30	114	105
chr17	28487750	28489750	Tead3	-0.14224443	0.00014068	hypomethylated	0.0056973	0.000051016	hypermethylated	22	97	89
chr17	28502088	28504088	Tulp1	-0.13690476	0.20534	insignificant	-0.081845	0.41204	insignificant	3	6	6
chr17	28623057	28625057	Fkbp5	0.10649415	0.029024	hypermethylated	0.033171	0.47439	insignificant	7	45	46
chr17	28666805	28668805	4930511I11Ril	0.0009855	0.41038	insignificant	0.024627	0.038588	hypermethylated	6	23	22
chr17	28685431	28687431	Gm749	-0.06085582	0.84385	insignificant	0.0047777	0.018831	hypermethylated	7	27	24
chr17	28697659	28699659	Clps	-0.27944139	1	insignificant	0.028	0.41887	insignificant	3	7	7
chr17	28711663	28713663	Lhfpl5	-0.05330062	1.9E-11	hypomethylated	0.017603	0.34215	insignificant	13	48	48
chr17	28759399	28761399	Srpk1	-0.14321108	0.000000237	hypomethylated	0.026494	0.26341	insignificant	14	48	49
chr17	28827286	28829286	Mapk14	-0.10149535	1.3E-27	hypomethylated	-0.013111	0.19591	insignificant	54	200	188
chr17	28828512	28830512	Mapk14	-0.11393478	7.22E-21	hypomethylated	-0.0047752	0.61019	insignificant	33	106	100
chr17	28905261	28907261	Mapk13	-0.13163886	6.98E-14	hypomethylated	-0.0035423	0.64714	insignificant	30	101	100
chr17	28937070	28939070	Brpf3	-0.08292181	0.00026652	hypomethylated	0.0040883	0.80239	insignificant	46	129	123
chr17	28994355	28996355	Pnpla1	-0.05124024	0.0013954	hypomethylated	0.019284	0.2049	insignificant	5	13	11
chr17	29089160	29091160	Kctd20	-0.10603477	4.38E-16	hypomethylated	-0.0032941	0.96536	insignificant	28	84	80
chr17	29144882	29146882	Stk38	-0.30900398	5.64E-09	hypomethylated	0.0095246	0.75614	insignificant	11	45	44
chr17	29168604	29170604	Srsf3	-0.15221288	3.5E-17	hypomethylated	-0.018629	0.51931	insignificant	39	101	96
chr17	29215906	29217906	Gm16197	-0.41571386	3.27E-09	stronglyHypometh	0.034757	0.03714	hypermethylated	5	32	32
chr17	29229716	29231716	Cdkn1a	-0.1573617	1.78E-13	hypomethylated	-0.015937	0.33577	insignificant	15	75	75
chr17	29271000	29273000	Rab44	0.02843446	0.43166	insignificant	-0.0010616	0.79239	insignificant	5	14	13
chr17	29374735	29376735	Cpne5	-0.12139972	3.37E-13	hypomethylated	-0.035418	0.48181	insignificant	23	104	97
chr17	29400916	29402916	Ppil1	-0.31751688	1.3E-13	hypomethylated	-0.012416	0.00000226	hypomethylated	12	44	42
chr17	29404732	29406732	BC004004	-0.12571923	0.00000516	hypomethylated	-0.009399	0.026094	hypomethylated	25	82	82
chr17	29484849	29486849	Mtch1	-0.22426822	0.000000437	hypomethylated	0.029483	0.35255	insignificant	4	21	20
chr17	29496858	29498858	Fgd2	-0.29017857	0.0051148	hypomethylated	-0.0055632	1	insignificant	3	8	8
chr17	29626989	29628989	Pim1	-0.09909625	6.53E-39	hypomethylated	0.0042774	0.45447	insignificant	76	272	278
chr17	29685746	29687746	Tbc1d22b	-0.16686261	6.58E-22	hypomethylated	-0.00045653	0.8411	insignificant	23	74	70
chr17	29686538	29688538	Tmem217	-0.15992049	6.52E-22	hypomethylated	0.0064856	0.80979	insignificant	23	71	70
chr17	29750775	29752775	Rnf8	-0.10892767	3.76E-11	hypomethylated	-0.0045325	0.91285	insignificant	21	106	99
chr17	29796545	29798545	Ftsjd2	-0.20804523	1.25E-17	hypomethylated	-0.017927	0.62761	insignificant	22	80	83
chr17	30024827	30026827	Mdga1	-0.07179359	7.03E-14	hypomethylated	0.0098981	0.10868	insignificant	40	141	137
chr17	30141031	30143031	Zfand3	-0.10633692	1.21E-20	hypomethylated	-0.00033548	0.82061	insignificant	45	198	193
chr17	30533125	30535125	Btbd9		1	noCoverage	0.11213	1	insignificant	0	11	12
chr17	30713232	30715232	Btbd9	-0.16198718	0.000068352	hypomethylated	-0.014968	0.68912	insignificant	6	72	71
chr17	30749604	30751604	Glo1	-0.57559226	0.0074428	stronglyHypometh	0.2531	0.22072	insignificant	2	9	8
chr17	30762880	30764880	Dnahc8	0.12121212	1	insignificant	0.021874	0.46352	insignificant	1	2	2
chr17	31037811	31039811	Glp1r	-0.06034159	0.000005356	hypomethylated	-0.0065025	0.71083	insignificant	15	59	64
chr17	31090627	31092627	Umodl1	-0.24141247	0.11971	insignificant	-0.058492	0.013818	hypomethylated	6	21	29
chr17	31193638	31195638	Abcg1	-0.1132547	8.03E-10	hypomethylated	-0.022652	0.68824	insignificant	20	62	53
chr17	31334694	31336694	Tmprss3		1	noCoverage	-0.10713	0.23579	insignificant	0	14	10
chr17	31335919	31337919	Tmprss3		1	noCoverage	-0.11293	0.18948	insignificant	0	14	14
chr17	31344010	31346010	Ubash3a	-0.54209207	0.47479	lowCoverage	-0.084174	0.19919	insignificant	1	15	12
chr17	31431427	31433427	Slc37a1	-0.14127844	3.04E-08	hypomethylated	-0.0010489	0.17179	insignificant	17	80	75
chr17	31522178	31524178	Pde9a	-0.1223717	8.78E-19	hypomethylated	-0.017709	0.051485	insignificant	15	42	43
chr17	31649432	31651432	Wdr4	-0.5	0.14349	insignificant	-0.033333	1	insignificant	1	2	2
chr17	31656059	31658059	Ndufv3	-0.16583801	0.00000602	hypomethylated	0.045527	0.70185	insignificant	2	34	28
chr17	31700717	31702717	Pknox1	-0.08225515	1.9E-35	hypomethylated	-0.0029668	0.82582	insignificant	63	266	265
chr17	31774076	31776076	Cbs	-0.05200787	0.15137	insignificant	0.024185	0.5562	insignificant	4	72	72
chr17	31774086	31776086	Cbs	-0.05200787	0.15137	insignificant	0.024185	0.5562	insignificant	4	72	72
chr17	31795699	31797699	U2af1	-0.09346434	0.0072664	hypomethylated	0.014891	0.6551	insignificant	1	41	41
chr17	31813889	31815889	Cryaa	-0.36852871	3.68E-09	stronglyHypometh	0.034012	0.95196	insignificant	4	38	38
chr17	31992737	31994737	Sik1	-0.16486958	0.00000032	hypomethylated	0.02294	0.15024	insignificant	21	81	73
chr17	32171453	32173453	Rrp1b	-0.08188943	3.95E-51	hypomethylated	0.017792	2.53E-17	hypermethylated	12	41	36
chr17	32172106	32174106	Rrp1b	-0.17603593	6.76E-23	hypomethylated	-0.023977	0.52266	insignificant	9	36	39
chr17	32184229	32186229	Rrp1b		1	noCoverage	-0.04326	0.68641	insignificant	0	10	10
chr17	32228368	32230368	Pdxk-ps		1	noCoverage	0.0085116	0.93448	insignificant	0	3	3
chr17	32303797	32305797	Notch3	-0.07908977	0.0070326	hypomethylated	0.011008	0.53504	insignificant	11	24	24
chr17	32421068	32423068	Brd4	-0.10114491	1.02E-35	hypomethylated	-0.0046221	0.2281	insignificant	55	146	146
chr17	32458098	32460098	Akap8	-0.17928521	1	insignificant	0.0085422	0.18719	insignificant	3	34	34
chr17	32524762	32526762	Wiz	-0.19783806	1.52E-20	hypomethylated	-0.0087469	0.69227	insignificant	10	35	35
chr17	32525895	32527895	Wiz	-0.19783806	1.52E-20	hypomethylated	-0.0087469	0.69227	insignificant	10	35	35
chr17	32538958	32540958	A530088E08R	-0.47774064	0.00016023	stronglyHypometh	0.049539	0.048328	hypermethylated	1	13	14
chr17	32540526	32542526	A530088E08R	0.03229989	1	insignificant	0.041189	0.63064	insignificant	2	11	12
chr17	32588667	32590667	Cyp4f39	-0.25260198	0.00000269	hypomethylated	0.042086	0.0059824	hypermethylated	7	28	28
chr17	32642406	32644406	Cyp4f17	-0.23001203	0.00074761	hypomethylated	0.054016	0.76643	insignificant	6	23	18
chr17	32795427	32797427	Cyp4f40		1	noCoverage	-0.027778	0.4191	insignificant	0	8	8
chr17	32925232	32927232	Zfp871		1	noCoverage	0.0079899	0.64482	insignificant	0	4	4
chr17	33101775	33103775	Zfp472	-0.17148088	0.000000929	hypomethylated	-0.0059644	0.44767	insignificant	13	56	56
chr17	33102661	33104661	Cyp4f41-ps	-0.18711217	0.000020987	hypomethylated	-0.0058828	0.39052	insignificant	8	34	34

chr17	33129083	33131083	Zfp952	-0.17043269	1	insignificant	0.025912	0.94599	insignificant	2	26	28
chr17	33170326	33172326	Zfp763		1	noCoverage	0.17687	0.65937	insignificant	0	10	7
chr17	33225311	33227311	Zfp563	-0.29689758	1.96E-10	hypomethylated	-0.015724	0.19943	insignificant	6	40	41
chr17	33276628	33278628	Morc2b		1	noCoverage	-0.13005	0.016888	hypomethylated	0	2	2
chr17	33495823	33497823	Zfp81	-0.32986395	0.010554	hypomethylated	0.070646	0.53873	insignificant	2	10	7
chr17	33568843	33570843	Actl9		1	noCoverage	0.097538	0.086941	insignificant	0	4	4
chr17	33660168	33662168	Adamts10	-0.13857992	0.000000116	hypomethylated	0.010433	0.61514	insignificant	11	44	43
chr17	33765036	33767036	Zfp414	-0.20309701	0.00000082	hypomethylated	0.01929	0.73742	insignificant	5	10	10
chr17	33822403	33824403	Hnrnpm	-0.11394992	3.18E-08	hypomethylated	-0.0058309	0.84553	insignificant	6	22	22
chr17	33918520	33920520	Angptl4		1	noCoverage	0.018227	0.92627	insignificant	0	10	9
chr17	33946467	33948467	Kank3	-0.07111537	0.1513	insignificant	0.025416	0.47728	insignificant	8	39	30
chr17	33960558	33962558	Ndufa7	0.02368817	0.059452	insignificant	0.11196	0.58408	insignificant	5	29	20
chr17	33961443	33963443	Rps28	0.04075896	1	insignificant	0.017947	0.70407	insignificant	4	17	16
chr17	33979035	33981035	Cd320		1	noCoverage	0.20034	0.52788	insignificant	0	12	6
chr17	34027578	34029578	Kifc1	-0.3324926	4.73E-15	hypomethylated	-0.078247	0.83574	insignificant	6	18	17
chr17	34045389	34047389	Daxx	-0.14756032	1.55E-25	hypomethylated	-0.0092368	0.30186	insignificant	29	133	132
chr17	34045545	34047545	Daxx	-0.14594838	1.62E-24	hypomethylated	-0.0088162	0.1795	insignificant	29	123	122
chr17	34052120	34054120	Zbtb22	-0.23297239	0.00000351	hypomethylated	0.0039241	0.000010523	hypermethylated	3	34	34
chr17	34055422	34057422	Tapbp	-0.17074586	1.03E-25	hypomethylated	0.0077695	0.14844	insignificant	22	88	84
chr17	34065838	34067838	Rgl2	-0.1566291	2.13E-32	hypomethylated	-0.0059273	0.086756	insignificant	36	76	76
chr17	34076667	34078667	Wdr46	-0.13490173	5.18E-17	hypomethylated	-0.0012133	0.78841	insignificant	30	109	109
chr17	34077275	34079275	H2-Ke2	-0.13490173	5.18E-17	hypomethylated	-0.0012133	0.78841	insignificant	30	109	109
chr17	34077288	34079288	H2-Ke2	-0.13490173	5.18E-17	hypomethylated	-0.0012133	0.78841	insignificant	30	109	109
chr17	34088433	34090433	B3galt4	-0.33500589	2.44E-11	stronglyHypometh	-0.038592	0.098499	insignificant	5	29	32
chr17	34091826	34093826	Vps52	-0.13140204	4.99E-42	hypomethylated	0.010803	0.025288	hypermethylated	35	138	134
chr17	34092586	34094586	Rps18	-0.24367905	1.16E-59	hypomethylated	0.018886	0.02238	hypermethylated	26	92	88
chr17	34115736	34117736	AA388235	-0.53571429	1	lowCoverage	-0.002381	0.14784	insignificant	1	2	2
chr17	34117436	34119436	AA388235	-0.30194805	0.1763	insignificant	-0.014448	0.85974	insignificant	3	8	8
chr17	34137278	34139278	H2-K1	-0.12804878	0.20869	insignificant	0.010573	0.80552	insignificant	3	8	8
chr17	34161625	34163625	Ring1	-0.18028839	1.02E-32	hypomethylated	0.00010166	0.0012305	hypermethylated	36	120	118
chr17	34162037	34164037	H2-Ke6	-0.23265536	3.53E-12	hypomethylated	0.018497	0.000050064	hypermethylated	11	58	56
chr17	34165000	34167000	H2-Ke6	-0.596719	2.09E-11	stronglyHypometh	0.043274	2.27E-14	hypermethylated	4	18	18
chr17	34167796	34169796	Rxrb	-0.11775211	3.02E-22	hypomethylated	0.011172	0.50057	insignificant	54	173	181
chr17	34168635	34170635	Rxrb	-0.09124875	3.27E-10	hypomethylated	0.013055	0.69416	insignificant	41	127	135
chr17	34175381	34177381	Col11a2	-0.34200798	1.45E-15	stronglyHypometh	-0.066766	0.000000348	hypomethylated	9	42	42
chr17	34228323	34230323	H2-Oa	-0.35	0.084294	insignificant	0.17273	0.69588	insignificant	3	6	8
chr17	34257328	34259328	Brd2	-0.1482625	3.04E-13	hypomethylated	0.01114	0.61474	insignificant	19	91	90
chr17	34258692	34260692	Brd2	-0.13921565	0.00041429	hypomethylated	0.029326	0.87343	insignificant	7	57	45
chr17	34271615	34273615	H2-DMa	-0.17631784	1.65E-17	hypomethylated	0.01417	0.76746	insignificant	9	47	46
chr17	34289135	34291135	H2-DMb1	-0.53897837	0.040964	stronglyHypometh	-0.16109	0.12347	insignificant	2	8	11
chr17	34323500	34325500	Tap1	-0.15135362	0.000000441	hypomethylated	0.01778	0.1845	insignificant	5	56	56
chr17	34324275	34326275	Tap1	-0.15135362	0.000000441	hypomethylated	0.01778	0.1845	insignificant	5	56	56
chr17	34334139	34336139	Psmb8	-0.15405633	0.00059386	hypomethylated	0.02203	0.10544	insignificant	8	62	62
chr17	34340423	34342423	Tap2	-0.13575255	0.00000186	hypomethylated	0.02113	0.16744	insignificant	8	34	32
chr17	34374849	34376849	H2-Ob		1	noCoverage	0.099756	1	insignificant	0	6	6
chr17	34399171	34401171	H2-Ab1	-0.1557971	0.35801	insignificant	0.025504	0.32197	insignificant	2	18	18
chr17	34424716	34426716	H2-Aa	-0.82719061	0.047237	stronglyHypometh	-0.085694	1	insignificant	1	6	6
chr17	34612882	34614882	Btn3a3		1	noCoverage	0.066667	0.78465	insignificant	0	2	2
chr17	34634374	34636374	Btnl5		1	noCoverage	-0.0037879	1	insignificant	0	3	3
chr17	34654297	34656297	Btnl6	0.03757353	0.099619	insignificant	0.078429	0.8813	insignificant	2	9	8
chr17	34700239	34702239	Notch4	0.35899742	1	lowCoverage	0.054755	0.64245	insignificant	1	13	17
chr17	34725750	34727750	Gpsm3	-0.3374995	0.0015588	stronglyHypometh	0.028071	0.94431	insignificant	3	21	21
chr17	34728415	34730415	Pbx2	-0.09921194	4.07E-21	hypomethylated	0.044965	0.16399	insignificant	35	94	105
chr17	34733806	34735806	Ager		1	noCoverage	0.07381	0.28743	insignificant	0	4	4
chr17	34740506	34742506	Rnf5	-0.14575041	0.000017415	hypomethylated	-0.020042	0.92134	insignificant	14	59	61
chr17	34741805	34743805	Agpat1	-0.1331325	3.73E-11	hypomethylated	-0.015672	0.25025	insignificant	13	54	53
chr17	34744958	34746958	Agpat1	-0.73577922	0.4183	lowCoverage	-0.019987	0.13036	insignificant	1	10	10
chr17	34752916	34754916	Egfl8	-0.91405178	0.00053263	stronglyHypometh	-0.015108	1	insignificant	2	16	16
chr17	34764042	34766042	Ppt2	-0.13523584	4.6E-22	hypomethylated	0.0006664	0.57952	insignificant	22	84	83
chr17	34765630	34767630	Prrt1		1	noCoverage	0.125	0.041802	hypermethylated	0	6	6
chr17	34780827	34782827	Fkbpl	-0.12133263	0.60608	insignificant	0.014034	0.015447	inconclusive	7	40	41
chr17	34783124	34785124	Atf6b	-0.1184771	0.27897	insignificant	0.027855	0.0073845	hypermethylated	40	116	113
chr17	34806479	34808479	Tnxb	0.00966646	0.50956	insignificant	0.053996	0.17501	insignificant	14	37	34
chr17	34941371	34943371	Cyp21a1		1	noCoverage	0.059704	0.82487	insignificant	0	9	9
chr17	34960399	34962399	Stk19		1	noCoverage	0.031313	0.6938	insignificant	0	10	11
chr17	34972963	34974963	Dom3z	-0.12571675	3.09E-31	hypomethylated	0.01075	0.022902	hypermethylated	49	197	201
chr17	34973848	34975848	Stk19	-0.13589261	3.89E-10	hypomethylated	-0.019669	0.025211	hypomethylated	29	150	150
chr17	34986335	34988335	Rdbp	-0.11104208	1.11E-24	hypomethylated	0.045786	0.21435	insignificant	33	124	113
chr17	34987149	34989149	Skiv2l	-0.12016251	1.69E-23	hypomethylated	0.041057	0.032805	hypermethylated	33	115	109
chr17	34999459	35001459	Cfb	-0.0859127	0.0045648	hypomethylated	-0.0049552	0.55269	insignificant	4	16	14
chr17	35030503	35032503	Zbtb12	-0.10621121	2.12E-27	hypomethylated	0.0039517	0.91393	insignificant	27	154	147
chr17	35034443	35036443	Ehmt2	-0.14167517	4.41E-33	hypomethylated	-0.016211	0.35425	insignificant	34	137	143
chr17	35034918	35036918	Ehmt2	-0.1535064	2.55E-40	hypomethylated	-0.018302	0.92933	insignificant	38	148	153
chr17	35050410	35052410	Slc44a4	-0.2581426	1	insignificant	-0.030944	0.15791	insignificant	4	25	23
chr17	35067197	35069197	Neu1	-0.02794245	0.40951	insignificant	-0.042532	0.029474	hypomethylated	2	38	38
chr17	35087953	35089953	1110038B12R	-0.14059	6.2E-12	hypomethylated	0.023793	0.7603	insignificant	13	65	62
chr17	35089413	35091413	1110038B12R	-0.32301208	0.0000059	hypomethylated	0.015098	0.10736	insignificant	5	22	19
chr17	35089416	35091416	1110038B12R	-0.32301208	0.0000059	hypomethylated	0.015098	0.10736	insignificant	5	22	19
chr17	35108647	35110647	Hspa1l	-0.02000823	0.347	insignificant	0.0076966	0.54974	insignificant	9	53	53
chr17	35109101	35111101	Hspa1a	-0.1466071	0.83375	insignificant	0.0030748	0.6463	insignificant	9	32	32
chr17	35117804	35119804	Lsm2	-0.0798784	0.00077831	hypomethylated	0.003894	0.25215	insignificant	16	96	96
chr17	35118074	35120074	Lsm2	-0.09415897	0.00050959	hypomethylated	0.00014261	0.33754	insignificant	16	98	98
chr17	35136851	35138851	Vars	-0.11191539	4.88E-38	hypomethylated	0.011265	0.15104	insignificant	43	196	186
chr17	35152523	35154523	D17H6S56E-3	-0.39339131	0.0050784	stronglyHypometh	-0.0013171	0.016718	hypomethylated	3	14	14
chr17	35183551	35185551	Msh5		1	noCoverage	0.04117	0.52138	insignificant	0	12	12

chr17	35183668	35185668	Msh5		1	noCoverage	0.04117	0.52138	insignificant	0	12	12
chr17	35186187	35188187	Clic1	-0.1620189	0.00000278	hypomethylated	0.037281	0.85936	insignificant	9	35	44
chr17	35194979	35196979	Ddah2	-0.29928326	1	insignificant	-0.016308	0.26595	insignificant	1	32	32
chr17	35203129	35205129	AU023871	-0.26303844	0.012261	hypomethylated	-0.053601	0.25521	insignificant	4	16	16
chr17	35203269	35205269	Ly6g6c	0.01410613	0.58608	insignificant	-0.021327	0.30863	insignificant	3	22	22
chr17	35212886	35214886	Ly6g6e		1	noCoverage	0.047523	0.026187	hypermethylated	0	5	5
chr17	35222540	35224540	Ly6g6f	0.33053315	0.57393	insignificant	0.1387	0.11058	insignificant	1	12	12
chr17	35225235	35227235	Abhd16a	-0.12379216	6.55E-09	hypomethylated	0.017096	0.1442	insignificant	22	101	96
chr17	35244244	35246244	Ly6g5c		1	noCoverage	-0.10766	0.86228	insignificant	0	18	14
chr17	35257440	35259440	Gpank1	-0.13207402	0.000015404	hypomethylated	0.0099073	0.49463	insignificant	18	124	124
chr17	35258392	35260392	Csnk2b	-0.31882014	0.00069305	hypomethylated	0.019192	0.97537	insignificant	6	63	62
chr17	35258896	35260896	Gpank1		1	noCoverage	0.031421	0.014507	hypermethylated	0	27	26
chr17	35263059	35265059	D17H6S53E	-0.11651867	2.22E-14	hypomethylated	0.0023306	0.0058435	hypermethylated	21	58	58
chr17	35268697	35270697	Apom	0.20810669	0.037838	hypermethylated	-0.013969	0.018692	inconclusive	2	35	39
chr17	35271186	35273186	Bag6	-0.14518299	7.71E-09	hypomethylated	0.051292	0.75041	insignificant	33	144	149
chr17	35301822	35303822	Prrc2a	-0.12461576	1.1E-26	hypomethylated	-0.0023686	0.81913	insignificant	15	71	67
chr17	35325385	35327385	Lst1	-0.61752013	0.00019876	stronglyHypometh	-0.069677	0.91382	insignificant	1	13	11
chr17	35330451	35332451	Ltb	0.08785323	0.000000122	inconclusive	0.032376	0.7153	insignificant	5	10	13
chr17	35338941	35340941	Tnf	-0.04822878	0.11647	insignificant	-0.060877	0.84989	insignificant	2	7	8
chr17	35342296	35344296	Lta	0.06554206	1	insignificant	0.1211	0.041363	hypermethylated	2	22	22
chr17	35372540	35374540	Atp6v1g2	-0.14478384	0.00000221	hypomethylated	0.03136	0.64331	insignificant	12	62	62
chr17	35372760	35374760	Nfkbil1	-0.13424934	0.00029226	hypomethylated	0.025511	0.56086	insignificant	9	56	56
chr17	35377690	35379690	Ddx39b	-0.11511092	2.79E-09	hypomethylated	0.00073197	0.70801	insignificant	26	117	117
chr17	35399038	35401038	H2-D1	-0.1255213	5.98E-12	hypomethylated	-0.023658	0.75032	insignificant	34	117	115
chr17	35456502	35458502	H2-Q1	-0.15577234	7.87E-20	hypomethylated	-0.000082366	0.42058	insignificant	24	96	96
chr17	35478277	35480277	H2-Q2	0.26481673	1	insignificant	-0.0080473	0.84685	insignificant	18	78	78
chr17	35515561	35517561	H2-Q4	-0.13643246	7.53E-44	hypomethylated	0.0091435	0.38288	insignificant	30	127	115
chr17	35530043	35532043	H2-Q8	0.04365499	1	insignificant	-0.0058207	0.78771	insignificant	6	80	75
chr17	35560821	35562821	H2-Q6	-0.15666453	2.17E-11	hypomethylated	-0.0082373	0.4636	insignificant	14	84	89
chr17	35575099	35577099	H2-Q7		1	noCoverage	-0.018427	0.43625	insignificant	0	77	64
chr17	35606033	35608033	H2-Q10	-0.18306699	3.59E-22	hypomethylated	-0.022744	0.86773	insignificant	24	92	87
chr17	35641983	35643983	Pou5f1	-0.23860029	0.00052914	hypomethylated	0.011879	0.72927	insignificant	7	14	14
chr17	35653060	35655060	Cchcr1	-0.20299297	0.000025956	hypomethylated	0.050507	0.34133	insignificant	12	49	42
chr17	35653769	35655769	Tcf19	-0.10885053	0.16701	insignificant	0.060259	0.21775	insignificant	15	55	48
chr17	35669145	35671145	Psors1c2	-0.68984127	0.000000199	stronglyHypometh	0.027421	0.075839	insignificant	2	15	14
chr17	35688072	35690072	Cdsn	-0.0694928	0.12431	insignificant	-0.071502	1	insignificant	9	26	26
chr17	35804537	35806537	Vars2	-0.15126309	2.85E-23	hypomethylated	0.065389	0.18774	insignificant	18	68	73
chr17	35810627	35812627	Gtf2h4		1	noCoverage	-0.12045	0.27186	insignificant	0	36	44
chr17	35837535	35839535	Ddr1	-0.47699817	1.21E-40	stronglyHypometh	0.0097347	0.90062	insignificant	9	38	38
chr17	35838989	35840989	Ddr1	-0.41443015	0.000016225	stronglyHypometh	0.020616	0.89111	insignificant	3	32	32
chr17	35957657	35959657	Ier3	-0.16770209	8.82E-09	hypomethylated	0.0066014	0.096358	insignificant	21	90	90
chr17	35959301	35961301	Flot1	-0.20435342	4.61E-10	hypomethylated	-0.0053051	0.26758	insignificant	8	77	71
chr17	35975246	35977246	Tubb5	0.13501401	1	lowCoverage	0.0066807	0.66375	insignificant	1	10	10
chr17	35977442	35979442	Mdc1	-0.15843111	5.05E-09	hypomethylated	0.021764	0.0028821	hypermethylated	15	83	91
chr17	35997262	35999262	Nrm	-0.09808137	0.000000333	hypomethylated	0.020933	0.40647	insignificant	14	64	58
chr17	36001539	36003539	Ppp1r18	-0.10651163	0.0048798	hypomethylated	0.020075	0.058538	insignificant	12	62	59
chr17	36002072	36004072	Ppp1r18	-0.15715417	5.62E-14	hypomethylated	0.026789	0.0055393	hypermethylated	16	80	77
chr17	36002559	36004559	Ppp1r18	-0.14423959	2.38E-14	hypomethylated	0.036764	0.05542	insignificant	17	103	96
chr17	36015722	36017722	Dhx16	-0.12424231	0.059847	insignificant	-0.0004307	0.58292	insignificant	20	117	115
chr17	36034323	36036323	Atat1	-0.07490359	0.000012301	hypomethylated	0.06316	0.86957	insignificant	3	30	34
chr17	36047013	36049013	Atat1	-0.58243168	6.52E-26	stronglyHypometh	-0.018984	0.32333	insignificant	3	23	24
chr17	36052855	36054855	Ppp1r10	-0.10912223	4.01E-20	hypomethylated	0.0039428	0.43543	insignificant	36	138	139
chr17	36053140	36055140	Ppp1r10	-0.10912223	4.01E-20	hypomethylated	-0.0016919	0.43515	insignificant	36	138	137
chr17	36053314	36055314	Mrps18b	-0.10890511	1.34E-20	hypomethylated	-0.0025954	0.42733	insignificant	36	126	125
chr17	36053833	36055833	Mir1894	-0.0944892	7.13E-08	hypomethylated	0.0036595	0.4823	insignificant	10	49	48
chr17	36097759	36099759	Abcf1		1	noCoverage	0.0012562	0.30397	insignificant	0	17	21
chr17	36106695	36108695	Abcf1	-0.41882746	1	lowCoverage	0.0083834	0.3324	insignificant	1	8	8
chr17	36115899	36117899	Gnl1	-0.09949454	4.23E-46	hypomethylated	0.014877	0.12848	insignificant	57	174	176
chr17	36115968	36117968	Prr3	-0.09949454	4.23E-46	hypomethylated	0.01555	0.12847	insignificant	57	174	175
chr17	36116412	36118412	Prr3	-0.13104475	1.72E-49	hypomethylated	0.010509	0.30069	insignificant	64	162	162
chr17	36116770	36118770	Prr3	-0.19292068	1.32E-87	hypomethylated	0.0051347	0.82891	insignificant	50	132	132
chr17	36130448	36132448	A930015D03R	-0.25311982	0.0070127	hypomethylated	-0.044041	0.5802	insignificant	8	40	24
chr17	36157505	36159505	H2-T24	-0.66883117	0.000018427	stronglyHypometh	0.046752	0.30926	insignificant	2	14	12
chr17	36169646	36171646	A930015D03Rik		1	noCoverage	0.16372	0.36329	insignificant	0	4	4
chr17	36178905	36180905	Gm6034	-0.26400641	0.56733	insignificant	0.0084295	1	insignificant	3	8	8
chr17	36280962	36282962	2410017I17Ril	-0.1159209	0.00000104	hypomethylated	0.0019257	0.54186	insignificant	11	79	76
chr17	36314721	36316721	Gm6623		1	noCoverage	0.00012268	1	insignificant	0	20	20
chr17	36408378	36410378	Trim39	-0.09967439	1.57E-11	hypomethylated	0.0079403	0.84009	insignificant	21	90	90
chr17	36599274	36601274	H2-M10.4		1	noCoverage	-0.015729	1	insignificant	0	6	6
chr17	36779589	36781589	H2-M9		1	noCoverage	0.1126	0.11998	insignificant	0	4	4
chr17	36948119	36950119	H2-M10.6	-0.0599537	0.68079	insignificant	-0.13491	0.89627	insignificant	3	8	9
chr17	36973085	36975085	Trim26	-0.10424683	0.0000054	hypomethylated	0.007859	0.079483	insignificant	26	87	88
chr17	37005518	37007518	Trim10	-0.23278525	0.50158	insignificant	0.042834	0.001076	hypermethylated	7	28	28
chr17	37078995	37080995	Rnf39	-0.14686713	2.39E-17	hypomethylated	-0.0091386	0.82134	insignificant	30	89	89
chr17	37094536	37096536	Znrd1as	-0.13445311	0.23834	insignificant	0.0024667	1	insignificant	9	46	46
chr17	37095373	37097373	Znrd1as	-0.12515679	0.3972	insignificant	0.024144	0.70978	insignificant	9	40	40
chr17	37126449	37128449	H2-M5	-0.3545695	1	insignificant	-0.02979	0.87098	insignificant	2	18	18
chr17	37138426	37140426	Zfp57	-0.1553435	0.000000045	hypomethylated	0.049264	0.77774	insignificant	13	48	55
chr17	37138469	37140469	Zfp57	-0.1553435	0.000000045	hypomethylated	0.049264	0.77774	insignificant	13	48	55
chr17	37139610	37141610	Zfp57	-0.13955594	0.000000307	hypomethylated	0.014752	0.7641	insignificant	13	45	49
chr17	37160343	37162343	Mog		1	noCoverage	0.082015	0.041922	hypermethylated	0	6	6
chr17	37181910	37183910	Gabbr1	-0.14865588	4.66E-10	hypomethylated	-0.00039833	0.41376	insignificant	20	81	67
chr17	37248925	37250925	Olfr92		1	noCoverage	0.19762	0.0547	insignificant	0	2	2
chr17	37369313	37371313	Olfr97	0.41363636	1	lowCoverage	0.1011	0.10738	insignificant	1	6	4
chr17	37406178	37408178	H2-M3	-0.18161572	0.000011722	hypomethylated	-0.0079173	0.94067	insignificant	5	22	22

chr17	37931390	37933390	Olfr123	0.08299124	0.62831	insignificant	0.089418	0.32494	insignificant	3	6	6
chr17	38203117	38205117	Olfr130	-0.725	0.082583	insignificant	0.15595	0.74685	insignificant	1	4	4
chr17	38690136	38692136	Gm20410		1	noCoverage	0.061905	0.04331	hypermethylated	0	4	4
chr17	39980080	39982080		-0.16814612	0	hypomethylated	-0.033625	0	hypomethylated	92	360	349
chr17	41070633	41072633	Mut	-0.08766519	8.74E-13	hypomethylated	0.0049868	0.55345	insignificant	23	77	76
chr17	41071500	41073500	Cenpq	-0.0223586	0.000000629	hypomethylated	0.010292	1	insignificant	13	36	36
chr17	42451895	42453895	3110082D06R	-0.18247705	0.000004337	hypomethylated	-0.0066436	0.81524	insignificant	11	109	95
chr17	42829233	42831233	Gpr115		1	noCoverage	-0.10417	0.57567	insignificant	0	2	2
chr17	43013373	43015373	Cd2ap	-0.13851127	0.00000762	hypomethylated	-0.016869	0.82968	insignificant	21	100	103
chr17	43152503	43154503	Tnfrsf21	-0.1222699	1.12E-16	hypomethylated	0.0058755	0.90766	insignificant	20	90	92
chr17	43639722	43641722	Mep1a	0.19618056	1	lowCoverage	0.12042	1	insignificant	1	6	6
chr17	43704399	43706399	Pla2g7	-0.260715	0.0033651	hypomethylated	0.0084987	0.86319	insignificant	5	36	36
chr17	43767248	43769248	Tdrd6	0.17741241	0.000080719	hypermethylated	0.037753	0.00097742	hypermethylated	8	47	46
chr17	43803373	43805373	Cyp39a1	-0.12257813	0.000000108	hypomethylated	0.012225	0.84308	insignificant	16	44	44
chr17	43803964	43805964	Cyp39a1	-0.12394915	0.00000223	hypomethylated	0.015371	0.74825	insignificant	13	34	34
chr17	43937799	43939799	Rcan2	-0.16521037	2.65E-17	hypomethylated	0.003274	0.002629	hypermethylated	27	112	113
chr17	44089139	44091139	Rcan2	0.085149	0.077664	insignificant	0.13971	0.028184	hypermethylated	9	24	22
chr17	44214794	44216794	Enpp5	-0.3014003	5.59E-08	hypomethylated	-0.104	0.87932	insignificant	2	26	24
chr17	44214796	44216796	Enpp5	-0.3014003	5.59E-08	hypomethylated	-0.104	0.87932	insignificant	2	26	24
chr17	44242757	44244757	Enpp4	-0.13784724	5.94E-08	hypomethylated	-0.036544	0.61403	insignificant	10	46	44
chr17	44324520	44326520	Clic5	-0.1620763	7.87E-11	hypomethylated	0.029549	0.33423	insignificant	6	28	24
chr17	44873597	44875597	Runx2		1	noCoverage	-0.039723	0.33109	insignificant	0	8	8
chr17	44913119	44915119	Supt3h	-0.08679203	8.79E-20	hypomethylated	0.014034	0.63	insignificant	28	103	115
chr17	45570656	45572656	Cdc5l	0.01277619	0.010079	hypermethylated	0.0050379	0.2414	insignificant	13	77	76
chr17	45642789	45644789	Aars2	-0.22615751	1.69E-16	hypomethylated	-0.0050877	0.86706	insignificant	10	34	34
chr17	45659382	45661382	Tcte1	0.4423711	2.02E-13	stronglyHypermeth	0.065169	0.023351	hypermethylated	3	18	18
chr17	45686626	45688626	Tmem151b	-0.32692308	0.33892	insignificant	0.067378	0.4178	insignificant	1	4	4
chr17	45691664	45693664	Nfkbie	-0.1169917	1.31E-08	hypomethylated	0.0082032	0.60289	insignificant	19	86	82
chr17	45700100	45702100	Slc35b2	-0.10742978	0.000000019	hypomethylated	0.0019733	0.032563	inconclusive	19	59	59
chr17	45710210	45712210	Hsp90ab1	-0.10543396	0.06045	insignificant	-0.025728	0.54595	insignificant	4	35	28
chr17	45730589	45732589	Slc29a1	0.03641744	0.31225	insignificant	0.023402	0.55916	insignificant	12	42	42
chr17	45732521	45734521	Slc29a1	-0.03360695	0.76147	insignificant	0.0051236	0.75661	insignificant	10	22	22
chr17	45736552	45738552	Gm7325	-0.03051268	0.000000152	inconclusive	-0.0044463	0.23951	insignificant	21	55	56
chr17	45822320	45824320	Mrpl14	-0.12041746	6.56E-18	hypomethylated	-0.0039532	0.13212	insignificant	33	163	165
chr17	45823167	45825167	Mrpl14	-0.0925641	0.000027247	hypomethylated	-0.010169	0.2006	insignificant	8	65	67
chr17	46169326	46171326	Vegfa	-0.17654062	0.18807	insignificant	0.1275	0.78836	insignificant	4	10	6
chr17	46246952	46248952	Mrps18a	-0.10596078	0.000024046	hypomethylated	-0.0037752	0.88997	insignificant	10	60	60
chr17	46290500	46292500	Mad2l1bp	-0.43933696	0.054115	insignificant	0.11217	0.63331	insignificant	2	16	16
chr17	46296980	46298980	Gtpbp2	-0.1846705	0.0028306	hypomethylated	0.00079058	0.34293	insignificant	10	77	76
chr17	46338803	46340803	Xpo5	-0.14116278	1.77E-19	hypomethylated	-0.011539	0.67283	insignificant	17	62	62
chr17	46339574	46341574	Polh	-0.14116278	1.77E-19	hypomethylated	-0.011539	0.67283	insignificant	17	62	62
chr17	46384028	46386028	Yipf3	-0.17258114	1.41E-16	hypomethylated	0.040009	0.000011839	hypermethylated	16	54	52
chr17	46384994	46386994	Polr1c	-0.12873943	9.43E-16	hypomethylated	0.043373	0.00000894	hypermethylated	17	56	54
chr17	46390113	46392113	Gm88	-0.10553421	1.01E-12	hypomethylated	-0.019682	0.76417	insignificant	24	111	108
chr17	46419962	46421962	Tjap1	0.04928738	0.27655	insignificant	0.040415	0.017535	hypermethylated	4	15	16
chr17	46433369	46435369	Dlk2	-0.13620696	4.25E-08	hypomethylated	0.003418	1	insignificant	6	49	49
chr17	46462715	46464715	Abcc10	0	1	insignificant	0	1	insignificant	2	4	4
chr17	46464972	46466972	Abcc10		1	noCoverage	-0.0049756	0.43505	insignificant	0	12	8
chr17	46519714	46521714	Zfp318	-0.07542165	3.68E-23	hypomethylated	0.00064721	0.51923	insignificant	66	209	199
chr17	46564890	46566890	Crip3	-0.52855478	0.48454	lowCoverage	0.13603	0.015728	hypermethylated	1	9	9
chr17	46624624	46626624	Ttbk1		1	noCoverage	-0.065489	0.010582	hypomethylated	0	16	16
chr17	46632737	46634737	BC048355	-0.11683657	1.09E-13	hypomethylated	0.0058405	0.52393	insignificant	14	69	67
chr17	46683337	46685337	Srf	-0.22046119	1.04E-11	hypomethylated	-0.037057	0.52933	insignificant	5	15	17
chr17	46693111	46695111	Srf	-0.06425251	0.67912	insignificant	0.040592	0.30522	insignificant	7	27	27
chr17	46766453	46768453	Ptk7		1	noCoverage	-0.05068	0.032432	inconclusive	0	11	20
chr17	46782093	46784093	Mrpl2	-0.19235934	2.3E-22	hypomethylated	-0.019233	0.86617	insignificant	26	100	99
chr17	46782196	46784196	Mrpl2	-0.18592089	5.58E-21	hypomethylated	-0.024324	0.79556	insignificant	25	98	97
chr17	46786286	46788286	Cul7	-0.1171088	4.53E-12	hypomethylated	0.0064398	0.063138	insignificant	14	103	102
chr17	46811204	46813204	Rrp36	-0.12037435	5.54E-13	hypomethylated	-0.0044025	0.60257	insignificant	8	18	18
chr17	46817085	46819085	Mea1	-0.11773023	7.88E-08	hypomethylated	0.0050037	0.93911	insignificant	21	100	96
chr17	46817879	46819879	Mea1	-0.30345176	0.0014323	hypomethylated	-0.001546	0.0094569	hypomethylated	7	86	87
chr17	46841951	46843951	Ppp2r5d	-0.32737664	0.0058045	hypomethylated	0.11502	0.49845	insignificant	2	23	17
chr17	46847411	46849411	Pex6	-0.10666508	3.25E-33	hypomethylated	0.014024	0.051382	insignificant	50	157	155
chr17	46866114	46868114	Gnmt	0.52785088	0.44271	lowCoverage	0.22736	0.000038246	hypermethylated	1	16	18
chr17	46889161	46891161	Cnpy3	-0.33802868	0.19053	insignificant	0.008085	0.47076	insignificant	11	31	31
chr17	46900661	46902661	Ptcra		1	noCoverage	0.063927	0.59593	insignificant	0	8	8
chr17	46908583	46910583	2310039H08R	-0.3576984	0.000060484	stronglyHypometh	0.030792	0.27486	insignificant	5	67	62
chr17	46919605	46921605	Rpl7l1	-0.18420565	1	insignificant	0.014293	0.17366	insignificant	6	47	47
chr17	46968362	46970362	BC032203	0.16775689	1	lowCoverage	0.075852	0.26046	insignificant	1	12	13
chr17	47026569	47028569	Tbcc	-0.17622671	5.02E-14	hypomethylated	-0.0090288	0.50948	insignificant	15	104	104
chr17	47046433	47048433	Prph2	0.09847277	0.79061	insignificant	-0.046722	0.53134	insignificant	9	50	50
chr17	47276890	47278890	Trerf1	-0.18102728	0.0011994	hypomethylated	0.032693	0.43711	insignificant	16	88	101
chr17	47366178	47368178	Trerf1	0.15506379	0.70063	insignificant	0.053379	0.011816	hypermethylated	3	21	18
chr17	47504835	47506835	Mrps10	-0.17987563	4.37E-18	hypomethylated	0.01762	0.023908	hypermethylated	15	60	59
chr17	47505145	47507145	Mrps10	-0.22384124	6.56E-20	hypomethylated	0.011193	0.027349	hypermethylated	15	65	65
chr17	47521341	47523341	Guca1b	-0.53996882	0.002807	stronglyHypometh	0.0041153	0.24286	insignificant	2	14	15
chr17	47551660	47553660	1700001C19Rik		1	noCoverage	-0.0066288	0.68731	insignificant	0	4	4
chr17	47572587	47574587	AI661453	-0.18180749	5.27E-22	hypomethylated	0.014866	0.43947	insignificant	17	74	75
chr17	47574324	47576324	AI661453	-0.33391608	0.0016898	stronglyHypometh	0.044611	0.62281	insignificant	2	4	4
chr17	47640999	47642999	Ccnd3	-0.01842016	0.36307	insignificant	0.010325	0.37389	insignificant	6	34	34
chr17	47729415	47731415	Ccnd3	-0.23635887	0.5964	insignificant	-0.073023	0.5157	insignificant	6	53	35
chr17	47747544	47749544	Med20	-0.13497789	1.49E-12	hypomethylated	-0.015937	0.72641	insignificant	26	131	130
chr17	47748441	47750441	Med20	-0.13557926	0.000006682	hypomethylated	-0.029803	0.33759	insignificant	14	48	47
chr17	47766638	47768638	Usp49	-0.08946057	3.41E-10	hypomethylated	0.024678	0.02347	hypermethylated	24	75	75
chr17	47825335	47827335	Tomm6		1	noCoverage	-0.072455	0.62879	insignificant	0	13	14

chr17	47831155	47833155	Frs3	-0.06380836	0.081893	insignificant	0.002108	0.83503	insignificant	38	118	112
chr17	47862790	47864790	Pgc	-0.83079382	0.21094	lowCoverage	-0.22518	0.87589	insignificant	1	17	13
chr17	47872985	47874985	Tfeb	-0.1298155	3.96E-31	hypomethylated	0.015246	0.35333	insignificant	35	108	103
chr17	47873879	47875879	Tfeb	-0.11759881	7.6E-38	hypomethylated	0.010248	0.34975	insignificant	47	196	190
chr17	47921692	47923692	Tfeb	0.07179438	1	insignificant	0.049534	0.54944	insignificant	3	30	33
chr17	47970207	47972207	Mdfi	-0.15601039	7.69E-18	hypomethylated	-0.014279	0.73961	insignificant	14	60	69
chr17	48061581	48063581	Foxp4	-0.14486781	1.83E-36	hypomethylated	0.042771	0.88128	insignificant	19	61	55
chr17	48225581	48227581	1700067P10Rik		1	noCoverage	0.044069	0.91377	insignificant	0	14	14
chr17	48304207	48306207	A530064D06R	0.1430163	1	lowCoverage	0.083492	0.057375	insignificant	1	17	16
chr17	48438362	48440362	Treml2		1	noCoverage	-0.27381	1	insignificant	0	4	7
chr17	48454486	48456486	B430306N03Rik		1	noCoverage	0.053957	0.15889	insignificant	0	10	7
chr17	48498240	48500240	Treml1	-0.17650973	0.066821	insignificant	0.041296	0.12474	insignificant	6	14	14
chr17	48548404	48550404	AI314976	-0.11456041	2.19E-48	hypomethylated	0.026985	0.098629	insignificant	50	154	154
chr17	48549145	48551145	AI314976	-0.07200153	0.2429	insignificant	0.069423	0.0031698	hypermethylated	8	34	34
chr17	48572053	48574053	Apobec2		1	noCoverage	-0.0021371	0.60024	insignificant	0	12	9
chr17	49070906	49072906	Lrfn2	-0.18942174	1.39E-10	hypomethylated	0.0096903	1	insignificant	12	30	30
chr17	49566688	49568688	Mocs1	-0.19491518	1.95E-08	hypomethylated	0.025857	1	insignificant	9	24	22
chr17	49703662	49705662	Daam2		1	noCoverage	0.072576	0.60502	insignificant	0	22	24
chr17	49753496	49755496	Kif6	-0.1346685	0.00000675	hypomethylated	-0.005454	0.58406	insignificant	3	28	28
chr17	50329822	50331822	Rftn1	-0.06944444	1	insignificant	0.033889	0.37115	insignificant	1	4	4
chr17	50432924	50434924	Dazl	0.0647927	1	insignificant	0.037379	0.13291	insignificant	1	54	52
chr17	50647871	50649871	Plcl2	-0.0811277	2.61E-23	hypomethylated	0.013849	0.66293	insignificant	68	210	179
chr17	51318674	51320674	Tbc1d5	-0.11762329	0.10548	insignificant	0.011527	0.32694	insignificant	11	40	40
chr17	51951379	51953379	Satb1	-0.1064126	1.71E-43	hypomethylated	0.0041042	0.015268	hypermethylated	50	181	174
chr17	51971972	51973972	Satb1	-0.10907149	1.65E-10	hypomethylated	-0.02287	0.5158	insignificant	30	86	84
chr17	52741087	52743087	Kcnh8	-0.18228827	3.35E-17	hypomethylated	-0.0038468	0.84497	insignificant	19	79	76
chr17	53617558	53619558	Rab5a	-0.1101155	2.83E-40	hypomethylated	0.0021452	0.51649	insignificant	61	199	184
chr17	53705295	53707295	Kat2b	-0.1798451	9.18E-15	hypomethylated	-0.079385	0.080882	insignificant	21	119	118
chr17	53722450	53724450	Kat2b	0.03398268	1	insignificant	0.0068021	0.86049	insignificant	2	14	16
chr17	53828640	53830640	Sgol1	-0.1382855	0.023519	hypomethylated	-0.021664	0.10407	insignificant	6	71	54
chr17	53985283	53987283	Sult1c2	-0.45138889	0.0024086	stronglyHypometh	-0.014881	0.68986	insignificant	1	2	2
chr17	55584014	55586014	St6gal2	-0.0966984	0.000005168	hypomethylated	-0.045366	0.02053	hypomethylated	15	92	105
chr17	55763970	55765970	Vmn2r118	0.15807399	0.62013	insignificant	0.065795	0.82694	insignificant	1	4	4
chr17	56018195	56020195	Zfp119a	-0.26984127	1	insignificant	0.12528	0.92704	insignificant	2	8	8
chr17	56030515	56032515	Zfp959	-0.23597093	0.031341	hypomethylated	-0.0030015	0.39147	insignificant	3	29	29
chr17	56084682	56086682	Zfp119b		1	noCoverage	0.041606	0.68884	insignificant	0	10	9
chr17	56097609	56099609	Ccdc94	-0.34387695	1.19E-10	stronglyHypometh	0.0046596	0.43312	insignificant	4	30	30
chr17	56108904	56110904	Shd	-0.09473935	0.000014695	hypomethylated	0.017957	0.002133	hypermethylated	16	82	74
chr17	56124933	56126933	Fsd1	-0.59632035	0.4065	lowCoverage	0.002638	0.90103	insignificant	1	4	4
chr17	56144991	56146991	Stap2		1	noCoverage	-0.069823	0.91373	insignificant	0	16	16
chr17	56147623	56149623	Mpnd	-0.11578849	1.26E-11	hypomethylated	-0.017376	0.59499	insignificant	17	73	71
chr17	56175999	56177999	Sh3gl1	-0.17452536	1.46E-09	hypomethylated	0.011008	1	insignificant	15	36	36
chr17	56178838	56180838	Chaf1a	-0.10555261	1	insignificant	-0.011645	0.74463	insignificant	12	82	86
chr17	56214412	56216412	Ubxn6	-0.25100619	0.00020518	hypomethylated	-0.012574	0.49903	insignificant	11	44	43
chr17	56218079	56220079	Hdgfrp2	-0.15939641	1.23E-08	hypomethylated	-0.0201	0.12344	insignificant	9	68	63
chr17	56256721	56258721	Plin5	-0.22628514	0.000051559	hypomethylated	0.015052	0.55847	insignificant	12	46	46
chr17	56256971	56258971	Plin5	-0.26431324	0.0010736	hypomethylated	0.050555	0.60079	insignificant	8	44	44
chr17	56261369	56263369	Lrg1	-0.07547886	0.39574	insignificant	0.0037534	0.40655	insignificant	3	33	33
chr17	56273275	56275275	Sema6b		1	noCoverage	0.040882	0.42955	insignificant	0	12	12
chr17	56279766	56281766	Sema6b	-0.24595009	0.00000183	hypomethylated	-0.0064645	0.73693	insignificant	12	83	82
chr17	56300913	56302913	Tnfaip8l1	-0.09595104	0.40344	insignificant	0.0091025	0.88428	insignificant	11	24	24
chr17	56323343	56325343	D17Wsu104e	0.22623923	3.14E-17	hypermethylated	-0.033953	0.029791	hypomethylated	2	48	48
chr17	56358312	56360312	Dpp9	0.06626252	0.000043647	hypermethylated	0.051139	0.66693	insignificant	4	39	39
chr17	56381410	56383410	Mir7b	-0.19706667	1.03E-09	hypomethylated	0.023156	0.47643	insignificant	8	31	32
chr17	56395215	56397215	Fem1a	-0.11938084	3.92E-09	hypomethylated	0.003811	0.47446	insignificant	24	184	182
chr17	56416190	56418190	Ticam1	-0.09343434	1	insignificant	0.019289	1	insignificant	4	8	8
chr17	56429934	56431934	Plin3	-0.71111111	0.0001526	stronglyHypometh	0.10969	0.68907	insignificant	1	6	6
chr17	56439709	56441709	Arrdc5	-0.0440115	0.43791	insignificant	0.032775	0.52706	insignificant	3	10	10
chr17	56441759	56443759	Uhrf1	-0.04219756	1.94E-15	inconclusive	-0.023104	0.021706	hypomethylated	14	81	80
chr17	56442735	56444735	Uhrf1	-0.13387706	0.00023446	hypomethylated	-0.033503	0.10143	insignificant	11	87	88
chr17	56464472	56466472	Kdm4b	-0.10052528	0.000000726	hypomethylated	-0.0098086	1	insignificant	16	112	116
chr17	56615903	56617903	Ptprs	0.1476702	0.000000602	hypermethylated	0.046808	0.37285	insignificant	9	65	58
chr17	56651906	56653906	Znrf4	0.2037037	1	insignificant	0.066317	0.12384	insignificant	1	8	8
chr17	56723404	56725404	Safb	-0.11554155	7.7E-34	hypomethylated	-0.016343	0.14792	insignificant	37	273	266
chr17	56724006	56726006	Safb2	-0.11207452	3.28E-23	hypomethylated	-0.017046	0.61431	insignificant	29	211	200
chr17	56749194	56751194	2410015M20F	-0.04937577	0.00050329	hypomethylated	0.1264	0.87528	insignificant	6	21	12
chr17	56751817	56753817	Rpl36	-0.09527501	9.05E-08	hypomethylated	0.024899	1	insignificant	23	108	109
chr17	56811679	56813679	Ranbp3	-0.16370176	8.54E-12	hypomethylated	-0.0063271	0.94243	insignificant	32	118	127
chr17	56856184	56858184	Ndufa11	-0.09621873	0.000000463	hypomethylated	0.0070284	0.30628	insignificant	26	86	86
chr17	56856344	56858344	Vmac	-0.11738521	5.71E-09	hypomethylated	0.0053971	0.79331	insignificant	19	67	67
chr17	56857122	56859122	Vmac	-0.10892851	0.24373	insignificant	-0.017061	0.74592	insignificant	11	39	38
chr17	56872650	56874650	Fut4-ps1	0.12684453	0.82357	insignificant	0.012746	0.8705	insignificant	3	10	10
chr17	56896953	56898953	Nrtn	-0.15532846	2.34E-16	hypomethylated	0.021478	0.37878	insignificant	12	39	34
chr17	56903173	56905173	Dus3l	-0.18491996	1.44E-09	hypomethylated	-0.025085	0.59801	insignificant	20	101	93
chr17	56908698	56910698	Prr22	-0.12418774	0.000023706	hypomethylated	0.02282	0.078687	insignificant	4	31	28
chr17	56970431	56972431	Rfx2	-0.10393427	0.1544	insignificant	-0.0012351	0.38239	insignificant	5	74	74
chr17	57014055	57016055	1700061G19Rik		1	noCoverage	0.08007	1	insignificant	0	8	6
chr17	57074811	57076811	Mllt1	-0.04788575	7.16E-18	hypomethylated	0.047299	0.68556	insignificant	26	85	66
chr17	57128686	57130686	Clpp	-0.14263133	5.45E-10	hypomethylated	-0.0048082	0.015697	inconclusive	15	38	44
chr17	57135761	57137761	Alkbh7		1	noCoverage	0.057142	0.8916	insignificant	0	24	16
chr17	57150711	57152711	Gtf2f1	0.62209838	0.00000072	stronglyHypermeth	-0.0021906	1	insignificant	2	45	45
chr17	57170930	57172930	Khsrp	-0.18945199	0.000000844	hypomethylated	-0.068535	0.45437	insignificant	10	37	35
chr17	57181077	57183077	Slc25a41	-0.15749608	0.00000176	hypomethylated	0.046559	0.82096	insignificant	2	6	6
chr17	57199286	57201286	Slc25a23		1	noCoverage	0.079904	0.25166	insignificant	0	32	38
chr17	57200699	57202699	Crb3	-0.20981646	4.24E-14	hypomethylated	0.019135	0.88217	insignificant	7	48	46

chr17	57227205	57229205	Tubb4a	-0.82071018	0.16514	lowCoverage	0.087587	0.28451	insignificant	1	6	4
chr17	57243807	57245807	Tnfsf9	-0.10970085	0.60792	insignificant	0.0075802	1	insignificant	4	20	20
chr17	57333609	57335609	Tnfsf14	0.54413319	0.49099	lowCoverage	-0.00032953	0.77283	insignificant	1	11	10
chr17	57367559	57369559	C3	0.023779	1	insignificant	0.0053807	0.66547	insignificant	2	4	4
chr17	57387064	57389064	Trip10	-0.12658307	1	insignificant	0.028515	0.44495	insignificant	2	33	44
chr17	57387873	57389873	Trip10	-0.16209428	0.2301	insignificant	0.01669	0.69414	insignificant	5	54	52
chr17	57417522	57419522	Vav1	-0.24721151	0.020254	hypomethylated	0.0094685	0.42482	insignificant	2	18	15
chr17	57497108	57499108	Emr1	0.15270936	1	lowCoverage	-0.012154	0.60042	insignificant	1	6	6
chr17	57907992	57909992	Cntnap5c	-0.25652658	0.0016878	hypomethylated	-0.015285	0.6752	insignificant	4	19	19
chr17	59152745	59154745	Nudt12	-0.27580128	0.33096	insignificant	0.11327	0.49651	insignificant	3	8	8
chr17	63230666	63232666	Efna5	-0.13551169	3.32E-27	hypomethylated	-0.0095073	0.51186	insignificant	49	185	153
chr17	63849929	63851929	Fbxl17		1	noCoverage	-0.066304	0.15911	insignificant	0	6	6
chr17	64286320	64288320	Fert2	0.01168015	0.57202	insignificant	0.032756	0.0068586	hypermethylated	8	20	20
chr17	64949988	64951988	Man2a1	-0.09872138	1.5E-47	hypomethylated	0.01466	0.08004	insignificant	65	223	206
chr17	65962895	65964895	Vapa	-0.10767703	9.48E-11	hypomethylated	-0.007396	0.40502	insignificant	24	93	93
chr17	65989096	65991096	Txndc2		1	noCoverage	-0.0015152	1	insignificant	0	8	4
chr17	66122092	66124092	Rab31	-0.04508315	0.000015451	hypomethylated	0.019856	0.37478	insignificant	8	26	26
chr17	66131694	66133694	Ppp4r1	-0.0780339	2.94E-33	hypomethylated	-0.0049717	0.24783	insignificant	73	253	273
chr17	66234263	66236263	Ralbp1	-0.23349653	1	insignificant	-0.007426	0.27164	insignificant	2	19	19
chr17	66235095	66237095	Ralbp1	-0.37273902	0.53683	insignificant	-0.0038211	1	insignificant	2	6	6
chr17	66426386	66428386	Ankrd12	-0.09284261	9.13E-19	hypomethylated	-0.0031907	0.79293	insignificant	29	90	90
chr17	66450831	66452831	Ndufv2	-0.21247904	7.18E-15	hypomethylated	0.017277	0.89706	insignificant	19	69	69
chr17	66459885	66461885	ORF19	-0.07691905	2.25E-11	hypomethylated	0.026042	0.021284	hypermethylated	14	48	48
chr17	66459984	66461984	ORF19	-0.07691905	2.25E-11	hypomethylated	0.026042	0.021284	hypermethylated	14	48	48
chr17	66471859	66473859	Ddx11	-0.17521998	1.62E-08	hypomethylated	0.049946	0.061784	insignificant	8	55	48
chr17	66799090	66801090	1110012J17Ri	-0.14133379	0.000051484	hypomethylated	-0.0012494	0.50203	insignificant	11	44	40
chr17	66869010	66871010	Rab12	-0.16722222	0.079761	insignificant	0.0044949	0.47247	insignificant	3	6	6
chr17	67703799	67705799	Ptprm	-0.08279052	8.37E-19	hypomethylated	-0.0053458	0.20615	insignificant	37	110	104
chr17	68045604	68047604	Lama1	-0.19394261	8.86E-08	hypomethylated	-0.0063264	0.71488	insignificant	9	22	22
chr17	68353448	68355448	Arhgap28		1	noCoverage	0.011265	0.8954	insignificant	0	14	14
chr17	68622136	68624136	L3mbtl4	-0.0952785	0.000016433	hypomethylated	0.060804	0.035519	hypermethylated	15	44	46
chr17	69505149	69507149	Epb4.1l3	-0.09170063	6.06E-16	hypomethylated	-0.0052987	0.86769	insignificant	33	139	134
chr17	69732317	69734317	Zfp161	-0.10096351	3.88E-36	hypomethylated	-0.0091226	0.62759	insignificant	59	200	201
chr17	69764786	69766786	C030034I22Ril	-0.159243	0.000000197	hypomethylated	0.0091485	0.21395	insignificant	9	45	42
chr17	69787665	69789665	A330050F15Ri	-0.14093599	6.63E-33	hypomethylated	0.017205	0.01665	hypermethylated	28	117	117
chr17	70870449	70872449	Dlgap1	-0.12012061	0.52661	insignificant	0.040998	0.33041	insignificant	1	16	14
chr17	71199130	71201130	Tgif1	-0.20965707	2.4E-16	hypomethylated	0.0177	0.21776	insignificant	19	79	78
chr17	71200550	71202550	Tgif1	-0.13011325	0.000000222	hypomethylated	0.022134	0.094543	insignificant	18	110	110
chr17	71201074	71203074	Tgif1	-0.08830748	0.00000364	hypomethylated	0.021606	0.15417	insignificant	26	148	144
chr17	71202872	71204872	Tgif1	-0.35394953	0.015156	stronglyHypometh	-0.031577	0.84741	insignificant	3	27	31
chr17	71339856	71341856	Myl12b	-0.11117624	9.77E-10	hypomethylated	-0.0087469	0.83562	insignificant	17	69	67
chr17	71351873	71353873	Myl12a	-0.12574458	0.000000283	hypomethylated	0.02356	0.82083	insignificant	6	40	40
chr17	71532317	71534317	Lpin2	-0.10888147	1.06E-10	hypomethylated	-0.0095317	0.13946	insignificant	46	160	153
chr17	71660305	71662305	Emilin2	-0.16302747	1.17E-13	hypomethylated	-0.032482	0.024167	hypomethylated	9	33	28
chr17	71691144	71693144	Gm4566		1	noCoverage	-0.058666	0.89941	insignificant	0	26	23
chr17	71824683	71826683	Smchd1	-0.0925247	5.61E-09	hypomethylated	-0.0031696	0.93504	insignificant	21	102	103
chr17	71876197	71878197	Ndc80	-0.08508114	0.0010139	hypomethylated	-0.015088	0.20327	insignificant	16	77	71
chr17	71900400	71902400	Spdya	-0.11824446	2.8E-29	hypomethylated	-0.015795	0.043045	hypomethylated	44	138	134
chr17	71947621	71949621	Trmt61b	-0.1941286	3.06E-34	hypomethylated	-0.011171	0.97312	insignificant	29	110	105
chr17	71948101	71950101	Trmt61b	-0.23484473	2.48E-17	hypomethylated	-0.0049662	0.57636	insignificant	15	59	55
chr17	71964554	71966554	Wdr43	-0.16235191	2.85E-25	hypomethylated	-0.03649	1	insignificant	41	157	165
chr17	72130286	72132286	Clip4	-0.15378973	1.93E-25	hypomethylated	0.0054872	0.96099	insignificant	22	68	60
chr17	72953647	72955647	Alk	-0.2285279	0.61339	insignificant	-0.077244	0.92961	insignificant	2	15	21
chr17	73185043	73187043	Ypel5	-0.13828412	1.67E-45	hypomethylated	-0.0023798	0.56588	insignificant	81	234	231
chr17	73266644	73268644	Lbh	-0.13033331	5.15E-16	hypomethylated	-0.00038588	0.37657	insignificant	18	146	137
chr17	73456324	73458324	Lclat1	-0.05996835	3.67E-17	hypomethylated	0.0050637	1	insignificant	17	111	107
chr17	74059791	74061791	Galnt14	-0.10130801	0.000000021	hypomethylated	-0.018897	0.37288	insignificant	19	58	58
chr17	74153180	74155180	Ehd3	-0.23704345	1.73E-09	hypomethylated	0.023657	0.00010646	hypermethylated	20	64	52
chr17	74694203	74696203	Memo1	-0.0749451	0.000041847	hypomethylated	0.0063269	0.076839	insignificant	28	135	136
chr17	74737326	74739326	Spast	-0.11677653	2.26E-18	hypomethylated	0.0072891	0.93429	insignificant	42	163	156
chr17	74793971	74795971	Slc30a6	-0.19853346	0.00000169	hypomethylated	-0.034054	0.27819	insignificant	13	40	40
chr17	74887853	74889853	Yipf4	-0.08539031	0.00033648	hypomethylated	0.028347	0.589	insignificant	17	66	66
chr17	74926634	74928634	Birc6	-0.12009542	3.37E-48	hypomethylated	0.0012748	0.22421	insignificant	53	205	202
chr17	75116089	75118089	Ttc27	-0.17706745	0.000029523	hypomethylated	-0.006718	0.72788	insignificant	5	46	40
chr17	75403868	75405868	Ltbp1	-0.14206796	7.86E-45	hypomethylated	0.0032797	0.56891	insignificant	58	175	175
chr17	75577221	75579221	Ltbp1	-0.49819625	0.13599	insignificant	-0.0035339	0.37171	insignificant	2	12	12
chr17	75951286	75953286	Fam98a	-0.1639701	0.000031055	hypomethylated	0.11167	0.64952	insignificant	2	7	4
chr17	78598587	78600587	Crim1	-0.10233096	3.26E-17	hypomethylated	-0.0068292	0.27476	insignificant	52	204	183
chr17	78906402	78908402	Vit		1	noCoverage	-0.1214	0.54018	insignificant	0	10	8
chr17	79135900	79137900	Strn	-0.18811435	8.85E-15	hypomethylated	0.0036011	0.86197	insignificant	16	49	40
chr17	79233855	79235855	Ccdc75	-0.10641166	1.72E-15	hypomethylated	0.00583	0.65942	insignificant	25	87	82
chr17	79234721	79236721	Heatr5b	-0.10749235	0.24225	insignificant	-0.0092017	0.43621	insignificant	6	26	26
chr17	79281912	79283912	Eif2ak2	-0.33512626	1.53E-09	stronglyHypometh	-0.086059	1	insignificant	6	24	21
chr17	79314838	79316838	1110001A16R	-0.16404417	0.000000561	hypomethylated	0.15401	0.87154	insignificant	5	27	14
chr17	79335474	79337474	2410091C18Ri	-0.10136139	2.53E-08	hypomethylated	0.064089	0.915	insignificant	12	42	42
chr17	79336410	79338410	2410091C18Ri	-0.20494505	0.061748	insignificant	-0.0033313	0.056138	insignificant	4	18	18
chr17	79420156	79422156	Prkd3	-0.12577525	2.29E-12	hypomethylated	0.019108	0.11973	insignificant	18	88	80
chr17	79754431	79756431	Cdc42ep3	-0.09961178	6.87E-13	hypomethylated	-0.0045878	0.051307	insignificant	18	124	129
chr17	80013239	80015239	Fam82a1	-0.24616463	7.49E-11	hypomethylated	-0.01313	0.20455	insignificant	30	92	93
chr17	80295368	80297368	Atl2	-0.0850635	0.00000013	hypomethylated	0.010755	0.53794	insignificant	13	56	50
chr17	80295463	80297463	Atl2	-0.04156379	0.0086	hypomethylated	0.043558	0.064723	insignificant	7	34	28
chr17	80461674	80463674	Hnrpll	-0.07586225	0.022242	hypomethylated	-0.0071823	0.72554	insignificant	8	52	52
chr17	80606645	80608645	Srsf7		1	noCoverage	0.072528	0.012448	hypermethylated	0	42	38
chr17	80622828	80624828	Gemin6		1	noCoverage	0.033464	0.79501	insignificant	0	21	19
chr17	80688551	80690551	Morn2	-0.1507516	9.09E-13	hypomethylated	0.0044736	0.83397	insignificant	11	55	55

chr17	80689816	80691816	Morn2	-0.65538194	0.00015948	stronglyHypometh	-0.20954	0.51707	insignificant	1	16	11
chr17	80705746	80707746	Arhgef33		1	noCoverage	0.065813	0.5934	insignificant	0	8	8
chr17	80772882	80774882	Gm10190	0.08268992	0.51462	insignificant	0.0057845	0.000038851	hypermethylated	13	88	88
chr17	80879793	80881793	Sos1	-0.09721426	0.010805	hypomethylated	-0.001098	0.59265	insignificant	9	26	27
chr17	80963174	80965174	Cdkl4		1	noCoverage	0.067003	0.029583	hypermethylated	0	27	21
chr17	81127433	81129433	Map4k3	0.03983595	0.74242	insignificant	0.023833	0.47107	insignificant	12	39	45
chr17	81342971	81344971	Tmem178	-0.10693079	4.14E-25	hypomethylated	-0.001379	0.2282	insignificant	49	152	152
chr17	81464425	81466425	Thumpd2	-0.43334386	4.15E-34	stronglyHypometh	-0.005943	0.000000192	hypomethylated	12	48	46
chr17	82137717	82139717	Slc8a1	0.08049242	1	insignificant	-0.020857	0.047582	hypomethylated	2	16	16
chr17	83613622	83615622	Pkdcc	-0.12429537	5.38E-34	hypomethylated	-0.0048653	0.15766	insignificant	63	168	171
chr17	83749270	83751270	Eml4	-0.11674736	2.23E-10	hypomethylated	-0.016185	0.4064	insignificant	21	101	74
chr17	83913673	83915673	Cox7a2l	-0.51923077	0.40625	lowCoverage	-0.02649	0.70069	insignificant	1	2	2
chr17	84031235	84033235	Kcng3	-0.13660907	6.76E-25	hypomethylated	-0.0032314	0.60502	insignificant	38	94	94
chr17	84104502	84106502	Mta3	-0.08965496	0.0070096	hypomethylated	-0.0048277	0.55985	insignificant	11	93	95
chr17	84587287	84589287	Zfp36l2	-0.15001178	0.00084215	hypomethylated	0.0085844	0.43673	insignificant	9	56	64
chr17	84910234	84912234	Plekhh2	-0.14149697	4.55E-34	hypomethylated	0.022564	0.64063	insignificant	30	104	93
chr17	85081470	85083470	Abcg8		1	noCoverage	0.041142	0.30468	insignificant	0	6	6
chr17	85082263	85084263	Abcg5	-0.46972222	0.49767	insignificant	0.035794	0.50841	insignificant	2	10	10
chr17	85190126	85192126	Lrpprc	-0.10380835	0.60871	insignificant	-0.0095179	0.83377	insignificant	3	10	10
chr17	85355080	85357080	Ppm1b	-0.3617576	0.00012689	stronglyHypometh	0.087762	0.85002	insignificant	5	27	27
chr17	85356340	85358340	Ppm1b	-0.09063117	3.38E-09	hypomethylated	0.0060673	0.37462	insignificant	30	158	157
chr17	85356763	85358763	1110020A21R	-0.07628831	0.000000102	hypomethylated	-0.0022591	0.79244	insignificant	27	146	145
chr17	85357050	85359050	1110020A21R	-0.07628831	0.000000102	hypomethylated	-0.0022591	0.79244	insignificant	27	146	145
chr17	85426686	85428686	Slc3a1	0.09419324	1	insignificant	-0.15506	0.70169	insignificant	2	14	14
chr17	85489039	85491039	1700106N22R	-0.11944492	0.00021497	hypomethylated	-0.0040026	0.50906	insignificant	12	98	93
chr17	85489607	85491607	1700106N22R	-0.12946716	0.00012487	hypomethylated	-0.0006481	1	insignificant	10	70	65
chr17	85489614	85491614	1700106N22R	-0.12946716	0.00012487	hypomethylated	-0.0006481	1	insignificant	10	68	63
chr17	86017615	86019615	Six3	-0.25215404	2.01E-16	hypomethylated	0.02932	0.88217	insignificant	8	45	47
chr17	86017705	86019705	Six3	-0.26792461	0.000011585	hypomethylated	0.03106	0.65224	insignificant	5	37	39
chr17	86018751	86020751	Six3	-0.31815235	0.000037024	hypomethylated	0.019544	0.47299	insignificant	2	17	23
chr17	86019173	86021173	Six3	-0.1718175	1.64E-17	hypomethylated	0.030679	0.024141	hypermethylated	21	59	65
chr17	86087594	86089594	Six2	-0.1200621	7.51E-49	hypomethylated	-0.0038579	0.92382	insignificant	35	136	132
chr17	86544515	86546515	Srbd1	-0.17455484	0.38942	insignificant	0.11536	0.25436	insignificant	9	24	17
chr17	86566124	86568124	Prkce	-0.10188898	7.55E-25	hypomethylated	0.00020469	0.22055	insignificant	74	261	243
chr17	87152203	87154203	Epas1	-0.08224937	0.011208	hypomethylated	0.027566	0.5131	insignificant	28	131	125
chr17	87347227	87349227	Atp6v1e2		1	noCoverage	0.12125	0.0049184	hypermethylated	0	9	9
chr17	87361450	87363450	Rhoq	-0.11858635	8.74E-33	hypomethylated	-0.020398	0.36244	insignificant	59	159	143
chr17	87423900	87425900	Cript	-0.15322865	3.44E-09	hypomethylated	0.048653	0.39938	insignificant	27	97	63
chr17	87424741	87426741	Pigf	-0.29858895	0.000028787	hypomethylated	-0.023816	0.10725	insignificant	5	31	35
chr17	87506018	87508018	Socs5	-0.12383589	6.04E-57	hypomethylated	0.004395	0.059801	insignificant	61	180	179
chr17	87665287	87667287	Mcfd2	-0.23351953	1.78E-18	hypomethylated	0.014081	0.054229	insignificant	7	32	29
chr17	87681225	87683225	Ttc7	-0.13890446	3.1E-31	hypomethylated	-0.0067179	0.66257	insignificant	52	177	176
chr17	87682154	87684154	4833418N02R	-0.13178797	8.65E-14	hypomethylated	-0.0025319	0.6881	insignificant	24	100	92
chr17	87846275	87848275	Calm2	-0.13043643	0.00058699	hypomethylated	-0.0070813	0.2598	insignificant	16	48	48
chr17	88034318	88036318	Epcam	0.26006883	0.0000055	hypermethylated	-0.14816	0.20189	insignificant	3	46	48
chr17	88070896	88072896	Msh2	-0.10030938	0.000000442	hypomethylated	0.0068296	0.45291	insignificant	22	109	102
chr17	88197334	88199334	Kcnk12	-0.1578384	1.98E-15	hypomethylated	-0.0057477	0.54693	insignificant	19	83	83
chr17	88373389	88375389	Msh6	-0.04209585	9.03E-09	hypomethylated	0.010794	1	insignificant	37	163	143
chr17	88464625	88466625	Fbxo11	-0.10050684	3.23E-13	hypomethylated	0.0042364	0.82576	insignificant	29	125	119
chr17	88839051	88841051	Foxn2	-0.13447387	1.59E-42	hypomethylated	-0.036587	0.0019444	hypomethylated	65	142	128
chr17	88928463	88930463	Klraq1	-0.11854955	1.34E-23	hypomethylated	-0.00072531	0.56199	insignificant	24	87	87
chr17	89024894	89026894	Ston1		1	noCoverage	-0.16158	0.20102	insignificant	0	8	8
chr17	89066999	89068999	Gtf2a1l	0.04270893	0.000090134	hypermethylated	-0.16098	0.82583	insignificant	9	28	35
chr17	89191316	89193316	Lhcgr	-0.2570239	0.00031442	hypomethylated	0.05754	1	insignificant	9	28	26
chr17	91492142	91494142	Nrxn1	-0.14818787	0.00038682	hypomethylated	0.042546	1	insignificant	5	17	14
chr17	93597761	93599761	Adcyap1	-0.30405812	0.0080348	hypomethylated	-0.030535	1	insignificant	4	10	10
chr17	95148439	95150439	2700099C18Ri	-0.11000446	1.57E-08	hypomethylated	-0.0059168	0.35382	insignificant	9	66	60
chr17	95149232	95151232	Mettl4	-0.06414345	0.0014152	hypomethylated	0.0075324	0.61578	insignificant	9	28	22
chr17	95233760	95235760	2610044O15R	-0.21198074	0.00029179	hypomethylated	-0.00079431	0.64032	insignificant	6	16	16
chr18	3335413	3337413	Gm6225	-0.09959442	7.38E-17	hypomethylated	-0.0051761	0.0076708	hypomethylated	25	96	99
chr18	3337587	3339587	Gm6225	-0.12720498	0.59641	insignificant	0.0017426	0.50862	insignificant	3	47	47
chr18	3382222	3384222	Cul2	-0.15646777	8.07E-25	hypomethylated	0.0017193	0.24328	insignificant	24	69	69
chr18	3506954	3508954	Bambi	-0.14307436	5.36E-21	hypomethylated	-0.020711	0.22209	insignificant	55	169	163
chr18	4352951	4354951	Map3k8	-0.16547402	1.54E-17	hypomethylated	0.0096855	0.77753	insignificant	17	69	66
chr18	4374589	4376589	Mtpap		1	noCoverage	-0.0069568	0.70726	insignificant	0	22	22
chr18	4633926	4635926	9430020K01Ri	-0.10720122	3.74E-37	hypomethylated	0.0066722	0.16577	insignificant	53	132	122
chr18	5045586	5047586	Svil		1	noCoverage	0.082748	0.59495	insignificant	0	4	6
chr18	5334437	5336437	Zfp438	-0.10091398	3.62E-08	hypomethylated	0.011717	0.29654	insignificant	17	57	57
chr18	5590888	5592888	Gm10125	-0.09785596	5.62E-32	hypomethylated	0.0035166	0.97381	insignificant	42	152	146
chr18	5592435	5594435	Gm10125	-0.12256776	6.11E-15	hypomethylated	-0.01619	0.96829	insignificant	32	104	105
chr18	6136096	6138096	Arhgap12	-0.34185464	0.000000327	stronglyHypometh	-0.051107	0.83094	insignificant	4	15	8
chr18	6241522	6243522	Kif5b	-0.09963889	0.000091	hypomethylated	0.028649	0.7751	insignificant	5	25	22
chr18	6490644	6492644	Epc1	-0.16506676	1.04E-22	hypomethylated	-0.0078531	0.0020354	inconclusive	26	116	116
chr18	6490854	6492854	Epc1	-0.1688201	2.5E-21	hypomethylated	0.004648	0.000045265	hypermethylated	24	100	90
chr18	6516085	6518085	Epc1	-0.12492726	0.026573	hypomethylated	0.0023131	1	insignificant	3	52	52
chr18	7004777	7006777	Mkx	-0.19251048	6.24E-26	hypomethylated	-0.004489	0.45225	insignificant	20	56	56
chr18	7867855	7869855	Wac	-0.11218404	5.69E-56	hypomethylated	-0.0094979	0.3801	insignificant	43	211	223
chr18	7868194	7870194	Wac	-0.11186834	1.3E-72	hypomethylated	-0.0089082	0.1125	insignificant	57	256	265
chr18	9211853	9213853	Fzd8	-0.10473023	3.1E-28	hypomethylated	0.0022853	0.69296	insignificant	48	181	180
chr18	9450148	9452148	Ccny	-0.13537182	2.17E-08	hypomethylated	-0.021284	0.21105	insignificant	16	35	42
chr18	9706645	9708645	Colec12	-0.11348168	4.02E-11	hypomethylated	0.006422	0.0037313	hypermethylated	23	106	111
chr18	9957177	9959177	Thoc1	-0.23806799	5.76E-11	hypomethylated	-0.018151	0.62867	insignificant	15	81	81
chr18	10181790	10183790	Rock1	-0.10492077	0.000010548	hypomethylated	0.017621	0.65627	insignificant	20	106	108
chr18	10324176	10326176	Greb1l	-0.13318913	3.45E-13	hypomethylated	0.049389	0.001866	hypermethylated	31	90	90
chr18	10610350	10612350	Esco1	-0.37159057	0.61755	insignificant	0.0064839	0.44969	insignificant	4	32	31

chr18	10616793	10618793	Snrpd1	-0.10440856	1.21E-19	hypomethylated	-0.0063753	0.57042	insignificant	30	104	92
chr18	10706694	10708694	Abhd3		1	noCoverage	-0.0089744	1	insignificant	0	4	4
chr18	10724622	10726622	Mib1	-0.0914288	4.85E-37	hypomethylated	0.0019626	0.063174	insignificant	59	193	180
chr18	11051507	11053507	Gata6	-0.15290907	0.0013539	hypomethylated	0.048256	0.90862	insignificant	7	52	44
chr18	11815350	11817350	Rbbp8	-0.14806513	2.32E-20	hypomethylated	-0.013197	0.62614	insignificant	34	134	133
chr18	11996782	11998782	Cables1	-0.10360503	6.3E-51	hypomethylated	0.0011306	0.674	insignificant	66	212	208
chr18	11998947	12000947	Cables1	-0.10692232	0.033259	hypomethylated	0.0071068	0.063803	insignificant	10	39	32
chr18	12279962	12281962	6030446N20R	-0.20838775	2.83E-12	hypomethylated	0.058784	0.2663	insignificant	6	18	21
chr18	12286358	12288358	Riok3	-0.13919517	9.48E-14	hypomethylated	0.0037355	0.15478	insignificant	32	98	94
chr18	12326238	12328238	3110002H16R	-0.2148711	1.36E-34	hypomethylated	-0.02785	0.34817	insignificant	31	118	105
chr18	12394895	12396895	Npc1	-0.1585518	0.000000255	hypomethylated	-0.013443	0.76739	insignificant	21	49	46
chr18	12464229	12466229	Ankrd29	-0.09821018	0.022211	hypomethylated	0.0043656	0.77412	insignificant	5	10	10
chr18	12491532	12493532	Lama3	-0.16531525	6.08E-11	hypomethylated	0.016372	0.96195	insignificant	12	44	44
chr18	12801041	12803041	Ttc39c	-0.15640391	3.6E-36	hypomethylated	0.014902	0.89619	insignificant	20	48	28
chr18	12872319	12874319	Mir1948	-0.08333433	0.040197	hypomethylated	0.095262	0.32943	insignificant	2	6	6
chr18	12898863	12900863	Cabyr	-0.16540463	0.00000134	hypomethylated	-0.0072115	0.72724	insignificant	20	53	53
chr18	13100229	13102229	Osbpl1a	-0.07776816	0.000018487	hypomethylated	-0.0089716	0.4012	insignificant	10	42	42
chr18	13129760	13131760	Impact	-0.25190929	0.0080264	hypomethylated	0.11335	0.00362	hypermethylated	3	17	19
chr18	14131242	14133242	Zfp521	-0.57907411	0.025281	stronglyHypometh	0.034125	0.08429	insignificant	3	12	11
chr18	14841423	14843423	Ss18	-0.11492533	0.000000156	hypomethylated	0.018962	0.54106	insignificant	13	58	41
chr18	14863659	14865659	Psma8		1	noCoverage	-0.10491	0.014719	hypomethylated	0	16	16
chr18	14940753	14942753	Taf4b	-0.14973697	1.06E-19	hypomethylated	-0.0096681	0.92074	insignificant	25	107	104
chr18	15222091	15224091	Kctd1	-0.20699562	0.0030471	hypomethylated	-0.012975	0.83818	insignificant	5	54	55
chr18	15562193	15564193	Aqp4		1	noCoverage	0.092593	0.17113	insignificant	0	6	6
chr18	16967558	16969558	Cdh2	-0.12062529	1.88E-23	hypomethylated	0.00052472	0.11631	insignificant	27	79	79
chr18	20404840	20406840	Dsg1c		1	noCoverage	0.018182	0.80736	insignificant	0	6	6
chr18	20715616	20717616	Dsg2	-0.17971454	0.012218	hypomethylated	0.019885	0.514	insignificant	2	51	42
chr18	20904905	20906905	B4galt6	-0.02151292	0.152	insignificant	0.0084782	0.22247	insignificant	8	20	20
chr18	21054579	21056579	Trappc8	-0.12576315	0.00018441	hypomethylated	0.0050652	0.43218	insignificant	24	84	83
chr18	21102125	21104125	Rnf125	0.00104023	0.19915	insignificant	0.082171	0.0050332	hypermethylated	3	31	30
chr18	21158841	21160841	Rnf138	-0.11008635	9.6E-43	hypomethylated	-0.0048795	0.78188	insignificant	40	122	122
chr18	21458640	21460640	Fam59a	-0.09327694	2.77E-10	hypomethylated	-0.012366	0.29188	insignificant	22	57	56
chr18	21810869	21812869	Klhl14	-0.15625657	0.00069812	hypomethylated	0.028652	0.10129	insignificant	5	81	87
chr18	22502589	22504589	Asxl3	-0.05790158	0.00000822	hypomethylated	-0.00026053	0.062224	insignificant	36	126	125
chr18	23197164	23199164	Nol4	-0.13920749	0.014131	hypomethylated	0.012167	0.39831	insignificant	7	67	67
chr18	23200154	23202154	Nol4	-0.29006734	0.0011112	hypomethylated	0.11521	0.52662	insignificant	2	21	14
chr18	23909833	23911833	Mapre2	-0.1509087	7.03E-14	hypomethylated	-0.0027305	0.18763	insignificant	19	70	69
chr18	23911225	23913225	Mapre2	-0.24194983	0.0010146	hypomethylated	0.0072958	0.6473	insignificant	5	22	19
chr18	23961470	23963470	Mapre2	-0.10281211	0.000000492	hypomethylated	0.0071816	0.3817	insignificant	22	79	73
chr18	24112188	24114188	Zfp397	-0.10623277	0.3709	insignificant	0.018976	0.81206	insignificant	7	88	87
chr18	24147134	24149134	Zfp35		1	noCoverage	-0.052698	1	insignificant	0	20	18
chr18	24179272	24181272	Zfp191	-0.08304562	2.7E-11	hypomethylated	-0.036953	0.19102	insignificant	10	40	37
chr18	24280320	24282320	Ino80c	-0.23768655	2.37E-11	hypomethylated	0.025094	1	insignificant	12	39	36
chr18	24362844	24364844	Galnt1	-0.07683556	9.7E-43	hypomethylated	0.0065056	0.092148	insignificant	80	236	218
chr18	24363494	24365494	Galnt1	-0.0803305	1.19E-45	hypomethylated	0.0063426	0.21912	insignificant	80	240	222
chr18	24628371	24630371	2700062C07Ri	-0.22831764	9.4E-14	hypomethylated	-0.0075025	0.80893	insignificant	13	45	47
chr18	24688705	24690705	Rprd1a	-0.29543651	0.050188	insignificant	0.025268	0.7003	insignificant	3	20	24
chr18	24761461	24763461	Elp2	-0.11209301	8.1E-21	hypomethylated	-0.010191	0.46681	insignificant	40	154	151
chr18	24762318	24764318	Elp2	-0.16299118	0.3541	insignificant	0.012582	0.43987	insignificant	7	27	27
chr18	24811191	24813191	Mocos	-0.17787485	1E-11	hypomethylated	-0.0035765	0.60154	insignificant	13	46	44
chr18	24866945	24868945	Fhod3	-0.14526441	9.78E-34	hypomethylated	-0.0042995	0.41288	insignificant	54	153	141
chr18	25326520	25328520	AW554918	-0.06772229	5.03E-14	hypomethylated	0.020425	0.1681	insignificant	51	127	129
chr18	25327378	25329378	5730494M16R	-0.09632548	0.00004141	hypomethylated	0.0095321	0.92032	insignificant	29	73	72
chr18	25912484	25914484	Celf4	-0.157847	1.49E-12	hypomethylated	-0.012748	0.88275	insignificant	29	122	117
chr18	30431549	30433549	Pik3c3		1	noCoverage	0.18767	0.0031567	hypermethylated	0	13	13
chr18	31793036	31795036	Sap130	-0.11200775	1.79E-23	hypomethylated	0.025935	0.72465	insignificant	28	134	129
chr18	31918533	31920533	Ammecr1l	-0.15441158	5.95E-12	hypomethylated	-0.00067644	0.44622	insignificant	16	84	88
chr18	31947812	31949812	Polr2d	-0.09544067	0.015133	hypomethylated	-0.0005994	0.3513	insignificant	8	44	42
chr18	31947829	31949829	Polr2d	-0.09544067	0.015133	hypomethylated	-0.0005994	0.3513	insignificant	8	44	42
chr18	31962710	31964710	Wdr33	-0.0979696	5.46E-13	hypomethylated	0.028507	0.42029	insignificant	17	92	67
chr18	32071557	32073557	Sft2d3	0.17336125	1	lowCoverage	0.039828	0.78035	insignificant	1	12	12
chr18	32090160	32092160	Lims2	-0.0706989	3.35E-10	hypomethylated	0.0035322	0.37621	insignificant	9	48	49
chr18	32196585	32198585	Myo7b		1	noCoverage	0.18958	0.52118	insignificant	0	6	6
chr18	32226387	32228387	Iws1	-0.1743235	7.88E-24	hypomethylated	0.0029626	0.089129	insignificant	20	73	73
chr18	32321742	32323742	Map3k2	-0.10817143	1.9E-18	hypomethylated	0.0085709	0.27204	insignificant	32	132	119
chr18	32398984	32400984	Ercc3	-0.19460033	3.21E-21	hypomethylated	0.0079201	0.35102	insignificant	11	44	46
chr18	32535870	32537870	Bin1	-0.10375477	0.000000427	hypomethylated	0.00014858	0.88803	insignificant	21	84	84
chr18	32975513	32977513	Tslp	-0.23432132	3.94E-10	hypomethylated	0.042734	0.70875	insignificant	2	26	24
chr18	32995878	32997878	Wdr36	-0.12035137	1.43E-27	hypomethylated	-0.011729	0.2219	insignificant	25	60	60
chr18	33097694	33099694	Camk4	-0.11050135	0.000008894	hypomethylated	0.0017727	0.19639	insignificant	23	92	87
chr18	33373470	33375470	Stard4	-0.65833333	0.35714	lowCoverage	0.053788	1	insignificant	1	4	6
chr18	33623075	33625075	D0H4S114	-0.13006884	1.98E-19	hypomethylated	-0.02134	0.45476	insignificant	20	34	34
chr18	33623288	33625288	D0H4S114	-0.13006884	1.98E-19	hypomethylated	-0.02134	0.45476	insignificant	20	34	34
chr18	33623401	33625401	D0H4S114	-0.13006884	1.98E-19	hypomethylated	-0.02134	0.45476	insignificant	20	34	34
chr18	33623683	33625683	D0H4S114	-0.15950899	0.000000037	hypomethylated	-0.02006	0.38878	insignificant	5	22	22
chr18	34166860	34168860	Epb4.1l4a	-0.25080613	1.88E-13	hypomethylated	0.02984	0.010507	hypermethylated	12	36	36
chr18	34379637	34381637	Apc	-0.12823001	3.08E-32	hypomethylated	0.012658	0.0070956	hypermethylated	36	114	112
chr18	34489798	34491798	Srp19	-0.19807793	1.05E-41	hypomethylated	-0.028457	0.34665	insignificant	42	113	113
chr18	34533069	34535069	Reep5	-0.47062159	2.92E-14	stronglyHypometh	-0.036608	0.0078885	hypomethylated	3	35	38
chr18	34568076	34570076	Pkd2l2	0.26050239	0.45199	insignificant	-0.0092246	0.84081	insignificant	3	16	16
chr18	34666477	34668477	Fam13b	-0.1353647	0.60493	insignificant	0.020189	0.34378	insignificant	3	44	36
chr18	34700981	34702981	Wnt8a	-0.60319412	0.00045571	stronglyHypometh	-0.018922	0.025285	hypomethylated	3	29	26
chr18	34738760	34740760	4933408B17R	-0.08730159	0.68685	insignificant	-0.025341	1	insignificant	3	6	6
chr18	34757122	34759122	4933408B17Rik		1	noCoverage	-0.14863	0.46738	insignificant	0	7	7
chr18	34783277	34785277	Kif20a	-0.19985142	0.000000011	hypomethylated	0.023819	0.056198	insignificant	9	67	67

chr18	34783589	34785589	Kif20a	-0.18578196	1.13E-08	hypomethylated	0.022331	0.030017	hypermethylated	9	64	64
chr18	34783686	34785686	Kif20a	-0.18578196	1.13E-08	hypomethylated	0.022331	0.030017	hypermethylated	9	64	64
chr18	34784464	34786464	Kif20a	0.10311331	0.47778	insignificant	-0.029759	0.052614	insignificant	5	38	38
chr18	34811390	34813390	Cdc23		1	noCoverage	-0.077349	0.4111	insignificant	0	27	29
chr18	34880041	34882041	Gfra3	-0.16236996	0.10535	insignificant	0.16535	0.25841	insignificant	6	16	12
chr18	34911187	34913187	Cdc25c		1	noCoverage	-0.19244	0.48631	insignificant	0	10	10
chr18	34917559	34919559	Fam53c	-0.21555214	1.47E-17	hypomethylated	0.013642	0.84241	insignificant	22	92	90
chr18	34935661	34937661	Kdm3b	-0.1241898	1.67E-33	hypomethylated	0.010462	0.27959	insignificant	43	146	137
chr18	34999311	35001311	Reep2	-0.16921157	0.000024791	hypomethylated	-0.039331	0.098144	insignificant	9	33	34
chr18	35019860	35021860	Egr1	-0.15544966	1.04E-33	hypomethylated	0.026688	1	insignificant	31	92	88
chr18	35091657	35093657	Etf1		1	noCoverage	-0.014061	0.67111	insignificant	0	66	66
chr18	35114005	35116005	Hspa9	-0.08763657	3.36E-19	hypomethylated	0.015506	0.22123	insignificant	32	130	127
chr18	35277565	35279565	Ctnna1	-0.17002544	6.16E-09	hypomethylated	0.015457	0.86386	insignificant	22	144	131
chr18	35374678	35376678	Lrrtm2	0.32857143	0.53297	insignificant	-0.013095	1	insignificant	2	7	6
chr18	35713220	35715220	Mir1949	-0.2002482	0.0040902	hypomethylated	0.04365	0.70515	insignificant	7	26	28
chr18	35720811	35722811	Matr3	0.0003188	0.017693	inconclusive	0.0021808	0.54922	insignificant	11	66	64
chr18	35757320	35759320	Paip2	-0.23698904	0.000016723	hypomethylated	-0.1206	0.0072122	hypomethylated	9	74	56
chr18	35786881	35788881	Slc23a1	0.22949101	1	insignificant	0.070996	0.44394	insignificant	1	14	12
chr18	35809021	35811021	2010001M09F	0.1649106	0.071965	insignificant	0.045173	0.33294	insignificant	3	6	9
chr18	35821840	35823840	Spata24	-0.2920149	0.0031441	hypomethylated	0.3288	0.64631	insignificant	0	11	4
chr18	35862798	35864798	Dnajc18		1	noCoverage	0.0070264	0.56952	insignificant	0	22	22
chr18	35881145	35883145	Ecscr	-0.50797165	0.0068835	stronglyHypometh	0.10715	0.39234	insignificant	2	10	10
chr18	35900208	35902208	Tmem173	0.2476754	0.10787	insignificant	0.17419	0.0011443	hypermethylated	2	13	10
chr18	35930212	35932212	Ube2d2	-0.13541268	1.19E-39	hypomethylated	0.0069384	0.43874	insignificant	45	158	163
chr18	35988471	35990471	Cxxc5	-0.07651924	1.71E-08	hypomethylated	0.014891	0.047348	hypermethylated	38	182	180
chr18	36123488	36125488	Psd2	-0.2436849	0.000033321	hypomethylated	0.029934	0.22285	insignificant	10	58	58
chr18	36356814	36358814	Nrg2	-0.15363282	0.0011088	hypomethylated	0.0014321	0.30135	insignificant	10	32	32
chr18	36439815	36441815	Pura	-0.1140014	7.64E-16	hypomethylated	0.00023055	0.84735	insignificant	38	108	105
chr18	36507277	36509277	0610010O12R	-0.12567436	2.72E-15	hypomethylated	0.0069135	0.54124	insignificant	19	107	100
chr18	36614149	36616149	Pfdn1	-0.16352298	0.1999	insignificant	-0.027402	0.27826	insignificant	5	14	14
chr18	36675459	36677459	Hbegf	-0.11230113	0.00059361	hypomethylated	0.047469	1	insignificant	4	27	18
chr18	36686805	36688805	Slc4a9	0.08333333	0.34311	insignificant	-0.055257	0.12679	insignificant	1	5	7
chr18	36719256	36721256	Ankhd1	-0.11233281	2.94E-32	hypomethylated	0.0048499	0.68108	insignificant	32	179	168
chr18	36822713	36824713	Eif4ebp3	0.09211304	0.27535	insignificant	0.0402	0.4462	insignificant	7	23	22
chr18	36829965	36831965	Sra1	-0.33138138	0.00000015	hypomethylated	0.066277	0.048883	hypermethylated	2	6	6
chr18	36837868	36839868	Slc35a4	-0.14138603	2.36E-09	hypomethylated	-0.015105	0.30782	insignificant	15	59	60
chr18	36893723	36895723	Tmco6	-0.12711728	0.29177	insignificant	-0.010917	0.97456	insignificant	14	71	71
chr18	36903309	36905309	Ik	-0.16519374	1	lowCoverage	0.0075338	0.95115	insignificant	1	59	54
chr18	36904202	36906202	Ik	-0.17026583	1	lowCoverage	0.014224	0.68522	insignificant	1	43	38
chr18	36918892	36920892	Wdr55	-0.20968276	0.000000289	hypomethylated	0.18539	0.55512	insignificant	4	43	35
chr18	36941933	36943933	Hars2	-0.14400765	8.41E-08	hypomethylated	-0.013335	0.33771	insignificant	9	74	73
chr18	36942859	36944859	Hars	-0.15735029	0.000096919	hypomethylated	-0.012762	0.62583	insignificant	8	42	39
chr18	36952576	36954576	Zmat2	-0.22917083	1	lowCoverage	0.016139	0.8415	insignificant	1	22	22
chr18	36960416	36962416	Vaultrc5	-0.10930259	0.00000105	hypomethylated	0.022199	0.52254	insignificant	8	30	28
chr18	37111342	37113342	Pcdha4	-0.18929173	0.000013944	hypomethylated	0.17356	0.000000526	hypermethylated	4	8	8
chr18	37111394	37113394	Pcdha4-g	-0.18929173	0.000013944	hypomethylated	0.17356	0.000000526	hypermethylated	4	8	8
chr18	37119093	37121093	Pcdha5		1	noCoverage	0.06685	0.79201	insignificant	0	14	20
chr18	37132577	37134577	Pcdha7		1	noCoverage	-0.0084175	1	insignificant	0	6	6
chr18	37151120	37153120	Pcdha8	0.28506705	1	insignificant	-0.053745	0.56667	insignificant	1	30	34
chr18	37156533	37158533	Pcdha9		1	noCoverage	0.0037942	0.22942	insignificant	0	21	15
chr18	37163973	37165973	Pcdha10		1	noCoverage	-0.082341	0.048797	hypomethylated	0	18	18
chr18	37169511	37171511	Pcdha11	0.0071653	0.56501	insignificant	-0.083094	0.030919	hypomethylated	4	10	10
chr18	37178883	37180883	Pcdha12		1	noCoverage	0.087549	0.20125	insignificant	0	28	29
chr18	37248789	37250789	Pcdhac1	-0.08310285	0.20687	insignificant	0.043958	0.46775	insignificant	11	80	80
chr18	37302622	37304622	Pcdhac2	-0.07964391	0.000070081	hypomethylated	-0.0030417	0.68229	insignificant	19	108	108
chr18	37423651	37425651	Pcdhb1	-0.43830644	0.038293	stronglyHypometh	0.044795	0.33398	insignificant	6	35	35
chr18	37453493	37455493	Pcdhb2	-0.51174255	1.53E-09	stronglyHypometh	-0.029648	0.000001563	hypomethylated	4	20	20
chr18	37459452	37461452	Pcdhb3	-0.08333333	0.14715	insignificant	0.13095	0.38856	insignificant	2	6	4
chr18	37479034	37481034	Pcdhb5	-0.29773301	0.13045	insignificant	0.033665	0.24276	insignificant	3	30	26
chr18	37492681	37494681	Pcdhb6	-0.12276215	0.4219	insignificant	-0.034429	1	insignificant	2	23	25
chr18	37500355	37502355	Pcdhb7		1	noCoverage	0.092805	0.21376	insignificant	0	16	12
chr18	37559508	37561508	Pcdhb9		1	noCoverage	0.2641	0.67032	insignificant	0	5	6
chr18	37580071	37582071	Pcdhb11	-0.76666667	0.34146	lowCoverage	0.033333	1	insignificant	1	5	3
chr18	37594274	37596274	Pcdhb12	0.32765152	0.23181	insignificant	0.004582	1	insignificant	2	4	4
chr18	37601170	37603170	Pcdhb13	-0.02359997	0.87259	insignificant	0.046048	0.17431	insignificant	4	25	25
chr18	37643674	37645674	Pcdhb17	0.0535387	0.37551	insignificant	0.022588	1	insignificant	5	23	24
chr18	37648118	37650118	Pcdhb18	-0.09659091	0.23735	insignificant	0.0068577	0.29607	insignificant	4	8	8
chr18	37677006	37679006	Pcdhb22	-0.00628839	1	insignificant	0.036225	0.68099	insignificant	4	17	17
chr18	37798377	37800377	Slc25a2		1	noCoverage	-0.026966	0.8655	insignificant	0	16	16
chr18	37820598	37822598	Pcdhga1	-0.24563955	0.033419	hypomethylated	-0.0034876	0.17118	insignificant	4	40	40
chr18	37827758	37829758	Pcdhga2	-0.04127032	0.22664	insignificant	0.10822	0.47283	insignificant	4	43	43
chr18	37832988	37834988	Pcdhga3	0.31284066	0.063041	insignificant	0.08781	0.82396	insignificant	2	52	44
chr18	37839111	37841111	Pcdhgb1	0.17679424	0.47627	insignificant	-0.013108	0.045787	inconclusive	5	21	25
chr18	37844053	37846053	Pcdhga4	0.17345628	0.17394	insignificant	0.03425	0.3424	insignificant	4	56	51
chr18	37848512	37850512	Pcdhgb2	-0.06376046	0.83902	insignificant	0.0082421	0.024571	inconclusive	5	27	27
chr18	37853154	37855154	Pcdhga5	-0.20102999	0.082921	insignificant	-0.022945	0.86255	insignificant	6	44	43
chr18	37879207	37881207	Pcdhgb4	-0.08911445	0.32977	insignificant	0.049119	0.62939	insignificant	3	26	30
chr18	37884359	37886359	Pcdhga8	0.04568751	0.000077344	inconclusive	0.094448	0.89578	insignificant	11	44	35
chr18	37889807	37891807	Pcdhgb5	-0.37525555	0.54401	insignificant	0.014254	0.0065071	hypermethylated	3	35	31
chr18	37895589	37897589	Pcdhga9	-0.30477077	0.092441	insignificant	-0.094849	0.52952	insignificant	2	35	33
chr18	37900747	37902747	Pcdhgb6		1	noCoverage		1	noCoverage	0	6	0
chr18	37905841	37907841	Pcdhga10	-0.31250507	1.32E-08	hypomethylated	0.058414	0.54735	insignificant	7	76	67
chr18	37910432	37912432	Pcdhgb7	-0.12423394	0.45104	insignificant	0.042129	0.45781	insignificant	3	16	12
chr18	37914426	37916426	Pcdhga11	-0.21230287	0.53646	insignificant	0.003309	0.052957	insignificant	9	67	61
chr18	37965063	37967063	Pcdhgc3	-0.13161331	6.69E-10	hypomethylated	-0.0019275	0.66417	insignificant	19	139	136

chr18	37973732	37975732	Pcdhgc4	-0.11462819	4.26E-09	hypomethylated	-0.0064683	0.87964	insignificant	9	52	52
chr18	37978199	37980199	Pcdhgc5	0.23941799	1	lowCoverage	0.16942	0.45952	insignificant	1	12	10
chr18	38095065	38097065	Diap1	-0.07733905	0.000074856	hypomethylated	0.011848	0.79587	insignificant	13	46	46
chr18	38114212	38116212	Rell2	-0.19187173	1.96E-24	hypomethylated	0.0024164	0.78331	insignificant	20	80	75
chr18	38114642	38116642	Hdac3	-0.1867796	3.91E-19	hypomethylated	0.0029858	0.7608	insignificant	16	72	67
chr18	38129385	38131385	Fchsd1	-0.15080932	0.000000039	hypomethylated	0.018142	0.51627	insignificant	8	52	52
chr18	38157228	38159228	Arap3	-0.27487233	9.9E-23	hypomethylated	-0.028224	0.4081	insignificant	10	34	30
chr18	38369416	38371416	Pcdh1	-0.18010751	4.4E-16	hypomethylated	0.0070266	0.39417	insignificant	15	77	72
chr18	38408902	38410902	0610009O20R	-0.15566338	0.60254	insignificant	-0.0038664	0.47815	insignificant	2	44	47
chr18	38409852	38411852	0610009O20R	-0.10863572	0.60036	insignificant	-0.016741	0.69568	insignificant	2	31	31
chr18	38455288	38457288	Rnf14	-0.17130303	5.82E-34	hypomethylated	0.008386	0.09234	insignificant	32	91	79
chr18	38455458	38457458	Rnf14	-0.17130303	5.82E-34	hypomethylated	0.008386	0.09234	insignificant	32	91	79
chr18	38498647	38500647	Gnpda1	-0.3541669	0.00000546	stronglyHypometh	-0.0017363	0.15348	insignificant	5	24	23
chr18	38577628	38579628	Ndfip1	-0.0558072	1	insignificant	0.0067826	0.7962	insignificant	41	147	148
chr18	38760922	38762922	Spry4	0.00533992	0.10383	insignificant	-0.02324	0.055099	insignificant	4	36	36
chr18	39151798	39153798	Arhgap26	-0.11399421	6.28E-51	hypomethylated	-0.0043839	0.34903	insignificant	51	141	142
chr18	39646899	39648899	Nr3c1	-0.09877746	0.38182	insignificant	0.031331	0.79998	insignificant	7	50	48
chr18	39932150	39934150	Pabpc2	0.05068453	0.26168	insignificant	-0.01027	0.48565	insignificant	5	42	42
chr18	40379053	40381053	Yipf5	-0.22785149	0.0083464	hypomethylated	-0.041152	0.35141	insignificant	13	36	36
chr18	40417014	40419014	Kctd16	0.02719577	0.63738	insignificant	0.052622	0.89474	insignificant	4	30	25
chr18	42110848	42112848	Prelid2	-0.55060018	3.81E-08	stronglyHypometh	-0.00085165	0.43936	insignificant	3	19	14
chr18	42212363	42214363	Sh3rf2	0.00548547	0.34418	insignificant	0.041237	0.00025428	hypermethylated	7	22	22
chr18	42421725	42423725	Lars	-0.13911754	0.0025038	hypomethylated	0.018051	0.50146	insignificant	10	39	36
chr18	42434006	42436006	Rbm27	-0.17923868	0.000000134	hypomethylated	0.015507	0.087365	insignificant	15	72	72
chr18	42434851	42436851	Gm4013	-0.23357433	0.000017233	hypomethylated	-0.0010578	0.0024135	hypomethylated	9	66	65
chr18	42553250	42555250	Pou4f3	-0.0708781	0.24313	insignificant	0.0019312	0.19923	insignificant	23	72	73
chr18	42670140	42672140	Tcerg1	-0.11339963	6.25E-24	hypomethylated	-0.0031597	0.4995	insignificant	50	156	158
chr18	42739306	42741306	Gpr151		1	noCoverage	-0.022222	1	insignificant	0	6	6
chr18	43058643	43060643	Ppp2r2b	0.36685135	0.0081436	stronglyHypermeth	-0.010784	0.21242	insignificant	8	34	34
chr18	43219125	43221125	Ppp2r2b	-0.57818445	0.00024179	stronglyHypometh	-0.063986	0.51156	insignificant	1	10	10
chr18	43366350	43368350	Stk32a	-0.13020544	0.000000151	hypomethylated	-0.0017232	0.90125	insignificant	11	56	56
chr18	43552985	43554985	Dpysl3	0.42901235	0.51784	insignificant	0.042528	0.094578	insignificant	2	9	9
chr18	43847427	43849427	Jakmip2		1	noCoverage	0.075069	0.74709	insignificant	0	14	14
chr18	44318957	44320957	Gm10267	-0.58333333	0.026807	stronglyHypometh	0.21667	0.038915	hypermethylated	2	4	4
chr18	44355831	44357831	Spink11		1	noCoverage	0.11078	0.15439	insignificant	0	7	4
chr18	44539153	44541153	Dcp2	-0.15746026	2.48E-48	hypomethylated	0.011416	0.97675	insignificant	31	98	90
chr18	44820318	44822318	A930012L18Ri	-0.17304318	2.09E-11	hypomethylated	0.00019893	0.70569	insignificant	16	78	75
chr18	44821920	44823920	A930012L18Rik		1	noCoverage	0.040278	0.63327	insignificant	0	6	6
chr18	44971836	44973836	Mcc	-0.15938381	0.23646	insignificant	0.042261	0.92708	insignificant	4	30	32
chr18	44987318	44989318	Ythdc2	-0.10521237	7.49E-22	hypomethylated	-0.0035815	0.25881	insignificant	32	103	103
chr18	45718807	45720807	Kcnn2	-0.1562004	1.55E-27	hypomethylated	-0.016824	0.37081	insignificant	45	160	160
chr18	46357674	46359674	1700018A14R	-0.14531365	3.52E-42	hypomethylated	0.0075034	0.061577	insignificant	64	159	155
chr18	46358472	46360472	1700018A14R	-0.13411419	0.0957	insignificant	0.0075202	0.11506	insignificant	28	77	75
chr18	46372261	46374261	Trim36	-0.19178277	0.0016649	hypomethylated	-0.037184	0.66864	insignificant	6	26	24
chr18	46440504	46442504	Pggt1b	-0.2495559	0.000017856	hypomethylated	0.032328	0.42298	insignificant	2	12	12
chr18	46471582	46473582	Ccdc112	-0.15018315	1	insignificant	-0.0081913	0.92162	insignificant	3	16	16
chr18	46625470	46627470	Mospd4	-0.43106618	0.019247	stronglyHypometh	0.029648	0.41734	insignificant	1	2	2
chr18	46685625	46687625	Fem1c		1	noCoverage	-0.11373	0.078236	insignificant	0	17	14
chr18	46756357	46758357	Eif1a	-0.17333839	3.57E-36	hypomethylated	0.0025246	0.00016527	hypermethylated	39	115	109
chr18	46757189	46759189	Eif1a	-0.24245929	5.82E-14	hypomethylated	-0.011313	0.95868	insignificant	13	51	51
chr18	46887996	46889996	Cdo1	-0.20959257	0.0011616	hypomethylated	-0.004458	1	insignificant	2	22	22
chr18	46900570	46902570	Ap3s1	-0.13706278	1.28E-39	hypomethylated	-0.0056992	0.13409	insignificant	42	130	133
chr18	46901233	46903233	Ap3s1	-0.1169755	1.51E-28	hypomethylated	0.0042289	0.19591	insignificant	37	116	117
chr18	47008692	47010692	4833403I15Ril	-0.18366235	0.013151	inconclusive	0.045754	0.00000207	hypermethylated	14	56	56
chr18	47080468	47082468	Gm949		1	noCoverage	0.025726	0.56481	insignificant	0	26	26
chr18	47117529	47119529	Commd10	-0.10809111	0.00013595	hypomethylated	0.011807	0.85985	insignificant	14	31	30
chr18	47528522	47530522	Sema6a	-0.1720535	0.038412	hypomethylated	-0.0095026	0.16743	insignificant	3	65	61
chr18	47696388	47698388	Gm5095	0.08815163	0.2595	insignificant	0.13354	0.31356	insignificant	3	12	12
chr18	49915255	49917255	Dtwd2	0.74422052	0.0023669	stronglyHypermeth	0.039835	0.60252	insignificant	2	35	32
chr18	49991666	49993666	Dmxl1	-0.0749864	2.17E-09	hypomethylated	0.013732	0.36158	insignificant	34	100	93
chr18	50138080	50140080	Tnfaip8	-0.12393909	1	insignificant	-0.017281	0.15205	insignificant	2	36	40
chr18	50211935	50213935	Tnfaip8	-0.17587411	6.76E-28	hypomethylated	0.010024	0.81046	insignificant	14	44	36
chr18	50286992	50288992	Hsd17b4	-0.07018627	0.039121	hypomethylated	0.0011493	1	insignificant	13	49	42
chr18	51276551	51278551	Prr16	-0.12805563	0.00000536	hypomethylated	0.0098209	0.0052298	hypermethylated	18	111	109
chr18	52490189	52492189	Ftmt	-0.26048691	0.68186	insignificant	0.029389	0.70491	insignificant	3	18	18
chr18	52624346	52626346	Srfbp1	0.01697344	0.00011373	hypermethylated	0.012265	0.025722	hypermethylated	23	83	78
chr18	52926464	52928464	Sncaip	-0.0853435	1.21E-30	hypomethylated	-0.0001146	0.067029	insignificant	39	115	115
chr18	52926746	52928746	Sncaip	-0.10032079	2.05E-37	hypomethylated	-0.0017786	0.081084	insignificant	44	135	135
chr18	52926797	52928797	Sncaip	-0.10502196	1.65E-37	hypomethylated	-0.0030977	0.083283	insignificant	44	139	139
chr18	53335018	53337018	Snx2	-0.11906411	1.25E-11	hypomethylated	0.014963	1	insignificant	20	106	113
chr18	53404315	53406315	Snx24	-0.12623814	9.22E-25	hypomethylated	0.0082768	0.73105	insignificant	43	130	129
chr18	53577661	53579661	Ppic	-0.15623936	4.98E-09	hypomethylated	0.012246	0.17042	insignificant	8	28	28
chr18	53623199	53625199	Prdm6	-0.0969601	2.89E-41	hypomethylated	0.0098643	0.28237	insignificant	57	220	211
chr18	53904201	53906201	Cep120	-0.14794319	0.00020493	hypomethylated	0.021463	0.05534	insignificant	8	43	50
chr18	54020766	54022766	Csnk1g3	-0.13675221	3.11E-47	hypomethylated	0.010151	0.17472	insignificant	54	167	147
chr18	55149834	55151834	Zfp608	-0.08863962	0.75916	insignificant	0.040255	0.045077	hypermethylated	8	39	31
chr18	56590785	56592785	Gramd3	-0.17903082	3.82E-19	hypomethylated	0.029258	0.34051	insignificant	17	57	48
chr18	56721222	56723222	Phax	-0.17079102	0.00001707	hypomethylated	-0.013523	0.45271	insignificant	19	62	58
chr18	56721952	56723952	Aldh7a1	0.10119048	0.60906	insignificant	0.0308	0.37677	insignificant	2	22	18
chr18	56731524	56733524	Phax	-0.05909516	5.81E-11	hypomethylated	-0.00084621	0.58897	insignificant	29	126	113
chr18	56732593	56734593	Phax	-0.0758527	0.000000012	hypomethylated	-0.0049425	0.48832	insignificant	21	100	87
chr18	56747001	56749001	1700065I17Ril	-0.50128205	0.11684	insignificant	0.0094924	1	insignificant	2	5	6
chr18	56866466	56868466	Lmnb1	-0.0680018	8.71E-12	hypomethylated	0.0075534	0.23574	insignificant	40	218	210
chr18	57085202	57087202	March3	-0.25858764	5.18E-08	hypomethylated	-0.094724	1	insignificant	6	16	14
chr18	57135033	57137033	C330018D20R	-0.32624087	0.00000294	hypomethylated	-0.21099	0.014633	hypomethylated	5	13	10

chr18	57291743	57293743	Megf10	-0.25012067	2.17E-15	hypomethylated	-0.030159	0.041609	hypomethylated	8	34	34
chr18	57513386	57515386	Prrc1	-0.09180143	1.19E-09	hypomethylated	0.013174	0.17408	insignificant	31	96	83
chr18	57711521	57713521	4930511M06F	-0.10984848	0.71753	insignificant	0.2299	0.12528	insignificant	2	4	4
chr18	57712049	57714049	4930511M06F	-0.10984848	0.71753	insignificant	0.2299	0.12528	insignificant	2	4	4
chr18	57712051	57714051	4930511M06F	-0.10984848	0.71753	insignificant	0.2299	0.12528	insignificant	2	4	4
chr18	58037331	58039331	Slc12a2	-0.0825292	1.19E-42	hypomethylated	0.0070989	0.68546	insignificant	78	283	257
chr18	58369580	58371580	Fbn2	-0.12156163	1.64E-18	hypomethylated	0.02492	0.65656	insignificant	18	98	104
chr18	58818135	58820135	Isoc1	-0.09726309	0.00000051	hypomethylated	-0.020003	1	insignificant	38	138	130
chr18	58995417	58997417	Adamts19	-0.12169962	1.43E-27	hypomethylated	0.0011009	0.87901	insignificant	41	128	130
chr18	59221034	59223034	A730017C20Rik		1	noCoverage	0.044575	0.0039297	hypermethylated	0	20	22
chr18	59333993	59335993	Chsy3	-0.07428526	9.09E-10	hypomethylated	-0.0010994	0.21832	insignificant	52	221	205
chr18	60661637	60663637	2010002N04R	-0.175	0.13589	insignificant	-0.225	0.058046	insignificant	2	4	4
chr18	60684874	60686874	Dctn4	-0.12338846	2.45E-16	hypomethylated	-0.0067243	0.21889	insignificant	15	49	49
chr18	60719439	60721439	Rbm22	-0.14441144	6.42E-17	hypomethylated	-0.0078348	0.27649	insignificant	24	70	58
chr18	60751370	60753370	Myoz3	-0.4118254	0.020308	stronglyHypometh	-0.029138	0.39011	insignificant	1	10	10
chr18	60769758	60771758	Synpo		1	noCoverage	-0.19121	0.9285	insignificant	0	12	14
chr18	60783959	60785959	Synpo	-0.08349578	0.00052665	hypomethylated	-0.042807	0.076491	insignificant	8	32	32
chr18	60933249	60935249	Rps14	-0.10934062	1.03E-08	hypomethylated	0.0063885	0.090911	insignificant	25	120	110
chr18	60962502	60964502	Cd74		1	noCoverage	0.029782	0.36978	insignificant	0	4	4
chr18	61008618	61010618	Tcof1	-0.34143791	0.0015138	stronglyHypometh	-0.02198	0.42182	insignificant	4	16	19
chr18	61070893	61072893	Arsi	-0.12610604	0.000000972	hypomethylated	0.034772	0.023895	hypermethylated	14	94	97
chr18	61084285	61086285	Camk2a	-0.3071219	0.0021483	hypomethylated	0.021187	0.09779	insignificant	7	16	16
chr18	61122207	61124207	Camk2a	-0.04390756	0.42367	insignificant	0.057306	0.23581	insignificant	2	4	4
chr18	61173853	61175853	Slc6a7	-0.05611111	0.26698	insignificant	-0.061018	0.47646	insignificant	2	10	10
chr18	61195853	61197853	Cdx1	-0.22830435	0.045877	hypomethylated	0.10241	0.0065511	hypermethylated	10	36	36
chr18	61203803	61205803	Pdgfrb	-0.38673959	0.30529	insignificant	-0.098083	0.10397	insignificant	4	14	17
chr18	61336703	61338703	Hmgxb3	-0.1091518	0.000000149	hypomethylated	0.030667	0.92922	insignificant	10	30	31
chr18	61336704	61338704	Hmgxb3	-0.1091518	0.000000149	hypomethylated	0.030667	0.92922	insignificant	10	29	30
chr18	61371250	61373250	Slc26a2	0.02236877	2.53E-16	inconclusive	0.0013233	0.96715	insignificant	11	46	46
chr18	61560085	61562085	Ppargc1b	-0.15584961	1.1E-26	hypomethylated	-0.0030022	0.29979	insignificant	18	45	51
chr18	61696190	61698190	Arhgef37	-0.34796194	0.16317	insignificant	0.028113	0.5024	insignificant	5	31	30
chr18	61714235	61716235	Csnk1a1	-0.09149334	2.26E-08	hypomethylated	0.0024956	0.80702	insignificant	40	157	154
chr18	61807548	61809548	E330013P06		1	noCoverage	-0.21907	0.29853	insignificant	0	12	16
chr18	61808912	61810912	E330013P06		1	noCoverage	0.021766	0.39213	insignificant	0	4	4
chr18	61867289	61869289	Pcyox1l	0.14278083	0.59024	insignificant	0.011008	0.49952	insignificant	2	13	10
chr18	61885043	61887043	1500015A07R	-0.11977579	3.7E-21	hypomethylated	-0.023265	1	insignificant	23	104	92
chr18	61885985	61887985	Grpel2	-0.1761231	0.00000291	hypomethylated	-0.021602	0.92692	insignificant	13	56	51
chr18	61946316	61948316	Afap1l1	-0.27582789	1.04E-25	hypomethylated	-0.0013162	0.51874	insignificant	11	29	27
chr18	62071478	62073478	Ablim3		1	noCoverage	-0.061681	0.79547	insignificant	0	27	26
chr18	62071506	62073506	Ablim3		1	noCoverage	-0.10507	0.77298	insignificant	0	15	14
chr18	62111728	62113728	Sh3tc2	-0.0085712	0.48515	insignificant	0.15696	0.4399	insignificant	1	34	36
chr18	62339613	62341613	Adrb2	-0.20241482	0.0000055	hypomethylated	0.026459	0.27823	insignificant	7	36	43
chr18	62482857	62484857	Htr4	-0.09458006	0.012261	hypomethylated	-0.0096505	0.35701	insignificant	3	46	47
chr18	62707564	62709564	Spink10	-0.11485587	2.91E-17	hypomethylated	-0.0078483	0.92023	insignificant	14	71	67
chr18	62708397	62710397	Fbxo38	-0.12136872	0.000046766	hypomethylated	0.0007843	0.88083	insignificant	6	32	32
chr18	63080980	63082980	Apcdd1	-0.14378292	1.09E-41	hypomethylated	-0.0030503	0.50432	insignificant	38	143	145
chr18	63136569	63138569	Napg	-0.26521842	0.000000254	hypomethylated	-0.043874	0.45843	insignificant	11	65	58
chr18	63546837	63548837	Fam38b	-0.22417475	0.0014788	hypomethylated	-0.075631	0.12797	insignificant	7	33	30
chr18	63852013	63854013	Txnl1	-0.22981408	0.0022207	hypomethylated	0.03098	0.012389	hypermethylated	5	40	40
chr18	63867348	63869348	Wdr7	-0.17881242	0.00022124	hypomethylated	-0.049119	0.66512	insignificant	8	19	18
chr18	64413012	64415012	St8sia3	-0.1508658	0.34566	insignificant	-0.040909	0.80445	insignificant	2	4	4
chr18	64499017	64501017	Onecut2	-0.07048794	5.44E-19	hypomethylated	0.011494	0.29572	insignificant	41	186	177
chr18	64648720	64650720	Fech	-0.1438902	0.000000472	hypomethylated	-0.0035153	0.41536	insignificant	2	22	22
chr18	64676211	64678211	Nars	-0.18600796	0.000000589	hypomethylated	0.014628	0.50951	insignificant	5	12	12
chr18	64820654	64822654	Atp8b1	-0.05914352	1.9E-26	hypomethylated	-0.027136	0.12232	insignificant	15	69	76
chr18	65046409	65048409	Nedd4l	-0.13236516	5.07E-43	hypomethylated	-0.00071419	0.3233	insignificant	48	180	169
chr18	65553542	65555542	Alpk2	0.22395833	1	lowCoverage	0.10505	0.50833	insignificant	1	4	4
chr18	65589650	65591650	Malt1	-0.10550424	2.13E-24	hypomethylated	0.011887	0.47349	insignificant	48	157	158
chr18	65738883	65740883	Zfp532	-0.08442273	3.76E-14	hypomethylated	0.012423	0.34828	insignificant	40	100	101
chr18	65959231	65961231	Sec11c	-0.1337171	0.000051166	hypomethylated	0.017539	0.69107	insignificant	9	43	43
chr18	66032147	66034147	Grp	-0.11500593	0.46383	insignificant	-0.0021396	0.60776	insignificant	11	50	50
chr18	66129832	66131832	Cplx4		1	noCoverage	0.020205	0.33662	insignificant	0	6	6
chr18	66162289	66164289	Lman1	-0.43246278	0.000000131	stronglyHypometh	-0.12042	4.6E-10	hypomethylated	1	14	19
chr18	66451492	66453492	Ccbe1	-0.16487918	0.6212	insignificant	-0.069573	0.74486	insignificant	3	27	24
chr18	66617257	66619257	Pmaip1	-0.17909555	0.000000178	hypomethylated	-0.005865	0.10477	insignificant	15	114	117
chr18	67020126	67022126	Mc4r	-0.35757576	0.10822	insignificant	-0.040693	0.87443	insignificant	2	12	11
chr18	67246989	67248989	Gnal	-0.10891678	2.97E-09	hypomethylated	0.0066182	0.2732	insignificant	38	139	139
chr18	67292479	67294479	Gnal	-0.1337646	1.76E-21	hypomethylated	0.020757	0.65432	insignificant	19	84	82
chr18	67364012	67366012	Chmp1b	-0.143445	1.25E-09	hypomethylated	-0.0050074	0.40132	insignificant	17	82	69
chr18	67405484	67407484	Mppe1	-0.44107256	0.000018668	stronglyHypometh	0.045408	0.093267	insignificant	3	24	22
chr18	67447876	67449876	Impa2	-0.11274752	1.19E-12	hypomethylated	0.011612	0.9635	insignificant	16	63	54
chr18	67502217	67504217	Cidea	-0.15497789	0.00000163	hypomethylated	0.011403	0.54599	insignificant	7	46	41
chr18	67503757	67505757	Cidea	-0.27253796	0.023442	hypomethylated	0.15349	0.0043505	hypermethylated	3	8	8
chr18	67549384	67551384	Tubb6	-0.14728872	1.41E-22	hypomethylated	0.012675	0.39191	insignificant	45	124	119
chr18	67608790	67610790	Afg3l2	0.00342866	0.18266	insignificant	0.022371	0.068484	insignificant	8	36	38
chr18	67623502	67625502	Slmo1	-0.08950394	0.012354	hypomethylated	-0.02386	0.45397	insignificant	9	88	80
chr18	67708827	67710827	Spire1	0.17940085	1	insignificant	-0.014518	0.26986	insignificant	2	18	12
chr18	67800252	67802252	Psmg2	-0.10809314	1.09E-41	hypomethylated	-0.00037202	0.80863	insignificant	66	213	210
chr18	67800990	67802990	Psmg2	-0.09363798	3.57E-15	hypomethylated	0.0013471	0.53293	insignificant	35	114	112
chr18	67884275	67886275	Ptpn2	-0.20821585	0.19886	insignificant	0.00048528	0.2269	insignificant	2	31	30
chr18	67933529	67935529	Seh1l	-0.09003324	3.2E-29	hypomethylated	0.0061581	0.10159	insignificant	47	94	94
chr18	67958760	67960760	Cep192	-0.23177442	7.27E-08	hypomethylated	0.00049962	0.60026	insignificant	16	100	95
chr18	68091910	68093910	D18Ertd653e	-0.11869268	3.51E-42	hypomethylated	0.0062746	0.21506	insignificant	54	169	165
chr18	68459008	68461008	Rnmt	-0.15554611	4.38E-10	hypomethylated	0.013229	0.93873	insignificant	23	88	88
chr18	68459987	68461987	4933403F05Ri	-0.26475015	0.001492	hypomethylated	0.0086261	0.90345	insignificant	6	26	26

chr18	69503145	69505145	Tcf4	-0.16316289	6.36E-15	hypomethylated	-0.013275	0.20846	insignificant	17	64	64
chr18	69504374	69506374	Tcf4	-0.12377482	2.78E-22	hypomethylated	0.00090112	0.034054	hypermethylated	41	109	116
chr18	70213023	70215023	Rab27b	-0.27608386	1	lowCoverage	-0.0059861	0.58274	insignificant	1	10	10
chr18	70631198	70633198	Stard6	-0.09415158	1.02E-30	hypomethylated	-0.00011304	0.019471	hypomethylated	28	124	108
chr18	70632134	70634134	4930503L19Ri	-0.18008299	2.28E-09	hypomethylated	0.047145	0.26401	insignificant	11	36	28
chr18	70689792	70691792	Poli	-0.34786514	2.33E-21	stronglyHypometh	-0.051859	0.099208	insignificant	3	18	18
chr18	70689975	70691975	Poli	0.16302624	0.60079	insignificant	-0.053	1	insignificant	1	14	14
chr18	70726945	70728945	Mbd2	-0.0714633	1.68E-18	hypomethylated	-0.022056	0.36366	insignificant	51	179	166
chr18	72510723	72512723	Dcc	-0.22222222	0.33259	insignificant	-0.13651	0.47571	insignificant	1	3	7
chr18	73731358	73733358	Mex3c	-0.09780967	2.14E-77	hypomethylated	0.0062167	0.0065525	hypermethylated	113	314	318
chr18	73863395	73865395	Smad4	-0.09688854	0.00051151	hypomethylated	0.0028566	0.52373	insignificant	22	70	70
chr18	73914133	73916133	Elac1	-0.25926187	6.52E-25	hypomethylated	0.026757	0.019295	hypermethylated	17	55	49
chr18	73975046	73977046	Me2	-0.14764815	1.56E-10	hypomethylated	-0.015274	0.26341	insignificant	15	60	60
chr18	74018040	74020040	Mro	0.20486111	1	insignificant	0.059028	0.70235	insignificant	2	8	8
chr18	74224603	74226603	Mapk4	-0.18256053	0.0031522	hypomethylated	-0.012455	0.13906	insignificant	10	39	38
chr18	74367472	74369472	Ska1		1	noCoverage	0.22917	1	insignificant	0	4	3
chr18	74374865	74376865	Cxxc1	-0.07189985	3.42E-13	hypomethylated	0.035519	0.0024941	hypermethylated	38	148	143
chr18	74426941	74428941	Mbd1	-0.20370025	3.29E-19	hypomethylated	0.00045104	0.5236	insignificant	16	93	84
chr18	74441753	74443753	Ccdc11	-0.76210826	0.11327	insignificant	-0.056099	0.88658	insignificant	1	9	6
chr18	74601272	74603272	Myo5b	-0.09754687	5.68E-20	hypomethylated	-0.012019	0.15328	insignificant	58	162	161
chr18	74937865	74939865	Acaa2	-0.12214195	0.14255	insignificant	0.0052375	0.68687	insignificant	3	29	26
chr18	74938109	74940109	Acaa2	-0.14737621	0.055146	insignificant	0.023968	0.51224	insignificant	5	39	36
chr18	75120917	75122917	Lipg	-0.12803004	1.62E-08	hypomethylated	0.030795	0.49114	insignificant	7	48	48
chr18	75159130	75161130	Rpl17	-0.18972523	9.87E-34	hypomethylated	-0.029216	0.92148	insignificant	37	111	111
chr18	75159721	75161721	Snord58b	-0.13605175	1.87E-30	hypomethylated	-0.026166	0.86147	insignificant	37	99	96
chr18	75164553	75166553	BC031181	-0.32854504	1.55E-17	hypomethylated	0.0005997	0.049268	inconclusive	17	63	59
chr18	75177425	75179425	Dym	-0.11950194	1.49E-21	hypomethylated	0.013534	0.0010482	hypermethylated	22	86	81
chr18	75526018	75528018	Smad7	-0.11973544	3.42E-56	hypomethylated	0.014851	0.23173	insignificant	50	202	187
chr18	75857350	75859350	Ctif	-0.17619048	0.075016	insignificant	-0.0050873	1	insignificant	4	14	14
chr18	75978831	75980831	Zbtb7c	-0.11510301	1.62E-17	hypomethylated	0.0015895	0.91127	insignificant	57	232	217
chr18	76400578	76402578	Smad2	-0.09293018	9.31E-49	hypomethylated	-0.0032888	0.33854	insignificant	93	280	277
chr18	77094143	77096143	Skor2	-0.09403289	0.0038157	hypomethylated	-0.000099147	0.97414	insignificant	14	75	66
chr18	77167765	77169765	Ier3ip1	-0.12425982	9.44E-21	hypomethylated	0.013567	0.13722	insignificant	23	61	57
chr18	77181853	77183853	Hdhd2	-0.12332532	1.68E-10	hypomethylated	-0.0067498	0.2581	insignificant	18	61	56
chr18	77182156	77184156	Hdhd2	-0.12332532	1.68E-10	hypomethylated	-0.0067498	0.2581	insignificant	18	61	56
chr18	77302946	77304946	Pias2	-0.13084991	1.5E-15	hypomethylated	0.012171	0.029772	hypermethylated	29	162	148
chr18	77303418	77305418	Pias2	-0.09906783	4.62E-11	hypomethylated	0.0027335	0.081651	insignificant	29	156	140
chr18	77423585	77425585	St8sia5	-0.13329413	9E-40	hypomethylated	0.01592	0.025319	hypermethylated	50	159	161
chr18	77519696	77521696	Loxhd1	0.15625376	0.39515	insignificant	0.061546	0.024943	hypermethylated	5	22	22
chr18	77803875	77805875	Rnf165	-0.1301998	9.78E-15	hypomethylated	0.034007	0.58468	insignificant	23	109	106
chr18	77951922	77953922	4930465K10Ri	-0.09596375	9.34E-35	hypomethylated	-0.0042226	0.31011	insignificant	68	251	243
chr18	77952749	77954749	8030462N17R	-0.10471588	2.52E-09	hypomethylated	-0.0092394	0.31108	insignificant	30	126	125
chr18	78006519	78008519	Haus1	-0.10069444	0.0067687	hypomethylated	0.019795	0.42706	insignificant	6	12	12
chr18	78011506	78013506	Atp5a1	-0.14699178	1.95E-21	hypomethylated	0.0085908	0.19246	insignificant	21	94	93
chr18	78032288	78034288	Pstpip2	-0.1885794	4.42E-61	hypomethylated	0.0073143	0.90232	insignificant	36	128	128
chr18	78134205	78136205	5430411K18Ri	-0.1599352	0.02425	hypomethylated	-0.0042768	0.41523	insignificant	15	50	47
chr18	78793689	78795689	Slc14a2		1	noCoverage	-0.0568	0.26147	insignificant	0	10	10
chr18	79306130	79308130	Setbp1	-0.08017696	9.63E-11	hypomethylated	0.010981	0.35726	insignificant	32	176	177
chr18	80242633	80244633	Pard6g	-0.08702069	0.000000196	hypomethylated	0.010072	0.038717	hypermethylated	29	107	107
chr18	80348221	80350221	Adnp2	-0.23897995	0.079218	insignificant	0.041031	0.11384	insignificant	2	18	18
chr18	80397358	80399358	Rbfa	-0.4234075	2.29E-10	stronglyHypometh	-0.037482	0.71528	insignificant	9	45	40
chr18	80402536	80404536	Txnl4a	-0.2725699	3.27E-22	hypomethylated	-0.0053737	0.60624	insignificant	7	77	69
chr18	80402584	80404584	Txnl4a	-0.27407913	2.69E-24	hypomethylated	-0.00073348	0.83525	insignificant	7	82	73
chr18	80402605	80404605	Txnl4a	-0.27407913	2.69E-24	hypomethylated	-0.00073348	0.83525	insignificant	7	82	73
chr18	80443841	80445841	Hsbp1l1	-0.2385468	0.0068731	hypomethylated	-0.0037584	0.10614	insignificant	1	8	8
chr18	80451964	80453964	Pqlc1	-0.10855679	0.0040227	hypomethylated	0.069038	0.87654	insignificant	8	30	27
chr18	80452044	80454044	Pqlc1	-0.10855679	0.0040227	hypomethylated	0.037407	0.9379	insignificant	8	30	26
chr18	80666406	80668406	Ctdp1	-0.08121839	0.00071101	hypomethylated	0.00094975	0.22796	insignificant	17	88	80
chr18	80904912	80906912	Nfatc1	-0.16479863	2.68E-11	hypomethylated	0.0091957	0.52973	insignificant	20	75	70
chr18	80909810	80911810	Nfatc1	-0.15968903	8.25E-17	hypomethylated	-0.023555	0.50108	insignificant	17	68	64
chr18	81130797	81132797	Atp9b	-0.53466738	0.1184	insignificant	0.020893	0.25824	insignificant	4	18	16
chr18	81183317	81185317	Sall3	-0.09377424	4.68E-23	hypomethylated	0.0020266	0.306	insignificant	67	264	266
chr18	82576169	82578169	Galr1	0.18292058	0.0014543	hypermethylated	0.10116	0.30562	insignificant	6	29	20
chr18	82643514	82645514	Mbp	-0.13513628	3.22E-15	hypomethylated	0.024483	0.69465	insignificant	18	74	72
chr18	82722854	82724854	Mbp	0.05122549	1	insignificant	0.0071078	0.79238	insignificant	2	8	8
chr18	82722899	82724899	Mbp	0.05122549	1	insignificant	0.0071078	0.79238	insignificant	2	8	8
chr18	82862126	82864126	Zfp236	-0.18263374	6.39E-13	hypomethylated	-0.015984	0.054534	insignificant	24	100	86
chr18	83079270	83081270	Zfp516	-0.11994838	5.77E-11	hypomethylated	0.010375	0.46441	insignificant	6	92	73
chr18	83083023	83085023	Zfp516	-0.12607561	0.0036333	hypomethylated	-0.0050091	0.63478	insignificant	5	101	112
chr18	83089952	83091952	Zfp516	0.27380952	0.32137	insignificant	-0.0068173	0.54037	insignificant	1	8	10
chr18	84255954	84257954	Zadh2	-0.12140896	1.65E-15	hypomethylated	-0.029055	0.45853	insignificant	39	137	138
chr18	84256549	84258549	Zadh2	-0.12140896	1.65E-15	hypomethylated	-0.029055	0.45853	insignificant	39	137	138
chr18	84758896	84760896	Zfp407	-0.20640911	3.05E-11	hypomethylated	-0.029575	0.63505	insignificant	12	32	30
chr18	84855025	84857025	Cndp2	-0.26679873	8.19E-20	hypomethylated	0.041424	0.24335	insignificant	7	45	45
chr18	84888633	84890633	Fam69c	-0.10183496	1.34E-28	hypomethylated	0.0051294	3.83E-08	hypermethylated	28	133	133
chr18	85019805	85021805	Cyb5	-0.23084469	3.01E-11	hypomethylated	0.0083351	0.75486	insignificant	9	56	58
chr18	85120916	85122916	Fbxo15	0.02253347	0.7652	insignificant	0.0043961	0.50371	insignificant	12	58	58
chr18	86563343	86565343	Neto1	-0.15978093	0.000000676	hypomethylated	-0.0084921	0.52641	insignificant	25	70	69
chr18	86881439	86883439	Cbln2	-0.10618969	0.36377	insignificant	-0.03151	0.89412	insignificant	8	29	28
chr18	89140181	89142181	Rttn	-0.2324853	2.01E-12	hypomethylated	-0.018574	0.47943	insignificant	10	30	30
chr18	89365818	89367818	Cd226		1	noCoverage	-0.058608	0.69942	insignificant	0	3	6
chr18	89938528	89940528	Dok6	-0.09867572	2.36E-16	hypomethylated	0.0077613	0.19603	insignificant	27	71	66
chr18	90678545	90680545	Tmx3	-0.13750629	4.82E-12	hypomethylated	0.0071749	0.96607	insignificant	13	68	68
chr19	3282046	3284046	Mrpl21	-0.14915101	4.2E-10	hypomethylated	0.041333	0.78199	insignificant	14	42	34
chr19	3283010	3285010	Ighmbp2	-0.03924698	0.000023254	hypomethylated	0.02955	0.74525	insignificant	13	32	28

chr19	3322300	3324300	Cpt1a	-0.14653535	1.96E-16	hypomethylated	-0.0083716	0.80146	insignificant	11	66	66
chr19	3387868	3389868	Mtl5	-0.07532885	2.27E-10	hypomethylated	-0.0067764	0.43413	insignificant	23	75	75
chr19	3414457	3416457	Gal	-0.11750177	0.00076022	hypomethylated	-0.0023512	0.82949	insignificant	6	34	34
chr19	3575749	3577749	Ppp6r3	-0.15498916	3.5E-10	hypomethylated	0.047953	0.0079746	hypermethylated	11	67	67
chr19	3686564	3688564	Lrp5	-0.19235267	0.0079763	hypomethylated	0.018298	0.43461	insignificant	11	43	42
chr19	3707332	3709332	1810055G02R	-0.10043741	0.000033336	hypomethylated	0.011265	0.6914	insignificant	10	76	69
chr19	3766420	3768420	Suv420h1	-0.10237165	2.3E-37	hypomethylated	0.0021232	0.62112	insignificant	49	162	162
chr19	3850772	3852772	Chka	-0.11065194	0.000000807	hypomethylated	0.020893	0.52527	insignificant	30	129	114
chr19	3905230	3907230	Tcirg1	-0.1501075	6.45E-10	hypomethylated	0.0037565	0.45925	insignificant	5	53	53
chr19	3906983	3908983	Tcirg1	0.74521986	0.0050607	stronglyHypermeth	0.0021586	0.89522	insignificant	2	29	29
chr19	3907133	3909133	Tcirg1	0.46546883	0.088166	insignificant	-0.023962	0.22404	insignificant	5	27	27
chr19	3912717	3914717	Ndufs8	-0.18055556	1	insignificant	0.0069444	0.54769	insignificant	3	6	6
chr19	3929716	3931716	Aldh3b1		1	noCoverage	0.011409	0.85954	insignificant	0	8	8
chr19	3934185	3936185	Unc93b1	-0.44036071	3.02E-10	stronglyHypometh	0.067366	0.19676	insignificant	5	33	30
chr19	3971327	3973327	Aldh3b2	-0.09679487	0.7036	insignificant	-0.004513	0.32627	insignificant	3	10	10
chr19	3985660	3987660	Acy3	-0.18378113	0.000096538	hypomethylated	0.00078515	0.81503	insignificant	6	14	14
chr19	3991751	3993751	Tbx10	0.12007168	1	lowCoverage	-0.020117	0.7638	insignificant	1	6	6
chr19	3999579	4001579	Nudt8	-0.14838435	7.61E-09	hypomethylated	0.010513	0.26923	insignificant	33	93	92
chr19	4002384	4004384	Doc2g	0.10611055	1	insignificant	0.030742	0.46128	insignificant	1	17	23
chr19	4012725	4014725	Ndufv1	-0.32914863	0.23329	insignificant	-0.06417	1	insignificant	5	14	14
chr19	4037912	4039912	Gstp1	0.66735044	0.0053688	stronglyHypermeth	0.0097342	0.62164	insignificant	2	28	16
chr19	4042221	4044221	Gstp2	-0.85714286	5.29E-17	stronglyHypometh	-0.19697	0.0058585	hypomethylated	3	6	6
chr19	4081487	4083487	Cabp2		1	noCoverage	-0.0060332	0.81491	insignificant	0	6	6
chr19	4082518	4084518	Cabp2	-0.0378997	0.057782	insignificant	0.022344	0.58261	insignificant	6	38	38
chr19	4096350	4098350	Cdk2ap2	-0.20947769	7.25E-40	hypomethylated	0.0042776	0.017055	hypermethylated	31	93	97
chr19	4099116	4101116	Pitpnm1	-0.1858714	5.68E-27	hypomethylated	0.024974	0.72801	insignificant	25	82	82
chr19	4099621	4101621	Pitpnm1	-0.15664241	2.11E-21	hypomethylated	0.026732	0.50034	insignificant	23	74	74
chr19	4124959	4126959	Tmem134	-0.22938557	3.67E-12	hypomethylated	-0.02589	0.57627	insignificant	15	54	52
chr19	4125827	4127827	Aip	-0.32102399	8.68E-16	hypomethylated	-0.048637	0.90107	insignificant	15	62	58
chr19	4147662	4149662	Coro1b	-0.13197535	5.92E-11	hypomethylated	0.0032847	0.43958	insignificant	7	38	38
chr19	4153645	4155645	Ptprcap	-0.38522121	1.12E-08	stronglyHypometh	-0.026516	0.54117	insignificant	4	12	12
chr19	4163245	4165245	Rps6kb2	-0.17327778	0.0015841	hypomethylated	0.015183	0.75273	insignificant	3	20	20
chr19	4175479	4177479	Carns1		1	noCoverage	0.10526	0.16795	insignificant	0	2	2
chr19	4191047	4193047	Tbc1d10c	-0.10138788	7.29E-23	hypomethylated	-0.0060155	0.77501	insignificant	45	148	146
chr19	4191173	4193173	Ppp1ca	-0.10225186	7.32E-23	hypomethylated	-0.0068795	0.77499	insignificant	45	147	146
chr19	4201603	4203603	Rad9	-0.18387182	9.21E-12	hypomethylated	0.01795	0.25668	insignificant	14	37	36
chr19	4213391	4215391	Clcf1	-0.19160601	8E-22	hypomethylated	0.0079263	0.3428	insignificant	28	108	96
chr19	4230898	4232898	Pold4		1	noCoverage	-0.028571	0.83001	insignificant	0	3	3
chr19	4305955	4307955	Adrbk1	-0.09524699	3.52E-14	hypomethylated	0.0022348	0.76698	insignificant	33	74	64
chr19	4397077	4399077	Kdm2a	-0.09732263	7.68E-24	hypomethylated	0.013966	0.00042782	hypermethylated	48	218	216
chr19	4439424	4441424	Rhod	-0.14249955	0.25423	insignificant	0.00256	0.0038677	hypermethylated	15	50	50
chr19	4477143	4479143	Syt12		1	noCoverage	0.061039	0.89456	insignificant	0	10	10
chr19	4509471	4511471	Pcx	-0.12323097	6.73E-19	hypomethylated	0.022852	0.062519	insignificant	20	111	108
chr19	4615667	4617667	Pcx	-0.35196243	1.48E-19	stronglyHypometh	-0.025024	0.24042	insignificant	5	37	33
chr19	4625617	4627617	Gm960	-0.0822248	2.6E-12	hypomethylated	-0.0016462	0.29394	insignificant	16	94	94
chr19	4710222	4712222	Spnb3	-0.05013148	0.0019789	hypomethylated	-0.011956	0.56552	insignificant	34	116	116
chr19	4755524	4757524	Rbm4b	-0.15394599	7.58E-24	hypomethylated	-0.008438	0.18369	insignificant	19	85	85
chr19	4793877	4795877	Rbm4	-0.28510363	1.43E-15	hypomethylated	0.039552	0.042258	hypermethylated	6	26	28
chr19	4811634	4813634	Rbm14	-0.21555583	0.000018757	hypomethylated	-0.0079163	0.96112	insignificant	11	38	37
chr19	4838365	4840365	Ccdc87	-0.25295109	4.35E-12	hypomethylated	-0.046557	0.10624	insignificant	13	58	68
chr19	4839322	4841322	Ccdc87	-0.26237572	6.66E-08	hypomethylated	0.011583	0.52913	insignificant	14	54	64
chr19	4854128	4856128	Ctsf	-0.16020844	0.000001	hypomethylated	0.0069363	0.211	insignificant	6	62	62
chr19	4877667	4879667	Zdhhc24	-0.1998231	3.85E-08	hypomethylated	-0.000000231	0.1047	insignificant	12	70	70
chr19	4877689	4879689	Zdhhc24	-0.1998231	3.85E-08	hypomethylated	-0.000000231	0.1047	insignificant	12	70	70
chr19	4877884	4879884	Zdhhc24	-0.14173619	0.00020173	hypomethylated	0.0072381	0.69324	insignificant	8	52	52
chr19	4906627	4908627	Dpp3	-0.38909319	0.000025787	stronglyHypometh	-0.092254	0.00031155	hypomethylated	4	21	19
chr19	4943092	4945092	Peli3	-0.24843697	1.13E-08	hypomethylated	-0.026487	0.51537	insignificant	3	27	28
chr19	4961305	4963305	Mrpl11	-0.21155245	3.69E-08	hypomethylated	-0.035698	0.9165	insignificant	6	15	12
chr19	4989971	4991971	Npas4	-0.36872203	1	insignificant	0.017279	0.71116	insignificant	2	36	42
chr19	5023005	5025005	Slc29a2	-0.2206208	0.00000233	hypomethylated	-0.08587	0.8579	insignificant	3	44	39
chr19	5037825	5039825	B3gnt1	-0.11455803	2.64E-08	hypomethylated	-0.0038125	0.32366	insignificant	13	126	126
chr19	5040403	5042403	Brms1	-0.19347677	6.27E-34	hypomethylated	-0.013567	0.37215	insignificant	26	80	78
chr19	5049807	5051807	Rin1	-0.20386313	0.000000735	hypomethylated	0.01736	0.94631	insignificant	7	18	18
chr19	5067077	5069077	Cd248	-0.15892718	1.53E-20	hypomethylated	0.023522	0.075972	insignificant	24	92	95
chr19	5085477	5087477	Tmem151a	-0.17222858	0.25192	insignificant	0.0042211	0.282	insignificant	21	49	49
chr19	5087552	5089552	Yif1a		1	noCoverage	-0.0329	0.50993	insignificant	0	10	10
chr19	5098418	5100418	Cnih2	-0.08829419	8.22E-22	hypomethylated	-0.0084584	1	insignificant	24	50	50
chr19	5106996	5108996	Klc2	0.42065846	5.03E-08	stronglyHypermeth	-0.039698	0.028599	inconclusive	7	47	45
chr19	5118408	5120408	Klc2	-0.18095191	0.020724	hypomethylated	-0.017432	0.3047	insignificant	10	32	31
chr19	5273119	5275119	Sf3b2	-0.07742065	0.000000195	hypomethylated	0.00068191	1	insignificant	19	66	66
chr19	5295455	5297455	Gal3st3	-0.08571913	8.72E-08	hypomethylated	0.003419	0.26685	insignificant	18	70	70
chr19	5297330	5299330	Gal3st3	-0.45212973	0.0070336	stronglyHypometh	0.029521	0.00000126	hypermethylated	10	33	33
chr19	5334740	5336740	Catsper1	0.22294372	1	insignificant	0.082002	0.024499	hypermethylated	2	8	8
chr19	5349574	5351574	Cst6	0.08547067	0.49543	insignificant	0.043586	0.91043	insignificant	5	24	21
chr19	5365812	5367812	Eif1ad	-0.12526575	8.08E-23	hypomethylated	-0.0024308	0.19157	insignificant	43	145	165
chr19	5366347	5368347	Banf1	-0.11097522	7.55E-23	hypomethylated	0.014726	0.21067	insignificant	43	135	140
chr19	5366645	5368645	Banf1	-0.14998858	3.75E-19	hypomethylated	0.049458	0.067027	insignificant	23	70	70
chr19	5387335	5389335	D330050I16Ri	-0.1633523	1.09E-34	hypomethylated	-0.029692	0.86848	insignificant	37	136	132
chr19	5388703	5390703	D330050I16Ri	-0.30954571	0.29875	insignificant	-0.041367	0.88196	insignificant	3	40	38
chr19	5424143	5426143	AI837181	-0.12909617	1.94E-33	hypomethylated	-0.0035445	0.52235	insignificant	48	147	168
chr19	5424916	5426916	Drap1	-0.09342132	0.00033392	hypomethylated	-0.031456	0.17164	insignificant	26	77	98
chr19	5446697	5448697	Fosl1	-0.15492159	2.47E-11	hypomethylated	0.025387	0.63873	insignificant	17	64	64
chr19	5457549	5459549	Ccdc85b	-0.14608358	0.21887	insignificant	0.00038939	0.94997	insignificant	7	28	28
chr19	5459693	5461693	Fibp	-0.16178681	0.0011129	hypomethylated	0.0099118	0.58657	insignificant	5	36	36
chr19	5468498	5470498	Ctsw	0.04833255	0.83453	insignificant	0.016878	0.59398	insignificant	2	10	10

chr19	5473689	5475689	Efemp2	-0.10644749	0.00074014	hypomethylated	-0.0068859	0.87797	insignificant	31	122	123
chr19	5473734	5475734	Efemp2	-0.10644749	0.00074014	hypomethylated	-0.0068859	0.87797	insignificant	31	122	123
chr19	5488336	5490336	Mus81	-0.29330152	0.013439	hypomethylated	0.003358	0.35184	insignificant	8	62	65
chr19	5489454	5491454	Cfl1	-0.12481682	1.27E-23	hypomethylated	0.012525	0.18612	insignificant	45	144	146
chr19	5503787	5505787	1700020D05R	-0.23257576	0.19172	insignificant	-0.012573	0.72157	insignificant	1	10	10
chr19	5510489	5512489	Snx32	-0.13013827	7.86E-12	hypomethylated	0.0068091	0.89868	insignificant	25	74	70
chr19	5560575	5562575	Ovol1	-0.01354392	0.0024851	hypomethylated	0.03145	0.032489	hypermethylated	18	99	85
chr19	5567073	5569073	Gm962	-0.12926402	1.89E-23	hypomethylated	-0.0014887	0.71473	insignificant	48	175	165
chr19	5600872	5602872	Rnaseh2c	-0.23635562	1.5E-14	hypomethylated	-0.061813	1	insignificant	14	56	43
chr19	5609707	5611707	Kat5	-0.11297937	2.08E-11	hypomethylated	0.020392	0.33558	insignificant	23	78	77
chr19	5610094	5612094	Kat5	-0.11681036	0.033749	hypomethylated	0.012555	0.04331	hypermethylated	7	22	21
chr19	5636489	5638489	Rela	-0.09959429	5.96E-14	hypomethylated	0.0004265	0.71085	insignificant	47	184	184
chr19	5663707	5665707	Sipa1	-0.18337763	0.14634	insignificant	-0.021996	0.0026496	hypomethylated	11	30	28
chr19	5688130	5690130	Map3k11	-0.13675137	2.17E-42	hypomethylated	0.00048632	0.46411	insignificant	50	183	178
chr19	5688908	5690908	Pcnxl3	-0.19419874	1.97E-29	hypomethylated	-0.0071338	0.60469	insignificant	38	122	120
chr19	5703475	5705475	Kcnk7	-0.4784334	0.1835	insignificant	0.063081	0.72197	insignificant	2	14	14
chr19	5726317	5728317	Ehbp1l1	-0.15776511	0.001243	hypomethylated	-0.023113	0.63877	insignificant	9	37	35
chr19	5729666	5731666	Fam89b	-0.07367038	0.00000382	inconclusive	-0.047072	0.070549	insignificant	14	69	71
chr19	5731721	5733721	Sssca1	-0.67130827	0.19963	insignificant	0.07444	0.49261	insignificant	0	15	18
chr19	5739903	5741903	Ltbp3	-0.21300466	9.86E-09	hypomethylated	0.035988	0.63602	insignificant	11	55	48
chr19	5771401	5773401	Scyl1	-0.11503623	0.22911	insignificant	0.0066414	0.71161	insignificant	2	12	12
chr19	5802671	5804671	Malat1	-0.2526531	0.00079702	hypomethylated	-0.073524	0.93038	insignificant	12	38	33
chr19	5845478	5847478	Neat1	-0.23139248	0.000000446	hypomethylated	0.0049772	0.45609	insignificant	8	40	30
chr19	5875208	5877208	Frmd8	-0.08240132	0.0044276	hypomethylated	0.0090146	0.40012	insignificant	7	28	28
chr19	5877465	5879465	Slc25a45	-0.24089835	0.38322	insignificant	-0.020065	0.62017	insignificant	1	10	8
chr19	5894107	5896107	Tigd3	-0.32735524	0.55785	insignificant	-0.010254	0.011715	hypomethylated	2	28	28
chr19	5912866	5914866	Dpf2	0.04568041	0.75613	insignificant	-0.025713	0.17312	insignificant	2	21	20
chr19	5964206	5966206	Pola2		1	noCoverage	0.018016	0.25221	insignificant	0	34	30
chr19	6015247	6017247	Capn1	-0.04325328	2.23E-08	hypomethylated	0.013075	0.078025	insignificant	4	69	65
chr19	6015825	6017825	Capn1	0.6162972	0.42202	lowCoverage	0.036761	0.012546	hypermethylated	1	39	35
chr19	6045575	6047575	Syvn1	-0.12006415	7.82E-14	hypomethylated	-0.027203	0.25325	insignificant	29	122	118
chr19	6046144	6048144	Syvn1	-0.08225685	6.43E-12	hypomethylated	0.0032878	0.54459	insignificant	30	122	122
chr19	6056887	6058887	Fau	-0.07352298	1.23E-08	hypomethylated	-0.024443	0.16043	insignificant	8	64	66
chr19	6056966	6058966	Fau	-0.07352298	1.23E-08	hypomethylated	-0.024443	0.16043	insignificant	8	64	66
chr19	6057751	6059751	Mrpl49	-0.15800501	0.00000099	hypomethylated	-0.01825	0.54464	insignificant	6	28	31
chr19	6060206	6062206	Znhit2-ps	-0.15259267	3.75E-31	hypomethylated	-0.00066739	0.87993	insignificant	30	158	159
chr19	6067850	6069850	1110014N23R	0.14885641	4.57E-08	hypermethylated	0.029973	0.00027614	hypermethylated	10	42	42
chr19	6077187	6079187	1110014N23R	-0.31156463	0.11451	insignificant	-0.010283	0.39064	insignificant	1	14	13
chr19	6084096	6086096	Cdca5	-0.07172849	0.04275	hypomethylated	-0.0058562	0.65519	insignificant	19	91	91
chr19	6084891	6086891	Zfpl1	-0.10880323	0.046664	hypomethylated	-0.0031397	0.85171	insignificant	21	103	103
chr19	6104797	6106797	Naaladl1		1	noCoverage	-0.010408	0.62783	insignificant	0	10	10
chr19	6118586	6120586	Snx15	-0.40320658	1	lowCoverage	-0.13638	0.29184	insignificant	1	12	10
chr19	6141137	6143137	Arl2	-0.30263132	0.000016985	hypomethylated	0.002595	0.29446	insignificant	4	47	47
chr19	6183409	6185409	1700123I01Ril	0.05845784	0.64564	insignificant	0.083031	0.052982	insignificant	3	32	32
chr19	6225400	6227400	Gpha2	-0.01064851	0.29136	insignificant	0.032182	0.095091	insignificant	2	14	14
chr19	6235840	6237840	Ppp2r5b	-0.37582708	5.74E-11	stronglyHypometh	-0.07859	0.7863	insignificant	5	18	16
chr19	6240667	6242667	Atg2a	-0.13514397	1	insignificant	0.15634	0.0066395	hypermethylated	2	37	28
chr19	6275895	6277895	Ehd1	-0.11528472	2.34E-34	hypomethylated	0.0005109	0.60924	insignificant	46	175	173
chr19	6305456	6307456	Cdc42bpg	-0.15378025	8.04E-22	hypomethylated	-0.03568	0.90826	insignificant	19	70	82
chr19	6333978	6335978	Men1	-0.14299803	7.34E-10	hypomethylated	0.025142	0.55294	insignificant	9	70	70
chr19	6334012	6336012	Men1	-0.14299803	7.34E-10	hypomethylated	0.025142	0.55294	insignificant	9	70	70
chr19	6334038	6336038	Men1	-0.14299803	7.34E-10	hypomethylated	0.025142	0.55294	insignificant	9	70	70
chr19	6340249	6342249	Map4k2	-0.08245513	0.000000056	hypomethylated	0.003634	0.36666	insignificant	29	90	90
chr19	6362689	6364689	Sf1	-0.09559563	1.42E-35	hypomethylated	-0.002163	0.89808	insignificant	68	231	229
chr19	6383428	6385428	Pygm	-0.24897002	0.55922	insignificant	0.0060902	0.68681	insignificant	3	26	26
chr19	6399582	6401582	Rasgrp2	-0.14220055	7.52E-08	hypomethylated	-0.0091647	0.90304	insignificant	24	60	69
chr19	6427015	6429015	Nrxn2	-0.09227995	1.68E-25	hypomethylated	-0.0044952	0.035958	hypomethylated	51	154	154
chr19	6915091	6917091	Rps6ka4	-0.19037281	2.49E-12	hypomethylated	-0.015966	0.40524	insignificant	13	33	33
chr19	6932701	6934701	Ccdc88b	-0.32107008	0.00023338	hypomethylated	0.066295	0.53907	insignificant	3	6	6
chr19	6983187	6985187	Trmt112	-0.07895531	2.18E-12	hypomethylated	0.0028122	0.4112	insignificant	32	153	153
chr19	6984135	6986135	Prdx5	-0.01859365	0.000000226	inconclusive	0.010348	0.57578	insignificant	25	142	142
chr19	6996298	6998298	Esrra	-0.20446156	0.00051054	hypomethylated	-0.024653	0.83439	insignificant	16	61	57
chr19	6999870	7001870	Kcnk4	-0.11154052	0.054906	insignificant	0.041187	0.52638	insignificant	14	90	86
chr19	7009005	7011005	Kcnk4		1	noCoverage	0.047235	0.64812	insignificant	0	2	2
chr19	7015344	7017344	Bad	-0.13059213	2.95E-23	hypomethylated	-0.0088218	0.96997	insignificant	33	137	127
chr19	7015683	7017683	Gpr137	-0.13059213	2.95E-23	hypomethylated	-0.0088218	0.96997	insignificant	33	137	127
chr19	7015997	7017997	Gpr137	-0.11577172	6.12E-13	hypomethylated	-0.001787	1	insignificant	26	104	99
chr19	7016940	7018940	Gpr137	-0.2366135	0.00019757	hypomethylated	-0.010589	0.52726	insignificant	6	14	14
chr19	7044242	7046242	Plcb3	-0.22836161	0.042291	hypomethylated	0.036527	0.019463	hypermethylated	4	31	34
chr19	7048537	7050537	Ppp1r14b	-0.12556072	1.1E-24	hypomethylated	0.0058928	0.010586	hypermethylated	24	128	126
chr19	7054930	7056930	Fkbp2	-0.1421283	0.024542	hypomethylated	-0.00041149	0.36202	insignificant	7	32	32
chr19	7054951	7056951	Fkbp2	-0.17208388	0.0087307	hypomethylated	-0.0019296	0.67745	insignificant	7	26	26
chr19	7062141	7064141	Vegfb	-0.22164237	0.048973	hypomethylated	0.0056175	0.0014874	hypermethylated	17	50	48
chr19	7066762	7068762	Dnajc4	-0.21622312	0.31132	insignificant	-0.012361	0.5669	insignificant	2	10	10
chr19	7069620	7071620	Trpt1	0.04083199	5.37E-13	inconclusive	0.020199	0.40577	insignificant	15	59	61
chr19	7070527	7072527	Nudt22	-0.23678865	9.05E-12	hypomethylated	0.0027179	0.69147	insignificant	9	36	36
chr19	7093959	7095959	Fermt3	-0.40851608	0.000000652	stronglyHypometh	-0.051423	0.44756	insignificant	2	6	6
chr19	7114516	7116516	Stip1	-0.05408223	4.93E-09	hypomethylated	0.056654	0.33935	insignificant	17	43	45
chr19	7130257	7132257	Macrod1	-0.12458995	0.00000216	hypomethylated	0.031222	0.39157	insignificant	19	86	93
chr19	7280774	7282774	Otub1	-0.07788022	0.40693	insignificant	0.03776	0.70576	insignificant	3	45	44
chr19	7292106	7294106	Cox8a	-0.49207591	0.0033556	stronglyHypometh	0.040887	0.90013	insignificant	3	40	45
chr19	7315712	7317712	Naa40	-0.16435698	7.65E-09	hypomethylated	0.034492	0.00010965	hypermethylated	8	40	38
chr19	7343253	7345253	Rcor2	-0.20854976	1.03E-22	hypomethylated	-0.0060564	0.4403	insignificant	18	75	71
chr19	7416334	7418334	Mark2	-0.28712674	0.00062949	hypomethylated	-0.12883	0.51117	insignificant	11	39	24
chr19	7457516	7459516	AI846148	-0.17719071	3.2E-17	hypomethylated	-0.044267	0.55289	insignificant	14	37	38

chr19	7491114	7493114	2700081O15R	-0.11696868	1.95E-36	hypomethylated	-0.026874	0.54405	insignificant	77	233	212
chr19	7567529	7569529	Atl3	-0.19791595	2.18E-16	hypomethylated	0.037645	0.54406	insignificant	24	104	92
chr19	7567927	7569927	Atl3	-0.20555485	2.24E-16	hypomethylated	0.030006	0.57137	insignificant	24	105	92
chr19	7630948	7632948	Pla2g16	-0.4017094	0.5298	insignificant	-0.068376	0.68012	insignificant	2	6	6
chr19	7686058	7688058	Hrasls5	-0.38995029	0.55627	insignificant	0.014519	0.23068	insignificant	3	20	20
chr19	8206472	8208472	Slc22a28		1	noCoverage	0.035897	0.76195	insignificant	0	5	5
chr19	8479595	8481595	Slc22a30		1	noCoverage	-0.14792	0.26975	insignificant	0	8	8
chr19	8666671	8668671	Slc22a8	0.39339827	1	lowCoverage	0.063312	0.6564	insignificant	1	4	4
chr19	8737494	8739494	Chrm1		1	noCoverage	0.33288	0.59343	insignificant	0	13	11
chr19	8788387	8790387	Slc3a2	-0.02682607	0.53593	insignificant	0.04514	0.22554	insignificant	5	44	42
chr19	8796976	8798976	Snhg1		1	noCoverage	0.045799	1	insignificant	0	30	26
chr19	8797859	8799859	Snord22		1	noCoverage	0.14403	0.3941	insignificant	0	14	10
chr19	8809328	8811328	Wdr74	-0.14588064	3.01E-11	hypomethylated	-0.00099473	0.73554	insignificant	10	43	44
chr19	8814207	8816207	1700092M07R	-0.14119781	0.0024995	inconclusive	0.0083805	0.57837	insignificant	7	32	32
chr19	8814913	8816913	Stx5a	-0.16795198	0.056291	insignificant	0.00090394	0.66614	insignificant	7	42	42
chr19	8815292	8817292	Stx5a	-0.1603178	0.056284	insignificant	0.0085381	0.66624	insignificant	7	44	42
chr19	8830592	8832592	Nxf1	-0.10635829	1.96E-14	hypomethylated	-0.0026831	0.032035	hypomethylated	34	104	104
chr19	8844485	8846485	Tmem223	-0.0166353	0.73657	insignificant	0.065907	0.0078307	hypermethylated	10	44	42
chr19	8847974	8849974	Gm2518	-0.10809147	2.84E-24	hypomethylated	0.0045845	0.87495	insignificant	44	153	148
chr19	8848957	8850957	Tmem179b	-0.09832341	1.67E-21	hypomethylated	0.0030222	0.56155	insignificant	40	129	124
chr19	8873047	8875047	Polr2g		1	noCoverage	-0.016208	1	insignificant	0	10	10
chr19	8875985	8877985	Zbtb3	-0.31028606	9.58E-09	hypomethylated	0.02526	0.28525	insignificant	5	17	14
chr19	8876019	8878019	Zbtb3	-0.31028606	9.58E-09	hypomethylated	0.02526	0.28525	insignificant	5	17	14
chr19	8892890	8894890	Hnrnpul2	-0.09347702	0.00000314	hypomethylated	0.00094952	0.67411	insignificant	18	141	146
chr19	8893784	8895784	Hnrnpul2	-0.11362364	4.06E-26	hypomethylated	-0.0060329	0.8416	insignificant	31	179	185
chr19	8910956	8912956	Bscl2	-0.38792373	0.016409	stronglyHypometh	0.010108	0.36026	insignificant	1	46	46
chr19	8913736	8915736	Gng3		1	noCoverage	-0.047258	0.35845	insignificant	0	16	16
chr19	8945048	8947048	Ubxn1	-0.23431622	8.53E-42	hypomethylated	0.0043395	0.60509	insignificant	23	81	83
chr19	8962392	8964392	1810009A15R	-0.15889832	0.000026992	hypomethylated	0.0038133	0.54202	insignificant	13	66	65
chr19	8963260	8965260	5730408K05R	-0.17078653	0.000011974	hypomethylated	-0.007783	0.43471	insignificant	6	27	27
chr19	8966476	8968476	Ints5	-0.17020476	2.55E-18	hypomethylated	-0.019169	0.75926	insignificant	16	71	68
chr19	8971600	8973600	Ganab	-0.20104748	1.9E-11	hypomethylated	0.0012331	0.70982	insignificant	15	76	70
chr19	8993882	8995882	B3gat3	-0.0647081	0.000012307	hypomethylated	0.0052645	0.76083	insignificant	17	57	48
chr19	9003183	9005183	Eml3	-0.14615512	2.27E-26	hypomethylated	0.070009	0.51639	insignificant	28	126	125
chr19	9003846	9005846	Rom1	-0.12347709	1.49E-25	hypomethylated	0.079761	0.50948	insignificant	28	112	111
chr19	9015409	9017409	Mta2	-0.12989773	6.18E-38	hypomethylated	0.0060349	0.88933	insignificant	54	151	151
chr19	9027339	9029339	Tut1	-0.17147436	1	insignificant	-0.012382	0.75261	insignificant	4	24	14
chr19	9040530	9042530	Eef1g	-0.23765144	0.34137	insignificant	0.025926	0.79509	insignificant	3	21	21
chr19	9062773	9064773	Ahnak	-0.09231238	4.02E-31	hypomethylated	0.024201	0.0067714	hypermethylated	31	112	98
chr19	9162446	9164446	Scgb1a1	-0.89983974	0.10891	lowCoverage	-0.090224	0.068077	insignificant	1	8	8
chr19	9974023	9976023	Incenp	-0.15716869	0.0029483	hypomethylated	0.023894	0.69372	insignificant	6	36	34
chr19	10056192	10058192	Fth1		1	noCoverage	0.00034501	1	insignificant	0	17	17
chr19	10091717	10093717	Rab3il1	-0.0725096	0.001103	hypomethylated	0.0018771	0.45093	insignificant	11	52	52
chr19	10115037	10117037	Fads3	-0.10807647	1.13E-24	hypomethylated	-0.012745	0.94392	insignificant	32	113	108
chr19	10175993	10177993	Fads2	-0.13791124	5.1E-12	hypomethylated	-0.0065792	0.42688	insignificant	13	64	61
chr19	10256377	10258377	Fads1	-0.13594075	7.33E-37	hypomethylated	-0.00555	0.97836	insignificant	34	130	130
chr19	10277691	10279691	1810006K21R	-0.16349956	1.16E-09	hypomethylated	0.035089	0.29717	insignificant	13	42	42
chr19	10278433	10280433	1810006K21R	-0.24777315	5.49E-13	hypomethylated	0.061326	0.11892	insignificant	9	26	26
chr19	10315238	10317238	Gm98	-0.09477633	0.10093	insignificant	0.022823	0.29466	insignificant	7	34	34
chr19	10379367	10381367	Dagla	-0.36759575	4.04E-22	stronglyHypometh	-0.034242	0.14524	insignificant	7	20	20
chr19	10462579	10464579	Syt7	-0.0935832	1.99E-40	hypomethylated	0.0099843	0.13202	insignificant	67	188	193
chr19	10531937	10533937	Lrrc10b	-0.01365379	1	insignificant	0.068527	0.00024171	hypermethylated	12	46	46
chr19	10557387	10559387	Ppp1r32	0.08214286	1	insignificant	0.033532	0.50812	insignificant	2	5	6
chr19	10598733	10600733	Cpsf7	-0.10976357	1.28E-24	hypomethylated	0.00054326	0.23343	insignificant	25	174	159
chr19	10599699	10601699	Sdhaf2	-0.11141748	8.49E-11	hypomethylated	0.00026465	0.86018	insignificant	18	136	123
chr19	10651212	10653212	Cybasc3	-0.2051808	1.03E-09	hypomethylated	0.00022416	0.81982	insignificant	7	55	55
chr19	10651587	10653587	Cybasc3	-0.23707983	2.98E-10	hypomethylated	0.0057704	0.82249	insignificant	7	48	48
chr19	10678748	10680748	Dak	-0.14278541	0.0037422	hypomethylated	0.19383	0.74523	insignificant	5	18	21
chr19	10679114	10681114	Ddb1	-0.14278541	0.0037422	hypomethylated	0.19383	0.74523	insignificant	5	18	21
chr19	10707722	10709722	Vwce	0.02686069	3.12E-21	inconclusive	0.028831	0.6391	insignificant	12	52	52
chr19	10762304	10764304	Vps37c	-0.08220375	2.16E-17	hypomethylated	0.021515	0.31061	insignificant	22	51	44
chr19	10813464	10815464	Cd5	0.125	1	insignificant	0.035819	0.6648	insignificant	1	4	4
chr19	10814436	10816436	A430093F15R	0.125	1	insignificant	0.035819	0.6648	insignificant	1	4	4
chr19	10904548	10906548	Cd6	-0.37112985	1.42E-09	stronglyHypometh	-0.03102	0.72375	insignificant	3	6	6
chr19	10916033	10918033	Slc15a3	-0.3614886	0.000000667	stronglyHypometh	0.072946	0.48702	insignificant	2	16	15
chr19	10944269	10946269	Tmem109	-0.31292676	0.00000687	hypomethylated	-0.034237	0.000062491	hypomethylated	5	15	15
chr19	10956233	10958233	Tmem109	-0.15946588	0.00024314	hypomethylated	-0.01297	0.94944	insignificant	5	34	34
chr19	10968781	10970781	Prpf19	-0.12388597	1.6E-20	hypomethylated	0.011026	0.25478	insignificant	21	80	78
chr19	11010649	11012649	Gpr44		1	noCoverage	0.033664	1	insignificant	0	4	2
chr19	11023756	11025756	Ccdc86	-0.20268458	0.0087901	hypomethylated	-0.032112	0.49345	insignificant	9	31	30
chr19	11239810	11241810	1700025F22R	-0.75430024	0.072727	insignificant	0.026431	0.4398	insignificant	2	8	6
chr19	11271213	11273213	4930526L06R	-0.14296537	0.2943	insignificant	-0.0293	0.89822	insignificant	1	20	20
chr19	11358303	11360303	Ms4a5		1	noCoverage	-0.065044	0.47928	insignificant	0	16	18
chr19	11821048	11823048	Gif	0.03455882	1	insignificant	-0.0084607	1	insignificant	1	8	8
chr19	11843904	11845904	Mrpl16	-0.40395585	1.99E-09	stronglyHypometh	-0.093437	0.33953	insignificant	7	16	16
chr19	11893383	11895383	Stx3	-0.19732741	1.92E-10	hypomethylated	0.018281	0.83362	insignificant	18	66	64
chr19	11893893	11895893	Stx3	-0.18036012	0.00000523	hypomethylated	0.021806	0.79064	insignificant	6	36	34
chr19	11945704	11947704	Olfr1419	-0.37599206	0.078618	insignificant	-0.14872	0.016814	hypomethylated	3	6	6
chr19	11985888	11987888	Patl1	-0.06494582	5.26E-10	hypomethylated	0.013374	0.72802	insignificant	21	119	94
chr19	12039333	12041333	Osbp	-0.10261519	3.6E-34	hypomethylated	0.026966	0.005811	hypermethylated	43	150	156
chr19	12184034	12186034	Olfr1428	0.13454169	0.60777	insignificant	-0.043569	0.010461	hypomethylated	1	25	21
chr19	12576486	12578486	Dtx4		1	noCoverage	0.072727	0.047829	hypermethylated	0	11	4
chr19	12978720	12980720	Gm5512		1	noCoverage	0.39686	0.000067903	stronglyhypermeth	0	16	14
chr19	13854037	13856037	Olfr1496		1	noCoverage	0.22078	0.18327	insignificant	0	4	4
chr19	14672473	14674473	Tle4	-0.09184387	5.11E-09	hypomethylated	0.0044201	0.57735	insignificant	26	135	135

chr19	15999515	16001515	Psat1	-0.54555423	0.0004145	stronglyHypometh	0.093165	0.92115	insignificant	1	19	16
chr19	16059479	16061479	Cep78	-0.05739629	0.0001153	hypomethylated	-0.00014336	0.14966	insignificant	8	44	44
chr19	16206320	16208320	Gnaq	-0.08337209	1.48E-48	hypomethylated	0.0059976	0.51881	insignificant	70	303	265
chr19	16237587	16239587	E030024N20R	0.15939911	0.36611	insignificant	0.14219	0.044066	hypermethylated	1	9	6
chr19	16509156	16511156	Gna14	-0.14638306	3.27E-24	hypomethylated	0.010843	0.33096	insignificant	24	84	84
chr19	16855417	16857417	Vps13a	-0.09096857	0.000000823	hypomethylated	-0.020826	0.28342	insignificant	19	57	50
chr19	16948320	16950320	Foxb2	-0.1462353	0.0053082	hypomethylated	-0.0025392	0.42628	insignificant	3	30	30
chr19	17431157	17433157	Gcnt1		1	noCoverage	0.016945	0.12024	insignificant	0	2	2
chr19	17467532	17469532	Rfk	-0.08546387	1.45E-12	hypomethylated	-0.0083568	0.93777	insignificant	19	88	84
chr19	17912122	17914122	Pcsk5	0.05314156	0.0037349	hypermethylated	-0.0028835	0.036717	hypomethylated	10	35	35
chr19	18705505	18707505	BC016495	-0.11005822	1.65E-27	hypomethylated	0.010123	0.43145	insignificant	22	106	100
chr19	18706303	18708303	BC016495	-0.16541421	0.36255	insignificant	0.043749	0.23825	insignificant	2	24	18
chr19	18744269	18746269	2410127L17Ri	-0.17494325	6.39E-08	hypomethylated	-0.0039584	0.000061757	hypomethylated	21	117	117
chr19	18786725	18788725	D030056L22Ri	-0.10923701	8.5E-10	hypomethylated	-0.018246	0.51887	insignificant	24	62	56
chr19	18823472	18825472	Trpm6	-0.168574	3.5E-20	hypomethylated	0.0094452	0.935	insignificant	14	58	58
chr19	21028692	21030692	Tmc1		1	noCoverage	0.025	1	insignificant	0	4	4
chr19	21345767	21347767	Zfand5	-0.09014106	8.48E-41	hypomethylated	0.010594	0.76758	insignificant	67	245	228
chr19	21726798	21728798	Fam108b	-0.09187863	2.27E-30	hypomethylated	-0.0014975	0.72053	insignificant	61	197	192
chr19	21727281	21729281	1110059E24Ri	-0.09117888	1.87E-30	hypomethylated	0.012043	0.75858	insignificant	60	187	185
chr19	21851831	21853831	Tmem2	-0.15876441	6.54E-33	hypomethylated	-0.010525	0.33002	insignificant	35	116	113
chr19	22212606	22214606	Trpm3	-0.24079807	0.00086491	hypomethylated	0.024754	0.20207	insignificant	6	18	18
chr19	22521697	22523697	Trpm3	-0.24086053	1	lowCoverage	0.12537	0.097681	insignificant	1	12	12
chr19	23214715	23216715	Klf9	-0.09640902	5.41E-29	hypomethylated	-0.00089332	0.93849	insignificant	41	212	210
chr19	23348367	23350367	Smc5	-0.09166667	0.38225	insignificant	0.012653	0.70086	insignificant	10	30	28
chr19	23760889	23762889	Ptar1	-0.09797716	1.05E-19	hypomethylated	0.011305	0.22689	insignificant	43	147	144
chr19	23832365	23834365	Apba1	-0.12999226	2.57E-23	hypomethylated	0.013323	0.24777	insignificant	20	104	105
chr19	24105509	24107509	Fam189a2	-0.09940908	4.42E-16	hypomethylated	-0.0016688	0.078686	insignificant	12	60	59
chr19	24248630	24250630	Tjp2	-0.13599359	2.16E-25	hypomethylated	0.014141	0.71967	insignificant	24	115	115
chr19	24299516	24301516	Tjp2	-0.29399137	1	insignificant	-0.0039642	0.14594	insignificant	2	35	30
chr19	24355076	24357076	Fxn	-0.42913442	0.49813	insignificant	0.19638	0.63257	insignificant	1	8	8
chr19	24551964	24553964	Fam122a	-0.22793832	0.0025171	hypomethylated	0.0046571	0.32897	insignificant	1	28	28
chr19	24630317	24632317	Pip5k1b	-0.09167136	1.43E-16	hypomethylated	-0.0033586	0.528	insignificant	43	159	150
chr19	24747497	24749497	E030010A14Rik		1	noCoverage	-0.1	0.41781	insignificant	0	14	6
chr19	24936332	24938332	Pgm5	-0.36359059	0.000011771	stronglyHypometh	-0.043188	0.33633	insignificant	1	2	2
chr19	24975799	24977799	Foxd4		1	noCoverage	0.046213	0.42073	insignificant	0	6	6
chr19	25036106	25038106	Cbwd1	-0.16655635	0.019406	hypomethylated	-0.0070063	1	insignificant	2	22	22
chr19	25073018	25075018	Dock8	-0.10560511	1.18E-61	hypomethylated	0.0080086	0.57764	insignificant	55	164	157
chr19	25310691	25312691	Kank1	-0.11520441	3.08E-17	hypomethylated	0.0049032	0.86228	insignificant	39	149	138
chr19	25579195	25581195	Dmrt1	-0.13261573	5.8E-10	hypomethylated	-0.0033205	0.27525	insignificant	19	76	72
chr19	25684026	25686026	Dmrt3	-0.12135934	7.21E-38	hypomethylated	-0.0007167	0.29108	insignificant	58	218	214
chr19	25745900	25747900	Dmrt2	-0.20913805	6.14E-08	hypomethylated	0.0043217	0.23085	insignificant	13	72	70
chr19	26678649	26680649	Smarca2	-0.13884171	8.93E-52	hypomethylated	0.017432	0.27197	insignificant	44	191	194
chr19	26821892	26823892	Smarca2	-0.29268756	0.34165	insignificant	-0.036269	0.82697	insignificant	3	15	14
chr19	27290509	27292509	Vldlr	-0.08312878	4.32E-24	hypomethylated	-0.0030841	0.47954	insignificant	36	165	165
chr19	27396108	27398108	Kcnv2	0.09630732	0.0065892	hypermethylated	0.05163	0.0026134	hypermethylated	3	18	18
chr19	27503526	27505526	C030016D13R	-0.12440018	1.33E-24	hypomethylated	0.0031321	0.50019	insignificant	27	100	96
chr19	27504310	27506310	D19Bwg1357e	-0.11270161	0.00000151	hypomethylated	-0.018266	0.02832	hypomethylated	8	50	48
chr19	28085656	28087656	Rfx3	-0.16245879	1.09E-13	hypomethylated	-0.020266	1	insignificant	25	93	87
chr19	28754567	28756567	Glis3	-0.13997114	0.00000112	hypomethylated	0.013793	0.92019	insignificant	5	21	21
chr19	28908655	28910655	Slc1a1	-0.16140754	1.68E-18	hypomethylated	0.038223	0.11348	insignificant	18	69	70
chr19	29037409	29039409	Ppapdc2	-0.12925773	1.04E-34	hypomethylated	0.031113	0.023334	hypermethylated	28	96	96
chr19	29038644	29040644	4430402I18Ril	-0.11697198	2.48E-20	hypomethylated	0.022262	0.028614	hypermethylated	21	52	52
chr19	29063983	29065983	Cdc37l1	-0.14878754	8.67E-17	hypomethylated	0.016242	0.66596	insignificant	25	99	84
chr19	29120972	29122972	1700018L02Ri	-0.16781966	0.000000772	hypomethylated	-0.0090222	0.86586	insignificant	20	70	70
chr19	29122392	29124392	1700018L02Ri	-0.1944803	0.00010785	hypomethylated	0.01514	0.33789	insignificant	9	18	18
chr19	29174864	29176864	Rcl1	-0.11419718	1.04E-24	hypomethylated	0.010128	0.31809	insignificant	39	122	118
chr19	29208768	29210768	Mir101b	-0.1386139	0.31942	insignificant	0.046435	1	insignificant	2	10	10
chr19	29325317	29327317	Jak2	-0.09375227	1.57E-31	hypomethylated	-0.0005437	0.46399	insignificant	72	213	204
chr19	29399808	29401808	Insl6	0.25931539	0.000042975	hypermethylated	-0.048253	0.0016565	inconclusive	3	21	21
chr19	29409160	29411160	Rln1	-0.53645833	0.0027906	stronglyHypometh	-0.0090074	0.17407	insignificant	0	0	0
chr19	29436361	29438361	5033414D02R	-0.28297719	0.01111	hypomethylated	0.016583	0.36442	insignificant	7	19	19
chr19	29440927	29442927	Cd274	-0.27439554	0.10063	insignificant	0.044202	0.63446	insignificant	1	14	14
chr19	29595771	29597771	C030046E11Ri	-0.11670817	1.01E-73	hypomethylated	0.0075271	0.93227	insignificant	93	260	249
chr19	29596477	29598477	C030046E11Ri	-0.09924985	9.52E-63	hypomethylated	0.009202	0.92747	insignificant	90	244	233
chr19	29771430	29773430	Mlana	0.19270833	1	insignificant	0.39062	0.043595	stronglyhypermeth	1	6	6
chr19	29880499	29882499	9930021J03Ri	-0.13479919	5.83E-16	hypomethylated	0.012739	0.41158	insignificant	14	62	57
chr19	29887464	29889464	Ranbp6	0.00291719	6.27E-09	inconclusive	-0.016096	0.30854	insignificant	9	38	38
chr19	30077279	30079279	Trpd52l3	0.03236347	0.56243	insignificant	0.050321	0.00040847	hypermethylated	3	24	24
chr19	30104002	30106002	Uhrf2	-0.08567046	3.95E-22	hypomethylated	-0.017263	0.056774	insignificant	52	149	149
chr19	30614169	30616169	Ppp1r2-ps3		1	noCoverage	0.0045239	0.76192	insignificant	0	34	34
chr19	31156330	31158330	Cstf2t	-0.22249818	5.12E-15	hypomethylated	-0.0023322	0.14492	insignificant	23	82	82
chr19	31738860	31740860	Prkg1	-0.14130745	4.99E-14	hypomethylated	-0.0040322	0.68197	insignificant	13	51	49
chr19	31942250	31944250	A1cf	-0.23201592	0.21117	insignificant	0.021454	0.070935	insignificant	3	22	22
chr19	32462705	32464705	2700046G09R	-0.10917246	4.11E-44	hypomethylated	0.010905	0.23361	insignificant	59	170	160
chr19	32462944	32464944	Sgms1	-0.11968664	1.13E-21	hypomethylated	-0.00068505	0.94705	insignificant	34	118	120
chr19	32559258	32561258	Minpp1	-0.12079789	9.01E-36	hypomethylated	0.0037999	0.94417	insignificant	40	112	106
chr19	32786788	32788788	Atad1	-0.18028373	0.000000392	hypomethylated	0.00032292	0.48995	insignificant	9	18	18
chr19	32831066	32833066	Pten	-0.10925776	4.59E-39	hypomethylated	0.0012413	0.19541	insignificant	66	232	231
chr19	34265759	34267759	Stambpl1	-0.11853466	1.12E-11	hypomethylated	0.0026004	0.86362	insignificant	14	65	65
chr19	34329826	34331826	Acta2	-0.11363636	0.068119	insignificant	0.059893	0.48259	insignificant	1	5	2
chr19	34364148	34366148	Fas	-0.22993181	0.029631	hypomethylated	0.05422	0.84927	insignificant	7	34	36
chr19	34549625	34551625	Ch25h	-0.48210304	0.49907	insignificant	0.063974	1	insignificant	1	8	8
chr19	34601964	34603964	Lipa	-0.375	0.12513	insignificant	0.16532	0.66523	insignificant	1	3	2
chr19	34624183	34626183	Ifit2		1	noCoverage	0.056128	0.33175	insignificant	0	6	8
chr19	34673917	34675917	Gm14446		1	noCoverage	0.025594	0.76554	insignificant	0	6	6

chr19	34676458	34678458	Gm14446		1	noCoverage	-0.047072	0.50766	insignificant	0	4	4
chr19	34821601	34823601	Slc16a12	-0.19869245	1.8E-16	hypomethylated	-0.0067817	0.39817	insignificant	16	36	34
chr19	34895263	34897263	Pank1	-0.52804709	0.016948	stronglyHypometh	0.019496	0.73426	insignificant	1	19	15
chr19	34952407	34954407	Pank1	-0.11065154	1.11E-36	hypomethylated	0.016238	0.184	insignificant	52	157	158
chr19	34953945	34955945	Pank1	-0.34408463	0.000000108	stronglyHypometh	-0.014479	1	insignificant	4	12	12
chr19	34995847	34997847	Kif20b	-0.2109425	9.39E-13	hypomethylated	0.027868	0.088548	insignificant	18	65	56
chr19	36131850	36133850	Htr7	-0.03549775	0.000025619	hypomethylated	0.021918	0.00091402	hypermethylated	28	105	98
chr19	36157205	36159205	Rpp30	-0.21367723	0.000000219	hypomethylated	0.011298	0.89245	insignificant	4	26	26
chr19	36628128	36630128	Hectd2	-0.0979897	4.17E-24	hypomethylated	0.006708	0.90348	insignificant	31	124	107
chr19	36811094	36813094	Ppp1r3c	-0.03280926	0.2374	insignificant	-0.075227	0.61423	insignificant	6	51	42
chr19	36907721	36909721	Tnks2	-0.12476212	5.99E-15	hypomethylated	-0.020934	0.31915	insignificant	31	116	116
chr19	36999568	37001568	Btaf1	-0.09874637	6.21E-47	hypomethylated	0.011062	0.020692	hypermethylated	52	162	156
chr19	37281034	37283034	March5	-0.12861482	2.27E-56	hypomethylated	0.019528	0.17675	insignificant	68	197	172
chr19	37281783	37283783	March5	-0.12924753	5.16E-40	hypomethylated	0.020564	0.1653	insignificant	52	140	121
chr19	37449892	37451892	Kif11	-0.08959184	0.0031093	hypomethylated	0.0061769	0.36382	insignificant	9	68	67
chr19	37508330	37510330	Hhex	-0.10276096	1.62E-29	hypomethylated	0.0099197	0.26401	insignificant	50	249	246
chr19	37623907	37625907	Exoc6	-0.13245629	5.05E-42	hypomethylated	-0.015895	0.18135	insignificant	41	104	104
chr19	37759169	37761169	Cyp26c1	-0.13325017	0.31658	insignificant	0.0069873	0.21438	insignificant	5	25	25
chr19	37771297	37773297	Cyp26a1	-0.14518122	1.08E-11	hypomethylated	-0.0029144	0.39323	insignificant	25	109	109
chr19	38118067	38120067	Myof	0.130238	0.00053654	hypermethylated	0.020781	0.2074	insignificant	3	18	18
chr19	38128514	38130514	Cep55	-0.09729655	0.002531	hypomethylated	0.025269	0.0072502	inconclusive	15	62	52
chr19	38128531	38130531	Cep55	-0.09729655	0.002531	hypomethylated	0.025269	0.0072502	inconclusive	15	62	52
chr19	38170568	38172568	O3far1	-0.06579428	0.00031981	hypomethylated	0.0019352	0.29176	insignificant	17	76	78
chr19	38199301	38201301	Rbp4	-0.04461868	0.006814	hypomethylated	0.0092813	0.67987	insignificant	11	34	39
chr19	38206270	38208270	Pde6c	-0.14403286	0.29521	insignificant	-0.0039564	0.080213	insignificant	4	15	12
chr19	38298622	38300622	5730455O13R	-0.15667911	0.000000269	hypomethylated	0.025853	0.62465	insignificant	9	20	23
chr19	38338271	38340271	Lgi1	-0.34593057	0.00057081	stronglyHypometh	-0.019489	0.66158	insignificant	3	6	6
chr19	38469469	38471469	Tmem20	-0.13636184	7.14E-15	hypomethylated	-0.0088181	0.79977	insignificant	23	78	72
chr19	38597686	38599686	Plce1	-0.0047619	1	insignificant	0.020202	0.53425	insignificant	5	14	14
chr19	38893727	38895727	Noc3l	-0.1278432	0.000000166	hypomethylated	0.03539	0.31432	insignificant	15	45	43
chr19	38910068	38912068	Tbc1d12	-0.09001403	1.16E-30	hypomethylated	0.004202	0.90338	insignificant	42	178	158
chr19	39004479	39006479	Hells	-0.12670623	5.35E-08	hypomethylated	0.0018595	0.79875	insignificant	27	87	90
chr19	39080508	39082508	Cyp2c55	0.09053919	1	insignificant	0.020103	0.46377	insignificant	1	13	14
chr19	40346106	40348106	Pdlim1	-0.1495333	2.97E-17	hypomethylated	0.062514	0.38178	insignificant	11	30	30
chr19	40588226	40590226	Sorbs1	-0.11671413	2.74E-08	hypomethylated	-0.0002379	0.81603	insignificant	6	18	18
chr19	40588302	40590302	Sorbs1	-0.27051158	0.000022829	hypomethylated	-0.012728	0.37289	insignificant	2	4	4
chr19	40662953	40664953	Aldh18a1		1	noCoverage	0.0048913	0.73324	insignificant	0	10	10
chr19	40686705	40688705	Tctn3		1	noCoverage	-0.0064103	0.57672	insignificant	0	10	10
chr19	40733283	40735283	Entpd1	-0.23487854	0.000022179	hypomethylated	0.05984	0.47942	insignificant	5	20	16
chr19	40904768	40906768	Ccnj	-0.1200303	1.21E-36	hypomethylated	-0.0028333	0.62723	insignificant	66	177	173
chr19	40968194	40970194	Zfp518a	-0.0868543	4.98E-10	hypomethylated	-0.022818	0.55034	insignificant	19	96	91
chr19	41069025	41071025	Blnk	-0.35015615	0.000017434	stronglyHypometh	-0.01072	0.76842	insignificant	5	34	34
chr19	41102764	41104764	Dntt	-0.38636364	0.059403	insignificant	-0.29614	0.023395	hypomethylated	1	4	4
chr19	41151603	41153603	Opalin	-0.31303489	0.15292	insignificant	-0.0074793	1	insignificant	2	7	6
chr19	41281264	41283264	Tll2	-0.15540423	0.0011868	hypomethylated	-0.0069545	0.95727	insignificant	7	32	32
chr19	41338494	41340494	Tm9sf3	-0.17540183	1.46E-10	hypomethylated	0.054176	0.043609	hypermethylated	13	40	40
chr19	41459560	41461560	Pik3ap1	-0.1344259	0.0992	insignificant	0.040117	0.76448	insignificant	6	34	36
chr19	41818346	41820346	Slit1	0.09796297	0.037195	hypermethylated	0.051497	0.066477	insignificant	4	20	20
chr19	41903459	41905459	Frat1	-0.08960366	4.46E-38	hypomethylated	0.0083064	0.077733	insignificant	44	193	179
chr19	41922622	41924622	Frat2	-0.10068595	0.020658	hypomethylated	0.007972	0.013661	inconclusive	7	73	66
chr19	41970643	41972643	Rrp12	0.00173889	0.62356	insignificant	-0.086793	0.040192	hypomethylated	5	29	26
chr19	41985360	41987360	Pgam1	-0.09774668	1.19E-09	hypomethylated	-0.012088	0.070757	insignificant	27	121	116
chr19	42006961	42008961	Zdhhc16	-0.15229745	1.72E-31	hypomethylated	0.01116	0.065319	insignificant	38	135	130
chr19	42007804	42009804	Exosc1	-0.16138267	5.89E-24	hypomethylated	0.0051122	0.23143	insignificant	16	66	64
chr19	42055252	42057252	Ubtd1	-0.10711725	2.25E-52	hypomethylated	-0.0091383	0.66913	insignificant	59	215	203
chr19	42055626	42057626	Ubtd1	-0.11325358	9.33E-48	hypomethylated	-0.0040255	0.95685	insignificant	56	189	170
chr19	42119340	42121340	Hoga1	0.10663919	0.5284	insignificant	-0.033984	0.55391	insignificant	3	20	20
chr19	42163924	42165924	Pi4k2a	-0.02784978	0.27846	insignificant	-0.018647	0.054217	insignificant	30	111	106
chr19	42203483	42205483	Avpi1	-0.09776432	0.035057	hypomethylated	0.0050026	0.77908	insignificant	4	47	45
chr19	42220878	42222878	Marveld1	-0.15236868	4.58E-15	hypomethylated	-0.031464	0.87645	insignificant	28	125	121
chr19	42244056	42246056	Zfyve27	-0.10486643	5.69E-21	hypomethylated	0.014628	0.075354	insignificant	30	96	92
chr19	42276742	42278742	Sfrp5	0.03795072	0.00036055	inconclusive	0.10045	0.71582	insignificant	3	19	10
chr19	42329228	42331228	Golga7b	-0.12612535	0.000019251	hypomethylated	0.016979	0.68471	insignificant	19	68	79
chr19	42354660	42356660	Mir3085		1	noCoverage	-0.057238	0.30059	insignificant	0	8	8
chr19	42506273	42508273	Crtac1	-0.07351197	0.41207	insignificant	0.0028014	0.040385	hypermethylated	19	104	92
chr19	42592294	42594294	D19Ertd386e	-0.07088339	0.18558	insignificant	-0.0054023	0.41073	insignificant	15	114	115
chr19	42687296	42689296	Loxl4	-0.22980854	0.000000375	hypomethylated	0.029936	0.45811	insignificant	11	26	26
chr19	42827265	42829265	Pyroxd2	-0.55323063	0.0019733	stronglyHypometh	0.083225	0.83245	insignificant	2	15	16
chr19	42854466	42856466	Hps1	-0.3398211	0.34539	insignificant	-0.053385	0.62485	insignificant	1	12	8
chr19	43513925	43515925	Cnnm1	-0.07339724	4.5E-44	hypomethylated	0.00055788	0.49969	insignificant	61	190	197
chr19	43599095	43601095	Got1	-0.08189966	0.050422	insignificant	-0.0077118	0.34773	insignificant	4	47	47
chr19	43685814	43687814	Nkx2-3	-0.15113875	5.87E-11	hypomethylated	0.012767	0.3866	insignificant	16	72	75
chr19	43749371	43751371	Slc25a28	-0.11945198	1.44E-14	hypomethylated	0.0021653	0.8543	insignificant	23	75	75
chr19	43763178	43765178	Entpd7	-0.17538051	1.02E-17	hypomethylated	0.094729	0.016522	hypermethylated	19	44	44
chr19	43826512	43828512	Cutc	-0.1381506	4.92E-22	hypomethylated	0.0062416	0.80407	insignificant	18	112	107
chr19	43827490	43829490	Cutc	-0.03021144	0.0252	hypomethylated	-0.0060428	0.21006	insignificant	5	58	53
chr19	44061010	44063010	Cpn1		1	noCoverage	-0.031593	0.81871	insignificant	0	6	6
chr19	44144176	44146176	Erlin1	-0.09320696	7.53E-18	hypomethylated	0.0040734	0.56372	insignificant	20	49	51
chr19	44144265	44146265	Erlin1	-0.07042083	0.00000106	hypomethylated	0.027443	0.57794	insignificant	13	38	42
chr19	44144275	44146275	Erlin1	-0.04165336	0.001155	hypomethylated	0.033718	0.050793	insignificant	12	36	40
chr19	44181967	44183967	Chuk	0.16842943	0.18783	insignificant	-0.03841	0.36876	insignificant	5	24	22
chr19	44220936	44222936	Bloc1s2	0.0625	1	insignificant	-0.021558	0.78498	insignificant	2	6	6
chr19	44367165	44369165	Scd2	-0.24919992	9.15E-11	hypomethylated	0.065602	0.71694	insignificant	8	31	22
chr19	44406815	44408815	Scd4	0.13862179	0.78722	insignificant	0.042308	0.28522	insignificant	2	7	7
chr19	44482199	44484199	Scd1	-0.24990503	0.000000726	hypomethylated	0.026929	0.55725	insignificant	6	18	20

chr19	44620338	44622338	Sec31b	-0.01940635	0.041987	hypomethylated	-0.047237	0.029191	hypomethylated	4	12	13
chr19	44629905	44631905	Ndufb8	-0.07396208	0.15188	insignificant	0.077818	0.06176	insignificant	6	14	15
chr19	44636343	44638343	Hif1an	-0.17702736	1.04E-11	hypomethylated	0.0059592	0.060696	insignificant	12	57	57
chr19	44830883	44832883	Pax2	-0.14429936	1.91E-42	hypomethylated	-0.0038211	0.14943	insignificant	45	122	122
chr19	45004608	45006608	Fam178a	-0.10887012	8.63E-22	hypomethylated	0.018106	0.43936	insignificant	17	78	70
chr19	45062833	45064833	Sema4g	-0.1324746	2.79E-24	hypomethylated	0.021765	0.073482	insignificant	37	106	102
chr19	45080047	45082047	Peo1	-0.08367028	9.16E-16	hypomethylated	0.0028207	0.8688	insignificant	35	169	163
chr19	45080932	45082932	Mrpl43	-0.11927538	0.029217	inconclusive	-0.01975	0.7698	insignificant	20	79	79
chr19	45088665	45090665	Lzts2	-0.18740975	4.32E-18	hypomethylated	0.0024303	0.11559	insignificant	16	51	50
chr19	45088699	45090699	Lzts2	-0.17178295	7.64E-18	hypomethylated	0.018057	0.10569	insignificant	16	50	50
chr19	45091624	45093624	Lzts2	-0.14913687	9.25E-25	hypomethylated	-0.015342	0.67978	insignificant	24	64	69
chr19	45120262	45122262	Sfxn3	-0.09646487	0.014131	hypomethylated	0.012853	0.36034	insignificant	3	14	18
chr19	45121065	45123065	Sfxn3	-0.32647123	0.014213	hypomethylated	-0.065719	0.60273	insignificant	2	18	18
chr19	45149628	45151628	Kazald1	-0.14034873	9.47E-10	hypomethylated	0.01161	0.30945	insignificant	23	70	69
chr19	45224204	45226204	Tlx1	-0.08767774	1.15E-09	hypomethylated	0.029834	0.97013	insignificant	24	76	70
chr19	45309726	45311726	Lbx1	-0.16877461	9.17E-26	hypomethylated	-0.039345	0.29817	insignificant	39	123	114
chr19	45437223	45439223	Btrc	-0.28912367	0.0043225	hypomethylated	-0.024926	1	insignificant	3	44	50
chr19	45634104	45636104	Dpcd	-0.20165078	6.3E-10	hypomethylated	0.0046722	0.65183	insignificant	7	44	41
chr19	45635033	45637033	Poll	-0.19271293	1.54E-09	hypomethylated	0.014715	0.90393	insignificant	6	34	33
chr19	45734683	45736683	Fbxw4	-0.17785085	1.63E-19	hypomethylated	-0.051523	0.68415	insignificant	15	62	60
chr19	45817374	45819374	Fgf8	0.19381356	0.10262	insignificant	0.057457	0.39729	insignificant	4	39	32
chr19	45824053	45826053	Npm3	-0.11013249	7.49E-09	hypomethylated	-0.014508	0.52962	insignificant	8	26	26
chr19	45857781	45859781	Mgea5	-0.10414479	0.0012095	hypomethylated	0.0082893	0.50648	insignificant	28	71	76
chr19	45890293	45892293	Kcnip2	-0.25254284	1.68E-15	hypomethylated	0.016702	0.42317	insignificant	19	61	63
chr19	46072978	46074978	9130011E15Ri	-0.25169939	2.05E-14	hypomethylated	0.10676	0.064791	insignificant	10	30	30
chr19	46076967	46078967	Hps6	-0.17018506	5.85E-09	hypomethylated	-0.00056874	0.061156	insignificant	24	86	78
chr19	46114111	46116111	Ldb1		1	noCoverage	-0.0083208	0.25515	insignificant	0	24	24
chr19	46119701	46121701	Ldb1	-0.11764844	2.08E-19	hypomethylated	0.00057683	0.37905	insignificant	25	83	86
chr19	46130028	46132028	Pprc1	-0.09487052	6.57E-23	hypomethylated	0.019614	0.13207	insignificant	28	109	86
chr19	46149352	46151352	Nolc1	-0.11253792	0.30425	insignificant	0.03316	0.22922	insignificant	26	108	99
chr19	46205388	46207388	Elovl3	-0.22201948	0.47393	insignificant	-0.042134	0.58059	insignificant	7	40	36
chr19	46222815	46224815	Pitx3	-0.15886909	8.31E-08	hypomethylated	-0.013852	0.76532	insignificant	7	51	49
chr19	46226047	46228047	Gbf1	-0.25402773	0.080342	insignificant	0.009017	0.55939	insignificant	3	18	18
chr19	46378226	46380226	Nfkb2	-0.14282394	4.62E-26	hypomethylated	-0.019025	0.23295	insignificant	37	135	133
chr19	46379184	46381184	Nfkb2	-0.13853305	1.42E-23	hypomethylated	-0.012412	0.22124	insignificant	29	119	110
chr19	46379419	46381419	Nfkb2	-0.12688929	1.24E-13	hypomethylated	-0.0082236	0.097305	insignificant	25	105	96
chr19	46401646	46403646	Psd	-0.12565703	6.5E-34	hypomethylated	0.0248	0.83807	insignificant	21	148	138
chr19	46401673	46403673	Fbxl15	-0.12367759	1.26E-33	hypomethylated	0.025133	0.57959	insignificant	22	150	140
chr19	46413150	46415150	Cuedc2		1	noCoverage	-0.022012	0.83127	insignificant	0	18	16
chr19	46430369	46432369	Tmem180	0.05516259	1	insignificant	0.028941	0.69765	insignificant	2	18	15
chr19	46470225	46472225	Sufu	-0.13392063	5.35E-32	hypomethylated	-0.023847	0.83415	insignificant	27	100	96
chr19	46470407	46472407	Sufu	-0.14649962	3.92E-10	hypomethylated	-0.031072	0.48068	insignificant	21	76	72
chr19	46575137	46577137	Trim8	-0.12250476	8.61E-47	hypomethylated	-0.00066599	0.95967	insignificant	58	221	217
chr19	46646854	46648854	Sfxn2	-0.15977906	5.11E-08	hypomethylated	0.030722	0.38727	insignificant	9	63	63
chr19	46647575	46649575	Arl3	-0.13583571	7.14E-08	hypomethylated	0.051734	0.45568	insignificant	9	54	55
chr19	46696890	46698890	D19Wsu162e	-0.02562249	0.03642	hypomethylated	-0.13734	0.00065371	hypomethylated	7	19	20
chr19	46763395	46765395	2010012O05R	-0.14269537	1.27E-12	hypomethylated	-0.0096113	0.34035	insignificant	6	27	27
chr19	46780932	46782932	As3mt		1	noCoverage	0.037006	0.38692	insignificant	0	10	10
chr19	46835098	46837098	Cnnm2	-0.12599167	3.58E-28	hypomethylated	0.010793	0.15776	insignificant	27	158	148
chr19	47036841	47038841	Nt5c2	-0.53125	0.000035683	stronglyHypometh	-0.10268	0.016915	hypomethylated	1	4	2
chr19	47044057	47046057	Nt5c2	-0.38026316	0.29324	insignificant	0.11764	0.044904	hypermethylated	1	8	16
chr19	47088187	47090187	Ina	-0.10316189	9.58E-08	hypomethylated	0.014325	0.16653	insignificant	27	114	114
chr19	47089679	47091679	Nt5c2	-0.26768095	0.000068094	hypomethylated	-0.037915	0.16372	insignificant	6	22	16
chr19	47125335	47127335	Pcgf6	-0.17188229	1	insignificant	-0.087853	0.77559	insignificant	2	32	28
chr19	47141237	47143237	Taf5	-0.20332537	2.84E-19	hypomethylated	0.026353	0.0006452	hypermethylated	28	90	90
chr19	47164255	47166255	Pdcd11	-0.10587298	0.000000154	hypomethylated	-0.01383	0.95981	insignificant	12	76	77
chr19	47165115	47167115	Pdcd11	-0.17300511	0.0000057	hypomethylated	-0.029598	0.06307	insignificant	7	56	50
chr19	47212784	47214784	Calhm2	-0.18002137	0.73156	insignificant	-0.11464	0.41703	insignificant	1	8	7
chr19	47252309	47254309	Neurl1a	-0.12687731	1.25E-32	hypomethylated	0.0065422	0.56439	insignificant	56	194	195
chr19	47302332	47304332	Neurl1a	0.0235123	0.00095506	inconclusive	-0.063339	0.014158	hypomethylated	8	28	31
chr19	47538901	47540901	Sh3pxd2a	-0.14867716	9.46E-19	hypomethylated	0.0071936	0.22138	insignificant	22	74	75
chr19	47611510	47613510	Obfc1	-0.19876995	1.18E-10	hypomethylated	-0.077107	1	insignificant	9	42	36
chr19	47653508	47655508	Slk	-0.11080962	8.13E-27	hypomethylated	-0.021303	0.61899	insignificant	39	100	122
chr19	47805245	47807245	Sfr1	-0.11975489	0.000024719	hypomethylated	0.037998	0.6625	insignificant	16	76	82
chr19	47911851	47913851	Wdr96	-0.06255729	0.057742	insignificant	0.059255	0.94612	insignificant	6	29	30
chr19	47928478	47930478	Gsto1	-0.23822193	1.69E-08	hypomethylated	-0.065038	0.9583	insignificant	13	56	52
chr19	47939034	47941034	Gsto2	-0.11992961	0.014843	hypomethylated	0.075108	0.0088188	hypermethylated	14	44	48
chr19	47939283	47941283	Gsto2	-0.17656218	0.0026803	hypomethylated	0.059786	0.044746	hypermethylated	13	43	46
chr19	47993789	47995789	Itprip	-0.19051464	0.0000075	hypomethylated	0.0043329	0.58969	insignificant	16	42	40
chr19	48011201	48013201	Ccdc147	-0.29933966	2.39E-26	hypomethylated	0.013217	1.54E-10	hypermethylated	7	26	26
chr19	48279514	48281514	Sorcs3	-0.15893059	8.95E-18	hypomethylated	-0.020005	0.42858	insignificant	39	142	152
chr19	50753102	50755102	Sorcs1	0.02126774	0.0032669	hypermethylated	-0.0050177	0.21956	insignificant	8	50	50
chr19	52337812	52339812	Ins1	-0.27026537	0.068129	insignificant	-0.037466	1	insignificant	2	8	6
chr19	52996670	52998670	AA387883	-0.35496615	0.25306	insignificant	-0.24573	0.82728	insignificant	4	12	21
chr19	53113032	53115032	Xpnpep1	-0.19165224	2.71E-13	hypomethylated	-0.0052036	5.54E-09	hypomethylated	18	54	54
chr19	53216245	53218245	Add3	-0.10839569	3.04E-10	hypomethylated	-0.0062252	0.37356	insignificant	24	92	88
chr19	53217486	53219486	Add3	-0.15205449	6.34E-08	hypomethylated	-0.031506	0.49401	insignificant	20	49	46
chr19	53329695	53331695	1700001K23Rik		1	noCoverage	-0.055	0.53427	insignificant	0	6	10
chr19	53383995	53385995	Mxi1	-0.11692267	0.0016778	hypomethylated	0.031988	0.39411	insignificant	22	92	91
chr19	53402902	53404902	Mxi1	-0.06177612	9.59E-13	hypomethylated	0.0086247	0.43206	insignificant	27	182	165
chr19	53403935	53405935	Mxi1	-0.05701947	3.1E-12	hypomethylated	0.0086865	0.45823	insignificant	22	169	152
chr19	53465063	53467063	Smndc1	-0.05695836	0.013534	inconclusive	-0.00091268	0.56028	insignificant	17	69	68
chr19	53602807	53604807	Dusp5	-0.10657204	1.25E-20	hypomethylated	0.012215	0.0026879	hypermethylated	40	198	200
chr19	53673885	53675885	Smc3	-0.17591169	6.4E-19	hypomethylated	-0.022153	0.30066	insignificant	38	120	112
chr19	53750795	53752795	Rbm20	-0.15236668	3.17E-30	hypomethylated	-0.0086195	0.91087	insignificant	34	113	113

chr19	53976840	53978840	Pdcd4	-0.10468721	2.42E-21	hypomethylated	0.020635	0.14219	insignificant	48	112	127
chr19	54017795	54019795	Shoc2	-0.16294516	6E-65	hypomethylated	0.0055542	0.11229	insignificant	55	134	134
chr19	54018365	54020365	Shoc2	-0.15863739	4.05E-63	hypomethylated	0.0031424	0.15584	insignificant	54	132	132
chr19	54019117	54021117	Shoc2	-0.20826214	1.94E-36	hypomethylated	-0.040848	0.66891	insignificant	31	72	70
chr19	54118671	54120671	Adra2a	-0.14040006	1.6E-29	hypomethylated	0.0063932	0.1894	insignificant	51	147	142
chr19	55173937	55175937	Gpam	-0.05075875	0.0000055	hypomethylated	0.01759	0.42198	insignificant	14	40	41
chr19	55254374	55256374	Tectb		1	noCoverage	0.12198	0.072388	insignificant	0	14	14
chr19	55326858	55328858	Acsl5	-0.20986022	0.00083213	hypomethylated	-0.00039312	0.37271	insignificant	11	48	48
chr19	55389840	55391840	Vti1a	-0.12058384	3.56E-16	hypomethylated	0.0072859	0.49106	insignificant	39	146	126
chr19	55390522	55392522	Vti1a	-0.12755889	3.43E-11	hypomethylated	0.035278	0.20634	insignificant	25	78	62
chr19	55815299	55817299	Tcf7l2	-0.10541839	1.2E-35	hypomethylated	0.05554	0.18532	insignificant	46	103	116
chr19	55968810	55970810	Tcf7l2	-0.08838286	0.00049745	hypomethylated	-0.0034517	0.64617	insignificant	12	45	42
chr19	56470618	56472618	Casp7	-0.19346367	0.000000104	hypomethylated	-0.01103	0.14034	insignificant	8	71	86
chr19	56535126	56537126	9930023K05Ri	-0.25985873	0.4493	insignificant	0.041367	0.83391	insignificant	2	7	9
chr19	56621750	56623750	Nhlrc2	-0.1310551	1.23E-19	hypomethylated	0.034283	0.14144	insignificant	27	106	98
chr19	56622516	56624516	Dclre1a	-0.1758213	3.39E-22	hypomethylated	0.015171	0.15979	insignificant	26	110	99
chr19	56795861	56797861	Adrb1	-0.10250727	7.85E-11	hypomethylated	0.024533	0.17575	insignificant	32	89	86
chr19	56899698	56901698	Tdrd1	-0.15739155	0.000000452	hypomethylated	-0.088586	0.0022106	hypomethylated	16	46	45
chr19	56899764	56901764	Tdrd1	-0.15739155	0.000000452	hypomethylated	-0.088586	0.0022106	hypomethylated	16	46	45
chr19	56899775	56901775	Tdrd1	-0.15739155	0.000000452	hypomethylated	-0.088586	0.0022106	hypomethylated	16	46	45
chr19	56947905	56949905	Vwa2	-0.11309633	1.12E-12	hypomethylated	0.016605	0.85507	insignificant	24	80	87
chr19	57083065	57085065	Afap1l2	-0.56956592	0.0079707	stronglyHypometh	-0.014244	0.0028283	inconclusive	4	35	35
chr19	57271982	57273982	Ablim1	-0.20184257	1.19E-12	hypomethylated	0.053065	1	insignificant	8	23	23
chr19	57290522	57292522	Ablim1		1	noCoverage	-0.050842	0.47243	insignificant	0	8	8
chr19	57434498	57436498	Fam160b1	-0.09865775	1.96E-22	hypomethylated	0.0023193	0.4796	insignificant	23	147	148
chr19	57526395	57528395	Trub1	-0.17453917	0.032605	hypomethylated	0.05663	0.92132	insignificant	4	14	15
chr19	57684523	57686523	Atrnl1	-0.09048231	6.85E-30	hypomethylated	0.0051003	0.41947	insignificant	53	186	189
chr19	58528956	58530956	Gfra1	-0.14506379	8.43E-15	hypomethylated	0.032955	0.11212	insignificant	26	72	67
chr19	58802376	58804376	Pnliprp1	-0.28571429	0.10213	insignificant	0.047619	1	insignificant	2	4	6
chr19	58833212	58835212	Pnliprp2	-0.18031153	0.027701	hypomethylated	-0.093382	0.079597	insignificant	6	14	14
chr19	58868904	58870904	1700019N19R	-0.06222235	0.000035165	hypomethylated	-0.012161	0.84806	insignificant	10	22	22
chr19	58935474	58937474	Hspa12a	-0.343513	1.74E-08	stronglyHypometh	-0.025454	0.0035234	inconclusive	5	26	24
chr19	59150559	59152559	4930506M07Rik		1	noCoverage	0.0010266	1	insignificant	0	14	14
chr19	59244519	59246519	Vax1	-0.19580879	0.0018866	hypomethylated	-0.0063654	0.17592	insignificant	9	60	62
chr19	59293137	59295137	Kcnk18	0.00155655	0.072327	insignificant	0.054706	0.069819	insignificant	6	34	34
chr19	59334367	59336367	Slc18a2	-0.14972572	4.84E-08	hypomethylated	-0.014938	0.42157	insignificant	13	45	45
chr19	59420270	59422270	Pdzd8	-0.05215552	0.000061419	hypomethylated	0.0068702	0.91558	insignificant	38	123	122
chr19	59532179	59534179	Emx2	-0.12424255	2.18E-17	hypomethylated	0.0057393	0.23647	insignificant	41	130	124
chr19	59533125	59535125	Emx2	-0.13382326	1.38E-20	hypomethylated	0.0017503	0.86937	insignificant	42	144	140
chr19	60019267	60021267	Rab11fip2	-0.1008975	0.000031998	hypomethylated	0.0085648	0.065078	insignificant	12	61	60
chr19	60019557	60021557	Rab11fip2	-0.09546371	0.0037863	hypomethylated	0.024972	0.27204	insignificant	4	24	24
chr19	60219577	60221577	E330013P04Rik		1	noCoverage	-0.083229	0.035953	hypomethylated	0	18	13
chr19	60302600	60304600	D19Ertd737e	0.01388889	1	insignificant	-0.072035	0.14687	insignificant	1	8	8
chr19	60830889	60832889	Nanos1	-0.08612567	4.76E-19	hypomethylated	0.018381	0.54428	insignificant	51	200	182
chr19	60866596	60868596	Eif3a	-0.1048211	0.23772	insignificant	0.021046	0.70118	insignificant	3	72	72
chr19	60886472	60888472	Fam45a	-0.17478658	1.03E-16	hypomethylated	0.019764	0.093964	insignificant	16	39	39
chr19	60964651	60966651	Grk5	-0.16665648	6.49E-16	hypomethylated	0.02036	0.23769	insignificant	36	115	109
chr19	61304321	61306321	Csf2ra	-0.15134126	0.00085591	hypomethylated	-0.020851	0.46526	insignificant	7	17	16
chr2	3034659	3036659	Fam171a1	-0.10893489	5.78E-11	hypomethylated	0.0027619	0.55017	insignificant	27	96	83
chr2	3200559	3202559	Nmt2	-0.09706129	0.00053546	hypomethylated	0.0031809	0.90981	insignificant	29	134	140
chr2	3249900	3251900	Rpp38		1	noCoverage	0.0052567	0.79252	insignificant	0	12	12
chr2	3339920	3341920	Meig1	-0.24954903	0.0015153	hypomethylated	-0.049463	0.53471	insignificant	4	22	20
chr2	3340402	3342402	Dclre1c	-0.27649188	0.000064183	hypomethylated	-0.083513	0.31321	insignificant	6	36	32
chr2	3392258	3394258	Suv39h2	-0.3239577	0.00095807	hypomethylated	-0.07602	0.0066638	hypomethylated	3	8	8
chr2	3429336	3431336	Cdnf	-0.18234379	1.95E-15	hypomethylated	-0.0090327	0.6999	insignificant	24	99	98
chr2	3430086	3432086	Hspa14	-0.17848141	5.96E-09	hypomethylated	-0.008017	0.96157	insignificant	12	53	52
chr2	3629729	3631729	Fam107b	-0.13793991	2.51E-20	hypomethylated	0.014947	0.43223	insignificant	25	73	68
chr2	4072908	4074908	Frmd4a	-0.31548185	1	lowCoverage	0.03338	0.54767	insignificant	1	13	12
chr2	4321021	4323021	Frmd4a	-0.11597806	0.0007548	hypomethylated	0.0014823	0.099653	insignificant	8	32	32
chr2	4479799	4481799	Frmd4a	-0.20047556	0.000000624	hypomethylated	0.050881	0.51722	insignificant	7	26	22
chr2	4573132	4575132	Prpf18	-0.19505724	1	lowCoverage	0.005865	0.81871	insignificant	1	16	16
chr2	4637876	4639876	Bend7	-0.05004174	8.17E-35	hypomethylated	-0.0040291	0.51703	insignificant	46	180	156
chr2	4801609	4803609	Sephs1	-0.10768616	2.29E-36	hypomethylated	-0.00065337	0.17776	insignificant	62	194	206
chr2	4839041	4841041	Phyh	-0.04700685	0.018183	hypomethylated	-0.022229	0.00014553	hypomethylated	11	57	56
chr2	4896167	4898167	Ucma	0.25178571	0.56246	insignificant	0.087903	0.30889	insignificant	1	16	16
chr2	4933837	4935837	Mcm10	-0.58752259	0.00002485	stronglyHypometh	-0.023829	0.90799	insignificant	2	15	17
chr2	4984984	4986984	Optn	-0.42355769	0.00070971	stronglyHypometh	-0.0075111	0.81205	insignificant	4	16	16
chr2	5057821	5059821	Ccdc3	-0.12294267	5.31E-21	hypomethylated	0.0011833	0.074979	insignificant	30	105	103
chr2	5635710	5637710	Camk1d	-0.14917049	3.46E-17	hypomethylated	0.0052178	0.5899	insignificant	25	82	80
chr2	5765079	5767079	Nudt5	-0.06585635	1	insignificant	0.013301	0.66974	insignificant	7	77	67
chr2	5766006	5768006	Cdc123	-0.03591164	1	insignificant	0.047888	0.40562	insignificant	6	39	30
chr2	5816399	5818399	Sec61a2		1	noCoverage	-0.077411	0.57899	insignificant	0	14	14
chr2	5871514	5873514	Upf2	-0.13278778	1.26E-12	hypomethylated	0.022747	0.22538	insignificant	33	133	129
chr2	6051231	6053231	Gm10857	-0.14847136	3.01E-10	hypomethylated	-0.03315	0.00058849	hypomethylated	9	28	27
chr2	6052914	6054914	Gm10857	-0.10567144	0.05164	insignificant	-0.062666	0.68061	insignificant	3	26	22
chr2	6052928	6054928	Gm10857	-0.10567144	0.05164	insignificant	-0.062666	0.68061	insignificant	3	26	22
chr2	6134040	6136040	A230108P19R	-0.33735711	0.11275	insignificant	-0.049942	0.37852	insignificant	4	41	41
chr2	6242802	6244802	Usp6nl	-0.11837694	2.29E-30	hypomethylated	-0.0038701	0.53778	insignificant	72	201	190
chr2	6272781	6274781	Usp6nl		1	noCoverage	0.10764	0.13999	insignificant	0	6	6
chr2	6849768	6851768	Celf2	0.05769231	1	insignificant	-0.040522	1	insignificant	2	4	4
chr2	7002348	7004348	Celf2	-0.21153415	0.38787	insignificant	0.15703	1	insignificant	2	4	8
chr2	9800227	9802227	Gata3	-0.15468833	1.99E-09	hypomethylated	0.014914	0.15139	insignificant	18	77	73
chr2	9801872	9803872	4930412O13R	-0.34992582	5.1E-26	stronglyHypometh	0.018984	0.070846	insignificant	13	59	45
chr2	9970236	9972236	Taf3	-0.15163402	2.12E-09	hypomethylated	0.030187	0.6273	insignificant	8	58	55
chr2	10001238	10003238	Kin	-0.17948758	1.08E-12	hypomethylated	0.08442	0.80959	insignificant	7	49	28

chr2	10291077	10293077	Sfmbt2		1	noCoverage	0.032917	0.77423	insignificant	0	12	12
chr2	10293062	10295062	Sfmbt2	-0.16588922	2.18E-13	hypomethylated	0.037309	0.3216	insignificant	14	109	111
chr2	11093008	11095008	Prkcq	-0.11050956	0.000013596	hypomethylated	-0.0075218	0.47954	insignificant	12	63	72
chr2	11423717	11425717	Pfkfb3	-0.28598906	1.43E-11	hypomethylated	-0.021162	0.85079	insignificant	18	56	56
chr2	11475556	11477556	Pfkfb3	-0.3085649	0.58804	insignificant	-0.04701	0.00024851	hypomethylated	4	32	33
chr2	11524826	11526826	Rbm17	-0.26720347	4.48E-11	hypomethylated	-0.022118	0.19166	insignificant	11	42	42
chr2	11626162	11628162	Il15ra	-0.14409655	3.24E-09	hypomethylated	0.027024	0.2067	insignificant	14	46	55
chr2	11626474	11628474	Il15ra	-0.18191619	6.14E-12	hypomethylated	0.033611	0.058636	insignificant	16	56	65
chr2	11698379	11700379	Ankrd16	-0.16163803	1.11E-20	hypomethylated	-0.016731	0.85545	insignificant	19	58	62
chr2	11699154	11701154	Fbxo18	-0.16941981	7.11E-21	hypomethylated	-0.0044947	0.058395	insignificant	14	62	62
chr2	12223547	12225547	E030013I19Ril	-0.11767831	2E-10	hypomethylated	-0.011502	0.21469	insignificant	9	32	32
chr2	12341087	12343087	Fam188a	-0.21338384	1	insignificant	-0.0063384	0.56554	insignificant	5	10	10
chr2	12844667	12846667	Pter	-0.35605603	0.0037837	stronglyHypometh	-0.0048729	0.954	insignificant	3	14	14
chr2	12932491	12934491	C1ql3	-0.1378372	9.87E-08	hypomethylated	0.033516	0.34256	insignificant	14	48	44
chr2	13192905	13194905	Rsu1		1	noCoverage	-0.042341	0.66448	insignificant	0	10	10
chr2	13466291	13468291	Trdmt1	-0.20882827	0.004469	hypomethylated	0.038879	0.77637	insignificant	2	12	11
chr2	13494937	13496937	Vim	-0.15901528	8.69E-12	hypomethylated	-0.0039959	0.74903	insignificant	24	82	80
chr2	13715147	13717147	St8sia6	-0.07120832	0.16466	insignificant	-0.015052	0.90348	insignificant	5	49	49
chr2	13977662	13979662	Ptpla	0.3189538	1	insignificant	-0.051041	0.14725	insignificant	1	16	26
chr2	13994738	13996738	Stam	-0.11106741	0.00000139	hypomethylated	0.0073453	0.46856	insignificant	19	88	88
chr2	14150040	14152040	Mrc1	0.1545068	0.36217	insignificant	-0.0072833	0.88907	insignificant	4	12	9
chr2	14524932	14526932	Cacnb2	-0.12455822	1.79E-20	hypomethylated	0.00053619	0.97557	insignificant	34	128	137
chr2	14970939	14972939	Nsun6	-0.32738095	0.15784	insignificant	0.073854	0.56423	insignificant	1	6	6
chr2	14975988	14977988	Arl5b	-0.12955456	1.8E-20	hypomethylated	0.017066	0.94246	insignificant	35	126	128
chr2	14976499	14978499	Nsun6	-0.10078294	4.57E-20	hypomethylated	0.010526	0.88417	insignificant	35	116	118
chr2	16276948	16278948	Plxdc2	-0.16904116	8.38E-17	hypomethylated	0.0021042	0.63339	insignificant	24	90	90
chr2	17652695	17654695	Nebl	-0.05619831	0.014045	hypomethylated	-0.0040749	0.55215	insignificant	7	35	35
chr2	17918573	17920573	H2afb1		1	noCoverage	-0.20245	0.00031851	hypomethylated	0	8	9
chr2	17947875	17949875	Gm17762	-0.14470092	0.002359	hypomethylated	0.0043146	0.5736	insignificant	5	100	104
chr2	17959368	17961368	A930004D18R	0.17098536	0.033847	hypermethylated	0.096272	0.014274	hypermethylated	5	14	14
chr2	17970076	17972076	2810030E01Ri	-0.34129648	0.17758	insignificant	0.039489	0.14428	insignificant	1	32	32
chr2	17975897	17977897	Mllt10	-0.32891618	1	insignificant	0.03472	0.82589	insignificant	1	14	14
chr2	18314457	18316457	Dnajc1	-0.34659091	6.34E-14	stronglyHypometh	-0.074369	0.58043	insignificant	4	9	10
chr2	18593088	18595088	Commd3	-0.1124286	0.000012526	hypomethylated	-0.0030656	0.71645	insignificant	19	84	78
chr2	18597644	18599644	Bmi1	-0.07987035	2.25E-31	hypomethylated	-0.0015775	0.703	insignificant	76	270	251
chr2	18619648	18621648	BC061194	-0.17288652	0.000071428	hypomethylated	-0.0084013	0.45358	insignificant	11	52	54
chr2	18918945	18920945	4930426L09Ri	-0.09938608	1.99E-23	hypomethylated	-0.0062174	0.010179	hypomethylated	49	187	186
chr2	18919748	18921748	Pip4k2a	-0.09460429	5.48E-12	hypomethylated	-0.011823	0.00053717	hypomethylated	22	85	85
chr2	19119744	19121744	Armc3	-0.43190638	0.00015383	stronglyHypometh	-0.082054	0.077614	insignificant	2	7	7
chr2	19292262	19294262	Msrb2	-0.2625	0.19642	insignificant	0.01376	1	insignificant	3	8	4
chr2	19366289	19368289	Ptf1a	-0.1355974	0.000077113	hypomethylated	-0.00074539	0.43279	insignificant	13	115	107
chr2	19475537	19477537	4921504E06Ri	-0.23401338	1	lowCoverage	-0.033159	0.7275	insignificant	1	23	22
chr2	19578688	19580688	Otud1	-0.08603152	3.72E-17	hypomethylated	0.0055299	0.0025589	hypermethylated	61	253	234
chr2	19579524	19581524	Gm3230	-0.08211378	6.05E-13	hypomethylated	0.010475	0.03256	hypermethylated	58	204	192
chr2	20430673	20432673	Etl4	0.0590625	1	lowCoverage	0.024506	0.09303	insignificant	1	16	15
chr2	20440139	20442139	Etl4	-0.24365456	1.83E-16	hypomethylated	0.03194	0.012441	hypermethylated	19	58	58
chr2	20889348	20891348	Gm13375	-0.0646726	8E-27	hypomethylated	-0.0091525	0.41299	insignificant	77	243	237
chr2	20889500	20891500	Gm13375	-0.06232119	7.61E-27	hypomethylated	-0.0096134	0.47785	insignificant	77	239	233
chr2	21126350	21128350	Thnsl1	-0.11260671	1.76E-12	hypomethylated	0.0061471	0.68327	insignificant	9	52	51
chr2	21126992	21128992	Thnsl1	-0.11260671	1.76E-12	hypomethylated	0.0061471	0.68327	insignificant	9	52	51
chr2	21288193	21290193	Gpr158		1	noCoverage	0.022601	0.32284	insignificant	0	25	25
chr2	22148129	22150129	Myo3a	-0.18602346	0.0093816	hypomethylated	0.07133	0.16077	insignificant	5	15	15
chr2	22476846	22478846	Gad2	-0.15682514	3.53E-11	hypomethylated	0.0068077	0.8	insignificant	16	50	50
chr2	22628846	22630846	Apbb1ip		1	noCoverage	0.017045	0.72373	insignificant	0	19	19
chr2	22750041	22752041	Pdss1	-0.10842193	3.08E-12	hypomethylated	0.014503	0.57357	insignificant	15	75	77
chr2	22895760	22897760	Abi1		1	noCoverage	0.30209	1	insignificant	0	10	6
chr2	22922720	22924720	Acbd5	-0.20384912	0.000000053	hypomethylated	0.006021	0.12261	insignificant	17	73	71
chr2	22923591	22925591	Acbd5	-0.15675134	6.44E-09	hypomethylated	0.014476	0.29474	insignificant	24	87	87
chr2	23011065	23013065	Mastl	-0.16461771	1.61E-26	hypomethylated	0.017692	0.55295	insignificant	22	62	61
chr2	23011544	23013544	Mastl	-0.32983098	7.25E-28	hypomethylated	0.026609	0.82635	insignificant	18	58	59
chr2	23427624	23429624	Spopl		1	noCoverage	0.2666	0.34942	insignificant	0	4	6
chr2	24007691	24009691	Il1f8	0.15725806	1	lowCoverage	-0.034409	0.33543	insignificant	1	4	4
chr2	24191379	24193379	Il1rn		1	noCoverage	-0.011257	0.088686	insignificant	0	4	4
chr2	24239916	24241916	Psd4	-0.44384322	0.27288	insignificant	0.066208	0.050615	insignificant	1	14	14
chr2	24618567	24620567	Cacna1b	-0.07752677	0.41908	insignificant	0.0094121	0.28133	insignificant	2	39	54
chr2	24618672	24620672	Cacna1b	-0.13516918	1	insignificant	0.01405	0.51988	insignificant	1	30	38
chr2	24775110	24777110	Ehmt1	-0.03104013	9.21E-29	hypomethylated	0.037815	1.92E-14	inconclusive	18	62	51
chr2	24790801	24792801	Arrdc1	-0.26963347	0.1813	insignificant	-0.066588	0.0020509	hypomethylated	12	56	54
chr2	24804321	24806321	Zmynd19	-0.11827062	3.98E-26	hypomethylated	0.01484	0.10071	insignificant	65	194	180
chr2	24816941	24818941	Wdr85	-0.25675806	0.57232	insignificant	-0.14996	0.60933	insignificant	1	27	30
chr2	24830552	24832552	Pnpla7	-0.16158418	1	insignificant	0.011289	0.050541	insignificant	1	73	75
chr2	24830618	24832618	Mrpl41	-0.16939017	1	insignificant	0.012701	0.030974	hypermethylated	1	69	71
chr2	24908898	24910898	Nelf	-0.19352692	2.78E-17	hypomethylated	0.068066	0.94439	insignificant	21	68	74
chr2	24950725	24952725	Noxa1	-0.18262363	0.035111	hypomethylated	0.016919	0.59115	insignificant	2	8	8
chr2	25035277	25037277	Nrarp	-0.10642186	7.51E-08	hypomethylated	-0.0010396	0.75684	insignificant	19	112	112
chr2	25052333	25054333	A830007P12Rik		1	noCoverage	0.045897	0.15262	insignificant	0	4	4
chr2	25067009	25069009	Cobra1	-0.37783034	2.41E-12	stronglyHypometh	0.014731	0.00044299	hypermethylated	11	42	42
chr2	25080222	25082222	Tubb4b		1	noCoverage	-0.045304	1	insignificant	0	38	32
chr2	25092307	25094307	Gm757	-0.47206899	0.00028946	stronglyHypometh	0.023642	0.86707	insignificant	4	19	18
chr2	25095417	25097417	2310002J15Ri	-0.18223048	8.63E-08	hypomethylated	0.0003891	0.73965	insignificant	6	36	36
chr2	25097448	25099448	Rnf208	-0.18928931	1.22E-53	hypomethylated	-0.011593	0.45937	insignificant	12	37	36
chr2	25109958	25111958	Tmem203	-0.13802871	2.35E-14	hypomethylated	0.0074871	0.71267	insignificant	29	121	121
chr2	25110934	25112934	Ndor1	-0.14674453	7.78E-11	hypomethylated	0.005975	0.49327	insignificant	19	78	78
chr2	25117117	25119117	Tprn	-0.16687807	2.27E-16	hypomethylated	-0.0054108	0.49778	insignificant	32	141	140
chr2	25126985	25128985	Anapc2	-0.08092118	0.00047318	inconclusive	0.012996	0.057503	insignificant	21	95	92

chr2	25127938	25129938	Anapc2	0.22703642	0.000011161	hypermethylated	-0.021773	0.34515	insignificant	5	35	35
chr2	25144430	25146430	Lrrc26	-0.12285277	7.4E-15	hypomethylated	0.0055977	0.6355	insignificant	27	90	90
chr2	25174683	25176683	Grin1	0.06251601	7.51E-15	hypermethylated	-0.0010327	0.687	insignificant	9	50	49
chr2	25187262	25189262	Man1b1	-0.2891431	2.71E-19	hypomethylated	-0.011051	0.91669	insignificant	12	81	79
chr2	25188091	25190091	AA543186	-0.42902148	1.92E-18	stronglyHypometh	0.0030109	0.080058	insignificant	6	22	22
chr2	25211852	25213852	Dpp7	-0.24424242	0.3437	insignificant	-0.0080591	0.89177	insignificant	3	10	10
chr2	25221146	25223146	Uap1l1	-0.49289846	0.0049347	stronglyHypometh	0.0075243	0.73134	insignificant	7	41	37
chr2	25226840	25228840	2010317E24Ri	-0.17030877	7.98E-23	hypomethylated	-0.000000771	0.0073034	inconclusive	22	80	83
chr2	25250393	25252393	Entpd2	-0.1574313	3.56E-10	hypomethylated	0.0068051	0.97451	insignificant	25	90	87
chr2	25257602	25259602	Npdc1	-0.12601358	4.36E-17	hypomethylated	-0.02118	0.15223	insignificant	18	56	42
chr2	25283193	25285193	Abca2	-0.11833562	7.73E-39	hypomethylated	-0.018278	0.45732	insignificant	56	139	124
chr2	25297298	25299298	Mir3087	0.09136596	0.39742	insignificant	0.0059289	0.039725	hypermethylated	4	35	35
chr2	25311362	25313362	Clic3		1	noCoverage	0.061905	0.21958	insignificant	0	2	2
chr2	25316614	25318614	BC029214	0.72741597	1.18E-18	stronglyHypermeth	0.043546	0.25405	insignificant	3	8	8
chr2	25355297	25357297	Fbxw5	-0.16174909	1.59E-11	hypomethylated	0.065186	0.27029	insignificant	13	50	45
chr2	25356026	25358026	C8g	-0.2454853	2.84E-14	hypomethylated	0.04925	0.24998	insignificant	13	56	51
chr2	25402414	25404414	Traf2	-0.11955241	0.000084589	hypomethylated	0.0056409	0.18929	insignificant	10	48	48
chr2	25412419	25414419	Edf1	-0.12686979	0.00001049	hypomethylated	-0.025524	0.32524	insignificant	21	98	96
chr2	25430391	25432391	Phpt1	-0.06598204	4.77E-15	hypomethylated	-0.0062565	0.00041709	inconclusive	11	47	48
chr2	25463966	25465966	B230208H17R	0.13914249	0.000013377	hypermethylated	0.045779	3.63E-13	hypermethylated	10	54	58
chr2	25473198	25475198	4921530D09R	-0.39910714	0.2299	insignificant	0.17609	0.015696	hypermethylated	2	8	8
chr2	25477522	25479522	Tmem141	-0.17668586	0.35637	insignificant	-0.036788	0.056678	insignificant	4	18	18
chr2	25477525	25479525	Tmem141	-0.17668586	0.35637	insignificant	-0.036788	0.056678	insignificant	4	18	18
chr2	25531305	25533305	Lcn6		1	noCoverage	0.023877	1	insignificant	0	4	4
chr2	25537245	25539245	Lcn10	-0.19153208	0.012223	hypomethylated	-0.034942	1	insignificant	2	16	16
chr2	25561398	25563398	Bmyc	-0.16838225	0.011246	hypomethylated	-0.031803	0.38475	insignificant	13	82	78
chr2	25677672	25679672	Lcn9	-0.06168825	0.41168	insignificant	0.13817	0.0049332	hypermethylated	6	13	12
chr2	25702768	25704768	Sohlh1		1	noCoverage	-0.062132	0.75917	insignificant	0	4	4
chr2	25718373	25720373	Kcnt1	-0.14476103	0.011148	hypomethylated	-0.016289	0.51999	insignificant	12	24	24
chr2	25732380	25734380	Kcnt1	-0.17887625	1.23E-20	hypomethylated	-0.008713	0.96426	insignificant	20	65	65
chr2	25838802	25840802	Camsap1	-0.16528544	7.73E-08	hypomethylated	0.027608	0.0064031	inconclusive	9	39	30
chr2	25877280	25879280	Ubac1	-0.2214484	0.00000217	hypomethylated	0.088593	0.094695	insignificant	6	23	17
chr2	25978331	25980331	Nacc2	-0.09860277	0.5981	insignificant	0.004848	0.18315	insignificant	3	39	39
chr2	26062076	26064076	Lhx3	-0.12644145	0.0084414	hypomethylated	0.055502	0.0052949	hypermethylated	12	47	47
chr2	26150077	26152077	4932418E24Ri	0.01592971	0.75533	insignificant	0.0098911	0.27113	insignificant	2	7	6
chr2	26170052	26172052	Gpsm1	-0.11497782	3.66E-17	hypomethylated	0.0016098	0.39708	insignificant	19	89	91
chr2	26174289	26176289	Gpsm1		1	noCoverage	0.06875	0.74513	insignificant	0	4	4
chr2	26207630	26209630	Dnlz	0.09923206	0.23311	insignificant	0.088131	1	insignificant	4	10	8
chr2	26215067	26217067	Card9	-0.55	0.038736	stronglyHypometh	-0.13542	0.50275	insignificant	1	4	4
chr2	26236173	26238173	Snapc4		1	noCoverage	-0.030112	0.36766	insignificant	0	14	11
chr2	26243867	26245867	Pmpca	-0.12975876	2.01E-20	hypomethylated	-0.0057556	0.90543	insignificant	36	141	139
chr2	26244836	26246836	Sdccag3	-0.14254042	0.054799	insignificant	0.0098329	0.54423	insignificant	5	28	28
chr2	26264708	26266708	Inpp5e	-0.42306771	0.53455	insignificant	-0.14102	0.28979	insignificant	2	5	4
chr2	26300736	26302736	Sec16a	-0.1268081	0.50346	insignificant	-0.0012716	0.42778	insignificant	12	77	77
chr2	26359342	26361342	Notch1	-0.0644081	0.7402	insignificant	0.055409	0.43522	insignificant	6	30	28
chr2	26435575	26437575	Egfl7	-0.39705882	0.039561	stronglyHypometh	-0.10084	0.46044	insignificant	2	4	4
chr2	26438383	26440383	Egfl7	-0.32895881	0.043106	hypomethylated	-0.058496	0.34506	insignificant	3	12	12
chr2	26441149	26443149	Egfl7	-0.21044398	9.1E-19	hypomethylated	-0.01457	0.74971	insignificant	22	80	74
chr2	26459730	26461730	Agpat2	-0.21824914	0.060955	insignificant	0.0049892	0.012497	inconclusive	7	63	63
chr2	26482976	26484976	Fam69b	-0.16598939	0.0056204	hypomethylated	-0.0033902	1	insignificant	8	34	34
chr2	26493505	26495505	Snora43		1	noCoverage	-0.014234	0.69295	insignificant	0	24	24
chr2	26494841	26496841	Snora17	-0.24664594	0.00001451	hypomethylated	-0.0056329	0.36461	insignificant	7	78	78
chr2	26495764	26497764	Snora17	0.62971331	0.30269	insignificant	-0.035814	0.1903	insignificant	2	26	25
chr2	26758333	26760333	Surf6	-0.14232048	3.99E-12	hypomethylated	0.023727	0.58819	insignificant	12	44	40
chr2	26765326	26767326	Rpl7a	-0.14664115	0.00000369	hypomethylated	0.032679	0.39804	insignificant	12	71	66
chr2	26766162	26768162	Med22	-0.08773239	0.00058927	inconclusive	0.026643	0.044011	inconclusive	4	48	48
chr2	26770940	26772940	Surf2	-0.26734688	2.77E-15	hypomethylated	-0.02299	0.32653	insignificant	17	70	74
chr2	26772050	26774050	Surf2	-0.12008987	0.00000285	hypomethylated	-0.052707	0.28703	insignificant	5	28	32
chr2	26788588	26790588	Gm711	-0.12425688	1	insignificant	-0.0075489	0.25778	insignificant	3	66	64
chr2	26789031	26791031	Surf4	-0.16357391	1	insignificant	-0.0021241	0.48757	insignificant	3	34	32
chr2	26819906	26821906	Rexo4	-0.28060449	0.65567	insignificant	-0.0023436	0.35397	insignificant	1	23	23
chr2	26864485	26866485	5930434B04R	-0.13354724	0.000000552	hypomethylated	0.038752	0.078154	insignificant	17	74	70
chr2	26883518	26885518	Slc2a6		1	noCoverage	0.087753	0.078792	insignificant	0	12	12
chr2	27020026	27022026	Dbh		1	noCoverage	0.014851	0.20009	insignificant	0	2	2
chr2	27282345	27284345	Vav2	-0.09997897	3.36E-10	hypomethylated	-0.0084612	0.52627	insignificant	22	52	52
chr2	27330918	27332918	Brd3	-0.09107898	3.24E-21	hypomethylated	-0.00014663	0.45868	insignificant	37	113	114
chr2	27331193	27333193	Brd3	-0.07928241	6.95E-15	hypomethylated	-0.0031932	0.7957	insignificant	20	72	72
chr2	27369666	27371666	Wdr5	-0.14525742	4.09E-22	hypomethylated	0.0039181	0.17536	insignificant	22	74	61
chr2	27531720	27533720	Rxra	-0.09492642	5.21E-13	hypomethylated	0.00017582	0.45093	insignificant	75	230	224
chr2	27740944	27742944	Col5a1	-0.10823093	4.18E-21	hypomethylated	-0.0046438	0.37399	insignificant	39	120	123
chr2	28047612	28049612	Olfm1	-0.1337294	6.78E-20	hypomethylated	0.011681	0.057832	insignificant	36	111	111
chr2	28060208	28062208	Olfm1	-0.13248694	2.13E-49	hypomethylated	0.014983	0.10858	insignificant	45	152	132
chr2	28302460	28304460	Ppp1r26	-0.12392996	6.6E-19	hypomethylated	-0.0013891	0.62965	insignificant	32	75	75
chr2	28321844	28323844	1700007K13Ri	-0.10060896	1.76E-25	hypomethylated	-0.0034155	0.3656	insignificant	28	80	80
chr2	28322585	28324585	Mrps2	-0.14633076	4.77E-35	hypomethylated	0.010567	0.00051843	hypermethylated	36	109	110
chr2	28367686	28369686	Ralgds	-0.10487448	8.58E-30	hypomethylated	0.0048958	0.17038	insignificant	44	158	150
chr2	28387983	28389983	Ralgds	-0.1659942	2.71E-25	hypomethylated	0.024212	0.060148	insignificant	16	67	63
chr2	28418882	28420882	Cel	-0.05910948	0.18337	insignificant	-0.032339	1	insignificant	2	4	4
chr2	28477502	28479502	Gfi1b	-0.11719577	0.45279	insignificant	0.013757	0.47001	insignificant	1	9	7
chr2	28495762	28497762	Tsc1	-0.09450437	1.93E-12	hypomethylated	0.010545	0.86313	insignificant	23	90	90
chr2	28554680	28556680	Ak8	-0.18082972	2.06E-14	hypomethylated	0.0084389	0.93542	insignificant	15	71	67
chr2	28555171	28557171	1700026L06Ri	-0.17865875	1.39E-13	hypomethylated	0.0076872	0.96613	insignificant	15	66	62
chr2	28694925	28696925	Ddx31	-0.14141946	1.63E-20	hypomethylated	0.0046565	0.74016	insignificant	25	111	103
chr2	28695880	28697880	Ddx31	-0.13232701	4.14E-20	hypomethylated	0.02261	0.3168	insignificant	17	60	60
chr2	28771941	28773941	Barhl1	-0.14394167	5.14E-18	hypomethylated	0.0090215	0.57339	insignificant	26	112	112

chr2	28771960	28773960	Barhl1	-0.15347156	3.3E-18	hypomethylated	0.009448	0.50921	insignificant	26	114	114
chr2	28910586	28912586	1700101E01Ri	-0.19086773	6.47E-10	hypomethylated	-0.0030161	0.37811	insignificant	11	37	37
chr2	28914782	28916782	Ttf1	-0.13691092	4.9E-10	hypomethylated	0.0083929	0.82115	insignificant	14	53	49
chr2	28979511	28981511	Setx	-0.14947648	1.15E-15	hypomethylated	-0.003099	0.090656	insignificant	16	104	104
chr2	29108513	29110513	6530402F18Ri	-0.23568581	1.5E-09	hypomethylated	-0.022624	0.86694	insignificant	8	19	20
chr2	29201355	29203355	Med27	-0.19059533	4.19E-22	hypomethylated	0.010167	0.00070912	hypermethylated	11	66	64
chr2	29474239	29476239	Rapgef1	-0.11630553	1.33E-39	hypomethylated	-0.0056223	0.14536	insignificant	40	118	132
chr2	29642782	29644782	Coq4	-0.21883211	1.55E-09	hypomethylated	-0.011528	0.49717	insignificant	8	58	49
chr2	29643191	29645191	Trub2	-0.06443043	1.02E-09	hypomethylated	-0.012594	0.95395	insignificant	7	52	40
chr2	29657199	29659199	Slc27a4	-0.10945834	7.57E-24	hypomethylated	-0.0053333	0.97601	insignificant	29	92	92
chr2	29681908	29683908	Urm1	-0.1897097	5.16E-28	hypomethylated	0.051009	0.61724	insignificant	12	56	53
chr2	29701247	29703247	Mir219-2	-0.2747225	7.65E-24	hypomethylated	-0.064426	0.070629	insignificant	29	101	99
chr2	29724013	29726013	Cercam	-0.06494636	0.0076237	hypomethylated	0.0056346	0.021783	inconclusive	13	58	58
chr2	29744239	29746239	Odf2	-0.09480291	2.95E-24	hypomethylated	0.0094357	0.049493	hypermethylated	38	159	153
chr2	29744582	29746582	Odf2	-0.09214038	1.28E-25	hypomethylated	0.0094652	0.040053	hypermethylated	37	157	151
chr2	29789928	29791928	Gle1	-0.23255337	0.35604	insignificant	-0.013076	0.52866	insignificant	7	40	39
chr2	29820079	29822079	Spna2	-0.08876186	1.76E-13	hypomethylated	0.0080552	0.90542	insignificant	21	127	135
chr2	29904399	29906399	Wdr34	-0.15604849	5.78E-25	hypomethylated	0.0064849	0.043321	inconclusive	14	54	52
chr2	29916562	29918562	Set	-0.08133785	3.29E-12	hypomethylated	-0.02702	0.5221	insignificant	22	73	70
chr2	29933285	29935285	Pkn3	-0.07871537	0.73938	insignificant	-0.00024259	0.3774	insignificant	15	42	42
chr2	29949155	29951155	Zdhhc12	-0.1421405	0.00010124	hypomethylated	-0.0099002	0.50614	insignificant	6	44	45
chr2	29979974	29981974	Zer1	-0.15338496	0.00000101	hypomethylated	-0.0066334	0.87025	insignificant	13	32	32
chr2	29988390	29990390	Tbc1d13	-0.17273722	0.00002342	hypomethylated	0.027135	0.2852	insignificant	13	36	40
chr2	30026043	30028043	Endog	-0.19078727	1.53E-13	hypomethylated	0.0096526	0.096746	insignificant	13	32	32
chr2	30033979	30035979	D2Wsu81e	-0.29588806	0.00000164	hypomethylated	-0.019102	0.3196	insignificant	2	4	4
chr2	30061219	30063219	Ccbl1	0.02454656	0.03489	hypermethylated	0.0071735	0.90877	insignificant	10	44	38
chr2	30092288	30094288	Lrrc8a	-0.08473728	7.91E-12	hypomethylated	0.0093979	0.97729	insignificant	34	124	124
chr2	30093151	30095151	1700084E18Ri	-0.07285176	4.23E-11	hypomethylated	0.015523	0.87833	insignificant	33	102	102
chr2	30140952	30142952	Nup188	-0.16033791	0.00041317	hypomethylated	-0.042449	0.36489	insignificant	12	65	71
chr2	30141874	30143874	Dolk	-0.20771657	0.013549	hypomethylated	-0.063881	0.7233	insignificant	7	42	47
chr2	30214751	30216751	Sh3glb2	0.04737459	0.000000653	inconclusive	0.027084	0.1078	insignificant	5	44	46
chr2	30218854	30220854	Fam73b	-0.19064308	1.59E-18	hypomethylated	-0.010274	0.58005	insignificant	15	58	58
chr2	30246935	30248935	Dolpp1	-0.06905493	0.000034407	hypomethylated	0.0013825	0.36893	insignificant	31	99	98
chr2	30270569	30272569	Ppp2r4	-0.11312003	4.58E-48	hypomethylated	0.018355	0.70522	insignificant	37	136	132
chr2	30271268	30273268	Crat	-0.17690944	8.59E-35	hypomethylated	-0.0016452	0.59881	insignificant	19	64	64
chr2	30329719	30331719	Ier5l	-0.15313526	0.00011085	hypomethylated	-0.0034378	0.36924	insignificant	15	62	62
chr2	30449563	30451563	Cstad	-0.20971081	0.000000233	hypomethylated	-0.0034284	0.8491	insignificant	14	39	39
chr2	30662496	30664496	Mettl11a		1	noCoverage	-0.0033892	0.18714	insignificant	0	34	34
chr2	30683820	30685820	Asb6	-0.23626374	0.000000144	hypomethylated	0.015248	0.72476	insignificant	5	14	10
chr2	30699886	30701886	Prrx2	-0.12626297	2.52E-10	hypomethylated	-0.0091379	0.71505	insignificant	35	124	124
chr2	30807520	30809520	Tor1b	-0.10283449	2.89E-13	hypomethylated	-0.0092508	0.79998	insignificant	19	78	86
chr2	30823438	30825438	Tor1a	-0.30645161	0.00000122	hypomethylated	0.03057	0.75015	insignificant	2	4	4
chr2	30836798	30838798	Usp20	-0.1335764	5.46E-16	hypomethylated	0.0015354	0.91408	insignificant	16	88	88
chr2	30837461	30839461	BC005624	-0.13595748	0.00054815	hypomethylated	0.0073212	0.48125	insignificant	4	42	42
chr2	30997528	30999528	Fnbp1	0.03384891	0.13161	insignificant	0.0045187	0.054262	insignificant	9	38	38
chr2	31006835	31008835	Gpr107	-0.14151637	0.000017897	hypomethylated	0.0094704	0.42887	insignificant	11	51	51
chr2	31100442	31102442	Ncs1	-0.10825257	5.16E-30	hypomethylated	0.0096109	0.89109	insignificant	58	184	182
chr2	31324789	31326789	Ass1	-0.12397937	0.000019942	hypomethylated	-0.022583	0.12626	insignificant	12	93	90
chr2	31427244	31429244	Fubp3	-0.12236573	1.61E-20	hypomethylated	-0.0075355	0.41279	insignificant	15	78	69
chr2	31494556	31496556	Prdm12	-0.18748866	1.24E-15	hypomethylated	0.014432	0.6373	insignificant	11	84	84
chr2	31525256	31527256	Exosc2	-0.13961664	1.95E-17	hypomethylated	0.017562	0.23317	insignificant	9	32	32
chr2	31543075	31545075	Abl1	-0.18718382	3.96E-25	hypomethylated	-0.016467	0.32554	insignificant	26	132	133
chr2	31614464	31616464	Abl1	-0.22149769	0.05615	insignificant	-0.014262	0.69745	insignificant	10	64	64
chr2	31666038	31668038	Qrfp	-0.59285714	0.42857	lowCoverage	-0.0078705	0.58489	insignificant	1	14	14
chr2	31701525	31703525	Fibcd1	-0.11092782	0.000000944	hypomethylated	-0.012498	0.97346	insignificant	24	116	100
chr2	31741800	31743800	Lamc3	-0.08102347	0.000049579	hypomethylated	0.019951	0.23301	insignificant	17	76	76
chr2	31804822	31806822	Aif1l	-0.11592704	5.94E-20	hypomethylated	0.012376	0.56499	insignificant	31	110	103
chr2	31828969	31830969	Nup214	-0.15557276	9.11E-12	hypomethylated	-0.0044151	0.65997	insignificant	20	74	76
chr2	31939225	31941225	Fam78a	-0.11161538	4.44E-29	hypomethylated	0.00053881	0.69296	insignificant	30	118	122
chr2	31950170	31952170	Ppapdc3		1	noCoverage	0.073183	0.55038	insignificant	0	20	17
chr2	32005667	32007667	Prrc2b	-0.11022302	3.78E-08	hypomethylated	-0.0058686	0.43537	insignificant	17	55	46
chr2	32091202	32093202	Pomt1	-0.11452363	3.45E-09	hypomethylated	0.026072	0.27881	insignificant	13	60	53
chr2	32126324	32128324		-0.03206525	0.34581	insignificant	0.062114	0.0011445	hypermethylated	5	30	30
chr2	32142772	32144772	Golga2	-0.2202071	0.00099808	hypomethylated	-0.034111	0.64777	insignificant	9	28	33
chr2	32143588	32145588	Swi5	-0.2202071	0.00099808	hypomethylated	-0.034111	0.64777	insignificant	9	28	33
chr2	32172979	32174979	Mir199b	-0.34000929	0.000000603	stronglyHypometh	-0.0040773	0.74399	insignificant	6	12	12
chr2	32217753	32219753	Ciz1	-0.1912598	0.000028539	hypomethylated	0.0064791	0.52468	insignificant	12	50	52
chr2	32237435	32239435	1110008P14Ri	-0.10333333	1	insignificant	0.12236	0.69737	insignificant	4	15	16
chr2	32250409	32252409	Ptges2	-0.12663583	1.69E-19	hypomethylated	0.00015894	1	insignificant	31	104	104
chr2	32286671	32288671	Slc25a25		1	noCoverage	-0.056209	0.11202	insignificant	0	6	6
chr2	32304976	32306976	Naif1	-0.16764881	9.99E-24	hypomethylated	0.015739	0.2679	insignificant	32	111	94
chr2	32306990	32308990	Naif1	-0.11498542	0.00000335	hypomethylated	-0.02109	0.48911	insignificant	9	28	24
chr2	32389878	32391878	Fam102a	-0.14850478	4.68E-40	hypomethylated	0.0086249	0.084385	insignificant	46	129	127
chr2	32425377	32427377	Dpm2	-0.05656566	0.000000412	hypomethylated	0.0091272	0.41945	insignificant	8	34	34
chr2	32442009	32444009	St6galnac4	-0.20278433	1.5E-26	hypomethylated	0.0024767	0.71483	insignificant	21	69	69
chr2	32461480	32463480	St6galnac6	-0.16508091	4.35E-24	hypomethylated	0.037689	0.11963	insignificant	17	85	85
chr2	32501114	32503114	Eng	-0.12976656	7.06E-16	hypomethylated	0.078944	0.19473	insignificant	10	32	30
chr2	32549695	32551695	Fpgs	-0.29899661	0.52291	insignificant	0.024126	0.15897	insignificant	5	41	43
chr2	32568304	32570304	Cdk9	-0.14911484	1.84E-41	hypomethylated	-0.010981	0.055914	insignificant	29	81	70
chr2	32568408	32570408	Cdk9	-0.11958085	3.02E-37	hypomethylated	-0.0042279	0.091707	insignificant	28	74	68
chr2	32575591	32577591	Sh2d3c	-0.60416667	3.8E-10	stronglyHypometh	0.074631	0.63262	insignificant	1	4	4
chr2	32611790	32613790	Tor2a	-0.15759268	5.63E-18	hypomethylated	-0.0062664	0.6432	insignificant	23	90	87
chr2	32630340	32632340	Ptrh1	-0.11200961	4.95E-08	hypomethylated	0.031911	0.0012122	hypermethylated	16	58	58
chr2	32630903	32632903	Ttc16	-0.15074711	0.0015375	hypomethylated	0.026079	0.0016013	hypermethylated	17	66	66
chr2	32702752	32704752	Stxbp1		1	noCoverage	-0.014093	0.21921	insignificant	0	18	18

chr2	32702757	32704757	Stxbp1		1	noCoverage	-0.014093	0.21921	insignificant	0	18	18
chr2	32730653	32732653	Fam129b	-0.12408129	0.0019793	hypomethylated	0.017394	0.55159	insignificant	20	92	89
chr2	32816231	32818231	Rpl12	0.02840314	0.3414	insignificant	0.016533	0.27916	insignificant	28	98	98
chr2	32816771	32818771	Rpl12	-0.00767987	0.10916	insignificant	0.011814	0.21942	insignificant	25	102	102
chr2	32817820	32819820	Snora65	0.01016061	0.67126	insignificant	0.053086	0.026773	hypermethylated	2	16	16
chr2	32837576	32839576	Slc2a8	-0.19040471	0.000017993	hypomethylated	0.0396	0.40998	insignificant	11	31	31
chr2	33070480	33072480	Angptl2	-0.40521974	0.0039064	stronglyHypometh	0.015069	0.7265	insignificant	3	10	8
chr2	33226998	33228998	Ralgps1	-0.18056838	6.76E-11	hypomethylated	0.065832	0.63854	insignificant	11	30	30
chr2	33286844	33288844	Zbtb34	-0.31727055	2.12E-16	hypomethylated	0.077739	0.64244	insignificant	16	61	61
chr2	33324052	33326052	Zbtb43	-0.17426235	0.00073997	hypomethylated	0.00064465	0.50726	insignificant	7	38	37
chr2	33496031	33498031	Lmx1b	-0.11310383	2.37E-16	hypomethylated	-0.0030493	0.37567	insignificant	39	172	154
chr2	34227556	34229556	Pbx3	-0.14636177	4.91E-34	hypomethylated	-0.0033611	0.61815	insignificant	53	214	208
chr2	34286544	34288544	Mapkap1	0.12534802	0.60361	insignificant	0.011176	0.098773	insignificant	4	16	15
chr2	34626609	34628609	Hspa5	-0.15206784	0.000000276	hypomethylated	0.0060196	0.2479	insignificant	28	103	108
chr2	34655311	34657311	Rabepk		1	noCoverage	0.029097	0.0067395	hypermethylated	0	12	6
chr2	34681755	34683755	Fbxw2	0.23901958	0.074575	insignificant	0.0033816	0.73881	insignificant	2	31	32
chr2	34726482	34728482	Psmd5	-0.12822945	0.0000025	hypomethylated	0.0096645	0.67454	insignificant	11	26	26
chr2	34728955	34730955	D730039F16R	-0.25525971	0.000015294	hypomethylated	0.33504	0.00013139	stronglyhypermeth	7	16	13
chr2	34769496	34771496	Phf19	-0.04297771	2.23E-10	hypomethylated	0.0012387	0.30813	insignificant	26	88	87
chr2	34964011	34966011	Cep110	-0.15561181	1.03E-17	hypomethylated	0.01365	1	insignificant	19	96	92
chr2	35056640	35058640	Rab14	-0.09123901	0.040507	hypomethylated	0.0069731	0.77236	insignificant	4	41	41
chr2	35136959	35138959	Gsn	-0.06602772	0.00008651	hypomethylated	-0.0092373	0.61168	insignificant	6	57	57
chr2	35192529	35194529	Stom	-0.06225777	0.76873	insignificant	0.054518	0.39654	insignificant	8	22	20
chr2	35316945	35318945	Ggta1	-0.16632529	2.85E-14	hypomethylated	-0.0076835	0.43131	insignificant	16	57	55
chr2	35412977	35414977	Dab2ip	0.08621918	0.48761	insignificant	0.07385	0.80355	insignificant	1	8	8
chr2	35476500	35478500	Dab2ip	-0.2025994	0.0096929	hypomethylated	-0.044427	0.73391	insignificant	5	28	28
chr2	35835144	35837144	Ttll11	-0.07296026	0.00027276	hypomethylated	-0.019959	0.15509	insignificant	17	58	50
chr2	35903992	35905992	Morn5	-0.15346895	0.000032066	hypomethylated	0.0050628	0.33868	insignificant	7	62	62
chr2	35904812	35906812	Ndufa8	-0.23739496	0.0016318	hypomethylated	-0.089633	0.11679	insignificant	1	17	16
chr2	35959580	35961580	Lhx6	-0.1493018	1.55E-11	hypomethylated	0.0020442	0.64011	insignificant	23	90	84
chr2	35960928	35962928	Lhx6	-0.33604577	0.000016696	stronglyHypometh	0.05139	0.39262	insignificant	2	4	4
chr2	35990917	35992917	Mrrf	-0.178737	0.00000027	hypomethylated	0.025264	0.41752	insignificant	13	51	52
chr2	35992224	35994224	Mrrf	-0.17503533	0.0066589	hypomethylated	0.033115	0.19093	insignificant	7	35	36
chr2	36084945	36086945	Ptgs1	-0.06092644	0.34657	insignificant	0.018277	1	insignificant	5	28	28
chr2	36244430	36246430	Mir684-1		1	noCoverage	0.13167	0.24293	insignificant	0	10	5
chr2	37055762	37057762	Olfr365		1	noCoverage	0.0625	0.55119	insignificant	0	2	2
chr2	37186268	37188268	Olfr368		1	noCoverage	-0.073286	0.3152	insignificant	0	6	6
chr2	37214852	37216852	Pdcl		1	noCoverage	-0.053707	0.033194	inconclusive	0	12	8
chr2	37286439	37288439	Zbtb6	-0.22113792	0.00000372	hypomethylated	0.0028466	0.32181	insignificant	7	20	20
chr2	37297804	37299804	Rabgap1	-0.22814151	0.000062243	hypomethylated	-0.077444	0.1698	insignificant	7	14	19
chr2	37298641	37300641	Zbtb26	-0.64324786	0.21517	insignificant	0.0026134	0.15641	insignificant	2	15	18
chr2	37306773	37308773	Rabgap1	-0.26624529	3.78E-27	hypomethylated	-0.012555	0.02414	hypomethylated	23	77	83
chr2	37630768	37632768	Crb2	-0.18551287	0.017164	hypomethylated	0.074273	0.1408	insignificant	8	56	51
chr2	38142904	38144904	Dennd1a	-0.06875	0.14245	insignificant	-0.00625	1	insignificant	5	16	17
chr2	38205827	38207827	Lhx2	-0.14742343	0.000042019	hypomethylated	-0.02106	0.90115	insignificant	15	97	95
chr2	38366216	38368216	Nek6	-0.0528653	0.027255	hypomethylated	-0.001296	0.28764	insignificant	24	120	120
chr2	38366395	38368395	Nek6	-0.0528653	0.027255	hypomethylated	-0.001296	0.28764	insignificant	24	120	120
chr2	38499426	38501426	Psmb7	-0.25357143	0.0022182	hypomethylated	0.038946	0.2899	insignificant	1	20	7
chr2	38570062	38572062	Nr5a1		1	noCoverage	0.073266	0.13291	insignificant	0	2	2
chr2	38781981	38783981	Nr6a1	-0.22333802	0.01016	hypomethylated	0.025405	0.77979	insignificant	4	24	24
chr2	38786499	38788499	Olfml2a	-0.06286351	0.117	insignificant	0.093547	0.76479	insignificant	5	23	16
chr2	38852828	38854828	Wdr38	0.06251174	0.000052543	inconclusive	-0.0082733	0.017476	inconclusive	6	44	47
chr2	38860651	38862651	Rpl35	-0.20877679	4.63E-12	hypomethylated	-0.074172	0.66678	insignificant	20	56	56
chr2	38862658	38864658	Arpc5l	-0.12774077	3.29E-18	hypomethylated	-0.0024294	0.80582	insignificant	34	96	92
chr2	38920926	38922926	Golga1	-0.11697417	3.67E-21	hypomethylated	-0.014178	0.30891	insignificant	17	64	64
chr2	39046250	39048250	Scai	-0.43114648	0.0058373	stronglyHypometh	-0.14027	0.19544	insignificant	3	23	30
chr2	39081858	39083858	Ppp6c	-0.1130056	0.49489	insignificant	-0.033729	0.4673	insignificant	4	20	18
chr2	44965657	44967657	Gm13476	-0.15585886	2.11E-14	hypomethylated	0.031906	0.16661	insignificant	17	68	67
chr2	44966779	44968779	Gm13476	-0.40040595	0.52168	insignificant	-0.029464	0.91056	insignificant	1	12	12
chr2	48668628	48670628	Acvr2a	-0.10837425	5.75E-12	hypomethylated	-0.0036028	0.90174	insignificant	52	169	159
chr2	48804027	48806027	Mbd5	-0.1124041	0.00000499	hypomethylated	0.0060733	0.19608	insignificant	15	61	60
chr2	48804787	48806787	Orc4	-0.16755051	0.55367	insignificant	0.0018525	0.93321	insignificant	2	22	22
chr2	49306005	49308005	Epc2	-0.09753223	1.16E-28	hypomethylated	-0.0067049	0.47533	insignificant	62	213	206
chr2	49473833	49475833	Kif5c	-0.13079644	5.99E-28	hypomethylated	0.0075414	0.89609	insignificant	40	110	110
chr2	49642205	49644205	Lypd6b	-0.10655639	0.000000523	hypomethylated	0.010186	0.51205	insignificant	21	67	62
chr2	49920981	49922981	Lypd6	-0.16638211	1.27E-08	hypomethylated	-0.017749	0.023687	hypomethylated	18	99	99
chr2	50152197	50154197	Mmadhc		1	noCoverage	-0.00168	1	insignificant	0	14	14
chr2	51482030	51484030	Tas2r134		1	noCoverage	-0.17402	0.25245	insignificant	0	4	4
chr2	51790164	51792164	Rbm43	-0.17027621	0.0072806	hypomethylated	-0.016069	0.67412	insignificant	16	72	67
chr2	51790529	51792529	Rbm43	-0.31209862	0.011143	hypomethylated	-0.029451	0.54154	insignificant	5	29	24
chr2	51892632	51894632	Tnfaip6	0.40697009	0.0051134	stronglyHypermeth	0.021501	0.017766	hypermethylated	3	15	14
chr2	51927356	51929356	Rif1	-0.10484415	2.66E-18	hypomethylated	0.0017909	0.71467	insignificant	45	146	140
chr2	52194318	52196318	Neb		1	noCoverage	0.2536	0.015914	hypermethylated	0	9	7
chr2	52280394	52282394	Arl5a	-0.30143709	1.39E-11	hypomethylated	0.036718	0.41659	insignificant	10	28	28
chr2	52532101	52534101	Cacnb4	0.54534314	0.42021	lowCoverage	0.01996	0.16206	insignificant	1	8	8
chr2	52601183	52603183	Stam2	-0.22066681	0.067189	insignificant	0.0010862	0.22078	insignificant	6	42	43
chr2	52715901	52717901	Fmnl2	-0.12677866	1.55E-35	hypomethylated	-0.0020365	1	insignificant	64	196	209
chr2	53050117	53052117	Arl6ip6	-0.09542258	2.02E-13	hypomethylated	-0.00067001	0.7218	insignificant	38	209	184
chr2	53050221	53052221	Prpf40a	-0.08915014	1.24E-09	hypomethylated	0.0012922	0.92431	insignificant	32	197	172
chr2	53937963	53939963	Rprm	-0.13393015	0.003594	hypomethylated	0.071492	0.74605	insignificant	2	13	12
chr2	54287797	54289797	Galnt13	-0.12925864	3.36E-10	hypomethylated	0.022286	3.11E-09	hypermethylated	46	175	170
chr2	55288566	55290566	Kcnj3	-0.19026675	0.000000257	hypomethylated	0.026829	0.14977	insignificant	12	62	67
chr2	56967449	56969449	Nr4a2	-0.16374762	4.63E-13	hypomethylated	0.069507	0.96794	insignificant	11	54	63
chr2	56976414	56978414	Nr4a2	-0.0832803	0.00000266	hypomethylated	0.027661	0.29376	insignificant	23	72	70
chr2	57089088	57091088	Gpd2	-0.1569364	4.57E-27	hypomethylated	0.0051494	0.19747	insignificant	31	84	77

chr2	57089792	57091792	Gpd2	-0.1628122	4.71E-27	hypomethylated	-0.00072644	0.21143	insignificant	31	87	77
chr2	57905163	57907163	Ermn	0.06944444	1	insignificant	-0.024306	0.34735	insignificant	3	8	8
chr2	58012533	58014533	Cytip	-0.31179036	0.085852	insignificant	0.14271	0.31795	insignificant	2	13	11
chr2	58210169	58212169	Acvr1c		1	noCoverage	-0.015441	0.47861	insignificant	0	27	27
chr2	58419239	58421239	Acvr1	-0.08967597	1.42E-24	hypomethylated	0.0041601	0.14822	insignificant	72	163	164
chr2	58606595	58608595	Upp2	0.04732639	0.73883	insignificant	0.058712	0.29413	insignificant	2	8	8
chr2	58997906	58999906	Pkp4	-0.10688153	5.06E-14	hypomethylated	0.010861	0.68117	insignificant	45	194	194
chr2	58998684	59000684	Pkp4	-0.11504679	4.76E-14	hypomethylated	0.0081208	0.52954	insignificant	49	200	200
chr2	59321709	59323709	Dapl1	-0.12631976	0.12833	insignificant	-0.1148	0.0382	hypomethylated	2	6	6
chr2	59449100	59451100	Tanc1	-0.1204983	3.49E-25	hypomethylated	0.0039695	0.56964	insignificant	48	127	98
chr2	59720663	59722663	Wdsub1		1	noCoverage	-0.00014489	0.52705	insignificant	0	16	16
chr2	60046992	60048992	March7	-0.06507728	5.22E-11	hypomethylated	0.0078958	0.97792	insignificant	46	174	176
chr2	60221288	60223288	Ly75	-0.07473817	0.00000161	hypomethylated	0.021208	0.11217	insignificant	7	34	26
chr2	60391318	60393318	Pla2r1		1	noCoverage	0.095856	0.19005	insignificant	0	12	12
chr2	60719495	60721495	Rbms1	-0.12392607	0.000078498	hypomethylated	0.024328	0.17247	insignificant	13	50	40
chr2	60801261	60803261	Rbms1	0.63329921	0.076932	insignificant	0.1333	0.085429	insignificant	2	26	23
chr2	61430153	61432153	Tank	-0.11479797	0.000000205	hypomethylated	-0.0070541	0.39777	insignificant	22	70	66
chr2	61548750	61550750	Psmd14	-0.26557856	5.67E-27	hypomethylated	0.025999	0.085286	insignificant	22	64	64
chr2	61641509	61643509	Tbr1	-0.20586881	6.9E-29	hypomethylated	0.0046808	0.3259	insignificant	15	64	64
chr2	61883596	61885596	Slc4a10	-0.78697881	0.004658	stronglyHypometh	-0.023903	0.5763	insignificant	1	8	8
chr2	62250288	62252288	Dpp4	-0.27389706	1	insignificant	0.17162	0.42942	insignificant	1	8	10
chr2	62412078	62414078	Fap	-0.4225553	0.00021023	stronglyHypometh	0.10346	1	insignificant	4	16	15
chr2	62484312	62486312	Ifih1	-0.06481481	0.32678	insignificant	0.13973	0.69048	insignificant	2	6	6
chr2	62501383	62503383	Gca	-0.11412958	0.28501	insignificant	0.0027688	0.89373	insignificant	20	63	66
chr2	63022344	63024344	Kcnh7	-0.22490886	0.17473	insignificant	0.006013	0.2444	insignificant	6	14	14
chr2	63936064	63938064	Fign	-0.17053679	1.04E-62	hypomethylated	-0.0032225	0.11041	insignificant	55	100	100
chr2	64860823	64862823	Grb14	-0.1120779	0.0075697	hypomethylated	0.041367	0.83976	insignificant	8	27	28
chr2	65076683	65078683	Cobll1	-0.09090622	5.83E-22	hypomethylated	-0.0014819	0.4692	insignificant	32	132	122
chr2	65405549	65407549	Scn3a	-0.03939799	0.13483	insignificant	-0.06324	0.018883	hypomethylated	3	15	14
chr2	65682823	65684823	Csrnp3		1	noCoverage	0.11111	0.21825	insignificant	0	6	6
chr2	65962850	65964850	Galnt3	-0.13026874	0.60462	insignificant	0.077546	0.14448	insignificant	7	58	50
chr2	66094674	66096674	Ttc21b		1	noCoverage	0.032215	1	insignificant	0	14	14
chr2	66277936	66279936	Gm13629	-0.45454545	0.000036058	stronglyHypometh	-0.035455	0.63823	insignificant	2	4	4
chr2	67954769	67956769	B3galt1	-0.20570286	1	insignificant	0.0067514	1	insignificant	3	9	6
chr2	68310038	68312038	Stk39	-0.12681099	1	insignificant	0.049369	0.69664	insignificant	2	46	46
chr2	68419469	68421469	4933409G03Rik		1	noCoverage	-0.033162	0.33576	insignificant	0	4	4
chr2	68698613	68700613	Lass6	-0.12202044	3.04E-26	hypomethylated	-0.00047634	0.41605	insignificant	34	119	119
chr2	68972856	68974856	Nostrin	-0.36108225	0.00027027	stronglyHypometh	-0.0090017	0.75365	insignificant	2	6	6
chr2	69424124	69426124	Lrp2	-0.1329051	0.000000908	hypomethylated	0.031932	0.69916	insignificant	8	58	59
chr2	69484311	69486311	Bbs5	-0.09309997	0.0072907	hypomethylated	0.045957	0.71054	insignificant	9	42	44
chr2	69507176	69509176	Kbtbd10	-0.34039398	0.004739	stronglyHypometh	0.021234	0.72663	insignificant	8	43	42
chr2	69550663	69552663	Fastkd1	-0.15680652	0.00020441	hypomethylated	0.019848	0.089007	insignificant	6	26	26
chr2	69560144	69562144	Ppig	-0.12101938	2.07E-12	hypomethylated	0.01531	0.91297	insignificant	24	131	125
chr2	69626793	69628793	Phospho2	-0.18010371	3.15E-11	hypomethylated	-0.013996	0.29573	insignificant	10	72	32
chr2	69627543	69629543	4930578N16R	-0.16578927	0.00073301	hypomethylated	-0.05437	0.049035	hypomethylated	7	58	18
chr2	69659426	69661426	Klhl23	-0.08915963	6.89E-17	hypomethylated	0.004239	0.29849	insignificant	47	151	144
chr2	69698618	69700618	Ssb	-0.12184009	7.4E-11	hypomethylated	0.0095618	0.39569	insignificant	7	68	73
chr2	69723661	69725661	Mettl5	-0.15439087	0.008968	hypomethylated	0.02261	0.015303	hypermethylated	7	37	34
chr2	69734302	69736302	Ubr3	-0.08622218	5.15E-19	hypomethylated	-0.0089691	0.67552	insignificant	48	169	169
chr2	69876182	69878182	Myo3b	-0.01759259	0.28933	insignificant	0.044734	0.14086	insignificant	3	8	8
chr2	70311979	70313979	Sp5	-0.19873997	0.000000617	hypomethylated	-0.038902	0.57545	insignificant	16	71	73
chr2	70345875	70347875	4933404M02F	-0.10479008	5.66E-12	hypomethylated	-0.010465	0.77261	insignificant	25	120	115
chr2	70399220	70401220	Gad1	-0.19768426	0.0000017	hypomethylated	0.0021115	0.63932	insignificant	8	50	44
chr2	70498565	70500565	Gorasp2	-0.06817445	7.8E-23	hypomethylated	0.0093221	0.59366	insignificant	52	131	118
chr2	70663537	70665537	Tlk1	-0.08076453	0.00000974	hypomethylated	-0.0083958	0.39298	insignificant	51	137	136
chr2	70892800	70894800	Dcaf17	-0.25088476	0.00000978	hypomethylated	-0.087941	0.23846	insignificant	10	54	48
chr2	70893663	70895663	Mettl8	-0.29058746	1.13E-08	hypomethylated	-0.094012	0.0034434	hypomethylated	11	52	46
chr2	70955110	70957110	Cybrd1	-0.17922347	7.54E-09	hypomethylated	-0.0081944	0.54782	insignificant	16	66	61
chr2	71048762	71050762	Dync1i2	-0.13807985	0.000014129	hypomethylated	0.0056214	0.37297	insignificant	15	66	66
chr2	71049002	71051002	Dync1i2	-0.13807985	0.000014129	hypomethylated	0.0056214	0.37297	insignificant	15	66	66
chr2	71205611	71207611	Slc25a12	-0.07110078	0.00014978	hypomethylated	0.010032	0.6714	insignificant	15	60	55
chr2	71226316	71228316	Hat1	-0.1070419	0.006941	hypomethylated	0.027398	0.33026	insignificant	9	30	32
chr2	71290394	71292394	Metap1d	-0.23454621	7.05E-10	hypomethylated	-0.033381	0.092077	insignificant	18	53	51
chr2	71366501	71368501	Dlx1	-0.11693322	1.3E-28	hypomethylated	-0.0041677	0.66405	insignificant	31	128	124
chr2	71375948	71377948	Dlx1as	-0.2460396	0.000071949	hypomethylated	-0.01383	0.54239	insignificant	3	44	43
chr2	71384811	71386811	Dlx2	-0.16467767	6.46E-11	hypomethylated	0.002068	0.32763	insignificant	10	41	41
chr2	71556473	71558473	Gm1631	0.13068182	1	insignificant	-0.074318	0.78767	insignificant	2	4	4
chr2	71624139	71626139	Itga6	-0.13291305	5.56E-39	hypomethylated	0.010956	0.44013	insignificant	56	183	174
chr2	71710328	71712328	Pdk1	-0.17041746	0.11471	insignificant	0.030671	0.7133	insignificant	13	60	64
chr2	71818343	71820343	Rapgef4	-0.1585063	0.70004	insignificant	0.066618	0.25474	insignificant	7	58	47
chr2	72122693	72124693	B230120H23R	-0.09363425	1.83E-09	hypomethylated	0.012194	0.039022	hypermethylated	35	125	111
chr2	72122757	72124757	B230120H23R	-0.09363425	1.83E-09	hypomethylated	0.012194	0.039022	hypermethylated	35	125	111
chr2	72313275	72315275	Cdca7	-0.11616481	4.99E-08	hypomethylated	-0.0097926	0.28339	insignificant	25	99	78
chr2	72817338	72819338	Sp3	-0.09675981	1.6E-25	hypomethylated	-0.0024352	0.23216	insignificant	55	174	194
chr2	72818503	72820503	1700011J10Ri	-0.11192576	9.75E-18	hypomethylated	0.0060666	0.83282	insignificant	25	92	88
chr2	73052504	73054504	Ola1	-0.35191717	0.000058104	stronglyHypometh	0.0069455	0.0036835	hypermethylated	5	32	26
chr2	73108982	73110982	Sp9	-0.09068265	0.0025811	hypomethylated	-0.017186	0.31148	insignificant	17	85	84
chr2	73149708	73151708	Scrn3	-0.12032325	0.000000966	hypomethylated	0.011192	0.94674	insignificant	27	88	88
chr2	73150649	73152649	Cir1	-0.09097573	0.002339	hypomethylated	-0.000077845	0.57817	insignificant	15	53	53
chr2	73224455	73226455	Gpr155	-0.18765799	2.67E-11	hypomethylated	0.0020462	0.85249	insignificant	6	32	37
chr2	73367467	73369467	Wipf1	-0.20037445	1.31E-09	hypomethylated	-0.0078773	0.85033	insignificant	16	50	46
chr2	73418363	73420363	Chrna1	0.22447735	0.00000563	hypermethylated	0.094485	0.00060495	hypermethylated	3	6	6
chr2	73613403	73615403	Chn1	-0.15814965	0.00065165	hypomethylated	0.056284	0.90496	insignificant	9	38	38
chr2	73730685	73732685	Atf2	-0.23970849	5.53E-08	hypomethylated	0.10449	0.23863	insignificant	8	41	36
chr2	73749351	73751351	Atp5g3	-0.08768716	0.000000333	hypomethylated	-0.0079344	0.93354	insignificant	7	26	26

chr2	74417005	74419005	Lnp	-0.10713386	0.000040929	hypomethylated	0.0093049	0.14435	insignificant	8	22	22
chr2	74497476	74499476	Evx2	-0.32084578	0.074313	insignificant	-0.061075	0.7432	insignificant	4	32	22
chr2	74505366	74507366	Hoxd13	-0.11150416	2.24E-21	hypomethylated	0.0063792	0.6877	insignificant	34	158	158
chr2	74512086	74514086	Hoxd12	-0.16414228	1.65E-10	hypomethylated	-0.0063729	0.90755	insignificant	23	104	104
chr2	74519449	74521449	Hoxd11	-0.08870836	3.22E-16	hypomethylated	0.010635	0.30785	insignificant	81	267	269
chr2	74529004	74531004	Hoxd10	-0.1068341	4.11E-25	hypomethylated	0.014827	0.4866	insignificant	26	114	114
chr2	74534819	74536819	Hoxd9	-0.100496	6.87E-22	hypomethylated	0.0067428	0.23451	insignificant	46	193	189
chr2	74542545	74544545	Hoxd8	-0.08935014	1.01E-14	hypomethylated	0.0084753	0.22549	insignificant	53	193	184
chr2	74549049	74551049	Hoxd3	-0.15000609	7.85E-13	hypomethylated	0.0042518	0.36858	insignificant	21	61	54
chr2	74559034	74561034	Hoxd4	-0.19147335	0.20096	insignificant	0.032792	0.63822	insignificant	3	24	24
chr2	74563126	74565126	Mir10b	-0.04037301	0.11726	insignificant	-0.03543	0.26407	insignificant	11	38	38
chr2	74600036	74602036	Hoxd1	-0.1431237	4.64E-24	hypomethylated	0.0021792	0.57932	insignificant	43	151	153
chr2	74662868	74664868	Mtx2	-0.08463288	0.00067599	hypomethylated	0.020606	0.28938	insignificant	15	92	78
chr2	75496315	75498315	Hnrnpa3	-0.09075096	2.7E-26	hypomethylated	-0.00027144	0.97667	insignificant	48	165	177
chr2	75496346	75498346	Gm6793	-0.09143872	2.69E-26	hypomethylated	-0.0009592	0.97667	insignificant	48	164	177
chr2	75542698	75544698	Nfe2l2	-0.16746408	6.16E-08	hypomethylated	0.018006	0.36614	insignificant	9	44	38
chr2	75669233	75671233	Agps	-0.03402598	0.15531	insignificant	-0.0049214	0.88687	insignificant	15	116	117
chr2	75776519	75778519	Ttc30b		1	noCoverage	-0.036111	0.15161	insignificant	0	28	31
chr2	76176710	76178710	Pde11a	-0.10963066	0.000034364	hypomethylated	-0.0089587	0.00010916	inconclusive	4	33	24
chr2	76207040	76209040	Rbm45	-0.21236012	8.4E-14	hypomethylated	-0.011298	0.19444	insignificant	13	82	78
chr2	76243594	76245594	Osbpl6	-0.11486102	1.12E-43	hypomethylated	0.0071105	0.50746	insignificant	73	197	194
chr2	76486051	76488051	Prkra	-0.16134908	3.15E-28	hypomethylated	0.081735	0.8805	insignificant	10	57	54
chr2	76511155	76513155	Plekha3	-0.41041873	0.00011219	stronglyHypometh	-0.017607	0.62586	insignificant	5	24	23
chr2	76512371	76514371	Plekha3	-0.12743708	9.42E-24	hypomethylated	0.0041963	0.13092	insignificant	49	160	159
chr2	76820604	76822604	Ttn	0.13873405	0.13403	insignificant	0.0061766	0.9123	insignificant	2	8	8
chr2	77008692	77010692	Ccdc141	0.27070303	0.12402	insignificant	0.00362	0.0047231	hypermethylated	6	27	26
chr2	77118682	77120682	Sestd1	-0.08359777	3.81E-22	hypomethylated	0.0032048	0.6407	insignificant	29	56	56
chr2	77654873	77656873	Zfp385b	-0.15947032	0.61141	insignificant	-0.00098422	0.49294	insignificant	6	50	50
chr2	78708203	78710203	Ube2e3	-0.09039803	2.63E-28	hypomethylated	0.0067077	0.28502	insignificant	61	255	237
chr2	79094582	79096582	Itga4	-0.07833904	0.00016862	hypomethylated	-0.00078542	0.60424	insignificant	6	31	30
chr2	79269145	79271145	Cerkl	-0.19223088	0.068395	insignificant	0.14948	0.61374	insignificant	1	14	14
chr2	79296793	79298793	Neurod1	0.2630747	1.26E-26	hypermethylated	0.0043485	0.86629	insignificant	6	19	26
chr2	79474581	79476581	Ssfa2	-0.10293807	2.82E-17	hypomethylated	0.012904	0.27627	insignificant	39	154	134
chr2	79546936	79548936	Ppp1r1c	0.18230519	1	lowCoverage	0.047344	0.2355	insignificant	1	20	20
chr2	80131628	80133628	Prdx6b		1	noCoverage	-0.073052	0.074988	insignificant	0	4	4
chr2	80154622	80156622	Dnajc10	-0.11756593	2.61E-12	hypomethylated	0.01174	0.10001	insignificant	24	88	89
chr2	80287553	80289553	Frzb	-0.22548286	0.34422	insignificant	0.032059	0.50907	insignificant	2	19	19
chr2	80421122	80423122	Nckap1	-0.10422991	0.00000229	hypomethylated	0.017212	0.48021	insignificant	30	104	109
chr2	80456370	80458370	Dusp19	-0.36781609	0.29097	insignificant	0.14301	0.58938	insignificant	3	6	7
chr2	80477968	80479968	Nup35	-0.18668562	0.000051854	hypomethylated	0.0093261	0.059971	insignificant	11	56	60
chr2	80478420	80480420	Nup35	-0.18668562	0.000051854	hypomethylated	0.0093261	0.059971	insignificant	11	56	60
chr2	81892814	81894814	Zfp804a	-0.01768269	1.92E-10	hypomethylated	0.0067139	0.77682	insignificant	34	89	89
chr2	83483734	83485734	Zc3h15	-0.10063454	1.14E-09	hypomethylated	-0.0083001	1	insignificant	29	88	90
chr2	83563553	83565553	Itgav	-0.10081639	4.43E-26	hypomethylated	-0.004481	0.1154	insignificant	49	144	142
chr2	83651884	83653884	Fam171b	-0.09624015	6.21E-11	hypomethylated	0.0039628	0.72656	insignificant	22	52	52
chr2	84498235	84500235	Gm13718	-0.13719416	0.0013244	hypomethylated	-0.018516	0.20936	insignificant	7	26	26
chr2	84510865	84512865	Tmx2		1	noCoverage	-0.057462	0.60974	insignificant	0	20	18
chr2	84517558	84519558	Med19	-0.16780018	0.35149	insignificant	0.0038736	0.69167	insignificant	3	44	44
chr2	84518204	84520204	Tmx2	-0.17350568	0.57875	insignificant	-0.02606	0.36551	insignificant	2	26	26
chr2	84555321	84557321	Zdhhc5	-0.20277819	0.022531	hypomethylated	0.018003	1	insignificant	5	23	23
chr2	84567425	84569425	Clp1		1	noCoverage	0.056086	0.00000738	hypermethylated	0	35	30
chr2	84573360	84575360	Ypel4	-0.41562314	8.75E-08	stronglyHypometh	-0.17916	0.61269	insignificant	3	6	10
chr2	84581335	84583335	Mir130a	-0.18443793	0.79773	insignificant	0.018099	0.033421	hypermethylated	6	20	19
chr2	84637984	84639984	Ube2l6	-0.28473164	1.33E-08	hypomethylated	0.010006	0.77137	insignificant	4	21	21
chr2	84666177	84668177	Timm10	-0.1776621	0.0072451	hypomethylated	0.0083753	0.15389	insignificant	5	54	49
chr2	84678564	84680564	Slc43a1	-0.15416023	8.04E-12	hypomethylated	-0.0027755	0.31706	insignificant	12	92	87
chr2	84679479	84681479	Slc43a1	-0.14907036	8.3E-10	hypomethylated	-0.0037921	0.24812	insignificant	15	95	97
chr2	84679782	84681782	Slc43a1	-0.13249798	0.000006403	hypomethylated	0.0026955	0.46496	insignificant	10	60	62
chr2	84726849	84728849	Rtn4rl2	-0.12443014	2E-13	hypomethylated	0.017935	0.57492	insignificant	21	80	80
chr2	84775812	84777812	Slc43a3	-0.30998884	3.23E-15	hypomethylated	0.04386	0.065438	insignificant	10	28	32
chr2	84875991	84877991	Ssrp1	-0.11787228	5.12E-11	hypomethylated	0.00097938	1	insignificant	10	48	48
chr2	84876357	84878357	Ssrp1	-0.11787228	5.12E-11	hypomethylated	0.00097938	1	insignificant	10	48	48
chr2	84876607	84878607	Ssrp1	-0.11787228	5.12E-11	hypomethylated	0.00097938	1	insignificant	10	48	48
chr2	84889616	84891616	Tnks1bp1	-0.14758353	0.53773	insignificant	0.09182	0.031072	hypermethylated	3	21	18
chr2	84975516	84977516	Aplnr	0.02537391	0.3616	insignificant	-0.0068439	0.13026	insignificant	7	16	14
chr2	85036856	85038856	Lrrc55	-0.33456018	1	insignificant	-0.10793	0.58016	insignificant	2	11	10
chr2	85429666	85431666	Olfr998		1	noCoverage	-0.23561	0.30318	insignificant	0	4	4
chr2	85504612	85506612	Olfr154		1	noCoverage	0.045719	0.32952	insignificant	0	4	5
chr2	85624630	85626630	Olfr1015		1	noCoverage	-0.058564	1	insignificant	0	7	7
chr2	85818468	85820468	Olfr1030	0.35614543	0.56696	insignificant	0.21079	0.18227	insignificant	1	7	7
chr2	85859796	85861796	Olfr1033		1	noCoverage	0.24583	0.25362	insignificant	0	10	5
chr2	85959380	85961380	Olfr1038-ps		1	noCoverage	-0.25404	0.50253	insignificant	0	4	5
chr2	86076990	86078990	Olfr1048	0.23848485	1	insignificant	0.18848	0.42415	insignificant	3	8	10
chr2	86667832	86669832	Olfr1094		1	noCoverage	0.21032	0.20848	insignificant	0	6	6
chr2	87498614	87500614	Olfr1134	0.16458333	1	lowCoverage	-0.11827	0.55653	insignificant	1	4	5
chr2	87934123	87936123	Olfr1164	-0.7	0.0013487	stronglyHypometh	-0.027586	1	insignificant	1	2	2
chr2	88252846	88254846	Olfr1180		1	noCoverage	-0.086174	0.69833	insignificant	0	4	4
chr2	89892479	89894479	Olfr140		1	noCoverage	-0.13617	0.65799	insignificant	0	4	2
chr2	90420804	90422804	Ptprj	-0.08841419	0.000000653	hypomethylated	0.0012961	0.5073	insignificant	16	52	52
chr2	90584526	90586526	Fnbp4	-0.10822423	1.06E-12	hypomethylated	0.012524	0.056898	insignificant	34	94	94
chr2	90621900	90623900	Agbl2	-0.16048462	0.02507	hypomethylated	-0.031212	0.50648	insignificant	11	54	53
chr2	90686311	90688311	Mtch2	-0.13724235	7.84E-16	hypomethylated	-0.023895	0.94994	insignificant	31	118	118
chr2	90724942	90726942	C1qtnf4	-0.19479084	2.91E-16	hypomethylated	-0.0051188	0.56944	insignificant	14	45	36
chr2	90743942	90745942	Kbtbd4	-0.07824717	0.38637	insignificant	-0.02705	0.13081	insignificant	9	36	36
chr2	90744878	90746878	Ndufs3	-0.10204944	1	insignificant	-0.016493	0.00000573	hypomethylated	12	48	48

chr2	90758207	90760207	Ptpmt1		1	noCoverage	-0.069048	0.55669	insignificant	0	4	4
chr2	90779614	90781614	Celf1	-0.10979616	2.78E-36	hypomethylated	-0.0076502	0.79778	insignificant	53	191	188
chr2	90874783	90876783	Rapsn	-0.05985577	0.14466	insignificant	-0.020109	0.53688	insignificant	4	8	8
chr2	90893172	90895172	Psmc3	-0.22668043	0.00000527	hypomethylated	-0.063766	0.86537	insignificant	4	24	28
chr2	90910378	90912378	Slc39a13	-0.11143435	2.45E-29	hypomethylated	-0.012561	1	insignificant	12	60	58
chr2	90935953	90937953	Sfpi1	-0.35783586	1.94E-10	stronglyHypometh	-0.052338	0.7475	insignificant	5	30	26
chr2	90957300	90959300	Mybpc3	-0.1216299	0.30122	insignificant	-0.032902	0.60009	insignificant	2	8	8
chr2	91019478	91021478	Madd	-0.27373063	0.16834	insignificant	0.11976	0.022154	hypermethylated	2	10	10
chr2	91023204	91025204	Madd	-0.27480159	0.00010671	hypomethylated	0.035921	0.50886	insignificant	2	6	9
chr2	91035192	91037192	Nr1h3	-0.26991525	0.36013	insignificant	0.14914	0.51121	insignificant	3	6	6
chr2	91035273	91037273	Nr1h3	-0.26991525	0.36013	insignificant	0.14914	0.51121	insignificant	3	6	6
chr2	91042068	91044068	Acp2	-0.28981563	4.37E-13	hypomethylated	-0.14657	0.63552	insignificant	6	41	37
chr2	91076302	91078302	A330069E16R	-0.2345696	0.00030858	hypomethylated	-0.028233	0.67613	insignificant	10	35	34
chr2	91077223	91079223	Ddb2	-0.31481481	1	insignificant	0.012636	0.1762	insignificant	1	18	17
chr2	91095969	91097969	Pacsin3	-0.11909529	1.27E-11	hypomethylated	-0.0035686	0.3133	insignificant	26	103	100
chr2	91096485	91098485	Pacsin3	-0.11250274	4.41E-14	hypomethylated	-0.0042223	0.17993	insignificant	16	74	72
chr2	91104271	91106271	Arfgap2	-0.16157331	8.46E-15	hypomethylated	0.083091	0.3854	insignificant	14	101	94
chr2	91284799	91286799	1110051M20F	-0.50067116	0.30431	insignificant	0.058714	0.073735	insignificant	2	20	20
chr2	91296687	91298687	Lrp4	-0.17067801	1.75E-21	hypomethylated	0.0006201	0.00014814	hypermethylated	37	130	121
chr2	91489274	91491274	Arhgap1	-0.18562549	5.16E-08	hypomethylated	0.0084989	0.35377	insignificant	15	74	73
chr2	91489948	91491948	Zfp408	-0.2827571	0.000000104	hypomethylated	0.0097283	0.94342	insignificant	11	62	62
chr2	91550106	91552106	Harbi1	-0.17585753	1.06E-37	hypomethylated	0.04611	0.0057572	hypermethylated	21	47	43
chr2	91550733	91552733	Harbi1	-0.27514851	9.59E-19	hypomethylated	-0.080877	0.24937	insignificant	6	15	12
chr2	91569294	91571294	Ambra1	-0.14173946	0.00026869	hypomethylated	0.012457	0.68101	insignificant	16	62	61
chr2	91761345	91763345	Chrm4	-0.16660453	5.35E-40	hypomethylated	0.0065388	0.59275	insignificant	47	134	134
chr2	91771856	91773856	Mdk	-0.00197049	0.6349	insignificant	-0.05366	0.28363	insignificant	4	57	51
chr2	91771950	91773950	Mdk		1	noCoverage	-0.016336	0.41816	insignificant	0	45	42
chr2	91772439	91774439	Dgkz		1	noCoverage	0.042063	0.88357	insignificant	0	8	8
chr2	91790505	91792505	Dgkz	-0.4886517	0.00013615	stronglyHypometh	0.067407	0.65922	insignificant	1	11	11
chr2	91803720	91805720	Dgkz	-0.04750886	0.000087876	hypomethylated	0.0014779	0.44775	insignificant	11	55	55
chr2	91864327	91866327	Creb3l1	-0.222132	0.000092841	hypomethylated	0.021234	0.55873	insignificant	9	32	29
chr2	92023338	92025338	Phf21a	-0.09865653	5.88E-30	hypomethylated	0.011828	0.7772	insignificant	46	142	138
chr2	92024639	92026639	Phf21a	-0.20685786	8.3E-14	hypomethylated	0.0033409	0.088582	insignificant	17	57	57
chr2	92031133	92033133	Mir1955	-0.04025689	0.32635	insignificant	0.039108	0.52322	insignificant	2	4	4
chr2	92211193	92213193	Gyltl1b	-0.18511728	0.00019692	hypomethylated	0.056625	0.77045	insignificant	5	26	26
chr2	92213832	92215832	Pex16	-0.31502672	7.22E-31	hypomethylated	0.028359	0.020662	inconclusive	9	66	66
chr2	92214395	92216395	Pex16	-0.19122409	1.61E-16	hypomethylated	0.0069946	0.73296	insignificant	9	48	48
chr2	92222074	92224074	1700029I15Ril	0.04	1	lowCoverage	0.017273	1	insignificant	1	5	4
chr2	92241420	92243420	Mapk8ip1	-0.26349309	2.54E-09	hypomethylated	0.0028605	0.000022534	hypermethylated	11	48	47
chr2	92274226	92276226	Cry2	-0.2050315	1.28E-16	hypomethylated	0.01188	0.96626	insignificant	8	52	52
chr2	92299951	92301951	Slc35c1	-0.22610937	3.11E-13	hypomethylated	0.042301	0.21786	insignificant	12	42	41
chr2	92438863	92440863	Chst1	-0.13495523	7.83E-24	hypomethylated	-0.0093984	0.08345	insignificant	36	126	124
chr2	92754257	92756257	Syt13	-0.17310654	0.00053579	hypomethylated	0.0030271	0.84706	insignificant	14	28	28
chr2	92886301	92888301	Prdm11	-0.04851533	0.57096	insignificant	-0.0017086	0.24838	insignificant	14	60	60
chr2	93026740	93028740	Trp53i11	-0.12277833	2.04E-24	hypomethylated	-0.0031318	0.61333	insignificant	43	155	155
chr2	93174644	93176644	Tspan18	-0.06539375	0.00013779	hypomethylated	0.019823	0.84232	insignificant	11	35	28
chr2	93302653	93304653	Gm10804	-0.12601741	0.29482	insignificant	-0.0088758	0.6968	insignificant	6	16	16
chr2	93303103	93305103	Gm10804	-0.1421649	0.038752	hypomethylated	-0.0043174	1	insignificant	7	20	20
chr2	93481590	93483590	Alx4	-0.12558339	1.13E-39	hypomethylated	-0.0012071	0.26022	insignificant	49	167	165
chr2	93662725	93664725	Ext2	-0.45396002	0.00068062	stronglyHypometh	-0.11811	0.010932	hypomethylated	1	28	28
chr2	93689933	93691933	Accs	-0.23056733	1.76E-08	hypomethylated	-0.050702	0.22024	insignificant	9	20	18
chr2	93709260	93711260	Accsl	-0.12884615	1	insignificant	0.26699	0.065345	insignificant	2	5	4
chr2	93797257	93799257	Gm13889	-0.13746062	0.23153	insignificant	0.0081841	0.3047	insignificant	1	19	22
chr2	93850887	93852887	Alkbh3	-0.21917074	2.95E-10	hypomethylated	-0.028089	0.74338	insignificant	8	27	26
chr2	94081610	94083610	Mir129-2	0.00620448	1	insignificant	0.0023931	1	insignificant	4	26	26
chr2	94245863	94247863	2810002D19R	-0.13281618	4.03E-18	hypomethylated	0.0096332	0.025697	hypermethylated	34	108	112
chr2	94246846	94248846	Ttc17	-0.14855021	0.0057651	hypomethylated	-0.0047109	0.22627	insignificant	10	30	35
chr2	94278304	94280304	Api5	-0.07337963	0.000014888	hypomethylated	-0.0056727	0.35847	insignificant	7	18	18
chr2	97306830	97308830	Lrrc4c	-0.16593602	0.000000261	hypomethylated	-0.040025	0.77856	insignificant	4	25	23
chr2	101517596	101519596	Traf6	-0.14307047	1.07E-35	hypomethylated	0.0021371	0.82116	insignificant	37	116	114
chr2	101637864	101639864	Prr5l	-0.30262787	0.0061574	hypomethylated	0.0061555	0.0099381	inconclusive	1	14	18
chr2	101725418	101727418	Commd9	-0.20199517	0.010093	hypomethylated	0.0087579	0.4962	insignificant	3	18	18
chr2	102026534	102028534	Ldlrad3	-0.2225733	0.000004704	hypomethylated	-0.024783	0.37456	insignificant	9	32	33
chr2	102241057	102243057	Trim44	-0.11463768	1.85E-15	hypomethylated	-0.005121	0.17495	insignificant	22	70	60
chr2	102291949	102293949	Fjx1	-0.13090218	0.00025956	hypomethylated	0.0023803	0.32978	insignificant	8	40	39
chr2	102389177	102391177	Pamr1	-0.27969151	0.0015124	hypomethylated	-0.062367	0.02009	hypomethylated	3	16	16
chr2	102497839	102499839	Slc1a2	-0.07531838	4.59E-23	hypomethylated	0.0035716	0.33975	insignificant	30	140	127
chr2	102912831	102914831	Apip	-0.14294292	0.00088645	hypomethylated	0.047503	0.75437	insignificant	10	58	52
chr2	102913670	102915670	Pdhx	-0.13146994	0.0031111	hypomethylated	0.044075	0.55898	insignificant	8	38	31
chr2	103143353	103145353	Ehf	-0.04670768	0.54219	insignificant	0.0744	0.16884	insignificant	1	6	6
chr2	103263224	103265224	Elf5	-0.28416375	0.44978	insignificant	-0.088466	0.035555	hypomethylated	4	12	12
chr2	103325310	103327310	Cat		1	noCoverage	-0.0061728	0.22953	insignificant	0	6	6
chr2	103405466	103407466	Abtb2	-0.13258994	1.24E-35	hypomethylated	0.039762	0.90943	insignificant	50	176	170
chr2	103601407	103603407	Nat10	-0.03559444	3.33E-08	hypomethylated	-0.0022625	0.8485	insignificant	14	48	48
chr2	103637235	103639235	Caprin1	-0.08042418	0.000000525	hypomethylated	0.018516	0.062535	insignificant	19	96	93
chr2	103637797	103639797	Caprin1	-0.33559542	0.040747	stronglyHypometh	0.026905	3.81E-09	hypermethylated	3	14	14
chr2	103797151	103799151	Lmo2	-0.14600817	0.058737	insignificant	0.010335	0.54878	insignificant	6	20	20
chr2	103808713	103810713	Lmo2	-0.2043719	2.17E-11	hypomethylated	0.040884	0.74383	insignificant	11	44	36
chr2	103809443	103811443	Lmo2	-0.18315539	2.33E-16	hypomethylated	0.02762	0.47096	insignificant	19	92	84
chr2	103866955	103868955	Fbxo3	-0.15991048	0.083668	insignificant	0.037324	0.4903	insignificant	21	66	68
chr2	103910166	103912166	Cd59b	0.10591194	1	insignificant	-0.032316	0.21995	insignificant	2	12	12
chr2	103934957	103936957	Cd59a	-0.06975724	0.01471	hypomethylated	0.20832	0.00000624	hypermethylated	4	18	19
chr2	103961925	103963925	A930018P22R	-0.11605913	6.56E-13	hypomethylated	0.019711	0.33457	insignificant	30	91	76
chr2	104250491	104252491	D430041D05R	-0.10498323	0.00034812	hypomethylated	-0.0056031	0.0045663	hypomethylated	13	70	70
chr2	104334646	104336646	Hipk3	-0.15433212	1.07E-11	hypomethylated	0.016231	0.053575	insignificant	12	60	60

chr2	104429640	104431640	Cstf3	-0.12271298	3.85E-11	hypomethylated	-0.0038653	0.52008	insignificant	12	57	55
chr2	104552319	104554319	Tcp11l1	-0.21258681	0.71034	insignificant	0.00029331	0.29983	insignificant	16	68	74
chr2	104582958	104584958	Depdc7	-0.19463798	0.00000186	hypomethylated	-0.049177	0.80722	insignificant	16	38	34
chr2	104656853	104658853	Qser1	-0.10323315	6.92E-09	hypomethylated	-0.007043	0.10828	insignificant	31	87	74
chr2	104690007	104692007	Prrg4		1	noCoverage	0.26549	0.54995	insignificant	0	6	1
chr2	104725480	104727480	Ccdc73	-0.29218559	0.0069953	hypomethylated	-0.029771	0.88261	insignificant	1	13	13
chr2	104857184	104859184	Eif3m	-0.09488562	1.87E-09	hypomethylated	0.1087	0.38507	insignificant	8	17	18
chr2	104965685	104967685	Wt1	-0.07521719	4.98E-09	hypomethylated	0.014668	0.81172	insignificant	31	114	118
chr2	104966667	104968667	Wt1	-0.07900993	2.32E-09	hypomethylated	0.023745	0.62586	insignificant	29	122	126
chr2	105063498	105065498	0610012H03R	-0.11953889	1.92E-09	hypomethylated	0.04037	0.029469	hypermethylated	8	37	30
chr2	105239476	105241476	Rcn1	-0.23812313	1.02E-16	hypomethylated	-0.013987	0.96542	insignificant	20	58	58
chr2	105515601	105517601	Pax6	-0.17437678	0.00000146	hypomethylated	-0.0066927	0.35512	insignificant	13	81	78
chr2	105743794	105745794	Immp1l	-0.18910392	6.25E-14	hypomethylated	-0.00027237	0.76272	insignificant	9	38	38
chr2	105744657	105746657	Elp4		1	noCoverage	0.01162	0.81203	insignificant	0	8	8
chr2	106532615	106534615	Mpped2	-0.14241725	2.11E-25	hypomethylated	-0.0093973	1	insignificant	42	130	126
chr2	106534757	106536757	Mpped2		1	noCoverage	0.065512	1	insignificant	0	14	18
chr2	106810548	106812548	2700007P21Ri	0.19400298	0.12091	insignificant	0.032945	0.75066	insignificant	5	16	18
chr2	106814554	106816554	2700007P21Ri	-0.08585859	1	insignificant	0.0068663	0.45355	insignificant	2	10	10
chr2	107129745	107131745	Kcna4	-0.18264235	0.19605	insignificant	0.071021	0.93795	insignificant	2	33	25
chr2	109119894	109121894	Kif18a	-0.15507688	9.64E-17	hypomethylated	-0.01445	0.46735	insignificant	13	43	43
chr2	109513856	109515856	Bdnf	-0.19069851	1.85E-10	hypomethylated	0.0044141	0.76942	insignificant	6	30	30
chr2	109515053	109517053	Bdnf	-0.24470085	1.83E-08	hypomethylated	-0.031582	0.032882	hypomethylated	11	39	41
chr2	109531592	109533592	Bdnf	0.16372801	0.65896	insignificant	0.039958	0.89129	insignificant	4	63	57
chr2	109532719	109534719	Bdnf	-0.12591312	5.49E-33	hypomethylated	0.0046642	0.60359	insignificant	30	149	143
chr2	109730034	109732034	Lin7c	-0.12640919	1.01E-19	hypomethylated	-0.0085045	0.88771	insignificant	22	112	106
chr2	109753476	109755476	Gm13939	-0.14312364	0.063061	insignificant	0.10661	0.60849	insignificant	3	10	10
chr2	109756803	109758803	Lgr4	-0.09579243	1.37E-25	hypomethylated	-0.00051253	0.3838	insignificant	60	170	168
chr2	111405599	111407599	Olfr1295	-0.78333333	0.17647	lowCoverage	-0.1	0.12166	insignificant	1	6	7
chr2	111619469	111621469	Olfr1302	-0.71454545	0.2807	lowCoverage	-0.16608	0.54681	insignificant	1	10	10
chr2	111714010	111716010	Olfr1305		1	noCoverage	0.46759	0.096974	insignificant	0	7	4
chr2	112078997	112080997	Lpcat4	-0.1113474	3.36E-30	hypomethylated	0.0027532	0.89709	insignificant	45	153	141
chr2	112099448	112101448	BC125332		1	noCoverage	0.015597	0.53342	insignificant	0	12	23
chr2	112101125	112103125	Nop10	-0.04373339	0.02507	hypomethylated	-0.0035852	0.45989	insignificant	6	22	27
chr2	112105470	112107470	Slc12a6	-0.14091336	1.13E-37	hypomethylated	-0.0018036	0.55864	insignificant	42	114	114
chr2	112123844	112125844	Slc12a6		1	noCoverage	-0.052117	0.86688	insignificant	0	6	6
chr2	112218367	112220367	2410042D21R	-0.1904021	1.14E-30	hypomethylated	-0.007673	0.11718	insignificant	38	84	92
chr2	112294181	112296181	2900064A13R	-0.10884911	0.000000102	hypomethylated	0.0032345	0.12636	insignificant	20	55	46
chr2	112332120	112334120	Aven	-0.06828274	3.35E-30	hypomethylated	0.0081886	0.70435	insignificant	53	127	133
chr2	113166892	113168892	Fmn1	-0.12315045	0.0042009	hypomethylated	-0.0062408	0.086174	insignificant	3	45	45
chr2	113598805	113600805	Grem1	-0.02278827	0.0018632	hypomethylated	-0.022927	0.32266	insignificant	15	39	42
chr2	113688818	113690818	Arhgap11a		1	noCoverage	0.091575	0.17661	insignificant	0	2	2
chr2	113838300	113840300	A530058N18R	-0.10502016	6.46E-14	hypomethylated	-0.0053409	0.18253	insignificant	13	67	65
chr2	113878547	113880547	Actc1	-0.03165377	0.7652	insignificant	-0.021006	0.5881	insignificant	2	17	16
chr2	113879131	113881131	C130080G10R	-0.03165377	0.7652	insignificant	-0.021006	0.5881	insignificant	2	17	16
chr2	114001075	114003075	Aqr		1	noCoverage	-0.021164	0.84686	insignificant	0	2	2
chr2	114027168	114029168	Zfp770	-0.14050344	0.017675	hypomethylated	0.022574	0.75983	insignificant	6	18	18
chr2	115337937	115339937	3110099E03Ri	0.15833333	1	insignificant	-0.066963	1	insignificant	2	6	6
chr2	115890357	115892357	Meis2	-0.09124767	0.000031269	hypomethylated	0.028006	0.30291	insignificant	16	57	59
chr2	115890794	115892794	Meis2	-0.10084205	0.000000606	hypomethylated	0.0086889	0.60789	insignificant	17	66	65
chr2	115901832	115903832	2810405F15Ri	-0.40499084	0.21603	insignificant	-0.14535	0.70099	insignificant	2	11	10
chr2	116946185	116948185	Spred1	-0.08471199	3.65E-36	hypomethylated	-0.0024986	0.87778	insignificant	58	287	287
chr2	117074474	117076474	Fam98b	-0.13749451	1	insignificant	0.0117	0.80903	insignificant	3	30	30
chr2	117168613	117170613	Rasgrp1	-0.15598069	1	insignificant	-0.053423	0.62813	insignificant	3	11	11
chr2	117936657	117938657	Thbs1	-0.26679326	1.7E-13	hypomethylated	0.0072521	0.50625	insignificant	5	24	21
chr2	118082702	118084702	Fsip1	-0.64980066	0.015343	stronglyHypometh	0.0090377	0.52739	insignificant	1	22	21
chr2	118199155	118201155	Gpr176	-0.27431704	0.1376	insignificant	-0.072078	0.25611	insignificant	7	35	33
chr2	118213352	118215352	Eif2ak4	-0.15160954	3.09E-13	hypomethylated	-0.00173	0.80757	insignificant	20	84	79
chr2	118213847	118215847	Eif2ak4	-0.15160954	3.09E-13	hypomethylated	-0.00173	0.80757	insignificant	20	84	79
chr2	118375414	118377414	Bmf	-0.17339513	1	lowCoverage	0.048368	1	insignificant	1	18	18
chr2	118422946	118424946	Bub1b	-0.18117645	3.63E-13	hypomethylated	0.054804	0.3657	insignificant	10	24	19
chr2	118488312	118490312	Pak6	-0.08257824	0.43171	insignificant	-0.00017727	0.52442	insignificant	6	42	34
chr2	118501981	118503981	Pak6	-0.16496619	2.8E-09	hypomethylated	0.0066861	0.58758	insignificant	16	106	106
chr2	118529699	118531699	Gm1337	-0.14846002	7.22E-08	hypomethylated	-0.0043726	0.89716	insignificant	6	20	20
chr2	118554174	118556174	Plcb2	-0.18611111	0.58744	insignificant	-0.0040598	0.28856	insignificant	4	12	13
chr2	118570497	118572497	5430417L22Ri	-0.11924928	2.53E-61	hypomethylated	0.017559	0.00057152	hypermethylated	76	234	230
chr2	118588397	118590397	A430105I19Ri	-0.05824306	0.051345	insignificant	0.037938	0.010284	hypermethylated	8	24	30
chr2	118597504	118599504	Phgr1	0.02633971	0.51256	insignificant	-0.025843	0.0055956	inconclusive	2	9	6
chr2	118604454	118606454	Disp2	-0.02199037	0.68955	insignificant	0.045875	0.010195	hypermethylated	4	38	38
chr2	118638738	118640738	D2Ertd750e	-0.1117904	1.05E-12	hypomethylated	-0.0079693	0.10208	insignificant	18	78	73
chr2	118686735	118688735	Ivd	-0.16303884	0.0017462	hypomethylated	0.015603	0.082772	insignificant	5	32	32
chr2	118726350	118728350	Bahd1	-0.08999323	2.69E-19	hypomethylated	0.0013239	0.2112	insignificant	81	283	274
chr2	118751232	118753232	Chst14	-0.14689491	0.000026715	hypomethylated	-0.017879	0.7897	insignificant	13	78	75
chr2	118855129	118857129	Ccdc32		1	noCoverage	0.25966	0.4598	insignificant	0	8	10
chr2	118859525	118861525	Rpusd2	-0.11275819	9.04E-10	hypomethylated	0.019333	0.93362	insignificant	13	67	67
chr2	118871854	118873854	Casc5	-0.13157774	9.11E-08	hypomethylated	0.048503	0.31966	insignificant	4	62	48
chr2	118937552	118939552	Rad51	-0.10066149	5.94E-13	hypomethylated	0.02171	0.97161	insignificant	22	89	71
chr2	118982770	118984770	Fam82a2	-0.2168527	6.37E-10	hypomethylated	-0.02172	0.50534	insignificant	10	48	46
chr2	118999244	119001244	Gm14137	-0.23616071	0.49083	insignificant	0.071628	0.49423	insignificant	4	16	15
chr2	119033455	119035455	Zfyve19	-0.29159544	0.0034747	hypomethylated	-0.025467	0.8284	insignificant	4	54	48
chr2	119034531	119036531	Dnajc17	-0.21844181	0.21911	insignificant	-0.013128	0.74232	insignificant	1	26	23
chr2	119055601	119057601	Ppp1r14d		1	noCoverage	0.17398	0.10462	insignificant	0	10	6
chr2	119062095	119064095	Spint1	-0.15173709	1.01E-16	hypomethylated	-0.0093929	0.58178	insignificant	24	121	117
chr2	119096962	119098962	Rhov	-0.15426357	0.031631	hypomethylated	-0.017131	0.9203	insignificant	5	16	12
chr2	119113477	119115477	Vps18	-0.16448584	0.11577	insignificant	0.0063854	0.14592	insignificant	10	58	39
chr2	119150519	119152519	Dll4	-0.14372308	2.02E-24	hypomethylated	0.0054226	0.72422	insignificant	33	121	117

chr2	119151933	119153933	Dll4	-0.14058937	1.06E-15	hypomethylated	0.0081463	0.0051619	hypermethylated	30	92	80
chr2	119175977	119177977	Chac1	-0.16359815	0.01389	hypomethylated	0.022682	0.25463	insignificant	14	40	38
chr2	119303365	119305365	Ino80	-0.22428239	9.46E-15	hypomethylated	-0.018792	7.72E-12	hypomethylated	20	76	69
chr2	119372442	119374442	1500003O03R	-0.12499636	5.33E-22	hypomethylated	0.001836	0.30245	insignificant	38	130	130
chr2	119373363	119375363	Exd1	-0.10547571	0.000000143	hypomethylated	0.0061911	0.96946	insignificant	16	72	72
chr2	119419031	119421031	1700020I14Ril	-0.09671255	1.4E-09	hypomethylated	0.014486	0.0012237	inconclusive	17	78	57
chr2	119488534	119490534	Ndufaf1	-0.15678633	0.11039	insignificant	-0.011209	0.03396	hypomethylated	6	20	18
chr2	119499803	119501803	Rtf1	-0.12894879	3.33E-44	hypomethylated	-0.0088435	0.11795	insignificant	47	157	159
chr2	119567072	119569072	Itpka	-0.10583329	6.41E-57	hypomethylated	0.0092611	0.62033	insignificant	59	191	180
chr2	119584261	119586261	Ltk	-0.17335186	0.000037478	hypomethylated	0.013617	0.34096	insignificant	18	87	91
chr2	119586167	119588167	Ltk	-0.1025641	0.6532	insignificant	0.058191	0.13779	insignificant	3	8	8
chr2	119624249	119626249	Tyro3	-0.12496454	1.15E-31	hypomethylated	0.008134	0.46288	insignificant	44	158	150
chr2	119721963	119723963	Mga	-0.21955694	0.0012457	hypomethylated	0.0055781	0.27202	insignificant	14	88	90
chr2	119797434	119799434	Mapkbp1	-0.16550073	0.10399	insignificant	-0.029401	0.3613	insignificant	5	43	34
chr2	119852218	119854218	Jmjd7	-0.01489899	0.86265	insignificant	0.40577	3.56E-21	stronglyhypermeth	5	30	8
chr2	119980311	119982311	Ehd4	-0.15966757	6.18E-15	hypomethylated	0.060239	0.012573	inconclusive	8	17	18
chr2	120114805	120116805	Pla2g4d	0.26623377	1	lowCoverage	0.18474	0.15956	insignificant	1	7	4
chr2	120228631	120230631	Ganc	-0.18351326	0.13642	insignificant	-0.0005998	0.59915	insignificant	3	30	30
chr2	120229852	120231852	Ganc		1	noCoverage	0.02042	0.40749	insignificant	0	10	10
chr2	120288328	120290328	Capn3	0.15576073	0.13134	insignificant	-0.12459	1	insignificant	3	12	12
chr2	120392406	120394406	Snap23	-0.13162433	0.040123	hypomethylated	0.0029262	0.15858	insignificant	8	50	48
chr2	120434171	120436171	Haus2	-0.26194863	1.23E-32	hypomethylated	0.024174	0.89559	insignificant	12	24	24
chr2	120435051	120437051	Lrrc57	-0.40477036	4.84E-34	stronglyHypometh	0.053886	0.00094162	hypermethylated	7	6	6
chr2	120435094	120437094	Lrrc57	-0.37326389	4.22E-08	stronglyHypometh	0.027778	0.56773	insignificant	1	6	6
chr2	120435244	120437244	Lrrc57		1	noCoverage	0.027778	0.56773	insignificant	0	6	6
chr2	120557253	120559253	Cdan1	-0.2455243	5.49E-14	hypomethylated	-0.093326	0.63501	insignificant	13	39	28
chr2	120676154	120678154	AV039307	-0.20089906	0.000064011	hypomethylated	0.032423	0.13667	insignificant	14	59	58
chr2	120676320	120678320	AV039307	-0.2496281	0.000038769	hypomethylated	0.017959	0.23612	insignificant	14	51	51
chr2	120796451	120798451	Ubr1	-0.09573262	0.0032314	hypomethylated	0.003809	0.17754	insignificant	9	30	27
chr2	120801797	120803797	Tmem62	-0.10585782	2.23E-12	hypomethylated	-0.0043728	0.35467	insignificant	23	78	71
chr2	120833143	120835143	Ccndbp1	-0.14048722	3.01E-11	hypomethylated	0.013615	0.88109	insignificant	16	54	57
chr2	120995941	120997941	Tubgcp4	-0.12716726	8.07E-31	hypomethylated	-0.0041754	0.064363	insignificant	37	161	130
chr2	120996885	120998885	Zscan29	-0.10813127	1.24E-13	hypomethylated	-0.031122	0.66032	insignificant	25	77	66
chr2	121114337	121116337	Mtap1a	-0.10101268	1.24E-23	hypomethylated	0.014099	0.94208	insignificant	14	56	54
chr2	121120189	121122189	Mtap1a	-0.26111111	1	insignificant	-0.016793	1	insignificant	2	8	8
chr2	121183376	121185376	Ckmt1	-0.26528226	1.23E-16	hypomethylated	-0.083164	0.095386	insignificant	11	46	39
chr2	121206676	121208676	Strc		1	noCoverage	-0.1996	0.017389	hypomethylated	0	4	4
chr2	121238637	121240637	Pdia3	-0.09804891	4.02E-32	hypomethylated	0.0021919	0.81046	insignificant	41	163	163
chr2	121239528	121241528	Catsper2	-0.09573549	2.34E-14	hypomethylated	0.0032657	0.6983	insignificant	29	121	121
chr2	121268337	121270337	Ell3	-0.36143015	0.0011879	stronglyHypometh	0.040178	0.65699	insignificant	2	20	13
chr2	121273963	121275963	Serf2	-0.1035497	1.88E-43	hypomethylated	0.0031919	0.1713	insignificant	54	176	178
chr2	121281823	121283823	2310003F16Ri	-0.19706536	0.0021016	hypomethylated	0.044846	0.96706	insignificant	9	56	54
chr2	121282500	121284500	2310003F16Ri	-0.1802132	0.0020832	hypomethylated	0.039274	0.86708	insignificant	9	50	50
chr2	121299759	121301759	Mfap1b	-0.29730148	0.32349	insignificant	0.16629	0.72311	insignificant	2	10	10
chr2	121331458	121333458	Wdr76	0.29822249	0.000000132	hypermethylated	-0.0060178	0.011792	hypomethylated	5	39	42
chr2	121332392	121334392	Mfap1a	-0.17633929	1	lowCoverage	-0.011771	0.31605	insignificant	1	16	14
chr2	121632793	121634793	Frmd5	-0.16494556	0.024229	hypomethylated	0.035484	0.39362	insignificant	4	16	16
chr2	121691705	121693705	Casc4	-0.14878469	7.81E-13	hypomethylated	-0.0045582	0.25129	insignificant	26	87	107
chr2	121781188	121783188	Ctdspl2	-0.10300698	3.12E-19	hypomethylated	0.004639	0.76075	insignificant	29	101	100
chr2	121781828	121783828	Mageb3	-0.11116052	0.000002603	hypomethylated	0.0057914	0.78659	insignificant	11	47	47
chr2	121853358	121855358	Eif3j	-0.11776792	5.21E-08	hypomethylated	0.0031502	0.015991	inconclusive	29	143	128
chr2	121944122	121946122	Spg11	-0.24738323	7.61E-08	hypomethylated	-0.13845	0.68644	insignificant	10	30	22
chr2	121972422	121974422	B2m	-0.27105	0.000025517	hypomethylated	-0.034592	0.6517	insignificant	6	36	34
chr2	122011007	122013007	4933406J08Rik		1	noCoverage	0.18252	1	insignificant	0	10	8
chr2	122059574	122061574	Sord	-0.20568826	8.79E-12	hypomethylated	-0.021011	0.051839	insignificant	9	62	65
chr2	122123635	122125635	Duoxa2		1	noCoverage	0.078248	0.81525	insignificant	0	20	17
chr2	122123901	122125901	Duox2		1	noCoverage	0.013703	0.90832	insignificant	0	23	17
chr2	122139466	122141466	Duoxa1	-0.15698872	0.62329	insignificant	0.17217	0.92332	insignificant	4	16	16
chr2	122194654	122196654	Shf	-0.10999577	0.38197	insignificant	-0.0040654	0.67642	insignificant	13	52	63
chr2	122293533	122295533	Bambi-ps1		1	noCoverage	-0.033797	1	insignificant	0	31	30
chr2	122437013	122439013	Gatm	0.08376747	0.61382	insignificant	-0.00055045	1	insignificant	4	24	17
chr2	122462622	122464622	AA467197	0.73361865	0.12181	insignificant	0.22695	0.10988	insignificant	4	44	30
chr2	122528399	122530399	Slc30a4	-0.07674526	0.000000427	hypomethylated	-0.0013862	0.39524	insignificant	16	58	55
chr2	122563240	122565240	Pldn	-0.10122406	0.010508	hypomethylated	0.037036	0.90649	insignificant	9	48	32
chr2	122590094	122592094	Sqrdl	-0.102209	4.46E-08	hypomethylated	0.042167	0.11653	insignificant	11	50	28
chr2	124435031	124437031	Sema6d	-0.11759109	1.12E-21	hypomethylated	-0.0014881	0.42711	insignificant	27	143	143
chr2	124949396	124951396	Myef2	-0.15606444	0.059574	insignificant	-0.0057443	0.13989	insignificant	3	26	25
chr2	125071983	125073983	Dut	-0.09125603	2.68E-09	hypomethylated	-0.0010355	0.2414	insignificant	23	155	161
chr2	125072250	125074250	Dut	-0.09125603	2.68E-09	hypomethylated	-0.0010355	0.2414	insignificant	23	155	161
chr2	125332174	125334174	Fbn1	-0.14708335	9.35E-10	hypomethylated	-0.032972	0.8711	insignificant	9	24	24
chr2	125450849	125452849	Cep152	-0.19796339	1	insignificant	0.012086	0.17169	insignificant	1	36	32
chr2	125497835	125499835	Eid1	-0.15694158	0.0040076	hypomethylated	-0.0029598	0.75476	insignificant	14	76	69
chr2	125549884	125551884	Shc4		1	noCoverage	0.040872	0.2732	insignificant	0	26	17
chr2	125608606	125610606	Secisbp2l		1	noCoverage	0.26776	0.046123	hypermethylated	0	6	6
chr2	125683953	125685953	Galk2	-0.16520074	0.000000175	hypomethylated	0.0090592	0.059197	insignificant	12	70	69
chr2	125684754	125686754	Cops2	-0.15103019	0.0086758	hypomethylated	0.041457	0.077003	insignificant	6	33	33
chr2	125976876	125978876	Dtwd1	-0.11665854	0.000051338	hypomethylated	-0.015262	0.71684	insignificant	8	61	60
chr2	126377759	126379759	Slc27a2	-0.23497118	2.08E-18	hypomethylated	-0.0085694	0.00085348	hypomethylated	17	96	108
chr2	126444401	126446401	Hdc	-0.03368133	1	insignificant	0.14541	0.065164	insignificant	1	26	24
chr2	126501222	126503222	Gabpb1	-0.25031631	1.81E-16	hypomethylated	-0.031328	0.074033	insignificant	23	69	68
chr2	126501223	126503223	Gabpb1	-0.25031631	1.81E-16	hypomethylated	-0.031328	0.074033	insignificant	23	69	68
chr2	126532098	126534098	Usp8	-0.16059214	0.00000026	hypomethylated	0.018342	0.22179	insignificant	27	112	114
chr2	126701997	126703997	Trpm7	-0.20496805	0.0017939	hypomethylated	-0.0023714	0.16465	insignificant	10	42	42
chr2	126758971	126760971	2010106G01Rik		1	noCoverage	0.0071429	0.28872	insignificant	0	10	10
chr2	126833446	126835446	Ap4e1	-0.12750548	2.49E-12	hypomethylated	0.023298	0.099394	insignificant	15	74	55

chr2	126895392	126897392	Blvra	-0.1342162	1.97E-13	hypomethylated	0.0032242	1	insignificant	9	59	53
chr2	126959690	126961690	Ncaph		1	noCoverage	0.12022	0.0090456	hypermethylated	0	23	14
chr2	126968326	126970326	Itpripl1	-0.13284182	0.000012878	hypomethylated	0.0074987	1	insignificant	12	51	47
chr2	126969193	126971193	Itpripl1		1	noCoverage	-0.032051	1	insignificant	0	6	6
chr2	127033139	127035139	Snrnp200	-0.26496353	4.1E-09	hypomethylated	-0.031894	0.68568	insignificant	14	50	49
chr2	127034016	127036016	1810024B03R	-0.24212111	6.82E-09	hypomethylated	-0.069688	0.55881	insignificant	13	51	44
chr2	127072710	127074710	Tmem127	-0.13792722	3.72E-18	hypomethylated	0.018881	0.077542	insignificant	22	91	79
chr2	127073552	127075552	Ciao1	-0.14606115	3.68E-18	hypomethylated	0.010747	0.077641	insignificant	22	86	79
chr2	127094964	127096964	Stard7	-0.10912607	1.06E-20	hypomethylated	0.014658	0.87405	insignificant	35	108	96
chr2	127160894	127162894	Dusp2	-0.10897915	4.42E-33	hypomethylated	0.0081609	0.47079	insignificant	42	140	126
chr2	127163374	127165374	Astl	-0.60344828	0.00014303	stronglyHypometh	-0.057994	0.62229	insignificant	1	2	2
chr2	127188021	127190021	Adra2b	-0.12493588	1.63E-17	hypomethylated	0.030423	0.74235	insignificant	18	99	96
chr2	127249934	127251934	Gpat2	-0.09187803	0.52737	insignificant	-0.024475	0.13469	insignificant	5	32	36
chr2	127270301	127272301	Fahd2a		1	noCoverage	-0.017435	0.15898	insignificant	0	11	11
chr2	127308235	127310235	Kcnip3	-0.2246235	0.000000189	hypomethylated	-0.038999	0.021291	inconclusive	6	63	76
chr2	127347106	127349106	Kcnip3	-0.50491281	0.00010843	stronglyHypometh	-0.087498	0.94956	insignificant	4	30	28
chr2	127367153	127369153	Prom2	-0.27214402	2.56E-19	hypomethylated	0.17103	0.64512	insignificant	6	21	20
chr2	127410413	127412413	Zfp661	-0.16734694	0.011817	hypomethylated	-0.022171	0.43856	insignificant	5	14	14
chr2	127412161	127414161	Mrps5	-0.12437106	6.91E-20	hypomethylated	0.0027712	0.25737	insignificant	18	98	97
chr2	127614590	127616590	Nphp1	0.02917774	0.20793	insignificant	0.033151	0.037963	hypermethylated	6	42	38
chr2	127618224	127620224	1500011K16Ri	-0.33879003	0.037116	stronglyHypometh	-0.021946	0.87956	insignificant	3	68	60
chr2	127657595	127659595	Bub1		1	noCoverage	0.16042	0.4362	insignificant	0	8	4
chr2	127679363	127681363	Acoxl	-0.31919879	0.37029	insignificant	0.006322	0.50407	insignificant	2	7	8
chr2	127950773	127952773	Bcl2l11	-0.11782925	0.00000494	hypomethylated	0.012387	0.67387	insignificant	24	160	159
chr2	128523732	128525732	Mertk	-0.12517506	5.73E-14	hypomethylated	-0.02143	0.53127	insignificant	20	62	61
chr2	128643038	128645038	Tmem87b	-0.24153119	1.25E-12	hypomethylated	-0.111	0.019188	hypomethylated	17	62	53
chr2	128688667	128690667	Fbln7	0.03340974	0.11662	insignificant	0.031828	0.45673	insignificant	2	12	21
chr2	128769756	128771756	Zc3h8	0.28309745	1	insignificant	0.18445	0.1562	insignificant	1	12	12
chr2	128792137	128794137	Zc3h6	-0.08749326	1.18E-23	hypomethylated	0.0046505	0.87265	insignificant	55	168	159
chr2	128890682	128892682	Ttl	-0.08737616	1.14E-13	hypomethylated	-0.014645	0.68857	insignificant	25	78	69
chr2	128925731	128927731	Polr1b	-0.26812376	3.73E-08	hypomethylated	0.044947	0.41664	insignificant	9	32	52
chr2	128954435	128956435	Chchd5	-0.22498754	7.34E-28	hypomethylated	-0.026556	1	insignificant	10	54	53
chr2	129023508	129025508	Slc20a1	-0.10043439	1.83E-30	hypomethylated	0.0073383	0.33582	insignificant	43	147	145
chr2	129122948	129124948	Gm14023	-0.24599359	0.12187	insignificant	0.033611	0.42535	insignificant	3	12	12
chr2	129417574	129419574	Sirpa	-0.11591014	0.00098488	hypomethylated	-0.015508	0.025814	hypomethylated	18	73	101
chr2	129417930	129419930	Sirpa	-0.13372744	0.00025776	hypomethylated	-0.011445	0.020914	hypomethylated	19	79	107
chr2	129625252	129627252	Stk35	-0.14660627	2.83E-18	hypomethylated	-0.014973	0.74559	insignificant	35	157	153
chr2	129627195	129629195	Stk35	-0.06345042	0.16338	insignificant	0.032507	0.086616	insignificant	7	36	38
chr2	129925129	129927129	AU015228	-0.03755773	0.033645	inconclusive	0.04871	0.65304	insignificant	5	19	20
chr2	130005100	130007100	Snrpb	-0.2827381	0.063557	insignificant	-0.0037246	0.4776	insignificant	1	12	11
chr2	130099147	130101147	Nop56	-0.10363235	3.82E-09	hypomethylated	-0.0032159	0.58751	insignificant	23	102	101
chr2	130100250	130102250	Snord110	-0.08924143	0.00046024	hypomethylated	-0.0010625	0.57742	insignificant	5	49	48
chr2	130110187	130112187	Idh3b	-0.82167023	8.22E-10	stronglyHypometh	0.022652	0.7074	insignificant	0	4	4
chr2	130120674	130122674	Ebf4	-0.09091518	2.16E-47	hypomethylated	0.0047136	0.23951	insignificant	64	192	193
chr2	130223365	130225365	Cpxm1	-0.15291757	0.27677	insignificant	-0.012263	0.010775	inconclusive	11	34	31
chr2	130229994	130231994	1700020A23R	-0.27789855	1	insignificant	-0.1175	0.051193	insignificant	2	12	12
chr2	130230031	130232031	1700020A23R	-0.27789855	1	insignificant	-0.1175	0.051193	insignificant	2	12	12
chr2	130231257	130233257	4933425O20R	-0.8057971	0.19598	lowCoverage	-0.058418	0.075977	insignificant	1	6	6
chr2	130249055	130251055	Vps16	-0.09464607	4.68E-12	hypomethylated	0.013332	0.011577	hypermethylated	12	68	68
chr2	130250024	130252024	Fam113a	-0.19787827	0.0012736	hypomethylated	-0.058444	0.16772	insignificant	7	50	45
chr2	130250377	130252377	Fam113a	-0.24888865	0.000182	hypomethylated	-0.09306	0.9582	insignificant	7	36	31
chr2	130275013	130277013	Ptpra	-0.08510448	1.39E-13	hypomethylated	0.015661	0.23437	insignificant	31	141	136
chr2	130388492	130390492	Mrps26	-0.10210897	4.89E-13	hypomethylated	0.048452	0.094571	insignificant	26	100	100
chr2	130400908	130402908	Oxt	-0.08852815	0.010508	hypomethylated	0.0020186	0.03392	inconclusive	7	36	38
chr2	130455722	130457722	Ubox5	-0.24134	1.76E-11	hypomethylated	0.073994	0.28071	insignificant	5	18	12
chr2	130455763	130457763	Ubox5	-0.22634866	0.10913	insignificant	0.11501	1	insignificant	3	13	8
chr2	130468539	130470539	Prosapip1	-0.24258118	2.39E-20	hypomethylated	-0.00097855	1	insignificant	13	32	41
chr2	130492576	130494576	Itpa	-0.33145835	4.63E-20	hypomethylated	-0.026622	0.77602	insignificant	11	43	40
chr2	130523255	130525255	Slc4a11	-0.59759862	0.0074449	stronglyHypometh	0.030859	0.60073	insignificant	1	14	14
chr2	130665846	130667846	4930402H24R	-0.06399336	4.63E-09	hypomethylated	0.0025617	0.52057	insignificant	19	60	60
chr2	130731231	130733231	Atrn	-0.13711902	1.22E-09	hypomethylated	-0.047822	0.32683	insignificant	25	106	96
chr2	130732132	130734132	A730017L22Ri	-0.1284573	7.27E-09	hypomethylated	-0.043735	0.2331	insignificant	21	91	84
chr2	130868418	130870418	Gfra4	0.03763441	1	insignificant	0.24041	0.052585	insignificant	1	4	4
chr2	130868824	130870824	Gfra4	-0.02899386	0.72584	insignificant	0.13545	0.025792	hypermethylated	2	10	10
chr2	130889550	130891550	Adam33		1	noCoverage	-0.020734	1	insignificant	0	16	16
chr2	130952147	130954147	Hspa12b	-0.09882427	0.000062543	hypomethylated	-0.0073893	0.050634	insignificant	5	26	26
chr2	130985756	130987756	1700037H04R	-0.19352934	0.056571	insignificant	0.022455	0.41316	insignificant	1	12	12
chr2	131005748	131007748	Cenpb	-0.10944067	0.00025373	hypomethylated	0.0095918	0.749	insignificant	8	36	36
chr2	131011686	131013686	Cdc25b	-0.16854406	0.0055165	hypomethylated	0.0117	0.10954	insignificant	14	54	54
chr2	131035175	131037175	2310035K24Ri	-0.21063763	0.61228	insignificant	-0.015857	0.52153	insignificant	4	26	26
chr2	131058873	131060873	Mavs	-0.15728497	3.72E-16	hypomethylated	0.010436	0.77223	insignificant	7	70	56
chr2	131087235	131089235	Pank2	-0.10684578	5.48E-14	hypomethylated	0.0026757	0.9256	insignificant	25	83	90
chr2	131178628	131180628	Rnf24	-0.13699187	0.036277	hypomethylated	0.081393	0.0039291	hypermethylated	7	39	39
chr2	131316597	131318597	Smox	0.1285629	5.95E-12	inconclusive	0.0037362	0.77576	insignificant	35	109	100
chr2	131388021	131390021	Adra1d	-0.160489	1.39E-13	hypomethylated	0.015311	0.09333	insignificant	18	79	68
chr2	131734663	131736663	Prnp	-0.12594905	1	insignificant	-0.010958	0.85647	insignificant	3	28	27
chr2	131775596	131777596	Prnd	0.26835331	1	insignificant	0.0032858	0.4054	insignificant	2	20	20
chr2	131855724	131857724	Rassf2	-0.75597576	2.86E-12	stronglyHypometh	0.033697	0.10266	insignificant	1	13	14
chr2	131970844	131972844	Slc23a2	-0.28594767	1.84E-08	hypomethylated	-0.059607	0.90274	insignificant	8	33	28
chr2	132073524	132075524	5730494N06R	-0.4127996	0.10299	insignificant	-0.024455	0.62261	insignificant	6	30	30
chr2	132078916	132080916	Pcna	0.00403625	1	insignificant	-0.0061158	0.45779	insignificant	4	24	23
chr2	132087992	132089992	Cds2	-0.09016385	0.0000079	hypomethylated	0.041552	0.60504	insignificant	11	120	69
chr2	132211183	132213183	Prokr2	-0.16290028	0.46699	insignificant	-0.15485	0.14332	insignificant	7	20	16
chr2	132403984	132405984	Gpcpd1	-0.50369402	0.00016702	stronglyHypometh	-0.079343	0.37658	insignificant	4	12	10
chr2	132422931	132424931	AU019990	-0.24221212	0.33773	insignificant	-0.032212	0.43593	insignificant	3	10	10

chr2	132606013	132608013	Chgb	-0.0968115	0.00067984	hypomethylated	0.013989	0.75294	insignificant	11	35	41
chr2	132641070	132643070	Mcm8	-0.13846076	9.53E-19	hypomethylated	-0.0011737	0.80796	insignificant	16	66	66
chr2	132641790	132643790	Trmt6	-0.10252443	5.48E-14	hypomethylated	-0.0038382	0.42008	insignificant	15	52	52
chr2	132671401	132673401	Crls1	-0.14595766	3.13E-16	hypomethylated	0.01765	0.70332	insignificant	35	125	124
chr2	132672469	132674469	Crls1	-0.1739003	9.21E-17	hypomethylated	0.00095769	0.000079411	inconclusive	37	119	118
chr2	132771772	132773772	Fermt1	-0.23151515	0.16662	insignificant	0.16015	0.000849	hypermethylated	2	10	10
chr2	133377934	133379934	Bmp2	-0.09761977	1.09E-41	hypomethylated	0.014289	0.0036194	hypermethylated	64	198	188
chr2	134469857	134471857	Tmx4	-0.11067821	1	insignificant	0.028391	0.59288	insignificant	4	11	11
chr2	134610899	134612899	Plcb1	-0.12056915	1.05E-14	hypomethylated	-0.031101	0.86205	insignificant	36	121	116
chr2	135566565	135568565	Plcb4	0.12643383	1	insignificant	0.051007	0.41566	insignificant	2	14	14
chr2	135882662	135884662	6330527O06R	-0.08472222	0.64947	insignificant	-0.18212	0.27301	insignificant	3	6	11
chr2	136538185	136540185	Snap25	-0.18980486	3.37E-10	hypomethylated	0.075054	0.026126	hypermethylated	7	39	39
chr2	136715953	136717953	2210009G21R	-0.02086892	0.34661	insignificant	-0.0095962	0.25648	insignificant	10	67	64
chr2	136716998	136718998	2210009G21R	-0.07403262	0.48694	insignificant	0.011112	0.44177	insignificant	7	58	58
chr2	136717142	136719142	2210009G21R	-0.12867975	0.090586	insignificant	0.013758	0.31375	insignificant	4	40	40
chr2	136942067	136944067	Jag1	-0.13072023	3.78E-34	hypomethylated	-0.0064012	0.23356	insignificant	47	142	142
chr2	138081319	138083319	Btbd3	-0.08092474	1.48E-08	hypomethylated	0.0048211	0.91847	insignificant	16	110	110
chr2	138103228	138105228	Btbd3	0.27790671	0.19524	insignificant	-0.1248	0.73911	insignificant	3	27	26
chr2	139502913	139504913	Ism1	-0.13162643	2.08E-21	hypomethylated	-0.0096611	0.27643	insignificant	53	154	144
chr2	139892496	139894496	Tasp1		1	noCoverage	0.03505	1	insignificant	0	8	8
chr2	139892521	139894521	Tasp1		1	noCoverage	0.009891	0.62342	insignificant	0	12	12
chr2	139995381	139997381	2310003L22Ri	-0.15902872	5.57E-12	hypomethylated	0.010625	0.16874	insignificant	15	67	67
chr2	139996294	139998294	Esf1	-0.18226726	0.000000185	hypomethylated	-0.007385	0.040538	inconclusive	12	55	56
chr2	140220165	140222165	Macrod2	-0.11702182	2.03E-54	hypomethylated	-0.0058566	0.019292	inconclusive	43	135	132
chr2	140220170	140222170	Macrod2	-0.11702182	2.03E-54	hypomethylated	-0.0058566	0.019292	inconclusive	43	135	132
chr2	142727200	142729200	Kif16b	-0.21030454	0.000000374	hypomethylated	0.038108	0.82268	insignificant	10	39	34
chr2	142887804	142889804	Snrpb2	-0.1045018	1.98E-09	hypomethylated	0.024543	1	insignificant	22	68	68
chr2	143370868	143372868	Pcsk2	-0.15618185	1.39E-22	hypomethylated	-0.0056832	0.78913	insignificant	33	103	103
chr2	143740066	143742066	Dstn	-0.12299295	9.08E-15	hypomethylated	0.021355	0.44314	insignificant	20	114	114
chr2	143836999	143838999	Rrbp1	0.4875319	2.49E-12	stronglyHypermeth	0.012903	0.27708	insignificant	2	46	46
chr2	144091938	144093938	Snord17	0.24754902	1	lowCoverage	-0.12745	0.37492	insignificant	1	4	4
chr2	144095770	144097770	8430406I07Ril	-0.15021968	6.99E-22	hypomethylated	-0.0076243	1	insignificant	27	104	107
chr2	144096308	144098308	Snx5	-0.03025247	0.21403	insignificant	-0.010645	0.3256	insignificant	17	56	53
chr2	144096638	144098638	Snx5	0.03173419	0.54453	insignificant	0.057452	0.038397	hypermethylated	5	20	17
chr2	144157098	144159098	Ovol2	-0.16291726	2.72E-28	hypomethylated	0.0082018	0.66046	insignificant	17	79	77
chr2	144157816	144159816	Ovol2	-0.27291223	1.12E-11	hypomethylated	0.040347	0.36068	insignificant	3	50	49
chr2	144193718	144195718	Gm20571	-0.19572306	6.83E-32	hypomethylated	0.0095472	0.85956	insignificant	34	98	94
chr2	144193767	144195767	Csrp2bp	-0.19572306	6.83E-32	hypomethylated	0.0095472	0.85956	insignificant	34	98	94
chr2	144193770	144195770	Csrp2bp	-0.19572306	6.83E-32	hypomethylated	0.0095472	0.85956	insignificant	34	98	94
chr2	144284015	144286015	LOC668917	-0.20375	2.83E-13	hypomethylated	0.1459	0.39859	insignificant	2	16	10
chr2	144352480	144354480	Polr3f	-0.21171649	8.32E-10	hypomethylated	0.029635	0.63307	insignificant	15	56	53
chr2	144353134	144355134	6330439K17Ri	-0.18040025	9.36E-09	hypomethylated	0.050623	0.7988	insignificant	14	50	49
chr2	144381012	144383012	Sec23b	-0.31145774	6.85E-50	hypomethylated	0.071168	0.94581	insignificant	16	14	14
chr2	144418800	144420800	Gm561	-0.34451828	0.10345	insignificant	0.05231	0.82167	insignificant	7	47	48
chr2	144424688	144426688	Dtd1	-0.12044282	0.00000158	hypomethylated	0.021148	0.40613	insignificant	13	48	48
chr2	145067346	145069346	Slc24a3		1	noCoverage	0.020029	0.028203	hypermethylated	0	34	36
chr2	145610851	145612851	Rin2		1	noCoverage	-0.12684	0.89421	insignificant	0	4	4
chr2	145727976	145729976	Naa20	-0.06244325	1.09E-16	hypomethylated	0.04117	0.036636	hypermethylated	32	107	96
chr2	145759519	145761519	4930529M08F	-0.31441652	1.34E-17	hypomethylated	0.02006	0.26234	insignificant	8	62	52
chr2	145760436	145762436	Crnkl1	-0.28010544	0.23685	insignificant	-0.01189	0.57761	insignificant	5	46	46
chr2	146046732	146048732	Insm1	-0.0846099	7.89E-10	hypomethylated	0.0033802	0.8812	insignificant	69	243	263
chr2	146337740	146339740	Ralgapa2	-0.10970217	4.18E-08	hypomethylated	-0.00026455	0.22336	insignificant	24	64	63
chr2	146680624	146682624	Plk1s1	-0.17223165	0.35946	insignificant	0.0071771	0.54937	insignificant	2	20	19
chr2	146837795	146839795	Xrn2	-0.12532985	4.07E-16	hypomethylated	-0.019946	0.30065	insignificant	31	133	123
chr2	146911081	146913081	Nkx2-4	-0.11728765	0.0001257	hypomethylated	0.012188	0.00000214	hypermethylated	13	44	29
chr2	147008818	147010818	Nkx2-2as	-0.15518391	5.39E-09	hypomethylated	-0.0022547	0.71914	insignificant	22	92	87
chr2	147012138	147014138	Nkx2-2as	-0.1576721	0.070152	insignificant	0.0054477	0.16072	insignificant	9	51	48
chr2	147189729	147191729	Pax1	-0.23663341	0.013627	hypomethylated	-0.033659	0.62825	insignificant	13	89	80
chr2	147872705	147874705	Foxa2	0.01071107	0.0073902	inconclusive	0.0245	0.26367	insignificant	8	49	48
chr2	148220112	148222112	Sstr4	-0.08729252	2.81E-21	hypomethylated	0.002116	0.21659	insignificant	35	90	90
chr2	148233924	148235924	Thbd	-0.18636308	1	insignificant	-0.0015635	0.77085	insignificant	3	25	21
chr2	148269271	148271271	Cd93	-0.28532268	1	lowCoverage	-0.014831	0.47577	insignificant	1	4	4
chr2	148497350	148499350	Nxt1	-0.1316852	3.16E-09	hypomethylated	-0.0033745	0.65384	insignificant	15	83	81
chr2	148497376	148499376	Nxt1	-0.1316852	3.16E-09	hypomethylated	-0.0033745	0.65384	insignificant	15	83	81
chr2	148505855	148507855	Gzf1	-0.22003105	1.23E-16	hypomethylated	0.0096039	0.89228	insignificant	16	71	69
chr2	148558156	148560156	Napb	-0.2127935	0.66341	insignificant	-0.031339	0.12412	insignificant	3	19	22
chr2	148575096	148577096	Cstl1	0.09747514	1	lowCoverage	0.028031	0.12238	insignificant	1	14	10
chr2	148606744	148608744	8030411F24Rik		1	noCoverage		1	noCoverage	0	6	0
chr2	148614096	148616096	Cst12	0.0817055	0.54852	insignificant	0.10353	0.00053795	hypermethylated	5	20	20
chr2	149655518	149657518	Tmem90b	-0.13727331	1.23E-26	hypomethylated	-0.01275	0.60439	insignificant	32	136	134
chr2	149962414	149964414	Zfp120	-0.56642512	0.043036	stronglyHypometh	-0.041541	0.64508	insignificant	1	4	4
chr2	150006490	150008490	Gm14139	0.21216681	0.34956	insignificant	-0.064129	0.58173	insignificant	1	10	10
chr2	150082252	150084252	Gm14124	-0.88333333	0.0029741	stronglyHypometh	0.054406	0.65044	insignificant	1	4	6
chr2	150310799	150312799	Zfp345		1	noCoverage	0.019548	1	insignificant	0	4	4
chr2	150434259	150436259	2310001A20R	-0.10973854	1	insignificant	-0.012321	0.91056	insignificant	5	10	12
chr2	150493976	150495976	E130215H24R	-0.22926337	0.0025147	hypomethylated	-0.029398	0.69451	insignificant	7	27	26
chr2	150573816	150575816	Entpd6	-0.08045244	2.79E-10	hypomethylated	0.015649	0.84909	insignificant	18	64	64
chr2	150611531	150613531	Pygb	-0.22224126	0.00000193	hypomethylated	-0.019477	0.49232	insignificant	12	54	49
chr2	150730467	150732467	Abhd12	-0.22833669	0.000081979	hypomethylated	0.05327	0.72779	insignificant	6	16	16
chr2	150734329	150736329	Gins1	-0.17317637	0.000011359	hypomethylated	0.021684	0.87304	insignificant	12	26	26
chr2	150865115	150867115	Nanp		1	noCoverage	0.12393	0.68231	insignificant	0	6	7
chr2	151301887	151303887	4921509C19Ri	0.25534972	0.0065672	hypermethylated	0.14335	0.00024075	hypermethylated	2	6	6
chr2	151319043	151321043	Nsfl1c	-0.12419325	0.39215	insignificant	0.0085556	0.67924	insignificant	7	38	33
chr2	151367234	151369234	Fkbp1a	-0.06755702	0.000029425	hypomethylated	-0.0001751	0.74904	insignificant	31	119	120
chr2	151458269	151460269	Snph		1	noCoverage	-0.12333	0.56087	insignificant	0	16	16

chr2	151494269	151496269	Rad21l	0.09047619	0.62458	insignificant	0.052395	0.19941	insignificant	4	10	10
chr2	151526743	151528743	5430405G05R	-0.1116303	1.89E-23	hypomethylated	-0.0084922	0.83581	insignificant	50	177	172
chr2	151567029	151569029	Psmf1		1	noCoverage	-0.0068362	1	insignificant	0	19	19
chr2	151667662	151669662	Rspo4	-0.10268116	2.15E-17	hypomethylated	-0.0034686	0.3902	insignificant	22	70	74
chr2	151736067	151738067	Angpt4	-0.02864774	1	insignificant	-0.080206	0.031617	hypomethylated	4	24	18
chr2	151799710	151801710	Fam110a	-0.33363619	2.54E-09	stronglyHypometh	-0.10174	0.0048473	hypomethylated	7	18	18
chr2	151805955	151807955	Fam110a	-0.37388863	3.39E-15	stronglyHypometh	-0.045203	0.61441	insignificant	6	17	16
chr2	151821246	151823246	2310046K01Rik		1	noCoverage	0.060524	0.73362	insignificant	0	38	34
chr2	151824601	151826601	2310046K01Ri	0.15616883	1	insignificant	0.022871	0.08487	insignificant	2	20	14
chr2	151906264	151908264	Scrt2	-0.13085354	6.64E-15	hypomethylated	0.0054635	0.65535	insignificant	27	120	121
chr2	151930465	151932465	Srxn1	-0.11865703	4.63E-20	hypomethylated	0.0042517	0.23754	insignificant	34	121	105
chr2	151968344	151970344	Tcf15	-0.08626524	2.47E-13	hypomethylated	0.01047	0.1668	insignificant	23	148	138
chr2	152051575	152053575	Csnk2a1	-0.24780515	5.27E-14	hypomethylated	-0.039371	0.24571	insignificant	11	26	22
chr2	152118607	152120607	Tbc1d20	-0.08977797	0.00048576	hypomethylated	-0.0045791	0.72973	insignificant	17	76	76
chr2	152158161	152160161	Rbck1	-0.2568135	0.0000102	hypomethylated	0.048396	0.40799	insignificant	6	45	46
chr2	152158375	152160375	Rbck1	-0.27893816	0.00048493	hypomethylated	0.065062	0.7391	insignificant	4	33	34
chr2	152169796	152171796	Trib3	-0.49273609	0.000000521	stronglyHypometh	0.060566	0.28053	insignificant	3	25	27
chr2	152202302	152204302	Nrsn2	-0.44947544	0.000000125	stronglyHypometh	-0.16704	0.065282	insignificant	5	21	18
chr2	152223782	152225782	Sox12	-0.08581384	5.33E-13	hypomethylated	0.018598	0.93703	insignificant	22	87	89
chr2	152240322	152242322	6820408C15R	-0.06233202	0.26735	insignificant	0.052194	0.096324	insignificant	6	57	33
chr2	152301798	152303798	Defb20		1	noCoverage	0.0050325	1	insignificant	0	6	6
chr2	152397479	152399479	Defb21	0.28426423	1	insignificant	0.091878	0.40192	insignificant	1	7	6
chr2	152406048	152408048	Defb19	0.13541667	0.59146	insignificant	0.052083	0.81618	insignificant	1	6	6
chr2	152429062	152431062	Defb36	0.12198428	0.37639	insignificant	0.031653	0.045333	hypermethylated	3	31	31
chr2	152451743	152453743	Rem1	-0.1570958	7.15E-28	hypomethylated	0.010938	0.90393	insignificant	29	107	99
chr2	152494196	152496196	H13	-0.18557425	0.0018588	hypomethylated	0.0061128	0.52162	insignificant	3	46	46
chr2	152511883	152513883	Mcts2	-0.03409594	0.014494	hypomethylated	0.028029	0.59867	insignificant	11	64	62
chr2	152561009	152563009	Id1	-0.26923077	0.0022014	hypomethylated	-0.014231	0.82865	insignificant	3	8	8
chr2	152578908	152580908	Cox4i2	0.09761355	0.60575	insignificant	-0.014291	0.48159	insignificant	4	18	17
chr2	152657418	152659418	Bcl2l1	-0.0733959	1.26E-16	hypomethylated	0.011324	0.15569	insignificant	11	34	34
chr2	152672699	152674699	Tpx2	-0.19460493	1.29E-11	hypomethylated	0.021964	0.71571	insignificant	8	21	21
chr2	152736087	152738087	Mylk2	-0.4478303	6.72E-08	stronglyHypometh	0.0070186	0.059393	insignificant	5	22	24
chr2	152758944	152760944	Foxs1		1	noCoverage	0.42857	0.50909	insignificant	0	2	2
chr2	152777141	152779141	Dusp15	-0.13459424	2.24E-19	hypomethylated	0.033028	0.86726	insignificant	16	60	60
chr2	152777318	152779318	Dusp15	-0.14438938	9.64E-08	hypomethylated	0.048003	0.37276	insignificant	9	38	38
chr2	152787220	152789220	Ttll9		1	noCoverage	0.12649	0.0055326	hypermethylated	0	20	13
chr2	152841119	152843119	Pdrg1	-0.5372805	0.00023412	stronglyHypometh	0.02232	0.41014	insignificant	2	11	11
chr2	152856587	152858587	Xkr7	-0.1136486	2.19E-17	hypomethylated	0.01513	0.17369	insignificant	26	120	115
chr2	152933203	152935203	Hck	-0.17307507	3.77E-11	hypomethylated	0.024193	0.5269	insignificant	6	38	38
chr2	152986036	152988036	Tm9sf4		1	noCoverage	0.047138	0.70929	insignificant	0	6	6
chr2	153066267	153068267	Pofut1	-0.10913703	5.26E-15	hypomethylated	0.0038495	0.6476	insignificant	29	139	127
chr2	153067094	153069094	Plagl2	-0.11323134	1.57E-10	hypomethylated	0.0017856	0.60347	insignificant	15	79	79
chr2	153116151	153118151	Kif3b	-0.11093167	2.23E-19	hypomethylated	0.01794	0.00084511	hypermethylated	42	108	102
chr2	153170874	153172874	Asxl1	-0.1210067	1.72E-19	hypomethylated	-0.0037983	0.42432	insignificant	38	160	146
chr2	153270215	153272215	8430427H17R	-0.16694956	8.86E-14	hypomethylated	-0.0068217	0.19256	insignificant	27	93	78
chr2	153355707	153357707	8430427H17R	-0.12621822	1.6E-15	hypomethylated	0.0032087	0.32599	insignificant	23	96	100
chr2	153458517	153460517	Commd7	-0.13715557	0.00000215	hypomethylated	-0.0078077	0.68887	insignificant	6	14	15
chr2	153474189	153476189	Dnmt3b	-0.09569919	7.93E-29	hypomethylated	0.00036296	0.23835	insignificant	33	117	117
chr2	153566022	153568022	Mapre1	-0.09630324	5.84E-15	hypomethylated	0.036257	1	insignificant	22	72	78
chr2	153699780	153701780	Bpifb2		1	noCoverage	-0.14762	0.19805	insignificant	0	4	7
chr2	153742965	153744965	Bpifb3	-0.12718938	0.15747	insignificant	-0.01211	0.30863	insignificant	1	6	4
chr2	153955082	153957082	Bpifa3	0.13425926	1	lowCoverage	0.020035	1	insignificant	1	6	4
chr2	154015553	154017553	Bpifb1	-0.24761905	0.20325	insignificant	-0.079658	0.29534	insignificant	2	5	4
chr2	154233820	154235820	Snta1	-0.35154361	5.1E-16	stronglyHypometh	0.040993	0.24176	insignificant	9	48	42
chr2	154261219	154263219	Cbfa2t2	-0.05071761	0.0001231	hypomethylated	-0.0050013	0.72882	insignificant	24	140	135
chr2	154373639	154375639	1700003F12Ri	-0.25304123	0.061891	insignificant	-0.028401	0.00021027	hypomethylated	4	57	50
chr2	154376511	154378511	1700007I08Ril	-0.23994759	0.085512	insignificant	0.036173	0.039489	hypermethylated	4	45	45
chr2	154384589	154386589	E2f1	0.25082166	0.4797	insignificant	0.050422	0.3276	insignificant	2	14	14
chr2	154395588	154397588	E2f1	-0.15803709	1.48E-10	hypomethylated	0.0018937	0.63847	insignificant	18	75	75
chr2	154429409	154431409	Pxmp4	-0.31151707	0.0058738	hypomethylated	0.12956	0.38495	insignificant	2	10	12
chr2	154438103	154440103	Zfp341	-0.10054584	0.00025485	hypomethylated	0.0023133	0.95381	insignificant	38	156	148
chr2	154481761	154483761	Chmp4b	-0.24057834	3E-24	hypomethylated	-0.04816	0.58905	insignificant	23	76	64
chr2	154615845	154617845	Raly	-0.08582427	1.34E-08	hypomethylated	0.005695	0.80609	insignificant	44	143	138
chr2	154718642	154720642	Eif2s2	-0.1696201	0.0013201	hypomethylated	0.012513	0.60208	insignificant	5	38	38
chr2	154900233	154902233	Ahcy	-0.1530196	0.3686	insignificant	0.040632	0.24323	insignificant	5	30	30
chr2	154958216	154960216	Itch	-0.11601817	5.48E-31	hypomethylated	0.0083748	0.64268	insignificant	35	107	101
chr2	155061268	155063268	Dynlrb1	-0.22429657	0.00000246	hypomethylated	0.0042986	0.51721	insignificant	7	42	37
chr2	155101179	155103179	Map1lc3a	-0.15582307	1.76E-40	hypomethylated	0.0001245	0.72962	insignificant	38	148	148
chr2	155206591	155208591	Trp53inp2	-0.18581395	3.2E-19	hypomethylated	-0.015294	0.43939	insignificant	22	66	66
chr2	155340582	155342582	Ggt7	-0.15764068	0.37579	insignificant	0.058815	0.19841	insignificant	3	8	8
chr2	155342778	155344778	Acss2	-0.18070702	7.03E-33	hypomethylated	0.14285	7.58E-39	hypermethylated	22	85	77
chr2	155418442	155420442	Gss	-0.05563576	0.066236	insignificant	0.012281	0.32213	insignificant	5	28	28
chr2	155447615	155449615	Mir499		1	noCoverage	-0.040051	0.58078	insignificant	0	13	12
chr2	155555211	155557211	Edem2		1	noCoverage	-0.061204	0.34226	insignificant	0	12	12
chr2	155575952	155577952	Procr	-0.37765782	0.049673	stronglyHypometh	-0.095209	0.15197	insignificant	4	32	32
chr2	155600079	155602079	Mmp24	-0.10702309	1.43E-37	hypomethylated	0.017287	0.000037019	hypermethylated	54	168	162
chr2	155652661	155654661	Eif6		1	noCoverage	0.23805	0.00000112	hypermethylated	0	7	5
chr2	155756046	155758046	Uqcc		1	noCoverage	0.1625	0.15476	insignificant	0	6	5
chr2	155781293	155783293	Cep250	-0.19374489	2.09E-16	hypomethylated	0.034895	0.000052827	hypermethylated	18	100	82
chr2	155832860	155834860	Ergic3	-0.32283126	1.27E-16	hypomethylated	0.051051	0.55419	insignificant	11	42	42
chr2	155833713	155835713	6430550D23R	-0.35180617	3.76E-17	stronglyHypometh	0.073157	0.61202	insignificant	7	35	34
chr2	155878683	155880683	Fer1l4		1	noCoverage	-0.087383	0.067013	insignificant	0	8	6
chr2	155889948	155891948	Spag4	-0.20840164	9.54E-24	hypomethylated	-0.038079	0.0052002	hypomethylated	17	72	68
chr2	155937701	155939701	Rbm12	-0.12909279	0.00000068	hypomethylated	0.070017	0.76826	insignificant	7	19	21
chr2	155968888	155970888	Romo1	-0.09362097	8.09E-13	hypomethylated	0.0046442	0.8768	insignificant	23	108	103

chr2	155968970	155970970	Romo1	-0.09362097	8.09E-13	hypomethylated	0.0046442	0.8768	insignificant	23	108	103
chr2	155969079	155971079	Romo1	-0.09362097	8.09E-13	hypomethylated	0.0046442	0.8768	insignificant	23	108	103
chr2	155969922	155971922	Nfs1	-0.098024	1.46E-12	hypomethylated	0.0085756	0.839	insignificant	19	74	69
chr2	156005976	156007976	Rbm39	-0.13055639	0.00000109	hypomethylated	0.039121	0.72663	insignificant	7	33	14
chr2	156021382	156023382	Phf20	-0.10201305	1.66E-12	hypomethylated	-0.0060553	0.1996	insignificant	28	86	86
chr2	156137208	156139208	4921517L17Ri	-0.14528063	2.95E-24	hypomethylated	0.020839	0.18531	insignificant	34	97	84
chr2	156138440	156140440	4921517L17Rik		1	noCoverage	0.14583	0.56069	insignificant	0	6	6
chr2	156245787	156247787	Epb4.1l1	-0.0658548	0.060462	insignificant	-0.018227	0.29797	insignificant	21	134	136
chr2	156300572	156302572	Epb4.1l1	-0.06355288	0.00014935	hypomethylated	0.021743	0.89412	insignificant	21	83	93
chr2	156372311	156374311	0610011L14Ri	-0.13539	0.000015796	hypomethylated	0.031378	0.69725	insignificant	12	31	32
chr2	156438440	156440440	Dlgap4	-0.08626311	1.58E-21	hypomethylated	0.018937	0.24841	insignificant	35	178	161
chr2	156545720	156547720	Dlgap4	-0.11977878	1.58E-32	hypomethylated	-0.0049427	0.30356	insignificant	39	116	102
chr2	156546014	156548014	Dlgap4	-0.14105727	9.27E-37	hypomethylated	-0.0051905	0.72378	insignificant	42	124	110
chr2	156600199	156602199	Myl9	-0.26369116	1.31E-18	hypomethylated	0.030621	0.21583	insignificant	17	56	48
chr2	156664812	156666812	Tgif2	-0.08881434	1.94E-11	hypomethylated	0.0038098	0.57962	insignificant	20	131	130
chr2	156687857	156689857	1110008F13Ri	-0.0968149	5.44E-34	hypomethylated	-0.0050324	0.0029067	hypomethylated	55	193	186
chr2	156688681	156690681	5430405H02R	-0.093582	8.81E-25	hypomethylated	-0.02273	0.0361	hypomethylated	41	154	148
chr2	156817847	156819847	Ndrg3	-0.23423443	0.0063641	hypomethylated	-0.01373	0.85698	insignificant	2	22	20
chr2	156832811	156834811	Dsn1	-0.25417576	0.00079297	hypomethylated	0.0098573	0.14846	insignificant	6	22	22
chr2	156905001	156907001	9830001H06R	-0.28199799	7.35E-09	hypomethylated	-0.00010881	0.67897	insignificant	15	49	49
chr2	156911790	156913790	Gm1332	-0.13553114	0.33857	insignificant	0.064469	0.3688	insignificant	1	8	8
chr2	156960958	156962958	Samhd1	-0.77485501	5.53E-15	stronglyHypometh	0.021405	0.91047	insignificant	0	10	11
chr2	157030270	157032270	Rbl1	-0.06581254	0.4078	insignificant	0.023942	0.012767	hypermethylated	18	58	58
chr2	157103833	157105833	Rpn2	-0.11498287	1.29E-40	hypomethylated	-0.0055854	0.36184	insignificant	49	152	139
chr2	157105285	157107285	Rpn2	-0.20512832	0.0021168	hypomethylated	-0.03245	0.90752	insignificant	10	34	33
chr2	157172391	157174391	Ghrh	-0.17641946	8.82E-10	hypomethylated	-0.0044817	0.60073	insignificant	5	30	28
chr2	157192329	157194329	Manbal	-0.11383477	4.25E-19	hypomethylated	0.014677	0.85944	insignificant	14	64	56
chr2	157249028	157251028	Src	-0.08923507	3.13E-17	hypomethylated	0.0019101	0.67863	insignificant	49	122	107
chr2	157384845	157386845	Nnat	-0.15912531	0.62557	insignificant	0.06827	0.00000436	hypermethylated	11	86	83
chr2	157392097	157394097	Blcap		1	noCoverage	-0.036901	0.77517	insignificant	0	19	18
chr2	157562136	157564136	Ctnnbl1	-0.22277145	1.26E-15	hypomethylated	0.0016552	0.27683	insignificant	9	41	36
chr2	157739388	157741388	Vstm2l	-0.12052731	0.00000967	hypomethylated	-0.0039235	0.67768	insignificant	24	142	142
chr2	157853529	157855529	Rprd1b	-0.19368157	8.63E-08	hypomethylated	-0.058814	0.038843	hypomethylated	4	66	64
chr2	158016743	158018743	1700060C20R	-0.0931967	0.62638	insignificant	-0.062055	0.8134	insignificant	2	11	10
chr2	158082976	158084976	Bpi	-0.67987167	0.000158	stronglyHypometh	-0.018932	0.35126	insignificant	2	26	23
chr2	158200373	158202373	Snhg11	-0.38800728	0.51186	insignificant	-0.0091322	0.24666	insignificant	2	9	9
chr2	158234588	158236588	Ralgapb	-0.10023915	2.65E-19	hypomethylated	0.0037886	0.89232	insignificant	43	115	115
chr2	158327347	158329347	Adig	0.06515262	1	insignificant	-0.020381	1	insignificant	4	12	12
chr2	158435493	158437493	Slc32a1	-0.18597361	6.44E-24	hypomethylated	-0.012249	0.79563	insignificant	13	32	32
chr2	158449648	158451648	Actr5	-0.12101909	1.39E-26	hypomethylated	-0.013695	1	insignificant	33	80	72
chr2	158463318	158465318	Mir3474	0.09239513	0.62751	insignificant	0.014467	0.5554	insignificant	2	32	32
chr2	158491468	158493468	Ppp1r16b	-0.19376159	1.59E-15	hypomethylated	0.014496	0.057139	insignificant	16	70	70
chr2	158491870	158493870	Ppp1r16b	-0.12471294	8.9E-20	hypomethylated	0.015739	0.096244	insignificant	29	124	124
chr2	158592834	158594834	Fam83d	-0.1135206	7.55E-10	hypomethylated	0.0011114	0.57163	insignificant	28	103	103
chr2	158619555	158621555	Dhx35	-0.11790685	0.000015677	hypomethylated	-0.026085	0.69971	insignificant	12	62	59
chr2	160192801	160194801	Mafb	-0.12957526	9.54E-09	hypomethylated	0.0016983	0.83459	insignificant	21	129	121
chr2	160470632	160472632	Top1	-0.16652209	6.06E-47	hypomethylated	-0.018934	0.89502	insignificant	42	151	144
chr2	160556045	160558045	Plcg1	-0.08907696	7.1E-10	hypomethylated	-0.014211	0.55362	insignificant	41	191	204
chr2	160698726	160700726	Zhx3	-0.15689181	0.000069177	hypomethylated	-0.071958	0.70164	insignificant	4	26	24
chr2	160705405	160707405	Lpin3		1	noCoverage	-0.2123	0.30346	insignificant	0	7	7
chr2	160712903	160714903	Lpin3	-0.35378386	2.75E-22	stronglyHypometh	0.04606	1	insignificant	3	6	6
chr2	160738064	160740064	Emilin3	-0.08342808	0.00078288	hypomethylated	0.017358	0.0086629	hypermethylated	8	44	44
chr2	160934792	160936792	Chd6	-0.17852348	2E-15	hypomethylated	-0.0075817	0.78946	insignificant	11	53	53
chr2	162485898	162487898	9430021M05F	-0.11375926	2.62E-19	hypomethylated	0.0046619	0.27731	insignificant	52	166	150
chr2	162486883	162488883	Ptprt	-0.11558927	7.68E-11	hypomethylated	-0.006185	0.5851	insignificant	12	50	50
chr2	162756243	162758243	Srsf6	-0.07337034	5.99E-16	hypomethylated	-0.0011411	0.71545	insignificant	56	222	219
chr2	162768200	162770200	L3mbtl1	-0.29174828	1.28E-08	hypomethylated	0.0094471	0.67457	insignificant	13	44	38
chr2	162812216	162814216	Sgk2	0.16666667	1	insignificant	-0.19267	0.67207	insignificant	2	6	6
chr2	162842207	162844207	Ift52	-0.10623695	0.8021	insignificant	0.021527	0.41741	insignificant	15	43	44
chr2	162879370	162881370	Mybl2	-0.09173677	0.00000354	hypomethylated	0.0042701	0.94837	insignificant	24	111	110
chr2	163050189	163052189	Tox2	-0.4207376	0.00000118	stronglyHypometh	-0.040544	1	insignificant	2	14	16
chr2	163223686	163225686	Jph2		1	noCoverage	0.11906	0.8896	insignificant	0	15	13
chr2	163245206	163247206	3230401D17R	-0.1515442	1.24E-10	hypomethylated	-0.0073127	0.78389	insignificant	21	89	92
chr2	163263202	163265202	Gdap1l1	-0.11301197	0.050256	insignificant	0.045968	0.011198	hypermethylated	14	66	65
chr2	163297280	163299280	2310001K24Ri	-0.1091787	0.004382	hypomethylated	0.0036977	0.92453	insignificant	11	46	46
chr2	163317053	163319053	R3hdml	-0.09625	0.37106	insignificant	0.13021	0.0055243	hypermethylated	5	16	16
chr2	163367457	163369457	0610008F07Ri	0.16590909	1	insignificant	0.0099871	0.87703	insignificant	1	4	4
chr2	163371923	163373923	Hnf4a	-0.10519148	0.0026373	hypomethylated	0.023569	0.46591	insignificant	6	16	16
chr2	163427049	163429049	Ttpal	-0.20327649	2.39E-27	hypomethylated	-0.0029986	0.11985	insignificant	20	74	73
chr2	163469585	163471585	0610039K10Ri	-0.10228816	1.43E-22	hypomethylated	-0.016882	0.42083	insignificant	27	118	105
chr2	163470879	163472879	0610039K10Rik		1	noCoverage	0.041695	0.8302	insignificant	0	28	28
chr2	163483121	163485121	Pkig	-0.09783296	3.99E-40	hypomethylated	-0.0051614	0.64833	insignificant	40	88	88
chr2	163483193	163485193	Pkig	-0.09783296	3.99E-40	hypomethylated	-0.0051614	0.64833	insignificant	40	88	88
chr2	163518772	163520772	Pkig	-0.08819876	0.53755	insignificant	-0.022744	0.80162	insignificant	1	4	5
chr2	163575913	163577913	Ada	-0.15118817	4.42E-18	hypomethylated	-0.0082618	0.89282	insignificant	14	53	53
chr2	163678485	163680485	Kcnk15	-0.12227253	2.84E-09	hypomethylated	-0.026261	0.65817	insignificant	26	114	104
chr2	163744419	163746419	Rims4	0.0452581	0.35308	insignificant	-0.027652	0.26505	insignificant	5	70	86
chr2	163819932	163821932	Ywhab	-0.08454486	0.000000037	hypomethylated	0.0094338	0.57429	insignificant	25	122	122
chr2	163896838	163898838	Tomm34	-0.24132513	1.77E-08	hypomethylated	0.013928	0.00041397	hypermethylated	14	36	36
chr2	163898913	163900913	Stk4	-0.16015928	4.64E-16	hypomethylated	0.0038655	0.27382	insignificant	24	59	58
chr2	163996849	163998849	Kcns1		1	noCoverage	0.17389	0.011527	hypermethylated	0	10	10
chr2	164008478	164010478	Wfdc5	-0.22420635	0.21464	insignificant	-0.085228	0.59971	insignificant	1	8	8
chr2	164157500	164159500	Svs5	-0.05298049	0.46154	insignificant	0.023423	0.040011	hypermethylated	3	14	10
chr2	164182243	164184243	Slpi	-0.41157289	0.017686	stronglyHypometh	0.086513	0.6499	insignificant	2	8	10
chr2	164227693	164229693	Rbpjl	-0.2900364	0.000000207	hypomethylated	-0.020653	0.40176	insignificant	6	26	18

chr2	164230114	164232114	Matn4	-0.08846154	1	insignificant	0.026564	0.63467	insignificant	1	4	4
chr2	164268688	164270688	Sdc4	-0.21166084	3.7E-11	hypomethylated	0.013604	0.49448	insignificant	12	40	40
chr2	164285470	164287470	Sys1	-0.14768511	2.71E-52	hypomethylated	0.031265	0.31986	insignificant	58	140	145
chr2	164310639	164312639	Dbndd2	-0.11596649	0.000000476	hypomethylated	0.0085107	0.16439	insignificant	26	120	112
chr2	164310954	164312954	Dbndd2	-0.12792783	0.000000189	hypomethylated	0.0076211	0.060927	insignificant	28	127	119
chr2	164311376	164313376	Dbndd2	-0.13701296	4.53E-08	hypomethylated	0.012375	0.037835	hypermethylated	28	131	123
chr2	164312610	164314610	Dbndd2	-0.36661077	0.53917	insignificant	0.036411	0.32062	insignificant	2	11	11
chr2	164322024	164324024	Pigt	-0.1164938	0.038761	hypomethylated	0.020409	0.85629	insignificant	4	39	36
chr2	164387215	164389215	Wfdc2	-0.2962521	0.0048866	hypomethylated	-0.10511	0.01645	hypomethylated	7	35	23
chr2	164439126	164441126	Wfdc8	0.17708333	1	insignificant	0.1055	0.050458	insignificant	2	4	4
chr2	164509606	164511606	Wfdc13	0.25608511	1	lowCoverage	0.044006	0.34674	insignificant	1	12	12
chr2	164568767	164570767	Dnttip1	0.1962963	1	lowCoverage	0.0095008	0.030998	inconclusive	1	6	6
chr2	164570514	164572514	Dnttip1	-0.12384693	9.57E-13	hypomethylated	-0.005464	0.7687	insignificant	15	47	44
chr2	164594428	164596428	Ube2c	-0.1956547	0.0049557	hypomethylated	-0.0058848	1	insignificant	11	100	107
chr2	164605234	164607234	Tnnc2	0.14947552	1	insignificant	0.10781	1	insignificant	3	13	8
chr2	164610520	164612520	Snx21	-0.12357862	1.95E-24	hypomethylated	-0.017866	0.81293	insignificant	44	143	140
chr2	164629613	164631613	Zswim3	-0.13893071	0.012875	hypomethylated	0.016441	0.84851	insignificant	15	46	44
chr2	164630381	164632381	Acot8	-0.10141986	0.289	insignificant	0.06749	0.33212	insignificant	4	25	22
chr2	164647185	164649185	Zswim1	-0.21132442	0.001105	hypomethylated	0.015	0.51827	insignificant	8	57	57
chr2	164654034	164656034	1700020C07Ri	0.10368046	1	insignificant	0.048956	0.019189	hypermethylated	2	8	9
chr2	164657372	164659372	Ctsa	-0.39467784	0.000012992	stronglyHypometh	0.025059	0.59989	insignificant	3	42	42
chr2	164659096	164661096	Ctsa	-0.27816008	2.7E-09	hypomethylated	-0.013793	0.69734	insignificant	8	39	39
chr2	164703867	164705867	Pcif1	-0.1249273	2.08E-34	hypomethylated	0.011123	0.49873	insignificant	51	164	164
chr2	164737250	164739250	Zfp335	-0.07592728	1	insignificant	0.027813	0.25173	insignificant	6	35	32
chr2	164772750	164774750	Mmp9	-0.2730425	0.41576	insignificant	0.020651	0.19016	insignificant	3	34	32
chr2	164792487	164794487	Slc12a5	-0.12307112	2.61E-13	hypomethylated	-0.003176	0.43776	insignificant	29	127	124
chr2	164860279	164862279	Ncoa5	-0.57902493	0.00084177	stronglyHypometh	0.08328	0.94843	insignificant	6	38	41
chr2	164880135	164882135	Cd40		1	noCoverage	-0.03538	0.91133	insignificant	0	6	8
chr2	164916250	164918250	1700025C18Rik		1	noCoverage	-0.026709	0.7191	insignificant	0	6	5
chr2	165060237	165062237	Cdh22		1	noCoverage	0.0021285	1	insignificant	0	32	30
chr2	165113327	165115327	Elmo2	-0.40578381	0.15557	insignificant	-0.04406	0.20293	insignificant	4	12	12
chr2	165142393	165144393	Elmo2	-0.45614035	0.15203	insignificant	0.086446	0.40674	insignificant	2	4	4
chr2	165151979	165153979	Elmo2	-0.15742555	0.00044594	hypomethylated	-0.0014617	0.87167	insignificant	16	69	61
chr2	165187619	165189619	Zfp663	-0.81878661	0.083602	insignificant	-0.05637	0.12792	insignificant	2	17	15
chr2	165213759	165215759	Zfp334		1	noCoverage	-0.028698	0.39528	insignificant	0	5	4
chr2	165298697	165300697	Slc13a3	0.01192766	1	insignificant	-0.0075124	0.16339	insignificant	4	14	14
chr2	165318814	165320814	2810408M09F	-0.00265911	0.50384	insignificant	0.0013797	0.00027971	inconclusive	12	42	38
chr2	165328477	165330477	Slc2a10	-0.21378635	0.00013871	hypomethylated	-0.037627	0.23885	insignificant	9	32	31
chr2	165479797	165481797	Eya2	-0.31746605	1.88E-17	hypomethylated	0.051454	0.43959	insignificant	4	13	12
chr2	165710188	165712188	Zmynd8		1	noCoverage	0.019746	0.89766	insignificant	0	26	24
chr2	165817136	165819136	Ncoa3	-0.09867278	1.76E-14	hypomethylated	0.012106	0.9367	insignificant	29	166	163
chr2	165981156	165983156	Sulf2	-0.16862463	0.5366	insignificant	-0.011691	0.95793	insignificant	3	30	30
chr2	166098407	166100407	Gm11468	0.1201087	0.74462	insignificant	0.096035	0.81235	insignificant	1	4	4
chr2	166539332	166541332	Prex1	-0.10775131	0.39347	insignificant	0.084269	0.8401	insignificant	8	31	28
chr2	166618266	166620266	Trp53rk	-0.14878665	0.019645	hypomethylated	-0.043394	3.37E-08	inconclusive	13	62	83
chr2	166630080	166632080	Arfgef2	-0.17238562	3.74E-13	hypomethylated	0.014608	0.052208	insignificant	18	62	67
chr2	166730595	166732595	Cse1l	-0.12222758	3.32E-25	hypomethylated	-0.0092216	0.023613	inconclusive	26	78	74
chr2	166821778	166823778	Stau1	-0.10092748	0.014511	hypomethylated	0.0099936	0.59738	insignificant	6	30	28
chr2	166839812	166841812	Ddx27	-0.18032301	1.25E-14	hypomethylated	0.015431	0.72958	insignificant	10	51	38
chr2	166887433	166889433	1500012F01Ri	-0.12276447	3.4E-55	hypomethylated	0.015565	0.00000221	hypermethylated	61	219	201
chr2	166888515	166890515	Snord12	-0.15591967	4.14E-27	hypomethylated	0.027355	0.20483	insignificant	20	94	89
chr2	166889792	166891792	Snord12	-0.40531229	0.12852	insignificant	-0.011082	0.95017	insignificant	2	16	16
chr2	167014299	167016299	Kcnb1	-0.1559856	5.55E-19	hypomethylated	-0.027361	0.21938	insignificant	35	168	150
chr2	167066037	167068037	Ptgis	-0.25546786	0.0007771	hypomethylated	-0.082751	0.68145	insignificant	2	13	12
chr2	167174678	167176678	B4galt5	-0.13394832	0.000000086	hypomethylated	0.047811	0.52779	insignificant	10	86	76
chr2	167246220	167248220	Slc9a8	-0.10617933	1.64E-10	hypomethylated	0.019761	0.7879	insignificant	25	74	75
chr2	167317144	167319144	Rnf114	-0.104761	2.71E-21	hypomethylated	0.014238	0.90695	insignificant	28	110	99
chr2	167318374	167320374	Rnf114	-0.12271668	1.13E-14	hypomethylated	0.019249	0.077206	insignificant	13	61	57
chr2	167362726	167364726	Snai1	-0.10665645	1.13E-08	hypomethylated	-0.015997	0.47465	insignificant	28	114	119
chr2	167457505	167459505	Ube2v1	-0.28611213	1.28E-18	hypomethylated	-0.002999	0.0002302	inconclusive	12	70	70
chr2	167487044	167489044	Tmem189	-0.11204693	1.85E-09	hypomethylated	0.020971	0.58092	insignificant	22	79	74
chr2	167513414	167515414	Cebpb	-0.08503384	2.31E-43	hypomethylated	-0.0017109	0.9823	insignificant	81	308	305
chr2	167515707	167517707	A530013C23Rik		1	noCoverage	-0.040119	0.44095	insignificant	0	14	13
chr2	167756826	167758826	Ptpn1	-0.13144843	4.23E-19	hypomethylated	0.009762	0.051673	insignificant	39	147	149
chr2	167836093	167838093	Fam65c		1	noCoverage	-0.013462	1	insignificant	0	4	4
chr2	167905503	167907503	Pard6b	-0.12205486	5.2E-36	hypomethylated	-0.0036955	0.83472	insignificant	44	121	118
chr2	168032562	168034562	Adnp		1	noCoverage	0.036848	0.06408	insignificant	0	9	8
chr2	168055121	168057121	Mocs3	-0.05878332	1.32E-22	hypomethylated	-0.006543	0.66489	insignificant	90	346	331
chr2	168055879	168057879	Dpm1	-0.05295211	1.48E-12	hypomethylated	-0.02028	0.44114	insignificant	77	298	283
chr2	168094831	168096831	Kcng1	-0.16522727	0.011002	hypomethylated	-0.22768	0.00048574	hypomethylated	5	16	13
chr2	168415691	168417691	Nfatc2	-0.11046819	4.99E-08	hypomethylated	0.00055564	0.85469	insignificant	7	60	60
chr2	168415783	168417783	Nfatc2	-0.13546827	0.000000301	hypomethylated	0.00013556	0.84522	insignificant	3	48	48
chr2	168415848	168417848	Nfatc2	-0.17032836	1.56E-09	hypomethylated	0.00094338	0.6619	insignificant	3	30	30
chr2	168567300	168569300	Atp9a	-0.09703556	1.2E-17	hypomethylated	-0.0025234	0.43364	insignificant	31	110	105
chr2	168592701	168594701	Sall4	-0.17953486	0.17253	insignificant	0.047301	0.11783	insignificant	4	14	14
chr2	168781087	168783087	Zfp64		1	noCoverage	-0.011865	0.56943	insignificant	0	10	10
chr2	169458145	169460145	Tshz2		1	noCoverage	0.026351	0.84815	insignificant	0	28	21
chr2	169956720	169958720	Zfp217	-0.09834735	0.064398	insignificant	0.014296	0.36035	insignificant	15	57	51
chr2	169968175	169970175	Zfp217	0.06702723	1	insignificant	0.02442	0.11374	insignificant	3	19	21
chr2	170253345	170255345	Bcas1	0.28414138	0.34223	insignificant	0.049453	0.12229	insignificant	2	14	14
chr2	170320927	170322927	Pfdn4	-0.0930746	0.000023058	hypomethylated	-0.01807	0.68281	insignificant	13	66	60
chr2	170322638	170324638	Cyp24a1	-0.16259424	7.67E-09	hypomethylated	-0.0031556	0.3272	insignificant	4	43	43
chr2	170335729	170337729	Pfdn4	-0.11605207	1.78E-10	hypomethylated	-0.011203	0.6867	insignificant	21	90	79
chr2	170556306	170558306	Dok5	-0.09366138	0.00089232	hypomethylated	-0.0094227	0.82117	insignificant	8	77	78
chr2	172072991	172074991	Mc3r	-0.94353922	2.35E-49	stronglyHypometh	-0.077718	0.57818	insignificant	5	8	6

chr2	172170076	172172076	2010011I20Ril	-0.15675149	2.58E-28	hypomethylated	-0.0046924	0.83182	insignificant	41	111	116
chr2	172195502	172197502	Cstf1	-0.13041792	1.56E-10	hypomethylated	-0.0082701	0.61152	insignificant	15	118	119
chr2	172196006	172198006	Aurka	-0.12460685	3.62E-09	hypomethylated	-0.010833	0.59149	insignificant	14	104	105
chr2	172265077	172267077	2410001C21Ri	-0.11879544	4.28E-11	hypomethylated	0.012126	1	insignificant	16	56	55
chr2	172297053	172299053	1700029J11Ri	0.07213218	0.75496	insignificant	-0.034246	0.68573	insignificant	5	16	16
chr2	172374092	172376092	Tfap2c	-0.08975981	2.92E-08	hypomethylated	0.00058183	0.83085	insignificant	21	81	79
chr2	172375490	172377490	Tfap2c	-0.15788549	1.61E-19	hypomethylated	-0.0038888	0.54024	insignificant	32	134	141
chr2	172765794	172767794	Bmp7	-0.18032057	3.51E-08	hypomethylated	0.021284	0.22941	insignificant	16	67	59
chr2	172804342	172806342	Spo11	-0.14810384	0.41289	insignificant	-0.0078272	0.93328	insignificant	7	40	40
chr2	172824637	172826637	Rae1	-0.09292311	5.7E-16	hypomethylated	0.01217	0.026446	hypermethylated	34	152	134
chr2	172846402	172848402	Rbm38	-0.0845671	8.95E-17	hypomethylated	0.012224	0.010917	inconclusive	38	127	136
chr2	172977573	172979573	Pck1	-0.12967154	0.38327	insignificant	0.0098102	0.2142	insignificant	4	16	16
chr2	173044423	173046423	Zbp1	-0.2654321	0.54019	insignificant	0.068609	0.81845	insignificant	2	12	12
chr2	173102034	173104034	Pmepa1	-0.08101076	0.00000011	hypomethylated	-0.010026	0.27142	insignificant	19	58	56
chr2	173347092	173349092	1700021F07Ri	0.01295583	0.19957	insignificant	-0.01544	0.49918	insignificant	6	22	25
chr2	173484345	173486345	Rab22a	-0.12501836	2.38E-20	hypomethylated	0.0016922	0.88952	insignificant	25	76	76
chr2	173485040	173487040	Ppp4r1l-ps	-0.2105298	1.24E-40	hypomethylated	0.013743	0.17704	insignificant	25	68	68
chr2	173562071	173564071	Vapb	-0.11370567	6.1E-21	hypomethylated	-0.014971	0.83022	insignificant	27	82	91
chr2	173901551	173903551	Stx16	-0.17950023	3.78E-19	hypomethylated	0.015167	0.47695	insignificant	17	52	54
chr2	173934851	173936851	Npepl1	-0.12224925	2.75E-24	hypomethylated	0.020496	0.17981	insignificant	41	136	121
chr2	174092626	174094626	Mir296		1	noCoverage	-0.012629	0.60057	insignificant	0	12	12
chr2	174108820	174110820	Gnas	-0.25243379	1.12E-17	hypomethylated	-0.061071	0.10047	insignificant	29	84	84
chr2	174120937	174122937	Gnas	-0.09060401	1	insignificant	0.010274	0.25604	insignificant	3	33	33
chr2	174122359	174124359	Gnas	0.02925324	0.25712	insignificant	-0.053628	0.027061	hypomethylated	14	75	79
chr2	174152414	174154414	Gnas	-0.01669923	0.45869	insignificant	0.019004	0.24481	insignificant	26	92	88
chr2	174154589	174156589	Gnas	-0.09491631	1.91E-25	hypomethylated	0.0043392	0.11782	insignificant	50	249	249
chr2	174154592	174156592	Gnas	-0.09491631	1.91E-25	hypomethylated	0.0043392	0.11782	insignificant	50	249	249
chr2	174240304	174242304	Th1l	-0.18355647	9.76E-13	hypomethylated	0.025758	0.30128	insignificant	14	66	56
chr2	174264493	174266493	Ctsz	-0.2035996	0.000019897	hypomethylated	0.032983	0.22332	insignificant	1	11	11
chr2	174289602	174291602	Atp5e	-0.13875246	2.68E-09	hypomethylated	0.014863	0.71645	insignificant	8	24	20
chr2	174298442	174300442	Slmo2	-0.11656165	0.024404	hypomethylated	0.0095747	0.7794	insignificant	3	20	20
chr2	174468034	174470034	Zfp831		1	noCoverage	0.0092971	0.28729	insignificant	0	7	6
chr2	174585273	174587273	Edn3	-0.10466796	1.35E-10	hypomethylated	0.038981	0.19539	insignificant	10	46	46
chr2	174893264	174895264	Gm14393	-0.34648265	0.41779	insignificant	-0.0031493	1	insignificant	1	7	6
chr2	177248446	177250446	Gm14420		1	noCoverage	-0.015201	0.5892	insignificant	0	25	28
chr2	177281930	177283930	Gm14403	-0.17235333	0.075594	insignificant	0.043243	0.072166	insignificant	2	19	19
chr2	177756988	177758988	Etohi1	-0.20261243	0.066168	insignificant	-0.02633	0.73938	insignificant	9	28	28
chr2	177852679	177854679	Phactr3	-0.19291732	1.78E-17	hypomethylated	0.051187	0.056218	insignificant	17	64	65
chr2	177875637	177877637	Phactr3	-0.25803596	0.0001352	hypomethylated	-0.06277	0.29625	insignificant	11	25	22
chr2	178148238	178150238	9030418K01Ri	-0.0818573	0.1039	insignificant	0.030153	0.50579	insignificant	37	139	136
chr2	178149177	178151177	9030418K01Ri	-0.14987248	0.000000148	hypomethylated	-0.025444	0.95414	insignificant	14	40	39
chr2	179176182	179178182	Cdh4	-0.09941196	8.31E-40	hypomethylated	0.00037681	0.83456	insignificant	49	202	196
chr2	179710557	179712557	4921531C22Ri	-0.09480253	3.73E-36	hypomethylated	0.0022244	0.2821	insignificant	69	221	202
chr2	179711351	179713351	Taf4a	-0.13746013	1.16E-18	hypomethylated	0.015238	0.31295	insignificant	22	50	50
chr2	179758691	179760691	Lsm14b	-0.08007828	1.68E-10	hypomethylated	-0.0074126	0.81184	insignificant	40	188	194
chr2	179776187	179778187	Ss18l1	-0.10240527	4.62E-36	hypomethylated	0.0040911	0.80806	insignificant	63	260	266
chr2	179777107	179779107	Psma7	-0.08271299	3.62E-09	hypomethylated	0.0077931	0.56877	insignificant	35	134	145
chr2	179804297	179806297	Gtpbp5	-0.14346855	0.0067225	hypomethylated	0.0067298	0.67802	insignificant	9	62	64
chr2	179838927	179840927	Hrh3	-0.21455348	0.000074736	hypomethylated	-0.0028957	0.53456	insignificant	6	42	42
chr2	179853070	179855070	Osbpl2	-0.04513696	0.088133	insignificant	-0.0033445	0.64422	insignificant	24	123	128
chr2	179905292	179907292	Adrm1	-0.11038982	1.13E-38	hypomethylated	-0.0084382	0.3977	insignificant	51	157	150
chr2	179960564	179962564	Lama5	-0.14115312	0.069462	insignificant	0.011246	0.66192	insignificant	15	63	60
chr2	179991083	179993083	Rps21	-0.16086855	0.00020174	hypomethylated	0.028146	0.5165	insignificant	22	79	70
chr2	179991240	179993240	Mir3091	-0.16086855	0.00020174	hypomethylated	0.028146	0.5165	insignificant	22	79	70
chr2	180008170	180010170	Cables2	-0.39351304	0.000013376	stronglyHypometh	-0.024504	0.027938	inconclusive	1	26	28
chr2	180024584	180026584	BC066135	0.2229266	0.014027	hypermethylated	0.043496	0.12368	insignificant	4	14	14
chr2	180069384	180071384	Gata5	-0.10599825	0.002123	hypomethylated	-0.010274	0.1723	insignificant	13	42	42
chr2	180194682	180196682	Slco4a1		1	noCoverage	0.12008	0.17839	insignificant	0	9	8
chr2	180233680	180235680	Ntsr1	-0.23810801	2.58E-08	hypomethylated	-0.022022	0.53092	insignificant	11	60	58
chr2	180323111	180325111	Ogfr	-0.1627333	1.34E-09	hypomethylated	0.012976	0.55279	insignificant	10	87	72
chr2	180331926	180333926	Col9a3	0.45435272	0.058847	insignificant	-0.014452	0.91084	insignificant	6	50	50
chr2	180377396	180379396	Tcfl5	0.11576355	1	insignificant	-0.0064587	0.40703	insignificant	0	1	1
chr2	180436805	180438805	Dido1	-0.21046136	1.74E-13	hypomethylated	-0.000024925	0.029808	hypomethylated	16	34	34
chr2	180444704	180446704	2310003C23Ri	-0.14091549	2.73E-09	hypomethylated	0.012638	0.94554	insignificant	26	113	119
chr2	180446250	180448250	2310003C23Rik		1	noCoverage	-0.047022	0.61999	insignificant	0	10	14
chr2	180511605	180513605	Bhlhe23	-0.23857759	0.0072694	hypomethylated	-0.012062	0.052684	insignificant	3	16	14
chr2	180627744	180629744	Mir124a-3	-0.12288607	7.89E-39	hypomethylated	-0.0010212	0.74762	insignificant	67	193	184
chr2	180655641	180657641	Ythdf1	-0.09164777	0.000017878	hypomethylated	0.00086567	0.94432	insignificant	16	40	40
chr2	180689404	180691404	Nkain4	0.28282828	1	lowCoverage	0.065769	0.23697	insignificant	1	4	4
chr2	180700929	180702929	Arfgap1	-0.17262791	7.98E-32	hypomethylated	-0.031888	0.099993	insignificant	26	110	112
chr2	180701004	180703004	Arfgap1	-0.17262791	7.98E-32	hypomethylated	-0.031888	0.099993	insignificant	26	110	112
chr2	180773882	180775882	Chrna4	-0.00721595	0.00017137	hypomethylated	0.00033065	0.55859	insignificant	9	42	42
chr2	180869930	180871930	Kcnq2	-0.16559947	0.00097647	hypomethylated	0.024516	0.38655	insignificant	7	25	18
chr2	180921047	180923047	Ppdpf	-0.14096689	1	insignificant	0.023122	0.59916	insignificant	4	43	39
chr2	180937494	180939494	Ptk6	0.23553827	0.0010935	hypermethylated	0.11156	0.00000455	hypermethylated	6	21	20
chr2	180953720	180955720	BC051628	-0.17726132	0.077753	insignificant	0.0097246	0.7714	insignificant	2	19	19
chr2	180976732	180978732	BC006779		1	noCoverage	0.26678	0.010179	hypermethylated	0	6	5
chr2	181022671	181024671	Gmeb2	-0.2536834	0.12803	insignificant	0.054997	0.000010459	hypermethylated	7	18	18
chr2	181049205	181051205	Stmn3	-0.39285714	0.047931	stronglyHypometh	0.13199	0.0060333	hypermethylated	1	4	4
chr2	181053428	181055428	Rtel1	-0.07240712	0.12213	insignificant	0.063766	0.12905	insignificant	10	53	55
chr2	181054510	181056510	Rtel1	-0.17862349	1	insignificant	0.052306	0.72434	insignificant	3	22	24
chr2	181098635	181100635	Zgpat	-0.11517221	1.52E-08	hypomethylated	-0.018394	0.34769	insignificant	23	77	81
chr2	181099127	181101127	Zgpat	-0.12428684	1.65E-11	hypomethylated	-0.004956	0.63872	insignificant	27	91	96
chr2	181100109	181102109	Arfrp1	-0.17073371	0.0000051	hypomethylated	0.010687	0.88851	insignificant	13	54	59
chr2	181114939	181116939	Lime1		1	noCoverage	-0.026839	0.66746	insignificant	0	6	6

chr2	181194131	181196131	Zbtb46	-0.13060764	3.42E-13	hypomethylated	0.0141	0.73895	insignificant	30	105	96
chr2	181226910	181228910	Abhd16b	0.05995434	0.62446	insignificant	0.0050191	0.68757	insignificant	5	19	20
chr2	181230957	181232957	Tpd52l2		1	noCoverage	-0.018915	0.92601	insignificant	0	17	17
chr2	181254209	181256209	Dnajc5	-0.10144731	0.000000887	hypomethylated	-0.011891	0.90246	insignificant	29	97	96
chr2	181316678	181318678	Uckl1		1	noCoverage	-0.0074511	0.33123	insignificant	0	17	14
chr2	181327166	181329166	Znf512b	-0.0840157	0.00000133	hypomethylated	-0.0090947	0.013634	hypomethylated	24	100	100
chr2	181333852	181335852	Samd10	-0.05520332	1	insignificant	-0.013462	0.76927	insignificant	12	32	32
chr2	181335023	181337023	Prpf6	-0.04027995	0.71794	insignificant	-0.0042897	0.90705	insignificant	19	76	76
chr2	181406345	181408345	Sox18	-0.21674507	8.09E-14	hypomethylated	0.0076556	0.23219	insignificant	3	16	16
chr2	181414014	181416014	Tcea2	-0.10960652	4.14E-12	hypomethylated	0.00056321	0.97297	insignificant	21	97	97
chr2	181428629	181430629	Rgs19	-0.14300973	7.35E-10	hypomethylated	0.01016	0.83073	insignificant	11	42	42
chr2	181571607	181573607	Pcmtd2		1	noCoverage	-0.029582	0.8315	insignificant	0	6	6
chr2	181598064	181600064	Polr3k	-0.16578536	0.000011714	hypomethylated	-0.056308	0.2475	insignificant	4	26	24
chr3	3507029	3509029	Hnf4g	-0.19756898	0.00003318	hypomethylated	-0.034086	0.67578	insignificant	7	20	20
chr3	5217553	5219553	Zfhx4	-0.16345053	0.0050985	hypomethylated	-0.0048682	0.10609	insignificant	9	43	49
chr3	5576150	5578150	Pxmp3	-0.14411556	0.00000105	hypomethylated	-0.015274	0.49725	insignificant	14	44	44
chr3	5576151	5578151	Pxmp3	-0.14411556	0.00000105	hypomethylated	-0.015274	0.49725	insignificant	14	44	44
chr3	5576239	5578239	Pxmp3	-0.07944812	0.024661	hypomethylated	-0.0023806	0.14251	insignificant	10	36	36
chr3	7365603	7367603	Pkia	-0.11572671	0.0000092	hypomethylated	-0.020922	0.90101	insignificant	12	65	63
chr3	7502425	7504425	Fam164a	-0.13630492	1.94E-15	hypomethylated	0.0083931	0.073595	insignificant	27	73	70
chr3	7613427	7615427	Il7	-0.40438871	0.00016027	stronglyHypometh	-0.028515	0.41585	insignificant	0	0	0
chr3	8463098	8465098	Gm6194		1	noCoverage	0.091667	0.3182	insignificant	0	4	4
chr3	8508526	8510526	Stmn2	-0.20688889	1	lowCoverage	0.11331	0.66883	insignificant	1	15	15
chr3	8667038	8669038	Hey1	-0.09064075	9.06E-23	hypomethylated	0.0018169	0.96019	insignificant	41	114	99
chr3	8923857	8925857	Mrps28	-0.24060194	0.031983	hypomethylated	-0.054204	0.061816	insignificant	3	24	22
chr3	8964054	8966054	Tpd52	-0.32929293	0.000027792	hypomethylated	-0.012218	0.061131	insignificant	3	10	10
chr3	9004515	9006515	Tpd52	0.48511905	0.4902	lowCoverage	0.080648	0.04283	hypermethylated	1	6	7
chr3	9249566	9251566	Zbtb10	-0.10668518	1.71E-15	hypomethylated	-0.032084	0.59407	insignificant	57	272	255
chr3	9402077	9404077	C030034L19Rik		1	noCoverage	-0.0060764	1	insignificant	0	8	8
chr3	9610085	9612085	Zfp704	-0.21273022	7.98E-11	hypomethylated	0.02157	0.16989	insignificant	6	16	18
chr3	9833679	9835679	Pag1	0.07536765	1	insignificant	-0.052757	0.52606	insignificant	1	4	4
chr3	10011605	10013605	Fabp5	-0.10144338	0.000000902	hypomethylated	-0.0041453	0.81056	insignificant	6	70	73
chr3	10331439	10333439	Impa1	-0.15340909	1	insignificant	-0.0027597	0.23632	insignificant	3	6	6
chr3	10351301	10353301	Zfand1	-0.1107268	0.15371	insignificant	-0.03448	0.21842	insignificant	8	39	28
chr3	10365972	10367972	Chmp4c	-0.31375334	1	lowCoverage	-0.00047495	0.90055	insignificant	1	8	8
chr3	10440124	10442124	Snx16	-0.27851038	0.000034558	hypomethylated	-0.061009	0.92885	insignificant	4	13	12
chr3	13470654	13472654	Ralyl	-0.07175781	0.35053	insignificant	0.0034827	0.39481	insignificant	4	35	30
chr3	14532787	14534787	Lrrcc1	-0.18636998	7.17E-11	hypomethylated	0.020157	0.12169	insignificant	9	40	37
chr3	14577670	14579670	E2f5	-0.05456315	0.40879	insignificant	0.0067688	0.088435	insignificant	12	86	86
chr3	14611256	14613256	1810022K09Ri	-0.11493018	3.88E-08	hypomethylated	0.0067654	0.42789	insignificant	16	64	64
chr3	14640726	14642726	Car13	0.0338133	1.06E-09	hypermethylated	0.023474	0.25957	insignificant	3	40	40
chr3	14862537	14864537	Car3	-0.10534181	6.92E-09	hypomethylated	0.029269	0.26684	insignificant	10	44	44
chr3	14885425	14887425	Car2	-0.10643564	6.2E-31	hypomethylated	0.0059932	0.56601	insignificant	40	129	123
chr3	16082182	16084182	Ythdf3	-0.09783665	1.94E-14	hypomethylated	-0.0031106	0.49814	insignificant	23	114	105
chr3	17694661	17696661	Mir124a-2	-0.15792427	0.13226	insignificant	0.0016986	0.78883	insignificant	5	31	31
chr3	17847443	17849443	Cypt12	0.04995331	0.69697	insignificant	0.13879	0.313	insignificant	2	15	15
chr3	17953324	17955324	Bhlhe22	-0.09162865	1.79E-26	hypomethylated	-0.00081078	0.28714	insignificant	46	135	128
chr3	19087243	19089243	Mtfr1	-0.13350118	1.33E-26	hypomethylated	0.00079948	0.81416	insignificant	15	73	71
chr3	19211322	19213322	Pde7a	-0.08071814	4.92E-25	hypomethylated	0.0044136	0.22433	insignificant	44	114	116
chr3	19430246	19432246	Dnajc5b	-0.02651515	0.0057238	hypomethylated	0.19574	0.59013	insignificant	4	11	8
chr3	19595396	19597396	Crh	-0.20467172	0.31703	insignificant	0.17127	0.0089011	hypermethylated	2	4	4
chr3	19793870	19795870	4632415L05Ri	-0.1543445	4.07E-11	hypomethylated	0.0064938	0.24936	insignificant	14	61	61
chr3	19935310	19937310	Hps3	-0.29339722	0.34583	insignificant	0.020268	0.41692	insignificant	6	21	20
chr3	19956810	19958810	Hltf	-0.13020673	3.17E-14	hypomethylated	0.016227	0.42337	insignificant	20	64	67
chr3	20054995	20056995	Gyg	-0.14873285	0.054436	insignificant	-0.05815	0.83582	insignificant	4	14	24
chr3	21974573	21976573	Tbl1xr1	-0.09607675	2.89E-21	hypomethylated	0.0019594	0.042181	hypermethylated	73	236	239
chr3	22148862	22150862	Rprl2	0.04137807	1	insignificant	0.1439	0.066487	insignificant	2	11	8
chr3	26052229	26054229	Nlgn1	-0.21739681	0.019419	hypomethylated	-0.02268	0.39751	insignificant	5	13	12
chr3	26230831	26232831	Nlgn1	-0.20595641	0.30265	insignificant	0.092033	0.65568	insignificant	2	12	15
chr3	27052776	27054776	Ect2	-0.10748717	0.6098	insignificant	-0.011432	0.10445	insignificant	6	11	11
chr3	27052800	27054800	Ect2	-0.10748717	0.6098	insignificant	-0.011432	0.10445	insignificant	5	11	11
chr3	27080925	27082925	Nceh1	-0.19063377	2.09E-17	hypomethylated	0.0194	0.77268	insignificant	11	64	65
chr3	27269272	27271272	Ghsr	-0.14893972	0.12733	insignificant	-0.043393	0.77516	insignificant	5	57	66
chr3	27609361	27611361	Fndc3b	-0.10986071	5.83E-31	hypomethylated	-0.0065449	0.90799	insignificant	49	135	135
chr3	27836601	27838601	Pld1	-0.12470983	6.52E-14	hypomethylated	-0.0016699	0.2	insignificant	17	78	78
chr3	27882092	27884092	Pld1	-0.56666667	1	lowCoverage	0.058333	0.72253	insignificant	1	4	4
chr3	28161135	28163135	Tnik	-0.16184719	2.28E-28	hypomethylated	0.0057486	0.6808	insignificant	15	101	97
chr3	28679232	28681232	Eif5a2	-0.17980463	1.29E-33	hypomethylated	0.033351	0.050272	insignificant	45	119	123
chr3	28703432	28705432	Rpl22l1	-0.08213392	4.3E-19	hypomethylated	0.0051027	0.14003	insignificant	15	85	86
chr3	28980498	28982498	Egfem1	-0.13937678	2.71E-26	hypomethylated	0.012214	0.026707	hypermethylated	31	126	129
chr3	29314744	29316744	Mir551b	0.0702381	0.60835	insignificant	0.096429	0.48969	insignificant	3	10	10
chr3	29912126	29914126	Mecom	-0.5	0.24786	insignificant	0.071429	0.31757	insignificant	1	2	2
chr3	30408409	30410409	Mecom	-0.31855685	7.94E-11	hypomethylated	-0.097855	0.47295	insignificant	5	15	14
chr3	30498792	30500792	Mynn	-0.22596535	0.0011876	hypomethylated	0.0051525	0.52909	insignificant	5	44	46
chr3	30500008	30502008	Mynn	-0.17327746	9E-17	hypomethylated	0.0067465	0.75105	insignificant	29	113	114
chr3	30546740	30548740	Lrrc34	-0.45509058	0.54185	insignificant	-0.11603	0.61576	insignificant	2	17	14
chr3	30690797	30692797	Sec62	-0.09228678	3.82E-14	hypomethylated	0.0085817	0.70819	insignificant	47	156	152
chr3	30753871	30755871	Gpr160	-0.09288455	0.000000534	hypomethylated	0.016659	0.22313	insignificant	20	72	79
chr3	30893692	30895692	Prkci	-0.12430581	0.0001018	hypomethylated	-0.0041842	0.6184	insignificant	15	128	120
chr3	30992982	30994982	Skil	-0.07947696	1.42E-47	hypomethylated	-0.00071915	0.17704	insignificant	103	318	333
chr3	31047841	31049841	Cldn11	-0.11073036	0.000000431	hypomethylated	-0.0087545	0.46921	insignificant	12	62	62
chr3	31209241	31211241	Slc7a14	-0.42361111	0.096258	insignificant	-0.078443	0.51204	insignificant	1	8	8
chr3	31800624	31802624	Kcnmb2	0.01007315	0.27255	insignificant	0.044551	0.054322	insignificant	3	20	20
chr3	32263410	32265410	4930429B21R	-0.19226769	2.08E-30	hypomethylated	-0.0053599	0.10212	insignificant	47	158	157
chr3	32264587	32266587	4930429B21R	-0.38632913	2.64E-28	stronglyHypometh	-0.029638	0.2598	insignificant	18	63	62

chr3	32390891	32392891	Kcnmb3	0.15277778	0.57977	insignificant	0.097222	0.78367	insignificant	2	4	4
chr3	32408471	32410471	Zfp639	-0.15706862	7.32E-37	hypomethylated	0.0025931	0.81622	insignificant	29	81	79
chr3	32408513	32410513	Zfp639	-0.15513813	7.31E-37	hypomethylated	0.0045236	0.81622	insignificant	29	82	79
chr3	32427403	32429403	Mfn1	-0.02436865	1.48E-27	hypomethylated	0.026962	0.76521	insignificant	16	48	48
chr3	32515457	32517457	Gnb4	-0.29148978	0.00000518	hypomethylated	-0.044576	0.063849	insignificant	14	42	34
chr3	32606467	32608467	Actl6a	-0.13487807	9.69E-21	hypomethylated	-0.0063127	0.93366	insignificant	31	97	93
chr3	32715547	32717547	Usp13	-0.09473886	3.66E-17	hypomethylated	0.025278	0.97825	insignificant	30	116	112
chr3	33042004	33044004	Pex5l	-0.13690754	5.29E-21	hypomethylated	-0.003898	0.055583	insignificant	24	70	70
chr3	33698116	33700116	Ttc14	-0.07803031	1.18E-12	hypomethylated	-0.0065196	0.32399	insignificant	42	121	114
chr3	33918000	33920000	Fxr1	-0.08033681	5.25E-27	hypomethylated	0.011496	0.16437	insignificant	51	206	184
chr3	33980248	33982248	Dnajc19	-0.17954903	0.69978	insignificant	-0.058211	0.29829	insignificant	3	12	10
chr3	34536385	34538385	Mir1897		1	noCoverage	0.11882	0.032928	hypermethylated	0	12	10
chr3	34547926	34549926	Sox2	-0.07855858	0.00000288	hypomethylated	0.0089546	0.057742	insignificant	49	157	149
chr3	35652059	35654059	Atp11b	-0.0435573	8.7E-13	hypomethylated	0.0076124	0.58026	insignificant	18	101	100
chr3	35828996	35830996	Dcun1d1		1	noCoverage	-0.046485	0.82385	insignificant	0	11	16
chr3	35897834	35899834	A330050B17R	-0.38723369	0.001292	stronglyHypometh	-0.010144	0.60294	insignificant	1	18	18
chr3	35899600	35901600	Mccc1	0.06006494	0.043712	inconclusive	-0.0074194	0.057661	insignificant	2	10	9
chr3	35963921	35965921	Acad9	-0.06838767	0.044474	hypomethylated	0.0088693	0.35808	insignificant	5	55	48
chr3	36049001	36051001	D3Ertd254e	-0.19523641	1.5E-31	hypomethylated	0.00057438	0.66322	insignificant	27	59	59
chr3	36450527	36452527	Exosc9	-0.08790598	0.051855	insignificant	-0.015287	0.3971	insignificant	10	26	24
chr3	36470918	36472918	Ccna2	-0.13851039	0.37286	insignificant	0.011193	0.027355	hypermethylated	13	40	42
chr3	36512311	36514311	Bbs7	-0.47080578	4E-14	stronglyHypometh	-0.047482	0.14069	insignificant	9	28	26
chr3	36589089	36591089	Trpc3		1	noCoverage	-0.031082	1	insignificant	0	8	8
chr3	36761027	36763027	4932438A13R	-0.10501715	3.02E-08	hypomethylated	-0.015138	0.89587	insignificant	26	85	94
chr3	36961577	36963577	Adad1	-0.3854867	0.000039842	stronglyHypometh	-0.0089314	0.069101	insignificant	18	88	88
chr3	37210475	37212475	Bbs12	-0.10384467	0.0011858	hypomethylated	-0.0022868	0.018112	hypomethylated	9	22	22
chr3	37211368	37213368	Cetn4	-0.10384467	0.0011858	hypomethylated	-0.0022868	0.018112	hypomethylated	9	22	22
chr3	37246574	37248574	Fgf2	-0.11207097	4.22E-25	hypomethylated	0.020138	0.12908	insignificant	33	88	89
chr3	37318201	37320201	Spata5	-0.10334981	2.4E-18	hypomethylated	0.011377	0.79057	insignificant	37	189	172
chr3	37318512	37320512	Spata5	-0.10932745	3.4E-15	hypomethylated	0.0060776	0.60035	insignificant	25	145	135
chr3	37537871	37539871	Spry1	-0.09312469	5.52E-36	hypomethylated	0.0075598	0.20785	insignificant	71	193	173
chr3	38383738	38385738	Ankrd50	-0.14041667	0.00071135	hypomethylated	0.010445	0.88776	insignificant	4	8	8
chr3	38784861	38786861	Fat4	-0.12323216	7.36E-29	hypomethylated	0.007406	0.044681	hypermethylated	48	193	193
chr3	40438688	40440688	Intu	-0.09857105	0.00000265	hypomethylated	-0.0034703	0.78642	insignificant	11	54	54
chr3	40511792	40513792	Slc25a31	-0.04085007	0.013401	hypomethylated	0.012634	0.42844	insignificant	10	59	54
chr3	40548534	40550534	Hspa4l	-0.08245658	4.36E-09	hypomethylated	0.0072619	0.070128	insignificant	19	110	123
chr3	40602872	40604872	Plk4	-0.09848956	1.48E-32	hypomethylated	-0.00099186	0.3963	insignificant	32	114	114
chr3	40650776	40652776	Gm2011		1	noCoverage	0.047807	0.74347	insignificant	0	4	4
chr3	40697198	40699198	3110057O12R	-0.13343917	0.000030463	hypomethylated	-0.0087673	0.65008	insignificant	18	69	69
chr3	40753552	40755552	Larp1b	-0.09006428	6E-27	hypomethylated	-0.022919	0.41736	insignificant	77	269	260
chr3	40886968	40888968	Pgrmc2	-0.11993505	2.84E-11	hypomethylated	0.02188	0.85843	insignificant	12	67	49
chr3	41366304	41368304	Phf17	-0.13896597	0.000000251	hypomethylated	0.089116	0.52366	insignificant	8	41	26
chr3	41366672	41368672	Phf17	-0.14213875	0.00000933	hypomethylated	0.062389	0.254	insignificant	8	49	34
chr3	41367801	41369801	Phf17	-0.09202155	1.18E-30	hypomethylated	-0.0010452	0.45482	insignificant	58	213	219
chr3	41545539	41547539	D3Ertd751e	-0.10499839	4.31E-18	hypomethylated	0.0013409	0.68652	insignificant	30	74	74
chr3	45181319	45183319	Pcdh10	-0.12217422	0.32076	insignificant	0.019788	0.070443	insignificant	3	47	46
chr3	46251863	46253863	Pabpc4l	-0.18493512	2.87E-09	hypomethylated	0.037795	0.22276	insignificant	10	32	35
chr3	48413022	48415022	1700018B24R	0.11904762	1	insignificant	0.035714	0.83507	insignificant	2	4	4
chr3	49561238	49563238	Pcdh18	-0.09155511	1	insignificant	-0.0079418	0.31041	insignificant	5	37	40
chr3	51027368	51029368	Ccrn4l	-0.09196283	1.57E-15	hypomethylated	-0.0049008	0.49424	insignificant	54	188	175
chr3	51200469	51202469	4930583H14R	-0.16959284	0.21256	insignificant	0.06539	0.54882	insignificant	3	14	16
chr3	51212877	51214877	Ndufc1	-0.09659995	0.0048078	hypomethylated	-0.0086757	0.34156	insignificant	6	22	22
chr3	51218937	51220937	Naa15	-0.11937225	1.78E-21	hypomethylated	0.007741	0.22615	insignificant	55	192	170
chr3	51286887	51288887	Rab33b	-0.09086899	1.82E-18	hypomethylated	0.031651	0.50108	insignificant	31	135	135
chr3	51362678	51364678	5031434O11R	-0.11581382	1.51E-15	hypomethylated	0.009464	0.51989	insignificant	29	122	105
chr3	51364745	51366745	Setd7	-0.17380038	5.42E-18	hypomethylated	0.014755	0.17957	insignificant	16	67	63
chr3	51908928	51910928	Maml3		1	noCoverage	0.1586	0.46952	insignificant	0	10	10
chr3	52071258	52073258	Foxo1	0.04391018	0.0050834	inconclusive	-0.023965	0.36146	insignificant	37	208	190
chr3	52821145	52823145	Cog6	-0.16645047	0.000044839	hypomethylated	0.10079	0.48855	insignificant	6	35	38
chr3	52844468	52846468	Lhfp	-0.18461669	4.56E-14	hypomethylated	-0.0023706	0.5541	insignificant	13	58	56
chr3	53266738	53268738	2810046L04Ri	-0.20187177	6.35E-11	hypomethylated	-0.047464	0.065534	insignificant	10	45	25
chr3	53267180	53269180	Nhlrc3	-0.21919041	3.93E-11	hypomethylated	0.057369	0.015591	hypermethylated	10	46	32
chr3	53291714	53293714	Stoml3	0.12334944	0.19024	insignificant	-0.048079	0.43458	insignificant	3	6	6
chr3	53461277	53463277	Frem2	-0.29898841	0.20732	insignificant	-0.063605	0.93742	insignificant	2	28	24
chr3	53667729	53669729	Ufm1	-0.31315783	3.5E-18	hypomethylated	0.04143	0.13238	insignificant	8	18	18
chr3	53959025	53961025	Trpc4	-0.24045551	0.0096604	hypomethylated	0.0019306	1	insignificant	2	16	16
chr3	54496026	54498026	Fam48a	-0.14528393	0.005155	hypomethylated	0.017798	0.33668	insignificant	7	60	60
chr3	54538460	54540460	Alg5	-0.08674452	4.21E-13	hypomethylated	0.0049277	1	insignificant	50	179	179
chr3	54539286	54541286	Exosc8	-0.1649252	0.000063856	hypomethylated	-0.0059678	0.88004	insignificant	25	83	82
chr3	54558503	54560503	Smad9	0.03639715	0.018273	hypermethylated	0.034245	0.03217	inconclusive	3	64	67
chr3	54611713	54613713	Rfxap	-0.15647187	1	insignificant	0.017654	0.64326	insignificant	3	28	29
chr3	54719809	54721809	6030405A18Rik		1	noCoverage	-0.019345	1	insignificant	0	0	0
chr3	54858977	54860977	Ccna1	-0.12552911	8.43E-09	hypomethylated	0.0051545	0.89543	insignificant	20	113	113
chr3	54915029	54917029	Spg20	-0.19722753	8.36E-12	hypomethylated	0.017496	0.4879	insignificant	22	94	80
chr3	54915087	54917087	Spg20	-0.19722753	8.36E-12	hypomethylated	0.017496	0.4879	insignificant	22	94	80
chr3	54984965	54986965	Sohlh2	-0.03797468	0.1796	insignificant	-0.015564	0.78053	insignificant	20	84	84
chr3	55045447	55047447	Dclk1	-0.135115	0.00000177	hypomethylated	-0.010066	0.46979	insignificant	8	29	29
chr3	55585431	55587431	Mab21l1	-0.18968713	0.00037457	hypomethylated	-0.000057053	0.87517	insignificant	10	44	44
chr3	55987623	55989623	Nbea	-0.06718142	1.34E-09	hypomethylated	-0.013969	0.13966	insignificant	14	69	69
chr3	57379798	57381798	Wwtr1	-0.08815108	6.48E-08	hypomethylated	-0.005633	0.61263	insignificant	7	36	36
chr3	57379832	57381832	Wwtr1	-0.01696618	0.00000548	hypomethylated	-0.0034497	0.66588	insignificant	6	32	32
chr3	57455606	57457606	Commd2	-0.1907225	0.00000254	hypomethylated	0.09142	0.81304	insignificant	4	13	8
chr3	57538987	57540987	Rnf13	-0.11910323	0.00000448	hypomethylated	0.024108	0.22632	insignificant	12	32	32
chr3	57651679	57653679	Pfn2	-0.09490209	6.74E-09	hypomethylated	-0.0086968	0.67569	insignificant	26	52	52
chr3	58218610	58220610	Tsc22d2	-0.08042898	7.22E-50	hypomethylated	-0.0060335	0.13683	insignificant	100	324	321

chr3	58328742	58330742	Eif2a	-0.13766652	2.11E-27	hypomethylated	0.018988	0.5782	insignificant	52	139	129
chr3	58329806	58331806	Eif2a	-0.12908591	2.68E-12	hypomethylated	-0.0089146	0.95759	insignificant	16	46	43
chr3	58379579	58381579	2810407C02Ri	-0.1527296	9.23E-23	hypomethylated	-0.0015279	0.53636	insignificant	35	108	103
chr3	58441129	58443129	Fam194a		1	noCoverage	0.22222	0.00092101	hypermethylated	0	6	6
chr3	58496310	58498310	Siah2	-0.13729569	0.000000115	hypomethylated	0.046806	0.80266	insignificant	10	46	40
chr3	58809899	58811899	Med12l	-0.10208829	9.34E-47	hypomethylated	0.019502	1	insignificant	59	189	189
chr3	58934546	58936546	Med12l	-0.28571429	0.12019	insignificant	0.26601	0.25308	insignificant	1	2	4
chr3	59066753	59068753	P2ry12	0.09386568	0.53582	insignificant	0.075016	0.13083	insignificant	2	8	8
chr3	59148178	59150178	Igsf10	0.15555556	1	insignificant	0.031929	0.081427	insignificant	1	9	14
chr3	60304173	60306173	Mbnl1	-0.10206227	8.59E-27	hypomethylated	0.0071836	0.067695	insignificant	51	128	126
chr3	60805716	60807716	P2ry1	-0.14022804	9.09E-24	hypomethylated	0.010388	0.75573	insignificant	18	92	82
chr3	61167428	61169428	Rap2b	-0.10900342	1.58E-15	hypomethylated	0.030529	0.15061	insignificant	25	125	94
chr3	62141698	62143698	Arhgef26	-0.13214474	8.75E-12	hypomethylated	-0.0034945	0.61501	insignificant	33	125	120
chr3	62310910	62312910	Dhx36	-0.06375767	0.0084255	hypomethylated	0.023721	0.24627	insignificant	8	34	34
chr3	63098793	63100793	Mme	-0.09495494	0.010114	hypomethylated	0.056166	0.15095	insignificant	10	38	29
chr3	63733307	63735307	E130311K13Ri	-0.244159	0.000000694	hypomethylated	-0.016836	0.031684	inconclusive	7	18	18
chr3	63768655	63770655	Slc33a1	-0.03731551	0.014928	inconclusive	0.013922	0.22836	insignificant	8	22	22
chr3	63779064	63781064	Gmps	-0.15983813	0.000018809	hypomethylated	0.0036891	0.93776	insignificant	34	95	88
chr3	64311607	64313607	Vmn2r5		1	noCoverage	-0.096658	1	insignificant	0	6	4
chr3	64912564	64914564	Kcnab1	-0.2579581	0.0066294	hypomethylated	0.15915	0.88894	insignificant	4	22	31
chr3	65196475	65198475	Ssr3		1	noCoverage	0.0035614	0.77137	insignificant	0	31	31
chr3	65331368	65333368	Tiparp	-0.07196115	4.08E-21	hypomethylated	0.0021059	0.26583	insignificant	62	271	265
chr3	65333336	65335336	Tiparp	-0.14868297	0.064407	insignificant	-0.054958	0.28278	insignificant	6	15	12
chr3	65469149	65471149	Lekr1	-0.06563356	0.000046261	hypomethylated	0.0068503	0.83999	insignificant	18	88	88
chr3	65469156	65471156	Lekr1	-0.06563356	0.000046261	hypomethylated	0.0068503	0.83999	insignificant	18	88	88
chr3	65762147	65764147	Ccnl1	-0.17825852	6.47E-21	hypomethylated	-0.014328	0.032922	hypomethylated	26	85	83
chr3	66100741	66102741	Veph1	-0.55	1	lowCoverage	-0.024747	0.00021434	hypomethylated	1	2	2
chr3	66785693	66787693	Shox2	-0.22297303	4.62E-10	hypomethylated	0.036849	0.39127	insignificant	6	44	37
chr3	66788593	66790593	Rsrc1	-0.14768939	0.37496	insignificant	0.011574	0.89649	insignificant	8	74	70
chr3	67177018	67179018	Mlf1	-0.09117636	0.00000065	hypomethylated	0.0045961	0.60001	insignificant	22	54	54
chr3	67233036	67235036	Gfm1	-0.28996084	6.7E-10	hypomethylated	0.026823	0.43686	insignificant	6	31	38
chr3	67267829	67269829	Gfm1		1	noCoverage	0.0125	0.85958	insignificant	0	12	10
chr3	67319445	67321445	Rarres1	-0.16291958	1.73E-08	hypomethylated	-0.063758	0.11692	insignificant	4	14	17
chr3	67385689	67387689	Mfsd1	-0.19861628	8.33E-12	hypomethylated	0.0063158	1	insignificant	7	27	27
chr3	67695141	67697141	Iqcj	-0.00486873	0.0024087	hypomethylated	0.050174	0.016442	hypermethylated	7	24	24
chr3	67867723	67869723	Schip1	0.18605664	1	insignificant	0.11454	0.032869	hypermethylated	2	18	16
chr3	68297114	68299114	Schip1	-0.15069714	4.27E-43	hypomethylated	-0.0051611	0.90537	insignificant	41	108	108
chr3	68493565	68495565	Il12a	-0.21858061	0.0017362	hypomethylated	-0.032276	0.24781	insignificant	8	31	28
chr3	68494345	68496345	Il12a	-0.20262065	0.001719	hypomethylated	-0.016316	0.24705	insignificant	8	29	28
chr3	68672507	68674507	1110032F04Ri	-0.0781246	6.28E-20	hypomethylated	-0.0010619	0.60836	insignificant	50	145	145
chr3	68807893	68809893	Smc4	-0.1388419	1.49E-18	hypomethylated	-0.001451	1	insignificant	20	100	78
chr3	68808492	68810492	Smc4	-0.12837882	7.23E-20	hypomethylated	-0.0040517	0.90691	insignificant	25	112	95
chr3	68848664	68850664	Trim59	-0.15187357	0.0032614	hypomethylated	-0.013469	0.016004	hypomethylated	14	70	71
chr3	68931014	68933014	Kpna4	-0.20699742	0.000029968	hypomethylated	0.0030078	0.58446	insignificant	11	54	54
chr3	69025340	69027340	Arl14	-0.21686231	0.000077808	hypomethylated	0.06651	0.050039	insignificant	4	10	8
chr3	69119839	69121839	Ppm1l	-0.10820019	1.17E-16	hypomethylated	0.0011359	0.0089243	hypermethylated	46	234	224
chr3	69402783	69404783	B3galnt1	-0.26018063	0.0049102	hypomethylated	0.0015476	0.43241	insignificant	2	22	22
chr3	69524976	69526976	Nmd3	-0.14166463	1.12E-29	hypomethylated	-0.0049757	0.23198	insignificant	40	129	124
chr3	69663818	69665818	1110032A04R	-0.16790525	0.00035202	hypomethylated	-0.0095594	0.65386	insignificant	6	43	43
chr3	72860865	72862865	Slitrk3	-0.18831259	0.000013834	hypomethylated	-0.037248	0.62126	insignificant	7	20	33
chr3	73512337	73514337	Bche		1	noCoverage	0.038462	0.76524	insignificant	0	3	3
chr3	75360454	75362454	Serpini1	-0.19270593	1.27E-22	hypomethylated	0.014646	0.26602	insignificant	21	54	53
chr3	75360721	75362721	Pdcd10	-0.32984281	0.00000301	hypomethylated	0.065899	0.45437	insignificant	6	20	19
chr3	75760753	75762753	Golim4	-0.26457583	0.00016648	hypomethylated	-0.049449	0.59367	insignificant	4	21	25
chr3	75877519	75879519	Fstl5		1	noCoverage	-0.010101	0.50789	insignificant	0	26	26
chr3	79148297	79150297	Gm17359	-0.2709134	0.00042425	hypomethylated	0.053143	0.017774	hypermethylated	3	12	12
chr3	79371601	79373601	Fnip2	-0.09169069	4.65E-14	hypomethylated	0.13105	0.30797	insignificant	21	85	94
chr3	79394310	79396310	Ppid	-0.10667943	2.49E-21	hypomethylated	0.0020525	0.64926	insignificant	28	84	82
chr3	79432000	79434000	4930579G24R	-0.24233728	0.0011395	hypomethylated	0.013583	0.088263	insignificant	9	38	40
chr3	79432689	79434689	Etfdh	-0.42294186	0.0016618	stronglyHypometh	0.0106	0.47903	insignificant	6	14	16
chr3	79646584	79648584	Tmem144	-0.32248914	0.000065446	hypomethylated	-0.14595	1	insignificant	6	16	16
chr3	79688851	79690851	Fam198b	-0.35897436	0.0028354	stronglyHypometh	0.0014423	1	insignificant	3	6	6
chr3	80606713	80608713	Gria2	-0.19583432	0.0033684	hypomethylated	0.026097	0.44359	insignificant	8	43	43
chr3	80839337	80841337	Pdgfc	-0.17365144	0.000075299	hypomethylated	-0.059328	0.9545	insignificant	10	64	69
chr3	81735537	81737537	Ctso	-0.24990442	1.39E-16	hypomethylated	0.012239	0.62698	insignificant	9	28	28
chr3	82160993	82162993	Mtap9	-0.09924698	4.01E-16	hypomethylated	0.02211	0.65998	insignificant	31	89	75
chr3	82680405	82682405	Rbm46		1	noCoverage	0.007113	0.76644	insignificant	0	8	8
chr3	82707896	82709896	4930564K09Ri	0.08310458	0.00000733	inconclusive	0.037115	0.068013	insignificant	5	30	26
chr3	82853712	82855712	Fgb	0.2613427	1	insignificant	0.15434	0.66039	insignificant	1	13	8
chr3	82858459	82860459	Plrg1	-0.22436402	8.1E-09	hypomethylated	0.028789	0.11228	insignificant	9	69	69
chr3	83569242	83571242	Sfrp2	-0.10842985	2.75E-24	hypomethylated	-0.0013147	0.36992	insignificant	38	144	145
chr3	83645530	83647530	Tlr2	-0.25	0.30826	insignificant	0.017912	0.18903	insignificant	2	4	4
chr3	83844083	83846083	D930015E06R	-0.12055291	2.28E-10	hypomethylated	0.0016862	0.82689	insignificant	14	53	53
chr3	83959708	83961708	Mnd1		1	noCoverage	-0.0040454	0.048904	inconclusive	0	10	10
chr3	84024799	84026799	Trim2	0.01260875	0.029645	hypermethylated	0.10932	0.93366	insignificant	2	16	17
chr3	84284351	84286351	Fhdc1	-0.27503837	7.18E-14	hypomethylated	0.0072678	0.3912	insignificant	16	37	37
chr3	84386547	84388547	Arfip1	-0.14887752	1.01E-09	hypomethylated	0.010413	0.049654	hypermethylated	24	73	70
chr3	84469113	84471113	Tmem154	-0.26960282	0.045916	hypomethylated	-0.0019718	0.16271	insignificant	7	22	20
chr3	84618498	84620498	Fbxw7	-0.12908988	1.91E-39	hypomethylated	0.015766	0.31207	insignificant	49	152	139
chr3	84755132	84757132	Fbxw7	0.09002976	1	insignificant	0.016815	0.69899	insignificant	4	8	8
chr3	85377050	85379050	Pet112l	-0.28485577	0.000067969	hypomethylated	-0.10612	0.27663	insignificant	6	16	15
chr3	85550131	85552131	Fam160a1	-0.26507937	0.01438	hypomethylated	0.078599	0.41189	insignificant	2	5	5
chr3	85691440	85693440	Glt28d2	-0.11192862	1	insignificant	0.033497	0.87042	insignificant	5	21	16
chr3	85806413	85808413	Prss48	-0.02461934	0.37132	insignificant	0.023261	0.33345	insignificant	9	40	40
chr3	85887355	85889355	Sh3d19	-0.25515968	0.000000785	hypomethylated	-0.12485	0.0018903	hypomethylated	6	38	37

chr3	85942780	85944780	Rnu73b		1	noCoverage	0.14376	0.8238	insignificant	0	12	10
chr3	85944609	85946609	Rnu73b	-0.10465223	4.06E-19	hypomethylated	-0.00019161	0.76666	insignificant	15	123	120
chr3	85946590	85948590	Rps3a	-0.69425019	1.01E-13	stronglyHypometh	-0.17216	0.01235	hypomethylated	1	3	2
chr3	86027611	86029611	Lrba	-0.10149835	1.39E-34	hypomethylated	0.0065445	0.07767	insignificant	57	204	201
chr3	86724806	86726806	Dclk2	0.06452981	0.00000417	hypermethylated	-0.067015	0.00000941	hypomethylated	4	43	37
chr3	86789507	86791507	Cd1d2	0.23020434	1	lowCoverage	-0.030351	1	insignificant	1	6	8
chr3	86803262	86805262	Cd1d1	-0.17090957	0.0008714	hypomethylated	0.0041169	0.30456	insignificant	2	20	20
chr3	86978669	86980669	Kirrel		1	noCoverage	0.020924	0.47776	insignificant	0	18	18
chr3	87328499	87330499	Etv3	-0.04013894	0.000000133	hypomethylated	-0.0098562	0.36625	insignificant	15	116	112
chr3	87421672	87423672	Arhgef11		1	noCoverage	-0.065859	0.89419	insignificant	0	6	6
chr3	87572875	87574875	Pear1	0.03902823	0.000000143	inconclusive	0.12458	0.13128	insignificant	10	32	20
chr3	87599084	87601084	Insrr	-0.22063394	2.58E-11	hypomethylated	-0.0018703	0.25832	insignificant	10	51	52
chr3	87599872	87601872	Insrr	-0.29042735	0.13447	insignificant	-0.012641	0.94043	insignificant	4	25	26
chr3	87689484	87691484	Prcc		1	noCoverage	0.17647	0.87087	insignificant	0	13	11
chr3	87709242	87711242	Hdgf	-0.10876491	8.61E-29	hypomethylated	0.0018948	0.27624	insignificant	33	136	127
chr3	87722465	87724465	Mrpl24		1	noCoverage	0.32251	0.19597	insignificant	0	11	7
chr3	87733235	87735235	Isg20l2	0.05402278	0.11919	insignificant	0.023701	0.11607	insignificant	9	61	67
chr3	87734117	87736117	Rrnad1	0.06784228	0.00000027	hypermethylated	0.024201	0.0056776	hypermethylated	11	51	56
chr3	87751614	87753614	Crabp2	-0.16666667	1	insignificant	0.053429	0.57233	insignificant	1	12	19
chr3	87774014	87776014	Nes	-0.18650551	1.57E-20	hypomethylated	-0.028179	0.89001	insignificant	32	124	123
chr3	87804278	87806278	Bcan	-0.26518096	0.00025082	hypomethylated	0.026739	0.32612	insignificant	3	31	32
chr3	87846029	87848029	Gpatch4	-0.02365703	0.00037743	inconclusive	0.053901	0.031453	hypermethylated	8	66	64
chr3	87862417	87864417	Apoa1bp	-0.27876985	0.12863	insignificant	0.018572	0.73081	insignificant	4	46	42
chr3	87884972	87886972	Iqgap3	-0.1091279	0.60649	insignificant	0.012321	0.84362	insignificant	4	20	20
chr3	87945316	87947316	Mef2d	-0.08980964	4.48E-24	hypomethylated	-0.00051157	0.58735	insignificant	43	182	182
chr3	88018092	88020092	Mir3093	-0.13803482	0.0014879	hypomethylated	0.0080384	0.871	insignificant	13	92	86
chr3	88018519	88020519	Mir9-1	-0.14454763	0.038199	hypomethylated	0.010622	0.64698	insignificant	10	80	74
chr3	88058606	88060606	Rhbg	-0.32283289	5.1E-14	hypomethylated	-0.042613	0.62026	insignificant	8	19	16
chr3	88100056	88102056	Cct3	-0.13820559	0.0012149	hypomethylated	-0.011277	0.62518	insignificant	9	18	18
chr3	88100760	88102760	Cct3	-0.23130503	0.00000625	hypomethylated	-0.0014572	0.29652	insignificant	9	22	22
chr3	88138355	88140355	Smg5	-0.08878848	3.07E-08	hypomethylated	0.0030073	0.37535	insignificant	10	39	38
chr3	88139181	88141181	Smg5	-0.16995134	3.78E-13	hypomethylated	0.020316	0.34379	insignificant	15	83	91
chr3	88167510	88169510	Paqr6	0.07124542	0.165	insignificant	0.018729	0.73051	insignificant	2	34	32
chr3	88173642	88175642	Bglap-rs1	0.16068267	1	insignificant	-0.059277	0.55861	insignificant	3	17	19
chr3	88214238	88216238	Pmf1	-0.11809227	0.24509	insignificant	0.027338	0.1141	insignificant	6	25	24
chr3	88229061	88231061	Slc25a44		1	noCoverage	-0.007147	0.70186	insignificant	0	10	10
chr3	88259683	88261683	Sema4a		1	noCoverage	-0.053631	0.57718	insignificant	0	4	4
chr3	88262806	88264806	Sema4a	0.06851122	0.69075	insignificant	-0.1096	0.6411	insignificant	3	9	9
chr3	88263023	88265023	Sema4a	0.06851122	0.69075	insignificant	-0.1096	0.6411	insignificant	3	9	9
chr3	88265104	88267104	Sema4a	-0.33056277	0.000060092	hypomethylated	0.057344	0.10696	insignificant	3	10	10
chr3	88297221	88299221	Lmna	-0.48046578	0.000004499	stronglyHypometh	0.012803	0.74736	insignificant	2	9	9
chr3	88307254	88309254	Lmna		1	noCoverage	-0.16585	0.0525	insignificant	0	13	11
chr3	88335316	88337316	Mex3a	-0.16355355	0.000000852	hypomethylated	-0.026894	0.50684	insignificant	22	96	93
chr3	88340304	88342304	Mir1905	0.04315666	0.020107	inconclusive	0.14016	0.56666	insignificant	4	41	34
chr3	88356637	88358637	Ubqln4	-0.14394901	1.47E-23	hypomethylated	-0.0043815	0.47831	insignificant	30	121	119
chr3	88356849	88358849	Ubqln4	-0.1399988	3.08E-18	hypomethylated	-0.0097185	0.69236	insignificant	32	113	113
chr3	88382592	88384592	Ssr2	-0.10836234	0.023075	hypomethylated	0.014689	0.69957	insignificant	2	32	32
chr3	88419128	88421128	Arhgef2	-0.20069528	0.11851	insignificant	0.042918	0.21801	insignificant	6	16	16
chr3	88424027	88426027	Arhgef2	-0.12118541	1.84E-28	hypomethylated	0.00075417	0.5202	insignificant	39	142	140
chr3	88424315	88426315	Arhgef2	-0.12118541	1.84E-28	hypomethylated	0.00075417	0.5202	insignificant	39	142	140
chr3	88488715	88490715	2810403A07R	-0.08049863	0.000000209	hypomethylated	0.0056464	0.96715	insignificant	21	69	81
chr3	88519775	88521775	Rit1	-0.22408921	7.9E-18	hypomethylated	0.015933	0.73532	insignificant	12	37	37
chr3	88638149	88640149	Gon4l	-0.18417279	0.093396	insignificant	0.024294	0.74133	insignificant	2	58	58
chr3	88754204	88756204	Dap3	-0.14977725	2.85E-45	hypomethylated	-0.011938	0.72469	insignificant	29	115	100
chr3	88768733	88770733	Ash1l		1	noCoverage	-0.10972	0.093853	insignificant	0	6	6
chr3	88893953	88895953	Rusc1	-0.16617609	1.45E-27	hypomethylated	-0.0052466	1	insignificant	30	108	108
chr3	88897285	88899285	Fdps		1	noCoverage	-0.010344	0.01221	hypomethylated	0	16	16
chr3	88905867	88907867	Fdps	-0.2194636	0.54398	insignificant	-0.031964	0.50367	insignificant	2	10	9
chr3	88939063	88941063	Pklr		1	noCoverage	0.07206	0.18369	insignificant	0	21	21
chr3	88939544	88941544	Pklr		1	noCoverage	0.003814	0.091517	insignificant	0	20	19
chr3	88964079	88966079	Hcn3	-0.40094484	1.86E-13	stronglyHypometh	-0.035651	0.78484	insignificant	2	8	6
chr3	88967726	88969726	Clk2	-0.10844091	1.14E-08	hypomethylated	0.020443	0.029212	hypermethylated	26	80	90
chr3	88980406	88982406	Scamp3	-0.26110912	6.2E-10	hypomethylated	0.029773	0.34097	insignificant	4	26	31
chr3	88986146	88988146	Fam189b	-0.31429208	0.022872	hypomethylated	0.05167	0.31767	insignificant	2	14	12
chr3	89018108	89020108	Thbs3	-0.16080204	0.39584	insignificant	-0.032482	0.11074	insignificant	5	48	45
chr3	89018257	89020257	Thbs3	-0.19683474	0.61657	insignificant	-0.05954	0.87269	insignificant	3	33	30
chr3	89031120	89033120	Mir92b	-0.21184509	0.004211	hypomethylated	-0.0097929	0.18409	insignificant	3	34	33
chr3	89031973	89033973	Muc1	-0.65972734	0.25824	lowCoverage	0.021766	0.89488	insignificant	1	19	14
chr3	89049121	89051121	Trim46	-0.24070655	5.42E-24	hypomethylated	0.012247	0.30817	insignificant	18	63	62
chr3	89049359	89051359	Krtcap2	-0.25038671	2.45E-24	hypomethylated	0.034761	0.24771	insignificant	18	56	55
chr3	89049819	89051819	Trim46	-0.2475884	3.84E-22	hypomethylated	0.028853	0.25462	insignificant	16	52	51
chr3	89069473	89071473	Dpm3	-0.14396455	0.00016715	hypomethylated	0.18881	0.48592	insignificant	3	28	20
chr3	89074492	89076492	Slc50a1		1	noCoverage	0.013994	0.60679	insignificant	0	21	14
chr3	89083567	89085567	Efna1	-0.14143028	3.39E-12	hypomethylated	0.023854	1	insignificant	8	73	74
chr3	89084873	89086873	Efna1	-0.1840748	0.000000688	hypomethylated	0.08316	0.28565	insignificant	8	58	63
chr3	89153932	89155932	Adam15	-0.22068438	3.92E-23	hypomethylated	0.065132	0.00344	hypermethylated	21	73	72
chr3	89169161	89171161	Dcst1	-0.19491758	0.076834	insignificant	0.042827	0.3513	insignificant	2	16	16
chr3	89197125	89199125	Zbtb7b	-0.14860798	1.43E-10	hypomethylated	0.015885	0.62683	insignificant	17	66	66
chr3	89215785	89217785	Flad1	-0.30524595	8.34E-15	hypomethylated	0.018095	0.25116	insignificant	10	28	29
chr3	89221472	89223472	Shc1	-0.12445823	1.21E-10	hypomethylated	-0.0085601	0.28919	insignificant	13	76	74
chr3	89222213	89224213	Cks1b	-0.16405137	0.22635	insignificant	-0.010856	0.94977	insignificant	4	38	37
chr3	89224542	89226542	Shc1	-0.3791464	0.000000135	stronglyHypometh	-0.03076	0.10327	insignificant	5	14	14
chr3	89233758	89235758	Pygo2	-0.09302568	1.19E-09	hypomethylated	-0.0011609	0.62632	insignificant	17	78	78
chr3	89239625	89241625	Pbxip1	-0.24172648	0.10553	insignificant	0.066054	0.45308	insignificant	3	12	12
chr3	89262039	89264039	Pmvk		1	noCoverage	0.013768	0.90601	insignificant	0	29	31

chr3	89262492	89264492	Pmvk		1	noCoverage	-0.00031062	1	insignificant	0	22	21
chr3	89323085	89325085	Kcnn3	-0.1031949	0.042351	hypomethylated	0.014339	0.41757	insignificant	10	37	36
chr3	89517943	89519943	Adar	-0.19860711	0.057064	insignificant	0.0055749	0.20562	insignificant	5	48	48
chr3	89533639	89535639	Adar	-0.20128205	0.0077706	hypomethylated	0.033629	0.89787	insignificant	4	18	18
chr3	89568554	89570554	Chrnb2	-0.40686813	0.0030306	stronglyHypometh	-0.11937	1	insignificant	1	2	2
chr3	89576530	89578530	Ube2q1	-0.12396504	2.3E-50	hypomethylated	0.0023441	0.20959	insignificant	46	154	142
chr3	89634291	89636291	She	-0.14439349	7.44E-38	hypomethylated	0.0078776	0.12728	insignificant	76	237	217
chr3	89717084	89719084	Il6ra	-0.15413625	4.38E-28	hypomethylated	0.014276	0.065984	insignificant	18	65	63
chr3	89767430	89769430	Atp8b2		1	noCoverage	0.0101	0.77993	insignificant	0	11	12
chr3	89802608	89804608	Hax1	-0.34845001	7.25E-27	stronglyHypometh	0.16058	0.0047402	hypermethylated	7	22	25
chr3	89855738	89857738	4933434E20Ri	-0.13906894	1.56E-11	hypomethylated	0.0048961	0.29206	insignificant	13	105	105
chr3	89856397	89858397	Ubap2l	-0.13485955	0.000022642	hypomethylated	-0.0065418	0.63359	insignificant	6	38	38
chr3	89856437	89858437	Ubap2l	-0.10532086	0.018543	hypomethylated	-0.0041777	0.48379	insignificant	5	36	36
chr3	89872941	89874941	Mir190b		1	noCoverage	-0.032531	0.51427	insignificant	0	14	14
chr3	89907053	89909053	Nup210l	-0.1365646	2.32E-08	hypomethylated	0.010875	0.17727	insignificant	5	19	19
chr3	90017569	90019569	Gm9846		1	noCoverage	-0.053483	0.22213	insignificant	0	17	15
chr3	90017570	90019570	Gm9846		1	noCoverage	-0.053483	0.22213	insignificant	0	17	15
chr3	90023738	90025738	Rab13	-0.24282202	4.74E-08	hypomethylated	-0.061839	0.14494	insignificant	10	38	42
chr3	90034518	90036518	Jtb	-0.11938366	3.98E-10	hypomethylated	0.02895	0.92875	insignificant	17	84	80
chr3	90047434	90049434	Creb3l4	-0.1205746	0.073132	insignificant	0.021925	0.88332	insignificant	2	26	28
chr3	90051113	90053113	Slc39a1	-0.1974889	0.0004752	hypomethylated	-0.02521	0.82767	insignificant	6	34	35
chr3	90057202	90059202	Crtc2	-0.12121387	7.2E-21	hypomethylated	-0.005601	0.82446	insignificant	17	104	99
chr3	90069455	90071455	Dennd4b	0.0029283	0.17534	insignificant	-0.0082195	0.97052	insignificant	13	67	66
chr3	90193849	90195849	Slc27a3	-0.28775711	1.52E-16	hypomethylated	0.0052569	0.19369	insignificant	9	22	18
chr3	90237558	90239558	Ints3	-0.27028514	1	insignificant	0.047348	0.71124	insignificant	4	34	34
chr3	90269788	90271788	Npr1	-0.34112351	0.00000118	stronglyHypometh	0.05933	0.20419	insignificant	4	23	23
chr3	90279122	90281122	Ilf2	-0.27270664	7.92E-08	hypomethylated	-0.0034141	0.11288	insignificant	6	46	40
chr3	90294935	90296935	Snapin	-0.25081701	0.000083248	hypomethylated	0.027364	0.29257	insignificant	2	20	20
chr3	90317681	90319681	S100a13	-0.23353893	0.00032218	hypomethylated	0.013076	0.43999	insignificant	9	27	32
chr3	90318252	90320252	S100a13	-0.2555057	0.000010986	hypomethylated	0.058885	0.39429	insignificant	4	12	14
chr3	90471992	90473992	S100a8	-0.21799517	0.086273	insignificant	-0.0081349	0.8583	insignificant	1	4	4
chr3	92119626	92121626	Sprr2b		1	noCoverage	0.12917	0.57541	insignificant	0	4	2
chr3	92425582	92427582	Lce6a		1	noCoverage	0.21667	0.087691	insignificant	0	4	4
chr3	92819608	92821608	Crct1	-0.38961039	0.028611	stronglyHypometh	-0.068182	0.66097	insignificant	1	2	2
chr3	93000194	93002194	Flg2		1	noCoverage	-0.01228	0.64465	insignificant	0	6	4
chr3	93196620	93198620	Rptn		1	noCoverage	-0.064106	0.089089	insignificant	0	6	6
chr3	93323417	93325417	S100a11	-0.17824563	9.74E-19	hypomethylated	0.032277	0.89134	insignificant	6	30	29
chr3	93599319	93601319	Tdpoz4		1	noCoverage	-0.0048469	0.48491	insignificant	0	7	8
chr3	93628941	93630941	Tdpoz3	-0.60930784	0.0039784	stronglyHypometh	0.12217	0.045826	hypermethylated	2	6	6
chr3	94113053	94115053	Them4	-0.10490106	1	insignificant	0.041489	0.22637	insignificant	8	45	49
chr3	94165365	94167365	C2cd4d	-0.08310794	0.020436	hypomethylated	0.032405	0.28861	insignificant	6	16	16
chr3	94202140	94204140	Lingo4	-0.14454249	1	insignificant	0.03297	1	insignificant	2	33	32
chr3	94216239	94218239	Tdrkh	-0.14699334	0.00013537	hypomethylated	0.059259	0.35423	insignificant	7	14	14
chr3	94246257	94248257	Mrpl9	-0.17970612	2.14E-09	hypomethylated	-0.0312	0.8649	insignificant	13	41	39
chr3	94281752	94283752	Celf3	-0.19377726	0.026656	hypomethylated	0.076746	0.84095	insignificant	4	24	24
chr3	94386624	94388624	Snx27	-0.48242556	0.25251	insignificant	-0.055138	0.69747	insignificant	3	7	6
chr3	94590437	94592437	Cgn	-0.14382765	0.000000585	hypomethylated	-0.015288	0.66977	insignificant	7	19	19
chr3	94640488	94642488	Pogz	-0.10167986	4.19E-14	hypomethylated	0.0087539	0.076295	insignificant	34	113	98
chr3	94690880	94692880	Psmb4	-0.56788744	0.0000056	stronglyHypometh	-0.036746	0.44883	insignificant	1	15	14
chr3	94757936	94759936	Rfx5	-0.23458001	1	insignificant	-0.036447	0.9393	insignificant	3	23	22
chr3	94777652	94779652	Pi4kb	-0.10282098	4.72E-19	hypomethylated	0.012309	0.96918	insignificant	23	84	80
chr3	94819160	94821160	Zfp687	-0.09252926	2.14E-08	hypomethylated	-0.0047376	0.18593	insignificant	11	100	96
chr3	94846467	94848467	Psmd4	-0.30257913	0.0004576	hypomethylated	0.075654	0.1055	insignificant	5	21	17
chr3	94910780	94912780	Pip5k1a	-0.15482326	1.47E-10	hypomethylated	-0.00031356	0.82286	insignificant	5	16	16
chr3	94913963	94915963	Vps72	-0.07963233	0.49423	insignificant	0.066875	0.0084553	hypermethylated	5	69	67
chr3	94937009	94939009	Lysmd1	0.13979951	1	insignificant	0.15001	0.49375	insignificant	1	16	14
chr3	94937934	94939934	Scnm1	-0.39178266	0.00000373	stronglyHypometh	0.049162	0.92528	insignificant	2	14	15
chr3	94946282	94948282	Tnfaip8l2		1	noCoverage	0.035096	0.095532	insignificant	0	17	15
chr3	95021864	95023864	Gabpb2	-0.10795698	0.0098393	hypomethylated	0.0030059	0.56884	insignificant	6	32	32
chr3	95031701	95033701	Cdc42se1	-0.17231915	1.68E-28	hypomethylated	-0.0049033	0.72254	insignificant	26	82	91
chr3	95031873	95033873	Cdc42se1	-0.17231915	1.68E-28	hypomethylated	-0.0049033	0.72254	insignificant	26	82	91
chr3	95032599	95034599	Cdc42se1	-0.18116274	1.37E-29	hypomethylated	-0.00024878	0.92655	insignificant	21	66	71
chr3	95045031	95047031	Gm128	-0.02904924	1	insignificant	0.049509	0.75751	insignificant	1	25	18
chr3	95085856	95087856	Fam63a	-0.1342986	0.000045085	hypomethylated	0.0061201	0.46785	insignificant	13	80	70
chr3	95085998	95087998	Fam63a	-0.1342986	0.000045085	hypomethylated	0.0061201	0.46785	insignificant	13	80	70
chr3	95111098	95113098	Anxa9	0.44924242	0.58052	insignificant	0.22559	0.86499	insignificant	3	14	13
chr3	95118173	95120173	Lass2	-0.1271344	4.79E-27	hypomethylated	0.014123	0.77845	insignificant	29	98	97
chr3	95161124	95163124	Setdb1	-0.49830867	0.023733	stronglyHypometh	0.03939	0.43618	insignificant	4	22	22
chr3	95230270	95232270	Gm4349	-0.26653068	1	lowCoverage	-0.027618	0.0017041	hypomethylated	1	21	23
chr3	95237311	95239311	Arnt	-0.23055627	0.00000179	hypomethylated	0.033439	0.70087	insignificant	4	34	32
chr3	95362598	95364598	Hormad1	0.00091508	1	insignificant	0.011072	0.45702	insignificant	21	50	50
chr3	95427901	95429901	Ensa	-0.20619421	0.072298	insignificant	0.00037376	0.43179	insignificant	21	78	78
chr3	95461642	95463642	Mcl1	-0.09063716	2.46E-42	hypomethylated	0.00032173	0.79559	insignificant	79	226	226
chr3	95491781	95493781	Adamtsl4	-0.1595228	3.21E-17	hypomethylated	-0.021489	0.027997	hypomethylated	16	54	50
chr3	95622876	95624876	Rprd2	-0.1852544	0.37935	insignificant	-0.010512	0.47562	insignificant	4	48	49
chr3	95659676	95661676	Prpf3	-0.10487502	0.000068478	hypomethylated	0.019152	0.71177	insignificant	18	64	64
chr3	95674542	95676542	Mrps21	-0.3278199	0.00021789	hypomethylated	-0.032748	0.90258	insignificant	2	24	24
chr3	95686151	95688151	C920021L13Ri	-0.1812723	2.46E-10	hypomethylated	0.016622	0.9247	insignificant	12	44	47
chr3	95696918	95698918	Aph1a	-0.17176217	1.5E-18	hypomethylated	0.0013247	0.46132	insignificant	8	38	38
chr3	95696977	95698977	Aph1a	-0.17176217	1.5E-18	hypomethylated	0.0013247	0.46132	insignificant	8	38	38
chr3	95708562	95710562	Car14	-0.31293461	0.00000099	hypomethylated	0.0028124	0.55089	insignificant	1	14	14
chr3	95732179	95734179	Anp32e	-0.01979397	0.000084927	hypomethylated	0.0071068	1	insignificant	39	150	142
chr3	95799762	95801762	Plekho1	-0.08548691	4.65E-19	hypomethylated	-0.0040736	0.11054	insignificant	39	111	111
chr3	95862378	95864378	Vps45	-0.43738027	2.65E-09	stronglyHypometh	-0.075061	0.80983	insignificant	2	8	6
chr3	95907449	95909449	Otud7b	-0.12538766	2.98E-26	hypomethylated	0.016614	0.13018	insignificant	36	92	92

chr3	95964892	95966892	Mtmr11	-0.05256892	1	insignificant	0.058359	0.23848	insignificant	1	14	11
chr3	95975472	95977472	Sf3b4	-0.13484302	9.16E-08	hypomethylated	0.0010151	0.51012	insignificant	15	56	50
chr3	95984149	95986149	Sv2a	-0.17252784	0.00000636	hypomethylated	0.027356	0.12597	insignificant	10	26	26
chr3	96001509	96003509	Bola1	-0.13416836	1.25E-08	hypomethylated	0.0044194	0.35132	insignificant	7	28	28
chr3	96022838	96024838	Hist2h2ab	-0.08838454	0.27997	insignificant	0.038832	0.30915	insignificant	2	22	20
chr3	96024043	96026043	Hist2h2be	-0.20170902	0.21996	insignificant	-0.014709	0.73485	insignificant	2	26	27
chr3	96024767	96026767	Hist2h2ac	-0.14300453	0.38913	insignificant	0.018089	0.8607	insignificant	4	34	35
chr3	96042999	96044999	Hist2h3c2-ps	-0.09575834	0.38461	insignificant	0.012862	0.97027	insignificant	8	43	43
chr3	96043050	96045050	Hist2h3c2-ps	-0.03556034	0.027338	inconclusive	0.0077044	0.94171	insignificant	10	41	41
chr3	96044173	96046173	Hist2h2aa2	0.27236087	0.00080451	hypermethylated	0.0029281	0.97002	insignificant	5	39	39
chr3	96044216	96046216	Hist2h2aa2	0.27236087	0.00080451	hypermethylated	0.0029281	0.97002	insignificant	5	39	39
chr3	96048460	96050460	Hist2h2aa1	-0.15913978	0.000000101	hypomethylated	0.011336	0.018578	inconclusive	9	31	31
chr3	96048503	96050503	Hist2h2aa2	-0.15913978	0.000000101	hypomethylated	0.011336	0.018578	inconclusive	9	31	31
chr3	96067240	96069240	Hist2h4	-0.22237585	0.00068247	hypomethylated	-0.0051466	1	insignificant	3	36	36
chr3	96071616	96073616	Hist2h3b	0.82310453	0.000039303	stronglyHypermeth	0.087388	0.55234	insignificant	1	40	34
chr3	96072622	96074622	Hist2h2bb		1	noCoverage	0.03171	0.58243	insignificant	0	61	52
chr3	96218756	96220756	Terc		1	noCoverage	0.05772	0.13098	insignificant	0	6	6
chr3	96328107	96330107	Hfe2	0.16336652	0.3471	insignificant	0.12166	0.063185	insignificant	3	21	23
chr3	96360879	96362879	Txnip	-0.14289065	0.2344	insignificant	0.0033535	0.18904	insignificant	7	75	75
chr3	96398081	96400081	Polr3gl		1	noCoverage	-0.0054757	0.15161	insignificant	0	21	18
chr3	96399558	96401558	Ankrd34a	-0.20857152	0.000000166	hypomethylated	-0.021269	0.53744	insignificant	12	37	34
chr3	96404055	96406055	Lix1l	-0.1219628	4.12E-28	hypomethylated	-0.0061454	0.093546	insignificant	22	72	74
chr3	96432850	96434850	Rbm8a	0.16396161	0.12037	insignificant	0.067772	0.010794	hypermethylated	3	38	38
chr3	96433742	96435742	6330549D23Rik		1	noCoverage	0.064007	0.2107	insignificant	0	14	14
chr3	96438279	96440279	Pex11b	-0.12110303	1.57E-10	hypomethylated	-0.0032737	0.93692	insignificant	25	80	80
chr3	96438352	96440352	Pex11b	-0.12110303	1.57E-10	hypomethylated	-0.0032737	0.93692	insignificant	25	80	80
chr3	96438769	96440769	Pex11b	-0.17894094	3.91E-12	hypomethylated	0.0086243	0.16658	insignificant	25	88	88
chr3	96448506	96450506	Itga10	-0.41309524	0.076589	insignificant	-0.070576	0.59914	insignificant	2	4	4
chr3	96473053	96475053	Ankrd35	0.38517648	0.0013811	stronglyHypermeth	-0.0078133	0.69132	insignificant	2	21	19
chr3	96499297	96501297	Pias3	-0.16610856	3.7E-20	hypomethylated	0.018218	0.52291	insignificant	27	96	96
chr3	96499998	96501998	Pias3	-0.11955013	4.95E-19	hypomethylated	0.033021	0.36737	insignificant	27	118	120
chr3	96512483	96514483	Nudt17	-0.13373735	0.0019974	hypomethylated	0.073374	0.59797	insignificant	4	10	11
chr3	96530533	96532533	Rnf115	-0.13755749	2.16E-14	hypomethylated	-0.022479	0.7353	insignificant	23	100	84
chr3	96531362	96533362	Rnf115	-0.1183114	3.42E-11	hypomethylated	-0.030627	0.69397	insignificant	21	88	72
chr3	96835338	96837338	Gja5	-0.18387446	0.010541	hypomethylated	0.020785	0.50709	insignificant	4	14	14
chr3	96961699	96963699	Acp6	-0.22460634	1.99E-14	hypomethylated	-0.020468	0.14502	insignificant	9	58	44
chr3	97414113	97416113	Chd1l	0.13257576	1	insignificant	-0.03548	0.19815	insignificant	2	8	8
chr3	97461134	97463134	Prkab2	-0.14848627	7.41E-40	hypomethylated	0.0077386	0.97008	insignificant	31	100	94
chr3	97571937	97573937	Pde4dip	-0.05527136	1	insignificant	-0.019431	1	insignificant	7	18	18
chr3	97672235	97674235	Pde4dip	0.39360269	0.033426	stronglyHypermeth	-0.042034	0.4647	insignificant	2	16	16
chr3	97692630	97694630	Pde4dip	0.20922869	0.54404	insignificant	-0.039993	0.030415	hypomethylated	2	4	4
chr3	97704149	97706149	Sec22b	-0.18294864	6.89E-20	hypomethylated	0.011071	0.41817	insignificant	11	52	52
chr3	97816460	97818460	Notch2	-0.09849565	8.4E-15	hypomethylated	-0.013163	0.55157	insignificant	27	112	120
chr3	98143892	98145892	Phgdh	-0.20322314	0.0033412	hypomethylated	0.0043031	0.66955	insignificant	3	14	12
chr3	98185403	98187403	Zfp697	-0.22155556	1	insignificant	-0.023015	0.51574	insignificant	3	10	10
chr3	98944012	98946012	Wars2	-0.11798907	0.00000033	hypomethylated	-0.02116	0.95483	insignificant	5	61	71
chr3	99056682	99058682	Tbx15	-0.13126999	1.46E-13	hypomethylated	-0.029676	0.33955	insignificant	21	121	111
chr3	99688339	99690339	Spag17	-0.04622812	0.69091	insignificant	0.032448	0.5267	insignificant	6	31	30
chr3	99965440	99967440	Gdap2	-0.02173881	0.65148	insignificant	0.010483	0.48158	insignificant	29	129	121
chr3	99966326	99968326	Wdr3	0.05260686	0.024426	hypermethylated	0.016762	0.10143	insignificant	16	89	81
chr3	100293115	100295115	Fam46c	-0.13188177	4.42E-12	hypomethylated	0.00071232	0.14987	insignificant	28	56	64
chr3	100489396	100491396	Man1a2	-0.11896716	1.41E-15	hypomethylated	-0.01668	0.2162	insignificant	38	117	118
chr3	100725415	100727415	Trim45	-0.14089779	4.29E-27	hypomethylated	0.034611	1	insignificant	13	44	55
chr3	100773586	100775586	Ttf2	-0.2156286	0.008512	hypomethylated	0.019459	0.71412	insignificant	5	20	22
chr3	100914089	100916089	Ptgfrn	-0.13949994	2.61E-30	hypomethylated	0.0022422	1	insignificant	29	87	87
chr3	101091862	101093862	Cd2	0.15652227	1	insignificant	0.050197	0.19051	insignificant	2	10	10
chr3	101180047	101182047	Igsf3	-0.12330161	9.22E-30	hypomethylated	0.0048262	0.77082	insignificant	37	126	115
chr3	101408580	101410580	Atp1a1	-0.16560479	9.81E-17	hypomethylated	0.0030324	0.85133	insignificant	13	41	41
chr3	101728376	101730376	Slc22a15	-0.10986883	0.61282	insignificant	0.000061259	0.83735	insignificant	2	40	40
chr3	101889432	101891432	Casq2	-0.04750479	0.82585	insignificant	0.010449	0.18689	insignificant	1	12	12
chr3	102007886	102009886	Vangl1	-0.10375003	0.000062876	hypomethylated	-0.00000616	1	insignificant	35	123	119
chr3	102008616	102010616	Vangl1	-0.03635841	0.074376	insignificant	-0.0022267	0.10089	insignificant	6	18	18
chr3	102272850	102274850	Ngf	-0.16857375	0.00083737	hypomethylated	0.050554	0.25925	insignificant	6	30	31
chr3	102537692	102539692	Tspan2	-0.1520396	6.77E-10	hypomethylated	0.0087036	0.65722	insignificant	24	79	77
chr3	102740023	102742023	Sycp1	-0.18551421	0.000049453	hypomethylated	-0.025888	0.093875	insignificant	10	25	24
chr3	102798662	102800662	Sike1	-0.11954886	0.00000018	hypomethylated	0.0035645	0.40617	insignificant	14	66	67
chr3	102823468	102825468	Csde1	-0.11238322	1.96E-16	hypomethylated	-0.013388	0.55496	insignificant	25	100	100
chr3	102861207	102863207	Nras	-0.10607652	0.094631	insignificant	0.013798	0.68401	insignificant	7	66	66
chr3	102876936	102878936	Ampd1	-0.28183024	0.005714	hypomethylated	0.12034	0.22785	insignificant	1	4	4
chr3	102930478	102932478	Dennd2c	0.04583333	1	insignificant	-0.012963	1	insignificant	1	4	4
chr3	102974633	102976633	Bcas2	-0.12269317	0.15403	insignificant	-0.0081551	0.86884	insignificant	7	69	68
chr3	103082215	103084215	Trim33	-0.05479476	0.0084432	hypomethylated	-0.0033007	0.032873	inconclusive	28	136	135
chr3	103378203	103380203	Syt6	-0.19038573	3.33E-28	hypomethylated	0.029108	0.01903	hypermethylated	25	80	65
chr3	103541924	103543924	Olfml3	-0.24303055	0.079719	insignificant	0.10141	0.042788	hypermethylated	1	6	6
chr3	103595198	103597198	Hipk1	-0.25264583	3.68E-27	hypomethylated	0.0089654	0.57102	insignificant	20	79	79
chr3	103612439	103614439	Ap4b1	-0.13309681	1.74E-16	hypomethylated	0.028768	0.35043	insignificant	13	85	86
chr3	103613310	103615310	Dclre1b	-0.12124671	9.65E-15	hypomethylated	0.027501	0.49245	insignificant	9	56	57
chr3	103717042	103719042	Rsbn1	-0.08922356	9.06E-37	hypomethylated	0.010924	0.24585	insignificant	31	141	141
chr3	103771032	103773032	Phtf1	-0.13718429	1.22E-12	hypomethylated	-0.0086015	0.63096	insignificant	20	80	78
chr3	103771180	103773180	Phtf1	-0.13718429	1.22E-12	hypomethylated	-0.0086015	0.63096	insignificant	20	80	78
chr3	103772239	103774239	Phtf1	-0.21134058	8.33E-12	hypomethylated	0.0090051	0.459	insignificant	12	60	60
chr3	104024329	104026329	Magi3	-0.24876984	0.57292	insignificant	0.15576	0.43942	insignificant	3	30	21
chr3	104315779	104317779	Lrig2	-0.18084884	0.000306	hypomethylated	0.012601	0.85773	insignificant	7	30	31
chr3	104441590	104443590	Slc16a1	-0.07257555	2.57E-55	hypomethylated	0.010428	0.34404	insignificant	64	221	213
chr3	104582973	104584973	Ppm1j	-0.13541685	1.16E-13	hypomethylated	0.0051285	0.164	insignificant	20	103	102

chr3	104590951	104592951	Rhoc	-0.20257135	7.14E-24	hypomethylated	0.038294	0.000001741	hypermethylated	23	62	65
chr3	104621223	104623223	Mov10	-0.17212302	0.32559	insignificant	-0.028528	0.02964	hypomethylated	1	14	11
chr3	104666998	104668998	Capza1	-0.03751071	0.099094	insignificant	0.0050214	0.8851	insignificant	31	126	123
chr3	104667423	104669423	Capza1	-0.11635188	0.000000011	hypomethylated	0.030871	0.92078	insignificant	21	95	95
chr3	104764627	104766627	Wnt2b	-0.07860772	1.97E-18	hypomethylated	0.023264	0.8513	insignificant	35	117	102
chr3	104855871	104857871	Cttnbp2nl	-0.21626581	2.72E-30	hypomethylated	0.051565	0.28877	insignificant	14	44	44
chr3	104856064	104858064	Cttnbp2nl	-0.29115599	1.65E-22	hypomethylated	0.10412	0.41676	insignificant	7	24	24
chr3	105254247	105256247	Kcnd3	-0.08702089	2.76E-28	hypomethylated	-0.016581	0.70065	insignificant	41	129	140
chr3	105260733	105262733	Kcnd3	-0.19301075	0.037738	hypomethylated	0.037199	0.14877	insignificant	2	31	31
chr3	105490489	105492489	Ddx20	-0.10416681	1.18E-11	hypomethylated	0.010124	0.0042839	hypermethylated	7	24	24
chr3	105506516	105508516	6530418L21Ri	-0.16244904	5.6E-15	hypomethylated	-0.010998	0.42884	insignificant	14	36	36
chr3	105507375	105509375	6530418L21Ri	-0.16244904	5.6E-15	hypomethylated	-0.010998	0.42884	insignificant	14	36	36
chr3	105604254	105606254	Rap1a	-0.14427435	0.000000766	hypomethylated	-0.021737	0.45653	insignificant	10	35	35
chr3	105706338	105708338	Adora3		1	noCoverage	0.040278	0.55215	insignificant	0	4	4
chr3	105761415	105763415	Wdr77	-0.12260657	4.15E-21	hypomethylated	0.0047737	0.41674	insignificant	26	76	76
chr3	106284348	106286348	Dennd2d	-0.21660402	1	insignificant	0.0013492	1	insignificant	1	34	34
chr3	106349744	106351744	Dram2	-0.05749649	3.08E-09	hypomethylated	-0.0054926	0.90747	insignificant	24	110	115
chr3	106350679	106352679	Dram2	-0.10062452	0.39297	insignificant	-0.0025565	0.8984	insignificant	4	42	42
chr3	106486904	106488904	4933421E11Ri	-0.13992351	0.0016023	hypomethylated	-0.00072545	0.88153	insignificant	7	18	18
chr3	106523599	106525599	4933421E11Ri	-0.15199868	0.6045	insignificant	0.083637	0.1371	insignificant	1	47	42
chr3	106838079	106840079	Kcna3	-0.08742953	1.28E-13	hypomethylated	-0.00017436	0.17984	insignificant	30	173	162
chr3	106903484	106905484	Kcna2	-0.13808971	7.77E-31	hypomethylated	-0.0050056	0.38135	insignificant	32	123	131
chr3	107080775	107082775	Hbxip	-0.13024272	1.78E-09	hypomethylated	0.0546	0.68215	insignificant	18	68	71
chr3	107136207	107138207	Rbm15	-0.1992152	8.66E-10	hypomethylated	-0.003381	1	insignificant	11	24	24
chr3	107261816	107263816	Kcnc4	-0.10151056	2.37E-10	hypomethylated	0.0016169	0.88032	insignificant	30	146	132
chr3	107320936	107322936	Slc6a17	-0.0628589	0.028091	hypomethylated	0.02871	0.37355	insignificant	7	20	15
chr3	107396948	107398948	Alx3	-0.14821847	2.91E-33	hypomethylated	0.00090177	0.24741	insignificant	35	150	130
chr3	107434628	107436628	Fam40a	-0.26316581	0.0014889	hypomethylated	0.08126	0.69327	insignificant	4	33	34
chr3	107499466	107501466	Ahcyl1		1	noCoverage	-0.034205	0.59143	insignificant	0	10	15
chr3	107697771	107699771	Gstm5	-0.14154313	0.00023734	hypomethylated	0.0075828	0.11074	insignificant	15	55	56
chr3	107699131	107701131	Gstm5	-0.16101972	0.5891	insignificant	0.021192	0.0020056	hypermethylated	5	11	11
chr3	107734663	107736663	Gstm7	-0.30952381	0.39003	insignificant	-0.13175	0.20755	insignificant	1	5	5
chr3	107746667	107748667	Gstm6	-0.23070696	0.11949	insignificant	0.013377	0.32489	insignificant	2	10	14
chr3	107789354	107791354	Gstm2	0.23148148	0.35673	insignificant	0.14034	0.54196	insignificant	1	9	5
chr3	107820891	107822891	Gstm1		1	noCoverage	0.037643	0.78716	insignificant	0	12	12
chr3	107847774	107849774	Gstm4	-0.13067334	0.000019084	hypomethylated	-0.0035877	0.85464	insignificant	7	24	24
chr3	107847777	107849777	Gstm4	-0.13067334	0.000019084	hypomethylated	-0.0035877	0.85464	insignificant	7	24	24
chr3	107889545	107891545	Ampd2	-0.11360446	0.00017005	hypomethylated	0.0084603	0.35796	insignificant	4	21	21
chr3	107894985	107896985	Gnat2	0.15547314	0.76692	insignificant	0.17182	0.22685	insignificant	1	13	16
chr3	107949032	107951032	Gnai3	-0.10294768	1	insignificant	-0.0044035	0.89945	insignificant	2	20	20
chr3	107988252	107990252	Amigo1	-0.11233374	4.31E-34	hypomethylated	0.0088101	0.00017133	hypermethylated	39	142	136
chr3	108003752	108005752	Cyb561d1	-0.10986279	0.00000156	hypomethylated	-0.029711	0.054747	insignificant	9	27	26
chr3	108013852	108015852	Atxn7l2		1	noCoverage	0.1014	0.16793	insignificant	0	15	12
chr3	108029517	108031517	Sypl2	0.09334099	1	insignificant	-0.02166	0.25449	insignificant	3	18	21
chr3	108058843	108060843	Psma5	-0.14572435	9.22E-19	hypomethylated	0.0077999	0.15356	insignificant	21	72	72
chr3	108086049	108088049	Sort1	-0.11611819	4.07E-33	hypomethylated	0.0035401	0.11136	insignificant	62	207	187
chr3	108185721	108187721	Psrc1	-0.18378906	0.00000002	hypomethylated	-0.056941	0.24892	insignificant	7	44	46
chr3	108185755	108187755	Psrc1	-0.18378906	0.00000002	hypomethylated	-0.056941	0.24892	insignificant	7	44	46
chr3	108218412	108220412	Celsr2	-0.14615671	2.73E-23	hypomethylated	0.010807	0.37025	insignificant	36	96	98
chr3	108248087	108250087	Sars	-0.30854618	1	insignificant	0.021353	0.55578	insignificant	4	16	14
chr3	108339440	108341440	5330417C22Ri	-0.1537077	0.0043249	hypomethylated	0.032984	0.19589	insignificant	10	31	32
chr3	108339501	108341501	1700013F07Ri	-0.1537077	0.0043249	hypomethylated	0.032984	0.19589	insignificant	10	31	32
chr3	108357320	108359320	Scarna2	-0.20570579	0.0035704	hypomethylated	-0.049834	0.8092	insignificant	13	38	36
chr3	108365384	108367384	Tmem167b	-0.24859522	0.01722	hypomethylated	0.053203	0.37384	insignificant	2	26	25
chr3	108373616	108375616	Taf13	-0.13924487	0.065299	insignificant	0.034861	0.67119	insignificant	18	52	52
chr3	108393195	108395195	Wdr47	-0.13659465	0.000000132	hypomethylated	0.019802	0.90964	insignificant	23	63	63
chr3	108455830	108457830	Clcc1	-0.04962388	0.0097645	hypomethylated	0.010445	0.24	insignificant	16	66	66
chr3	108455857	108457857	Clcc1	-0.04962388	0.0097645	hypomethylated	0.010445	0.24	insignificant	16	66	66
chr3	108455907	108457907	Clcc1	-0.04962388	0.0097645	hypomethylated	0.010445	0.24	insignificant	16	66	66
chr3	108525217	108527217	Gpsm2	-0.13715171	1	insignificant	0.059493	0.65246	insignificant	4	26	28
chr3	108692926	108694926	Fndc7	0.16616287	1	insignificant	-0.23454	0.21857	insignificant	1	16	6
chr3	108714622	108716622	Prpf38b	-0.16670639	0.000000116	hypomethylated	-0.015916	0.54815	insignificant	17	56	54
chr3	108742001	108744001	4921515J06Ri	0.13807054	0.41236	insignificant	0.087321	0.00095602	hypermethylated	11	37	37
chr3	108742474	108744474	4921515J06Ri	0.13807054	0.41236	insignificant	0.087321	0.00095602	hypermethylated	11	37	37
chr3	108830525	108832525	Fam102b	-0.11137452	1.61E-08	hypomethylated	0.0098753	1	insignificant	22	46	46
chr3	108882416	108884416	4930443G12Rik		1	noCoverage	0.079785	0.16957	insignificant	0	6	6
chr3	108925066	108927066	Slc25a24	-0.38347884	6.23E-14	stronglyHypometh	0.0033156	0.1404	insignificant	9	39	39
chr3	109142600	109144600	Vav3	-0.0957015	5.71E-35	hypomethylated	-0.0034461	0.79816	insignificant	66	156	156
chr3	109946390	109948390	Ntng1		1	noCoverage	-0.0020085	1	insignificant	0	34	34
chr3	110053916	110055916	Prmt6	-0.29475945	9.4E-12	hypomethylated	0.0048716	0.14219	insignificant	6	24	24
chr3	113277680	113279680	Amy1	-0.78809524	0.0061597	stronglyHypometh	-0.13185	0.69736	insignificant	1	5	4
chr3	113333067	113335067	Rnpc3	-0.09594705	0.14977	insignificant	-0.043863	0.50219	insignificant	5	23	22
chr3	113732457	113734457	Col11a1	-0.26658042	0.00000143	hypomethylated	0.0084649	0.18587	insignificant	3	23	24
chr3	115417973	115419973	S1pr1	-0.14448148	2.27E-08	hypomethylated	0.0041651	0.67645	insignificant	6	22	22
chr3	115590100	115592100	Dph5	-0.14942831	2.93E-09	hypomethylated	-0.010218	0.73211	insignificant	10	40	40
chr3	115590977	115592977	A930005H10Rik		1	noCoverage	0.027909	0.6899	insignificant	0	17	16
chr3	115591038	115593038	A930005H10Rik		1	noCoverage	0.03625	0.78538	insignificant	0	13	12
chr3	115591048	115593048	A930005H10Rik		1	noCoverage	0.03625	0.78538	insignificant	0	13	12
chr3	115709366	115711366	Extl2	-0.16419772	9.72E-14	hypomethylated	-0.017703	0.26796	insignificant	14	79	78
chr3	115710214	115712214	Extl2	-0.15051273	1.51E-09	hypomethylated	-0.035633	0.037809	hypomethylated	12	55	54
chr3	115710324	115712324	Slc30a7	-0.19758924	7.32E-09	hypomethylated	-0.045155	0.021636	hypomethylated	7	31	30
chr3	115956402	115958402	Gpr88		1	noCoverage	0.13631	0.017034	hypermethylated	0	12	12
chr3	116126950	116128950	Cdc14a	-0.0875	0.00052862	hypomethylated	0.0089452	0.3704	insignificant	2	22	22
chr3	116211093	116213093	Rtcd1		1	noCoverage	-0.15793	0.17561	insignificant	0	9	6
chr3	116214996	116216996	Dbt	-0.16312209	2.22E-16	hypomethylated	-0.021588	0.56777	insignificant	10	51	51

chr3	116296925	116298925	Sass6	-0.12829682	0.0002901	hypomethylated	0.01354	0.609	insignificant	19	94	94
chr3	116415198	116417198	Slc35a3	-0.10053435	0.0029047	hypomethylated	-0.0075703	1	insignificant	12	34	34
chr3	116511084	116513084	Agl	-0.08049543	0.000017719	hypomethylated	0.021356	0.28466	insignificant	6	16	12
chr3	116561484	116563484	Frrs1		1	noCoverage	0.017512	1	insignificant	0	8	8
chr3	116563597	116565597	Frrs1	-0.18406526	2.69E-25	hypomethylated	0.024124	0.3747	insignificant	12	30	30
chr3	116671870	116673870	4930455H04R	0.09953704	1	lowCoverage	0.053131	0.15013	insignificant	1	6	6
chr3	117277378	117279378	4833424O15R	-0.10943948	1.02E-14	hypomethylated	0.0038205	0.13308	insignificant	31	129	127
chr3	117571854	117573854	Snx7	-0.04193265	0.11352	insignificant	-0.0094631	0.85015	insignificant	14	36	37
chr3	118135774	118137774	Mir137	-0.12896296	0.69264	insignificant	0.056023	0.70978	insignificant	3	40	41
chr3	118264095	118266095	Dpyd	-0.23573201	0.0015665	hypomethylated	0.060959	0.68917	insignificant	5	31	32
chr3	119486306	119488306	Ptbp2	-0.18399253	1	insignificant	-0.017903	0.73445	insignificant	4	21	17
chr3	120874613	120876613	Rwdd3		1	noCoverage	0.11827	0.50856	insignificant	0	14	17
chr3	120966234	120968234	Tmem56	-0.20354772	0.086658	insignificant	0.0029437	0.14069	insignificant	5	26	26
chr3	120993734	120995734	Alg14	-0.17961952	0.58534	insignificant	0.044382	1	insignificant	2	29	25
chr3	121128458	121130458	Cnn3	-0.07148894	0.00000668	hypomethylated	-0.010976	0.21862	insignificant	47	194	195
chr3	121235262	121237262	Slc44a3		1	noCoverage	-0.075989	0.18208	insignificant	0	7	6
chr3	121425454	121427454	F3	-0.16217039	0.0031448	hypomethylated	-0.0101	0.56485	insignificant	15	80	80
chr3	121518133	121520133	Abcd3	-0.1075203	1.87E-08	hypomethylated	0.011555	0.72132	insignificant	11	54	54
chr3	121655243	121657243	Arhgap29	-0.15110741	4.76E-22	hypomethylated	-0.012762	0.39831	insignificant	35	88	94
chr3	121746377	121748377	Abca4	-0.14455503	0.02214	hypomethylated	0.031961	0.49205	insignificant	3	14	14
chr3	121947509	121949509	Gclm	-0.08522946	2.6E-26	hypomethylated	-0.0048045	0.94841	insignificant	38	161	152
chr3	121976331	121978331	Dnttip2	-0.13470409	3.46E-10	hypomethylated	-0.0030104	0.024483	inconclusive	23	83	82
chr3	121996621	121998621	Mir760	-0.11061862	0.0012561	hypomethylated	0.046422	0.60377	insignificant	2	64	58
chr3	122121697	122123697	Bcar3	-0.18728106	2.44E-27	hypomethylated	-0.015983	0.77732	insignificant	22	63	70
chr3	122322585	122324585	Fnbp1l	-0.19449082	0.00000171	hypomethylated	0.046977	0.48625	insignificant	15	57	50
chr3	122431075	122433075	Pde5a	-0.13868343	9.1E-22	hypomethylated	0.0073417	0.43238	insignificant	42	122	112
chr3	122597309	122599309	Fabp2	0.10595238	1	lowCoverage	0.025366	0.64246	insignificant	1	8	12
chr3	122626314	122628314	1810037I17Ril	-0.12769165	6.59E-11	hypomethylated	-0.0028047	0.76763	insignificant	23	53	58
chr3	122687365	122689365	Usp53	-0.12197273	5.44E-14	hypomethylated	0.02239	0.43728	insignificant	14	44	51
chr3	122969413	122971413	Sec24d	-0.03897568	0.001257	inconclusive	0.00038874	0.14901	insignificant	6	98	91
chr3	123148830	123150830	Prss12	-0.11233335	2.96E-27	hypomethylated	0.0045745	0.68715	insignificant	42	133	130
chr3	123210984	123212984	Snora24	-0.1270079	0.0062176	hypomethylated	0.010014	0.89529	insignificant	4	54	54
chr3	123211254	123213254	Snora24	-0.1270079	0.0062176	hypomethylated	0.010014	0.89529	insignificant	4	54	54
chr3	124022954	124024954	Tram1l1	-0.13474958	0.17429	insignificant	0.034333	0.00013348	hypermethylated	30	96	96
chr3	125106008	125108008	Ndst4	-0.65151886	0.39267	lowCoverage	0.089953	0.54826	insignificant	1	10	14
chr3	125641468	125643468	Ugt8a	-0.0701494	7.28E-08	hypomethylated	-0.0053113	0.93003	insignificant	19	87	91
chr3	126065769	126067769	Arsj		1	noCoverage	0.1233	0.19342	insignificant	0	40	43
chr3	126298890	126300890	Camk2d	-0.07545101	5.61E-14	hypomethylated	0.013107	0.6532	insignificant	29	167	163
chr3	126646304	126648304	Ank2		1	noCoverage	0.026472	0.16098	insignificant	0	20	20
chr3	127255406	127257406	4930422G04R	-0.11856922	0.037391	hypomethylated	-0.010847	0.052563	insignificant	8	44	38
chr3	127256267	127258267	Larp7	-0.09849213	0.036642	hypomethylated	-0.0011485	0.043117	inconclusive	8	26	26
chr3	127335062	127337062	Neurog2	-0.08167301	0.00065255	hypomethylated	-0.0072403	0.72563	insignificant	29	148	137
chr3	127483445	127485445	Alpk1		1	noCoverage	0.004188	0.84924	insignificant	0	10	10
chr3	127491830	127493830	Tifa	-0.12456808	0.000033429	hypomethylated	-0.011731	0.05052	insignificant	18	75	76
chr3	127540410	127542410	Ap1ar	-0.10085036	1.19E-08	hypomethylated	0.011595	0.091457	insignificant	7	45	35
chr3	127599241	127601241	5730508B09R	-0.41666667	0.0034934	stronglyHypometh	-0.072917	0.45965	insignificant	3	10	10
chr3	128901835	128903835	Pitx2	-0.18465242	6.07E-11	hypomethylated	0.032521	0.75183	insignificant	8	70	63
chr3	128901841	128903841	Pitx2	-0.18465242	6.07E-11	hypomethylated	0.032521	0.75183	insignificant	8	70	63
chr3	128915854	128917854	Pitx2	-0.2546729	0.00000137	hypomethylated	0.00042798	0.28246	insignificant	10	54	52
chr3	129035667	129037667	Enpep		1	noCoverage	0.074561	1	insignificant	0	3	6
chr3	129135590	129137590	Gm5712	-0.16982993	0.25815	insignificant	0.017728	1	insignificant	5	18	18
chr3	129234303	129236303	Elovl6	-0.15234132	0.0050938	hypomethylated	0.02898	0.3078	insignificant	9	31	35
chr3	129534314	129536314	Gar1	-0.08170645	1	insignificant	0.0024502	0.52126	insignificant	3	44	44
chr3	129538656	129540656	Cfi		1	noCoverage	-0.0025575	0.88737	insignificant	0	6	6
chr3	129580539	129582539	Pla2g12a	-0.07243994	0.000000121	hypomethylated	0.015857	0.55342	insignificant	15	70	54
chr3	129603342	129605342	Casp6	-0.07187377	0.41113	insignificant	-0.001118	0.82142	insignificant	7	48	48
chr3	129673124	129675124	Ccdc109b		1	noCoverage	0.0035454	0.36185	insignificant	0	24	24
chr3	129763825	129765825	Sec24b	-0.13765161	0.00091802	hypomethylated	-0.0065934	0.9316	insignificant	7	35	35
chr3	129882795	129884795	Col25a1	-0.17287077	0.059778	insignificant	0.064424	0.87057	insignificant	5	30	36
chr3	130319365	130321365	Agxt2l1	-0.29256309	0.53821	insignificant	-0.065679	0.047708	hypomethylated	3	22	22
chr3	130319492	130321492	Agxt2l1	-0.28705237	0.54394	insignificant	-0.056091	0.096972	insignificant	3	20	20
chr3	130432222	130434222	Rpl34	-0.10280951	1.47E-18	hypomethylated	0.0017765	0.37356	insignificant	21	118	114
chr3	130433226	130435226	Rpl34	-0.23858488	0.23576	insignificant	0.12034	0.83809	insignificant	6	64	63
chr3	130433247	130435247	Rpl34	-0.24628116	0.38459	insignificant	0.12395	0.83782	insignificant	5	62	61
chr3	130812388	130814388	Lef1	-0.14375951	1.32E-28	hypomethylated	-0.0042543	0.15251	insignificant	37	108	96
chr3	130974955	130976955	Hadh	-0.25417988	0.63327	insignificant	-0.016785	0.3712	insignificant	7	28	30
chr3	131006145	131008145	Cyp2u1		1	noCoverage	-0.028319	0.86403	insignificant	0	6	6
chr3	131047841	131049841	Sgms2		1	noCoverage	0.083333	0.75207	insignificant	0	6	4
chr3	131226731	131228731	Papss1	-0.0759901	3.71E-10	hypomethylated	0.017748	0.35528	insignificant	27	103	108
chr3	131747255	131749255	Dkk2	-0.22124021	0.33389	insignificant	0.038477	0.61661	insignificant	1	19	15
chr3	132346107	132348107	Tbck	-0.20539577	0.000047045	hypomethylated	-0.036612	0.017363	hypomethylated	7	56	47
chr3	132346843	132348843	Tbck	-0.18499911	0.00052244	hypomethylated	-0.032875	0.089809	insignificant	5	40	39
chr3	132613255	132615255	Npnt	-0.15938655	0.000054283	hypomethylated	-0.0036762	0.78568	insignificant	15	43	43
chr3	132753916	132755916	Ints12	-0.14654137	2.62E-13	hypomethylated	-0.0073703	0.2606	insignificant	22	102	102
chr3	132754704	132756704	Gstcd	-0.16756218	3.83E-14	hypomethylated	-0.0055258	0.33397	insignificant	22	74	74
chr3	132972079	132974079	Ppa2	-0.14540906	6.08E-26	hypomethylated	0.057628	0.083189	insignificant	16	111	114
chr3	133207354	133209354	Tet2	-0.06781265	4.45E-15	hypomethylated	-0.0029791	0.68551	insignificant	22	143	143
chr3	133902111	133904111	Cxxc4	-0.10289707	0.00000276	hypomethylated	0.0073222	0.31675	insignificant	13	87	73
chr3	134490970	134492970	Tacr3	-0.39173458	1.09E-08	stronglyHypometh	0.01341	1	insignificant	8	48	48
chr3	134874526	134876526	Cenpe	-0.10777673	0.60592	insignificant	0.011259	0.68471	insignificant	8	49	47
chr3	134969663	134971663	Nhedc2		1	noCoverage	-0.0086034	0.34078	insignificant	0	12	10
chr3	135086397	135088397	Cisd2	-0.06899927	0.000000122	hypomethylated	-0.011647	0.89714	insignificant	16	47	38
chr3	135100722	135102722	Ube2d3	-0.1247496	1.11E-17	hypomethylated	0.012489	0.50067	insignificant	51	225	220
chr3	135101629	135103629	4930539J05Ri	-0.13848665	8.01E-24	hypomethylated	0.011342	0.53261	insignificant	37	158	150
chr3	135147574	135149574	Manba	-0.16935739	1.16E-12	hypomethylated	0.010363	0.11616	insignificant	24	96	94

chr3	135354511	135356511	Nfkb1	-0.18384329	0.0017044	hypomethylated	-0.036052	0.83225	insignificant	8	63	53
chr3	135487454	135489454	Slc39a8	-0.22067901	4.56E-12	hypomethylated	0.027715	0.21521	insignificant	9	30	23
chr3	135487620	135489620	Slc39a8	-0.16173518	4.39E-17	hypomethylated	0.0078295	0.13846	insignificant	17	58	51
chr3	135487753	135489753	Slc39a8	-0.15685054	4.75E-17	hypomethylated	-0.00035461	0.16702	insignificant	17	62	55
chr3	136332733	136334733	Ppp3ca	-0.09903023	7.53E-37	hypomethylated	-0.0019013	0.92703	insignificant	95	297	284
chr3	137003041	137005041	Emcn		1	noCoverage	0.054861	0.018962	hypermethylated	0	6	6
chr3	137215586	137217586	Gm4861		1	noCoverage	0.0063492	0.33473	insignificant	0	4	4
chr3	137285635	137287635	Ddit4l	-0.19847406	0.000045662	hypomethylated	0.019625	0.96989	insignificant	13	64	58
chr3	137526562	137528562	H2afz	-0.09589714	2.25E-32	hypomethylated	-0.0062386	0.52944	insignificant	41	238	224
chr3	137529638	137531638	Dnajb14	-0.07646121	2.51E-16	hypomethylated	0.0054033	0.91434	insignificant	37	143	133
chr3	137580518	137582518	Lamtor3	0.21936318	1.45E-10	hypermethylated	0.051051	0.32563	insignificant	2	40	34
chr3	137644513	137646513	Dapp1	-0.18850535	0.19333	insignificant	-0.050807	0.08711	insignificant	2	19	10
chr3	137730352	137732352	Gm5105	0.09611678	0.61273	insignificant	-0.006725	0.038957	hypomethylated	6	34	28
chr3	137805501	137807501	Rg9mtd2	-0.1013602	2.18E-10	hypomethylated	-0.0021447	0.74946	insignificant	33	127	125
chr3	137806352	137808352	Mttp	-0.09946626	0.000000152	hypomethylated	-0.006873	0.806	insignificant	30	101	101
chr3	138105127	138107127	Adh5	-0.12664064	6.77E-33	hypomethylated	0.0092593	0.93651	insignificant	31	100	95
chr3	138152346	138154346	Metap1	-0.17689707	6.92E-25	hypomethylated	0.0039615	0.88774	insignificant	16	76	75
chr3	138188154	138190154	Eif4e	-0.08096215	0.60759	insignificant	0.009409	0.91875	insignificant	2	28	32
chr3	138188384	138190384	Mir1956	-0.12626484	1.69E-16	hypomethylated	0.0057845	0.95653	insignificant	11	60	64
chr3	138404158	138406158	Tspan5	-0.12318756	7.66E-38	hypomethylated	-0.0057403	0.29024	insignificant	52	137	137
chr3	138738163	138740163	Rap1gds1	-0.08258252	0.000093847	hypomethylated	-0.025361	0.81896	insignificant	24	102	98
chr3	138867856	138869856	B930007M17R	-0.18287849	0.36673	insignificant	-0.014038	0.11321	insignificant	4	49	46
chr3	141127527	141129527	Unc5c	-0.10463814	9.35E-10	hypomethylated	0.0069857	0.32446	insignificant	21	150	150
chr3	142058660	142060660	Pdlim5	-0.1396795	0.000000136	hypomethylated	0.014398	0.26775	insignificant	9	38	33
chr3	142192301	142194301	Gbp7	-0.24137099	0.0010757	hypomethylated	0.031	0.67237	insignificant	2	17	17
chr3	142192305	142194305	Gbp7	-0.24137099	0.0010757	hypomethylated	0.031	0.67237	insignificant	2	17	17
chr3	142222015	142224015	Gbp3		1	noCoverage	0.046094	0.81285	insignificant	0	17	12
chr3	142282626	142284626	Gbp2		1	noCoverage	0.0016164	0.84121	insignificant	0	2	2
chr3	142363043	142365043	Ccbl2	-0.15657469	0.000010641	hypomethylated	0.0019802	0.69883	insignificant	14	56	63
chr3	142427210	142429210	Gtf2b	-0.12298815	0.0001513	hypomethylated	-0.018707	1	insignificant	15	96	88
chr3	142544968	142546968	Pkn2	-0.09276276	1.54E-08	hypomethylated	-0.0011394	0.67438	insignificant	24	83	84
chr3	143865296	143867296	Lmo4	-0.22025095	0.34067	insignificant	0.02987	0.91196	insignificant	3	20	18
chr3	143865360	143867360	Lmo4	-0.22025095	0.34067	insignificant	0.02987	0.91196	insignificant	3	20	18
chr3	143868219	143870219	Lmo4	-0.25152001	0.000048254	hypomethylated	-0.10549	0.90741	insignificant	10	71	58
chr3	144232390	144234390	###########	-0.12423754	1.89E-26	hypomethylated	-0.013077	0.063249	insignificant	40	178	166
chr3	144233180	144235180	###########	-0.1677407	1.38E-10	hypomethylated	-0.03752	0.026663	inconclusive	17	95	88
chr3	144383287	144385287	Sh3glb1	0.0590232	0.51717	insignificant	-0.061211	0.35534	insignificant	2	12	12
chr3	144762005	144764005	Clca5	-0.38005051	0.040583	stronglyHypometh	0.013889	1	insignificant	1	4	4
chr3	144780552	144782552	Odf2l	-0.12652379	0.00032879	hypomethylated	-0.021988	0.043451	hypomethylated	20	80	89
chr3	144780563	144782563	Odf2l	-0.12652379	0.00032879	hypomethylated	-0.021988	0.043451	hypomethylated	20	80	89
chr3	144954435	144956435	Col24a1	-0.26126984	1	insignificant	0.0099852	0.20528	insignificant	2	9	9
chr3	145238171	145240171	Znhit6	-0.25812411	3.76E-14	hypomethylated	0.016743	0.49874	insignificant	9	39	39
chr3	145312949	145314949	Cyr61	-0.27108122	0.00043128	hypomethylated	0.037649	0.49023	insignificant	3	29	26
chr3	145420655	145422655	Ddah1	-0.08788552	1.79E-20	hypomethylated	0.01948	0.067799	insignificant	34	116	109
chr3	145586341	145588341	Bcl10	-0.13230955	9.6E-12	hypomethylated	-0.0018641	0.54505	insignificant	24	76	76
chr3	145599995	145601995	2410004B18R	-0.15797129	2.41E-24	hypomethylated	0.010027	0.00000189	hypermethylated	34	78	76
chr3	145649833	145651833	Syde2	-0.08541783	1.36E-42	hypomethylated	0.0014015	0.2169	insignificant	99	319	317
chr3	145771000	145773000	Wdr63	0.31589894	0.67485	insignificant	-0.0070368	0.28753	insignificant	2	16	12
chr3	145811796	145813796	Mcoln2	-0.11326459	0.000000358	hypomethylated	0.026603	0.69469	insignificant	25	104	94
chr3	145812137	145814137	Mcoln2	-0.11998994	0.000000122	hypomethylated	0.019626	0.43879	insignificant	26	106	96
chr3	145882924	145884924	Lpar3	-0.1363793	2.33E-25	hypomethylated	0.016779	0.00000722	hypermethylated	44	116	113
chr3	146041908	146043908	Gm10636		1	noCoverage	-0.125	0.40743	insignificant	0	2	4
chr3	146066650	146068650	Ssx2ip	-0.10349845	1.93E-24	hypomethylated	0.012134	0.5922	insignificant	45	146	146
chr3	146112456	146114456	Ctbs	-0.14964381	9.05E-15	hypomethylated	0.02432	0.057669	insignificant	5	40	44
chr3	146161799	146163799	Gng5	-0.08801133	2.4E-24	hypomethylated	0.015975	0.18424	insignificant	61	166	157
chr3	146162717	146164717	Spata1	-0.09072907	7.82E-19	hypomethylated	0.0079781	0.5574	insignificant	46	114	114
chr3	146184387	146186387	Rpf1	-0.52767262	0.00000002	stronglyHypometh	-0.13416	1	insignificant	3	28	28
chr3	146259112	146261112	Uox		1	noCoverage	0.090455	0.020721	hypermethylated	0	10	10
chr3	146475910	146477910	Prkacb	-0.1007874	0.00000666	hypomethylated	0.033081	0.63999	insignificant	11	50	45
chr3	146514330	146516330	Ttll7	-0.16222111	0.00000004	hypomethylated	0.011705	0.71825	insignificant	12	32	32
chr3	151099845	151101845	Eltd1	-0.12777287	0.00044058	hypomethylated	-0.026724	0.46195	insignificant	12	41	42
chr3	151500492	151502492	Ptgfr	-0.27047454	2.52E-08	hypomethylated	-0.01924	0.70292	insignificant	5	19	18
chr3	151828864	151830864	Gipc2	-0.18509342	0.15325	insignificant	-0.069185	0.18789	insignificant	7	35	32
chr3	151872421	151874421	Fubp1	-0.10683857	2.03E-25	hypomethylated	0.0081618	0.50197	insignificant	53	202	155
chr3	151873263	151875263	Dnajb4	-0.12424385	2.56E-24	hypomethylated	0.0093309	0.80718	insignificant	38	141	114
chr3	151929284	151931284	Nexn	-0.36125649	0.026577	stronglyHypometh	-0.13278	0.20041	insignificant	3	27	20
chr3	152003371	152005371	Fam73a	-0.32917777	0.0091482	hypomethylated	0.051092	0.49869	insignificant	3	11	11
chr3	152008444	152010444	Usp33	-0.09457231	0.000000201	hypomethylated	0.0051215	0.85256	insignificant	14	92	73
chr3	152057973	152059973	Zzz3	-0.08373642	2.12E-37	hypomethylated	0.019527	0.34533	insignificant	77	259	219
chr3	152058657	152060657	Zzz3	-0.08852008	3.23E-39	hypomethylated	0.01702	0.52438	insignificant	81	275	235
chr3	152331104	152333104	Ak5	-0.10800139	0.34925	insignificant	0.12665	0.22633	insignificant	9	41	39
chr3	152376063	152378063	Pigk	-0.08432261	0.10719	insignificant	0.005566	0.70299	insignificant	9	71	71
chr3	152645171	152647171	St6galnac5	-0.5	0.0038241	stronglyHypometh	0.28846	0.31029	insignificant	1	3	4
chr3	153388097	153390097	St6galnac3	-0.15131214	1.81E-08	hypomethylated	0.012788	0.082259	insignificant	2	22	22
chr3	153506210	153508210	Asb17		1	noCoverage	0.03606	0.72046	insignificant	0	13	12
chr3	153575153	153577153	Rabggtb	-0.12197761	0.000000119	hypomethylated	-0.0099492	0.85286	insignificant	11	42	41
chr3	153575167	153577167	Rabggtb	-0.12197761	0.000000119	hypomethylated	-0.0099492	0.85286	insignificant	11	42	41
chr3	153575930	153577930	Rabggtb	-0.12197761	0.000000119	hypomethylated	-0.0042042	0.85289	insignificant	11	42	39
chr3	153607396	153609396	Acadm	-0.23402259	0.0055798	hypomethylated	-0.012681	0.93563	insignificant	6	23	23
chr3	153635399	153637399	Slc44a5		1	noCoverage	0.02417	0.91558	insignificant	0	37	45
chr3	153993524	153995524	AI606473	-0.12894345	0.013903	hypomethylated	-0.044021	0.24358	insignificant	3	16	17
chr3	154258975	154260975	Cryz	-0.1446756	1.46E-11	hypomethylated	-0.0041552	0.61524	insignificant	20	92	88
chr3	154260062	154262062	Cryz	-0.19101025	5.21E-09	hypomethylated	0.0068462	0.33608	insignificant	8	30	30
chr3	156223757	156225757	Negr1	-0.12458946	1.24E-19	hypomethylated	0.0077033	0.078757	insignificant	39	120	114
chr3	156223922	156225922	Negr1	-0.12458946	1.24E-19	hypomethylated	0.0077033	0.078757	insignificant	39	120	114

chr3	157196360	157198360	Zranb2	-0.12294147	1.16E-38	hypomethylated	0.0097123	0.29016	insignificant	50	171	166
chr3	157228855	157230855	Ptger3	-0.12261761	6.64E-30	hypomethylated	-0.0010084	0.90853	insignificant	26	108	106
chr3	157588027	157590027	Cth		1	noCoverage	-0.092677	0.84444	insignificant	0	15	12
chr3	157609429	157611429	Ankrd13c	-0.12126529	2.58E-39	hypomethylated	0.0022698	0.2623	insignificant	54	174	165
chr3	157694718	157696718	Srsf11	-0.67501682	1.59E-50	stronglyHypometh	-0.25921	0.51468	insignificant	7	14	14
chr3	157698666	157700666	Lrrc40	-0.08574809	0.000046268	hypomethylated	0.0015145	0.85432	insignificant	19	71	71
chr3	157699603	157701603	Srsf11	0.03691919	0.8044	insignificant	0.028659	0.2734	insignificant	4	12	12
chr3	158225185	158227185	Lrrc7	-0.52529762	0.4698	lowCoverage	0.0074266	1	insignificant	1	12	12
chr3	159157396	159159396	Depdc1a		1	noCoverage	-0.059951	0.87001	insignificant	0	9	4
chr3	159501658	159503658	Wls		1	noCoverage	0.020112	0.89846	insignificant	0	14	20
chr4	3501025	3503025	Tgs1	-0.13017755	8.36E-13	hypomethylated	-0.0011034	0.89312	insignificant	27	80	74
chr4	3501915	3503915	Tmem68	-0.15391815	6.96E-13	hypomethylated	0.0039022	0.39288	insignificant	21	67	64
chr4	3604267	3606267	Lyn	-0.14733027	4.71E-19	hypomethylated	-0.0048531	0.65114	insignificant	25	82	83
chr4	3762747	3764747	Rps20	-0.07540291	0.000000543	hypomethylated	-0.013948	0.37806	insignificant	9	36	43
chr4	3865034	3867034	Chchd7	-0.09037352	0.00031652	hypomethylated	0.003837	0.38225	insignificant	23	106	110
chr4	3865060	3867060	Chchd7	-0.09037352	0.00031652	hypomethylated	0.003837	0.38225	insignificant	23	106	110
chr4	3865552	3867552	Plag1	-0.09484746	0.00052246	hypomethylated	-0.0006369	0.44038	insignificant	22	101	110
chr4	4065592	4067592	Penk	-0.12345275	0.0062974	hypomethylated	-0.062077	0.06216	insignificant	16	85	68
chr4	4720453	4722453	Impad1	-0.41545635	0.000053248	stronglyHypometh	-0.076824	0.92254	insignificant	4	9	8
chr4	5570325	5572325	Fam110b	-0.14827351	0.000000111	hypomethylated	0.0020345	0.84579	insignificant	21	78	77
chr4	6117251	6119251	Ubxn2b	-0.15420681	8.13E-21	hypomethylated	0.0063699	0.45576	insignificant	15	54	50
chr4	6291826	6293826	Sdcbp	-0.10958729	9.96E-09	hypomethylated	0.071304	0.80088	insignificant	7	53	43
chr4	6381418	6383418	Nsmaf	-0.30413795	0.015694	hypomethylated	-0.0042267	0.0071574	hypomethylated	2	23	23
chr4	6917870	6919870	Tox	-0.11364092	0.010123	hypomethylated	0.019372	0.093288	insignificant	14	47	48
chr4	8166188	8168188	Car8	-0.10836938	0.010294	hypomethylated	0.070774	0.0018467	hypermethylated	8	17	18
chr4	8461790	8463790	Rab2a	-0.09304757	0.00000412	hypomethylated	0.024444	0.59123	insignificant	28	100	95
chr4	8617067	8619067	Chd7	-0.07238113	1.55E-42	hypomethylated	-0.0016109	0.35477	insignificant	135	437	422
chr4	9195463	9197463	Clvs1		1	noCoverage	0.052381	0.13879	insignificant	0	10	10
chr4	9596309	9598309	Asph	-0.34152764	0.53483	insignificant	-0.046361	0.040381	inconclusive	1	22	24
chr4	9696731	9698731	4930412C18Ri	-0.13629566	2.16E-09	hypomethylated	-0.01824	0.39408	insignificant	5	27	28
chr4	9770518	9772518	Gdf6	-0.10066637	3.34E-16	hypomethylated	0.016494	0.0012459	hypermethylated	33	135	128
chr4	10800644	10802644	2610301B20R	-0.12168643	2.77E-19	hypomethylated	-0.0037408	0.17814	insignificant	42	166	167
chr4	10934766	10936766	Plekhf2	-0.05130511	0.0017234	hypomethylated	-0.044967	0.37416	insignificant	16	54	54
chr4	11003351	11005351	2310030N02R	-0.58739351	0.040817	stronglyHypometh	0.026312	0.17986	insignificant	2	21	20
chr4	11060243	11062243	Mir684-2		1	noCoverage	0.027709	0.60562	insignificant	0	8	9
chr4	11082587	11084587	Trp53inp1	-0.14293358	0.00000725	hypomethylated	0.030522	0.058763	insignificant	17	84	79
chr4	11117500	11119500	Ccne2	-0.10564875	7.78E-39	hypomethylated	-0.0055133	0.39864	insignificant	60	215	197
chr4	11117855	11119855	Ccne2	-0.10746193	4.49E-33	hypomethylated	-0.018059	0.72213	insignificant	46	167	158
chr4	11181406	11183406	Ints8	0.65590653	8.17E-08	stronglyHypermeth	0.097635	1	insignificant	2	22	22
chr4	11249278	11251278	Dpy19l4	-0.17853009	0.00017463	hypomethylated	-0.028278	1	insignificant	4	24	22
chr4	11313930	11315930	Esrp1	-0.1420378	5.13E-26	hypomethylated	-0.00076855	0.85033	insignificant	38	119	129
chr4	11412104	11414104	1110037F02Ri	-0.1273001	0.00014143	hypomethylated	0.0064472	0.66377	insignificant	19	71	80
chr4	11485118	11487118	Rad54b	-0.11709481	0.000012716	hypomethylated	-0.005842	0.55912	insignificant	14	48	42
chr4	11630593	11632593	Gem	-0.09658308	2.53E-09	hypomethylated	-0.0035945	0.54381	insignificant	11	62	62
chr4	11892860	11894860	Pdp1	-0.08150264	1.69E-31	hypomethylated	0.0088923	0.054691	insignificant	46	129	120
chr4	11893597	11895597	Pdp1	-0.08511905	0.21621	insignificant	0.026137	0.40175	insignificant	4	14	14
chr4	12015104	12017104	Tmem67	-0.32194617	1.82E-08	hypomethylated	-0.0014966	0.11896	insignificant	2	12	12
chr4	12015516	12017516	C430048L16Ri	-0.34738243	0.000000252	stronglyHypometh	-0.086104	0.19702	insignificant	2	14	14
chr4	12066263	12068263	Rbm12b	-0.21022727	0.13697	insignificant	-0.18195	0.11034	insignificant	2	12	32
chr4	12099162	12101162	Fam92a		1	noCoverage	0.27962	0.00070888	hypermethylated	0	4	4
chr4	12832983	12834983	Gm11818	-0.18275644	0.002983	hypomethylated	0.030227	1	insignificant	7	32	24
chr4	13669448	13671448	Runx1t1	-0.06754784	3.36E-13	hypomethylated	0.011406	0.56549	insignificant	32	155	149
chr4	13677443	13679443	Runx1t1	-0.20339801	0.0071737	hypomethylated	0.092531	0.000013024	hypermethylated	13	26	25
chr4	13710928	13712928	Runx1t1	-0.17982456	1	insignificant	0.00084746	1	insignificant	3	12	12
chr4	14753734	14755734	Otud6b	-0.52114695	0.000000122	stronglyHypometh	0.17123	1	insignificant	1	2	3
chr4	14790365	14792365	Tmem55a	-0.06871413	3.29E-08	hypomethylated	-0.020581	1	insignificant	10	41	40
chr4	15076278	15078278	Necab1	-0.27625418	0.053446	insignificant	0.12128	0.010131	hypermethylated	2	12	12
chr4	15191966	15193966	Tmem64	-0.07781696	3.55E-19	hypomethylated	0.0068343	0.70305	insignificant	47	169	166
chr4	15807410	15809410	Calb1	-0.04912842	1.77E-11	hypomethylated	0.020604	0.83735	insignificant	6	42	38
chr4	15872654	15874654	Decr1		1	noCoverage	-0.0038931	0.68821	insignificant	0	4	4
chr4	15884113	15886113	Nbn	-0.11854228	4.74E-28	hypomethylated	0.012053	0.61769	insignificant	39	94	96
chr4	15941024	15943024	Osgin2	-0.07277697	7.39E-17	hypomethylated	-0.0025894	0.71089	insignificant	23	90	90
chr4	16090645	16092645	Ripk2	-0.22317618	1.25E-55	hypomethylated	-0.046619	0.21962	insignificant	25	70	67
chr4	17779628	17781628	Mmp16	-0.17232831	0.000000166	hypomethylated	0.01272	0.36114	insignificant	9	53	53
chr4	19497251	19499251	Cpne3	-0.12481203	1	insignificant	-0.011003	1	insignificant	4	8	8
chr4	19501213	19503213	Fam82b	-0.33288241	6.62E-09	hypomethylated	-0.060038	0.20118	insignificant	9	38	27
chr4	19636140	19638140	Wwp1	-0.23609688	2.06E-23	hypomethylated	-0.11189	0.60975	insignificant	19	59	58
chr4	19934574	19936574	Ttpa	-0.13159036	1.5E-18	hypomethylated	0.011073	0.80772	insignificant	25	88	88
chr4	19968198	19970198	Ggh	-0.13357224	3.76E-34	hypomethylated	0.023578	0.097546	insignificant	23	74	86
chr4	21613110	21615110	Prdm13	-0.12842419	0.50577	insignificant	0.037797	0.82609	insignificant	7	32	35
chr4	21653849	21655849	Ccnc	-0.1544448	4.48E-16	hypomethylated	0.0028251	1	insignificant	17	68	68
chr4	21694358	21696358	2610029I01Ril	-0.51701223	0.00000621	stronglyHypometh	-0.070723	0.00012198	hypomethylated	3	11	11
chr4	21702416	21704416	Usp45	-0.10248462	0.00055334	hypomethylated	0.0075606	0.86497	insignificant	12	51	51
chr4	21773729	21775729	Sfrs18	-0.14328153	1.35E-12	hypomethylated	-0.0026347	0.80192	insignificant	19	92	83
chr4	21775595	21777595	Sfrs18	0.4047619	0.010616	stronglyHypermeth	0.13328	0.80101	insignificant	3	21	14
chr4	21805821	21807821	Coq3	-0.2265186	1.31E-08	hypomethylated	-0.031207	0.23428	insignificant	12	72	76
chr4	21857472	21859472	6230409E13Ri	-0.08658985	0.00000723	hypomethylated	0.0062414	0.87906	insignificant	38	141	137
chr4	22283711	22285711	Fbxl4	-0.10773573	6.15E-20	hypomethylated	-0.0020627	0.57195	insignificant	34	93	90
chr4	22415278	22417278	Pou3f2	-0.22235038	1.03E-24	hypomethylated	0.015201	0.75001	insignificant	13	73	70
chr4	24422608	24424608	Mms22l	-0.1237406	3.78E-28	hypomethylated	0.018079	0.92022	insignificant	40	115	110
chr4	24778233	24780233	Klhl32	-0.08955831	5.51E-15	hypomethylated	-0.014185	0.16705	insignificant	13	26	26
chr4	24824229	24826229	Ndufaf4	-0.19833427	6.28E-19	hypomethylated	-0.011784	0.7578	insignificant	21	62	62
chr4	24899565	24901565	Gpr63		1	noCoverage	0.058333	1	insignificant	0	4	4
chr4	25208968	25210968	1810074P20Ri	-0.55555556	0.0012664	stronglyHypometh	-0.036616	1	insignificant	0	0	0
chr4	25727150	25729150	Fut9	-0.11419297	1	insignificant	0.048225	0.096704	insignificant	3	6	6

chr4	26273799	26275799	Manea	-0.13546924	0.00000116	hypomethylated	-0.028287	0.30347	insignificant	7	21	21
chr4	28739294	28741294	Epha7	-0.0900324	1.39E-08	hypomethylated	-0.0043555	0.75704	insignificant	27	178	164
chr4	32050081	32052081	Map3k7	-0.11147566	0.000046062	hypomethylated	-0.0041267	0.095963	insignificant	31	99	92
chr4	32503409	32505409	Bach2		1	noCoverage	-0.068591	1	insignificant	0	4	4
chr4	32701447	32703447	Casp8ap2	-0.23332255	3.74E-09	hypomethylated	0.016653	0.8635	insignificant	9	54	54
chr4	32743093	32745093	Mdn1	-0.12053827	4.09E-26	hypomethylated	-0.0013449	0.59383	insignificant	33	77	77
chr4	33010480	33012480	Ankrd6	-0.23556771	0.0075619	hypomethylated	-0.057036	0.17732	insignificant	3	15	16
chr4	33037801	33039801	Ankrd6	-0.1370759	1.33E-16	hypomethylated	-0.026529	0.85944	insignificant	9	59	56
chr4	33068972	33070972	Rragd	-0.10074012	4.99E-23	hypomethylated	0.022128	0.12037	insignificant	38	112	116
chr4	33117399	33119399	Ube2j1	-0.09044764	3.03E-17	hypomethylated	-0.0036978	0.49463	insignificant	45	161	145
chr4	33118298	33120298	4933421O10Rik	-0.08863259	5.59E-11	hypomethylated	0.0046619	0.67253	insignificant	26	90	78
chr4	33218530	33220530	Gabrr1	0.0341189	0.3564	insignificant	0.056276	0.63096	insignificant	3	6	6
chr4	33276712	33278712	Pm20d2		1	noCoverage	0.050422	0.65871	insignificant	0	8	8
chr4	33294965	33296965	Srsf12	-0.19305885	1.79E-27	hypomethylated	0.0044256	0.02776	hypermethylated	38	133	127
chr4	33335762	33337762	Pnrc1		1	noCoverage	-0.0061041	0.17571	insignificant	0	14	11
chr4	33396285	33398285	Rngtt	-0.23678395	1.59E-09	hypomethylated	-0.034261	0.20574	insignificant	22	86	82
chr4	34010606	34012606	Cnr1	-0.11875413	6.57E-17	hypomethylated	-0.01505	0.44382	insignificant	38	192	195
chr4	34137042	34139042	Spaca1		1	noCoverage	0.021716	0.94385	insignificant	0	26	26
chr4	34496863	34498863	Akirin2	-0.08539814	2.15E-35	hypomethylated	0.0080699	0.016615	hypermethylated	54	174	170
chr4	34561206	34563206	Rars2	-0.10053713	2.73E-21	hypomethylated	0.00022637	0.31642	insignificant	20	67	75
chr4	34562191	34564191	Orc3	-0.09041659	2.93E-12	hypomethylated	0.055351	0.095327	insignificant	23	55	68
chr4	34634687	34636687	Slc35a1		1	noCoverage	-0.014546	1	insignificant	0	16	16
chr4	34830197	34832197	Zfp292	-0.19247942	0.000000113	hypomethylated	0.05343	0.050959	insignificant	10	45	37
chr4	35104715	35106715	Mob3b		1	noCoverage	-0.039249	0.020536	inconclusive	0	14	14
chr4	35173129	35175129	3110043O21Rik	-0.18787879	0.0076328	hypomethylated	-0.12403	0.21833	insignificant	3	16	16
chr4	36615619	36617619	Lingo2	-0.29375	0.43367	insignificant	0.12292	0.2837	insignificant	2	4	4
chr4	36898777	36900777	Lingo2	-0.13622564	0.0056267	hypomethylated	0.028437	0.16677	insignificant	13	57	57
chr4	40089297	40091297	Aco1	-0.14521634	5.4E-13	hypomethylated	0.0041985	0.92926	insignificant	19	72	67
chr4	40186858	40188858	Ddx58		1	noCoverage	-0.0025638	0.48203	insignificant	0	22	20
chr4	40216874	40218874	2010003O02Rik	-0.11725328	1	insignificant	-0.011631	0.000048059	inconclusive	6	23	26
chr4	40226401	40228401	Ndufb6		1	noCoverage	-0.013095	0.82723	insignificant	0	12	12
chr4	40418212	40420212	Tmem215	-0.04218545	0.000000387	hypomethylated	-0.012558	0.63078	insignificant	4	28	26
chr4	40419162	40421162	Tmem215	-0.11665794	0.29207	insignificant	-0.0055157	0.96516	insignificant	9	42	42
chr4	40649949	40651949	Aptx	-0.16448302	1.15E-12	hypomethylated	0.0035048	0.75743	insignificant	13	47	47
chr4	40650220	40652220	Aptx	-0.18083403	0.02805	hypomethylated	-0.000044189	0.51746	insignificant	4	17	17
chr4	40668500	40670500	Dnaja1	-0.15694336	7.61E-16	hypomethylated	-0.007677	0.62803	insignificant	42	142	140
chr4	40668949	40670949	Mir207	-0.15694336	7.61E-16	hypomethylated	-0.007677	0.62803	insignificant	42	142	140
chr4	40668954	40670954	Dnaja1	-0.15694336	7.61E-16	hypomethylated	-0.007677	0.62803	insignificant	42	142	140
chr4	40668962	40670962	Dnaja1	-0.15694336	7.61E-16	hypomethylated	-0.007677	0.62803	insignificant	42	142	140
chr4	40801031	40803031	B4galt1	-0.2508831	3.96E-08	hypomethylated	-0.0036592	0.298	insignificant	7	38	38
chr4	40866088	40868088	Spink4		1	noCoverage	0.042619	0.40694	insignificant	0	10	10
chr4	40894585	40896585	Chmp5	-0.09393043	7.19E-16	hypomethylated	0.0093519	0.0068546	hypermethylated	33	112	107
chr4	40895327	40897327	Bag1	-0.09299185	0.002672	hypomethylated	0.0095884	0.79556	insignificant	2	30	30
chr4	40916975	40918975	Nfx1	-0.14525163	2.17E-15	hypomethylated	-0.010301	0.64669	insignificant	19	83	77
chr4	40939727	40941727	Mir3094		1	noCoverage	0.12154	0.20128	insignificant	0	14	15
chr4	40995169	40997169	Aqp7	-0.29052444	0.071159	insignificant	0.087235	0.015207	hypermethylated	3	12	12
chr4	41045216	41047216	Aqp3	-0.63203878	5.72E-11	stronglyHypometh	0.0242	0.29945	insignificant	2	9	8
chr4	41071372	41073372	Nol6	-0.18594549	0.000000404	hypomethylated	-0.011977	0.54027	insignificant	7	21	21
chr4	41082053	41084053	Ube2r2	-0.09547575	0.00049995	hypomethylated	-0.017236	0.52723	insignificant	29	141	125
chr4	41222168	41224168	Ubap2	-0.12702291	1	lowCoverage	0.082406	0.36732	insignificant	1	30	30
chr4	41295028	41297028	Ubap1	-0.1234767	0.00000207	hypomethylated	0.0082483	0.67129	insignificant	10	69	69
chr4	41411180	41413180	Nudt2	-0.24545569	5.29E-17	hypomethylated	-0.020062	1	insignificant	20	103	77
chr4	41411881	41413881	Kif24	-0.38365004	1.3E-10	stronglyHypometh	0.076511	0.50486	insignificant	2	48	22
chr4	41450109	41452109	AI464131	-0.52371744	1.84E-08	stronglyHypometh	0.011327	0.29997	insignificant	4	22	20
chr4	41454797	41456797	1110017D15Rik	-0.25845915	0.0076877	inconclusive	-0.014841	0.94906	insignificant	6	22	22
chr4	41515826	41517826	Dnaic1	-0.06548199	4.29E-31	hypomethylated	-0.011059	0.49835	insignificant	35	118	121
chr4	41516560	41518560	2310028H24Rik	-0.15544198	1	insignificant	0.01905	0.40806	insignificant	2	28	30
chr4	41587335	41589335	Enho		1	noCoverage	0.014069	1	insignificant	0	8	8
chr4	41642477	41644477	Cntfr	-0.17090216	0.37665	insignificant	0.024967	0.58978	insignificant	3	16	26
chr4	41644123	41646123	Cntfr	-0.2306313	1.14E-20	hypomethylated	-0.0028029	0.074509	insignificant	11	43	44
chr4	41660549	41662549	2810432D09Rik	-0.14811505	3.38E-20	hypomethylated	-0.00014737	0.82561	insignificant	23	76	73
chr4	41678174	41680174	Arid3c	-0.16217305	0.0011264	hypomethylated	-0.034801	0.50483	insignificant	9	57	54
chr4	41688186	41690186	Sigmar1		1	noCoverage	0.035857	0.3353	insignificant	0	10	10
chr4	41701125	41703125	Galt		1	noCoverage	0.035668	0.65541	insignificant	0	29	24
chr4	41706315	41708315	Il11ra1	-0.21098296	4.41E-10	hypomethylated	-0.023869	0.013756	hypomethylated	4	18	16
chr4	41708182	41710182	Il11ra1	-0.15359477	1	insignificant	-0.015383	0.61114	insignificant	2	6	6
chr4	41720974	41722974	Ccl27a	-0.21405229	0.000017305	hypomethylated	-0.043417	0.43661	insignificant	6	12	12
chr4	41721007	41723007	Ccl27a	-0.21405229	0.000017305	hypomethylated	-0.043417	0.43661	insignificant	6	12	12
chr4	41721049	41723049	Ccl27a	-0.21977124	0.0034455	hypomethylated	-0.029295	0.57129	insignificant	3	6	6
chr4	41811375	41813375	Gm13308		1	noCoverage	-0.046131	0.73901	insignificant	0	24	14
chr4	42929122	42931122	N28178	-0.09215585	1.58E-27	hypomethylated	0.0035917	0.91635	insignificant	50	149	120
chr4	42964965	42966965	Dnajb5	0.09343434	1	insignificant	-0.086234	0.78651	insignificant	2	4	7
chr4	42981831	42983831	1700022I11Rik	-0.3167578	0.30602	insignificant	-0.085437	0.063647	insignificant	2	14	14
chr4	43013379	43015379	Vcp		1	noCoverage	-0.054475	0.083074	insignificant	0	22	22
chr4	43023173	43025173	Fancg		1	noCoverage	0.020792	0.029226	hypermethylated	0	8	8
chr4	43038628	43040628	Pigo	-0.20514403	0.00000901	hypomethylated	-0.073021	0.82887	insignificant	7	21	18
chr4	43044256	43046256	Stoml2	-0.13274671	0.000060969	hypomethylated	-0.010634	1	insignificant	4	20	20
chr4	43059075	43061075	B230312A22Rik	-0.34655067	0.18377	insignificant	-0.066533	0.86976	insignificant	2	20	20
chr4	43070855	43072855	Unc13b	-0.14974096	2.34E-30	hypomethylated	0.032732	0.027088	hypermethylated	28	92	92
chr4	43070944	43072944	Unc13b	-0.14974096	2.34E-30	hypomethylated	0.032732	0.027088	hypermethylated	28	92	92
chr4	43393853	43395853	Rusc2	-0.06244798	0.0073724	hypomethylated	0.0065014	0.0014047	hypermethylated	24	115	112
chr4	43418445	43420445	Rusc2	0.26906566	0.0010073	hypermethylated	0.076965	0.53454	insignificant	6	16	36
chr4	43442006	43444006	Fam166b	-0.03876812	0.54902	insignificant	-0.084632	1	insignificant	2	12	12
chr4	43454148	43456148	Tesk1	-0.16595094	1.13E-46	hypomethylated	-0.0076986	0.28307	insignificant	32	75	89
chr4	43505275	43507275	Rmrp	-0.1615902	1.43E-13	hypomethylated	0.009168	0.96615	insignificant	11	108	108

chr4	43505931	43507931	Rmrp	-0.13428168	1.14E-09	hypomethylated	-0.0085009	0.44958	insignificant	5	62	62
chr4	43512532	43514532	E130306D19R	-0.05729718	0.63504	insignificant	0.0088754	0.22189	insignificant	5	24	24
chr4	43518897	43520897	Car9	-0.02957485	0.44889	insignificant	0.1263	0.88538	insignificant	2	6	8
chr4	43536260	43538260	Tpm2	-0.11631961	0.0019661	hypomethylated	0.017405	0.29137	insignificant	11	97	95
chr4	43574505	43576505	Creb3	-0.14398761	7.21E-24	hypomethylated	-0.014457	0.54636	insignificant	20	86	100
chr4	43575455	43577455	Tln1	-0.27501533	0.1286	insignificant	-0.077575	0.82365	insignificant	6	24	22
chr4	43590606	43592606	Rgp1	-0.12060178	4.64E-15	hypomethylated	0.002097	0.61756	insignificant	28	121	118
chr4	43591736	43593736	Rgp1	-0.15142254	0.00000481	hypomethylated	-0.00042894	1	insignificant	12	55	54
chr4	43597366	43599366	Rgp1	-0.55663082	0.079768	insignificant	-0.032689	0.7939	insignificant	2	6	6
chr4	43643806	43645806	Npr2	-0.12810147	2.99E-64	hypomethylated	0.026404	2.4E-15	hypermethylated	51	156	159
chr4	43666424	43668424	Spag8	-0.13862746	0.31435	insignificant	0.0075572	0.8491	insignificant	7	27	28
chr4	43680842	43682842	Tmem8b	-0.12544952	5.08E-23	hypomethylated	-0.0017881	0.27727	insignificant	19	78	70
chr4	43681731	43683731	4930412F15Ri	-0.19056605	6.28E-26	hypomethylated	-0.013546	0.19878	insignificant	19	86	76
chr4	43739069	43741069	Hrct1	-0.0436444	1	insignificant	-0.015127	0.33488	insignificant	4	13	13
chr4	43741905	43743905	5430416O09R	-0.0956439	0.18207	insignificant	0.15544	0.00047939	hypermethylated	3	12	12
chr4	43849389	43851389	Olfr157	-0.04082492	0.50451	insignificant	-0.0084175	0.81835	insignificant	2	6	6
chr4	43866163	43868163	Olfr155	-0.23611111	0.60904	insignificant	-0.19031	0.01417	hypomethylated	3	8	11
chr4	43887401	43889401	Reck	-0.13392098	7.74E-36	hypomethylated	0.0094551	0.68241	insignificant	46	168	168
chr4	43969573	43971573	Glipr2	-0.05053745	0.069387	insignificant	0.0046998	0.90331	insignificant	8	73	69
chr4	43995375	43997375	Ccin	0.16870051	0.000009474	hypermethylated	0.0098373	0.9169	insignificant	14	71	87
chr4	44024514	44026514	Clta	-0.15576153	7.7E-15	hypomethylated	0.013837	0.8153	insignificant	24	64	64
chr4	44180410	44182410	Rnf38	-0.0688189	4.65E-18	hypomethylated	0.0021342	0.40452	insignificant	50	178	172
chr4	44181155	44183155	Rnf38	-0.09091694	0.000000137	hypomethylated	0.00045753	0.87243	insignificant	17	54	54
chr4	44312788	44314788	Melk	-0.15600563	8.72E-13	hypomethylated	0.015223	0.89676	insignificant	20	62	63
chr4	44723312	44725312	Pax5	-0.22632108	1.86E-11	hypomethylated	0.021137	0.78697	insignificant	14	44	44
chr4	44768430	44770430	Zcchc7	-0.16560739	1.08E-28	hypomethylated	-0.0037691	0.030842	hypomethylated	13	94	94
chr4	44993282	44995282	Grhpr	-0.23097676	4.21E-18	hypomethylated	-0.038701	0.96886	insignificant	12	56	54
chr4	45025284	45027284	Zbtb5	-0.11950904	1	insignificant	-0.0014438	0.22633	insignificant	3	10	10
chr4	45030496	45032496	Polr1e	-0.18184783	1.27E-14	hypomethylated	0.0055987	0.18723	insignificant	7	40	40
chr4	45097476	45099476	Fbxo10	0.28444724	0.75674	insignificant	0.0010902	0.36954	insignificant	1	25	25
chr4	45120985	45122985	Tomm5	0.41243694	0.16497	insignificant	0.16908	0.22024	insignificant	1	20	20
chr4	45196777	45198777	Frmpd1	-0.12111174	1.47E-21	hypomethylated	0.0035291	0.75279	insignificant	42	121	112
chr4	45333475	45335475	Exosc3	-0.14872928	6.36E-19	hypomethylated	-0.029641	0.0023236	inconclusive	8	21	20
chr4	45353972	45355972	Dcaf10	-0.11240593	6.34E-24	hypomethylated	-0.01174	0.84191	insignificant	41	148	132
chr4	45421638	45423638	Mcart1	-0.10791698	0.14487	insignificant	-0.068195	0.94533	insignificant	16	35	35
chr4	45543700	45545700	Shb	-0.16035194	0.024772	hypomethylated	-0.029753	0.53109	insignificant	4	58	56
chr4	45695460	45697460	Gm829	-0.09259636	0.078905	insignificant	-0.019047	0.30402	insignificant	4	13	13
chr4	45810893	45812893	Aldh1b1	-0.10696954	0.85399	insignificant	0.021589	0.90111	insignificant	12	54	54
chr4	45839699	45841699	Igfbpl1	-0.10223023	0.010336	hypomethylated	0.0167	0.33659	insignificant	7	18	18
chr4	46051093	46053093	Tmod1	-0.10425715	6.79E-27	hypomethylated	-0.0064624	0.57677	insignificant	40	127	128
chr4	46150382	46152382	Ncbp1	-0.1314786	2.35E-29	hypomethylated	0.010457	1	insignificant	30	96	75
chr4	46151347	46153347	Tstd2	-0.14145319	3.67E-19	hypomethylated	-0.017482	0.87826	insignificant	22	86	69
chr4	46209183	46211183	Xpa	-0.22537915	0.0067687	hypomethylated	-0.042957	0.83716	insignificant	4	18	18
chr4	46356065	46358065	Foxe1	-0.09654834	1.91E-27	hypomethylated	0.0058486	0.0015592	hypermethylated	83	265	264
chr4	46402295	46404295	5830415F09Ri	-0.10674849	0.0012006	hypomethylated	0.0049813	0.87779	insignificant	11	40	40
chr4	46417055	46419055	Hemgn	-0.08326118	0.38327	insignificant	-0.0052089	0.60634	insignificant	2	7	7
chr4	46462988	46464988	Anp32b	-0.10617779	4.87E-46	hypomethylated	0.00061103	0.44191	insignificant	79	229	226
chr4	46501200	46503200	Nans	-0.2388639	6E-23	hypomethylated	0.011382	0.90697	insignificant	13	66	64
chr4	46549016	46551016	Trim14	0.24606721	1	insignificant	0.036543	0.55527	insignificant	2	12	10
chr4	46579312	46581312	Coro2a	-0.32495778	0.039611	hypomethylated	-0.039547	0.37164	insignificant	2	10	10
chr4	46614801	46616801	Coro2a	-0.12592552	2.95E-18	hypomethylated	0.013716	0.63139	insignificant	12	46	35
chr4	46663071	46665071	Tbc1d2	-0.16776377	0.000086259	hypomethylated	0.015392	0.81244	insignificant	6	18	18
chr4	47004586	47006586	Gabbr2	-0.13696967	0.00000147	hypomethylated	-0.013296	0.83002	insignificant	16	86	86
chr4	47070178	47072178	Anks6	-0.14824615	8.96E-17	hypomethylated	-0.037974	0.30949	insignificant	15	79	76
chr4	47103824	47105824	Galnt12	-0.12383595	2.91E-09	hypomethylated	0.0089601	0.12832	insignificant	16	64	64
chr4	47219883	47221883	Col15a1	-0.09874884	0.000000617	hypomethylated	0.0043577	0.48295	insignificant	25	105	104
chr4	47365176	47367176	Tgfbr1	-0.14133971	2.31E-14	hypomethylated	0.0081166	0.46707	insignificant	38	106	106
chr4	47486532	47488532	Sec61b	-0.13174471	0.017619	hypomethylated	0.0028546	0.60553	insignificant	4	46	47
chr4	47487239	47489239	Alg2		1	noCoverage	0.0079815	0.74056	insignificant	0	16	16
chr4	48063119	48065119	Nr4a3	-0.09680325	0.00028542	hypomethylated	0.036623	0.39409	insignificant	8	49	53
chr4	48136790	48138790	Stx17	-0.12764192	5.84E-11	hypomethylated	-0.019647	0.8417	insignificant	27	81	72
chr4	48291673	48293673	Invs	-0.1473457	0.00000147	hypomethylated	0.0021218	0.53135	insignificant	14	68	68
chr4	48292461	48294461	Erp44	-0.11868219	0.15622	insignificant	0.0025524	0.78445	insignificant	12	46	46
chr4	48486294	48488294	Tex10	-0.06580065	0.00021094	hypomethylated	0.011021	0.074236	insignificant	14	36	37
chr4	48551817	48553817	5730528L13Ri	-0.10045643	8.75E-35	hypomethylated	0.0093951	0.40515	insignificant	51	164	166
chr4	48551952	48553952	5730528L13Ri	-0.10045643	8.75E-35	hypomethylated	0.0093951	0.40515	insignificant	51	164	166
chr4	48552370	48554370	5730528L13Ri	-0.10045643	8.75E-35	hypomethylated	0.0093951	0.40515	insignificant	51	164	166
chr4	48597064	48599064	Tmeff1	-0.12438234	3.03E-29	hypomethylated	-0.031991	0.40936	insignificant	63	189	182
chr4	48675385	48677385	Murc	0.06322844	1	insignificant	0.020457	0.33089	insignificant	2	26	22
chr4	49071333	49073333	E130309F12Ri	-0.1818596	8.47E-08	hypomethylated	0.011955	0.95676	insignificant	13	66	66
chr4	49533088	49535088	Zfp189	-0.09638334	0.000000819	hypomethylated	0.027669	1	insignificant	7	41	53
chr4	49533955	49535955	Mrpl50	-0.03684861	0.93061	insignificant	-0.083239	0.023353	hypomethylated	8	35	44
chr4	49643931	49645931	Rnf20	-0.18583189	0.0043027	hypomethylated	-0.023653	0.29461	insignificant	3	39	36
chr4	49857953	49859953	Grin3a	-0.14933559	8.56E-18	hypomethylated	0.039905	0.24999	insignificant	24	86	84
chr4	52451120	52453120	Smc2	-0.17847922	0.00002172	hypomethylated	-0.00091422	0.35701	insignificant	12	52	58
chr4	52924664	52926664	Olfr272	-0.08499373	0.21575	insignificant	-0.034362	0.7084	insignificant	6	14	14
chr4	53001155	53003155	Nipsnap3a		1	noCoverage	-0.007815	0.86467	insignificant	0	12	12
chr4	53023795	53025795	Nipsnap3b	-0.08181141	0.018896	hypomethylated	0.049517	0.0021877	hypermethylated	12	35	53
chr4	53172767	53174767	Abca1	-0.15230475	0.03167	hypomethylated	-0.0065597	0.86559	insignificant	11	40	42
chr4	53283104	53285104	AI427809	-0.03366157	0.82456	insignificant	-0.022564	0.93288	insignificant	8	29	28
chr4	53452284	53454284	Slc44a1	-0.08118001	6.36E-31	hypomethylated	0.016954	0.036473	hypermethylated	60	204	198
chr4	53643342	53645342	Fsd1l	-0.11060063	0.0010887	hypomethylated	-0.00064219	0.79594	insignificant	15	88	98
chr4	53726053	53728053	Fktn	-0.14322243	2.12E-08	hypomethylated	-0.0028955	0.41891	insignificant	12	73	73
chr4	53791576	53793576	Tal2	-0.15016329	0.049449	hypomethylated	0.038213	0.075532	insignificant	5	18	18
chr4	53837916	53839916	Tmem38b	-0.12325225	0.010867	hypomethylated	-0.0032864	0.9543	insignificant	11	69	67

chr4	54959816	54961816	Zfp462	-0.34234694	0.00001571	stronglyHypometh	-0.046001	0.60026	insignificant	6	14	14
chr4	55361913	55363913	Rad23b	-0.11181412	3.42E-48	hypomethylated	-0.00021598	0.11617	insignificant	76	226	225
chr4	55545347	55547347	Klf4	-0.13419656	0.000062917	hypomethylated	0.014586	0.01109	hypermethylated	29	128	137
chr4	56814200	56816200	BC026590	-0.15717475	6.16E-11	hypomethylated	-0.00022279	0.2647	insignificant	18	103	96
chr4	56815203	56817203	BC026590	-0.29428652	6.42E-16	hypomethylated	0.02158	0.41284	insignificant	7	35	28
chr4	56878083	56880083	Ctnnal1		1	noCoverage	0.0070941	0.13622	insignificant	0	13	13
chr4	56908170	56910170	D730040F13R	-0.09470899	0.4131	insignificant	0.06049	0.089027	insignificant	3	9	8
chr4	57003263	57005263	6430704M03F	-0.17602768	0.1852	insignificant	-0.090313	0.84255	insignificant	1	6	6
chr4	57156028	57158028	Epb4.1l4b	-0.04088872	3E-14	hypomethylated	0.0085051	0.27071	insignificant	67	160	146
chr4	57314709	57316709	Ptpn3	-0.12816757	1	insignificant	0.012552	1	insignificant	1	17	16
chr4	57580119	57582119	Palm2	-0.20867208	0.00003817	hypomethylated	-0.04736	0.049792	hypomethylated	11	36	33
chr4	57857119	57859119	Akap2	0.02787342	0.213	insignificant	0.024288	0.096827	insignificant	4	10	8
chr4	57866433	57868433	Akap2	-0.08574507	1	insignificant	-0.062947	0.010084	hypomethylated	4	8	8
chr4	57969283	57971283	Txn1		1	noCoverage	-0.013492	0.70058	insignificant	0	10	10
chr4	58297833	58299833	Musk	0.04086538	1	insignificant	-0.057174	0.54451	insignificant	4	8	8
chr4	58566165	58568165	Lpar1	-0.11092093	4.12E-08	hypomethylated	-0.0016392	0.40947	insignificant	14	42	42
chr4	58566363	58568363	Lpar1	-0.35645273	0.00003382	stronglyHypometh	-0.012301	1	insignificant	3	6	6
chr4	58925597	58927597	AI314180	-0.11412037	0.064843	insignificant	0.0090191	0.69686	insignificant	11	40	36
chr4	58955499	58957499	Zkscan16		1	noCoverage	0.13292	0.16139	insignificant	0	10	6
chr4	59015064	59017064	Dnajc25	-0.11770828	1.6E-15	hypomethylated	-0.010705	0.92107	insignificant	35	106	109
chr4	59047027	59049027	Gng10	-0.04268706	0.0000021	hypomethylated	0.009243	0.043502	hypermethylated	45	101	110
chr4	59201421	59203421	Ugcg	-0.08520088	1.17E-33	hypomethylated	0.012885	0.016747	hypermethylated	28	146	139
chr4	59451505	59453505	Susd1	-0.18756986	0.000053175	hypomethylated	0.0019248	0.27879	insignificant	4	18	18
chr4	59562236	59564236	Rod1	-0.11689367	0.0045755	hypomethylated	-0.011379	0.90318	insignificant	7	50	50
chr4	59593434	59595434	Hsdl2	-0.13923543	0.0019517	hypomethylated	-0.0076755	0.43166	insignificant	13	72	73
chr4	59638092	59640092	E130308A19R	-0.1030331	7.92E-26	hypomethylated	-0.0052183	0.30678	insignificant	37	180	160
chr4	59638198	59640198	E130308A19R	-0.1030331	7.92E-26	hypomethylated	-0.0052183	0.30678	insignificant	37	180	160
chr4	59796727	59798727	1110054O05R	-0.1035919	0.000047301	hypomethylated	-0.014641	0.2663	insignificant	6	24	24
chr4	59817521	59819521	Snx30	-0.07730124	2.05E-18	hypomethylated	-0.0031721	0.65834	insignificant	18	96	96
chr4	61811875	61813875	Mup21	0.21586322	0.64369	insignificant	0.20347	0.19014	insignificant	2	19	14
chr4	61869580	61871580	Zfp37	-0.09306518	0.10502	insignificant	0.011647	0.36723	insignificant	8	26	24
chr4	61946478	61948478	Slc31a2	-0.15104386	7.33E-16	hypomethylated	0.016498	0.33515	insignificant	13	57	48
chr4	62020734	62022734	Slc31a1	-0.14024538	0.14629	insignificant	0.010806	0.47036	insignificant	7	75	64
chr4	62021582	62023582	Slc31a1	-0.14135026	0.20766	insignificant	0.044702	0.56248	insignificant	4	43	33
chr4	62068816	62070816	Prpf4	-0.17089608	1.82E-26	hypomethylated	0.045251	0.016646	hypermethylated	18	71	68
chr4	62069657	62071657	Cdc26	-0.18757608	2.15E-27	hypomethylated	0.036587	0.0175	hypermethylated	19	65	66
chr4	62131906	62133906	Wdr31	-0.09387755	0.014174	hypomethylated	0.01777	0.34237	insignificant	5	14	14
chr4	62140100	62142100	Bspry	-0.1122422	0.0026516	hypomethylated	0.019873	0.80583	insignificant	8	62	26
chr4	62163234	62165234	Hdhd3	-0.16306309	7.16E-08	hypomethylated	0.13043	0.071158	insignificant	2	15	10
chr4	62181097	62183097	Alad		1	noCoverage	0.037843	0.087473	insignificant	0	10	9
chr4	62185402	62187402	4933430I17Ril	-0.12489726	0.0063218	hypomethylated	0.0093961	0.42761	insignificant	4	42	42
chr4	62186048	62188048	Pole3	-0.10279475	0.24378	insignificant	0.005868	0.64653	insignificant	2	28	28
chr4	62219880	62221880	Rgs3	-0.52758621	0.0071877	stronglyHypometh	0.18011	0.18136	insignificant	1	5	8
chr4	62279705	62281705	Rgs3	0.63813573	0.46835	lowCoverage	0.056244	0.42991	insignificant	1	14	14
chr4	62625607	62627607	Zfp618	-0.09339137	6.22E-26	hypomethylated	0.0076884	0.77367	insignificant	53	157	162
chr4	62815176	62817176	Ambp	0.13616071	0.59076	insignificant	-0.05428	0.26676	insignificant	1	4	4
chr4	62875445	62877445	Col27a1	-0.08450367	1.16E-13	hypomethylated	0.012878	0.061582	insignificant	34	135	114
chr4	62916884	62918884	Mir455	-0.12548983	0.27587	insignificant	0.012729	0.92915	insignificant	4	30	34
chr4	63022482	63024482	Orm2	-0.78819444	0.053908	insignificant	0.059028	0.29252	insignificant	2	8	8
chr4	63064479	63066479	Akna	-0.19851456	0.2053	insignificant	0.067585	0.50583	insignificant	3	6	10
chr4	63156985	63158985	Whrn	-0.1166027	0.000000171	hypomethylated	-0.0061335	0.37531	insignificant	10	33	33
chr4	63204798	63206798	Atp6v1g1	-0.27502378	2.55E-17	hypomethylated	-0.029136	0.069533	insignificant	12	49	51
chr4	63261798	63263798	Gm11213	0.14905791	1	insignificant	0.026729	0.83693	insignificant	1	9	9
chr4	63406147	63408147	Tnfsf15		1	noCoverage	-0.1254	0.38921	insignificant	0	4	5
chr4	63522318	63524318	Tnfsf8		1	noCoverage	0.014669	1	insignificant	0	8	7
chr4	64784207	64786207	Pappa	-0.13043809	1.1E-22	hypomethylated	-0.020341	0.12328	insignificant	29	83	96
chr4	65265019	65267019	Trim32		1	noCoverage	0.0041301	0.74769	insignificant	0	8	8
chr4	66065517	66067517	Astn2	-0.24580273	5.14E-10	hypomethylated	-0.030564	0.30379	insignificant	6	28	36
chr4	68615431	68617431	Dbc1	-0.34293949	0.000002847	stronglyHypometh	0.066283	0.91095	insignificant	2	8	10
chr4	70071401	70073401	Cdk5rap2	-0.00243313	0.000000381	hypomethylated	-0.0031327	0.29644	insignificant	11	50	50
chr4	70195962	70197962	Megf9	-0.10535509	5.51E-20	hypomethylated	0.0070345	0.049969	hypermethylated	35	96	96
chr4	71861277	71863277	C630043F03Ri	-0.12124288	1.01E-08	hypomethylated	0.01109	0.44523	insignificant	30	123	123
chr4	71861953	71863953	Tle1	-0.25510096	0.00081341	hypomethylated	-0.056092	0.54157	insignificant	8	33	30
chr4	73436506	73438506	Rasef	-0.0574053	0.56092	insignificant	-0.021957	0.017146	hypomethylated	29	101	89
chr4	73658506	73660506	Frmd3	-0.09712017	0.12301	insignificant	0.022879	1	insignificant	4	23	22
chr4	73887400	73889400	Kdm4c		1	noCoverage	0.20865	0.17076	insignificant	0	5	4
chr4	73896782	73898782	Kdm4c	-0.1073807	3.3E-15	hypomethylated	-0.0017001	0.62877	insignificant	27	103	110
chr4	74924190	74926190	3110001D03R	-0.15965376	0.086276	insignificant	0.035973	0.10239	insignificant	5	23	22
chr4	80479133	80481133	Tyrp1		1	noCoverage	0.066896	0.1728	insignificant	0	10	10
chr4	80555589	80557589	D4Bwg0951e		1	noCoverage	-0.0036171	0.2678	insignificant	0	41	37
chr4	81088709	81090709	Mpdz	-0.16559508	1.19E-11	hypomethylated	0.03598	0.42255	insignificant	14	52	52
chr4	82151212	82153212	Nfib	-0.11934112	9.7E-31	hypomethylated	-0.00057807	0.6116	insignificant	75	199	202
chr4	82505565	82507565	Zdhhc21	-0.12370768	0.0012515	hypomethylated	-0.0040488	0.8451	insignificant	8	26	26
chr4	82970093	82972093	Ttc39b	-0.08888037	0.0041926	hypomethylated	0.0025786	0.6873	insignificant	9	26	26
chr4	83062647	83064647	Snapc3	-0.15154905	7.53E-12	hypomethylated	-0.0040034	0.75962	insignificant	16	74	74
chr4	83132294	83134294	Psip1	-0.09311065	0.00087174	hypomethylated	0.018547	1	insignificant	7	24	24
chr4	83170448	83172448	4930473A06R	-0.10251486	6.87E-10	hypomethylated	-0.0055947	0.86318	insignificant	33	127	127
chr4	84320990	84322990	Bnc2	-0.06229255	1.54E-08	hypomethylated	0.0019121	0.70241	insignificant	19	134	110
chr4	84529230	84531230	Cntln	-0.0731306	3.72E-12	hypomethylated	0.018399	0.15958	insignificant	36	136	136
chr4	84850359	84852359	Sh3gl2	-0.13960309	0.016633	hypomethylated	0.037112	0.89874	insignificant	5	44	42
chr4	86204207	86206207	Fam154a	-0.38257576	0.0033079	stronglyHypometh	-0.099418	0.34575	insignificant	2	4	6
chr4	86220576	86222576	Rraga	-0.13355977	7.02E-13	hypomethylated	0.032814	0.33896	insignificant	14	96	97
chr4	86257926	86259926	Haus6	-0.28169914	0.000026061	hypomethylated	-0.01538	0.96605	insignificant	10	47	48
chr4	86393458	86395458	Dennd4c	-0.11838174	4.25E-11	hypomethylated	-0.000039739	0.4822	insignificant	28	81	73
chr4	86503271	86505271	Rps6	-0.1481736	6.89E-18	hypomethylated	0.0082267	0.8386	insignificant	16	41	41

chr4	86519317	86521317	Acer2	-0.26555473	4.43E-25	hypomethylated	-0.057828	0.000003111	inconclusive	17	50	37
chr4	86873867	86875867	Slc24a2	-0.37261383	0.0021408	stronglyHypometh	-0.010879	0.77018	insignificant	2	8	8
chr4	86876444	86878444	Slc24a2	-0.10966422	1	insignificant	0.00087862	0.35206	insignificant	3	12	12
chr4	87679311	87681311	Mllt3	-0.14286616	0.023011	hypomethylated	0.0046068	0.92165	insignificant	12	42	43
chr4	87739533	87741533	BC057079	-0.15696515	0.000004608	hypomethylated	-0.0031059	0.54169	insignificant	16	42	42
chr4	88368412	88370412	Klhl9		1	noCoverage	-0.014352	0.66612	insignificant	0	9	9
chr4	88399771	88401771	Gm13271	0.36333333	1	insignificant	0.24615	0.0084912	hypermethylated	3	10	8
chr4	88418897	88420897	Gm13285		1	noCoverage	-0.035817	0.8698	insignificant	0	35	33
chr4	88424753	88426753	Gm13285		1	noCoverage	-0.063615	0.00000432	hypomethylated	0	32	48
chr4	88427034	88429034	Ifnz		1	noCoverage	-0.0092498	0.31568	insignificant	0	33	36
chr4	88432880	88434880	Gm13277		1	noCoverage	0.034343	0.41942	insignificant	0	9	9
chr4	88433535	88435535	Gm13285		1	noCoverage	0.25571	0.093533	insignificant	0	9	13
chr4	88435798	88437798	Gm13278		1	noCoverage	0.01065	0.86699	insignificant	0	20	20
chr4	88436453	88438453	Gm13285		1	noCoverage	0.01065	0.86699	insignificant	0	20	20
chr4	88438645	88440645	Gm13275		1	noCoverage	0.007122	0.45052	insignificant	0	19	38
chr4	88441645	88443645	Gm13278		1	noCoverage	0.022737	1	insignificant	0	20	9
chr4	88442300	88444300	Gm13285		1	noCoverage	0.10543	1	insignificant	0	24	13
chr4	88444478	88446478	Gm13275		1	noCoverage	0.006456	0.75448	insignificant	0	18	18
chr4	88445223	88447223	Gm13285		1	noCoverage	0.13069	0.60546	insignificant	0	18	21
chr4	88448157	88450157	Gm13285		1	noCoverage	0.10833	0.76395	insignificant	0	15	20
chr4	88451089	88453089	Gm13285	-0.49215986	7.75E-70	stronglyHypometh	-0.023459	4.2E-37	hypomethylated	8	25	25
chr4	88472319	88474319	Ifna6	-0.81547619	0.33333	lowCoverage	-0.057143	0.4202	insignificant	1	4	4
chr4	88782273	88784273	Mtap	-0.11658021	0.00019602	hypomethylated	0.029144	0.056979	insignificant	17	49	56
chr4	88928096	88930096	Cdkn2a		1	noCoverage	0.019841	0.58925	insignificant	0	8	10
chr4	88940523	88942523	Cdkn2a	-0.10066907	0.00000382	hypomethylated	0.056648	0.0090464	hypermethylated	54	174	174
chr4	88956941	88958941	Cdkn2b		1	noCoverage	0.0061659	0.53358	insignificant	0	8	8
chr4	89353888	89355888	Dmrta1	-0.11590429	0.00028973	hypomethylated	0.012668	0.027787	hypermethylated	23	64	64
chr4	91038746	91040746	Elavl2	-0.12518933	0.016036	hypomethylated	0.039063	0.000765	hypermethylated	9	46	46
chr4	91043187	91045187	Elavl2		1	noCoverage	0.029972	0.17564	insignificant	0	16	20
chr4	91066675	91068675	Elavl2	-0.04074238	0.35318	insignificant	-0.02751	0.025675	hypomethylated	11	47	46
chr4	93002202	93004202	Tusc1	-0.12811627	0.1504	insignificant	0.0037325	0.094944	insignificant	3	29	22
chr4	94269938	94271938	Plaa	-0.11687701	9.12E-16	hypomethylated	0.0083237	0.00070377	inconclusive	12	48	48
chr4	94280210	94282210	Ift74		1	noCoverage	-0.028928	0.91725	insignificant	0	12	12
chr4	94330847	94332847	Mir872	0.12316176	0.31465	insignificant	0.11306	0.12141	insignificant	2	4	6
chr4	94645791	94647791	Mysm1	-0.13727273	0.010263	hypomethylated	0.031352	0.75546	insignificant	5	13	11
chr4	94718913	94720913	Jun	-0.14183914	6.13E-12	hypomethylated	-0.019931	0.16374	insignificant	13	76	73
chr4	95223197	95225197	Fggy	-0.02139992	0.6305	insignificant	-0.048293	0.60033	insignificant	2	16	22
chr4	95633069	95635069	Hook1	-0.08797252	0.000000262	hypomethylated	0.0056646	0.8514	insignificant	24	102	94
chr4	97247633	97249633	Nfia	-0.90288462	0.013462	stronglyHypometh	-0.0080433	1	insignificant	1	8	8
chr4	97251282	97253282	Nfia	-0.26963264	0.000000832	hypomethylated	-0.030489	0.36182	insignificant	4	8	8
chr4	97443316	97445316	Nfia	-0.0851526	0.0060182	hypomethylated	-0.0097401	0.48558	insignificant	28	117	113
chr4	98049956	98051956	Tm2d1		1	noCoverage	0.019877	0.40594	insignificant	0	8	10
chr4	98061516	98063516	Inadl	-0.13044096	0.000000212	hypomethylated	-0.0074249	0.40229	insignificant	20	82	79
chr4	98392444	98394444	L1td1	-0.33429831	9.84E-10	stronglyHypometh	-0.014685	0.10064	insignificant	6	35	30
chr4	98484228	98486228	Kank4	-0.34312556	2.3E-15	stronglyHypometh	-0.030893	0.0070898	inconclusive	11	32	32
chr4	98589500	98591500	Usp1	-0.11326498	4.6E-23	hypomethylated	0.0021401	0.7786	insignificant	52	163	162
chr4	98787606	98789606	Dock7	-0.14319166	0.021489	hypomethylated	0.11124	1	insignificant	7	22	25
chr4	98859624	98861624	Atg4c	-0.15162166	9.62E-11	hypomethylated	0.0095713	0.7855	insignificant	7	47	45
chr4	98859837	98861837	Atg4c	-0.15162166	9.62E-11	hypomethylated	0.0095713	0.7855	insignificant	7	47	45
chr4	99321989	99323989	Foxd3	-0.17354826	5.52E-48	hypomethylated	0.03504	0.006699	hypermethylated	48	192	191
chr4	99381320	99383320	Alg6	-0.28353081	0.000020458	hypomethylated	0.025993	0.34602	insignificant	5	19	19
chr4	99495190	99497190	Efcab7	-0.13051348	3.56E-09	hypomethylated	0.009343	0.36389	insignificant	8	41	41
chr4	99495809	99497809	Itgb3bp	-0.13051348	3.56E-09	hypomethylated	0.009343	0.36389	insignificant	8	41	41
chr4	99601055	99603055	Pgm2	-0.13126544	0.0023423	hypomethylated	-0.0042296	0.55894	insignificant	23	129	126
chr4	99767395	99769395	Ror1	-0.11785998	3.81E-18	hypomethylated	-0.0086385	0.20661	insignificant	20	94	88
chr4	100150471	100152471	Ube2u	0.27334194	1	lowCoverage	-0.26639	0.27549	insignificant	1	18	18
chr4	100448283	100450283	Cachd1	-0.12199097	1.24E-51	hypomethylated	0.031026	0.04959	hypermethylated	33	82	86
chr4	100740642	100742642	Raver2	-0.08361786	4.14E-44	hypomethylated	0.013881	0.40991	insignificant	44	173	176
chr4	101019632	101021632	Mir101a		1	noCoverage	-0.064124	0.1108	insignificant	0	8	8
chr4	101090893	101092893	Ak4	-0.14357642	1.41E-30	hypomethylated	0.01822	0.71202	insignificant	35	125	123
chr4	101090916	101092916	Ak4	-0.14357642	1.41E-30	hypomethylated	0.01822	0.71202	insignificant	35	125	123
chr4	101090925	101092925	Ak4	-0.14357642	1.41E-30	hypomethylated	0.01822	0.71202	insignificant	35	125	123
chr4	101091307	101093307	Ak4	-0.1297064	4.42E-29	hypomethylated	0.018292	0.70624	insignificant	35	123	121
chr4	101168252	101170252	Dnajc6	-0.09656701	6.95E-18	hypomethylated	-0.00089449	0.24065	insignificant	45	128	125
chr4	101179580	101181580	Dnajc6	-0.17655668	0.014169	hypomethylated	0.010807	0.35164	insignificant	4	26	24
chr4	101222198	101224198	Dnajc6	-0.08902601	0.000038851	hypomethylated	0.084043	0.12631	insignificant	21	67	73
chr4	101389011	101391011	Lepr	-0.23613368	9.7E-11	hypomethylated	-0.036742	0.35743	insignificant	3	32	25
chr4	101658444	101660444	Gm12789		1	noCoverage	0.076416	0.47647	insignificant	0	8	8
chr4	102241664	102243664	Pde4b	-0.21537062	0.0019681	hypomethylated	0.023713	0.73985	insignificant	4	24	24
chr4	102261447	102263447	Pde4b		1	noCoverage	-0.080952	0.62812	insignificant	0	10	14
chr4	102431967	102433967	Sgip1	-0.05608492	0.027601	hypomethylated	0.089346	0.040115	hypermethylated	2	18	18
chr4	102786069	102788069	Mier1	-0.08029212	1.04E-44	hypomethylated	0.001428	0.29089	insignificant	43	158	155
chr4	102786904	102788904	Wdr78	-0.06499449	5.52E-34	hypomethylated	0.010457	0.075223	insignificant	31	105	108
chr4	102790994	102792994	Mier1	-0.09549729	1.59E-15	hypomethylated	0.015406	0.82393	insignificant	25	110	100
chr4	102887489	102889489	Slc35d1	-0.01334063	0.50951	insignificant	0.0067176	0.82607	insignificant	15	84	84
chr4	102963468	102965468	4921539E11Ri	0.06777778	0.67662	insignificant	0.10721	0.14682	insignificant	1	5	5
chr4	102985450	102987450	Oma1	-0.1101082	0.000012657	hypomethylated	0.0064052	0.61765	insignificant	22	77	77
chr4	104039164	104041164	Dab1	-0.13258682	7.87E-18	hypomethylated	0.017189	0.55595	insignificant	32	139	137
chr4	104039175	104041175	Dab1	-0.13258682	7.87E-18	hypomethylated	0.017189	0.55595	insignificant	32	141	139
chr4	104437921	104439921	C8b	0.15570175	0.35171	insignificant	0.12262	0.0011432	hypermethylated	2	8	8
chr4	104585060	104587060	1700024P16Ri	-0.29301645	0.000027885	hypomethylated	-0.019675	0.48942	insignificant	4	20	22
chr4	104782503	104784503	Prkaa2	0.15013504	0.74163	insignificant	0.15303	0.25281	insignificant	2	9	8
chr4	104828951	104830951	Ppap2b	-0.12554877	4.75E-09	hypomethylated	0.014274	0.80455	insignificant	32	133	133
chr4	105987817	105989817	Usp24	-0.10462815	2.56E-39	hypomethylated	0.0071154	0.68655	insignificant	73	216	207
chr4	106136930	106138930	Pcsk9	-0.27631579	1	insignificant	-0.012427	0.56377	insignificant	2	4	4

chr4	106164848	106166848	Bsnd		1	noCoverage	0.033208	1	insignificant	0	2	2
chr4	106232642	106234642	Dhcr24	-0.13255008	7.47E-32	hypomethylated	-0.0049721	0.41843	insignificant	24	123	120
chr4	106289843	106291843	BC055111	-0.16531714	1.43E-16	hypomethylated	-0.0035673	0.80617	insignificant	6	14	14
chr4	106294053	106296053	Ttc22	0.02224037	0.065997	insignificant	0.027619	0.25133	insignificant	11	67	72
chr4	106322673	106324673	Pars2	-0.24393939	0.000018658	hypomethylated	-0.02261	0.70805	insignificant	10	20	20
chr4	106351291	106353291	Ttc4	0.02382482	0.47396	insignificant	-0.080946	0.010221	hypomethylated	6	31	28
chr4	106351549	106353549	Ttc4	0.04166667	1	insignificant	-0.010554	0.77328	insignificant	2	6	4
chr4	106472436	106474436	Acot11	-0.36852522	5.98E-11	stronglyHypometh	0.040505	0.83703	insignificant	6	24	24
chr4	106583074	106585074	Ssbp3	-0.07036017	5.28E-19	hypomethylated	0.0095638	0.27105	insignificant	48	267	267
chr4	106739471	106741471	Mrpl37	-0.13242907	1.53E-20	hypomethylated	0.0051406	0.7463	insignificant	13	56	55
chr4	106741772	106743772	Cyb5rl	0.09280163	1	insignificant	0.070214	0.00063345	hypermethylated	2	8	8
chr4	106850234	106852234	Tmem59	-0.16903223	5.49E-28	hypomethylated	0.026337	0.063541	insignificant	25	100	88
chr4	106850971	106852971	2210012G02R	-0.12383438	8.69E-19	hypomethylated	0.043622	0.20753	insignificant	21	74	62
chr4	106925538	106927538	Hspb11	-0.11616852	1.82E-22	hypomethylated	0.012663	0.67674	insignificant	21	90	79
chr4	106926138	106928138	Lrrc42	-0.15095063	0.000000867	hypomethylated	0.0026742	0.48613	insignificant	12	28	28
chr4	106979748	106981748	Dio1		1	noCoverage	-0.017076	0.7286	insignificant	0	6	3
chr4	106986035	106988035	Yipf1	-0.38258892	0.057789	insignificant	-0.077015	0.76516	insignificant	3	31	23
chr4	107039388	107041388	Tmem48	-0.11895296	2.85E-15	hypomethylated	0.0018212	0.093297	insignificant	17	56	56
chr4	107106323	107108323	Glis1	-0.10917285	8.91E-22	hypomethylated	-0.0072479	0.25294	insignificant	47	178	192
chr4	107356767	107358767	Dmrtb1	0.00573856	1	insignificant	-0.10818	0.051668	insignificant	3	25	24
chr4	107473863	107475863	Lrp8	-0.10443243	1.67E-22	hypomethylated	0.00064254	0.50866	insignificant	52	154	154
chr4	107551417	107553417	Magoh	-0.29097407	0.0040416	hypomethylated	-0.00021932	0.62831	insignificant	8	35	43
chr4	107561503	107563503	0610037L13Ri	-0.20691374	0.000000407	hypomethylated	-0.018832	0.7813	insignificant	19	56	55
chr4	107596194	107598194	Cpt2	-0.11053355	0.000000293	hypomethylated	0.050597	0.33536	insignificant	5	18	19
chr4	107639938	107641938	Slc1a7	0.18463225	0.080451	insignificant	0.038728	0.0018087	hypermethylated	2	13	13
chr4	107704695	107706695	Podn	-0.0865865	0.46641	insignificant	-0.011543	0.053589	insignificant	6	28	28
chr4	107791104	107793104	Scp2	-0.1530303	0.089413	insignificant	-0.040001	0.7049	insignificant	6	20	24
chr4	107837070	107839070	Echdc2	-0.08067031	5.97E-09	hypomethylated	0.0027527	0.094367	insignificant	11	46	46
chr4	107890527	107892527	Zyg11a	-0.13295585	0.040746	hypomethylated	-0.012892	0.94065	insignificant	7	20	14
chr4	107999756	108001756	2010305A19R	-0.17554716	0.58937	insignificant	-0.029104	0.11462	insignificant	5	34	42
chr4	108055954	108057954	Fam159a	-0.11074641	1.86E-13	hypomethylated	-0.018911	0.37894	insignificant	15	54	54
chr4	108079318	108081318	Gpx7		1	noCoverage	0.017085	0.52546	insignificant	0	21	20
chr4	108131030	108133030	Zcchc11	-0.10451838	4.97E-15	hypomethylated	0.0081667	0.70595	insignificant	22	122	129
chr4	108251058	108253058	Orc1	-0.17001535	0.000011314	hypomethylated	-0.0032789	0.23351	insignificant	28	96	92
chr4	108251941	108253941	Prpf38a	-0.16140018	1.31E-08	hypomethylated	-0.010137	0.28128	insignificant	17	53	53
chr4	108291560	108293560	Cc2d1b	-0.10939279	1.37E-33	hypomethylated	-0.0010063	0.39632	insignificant	44	124	124
chr4	108506189	108508189	Btf3l4	-0.15416983	0.0016809	hypomethylated	-0.024495	0.41834	insignificant	14	84	86
chr4	108506282	108508282	Txndc12	-0.19917383	0.00015682	hypomethylated	-0.039572	0.43187	insignificant	14	57	59
chr4	108519461	108521461	Kti12	-0.14255101	2.53E-31	hypomethylated	-0.016723	0.18265	insignificant	30	101	103
chr4	108550674	108552674	Rab3b	-0.15675418	8.36E-15	hypomethylated	0.041485	0.53652	insignificant	9	45	45
chr4	108672409	108674409	Nrd1	-0.17259495	0.000012557	hypomethylated	0.028884	0.97532	insignificant	19	78	75
chr4	108951879	108953879	Eps15	0.01361978	7.98E-27	inconclusive	0.02605	0.76235	insignificant	53	119	105
chr4	109078704	109080704	Ttc39a	-0.13386483	1.02E-18	hypomethylated	0.00386	0.63285	insignificant	20	74	76
chr4	109087251	109089251	Ttc39a		1	noCoverage	-0.029167	0.40483	insignificant	0	6	6
chr4	109149110	109151110	Rnf11	-0.17297543	2.39E-22	hypomethylated	-0.0093928	0.37839	insignificant	20	70	70
chr4	109337977	109339977	Cdkn2c	-0.08694801	1.14E-23	hypomethylated	0.0082166	0.54815	insignificant	51	205	197
chr4	109348231	109350231	Faf1	-0.1018628	1.36E-28	hypomethylated	0.01476	0.065758	insignificant	50	131	127
chr4	109649629	109651629	Dmrta2	-0.09735354	0.02542	hypomethylated	0.0017996	0.77867	insignificant	32	128	134
chr4	110069395	110071395	Agbl4	-0.11908248	0.00012104	hypomethylated	-0.0092318	0.39538	insignificant	5	20	20
chr4	111086610	111088610	Bend5	-0.14083969	3.88E-13	hypomethylated	0.010344	0.39844	insignificant	29	94	93
chr4	111391614	111393614	Spata6	-0.10260031	1.97E-11	hypomethylated	-0.010838	0.85039	insignificant	24	66	66
chr4	114078328	114080328	Gm12824	-0.09725231	5.01E-16	hypomethylated	0.013026	0.058588	insignificant	38	139	139
chr4	114493324	114495324	Gm12830	-0.1238423	0.000000718	hypomethylated	0.026944	0.14505	insignificant	12	44	44
chr4	114580893	114582893	9130206I24Ril	-0.11053884	1.99E-29	hypomethylated	-0.0005975	0.87981	insignificant	43	195	190
chr4	114581503	114583503	9130206I24Ril	-0.154022	1E-15	hypomethylated	0.05791	0.26551	insignificant	21	84	88
chr4	114598618	114600618	Foxe3	-0.25344655	1	insignificant	0.012275	0.7557	insignificant	1	18	18
chr4	114659833	114661833	Cmpk1	-0.16427279	1.41E-09	hypomethylated	-0.038437	0.73021	insignificant	13	29	28
chr4	114671722	114673722	Stil	-0.06568961	0.025204	hypomethylated	0.029632	0.94333	insignificant	7	48	41
chr4	114731131	114733131	Tal1	-0.29648496	0.0097091	hypomethylated	-0.042457	1	insignificant	6	22	22
chr4	115255732	115257732	Gm12839	0.20241064	1	insignificant	0.027091	0.27375	insignificant	2	34	29
chr4	115409677	115411677	4732418C07Rl	-0.12362697	3.08E-12	hypomethylated	-0.024695	0.39936	insignificant	7	45	44
chr4	115456589	115458589	Atpaf1	-0.0642811	0.00052798	hypomethylated	0.0049959	0.97204	insignificant	17	81	80
chr4	115499696	115501696	Mob3c	-0.10922123	7.43E-13	hypomethylated	-0.0058055	0.44507	insignificant	19	75	75
chr4	115510850	115512850	Mknk1	-0.17854247	0.0020243	hypomethylated	-0.0082909	0.11838	insignificant	4	40	40
chr4	115556004	115558004	Kncn	0.07849384	1	insignificant	0.047812	0.37164	insignificant	2	14	13
chr4	115612531	115614531	Dmbx1	-0.08876448	1.32E-13	hypomethylated	0.074557	1	insignificant	15	32	35
chr4	115690507	115692507	Faah	-0.17219896	0.0057051	hypomethylated	-0.077233	0.89619	insignificant	5	13	12
chr4	115747069	115749069	Lrrc41	-0.12460895	1.16E-19	hypomethylated	0.012114	0.020692	hypermethylated	30	163	154
chr4	115747675	115749675	Uqcrh	-0.10790578	5.68E-22	hypomethylated	0.0086612	0.47047	insignificant	35	167	158
chr4	115796294	115798294	Rad54l	-0.24652893	0.0000012	hypomethylated	0.081512	0.65068	insignificant	3	11	12
chr4	115796295	115798295	Rad54l	-0.24652893	0.0000012	hypomethylated	0.081512	0.65068	insignificant	3	11	12
chr4	115817221	115819221	1520402A15Rik		1	noCoverage	0.016574	0.70406	insignificant	0	11	13
chr4	115822122	115824122	Pomgnt1	-0.12214076	1.24E-11	hypomethylated	0.0052238	0.72452	insignificant	29	129	125
chr4	115840203	115842203	Tspan1	0.20833333	1	insignificant	0.023918	0.34164	insignificant	1	10	10
chr4	115844976	115846976	1700042G07R	-0.0745604	0.20204	insignificant	0.035984	0.012714	hypermethylated	6	17	16
chr4	115893518	115895518	Pik3r3	-0.18890704	2.26E-24	hypomethylated	0.0031862	0.43699	insignificant	24	91	90
chr4	116136788	116138788	Mast2	-0.08420953	0.0091483	hypomethylated	-0.0096632	0.026556	hypomethylated	14	58	52
chr4	116179153	116181153	Ipp	-0.14079019	2.77E-11	hypomethylated	-0.0011304	0.57234	insignificant	28	79	79
chr4	116228548	116230548	Tmem69	-0.15706534	1.83E-37	hypomethylated	0.0095376	0.2489	insignificant	40	101	94
chr4	116229331	116231331	Gpbp1l1	-0.14616313	2.83E-36	hypomethylated	0.01042	0.077739	insignificant	43	130	116
chr4	116268334	116270334	Ccdc17	-0.12108829	0.00065021	hypomethylated	-0.014569	0.7662	insignificant	12	44	40
chr4	116270235	116272235	C530005A16R	-0.13356205	0.016377	hypomethylated	0.049647	0.52522	insignificant	6	29	30
chr4	116300102	116302102	Nasp	-0.58646617	0.56261	insignificant	-0.020684	0.84933	insignificant	2	7	7
chr4	116324256	116326256	Akr1a1	-0.174105	0.00025241	hypomethylated	-0.00042571	1	insignificant	3	6	6
chr4	116357203	116359203	Prdx1	-0.11686858	0.0021642	hypomethylated	-0.0096637	0.85433	insignificant	22	88	86

chr4	116380534	116382534	Ccdc163	-0.35361237	0.10299	insignificant	-0.014546	0.19563	insignificant	2	22	24
chr4	116380990	116382990	Mmachc		1	noCoverage	-0.001853	0.601	insignificant	0	12	12
chr4	116392559	116394559	Tesk2	-0.23966765	9.36E-28	hypomethylated	-0.0088399	0.29268	insignificant	20	86	87
chr4	116479338	116481338	Mutyh	-0.48763981	2.12E-15	stronglyHypometh	0.058964	0.03147	inconclusive	6	32	32
chr4	116480164	116482164	Toe1		1	noCoverage	0.11472	0.74735	insignificant	0	16	19
chr4	116494113	116496113	Hpdl	-0.29088715	0.15726	insignificant	0.033041	0.85523	insignificant	4	15	15
chr4	116549006	116551006	Zswim5	-0.11352062	1.16E-87	hypomethylated	0.0062434	0.0030199	hypermethylated	80	224	225
chr4	116666952	116668952	Hectd3	-0.13308769	1.98E-13	hypomethylated	-0.00068816	0.49633	insignificant	45	134	134
chr4	116666980	116668980	Hectd3	-0.13308769	1.98E-13	hypomethylated	-0.00068816	0.49633	insignificant	45	134	134
chr4	116691012	116693012	Eif2b3	-0.099925	0.00003444	hypomethylated	-0.033682	0.24177	insignificant	27	91	94
chr4	116767960	116769960	Ptch2	-0.19976158	0.00000161	hypomethylated	0.018734	0.27398	insignificant	12	32	33
chr4	116798330	116800330	Btbd19		1	noCoverage	0.19934	0.33111	insignificant	0	5	4
chr4	116798417	116800417	Tctex1d4		1	noCoverage	0.19934	0.33111	insignificant	0	5	4
chr4	116806557	116808557	Plk3	-0.24612181	0.1369	insignificant	-0.026146	0.44034	insignificant	5	35	34
chr4	116827179	116829179	Snord38a	-0.11788788	0.000000661	hypomethylated	-0.0029974	0.34118	insignificant	17	95	84
chr4	116828453	116830453	Snord55	-0.21913101	0.0008252	hypomethylated	-0.031349	0.87619	insignificant	13	75	58
chr4	116828737	116830737	Snord55	0.48877666	0.55295	insignificant	0.092788	0.013859	hypermethylated	1	36	18
chr4	116855229	116857229	Kif2c	-0.49337579	0.028761	stronglyHypometh	0.0025028	0.45723	insignificant	3	28	28
chr4	116923593	116925593	Tmem53	-0.13219614	1.51E-08	hypomethylated	0.0051686	0.95019	insignificant	14	43	43
chr4	116924522	116926522	Gm1661	-0.14569603	0.0010481	hypomethylated	0.0091885	1	insignificant	8	31	31
chr4	117354830	117356830	Dmap1	-0.20627798	0.19451	insignificant	0.0075032	0.84661	insignificant	3	12	12
chr4	117506862	117508862	Slc6a9	-0.11586988	5.45E-36	hypomethylated	-0.0008377	0.54502	insignificant	29	75	70
chr4	117545075	117547075	B4galt2	-0.1365281	0.37621	insignificant	-0.15168	0.00030788	hypomethylated	6	24	24
chr4	117556074	117558074	Atp6v0b	-0.09933698	0.031763	hypomethylated	-0.012275	0.53524	insignificant	1	26	26
chr4	117559934	117561934	Atp6v0b	-0.2416754	1.44E-08	hypomethylated	0.029533	0.36911	insignificant	8	34	34
chr4	117564608	117566608	Dph2	-0.41071429	0.0032188	stronglyHypometh	0.28159	0.17105	insignificant	2	9	5
chr4	117587604	117589604	Ipo13	-0.2010615	2.64E-09	hypomethylated	-0.010068	0.44803	insignificant	16	46	58
chr4	117602368	117604368	Artn	0.20518942	0.0072634	hypermethylated	-0.037627	0.10117	insignificant	3	10	11
chr4	117807495	117809495	St3gal3	-0.14450387	9.43E-10	hypomethylated	0.011416	0.7697	insignificant	25	54	57
chr4	117807519	117809519	St3gal3	-0.15939519	3.27E-11	hypomethylated	0.011491	0.54788	insignificant	23	50	53
chr4	117852648	117854648	Kdm4a	-0.30834142	1	insignificant	-0.033046	0.57995	insignificant	1	17	18
chr4	117964002	117966002	Ptprf	-0.21585241	0.000032194	hypomethylated	0.0031555	0.0042577	hypermethylated	10	34	32
chr4	118031618	118033618	Hyi	-0.08179189	0.10185	insignificant	0.010614	0.9476	insignificant	5	53	46
chr4	118080941	118082941	Med8	-0.14441364	1.29E-28	hypomethylated	-0.0015512	0.90302	insignificant	22	92	90
chr4	118081868	118083868	Szt2	-0.14271321	1.36E-28	hypomethylated	0.023116	0.90185	insignificant	19	68	66
chr4	118099697	118101697	Elovl1	-0.23035892	1.66E-10	hypomethylated	0.01182	0.019402	hypermethylated	15	40	40
chr4	118101305	118103305	Elovl1	-0.28184889	0.15555	insignificant	-0.041908	0.43608	insignificant	1	12	12
chr4	118109948	118111948	Cdc20	-0.41458806	0.000062143	stronglyHypometh	0.028894	0.1699	insignificant	3	56	58
chr4	118130100	118132100	Mpl	0.10730605	0.000000027	inconclusive	-0.11329	0.73199	insignificant	5	22	22
chr4	118162454	118164454	Tie1		1	noCoverage	0.0018691	0.86637	insignificant	0	6	6
chr4	118198879	118200879	2610528J11Rik		1	noCoverage	0.045714	0.26637	insignificant	0	8	8
chr4	118216331	118218331	Tmem125	-0.50383419	0.00013972	stronglyHypometh	0.16102	0.4121	insignificant	4	16	23
chr4	118292403	118294403	Ebna1bp2	-0.16863246	0.000036282	hypomethylated	0.0080499	0.64603	insignificant	6	35	41
chr4	118293010	118295010	Wdr65	-0.13701565	0.000020303	hypomethylated	0.0043769	0.95216	insignificant	6	30	36
chr4	118298008	118300008	D4Ertd617e	-0.13333333	0.040659	hypomethylated	0.0075758	0.7407	insignificant	2	4	4
chr4	118397853	118399853	Olfr1340		1	noCoverage	0.013393	0.80713	insignificant	0	2	2
chr4	118406135	118408135	Olfr1339		1	noCoverage	-0.067775	0.23931	insignificant	0	6	3
chr4	118427141	118429141	Olfr1338	-0.30530303	0.049687	hypomethylated	-0.03469	0.78769	insignificant	1	4	2
chr4	118780349	118782349	Slc2a1	-0.1084864	0.11462	insignificant	0.005283	0.84772	insignificant	8	92	90
chr4	118846461	118848461	Zfp691	-0.13944368	0.0033069	hypomethylated	0.018606	1	insignificant	12	43	42
chr4	118846800	118848800	Zfp691	-0.3	0.037916	hypomethylated	0.053788	0.31051	insignificant	4	10	10
chr4	118862616	118864616	Ermap	-0.00913421	0.74393	insignificant	-0.14262	0.000027628	hypomethylated	1	12	12
chr4	118866914	118868914	Ccdc23	-0.04012027	1	insignificant	-0.018535	0.78028	insignificant	25	100	104
chr4	118867129	118869129	Ccdc23	-0.07184699	0.19955	insignificant	-0.035262	0.46079	insignificant	25	104	106
chr4	118904519	118906519	Lepre1	-0.12274366	1.47E-12	hypomethylated	0.014981	0.10404	insignificant	24	102	102
chr4	118905329	118907329	AU022252	-0.10969459	8.78E-09	hypomethylated	0.017727	0.12192	insignificant	16	78	78
chr4	118905433	118907433	Lepre1	-0.10969459	8.78E-09	hypomethylated	0.017727	0.12192	insignificant	16	78	78
chr4	118927045	118929045	Cldn19	0.04784509	0.053377	insignificant	-0.022103	0.92827	insignificant	7	17	17
chr4	118927073	118929073	Cldn19	0.04784509	0.053377	insignificant	-0.022103	0.92827	insignificant	7	17	17
chr4	118967118	118969118	Ybx1	-0.12009152	2.12E-14	hypomethylated	-0.0036376	0.50623	insignificant	25	84	77
chr4	118993128	118995128	Ppih	-0.16338147	4.55E-08	hypomethylated	0.040785	0.38086	insignificant	9	19	20
chr4	119088126	119090126	Ccdc30	-0.16815723	0.00030892	hypomethylated	0.0080123	0.82669	insignificant	10	28	26
chr4	119094288	119096288	Zmynd12	-0.10586694	6.57E-13	hypomethylated	0.012799	0.38968	insignificant	22	142	139
chr4	119095025	119097025	Zmynd12	-0.11470625	1.22E-09	hypomethylated	0.0025083	0.90871	insignificant	16	80	80
chr4	119165203	119167203	Rimkla	-0.03455455	0.00051706	hypomethylated	0.013101	0.77328	insignificant	11	30	32
chr4	119211292	119213292	Foxj3	-0.07640358	6.32E-23	hypomethylated	0.006776	0.0020851	hypermethylated	100	285	285
chr4	119211374	119213374	AA415398	-0.07640358	6.32E-23	hypomethylated	0.006776	0.0020851	hypermethylated	100	285	285
chr4	119309336	119311336	Guca2a	-0.21067243	1	insignificant	0.0063673	0.94293	insignificant	3	16	16
chr4	119486282	119488282	Hivep3	-0.26666887	0.57686	insignificant	-0.055824	0.25979	insignificant	4	24	19
chr4	119833028	119835028	Edn2	-0.19961526	0.46086	insignificant	0.026386	0.0069359	hypermethylated	3	29	31
chr4	119959866	119961866	Foxo6	-0.11416518	1.03E-29	hypomethylated	-0.019239	0.94758	insignificant	47	139	124
chr4	120076885	120078885	Scmh1	-0.1145235	1.14E-14	hypomethylated	0.0004826	0.80346	insignificant	33	104	101
chr4	120203835	120205835	Slfnl1		1	noCoverage	0.20468	0.091007	insignificant	0	10	11
chr4	120242881	120244881	Ctps	-0.12674066	6.89E-18	hypomethylated	-0.00087402	0.84823	insignificant	14	37	41
chr4	120260349	120262349	Gm8439	0.31818182	1	lowCoverage	0.16434	0.82679	insignificant	1	4	8
chr4	120338167	120340167	Cited4	-0.14715439	1.43E-30	hypomethylated	0.0070136	0.48524	insignificant	39	119	102
chr4	120419781	120421781	Kcnq4	-0.12847498	4.84E-49	hypomethylated	-0.0034057	0.84979	insignificant	60	163	155
chr4	120445302	120447302	Nfyc	0.12962963	1	insignificant	0.12086	0.0056744	hypermethylated	2	6	6
chr4	120498320	120500320	Nfyc		1	noCoverage	0.070984	1	insignificant	0	12	12
chr4	120597610	120599610	Dem1	0.33132627	0.0016316	hypermethylated	0.030953	0.60643	insignificant	2	28	28
chr4	120624306	120626306	Zfp69	-0.20205172	1.14E-23	hypomethylated	0.030016	0.03714	inconclusive	11	37	34
chr4	120689852	120691852	Smap2	-0.13547479	0.000010005	hypomethylated	0.044865	0.10655	insignificant	8	25	23
chr4	120711170	120713170	Col9a2	-0.1998085	2.85E-26	hypomethylated	0.00018829	0.016229	hypermethylated	16	46	46
chr4	120770848	120772848	Zmpste24	-0.26915879	0.042019	hypomethylated	-0.046587	0.15601	insignificant	6	20	20
chr4	122512469	122514469	Ppt1	0.04747306	0.69229	insignificant	-0.079308	0.18292	insignificant	1	6	6

chr4	122563124	122565124	Cap1	-0.1583919	0.00095099	hypomethylated	0.069139	0.30721	insignificant	11	57	36
chr4	122638431	122640431	Mfsd2a	-0.14732452	0.0034691	hypomethylated	0.0064214	0.6495	insignificant	6	26	26
chr4	122672341	122674341	Mycl1	-0.12527001	8.71E-25	hypomethylated	0.0015263	0.50766	insignificant	39	161	140
chr4	122692839	122694839	Trit1	-0.23095983	2.71E-10	hypomethylated	0.0089211	1	insignificant	10	31	29
chr4	122781407	122783407	Bmp8b	-0.14206884	1.45E-10	hypomethylated	0.026761	0.000093483	hypermethylated	36	146	146
chr4	122792490	122794490	Oxct2b		1	noCoverage	-0.016046	0.35195	insignificant	0	27	27
chr4	122817184	122819184	Ppie	-0.14895632	0.00006656	hypomethylated	-0.017592	0.89798	insignificant	9	28	25
chr4	122859746	122861746	Hpcal4	-0.15949511	0.0019269	hypomethylated	0.011876	0.23202	insignificant	18	71	71
chr4	122877795	122879795	Nt5c1a	-0.06765853	0.27424	insignificant	0.057582	0.048125	hypermethylated	12	34	34
chr4	122909798	122911798	Heyl	-0.12831535	2.08E-16	hypomethylated	0.0023246	0.5277	insignificant	25	68	68
chr4	122959153	122961153	Pabpc4	-0.08138486	2.57E-11	hypomethylated	0.020454	0.72502	insignificant	20	54	52
chr4	122959527	122961527	Pabpc4	-0.08138486	2.57E-11	hypomethylated	0.020454	0.72502	insignificant	20	54	52
chr4	123000877	123002877	Bmp8a		1	noCoverage	0.015695	1	insignificant	0	3	3
chr4	123020046	123022046	Bmp8a	-0.27765984	1.18E-09	hypomethylated	-0.042571	0.79659	insignificant	5	22	26
chr4	123089154	123091154	D830031N03R	-0.14711453	1.14E-20	hypomethylated	-0.0059127	0.26112	insignificant	28	67	67
chr4	123395404	123397404	BC002163	-0.09693129	0.00022305	hypomethylated	0.01831	0.86737	insignificant	9	48	49
chr4	123395429	123397429	BC002163	-0.11380202	0.000028385	hypomethylated	0.02333	0.82088	insignificant	9	40	41
chr4	123427542	123429542	Akirin1	-0.13055008	1.96E-12	hypomethylated	-0.0072314	0.17805	insignificant	19	76	74
chr4	123581255	123583255	Mycbp	-0.08186727	4.7E-12	hypomethylated	0.0095529	0.21123	insignificant	34	94	86
chr4	123593675	123595675	Rragc	-0.08261475	3.22E-20	hypomethylated	0.00098446	0.79337	insignificant	34	174	171
chr4	124333888	124335888	Pou3f1	-0.10563511	1.27E-28	hypomethylated	0.0077423	0.11456	insignificant	88	273	263
chr4	124370798	124372798	Utp11l	-0.17577977	0.020926	hypomethylated	-0.041634	0.41825	insignificant	6	17	16
chr4	124376942	124378942	Fhl3	-0.13848494	2E-24	hypomethylated	-0.0063481	0.90172	insignificant	35	142	145
chr4	124391104	124393104	Sf3a3	-0.13630975	4.33E-18	hypomethylated	0.020314	0.96151	insignificant	8	36	34
chr4	124420024	124422024	Mir698	0.0754085	0.35393	insignificant	0.13914	0.35292	insignificant	6	17	16
chr4	124478792	124480792	Mtf1	-0.20165507	6.68E-16	hypomethylated	-0.0029109	0.36724	insignificant	19	74	74
chr4	124527002	124529002	Yrdc	-0.11568758	3.14E-63	hypomethylated	0.0065807	0.38358	insignificant	53	205	222
chr4	124527974	124529974	1110065P20Ri	-0.09115702	6.17E-25	hypomethylated	0.0069354	0.18772	insignificant	34	130	144
chr4	124539415	124541415	Maneal	-0.22032536	9.02E-53	hypomethylated	0.014506	0.0011547	hypermethylated	23	84	76
chr4	124558028	124560028	Epha10	-0.16639031	1.08E-12	hypomethylated	-0.010207	0.011479	inconclusive	23	93	93
chr4	124613291	124615291	9930104L06Ri	-0.19697047	1.09E-11	hypomethylated	-0.00028563	0.67947	insignificant	7	64	60
chr4	124614161	124616161	Cdca8	0.35307018	1	insignificant	0.089877	0.54213	insignificant	1	18	14
chr4	124662673	124664673	Rspo1	-0.11181265	7.92E-22	hypomethylated	0.0025448	0.34208	insignificant	22	106	106
chr4	124706257	124708257	Gnl2	-0.12295291	3.35E-13	hypomethylated	-0.0020105	0.6606	insignificant	23	76	74
chr4	124742901	124744901	Dnali1	-0.06326163	1.87E-13	hypomethylated	0.0026155	0.63066	insignificant	36	122	122
chr4	124742936	124744936	Snip1	-0.06326163	1.87E-13	hypomethylated	0.0026155	0.63066	insignificant	36	122	122
chr4	124761390	124763390	Meaf6	-0.11279688	1.25E-29	hypomethylated	0.018464	0.34863	insignificant	43	149	142
chr4	124805125	124807125	Zc3h12a	-0.14347201	9.77E-24	hypomethylated	0.056777	0.65523	insignificant	14	47	49
chr4	125167074	125169074	Grik3	-0.11098936	2.63E-30	hypomethylated	0.0020479	0.25218	insignificant	82	235	239
chr4	125700902	125702902	Csf3r		1	noCoverage	-0.059764	0.34302	insignificant	0	9	8
chr4	125723171	125725171	Mrps15	-0.11537248	4.27E-15	hypomethylated	0.018037	0.0072805	hypermethylated	9	64	64
chr4	125734808	125736808	Oscp1	-0.13296765	1.13E-18	hypomethylated	-0.0090663	0.80073	insignificant	39	114	112
chr4	125772896	125774896	Lsm10	-0.11179938	2.35E-08	hypomethylated	-0.012125	0.23021	insignificant	4	44	34
chr4	125780200	125782200	Stk40	-0.18469813	1.31E-23	hypomethylated	-0.013115	0.29792	insignificant	32	95	98
chr4	125824246	125826246	Fam176b	-0.32179487	1.56E-59	hypomethylated	0.00021327	0.59831	insignificant	13	40	40
chr4	125839909	125841909	1700029G01R	-0.09002492	2.69E-11	hypomethylated	-0.0032364	0.9562	insignificant	21	52	52
chr4	125879954	125881954	Thrap3	-0.12479999	8.61E-08	hypomethylated	0.030017	0.02559	hypermethylated	12	55	53
chr4	125933563	125935563	Mtap7d1	-0.00424415	0.64549	insignificant	-0.054462	0.04965	hypomethylated	10	53	49
chr4	125938648	125940648	Trappc3	-0.2335416	4.81E-42	hypomethylated	-0.0076307	0.0052115	inconclusive	18	71	70
chr4	125963037	125965037	Col8a2	-0.14206781	3.32E-11	hypomethylated	0.034027	0.0051574	hypermethylated	24	80	76
chr4	125998947	126000947	Tekt2	-0.08966435	5.73E-55	hypomethylated	0.035161	0.18466	insignificant	9	34	33
chr4	126106786	126108786	Eif2c3	-0.13066179	9.22E-13	hypomethylated	0.040323	0.58954	insignificant	10	47	39
chr4	126145665	126147665	Eif2c1	-0.08359449	0.012835	hypomethylated	-0.0041338	0.54095	insignificant	18	71	71
chr4	126210702	126212702	Eif2c4	-0.09343455	1.89E-10	hypomethylated	-0.0056005	0.50431	insignificant	29	84	84
chr4	126233223	126235223	Clspn	-0.14961705	1.89E-16	hypomethylated	-0.016332	0.76683	insignificant	26	96	92
chr4	126286097	126288097	5730409E04Ri	-0.10849193	2.84E-21	hypomethylated	-0.004553	0.75683	insignificant	20	48	50
chr4	126353886	126355886	Psmb2	-0.23483557	0.000000114	hypomethylated	0.0025277	0.23754	insignificant	8	43	43
chr4	126413513	126415513	Tfap2e	-0.41346965	0.1563	insignificant	0.014754	0.34107	insignificant	2	16	16
chr4	126429798	126431798	AU040320	-0.14848227	6.88E-39	hypomethylated	0.021934	0.14748	insignificant	38	176	170
chr4	126430013	126432013	AU040320	-0.14848227	6.88E-39	hypomethylated	0.021934	0.14748	insignificant	38	176	170
chr4	126430673	126432673	Ncdn	-0.19755728	2.29E-36	hypomethylated	0.029747	0.58244	insignificant	27	126	127
chr4	126645167	126647167	Zmym4	-0.21313364	0.00000016	hypomethylated	-0.033457	0.57592	insignificant	6	48	48
chr4	126697544	126699544	Sfpq	-0.14714264	1.22E-30	hypomethylated	0.011819	0.022996	hypermethylated	37	118	110
chr4	126738376	126740376	Zmym1	0.23547776	1	insignificant	0.044608	0.67604	insignificant	2	28	25
chr4	126753626	126755626	Zmym6	-0.10782292	0.24064	insignificant	0.0078331	0.061949	insignificant	5	58	60
chr4	126802289	126804289	Gm12942	-0.12279982	0.000000048	hypomethylated	0.01337	0.4642	insignificant	7	32	30
chr4	126845514	126847514	Dlgap3	-0.07038209	0.00013032	hypomethylated	0.00072404	0.91192	insignificant	44	128	118
chr4	126920027	126922027	BC003266	0.16938776	1	insignificant	0.14468	0.78069	insignificant	2	14	10
chr4	126991222	126993222	Gja4	-0.175	0.3192	insignificant	0.056965	0.73483	insignificant	3	10	10
chr4	127006369	127008369	Gjb3	-0.23775004	0.0002063	hypomethylated	-0.0045936	0.010593	inconclusive	7	52	46
chr4	127031325	127033325	Gjb4	0.1977918	0.31614	insignificant	0.11679	0.015072	hypermethylated	4	14	14
chr4	127648085	127650085	CK137956	-0.19367731	0.14213	insignificant	-0.0044639	0.68857	insignificant	2	36	40
chr4	128287342	128289342	Zscan20	-0.307541	0.00000277	hypomethylated	-0.15742	0.49185	insignificant	6	22	18
chr4	128295863	128297863	Tlr12		1	noCoverage	-0.26811	1	insignificant	0	16	13
chr4	128330945	128332945	Phc2	-0.1408298	1.76E-23	hypomethylated	0.012988	0.36139	insignificant	39	155	141
chr4	128364981	128366981	Phc2	-0.11955786	0.6607	insignificant	0.25483	3.03E-09	hypermethylated	3	8	9
chr4	128403814	128405814	Phc2	-0.10156004	1.95E-17	hypomethylated	-0.011045	0.88956	insignificant	25	115	109
chr4	128483356	128485356	Zfp362	-0.09286003	2.48E-26	hypomethylated	0.0065312	0.004378	hypermethylated	49	158	140
chr4	128560383	128562383	Trim62	-0.17687255	4.89E-25	hypomethylated	-0.03812	1	insignificant	29	145	127
chr4	128639661	128641661	Adc	-0.19954629	1.45E-28	hypomethylated	0.0092807	1	insignificant	12	78	78
chr4	128669508	128671508	Ak2	-0.13442013	0.000000522	hypomethylated	-0.013843	0.73406	insignificant	6	32	32
chr4	128669557	128671557	Ak2	-0.13442013	0.000000522	hypomethylated	-0.013843	0.73406	insignificant	6	32	32
chr4	128734514	128736514	Rnf19b	-0.09571162	3.2E-36	hypomethylated	0.0052173	0.12332	insignificant	65	203	207
chr4	128781859	128783859	Tmem54	-0.14937257	3.53E-11	hypomethylated	0.036987	0.27612	insignificant	11	70	70
chr4	128798535	128800535	Hpca	-0.18818249	0.0032871	hypomethylated	0.015227	1	insignificant	14	44	49

chr4	128798984	128800984	Hpca	-0.07879379	0.0021665	hypomethylated	0.0062446	0.76194	insignificant	2	6	9
chr4	128813303	128815303	Fndc5	0.06204527	0.15486	insignificant	-0.0046081	0.85861	insignificant	5	48	47
chr4	128866038	128868038	Yars	-0.12890343	0.0000015	hypomethylated	0.026461	0.6162	insignificant	20	66	56
chr4	128866726	128868726	S100pbp	-0.13499282	0.000032113	hypomethylated	0.054441	0.37034	insignificant	11	42	32
chr4	128925687	128927687	C77080	-0.21567182	0.000000905	hypomethylated	-0.017296	0.24935	insignificant	22	67	62
chr4	128963864	128965864	Sync	-0.2249214	0.000000518	hypomethylated	0.039016	0.74235	insignificant	9	37	32
chr4	129012269	129014269	Zbtb8os	-0.13496229	2.34E-12	hypomethylated	0.024881	0.67326	insignificant	28	135	152
chr4	129012614	129014614	Rbbp4	-0.17981014	0.0066115	hypomethylated	0.030859	0.66755	insignificant	9	77	93
chr4	129055272	129057272	Zbtb8a	-0.10694444	0.32911	insignificant	-0.01113	0.83984	insignificant	2	12	12
chr4	129118062	129120062	Zbtb8b	0.05307757	0.24429	insignificant	0.0026059	0.20965	insignificant	5	41	38
chr4	129137922	129139922	Bsdc1	-0.21842491	6.94E-31	hypomethylated	0.011615	0.40638	insignificant	20	56	57
chr4	129167433	129169433	1700125D06R	-0.08031113	0.56671	insignificant	0.021675	0.022178	hypermethylated	30	100	100
chr4	129168014	129170014	Tssk3	-0.0369105	0.32167	insignificant	0.028788	0.0014284	hypermethylated	30	92	94
chr4	129189824	129191824	Marcksl1	-0.06615533	4.23E-17	hypomethylated	-0.005417	0.79405	insignificant	31	162	163
chr4	129219890	129221890	Hdac1	-0.21488186	0.00076324	hypomethylated	-0.053117	0.090313	insignificant	10	56	54
chr4	129235616	129237616	Lck		1	noCoverage	-0.2139	0.72233	insignificant	0	4	4
chr4	129250885	129252885	Lck		1	noCoverage	-0.036424	0.020302	hypomethylated	0	14	14
chr4	129255824	129257824	Fam167b	-0.26944444	0.653	insignificant	0.19537	0.050956	insignificant	3	7	6
chr4	129276950	129278950	2510006D16R	-0.18982778	2.51E-18	hypomethylated	0.0043697	0.94773	insignificant	23	86	81
chr4	129277892	129279892	Eif3i	-0.2548715	2.33E-14	hypomethylated	-0.020568	0.93841	insignificant	14	53	47
chr4	129296337	129298337	Iqcc	0.60697145	0.14095	insignificant	0.022682	0.74874	insignificant	1	20	16
chr4	129301152	129303152	Ccdc28b	-0.18923611	0.20005	insignificant	0.029317	0.37549	insignificant	3	16	14
chr4	129350011	129352011	Kpna6	-0.19583333	0.026737	hypomethylated	0.12113	1	insignificant	2	12	12
chr4	129374082	129376082	Tmem39b	-0.00232093	1	insignificant	0.018377	0.747	insignificant	4	14	14
chr4	129419526	129421526	Khdrbs1	-0.10180976	1.49E-09	hypomethylated	0.0080096	0.46968	insignificant	21	72	73
chr4	129496722	129498722	Ptp4a2	-0.09070463	2.63E-19	hypomethylated	-0.0043502	0.061784	insignificant	49	233	216
chr4	129496951	129498951	Ptp4a2	-0.08611337	9.04E-19	hypomethylated	-0.0051271	0.084261	insignificant	48	231	214
chr4	129661321	129663321	Bai2	-0.11971214	7.59E-42	hypomethylated	-0.013353	0.46304	insignificant	58	165	165
chr4	129724083	129726083	Col16a1	-0.11826153	1.58E-10	hypomethylated	-0.011761	0.51687	insignificant	17	62	62
chr4	129783799	129785799	Pef1	-0.16660801	4.95E-10	hypomethylated	0.0049925	0.78619	insignificant	9	46	46
chr4	129815795	129817795	Hcrtr1	-0.09276462	2.15E-13	hypomethylated	0.026164	0.60995	insignificant	21	96	85
chr4	129816406	129818406	Hcrtr1	0.05871693	4.97E-12	inconclusive	0.036994	0.11813	insignificant	8	40	38
chr4	129852046	129854046	Tinagl1	-0.21460433	0.000004848	hypomethylated	-0.0039221	0.32661	insignificant	5	16	16
chr4	129852366	129854366	Tinagl1		1	noCoverage	-0.0037653	1	insignificant	0	6	6
chr4	129952830	129954830	Serinc2		1	noCoverage	0.096296	1	insignificant	0	6	12
chr4	129985021	129987021	Fabp3	-0.20616502	0.0020282	hypomethylated	-0.050381	0.81213	insignificant	9	64	56
chr4	130036378	130038378	Snrnp40	-0.14828927	3.66E-08	hypomethylated	0.0027833	0.36316	insignificant	17	64	64
chr4	130037190	130039190	Zcchc17	-0.11775722	0.0070591	hypomethylated	0.02179	0.26702	insignificant	12	42	43
chr4	130124704	130126704	Nkain1	-0.16694498	3.75E-17	hypomethylated	-0.064309	0.50716	insignificant	28	105	96
chr4	130218273	130220273	Pum1	-0.09437583	1.38E-18	hypomethylated	-0.012597	0.61079	insignificant	36	119	130
chr4	130347451	130349451	Sdc3	-0.09270314	1.32E-12	hypomethylated	-0.0045396	0.92568	insignificant	38	151	150
chr4	130499299	130501299	Matn1	0.3765435	0.05352	insignificant	0.067831	0.48083	insignificant	2	7	8
chr4	131394193	131396193	Ptpru	-0.38605539	0.11256	insignificant	0.018726	0.65894	insignificant	6	24	31
chr4	131398385	131400385	Mecr	-0.24457689	5.24E-19	hypomethylated	-0.020888	0.12708	insignificant	16	82	79
chr4	131428553	131430553	Srsf4	-0.09362087	3.77E-27	hypomethylated	0.010233	0.26522	insignificant	62	221	208
chr4	131604993	131606993	Epb4.1	-0.13401447	0.079725	insignificant	-0.0012275	0.66653	insignificant	13	36	35
chr4	131628012	131630012	Epb4.1		1	noCoverage	-0.041522	0.60913	insignificant	0	2	2
chr4	131631228	131633228	Epb4.1	-0.26582932	7.7E-14	hypomethylated	0.021771	0.85937	insignificant	8	22	22
chr4	131700401	131702401	Oprd1	-0.15226725	1.35E-12	hypomethylated	0.001918	0.70441	insignificant	14	38	42
chr4	131768171	131770171	Ythdf2	-0.19084776	0.15075	insignificant	0.040019	0.53672	insignificant	3	35	38
chr4	131817464	131819464	Gmeb1	-0.73565416	0.0045837	stronglyHypometh	-0.050895	0.46964	insignificant	2	11	11
chr4	131826101	131828101	Rnu11	-0.36814957	0.0015658	stronglyHypometh	-0.096194	0.29916	insignificant	1	30	30
chr4	131829289	131831289	Taf12	-0.19827749	0.345	insignificant	0.012078	1	insignificant	6	45	47
chr4	131859271	131861271	Rab42-ps	-0.0401522	0.18443	insignificant	-0.015994	1	insignificant	3	14	12
chr4	131863592	131865592	Snhg12	-0.21681096	0.051369	insignificant	0.028014	0.21732	insignificant	3	38	40
chr4	131864379	131866379	Snora16a	-0.31757677	0.036353	hypomethylated	-0.067086	0.029671	hypomethylated	2	28	28
chr4	131885453	131887453	Trnau1ap	-0.2247018	0.13158	insignificant	0.0049577	0.00000117	inconclusive	6	20	20
chr4	131909601	131911601	Snhg3	-0.35527107	1.77E-14	stronglyHypometh	0.032264	0.032179	hypermethylated	7	22	22
chr4	131978361	131980361	Phactr4	-0.01049548	0.00020185	hypomethylated	0.0060649	0.46179	insignificant	10	53	53
chr4	132019836	132021836	Med18	0.10297606	2.65E-14	hypermethylated	0.018859	0.43461	insignificant	6	35	35
chr4	132066371	132068371	Sesn2	-0.23576436	2.03E-08	hypomethylated	-0.02402	0.58709	insignificant	8	18	18
chr4	132089574	132091574	Atpif1	-0.22474529	4.07E-11	hypomethylated	-0.025862	0.45072	insignificant	8	42	42
chr4	132090473	132092473	Dnajc8	-0.16185858	8.02E-27	hypomethylated	-0.0047466	0.93538	insignificant	26	78	68
chr4	132193960	132195960	Eya3	-0.18375453	1.76E-12	hypomethylated	0.015073	0.74507	insignificant	19	62	64
chr4	132193970	132195970	Eya3	-0.18375453	1.76E-12	hypomethylated	0.015073	0.74507	insignificant	19	62	64
chr4	132288461	132290461	Xkr8	-0.24469292	1.04E-42	hypomethylated	0.0055251	0.000000441	hypermethylated	11	65	65
chr4	132313086	132315086	Smpdl3b	-0.40064103	0.31303	insignificant	-0.046474	0.50524	insignificant	2	6	6
chr4	132323274	132325274	Rpa2	0.10330469	0.90501	insignificant	-0.016013	0.9138	insignificant	12	111	110
chr4	132352279	132354279	BC013712	-0.25752575	0.00000985	hypomethylated	0.20912	0.20015	insignificant	2	17	8
chr4	132440373	132442373	Stx12	-0.1795753	5.28E-20	hypomethylated	-0.030587	0.67194	insignificant	14	68	63
chr4	132478466	132480466	Fam76a	-0.10347773	7.05E-08	hypomethylated	0.013064	0.32281	insignificant	20	70	71
chr4	132529009	132531009	Fgr	-0.1899102	0.0000001	hypomethylated	0.038966	0.88238	insignificant	6	38	38
chr4	132566420	132568420	Ahdc1	-0.15523861	1.14E-19	hypomethylated	0.0352	0.86725	insignificant	37	82	74
chr4	132685547	132687547	Wasf2	-0.12876573	4.09E-20	hypomethylated	0.010789	0.20561	insignificant	24	70	74
chr4	132768451	132770451	Gpr3	-0.11640623	0.024985	hypomethylated	0.025667	0.34551	insignificant	14	46	57
chr4	132775723	132777723	Cd164l2	-0.13949661	0.19452	insignificant	0.011451	0.14166	insignificant	5	37	34
chr4	132795732	132797732	Map3k6	-0.1179495	1.93E-15	hypomethylated	0.0083071	0.0059977	hypermethylated	23	72	60
chr4	132833705	132835705	Tmem222	-0.51126686	0.01188	stronglyHypometh	0.019586	0.00023616	hypermethylated	3	18	18
chr4	132895230	132897230	Wdtc1	-0.12136242	1.99E-11	hypomethylated	-0.0059769	0.79205	insignificant	20	73	76
chr4	132924686	132926686	Slc9a1	-0.19550447	1.97E-11	hypomethylated	-0.013243	0.9617	insignificant	8	30	30
chr4	133035047	133037047	Fam46b	-0.37355832	9.52E-29	stronglyHypometh	-0.021878	0.11435	insignificant	15	49	52
chr4	133054465	133056465	Trnp1	-0.19526307	1.76E-08	hypomethylated	0.21491	0.26002	insignificant	4	10	6
chr4	133073877	133075877	1810019J16Ri	-0.03307885	0.37458	insignificant	0.033937	0.000000833	hypermethylated	13	100	77
chr4	133101942	133103942	Nudc	-0.25536301	6.05E-18	hypomethylated	0.050524	0.85302	insignificant	5	11	11
chr4	133108304	133110304	Nr0b2	-0.09072384	0.19968	insignificant	-0.033757	0.78261	insignificant	3	6	6

chr4	133129659	133131659	Gpatch3	-0.21853216	2.69E-11	hypomethylated	0.016811	0.49605	insignificant	23	80	80
chr4	133139287	133141287	Gpn2	-0.16574678	0.0013122	hypomethylated	-0.0033323	0.064492	insignificant	13	56	56
chr4	133158083	133160083	Sfn	0.06832579	1	insignificant	-0.051674	0.16967	insignificant	3	10	10
chr4	133189344	133191344	Zdhhc18	-0.16558704	0.024307	hypomethylated	-0.048716	0.8232	insignificant	14	39	37
chr4	133228562	133230562	Pigv	-0.10036751	1.1E-12	hypomethylated	0.013218	0.59201	insignificant	20	46	46
chr4	133309526	133311526	Arid1a	-0.11047524	4.18E-25	hypomethylated	0.0037587	0.20712	insignificant	55	127	127
chr4	133443714	133445714	Rps6ka1	-0.10964997	1.26E-11	hypomethylated	0.02374	0.91228	insignificant	10	39	34
chr4	133523906	133525906	Hmgn2	-0.12765581	4.16E-10	hypomethylated	-0.0051515	0.14919	insignificant	17	60	72
chr4	133574731	133576731	Lin28a	-0.32524933	0.038023	hypomethylated	-0.0018367	0.50756	insignificant	2	20	20
chr4	133623366	133625366	Aim1l	0.11232531	0.0071461	hypermethylated	-0.045332	0.44036	insignificant	5	16	16
chr4	133650988	133652988	Cd52		1	noCoverage		1	noCoverage	0	6	0
chr4	133657513	133659513	Ubxn11	0.06717367	1	insignificant	0.023134	0.61139	insignificant	2	27	35
chr4	133684668	133686668	Sh3bgrl3	-0.19508205	6.22E-10	hypomethylated	0.072848	0.20133	insignificant	7	30	30
chr4	133743000	133745000	Ccdc21	-0.16380454	0.10161	insignificant	-0.087091	0.94082	insignificant	9	27	26
chr4	133794314	133796314	Cnksr1	0.24169693	0.10376	insignificant	-0.024609	0.88956	insignificant	1	23	22
chr4	133801506	133803506	Zfp593	-0.14248148	2.44E-12	hypomethylated	-0.027904	0.6585	insignificant	9	34	28
chr4	133843168	133845168	Pdik1l	-0.11156215	2.23E-34	hypomethylated	0.0079156	0.29762	insignificant	44	125	122
chr4	133843761	133845761	Pdik1l	-0.21326754	3E-14	hypomethylated	-0.022856	0.86009	insignificant	9	28	28
chr4	133870034	133872034	Trim63	-0.00231766	0.58401	insignificant	0.0093033	0.4618	insignificant	3	13	10
chr4	133897984	133899984	Slc30a2	-0.15029439	0.00038522	hypomethylated	0.0075745	0.24817	insignificant	19	85	78
chr4	133928462	133930462	Extl1	-0.18495344	0.00000218	hypomethylated	-0.0060952	0.66809	insignificant	2	10	10
chr4	133951274	133953274	Pafah2		1	noCoverage	0.012936	0.38774	insignificant	0	12	12
chr4	134023234	134025234	Stmn1	-0.11993105	0.000000561	hypomethylated	0.0012599	0.57208	insignificant	17	142	142
chr4	134051892	134053892	Paqr7	-0.18501239	1.27E-16	hypomethylated	-0.0047342	0.96118	insignificant	18	49	49
chr4	134065913	134067913	2610002D18R	-0.06525807	0.14716	insignificant	-0.0448	0.31294	insignificant	13	41	39
chr4	134088667	134090667	Fam54b	-0.1792713	0.057463	insignificant	-0.0085933	0.52864	insignificant	11	42	45
chr4	134108081	134110081	Sepn1	-0.13690476	0.35243	insignificant	0.02004	0.81455	insignificant	2	22	22
chr4	134260205	134262205	Man1c1	-0.18930309	0.87093	insignificant	0.046045	0.21368	insignificant	12	43	45
chr4	134323919	134325919	Ldlrap1	-0.08312814	2.02E-16	hypomethylated	-0.039902	0.026988	inconclusive	16	47	47
chr4	134409260	134411260	Tmem57	-0.15166137	1E-24	hypomethylated	-0.0023933	0.30687	insignificant	33	146	142
chr4	134419450	134421450	Rhd	-0.0550438	0.64084	insignificant	-0.033968	0.019011	inconclusive	5	27	27
chr4	134470831	134472831	Tmem50a	-0.19964332	0.000000157	hypomethylated	-0.089	0.59447	insignificant	7	34	31
chr4	134478539	134480539	D4Wsu53e	-0.09613105	2.64E-41	hypomethylated	0.0038871	0.68366	insignificant	36	199	195
chr4	134485894	134487894	Syf2	-0.18201496	8.8E-33	hypomethylated	-0.01787	0.54862	insignificant	20	88	97
chr4	134828675	134830675	Clic4	-0.11910177	0.000000294	hypomethylated	-0.0052622	0.61988	insignificant	8	71	71
chr4	134909129	134911129	Srrm1	-0.10722971	2.24E-08	hypomethylated	0.03052	0.77327	insignificant	10	28	29
chr4	134941587	134943587	A330049M08Rik		1	noCoverage	0.052822	0.098138	insignificant	0	14	12
chr4	134954142	134956142	A330049M08F	-0.49803131	0.0011783	stronglyHypometh	0.023	0.1567	insignificant	4	24	28
chr4	134989720	134991720	Rcan3	-0.1448406	3.29E-23	hypomethylated	0.01646	0.24321	insignificant	12	30	34
chr4	135050419	135052419	Nipal3	-0.1755463	0.000076909	hypomethylated	0.030035	0.072311	insignificant	20	72	72
chr4	135050901	135052901	4930555I21Ril	-0.20764702	0.017156	hypomethylated	0.046982	0.013733	hypermethylated	12	42	42
chr4	135129535	135131535	Grhl3	-0.51723355	0.024571	stronglyHypometh	-0.030008	0.21667	insignificant	2	22	24
chr4	135181569	135183569	1700029M20F	-0.31554202	0.14926	insignificant	0.033622	0.62612	insignificant	3	28	28
chr4	135241371	135243371	Il28ra	-0.16235237	2.07E-09	hypomethylated	0.020705	0.83478	insignificant	21	77	69
chr4	135283134	135285134	Il22ra1	-0.49150466	0.00026842	stronglyHypometh	0.026899	0.276	insignificant	2	21	23
chr4	135314629	135316629	Myom3	-0.21932367	0.043031	hypomethylated	0.042976	0.20093	insignificant	1	6	6
chr4	135411006	135413006	Srsf10	-0.1001549	6.73E-29	hypomethylated	0.012533	0.12787	insignificant	47	198	201
chr4	135429761	135431761	Pnrc2	-0.21536524	0.010554	hypomethylated	0.01535	0.23254	insignificant	3	38	38
chr4	135450318	135452318	Cnr2	-0.53452381	0.22291	insignificant	0.016211	0.7397	insignificant	1	4	4
chr4	135475640	135477640	Fuca1	-0.14086915	8.41E-18	hypomethylated	0.0012277	0.2346	insignificant	23	71	70
chr4	135501367	135503367	Hmgcl	-0.16717555	3.63E-12	hypomethylated	0.0076308	0.55525	insignificant	15	86	83
chr4	135528509	135530509	Lypla2	-0.1323184	0.23687	insignificant	0.1068	0.12762	insignificant	3	8	6
chr4	135543159	135545159	1110049F12Ri	-0.2156982	0.75011	insignificant	0.030578	1	insignificant	9	58	52
chr4	135577564	135579564	Tceb3	-0.13207688	2.31E-08	hypomethylated	0.048834	0.43379	insignificant	8	44	44
chr4	135609286	135611286	Rpl11		1	noCoverage	-0.042445	0.38335	insignificant	0	29	27
chr4	135698736	135700736	Id3	-0.12679032	8.94E-14	hypomethylated	-0.013448	0.58441	insignificant	18	107	107
chr4	135727308	135729308	E2f2	-0.10204171	4.6E-16	hypomethylated	0.002616	0.77567	insignificant	30	108	106
chr4	135761279	135763279	Asap3	-0.14852253	0.000026378	hypomethylated	-0.030588	1	insignificant	12	63	69
chr4	135802871	135804871	Tcea3	-0.16084075	0.27743	insignificant	-0.016279	0.17897	insignificant	17	87	86
chr4	135840983	135842983	Zfp46	-0.20104133	5.1E-26	hypomethylated	-0.012811	0.74553	insignificant	21	66	57
chr4	135865890	135867890	Hnrnpr	-0.12804475	6.32E-19	hypomethylated	-0.001562	0.37183	insignificant	40	150	147
chr4	135978438	135980438	Htr1d	-0.23117592	0.69046	insignificant	0.016509	0.10578	insignificant	8	34	39
chr4	136024675	136026675	Luzp1	-0.1041648	9.55E-35	hypomethylated	-0.0042653	0.017562	inconclusive	41	132	124
chr4	136391850	136393850	Ephb2	-0.10176296	1.92E-14	hypomethylated	0.011416	0.76777	insignificant	28	107	107
chr4	136442092	136444092	C1qb	-0.15555556	0.50073	insignificant	0.0098485	0.76657	insignificant	3	7	6
chr4	136454759	136456759	C1qa	0.25492832	0.3413	insignificant	0.085821	0.24678	insignificant	1	10	9
chr4	136512731	136514731	Epha8	-0.1783189	2.45E-08	hypomethylated	-0.039238	0.00003915	inconclusive	22	60	60
chr4	136832549	136834549	Wnt4	-0.10776017	1.94E-22	hypomethylated	0.0012189	0.47327	insignificant	51	192	180
chr4	136913652	136915652	Cdc42	-0.09669118	0.089243	insignificant	-0.14861	0.0025053	hypomethylated	3	14	16
chr4	137023717	137025717	Hspg2	-0.15629897	3.43E-12	hypomethylated	-0.022127	0.37988	insignificant	21	83	91
chr4	137149103	137151103	Usp48	-0.05231074	3.45E-13	hypomethylated	-0.010047	0.68261	insignificant	23	102	102
chr4	137236617	137238617	Rap1gap	-0.09443362	9.01E-16	hypomethylated	0.010956	0.000049843	hypermethylated	19	86	84
chr4	137352292	137354292	Alpl	-0.2140541	7.39E-08	hypomethylated	0.012798	0.42909	insignificant	7	22	18
chr4	137417151	137419151	Ece1	-0.18055794	3.09E-12	hypomethylated	0.0079387	0.72848	insignificant	22	84	84
chr4	137548384	137550384	Eif4g3	-0.08083857	2.35E-15	hypomethylated	0.0029491	0.096716	insignificant	55	236	211
chr4	137771541	137773541	Hp1bp3	-0.08533457	1.45E-41	hypomethylated	0.0014082	0.5678	insignificant	67	232	227
chr4	137772045	137774045	Hp1bp3	-0.08040058	2.61E-42	hypomethylated	0.00056497	1	insignificant	70	239	233
chr4	137805325	137807325	Sh2d5	-0.13922032	4.99E-18	hypomethylated	-0.011548	0.6442	insignificant	12	54	53
chr4	137817165	137819165	Kif17	-0.12563146	3.33E-33	hypomethylated	-0.017784	0.23702	insignificant	43	124	132
chr4	137859652	137861652	Ddost	-0.12297389	1.16E-14	hypomethylated	-0.0012249	1	insignificant	33	101	101
chr4	137882211	137884211	Pink1	-0.164807	0.67742	insignificant	0.019365	0.1231	insignificant	6	33	37
chr4	137923870	137925870	Cda	-0.1831153	3.32E-20	hypomethylated	0.015452	0.000052829	hypermethylated	12	43	43
chr4	137989586	137991586	Mul1	-0.22612487	1.81E-31	hypomethylated	0.028526	0.72421	insignificant	26	97	91
chr4	138010062	138012062	Camk2n1	-0.08670008	1.54E-25	hypomethylated	0.00534	0.80598	insignificant	51	207	197
chr4	138179907	138181907	Vwa5b1	0.39441196	0.32966	insignificant	0.39441	0.014275	stronglyhypermeth	1	9	6

chr4	138280239	138282239	Pla2g2c	-0.2804271	5.03E-14	hypomethylated	0.022181	0.9466	insignificant	9	28	28
chr4	138281166	138283166	Ubxn10	-0.1795809	0.0001325	hypomethylated	0.048345	1	insignificant	3	6	6
chr4	138313513	138315513	Pla2g2f	-0.27134583	0.19427	insignificant	-0.04943	0.68132	insignificant	1	12	12
chr4	138375172	138377172	Pla2g5	-0.26388889	0.5657	insignificant	0.013889	0.86054	insignificant	1	6	6
chr4	138469862	138471862	Otud3	-0.27696102	1.64E-11	hypomethylated	0.091256	0.13229	insignificant	9	38	41
chr4	138522026	138524026	Rnf186	-0.23928614	0.00011698	hypomethylated	0.020954	0.23464	insignificant	5	62	62
chr4	138527819	138529819	Tmco4	-0.17498026	0.000069597	hypomethylated	-0.0010354	0.64972	insignificant	10	67	67
chr4	138630704	138632704	Htr6	-0.10033527	2.67E-17	hypomethylated	0.005123	0.72361	insignificant	46	143	143
chr4	138648885	138650885	Nbl1	-0.07007028	0.016998	hypomethylated	-0.021923	0.96274	insignificant	2	58	58
chr4	138686952	138688952	Minos1	-0.13906779	5.76E-24	hypomethylated	0.018104	0.81003	insignificant	11	24	24
chr4	138687028	138689028	Minos1	-0.14696488	2.71E-13	hypomethylated	0.044454	0.065664	insignificant	6	14	14
chr4	138747845	138749845	Capzb	-0.14573415	5.36E-08	hypomethylated	-0.010758	0.77327	insignificant	19	106	101
chr4	138747893	138749893	Capzb	-0.14573415	5.36E-08	hypomethylated	-0.010758	0.77327	insignificant	19	106	101
chr4	138865658	138867658	Akr7a5	-0.1588066	1.63E-32	hypomethylated	0.0092972	0.87286	insignificant	37	130	129
chr4	138866615	138868615	Pqlc2	-0.15420859	1.38E-14	hypomethylated	0.018348	0.90646	insignificant	20	80	80
chr4	138907507	138909507	C230096C10R	-0.12358616	0.26886	insignificant	0.020724	0.87011	insignificant	13	79	69
chr4	138908213	138910213	Mrto4	-0.19357137	0.43233	insignificant	0.051629	1	insignificant	13	59	59
chr4	138935573	138937573	Ubr4	-0.13799436	8.16E-25	hypomethylated	0.010852	0.18137	insignificant	29	79	79
chr4	139130050	139132050	Iffo2	-0.11247287	1.42E-33	hypomethylated	-0.0042492	0.3615	insignificant	54	196	195
chr4	139177808	139179808	Aldh4a1	-0.25878994	0.00272	hypomethylated	-0.022903	0.28073	insignificant	4	34	30
chr4	139208452	139210452	Tas1r2	0.09116091	0.46818	insignificant	0.05992	0.25844	insignificant	3	6	6
chr4	139388883	139390883	Pax7	-0.23202044	9.62E-38	hypomethylated	-0.01187	0.61884	insignificant	15	64	59
chr4	139802726	139804726	Igsf21	-0.27699885	0.29527	insignificant	-0.016954	0.53988	insignificant	6	26	24
chr4	140204671	140206671	Arhgef10l	-0.14448052	0.0056811	hypomethylated	-0.17506	0.000031161	hypomethylated	1	18	26
chr4	140221820	140223820	Arhgef10l	-0.72252881	0.28222	lowCoverage	0.0065762	0.15356	insignificant	1	14	14
chr4	140256387	140258387	Rcc2	-0.11143218	6.01E-15	hypomethylated	-0.00039416	0.57842	insignificant	23	135	144
chr4	140366563	140368563	Padi3	-0.47264957	0.14046	insignificant	-0.3582	0.04042	stronglyhypometh	3	6	7
chr4	140401693	140403693	Padi1		1	noCoverage	0.2204	0.85943	insignificant	0	2	5
chr4	140461274	140463274	Padi2	-0.52075415	4.68E-24	stronglyHypometh	0.010524	0.0043426	hypermethylated	3	18	18
chr4	140516185	140518185	Sdhb	-0.16271263	6.36E-27	hypomethylated	0.022131	0.20294	insignificant	22	83	80
chr4	140541787	140543787	Atp13a2	-0.12995663	2.4E-33	hypomethylated	-0.001667	0.03079	inconclusive	29	73	71
chr4	140565338	140567338	Mfap2	-0.18940433	4.42E-11	hypomethylated	0.034367	0.31628	insignificant	9	32	32
chr4	140565551	140567551	Mfap2	-0.17609107	4.56E-13	hypomethylated	0.038526	0.7599	insignificant	10	34	34
chr4	140609576	140611576	Crocc	-0.03793428	0.0046372	hypomethylated	0.024007	0.26534	insignificant	6	36	38
chr4	140616460	140618460	Crocc	-0.21944005	0.000017546	hypomethylated	-0.03877	0.054577	insignificant	4	39	36
chr4	140634260	140636260	Necap2	-0.49037065	4.35E-33	stronglyHypometh	0.075063	0.3594	insignificant	9	36	38
chr4	140643259	140645259	Spata21	-0.20301717	0.16691	insignificant	-0.043992	0.28527	insignificant	5	12	12
chr4	140702836	140704836	Fbxo42	-0.08875849	2.69E-31	hypomethylated	-0.00042181	0.40739	insignificant	26	153	160
chr4	140768870	140770870	6330545A04R	-0.12683698	1	insignificant	0.049583	0.42796	insignificant	7	41	38
chr4	140797798	140799798	Arhgef19	-0.05883617	1	insignificant	0.031442	0.093755	insignificant	3	14	14
chr4	140856154	140858154	Epha2	-0.16068292	0.000000518	hypomethylated	-0.044045	0.96886	insignificant	19	90	86
chr4	140923134	140925134	Fam131c	-0.06111657	2.49E-15	hypomethylated	-0.0032369	0.18347	insignificant	15	90	92
chr4	140954014	140956014	Clcnka	-0.10341707	0.32518	insignificant	-0.045947	1	insignificant	4	10	10
chr4	140954621	140956621	Clcnka	0.05006339	0.36957	insignificant	0.023128	0.20975	insignificant	6	28	25
chr4	140975693	140977693	Hspb7	0.07142857	0.032666	hypermethylated	-0.29464	0.27115	insignificant	2	13	8
chr4	140992020	140994020	Gm694	-0.33827083	0.39074	insignificant	0.034299	0.3941	insignificant	2	16	17
chr4	140999587	141001587	Zbtb17	-0.12049149	6.52E-45	hypomethylated	-0.0042056	0.42673	insignificant	46	112	112
chr4	141094512	141096512	Spen	-0.09419678	1.18E-20	hypomethylated	0.012	0.029232	inconclusive	29	88	80
chr4	141101076	141103076	B330016D10R	-0.10552135	1.34E-20	hypomethylated	-0.0027378	0.65605	insignificant	53	148	157
chr4	141155839	141157839	Fblim1	0.06083485	0.72714	insignificant	0.02614	0.01273	hypermethylated	7	34	33
chr4	141169284	141171284	AI507597	-0.13581313	0.053121	insignificant	0.010077	0.84487	insignificant	4	28	27
chr4	141179749	141181749	Slc25a34	0.00879396	0.79547	insignificant	0.1491	0.00017931	hypermethylated	3	20	20
chr4	141220030	141222030	Plekhm2	-0.21214656	2.93E-19	hypomethylated	-0.0043872	0.4746	insignificant	22	76	71
chr4	141279334	141281334	Ddi2	-0.11626684	7.97E-30	hypomethylated	-0.005209	0.86546	insignificant	14	62	62
chr4	141301589	141303589	Agmat		1	noCoverage	-0.012434	1	insignificant	0	21	18
chr4	141346559	141348559	Dnajc16	-0.22531703	0.000025083	hypomethylated	0.021361	0.30545	insignificant	10	46	49
chr4	141348526	141350526	Casp9	-0.13360981	2.71E-14	hypomethylated	0.020368	0.20048	insignificant	15	32	32
chr4	141430835	141432835	Efhd2	-0.05992724	0.0013313	hypomethylated	0.0052714	0.00000286	inconclusive	7	38	32
chr4	141639212	141641212	Gm10565	-0.1711465	6.56E-24	hypomethylated	-0.014186	0.8122	insignificant	41	127	134
chr4	141640219	141642219	Gm10565	-0.21672849	0.00000268	hypomethylated	-0.015783	0.65589	insignificant	11	66	66
chr4	141766123	141768123	Kazn	-0.6	0.034706	stronglyHypometh	0	0.21473	insignificant	1	2	2
chr4	142802612	142804612	Prdm2	-0.17875652	0.000010361	hypomethylated	-0.049585	0.3943	insignificant	4	58	55
chr4	142889410	142891410	Pdpn	-0.31124527	0.00015363	hypomethylated	0.098576	0.24049	insignificant	3	6	6
chr4	142938652	142940652	Lrrc38	-0.1097939	5.25E-43	hypomethylated	0.014635	0.11708	insignificant	34	119	119
chr4	142983343	142985343	Pramel1	0.02679588	0.74272	insignificant	-0.024157	0.22028	insignificant	6	39	46
chr4	143001328	143003328	Pramef8	-0.1508088	0.38165	insignificant	0.014911	0.45964	insignificant	5	49	40
chr4	143998367	144000367	Pramef12		1	noCoverage	0.070032	0.51245	insignificant	0	12	14
chr4	144481729	144483729	Dhrs3	-0.19242859	9.83E-21	hypomethylated	-0.027989	0.38748	insignificant	19	58	56
chr4	144482039	144484039	Dhrs3	-0.19242859	9.83E-21	hypomethylated	-0.027989	0.38748	insignificant	19	58	56
chr4	144905050	144907050	Tnfrsf8	-0.32142857	1	lowCoverage	-0.0093515	1	insignificant	1	14	18
chr4	145053112	145055112	Smarca5-ps	-0.07334987	0.02093	hypomethylated	-0.023648	0.0027448	hypomethylated	9	108	92
chr4	145259599	145261599	Gm13242		1	noCoverage	0.04058	0.72721	insignificant	0	9	10
chr4	145426121	145428121	Gm13238	0.27439457	0.090338	insignificant	0.018112	0.29414	insignificant	6	42	45
chr4	146033924	146035924	Gm13034	-0.24816434	1.91E-09	hypomethylated	-0.049276	0.00044842	hypomethylated	6	156	147
chr4	146548974	146550974	Gm13152		1	noCoverage	0.11217	0.0059903	hypermethylated	0	8	8
chr4	147222484	147224484	Zfp933	-0.18566037	0.38588	insignificant	-0.011888	0.90935	insignificant	7	20	20
chr4	147242087	147244087	Fv1	-0.20964414	0.000000417	hypomethylated	-0.011195	0.17774	insignificant	11	66	66
chr4	147242828	147244828	Fv1	0.09575241	4.75E-14	hypermethylated	-0.023466	0.13777	insignificant	7	32	32
chr4	147310885	147312885	Plod1	-0.21717607	0.00017087	hypomethylated	0.0069763	0.82266	insignificant	12	28	28
chr4	147314003	147316003	2510039O18R	-0.1094716	1.35E-17	hypomethylated	-0.012992	0.54248	insignificant	41	159	148
chr4	147358897	147360897	Nppb	-0.3137598	0.53927	insignificant	-0.036352	0.8209	insignificant	3	10	10
chr4	147412185	147414185	Mthfr	-0.16068877	1.99E-36	hypomethylated	-0.0043509	0.50499	insignificant	18	88	80
chr4	147412876	147414876	Mthfr	-0.13088448	3.65E-44	hypomethylated	0.035577	0.018002	inconclusive	15	72	71
chr4	147414297	147416297	Mthfr	-0.05710146	0.25663	insignificant	-0.018108	0.060479	insignificant	5	33	32
chr4	147462173	147464173	Agtrap	-0.23981614	0.47833	insignificant	0.054171	1	insignificant	5	24	25

chr4	147504807	147506807	2610109H07R	-0.11358922	5.01E-10	hypomethylated	0.049481	0.85668	insignificant	15	77	61
chr4	147513598	147515598	Mad2l2	-0.21892507	4.8E-21	hypomethylated	-0.016464	0.31349	insignificant	13	65	60
chr4	147526034	147528034	Fbxo6	0.18556568	1.28E-24	hypermethylated	-0.0079491	0.037776	inconclusive	7	23	23
chr4	147526138	147528138	Fbxo6	0.08937919	2.69E-11	hypermethylated	-0.0097157	0.029173	inconclusive	7	20	20
chr4	147526244	147528244	Fbxo6	0.20233026	0.00000156	hypermethylated	-0.0099845	0.0021668	inconclusive	6	20	20
chr4	147533704	147535704	Fbxo44	-0.12373177	1.19E-15	hypomethylated	0.026252	0.54276	insignificant	19	104	88
chr4	147533776	147535776	Fbxo2	-0.12373177	1.19E-15	hypomethylated	0.026252	0.54276	insignificant	19	104	88
chr4	147534173	147536173	Fbxo44	-0.11072359	3.45E-13	hypomethylated	0.046645	0.29871	insignificant	18	88	66
chr4	147662074	147664074	Ptchd2	-0.1282163	0.11069	insignificant	0.11931	0.2268	insignificant	9	20	24
chr4	147818860	147820860	Ubiad1		1	noCoverage	-0.020408	1	insignificant	0	12	6
chr4	147821690	147823690	Mtor	-0.17078503	6.92E-11	hypomethylated	-0.014518	0.70769	insignificant	22	134	129
chr4	147931535	147933535	Exosc10	-0.13023308	1.04E-22	hypomethylated	-0.0029697	0.40269	insignificant	26	56	56
chr4	147964621	147966621	Srm	-0.12305968	4.23E-14	hypomethylated	-0.0019694	0.93793	insignificant	24	91	91
chr4	148001105	148003105	Tardbp	-0.63676649	5.96E-59	stronglyHypometh	0.16272	0.18865	insignificant	11	15	10
chr4	148177500	148179500	Casz1	0.05487286	7.74E-11	inconclusive	-0.0071426	0.69467	insignificant	38	120	120
chr4	148473921	148475921	Pex14	-0.48409397	0.0041518	stronglyHypometh	0.042649	0.000050837	inconclusive	1	26	29
chr4	148477261	148479261	Dffa	-0.14051483	0.000000222	hypomethylated	-0.028094	0.76275	insignificant	8	44	38
chr4	148500850	148502850	Cort	-0.03103777	0.42443	insignificant	0.046105	0.54216	insignificant	3	30	32
chr4	148511709	148513709	Apitd1	-0.21017542	1	insignificant	-0.001474	0.11887	insignificant	5	23	21
chr4	148540816	148542816	Pgd		1	noCoverage	0.060736	0.51016	insignificant	0	23	17
chr4	148681807	148683807	Kif1b	-0.11037037	1.06E-24	hypomethylated	-0.0027898	0.41127	insignificant	22	96	96
chr4	148800740	148802740	Ube4b		1	noCoverage	-0.057389	0.8632	insignificant	0	2	2
chr4	148829077	148831077	Rbp7		1	noCoverage	-0.13462	0.1687	insignificant	0	2	2
chr4	148858441	148860441	Lzic	-0.11539382	9.78E-17	hypomethylated	0.0095422	0.21758	insignificant	29	83	83
chr4	148859251	148861251	Nmnat1	-0.12778041	1.49E-21	hypomethylated	0.0054832	0.19011	insignificant	23	69	69
chr4	148891349	148893349	Ctnnbip1	-0.10873076	0.000016962	hypomethylated	-0.0078623	0.40979	insignificant	15	40	32
chr4	148918225	148920225	Ctnnbip1	-0.058743	0.0035454	hypomethylated	-0.073416	0.000090815	hypomethylated	10	24	24
chr4	148959746	148961746	Clstn1	-0.08379394	8.3E-48	hypomethylated	-0.0043476	0.74047	insignificant	60	208	228
chr4	149050198	149052198	Pik3cd	-0.10316809	0.74641	insignificant	0.071569	0.86957	insignificant	9	33	33
chr4	149072817	149074817	Pik3cd	-0.4125	0.49231	insignificant	0.031944	0.60835	insignificant	2	10	10
chr4	149075738	149077738	Pik3cd	-0.28496206	0.55448	insignificant	0.023502	0.37496	insignificant	2	23	13
chr4	149112143	149114143	Tmem201	-0.15222222	0.07916	insignificant	0.066594	0.11442	insignificant	3	6	6
chr4	149112145	149114145	Tmem201	-0.15222222	0.07916	insignificant	0.066594	0.11442	insignificant	2	4	4
chr4	149148376	149150376	Slc25a33	-0.23410474	4.28E-15	hypomethylated	-0.058506	0.78833	insignificant	11	20	10
chr4	149329115	149331115	Spsb1	-0.16871434	0.000019791	hypomethylated	-0.0049672	0.082457	insignificant	9	24	23
chr4	149441562	149443562	Mir34a		1	noCoverage	0.026474	0.48192	insignificant	0	7	4
chr4	149492452	149494452	Slc2a5	-0.034032	0.083695	insignificant	0.048625	0.43066	insignificant	3	17	12
chr4	149522080	149524080	Slc2a7		1	noCoverage	0.26696	1.49E-08	hypermethylated	0	10	10
chr4	149610305	149612305	Eno1	-0.11859862	1.1E-21	hypomethylated	0.0020261	0.17698	insignificant	23	97	97
chr4	149610432	149612432	Gm5506	-0.12174954	1.3E-21	hypomethylated	0.007773	0.8641	insignificant	21	89	89
chr4	149655024	149657024	Rere	-0.09048875	0.00012185	hypomethylated	-0.0042069	0.426	insignificant	19	88	88
chr4	150026283	150028283	Slc45a1	-0.0988378	0.068568	insignificant	0.069595	0.04182	hypermethylated	4	17	16
chr4	150228199	150230199	Errfi1	-0.09318494	3.08E-25	hypomethylated	-0.0083959	0.07106	insignificant	42	158	149
chr4	150284030	150286030	Park7	-0.37074421	0.003566	stronglyHypometh	0.026533	0.039881	inconclusive	5	28	28
chr4	150301107	150303107	Tnfrsf9		1	noCoverage	-0.23333	0.11759	insignificant	0	4	3
chr4	150418731	150420731	Per3	-0.05659356	0.34576	insignificant	0.017379	0.61565	insignificant	14	44	43
chr4	150432062	150434062	Vamp3	-0.11328074	0.38215	insignificant	0.0034581	0.90482	insignificant	8	37	28
chr4	151235877	151237877	Camta1	-0.11030426	3.92E-16	hypomethylated	-0.0043142	0.59598	insignificant	37	171	148
chr4	151306828	151308828	Dnajc11	-0.11006192	1.71E-18	hypomethylated	-0.00068708	0.45574	insignificant	48	154	143
chr4	151370288	151372288	Phf13	-0.08671045	4.91E-30	hypomethylated	0.0064373	0.61198	insignificant	45	181	165
chr4	151381999	151383999	Klhl21	-0.10588262	4E-45	hypomethylated	-0.003838	0.87076	insignificant	27	93	93
chr4	151401780	151403780	Zbtb48	-0.40189961	1	lowCoverage	-0.031024	0.7299	insignificant	1	48	44
chr4	151412435	151414435	Nol9	-0.12075681	1.62E-12	hypomethylated	0.00066541	1	insignificant	52	160	154
chr4	151412599	151414599	Nol9	-0.12075681	1.62E-12	hypomethylated	0.00066541	1	insignificant	52	160	154
chr4	151469827	151471827	Plekhg5	-0.47380952	0.00070301	stronglyHypometh	0.072024	0.27762	insignificant	2	10	8
chr4	151489451	151491451	Tnfrsf25	-0.12718371	0.000000229	hypomethylated	0.0076589	0.063623	insignificant	17	42	42
chr4	151503034	151505034	Espn	-0.14796181	0.13167	insignificant	0.00077227	0.015562	hypermethylated	9	49	48
chr4	151510434	151512434	Espn		1	noCoverage	-0.024061	0.19807	insignificant	0	8	10
chr4	151526316	151528316	Espn	-0.14499239	2.22E-11	hypomethylated	-0.0036672	0.81153	insignificant	21	56	56
chr4	151531975	151533975	Hes2	-0.15642025	0.025971	hypomethylated	0.018454	0.38592	insignificant	5	34	34
chr4	151551208	151553208	Acot7	-0.11889786	5.26E-51	hypomethylated	0.0036834	0.81056	insignificant	56	144	144
chr4	151559242	151561242	Acot7	-0.3488635	0.001194	stronglyHypometh	0.070938	0.024784	hypermethylated	3	10	10
chr4	151647470	151649470	Gpr153	-0.0643191	4.85E-12	hypomethylated	-0.0064187	0.83151	insignificant	33	130	124
chr4	151665771	151667771	Hes3		1	noCoverage	0.06756	0.16153	insignificant	0	14	14
chr4	151670459	151672459	Icmt	-0.12606126	3.42E-65	hypomethylated	-0.0046332	0.25307	insignificant	58	155	153
chr4	151692734	151694734	Rnf207	-0.30984718	0.00092741	hypomethylated	0.0051969	0.10197	insignificant	5	45	43
chr4	151698986	151700986	Rpl22	-0.08285599	9.67E-28	hypomethylated	0.0013602	0.000027023	hypermethylated	21	83	96
chr4	151711759	151713759	Chd5	-0.1013186	3.56E-41	hypomethylated	0.0035789	0.19875	insignificant	63	187	187
chr4	151851250	151853250	Nphp4	-0.12789188	6.09E-44	hypomethylated	0.00086314	1	insignificant	30	114	114
chr4	151851588	151853588	Kcnab2	-0.13319409	2.25E-38	hypomethylated	-0.0053504	0.50647	insignificant	28	88	89
chr4	152070648	152072648	Gm833	0.12296528	0.54663	insignificant	0.0040879	0.10259	insignificant	6	30	24
chr4	152856939	152858939	Ajap1	-0.05604794	0.0015588	hypomethylated	-0.018533	1	insignificant	12	92	92
chr4	153330345	153332345	A430005L14Ri	-0.09114354	0.0025605	hypomethylated	-0.0041704	0.20577	insignificant	19	112	112
chr4	153348669	153350669	BC046331	-0.09920216	1.5E-19	hypomethylated	0.015632	0.9069	insignificant	35	127	111
chr4	153349190	153351190	Dffb	-0.11821017	4.03E-11	hypomethylated	0.021876	0.69936	insignificant	18	63	51
chr4	153384911	153386911	Lrrc47	-0.1528882	1.53E-32	hypomethylated	-0.041183	0.4467	insignificant	50	148	138
chr4	153416786	153418786	Ccdc27	0.12546286	0.093701	insignificant	0.037394	0.19449	insignificant	7	32	32
chr4	153471282	153473282	Trp73	0.14155115	0.70642	insignificant	0.028819	0.87107	insignificant	1	19	18
chr4	153514317	153516317	Trp73	0.00510007	0.1625	insignificant	0.0010992	1	insignificant	11	107	106
chr4	153515480	153517480	Wdr8	-0.05175194	0.000053739	hypomethylated	0.001007	0.61889	insignificant	17	82	80
chr4	153534793	153536793	Tprgl	-0.15940458	9.02E-11	hypomethylated	0.01811	0.00059917	hypermethylated	12	63	66
chr4	153543821	153545821	Megf6	-0.11329115	1.43E-08	hypomethylated	-0.0084064	0.31071	insignificant	15	101	107
chr4	153674004	153676004	Arhgef16	-0.13576212	4.55E-21	hypomethylated	0.013395	0.0069539	hypermethylated	23	66	62
chr4	154010982	154012982	Prdm16	-0.10067404	7.43E-45	hypomethylated	0.031857	0.48387	insignificant	34	133	116
chr4	154229308	154231308	Ttc34	-0.09903267	1.21E-10	hypomethylated	0.0040931	0.28046	insignificant	18	134	134

chr4	154242693	154244693	Mmel1	-0.09877671	0.00071908	hypomethylated	0.057478	0.48617	insignificant	4	10	9
chr4	154334031	154336031	Hes5	-0.09668454	4.91E-57	hypomethylated	0.0081529	0.077634	insignificant	67	162	153
chr4	154337241	154339241	Pank4	-0.10307622	3.74E-19	hypomethylated	0.030694	0.00000102	hypermethylated	22	100	91
chr4	154385093	154387093	Plch2	-0.09695595	0.28738	insignificant	-0.015349	1	insignificant	3	7	5
chr4	154440138	154442138	Pex10	-0.10479494	2.46E-08	hypomethylated	-0.011682	0.0012591	hypomethylated	19	87	87
chr4	154459685	154461685	Morn1	-0.10685132	1.29E-19	hypomethylated	0.0010374	0.56397	insignificant	46	135	134
chr4	154460406	154462406	Rer1	-0.15208599	1.05E-08	hypomethylated	-0.032868	0.14753	insignificant	23	55	57
chr4	154596644	154598644	Ski	-0.07708632	5.88E-42	hypomethylated	0.010387	0.063381	insignificant	72	191	167
chr4	154623074	154625074	2610002J02Ri	-0.19493698	0.000051237	hypomethylated	-0.010662	0.42144	insignificant	6	51	51
chr4	154719881	154721881	Prkcz	-0.10855347	0.00016683	hypomethylated	0.0051117	0.40449	insignificant	5	15	14
chr4	154735500	154737500	Prkcz	-0.12123782	0.000011436	hypomethylated	-0.0083296	0.87498	insignificant	9	65	66
chr4	154772178	154774178	Gabrd	-0.38657407	0.73334	insignificant	-0.095614	0.039677	inconclusive	8	27	24
chr4	154842223	154844223	Tmem52	-0.04771721	0.50555	insignificant	-0.016595	0.11741	insignificant	3	10	16
chr4	154864469	154866469	Gnb1	-0.12770699	1.15E-20	hypomethylated	0.014421	0.91081	insignificant	40	113	108
chr4	154936923	154938923	Nadk	-0.14212341	0.00001817	hypomethylated	0.0063019	0.59116	insignificant	17	70	65
chr4	154974524	154976524	Slc35e2	-0.12910152	0.00000316	hypomethylated	0.039678	0.62883	insignificant	10	104	77
chr4	154997977	154999977	Cdk11b	-0.10054792	6.92E-09	hypomethylated	0.0065012	0.9009	insignificant	18	90	90
chr4	155027493	155029493	Mmp23	-0.20965241	2.23E-10	hypomethylated	-0.01252	0.0081534	inconclusive	18	88	89
chr4	155043336	155045336	Mib2	-0.0307041	0.48756	insignificant	-0.15584	0.06162	insignificant	3	8	8
chr4	155067580	155069580	B930041F14Ri	-0.08355131	2.05E-17	hypomethylated	-0.0098599	0.69173	insignificant	30	182	178
chr4	155077923	155079923	Ssu72	-0.1302226	5.21E-11	hypomethylated	0.030857	0.29991	insignificant	28	159	142
chr4	155107912	155109912	Gm5151	-0.10865816	2.1E-43	hypomethylated	0.021262	1	insignificant	54	176	170
chr4	155134300	155136300	2610204G22R	-0.10824391	1.06E-12	hypomethylated	0.0078423	0.32672	insignificant	18	72	82
chr4	155135207	155137207	Atad3a	-0.16021189	0.000000102	hypomethylated	-0.0041716	0.56897	insignificant	7	30	31
chr4	155148670	155150670	Vwa1	-0.34761905	0.12662	insignificant	0.029631	0.020296	inconclusive	4	15	15
chr4	155176726	155178726	Mrpl20	-0.23337054	0.0065227	hypomethylated	0.028088	0.044167	inconclusive	9	32	32
chr4	155185597	155187597	Ccnl2	-0.10225589	1.05E-13	hypomethylated	0.00027636	0.045112	hypermethylated	31	141	138
chr4	155204754	155206754	Aurkaip1	-0.21217899	1.61E-14	hypomethylated	0.025305	0.011937	hypermethylated	19	66	51
chr4	155212788	155214788	Mxra8	-0.26792957	0.058094	insignificant	-0.096554	0.003385	hypomethylated	5	17	17
chr4	155220520	155222520	Dvl1	-0.11230888	4.83E-26	hypomethylated	0.0042428	0.54493	insignificant	46	167	160
chr4	155242675	155244675	Cpsf3l	-0.11295389	5.78E-17	hypomethylated	0.0067741	0.20797	insignificant	26	126	128
chr4	155243549	155245549	Gltpd1	-0.12115933	4.13E-13	hypomethylated	0.0037353	0.15432	insignificant	25	114	116
chr4	155264983	155266983	Acap3	-0.09645393	7.04E-17	hypomethylated	0.0040336	0.50306	insignificant	52	196	196
chr4	155265871	155267871	Pusl1	-0.07129036	1.25E-13	hypomethylated	-0.00089712	0.59682	insignificant	42	158	154
chr4	155316939	155318939	Ube2j2	-0.09577724	1.51E-09	hypomethylated	0.0037918	0.53291	insignificant	39	142	138
chr4	155319361	155321361	Gm5801		1	noCoverage	0.0023016	0.63045	insignificant	0	10	9
chr4	155335426	155337426	Fam132a	-0.19299048	0.0088438	hypomethylated	-0.0022761	0.81549	insignificant	7	43	43
chr4	155366022	155368022	Sdf4	-0.07996852	1.71E-25	hypomethylated	0.0035246	0.43077	insignificant	62	296	285
chr4	155366787	155368787	B3galt6	-0.10283516	8.66E-20	hypomethylated	0.014532	0.93441	insignificant	33	164	159
chr4	155399450	155401450	Tnfrsf18	-0.77458973	0.000036322	stronglyHypometh	-0.032205	0.53807	insignificant	1	18	18
chr4	155483106	155485106	9430015G10R	-0.11367119	0.0011599	hypomethylated	0.042305	0.6645	insignificant	9	52	45
chr4	155559244	155561244	Agrn		1	noCoverage	0.051912	1	insignificant	0	6	6
chr4	155576392	155578392	AW011738	-0.12270457	0.000014246	hypomethylated	0.019982	1	insignificant	15	82	66
chr4	155589035	155591035	2310042D19R	-0.06217949	0.65558	insignificant	-0.020513	1	insignificant	2	4	4
chr4	155608966	155610966	Klhl17	-0.09487971	3.92E-10	hypomethylated	-0.013432	0.1459	insignificant	28	134	126
chr5	3343311	3345311	Cdk6	-0.1542074	3.51E-31	hypomethylated	0.0046641	0.61578	insignificant	23	97	98
chr5	3542832	3544832	Fam133b	-0.03684391	0.33873	insignificant	0.011371	0.16641	insignificant	32	120	120
chr5	3595065	3597065	Pex1	-0.07392589	1.48E-08	hypomethylated	0.0049302	0.26751	insignificant	11	71	71
chr5	3596547	3598547	C030048B08R	-0.1446529	0.0023334	hypomethylated	0.087346	0.29643	insignificant	3	17	15
chr5	3647936	3649936	Gatad1	-0.23406863	0.000698	hypomethylated	-0.076376	0.74923	insignificant	6	12	13
chr5	3802164	3804164	Krit1	-0.08764152	2.92E-35	hypomethylated	0.0032817	0.33587	insignificant	44	154	154
chr5	3802180	3804180	Krit1	-0.08764152	2.92E-35	hypomethylated	0.0032817	0.33587	insignificant	44	154	154
chr5	3803109	3805109	Ankib1	-0.13190418	0.0000034	hypomethylated	-0.0072677	0.11805	insignificant	19	63	63
chr5	3844172	3846172	4932412H11R	-0.92791005	0.0080164	stronglyHypometh	0.051852	0.046918	hypermethylated	2	6	6
chr5	3927185	3929185	Akap9	-0.13456788	3.8E-14	hypomethylated	0.022346	0.63751	insignificant	37	98	103
chr5	4104697	4106697	Cyp51	-0.12193531	1	insignificant	0.026589	0.26162	insignificant	4	10	10
chr5	4758216	4760216	Fzd1	-0.30963558	0.000038097	hypomethylated	0.025546	0.082467	insignificant	5	24	25
chr5	5380251	5382251	Cdk14	-0.09912674	1.13E-31	hypomethylated	-0.0013757	0.61285	insignificant	54	118	118
chr5	5514789	5516789	Cldn12	-0.03907678	2.97E-10	hypomethylated	-0.0026332	0.295	insignificant	14	42	42
chr5	5514849	5516849	Cldn12	-0.10056598	5.62E-09	hypomethylated	-0.0023908	0.14829	insignificant	11	28	28
chr5	5514976	5516976	Cldn12	-0.01430312	0.00023484	hypomethylated	0.0385	0.019884	hypermethylated	3	8	8
chr5	5559501	5561501	Gtpbp10		1	noCoverage	-0.013002	0.35481	insignificant	0	38	38
chr5	5572798	5574798	Gm8773	-0.26391279	0.00000299	hypomethylated	0.013165	0.047764	inconclusive	10	51	50
chr5	5694077	5696077	Steap2	-0.14058343	2.35E-19	hypomethylated	0.0057704	0.5716	insignificant	15	72	72
chr5	5694568	5696568	Steap2		1	noCoverage	-0.012037	1	insignificant	0	6	6
chr5	5749317	5751317	Steap1	-0.19918194	5.25E-13	hypomethylated	-0.041395	0.85996	insignificant	15	57	52
chr5	7344378	7346378	Zfp804b	-0.18880629	0.60699	insignificant	-0.005937	0.61404	insignificant	4	37	37
chr5	7959471	7961471	Steap4	-0.20128928	0.0072037	hypomethylated	0.065256	0.0045477	hypermethylated	10	27	26
chr5	8045079	8047079	Sri		1	noCoverage	-0.078738	1	insignificant	0	6	6
chr5	8055541	8057541	Sri	-0.1390377	0.000019661	hypomethylated	0.015896	0.14491	insignificant	9	72	64
chr5	8368081	8370081	Adam22	-0.16305065	0.00013153	hypomethylated	-0.01555	0.21978	insignificant	9	18	18
chr5	8421849	8423849	Slc25a40	-0.12951548	5.11E-34	hypomethylated	0.007837	0.86965	insignificant	54	138	128
chr5	8422716	8424716	Dbf4	-0.12290097	1E-20	hypomethylated	0.0046032	0.73162	insignificant	39	88	79
chr5	8622952	8624952	Rundc3b		1	noCoverage	0.027361	0.31347	insignificant	0	4	4
chr5	8659091	8661091	Abcb1a	-0.20208406	0.00010332	hypomethylated	-0.0084967	0.51608	insignificant	4	32	31
chr5	8797146	8799146	Abcb1b	-0.19424092	0.0014133	hypomethylated	0.004887	0.75671	insignificant	5	44	44
chr5	8892720	8894720	Abcb4	-0.13208304	1.5E-14	hypomethylated	0.034832	0.74955	insignificant	19	100	94
chr5	8997146	8999146	Crot	-0.15908853	0.0011762	hypomethylated	-0.052063	0.01868	hypomethylated	6	38	36
chr5	9099736	9101736	4930420K17Ri	-0.08377366	1.48E-15	hypomethylated	0.0036915	0.97184	insignificant	37	163	156
chr5	9161776	9163776	Dmtf1		1	noCoverage	0.0042929	0.90022	insignificant	0	11	11
chr5	9265192	9267192	9330182L06Ri	-0.09654632	6.57E-11	hypomethylated	-0.014991	0.52866	insignificant	29	87	103
chr5	9725352	9727352	Grm3	-0.29379509	1	lowCoverage	-0.015045	0.86581	insignificant	1	11	10
chr5	12382165	12384165	Sema3d	-0.20095829	0.031372	hypomethylated	-0.019388	0.77044	insignificant	5	34	34
chr5	13398308	13400308	Sema3a	-0.30152979	0.56256	insignificant	-0.035627	0.55432	insignificant	1	18	18
chr5	13790618	13792618	Speer3		1	noCoverage	0.0086592	1	insignificant	0	4	4

chr5	14024275	14026275	Sema3e	-0.09059019	2.12E-08	hypomethylated	-0.0012807	0.73751	insignificant	10	64	64
chr5	14513917	14515917	Pclo	-0.11583226	5.83E-16	hypomethylated	0.019611	0.10452	insignificant	23	100	90
chr5	14944293	14946293	Speer8-ps1		1	noCoverage	-0.13302	0.44667	insignificant	0	4	4
chr5	15439508	15441508	Cacna2d1	-0.11608251	6.12E-15	hypomethylated	-0.0037945	0.48383	insignificant	52	196	182
chr5	17079633	17081633	Sema3c	-0.18859165	1.16E-35	hypomethylated	0.036452	0.069824	insignificant	24	56	56
chr5	18731863	18733863	Magi2	-0.14929363	6.24E-12	hypomethylated	0.040631	0.03667	hypermethylated	14	75	82
chr5	19412335	19414335	Magi2	-0.38988095	0.28403	insignificant	-0.0047527	0.91236	insignificant	1	6	6
chr5	20387270	20389270	Tmem60	-0.0919555	1.37E-28	hypomethylated	-0.017268	0.375	insignificant	55	271	267
chr5	20387942	20389942	Phtf2	-0.13174371	3.6E-10	hypomethylated	-0.033189	0.14237	insignificant	8	86	82
chr5	20455806	20457806	A630072M18R	-0.07329421	1.06E-29	hypomethylated	0.00042315	0.93483	insignificant	33	171	171
chr5	20457640	20459640	A630072M18R	-0.05236026	0.026909	hypomethylated	0.010319	0.86369	insignificant	10	57	57
chr5	20561615	20563615	Ptpn12	-0.133702	3.37E-13	hypomethylated	-0.016743	0.76963	insignificant	31	103	113
chr5	20691084	20693084	Pion	-0.13636771	0.000016826	hypomethylated	-0.014293	0.89402	insignificant	12	46	46
chr5	20929720	20931720	Fam185a	-0.13807761	0.00002361	hypomethylated	0.016756	0.59786	insignificant	15	68	68
chr5	20930495	20932495	Fam185a	-0.13588853	0.000014034	hypomethylated	-0.0038885	0.84205	insignificant	15	66	66
chr5	21150801	21152801	Armc10	-0.08400557	5.25E-14	hypomethylated	0.014949	0.15176	insignificant	24	92	94
chr5	21151423	21153423	Armc10	-0.11878614	3.36E-10	hypomethylated	0.014136	0.46274	insignificant	22	79	79
chr5	21207163	21209163	Napepld	-0.13229827	3.7E-10	hypomethylated	0.0059919	0.33516	insignificant	17	72	68
chr5	21241977	21243977	Pmpcb	-0.07702495	0.81309	insignificant	-0.013471	0.59838	insignificant	15	38	38
chr5	21290100	21292100	Psmc2	-0.11041609	2.26E-15	hypomethylated	0.026746	0.55247	insignificant	32	119	123
chr5	21290983	21292983	Psmc2	-0.12367788	1.4E-16	hypomethylated	0.033212	0.65948	insignificant	24	98	94
chr5	21371422	21373422	Slc26a5		1	noCoverage	-0.094694	0.7243	insignificant	0	9	6
chr5	21850523	21852523	Reln	-0.14010607	1.97E-23	hypomethylated	-0.0085955	0.48574	insignificant	31	123	122
chr5	22056149	22058149	Orc5	-0.32619964	0.38102	insignificant	-0.051729	0.075217	insignificant	3	8	7
chr5	22251010	22253010	Lhfpl3	-0.17249043	0.00000114	hypomethylated	-0.033409	1	insignificant	24	71	81
chr5	22252702	22254702	Lhfpl3	-0.29089742	0.071627	insignificant	0.10077	0.93629	insignificant	2	19	18
chr5	22939246	22941246	Mll5	-0.12062141	0.000078227	hypomethylated	0.005446	0.50216	insignificant	13	102	101
chr5	23216894	23218894	Mir3096		1	noCoverage	-0.077245	0.77414	insignificant	0	24	26
chr5	23218484	23220484	AI506816		1	noCoverage	0.00020621	0.71132	insignificant	0	10	14
chr5	23289479	23291479	Pus7	-0.42759565	0.50824	insignificant	0.0040878	0.67359	insignificant	2	5	5
chr5	23292561	23294561	Rint1	-0.27470864	8.63E-18	hypomethylated	0.0012988	1	insignificant	22	63	59
chr5	23349963	23351963	Tomm7	-0.16139688	0.61929	insignificant	-0.029437	1	insignificant	5	32	27
chr5	23536501	23538501	Fam126a		1	noCoverage	-0.015295	0.49451	insignificant	0	20	20
chr5	23605407	23607407	Klhl7	-0.23214477	0.00046785	hypomethylated	-0.017183	0.52374	insignificant	5	41	27
chr5	23669780	23671780	Nupl2	-0.1429777	0.000035582	hypomethylated	0.0063971	0.68912	insignificant	17	47	47
chr5	23857422	23859422	Kcnh2	-0.15761671	0.000000194	hypomethylated	-0.0055326	0.016731	inconclusive	23	92	92
chr5	23869636	23871636	Nos3	-0.11288152	0.21794	insignificant	-0.17392	0.0035798	hypomethylated	7	28	29
chr5	23898973	23900973	Abcb8	-0.11161224	0.042064	hypomethylated	0.013676	0.40823	insignificant	9	20	28
chr5	23918268	23920268	Accn3	0.00412977	0.83407	insignificant	0.00098373	0.70991	insignificant	3	28	28
chr5	23929348	23931348	Cdk5	-0.11908573	7.67E-14	hypomethylated	0.0060323	0.61689	insignificant	37	115	110
chr5	23930049	23932049	Slc4a2	-0.10258339	1.81E-09	hypomethylated	0.0096189	0.54386	insignificant	36	99	94
chr5	23951053	23953053	Tmub1	-0.01497041	0.024176	inconclusive	0.020471	0.67729	insignificant	11	38	40
chr5	23953664	23955664	Tmub1	-0.18410159	0.000000208	hypomethylated	-0.022292	0.31006	insignificant	6	14	14
chr5	23956994	23958994	Agap3	-0.12977232	2.17E-38	hypomethylated	-0.0059094	0.090656	insignificant	64	172	176
chr5	24032666	24034666	Gbx1	-0.10796753	9.07E-17	hypomethylated	-0.0036542	0.45184	insignificant	16	72	72
chr5	24083285	24085285	Abcf2	-0.14857292	0.05271	insignificant	0.025833	0.58193	insignificant	3	34	32
chr5	24091567	24093567	Chpf2	0.05622027	0.040299	hypermethylated	0.0014439	0.92807	insignificant	3	58	58
chr5	24096931	24098931	Mir671	-0.27535216	1.18E-19	hypomethylated	-0.013037	0.27744	insignificant	16	73	71
chr5	24107820	24109820	Smarcd3	-0.10241	1.92E-12	hypomethylated	-0.006493	0.767	insignificant	13	36	36
chr5	24150270	24152270	1700022A21R	-0.83333333	0.19737	lowCoverage	0.069231	0.32571	insignificant	1	6	6
chr5	24190632	24192632	Nub1	-0.09406962	2.29E-15	hypomethylated	0.042573	0.30729	insignificant	17	85	77
chr5	24236498	24238498	Wdr86	-0.36797737	0.0057606	stronglyHypometh	0.050692	0.78147	insignificant	2	35	35
chr5	24263661	24265661	Crygn	0.04758014	0.68963	insignificant	0.035895	0.010335	hypermethylated	4	17	16
chr5	24348179	24350179	Rheb	-0.0730357	9.05E-10	hypomethylated	0.0015724	0.88722	insignificant	24	101	93
chr5	24414327	24416327	Prkag2	-0.10068928	0.0060785	hypomethylated	0.00072441	0.66522	insignificant	1	51	50
chr5	24605792	24607792	2900005J15Ri	-0.13894569	1.06E-27	hypomethylated	0.018869	1	insignificant	23	106	99
chr5	24606460	24608460	Prkag2	-0.14393479	7.94E-24	hypomethylated	0.035579	0.86706	insignificant	17	59	50
chr5	24727710	24729710	Galnt11	-0.0674734	1.85E-10	hypomethylated	0.0044998	0.31664	insignificant	32	89	86
chr5	25004601	25006601	Mll3	-0.09871821	5.51E-13	hypomethylated	-0.005712	0.58455	insignificant	48	223	210
chr5	25004614	25006614	4831440E17Ri	-0.10095654	2.37E-11	hypomethylated	-0.0058943	0.47099	insignificant	46	219	206
chr5	25020884	25022884	Cct8l1	0.03550257	1	insignificant	-0.034898	0.29289	insignificant	5	14	17
chr5	25034835	25036835	1700096K18Ri	-0.13643391	2.69E-24	hypomethylated	0.014992	0.065844	insignificant	31	90	88
chr5	25211615	25213615	Xrcc2		1	noCoverage	-0.026974	0.8191	insignificant	0	11	10
chr5	25264843	25266843	Actr3b	-0.30495158	7.21E-12	hypomethylated	-0.039618	0.65474	insignificant	12	49	40
chr5	27142896	27144896	Dpp6	-0.09269695	0.00073147	hypomethylated	0.024507	0.41642	insignificant	12	85	82
chr5	27374738	27376738	Dpp6	-0.0938342	0.029044	hypomethylated	-0.032809	0.63412	insignificant	8	67	63
chr5	27587474	27589474	Dpp6	-0.21087868	0.58609	insignificant	0.0048675	0.69659	insignificant	7	32	31
chr5	28117879	28119879	Paxip1	-0.12557914	3.91E-27	hypomethylated	0.0066314	0.53696	insignificant	15	65	65
chr5	28167486	28169486	Htr5a	-0.31085974	0.51162	insignificant	0.0020388	0.24077	insignificant	2	25	20
chr5	28396951	28398951	Insig1	-0.06885411	1.69E-10	hypomethylated	-0.0022734	0.029007	hypomethylated	25	110	110
chr5	28491235	28493235	En2	-0.09665166	1.64E-37	hypomethylated	-0.00027988	0.31721	insignificant	71	252	239
chr5	28642728	28644728	Rbm33	-0.09579706	5.05E-57	hypomethylated	-0.0015522	0.40762	insignificant	60	206	211
chr5	28792523	28794523	9530036O11R	-0.25854474	0.000025607	hypomethylated	0.02071	0.40983	insignificant	3	19	19
chr5	29704930	29706930	Lmbr1	-0.5131302	0.00000154	stronglyHypometh	0.053482	0.6572	insignificant	4	17	16
chr5	29760206	29762206	Nom1	0.05276306	0.010059	hypermethylated	-0.0002491	0.7826	insignificant	12	62	62
chr5	29805010	29807010	Mnx1	-0.16731882	0.00000219	hypomethylated	0.015366	0.58136	insignificant	10	70	71
chr5	29894781	29896781	Ube3c	-0.03512713	1.58E-11	hypomethylated	0.0089104	0.40284	insignificant	38	149	146
chr5	30061437	30063437	Dnajb6	-0.11397524	6.42E-15	hypomethylated	-0.00030937	0.18021	insignificant	31	123	122
chr5	30062476	30064476	Dnajb6	-0.14642062	7.44E-08	hypomethylated	-0.024004	0.11215	insignificant	24	94	90
chr5	30338700	30340700	Il6	-0.31714286	0.10676	insignificant	-0.011821	0.90634	insignificant	2	10	7
chr5	30430735	30432735	Fam59b	-0.11568712	1.53E-34	hypomethylated	0.018282	0.13748	insignificant	28	123	127
chr5	30480861	30482861	Hadhb	-0.18354826	0.0018611	hypomethylated	0.0072022	0.53699	insignificant	4	30	30
chr5	30481520	30483520	Hadha	-0.3197995	0.29927	insignificant	-0.0016177	0.47555	insignificant	2	7	7
chr5	30532262	30534262	Gpr113	-0.15430403	0.1863	insignificant	0.096154	0.44266	insignificant	1	13	13
chr5	30558157	30560157	Ept1	-0.24066651	1.65E-11	hypomethylated	-0.038657	0.95814	insignificant	7	52	38

chr5	30606913	30608913	Ccdc164	-0.15780094	0.33534	insignificant	-0.027573	0.045563	inconclusive	12	40	36
chr5	30764305	30766305	Otof	-0.65918687	0.41196	lowCoverage	-0.17351	0.80231	insignificant	1	6	10
chr5	30767449	30769449	1700001C02R	-0.14789275	5.1E-11	hypomethylated	0.037061	0.77142	insignificant	16	53	53
chr5	30848209	30850209	Cib4		1	noCoverage	-0.07105	0.8904	insignificant	0	5	6
chr5	30889542	30891542	Kcnk3	-0.12188472	6.32E-26	hypomethylated	-0.0099696	0.043399	hypomethylated	45	142	148
chr5	30949311	30951311	4930471M23R	-0.16878383	3.49E-21	hypomethylated	-0.0013141	0.7968	insignificant	27	68	66
chr5	30968274	30970274	Cenpa	-0.17356436	0.000071213	hypomethylated	0.00083548	0.60121	insignificant	8	54	54
chr5	31013267	31015267	Dpysl5	-0.19437567	9.51E-11	hypomethylated	0.016723	0.60319	insignificant	7	67	70
chr5	31116128	31118128	Mapre3	-0.10047133	7.58E-25	hypomethylated	0.046792	0.093313	insignificant	23	60	67
chr5	31171019	31173019	Tmem214	-0.14278211	3.97E-17	hypomethylated	0.00010039	0.55123	insignificant	14	60	61
chr5	31190224	31192224	Agbl5	-0.14054825	1.18E-23	hypomethylated	0.013441	0.21396	insignificant	24	108	106
chr5	31190378	31192378	Agbl5	-0.14715349	2.53E-21	hypomethylated	0.017567	0.22932	insignificant	21	94	92
chr5	31210095	31212095	Ost4	-0.09141704	1.82E-09	hypomethylated	0.0034496	0.86454	insignificant	10	44	44
chr5	31210161	31212161	Ost4	-0.09141704	1.82E-09	hypomethylated	0.0034496	0.86454	insignificant	10	44	44
chr5	31215158	31217158	Emilin1	-0.40723982	0.5125	insignificant	-0.10863	0.86996	insignificant	1	13	12
chr5	31223267	31225267	Khk	-0.0925561	0.00097476	hypomethylated	0.021922	0.48668	insignificant	8	42	36
chr5	31247800	31249800	Cgref1	0.03125	1	insignificant	-0.19718	0.3877	insignificant	2	8	10
chr5	31247853	31249853	Cgref1	0.03125	1	insignificant	-0.19718	0.3877	insignificant	2	8	10
chr5	31251438	31253438	Abhd1		1	noCoverage	0.00036389	0.30707	insignificant	0	24	24
chr5	31251478	31253478	Abhd1		1	noCoverage	0.00036389	0.30707	insignificant	0	24	24
chr5	31262734	31264734	Preb	-0.15101804	0.044074	hypomethylated	-0.025093	0.000052564	hypomethylated	9	34	34
chr5	31350012	31352012	0610007C21R	-0.18679886	5.5E-19	hypomethylated	0.01663	0.68314	insignificant	18	61	61
chr5	31350452	31352452	Slc5a6	-0.19674175	9.75E-20	hypomethylated	0.020637	0.92955	insignificant	19	63	63
chr5	31350935	31352935	Slc5a6	-0.37116138	3.82E-17	stronglyHypometh	0.024011	0.94042	insignificant	5	14	14
chr5	31351297	31353297	Slc5a6	-0.3702805	1.94E-13	stronglyHypometh	0.039722	0.58978	insignificant	2	8	8
chr5	31356183	31358183	Cad	-0.12887208	2.41E-20	hypomethylated	0.01391	0.62491	insignificant	28	99	91
chr5	31395900	31397900	Slc30a3	-0.17859206	9.92E-09	hypomethylated	-0.0031451	0.054635	insignificant	15	71	68
chr5	31409691	31411691	Dnajc5g	0.2345063	0.20208	insignificant	0.17721	6.91E-10	hypermethylated	5	16	16
chr5	31441268	31443268	Ucn	-0.06745618	0.0037427	hypomethylated	0.061967	0.042922	hypermethylated	4	22	22
chr5	31482517	31484517	Gtf3c2	0.75899101	0.0024268	stronglyHypermeth	0.0227	0.83311	insignificant	2	22	22
chr5	31494676	31496676	Snx17	-0.14748411	6.5E-55	hypomethylated	-0.00099607	0.6881	insignificant	32	116	116
chr5	31495512	31497512	Eif2b4	-0.14394853	6.84E-33	hypomethylated	-0.0077261	0.39551	insignificant	17	56	56
chr5	31504415	31506415	Ppm1g	-0.20271321	8.35E-12	inconclusive	-0.022715	0.14947	insignificant	13	49	48
chr5	31504676	31506676	Ppm1g	-0.26426395	2.6E-23	inconclusive	-0.019634	0.17048	insignificant	4	14	14
chr5	31522918	31524918	Ppm1g	-0.17777778	0.22038	insignificant	-0.0049026	0.81118	insignificant	5	13	13
chr5	31542290	31544290	Nrbp1	-0.04965943	3.13E-20	hypomethylated	0.009955	0.95314	insignificant	52	166	161
chr5	31553078	31555078	Krtcap3	0.21252594	1	lowCoverage	-0.13512	0.00010159	hypomethylated	1	14	14
chr5	31593487	31595487	Ift172	-0.36064815	2.45E-09	stronglyHypometh	-0.070064	1	insignificant	2	9	8
chr5	31598250	31600250	Fndc4	-0.26210489	0.0023205	hypomethylated	-0.020774	0.94074	insignificant	3	37	31
chr5	31598953	31600953	Gckr		1	noCoverage	-0.08181	0.27736	insignificant	0	14	12
chr5	31753808	31755808	Zfp512	-0.29534418	0.00069631	hypomethylated	-0.075393	0.86062	insignificant	5	15	22
chr5	31796133	31798133	Gpn1	-0.18375963	5.2E-17	hypomethylated	0.00049411	0.68309	insignificant	26	78	78
chr5	31828367	31830367	Slc4a1ap	-0.20880587	2.37E-30	hypomethylated	-0.039288	0.82768	insignificant	26	93	90
chr5	31829135	31831135	Supt7l	-0.23414493	5.03E-23	hypomethylated	-0.065255	0.65556	insignificant	16	70	68
chr5	31915323	31917323	Mrpl33	-0.11222797	0.00095305	hypomethylated	0.0050805	0.29028	insignificant	12	46	40
chr5	31999422	32001422	Bre	-0.02894112	0.000020592	hypomethylated	-0.0014442	0.69717	insignificant	32	82	86
chr5	31999983	32001983	Rbks	-0.01375713	0.26538	insignificant	-0.0026693	0.053932	insignificant	28	64	66
chr5	32437844	32439844	Fosl2	-0.09393135	1.54E-14	hypomethylated	-0.014101	0.3406	insignificant	38	164	156
chr5	32534080	32536080	Plb1	0.08571429	1	insignificant	-0.27381	0.33773	insignificant	1	4	7
chr5	32760342	32762342	Ppp1cb	-0.11513767	0.00048082	hypomethylated	-0.0047729	0.69932	insignificant	20	116	113
chr5	32912605	32914605	Yes1	-0.11522341	1.39E-52	hypomethylated	0.003962	0.82134	insignificant	57	136	136
chr5	33128275	33130275	Pisd	-0.23119969	0.0047425	hypomethylated	0.096695	0.68967	insignificant	6	39	30
chr5	33196879	33198879	C330019G07Rik		1	noCoverage	0.016667	1	insignificant	0	2	2
chr5	33205369	33207369	Depdc5	-0.14316832	0.000067843	hypomethylated	0.0063218	0.058042	insignificant	10	79	74
chr5	33360464	33362464	Ywhah	-0.10035591	1.49E-23	hypomethylated	0.0058182	0.30101	insignificant	31	125	126
chr5	33445867	33447867	Slc5a1	-0.20957361	0.001491	hypomethylated	-0.068869	0.21566	insignificant	3	20	25
chr5	33560887	33562887	Spon2	-0.56073079	0.1239	insignificant	-0.029481	0.26966	insignificant	1	8	8
chr5	33617653	33619653	Ctbp1	-0.09011992	0.0028474	hypomethylated	-0.013897	0.79038	insignificant	14	46	42
chr5	33677220	33679220	Maea	-0.14395241	6.39E-44	hypomethylated	-0.0016495	0.28268	insignificant	56	158	157
chr5	33720344	33722344	4933407H18R	-0.13405577	0.00002733	hypomethylated	0.0080446	0.86175	insignificant	9	74	74
chr5	33972284	33974284	Fam53a	-0.09655727	1.24E-11	hypomethylated	-0.00087841	0.87047	insignificant	17	74	74
chr5	33999795	34001795	Tacc3	-0.12704181	0.017404	hypomethylated	-0.0069271	0.73529	insignificant	8	85	78
chr5	34000481	34002481	Tmem129	-0.12119974	0.039631	hypomethylated	0.00001564	0.69115	insignificant	6	61	58
chr5	34063372	34065372	Fgfr3	-0.09480309	5.24E-28	hypomethylated	0.00074424	0.71148	insignificant	55	155	152
chr5	34063408	34065408	Fgfr3	-0.093029	1.33E-28	hypomethylated	0.0038872	0.37254	insignificant	55	167	164
chr5	34063954	34065954	Fgfr3	-0.10553736	7.84E-31	hypomethylated	-0.0015883	0.79535	insignificant	61	203	200
chr5	34125353	34127353	Letm1	-0.13596439	0.000035733	hypomethylated	0.0019752	0.91883	insignificant	7	16	16
chr5	34278907	34280907	Whsc2	-0.14823287	3.12E-15	hypomethylated	0.0096519	0.83208	insignificant	15	56	50
chr5	34325122	34327122	Gm1673	-0.08138284	0.000096648	hypomethylated	0.0050646	0.76105	insignificant	13	115	115
chr5	34337632	34339632	Nat8l	-0.10419187	4.33E-09	hypomethylated	0.0019448	0.00025867	hypermethylated	47	182	179
chr5	34512073	34514073	Haus3	-0.13537736	8.55E-09	hypomethylated	0.026056	0.39409	insignificant	11	41	41
chr5	34512097	34514097	Haus3	-0.13537736	8.55E-09	hypomethylated	0.026056	0.39409	insignificant	11	41	41
chr5	34530359	34532359	Mxd4	-0.14641433	5.55E-08	hypomethylated	-0.00065435	0.3778	insignificant	10	31	31
chr5	34630973	34632973	Zfyve28	-0.03223614	0.00028276	inconclusive	-0.041426	0.091679	insignificant	24	77	74
chr5	34678038	34680038	Rnf4	-0.08769014	0.78566	insignificant	0.0029332	0.080417	insignificant	33	106	103
chr5	34856628	34858628	Tnip2	-0.11188838	4.79E-10	hypomethylated	0.03678	0.16891	insignificant	7	22	22
chr5	34867432	34869432	Sh3bp2		1	noCoverage	0.00030812	1	insignificant	0	17	17
chr5	34885013	34887013	Sh3bp2	0.10997024	0.25061	insignificant	0.012054	0.5448	insignificant	3	6	6
chr5	34891224	34893224	Sh3bp2	-0.18010845	3.92E-10	hypomethylated	0.0067988	0.70815	insignificant	15	70	71
chr5	34891336	34893336	Sh3bp2	-0.15925775	0.000000015	hypomethylated	0.0056228	0.46606	insignificant	15	68	69
chr5	34915362	34917362	Add1	-0.17967276	5.2E-23	hypomethylated	0.0041925	0.84751	insignificant	33	92	92
chr5	34979763	34981763	Mfsd10	-0.18452381	1	insignificant	0.029365	0.58601	insignificant	1	6	6
chr5	35002027	35004027	Grk4	-0.13012586	1.16E-16	hypomethylated	0.011358	0.021598	hypermethylated	29	141	138
chr5	35002797	35004797	Nop14	-0.05785092	8.12E-14	hypomethylated	0.010819	0.023868	hypermethylated	29	135	132
chr5	35103388	35105388	Htt	-0.153352	0.000027798	hypomethylated	0.018109	0.88818	insignificant	16	71	68

chr5	35257563	35259563	A930005I04Ri	-0.88	0.15909	lowCoverage	0.021449	0.76828	insignificant	1	2	2
chr5	35291096	35293096	Rgs12	-0.1321742	5.86E-16	hypomethylated	0.0072074	0.19875	insignificant	21	107	85
chr5	35398732	35400732	Dok7	-0.11738088	4.24E-10	hypomethylated	0.0086964	0.038598	hypermethylated	18	58	58
chr5	35448346	35450346	Lrpap1	-0.0839207	2.02E-10	hypomethylated	0.018447	0.032207	hypermethylated	9	22	22
chr5	35620214	35622214	Adra2c	-0.1007985	8.09E-25	hypomethylated	-0.001994	0.56556	insignificant	38	168	159
chr5	35730765	35732765	Hmx1	-0.11150543	1.5E-23	hypomethylated	0.018132	0.02417	hypermethylated	68	179	190
chr5	35924708	35926708	Acox3	-0.1706095	7.97E-08	hypomethylated	-0.0065478	0.69104	insignificant	17	66	66
chr5	36022431	36024431	Htra3	-0.89732143	0.15493	lowCoverage	-0.073988	0.64458	insignificant	1	10	10
chr5	36099528	36101528	Ablim2	-0.12795453	5.16E-15	hypomethylated	0.013968	0.39365	insignificant	28	108	100
chr5	36234967	36236967	Afap1	-0.15778283	1.14E-14	hypomethylated	0.012839	1	insignificant	27	126	126
chr5	36545669	36547669	Psapl1	0.1047619	1	insignificant	0.038474	0.5103	insignificant	1	4	4
chr5	36740788	36742788	Sorcs2	-0.34083217	0.069959	insignificant	-0.056661	0.87861	insignificant	5	32	29
chr5	36806833	36808833	Grpel1	-0.14344784	5.57E-09	hypomethylated	0.0031555	0.82595	insignificant	14	92	97
chr5	36826236	36828236	Ccdc96	-0.1061609	2.8E-24	hypomethylated	-0.0003362	0.74003	insignificant	55	195	189
chr5	36826934	36828934	Tada2b	-0.15812033	3.73E-20	hypomethylated	-0.0049508	0.48702	insignificant	36	116	120
chr5	36925046	36927046	Tbc1d14	-0.19999475	0.36333	insignificant	-0.048598	0.64886	insignificant	1	22	20
chr5	37087208	37089208	D5Ertd579e	-0.20210945	0.015849	hypomethylated	-0.016653	0.72841	insignificant	7	39	39
chr5	37258808	37260808	Ppp2r2c	-0.10891089	3.17E-38	hypomethylated	-0.0047002	0.31362	insignificant	66	165	159
chr5	37380221	37382221	Wfs1	-0.10827307	0.0042341	hypomethylated	-0.000061447	1	insignificant	11	46	48
chr5	37632318	37634318	Crmp1	-0.08989876	2.74E-17	hypomethylated	0.034855	0.000007522	hypermethylated	46	186	165
chr5	37636030	37638030	Crmp1	-0.18091894	7.36E-11	hypomethylated	0.01297	0.66498	insignificant	25	110	109
chr5	37728120	37730120	Evc	-0.27257222	3.35E-09	hypomethylated	0.0081764	0.00076316	hypermethylated	10	49	48
chr5	37728716	37730716	Evc2		1	noCoverage	-0.040126	0.32959	insignificant	0	19	18
chr5	38108392	38110392	Stk32b	-0.3013138	0.0019818	hypomethylated	-0.078105	0.92346	insignificant	3	43	39
chr5	38125757	38127757	Cytl1	0.27380952	1	lowCoverage	-0.00075188	0.42734	insignificant	1	8	8
chr5	38210802	38212802	Msx1as		1	noCoverage	-0.11509	0.12509	insignificant	0	12	12
chr5	38215824	38217824	Msx1	-0.13733387	0.00078257	hypomethylated	0.018403	0.018225	hypermethylated	18	72	75
chr5	38429473	38431473	Stx18	-0.16023654	1.13E-11	hypomethylated	-0.031793	0.85188	insignificant	11	57	54
chr5	38550706	38552706	Nsg1	-0.17618129	1.44E-22	hypomethylated	0.014685	0.73866	insignificant	20	87	82
chr5	38610720	38612720	Lyar	-0.12404024	0.000062128	hypomethylated	0.02112	0.69416	insignificant	25	110	107
chr5	38611667	38613667	Zbtb49	-0.16444226	0.00090709	hypomethylated	0.013414	0.17636	insignificant	21	55	55
chr5	38650613	38652613	Tmem128	-0.23866641	0.023029	hypomethylated	0.050191	0.80327	insignificant	8	47	44
chr5	38667642	38669642	Otop1	-0.16185433	1.22E-09	hypomethylated	-0.029656	0.94566	insignificant	21	77	82
chr5	38709747	38711747	Drd5	-0.16784807	0.19751	insignificant	0.0049942	0.018947	hypermethylated	16	123	122
chr5	38874624	38876624	Slc2a9	0.05416667	1	lowCoverage	0.018452	1	insignificant	1	4	4
chr5	38952834	38954834	Wdr1	-0.11137717	3.92E-16	hypomethylated	-0.027733	0.56808	insignificant	31	84	82
chr5	40035870	40037870	Hs3st1	-0.25572084	0.00000209	hypomethylated	-0.064237	0.79258	insignificant	5	19	16
chr5	42099394	42101394	Rab28	-0.07615402	0.64129	insignificant	0.0094525	0.81191	insignificant	5	22	20
chr5	42155459	42157459	Nkx3-2	-0.23221074	7.02E-21	hypomethylated	-0.052097	0.08797	insignificant	16	61	56
chr5	42235554	42237554	Bod1l	-0.13811314	0.00026183	hypomethylated	0.000021458	0.40484	insignificant	7	24	24
chr5	43623701	43625701	Cpeb2	-0.08334839	1.74E-43	hypomethylated	0.010036	0.071246	insignificant	64	284	277
chr5	43626593	43628593	Gm7854	-0.0202412	0.58067	insignificant	0.048776	0.46677	insignificant	6	44	44
chr5	43992160	43994160	C1qtnf7	0.13942308	1	insignificant	0.024038	1	insignificant	1	4	4
chr5	44052684	44054684	Cc2d2a	-0.34362674	0.00000689	stronglyHypometh	0.014044	0.37233	insignificant	3	11	10
chr5	44173388	44175388	Fbxl5	-0.08298195	2.57E-08	hypomethylated	0.010927	0.95027	insignificant	13	60	60
chr5	44492975	44494975	Prom1	-0.32959818	0.30349	insignificant	-0.0055517	0.82154	insignificant	2	12	12
chr5	44617845	44619845	Tapt1	-0.09438889	1.9E-12	hypomethylated	-0.0041902	0.54526	insignificant	30	92	86
chr5	45841468	45843468	Qdpr		1	noCoverage	0	1	insignificant	0	4	4
chr5	45883612	45885612	Lap3	-0.07527472	0.00016756	hypomethylated	0.0076148	0.20041	insignificant	19	104	98
chr5	45910467	45912467	Med28	-0.11190703	1.42E-23	hypomethylated	0.013925	0.10105	insignificant	23	72	72
chr5	46060163	46062163	Ncapg	-0.10567273	0.000021125	hypomethylated	-0.0021052	0.033762	inconclusive	27	150	151
chr5	46247791	46249791	Lcorl	-0.08078949	4.13E-08	hypomethylated	0.0095255	0.81838	insignificant	43	164	160
chr5	46248779	46250779	Lcorl	-0.08129216	0.62181	insignificant	0.07115	0.82813	insignificant	7	26	27
chr5	48373393	48375393	Slit2	0.02750963	0.0080384	hypermethylated	0.0013749	0.2534	insignificant	24	100	100
chr5	48614180	48616180	Mir218-1		1	noCoverage	0.066667	0.40914	insignificant	0	5	6
chr5	48762630	48764630	Pacrgl	-0.11253007	0.36282	insignificant	-0.0024154	0.91319	insignificant	2	32	32
chr5	48990921	48992921	Kcnip4	-0.01297126	0.68422	insignificant	-0.0084	0.43234	insignificant	1	10	10
chr5	49280796	49282796	Kcnip4	-0.31172137	0.000051361	hypomethylated	0.066208	0.62775	insignificant	4	10	13
chr5	50450235	50452235	Gpr125	0.13864543	0.0011786	hypermethylated	0.00018678	0.11597	insignificant	3	28	28
chr5	52580919	52582919	9230114K14Ri	-0.104152	3.15E-12	hypomethylated	-0.0036402	0.96617	insignificant	36	115	111
chr5	52581758	52583758	Dhx15	-0.07372952	1	insignificant	-0.0062438	1	insignificant	8	16	16
chr5	52754042	52756042	Sod3	-0.39530763	0.0018737	stronglyHypometh	-0.080386	0.33708	insignificant	3	10	8
chr5	52957519	52959519	Lgi2	-0.08946799	0.00000438	hypomethylated	0.031093	0.55037	insignificant	5	34	43
chr5	53060940	53062940	Sepsecs	0.08649919	3.96E-08	inconclusive	0.0095613	0.022095	hypermethylated	7	40	40
chr5	53131812	53133812	Pi4k2b	-0.19714392	6.29E-20	hypomethylated	0.025053	0.027129	inconclusive	31	81	83
chr5	53173305	53175305	Zcchc4	-0.12145891	1.9E-09	hypomethylated	0.024899	0.089024	insignificant	7	30	30
chr5	53224373	53226373	Anapc4	-0.13157951	1.94E-12	hypomethylated	-0.0027415	0.55619	insignificant	14	52	52
chr5	53439591	53441591	Slc34a2	-0.07102714	0.84213	insignificant	0.026768	0.4259	insignificant	20	50	52
chr5	53604691	53606691	Sel1l3	-0.07131772	0.0024713	hypomethylated	0.00091703	0.54951	insignificant	13	38	38
chr5	53657344	53659344	1810013D10R	-0.14782675	8.77E-16	hypomethylated	0.031901	0.65147	insignificant	20	64	68
chr5	53946017	53948017	Rbpj	0.06699684	1	insignificant	0.030814	0.6195	insignificant	2	11	10
chr5	53980453	53982453	Rbpj	-0.1244526	9.78E-59	hypomethylated	0.004557	0.082655	insignificant	69	214	204
chr5	53980812	53982812	Rbpj	-0.13336746	2.26E-50	hypomethylated	-0.0023579	0.79333	insignificant	48	153	146
chr5	54199865	54201865	Tbc1d19	-0.10558514	0.0018326	hypomethylated	0.012509	0.44236	insignificant	11	43	42
chr5	54388761	54390761	Stim2	-0.11797724	1.16E-17	hypomethylated	0.020122	0.14058	insignificant	42	130	114
chr5	58108259	58110259	Pcdh7	-0.12668219	6.16E-13	hypomethylated	0.010156	0.065987	insignificant	28	111	111
chr5	58109158	58111158	4932441J04Ri	-0.12369393	1.96E-11	hypomethylated	0.0015713	0.058085	insignificant	44	177	175
chr5	62199081	62201081	G6pd2	0.1094861	1	lowCoverage	0.026153	1	insignificant	1	4	4
chr5	63157416	63159416	Arap2	-0.06802285	0.000003854	hypomethylated	-0.00081183	0.37961	insignificant	15	53	42
chr5	64039341	64041341	3110047P20Ri	-0.15256711	0.000093496	hypomethylated	0.10054	0.21376	insignificant	6	41	36
chr5	64202733	64204733	0610040J01Ri	-0.12820844	3.3E-10	hypomethylated	-0.04397	0.7033	insignificant	24	93	87
chr5	64360136	64362136	Rell1	-0.11666667	0.076234	insignificant	0.0043478	0.87299	insignificant	5	10	10
chr5	64483188	64485188	Pgm1	-0.14406964	4.78E-32	hypomethylated	0.0013579	0.43498	insignificant	35	101	92
chr5	64550449	64552449	Tbc1d1	-0.14466328	7.66E-19	hypomethylated	-0.0017202	0.75467	insignificant	21	122	121
chr5	65193761	65195761	Klf3	-0.1100434	3.31E-38	hypomethylated	0.0013461	0.74509	insignificant	63	185	185

chr5	65323938	65325938	Tlr1		1	noCoverage	-0.10644	0.86585	insignificant	0	7	7
chr5	65351273	65353273	Tlr6		1	noCoverage	-0.11234	0.72919	insignificant	0	11	10
chr5	65360313	65362313	Fam114a1	-0.14959892	1.24E-12	hypomethylated	-0.035845	0.65798	insignificant	8	80	60
chr5	65360556	65362556	Mir574	-0.14959892	1.24E-12	hypomethylated	-0.035845	0.65798	insignificant	8	80	60
chr5	65589934	65591934	Wdr19	-0.23406555	3.26E-11	hypomethylated	-0.012469	0.67965	insignificant	6	30	30
chr5	65726878	65728878	Rfc1	-0.12396102	0.0003198	hypomethylated	0.0031776	0.88918	insignificant	9	31	31
chr5	65738649	65740649	Klb	0.04550448	0.57422	insignificant	0.051768	0.0019481	hypermethylated	11	41	39
chr5	65781735	65783735	Lias	-0.13619022	3.84E-12	hypomethylated	0.000047278	0.48593	insignificant	21	104	95
chr5	65782670	65784670	Rpl9	-0.12074366	0.00000011	hypomethylated	-0.010454	0.82035	insignificant	16	49	49
chr5	65827081	65829081	Ugdh	-0.18984297	0.0000016	hypomethylated	0.011517	0.49712	insignificant	10	36	36
chr5	65884074	65886074	1110003E01Ri	-0.10812951	1.54E-34	hypomethylated	0.0015678	0.3989	insignificant	26	89	82
chr5	65927499	65929499	Ube2k	-0.1432137	9.46E-30	hypomethylated	0.0093873	0.90407	insignificant	43	132	121
chr5	66089095	66091095	Pds5a	-0.11193305	7.27E-32	hypomethylated	0.028106	0.8028	insignificant	61	229	239
chr5	66153759	66155759	N4bp2	-0.07739184	0.000010146	hypomethylated	0.0030099	0.64656	insignificant	43	194	181
chr5	66253807	66255807	Rhoh		1	noCoverage	-0.021817	0.69408	insignificant	0	12	12
chr5	66357362	66359362	Chrna9		1	noCoverage	-0.031842	0.58933	insignificant	0	11	8
chr5	66542207	66544207	Rbm47	-0.16815273	0.38866	insignificant	0.0069933	0.40877	insignificant	6	36	37
chr5	66543163	66545163	Rbm47	-0.67273206	0.32447	lowCoverage	0.043224	0.00053835	hypermethylated	1	17	18
chr5	66650363	66652363	Nsun7	-0.15435991	3.31E-29	hypomethylated	0.014996	0.5684	insignificant	30	86	87
chr5	67009910	67011910	Apbb2	-0.1112866	9.64E-11	hypomethylated	0.0017165	0.76321	insignificant	17	94	92
chr5	67066359	67068359	Uchl1	-0.16682005	1.4E-14	hypomethylated	0.037434	0.92721	insignificant	10	46	40
chr5	67136078	67138078	Limch1	-0.20705275	2.6E-25	hypomethylated	-0.012051	0.2624	insignificant	26	105	98
chr5	67490365	67492365	Phox2b	-0.17597462	0.085393	insignificant	0.0059932	0.63932	insignificant	5	14	14
chr5	67650890	67652890	Tmem33	-0.13979345	5.46E-22	hypomethylated	0.0059818	0.44006	insignificant	32	105	104
chr5	67697195	67699195	Slc30a9	-0.11965979	2.89E-44	hypomethylated	0.0014272	0.33104	insignificant	37	129	118
chr5	67819038	67821038	Bend4	-0.00098779	0.79154	insignificant	0.036928	0.76798	insignificant	23	78	76
chr5	67854136	67856136	C330024D21R	0.05738993	0.75444	insignificant	0.08762	0.025581	hypermethylated	5	25	24
chr5	67998121	68000121	Shisa3	-0.14088015	5.06E-29	hypomethylated	-0.00038858	0.35841	insignificant	37	169	163
chr5	68238670	68240670	Atp8a1	-0.23249519	0.00040036	hypomethylated	-0.017761	0.000017088	hypomethylated	9	46	47
chr5	69947180	69949180	Guf1	-0.0716976	1.72E-14	hypomethylated	0.00083342	0.69054	insignificant	33	125	128
chr5	69983524	69985524	Gnpda2		1	noCoverage	-0.18598	0.83126	insignificant	0	8	17
chr5	71233856	71235856	Gabrg1		1	noCoverage	-0.0058824	0.60992	insignificant	0	10	8
chr5	71487088	71489088	Gabra2		1	noCoverage	0.043697	0.17845	insignificant	0	26	26
chr5	72049547	72051547	Gabra4		1	noCoverage	0.042742	0.70032	insignificant	0	10	10
chr5	72090254	72092254	Gabrb1	-0.18318825	0.0075403	hypomethylated	0.034751	0.60144	insignificant	3	18	19
chr5	72559422	72561422	Commd8	-0.11577982	0.22603	insignificant	0.0027139	0.62455	insignificant	4	18	17
chr5	72593567	72595567	Atp10d		1	noCoverage	0.044711	0.72289	insignificant	0	43	33
chr5	72593581	72595581	Atp10d		1	noCoverage	0.044711	0.72289	insignificant	0	43	33
chr5	72895447	72897447	Corin	-0.32899306	0.00062841	hypomethylated	-0.054754	0.097995	insignificant	7	16	17
chr5	72950884	72952884	Nfxl1	0.03546175	0.56033	insignificant	0.0052396	0.32792	insignificant	5	36	36
chr5	72978789	72980789	Gm5868	-0.12	1	insignificant	-0.074791	0.90391	insignificant	2	4	5
chr5	73033991	73035991	Cnga1	-0.6969697	0.0017532	stronglyHypometh	0.1897	0.23952	insignificant	1	6	6
chr5	73038034	73040034	Nipal1	-0.24545766	0.000076469	hypomethylated	-0.03653	0.38552	insignificant	7	35	34
chr5	73127744	73129744	Txk		1	noCoverage	-0.096726	0.66826	insignificant	0	4	4
chr5	73259687	73261687	Tec	-0.12393623	8.14E-12	hypomethylated	0.015013	0.73659	insignificant	23	65	65
chr5	73304555	73306555	Slain2	-0.09562762	9.55E-46	hypomethylated	-0.0015492	0.30973	insignificant	55	202	203
chr5	73397142	73399142	Slc10a4	-0.14938131	0.0018631	hypomethylated	-0.026984	0.0059724	inconclusive	13	75	76
chr5	73647857	73649857	Fryl	-0.17115834	3.8E-09	hypomethylated	-0.011802	0.59189	insignificant	8	37	39
chr5	73683032	73685032	Ociad1	-0.11142993	0.000017881	hypomethylated	0.033425	0.57153	insignificant	17	73	67
chr5	73796316	73798316	Cwh43	-0.24857642	0.000055972	hypomethylated	-0.0084311	0.26975	insignificant	8	40	37
chr5	73871293	73873293	Dcun1d4	-0.0375817	1	insignificant	-0.082743	0.52895	insignificant	2	4	4
chr5	73881264	73883264	Dcun1d4	-0.12640421	2.82E-16	hypomethylated	-0.0067354	0.65965	insignificant	29	150	133
chr5	74038970	74040970	Sgcb	-0.25164689	0.23023	insignificant	0.003115	1	insignificant	2	8	8
chr5	74041618	74043618	Spata18	-0.19980673	1.15E-12	hypomethylated	-0.043262	0.28769	insignificant	10	72	73
chr5	74464436	74466436	Usp46	-0.10025517	1.16E-22	hypomethylated	-0.0042077	0.74309	insignificant	26	79	79
chr5	74488107	74490107	2700023E23Ri	-0.1330242	2.27E-38	hypomethylated	0.01064	0.03035	hypermethylated	31	92	92
chr5	74488554	74490554	Snora26	-0.1330242	2.27E-38	hypomethylated	0.01064	0.03035	hypermethylated	31	92	92
chr5	74590350	74592350	Rasl11b	-0.1242851	2.14E-12	hypomethylated	-0.017355	0.36797	insignificant	19	108	89
chr5	74927774	74929774	Scfd2	0.45079984	5.3E-10	stronglyHypermeth	0.05928	0.24531	insignificant	2	14	14
chr5	74930506	74932506	Fip1l1	-0.11109019	2.33E-26	hypomethylated	-0.00048072	0.24562	insignificant	20	64	64
chr5	75098928	75100928	Lnx1		1	noCoverage	0.0081829	0.88549	insignificant	0	9	8
chr5	75440651	75442651	Chic2	-0.22699001	8.38E-11	hypomethylated	0.015957	0.20456	insignificant	13	49	45
chr5	75470625	75472625	Gsx2	-0.07061087	0.00000212	hypomethylated	-0.0042104	0.057639	insignificant	24	120	113
chr5	75547315	75549315	Pdgfra		1	noCoverage	-0.064183	0.67275	insignificant	0	31	36
chr5	75551190	75553190	Pdgfra	-0.20150556	0.000000112	hypomethylated	-0.04551	0.8436	insignificant	9	54	53
chr5	75970011	75972011	Kit	-0.18885817	0.00000288	hypomethylated	-0.01161	0.90177	insignificant	8	54	54
chr5	75970015	75972015	Kit	-0.18885817	0.00000288	hypomethylated	-0.01161	0.90177	insignificant	8	54	54
chr5	76374453	76376453	Kdr		1	noCoverage	-0.014769	1	insignificant	0	10	10
chr5	76568301	76570301	Srd5a3	-0.18145109	3.03E-10	hypomethylated	0.0040532	0.65572	insignificant	15	58	63
chr5	76611904	76613904	Tmem165	-0.15428372	2.68E-45	hypomethylated	-0.0030583	0.4167	insignificant	38	125	116
chr5	76733573	76735573	Clock	-0.10619164	3.57E-47	hypomethylated	0.00041021	0.8048	insignificant	52	98	98
chr5	76792802	76794802	Nmu	-0.06321784	0.85064	insignificant	-0.036499	1	insignificant	8	20	20
chr5	76912101	76914101	Gm7271	-0.34778444	0.000044017	stronglyHypometh	0.099537	0.01994	hypermethylated	4	23	22
chr5	76957347	76959347	Exoc1	-0.20579191	4.11E-08	hypomethylated	-0.017987	0.36914	insignificant	10	29	20
chr5	77379435	77381435	Paics	-0.12885661	7.25E-13	hypomethylated	0.0038918	0.96635	insignificant	14	69	66
chr5	77380603	77382603	Paics	-0.15191282	0.00011158	hypomethylated	0.00019085	0.75008	insignificant	7	35	35
chr5	77402725	77404725	Srp72	-0.16125269	3.89E-14	hypomethylated	-0.017879	0.56939	insignificant	21	92	93
chr5	77432079	77434079	Arl9	0.07020375	0.085923	insignificant	0.052759	0.00073464	hypermethylated	3	14	14
chr5	77444047	77446047	1700023E05Ri	-0.14295351	2.76E-09	hypomethylated	0.036642	0.54323	insignificant	21	58	58
chr5	77524292	77526292	Hopx	-0.09251794	0.11596	insignificant	-0.0061104	0.59788	insignificant	14	30	30
chr5	77544148	77546148	Hopx	0.15767196	1	lowCoverage	-0.3196	0.00052547	hypomethylated	1	9	8
chr5	77640478	77642478	Spink2		1	noCoverage	0.10781	0.0062797	hypermethylated	0	10	12
chr5	77693518	77695518	Rest	-0.09218492	4.19E-19	hypomethylated	-0.0022758	0.39376	insignificant	77	259	224
chr5	77738508	77740508	Polr2b	-0.13703429	1.31E-20	hypomethylated	-0.005371	0.60857	insignificant	32	115	110
chr5	77739111	77741111	Noa1	-0.14533784	0.092329	insignificant	-0.00049769	0.49199	insignificant	7	32	32

chr5	77837070	77839070	Igfbp7	-0.38948624	1.5E-13	stronglyHypometh	0.005107	0.017381	inconclusive	13	46	46
chr5	77940773	77942773	Pea15b		1	noCoverage	-0.28125	0.05925	insignificant	0	4	4
chr5	81449617	81451617	Lphn3	-0.1929352	4.27E-09	hypomethylated	-0.01768	0.50205	insignificant	11	75	76
chr5	84846407	84848407	Epha5		1	noCoverage	0.17816	0.69427	insignificant	0	10	9
chr5	86494608	86496608	Cenpc1	-0.21082695	6.33E-08	hypomethylated	-0.0011959	0.83311	insignificant	7	57	62
chr5	86601768	86603768	Uba6		1	noCoverage	-0.041592	0.78496	insignificant	0	26	25
chr5	87061449	87063449	Tmprss11f	0.05	1	lowCoverage	0.05	1	insignificant	1	4	2
chr5	87232514	87234514	Ythdc1	-0.1242541	4.6E-30	hypomethylated	0.014612	0.72512	insignificant	42	118	127
chr5	87766220	87768220	Ugt2a3	0.25801939	1	insignificant	0.02811	0.049019	hypermethylated	1	14	15
chr5	87911283	87913283	Ugt2a2		1	noCoverage	0.17652	0.13945	insignificant	0	4	4
chr5	87967220	87969220	Sult1b1		1	noCoverage	0.26068	0.59356	insignificant	0	5	4
chr5	88353657	88355657	Csn3	-0.0803651	0.13684	insignificant	0.044564	0.18693	insignificant	3	8	8
chr5	88407475	88409475	4931407G18R	0.24252104	1	insignificant	0.016824	0.26049	insignificant	2	15	14
chr5	88804132	88806132	Amtn		1	noCoverage	-0.040043	0.804	insignificant	0	6	6
chr5	88884035	88886035	Ambn	0.17773893	1	insignificant	-0.069201	0.50326	insignificant	2	4	4
chr5	88982507	88984507	Utp3	-0.11552847	6.15E-14	hypomethylated	-0.0037415	0.69825	insignificant	26	122	122
chr5	89011598	89013598	Rufy3	0.10887531	0.61852	insignificant	-0.2112	0.030156	hypomethylated	3	11	15
chr5	89104605	89106605	Grsf1	-0.06971612	7.49E-12	hypomethylated	-0.0040993	0.41107	insignificant	37	189	180
chr5	89105196	89107196	Grsf1	-0.12639395	0.000012881	hypomethylated	-0.0026104	0.94257	insignificant	9	41	36
chr5	89148895	89150895	Mob1b	-0.21070987	6.41E-78	hypomethylated	0.022673	1	insignificant	30	99	84
chr5	89193037	89195037	Dck	-0.12341976	1.1E-10	hypomethylated	0.0060426	0.67402	insignificant	29	85	86
chr5	89315284	89317284	Slc4a4	-0.09883417	0.000000708	hypomethylated	0.00048142	0.53806	insignificant	34	119	109
chr5	89955453	89957453	Npffr2		1	noCoverage	0.054383	0.095447	insignificant	0	6	8
chr5	90044460	90046460	Gm7056		1	noCoverage	0.18152	0.58018	insignificant	0	5	4
chr5	90312359	90314359	Adamts3	-0.31836344	0.015166	hypomethylated	0.005217	1	insignificant	6	58	48
chr5	90653021	90655021	Cox18		1	noCoverage	0.05497	0.30079	insignificant	0	18	18
chr5	90795211	90797211	Ankrd17	-0.0833953	0.00016336	hypomethylated	0.011777	0.46451	insignificant	7	78	74
chr5	90946974	90948974	Afm		1	noCoverage	-0.12537	1	insignificant	0	4	4
chr5	91187324	91189324	Cxcl5	-0.30321068	1	insignificant	-0.060389	0.051787	insignificant	3	22	22
chr5	91200460	91202460	Pf4		1	noCoverage	0.057698	0.34654	insignificant	0	15	15
chr5	91214128	91216128	Cxcl3	-0.45462653	0.10775	insignificant	-0.017762	0.39312	insignificant	7	36	36
chr5	91222559	91224559	Cxcl15		1	noCoverage	0.0037296	0.38186	insignificant	0	4	4
chr5	91331924	91333924	Cxcl2	-0.21153846	0.009256	hypomethylated	-0.013478	0.51734	insignificant	2	10	10
chr5	91359221	91361221	Mthfd2l	-0.10068432	2.12E-35	hypomethylated	0.0080591	0.57088	insignificant	35	95	74
chr5	91502642	91504642	Ereg	0.07804683	0.8481	insignificant	0.010304	0.66274	insignificant	10	45	49
chr5	91567640	91569640	Areg	-0.19300078	5.51E-19	hypomethylated	0.076097	0.5374	insignificant	9	59	46
chr5	91831939	91833939	Btc	-0.57894737	0.0015918	stronglyHypometh	0.032164	1	insignificant	1	2	2
chr5	91945725	91947725	Parm1	-0.16372365	0.0053247	hypomethylated	-0.00068606	0.65388	insignificant	8	88	88
chr5	92390907	92392907	Thap6	-0.14413297	4.75E-17	hypomethylated	-0.010641	0.37912	insignificant	25	80	80
chr5	92392094	92394094	Thap6	-0.1531927	3.91E-08	hypomethylated	-0.015774	0.18423	insignificant	15	42	42
chr5	92415498	92417498	Gm1045	-0.28333333	0.32262	insignificant	0.27607	0.00012307	hypermethylated	1	6	6
chr5	92472032	92474032	Cdkl2	-0.0455785	0.000000702	hypomethylated	0.019765	0.28899	insignificant	9	32	31
chr5	92472044	92474044	Cdkl2	-0.07896076	4.42E-09	hypomethylated	0.023313	0.32378	insignificant	9	26	25
chr5	92511943	92513943	G3bp2	-0.0641614	3.07E-10	hypomethylated	0.003258	0.33769	insignificant	27	94	94
chr5	92512583	92514583	G3bp2		1	noCoverage	0.0032526	0.38896	insignificant	0	14	14
chr5	92512761	92514761	G3bp2		1	noCoverage	0.01955	0.16822	insignificant	0	4	4
chr5	92565963	92567963	Uso1	-0.13976201	6.22E-25	hypomethylated	-0.014879	0.96679	insignificant	48	113	106
chr5	92707207	92709207	Naaa	-0.09923003	0.099157	insignificant	0.048808	0.4802	insignificant	3	28	30
chr5	92739050	92741050	Sdad1	-0.1682825	0.072423	insignificant	-0.080374	0.00011521	hypomethylated	5	16	16
chr5	92777880	92779880	Art3	-0.16573099	0.000097311	hypomethylated	0.049763	0.31187	insignificant	3	6	6
chr5	92792303	92794303	Cxcl11	-0.05062884	0.83421	insignificant	0.10457	0.17922	insignificant	3	16	9
chr5	92864225	92866225	Nup54	-0.29849348	1	lowCoverage	-0.0057693	0.77458	insignificant	1	28	29
chr5	92934634	92936634	Scarb2	-0.13551921	0.008935	hypomethylated	-0.0081403	0.36509	insignificant	11	28	28
chr5	92983113	92985113		-0.0357806	0.6381	insignificant	0.0011755	0.71973	insignificant	4	10	10
chr5	92999562	93001562	Fam47e	0.03508191	0.14155	insignificant	0.022495	0.23744	insignificant	3	12	12
chr5	93031076	93033076	Stbd1	-0.15217166	0.00000248	hypomethylated	-0.0045646	0.90777	insignificant	12	41	34
chr5	93104153	93106153	Ccdc158	-0.13889395	0.000011736	hypomethylated	-0.028041	0.95814	insignificant	14	64	68
chr5	93111460	93113460	Shroom3	-0.21067608	0.60079	insignificant	0.058179	0.0065711	hypermethylated	5	26	26
chr5	93217588	93219588	Mir1961		1	noCoverage	0.072294	0.71463	insignificant	0	6	6
chr5	93237413	93239413	Shroom3	-0.3061709	0.00010697	hypomethylated	0.003652	0.031863	hypermethylated	8	44	47
chr5	93326023	93328023	Shroom3	-0.08163689	0.000000602	hypomethylated	0.017787	0.45979	insignificant	27	87	91
chr5	93474048	93476048	Ankrd56	-0.11211335	0.000000319	hypomethylated	0.010004	0.33686	insignificant	20	80	83
chr5	93521482	93523482	###########	-0.0961788	0.0069555	hypomethylated	0.011697	0.31719	insignificant	27	149	145
chr5	93635521	93637521	Ccni	-0.07206937	0.00013478	hypomethylated	0.01488	0.5445	insignificant	6	41	41
chr5	93695598	93697598	Ccng2	-0.13033682	8.1E-32	hypomethylated	0.011	0.55505	insignificant	38	138	141
chr5	96590774	96592774	Cnot6l	-0.12241177	1.67E-29	hypomethylated	-0.0084141	0.45752	insignificant	28	142	139
chr5	96591009	96593009	Cnot6l	-0.14527195	5.92E-22	hypomethylated	-0.010039	0.19941	insignificant	22	93	89
chr5	96638133	96640133	Mrpl1	-0.23871676	0.000000793	hypomethylated	-0.027918	0.060359	insignificant	6	20	20
chr5	96801973	96803973	Fras1	-0.1041636	0.000578	hypomethylated	-0.0072926	1	insignificant	16	65	60
chr5	97221403	97223403	Anxa3	-0.34848485	0.092382	insignificant	0.040159	0.44785	insignificant	3	13	13
chr5	97425707	97427707	Bmp2k	-0.09881363	2.37E-23	hypomethylated	-0.0023902	0.29381	insignificant	59	194	185
chr5	97540615	97542615	Paqr3	-0.18402778	0.000000341	hypomethylated	-0.086079	0.53635	insignificant	3	16	26
chr5	97821349	97823349	Naa11	0.05317706	0.15309	insignificant	0.059693	0.013414	hypermethylated	7	16	16
chr5	98459981	98461981	Antxr2	-0.10610705	1	insignificant	0.047602	0.63183	insignificant	3	32	34
chr5	98608887	98610887	Prdm8	-0.23228892	0.000000014	hypomethylated	-0.016118	0.37649	insignificant	9	24	24
chr5	98682202	98684202	Fgf5	-0.12662573	3.37E-09	hypomethylated	-0.001388	0.94948	insignificant	38	119	119
chr5	98757322	98759322	1700007G11R	-0.1284364	5.89E-11	hypomethylated	-0.0052855	0.69742	insignificant	20	69	68
chr5	99282457	99284457	Bmp3	-0.11901722	5.06E-17	hypomethylated	0.0021884	0.15723	insignificant	40	127	131
chr5	99466098	99468098	Prkg2	-0.11253168	0.000000353	hypomethylated	0.0083076	0.8672	insignificant	23	58	58
chr5	99681946	99683946	Rasgef1b	-0.17492284	0.00011282	hypomethylated	0.048731	0.04504	hypermethylated	11	30	20
chr5	100158079	100160079	A930011G23Rik		1	noCoverage	0.02811	0.32326	insignificant	0	27	26
chr5	100407957	100409957	Hnrnpd	-0.09517737	6.66E-18	hypomethylated	0.0017308	0.05377	insignificant	22	99	100
chr5	100468012	100470012	Enoph1	-0.08858163	1.43E-46	hypomethylated	-0.000059993	0.17333	insignificant	50	207	207
chr5	100468241	100470241	Hnrpdl	-0.09138383	0.00000345	hypomethylated	-0.0045116	0.54674	insignificant	31	152	151
chr5	100845253	100847253	Sec31a	-0.27065085	9.71E-25	hypomethylated	-0.0023304	0.10501	insignificant	22	68	66

chr5	100858554	100860554	5430416N02R	-0.28270851	8.31E-18	hypomethylated	-0.10227	0.87039	insignificant	13	57	48
chr5	100927592	100929592	Lin54	-0.13017593	1.57E-32	hypomethylated	-0.0036628	0.78749	insignificant	42	96	102
chr5	100946327	100948327	Cops4	-0.16241853	0.000034491	hypomethylated	0.0096101	0.9142	insignificant	10	51	51
chr5	101103275	101105275	Coq2	-0.18450155	0.00000352	hypomethylated	-0.042507	0.5238	insignificant	6	34	30
chr5	101148702	101150702	Hpse	-0.16936418	9.39E-08	hypomethylated	-0.034824	0.52827	insignificant	4	23	27
chr5	101226777	101228777	Mrps18c	-0.13917458	2.03E-25	hypomethylated	0.0021102	0.47033	insignificant	29	98	98
chr5	101227619	101229619	Helq	-0.1457338	1	insignificant	0.005224	0.34761	insignificant	2	26	26
chr5	101249954	101251954	Fam175a	-0.27674428	1	insignificant	0.10791	0.052293	insignificant	1	19	22
chr5	101274247	101276247	Agpat9	-0.12252728	8.23E-10	hypomethylated	0.0054762	0.59548	insignificant	17	63	62
chr5	102093730	102095730	Nkx6-1	-0.19532816	4.14E-09	hypomethylated	-0.021916	0.30719	insignificant	9	38	40
chr5	102193148	102195148	Cds1	-0.06042927	2.22E-14	hypomethylated	0.0052103	0.92687	insignificant	59	185	188
chr5	102498940	102500940	Wdfy3	-0.10086366	2.3E-10	hypomethylated	0.0096051	0.83245	insignificant	14	63	59
chr5	102909409	102911409	Arhgap24	-0.1896529	0.23075	insignificant	0.013133	0.96945	insignificant	5	36	36
chr5	103853210	103855210	Ptpn13	-0.09586872	8.64E-19	hypomethylated	0.0045122	0.11009	insignificant	46	216	211
chr5	104058422	104060422	Slc10a6		1	noCoverage	0.029078	0.71037	insignificant	0	11	11
chr5	104084743	104086743	1700016H13R	-0.17016384	1.47E-12	hypomethylated	0.020535	0.91916	insignificant	7	30	30
chr5	104084751	104086751	1700016H13R	-0.17016384	1.47E-12	hypomethylated	0.020535	0.91916	insignificant	7	30	30
chr5	104182180	104184180	Aff1	-0.1488374	1.27E-23	hypomethylated	-0.030925	0.00092279	hypomethylated	36	145	131
chr5	104182591	104184591	Aff1	-0.16033247	4.95E-23	hypomethylated	-0.047417	0.0016007	hypomethylated	28	123	112
chr5	104450815	104452815	Hsd17b11	-0.26116265	0.012352	hypomethylated	0.0025086	0.951	insignificant	2	14	16
chr5	104475029	104477029	Nudt9	-0.16102052	2.31E-29	hypomethylated	-0.013507	0.65548	insignificant	23	79	79
chr5	104543107	104545107	Sparcl1	0.01756231	0.26824	insignificant	0.079089	0.85135	insignificant	5	20	16
chr5	104630635	104632635	Dmp1	-0.39648623	1	lowCoverage	0.030905	0.32005	insignificant	1	6	6
chr5	104863136	104865136	Spp1		1	noCoverage	-0.025397	1	insignificant	0	4	4
chr5	104887475	104889475	Pkd2	-0.0836773	2.44E-21	hypomethylated	0.014633	0.1435	insignificant	52	152	162
chr5	104936370	104938370	BC005561	-0.09994074	2.42E-14	hypomethylated	-0.0098835	0.39029	insignificant	22	105	104
chr5	105289087	105291087	Zfp951		1	noCoverage	-0.22179	0.061125	insignificant	0	10	9
chr5	105411736	105413736	Abcg3	0.10761715	0.60909	insignificant	0.011137	0.69563	insignificant	4	38	35
chr5	105843793	105845793	Lrrc8b	-0.11529459	1.5E-18	hypomethylated	0.004019	0.27256	insignificant	22	121	107
chr5	105947489	105949489	Lrrc8c	-0.08673485	5.52E-32	hypomethylated	0.0044381	0.26599	insignificant	43	148	148
chr5	106127987	106129987	Lrrc8d	-0.11067517	6.15E-44	hypomethylated	-0.00041552	0.19319	insignificant	93	227	229
chr5	106128828	106130828	Lrrc8d	-0.12299923	3.15E-45	hypomethylated	-0.00034338	0.68274	insignificant	90	237	238
chr5	106304586	106306586	Zfp326	-0.10382504	6.15E-08	hypomethylated	0.0057422	0.14809	insignificant	25	121	120
chr5	106887185	106889185	Barhl2	-0.11328528	2.84E-40	hypomethylated	-0.033054	0.44536	insignificant	36	179	163
chr5	107125849	107127849	Zfp644	-0.24801587	0.040711	hypomethylated	0.045499	0.13732	insignificant	2	4	4
chr5	107354909	107356909	Hfm1	-0.06698898	0.0063558	hypomethylated	0.011508	0.56376	insignificant	15	66	72
chr5	107392340	107394340	Cdc7	-0.14203301	1.34E-14	hypomethylated	0.0037599	1	insignificant	19	111	101
chr5	107718614	107720614	Tgfbr3		1	noCoverage	0.10235	0.077064	insignificant	0	12	12
chr5	107759212	107761212	Brdt	-0.15027333	0.079877	insignificant	0.025612	0.33367	insignificant	4	21	20
chr5	107831531	107833531	Ephx4	-0.07973846	7.88E-08	hypomethylated	0.015541	0.031508	inconclusive	24	108	111
chr5	107859567	107861567	Lpcat2b	0.04758368	0.2304	insignificant	0.16111	0.00036975	hypermethylated	3	6	6
chr5	107962729	107964729	1700028K03Ri	-0.00678249	0.44238	insignificant	-0.020166	0.77784	insignificant	9	32	30
chr5	108025398	108027398	Rpap2	-0.17630681	2.48E-10	hypomethylated	-0.045024	0.12214	insignificant	11	51	49
chr5	108025735	108027735	Rpap2	-0.19009398	2.52E-10	hypomethylated	-0.044352	0.14433	insignificant	11	53	51
chr5	108026658	108028658	Rpap2	-0.22361076	5.04E-11	hypomethylated	-0.045633	0.15622	insignificant	11	54	53
chr5	108026907	108028907	Rpap2	-0.29669299	4.22E-08	hypomethylated	-0.064951	0.77427	insignificant	6	24	23
chr5	108153363	108155363	Gfi1	-0.13376857	4.7E-09	hypomethylated	-0.0050039	0.065305	insignificant	9	98	98
chr5	108258271	108260271	1700013N18R	0.06962332	0.259	insignificant	-0.011733	0.62662	insignificant	3	24	23
chr5	108328608	108330608	Rpl5	-0.18068347	2.45E-41	hypomethylated	0.018936	0.30348	insignificant	33	131	124
chr5	108416096	108418096	Fam69a	-0.12508045	0.11757	insignificant	0.042164	0.055438	insignificant	6	73	58
chr5	108493760	108495760	Mtf2	-0.10820499	0.000000037	hypomethylated	0.01285	0.31184	insignificant	14	69	69
chr5	108560932	108562932	Ccdc18	-0.13417629	5.41E-12	hypomethylated	0.013075	0.053764	insignificant	18	94	90
chr5	108561610	108563610	Tmed5	-0.10135919	0.0051484	hypomethylated	0.029363	0.29271	insignificant	10	52	50
chr5	108696915	108698915	Dr1	-0.11449553	7.08E-23	hypomethylated	0.0078804	0.67631	insignificant	34	97	97
chr5	108740943	108742943	Pigg	-0.15825251	4.46E-14	hypomethylated	0.013021	0.52782	insignificant	11	63	54
chr5	108816391	108818391	Pde6b	-0.06144528	0.24476	insignificant	-0.071413	0.010728	hypomethylated	3	6	6
chr5	108863397	108865397	Atp5k	0.54556729	0.34132	lowCoverage	-0.072958	0.91551	insignificant	1	18	18
chr5	108889350	108891350	Pcgf3	-0.11963275	1.92E-11	hypomethylated	0.024789	0.94262	insignificant	28	81	75
chr5	108979046	108981046	Cplx1	0.0420729	0.071631	insignificant	0.019735	0.78538	insignificant	8	48	48
chr5	109057828	109059828	Tmem175		1	noCoverage	0.0081651	0.60941	insignificant	0	15	16
chr5	109089788	109091788	Dgkq	-0.28947368	0.17306	insignificant	0.09386	0.75977	insignificant	2	4	6
chr5	109122247	109124247	Fgfrl1	-0.07769706	8.95E-09	hypomethylated	0.014681	0.32411	insignificant	46	149	149
chr5	109237773	109239773	Vmn2r8	0.04807692	1	insignificant	0.19451	0.4022	insignificant	1	4	4
chr5	109653641	109655641	Vmn2r14	0.24166667	0.41456	insignificant	0.13125	0.27206	insignificant	2	4	4
chr5	109758399	109760399	Vmn2r16	-0.27234927	0.06662	insignificant	-0.05421	0.58612	insignificant	1	2	4
chr5	109988012	109990012	Crlf2	-0.21309716	0.000080062	hypomethylated	-0.025735	0.078276	insignificant	7	30	30
chr5	110263161	110265161	Gm10416	0.10102708	1	lowCoverage	-0.031748	1	insignificant	1	11	11
chr5	110424545	110426545	Zfp932		1	noCoverage	0.054712	0.19888	insignificant	0	35	35
chr5	110527987	110529987	Plcxd1		1	noCoverage	0.12075	0.37052	insignificant	0	10	10
chr5	110528577	110530577	Plcxd1	-0.03310942	0.00063121	hypomethylated	0.027596	0.94243	insignificant	12	43	49
chr5	110537216	110539216	Gtpbp6	-0.21831301	0.02824	hypomethylated	-0.026835	0.84927	insignificant	1	16	16
chr5	110538110	110540110	Zfp605	-0.30978237	0.0014256	hypomethylated	-0.063778	1	insignificant	1	14	14
chr5	110563858	110565858	Chfr	-0.2045391	0.000026279	hypomethylated	0.0083195	0.94582	insignificant	6	46	46
chr5	110604719	110606719	Golga3	-0.15832925	1.8E-26	hypomethylated	0.010196	0.68466	insignificant	22	80	75
chr5	110659059	110661059	Ankle2	-0.08499081	7.16E-17	hypomethylated	0.013726	0.87477	insignificant	27	85	87
chr5	110698918	110700918	Pgam5	-0.20410398	0.050889	insignificant	0.23446	0.093165	insignificant	4	22	20
chr5	110714337	110716337	Pole	-0.09836146	4.08E-23	hypomethylated	0.0057559	0.93532	insignificant	33	123	88
chr5	110715187	110717187	Pxmp2	-0.10200881	4.86E-10	hypomethylated	0.026988	0.77132	insignificant	15	75	42
chr5	110782114	110784114	Gm1679	-0.24214173	0.81013	insignificant	0.07693	0.13019	insignificant	5	29	28
chr5	110816146	110818146	Fbrsl1	-0.19690856	0.0073994	hypomethylated	-0.018165	0.28143	insignificant	7	22	18
chr5	110877522	110879522	Fbrsl1	-0.19075001	8.76E-17	hypomethylated	0.01643	0.4555	insignificant	11	40	40
chr5	110972363	110974363	Galnt9	-0.09915841	2.16E-13	hypomethylated	0.0042226	0.30256	insignificant	16	129	122
chr5	111081469	111083469	Ddx51	-0.05364857	0.000042315	hypomethylated	0.0043595	0.0015584	hypermethylated	35	130	130
chr5	111082401	111084401	Noc4l	-0.1711176	1.74E-09	hypomethylated	-0.021769	0.64221	insignificant	23	82	80
chr5	111199736	111201736	Ep400	-0.09932359	0.15093	insignificant	0.097076	0.51011	insignificant	4	24	19

chr5	111239100	111241100	Ulk1	-0.1339496	0.002082	hypomethylated	-0.0010651	0.039748	hypomethylated	9	35	35
chr5	111268035	111270035	Chek2	-0.13490952	0.0028969	hypomethylated	0.046494	0.45063	insignificant	5	23	26
chr5	111268796	111270796	Hscb	-0.20961278	0.66567	insignificant	0.00081651	0.0010556	hypermethylated	7	43	46
chr5	111307821	111309821	Ttc28	-0.1567407	0.00000154	hypomethylated	-0.014339	0.00010929	hypomethylated	21	125	125
chr5	111758782	111760782	Pitpnb	-0.14922765	5.77E-37	hypomethylated	-0.000887	0.29954	insignificant	27	78	78
chr5	111846185	111848185	Mn1	-0.12655366	1.15E-44	hypomethylated	0.02137	0.00000251	hypermethylated	92	301	278
chr5	112009580	112011580	C130026L21Ri	-0.17416322	0.0028139	hypomethylated	-0.039819	0.32184	insignificant	13	42	42
chr5	112657968	112659968	Miat	-0.1071155	0.00010983	hypomethylated	0.091704	0.2826	insignificant	6	12	14
chr5	112683840	112685840	Crybb1	-0.48653575	0.28923	insignificant	0.069153	0.21149	insignificant	3	14	14
chr5	112704726	112706726	Tpst2	-0.15513012	7.23E-12	hypomethylated	-0.013133	0.013358	inconclusive	13	74	73
chr5	112754388	112756388	Tfip11	-0.13667125	1.61E-29	hypomethylated	0.015493	0.72159	insignificant	29	97	100
chr5	112771114	112773114	Hps4	-0.10286344	4.25E-25	hypomethylated	0.039397	0.97338	insignificant	36	94	91
chr5	112772060	112774060	Srrd	-0.10325249	1.62E-10	hypomethylated	0.052012	0.83714	insignificant	12	34	29
chr5	112785053	112787053	Hps4	0.13465761	1	insignificant	0.032494	0.03714	hypermethylated	2	16	12
chr5	112821233	112823233	Asphd2	-0.23473452	0.10618	insignificant	0.090364	0.010827	hypermethylated	4	14	16
chr5	112877445	112879445	Gm6588	-0.01041449	1	insignificant	0.0099012	0.63903	insignificant	8	48	45
chr5	113006205	113008205	Sez6l	0.09472657	0.00020617	hypermethylated	0.12865	0.3781	insignificant	9	51	52
chr5	113444534	113446534	Adrbk2	-0.1369946	1	insignificant	-0.012393	0.50934	insignificant	6	23	22
chr5	113510604	113512604	Crybb3		1	noCoverage	-0.075036	0.1175	insignificant	0	6	7
chr5	113592333	113594333	2900026A02R	0.0005576	0.0014822	hypermethylated	0.081929	0.5926	insignificant	9	43	46
chr5	113654928	113656928	Tmem211	-0.41764706	0.000015394	stronglyHypometh	0.050289	0.74164	insignificant	4	15	16
chr5	113739806	113741806	Sgsm1		1	noCoverage	0.11773	0.091565	insignificant	0	8	10
chr5	113771105	113773105		0.18676388	1	insignificant	-0.010226	0.16419	insignificant	1	13	10
chr5	113785313	113787313	Aym1	-0.39597035	0.046355	stronglyHypometh	-0.0043674	0.10092	insignificant	3	14	14
chr5	113918466	113920466	Wscd2	-0.18846482	4.98E-19	hypomethylated	-0.0014609	0.49054	insignificant	32	90	90
chr5	114174781	114176781	1700069L16Ri	-0.03337307	0.32339	insignificant	0.017038	0.55545	insignificant	4	18	18
chr5	114184790	114186790	Ficd	-0.1324324	2.81E-15	hypomethylated	-0.0096274	0.59858	insignificant	26	128	125
chr5	114221658	114223658	Sart3	-0.11030965	1.68E-16	hypomethylated	-0.0016141	0.10526	insignificant	26	168	168
chr5	114221820	114223820	Iscu	-0.11984874	8.09E-15	hypomethylated	-0.0020223	0.08527	insignificant	21	146	146
chr5	114250361	114252361	Tmem119	-0.21190476	1	insignificant	-0.045238	0.083571	insignificant	2	6	6
chr5	114280510	114282510	Selplg	-0.18460383	0.0077433	hypomethylated	-0.015391	0.83948	insignificant	3	6	6
chr5	114358715	114360715	Coro1c	-0.15002294	1.05E-14	hypomethylated	-0.044984	0.58301	insignificant	23	65	70
chr5	114443766	114445766	Ssh1	-0.13108533	4.34E-13	hypomethylated	-0.015759	0.29995	insignificant	22	66	77
chr5	114452834	114454834	Dao	0.07490543	1	insignificant	-0.083107	0.60921	insignificant	3	13	13
chr5	114549341	114551341	Usp30	-0.10365619	1.26E-23	hypomethylated	-0.004338	0.33813	insignificant	34	134	126
chr5	114578185	114580185	Alkbh2	-0.36641226	1.52E-21	stronglyHypometh	-0.045162	0.79142	insignificant	8	30	30
chr5	114579443	114581443	Ung	-0.13440578	4.38E-12	hypomethylated	-0.006093	0.092996	insignificant	28	102	103
chr5	114580164	114582164	Ung	-0.08894522	1.99E-14	hypomethylated	-0.001097	0.25164	insignificant	38	134	135
chr5	114614526	114616526	Acacb	0.13122172	1	insignificant	0.053147	0.55978	insignificant	1	4	5
chr5	114723770	114725770	Foxn4	-0.18311227	8.87E-19	hypomethylated	-0.021061	0.74361	insignificant	21	108	103
chr5	114763949	114765949	Myo1h	-0.35423687	0.038408	stronglyHypometh	0.021531	0.72351	insignificant	2	12	12
chr5	114829615	114831615	Ube3b	-0.09815485	4.99E-12	hypomethylated	0.0049914	0.93414	insignificant	23	84	84
chr5	114830514	114832514	Kctd10	-0.16530334	5.08E-09	hypomethylated	0.0096383	0.71239	insignificant	13	28	28
chr5	114893314	114895314	Mvk	-0.15887368	1.12E-10	hypomethylated	0.0063918	0.92191	insignificant	7	36	36
chr5	114894036	114896036	Mmab	-0.31687021	9.93E-19	hypomethylated	-0.021046	0.4465	insignificant	7	46	46
chr5	115017259	115019259	BC057022	-0.07330269	1.42E-41	hypomethylated	-0.0033456	0.80846	insignificant	59	180	177
chr5	115108430	115110430	Trpv4	-0.12576424	0.000029968	hypomethylated	-0.029752	0.13634	insignificant	17	65	66
chr5	115140944	115142944	Gltp	-0.18024207	3.76E-21	hypomethylated	-0.017854	0.33652	insignificant	23	89	91
chr5	115156787	115158787	Tchp	-0.14915338	1.07E-11	hypomethylated	-0.014094	0.028268	inconclusive	14	54	55
chr5	115223500	115225500	Git2	-0.09267316	5.28E-20	hypomethylated	-0.0042596	0.63531	insignificant	44	172	174
chr5	115223501	115225501	Git2	-0.09267316	5.28E-20	hypomethylated	-0.0042596	0.63531	insignificant	44	172	174
chr5	115224148	115226148	Ankrd13a	-0.09103003	5.18E-19	hypomethylated	-0.0055154	0.69968	insignificant	40	148	151
chr5	115224992	115226992	4930515G01R	-0.07886315	3.55E-08	hypomethylated	-0.0031418	0.33243	insignificant	21	82	82
chr5	115263985	115265985	1500011B03R	0.15108225	1	insignificant	-0.034048	0.55354	insignificant	1	14	13
chr5	115300446	115302446			1	noCoverage	-0.00027211	0.44289	insignificant	0	15	7
chr5	115345942	115347942	Oasl2		1	noCoverage	0.014688	0.0027029	hypermethylated	0	27	25
chr5	115372248	115374248	Oasl1	-0.21567299	0.000015626	hypomethylated	0.0087253	0.85154	insignificant	8	41	42
chr5	115391210	115393210	2210016L21Ri	-0.19629839	0.090599	insignificant	0.027612	0.85077	insignificant	3	8	8
chr5	115421071	115423071	Hnf1a	-0.21256039	0.15945	insignificant	0.07543	1	insignificant	4	23	24
chr5	115460532	115462532	Sppl3	-0.09719651	2.48E-32	hypomethylated	0.0025534	0.96316	insignificant	75	256	256
chr5	115569322	115571322	Acads	-0.07283418	0.018889	hypomethylated	0.062618	0.88517	insignificant	8	15	12
chr5	115584984	115586984	Unc119b	-0.06279762	0.49213	insignificant	0.0041564	0.42584	insignificant	3	8	8
chr5	115608185	115610185	Mlec	-0.14643856	2.27E-22	hypomethylated	0.019405	0.93084	insignificant	30	81	80
chr5	115636130	115638130	Cabp1		1	noCoverage	0.021832	0.54175	insignificant	0	16	16
chr5	115684859	115686859	Pop5	-0.27037799	4.56E-21	hypomethylated	0.015756	0.83108	insignificant	29	94	93
chr5	115722904	115724904	Rnf10	-0.42688908	0.00000181	stronglyHypometh	0.082465	0.59051	insignificant	4	20	18
chr5	115728710	115730710	Coq5	-0.13417831	1.62E-14	hypomethylated	0.0078539	0.30659	insignificant	13	68	57
chr5	115750999	115752999	Dynll1	-0.14998332	0.00049619	hypomethylated	0.0076605	1	insignificant	16	62	68
chr5	115776185	115778185	Srsf9	-0.06886366	0.00019443	hypomethylated	0.0029747	0.82902	insignificant	15	125	110
chr5	115790255	115792255	Triap1	-0.13764569	3.28E-25	hypomethylated	0.01737	0.072912	insignificant	22	89	94
chr5	115791170	115793170	Gatc	-0.1389689	1.5E-14	hypomethylated	0.025749	0.83322	insignificant	13	52	57
chr5	115798964	115800964	Cox6a1	-0.17604838	0.00040517	hypomethylated	-0.030372	3.14E-20	hypomethylated	1	41	37
chr5	115878693	115880693	Msi1	-0.1101994	2.26E-62	hypomethylated	-0.0036321	0.88771	insignificant	78	183	182
chr5	115915274	115917274	Pla2g1b		1	noCoverage	-0.030397	0.84379	insignificant	0	15	12
chr5	115934306	115936306	Sirt4	-0.68597884	0.0072455	stronglyHypometh	-0.051598	0.5189	insignificant	2	27	25
chr5	115934493	115936493	Sirt4		1	noCoverage	-0.030881	0.76769	insignificant	0	21	19
chr5	115955710	115957710	Pxn	-0.18627495	1.48E-13	hypomethylated	0.018557	0.075865	insignificant	23	82	63
chr5	116008475	116010475	Rplp0	-0.1787815	1.23E-31	hypomethylated	0.015271	0.21642	insignificant	18	106	102
chr5	116014271	116016271	Gcn1l1	-0.12814872	2.5E-47	hypomethylated	0.011536	0.87584	insignificant	49	122	122
chr5	116080995	116082995	Rab35	-0.07606902	4.65E-41	hypomethylated	0.0040842	0.56577	insignificant	68	249	247
chr5	116081825	116083825	1110006O24R	-0.06115527	3.64E-20	hypomethylated	0.0064475	0.61828	insignificant	44	167	165
chr5	116181568	116183568	Ccdc64	-0.05793014	1.9E-22	hypomethylated	0.0096708	0.9605	insignificant	76	267	270
chr5	116294664	116296664	Cit	-0.26256232	0.010134	hypomethylated	0.010783	0.93844	insignificant	5	18	18
chr5	116474437	116476437	Prkab1	-0.22019379	0.00000369	hypomethylated	0.010282	0.28163	insignificant	5	82	79
chr5	116533255	116535255	Tmem233	0.03792343	0.034519	hypermethylated	-0.013407	0.1347	insignificant	4	22	20

chr5	116738994	116740994	Ccdc60	-0.11605071	1.84E-10	hypomethylated	0.06115	0.048052	hypermethylated	11	21	24
chr5	117041826	117043826	Srrm4	-0.26520631	5.16E-24	hypomethylated	0.063543	0.66194	insignificant	4	35	28
chr5	117566002	117568002	Suds3	-0.18515792	0.00035145	hypomethylated	-0.011099	0.16309	insignificant	12	34	34
chr5	117569137	117571137	Taok3	-0.10373891	5.38E-10	hypomethylated	0.011414	0.081016	insignificant	18	116	116
chr5	117582596	117584596	Taok3		1	noCoverage	0.024074	0.12758	insignificant	0	18	18
chr5	117737573	117739573	Pebp1	-0.02363661	0.000000135	hypomethylated	0.038337	0.091757	insignificant	19	67	70
chr5	117768274	117770274	Vsig10	-0.11990301	2.82E-23	hypomethylated	0.0012854	0.40476	insignificant	47	136	133
chr5	117806313	117808313	Wsb2	-0.13853491	3.1E-21	hypomethylated	-0.011478	0.16755	insignificant	36	149	147
chr5	117839032	117841032	Rfc5	-0.33368084	2.45E-09	stronglyHypometh	-0.035571	0.69243	insignificant	12	38	37
chr5	117863008	117865008	Ksr2	-0.08327685	0.000021985	hypomethylated	-0.0038972	1	insignificant	27	96	98
chr5	117863837	117865837	Ksr2	-0.09607863	0.00000191	hypomethylated	-0.0020402	0.73543	insignificant	22	72	72
chr5	118316127	118318127	Nos1	0.12484396	0.00003262	hypermethylated	0.031179	0.000073194	hypermethylated	7	61	64
chr5	118425778	118427778	Fbxo21	-0.21535533	4.98E-21	hypomethylated	-0.025469	0.2513	insignificant	26	94	88
chr5	118476832	118478832	Tesc	-0.14611874	4.08E-46	hypomethylated	-0.0061447	0.67586	insignificant	34	143	143
chr5	118618772	118620772	Hrk	-0.10727539	1.87E-22	hypomethylated	0.0036215	0.17386	insignificant	47	183	162
chr5	118694235	118696235	2410131K14Ri	-0.0898872	0.000079162	hypomethylated	0.0047469	0.79518	insignificant	24	90	86
chr5	118694870	118696870	Rnft2	-0.0898872	0.000079162	hypomethylated	0.0047469	0.79518	insignificant	24	90	86
chr5	118695034	118697034	Rnft2	-0.09337699	0.000028522	hypomethylated	0.0057653	0.76979	insignificant	24	76	72
chr5	119009727	119011727	Med13l	-0.07033948	3.03E-33	hypomethylated	0.0032521	0.95826	insignificant	72	334	299
chr5	120119677	120121677	Tbx3	-0.15104274	3.77E-14	hypomethylated	-0.017054	0.63548	insignificant	26	147	136
chr5	120283671	120285671	Tbx5	-0.12232533	0.000013462	hypomethylated	0.052429	0.10521	insignificant	9	39	40
chr5	120565521	120567521	Rbm19	-0.14915576	1.33E-14	hypomethylated	-0.005458	0.1155	insignificant	17	66	64
chr5	120880894	120882894	Lhx5	-0.15822262	6.53E-14	hypomethylated	0.005623	0.21511	insignificant	19	90	90
chr5	120922772	120924772	Sdsl	0.04587302	0.53643	insignificant	0.1434	0.099086	insignificant	4	10	8
chr5	120925555	120927555	Sds	-0.19994196	0.0036483	hypomethylated	-0.013622	0.75704	insignificant	11	22	23
chr5	120953632	120955632	Plbd2	-0.24708333	0.00043156	hypomethylated	0.032183	0.024217	hypermethylated	4	16	22
chr5	120960200	120962200	Slc24a6		1	noCoverage	-0.025833	0.85505	insignificant	0	20	20
chr5	121038031	121040031	Iqcd	-0.18359219	5.36E-12	hypomethylated	0.0051872	0.75768	insignificant	16	48	48
chr5	121038622	121040622	Tpcn1	-0.77482993	0.05418	insignificant	-0.0088348	0.77459	insignificant	2	14	14
chr5	121062138	121064138	Ddx54	-0.12492258	1.74E-08	hypomethylated	0.012678	0.16572	insignificant	28	125	126
chr5	121062598	121064598	1110008J03Ri	-0.18206851	0.00050524	hypomethylated	0.02788	0.01167	hypermethylated	22	109	110
chr5	121084244	121086244	Ccdc42b	0.54722222	0.46996	lowCoverage	-0.023333	1	insignificant	1	6	6
chr5	121097830	121099830	Rasal1	-0.19211366	4.95E-35	hypomethylated	-0.0022011	0.48864	insignificant	29	96	95
chr5	121161678	121163678	Dtx1	0.52952366	1.15E-09	stronglyHypermeth	0.053075	0.1492	insignificant	1	20	20
chr5	121245539	121247539	Oas1e		1	noCoverage	-0.0049087	1	insignificant	0	6	6
chr5	121261643	121263643	Oas1b	-0.31056698	0.00014274	hypomethylated	0.070978	0.63301	insignificant	6	19	18
chr5	121261646	121263646	Oas1b	-0.31056698	0.00014274	hypomethylated	0.070978	0.63301	insignificant	6	19	18
chr5	121262523	121264523	Oas1c	-0.24959829	0.0025173	hypomethylated	0.07014	0.5804	insignificant	5	15	14
chr5	121459527	121461527	Rph3a	-0.10586957	0.14508	insignificant	0.037046	0.82712	insignificant	4	10	10
chr5	121641406	121643406	Ptpn11	-0.27884796	2.61E-25	hypomethylated	0.028297	0.83824	insignificant	14	58	61
chr5	121653509	121655509	Rpl6	-0.14512666	0.026842	hypomethylated	-0.018956	0.80022	insignificant	16	66	52
chr5	121846990	121848990	Naa25	-0.09217563	4.08E-23	hypomethylated	0.0013409	0.89045	insignificant	41	116	116
chr5	121901717	121903717	Tmem116	-0.11585133	7.99E-11	hypomethylated	0.010404	0.47529	insignificant	22	134	130
chr5	121902483	121904483	Erp29	-0.12629076	0.053279	insignificant	0.0086913	1	insignificant	1	48	44
chr5	121995901	121997901	Mapkapk5	-0.16849552	0.022866	hypomethylated	0.035884	0.0358	inconclusive	7	42	38
chr5	122043833	122045833	Aldh2	-0.21423077	0.00000292	hypomethylated	0.0072358	0.89213	insignificant	7	22	22
chr5	122068947	122070947	Acad12	-0.12126022	5.31E-24	hypomethylated	-0.012736	0.59731	insignificant	14	52	52
chr5	122109594	122111594	Brap	-0.119362	0.000016484	hypomethylated	-0.017341	0.97113	insignificant	17	98	94
chr5	122110519	122112519	Acad10	-0.10778599	0.00009584	hypomethylated	-0.014275	1	insignificant	17	81	72
chr5	122160617	122162617	Atxn2	-0.08089138	3.08E-35	hypomethylated	0.0058497	0.32033	insignificant	73	265	248
chr5	122286810	122288810	Sh2b3	-0.11896662	1	insignificant	0.021134	0.62093	insignificant	4	67	60
chr5	122298036	122300036	Fam109a	-0.16487576	2.45E-42	hypomethylated	0.00044987	0.00000035	hypermethylated	29	132	124
chr5	122497834	122499834	Cux2	-0.13909137	1.39E-12	hypomethylated	-0.027256	0.38598	insignificant	17	78	71
chr5	122588071	122590071	Ccdc63	0.07252967	1	insignificant	-0.039366	0.70138	insignificant	2	14	13
chr5	122607287	122609287	Ppp1cc	-0.12065525	1.69E-09	hypomethylated	0.0048557	1	insignificant	27	141	155
chr5	122658745	122660745	Hvcn1	-0.1792379	5.3E-11	hypomethylated	-0.024824	0.069664	insignificant	12	40	40
chr5	122659306	122661306	Hvcn1	-0.17683268	0.000000437	hypomethylated	-0.041588	0.74742	insignificant	8	30	30
chr5	122714469	122716469	Tctn1		1	noCoverage	-0.074845	0.37119	insignificant	0	15	13
chr5	122733406	122735406	Pptc7	-0.1259197	1.74E-27	hypomethylated	0.0056444	0.14201	insignificant	24	94	102
chr5	122803421	122805421	Vps29	-0.1297552	5.06E-52	hypomethylated	0.0075756	0.44795	insignificant	38	143	139
chr5	122804204	122806204	Rad9b	-0.03933123	4.1E-13	hypomethylated	0.0084034	0.025325	hypermethylated	41	125	124
chr5	122821516	122823516	Gpn3	-0.1068947	1.26E-10	hypomethylated	-0.013338	0.058879	insignificant	16	94	82
chr5	122821972	122823972	1500011H22R	-0.12209686	1.25E-10	hypomethylated	-0.02854	0.047585	hypomethylated	16	83	82
chr5	122840936	122842936	Arpc3	-0.20143745	8.01E-10	hypomethylated	0.030643	0.97053	insignificant	20	78	78
chr5	122871452	122873452	Anapc7	-0.13601874	1.49E-18	hypomethylated	0.0028562	1	insignificant	31	95	98
chr5	122952234	122954234	Atp2a2	-0.11912996	1	lowCoverage	0.0034257	0.063418	insignificant	1	88	84
chr5	123064527	123066527	Ift81	0.14460784	1	insignificant	-0.047658	0.75935	insignificant	1	12	18
chr5	123092919	123094919	P2rx7	-0.36460147	1.79E-08	stronglyHypometh	0.067941	0.072921	insignificant	3	31	26
chr5	123156565	123158565	P2rx4	-0.10252471	4.31E-11	hypomethylated	0.039563	0.70537	insignificant	29	79	80
chr5	123229419	123231419	Camkk2	-0.43664958	2.07E-65	stronglyHypometh	0.073589	0.19456	insignificant	20	28	28
chr5	123271348	123273348	Anapc5	0.01876658	0.60307	insignificant	-0.03389	0.31215	insignificant	2	13	11
chr5	123299196	123301196	Rnf34	-0.1444553	7.79E-12	hypomethylated	-0.021921	0.43136	insignificant	18	72	49
chr5	123350359	123352359	Kdm2b	-0.13912139	2.04E-10	hypomethylated	-0.015165	0.018368	inconclusive	8	58	63
chr5	123438542	123440542	Kdm2b	-0.1321159	1.61E-09	hypomethylated	0.025887	0.63714	insignificant	17	75	75
chr5	123439101	123441101	Kdm2b	-0.10959323	1.75E-08	hypomethylated	0.012935	0.68691	insignificant	16	71	71
chr5	123464082	123466082	Orai1	-0.12697506	3.86E-31	hypomethylated	0.005858	0.41804	insignificant	40	131	128
chr5	123525283	123527283	Tmem120b	-0.12724643	3.35E-21	hypomethylated	-0.0095783	0.041114	inconclusive	28	91	82
chr5	123582638	123584638	Rhof	-0.24396472	1.09E-09	hypomethylated	0.039839	0.20955	insignificant	7	38	34
chr5	123677198	123679198	Psmd9	-0.11432638	7.29E-18	hypomethylated	0.0311	0.46851	insignificant	13	63	51
chr5	123793456	123795456	Bcl7a	-0.11352761	2.98E-41	hypomethylated	0.0077226	0.67784	insignificant	76	230	205
chr5	123843827	123845827	Mlxip	-0.13668157	1.19E-43	hypomethylated	0.0053292	0.69579	insignificant	45	167	157
chr5	123932110	123934110	Il31		1	noCoverage	0.10694	0.87341	insignificant	0	10	12
chr5	123938333	123940333	Lrrc43		1	noCoverage	-0.019298	0.74576	insignificant	0	5	6
chr5	123959468	123961468	B3gnt4	0.07323578	0.15807	insignificant	-0.0079953	0.042593	inconclusive	3	27	28
chr5	123974173	123976173	Diablo	-0.08034598	4.37E-12	hypomethylated	0.014197	0.0014198	hypermethylated	27	100	96

chr5	124023024	124025024	Vps33a		1	noCoverage	0.11112	0.0414	hypermethylated	0	16	18
chr5	124134300	124136300	Clip1	-0.11377922	0.00000356	hypomethylated	-0.010787	0.75207	insignificant	22	76	76
chr5	124198734	124200734	Kntc1	-0.16594392	0.000010772	hypomethylated	0.00084441	0.79161	insignificant	12	88	87
chr5	124199421	124201421	Rsrc2	-0.183169	0.000058809	hypomethylated	0.0001289	0.58018	insignificant	7	73	73
chr5	124330029	124332029	Gpr81	-0.3453125	0.0046642	stronglyHypometh	0.049443	0.43957	insignificant	4	8	8
chr5	124356283	124358283	Denr	-0.18556305	8.46E-34	hypomethylated	0.015264	0.013294	hypermethylated	38	124	119
chr5	124379697	124381697	Ccdc62	-0.08315385	0.027632	inconclusive	0.015178	1	insignificant	22	88	88
chr5	124422636	124424636	Hip1r	-0.08722291	0.000024439	hypomethylated	0.0085132	0.21038	insignificant	12	93	93
chr5	124545807	124547807	Abcb9	-0.12836749	1.26E-10	hypomethylated	0.06439	0.28453	insignificant	11	35	37
chr5	124561346	124563346	Ogfod2	-0.16510514	0.0029565	hypomethylated	-0.0018408	0.31564	insignificant	3	44	54
chr5	124565116	124567116	Arl6ip4	-0.09953697	0.041698	hypomethylated	0.023965	0.833	insignificant	27	89	89
chr5	124666427	124668427	Pitpnm2	-0.12181818	0.60107	insignificant	0.01	0.73638	insignificant	1	5	4
chr5	124777097	124779097	2810006K23Ri	-0.09917492	7.03E-13	hypomethylated	0.0080541	0.56963	insignificant	30	113	114
chr5	124804637	124806637	Cdk2ap1	-0.08695234	0.25471	insignificant	0.0066186	0.87284	insignificant	8	40	40
chr5	124875923	124877923	Sbno1	-0.23523698	3.14E-11	hypomethylated	0.017274	0.95293	insignificant	8	47	50
chr5	124888938	124890938	Setd8	-0.14335545	1.83E-10	hypomethylated	0.020587	0.96739	insignificant	12	74	72
chr5	124932163	124934163	Snrnp35	-0.13516587	3.28E-19	hypomethylated	0.040491	0.62259	insignificant	22	93	85
chr5	124981400	124983400	Rilpl1	-0.00452272	0.000000976	hypomethylated	-0.027742	0.95546	insignificant	4	35	33
chr5	124989799	124991799	Tmed2	-0.05064519	0.00012199	hypomethylated	0.0020535	1	insignificant	37	131	124
chr5	125001872	125003872	Ddx55	-0.13421184	1.15E-22	hypomethylated	-0.0016737	0.70268	insignificant	28	100	105
chr5	125028156	125030156	Gtf2h3	-0.09352338	4.5E-13	hypomethylated	0.010304	0.78828	insignificant	15	70	70
chr5	125029066	125031066	Eif2b1	-0.09020026	9.75E-08	hypomethylated	0.010165	0.36818	insignificant	7	38	38
chr5	125047757	125049757	Tctn2	-0.19759474	7.97E-08	hypomethylated	-0.0068427	0.62495	insignificant	14	48	50
chr5	125078647	125080647	Atp6v0a2		1	noCoverage	-0.091843	0.73722	insignificant	0	15	19
chr5	125204454	125206454	Dnahc10	-0.16925058	1	insignificant	0.065451	0.60844	insignificant	2	24	28
chr5	125342074	125344074	Zfp664	-0.09599346	3.67E-66	hypomethylated	-0.0034702	0.93999	insignificant	104	378	371
chr5	125342591	125344591	Ccdc92	-0.11781525	9.65E-31	hypomethylated	0.0045036	0.78041	insignificant	74	298	294
chr5	125482844	125484844	Fam101a	0.08620408	0.25715	insignificant	0.019771	1	insignificant	4	26	26
chr5	125659584	125661584	Ncor2	0.1280118	0.087951	insignificant	0.016441	0.93141	insignificant	3	42	42
chr5	125821444	125823444	Scarb1	-0.07128238	0.016331	hypomethylated	0.015109	0.42964	insignificant	5	40	40
chr5	125870387	125872387	Ubc	-0.23774838	0.58865	insignificant	0.057037	0.16763	insignificant	1	35	35
chr5	125914418	125916418	Dhx37	-0.14607079	1.64E-09	hypomethylated	0.03601	0.89662	insignificant	11	25	28
chr5	125920937	125922937	Bri3bp	-0.10725028	4.67E-19	hypomethylated	0.010946	0.16701	insignificant	34	135	136
chr5	125955242	125957242	Aacs	-0.18908021	4.37E-09	hypomethylated	0.053449	0.87003	insignificant	21	89	86
chr5	126011787	126013787	Tmem132b	-0.12621811	4.11E-33	hypomethylated	0.011346	0.2708	insignificant	51	144	136
chr5	127721195	127723195	Tmem132c	-0.11020274	5.34E-18	hypomethylated	0.019376	0.016349	hypermethylated	39	149	146
chr5	128097762	128099762	Slc15a4	-0.29986916	4.86E-13	hypomethylated	-0.002354	0.017763	hypomethylated	8	47	45
chr5	128111631	128113631	Glt1d1	-0.11787984	0.0071164	hypomethylated	0.0070695	0.79529	insignificant	8	42	42
chr5	128938937	128940937	Tmem132d		1	noCoverage	-0.1153	0.030696	hypomethylated	0	20	25
chr5	129105980	129107980	Fzd10	-0.02125203	8.68E-16	hypomethylated	0.010291	0.74061	insignificant	69	236	244
chr5	129106562	129108562	5930412G12R	-0.00173646	5.49E-15	hypomethylated	0.017408	0.70188	insignificant	58	209	216
chr5	129525030	129527030	Ran	-0.09478062	5.87E-36	hypomethylated	-0.012812	0.02507	hypomethylated	62	231	240
chr5	130006105	130008105	Sfswap	-0.10718775	0.000000132	hypomethylated	0.0067999	0.93723	insignificant	35	109	106
chr5	130089088	130091088	Mmp17	-0.16811103	2.38E-44	hypomethylated	-0.024316	0.77414	insignificant	43	155	144
chr5	130175963	130177963	Zfp11	-0.38960114	0.000076116	stronglyHypometh	-0.017307	1	insignificant	3	6	6
chr5	130214386	130216386	###########	-0.28323895	0.17966	insignificant	0.023811	0.47066	insignificant	2	12	12
chr5	130220402	130222402	Mrps17	-0.17748793	0.000000208	hypomethylated	0.00035473	0.31372	insignificant	7	45	44
chr5	130229949	130231949	Gbas	-0.1028528	1.62E-12	hypomethylated	0.018939	0.27572	insignificant	28	88	89
chr5	130292260	130294260	Cct6a	-0.17330994	1.3E-13	hypomethylated	-0.0036056	0.967	insignificant	11	60	56
chr5	130293129	130295129	Cct6a	-0.18536677	2.35E-10	hypomethylated	-0.029421	0.83996	insignificant	7	46	42
chr5	130321865	130323865	Sumf2	-0.09849206	0.24095	insignificant	-0.039866	0.39336	insignificant	5	10	10
chr5	130363340	130365340	Chchd2	-0.27872666	0.00000706	hypomethylated	0.041541	0.82338	insignificant	4	20	20
chr5	130370592	130372592	2410018M08R	-0.26915745	0.00049285	hypomethylated	-0.081741	0.011901	hypomethylated	6	47	43
chr5	130383409	130385409	4930579G22R	-0.1586577	0.000000963	hypomethylated	-0.0054031	0.19002	insignificant	18	58	59
chr5	130416982	130418982	Vkorc1l1	-0.12104753	0.0002219	hypomethylated	-0.0030138	0.29279	insignificant	16	69	74
chr5	130478698	130480698	Gusb	-0.53571429	0.041698	stronglyHypometh	-0.19583	0.44738	insignificant	2	4	8
chr5	130500201	130502201	Asl	-0.35183081	0.71135	insignificant	-0.14161	0.042806	hypomethylated	3	16	23
chr5	130504175	130506175	Crcp	-0.22901111	5.7E-13	hypomethylated	-0.023228	1	insignificant	22	62	58
chr5	130554355	130556355	Tpst1	-0.12019372	1.36E-28	hypomethylated	0.019896	0.46558	insignificant	42	118	105
chr5	130619757	130621757	Kctd7	-0.11208851	1.65E-08	hypomethylated	-0.0022546	0.36362	insignificant	19	89	82
chr5	130646688	130648688	Rabgef1	-0.0965952	1.68E-20	hypomethylated	0.01128	0.62226	insignificant	31	70	59
chr5	130662049	130664049	Rabgef1	-0.28594771	1	insignificant	0.094072	0.29294	insignificant	2	6	6
chr5	130694613	130696613	0610007L01Ri	0.11697531	1	insignificant	0.10128	0.0032544	hypermethylated	1	6	10
chr5	130697204	130699204	0610007L01Ri	-0.0942569	9.52E-20	hypomethylated	0.014357	0.19745	insignificant	44	118	118
chr5	130730488	130732488	Tyw1	-0.17036669	0.000030852	hypomethylated	-0.0033044	0.64411	insignificant	11	89	90
chr5	130731332	130733332	Tyw1	-0.39438339	0.36895	insignificant	-0.078921	1	insignificant	3	13	13
chr5	130731906	130733906	Tyw1	-0.28744113	0.18131	insignificant	-0.044516	0.56503	insignificant	4	21	21
chr5	130844327	130846327	Caln1	-0.24511033	0.365	insignificant	-0.21269	0.07318	insignificant	5	20	20
chr5	130923661	130925661	Caln1	-0.1735688	4.23E-19	hypomethylated	-0.025525	0.074206	insignificant	25	119	104
chr5	131783392	131785392	Wbscr17	-0.22285634	0.00051903	hypomethylated	0.022793	0.78388	insignificant	6	21	21
chr5	133018213	133020213	Auts2	-0.06452829	0.000000746	hypomethylated	0.0025562	0.17101	insignificant	47	193	190
chr5	134574617	134576617	Gatsl2	-0.13991499	1.79E-09	hypomethylated	0.0057231	0.094325	insignificant	14	86	86
chr5	134658907	134660907	Gtf2ird2	-0.06731354	7.57E-21	hypomethylated	0.0011923	0.47655	insignificant	26	115	108
chr5	134790616	134792616	Gtf2i	-0.17957532	2.72E-16	hypomethylated	0.013421	0.017016	inconclusive	22	69	70
chr5	134932132	134934132	Gtf2ird1	-0.13311441	1.73E-26	hypomethylated	-0.030776	0.094594	insignificant	21	77	70
chr5	134932581	134934581	Gtf2ird1	-0.18077443	5.54E-19	hypomethylated	-0.055275	0.11458	insignificant	13	51	48
chr5	135028304	135030304	Clip2	-0.21587594	2.48E-15	hypomethylated	0.023699	1	insignificant	7	25	25
chr5	135035963	135037963	Syna	0.04305305	1	insignificant	-0.10488	0.057165	insignificant	3	19	12
chr5	135057559	135059559	Rfc2	-0.24821877	6.59E-10	hypomethylated	0.034884	0.15206	insignificant	12	61	61
chr5	135090893	135092893	Lat2	0.08449821	0.13501	insignificant	0.10553	0.22935	insignificant	2	24	21
chr5	135115198	135117198	Eif4h	-0.14093599	1.87E-11	hypomethylated	-0.0045119	0.56344	insignificant	14	42	42
chr5	135164460	135166460	Limk1	-0.13807081	2.04E-14	hypomethylated	0.020654	0.49263	insignificant	22	89	84
chr5	135407242	135409242	Wbscr27	-0.26533421	0.00019379	hypomethylated	0.014784	0.025502	inconclusive	10	60	60
chr5	135422804	135424804	Cldn4	-0.0881339	0.37798	insignificant	0.050966	0.15682	insignificant	2	18	20
chr5	135461083	135463083	Cldn3	-0.13788588	0.0072651	hypomethylated	-0.035692	0.39047	insignificant	9	105	111

chr5	135477220	135479220	Wbscr25	-0.25217067	0.00003029	hypomethylated	0.021557	0.7421	insignificant	2	19	19
chr5	135484021	135486021	Abhd11	-0.20557524	2.03E-24	hypomethylated	-0.0052272	0.79894	insignificant	7	55	52
chr5	135484168	135486168	Abhd11	-0.20557524	2.03E-24	hypomethylated	-0.0052272	0.79894	insignificant	7	56	53
chr5	135484325	135486325	Abhd11	-0.18660924	5.24E-22	hypomethylated	-0.0023695	0.493	insignificant	7	54	51
chr5	135498441	135500441	Stx1a	-0.19119661	3.61E-19	hypomethylated	-0.022167	0.20571	insignificant	30	82	88
chr5	135539075	135541075	Dnajc30	-0.11281651	1.37E-17	hypomethylated	0.034327	0.6142	insignificant	33	122	127
chr5	135539980	135541980	Wbscr22	-0.10161041	7.17E-14	hypomethylated	0.00033915	0.76755	insignificant	25	101	101
chr5	135554136	135556136	Vps37d	-0.23615937	0.00035102	hypomethylated	0.058658	0.16143	insignificant	12	42	43
chr5	135581782	135583782	Mlxipl	-0.06790551	8.13E-08	hypomethylated	0.0098443	0.0052229	hypermethylated	8	41	41
chr5	135624580	135626580	Tbl2	-0.16539986	3.51E-13	hypomethylated	0.016729	0.71275	insignificant	16	75	84
chr5	135643241	135645241	Bcl7b	-0.13845802	8.19E-13	hypomethylated	-0.0024038	1	insignificant	15	81	81
chr5	135662192	135664192	Baz1b	-0.09547488	9.6E-27	hypomethylated	0.0088357	0.21944	insignificant	48	226	212
chr5	135726917	135728917	Fzd9	-0.12216593	3.33E-24	hypomethylated	0.0045095	0.33496	insignificant	24	89	89
chr5	135825861	135827861	Fkbp6	-0.01097482	0.000016898	inconclusive	-0.04913	0.000011317	hypomethylated	10	38	38
chr5	135828165	135830165	Trim50		1	noCoverage	0.027269	0.6839	insignificant	0	6	6
chr5	135844822	135846822	Nsun5	-0.24764035	2.46E-11	hypomethylated	0.012643	0.014578	hypermethylated	12	39	40
chr5	135870416	135872416	Pom121	0.19834919	0.14701	insignificant	-0.035685	0.12085	insignificant	4	39	36
chr5	136020992	136022992	Hip1	-0.15858414	4.87E-10	hypomethylated	0.021043	0.84238	insignificant	7	34	37
chr5	136107523	136109523	Rhbdd2	-0.10536707	0.00026503	hypomethylated	-0.0086308	0.79696	insignificant	1	41	44
chr5	136164083	136166083	Por	-0.1095588	0.000029686	hypomethylated	0.013278	0.94246	insignificant	14	84	70
chr5	136220042	136222042	Tmem120a	-0.22801468	2.57E-13	hypomethylated	-0.0090726	0.05993	insignificant	11	34	34
chr5	136253518	136255518	Mdh2	-0.16053525	1.18E-36	hypomethylated	0.0066737	0.76443	insignificant	45	146	146
chr5	136254255	136256255	Styxl1	-0.12174373	9.81E-20	hypomethylated	0.017673	0.87874	insignificant	34	119	118
chr5	136362788	136364788	Hspb1	-0.27158098	0.00079268	hypomethylated	0.021513	0.097276	insignificant	2	52	53
chr5	136410511	136412511	Ywhag	-0.2602345	0.026133	hypomethylated	-0.016079	0.42717	insignificant	8	24	22
chr5	136450346	136452346	Srcrb4d	-0.62816327	0.14632	insignificant	-0.274	0.8817	insignificant	2	7	4
chr5	136454974	136456974	Zp3	0.1893892	1	lowCoverage	-0.10246	0.90081	insignificant	1	13	12
chr5	136469746	136471746	Dtx2		1	noCoverage	0.028259	0.5667	insignificant	0	16	16
chr5	136558785	136560785	Rasa4	-0.22809524	0.021803	hypomethylated	0.030833	0.78088	insignificant	3	10	8
chr5	136591560	136593560	Polr2j	-0.1024376	0.00000166	hypomethylated	0.0053383	0.96156	insignificant	5	60	60
chr5	136611944	136613944	Lrwd1	-0.17989148	0.31705	insignificant	0.065739	0.4315	insignificant	6	27	32
chr5	136613650	136615650	Alkbh4	0.15108225	1	lowCoverage	-0.17715	0.5041	insignificant	1	5	10
chr5	136674824	136676824	Prkrip1	-0.18715045	0.000000187	hypomethylated	0.0008906	0.043345	hypermethylated	9	40	40
chr5	136720773	136722773	Sh2b2	-0.14956749	1	insignificant	0.030479	0.34603	insignificant	2	24	24
chr5	137043275	137045275	Cux1	-0.10985449	0.000000058	hypomethylated	0.014571	0.64905	insignificant	17	117	112
chr5	137043301	137045301	Cux1	-0.10985449	0.000000058	hypomethylated	0.014571	0.64905	insignificant	17	117	112
chr5	137358977	137360977	Emid2	-0.3336096	0.014885	stronglyHypometh	-0.13588	0.68806	insignificant	7	36	28
chr5	137383019	137385019	Rabl5	-0.27407899	1	insignificant	0.022169	0.65772	insignificant	3	14	15
chr5	137394015	137396015	4933404O12R	-0.14836729	9.6E-15	hypomethylated	0.000054419	0.75649	insignificant	15	52	51
chr5	137428144	137430144	Fis1	0.26388668	0.0099048	hypermethylated	0.07516	0.013807	hypermethylated	3	59	42
chr5	137436946	137438946	Fis1	-0.19221491	0.0045764	hypomethylated	0.06943	0.12126	insignificant	5	20	24
chr5	137461888	137463888	Plod3	-0.11493147	5.61E-23	hypomethylated	-0.012657	0.22843	insignificant	36	142	130
chr5	137463752	137465752	Plod3		1	noCoverage	0.049621	0.027748	hypermethylated	0	22	22
chr5	137466302	137468302	Mir702	0.09334611	0.055086	insignificant	0.025289	0.063455	insignificant	4	44	44
chr5	137505164	137507164	Vgf	-0.11836956	5.94E-53	hypomethylated	0.016366	0.17102	insignificant	37	124	113
chr5	137521930	137523930	Ap1s1	-0.1863893	1.64E-15	hypomethylated	-0.047302	0.076864	insignificant	13	45	42
chr5	137548142	137550142	Serpine1	-0.13532202	0.00000293	hypomethylated	0.020445	0.14344	insignificant	6	21	21
chr5	137592077	137594077	Trim56	-0.45470085	0.00000015	stronglyHypometh	-0.0098838	0.03055	hypomethylated	1	26	20
chr5	137728504	137730504	Ache	-0.1668003	6.77E-13	hypomethylated	0.024241	0.12943	insignificant	25	69	69
chr5	137734896	137736896	Ufsp1	-0.17466112	1.07E-33	hypomethylated	-0.0081227	0.88528	insignificant	22	59	62
chr5	137755469	137757469	Slc12a9	-0.22811363	0.00039601	inconclusive	0.021649	0.085481	insignificant	15	52	48
chr5	137774810	137776810	Slc12a9	0.00310139	0.57012	insignificant	-0.044995	1	insignificant	4	30	25
chr5	137790336	137792336	Ephb4	-0.18916886	7.41E-08	hypomethylated	0.016631	0.94086	insignificant	5	47	34
chr5	137918292	137920292	Zan	-0.10084886	0.49148	insignificant	-0.024708	0.81232	insignificant	1	26	24
chr5	137927044	137929044	Epo		1	noCoverage	-0.0052334	0.91939	insignificant	0	6	6
chr5	137943657	137945657	Pop7	-0.15605062	3.51E-11	hypomethylated	0.011788	0.8312	insignificant	22	87	85
chr5	137959107	137961107	Gigyf1		1	noCoverage	-0.0061438	0.85522	insignificant	0	15	15
chr5	137974457	137976457	Gnb2	-0.23109244	0.0056904	hypomethylated	0.026514	1	insignificant	4	17	15
chr5	137993782	137995782	Actl6b	-0.08753045	0.0051455	hypomethylated	0.017399	0.82922	insignificant	23	54	56
chr5	138042268	138044268	Mospd3	-0.25075155	0.00053441	hypomethylated	-0.0018396	0.076723	insignificant	9	47	47
chr5	138052632	138054632	Pcolce	-0.05899471	0.72879	insignificant	0.066494	1	insignificant	4	9	12
chr5	138069350	138071350	Lrch4-sap25	-0.15480222	0.00034388	hypomethylated	-0.0012744	0.052225	insignificant	13	58	57
chr5	138086942	138088942	Irs3		1	noCoverage	0.11382	1	insignificant	0	17	14
chr5	138125845	138127845	Agfg2	-0.10460395	0.00000126	hypomethylated	0.012431	0.27428	insignificant	6	42	42
chr5	138125921	138127921	Agfg2		1	noCoverage	0.029234	0.49064	insignificant	0	26	26
chr5	138181226	138183226	6430598A04R	-0.21901318	6.31E-08	hypomethylated	-0.065178	0.35767	insignificant	4	40	36
chr5	138186223	138188223	Tsc22d4	-0.24978536	0.000000561	hypomethylated	-0.012215	0.41474	insignificant	7	17	26
chr5	138219145	138221145	Ppp1r35	-0.22609984	1.59E-39	hypomethylated	-0.029281	0.069344	insignificant	37	108	110
chr5	138227929	138229929	Mepce	-0.48861667	0.0036004	stronglyHypometh	-0.030106	0.025577	inconclusive	9	53	48
chr5	138228029	138230029	Zcwpw1	-0.48861667	0.0036004	stronglyHypometh	-0.030106	0.025577	inconclusive	9	53	48
chr5	138277506	138279506	Pilra	0.008821	0.68502	insignificant	0.10134	0.000015467	hypermethylated	5	27	22
chr5	138299277	138301277	Pilrb1		1	noCoverage	-0.091667	0.35209	insignificant	0	4	4
chr5	138421748	138423748	Azgp1	-0.48462567	0.0024807	stronglyHypometh	-0.12219	0.20237	insignificant	1	4	3
chr5	138525311	138527311	Zkscan1	-0.14929845	1.24E-11	hypomethylated	-0.0013031	0.67281	insignificant	16	62	60
chr5	138557132	138559132	Zscan21	-0.18028858	0.00000229	hypomethylated	-0.017482	0.1107	insignificant	10	60	57
chr5	138596972	138598972	Zfp113	-0.4083833	0.0006345	stronglyHypometh	-0.086594	0.35277	insignificant	3	10	11
chr5	138601329	138603329	Cops6	-0.46245421	0.082406	insignificant	-0.093407	0.86131	insignificant	2	7	6
chr5	138606632	138608632	Mcm7	-0.07777778	0.22846	insignificant	-0.13743	0.053219	insignificant	2	4	2
chr5	138612248	138614248	Ap4m1	-0.12187684	0.0019875	hypomethylated	0.0072042	0.19158	insignificant	13	93	94
chr5	138613090	138615090	Mcm7	-0.13367564	0.14324	insignificant	0.030323	0.082416	insignificant	4	38	39
chr5	138627762	138629762	Cnpy4	-0.18099905	0.000000111	hypomethylated	-0.05635	0.24776	insignificant	6	15	20
chr5	138628414	138630414	Taf6	-0.53448276	0.000000176	stronglyHypometh	-0.006705	0.030889	inconclusive	1	2	2
chr5	138634541	138636541	Mblac1	-0.10156108	1.52E-09	hypomethylated	0.018077	0.65976	insignificant	11	44	50
chr5	138695709	138697709	0910001L09Ri	-0.13162857	0.000061565	hypomethylated	0.019047	0.5033	insignificant	7	34	34
chr5	138705280	138707280	BC037034	0.47973485	0.055013	insignificant	-0.0203	0.16917	insignificant	1	10	10

chr5	138713982	138715982	Gpc2	-0.0829539	0.00006137	hypomethylated	0.025642	0.93351	insignificant	5	12	14
chr5	138720736	138722736	Stag3	-0.30099337	0.0013354	hypomethylated	-0.0058301	0.73109	insignificant	10	84	83
chr5	138721165	138723165	Gpc2	-0.3765897	0.018871	stronglyHypometh	-0.014719	0.43552	insignificant	7	58	58
chr5	138881703	138883703	Zfp157	-0.16940687	0.0032007	hypomethylated	0.0020789	0.48143	insignificant	7	22	22
chr5	139005922	139007922	1700123K08Rik		1	noCoverage	-0.037446	0.44047	insignificant	0	2	2
chr5	139060971	139062971	Zfp68	-0.18865323	1	insignificant	0.11885	0.17296	insignificant	1	10	10
chr5	139063086	139065086	A430033K04Rik		1	noCoverage	-0.04499	0.59825	insignificant	0	13	12
chr5	139230034	139232034	Fam20c	-0.10052464	2.15E-60	hypomethylated	0.0036962	1	insignificant	73	236	234
chr5	139295033	139297033	Gm5294	-0.06729574	0.86188	insignificant	-0.0040309	0.071257	insignificant	6	15	18
chr5	139470907	139472907	Pdgfa	-0.09998473	3.05E-22	hypomethylated	-0.0021892	0.67376	insignificant	71	208	205
chr5	139605706	139607706	Prkar1b	-0.10352212	0.00016584	hypomethylated	0.025511	0.0098918	hypermethylated	10	52	52
chr5	139625176	139627176	Heatr2	-0.09219466	5.64E-33	hypomethylated	0.0043805	0.0015626	hypermethylated	42	167	136
chr5	139675623	139677623	Sun1	-0.10327187	7.83E-17	hypomethylated	0.0040238	0.88025	insignificant	29	128	132
chr5	139727277	139729277	Get4	-0.06392742	8.51E-09	hypomethylated	-0.0095191	0.14274	insignificant	12	89	89
chr5	139728450	139730450	Get4		1	noCoverage	0.0083823	0.61244	insignificant	0	21	21
chr5	139801418	139803418	Adap1	-0.10189415	1.27E-20	hypomethylated	0.0017963	0.3437	insignificant	23	76	72
chr5	139821120	139823120	Cox19	0.07011675	0.78315	insignificant	-0.0081324	0.085852	insignificant	4	34	35
chr5	139827570	139829570	Cyp2w1	-0.19525659	0.011021	hypomethylated	-0.0027108	1	insignificant	3	14	14
chr5	139845699	139847699	Mir339	-0.06525974	0.28054	insignificant	-0.020125	0.53248	insignificant	2	6	6
chr5	139852650	139854650	D830046C22R	-0.05680515	1	insignificant	0.0084122	0.28194	insignificant	1	6	6
chr5	139855620	139857620	Gpr146	-0.22236428	0.00000362	hypomethylated	-0.035224	0.088732	insignificant	21	90	89
chr5	139881340	139883340	C130050O18Rik		1	noCoverage	0.11608	0.73546	insignificant	0	7	4
chr5	139936488	139938488	3110082I17Rik		1	noCoverage	-0.0062799	1	insignificant	0	10	10
chr5	139960445	139962445	Zfand2a		1	noCoverage	0.037239	0.024897	inconclusive	0	42	42
chr5	140018851	140020851	Uncx	-0.133885	6.45E-17	hypomethylated	-0.016556	0.33288	insignificant	32	164	162
chr5	140212287	140214287	Micall2	0.05616499	0.65079	insignificant	0.042637	1	insignificant	8	32	27
chr5	140251632	140253632	Ints1	-0.2173434	3.68E-21	hypomethylated	-0.013498	0.88816	insignificant	6	34	34
chr5	140266489	140268489	Mafk	-0.12840825	2.2E-12	hypomethylated	0.010198	0.521	insignificant	24	105	96
chr5	140289197	140291197	Tmem184a	-0.55113484	0.0068077	stronglyHypometh	0.062462	0.13826	insignificant	1	14	14
chr5	140302797	140304797	Psmg3	-0.52938517	0.11475	insignificant	0.03371	0.54561	insignificant	1	2	2
chr5	140382896	140384896	Elfn1	0.0461351	0.33035	insignificant	-0.019563	0.63315	insignificant	9	132	100
chr5	140797506	140799506	Mad1l1	-0.20615079	0.00000487	hypomethylated	-0.028347	0.17832	insignificant	12	36	36
chr5	140806875	140808875	Nudt1	-0.42539901	0.00000313	stronglyHypometh	0.01617	0.0044751	inconclusive	4	38	38
chr5	140807852	140809852	Ftsj2	0.35895504	0.5243	insignificant	0.041668	0.52381	insignificant	3	14	15
chr5	140865201	140867201	Snx8	-0.16616337	4.31E-15	hypomethylated	-0.031944	0.68298	insignificant	20	55	53
chr5	140894258	140896258	Eif3b	-0.08366765	5.91E-15	hypomethylated	-0.0018056	0.66201	insignificant	33	150	140
chr5	140980562	140982562	Chst12		1	noCoverage	0.00972	0.48336	insignificant	0	26	26
chr5	141041021	141043021	Grifin	-0.35789928	0.62235	insignificant	0.018584	0.59557	insignificant	2	10	13
chr5	141082294	141084294	Lfng	-0.124692	4.7E-15	hypomethylated	-0.0051568	0.91156	insignificant	25	139	125
chr5	141124985	141126985	Ttyh3	-0.15858744	0.00017938	hypomethylated	-0.039348	0.88563	insignificant	7	26	23
chr5	141178332	141180332	Baat1	-0.20033078	5.43E-08	hypomethylated	-0.012887	0.1726	insignificant	7	26	23
chr5	141179976	141181976	Baat1	-0.2027672	0.00018344	hypomethylated	-0.016745	0.76602	insignificant	10	44	41
chr5	141306385	141308385	Gna12	-0.13326613	0.000000752	hypomethylated	0.026576	0.19509	insignificant	3	31	29
chr5	141716487	141718487	Sdk1	-0.09521353	6.6E-42	hypomethylated	0.0014913	0.16652	insignificant	96	263	252
chr5	142876450	142878450	Foxk1	-0.1402083	3.8E-24	hypomethylated	-0.016903	0.26986	insignificant	42	140	122
chr5	142938884	142940884	C330006K01R	-0.1617954	3.16E-08	hypomethylated	-0.006718	0.49699	insignificant	23	84	78
chr5	143006030	143008030	Radil	0.04710073	0.49891	insignificant	0.071148	0.34154	insignificant	11	37	36
chr5	143027031	143029031	Radil	-0.12773319	1	insignificant	-0.010165	0.62879	insignificant	3	30	33
chr5	143084706	143086706	Mmd2	0.09876447	0.81073	insignificant	0.013851	0.84475	insignificant	4	23	23
chr5	143104537	143106537	Wipi2	-0.13299451	3.86E-11	hypomethylated	-0.011009	0.71484	insignificant	17	154	160
chr5	143177054	143179054	Slc29a4	-0.08734718	7.23E-35	hypomethylated	0.019903	0.71299	insignificant	31	97	96
chr5	143579066	143581066	Tnrc18	-0.18362236	2.25E-12	hypomethylated	0.031	0.53995	insignificant	14	73	73
chr5	143656917	143658917	Fbxl18	-0.46992174	2.36E-18	stronglyHypometh	0.081182	0.00051853	inconclusive	6	36	31
chr5	143668403	143670403	Actb	-0.18971662	0.003381	hypomethylated	0.017191	0.85199	insignificant	10	36	36
chr5	143721033	143723033	Fscn1	-0.13923176	2.92E-30	hypomethylated	0.004959	0.000073845	hypermethylated	44	132	132
chr5	143874699	143876699	Rnf216		1	noCoverage	-0.0035025	1	insignificant	0	10	10
chr5	143941695	143943695	4933411G11R	-0.05240793	9.18E-08	inconclusive	0.0094327	0.000002678	inconclusive	22	65	62
chr5	143942422	143944422	Rbak	0.12237614	7.4E-14	hypermethylated	0.020844	8.25E-10	inconclusive	10	33	34
chr5	143995886	143997886	Zfp12	0.00271989	0.00000693	hypermethylated	-0.16679	0.048517	hypomethylated	8	46	40
chr5	144077039	144079039	E130309D02R	-0.14394557	0.075571	insignificant	-0.026858	0.010166	hypomethylated	12	32	32
chr5	144089986	144091986	0610040B10R	-0.02809168	0.00052914	hypomethylated	0.0036643	0.65162	insignificant	32	94	91
chr5	144090905	144092905	Zdhhc4	0.12222472	0.11699	insignificant	0.0072329	0.76242	insignificant	12	28	28
chr5	144118016	144120016	Grid2ip	-0.15346309	0.00053054	hypomethylated	0.086983	0.30639	insignificant	9	40	43
chr5	144124586	144126586	Grid2ip	-0.13711974	8.79E-09	hypomethylated	0.04151	0.0057636	hypermethylated	7	60	64
chr5	144164498	144166498	Kdelr2	-0.09745977	2.13E-28	hypomethylated	0.0017194	0.4432	insignificant	19	69	69
chr5	144224360	144226360	Daglb	-0.14133416	2.49E-09	hypomethylated	0.017498	0.94418	insignificant	23	96	92
chr5	144288861	144290861	Rac1	-0.11410123	1.24E-10	hypomethylated	-0.020433	0.51605	insignificant	26	61	60
chr5	144308573	144310573	2810453I06Ril	-0.10872232	7.86E-27	hypomethylated	0.015985	0.25372	insignificant	20	57	66
chr5	144382315	144384315	Cyth3	-0.09906612	7.76E-10	hypomethylated	0.027987	0.065324	insignificant	16	95	95
chr5	144493149	144495149	Usp42	-0.07971206	4.61E-10	hypomethylated	0.016116	0.31139	insignificant	25	77	72
chr5	144518222	144520222	D130017N08R	-0.30334438	3.69E-08	hypomethylated	0.060468	0.15387	insignificant	13	48	46
chr5	144577660	144579660	Eif2ak1	-0.11093239	0.0067277	hypomethylated	-0.0007421	0.60055	insignificant	8	68	68
chr5	144655936	144657936	Ankrd61	0.06577965	1	insignificant	0.027152	0.050293	insignificant	3	12	12
chr5	144669869	144671869	Pms2	-0.15029791	2.94E-33	hypomethylated	0.024741	0.048038	inconclusive	24	59	54
chr5	144670708	144672708	Pms2	-0.30211962	2.7E-54	hypomethylated	0.026726	0.77667	insignificant	17	26	25
chr5	144775722	144777722	Ccz1	-0.24101848	0.000798	hypomethylated	0.12622	0.81645	insignificant	4	19	17
chr5	144860304	144862304	Lmtk2	-0.16069016	1.62E-45	hypomethylated	-0.011887	0.088417	insignificant	28	88	95
chr5	144950154	144952154	Bhlha15	-0.07205889	0.051046	insignificant	0.025308	0.27967	insignificant	11	26	26
chr5	144984447	144986447	Tecpr1	-0.19142382	0.0027803	hypomethylated	-0.0094769	0.022754	hypomethylated	11	44	42
chr5	145015093	145017093	Bri3	-0.17844612	2.64E-22	hypomethylated	-0.015963	0.40871	insignificant	26	98	99
chr5	145118981	145120981	Baiap2l1	-0.05615799	2.14E-10	hypomethylated	-0.039369	0.00065209	hypomethylated	5	41	51
chr5	145305755	145307755	Nptx2	-0.10951563	3.83E-29	hypomethylated	0.037128	0.022893	hypermethylated	50	293	279
chr5	145522447	145524447	Tmem130	0.14122596	0.3827	insignificant	0.025245	0.55449	insignificant	4	8	8
chr5	145528660	145530660	Trrap	-0.06684183	0.00000665	hypomethylated	-0.011259	0.79703	insignificant	42	147	143
chr5	145726699	145728699	Smurf1	-0.12413382	8.13E-13	hypomethylated	0.01507	0.51433	insignificant	18	58	58

chr5	145793633	145795633	Gm4871		1	noCoverage	0.31538	0.028928	hypermethylated	0	4	4
chr5	145843737	145845737	Arpc1a	-0.17566174	4.89E-09	hypomethylated	-0.033638	1	insignificant	9	43	40
chr5	145874124	145876124	Arpc1b	-0.12885864	2.21E-16	hypomethylated	0.0047171	0.84874	insignificant	25	139	139
chr5	145900265	145902265	Bud31	-0.12865386	2.26E-18	hypomethylated	-0.0070559	0.098787	insignificant	29	127	120
chr5	145900958	145902958	Pdap1	-0.11700529	1.12E-10	hypomethylated	-0.0044259	0.69773	insignificant	15	73	66
chr5	145927092	145929092	Cpsf4	-0.14881198	4.43E-27	hypomethylated	0.0037534	0.77648	insignificant	34	121	121
chr5	145927973	145929973	Ptcd1	-0.16156935	5.73E-28	hypomethylated	0.023854	1	insignificant	27	93	95
chr5	145952461	145954461	Atp5j2		1	noCoverage	0.075	0.62353	insignificant	0	2	2
chr5	145962751	145964751	Zkscan5	0.0712482	1	insignificant	0.012143	0.45088	insignificant	4	12	12
chr5	145964427	145966427	Zkscan5	-0.15871643	0.00022419	hypomethylated	-0.034618	0.16495	insignificant	12	42	44
chr5	145991583	145993583	Zfp655	-0.08378163	1.2E-19	hypomethylated	0.0088438	0.45256	insignificant	35	131	124
chr5	145991627	145993627	Zfp655	-0.08378163	1.2E-19	hypomethylated	0.0088438	0.45256	insignificant	35	131	124
chr5	146043211	146045211	Zfp498	0.0364701	0.50074	insignificant	-0.076655	0.0027614	hypomethylated	9	32	30
chr5	147033013	147035013	Rnf6	-0.1224295	8.05E-16	hypomethylated	0.0039093	0.85514	insignificant	11	36	36
chr5	147042250	147044250	Cdk8	-0.11351249	7.16E-23	hypomethylated	0.011101	0.47568	insignificant	36	154	135
chr5	147195581	147197581	Wasf3	-0.18535203	1.18E-17	hypomethylated	0.0063184	0.26126	insignificant	27	80	69
chr5	147395980	147397980	Gpr12	-0.09064246	0.014792	hypomethylated	0.079585	0.62076	insignificant	19	88	87
chr5	147606532	147608532	Usp12	-0.13053375	0.00000989	hypomethylated	0.016224	0.43053	insignificant	7	42	42
chr5	147643465	147645465	Rpl21	-0.19142856	2.68E-11	hypomethylated	0.014728	0.33525	insignificant	13	115	115
chr5	147655646	147657646	Rasl11a	-0.10963933	0.00000183	hypomethylated	-0.012914	0.4497	insignificant	19	85	85
chr5	147759232	147761232	Gtf3a	-0.23566919	0.000002888	hypomethylated	0.051972	0.65323	insignificant	10	52	58
chr5	147775350	147777350	Mtif3	-0.63611111	1.32E-12	stronglyHypometh	0.056097	0.63823	insignificant	1	8	8
chr5	147888116	147890116	Polr1d	-0.1328466	2.53E-16	hypomethylated	0.003614	0.63604	insignificant	25	90	90
chr5	147888143	147890143	Polr1d	-0.13563393	7.63E-17	hypomethylated	0.0037354	0.60085	insignificant	25	88	88
chr5	147888148	147890148	Lnx2	-0.13563393	7.63E-17	hypomethylated	0.0037354	0.60085	insignificant	25	88	88
chr5	147999271	148001271	Gsx1	-0.11928067	4.14E-43	hypomethylated	-0.0016077	0.12761	insignificant	54	159	153
chr5	148080706	148082706	Pdx1	-0.15363216	7.52E-25	hypomethylated	0.014414	0.89024	insignificant	17	69	66
chr5	148118825	148120825	Cdx2		1	noCoverage	0.021724	0.783	insignificant	0	4	4
chr5	148134016	148136016	Prhoxnb	-0.69455004	1.79E-13	stronglyHypometh	0.065011	1	insignificant	2	13	9
chr5	148212065	148214065	Flt3	-0.22167207	1.54E-10	hypomethylated	0.0018338	0.55295	insignificant	8	27	27
chr5	148241155	148243155	Pan3	-0.10576213	2.37E-27	hypomethylated	-0.008384	0.13821	insignificant	56	234	246
chr5	148537564	148539564	Flt1	-0.13791837	9.29E-31	hypomethylated	0.0010944	0.027169	hypermethylated	27	106	106
chr5	148671203	148673203	Pomp	-0.21595219	3.08E-18	hypomethylated	0.020218	0.68741	insignificant	13	76	74
chr5	148706378	148708378	Slc46a3	-0.11460306	0.00039063	hypomethylated	0.01952	0.8331	insignificant	9	45	49
chr5	148767895	148769895	Mtus2	-0.09720902	3.2E-29	hypomethylated	0.023848	0.084193	insignificant	44	122	112
chr5	149211480	149213480	Slc7a1	-0.18782769	0.00066531	hypomethylated	0.085909	0.71814	insignificant	5	24	27
chr5	149364364	149366364	Ubl3		1	noCoverage	-0.11905	1	insignificant	0	7	6
chr5	149552415	149554415	2210417A02R	0.09568798	1	insignificant	-0.0067765	0.27679	insignificant	2	12	14
chr5	149740223	149742223	Katnal1	-0.28115648	6.9E-36	hypomethylated	-0.010602	0.00061043	hypomethylated	21	99	94
chr5	149864613	149866613	Hmgb1	-0.13671171	0.0024351	hypomethylated	0.012059	0.76249	insignificant	16	36	32
chr5	149995135	149997135	Uspl1	-0.2084541	2.9E-10	hypomethylated	-0.011359	0.31013	insignificant	3	24	24
chr5	149995639	149997639	Uspl1	-0.24726923	1.77E-12	hypomethylated	0.0077405	0.054149	insignificant	3	30	30
chr5	150213380	150215380	6330406I15Ril	-0.17631392	0.017162	hypomethylated	0.00088874	0.57376	insignificant	3	16	16
chr5	150241280	150243280	4930588N13R	-0.11887203	0.16559	insignificant	-0.035276	0.61693	insignificant	3	24	24
chr5	150330253	150332253	Wdr95		1	noCoverage	0.067187	0.25676	insignificant	0	8	8
chr5	150438890	150440890	Hsph1	-0.25338034	2.25E-18	hypomethylated	-0.014243	0.56562	insignificant	18	57	53
chr5	150479831	150481831	B3galtl	-0.11682645	3.93E-25	hypomethylated	0.031849	0.032844	hypermethylated	31	90	83
chr5	150820249	150822249	Rxfp2	-0.82142857	0.065789	insignificant	-0.2022	0.51722	insignificant	1	7	7
chr5	151061504	151063504	Fry	0.02819581	0.84864	insignificant	-0.018611	0.075011	insignificant	6	35	35
chr5	151324197	151326197	Brca2	-0.07693776	1.01E-10	hypomethylated	-0.017536	0.53233	insignificant	23	52	45
chr5	151324204	151326204	Brca2	-0.07693776	1.01E-10	hypomethylated	-0.017536	0.53233	insignificant	23	52	45
chr5	151397100	151399100	N4bp2l1	-0.2435302	2.8E-19	hypomethylated	0.02498	0.049534	hypermethylated	18	48	48
chr5	151468187	151470187	N4bp2l2	-0.32124914	1	insignificant	-0.031362	0.11839	insignificant	4	10	10
chr5	151475401	151477401	Pds5b	-0.08718029	1.48E-11	hypomethylated	-0.001076	0.89803	insignificant	47	160	174
chr5	151754181	151756181	Kl	-0.13661022	0.0000236	hypomethylated	0.0041592	0.44932	insignificant	15	72	71
chr5	151992768	151994768	Stard13		1	noCoverage	-0.026179	0.51147	insignificant	0	18	18
chr5	152453783	152455783	Rfc3	-0.20316318	6.04E-13	hypomethylated	-0.0094409	0.58878	insignificant	11	22	22
chr6	3237518	3239518	Gm8579		1	noCoverage	0.051177	0.10353	insignificant	0	16	16
chr6	3447392	3449392	Ccdc132	-0.17895379	0.0014542	hypomethylated	0.048622	0.51222	insignificant	8	36	36
chr6	3713623	3715623	Calcr	-0.19174236	0.000016818	hypomethylated	0.033322	0.88576	insignificant	12	50	51
chr6	3714713	3716713	Calcr		1	noCoverage	0.11478	0.57239	insignificant	0	6	4
chr6	3952986	3954986	Gng11	0.20902055	0.46886	insignificant	-0.0076606	0.33146	insignificant	4	36	36
chr6	4036927	4038927	Bet1		1	noCoverage	0.025327	0.40564	insignificant	0	24	24
chr6	4454696	4456696	Col1a2	-0.2609459	0.00000272	hypomethylated	0.037946	0.23807	insignificant	6	22	22
chr6	4550065	4552065	Casd1	-0.13069199	8.38E-29	hypomethylated	-0.0025549	0.05267	insignificant	38	92	103
chr6	4696305	4698305	Peg10	0.18646903	0.024736	hypermethylated	0.017187	0.095288	insignificant	5	30	30
chr6	4697204	4699204	Sgce	0.18106391	0.035002	hypermethylated	0.13825	0.0015358	hypermethylated	5	18	18
chr6	4852319	4854319	Ppp1r9a	-0.10564839	9.86E-18	hypomethylated	-0.0013517	0.5964	insignificant	30	137	150
chr6	5248373	5250373	Pon2	-0.31747298	6.63E-18	hypomethylated	-0.10895	0.00037928	hypomethylated	7	21	19
chr6	5332385	5334385	Asb4	-0.14130767	0.033883	hypomethylated	0.074484	0.000083975	hypermethylated	7	48	47
chr6	5674638	5676638	Dync1i1		1	noCoverage	0.0037865	0.88272	insignificant	0	24	24
chr6	6167173	6169173	Slc25a13	-0.21322462	2.17E-10	hypomethylated	-0.016531	0.33147	insignificant	7	18	18
chr6	6812333	6814333	Dlx6	-0.0917414	6.94E-18	hypomethylated	0.0069678	0.34422	insignificant	22	106	104
chr6	6815150	6817150	Dlx6as2	-0.43154762	0.50512	insignificant	0.018239	0.73054	insignificant	2	8	5
chr6	6832068	6834068	Dlx5		1	noCoverage	0.21095	0.0034957	hypermethylated	0	8	9
chr6	6905017	6907017	Acn9	-0.12678327	0.0004547	hypomethylated	0.0044246	0.18828	insignificant	23	70	70
chr6	7504070	7506070	Tac1	-0.23151015	0.034699	hypomethylated	0.036415	0.029624	hypermethylated	2	38	38
chr6	7643182	7645182	Asns	0.36035112	0.5283	insignificant	0.011871	0.74853	insignificant	1	6	6
chr6	7794223	7796223	C1galt1	-0.102236	5.9E-27	hypomethylated	0.0020338	0.97072	insignificant	54	116	116
chr6	8158226	8160226	Mios	-0.12811507	1.41E-19	hypomethylated	0.0061201	0.70782	insignificant	33	135	134
chr6	8208287	8210287	Gm16039	-0.12547175	0.000000276	hypomethylated	-0.003631	0.90597	insignificant	23	69	69
chr6	8209141	8211141	Rpa3	-0.11460701	0.000000214	hypomethylated	-0.010528	0.75287	insignificant	23	55	55
chr6	8458595	8460595	Glcci1	-0.0816432	8.64E-38	hypomethylated	0.012176	0.19242	insignificant	70	243	241
chr6	8899018	8901018	Nxph1	-0.2231494	0.00000227	hypomethylated	-0.011869	0.91297	insignificant	13	40	34
chr6	11857446	11859446	Ndufa4	-0.10862326	9E-19	hypomethylated	-0.0079662	0.36239	insignificant	24	75	75

chr6	11874880	11876880	Phf14	-0.12474645	1.93E-13	hypomethylated	0.0084123	0.075476	insignificant	13	75	75
chr6	12699253	12701253	Thsd7a	-0.15116596	0.00000536	hypomethylated	0.022472	0.46365	insignificant	8	24	24
chr6	13018758	13020758	Tmem106b	-0.13970641	2.19E-20	hypomethylated	0.035189	0.68378	insignificant	25	79	75
chr6	13558063	13560063	Tmem168	-0.13966955	0.05515	insignificant	-0.015887	0.72603	insignificant	6	16	16
chr6	13627966	13629966	B630005N14R	-0.1123283	0.00044764	hypomethylated	-0.0051702	0.417	insignificant	4	24	24
chr6	13789848	13791848	Gpr85	0.28154762	1	insignificant	0.062783	0.38821	insignificant	1	16	19
chr6	14850348	14852348	Foxp2	-0.12731563	1.74E-09	hypomethylated	0.038434	0.39756	insignificant	28	87	90
chr6	15669660	15671660	Mdfic	-0.11891932	4.35E-11	hypomethylated	0.0030094	0.71404	insignificant	20	64	58
chr6	17014148	17016148	Tes	-0.11704072	7.66E-09	hypomethylated	0.0047199	0.50885	insignificant	12	41	41
chr6	17230340	17232340	Cav2	-0.13070889	0.00040208	hypomethylated	0.032645	0.7143	insignificant	6	42	38
chr6	17255369	17257369	Cav1	-0.20973224	3.1E-13	hypomethylated	0.03171	0.48606	insignificant	9	28	28
chr6	17412956	17414956	Met	-0.13500125	1.36E-14	hypomethylated	0.00518	0.25821	insignificant	24	101	97
chr6	17586097	17588097	Capza2	-0.11023667	1.2E-25	hypomethylated	-0.002741	0.93237	insignificant	39	118	113
chr6	17698215	17700215	St7	0.25298253	1	insignificant	0.018399	0.46014	insignificant	2	21	19
chr6	17980445	17982445	Wnt2	-0.17519227	0.000000565	hypomethylated	0.01765	0.40396	insignificant	20	81	71
chr6	18059061	18061061	Asz1	-0.29419076	0.00054054	hypomethylated	0.0090239	0.76849	insignificant	6	16	16
chr6	18119686	18121686	Cftr	-0.19340909	0.00024361	hypomethylated	-0.049436	0.28625	insignificant	3	20	16
chr6	18464825	18466825	Cttnbp2	-0.14038921	0.60671	insignificant	-0.0082581	0.23533	insignificant	4	44	44
chr6	18797634	18799634	Naa38	-0.15485661	2.64E-08	hypomethylated	0.018593	0.17349	insignificant	31	100	92
chr6	18815317	18817317	Ankrd7	0.15657852	1	lowCoverage	0.018574	0.54853	insignificant	1	14	16
chr6	21165108	21167108	Kcnd2	-0.26892622	1.58E-08	hypomethylated	0.038621	0.065501	insignificant	3	28	28
chr6	21802515	21804515	Tspan12	-0.28376688	0.0013486	hypomethylated	0.073791	0.074298	insignificant	3	8	8
chr6	21898614	21900614	Ing3	-0.10087534	0.0038024	hypomethylated	-0.00040673	0.56717	insignificant	15	65	65
chr6	21934909	21936909	A430107O13R	-0.22058595	0.000008378	hypomethylated	0.0053991	0.8277	insignificant	7	26	26
chr6	22306081	22308081	Fam3c	-0.05804985	0.00019261	hypomethylated	-0.000077065	0.57536	insignificant	20	72	72
chr6	22824501	22826501	Ptprz1	-0.10263008	7.83E-10	hypomethylated	0.0094498	0.33372	insignificant	19	74	64
chr6	23198264	23200264	Fezf1	-0.10200765	0.012244	hypomethylated	-0.0053217	0.85111	insignificant	21	67	70
chr6	23789300	23791300	Cadps2	-0.11274111	1.19E-22	hypomethylated	0.0078737	0.77515	insignificant	22	152	151
chr6	24465067	24467067	Iqub	0.34090909	1	insignificant	0.079545	0.74474	insignificant	1	4	4
chr6	24477143	24479143	Asb15	-0.22721417	3.22E-09	hypomethylated	0.0088193	0.84654	insignificant	5	10	10
chr6	24614995	24616995	Wasl	-0.14565458	8.68E-10	hypomethylated	0.0055824	0.005425	hypermethylated	22	71	74
chr6	24682244	24684244	Hyal6	-0.24255028	0.011134	hypomethylated	0.038988	0.24321	insignificant	4	10	10
chr6	24697366	24699366	Hyal4		1	noCoverage	-0.011628	1	insignificant	0	3	2
chr6	24906125	24908125	Tmem229a	-0.12356493	1.72E-08	hypomethylated	0.093577	0.3185	insignificant	11	36	26
chr6	25639980	25641980	Gpr37	-0.15466012	0.048066	hypomethylated	-0.004595	0.50878	insignificant	6	31	28
chr6	25759226	25761226	Pot1a		1	noCoverage	0.084989	0.5376	insignificant	0	16	18
chr6	27886750	27888750	Grm8		1	noCoverage	-0.0055556	1	insignificant	0	3	3
chr6	28084369	28086369	Grm8	-0.12889132	5.3E-09	hypomethylated	0.00089658	0.44639	insignificant	22	69	69
chr6	28211601	28213601	Zfp800	-0.12971067	4.19E-19	hypomethylated	0.042692	0.07402	insignificant	33	110	78
chr6	28371724	28373724	Arf5	-0.24083392	3.31E-20	hypomethylated	-0.03813	0.69976	insignificant	20	43	58
chr6	28372639	28374639	Arf5	-0.1867511	2.89E-18	hypomethylated	-0.0066781	0.85788	insignificant	27	79	94
chr6	28376900	28378900	Fscn3	-0.05969766	0.36832	insignificant	0.0028166	0.75622	insignificant	2	4	6
chr6	28399340	28401340	Pax4	-0.52708333	0.23265	insignificant	0.022093	0.7122	insignificant	1	4	4
chr6	28429347	28431347	Snd1	-0.1365097	8.39E-08	hypomethylated	0.01341	0.36882	insignificant	22	92	86
chr6	28781747	28783747	Snd1	-0.10276025	1.04E-19	hypomethylated	-0.0085277	0.87043	insignificant	31	84	76
chr6	29009220	29011220	Lep	0.15977653	1	insignificant	0.023673	0.35683	insignificant	5	28	26
chr6	29114724	29116724	Rbm28		1	noCoverage	-0.07973	0.17379	insignificant	0	20	22
chr6	29162271	29164271	Impdh1	-0.1299891	0.00001411	hypomethylated	0.00040222	0.19029	insignificant	18	51	50
chr6	29221487	29223487	Hilpda	-0.17457469	0.000001555	hypomethylated	0.028745	1	insignificant	12	38	38
chr6	29221625	29223625	Hilpda	-0.20608451	0.00000743	hypomethylated	0.069346	0.87323	insignificant	8	26	28
chr6	29268139	29270139	Fam71f1	-0.85473295	0.069885	insignificant	-0.1032	0.83048	insignificant	1	10	11
chr6	29297118	29299118	Calu	-0.14635763	4.3E-47	hypomethylated	0.0059706	0.60091	insignificant	36	140	139
chr6	29345635	29347635		-0.12211196	5.58E-08	hypomethylated	-0.0098347	0.00000651	inconclusive	33	108	104
chr6	29382152	29384152	Flnc	-0.09305262	1.17E-09	hypomethylated	0.0086255	0.077047	insignificant	19	91	95
chr6	29416782	29418782	Atp6v1f	-0.15817457	0.0020936	hypomethylated	0.00376	0.36853	insignificant	9	49	48
chr6	29457934	29459934	Kcp		1	noCoverage	-0.011929	0.7578	insignificant	0	0	0
chr6	29475732	29477732	Irf5	-0.14230677	4.27E-44	hypomethylated	0.0015819	0.59125	insignificant	42	166	161
chr6	29559607	29561607	Tnpo3	-0.40403726	8.85E-18	stronglyHypometh	-0.01637	0.63554	insignificant	13	56	66
chr6	29643255	29645255	Tspan33	-0.13056539	0.028939	hypomethylated	0.0084818	0.064207	insignificant	9	58	58
chr6	29684496	29686496	Smo	-0.09910858	1.82E-24	hypomethylated	0.0047761	0.32544	insignificant	42	154	135
chr6	29717442	29719442	Ahcyl2	-0.12641848	8.28E-17	hypomethylated	0.0021572	0.73044	insignificant	19	96	100
chr6	29808595	29810595	Ahcyl2	-0.54980519	0.00026811	stronglyHypometh	-0.053214	0.65824	insignificant	2	5	4
chr6	29866012	29868012	Fam40b	-0.12985631	1.64E-16	hypomethylated	-0.0088724	0.57498	insignificant	27	124	124
chr6	29996987	29998987	Nrf1	-0.10583022	5.17E-24	hypomethylated	0.015656	0.49201	insignificant	41	152	151
chr6	30115992	30117992	Mir182	-0.57189542	0.0089095	stronglyHypometh	0.032432	0.74747	insignificant	2	4	4
chr6	30254517	30256517	Gm2058	-0.14211896	0.00010007	inconclusive	-0.15942	0.062046	insignificant	6	20	32
chr6	30254539	30256539	Gm2058	-0.18629075	0.00057944	inconclusive	-0.18652	0.047191	hypomethylated	6	18	30
chr6	30341010	30343010	Zc3hc1	-0.56	0.33725	insignificant	-0.06761	0.56738	insignificant	0	0	0
chr6	30350908	30352908	Klhdc10	-0.16493964	1.46E-28	hypomethylated	0.019661	0.16581	insignificant	23	99	99
chr6	30459706	30461706	1700025E21Ri	0.01316366	0.21489	insignificant	0.10365	0.066416	insignificant	2	16	16
chr6	30461285	30463285	1700025E21Ri	0.02821633	0.37442	insignificant	0.077387	0.0017472	hypermethylated	3	18	18
chr6	30490641	30492641	Cpa2	0.27857143	1	insignificant	0.16039	1	insignificant	1	5	5
chr6	30517375	30519375	Cpa4	-0.09868453	0.1332	insignificant	-0.0095142	0.50614	insignificant	6	16	16
chr6	30643682	30645682	Tsga14	-0.08929602	1	insignificant	-0.0013721	0.015973	hypomethylated	3	20	21
chr6	30846760	30848760	Tsga13	-0.11878887	0.00000183	hypomethylated	0.004363	0.94965	insignificant	7	29	29
chr6	30908990	30910990	Klf14	-0.12269459	0.015447	hypomethylated	-0.011953	0.018363	hypomethylated	4	10	10
chr6	31347827	31349827	Mkln1	-0.09719754	2.01E-11	hypomethylated	0.008007	0.44597	insignificant	42	133	135
chr6	31513937	31515937	Podxl		1	noCoverage	-0.13759	0.4058	insignificant	0	48	40
chr6	32538192	32540192	Plxna4	-0.17844604	0.00070524	hypomethylated	0.040311	0.21342	insignificant	2	16	16
chr6	33010152	33012152	Chchd3	-0.40461842	0.000011147	stronglyHypometh	0.047001	0.74669	insignificant	5	37	36
chr6	33198149	33200149	Exoc4	-0.20472794	0.00013999	hypomethylated	0.043833	0.94905	insignificant	8	46	47
chr6	34267489	34269489	Akr1b3	-0.18136521	0.000032844	hypomethylated	-0.0074816	0.81624	insignificant	5	28	28
chr6	34303163	34305163	Akr1b8	-0.06146253	0.79117	insignificant	-0.088649	0.16109	insignificant	4	14	14
chr6	34333246	34335246	Akr1b10	-0.44849519	0.046839	stronglyHypometh	-0.087161	1	insignificant	3	10	11
chr6	34425355	34427355	Bpgm	-0.19112072	7.54E-13	hypomethylated	0.026313	0.29156	insignificant	13	55	50

chr6	34729431	34731431	Agbl3	-0.08143509	4.94E-09	hypomethylated	-0.007886	0.86955	insignificant	25	109	117
chr6	34828065	34830065	3110062M04R	-0.15545238	1.24E-09	hypomethylated	-0.014998	0.85339	insignificant	7	32	32
chr6	34860831	34862831	Wdr91	-0.40031425	6.51E-13	stronglyHypometh	-0.0014145	0.15867	insignificant	4	23	22
chr6	34869959	34871959	Stra8	0.15074829	1	lowCoverage	0.042791	0.0015352	hypermethylated	1	27	27
chr6	35083737	35085737	Cnot4	-0.18592664	1.76E-11	hypomethylated	-0.017797	0.95257	insignificant	18	46	46
chr6	35126615	35128615	Nup205	-0.07621413	1.85E-18	hypomethylated	0.0022235	0.83225	insignificant	48	166	161
chr6	35201698	35203698	1810058I24Ril	-0.19285915	0.0094271	hypomethylated	0.016486	1	insignificant	15	46	46
chr6	35201731	35203731	1810058I24Ril	-0.19285915	0.0094271	hypomethylated	0.016486	1	insignificant	15	46	46
chr6	35489888	35491888	Mtpn	-0.41251306	0.24433	insignificant	-0.14735	0.17187	insignificant	3	10	8
chr6	36338234	36340234	Chrm2	-0.24491366	6.78E-13	hypomethylated	-0.014383	0.5395	insignificant	5	14	14
chr6	36370741	36372741	Mir490	-0.1485576	0.00000135	hypomethylated	-0.046702	0.20216	insignificant	7	12	16
chr6	36472169	36474169	Chrm2	0.04529617	0.76887	insignificant	0.088112	0.014794	hypermethylated	2	6	6
chr6	37249976	37251976	Dgki	-0.14639875	1.46E-08	hypomethylated	0.056253	0.82969	insignificant	9	30	26
chr6	37392148	37394148	Creb3l2	-0.30328821	0.35671	insignificant	0.018244	0.0073055	hypermethylated	7	31	31
chr6	37819810	37821810	Trim24	-0.09926279	1.87E-34	hypomethylated	0.006804	0.43347	insignificant	52	189	183
chr6	38204009	38206009	D630045J12Ri	-0.08874612	0.00000842	hypomethylated	0.00099687	0.66258	insignificant	15	32	32
chr6	38249259	38251259	Zc3hav1l	-0.52	2.44E-09	stronglyHypometh	0.078855	0.28609	insignificant	2	4	4
chr6	38304603	38306603	Zc3hav1		1	noCoverage	-0.067829	0.66604	insignificant	0	14	13
chr6	38382924	38384924	Ubn2	-0.0818227	2.04E-30	hypomethylated	-0.0055671	0.9801	insignificant	68	244	244
chr6	38483860	38485860	1110001J03Ri	-0.1752666	1.63E-09	hypomethylated	0.026222	0.29683	insignificant	8	37	37
chr6	38500443	38502443	Luc7l2	-0.10403906	0.000026165	hypomethylated	0.031428	0.42209	insignificant	11	54	43
chr6	38587239	38589239	Klrg2		1	noCoverage	-0.059924	0.038104	hypomethylated	0	14	15
chr6	38612068	38614068	Clec2l	-0.10454644	1.1E-33	hypomethylated	-0.016428	0.9379	insignificant	43	84	93
chr6	38787223	38789223	Hipk2		1	noCoverage	-0.025592	0.33862	insignificant	0	10	10
chr6	38826189	38828189	Hipk2	-0.0744226	0.00046814	hypomethylated	-0.0054342	0.035876	hypomethylated	24	101	109
chr6	39068348	39070348	Parp12	-0.16948361	0.61738	insignificant	-0.063752	0.059212	insignificant	15	89	83
chr6	39156772	39158772	Jhdm1d	-0.07174731	0.000067069	hypomethylated	-0.017135	0.40706	insignificant	17	83	78
chr6	39327706	39329706	Slc37a3	-0.45069178	0.01109	stronglyHypometh	0.33392	0.01786	stronglyhypermeth	0	10	6
chr6	39330426	39332426	Rab19		1	noCoverage	0.015326	0.84798	insignificant	0	8	13
chr6	39370368	39372368	Mkrn1	0.48198273	0.34122	lowCoverage	0.012296	0.59123	insignificant	1	21	21
chr6	39507833	39509833	Dennd2a	-0.19861188	0.036813	hypomethylated	-0.0060349	0.72515	insignificant	7	49	45
chr6	39522874	39524874	Adck2	-0.20675352	6.95E-13	hypomethylated	-0.0075813	0.63378	insignificant	18	59	59
chr6	39541581	39543581	Ndufb2	0.07310413	1.35E-29	hypermethylated	0.0068182	4.11E-13	hypermethylated	29	94	94
chr6	39675462	39677462	Braf		1	noCoverage	-0.04501	0.13506	insignificant	0	19	20
chr6	39760935	39762935	Mrps33	-0.10452444	0.00022605	hypomethylated	0.02446	0.73032	insignificant	8	34	37
chr6	40059251	40061251	Gm5567	-0.44862657	0.00000902	stronglyHypometh	-0.082993	0.85292	insignificant	3	10	9
chr6	40274476	40276476	Agk	-0.0648421	2.76E-10	hypomethylated	0.044719	0.46996	insignificant	20	52	52
chr6	40386132	40388132	E330009J07Ril	-0.12421637	2.57E-12	hypomethylated	0.0046999	0.35087	insignificant	15	34	30
chr6	40420413	40422413	Ssbp1	-0.22796721	1.81E-14	hypomethylated	-0.023145	0.52766	insignificant	13	48	48
chr6	40520386	40522386	Olfr460		1	noCoverage	-0.07554	0.53465	insignificant	0	12	10
chr6	41554480	41556480	Ephb6	0.05300454	0.0021713	inconclusive	-0.0076273	0.13122	insignificant	18	95	92
chr6	41796547	41798547	Pip		1	noCoverage	-0.21667	0.016329	hypomethylated	0	2	2
chr6	42164326	42166326	Tas2r144	0.25365854	1	insignificant	0.083962	0.56453	insignificant	1	5	4
chr6	42194933	42196933	Gstk1	-0.15028119	3.12E-13	hypomethylated	-0.018072	0.69483	insignificant	12	45	45
chr6	42210968	42212968	Tmem139		1	noCoverage	0.097082	0.45979	insignificant	0	6	8
chr6	42214037	42216037	Casp2	-0.10172397	0.000029587	hypomethylated	0.0099366	1	insignificant	5	55	47
chr6	42274639	42276639	Fam131b	-0.11199749	6.79E-13	hypomethylated	0.016604	0.57352	insignificant	22	83	83
chr6	42298826	42300826	Zyx	-0.1099852	8.26E-16	hypomethylated	0.0026057	0.88144	insignificant	41	178	178
chr6	42323267	42325267	2010310C07Ri	-0.33097178	0.059893	insignificant	-0.078879	1	insignificant	4	15	14
chr6	42354527	42356527	Tas2r135	0.00039642	0.7683	insignificant	0.10605	0.37008	insignificant	1	6	6
chr6	42595040	42597040	Fam115c	-0.33231365	9.95E-13	hypomethylated	0.022902	0.82861	insignificant	4	24	25
chr6	42643058	42645058	Fam115a		1	noCoverage	0.0045802	0.9118	insignificant	0	8	9
chr6	43122986	43124986	Olfr13		1	noCoverage	0.0089686	0.5047	insignificant	0	6	6
chr6	43165913	43167913	Olfr434	-0.25252525	0.30459	insignificant	0.061277	0.040147	hypermethylated	1	5	4
chr6	43184608	43186608	Olfr47		1	noCoverage	0.11719	0.0053893	hypermethylated	0	6	6
chr6	43214642	43216642	Arhgef5	-0.23930612	9.96E-11	hypomethylated	-0.012512	0.4057	insignificant	24	56	56
chr6	43259553	43261553	Nobox	-0.0162963	1	insignificant	0.019352	0.74952	insignificant	2	12	12
chr6	43616174	43618174	Tpk1	-0.06974048	0.00049995	hypomethylated	0.052408	0.4685	insignificant	8	38	40
chr6	47403322	47405322	Cul1	-0.09542197	5.43E-34	hypomethylated	0.011605	0.0075644	hypermethylated	79	261	256
chr6	47545029	47547029	Ezh2	-0.15546739	0.000000124	hypomethylated	-0.016566	0.76068	insignificant	8	40	45
chr6	47618090	47620090	Rn4.5s		1	noCoverage	-0.010223	3.78E-10	hypomethylated	0	39	35
chr6	47635375	47637375	Rn4.5s		1	noCoverage	0.031751	0.2784	insignificant	0	23	3
chr6	47702924	47704924	Rn4.5s		1	noCoverage	-0.017434	0.053063	insignificant	0	41	18
chr6	47780504	47782504	Zfp786	-0.12875817	0.012579	hypomethylated	0.088414	0.84399	insignificant	5	10	11
chr6	47784659	47786659	Zfp398	-0.09369209	2.85E-20	hypomethylated	-0.003835	0.11415	insignificant	28	123	116
chr6	47826553	47828553	Zfp282	-0.10142918	6.42E-13	hypomethylated	-0.0025868	0.80535	insignificant	35	128	124
chr6	47869566	47871566	Zfp212	-0.10757807	0.00015672	hypomethylated	0.002129	0.31939	insignificant	21	63	68
chr6	47892173	47894173	Zfp783	-0.11487801	0.000005352	hypomethylated	-0.046576	0.95392	insignificant	21	110	100
chr6	47902388	47904388	Zfp956	-0.18599637	0.00000165	hypomethylated	0.005691	0.84732	insignificant	7	34	34
chr6	47998113	48000113	Zfp777	-0.09319447	6.57E-44	hypomethylated	-0.0033649	0.17156	insignificant	68	217	192
chr6	48036592	48038592	Zfp746		1	noCoverage	-0.0066616	0.49308	insignificant	0	31	31
chr6	48344584	48346584	Krba1	-0.13518522	5.37E-10	hypomethylated	0.018168	0.73555	insignificant	11	65	77
chr6	48395089	48397089	Zfp467	-0.12403596	0.000060856	hypomethylated	0.015433	1	insignificant	10	58	59
chr6	48395824	48397824	Zfp467	0.09796544	0.62074	insignificant	0.020973	0.35829	insignificant	4	8	8
chr6	48397227	48399227	Sspo	0.22709734	0.37278	insignificant	0.033945	0.68724	insignificant	2	9	9
chr6	48453337	48455337	Zfp862	-0.19549883	0.0000068	hypomethylated	-0.00034008	0.38895	insignificant	6	22	22
chr6	48503797	48505797	Lrrc61	-0.11980305	1	insignificant	0.044457	0.6904	insignificant	2	20	14
chr6	48522669	48524669	Rarres2		1	noCoverage	-0.17614	0.72752	insignificant	0	4	2
chr6	48538443	48540443	Gm5111		1	noCoverage	-0.029605	0.69724	insignificant	0	6	6
chr6	48542881	48544881	Repin1	-0.13718734	5.82E-14	hypomethylated	0.0046132	0.15081	insignificant	22	84	84
chr6	48577165	48579165	AI854703	-0.13836144	2.59E-14	hypomethylated	-0.018194	0.22633	insignificant	14	28	28
chr6	48688045	48690045	Gimap1	-0.22772234	0.062902	insignificant	0.037682	0.67987	insignificant	3	16	16
chr6	48695195	48697195	Gimap5	-0.39831691	1.6E-18	stronglyHypometh	0.13678	0.29387	insignificant	2	5	8
chr6	49022793	49024793	2410003K15Ri	-0.09914235	0.0059283	hypomethylated	-0.0073121	1	insignificant	10	77	80
chr6	49164953	49166953	Igf2bp3	-0.11896671	0.47254	insignificant	0.035987	0.75964	insignificant	21	86	85

chr6	49214051	49216051	Tra2a	-0.17456426	4.24E-18	hypomethylated	0.045906	0.0019635	inconclusive	14	46	47
chr6	49268351	49270351	Ccdc126	-0.13145045	1	insignificant	0.011094	0.92945	insignificant	5	44	44
chr6	49316737	49318737	D330028D13R	-0.14831546	6.92E-08	hypomethylated	0.02813	0.84471	insignificant	5	49	63
chr6	49344602	49346602	Stk31	-0.92098315	0.0066825	stronglyHypometh	-0.0046605	0.49893	insignificant	2	22	22
chr6	49771727	49773727	Npy	-0.19000364	0.00000893	hypomethylated	-0.041411	0.41775	insignificant	17	97	93
chr6	50059239	50061239	Mpp6	-0.10364797	1.28E-32	hypomethylated	-0.0012992	0.92082	insignificant	66	174	181
chr6	50211768	50213768	Dfna5	0.02806628	0.80346	insignificant	0.003555	0.61079	insignificant	7	32	30
chr6	50406169	50408169	Osbpl3	-0.35785216	0.011467	stronglyHypometh	0.022458	0.0060416	hypermethylated	7	24	24
chr6	50515641	50517641	5430402O13R	-0.23539337	6.84E-12	hypomethylated	-0.032492	0.90375	insignificant	11	50	49
chr6	50516473	50518473	Cycs	-0.25462963	0.000063695	hypomethylated	-0.070316	0.88085	insignificant	5	18	17
chr6	50546589	50548589	4921507P07Ri	-0.16433098	1	insignificant	-0.029705	0.77142	insignificant	2	29	28
chr6	51219909	51221909	Mir148a	-0.12749593	1.32E-26	hypomethylated	0.0029031	0.5293	insignificant	29	134	134
chr6	51381668	51383668	Nfe2l3	-0.12937192	0.024159	hypomethylated	0.0061284	1	insignificant	16	47	47
chr6	51419614	51421614	Cbx3	-0.12429477	6.2E-17	hypomethylated	-0.02571	0.20203	insignificant	49	189	203
chr6	51419893	51421893	Cbx3	-0.12301293	1.02E-16	hypomethylated	-0.024358	0.22406	insignificant	49	179	193
chr6	51472901	51474901	Snx10	-0.20562861	0.00055397	hypomethylated	-0.042414	0.70686	insignificant	10	26	26
chr6	51962548	51964548	Skap2	-0.18962019	7.82E-13	hypomethylated	-0.039534	0.44349	insignificant	14	41	42
chr6	52108316	52110316	Hoxa1	-0.22099346	1.11E-18	hypomethylated	-0.017995	0.16433	insignificant	12	52	50
chr6	52114830	52116830	Hoxa2	-0.01916854	0.54657	insignificant	0.071702	0.000000475	hypermethylated	9	20	18
chr6	52141702	52143702	Hoxa3	-0.16418287	0.000015775	hypomethylated	-0.0084834	0.021935	hypomethylated	14	56	68
chr6	52150122	52152122	2700086A05R	-0.13371911	0.0061296	hypomethylated	-0.13639	1	insignificant	6	28	24
chr6	52154586	52156586	Hoxa5	-0.07137291	0.000095949	hypomethylated	-0.052025	0.00000256	hypomethylated	14	56	56
chr6	52158623	52160623	Hoxa3	-0.11612101	6.04E-10	hypomethylated	0.023641	0.30997	insignificant	27	66	56
chr6	52168572	52170572	Hoxa7	-0.01176471	0.50086	insignificant	0.051507	0.86017	insignificant	1	4	4
chr6	52177369	52179369	Hoxa9	-0.2522256	0.32593	insignificant	-0.03684	0.10676	insignificant	3	39	38
chr6	52180164	52182164	Mir196b	-0.15852162	1.05E-12	hypomethylated	0.010593	0.1248	insignificant	12	48	55
chr6	52194241	52196241	Hoxa11as	-0.11690444	4.42E-13	hypomethylated	0.034945	0.15626	insignificant	32	142	150
chr6	52195766	52197766	Hoxa11	-0.17170782	1.12E-09	hypomethylated	0.046236	0.05101	insignificant	21	82	77
chr6	52210874	52212874	Hoxa13	-0.14480052	0.10266	insignificant	0.00055925	0.35925	insignificant	6	33	35
chr6	52262491	52264491	Evx1	-0.2063257	0.0016492	hypomethylated	0.017003	0.97307	insignificant	13	66	66
chr6	52590294	52592294	Hibadh	-0.09691639	1.31E-08	hypomethylated	0.0037381	0.63394	insignificant	11	38	38
chr6	52662722	52664722	Tax1bp1	-0.09176756	3.02E-14	hypomethylated	-0.0021426	0.15332	insignificant	35	118	119
chr6	53018618	53020618	Jazf1	-0.32927802	3.79E-19	hypomethylated	0.019864	0.86578	insignificant	9	32	27
chr6	53236288	53238288	9430076C15Ri	-0.13147529	7E-13	hypomethylated	0.0045116	0.98033	insignificant	36	163	153
chr6	53522367	53524367	Creb5	-0.22222244	0.53086	insignificant	0.11929	0.84836	insignificant	2	10	10
chr6	53770819	53772819	Tril	-0.20588779	6.97E-26	hypomethylated	0.013437	0.39578	insignificant	24	68	70
chr6	53988925	53990925	Chn2	-0.08841316	1.55E-14	hypomethylated	0.018173	0.08301	insignificant	42	134	135
chr6	54221815	54223815	Chn2		1	noCoverage	0.0062092	0.062083	insignificant	0	5	4
chr6	54276005	54278005	Prr15	-0.33512052	0.0057097	stronglyHypometh	0.017672	0.099422	insignificant	4	31	36
chr6	54401876	54403876	Wipf3	0.05380427	1	insignificant	0.052856	0.12565	insignificant	5	14	14
chr6	54516376	54518376	Scrn1	-0.20345411	5.62E-12	hypomethylated	0.022353	0.71031	insignificant	6	16	16
chr6	54543122	54545122	Fkbp14	-0.11927717	0.00000587	hypomethylated	-0.024227	0.94176	insignificant	10	26	22
chr6	54544104	54546104	Plekha8	-0.1079273	1.36E-18	hypomethylated	-0.014729	0.18483	insignificant	29	100	96
chr6	54630765	54632765	2410066E13Ri	-0.12863643	5.88E-09	hypomethylated	0.0037133	0.059998	insignificant	33	100	100
chr6	54765909	54767909	Znrf2	-0.08359391	1E-24	hypomethylated	0.0028003	0.51584	insignificant	63	208	206
chr6	54921655	54923655	Nod1	-0.14018642	0.00000583	hypomethylated	-0.015577	0.83365	insignificant	14	59	57
chr6	54922606	54924606	Nod1		1	noCoverage	0.021474	1	insignificant	0	5	5
chr6	54942861	54944861	Ggct	-0.16277441	1	insignificant	-0.024722	0.66279	insignificant	4	28	28
chr6	54986994	54988994	Gars	-0.07818662	0.0014869	hypomethylated	0.0034828	0.79082	insignificant	14	85	84
chr6	55285292	55287292	Aqp1		1	noCoverage	-0.0097909	0.0056658	hypomethylated	0	14	14
chr6	55400973	55402973	Adcyap1r1	-0.14222226	0.00000296	hypomethylated	0.063485	0.52074	insignificant	10	45	40
chr6	55966508	55968508	Ppp1r17		1	noCoverage	-0.060648	1	insignificant	0	6	4
chr6	56319628	56321628	Pde1c		1	noCoverage	0.13863	1	insignificant	0	6	4
chr6	56654693	56656693	Lsm5	-0.11165971	0.23471	insignificant	0.010792	0.34509	insignificant	5	18	18
chr6	56663898	56665898	Avl9	-0.15469076	8.56E-29	hypomethylated	0.014325	0.018963	hypermethylated	36	136	128
chr6	56747807	56749807	Kbtbd2	-0.22857637	0.0072951	hypomethylated	0.058821	0.46529	insignificant	3	20	12
chr6	56781052	56783052	Fkbp9	-0.14722695	4.08E-27	hypomethylated	0.047942	0.47848	insignificant	22	32	34
chr6	56873926	56875926	Nt5c3	0.19311394	0.062462	insignificant	0.036638	0.33558	insignificant	8	36	36
chr6	57485420	57487420	Ppm1k	-0.08853027	0.16958	insignificant	-0.030977	0.38338	insignificant	5	26	26
chr6	57642072	57644072	Pigy	-0.27505162	0.00000109	hypomethylated	0.097797	0.45197	insignificant	7	22	22
chr6	57651448	57653448	Lancl2	-0.09174092	0.000000835	hypomethylated	-0.00014815	0.17285	insignificant	13	57	57
chr6	57775119	57777119	Vopp1		1	noCoverage	0.0125	0.86367	insignificant	0	14	14
chr6	59157863	59159863	Tigd2	-0.11126716	2.81E-25	hypomethylated	0.029775	0.081405	insignificant	32	99	99
chr6	60778990	60780990	Snca		1	noCoverage	-0.043163	0.712	insignificant	0	13	12
chr6	61129318	61131318	Fam190a	-0.07836035	1.58E-23	hypomethylated	-0.007884	0.97536	insignificant	38	146	152
chr6	63205850	63207850	Grid2	-0.08615935	7.55E-08	hypomethylated	0.0072322	0.12073	insignificant	35	156	142
chr6	64678139	64680139	Atoh1	-0.15559947	5.1E-20	hypomethylated	0.00017296	0.70511	insignificant	25	81	94
chr6	64991660	64993660	Smarcad1	-0.07587192	0.0055581	hypomethylated	-0.0071871	0.091989	insignificant	34	121	112
chr6	65620604	65622604	A930038C07R	-0.153577	2.8E-15	hypomethylated	0.0099356	0.0543	insignificant	28	111	111
chr6	65727955	65729955	Prdm5	-0.15826136	7.84E-12	hypomethylated	-0.0020657	0.14187	insignificant	18	60	60
chr6	65901564	65903564	4930544G11R	-0.0987061	0.000000145	hypomethylated	-0.011491	0.37099	insignificant	6	30	30
chr6	66484461	66486461	Mad2l1	-0.10490523	0.011368	hypomethylated	-0.012321	0.014134	hypomethylated	21	75	74
chr6	66666883	66668883	Vmn1r36	-0.81944444	0.2093	lowCoverage	0.041667	0.76168	insignificant	1	4	2
chr6	66845390	66847390	Gng12	-0.08639278	3.91E-14	hypomethylated	0.0038411	0.30895	insignificant	34	126	126
chr6	66845884	66847884	Gng12	-0.10063113	4.08E-14	hypomethylated	-0.0014273	0.29521	insignificant	34	128	128
chr6	66987401	66989401	E230016M11F	-0.37526284	0.049791	stronglyHypometh	0.024864	0.59708	insignificant	2	27	28
chr6	67215972	67217972	Serbp1	-0.12221895	8.71E-18	hypomethylated	0.014847	0.36068	insignificant	26	139	129
chr6	67485816	67487816	Tacstd2	-0.32794118	0.56872	insignificant	-0.088879	0.71136	insignificant	4	8	8
chr6	69277511	69279511	Rprl1		1	noCoverage	-0.14301	0.06931	insignificant	0	17	16
chr6	70742169	70744169	Rpia	-0.28736552	9.13E-11	hypomethylated	0.00934	0.044216	hypermethylated	10	38	38
chr6	70793520	70795520	Eif2ak3	-0.16951764	0.59748	insignificant	-0.023098	0.36697	insignificant	3	117	109
chr6	70905599	70907599	Foxi3	-0.1744306	6.41E-37	hypomethylated	0.021656	0.27176	insignificant	36	129	116
chr6	71094370	71096370	Thnsl2	0.35663559	0.00028862	stronglyHypermeth	-0.1451	0.92964	insignificant	2	13	16
chr6	71148881	71150881	Fabp1		1	noCoverage	0.12576	0.20675	insignificant	0	6	8
chr6	71212275	71214275	Smyd1		1	noCoverage	0	0.34823	insignificant	0	4	4

chr6	71221012	71223012	Krcc1	-0.15332492	0.00021914	hypomethylated	-0.029445	0.028044	hypomethylated	13	70	81
chr6	71271805	71273805	Cd8b1	-0.05584601	1	insignificant	0.09759	0.00000228	hypermethylated	2	14	14
chr6	71442887	71444887	Rnf103	-0.14768364	6.06E-32	hypomethylated	0.015949	0.78421	insignificant	23	145	145
chr6	71492847	71494847	Chmp3	-0.11993851	7.51E-12	hypomethylated	0.038138	0.88801	insignificant	25	59	62
chr6	71582680	71584680	Kdm3a	-0.10188793	1	insignificant	-0.016389	0.66167	insignificant	6	59	59
chr6	71582899	71584899	Kdm3a	-0.09637284	1	insignificant	-0.018595	0.65427	insignificant	6	52	52
chr6	71656853	71658853	Reep1	-0.10810747	7.93E-26	hypomethylated	0.029609	0.85866	insignificant	26	79	78
chr6	71780324	71782324	Immt	-0.10546459	4.47E-08	hypomethylated	0.00021603	0.43083	insignificant	26	122	118
chr6	71858046	71860046	Polr1a	-0.276417	0.030043	hypomethylated	-0.00923	0.24811	insignificant	4	37	48
chr6	71858756	71860756	Ptcd3	-0.27961906	0.030088	hypomethylated	0.016005	0.16672	insignificant	4	32	38
chr6	72046606	72048606	St3gal5	-0.11796556	0.000000994	hypomethylated	-0.02867	0.71843	insignificant	14	68	67
chr6	72185571	72187571	Atoh8		1	noCoverage	-0.014777	0.2689	insignificant	0	8	8
chr6	72295169	72297169	Usp39		1	noCoverage	-0.029634	0.70188	insignificant	0	29	28
chr6	72296310	72298310	0610030E20Ri	-0.11848412	3.71E-12	hypomethylated	0.0058582	0.28361	insignificant	18	77	76
chr6	72304476	72306476	Tmem150a	-0.12227361	0.000014486	hypomethylated	-0.0078069	0.23143	insignificant	30	104	102
chr6	72312375	72314375	Rnf181	-0.05263835	0.3481	insignificant	0.051504	0.37742	insignificant	3	19	21
chr6	72330462	72332462	Vamp5	-0.41368329	2.49E-08	stronglyHypometh	-0.081294	0.0019287	inconclusive	8	41	36
chr6	72340661	72342661	Vamp8		1	noCoverage	-0.011562	0.70994	insignificant	0	12	11
chr6	72363326	72365326	Ggcx	-0.28219195	0.000000666	hypomethylated	-0.077733	0.32048	insignificant	3	36	26
chr6	72389552	72391552	Mat2a	-0.09633766	0.00029194	hypomethylated	-0.0017169	0.83599	insignificant	19	122	122
chr6	72493432	72495432	Capg		1	noCoverage	-0.02413	0.000083723	inconclusive	0	16	27
chr6	72498442	72500442	Capg	0.24622101	0.00023772	hypermethylated	0.08845	0.75668	insignificant	2	16	16
chr6	72566742	72568742	Tgoln1	-0.04634448	6.7E-14	hypomethylated	0.040723	0.043216	hypermethylated	10	39	36
chr6	72566994	72568994	Tgoln1	-0.05724953	6.55E-08	hypomethylated	0.06728	0.00000729	hypermethylated	9	21	18
chr6	72738950	72740950	Tcf7l1	-0.11524643	4.23E-14	hypomethylated	-0.0064891	0.50553	insignificant	25	81	76
chr6	72849973	72851973	Kcmf1	-0.11023388	0.10541	insignificant	-0.0037313	0.038877	inconclusive	24	100	100
chr6	72908226	72910226	Tmsb10	-0.1363909	0.0010044	hypomethylated	-0.022302	0.37004	insignificant	4	18	18
chr6	72908477	72910477	Tmsb10	-0.25	0.092348	insignificant	0.11667	0.088799	insignificant	2	6	6
chr6	72908742	72910742	Tmsb10	-0.25	0.092348	insignificant	0.11667	0.088799	insignificant	2	6	6
chr6	73171625	73173625	Dnahc6	-0.22261905	0.58025	insignificant	0.0020597	0.28624	insignificant	2	12	14
chr6	73197498	73199498	Suclg1	-0.21501604	5.93E-20	hypomethylated	-0.016163	0.65885	insignificant	21	80	81
chr6	77191710	77193710	Lrrtm1	-0.1287744	3.27E-17	hypomethylated	0.029395	0.12813	insignificant	23	74	66
chr6	77929661	77931661	Ctnna2	-0.14015986	0.24795	insignificant	-0.060889	0.0078483	inconclusive	7	39	35
chr6	81872662	81874662	AW146020	-0.11612562	2.6E-17	hypomethylated	0.0093446	0.8466	insignificant	31	87	82
chr6	81915943	81917943	Mrpl19	-0.16666667	0.53579	insignificant	0.13333	0.86459	insignificant	2	6	4
chr6	81990621	81992621	Fam176a	0.01594607	0.0030544	inconclusive	0.011052	0.59343	insignificant	6	53	56
chr6	82351468	82353468	Tacr1	-0.226738	0.00000283	hypomethylated	-0.014492	0.40731	insignificant	18	58	58
chr6	82602859	82604859	Pole4	-0.16021464	0.000000298	hypomethylated	-0.041606	0.68699	insignificant	21	59	59
chr6	82724448	82726448	Hk2	-0.76096631	0.00000775	stronglyHypometh	0.0056007	0.44958	insignificant	2	11	10
chr6	82889744	82891744	Sema4f	-0.29824561	0.56385	insignificant	0.0083582	0.66293	insignificant	1	4	4
chr6	82983217	82985217	Loxl3	-0.19625979	0.000000101	hypomethylated	-0.0026715	0.80689	insignificant	11	54	54
chr6	82983465	82985465	Dok1	-0.24799054	0.000000175	hypomethylated	0.0008764	0.92228	insignificant	7	32	32
chr6	83003646	83005646	Aup1	-0.12482317	1.78E-45	hypomethylated	0.0042654	0.94183	insignificant	48	178	177
chr6	83004565	83006565	Aup1	-0.15044802	1.04E-30	hypomethylated	-0.014962	0.71509	insignificant	32	126	125
chr6	83020219	83022219	Tlx2	-0.13182196	1.95E-26	hypomethylated	0.01974	0.36165	insignificant	23	89	84
chr6	83027383	83029383	Pcgf1	-0.09124601	8.85E-08	hypomethylated	0.0042887	0.26372	insignificant	20	89	82
chr6	83035358	83037358	Lbx2	-0.2385401	0.000000666	hypomethylated	-0.028034	0.52394	insignificant	6	15	15
chr6	83050509	83052509	Ccdc142	-0.115986	1.63E-41	hypomethylated	-0.0056743	0.063123	insignificant	30	119	129
chr6	83058101	83060101	Mrpl53	-0.07993213	0.00000985	hypomethylated	0.027057	0.78517	insignificant	11	65	48
chr6	83064499	83066499	Mogs	-0.11657505	2.74E-33	hypomethylated	-0.0071405	0.55279	insignificant	41	133	130
chr6	83071455	83073455	Wbp1	-0.12690754	3.83E-29	hypomethylated	0.055227	0.38772	insignificant	22	79	61
chr6	83075023	83077023	Ino80b	-0.15546863	0.0087262	hypomethylated	0.042576	0.76935	insignificant	5	28	28
chr6	83084801	83086801	Rtkn	-0.23060698	1.93E-17	hypomethylated	0.032086	0.043718	hypermethylated	16	53	59
chr6	83086077	83088077	Rtkn	-0.27695106	0.1213	insignificant	-0.0032319	0.90583	insignificant	1	12	12
chr6	83105397	83107397	1700003E16Rik		1	noCoverage	0.027428	0.45805	insignificant	0	4	4
chr6	83106373	83108373	Wdr54		1	noCoverage	-0.015445	0.63618	insignificant	0	8	6
chr6	83114917	83116917	Dctn1	-0.0740689	1	insignificant	0.025333	0.56523	insignificant	5	49	51
chr6	83128582	83130582	Dctn1	-0.35352941	0.018096	stronglyHypometh	0.087516	0.0096741	hypermethylated	2	6	6
chr6	83267598	83269598	Mthfd2	-0.10779596	0.000084106	hypomethylated	0.0083773	0.89992	insignificant	5	10	10
chr6	83275032	83277032	Mob1a	-0.09755464	4.66E-18	hypomethylated	-0.0075991	0.45506	insignificant	21	73	73
chr6	83298477	83300477	Bola3	-0.16651243	3.69E-32	hypomethylated	0.0060896	0.96292	insignificant	23	64	64
chr6	83390748	83392748	B230319C09R	-0.17679624	2.15E-23	hypomethylated	0.010827	0.25547	insignificant	32	85	82
chr6	83391672	83393672	Tet3	-0.17766304	0.00000234	hypomethylated	0.0034193	0.8574	insignificant	13	34	34
chr6	83660257	83662257	Vax2	-0.1364451	2.07E-19	hypomethylated	0.044527	0.29601	insignificant	14	59	62
chr6	83660926	83662926	Vax2os2	-0.11670593	0.000000035	hypomethylated	0.058767	0.27035	insignificant	8	41	44
chr6	83662195	83664195	Vax2os2	-0.26115776	0.46158	insignificant	0.055408	0.38926	insignificant	2	14	14
chr6	83711639	83713639	Ankrd53	-0.10848861	0.4603	insignificant	0.029279	0.71699	insignificant	2	12	10
chr6	83725806	83727806	Tex261	-0.06390977	0.61492	insignificant	0.0097794	0.15577	insignificant	7	19	19
chr6	83744050	83746050	Nagk	-0.10260115	3.28E-08	hypomethylated	0.026934	0.11548	insignificant	13	103	90
chr6	83744151	83746151	Nagk	-0.12556104	2.04E-08	hypomethylated	0.051866	0.20401	insignificant	13	108	99
chr6	83781735	83783735	Paip2b	-0.31136497	0.00020827	hypomethylated	-0.041468	0.19395	insignificant	4	42	42
chr6	83863346	83865346	Zfml	-0.08268256	8.59E-12	hypomethylated	0.011704	0.0027544	hypermethylated	22	94	94
chr6	83957583	83959583	Dysf	-0.10844574	0.022501	hypomethylated	0.022604	0.17803	insignificant	11	47	47
chr6	85019507	85021507	Exoc6b	-0.12346456	1	insignificant	0.27063	0.8596	insignificant	3	15	8
chr6	85025134	85027134	Npm3-ps1		1	noCoverage	-0.0078673	0.11712	insignificant	0	39	43
chr6	85076088	85078088	Gm5878	-0.08320543	0.031465	inconclusive	-0.0027925	0.01989	inconclusive	17	48	43
chr6	85087758	85089758	Spr		1	noCoverage	0.027199	0.059477	insignificant	0	4	4
chr6	85136924	85138924	Emx1	-0.12907101	0.0098992	hypomethylated	0.013551	0.55941	insignificant	13	93	89
chr6	85283416	85285416	Sfxn5		1	noCoverage	-0.021939	0.70962	insignificant	0	14	14
chr6	85324628	85326628	Rab11fip5	-0.10469396	0.00000129	hypomethylated	0.007863	0.31302	insignificant	9	42	50
chr6	85372879	85374879	Noto	-0.23185224	0.02289	hypomethylated	0.067447	0.00000133	hypermethylated	7	30	30
chr6	85380969	85382969	Smyd5	0.03012706	0.13467	insignificant	0.019289	0.6071	insignificant	16	57	47
chr6	85400498	85402498	Cct7	-0.10787289	1.42E-15	hypomethylated	0.0018552	0.74353	insignificant	27	175	170
chr6	85401296	85403296	1700040I03Ril	-0.09612691	4.77E-09	hypomethylated	0.0035873	0.7634	insignificant	16	123	118
chr6	85401964	85403964	1700040I03Rik		1	noCoverage	0.0084584	0.69609	insignificant	0	41	36

chr6	85452880	85454880	Fbxo41	-0.10649412	0.000003452	hypomethylated	0.0097399	1	insignificant	9	26	24
chr6	85463536	85465536	Egr4	-0.10912975	3.63E-12	hypomethylated	0.011445	0.3702	insignificant	18	68	68
chr6	85536524	85538524	Alms1	-0.17351747	0.024558	hypomethylated	-0.040156	0.58886	insignificant	2	32	31
chr6	85819131	85821131	Cml2	-0.9125	0.22222	lowCoverage	-0.015064	1	insignificant	1	8	6
chr6	85864712	85866712	Tprkb	-0.0987593	5.93E-12	hypomethylated	-0.013658	0.88194	insignificant	22	96	101
chr6	85865671	85867671	Cml1	-0.07580278	1.1E-09	hypomethylated	-0.0088971	0.58708	insignificant	19	82	82
chr6	85911661	85913661	Dusp11	-0.31415076	0.00043446	hypomethylated	0.016208	0.75764	insignificant	4	36	35
chr6	85966184	85968184	Figla	-0.41250988	0.0056307	stronglyHypometh	-0.052415	0.082746	insignificant	2	24	24
chr6	86144244	86146244	Tgfa	-0.16630198	2.45E-39	hypomethylated	0.0057219	0.20689	insignificant	41	138	134
chr6	86314676	86316676	Fam136a	-0.08924451	0.13488	insignificant	0.0006317	0.26324	insignificant	32	104	104
chr6	86320533	86322533	Snrpg	-0.13293728	2.16E-11	hypomethylated	0.0025719	0.32449	insignificant	8	56	56
chr6	86353212	86355212	Tia1	-0.19502936	4.55E-21	hypomethylated	0.025215	0.54697	insignificant	17	67	67
chr6	86387391	86389391	C87436	-0.19187267	1.89E-25	hypomethylated	-0.0090588	0.81791	insignificant	18	81	81
chr6	86387639	86389639	C87436	-0.19484337	4.19E-26	hypomethylated	-0.0051965	0.93998	insignificant	20	85	83
chr6	86476159	86478159	Pcbp1	-0.08577338	9.48E-17	hypomethylated	-0.001507	0.67057	insignificant	50	198	200
chr6	86577167	86579167	Asprv1	0.11097465	0.74682	insignificant	0.064361	1	insignificant	2	18	23
chr6	86619153	86621153	Mxd1	-0.10074441	5.33E-15	hypomethylated	0.00044848	1	insignificant	35	107	108
chr6	86683372	86685372	Gmcl1	-0.17401041	0.00000166	hypomethylated	-0.0011772	0.53105	insignificant	13	57	57
chr6	86743578	86745578	Anxa4	-0.51	0.16384	insignificant	-0.34392	0.52617	insignificant	1	5	15
chr6	86798510	86800510	Aak1	-0.13606023	1.88E-21	hypomethylated	-0.015144	0.10391	insignificant	37	115	123
chr6	86799434	86801434	2610306M01F	-0.13035726	3E-20	hypomethylated	-0.0070795	0.057376	insignificant	34	88	87
chr6	86958829	86960829	Nfu1	-0.14059039	0.00046417	hypomethylated	0.0038723	0.4691	insignificant	13	45	40
chr6	86991839	86993839	Gfpt1	-0.1551515	7.17E-18	hypomethylated	-0.044452	0.025631	hypomethylated	17	82	104
chr6	87300909	87302909	Gkn1	-0.03079044	0.53251	insignificant	0.11147	0.0096956	hypermethylated	2	8	8
chr6	87322358	87324358	Gkn2		1	noCoverage	0.037276	0.24213	insignificant	0	14	14
chr6	87377995	87379995	Bmp10	0.20121592	0.37528	insignificant	0.0082087	0.59494	insignificant	3	33	30
chr6	87540695	87542695	Prokr1	-0.63987287	0.29851	lowCoverage	-0.061756	0.023321	hypomethylated	1	24	24
chr6	87622162	87624162	Aplf		1	noCoverage	0.00047561	0.56551	insignificant	0	8	8
chr6	87679862	87681862	Ccdc48	-0.07057195	0.00000167	hypomethylated	-0.0062644	0.2817	insignificant	20	83	87
chr6	87759748	87761748	Rab43	-0.09264487	6.25E-21	hypomethylated	0.01134	0.061047	insignificant	36	142	137
chr6	87761773	87763773	Rab43	-0.15345144	0.053608	insignificant	0.12614	0.827	insignificant	2	17	21
chr6	87788753	87790753	Isy1		1	noCoverage	0.041476	0.014805	hypermethylated	0	16	15
chr6	87801100	87803100	Cnbp	-0.10937797	0.0059452	hypomethylated	-0.022841	0.56065	insignificant	10	24	24
chr6	87836933	87838933	Copg	-0.18688594	1	insignificant	-0.0121	0.5246	insignificant	1	20	20
chr6	87836965	87838965	Copg	-0.18688594	1	insignificant	-0.0121	0.5246	insignificant	1	20	20
chr6	87862969	87864969	8430410A17R	-0.1049155	1.71E-11	hypomethylated	-0.011241	0.51504	insignificant	11	73	72
chr6	87930676	87932676	Gm5577	-0.0773644	1.03E-21	hypomethylated	-0.0011069	0.31135	insignificant	43	198	196
chr6	87931476	87933476	H1fx	-0.02585732	0.00023534	hypomethylated	-0.0050435	0.53862	insignificant	11	71	71
chr6	87963684	87965684	Rab7	0.19223928	0.20636	insignificant	-0.025935	1	insignificant	3	24	19
chr6	88033466	88035466	Rpn1	-0.12196203	1.85E-15	hypomethylated	-0.030506	0.011618	hypomethylated	16	80	80
chr6	88147657	88149657	Gata2	-0.13762596	6.33E-23	hypomethylated	-0.0013626	0.35739	insignificant	25	107	96
chr6	88171261	88173261	Dnajb8	-0.2000893	0.0024483	hypomethylated	-0.0030223	0.1196	insignificant	7	41	40
chr6	88396533	88398533	Eefsec	-0.11881653	0.044425	hypomethylated	0.053308	1	insignificant	15	33	32
chr6	88468794	88470794	Sec61a1	-0.11958273	3.14E-08	hypomethylated	0.054153	1	insignificant	16	37	40
chr6	88577439	88579439	Kbtbd12	-0.12358277	0.06773	insignificant	0.059575	0.3709	insignificant	1	18	19
chr6	88673405	88675405	Mgll	-0.09802893	6.91E-37	hypomethylated	-0.00047396	0.59549	insignificant	31	135	126
chr6	88673697	88675697	Mgll	-0.09802893	6.91E-37	hypomethylated	-0.00047396	0.59549	insignificant	31	135	126
chr6	88791929	88793929	Podxl2	-0.12132677	1.36E-14	hypomethylated	0.0037399	0.000014069	hypermethylated	18	106	105
chr6	88824038	88826038	Podxl2	-0.10526711	4.1E-35	hypomethylated	0.0073134	0.10036	insignificant	37	176	175
chr6	88851244	88853244	Tpra1	-0.10804936	1.62E-16	hypomethylated	-0.0092083	0.9033	insignificant	12	59	74
chr6	89045108	89047108	4933427D06Rik		1	noCoverage	0.058528	0.69635	insignificant	0	7	10
chr6	89312607	89314607	Plxna1	-0.09094827	2.02E-12	hypomethylated	0.016135	0.16406	insignificant	22	72	81
chr6	89592981	89594981	Txnrd3	-0.10309355	6.97E-32	hypomethylated	0.00035557	0.81249	insignificant	40	124	131
chr6	89695475	89697475	Vmn1r41	-0.84292763	5.61E-17	stronglyHypometh	-0.078125	1	insignificant	1	4	2
chr6	89795531	89797531	Vmn1r42		1	noCoverage	-0.094737	0.78773	insignificant	0	4	4
chr6	90275179	90277179	Chst13	-0.13787879	0.047728	hypomethylated	0.022466	0.16353	insignificant	2	4	4
chr6	90318487	90320487	Zxdc	-0.10302515	1.05E-29	hypomethylated	-0.010615	0.3307	insignificant	50	154	154
chr6	90378474	90380474	Ccdc37	-0.21954509	1.77E-09	hypomethylated	0.080002	0.50187	insignificant	13	32	36
chr6	90411619	90413619	Klf15	-0.05839775	0.0018691	hypomethylated	0.0043294	0.97949	insignificant	34	123	127
chr6	90499841	90501841	Aldh1l1	-0.19928833	0.000023595	hypomethylated	-0.0030347	0.70804	insignificant	6	35	37
chr6	90553873	90555873	Slc41a3	0.41155858	0.00023487	stronglyHypermeth	-0.096565	0.51061	insignificant	2	14	22
chr6	90568209	90570209	Slc41a3	-0.00419168	0.27707	insignificant	0.088409	0.00011755	hypermethylated	6	30	30
chr6	90666523	90668523	Iqsec1	-0.15384615	0.018028	hypomethylated	0.054487	0.43285	insignificant	6	6	12
chr6	90760117	90762117	Iqsec1		1	noCoverage	0.31581	0.037044	hypermethylated	0	4	2
chr6	91066820	91068820	Nup210	-0.07842099	6.59E-20	hypomethylated	0.018136	0.22865	insignificant	18	68	61
chr6	91105808	91107808	Hdac11	-0.19453125	0.57511	insignificant	0.08493	0.88526	insignificant	1	16	11
chr6	91161757	91163757	Fbln2	-0.06458398	0.00015969	hypomethylated	0.014946	0.86227	insignificant	8	54	52
chr6	91422744	91424744	Tmem43	-0.12650145	0.0010304	hypomethylated	-0.0063388	0.22378	insignificant	13	83	83
chr6	91423417	91425417	Chchd4	-0.12233453	0.0098663	hypomethylated	-0.0059515	0.76261	insignificant	8	55	55
chr6	91465028	91467028	Lsm3	-0.05491528	1.87E-11	hypomethylated	0.0063307	0.25802	insignificant	18	103	102
chr6	91465882	91467882	Lsm3	-0.07616666	0.091868	insignificant	-0.008158	0.21913	insignificant	5	52	52
chr6	91633060	91635060	Slc6a6	-0.08864366	2.76E-11	hypomethylated	0.00172	0.46703	insignificant	25	82	82
chr6	91757387	91759387	Grip2		1	noCoverage	-0.0025183	0.7005	insignificant	0	7	8
chr6	91827046	91829046	C130022K22R	-0.16478909	2.77E-10	hypomethylated	-0.0045516	0.84364	insignificant	6	56	56
chr6	91936103	91938103	Fgd5	-0.0104009	0.39488	insignificant	0.071217	0.0072525	hypermethylated	6	16	16
chr6	92040411	92042411	Nr2c2	-0.10304171	1.17E-35	hypomethylated	-0.0032666	0.281	insignificant	68	235	235
chr6	92134017	92136017	Mrps25	-0.11690228	0.00000283	hypomethylated	0.010424	0.48307	insignificant	8	16	16
chr6	92164805	92166805	Zfyve20	-0.16233645	0.38523	insignificant	0.0059447	0.21591	insignificant	3	24	24
chr6	92194642	92196642	Trh	-0.16010728	3.93E-12	hypomethylated	-0.03015	0.96267	insignificant	15	47	46
chr6	92431386	92433386	Prickle2		1	noCoverage	0.11871	0.1413	insignificant	0	4	4
chr6	92484855	92486855	Prickle2		1	noCoverage	-0.04375	0.83056	insignificant	0	4	4
chr6	92656178	92658178	Prickle2	-0.22520397	0.00023051	hypomethylated	-0.087803	0.70363	insignificant	4	9	8
chr6	92889575	92891575	9530026P05Ri	-0.28647661	0.5579	insignificant	0.078292	0.16927	insignificant	1	9	9
chr6	94233898	94235898	Magi1	-0.11757509	0.00000448	hypomethylated	0.014367	0.84738	insignificant	11	45	44
chr6	94449307	94451307	Slc25a26	-0.16552012	0.023079	hypomethylated	-0.002182	1	insignificant	6	44	41

chr6	94650139	94652139	Lrig1	-0.04525441	0.15846	insignificant	0.0084821	0.79904	insignificant	8	20	20
chr6	95066899	95068899	Kbtbd8	-0.07273989	1.11E-18	hypomethylated	0.023217	0.73063	insignificant	25	98	103
chr6	97098877	97100877	A130022J15Ri	-0.33971128	0.0011803	stronglyHypometh	-0.00000927	0.62812	insignificant	7	20	20
chr6	97129118	97131118	Tmf1	-0.10749288	9.41E-24	hypomethylated	-0.01491	0.31478	insignificant	14	53	48
chr6	97159785	97161785	Arl6ip5	-0.22182009	0.0011945	hypomethylated	0.011067	1	insignificant	2	14	14
chr6	97202774	97204774	Lmod3	-0.06644671	0.000045526	hypomethylated	-0.001344	0.90266	insignificant	7	15	15
chr6	97567651	97569651	Frmd4b	-0.4446356	0.01117	stronglyHypometh	-0.097644	0.35669	insignificant	0	20	22
chr6	97756051	97758051	Mitf	-0.11977237	2.85E-09	hypomethylated	0.0069225	0.52464	insignificant	19	57	56
chr6	97878810	97880810	Mitf	-0.62456229	0.45622	lowCoverage	-0.21457	0.5599	insignificant	1	10	10
chr6	98978292	98980292	Foxp1	-0.09074991	0.000050554	hypomethylated	0.012678	0.38213	insignificant	9	78	77
chr6	99385339	99387339	Foxp1		1	noCoverage	-0.13839	0.38049	insignificant	0	6	2
chr6	99616765	99618765	Eif4e3	-0.1188537	0.00039814	hypomethylated	-0.012807	0.68457	insignificant	10	10	10
chr6	99641672	99643672	Gpr27	-0.06051051	6.11E-52	hypomethylated	0.0037265	0.70318	insignificant	96	337	322
chr6	99676386	99678386	Prok2		1	noCoverage	0.16706	0.013035	hypermethylated	0	11	10
chr6	100237352	100239352	Rybp	-0.10630711	4.96E-20	hypomethylated	0.002203	0.10118	insignificant	48	153	146
chr6	100621151	100623151	Shq1	-0.13168772	1	insignificant	0.15387	0.8173	insignificant	3	17	15
chr6	100653727	100655727	Gxylt2	-0.08387951	8.02E-08	hypomethylated	0.0095658	0.74169	insignificant	17	55	61
chr6	100782631	100784631	Ppp4r2	-0.10428803	8.62E-18	hypomethylated	0.0036318	0.74908	insignificant	25	142	129
chr6	101327891	101329891	Pdzrn3	-0.19598954	1.3E-09	hypomethylated	0.0049725	0.68691	insignificant	10	28	28
chr6	103459869	103461869	Chl1	-0.26860529	0.32555	insignificant	0.0021762	0.57247	insignificant	1	22	22
chr6	105626738	105628738	Cntn4	-0.27010826	0.0016966	hypomethylated	0.020646	0.81727	insignificant	4	40	38
chr6	106067758	106069758	Cntn4		1	noCoverage	0.097718	1	insignificant	0	8	3
chr6	106718165	106720165	Trnt1	-0.14852559	1.02E-16	hypomethylated	0.0085634	0.72483	insignificant	19	46	46
chr6	106750068	106752068	Crbn	-0.45070345	0.00000183	stronglyHypometh	-0.094453	0.56497	insignificant	8	16	4
chr6	107478778	107480778	Lrrn1	-0.18061388	0.0078879	hypomethylated	0.10984	0.21539	insignificant	10	38	48
chr6	108014039	108016039	Setmar	-0.1678013	0.58559	insignificant	0.035057	0.62007	insignificant	2	17	18
chr6	108162089	108164089	Itpr1	-0.21953068	7.87E-27	hypomethylated	-0.029197	1	insignificant	16	66	76
chr6	108609622	108611622	Bhlhe40	-0.16835054	1.53E-11	hypomethylated	0.00053652	0.097214	insignificant	10	50	52
chr6	108732052	108734052	Arl8b	-0.06345837	6.71E-09	hypomethylated	-0.00093089	0.33164	insignificant	31	90	90
chr6	108777634	108779634	Edem1	-0.10199173	1.87E-08	hypomethylated	-0.00081874	1	insignificant	33	135	135
chr6	110594591	110596591	Grm7	-0.16358321	0.000000169	hypomethylated	0.0087355	0.054327	insignificant	17	93	98
chr6	112222751	112224751	Lmcd1		1	noCoverage	-0.0023606	0.00000696	hypomethylated	0	22	22
chr6	112408498	112410498	Cav3	0.18230079	1	insignificant	0.074482	0.099964	insignificant	2	14	12
chr6	112439802	112441802	Oxtr	-0.15406301	3.48E-09	hypomethylated	0.034556	0.589	insignificant	6	28	24
chr6	112897260	112899260	Srgap3	-0.13613966	0.00000378	hypomethylated	0.024838	0.3506	insignificant	4	10	10
chr6	112995320	112997320	Thumpd3	-0.16948461	1.51E-27	hypomethylated	0.020834	1	insignificant	18	48	46
chr6	113026632	113028632	Setd5	-0.10954727	7.33E-20	hypomethylated	0.0042774	0.38687	insignificant	33	167	165
chr6	113027238	113029238	Gt(ROSA)26So	-0.11563462	0.000015011	hypomethylated	0.001694	0.4384	insignificant	14	91	93
chr6	113145378	113147378	Lhfpl4		1	noCoverage	0.013275	0.50096	insignificant	0	16	16
chr6	113186836	113188836	Mtmr14	-0.14407464	9.61E-17	hypomethylated	-0.0038168	0.72471	insignificant	32	96	93
chr6	113231300	113233300	Cpne9	-0.24044488	1.14E-14	hypomethylated	-0.012043	0.30343	insignificant	22	90	93
chr6	113256190	113258190	Brpf1	-0.14767535	8.08E-26	hypomethylated	-0.00026805	0.097024	insignificant	28	121	128
chr6	113275969	113277969	Ogg1	-0.20102807	1.01E-36	hypomethylated	0.017169	0.89073	insignificant	21	86	77
chr6	113327106	113329106	Arpc4	-0.16896138	6.59E-16	hypomethylated	0.02761	0.42786	insignificant	21	88	93
chr6	113327514	113329514	Tada3	-0.17823684	2.63E-18	hypomethylated	0.018866	0.35568	insignificant	24	94	97
chr6	113338253	113340253	Ttll3	0.0611348	0.065595	insignificant	-0.01449	0.42221	insignificant	5	31	22
chr6	113341490	113343490	Ttll3	-0.17173622	1.4E-20	hypomethylated	-0.023758	0.33687	insignificant	17	84	81
chr6	113369334	113371334	Rpusd3	-0.37860326	0.003156	stronglyHypometh	0.049297	0.24025	insignificant	6	34	35
chr6	113385749	113387749	Cidec	0.27607373	1	insignificant	0.055011	0.045505	hypermethylated	2	24	25
chr6	113391628	113393628	Jagn1	-0.12316595	1.04E-14	hypomethylated	0.00058922	0.39244	insignificant	13	59	56
chr6	113407477	113409477	Il17re		1	noCoverage	-0.0008559	0.61922	insignificant	0	7	6
chr6	113407888	113409888	Il17re		1	noCoverage	-0.0008559	0.61922	insignificant	0	7	6
chr6	113420448	113422448	Il17rc	0.19740058	0.083236	insignificant	0.15443	0.0085345	hypermethylated	1	25	24
chr6	113432562	113434562	Creld1	-0.2047929	1.32E-34	hypomethylated	-0.0092178	0.42667	insignificant	27	120	118
chr6	113451812	113453812	Prrt3	-0.32125924	5.03E-08	hypomethylated	0.025934	0.92728	insignificant	8	16	16
chr6	113480675	113482675	Fancd2	-0.08925516	0.0074859	hypomethylated	0.0039162	0.24163	insignificant	6	56	46
chr6	113481632	113483632	Tmem111	-0.07382256	0.6313	insignificant	0.0040722	0.57211	insignificant	1	26	26
chr6	113550709	113552709	4931417G12R	0.16445075	1	insignificant	0.055936	0.56333	insignificant	1	16	21
chr6	113553765	113555765	6720456B07R	-0.18433851	1.66E-11	hypomethylated	-0.001978	0.30195	insignificant	14	113	120
chr6	113573014	113575014	Vhl	-0.11494113	7.92E-16	hypomethylated	0.0017724	0.12671	insignificant	28	118	113
chr6	113587460	113589460	Irak2		1	noCoverage	-0.063275	0.017103	hypomethylated	0	40	39
chr6	113646492	113648492	Tatdn2	-0.09011642	6E-16	hypomethylated	-0.0063298	0.65355	insignificant	41	177	199
chr6	113690675	113692675	Sec13		1	noCoverage	0.0041908	0.057088	insignificant	0	12	14
chr6	113841370	113843370	Atp2b2	-0.13374819	0.041891	hypomethylated	0.27112	3.03E-34	hypermethylated	5	36	20
chr6	114080234	114082234	Slc6a11	-0.15444326	9.6E-13	hypomethylated	0.0088387	0.82203	insignificant	8	16	16
chr6	114231628	114233628	Slc6a1	-0.14092253	0.000092273	hypomethylated	-0.00079245	0.5484	insignificant	16	49	49
chr6	114346929	114348929	Hrh1	-0.1801306	1.28E-16	hypomethylated	0.0082125	0.79704	insignificant	24	84	84
chr6	114592142	114594142	Atg7	-0.21313349	1.54E-23	hypomethylated	-0.024705	0.28755	insignificant	10	39	48
chr6	114987892	114989892	1500001M20R	-0.15480465	5.06E-09	hypomethylated	-0.0042705	0.0033986	hypomethylated	10	28	28
chr6	115083919	115085919	Syn2	-0.11141177	2.69E-27	hypomethylated	0.02604	0.00037412	hypermethylated	27	117	114
chr6	115201867	115203867	Timp4		1	noCoverage	0.019769	0.24677	insignificant	0	6	9
chr6	115310238	115312238	Pparg	-0.11508822	1.78E-08	hypomethylated	0.02405	0.000017874	hypermethylated	16	127	118
chr6	115493721	115495721	Tsen2	-0.10273081	1	insignificant	0.035108	0.80279	insignificant	4	26	25
chr6	115550955	115552955	Mkrn2	-0.12145733	0.000000712	hypomethylated	-0.012214	0.83236	insignificant	9	22	21
chr6	115626653	115628653	Raf1	-0.15721958	0.000059481	hypomethylated	0.11139	0.47122	insignificant	7	32	39
chr6	115709017	115711017	Tmem40	-0.54029569	1	insignificant	-0.09606	0.011135	inconclusive	1	6	6
chr6	115723574	115725574	Cand2	-0.20257576	0.00081421	hypomethylated	0.0152	0.55781	insignificant	6	30	33
chr6	115758121	115760121	Rpl32	-0.18499036	3.43E-19	hypomethylated	0.028183	0.12246	insignificant	21	93	92
chr6	115758761	115760761	Rpl32		1	noCoverage	0.039855	0.7492	insignificant	0	20	19
chr6	115802545	115804545	Ift122	-0.15567279	3.1E-22	hypomethylated	-0.024218	0.10262	insignificant	15	80	78
chr6	115803359	115805359	Mbd4	-0.1209832	8.73E-17	hypomethylated	-0.038238	0.95799	insignificant	9	54	52
chr6	115880944	115882944	Rho	-0.14956836	0.34781	insignificant	0.005941	1	insignificant	2	33	35
chr6	115945023	115947023	Plxnd1	-0.1668953	1.05E-08	hypomethylated	-0.019547	0.78268	insignificant	11	32	31
chr6	116143392	116145392	Tmcc1	-0.2726431	5.62E-12	hypomethylated	-0.18771	8.72E-08	hypomethylated	13	33	28
chr6	116157050	116159050	D6Wsu116e	-0.30260463	4.44E-10	hypomethylated	-0.0099232	0.41148	insignificant	12	41	38

chr6	116213252	116215252	Anubl1	-0.08430865	5.24E-08	hypomethylated	-0.011583	0.62264	insignificant	28	120	126
chr6	116287140	116289140	March8	-0.08485826	0.00068307	hypomethylated	-0.0099584	0.96946	insignificant	22	112	106
chr6	116455533	116457533	Olfr212		1	noCoverage	-0.013563	0.81853	insignificant	0	12	12
chr6	116532962	116534962	Olfr215	0.07336957	1	insignificant	0.037655	0.68296	insignificant	1	2	2
chr6	116599701	116601701	8430408G22Rik		1	noCoverage	-0.085927	0.11781	insignificant	0	8	12
chr6	116623854	116625854	Rassf4	-0.01749026	0.47759	insignificant	-0.031935	0.14839	insignificant	1	20	20
chr6	116666776	116668776	Tmem72	-0.03290236	1	insignificant	0.025052	0.21328	insignificant	3	17	17
chr6	117117552	117119552	Cxcl12	-0.12403432	2.87E-10	hypomethylated	0.0026211	0.59648	insignificant	37	144	128
chr6	117790259	117792259	Zfp637	-0.22737619	7.25E-26	hypomethylated	0.0053187	0.57984	insignificant	9	66	63
chr6	117812094	117814094	Zfp239	-0.35686275	3.77E-08	stronglyHypometh	-0.1772	0.84757	insignificant	4	15	13
chr6	117849357	117851357	Hnrnpf	-0.36012841	0.54424	insignificant	0.062387	0.64414	insignificant	1	6	5
chr6	117855799	117857799	Hnrnpf	-0.11252809	2.76E-73	hypomethylated	0.0058153	0.48907	insignificant	64	209	195
chr6	117856821	117858821	Hnrnpf	-0.11077024	2.26E-65	hypomethylated	0.0064407	0.4207	insignificant	67	229	215
chr6	117866311	117868311	Hnrnpf	-0.1760863	7.99E-18	hypomethylated	0.013539	0.89245	insignificant	16	88	88
chr6	118015402	118017402	Rasgef1a	0.27619523	0.43436	insignificant	0.0034032	0.92769	insignificant	2	16	16
chr6	118089158	118091158	Csgalnact2		1	noCoverage	0.024892	0.90995	insignificant	0	8	7
chr6	118147762	118149762	Ret	-0.43382728	0.00030722	stronglyHypometh	0.044234	0.66173	insignificant	4	10	10
chr6	118369435	118371435	Bms1	-0.13207864	0.038122	hypomethylated	-0.029981	0.046447	hypomethylated	1	18	18
chr6	118429291	118431291	Zfp9	-0.36262626	1	insignificant	0.024648	0.43588	insignificant	3	9	11
chr6	118512274	118514274	Ankrd26	-0.27503758	0.038051	hypomethylated	-0.084757	0.20055	insignificant	7	23	20
chr6	119058207	119060207	Cacna1c	-0.07501065	1.79E-19	hypomethylated	-0.001866	0.68397	insignificant	25	169	159
chr6	119124270	119126270	Dcp1b	-0.15441578	2.92E-11	hypomethylated	-0.042652	0.55463	insignificant	11	52	50
chr6	119146427	119148427	Dcp1b		1	noCoverage	0.12247	0.044728	hypermethylated	0	6	6
chr6	119279222	119281222	Cacna2d4	-0.10048645	2.21E-09	hypomethylated	0.0018678	0.13287	insignificant	10	56	56
chr6	119280784	119282784	Cacna2d4		1	noCoverage	0.01618	0.37323	insignificant	0	24	24
chr6	119428685	119430685	Fbxl14	-0.0882224	1.24E-23	hypomethylated	-0.0011532	0.31748	insignificant	74	254	243
chr6	119494365	119496365	Wnt5b	-0.12866623	0.10255	insignificant	-0.011492	0.40139	insignificant	10	52	51
chr6	119797210	119799210	3110021A11R	-0.08528633	3.8E-27	hypomethylated	-0.00599	0.87879	insignificant	36	138	151
chr6	119798168	119800168	Erc1	-0.03509475	0.070916	insignificant	-0.011189	0.56007	insignificant	11	62	75
chr6	119851715	119853715	Rad52	-0.15581962	2.31E-13	hypomethylated	0.018102	0.082727	insignificant	24	60	60
chr6	119984329	119986329	Mir706	-0.78578304	0.00024225	stronglyHypometh	0.0018916	0.47886	insignificant	3	17	16
chr6	119988673	119990673	Wnk1	-0.06781446	0.16196	insignificant	0.0059538	0.6922	insignificant	10	70	72
chr6	120244577	120246577	B4galnt3	-0.16350999	4.83E-14	hypomethylated	-0.004183	0.47908	insignificant	16	55	49
chr6	120307378	120309378	Ccdc77	0.16998991	0.67675	insignificant	0.0003824	0.11863	insignificant	1	23	27
chr6	120313116	120315116	Kdm5a	-0.13876638	8.55E-42	hypomethylated	0.021809	0.26258	insignificant	33	149	155
chr6	120412214	120414214	Il17ra	-0.10164844	1.32E-17	hypomethylated	0.0025106	0.84614	insignificant	27	94	94
chr6	120443825	120445825	Cecr6	-0.16099141	1.14E-30	hypomethylated	0.012712	0.0038029	hypermethylated	21	50	52
chr6	120481317	120483317	Cecr5	-0.10673946	0.0052888	hypomethylated	0.006945	0.1307	insignificant	5	52	53
chr6	120615438	120617438	Cecr2	-0.09847427	7.65E-40	hypomethylated	-0.014616	0.20296	insignificant	98	324	334
chr6	120772703	120774703	Atp6v1e1	-0.41666667	0.081952	insignificant	0.18429	0.89892	insignificant	2	12	16
chr6	120785247	120787247	Bcl2l13	-0.10344892	0.00000163	hypomethylated	-0.0077908	0.53559	insignificant	21	78	78
chr6	120866838	120868838	Bid	-0.22522385	0.02244	hypomethylated	0.048965	0.93009	insignificant	4	26	20
chr6	121132684	121134684	Pex26	-0.25706886	0.068952	insignificant	0.014886	0.67135	insignificant	5	22	29
chr6	121159787	121161787	Tuba8	-0.31755359	0.34598	insignificant	-0.0075753	0.8636	insignificant	3	16	16
chr6	121194923	121196923	Usp18	-0.13925603	0.000016453	hypomethylated	0.029767	0.29504	insignificant	13	38	38
chr6	121249313	121251313	Slc6a13	-0.4202362	0.012428	stronglyHypometh	-0.031982	0.014872	hypomethylated	3	22	22
chr6	121295714	121297714	Slc6a12	-0.23940112	0.084149	insignificant	0.093136	0.000032129	hypermethylated	4	12	12
chr6	121423696	121425696	Iqsec3		1	noCoverage	-0.00016447	0.82582	insignificant	0	20	20
chr6	121585190	121587190	A2m		1	noCoverage	0.17247	0.035687	hypermethylated	0	6	3
chr6	122258027	122260027	M6pr	-0.17917382	8.94E-16	hypomethylated	0.0034677	0.53373	insignificant	7	31	28
chr6	122436323	122438323	Rimklb	-0.16687686	0.000000073	hypomethylated	-0.048753	0.12007	insignificant	8	59	73
chr6	122502826	122504826	Aicda	0.13541667	0.69633	insignificant	0.16383	1	insignificant	2	8	4
chr6	122560089	122562089	Gdf3		1	noCoverage	0.025	0.68139	insignificant	0	4	4
chr6	122575441	122577441	Dppa3	-0.04868465	0.00070076	hypomethylated	0.081218	0.034765	hypermethylated	7	34	28
chr6	122692763	122694763	Slc2a3	-0.30081968	0.83153	insignificant	0.0041184	0.066335	insignificant	3	21	24
chr6	122769201	122771201	Foxj2	-0.11758258	4.66E-25	hypomethylated	0.00013551	0.59027	insignificant	38	136	122
chr6	122823574	122825574	Necap1	-0.13575592	0.000042185	hypomethylated	-0.049868	0.49437	insignificant	7	40	41
chr6	124365085	124367085	Pex5	-0.15975275	0.35018	insignificant	0.01992	0.33854	insignificant	3	14	14
chr6	124414802	124416802	Clstn3		1	noCoverage	0.079409	0.74004	insignificant	0	8	8
chr6	124442130	124444130	C1rl	0.33993506	1	lowCoverage	0.2566	0.1497	insignificant	1	8	6
chr6	124461638	124463638	C1ra	-0.30532213	0.25879	insignificant	-0.078049	0.66885	insignificant	2	6	6
chr6	124492339	124494339	C1s		1	noCoverage	0.097727	0.46081	insignificant	0	4	4
chr6	124492377	124494377	C1s		1	noCoverage	0.097727	0.46081	insignificant	0	4	4
chr6	124519447	124521447	C1rb		1	noCoverage	0.022222	1	insignificant	0	6	6
chr6	124612121	124614121	Lpcat3	-0.1157149	0.00000333	hypomethylated	-0.0028023	0.15992	insignificant	31	137	144
chr6	124661306	124663306	Phb2	-0.16885275	3.64E-10	hypomethylated	0.0027204	0.73874	insignificant	9	56	55
chr6	124662196	124664196	Emg1	-0.15758229	0.0000002	hypomethylated	0.013006	0.62916	insignificant	3	35	34
chr6	124668003	124670003	Mir141	0.08248405	0.013061	hypermethylated	0.018332	0.78861	insignificant	10	28	28
chr6	124668408	124670408	Mir141	0.08202651	0.012543	hypermethylated	0.0102	0.92748	insignificant	10	26	26
chr6	124688727	124690727	Ptpn6	0.05231481	1	insignificant	0.047327	0.43637	insignificant	1	6	6
chr6	124691097	124693097	Grcc10	-0.23658865	0.00018139	hypomethylated	0.0050161	0.36998	insignificant	2	15	15
chr6	124693852	124695852	Atn1	-0.03815139	0.00000058	inconclusive	0.0021397	0.44832	insignificant	10	46	44
chr6	124706505	124708505	Atn1	-0.09460181	0.0059175	hypomethylated	-0.029456	1	insignificant	32	141	139
chr6	124719527	124721527	Lrrc23	0.2280835	1.66E-10	hypermethylated	0.005641	0.000054623	inconclusive	6	56	56
chr6	124729736	124731736	Lrrc23	-0.32291667	0.16544	insignificant	0.043252	0.84423	insignificant	1	12	12
chr6	124757958	124759958	Spsb2	-0.15314995	3.32E-08	hypomethylated	0.0068863	0.36422	insignificant	16	69	69
chr6	124764314	124766314	Tpi1	-0.21112594	0.8783	insignificant	-0.044673	0.93017	insignificant	4	15	16
chr6	124779193	124781193	Cdca3	-0.17763255	1.23E-20	hypomethylated	-0.0027571	0.79619	insignificant	18	72	72
chr6	124779465	124781465	Usp5	-0.20050055	0.00000193	hypomethylated	-0.000064928	1	insignificant	10	44	44
chr6	124807705	124809705	Leprel2	-0.0849653	0.050017	insignificant	0.021353	0.12548	insignificant	13	42	40
chr6	124813935	124815935	Cd4		1	noCoverage	0.04	0.62499	insignificant	0	10	10
chr6	124861723	124863723	Lag3	-0.54945055	0.033114	stronglyHypometh	0.03694	0.74327	insignificant	3	13	13
chr6	124867964	124869964	Ptms	-0.09061174	3.15E-09	hypomethylated	0.038158	0.014795	inconclusive	2	26	15
chr6	124880405	124882405	Mlf2	-0.09752769	9.38E-16	hypomethylated	0.027591	0.56722	insignificant	25	107	108
chr6	124915415	124917415	Cops7a	-0.12696722	2.3E-12	hypomethylated	-0.010017	0.5384	insignificant	24	77	75

chr6	124915547	124917547	Cops7a	-0.14670031	0.000000893	hypomethylated	-0.0034875	0.49813	insignificant	13	49	47
chr6	124945737	124947737	C530028O21R	-0.13860903	4.95E-12	hypomethylated	0.12032	1.32E-13	hypermethylated	20	47	13
chr6	124947454	124949454	C530028O21R	-0.34902645	0.43836	insignificant	-0.086081	0.0014894	hypomethylated	3	14	15
chr6	124947627	124949627	C530028O21R	-0.34902645	0.43836	insignificant	-0.086081	0.0014894	hypomethylated	3	14	15
chr6	124958822	124960822	Zfp384	-0.13083928	6.87E-17	hypomethylated	-0.0064144	0.56319	insignificant	24	117	126
chr6	124988865	124990865	Ing4	-0.20116224	1	insignificant	-0.020506	0.42915	insignificant	3	34	28
chr6	124998944	125000944	Acrbp	-0.07222222	0.18834	insignificant	-0.003869	0.57647	insignificant	6	12	12
chr6	125016937	125018937	Lpar5	0.13429071	0.57975	insignificant	0.096791	0.45697	insignificant	2	11	12
chr6	125020294	125022294	Lpar5		1	noCoverage	0.095238	1	insignificant	0	5	7
chr6	125045180	125047180	Chd4	-0.1389897	5.98E-24	hypomethylated	0.053177	0.084661	insignificant	34	147	133
chr6	125080900	125082900	Nop2	-0.15489772	3.95E-12	hypomethylated	-0.0039604	0.97008	insignificant	13	77	68
chr6	125094258	125096258	Iffo1	-0.11535213	7.49E-11	hypomethylated	-0.0099871	0.023133	inconclusive	15	96	83
chr6	125094268	125096268	Iffo1	-0.11535213	7.49E-11	hypomethylated	-0.0099871	0.023133	inconclusive	15	96	83
chr6	125115601	125117601	Gapdh	-0.22624749	0.042638	hypomethylated	-0.0014151	0.77455	insignificant	2	14	14
chr6	125136449	125138449	Ncapd2	-0.4903044	0.032639	stronglyHypometh	0.012756	0.47999	insignificant	2	24	24
chr6	125141217	125143217	Mrpl51	-0.11079109	3.3E-14	hypomethylated	0.025328	0.37135	insignificant	19	107	104
chr6	125141604	125143604	Ncapd2	-0.11376418	1.79E-09	hypomethylated	0.002653	0.62553	insignificant	14	89	86
chr6	125164598	125166598	Vamp1	-0.10382271	0.00000621	hypomethylated	0.0037088	0.46307	insignificant	6	32	32
chr6	125180293	125182293	E130112N10R	-0.55277778	0.00053954	stronglyHypometh	0.052614	1	insignificant	1	4	4
chr6	125181878	125183878	E130112N10R	-0.06101136	0.057372	insignificant	0.010279	0.83127	insignificant	3	18	19
chr6	125187045	125189045	E130112N10R	-0.39761905	0.023735	stronglyHypometh	0.0057143	1	insignificant	3	6	6
chr6	125214542	125216542	4930417O13R	-0.81341108	0.11111	lowCoverage	0.14666	0.37948	insignificant	1	7	6
chr6	125236060	125238060	Tuba3a	-0.08328871	0.059927	insignificant	0.011614	0.30181	insignificant	4	12	12
chr6	125263888	125265888	Ltbr		1	noCoverage	-0.022799	0.85368	insignificant	0	14	7
chr6	125270357	125272357	Scnn1a	0.24627227	0.57692	insignificant	0.028858	0.33833	insignificant	2	16	16
chr6	125298740	125300740	Tnfrsf1a	-0.20039907	0.000000595	hypomethylated	-0.020665	0.76533	insignificant	15	54	52
chr6	125330522	125332522	Plekhg6	-0.08044881	0.19963	insignificant	0.027185	0.01028	hypermethylated	3	26	26
chr6	125444773	125446773	Cd9	-0.41425773	0.018531	stronglyHypometh	-0.10204	0.0372	inconclusive	8	25	22
chr6	125639436	125641436	Ano2	-0.17577681	0.012091	hypomethylated	-0.044484	0.13404	insignificant	3	8	8
chr6	126114978	126116978	Ntf3	-0.21657897	0.0017068	hypomethylated	-0.00011208	0.3442	insignificant	7	46	47
chr6	126116762	126118762	Ntf3	-0.23494058	0.00044099	hypomethylated	-0.011202	0.93429	insignificant	7	20	20
chr6	126690692	126692692	Kcna6		1	noCoverage	0.14641	1	insignificant	0	4	4
chr6	126799162	126801162	Ndufa9		1	noCoverage	0.094284	1	insignificant	0	10	5
chr6	126964559	126966559	Fgf6	-0.1157672	0.0425	hypomethylated	-0.0070625	0.024125	inconclusive	4	30	32
chr6	127059570	127061570	9630033F20Ri	-0.20587864	1.24E-10	hypomethylated	0.02463	0.034384	hypermethylated	7	16	16
chr6	127101066	127103066	Ccnd2	-0.21199747	0.19773	insignificant	-0.035953	1	insignificant	2	27	18
chr6	127402740	127404740	Parp11	-0.21238495	2.4E-21	hypomethylated	0.057466	0.488	insignificant	12	92	74
chr6	127718747	127720747	Prmt8	-0.11051211	2.3E-11	hypomethylated	0.012291	0.35048	insignificant	31	130	141
chr6	127836639	127838639	Tspan11	-0.32008003	0.00011421	hypomethylated	-0.056868	0.22108	insignificant	6	23	17
chr6	128093596	128095596	Tspan9	-0.17405229	0.00014767	hypomethylated	-0.036931	0.91775	insignificant	5	28	28
chr6	128250831	128252831	Tead4	-0.16329365	0.085817	insignificant	0.002731	0.94136	insignificant	5	24	24
chr6	128305869	128307869	Tulp3	0.08146836	0.38531	insignificant	0.021485	0.088044	insignificant	4	25	22
chr6	128312011	128314011	Foxm1	-0.12832439	7.36E-35	hypomethylated	-0.022869	0.34531	insignificant	35	134	120
chr6	128312840	128314840	5930416I19Ril	-0.16094237	3.41E-33	hypomethylated	-0.037157	0.38765	insignificant	18	51	48
chr6	128312915	128314915	5930416I19Ril	-0.16094237	3.41E-33	hypomethylated	-0.037157	0.38765	insignificant	18	51	48
chr6	128348783	128350783	Nrip2	-0.0320864	0.23725	insignificant	0.044582	0.0073933	hypermethylated	6	18	20
chr6	128387774	128389774	Gm10069	-0.10666084	1.21E-34	hypomethylated	-0.0021717	0.55317	insignificant	50	171	166
chr6	128388649	128390649	Fkbp4	-0.16175811	2.49E-17	hypomethylated	0.011453	0.099834	insignificant	12	32	28
chr6	128572952	128574952	Klrb1a		1	noCoverage	-0.175	1	insignificant	0	2	2
chr6	129225387	129227387	Cd69		1	noCoverage	-0.11161	0.62024	insignificant	0	5	2
chr6	129346314	129348314	Clec1b		1	noCoverage	0.041703	0.23475	insignificant	0	8	8
chr6	129402018	129404018	Clec1a		1	noCoverage	0.11484	1	insignificant	0	6	6
chr6	129482209	129484209	Gabarapl1	-0.41205299	0.016874	stronglyHypometh	-0.084559	0.66282	insignificant	2	20	28
chr6	129640552	129642552	Mir680-1		1	noCoverage	-0.037566	0.45158	insignificant	0	4	6
chr6	129725867	129727867	Gm156		1	noCoverage	0.16667	0.56142	insignificant	0	6	4
chr6	130079853	130081853	Klra15		1	noCoverage	0.13415	0.66695	insignificant	0	13	7
chr6	130079897	130081897	Klra15		1	noCoverage	0.13415	0.66695	insignificant	0	13	7
chr6	130079916	130081916	Klra15		1	noCoverage	0.13415	0.66695	insignificant	0	13	7
chr6	130110766	130112766	Klra15		1	noCoverage		1	noCoverage	0	4	0
chr6	130330680	130332680	Klra15	-0.29166667	0.41538	insignificant	-0.0059524	1	insignificant	1	2	2
chr6	130330826	130332826	Klra15	-0.29166667	0.41538	insignificant	-0.0059524	1	insignificant	1	2	2
chr6	131243262	131245262	Magohb	-0.18477831	0.00095204	hypomethylated	0.0047247	0.10111	insignificant	8	40	40
chr6	131338468	131340468	Csda	-0.16139657	1	insignificant	0.07523	0.00000818	hypermethylated	7	23	24
chr6	132264761	132266761	Prpmp5		1	noCoverage	0.05	1	insignificant	0	4	4
chr6	133054256	133056256	2700089E24Ri	-0.11876381	2.71E-09	hypomethylated	0.014032	0.33299	insignificant	15	63	59
chr6	133984724	133986724	Etv6	-0.08167832	0.013997	hypomethylated	0.032448	0.53885	insignificant	13	114	117
chr6	134345346	134347346	Bcl2l14		1	noCoverage	-0.16143	0.72395	insignificant	0	8	8
chr6	134516931	134518931	Lrp6	-0.0903554	0.03907	hypomethylated	0.00085128	0.39073	insignificant	4	66	42
chr6	134589957	134591957	Loh12cr1	-0.11670522	0.00023121	hypomethylated	-0.0032013	0.14522	insignificant	13	93	81
chr6	134742646	134744646	Dusp16	-0.32289626	5.25E-30	hypomethylated	0.016899	7.29E-22	hypermethylated	17	83	67
chr6	134779216	134781216	Crebl2	-0.10817235	0.60128	insignificant	0.018291	0.7018	insignificant	4	55	48
chr6	134846978	134848978	2810454H06R	0.0063856	0.029878	hypermethylated	0.0059352	0.87581	insignificant	17	95	77
chr6	134847833	134849833	Gpr19	-0.10004152	0.00000178	hypomethylated	-0.0045106	0.79754	insignificant	14	89	71
chr6	134847943	134849943	Gpr19	-0.13041378	0.000000506	hypomethylated	-0.01886	0.71144	insignificant	9	69	55
chr6	134869418	134871418	Cdkn1b	-0.11683653	3.15E-15	hypomethylated	-0.0023652	0.047778	hypomethylated	10	129	133
chr6	134931018	134933018	Apold1	0.08152357	1	insignificant	0.00081142	1	insignificant	1	12	12
chr6	134960629	134962629	Ddx47		1	noCoverage	0.059591	0.096063	insignificant	0	28	21
chr6	135014679	135016679	Gprc5a		1	noCoverage	0.016058	0.74024	insignificant	0	46	47
chr6	135068301	135070301	Gprc5d		1	noCoverage	0.068858	0.52594	insignificant	0	8	8
chr6	135118233	135120233	Hebp1	-0.18202614	0.36375	insignificant	0.026748	0.12904	insignificant	2	4	4
chr6	135147004	135149004	8430419L09Ri	-0.10900224	1.93E-23	hypomethylated	-0.026289	0.24076	insignificant	16	90	78
chr6	135311948	135313948	Emp1	-0.46533613	1	lowCoverage	0.020678	0.74015	insignificant	1	8	6
chr6	136466371	136468371	Atf7ip	-0.15907372	0.0033886	hypomethylated	0.017242	0.71227	insignificant	10	83	82
chr6	136610414	136612414	Plbd1		1	noCoverage	-0.034736	0.13613	insignificant	0	12	10
chr6	136730263	136732263	Gucy2c		1	noCoverage	0.0084838	0.62239	insignificant	0	8	10

chr6	136752900	136754900	Hist4h4	0.11300882	0.73849	insignificant	-0.012137	0.56434	insignificant	2	7	6
chr6	136755768	136757768	H2afj	-0.10447327	0.76541	insignificant	-0.075278	0.0010374	inconclusive	10	57	42
chr6	136776363	136778363	BC049715	-0.15248557	8.48E-15	hypomethylated	0.004071	0.60385	insignificant	18	49	45
chr6	136824326	136826326	Mgp	0.174415	1	insignificant	0.055825	0.0092285	hypermethylated	1	28	21
chr6	136870701	136872701	Erp27	0.22815693	0.026587	hypermethylated	0.005574	0.20091	insignificant	2	19	22
chr6	137118239	137120239	Rerg	-0.15116358	0.00000132	hypomethylated	0.038515	0.0041816	hypermethylated	5	32	32
chr6	137199819	137201819	Ptpro	-0.14499864	0.032132	hypomethylated	-0.028752	0.73241	insignificant	11	48	45
chr6	137597641	137599641	Eps8	-0.11721233	1	insignificant	0.054522	0.66442	insignificant	1	62	53
chr6	137682602	137684602	Strap	-0.18552036	0.00043053	hypomethylated	-0.031817	1	insignificant	8	17	16
chr6	137702097	137704097	Dera	-0.15802171	0.000044606	hypomethylated	-0.015305	0.6666	insignificant	8	28	24
chr6	138530489	138532489	Lmo3		1	noCoverage	-0.39133	0.0049286	stronglyhypometh	0	17	10
chr6	138606965	138608965	Igbp1b	0.07256401	1	insignificant	-0.02313	0.29981	insignificant	1	8	8
chr6	139450430	139452430	Rergl	-0.76638177	0.28358	lowCoverage	-0.021432	0.69115	insignificant	1	4	4
chr6	139534771	139536771	Pik3c2g		1	noCoverage	-0.04761	0.0011068	inconclusive	0	20	14
chr6	139788965	139790965	Pik3c2g	0.18108974	0.11604	insignificant	0.060897	0.46755	insignificant	4	8	8
chr6	140371619	140373619	Plekha5	-0.08985853	1.26E-16	hypomethylated	0.0054843	0.17232	insignificant	60	193	189
chr6	140570183	140572183	Aebp2	-0.01634173	0.090752	insignificant	0.00078592	0.24567	insignificant	16	85	85
chr6	140571209	140573209	Aebp2	-0.03874455	0.18683	insignificant	0.013522	0.090453	insignificant	25	158	148
chr6	140571818	140573818	Aebp2	-0.09154987	8.36E-17	hypomethylated	0.0085674	0.039697	hypermethylated	31	159	137
chr6	141196789	141198789	Pde3a	-0.12051552	3.27E-19	hypomethylated	0.020904	0.332	insignificant	28	97	101
chr6	142293216	142295216	Pyroxd1	-0.21301549	0.024735	hypomethylated	0.040092	0.0024612	hypermethylated	13	79	81
chr6	142334762	142336762	Golt1b	-0.11491199	3.6E-40	hypomethylated	0.0052916	0.33374	insignificant	49	153	144
chr6	142335607	142337607	Golt1b	-0.10740932	1.28E-31	hypomethylated	-0.0032445	0.49154	insignificant	33	110	101
chr6	142519876	142521876	Kcnj8	-0.14596427	2.25E-11	hypomethylated	0.060837	0.60112	insignificant	6	18	18
chr6	142650794	142652794	Abcc9	-0.39906548	1.24E-11	stronglyHypometh	0.046176	0.44033	insignificant	6	14	14
chr6	142704205	142706205	Cmas	-0.11366824	3.74E-12	hypomethylated	0.028067	0.0087045	hypermethylated	27	107	107
chr6	142752986	142754986	Gm766	0.2140158	1	lowCoverage	0.0033564	0.83537	insignificant	1	5	5
chr6	142912972	142914972	St8sia1		1	noCoverage	0.027976	0.0019234	inconclusive	0	28	28
chr6	143048627	143050627	5730419I09Ril	-0.15333416	0.0054973	hypomethylated	0.0062238	1	insignificant	5	35	35
chr6	143114749	143116749	Etnk1	-0.09450607	2.89E-21	hypomethylated	0.0050098	0.4665	insignificant	45	193	203
chr6	143193407	143195407	D6Ertd474e	-0.14424642	0.69473	insignificant	0.055059	0.29723	insignificant	2	6	6
chr6	145069258	145071258	Lrmp	-0.31434783	0.29594	insignificant	0.13062	0.15681	insignificant	1	10	10
chr6	145158653	145160653	Lyrm5	-0.08316547	0.10892	insignificant	-0.005833	0.57654	insignificant	9	78	75
chr6	145158666	145160666	Lyrm5	-0.08316547	0.10892	insignificant	-0.005833	0.57654	insignificant	9	78	75
chr6	145159490	145161490	Casc1	-0.04757663	0.24033	insignificant	-0.013719	0.15941	insignificant	8	54	51
chr6	145198751	145200751	Kras	-0.13854818	1.16E-15	hypomethylated	0.012001	0.0023124	inconclusive	22	104	104
chr6	145335144	145337144	1700073E17Rik		1	noCoverage	-0.0035795	0.62755	insignificant	0	13	10
chr6	145563482	145565482	Tuba3b	0.01919948	0.37464	insignificant	0.1014	0.020451	hypermethylated	7	44	44
chr6	145813860	145815860	Bhlhe41	-0.08035564	2.98E-21	hypomethylated	-0.0093827	0.28675	insignificant	38	121	120
chr6	146450434	146452434	Itpr2	-0.10789156	0.00027599	hypomethylated	0.012503	0.66446	insignificant	11	106	78
chr6	146525432	146527432	Fgfr1op2	-0.10765425	0.00000213	hypomethylated	0.02288	0.07482	insignificant	27	138	124
chr6	146526357	146528357	4933424B01R	-0.11347885	0.000000458	hypomethylated	0.01876	0.26124	insignificant	13	74	73
chr6	146590100	146592100	Med21	-0.2732016	5.67E-08	hypomethylated	-0.021411	0.0089446	hypomethylated	10	66	71
chr6	146672451	146674451	Stk38l	-0.08426204	0.12491	insignificant	-0.016835	0.18379	insignificant	12	60	59
chr6	146753051	146755051	Arntl2		1	noCoverage	0.059028	0.59929	insignificant	0	6	6
chr6	146836015	146838015	Ppfibp1	-0.10567475	6.74E-18	hypomethylated	-0.0027755	0.62276	insignificant	26	133	129
chr6	146990291	146992291	Mrps35	-0.15381099	0.00000598	hypomethylated	-0.045244	0.31478	insignificant	15	103	95
chr6	147035554	147037554	Gm5887	0.34245939	0.34235	insignificant	-0.65754	2.59E-16	stronglyhypometh	2	18	3
chr6	147038596	147040596	Klhdc5	-0.11444708	0.38818	insignificant	0.0076329	0.2395	insignificant	9	78	36
chr6	147212607	147214607	Pthlh	-0.15309897	0.000000705	hypomethylated	-0.013794	0.72783	insignificant	16	41	38
chr6	147423392	147425392	Ccdc91	-0.12723541	5.13E-17	hypomethylated	-0.010856	0.0051799	inconclusive	32	96	94
chr6	147994937	147996937	Far2	-0.17373737	0.35584	insignificant	-0.038023	0.29256	insignificant	5	15	15
chr6	148159809	148161809	4732416N19R	-0.1063539	3.64E-10	hypomethylated	0.019946	0.0078625	hypermethylated	30	135	132
chr6	148160896	148162896	Ergic2	-0.46686711	1.78E-08	stronglyHypometh	0.043305	0.94449	insignificant	4	16	16
chr6	148302177	148304177	Rps4y2	-0.00334229	0.84295	insignificant	0.023008	0.60372	insignificant	24	94	91
chr6	148392874	148394874	Tmtc1	-0.11357372	3.3E-20	hypomethylated	0.03101	0.081548	insignificant	14	61	67
chr6	148779989	148781989	Ipo8	-0.15431603	0.1413	insignificant	0.0050943	0.66185	insignificant	5	20	20
chr6	148844648	148846648	Caprin2	-0.24442151	1.36E-25	hypomethylated	-0.0035698	0.21147	insignificant	9	50	56
chr6	148894954	148896954	Fam60a		1	noCoverage	-0.01371	0.083764	insignificant	0	40	36
chr6	149050202	149052202	Dennd5b	-0.29193277	3.26E-11	hypomethylated	0.022188	0.60873	insignificant	5	10	10
chr6	149089118	149091118	4833442J19Rik		1	noCoverage	-0.014506	0.68968	insignificant	0	31	24
chr6	149137234	149139234	Amn1	-0.13333333	0.014289	hypomethylated	-0.071458	0.0088056	hypomethylated	4	13	13
chr6	149256935	149258935	2810474O19R	-0.10349733	5.84E-12	hypomethylated	0.018469	0.96527	insignificant	23	74	64
chr6	149356407	149358407	Bicd1	-0.17625	0.051521	insignificant	0.018798	0.93931	insignificant	5	14	14
chr7	3169204	3171204	AU018091	0.13231456	0.028882	hypermethylated	-0.055307	0.38556	insignificant	5	16	16
chr7	3217626	3219626	Mir290	-0.18248621	0.003973	hypomethylated	-0.017667	0.87139	insignificant	9	47	53
chr7	3217784	3219784	D7Ertd143e	-0.1873144	0.0058072	hypomethylated	-0.011738	0.49653	insignificant	9	50	55
chr7	3217919	3219919	Mir291a	-0.21609714	0.0023511	hypomethylated	-0.020973	0.20078	insignificant	9	45	49
chr7	3218189	3220189	Mir292	-0.21609714	0.0023511	hypomethylated	-0.020973	0.20078	insignificant	9	45	49
chr7	3218482	3220482	Mir291b	-0.21609714	0.0023511	hypomethylated	-0.020973	0.20078	insignificant	9	45	49
chr7	3219343	3221343	Mir293	-0.03021042	0.089856	insignificant	-0.0082794	0.15773	insignificant	3	30	32
chr7	3219641	3221641	Mir294		1	noCoverage	0.015508	0.072353	insignificant	0	6	4
chr7	3249741	3251741	Nlrp12	-0.18064713	0.48646	insignificant	0.16752	0.0018238	hypermethylated	1	18	17
chr7	3288038	3290038	Myadm	-0.09167185	0.0018014	hypomethylated	0.039175	0.52965	insignificant	8	40	40
chr7	3289555	3291555	Myadm	-0.19804004	0.12125	insignificant	0.0051791	0.67895	insignificant	7	16	16
chr7	3289556	3291556	Myadm	-0.19804004	0.12125	insignificant	0.0051791	0.67895	insignificant	7	16	16
chr7	3292028	3294028	Myadm	-0.45313971	0.000001226	stronglyHypometh	-0.014924	0.61487	insignificant	3	20	16
chr7	3393458	3395458	Cacng8	0.17156863	1	insignificant	0.28824	0.63921	insignificant	3	6	6
chr7	3424422	3426422	Cacng6	-0.24878429	0.00013782	hypomethylated	-0.014919	0.60279	insignificant	11	30	27
chr7	3567735	3569735	Oscar	-0.15919894	0.00046113	hypomethylated	0.0078956	0.95492	insignificant	7	34	34
chr7	3567974	3569974	Ndufa3	-0.15919894	0.00046113	hypomethylated	0.0078956	0.95492	insignificant	7	34	34
chr7	3580586	3582586	Prpf31	-0.18002426	2.04E-11	hypomethylated	0.030391	0.33631	insignificant	13	66	57
chr7	3581486	3583486	Tfpt	-0.20864236	5.33E-08	hypomethylated	0.016494	0.20268	insignificant	9	47	43
chr7	3595870	3597870	Cnot3	-0.1042135	1.6E-16	hypomethylated	0.012927	0.36093	insignificant	38	132	134
chr7	3617442	3619442	Tmc4	-0.22306661	0.77334	insignificant	-0.047705	0.12327	insignificant	4	31	31

chr7	3629155	3631155	Tmc4	-0.06376826	0.16116	insignificant	0.03326	0.4903	insignificant	2	23	24
chr7	3644211	3646211	Tsen34	-0.07943284	1.01E-14	hypomethylated	-0.0042266	0.6086	insignificant	27	183	178
chr7	3645109	3647109	Tsen34	-0.11313717	3.52E-17	hypomethylated	0.0055613	0.85509	insignificant	19	102	102
chr7	3645127	3647127	Tsen34	-0.11313717	3.52E-17	hypomethylated	0.0055613	0.85509	insignificant	19	102	102
chr7	3654642	3656642	Rps9	-0.17859613	1	insignificant	0.02578	0.48307	insignificant	2	59	54
chr7	4014806	4016806	Lair1		1	noCoverage	0.24091	0.49058	insignificant	0	8	8
chr7	4070134	4072134	Ttyh1	-0.17934574	2.22E-22	hypomethylated	0.024499	0.95863	insignificant	22	52	50
chr7	4072330	4074330	9430041J12Ri	-0.33332612	0.031024	hypomethylated	-0.037605	0.043096	hypomethylated	1	10	10
chr7	4087657	4089657	Leng8	-0.10091067	0.000019804	hypomethylated	0.0039053	0.50104	insignificant	17	78	80
chr7	4088528	4090528	D030047H15R	-0.09045676	0.00053584	hypomethylated	0.0042546	0.33737	insignificant	15	74	76
chr7	4101474	4103474	Leng9	-0.09510808	0.050546	insignificant	0.013004	0.18769	insignificant	5	30	29
chr7	4188355	4190355	Lilra5	-0.13198076	0.19854	insignificant	0.030535	0.22921	insignificant	1	18	20
chr7	4288325	4290325	Ncr1	-0.72916667	0.13904	insignificant	0.074405	0.79862	insignificant	1	4	4
chr7	4415343	4417343	Eps8l1		1	noCoverage	-0.0042017	0.73135	insignificant	0	2	2
chr7	4422486	4424486	Eps8l1	0.08417548	0.49382	insignificant	0.03426	0.87105	insignificant	3	21	14
chr7	4453282	4455282	Ppp1r12c	-0.20122887	1.36E-08	hypomethylated	0.060688	0.11584	insignificant	10	62	54
chr7	4474045	4476045	Tnni3	-0.19746578	0.12262	insignificant	-0.069568	0.00092086	hypomethylated	6	43	38
chr7	4498169	4500169	Syt5	-0.17330263	2.66E-10	hypomethylated	0.014295	0.60584	insignificant	11	61	60
chr7	4610552	4612552	Ppp6r1	-0.1483402	0.00025556	hypomethylated	-0.00068753	0.29465	insignificant	6	62	57
chr7	4636565	4638565	Hspbp1	-0.09519498	0.016685	hypomethylated	-0.017287	0.012409	hypomethylated	2	26	26
chr7	4641529	4643529	Brsk1	-0.22581114	0.0096895	hypomethylated	0.032383	0.6549	insignificant	4	33	33
chr7	4676684	4678684	Tmem150b		1	noCoverage	0.071758	0.91616	insignificant	0	10	10
chr7	4676853	4678853	Tmem150b		1	noCoverage	-0.0026899	0.27197	insignificant	0	8	8
chr7	4690728	4692728	Suv420h2	-0.10372946	0.16067	insignificant	0.024942	0.68697	insignificant	8	34	34
chr7	4704504	4706504	Cox6b2	-0.31039288	5.09E-12	hypomethylated	0.062861	0.48035	insignificant	3	10	10
chr7	4704696	4706696	Cox6b2	-0.31039288	5.09E-12	hypomethylated	0.062861	0.48035	insignificant	3	10	10
chr7	4722872	4724872	Fam71e2	-0.28324701	0.80346	insignificant	-0.022701	0.23001	insignificant	2	14	14
chr7	4729743	4731743	Il11	-0.64080386	0.0081888	stronglyHypometh	-0.043961	0.039017	hypomethylated	2	17	17
chr7	4733541	4735541	Tmem190	0.2075	1	lowCoverage	0.0825	0.30433	insignificant	1	2	2
chr7	4741162	4743162	2210411K11Ri	-0.49314988	1.33E-28	stronglyHypometh	0.06329	0.32279	insignificant	4	32	32
chr7	4743566	4745566	Rpl28	-0.15553615	0.000063746	hypomethylated	0.0031944	0.70399	insignificant	11	101	99
chr7	4763942	4765942	Ube2s	-0.15938681	0.026228	hypomethylated	-0.016612	0.19389	insignificant	4	100	95
chr7	4796298	4798298	Isoc2b	-0.21700362	0.00071289	hypomethylated	-0.066777	0.72606	insignificant	7	28	26
chr7	4817781	4819781	Isoc2b	-0.20038925	0.027363	hypomethylated	0.056506	0.29785	insignificant	1	46	45
chr7	4827654	4829654	Isoc2a	-0.36413043	1.08E-10	stronglyHypometh	-0.0077202	0.8431	insignificant	3	6	6
chr7	4865818	4867818	Zfp628	-0.16193392	0.000011081	hypomethylated	-0.021094	0.32823	insignificant	18	64	61
chr7	4872852	4874852	Nat14	-0.41990313	2.25E-23	stronglyHypometh	0.0095956	0.94332	insignificant	11	39	38
chr7	4915950	4917950	Sbk2	-0.06372054	0.00000755	hypomethylated	0.099161	0.30688	insignificant	2	10	10
chr7	4947703	4949703	Zfp579	-0.34496374	1.12E-36	stronglyHypometh	0.011846	0.2437	insignificant	5	22	22
chr7	4964844	4966844	Fiz1	-0.31191626	1	insignificant	0.015615	0.67879	insignificant	3	24	26
chr7	4965855	4967855	Fiz1	-0.11644125	1.12E-11	hypomethylated	-0.0065655	0.47617	insignificant	25	115	105
chr7	4966109	4968109	Zfp524	-0.10911879	2.94E-10	hypomethylated	-0.0074695	0.94246	insignificant	24	113	103
chr7	4966299	4968299	Fiz1	-0.0932267	6.54E-09	hypomethylated	-0.012123	0.93711	insignificant	22	97	87
chr7	4966330	4968330	Fiz1	-0.0932267	6.54E-09	hypomethylated	-0.012123	0.93711	insignificant	22	97	87
chr7	4970977	4972977	Zfp865	-0.19224647	9.81E-45	hypomethylated	-0.002999	0.43601	insignificant	24	100	94
chr7	4990048	4992048	Zfp784		1	noCoverage	0.25027	0.078384	insignificant	0	30	16
chr7	5002133	5004133	Zfp580		1	noCoverage	-0.0030944	0.68665	insignificant	0	34	29
chr7	5007327	5009327	Ccdc106	-0.13580717	0.83272	insignificant	-0.0069735	0.40682	insignificant	7	34	34
chr7	5012783	5014783	U2af2	0.1530674	0.0011721	inconclusive	0.013226	0.05968	insignificant	18	143	137
chr7	5030906	5032906	Epn1	-0.11369039	1.01E-20	hypomethylated	0.026833	0.25225	insignificant	33	85	87
chr7	5068513	5070513	Rfpl4	-0.38391031	0.0065298	stronglyHypometh	-0.064742	0.47499	insignificant	1	14	11
chr7	5077552	5079552	Rasl2-9-ps	-0.27506086	0.00021348	hypomethylated	0.1304	0.94993	insignificant	7	34	32
chr7	5301142	5303142	Gm5065		1	noCoverage	0.20501	0.011883	hypermethylated	0	6	6
chr7	5302637	5304637	Nlrp2		1	noCoverage	0.20501	0.011883	hypermethylated	0	6	6
chr7	5562915	5564915	Vmn1r61	-0.37344173	0.47739	insignificant	-0.011782	0.80785	insignificant	2	4	4
chr7	5625923	5627923	Vmn1r186		1	noCoverage	0.089286	0.47437	insignificant	0	4	4
chr7	5995762	5997762	Nlrp4c	0.16312322	1	insignificant	0.061104	0.45949	insignificant	2	12	12
chr7	6107573	6109573	Zfp787	-0.1491934	4.34E-15	hypomethylated	0.0036496	0.39295	insignificant	20	78	74
chr7	6123114	6125114	Zfp444	-0.18219669	1.17E-19	hypomethylated	-0.01418	0.72262	insignificant	21	64	64
chr7	6147691	6149691	Galp	-0.20619048	0.46636	insignificant	0.05125	0.24665	insignificant	1	4	4
chr7	6172879	6174879	Zscan5b	0.08162391	0.34654	insignificant	0.068495	0.77206	insignificant	5	28	23
chr7	6203311	6205311	Gm6792		1	noCoverage	-0.011197	0.85851	insignificant	0	22	19
chr7	6206523	6208523	Gm6792		1	noCoverage	0.048822	0.18914	insignificant	0	2	2
chr7	6237181	6239181	Zfp667	-0.13759408	1.83E-15	hypomethylated	-0.010478	0.5138	insignificant	17	38	40
chr7	6282036	6284036	Zfp583		1	noCoverage	0.050845	0.39692	insignificant	0	26	26
chr7	6335027	6337027	Zfp28	-0.25839119	1.09E-08	hypomethylated	-0.019869	0.072089	insignificant	14	60	59
chr7	6366885	6368885	Gm16532	-0.213882	0.12683	insignificant	-0.03728	0.41007	insignificant	2	14	14
chr7	6391612	6393612	Olfr1344		1	noCoverage	-0.021119	0.52917	insignificant	0	12	12
chr7	6454935	6456935	Olfr1348		1	noCoverage	-0.16574	0.14863	insignificant	0	4	5
chr7	6649143	6651143	Zim1	0.00978884	0.47998	insignificant	0.023503	0.27705	insignificant	5	14	14
chr7	6658470	6660470	Peg3as		1	noCoverage	0.11911	1	insignificant	0	10	8
chr7	6682451	6684451	Usp29	-0.06427317	1	insignificant	0.061072	0.29318	insignificant	5	12	16
chr7	6755297	6757297	Mir3099		1	noCoverage	0.13543	0.024363	hypermethylated	0	10	10
chr7	6929373	6931373	Zim3	0.0553436	0.86286	insignificant	0.10251	0.00079695	hypermethylated	16	44	44
chr7	6947095	6949095	Aurkc	0.17251432	1	insignificant	-0.007981	0.60498	insignificant	2	20	19
chr7	7074187	7076187	Zfp954		1	noCoverage	0.20733	0.00063764	hypermethylated	0	6	6
chr7	7123063	7125063	Zfp418	0.19245067	0.7153	insignificant	0.015051	0.5328	insignificant	7	20	20
chr7	7231000	7233000	Vmn2r29	-0.10898808	0.21563	insignificant	-0.048107	0.00021854	hypomethylated	14	39	38
chr7	7252222	7254222	Clcn4-2	-0.2975715	9.08E-71	hypomethylated	0.059922	0.25061	insignificant	12	41	36
chr7	11080730	11082730	Zik1	-0.71736229	0.086878	insignificant	-0.073523	0.60501	insignificant	2	10	9
chr7	12255868	12257868	Vmn1r72	-0.29464286	0.19769	insignificant	0.0074405	0.4947	insignificant	1	4	4
chr7	12907477	12909477	Vmn1r83		1	noCoverage	0.022661	0.78836	insignificant	0	4	4
chr7	13062653	13064653	Zfp606	-0.1258094	5.49E-09	hypomethylated	0.0061382	0.4209	insignificant	13	80	80
chr7	13419158	13421158	Zfp110	-0.13844655	3.49E-08	hypomethylated	0.0041683	0.73918	insignificant	10	63	63
chr7	13465526	13467526	Zfp128		1	noCoverage	0.038435	0.54476	insignificant	0	14	14

chr7	13482163	13484163	Zscan22	-0.21107447	0.000000208	hypomethylated	-0.0047172	0.71081	insignificant	9	50	50
chr7	13506659	13508659	Rps5	-0.1089379	0.000035862	hypomethylated	-0.0088735	0.92044	insignificant	8	60	60
chr7	13511764	13513764	2310014L17Ri	-0.12680287	7.8E-14	hypomethylated	-0.0054788	0.36986	insignificant	28	75	74
chr7	13550212	13552212	Zfp324	-0.12334764	0.00037919	hypomethylated	-0.0068491	0.71532	insignificant	6	54	53
chr7	13562196	13564196	Zfp446	-0.1097967	1	insignificant	0.018986	0.14335	insignificant	6	50	44
chr7	13583541	13585541	Slc27a5		1	noCoverage	0.54167	0.000028341	stronglyhypermeth	0	2	4
chr7	13595149	13597149	Zbtb45	-0.26098395	7.2E-09	hypomethylated	-0.068251	0.56023	insignificant	7	26	26
chr7	13608500	13610500	Trim28	-0.10648573	0.000011133	hypomethylated	-0.013638	0.47307	insignificant	15	62	60
chr7	13620126	13622126	Ube2m	-0.11854964	1.16E-11	inconclusive	0.041934	0.32078	insignificant	13	33	32
chr7	13623327	13625327	Ube2m	-0.17614934	0.000031206	hypomethylated	-0.029498	0.25735	insignificant	11	58	56
chr7	13623619	13625619	Ube2m	-0.29607458	0.000000539	hypomethylated	-0.051859	0.95748	insignificant	8	26	24
chr7	13640113	13642113	Mzf1	-0.30723062	1	insignificant	0.01966	0.62154	insignificant	3	31	30
chr7	13762067	13764067	Vmn1r88		1	noCoverage	0.0048669	0.20059	insignificant	0	11	9
chr7	13861661	13863661	Lig1	-0.17006803	0.17835	insignificant	-0.010068	0.9034	insignificant	3	6	6
chr7	13863174	13865174	Lig1	-0.15278641	4.03E-18	hypomethylated	-0.0060187	0.77699	insignificant	32	61	75
chr7	13863613	13865613	Lig1	-0.15278641	4.03E-18	hypomethylated	-0.0060187	0.77699	insignificant	32	61	75
chr7	13864070	13866070	Lig1	-0.12153163	0.14999	insignificant	-0.021972	0.43771	insignificant	14	17	31
chr7	14035189	14037189	Bsph1		1	noCoverage	0.034034	1	insignificant	0	4	5
chr7	14574937	14576937	Sult2a4	-0.10807121	0.0018111	hypomethylated	-0.070482	0.023008	hypomethylated	4	54	58
chr7	15208415	15210415	Gm18756	-0.02218246	0.0263	hypomethylated	0.025762	0.00099176	hypermethylated	22	80	76
chr7	16334718	16336718	Obox5		1	noCoverage	0.025232	1	insignificant	0	7	7
chr7	16457734	16459734	Crx		1	noCoverage	0.09697	0.75994	insignificant	0	10	11
chr7	16507720	16509720	Sepw1	0.33499288	0.018342	stronglyHypermeth	0.02441	0.42312	insignificant	3	30	30
chr7	16524209	16526209	Gltscr2	-0.01858697	0.023339	hypomethylated	0.010268	0.80211	insignificant	9	28	25
chr7	16531457	16533457	Gltscr2	-0.49405275	0.086042	insignificant	-0.021872	0.70635	insignificant	1	31	32
chr7	16552884	16554884	Ehd2	0.15918368	0.043582	hypermethylated	0.043743	0.18456	insignificant	1	7	7
chr7	16682991	16684991	Napa	-0.09380438	4.98E-09	hypomethylated	0.013098	0.14256	insignificant	21	62	62
chr7	16704224	16706224	Kptn	-0.26617972	0.000001214	hypomethylated	-0.015016	0.50404	insignificant	5	14	14
chr7	16714648	16716648	Slc8a2	-0.20686328	7.1E-22	hypomethylated	-0.002514	0.24304	insignificant	12	32	28
chr7	16759728	16761728	Meis3	-0.0840275	0.000062183	hypomethylated	0.0098111	0.81369	insignificant	14	43	42
chr7	16827680	16829680	Gpr77	-0.48400814	0.036958	stronglyHypometh	0.096239	0.0096898	hypermethylated	2	14	14
chr7	16844687	16846687	C5ar1	0.01139817	0.74543	insignificant	0.018626	0.7518	insignificant	4	8	8
chr7	16844889	16846889	C5ar1	0.01139817	0.74543	insignificant	0.018626	0.7518	insignificant	4	8	8
chr7	16859041	16861041	Ccdc9	-0.20141699	0.000031175	hypomethylated	-0.04118	0.6367	insignificant	7	40	38
chr7	16870008	16872008	Ccdc9	-0.1820064	4.98E-09	hypomethylated	0.019952	0.5341	insignificant	6	43	44
chr7	16893931	16895931	Bbc3	-0.088676	1.64E-26	hypomethylated	0.0010918	0.76431	insignificant	44	163	174
chr7	16973134	16975134	Sae1	-0.08317246	5.32E-09	hypomethylated	0.02758	0.33871	insignificant	7	36	36
chr7	16985544	16987544	Zc3h4	-0.10637287	2.12E-21	hypomethylated	0.023411	0.14717	insignificant	38	174	174
chr7	17037127	17039127	Tmem160	-0.08238194	0.000000287	hypomethylated	-0.0039305	0.37649	insignificant	12	90	88
chr7	17062129	17064129	Npas1	0.12754091	0.0040826	inconclusive	0.14376	0.000000957	hypermethylated	5	17	20
chr7	17200342	17202342	Grlf1	-0.26089315	0.65398	insignificant	-0.087088	0.31979	insignificant	5	40	37
chr7	17306277	17308277	Ceacam9		1	noCoverage	0.31429	0.18098	insignificant	0	2	4
chr7	17322792	17324792	Ap2s1	-0.19165974	1.25E-17	hypomethylated	0.027687	0.67659	insignificant	19	61	60
chr7	17365694	17367694	Slc1a5	-0.11401327	0.000019743	hypomethylated	0.0026076	0.23955	insignificant	14	74	85
chr7	17400237	17402237	Strn4	-0.08950149	7.4E-10	hypomethylated	-0.0072957	0.15726	insignificant	30	136	136
chr7	17402081	17404081	Strn4	-0.1241395	3.93E-08	hypomethylated	0.014575	0.00090498	hypermethylated	15	34	33
chr7	17427413	17429413	Prkd2	-0.19036668	7.15E-13	hypomethylated	0.0077902	0.030151	hypermethylated	11	56	57
chr7	17459665	17461665	Dact3	-0.18811612	5.83E-09	hypomethylated	0.073496	0.64726	insignificant	9	51	47
chr7	17476134	17478134	Gng8		1	noCoverage	-0.011753	0.90151	insignificant	0	13	13
chr7	17490838	17492838	Ptgir		1	noCoverage	0.10897	0.91267	insignificant	0	8	8
chr7	17509381	17511381	Calm3	-0.09090377	0.00011923	hypomethylated	-0.0041955	0.28173	insignificant	6	49	49
chr7	17529030	17531030	Pnmal2	-0.14443071	8.05E-12	hypomethylated	0.014212	0.65145	insignificant	20	63	58
chr7	17578936	17580936	Ccdc8	0.39552065	0.59995	insignificant	-0.010086	1	insignificant	3	33	33
chr7	17613263	17615263	Ppp5c	-0.40079161	0.041266	stronglyHypometh	0.02178	0.034087	hypermethylated	3	17	17
chr7	17642028	17644028	Hif3a	-0.25044541	0.00000256	hypomethylated	0.038806	0.13754	insignificant	4	24	24
chr7	18594237	18596237	Ceacam13		1	noCoverage	0.028431	1	insignificant	0	4	4
chr7	19271341	19273341	Psg20	-0.68080357	0.0019964	stronglyHypometh	-0.015968	1	insignificant	1	4	4
chr7	19406940	19408940	Psg17	-0.48153431	0.00041204	stronglyHypometh	0.13781	0.0033662	hypermethylated	4	8	8
chr7	19424314	19426314	Mill2		1	noCoverage	0.020869	0.16308	insignificant	0	21	21
chr7	19469038	19471038	Pglyrp1	-0.22275641	0.025439	hypomethylated	0.057763	0.16063	insignificant	3	8	8
chr7	19495753	19497753	Ccdc61	-0.28947368	0.00020043	hypomethylated	0.016082	0.034748	hypermethylated	1	2	2
chr7	19510236	19512236	Nova2	-0.19530924	0.20925	insignificant	0.0097026	0.049597	hypermethylated	3	59	72
chr7	19571872	19573872	Nanos2		1	noCoverage	-0.16326	0.57765	insignificant	0	8	10
chr7	19575593	19577593	Mypop	-0.07125086	0.000013088	hypomethylated	0.02006	0.24993	insignificant	18	75	67
chr7	19588413	19590413	Irf2bp1	-0.18840682	1.84E-92	hypomethylated	0.028174	0.026749	hypermethylated	44	138	139
chr7	19608725	19610725	Sympk	-0.13308919	1.9E-57	hypomethylated	0.0053952	0.32836	insignificant	52	160	158
chr7	19608888	19610888	Foxa3	-0.12993728	3.01E-51	hypomethylated	0.00561	0.39143	insignificant	48	148	144
chr7	19639035	19641035	Rsph6a	0.08998866	0.23881	insignificant	0.0032942	0.90361	insignificant	6	31	30
chr7	19660548	19662548	Dmwd	-0.14543909	3.01E-50	hypomethylated	-0.00065637	0.30137	insignificant	38	113	79
chr7	19668197	19670197	Dmpk	-0.08663743	0.61242	insignificant	-0.010094	0.8652	insignificant	1	6	6
chr7	19671176	19673176	Mir3100	-0.13524424	1.95E-10	hypomethylated	0.030407	0.74014	insignificant	8	34	34
chr7	19678892	19680892	Six5	-0.11864653	4E-41	hypomethylated	0.015423	0.21218	insignificant	47	139	134
chr7	19704207	19706207	Fbxo46	0.09996435	0.049771	inconclusive	0.016718	0.58358	insignificant	3	38	36
chr7	19734186	19736186	Snrpd2	-0.1640793	1.01E-15	hypomethylated	-0.012277	0.70448	insignificant	7	47	59
chr7	19734545	19736545	Qpctl	-0.1545231	1.76E-15	hypomethylated	-0.0012276	0.83252	insignificant	5	35	44
chr7	19765518	19767518	Eml2	-0.10339894	0.019122	hypomethylated	0.017937	0.47718	insignificant	11	48	48
chr7	19765813	19767813	Mir330	-0.00350368	0.049586	inconclusive	0.014263	0.00039654	hypermethylated	16	58	58
chr7	19796886	19798886	Gpr4	-0.25072783	0.000045882	hypomethylated	-0.073149	0.003687	inconclusive	9	50	54
chr7	19812737	19814737	Opa3	-0.10479075	0.000000874	hypomethylated	0.0039889	0.83768	insignificant	9	83	80
chr7	19857203	19859203	Vasp	-0.18807283	6.14E-11	hypomethylated	-0.0049547	0.48927	insignificant	12	53	49
chr7	19867015	19869015	Rtn2	-0.11603522	0.0010014	hypomethylated	0.0082657	0.24436	insignificant	14	71	70
chr7	19875447	19877447	Rtn2	-0.20555753	0.051771	insignificant	0.045488	0.78444	insignificant	4	14	15
chr7	19895394	19897394	Fosb	-0.20969268	0.010897	hypomethylated	0.0070122	0.92855	insignificant	11	38	38
chr7	19929419	19931419	Ercc1	-0.1999543	0.0004892	hypomethylated	0.012973	0.83817	insignificant	9	60	55
chr7	19944832	19946832	Cd3eap	-0.05289462	0.17617	insignificant	0.0070088	0.5483	insignificant	11	76	79

chr7	19945564	19947564	Ppp1r13l	-0.18089774	1.56E-08	hypomethylated	-0.0019187	0.57333	insignificant	11	76	79
chr7	19966387	19968387	Ercc2	-0.15644035	7.24E-17	hypomethylated	-0.0057379	0.0045902	hypomethylated	24	72	72
chr7	19970991	19972991	Mir343	-0.19787449	0.041314	hypomethylated	-0.04296	0.33514	insignificant	1	8	8
chr7	19985205	19987205	Klc3	-0.53224206	0.0021711	stronglyHypometh	-0.26046	0.25482	insignificant	2	9	4
chr7	19995442	19997442	Ckm	-0.0023348	0.50773	insignificant	0.11272	0.11548	insignificant	8	29	28
chr7	20043843	20045843	Mark4	-0.14321196	0.00000286	hypomethylated	0.022731	0.81456	insignificant	15	37	37
chr7	20093077	20095077	Trappc6a	-0.1719566	3.77E-28	hypomethylated	0.0033558	0.62901	insignificant	25	73	77
chr7	20093680	20095680	Bloc1s3	-0.18602373	2.5E-21	hypomethylated	0.030409	0.73509	insignificant	20	58	62
chr7	20103079	20105079	Nkpd1	-0.60904849	0.17698	insignificant	0.035959	0.30799	insignificant	1	14	17
chr7	20147747	20149747	Ppp1r37	-0.2835836	1.17E-10	hypomethylated	-0.041751	0.084855	insignificant	5	20	18
chr7	20158692	20160692	Gemin7	-0.27395605	1.58E-17	hypomethylated	-0.019413	0.60016	insignificant	13	72	72
chr7	20161635	20163635	Zfp296	-0.09671163	0.0010572	hypomethylated	0.042816	0.18465	insignificant	15	80	82
chr7	20189817	20191817	Clasrp	0.71920024	0.00010699	stronglyHypermeth	0.11217	1	insignificant	2	30	33
chr7	20214787	20216787	Relb	-0.11050122	1.52E-10	hypomethylated	0.011031	0.29004	insignificant	19	34	34
chr7	20250379	20252379	Clptm1	-0.11759781	0.0025201	hypomethylated	-0.034561	0.0081756	hypomethylated	6	68	65
chr7	20262213	20264213	Apoc2	0.11002356	0.78194	insignificant	0.11438	0.1357	insignificant	4	16	15
chr7	20300778	20302778	Tomm40	-0.11049073	0.025735	hypomethylated	0.0058727	0.63863	insignificant	9	55	55
chr7	20334922	20336922	Pvrl2	-0.28474617	0.015292	hypomethylated	-0.0076849	1	insignificant	5	21	21
chr7	20355881	20357881	Bcam	-0.32318236	0.30263	insignificant	-0.056782	0.3734	insignificant	4	18	22
chr7	20382158	20384158	Cblc	-0.02041722	1	insignificant	0.07897	0.27169	insignificant	1	19	19
chr7	20408104	20410104	Bcl3	-0.22050035	9.21E-14	hypomethylated	-0.0138	0.785	insignificant	13	52	46
chr7	20446648	20448648	Ceacam16	0.10555556	1	insignificant	0.052991	0.48932	insignificant	1	2	2
chr7	20473314	20475314	Ceacam19	0.37591991	0.53893	insignificant	0.22314	0.022036	hypermethylated	1	14	6
chr7	20506492	20508492	Pvr	-0.36291164	0.57089	insignificant	-0.044117	0.021281	hypomethylated	3	32	30
chr7	20536092	20538092	2210010C17Ri	0.32782313	0.17127	insignificant	-0.0029735	0.90563	insignificant	4	14	14
chr7	24169915	24171915	Nlrp5	0.12801557	0.66972	insignificant	0.086297	0.012522	hypermethylated	4	13	13
chr7	24537929	24539929	Vmn1r174		1	noCoverage	-0.25952	0.21446	insignificant	0	8	4
chr7	24865962	24867962	Zfp180	-0.19101455	0.0000077	hypomethylated	0.072582	0.014158	hypermethylated	11	43	48
chr7	24896338	24898338	Zfp112	-0.34093006	0.17281	insignificant	0.10561	0.64897	insignificant	3	18	16
chr7	24918181	24920181	Zfp235	-0.19121051	0.13941	insignificant	-0.015538	0.16802	insignificant	4	32	22
chr7	25022617	25024617	Zfp109	-0.01718705	0.012673	inconclusive	-0.012838	0.082107	insignificant	4	22	22
chr7	25038812	25040812	Zfp108	0.39347662	0.087124	insignificant	-0.029759	0.52753	insignificant	5	16	12
chr7	25054436	25056436	Zfp93		1	noCoverage	0.066198	0.30079	insignificant	0	22	21
chr7	25084568	25086568	Zfp61	-0.22509626	0.00033477	hypomethylated	-0.053148	0.33603	insignificant	7	28	30
chr7	25101685	25103685	Zfp94	-0.12616321	0.025334	hypomethylated	-0.012404	0.37697	insignificant	3	30	28
chr7	25154281	25156281	Kcnn4		1	noCoverage	0.022111	0.60338	insignificant	0	7	7
chr7	25183646	25185646	1500002O20R	-0.16498682	0.000020665	hypomethylated	-0.050124	0.29337	insignificant	4	14	14
chr7	25246518	25248518	Plaur	-0.26418435	5.54E-12	hypomethylated	-0.021993	0.60048	insignificant	8	30	28
chr7	25266041	25268041	Cadm4	-0.07532396	3.3E-13	hypomethylated	0.0084748	0.061498	insignificant	31	150	150
chr7	25291105	25293105	Zfp428	-0.10483545	8.11E-10	hypomethylated	0.00087122	0.069596	insignificant	23	139	145
chr7	25314666	25316666	Irgq	-0.15245837	3E-12	hypomethylated	0.014358	0.33141	insignificant	11	77	76
chr7	25331024	25333024	Xrcc1	-0.14775164	1.71E-23	hypomethylated	-0.027025	0.52147	insignificant	26	110	106
chr7	25331168	25333168	Xrcc1	-0.16231295	7.01E-24	hypomethylated	-0.027528	0.48124	insignificant	26	112	108
chr7	25371561	25373561	Ethe1	-0.49023836	1	lowCoverage	-0.051215	0.60315	insignificant	1	29	30
chr7	25372660	25374660	Ethe1	-0.61088683	1	lowCoverage	-0.099285	0.3883	insignificant	0	27	27
chr7	25395346	25397346	Phldb3	-0.15610766	8.56E-12	hypomethylated	0.02621	0.63284	insignificant	22	80	85
chr7	25420588	25422588	Lypd3	-0.12648155	0.031741	hypomethylated	0.083066	6.99E-13	hypermethylated	11	55	53
chr7	25445312	25447312	Gm4598	-0.10516591	0.55314	insignificant	0.054408	0.95903	insignificant	8	35	33
chr7	25457069	25459069	Tex101	-0.02368968	0.19802	insignificant	0.048938	0.00053089	hypermethylated	7	24	24
chr7	25493277	25495277	BC049730	0.03896801	0.46134	insignificant	0.061991	0.019196	hypermethylated	3	12	16
chr7	25654075	25656075	Dmrtc2	-0.74696891	0.0034985	stronglyHypometh	-0.0046725	0.50787	insignificant	4	66	60
chr7	25654710	25656710	Lypd4	-0.72678479	0.0035965	stronglyHypometh	0.0098593	0.59424	insignificant	4	56	56
chr7	25668732	25670732	Rps19	-0.10700306	1.88E-26	hypomethylated	0.010402	0.25916	insignificant	33	110	109
chr7	25681529	25683529	Cd79a		1	noCoverage	-0.23477	0.0047879	hypomethylated	0	12	14
chr7	25687004	25689004	Arhgef1	-0.15551037	1.19E-36	hypomethylated	0.015803	0.73077	insignificant	33	115	116
chr7	25688364	25690364	Arhgef1	-0.18645388	6.92E-12	hypomethylated	-0.00052842	0.71109	insignificant	10	46	46
chr7	25757747	25759747	Rabac1		1	noCoverage	-0.04211	0.11615	insignificant	0	12	12
chr7	25790914	25792914	Atp1a3	-0.10647832	0.000086923	hypomethylated	0.024302	0.92434	insignificant	11	27	31
chr7	25857388	25859388	Grik5	-0.20491716	3.44E-16	hypomethylated	-0.030944	0.33893	insignificant	10	31	31
chr7	25861223	25863223	Zfp574	-0.13850443	1.73E-24	hypomethylated	0.010429	0.14335	insignificant	19	117	113
chr7	25861264	25863264	Zfp574	-0.13850443	1.73E-24	hypomethylated	0.010429	0.14335	insignificant	19	117	113
chr7	25917479	25919479	Pou2f2	-0.1615746	0.000042808	hypomethylated	0.02219	0.24359	insignificant	7	23	23
chr7	26022870	26024870	Gsk3a	-0.15071169	0.00000242	hypomethylated	-0.014762	0.58802	insignificant	6	35	42
chr7	26035777	26037777	Erf	-0.16485553	5.23E-08	hypomethylated	0.041927	0.6948	insignificant	11	45	44
chr7	26066197	26068197	Cic	-0.09448602	0.000097207	hypomethylated	0.01434	0.8989	insignificant	26	100	121
chr7	26085377	26087377	Prr19	-0.38002083	0.3577	insignificant	-0.017671	0.12577	insignificant	2	20	20
chr7	26090126	26092126	Tmem145	-0.13094328	3.73E-11	hypomethylated	-0.0074731	0.44995	insignificant	23	68	66
chr7	26101182	26103182	Megf8	-0.21305433	0.30373	insignificant	0.041133	0.026213	inconclusive	15	64	59
chr7	26179432	26181432	4732471J01Ri	-0.27645675	1.79E-12	hypomethylated	0.028839	0.020839	hypermethylated	6	16	16
chr7	26181006	26183006	4732471J01Ri	-0.15963691	0.00009439	hypomethylated	-0.0087516	0.09869	insignificant	8	30	28
chr7	26262644	26264644	4732471J01Rik		1	noCoverage	0.093357	0.39761	insignificant	0	13	6
chr7	26325023	26327023	Ceacam2		1	noCoverage	-0.11896	0.36221	insignificant	0	15	20
chr7	26400911	26402911	Gm7092	-0.0575305	1	insignificant	0.0092389	0.77472	insignificant	3	9	9
chr7	26403511	26405511	Atp5sl	-0.45954425	0.000063547	stronglyHypometh	-0.27339	0.90008	insignificant	3	31	20
chr7	26411642	26413642	B3gnt8	-0.14554889	1.42E-10	hypomethylated	0.02655	0.40368	insignificant	13	66	63
chr7	26443171	26445171	Exosc5	-0.12844821	0.40686	insignificant	0.0029888	0.8562	insignificant	6	58	56
chr7	26443780	26445780	Bckdha	-0.01551581	0.78545	insignificant	0.0037534	0.79055	insignificant	7	60	58
chr7	26460185	26462185	Tmem91	0.2465512	0.068266	insignificant	0.14068	0.28845	insignificant	3	20	28
chr7	26465176	26467176	B9d2		1	noCoverage	0.018519	0.92662	insignificant	0	6	6
chr7	26471020	26473020	Tgfb1	-0.10038415	0.036532	hypomethylated	-0.0027249	0.93912	insignificant	9	73	65
chr7	26504072	26506072	Ccdc97	-0.38570134	8.16E-25	stronglyHypometh	-0.013912	0.19454	insignificant	6	22	22
chr7	26539720	26541720	Hnrnpul1	-0.28082017	0.00000165	hypomethylated	-0.10496	0.27718	insignificant	12	51	44
chr7	26539739	26541739	Hnrnpul1	-0.28082017	0.00000165	hypomethylated	-0.10496	0.27718	insignificant	12	51	44
chr7	26573752	26575752	Axl	-0.00694444	0.54386	insignificant	0.12153	0.043904	hypermethylated	2	4	4
chr7	26601549	26603549	Cyp2s1	-0.29940476	0.041361	hypomethylated	-0.019441	0.57246	insignificant	2	4	4

chr7	27219131	27221131	Nlrp4a	-0.40641026	0.26779	insignificant	-0.070994	0.59635	insignificant	2	4	4
chr7	27937732	27939732	Cyp2t4	-0.16787749	0.37349	insignificant	-0.022639	1	insignificant	2	6	6
chr7	27951821	27953821	Egln2	-0.19406355	0.033924	hypomethylated	-0.051618	0.15256	insignificant	2	28	33
chr7	27963902	27965902	Rab4b	-0.16282895	0.2144	insignificant	0.088453	1	insignificant	4	8	13
chr7	27980779	27982779	BC024978	-0.11448954	8E-12	hypomethylated	0.021742	0.28066	insignificant	20	71	70
chr7	28013616	28015616	Itpkc	-0.02738209	0.00000861	hypomethylated	-0.038603	0.42383	insignificant	6	30	26
chr7	28017031	28019031	Adck4		1	noCoverage	0.074618	0.44247	insignificant	0	7	3
chr7	28042779	28044779	Numbl	-0.13940038	3.63E-34	hypomethylated	-0.016116	0.37093	insignificant	34	101	96
chr7	28118667	28120667	Ltbp4	-0.15817816	4.67E-10	hypomethylated	-0.0018477	0.4715	insignificant	17	83	67
chr7	28122631	28124631	Ltbp4	-0.22479409	0.49772	insignificant	-0.010612	0.38208	insignificant	7	30	30
chr7	28141027	28143027	Spnb4	-0.21279361	0.00001435	hypomethylated	0.012624	0.2558	insignificant	6	32	32
chr7	28179525	28181525	Spnb4	-0.13415264	0.38115	insignificant	0.036965	0.085929	insignificant	2	30	30
chr7	28181217	28183217	Spnb4	-0.03464616	0.0049741	inconclusive	0.027334	0.078412	insignificant	8	76	76
chr7	28181572	28183572	Spnb4	-0.06371102	0.05488	insignificant	0.026994	0.0046765	hypermethylated	8	52	52
chr7	28231608	28233608	Spnb4	-0.12203766	5.23E-24	hypomethylated	-0.024898	0.080394	insignificant	30	97	90
chr7	28231996	28233996	Blvrb	-0.18955931	7.48E-12	hypomethylated	-0.023955	0.56967	insignificant	15	65	58
chr7	28257858	28259858	Sertad3	-0.17473207	1.89E-08	hypomethylated	0.0019566	0.45421	insignificant	17	69	68
chr7	28270971	28272971	Sertad1	-0.1941501	1.06E-39	hypomethylated	0.068165	0.47297	insignificant	26	52	52
chr7	28283342	28285342	Prx	-0.81077037	0.00000543	stronglyHypometh	-0.023916	0.55548	insignificant	1	4	4
chr7	28307279	28309279	Hipk4	-0.21284191	0.24016	insignificant	0.06606	0.28891	insignificant	4	24	22
chr7	28337313	28339313	2310022A10R	-0.09640879	1.77E-08	hypomethylated	0.0052373	0.7468	insignificant	7	32	32
chr7	28338131	28340131	Pld3	-0.09640879	1.77E-08	hypomethylated	0.0052373	0.7468	insignificant	7	32	32
chr7	28375578	28377578	Akt2	-0.12752159	0.23746	insignificant	0.022118	0.60875	insignificant	2	26	28
chr7	28437942	28439942	Ttc9b	-0.09242852	0.0041968	hypomethylated	-0.0015297	0.045103	inconclusive	26	100	108
chr7	28458679	28460679	C030039L03Ri	-0.16379487	9.94E-21	hypomethylated	-0.042026	0.2733	insignificant	32	95	82
chr7	28459617	28461617	C030039L03Ri	-0.67819549	0.079425	insignificant	-0.15042	0.57718	insignificant	2	5	4
chr7	28473358	28475358	C030039L03Ri	-0.61637154	0.00000167	stronglyHypometh	-0.027121	0.67053	insignificant	3	12	14
chr7	28515427	28517427	Zfp60	-0.23838307	0.000000012	hypomethylated	-0.012162	0.016479	inconclusive	6	40	42
chr7	28549691	28551691	Gm10046	-0.39532239	0.50166	insignificant	0.011496	0.608	insignificant	1	33	33
chr7	28622602	28624602	Zfp59	-0.28148847	7.4E-19	hypomethylated	-0.031664	0.05804	insignificant	10	28	28
chr7	28644603	28646603	Zfp607	-0.21633157	0.34219	insignificant	-0.0073296	0.91306	insignificant	2	26	26
chr7	28714449	28716449	1700049G17R	-0.16677334	1	insignificant	-0.022354	0.7165	insignificant	2	15	16
chr7	28835111	28837111	Psmc4	-0.06341575	0.23635	insignificant	0.040879	0.10701	insignificant	6	16	16
chr7	28855254	28857254	Fcgbp	-0.13431148	0.000057687	hypomethylated	-0.076005	0.72184	insignificant	4	29	29
chr7	28913484	28915484	9530053A07R	0.06714286	1	insignificant	-0.10944	0.71997	insignificant	1	6	6
chr7	28953766	28955766	Fbl	-0.12837718	3.32E-34	hypomethylated	0.010795	0.36169	insignificant	21	85	87
chr7	28963501	28965501	Dyrk1b	-0.08716934	1.01E-16	hypomethylated	0.0006341	0.96991	insignificant	27	134	127
chr7	28963512	28965512	Dyrk1b	-0.08716934	1.01E-16	hypomethylated	0.0006341	0.96991	insignificant	27	134	127
chr7	29051899	29053899	Eid2	-0.13061661	1.05E-21	hypomethylated	-0.0046257	0.86864	insignificant	36	115	115
chr7	29061724	29063724	Eid2b	-0.0711157	4.68E-17	hypomethylated	0.014995	0.12334	insignificant	21	111	109
chr7	29076153	29078153	BC089491	-0.09207302	0.2257	insignificant	0.01263	0.95285	insignificant	7	27	27
chr7	29086804	29088804	Dll3	-0.32846627	2.52E-11	hypomethylated	-0.062601	0.43818	insignificant	4	28	32
chr7	29123738	29125738	Supt5h	0.0476204	6.99E-12	hypermethylated	-0.066664	0.50719	insignificant	6	42	41
chr7	29134707	29136707	Rps16	-0.13876829	0.000000099	hypomethylated	0.019131	0.48434	insignificant	12	69	61
chr7	29135558	29137558	LOC10030256	-0.13424389	5.52E-08	hypomethylated	0.020368	0.53318	insignificant	10	63	55
chr7	29157681	29159681	Plekhg2	-0.0882906	0.68424	insignificant	0.010282	0.60408	insignificant	15	80	80
chr7	29164247	29166247	Zfp36	-0.3189959	0.00000572	hypomethylated	0.059775	0.081121	insignificant	5	41	40
chr7	29177014	29179014	Paf1	-0.14967324	9.28E-19	hypomethylated	-0.02826	0.19434	insignificant	16	61	57
chr7	29177709	29179709	Med29	0.09233259	0.0002843	inconclusive	0.071313	0.028795	hypermethylated	3	13	10
chr7	29221210	29223210	Samd4b	-0.13437553	7.16E-23	hypomethylated	0.0030402	0.090089	insignificant	17	79	71
chr7	29221465	29223465	Gmfg	-0.1627166	3.2E-23	hypomethylated	0.000223	0.064888	insignificant	17	58	58
chr7	29236003	29238003	Lrfn1	-0.14556776	9.17E-17	hypomethylated	-0.027026	0.3304	insignificant	38	113	121
chr7	29236256	29238256	Lrfn1	-0.15209182	5.38E-18	hypomethylated	-0.023391	0.11223	insignificant	39	115	123
chr7	29280959	29282959	1700028B04R	-0.06576518	0.73125	insignificant	-0.021244	0.29625	insignificant	3	28	26
chr7	29295379	29297379	Il28a	-0.44760101	0.0021879	stronglyHypometh	-0.0074301	1	insignificant	1	2	2
chr7	29324903	29326903	Sycn	0.040625	1	insignificant	0.02557	0.019201	hypermethylated	4	20	20
chr7	29332273	29334273	Nccrp1	-0.27858463	0.000001396	hypomethylated	-0.035843	0.38376	insignificant	4	8	9
chr7	29383203	29385203	Pak4	-0.36716766	0.0002572	stronglyHypometh	-0.023762	0.4914	insignificant	3	14	15
chr7	29416034	29418034	C330005M16F	-0.51844815	8.76E-08	stronglyHypometh	0.039039	0.65136	insignificant	3	26	21
chr7	29476867	29478867	Fbxo27	-0.52740669	1.6E-16	stronglyHypometh	-0.0020815	0.00092206	inconclusive	4	46	44
chr7	29477162	29479162	Fbxo27	-0.50914077	4.56E-15	stronglyHypometh	-0.01867	0.0002375	inconclusive	4	40	38
chr7	29500808	29502808	Fbxo17	-0.22864496	9.59E-15	hypomethylated	0.10702	0.79206	insignificant	14	20	25
chr7	29526009	29528009	Mrps12	-0.13892971	2.78E-17	hypomethylated	0.0087169	0.21971	insignificant	19	73	73
chr7	29526800	29528800	Mrps12	-0.29364907	8.9E-18	hypomethylated	0.0067697	0.68981	insignificant	15	63	63
chr7	29540192	29542192	Gm6537	-0.25687646	1	insignificant	0.025175	0.56214	insignificant	2	13	13
chr7	29550770	29552770	Sirt2	-0.15891371	1.36E-10	hypomethylated	-0.0032036	0.74032	insignificant	20	76	76
chr7	29551543	29553543	Sirt2	-0.13974636	0.000053583	hypomethylated	-0.012201	0.0015183	inconclusive	12	58	58
chr7	29572987	29574987	Rinl		1	noCoverage	-0.082096	0.5539	insignificant	0	12	12
chr7	29594908	29596908	Hnrnpl	-0.14315418	7.57E-18	hypomethylated	-0.0082575	0.3664	insignificant	22	62	63
chr7	29609356	29611356	Ech1	-0.18126462	0.022686	hypomethylated	0.00072581	0.16575	insignificant	6	24	25
chr7	29665674	29667674	Capn12	0.2844623	0.012036	hypermethylated	-0.013428	0.39784	insignificant	3	30	28
chr7	29747299	29749299	Actn4	-0.22766155	0.000000481	hypomethylated	0.024329	0.000007491	inconclusive	16	66	65
chr7	29766833	29768833	Eif3k	-0.2157186	5.66E-08	hypomethylated	-0.0052236	0.52402	insignificant	8	34	34
chr7	29766872	29768872	Map4k1	-0.27607601	0.000000122	hypomethylated	-0.0055501	0.52631	insignificant	6	32	32
chr7	29910170	29912170	Ryr1	0.1	0.68213	insignificant	0.25238	0.049676	hypermethylated	1	2	3
chr7	29918951	29920951	Rasgrp4	-0.01710139	0.017416	hypomethylated	-0.02166	0.14814	insignificant	2	20	24
chr7	29941229	29943229	Fam98c	0.1031968	1	lowCoverage	-0.027079	0.061953	insignificant	1	14	14
chr7	29953666	29955666	Spred3	-0.20203335	0.0070301	hypomethylated	-0.0030966	1	insignificant	4	46	42
chr7	29954228	29956228	Ggn	-0.18717518	0.031234	hypomethylated	-0.00027023	0.73275	insignificant	5	70	66
chr7	29954408	29956408	Ggn	-0.18717518	0.031234	hypomethylated	-0.00027023	0.73275	insignificant	5	70	66
chr7	29999052	30001052	Catsperg1	-0.16054156	0.013057	hypomethylated	-0.064644	0.11198	insignificant	4	16	16
chr7	30017541	30019541	Kcnk6	-0.19430656	0.000000239	hypomethylated	0.022799	0.21691	insignificant	7	29	26
chr7	30022341	30024341	Yif1b	-0.1788228	8.45E-24	hypomethylated	0.0011477	0.45198	insignificant	23	70	76
chr7	30033486	30035486	2200002D01R	-0.24219779	1.85E-27	hypomethylated	-0.11823	0.0068868	hypomethylated	6	12	12
chr7	30066996	30068996	Spint2	-0.28276159	0.000000355	hypomethylated	0.028389	0.56701	insignificant	4	19	20

chr7	30073338	30075338	Ppp1r14a	-0.04976815	0.000000108	hypomethylated	0.027144	0.76795	insignificant	27	85	88
chr7	30088023	30090023	Dpf1	-0.18827051	1.01E-25	hypomethylated	0.016049	0.33302	insignificant	35	126	112
chr7	30552570	30554570	Zfp84	-0.30399398	0.000013763	hypomethylated	-0.01625	0.72503	insignificant	6	22	23
chr7	30557312	30559312	Mir1964	-0.02083333	0.74909	insignificant	0.0625	0.060505	insignificant	2	4	4
chr7	30568808	30570808	Zfp30	0.02028737	0.098869	insignificant	0.036297	0.58597	insignificant	13	49	46
chr7	30600090	30602090	Zfp790	-0.18367563	0.31857	insignificant	-0.0064706	0.86952	insignificant	5	36	42
chr7	30643997	30645997	Zfp420	-0.16396104	0.00005289	hypomethylated	-0.013261	0.64723	insignificant	6	14	14
chr7	30691123	30693123	Zfp27	-0.10687992	0.00075183	inconclusive	0.026086	0.30922	insignificant	5	18	18
chr7	30736912	30738912	Zfp74	-0.14612069	1	insignificant	-0.0072318	0.10641	insignificant	2	8	8
chr7	30767973	30769973	Zfp568	-0.09392258	7.5E-17	hypomethylated	0.0058734	0.83362	insignificant	18	66	66
chr7	30836415	30838415	Zfp14	-0.91955637	0.023302	stronglyHypometh	-0.038627	0.83121	insignificant	1	12	12
chr7	30857842	30859842	Zfp82		1	noCoverage	0.0026292	1	insignificant	0	12	12
chr7	30879094	30881094	Zfp260	-0.23991382	0.054098	insignificant	-0.018401	0.414	insignificant	3	20	20
chr7	30905966	30907966	Zfp382	-0.17355261	5.61E-24	hypomethylated	0.0097471	0.182	insignificant	18	53	50
chr7	30953940	30955940	Gm5113	-0.28889675	0.0038086	hypomethylated	0.010514	1	insignificant	3	16	20
chr7	30954746	30956746	Zfp146	-0.08349814	0.42934	insignificant	0.068718	0.57529	insignificant	1	12	12
chr7	30968189	30970189	Cox7a1		1	noCoverage	-0.0011268	0.44436	insignificant	0	8	8
chr7	30980067	30982067	Capns1	-0.36795426	1.18E-16	stronglyHypometh	0.025758	0.12625	insignificant	3	32	33
chr7	31016092	31018092	Polr2i	-0.09157388	0.0006385	hypomethylated	0.0033528	0.52157	insignificant	9	120	109
chr7	31017048	31019048	Tbcb	0.0809871	0.0035843	hypermethylated	0.030859	0.0076731	inconclusive	12	74	65
chr7	31065440	31067440	Wdr62	-0.3350282	0.30125	insignificant	0.035763	0.75313	insignificant	2	18	12
chr7	31092771	31094771	Alkbh6		1	noCoverage	-0.011966	1	insignificant	0	12	12
chr7	31098834	31100834	AI428936	-0.08609446	0.22587	insignificant	0.055426	0.0019599	hypermethylated	12	29	29
chr7	31107394	31109394	Sdhaf1	-0.11796731	0.000000326	hypomethylated	0.058337	0.36773	insignificant	6	24	25
chr7	31147791	31149791	Lrfn3	-0.22991104	0.000088295	hypomethylated	-0.031203	0.49938	insignificant	11	43	43
chr7	31197806	31199806	Tyrobp	-0.48518519	0.000000588	stronglyHypometh	0.017251	0.66826	insignificant	1	4	4
chr7	31204873	31206873	Hcst	-0.14093554	0.000051454	hypomethylated	-0.027638	0.51801	insignificant	7	45	42
chr7	31207322	31209322	Nfkbid	-0.18870768	0.036993	hypomethylated	0.010065	0.0016927	hypermethylated	3	54	52
chr7	31230580	31232580	Aplp1	-0.50739436	2.06E-30	stronglyHypometh	0.0033495	0.00073975	hypermethylated	9	35	35
chr7	31242534	31244534	Nphs1	-0.25397112	2.28E-08	hypomethylated	-0.043597	1	insignificant	8	19	18
chr7	31244076	31246076	Nphs1	-0.38555627	0.23924	insignificant	0.032362	0.014851	hypermethylated	5	21	20
chr7	31250610	31252610	Nphs1	-0.05143478	0.80766	insignificant	-0.0015008	0.31833	insignificant	9	31	31
chr7	31277676	31279676	Prodh2	-0.38609879	0.080259	insignificant	0.086488	0.44895	insignificant	2	5	8
chr7	31299140	31301140	Gm1082	-0.22732562	1	insignificant	0.024745	0.0001696	hypermethylated	4	18	12
chr7	31320028	31322028	Arhgap33	-0.11708579	0.00026876	hypomethylated	-0.0005785	0.057662	insignificant	9	26	26
chr7	31337291	31339291	BC053749	-0.30151623	5.41E-11	hypomethylated	0.026327	0.59245	insignificant	3	44	44
chr7	31337320	31339320	Hspb6	-0.30151623	5.41E-11	hypomethylated	0.026327	0.59245	insignificant	3	44	44
chr7	31344665	31346665	Lin37		1	noCoverage	-0.0006218	0.0093446	inconclusive	0	10	8
chr7	31347358	31349358	U2af1l4	-0.14467886	2.43E-14	hypomethylated	0.02448	0.21444	insignificant	13	70	70
chr7	31348203	31350203	Psenen	-0.18995953	7.57E-08	hypomethylated	-0.0081393	0.022396	inconclusive	17	95	95
chr7	31349445	31351445	Tmem149	-0.04715916	1	insignificant	-0.04742	0.817	insignificant	5	37	37
chr7	31373745	31375745	Zbtb32	-0.14755111	1.13E-22	hypomethylated	0.015748	0.011031	inconclusive	19	152	144
chr7	31397493	31399493	Upk1a	-0.24644886	0.39132	insignificant	-0.039604	0.25649	insignificant	2	16	16
chr7	31411170	31413170	Cox6b1	-0.50964688	2.98E-08	stronglyHypometh	0.033221	0.00023615	hypermethylated	5	32	32
chr7	31435247	31437247	Rbm42	-0.31955489	0.11468	insignificant	0.0048309	0.02355	inconclusive	5	37	36
chr7	31450013	31452013	Haus5		1	noCoverage	-0.016667	0.044069	hypomethylated	0	10	10
chr7	31482944	31484944	2200002J24Ri	-0.59118191	0.3039	lowCoverage	0.043079	0.61929	insignificant	1	12	12
chr7	31496250	31498250	Atp4a		1	noCoverage	-0.014069	0.79083	insignificant	0	7	6
chr7	31514553	31516553	Gapdhs	-0.13953464	0.000028335	hypomethylated	-0.020746	0.69226	insignificant	4	28	28
chr7	31547774	31549774	Dmkn	-0.47312063	0.13655	insignificant	0.00091492	0.12027	insignificant	7	16	16
chr7	31571923	31573923	Krtdap	-0.04413152	0.41657	insignificant	-0.070747	0.80093	insignificant	8	35	34
chr7	31664047	31666047	Cd22	-0.14605036	0.094086	insignificant	-0.045986	1	insignificant	5	12	12
chr7	31665361	31667361	Cd22	-0.11756769	0.021338	hypomethylated	-0.032201	0.80729	insignificant	2	4	4
chr7	31699851	31701851	Mag	-0.23630871	0.055157	insignificant	0.097689	0.002091	hypermethylated	3	8	9
chr7	31729036	31731036	Hamp	0.17600733	1	insignificant	0.078811	0.075754	insignificant	2	6	6
chr7	31741822	31743822	Usf2	-0.14883355	1.11E-09	hypomethylated	0.027279	0.28937	insignificant	28	154	154
chr7	31758488	31760488	Lsr	-0.15942679	3.99E-11	hypomethylated	0.069154	0.62638	insignificant	10	44	48
chr7	31826837	31828837	Fxyd5	-0.20768458	0.57622	insignificant	0.058837	0.22438	insignificant	1	31	29
chr7	31827341	31829341	Fxyd5	-0.37876603	0.043376	stronglyHypometh	0.22512	0.68559	insignificant	2	8	8
chr7	31836473	31838473	Fxyd7	-0.17722056	0.00032429	hypomethylated	0.092282	0.00037427	hypermethylated	5	48	43
chr7	31861716	31863716	Fxyd3	-0.00212766	0.011787	hypomethylated	0.061607	0.83654	insignificant	2	4	4
chr7	31900309	31902309	Hpn	-0.28308607	0.029429	hypomethylated	0.040995	0.21736	insignificant	10	36	35
chr7	31911964	31913964	Scn1b	-0.13589894	4.91E-24	hypomethylated	0.014943	1	insignificant	15	42	47
chr7	31936069	31938069	Gramd1a	-0.16398633	4.28E-09	hypomethylated	0.017392	0.24782	insignificant	9	36	36
chr7	34918287	34920287	Wtip	-0.15182744	0.60459	insignificant	0.020799	0.5707	insignificant	5	46	46
chr7	34953548	34955548	Uba2	-0.11750168	0.002765	hypomethylated	0.020565	0.96025	insignificant	12	39	39
chr7	34981666	34983666	Pdcd2l		1	noCoverage	0.15126	0.092463	insignificant	0	17	17
chr7	35015324	35017324	Gpi1	-0.1034188	6.92E-17	hypomethylated	-0.0024735	0.25579	insignificant	5	26	29
chr7	35174559	35176559	Lsm14a	-0.09110966	1.94E-15	hypomethylated	0.0075803	0.48775	insignificant	22	119	124
chr7	35437860	35439860	Kctd15	-0.1176994	2.27E-08	hypomethylated	0.0071478	0.40661	insignificant	34	148	149
chr7	35597730	35599730	Chst8	0.14733011	0.16546	insignificant	0.15291	0.85128	insignificant	4	17	12
chr7	35841585	35843585	Cebpg	-0.13159885	0.00014922	hypomethylated	-0.037838	0.40756	insignificant	12	46	42
chr7	35903311	35905311	Cebpa	-0.08345913	4.84E-26	hypomethylated	0.011854	0.25988	insignificant	58	273	253
chr7	35970403	35972403	Slc7a10	-0.19417498	3.87E-22	hypomethylated	-0.054869	0.008186	inconclusive	25	108	90
chr7	36000364	36002364	Lrp3	-0.07679056	3.08E-10	hypomethylated	0.0098205	0.58359	insignificant	17	62	62
chr7	36103459	36105459	Gpatch1	0.0313541	0.13995	insignificant	0.013658	0.75633	insignificant	3	16	16
chr7	36118255	36120255	Rhpn2	-0.14851499	0.000001439	hypomethylated	0.002906	0.64687	insignificant	14	72	67
chr7	36181111	36183111	Ccdc123	-0.21368815	0.003187	hypomethylated	-0.013197	0.27032	insignificant	7	32	28
chr7	36181786	36183786	C230052I12Ril	-0.2362782	0.000073156	hypomethylated	-0.013963	0.21171	insignificant	5	22	18
chr7	36233110	36235110	Slc7a9	-0.4141129	0.0098804	stronglyHypometh	-0.076542	0.066997	insignificant	1	8	8
chr7	36233116	36235116	Slc7a9	-0.4141129	0.0098804	stronglyHypometh	-0.076542	0.066997	insignificant	1	8	8
chr7	36233197	36235197	Slc7a9	-0.4141129	0.0098804	stronglyHypometh	-0.076542	0.066997	insignificant	1	8	8
chr7	36322763	36324763	Tdrd12	0.18215832	0.34905	insignificant	-0.017842	0.70933	insignificant	2	14	14
chr7	36340947	36342947	Nudt19	-0.13916823	0.0078268	hypomethylated	-0.0041142	0.48847	insignificant	3	8	8
chr7	36370265	36372265	Ankrd27	-0.16813008	2.29E-13	hypomethylated	-0.0011285	0.90737	insignificant	21	93	95

chr7	36370601	36372601	Rgs9bp	-0.18846683	2.15E-09	hypomethylated	-0.004156	0.46094	insignificant	14	58	60
chr7	36432501	36434501	Pdcd5		1	noCoverage	-0.03358	0.54149	insignificant	0	12	12
chr7	36539473	36541473	Dpy19l3	-0.13567444	0.000027904	hypomethylated	-0.018073	0.45821	insignificant	13	32	32
chr7	36586610	36588610	E130304I02Ril	-0.10342186	0.000000266	hypomethylated	-0.0031986	0.92532	insignificant	23	70	64
chr7	36588008	36590008	E130304I02Ril	-0.1251768	0.24175	insignificant	-0.02601	0.1975	insignificant	4	8	8
chr7	37482136	37484136	Tshz3	-0.10710008	1.63E-27	hypomethylated	0.01837	0.74893	insignificant	44	189	188
chr7	38554771	38556771	Zfp536	-0.13625901	1.52E-24	hypomethylated	0.00079402	0.43367	insignificant	52	152	152
chr7	38804571	38806571	C80913	-0.12568288	9.13E-09	hypomethylated	-0.009504	0.5186	insignificant	9	20	21
chr7	38892509	38894509	Ccne1	-0.11564277	1.18E-27	hypomethylated	-0.0068852	0.1814	insignificant	39	126	127
chr7	38967235	38969235	1600014C10Ri	-0.16904649	2.08E-09	hypomethylated	-0.007366	0.76056	insignificant	10	40	40
chr7	38967805	38969805	1600014C10Ri	-0.20051747	6.93E-10	hypomethylated	-0.01106	0.60632	insignificant	10	33	33
chr7	39013013	39015013	Plekhf1	-0.07612622	0.034204	hypomethylated	0.02049	1	insignificant	7	28	33
chr7	39056367	39058367	Pop4		1	noCoverage	0.033384	0.54079	insignificant	0	13	12
chr7	46519904	46521904	Gm16387		1	noCoverage	0.0069444	0.62222	insignificant	0	4	4
chr7	46772135	46774135	Zfp619	-0.09022706	0.0059311	hypomethylated	0.0085594	0.84909	insignificant	16	56	60
chr7	46842887	46844887	Gm2058	-0.11124835	0.000074143	hypomethylated	0.005429	0.8169	insignificant	16	57	57
chr7	48153647	48155647	Vstm2b	-0.17518592	1.03E-19	hypomethylated	0.0087337	0.0070532	hypermethylated	35	110	110
chr7	48287088	48289088	Gm4884		1	noCoverage	0.12867	0.18502	insignificant	0	4	4
chr7	48648630	48650630	AI987944	-0.22927086	0.57315	insignificant	0.017169	0.51405	insignificant	3	15	15
chr7	48648749	48650749	AI987944	-0.22927086	0.57315	insignificant	0.017169	0.51405	insignificant	3	15	15
chr7	48755260	48757260	AW146154		1	noCoverage	0.11635	0.055766	insignificant	0	17	12
chr7	48887900	48889900	Zfp788		1	noCoverage	0.035672	0.52685	insignificant	0	28	22
chr7	48922383	48924383	Vmn2r-ps54	-0.1042	0.0072399	hypomethylated	0.11519	0.33256	insignificant	1	18	14
chr7	49703450	49705450	4933421I07Rik		1	noCoverage	0.0077948	1	insignificant	0	7	7
chr7	49948088	49950088	Gm5595	-0.41435185	2.58E-09	stronglyHypometh	-0.041088	0.60693	insignificant	3	6	6
chr7	50189159	50191159	Vmn2r63	0.12792642	1	insignificant	-0.022074	1	insignificant	1	4	4
chr7	50431957	50433957	Zfp936	-0.18278293	1	insignificant	0.10424	0.37193	insignificant	3	9	8
chr7	50568631	50570631	Zfp715	-0.10529579	0.00005437	hypomethylated	0.066536	0.55844	insignificant	8	16	17
chr7	50605774	50607774	Siglec5	-0.3676983	0.0024968	stronglyHypometh	-0.029776	0.4084	insignificant	3	12	12
chr7	50684470	50686470	Lim2	0.14776786	1	insignificant	0.089797	0.804	insignificant	1	8	6
chr7	50698512	50700512	Etfb	-0.17780101	2.97E-11	hypomethylated	0.011975	0.73212	insignificant	16	38	39
chr7	50745393	50747393	Iglon5	-0.17944545	0.0068415	hypomethylated	0.00089494	0.78306	insignificant	7	41	41
chr7	50788541	50790541	Cd33		1	noCoverage	-0.0046992	1	insignificant	0	4	4
chr7	50816739	50818739	Zfp658	-0.1245098	1	insignificant	0.041699	0.79735	insignificant	1	17	14
chr7	50833955	50835955	Zfp719		1	noCoverage	0.0021166	1	insignificant	0	20	17
chr7	50861538	50863538	Zfp819	-0.23677527	1.84E-20	hypomethylated	-0.018548	0.57878	insignificant	10	41	40
chr7	50926400	50928400	Ctu1	0.08214534	0.0018715	hypermethylated	0.11685	0.33063	insignificant	6	21	17
chr7	50966936	50968936	Klk13	0.12275407	0.69117	insignificant	0.013237	0.52688	insignificant	2	8	8
chr7	51023469	51025469	Klk12	-0.40833333	0.26886	insignificant	-0.032143	1	insignificant	1	6	8
chr7	51046260	51048260	Klk9	0.04671082	0.44883	insignificant	-0.033996	0.19271	insignificant	5	20	21
chr7	51051946	51053946	Klk8	-0.15979185	0.03399	hypomethylated	-0.023177	0.95324	insignificant	7	36	36
chr7	51078913	51080913	Klk6		1	noCoverage	0.092312	0.3609	insignificant	0	6	9
chr7	51079819	51081819	Klk6		1	noCoverage	0.068903	0.36527	insignificant	0	6	7
chr7	51096638	51098638	Klk5		1	noCoverage	0.027602	0.89231	insignificant	0	6	7
chr7	51491492	51493492	1700028J19Ri	-0.16691922	0.090303	insignificant	0.063085	0.068857	insignificant	5	18	19
chr7	51501091	51503091	2410002F23Ri	-0.08264847	1.46E-19	hypomethylated	-0.0025097	0.63698	insignificant	21	95	90
chr7	51564633	51566633	Shank1	-0.12927072	5.53E-12	hypomethylated	0.0017965	0.92985	insignificant	11	90	95
chr7	51630400	51632400	1700008O03R	-0.33068955	0.55826	insignificant	0.063407	0.00099943	hypermethylated	1	8	8
chr7	51638495	51640495	Syt3	-0.16654491	0.21914	insignificant	0.019944	0.56119	insignificant	4	24	24
chr7	51696856	51698856	Lrrc4b	-0.31613719	0.00034458	hypomethylated	-0.0028937	0.25738	insignificant	4	19	18
chr7	51722425	51724425	Josd2	-0.06763653	1.02E-31	hypomethylated	0.070851	0.001245	hypermethylated	23	60	51
chr7	51750957	51752957	Fam71e1	-0.12168319	1.92E-14	hypomethylated	-0.018071	0.42642	insignificant	23	101	101
chr7	51751883	51753883	2310044H10R	-0.09033846	8.57E-10	hypomethylated	-0.024582	0.78102	insignificant	16	70	70
chr7	51780039	51782039	Mybpc2	-0.3909559	0.0000024	stronglyHypometh	0.080303	0.00045896	hypermethylated	8	23	16
chr7	51787441	51789441	Spib	-0.23621607	0.02347	hypomethylated	-0.014829	0.45899	insignificant	4	13	13
chr7	51804185	51806185	Nr1h2	0.29208183	1	insignificant	0.45798	0.00071972	stronglyhypermeth	2	10	6
chr7	51809293	51811293	Nr1h2	-0.55006876	4.45E-17	stronglyHypometh	0.14653	0.57015	insignificant	3	30	22
chr7	51845255	51847255	Kcnc3	-0.15669039	4.87E-22	hypomethylated	-0.017011	0.34404	insignificant	50	160	158
chr7	51963112	51965112	Izumo2		1	noCoverage	0.026035	0.64238	insignificant	0	28	25
chr7	52002998	52004998	Vrk3	-0.19187482	4.34E-12	hypomethylated	-0.020991	0.33727	insignificant	18	56	56
chr7	52003987	52005987	Zfp473	-0.19187351	2.11E-09	hypomethylated	-0.011543	0.70314	insignificant	9	30	30
chr7	52070739	52072739	Nup62-il4i1	-0.10752648	5.7E-14	hypomethylated	0.0086621	0.77591	insignificant	21	66	63
chr7	52070789	52072789	Nup62	-0.10752648	5.7E-14	hypomethylated	0.0086621	0.77591	insignificant	21	66	63
chr7	52071028	52073028	Atf5	-0.10752648	5.7E-14	hypomethylated	0.0086621	0.77591	insignificant	21	66	63
chr7	52072028	52074028	Nup62-il4i1	-0.11807528	0.00000775	hypomethylated	-0.0083562	0.10078	insignificant	10	30	30
chr7	52103596	52105596	Akt1s1	-0.16676993	0.0077289	hypomethylated	-0.0066444	0.6426	insignificant	5	55	48
chr7	52104068	52106068	Mir707	-0.15833659	0.60638	insignificant	-0.014743	0.4987	insignificant	2	38	38
chr7	52104449	52106449	Akt1s1		1	noCoverage	-0.0093345	0.35865	insignificant	0	25	25
chr7	52111550	52113550	Pnkp	-0.14744976	3.36E-12	hypomethylated	0.01272	0.69154	insignificant	11	41	40
chr7	52125158	52127158	Ptov1	-0.28902232	1	insignificant	-0.091134	0.47889	insignificant	2	24	20
chr7	52147736	52149736	Med25	-0.08430068	5.9E-11	hypomethylated	0.01893	0.19652	insignificant	23	54	58
chr7	52150448	52152448	Fuz	-0.15796791	0.0005799	hypomethylated	0.010158	0.90149	insignificant	9	36	36
chr7	52184860	52186860	Ap2a1	-0.11650322	4.74E-13	hypomethylated	0.027391	0.21735	insignificant	12	68	68
chr7	52197609	52199609	Tsks	0.01446046	1	insignificant	0.041637	0.40365	insignificant	4	36	36
chr7	52241702	52243702	Prmt1	-0.4116508	0.00000988	stronglyHypometh	0.20574	0.20429	insignificant	1	12	6
chr7	52252029	52254029	Irf3	-0.13357296	0.089722	insignificant	0.014164	0.57363	insignificant	5	48	48
chr7	52252949	52254949	Irf3	-0.11261589	0.60242	insignificant	0.013009	1	insignificant	3	27	27
chr7	52271619	52273619	Rras	-0.14144683	1.48E-10	hypomethylated	0.0142	0.18184	insignificant	18	82	71
chr7	52272376	52274376	Rras	-0.16486189	9.83E-08	hypomethylated	-0.004188	0.41697	insignificant	13	63	61
chr7	52308251	52310251	Prr12	-0.14789007	0.000014953	hypomethylated	-0.0012728	0.062857	insignificant	8	53	47
chr7	52316798	52318798	Nosip	-0.23938782	0.067377	insignificant	-0.047815	0.60555	insignificant	8	34	28
chr7	52317022	52319022	Prrg2	-0.23938782	0.067377	insignificant	-0.047815	0.60555	insignificant	8	34	28
chr7	52347583	52349583	Rcn3	-0.1047619	0.0035253	hypomethylated	0.065893	1	insignificant	3	6	6
chr7	52359192	52361192	Fcgrt	-0.30950086	0.0083755	hypomethylated	0.069956	0.00046986	hypermethylated	5	26	25
chr7	52378481	52380481	Rps11	-0.15440325	6.99E-19	hypomethylated	-0.01221	0.77763	insignificant	22	101	98

chr7	52379759	52381759	Rps11	-0.14583333	0.36678	insignificant	0.021825	0.50069	insignificant	3	8	8
chr7	52381805	52383805	Snord35a	0.25581017	1	insignificant	-0.044848	0.021998	hypomethylated	3	15	15
chr7	52382038	52384038	Snord35a	0.25581017	1	insignificant	-0.044848	0.021998	hypomethylated	3	15	15
chr7	52382315	52384315	Snord35a	0.02903486	0.11542	insignificant	-0.031257	0.7529	insignificant	9	45	47
chr7	52382833	52384833	Snord34	-0.13089299	1.59E-09	hypomethylated	-0.012728	0.4052	insignificant	14	50	52
chr7	52384105	52386105	Rpl13a	-0.13903878	3.74E-09	hypomethylated	-0.020319	0.25452	insignificant	8	36	38
chr7	52391802	52393802	Flt3l	-0.15801282	0.1801	insignificant	0.061285	0.00000117	hypermethylated	2	20	19
chr7	52408767	52410767	Pih1d1	-0.18681086	0.000000343	hypomethylated	0.043755	0.41084	insignificant	10	47	38
chr7	52409908	52411908	Pih1d1	-0.59906292	0.013549	stronglyHypometh	-0.040288	0.041587	hypomethylated	4	33	27
chr7	52418290	52420290	Slc17a7	-0.22632564	2.45E-18	hypomethylated	0.061353	0.18111	insignificant	18	53	55
chr7	52434967	52436967	Gm581	-0.40608944	0.5333	insignificant	-0.036303	0.83759	insignificant	2	16	25
chr7	52435364	52437364	Pth2	-0.32086112	0.000024751	hypomethylated	0.013571	0.026701	hypermethylated	4	28	38
chr7	52460262	52462262	Ccdc155		1	noCoverage	-0.0012712	0.19947	insignificant	0	21	16
chr7	52467253	52469253	Dkkl1	-0.21206492	0.00063505	hypomethylated	-0.0073593	0.011954	hypomethylated	5	22	22
chr7	52470122	52472122	Tead2	-0.14280564	2.45E-14	hypomethylated	0.064309	0.0012186	hypermethylated	26	68	77
chr7	52494209	52496209	Cd37	0.12814996	0.24421	insignificant	0.048671	0.000032624	hypermethylated	6	33	33
chr7	52537242	52539242	1700039E15Ri	-0.02721074	0.2796	insignificant	0.062675	0.0073981	hypermethylated	8	26	26
chr7	52589150	52591150	Trpm4	-0.10366847	1	insignificant	-0.43641	0.092117	insignificant	2	6	14
chr7	52589638	52591638	Hrc	-0.13191802	0.69974	insignificant	-0.023547	0.067353	insignificant	2	8	8
chr7	52621602	52623602	Mtag2	-0.14582565	1.29E-20	hypomethylated	0.0011142	0.8068	insignificant	27	146	138
chr7	52622389	52624389	Lin7b	-0.12530208	0.00000126	hypomethylated	0.0032967	0.81866	insignificant	9	81	75
chr7	52625934	52627934	Lin7b	-0.1171841	0.033587	hypomethylated	-0.024999	0.73123	insignificant	1	18	18
chr7	52651017	52653017	Snrnp70	-0.0741161	0.0006834	hypomethylated	0.0045148	0.77939	insignificant	8	64	62
chr7	52660329	52662329	Kcna7	-0.12246573	6.8E-28	hypomethylated	-0.010143	0.41141	insignificant	32	144	141
chr7	52668064	52670064	Ntf5	-0.18163378	6.44E-31	hypomethylated	0.03064	0.0045111	hypermethylated	18	57	44
chr7	52675315	52677315	Lhb	-0.07976679	6.57E-14	hypomethylated	0.010531	0.0065494	hypermethylated	21	60	56
chr7	52689208	52691208	Gys1	-0.18292522	7.46E-09	hypomethylated	0.005354	0.40633	insignificant	9	30	30
chr7	52689834	52691834	Ruvbl2	-0.19367922	5.47E-08	hypomethylated	0.0044335	0.63042	insignificant	3	16	16
chr7	52715256	52717256	Ftl1	0.19755853	1	insignificant	0.00091914	0.27355	insignificant	4	32	32
chr7	52722268	52724268	Bax	0.51269819	0.045488	stronglyHypermeth	-0.090029	0.53882	insignificant	1	40	41
chr7	52744166	52746166	Dhdh		1	noCoverage	-0.085469	0.25351	insignificant	0	13	16
chr7	52780699	52782699	Plekha4	-0.10730164	0.45879	insignificant	0.0018456	0.21137	insignificant	12	78	74
chr7	52781638	52783638	Ppp1r15a	-0.06956064	0.18212	insignificant	0.0064768	0.57104	insignificant	3	23	23
chr7	52809297	52811297	Hsd17b14		1	noCoverage	-0.018191	0.29612	insignificant	0	12	14
chr7	52824732	52826732	0610005C13Ri	-0.1937865	0.00079181	hypomethylated	-0.0044276	0.21633	insignificant	15	70	66
chr7	52870860	52872860	Fut1	-0.54544347	0.0041701	stronglyHypometh	0.029934	0.93376	insignificant	2	12	12
chr7	52871975	52873975	Fut1	-0.33496806	3.75E-12	stronglyHypometh	0.022214	0.66536	insignificant	9	30	30
chr7	52876180	52878180	Izumo1	0.19017857	1	insignificant	0.078715	0.089454	insignificant	3	8	7
chr7	52881906	52883906	Rasip1	-0.00555556	1	insignificant	0.039316	0.49499	insignificant	2	6	6
chr7	52894346	52896346	Mamstr	0.17141358	0.0083639	hypermethylated	0.022404	0.18641	insignificant	8	39	36
chr7	52934928	52936928	Sec1	-0.42070219	0.032314	stronglyHypometh	0.063028	0.13394	insignificant	3	20	20
chr7	52938783	52940783	Ntn5	-0.37608225	0.47562	insignificant	0.12447	0.51546	insignificant	2	10	10
chr7	52954336	52956336	Car11	-0.08575054	0.0018151	hypomethylated	0.087603	0.10723	insignificant	11	52	44
chr7	52959616	52961616	Dbp	-0.18411997	6.15E-15	hypomethylated	-0.005495	0.27109	insignificant	16	47	47
chr7	52970577	52972577	Sphk2	-0.14105745	0.090358	insignificant	0.00090846	1	insignificant	7	24	24
chr7	52972440	52974440	Rpl18	-0.1312336	1.31E-15	hypomethylated	-0.012335	0.73733	insignificant	30	117	112
chr7	52973372	52975372	Sphk2	-0.21896238	7.91E-17	hypomethylated	0.0047199	0.3146	insignificant	15	69	64
chr7	52975589	52977589	Fam83e	-0.00713657	0.54715	insignificant	0.047647	0.036736	hypermethylated	6	17	17
chr7	52981186	52983186	Spaca4	-0.00820663	0.62876	insignificant	-0.00056774	1	insignificant	3	15	8
chr7	53014925	53016925	Sult2b1	-0.26773892	3.89E-08	hypomethylated	-0.052054	0.82609	insignificant	7	27	23
chr7	53038316	53040316	Lmtk3	-0.19011429	1	insignificant	-0.0082962	0.37466	insignificant	7	37	42
chr7	53069686	53071686	Cyth2		1	noCoverage	-0.11588	0.67908	insignificant	0	5	8
chr7	53080117	53082117	Grwd1	-0.40567766	0.23803	insignificant	0.22527	0.0014698	hypermethylated	1	4	4
chr7	53122051	53124051	Grin2d		1	noCoverage	0.041765	0.665	insignificant	0	4	8
chr7	53127209	53129209	Kdelr1	-0.23241897	1.83E-16	hypomethylated	0.015944	0.095501	insignificant	10	57	57
chr7	53151438	53153438	Tmem143		1	noCoverage	0.0058484	0.73413	insignificant	0	20	20
chr7	53152081	53154081	Syngr4		1	noCoverage	-0.01202	0.048546	hypomethylated	0	16	16
chr7	53176219	53178219	Emp3	-0.23295455	0.046254	hypomethylated	0.11156	0.92906	insignificant	4	8	8
chr7	53176796	53178796	Emp3	-0.12727273	1	insignificant	0.13384	0.81887	insignificant	3	6	6
chr7	53182767	53184767	Ccdc114	-0.21788103	0.00025482	hypomethylated	-0.012464	0.35839	insignificant	6	18	18
chr7	53285656	53287656	Abcc6		1	noCoverage	-0.10852	0.75724	insignificant	0	2	2
chr7	53288065	53290065	Nomo1	-0.1304078	1.45E-17	hypomethylated	-0.005088	0.88763	insignificant	29	95	91
chr7	53355607	53357607	Kcnj11	-0.20919172	0.000000274	hypomethylated	-0.031539	0.15686	insignificant	2	24	36
chr7	53435403	53437403	Abcc8	-0.2037935	0.22802	insignificant	-0.071108	0.16034	insignificant	4	29	23
chr7	53493860	53495860	Ush1c	-0.34250356	1	insignificant	-0.086858	1	insignificant	1	12	10
chr7	53495356	53497356	Otog	0.05234395	1	insignificant	0.0043831	0.16656	insignificant	3	15	16
chr7	53630843	53632843	Myod1	-0.04513465	0.67163	insignificant	-0.017595	0.20352	insignificant	10	73	73
chr7	53650837	53652837	Kcnc1	-0.10515288	9.92E-25	hypomethylated	0.0066498	0.35785	insignificant	43	160	160
chr7	53650866	53652866	Kcnc1	-0.10515288	9.92E-25	hypomethylated	0.0066498	0.35785	insignificant	43	160	160
chr7	53895177	53897177	Sergef	-0.0100012	0.00083814	hypomethylated	-0.035163	0.33695	insignificant	11	38	36
chr7	53922718	53924718	Tph1	-0.14410714	0.22585	insignificant	0.027864	0.65009	insignificant	4	18	16
chr7	53927907	53929907	Tph1		1	noCoverage	0.12463	0.3354	insignificant	0	6	6
chr7	53966021	53968021	Saal1	-0.14716931	0.000033849	hypomethylated	0.024186	0.081777	insignificant	12	30	30
chr7	53998350	54000350	Saa1		1	noCoverage	0.013229	1	insignificant	0	19	19
chr7	54006202	54008202	Saa2		1	noCoverage	-0.1128	1	insignificant	0	5	5
chr7	54050463	54052463	Gtf2h1	-0.13769331	3.66E-21	hypomethylated	0.0031775	0.60784	insignificant	26	120	110
chr7	54051251	54053251	Hps5	-0.15617124	3.09E-21	hypomethylated	-0.0067071	0.93639	insignificant	27	107	96
chr7	54100173	54102173	Ldha	-0.17521416	0.000004809	hypomethylated	0.024489	0.43738	insignificant	16	73	75
chr7	54101451	54103451	Ldha	0.54589372	5.06E-13	stronglyHypermeth	0.013907	0.0068646	hypermethylated	4	23	28
chr7	54175300	54177300	Tsg101	-0.0966641	7.67E-21	hypomethylated	0.0067552	0.82121	insignificant	11	44	44
chr7	54213888	54215888	Uevld	-0.11863108	1	insignificant	0.01626	0.066693	insignificant	2	30	36
chr7	54263784	54265784	Spty2d1	-0.30602355	9.16E-08	hypomethylated	-0.00069756	0.89607	insignificant	4	8	11
chr7	54305009	54307009	Tmem86a	-0.13525072	1.67E-25	hypomethylated	0.0087615	0.15401	insignificant	27	82	82
chr7	54387968	54389968	Ptpn5	0.80902151	0.18985	lowCoverage	-0.01466	0.61715	insignificant	1	34	30
chr7	54389054	54391054	Ptpn5	-0.05456476	0.4513	insignificant	0.013533	0.89121	insignificant	2	11	10

chr7	56043372	56045372	Zdhhc13	-0.06567383	1.26E-10	hypomethylated	0.0039189	0.52622	insignificant	8	66	66
chr7	56136411	56138411	E2f8	-0.09730088	7.29E-44	hypomethylated	0.064549	0.5283	insignificant	42	180	176
chr7	56213442	56215442	Nav2	-0.22080143	1.92E-08	hypomethylated	0.010898	0.15511	insignificant	15	49	47
chr7	56500558	56502558	Nav2	-0.12849904	1.96E-10	hypomethylated	-0.0087385	0.44263	insignificant	37	176	172
chr7	56892205	56894205	Dbx1	-0.31072958	4.3E-10	hypomethylated	0.038926	0.75985	insignificant	8	54	55
chr7	57013475	57015475	Htatip2	-0.14728171	2.65E-12	hypomethylated	-0.001627	0.19703	insignificant	16	61	60
chr7	57013881	57015881	Htatip2	-0.14728171	2.65E-12	hypomethylated	-0.001627	0.19703	insignificant	16	61	60
chr7	57032727	57034727	Prmt3	-0.09533419	1.72E-14	hypomethylated	-0.0082242	0.84394	insignificant	37	105	101
chr7	57164668	57166668	Slc6a5	-0.09836978	0.060228	insignificant	0.050148	0.85385	insignificant	6	27	25
chr7	57166069	57168069	Slc6a5	-0.16984988	1.21E-18	hypomethylated	0.0097553	0.85034	insignificant	27	69	71
chr7	57229719	57231719	Nell1	-0.10200551	0.0011691	hypomethylated	-0.0031458	0.66915	insignificant	20	129	129
chr7	58765398	58767398	Ano5	0.24667243	0.14571	insignificant	0.021696	0.034135	hypermethylated	3	24	21
chr7	58876199	58878199	Slc17a6	-0.15630521	0.000000821	hypomethylated	0.018168	0.9619	insignificant	14	45	44
chr7	59117637	59119637	Fancf	-0.5	0.2963	lowCoverage	0.2547	0.19325	insignificant	1	6	4
chr7	59261084	59263084	Svip	-0.16235646	2.32E-08	hypomethylated	-0.035744	0.3908	insignificant	9	19	19
chr7	62089614	62091614	Luzp2	-0.27616334	0.026441	hypomethylated	-0.0097244	0.65739	insignificant	4	18	19
chr7	63048517	63050517	Tubgcp5	-0.19906693	8.26E-15	hypomethylated	0.0087241	0.0041971	hypermethylated	22	68	68
chr7	63096440	63098440	Cyfip1	-0.11410784	3.93E-14	hypomethylated	0.0231	0.88904	insignificant	25	75	68
chr7	63216900	63218900	A230056P14R	-0.07895643	7.95E-18	hypomethylated	-0.00067923	0.83244	insignificant	23	108	108
chr7	63217820	63219820	Nipa2	-0.07709916	0.0012932	hypomethylated	-0.0036649	0.29103	insignificant	5	34	34
chr7	63274943	63276943	Nipa1	-0.08721005	0.000000108	inconclusive	-0.081943	0.13796	insignificant	7	26	26
chr7	63304524	63306524	Herc2	-0.11987839	2.03E-12	hypomethylated	-0.0034948	0.77923	insignificant	27	121	123
chr7	63494140	63496140	Oca2		1	noCoverage	-0.0028046	0.23097	insignificant	0	14	10
chr7	64641641	64643641	Gm9962	-0.14254501	0.00024575	hypomethylated	-0.012763	0.11582	insignificant	18	116	110
chr7	64642241	64644241	Gabrg3	-0.13658607	0.0036341	hypomethylated	-0.023615	0.27348	insignificant	15	72	72
chr7	64765379	64767379	Gabra5	-0.10326026	0.23565	insignificant	0.015395	0.099527	insignificant	3	10	10
chr7	64844903	64846903	Gabrb3	-0.09304044	4.02E-20	hypomethylated	0.015294	0.48981	insignificant	47	111	102
chr7	64845542	64847542	Gabrb3	-0.11339219	1.83E-29	hypomethylated	0.0094447	0.92795	insignificant	56	144	132
chr7	65912571	65914571	Atp10a	-0.10506324	3.8E-10	hypomethylated	-0.00057843	0.80109	insignificant	15	70	73
chr7	66483119	66485119	Ube3a	-0.10986503	0.00089069	hypomethylated	-0.047706	0.4903	insignificant	17	108	104
chr7	66483121	66485121	Ube3a	-0.10986503	0.00089069	hypomethylated	-0.047706	0.4903	insignificant	17	108	104
chr7	66931449	66933449	Snord116		1	noCoverage	0.094385	0.57982	insignificant	0	11	8
chr7	67006708	67008708	Snord116l2		1	noCoverage	0.031361	0.056244	insignificant	0	14	16
chr7	67011765	67013765	Snord116l2		1	noCoverage	0.28018	0.13945	insignificant	0	9	9
chr7	67150042	67152042	Snrpn	0.053432	1	insignificant	0.066766	0.51458	insignificant	2	6	6
chr7	69492162	69494162	Ndn	-0.35506643	0.55477	insignificant	-0.055743	0.11925	insignificant	3	16	16
chr7	69520864	69522864	Magel2	0.02899024	1.89E-08	inconclusive	0.038014	0.0022358	hypermethylated	15	44	48
chr7	69565025	69567025	Mkrn3	-0.24849736	0.016849	hypomethylated	-0.020939	0.12591	insignificant	9	18	18
chr7	70357412	70359412	Chrna7	-0.01790752	1	insignificant	0.0032641	0.78262	insignificant	6	26	26
chr7	70588657	70590657	Otud7a	-0.05989737	1.22E-10	hypomethylated	0.016695	0.23519	insignificant	38	109	109
chr7	71083801	71085801	Klf13	-0.12460194	6.92E-15	hypomethylated	0.0059982	0.52987	insignificant	25	106	105
chr7	71349691	71351691	Mir211		1	noCoverage	-0.073901	0.010186	hypomethylated	0	9	9
chr7	71431555	71433555	Mtmr10	-0.11103467	5.35E-15	hypomethylated	-0.0049684	0.67917	insignificant	23	121	116
chr7	71518981	71520981	Fan1		1	noCoverage	0.0028655	0.2602	insignificant	0	10	10
chr7	71536656	71538656	Mcee	-0.14754814	7.11E-19	hypomethylated	-0.006596	0.84224	insignificant	28	101	100
chr7	71537122	71539122	Mphosph10	-0.16030682	3.1E-09	hypomethylated	-0.0085626	0.93686	insignificant	16	68	67
chr7	71645591	71647591	Apba2	-0.14922605	6.71E-16	hypomethylated	-0.012473	0.52883	insignificant	46	171	168
chr7	72017926	72019926	Ndnl2	-0.42174492	0.24707	insignificant	0.18014	0.77991	insignificant	1	26	17
chr7	72301414	72303414	Fam189a1	-0.08885245	2.84E-09	hypomethylated	-0.018094	0.97391	insignificant	35	94	94
chr7	72516130	72518130	Tjp1	-0.33763034	3.04E-09	stronglyHypometh	0.015391	0.7592	insignificant	10	35	34
chr7	72837302	72839302	Tm2d3	-0.12103776	9.59E-16	hypomethylated	0.0062076	0.81904	insignificant	21	77	76
chr7	73006021	73008021	Pcsk6	-0.10951564	7.03E-20	hypomethylated	-0.0080891	0.77415	insignificant	42	123	125
chr7	73204221	73206221	Snrpa1	-0.11398924	1.08E-14	hypomethylated	-0.0027653	0.092374	insignificant	20	106	106
chr7	73223534	73225534	H47	-0.14322516	3.78E-10	hypomethylated	-0.014678	0.077625	insignificant	25	93	91
chr7	73253400	73255400	Chsy1	-0.07606439	2.14E-14	hypomethylated	0.010075	0.076876	insignificant	65	246	225
chr7	73533227	73535227	Lrrk1	0.3662892	1	insignificant	0.048362	0.81907	insignificant	1	8	17
chr7	73572363	73574363	Aldh1a3	-0.14222443	6.13E-19	hypomethylated	0.02345	0.62519	insignificant	18	55	42
chr7	73833774	73835774	Lins	-0.17546154	1.01E-16	hypomethylated	0.054919	0.20099	insignificant	15	48	50
chr7	73834447	73836447	Lins	-0.13998833	1.28E-13	hypomethylated	0.05555	0.17133	insignificant	12	38	38
chr7	73983620	73985620	Adamts17	-0.14738278	3.59E-27	hypomethylated	-0.014525	0.95612	insignificant	43	125	123
chr7	74366429	74368429	Lysmd4	-0.16585043	0.000011325	hypomethylated	0.020848	0.3362	insignificant	7	58	58
chr7	74366501	74368501	Lysmd4	-0.16585043	0.000011325	hypomethylated	0.020848	0.3362	insignificant	7	58	58
chr7	74517744	74519744	Mef2a	-0.12856224	1.36E-26	hypomethylated	-0.01053	0.80742	insignificant	28	117	110
chr7	74790122	74792122	Lrrc28	-0.26836219	1.11E-09	hypomethylated	0.035052	0.24688	insignificant	4	36	36
chr7	74904628	74906628	Synm	-0.09385061	1.99E-12	hypomethylated	0.0058622	0.95202	insignificant	10	42	42
chr7	75096142	75098142	Igf1r	-0.0926086	1.17E-51	hypomethylated	0.0084159	0.16772	insignificant	64	258	253
chr7	75409119	75411119	Pgpep1l	-0.17609126	0.61092	insignificant	-0.024636	0.76525	insignificant	3	16	16
chr7	75894124	75896124	Arrdc4	-0.03246408	0.38341	insignificant	0.051111	0.43536	insignificant	3	6	6
chr7	77505479	77507479	Nr2f2	-0.12940882	3.74E-38	hypomethylated	0.0059977	0.18986	insignificant	50	207	183
chr7	77511632	77513632	Nr2f2	-0.44652484	0.00000102	stronglyHypometh	0.083058	0.021342	hypermethylated	1	10	12
chr7	80519405	80521405	Rgma	-0.11612419	2.96E-34	hypomethylated	0.021224	0.151	insignificant	55	137	138
chr7	80686632	80688632	Chd2	0.025	1	insignificant	-0.00625	1	insignificant	1	4	4
chr7	80703281	80705281	1810026B05R	-0.0725216	0.045172	hypomethylated	-0.018067	0.78751	insignificant	5	50	46
chr7	80884192	80886192	Fam174b	-0.09830398	0.000000536	hypomethylated	0.0018061	0.80429	insignificant	15	75	62
chr7	81158568	81160568	St8sia2	-0.11452836	0.00019217	hypomethylated	-0.0085077	0.87691	insignificant	8	31	32
chr7	81699666	81701666	Slco3a1	-0.10893086	0.000000201	hypomethylated	0.0016851	0.72484	insignificant	18	58	58
chr7	82453272	82455272	Sv2b	-0.21931435	0.36663	insignificant	0.12153	0.70662	insignificant	6	37	42
chr7	82454148	82456148	Sv2b		1	noCoverage	0.19722	0.32191	insignificant	0	9	14
chr7	82599419	82601419	Akap13	-0.00206794	0.88688	insignificant	0.014858	0.19222	insignificant	29	113	113
chr7	82992223	82994223	Klhl25	-0.13243616	1.02E-24	hypomethylated	-0.0089723	0.039377	hypomethylated	39	131	125
chr7	85722724	85724724	Ntrk3	-0.15091792	0.00036407	hypomethylated	0.034636	0.22506	insignificant	7	63	63
chr7	85927016	85929016	Mrps11	-0.15557796	3.01E-10	hypomethylated	0.0061961	0.27772	insignificant	9	72	72
chr7	85927975	85929975	Mrpl46	-0.20105016	6.45E-11	hypomethylated	-0.0086043	0.53536	insignificant	5	34	34
chr7	85992097	85994097	Det1	-0.15537165	4.47E-13	hypomethylated	-0.0040307	1	insignificant	5	14	15
chr7	86039812	86041812	Aen	-0.14123655	5.35E-11	hypomethylated	-0.0060718	0.63607	insignificant	13	48	42

chr7	86197368	86199368	Acan	-0.10225238	0.0035768	hypomethylated	0.015672	0.2969	insignificant	24	99	86
chr7	86275904	86277904	Hapln3	-0.32222622	0.00000152	hypomethylated	-0.018172	0.17867	insignificant	7	14	14
chr7	86293946	86295946	Mfge8	-0.05748461	0.0038517	hypomethylated	0.0030666	0.32424	insignificant	9	26	26
chr7	86417151	86419151	Abhd2	-0.18490717	4.53E-29	hypomethylated	0.017348	0.0011033	hypermethylated	17	73	70
chr7	86536223	86538223	Fanci	-0.17077128	2.75E-13	hypomethylated	-0.0037852	0.6955	insignificant	14	30	30
chr7	86611159	86613159	Polg	-0.15997103	1.44E-19	hypomethylated	0.0064371	0.59489	insignificant	14	80	80
chr7	86649149	86651149	Mir9-3	-0.16327769	9.94E-27	hypomethylated	0.012902	0.4681	insignificant	34	111	98
chr7	86762543	86764543	Rhcg	-0.22899262	0.00093251	hypomethylated	0.014912	0.32342	insignificant	1	22	24
chr7	86804081	86806081	5730590G19R	-0.14162091	3.9E-12	hypomethylated	0.0038721	0.1797	insignificant	29	86	90
chr7	86859072	86861072	Kif7	-0.11992389	4.83E-13	hypomethylated	-0.0076932	0.77794	insignificant	16	80	78
chr7	86887048	86889048	Wdr93	-0.06353032	0.0011446	hypomethylated	0.0013334	0.90668	insignificant	16	76	76
chr7	86887911	86889911	Pex11a	-0.0638422	0.3264	insignificant	-0.028399	0.55304	insignificant	6	16	16
chr7	86954612	86956612	Mesp2	-0.13099257	0.49707	insignificant	0.00023626	0.19377	insignificant	11	57	58
chr7	86987238	86989238	Anpep	0.13553114	0.39772	insignificant	-0.028796	0.057661	insignificant	4	12	12
chr7	87065526	87067526	Ap3s2	-0.14165522	0.0014877	hypomethylated	0.0006634	0.55354	insignificant	2	16	16
chr7	87080150	87082150	2610034B18R	-0.01544426	0.14871	insignificant	0.031728	0.43185	insignificant	7	30	31
chr7	87168699	87170699	Zfp710	-0.08758086	0.00000107	hypomethylated	0.064566	0.41878	insignificant	15	36	40
chr7	87170113	87172113	Zfp710	-0.12389361	4.26E-17	hypomethylated	0.011215	0.33209	insignificant	46	141	135
chr7	87260236	87262236	Idh2	-0.05776025	0.0018188	hypomethylated	-0.0063675	0.40144	insignificant	2	24	24
chr7	87297901	87299901	Mir1965	-0.1826572	0.038719	hypomethylated	0.012974	0.5864	insignificant	1	4	4
chr7	87330726	87332726	Sema4b	-0.22685589	1.41E-18	hypomethylated	-0.023178	0.24577	insignificant	15	65	65
chr7	87376778	87378778	D330012F22R	-0.13335244	2.16E-15	hypomethylated	0.0039506	0.5254	insignificant	28	81	83
chr7	87377502	87379502	Cib1	-0.12757883	2.46E-13	hypomethylated	0.0071453	0.037872	hypermethylated	29	85	87
chr7	87390261	87392261	Ttll13	-0.37526559	0.0001698	stronglyHypometh	-0.0069223	0.052108	insignificant	5	30	28
chr7	87405100	87407100	Ngrn	-0.15974522	0.00000592	hypomethylated	0.025663	0.35994	insignificant	20	58	56
chr7	87413540	87415540	Vps33b	-0.20234698	3.18E-15	hypomethylated	0.018417	0.77658	insignificant	18	56	56
chr7	87438350	87440350	Prc1	-0.23354577	4.19E-18	hypomethylated	0.0013896	0.5796	insignificant	18	100	101
chr7	87469340	87471340	Unc45a	0.00723519	0.29638	insignificant	-0.021135	0.94806	insignificant	8	42	38
chr7	87485105	87487105	Unc45a	-0.36728395	0.024818	stronglyHypometh	-0.0048895	1	insignificant	2	6	6
chr7	87487022	87489022	Hddc3	-0.33337439	0.000081491	stronglyHypometh	0.033711	0.26686	insignificant	4	13	13
chr7	87516012	87518012	Man2a2	-0.08959006	1.97E-21	hypomethylated	0.0068072	0.70168	insignificant	24	98	98
chr7	87532832	87534832	Fes		1	noCoverage	-0.0048899	0.60863	insignificant	0	18	18
chr7	87547648	87549648	Furin	-0.09630532	0.000002463	hypomethylated	0.0068687	0.69266	insignificant	21	85	90
chr7	87679884	87681884	Blm	-0.14234067	0.16419	insignificant	-0.0035005	0.03756	inconclusive	8	54	52
chr7	87680005	87682005	Blm	-0.24728229	0.0046683	hypomethylated	0.0059924	0.19464	insignificant	3	26	24
chr7	87833763	87835763	Crtc3	-0.0856501	0.00000789	hypomethylated	-0.0095717	0.56517	insignificant	15	30	30
chr7	87948217	87950217	Iqgap1	-0.09569816	0.59961	insignificant	-0.0060144	0.88217	insignificant	3	29	29
chr7	88005993	88007993	Zscan2	-0.12362773	0.14283	insignificant	0.0088009	0.3263	insignificant	3	34	29
chr7	88046155	88048155	Nmb	-0.32310063	1	lowCoverage	0.0070925	0.93016	insignificant	1	12	12
chr7	88049957	88051957	Nmb	-0.31714286	0.00000401	hypomethylated	0.0036478	0.38294	insignificant	3	10	10
chr7	88092436	88094436	Sec11a	-0.181228	0.000000292	hypomethylated	-0.061165	0.86154	insignificant	4	40	32
chr7	88137569	88139569	Zfp592	-0.09462165	2.73E-50	hypomethylated	0.0070282	0.45672	insignificant	78	199	189
chr7	88201485	88203485	Alpk3	-0.04522412	0.0028167	hypomethylated	0.058418	0.04577	hypermethylated	12	28	28
chr7	88357689	88359689	Pde8a	-0.06877371	4.63E-11	hypomethylated	0.0041756	0.43202	insignificant	51	190	207
chr7	88490120	88492120	Rps17	-0.27786744	1.4E-19	hypomethylated	0.00076501	0.1709	insignificant	8	20	20
chr7	88599562	88601562	Cpeb1	-0.08539515	6.41E-14	hypomethylated	-0.010665	0.21467	insignificant	28	104	104
chr7	88643216	88645216	BC048679		1	noCoverage	0.028836	0.087269	insignificant	0	8	9
chr7	88715177	88717177	Whamm	-0.07359885	1.47E-13	hypomethylated	0.00028502	0.38919	insignificant	40	127	127
chr7	88851811	88853811	Homer2	-0.10713423	0.00082645	hypomethylated	-0.032218	0.74842	insignificant	14	43	35
chr7	88906838	88908838	Fam103a1	-0.14580129	1.42E-25	hypomethylated	-0.013397	0.070789	insignificant	31	131	128
chr7	88934339	88936339	3110040N11R	-0.08250972	1	insignificant	0.0014697	0.34729	insignificant	6	16	16
chr7	88974317	88976317	Btbd1	-0.06455343	0.090872	insignificant	-0.01757	0.9462	insignificant	12	45	44
chr7	89002907	89004907	Tm6sf1	-0.23256597	9.17E-09	hypomethylated	0.00013076	0.53587	insignificant	12	48	48
chr7	89079345	89081345	Hdgfrp3	0.03239028	1.43E-08	inconclusive	0.10029	0.0001053	hypermethylated	21	59	73
chr7	89137185	89139185	Bnc1	-0.16163908	5.92E-33	hypomethylated	0.02702	0.00076969	hypermethylated	29	86	86
chr7	89318727	89320727	Sh3gl3	-0.01874226	0.00012513	inconclusive	0.019532	0.78766	insignificant	21	68	64
chr7	89483203	89485203	Adamtsl3	-0.09971745	7.49E-09	hypomethylated	0.024785	0.15601	insignificant	22	96	96
chr7	89796123	89798123	Eftud1	-0.11992068	1.98E-15	hypomethylated	0.0065344	0.79601	insignificant	26	84	84
chr7	89797038	89799038	Fam154b	-0.14290537	1.71E-12	hypomethylated	-0.0008435	0.48217	insignificant	17	54	54
chr7	90014842	90016842	Mex3b	-0.08617566	4.02E-09	hypomethylated	-0.000023692	0.69374	insignificant	64	195	202
chr7	91032851	91034851	Mesdc1	-0.1904069	1.32E-17	hypomethylated	0.013034	0.96598	insignificant	23	64	64
chr7	91039509	91041509	Mesdc2	-0.20318753	0.2938	insignificant	0.0037633	0.17733	insignificant	12	54	50
chr7	91235015	91237015	9930013L23Ri	-0.11421757	0.00049702	hypomethylated	-0.00091587	0.53287	insignificant	14	30	30
chr7	91300403	91302403	Fam108c	-0.07748306	1.84E-10	hypomethylated	-0.012366	0.92996	insignificant	23	106	106
chr7	91558469	91560469	Arnt2	-0.10995253	0.68057	insignificant	-0.0033623	0.0050357	hypomethylated	15	57	52
chr7	91754452	91756452	Fah	-0.24220966	4.54E-36	hypomethylated	0.0033156	0.76819	insignificant	14	45	45
chr7	92564186	92566186	Vmn2r69	-0.11493698	0.00028133	hypomethylated	-0.18798	0.023246	hypomethylated	5	19	19
chr7	94394304	94396304	Nox4	-0.16870196	5.43E-18	hypomethylated	-0.00029756	0.78906	insignificant	20	61	60
chr7	95425602	95427602	Ctsc	-0.2809694	0.000040055	hypomethylated	-0.090366	0.33988	insignificant	2	9	11
chr7	95577782	95579782	Rab38	-0.17439394	0.000029183	hypomethylated	0.0087042	0.85559	insignificant	12	45	45
chr7	96487297	96489297	Tmem135	-0.08096238	0.15151	insignificant	0.010949	0.36853	insignificant	10	26	26
chr7	96551875	96553875	Fzd4	-0.16424637	4.3E-20	hypomethylated	-0.00067522	0.58064	insignificant	35	115	115
chr7	96666096	96668096	Prss23	-0.15204212	4.47E-11	hypomethylated	0.0084367	1	insignificant	10	32	32
chr7	96780327	96782327	Me3	-0.12280078	0.057965	insignificant	0.044557	0.29632	insignificant	1	28	28
chr7	97089695	97091695	l7Rn6	-0.38621534	0.00021225	stronglyHypometh	0.026188	0.79243	insignificant	8	49	50
chr7	97129486	97131486	Eed	-0.14699078	0.58736	insignificant	0.058694	0.041245	hypermethylated	3	41	35
chr7	97277741	97279741	Picalm	-0.09642374	4.36E-41	hypomethylated	0.039068	0.0036199	hypermethylated	46	167	168
chr7	97413942	97415942	Ccdc83	-0.66238248	0.0034875	stronglyHypometh	-0.15427	0.70149	insignificant	1	16	16
chr7	97534490	97536490	Sytl2	-0.25860321	0.0031185	hypomethylated	-0.050908	0.48945	insignificant	6	12	12
chr7	97590290	97592290	Crebzf	-0.08603395	1.25E-35	hypomethylated	0.0097911	0.59967	insignificant	67	239	201
chr7	97605718	97607718	Tmem126a	-0.26332784	0.00000844	hypomethylated	0.0099182	0.20347	insignificant	7	25	22
chr7	97624505	97626505	Tmem126b	-0.16517729	1.07E-13	hypomethylated	-0.012199	0.19416	insignificant	6	38	38
chr7	98238295	98240295	Dlg2	0.34702295	0.0023248	stronglyHypermeth	-0.038518	0.18043	insignificant	2	20	20
chr7	99785652	99787652	Ankrd42	-0.46246777	0.0074712	stronglyHypometh	0.040129	0.63703	insignificant	3	14	14
chr7	99818422	99820422	Pcf11	-0.12774924	0.00053079	hypomethylated	0.013049	0.78569	insignificant	9	46	46

chr7	99889223	99891223	Rab30	-0.16618306	1.24E-31	hypomethylated	0.013614	0.023869	inconclusive	12	48	40
chr7	99889969	99891969	4632427E13Ri	-0.15946429	0.000062529	hypomethylated	-0.012589	0.030671	hypomethylated	3	8	8
chr7	100022742	100024742	4632434I11Ril	-0.14532007	2.44E-31	hypomethylated	0.031853	1	insignificant	20	83	67
chr7	100022762	100024762	Prcp	-0.13495633	3.81E-31	hypomethylated	0.030283	1	insignificant	19	81	65
chr7	100227388	100229388	Fam181b	-0.09488447	1.01E-21	hypomethylated	-0.0037336	0.14791	insignificant	32	140	151
chr7	103358146	103360146	Odz4	-0.10414798	1.71E-25	hypomethylated	-0.0080518	0.0083822	inconclusive	47	164	161
chr7	104099036	104101036	Nars2	-0.11098292	1.09E-08	hypomethylated	0.0089099	0.80266	insignificant	6	40	40
chr7	104229260	104231260	Gab2	-0.1058398	2.2E-33	hypomethylated	0.0078965	0.46269	insignificant	65	154	144
chr7	104479832	104481832	Kctd21	-0.14384641	0.005754	hypomethylated	0.01992	0.78661	insignificant	11	53	49
chr7	104547512	104549512	Ndufc2	-0.29473898	0.00000164	hypomethylated	-0.10089	2.42E-12	hypomethylated	12	60	55
chr7	104566020	104568020	Thrsp	-0.26134564	0.0029158	hypomethylated	0.026827	0.53591	insignificant	2	5	5
chr7	104600713	104602713	Kctd14	-0.26492325	5.06E-09	hypomethylated	0.0018584	0.96744	insignificant	9	36	36
chr7	104628465	104630465	Ints4	-0.15303141	0.000000164	hypomethylated	0.0092548	0.87901	insignificant	7	36	34
chr7	104727405	104729405	Rsf1	-0.10981525	2.15E-17	hypomethylated	-0.012787	0.75671	insignificant	36	136	130
chr7	104728007	104730007	1810020D17R	-0.11063308	2.15E-17	hypomethylated	-0.013605	0.75669	insignificant	36	134	130
chr7	104844200	104846200	Clns1a	-0.07694649	3.55E-11	hypomethylated	-0.030953	0.77504	insignificant	40	115	115
chr7	104886757	104888757	Aqp11	0.36033549	0.23315	insignificant	0.069601	0.40998	insignificant	7	55	49
chr7	104990448	104992448	Pak1	-0.35558922	0.027422	stronglyHypometh	-0.032632	0.68788	insignificant	4	19	18
chr7	105268003	105270003	Myo7a	-0.52785146	0.47445	lowCoverage	0.030972	0.71502	insignificant	1	4	4
chr7	105326703	105328703	Capn5	-0.16601772	0.0069798	hypomethylated	-0.011191	0.011687	inconclusive	12	48	48
chr7	105458037	105460037	Acer3	-0.14160654	0.14168	insignificant	-0.0062982	0.49929	insignificant	2	23	23
chr7	105509798	105511798	Tsku	-0.15732337	1.34E-20	hypomethylated	-0.011935	0.0046278	inconclusive	19	48	50
chr7	105509838	105511838	Tsku	-0.14351143	1.73E-16	hypomethylated	-0.024654	0.21136	insignificant	13	36	38
chr7	105641731	105643731	Lrrc32		1	noCoverage	0.097637	0.24477	insignificant	0	4	10
chr7	105805079	105807079	2210018M11F	-0.15913309	0.39294	insignificant	0.00068809	0.44796	insignificant	2	37	42
chr7	105850872	105852872	Prkrir	-0.0915577	7.11E-19	hypomethylated	0.0061533	0.7061	insignificant	41	162	183
chr7	105986354	105988354	Wnt11	-0.12520359	2.96E-09	hypomethylated	-0.022027	0.16545	insignificant	23	80	91
chr7	106289654	106291654	Uvrag	-0.05991198	0.000052658	hypomethylated	-0.0066067	0.73487	insignificant	5	23	23
chr7	106331223	106333223	Dgat2	-0.1662305	0.000092275	hypomethylated	-0.0010738	0.45187	insignificant	10	39	39
chr7	106414956	106416956	Mtap6	-0.21234301	8.11E-16	hypomethylated	-0.003924	0.73261	insignificant	17	67	57
chr7	106415855	106417855	Mtap6	-0.14105535	5.85E-31	hypomethylated	0.016428	0.44313	insignificant	30	151	129
chr7	106529058	106531058	Gdpd5	-0.07906127	1.98E-11	hypomethylated	0.014139	0.73393	insignificant	33	106	105
chr7	106613513	106615513	Klhl35		1	noCoverage	-0.15	1	insignificant	0	4	6
chr7	106631442	106633442	Snord15a	-0.18618152	0.00022341	hypomethylated	-0.012624	0.61048	insignificant	7	38	40
chr7	106632219	106634219	Snord15a	-0.38492063	0.007103	stronglyHypometh	0.072596	0.76455	insignificant	2	6	9
chr7	106682995	106684995	Arrb1	-0.11863825	4.12E-16	hypomethylated	-0.0050897	0.11651	insignificant	20	87	84
chr7	106775613	106777613	Gm4980	-0.63928571	0.36	lowCoverage	0.070089	0.66908	insignificant	1	4	4
chr7	106859850	106861850	Slco2b1	-0.84583962	0.033211	stronglyHypometh	-0.12891	0.27908	insignificant	1	6	6
chr7	106976927	106978927	Neu3		1	noCoverage	0.27764	0.69946	insignificant	0	6	9
chr7	107153913	107155913	Chrdl2	-0.09309851	0.1149	insignificant	-0.0016674	0.85701	insignificant	3	20	20
chr7	107270010	107272010	Pold3	-0.10768347	0.60819	insignificant	0.017289	0.93746	insignificant	3	10	10
chr7	107306786	107308786	Lipt2	-0.11117725	0.38682	insignificant	0.0075971	0.90303	insignificant	3	63	63
chr7	107326192	107328192	Kcne3	-0.21656954	0.19364	insignificant	0.026255	0.79743	insignificant	6	34	37
chr7	107375116	107377116	Pgm2l1	-0.12522986	1.13E-12	hypomethylated	-0.025873	0.30844	insignificant	17	78	92
chr7	107412053	107414053	Gpx2-ps1	-0.1558908	0.30649	insignificant	0.20383	0.88132	insignificant	2	12	8
chr7	107433029	107435029	P4ha3	-0.81666667	0.18584	lowCoverage	-0.00050777	0.20841	insignificant	1	12	12
chr7	107519742	107521742	C2cd3	-0.11549811	5.04E-33	hypomethylated	0.035291	0.97367	insignificant	25	98	98
chr7	107520406	107522406	Ppme1	-0.09349855	4.55E-21	hypomethylated	0.04594	0.42256	insignificant	17	74	74
chr7	107620499	107622499	Ucp3	-0.19930269	0.20162	insignificant	0.0092511	0.67128	insignificant	4	16	18
chr7	107640853	107642853	Ucp2	-0.20468997	3.7E-11	hypomethylated	0.01574	0.077259	insignificant	18	84	82
chr7	107684613	107686613	Chchd8		1	noCoverage	0.038379	0.77149	insignificant	0	16	16
chr7	107731644	107733644	Mrpl48	-0.19650206	0.012179	hypomethylated	0.31878	0.89293	insignificant	2	4	8
chr7	107755099	107757099	Rab6a	-0.11905952	1.29E-26	hypomethylated	0.010971	0.0099292	hypermethylated	37	133	131
chr7	107756510	107758510	Mrpl48	-0.12540896	6.63E-20	hypomethylated	0.019845	0.13862	insignificant	25	95	93
chr7	107805479	107807479	Plekhb1	-0.06087188	0.0029264	hypomethylated	0.016277	0.096972	insignificant	4	23	22
chr7	107806970	107808970	Plekhb1	-0.0422615	0.7565	insignificant	-0.073996	0.09448	insignificant	2	17	15
chr7	107810908	107812908	Plekhb1	-0.55163091	2.34E-19	stronglyHypometh	0.014526	0.44465	insignificant	3	34	36
chr7	107854215	107856215	Fam168a	-0.18727209	6.43E-12	hypomethylated	0.0067417	0.39909	insignificant	7	17	17
chr7	108030923	108032923	Mir3102	-0.15545162	0.023126	hypomethylated	-0.013267	0.69415	insignificant	2	28	26
chr7	108080675	108082675	Arhgef17	-0.3317531	2.15E-14	hypomethylated	-0.098571	1	insignificant	3	16	10
chr7	108160505	108162505	P2ry2	-0.13749156	0.00000675	hypomethylated	-0.012893	0.67729	insignificant	7	18	35
chr7	108256288	108258288	Fchsd2	-0.11109399	5.97E-26	hypomethylated	0.014299	0.81325	insignificant	37	153	139
chr7	108450602	108452602	Atg16l2	-0.2357701	0.0022229	hypomethylated	0.0022266	0.9001	insignificant	6	18	18
chr7	108468832	108470832	Stard10	-0.13709996	1.15E-31	hypomethylated	0.0018687	0.19399	insignificant	51	157	152
chr7	108495582	108497582	Arap1	-0.14934422	1.64E-48	hypomethylated	0.016136	0.30708	insignificant	18	64	68
chr7	108533039	108535039	Arap1	-0.1	0.41372	insignificant	0.078438	0.05542	insignificant	1	5	6
chr7	108541901	108543901	Arap1	-0.05299495	0.16691	insignificant	0.0037062	1	insignificant	5	10	10
chr7	108598765	108600765	Pde2a	-0.09963051	1.31E-10	hypomethylated	-0.0053281	0.2946	insignificant	27	73	70
chr7	108598821	108600821	Pde2a	-0.09963051	1.31E-10	hypomethylated	-0.0053281	0.2946	insignificant	27	73	70
chr7	108622889	108624889	Mir139	-0.11864291	0.29531	insignificant	-0.04599	0.045547	hypomethylated	2	6	6
chr7	108704316	108706316	Art2a-ps	0.07460317	0.68333	insignificant	0.13294	0.010041	hypermethylated	1	6	6
chr7	108709379	108711379	Art2a-ps		1	noCoverage	0.048774	0.038948	hypermethylated	0	8	8
chr7	108811281	108813281	Clpb	-0.1307762	2.29E-10	hypomethylated	-0.0037824	0.5666	insignificant	21	70	70
chr7	108965826	108967826	Phox2a	0.11448634	0.34672	insignificant	0.16618	0.028299	hypermethylated	6	42	24
chr7	108986338	108988338	Inppl1	-0.08665065	0.00000265	hypomethylated	-0.0066485	0.14056	insignificant	18	76	76
chr7	108986740	108988740	Inppl1	-0.40150183	0.5135	insignificant	-0.076228	0.2509	insignificant	2	4	4
chr7	109019197	109021197	Folr1	0.32120017	1	lowCoverage	0.057624	0.4856	insignificant	1	12	12
chr7	109043928	109045928	3200002M19F	-0.15254171	0.00224	hypomethylated	-0.01644	0.0060465	inconclusive	19	66	63
chr7	109053350	109055350	2400001E08Ri	-0.14116755	1.74E-17	hypomethylated	0.0042534	0.78072	insignificant	18	69	69
chr7	109054873	109056873	2400001E08Ri	-0.52786799	1.05E-11	stronglyHypometh	0.03815	0.36689	insignificant	5	32	26
chr7	109082371	109084371	Lrrc51	-0.14190682	0.029267	hypomethylated	0.026712	0.00049625	hypermethylated	16	80	70
chr7	109117356	109119356	Numa1	-0.10580731	0.000205	hypomethylated	-0.022171	0.64974	insignificant	10	49	48
chr7	109166669	109168669	Il18bp	0.03271268	0.52003	insignificant	-0.083071	1	insignificant	2	4	4
chr7	109213226	109215226	Trpc2	-0.12842988	3.31E-26	hypomethylated	0.038709	0.10028	insignificant	17	40	53
chr7	109213646	109215646	Rnf121	-0.12432125	1.82E-11	hypomethylated	-0.00043685	0.87056	insignificant	17	42	53

chr7	109248743	109250743	Art5		1	noCoverage	-0.0088501	0.88125	insignificant	0	8	8
chr7	109265227	109267227	Chrna10	-0.07362759	0.43696	insignificant	0.026324	0.12235	insignificant	2	29	26
chr7	109358634	109360634	Nup98	-0.13977411	0.010749	hypomethylated	-0.0046252	0.036454	inconclusive	8	50	52
chr7	109370587	109372587	Pgap2	-0.21818778	1.83E-10	hypomethylated	-0.004482	0.28844	insignificant	18	59	58
chr7	109398632	109400632	Rhog	0.35045492	0.39652	insignificant	0.0092475	0.841	insignificant	2	31	40
chr7	109415337	109417337	Stim1	-0.14215668	2.76E-14	hypomethylated	-0.012367	0.75134	insignificant	11	83	75
chr7	109589208	109591208	Rrm1	-0.1082552	0.000035507	hypomethylated	0.0067054	0.57093	insignificant	4	51	44
chr7	109701799	109703799	Olfr549	0.05952385	1	insignificant	-0.032847	1	insignificant	3	12	8
chr7	109713983	109715983	Trim21		1	noCoverage	0.011914	0.089643	insignificant	0	16	21
chr7	109725982	109727982	Olfr550	0.01160917	0.68057	insignificant	0.062525	0.69417	insignificant	7	24	22
chr7	109751869	109753869	Olfr552		1	noCoverage	0.062536	0.67426	insignificant	0	8	8
chr7	109787761	109789761	Olfr554	-0.08313412	0.028645	hypomethylated	0.13785	0.041122	hypermethylated	2	6	6
chr7	109806336	109808336	Olfr555		1	noCoverage	-0.13194	0.58074	insignificant	0	8	5
chr7	109835840	109837840	Trim68	-0.1440404	0.00050432	hypomethylated	0.017184	0.7186	insignificant	6	15	14
chr7	109922039	109924039	Olfr561	-0.42109661	0.000001051	stronglyHypometh	0.1051	0.022918	hypermethylated	3	12	12
chr7	110024635	110026635	Olfr568	0.14141414	0.44603	insignificant	0.066162	0.10824	insignificant	3	6	6
chr7	110304259	110306259	Olfr589		1	noCoverage	0.079733	0.22655	insignificant	0	2	2
chr7	110617554	110619554	Olfr608		1	noCoverage	0.21364	0.082915	insignificant	0	9	4
chr7	111084895	111086895	Olfr632	0.07463618	1	insignificant	-0.01703	0.53467	insignificant	2	10	8
chr7	111094081	111096081	Olfr633		1	noCoverage	0.00067303	1	insignificant	0	8	8
chr7	111187311	111189311	Olfr641	-0.8699553	0.13198	lowCoverage	-0.017792	0.45778	insignificant	1	6	6
chr7	111198866	111200866	Olfr642	0.06231652	1	insignificant	0.011056	0.45346	insignificant	1	18	18
chr7	111253794	111255794	Olfr646		1	noCoverage	0.02153	0.1956	insignificant	0	10	10
chr7	111328920	111330920	Olfr648		1	noCoverage	0.11026	1	insignificant	0	13	8
chr7	111338719	111340719	Olfr649	-0.20598291	0.038538	hypomethylated	0.10958	0.55928	insignificant	1	4	4
chr7	111366308	111368308	Trim6	-0.25688056	4.54E-10	hypomethylated	-0.0073974	0.21622	insignificant	9	26	29
chr7	111735268	111737268	Olfr654	0.1	0.5724	insignificant	-0.09375	0.73585	insignificant	1	4	4
chr7	111765170	111767170	Olfr656	-0.0365353	1	insignificant	0.060079	0.0181	hypermethylated	1	3	4
chr7	111795819	111797819	Olfr658	-0.13116883	0.65625	insignificant	-0.050487	0.76603	insignificant	1	11	11
chr7	112028222	112030222	Olfr665		1	noCoverage	0.066698	0.74967	insignificant	0	4	4
chr7	112203761	112205761	Olfr677	-0.31730769	0.0011486	hypomethylated	-0.065476	0.053074	insignificant	2	4	4
chr7	112216982	112218982	Olfr678	-0.39460317	0.060998	insignificant	0.057708	0.061625	insignificant	3	6	6
chr7	112292823	112294823	Olfr683		1	noCoverage	-0.015464	0.22387	insignificant	0	10	10
chr7	112548525	112550525	Fam160a2	-0.10012227	0.0047165	hypomethylated	0.018539	0.55815	insignificant	11	44	43
chr7	112573333	112575333	Cckbr	-0.14746947	0.68857	insignificant	0.024767	0.32427	insignificant	2	22	22
chr7	112630711	112632711	Prkcdbp	0.00243219	0.80569	insignificant	0.011121	0.67523	insignificant	3	20	20
chr7	112701873	112703873	Smpd1	-0.23120326	0.11231	insignificant	-0.026795	0.28622	insignificant	2	42	42
chr7	112730049	112732049	Apbb1	-0.24310345	0.00077736	hypomethylated	0.037669	0.79883	insignificant	2	12	12
chr7	112748630	112750630	Hpx	0.20526316	1	insignificant	0.045007	0.59934	insignificant	1	4	4
chr7	112782013	112784013	Arfip2	-0.12622128	9.16E-10	hypomethylated	-0.021429	0.74969	insignificant	19	55	53
chr7	112788053	112790053	Fxc1	-0.103575	0.01469	hypomethylated	0.03768	0.94052	insignificant	6	49	45
chr7	112788930	112790930	Arfip2		1	noCoverage	0.027514	0.64505	insignificant	0	22	21
chr7	112884103	112886103	Ilk	-0.17313752	5.23E-17	hypomethylated	-0.000062358	0.65882	insignificant	27	115	115
chr7	112884238	112886238	Ilk	-0.16161086	1.38E-16	hypomethylated	0.0014115	0.63466	insignificant	27	113	113
chr7	112885014	112887014	Rrp8	-0.17479893	0.00019367	hypomethylated	0.0051185	0.21809	insignificant	13	55	55
chr7	112900721	112902721	Dchs1	-0.12526205	0.45116	insignificant	0.097416	0.010763	hypermethylated	1	6	6
chr7	112936064	112938064	Dchs1	-0.11403257	0.58355	insignificant	0.0025114	0.46219	insignificant	10	22	39
chr7	112959601	112961601	Mrpl17	-0.14964226	0.14232	insignificant	-0.011394	0.81047	insignificant	3	12	12
chr7	113833243	113835243	Olfr694		1	noCoverage	0.1098	0.65723	insignificant	0	5	5
chr7	113896900	113898900	Olfr698		1	noCoverage	0.011948	0.65723	insignificant	0	5	6
chr7	113987126	113989126	Olfr703	0.11665747	1	lowCoverage	0.0084882	0.3597	insignificant	1	7	6
chr7	114035621	114037621	Olfr707		1	noCoverage	0.12438	0.039848	hypermethylated	0	9	8
chr7	114146119	114148119	Olfr2	-0.15833333	0.018462	hypomethylated	0.046212	1	insignificant	2	4	4
chr7	114351958	114353958	Rbmxl2	-0.04157431	0.00098446	hypomethylated	0.010663	0.49385	insignificant	73	265	252
chr7	114513303	114515303	Syt9	-0.10431613	3.94E-24	hypomethylated	-0.0024452	0.41529	insignificant	48	180	180
chr7	114709946	114711946	Olfml1	-0.0874475	1	insignificant	-0.0066017	0.20525	insignificant	4	17	17
chr7	114737564	114739564	Ppfibp2	-0.20683365	1.25E-21	hypomethylated	0.010908	0.93269	insignificant	11	54	54
chr7	114901510	114903510	Cyb5r2	-0.30491751	0.025416	hypomethylated	0.025441	0.58767	insignificant	4	23	28
chr7	115303385	115305385	Olfr485	-0.09444444	0.47702	insignificant	-0.011111	0.80577	insignificant	1	6	6
chr7	115754822	115756822	Olfr506	-0.00545635	0.32999	insignificant	0.020543	0.56549	insignificant	2	6	6
chr7	115855868	115857868	Olfr512	0.21031746	1	insignificant	0.072419	0.3451	insignificant	1	4	4
chr7	116012666	116014666	Olfr517	-0.65625	0.4	lowCoverage	0.17708	0.19082	insignificant	1	2	2
chr7	116076928	116078928	Eif3f	-0.24333465	2.09E-32	hypomethylated	-0.020937	0.60629	insignificant	8	33	20
chr7	116153393	116155393	Tub	-0.10774885	5.02E-27	hypomethylated	-0.0062771	1	insignificant	37	112	112
chr7	116313822	116315822	Lmo1		1	noCoverage	0.040423	0.87851	insignificant	0	12	14
chr7	116582567	116584567	Stk33	-0.14976428	8.81E-10	hypomethylated	0.010559	0.56343	insignificant	20	62	61
chr7	116637252	116639252	Trim66	0.05052552	1	insignificant	-0.057493	0.19654	insignificant	2	10	10
chr7	116651648	116653648	Trim66	-0.05909481	0.82608	insignificant	-0.04168	1	insignificant	3	10	11
chr7	116661708	116663708	Rpl27a	-0.23326395	0.000035656	hypomethylated	-0.036623	0.48633	insignificant	6	20	19
chr7	116662645	116664645	Snora3	-0.36415816	0.005591	stronglyHypometh	-0.066319	0.41094	insignificant	3	7	6
chr7	116760082	116762082	St5	-0.13974227	0.0028357	hypomethylated	-0.00050883	0.94023	insignificant	8	18	18
chr7	116846250	116848250	D930014E17R	-0.15379407	8.29E-16	hypomethylated	-0.006204	0.90925	insignificant	28	94	93
chr7	116867285	116869285	BC051019		1	noCoverage	0.12469	0.083123	insignificant	0	2	4
chr7	116895777	116897777	Tmem9b	-0.18019868	2.68E-31	hypomethylated	0.012579	0.87484	insignificant	24	49	49
chr7	116925059	116927059	Nrip3	-0.00264657	8.94E-11	hypomethylated	0.017419	0.43785	insignificant	9	62	60
chr7	117009193	117011193	Scube2	-0.16132733	0.016235	hypomethylated	-0.025266	0.49399	insignificant	14	27	33
chr7	117103936	117105936	Dennd5a	-0.15993032	1	insignificant	0.044814	1	insignificant	1	17	18
chr7	117129744	117131744	Tmem41b	0.03903838	1	insignificant	0.0041839	0.076422	insignificant	7	50	50
chr7	117160938	117162938	Ipo7	-0.13246476	6.38E-11	hypomethylated	0.06036	0.11941	insignificant	23	79	59
chr7	117204215	117206215	Zfp143	-0.12823542	3.69E-20	hypomethylated	0.00026354	0.24816	insignificant	37	114	118
chr7	117264572	117266572	Wee1	-0.08647171	1.1E-37	hypomethylated	-0.00031992	0.1586	insignificant	64	276	267
chr7	117364216	117366216	Swap70	-0.09305599	1.58E-16	hypomethylated	0.0024799	0.79862	insignificant	21	83	80
chr7	117758434	117760434	Sbf2	-0.18236227	8.02E-11	hypomethylated	0.059729	0.11077	insignificant	8	41	41
chr7	117770182	117772182	Adm	-0.22124648	0.0032918	hypomethylated	-0.0010643	0.48458	insignificant	7	22	22
chr7	118171464	118173464	Ctr9	-0.15630433	1.39E-16	hypomethylated	0.0019697	0.43872	insignificant	8	60	56

chr7	118226544	118228544	Eif4g2	-0.23108086	0.010146	hypomethylated	-0.092576	0.93487	insignificant	11	74	56
chr7	118266189	118268189	1700012D14R	-0.13585754	0.010829	hypomethylated	0.054781	1	insignificant	7	30	17
chr7	118923491	118925491	Galntl4	-0.05695271	1.03E-09	inconclusive	0.0062989	0.000021029	inconclusive	11	66	60
chr7	119166019	119168019	Usp47	-0.12877076	9.66E-30	hypomethylated	-0.0011119	0.44655	insignificant	27	110	117
chr7	119302571	119304571	Dkk3	-0.10774115	0.315	insignificant	-0.11241	0.0067629	hypomethylated	4	8	8
chr7	119368349	119370349	Mical2	-0.21145484	1.53E-09	hypomethylated	0.033552	0.0277	hypermethylated	13	75	72
chr7	119570219	119572219	Parva	-0.09642309	0.39431	insignificant	0.048875	0.33105	insignificant	3	22	21
chr7	119821833	119823833	Tead1	-0.0914492	1.61E-11	hypomethylated	0.020257	0.090158	insignificant	33	133	135
chr7	120096475	120098475	Rassf10	-0.1144012	8.5E-25	hypomethylated	0.01663	0.16718	insignificant	44	175	175
chr7	120349978	120351978	Arntl	-0.10449339	5.21E-13	hypomethylated	-0.00050734	0.38029	insignificant	40	170	178
chr7	120512853	120514853	Btbd10	-0.10641192	1.27E-16	hypomethylated	0.003845	0.69113	insignificant	25	101	113
chr7	120656375	120658375	Far1	-0.08688191	3.53E-08	hypomethylated	-0.003835	0.73751	insignificant	27	153	153
chr7	120908511	120910511	Spon1	-0.12749494	0.000000138	hypomethylated	0.019762	0.54049	insignificant	14	77	77
chr7	121261295	121263295	Rras2	-0.21911184	0.34681	insignificant	0.086631	0.27608	insignificant	1	10	11
chr7	121419630	121421630	Psma1		1	noCoverage	0.025	1	insignificant	0	8	8
chr7	121557767	121559767	Pde3b	-0.08649496	2.56E-34	hypomethylated	-0.0079633	0.54564	insignificant	78	230	223
chr7	121558675	121560675	4933406I18Ril	-0.09212722	6E-23	hypomethylated	-0.0067084	0.69923	insignificant	68	254	244
chr7	121706486	121708486	Cyp2r1	-0.24989336	2.18E-12	hypomethylated	-0.072064	0.058983	insignificant	7	30	36
chr7	121861156	121863156	Calcb		1	noCoverage	-0.02343	0.64561	insignificant	0	6	6
chr7	121888279	121890279	Insc	-0.02958329	0.68985	insignificant	0.01409	0.50741	insignificant	7	18	18
chr7	123174561	123176561	Sox6	-0.19227927	1.79E-56	hypomethylated	0.0058758	0.94795	insignificant	28	91	85
chr7	123182258	123184258	Sox6	-0.11780215	0.016686	hypomethylated	0.011411	0.37273	insignificant	9	40	39
chr7	123235893	123237893	1110004F10Ri	-0.00117485	0.61715	insignificant	0.07878	0.31037	insignificant	14	53	53
chr7	123236663	123238663	1700003G18R	-0.00117485	0.61715	insignificant	0.07878	0.31037	insignificant	14	53	53
chr7	123333355	123335355	Plekha7		1	noCoverage	0.025174	0.4171	insignificant	0	8	8
chr7	123477704	123479704	Rps13	-0.12558377	7.34E-11	hypomethylated	-0.00017928	1	insignificant	14	75	75
chr7	123586972	123588972	Pik3c2a	-0.15579606	0.61149	insignificant	-0.034485	0.42284	insignificant	4	52	46
chr7	123646887	123648887	Nucb2	-0.12866862	0.00039967	hypomethylated	0.015785	0.42169	insignificant	12	77	73
chr7	124523492	124525492	Xylt1	-0.06411993	3.09E-15	hypomethylated	0.0064926	0.76186	insignificant	31	133	133
chr7	125259661	125261661	Rps15a	-0.40230902	0.000000305	stronglyHypometh	-0.0035236	0.39288	insignificant	2	12	12
chr7	125273139	125275139	Arl6ip1	-0.12160494	0.050698	insignificant	-0.017785	0.49521	insignificant	6	18	18
chr7	125386420	125388420	4930583K01Ri	-0.0902975	4.42E-38	hypomethylated	0.0053532	0.51481	insignificant	41	181	175
chr7	125387151	125389151	Smg1	-0.15528987	1.25E-23	hypomethylated	-0.013073	0.22631	insignificant	14	76	69
chr7	125587066	125589066	Syt17		1	noCoverage	0.018687	0.85457	insignificant	0	2	2
chr7	125635489	125637489	Itpripl2	-0.34411765	0.049721	stronglyHypometh	0.098739	0.81345	insignificant	0	0	0
chr7	125676827	125678827	Coq7	-0.08705357	1	insignificant	0.0017743	0.87621	insignificant	3	14	14
chr7	125728200	125730200	Tmc7	-0.16053095	6.43E-12	hypomethylated	-0.050823	0.010731	hypomethylated	8	27	24
chr7	125739810	125741810	Tmc5	0.72353085	0.28143	lowCoverage	0.035015	0.1767	insignificant	1	50	56
chr7	125776249	125778249	Tmc5		1	noCoverage	-0.026653	0.81086	insignificant	0	6	7
chr7	125849252	125851252	Gde1	-0.30648375	0.0019516	hypomethylated	0.018683	0.32131	insignificant	3	26	26
chr7	125855124	125857124	6330503K22Ri	-0.14192602	1.35E-12	hypomethylated	0.011343	0.6233	insignificant	15	60	59
chr7	125882784	125884784	9030624J02Rik		1	noCoverage	0.059222	0.39783	insignificant	0	20	20
chr7	125998288	126000288	Iqck	-0.12198802	7E-21	hypomethylated	-0.010628	0.51965	insignificant	42	99	118
chr7	125999127	126001127	2310008H09R	-0.10731579	6.93E-17	hypomethylated	-0.0042126	0.56968	insignificant	40	85	98
chr7	125999512	126001512	2310008H09R	-0.13268909	2.25E-13	hypomethylated	-0.0042392	0.8488	insignificant	15	39	38
chr7	126138725	126140725	Gprc5b	-0.14860877	0.0010027	hypomethylated	0.010521	0.77104	insignificant	5	29	26
chr7	126327888	126329888	Gpr139	-0.15181638	0.00000177	hypomethylated	0.028868	0.17834	insignificant	5	57	58
chr7	126602786	126604786	Gp2		1	noCoverage	0.039265	0.53204	insignificant	0	6	6
chr7	126666996	126668996	Pdilt	0	0.075729	insignificant	0.046296	0.4086	insignificant	2	4	4
chr7	126668778	126670778	Acsm5	0.05462276	0.17134	insignificant	-0.032931	0.67862	insignificant	2	14	14
chr7	126704221	126706221	Acsm2		1	noCoverage	-0.25	0.6315	insignificant	0	7	7
chr7	126864279	126866279	Thumpd1	-0.09934225	1	insignificant	-0.013705	0.097079	insignificant	4	14	14
chr7	126903436	126905436	Acsm3		1	noCoverage	0.028772	0.22465	insignificant	0	14	14
chr7	126936643	126938643	2610020H08R	-0.07756971	0.000000232	hypomethylated	-0.0040383	0.70806	insignificant	15	52	52
chr7	126936700	126938700	2610020H08R	-0.07756971	0.000000232	hypomethylated	-0.0040383	0.70806	insignificant	15	52	52
chr7	126937572	126939572	Eri2	-0.14442204	0.70471	insignificant	0.01746	0.54105	insignificant	4	12	12
chr7	127038805	127040805	Lyrm1	-0.13210668	4.02E-20	hypomethylated	0.0042108	0.59026	insignificant	19	89	89
chr7	127038972	127040972	Dcun1d3	-0.1277948	7.37E-19	hypomethylated	0.0050122	0.76317	insignificant	19	83	83
chr7	127039259	127041259	Dcun1d3	-0.16910839	9.57E-13	hypomethylated	0.0007299	0.70351	insignificant	10	41	41
chr7	127244939	127246939	Tmem159	-0.13457742	0.00046339	hypomethylated	0.012689	0.80942	insignificant	10	54	50
chr7	127316371	127318371	Anks4b	0.18863636	1	lowCoverage	0.11627	0.42832	insignificant	1	4	4
chr7	127345502	127347502	Crym	-0.19078043	6.65E-08	hypomethylated	0.079731	0.000000323	hypermethylated	7	18	16
chr7	127346477	127348477	Abca14	-0.20532992	3.91E-12	hypomethylated	0.086745	8.07E-08	hypermethylated	9	22	20
chr7	127740138	127742138	E130201H02R	-0.18826513	1.53E-23	hypomethylated	-0.0052346	0.51198	insignificant	14	58	58
chr7	127777702	127779702	Uqcrc2	-0.16227112	8.92E-17	hypomethylated	0.0012197	0.51024	insignificant	19	70	63
chr7	127813764	127815764	Pdzd9		1	noCoverage	0.071462	0.5536	insignificant	0	6	6
chr7	127813857	127815857	Pdzd9		1	noCoverage	0.071462	0.5536	insignificant	0	6	6
chr7	127820133	127822133	BC030336	-0.07775127	0.000081705	hypomethylated	-0.0040597	0.20156	insignificant	24	74	74
chr7	127882070	127884070	Vwa3a	0.40909091	1	lowCoverage	0.10261	0.46264	insignificant	1	4	5
chr7	127985396	127987396	Eef2k	-0.1211923	2.33E-08	hypomethylated	0.014282	0.82818	insignificant	10	43	42
chr7	128060257	128062257	Polr3e	-0.11518884	1.07E-63	hypomethylated	0.0054538	0.29838	insignificant	61	191	184
chr7	128125826	128127826	Cdr2	-0.22585034	0.55391	insignificant	0.016437	0.67804	insignificant	1	21	13
chr7	128176958	128178958	Mettl9	-0.11636953	1.69E-32	hypomethylated	-0.0048172	0.14902	insignificant	49	175	174
chr7	128534809	128536809	Hs3st2	-0.05668854	7.27E-14	hypomethylated	0.021712	8.16E-12	hypermethylated	42	173	162
chr7	128850767	128852767	Usp31	-0.06953749	0.00000091	hypomethylated	-0.0061696	0.14043	insignificant	15	38	38
chr7	128877020	128879020	Scnn1g	-0.13595889	0.00000191	hypomethylated	0.053787	0.94357	insignificant	7	41	38
chr7	129007624	129009624	Scnn1b	-0.10738355	0.00059776	hypomethylated	0.022108	0.84362	insignificant	8	20	20
chr7	129125207	129127207	Cog7	-0.25749031	6.3E-22	hypomethylated	0.077333	0.44736	insignificant	10	30	41
chr7	129164712	129166712	Gga2	-0.44255051	1	insignificant	-0.20323	0.16655	insignificant	3	12	9
chr7	129209711	129211711	Ubfd1	-0.14140987	8.05E-19	hypomethylated	0.0016528	0.78894	insignificant	23	96	96
chr7	129210577	129212577	Ubfd1	-0.14360242	5.25E-16	hypomethylated	0.0034827	0.67585	insignificant	20	78	78
chr7	129245362	129247362	Ndufab1	-0.10543821	0.25213	insignificant	0.015604	0.72163	insignificant	8	20	20
chr7	129275554	129277554	Dctn5	-0.15504701	0.0021357	hypomethylated	-0.025985	0.1017	insignificant	9	37	52
chr7	129276460	129278460	Palb2	-0.14428578	0.0021169	hypomethylated	-0.024653	0.087763	insignificant	9	33	40
chr7	129301950	129303950	Plk1	-0.24551213	5.61E-16	hypomethylated	-0.019425	0.11297	insignificant	19	73	79

chr7	129362009	129364009	2010110P09Ri	-0.64077144	0.087657	insignificant	0.10422	0.099627	insignificant	1	28	28
chr7	129431638	129433638	Prkcb	-0.1359304	2.96E-16	hypomethylated	-0.0045502	0.60547	insignificant	25	91	86
chr7	129814257	129816257	Cacng3	-0.15596874	0.00061375	hypomethylated	0.031903	0.34181	insignificant	8	26	28
chr7	130113077	130115077	Rbbp6	-0.01984122	0.034643	inconclusive	0.021664	0.0028288	hypermethylated	57	193	189
chr7	130266398	130268398	Tnrc6a	-0.11152215	4.36E-14	hypomethylated	0.0088662	0.73698	insignificant	16	80	80
chr7	130513429	130515429	Arhgap17	-0.20253305	0.058019	insignificant	-0.078481	0.47545	insignificant	3	44	45
chr7	130520495	130522495	Lcmt1	-0.1427992	0.00000407	hypomethylated	-0.0017398	0.7261	insignificant	8	28	28
chr7	130643963	130645963	Zkscan2		1	noCoverage	0.000076897	0.79604	insignificant	0	24	22
chr7	132511374	132513374	4930571K23Ri	-0.05973193	0.28305	insignificant	-0.29216	0.38277	insignificant	4	8	8
chr7	132587189	132589189	Jmjd5	-0.15740283	0.000000273	hypomethylated	0.021293	0.53277	insignificant	21	94	97
chr7	132635056	132637056	Nsmce1	0.10122616	0.00000482	hypermethylated	-0.017902	0.0001249	hypomethylated	12	45	47
chr7	132694795	132696795	Il4ra	-0.15959106	0.14123	insignificant	0.016565	0.80385	insignificant	2	40	40
chr7	132850389	132852389	D430042O09R	-0.1139798	2.97E-20	hypomethylated	-0.013818	1	insignificant	22	108	107
chr7	132851202	132853202	Gtf3c1	-0.00760072	0.94094	insignificant	-0.0072256	0.004764	hypomethylated	18	70	70
chr7	133225925	133227925	Gsg1l	-0.13359194	0.50562	insignificant	0.0069966	0.02107	inconclusive	6	21	21
chr7	133343922	133345922	Xpo6	-0.15822072	0.00010549	hypomethylated	-0.046321	0.0007125	hypomethylated	11	45	38
chr7	133415132	133417132	Sbk1	-0.10385156	1.25E-30	hypomethylated	0.0011648	0.28747	insignificant	49	149	146
chr7	133513048	133515048	Spns1	-0.14266745	0.0000042	hypomethylated	-0.043282	0.018852	hypomethylated	9	28	28
chr7	133521448	133523448	Spns1	-0.29744817	1	lowCoverage	0.011188	0.70004	insignificant	1	11	11
chr7	133540251	133542251	Nfatc2ip	-0.21864485	1.36E-13	hypomethylated	0.0085065	0.59352	insignificant	6	22	22
chr7	133571280	133573280	Rabep2	-0.09268852	1.24E-20	hypomethylated	0.0087931	0.12512	insignificant	28	132	132
chr7	133629868	133631868	Tufm	-0.15633537	7.45E-09	hypomethylated	0.0067092	0.6174	insignificant	17	40	40
chr7	133646816	133648816	Atxn2l	-0.11013878	1.38E-19	hypomethylated	0.00048293	0.6732	insignificant	29	104	105
chr7	133709880	133711880	Eif3c	-0.26326846	5.56E-09	hypomethylated	-0.052622	0.7295	insignificant	8	24	23
chr7	133726750	133728750	Cln3	-0.33447241	0.000040106	stronglyHypometh	-0.024577	0.55017	insignificant	3	16	16
chr7	133727521	133729521	Apobr	-0.57077991	0.000002628	stronglyHypometh	0.030807	0.3069	insignificant	1	6	6
chr7	133727794	133729794	Cln3		1	noCoverage	-0.0045975	0.16815	insignificant	0	6	6
chr7	133738424	133740424	Il27		1	noCoverage	0.049207	0.723	insignificant	0	4	4
chr7	133791822	133793822	Ccdc101	-0.10308042	5.26E-10	hypomethylated	0.054808	0.047513	hypermethylated	16	83	80
chr7	133838513	133840513	Bola2	-0.11251475	4.13E-17	hypomethylated	-0.0031303	0.82983	insignificant	15	126	127
chr7	133839297	133841297	Slx1b	-0.13051003	1.49E-09	hypomethylated	-0.0085855	0.38768	insignificant	7	79	79
chr7	133848268	133850268	Coro1a		1	noCoverage	-0.012616	0.92809	insignificant	0	32	32
chr7	133902139	133904139	Mapk3	-0.10032598	0.00000135	hypomethylated	-0.0022784	0.40042	insignificant	18	68	68
chr7	133908927	133910927	Gdpd3	-0.0345409	1	insignificant	-0.010408	0.32574	insignificant	5	22	21
chr7	133919488	133921488	Ypel3	-0.13964571	0.00000213	hypomethylated	0.00561	0.23485	insignificant	22	78	68
chr7	133923996	133925996	Tbx6	-0.24886653	0.00021251	hypomethylated	0.0092681	0.028941	hypermethylated	6	20	20
chr7	133935985	133937985	Ppp4c	-0.16620787	2.66E-11	hypomethylated	0.032626	0.35942	insignificant	14	20	21
chr7	133942692	133944692	Aldoa	-0.02023801	0.000015688	hypomethylated	0.027632	0.67884	insignificant	13	79	73
chr7	133942768	133944768	Aldoa	-0.04348842	0.000008	hypomethylated	0.034119	0.581	insignificant	10	67	61
chr7	133943961	133945961	Aldoa		1	noCoverage	0.035381	0.61784	insignificant	0	4	5
chr7	133959398	133961398	Fam57b	-0.14347071	0.062761	insignificant	0.11121	0.0056591	hypermethylated	3	10	10
chr7	133965816	133967816	Fam57b	-0.30193695	0.0057584	hypomethylated	-0.10037	1	insignificant	2	7	10
chr7	133967166	133969166	Fam57b	-0.44024864	0.00067826	stronglyHypometh	0.031439	0.86229	insignificant	2	4	4
chr7	133975010	133977010	4930451I11Ri	-0.18609237	0.00038942	hypomethylated	0.097386	0.56284	insignificant	2	20	13
chr7	133990066	133992066	Doc2a	-0.10191747	1.47E-19	hypomethylated	0.027376	0.000010857	hypermethylated	30	126	131
chr7	134004485	134006485	Hirip3	-0.09621873	0.058205	insignificant	0.017581	0.18842	insignificant	5	58	58
chr7	134004977	134006977	Hirip3	-0.18313505	0.086806	insignificant	0.0042963	0.88676	insignificant	2	40	40
chr7	134041792	134043792	Tmem219	-0.10154709	4.63E-09	hypomethylated	0.0075757	0.81917	insignificant	7	26	26
chr7	134071392	134073392	Kctd13	-0.14137421	9.24E-17	hypomethylated	0.021116	0.69133	insignificant	33	97	100
chr7	134093095	134095095	Asphd1	-0.2046666	0.00018136	hypomethylated	0.038192	0.88821	insignificant	8	35	30
chr7	134093242	134095242	Asphd1	-0.2046666	0.00018136	hypomethylated	0.038192	0.88821	insignificant	8	35	30
chr7	134118862	134120862	Cdipt	-0.17279718	1.9E-24	hypomethylated	0.013504	0.28265	insignificant	17	88	81
chr7	134118901	134120901	Cdipt	-0.17279718	1.9E-24	hypomethylated	0.013504	0.28265	insignificant	17	88	81
chr7	134158108	134160108	2900092E17Ri	-0.00881043	0.32553	insignificant	0.013461	0.72185	insignificant	7	31	34
chr7	134164725	134166725	Maz	-0.12887427	2.89E-10	hypomethylated	0.01756	0.80036	insignificant	11	30	30
chr7	134169993	134171993	Maz	-0.02710306	0.028686	hypomethylated	0.013241	0.10367	insignificant	24	121	89
chr7	134185934	134187934	Kif22	0.0165898	0.48763	insignificant	0.09747	0.26678	insignificant	7	24	19
chr7	134233949	134235949	AI467606		1	noCoverage	-0.22012	0.94839	insignificant	0	10	5
chr7	134265543	134267543	Qprt		1	noCoverage	-0.06241	0.23477	insignificant	0	7	6
chr7	134281331	134283331	Spn	-0.77250446	0.22449	lowCoverage	0.0098485	1	insignificant	1	4	4
chr7	134339514	134341514	Cd2bp2	0.28957205	1	insignificant	0.1791	1	insignificant	1	20	16
chr7	134351982	134353982	Tbc1d10b	-0.14684657	2.75E-09	hypomethylated	0.0065096	0.61367	insignificant	15	57	57
chr7	134375956	134377956	Zfp553	-0.09299043	2.37E-24	hypomethylated	0.0047186	0.3397	insignificant	32	97	97
chr7	134376644	134378644	Gm4532	-0.10379644	2.99E-09	hypomethylated	0.0061299	0.77477	insignificant	19	65	65
chr7	134387039	134389039	Zfp771	-0.12805516	1.29E-26	hypomethylated	-0.0037272	0.69453	insignificant	32	93	88
chr7	134404181	134406181	Dctpp1	-0.25435121	0.000029166	hypomethylated	-0.0039697	0.14912	insignificant	9	22	21
chr7	134417573	134419573	Sephs2	-0.10028446	1	insignificant	-0.022469	0.0011581	hypomethylated	2	15	14
chr7	134438861	134440861	Itgal	-0.31465458	0.022211	hypomethylated	-0.046575	0.23991	insignificant	1	18	18
chr7	134488828	134490828	Zfp768	-0.13443057	3.66E-20	hypomethylated	-0.011494	0.74014	insignificant	40	118	129
chr7	134541429	134543429	Mir684-1		1	noCoverage	-0.13096	0.44247	insignificant	0	12	10
chr7	134565548	134567548	Zfp688	-0.2650463	0.0025813	hypomethylated	0.033897	0.64782	insignificant	2	4	4
chr7	134592672	134594672	Zfp689	0.23483865	0.52678	insignificant	0.050695	0.39582	insignificant	2	11	10
chr7	134614127	134616127	Prr14	-0.22016651	4.31E-17	hypomethylated	-0.013779	0.86582	insignificant	25	92	96
chr7	134627734	134629734	Fbrs	0.03211309	0.1103	insignificant	-0.10047	0.2354	insignificant	8	24	24
chr7	134656383	134658383	1700008J07Ri	-0.25183122	3.5E-09	hypomethylated	-0.028118	0.23891	insignificant	8	22	21
chr7	134715861	134717861	Phkg2	-0.15795352	0.000000102	hypomethylated	-0.01077	0.9359	insignificant	10	39	40
chr7	134731211	134733211	Rnf40	-0.17887279	5.18E-32	hypomethylated	0.00038352	0.17638	insignificant	26	91	91
chr7	134732109	134734109	Gm166	-0.22634694	1.73E-15	hypomethylated	0.0096124	0.33797	insignificant	26	95	94
chr7	134757947	134759947	Zfp629	-0.14389783	5.19E-19	hypomethylated	0.019814	0.67466	insignificant	29	99	84
chr7	134851000	134853000	Mir762	-0.12377435	1.91E-19	hypomethylated	-0.020459	0.84295	insignificant	34	98	102
chr7	134852280	134854280	Mir762	-0.07387468	0.00020466	hypomethylated	-0.014564	0.7592	insignificant	16	34	40
chr7	134855249	134857249	Ctf1	-0.3258101	0.55645	insignificant	0.041074	0.21782	insignificant	3	37	38
chr7	134869130	134871130	Ctf2		1	noCoverage	0.056457	0.88653	insignificant	0	13	12
chr7	134889288	134891288	Fbxl19	-0.12250857	0.25222	insignificant	0.054891	0.48861	insignificant	6	48	38
chr7	134912328	134914328	Orai3	-0.20968941	1E-17	hypomethylated	0.0011178	0.36863	insignificant	12	51	48

chr7	134919902	134921902	Setd1a	-0.0752503	1.78E-26	hypomethylated	-0.00082115	0.50479	insignificant	37	167	167
chr7	134943122	134945122	Hsd3b7	-0.53888889	0.0054708	stronglyHypometh	0.01453	0.11162	insignificant	2	6	6
chr7	134984321	134986321	Stx4a	-0.14034866	9.09E-18	hypomethylated	-0.0036543	0.16956	insignificant	25	100	99
chr7	135020214	135022214	Zfp646	-0.21640844	1.15E-13	hypomethylated	-0.012537	0.40504	insignificant	17	66	64
chr7	135020337	135022337	Zfp646	-0.18009104	0.00000275	hypomethylated	-0.0088213	0.14723	insignificant	15	66	63
chr7	135039131	135041131	Vkorc1	-0.3139129	0.0000023	hypomethylated	-0.032922	0.00036418	hypomethylated	6	40	39
chr7	135046586	135048586	Bckdk	-0.48293733	0.0037037	stronglyHypometh	0.032607	0.08907	insignificant	5	36	36
chr7	135055030	135057030	Myst1	-0.32925148	4.59E-25	hypomethylated	0.0039492	0.070696	insignificant	16	41	44
chr7	135073627	135075627	Prss8	0.20313918	0.3123	insignificant	0.087417	0.041417	hypermethylated	3	12	10
chr7	135109992	135111992	Fus	-0.17167153	3.4E-18	hypomethylated	0.0019141	0.50528	insignificant	21	96	91
chr7	135146891	135148891	Trim72	0.0268672	0.1337	insignificant	0.023373	0.000017255	hypermethylated	8	30	29
chr7	135205153	135207153	Itgam	-0.22932449	0.37869	insignificant	-0.022327	0.88194	insignificant	3	16	16
chr7	135379870	135381870	Armc5	-0.02918651	2.65E-16	hypomethylated	0.010039	0.013239	hypermethylated	43	160	167
chr7	135381545	135383545	9130023H24R	-0.05346205	0.000027385	hypomethylated	0.011243	0.041953	hypermethylated	15	57	65
chr7	135389384	135391384	Tgfb1i1	-0.23550725	0.000017852	hypomethylated	0.042929	0.87146	insignificant	6	12	12
chr7	135561686	135563686	Rgs10	-0.12794408	1.02E-32	hypomethylated	0.006201	0.24414	insignificant	28	111	108
chr7	135605027	135607027	Tial1	-0.16095401	2.86E-09	hypomethylated	0.022876	0.13266	insignificant	28	94	98
chr7	135666096	135668096	Bag3	-0.1151762	2.28E-18	hypomethylated	0.0018606	0.68819	insignificant	32	133	129
chr7	135753878	135755878	Inpp5f	-0.15373616	4.32E-40	hypomethylated	-0.011421	0.79747	insignificant	30	106	94
chr7	135883943	135885943	Mcmbp	-0.30378027	0.000000424	hypomethylated	-0.0069886	0.0097705	inconclusive	7	36	36
chr7	135887383	135889383	Sec23ip	-0.15218796	5.26E-21	hypomethylated	0.002644	0.1076	insignificant	18	71	69
chr7	136399607	136401607	Ppapdc1a	-0.14658803	2.05E-18	hypomethylated	0.0098336	0.056346	insignificant	19	68	68
chr7	136734376	136736376	Wdr11	-0.13561142	3.94E-30	hypomethylated	-0.0056881	0.37217	insignificant	23	57	57
chr7	137663044	137665044	Ate1	-0.10289437	0.000000137	hypomethylated	-0.011334	1	insignificant	28	103	103
chr7	137663053	137665053	Ate1	-0.10289437	0.000000137	hypomethylated	-0.011334	1	insignificant	28	103	103
chr7	137663475	137665475	Ate1	-0.06003098	0.39818	insignificant	-0.0054915	0.74177	insignificant	10	36	36
chr7	137835069	137837069	Tacc2	-0.14763918	0.0051073	hypomethylated	0.024388	0.018458	hypermethylated	14	53	53
chr7	138008423	138010423	Plekha1	-0.08054168	5.96E-32	hypomethylated	0.0039383	0.206	insignificant	45	178	172
chr7	138078716	138080716	Htra1	-0.12229573	2.64E-15	hypomethylated	0.0055054	0.43953	insignificant	28	183	161
chr7	138316915	138318915	5430419D17Rik		1	noCoverage	0.0098214	0.26814	insignificant	0	6	6
chr7	138477135	138479135	Fam24a	0.22852564	1	lowCoverage	0.18158	0.0024112	hypermethylated	1	8	12
chr7	138506212	138508212	2310057M21F	0.20758587	0.88634	insignificant	-0.009715	0.15354	insignificant	3	22	21
chr7	138513659	138515659	Pstk	-0.10623944	7.28E-08	hypomethylated	-0.0041961	0.67875	insignificant	9	55	52
chr7	138553114	138555114	Acadsb	-0.18632679	6.16E-39	hypomethylated	0.039531	0.47564	insignificant	18	88	88
chr7	138553992	138555992	Ikzf5	-0.29607785	1	lowCoverage	0.098904	0.4453	insignificant	1	24	24
chr7	138685476	138687476	Hmx3	-0.13283455	6.43E-21	hypomethylated	0.0085059	0.38834	insignificant	33	175	168
chr7	138696575	138698575	Hmx2	-0.16285991	4.16E-27	hypomethylated	0.024119	0.23915	insignificant	28	113	105
chr7	138702904	138704904	Bub3	-0.15199422	4.26E-11	hypomethylated	0.015662	0.60156	insignificant	10	50	48
chr7	139157142	139159142	Gpr26	-0.11476761	0.000000166	hypomethylated	0.0052404	0.69103	insignificant	27	123	115
chr7	139346424	139348424	Cpxm2	-0.6	0.23214	lowCoverage	-0.1	0.77753	insignificant	1	2	2
chr7	139506346	139508346	Gm10584	-0.14109599	1.23E-17	hypomethylated	-0.0028686	0.97602	insignificant	31	107	108
chr7	139508838	139510838	Chst15	-0.3449642	0.58119	insignificant	-0.03438	0.55698	insignificant	2	22	22
chr7	139768081	139770081	Oat	-0.00781932	0.38241	insignificant	0.019855	0.42352	insignificant	3	11	13
chr7	139791320	139793320	Nkx1-2	-0.1467323	0.0036372	hypomethylated	-0.023522	0.52203	insignificant	8	16	16
chr7	139801325	139803325	Lhpp	-0.15806059	0.3939	insignificant	0.0012548	0.17766	insignificant	7	44	44
chr7	139978618	139980618	Fam53b	0.075	1	insignificant	0.075	0.25895	insignificant	2	4	4
chr7	140004879	140006879	Fam53b	-0.12182014	5.47E-21	hypomethylated	0.0060223	0.2467	insignificant	31	99	100
chr7	140044330	140046330	Mettl10	0.00534754	0.54162	insignificant	-0.072352	0.67817	insignificant	3	22	18
chr7	140049907	140051907	Fam175b	-0.11318783	2.67E-23	hypomethylated	0.00076622	0.4333	insignificant	19	79	79
chr7	140140304	140142304	Zranb1		1	noCoverage	0.041667	1	insignificant	0	8	8
chr7	140315166	140317166	Ctbp2	-0.12530534	7.42E-42	hypomethylated	0.020109	0.23061	insignificant	41	155	155
chr7	140828357	140830357	2700050L05Ri	-0.17580842	0.000016006	hypomethylated	-0.083544	0.9047	insignificant	20	47	41
chr7	140900015	140902015	Bccip	-0.13911127	3.49E-33	hypomethylated	-0.00043289	0.46431	insignificant	31	101	96
chr7	140900978	140902978	Uros	-0.25868251	8.39E-16	hypomethylated	0.055571	0.0040569	hypermethylated	9	29	24
chr7	140967573	140969573	Fank1	-0.0819349	3.05E-27	hypomethylated	-0.0020131	1	insignificant	41	147	147
chr7	140968486	140970486	Dhx32	-0.07897486	4.37E-19	hypomethylated	-0.0039446	0.52735	insignificant	18	48	48
chr7	141416780	141418780	Adam12	-0.05565905	0.064665	insignificant	-0.002259	0.38123	insignificant	15	48	48
chr7	141568511	141570511	D7Ertd443e	-0.75320513	0.17308	lowCoverage	0.20513	0.26774	insignificant	1	6	6
chr7	141861369	141863369	Dock1	-0.11796512	5.32E-23	hypomethylated	0.0091669	0.52773	insignificant	29	93	68
chr7	142459301	142461301	Nps	-0.8607443	0.0049128	stronglyHypometh	-0.25899	0.000039774	hypomethylated	2	7	14
chr7	142601049	142603049	Foxi2	-0.08924562	0.000000031	hypomethylated	0.036933	0.48106	insignificant	17	48	38
chr7	142728506	142730506	Ptpre	-0.10385304	4.86E-40	hypomethylated	0.021339	0.15158	insignificant	30	105	97
chr7	142908062	142910062	Mki67	0.03786875	0.39632	insignificant	0.0079899	0.77706	insignificant	5	10	10
chr7	144085293	144087293	Mgmt		1	noCoverage	-0.014511	0.57977	insignificant	0	12	12
chr7	144506128	144508128	Ebf3	-0.04697207	0.59032	insignificant	0.0099112	1	insignificant	28	133	135
chr7	144602439	144604439	9430038I01Ril	-0.16666523	0.00017249	hypomethylated	0.043532	0.78567	insignificant	2	21	18
chr7	144628330	144630330	Glrx3	-0.12561225	4.24E-14	hypomethylated	-0.0072165	0.36327	insignificant	29	109	111
chr7	144628356	144630356	Gm12669	-0.12561225	4.24E-14	hypomethylated	-0.0072165	0.36327	insignificant	29	109	111
chr7	145589413	145591413	Tcerg1l	-0.19008304	1.86E-13	hypomethylated	0.02489	0.30618	insignificant	20	84	81
chr7	146037068	146039068	Ppp2r2d	-0.11955183	1.32E-21	hypomethylated	0.0016673	0.17695	insignificant	44	192	182
chr7	146037950	146039950	Mapk1ip1	-0.13225907	1.05E-13	hypomethylated	-0.012477	0.34223	insignificant	33	147	136
chr7	146101189	146103189	Bnip3	-0.2416886	1.14E-10	hypomethylated	-0.013205	0.6422	insignificant	11	47	48
chr7	146131432	146133432	Jakmip3	-0.00509312	0.000000637	hypomethylated	0.016617	0.28563	insignificant	24	125	123
chr7	146270899	146272899	Dpysl4	-0.1463039	2.99E-09	hypomethylated	0.035686	0.00000271	hypermethylated	24	118	124
chr7	146374393	146376393	Stk32c	-0.12717193	9.49E-14	hypomethylated	0.00047673	0.16767	insignificant	10	56	56
chr7	146397886	146399886	Lrrc27	-0.11645976	0.0041282	hypomethylated	0.0052839	0.61076	insignificant	7	60	60
chr7	146398173	146400173	Lrrc27	-0.11645976	0.0041282	hypomethylated	0.0052839	0.61076	insignificant	7	60	60
chr7	146399206	146401206	Lrrc27		1	noCoverage	-0.0062297	1	insignificant	0	30	30
chr7	146433380	146435380	Pwwp2b	-0.17516958	1.29E-56	hypomethylated	0.011988	0.0092648	hypermethylated	80	196	174
chr7	146574007	146576007	Inpp5a	-0.13738629	3.42E-42	hypomethylated	-0.0038623	0.5177	insignificant	43	118	117
chr7	146768696	146770696	Nkx6-2	-0.10721501	0.00070498	hypomethylated	0.040294	0.33144	insignificant	6	39	32
chr7	147019072	147021072	Gpr123	-0.11668668	0.00079725	hypomethylated	0.027646	0.3142	insignificant	13	37	36
chr7	147079594	147081594	Kndc1	-0.17650443	7.34E-20	hypomethylated	-0.021901	1	insignificant	25	65	56
chr7	147128754	147130754	Utf1	-0.12931664	4.69E-34	hypomethylated	0.013199	0.054871	insignificant	39	100	100
chr7	147164654	147166654	Adam8		1	noCoverage	-0.15514	0.61513	insignificant	0	14	10

chr7	147221289	147223289	Zfp511	-0.21012839	5.16E-17	hypomethylated	-0.018273	0.73018	insignificant	18	98	93
chr7	147222249	147224249	Tubgcp2	-0.19474016	1.09E-09	hypomethylated	0.0098628	0.78078	insignificant	14	80	78
chr7	147234987	147236987	Msx3		1	noCoverage	0.003928	0.31814	insignificant	0	18	16
chr7	147268447	147270447	Caly		1	noCoverage	-0.0013799	1	insignificant	0	8	8
chr7	147310583	147312583	Paox	-0.15031124	0.000000186	hypomethylated	-0.00016243	0.93331	insignificant	5	16	16
chr7	147322462	147324462	Mtg1	-0.17478072	5.71E-25	hypomethylated	-0.013077	0.4639	insignificant	16	49	56
chr7	147340558	147342558	Sprn	-0.11173633	0.010352	hypomethylated	0.013697	0.0064355	hypermethylated	6	22	19
chr7	147348847	147350847	Olfr522		1	noCoverage	0.10418	0.42115	insignificant	0	12	11
chr7	147361002	147363002	Olfr523		1	noCoverage	-0.17668	1	insignificant	0	10	12
chr7	147403165	147405165	Cd163l1	-0.49444444	0.0012445	stronglyHypometh	-0.090278	1	insignificant	2	9	8
chr7	147531886	147533886	Olfr60	-0.16333333	1	insignificant	-0.052222	0.35613	insignificant	1	5	5
chr7	147559507	147561507	Olfr530		1	noCoverage	0.11349	0.16239	insignificant	0	4	4
chr7	148020394	148022394	1190003J15Ri	-0.04427195	0.37164	insignificant	-0.073485	0.61269	insignificant	1	8	8
chr7	148030463	148032463	Scgb1c1	0.1367619	0.13756	insignificant	0.056461	0.29781	insignificant	6	18	18
chr7	148032814	148034814	Odf3		1	noCoverage	0.13895	0.87661	insignificant	0	20	18
chr7	148042282	148044282	Ric8	-0.18318202	6.84E-18	hypomethylated	0.0030157	0.96742	insignificant	19	75	72
chr7	148042295	148044295	Ric8	-0.18318202	6.84E-18	hypomethylated	0.0030157	0.96742	insignificant	19	75	72
chr7	148067292	148069292	Psmd13	-0.14218586	4.12E-13	hypomethylated	0.00079914	0.40357	insignificant	23	103	94
chr7	148067768	148069768	Psmd13	-0.15163977	3.19E-10	hypomethylated	-0.004248	0.3563	insignificant	20	76	69
chr7	148068208	148070208	Psmd13	-0.14265239	0.00013574	hypomethylated	0.0041639	0.73375	insignificant	12	38	31
chr7	148090962	148092962	BC024386		1	noCoverage	0.052912	0.6205	insignificant	0	13	11
chr7	148105800	148107800	Nlrp6	-0.47251987	0.00054173	stronglyHypometh	0.076389	0.92915	insignificant	2	25	23
chr7	148126479	148128479	Athl1	-0.12934943	5.87E-11	hypomethylated	-0.0076992	0.95753	insignificant	5	32	32
chr7	148141860	148143860	Ifitm2	0.04243697	1	insignificant	0.04107	0.48602	insignificant	3	17	16
chr7	148196643	148198643	Ifitm3		1	noCoverage	0.0057908	0.075446	insignificant	0	7	10
chr7	148202791	148204791	Ifitm6	-0.20688124	0.61121	insignificant	0.14601	0.000014236	hypermethylated	2	14	14
chr7	148246172	148248172	B4galnt4	-0.12053405	1.86E-52	hypomethylated	0.002814	0.0063479	hypermethylated	44	136	137
chr7	148263127	148265127	Pkp3	-0.16288732	0.068856	insignificant	-0.017361	0.82998	insignificant	7	19	18
chr7	148264667	148266667	Pkp3	0.00594394	0.71827	insignificant	0.029655	0.7996	insignificant	7	22	23
chr7	148303705	148305705	Ano9	-0.14175635	0.41987	insignificant	-0.017637	0.4717	insignificant	3	14	14
chr7	148316184	148318184	Ptdss2	-0.24130232	4.42E-46	hypomethylated	-0.018953	0.18983	insignificant	18	68	67
chr7	148358750	148360750	Rnh1	0.10457516	0.38293	insignificant	-0.0014522	0.088177	insignificant	5	18	18
chr7	148379028	148381028	Lrrc56	-0.13265415	9.34E-53	hypomethylated	-0.00012324	0.27153	insignificant	64	170	169
chr7	148379903	148381903	Lrrc56	-0.13582446	1.73E-12	hypomethylated	-0.0015106	0.47659	insignificant	28	96	95
chr7	148380041	148382041	Lrrc56	-0.14959536	5.3E-11	hypomethylated	-0.002086	0.27009	insignificant	24	70	69
chr7	148399979	148401979	1600016N20R	-0.01484406	8.76E-10	hypomethylated	0.0022163	0.45168	insignificant	9	56	65
chr7	148400758	148402758	Rassf7	-0.1039025	8.07E-12	hypomethylated	0.0017662	0.93966	insignificant	13	94	103
chr7	148407392	148409392	Mir210	-0.19206509	2.96E-13	hypomethylated	0.011748	0.74265	insignificant	11	60	60
chr7	148413686	148415686	Phrf1	-0.19342994	8.31E-38	hypomethylated	0.0040992	0.21402	insignificant	19	74	74
chr7	148462685	148464685	Sct	-0.50835866	0.19634	insignificant	0.020852	0.78681	insignificant	2	4	4
chr7	148465030	148467030	Sct	-0.19878003	0.000022805	hypomethylated	0.0011358	0.76086	insignificant	5	18	18
chr7	148476904	148478904	Drd4	-0.23765129	5.39E-13	hypomethylated	-0.0015402	0.85125	insignificant	5	23	10
chr7	148513028	148515028	Tmem80	-0.07632298	0.000092557	hypomethylated	-0.0059083	0.020255	inconclusive	21	115	119
chr7	148513624	148515624	Deaf1	-0.10804716	0.000044647	hypomethylated	0.00018886	0.000019793	hypermethylated	12	55	56
chr7	148523900	148525900	Eps8l2	-0.11753964	4.18E-37	hypomethylated	-0.039213	0.25369	insignificant	13	48	46
chr7	148524682	148526682	Deaf1	-0.09806412	5.79E-23	hypomethylated	-0.019692	0.35594	insignificant	17	46	44
chr7	148551009	148553009	B230206H07Rik		1	noCoverage	-0.02402	1	insignificant	0	4	4
chr7	148577058	148579058	Taldo1	-0.25666039	1.73E-27	hypomethylated	-0.0035443	0.47181	insignificant	10	40	40
chr7	148591433	148593433		0.11101651	1	insignificant	0.041336	0.65885	insignificant	1	19	18
chr7	148600024	148602024	Pddc1	-0.31398088	1.37E-11	hypomethylated	0.00046359	0.384	insignificant	6	38	36
chr7	148615319	148617319	Slc25a22	-0.19143092	0.15552	insignificant	-0.00026858	0.73315	insignificant	3	14	14
chr7	148623773	148625773	Lrdd	0.01204533	0.10549	insignificant	0.0063218	0.86738	insignificant	5	19	19
chr7	148629254	148631254	Lrdd	-0.55720032	0.000096317	stronglyHypometh	-0.070373	0.6001	insignificant	4	17	14
chr7	148632548	148634548	Rplp2	-0.09175746	2.09E-20	hypomethylated	-0.0030034	0.37954	insignificant	20	88	86
chr7	148633731	148635731	Snora52	-0.15845096	0.012771	hypomethylated	-0.0010764	1	insignificant	3	20	20
chr7	148640086	148642086	Pnpla2	-0.26475685	0.0010364	hypomethylated	0.04397	0.001253	hypermethylated	8	37	37
chr7	148645992	148647992	Efcab4a	-0.17795303	0.000000503	hypomethylated	-0.0079242	0.041937	inconclusive	19	93	90
chr7	148652260	148654260	Cd151	-0.20936321	2.53E-14	hypomethylated	-0.023572	0.96801	insignificant	15	62	66
chr7	148653687	148655687	Cd151	-0.37907782	0.22525	insignificant	-0.065075	0.51187	insignificant	1	12	15
chr7	148660138	148662138	Polr2l	-0.13721864	2.82E-30	hypomethylated	0.014399	0.7933	insignificant	41	159	135
chr7	148661052	148663052	Polr2l	-0.11374555	1.33E-22	hypomethylated	0.0066549	0.80964	insignificant	32	130	106
chr7	148725756	148727756	Chid1	-0.05098372	0.49494	insignificant	0.0063957	0.94296	insignificant	5	23	23
chr7	148747078	148749078	Ap2a2	-0.10010214	1.37E-12	hypomethylated	0.018846	0.04045	hypermethylated	39	114	108
chr7	148841207	148843207	Muc6	-0.34519231	0.079063	insignificant	0.074126	0.2293	insignificant	1	5	5
chr7	149023976	149025976	Muc5b		1	noCoverage	0.072578	0.42884	insignificant	0	6	6
chr7	149088311	149090311	Tollip	-0.08723344	1.05E-08	hypomethylated	0.01453	1	insignificant	9	34	34
chr7	149134655	149136655	Brsk2	-0.13571812	2.55E-68	hypomethylated	0.010704	0.98119	insignificant	61	191	179
chr7	149134990	149136990	Brsk2	-0.13007047	1.29E-64	hypomethylated	0.01113	0.94246	insignificant	61	189	177
chr7	149246939	149248939	Mob2	-0.12282925	0.00054049	hypomethylated	0.033771	0.37595	insignificant	7	40	42
chr7	149488406	149490406	Krtap5-4	0.01487292	1	insignificant	-0.13034	0.059707	insignificant	3	6	6
chr7	149558164	149560164	6330512M04F	-0.0645372	0.047515	inconclusive	0.018469	0.0052654	hypermethylated	15	46	46
chr7	149573775	149575775	Ctsd	-0.07822727	1.74E-08	hypomethylated	0.0040658	0.34942	insignificant	12	43	43
chr7	149619881	149621881	Syt8	-0.32937182	0.10223	insignificant	0.055497	0.89144	insignificant	2	4	4
chr7	149627372	149629372	Tnni2	-0.15377799	0.020363	hypomethylated	-0.020147	0.1942	insignificant	2	16	16
chr7	149645774	149647774	Lsp1	-0.42817211	0.00033392	stronglyHypometh	0.08225	0.0024129	hypermethylated	1	8	8
chr7	149656747	149658747	Lsp1	-0.71114175	0.000026296	stronglyHypometh	-0.17924	0.11611	insignificant	2	8	8
chr7	149683740	149685740	Tnnt3	-0.22836257	0.090245	insignificant	0.19497	0.097951	insignificant	2	4	5
chr7	149718021	149720021	Mrpl23	-0.10113667	0.000000681	hypomethylated	0.0065855	0.96623	insignificant	13	66	74
chr7	149744503	149746503	Nctc1	-0.23173844	0.26042	insignificant	0.055467	0.31408	insignificant	1	6	6
chr7	149763052	149765052	Mir675	-0.18538703	0.3837	insignificant	0.078455	4.79E-09	hypermethylated	9	37	32
chr7	149764051	149766051	Mir675	-0.36764291	0.01963	stronglyHypometh	0.096022	0.000012226	hypermethylated	4	25	22
chr7	149840901	149842901	Mir483		1	noCoverage	0.066297	0.051871	insignificant	0	15	18
chr7	149843386	149845386	Igf2	-0.17734318	1.94E-27	hypomethylated	0.009521	0.21275	insignificant	36	99	92
chr7	149844597	149846597	Igf2as	-0.15300308	7.93E-44	hypomethylated	-0.0060059	0.21289	insignificant	60	178	170
chr7	149844709	149846709	Igf2	-0.15300308	7.93E-44	hypomethylated	-0.0060059	0.21289	insignificant	60	178	170

chr7	149846940	149848940	Igf2as		1	noCoverage	-0.18632	0.3411	insignificant	0	5	11
chr7	150085871	150087871	Th		1	noCoverage	-0.020944	0.71048	insignificant	0	4	2
chr7	150155169	150157169	Ascl2	-0.13550373	0.00000283	hypomethylated	0.034615	0.72911	insignificant	9	40	43
chr7	150189950	150191950	Tspan32	-0.49170274	0.0014422	stronglyHypometh	0.11238	0.70289	insignificant	2	7	7
chr7	150190563	150192563	Tspan32	-0.49170274	0.0014422	stronglyHypometh	0.11238	0.70289	insignificant	2	7	7
chr7	150237654	150239654	Cd81	-0.17413244	9.03E-19	hypomethylated	0.013012	0.42328	insignificant	18	94	94
chr7	150239273	150241273	Cd81	-0.24178963	0.03741	hypomethylated	-0.035179	0.64976	insignificant	9	50	50
chr7	150254272	150256272	Tssc4	-0.15273371	0.00000138	hypomethylated	0.01113	0.25476	insignificant	13	95	95
chr7	150280547	150282547	Trpm5		1	noCoverage	0.0055556	0.62822	insignificant	0	5	4
chr7	150292158	150294158	Kcnq1	-0.08153348	0.22076	insignificant	-0.013448	0.71113	insignificant	10	90	90
chr7	150646955	150648955	Cdkn1c	0.0678135	0.27862	insignificant	0.059357	0.69997	insignificant	16	61	61
chr7	150660020	150662020	Slc22a18	-0.07186309	0.27798	insignificant	-0.081566	0.22263	insignificant	7	22	22
chr7	150688429	150690429	Phlda2	-0.02983688	0.019966	hypomethylated	0.0082828	0.52604	insignificant	11	56	53
chr7	150734995	150736995	Nap1l4	-0.00294843	0.00000233	hypomethylated	0.0078745	0.07075	insignificant	7	51	51
chr7	150785947	150787947	Cars	-0.154445	0.0018961	hypomethylated	0.012909	0.64869	insignificant	7	20	20
chr7	150926265	150928265	Osbpl5	-0.11776892	0.059743	insignificant	0.019861	0.041195	hypermethylated	2	20	20
chr7	150927868	150929868	Osbpl5		1	noCoverage	0.15476	1	insignificant	0	4	2
chr7	150952898	150954898	Mrgprg	-0.27049731	0.32543	insignificant	0.0061138	0.79185	insignificant	1	4	4
chr7	151008071	151010071	Dhcr7	-0.31143981	8.1E-13	hypomethylated	-0.0064523	0.010758	inconclusive	7	30	30
chr7	151008746	151010746	Nadsyn1	-0.38177083	1.45E-08	stronglyHypometh	-0.057265	0.15106	insignificant	3	16	16
chr7	151360424	151362424	Shank2	-0.20335423	0.0017197	hypomethylated	0.042163	0.026101	hypermethylated	10	60	57
chr7	151469289	151471289	Shank2	-0.20286728	2.12E-16	hypomethylated	-0.0017197	0.30021	insignificant	13	56	50
chr7	151656646	151658646	Cttn	-0.13244479	0.032867	hypomethylated	0.15905	0.57493	insignificant	1	7	2
chr7	151739634	151741634	Ppfia1	-0.18924989	0.000000835	hypomethylated	-0.042121	0.48271	insignificant	19	67	47
chr7	151768341	151770341	Fadd	-0.09106606	0.043852	hypomethylated	0.01881	1	insignificant	5	20	20
chr7	151924383	151926383	Ano1	-0.19091263	1.02E-11	hypomethylated	0.023167	0.94101	insignificant	10	20	23
chr7	152023516	152025516	Fgf3	-0.10240331	0.000000049	hypomethylated	0.013034	0.56217	insignificant	34	159	161
chr7	152046290	152048290	Fgf4	-0.08339983	1.63E-20	hypomethylated	0.017345	0.3384	insignificant	37	164	159
chr7	152081436	152083436	Fgf15	-0.18806523	3.8E-12	hypomethylated	-0.018674	0.43034	insignificant	24	87	86
chr7	152100098	152102098	Oraov1	-0.1425673	0.00000334	hypomethylated	-0.0078505	0.43034	insignificant	13	46	50
chr7	152125830	152127830	Ccnd1	-0.13997769	1.01E-09	hypomethylated	0.0047751	0.35054	insignificant	15	73	73
chr7	152469832	152471832	Tpcn2	-0.15972222	0.00035529	hypomethylated	0.53075	0.0028935	stronglyhypermeth	1	6	2
chr8	3279617	3281617	Insr	-0.22881834	0.58695	insignificant	-0.054567	0.39897	insignificant	4	21	14
chr8	3352414	3354414	A430078G23R	-0.28223214	1	insignificant	0.024905	0.87715	insignificant	2	17	15
chr8	3392007	3394007	Arhgef18	-0.14746882	9.84E-19	hypomethylated	-0.063175	0.00000565	hypomethylated	15	38	37
chr8	3492137	3494137	Zfp358	-0.1661698	8.63E-24	hypomethylated	-0.0045159	1	insignificant	25	59	61
chr8	3499518	3501518	Mcoln1	-0.06358494	0.39044	insignificant	-0.00044105	0.86268	insignificant	6	59	66
chr8	3514383	3516383	Pnpla6	-0.21497778	4.47E-23	hypomethylated	0.011556	1	insignificant	14	56	56
chr8	3586449	3588449	Camsap3	-0.09207133	0.000066308	hypomethylated	-0.0064138	0.19361	insignificant	29	90	90
chr8	3620550	3622550	2900053A13R	-0.17661161	1.62E-16	hypomethylated	-0.013075	0.77156	insignificant	15	78	76
chr8	3621296	3623296	Xab2	-0.14358791	0.0002822	hypomethylated	-0.027283	0.62742	insignificant	10	46	44
chr8	3630159	3632159	Stxbp2	-0.11495013	0.000022587	hypomethylated	0.011603	0.29028	insignificant	8	64	64
chr8	3664761	3666761	1810033B17R	0.22916667	0.20629	insignificant	0.05	0.5587	insignificant	1	6	6
chr8	3675476	3677476	Trappc5	-0.24183141	0.0032174	hypomethylated	0.022125	0.20284	insignificant	8	39	34
chr8	3803689	3805689	Rprl3		1	noCoverage	0.16234	0.4557	insignificant	0	19	12
chr8	4165566	4167566	Evi5l	-0.1205405	0.00031264	hypomethylated	-0.0033975	1	insignificant	12	54	54
chr8	4225826	4227826	Lrrc8e	-0.24053653	9.08E-23	hypomethylated	0.00097065	0.26487	insignificant	18	47	52
chr8	4237739	4239739	Map2k7	-0.20924075	0.37317	insignificant	-0.007198	0.96271	insignificant	5	50	55
chr8	4252101	4254101	Snapc2	-0.21760295	1	insignificant	-0.018813	0.39289	insignificant	3	45	54
chr8	4259274	4261274	Timm44	-0.34223807	0.000001185	stronglyHypometh	0.029158	0.61401	insignificant	5	30	30
chr8	4275905	4277905	Timm44		1	noCoverage	-0.037666	1	insignificant	0	11	11
chr8	4324209	4326209	Ccl25	-0.10959186	1.38E-11	hypomethylated	-0.021497	0.5493	insignificant	29	140	154
chr8	4325100	4327100	Elavl1	-0.19056922	1	insignificant	-0.074141	0.95195	insignificant	2	42	48
chr8	4348587	4350587	Ccl25	-0.26206402	0.00056708	hypomethylated	0.032299	0.79124	insignificant	2	4	4
chr8	4492404	4494404	Lass4	-0.04486536	0.7999	insignificant	0.0060025	0.38885	insignificant	3	45	40
chr8	4612169	4614169	Zfp958		1	noCoverage	-0.14145	0.47925	insignificant	0	23	19
chr8	4779534	4781534	Shcbp1	-0.20909052	0.00099422	hypomethylated	-0.061878	1	insignificant	5	17	16
chr8	8660773	8662773	Efnb2	-0.1269646	1.04E-50	hypomethylated	-0.0069402	0.88361	insignificant	41	149	161
chr8	9771023	9773023	Fam155a	-0.04963476	0.00000147	hypomethylated	0.014614	0.24713	insignificant	19	81	93
chr8	9976323	9978323	Lig4	-0.08401258	5.67E-14	hypomethylated	0.0097221	0.60213	insignificant	26	105	88
chr8	9976716	9978716	Abhd13	-0.00830604	0.00000155	hypomethylated	0.0024316	0.3844	insignificant	17	85	68
chr8	10005632	10007632	Tnfsf13b	-0.35969134	0.087669	insignificant	0.062503	0.37761	insignificant	2	27	24
chr8	10152922	10154922	Myo16	-0.03001016	0.29546	insignificant	-0.11439	0.089937	insignificant	7	18	18
chr8	10928457	10930457	3930402G23R	0.00907362	0.057937	insignificant	0.022587	0.8613	insignificant	5	14	14
chr8	11008430	11010430	Irs2	-0.08514217	1.7E-14	hypomethylated	-0.0044898	0.80929	insignificant	46	239	228
chr8	11311828	11313828	Col4a2	-0.10924576	1.93E-14	hypomethylated	0.010876	0.33992	insignificant	26	110	108
chr8	11312826	11314826	Col4a1	-0.10342632	1.38E-14	hypomethylated	0.019557	0.19208	insignificant	13	56	56
chr8	11478499	11480499	Rab20	-0.12858622	0.11432	insignificant	0.07215	0.15527	insignificant	7	42	36
chr8	11496505	11498505	Carkd	-0.07218805	0.000010166	hypomethylated	-0.037144	0.092808	insignificant	16	84	100
chr8	11496567	11498567	Carkd	-0.08752086	0.000000539	hypomethylated	-0.041279	0.017671	hypomethylated	16	88	104
chr8	11550771	11552771	Cars2	-0.4167911	1.66E-12	stronglyHypometh	0.048941	1	insignificant	8	36	33
chr8	11555065	11557065	Ing1	-0.10002898	2.54E-33	hypomethylated	0.0089942	0.028667	hypermethylated	67	209	209
chr8	11635036	11637036	Ankrd10	-0.1360559	4E-10	hypomethylated	-0.028452	0.97668	insignificant	20	111	114
chr8	11635754	11637754	Ankrd10	-0.29273208	0.0057694	hypomethylated	-0.06232	0.70661	insignificant	8	56	62
chr8	11694514	11696514	Gm6524	0.28963845	1	insignificant	0.019719	1	insignificant	1	12	12
chr8	11727104	11729104	Arhgef7	-0.08670267	7.61E-34	hypomethylated	-0.014942	0.18059	insignificant	63	244	234
chr8	11757329	11759329	Arhgef7	-0.12166812	5.16E-16	hypomethylated	0.0010314	0.73977	insignificant	35	115	111
chr8	12384770	12386770	Gm5607	-0.1130567	2.49E-18	hypomethylated	-0.009254	0.59969	insignificant	31	156	164
chr8	12394518	12396518	Sox1	-0.14412885	3.51E-09	hypomethylated	0.021666	0.81314	insignificant	10	47	37
chr8	12672100	12674100	Tubgcp3	-0.1207185	0.0010693	hypomethylated	0.022213	0.18075	insignificant	9	48	42
chr8	12756015	12758015	Atp11a	-0.10327767	4.33E-43	hypomethylated	0.0044567	0.00027937	hypermethylated	75	201	194
chr8	12914892	12916892	Mcf2l	-0.2336478	0.078102	insignificant	0.080255	0.075616	insignificant	5	10	10
chr8	12948417	12950417	Mcf2l	-0.33016138	0.32294	insignificant	-0.023509	0.78299	insignificant	1	12	12
chr8	13025033	13027033	F7	-0.38901764	3.09E-11	stronglyHypometh	0.041028	0.61917	insignificant	4	10	12
chr8	13036307	13038307	F10	-0.31486742	0.030923	hypomethylated	-0.18402	0.060617	insignificant	4	8	9

chr8	13059907	13061907	Proz	-0.22850864	0.56976	insignificant	-0.006959	0.05669	insignificant	7	26	24
chr8	13104720	13106720	Cul4a	-0.10123107	1.6E-32	hypomethylated	0.018977	0.12379	insignificant	55	207	214
chr8	13105343	13107343	Pcid2	-0.15695617	2.33E-19	hypomethylated	0.0031195	0.87933	insignificant	35	133	136
chr8	13158134	13160134	Lamp1	-0.20624199	0.00076328	hypomethylated	-0.04638	0.22513	insignificant	8	69	55
chr8	13189098	13191098	Grtp1		1	noCoverage	-0.12822	0.58106	insignificant	0	10	10
chr8	13287012	13289012	Tmco3	-0.09199408	4.66E-18	hypomethylated	0.001941	0.68756	insignificant	31	120	118
chr8	13288126	13290126	Tmco3	-0.0908509	0.2372	insignificant	-0.0009538	0.41417	insignificant	11	40	40
chr8	13338673	13340673	Tfdp1	-0.12658339	6.56E-18	hypomethylated	-0.0075916	0.71608	insignificant	34	125	115
chr8	13396778	13398778	Atp4b	0.09740991	0.67428	insignificant	0.037712	0.51175	insignificant	1	6	6
chr8	13404080	13406080	Grk1	0.06631373	0.090191	insignificant	0.013132	0.59835	insignificant	5	10	10
chr8	13434458	13436458	Fam70b		1	noCoverage	-0.021556	0.62959	insignificant	0	10	10
chr8	13562448	13564448	1700029H14R	0.06716721	0.1981	insignificant	-0.023471	0.82272	insignificant	5	16	17
chr8	13677587	13679587	Rasa3	-0.36756683	0.00012492	stronglyHypometh	-0.077746	0.7734	insignificant	0	0	0
chr8	13704888	13706888	4932443I19Ril	0.03508758	1	insignificant	0.020856	0.72774	insignificant	5	24	23
chr8	13756689	13758689	Cdc16	-0.11390631	2.1E-11	hypomethylated	-0.0005296	0.80969	insignificant	24	81	81
chr8	13784614	13786614	Upf3a	-0.11699058	2.95E-30	hypomethylated	-0.00050403	1	insignificant	42	145	151
chr8	13868640	13870640	Zfp828	-0.12634722	8.11E-10	hypomethylated	0.0020531	0.88551	insignificant	26	89	84
chr8	13890271	13892271	2410022L05Ri	-0.08654172	0.000000818	hypomethylated	0.011131	0.2158	insignificant	13	50	50
chr8	13906805	13908805	Fbxo25	-0.09886161	2.42E-14	hypomethylated	-0.015476	0.38586	insignificant	19	89	85
chr8	13974777	13976777	2610019F03Rik		1	noCoverage	0.032667	0.043221	hypermethylated	0	20	24
chr8	14090327	14092327	Erich1	-0.12487677	2.73E-11	hypomethylated	0.050582	0.5412	insignificant	8	28	30
chr8	14094874	14096874	Dlgap2	-0.13237821	2.91E-15	hypomethylated	-0.0049382	0.31948	insignificant	42	187	180
chr8	14887535	14889535	Cln8	-0.19187675	0.00054589	hypomethylated	0.0055604	0.50599	insignificant	5	12	13
chr8	14910716	14912716	Arhgef10	-0.09601244	0.62297	insignificant	0.021051	1	insignificant	7	34	42
chr8	15010024	15012024	Kbtbd11	-0.07712993	1.03E-08	hypomethylated	-0.0043529	0.6041	insignificant	39	125	125
chr8	15046069	15048069	BB014433		1	noCoverage	0.025765	0.44007	insignificant	0	4	4
chr8	17535385	17537385	Csmd1	-0.17944869	1	insignificant	0.01063	0.60815	insignificant	3	15	15
chr8	18594172	18596172	Mcph1	-0.09136839	6.46E-11	hypomethylated	-0.00076966	0.76941	insignificant	18	118	119
chr8	18845278	18847278	Agpat5	-0.11319977	6.48E-36	hypomethylated	0.0024687	0.49458	insignificant	50	186	177
chr8	18978116	18980116	Defb40		1	noCoverage	-0.083416	0.43482	insignificant	0	10	10
chr8	19193327	19195327	Defb14	-0.08224638	0.012788	hypomethylated	0.070833	0.3341	insignificant	2	4	4
chr8	19893010	19895010	6820431F20Rik		1	noCoverage	0.15762	0.088927	insignificant	0	24	24
chr8	20020278	20022278	6820431F20Rik		1	noCoverage	0.012065	0.00000129	inconclusive	0	25	18
chr8	20020392	20022392	6820431F20Rik		1	noCoverage	0.14791	0.047282	inconclusive	0	13	9
chr8	22041664	22043664	Gm15056	-0.35	0.11342	insignificant	0.034615	0.48498	insignificant	1	16	13
chr8	22084427	22086427	AY761185		1	noCoverage	0.090551	0.38183	insignificant	0	17	28
chr8	22340282	22342282	Gm15293		1	noCoverage	0.024457	0.73522	insignificant	0	3	3
chr8	22436072	22438072	Defa5		1	noCoverage	0.046053	0.66407	insignificant	0	3	3
chr8	22674857	22676857	Gm14850		1	noCoverage	-0.10714	0.43438	insignificant	0	2	2
chr8	22775268	22777268	Gm15315		1	noCoverage	0.14069	0.0054675	hypermethylated	0	3	3
chr8	23056233	23058233	Defb13	-0.06212121	0.50323	insignificant	0.046369	0.885	insignificant	1	8	8
chr8	23170191	23172191	Alg11	-0.1487191	0.23944	insignificant	0.010648	1	insignificant	3	69	59
chr8	23170546	23172546	Alg11		1	noCoverage	0.0029133	0.33197	insignificant	0	67	57
chr8	23235525	23237525	Nek5	-0.22632614	1	lowCoverage	0.0035548	0.14072	insignificant	1	10	10
chr8	23276907	23278907	Nek3		1	noCoverage	0.020682	0.32695	insignificant	0	7	4
chr8	23296291	23298291	Ckap2	-0.13157895	1	insignificant	-0.067251	0.26886	insignificant	3	19	18
chr8	23302331	23304331	Vps36		1	noCoverage	0.022944	0.43675	insignificant	0	39	39
chr8	23392912	23394912	Tpte	-0.04115868	0.85437	insignificant	-0.015981	1	insignificant	4	14	11
chr8	23509093	23511093	Slc25a15	-0.11111111	0.60764	insignificant	0.012515	0.61598	insignificant	7	18	18
chr8	23520811	23522811	Mrps31	-0.24853776	1.14E-21	hypomethylated	-0.0055261	0.78897	insignificant	12	50	44
chr8	23586171	23588171	Slc20a2	-0.06243247	7.91E-12	hypomethylated	-0.0015523	0.46825	insignificant	41	152	146
chr8	23587351	23589351	Slc20a2	-0.08185508	3.63E-08	hypomethylated	0.0089128	0.60378	insignificant	14	55	48
chr8	23704285	23706285	Vdac3	-0.04446233	0.39157	insignificant	0.01325	0.24072	insignificant	11	40	40
chr8	23733514	23735514	Dkk4		1	noCoverage	-0.13034	1	insignificant	0	9	10
chr8	23747281	23749281	A930013F10Rik		1	noCoverage	-0.16252	0.77019	insignificant	0	6	7
chr8	23867215	23869215	Plat	-0.28437282	0.00017778	hypomethylated	0.0028602	0.021849	inconclusive	4	28	30
chr8	23916126	23918126	Ap3m2	-0.17453894	1.08E-18	hypomethylated	0.0022692	0.5146	insignificant	21	68	68
chr8	23969010	23971010	Myst3	-0.10905896	2.19E-34	hypomethylated	-0.00437	0.69659	insignificant	58	212	211
chr8	24084353	24086353	Ank1	-0.11680482	2.74E-57	hypomethylated	0.0073064	0.63497	insignificant	54	197	168
chr8	24167746	24169746	Ank1	-0.15561488	2.14E-33	hypomethylated	0.0066194	1	insignificant	40	109	109
chr8	24252026	24254026	Mir486	0.11802552	1	insignificant	0.054069	0.082111	insignificant	3	12	12
chr8	24253134	24255134	Ank1	0.11802552	1	insignificant	0.054069	0.082111	insignificant	3	12	12
chr8	24262742	24264742	Nkx6-3	-0.09119929	0.29169	insignificant	-0.046138	0.77078	insignificant	5	31	26
chr8	24318925	24320925	Agpat6	-0.1422977	1.43E-13	hypomethylated	-0.0065878	0.011055	hypomethylated	19	47	38
chr8	24348140	24350140	Gins4	-0.06809678	0.064669	insignificant	-0.036666	0.10257	insignificant	5	21	20
chr8	24367552	24369552	Golga7	-0.14372797	0.05914	insignificant	0.037594	0.88414	insignificant	5	32	36
chr8	24520973	24522973	Sfrp1	-0.07367466	1.65E-10	hypomethylated	-0.0035358	0.87897	insignificant	25	121	133
chr8	24779132	24781132	Zmat4	-0.20996702	0.0034685	hypomethylated	-0.017784	0.64467	insignificant	5	36	36
chr8	25707481	25709481	Ido1		1	noCoverage	-0.049632	1	insignificant	0	4	2
chr8	26059276	26061276	Adam9		1	noCoverage	-0.091766	0.76414	insignificant	0	6	8
chr8	26126682	26128682	Tm2d2	-0.11249746	3.41E-18	hypomethylated	0.020423	0.87533	insignificant	28	137	130
chr8	26127394	26129394	Adam9	-0.1475777	3.45E-14	hypomethylated	0.018113	0.7786	insignificant	22	95	97
chr8	26212283	26214283	Plekha2	-0.15685502	5.33E-30	hypomethylated	0.015319	0.30364	insignificant	19	94	107
chr8	26311921	26313921	Tacc1	0.18773931	1.61E-09	inconclusive	0.010048	0.40726	insignificant	4	57	57
chr8	26628243	26630243	Fgfr1	-0.09474092	2.17E-21	hypomethylated	-0.0085904	0.87719	insignificant	48	170	167
chr8	26707959	26709959	Letm2		1	noCoverage	0.254	0.47132	insignificant	0	12	10
chr8	26711777	26713777	Whsc1l1	-0.13400695	6.55E-59	hypomethylated	0.0029324	0.33212	insignificant	53	151	147
chr8	26829519	26831519	Ppapdc1b	-0.37348008	0.007855	stronglyHypometh	-0.073149	0.10295	insignificant	5	24	20
chr8	26864752	26866752	Ddhd2	-0.10007985	6.81E-09	hypomethylated	-0.010331	0.2335	insignificant	29	70	70
chr8	26895062	26897062	Lsm1	-0.10066362	6.92E-32	hypomethylated	-0.0020302	0.26181	insignificant	29	114	116
chr8	26895681	26897681	Bag4	-0.10120512	3.12E-29	hypomethylated	-0.0094549	0.76307	insignificant	21	86	88
chr8	26917984	26919984	Star	-0.24115695	0.0045001	hypomethylated	0.034571	0.65616	insignificant	2	3	4
chr8	26951251	26953251	Ash2l	-0.15643897	0.6036	insignificant	0.0013017	0.48649	insignificant	4	21	14
chr8	26959094	26961094	Kcnu1	0.15104167	1	insignificant	-0.00047348	1	insignificant	4	8	8
chr8	27126073	27128073	Fnta	0.56407065	0.24763	insignificant	0.15316	0.14328	insignificant	2	28	22

chr8	27228851	27230851	Rnf170	-0.11131802	4.15E-27	hypomethylated	0.0057345	0.68317	insignificant	41	192	155
chr8	27229696	27231696	Rnf170	-0.12105758	3.47E-18	hypomethylated	0.012019	0.048411	inconclusive	27	134	111
chr8	27267640	27269640	Thap1	-0.07437114	8.79E-16	hypomethylated	0.0048426	0.11086	insignificant	47	161	158
chr8	28086807	28088807	Zfp703	-0.18196653	1.72E-24	hypomethylated	-0.0032279	0.85018	insignificant	30	84	91
chr8	28133330	28135330	Erlin2	-0.11904993	0.00023771	hypomethylated	-0.0085705	0.77586	insignificant	21	88	88
chr8	28152026	28154026	Prosc	-0.13851257	0.0028934	hypomethylated	0.003962	0.62695	insignificant	14	78	75
chr8	28195312	28197312	Gpr124	-0.15076523	2.14E-43	hypomethylated	0.0054866	0.11731	insignificant	47	131	122
chr8	28239104	28241104	Brf2	0.06241106	0.71518	insignificant	0.05884	0.17731	insignificant	3	12	6
chr8	28285118	28287118	Rab11fip1	-0.29274295	0.010504	hypomethylated	-0.0042814	0.5333	insignificant	1	2	2
chr8	28369798	28371798	Eif4ebp1	-0.15505849	9.38E-36	hypomethylated	-0.009284	0.19408	insignificant	20	108	114
chr8	28405516	28407516	Gm9731	-0.00313099	0.30759	insignificant	0.057684	0.17573	insignificant	8	30	39
chr8	28453865	28455865	Tex24		1	noCoverage	0.17995	0.14041	insignificant	0	2	2
chr8	28478182	28480182	Chrnb3	0.11833751	1	insignificant	-0.061938	0.60178	insignificant	2	9	8
chr8	28557141	28559141	Poteg		1	noCoverage	0.14619	0.098968	insignificant	0	6	6
chr8	29195325	29197325	Gm8096	-0.15653941	0.04681	hypomethylated	-0.082962	0.02388	hypomethylated	1	6	6
chr8	30330108	30332108	Unc5d	-0.13032191	9.65E-08	hypomethylated	0.12939	0.29975	insignificant	19	50	58
chr8	32199133	32201133	Dusp26	-0.05948443	0.094318	insignificant	0.018368	0.39225	insignificant	12	41	41
chr8	32221317	32223317	Rnf122	-0.13617129	6.08E-21	hypomethylated	0.016585	0.64822	insignificant	28	106	100
chr8	32259787	32261787	BC019943	-0.16598763	9.83E-11	hypomethylated	0.01141	0.49848	insignificant	13	72	68
chr8	32279196	32281196	Mak16	-0.17063534	3.12E-12	hypomethylated	0.066664	0.86082	insignificant	12	35	38
chr8	32296803	32298803	Fut10	-0.18182227	0.00000444	hypomethylated	0.0072086	0.51225	insignificant	14	48	48
chr8	32296849	32298849	Fut10	-0.18182227	0.00000444	hypomethylated	0.0072086	0.51225	insignificant	14	48	48
chr8	33028675	33030675	Nrg1	0.39495592	0.54562	insignificant	0.025084	0.66588	insignificant	1	11	10
chr8	34495796	34497796	Purg	-0.04293451	0.57836	insignificant	-0.0111	0.50912	insignificant	33	109	109
chr8	34495999	34497999	Purg	-0.04293451	0.57836	insignificant	-0.0111	0.50912	insignificant	33	109	109
chr8	34593782	34595782	Hmgb1-rs17	-0.03546793	0.78307	insignificant	0.008714	0.17737	insignificant	3	18	17
chr8	34709092	34711092	Ppp2cb	-0.08684684	2.94E-14	hypomethylated	-0.0082477	0.39138	insignificant	34	149	142
chr8	34752448	34754448	Ubxn8	-0.15706354	3.8E-09	hypomethylated	0.0092048	0.39573	insignificant	5	28	28
chr8	34762709	34764709	Gsr	-0.17030947	4.07E-24	hypomethylated	-0.046783	0.19851	insignificant	25	86	86
chr8	34841385	34843385	Gtf2e2	-0.12108104	8.37E-16	hypomethylated	-0.0089847	0.067924	insignificant	19	87	93
chr8	34841538	34843538	Gtf2e2	-0.15789047	3.57E-16	hypomethylated	-0.0041942	0.28504	insignificant	18	85	91
chr8	34841810	34843810	Gtf2e2	-0.15789047	3.57E-16	hypomethylated	-0.0041942	0.28504	insignificant	18	85	91
chr8	35040313	35042313	Rbpms	-0.14860781	0.0070154	hypomethylated	0.0016446	0.43373	insignificant	17	78	78
chr8	35171565	35173565	Dctn6		1	noCoverage	0.11954	0.019325	hypermethylated	0	6	6
chr8	35177083	35179083	Mboat4		1	noCoverage	-0.086226	0.55022	insignificant	0	8	8
chr8	35209793	35211793	Leprotl1	-0.14053885	1.03E-20	hypomethylated	-0.016428	0.53864	insignificant	14	34	27
chr8	35216616	35218616	Tmem66	-0.24617509	6.92E-21	hypomethylated	0.016182	0.47619	insignificant	9	20	23
chr8	35869663	35871663	Dusp4	-0.12286699	8.57E-37	hypomethylated	-0.0017403	0.72351	insignificant	54	177	170
chr8	36028744	36030744	Tnks	0.08418367	1	insignificant	0.021796	0.49506	insignificant	6	14	12
chr8	36437794	36439794	Ppp1r3b	-0.14194619	5.34E-22	hypomethylated	0.0084736	0.83479	insignificant	28	96	96
chr8	36558587	36560587	Eri1	-0.04824805	0.41102	insignificant	0.058532	0.0023767	hypermethylated	14	42	44
chr8	36649851	36651851	Mfhas1	-0.11316551	2.39E-34	hypomethylated	-0.0083466	0.88005	insignificant	64	203	198
chr8	36889613	36891613	Cldn23	-0.161057	2.33E-13	hypomethylated	-0.012616	0.86633	insignificant	12	46	46
chr8	37156881	37158881	D8Ertd82e	-0.14935011	9.67E-25	hypomethylated	-0.0010382	0.8883	insignificant	33	125	126
chr8	37312570	37314570	Lonrf1		1	noCoverage	-0.064992	1	insignificant	0	20	19
chr8	37796108	37798108	Dlc1	0.27497666	1	lowCoverage	0.14279	0.15525	insignificant	1	9	10
chr8	38015490	38017490	A730069N07R	-0.39585194	1	insignificant	-0.078857	0.17107	insignificant	2	8	10
chr8	38055628	38057628	AI429214	-0.22034334	0.10363	insignificant	0.019115	0.32579	insignificant	9	21	20
chr8	40067920	40069920	Tusc3	-0.01800076	0.031623	hypomethylated	-0.0090454	0.41557	insignificant	13	80	80
chr8	41372307	41374307	Fgf20	-0.20391209	0.044009	hypomethylated	0.016907	0.64977	insignificant	5	10	10
chr8	41392404	41394404	Efha2	-0.05716391	1.2E-09	hypomethylated	0.012535	0.39369	insignificant	16	114	113
chr8	41508168	41510168	Zdhhc2	-0.09421384	2.75E-22	hypomethylated	0.00065877	0.55224	insignificant	53	178	177
chr8	41596136	41598136	Vps37a	-0.10811742	2.78E-30	hypomethylated	0.018745	0.20181	insignificant	47	178	164
chr8	41596658	41598658	Vps37a	-0.11523317	4.96E-29	hypomethylated	0.019592	0.34716	insignificant	44	146	143
chr8	41720146	41722146	Mtmr7	0.04373997	0.15825	insignificant	0.079358	0.0064243	hypermethylated	10	28	26
chr8	41946720	41948720	Slc7a2	-0.10106312	0.00042032	hypomethylated	0.030504	0.11793	insignificant	8	18	26
chr8	41946746	41948746	Slc7a2	-0.10106312	0.00042032	hypomethylated	0.030504	0.11793	insignificant	8	18	26
chr8	42010586	42012586	Pdgfrl	-0.17027762	0.000027327	hypomethylated	0.031474	0.3483	insignificant	4	20	20
chr8	42101821	42103821	B430010I23Ril	-0.35212267	5.27E-10	stronglyHypometh	-0.14201	0.093541	insignificant	4	21	18
chr8	42140148	42142148	Mtus1	-0.79166667	0.22034	lowCoverage	-0.024524	1	insignificant	1	4	4
chr8	42219080	42221080	Mtus1	-0.17575758	0.0026339	hypomethylated	-0.070758	0.41946	insignificant	2	22	20
chr8	42300510	42302510	Fgl1	-0.10277778	0.22569	insignificant	-0.041484	0.2314	insignificant	3	8	7
chr8	42324112	42326112	Pcm1	-0.1028249	0.00024444	hypomethylated	0.0086843	0.31077	insignificant	15	92	96
chr8	42460051	42462051	Asah1	-0.18426178	0.000014936	hypomethylated	-0.080761	0.80707	insignificant	13	43	38
chr8	42502472	42504472	Frg1	-0.12504456	0.0067837	hypomethylated	-0.0014178	0.013067	hypomethylated	5	28	28
chr8	44267475	44269475	Triml2	0.01948343	1	insignificant	0.0076663	0.77413	insignificant	2	14	14
chr8	44392363	44394363	Zfp42	0.15373768	1	insignificant	-0.014551	0.0066908	hypomethylated	4	28	28
chr8	46034561	46036561	Fat1		1	noCoverage	-0.016975	0.68071	insignificant	0	9	9
chr8	46153563	46155563	Mtnr1a	-0.19474178	0.000043525	hypomethylated	0.061348	0.64916	insignificant	3	24	24
chr8	46347385	46349385	F11		1	noCoverage	0.040826	0.74497	insignificant	0	4	4
chr8	46380189	46382189	Klkb1	0.00797563	0.81814	insignificant	0.098073	0.10949	insignificant	1	12	12
chr8	46418550	46420550	Cyp4v3	-0.23015204	0.000069782	hypomethylated	0.0014221	0.95684	insignificant	9	39	39
chr8	46495893	46497893	Tlr3		1	noCoverage	-0.033333	0.080245	insignificant	0	2	2
chr8	46969838	46971838	Pdlim3	-0.2323242	0.00000131	hypomethylated	-0.029763	0.21296	insignificant	5	16	16
chr8	47019002	47021002	Ccdc110	-0.37618164	0.5557	insignificant	0.061146	0.061018	insignificant	3	19	19
chr8	47059892	47061892	Ufsp2	-0.1181881	0.063333	insignificant	0.00054439	0.13352	insignificant	9	70	70
chr8	47060606	47062606	1700029J07Ri	-0.12301959	0.15009	insignificant	0.00017687	0.1125	insignificant	6	58	58
chr8	47085204	47087204	Ankrd37	-0.15901626	0.25262	insignificant	-0.0010765	0.61944	insignificant	5	22	22
chr8	47094955	47096955	Lrp2bp		1	noCoverage	0.10263	0.51941	insignificant	0	4	4
chr8	47209500	47211500	Snx25		1	noCoverage	0.0047619	1	insignificant	0	4	4
chr8	47296363	47298363	Slc25a4	-0.08620063	0.000054508	hypomethylated	0.011594	0.41148	insignificant	13	40	36
chr8	47380025	47382025	Helt	-0.14128011	0.000014665	hypomethylated	-0.017329	0.8025	insignificant	8	60	56
chr8	47555395	47557395	Acsl1	-0.11572493	4.53E-22	hypomethylated	-0.022288	0.43489	insignificant	35	152	152
chr8	47636422	47638422	Mlf1ip	-0.15355078	3.27E-20	hypomethylated	0.0052461	1	insignificant	22	68	68
chr8	47701802	47703802	Casp3	-0.17842427	0.000000015	hypomethylated	0.0069869	0.97207	insignificant	19	85	85

chr8	47702554	47704554	Ccdc111	-0.14133301	0.000004367	hypomethylated	0.0097942	0.57533	insignificant	16	61	56
chr8	47824098	47826098	Irf2	-0.12485625	6.82E-14	hypomethylated	0.014091	0.18247	insignificant	30	118	128
chr8	48071278	48073278	Enpp6		1	noCoverage	0.26748	0.0051987	hypermethylated	0	11	6
chr8	48374756	48376756	Stox2	-0.06706229	1	insignificant	0.00029515	0.69807	insignificant	6	18	18
chr8	48437702	48439702	Stox2		1	noCoverage	-0.19676	0.21201	insignificant	0	4	4
chr8	48617998	48619998	Rwdd4a	-0.13545782	1.25E-14	hypomethylated	0.0039107	0.28807	insignificant	22	104	100
chr8	48618824	48620824	D030016E14R	-0.1126193	5.52E-10	hypomethylated	0.0013532	0.79139	insignificant	19	82	78
chr8	48760513	48762513	Ing2	-0.0747086	0.46116	insignificant	-0.0024918	0.19826	insignificant	38	149	142
chr8	48799285	48801285	Cdkn2aip	-0.10593181	1E-41	hypomethylated	0.0030462	0.61048	insignificant	38	144	143
chr8	48908835	48910835	Cldn22	-0.40469002	0.0000354	stronglyHypometh	-0.10384	0.14064	insignificant	1	7	4
chr8	49075905	49077905	Wwc2	-0.16487265	2.14E-18	hypomethylated	0.0027418	0.97105	insignificant	15	89	96
chr8	49183445	49185445	Dctd		1	noCoverage	-0.16523	0.40697	insignificant	0	5	4
chr8	49194311	49196311	Dctd	-0.08407057	0.00000112	hypomethylated	0.012533	0.96529	insignificant	18	89	89
chr8	49194366	49196366	Dctd	-0.08407057	0.00000112	hypomethylated	0.012533	0.96529	insignificant	18	89	89
chr8	49760044	49762044	Odz3		1	noCoverage	0.046432	1	insignificant	0	28	28
chr8	54596079	54598079	Aga	-0.20684216	1.79E-13	hypomethylated	0.002749	0.22336	insignificant	9	22	22
chr8	54724419	54726419	Neil3	-0.70746414	0.002093	stronglyHypometh	0.024203	0.02464	hypermethylated	2	15	15
chr8	55161885	55163885	Vegfc	-0.12002591	1.04E-11	hypomethylated	0.0023419	0.20388	insignificant	29	83	82
chr8	55615351	55617351	Spcs3	-0.07665062	0.00000203	hypomethylated	0.00685	0.51794	insignificant	10	30	30
chr8	55634683	55636683	Asb5		1	noCoverage	0.049348	0.048502	hypermethylated	0	9	12
chr8	55685133	55687133	Spata4	0.05119943	1	insignificant	0.082537	0.12166	insignificant	3	7	7
chr8	55809721	55811721	Wdr17		1	noCoverage	-0.023537	0.92748	insignificant	0	12	12
chr8	58772348	58774348	Hpgd	-0.08990975	0.063449	insignificant	-0.023488	0.34823	insignificant	8	18	18
chr8	59028930	59030930	Fbxo8	-0.12989822	0.038406	hypomethylated	0.053041	0.14692	insignificant	11	99	106
chr8	59029363	59031363	Fbxo8	-0.11493006	0.063808	insignificant	0.056165	0.14187	insignificant	11	93	100
chr8	59798779	59800779	Hand2	-0.18424362	1.14E-15	hypomethylated	-0.020993	0.0019549	inconclusive	14	93	63
chr8	59966657	59968657	Sap30	-0.16957509	1	insignificant	-0.08166	0.46443	insignificant	1	25	23
chr8	59989639	59991639	Hmgb2	-0.10775914	9.39E-32	hypomethylated	0.014212	0.08562	insignificant	42	204	201
chr8	60131852	60133852	Galnt7	-0.17064384	1.82E-09	hypomethylated	0.0060183	0.30641	insignificant	9	36	34
chr8	61179593	61181593	Galntl6		1	noCoverage	0.27819	0.27158	insignificant	0	3	2
chr8	61389551	61391551	BC030500	-0.21898771	0.00021931	hypomethylated	-0.0078284	0.92238	insignificant	11	30	32
chr8	61390424	61392424	Galntl6	-0.23375036	0.000000297	hypomethylated	0.034956	0.28806	insignificant	16	44	46
chr8	62983920	62985920	Aadat	-0.48854973	3.5E-10	stronglyHypometh	0.1196	0.26008	insignificant	4	17	12
chr8	63110656	63112656	Mfap3l	-0.12559506	3.31E-22	hypomethylated	0.0092224	0.25543	insignificant	37	157	144
chr8	63368247	63370247	2700029M09F	-0.08151411	0.00028024	hypomethylated	-0.034483	0.86845	insignificant	23	102	92
chr8	63433596	63435596	Clcn3	-0.57142857	0.058559	insignificant	0.21561	0.64459	insignificant	1	2	4
chr8	63462108	63464108	Clcn3	-0.1678779	1.33E-08	hypomethylated	-0.021517	0.24128	insignificant	16	51	49
chr8	63471016	63473016	Nek1	-0.12853173	1.31E-11	hypomethylated	0.024787	0.76889	insignificant	22	119	120
chr8	63701967	63703967	Sh3rf1	-0.1212727	1.19E-10	hypomethylated	0.018036	0.85237	insignificant	41	163	147
chr8	63965530	63967530	Cbr4	-0.14457395	7.63E-09	hypomethylated	0.025429	1	insignificant	12	42	44
chr8	64405885	64407885	Ddx60	-0.11755684	0.0007901	hypomethylated	0.025268	0.50638	insignificant	3	20	18
chr8	65429242	65431242	Spock3	-0.05843564	0.0050422	hypomethylated	0.053977	0.0012037	hypermethylated	11	85	75
chr8	66430967	66432967	Gm4975	0.02127719	0.61086	insignificant	-0.03599	0.30016	insignificant	2	17	17
chr8	66684790	66686790	Tll1		1	noCoverage	-0.030557	0.84933	insignificant	0	25	31
chr8	67171837	67173837	Cpe	-0.07081229	0.15104	insignificant	0.0058001	0.79716	insignificant	7	18	18
chr8	67212375	67214375	Sc4mol	-0.08845566	0.0023318	hypomethylated	0.0030553	0.50125	insignificant	10	64	63
chr8	67373716	67375716	Klhl2	-0.0570913	0.000030871	hypomethylated	0.0073976	0.23886	insignificant	28	103	103
chr8	67470083	67472083	Tmem192	-0.1063009	1.65E-16	hypomethylated	0.0057799	0.38601	insignificant	41	131	131
chr8	67511543	67513543	Trim75	-0.23550725	0.29128	insignificant	0.060393	0.32562	insignificant	1	7	6
chr8	68140938	68142938	March1	-0.32053571	0.000067501	hypomethylated	-0.01705	0.91624	insignificant	4	8	8
chr8	68141423	68143423	March1	-0.32053571	0.000067501	hypomethylated	-0.01705	0.91624	insignificant	4	8	8
chr8	69010406	69012406	1810029B16R	-0.27313994	3.04E-08	hypomethylated	-0.039159	0.89717	insignificant	8	25	24
chr8	69034638	69036638	Tktl2	0.06936101	1	insignificant	-0.087437	1	insignificant	1	18	20
chr8	69211993	69213993	Npy5r	-0.28767699	0.00026065	hypomethylated	0.038645	0.12469	insignificant	6	16	17
chr8	69220320	69222320	Npy1r	-0.1764758	0.012377	hypomethylated	0.027869	0.28996	insignificant	5	11	10
chr8	69383115	69385115	Naf1	-0.10117103	1.69E-18	hypomethylated	0.0055009	0.37348	insignificant	52	200	176
chr8	70017773	70019773	Nat2	-0.22986153	0.022957	hypomethylated	0.029935	0.2237	insignificant	10	22	22
chr8	70017846	70019846	Nat2	-0.22986153	0.022957	hypomethylated	0.029935	0.2237	insignificant	10	22	22
chr8	70046752	70048752	Nat3	0.16433566	0.30708	insignificant	-0.081197	0.36504	insignificant	6	13	13
chr8	70434845	70436845	Psd3	0.1	1	insignificant	-0.025	0.17549	insignificant	2	4	4
chr8	70498473	70500473	Psd3	0.16974089	1	insignificant	0.092238	0.34466	insignificant	2	9	11
chr8	70799426	70801426	Sh2d4a	-0.20906518	0.0015211	hypomethylated	-0.0040647	0.54458	insignificant	11	38	42
chr8	71259041	71261041	Csgalnact1	-0.35579054	2.23E-16	stronglyHypometh	-0.036223	0.33378	insignificant	9	29	26
chr8	71316852	71318852	Ints10	-0.09192306	0.000000535	hypomethylated	0.019672	0.36946	insignificant	23	90	90
chr8	71403453	71405453	Lpl	-0.15442734	0.21786	insignificant	0.012336	0.70993	insignificant	3	56	53
chr8	71611634	71613634	Atp6v1b2	-0.17612783	0.010644	hypomethylated	-0.029068	0.11009	insignificant	11	50	47
chr8	71613121	71615121	Slc18a1	-0.18928827	0.57639	insignificant	0.0075542	0.29323	insignificant	4	16	16
chr8	71793978	71795978	D130040H23Rik		1	noCoverage	0.054029	0.36046	insignificant	0	8	8
chr8	72149074	72151074	Zfp868	-0.23931314	1	lowCoverage	0.05217	0.16614	insignificant	1	29	31
chr8	72149447	72151447	Zfp868		1	noCoverage	0.07624	0.089415	insignificant	0	6	8
chr8	72177454	72179454	Zfp964	-0.14055968	0.0041392	hypomethylated	0.027032	0.90572	insignificant	7	47	45
chr8	72240385	72242385	Zfp869	-0.11736917	0.000012392	hypomethylated	0.02862	0.83439	insignificant	11	40	40
chr8	72298810	72300810	Zfp866	-0.72826087	3.98E-08	stronglyHypometh	0.15251	1	insignificant	1	4	4
chr8	72314061	72316061	Atp13a1	-0.14389219	8.99E-25	hypomethylated	0.0096751	0.039608	hypermethylated	27	60	62
chr8	72331585	72333585	Gmip	-0.12908105	0.00000751	hypomethylated	-0.0081334	0.57662	insignificant	15	67	68
chr8	72345463	72347463	Lpar2	-0.16742621	0.00000104	hypomethylated	0.013986	0.82243	insignificant	11	39	34
chr8	72355602	72357602	Pbx4		1	noCoverage	-0.015682	0.14201	insignificant	0	28	22
chr8	72411291	72413291	Cilp2	-0.21640332	0.040598	hypomethylated	-0.021217	0.44043	insignificant	5	45	45
chr8	72425113	72427113	Tssk6	-0.18791961	1.05E-32	hypomethylated	0.021294	0.1286	insignificant	38	142	131
chr8	72426457	72428457	Tssk6	-0.19685937	5.46E-17	hypomethylated	0.024409	0.17648	insignificant	12	54	50
chr8	72498282	72500282	Gatad2a	-0.03934146	0.39013	insignificant	0.0079934	0.19156	insignificant	10	45	45
chr8	72520278	72522278	Gatad2a	-0.15491643	0.000000092	hypomethylated	-0.055668	1	insignificant	9	61	52
chr8	72565711	72567711	Sugp1	-0.12097707	0.00089039	hypomethylated	0.021481	0.034127	hypermethylated	24	83	80
chr8	72566633	72568633	Sugp1	-0.17359632	0.000000351	hypomethylated	0.031245	0.36567	insignificant	15	35	32
chr8	72595830	72597830	Tm6sf2		1	noCoverage	0.047083	0.37139	insignificant	0	6	6

chr8	72606427	72608427	Hapln4	-0.18328671	1.03E-23	hypomethylated	0.064697	0.16816	insignificant	12	24	24
chr8	72644743	72646743	Ncan	-0.20376272	0.00012319	inconclusive	-0.10487	0.43358	insignificant	4	10	12
chr8	72654231	72656231	Nr2c2ap	-0.19514591	1.49E-25	hypomethylated	0.0057632	0.53194	insignificant	22	63	66
chr8	72662611	72664611	2310045N01R	-0.29150439	0.21575	insignificant	-0.030284	0.73396	insignificant	4	16	16
chr8	72663096	72665096	Rfxank	-0.29150439	0.21575	insignificant	-0.030284	0.73396	insignificant	4	16	16
chr8	72695309	72697309	Tmem161a	0.18441738	1	insignificant	0.062686	0.89189	insignificant	1	10	8
chr8	72736180	72738180	Slc25a42	-0.11800386	0.37299	insignificant	0.046644	0.71954	insignificant	2	44	37
chr8	72757124	72759124	Sugp2	-0.08966008	9.68E-12	hypomethylated	0.0061396	0.056196	insignificant	18	119	118
chr8	72758321	72760321	Armc6	-0.09879924	0.000000151	hypomethylated	-0.0020588	0.069056	insignificant	11	77	76
chr8	72805897	72807897	Homer3	-0.11912451	4.09E-25	hypomethylated	-0.0094224	0.87236	insignificant	30	104	100
chr8	72825683	72827683	Cope	-0.23383345	0.000000675	hypomethylated	-0.11758	0.77749	insignificant	14	48	56
chr8	72826351	72828351	Cope	-0.24624456	0.87169	insignificant	-0.17063	0.30605	insignificant	7	34	42
chr8	72838673	72840673	Lass1	-0.22041216	0.051116	insignificant	-0.032669	0.13909	insignificant	12	37	34
chr8	72852663	72854663	Gdf1	-0.27830802	0.000004226	hypomethylated	-0.073636	0.00018686	hypomethylated	13	69	69
chr8	72877172	72879172	Upf1	-0.11877337	0.15849	insignificant	-0.012047	0.76171	insignificant	11	57	60
chr8	72896446	72898446	Comp	-0.01986351	0.64723	insignificant	0.035214	0.47783	insignificant	14	50	50
chr8	72963472	72965472	Crtc1	-0.1109499	2.71E-09	hypomethylated	0.013506	0.11989	insignificant	12	44	44
chr8	73000842	73002842	Klhl26	-0.21470681	0.37539	insignificant	-0.051226	0.44821	insignificant	4	16	14
chr8	73011257	73013257	Tmem59l	-0.03704358	0.84244	insignificant	0.021106	0.030454	hypermethylated	23	83	82
chr8	73016054	73018054	Crlf1	-0.0899341	1.11E-20	hypomethylated	0.013019	0.49737	insignificant	50	164	162
chr8	73030638	73032638	2810428I15Ril	-0.21349206	0.31576	insignificant	-0.030612	0.12783	insignificant	4	18	26
chr8	73034266	73036266	Uba52	-0.26740683	2.84E-32	hypomethylated	-0.043973	0.044773	hypomethylated	14	90	84
chr8	73047079	73049079	2810422J05Ri	-0.17786132	0.67593	insignificant	-0.093513	0.74641	insignificant	1	14	12
chr8	73050641	73052641	Fkbp8	-0.03119331	2.93E-11	hypomethylated	-0.018121	0.35052	insignificant	7	56	56
chr8	73050744	73052744	Fkbp8	-0.03119331	2.93E-11	hypomethylated	-0.018121	0.35052	insignificant	7	56	56
chr8	73062573	73064573	Ell	-0.10637014	0.00066037	hypomethylated	0.031947	0.62264	insignificant	12	59	57
chr8	73117379	73119379	Isyna1	-0.10455492	7.72E-09	hypomethylated	0.0097835	0.036588	hypermethylated	23	134	134
chr8	73132213	73134213	Ssbp4	-0.14304018	5.83E-23	hypomethylated	0.018746	0.34158	insignificant	24	120	118
chr8	73139742	73141742	Lrrc25		1	noCoverage	-0.025512	0.27502	insignificant	0	2	2
chr8	73183580	73185580	Pgpep1	-0.19515031	4.53E-17	hypomethylated	0.015031	0.0010487	hypermethylated	20	56	56
chr8	73196129	73198129	Lsm4	-0.15559243	0.0093692	hypomethylated	0.023339	0.36548	insignificant	16	49	52
chr8	73220637	73222637	Jund	-0.21933622	0.089329	insignificant	-0.0041847	1	insignificant	4	19	19
chr8	73241441	73243441	Gm3336	0.07641154	0.36902	insignificant	0.10416	0.40051	insignificant	4	31	28
chr8	73246962	73248962	Pde4c	0.37352941	1	lowCoverage	0.28529	0.072093	insignificant	1	4	2
chr8	73277577	73279577	Rab3a	-0.14388622	1.64E-11	hypomethylated	-0.0075761	0.801	insignificant	9	98	98
chr8	73284820	73286820	Mpv17l2	-0.31540711	0.33247	insignificant	-0.036527	0.17249	insignificant	1	16	16
chr8	73290562	73292562	Ifi30	-0.3038187	0.033365	hypomethylated	-0.024118	0.19531	insignificant	8	17	17
chr8	73299760	73301760	2010320M18F	-0.12296886	3.53E-31	hypomethylated	0.0033289	0.049408	hypermethylated	37	128	112
chr8	73300611	73302611	Pik3r2	-0.08342117	0.00041947	hypomethylated	0.0063765	0.39711	insignificant	12	48	48
chr8	73316332	73318332	Mast3	-0.18984012	0.12357	insignificant	0.013592	0.82374	insignificant	0	10	10
chr8	73331347	73333347	Il12rb1	-0.22365178	0.43834	insignificant	0.032494	0.087989	insignificant	1	14	14
chr8	73363619	73365619	Arrdc2	-0.25599947	3.6E-11	hypomethylated	0.021664	0.68068	insignificant	11	22	22
chr8	73380907	73382907	Kcnn1		1	noCoverage	-0.051763	0.86743	insignificant	0	6	6
chr8	73397389	73399389	Ccdc124	-0.03111095	5.41E-11	hypomethylated	0.027681	0.033588	inconclusive	20	60	60
chr8	73416656	73418656	Slc5a5	-0.18942689	0.0077275	hypomethylated	-0.10664	0.073966	insignificant	5	18	17
chr8	73419755	73421755	Rpl18a	-0.14660847	0.57531	insignificant	-0.017697	0.0093711	hypomethylated	28	92	94
chr8	73421311	73423311	Rpl18a	-0.08705571	0.1084	insignificant	-0.013674	0.80339	insignificant	11	25	21
chr8	73421342	73423342	Rpl18a	-0.08705571	0.1084	insignificant	-0.013674	0.80339	insignificant	11	25	21
chr8	73428872	73430872	Mtap1s	-0.12687094	2.47E-15	hypomethylated	0.0017556	0.30444	insignificant	35	110	113
chr8	73795612	73797612	Myo9b	-0.11929179	7.69E-19	hypomethylated	-0.0077426	0.58449	insignificant	33	100	100
chr8	73796489	73798489	Haus8	-0.12159161	2.19E-16	hypomethylated	-0.0075123	0.44931	insignificant	26	78	78
chr8	73889746	73891746	Use1	-0.0952868	0.7831	insignificant	0.016118	0.56587	insignificant	7	51	49
chr8	73890115	73892115	Use1	-0.0952868	0.7831	insignificant	0.016118	0.56587	insignificant	7	51	49
chr8	73894196	73896196	Ocel1	-0.13245364	3.27E-11	hypomethylated	0.0054192	0.2864	insignificant	27	102	99
chr8	73905851	73907851	Nr2f6	-0.15858203	3.14E-21	hypomethylated	-0.011408	0.75808	insignificant	30	104	104
chr8	73919700	73921700	Ushbp1	0.00404663	0.083417	insignificant	0.022057	0.13407	insignificant	9	77	72
chr8	73919753	73921753	5430437P03Ri	0.01408321	0.000018063	hypermethylated	0.019807	0.038717	hypermethylated	9	77	72
chr8	73928910	73930910	Ankle1	-0.21194749	0.000000727	hypomethylated	-0.015496	0.63456	insignificant	5	73	68
chr8	73987556	73989556	Mrpl34	-0.10342527	0.00009064	hypomethylated	0.0051281	0.052092	insignificant	15	65	65
chr8	73987824	73989824	Mrpl34	-0.09665534	0.00013272	hypomethylated	0.0054643	0.054245	insignificant	15	61	61
chr8	73992092	73994092	Dda1	-0.08806843	6.47E-12	hypomethylated	0.010528	0.041012	hypermethylated	27	119	112
chr8	74009966	74011966	Gtpbp3	-0.03319658	0.00022675	hypomethylated	0.066339	0.60153	insignificant	9	46	49
chr8	74011001	74013001	Gtpbp3	-0.26680918	1.36E-08	hypomethylated	0.031575	0.16197	insignificant	6	45	43
chr8	74035668	74037668	Plvap	-0.33181042	0.000015344	hypomethylated	0.026002	0.39118	insignificant	7	35	38
chr8	74061336	74063336	Bst2	-0.3296702	3.61E-08	hypomethylated	0.0023836	0.4981	insignificant	4	24	19
chr8	74065828	74067828	Fam125a	-0.1545325	0.00000946	hypomethylated	0.020479	0.085994	insignificant	11	88	91
chr8	74082770	74084770	Tmem221	0.23882023	0.68036	insignificant	0.011074	0.018666	hypermethylated	2	23	22
chr8	74090548	74092548	Nxnl1	-0.01925926	1	insignificant	0.023781	0.60789	insignificant	3	6	6
chr8	74091825	74093825	Slc27a1	-0.18950008	8.67E-18	hypomethylated	0.018385	0.64229	insignificant	10	31	31
chr8	74115082	74117082	Pgls	-0.10961779	0.00000077	hypomethylated	-0.0025817	0.2845	insignificant	23	99	96
chr8	74120544	74122544	Fam129c		1	noCoverage	-0.0017094	1	insignificant	0	6	6
chr8	74133922	74135922	Glt25d1	-0.22816005	0.60218	insignificant	-0.064906	0.3426	insignificant	2	61	53
chr8	74195656	74197656	Unc13a	-0.10604703	1.42E-10	hypomethylated	0.051123	0.51686	insignificant	11	36	31
chr8	74199281	74201281	Jak3	-0.50577226	1	lowCoverage	-0.11075	0.39251	insignificant	1	12	10
chr8	74212150	74214150	Insl3	-0.07756099	0.57251	insignificant	0.0045459	0.49231	insignificant	8	16	16
chr8	74249580	74251580	Fcho1	0.06254902	0.15104	insignificant	-0.064074	0.63772	insignificant	1	5	7
chr8	74404966	74406966	Zfp709	-0.1632105	2.04E-19	hypomethylated	-0.011085	0.053094	insignificant	11	56	52
chr8	74431504	74433504	Zfp882	-0.20533412	0.12878	insignificant	-0.041175	0.18501	insignificant	5	34	32
chr8	74445723	74447723	Zfp617	-0.19728638	1.01E-18	hypomethylated	0.029158	0.26465	insignificant	24	107	109
chr8	74473964	74475964	Zfp961	-0.27685809	0.077745	insignificant	-0.012827	0.88164	insignificant	6	31	31
chr8	74580558	74582558	Olfr372	0.03227931	0.51972	insignificant	-0.019173	0.68849	insignificant	2	6	6
chr8	74622660	74624660	Olfr373	-0.15568182	0.60775	insignificant	0.047203	0.6088	insignificant	1	4	4
chr8	74658190	74660190	Tpm4	-0.10840861	0.000000786	hypomethylated	0.0064735	0.31101	insignificant	27	107	99
chr8	74684098	74686098	Rab8a	-0.16881926	4.56E-30	hypomethylated	0.0087831	0.044715	inconclusive	24	100	98
chr8	74712566	74714566	Hsh2d	-0.58585859	5.91E-09	stronglyHypometh	-0.20993	0.4002	insignificant	2	9	6

chr8	74742628	74744628	Fam32a	-0.08153686	0.5144	insignificant	0.090995	0.0018492	hypermethylated	15	51	48
chr8	74763030	74765030	Ap1m1	-0.19818815	5.33E-14	hypomethylated	0.023775	0.15031	insignificant	14	83	84
chr8	74841960	74843960	Klf2	-0.12821523	1.91E-29	hypomethylated	-0.015178	0.31556	insignificant	43	125	136
chr8	74945373	74947373	Eps15l1	-0.11609734	0.14569	insignificant	-0.0000869	1	insignificant	6	22	22
chr8	74966778	74968778	1700030K09Ri	-0.48673414	0.000000259	stronglyHypometh	0.016911	0.55127	insignificant	6	22	22
chr8	74967677	74969677	Calr3	-0.26216573	0.014337	hypomethylated	-0.11215	0.47675	insignificant	5	12	10
chr8	74999132	75001132	Cherp	-0.27020219	4.7E-18	hypomethylated	0.064411	0.2128	insignificant	10	44	38
chr8	75016513	75018513	Slc35e1	-0.10531907	3.93E-12	hypomethylated	0.0042865	0.77924	insignificant	25	79	78
chr8	75072209	75074209	Med26	-0.16214447	1	insignificant	0.01448	0.25588	insignificant	3	20	20
chr8	75094947	75096947	9130011J15Ri	-0.11605038	0.000066754	hypomethylated	0.022508	0.48393	insignificant	9	81	71
chr8	75095001	75097001	Tmem38a	-0.12761114	0.000033362	hypomethylated	0.032292	0.17009	insignificant	8	71	61
chr8	75169609	75171609	Nwd1	-0.29742063	0.00000124	hypomethylated	0.10028	0.64323	insignificant	3	12	6
chr8	75246186	75248186	Sin3b	-0.09794028	0.0083746	hypomethylated	0.0092549	0.37082	insignificant	8	54	54
chr8	75284778	75286778	F2rl3	-0.38333333	0.51939	insignificant	0.25776	0.79078	insignificant	2	6	10
chr8	75876455	75878455	Large	-0.11859896	1.65E-29	hypomethylated	0.010816	0.20475	insignificant	61	204	199
chr8	77516601	77518601	Hmgxb4	-0.12764691	4.59E-39	hypomethylated	0.017169	0.31106	insignificant	44	144	124
chr8	77556584	77558584	Tom1	-0.16444791	7.88E-33	hypomethylated	0.016175	0.93746	insignificant	22	96	80
chr8	77616516	77618516	Hmox1	-0.17095356	7.31E-08	hypomethylated	-0.020649	0.72355	insignificant	10	31	30
chr8	77632426	77634426	Mcm5	-0.21527823	1.95E-14	hypomethylated	0.012672	1	insignificant	15	60	60
chr8	77736842	77738842	Rasd2	-0.06973023	5.13E-15	hypomethylated	-0.008783	0.64095	insignificant	36	185	183
chr8	79424072	79426072	Gm10649	-0.14391052	7.62E-29	hypomethylated	0.010567	0.13785	insignificant	30	124	118
chr8	79425406	79427406	Nr3c2	-0.16135187	2.36E-08	hypomethylated	-0.0047159	0.53455	insignificant	10	71	69
chr8	80039954	80041954	0610038B21R	-0.1604574	0.000019876	hypomethylated	0.020369	0.044824	hypermethylated	14	72	71
chr8	80041806	80043806	Arhgap10	-0.09009957	0.00021777	hypomethylated	-0.0037614	0.28964	insignificant	11	56	56
chr8	80072295	80074295	Prmt10	-0.25446429	0.25311	insignificant	-0.081299	0.48893	insignificant	4	16	14
chr8	80134552	80136552	Tmem184c	-0.875	0.14286	lowCoverage	0.017105	0.50417	insignificant	1	4	4
chr8	80960551	80962551	Pou4f2	-0.20219251	1.11E-36	hypomethylated	-0.002973	0.21076	insignificant	18	59	58
chr8	81032233	81034233	Slc10a7	-0.08099804	3.94E-14	hypomethylated	0.0064263	0.95035	insignificant	26	184	180
chr8	81032827	81034827	Slc10a7	-0.11349656	7.44E-10	hypomethylated	0.0037722	0.6286	insignificant	13	81	81
chr8	81345051	81347051	Lsm6	-0.51817468	8.85E-17	stronglyHypometh	0.032346	0.11137	insignificant	3	12	12
chr8	81551335	81553335	Zfp827	-0.11833195	2.9E-18	hypomethylated	0.0044853	0.21983	insignificant	13	94	74
chr8	81818855	81820855	Mmaa	-0.07862826	1.99E-09	hypomethylated	0.003479	0.18145	insignificant	7	59	59
chr8	81923367	81925367	Smad1	-0.14345421	2.15E-09	hypomethylated	0.014607	0.14644	insignificant	9	52	52
chr8	82162574	82164574	Otud4	-0.08327431	1.65E-35	hypomethylated	0.0015226	0.69733	insignificant	64	237	243
chr8	82234718	82236718	Anapc10	-0.1087326	0.0031057	hypomethylated	0.0061471	0.22911	insignificant	10	74	74
chr8	82235639	82237639	Abce1	0.16112561	0.000000109	hypermethylated	0.039845	0.040356	hypermethylated	3	34	34
chr8	82581907	82583907	Hhip	-0.14071658	1	insignificant	0.013319	0.41751	insignificant	6	26	26
chr8	83016943	83018943	Gypa	-0.32900641	0.10661	insignificant	0.026573	0.11628	insignificant	1	24	18
chr8	83133937	83135937	Frem3	-0.17035603	1.72E-09	hypomethylated	0.0045253	0.96194	insignificant	8	28	28
chr8	83263358	83265358	Smarca5		1	noCoverage	-0.092885	0.000039458	hypomethylated	0	34	42
chr8	83404378	83406378	Gab1	-0.17032001	0.00022124	hypomethylated	0.00018454	0.52002	insignificant	12	34	34
chr8	83538805	83540805	Usp38	-0.1560627	3.57E-09	hypomethylated	-0.031939	0.3947	insignificant	17	65	63
chr8	84926444	84928444	Il15	-0.3019384	1.12E-11	hypomethylated	-0.020397	0.083138	insignificant	3	22	22
chr8	85298025	85300025	Zfp330	-0.14645936	0.012436	hypomethylated	0.0026289	0.47728	insignificant	6	18	16
chr8	85386254	85388254	Rnf150	-0.11382979	1.48E-20	hypomethylated	-0.016692	0.45271	insignificant	25	113	101
chr8	85688250	85690250	Tbc1d9	-0.10570782	2.08E-17	hypomethylated	-0.0079561	0.93481	insignificant	39	157	157
chr8	85813246	85815246	Ucp1	-0.49251338	3.37E-11	stronglyHypometh	-0.034938	0.055609	insignificant	3	16	14
chr8	85856385	85858385	Elmod2	-0.15646147	0.001854	hypomethylated	-0.004157	0.084813	insignificant	6	51	49
chr8	85912789	85914789	Clgn	-0.14933468	0.011362	hypomethylated	0.022804	0.036584	hypermethylated	6	59	51
chr8	85982290	85984290	Scoc		1	noCoverage	-0.059685	0.033699	inconclusive	0	4	4
chr8	86089656	86091656	Ndufb7	-0.28557386	2.02E-13	hypomethylated	-0.034078	0.92901	insignificant	16	68	64
chr8	86118390	86120390	Tecr	-0.15433485	0.095305	insignificant	-0.029418	1	insignificant	1	33	32
chr8	86131073	86133073	Dnajb1	-0.09631409	1.73E-20	hypomethylated	-0.0006301	0.9273	insignificant	45	164	161
chr8	86175576	86177576	Gipc1	-0.18255766	2.87E-13	hypomethylated	-0.049055	0.12316	insignificant	21	89	84
chr8	86189538	86191538	Ptger1		1	noCoverage	0.0079657	0.82757	insignificant	0	4	4
chr8	86223078	86225078	Pkn1	-0.15068758	9.64E-08	hypomethylated	0.0026458	1	insignificant	7	31	31
chr8	86238075	86240075	Ddx39	-0.1515366	5.06E-21	hypomethylated	-0.004505	0.41315	insignificant	35	142	141
chr8	86265210	86267210	Cd97	-0.57284584	0.057768	insignificant	0.033627	0.8665	insignificant	2	25	25
chr8	86422996	86424996	Lphn1	-0.09594105	7.54E-33	hypomethylated	0.002769	0.64723	insignificant	75	262	253
chr8	86478592	86480592	Asf1b	-0.11177433	1.29E-10	hypomethylated	0.0083426	0.85143	insignificant	29	151	148
chr8	86495876	86497876	Prkaca	-0.07921317	2.18E-30	hypomethylated	0.0019708	0.7754	insignificant	34	129	129
chr8	86520570	86522570	Samd1	-0.08373163	3.86E-19	hypomethylated	0.0065387	0.099188	insignificant	57	246	239
chr8	86524604	86526604	1700067K01Ri	-0.00383267	0.079216	insignificant	0.053893	0.44592	insignificant	11	36	30
chr8	86535532	86537532	Mir1199	-0.15663471	7.79E-13	hypomethylated	0.0031969	0.46104	insignificant	35	121	118
chr8	86544372	86546372	Palm3	0.12286771	0.22986	insignificant	0.09997	0.000000392	hypermethylated	11	35	37
chr8	86566474	86568474	Il27ra		1	noCoverage	0.014261	1	insignificant	0	12	9
chr8	86568878	86570878	Rln3		1	noCoverage	-0.16114	0.070511	insignificant	0	15	14
chr8	86589734	86591734	Rfx1	-0.13319971	2.69E-17	hypomethylated	-0.0034331	0.22146	insignificant	40	143	128
chr8	86628661	86630661	Dcaf15	-0.35800773	0.0001114	stronglyHypometh	0.043565	0.25683	insignificant	4	18	14
chr8	86670936	86672936	4930432K21Ri	-0.19301159	0.000000382	hypomethylated	-0.019144	0.85056	insignificant	6	32	32
chr8	86671652	86673652	Cc2d1a	-0.16513829	0.00000164	hypomethylated	-0.013013	0.45116	insignificant	5	30	30
chr8	86700451	86702451	Nanos3		1	noCoverage	-0.002825	0.6892	insignificant	0	12	12
chr8	86702686	86704686	Mir181d	-0.4451636	1.18E-08	stronglyHypometh	-0.026552	0.55777	insignificant	2	15	15
chr8	86702860	86704860	Mir181d	-0.63943381	0.00000194	stronglyHypometh	-0.13914	0.043499	hypomethylated	1	5	5
chr8	86732806	86734806	Mir3074-2	-0.32921168	0.18422	insignificant	0.016926	0.1949	insignificant	3	12	12
chr8	86760941	86762941	Zswim4	-0.09661755	0.0044943	hypomethylated	0.021933	0.098771	insignificant	10	56	57
chr8	86781223	86783223	Ccdc130	-0.36363636	1	lowCoverage	-0.010695	0.36228	insignificant	1	3	3
chr8	86794259	86796259	Ccdc130	-0.26953028	5.31E-20	hypomethylated	0.04912	0.12648	insignificant	12	55	55
chr8	86938262	86940262	Cacna1a	-0.11199157	4.81E-21	hypomethylated	0.01702	0.72194	insignificant	39	118	118
chr8	87186751	87188751	Ier2	-0.18069902	6.21E-40	hypomethylated	-0.0078526	1	insignificant	30	82	82
chr8	87211761	87213761	Nacc1	-0.17305929	5.69E-08	hypomethylated	0.009343	0.8191	insignificant	20	80	82
chr8	87212145	87214145	Trmt1	-0.2023343	6.11E-20	hypomethylated	0.024636	0.79448	insignificant	22	82	84
chr8	87224355	87226355	Lyl1	-0.12735537	0.00012415	hypomethylated	0.0046998	0.80831	insignificant	11	46	42
chr8	87324239	87326239	Nfix	-0.06598853	2.19E-08	hypomethylated	0.00045801	0.69563	insignificant	18	72	70
chr8	87355180	87357180	Gadd45gip1	-0.20255651	0.00000318	hypomethylated	-0.0058682	0.46838	insignificant	23	75	76

chr8	87356164	87358164	Dand5	-0.20985837	0.000000823	hypomethylated	-0.015221	0.65503	insignificant	17	49	50
chr8	87364540	87366540	Rad23a	-0.11606244	0.000074257	hypomethylated	0.0036782	0.6736	insignificant	5	10	10
chr8	87379884	87381884	Farsa	-0.21360291	0.35694	insignificant	0.016425	0.78541	insignificant	4	45	45
chr8	87395009	87397009	Syce2	-0.13158904	1.09E-17	hypomethylated	-0.013927	0.15699	insignificant	29	128	128
chr8	87395156	87397156	Syce2	-0.13158904	1.09E-17	hypomethylated	-0.013927	0.15699	insignificant	29	128	128
chr8	87424826	87426826	Klf1	-0.21079208	4.11E-14	hypomethylated	-0.015155	0.084736	insignificant	6	18	18
chr8	87431522	87433522	Dnase2a	-0.20542012	2.53E-12	hypomethylated	-0.047467	0.97121	insignificant	15	93	86
chr8	87469889	87471889	Rtbdn	-0.22529992	1.17E-15	hypomethylated	0.01588	0.9646	insignificant	11	53	55
chr8	87489910	87491910	Rnaseh2a	-0.23631579	0.61774	insignificant	0.078667	0.023894	hypermethylated	3	25	23
chr8	87492546	87494546	Prdx2	-0.06954039	0.0050949	inconclusive	0.024387	0.040724	hypermethylated	23	75	68
chr8	87502617	87504617	Junb	-0.12454831	6.3E-14	hypomethylated	0.017017	0.76142	insignificant	9	57	57
chr8	87513493	87515493	Hook2	-0.16695233	0.055525	insignificant	0.0055002	1	insignificant	4	26	26
chr8	87549177	87551177	2310036O22R	-0.22203682	1.82E-13	hypomethylated	0.024257	0.51629	insignificant	14	86	71
chr8	87549731	87551731	2310036O22R	-0.2409482	3.13E-16	hypomethylated	0.030209	0.81085	insignificant	17	105	85
chr8	87559813	87561813	Tnpo2	-0.16004826	1.09E-17	hypomethylated	0.041005	0.010924	hypermethylated	18	49	42
chr8	87560066	87562066	Tnpo2	-0.1432975	5.28E-20	hypomethylated	0.023838	0.0074568	hypermethylated	21	72	66
chr8	87583017	87585017	Fbxw9	-0.25783375	0.000041179	hypomethylated	0.035239	0.63425	insignificant	4	74	71
chr8	87594655	87596655	Dhps	-0.04618745	0.68291	insignificant	0.009847	0.64018	insignificant	5	60	54
chr8	87603861	87605861	BC056474	-0.15586894	0.0015906	hypomethylated	-0.0088481	0.5297	insignificant	14	68	62
chr8	87604645	87606645	BC056474	-0.17094648	0.045074	hypomethylated	0.0010795	0.93249	insignificant	3	22	16
chr8	87822530	87824530	Orc6	-0.18411569	4.98E-19	hypomethylated	0.019853	0.12275	insignificant	15	112	98
chr8	87823396	87825396	Vps35	-0.20802446	1.36E-20	hypomethylated	0.025034	0.010348	inconclusive	9	68	57
chr8	87956740	87958740	4921524J17Ri	-0.00833333	1	insignificant	-0.036607	0.77401	insignificant	2	8	8
chr8	88015515	88017515	Gpt2	-0.12173227	8.41E-11	hypomethylated	0.011026	0.72901	insignificant	18	80	71
chr8	88079170	88081170	Dnaja2	-0.1104586	0.00000255	hypomethylated	0.0035997	0.21757	insignificant	18	71	62
chr8	88363900	88365900	Phkb	-0.11524035	0.000000147	hypomethylated	-0.0017356	1	insignificant	26	94	79
chr8	89090489	89092489	Abcc12	0.22082148	0.57638	insignificant	0.031745	0.71391	insignificant	1	30	26
chr8	89146941	89148941	Lonp2	-0.16726801	9.63E-21	hypomethylated	0.025056	0.18405	insignificant	16	57	40
chr8	89268597	89270597	Gm10638	-0.08958833	1.7E-40	hypomethylated	-0.0031045	0.6757	insignificant	84	227	218
chr8	89269905	89271905	Gm10638	-0.13119548	1.35E-16	hypomethylated	-0.015645	0.26498	insignificant	31	107	102
chr8	89995710	89997710	Gm2694	-0.11929512	1.11E-46	hypomethylated	0.0042413	0.60895	insignificant	41	209	220
chr8	89996491	89998491	Cbln1	-0.14899291	1.14E-25	hypomethylated	-0.0010338	0.41552	insignificant	20	114	125
chr8	90483494	90485494	Zfp423	-0.16765049	1.39E-48	hypomethylated	-0.01885	0.29153	insignificant	46	179	158
chr8	90641657	90643657	Tmem188	-0.00748925	1.81E-10	hypomethylated	0.03481	0.4649	insignificant	9	63	60
chr8	90660783	90662783	Heatr3	-0.09354142	1.28E-08	hypomethylated	0.0083772	0.54802	insignificant	28	103	100
chr8	90722111	90724111	Papd5	-0.07373313	3.71E-22	hypomethylated	0.007837	0.19759	insignificant	63	251	241
chr8	90795301	90797301	Adcy7	-0.16007321	7.9E-10	hypomethylated	0.0074324	0.61542	insignificant	16	78	78
chr8	90811964	90813964	Adcy7	-0.54825857	0.46113	lowCoverage	-0.0068675	0.17914	insignificant	1	17	18
chr8	90886090	90888090	Brd7	-0.12346764	0.22543	insignificant	-0.016284	0.78738	insignificant	8	30	30
chr8	91044242	91046242	Nkd1	-0.10875163	3.6E-27	hypomethylated	0.0013916	0.53386	insignificant	66	216	209
chr8	91170245	91172245	Nod2	-0.30539535	0.00000184	hypomethylated	0.014045	0.29211	insignificant	3	14	14
chr8	91219926	91221926	Cyld	-0.10713359	0.39557	insignificant	0.0033091	0.8851	insignificant	11	65	65
chr8	91219942	91221942	Cyld	-0.10713359	0.39557	insignificant	0.0033091	0.8851	insignificant	11	65	65
chr8	91568061	91570061	Sall1	-0.11268537	0.26541	insignificant	-0.0035454	0.95662	insignificant	4	41	41
chr8	92872151	92874151	Tox3	-0.17247485	4.98E-15	hypomethylated	-0.051532	0.85874	insignificant	30	81	78
chr8	93351733	93353733	Chd9	-0.05184541	0.86013	insignificant	-0.00069761	0.17024	insignificant	11	33	33
chr8	93592992	93594992	Rbl2	-0.15127149	0.15211	insignificant	-0.025658	0.083588	insignificant	11	72	71
chr8	93836423	93838423	Fto	-0.10788517	0.0020267	hypomethylated	0.014613	0.44938	insignificant	4	36	34
chr8	93837121	93839121	Fto	-0.11868813	0.21951	insignificant	0.021536	0.67018	insignificant	1	22	22
chr8	94325273	94327273	Irx3	-0.18549497	7.27E-34	hypomethylated	0.023659	0.060572	insignificant	21	85	85
chr8	94880019	94882019	4933436C20Ri	-0.12819105	4.45E-11	hypomethylated	0.013034	0.39309	insignificant	25	136	131
chr8	94880694	94882694	Irx5	-0.11605153	5.39E-12	hypomethylated	-0.0017047	0.47972	insignificant	38	203	183
chr8	95197300	95199300	Irx6	-0.17511007	1.35E-16	hypomethylated	0.02139	0.56212	insignificant	21	94	86
chr8	95350226	95352226	Mmp2	-0.1388597	4.59E-10	hypomethylated	-0.027972	0.38766	insignificant	13	53	53
chr8	95378248	95380248	Lpcat2	-0.18474211	1.62E-22	hypomethylated	-0.013408	2.77E-09	inconclusive	21	61	58
chr8	95483945	95485945	Slc6a2	-0.18909958	4.36E-19	hypomethylated	-0.0026575	0.91326	insignificant	39	107	105
chr8	96333737	96335737	Gnao1	-0.10382317	5.71E-39	hypomethylated	0.017535	0.58412	insignificant	71	237	235
chr8	96337455	96339455	Gnao1	-0.05313942	0.063798	insignificant	0.095009	0.1812	insignificant	5	16	10
chr8	96536540	96538540	Amfr	-0.08616568	3.82E-13	hypomethylated	0.025222	0.71688	insignificant	14	54	52
chr8	96560097	96562097	Ogfod1	-0.15759819	8.24E-14	hypomethylated	-0.014274	0.48351	insignificant	21	77	73
chr8	96560939	96562939	Nudt21	-0.15719019	0.00000115	hypomethylated	-0.019493	0.58439	insignificant	12	38	39
chr8	96660103	96662103	Mt4	-0.11842105	1	insignificant	-0.024671	0.62038	insignificant	2	4	4
chr8	96675506	96677506	Mt3	-0.29299168	1.65E-10	hypomethylated	0.16286	0.54369	insignificant	2	16	16
chr8	96695517	96697517	Mt2	-0.16771623	2.15E-40	hypomethylated	-0.017454	0.42684	insignificant	19	93	103
chr8	96701988	96703988	Mt1	-0.17156056	0.000000578	hypomethylated	0.01205	0.38139	insignificant	13	59	59
chr8	96737500	96739500	Nup93	-0.05392007	0.22658	insignificant	0.0061101	0.18443	insignificant	17	76	76
chr8	96847210	96849210	Mir138-2	-0.02333956	0.32566	insignificant	0.15703	0.00065394	hypermethylated	3	16	16
chr8	96909399	96911399	Herpud1	-0.25458126	6.42E-14	hypomethylated	-0.11028	0.24999	insignificant	12	64	71
chr8	96959003	96961003	9330175E14Ri	-0.28837865	0.15146	insignificant	0.024685	0.68467	insignificant	3	22	22
chr8	96995662	96997662	Nlrc5	0.16481481	1	insignificant	0.055118	0.45274	insignificant	1	18	10
chr8	97055927	97057927	Cpne2	-0.08228213	9.92E-19	hypomethylated	0.012339	0.016148	hypermethylated	43	118	118
chr8	97124840	97126840	Rspry1	-0.08716875	1.95E-25	hypomethylated	0.011372	0.3563	insignificant	37	162	149
chr8	97125626	97127626	Fam192a	-0.09483359	1.89E-20	hypomethylated	-0.0021127	0.75887	insignificant	17	91	91
chr8	97189654	97191654	Arl2bp	-0.13686472	5.79E-21	hypomethylated	-0.020673	0.85538	insignificant	22	121	109
chr8	97190005	97192005	Arl2bp	-0.13686472	5.79E-21	hypomethylated	-0.020673	0.85538	insignificant	22	121	109
chr8	97268583	97270583	Ccl22	0.12409812	1	insignificant	-0.17479	1	insignificant	2	14	14
chr8	97295079	97297079	Cx3cl1	-0.18582424	0.000000137	hypomethylated	0.010841	0.6899	insignificant	6	28	28
chr8	97333352	97335352	Ccl17	-0.25393357	0.082679	insignificant	0.057765	1	insignificant	2	4	4
chr8	97361316	97363316	Coq9	-0.11179282	7.83E-11	hypomethylated	0.020338	0.64452	insignificant	28	135	122
chr8	97362240	97364240	Ciapin1	-0.1101262	1.11E-08	hypomethylated	0.027627	0.67102	insignificant	17	62	62
chr8	97380349	97382349	Polr2c	-0.14402808	0.1616	insignificant	-0.012442	0.25161	insignificant	21	84	81
chr8	97400212	97402212	Dok4		1	noCoverage	0.017467	0.60206	insignificant	0	16	18
chr8	97441998	97443998	Ccdc102a		1	noCoverage	0.017999	0.26293	insignificant	0	14	14
chr8	97446593	97448593	Gpr114	-0.48489148	0.23101	insignificant	-0.0052804	0.55692	insignificant	2	14	10
chr8	97500008	97502008	Gpr56	-0.26976363	0.066383	insignificant	-0.01478	0.80123	insignificant	3	14	14

chr8	97507298	97509298	Gpr56		1	noCoverage	-0.074234	0.86122	insignificant	0	12	14
chr8	97604100	97606100	Katnb1	-0.19329271	3.27E-10	hypomethylated	0.011249	0.72377	insignificant	12	48	42
chr8	97666440	97668440	Kifc3	-0.36569733	0.53357	insignificant	0.032822	0.76383	insignificant	2	16	16
chr8	97855183	97857183	AA960436	-0.10683216	2.69E-38	hypomethylated	0.018511	0.97701	insignificant	57	163	157
chr8	97855850	97857850	Zfp319	-0.15721649	2.86E-10	hypomethylated	0.035801	0.50153	insignificant	23	81	87
chr8	97875236	97877236	Mmp15	-0.13774086	5.73E-50	hypomethylated	0.0071642	0.32723	insignificant	44	164	164
chr8	97958769	97960769	Gtl3	-0.21163217	9.4E-09	hypomethylated	-0.058438	0.4849	insignificant	8	46	44
chr8	98012720	98014720	Csnk2a2	-0.2279322	5.78E-52	hypomethylated	0.056635	0.0086915	hypermethylated	12	26	29
chr8	98056999	98058999	Ccdc113	0.06050014	0.73061	insignificant	0.063613	0.0071726	hypermethylated	5	22	20
chr8	98099097	98101097	Prss54		1	noCoverage	0.063835	0.42441	insignificant	0	4	4
chr8	98156458	98158458	Gins3	-0.15151514	1.26E-12	hypomethylated	0.0090978	0.10211	insignificant	16	88	90
chr8	98225936	98227936	Ndrg4	-0.24033044	0.043148	hypomethylated	0.012831	1	insignificant	4	42	40
chr8	98238812	98240812	Setd6	-0.1135453	0.00011826	hypomethylated	0.016797	0.28481	insignificant	17	50	48
chr8	98329729	98331729	4930513N10R	-0.1325815	3.1E-21	hypomethylated	0.0082421	0.9016	insignificant	34	156	145
chr8	98331366	98333366	4930513N10R	-0.13502889	0.000047135	hypomethylated	0.012906	0.44827	insignificant	10	28	28
chr8	98377391	98379391	Slc38a7	0.23183681	0.39586	insignificant	-0.10511	0.35171	insignificant	3	17	21
chr8	101940357	101942357	Cdh8	0.13208285	1	insignificant	0.16809	0.0045208	hypermethylated	1	32	27
chr8	105309011	105311011	Cdh11	-0.24175691	1.12E-25	hypomethylated	0.031522	1	insignificant	11	52	52
chr8	106624524	106626524	Cdh5		1	noCoverage	0.27518	0.26991	insignificant	0	7	12
chr8	106693412	106695412	Bean1	-0.13447719	5.24E-14	hypomethylated	0.003488	0.039594	hypermethylated	23	81	96
chr8	106772458	106774458	Cklf	-0.45552725	2.68E-08	stronglyHypometh	0.018052	0.51703	insignificant	9	25	25
chr8	106773802	106775802	Cklf	-0.27095335	9.83E-19	hypomethylated	0.018928	0.029085	hypermethylated	11	39	39
chr8	106773831	106775831	Cklf	-0.27095335	9.83E-19	hypomethylated	0.018928	0.029085	hypermethylated	11	39	39
chr8	106817081	106819081	Cmtm1		1	noCoverage	-0.067974	0.878	insignificant	0	9	9
chr8	106845136	106847136	Cmtm2b	0.08474905	0.025115	hypermethylated	0.031373	0.012142	hypermethylated	7	27	27
chr8	106863493	106865493	Cmtm3	-0.15912947	3.49E-16	hypomethylated	-0.0021626	0.22079	insignificant	24	121	124
chr8	106919596	106921596	Cmtm4	-0.23460875	7.86E-10	hypomethylated	-0.030025	0.56918	insignificant	6	52	52
chr8	107033787	107035787	Ccdc79	-0.17385546	0.59645	insignificant	0.038697	0.1678	insignificant	3	14	14
chr8	107058537	107060537	Nae1	-0.11597405	1	insignificant	0.017983	0.36608	insignificant	6	18	18
chr8	107063706	107065706	Car7	-0.13200235	1.81E-12	hypomethylated	0.0018574	0.61419	insignificant	15	67	71
chr8	107114367	107116367	Pdp2	0.1391165	1	insignificant	0.033422	0.72224	insignificant	2	18	15
chr8	107165628	107167628	Fam96b	-0.06545776	1	insignificant	0.0096632	0.27841	insignificant	3	19	19
chr8	107354543	107356543	Ces2b	0.07332041	0.41369	insignificant	-0.15777	0.82661	insignificant	1	8	6
chr8	107470255	107472255	Ces2f	-0.85160256	0.19231	lowCoverage	-0.045157	0.29242	insignificant	1	12	12
chr8	107693573	107695573	Cbfb	-0.08391488	1.98E-41	hypomethylated	0.0028268	0.35262	insignificant	73	198	176
chr8	107748087	107750087	D230025D16R	-0.18662343	4.24E-23	hypomethylated	-0.0096088	1	insignificant	18	64	71
chr8	107779051	107781051	B3gnt9-ps	0.22853147	1	lowCoverage	-0.018328	1	insignificant	1	5	6
chr8	107787547	107789547	Fbxl8	-0.0477491	3.64E-11	hypomethylated	-0.0037609	0.040169	hypomethylated	18	68	65
chr8	107788494	107790494	Fbxl8	-0.12226007	1.66E-09	hypomethylated	0.025038	0.48468	insignificant	8	27	22
chr8	107792773	107794773	Hsf4	-0.17742881	2.34E-29	hypomethylated	-0.0084213	0.62636	insignificant	31	118	114
chr8	107799346	107801346	Nol3	-0.24264792	0.016293	hypomethylated	0.04671	0.56188	insignificant	4	24	24
chr8	107813428	107815428	4931428F04Ri	-0.14692202	0.00011756	hypomethylated	0.02731	0.022228	inconclusive	9	76	68
chr8	107819998	107821998	E2f4	-0.15847094	1.42E-27	hypomethylated	0.017031	0.16133	insignificant	33	90	83
chr8	107820562	107822562	E2f4	-0.18562938	2.74E-22	hypomethylated	0.016694	0.016286	hypermethylated	31	93	86
chr8	107828500	107830500	Elmo3	-0.31096308	0.10786	insignificant	0.017309	0.0792	insignificant	4	14	14
chr8	107849263	107851263	Tmem208	-0.21819176	0.001392	hypomethylated	-0.034941	0.55223	insignificant	8	40	35
chr8	107850176	107852176	Tmem208	-0.39121698	0.053729	insignificant	-0.011731	0.75663	insignificant	4	32	32
chr8	107871157	107873157	Slc9a5	-0.15367822	1.96E-13	hypomethylated	-0.00085994	0.26631	insignificant	14	95	94
chr8	107871870	107873870	Fhod1	-0.1271622	1.72E-19	hypomethylated	-0.0053991	0.32422	insignificant	27	97	101
chr8	107898280	107900280	Plekhg4	0.06016192	0.00092176	inconclusive	0.027896	0.13186	insignificant	24	90	90
chr8	107936521	107938521	Lrrc36	0.07397025	0.043329	hypermethylated	0.010045	0.08957	insignificant	4	23	29
chr8	107937387	107939387	Lrrc36	0.05005482	0.043688	hypermethylated	-0.01387	0.097779	insignificant	4	24	29
chr8	107995322	107997322	Tppp3	0.05919403	0.52839	insignificant	-0.020945	1	insignificant	5	26	26
chr8	108020770	108022770	Zdhhc1	-0.1524822	0.015227	hypomethylated	0.027381	0.28846	insignificant	9	21	21
chr8	108041645	108043645	Hsd11b2	-0.13871635	3.02E-11	hypomethylated	0.0047595	0.36261	insignificant	24	82	88
chr8	108089940	108091940	Atp6v0d1	-0.18546216	2.94E-12	hypomethylated	0.0047789	0.2095	insignificant	11	54	50
chr8	108092198	108094198	Agrp	-0.77066642	0.20364	lowCoverage	0.02309	0.75718	insignificant	1	16	16
chr8	108128128	108130128	Fam65a	-0.07011168	4.1E-12	hypomethylated	0.011498	0.071219	insignificant	39	118	124
chr8	108138365	108140365	Mir1966	0.07357232	1	insignificant	-0.0067209	0.59153	insignificant	4	26	26
chr8	108159437	108161437	Ctcf	-0.05449343	8.27E-26	hypomethylated	0.00022453	0.15994	insignificant	93	309	301
chr8	108213805	108215805	Rltpr	-0.12356464	1	insignificant	-0.084307	0.29946	insignificant	3	20	14
chr8	108224053	108226053	Pard6a	-0.17973526	2.07E-31	hypomethylated	0.0061449	0.85645	insignificant	20	110	104
chr8	108224548	108226548	Pard6a	-0.17415299	1.05E-30	hypomethylated	0.0065794	0.58024	insignificant	20	86	87
chr8	108224995	108226995	Pard6a	-0.2336898	7.35E-34	hypomethylated	-0.019216	0.4766	insignificant	12	68	69
chr8	108231202	108233202	4933405L10Ri	-0.23146811	1	insignificant	0.010955	0.036993	hypermethylated	2	42	42
chr8	108232068	108234068	E130303B06Ri	-0.54428967	0.84723	insignificant	0.012651	0.029597	hypermethylated	3	23	29
chr8	108282507	108284507	Gfod2	-0.31291039	0.0029234	hypomethylated	0.050668	0.86722	insignificant	8	44	45
chr8	108350643	108352643	Tsnaxip1	-0.17109909	0.00000014	hypomethylated	0.0058326	0.14025	insignificant	9	48	46
chr8	108351250	108353250	Tsnaxip1	-0.15159008	0.010981	hypomethylated	0.0060453	0.337	insignificant	2	14	12
chr8	108375908	108377908	Cenpt		1	noCoverage	0.090067	0.36385	insignificant	0	16	20
chr8	108378002	108380002	Thap11	-0.15427226	3.92E-39	hypomethylated	0.010056	0.24902	insignificant	36	88	88
chr8	108383533	108385533	Nutf2	-0.16531791	1.99E-17	hypomethylated	0.0032026	1	insignificant	23	64	64
chr8	108383559	108385559	Nutf2-ps1	-0.16531791	1.99E-17	hypomethylated	0.0032026	1	insignificant	23	64	64
chr8	108403850	108405850	Edc4	-0.12149804	1.16E-08	hypomethylated	0.019772	0.51711	insignificant	12	50	50
chr8	108416469	108418469	Nrn1l	-0.10124049	0.10285	insignificant	-0.047136	1	insignificant	4	12	12
chr8	108423373	108425373	Pskh1	-0.23546036	0.00003005	hypomethylated	-0.093136	0.83114	insignificant	8	40	38
chr8	108457762	108459762	Ctrl	-0.22928807	0.000000574	hypomethylated	-0.02106	0.4735	insignificant	3	10	12
chr8	108467302	108469302	Lcat	-0.19292056	0.0011174	hypomethylated	0.0058462	0.71953	insignificant	8	18	18
chr8	108489939	108491939	Slc12a4	-0.09765967	1.06E-09	hypomethylated	-0.018303	0.80242	insignificant	14	52	50
chr8	108520323	108522323	Dpep2		1	noCoverage	-0.0085784	0.52609	insignificant	0	4	4
chr8	108534406	108536406	Dus2l	-0.13714336	8.31E-19	hypomethylated	0.0086241	0.32436	insignificant	22	122	116
chr8	108535386	108537386	Ddx28	-0.41321365	0.000075774	stronglyHypometh	-0.045562	0.23252	insignificant	7	40	36
chr8	108582502	108584502	Nfatc3	-0.15283373	1.64E-22	hypomethylated	0.0013018	0.17125	insignificant	33	144	134
chr8	108660874	108662874	Esrp2	-0.19320635	0.00000181	hypomethylated	0.016861	0.77976	insignificant	11	54	55
chr8	108673298	108675298	Pla2g15	-0.23538224	7.48E-26	hypomethylated	0.023186	0.64734	insignificant	12	50	49

chr8	108691774	108693774	Slc7a6	-0.11585712	7.13E-23	hypomethylated	0.020876	0.1561	insignificant	21	109	90
chr8	108733953	108735953	Prmt7	-0.08231639	6.14E-15	hypomethylated	0.00059687	0.82254	insignificant	40	149	149
chr8	108734833	108736833	Slc7a6os	-0.10102408	0.1804	insignificant	0.0019726	0.9699	insignificant	26	74	74
chr8	108861888	108863888	Smpd3	-0.11846171	0.087155	insignificant	0.020697	0.79261	insignificant	4	10	10
chr8	108938238	108940238	Zfp90	-0.23818124	8.72E-12	hypomethylated	0.069734	0.47294	insignificant	13	54	46
chr8	109033790	109035790	Cdh3	-0.17563419	6.52E-11	hypomethylated	-0.010977	0.5956	insignificant	17	87	87
chr8	109033812	109035812	Cdh3	-0.17519173	3.1E-13	hypomethylated	-0.01005	0.68446	insignificant	18	89	89
chr8	109126267	109128267	Cdh1	-0.12524083	5.77E-19	hypomethylated	0.0051888	0.53914	insignificant	35	123	120
chr8	109205967	109207967	Tmco7	-0.08590743	0.0065508	hypomethylated	-0.0044604	0.75672	insignificant	14	58	58
chr8	109393141	109395141	Has3	-0.13652451	1.67E-20	hypomethylated	0.016085	0.56996	insignificant	23	78	83
chr8	109416539	109418539	Cirh1a	-0.11996833	5.2E-23	hypomethylated	0.025208	0.46681	insignificant	31	149	126
chr8	109417493	109419493	Chtf8	-0.38872953	0.00029007	stronglyHypometh	0.10582	0.0027198	inconclusive	2	29	20
chr8	109458649	109460649	Sntb2	-0.1259783	4.26E-17	hypomethylated	0.0013555	0.93072	insignificant	27	154	151
chr8	109554225	109556225	Vps4a	-0.11910929	1.26E-29	hypomethylated	-0.0034373	0.63655	insignificant	39	120	122
chr8	109572514	109574514	Cog8	-0.09936205	0.0052908	hypomethylated	0.011449	0.25151	insignificant	5	10	10
chr8	109579776	109581776	Nip7	-0.15113014	6.75E-27	hypomethylated	-0.0015012	0.62635	insignificant	55	200	200
chr8	109580637	109582637	Cog8	-0.19154399	1.26E-20	hypomethylated	-0.0057402	0.55507	insignificant	29	116	116
chr8	109673560	109675560	Cyb5b	-0.16236037	3.49E-11	hypomethylated	0.0010858	0.4886	insignificant	20	87	75
chr8	109816369	109818369	Nfat5	-0.11968412	4.09E-31	hypomethylated	0.0091802	0.77628	insignificant	60	200	200
chr8	109927105	109929105	Nqo1	-0.60619782	1	lowCoverage	0.022225	0.61081	insignificant	1	18	18
chr8	109948938	109950938	Nob1	-0.3525528	2.97E-45	stronglyHypometh	-0.069647	0.16845	insignificant	11	69	64
chr8	109959297	109961297	Wwp2	-0.14680401	8.06E-11	hypomethylated	-0.033453	0.055062	insignificant	17	65	66
chr8	111237543	111239543	Zfhx3	-0.129146	2.27E-38	hypomethylated	0.0090777	0.0016221	hypermethylated	62	170	170
chr8	111459759	111461759	Mir3108	0.0656586	0.080778	insignificant	-0.00625	0.86486	insignificant	2	8	8
chr8	111864808	111866808	Gm1943	-0.27825746	0.2296	insignificant	0.016385	1	insignificant	1	7	8
chr8	112088885	112090885	Txnl4b	-0.11829193	0.10138	insignificant	-0.0006165	0.90398	insignificant	9	93	92
chr8	112089501	112091501	Dhx38	-0.12087599	1	insignificant	-0.0012739	0.73097	insignificant	3	50	50
chr8	112132573	112134573	Dhodh	-0.14952271	0.0001064	hypomethylated	0.0024978	1	insignificant	8	23	22
chr8	112137416	112139416	Pkd1l3		1	noCoverage	0.13954	0.15507	insignificant	0	30	31
chr8	112217194	112219194	2400003C14Ri	-0.13322835	8.92E-11	hypomethylated	0.021775	0.00029884	hypermethylated	13	27	27
chr8	112228448	112230448	Zfp821	-0.09640089	6.86E-22	hypomethylated	0.0048168	0.4303	insignificant	32	129	129
chr8	112228987	112230987	Zfp821	-0.09640089	6.86E-22	hypomethylated	0.0048168	0.4303	insignificant	32	129	129
chr8	112301539	112303539	Ap1g1	-0.10776958	7.42E-35	hypomethylated	0.015374	0.26683	insignificant	45	140	140
chr8	112391502	112393502	Phlpp2	-0.1185114	1.66E-27	hypomethylated	0.013985	0.56826	insignificant	53	154	157
chr8	112486105	112488105	Marveld3	-0.54298139	4.65E-22	stronglyHypometh	0.043933	0.28948	insignificant	5	30	30
chr8	112602633	112604633	Zfp612	-0.09187978	3.71E-08	hypomethylated	0.032502	0.35889	insignificant	6	61	61
chr8	112692106	112694106	Calb2	-0.14147546	0.00000587	hypomethylated	0.019164	0.20213	insignificant	13	62	56
chr8	112740859	112742859	Ftsjd1	-0.11401234	0.00000273	hypomethylated	0.060105	0.3217	insignificant	9	34	26
chr8	113141537	113143537	Vac14	-0.19947283	1.37E-13	hypomethylated	0.012671	0.41817	insignificant	19	71	67
chr8	113244383	113246383	Mtss1l	-0.10744223	5.38E-29	hypomethylated	-0.0099296	0.44942	insignificant	41	102	102
chr8	113329803	113331803	Il34	-0.64285714	0.28261	lowCoverage	0.040476	0.3877	insignificant	1	2	2
chr8	113369923	113371923	Cog4	-0.18682653	9.43E-15	hypomethylated	0.026474	0.060347	insignificant	14	70	66
chr8	113370703	113372703	Sf3b3	-0.15157086	2.56E-11	hypomethylated	0.042741	0.81976	insignificant	5	22	18
chr8	113426359	113428359	Fuk		1	noCoverage	0.014827	0.83138	insignificant	0	9	9
chr8	113442764	113444764	St3gal2	-0.15933611	0.0034475	hypomethylated	0.0073956	0.22342	insignificant	9	64	66
chr8	113551670	113553670	Ddx19b	-0.13310082	0.1135	insignificant	0.012072	0.062941	insignificant	5	22	22
chr8	113555651	113557651	Ddx19b	0.06010953	0.627	insignificant	-0.034169	0.42412	insignificant	2	16	19
chr8	113556866	113558866	Aars	-0.18026001	0.18587	insignificant	0.0077702	0.073245	insignificant	15	52	52
chr8	113579238	113581238	Exosc6	-0.08365829	2.72E-24	hypomethylated	-0.0013641	0.13387	insignificant	52	155	149
chr8	113617644	113619644	Pdpr	-0.12698419	1.3E-09	hypomethylated	-0.0010236	0.31224	insignificant	25	135	148
chr8	113783102	113785102	Glg1	-0.25346275	0.00018107	hypomethylated	0.016571	0.48259	insignificant	14	74	74
chr8	113824122	113826122	Rfwd3		1	noCoverage	-0.010875	0.30992	insignificant	0	30	33
chr8	113861803	113863803	Mlkl	-0.15095924	1.55E-21	hypomethylated	0.00011233	0.42321	insignificant	13	44	44
chr8	113917721	113919721	Fa2h	-0.14517835	2.76E-17	hypomethylated	0.012011	0.7321	insignificant	11	30	30
chr8	114046007	114048007	Wdr59		1	noCoverage	-0.077838	0.025237	hypomethylated	0	9	9
chr8	114059539	114061539	Znrf1	-0.11061511	2.45E-38	hypomethylated	-0.00022976	0.84672	insignificant	62	187	175
chr8	114061242	114063242	Znrf1	-0.12242243	3.88E-20	hypomethylated	0.015055	0.8284	insignificant	14	47	47
chr8	114154222	114156222	Ldhd	-0.25161466	0.18439	insignificant	-0.062168	0.02374	hypomethylated	9	22	22
chr8	114166342	114168342	Zfp1	-0.1369483	0.018141	hypomethylated	0.0010403	1	insignificant	5	42	42
chr8	114256036	114258036	Bcar1	-0.29850324	0.00025132	hypomethylated	-0.03712	0.46924	insignificant	8	26	24
chr8	114267749	114269749	Bcar1	-0.15132442	0.00000454	hypomethylated	-0.0072688	0.85136	insignificant	18	55	54
chr8	114378210	114380210	Cfdp1	0.31077197	0.31428	insignificant	0.15096	0.0047768	hypermethylated	2	24	34
chr8	114400575	114402575	Tmem170	-0.23984482	0.000000706	hypomethylated	0.015453	0.53089	insignificant	5	42	42
chr8	114434099	114436099	Chst5	-0.18499984	0.00075844	hypomethylated	0.14507	0.0097043	hypermethylated	7	26	29
chr8	114457691	114459691	Tmem231		1	noCoverage	-0.046683	0.93758	insignificant	0	19	19
chr8	114463617	114465617	Gabarapl2	-0.11762437	0.00030367	hypomethylated	-0.021208	0.93742	insignificant	17	116	112
chr8	114516202	114518202	Adat1	-0.58461538	0.000026361	stronglyHypometh	-0.30722	0.00013363	hypomethylated	2	10	4
chr8	114534258	114536258	Terf2ip	-0.10258483	9.86E-12	hypomethylated	-0.005871	0.70307	insignificant	17	110	110
chr8	114535205	114537205	Kars	-0.09210982	2.63E-09	hypomethylated	-0.0082545	0.58513	insignificant	16	100	100
chr8	115092942	115094942	Cntnap4	-0.49687751	0.09159	insignificant	-0.062881	0.77693	insignificant	5	17	14
chr8	116158485	116160485	Mon1b	-0.21693485	4.71E-17	hypomethylated	0.010161	0.24989	insignificant	24	67	61
chr8	116158698	116160698	Mon1b	-0.22845052	2.85E-17	hypomethylated	0.011111	0.44994	insignificant	24	68	62
chr8	116372739	116374739	Adamts18	-0.20300225	0.0018015	hypomethylated	-0.025804	0.39618	insignificant	7	14	14
chr8	116656478	116658478	Nudt7	-0.42276041	2.22E-12	stronglyHypometh	0.14935	0.65339	insignificant	2	19	11
chr8	116728539	116730539	Vat1l	-0.22341441	0.00000939	hypomethylated	-0.047784	0.77646	insignificant	6	50	48
chr8	116962551	116964551	Wwox	-0.16787712	4.03E-18	hypomethylated	0.0088274	0.65317	insignificant	7	48	48
chr8	118230794	118232794	Maf	-0.16579648	1.23E-27	hypomethylated	0.033388	0.31981	insignificant	30	108	104
chr8	119027914	119029914	Dynlrb2	-0.36355219	0.2896	insignificant	-0.0117	0.79084	insignificant	1	6	6
chr8	119256891	119258891	Cdyl2	-0.07690726	5.31E-10	hypomethylated	0.0023397	0.87179	insignificant	16	58	55
chr8	119444639	119446639	Cenpn	-0.10499241	9.17E-08	hypomethylated	-0.000065041	1	insignificant	22	84	84
chr8	119445336	119447336	Cenpn	-0.20140719	1.81E-11	hypomethylated	-0.014152	0.89066	insignificant	13	43	40
chr8	119466292	119468292	Atmin	-0.08825368	2.71E-13	hypomethylated	0.0015293	0.2901	insignificant	19	108	108
chr8	119502843	119504843	1700030J22Ri	-0.07824341	1.8E-09	hypomethylated	-0.012024	0.57641	insignificant	9	31	31
chr8	119517349	119519349	Gcsh	-0.24584423	0.000024631	hypomethylated	0.11115	0.026833	hypermethylated	14	31	34
chr8	119606349	119608349	Pkd1l2	-0.13134746	0.51429	insignificant	-0.047548	0.425	insignificant	5	16	16

chr8	119618764	119620764	Bcmo1		1	noCoverage	-0.049051	0.25871	insignificant	0	10	10
chr8	119681034	119683034	Gan	-0.10759914	1.13E-17	hypomethylated	-0.0026655	0.79984	insignificant	41	163	159
chr8	119779918	119781918	4933407C03Ri	-0.09891717	6.22E-27	hypomethylated	-0.0047942	1	insignificant	71	243	242
chr8	120021190	120023190	Plcg2	-0.16078448	6.76E-20	hypomethylated	0.023901	0.19833	insignificant	24	80	80
chr8	120325829	120327829	Mphosph6	-0.16609906	0.00030031	hypomethylated	-0.00083505	0.23073	insignificant	10	47	46
chr8	120806654	120808654	Cdh13	-0.19762531	6.65E-27	hypomethylated	-0.017787	0.032591	hypomethylated	20	70	66
chr8	121867437	121869437	Hsbp1	-0.1371923	0.0023607	hypomethylated	-0.0012232	0.1043	insignificant	27	88	88
chr8	121917791	121919791	Mlycd	-0.10471293	8.07E-15	hypomethylated	0.0041994	0.27149	insignificant	57	160	156
chr8	121960061	121962061	Osgin1	0.19455002	1	insignificant	-0.12694	0.59203	insignificant	3	13	10
chr8	121969618	121971618	Necab2	-0.10348934	2.73E-18	hypomethylated	-0.0015728	0.21656	insignificant	55	165	154
chr8	122025598	122027598	Slc38a8	-0.31699346	0.0068581	hypomethylated	-0.092387	0.75301	insignificant	2	11	12
chr8	122082661	122084661	Mbtps1	-0.09304089	0.47493	insignificant	0.020806	0.00017272	hypermethylated	16	44	44
chr8	122098134	122100134	Lrrc50	-0.10444436	2.86E-24	hypomethylated	-0.012724	0.88373	insignificant	28	139	140
chr8	122099100	122101100	Hsdl1	0.07017473	0.000022152	inconclusive	0.018313	0.91873	insignificant	13	52	52
chr8	122135646	122137646	Adad2	-0.03085519	0.078839	insignificant	-0.0090285	0.61533	insignificant	3	30	30
chr8	122189264	122191264	Wfdc1		1	noCoverage	0.012111	0.81147	insignificant	0	9	9
chr8	122222908	122224908	Atp2c2	-0.03234173	0.00022515	hypomethylated	0.02543	0.0011835	hypermethylated	16	42	47
chr8	122302316	122304316	4632415K11Ri	-0.32452487	0.20971	insignificant	-0.0015371	0.1045	insignificant	4	27	24
chr8	122364479	122366479	Cotl1		1	noCoverage	-0.0010598	0.89923	insignificant	0	12	12
chr8	122385204	122387204	Klhl36	-0.23096885	4.36E-39	hypomethylated	-0.019697	0.0046497	inconclusive	21	80	83
chr8	122433751	122435751	Usp10	-0.11430259	2.43E-08	hypomethylated	-0.0069174	0.017709	hypomethylated	33	136	124
chr8	122515369	122517369	Crispld2	-0.2379849	4.95E-10	hypomethylated	0.060712	0.020277	hypermethylated	4	53	51
chr8	122625372	122627372	Zdhhc7	-0.08307929	1.98E-21	hypomethylated	0.014399	0.015597	hypermethylated	16	82	82
chr8	122637051	122639051	6430548M08F	-0.08617211	3.95E-26	hypomethylated	-0.0043498	0.74603	insignificant	48	192	191
chr8	122667828	122669828	6430548M08F	0.01041667	0.57868	insignificant	0.082966	0.12865	insignificant	2	4	4
chr8	122701369	122703369	Fam92b	-0.2930757	0.2774	insignificant	-0.048539	0.42575	insignificant	1	10	10
chr8	123011765	123013765	Gse1	-0.10942082	1.18E-22	hypomethylated	0.004517	0.86685	insignificant	32	134	131
chr8	123058756	123060756	Gse1	-0.10178229	6.06E-38	hypomethylated	-0.0036453	0.75304	insignificant	46	180	180
chr8	123060308	123062308	Gse1	-0.10055779	2.52E-26	hypomethylated	-0.0069061	1	insignificant	56	202	206
chr8	123112975	123114975	Gins2	-0.16716592	0.000057631	hypomethylated	0.013683	0.67512	insignificant	9	42	42
chr8	123158282	123160282	1190005I06Ril	-0.17368143	8.48E-43	hypomethylated	-0.022304	0.00005635	inconclusive	12	51	49
chr8	123191189	123193189	Cox4i1	-0.10748946	4.64E-32	hypomethylated	0.0057616	0.40808	insignificant	47	147	150
chr8	123192012	123194012	Cox4nb	-0.16942043	4.69E-20	hypomethylated	0.026517	0.13325	insignificant	30	91	96
chr8	123259275	123261275	Irf8	-0.03469111	0.0074124	hypomethylated	-0.070494	0.20598	insignificant	4	52	60
chr8	123607373	123609373	Foxf1a	-0.12894668	3.13E-16	hypomethylated	0.007119	0.55332	insignificant	50	224	236
chr8	123639070	123641070	Foxc2	-0.12373738	0.00000095	hypomethylated	-0.00051411	0.26727	insignificant	31	135	131
chr8	123650584	123652584	Foxl1	-0.12249801	0.000012442	hypomethylated	-0.014666	0.80972	insignificant	16	65	64
chr8	124065854	124067854	1700018B08R	-0.02945266	0.20643	insignificant	0.059684	0.64215	insignificant	6	52	49
chr8	124102706	124104706	Fbxo31		1	noCoverage	-0.0016447	1	insignificant	0	8	8
chr8	124113367	124115367	Map1lc3b	-0.07528034	0.026673	hypomethylated	-0.00062635	0.75871	insignificant	14	58	63
chr8	124175833	124177833	Zcchc14	-0.10213416	4.83E-31	hypomethylated	-0.0021512	0.052704	insignificant	74	326	282
chr8	124253462	124255462	Jph3	-0.10971179	1.77E-35	hypomethylated	0.0046437	0.17768	insignificant	53	171	168
chr8	124353469	124355469	Klhdc4	-0.10701235	0.0014822	hypomethylated	0.020396	0.84568	insignificant	5	14	12
chr8	124430732	124432732	BC048644	-0.11714719	3.15E-19	hypomethylated	-0.0076934	0.64201	insignificant	29	116	116
chr8	124431586	124433586	Slc7a5	-0.22474747	1	insignificant	-0.040404	0.32158	insignificant	1	22	22
chr8	124468812	124470812	Car5a	0.13557902	1	insignificant	0.013357	0.68468	insignificant	3	9	11
chr8	124473432	124475432	Banp	-0.10888083	2.45E-26	hypomethylated	0.002458	0.00090288	hypermethylated	50	156	154
chr8	124805040	124807040	Zfpm1	-0.07880602	4.38E-29	hypomethylated	-0.0037225	0.18247	insignificant	54	235	218
chr8	124899515	124901515	Zc3h18	-0.08512051	6.41E-28	hypomethylated	0.01698	0.027429	hypermethylated	46	218	190
chr8	124944984	124946984	Il17c	-0.26215698	0.020951	hypomethylated	0.034836	0.24924	insignificant	4	16	16
chr8	124956840	124958840	Cyba	0.05040311	0.85446	insignificant	0.026231	0.29755	insignificant	4	27	27
chr8	124967322	124969322	Mvd	-0.19995719	2.69E-09	hypomethylated	-0.003525	0.87601	insignificant	11	44	43
chr8	124975222	124977222	LOC319574		1	noCoverage	-0.064835	0.61347	insignificant	0	14	14
chr8	124984592	124986592	Snai3		1	noCoverage	-0.0039683	0.7663	insignificant	0	24	24
chr8	124999042	125001042	Ctu2	-0.07233109	1.21E-21	hypomethylated	-0.0038353	0.62656	insignificant	68	157	154
chr8	124999964	125001964	Rnf166	-0.04606601	3.15E-14	hypomethylated	-0.00034466	0.60736	insignificant	44	100	98
chr8	125075229	125077229	Fam38a	-0.02427653	0.18015	insignificant	-0.012772	0.000000117	hypomethylated	13	53	52
chr8	125090914	125092914	Cdt1	-0.13359493	3.77E-35	hypomethylated	0.024241	0.5834	insignificant	17	82	82
chr8	125100807	125102807	Aprt	0.1729798	0.65787	insignificant	-0.23611	0.18868	insignificant	1	6	6
chr8	125134525	125136525	Trappc2l	-0.13076613	2.22E-19	hypomethylated	0.0088926	0.19597	insignificant	12	50	45
chr8	125135387	125137387	Galns	-0.15825115	2.79E-26	hypomethylated	0.010926	0.0020756	hypermethylated	12	52	47
chr8	125202075	125204075	Cbfa2t3	-0.12589046	1.24E-28	hypomethylated	0.0015327	0.69417	insignificant	35	140	138
chr8	125298404	125300404	Acsf3	-0.16570247	5.65E-09	hypomethylated	-0.031805	0.79601	insignificant	7	58	57
chr8	125565897	125567897	Ankrd11	-0.09206281	4.12E-60	hypomethylated	0.0037956	0.28738	insignificant	83	225	222
chr8	125588407	125590407	Spg7	-0.13575371	2.4E-37	hypomethylated	0.0051032	0.76204	insignificant	36	107	105
chr8	125625249	125627249	Rpl13	-0.12266282	6.57E-17	hypomethylated	0.059651	0.53638	insignificant	37	132	132
chr8	125625957	125627957	Snord68	-0.10045128	7.29E-17	hypomethylated	0.061135	0.87231	insignificant	39	138	138
chr8	125640273	125642273	Cpne7	-0.14009825	3.22E-19	hypomethylated	0.016642	5.21E-16	hypermethylated	27	137	133
chr8	125682180	125684180	Sult5a1	0.08743687	1	insignificant	-0.095764	0.20852	insignificant	2	12	12
chr8	125709134	125711134	Dpep1	0.12401226	0.78363	insignificant	0.066958	0.22143	insignificant	4	37	36
chr8	125735757	125737757	4732415M23F	-0.11138107	0.00002064	hypomethylated	0.010664	1	insignificant	29	103	100
chr8	125736688	125738688	Chmp1a	-0.18121372	3.36E-12	hypomethylated	0.036598	4.96E-08	hypermethylated	15	40	40
chr8	125747740	125749740	Cdk10		1	noCoverage	-0.0042818	0.28082	insignificant	0	67	67
chr8	125760109	125762109	Spata2L	-0.56276914	0.000000041	stronglyHypometh	-0.087454	0.15894	insignificant	2	16	16
chr8	125777094	125779094	Zfp276	-0.04868401	1.34E-24	hypomethylated	0.0042957	0.43079	insignificant	70	211	220
chr8	125778075	125780075	Zfp276	-0.04959217	0.000000687	hypomethylated	-0.021089	0.42287	insignificant	44	152	143
chr8	125842476	125844476	Fanca	-0.08396067	1	insignificant	0.038454	0.48317	insignificant	5	17	20
chr8	125855612	125857612	Spire2	-0.1130939	7.84E-21	hypomethylated	0.041081	0.000019417	hypermethylated	26	124	123
chr8	125896652	125898652	Tcf25	-0.12268681	7.18E-22	hypomethylated	-0.006119	0.89565	insignificant	21	103	91
chr8	125896723	125898723	Tcf25	-0.12268681	7.18E-22	hypomethylated	-0.006119	0.89565	insignificant	21	103	91
chr8	125896734	125898734	Tcf25	-0.12268681	7.18E-22	hypomethylated	-0.006119	0.89565	insignificant	21	103	91
chr8	125934463	125936463	Tubb3	-0.14047949	2.08E-13	hypomethylated	-0.023891	0.78513	insignificant	13	51	50
chr8	125965885	125967885	Def8	-0.21777593	3.72E-18	hypomethylated	0.00093581	0.40956	insignificant	13	50	46
chr8	126000761	126002761	Afg3l1	-0.061003	0.00082535	hypomethylated	0.032055	0.00010195	hypermethylated	11	84	36
chr8	126039355	126041355	Dbndd1	-0.33479197	0.27507	insignificant	0.025541	0.61243	insignificant	7	29	28

chr8	126041734	126043734	Gas8	-0.1141633	0.38244	insignificant	-0.015862	0.12027	insignificant	10	43	40
chr8	126176828	126178828	Rhou	-0.07946724	8.48E-30	hypomethylated	0.0022646	0.45757	insignificant	62	167	168
chr8	126328895	126330895	Rab4a	-0.19374628	1.26E-21	hypomethylated	0.013159	0.63736	insignificant	20	64	64
chr8	126418636	126420636	Acta1	-0.20764097	0.047127	hypomethylated	0.065628	1	insignificant	7	20	23
chr8	126473165	126475165	Nup133	-0.0673166	0.0019126	hypomethylated	-0.0071501	0.94086	insignificant	9	61	62
chr8	126507022	126509022	Abcb10	-0.14619342	0.00039909	hypomethylated	-0.019659	0.35122	insignificant	11	34	38
chr8	126545209	126547209	Taf5l	-0.08475184	1.23E-13	hypomethylated	-0.025624	0.47513	insignificant	19	97	94
chr8	126545540	126547540	Mir1967		1	noCoverage	-0.054329	0.58731	insignificant	0	16	14
chr8	126754293	126756293	Galnt2	-0.05765994	6.51E-13	hypomethylated	-0.00072052	0.034952	inconclusive	45	211	199
chr8	126957836	126959836	Pgbd5	-0.08837691	3.31E-31	hypomethylated	-0.0059289	0.53237	insignificant	62	182	180
chr8	127043666	127045666	Cog2	-0.08638438	1	insignificant	0.0011453	1	insignificant	4	28	28
chr8	127099010	127101010	Capn9	-0.11618172	1	insignificant	0.034352	0.34256	insignificant	4	16	14
chr8	127187269	127189269	2310022B05R	-0.12727273	1	insignificant	0.039394	1	insignificant	2	10	12
chr8	127245038	127247038	Arv1	-0.13909687	2.56E-34	hypomethylated	-0.011644	0.37606	insignificant	37	131	120
chr8	127245875	127247875	Ttc13	-0.09942518	6.16E-11	hypomethylated	-0.0068769	0.12312	insignificant	19	79	73
chr8	127275709	127277709	Fam89a	0.07808579	0.26491	insignificant	-0.032228	0.00000162	hypomethylated	8	47	45
chr8	127315918	127317918	Trim67	-0.09089619	8.5E-13	hypomethylated	0.013093	0.0054317	hypermethylated	36	138	138
chr8	127385932	127387932	Gnpat	-0.19595081	0.065918	insignificant	-0.0089562	0.66077	insignificant	5	30	30
chr8	127386929	127388929	2810004N23R	-0.1102659	0.10337	insignificant	0.029366	0.52178	insignificant	5	22	23
chr8	127420785	127422785	Gm505	-0.07408522	7.1E-27	hypomethylated	0.022446	0.097267	insignificant	44	217	170
chr8	127421605	127423605	Exoc8	-0.13168536	3.35E-12	hypomethylated	0.053121	0.68262	insignificant	19	112	73
chr8	127473154	127475154	Egln1	-0.13730267	4.03E-59	hypomethylated	-0.0021854	0.75316	insignificant	40	122	118
chr8	127535896	127537896	Tsnax	-0.11520224	1.2E-36	hypomethylated	0.010691	0.00000701	hypermethylated	52	143	126
chr8	127577094	127579094	Disc1	-0.11824974	6.48E-19	hypomethylated	0.019464	0.22463	insignificant	21	84	94
chr8	128016610	128018610	Sipa1l2	-0.30922865	0.27879	insignificant	0.068044	0.66709	insignificant	1	11	8
chr8	128192717	128194717	4933403G14R	-0.11900994	1.16E-09	hypomethylated	-0.034616	0.087705	insignificant	30	98	95
chr8	128257102	128259102	Ntpcr	-0.29379238	1.81E-25	hypomethylated	0.0026161	0.75183	insignificant	16	38	38
chr8	128422193	128424193	Pcnxl2	-0.12789352	0.64478	insignificant	0.063283	0.64961	insignificant	2	4	4
chr8	128433349	128435349	BC021891	-0.13442643	1.33E-39	hypomethylated	0.019243	0.000084361	hypermethylated	46	204	196
chr8	128518001	128520001	Kcnk1	-0.09359867	3.29E-10	hypomethylated	0.00035503	0.7398	insignificant	25	63	59
chr8	128821478	128823478	Slc35f3	-0.10294916	1.49E-23	hypomethylated	0.010207	0.07423	insignificant	20	100	93
chr8	128945400	128947400	1810063B05R	-0.17981675	3.52E-12	hypomethylated	-0.00038893	1	insignificant	25	84	81
chr8	128998965	129000965	Gm17296	-0.09116327	2.57E-21	hypomethylated	0.017299	0.075668	insignificant	14	48	48
chr8	129117336	129119336	Irf2bp2	-0.07951318	1.28E-37	hypomethylated	0.00031256	0.6518	insignificant	67	281	277
chr8	129469730	129471730	Tomm20	-0.09941003	0.0002705	hypomethylated	0.0055315	0.00082827	hypermethylated	6	28	27
chr8	129494979	129496979	Rbm34	-0.11327031	0.020801	hypomethylated	0.042247	0.55013	insignificant	3	6	6
chr8	129586955	129588955	Pard3	-0.10691463	1.52E-34	hypomethylated	-0.0064556	0.12161	insignificant	52	209	212
chr8	129822511	129824511	Pard3	0.01175595	0.15999	insignificant	0.088371	0.019791	hypermethylated	7	12	12
chr8	129822551	129824551	Pard3	0.01175595	0.15999	insignificant	0.088371	0.019791	hypermethylated	7	12	12
chr8	130881972	130883972	Nrp1	-0.19930657	0.000000609	hypomethylated	-0.029005	0.060328	insignificant	17	68	74
chr8	130882140	130884140	Mir1903	-0.19613823	0.00000107	hypomethylated	-0.030391	0.090911	insignificant	17	64	70
chr8	131208553	131210553	Itgb1	-0.06715492	1.12E-11	hypomethylated	0.0065491	0.087298	insignificant	35	155	157
chr9	3199813	3201813	4930433N12R	-0.27040969	0.00047489	hypomethylated	0.054236	0.13137	insignificant	2	14	21
chr9	3403084	3405084	Cwf19l2	-0.23395744	0.01959	hypomethylated	-0.0013929	0.56171	insignificant	4	30	26
chr9	3531353	3533353	Gucy1a2	-0.37156385	0.0027385	stronglyHypometh	-0.034407	0.58124	insignificant	2	8	8
chr9	4308742	4310742	Kbtbd3	-0.12066911	7E-48	hypomethylated	0.0076314	0.43423	insignificant	43	140	142
chr9	4308815	4310815	Kbtbd3	-0.12066911	7E-48	hypomethylated	0.0076314	0.43423	insignificant	43	140	142
chr9	4309494	4311494	Aasdhppt	-0.13321547	1.36E-23	hypomethylated	0.019473	0.10191	insignificant	23	78	80
chr9	6167611	6169611	Pdgfd	-0.27724131	0.34529	insignificant	-0.03332	0.50203	insignificant	3	18	24
chr9	7177046	7179046	Dync2h1	-0.24728419	0.000016926	hypomethylated	-0.049912	0.19916	insignificant	2	16	16
chr9	7183565	7185565	Dcun1d5	-0.17055192	1.39E-15	hypomethylated	-0.00090054	0.37343	insignificant	16	56	56
chr9	7346373	7348373	Mmp12	-0.14697712	0.30504	insignificant	-0.096021	0.32652	insignificant	2	6	6
chr9	7444821	7446821	Mmp3	0.1520979	1	lowCoverage	0.13739	0.0063539	hypermethylated	1	4	4
chr9	7570457	7572457	Mmp27		1	noCoverage	0.08456	0.3194	insignificant	0	6	6
chr9	7763076	7765076	Tmem123	-0.12655075	0.0025923	hypomethylated	0.0030781	0.69536	insignificant	3	28	28
chr9	7835255	7837255	Birc2	-0.19040731	6.99E-16	hypomethylated	0.020325	0.12625	insignificant	16	41	38
chr9	8004596	8006596	Yap1	-0.12125251	9.98E-09	hypomethylated	0.053714	0.40945	insignificant	11	71	54
chr9	8042823	8044823	9230110C19R	0.13088335	1	insignificant	0.18442	0.037411	hypermethylated	1	19	18
chr9	8134294	8136294	AK129341	-0.05150901	0.0011091	hypomethylated	-0.00054854	0.1947	insignificant	5	46	46
chr9	8220888	8222888	1700128F08R	0.12078991	1	insignificant	0.016447	0.073586	insignificant	1	20	20
chr9	8543141	8545141	Trpc6	-0.11586413	0.00000522	hypomethylated	0.016019	0.48087	insignificant	7	29	29
chr9	8898832	8900832	Pgr	-0.20117932	1.04E-08	hypomethylated	0.052169	0.18253	insignificant	5	30	30
chr9	9239013	9241013	Arhgap42	-0.20433135	0.00010891	hypomethylated	0.021347	0.85518	insignificant	11	45	43
chr9	13233580	13235580	Phxr4	-0.61282051	0.010896	stronglyHypometh	-0.092754	0.82193	insignificant	1	3	2
chr9	13552624	13554624	Mtmr2	-0.07794662	4.42E-15	hypomethylated	0.0095834	0.53331	insignificant	16	91	83
chr9	13631170	13633170	Fam76b	-0.11429689	1.31E-17	hypomethylated	0.0042486	0.45473	insignificant	18	93	96
chr9	13631497	13633497	Cep57	-0.14378411	3.96E-18	hypomethylated	0.010017	0.21542	insignificant	15	57	60
chr9	13631551	13633551	Cep57	-0.14378411	3.96E-18	hypomethylated	0.010017	0.21542	insignificant	15	57	60
chr9	14079744	14081744	Sesn3	-0.15738216	3.98E-28	hypomethylated	0.053671	0.00034492	hypermethylated	21	109	84
chr9	14185686	14187686	Endod1	-0.19787892	0.0011992	hypomethylated	-0.023719	0.10574	insignificant	3	18	18
chr9	14304062	14306062	Cwc15	-0.13874386	1.38E-12	hypomethylated	0.016624	0.20065	insignificant	29	88	86
chr9	14304926	14306926	Kdm4d	-0.14605762	1.51E-11	hypomethylated	0.024341	0.12883	insignificant	24	62	60
chr9	14419444	14421444	Amotl1	-0.16796968	9.23E-13	hypomethylated	-0.0076482	0.3148	insignificant	11	24	24
chr9	14556566	14558566	Fut4	-0.16290043	0.019426	hypomethylated	-0.01325	0.6601	insignificant	3	14	14
chr9	14575474	14577474	1700012B09R	-0.28401361	0.000000296	hypomethylated	0.052199	0.00000332	inconclusive	2	7	4
chr9	14587408	14589408	Ankrd49	-0.12167613	0.000000262	hypomethylated	0.011531	0.86186	insignificant	14	42	38
chr9	14588150	14590150	Mre11a	-0.12167613	0.000000262	hypomethylated	0.011531	0.86186	insignificant	14	42	38
chr9	14663697	14665697	Gpr83	-0.16869727	0.0027189	hypomethylated	0.04403	0.21431	insignificant	16	77	79
chr9	14708395	14710395	Folr4	0.36746032	0.27676	insignificant	0.010136	0.036157	hypermethylated	1	7	8
chr9	14849922	14851922	Panx1	-0.26044842	5.23E-08	hypomethylated	-0.0061393	0.43067	insignificant	14	36	35
chr9	14916552	14918552	Hephl1		1	noCoverage	0.15	0.63282	insignificant	0	2	2
chr9	15084311	15086311	Med17	-0.11016403	0.00000163	hypomethylated	-0.0011896	0.84602	insignificant	9	35	35
chr9	15109657	15111657	Taf1d	-0.21862417	1.08E-17	hypomethylated	0.0038127	0.85388	insignificant	20	130	129
chr9	15110611	15112611	4931406C07R	-0.12938838	2.88E-11	hypomethylated	0.0028001	0.66929	insignificant	16	107	107
chr9	15110892	15112892	4931406C07R	-0.18826715	0.0078489	hypomethylated	0.004891	0.69808	insignificant	7	60	60

chr9	15162232	15164232	5830418K08Ri	-0.09904102	0.061056	insignificant	0.0090588	0.74717	insignificant	6	24	24
chr9	15513212	15515212	Slc36a4	-0.09754232	9.86E-22	hypomethylated	0.0089009	0.066721	insignificant	32	97	92
chr9	18095710	18097710	Chordc1	-0.12296149	1.3E-12	hypomethylated	-0.0069629	0.85101	insignificant	21	89	80
chr9	18278003	18280003	Zfp558	-0.1165466	0.000000292	hypomethylated	0.02036	0.84563	insignificant	8	31	28
chr9	18791433	18793433	Olfr834	-0.38131868	0.0043978	stronglyHypometh	-0.025784	0.67542	insignificant	1	5	2
chr9	19425534	19427534	Zfp317	-0.11429391	0.61959	insignificant	0.017179	0.9468	insignificant	3	31	31
chr9	20189602	20191602	Zfp560	-0.05311108	0.0020832	hypomethylated	0.017673	0.10778	insignificant	14	53	52
chr9	20297190	20299190	Zfp426	-0.15480673	0.00000796	hypomethylated	-0.00067453	0.1783	insignificant	6	28	28
chr9	20325863	20327863	Zfp266	-0.21680827	0.000099747	hypomethylated	0.010923	0.80861	insignificant	9	50	44
chr9	20384771	20386771	Zfp846	-0.18337098	0.13469	insignificant	-0.035627	0.27942	insignificant	6	14	14
chr9	20410918	20412918	5730577I03Ril	-0.12697814	7.86E-10	hypomethylated	-0.026787	0.58659	insignificant	17	39	38
chr9	20446761	20448761	Ubl5	-0.15074487	0.0065671	hypomethylated	0.027733	0.068112	insignificant	10	53	50
chr9	20447250	20449250	Fbxl12	-0.2492952	0.00000352	hypomethylated	0.0043413	0.72566	insignificant	10	43	40
chr9	20449211	20451211	Ubl5	-0.56378814	1.61E-32	stronglyHypometh	-0.042596	1	insignificant	12	26	27
chr9	20455573	20457573	Pin1	-0.17680407	5.86E-09	hypomethylated	0.03693	0.19585	insignificant	18	56	56
chr9	20619478	20621478	Col5a3	-0.17731933	0.10641	insignificant	0.0035322	0.060215	insignificant	8	34	32
chr9	20672085	20674085	A230050P20R	0.01796592	0.18423	insignificant	0.01419	0.55409	insignificant	19	82	81
chr9	20691618	20693618	Ppan	-0.03575468	0.31744	insignificant	0.027152	3.6E-28	hypermethylated	19	92	86
chr9	20757067	20759067	Dnmt1	0.00706807	0.00064997	hypermethylated	0.098447	1.04E-15	hypermethylated	15	36	36
chr9	20781237	20783237	S1pr2	-0.22747036	0.0050715	hypomethylated	-0.076294	0.0059365	inconclusive	2	11	10
chr9	20806180	20808180	Mrpl4	-0.18167215	2.61E-16	hypomethylated	0.0049947	0.75762	insignificant	21	90	81
chr9	20819403	20821403	Icam1	-0.23626071	0.00010329	hypomethylated	0.027317	0.51872	insignificant	8	38	31
chr9	20832816	20834816	Icam4	-0.26677418	5.71E-29	hypomethylated	0.0262	0.0060801	hypermethylated	13	52	51
chr9	20835481	20837481	Icam5	-0.2060129	1.38E-09	hypomethylated	-0.033053	0.35463	insignificant	12	41	40
chr9	20836772	20838772	Icam5	-0.19920864	1.45E-13	hypomethylated	-0.05406	0.47822	insignificant	11	62	58
chr9	20871537	20873537	Fdx1l	-0.00284621	0.44012	insignificant	0.0048996	0.32415	insignificant	2	36	36
chr9	20877958	20879958	Fdx1l	-0.15712821	0.000060461	hypomethylated	0.091212	0.60631	insignificant	5	15	22
chr9	20896432	20898432	Raver1	-0.2457265	0.0016242	hypomethylated	0.048601	0.909	insignificant	6	24	25
chr9	20954350	20956350	Cdc37	-0.2145509	1	insignificant	-0.074677	0.94724	insignificant	5	32	29
chr9	20969157	20971157	Pde4a	-0.11051089	3.8E-16	hypomethylated	0.013715	0.88278	insignificant	34	107	97
chr9	21043776	21045776	Keap1	-0.36231488	0.00064777	stronglyHypometh	0.0474	0.3389	insignificant	2	8	8
chr9	21052887	21054887	S1pr5	-0.31360065	0.00000115	hypomethylated	-0.031341	0.20358	insignificant	11	48	50
chr9	21068742	21070742	Atg4d	-0.16310869	7.02E-35	hypomethylated	-0.0051946	0.41846	insignificant	22	36	36
chr9	21116777	21118777	Ap1m2	0.02789463	0.83063	insignificant	0.05979	0.27549	insignificant	5	35	35
chr9	21124162	21126162	Slc44a2	-0.17483779	0.0032939	hypomethylated	-0.0053278	1	insignificant	8	32	32
chr9	21141271	21143271	Slc44a2	-0.18458774	0.0026948	hypomethylated	-0.028521	0.38904	insignificant	3	14	18
chr9	21171446	21173446	Ilf3	-0.11425525	1.82E-10	hypomethylated	-0.00056305	0.28454	insignificant	21	97	97
chr9	21215280	21217280	Qtrt1	-0.1970267	0.000033925	hypomethylated	0.042154	0.14278	insignificant	14	76	74
chr9	21228388	21230388	Dnm2	-0.21376263	0.014032	hypomethylated	0.042639	0.004418	hypermethylated	15	92	67
chr9	21314630	21316630	Tmed1	-0.46322805	0.00035	stronglyHypometh	0.095629	0.0011974	inconclusive	3	29	24
chr9	21329620	21331620	AB124611		1	noCoverage	-0.021778	0.64043	insignificant	0	9	10
chr9	21350337	21352337	Carm1	-0.09891714	3.12E-21	hypomethylated	0.0034992	0.0052404	hypermethylated	55	226	190
chr9	21396165	21398165	1810026J23Ri	-0.11679846	7.57E-64	hypomethylated	0.0075715	0.36039	insignificant	37	156	139
chr9	21397190	21399190	1810026J23Ri	-0.12448209	1.82E-74	hypomethylated	0.015702	0.36279	insignificant	40	166	151
chr9	21412773	21414773	Gm7904	0.0324452	0.62897	insignificant	0.038326	0.197	insignificant	3	14	15
chr9	21419612	21421612	Smarca4	-0.23366979	0.0057303	hypomethylated	-0.017921	0.0019255	hypomethylated	10	58	68
chr9	21527037	21529037	Ldlr	-0.16709875	9.53E-10	hypomethylated	-0.0088024	0.75336	insignificant	13	71	71
chr9	21602990	21604990	Kank2	-0.09185795	0.00000154	hypomethylated	0.0099958	0.63813	insignificant	12	53	57
chr9	21638953	21640953	Gm6484	-0.51623932	7.28E-11	stronglyHypometh	-0.10348	0.082984	insignificant	4	9	9
chr9	21657079	21659079	Dock6	-0.06685399	6.33E-08	hypomethylated	0.0059961	0.73866	insignificant	15	47	48
chr9	21722565	21724565	Rab3d	-0.28025472	1.79E-09	hypomethylated	0.059556	0.48521	insignificant	5	28	28
chr9	21730914	21732914	Ccdc159	-0.35966509	1.32E-10	stronglyHypometh	-0.031826	0.16446	insignificant	9	35	34
chr9	21731906	21733906	Tmem205	-0.65257263	0.0081857	stronglyHypometh	-0.097365	0.014048	hypomethylated	4	22	20
chr9	21759196	21761196	2310047B19R	-0.22427551	0.017917	hypomethylated	-0.053364	0.52349	insignificant	3	43	45
chr9	21793897	21795897	Rgl3	-0.22693837	1.16E-11	hypomethylated	-0.024029	0.055215	insignificant	13	62	69
[truncated: 172,492 more chars]
